# Supplementary figures and images for: Dissemination of antibiotic resistance genes associated with the sporobiota in sediments impacted by wastewater
Source: PeerJ. 2018 Jun 20;6:e4989. doi: 10.7717/peerj.4989 (PMC6015491; doi:10.7717/peerj.4989)

A.

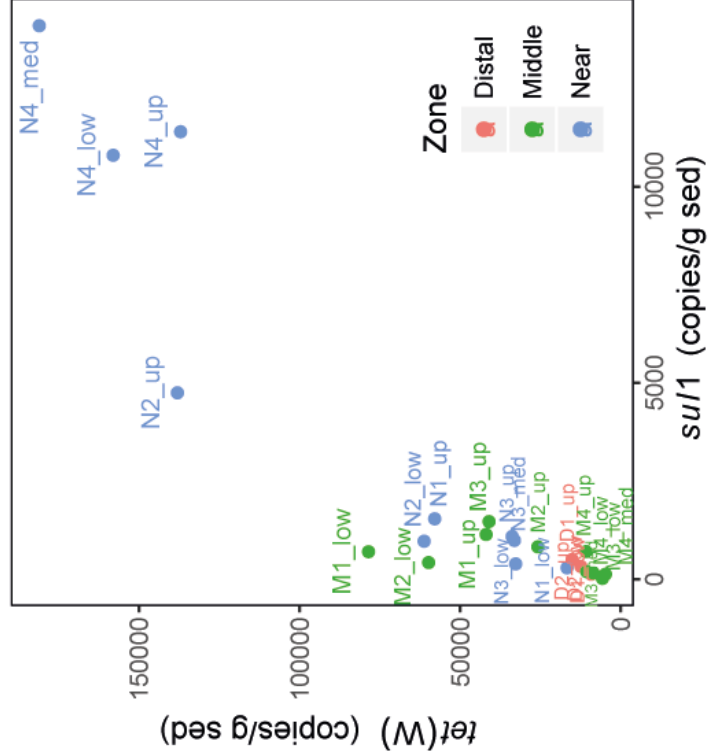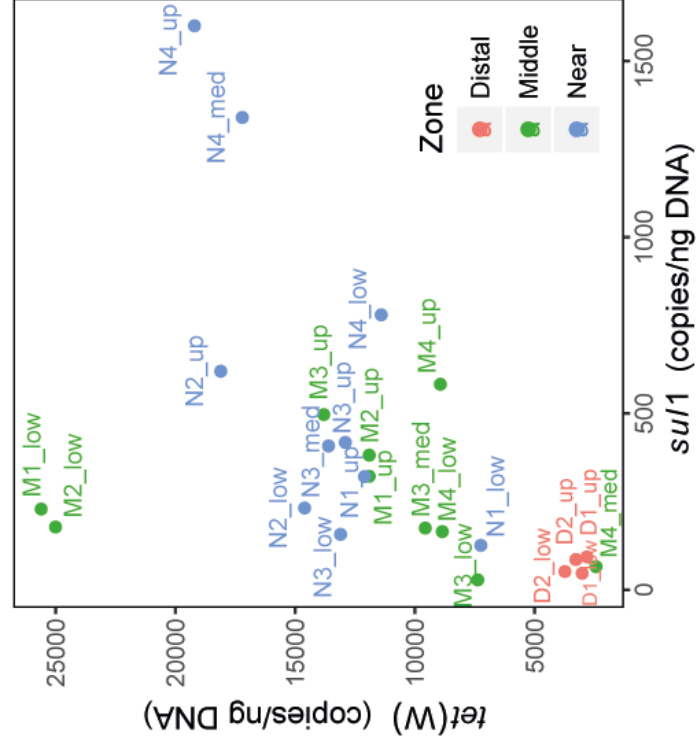

B.

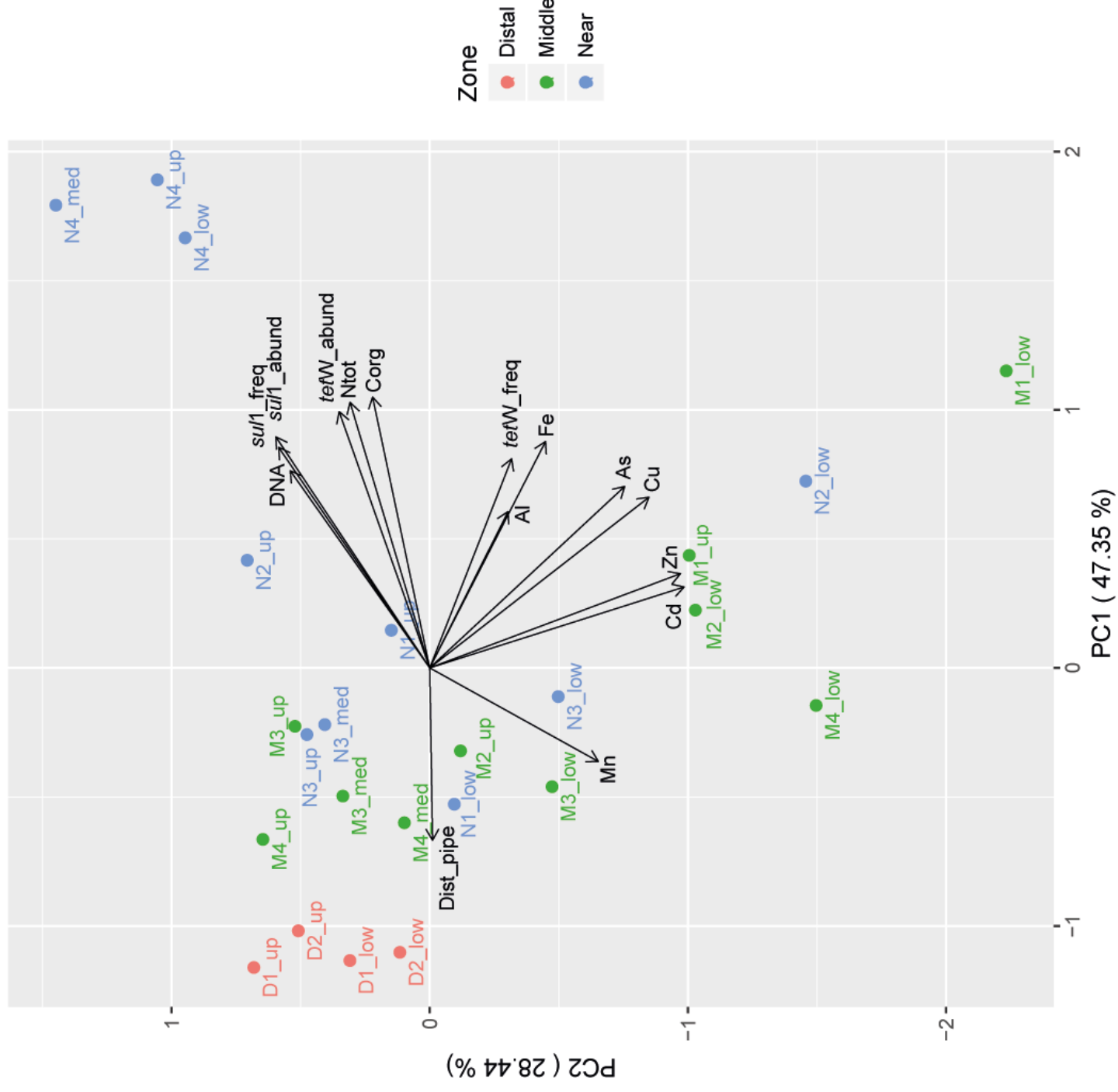

Supplement: Figure S1 — (A) Biplot representing the ARG abundance (copies/g sediment, above) and frequency (copies/ng DNA, below) in each samples. (B) Principal component analysis (PCA, scaling 2) on the environmental parameters, including Corg, Ntot, trace and heavy metals (TMs), the distance to the outlet pipe (Dist_pipe), DNA abundance in ng/g sediment (DNA_ng_gSed), and ARG abundance (cp_gSed) and frequency (cp_ng). Arrows represent the contribution of variables to the axis, and angles between variables reflect their correlations. Colors correspond to the three sampling zone: near, middle and distal. Values for Corg, Ntot, and TMs were obtained from previous studies (Bueche, 2014; Sauvain et al., 2014) . [file peerj-06-4989-s001.pdf]

## A. *tet(W)*

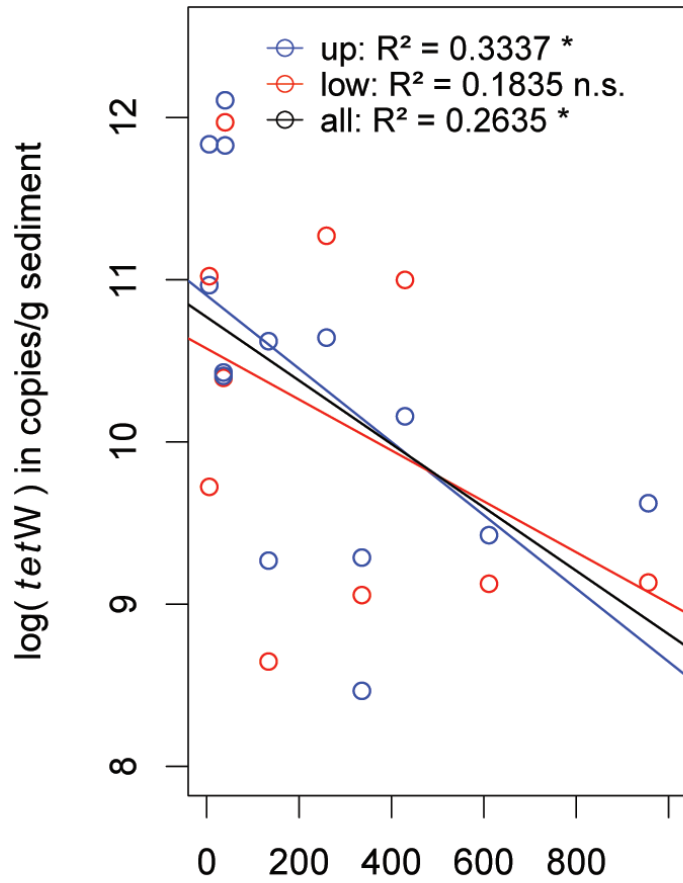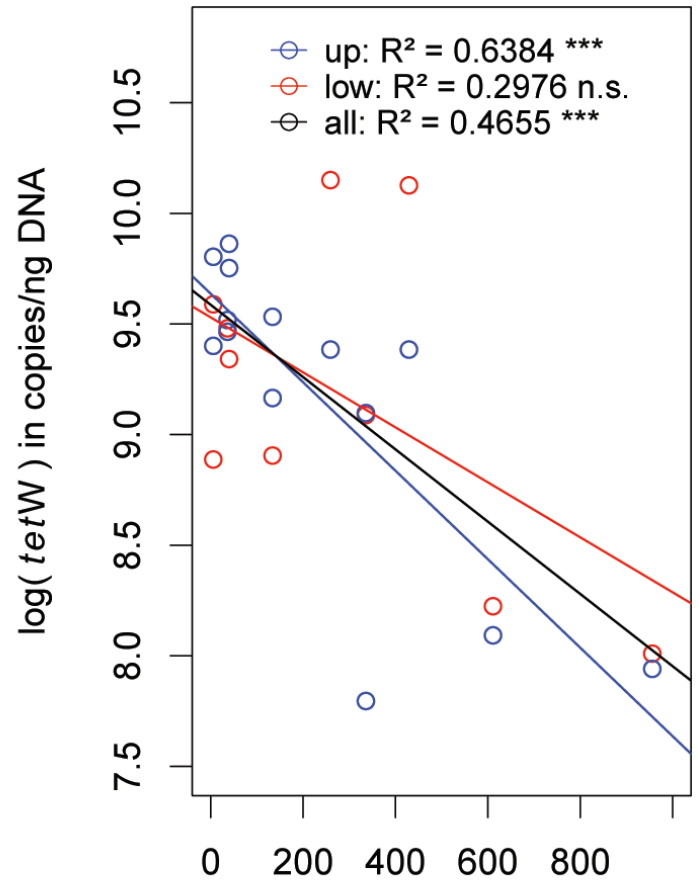

## B. *su1*

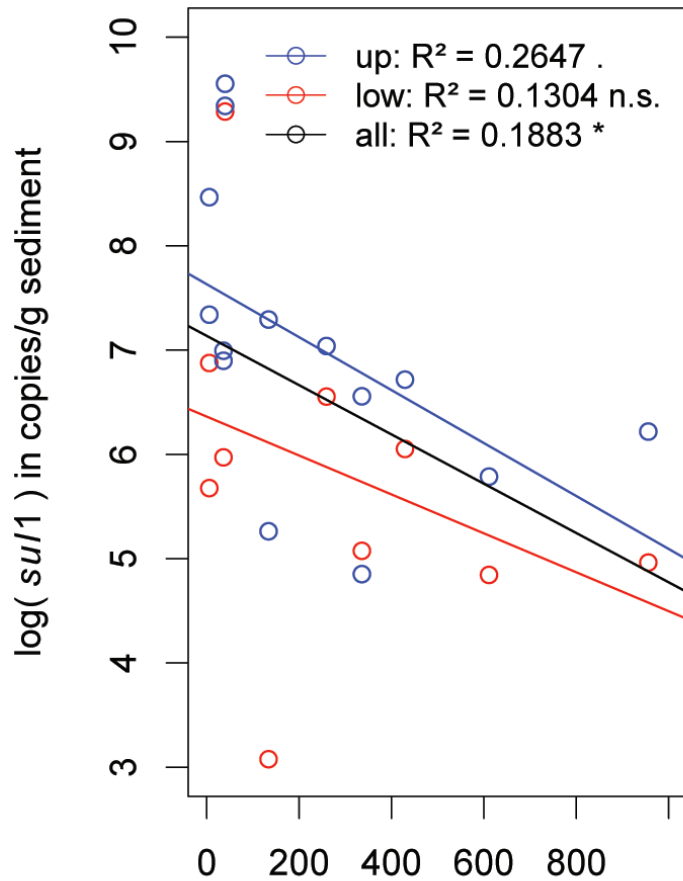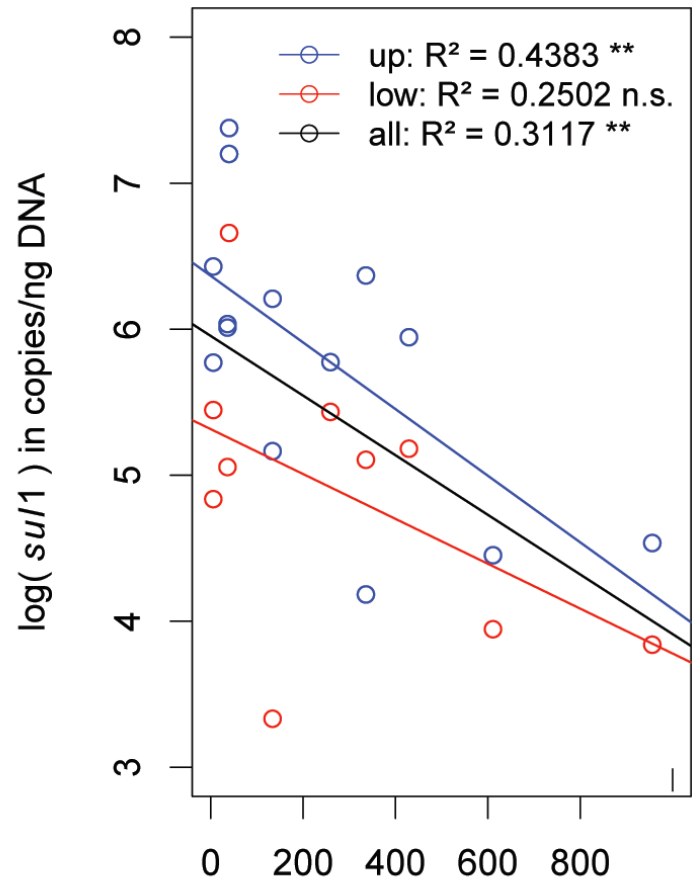

Distance to the pipe - all directions (m)

Supplement: Figure S2 — Linear regressions of log-transformed ARG abundance (left) and frequency (right), on the distance to the WWTP outlet pipe (m). Linear regressions calculated for (A) tet(W) and (B) sul1. Blue circles and lines correspond to the samples from the upper sediment. Red circles and lines correspond to the samples from the lower sediment. Black lines correspond to samples all together. Significance of the models was tested by analysis of variance (ANOVA). Significance codes of p-values: 0 < *** < 0.00. (n.s.) stands for “not significant”. [file peerj-06-4989-s002.pdf]

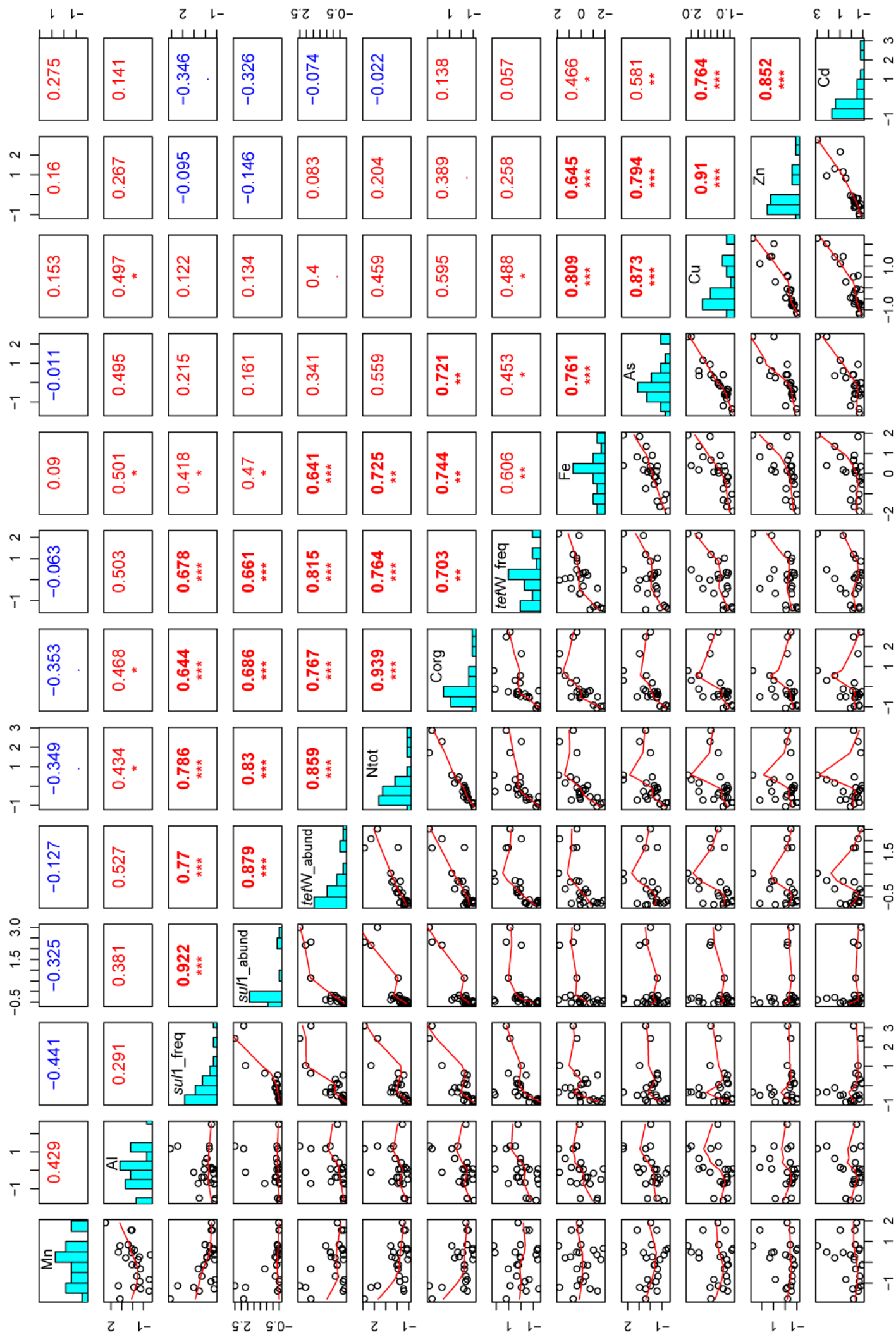

Supplement: Figure S3 — Values for Corg, Ntot, and TMs were obtained from previous studies (Bueche, 2014; Sauvain et al., 2014). Significance codes of p-values: 0 < ∗∗∗ < 0.001 < ∗∗ < 0.01 < ∗ < 0.05 < . < 0.1. [file peerj-06-4989-s003.pdf]

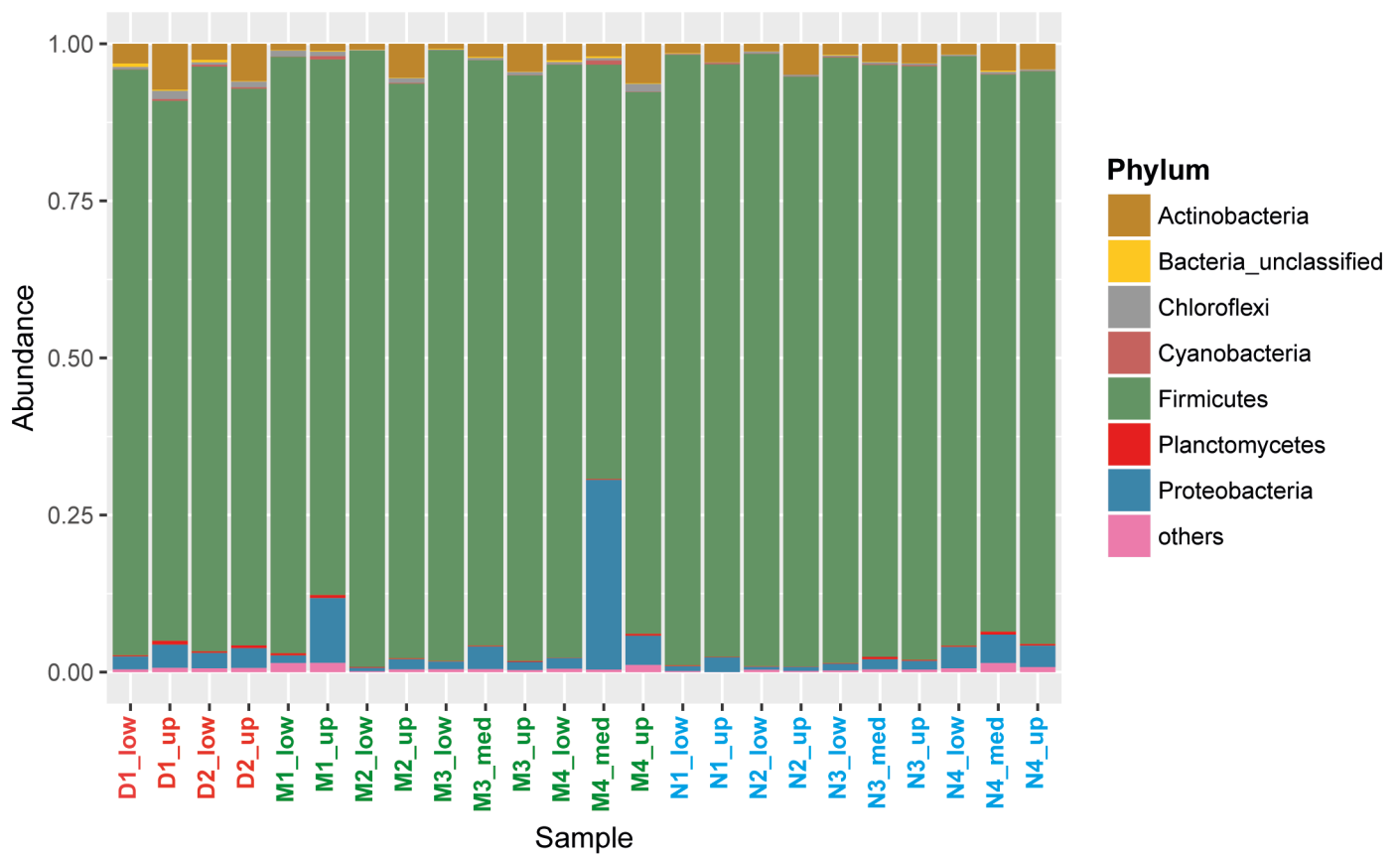

Supplement: Figure S4 — Composition based on 16S rRNA sequencing on spores DNA. Only taxa accounting for at least 0.1% of the total community are shown. Firmicutes was the dominant phylum accounting for at least 85.2% of the sporobiome community. The other main phyla detected corresponded to Proteobacteria (3.81%), Actinobacteria (2.95%), Chloroflexi (0.44%), Planctomycetes (0.22%), and Cyanobacteria (0.16%). [file peerj-06-4989-s004.pdf]
