# Supplementary material for: Dissemination of antibiotic resistance genes associated with the sporobiota in sediments impacted by wastewater
Source: PeerJ. 2018 Jun 20;6:e4989. doi: 10.7717/peerj.4989 (PMC6015491; doi:10.7717/peerj.4989)
Supplement: Supplemental Information 1 — Organic carbon and nitrogen content. Particulate cadmium, copper, iron, manganese, aluminium, zinc and arsenic concentrations in sediments. [file peerj-06-4989-s006.docx]

|  | **Sample** | **Sediment depth** | **Dist. to the pipe** | **Corg** | **Ntot** | **Cd** | **Cu** | **Fe** | **Mn** | **Al** | **Zn** | **As** |
| --- | --- | --- | --- | --- | --- | --- | --- | --- | --- | --- | --- | --- |
|  |  | (cm) | (m) | (%) | (%) | (mg/kg) | (mg/kg) | (g/kg) | (mg/kg) | (g/kg) | (mg/kg) | (mg/kg) |
|  |  |  |  |  |  |  |  |  |  |  |  |  |
|  | D1_low | 3-9 | 955.89 | 2.64 | 0.21 | 1.90 | 114.0 | 19.9 | 387.0 | 13.9 | 322.0 | 10.4 |
|  | D1_up | 0-3 | 955.89 | 2.67 | 0.23 | 0.74 | 75.0 | 18.2 | 408.0 | 16.3 | 170.0 | 7.8 |
|  | D2_low | 3-9 | 610.99 | 2.36 | 0.17 | 2.76 | 137.0 | 22.5 | 396.0 | 9.2 | 542.0 | 9.1 |
|  | D2_up | 0-3 | 610.99 | 2.78 | 0.23 | 0.81 | 81.0 | 24.8 | 405.0 | 15.3 | 205.0 | 8.5 |
|  | M1_low | 3-9 | 259.15 | 7.72 | 0.65 | 10.13 | 362.0 | 47.9 | 413.0 | 20.8 | 1564.0 | 22.0 |
|  | M1_up | 0-3 | 259.15 | 7.00 | 0.53 | 6.27 | 292.0 | 43.5 | 390.0 | 18.6 | 1032.0 | 14.7 |
|  | M2_low | 3-9 | 429.04 | 5.08 | 0.40 | 4.38 | 293.0 | 34.3 | 400.0 | 16.3 | 974.0 | 15.6 |
|  | M2_up | 0-3 | 429.04 | 4.01 | 0.38 | 1.66 | 165.0 | 33.1 | 415.0 | 17.7 | 399.0 | 13.0 |
|  | M3_low | 3-9 | 133.76 | 3.97 | 0.28 | 3.79 | 197.0 | 33.5 | 349.0 | 9.6 | 858.0 | 14.3 |
|  | M3_med | 1.5-3 | 133.76 | 4.40 | 0.41 | 0.77 | 111.0 | 31.6 | 345.0 | 13.2 | 341.0 | 11.7 |
|  | M3_up | 0-1.5 | 133.76 | 4.30 | 0.43 | 0.66 | 116.0 | 30.0 | 355.0 | 14.4 | 338.0 | 11.0 |
|  | M4_low | 3-9 | 335.80 | 3.97 | 0.27 | 5.03 | 266.0 | 39.4 | 443.0 | 16.6 | 1343.0 | 17.6 |
|  | M4_med | 1.5-3 | 335.80 | 4.82 | 0.32 | 2.22 | 137.0 | 27.0 | 331.0 | 12.9 | 526.0 | 14.9 |
|  | M4_up | 0-1.5 | 335.80 | 3.96 | 0.31 | 1.00 | 109.0 | 22.1 | 327.0 | 9.7 | 364.0 | 11.3 |
|  | N1_low | 3-9 | 5.39 | 3.79 | 0.31 | 1.76 | 123.0 | 33.9 | 443.0 | 16.2 | 349.0 | 11.4 |
|  | N1_up | 0-3 | 5.39 | 5.96 | 0.62 | 1.88 | 155.0 | 40.0 | 392.0 | 13.4 | 416.0 | 12.8 |
|  | N2_low | 3-9 | 5.39 | 6.30 | 0.58 | 8.06 | 341.0 | 36.0 | 401.0 | 21.4 | 902.0 | 22.0 |
|  | N2_up | 0-3 | 5.39 | 4.69 | 0.46 | 1.59 | 129.0 | 35.8 | 369.0 | 13.0 | 346.0 | 11.2 |
|  | N3_low | 3-9 | 36.14 | 4.33 | 0.35 | 1.39 | 171.0 | 34.0 | 457.0 | 26.3 | 493.0 | 13.3 |
|  | N3_med | 1.5-3 | 36.14 | 4.69 | 0.45 | 0.11 | 105.0 | 31.5 | 377.0 | 16.2 | 277.0 | 12.1 |
|  | N3_up | 0-1.5 | 36.14 | 4.70 | 0.49 | 0.18 | 111.0 | 28.7 | 376.0 | 14.4 | 268.0 | 12.2 |
|  | N4_low | 3-9 | 39.61 | 10.10 | 1.16 | 1.06 | 221.0 | 47.3 | 334.0 | 15.5 | 489.0 | 16.8 |
|  | N4_med | 1.5-3 | 39.61 | 13.20 | 0.99 | 0.42 | 168.0 | 35.7 | 337.0 | 21.4 | 381.0 | 13.4 |
|  | N4_up | 0-1.5 | 39.61 | 12.50 | 1.33 | 2.04 | 216.0 | 38.2 | 312.0 | 20.8 | 478.0 | 14.9 |
|  |  |  |  |  |  |  |  |  |  |  |  |  |
