# Supplementary material for: Dissemination of antibiotic resistance genes associated with the sporobiota in sediments impacted by wastewater
Source: PeerJ. 2018 Jun 20;6:e4989. doi: 10.7717/peerj.4989 (PMC6015491; doi:10.7717/peerj.4989)
Supplement: Figure S5 — OTUs including less than 4 sequences in the whole dataset were removed from the analysis. OTUs were classified at genus level (or higher taxonomic rank if not possible). Colors correspond to different classes. The 500 most abundant OTUs are shown. [file peerj-06-4989-s005.pdf]

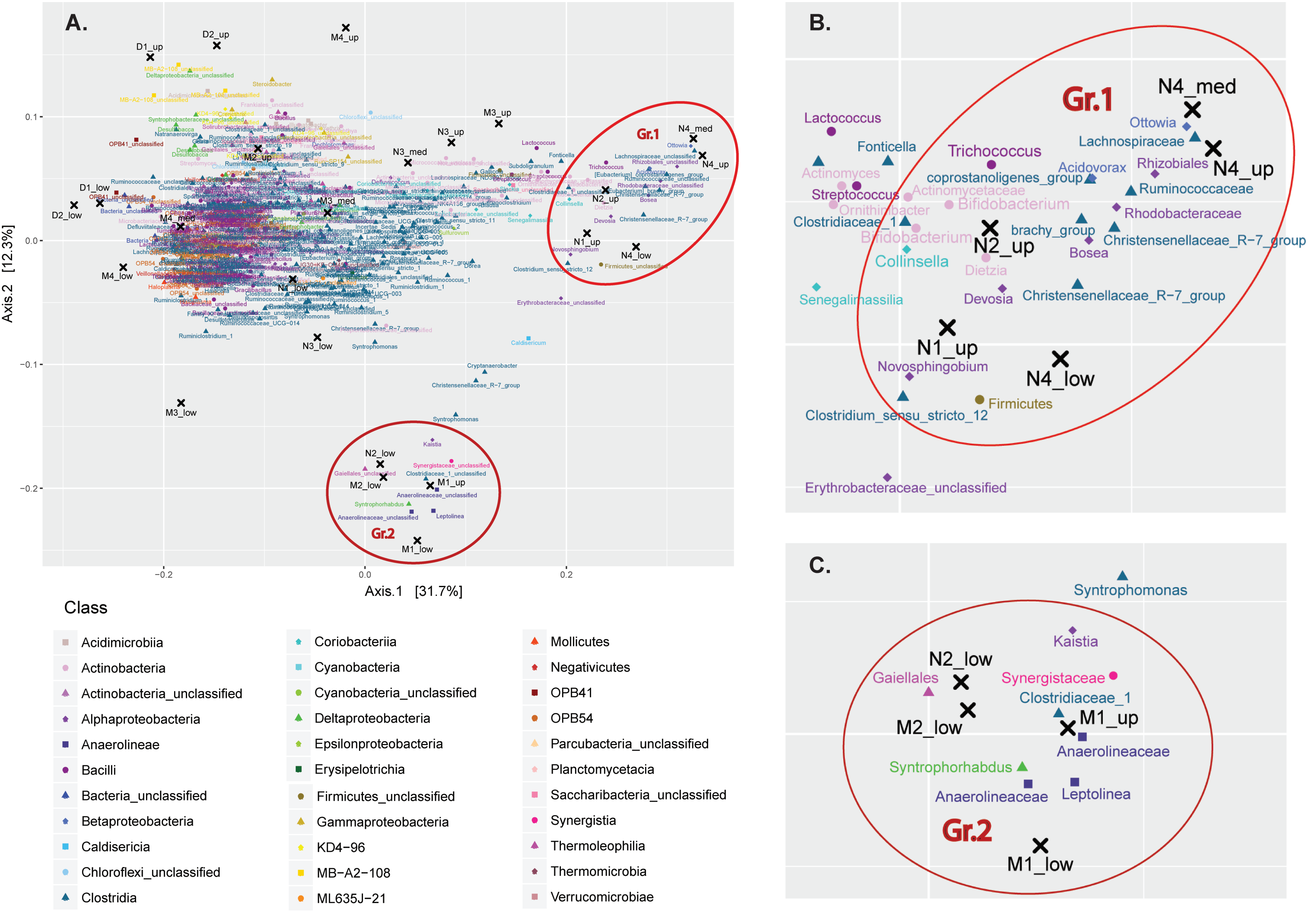
A.
B.
C.
Axis.1 [31.7%]
Axis.2 [12.3%]
Gr.1
Gr.2
N4_med
N4_up
N2_up
N1_up
N4_low
N2_low
M2_low
M1_up
M1_low
Lactococcus
Fonticella
Actinomyces
Streptococcus
Ornithinibacter
Clostridiaceae_1
Trichococcus
coprostanoligenes_group
Actinomycetaceae
Bifidobacterium
Collinsella
Dietzia
Devosia
Senegalimassilia
Ottowia
Lachnospiraceae
Acidovorax
Rhizobiales
Ruminococcaceae
Rhodobacteraceae
brachy_group
Bosea
Christensenellaceae_R-7_group
Novosphingobium
Firmicutes
Clostridium_sensu_stricto_12
Erythrobacteraceae_unclassified
Syntrophomonas
Kaistia
Gaiellales
Synergistaceae
Anaerolineaceae
Syntrophorhabdus
Leptolinea
Class
Acidimicrobiia
Actinobacteria
Actinobacteria_unclassified
Alphaproteobacteria
Anaerolineae
Bacilli
Bacteria_unclassified
Betaproteobacteria
Caldisericia
Chloroflexi_unclassified
Clostridia
Coriobacteriia
Cyanobacteria
Cyanobacteria_unclassified
Deltaproteobacteria
Epsilonproteobacteria
Erysipelotrichia
Firmicutes_unclassified
Gammaproteobacteria
KD4-96
MB-A2-108
ML635J-21
Mollicutes
Negativicutes
OPB41
OPB54
Parcubacteria_unclassified
Planctomycetacia
Saccharibacteria_unclassified
Synergistia
Thermoleophilia
Thermomicrobia
Verrucomicrobiae
