# Supplementary material for: Dissemination of antibiotic resistance genes associated with the sporobiota in sediments impacted by wastewater
Source: PeerJ. 2018 Jun 20;6:e4989. doi: 10.7717/peerj.4989 (PMC6015491; doi:10.7717/peerj.4989)
Supplement: Supplemental Information 2 — Summary of the Blastp results for the presence of Tet(W) in the genomes of the close relatives to the 10-most positively correlated OTUs in the dataset (Table 2). [file peerj-06-4989-s007.pdf]

BLASTP 2.8.0+
Reference: Stephen F. Altschul, Thomas L. Madden, Alejandro A. Schaffer, Jinghui Zhang, Zheng Zhang, Webb Miller, and David J. Lipman (1997), "Gapped BLAST and PSI-BLAST: a new generation of protein database search programs", Nucleic Acids Res. 25:3389-3402.

Reference for compositional score matrix adjustment: Stephen F. Altschul, John C. Wootton, E. Michael Gertz, Richa Agarwala, Aleksandr Morgulis, Alejandro A. Schaffer, and Yi-Kuo Yu (2005) "Protein database searches using compositionally adjusted substitution matrices", FEBS J. 272:5101-5109.

RID: FP78XMRD016

Database: All non-redundant GenBank CDS translations+PDB+SwissProt+PIR+PRF excluding environmental samples from WGS projects
           152,513,734 sequences; 55,875,586,980 total letters
Query= CAO78565.1 tetracycline resistance protein [Clostridioides difficile]

Length=639

```
Score     E
Sequences producing significant alignments:
(Bits)  Value

WP_063856423.1  MULTISPECIES: tetracycline resistance ribosoma...
1313    0.0
WP_075877655.1  TetM/TetW/TetO/TetS family tetracycline resist...
1311    0.0
WP_087325445.1  TetM/TetW/TetO/TetS family tetracycline resist...
1310    0.0
AMP42199.1  tetracycline resistance protein Tet(W) [uncultured...
1308    0.0
WP_043170315.1  TetM/TetW/TetO/TetS family tetracycline resist...
1308    0.0
WP_033126273.1  MULTISPECIES: TetM/TetW/TetO/TetS family tetra...
1307    0.0
WP_078934043.1  TetM/TetW/TetO/TetS family tetracycline resist...
1305    0.0
WP_063856418.1  tetracycline resistance ribosomal protection p...
1305    0.0
WP_087193190.1  TetM/TetW/TetO/TetS family tetracycline resist...
1302    0.0
WP_032746258.1  TetM/TetW/TetO/TetS family tetracycline resist...
1302    0.0
```

WP_063856417.1 tetracycline resistance ribosomal protection p...
1301   0.0
WP_005876003.1 TetM/TetW/TetO/TetS family tetracycline resist...
1301   0.0
WP_033523205.1 MULTISPECIES: TetM/TetW/TetO/TetS family tetra...
1301   0.0
DAB17102.1 TPA: tetracycline resistance ribosomal protection ...
1300   0.0
WP_066647691.1 TetM/TetW/TetO/TetS family tetracycline resist...
1300   0.0
WP_005924790.1 TetM/TetW/TetO/TetS family tetracycline resist...
1299   0.0
WP_063856414.1 tetracycline resistance ribosomal protection p...
1298   0.0
APO35064.1 ferrous iron transport protein B [uncultured bacte...
1298   0.0
AMP53489.1 TetM-TetW-TetO-TetS [uncultured bacterium]
1298   0.0
AMP51295.1 TetM-TetW-TetO-TetS [uncultured bacterium]
1298   0.0
WP_035010862.1 TetM/TetW/TetO/TetS family tetracycline resist...
1298   0.0
CBL36335.1 small GTP-binding protein domain [butyrate-produci...
1298   0.0
AMP47980.1 TetM-TetW-TetO-TetS [uncultured bacterium]
1297   0.0
WP_013702080.1 TetM/TetW/TetO/TetS family tetracycline resist...
1297   0.0
WP_033493020.1 MULTISPECIES: tetracycline resistance ribosoma...
1297   0.0
APO28712.1 ferrous iron transport protein B [uncultured bacte...
1297   0.0
AMJ37860.1 TetM-TetW-TetO-TetS [uncultured bacterium]
1297   0.0
WP_013411012.1 TetM/TetW/TetO/TetS family tetracycline resist...
1297   0.0
APO30252.1 Ferrous iron transport protein B [uncultured bacte...
1296   0.0
WP_090548148.1 MULTISPECIES: TetM/TetW/TetO/TetS family tetra...
1296   0.0
WP_000691721.1 MULTISPECIES: tetracycline resistance ribosoma...
1296   0.0
APO34064.1 ferrous iron transport protein B [uncultured bacte...
1296   0.0
APO32472.1 ferrous iron transport protein B [uncultured bacte...
1296   0.0
SCJ49094.1 Tetracycline resistance protein tetM from transpos...
1296   0.0
WP_058985878.1 TetM/TetW/TetO/TetS family tetracycline resist...
1296   0.0
WP_058966934.1 TetM/TetW/TetO/TetS family tetracycline resist...
1296   0.0
WP_033163194.1 TetM/TetW/TetO/TetS family tetracycline resist...
1295   0.0

WP_002586627.1 MULTISPECIES: tetracycline resistance ribosoma...
1295   0.0
WP_039689012.1 TetM/TetW/TetO/TetS family tetracycline resist...
1295   0.0
CCA30603.1 tetracycline resistance protein [Clostridioides di...
1295   0.0
WP_087988639.1 TetM/TetW/TetO/TetS family tetracycline resist...
1295   0.0
WP_075576245.1 TetM/TetW/TetO/TetS family tetracycline resist...
1295   0.0
SCI66756.1 Tetracycline resistance protein tetM from transpos...
1295   0.0
SCH27787.1 Tetracycline resistance protein tetM from transpos...
1295   0.0
AMP49021.1 TetM-TetW-TetO-TetS [uncultured bacterium]
1295   0.0
WP_060941960.1 TetM/TetW/TetO/TetS family tetracycline resist...
1295   0.0
CUN67961.1 Tetracycline resistance protein tetM from transpos...
1295   0.0
WP_039199418.1 MULTISPECIES: TetM/TetW/TetO/TetS family tetra...
1295   0.0
WP_063856424.1 tetracycline resistance ribosomal protection p...
1294   0.0
APO30266.1 Ferrous iron transport protein B [uncultured bacte...
1294   0.0
WP_070570683.1 TetM/TetW/TetO/TetS family tetracycline resist...
1294   0.0
AMP52575.1 TetM-TetW-TetO-TetS [uncultured bacterium]
1294   0.0
AMP49255.1 TetM-TetW-TetO-TetS [uncultured bacterium]
1294   0.0
WP_060812403.1 TetM/TetW/TetO/TetS family tetracycline resist...
1294   0.0
WP_056994958.1 MULTISPECIES: TetM/TetW/TetO/TetS family tetra...
1294   0.0
WP_013136942.1 TetM/TetW/TetO/TetS family tetracycline resist...
1294   0.0
WP_008863485.1 TetM/TetW/TetO/TetS family tetracycline resist...
1294   0.0
ABF72129.1 TetW [Trueperella pyogenes]
1294   0.0
WP_102343532.1 TetM/TetW/TetO/TetS family tetracycline resist...
1294   0.0
WP_097800170.1 TetM/TetW/TetO/TetS family tetracycline resist...
1294   0.0
WP_087248211.1 TetM/TetW/TetO/TetS family tetracycline resist...
1294   0.0
WP_078931819.1 TetM/TetW/TetO/TetS family tetracycline resist...
1294   0.0
APO29840.1 Ferrous iron transport protein B [uncultured bacte...
1294   0.0
WP_070739220.1 MULTISPECIES: TetM/TetW/TetO/TetS family tetra...
1294   0.0

AMP47119.1 TetM–TetW–TetO–TetS [uncultured bacterium]
1294   0.0
WP_014078528.1 MULTISPECIES: tetracycline resistance ribosoma...
1294   0.0
AIW80467.1 TetM–TetW–TetO–TetS [uncultured bacterium]
1294   0.0
ACT97610.1 TetW [uncultured organism]
1294   0.0
WP_097782597.1 TetM/TetW/TetO/TetS family tetracycline resist...
1293   0.0
WP_074754411.1 TetM/TetW/TetO/TetS family tetracycline resist...
1293   0.0
WP_071129837.1 TetM/TetW/TetO/TetS family tetracycline resist...
1293   0.0
WP_061870345.1 MULTISPECIES: TetM/TetW/TetO/TetS family tetra...
1293   0.0
AMP57701.1 TetM–TetW–TetO–TetS [uncultured bacterium]
1293   0.0
AMJ35140.1 TetM–TetW–TetO–TetS [uncultured bacterium]
1293   0.0
WP_052792087.1 TetM/TetW/TetO/TetS family tetracycline resist...
1293   0.0
WP_052829461.1 TetM/TetW/TetO/TetS family tetracycline resist...
1293   0.0
WP_034502607.1 TetM/TetW/TetO/TetS family tetracycline resist...
1293   0.0
WP_021612625.1 TetM/TetW/TetO/TetS family tetracycline resist...
1293   0.0
WP_098924880.1 TetM/TetW/TetO/TetS family tetracycline resist...
1293   0.0
WP_070363278.1 TetM/TetW/TetO/TetS family tetracycline resist...
1293   0.0
WP_066860834.1 TetM/TetW/TetO/TetS family tetracycline resist...
1293   0.0
WP_033507052.1 TetM/TetW/TetO/TetS family tetracycline resist...
1293   0.0
WP_009305318.1 MULTISPECIES: tetracycline resistance ribosoma...
1293   0.0
WP_087376933.1 TetM/TetW/TetO/TetS family tetracycline resist...
1293   0.0
WP_090529887.1 TetM/TetW/TetO/TetS family tetracycline resist...
1293   0.0
WP_090152943.1 TetM/TetW/TetO/TetS family tetracycline resist...
1293   0.0
AMP57752.1 TetM–TetW–TetO–TetS [uncultured bacterium]
1293   0.0
AMP53595.1 TetM–TetW–TetO–TetS [uncultured bacterium]
1293   0.0
AMP48954.1 TetM–TetW–TetO–TetS [uncultured bacterium]
1293   0.0
AHH55322.1 tetracycline resistance protein [Streptococcus suis]
1293   0.0
ASQ15577.1 TetW [Bifidobacterium longum subsp. longum]
1292   0.0

WP_087253332.1 TetM/TetW/TetO/TetS family tetracycline resist...
1292   0.0
WP_087384078.1 TetM/TetW/TetO/TetS family tetracycline resist...
1292   0.0
AMP49401.1 TetM-TetW-TetO-TetS [uncultured bacterium]
1292   0.0
WP_025299865.1 TetM/TetW/TetO/TetS family tetracycline resist...
1292   0.0
WP_063856416.1 tetracycline resistance ribosomal protection p...
1292   0.0
APO30931.1 ferrous iron transport protein B [uncultured bacte...
1292   0.0
WP_066661399.1 TetM/TetW/TetO/TetS family tetracycline resist...
1292   0.0
WP_022542861.1 TetM/TetW/TetO/TetS family tetracycline resist...
1292   0.0
APO28864.1 Ferrous iron transport protein B [uncultured bacte...
1291   0.0
WP_072574872.1 TetM/TetW/TetO/TetS family tetracycline resist...
1291   0.0
AMP56620.1 TetM-TetW-TetO-TetS [uncultured bacterium]
1291   0.0
WP_024729649.1 TetM/TetW/TetO/TetS family tetracycline resist...
1291   0.0
WP_087236340.1 TetM/TetW/TetO/TetS family tetracycline resist...
1291   0.0
WP_086131634.1 TetM/TetW/TetO/TetS family tetracycline resist...
1291   0.0
WP_091695423.1 TetM/TetW/TetO/TetS family tetracycline resist...
1291   0.0
AMP49509.1 TetM-TetW-TetO-TetS [uncultured bacterium]
1291   0.0
WP_003672618.1 tetracycline resistance ribosomal protection p...
1291   0.0
WP_003819032.1 TetM/TetW/TetO/TetS family tetracycline resist...
1291   0.0
WP_105305034.1 TetM/TetW/TetO/TetS family tetracycline resist...
1291   0.0
WP_099570919.1 TetM/TetW/TetO/TetS family tetracycline resist...
1291   0.0
WP_063856425.1 tetracycline resistance ribosomal protection p...
1291   0.0
APO35541.1 ferrous iron transport protein B [uncultured bacte...
1291   0.0
AMP56769.1 TetM-TetW-TetO-TetS [uncultured bacterium]
1291   0.0
AMP56061.1 TetM-TetW-TetO-TetS [uncultured bacterium]
1291   0.0
AMP51613.1 TetM-TetW-TetO-TetS [uncultured bacterium]
1291   0.0
WP_004217777.1 tetracycline resistance ribosomal protection p...
1290   0.0
WP_085380940.1 TetM/TetW/TetO/TetS family tetracycline resist...
1290   0.0

AMP55046.1 TetM-TetW-TetO-TetS [uncultured bacterium]
1290   0.0
WP_098045504.1 MULTISPECIES: TetM/TetW/TetO/TetS family tetra...
1290   0.0
WP_098036734.1 MULTISPECIES: TetM/TetW/TetO/TetS family tetra...
1290   0.0
WP_087236767.1 TetM/TetW/TetO/TetS family tetracycline resist...
1290   0.0
WP_087363777.1 TetM/TetW/TetO/TetS family tetracycline resist...
1290   0.0
AMJ37421.1 TetM-TetW-TetO-TetS [uncultured bacterium]
1290   0.0
WP_080907810.1 TetM/TetW/TetO/TetS family tetracycline resist...
1289   0.0
WP_071573697.1 TetM/TetW/TetO/TetS family tetracycline resist...
1289   0.0
WP_069988272.1 MULTISPECIES: TetM/TetW/TetO/TetS family tetra...
1289   0.0
SCI17595.1 Tetracycline resistance protein tetM from transpos...
1289   0.0
ACT97618.1 tetracycline resistance protein tetW [uncultured o...
1289   0.0
ACT97617.1 tetracycline resistance protein tetW [uncultured o...
1289   0.0
APO28173.1 ferrous iron transport protein B [uncultured bacte...
1289   0.0
WP_072369412.1 TetM/TetW/TetO/TetS family tetracycline resist...
1289   0.0
WP_063164311.1 TetM/TetW/TetO/TetS family tetracycline resist...
1289   0.0
AMP53586.1 TetM-TetW-TetO-TetS [uncultured bacterium]
1289   0.0
CUR43792.1 Tetracycline resistance protein TetW [Lactobacillu...
1289   0.0
WP_087284258.1 TetM/TetW/TetO/TetS family tetracycline resist...
1288   0.0
ACA23181.1 TetW [Bifidobacterium longum]
1288   0.0
WP_070691289.1 TetM/TetW/TetO/TetS family tetracycline resist...
1288   0.0
AMP58041.1 TetM-TetW-TetO-TetS [uncultured bacterium]
1288   0.0
AMP55465.1 TetM-TetW-TetO-TetS [uncultured bacterium]
1288   0.0
WP_046921744.1 TetM/TetW/TetO/TetS family tetracycline resist...
1288   0.0
WP_098045951.1 TetM/TetW/TetO/TetS family tetracycline resist...
1288   0.0
AMP54790.1 TetM-TetW-TetO-TetS [uncultured bacterium]
1288   0.0
AMP53110.1 TetM-TetW-TetO-TetS [uncultured bacterium]
1288   0.0
AMP52280.1 TetM-TetW-TetO-TetS [uncultured bacterium]
1288   0.0

WP_014566327.1 TetM/TetW/TetO/TetS family tetracycline resist...
1288   0.0
WP_107208979.1 TetM/TetW/TetO/TetS family tetracycline resist...
1288   0.0
WP_101693181.1 TetM/TetW/TetO/TetS family tetracycline resist...
1288   0.0
WP_098047066.1 TetM/TetW/TetO/TetS family tetracycline resist...
1288   0.0
WP_087713428.1 MULTISPECIES: TetM/TetW/TetO/TetS family tetra...
1288   0.0
WP_087190428.1 TetM/TetW/TetO/TetS family tetracycline resist...
1288   0.0
AMP54855.1 TetM-TetW-TetO-TetS [uncultured bacterium]
1288   0.0
WP_098035490.1 MULTISPECIES: TetM/TetW/TetO/TetS family tetra...
1287   0.0
WP_086118050.1 TetM/TetW/TetO/TetS family tetracycline resist...
1287   0.0
AMP52265.1 TetM-TetW-TetO-TetS [uncultured bacterium]
1287   0.0
WP_105303201.1 TetM/TetW/TetO/TetS family tetracycline resist...
1287   0.0
WP_006500706.1 TetM/TetW/TetO/TetS family tetracycline resist...
1287   0.0
WP_013642473.1 TetM/TetW/TetO/TetS family tetracycline resist...
1287   0.0
WP_098039738.1 TetM/TetW/TetO/TetS family tetracycline resist...
1286   0.0
WP_095183509.1 TetM/TetW/TetO/TetS family tetracycline resist...
1286   0.0
WP_089145870.1 MULTISPECIES: TetM/TetW/TetO/TetS family tetra...
1286   0.0
WP_089150173.1 TetM/TetW/TetO/TetS family tetracycline resist...
1286   0.0
WP_052543100.1 tetracycline resistance ribosomal protection p...
1286   0.0
WP_065991006.1 TetM/TetW/TetO/TetS family tetracycline resist...
1286   0.0
WP_063856415.1 tetracycline resistance ribosomal protection p...
1285   0.0
WP_016497304.1 TetM/TetW/TetO/TetS family tetracycline resist...
1285   0.0
WP_090265483.1 TetM/TetW/TetO/TetS family tetracycline resist...
1285   0.0
AMP50237.1 TetM-TetW-TetO-TetS [uncultured bacterium]
1285   0.0
WP_086143532.1 TetM/TetW/TetO/TetS family tetracycline resist...
1285   0.0
WP_003695455.1 TetM/TetW/TetO/TetS family tetracycline resist...
1285   0.0
WP_089150426.1 TetM/TetW/TetO/TetS family tetracycline resist...
1284   0.0
WP_086120764.1 TetM/TetW/TetO/TetS family tetracycline resist...
1284   0.0

WP_090862004.1 TetM/TetW/TetO/TetS family tetracycline resist...
1284   0.0
WP_070628949.1 TetM/TetW/TetO/TetS family tetracycline resist...
1284   0.0
WP_086132636.1 TetM/TetW/TetO/TetS family tetracycline resist...
1284   0.0
ABF72138.1 TetW [Trueperella pyogenes]
1284   0.0
WP_089147928.1 MULTISPECIES: TetM/TetW/TetO/TetS family tetra...
1283   0.0
WP_065436612.1 TetM/TetW/TetO/TetS family tetracycline resist...
1283   0.0
WP_089143419.1 MULTISPECIES: TetM/TetW/TetO/TetS family tetra...
1283   0.0
WP_086141728.1 TetM/TetW/TetO/TetS family tetracycline resist...
1283   0.0
WP_086141437.1 TetM/TetW/TetO/TetS family tetracycline resist...
1283   0.0
APO34595.1 ferrous iron transport protein B Edit: TetW [uncul...
1283   0.0
WP_033524005.1 TetM/TetW/TetO/TetS family tetracycline resist...
1283   0.0
APO33185.1 ferrous iron transport protein B [uncultured bacte...
1283   0.0
WP_033510437.1 TetM/TetW/TetO/TetS family tetracycline resist...
1282   0.0
WP_031474227.1 MULTISPECIES: TetM/TetW/TetO/TetS family tetra...
1282   0.0
WP_049342005.1 TetM/TetW/TetO/TetS family tetracycline resist...
1282   0.0
AMP58106.1 TetM-TetW-TetO-TetS [uncultured bacterium]
1281   0.0
AMP55886.1 TetM-TetW-TetO-TetS [uncultured bacterium]
1281   0.0
WP_050618045.1 MULTISPECIES: tetracycline resistance ribosoma...
1281   0.0
WP_086143304.1 TetM/TetW/TetO/TetS family tetracycline resist...
1281   0.0
WP_004010384.1 MULTISPECIES: TetM/TetW/TetO/TetS family tetra...
1281   0.0
WP_024532698.1 TetM/TetW/TetO/TetS family tetracycline resist...
1280   0.0
WP_049362095.1 MULTISPECIES: TetM/TetW/TetO/TetS family tetra...
1279   0.0
SCI53441.1 Tetracycline resistance protein tetM from transpos...
1278   0.0
AMP54005.1 TetM-TetW-TetO-TetS [uncultured bacterium]
1278   0.0
WP_055251078.1 TetM/TetW/TetO/TetS family tetracycline resist...
1278   0.0
WP_070648983.1 TetM/TetW/TetO/TetS family tetracycline resist...
1278   0.0
WP_070593944.1 TetM/TetW/TetO/TetS family tetracycline resist...
1277   0.0

```
WP_049353247.1  TetM/TetW/TetO/TetS family tetracycline resist...
1277    0.0
WP_070643840.1  TetM/TetW/TetO/TetS family tetracycline resist...
1276    0.0
WP_019541681.1  TetM/TetW/TetO/TetS family tetracycline resist...
1276    0.0
AMP54009.1  TetM-TetW-TetO-TetS [uncultured bacterium]
1275    0.0
WP_070824660.1  MULTISPECIES: TetM/TetW/TetO/TetS family tetra...
1274    0.0
WP_066305045.1  TetM/TetW/TetO/TetS family tetracycline resist...
1274    0.0
WP_042433210.1  TetM/TetW/TetO/TetS family tetracycline resist...
1274    0.0
WP_070738148.1  TetM/TetW/TetO/TetS family tetracycline resist...
1273    0.0
WP_075843486.1  MULTISPECIES: TetM/TetW/TetO/TetS family tetra...
1272    0.0
AMP42228.1  tetracycline resistance protein Tet(W) [uncultured...
1272    0.0
WP_009141881.1  TetM/TetW/TetO/TetS family tetracycline resist...
1272    0.0
WP_075279296.1  TetM/TetW/TetO/TetS family tetracycline resist...
1271    0.0
WP_070633855.1  TetM/TetW/TetO/TetS family tetracycline resist...
1271    0.0
WP_099432720.1  TetM/TetW/TetO/TetS family tetracycline resist...
1270    0.0
WP_068539726.1  MULTISPECIES: TetM/TetW/TetO/TetS family tetra...
1270    0.0
WP_005987406.1  TetM/TetW/TetO/TetS family tetracycline resist...
1270    0.0
APO33657.1  ferrous iron transport protein B [uncultured bacte...
1269    0.0
WP_102240720.1  TetM/TetW/TetO/TetS family tetracycline resist...
1269    0.0
APO35550.1  ferrous iron transport protein B [uncultured bacte...
1269    0.0
WP_063856422.1  MULTISPECIES: tetracycline resistance ribosoma...
1268    0.0
AMP49825.1  TetM-TetW-TetO-TetS [uncultured bacterium]
1268    0.0
WP_029731081.1  TetM/TetW/TetO/TetS family tetracycline resist...
1267    0.0
WP_073324048.1  TetM/TetW/TetO/TetS family tetracycline resist...
1266    0.0
APO33653.1  ferrous iron transport protein B [uncultured bacte...
1262    0.0
AAR29969.1  mosaic tetracycline-resistance protein [Megasphaer...
1261    0.0
WP_066743970.1  TetM/TetW/TetO/TetS family tetracycline resist...
1259    0.0
AMP53584.1  TetM-TetW-TetO-TetS [uncultured bacterium]
1248    0.0
```

CAP09895.1 mosaic tetracycline resistance protein [Bifidobact...
1247   0.0
CAP09892.1 mosaic tetracycline resistance protein [Bifidobact...
1246   0.0
AMP42147.1 tetracycline resistance protein Tet(W/N/W) [uncult...
1242   0.0
AMP51298.1 TetM-TetW-TetO-TetS [uncultured bacterium]
1242   0.0
CAP09894.1 mosaic tetracycline resistance protein [Bifidobact...
1242   0.0
ALE37170.1 tetracycline resistance protein TetW [Bifidobacter...
1241   0.0
CAP09896.1 mosaic tetracycline resistance protein [Bifidobact...
1241   0.0
AMP55635.1 TetM-TetW-TetO-TetS [uncultured bacterium]
1239   0.0
WP_080908661.1 TetM/TetW/TetO/TetS family tetracycline resist...
1239   0.0
WP_102815896.1 TetM/TetW/TetO/TetS family tetracycline resist...
1238   0.0
WP_052822407.1 TetM/TetW/TetO/TetS family tetracycline resist...
1237   0.0
WP_075704061.1 TetM/TetW/TetO/TetS family tetracycline resist...
1237   0.0
WP_055244167.1 MULTISPECIES: TetM/TetW/TetO/TetS family tetra...
1237   0.0
WP_070637913.1 TetM/TetW/TetO/TetS family tetracycline resist...
1235   0.0
CAP09893.1 mosaic tetracycline resistance protein [Bifidobact...
1234   0.0
WP_101549584.1 TetM/TetW/TetO/TetS family tetracycline resist...
1229   0.0
WP_026385517.1 TetM/TetW/TetO/TetS family tetracycline resist...
1222   0.0
KFI80889.1 tetracycline resistance protein [Bifidobacterium p...
1219   0.0
WP_055161581.1 MULTISPECIES: TetM/TetW/TetO/TetS family tetra...
1217   0.0
WP_077597359.1 TetM/TetW/TetO/TetS family tetracycline resist...
1216   0.0
WP_054336482.1 tetracycline resistance ribosomal protection p...
1215   0.0
PKU93883.1 tetracycline resistance protein [Bifidobacterium p...
1210   0.0
WP_063856421.1 tetracycline resistance ribosomal protection p...
1207   0.0
PKU99030.1 tetracycline resistance protein [Bifidobacterium p...
1206   0.0
KOP61953.1 tetracycline resistance protein [Bifidobacterium l...
1206   0.0
WP_086129278.1 TetM/TetW/TetO/TetS family tetracycline resist...
1197   0.0
WP_024963478.1 tetracycline resistance ribosomal protection p...
1189   0.0

AAO42741.1 tetracycline resistance protein [Megasphaera elsde...
1184   0.0
WP_016230557.1 TetM/TetW/TetO/TetS family tetracycline resist...
1174   0.0
WP_031583918.1 TetM/TetW/TetO/TetS family tetracycline resist...
1172   0.0
WP_050625029.1 MULTISPECIES: TetM/TetW/TetO/TetS family tetra...
1170   0.0
WP_011526261.1 MULTISPECIES: tetracycline resistance ribosoma...
1169   0.0
WP_070835237.1 TetM/TetW/TetO/TetS family tetracycline resist...
1166   0.0
WP_096920879.1 TetM/TetW/TetO/TetS family tetracycline resist...
1165   0.0
ACX71625.1 tetracycline resistance protein [Streptococcus suis]
1162   0.0
CBK96064.1 small GTP-binding protein domain [[[Eubacterium] ...
1159   0.0
AMP53220.1 TetM-TetW-TetO-TetS [uncultured bacterium]
1142   0.0
EQI16708.1 tetracycline resistance protein tetM [Clostridioid...
1141   0.0
CAM97383.1 mosaic tetracycline resistance protein [Bifidobact...
1134   0.0
APO27506.1 EF-G: translation elongation factor [uncultured ba...
1125   0.0
WP_063856419.1 tetracycline resistance ribosomal protection p...
1122   0.0
KFI71207.1 TetW [Bifidobacterium merycicum]
1120   0.0
WP_063856420.1 tetracycline resistance ribosomal protection p...
1119   0.0
WP_044674422.1 TetM/TetW/TetO/TetS family tetracycline resist...
1116   0.0
ACJ38544.1 tetracycline resistance protein [Trueperella pyoge...
1116   0.0
WP_105136801.1 TetM/TetW/TetO/TetS family tetracycline resist...
1115   0.0
WP_027971259.1 MULTISPECIES: TetM/TetW/TetO/TetS family tetra...
1114   0.0
KFI69516.1 Tetracycline resistance protein tetW [Bifidobacter...
1112   0.0
WP_029176297.1 TetM/TetW/TetO/TetS family tetracycline resist...
1112   0.0
WP_038817059.1 MULTISPECIES: TetM/TetW/TetO/TetS family tetra...
1112   0.0
WP_029177869.1 TetM/TetW/TetO/TetS family tetracycline resist...
1112   0.0
WP_105207356.1 TetM/TetW/TetO/TetS family tetracycline resist...
1111   0.0
WP_107209023.1 TetM/TetW/TetO/TetS family tetracycline resist...
1110   0.0
WP_024408858.1 TetM/TetW/TetO/TetS family tetracycline resist...
1109   0.0

WP_013646121.1 MULTISPECIES: TetM/TetW/TetO/TetS family tetra...
1108   0.0
ATD53312.1 ribosomal protection tetracycline resistance prote...
1108   0.0
WP_105160480.1 TetM/TetW/TetO/TetS family tetracycline resist...
1107   0.0
WP_105126631.1 TetM/TetW/TetO/TetS family tetracycline resist...
1107   0.0
WP_105211840.1 TetM/TetW/TetO/TetS family tetracycline resist...
1106   0.0
ABN79416.1 mosaic tetracycline resistance protein [uncultured...
1106   0.0
WP_052777607.1 TetM/TetW/TetO/TetS family tetracycline resist...
1105   0.0
AMP53218.1 TetM-TetW-TetO-TetS [uncultured bacterium]
1091   0.0
AMP53219.1 TetM-TetW-TetO-TetS [uncultured bacterium]
1082   0.0
AMP49819.1 TetM-TetW-TetO-TetS [uncultured bacterium]
1077   0.0
KXB53642.1 elongation factor Tu GTP binding domain protein [C...
1065   0.0
WP_035300359.1 MULTISPECIES: TetM/TetW/TetO/TetS family tetra...
1061   0.0
ERI78723.1 putative translation elongation factor G [Clostrid...
1060   0.0
APO34062.1 ferrous iron transport protein B [uncultured bacte...
1058   0.0
ALB05595.1 tetracycline resistance protein W [Bifidobacterium...
1055   0.0
ACX71626.1 tetracycline resistance protein [Streptococcus suis]
1034   0.0
AMP50917.1 TetM-TetW-TetO-TetS [uncultured bacterium]
1025   0.0
KFI74536.1 tetracycline resistance protein tetW [Bifidobacter...
1023   0.0
AMP56474.1 TetM-TetW-TetO-TetS [uncultured bacterium]
1013   0.0
WP_070087585.1 MULTISPECIES: TetM/TetW/TetO/TetS family tetra...
972   0.0
APO27487.1 ferrous iron transport protein B [uncultured bacte...
971   0.0
AMP50126.1 TetM-TetW-TetO-TetS [uncultured bacterium]
971   0.0
WP_009266810.1 TetM/TetW/TetO/TetS family tetracycline resist...
966   0.0
APO27459.1 EF-G: translation elongation factor [uncultured ba...
964   0.0
WP_059430671.1 TetM/TetW/TetO/TetS family tetracycline resist...
963   0.0
APO28175.1 ferrous iron transport protein B [uncultured bacte...
959   0.0
SCJ12353.1 Tetracycline resistance protein tetM from transpos...
945   0.0

```
AMP50067.1  TetM-TetW-TetO-TetS [uncultured bacterium]
942     0.0
WP_074390203.1  TetM/TetW/TetO/TetS family tetracycline resist...
941     0.0
WP_055195147.1  TetM/TetW/TetO/TetS family tetracycline resist...
941     0.0
WP_107435213.1  TetM/TetW/TetO/TetS family tetracycline resist...
940     0.0
APO27580.1  ferrous iron transport protein B [uncultured bacte...
939     0.0
WP_053865959.1  TetM/TetW/TetO/TetS family tetracycline resist...
939     0.0
WP_074924842.1  TetM/TetW/TetO/TetS family tetracycline resist...
938     0.0
WP_105310307.1  TetM/TetW/TetO/TetS family tetracycline resist...
937     0.0
WP_078768811.1  TetM/TetW/TetO/TetS family tetracycline resist...
937     0.0
APO28015.1  ferrous iron transport protein B [uncultured bacte...
936     0.0
WP_002602099.1  MULTISPECIES: tetracycline resistance ribosoma...
936     0.0
WP_044761229.1  TetM/TetW/TetO/TetS family tetracycline resist...
935     0.0
WP_055065785.1  TetM/TetW/TetO/TetS family tetracycline resist...
934     0.0
WP_002584856.1  MULTISPECIES: TetM/TetW/TetO/TetS family tetra...
933     0.0
WP_073282779.1  TetM/TetW/TetO/TetS family tetracycline resist...
932     0.0
WP_063725249.1  TetM/TetW/TetO/TetS family tetracycline resist...
932     0.0
AMP47017.1  TetM-TetW-TetO-TetS [uncultured bacterium]
932     0.0
WP_002605705.1  TetM/TetW/TetO/TetS family tetracycline resist...
932     0.0
WP_055257520.1  TetM/TetW/TetO/TetS family tetracycline resist...
932     0.0
WP_105105938.1  TetM/TetW/TetO/TetS family tetracycline resist...
932     0.0
AMP50690.1  TetM-TetW-TetO-TetS [uncultured bacterium]
932     0.0
WP_003505402.1  MULTISPECIES: tetracycline resistance ribosoma...
932     0.0
WP_105153139.1  TetM/TetW/TetO/TetS family tetracycline resist...
931     0.0
WP_075759481.1  TetM/TetW/TetO/TetS family tetracycline resist...
931     0.0
APO32499.1  feoB: ferrous iron transport protein [uncultured b...
931     0.0
WP_070432258.1  TetM/TetW/TetO/TetS family tetracycline resist...
931     0.0
WP_055159350.1  MULTISPECIES: TetM/TetW/TetO/TetS family tetra...
931     0.0
```

WP_044774256.1 TetM/TetW/TetO/TetS family tetracycline resist...
931   0.0
WP_044770894.1 TetM/TetW/TetO/TetS family tetracycline resist...
931   0.0
WP_044677300.1 TetM/TetW/TetO/TetS family tetracycline resist...
931   0.0
WP_024377848.1 TetM/TetW/TetO/TetS family tetracycline resist...
931   0.0
WP_024397217.1 TetM/TetW/TetO/TetS family tetracycline resist...
931   0.0
CDD17739.1 small GTP-binding protein domain [Clostridium sp. ...
931   0.0
WP_000691759.1 MULTISPECIES: tetracycline resistance ribosoma...
931   0.0
SCI83194.1 Tetracycline resistance protein tetM from transpos...
931   0.0
WP_044991838.1 TetM/TetW/TetO/TetS family tetracycline resist...
931   0.0
WP_006858004.1 TetM/TetW/TetO/TetS family tetracycline resist...
931   0.0
WP_002935081.1 TetM/TetW/TetO/TetS family tetracycline resist...
931   0.0
WP_009440328.1 TetM/TetW/TetO/TetS family tetracycline resist...
931   0.0
WP_014636938.1 TetM/TetW/TetO/TetS family tetracycline resist...
931   0.0
WP_014636291.1 MULTISPECIES: tetracycline resistance ribosoma...
931   0.0
APO28702.1 ferrous iron transport protein B [uncultured bacte...
931   0.0
AMP50099.1 TetM-TetW-TetO-TetS [uncultured bacterium]
931   0.0
AMP49403.1 TetM-TetW-TetO-TetS [uncultured bacterium]
931   0.0
WP_055182589.1 TetM/TetW/TetO/TetS family tetracycline resist...
931   0.0
WP_105157116.1 TetM/TetW/TetO/TetS family tetracycline resist...
930   0.0
WP_105145235.1 TetM/TetW/TetO/TetS family tetracycline resist...
930   0.0
AUS90925.1 tetracycline resistance protein TetO [Streptococcu...
930   0.0
SCJ84798.1 Tetracycline resistance protein tetM from transpos...
930   0.0
AMP57970.1 TetM-TetW-TetO-TetS [uncultured bacterium]
930   0.0
AMP51260.1 TetM-TetW-TetO-TetS [uncultured bacterium]
930   0.0
WP_055284540.1 TetM/TetW/TetO/TetS family tetracycline resist...
930   0.0
WP_044776625.1 TetM/TetW/TetO/TetS family tetracycline resist...
930   0.0
WP_008399617.1 MULTISPECIES: TetM/TetW/TetO/TetS family tetra...
930   0.0

```
WP_105158873.1  TetM/TetW/TetO/TetS family tetracycline resist...
930     0.0
WP_087378658.1  MULTISPECIES: TetM/TetW/TetO/TetS family tetra...
930     0.0
WP_076773831.1  TetM/TetW/TetO/TetS family tetracycline resist...
930     0.0
AMP55043.1  TetM-TetW-TetO-TetS [uncultured bacterium]
930     0.0
WP_063856401.1  tetracycline resistance ribosomal protection p...
930     0.0
WP_044772610.1  TetM/TetW/TetO/TetS family tetracycline resist...
930     0.0
WP_044769057.1  TetM/TetW/TetO/TetS family tetracycline resist...
930     0.0
WP_044762341.1  TetM/TetW/TetO/TetS family tetracycline resist...
930     0.0
WP_024413659.1  TetM/TetW/TetO/TetS family tetracycline resist...
930     0.0
WP_024402538.1  TetM/TetW/TetO/TetS family tetracycline resist...
930     0.0
WP_105135974.1  TetM/TetW/TetO/TetS family tetracycline resist...
929     0.0
WP_099778982.1  TetM/TetW/TetO/TetS family tetracycline resist...
929     0.0
WP_074413645.1  TetM/TetW/TetO/TetS family tetracycline resist...
929     0.0
AMP50505.1  TetM-TetW-TetO-TetS [uncultured bacterium]
929     0.0
WP_006427719.1  MULTISPECIES: TetM/TetW/TetO/TetS family tetra...
929     0.0
WP_057485305.1  tetracycline resistance protein tetM [Streptoc...
929     0.0
WP_055160143.1  TetM/TetW/TetO/TetS family tetracycline resist...
929     0.0
WP_029943221.1  TetM/TetW/TetO/TetS family tetracycline resist...
929     0.0
WP_014638231.1  TetM/TetW/TetO/TetS family tetracycline resist...
929     0.0
AUS91022.1  tetracycline resistance protein tetM [Streptococcu...
929     0.0
WP_099779911.1  TetM/TetW/TetO/TetS family tetracycline resist...
929     0.0
AFK31665.1  tetracycline resistance protein O/32/O [Streptococ...
929     0.0
ALA07062.1  tetracycline resistance protein TetO [Streptococcu...
929     0.0
AMP55640.1  TetM-TetW-TetO-TetS [uncultured bacterium]
929     0.0
WP_049496346.1  TetM/TetW/TetO/TetS family tetracycline resist...
929     0.0
WP_028086456.1  MULTISPECIES: TetM/TetW/TetO/TetS family tetra...
929     0.0
WP_002594219.1  TetM/TetW/TetO/TetS family tetracycline resist...
929     0.0
```

WP_105146473.1 TetM/TetW/TetO/TetS family tetracycline resist...
929   0.0
WP_105124962.1 TetM/TetW/TetO/TetS family tetracycline resist...
929   0.0
WP_105115755.1 TetM/TetW/TetO/TetS family tetracycline resist...
929   0.0
APO27460.1 ferrous iron transport protein B [uncultured bacte...
929   0.0
AMP50086.1 TetM-TetW-TetO-TetS [uncultured bacterium]
929   0.0
WP_044977943.1 TetM/TetW/TetO/TetS family tetracycline resist...
929   0.0
WP_044681968.1 TetM/TetW/TetO/TetS family tetracycline resist...
929   0.0
WP_031912846.1 MULTISPECIES: TetM/TetW/TetO/TetS family tetra...
929   0.0
WP_009300547.1 MULTISPECIES: TetM/TetW/TetO/TetS family tetra...
929   0.0
WP_100664818.1 TetM/TetW/TetO/TetS family tetracycline resist...
928   0.0
WP_074392924.1 TetM/TetW/TetO/TetS family tetracycline resist...
928   0.0
WP_074392551.1 TetM/TetW/TetO/TetS family tetracycline resist...
928   0.0
AMP52239.1 TetM-TetW-TetO-TetS [uncultured bacterium]
928   0.0
WP_029180377.1 TetM/TetW/TetO/TetS family tetracycline resist...
928   0.0
WP_024418038.1 TetM/TetW/TetO/TetS family tetracycline resist...
928   0.0
ACT76129.1 TetO [Streptococcus suis]
928   0.0
ACT76128.1 TetO [Streptococcus suis]
928   0.0
WP_055169801.1 TetM/TetW/TetO/TetS family tetracycline resist...
928   0.0
WP_054705475.1 TetM/TetW/TetO/TetS family tetracycline resist...
928   0.0
WP_015573056.1 tetracycline resistance ribosomal protection p...
928   0.0
WP_105123813.1 TetM/TetW/TetO/TetS family tetracycline resist...
927   0.0
WP_105122866.1 TetM/TetW/TetO/TetS family tetracycline resist...
927   0.0
WP_086301235.1 TetM/TetW/TetO/TetS family tetracycline resist...
927   0.0
WP_073930560.1 TetM/TetW/TetO/TetS family tetracycline resist...
927   0.0
WP_073689108.1 tetracycline resistance ribosomal protection p...
927   0.0
SCJ39489.1 Tetracycline resistance protein tetM from transpos...
927   0.0
WP_024415696.1 TetM/TetW/TetO/TetS family tetracycline resist...
927   0.0

```
WP_105141148.1  TetM/TetW/TetO/TetS family tetracycline resist...
927     0.0
WP_105130533.1  TetM/TetW/TetO/TetS family tetracycline resist...
927     0.0
WP_105095358.1  TetM/TetW/TetO/TetS family tetracycline resist...
927     0.0
WP_095122938.1  TetM/TetW/TetO/TetS family tetracycline resist...
927     0.0
WP_063856404.1  tetracycline resistance ribosomal protection p...
927     0.0
AMP46920.1  TetM-TetW-TetO-TetS [uncultured bacterium]
927     0.0
WP_047205445.1  tetracycline resistance ribosomal protection p...
927     0.0
WP_024395804.1  TetM/TetW/TetO/TetS family tetracycline resist...
927     0.0
WP_017144211.1  TetM/TetW/TetO/TetS family tetracycline resist...
927     0.0
WP_009321211.1  TetM/TetW/TetO/TetS family tetracycline resist...
927     0.0
WP_012775603.1  tetracycline resistance ribosomal protection p...
927     0.0
WP_009015508.1  TetM/TetW/TetO/TetS family tetracycline resist...
927     0.0
WP_000691746.1  MULTISPECIES: tetracycline resistance ribosoma...
927     0.0
AMP54850.1  TetM-TetW-TetO-TetS [uncultured bacterium]
926     0.0
EFM82549.1  putative translation elongation factor G [Enteroco...
926     0.0
ACT76131.1  TetO [Streptococcus suis]
926     0.0
WP_105155653.1  TetM/TetW/TetO/TetS family tetracycline resist...
926     0.0
P23835.1  RecName: Full=Tetracycline resistance protein TetO; ...
926     0.0
WP_105129783.1  TetM/TetW/TetO/TetS family tetracycline resist...
926     0.0
WP_105108102.1  TetM/TetW/TetO/TetS family tetracycline resist...
926     0.0
WP_099806636.1  TetM/TetW/TetO/TetS family tetracycline resist...
926     0.0
AMP54781.1  TetM-TetW-TetO-TetS [uncultured bacterium]
926     0.0
WP_044666383.1  MULTISPECIES: tetracycline resistance ribosoma...
926     0.0
WP_028254891.1  TetM/TetW/TetO/TetS family tetracycline resist...
926     0.0
CCY07492.1  small GTP-binding protein domain protein [Coprobac...
926     0.0
WP_014636626.1  TetM/TetW/TetO/TetS family tetracycline resist...
926     0.0
ACT76126.1  TetO [Streptococcus suis]
926     0.0
```

WP_063856395.1 tetracycline resistance ribosomal protection p...
926   0.0
WP_075680358.1 TetM/TetW/TetO/TetS family tetracycline resist...
925   0.0
APO27493.1 ferrous iron transport protein B [uncultured bacte...
925   0.0
WP_065736305.1 tetracycline resistance ribosomal protection p...
925   0.0
WP_029173152.1 TetM/TetW/TetO/TetS family tetracycline resist...
925   0.0
WP_024415386.1 TetM/TetW/TetO/TetS family tetracycline resist...
925   0.0
WP_002846154.1 MULTISPECIES: TetM/TetW/TetO/TetS family tetra...
925   0.0
AVY51757.1 tetracycline resistance ribosomal protection prote...
925   0.0
WP_098923458.1 TetM/TetW/TetO/TetS family tetracycline resist...
925   0.0
WP_087367883.1 tetracycline resistance ribosomal protection p...
925   0.0
WP_065736257.1 tetracycline resistance ribosomal protection p...
925   0.0
AMP52947.1 TetM-TetW-TetO-TetS [uncultured bacterium]
925   0.0
CUP58281.1 Tetracycline resistance protein tetM from transpos...
925   0.0
WP_029998948.1 TetM/TetW/TetO/TetS family tetracycline resist...
925   0.0
ACT76127.1 TetO [Streptococcus suis]
925   0.0
APO27484.1 ferrous iron transport protein B [uncultured bacte...
924   0.0
WP_069168329.1 MULTISPECIES: tetracycline resistance ribosoma...
924   0.0
SCI82746.1 Tetracycline resistance protein tetM from transpos...
924   0.0
WP_049523834.1 TetM/TetW/TetO/TetS family tetracycline resist...
924   0.0
WP_052782741.1 TetM/TetW/TetO/TetS family tetracycline resist...
924   0.0
WP_032510825.1 TetM/TetW/TetO/TetS family tetracycline resist...
924   0.0
WP_057036446.1 TetM/TetW/TetO/TetS family tetracycline resist...
924   0.0
WP_055940423.1 TetM/TetW/TetO/TetS family tetracycline resist...
924   0.0
WP_052659138.1 MULTISPECIES: TetM/TetW/TetO/TetS family tetra...
924   0.0
CDL74663.1 conjugative transposon tetracycline resistance pro...
924   0.0
WP_102743419.1 TetM/TetW/TetO/TetS family tetracycline resist...
924   0.0
APO28170.1 ferrous iron transport protein B [uncultured bacte...
924   0.0

AMP48943.1 TetM-TetW-TetO-TetS [uncultured bacterium]
924   0.0
WP_063668083.1 TetM/TetW/TetO/TetS family tetracycline resist...
924   0.0
WP_024478494.1 tetracycline resistance ribosomal protection p...
924   0.0
WP_015521635.1 MULTISPECIES: TetM/TetW/TetO/TetS family tetra...
924   0.0
WP_017766893.1 TetM/TetW/TetO/TetS family tetracycline resist...
924   0.0
WP_105309243.1 TetM/TetW/TetO/TetS family tetracycline resist...
923   0.0
WP_084588712.1 tetracycline resistance ribosomal protection p...
923   0.0
APO34055.1 ferrous iron transport protein B [uncultured bacte...
923   0.0
WP_065465655.1 TetM/TetW/TetO/TetS family tetracycline resist...
923   0.0
WP_059429510.1 TetM/TetW/TetO/TetS family tetracycline resist...
923   0.0
WP_052855148.1 MULTISPECIES: TetM/TetW/TetO/TetS family tetra...
923   0.0
WP_052805581.1 TetM/TetW/TetO/TetS family tetracycline resist...
923   0.0
WP_052785290.1 TetM/TetW/TetO/TetS family tetracycline resist...
923   0.0
WP_050117422.1 tetracycline resistance ribosomal protection p...
923   0.0
WP_029171436.1 TetM/TetW/TetO/TetS family tetracycline resist...
923   0.0
WP_024376808.1 TetM/TetW/TetO/TetS family tetracycline resist...
923   0.0
AHZ86999.1 Tet(M) [Streptococcus mitis]
923   0.0
WP_002576223.1 TetM/TetW/TetO/TetS family tetracycline resist...
923   0.0
WP_002904444.1 TetM/TetW/TetO/TetS family tetracycline resist...
923   0.0
WP_002823161.1 MULTISPECIES: TetM/TetW/TetO/TetS family tetra...
923   0.0
WP_005825846.1 TetM/TetW/TetO/TetS family tetracycline resist...
923   0.0
WP_094130791.1 tetracycline resistance ribosomal protection p...
923   0.0
WP_094141197.1 TetM/TetW/TetO/TetS family tetracycline resist...
923   0.0
WP_087203225.1 TetM/TetW/TetO/TetS family tetracycline resist...
923   0.0
WP_075478885.1 TetM/TetW/TetO/TetS family tetracycline resist...
923   0.0
WP_072447158.1 MULTISPECIES: TetM/TetW/TetO/TetS family tetra...
923   0.0
WP_074664471.1 TetM/TetW/TetO/TetS family tetracycline resist...
923   0.0

WP_070043520.1 TetM/TetW/TetO/TetS family tetracycline resist...
923   0.0
WP_061825287.1 tetracycline resistance ribosomal protection p...
923   0.0
WP_059434794.1 TetM/TetW/TetO/TetS family tetracycline resist...
923   0.0
WP_057827665.1 TetM/TetW/TetO/TetS family tetracycline resist...
923   0.0
KPA68082.1 tetracycline resistance protein tetM [Streptococcu...
923   0.0
WP_029172349.1 TetM/TetW/TetO/TetS family tetracycline resist...
923   0.0
WP_031794799.1 tetracycline resistance ribosomal protection p...
923   0.0
WP_035401840.1 TetM/TetW/TetO/TetS family tetracycline resist...
923   0.0
WP_006191702.1 tetracycline resistance ribosomal protection p...
923   0.0
WP_002812495.1 TetM/TetW/TetO/TetS family tetracycline resist...
923   0.0
WP_010729924.1 MULTISPECIES: tetracycline resistance ribosoma...
923   0.0
WP_101479534.1 TetM/TetW/TetO/TetS family tetracycline resist...
922   0.0
WP_100881386.1 TetM/TetW/TetO/TetS family tetracycline resist...
922   0.0
prf||1408188A tetracycline resistance gene tetO
922   0.0
WP_000691747.1 tetracycline resistance ribosomal protection p...
922   0.0
WP_075721707.1 TetM/TetW/TetO/TetS family tetracycline resist...
922   0.0
AMP53111.1 TetM-TetW-TetO-TetS [uncultured bacterium]
922   0.0
KXB43475.1 hypothetical protein HMPREF3188_01537 [Tissierelli...
922   0.0
WP_002596250.1 MULTISPECIES: TetM/TetW/TetO/TetS family tetra...
922   0.0
WP_002843300.1 MULTISPECIES: tetracycline resistance ribosoma...
922   0.0
EEN74261.1 putative translation elongation factor G [Enteroco...
922   0.0
WP_005357924.1 MULTISPECIES: TetM/TetW/TetO/TetS family tetra...
922   0.0
ABF36201.1 Tetracycline resistance protein tetM [Streptococcu...
922   0.0
WP_095761997.1 tetracycline resistance ribosomal protection p...
922   0.0
WP_088868051.1 TetM/TetW/TetO/TetS family tetracycline resist...
922   0.0
WP_000691758.1 MULTISPECIES: TetM/TetW/TetO/TetS family tetra...
922   0.0
KXB72354.1 putative translation elongation factor G [Peptostr...
922   0.0

WP_057498847.1 tetracycline resistance ribosomal protection p...
922   0.0
WP_024406177.1 MULTISPECIES: tetracycline resistance ribosoma...
922   0.0
WP_032593159.1 TetM/TetW/TetO/TetS family tetracycline resist...
922   0.0
WP_016398734.1 tetracycline resistance ribosomal protection p...
922   0.0
WP_000691731.1 tetracycline resistance ribosomal protection p...
922   0.0
WP_002569200.1 TetM/TetW/TetO/TetS family tetracycline resist...
922   0.0
WP_104846472.1 tetracycline resistance ribosomal protection p...
922   0.0
WP_101248398.1 tetracycline resistance ribosomal protection p...
922   0.0
WP_095761390.1 tetracycline resistance ribosomal protection p...
922   0.0
APO27488.1 ferrous iron transport protein B [uncultured bacte...
922   0.0
WP_073053263.1 MULTISPECIES: TetM/TetW/TetO/TetS family tetra...
922   0.0
WP_011187228.1 tetracycline resistance ribosomal protection p...
922   0.0
AMP50063.1 TetM-TetW-TetO-TetS [uncultured bacterium]
922   0.0
WP_043029018.1 tetracycline resistance ribosomal protection p...
922   0.0
WP_058652115.1 TetM/TetW/TetO/TetS family tetracycline resist...
922   0.0
WP_060473528.1 MULTISPECIES: TetM/TetW/TetO/TetS family tetra...
922   0.0
WP_052802401.1 TetM/TetW/TetO/TetS family tetracycline resist...
922   0.0
WP_052782483.1 TetM/TetW/TetO/TetS family tetracycline resist...
922   0.0
WP_044773055.1 TetM/TetW/TetO/TetS family tetracycline resist...
922   0.0
WP_043035760.1 TetM/TetW/TetO/TetS family tetracycline resist...
922   0.0
WP_033655463.1 tetracycline resistance ribosomal protection p...
922   0.0
WP_029188156.1 TetM/TetW/TetO/TetS family tetracycline resist...
922   0.0
EPI45798.1 putative translation elongation factor G [Gardnere...
922   0.0
WP_008976815.1 TetM/TetW/TetO/TetS family tetracycline resist...
922   0.0
ACT76130.1 TetO [Streptococcus suis]
922   0.0
ACS92754.1 tetracycline resistance determinant [Streptococcus...
922   0.0
APO27574.1 ferrous iron transport protein B [uncultured bacte...
921   0.0

```
WP_072510068.1  TetM/TetW/TetO/TetS family tetracycline resist...
921     0.0
WP_070811548.1  MULTISPECIES: TetM/TetW/TetO/TetS family tetra...
921     0.0
WP_070310173.1  TetM/TetW/TetO/TetS family tetracycline resist...
921     0.0
AMJ37901.1  TetM-TetW-TetO-TetS [uncultured bacterium]
921     0.0
WP_063856405.1  tetracycline resistance ribosomal protection p...
921     0.0
WP_057096870.1  TetM/TetW/TetO/TetS family tetracycline resist...
921     0.0
WP_057043774.1  TetM/TetW/TetO/TetS family tetracycline resist...
921     0.0
WP_049545748.1  tetracycline resistance ribosomal protection p...
921     0.0
WP_049527354.1  tetracycline resistance ribosomal protection p...
921     0.0
WP_052797194.1  TetM/TetW/TetO/TetS family tetracycline resist...
921     0.0
WP_052780628.1  TetM/TetW/TetO/TetS family tetracycline resist...
921     0.0
WP_044681400.1  TetM/TetW/TetO/TetS family tetracycline resist...
921     0.0
WP_032812904.1  MULTISPECIES: tetracycline resistance ribosoma...
921     0.0
WP_019107244.1  MULTISPECIES: tetracycline resistance ribosoma...
921     0.0
EJX98572.1  putative translation elongation factor G [Enteroco...
921     0.0
WP_010248735.1  tetracycline resistance ribosomal protection p...
921     0.0
WP_002896813.1  TetM/TetW/TetO/TetS family tetracycline resist...
921     0.0
WP_002371500.1  MULTISPECIES: tetracycline resistance ribosoma...
921     0.0
WP_101636627.1  tetracycline resistance ribosomal protection p...
921     0.0
WP_086228892.1  MULTISPECIES: TetM/TetW/TetO/TetS family tetra...
921     0.0
WP_081123660.1  TetM/TetW/TetO/TetS family tetracycline resist...
921     0.0
APO26810.1  ferrous iron transport protein B [uncultured bacte...
921     0.0
WP_074470838.1  TetM/TetW/TetO/TetS family tetracycline resist...
921     0.0
WP_070315526.1  TetM/TetW/TetO/TetS family tetracycline resist...
921     0.0
AMP50395.1  TetM-TetW-TetO-TetS [uncultured bacterium]
921     0.0
WP_052790168.1  tetracycline resistance protein tetM [Campylob...
921     0.0
WP_047207765.1  tetracycline resistance ribosomal protection p...
921     0.0
```

WP_039931034.1 MULTISPECIES: tetracycline resistance ribosoma...
921   0.0
WP_002860782.1 MULTISPECIES: TetM/TetW/TetO/TetS family tetra...
921   0.0
EFX39748.1 putative translation elongation factor G [Streptoc...
921   0.0
WP_041972879.1 MULTISPECIES: tetracycline resistance ribosoma...
921   0.0
WP_003125887.1 MULTISPECIES: tetracycline resistance ribosoma...
921   0.0
ACS92759.1 tetracycline resistance determinant [Streptococcus...
921   0.0
WP_002909882.1 MULTISPECIES: tetracycline resistance ribosoma...
921   0.0
WP_063856113.1 tetracycline resistance ribosomal protection p...
921   0.0
WP_063856111.1 tetracycline resistance ribosomal protection p...
921   0.0
WP_105209091.1 TetM/TetW/TetO/TetS family tetracycline resist...
920   0.0
WP_086251283.1 TetM/TetW/TetO/TetS family tetracycline resist...
920   0.0
WP_081201664.1 TetM/TetW/TetO/TetS family tetracycline resist...
920   0.0
WP_072237618.1 MULTISPECIES: TetM/TetW/TetO/TetS family tetra...
920   0.0
AMP58109.1 TetM-TetW-TetO-TetS [uncultured bacterium]
920   0.0
AMP56778.1 TetM-TetW-TetO-TetS [uncultured bacterium]
920   0.0
AMP49313.1 TetM-TetW-TetO-TetS [uncultured bacterium]
920   0.0
AMP47007.1 TetM-TetW-TetO-TetS [uncultured bacterium]
920   0.0
WP_061329395.1 tetracycline resistance ribosomal protection p...
920   0.0
WP_012669445.1 MULTISPECIES: tetracycline resistance ribosoma...
920   0.0
WP_057993037.1 TetM/TetW/TetO/TetS family tetracycline resist...
920   0.0
WP_044685046.1 TetM/TetW/TetO/TetS family tetracycline resist...
920   0.0
CDO19407.1 tetM protein [Streptococcus pneumoniae]
920   0.0
WP_031912030.1 tetracycline resistance ribosomal protection p...
920   0.0
WP_022864100.1 TetM/TetW/TetO/TetS family tetracycline resist...
920   0.0
WP_001668880.1 tetracycline resistance ribosomal protection p...
920   0.0
WP_000691749.1 MULTISPECIES: tetracycline resistance ribosoma...
920   0.0
WP_000691744.1 MULTISPECIES: tetracycline resistance ribosoma...
920   0.0

EFQ13124.1 putative translation elongation factor G [Enteroco...
920   0.0
EFM26596.1 putative translation elongation factor G [Streptoc...
920   0.0
EEI87258.1 putative translation elongation factor G [Anaeroco...
920   0.0
WP_003454408.1 tetracycline resistance ribosomal protection p...
920   0.0
WP_050824634.1 MULTISPECIES: tetracycline resistance ribosoma...
920   0.0
WP_050232631.1 tetracycline resistance ribosomal protection p...
920   0.0
WP_105135456.1 tetracycline resistance ribosomal protection p...
920   0.0
WP_104774283.1 MULTISPECIES: tetracycline resistance ribosoma...
920   0.0
WP_096013982.1 TetM/TetW/TetO/TetS family tetracycline resist...
920   0.0
WP_086233925.1 TetM/TetW/TetO/TetS family tetracycline resist...
920   0.0
WP_078755722.1 tetracycline resistance ribosomal protection p...
920   0.0
WP_072230499.1 TetM/TetW/TetO/TetS family tetracycline resist...
920   0.0
WP_072215556.1 MULTISPECIES: TetM/TetW/TetO/TetS family tetra...
920   0.0
WP_069159276.1 MULTISPECIES: tetracycline resistance ribosoma...
920   0.0
WP_002779752.1 MULTISPECIES: tetracycline resistance ribosoma...
920   0.0
AMP52275.1 TetM-TetW-TetO-TetS [uncultured bacterium]
920   0.0
AMP49243.1 TetM-TetW-TetO-TetS [uncultured bacterium]
920   0.0
WP_061461548.1 tetracycline resistance ribosomal protection p...
920   0.0
WP_057032384.1 TetM/TetW/TetO/TetS family tetracycline resist...
920   0.0
WP_057043153.1 TetM/TetW/TetO/TetS family tetracycline resist...
920   0.0
WP_049552042.1 tetracycline resistance ribosomal protection p...
920   0.0
WP_050224152.1 tetracycline resistance ribosomal protection p...
920   0.0
AKO48464.1 tetracycline resistance protein tetM [[Haemophilus...
920   0.0
WP_044979124.1 tetracycline resistance ribosomal protection p...
920   0.0
WP_000691739.1 MULTISPECIES: tetracycline resistance ribosoma...
920   0.0
WP_024052575.1 MULTISPECIES: tetracycline resistance ribosoma...
920   0.0
WP_000691736.1 MULTISPECIES: tetracycline resistance ribosoma...
920   0.0

WP_023890664.1 MULTISPECIES: TetM/TetW/TetO/TetS family tetra...
920   0.0
WP_021414738.1 tetracycline resistance ribosomal protection p...
920   0.0
WP_019117172.1 MULTISPECIES: tetracycline resistance ribosoma...
920   0.0
WP_016456422.1 TetM/TetW/TetO/TetS family tetracycline resist...
920   0.0
WP_000691725.1 tetracycline resistance ribosomal protection p...
920   0.0
WP_002808760.1 MULTISPECIES: TetM/TetW/TetO/TetS family tetra...
920   0.0
WP_004007597.1 MULTISPECIES: TetM/TetW/TetO/TetS family tetra...
920   0.0
WP_063856112.1 tetracycline resistance ribosomal protection p...
920   0.0
WP_100425802.1 TetM/TetW/TetO/TetS family tetracycline resist...
920   0.0
WP_002989474.1 MULTISPECIES: tetracycline resistance ribosoma...
920   0.0
WP_086290661.1 MULTISPECIES: TetM/TetW/TetO/TetS family tetra...
920   0.0
WP_070654311.1 MULTISPECIES: tetracycline resistance ribosoma...
920   0.0
WP_070579738.1 TetM/TetW/TetO/TetS family tetracycline resist...
920   0.0
WP_000691727.1 MULTISPECIES: tetracycline resistance ribosoma...
920   0.0
WP_061853025.1 tetracycline resistance ribosomal protection p...
920   0.0
AMJ38195.1 TetM-TetW-TetO-TetS [uncultured bacterium]
920   0.0
AMJ34666.1 TetM-TetW-TetO-TetS [uncultured bacterium]
920   0.0
CWI49647.1 tetracycline resistance protein TetM [Streptococcu...
920   0.0
WP_057980547.1 TetM/TetW/TetO/TetS family tetracycline resist...
920   0.0
WP_049098680.1 tetracycline resistance ribosomal protection p...
920   0.0
CTP13009.1 putative conjugative element protein [Streptococcu...
920   0.0
WP_055348549.1 tetracycline resistance protein tetM [Streptoc...
920   0.0
WP_044601084.1 TetM/TetW/TetO/TetS family tetracycline resist...
920   0.0
WP_002403674.1 tetracycline resistance ribosomal protection p...
920   0.0
WP_031919647.1 tetracycline resistance ribosomal protection p...
920   0.0
WP_019213884.1 tetracycline resistance ribosomal protection p...
920   0.0
WP_002818118.1 TetM/TetW/TetO/TetS family tetracycline resist...
920   0.0

WP_003831706.1 TetM/TetW/TetO/TetS family tetracycline resist...
920   0.0
WP_004137776.1 tetracycline resistance ribosomal protection p...
920   0.0
WP_010790859.1 MULTISPECIES: TetM/TetW/TetO/TetS family tetra...
920   0.0
EFM24959.1 putative translation elongation factor G [Peptonip...
920   0.0
EFH06235.1 putative translation elongation factor G [Clostrid...
920   0.0
EEI57513.1 putative translation elongation factor G [Enteroco...
920   0.0
WP_061364406.1 tetracycline resistance ribosomal protection p...
919   0.0
WP_061815145.1 tetracycline resistance ribosomal protection p...
919   0.0
WP_096421997.1 tetracycline resistance ribosomal protection p...
919   0.0
WP_088666504.1 TetM/TetW/TetO/TetS family tetracycline resist...
919   0.0
WP_071315221.1 TetM/TetW/TetO/TetS family tetracycline resist...
919   0.0
WP_075227092.1 tetracycline resistance ribosomal protection p...
919   0.0
WP_070301896.1 TetM/TetW/TetO/TetS family tetracycline resist...
919   0.0
AMP42232.1 tetracycline resistance protein Tet(O) [uncultured...
919   0.0
KXA54623.1 putative translation elongation factor G [Streptoc...
919   0.0
WP_052817824.1 TetM/TetW/TetO/TetS family tetracycline resist...
919   0.0
WP_052793287.1 TetM/TetW/TetO/TetS family tetracycline resist...
919   0.0
WP_052783935.1 TetM/TetW/TetO/TetS family tetracycline resist...
919   0.0
WP_050971349.1 tetracycline resistance ribosomal protection p...
919   0.0
CTO84842.1 putative conjugative element protein [Streptococcu...
919   0.0
CTO36092.1 putative conjugative element protein [Streptococcu...
919   0.0
WP_063856109.1 tetracycline resistance ribosomal protection p...
919   0.0
WP_050272947.1 tetracycline resistance ribosomal protection p...
919   0.0
WP_050247506.1 tetracycline resistance ribosomal protection p...
919   0.0
WP_050234184.1 tetracycline resistance ribosomal protection p...
919   0.0
WP_041981115.1 tetracycline resistance ribosomal protection p...
919   0.0
WP_038851197.1 TetM/TetW/TetO/TetS family tetracycline resist...
919   0.0

CDL74246.1 conjugative transposon tetracycline resistance pro...
919   0.0
WP_024477701.1 tetracycline resistance ribosomal protection p...
919   0.0
WP_020998529.1 MULTISPECIES: tetracycline resistance ribosoma...
919   0.0
WP_016397077.1 tetracycline resistance ribosomal protection p...
919   0.0
WP_003711229.1 tetracycline resistance ribosomal protection p...
919   0.0
WP_000691745.1 MULTISPECIES: tetracycline resistance ribosoma...
919   0.0
WP_063855884.1 tetracycline resistance ribosomal protection p...
919   0.0
ADH21996.1 Tn916, tetracycline resistance protein [synthetic ...
919   0.0
WP_015646599.1 tetracycline resistance ribosomal protection p...
919   0.0
WP_107974002.1 tetracycline resistance ribosomal protection p...
919   0.0
WP_061841616.1 tetracycline resistance ribosomal protection p...
919   0.0
WP_100004275.1 tetracycline resistance ribosomal protection p...
919   0.0
WP_086268246.1 TetM/TetW/TetO/TetS family tetracycline resist...
919   0.0
WP_086234301.1 MULTISPECIES: TetM/TetW/TetO/TetS family tetra...
919   0.0
WP_086232400.1 TetM/TetW/TetO/TetS family tetracycline resist...
919   0.0
WP_085613781.1 tetracycline resistance ribosomal protection p...
919   0.0
AMP55627.1 TetM-TetW-TetO-TetS [uncultured bacterium]
919   0.0
WP_057980088.1 TetM/TetW/TetO/TetS family tetracycline resist...
919   0.0
WP_057036876.1 TetM/TetW/TetO/TetS family tetracycline resist...
919   0.0
WP_052802847.1 TetM/TetW/TetO/TetS family tetracycline resist...
919   0.0
WP_052799797.1 TetM/TetW/TetO/TetS family tetracycline resist...
919   0.0
WP_050313386.1 tetracycline resistance ribosomal protection p...
919   0.0
KLS39627.1 tetracycline resistance protein tetM [Neisseria go...
919   0.0
WP_047339182.1 tetracycline resistance ribosomal protection p...
919   0.0
WP_047241744.1 tetracycline resistance ribosomal protection p...
919   0.0
AIW80587.1 TetM-TetW-TetO-TetS [uncultured bacterium]
919   0.0
WP_050091355.1 tetracycline resistance ribosomal protection p...
919   0.0

WP_047922456.1 tetracycline resistance ribosomal protection p...
919   0.0
WP_050242154.1 tetracycline resistance ribosomal protection p...
919   0.0
WP_039975152.1 tetracycline resistance ribosomal protection p...
919   0.0
WP_039095955.1 TetM/TetW/TetO/TetS family tetracycline resist...
919   0.0
ADF36620.1 TetM [Neisseria gonorrhoeae]
919   0.0
WP_002834238.1 TetM/TetW/TetO/TetS family tetracycline resist...
919   0.0
EFX53501.1 putative translation elongation factor G [Streptoc...
919   0.0
WP_000691742.1 tetracycline resistance ribosomal protection p...
919   0.0
WP_063856397.1 MULTISPECIES: tetracycline resistance ribosoma...
919   0.0
WP_061654034.1 MULTISPECIES: tetracycline resistance ribosoma...
919   0.0
WP_104722935.1 tetracycline resistance ribosomal protection p...
919   0.0
WP_101882586.1 TetM/TetW/TetO/TetS family tetracycline resist...
919   0.0
WP_086252249.1 TetM/TetW/TetO/TetS family tetracycline resist...
919   0.0
WP_076995854.1 TetM/TetW/TetO/TetS family tetracycline resist...
919   0.0
WP_075213493.1 tetracycline resistance ribosomal protection p...
919   0.0
WP_072236497.1 TetM/TetW/TetO/TetS family tetracycline resist...
919   0.0
WP_071705138.1 TetM/TetW/TetO/TetS family tetracycline resist...
919   0.0
BAV93039.1 tetracycline resistance protein TetM [Haemophilus ...
919   0.0
WP_070852878.1 tetracycline resistance ribosomal protection p...
919   0.0
AOV09463.1 tetracycline resistance protein [Campylobacter jej...
919   0.0
WP_070299010.1 TetM/TetW/TetO/TetS family tetracycline resist...
919   0.0
WP_061457615.1 tetracycline resistance ribosomal protection p...
919   0.0
WP_060812114.1 tetracycline resistance ribosomal protection p...
919   0.0
WP_060472723.1 TetM/TetW/TetO/TetS family tetracycline resist...
919   0.0
CTF67619.1 putative conjugative element protein [Streptococcu...
919   0.0
WP_050142810.1 tetracycline resistance ribosomal protection p...
919   0.0
WP_050274428.1 tetracycline resistance ribosomal protection p...
919   0.0

AKO45297.1 tetracycline resistance protein tetM [[Haemophilus...
919   0.0
WP_055347831.1 tetracycline resistance ribosomal protection p...
919   0.0
WP_044766725.1 TetM/TetW/TetO/TetS family tetracycline resist...
919   0.0
WP_041974943.1 tetracycline resistance ribosomal protection p...
919   0.0
WP_044908248.1 tetracycline resistance ribosomal protection p...
919   0.0
WP_047511001.1 tetracycline resistance ribosomal protection p...
919   0.0
AID58520.1 tet(M) [Haemophilus parainfluenzae]
919   0.0
ADE19326.1 tetM [Tri-shuttle cloning vector pmycYACTn]
919   0.0
WP_063856406.1 tetracycline resistance ribosomal protection p...
919   0.0
WP_016441094.1 TetM/TetW/TetO/TetS family tetracycline resist...
919   0.0
WP_001614674.1 tetracycline resistance ribosomal protection p...
919   0.0
WP_002807700.1 MULTISPECIES: TetM/TetW/TetO/TetS family tetra...
919   0.0
WP_007392011.1 TetM/TetW/TetO/TetS family tetracycline resist...
919   0.0
WP_004026801.1 tetracycline resistance ribosomal protection p...
919   0.0
WP_063856398.1 tetracycline resistance ribosomal protection p...
919   0.0
WP_105249165.1 tetracycline resistance ribosomal protection p...
918   0.0
WP_105211910.1 tetracycline resistance ribosomal protection p...
918   0.0
WP_104885338.1 tetracycline resistance ribosomal protection p...
918   0.0
WP_104835953.1 tetracycline resistance ribosomal protection p...
918   0.0
WP_063856391.1 tetracycline resistance ribosomal protection p...
918   0.0
WP_063856392.1 tetracycline resistance ribosomal protection p...
918   0.0
WP_095256818.1 TetM/TetW/TetO/TetS family tetracycline resist...
918   0.0
WP_002857791.1 MULTISPECIES: TetM/TetW/TetO/TetS family tetra...
918   0.0
WP_086239295.1 TetM/TetW/TetO/TetS family tetracycline resist...
918   0.0
WP_072234042.1 TetM/TetW/TetO/TetS family tetracycline resist...
918   0.0
WP_070663994.1 tetracycline resistance ribosomal protection p...
918   0.0
AMP58099.1 TetM-TetW-TetO-TetS [uncultured bacterium]
918   0.0

WP_061824231.1 tetracycline resistance ribosomal protection p...
918   0.0
WP_000691743.1 MULTISPECIES: tetracycline resistance ribosoma...
918   0.0
WP_057991785.1 MULTISPECIES: TetM/TetW/TetO/TetS family tetra...
918   0.0
WP_052943236.1 TetM/TetW/TetO/TetS family tetracycline resist...
918   0.0
WP_050308251.1 tetracycline resistance ribosomal protection p...
918   0.0
WP_050131553.1 tetracycline resistance ribosomal protection p...
918   0.0
WP_063856107.1 tetracycline resistance ribosomal protection p...
918   0.0
WP_063856115.1 tetracycline resistance ribosomal protection p...
918   0.0
WP_060458520.1 tetracycline resistance ribosomal protection p...
918   0.0
WP_032587445.1 MULTISPECIES: TetM/TetW/TetO/TetS family tetra...
918   0.0
WP_031904673.1 tetracycline resistance ribosomal protection p...
918   0.0
WP_031774827.1 tetracycline resistance ribosomal protection p...
918   0.0
WP_037612731.1 tetracycline resistance ribosomal protection p...
918   0.0
WP_023916197.1 MULTISPECIES: tetracycline resistance ribosoma...
918   0.0
WP_017647393.1 MULTISPECIES: tetracycline resistance ribosoma...
918   0.0
WP_002804228.1 MULTISPECIES: TetM/TetW/TetO/TetS family tetra...
918   0.0
WP_002785822.1 TetM/TetW/TetO/TetS family tetracycline resist...
918   0.0
WP_000691728.1 tetracycline resistance ribosomal protection p...
918   0.0
ADI70435.1 putative translation elongation factor G [Streptoc...
918   0.0
WP_061748924.1 tetracycline resistance ribosomal protection p...
918   0.0
WP_061367944.1 tetracycline resistance ribosomal protection p...
918   0.0
WP_061847427.1 tetracycline resistance ribosomal protection p...
918   0.0
WP_100608496.1 TetM/TetW/TetO/TetS family tetracycline resist...
918   0.0
WP_105577399.1 tetracycline resistance ribosomal protection p...
918   0.0
WP_006155610.1 MULTISPECIES: tetracycline resistance ribosoma...
918   0.0
WP_083977868.1 tetracycline resistance ribosomal protection p...
918   0.0
APO27237.1 ferrous iron transport protein B [uncultured bacte...
918   0.0

WP_072661552.1 tetracycline resistance ribosomal protection p...
918   0.0
AMP51392.1 TetM-TetW-TetO-TetS [uncultured bacterium]
918   0.0
WP_060811023.1 tetracycline resistance ribosomal protection p...
918   0.0
WP_063674638.1 TetM/TetW/TetO/TetS family tetracycline resist...
918   0.0
WP_058692378.1 tetracycline resistance ribosomal protection p...
918   0.0
ALT05248.1 tetracycline resistance protein [Staphylococcus au...
918   0.0
WP_053794614.1 tetracycline resistance ribosomal protection p...
918   0.0
WP_049532577.1 tetracycline resistance ribosomal protection p...
918   0.0
WP_052793722.1 TetM/TetW/TetO/TetS family tetracycline resist...
918   0.0
WP_050216613.1 tetracycline resistance ribosomal protection p...
918   0.0
WP_050305062.1 tetracycline resistance ribosomal protection p...
918   0.0
WP_055348096.1 tetracycline resistance ribosomal protection p...
918   0.0
WP_050093829.1 tetracycline resistance ribosomal protection p...
918   0.0
WP_024721683.1 TetM/TetW/TetO/TetS family tetracycline resist...
918   0.0
ADB66722.1 ribosomal protection protein [Streptococcus agalac...
918   0.0
ACH85962.1 tetracycline resistance protein [Staphylococcus au...
918   0.0
WP_021382096.1 MULTISPECIES: tetracycline resistance ribosoma...
918   0.0
WP_000691729.1 MULTISPECIES: tetracycline resistance ribosoma...
918   0.0
3J25_A Chain A, Structural Basis For Tetm-Mediated Tetracycli...
918   0.0
WP_002836924.1 TetM/TetW/TetO/TetS family tetracycline resist...
918   0.0
WP_000691735.1 tetracycline resistance ribosomal protection p...
918   0.0
CBW39416.1 conjugative transposon tetracycline resistance pro...
918   0.0
EFX36382.1 putative translation elongation factor G [Streptoc...
918   0.0
EFU09414.1 putative translation elongation factor G [Enteroco...
918   0.0
WP_063856393.1 tetracycline resistance ribosomal protection p...
918   0.0
WP_102170272.1 tetracycline resistance ribosomal protection p...
917   0.0
WP_099875793.1 tetracycline resistance ribosomal protection p...
917   0.0

WP_088849421.1 tetracycline resistance ribosomal protection p...
917   0.0
WP_088792637.1 tetracycline resistance ribosomal protection p...
917   0.0
WP_086032702.1 tetracycline resistance ribosomal protection p...
917   0.0
WP_070544140.1 MULTISPECIES: tetracycline resistance ribosoma...
917   0.0
AMP46943.1 TetM-TetW-TetO-TetS [uncultured bacterium]
917   0.0
WP_057099610.1 TetM/TetW/TetO/TetS family tetracycline resist...
917   0.0
WP_049513432.1 tetracycline resistance ribosomal protection p...
917   0.0
WP_052853097.1 TetM/TetW/TetO/TetS family tetracycline resist...
917   0.0
WP_052778388.1 MULTISPECIES: TetM/TetW/TetO/TetS family tetra...
917   0.0
WP_050308000.1 tetracycline resistance ribosomal protection p...
917   0.0
CRH83910.1 Protein translation elongation factor G (EF-G) [Ch...
917   0.0
WP_050079309.1 tetracycline resistance ribosomal protection p...
917   0.0
WP_050212334.1 tetracycline resistance ribosomal protection p...
917   0.0
CDL74203.1 conjugative transposon tetracycline resistance pro...
917   0.0
WP_031912118.1 tetracycline resistance ribosomal protection p...
917   0.0
CDF09859.1 tet(M) [Eubacterium sp. CAG:76]
917   0.0
WP_002819063.1 MULTISPECIES: TetM/TetW/TetO/TetS family tetra...
917   0.0
WP_002843722.1 MULTISPECIES: TetM/TetW/TetO/TetS family tetra...
917   0.0
WP_095906095.1 tetracycline resistance ribosomal protection p...
917   0.0
WP_081526881.1 tetracycline resistance ribosomal protection p...
917   0.0
WP_070709775.1 tetracycline resistance ribosomal protection p...
917   0.0
WP_061763260.1 tetracycline resistance ribosomal protection p...
917   0.0
WP_061646118.1 tetracycline resistance ribosomal protection p...
917   0.0
WP_060793390.1 tetracycline resistance ribosomal protection p...
917   0.0
KXA16596.1 putative translation elongation factor G [Fusobact...
917   0.0
WP_057491875.1 TetM/TetW/TetO/TetS family tetracycline resist...
917   0.0
WP_050974015.1 tetracycline resistance ribosomal protection p...
917   0.0

WP_050971803.1 tetracycline resistance ribosomal protection p...
917   0.0
WP_050201474.1 tetracycline resistance ribosomal protection p...
917   0.0
WP_050072437.1 MULTISPECIES: tetracycline resistance ribosoma...
917   0.0
WP_044329681.1 tetracycline resistance ribosomal protection p...
917   0.0
AJE60963.1 TetM [Enterococcus faecium]
917   0.0
WP_025187938.1 tetracycline resistance ribosomal protection p...
917   0.0
WP_031793925.1 tetracycline resistance ribosomal protection p...
917   0.0
ELU61350.1 putative translation elongation factor G [Streptoc...
917   0.0
AEX58674.1 tetracycline resistance protein [Streptococcus pyo...
917   0.0
WP_001820713.1 tetracycline resistance ribosomal protection p...
917   0.0
WP_063856400.1 tetracycline resistance ribosomal protection p...
917   0.0
WP_105142289.1 tetracycline resistance ribosomal protection p...
917   0.0
WP_104929836.1 TetM/TetW/TetO/TetS family tetracycline resist...
917   0.0
WP_088804464.1 tetracycline resistance ribosomal protection p...
917   0.0
WP_070583403.1 MULTISPECIES: tetracycline resistance ribosoma...
917   0.0
WP_070309696.1 TetM/TetW/TetO/TetS family tetracycline resist...
917   0.0
AMP54192.1 TetM-TetW-TetO-TetS [uncultured bacterium]
917   0.0
AMP50513.1 TetM-TetW-TetO-TetS [uncultured bacterium]
917   0.0
WP_061465177.1 tetracycline resistance ribosomal protection p...
917   0.0
WP_061763772.1 tetracycline resistance ribosomal protection p...
917   0.0
WP_057032430.1 TetM/TetW/TetO/TetS family tetracycline resist...
917   0.0
WP_057030943.1 TetM/TetW/TetO/TetS family tetracycline resist...
917   0.0
WP_057037575.1 TetM/TetW/TetO/TetS family tetracycline resist...
917   0.0
WP_057036252.1 MULTISPECIES: TetM/TetW/TetO/TetS family tetra...
917   0.0
WP_054384447.1 tetracycline resistance ribosomal protection p...
917   0.0
WP_050253426.1 tetracycline resistance ribosomal protection p...
917   0.0
CTG81086.1 putative conjugative element protein [Streptococcu...
917   0.0

WP_050168486.1 tetracycline resistance ribosomal protection p...
917   0.0
WP_031910311.1 tetracycline resistance ribosomal protection p...
917   0.0
WP_050203260.1 tetracycline resistance ribosomal protection p...
917   0.0
WP_050235045.1 tetracycline resistance ribosomal protection p...
917   0.0
WP_050205550.1 tetracycline resistance ribosomal protection p...
917   0.0
WP_057486053.1 tetracycline resistance ribosomal protection p...
917   0.0
WP_057518699.1 tetracycline resistance ribosomal protection p...
917   0.0
WP_044764178.1 tetracycline resistance ribosomal protection p...
917   0.0
WP_044761286.1 tetracycline resistance ribosomal protection p...
917   0.0
CDL74131.1 conjugative transposon tetracycline resistance pro...
917   0.0
WP_030127075.1 MULTISPECIES: tetracycline resistance ribosoma...
917   0.0
WP_003701913.1 tetracycline resistance ribosomal protection p...
917   0.0
WP_000691741.1 MULTISPECIES: tetracycline resistance ribosoma...
917   0.0
WP_019109213.1 MULTISPECIES: TetM/TetW/TetO/TetS family tetra...
917   0.0
WP_010777232.1 tetracycline resistance ribosomal protection p...
917   0.0
WP_000691730.1 tetracycline resistance ribosomal protection p...
917   0.0
WP_000691734.1 MULTISPECIES: tetracycline resistance ribosoma...
917   0.0
WP_025456273.1 tetracycline resistance ribosomal protection p...
917   0.0
WP_063856399.1 tetracycline resistance ribosomal protection p...
917   0.0
WP_101928442.1 tetracycline resistance ribosomal protection p...
916   0.0
WP_000691726.1 tetracycline resistance ribosomal protection p...
916   0.0
WP_081538458.1 MULTISPECIES: tetracycline resistance ribosoma...
916   0.0
WP_069288536.1 tetracycline resistance ribosomal protection p...
916   0.0
WP_061088007.1 tetracycline resistance ribosomal protection p...
916   0.0
WP_032490535.1 tetracycline resistance ribosomal protection p...
916   0.0
WP_057992781.1 TetM/TetW/TetO/TetS family tetracycline resist...
916   0.0
CTG46355.1 putative conjugative element protein [Streptococcu...
916   0.0

```
WP_050280708.1  tetracycline resistance ribosomal protection p...
916     0.0
WP_050218283.1  tetracycline resistance ribosomal protection p...
916     0.0
WP_050211189.1  tetracycline resistance ribosomal protection p...
916     0.0
WP_050237755.1  tetracycline resistance ribosomal protection p...
916     0.0
WP_031797197.1  tetracycline resistance ribosomal protection p...
916     0.0
WP_017646602.1  tetracycline resistance ribosomal protection p...
916     0.0
WP_010729706.1  tetracycline resistance ribosomal protection p...
916     0.0
WP_002781029.1  MULTISPECIES: TetM/TetW/TetO/TetS family tetra...
916     0.0
WP_103219860.1  tetracycline resistance ribosomal protection p...
916     0.0
WP_094372133.1  tetracycline resistance ribosomal protection p...
916     0.0
WP_086241454.1  TetM/TetW/TetO/TetS family tetracycline resist...
916     0.0
WP_084879979.1  tetracycline resistance ribosomal protection p...
916     0.0
WP_081573876.1  tetracycline resistance ribosomal protection p...
916     0.0
APO31175.1  Ferrous iron transport protein B [uncultured bacte...
916     0.0
AMP53771.1  TetM-TetW-TetO-TetS [uncultured bacterium]
916     0.0
BAU68108.1  tetracycline resistance protein TetO [Streptococcu...
916     0.0
WP_060470565.1  tetracycline resistance ribosomal protection p...
916     0.0
WP_057100161.1  TetM/TetW/TetO/TetS family tetracycline resist...
916     0.0
WP_049366043.1  tetracycline resistance ribosomal protection p...
916     0.0
WP_052774203.1  TetM/TetW/TetO/TetS family tetracycline resist...
916     0.0
WP_050247433.1  tetracycline resistance ribosomal protection p...
916     0.0
WP_050101597.1  tetracycline resistance ribosomal protection p...
916     0.0
WP_049510748.1  MULTISPECIES: tetracycline resistance ribosoma...
916     0.0
WP_050077833.1  tetracycline resistance ribosomal protection p...
916     0.0
WP_050200822.1  tetracycline resistance ribosomal protection p...
916     0.0
WP_038835108.1  TetM/TetW/TetO/TetS family tetracycline resist...
916     0.0
CDL73864.1  conjugative transposon tetracycline resistance pro...
916     0.0
```

EJX44727.1 putative translation elongation factor G [Enteroco...
916   0.0
WP_011117014.1 tetracycline resistance ribosomal protection p...
916   0.0
WP_061702631.1 tetracycline resistance ribosomal protection p...
915   0.0
WP_106775878.1 tetracycline resistance ribosomal protection p...
915   0.0
WP_105184076.1 tetracycline resistance ribosomal protection p...
915   0.0
WP_105139211.1 tetracycline resistance ribosomal protection p...
915   0.0
WP_101785285.1 tetracycline resistance ribosomal protection p...
915   0.0
WP_100059493.1 TetM/TetW/TetO/TetS family tetracycline resist...
915   0.0
WP_086246158.1 TetM/TetW/TetO/TetS family tetracycline resist...
915   0.0
WP_077327446.1 tetracycline resistance ribosomal protection p...
915   0.0
APO28016.1 ferrous iron transport protein B [uncultured bacte...
915   0.0
ABP89888.1 Translation elongation factor (GTPases) [Streptoco...
915   0.0
WP_060936428.1 MULTISPECIES: tetracycline resistance ribosoma...
915   0.0
WP_050117940.1 tetracycline resistance ribosomal protection p...
915   0.0
WP_050089233.1 tetracycline resistance ribosomal protection p...
915   0.0
WP_039234362.1 tetracycline resistance ribosomal protection p...
915   0.0
WP_002915791.1 MULTISPECIES: TetM/TetW/TetO/TetS family tetra...
915   0.0
WP_010727156.1 MULTISPECIES: tetracycline resistance ribosoma...
915   0.0
WP_002345005.1 MULTISPECIES: tetracycline resistance ribosoma...
915   0.0
WP_063856110.1 tetracycline resistance ribosomal protection p...
915   0.0
WP_002934567.1 TetM/TetW/TetO/TetS family tetracycline resist...
915   0.0
ADK91600.1 TetS/M [Streptococcus equinus]
915   0.0
WP_061842897.1 tetracycline resistance ribosomal protection p...
915   0.0
WP_061761042.1 tetracycline resistance ribosomal protection p...
915   0.0
WP_063856390.1 tetracycline resistance ribosomal protection p...
915   0.0
WP_087302314.1 MULTISPECIES: TetM/TetW/TetO/TetS family tetra...
915   0.0
KXB37895.1 putative translation elongation factor G [Aerococc...
915   0.0

WP_059280319.1 TetM/TetW/TetO/TetS family tetracycline resist...
915   0.0
WP_052800762.1 TetM/TetW/TetO/TetS family tetracycline resist...
915   0.0
WP_050967045.1 tetracycline resistance ribosomal protection p...
915   0.0
WP_001574275.1 MULTISPECIES: tetracycline resistance ribosoma...
915   0.0
WP_023042854.1 TetM/TetW/TetO/TetS family tetracycline resist...
915   0.0
WP_021034343.1 MULTISPECIES: TetM/TetW/TetO/TetS family tetra...
915   0.0
WP_000691740.1 tetracycline resistance ribosomal protection p...
915   0.0
WP_000691724.1 tetracycline resistance ribosomal protection p...
915   0.0
WP_088819470.1 tetracycline resistance ribosomal protection p...
915   0.0
WP_086309543.1 tetracycline resistance ribosomal protection p...
915   0.0
WP_081529600.1 tetracycline resistance ribosomal protection p...
915   0.0
AQM74965.1 Tetracycline resistance ribosomal protection prote...
915   0.0
WP_069469652.1 tetracycline resistance ribosomal protection p...
915   0.0
WP_050364126.1 tetracycline resistance ribosomal protection p...
915   0.0
WP_047389487.1 tetracycline resistance ribosomal protection p...
915   0.0
AIF29636.1 tetracycline resistance protein [Campylobacter coli]
915   0.0
WP_024388620.1 TetM/TetW/TetO/TetS family tetracycline resist...
915   0.0
WP_063856403.1 tetracycline resistance ribosomal protection p...
915   0.0
CBW38865.1 conjugative transposon tetracycline resistance pro...
915   0.0
WP_061642374.1 tetracycline resistance ribosomal protection p...
914   0.0
WP_100728246.1 tetracycline resistance ribosomal protection p...
914   0.0
WP_098183854.1 tetracycline resistance ribosomal protection p...
914   0.0
WP_086097496.1 tetracycline resistance ribosomal protection p...
914   0.0
WP_081535763.1 tetracycline resistance ribosomal protection p...
914   0.0
ACB89423.1 tetracycline resistance protein TetM [Streptococcu...
914   0.0
APO27467.1 ferrous iron transport protein B [uncultured bacte...
914   0.0
CTN85508.1 putative conjugative element protein [Streptococcu...
914   0.0

WP_050116747.1 tetracycline resistance ribosomal protection p...
914   0.0
WP_050198644.1 tetracycline resistance ribosomal protection p...
914   0.0
WP_050203222.1 tetracycline resistance ribosomal protection p...
914   0.0
EFH13796.1 putative translation elongation factor G [Clostrid...
914   0.0
WP_012775613.1 tetracycline resistance ribosomal protection p...
914   0.0
WP_078122686.1 tetracycline resistance ribosomal protection p...
914   0.0
WP_002414694.1 MULTISPECIES: tetracycline resistance ribosoma...
914   0.0
WP_097698785.1 tetracycline resistance ribosomal protection p...
914   0.0
WP_083989376.1 tetracycline resistance ribosomal protection p...
914   0.0
APO34026.1 ferrous iron transport protein B [uncultured bacte...
914   0.0
WP_055275199.1 MULTISPECIES: tetracycline resistance ribosoma...
914   0.0
WP_057531665.1 tetracycline resistance ribosomal protection p...
914   0.0
WP_050236762.1 tetracycline resistance ribosomal protection p...
914   0.0
WP_063856402.1 tetracycline resistance ribosomal protection p...
914   0.0
WP_000691733.1 tetracycline resistance ribosomal protection p...
914   0.0
WP_000691732.1 tetracycline resistance ribosomal protection p...
914   0.0
WP_002343718.1 tetracycline resistance ribosomal protection p...
914   0.0
WP_061648168.1 tetracycline resistance ribosomal protection p...
914   0.0
WP_104670782.1 tetracycline resistance ribosomal protection p...
914   0.0
WP_086131656.1 MULTISPECIES: tetracycline resistance ribosoma...
914   0.0
AQM75603.1 Tetracycline resistance protein TetO [Campylobacte...
914   0.0
APO33640.1 ferrous iron transport protein B [uncultured bacte...
914   0.0
WP_071132114.1 TetM/TetW/TetO/TetS family tetracycline resist...
914   0.0
WP_050219862.1 tetracycline resistance ribosomal protection p...
914   0.0
WP_025989258.1 MULTISPECIES: tetracycline resistance ribosoma...
914   0.0
WP_020224358.1 MULTISPECIES: tetracycline resistance ribosoma...
914   0.0
WP_061746200.1 tetracycline resistance ribosomal protection p...
913   0.0

WP_084357849.1 tetracycline resistance ribosomal protection p...
913   0.0
WP_061857705.1 tetracycline resistance ribosomal protection p...
913   0.0
AMP50910.1 TetM-TetW-TetO-TetS [uncultured bacterium]
913   0.0
WP_061758507.1 tetracycline resistance ribosomal protection p...
913   0.0
WP_057531327.1 tetracycline resistance ribosomal protection p...
913   0.0
AIJ27535.1 TetO [Campylobacter jejuni]
913   0.0
CDQ30209.1 putative integrative and conjugative element prote...
913   0.0
WP_018368128.1 tetracycline resistance ribosomal protection p...
913   0.0
WP_089144890.1 tetracycline resistance ribosomal protection p...
913   0.0
WP_084588758.1 tetracycline resistance ribosomal protection p...
913   0.0
APO33206.1 ferrous iron transport protein B [uncultured bacte...
913   0.0
WP_066127145.1 tetracycline resistance ribosomal protection p...
913   0.0
WP_061573585.1 tetracycline resistance ribosomal protection p...
913   0.0
WP_050356456.1 tetracycline resistance ribosomal protection p...
913   0.0
WP_044667602.1 tetracycline resistance ribosomal protection p...
913   0.0
WP_043027138.1 tetracycline resistance ribosomal protection p...
913   0.0
WP_044812736.1 tetracycline resistance ribosomal protection p...
913   0.0
WP_002206236.1 tetracycline resistance ribosomal protection p...
913   0.0
WP_004632336.1 MULTISPECIES: tetracycline resistance ribosoma...
913   0.0
WP_104884981.1 tetracycline resistance ribosomal protection p...
912   0.0
WP_098034699.1 tetracycline resistance ribosomal protection p...
912   0.0
WP_105208278.1 tetracycline resistance ribosomal protection p...
912   0.0
AMJ37079.1 TetM-TetW-TetO-TetS [uncultured bacterium]
912   0.0
KRL89337.1 tetracycline resistance protein tetm [Lactobacillu...
912   0.0
WP_050392063.1 tetracycline resistance ribosomal protection p...
912   0.0
WP_099987656.1 tetracycline resistance ribosomal protection p...
912   0.0
WP_096920339.1 GTP-binding protein [Eubacterium sp. YI]
912   0.0

WP_057041522.1 TetM/TetW/TetO/TetS family tetracycline resist...
912   0.0
WP_052776466.1 TetM/TetW/TetO/TetS family tetracycline resist...
912   0.0
WP_050246763.1 tetracycline resistance ribosomal protection p...
912   0.0
WP_033626236.1 tetracycline resistance ribosomal protection p...
912   0.0
WP_018659523.1 tetracycline resistance ribosomal protection p...
912   0.0
WP_041160408.1 tetracycline resistance ribosomal protection p...
912   0.0
WP_059443098.1 TetM/TetW/TetO/TetS family tetracycline resist...
912   0.0
WP_056955036.1 tetracycline resistance ribosomal protection p...
912   0.0
WP_049515420.1 tetracycline resistance ribosomal protection p...
912   0.0
WP_070044453.1 TetM/TetW/TetO/TetS family tetracycline resist...
911   0.0
WP_055387115.1 tetracycline resistance ribosomal protection p...
911   0.0
WP_044681819.1 tetracycline resistance ribosomal protection p...
911   0.0
WP_031929533.1 tetracycline resistance ribosomal protection p...
911   0.0
WP_021137831.1 MULTISPECIES: TetM/TetW/TetO/TetS family tetra...
911   0.0
WP_063856114.1 tetracycline resistance ribosomal protection p...
911   0.0
AFD04636.1 tetracycline resistance protein [Veillonella parvula]
911   0.0
WP_002872163.1 MULTISPECIES: tetracycline resistance ribosoma...
911   0.0
WP_074626734.1 tetracycline resistance ribosomal protection p...
911   0.0
BAU68106.1 tetracycline resistance protein TetO [Streptococcu...
911   0.0
WP_019418832.1 MULTISPECIES: tetracycline resistance ribosoma...
911   0.0
WP_000691751.1 tetracycline resistance ribosomal protection p...
911   0.0
WP_099832549.1 tetracycline resistance ribosomal protection p...
910   0.0
WP_099971156.1 tetracycline resistance ribosomal protection p...
910   0.0
WP_026389858.1 MULTISPECIES: tetracycline resistance ribosoma...
910   0.0
WP_000691750.1 tetracycline resistance ribosomal protection p...
910   0.0
WP_002317540.1 tetracycline resistance ribosomal protection p...
910   0.0
ELU79162.1 putative translation elongation factor G [Streptoc...
910   0.0

WP_063856396.1 tetracycline resistance ribosomal protection p...
910   0.0
WP_101514745.1 tetracycline resistance ribosomal protection p...
910   0.0
WP_099460258.1 tetracycline resistance ribosomal protection p...
910   0.0
WP_081571465.1 tetracycline resistance ribosomal protection p...
910   0.0
BAU68111.1 tetracycline resistance protein TetO [Streptococcu...
910   0.0
CBW38807.1 Tetracycline resistance protein [Streptococcus pne...
910   0.0
WP_070439364.1 tetracycline resistance ribosomal protection p...
910   0.0
WP_061756818.1 tetracycline resistance ribosomal protection p...
910   0.0
WP_060809800.1 TetM/TetW/TetO/TetS family tetracycline resist...
910   0.0
WP_057095767.1 TetM/TetW/TetO/TetS family tetracycline resist...
910   0.0
WP_018574723.1 tetracycline resistance ribosomal protection p...
910   0.0
EFT39102.1 putative translation elongation factor G [Enteroco...
910   0.0
WP_039136739.1 tetracycline resistance ribosomal protection p...
909   0.0
AIF29622.1 tetracycline resistance protein [Campylobacter coli]
909   0.0
WP_004070750.1 TetM/TetW/TetO/TetS family tetracycline resist...
909   0.0
WP_002364936.1 MULTISPECIES: tetracycline resistance ribosoma...
909   0.0
WP_063856394.1 tetracycline resistance ribosomal protection p...
909   0.0
WP_105127927.1 tetracycline resistance ribosomal protection p...
909   0.0
WP_050201754.1 tetracycline resistance ribosomal protection p...
909   0.0
WP_000691723.1 tetracycline resistance ribosomal protection p...
909   0.0
WP_099958901.1 tetracycline resistance ribosomal protection p...
909   0.0
WP_050207143.1 tetracycline resistance ribosomal protection p...
909   0.0
WP_002335119.1 tetracycline resistance ribosomal protection p...
909   0.0
WP_002324549.1 tetracycline resistance ribosomal protection p...
909   0.0
ADV76307.1 TetM [Streptococcus oralis]
909   0.0
WP_014386813.1 MULTISPECIES: tetracycline resistance ribosoma...
909   0.0
APO27569.1 ferrous iron transport protein B [uncultured bacte...
908   0.0

WP_045773567.1 tetracycline resistance ribosomal protection p...
908   0.0
WP_044793436.1 tetracycline resistance ribosomal protection p...
908   0.0
WP_032582765.1 MULTISPECIES: TetM/TetW/TetO/TetS family tetra...
908   0.0
EOJ57657.1 tetracycline resistance protein tetM transposon [E...
908   0.0
WP_076005168.1 tetracycline resistance ribosomal protection p...
908   0.0
BAU68114.1 tetracycline resistance protein TetO [Streptococcu...
908   0.0
KXA54208.1 putative translation elongation factor G [Streptoc...
908   0.0
WP_050111690.1 tetracycline resistance ribosomal protection p...
908   0.0
WP_000691753.1 MULTISPECIES: tetracycline resistance ribosoma...
908   0.0
BAU68109.1 tetracycline resistance protein TetO [Streptococcu...
907   0.0
WP_049487775.1 MULTISPECIES: tetracycline resistance ribosoma...
907   0.0
WP_032581261.1 TetM/TetW/TetO/TetS family tetracycline resist...
907   0.0
WP_096541504.1 tetracycline resistance ribosomal protection p...
907   0.0
WP_094754176.1 tetracycline resistance ribosomal protection p...
907   0.0
WP_049474483.1 tetracycline resistance ribosomal protection p...
907   0.0
WP_033842223.1 tetracycline resistance ribosomal protection p...
907   0.0
WP_044116373.1 tetracycline resistance ribosomal protection p...
907   0.0
WP_010726404.1 tetracycline resistance ribosomal protection p...
907   0.0
BAU68107.1 tetracycline resistance protein TetO [Streptococcu...
906   0.0
WP_052996649.1 tetracycline resistance ribosomal protection p...
906   0.0
ADL64463.1 tetracycline resistance protein TetM [Staphylococc...
906   0.0
WP_000691748.1 tetracycline resistance ribosomal protection p...
906   0.0
WP_065736057.1 tetracycline resistance ribosomal protection p...
906   0.0
WP_000691737.1 MULTISPECIES: tetracycline resistance ribosoma...
906   0.0
WP_047200574.1 tetracycline resistance ribosomal protection p...
906   0.0
ACZ73819.1 TetO [Clostridium butyricum]
905   0.0
ADB66720.1 ribosomal protection protein [Streptococcus agalac...
905   0.0

```
WP_010724539.1  tetracycline resistance ribosomal protection p...
905     0.0
WP_094969908.1  tetracycline resistance ribosomal protection p...
905     0.0
WP_063284549.1  tetracycline resistance ribosomal protection p...
905     0.0
WP_053015161.1  tetracycline resistance ribosomal protection p...
905     0.0
WP_010706106.1  tetracycline resistance ribosomal protection p...
905     0.0
AIU85825.1  Tetracycline resistance protein TetM [Staphylococc...
904     0.0
WP_042857111.1  tetracycline resistance ribosomal protection p...
904     0.0
WP_107372276.1  tetracycline resistance ribosomal protection p...
903     0.0
WP_060544734.1  tetracycline resistance ribosomal protection p...
903     0.0
WP_103242225.1  TetM/TetW/TetO/TetS family tetracycline resist...
903     0.0
APO28030.1  ferrous iron transport protein B [uncultured bacte...
903     0.0
WP_053015717.1  tetracycline resistance ribosomal protection p...
903     0.0
WP_063856108.1  tetracycline resistance ribosomal protection p...
903     0.0
WP_000691738.1  tetracycline resistance ribosomal protection p...
903     0.0
WP_063856410.1  tetracycline resistance ribosomal protection p...
903     0.0
WP_073120086.1  tetracycline resistance ribosomal protection p...
902     0.0
SCT38110.1  tetracycline resistance protein TetM [Staphylococc...
902     0.0
ACZ73820.1  TetO [Clostridium butyricum]
902     0.0
WP_016227219.1  TetM/TetW/TetO/TetS family tetracycline resist...
902     0.0
WP_086325430.1  tetracycline resistance ribosomal protection p...
902     0.0
WP_084099453.1  tetracycline resistance ribosomal protection p...
902     0.0
KXW28397.1  tetracycline resistance protein tetM [Streptococcu...
901     0.0
WP_079042646.1  TetM/TetW/TetO/TetS family tetracycline resist...
900     0.0
APO28881.1  Ferrous iron transport protein B [uncultured bacte...
897     0.0
ADO27721.1  tetracycline-resistance protein [Streptococcus suis]
896     0.0
APO30942.1  ferrous iron transport protein B [uncultured bacte...
895     0.0
WP_002405437.1  MULTISPECIES: tetracycline resistance ribosoma...
895     0.0
```

WP_000691722.1 tetracycline resistance ribosomal protection p...
895   0.0
WP_095803332.1 TetM/TetW/TetO/TetS family tetracycline resist...
895   0.0
APO29877.1 Ferrous iron transport protein B [uncultured bacte...
895   0.0
AMP54002.1 TetM-TetW-TetO-TetS [uncultured bacterium]
894   0.0
EOL83668.1 tetracycline resistance protein tetS [Enterococcus...
894   0.0
WP_021401470.1 MULTISPECIES: tetracycline resistance ribosoma...
894   0.0
WP_080115046.1 TetM/TetW/TetO/TetS family tetracycline resist...
894   0.0
WP_101912895.1 tetracycline resistance ribosomal protection p...
893   0.0
WP_011997530.1 tetracycline resistance ribosomal protection p...
893   0.0
WP_063856411.1 tetracycline resistance ribosomal protection p...
893   0.0
WP_014262666.1 MULTISPECIES: tetracycline resistance ribosoma...
892   0.0
WP_005228367.1 TetM/TetW/TetO/TetS family tetracycline resist...
892   0.0
WP_011860841.1 MULTISPECIES: tetracycline resistance ribosoma...
890   0.0
AMP55630.1 TetM-TetW-TetO-TetS [uncultured bacterium]
890   0.0
WP_050975574.1 tetracycline resistance ribosomal protection p...
889   0.0
WP_031255279.1 tetracycline resistance ribosomal protection p...
889   0.0
AAL27026.1 tetracycline resistance protein TetM [Staphylococc...
887   0.0
AAL27025.1 tetracycline resistance protein TetM [Staphylococc...
884   0.0
KWW83509.1 tetracycline resistance protein tetM [Enterococcus...
882   0.0
WP_065735440.1 tetracycline resistance ribosomal protection p...
877   0.0
AAO24820.1 TetM [Clostridioides difficile]
877   0.0
APO28168.1 ferrous iron transport protein B [uncultured bacte...
876   0.0
AAO16478.1 putative tetracycline resistance protein [uncultur...
871   0.0
AAL27024.1 tetracycline resistance protein TetM [Staphylococc...
870   0.0
APO32506.1 ferrous iron transport protein B [uncultured bacte...
863   0.0
WP_078154654.1 tetracycline resistance ribosomal protection p...
863   0.0
WP_085826518.1 tetracycline resistance ribosomal protection p...
860   0.0

WP_044769927.1 TetM/TetW/TetO/TetS family tetracycline resist...
859   0.0
ASQ41002.1 TetO [Campylobacter coli]
858   0.0
EPX10696.1 tetracycline resistance protein tetM [Streptococcu...
856   0.0
WP_080440411.1 tetracycline resistance ribosomal protection p...
855   0.0
SMJ13358.1 tetracycline resistance protein TetP [Enterococcus...
853   0.0
CIR68804.1 tetracycline resistance protein TetM [Streptococcu...
849   0.0
AIG19304.1 TetM [Escherichia coli]
849   0.0
WP_081291434.1 elongation factor G [Bifidobacterium longum]
848   0.0
AIG19305.1 TetM [Escherichia coli]
847   0.0
AMP50231.1 TetM-TetW-TetO-TetS [uncultured bacterium]
847   0.0
CYU66281.1 tetracycline resistance protein tetM [Streptococcu...
845   0.0
WP_101527334.1 TetM/TetW/TetO/TetS family tetracycline resist...
843   0.0
OBV22681.1 putative tetracycline resistance protein TetM [Sta...
843   0.0
WP_070673567.1 TetM/TetW/TetO/TetS family tetracycline resist...
841   0.0
CAJ09686.1 tetracycline resistance protein [Lactococcus garvi...
839   0.0
CAJ09688.1 tetracycline resistance protein [Enterococcus faec...
839   0.0
CAJ09687.1 tetracycline resistance protein [Enterococcus durans]
837   0.0
AHN92781.1 elongation factor Tu GTP binding domain-containing...
837   0.0
AAO16479.1 putative tetracycline resistance protein [uncultur...
834   0.0
AMP57965.1 TetM-TetW-TetO-TetS [uncultured bacterium]
833   0.0
AMP53019.1 TetM-TetW-TetO-TetS [uncultured bacterium]
829   0.0
CAJ09689.1 tetracycline resistance protein [Enterococcus faec...
826   0.0
ACL29762.1 tetracycline resistance protein TetW [Bifidobacter...
823   0.0
SCP96838.1 translation elongation factor 2 (EF-2/EF-G) [Anaer...
823   0.0
APO33198.1 EF-G: translation elongation factor [uncultured ba...
821   0.0
WP_070604146.1 GTP-binding protein [Rothia sp. HMSC062H08]
816   0.0
CAJ09685.1 tetracycline resistance protein [Lactobacillus pla...
816   0.0

WP_050282912.1 TetM/TetW/TetO/TetS family tetracycline resist...
814   0.0
WP_081636729.1 TetM/TetW/TetO/TetS family tetracycline resist...
811   0.0
KOA53398.1 elongation factor G [Bifidobacterium breve MCC 0305]
811   0.0
AVE02224.1 TetM [Neisseria gonorrhoeae]
810   0.0
AVE02236.1 TetM [Neisseria gonorrhoeae]
810   0.0
AVE02243.1 TetM [Neisseria gonorrhoeae]
810   0.0
AVE02230.1 TetM [Neisseria gonorrhoeae]
809   0.0
AVE02216.1 TetM [Neisseria gonorrhoeae]
808   0.0
AVE02221.1 TetM [Neisseria gonorrhoeae]
808   0.0
ACI02031.1 tetracycline resistance protein [uncultured bacter...
807   0.0
AVE02231.1 TetM [Neisseria gonorrhoeae]
807   0.0
AMJ37087.1 TetM-TetW-TetO-TetS [uncultured bacterium]
807   0.0
AMP50915.1 TetM-TetW-TetO-TetS [uncultured bacterium]
806   0.0
AVE02223.1 TetM [Neisseria gonorrhoeae]
805   0.0
WP_087663472.1 TetM/TetW/TetO/TetS family tetracycline resist...
805   0.0
WP_070015747.1 TetM/TetW/TetO/TetS family tetracycline resist...
805   0.0
WP_086141897.1 hypothetical protein [Lactobacillus reuteri]
805   0.0
WP_102790109.1 TetM/TetW/TetO/TetS family tetracycline resist...
803   0.0
WP_070032070.1 TetM/TetW/TetO/TetS family tetracycline resist...
800   0.0
WP_070047116.1 TetM/TetW/TetO/TetS family tetracycline resist...
799   0.0
WP_069988658.1 TetM/TetW/TetO/TetS family tetracycline resist...
796   0.0
AMP57957.1 TetM-TetW-TetO-TetS [uncultured bacterium]
792   0.0
WP_080610153.1 TetM/TetW/TetO/TetS family tetracycline resist...
791   0.0
EPD48595.1 tetracycline resistance protein tetO [Corynebacter...
791   0.0
WP_050209792.1 TetM/TetW/TetO/TetS family tetracycline resist...
788   0.0
CQH34122.1 tetracycline resistance protein TetM [Streptococcu...
787   0.0
WP_100618510.1 TetM/TetW/TetO/TetS family tetracycline resist...
786   0.0

CFP99304.1 tetracycline resistance protein TetM [Streptococcu...
786   0.0
WP_078127095.1 TetM/TetW/TetO/TetS family tetracycline resist...
784   0.0
WP_050269353.1 TetM/TetW/TetO/TetS family tetracycline resist...
783   0.0
CAR48432.1 tetracycline resistance protein Tet(M) [Enterococc...
783   0.0
CAR48433.1 tetracycline resistance protein Tet(M) [Enterococc...
780   0.0
EEG70485.1 GTP-binding domain protein [Bifidobacterium pseudo...
779   0.0
CJI69577.1 tetracycline resistance protein TetM [Streptococcu...
778   0.0
ABC61394.1 tetracycline resistance [uncultured organism]
778   0.0
ACY03204.1 tetracycline resistance protein [uncultured organism]
777   0.0
ACY03229.1 tetracycline resistance protein [uncultured organism]
776   0.0
ACY03216.1 tetracycline resistance protein [uncultured organism]
776   0.0
ACY03231.1 tetracycline resistance protein [uncultured organism]
776   0.0
ACY03224.1 tetracycline resistance protein [uncultured organism]
776   0.0
ACY03161.1 tetracycline resistance protein [uncultured organism]
776   0.0
ACY03156.1 tetracycline resistance protein [uncultured organism]
776   0.0
ACY03110.1 tetracycline resistance protein [uncultured organism]
776   0.0
ACY03109.1 tetracycline resistance protein [uncultured organism]
776   0.0
ACY03096.1 tetracycline resistance protein [uncultured organism]
776   0.0
ACY03093.1 tetracycline resistance protein [uncultured organism]
776   0.0
ACY03086.1 tetracycline resistance protein [uncultured organism]
776   0.0
ACY03059.1 tetracycline resistance protein [uncultured organism]
776   0.0
ACY03053.1 tetracycline resistance protein [uncultured organism]
776   0.0
ACY03049.1 tetracycline resistance protein [uncultured organism]
776   0.0
ACY03044.1 tetracycline resistance protein [uncultured organism]
776   0.0
ACY03024.1 tetracycline resistance protein [uncultured organism]
776   0.0
ABC61437.1 tetracycline resistance [uncultured organism]
776   0.0
ABC61396.1 tetracycline resistance [uncultured organism]
776   0.0

ACY03177.1 tetracycline resistance protein [uncultured organism]
775   0.0
ACY03167.1 tetracycline resistance protein [uncultured organism]
775   0.0
ACY03056.1 tetracycline resistance protein [uncultured organism]
775   0.0
ABC61443.1 tetracycline resistance [uncultured organism]
775   0.0
ABC61401.1 tetracycline resistance [uncultured organism]
775   0.0
ABC61400.1 tetracycline resistance [uncultured organism]
775   0.0
ABC61438.1 tetracycline resistance [uncultured organism]
775   0.0
ACY03198.1 tetracycline resistance protein [uncultured organism]
775   0.0
ACY03026.1 tetracycline resistance protein [uncultured organism]
775   0.0
ABC61393.1 tetracycline resistance [uncultured organism]
775   0.0
ABC61398.1 tetracycline resistance [uncultured organism]
775   0.0
ACY03265.1 tetracycline resistance protein [uncultured organism]
774   0.0
ACY03213.1 tetracycline resistance protein [uncultured organism]
774   0.0
ACY03112.1 tetracycline resistance protein [uncultured organism]
774   0.0
ACY03088.1 tetracycline resistance protein [uncultured organism]
774   0.0
ACY03039.1 tetracycline resistance protein [uncultured organism]
774   0.0
ACY03034.1 tetracycline resistance protein [uncultured organism]
774   0.0
CAR48431.1 tetracycline resistance protein Tet(M) [Enterococc...
774   0.0
ABC61428.1 tetracycline resistance [uncultured organism]
774   0.0
ACY03272.1 tetracycline resistance protein [uncultured organism]
774   0.0
ACY03233.1 tetracycline resistance protein [uncultured organism]
774   0.0
ACY03202.1 tetracycline resistance protein [uncultured organism]
774   0.0
ACY03089.1 tetracycline resistance protein [uncultured organism]
774   0.0
ABC61432.1 tetracycline resistance [uncultured organism]
774   0.0
ABC61421.1 tetracycline resistance [uncultured organism]
774   0.0
ABC61452.1 tetracycline resistance [uncultured organism]
774   0.0
ACY03259.1 tetracycline resistance protein [uncultured organism]
773   0.0

ACY03242.1 tetracycline resistance protein [uncultured organism]
773   0.0
ACY03241.1 tetracycline resistance protein [uncultured organism]
773   0.0
ACY03218.1 tetracycline resistance protein [uncultured organism]
773   0.0
ACY03138.1 tetracycline resistance protein [uncultured organism]
773   0.0
ACY03122.1 tetracycline resistance protein [uncultured organism]
773   0.0
ACY03235.1 tetracycline resistance protein [uncultured organism]
773   0.0
ACY03152.1 tetracycline resistance protein [uncultured organism]
773   0.0
ACY03166.1 tetracycline resistance protein [uncultured organism]
773   0.0
ABC61446.1 tetracycline resistance [uncultured organism]
773   0.0
ACY03197.1 tetracycline resistance protein [uncultured organism]
772   0.0
ACY03188.1 tetracycline resistance protein [uncultured organism]
772   0.0
ACY03215.1 tetracycline resistance protein [uncultured organism]
772   0.0
ACY03148.1 tetracycline resistance protein [uncultured organism]
772   0.0
ACY03232.1 tetracycline resistance protein [uncultured organism]
771   0.0
ACY03169.1 tetracycline resistance protein [uncultured organism]
771   0.0
ACY03038.1 tetracycline resistance protein [uncultured organism]
771   0.0
ACY03033.1 tetracycline resistance protein [uncultured organism]
771   0.0
ABC61444.1 tetracycline resistance [uncultured organism]
771   0.0
ABC61464.1 tetracycline resistance [uncultured organism]
771   0.0
ACY03251.1 tetracycline resistance protein [uncultured organism]
771   0.0
ACY03244.1 tetracycline resistance protein [uncultured organism]
771   0.0
ACY03238.1 tetracycline resistance protein [uncultured organism]
771   0.0
ACY03228.1 tetracycline resistance protein [uncultured organism]
771   0.0
ACY03222.1 tetracycline resistance protein [uncultured organism]
771   0.0
ACY03046.1 tetracycline resistance protein [uncultured organism]
771   0.0
ABC61453.1 tetracycline resistance [uncultured organism]
771   0.0
ACY03264.1 tetracycline resistance protein [uncultured organism]
771   0.0

ABC61434.1 tetracycline resistance [uncultured organism]
771   0.0
AMP55651.1 TetM-TetW-TetO-TetS [uncultured bacterium]
770   0.0
ACY03267.1 tetracycline resistance protein [uncultured organism]
770   0.0
ACY03128.1 tetracycline resistance protein [uncultured organism]
770   0.0
ACY03023.1 tetracycline resistance protein [uncultured organism]
770   0.0
ACY03203.1 tetracycline resistance protein [uncultured organism]
769   0.0
ACY03214.1 tetracycline resistance protein [uncultured organism]
769   0.0
ACY03035.1 tetracycline resistance protein [uncultured organism]
769   0.0
ACY03070.1 tetracycline resistance protein [uncultured organism]
769   0.0
ACY03041.1 tetracycline resistance protein [uncultured organism]
769   0.0
ACY03040.1 tetracycline resistance protein [uncultured organism]
769   0.0
ABC61439.1 tetracycline resistance [uncultured organism]
769   0.0
ACY03126.1 tetracycline resistance protein [uncultured organism]
768   0.0
ACY03067.1 tetracycline resistance protein [uncultured organism]
768   0.0
ABC61431.1 tetracycline resistance [uncultured organism]
768   0.0
ABC61442.1 tetracycline resistance [uncultured organism]
768   0.0
ABC61399.1 tetracycline resistance [uncultured organism]
768   0.0
ABC61430.1 tetracycline resistance [uncultured organism]
768   0.0
ACY03127.1 tetracycline resistance protein [uncultured organism]
767   0.0
ACY03083.1 tetracycline resistance protein [uncultured organism]
767   0.0
ABC61433.1 tetracycline resistance [uncultured organism]
767   0.0
ACY03226.1 tetracycline resistance protein [uncultured organism]
766   0.0
ACY03130.1 tetracycline resistance protein [uncultured organism]
766   0.0
ACY03117.1 tetracycline resistance protein [uncultured organism]
766   0.0
ACY03257.1 tetracycline resistance protein [uncultured organism]
765   0.0
ACY03190.1 tetracycline resistance protein [uncultured organism]
765   0.0
ACY03055.1 tetracycline resistance protein [uncultured organism]
764   0.0

ACY03240.1 tetracycline resistance protein [uncultured organism]
764   0.0
ACY03136.1 tetracycline resistance protein [uncultured organism]
764   0.0
ACY03123.1 tetracycline resistance protein [uncultured organism]
764   0.0
ACY03146.1 tetracycline resistance protein [uncultured organism]
764   0.0
ACY03078.1 tetracycline resistance protein [uncultured organism]
764   0.0
ACY03072.1 tetracycline resistance protein [uncultured organism]
764   0.0
ACY03029.1 tetracycline resistance protein [uncultured organism]
764   0.0
ACY03025.1 tetracycline resistance protein [uncultured organism]
763   0.0
APO32512.1 EF-G: translation elongation factor [uncultured ba...
763   0.0
ACY03042.1 tetracycline resistance protein [uncultured organism]
763   0.0
ABC61429.1 tetracycline resistance [uncultured organism]
763   0.0
AAO16480.1 putative tetracycline resistance protein [uncultur...
763   0.0
APO35565.1 EF-G: translation elongation factor [uncultured ba...
763   0.0
ACY03061.1 tetracycline resistance protein [uncultured organism]
763   0.0
ACY03223.1 tetracycline resistance protein [uncultured organism]
762   0.0
ABC61449.1 tetracycline resistance [uncultured organism]
762   0.0
ACY03178.1 tetracycline resistance protein [uncultured organism]
762   0.0
ACY03071.1 tetracycline resistance protein [uncultured organism]
762   0.0
ACY03069.1 tetracycline resistance protein [uncultured organism]
762   0.0
ACY03211.1 tetracycline resistance protein [uncultured organism]
761   0.0
EMG24351.1 Tetracycline resistance protein tetS [Streptococcu...
761   0.0
ACY03150.1 tetracycline resistance protein [uncultured organism]
761   0.0
ACY03103.1 tetracycline resistance protein [uncultured organism]
761   0.0
ACY03084.1 tetracycline resistance protein [uncultured organism]
761   0.0
ACY03079.1 tetracycline resistance protein [uncultured organism]
761   0.0
ACY03062.1 tetracycline resistance protein [uncultured organism]
761   0.0
ABC61440.1 tetracycline resistance [uncultured organism]
761   0.0

ACY03186.1 tetracycline resistance protein [uncultured organism]
761   0.0
ACY03181.1 tetracycline resistance protein [uncultured organism]
761   0.0
ACY03160.1 tetracycline resistance protein [uncultured organism]
761   0.0
ACY03140.1 tetracycline resistance protein [uncultured organism]
761   0.0
ACY03124.1 tetracycline resistance protein [uncultured organism]
761   0.0
ACY03101.1 tetracycline resistance protein [uncultured organism]
761   0.0
ACY03097.1 tetracycline resistance protein [uncultured organism]
761   0.0
ABC61460.1 tetracycline resistance [uncultured organism]
761   0.0
ABC61459.1 tetracycline resistance [uncultured organism]
761   0.0
ACY03270.1 tetracycline resistance protein [uncultured organism]
760   0.0
ACY03217.1 tetracycline resistance protein [uncultured organism]
760   0.0
ACY03205.1 tetracycline resistance protein [uncultured organism]
760   0.0
ACY03196.1 tetracycline resistance protein [uncultured organism]
760   0.0
ACY03173.1 tetracycline resistance protein [uncultured organism]
760   0.0
ACY03147.1 tetracycline resistance protein [uncultured organism]
760   0.0
ACY03105.1 tetracycline resistance protein [uncultured organism]
760   0.0
ACY03091.1 tetracycline resistance protein [uncultured organism]
760   0.0
ACY03065.1 tetracycline resistance protein [uncultured organism]
760   0.0
ACY03063.1 tetracycline resistance protein [uncultured organism]
760   0.0
ACY03037.1 tetracycline resistance protein [uncultured organism]
760   0.0
ABC61424.1 tetracycline resistance [uncultured organism]
760   0.0
AMP51880.1 TetM-TetW-TetO-TetS [uncultured bacterium]
760   0.0
ACY03227.1 tetracycline resistance protein [uncultured organism]
760   0.0
ACY03179.1 tetracycline resistance protein [uncultured organism]
760   0.0
ACY03135.1 tetracycline resistance protein [uncultured organism]
760   0.0
ACY03154.1 tetracycline resistance protein [uncultured organism]
759   0.0
ABC61467.1 tetracycline resistance [uncultured organism]
759   0.0

ABC61376.1 tetracycline resistance [uncultured organism]
759   0.0
ACY03263.1 tetracycline resistance protein [uncultured organism]
759   0.0
ACY03254.1 tetracycline resistance protein [uncultured organism]
759   0.0
ACY03185.1 tetracycline resistance protein [uncultured organism]
759   0.0
ACY03098.1 tetracycline resistance protein [uncultured organism]
759   0.0
ABC61397.1 tetracycline resistance [uncultured organism]
759   0.0
WP_050273534.1 TetM/TetW/TetO/TetS family tetracycline resist...
759   0.0
ACY03266.1 tetracycline resistance protein [uncultured organism]
759   0.0
ACY03261.1 tetracycline resistance protein [uncultured organism]
759   0.0
ACY03250.1 tetracycline resistance protein [uncultured organism]
759   0.0
ACY03210.1 tetracycline resistance protein [uncultured organism]
759   0.0
ABC61450.1 tetracycline resistance [uncultured organism]
759   0.0
ABC61458.1 tetracycline resistance [uncultured organism]
759   0.0
ACY03195.1 tetracycline resistance protein [uncultured organism]
758   0.0
ACY03174.1 tetracycline resistance protein [uncultured organism]
758   0.0
ACY03171.1 tetracycline resistance protein [uncultured organism]
758   0.0
ACY03151.1 tetracycline resistance protein [uncultured organism]
758   0.0
ACY03120.1 tetracycline resistance protein [uncultured organism]
758   0.0
ACY03047.1 tetracycline resistance protein [uncultured organism]
758   0.0
ACY03028.1 tetracycline resistance protein [uncultured organism]
758   0.0
CAR48430.1 tetracycline resistance protein Tet(M) [Enterococc...
758   0.0
ACY03208.1 tetracycline resistance protein [uncultured organism]
758   0.0
ACY03159.1 tetracycline resistance protein [uncultured organism]
758   0.0
ACY03104.1 tetracycline resistance protein [uncultured organism]
758   0.0
ABC61381.1 tetracycline resistance [uncultured organism]
758   0.0
ACY03077.1 tetracycline resistance protein [uncultured organism]
758   0.0
ACY03050.1 tetracycline resistance protein [uncultured organism]
758   0.0

ABC61383.1 tetracycline resistance [uncultured organism]
758   0.0
ABC61390.1 tetracycline resistance [uncultured organism]
758   0.0
AAP21776.1 putative ribosomal protection protein [uncultured ...
758   0.0
ACY03268.1 tetracycline resistance protein [uncultured organism]
757   0.0
ACY03107.1 tetracycline resistance protein [uncultured organism]
757   0.0
ACY03192.1 tetracycline resistance protein [uncultured organism]
757   0.0
ACY03144.1 tetracycline resistance protein [uncultured organism]
757   0.0
ACY03057.1 tetracycline resistance protein [uncultured organism]
757   0.0
ABC61388.1 tetracycline resistance [uncultured organism]
757   0.0
ACY03225.1 tetracycline resistance protein [uncultured organism]
756   0.0
ACY03043.1 tetracycline resistance protein [uncultured organism]
756   0.0
ACY03219.1 tetracycline resistance protein [uncultured organism]
756   0.0
ABC61426.1 tetracycline resistance [uncultured organism]
756   0.0
ABC61375.1 tetracycline resistance [uncultured organism]
756   0.0
ABC61391.1 tetracycline resistance [uncultured organism]
756   0.0
ACY03194.1 tetracycline resistance protein [uncultured organism]
755   0.0
ACY03051.1 tetracycline resistance protein [uncultured organism]
755   0.0
ACY03247.1 tetracycline resistance protein [uncultured organism]
754   0.0
ABC61384.1 tetracycline resistance [uncultured organism]
754   0.0
ABC61448.1 tetracycline resistance [uncultured organism]
754   0.0
ACY03189.1 tetracycline resistance protein [uncultured organism]
754   0.0
ACY03153.1 tetracycline resistance protein [uncultured organism]
754   0.0
ABC61386.1 tetracycline resistance [uncultured organism]
754   0.0
ACY03256.1 tetracycline resistance protein [uncultured organism]
754   0.0
ACY03249.1 tetracycline resistance protein [uncultured organism]
753   0.0
ACY03191.1 tetracycline resistance protein [uncultured organism]
753   0.0
ACY03074.1 tetracycline resistance protein [uncultured organism]
753   0.0

ACY03132.1 tetracycline resistance protein [uncultured organism]
752   0.0
ABC61441.1 tetracycline resistance [uncultured organism]
751   0.0
ABC61413.1 tetracycline resistance [uncultured organism]
751   0.0
ACY03113.1 tetracycline resistance protein [uncultured organism]
751   0.0
ACY03187.1 tetracycline resistance protein [uncultured organism]
750   0.0
AHF25349.1 tetracycline resistance [uncultured bacterium Cont...
750   0.0
ACY03157.1 tetracycline resistance protein [uncultured organism]
749   0.0
ABC61463.1 tetracycline resistance [uncultured organism]
748   0.0
OQM49056.1 tetracycline resistance protein tetM [Bifidobacter...
748   0.0
ACY03022.1 tetracycline resistance protein [uncultured organism]
748   0.0
ACY03073.1 tetracycline resistance protein [uncultured organism]
747   0.0
ABC61445.1 tetracycline resistance [uncultured organism]
747   0.0
WP_095296275.1 tetracycline resistance ribosomal protection p...
744   0.0
ACY03182.1 tetracycline resistance protein [uncultured organism]
744   0.0
WP_085811604.1 GTP-binding protein [Enterococcus faecium]
743   0.0
ACY03184.1 tetracycline resistance protein [uncultured organism]
743   0.0
ACY03082.1 tetracycline resistance protein [uncultured organism]
741   0.0
ABC61427.1 tetracycline resistance [uncultured organism]
741   0.0
ACY03058.1 tetracycline resistance protein [uncultured organism]
740   0.0
WP_085842501.1 GTP-binding protein [Enterococcus faecium]
739   0.0
ABC61409.1 tetracycline resistance [uncultured organism]
739   0.0
WP_085852053.1 GTP-binding protein [Enterococcus faecium]
738   0.0
ACY03066.1 tetracycline resistance protein [uncultured organism]
738   0.0
ACY03064.1 tetracycline resistance protein [uncultured organism]
738   0.0
ACY03142.1 tetracycline resistance protein [uncultured organism]
738   0.0
ACY03175.1 tetracycline resistance protein [uncultured organism]
738   0.0
ACY03121.1 tetracycline resistance protein [uncultured organism]
738   0.0

ABC61416.1 tetracycline resistance [uncultured organism]
737   0.0
ABC61425.1 tetracycline resistance [uncultured organism]
737   0.0
ABC61462.1 tetracycline resistance [uncultured organism]
737   0.0
OUK46079.1 tetracycline resistance ribosomal protection prote...
737   0.0
WP_085815752.1 GTP-binding protein [Enterococcus faecium]
737   0.0
ACY03271.1 tetracycline resistance protein [uncultured organism]
737   0.0
ABC61402.1 tetracycline resistance [uncultured organism]
736   0.0
ACY03221.1 tetracycline resistance protein [uncultured organism]
736   0.0
ACY03170.1 tetracycline resistance protein [uncultured organism]
736   0.0
ABC61412.1 tetracycline resistance [uncultured organism]
736   0.0
APO27858.1 ferrous iron transport protein B [uncultured bacte...
735   0.0
ACY03116.1 tetracycline resistance protein [uncultured organism]
735   0.0
ABC61415.1 tetracycline resistance [uncultured organism]
735   0.0
WP_050273543.1 GTP-binding protein [Streptococcus pneumoniae]
734   0.0
ABC61414.1 tetracycline resistance [uncultured organism]
734   0.0
ABC61420.1 tetracycline resistance [uncultured organism]
734   0.0
ABC61410.1 tetracycline resistance [uncultured organism]
733   0.0
ABC61407.1 tetracycline resistance [uncultured organism]
733   0.0
ABC61408.1 tetracycline resistance [uncultured organism]
732   0.0
ACY03085.1 tetracycline resistance protein [uncultured organism]
732   0.0
ABC61417.1 tetracycline resistance [uncultured organism]
732   0.0
ABC61423.1 tetracycline resistance [uncultured organism]
731   0.0
ABC61457.1 tetracycline resistance [uncultured organism]
731   0.0
WP_105166162.1 GTP-binding protein [Escherichia coli]
731   0.0
AMJ37904.1 TetM-TetW-TetO-TetS [uncultured bacterium]
731   0.0
ACY03237.1 tetracycline resistance protein [uncultured organism]
730   0.0
ACY03115.1 tetracycline resistance protein [uncultured organism]
729   0.0

ACY03076.1 tetracycline resistance protein [uncultured organism]
729   0.0
ABC61455.1 tetracycline resistance [uncultured organism]
729   0.0
APO34029.1 ferrous iron transport protein B [uncultured bacte...
729   0.0
ABC61422.1 tetracycline resistance [uncultured organism]
728   0.0
ACY03027.1 tetracycline resistance protein [uncultured organism]
727   0.0
ACY03131.1 tetracycline resistance protein [uncultured organism]
726   0.0
ACY03045.1 tetracycline resistance protein [uncultured organism]
726   0.0
ACY03172.1 tetracycline resistance protein [uncultured organism]
725   0.0
EFT49119.1 GTP-binding domain protein [Enterococcus faecalis ...
724   0.0
WP_025187974.1 TetM/TetW/TetO/TetS family tetracycline resist...
724   0.0
ACY03137.1 tetracycline resistance protein [uncultured organism]
724   0.0
WP_061099911.1 GTP-binding protein [Enterococcus faecium]
723   0.0
ACY03134.1 tetracycline resistance protein [uncultured organism]
721   0.0
ABC61461.1 tetracycline resistance [uncultured organism]
717   0.0
AMP53599.1 TetM-TetW-TetO-TetS [uncultured bacterium]
717   0.0
AMJ32412.1 TetM-TetW-TetO-TetS [uncultured bacterium]
717   0.0
ACY03258.1 tetracycline resistance protein [uncultured organism]
717   0.0
ACY03200.1 tetracycline resistance protein [uncultured organism]
716   0.0
ACY03180.1 tetracycline resistance protein [uncultured organism]
715   0.0
AMJ33417.1 TetM-TetW-TetO-TetS [uncultured bacterium]
714   0.0
ACY03036.1 tetracycline resistance protein [uncultured organism]
713   0.0
ACD97480.1 truncated tetracycline resistance protein [Bifidob...
711   0.0
WP_060786374.1 tetracycline resistance protein tetM [Campylob...
708   0.0
OUZ18231.1 tetracycline resistance protein TetM transposon [E...
707   0.0
AKS25200.1 TetM [Ureaplasma parvum]
705   0.0
AAR22500.1 tetracycline resistance protein [Lactobacillus sakei]
701   0.0
ACY03165.1 tetracycline resistance protein [uncultured organism]
699   0.0

ABC61465.1 tetracycline resistance [uncultured organism]
698   0.0
WP_050305670.1 GTP-binding protein [Streptococcus agalactiae]
693   0.0
WP_105223001.1 GTP-binding protein [Enterococcus faecium]
690   0.0
CCH26394.1 tetracycline-resistance protein [Streptococcus pyo...
689   0.0
WP_104760463.1 GTP-binding protein [Enterococcus faecium]
688   0.0
ACY03168.1 tetracycline resistance protein [uncultured organism]
687   0.0
WP_104771991.1 GTP-binding protein [Enterococcus faecium]
686   0.0
EEA80120.1 elongation factor Tu domain 2 [Tyzzerella nexilis ...
686   0.0
WP_072419560.1 tetracycline resistance protein [Ruminococcus ...
683   0.0
APO26792.1 ferrous iron transport protein B [uncultured bacte...
682   0.0
WP_057557796.1 GTP-binding protein [Streptococcus pneumoniae]
679   0.0
WP_100042644.1 tetracycline resistance ribosomal protection p...
674   0.0
WP_086142194.1 elongation factor G [Lactobacillus reuteri]
672   0.0
APO28025.1 ferrous iron transport protein B [uncultured bacte...
672   0.0
CUO81801.1 Tetracycline resistance protein tetM from transpos...
671   0.0
WP_050696799.1 tetracycline resistance ribosomal protection p...
671   0.0
WP_025234518.1 TetM/TetW/TetO/TetS family tetracycline resist...
671   0.0
ACY03030.1 tetracycline resistance protein [uncultured organism]
669   0.0
WP_092244206.1 tetracycline resistance ribosomal protection p...
669   0.0
WP_002323361.1 TetM/TetW/TetO/TetS family tetracycline resist...
669   0.0
CAJ57437.1 tetracycline resistance protein [Bifidobacterium p...
669   0.0
EFY04904.1 GTP-binding domain protein [Phascolarctobacterium ...
665   0.0
KZK31390.1 tetracycline resistance protein tetM [Enterococcus...
664   0.0
APO31176.1 EF-G: translation elongation factor [uncultured ba...
662   0.0
SCG90217.1 Tetracycline resistance protein tetM from transpos...
662   0.0
AMP47123.1 TetM-TetW-TetO-TetS [uncultured bacterium]
661   0.0
CAJ57440.1 tetracycline resistance protein [Bifidobacterium a...
661   0.0

AMP55463.1 TetM-TetW-TetO-TetS [uncultured bacterium]
660   0.0
CAJ57439.1 tetracycline resistance protein [Bifidobacterium a...
660   0.0
CAJ57441.1 tetracycline resistance protein [Bifidobacterium a...
659   0.0
ACT97609.1 TetW [uncultured organism]
658   0.0
WP_026891211.1 TetM/TetW/TetO/TetS family tetracycline resist...
657   0.0
AMP54011.1 TetM-TetW-TetO-TetS [uncultured bacterium]
654   0.0
WP_085910368.1 elongation factor G [Enterococcus faecium]
653   0.0
WP_100303572.1 tetracycline resistance ribosomal protection p...
653   0.0
WP_081140374.1 GTP-binding protein [Enterococcus faecium]
652   0.0
WP_084913399.1 elongation factor G [Streptococcus salivarius]
652   0.0
WP_044755690.1 TetM/TetW/TetO/TetS family tetracycline resist...
652   0.0
WP_013273781.1 TetM/TetW/TetO/TetS family tetracycline resist...
650   0.0
AAN84500.1 tetracycline resistance protein [Lactobacillus sak...
650   0.0
AIW80491.1 TetM-TetW-TetO-TetS [uncultured bacterium]
649   0.0
AAN84489.1 tetracycline resistance protein [Lactobacillus ali...
648   0.0
CKE26186.1 tetracycline resistance protein TetM [Streptococcu...
648   0.0
AAN84498.1 tetracycline resistance protein [Lactobacillus pla...
647   0.0
AAN84481.1 tetracycline resistance protein [Lactobacillus sak...
646   0.0
AAN84484.1 tetracycline resistance protein [Lactobacillus cur...
646   0.0
CAG28845.1 tetracycline resistance protein tetM [Listeria mon...
646   0.0
AAN84492.1 tetracycline resistance protein [Lactobacillus pla...
646   0.0
AAN84486.1 tetracycline resistance protein [Lactobacillus sak...
645   0.0
AAN84482.1 tetracycline resistance protein [Lactobacillus sak...
645   0.0
AAN84478.1 tetracycline resistance protein [Lactobacillus pla...
644   0.0
AAN84496.1 tetracycline resistance protein [Lactobacillus sak...
644   0.0
CAG28846.1 tetracycline resistance protein tetM [Listeria inn...
644   0.0
WP_049099061.1 GTP-binding protein [Enterococcus faecalis]
644   0.0

EGG59021.1 elongation factor Tu domain 2 [Enterococcus faecal...
644   0.0
CAE51931.1 tetracycline resistance protein TetM [Enterococcus...
644   0.0
AAN84501.1 tetracycline resistance protein [Lactobacillus pla...
644   0.0
AAN84493.1 tetracycline resistance protein [Lactobacillus pla...
644   0.0
CAE51932.1 tetracycline resistance protein TetM [Enterococcus...
643   0.0
CAE51930.1 tetracycline resistance protein TetM [Enterococcus...
643   0.0
WP_080466125.1 elongation factor G [Enterococcus faecium]
642   0.0
APO27239.1 ferrous iron transport protein B [uncultured bacte...
642   0.0
AAN84480.1 tetracycline resistance protein [Lactobacillus cur...
642   0.0
AAN84485.1 tetracycline resistance protein [Lactobacillus sak...
642   0.0
ANC47981.1 TetW [Haemophilus parainfluenzae]
641   0.0
AAN84491.1 tetracycline resistance protein [Lactobacillus ali...
641   0.0
AAN84488.1 tetracycline resistance protein [Lactobacillus sak...
641   0.0
AAN84479.1 tetracycline resistance protein [Lactobacillus sak...
641   0.0
ACY03114.1 tetracycline resistance protein [uncultured organism]
640   0.0
AAN84497.1 tetracycline resistance protein [Lactobacillus pla...
640   0.0
WP_085851141.1 elongation factor G [Enterococcus faecium]
639   0.0
AAN84483.1 tetracycline resistance protein [Lactobacillus sak...
639   0.0
AAN84487.1 tetracycline resistance protein [Lactobacillus sak...
639   0.0
AAN84494.1 tetracycline resistance protein [Lactobacillus pla...
638   0.0
WP_100626586.1 tetracycline resistance ribosomal protection p...
637   0.0
CAG28847.1 tetracycline resistance protein tetM [Listeria mon...
637   0.0
AMJ37115.1 TetM-TetW-TetO-TetS [uncultured bacterium]
637   0.0
AAN84499.1 tetracycline resistance protein [Lactobacillus cur...
637   0.0
AMP48008.1 TetM-TetW-TetO-TetS [uncultured bacterium]
636   0.0
WP_074400133.1 elongation factor G [Enterococcus faecium]
635   0.0
WP_065429291.1 TetM/TetW/TetO/TetS family tetracycline resist...
635   0.0

AMP55037.1 TetM-TetW-TetO-TetS [uncultured bacterium]
635   0.0
WP_085837565.1 tetracycline resistance ribosomal protection p...
634   0.0
ABP01799.1 TetW [Bifidobacterium longum]
632   0.0
WP_085812146.1 GTP-binding protein [Enterococcus faecium]
630   0.0
WP_024411341.1 TetM/TetW/TetO/TetS family tetracycline resist...
630   0.0
KWW59413.1 tetracycline resistance protein tetM [Enterococcus...
629   0.0
KWW87505.1 tetracycline resistance protein tetM [Enterococcus...
628   0.0
EFY05111.1 elongation factor G domain protein [Phascolarctoba...
627   0.0
WP_050306474.1 tetracycline resistance protein tetM [Streptoc...
627   0.0
WP_040565034.1 tetracycline resistance protein tetM [Phascola...
627   0.0
WP_057030721.1 tetracycline resistance protein tetM [Campylob...
626   0.0
WP_002347658.1 tetracycline resistance protein tetM transposo...
625   0.0
WP_101706231.1 elongation factor G [Enterococcus faecium]
625   0.0
KWX94480.1 tetracycline resistance protein tetM [Enterococcus...
625   0.0
APO35061.1 EF-G: translation elongation factor [uncultured ba...
624   0.0
AUT06869.1 Tetracycline resistance protein TetS [Streptococcu...
624   0.0
ELB79897.1 tetracycline resistance protein tetM transposon [E...
624   0.0
WP_086322141.1 elongation factor G [Enterococcus faecium]
623   0.0
WP_080440977.1 elongation factor G [Enterococcus faecium]
623   0.0
WP_080440513.1 elongation factor G [Enterococcus faecium]
623   0.0
WP_080032870.1 elongation factor G [Enterococcus faecium]
623   0.0
WP_033644845.1 TetM/TetW/TetO/TetS family tetracycline resist...
623   0.0
WP_057034403.1 tetracycline resistance protein tetM [Campylob...
622   0.0
BAG12531.1 tetracycline resistance protein [Streptococcus pne...
621   0.0
WP_060806737.1 tetracycline resistance protein tetM [Enteroco...
621   0.0
KLO68607.1 tetracycline resistance protein tetM [Enterococcus...
621   0.0
BAG12549.1 tetracycline resistance protein [Streptococcus pne...
620   0.0

CAJ57438.1 tetracycline resistance protein, partial [Bifidoba...
620   0.0
DAB15977.1 TPA: tetracycline resistance ribosomal protection ...
620   0.0
WP_060776014.1 tetracycline resistance protein tetM [Enteroco...
619   0.0
CNE17273.1 tetracycline resistance protein TetM [Streptococcu...
619   0.0
CTO47219.1 putative conjugative element protein [Streptococcu...
618   0.0
CVK50895.1 tetracycline resistance protein TetM [Streptococcu...
616   0.0
WP_047341729.1 tetracycline resistance protein tetM [Enteroco...
614   0.0
APO35070.1 Elongation factor G, domain IV [uncultured bacterium]
614   0.0
AMP49329.1 TetM-TetW-TetO-TetS [uncultured bacterium]
613   0.0
ACT97608.1 TetW [uncultured organism]
613   0.0
KFI81886.1 TetW [Bifidobacterium pullorum]
611   0.0
APO32484.1 EF-G: translation elongation factor [uncultured ba...
610   0.0
EWH39648.1 tetracycline resistance protein tetW [Bifidobacter...
610   0.0
AMP50714.1 TetM-TetW-TetO-TetS [uncultured bacterium]
607   0.0
APO35111.1 Elongation factor G, domain IV [uncultured bacterium]
602   0.0
ASQ41001.1 TetO [Campylobacter coli]
602   0.0
SFW54395.1 Elongation Factor G, domain II [Ruminococcus sp. Y...
602   0.0
WP_060793231.1 GTP-binding protein [Campylobacter coli]
600   0.0
WP_060787652.1 GTP-binding protein [Campylobacter coli]
598   0.0
AIF29591.1 truncated tetracycline resistance protein [Campylo...
598   0.0
WP_024413125.1 TetM/TetW/TetO/TetS family tetracycline resist...
598   0.0
APO28026.1 Elongation factor G, domain IV [uncultured bacterium]
598   0.0
AFJ97266.1 truncated tetracycline resistant protein [Campylob...
597   0.0
AGN49056.1 truncated tetracycline resistance protein [Campylo...
597   0.0
AIF29618.1 truncated tetracycline resistance protein [Campylo...
597   0.0
AMP50518.1 TetM-TetW-TetO-TetS [uncultured bacterium]
596   0.0
WP_069285002.1 GTP-binding protein [Campylobacter jejuni]
593   0.0

ABW36895.1 TetW [Bifidobacterium adolescentis]
593   0.0
WP_007391378.1 TetM/TetW/TetO/TetS family tetracycline resist...
591   0.0
AIF29604.1 truncated tetracycline resistance protein [Campylo...
590   0.0
WP_070059241.1 tetracycline resistance ribosomal protection p...
587   0.0
WP_070014217.1 tetracycline resistance ribosomal protection p...
587   0.0
AAO16469.1 putative tetracycline resistance protein [uncultur...
586   0.0
CNA02064.1 tetracycline resistance protein TetM [Streptococcu...
585   0.0
AAP21775.1 putative ribosomal protection protein [uncultured ...
585   0.0
AAO16470.1 putative tetracycline resistance protein [uncultur...
584   0.0
AAO16471.1 putative tetracycline resistance protein [uncultur...
583   0.0
BAC99974.1 ribosomal protection protein Tet(M) [Vibrio sp. KR...
580   0.0
BAC99972.1 ribosomal protection protein Tet(M) [Gram-positive...
579   0.0
WP_055170010.1 GTP-binding protein [[Ruminococcus] gnavus]
579   0.0
BAC99975.1 ribosomal protection protein Tet(M) [Lactococcus g...
577   0.0
AAO16472.1 putative tetracycline resistance protein [uncultur...
577   0.0
BAC99973.1 ribosomal protection protein Tet(M) [Photobacteriu...
577   0.0
WP_057037234.1 tetracycline resistance protein tetM [Campylob...
576   0.0
AMP50909.1 TetM-TetW-TetO-TetS [uncultured bacterium]
573   0.0
WP_080440083.1 elongation factor G [Enterococcus faecium]
573   0.0
AMP51376.1 TetM-TetW-TetO-TetS [uncultured bacterium]
573   0.0
APR29429.1 elongation factor G [Pediococcus acidilactici]
571   0.0
BAC99976.1 ribosomal protection protein Tet(M) [Lactococcus g...
569   0.0
WP_080961993.1 elongation factor G [Enterococcus cecorum]
568   0.0
EVK37104.1 tetracycline resistance protein tetM transposon Tn...
568   0.0
KFI81887.1 tetracycline resistance protein TetW [Bifidobacter...
567   0.0
WP_085839204.1 elongation factor G [Enterococcus faecium]
566   0.0
WP_074399838.1 elongation factor G [Enterococcus faecium]
563   0.0

WP_087704884.1 GTP-binding protein [Campylobacter jejuni]
560   0.0
WP_094850703.1 GTP-binding protein [Enterococcus faecium]
558   0.0
WP_098381401.1 elongation factor G [Enterococcus faecium]
556   0.0
GAK48687.1 putative tetracycline resistance protein tetM [Lac...
555   0.0
WP_087121934.1 elongation factor G [Enterococcus faecium]
554   0.0
WP_010731388.1 tetracycline resistance protein tetM [Enteroco...
553   0.0
WP_013668947.1 tetracycline resistance ribosomal protection p...
553   0.0
WP_063856413.1 tetracycline resistance ribosomal protection p...
552   0.0
AFC62153.1 translation elongation factor G [Enterococcus faec...
552   0.0
WP_077149101.1 MULTISPECIES: elongation factor G [Bacilli]
552   0.0
EJX67401.1 elongation factor Tu domain 2 [Enterococcus faeciu...
551   0.0
WP_095257136.1 tetracycline resistance ribosomal protection p...
551   0.0
WP_045078625.1 tetracycline resistance ribosomal protection p...
550   0.0
WP_010730930.1 tetracycline resistance protein tetM [Enteroco...
550   0.0
WP_095283056.1 tetracycline resistance ribosomal protection p...
550   0.0
WP_025642386.1 MULTISPECIES: tetracycline resistance ribosoma...
550   0.0
WP_054252248.1 tetracycline resistance ribosomal protection p...
550   0.0
WP_063856412.1 tetracycline resistance ribosomal protection p...
550   0.0
ANZ79471.1 tetracycline resistance protein TetQ [Helcococcus ...
549   0.0
SOD12959.1 small GTP-binding protein domain-containing protei...
548   0.0
AMP53212.1 elongation factor Tu GTP binding domain protein [u...
548   0.0
WP_095236811.1 tetracycline resistance ribosomal protection p...
547   0.0
AMP50920.1 elongation factor Tu GTP binding domain protein [u...
547   0.0
WP_039256333.1 tetracycline resistance ribosomal protection p...
547   0.0
WP_100630146.1 GTP-binding protein [Campylobacter jejuni]
547   0.0
WP_095335272.1 tetracycline resistance ribosomal protection p...
546   0.0
WP_075660133.1 tetracycline resistance ribosomal protection p...
546   0.0

WP_095307524.1 tetracycline resistance ribosomal protection p...
546   0.0
WP_095286326.1 tetracycline resistance ribosomal protection p...
546   0.0
WP_095341175.1 tetracycline resistance ribosomal protection p...
545   0.0
PAH56484.1 tetracycline resistance protein tetQ [Staphylococc...
545   0.0
WP_095258580.1 tetracycline resistance ribosomal protection p...
545   0.0
WP_018579533.1 tetracycline resistance ribosomal protection p...
545   0.0
WP_009247736.1 hypothetical protein [Lachnospiraceae bacteriu...
545   0.0
WP_095279100.1 tetracycline resistance ribosomal protection p...
544   0.0
WP_095239735.1 tetracycline resistance ribosomal protection p...
544   0.0
APO34030.1 Elongation factor G, domain IV [uncultured bacterium]
544   0.0
WP_095257003.1 tetracycline resistance ribosomal protection p...
543   0.0
WP_095243793.1 tetracycline resistance ribosomal protection p...
543   0.0
WP_095339879.1 tetracycline resistance ribosomal protection p...
543   0.0
WP_095259616.1 tetracycline resistance ribosomal protection p...
543   0.0
WP_095251357.1 tetracycline resistance ribosomal protection p...
543   0.0
WP_095337211.1 tetracycline resistance ribosomal protection p...
542   0.0
WP_095339683.1 tetracycline resistance ribosomal protection p...
542   0.0
WP_095337399.1 tetracycline resistance ribosomal protection p...
541   0.0
WP_095280129.1 tetracycline resistance ribosomal protection p...
541   0.0
WP_095341257.1 tetracycline resistance ribosomal protection p...
540   0.0
EFW05334.1 Tet(32) protein [Coprobacillus sp. 29_1]
540   0.0
EGN48717.1 hypothetical protein HMPREF0990_00618 [Lachnospira...
538   0.0
KQI48375.1 tetracycline resistance protein tetM [Campylobacte...
536   0.0
KQI04765.1 tetracycline resistance protein tetM [Campylobacte...
536   0.0
KQI56696.1 tetracycline resistance protein tetM [Campylobacte...
535   0.0
APO31155.1 Elongation factor G, domain IV [uncultured bacterium]
532   0.0
CMY16834.1 tetracycline resistance protein TetM [Streptococcu...
531   0.0

WP_080440770.1 elongation factor G [Enterococcus faecium]
530   0.0
EQC82011.1 hypothetical protein HSIEG1_3466 [Enterococcus sp....
530   0.0
AFC92984.1 tetracycline resistance protein TetM [Streptococcu...
530   0.0
WP_084744561.1 elongation factor G [Dielma fastidiosa]
530   0.0
WP_001827031.1 TetM/TetW/TetO/TetS family tetracycline resist...
530   0.0
WP_001842401.1 MULTISPECIES: TetM/TetW/TetO/TetS family tetra...
530   0.0
EFO68117.1 elongation factor G domain protein [Lactobacillus ...
530   0.0
WP_087449168.1 elongation factor G [Lactobacillus salivarius]
529   0.0
EFQ49657.1 elongation factor G domain protein [Lactobacillus ...
529   0.0
WP_001818474.1 TetM/TetW/TetO/TetS family tetracycline resist...
529   0.0
WP_000288739.1 TetM/TetW/TetO/TetS family tetracycline resist...
528   0.0
WP_005592190.1 TetM/TetW/TetO/TetS family tetracycline resist...
528   0.0
EFO69476.1 elongation factor G domain protein [Lactobacillus ...
528   0.0
EZM54652.1 tetracycline resistance protein tetM [Streptococcu...
527   0.0
WP_001826584.1 MULTISPECIES: TetM/TetW/TetO/TetS family tetra...
526   0.0
WP_088745803.1 elongation factor G [Enterococcus hirae]
524   1e-180
EGL86972.1 elongation factor G, domain IV [Streptococcus oral...
524   1e-180
WP_095306029.1 tetracycline resistance ribosomal protection p...
536   2e-180
WP_078846499.1 elongation factor G [Streptococcus pneumoniae]
524   2e-180
WP_088775667.1 elongation factor G [Enterococcus hirae]
523   2e-180
PAF98180.1 tetracycline resistance protein tetQ [Staphylococc...
535   3e-180
ALQ80975.1 truncated tetracycline resistance protein TetO [Ca...
523   5e-180
WP_095338440.1 tetracycline resistance ribosomal protection p...
534   6e-180
AMP50106.1 TetM-TetW-TetO-TetS [uncultured bacterium]
523   2e-179
WP_095303420.1 tetracycline resistance ribosomal protection p...
532   4e-179
AMP52936.1 TetM-TetW-TetO-TetS [uncultured bacterium]
525   4e-179
WP_095337545.1 tetracycline resistance ribosomal protection p...
531   8e-179

WP_082425725.1 GTP-binding protein [[Eubacterium] hallii]
521   1e-178
AMP55021.1 elongation factor G, domain IV [uncultured bacterium]
518   8e-178
WP_095314941.1 tetracycline resistance ribosomal protection p...
528   9e-178
WP_001817446.1 MULTISPECIES: TetM/TetW/TetO/TetS family tetra...
516   1e-177
WP_080441669.1 elongation factor G [Enterococcus faecium]
516   2e-177
EJG80815.1 elongation factor G family protein [Streptococcus ...
516   2e-177
EHT67641.1 elongation factor G family protein [Staphylococcus...
514   1e-176
WP_095242221.1 tetracycline resistance ribosomal protection p...
525   2e-176
KQI39558.1 tetracycline resistance protein tetM [Campylobacte...
512   5e-176
WP_104885109.1 elongation factor G [Enterococcus faecalis]
511   1e-175
WP_095276488.1 tetracycline resistance ribosomal protection p...
523   1e-175
KQI16481.1 tetracycline resistance protein tetM [Campylobacte...
510   2e-175
APO35579.1 ferrous iron transport protein B [uncultured bacte...
512   3e-175
CRH79370.1 Protein translation elongation factor G (EF-G) [Ch...
509   5e-175
WP_095231744.1 tetracycline resistance ribosomal protection p...
521   6e-175
AMP47006.1 TetM-TetW-TetO-TetS [uncultured bacterium]
508   7e-175
CMZ52844.1 tetracycline resistance protein TetM [Streptococcu...
506   5e-174
ABN50376.1 TetW [Bifidobacterium longum subsp. longum]
503   6e-174
WP_095157387.1 elongation factor G [Escherichia coli]
505   1e-173
AMP52945.1 elongation factor Tu GTP binding domain protein [u...
508   1e-173
WP_095239837.1 tetracycline resistance ribosomal protection p...
517   3e-173
SMH82925.1 tetracycline resistance protein TetP [Enterococcus...
508   3e-173
WP_095248115.1 tetracycline resistance ribosomal protection p...
516   7e-173
WP_095315064.1 tetracycline resistance ribosomal protection p...
516   7e-173
WP_018109503.1 tetracycline resistance ribosomal protection p...
516   7e-173
WP_088656287.1 tetracycline resistance ribosomal protection p...
515   1e-172
CJG39265.1 tetracycline resistance protein TetM [Streptococcu...
503   1e-172

ABN50371.1 TetW [Bifidobacterium bifidum]
500   1e-172
ABN50372.1 TetW [Bifidobacterium bifidum]
499   2e-172
WP_006744283.1 tetracycline resistance ribosomal protection p...
514   3e-172
ABN50378.1 TetW [Bifidobacterium animalis subsp. lactis]
499   4e-172
WP_086980648.1 MULTISPECIES: tetracycline resistance ribosoma...
513   6e-172
WP_095237498.1 tetracycline resistance ribosomal protection p...
510   1e-171
ABN50373.1 TetW [Bifidobacterium bifidum]
497   1e-171
SDA33454.1 small GTP-binding protein domain-containing protei...
497   2e-171
ABN50370.1 TetW [Bifidobacterium longum subsp. longum]
496   4e-171
CJD86533.1 tetracycline resistance protein TetM [Streptococcu...
498   7e-171
WP_095316442.1 tetracycline resistance ribosomal protection p...
508   6e-170
CJE23849.1 tetracycline resistance protein TetM [Streptococcu...
497   3e-169
WP_095235796.1 tetracycline resistance ribosomal protection p...
506   6e-169
EWH39649.1 tetracycline resistance protein tetW [Bifidobacter...
490   1e-168
PAJ44070.1 tetracycline resistance protein tetQ [Staphylococc...
505   2e-168
CMV04022.1 tetracycline resistance protein TetM [Streptococcu...
494   6e-168
ACI02008.1 tetracycline resistance protein [uncultured bacter...
487   7e-168
EFO70334.1 elongation factor G domain protein [Lactobacillus ...
490   1e-167
APO31201.1 Elongation factor G, domain IV [uncultured bacterium]
490   1e-167
EHZ99044.1 elongation factor G, domain IV family protein [Str...
489   1e-167
ACD97481.1 truncated tetracycline resistance protein [Bifidob...
488   2e-167
WP_001825260.1 elongation factor G [Streptococcus pneumoniae]
489   2e-167
AHF25350.1 ribosomal protection tetracycline resistance prote...
485   9e-167
AMJ37888.1 elongation factor G, domain IV [uncultured bacterium]
487   1e-166
KQI20590.1 tetracycline resistance protein tetM [Campylobacte...
487   2e-165
WP_100356330.1 tetracycline resistance ribosomal protection p...
494   2e-164
ABN50377.1 TetW [Bifidobacterium animalis subsp. lactis]
478   6e-164

WP_005799729.1 tetracycline resistance ribosomal protection p...
492   9e-164
ABI26642.1 TetM [Streptococcus sp. CJ]
482   1e-163
PQF67328.1 tetracycline resistance ribosomal protection prote...
483   1e-163
WP_005794074.1 tetracycline resistance ribosomal protection p...
492   1e-163
WP_095337845.1 tetracycline resistance ribosomal protection p...
491   3e-163
ABN50379.1 TetW [Bifidobacterium animalis subsp. lactis]
476   3e-163
WP_008645586.1 MULTISPECIES: tetracycline resistance ribosoma...
491   4e-163
AEW22283.1 elongation factor Tu GTP binding domain protein [T...
490   6e-163
WP_007366526.1 MULTISPECIES: tetracycline resistance ribosoma...
490   6e-163
OWW57410.1 tetracycline resistance protein tetM [Enterococcus...
480   9e-163
AMP49708.1 tet_ribosomoal_protect [uncultured bacterium]
489   1e-162
AMP55332.1 tet_ribosomoal_protect [uncultured bacterium]
489   1e-162
AMP52289.1 tet_ribosomoal_protect [uncultured bacterium]
489   2e-162
WP_016278665.1 tetracycline resistance ribosomal protection p...
489   3e-162
WP_049702038.1 tetracycline resistance ribosomal protection p...
488   3e-162
WP_074705912.1 tetracycline resistance ribosomal protection p...
488   6e-162
APO33635.1 feoB: ferrous iron transport protein [uncultured b...
488   7e-162
WP_005861693.1 MULTISPECIES: tetracycline resistance ribosoma...
487   8e-162
WP_094380917.1 tetracycline resistance ribosomal protection p...
487   1e-161
WP_036865782.1 tetracycline resistance ribosomal protection p...
487   1e-161
WP_044066144.1 tetracycline resistance ribosomal protection p...
487   2e-161
EEU51539.1 putative translation elongation factor G [Parabact...
487   2e-161
WP_074708051.1 tetracycline resistance ribosomal protection p...
486   2e-161
WP_095264430.1 tetracycline resistance ribosomal protection p...
486   2e-161
EFV16581.1 elongation factor G [Lachnospiraceae bacterium 5_1...
473   2e-161
WP_005816789.1 tetracycline resistance ribosomal protection p...
486   2e-161
WP_006255810.1 tetracycline resistance ribosomal protection p...
486   3e-161

WP_004310568.1 MULTISPECIES: tetracycline resistance ribosoma...
486   3e-161
WP_036887565.1 tetracycline resistance ribosomal protection p...
486   3e-161
WP_042987717.1 tetracycline resistance ribosomal protection p...
486   3e-161
WP_042520198.1 tetracycline resistance ribosomal protection p...
486   4e-161
WP_044080776.1 tetracycline resistance ribosomal protection p...
486   4e-161
WP_002560998.1 MULTISPECIES: tetracycline resistance ribosoma...
486   4e-161
CAA79727.1 TetA(Q)2 protein [Bacteroides fragilis]
486   5e-161
WP_032557833.1 tetracycline resistance ribosomal protection p...
485   5e-161
OYN69742.1 tetracycline resistance ribosomal protection prote...
471   6e-161
WP_005796948.1 tetracycline resistance ribosomal protection p...
485   6e-161
WP_005802293.1 tetracycline resistance ribosomal protection p...
485   6e-161
WP_089543219.1 tetracycline resistance ribosomal protection p...
485   6e-161
WP_102187702.1 tetracycline resistance ribosomal protection p...
485   6e-161
ABN50369.1 TetW [Bifidobacterium breve]
470   6e-161
APO35112.1 ferrous iron transport protein B [uncultured bacte...
473   7e-161
WP_106813142.1 tetracycline resistance ribosomal protection p...
485   8e-161
WP_094423033.1 tetracycline resistance ribosomal protection p...
484   9e-161
WP_095239968.1 tetracycline resistance ribosomal protection p...
485   1e-160
WP_005799343.1 tetracycline resistance ribosomal protection p...
484   1e-160
WP_077195545.1 tetracycline resistance ribosomal protection p...
484   1e-160
WP_032589565.1 tetracycline resistance ribosomal protection p...
484   1e-160
WP_057058907.1 tetracycline resistance ribosomal protection p...
484   1e-160
WP_063856408.1 tetracycline resistance ribosomal protection p...
485   1e-160
EFI05973.1 tetracycline resistance protein [Bacteroides sp. 1...
485   1e-160
WP_063856409.1 tetracycline resistance ribosomal protection p...
484   1e-160
WP_009127153.1 tetracycline resistance ribosomal protection p...
484   1e-160
AUA17601.1 truncated TetO [Campylobacter coli]
471   1e-160

WP_079046502.1 elongation factor G [Streptococcus pneumoniae]
473   2e-160
AMP55648.1 tet_ribosomoal_protect [uncultured bacterium]
484   2e-160
WP_004293868.1 MULTISPECIES: tetracycline resistance ribosoma...
484   2e-160
WP_074783792.1 tetracycline resistance ribosomal protection p...
484   2e-160
WP_074635749.1 tetracycline resistance ribosomal protection p...
484   2e-160
AAB51122.1 tetracycline resistance protein [Prevotella interm...
484   2e-160
WP_044075064.1 tetracycline resistance ribosomal protection p...
483   3e-160
EFA20356.1 putative translation elongation factor G [Bacteroi...
484   3e-160
WP_055236246.1 tetracycline resistance ribosomal protection p...
483   3e-160
AAZ79478.1 TetQ [Bifidobacterium longum]
484   3e-160
WP_032538282.1 tetracycline resistance ribosomal protection p...
483   3e-160
WP_049139058.1 tetracycline resistance ribosomal protection p...
483   4e-160
EKA87903.1 tetracycline resistance protein tetQ [Bacteroides ...
483   4e-160
WP_106504845.1 elongation factor G [Escherichia coli]
471   5e-160
WP_071148955.1 tetracycline resistance ribosomal protection p...
483   5e-160
WP_018676468.1 tetracycline resistance ribosomal protection p...
483   5e-160
WP_005680575.1 tetracycline resistance ribosomal protection p...
483   5e-160
WP_065739727.1 MULTISPECIES: tetracycline resistance ribosoma...
483   5e-160
WP_044073065.1 tetracycline resistance ribosomal protection p...
483   5e-160
WP_005944279.1 tetracycline resistance ribosomal protection p...
482   7e-160
CRH91225.1 Protein translation elongation factor G (EF-G) [Ch...
469   7e-160
WP_094466893.1 tetracycline resistance ribosomal protection p...
482   9e-160
WP_049129063.1 tetracycline resistance ribosomal protection p...
482   1e-159
WP_072831140.1 MULTISPECIES: tetracycline resistance ribosoma...
482   1e-159
AMP54775.1 tet_ribosomoal_protect [uncultured bacterium]
482   1e-159
WP_006849477.1 tetracycline resistance ribosomal protection p...
482   1e-159
WP_074560483.1 tetracycline resistance ribosomal protection p...
482   1e-159

WP_063856407.1 tetracycline resistance ribosomal protection p...
482   1e-159
WP_074558868.1 tetracycline resistance ribosomal protection p...
482   1e-159
WP_004371707.1 MULTISPECIES: tetracycline resistance ribosoma...
481   2e-159
WP_049100682.1 tetracycline resistance ribosomal protection p...
481   2e-159
WP_032846090.1 tetracycline resistance ribosomal protection p...
481   2e-159
WP_032602870.1 tetracycline resistance ribosomal protection p...
481   2e-159
AMP53998.1 tet_ribosomoal_protect [uncultured bacterium]
481   2e-159
AMP47132.1 tet_ribosomoal_protect [uncultured bacterium]
481   2e-159
SHH94198.1 ribosomal protection tetracycline resistance prote...
481   2e-159
AMP47005.1 tet_ribosomoal_protect [uncultured bacterium]
481   2e-159
WP_044225003.1 tetracycline resistance ribosomal protection p...
481   2e-159
OWT31781.1 teto [Campylobacter coli HN-CCD07046]
479   2e-159
WP_023056813.1 MULTISPECIES: tetracycline resistance ribosoma...
481   2e-159
EXZ93467.1 tetracycline resistance protein tetM [Bacteroides ...
481   2e-159
WP_005850524.1 tetracycline resistance ribosomal protection p...
481   3e-159
CBK68392.1 small GTP-binding protein domain [Bacteroides xyla...
481   3e-159
WP_032586787.1 tetracycline resistance ribosomal protection p...
481   3e-159
WP_004291466.1 MULTISPECIES: tetracycline resistance ribosoma...
481   3e-159
WP_036878672.1 tetracycline resistance ribosomal protection p...
481   3e-159
WP_036630022.1 tetracycline resistance ribosomal protection p...
481   4e-159
WP_073342757.1 tetracycline resistance ribosomal protection p...
481   4e-159
WP_087382020.1 tetracycline resistance ribosomal protection p...
480   4e-159
AMP53480.1 tet_ribosomoal_protect [uncultured bacterium]
480   5e-159
PCS24729.1 tetracycline resistance ribosomal protection prote...
464   5e-159
WP_086325387.1 elongation factor G [Enterococcus faecium]
467   6e-159
WP_102697449.1 tetracycline resistance ribosomal protection p...
480   6e-159
WP_060933101.1 tetracycline resistance ribosomal protection p...
480   6e-159

EXZ85794.1 tetracycline resistance protein tetM [Bacteroides ...
480   7e-159
WP_032543185.1 MULTISPECIES: tetracycline resistance ribosoma...
480   7e-159
WP_096920801.1 elongation factor G [Eubacterium sp. YI]
464   8e-159
WP_070706770.1 tetracycline resistance ribosomal protection p...
480   8e-159
WP_032595419.1 tetracycline resistance ribosomal protection p...
480   8e-159
WP_101603347.1 tetracycline resistance ribosomal protection p...
480   8e-159
WP_007896729.1 tetracycline resistance ribosomal protection p...
479   8e-159
WP_008148693.1 MULTISPECIES: tetracycline resistance ribosoma...
480   9e-159
WP_004331540.1 tetracycline resistance ribosomal protection p...
480   9e-159
WP_101542859.1 tetracycline resistance ribosomal protection p...
479   1e-158
AMP57743.1 tet_ribosomoal_protect [uncultured bacterium]
479   1e-158
CFA01818.1 tetracycline resistance protein TetM [Streptococcu...
465   1e-158
WP_095231440.1 tetracycline resistance ribosomal protection p...
479   2e-158
ABP01800.1 TetW [Bifidobacterium longum]
463   2e-158
AMP57708.1 tet_ribosomoal_protect [uncultured bacterium]
479   2e-158
EEG70484.1 elongation factor G domain protein [Bifidobacteriu...
463   2e-158
ABN50380.1 TetW [Bifidobacterium animalis]
463   2e-158
AFH53885.1 TetW tetracycline resistance protein [uncultured o...
462   3e-158
WP_071122301.1 tetracycline resistance ribosomal protection p...
477   4e-158
WP_036926136.1 tetracycline resistance ribosomal protection p...
478   4e-158
WP_066041057.1 tetracycline resistance ribosomal protection p...
478   4e-158
APO29854.1 ferrous iron transport protein B [uncultured bacte...
468   5e-158
WP_032571255.1 tetracycline resistance ribosomal protection p...
478   5e-158
AMP53022.1 tet_ribosomoal_protect [uncultured bacterium]
476   6e-158
OKZ42324.1 tetracycline resistance ribosomal protection prote...
477   9e-158
AMP57961.1 tet_ribosomoal_protect [uncultured bacterium]
477   9e-158
WP_095341089.1 tetracycline resistance ribosomal protection p...
476   3e-157

AP026791.1 Elongation factor G, domain IV [uncultured bacterium]
459   5e-157
WP_044656661.1 tetracycline resistance ribosomal protection p...
471   5e-156
WP_095337479.1 tetracycline resistance ribosomal protection p...
473   6e-156
WP_015391965.1 tetracycline resistance ribosomal protection p...
472   2e-155
AMP54852.1 tet_ribosomoal_protect [uncultured bacterium]
471   2e-155
WP_027631504.1 tetracycline resistance ribosomal protection p...
471   2e-155
PLK29559.1 tetracycline resistance ribosomal protection prote...
453   1e-154
WP_077829768.1 tetracycline resistance ribosomal protection p...
469   2e-154
AMP57963.1 elongation factor Tu GTP binding domain protein [u...
458   3e-154
AMP56085.1 elongation factor Tu domain 2 [uncultured bacterium]
453   3e-154
WP_017210340.1 tetracycline resistance ribosomal protection p...
468   3e-154
OUN15238.1 tetracycline resistance ribosomal protection prote...
451   8e-154
AMP53228.1 tet_ribosomoal_protect [uncultured bacterium]
466   9e-154
AMP56777.1 elongation factor G, domain IV [uncultured bacterium]
450   1e-153
AP028708.1 Elongation factor G, domain IV [uncultured bacterium]
453   2e-153
AMP52570.1 tet_ribosomoal_protect [uncultured bacterium]
465   3e-153
WP_006440995.1 MULTISPECIES: tetracycline resistance ribosoma...
466   3e-153
WP_068759696.1 tetracycline resistance ribosomal protection p...
464   1e-152
KQI09701.1 tetracycline resistance protein tetM [Campylobacte...
451   1e-152
WP_023974176.1 tetracycline resistance ribosomal protection p...
464   1e-152
WP_083303604.1 elongation factor G [Rothia sp. HMSC062H08]
447   2e-152
WP_039634701.1 tetracycline resistance ribosomal protection p...
463   3e-152
WP_026885591.1 tetracycline resistance ribosomal protection p...
463   3e-152
AMP48947.1 tet_ribosomoal_protect [uncultured bacterium]
462   3e-152
WP_078116767.1 tetracycline resistance ribosomal protection p...
463   4e-152
KIE45688.1 small GTP-binding domain protein [Clostridium arge...
463   5e-152
AMP56792.1 elongation factor G, domain IV [uncultured bacterium]
456   5e-152

PQF66632.1 hypothetical protein CUS68_13010 [Enterococcus fae...
448   6e-152
AMP55022.1 elongation factor G, domain IV [uncultured bacterium]
448   6e-152
PJP40542.1 tetracycline resistance ribosomal protection prote...
448   8e-152
WP_077855104.1 tetracycline resistance ribosomal protection p...
462   8e-152
WP_002405377.1 MULTISPECIES: elongation factor G [Enterococcus]
447   1e-151
WP_077843733.1 tetracycline resistance ribosomal protection p...
462   1e-151
WP_010964757.1 MULTISPECIES: tetracycline resistance ribosoma...
461   1e-151
WP_032122132.1 tetracycline resistance ribosomal protection p...
461   1e-151
WP_055342657.1 tetracycline resistance ribosomal protection p...
461   2e-151
AMP53115.1 tet_ribosomoal_protect [uncultured bacterium]
461   2e-151
WP_023050237.1 MULTISPECIES: tetracycline resistance ribosoma...
459   7e-151
EFV16582.1 elongation factor Tu GTP binding domain-containing...
449   8e-151
KQI09521.1 tetracycline resistance protein tetM [Campylobacte...
448   8e-151
WP_103698728.1 tetracycline resistance ribosomal protection p...
459   1e-150
AMJ37424.1 tet_ribosomoal_protect [uncultured bacterium]
458   1e-150
AMP58094.1 tet_ribosomoal_protect [uncultured bacterium]
458   1e-150
WP_004456831.1 tetracycline resistance ribosomal protection p...
458   3e-150
SMM96685.1 tetracycline resistance protein TetP [Enterococcus...
443   4e-150
AMP54171.1 tet_ribosomoal_protect [uncultured bacterium]
457   4e-150
WP_003479689.1 tetracycline resistance ribosomal protection p...
457   4e-150
KKJ73154.1 tetracycline resistance protein tetM [Enterococcus...
447   5e-150
COK17373.1 tetracycline resistance protein TetM [Streptococcu...
442   1e-149
WP_057258411.1 tetracycline resistance ribosomal protection p...
456   2e-149
WP_095237487.1 tetracycline resistance ribosomal protection p...
454   2e-149
WP_012478245.1 tetracycline resistance ribosomal protection p...
454   7e-149
WP_102013996.1 hypothetical protein [Klebsiella pneumoniae]
443   7e-149
AFQ32088.1 tetracycline resistance protein [Enterococcus sp. ...
441   9e-149

WP_107196944.1 hypothetical protein [Megasphaera sp. AJH120]
439   9e-149
WP_002581202.1 tetracycline resistance ribosomal protection p...
454   9e-149
WP_008627130.1 tetracycline resistance ribosomal protection p...
453   1e-148
SJT57314.1 Tetracycline resistance protein tetM from transpos...
453   2e-148
KQI39023.1 tetracycline resistance protein tetM [Campylobacte...
441   4e-148
WP_043664816.1 tetracycline resistance ribosomal protection p...
451   1e-147
KQI19705.1 tetracycline resistance protein tetM [Campylobacte...
436   3e-147
EIA67973.1 teto [Campylobacter coli 1417]
436   3e-147
WP_021656448.1 tetracycline resistance ribosomal protection p...
450   3e-147
AMP53986.1 elongation factor G, domain IV [uncultured bacterium]
436   3e-147
AMJ37436.1 elongation factor Tu GTP binding domain protein [u...
446   7e-147
AMP58049.1 tet_ribosomoal_protect [uncultured bacterium]
448   9e-147
WP_088764565.1 hypothetical protein [Enterococcus hirae]
434   1e-146
WP_050356083.1 tetracycline resistance ribosomal protection p...
448   2e-146
WP_083599922.1 tetracycline resistance ribosomal protection p...
447   6e-146
AMJ37843.1 elongation factor Tu GTP binding domain protein [u...
446   7e-146
SNO84620.1 tetracycline resistance protein TetM [Streptococcu...
435   1e-145
ACZ73821.1 TetO/32/O [Clostridium butyricum]
433   6e-145
CFA01815.1 tetracycline resistance protein TetM [Streptococcu...
432   9e-145
OWW58801.1 tetracycline resistance protein tetM [Enterococcus...
427   5e-144
WP_094436070.1 tetracycline resistance ribosomal protection p...
436   2e-143
CVO25194.1 tetracycline resistance protein TetM [Streptococcu...
429   3e-143
WP_094435820.1 tetracycline resistance ribosomal protection p...
436   4e-143
KFI85012.1 elongation factor G [Bifidobacterium reuteri DSM 2...
423   7e-143
WP_051685219.1 GTP-binding protein [Clostridium sp. KNHs205]
438   1e-142
WP_101368346.1 GTP-binding protein [Plantactinospora sp. CNZ320]
438   2e-142
EHZ07660.1 small GTP-binding domain protein [Streptococcus pn...
426   3e-142

WP_049130509.1 tetracycline resistance ribosomal protection p...
432   9e-142
KKJ72448.1 tetracycline resistance protein tetM [Enterococcus...
421   1e-141
WP_069876276.1 GTP-binding protein [Fusibacter sp. 3D3]
436   1e-141
WP_035130938.1 tetracycline resistance ribosomal protection p...
432   5e-141
ABN50375.1 TetW [Bifidobacterium animalis]
418   5e-141
WP_001878374.1 hypothetical protein [Enterococcus faecium]
417   2e-140
WP_077073062.1 GTP-binding protein [Mailhella massiliensis]
416   2e-140
AMP54797.1 elongation factor Tu GTP binding domain protein [u...
416   3e-140
AMP53491.1 elongation factor Tu GTP binding domain protein [u...
428   2e-139
AMP57964.1 elongation factor Tu GTP binding domain protein [u...
415   3e-139
CMV03996.1 tetracycline resistance protein TetM [Streptococcu...
414   3e-139
GAK48696.1 putative tetracycline resistance protein tetM [Lac...
415   4e-138
WP_093550044.1 GTP-binding protein [Streptomyces sp. DvalAA-43]
427   4e-138
WP_055742361.1 tetracycline resistance ribosomal protection p...
427   4e-138
KID49811.1 tetracycline resistance protein tetQ [Fusobacteriu...
424   5e-138
WP_072665882.1 hypothetical protein [Escherichia coli]
410   1e-137
WP_093258249.1 GTP-binding protein [Thermostaphylospora chrom...
424   3e-137
WP_019421067.1 tetracycline resistance ribosomal protection p...
424   7e-137
AMP50402.1 elongation factor G, domain IV [uncultured bacterium]
409   1e-136
WP_089977705.1 GTP-binding protein [Lachnospiraceae bacterium...
422   2e-136
WP_072483911.1 GTP-binding protein [Streptomyces atratus]
422   2e-136
APO29855.1 Elongation factor G, domain IV [uncultured bacterium]
407   2e-136
WP_071699236.1 GTP-binding protein [Eisenbergiella tayi]
421   2e-136
AMP55028.1 elongation factor Tu GTP binding domain protein [u...
405   3e-136
WP_009250390.1 MULTISPECIES: GTP-binding protein [Clostridiales]
421   4e-136
WP_069431425.1 GTP-binding protein [Eisenbergiella tayi]
421   5e-136
WP_069430568.1 GTP-binding protein [Eisenbergiella tayi]
419   1e-135

WP_021639419.1 GTP-binding protein [Clostridium sp. KLE 1755]
419   1e-135
WP_069158534.1 GTP-binding protein [Eisenbergiella tayi]
419   1e-135
WP_102286891.1 GTP-binding protein [Eisenbergiella massiliensis]
419   2e-135
WP_016131565.1 tetracycline resistance ribosomal protection p...
420   3e-135
WP_069154626.1 GTP-binding protein [Eisenbergiella tayi]
419   3e-135
WP_073587271.1 GTP-binding protein [Anaerocolumna xylanovorans]
418   4e-135
WP_040020662.1 GTP-binding protein [Streptomyces sp. 150FB]
419   8e-135
WP_069409953.1 MULTISPECIES: GTP-binding protein [Lachnospira...
417   1e-134
EEA80788.1 hypothetical protein CLONEX_03343, partial [Tyzzer...
406   1e-133
WP_090666926.1 GTP-binding protein [Paenibacillus tianmuensis]
415   1e-133
WP_025846249.1 GTP-binding protein [Paenibacillus ehimensis]
415   2e-133
WP_098360021.1 tetracycline resistance ribosomal protection p...
414   3e-133
WP_088833155.1 GTP-binding protein [Paenibacillus elgii]
414   4e-133
WP_098777649.1 tetracycline resistance ribosomal protection p...
413   6e-133
WP_096541513.1 hypothetical protein [Enterococcus faecium]
398   6e-133
WP_088961973.1 GTP-binding protein [Micromonospora purpureoch...
414   9e-133
WP_098157013.1 tetracycline resistance ribosomal protection p...
413   9e-133
WP_042511105.1 tetracycline resistance ribosomal protection p...
413   1e-132
WP_098604074.1 tetracycline resistance ribosomal protection p...
413   2e-132
WP_097833579.1 tetracycline resistance ribosomal protection p...
412   2e-132
WP_016115276.1 MULTISPECIES: tetracycline resistance ribosoma...
412   2e-132
WP_000207835.1 tetracycline resistance ribosomal protection p...
412   2e-132
AMP50704.1 elongation factor Tu GTP binding domain protein [u...
399   2e-132
WP_001982467.1 tetracycline resistance ribosomal protection p...
412   3e-132
WP_088094443.1 tetracycline resistance ribosomal protection p...
412   4e-132
WP_003198288.1 MULTISPECIES: tetracycline resistance ribosoma...
412   4e-132
WP_044438193.1 tetracycline resistance ribosomal protection p...
411   4e-132

WP_016127190.1 MULTISPECIES: GTP-binding protein [Bacillus ce...
411   5e-132
WP_000207834.1 tetracycline resistance ribosomal protection p...
411   5e-132
WP_097787011.1 tetracycline resistance ribosomal protection p...
411   6e-132
WP_098719375.1 tetracycline resistance ribosomal protection p...
411   6e-132
WP_098632468.1 GTP-binding protein [Bacillus mycoides]
411   6e-132
WP_097969867.1 tetracycline resistance ribosomal protection p...
411   6e-132
WP_098114173.1 tetracycline resistance ribosomal protection p...
411   7e-132
WP_098040140.1 tetracycline resistance ribosomal protection p...
411   8e-132
WP_098610209.1 tetracycline resistance ribosomal protection p...
411   8e-132
WP_033797794.1 MULTISPECIES: tetracycline resistance ribosoma...
411   8e-132
WP_097898825.1 MULTISPECIES: tetracycline resistance ribosoma...
411   9e-132
WP_087962609.1 GTP-binding protein [Bacillus mycoides]
410   9e-132
WP_098716262.1 tetracycline resistance ribosomal protection p...
410   9e-132
WP_000207804.1 GTP-binding protein [Bacillus cereus]
410   1e-131
WP_097849062.1 tetracycline resistance ribosomal protection p...
410   1e-131
WP_098165280.1 tetracycline resistance ribosomal protection p...
410   1e-131
WP_069627467.1 GTP-binding protein [Streptomyces niveus]
410   1e-131
WP_098270667.1 GTP-binding protein [Bacillus cereus]
410   1e-131
WP_098784134.1 tetracycline resistance ribosomal protection p...
410   1e-131
WP_072180934.1 tetracycline resistance ribosomal protection p...
410   1e-131
WP_098801556.1 tetracycline resistance ribosomal protection p...
410   1e-131
WP_098136043.1 tetracycline resistance ribosomal protection p...
410   1e-131
WP_098666379.1 GTP-binding protein [Bacillus cereus]
410   1e-131
WP_003190174.1 MULTISPECIES: GTP-binding protein [Bacillus]
410   1e-131
WP_088311840.1 MULTISPECIES: GTP-binding protein [Bacillus]
410   1e-131
WP_078203520.1 tetracycline resistance ribosomal protection p...
410   1e-131
WP_098159301.1 tetracycline resistance ribosomal protection p...
410   2e-131

WP_040118804.1 tetracycline resistance ribosomal protection p...
410   2e-131
WP_098328240.1 tetracycline resistance ribosomal protection p...
409   2e-131
WP_063225765.1 tetracycline resistance ribosomal protection p...
409   2e-131
WP_078214468.1 tetracycline resistance ribosomal protection p...
409   2e-131
WP_098206819.1 tetracycline resistance ribosomal protection p...
410   2e-131
WP_018780740.1 MULTISPECIES: tetracycline resistance ribosoma...
409   3e-131
WP_000207829.1 GTP-binding protein [Bacillus cereus]
409   3e-131
WP_016101144.1 tetracycline resistance ribosomal protection p...
409   3e-131
WP_098217427.1 tetracycline resistance ribosomal protection p...
409   3e-131
WP_061688473.1 GTP-binding protein [Bacillus cereus]
409   3e-131
WP_000207828.1 GTP-binding protein [Bacillus thuringiensis]
409   3e-131
WP_088045037.1 MULTISPECIES: tetracycline resistance ribosoma...
409   4e-131
WP_023542570.1 GTP-binding protein [Streptomyces niveus]
409   4e-131
WP_098806626.1 tetracycline resistance ribosomal protection p...
409   4e-131
WP_090690119.1 MULTISPECIES: tetracycline resistance ribosoma...
409   5e-131
WP_107886692.1 GTP-binding protein [Bacillus sp. OV752]
408   5e-131
WP_091258620.1 GTP-binding protein [Micromonospora chaiyaphum...
409   6e-131
WP_087885812.1 GTP-binding protein [Streptomyces alboflavus]
409   6e-131
WP_107078481.1 GTP-binding protein [Micromonospora sp. MH33]
409   6e-131
WP_078437912.1 tetracycline resistance ribosomal protection p...
408   6e-131
KQI57121.1 tetracycline resistance protein tetM [Campylobacte...
395   6e-131
WP_026592882.1 tetracycline resistance ribosomal protection p...
408   8e-131
WP_070139576.1 GTP-binding protein [Bacillus mycoides]
408   1e-130
WP_071727303.1 GTP-binding protein [Bacillus anthracis]
407   1e-130
WP_098160195.1 tetracycline resistance ribosomal protection p...
408   1e-130
WP_098198108.1 tetracycline resistance ribosomal protection p...
408   1e-130
WP_098475563.1 GTP-binding protein [Bacillus cereus]
407   1e-130

WP_070168131.1 GTP-binding protein [Bacillus mycoides]
407   1e-130
WP_000207826.1 GTP-binding protein [Bacillus cereus]
407   2e-130
WP_000207831.1 MULTISPECIES: tetracycline resistance ribosoma...
407   2e-130
WP_070146670.1 GTP-binding protein [Bacillus mycoides]
407   2e-130
KQI04128.1 tetracycline resistance protein tetM [Campylobacte...
394   2e-130
WP_093772799.1 GTP-binding protein [Streptomyces sp. 136MFCol...
408   2e-130
WP_078178770.1 GTP-binding protein [Bacillus mycoides]
407   2e-130
WP_098362301.1 GTP-binding protein [Bacillus cereus]
407   3e-130
WP_087932007.1 tetracycline resistance ribosomal protection p...
407   3e-130
WP_071151433.1 GTP-binding protein [Bacillus mycoides]
407   3e-130
WP_036681001.1 GTP-binding protein [Paenibacillus tyrfis]
407   3e-130
WP_003207541.1 tetracycline resistance ribosomal protection p...
407   3e-130
WP_002127951.1 GTP-binding protein [Bacillus cereus]
406   4e-130
WP_006095187.1 tetracycline resistance ribosomal protection p...
407   4e-130
WP_033730733.1 GTP-binding protein [Bacillus mycoides]
406   4e-130
WP_088292138.1 tetracycline resistance ribosomal protection p...
406   4e-130
WP_002110574.1 GTP-binding protein [Bacillus cereus]
406   5e-130
WP_074543706.1 GTP-binding protein [Bacillus cereus]
406   5e-130
WP_061686980.1 tetracycline resistance ribosomal protection p...
406   5e-130
OJV64369.1 hypothetical protein BGO41_12150 [Clostridiales ba...
405   5e-130
WP_085311774.1 GTP-binding protein [Bacillus mycoides]
406   5e-130
WP_017561331.1 tetracycline resistance ribosomal protection p...
406   5e-130
WP_070128573.1 GTP-binding protein [Bacillus mycoides]
406   5e-130
WP_063179400.1 GTP-binding protein [Paenibacillus elgii]
406   6e-130
WP_060750414.1 GTP-binding protein [Bacillus mycoides]
405   6e-130
WP_100536632.1 GTP-binding protein [Paenibacillus sp. GM2FR]
405   6e-130
WP_061674877.1 tetracycline resistance ribosomal protection p...
405   6e-130

WP_007130363.1 GTP-binding protein [Paenibacillus lactis]
405   6e-130
KQI58253.1 tetracycline resistance protein tetM [Campylobacte...
393   6e-130
WP_002168116.1 tetracycline resistance ribosomal protection p...
405   6e-130
WP_043446900.1 GTP-binding protein [Streptomyces nodosus]
406   6e-130
WP_053493716.1 GTP-binding protein [Bacillus sp. FJAT-18019]
405   7e-130
WP_028405524.1 GTP-binding protein [Bacillus sp. J13]
405   8e-130
WP_093929439.1 GTP-binding protein [Streptomyces sp. ok210]
406   9e-130
WP_098947067.1 tetracycline resistance ribosomal protection p...
405   1e-129
WP_098608772.1 GTP-binding protein [Bacillus cereus]
405   1e-129
WP_098264717.1 GTP-binding protein [Bacillus cereus]
405   1e-129
WP_049144675.1 tetracycline resistance protein tetM [Enteroco...
393   1e-129
WP_098321019.1 tetracycline resistance ribosomal protection p...
405   1e-129
WP_088078813.1 GTP-binding protein [Bacillus mycoides]
405   1e-129
COF14579.1 tetracycline resistance protein tetM [Streptococcu...
405   1e-129
WP_098635641.1 GTP-binding protein [Bacillus mycoides]
405   2e-129
WP_098124708.1 tetracycline resistance ribosomal protection p...
405   2e-129
WP_078175727.1 GTP-binding protein [Bacillus mycoides]
404   2e-129
WP_088099180.1 GTP-binding protein [Bacillus mycoides]
404   2e-129
WP_089547083.1 GTP-binding protein [Paenibacillus sp. SSG-1]
404   2e-129
WP_062745233.1 GTP-binding protein [Bacillus clausii]
404   2e-129
WP_000207769.1 GTP-binding protein [Bacillus anthracis]
404   2e-129
WP_087996040.1 GTP-binding protein [Bacillus mycoides]
404   2e-129
WP_070143784.1 MULTISPECIES: GTP-binding protein [Bacillus ce...
404   2e-129
WP_098773602.1 tetracycline resistance ribosomal protection p...
404   2e-129
WP_088994432.1 GTP-binding protein [Micromonospora echinauran...
404   3e-129
WP_000207832.1 GTP-binding protein [Bacillus cereus]
404   3e-129
WP_016105246.1 GTP-binding protein [Bacillus cereus]
404   3e-129

WP_061403017.1 GTP-binding protein [Bacillus thuringiensis]
404   3e-129
WP_098953021.1 GTP-binding protein [Bacillus cereus]
404   3e-129
WP_048374724.1 tetracycline resistance ribosomal protection p...
404   3e-129
WP_002161322.1 GTP-binding protein [Bacillus cereus]
404   4e-129
WP_082527939.1 GTP-binding protein [Devosia sp. Root105]
405   4e-129
WP_098586771.1 GTP-binding protein [Bacillus cereus]
403   5e-129
WP_038813258.1 GTP-binding protein [Bacillus anthracis]
403   5e-129
WP_095294276.1 GTP-binding protein [Bacillus clausii]
403   5e-129
WP_058921329.1 GTP-binding protein [Streptomyces sp. CdTB01]
404   5e-129
WP_002142307.1 MULTISPECIES: GTP-binding protein [Bacillus ce...
403   5e-129
WP_029442677.1 GTP-binding protein [Bacillus thuringiensis]
403   5e-129
WP_028798584.1 GTP-binding protein [Streptomyces sp. 142MFCol...
404   6e-129
WP_000207827.1 GTP-binding protein [Bacillus cereus]
403   6e-129
WP_088031470.1 GTP-binding protein [Bacillus thuringiensis]
403   6e-129
WP_095296390.1 hypothetical protein [Staphylococcus aureus]
387   6e-129
OQR57093.1 tetracycline resistance protein [Bacillus sp. CDB3]
403   6e-129
WP_061981245.1 GTP-binding protein [Amycolatopsis regifaucium]
403   7e-129
WP_063346325.1 GTP-binding protein [Streptomyces sp. MJM8645]
404   7e-129
WP_075395781.1 MULTISPECIES: tetracycline resistance ribosoma...
403   8e-129
WP_098835740.1 tetracycline resistance ribosomal protection p...
403   8e-129
WP_002146954.1 GTP-binding protein [Bacillus cereus]
403   8e-129
WP_033677936.1 tetracycline resistance ribosomal protection p...
403   9e-129
WP_055617616.1 GTP-binding protein [Streptomyces phaeochromog...
403   9e-129
WP_071725854.1 MULTISPECIES: tetracycline resistance ribosoma...
402   9e-129
EFI65603.1 small GTP-binding protein [Bacillus cereus SJ1]
402   1e-128
WP_030915314.1 GTP-binding protein [Streptomyces sp. NRRL B-2...
404   1e-128
WP_071224254.1 GTP-binding protein [Paenibacillus sp. LC231]
402   1e-128

WP_098870698.1 GTP-binding protein [Bacillus cereus]
402   1e-128
WP_098561585.1 GTP-binding protein [Bacillus cereus]
402   1e-128
WP_011248562.1 GTP-binding protein [Bacillus clausii]
402   1e-128
WP_098743630.1 GTP-binding protein [Paenibacillus sp. EZ-K15]
402   1e-128
WP_088115473.1 GTP-binding protein [Bacillus thuringiensis]
402   1e-128
WP_075720334.1 tetracycline resistance ribosomal protection p...
402   1e-128
WP_038414160.1 GTP-binding protein [Bacillus anthracis]
402   1e-128
WP_101194573.1 GTP-binding protein [Bacillus sp. HBCD-sjtu]
402   1e-128
WP_071734271.1 MULTISPECIES: GTP-binding protein [Bacillus]
402   1e-128
WP_097950242.1 GTP-binding protein [Bacillus cereus]
402   1e-128
WP_000207770.1 GTP-binding protein [Bacillus anthracis]
402   1e-128
WP_108530622.1 GTP-binding protein [Paenibacillus elgii]
402   2e-128
WP_035206202.1 GTP-binding protein [Bacillus clausii]
402   2e-128
WP_002172516.1 GTP-binding protein [Bacillus cereus]
402   2e-128
WP_098228202.1 GTP-binding protein [Bacillus anthracis]
402   2e-128
WP_076870557.1 tetracycline resistance ribosomal protection p...
402   2e-128
KOS27869.1 tetracycline resistance protein [Bacillus anthracis]
402   2e-128
WP_010494296.1 GTP-binding protein [Paenibacillus elgii]
402   2e-128
WP_016129768.1 GTP-binding protein [Bacillus cereus]
402   2e-128
WP_002032917.1 MULTISPECIES: GTP-binding protein [Bacillus]
402   2e-128
WP_042978785.1 GTP-binding protein [Bacillus mycoides]
402   2e-128
WP_103626420.1 GTP-binding protein [Bacillus thuringiensis]
402   2e-128
WP_098250590.1 tetracycline resistance ribosomal protection p...
402   2e-128
WP_071759057.1 tetracycline resistance ribosomal protection p...
402   2e-128
WP_098991876.1 GTP-binding protein [Bacillus cereus]
402   2e-128
WP_016091670.1 GTP-binding protein [Bacillus cereus]
402   2e-128
WP_044856292.1 GTP-binding protein [Amycolatopsis orientalis]
401   3e-128

ABN50374.1 TetW [Bifidobacterium longum subsp. infantis]
385   3e-128
WP_042516359.1 GTP-binding protein [Bacillus cereus]
401   3e-128
WP_098225754.1 MULTISPECIES: GTP-binding protein [Bacillus ce...
401   3e-128
WP_098882388.1 GTP-binding protein [Bacillus cereus]
401   3e-128
WP_095254472.1 GTP-binding protein [Bacillus clausii]
401   3e-128
WP_095236425.1 GTP-binding protein [Bacillus clausii]
401   3e-128
WP_016362130.1 GTP-binding protein [Bacillus cereus]
401   3e-128
WP_088314844.1 GTP-binding protein [Bacillus cereus]
401   3e-128
WP_087964529.1 GTP-binding protein [Bacillus thuringiensis]
401   3e-128
WP_026877278.1 GTP-binding protein [Jiangella gansuensis]
402   3e-128
WP_002013560.1 GTP-binding protein [Bacillus cereus]
401   3e-128
WP_063245371.1 GTP-binding protein [Bacillus cereus]
401   3e-128
WP_063218313.1 GTP-binding protein [Bacillus cereus]
401   4e-128
WP_070170321.1 GTP-binding protein [Bacillus mycoides]
401   4e-128
WP_095326647.1 GTP-binding protein [Bacillus clausii]
401   4e-128
WP_001988924.1 MULTISPECIES: GTP-binding protein [Bacillus ce...
401   4e-128
WP_099476583.1 GTP-binding protein [Paenibacillus ihbetae]
401   4e-128
WP_093897522.1 GTP-binding protein [Streptomyces sp. Ncost-T1...
402   4e-128
WP_099933493.1 GTP-binding protein [Streptomyces sp. 70]
401   4e-128
WP_078184917.1 GTP-binding protein [Bacillus mycoides]
401   4e-128
WP_078181859.1 GTP-binding protein [Bacillus cereus]
401   4e-128
WP_037307843.1 GTP-binding protein [Amycolatopsis orientalis]
400   5e-128
WP_000207836.1 MULTISPECIES: GTP-binding protein [Bacillus ce...
401   5e-128
WP_098305786.1 GTP-binding protein [Bacillus cereus]
401   5e-128
WP_000207837.1 GTP-binding protein [Bacillus cereus]
400   6e-128
WP_016331722.1 GTP-binding protein [Amycolatopsis orientalis]
400   6e-128
WP_103240809.1 GTP-binding protein [Acetatifactor muris]
400   6e-128

WP_071746398.1 GTP-binding protein [Bacillus sp. TD42]
400   6e-128
WP_055998563.1 GTP-binding protein [Devosia sp. Root413D1]
400   6e-128
WP_000207830.1 MULTISPECIES: GTP-binding protein [Bacillus]
400   6e-128
WP_098209806.1 GTP-binding protein [Bacillus toyonensis]
400   6e-128
WP_016124781.1 GTP-binding protein [Bacillus cereus]
400   6e-128
WP_009247735.1 GTP-binding protein [Lachnospiraceae bacterium...
387   7e-128
WP_105585111.1 GTP-binding protein [Bacillus sp. MYb209]
400   7e-128
WP_098569164.1 GTP-binding protein [Bacillus thuringiensis]
400   7e-128
OOG94265.1 hypothetical protein BTH41_01964 [Bacillus mycoides]
400   7e-128
WP_076326053.1 GTP-binding protein [Paenibacillus lautus]
400   7e-128
WP_063220522.1 MULTISPECIES: GTP-binding protein [Bacillus]
400   7e-128
WP_078986107.1 tetracycline resistance ribosomal protection p...
400   7e-128
WP_050844106.1 tetracycline resistance ribosomal protection p...
400   7e-128
WP_108015583.1 GTP-binding protein [Streptomyces sp. VMFN-G11Ma]
401   7e-128
WP_041993552.1 GTP-binding protein [Streptomyces sp. AcH 505]
400   8e-128
WP_097808112.1 GTP-binding protein [Bacillus cereus]
400   8e-128
WP_002200055.1 GTP-binding protein [Bacillus cereus]
400   8e-128
WP_016081116.1 GTP-binding protein [Bacillus cereus]
400   8e-128
WP_077317067.1 GTP-binding protein [Bacillus cereus]
400   8e-128
WP_088039658.1 GTP-binding protein [Bacillus mycoides]
400   8e-128
WP_076166380.1 GTP-binding protein [Paenibacillus rhizosphaerae]
400   9e-128
WP_002190029.1 GTP-binding protein [Bacillus cereus]
400   9e-128
WP_000207771.1 GTP-binding protein [Bacillus anthracis]
400   9e-128
WP_030969548.1 GTP-binding protein [Streptomyces sp. NRRL S-1...
401   9e-128
ATI53282.1 tetracycline resistance protein [Bacillus cereus]
400   9e-128
WP_098096393.1 tetracycline resistance ribosomal protection p...
400   9e-128
WP_071719171.1 GTP-binding protein [Bacillus sp. NH24A2]
400   9e-128

PJN59947.1 Tetracycline resistance protein TetO [Bacillus myc...
399   1e-127
WP_105989931.1 GTP-binding protein [Bacillus sp. M21]
400   1e-127
WP_098431652.1 GTP-binding protein [Bacillus cereus]
400   1e-127
WP_081143295.1 MULTISPECIES: GTP-binding protein [Bacillus ce...
400   1e-127
WP_042512565.1 GTP-binding protein [Bacillus cereus]
400   1e-127
WP_098679128.1 GTP-binding protein [Bacillus thuringiensis]
400   1e-127
WP_088118606.1 GTP-binding protein [Bacillus thuringiensis]
400   1e-127
WP_051811298.1 MULTISPECIES: GTP-binding protein [Streptomyces]
400   1e-127
WP_098487293.1 GTP-binding protein [Bacillus cereus]
400   1e-127
WP_088327538.1 tetracycline resistance ribosomal protection p...
400   1e-127
WP_002192815.1 GTP-binding protein [Bacillus cereus]
400   1e-127
WP_044782840.1 GTP-binding protein [Bacillus thuringiensis]
399   1e-127
WP_041046650.1 GTP-binding protein [Paenibacillus sp. VKM B-2...
400   2e-127
WP_054402068.1 GTP-binding protein [Paenibacillus solani]
399   2e-127
WP_000207810.1 MULTISPECIES: GTP-binding protein [Bacillus ce...
399   2e-127
WP_098137662.1 GTP-binding protein [Bacillus cereus]
399   2e-127
WP_098874532.1 GTP-binding protein [Bacillus cereus]
399   2e-127
WP_087953267.1 tetracycline resistance ribosomal protection p...
399   2e-127
WP_070178899.1 GTP-binding protein [Bacillus mycoides]
399   2e-127
WP_098566928.1 GTP-binding protein [Bacillus thuringiensis]
399   2e-127
WP_065212576.1 MULTISPECIES: GTP-binding protein [Bacillus ce...
399   2e-127
WP_097795505.1 tetracycline resistance ribosomal protection p...
399   2e-127
WP_097947204.1 GTP-binding protein [Bacillus thuringiensis]
399   2e-127
WP_088351260.1 GTP-binding protein [Bacillus cereus]
399   2e-127
WP_098889288.1 GTP-binding protein [Bacillus cereus]
399   2e-127
WP_071711782.1 GTP-binding protein [Bacillus sp. TD41]
399   2e-127
WP_098055673.1 GTP-binding protein [Bacillus toyonensis]
399   3e-127

WP_106101995.1 tetracycline resistance ribosomal protection p...
399   3e-127
WP_074625853.1 GTP-binding protein [Bacillus cereus]
399   3e-127
WP_002137036.1 GTP-binding protein [Bacillus cereus]
399   3e-127
WP_098169382.1 tetracycline resistance ribosomal protection p...
399   3e-127
WP_042875224.1 MULTISPECIES: GTP-binding protein [Bacillus ce...
399   3e-127
WP_073304529.1 GTP-binding protein [Bacillus rhizosphaerae]
399   3e-127
WP_073493684.1 GTP-binding protein [Streptomyces phaeoluteigr...
399   3e-127
WP_048565472.1 GTP-binding protein [Bacillus cereus]
399   3e-127
WP_001826586.1 GTP-binding protein [Streptococcus mitis]
386   3e-127
WP_065224575.1 GTP-binding protein [Bacillus cereus]
399   3e-127
WP_063675491.1 GTP-binding protein [Bacillus thuringiensis]
399   3e-127
WP_030675138.1 GTP-binding protein [Streptomyces cellulosae]
399   3e-127
WP_098630095.1 GTP-binding protein [Bacillus cereus]
399   3e-127
WP_098791130.1 GTP-binding protein [Bacillus cereus]
399   3e-127
WP_043716335.1 GTP-binding protein [Kutzneria sp. 744]
399   3e-127
WP_071736035.1 tetracycline resistance ribosomal protection p...
399   3e-127
WP_000207820.1 tetracycline resistance ribosomal protection p...
399   4e-127
WP_097855462.1 GTP-binding protein [Bacillus cereus]
399   4e-127
WP_005592232.1 GTP-binding protein [Streptococcus cristatus]
385   4e-127
WP_048558405.1 MULTISPECIES: GTP-binding protein [Bacillus ce...
398   4e-127
WP_065703803.1 MULTISPECIES: GTP-binding protein [Bacillus ce...
398   4e-127
WP_098488863.1 GTP-binding protein [Bacillus cereus]
398   4e-127
WP_000207838.1 GTP-binding protein [Bacillus thuringiensis]
398   4e-127
WP_073541995.1 GTP-binding protein [Bacillus cereus]
398   5e-127
WP_098339567.1 tetracycline resistance ribosomal protection p...
399   5e-127
WP_088068882.1 GTP-binding protein [Bacillus thuringiensis]
398   5e-127
WP_098453222.1 GTP-binding protein [Bacillus cereus]
398   5e-127

WP_086403602.1 GTP-binding protein [Bacillus thuringiensis]
398   5e-127
WP_098363740.1 GTP-binding protein [Bacillus anthracis]
398   5e-127
WP_061659307.1 GTP-binding protein [Bacillus cereus]
398   5e-127
WP_098216642.1 GTP-binding protein [Bacillus cereus]
398   5e-127
WP_094424885.1 GTP-binding protein [Bacillus clausii]
398   5e-127
WP_097999643.1 GTP-binding protein [Bacillus cereus]
398   5e-127
WP_071739201.1 MULTISPECIES: GTP-binding protein [Bacillus ce...
398   5e-127
WP_033695866.1 MULTISPECIES: GTP-binding protein [Bacillus ce...
398   5e-127
WP_000207814.1 MULTISPECIES: GTP-binding protein [Bacillus ce...
398   6e-127
WP_097881992.1 GTP-binding protein [Bacillus cereus]
398   6e-127
WP_098080641.1 GTP-binding protein [Bacillus wiedmannii]
398   6e-127
WP_098601565.1 GTP-binding protein [Bacillus cereus]
398   6e-127
WP_098652469.1 GTP-binding protein [Bacillus toyonensis]
398   6e-127
WP_027772293.1 GTP-binding protein [Streptomyces sp. CNQ329]
399   6e-127
WP_098156070.1 GTP-binding protein [Bacillus wiedmannii]
398   7e-127
WP_088009402.1 GTP-binding protein [Bacillus mycoides]
398   7e-127
WP_000207813.1 GTP-binding protein [Bacillus cereus]
398   7e-127
WP_001977398.1 GTP-binding protein [Bacillus cereus]
398   7e-127
WP_048538277.1 GTP-binding protein [Bacillus cereus]
398   7e-127
WP_098603752.1 GTP-binding protein [Bacillus cereus]
398   7e-127
WP_098938717.1 GTP-binding protein [Bacillus cereus]
398   7e-127
WP_016091155.1 tetracycline resistance ribosomal protection p...
398   7e-127
WP_098288925.1 GTP-binding protein [Bacillus cereus]
398   8e-127
WP_098913240.1 GTP-binding protein [Bacillus cereus]
397   8e-127
WP_086601557.1 GTP-binding protein [Streptomyces swartbergensis]
398   8e-127
WP_061655559.1 GTP-binding protein [Bacillus cereus]
397   8e-127
WP_000207797.1 GTP-binding protein [Bacillus cereus]
397   8e-127

WP_098125916.1 GTP-binding protein [Bacillus cereus]
397   8e-127
WP_044792915.1 GTP-binding protein [Bacillus thuringiensis]
397   8e-127
WP_098270357.1 GTP-binding protein [Bacillus anthracis]
397   8e-127
EGN48718.1 hypothetical protein HMPREF0990_00619 [Lachnospira...
385   9e-127
WP_098880723.1 GTP-binding protein [Bacillus cereus]
397   9e-127
WP_089171336.1 MULTISPECIES: GTP-binding protein [Bacillus ce...
397   9e-127
WP_098144011.1 GTP-binding protein [Bacillus wiedmannii]
397   9e-127
WP_098768440.1 GTP-binding protein [Bacillus cereus]
397   9e-127
WP_073939854.1 GTP-binding protein [Streptomyces sp. TSRI0107]
398   9e-127
WP_098072013.1 GTP-binding protein [Bacillus toyonensis]
397   9e-127
WP_077016163.1 GTP-binding protein [Kribbella sp. ALI-6-A]
398   1e-126
WP_098281203.1 GTP-binding protein [Bacillus cereus]
397   1e-126
WP_059194595.1 GTP-binding protein [Streptomyces antibioticus]
397   1e-126
WP_001826764.1 GTP-binding protein [Streptococcus pneumoniae]
384   1e-126
WP_000207801.1 GTP-binding protein [Bacillus cereus]
397   1e-126
WP_074563084.1 GTP-binding protein [Bacillus cereus]
397   1e-126
WP_098235357.1 GTP-binding protein [Bacillus thuringiensis]
397   1e-126
WP_100061905.1 GTP-binding protein [Bacillus toyonensis]
397   1e-126
WP_071679760.1 MULTISPECIES: GTP-binding protein [Bacillus]
397   1e-126
WP_042514915.1 MULTISPECIES: GTP-binding protein [Bacillus ce...
397   1e-126
WP_098431991.1 GTP-binding protein [Bacillus cereus]
397   1e-126
WP_076137651.1 GTP-binding protein [Paenibacillus odorifer]
397   1e-126
WP_098375902.1 GTP-binding protein [Bacillus cereus]
397   1e-126
WP_098380667.1 GTP-binding protein [Bacillus cereus]
397   1e-126
WP_000207815.1 MULTISPECIES: GTP-binding protein [Bacillus ce...
397   1e-126
WP_074602035.1 GTP-binding protein [Bacillus cereus]
397   1e-126
WP_000207796.1 GTP-binding protein [Bacillus cereus]
397   1e-126

WP_001817445.1 MULTISPECIES: GTP-binding protein [Bacilli]
384   1e-126
WP_061457388.1 GTP-binding protein [Bacillus cereus]
397   1e-126
WP_016110228.1 GTP-binding protein [Bacillus cereus]
397   1e-126
WP_049563573.1 GTP-binding protein [Nonomuraea sp. SBT364]
397   1e-126
WP_098852368.1 GTP-binding protein [Bacillus thuringiensis]
397   1e-126
EHD87858.1 small GTP-binding domain protein [Streptococcus pn...
384   1e-126
WP_098298136.1 GTP-binding protein [Bacillus cereus]
397   1e-126
WP_075677246.1 MULTISPECIES: GTP-binding protein [Bacillus]
397   1e-126
WP_098015982.1 GTP-binding protein [Bacillus toyonensis]
397   1e-126
WP_000207793.1 GTP-binding protein [Bacillus cereus]
397   1e-126
WP_044584586.1 GTP-binding protein [Bacillus bombysepticus]
397   1e-126
WP_001821424.1 MULTISPECIES: GTP-binding protein [Streptococcus]
384   1e-126
WP_003068336.1 MULTISPECIES: GTP-binding protein [Lactobacill...
384   1e-126
WP_107724531.1 GTP-binding protein [Desmospora activa]
397   1e-126
WP_022524490.1 GTP-binding protein [Streptococcus constellatus]
384   1e-126
WP_000207787.1 MULTISPECIES: GTP-binding protein [Bacillus ce...
397   1e-126
WP_098767570.1 GTP-binding protein [Bacillus cereus]
397   2e-126
WP_095938230.1 GTP-binding protein [Streptomyces sp. Tue6028]
397   2e-126
WP_098286740.1 GTP-binding protein [Bacillus cereus]
397   2e-126
WP_098069168.1 GTP-binding protein [Bacillus wiedmannii]
397   2e-126
WP_048555101.1 GTP-binding protein [Bacillus cereus]
397   2e-126
WP_098423546.1 GTP-binding protein [Bacillus anthracis]
397   2e-126
WP_100618842.1 GTP-binding protein [Bacillus cereus]
397   2e-126
WP_070173394.1 MULTISPECIES: GTP-binding protein [Bacillus ce...
397   2e-126
WP_077565691.1 GTP-binding protein [Paenibacillus ihbetae]
397   2e-126
PJP40604.1 tetracycline resistance ribosomal protection prote...
384   2e-126
WP_097854653.1 GTP-binding protein [Bacillus toyonensis]
397   2e-126

WP_000207794.1 MULTISPECIES: GTP-binding protein [Bacillus ce...
397   2e-126
EFO69518.1 GTP-binding domain protein [Lactobacillus iners La...
384   2e-126
WP_099935351.1 GTP-binding protein [Streptomyces sp. 1121.2]
397   2e-126
WP_071740767.1 GTP-binding protein [Bacillus cereus]
397   2e-126
WP_098319669.1 GTP-binding protein [Bacillus anthracis]
397   2e-126
WP_098184567.1 GTP-binding protein [Bacillus toyonensis]
397   2e-126
WP_098180473.1 GTP-binding protein [Bacillus toyonensis]
397   2e-126
WP_078438923.1 GTP-binding protein [Bacillus cereus]
397   2e-126
WP_098296801.1 GTP-binding protein [Bacillus cereus]
397   2e-126
WP_076207755.1 GTP-binding protein [Paenibacillus odorifer]
397   2e-126
WP_030931205.1 GTP-binding protein [Streptomyces violaceoruber]
397   2e-126
WP_030282618.1 GTP-binding protein [Streptomyces sp. NRRL B-5...
397   2e-126
WP_098300198.1 GTP-binding protein [Bacillus cereus]
397   2e-126
WP_048535731.1 GTP-binding protein [Bacillus cereus]
397   2e-126
WP_080326834.1 tetracycline resistance ribosomal protection p...
396   2e-126
WP_070184032.1 MULTISPECIES: GTP-binding protein [Bacillus]
396   2e-126
WP_016118171.1 MULTISPECIES: GTP-binding protein [Bacillus ce...
396   2e-126
WP_097980569.1 GTP-binding protein [Bacillus toyonensis]
396   2e-126
WP_098379193.1 GTP-binding protein [Bacillus cereus]
396   2e-126
WP_098349869.1 GTP-binding protein [Bacillus anthracis]
396   2e-126
WP_098887702.1 GTP-binding protein [Bacillus cereus]
396   2e-126
WP_091306093.1 GTP-binding protein [Micromonospora chersina]
397   2e-126
WP_098826159.1 GTP-binding protein [Bacillus toyonensis]
396   2e-126
WP_000207773.1 GTP-binding protein [Bacillus cereus]
396   2e-126
WP_048568594.1 GTP-binding protein [Bacillus cereus]
396   3e-126
WP_098785967.1 GTP-binding protein [Bacillus cereus]
396   3e-126
WP_098581792.1 GTP-binding protein [Bacillus cereus]
396   3e-126

WP_097954308.1 GTP-binding protein [Bacillus toyonensis]
396   3e-126
WP_025147870.1 GTP-binding protein [Bacillus sp. H1a]
396   3e-126
WP_098681228.1 GTP-binding protein [Bacillus wiedmannii]
396   3e-126
WP_098880230.1 GTP-binding protein [Bacillus anthracis]
396   3e-126
WP_000207784.1 MULTISPECIES: tetracycline resistance ribosoma...
396   3e-126
WP_044796897.1 GTP-binding protein [Bacillus cereus]
396   3e-126
WP_098238255.1 GTP-binding protein [Bacillus cereus]
396   3e-126
WP_098541970.1 GTP-binding protein [Bacillus cereus]
396   3e-126
WP_098876292.1 GTP-binding protein [Bacillus cereus]
396   3e-126
WP_098616777.1 GTP-binding protein [Bacillus cereus]
396   3e-126
WP_000207807.1 GTP-binding protein [Bacillus cereus]
396   3e-126
WP_009593478.1 GTP-binding protein [Paenibacillus sp. HGF5]
396   3e-126
WP_000207795.1 MULTISPECIES: GTP-binding protein [Bacillus]
396   3e-126
WP_078110412.1 tetracycline resistance ribosomal protection p...
396   3e-126
WP_097882251.1 GTP-binding protein [Bacillus toyonensis]
396   3e-126
WP_088115829.1 GTP-binding protein [Bacillus cereus]
395   3e-126
WP_097996165.1 GTP-binding protein [Bacillus anthracis]
396   3e-126
WP_069604671.1 GTP-binding protein [Bacillus cereus]
396   3e-126
WP_098487951.1 GTP-binding protein [Bacillus cereus]
396   4e-126
WP_098355296.1 GTP-binding protein [Bacillus cereus]
396   4e-126
WP_098132503.1 GTP-binding protein [Bacillus wiedmannii]
396   4e-126
WP_000207790.1 MULTISPECIES: GTP-binding protein [Bacillus ce...
396   4e-126
WP_098581325.1 GTP-binding protein [Bacillus cereus]
396   4e-126
WP_000207806.1 MULTISPECIES: GTP-binding protein [Bacillus]
396   4e-126
WP_028539038.1 MULTISPECIES: GTP-binding protein [Paenibacillus]
397   4e-126
EOS55661.1 translation elongation factor G [Paenibacillus bar...
397   4e-126
WP_098123664.1 GTP-binding protein [Bacillus wiedmannii]
396   4e-126

WP_088091599.1 MULTISPECIES: GTP-binding protein [Bacillus ce...
396   4e-126
CJV61492.1 tetracycline resistance protein TetM [Streptococcu...
380   4e-126
WP_098274036.1 GTP-binding protein [Bacillus cereus]
396   4e-126
WP_030026231.1 MULTISPECIES: GTP-binding protein [Bacillus ce...
396   4e-126
WP_000207785.1 MULTISPECIES: GTP-binding protein [Bacillus]
395   4e-126
WP_098718789.1 GTP-binding protein [Bacillus toyonensis]
395   4e-126
WP_088046323.1 GTP-binding protein [Bacillus thuringiensis]
395   4e-126
WP_072655305.1 GTP-binding protein [Streptomyces viridifaciens]
396   4e-126
WP_097879342.1 tetracycline resistance ribosomal protection p...
395   4e-126
WP_063536710.1 GTP-binding protein [Bacillus cereus]
395   4e-126
WP_098631347.1 GTP-binding protein [Bacillus wiedmannii]
395   5e-126
AFT65577.1 TetM, partial [Clostridioides difficile]
384   5e-126
WP_050821928.1 MULTISPECIES: GTP-binding protein [Bacillus]
395   5e-126
SME99119.1 ribosomal protection tetracycline resistance prote...
396   5e-126
WP_085169366.1 GTP-binding protein [Paenibacillus sp. J6]
396   5e-126
WP_053564128.1 MULTISPECIES: GTP-binding protein [Bacillus ce...
395   5e-126
WP_088299050.1 GTP-binding protein [Bacillus cereus]
395   5e-126
WP_058004226.1 tetracycline resistance ribosomal protection p...
396   5e-126
WP_097995016.1 GTP-binding protein [Bacillus cereus]
395   5e-126
WP_087981043.1 GTP-binding protein [Bacillus cereus]
395   5e-126
WP_106074913.1 tetracycline resistance ribosomal protection p...
395   5e-126
WP_098771490.1 GTP-binding protein [Bacillus cereus]
395   5e-126
WP_098822820.1 GTP-binding protein [Bacillus toyonensis]
395   5e-126
CUB51017.1 Tetracycline resistance protein TetO [Bacillus sub...
395   5e-126
AMP51306.1 elongation factor Tu GTP binding domain protein [u...
393   5e-126
WP_073523415.1 GTP-binding protein [Bacillus cereus]
395   5e-126
WP_098174194.1 GTP-binding protein [Bacillus toyonensis]
395   6e-126

WP_078186747.1 GTP-binding protein [Bacillus cereus]
395   6e-126
WP_098651237.1 GTP-binding protein [Bacillus toyonensis]
395   6e-126
WP_098387102.1 GTP-binding protein [Bacillus cereus]
395   6e-126
CUB13758.1 Tetracycline resistance protein TetO [Bacillus cer...
395   6e-126
WP_098358159.1 MULTISPECIES: GTP-binding protein [Bacillus]
395   6e-126
WP_078421523.1 GTP-binding protein [Bacillus cereus]
395   6e-126
WP_061139140.1 MULTISPECIES: GTP-binding protein [Bacillus]
395   6e-126
WP_053512514.1 MULTISPECIES: GTP-binding protein [Bacillus ce...
395   6e-126
WP_063225298.1 MULTISPECIES: GTP-binding protein [Bacillus ce...
395   6e-126
KOS27491.1 tetracycline resistance protein [Bacillus anthracis]
395   6e-126
WP_086403399.1 GTP-binding protein [Bacillus thuringiensis]
395   6e-126
WP_098627842.1 tetracycline resistance ribosomal protection p...
395   6e-126
WP_088106656.1 GTP-binding protein [Bacillus cereus]
395   6e-126
WP_098520520.1 GTP-binding protein [Bacillus cereus]
395   7e-126
WP_097918509.1 GTP-binding protein [Bacillus cereus]
395   7e-126
WP_000207786.1 MULTISPECIES: GTP-binding protein [Bacillus]
395   7e-126
WP_071381934.1 GTP-binding protein [Streptomyces sp. MUSC 1]
395   7e-126
WP_097905485.1 GTP-binding protein [Bacillus cereus]
395   7e-126
WP_098390105.1 GTP-binding protein [Bacillus thuringiensis]
395   7e-126
WP_098207495.1 GTP-binding protein [Bacillus cereus]
395   7e-126
WP_058083215.1 GTP-binding protein [Streptomyces hygroscopicus]
396   7e-126
WP_061182873.1 GTP-binding protein [Bacillus cereus]
395   7e-126
WP_070806371.1 GTP-binding protein [Bacillus sp. ABP14]
395   8e-126
WP_103652782.1 GTP-binding protein [Bacillus thuringiensis]
395   8e-126
WP_097861031.1 GTP-binding protein [Bacillus toyonensis]
395   8e-126
WP_087970853.1 GTP-binding protein [Bacillus thuringiensis]
395   8e-126
WP_098202919.1 MULTISPECIES: GTP-binding protein [Bacillus ce...
395   8e-126

WP_042598191.1 GTP-binding protein [Bacillus thuringiensis]
395   8e-126
WP_098294807.1 GTP-binding protein [Bacillus cereus]
395   8e-126
WP_005164048.1 GTP-binding protein [Amycolatopsis azurea]
395   8e-126
WP_098814471.1 GTP-binding protein [Bacillus wiedmannii]
395   8e-126
WP_071772934.1 GTP-binding protein [Bacillus sp. NH11B]
395   8e-126
WP_044090702.1 GTP-binding protein [Bifidobacterium reuteri]
378   9e-126
WP_091344501.1 GTP-binding protein [Micromonospora rhizosphae...
395   9e-126
WP_098878908.1 GTP-binding protein [Bacillus cereus]
395   9e-126
WP_071977357.1 GTP-binding protein [Bacillus obstructivus]
395   9e-126
WP_000207799.1 MULTISPECIES: GTP-binding protein [Bacillus]
395   9e-126
WP_016430685.1 GTP-binding protein [Streptomyces sp. HGB0020]
395   9e-126
WP_088046657.1 tetracycline resistance ribosomal protection p...
395   9e-126
WP_086390623.1 GTP-binding protein [Bacillus thuringiensis]
395   9e-126
WP_070179873.1 GTP-binding protein [Bacillus thuringiensis]
395   9e-126
WP_098126762.1 GTP-binding protein [Bacillus toyonensis]
395   9e-126
COS31641.1 putative tetracycline resistance protein tet(O/W/3...
393   1e-125
WP_098587722.1 GTP-binding protein [Bacillus thuringiensis]
395   1e-125
WP_097820242.1 GTP-binding protein [Bacillus cereus]
395   1e-125
WP_016108676.1 GTP-binding protein [Bacillus cereus]
395   1e-125
WP_002066047.1 GTP-binding protein [Bacillus cereus]
395   1e-125
WP_098103118.1 tetracycline resistance ribosomal protection p...
395   1e-125
WP_000207821.1 MULTISPECIES: GTP-binding protein [Bacillus]
395   1e-125
WP_097864155.1 GTP-binding protein [Bacillus cereus]
395   1e-125
ATI60177.1 tetracycline resistance protein [Bacillus cereus]
395   1e-125
WP_000207800.1 MULTISPECIES: GTP-binding protein [Bacillus ce...
394   1e-125
WP_016136730.1 MULTISPECIES: GTP-binding protein [Bacillus]
394   1e-125
WP_030549954.1 MULTISPECIES: GTP-binding protein [Streptomyce...
395   1e-125

WP_000207772.1 tetracycline resistance ribosomal protection p...
394   1e-125
WP_000207839.1 GTP-binding protein [Bacillus cereus]
394   1e-125
WP_098266313.1 GTP-binding protein [Bacillus cereus]
394   1e-125
WP_053849966.1 GTP-binding protein [Streptomyces sp. NRRL B-2...
395   1e-125
WP_033348106.1 GTP-binding protein [Kitasatospora aureofaciens]
395   1e-125
WP_016717851.1 GTP-binding protein [Bacillus cereus]
394   1e-125
WP_098651355.1 GTP-binding protein [Bacillus toyonensis]
394   1e-125
WP_074650727.1 GTP-binding protein [Bacillus cereus]
394   1e-125
WP_064471179.1 GTP-binding protein [Bacillus wiedmannii]
394   1e-125
WP_078498386.1 GTP-binding protein [Paenibacillus selenitired...
394   1e-125
WP_087949617.1 GTP-binding protein [Bacillus thuringiensis]
394   2e-125
WP_076538512.1 GTP-binding protein [Bacillus cereus]
394   2e-125
WP_064057545.1 GTP-binding protein [Bacillus wiedmannii]
394   2e-125
WP_061530631.1 MULTISPECIES: GTP-binding protein [Bacillus ce...
394   2e-125
WP_099919045.1 GTP-binding protein [Streptomyces sp. 94]
395   2e-125
WP_036663538.1 GTP-binding protein [Paenibacillus sp. FSL H8-...
394   2e-125
WP_000207775.1 GTP-binding protein [Bacillus cereus]
394   2e-125
WP_098395841.1 MULTISPECIES: GTP-binding protein [Bacillus ce...
394   2e-125
WP_098213081.1 GTP-binding protein [Bacillus cereus]
394   2e-125
WP_026931358.1 GTP-binding protein [Glycomyces tenuis]
395   2e-125
WP_062821705.1 GTP-binding protein [Bacillus cereus]
394   2e-125
WP_061682005.1 GTP-binding protein [Bacillus cereus]
394   2e-125
WP_098935345.1 GTP-binding protein [Bacillus toyonensis]
394   2e-125
WP_087984601.1 GTP-binding protein [Bacillus thuringiensis]
394   2e-125
WP_054711139.1 GTP-binding protein [Bacillus sp. JCM 19041]
394   2e-125
WP_076303356.1 GTP-binding protein [Paenibacillus odorifer]
394   2e-125
WP_000207819.1 GTP-binding protein [Bacillus cereus]
394   2e-125

WP_021727846.1 MULTISPECIES: GTP-binding protein [Bacillus ce...
394   2e-125
WP_098840828.1 GTP-binding protein [Bacillus sp. AFS033286]
394   2e-125
WP_060487229.1 GTP-binding protein [Bacillus cereus]
394   2e-125
WP_000207791.1 GTP-binding protein [Bacillus thuringiensis]
394   2e-125
WP_098356146.1 GTP-binding protein [Bacillus cereus]
394   2e-125
WP_087875509.1 GTP-binding protein [Bacillus thuringiensis]
394   2e-125
WP_048656357.1 GTP-binding protein [Bacillus cereus]
394   2e-125
WP_074554723.1 GTP-binding protein [Bacillus cereus]
394   2e-125
WP_102956528.1 GTP-binding protein [Bacillus thuringiensis]
394   2e-125
WP_070756619.1 GTP-binding protein [Bacillus sp. RZ2MS9]
394   2e-125
WP_048566143.1 MULTISPECIES: GTP-binding protein [Bacillus]
394   2e-125
WP_071723687.1 GTP-binding protein [Bacillus sp. 0711P9-1]
394   2e-125
WP_098688864.1 GTP-binding protein [Bacillus toyonensis]
394   2e-125
WP_000207776.1 GTP-binding protein [Bacillus thuringiensis]
394   2e-125
WP_097223686.1 GTP-binding protein [Streptomyces sp. OV198]
394   2e-125
WP_097858981.1 GTP-binding protein [Bacillus wiedmannii]
394   2e-125
WP_071732015.1 GTP-binding protein [Bacillus thuringiensis]
394   2e-125
WP_041183863.1 GTP-binding protein [Bacillus cereus]
394   2e-125
WP_063272418.1 GTP-binding protein [Amycolatopsis keratiniphila]
394   2e-125
WP_098341682.1 GTP-binding protein [Bacillus cereus]
394   2e-125
WP_000207817.1 GTP-binding protein [Bacillus cereus]
394   2e-125
WP_067360068.1 GTP-binding protein [Streptomyces olivochromog...
394   2e-125
WP_098577319.1 GTP-binding protein [Bacillus wiedmannii]
394   2e-125
WP_098560664.1 GTP-binding protein [Bacillus cereus]
394   2e-125
WP_086388295.1 GTP-binding protein [Bacillus thuringiensis]
394   2e-125
WP_085927335.1 GTP-binding protein [Streptomyces platensis]
395   2e-125
WP_033318981.1 GTP-binding protein [Streptomyces yerevanensis]
394   2e-125

WP_098063664.1 GTP-binding protein [Bacillus toyonensis]
394   2e-125
WP_079657901.1 GTP-binding protein [Streptomyces sp. 3214.6]
394   2e-125
WP_100633937.1 GTP-binding protein [Bacillus cereus]
394   3e-125
WP_088096824.1 GTP-binding protein [Bacillus cereus]
394   3e-125
WP_095850877.1 GTP-binding protein [Streptomyces sp. Ag82_O1-15]
394   3e-125
WP_098688062.1 GTP-binding protein [Bacillus toyonensis]
394   3e-125
AMP46867.1 elongation factor Tu GTP binding domain protein [u...
380   3e-125
WP_098759426.1 GTP-binding protein [Bacillus anthracis]
394   3e-125
WP_097830202.1 GTP-binding protein [Bacillus cereus]
394   3e-125
WP_098298894.1 GTP-binding protein [Bacillus anthracis]
394   3e-125
WP_097822362.1 GTP-binding protein [Bacillus thuringiensis]
394   3e-125
WP_072028650.1 GTP-binding protein [Amycolatopsis keratiniphila]
393   3e-125
WP_086385810.1 GTP-binding protein [Bacillus thuringiensis]
394   3e-125
WP_016134813.1 GTP-binding protein [Bacillus cereus]
394   3e-125
WP_074540974.1 GTP-binding protein [Bacillus cereus]
394   3e-125
WP_087966943.1 GTP-binding protein [Bacillus thuringiensis]
394   3e-125
WP_084064178.1 MULTISPECIES: GTP-binding protein [Bacillus]
394   3e-125
WP_098115864.1 GTP-binding protein [Bacillus wiedmannii]
394   3e-125
WP_061671897.1 GTP-binding protein [Bacillus wiedmannii]
394   3e-125
WP_098130182.1 GTP-binding protein [Bacillus wiedmannii]
394   3e-125
OEV31870.1 GTP-binding protein [Streptomyces nanshensis]
394   3e-125
WP_098099331.1 GTP-binding protein [Bacillus toyonensis]
393   4e-125
WP_097960934.1 GTP-binding protein [Bacillus thuringiensis]
393   4e-125
WP_088229516.1 MULTISPECIES: GTP-binding protein [Bacillus]
393   4e-125
WP_064449298.1 GTP-binding protein [Bacillus wiedmannii]
393   4e-125
WP_030986857.1 GTP-binding protein [Streptomyces sp. NRRL WC-...
394   4e-125
WP_059262515.1 GTP-binding protein [Streptomyces corchorusii]
394   4e-125

WP_000207803.1 GTP-binding protein [Bacillus cereus]
393   4e-125
AMJ35357.1 elongation factor Tu GTP binding domain protein [u...
380   4e-125
WP_070172510.1 GTP-binding protein [Bacillus anthracis]
393   4e-125
WP_020673402.1 GTP-binding protein [Amycolatopsis nigrescens]
393   4e-125
WP_044307263.1 GTP-binding protein [Bacillus cereus]
393   4e-125
WP_097987378.1 GTP-binding protein [Bacillus thuringiensis]
393   4e-125
WP_048557758.1 GTP-binding protein [Bacillus cereus]
393   4e-125
WP_098985922.1 GTP-binding protein [Bacillus cereus]
393   4e-125
WP_106103319.1 GTP-binding protein [Bacillus thuringiensis]
393   4e-125
WP_098253599.1 GTP-binding protein [Bacillus anthracis]
393   4e-125
WP_076277910.1 GTP-binding protein [Paenibacillus odorifer]
394   4e-125
WP_098048452.1 GTP-binding protein [Bacillus wiedmannii]
393   4e-125
WP_097936194.1 GTP-binding protein [Bacillus toyonensis]
393   4e-125
WP_016087331.1 GTP-binding protein [Bacillus cereus]
393   4e-125
WP_038518607.1 GTP-binding protein [Amycolatopsis japonica]
393   4e-125
WP_106081949.1 GTP-binding protein [Bacillus thuringiensis]
393   4e-125
WP_098009604.1 GTP-binding protein [Bacillus toyonensis]
393   4e-125
WP_106824753.1 GTP-binding protein [Bacillus cereus]
393   4e-125
WP_000207782.1 GTP-binding protein [Bacillus cereus]
393   4e-125
WP_098781735.1 GTP-binding protein [Bacillus cereus]
393   4e-125
WP_000207812.1 GTP-binding protein [Bacillus cereus]
393   5e-125
WP_074615634.1 GTP-binding protein [Bacillus cereus]
393   5e-125
WP_094594591.1 GTP-binding protein [Paenibacillus sp. XY044]
393   5e-125
WP_096775460.1 GTP-binding protein [Paenibacillus lautus]
393   5e-125
WP_074083232.1 GTP-binding protein [Paenibacillus sp. P32E]
394   5e-125
WP_091288457.1 GTP-binding protein [Amycolatopsis xylanica]
392   5e-125
WP_098578189.1 GTP-binding protein [Bacillus thuringiensis]
393   5e-125

WP_014676696.1 GTP-binding protein [Streptomyces hygroscopicus]
394   5e-125
WP_079107999.1 GTP-binding protein [Streptococcus pneumoniae]
380   5e-125
WP_060631344.1 GTP-binding protein [Bacillus thuringiensis]
393   5e-125
WP_076145470.1 GTP-binding protein [Paenibacillus odorifer]
393   5e-125
WP_098661250.1 GTP-binding protein [Bacillus toyonensis]
393   5e-125
WP_098179351.1 GTP-binding protein [Bacillus toyonensis]
393   5e-125
WP_000209604.1 MULTISPECIES: GTP-binding protein [Bacillus]
393   5e-125
WP_006301404.1 GTP-binding protein [Aminomonas paucivorans]
393   5e-125
WP_015734827.1 GTP-binding protein [Paenibacillus sp. Y412MC10]
393   5e-125
WP_098582829.1 GTP-binding protein [Bacillus cereus]
393   5e-125
WP_098597779.1 GTP-binding protein [Bacillus cereus]
393   5e-125
WP_060851999.1 GTP-binding protein [Bacillus thuringiensis]
393   5e-125
WP_098347023.1 GTP-binding protein [Bacillus anthracis]
393   6e-125
WP_098405331.1 GTP-binding protein [Bacillus cereus]
393   6e-125
WP_097914093.1 GTP-binding protein [Bacillus thuringiensis]
393   6e-125
WP_098645458.1 GTP-binding protein [Bacillus cereus]
393   6e-125
WP_098326990.1 GTP-binding protein [Bacillus cereus]
393   6e-125
WP_078993835.1 GTP-binding protein [Bacillus thuringiensis]
393   6e-125
WP_074101168.1 GTP-binding protein [Paenibacillus sp. P3E]
393   6e-125
OUB20038.1 tetracycline resistance protein [Bacillus thuringi...
393   6e-125
WP_025966074.1 GTP-binding protein [Bacillus cereus]
393   6e-125
WP_023522319.1 GTP-binding protein [Bacillus thuringiensis]
393   6e-125
WP_043936983.1 MULTISPECIES: GTP-binding protein [Bacillus]
393   6e-125
WP_098276660.1 GTP-binding protein [Bacillus thuringiensis]
393   6e-125
WP_097920775.1 GTP-binding protein [Bacillus thuringiensis]
393   6e-125
WP_087955843.1 GTP-binding protein [Bacillus thuringiensis]
393   6e-125
WP_076856476.1 GTP-binding protein [Bacillus cereus]
392   7e-125

WP_046199296.1 MULTISPECIES: GTP-binding protein [Bacillus]
392   7e-125
WP_061660011.1 GTP-binding protein [Bacillus cereus]
392   7e-125
WP_037750473.1 GTP-binding protein [Streptomyces mirabilis]
393   8e-125
WP_063547740.1 MULTISPECIES: GTP-binding protein [Bacillus ce...
392   8e-125
WP_000207778.1 GTP-binding protein [Bacillus cereus]
392   8e-125
WP_095755238.1 GTP-binding protein [Streptomyces sp. SA15]
393   8e-125
CGG62230.1 putative tetracycline resistance protein tet(O/W/3...
392   8e-125
WP_098914269.1 GTP-binding protein [Bacillus cereus]
392   8e-125
WP_046262628.1 GTP-binding protein [Streptomyces sp. WM6386]
393   9e-125
WP_000207802.1 GTP-binding protein [Bacillus cereus]
392   9e-125
WP_076309578.1 GTP-binding protein [Paenibacillus odorifer]
393   9e-125
WP_046110059.1 GTP-binding protein [Devosia geojensis]
392   9e-125
WP_098204289.1 GTP-binding protein [Bacillus wiedmannii]
392   9e-125
WP_000207777.1 GTP-binding protein [Bacillus cereus]
392   1e-124
WP_073935346.1 GTP-binding protein [Streptomyces sp. CB02400]
392   1e-124
WP_098785410.1 GTP-binding protein [Bacillus cereus]
392   1e-124
WP_030244424.1 GTP-binding protein [Streptomyces sp. NRRL S-350]
392   1e-124
WP_088745802.1 GTP-binding protein [Enterococcus hirae]
379   1e-124
WP_070011477.1 GTP-binding protein [Streptomyces abyssalis]
393   1e-124
WP_046946010.1 GTP-binding protein [Bacillus cereus]
392   1e-124
WP_076316927.1 GTP-binding protein [Paenibacillus odorifer]
392   1e-124
WP_000207774.1 GTP-binding protein [Bacillus cereus]
392   1e-124
WP_074789888.1 GTP-binding protein [Bacillus thuringiensis]
392   1e-124
EGL86977.1 GTP-binding domain protein [Streptococcus oralis S...
380   1e-124
WP_084744559.1 GTP-binding protein [Dielma fastidiosa]
379   1e-124
WP_098227245.1 GTP-binding protein [Bacillus toyonensis]
392   1e-124
WP_098091366.1 GTP-binding protein [Bacillus wiedmannii]
392   1e-124

WP_076129526.1 GTP-binding protein [Paenibacillus odorifer]
392   1e-124
WP_098670710.1 GTP-binding protein [Bacillus sp. AFS059628]
392   1e-124
WP_098106955.1 GTP-binding protein [Bacillus toyonensis]
392   1e-124
WP_098925948.1 GTP-binding protein [Bacillus anthracis]
392   1e-124
WP_097997376.1 GTP-binding protein [Bacillus thuringiensis]
392   2e-124
WP_048526200.1 GTP-binding protein [Bacillus cereus]
392   2e-124
WP_000207841.1 GTP-binding protein [Bacillus cereus]
392   2e-124
WP_098722196.1 GTP-binding protein [Bacillus wiedmannii]
392   2e-124
WP_098451801.1 GTP-binding protein [Bacillus thuringiensis]
392   2e-124
WP_101787645.1 GTP-binding protein [Nonomuraea indica]
392   2e-124
WP_065229002.1 MULTISPECIES: GTP-binding protein [Bacillus ce...
392   2e-124
WP_000207798.1 GTP-binding protein [Bacillus cereus]
392   2e-124
WP_098259317.1 GTP-binding protein [Bacillus cereus]
392   2e-124
WP_076282042.1 GTP-binding protein [Paenibacillus odorifer]
392   2e-124
WP_000207779.1 MULTISPECIES: GTP-binding protein [Bacillus]
392   2e-124
WP_078418104.1 GTP-binding protein [Bacillus cereus]
392   2e-124
WP_030845064.1 GTP-binding protein [Streptomyces sp. NRRL S-475]
392   2e-124
WP_093698327.1 GTP-binding protein [Streptomyces sp. 2231.1]
392   2e-124
WP_098672105.1 GTP-binding protein [Bacillus cereus]
391   2e-124
WP_097981879.1 MULTISPECIES: GTP-binding protein [Bacillus]
391   2e-124
WP_098953430.1 GTP-binding protein [Bacillus wiedmannii]
391   2e-124
WP_098252462.1 GTP-binding protein [Bacillus cereus]
391   2e-124
WP_061130392.1 GTP-binding protein [Bacillus cereus]
391   2e-124
AMP54771.1 elongation factor Tu GTP binding domain protein [u...
379   2e-124
WP_098366028.1 GTP-binding protein [Bacillus thuringiensis]
391   2e-124
WP_098815310.1 GTP-binding protein [Bacillus wiedmannii]
391   2e-124
WP_062921122.1 MULTISPECIES: GTP-binding protein [Bacillus]
391   2e-124

WP_000207818.1 GTP-binding protein [Bacillus thuringiensis]
391   2e-124
WP_097841252.1 MULTISPECIES: GTP-binding protein [Bacillus]
391   2e-124
WP_000207781.1 MULTISPECIES: GTP-binding protein [Bacillus ce...
391   2e-124
WP_016095714.1 GTP-binding protein [Bacillus cereus]
391   2e-124
WP_098684107.1 GTP-binding protein [Bacillus thuringiensis]
391   2e-124
WP_079245696.1 GTP-binding protein [Bacillus thuringiensis]
391   2e-124
WP_000207809.1 GTP-binding protein [Bacillus cereus]
391   2e-124
WP_000207816.1 GTP-binding protein [Bacillus cereus]
391   2e-124
WP_000207811.1 MULTISPECIES: GTP-binding protein [Bacillus ce...
391   3e-124
WP_098162334.1 GTP-binding protein [Bacillus toyonensis]
391   3e-124
WP_061884320.1 GTP-binding protein [Bacillus thuringiensis]
391   3e-124
WP_103622490.1 GTP-binding protein [Bacillus thuringiensis]
391   3e-124
WP_098284721.1 GTP-binding protein [Bacillus thuringiensis]
391   3e-124
WP_088867818.1 GTP-binding protein [Bacillus cereus]
391   3e-124
WP_098911125.1 GTP-binding protein [Bacillus thuringiensis]
391   3e-124
WP_064475662.1 GTP-binding protein [Bacillus wiedmannii]
391   3e-124
WP_076305359.1 GTP-binding protein [Paenibacillus odorifer]
391   3e-124
AFT65576.1 TetM, partial [Clostridioides difficile]
380   3e-124
WP_000207792.1 GTP-binding protein [Bacillus cereus]
391   3e-124
WP_074533382.1 GTP-binding protein [Bacillus cereus]
391   3e-124
WP_074110803.1 GTP-binding protein [Paenibacillus sp. P46E]
391   3e-124
WP_000691752.1 GTP-binding protein [Streptococcus pneumoniae]
378   3e-124
WP_089149480.1 GTP-binding protein [Bacillus sp. M13(2017)]
391   4e-124
WP_090911413.1 GTP-binding protein [Paenibacillus sp. cl141a]
391   4e-124
WP_097271842.1 GTP-binding protein [Streptomyces sp. TLI_55]
391   4e-124
WP_098834199.1 GTP-binding protein [Bacillus thuringiensis]
391   4e-124
WP_098926545.1 GTP-binding protein [Bacillus cereus]
391   4e-124

WP_074608511.1 GTP-binding protein [Bacillus cereus]
390   4e-124
WP_098022276.1 GTP-binding protein [Bacillus cereus]
390   4e-124
WP_106535987.1 GTP-binding protein [Haloactinopolyspora alba]
391   4e-124
WP_054236553.1 GTP-binding protein [Actinobacteria bacterium ...
391   4e-124
WP_098977837.1 GTP-binding protein [Bacillus wiedmannii]
390   4e-124
WP_000207808.1 GTP-binding protein [Bacillus cereus]
390   4e-124
WP_016122745.1 MULTISPECIES: GTP-binding protein [Bacillus ce...
390   4e-124
WP_088059261.1 GTP-binding protein [Bacillus mycoides]
390   4e-124
WP_098057015.1 GTP-binding protein [Bacillus wiedmannii]
390   4e-124
WP_098686565.1 GTP-binding protein [Bacillus cereus]
390   5e-124
WP_098430929.1 GTP-binding protein [Bacillus cereus]
390   5e-124
WP_018759654.1 GTP-binding protein [Paenibacillus terrigena]
390   5e-124
WP_035700787.1 GTP-binding protein [Glycomyces tenuis]
390   5e-124
WP_076307103.1 GTP-binding protein [Paenibacillus odorifer]
391   5e-124
WP_006677586.1 GTP-binding protein [Paenibacillus dendritifor...
391   5e-124
WP_093782400.1 GTP-binding protein [Streptomyces guanduensis]
391   5e-124
WP_086387007.1 GTP-binding protein [Bacillus thuringiensis]
390   5e-124
WP_098147955.1 GTP-binding protein [Bacillus wiedmannii]
390   5e-124
WP_037346117.1 GTP-binding protein [Amycolatopsis sp. MJM2582]
390   5e-124
WP_060895799.1 GTP-binding protein [Streptomyces diastatochro...
390   5e-124
WP_059128016.1 GTP-binding protein [Streptomyces sp. NRRL F-5...
391   5e-124
WP_103591471.1 GTP-binding protein [Bacillus thuringiensis]
390   5e-124
WP_012923280.1 GTP-binding protein [Kribbella flavida]
390   6e-124
WP_098280350.1 GTP-binding protein [Bacillus cereus]
390   6e-124
WP_098596160.1 GTP-binding protein [Bacillus cereus]
390   6e-124
WP_051855603.1 GTP-binding protein [Streptomyces sp. NRRL B-1...
391   6e-124
WP_061536650.1 MULTISPECIES: GTP-binding protein [Bacillus]
390   6e-124

WP_098868136.1 GTP-binding protein [Bacillus thuringiensis]
390   6e-124
WP_026923627.1 GTP-binding protein [Glycomyces arizonensis]
390   7e-124
WP_045862942.1 GTP-binding protein [Streptomyces sp. WMMB 714]
391   7e-124
WP_020272080.1 GTP-binding protein [Streptomyces afghaniensis]
390   7e-124
WP_088084525.1 GTP-binding protein [Bacillus cereus]
390   7e-124
WP_098639898.1 GTP-binding protein [Bacillus wiedmannii]
390   7e-124
WP_012921159.1 GTP-binding protein [Kribbella flavida]
390   8e-124
WP_098872226.1 GTP-binding protein [Bacillus cereus]
390   8e-124
WP_088363862.1 GTP-binding protein [Bacillus cereus]
390   8e-124
WP_098650532.1 GTP-binding protein [Bacillus wiedmannii]
390   8e-124
WP_000207825.1 MULTISPECIES: GTP-binding protein [Bacillus ce...
390   8e-124
WP_006927594.1 GTP-binding protein [Bacillus sp. GeD10]
390   8e-124
WP_098911284.1 GTP-binding protein [Bacillus toyonensis]
390   8e-124
WP_000207805.1 GTP-binding protein [Bacillus thuringiensis]
390   8e-124
WP_098855141.1 GTP-binding protein [Bacillus thuringiensis]
390   8e-124
WP_001992644.1 GTP-binding protein [Bacillus cereus]
390   9e-124
WP_099683621.1 GTP-binding protein [Bacillus sp. 100374]
390   9e-124
WP_098509032.1 GTP-binding protein [Bacillus cereus]
390   9e-124
WP_098137940.1 GTP-binding protein [Bacillus toyonensis]
390   9e-124
WP_078077952.1 GTP-binding protein [Streptomyces niveus]
390   9e-124
WP_103141466.1 GTP-binding protein [Bacillus cereus]
390   9e-124
WP_098084642.1 GTP-binding protein [Bacillus wiedmannii]
390   9e-124
WP_010724625.1 transposase [Enterococcus faecium]
374   9e-124
WP_098116165.1 GTP-binding protein [Bacillus wiedmannii]
390   9e-124
WP_098843072.1 GTP-binding protein [Bacillus thuringiensis]
390   9e-124
WP_098902478.1 GTP-binding protein [Bacillus thuringiensis]
390   1e-123
WP_016514015.1 MULTISPECIES: GTP-binding protein [Bacillus ce...
390   1e-123

WP_093932369.1 GTP-binding protein [Amycolatopsis thailandensis]
389   1e-123
WP_031027523.1 GTP-binding protein [Streptomyces sp. NRRL WC-...
390   1e-123
WP_101169116.1 GTP-binding protein [Bacillus sp. SN10]
390   1e-123
WP_097960038.1 MULTISPECIES: GTP-binding protein [Bacillus ce...
389   1e-123
WP_097843615.1 GTP-binding protein [Bacillus cereus]
389   1e-123
WP_057294845.1 GTP-binding protein [Nocardioides sp. Soil796]
390   1e-123
WP_056889944.1 GTP-binding protein [Nocardioides sp. Root151]
390   1e-123
WP_098541581.1 GTP-binding protein [Bacillus cereus]
389   1e-123
WP_098001540.1 GTP-binding protein [Bacillus toyonensis]
389   1e-123
WP_098711493.1 GTP-binding protein [Bacillus toyonensis]
389   1e-123
AFT65575.1 TetM, partial [Clostridioides difficile]
378   1e-123
WP_003994940.1 GTP-binding protein [Streptomyces viridochromo...
390   1e-123
WP_067127113.1 GTP-binding protein [Microtetraspora malaysien...
390   1e-123
WP_000209603.1 GTP-binding protein [Bacillus thuringiensis]
389   1e-123
WP_097908587.1 GTP-binding protein [Bacillus wiedmannii]
389   1e-123
WP_098821275.1 GTP-binding protein [Bacillus toyonensis]
389   2e-123
WP_061676917.1 GTP-binding protein [Bacillus wiedmannii]
389   2e-123
WP_010040474.1 GTP-binding protein [Streptomyces chartreusis]
389   2e-123
WP_097959893.1 GTP-binding protein [Bacillus toyonensis]
389   2e-123
WP_076875058.1 GTP-binding protein [Bacillus cereus]
389   2e-123
WP_098805272.1 GTP-binding protein [Bacillus toyonensis]
389   2e-123
WP_046954224.1 GTP-binding protein [Bacillus cereus]
389   2e-123
WP_106382945.1 GTP-binding protein [Bacillus wiedmannii]
389   2e-123
WP_089007929.1 GTP-binding protein [Micromonospora viridifaci...
389   2e-123
WP_098954518.1 GTP-binding protein [Bacillus toyonensis]
389   2e-123
WP_030659124.1 MULTISPECIES: GTP-binding protein [Streptomyces]
390   2e-123
WP_097797620.1 MULTISPECIES: GTP-binding protein [Bacillus ce...
389   2e-123

WP_098222129.1 GTP-binding protein [Bacillus wiedmannii]
389   2e-123
WP_098709482.1 GTP-binding protein [Bacillus toyonensis]
389   2e-123
WP_000207823.1 GTP-binding protein [Bacillus cereus]
389   2e-123
WP_010706236.1 transposase [Enterococcus faecium]
373   3e-123
WP_098607789.1 GTP-binding protein [Bacillus cereus]
389   3e-123
WP_098906152.1 GTP-binding protein [Bacillus cereus]
388   3e-123
WP_028433974.1 GTP-binding protein [Streptomyces sp. TAA486]
389   3e-123
WP_075307713.1 GTP-binding protein [Bacillus cereus]
388   3e-123
WP_106583421.1 GTP-binding protein [Murinocardiopsis flavida]
389   3e-123
WP_062930886.1 GTP-binding protein [Streptomyces sp. S10(2016)]
389   3e-123
WP_000207822.1 GTP-binding protein [Bacillus cereus]
388   3e-123
WP_098209106.1 GTP-binding protein [Bacillus wiedmannii]
388   4e-123
WP_027346317.1 GTP-binding protein [Hamadaea tsunoensis]
388   4e-123
WP_000207780.1 GTP-binding protein [Bacillus cereus]
388   4e-123
WP_059413886.1 GTP-binding protein [Streptomyces azureus]
388   4e-123
WP_074108390.1 GTP-binding protein [Paenibacillus sp. P26E]
388   4e-123
WP_088054828.1 GTP-binding protein [Bacillus thuringiensis]
388   4e-123
WP_036638349.1 MULTISPECIES: GTP-binding protein [Paenibacillus]
388   5e-123
WP_067026426.1 GTP-binding protein [Streptomyces sp. RV15]
388   5e-123
ANN35368.1 tetracycline resistance protein [Bacillus thuringi...
388   5e-123
WP_007386640.1 GTP-binding protein [Streptomyces sviceus]
388   6e-123
WP_097287913.1 GTP-binding protein [Streptomyces sp. OK228]
388   6e-123
WP_098079316.1 GTP-binding protein [Bacillus wiedmannii]
387   6e-123
WP_076273581.1 GTP-binding protein [Paenibacillus odorifer]
388   6e-123
WP_099944941.1 GTP-binding protein [Streptomyces sp. 93]
388   6e-123
WP_098509130.1 MULTISPECIES: GTP-binding protein [Bacillus ce...
387   6e-123
WP_030252322.1 GTP-binding protein [Streptacidiphilus jeojiense]
388   6e-123

WP_030605290.1 GTP-binding protein [Streptomyces fulvoviolaceus]
388   6e-123
WP_104784234.1 GTP-binding protein [Streptomyces sp. 46]
388   7e-123
WP_034317801.1 GTP-binding protein [Amycolatopsis lurida]
387   7e-123
WP_007914803.1 GTP-binding protein [Ktedonobacter racemifer]
388   7e-123
AFT65574.1 TetM, partial [Clostridioides difficile]
376   7e-123
ABM65169.1 TetW [Bifidobacterium thermophilum]
370   7e-123
WP_020633936.1 GTP-binding protein [Amycolatopsis alba]
387   7e-123
WP_088029897.1 GTP-binding protein [Bacillus cereus]
387   8e-123
WP_031098384.1 MULTISPECIES: GTP-binding protein [Streptomyces]
388   8e-123
WP_068782386.1 GTP-binding protein [Paenibacillus sp. GM2]
389   8e-123
WP_053757057.1 GTP-binding protein [Streptomyces sp. AS58]
387   8e-123
WP_103492161.1 MULTISPECIES: GTP-binding protein [Streptomyces]
388   8e-123
WP_080041786.1 GTP-binding protein [Nonomuraea sp. ATCC 55076]
388   8e-123
WP_006209881.1 GTP-binding protein [Paenibacillus vortex]
387   8e-123
WP_068620994.1 GTP-binding protein [Paenibacillus tuaregi]
387   8e-123
WP_076117876.1 GTP-binding protein [Paenibacillus odorifer]
387   9e-123
WP_000207783.1 GTP-binding protein [Bacillus cereus]
387   9e-123
WP_031046543.1 MULTISPECIES: GTP-binding protein [Streptomyces]
387   9e-123
WP_062321889.1 GTP-binding protein [Paenibacillus pabuli]
387   1e-122
WP_055485788.1 GTP-binding protein [Streptomyces sp. WMMB 322]
388   1e-122
WP_048547720.1 MULTISPECIES: GTP-binding protein [Bacillus ce...
387   1e-122
WP_098220272.1 GTP-binding protein [Bacillus cereus]
387   1e-122
WP_073847927.1 GTP-binding protein [Amycolatopsis sp. CB00013]
387   1e-122
WP_038569059.1 GTP-binding protein [Paenibacillus odorifer]
387   1e-122
WP_094903857.1 GTP-binding protein [Paenibacillus odorifer]
387   1e-122
WP_033364982.1 GTP-binding protein [Dactylosporangium auranti...
387   1e-122
WP_069768779.1 GTP-binding protein [Streptomyces sp. LUP30]
387   1e-122

WP_098082541.1 GTP-binding protein [Bacillus wiedmannii]
387   1e-122
WP_107134524.1 GTP-binding protein [Bacillus wiedmannii]
387   1e-122
KMS88978.1 GTP-binding protein [Streptomyces regensis]
387   1e-122
WP_042266215.1 GTP-binding protein [Paenibacillus graminis]
387   1e-122
WP_030350511.1 GTP-binding protein [Streptomyces scopuliridis]
387   2e-122
WP_098617594.1 GTP-binding protein [Bacillus thuringiensis]
387   2e-122
WP_076225049.1 GTP-binding protein [Paenibacillus odorifer]
387   2e-122
WP_046587741.1 GTP-binding protein [Bacillus sp. SA1-12]
387   2e-122
WP_031133712.1 MULTISPECIES: GTP-binding protein [Streptomyces]
387   2e-122
WP_098127963.1 GTP-binding protein [Bacillus wiedmannii]
386   2e-122
WP_098200540.1 GTP-binding protein [Bacillus wiedmannii]
386   2e-122
WP_042124754.1 GTP-binding protein [Paenibacillus sp. FSL R5-...
387   2e-122
WP_098201457.1 GTP-binding protein [Bacillus wiedmannii]
386   2e-122
WP_086407383.1 MULTISPECIES: GTP-binding protein [Bacillus]
386   2e-122
WP_067443508.1 GTP-binding protein [Streptomyces lincolnensis]
386   2e-122
WP_046563544.1 GTP-binding protein [Micromonospora sp. HK10]
387   2e-122
WP_037714123.1 GTP-binding protein [Streptomyces mirabilis]
386   2e-122
WP_061666951.1 MULTISPECIES: GTP-binding protein [Bacillus ce...
386   3e-122
WP_098867149.1 GTP-binding protein [Bacillus thuringiensis]
386   3e-122
WP_088947873.1 GTP-binding protein [Micromonospora zamorensis]
386   3e-122
WP_088122469.1 GTP-binding protein [Bacillus cereus]
386   3e-122
WP_098133135.1 GTP-binding protein [Bacillus wiedmannii]
386   3e-122
PJQ99820.1 tetracycline resistance ribosomal protection prote...
370   3e-122
WP_030172602.1 GTP-binding protein [Streptomyces sp. NRRL S-813]
386   3e-122
WP_000207789.1 GTP-binding protein [Bacillus cereus]
385   4e-122
KOV87046.1 GTP-binding protein [Streptomyces sp. NRRL WC-3723]
386   4e-122
WP_098641881.1 GTP-binding protein [Bacillus wiedmannii]
385   4e-122

WP_088113343.1 GTP-binding protein [Bacillus cereus]
385   4e-122
WP_076100045.1 GTP-binding protein [Paenibacillus odorifer]
385   5e-122
WP_101612711.1 GTP-binding protein [Amycolatopsis sp. BJA-103]
385   5e-122
WP_093472534.1 MULTISPECIES: GTP-binding protein [Streptomyces]
386   5e-122
WP_073890912.1 GTP-binding protein [Streptomyces sp. CB01883]
386   5e-122
WP_048560638.1 MULTISPECIES: GTP-binding protein [Bacillus ce...
385   5e-122
WP_025117848.1 MULTISPECIES: GTP-binding protein [Bacillus]
385   5e-122
KOV72169.1 GTP-binding protein [Streptomyces sp. AS58]
385   5e-122
WP_086734279.1 GTP-binding protein [Streptomyces glaucescens]
385   5e-122
WP_098517190.1 GTP-binding protein [Bacillus thuringiensis]
385   6e-122
WP_020525111.1 GTP-binding protein [Catelliglobosispora koree...
385   6e-122
WP_060862270.1 GTP-binding protein [Paenibacillus riograndensis]
385   6e-122
WP_098374038.1 GTP-binding protein [Bacillus cereus]
385   6e-122
WP_072938814.1 GTP-binding protein [Bacillus sp. bc15]
385   6e-122
KWX54318.1 tetracycline resistance protein tetM [Campylobacte...
369   6e-122
WP_076284038.1 GTP-binding protein [Paenibacillus odorifer]
385   7e-122
WP_068778043.1 GTP-binding protein [Paenibacillus sp. GM2]
385   7e-122
WP_063260315.1 GTP-binding protein [Bacillus cereus]
385   8e-122
WP_036686792.1 MULTISPECIES: GTP-binding protein [Paenibacillus]
385   8e-122
WP_090993395.1 GTP-binding protein [Paenibacillus sp. OK003]
385   9e-122
WP_016512644.1 MULTISPECIES: GTP-binding protein [Bacillus]
384   9e-122
WP_015622942.1 GTP-binding protein [Actinoplanes sp. N902-109]
385   9e-122
WP_075028184.1 GTP-binding protein [Streptomyces mirabilis]
385   9e-122
WP_103816462.1 GTP-binding protein [Streptomyces sp. Ru62]
385   1e-121
WP_067232841.1 GTP-binding protein [Streptomyces longwoodensis]
385   1e-121
WP_091406681.1 GTP-binding protein [Micromonospora saelicesen...
385   1e-121
WP_089153402.1 GTP-binding protein [Paenibacillus rubinfantis]
385   1e-121

WP_086870746.1 GTP-binding protein [Streptomyces viridochromo...
385   1e-121
WP_103842088.1 GTP-binding protein [Streptomyces sp. Ru72]
385   1e-121
WP_036324150.1 GTP-binding protein [Microbispora sp. ATCC PTA...
384   1e-121
WP_078636267.1 GTP-binding protein [Streptomyces antibioticus]
384   1e-121
WP_093506731.1 GTP-binding protein [Streptomyces sp. Ag109_O5...
384   1e-121
WP_067279567.1 GTP-binding protein [Streptomyces jeddahensis]
385   1e-121
WP_094876316.1 GTP-binding protein [Paenibacillus sp. VTT E-1...
384   2e-121
WP_000207788.1 GTP-binding protein [Bacillus cereus]
384   2e-121
WP_094874042.1 GTP-binding protein [Paenibacillus sp. VTT E-1...
384   2e-121
WP_098945939.1 GTP-binding protein [Bacillus sp. AFS029637]
384   2e-121
WP_103623351.1 GTP-binding protein [Bacillus thuringiensis]
382   2e-121
WP_042210947.1 GTP-binding protein [Paenibacillus borealis]
384   2e-121
SNT39461.1 ribosomal protection tetracycline resistance prote...
384   2e-121
WP_030937792.1 GTP-binding protein [Streptomyces sp. NRRL S-646]
385   2e-121
CCX36685.1 small GTP-binding protein [Clostridium sp. CAG:1013]
384   2e-121
WP_015655786.1 GTP-binding protein [Streptomyces davaonensis]
384   2e-121
WP_042233132.1 GTP-binding protein [Paenibacillus sp. FSL R5-...
384   2e-121
WP_066367314.1 GTP-binding protein [Bacillus sp. FJAT-27264]
384   2e-121
WP_030344712.1 GTP-binding protein [Streptomyces sp. NRRL S-1...
384   2e-121
WP_090891717.1 GTP-binding protein [Bacillus caseinilyticus]
384   2e-121
WP_063564692.1 GTP-binding protein [Paenibacillus sp. O199]
384   2e-121
WP_031110204.1 GTP-binding protein [Streptomyces sp. NRRL S-146]
384   2e-121
WP_053193661.1 GTP-binding protein [Streptomyces resistomycif...
384   2e-121
WP_085206754.1 GTP-binding protein [Streptomyces sp. Amel2xC10]
384   2e-121
WP_000207824.1 GTP-binding protein [Bacillus cereus]
384   2e-121
WP_024492581.1 GTP-binding protein [Streptomyces sp. AW19M42]
384   2e-121
WP_086730612.1 GTP-binding protein [Streptomyces carpinensis]
384   3e-121

WP_076151083.1 GTP-binding protein [Paenibacillus glucanolyti...
383   3e-121
WP_062665501.1 MULTISPECIES: GTP-binding protein [Streptomyces]
384   3e-121
WP_098230648.1 GTP-binding protein [Bacillus wiedmannii]
383   3e-121
WP_097890251.1 GTP-binding protein [Bacillus wiedmannii]
383   3e-121
WP_018384796.1 GTP-binding protein [Streptomyces vitaminophilus]
384   4e-121
WP_019912996.1 GTP-binding protein [Paenibacillus sp. HW567]
383   4e-121
WP_026162623.1 GTP-binding protein [Kribbella catacumbae]
383   4e-121
WP_042134229.1 GTP-binding protein [Paenibacillus sp. FSL P4-...
383   4e-121
WP_063479332.1 GTP-binding protein [Paenibacillus glucanolyti...
383   4e-121
WP_076298378.1 GTP-binding protein [Paenibacillus odorifer]
383   5e-121
WP_046959814.1 GTP-binding protein [Bacillus cereus]
382   5e-121
WP_097923941.1 GTP-binding protein [Bacillus thuringiensis]
382   5e-121
WP_086170746.1 GTP-binding protein [Streptomyces pharetrae]
383   6e-121
WP_088609194.1 GTP-binding protein [Bacillus sp. K2I17]
382   6e-121
WP_024630144.1 MULTISPECIES: GTP-binding protein [Paenibacillus]
383   6e-121
WP_096189851.1 GTP-binding protein [Bacillus sp. FJAT-44876]
382   6e-121
WP_046503944.1 GTP-binding protein [Streptomyces odonnellii]
384   6e-121
WP_053196001.1 GTP-binding protein [Streptomyces viridochromo...
383   7e-121
WP_099852807.1 GTP-binding protein [Micromonospora sp. CNZ299]
383   7e-121
WP_090805321.1 GTP-binding protein [Paenibacillus sp. 276b]
383   7e-121
WP_074309255.1 GTP-binding protein [Micromonospora cremea]
383   7e-121
GAK38578.1 small GTP-binding protein [Paenibacillus sp. TCA20]
382   8e-121
WP_042185495.1 GTP-binding protein [Paenibacillus sp. FSL H7-...
382   8e-121
WP_076242340.1 GTP-binding protein [Paenibacillus sp. FSL H8-...
382   8e-121
WP_036649654.1 GTP-binding protein [Paenibacillus wynnii]
382   8e-121
WP_089210847.1 GTP-binding protein [Streptosporangium subroseum]
382   1e-120
WP_062833737.1 GTP-binding protein [Paenibacillus amylolyticus]
382   1e-120

WP_052511656.1 GTP-binding protein [Paenibacillus sp. TCA20]
382   1e-120
WP_101402599.1 GTP-binding protein [Streptomyces sp. OK885]
382   1e-120
WP_088063841.1 GTP-binding protein [Bacillus cereus]
382   1e-120
WP_090949071.1 GTP-binding protein [Paenibacillus sp. OK060]
382   1e-120
WP_098858081.1 GTP-binding protein [Bacillus wiedmannii]
381   1e-120
WP_039301914.1 GTP-binding protein [Paenibacillus sp. IHB B 3...
382   1e-120
AMJ36824.1 elongation factor G, domain IV [uncultured bacterium]
379   1e-120
WP_046501621.1 GTP-binding protein [Paenibacillus riograndensis]
382   2e-120
WP_086576182.1 tetracycline resistance ribosomal protection p...
382   2e-120
WP_102638281.1 GTP-binding protein [Streptomyces sp. SMS_SU21]
381   2e-120
WP_079347056.1 GTP-binding protein [Paenibacillus sp. VT-16-81]
381   2e-120
WP_038694233.1 GTP-binding protein [Paenibacillus stellifer]
381   2e-120
WP_073515491.1 GTP-binding protein [Bacillus cereus]
381   2e-120
WP_037885331.1 GTP-binding protein [Streptomyces viridochromo...
381   2e-120
WP_104791053.1 GTP-binding protein [Streptomyces sp. QL37]
382   3e-120
WP_079694783.1 MULTISPECIES: GTP-binding protein [Paenibacillus]
381   3e-120
WP_029137645.1 GTP-binding protein [Nakamurella lactea]
381   3e-120
WP_094095010.1 GTP-binding protein [Paenibacillus physcomitre...
382   3e-120
WP_089014005.1 GTP-binding protein [Micromonospora inositola]
381   3e-120
WP_098830153.1 GTP-binding protein [Bacillus wiedmannii]
380   3e-120
WP_043350686.1 GTP-binding protein [Beutenbergia cavernae]
381   4e-120
WP_073728811.1 GTP-binding protein [Streptomyces sp. TSRI0281]
381   4e-120
WP_098625819.1 GTP-binding protein [Bacillus wiedmannii]
380   4e-120
WP_031165186.1 tetracycline resistance ribosomal protection p...
380   4e-120
WP_093909984.1 GTP-binding protein [Streptomyces sp. cf386]
381   4e-120
WP_076111228.1 GTP-binding protein [Paenibacillus borealis]
380   4e-120
WP_107459851.1 GTP-binding protein [Streptomyces sp. A244]
380   5e-120

WP_031487082.1 GTP-binding protein [Streptomyces bicolor]
380   6e-120
WP_086564096.1 GTP-binding protein [Streptomyces africanus]
380   7e-120
CUQ11287.1 Tetracycline resistance protein tetM from transpos...
366   7e-120
WP_053783744.1 GTP-binding protein [Paenibacillus sp. A59]
380   7e-120
WP_010988902.1 GTP-binding protein [Streptomyces avermitilis]
380   8e-120
ACQ82047.1 small GTP-binding protein [Beutenbergia cavernae D...
380   8e-120
WP_091154973.1 GTP-binding protein [Paenibacillus sp. 453mf]
380   1e-119
WP_083180455.1 GTP-binding protein [Paenibacillus yonginensis]
381   1e-119
SFS47170.1 ribosomal protection tetracycline resistance prote...
380   1e-119
WP_048578961.1 GTP-binding protein [Streptomyces viridochromo...
380   1e-119
WP_076289457.1 GTP-binding protein [Paenibacillus pabuli]
379   1e-119
WP_064639114.1 GTP-binding protein [Paenibacillus sp. AD87]
379   2e-119
WP_058849791.1 GTP-binding protein [Streptomyces silvensis]
379   2e-119
WP_038586981.1 GTP-binding protein [Paenibacillus sp. FSL H7-...
379   2e-119
WP_019544083.1 tetracycline resistance ribosomal protection p...
379   2e-119
WP_053752191.1 GTP-binding protein [Streptomyces sp. MMG1533]
379   2e-119
WP_067455119.1 GTP-binding protein [Actinomadura macra]
378   2e-119
WP_090895831.1 GTP-binding protein [Paenibacillus sp. OK076]
379   2e-119
ACC86068.1 putative ribosomal protection protein [Clostridiac...
366   2e-119
WP_086421795.1 GTP-binding protein [Bacillus thuringiensis]
378   2e-119
WP_077015246.1 GTP-binding protein [Kribbella sp. ALI-6-A]
379   2e-119
WP_094056801.1 GTP-binding protein [Streptomyces sp. XY006]
379   2e-119
ELS58735.1 putative Translation elongation factor G [Streptom...
379   2e-119
SDK43429.1 ribosomal protection tetracycline resistance prote...
379   2e-119
WP_068580501.1 GTP-binding protein [Paenibacillus macquariensis]
379   3e-119
WP_030045349.1 GTP-binding protein [Streptomyces resistomycif...
375   3e-119
WP_108467308.1 GTP-binding protein [Paenibacillus sp. CAA11]
379   3e-119

WP_098190439.1 GTP-binding protein [Bacillus wiedmannii]
378   3e-119
AMJ37778.1 elongation factor G, domain IV [uncultured bacterium]
376   3e-119
WP_092882214.1 GTP-binding protein [Actinopolymorpha cephalot...
378   3e-119
WP_054939188.1 GTP-binding protein [Paenibacillus ihuae]
378   3e-119
WP_099501283.1 MULTISPECIES: GTP-binding protein [Streptomyces]
379   4e-119
WP_056919357.1 GTP-binding protein [Phycicoccus sp. Root101]
377   4e-119
WP_099853395.1 GTP-binding protein [Paenibacillus polymyxa]
378   4e-119
AMP52929.1 elongation factor G, domain IV [uncultured bacterium]
374   4e-119
WP_037571424.1 GTP-binding protein [Streptacidiphilus oryzae]
379   5e-119
WP_097915395.1 GTP-binding protein [Bacillus wiedmannii]
377   5e-119
AHN92783.1 hypothetical protein [uncultured bacterium DCM005T...
362   6e-119
WP_020393203.1 GTP-binding protein [Kribbella catacumbae]
377   6e-119
WP_056916266.1 GTP-binding protein [Phycicoccus sp. Root563]
377   7e-119
WP_087443748.1 GTP-binding protein [Paenibacillus thiaminolyt...
377   7e-119
WP_093889762.1 GTP-binding protein [Streptosporangium canum]
377   7e-119
PKC90356.1 tetracycline resistance protein tetW [Bifidobacter...
361   7e-119
WP_098802486.1 GTP-binding protein [Bacillus wiedmannii]
377   8e-119
WP_062239896.1 GTP-binding protein [Streptomyces griseorubigi...
377   8e-119
WP_089021306.1 GTP-binding protein [Micromonospora coriariae]
377   8e-119
WP_062020304.1 GTP-binding protein [Streptomyces phaeopurpureus]
377   9e-119
WP_085922271.1 tetracycline resistance ribosomal protection p...
377   1e-118
AFT65578.1 TetM, partial [Clostridioides difficile]
366   1e-118
WP_046587315.1 GTP-binding protein [Streptomyces mangrovisoli]
377   1e-118
WP_097318893.1 GTP-binding protein [Actinoplanes atraurantiacus]
377   1e-118
WP_054977783.1 GTP-binding protein [Paenibacillus sp. A3]
376   1e-118
OME83711.1 hypothetical protein BK122_09865 [Paenibacillus pa...
377   1e-118
WP_103515238.1 GTP-binding protein [Streptomyces sp. SM10]
377   1e-118

WP_076082619.1 GTP-binding protein [Streptomyces sp. IMTB 2501]
377   1e-118
WP_077010096.1 GTP-binding protein [Saccharothrix sp. ALI-22-I]
376   1e-118
KOU61854.1 GTP-binding protein [Streptomyces sp. MMG1533]
376   1e-118
WP_023958886.1 GTP-binding protein [Paenibacillus sp. JCM 10914]
376   2e-118
WP_055624457.1 GTP-binding protein [Streptomyces sp. JHA19]
377   2e-118
AFT65573.1 TetM, partial [Clostridioides difficile]
365   2e-118
WP_052589207.1 GTP-binding protein [Luteipulveratus mongolien...
376   2e-118
CDD30461.1 small GTP-binding protein [Firmicutes bacterium CA...
376   2e-118
WP_087986521.1 GTP-binding protein [Bacillus thuringiensis]
375   2e-118
WP_076168364.1 GTP-binding protein [Paenibacillus rhizosphaerae]
376   3e-118
WP_050671488.1 GTP-binding protein [Luteipulveratus halotoler...
375   3e-118
WP_030790530.1 GTP-binding protein [Streptomyces sp. NRRL S-920]
376   3e-118
WP_026257429.1 GTP-binding protein [Actinopolymorpha alba]
375   3e-118
WP_093623424.1 GTP-binding protein [Streptomyces sp. 3213.3]
375   3e-118
WP_070018520.1 GTP-binding protein [Streptomyces nanshensis]
376   4e-118
AFD32972.1 TetS [Enterococcus gallinarum]
362   4e-118
WP_053804350.1 tetracycline resistance ribosomal protection p...
375   4e-118
WP_094593705.1 GTP-binding protein [Paenibacillus sp. XY044]
375   5e-118
WP_031187249.1 MULTISPECIES: tetracycline resistance ribosoma...
375   5e-118
WP_030596573.1 MULTISPECIES: tetracycline resistance ribosoma...
375   5e-118
WP_026211992.1 GTP-binding protein [Longispora albida]
375   6e-118
WP_003981038.1 MULTISPECIES: tetracycline resistance ribosoma...
375   6e-118
WP_073749651.1 GTP-binding protein [Streptomyces sp. CB02058]
375   6e-118
SDW17994.1 ribosomal protection tetracycline resistance prote...
375   6e-118
WP_053673433.1 GTP-binding protein [Streptomyces sp. NRRL B-1...
375   7e-118
WP_028279377.1 GTP-binding protein [Arthrobacter sp. H5]
374   7e-118
WP_073736610.1 GTP-binding protein [Streptomyces sp. CB02488]
375   8e-118

WP_068728233.1 GTP-binding protein [Paenibacillus sp. DMB5]
375   8e-118
WP_030629387.1 tetracycline resistance ribosomal protection p...
375   8e-118
SMF81208.1 ribosomal protection tetracycline resistance prote...
375   8e-118
WP_090715404.1 GTP-binding protein [Paenibacillus typhae]
375   9e-118
WP_087913507.1 GTP-binding protein [Paenibacillus donghaensis]
375   9e-118
AKJ08989.1 GTP-binding protein [Streptomyces incarnatus]
374   9e-118
WP_052482014.1 GTP-binding protein [Streptomyces albus]
376   1e-117
WP_027750437.1 GTP-binding protein [Streptomyces sp. CNH287]
375   1e-117
WP_106614004.1 GTP-binding protein [Saccharothrix carnea]
374   1e-117
WP_039870362.1 GTP-binding protein [Paenibacillus sp. FSL R7-...
374   1e-117
WP_099171569.1 GTP-binding protein [Streptomyces sp. LamerLS-...
375   1e-117
WP_090723024.1 GTP-binding protein [Paenibacillus sp. PDC88]
374   1e-117
WP_027743837.1 GTP-binding protein [Streptomyces sp. CNT371]
375   1e-117
WP_068597041.1 GTP-binding protein [Paenibacillus macquariensis]
374   1e-117
WP_024883416.1 GTP-binding protein [Streptomyces sp. CNH189]
374   1e-117
WP_074648684.1 GTP-binding protein [Paenibacillus jilunlii]
374   1e-117
SHN59487.1 ribosomal protection tetracycline resistance prote...
374   1e-117
WP_107910239.1 GTP-binding protein [Streptomyces chartreusis]
375   2e-117
WP_042199732.1 GTP-binding protein [Paenibacillus camerounensis]
374   2e-117
WP_076330539.1 GTP-binding protein [Paenibacillus amylolyticus]
374   2e-117
WP_030372719.1 tetracycline resistance ribosomal protection p...
374   2e-117
KOY14136.1 hypothetical protein AMS66_23215 [Paenibacillus sp...
374   2e-117
AMP53612.1 elongation factor Tu GTP binding domain protein [u...
368   2e-117
WP_099518086.1 GTP-binding protein [Paenibacillus sp. BIHB4019]
374   2e-117
WP_106246637.1 GTP-binding protein [Allonocardiopsis opalescens]
374   2e-117
WP_037698501.1 GTP-binding protein [Streptomyces scabiei]
374   2e-117
ALP02588.1 Tetracycline resistance protein TetM [Clostridioid...
360   3e-117

WP_033031213.1 tetracycline resistance ribosomal protection p...
373   3e-117
WP_030879823.1 tetracycline resistance ribosomal protection p...
373   3e-117
OMC71145.1 GTP-binding protein [Paenibacillus sp. FSL H7-0326]
373   3e-117
WP_062714720.1 GTP-binding protein [Streptomyces regalis]
374   3e-117
OZH72043.1 elongation factor G [Enterococcus faecium]
357   3e-117
WP_019057847.1 GTP-binding protein [Streptomyces prunicolor]
373   4e-117
WP_010731387.1 GTP-binding protein [Enterococcus faecium]
359   4e-117
WP_083655696.1 GTP-binding protein [Paenibacillus sp. FSL H7-...
373   4e-117
WP_079910133.1 GTP-binding protein [Paenibacillus sp. 32352]
373   4e-117
WP_075124903.1 GTP-binding protein [Actinophytocola xanthii]
372   4e-117
WP_030641547.1 tetracycline resistance ribosomal protection p...
373   4e-117
EJX67630.1 GTP-binding domain protein [Enterococcus faecium P...
359   4e-117
WP_089546508.1 GTP-binding protein [Paenibacillus sp. SSG-1]
373   5e-117
WP_092885270.1 GTP-binding protein [Actinopolymorpha cephalot...
372   5e-117
ETK35607.1 GTP-binding protein [Microbispora sp. ATCC PTA-5024]
372   5e-117
WP_018844188.1 GTP-binding protein [Streptomyces sp. CNS335]
373   5e-117
WP_056884328.1 GTP-binding protein [Phycicoccus sp. Soil748]
372   5e-117
EJX78721.1 elongation factor Tu domain 2 [Enterococcus faeciu...
358   5e-117
WP_002345019.1 MULTISPECIES: GTP-binding protein [Bacilli]
358   5e-117
PSL58321.1 ribosomal protection tetracycline resistance prote...
372   5e-117
SEA63444.1 ribosomal protection tetracycline resistance prote...
372   6e-117
WP_043670067.1 GTP-binding protein [Streptomyces xylophagus]
373   6e-117
WP_053709350.1 GTP-binding protein [Streptomyces sp. NRRL B-3...
372   7e-117
WP_053699798.1 tetracycline resistance ribosomal protection p...
372   7e-117
WP_097222482.1 GTP-binding protein [Streptomyces sp. Ag82_G6-1]
372   7e-117
WP_092657465.1 GTP-binding protein [Actinopolymorpha singapor...
372   8e-117
WP_020521069.1 GTP-binding protein [Catelliglobosispora koree...
371   8e-117

WP_030663885.1 tetracycline resistance ribosomal protection p...
372   8e-117
KOV97088.1 GTP-binding protein [Streptomyces sp. NRRL B-1140]
372   8e-117
WP_103889659.1 GTP-binding protein [Streptomyces yanglinensis]
373   8e-117
WP_060731365.1 tetracycline resistance ribosomal protection p...
372   8e-117
WP_093722930.1 GTP-binding protein [Streptomyces sp. DI166]
372   9e-117
PKU99047.1 TetW [Bifidobacterium pseudolongum subsp. pseudolo...
354   1e-116
WP_079415894.1 GTP-binding protein [Paenibacillus ferrarius]
372   1e-116
WP_077073063.1 (d)CMP kinase [Mailhella massiliensis]
360   1e-116
AFD29105.1 TetS [Enterococcus faecalis]
358   1e-116
WP_062519581.1 GTP-binding protein [Paenibacillus jilunlii]
372   1e-116
AFD32983.1 TetS [Enterococcus faecium]
357   1e-116
WP_095358740.1 GTP-binding protein [Paenibacillus sp. 7523-1]
371   1e-116
WP_080695809.1 tetracycline resistance ribosomal protection p...
368   1e-116
WP_042173515.1 GTP-binding protein [Paenibacillus sp. FSL R7-...
371   2e-116
WP_073501082.1 GTP-binding protein [Streptomyces paucisporeus]
372   2e-116
WP_030630763.1 MULTISPECIES: tetracycline resistance ribosoma...
371   2e-116
SLJ87924.1 ribosomal protection tetracycline resistance prote...
371   2e-116
WP_092655347.1 GTP-binding protein [Actinopolymorpha singapor...
372   2e-116
WP_085809117.1 hypothetical protein [Enterococcus faecium]
355   2e-116
WP_093748296.1 GTP-binding protein [Streptomyces sp. PAN_FS17]
372   2e-116
WP_020575777.1 GTP-binding protein [Actinopolymorpha alba]
372   2e-116
SDD10449.1 ribosomal protection tetracycline resistance prote...
370   2e-116
WP_019068592.1 GTP-binding protein [Streptomyces hokutonensis]
371   2e-116
WP_049580573.1 GTP-binding protein [Streptomyces sp. SBT349]
371   2e-116
WP_105410658.1 GTP-binding protein [Paenibacillus sp. PCH8]
371   2e-116
WP_091451254.1 GTP-binding protein [Alloactinosynnema iranicum]
370   3e-116
WP_029197645.1 GTP-binding protein [Paenibacillus alginolyticus]
371   3e-116

CMY16859.1 tetracycline resistance protein TetM [Streptococcu...
358   3e-116
WP_015847478.1 GTP-binding protein [Paenibacillus sp. JDR-2]
370   3e-116
OAX48496.1 Tetracycline resistance protein TetO [Paenibacillu...
370   3e-116
WP_023362944.1 GTP-binding protein [Actinoplanes friuliensis]
370   3e-116
WP_093868422.1 GTP-binding protein [Streptomyces sp. KS_5]
371   3e-116
KOV96771.1 GTP-binding protein [Streptomyces sp. NRRL B-3648]
370   3e-116
WP_090951708.1 GTP-binding protein [Paenibacillus sp. OK060]
370   3e-116
WP_054968289.1 GTP-binding protein [Alicyclobacillus ferrooxy...
370   3e-116
WP_046231214.1 GTP-binding protein [Paenibacillus algorifonti...
370   3e-116
WP_030560687.1 GTP-binding protein [Streptomyces aureocircula...
371   4e-116
WP_099161420.1 GTP-binding protein [Micromonospora sp. WMMA2032]
370   4e-116
WP_037780130.1 GTP-binding protein [Streptomyces sp. CNH099]
371   4e-116
WP_088645945.1 GTP-binding protein [Micromonospora wenchangen...
370   4e-116
WP_098381402.1 GTP-binding protein [Enterococcus faecium]
357   4e-116
SDS20277.1 ribosomal protection tetracycline resistance prote...
370   5e-116
SOD49833.1 ribosomal protection tetracycline resistance prote...
370   5e-116
WP_014444434.1 GTP-binding protein [Actinoplanes missouriensis]
370   6e-116
WP_036670278.1 GTP-binding protein [Paenibacillus sp. FSL R5-...
370   6e-116
AKJ08846.1 GTP-binding protein [Streptomyces incarnatus]
370   6e-116
WP_012893562.1 tetracycline resistance ribosomal protection p...
370   6e-116
WP_040950387.1 GTP-binding protein [Gorillibacterium massilie...
370   6e-116
WP_083678877.1 MULTISPECIES: GTP-binding protein [Paenibacillus]
369   7e-116
EEL45061.1 GTP-binding elongation factor protein, TetM/TetO [...
369   7e-116
WP_091069160.1 GTP-binding protein [Paenibacillus sp. NFR01]
369   8e-116
WP_073827900.1 GTP-binding protein [Micromonospora sp. TSRI0369]
369   8e-116
WP_042187779.1 GTP-binding protein [Kibdelosporangium sp. MJ1...
368   8e-116
WP_025510906.1 GTP-binding protein [Lachnospiraceae bacterium...
365   9e-116

WP_091576591.1 GTP-binding protein [Micromonospora sediminicola]
369   9e-116
AGS74493.1 translation elongation factor G [Enterococcus faec...
356   1e-115
OLE28133.1 GTP-binding protein [Actinobacteria bacterium 13_1...
369   1e-115
EJG80816.1 small GTP-binding domain protein [Streptococcus pn...
357   1e-115
EQC82012.1 Tetracycline resistance protein TetM [Enterococcus...
356   1e-115
WP_010730929.1 GTP-binding protein [Enterococcus faecium]
355   1e-115
EHT67642.1 small GTP-binding domain protein [Staphylococcus a...
356   1e-115
WP_085815349.1 hypothetical protein [Enterococcus faecium]
352   1e-115
WP_067219782.1 GTP-binding protein [Marinomonas gallaica]
369   1e-115
WP_013862151.1 GTP-binding protein [Microlunatus phosphovorus]
369   2e-115
WP_037754408.1 GTP-binding protein [Streptomyces sp. CNQ-525]
369   2e-115
WP_007917907.1 GTP-binding protein [Ktedonobacter racemifer]
369   2e-115
WP_081220688.1 GTP-binding protein [Streptomyces sp. M41(2017)]
369   2e-115
WP_091032003.1 GTP-binding protein [Paenibacillus sp. CF095]
369   2e-115
ESA52577.1 50S ribosome-binding GTPase [Streptococcus pyogene...
356   2e-115
WP_020943791.1 GTP-binding protein [Streptomyces collinus]
369   2e-115
WP_095287887.1 GTP-binding protein [Paenibacillus sp. 7516]
369   2e-115
WP_078981364.1 GTP-binding protein [Streptomyces scabrisporus]
369   2e-115
KIF02847.1 GTP-binding protein [Streptomyces sp. RSD-27]
368   2e-115
WP_097867307.1 GTP-binding protein [Streptomyces sp. rh34]
369   2e-115
WP_056036530.1 GTP-binding protein [Paenibacillus sp. Leaf72]
368   2e-115
EGI85972.1 small GTP-binding domain protein [Streptococcus pn...
355   2e-115
WP_107732006.1 GTP-binding protein [Chromobacterium haemolyti...
368   3e-115
ACT97612.1 TetA(Q) [uncultured organism]
365   3e-115
SPL96878.1 Oxytetracycline resistance protein OtrA [Actinomad...
368   3e-115
EVA35755.1 tetracycline resistance protein tetM [Staphylococc...
354   3e-115
WP_100529364.1 GTP-binding protein [Paenibacillus sp. GM1FR]
368   3e-115

WP_052581730.1 GTP-binding protein [Actinosporangium sp. NRRL...
368   3e-115
WP_059246843.1 MULTISPECIES: GTP-binding protein [Streptomyces]
368   3e-115
WP_076318479.1 GTP-binding protein [Paenibacillus amylolyticus]
368   4e-115
KJK33637.1 GTP-binding protein [Lechevalieria aerocolonigenes]
365   4e-115
WP_086698833.1 GTP-binding protein [Streptomyces tricolor]
368   4e-115
WP_091027997.1 GTP-binding protein [Glycomyces harbinensis]
368   4e-115
OFE17018.1 hypothetical protein BA895_17680 [Humibacillus sp....
367   4e-115
WP_091189770.1 GTP-binding protein [Paenibacillus catalpae]
368   4e-115
WP_082704145.1 GTP-binding protein [Paenibacillus pabuli]
367   5e-115
WP_097248842.1 GTP-binding protein [Streptomyces sp. 1222.2]
368   5e-115
WP_067044099.1 GTP-binding protein [Streptomyces sp. FXJ1.172]
369   5e-115
WP_068661669.1 GTP-binding protein [Paenibacillus sp. 1ZS3-15]
367   6e-115
WP_046769230.1 GTP-binding protein [Jiangella alkaliphila]
367   7e-115
EHE01245.1 elongation factor G, domain IV family protein [Str...
352   7e-115
WP_092884428.1 GTP-binding protein [Actinopolymorpha cephalot...
368   7e-115
WP_056921987.1 GTP-binding protein [Phycicoccus sp. Soil802]
367   7e-115
WP_036609508.1 MULTISPECIES: GTP-binding protein [Paenibacillus]
367   7e-115
WP_090980187.1 GTP-binding protein [Paenibacillus sp. CF384]
367   7e-115
WP_076208951.1 GTP-binding protein [Paenibacillus sp. FSL R5-...
367   7e-115
WP_037282496.1 GTP-binding protein [Saccharibacillus sacchari]
367   7e-115
WP_091083523.1 GTP-binding protein [Nonomuraea wenchangensis]
367   8e-115
WP_046915797.1 GTP-binding protein [Streptomyces stelliscabiei]
367   8e-115
WP_091668895.1 GTP-binding protein [Micromonospora auratinigra]
367   9e-115
WP_079571409.1 GTP-binding protein [Krasilnikoviella flava]
366   9e-115
WP_030671742.1 GTP-binding protein [Streptomyces sp. NRRL B-1...
367   1e-114
WP_098247510.1 GTP-binding protein [Streptomyces formicae]
368   1e-114
WP_082350871.1 GTP-binding protein [Paenibacillus sp. A59]
366   1e-114

WP_042169003.1 GTP-binding protein [Paenibacillus gorillae]
367   1e-114
KNB70578.1 hypothetical protein ADS79_16860 [Brevibacillus re...
367   1e-114
WP_083283123.1 GTP-binding protein [Humibacillus sp. DSM 29435]
368   1e-114
ATL30409.1 Oxytetracycline resistance protein OtrA [Streptomy...
367   1e-114
EEL33958.1 GTP-binding elongation factor protein, TetM/TetO [...
365   1e-114
WP_013017024.1 GTP-binding protein [Stackebrandtia nassauensis]
367   1e-114
PLX04511.1 GTP-binding protein [Marinilabiliales bacterium]
366   1e-114
WP_010733535.1 tetracycline resistance protein tetM transposo...
350   1e-114
WP_063810217.1 GTP-binding protein [Kibdelosporangium phytoha...
364   1e-114
WP_095206223.1 GTP-binding protein [Paenibacillus sp. O199]
366   2e-114
WP_018156866.1 GTP-binding protein [Demetria terragena]
366   2e-114
WP_086790078.1 GTP-binding protein [Crossiella equi]
366   2e-114
WP_105598174.1 MULTISPECIES: GTP-binding protein [Paenibacillus]
366   2e-114
WP_091017379.1 GTP-binding protein [Paenibacillus amylolyticus]
366   2e-114
WP_043627550.1 GTP-binding protein [Nonomuraea candida]
367   2e-114
WP_068650680.1 GTP-binding protein [Paenibacillus antarcticus]
365   3e-114
WP_055709911.1 GTP-binding protein [Streptomyces puniciscabiei]
365   3e-114
WP_086750285.1 GTP-binding protein [Streptomyces scabiei]
366   3e-114
WP_091064578.1 GTP-binding protein [Micromonospora humi]
365   3e-114
WP_083584658.1 GTP-binding protein [Paenibacillus sp. ov031]
365   3e-114
WP_091249948.1 GTP-binding protein [Micromonospora matsumotoe...
365   4e-114
WP_037060640.1 GTP-binding protein [Rhizobium leguminosarum]
365   4e-114
WP_067099538.1 GTP-binding protein [Marinomonas atlantica]
365   4e-114
WP_091328730.1 GTP-binding protein [Micromonospora aurantiaca]
365   4e-114
SFF66120.1 ribosomal protection tetracycline resistance prote...
366   4e-114
BAR83764.1 GTP-binding elongation factor protein, TetM/TetO f...
364   4e-114
WP_060647963.1 GTP-binding protein [Paenibacillus sp. Soil724D2]
365   5e-114

WP_020134762.1 GTP-binding protein [Streptomyces sp. 351MFTsu...
364   5e-114
WP_094212894.1 GTP-binding protein [Streptomyces sp. 2R]
365   6e-114
WP_090807525.1 GTP-binding protein [Paenibacillus sp. 276b]
364   6e-114
ADK05618.1 tetracycline resistance protein, GTP-binding elong...
363   7e-114
WP_096761568.1 GTP-binding protein [Micromonospora sp. Rc5]
364   7e-114
WP_079694252.1 MULTISPECIES: GTP-binding protein [Paenibacillus]
364   7e-114
WP_067498620.1 GTP-binding protein [Actinoplanes sp. TFC3]
364   8e-114
WP_056622295.1 GTP-binding protein [Paenibacillus sp. Soil750]
365   8e-114
WP_090822327.1 GTP-binding protein [Paenibacillus sp. yr247]
365   8e-114
WP_046706840.1 GTP-binding protein [Streptomyces europaeiscab...
365   8e-114
WP_067037992.1 GTP-binding protein [Marinomonas gallaica]
365   9e-114
WP_076326844.1 GTP-binding protein [Paenibacillus amylolyticus]
364   9e-114
WP_057376394.1 GTP-binding protein [Phycicoccus sp. Soil803]
363   9e-114
SBT21816.1 Tetracycline resistance protein TetO [Marinomonas ...
364   9e-114
WP_017644183.1 GTP-binding protein [Streptococcus agalactiae]
350   1e-113
WP_075132401.1 GTP-binding protein [Actinophytocola xinjiange...
363   1e-113
WP_085816978.1 hypothetical protein [Enterococcus faecium]
347   1e-113
WP_098892387.1 GTP-binding protein [Streptomyces sp. or3]
363   1e-113
WP_019423546.1 GTP-binding protein [Paenibacillus sp. OSY-SE]
363   2e-113
WP_074094524.1 GTP-binding protein [Paenibacillus xylanexedens]
363   2e-113
WP_030268770.1 GTP-binding protein [Micromonospora globosa]
363   2e-113
WP_009155796.1 GTP-binding protein [Saccharomonospora marina]
363   2e-113
APO27433.1 ferrous iron transport protein B [uncultured bacte...
346   2e-113
WP_060890206.1 GTP-binding protein [Streptomyces europaeiscab...
363   2e-113
WP_103954083.1 GTP-binding protein [Nonomuraea solani]
363   2e-113
WP_076249849.1 MULTISPECIES: GTP-binding protein [Paenibacillus]
363   2e-113
WP_047683683.1 GTP-binding protein [Paenibacillus chondroitinus]
363   2e-113

WP_033308524.1 GTP-binding protein [Streptomyces iakyrus]
363   2e-113
KZD40308.1 Ribosome protection-type tetracycline resistance r...
362   2e-113
WP_054212791.1 GTP-binding protein [Actinobacteria bacterium ...
363   2e-113
WP_033278824.1 GTP-binding protein [Streptomyces sp. NRRL F-525]
363   2e-113
WP_082935267.1 GTP-binding protein [Paenibacillus sp. AD87]
363   3e-113
WP_018348410.1 GTP-binding protein [Longispora albida]
362   3e-113
WP_093716798.1 GTP-binding protein [Streptomyces alni]
364   3e-113
WP_071095573.1 GTP-binding protein [Micromonospora sp. WMMB235]
362   3e-113
CKH18343.1 tetracycline resistance protein TetM [Streptococcu...
349   3e-113
WP_078174720.1 GTP-binding protein [Bacillus cereus]
362   3e-113
WP_091530565.1 GTP-binding protein [Microlunatus soli]
363   3e-113
WP_073960390.1 GTP-binding protein [Streptomyces sp. TSRI0395]
362   4e-113
WP_094013456.1 GTP-binding protein [Paenibacillus rigui]
363   4e-113
WP_046724499.1 GTP-binding protein [Streptomyces xiamenensis]
362   5e-113
WP_033172074.1 GTP-binding protein [Streptomyces sp. URHA0041]
363   5e-113
WP_021653378.1 GTP-binding protein [Clostridiales bacterium o...
362   5e-113
AKN68844.1 GTP-binding protein [Streptomyces sp. PBH53]
363   5e-113
WP_028608445.1 GTP-binding protein [Paenibacillus harenae]
362   5e-113
WP_090929080.1 GTP-binding protein [Nonomuraea jiangxiensis]
362   5e-113
WP_057661166.1 GTP-binding protein [Streptomyces anulatus]
362   5e-113
WP_106681444.1 GTP-binding protein [Streptosporangium nondias...
362   6e-113
WP_050362714.1 GTP-binding protein [Streptomyces europaeiscab...
361   7e-113
WP_079074500.1 GTP-binding protein [Streptomyces atriruber]
363   7e-113
WP_073805610.1 GTP-binding protein [Streptomyces sp. TSRI0261]
362   7e-113
AVR54585.1 tetracycline resistance protein TetM [Actinobacill...
346   8e-113
WP_007455273.1 GTP-binding protein [Streptomyces sp. W007]
362   8e-113
WP_067367508.1 GTP-binding protein [Micromonospora rosaria]
362   8e-113

WP_018838230.1 GTP-binding protein [Streptomyces sp. CNQ766]
362   9e-113
WP_097912592.1 GTP-binding protein [Streptomyces sp. b84]
362   9e-113
WP_057302783.1 GTP-binding protein [Paenibacillus sp. Root444D2]
362   9e-113
ACH92687.1 tetracycline resistance protein [Enterococcus durans]
346   1e-112
WP_018681915.1 GTP-binding protein [Actinokineospora enzanensis]
361   1e-112
WP_018853606.1 GTP-binding protein [Streptomyces sp. CNY243]
362   1e-112
WP_103547400.1 GTP-binding protein [Streptomyces sp. A249]
361   1e-112
WP_091786258.1 GTP-binding protein [Phycicoccus dokdonensis]
360   1e-112
WP_056700192.1 GTP-binding protein [Streptomyces sp. Root1295]
361   1e-112
WP_017582536.1 GTP-binding protein [Nocardiopsis valliformis]
361   1e-112
WP_037617651.1 GTP-binding protein [Streptomyces aureus]
361   1e-112
WP_035033836.1 GTP-binding protein [Devosia sp. DBB001]
361   1e-112
WP_093615859.1 GTP-binding protein [Actinoplanes philippinensis]
361   1e-112
PID94477.1 GTP-binding protein [Bacteroidetes bacterium]
361   2e-112
WP_065851019.1 GTP-binding protein [Paenibacillus pectinilyti...
361   2e-112
ASM62156.1 tetracycline resistance protein tetM [Enterococcus...
347   2e-112
WP_035090097.1 GTP-binding protein [Devosia sp. 17-2-E-8]
360   3e-112
WP_098513541.1 GTP-binding protein [Amycolatopsis sulphurea]
360   3e-112
SDH01799.1 ribosomal protection tetracycline resistance prote...
360   3e-112
WP_055559772.1 GTP-binding protein [Streptomyces luridiscabiei]
360   3e-112
WP_013285700.1 GTP-binding protein [Micromonospora aurantiaca]
360   4e-112
WP_056908261.1 GTP-binding protein [Nocardioides sp. Root122]
360   4e-112
WP_030430361.1 GTP-binding protein [Allokutzneria albata]
358   4e-112
AIA10470.1 elongation factor Tu GTP binding domain protein [u...
358   4e-112
WP_012998773.1 GTP-binding protein [Streptomyces scabiei]
360   5e-112
WP_106581006.1 GTP-binding protein [Murinocardiopsis flavida]
360   5e-112
WP_086829108.1 GTP-binding protein [Allokutzneria sp. NRRL B-...
358   5e-112

WP_087767437.1 GTP-binding protein [Streptomyces sp. CS057]
359   5e-112
WP_037726743.1 GTP-binding protein [Streptomyces scabiei]
360   5e-112
OMF07155.1 GTP-binding protein [Paenibacillus sp. FSL H7-0331]
360   5e-112
PLW96498.1 GTP-binding protein [Marinilabiliales bacterium]
360   6e-112
SEF74622.1 ribosomal protection tetracycline resistance prote...
359   6e-112
WP_083689717.1 GTP-binding protein [Paenibacillus sp. FSL H7-...
360   6e-112
WP_013477235.1 GTP-binding protein [Micromonospora sp. L5]
359   7e-112
ACH92689.1 tetracycline resistance protein [Enterococcus faec...
343   7e-112
WP_005483987.1 GTP-binding protein [Streptomyces bottropensis]
360   8e-112
WP_031126850.1 GTP-binding protein [Streptomyces sp. NRRL S-623]
359   8e-112
WP_099126970.1 GTP-binding protein [Streptomyces sp. ScaeMP-e48]
359   8e-112
WP_093151315.1 GTP-binding protein [Saccharopolyspora antimic...
357   1e-111
ASM62155.1 tetracycline resistance protein tetM [Listeria mon...
345   1e-111
WP_043177079.1 GTP-binding protein [Streptomyces sp. NRRL F-2...
358   1e-111
WP_018539074.1 tetracycline resistance ribosomal protection p...
358   1e-111
AKG44647.1 gtp-binding protein [Streptomyces xiamenensis]
358   1e-111
EEK78367.1 GTP-binding elongation factor protein, TetM/TetO [...
357   2e-111
WP_027767877.1 GTP-binding protein [Streptomyces sp. CNQ865]
358   2e-111
WP_073781949.1 tetracycline resistance ribosomal protection p...
358   2e-111
SBO93662.1 Oxytetracycline resistance protein OtrA [Nonomurae...
359   2e-111
WP_047175224.1 GTP-binding protein [Streptomyces sp. MNU77]
358   2e-111
WP_060877830.1 GTP-binding protein [Streptomyces scabiei]
356   2e-111
WP_069631354.1 GTP-binding protein [Streptomyces griseus]
358   2e-111
WP_061334743.1 GTP-binding protein [Streptomyces scabiei]
356   2e-111
WP_094928628.1 GTP-binding protein [Pseudonocardia sp. MH-G8]
359   2e-111
WP_051569350.1 GTP-binding protein [Alkaliphilus transvaalensis]
357   2e-111
WP_036745743.1 GTP-binding protein [Paenibacillus sp. UNC451MF]
358   2e-111

WP_073343070.1 GTP-binding protein [Caldanaerobius fijiensis]
357   3e-111
CDP52193.1 Ribosome protection-type tetracycline resistance r...
357   3e-111
WP_049741027.1 GTP-binding protein [Brevibacillus reuszeri]
357   3e-111
WP_061927785.1 GTP-binding protein [Streptomyces bungoensis]
358   3e-111
WP_003970925.1 GTP-binding protein [Streptomyces griseus]
358   3e-111
WP_091224856.1 GTP-binding protein [Paenibacillus sp. BC26]
358   3e-111
WP_088971135.1 GTP-binding protein [Micromonospora siamensis]
357   3e-111
WP_030323194.1 GTP-binding protein [Streptomyces sp. NRRL B-3...
357   4e-111
WP_069886168.1 GTP-binding protein [Streptomyces luteocolor]
358   4e-111
WP_091174333.1 GTP-binding protein [Paenibacillus sp. 1_12]
357   4e-111
WP_049714089.1 GTP-binding protein [Streptomyces caatingaensis]
357   5e-111
WP_035154545.1 GTP-binding protein [Cohnella thermotolerans]
357   6e-111
WP_106326020.1 GTP-binding protein [Actinoplanes italicus]
357   7e-111
WP_028683953.1 MULTISPECIES: GTP-binding protein [Pseudomonas]
357   7e-111
WP_056628693.1 GTP-binding protein [Paenibacillus sp. Soil522]
357   7e-111
OSC85072.1 tetracycline resistance protein tetM [Enterococcus...
342   8e-111
WP_015606904.1 GTP-binding protein [Streptomyces fulvissimus]
357   9e-111
WP_093561750.1 GTP-binding protein [Streptomyces sp. OspMP-M43]
357   9e-111
WP_067343820.1 GTP-binding protein [Streptomyces noursei]
357   9e-111
WP_020550843.1 GTP-binding protein [Streptomyces scabrisporus]
356   1e-110
WP_059078408.1 GTP-binding protein [Streptomyces scabiei]
357   1e-110
WP_051861185.1 GTP-binding protein [Streptomyces ochraceiscle...
356   1e-110
WP_030703094.1 GTP-binding protein [Streptomyces griseus]
356   1e-110
WP_106767107.1 GTP-binding protein [Paenibacillus sp. Marseil...
356   1e-110
WP_079036352.1 GTP-binding protein [Streptomyces silaceus]
357   1e-110
WP_057470182.1 GTP-binding protein [Rhizobium sp. Root1203]
355   1e-110
WP_097932402.1 MULTISPECIES: GTP-binding protein [Streptomyces]
356   2e-110

WP_102924936.1 tetracycline resistance ribosomal protection p...
356   2e-110
WP_028555190.1 GTP-binding protein [Paenibacillus sp. URHA0014]
356   2e-110
AHH94550.1 Oxytetracycline resistance protein [Kutzneria albi...
355   2e-110
AMP54018.1 elongation factor Tu domain 2 [uncultured bacterium]
339   2e-110
WP_079043944.1 GTP-binding protein [Streptomyces kanamyceticus]
356   2e-110
WP_061982171.1 GTP-binding protein [Amycolatopsis regifaucium]
355   3e-110
WP_093683803.1 GTP-binding protein [Streptomyces sp. MnatMP-M77]
355   3e-110
WP_107155380.1 GTP-binding protein [Micromonospora sp. RP3T]
355   3e-110
WP_081139858.1 hypothetical protein [Enterococcus faecium]
339   3e-110
WP_032754132.1 GTP-binding protein [Streptomyces alboviridis]
355   3e-110
WP_020574476.1 GTP-binding protein [Actinopolymorpha alba]
355   3e-110
WP_067687215.1 GTP-binding protein [Actinoplanes awajinensis]
355   3e-110
AHF55841.1 tetracycline resistance protein [bacterium KGJ-3-10]
339   4e-110
WP_067904776.1 GTP-binding protein [Actinomadura chibensis]
354   4e-110
WP_018956676.1 GTP-binding protein [Streptomyces sp. CNB091]
355   4e-110
WP_091289612.1 GTP-binding protein [Amycolatopsis xylanica]
353   5e-110
WP_067589256.1 GTP-binding protein [Nocardia terpenica]
353   5e-110
WP_094045244.1 MULTISPECIES: GTP-binding protein [Cohnella]
355   5e-110
WP_016702946.1 GTP-binding protein [Pseudomonas chlororaphis]
354   5e-110
WP_060888820.1 GTP-binding protein [Streptomyces scabiei]
352   5e-110
WP_076260549.1 GTP-binding protein [Monashia flava]
355   5e-110
WP_070202857.1 GTP-binding protein [Streptomyces nanshensis]
354   6e-110
OQX97406.1 hypothetical protein B6I20_12670 [Bacteroidetes ba...
354   6e-110
SMD19259.1 ribosomal protection tetracycline resistance prote...
352   7e-110
KUO75292.1 hypothetical protein APF81_17550 [Desulfosporosinu...
353   8e-110
WP_091304439.1 GTP-binding protein [Amycolatopsis tolypomycina]
353   8e-110
WP_060881072.1 GTP-binding protein [Streptomyces scabiei]
352   8e-110

WP_014651989.1 GTP-binding protein [Paenibacillus mucilaginosus]
354   8e-110
WP_034263388.1 GTP-binding protein [Actinospica robiniae]
355   8e-110
WP_097231815.1 GTP-binding protein [Streptomyces zhaozhouensis]
354   1e-109
WP_081177270.1 GTP-binding protein [Rhizobium rhizosphaerae]
353   1e-109
WP_051115578.1 GTP-binding protein [Actinoplanes globisporus]
353   1e-109
WP_044856247.1 GTP-binding protein [Amycolatopsis orientalis]
353   1e-109
WP_090799009.1 GTP-binding protein [Asanoa ishikariensis]
353   1e-109
AHF55847.1 tetracycline resistance protein [bacterium KGJ-3-22]
338   1e-109
WP_075637212.1 GTP-binding protein [Rhizobium rhizosphaerae]
353   1e-109
WP_077601884.1 GTP-binding protein [Oceanobacillus sojae]
352   1e-109
WP_076994264.1 GTP-binding protein [Actinosynnema sp. ALI-1.44]
351   2e-109
WP_026241993.1 GTP-binding protein [Streptomyces sp. ScaeMP-e10]
353   2e-109
WP_076335606.1 GTP-binding protein [Paenibacillus sp. FSL H8-...
353   2e-109
WP_028059603.1 GTP-binding protein [Solirubrobacter sp. URHD0...
352   2e-109
WP_053562322.1 GTP-binding protein [Streptomyces sp. CFMR 7]
353   2e-109
WP_076966976.1 GTP-binding protein [Streptomyces sp. IB2014 0...
353   3e-109
WP_052394416.1 GTP-binding protein [Kutzneria sp. 744]
351   3e-109
WP_030566475.1 GTP-binding protein [Streptomyces cyaneofuscatus]
353   3e-109
WP_076220604.1 GTP-binding protein [Paenibacillus odorifer]
348   3e-109
AHF55828.1 tetracycline resistance protein [bacterium KGJ-3-14]
337   3e-109
WP_090294166.1 GTP-binding protein [Ensifer sp. YR511]
352   4e-109
WP_030442672.1 GTP-binding protein [Actinoplanes subtropicus]
351   4e-109
WP_019527195.1 GTP-binding protein [Streptomyces sp. FxanaD5]
352   5e-109
WP_055633440.1 GTP-binding protein [Streptomyces griseoruber]
352   5e-109
WP_098754677.1 GTP-binding protein [Streptomyces sp. Ru87]
352   5e-109
WP_099213815.1 GTP-binding protein [Tenacibaculum sp. 4G03]
352   5e-109
ASM62154.1 tetracycline resistance protein tetM [Listeria mon...
338   5e-109

WP_093752766.1 GTP-binding protein [Streptomyces sp. DvalAA-19]
352   6e-109
WP_090642395.1 GTP-binding protein [Paenibacillus sp. UNC496MF]
352   6e-109
WP_068925930.1 GTP-binding protein [Planobispora rosea]
351   6e-109
AHF55837.1 tetracycline resistance protein [bacterium KWD-2-2]
336   7e-109
WP_098694911.1 GTP-binding protein [Nocardia terpenica]
350   7e-109
WP_037341661.1 GTP-binding protein [Amycolatopsis sp. MJM2582]
350   8e-109
WP_073744089.1 GTP-binding protein [Streptomyces sp. CB02115]
351   8e-109
WP_069911606.1 GTP-binding protein [Devosia insulae]
350   9e-109
AHF55848.1 tetracycline resistance protein [bacterium KGJ-3-23]
335   1e-108
WP_038579068.1 GTP-binding protein [Pseudomonas chlororaphis]
351   1e-108
WP_025354783.1 GTP-binding protein [Kutzneria albida]
350   1e-108
KQI00749.1 tetracycline resistance protein tetM [Campylobacte...
337   1e-108
WP_088939695.1 GTP-binding protein [Rhizobium sp. N122]
350   1e-108
WP_020134823.1 tetracycline resistance ribosomal protection p...
349   1e-108
WP_097963679.1 GTP-binding protein [Streptomyces sp. or20]
350   1e-108
KQI16217.1 tetracycline resistance protein tetM [Campylobacte...
336   2e-108
AHF55846.1 tetracycline resistance protein [bacterium KGJ-3-18]
335   2e-108
WP_032123147.1 GTP-binding protein [Clostridium amazonitimone...
350   2e-108
WP_018765788.1 GTP-binding protein [Bacillus sp. 105MF]
349   2e-108
SDJ58151.1 ribosomal protection tetracycline resistance prote...
351   2e-108
WP_097534149.1 GTP-binding protein [Rhizobium hidalgonense]
350   2e-108
WP_076266373.1 GTP-binding protein [Paenibacillus sp. FSL A5-...
350   2e-108
PVD01959.1 GTP-binding protein [Streptomyces sp. CS090A]
350   2e-108
AEP33194.1 TetS [Enterococcus faecium]
336   2e-108
WP_093607794.1 GTP-binding protein [Streptomyces indicus]
351   2e-108
WP_020631031.1 GTP-binding protein [Amycolatopsis alba]
349   3e-108
WP_073870060.1 GTP-binding protein [Streptomyces sp. CB00072]
350   3e-108

WP_053660898.1 GTP-binding protein [Micromonospora sp. NRRL B...
350   3e-108
AHF55845.1 tetracycline resistance protein [bacterium KGJ-3-16]
334   3e-108
AHF55827.1 tetracycline resistance protein [bacterium KGJ-3-1]
334   3e-108
WP_054025580.1 GTP-binding protein [Bacillus sp. FJAT-28004]
350   3e-108
WP_030431697.1 GTP-binding protein [Allokutzneria albata]
348   3e-108
WP_017965008.1 GTP-binding protein [Rhizobium leguminosarum]
350   3e-108
WP_103419141.1 GTP-binding protein [Streptomyces sp. ZL-24]
350   3e-108
WP_037308011.1 GTP-binding protein [Amycolatopsis orientalis]
349   3e-108
WP_089243950.1 GTP-binding protein [Asanoa hainanensis]
349   3e-108
WP_047019259.1 GTP-binding protein [Streptomyces sp. CNQ-509]
350   4e-108
WP_028735758.1 MULTISPECIES: GTP-binding protein [Rhizobium]
349   4e-108
WP_031521737.1 GTP-binding protein [Streptomyces sp. NRRL F-5...
350   4e-108
WP_043831916.1 GTP-binding protein [Amycolatopsis orientalis]
349   4e-108
WP_029451945.1 GTP-binding protein [Clostridium algidicarnis]
350   4e-108
AHF55824.1 tetracycline resistance protein [bacterium KWD-2-3]
334   4e-108
WP_041048094.1 GTP-binding protein [Paenibacillus sp. VKM B-2...
350   4e-108
WP_079431487.1 GTP-binding protein [Streptomyces katrae]
349   4e-108
OFY36837.1 hypothetical protein A2W91_00175 [Bacteroidetes ba...
349   4e-108
WP_013919165.1 GTP-binding protein [Paenibacillus mucilaginosus]
350   5e-108
WP_088490834.1 GTP-binding protein [Saccharibacillus sp. O23]
350   5e-108
WP_069464604.1 GTP-binding protein [Streptomyces rubidus]
350   5e-108
WP_020517034.1 GTP-binding protein [Actinoplanes globisporus]
348   5e-108
WP_062369874.1 GTP-binding protein [Rhizobium altiplani]
349   5e-108
AHF55842.1 tetracycline resistance protein [bacterium KGJ-3-11]
333   5e-108
WP_089296998.1 GTP-binding protein [Actinoplanes regularis]
349   6e-108
WP_090116095.1 GTP-binding protein [Cohnella sp. OV330]
349   6e-108
AHF55839.1 tetracycline resistance protein [bacterium KGJ-3-5]
333   6e-108

AHF55836.1 tetracycline resistance protein [bacterium KWD-2-18]
333   6e-108
AHF55826.1 tetracycline resistance protein [bacterium KWD-2-5]
333   6e-108
WP_057311173.1 GTP-binding protein [Paenibacillus sp. Soil766]
349   6e-108
OIV36439.1 GTP-binding protein [Streptomyces gilvigriseus]
348   7e-108
WP_060516073.1 MULTISPECIES: GTP-binding protein [Ensifer]
348   7e-108
WP_104410087.1 GTP-binding protein [Clostridium algidicarnis]
349   7e-108
EPH39370.1 putative Oxytetracycline resistance protein [Strep...
351   1e-107
WP_042157825.1 tetracycline resistance ribosomal protection p...
348   1e-107
WP_079422478.1 GTP-binding protein [Clostridium oryzae]
348   1e-107
WP_047508868.1 GTP-binding protein [Rhizobium sp. YR528]
348   1e-107
WP_056760020.1 GTP-binding protein [Terrabacter sp. Root181]
350   1e-107
AHF55838.1 tetracycline resistance protein [bacterium KGJ-3-3]
333   1e-107
WP_102413038.1 GTP-binding protein [Clostridiales bacterium M...
348   1e-107
AHF55832.1 tetracycline resistance protein [bacterium KWD-2-4]
333   1e-107
WP_034793153.1 MULTISPECIES: GTP-binding protein [Ensifer]
348   1e-107
ANC47978.1 TetM [Butyrivibrio sp. 268.8]
336   1e-107
AHF55825.1 tetracycline resistance protein [bacterium KGJ-3-4]
332   1e-107
WP_053373614.1 GTP-binding protein [Paenibacillus sp. FJAT-27...
348   1e-107
AHF55852.1 tetracycline resistance protein [bacterium KGJ-3-30]
332   1e-107
WP_013017334.1 GTP-binding protein [Stackebrandtia nassauensis]
348   1e-107
WP_028958988.1 GTP-binding protein [Streptomyces sp. UNC401CL...
348   2e-107
WP_027087725.1 GTP-binding protein [Cohnella panacarvi]
348   2e-107
WP_047520919.1 GTP-binding protein [Rhizobium sp. CF048]
347   2e-107
WP_077836582.1 GTP-binding protein [Enterococcus faecium]
333   2e-107
AHF55843.1 tetracycline resistance protein [bacterium KGJ-3-13]
332   2e-107
WP_037124502.1 GTP-binding protein [Rhizobium sp. OV201]
347   2e-107
WP_079170395.1 GTP-binding protein [Streptomyces gilvigriseus]
347   2e-107

PKP01044.1 GTP-binding protein [Bacteroidetes bacterium HGW-B...
348   2e-107
ACQ85279.1 TetO [Campylobacter jejuni]
335   2e-107
AHF55851.1 tetracycline resistance protein [bacterium KGJ-3-29]
332   2e-107
WP_101046161.1 GTP-binding protein [Tenacibaculum sp. Bg11-29]
348   2e-107
WP_082942297.1 GTP-binding protein [Neglecta timonensis]
350   2e-107
AHF55833.1 tetracycline resistance protein [bacterium KWD-2-6]
332   2e-107
AHF55835.1 tetracycline resistance protein [bacterium KWD-2-12]
332   2e-107
WP_065387043.1 GTP-binding protein [Bacillus sp. FJAT-26390]
347   3e-107
WP_028937230.1 GTP-binding protein [Pseudonocardia spinosispora]
346   3e-107
PKB00878.1 ribosomal protection tetracycline resistance prote...
347   3e-107
ACQ85278.1 TetO [Campylobacter jejuni]
334   4e-107
WP_100864842.1 GTP-binding protein [Streptomyces sp. Ag109_G2-1]
347   4e-107
BAQ18917.1 tetracycline-resistance protein [Enterococcus faec...
331   4e-107
WP_102715800.1 GTP-binding protein [Paenibacillus castaneae]
347   4e-107
WP_076688896.1 GTP-binding protein [Streptomyces alfalfae]
348   4e-107
KND42633.1 GTP-binding protein [Streptomyces stelliscabiei]
344   4e-107
WP_038509069.1 GTP-binding protein [Amycolatopsis japonica]
346   4e-107
WP_067990619.1 GTP-binding protein [Nocardia pseudobrasiliensis]
345   5e-107
ACK58049.1 tetracycline resistance protein [Vibrio tasmaniensis]
331   5e-107
AHF55834.1 tetracycline resistance protein [bacterium KWD-2-10]
331   5e-107
WP_062782474.1 GTP-binding protein [Streptomyces sp. NRRL S-1...
347   5e-107
WP_095435748.1 GTP-binding protein [Rhizobium sp. 10195]
346   6e-107
ABR22527.1 tetracycline resistance protein [Vibrio tasmaniensis]
331   7e-107
WP_057586367.1 GTP-binding protein [Terrabacter sp. Soil810]
348   7e-107
OKP70014.1 GTP-binding protein [Ensifer adhaerens]
346   7e-107
WP_030156384.1 GTP-binding protein [Glycomyces sp. NRRL B-16210]
346   9e-107
WP_091587648.1 GTP-binding protein [Micromonospora krabiensis]
346   9e-107

WP_037815988.1 GTP-binding protein [Streptomyces sp. NRRL F-3...
345   1e-106
WP_005165950.1 GTP-binding protein [Amycolatopsis azurea]
345   1e-106
WP_049144669.1 GTP-binding protein [Enterococcus faecium]
332   1e-106
OFY07980.1 hypothetical protein A2W94_08540 [Bacteroidetes ba...
345   1e-106
ACQ85281.1 TetO [Campylobacter coli]
333   1e-106
KGK88547.1 GTP-binding protein [Clostridium sp. HMP27]
345   2e-106
WP_015797426.1 GTP-binding protein [Catenulispora acidiphila]
345   2e-106
WP_047789600.1 GTP-binding protein [Tenacibaculum mesophilum]
345   2e-106
KSV70644.1 hypothetical protein N182_05010 [Sinorhizobium sp....
345   2e-106
ABR21775.1 TetM [Pseudoalteromonas marina]
329   2e-106
ACQ85276.1 TetO [Campylobacter jejuni]
332   2e-106
WP_025416584.1 GTP-binding protein [Rhizobium leguminosarum]
345   2e-106
WP_072830076.1 GTP-binding protein [Clostridium collagenovorans]
345   2e-106
WP_000418263.1 GTP-binding protein [Bacillus cereus]
343   3e-106
WP_033293268.1 GTP-binding protein [Amycolatopsis jejuensis]
343   3e-106
WP_039629441.1 tetracycline resistance ribosomal protection p...
345   3e-106
WP_053249997.1 GTP-binding protein [Ensifer adhaerens]
344   3e-106
WP_092712344.1 GTP-binding protein [Rhizobium multihospitium]
344   3e-106
WP_037365918.1 GTP-binding protein [Amycolatopsis orientalis]
343   3e-106
WP_035748704.1 GTP-binding protein [Glycomyces sp. NRRL B-16210]
343   4e-106
WP_093948742.1 GTP-binding protein [Amycolatopsis sp. H5]
343   4e-106
WP_065656397.1 GTP-binding protein [Agrobacterium tumefaciens]
344   4e-106
ABQ23358.1 tetracycline resistance efflux pump [Edwardsiella ...
328   4e-106
WP_072474510.1 GTP-binding protein [Amycolatopsis australiensis]
343   4e-106
WP_093350263.1 MULTISPECIES: GTP-binding protein [Saccharopol...
343   4e-106
WP_079110165.1 GTP-binding protein [Streptomyces sp. MBT76]
347   5e-106
WP_073848196.1 GTP-binding protein [Amycolatopsis sp. CB00013]
343   5e-106

WP_104679733.1 GTP-binding protein [Agrobacterium tumefaciens]
343   5e-106
WP_090575047.1 GTP-binding protein [Paenibacillus sp. OV219]
344   6e-106
WP_073183306.1 GTP-binding protein [Tenacibaculum mesophilum]
343   8e-106
WP_084428751.1 GTP-binding protein [Kibdelosporangium aridum]
342   9e-106
ABQ23360.1 tetracycline resistance efflux pump, partial [Aero...
327   1e-105
BAF95550.1 tetracycline resistance protein [Streptomyces sp. ...
327   1e-105
WP_092748867.1 GTP-binding protein [Rhizobium aethiopicum]
343   1e-105
EFR32593.1 GTP-binding domain protein [Peptoniphilus harei AC...
328   1e-105
WP_003580605.1 GTP-binding protein [Rhizobium leguminosarum]
343   1e-105
WP_047623375.1 GTP-binding protein [Rhizobium leguminosarum]
343   1e-105
WP_033357111.1 GTP-binding protein [Dactylosporangium auranti...
343   1e-105
EGL39683.1 GTP-binding domain protein [Megasphaera sp. UPII 1...
328   1e-105
ABQ23359.1 tetracycline resistance efflux pump, partial [Aero...
327   2e-105
WP_069814409.1 GTP-binding protein [Streptomyces sp. TP-A0874]
342   2e-105
WP_063854497.1 tetracycline resistance ribosomal protection p...
343   2e-105
WP_097598021.1 GTP-binding protein [Rhizobium sp. L9]
342   2e-105
WP_006731900.1 GTP-binding protein [Lactobacillus iners]
328   2e-105
WP_016336704.1 GTP-binding protein [Amycolatopsis orientalis]
342   2e-105
WP_091529320.1 GTP-binding protein [Microlunatus soli]
342   2e-105
WP_062240025.1 tetracycline resistance ribosomal protection p...
341   2e-105
WP_028890491.1 GTP-binding protein [Tenacibaculum ovolyticum]
342   3e-105
WP_043841447.1 GTP-binding protein [Roseivivax atlanticus]
342   3e-105
WP_023363761.1 GTP-binding protein [Actinoplanes friuliensis]
342   3e-105
WP_093575845.1 GTP-binding protein [Amycolatopsis rubida]
341   3e-105
WP_091609574.1 GTP-binding protein [Micromonospora mirobrigen...
342   3e-105
WP_054182740.1 GTP-binding protein [Rhizobium acidisoli]
342   3e-105
WP_106998838.1 GTP-binding protein [Rhizobium sp. JAB6]
341   4e-105

OLE01321.1 hypothetical protein AUG91_01940 [Actinobacteria b...
340   4e-105
WP_067698781.1 GTP-binding protein [Actinoplanes awajinensis]
341   4e-105
WP_064249559.1 GTP-binding protein [Rhizobium leguminosarum]
341   4e-105
WP_077690258.1 GTP-binding protein [Nocardiopsis sinuspersici]
342   4e-105
WP_010074806.1 GTP-binding protein [Clostridium cellulovorans]
342   4e-105
WP_060603596.1 GTP-binding protein [Sinorhizobium sp. GW3]
341   4e-105
WP_007890980.1 GTP-binding protein [Streptococcus pseudoporci...
327   5e-105
WP_064705513.1 GTP-binding protein [Rhizobium bangladeshense]
341   5e-105
WP_047614130.1 GTP-binding protein [Rhizobium leguminosarum]
341   6e-105
WP_100445478.1 GTP-binding protein [Glycomyces sp. TRM 41368]
340   7e-105
WP_051308702.1 GTP-binding protein [Streptomyces sp. CNS606]
342   7e-105
WP_011722765.1 GTP-binding protein [Clostridium novyi]
341   1e-104
BAF02677.1 tetracycline resistance protein [Vibrio sp. 04Ya057]
325   1e-104
WP_072030488.1 GTP-binding protein [Amycolatopsis keratiniphila]
340   1e-104
WP_097542187.1 GTP-binding protein [Rhizobium anhuiense]
340   1e-104
WP_017585393.1 GTP-binding protein [Nocardiopsis ganjiahuensis]
340   1e-104
WP_101917382.1 GTP-binding protein [Tenacibaculum sp. TNO020]
341   1e-104
WP_080277886.1 GTP-binding protein [Bacillus anthracis]
338   1e-104
BAF95547.1 tetracycline resistance protein [Brachybacterium s...
325   1e-104
WP_049868737.1 GTP-binding protein [Paenibacillus sp. D9]
341   1e-104
WP_047610790.1 GTP-binding protein [Rhizobium tropici]
340   1e-104
KUJ70082.1 GTP-binding protein [Streptomyces albus subsp. albus]
340   1e-104
SDS83464.1 ribosomal protection tetracycline resistance prote...
340   1e-104
AMP51625.1 hypothetical protein [uncultured bacterium]
323   1e-104
WP_086082386.1 GTP-binding protein [Rhizobium etli]
340   1e-104
WP_037771319.1 tetracycline resistance ribosomal protection p...
340   2e-104
WP_017988897.1 GTP-binding protein [Rhizobium leguminosarum]
340   2e-104

WP_039283559.1 GTP-binding protein [Clostridium botulinum]
340   2e-104
ABP96836.1 tetracycline resistance protein [Vibrio splendidus]
324   2e-104
ABM65168.1 TetW [Bifidobacterium pseudolongum subsp. globosum]
322   2e-104
WP_091425021.1 GTP-binding protein [Micromonospora tulbaghiae]
340   2e-104
WP_039257044.1 GTP-binding protein [Clostridium botulinum]
340   2e-104
WP_062213991.1 GTP-binding protein [Streptomyces sp. NBRC 109...
340   2e-104
WP_105734727.1 GTP-binding protein [Phyllobacterium myrsinace...
339   2e-104
WP_093939169.1 GTP-binding protein [Amycolatopsis thailandensis]
338   3e-104
WP_017591673.1 GTP-binding protein [Nocardiopsis potens]
340   3e-104
WP_069611866.1 GTP-binding protein [Rhizobium sp. YK2]
339   3e-104
WP_086857843.1 GTP-binding protein [Amycolatopsis lexingtonen...
338   3e-104
WP_039245283.1 GTP-binding protein [Clostridium novyi]
340   3e-104
WP_003541224.1 GTP-binding protein [Rhizobium leguminosarum]
339   4e-104
WP_039259388.1 GTP-binding protein [Clostridium botulinum]
339   4e-104
WP_091413159.1 GTP-binding protein [Friedmanniella luteola]
340   4e-104
AHI45106.1 TetO [Campylobacter jejuni]
326   4e-104
WP_025395228.1 GTP-binding protein [Rhizobium leguminosarum]
338   5e-104
WP_052865430.1 tetracycline resistance ribosomal protection p...
338   5e-104
WP_095565394.1 GTP-binding protein [Plantactinospora sp. KBS50]
339   5e-104
WP_039276861.1 GTP-binding protein [Clostridium botulinum]
339   5e-104
WP_026413758.1 GTP-binding protein [Actinomadura oligospora]
338   6e-104
AUI41095.1 tetracycline resistance ribosomal protection prote...
322   6e-104
WP_063274413.1 GTP-binding protein [Amycolatopsis keratiniphila]
338   6e-104
BAF95546.1 tetracycline resistance protein [Brachybacterium s...
323   7e-104
WP_095315242.1 tetracycline resistance ribosomal protection p...
333   7e-104
BAF02689.1 tetracycline resistance protein [Vibrio sp. 04Ya004]
323   7e-104
WP_037671305.1 tetracycline resistance ribosomal protection p...
338   7e-104

WP_091039199.1 GTP-binding protein [Glycomyces harbinensis]
338   8e-104
WP_064694617.1 GTP-binding protein [Rhizobium aegyptiacum]
338   8e-104
WP_077978721.1 GTP-binding protein [Rhizobium laguerreae]
338   9e-104
WP_064536790.1 GTP-binding protein [Streptomyces sp. SAT1]
338   9e-104
WP_064810768.1 MULTISPECIES: GTP-binding protein [Rhizobium]
338   9e-104
AFU65884.1 DacR1 [Dactylosporangium sp. SC14051]
338   1e-103
WP_098215846.1 GTP-binding protein [Bacillus pseudomycoides]
336   1e-103
WP_085015334.1 GTP-binding protein [Frankia sp. KB5]
338   1e-103
WP_039251942.1 GTP-binding protein [Clostridium novyi]
338   1e-103
WP_081633949.1 GTP-binding protein [Terracoccus sp. 273MFTsu3.1]
342   1e-103
WP_068705992.1 GTP-binding protein [Tenacibaculum soleae]
338   1e-103
WP_067589893.1 GTP-binding protein [Amycolatopsis sp. M39]
337   1e-103
AMP52232.1 elongation factor Tu domain 2 [uncultured bacterium]
322   1e-103
EJB07701.1 LOW QUALITY PROTEIN: small GTP-binding protein dom...
337   1e-103
WP_069113481.1 GTP-binding protein [Jiangella alba]
338   1e-103
WP_053852026.1 tetracycline resistance ribosomal protection p...
336   1e-103
WP_090940822.1 GTP-binding protein [Nonomuraea jiangxiensis]
337   1e-103
WP_085778704.1 GTP-binding protein [Rhizobium sp. NXC14]
337   1e-103
WP_064839413.1 MULTISPECIES: GTP-binding protein [Rhizobium]
337   2e-103
WP_047930292.1 GTP-binding protein [Clostridium botulinum]
338   2e-103
AHI45110.1 TetO [Campylobacter jejuni]
324   2e-103
PLX01135.1 GTP-binding protein [Marinilabiliales bacterium]
337   2e-103
WP_063474642.1 GTP-binding protein [Rhizobium anhuiense]
337   2e-103
WP_057468060.1 GTP-binding protein [Rhizobium sp. Root1203]
337   2e-103
WP_028740902.1 GTP-binding protein [Rhizobium leguminosarum]
337   2e-103
BAF95552.1 tetracycline resistance protein [Arthrobacter sp. ...
322   2e-103
WP_030367961.1 GTP-binding protein [Streptomyces roseovertici...
337   2e-103

WP_006733447.1 GTP-binding protein [Lactobacillus iners]
322   2e-103
WP_056823271.1 GTP-binding protein [Rhizobium sp. Root708]
337   2e-103
WP_017961207.1 GTP-binding protein [Rhizobium leguminosarum]
337   2e-103
WP_011652714.1 GTP-binding protein [Rhizobium leguminosarum]
337   2e-103
ANP86357.1 GTP-binding protein [Rhizobium leguminosarum]
337   2e-103
ANZ45594.1 hypothetical protein BED41_11235 [Cloacibacillus p...
337   2e-103
WP_075224069.1 GTP-binding protein [Rhizobium leguminosarum]
337   2e-103
WP_101435808.1 GTP-binding protein [Amycolatopsis niigatensis]
336   2e-103
WP_020662538.1 GTP-binding protein [Amycolatopsis benzoatilyt...
337   2e-103
WP_064803668.1 MULTISPECIES: GTP-binding protein [Rhizobium]
337   2e-103
WP_018074229.1 GTP-binding protein [Rhizobium leguminosarum]
337   2e-103
ACH43046.1 tetracycline resistance protein Tet(M) [Enterococc...
321   2e-103
WP_088687521.1 GTP-binding protein [Rhizobium sp. R634]
337   3e-103
WP_069812828.1 GTP-binding protein [Streptomyces sp. TP-A0874]
337   3e-103
WP_013726215.1 GTP-binding protein [Clostridium botulinum]
337   3e-103
WP_099161828.1 GTP-binding protein [Micromonospora sp. WMMA2032]
336   3e-103
WP_053284784.1 GTP-binding protein [Clostridium botulinum]
337   3e-103
WP_015341029.1 GTP-binding protein [Rhizobium tropici]
336   4e-103
WP_009072799.1 MULTISPECIES: GTP-binding protein [Actinobacte...
336   4e-103
WP_024320831.1 GTP-binding protein [Rhizobium leguminosarum]
336   5e-103
WP_029870343.1 GTP-binding protein [Rhizobium leguminosarum]
336   5e-103
WP_039240195.1 GTP-binding protein [Clostridium botulinum]
336   5e-103
BAF95553.1 tetracycline resistance protein [Brachybacterium s...
320   5e-103
WP_039250656.1 GTP-binding protein [Clostridium novyi]
337   5e-103
AAP21774.1 putative ribosomal protection protein [uncultured ...
329   6e-103
WP_097630451.1 GTP-binding protein [Rhizobium anhuiense]
336   6e-103
WP_064682644.1 GTP-binding protein [Rhizobium bangladeshense]
336   7e-103

WP_003366748.1 GTP-binding protein [Clostridium botulinum]
336   7e-103
AHI45108.1 TetO [Campylobacter jejuni]
323   7e-103
WP_086341339.1 GTP-binding protein [Enterococcus sp. 6C8_DIV0...
335   8e-103
BAG23437.1 putative tetracycline resistance protein [Streptom...
336   8e-103
WP_085828953.1 GTP-binding protein [Clostridium massiliodielm...
336   8e-103
AHI45109.1 TetO [Campylobacter jejuni]
322   8e-103
WP_042180585.1 GTP-binding protein [Kibdelosporangium sp. MJ1...
334   9e-103
WP_030248611.1 GTP-binding protein [Streptacidiphilus jeojiense]
336   9e-103
WP_012758195.1 GTP-binding protein [Rhizobium leguminosarum]
335   9e-103
WP_017994661.1 GTP-binding protein [Rhizobium leguminosarum]
335   1e-102
WP_025662269.1 GTP-binding protein [Rhizobium sp. IBUN]
335   1e-102
WP_086683054.1 GTP-binding protein [Amycolatopsis pretoriensis]
334   1e-102
WP_071613075.1 GTP-binding protein [Clostridium estertheticum]
335   1e-102
WP_097592648.1 GTP-binding protein [Rhizobium sp. J15]
335   1e-102
WP_079035534.1 GTP-binding protein [Streptomyces flocculus]
336   1e-102
SBO97526.1 Ribosome protection-type tetracycline resistance r...
335   1e-102
WP_100605928.1 tetracycline resistance ribosomal protection p...
335   1e-102
BAL04138.1 tetracycline resistance protein [Vibrio ichthyoent...
319   1e-102
WP_076359067.1 MULTISPECIES: GTP-binding protein [Paenibacillus]
335   2e-102
WP_054292861.1 GTP-binding protein [Kibdelosporangium phytoha...
333   2e-102
BAF95544.1 tetracycline resistance protein [Bacillus sp. APFT...
319   2e-102
KJK11378.1 hypothetical protein UB45_14010 [Terrabacter sp. 28]
336   2e-102
WP_017621924.1 GTP-binding protein [Nocardiopsis gilva]
335   2e-102
AHI45107.1 TetO [Campylobacter jejuni]
322   2e-102
WP_007028189.1 GTP-binding protein [Amycolatopsis decaplanina]
333   2e-102
WP_056826659.1 GTP-binding protein [Terrabacter sp. Root85]
336   2e-102
WP_067360275.1 tetracycline resistance ribosomal protection p...
333   3e-102

BAF95545.1 tetracycline resistance protein [Bacillus sp. APFT...
318   3e-102
WP_084958049.1 GTP-binding protein [Thermoactinospora rubra]
333   3e-102
WP_017624595.1 GTP-binding protein [Nocardiopsis chromatogenes]
334   3e-102
WP_018446486.1 GTP-binding protein [Rhizobium gallicum]
334   3e-102
WP_086571522.1 tetracycline resistance ribosomal protection p...
335   3e-102
AHI45112.1 TetO [Campylobacter jejuni]
321   3e-102
WP_088683289.1 GTP-binding protein [Rhizobium sp. R635]
333   4e-102
WP_047629084.1 GTP-binding protein [Rhizobium tropici]
333   4e-102
WP_056773652.1 GTP-binding protein [Terrabacter sp. Soil811]
335   4e-102
WP_018482862.1 GTP-binding protein [Rhizobium leguminosarum]
333   6e-102
WP_097629337.1 GTP-binding protein [Rhizobium sp. L18]
333   7e-102
WP_028732787.1 GTP-binding protein [Rhizobium leguminosarum]
333   7e-102
WP_037091390.1 GTP-binding protein [Rhizobium leguminosarum]
333   8e-102
WP_013454445.1 GTP-binding protein [Marivirga tractuosa]
333   9e-102
SMI87769.1 tetracycline resistance protein TetP [Enterococcus...
316   9e-102
WP_087925631.1 GTP-binding protein [Streptomyces albireticuli]
333   1e-101
WP_106193694.1 GTP-binding protein [Umezawaea tangerina]
331   1e-101
WP_004950796.1 GTP-binding protein [Streptomyces mobaraensis]
334   1e-101
PHS08958.1 GTP-binding protein [Kordia sp.]
333   1e-101
WP_034476055.1 GTP-binding protein [Agrobacterium rhizogenes]
332   1e-101
WP_094085597.1 GTP-binding protein [Rhizobium leguminosarum]
332   1e-101
WP_101610104.1 GTP-binding protein [Amycolatopsis sp. BJA-103]
332   1e-101
WP_085515285.1 GTP-binding protein [Marivirga sericea]
333   1e-101
WP_091578945.1 GTP-binding protein [Micromonospora sediminicola]
332   2e-101
WP_035737742.1 GTP-binding protein [Glycomyces arizonensis]
332   2e-101
WP_026930414.1 GTP-binding protein [Glycomyces tenuis]
332   2e-101
WP_096764780.1 GTP-binding protein [Rhizobium sophoriradicis]
332   2e-101

WP_071087632.1 GTP-binding protein [Rhizobium sp. RSm-3]
332   2e-101
WP_043619619.1 GTP-binding protein [Ensifer sp. ZNC0028]
332   2e-101
CNA02039.1 tetracycline resistance protein TetM [Streptococcu...
315   2e-101
WP_085737717.1 GTP-binding protein [Rhizobium sp. CIAT894]
332   2e-101
WP_097223733.1 tetracycline resistance ribosomal protection p...
330   3e-101
WP_028738651.1 MULTISPECIES: GTP-binding protein [Rhizobium]
331   3e-101
WP_020050745.1 GTP-binding protein [Rhizobium leguminosarum]
331   3e-101
EJZ18119.1 tetracycline resistance protein [Rhizobium sp. Pop5]
331   3e-101
WP_047468645.1 GTP-binding protein [Agrobacterium rhizogenes]
331   4e-101
AMP46949.1 elongation factor G, domain IV [uncultured bacterium]
317   4e-101
EJC75820.1 small GTP-binding protein domain protein [Rhizobiu...
332   5e-101
WP_007630281.1 GTP-binding protein [Rhizobium sp. CCGE 510]
331   5e-101
WP_095850965.1 tetracycline resistance ribosomal protection p...
330   5e-101
AMP51668.1 elongation factor G, domain IV [uncultured bacterium]
326   5e-101
WP_058884870.1 GTP-binding protein [Tenacibaculum dicentrarchi]
331   5e-101
ANM05045.1 translation elongation factor/tetracycline resista...
331   6e-101
WP_049733560.1 GTP-binding protein [Rhizobium ecuadorense]
330   6e-101
WP_085742851.1 GTP-binding protein [Rhizobium phaseoli]
330   7e-101
WP_090791959.1 GTP-binding protein [Asanoa ishikariensis]
330   7e-101
WP_018242774.1 GTP-binding protein [Rhizobium leguminosarum]
330   7e-101
WP_098745613.1 GTP-binding protein [Micromonospora sp. WMMA1996]
330   7e-101
AMP49507.1 elongation factor Tu GTP binding domain protein [u...
316   9e-101
WP_078875480.1 GTP-binding protein [Streptomyces sp. NRRL F-5...
331   1e-100
WP_030899138.1 GTP-binding protein [Streptomyces sp. NRRL F-5...
331   1e-100
WP_046628486.1 GTP-binding protein [Neorhizobium galegae]
330   1e-100
WP_099944884.1 tetracycline resistance ribosomal protection p...
329   1e-100
WP_097621729.1 GTP-binding protein [Rhizobium sp. L43]
330   1e-100

WP_078872690.1 GTP-binding protein [Streptomyces sp. NRRL S-1...
331   1e-100
AAO16476.1 putative tetracycline resistance protein [uncultur...
323   2e-100
WP_007693710.1 GTP-binding protein [Rhizobium sp. AP16]
330   2e-100
WP_075851581.1 GTP-binding protein [Rhizobium hainanense]
329   2e-100
WP_091049845.1 GTP-binding protein [Glycomyces sambucus]
328   2e-100
WP_086817188.1 GTP-binding protein [Streptomyces cacaoi]
330   2e-100
WP_016471516.1 GTP-binding protein [Streptomyces sp. HPH0547]
330   3e-100
WP_080790836.1 GTP-binding protein [Agrobacterium genomosp. 9]
328   3e-100
WP_101914537.1 GTP-binding protein [Tenacibaculum finnmarkense]
329   3e-100
WP_088948994.1 GTP-binding protein [Micromonospora zamorensis]
329   3e-100
AAO16475.1 putative tetracycline resistance protein [uncultur...
322   4e-100
WP_033180886.1 GTP-binding protein [Rhizobium leguminosarum]
328   4e-100
WP_108493615.1 GTP-binding protein [Promicromonospora sp. AC04]
327   4e-100
WP_106366688.1 GTP-binding protein [Glycomyces artemisiae]
328   4e-100
WP_038689909.1 GTP-binding protein [Rhizobium sp. IE4771]
328   4e-100
WP_088673344.1 GTP-binding protein [Rhizobium sp. R339]
328   4e-100
WP_057216731.1 GTP-binding protein [Ensifer sp. Root127]
328   5e-100
WP_072380130.1 GTP-binding protein [Rhizobium tibeticum]
328   5e-100
PCI34160.1 GTP-binding protein [Flavobacteriaceae bacterium]
328   5e-100
WP_097667096.1 GTP-binding protein [Rhizobium sp. M1]
328   5e-100
WP_037107199.1 GTP-binding protein [Rhizobium sp. OK665]
328   7e-100
WP_034315358.1 GTP-binding protein [Amycolatopsis lurida]
327   7e-100
WP_035103788.1 GTP-binding protein [Devosia sp. LC5]
328   7e-100
WP_052589716.1 GTP-binding protein [Luteipulveratus mongolien...
327   9e-100
WP_084814088.1 GTP-binding protein [Ensifer sp. 1H6]
328   9e-100
WP_057206998.1 GTP-binding protein [Ensifer sp. Root31]
328   9e-100
WP_030408550.1 GTP-binding protein [Streptomyces sp. NRRL F-5...
328   1e-99

PKP17033.1 GTP-binding protein [Bacteroidetes bacterium HGW-B...
328   1e-99
PHR24012.1 GTP-binding protein [Fluviicola sp.]
328   1e-99
WP_080866109.1 GTP-binding protein [Agrobacterium tumefaciens]
327   1e-99
WP_012649611.1 GTP-binding protein [Agrobacterium tumefaciens]
327   1e-99
WP_012558297.1 GTP-binding protein [Rhizobium leguminosarum]
327   1e-99
WP_093871562.1 GTP-binding protein [Tenacibaculum sp. MAR_201...
327   1e-99
WP_057226042.1 GTP-binding protein [Ensifer sp. Root142]
327   2e-99
WP_084070521.1 GTP-binding protein [Desulfobacterium vacuolatum]
327   2e-99
WP_101954789.1 GTP-binding protein [Tenacibaculum finnmarkense]
327   2e-99
WP_101932033.1 GTP-binding protein [Tenacibaculum dicentrarchi]
327   2e-99
WP_101901742.1 GTP-binding protein [Tenacibaculum dicentrarchi]
327   2e-99
WP_103785291.1 tetracycline resistance ribosomal protection p...
325   2e-99
WP_025430445.1 GTP-binding protein [Ensifer adhaerens]
327   2e-99
WP_034087015.1 GTP-binding protein [Streptacidiphilus albus]
325   2e-99
WP_099919107.1 tetracycline resistance ribosomal protection p...
325   3e-99
WP_092620003.1 GTP-binding protein [Jiangella sp. DSM 45060]
326   3e-99
WP_087743847.1 GTP-binding protein [Agrobacterium tumefaciens]
326   3e-99
WP_031028518.1 GTP-binding protein [Streptomyces sp. NRRL F-5...
327   3e-99
WP_030323068.1 tetracycline resistance ribosomal protection p...
325   3e-99
WP_020809762.1 GTP-binding protein [Agrobacterium tumefaciens]
326   3e-99
WP_053202290.1 GTP-binding protein [Jiangella muralis]
326   4e-99
AAO16473.1 putative tetracycline resistance protein [uncultur...
319   4e-99
WP_077150566.1 hypothetical protein [Enterococcus faecium]
309   4e-99
KPC96456.1 GTP-binding protein [Streptomyces sp. NRRL F-6602]
325   4e-99
WP_062945390.1 GTP-binding protein [Rhizobium leguminosarum]
326   4e-99
WP_003502521.1 GTP-binding protein [Agrobacterium tumefaciens]
325   5e-99
WP_057221147.1 MULTISPECIES: GTP-binding protein [Ensifer]
325   6e-99

WP_007457364.1 GTP-binding protein [Micromonospora lupini]
325   6e-99
WP_064965718.1 GTP-binding protein [Tenacibaculum ovolyticum]
325   6e-99
WP_101907180.1 GTP-binding protein [Tenacibaculum dicentrarchi]
326   6e-99
WP_080864274.1 GTP-binding protein [Agrobacterium tumefaciens]
325   6e-99
WP_099085457.1 GTP-binding protein [Agrobacterium tumefaciens]
325   7e-99
AMP56067.1 elongation factor G, domain IV [uncultured bacterium]
310   7e-99
WP_107027067.1 GTP-binding protein [Ensifer sp. NM-2]
325   8e-99
WP_103836572.1 tetracycline resistance ribosomal protection p...
325   8e-99
WP_091877371.1 GTP-binding protein [Phyllobacterium sp. OV277]
325   8e-99
WP_068400263.1 GTP-binding protein [Rhizobium sp. AC27/96]
325   9e-99
WP_028870517.1 GTP-binding protein [Psychroserpens burtonensis]
325   9e-99
WP_018494747.1 GTP-binding protein [Rhizobium leguminosarum]
325   1e-98
OWT31778.1 mosaic tetracycline resistance protein [Campylobac...
308   1e-98
WP_016430778.1 tetracycline resistance ribosomal protection p...
323   1e-98
WP_094923030.1 GTP-binding protein [Pseudonocardia sp. MH-G8]
323   2e-98
WP_076988305.1 GTP-binding protein [Actinosynnema sp. ALI-1.44]
323   2e-98
WP_018528928.1 tetracycline resistance ribosomal protection p...
323   2e-98
WP_049571435.1 GTP-binding protein [Nonomuraea sp. SBT364]
324   2e-98
WP_068953105.1 GTP-binding protein [Pararhizobium polonicum]
324   3e-98
WP_055508442.1 tetracycline resistance ribosomal protection p...
323   3e-98
WP_051823248.1 tetracycline resistance ribosomal protection p...
323   5e-98
OJU39843.1 tetracycline resistance ribosomal protection prote...
319   6e-98
WP_014151327.1 GTP-binding protein [Streptomyces cattleya]
323   8e-98
WP_095126029.1 GTP-binding protein [Pseudomonas sp. Irchel s3...
322   9e-98
WP_077062765.1 GTP-binding protein [Streptomyces sp. MP131-18]
323   9e-98
WP_016733539.1 GTP-binding protein [Rhizobium phaseoli]
322   1e-97
WP_064818588.1 GTP-binding protein [Rhizobium phaseoli]
322   1e-97

WP_015023938.1 GTP-binding protein [Psychroflexus torquis]
322   1e-97
WP_038524612.1 GTP-binding protein [Streptomyces albulus]
322   2e-97
WP_015796768.1 GTP-binding protein [Catenulispora acidiphila]
322   2e-97
WP_053165149.1 GTP-binding protein [Streptomyces albulus]
322   2e-97
WP_091416501.1 GTP-binding protein [Friedmanniella luteola]
322   2e-97
WP_077982397.1 GTP-binding protein [Agrobacterium salinitoler...
322   2e-97
WP_086950907.1 GTP-binding protein [Vagococcus fluvialis]
320   2e-97
WP_047461124.1 GTP-binding protein [Agrobacterium rhizogenes]
322   2e-97
AQZ61532.1 GTP-binding protein [Nonomuraea sp. ATCC 55076]
322   2e-97
WP_046664630.1 GTP-binding protein [Neorhizobium galegae]
321   2e-97
WP_086158027.1 GTP-binding protein [Streptomyces sp. SCSIO 03...
322   2e-97
SDR78662.1 ribosomal protection tetracycline resistance prote...
322   2e-97
WP_064827966.1 GTP-binding protein [Rhizobium phaseoli]
321   3e-97
WP_080046591.1 GTP-binding protein [Nonomuraea sp. ATCC 55076]
322   3e-97
WP_027668604.1 GTP-binding protein [Rhizobium leguminosarum]
321   3e-97
WP_106517566.1 tetracycline resistance ribosomal protection p...
320   3e-97
WP_028798647.1 tetracycline resistance ribosomal protection p...
320   3e-97
WP_095049040.1 GTP-binding protein [Pseudomonas sp. Irchel s3h9]
321   4e-97
WP_055584822.1 GTP-binding protein [Streptomyces diastatochro...
321   4e-97
CNE17313.1 tetracycline resistance protein TetM [Streptococcu...
306   4e-97
WP_030869320.1 tetracycline resistance ribosomal protection p...
320   4e-97
WP_103963050.1 GTP-binding protein [Nonomuraea solani]
320   4e-97
CTO47212.1 putative conjugative element protein [Streptococcu...
306   5e-97
WP_020931295.1 GTP-binding protein [Streptomyces albulus]
320   5e-97
PLX13322.1 GTP-binding protein [Marinilabiliales bacterium]
319   5e-97
KUL36925.1 GTP-binding protein [Streptomyces sp. NRRL F-4489]
321   5e-97
SPL98248.1 Ribosome protection-type tetracycline resistance r...
322   6e-97

WP_051798217.1 tetracycline resistance ribosomal protection p...
320   6e-97
WP_011027352.1 tetracycline resistance ribosomal protection p...
320   6e-97
WP_024316514.1 GTP-binding protein [Rhizobium favelukesii]
320   7e-97
WP_045016004.1 GTP-binding protein [Agrobacterium arsenijevicii]
320   7e-97
AOS89055.1 TetM [Lactococcus garvieae]
304   7e-97
PKP50826.1 GTP-binding protein [Bacteroidetes bacterium HGW-B...
320   8e-97
WP_057612944.1 tetracycline resistance ribosomal protection p...
318   9e-97
WP_059204263.1 tetracycline resistance ribosomal protection p...
318   1e-96
WP_105429325.1 GTP-binding protein [Neorhizobium sp. T6_25]
319   1e-96
WP_027420549.1 GTP-binding protein [Crocinitomix catalasitica]
320   1e-96
WP_079064357.1 GTP-binding protein [Streptomyces sp. NRRL F-4...
321   1e-96
WP_107156361.1 GTP-binding protein [Micromonospora sp. RP3T]
319   1e-96
CAM59625.1 tetracycline resistance protein [Lactobacillus par...
307   1e-96
WP_099276621.1 GTP-binding protein [Rhodobacteraceae bacteriu...
315   1e-96
WP_046640980.1 GTP-binding protein [Neorhizobium galegae]
319   2e-96
AMJ37914.1 elongation factor G, domain IV [uncultured bacterium]
313   2e-96
SMH99378.1 translation elongation factor G [Enterococcus faec...
303   2e-96
WP_105434621.1 GTP-binding protein [Neorhizobium sp. T20_22]
318   2e-96
WP_010971555.1 MULTISPECIES: GTP-binding protein [Agrobacteri...
319   2e-96
WP_089253453.1 GTP-binding protein [Asanoa hainanensis]
319   2e-96
AMP55632.1 elongation factor Tu GTP binding domain protein [u...
303   2e-96
WP_020014263.1 GTP-binding protein [Promicromonospora sukumoe]
318   2e-96
WP_031175002.1 MULTISPECIES: GTP-binding protein [Streptomyces]
319   2e-96
EHL64011.1 small GTP-binding protein domain protein [Synergis...
318   2e-96
WP_062715834.1 tetracycline resistance ribosomal protection p...
318   2e-96
WP_099933415.1 tetracycline resistance ribosomal protection p...
317   3e-96
WP_009949137.1 GTP-binding protein [Saccharopolyspora erythraea]
317   3e-96

WP_071655982.1 GTP-binding protein [Streptomyces gilvigriseus]
318   3e-96
WP_105426565.1 GTP-binding protein [Neorhizobium sp. T17_20]
318   3e-96
WP_108015656.1 tetracycline resistance ribosomal protection p...
317   4e-96
CDZ57213.1 GTP-binding elongation factor protein, TetM/TetO [...
315   4e-96
WP_083829741.1 GTP-binding protein [Synergistes sp. 3_1_syn1]
318   4e-96
WP_106017159.1 GTP-binding protein [Enterococcus faecium]
303   4e-96
WP_080440097.1 GTP-binding protein [Enterococcus faecium]
302   5e-96
WP_029681450.1 MULTISPECIES: GTP-binding protein [Enterococcus]
302   5e-96
EPI26045.1 GTP-binding domain protein [Enterococcus faecium S...
303   5e-96
WP_053615648.1 GTP-binding protein [Nocardiopsis sp. NRRL B-1...
318   5e-96
AMP49519.1 elongation factor Tu GTP binding domain protein [u...
310   6e-96
EJX65240.1 GTP-binding domain protein [Enterococcus faecium P...
303   6e-96
WP_095938152.1 tetracycline resistance ribosomal protection p...
317   6e-96
WP_033651157.1 MULTISPECIES: GTP-binding protein [Enterococcus]
302   6e-96
WP_037196453.1 GTP-binding protein [Rhizobium sp. OK494]
317   7e-96
WP_037714257.1 tetracycline resistance ribosomal protection p...
316   7e-96
EJX82487.1 GTP-binding domain protein [Enterococcus faecium E...
302   7e-96
WP_033658535.1 MULTISPECIES: GTP-binding protein [Enterococcus]
302   8e-96
WP_105402079.1 GTP-binding protein [Neorhizobium sp. T7_12]
317   9e-96
ONN39273.1 hypothetical protein BTN91_17075 [Enterococcus fae...
302   9e-96
WP_026197042.1 GTP-binding protein [Sciscionella marina]
315   1e-95
SNT61137.1 ribosomal protection tetracycline resistance prote...
317   1e-95
CVK50870.1 tetracycline resistance protein TetM [Streptococcu...
301   2e-95
WP_062040124.1 tetracycline resistance ribosomal protection p...
315   2e-95
WP_037085515.1 GTP-binding protein [Rhizobium vignae]
316   2e-95
WP_044398207.1 GTP-binding protein [Lacinutrix sp. Hel_I_90]
316   2e-95
WP_049134477.1 tetracycline resistance ribosomal protection p...
309   3e-95

WP_087563939.1 GTP-binding protein [Polaribacter sp. SA4-10]
316   3e-95
WP_105436481.1 GTP-binding protein [Neorhizobium sp. T11_12]
315   4e-95
WP_063775610.1 GTP-binding protein [Actinoplanes rectilineatus]
315   4e-95
WP_046800259.1 MULTISPECIES: GTP-binding protein [Rhizobium/A...
315   4e-95
AUT30819.1 TetM [Enterococcus faecalis]
301   4e-95
OJX16406.1 GTP-binding protein [Devosia sp. 67-54]
315   4e-95
AOS89057.1 TetM [Lactococcus garvieae]
299   5e-95
WP_031046115.1 MULTISPECIES: tetracycline resistance ribosoma...
314   5e-95
WP_102919458.1 tetracycline resistance ribosomal protection p...
315   6e-95
WP_093455492.1 tetracycline resistance ribosomal protection p...
314   6e-95
WP_016325250.1 MULTISPECIES: tetracycline resistance ribosoma...
314   7e-95
WP_105154317.1 GTP-binding protein [Streptococcus suis]
300   7e-95
PCH76293.1 GTP-binding protein [Flavobacteriaceae bacterium]
315   7e-95
WP_045534099.1 MULTISPECIES: GTP-binding protein [Rhizobium]
315   8e-95
AMP49322.1 elongation factor Tu GTP binding domain protein [u...
299   1e-94
WP_095683712.1 GTP-binding protein [Streptomyces sp. CLI2509]
315   1e-94
WP_030607306.1 tetracycline resistance ribosomal protection p...
313   1e-94
WP_064831270.1 GTP-binding protein [Rhizobium phaseoli]
314   2e-94
WP_086829605.1 GTP-binding protein [Allokutzneria sp. NRRL B-...
312   2e-94
WP_073900276.1 GTP-binding protein [Saccharothrix sp. CB00851]
314   2e-94
WP_020117114.1 tetracycline resistance ribosomal protection p...
312   2e-94
BAF95548.1 tetracycline resistance protein [Bacillus sp. APFT...
298   2e-94
WP_007830218.1 GTP-binding protein [Streptomyces sp. Tu6071]
314   2e-94
WP_027735987.1 GTP-binding protein [Streptomyces sp. CNT360]
315   2e-94
WP_106504825.1 GTP-binding protein [Escherichia coli]
297   3e-94
ATU88011.1 TetM [Enterococcus faecalis]
300   3e-94
WP_078983558.1 tetracycline resistance ribosomal protection p...
313   3e-94

WP_084482346.1 GTP-binding protein [Cloacibacillus evryensis]
313   3e-94
WP_047555357.1 GTP-binding protein [Rhizobium leguminosarum]
313   3e-94
WP_002359507.1 GTP-binding protein [Enterococcus faecium]
298   4e-94
WP_034271652.1 GTP-binding protein [Actinospica robiniae]
311   4e-94
WP_089965059.1 GTP-binding protein [Clostridium gasigenes]
313   5e-94
WP_037738227.1 tetracycline resistance ribosomal protection p...
311   5e-94
ONN47801.1 elongation factor G [Enterococcus faecium]
296   6e-94
WP_093816482.1 GTP-binding protein [Streptomyces sp. TverLS-915]
313   6e-94
WP_093583229.1 GTP-binding protein [Streptomyces sp. LcepLS]
313   7e-94
WP_105614890.1 GTP-binding protein [Vallitalea sp. S15]
312   7e-94
WP_077469487.1 GTP-binding protein [Rhizobium sp. P44RR-XXIV]
312   7e-94
WP_033699270.1 GTP-binding protein [Enterococcus faecium]
297   9e-94
WP_046611450.1 GTP-binding protein [Neorhizobium galegae]
311   1e-93
WP_010267699.1 MULTISPECIES: GTP-binding protein [Streptomyces]
311   2e-93
WP_052842639.1 tetracycline resistance ribosomal protection p...
311   2e-93
WP_046603362.1 GTP-binding protein [Neorhizobium galegae]
311   2e-93
WP_063736000.1 tetracycline resistance ribosomal protection p...
310   2e-93
WP_093737480.1 GTP-binding protein [Streptomyces sp. DvalAA-14]
311   2e-93
WP_063854498.1 tetracycline resistance ribosomal protection p...
310   2e-93
SMJ68293.1 translation elongation factor G [Enterococcus faec...
296   2e-93
WP_049099151.1 tetracycline resistance protein tetM [Enteroco...
294   2e-93
WP_073764118.1 tetracycline resistance ribosomal protection p...
311   3e-93
AIA10937.1 elongation factor Tu GTP binding domain protein [u...
310   4e-93
WP_050503017.1 tetracycline resistance ribosomal protection p...
310   4e-93
PLW92762.1 GTP-binding protein [Marinilabiliales bacterium]
308   1e-92
WP_097271705.1 tetracycline resistance ribosomal protection p...
308   1e-92
WP_037948005.1 tetracycline resistance ribosomal protection p...
309   1e-92

WP_012484426.1 GTP-binding protein [Rhizobium etli]
309   1e-92
WP_090765475.1 GTP-binding protein [Nonomuraea maritima]
309   1e-92
WP_010726522.1 transposase [Enterococcus faecium]
292   1e-92
WP_079046514.1 GTP-binding protein [Streptococcus pneumoniae]
293   1e-92
AGP55678.1 tetracycline resistance protein tetM [Streptomyces...
309   1e-92
APO32513.1 ferrous iron transport protein B [uncultured bacte...
292   1e-92
AHK46947.1 peptide chain release factor 3 [Ensifer adhaerens ...
308   2e-92
SEG75335.1 ribosomal protection tetracycline resistance prote...
301   2e-92
AMP55645.1 elongation factor Tu domain 2 [uncultured bacterium]
291   2e-92
WP_092769184.1 MULTISPECIES: GTP-binding protein [Rhizobium]
308   2e-92
ACJ38543.1 tetracycline resistance protein [Trueperella pyoge...
294   3e-92
WP_069760792.1 tetracycline resistance ribosomal protection p...
307   3e-92
APO35566.1 ferrous iron transport protein B [uncultured bacte...
292   3e-92
WP_086882709.1 tetracycline resistance ribosomal protection p...
308   4e-92
WP_053752111.1 tetracycline resistance ribosomal protection p...
306   4e-92
WP_105439003.1 GTP-binding protein [Neorhizobium sp. T25_13]
307   4e-92
WP_106618717.1 GTP-binding protein [Saccharothrix carnea]
308   5e-92
WP_102910277.1 tetracycline resistance ribosomal protection p...
306   5e-92
WP_009715689.1 tetracycline resistance ribosomal protection p...
306   6e-92
WP_006312585.1 GTP-binding protein [Agrobacterium sp. ATCC 31...
307   6e-92
PLX23049.1 GTP-binding protein [Salinivirgaceae bacterium]
306   8e-92
PSL52798.1 ribosomal protection tetracycline resistance prote...
307   9e-92
WP_046630937.1 GTP-binding protein [Neorhizobium galegae]
306   1e-91
WP_044367550.1 tetracycline resistance ribosomal protection p...
306   2e-91
KFI82701.1 tetracycline resistance protein [Bifidobacterium r...
288   2e-91
WP_053923900.1 tetracycline resistance ribosomal protection p...
305   2e-91
WP_061440853.1 tetracycline resistance ribosomal protection p...
305   2e-91

WP_107478756.1 tetracycline resistance ribosomal protection p...
305   2e-91
PCI04775.1 GTP-binding protein [Flavobacteriaceae bacterium]
305   3e-91
WP_100045794.1 tetracycline resistance ribosomal protection p...
304   6e-91
WP_073728628.1 tetracycline resistance ribosomal protection p...
303   6e-91
WP_072664661.1 hypothetical protein [Escherichia coli]
288   6e-91
EEM59365.1 GTP-binding elongation factor protein, TetM/TetO [...
297   8e-91
AMP50125.1 elongation factor Tu GTP binding domain protein [u...
287   8e-91
PRX69439.1 ribosomal protection tetracycline resistance prote...
301   1e-90
WP_030185600.1 tetracycline resistance ribosomal protection p...
303   2e-90
WP_044571383.1 GTP-binding protein [Streptomyces iranensis]
303   3e-90
AOS89056.1 truncated TetM [Lactococcus garvieae]
286   5e-90
WP_084913470.1 GTP-binding protein [Streptococcus salivarius]
286   6e-90
WP_020539169.1 GTP-binding protein [Lewinella cohaerens]
302   6e-90
APO28710.1 feoB: ferrous iron transport protein [uncultured b...
285   8e-90
WP_030937601.1 tetracycline resistance ribosomal protection p...
300   1e-89
AMP50695.1 elongation factor G, domain IV [uncultured bacterium]
296   1e-89
AEN80267.1 TetM [Enterococcus sp. R41]
284   1e-89
OSC69059.1 GTP-binding protein [Streptomyces sp. 4F]
300   2e-89
WP_069571338.1 tetracycline resistance ribosomal protection p...
301   2e-89
WP_053745107.1 tetracycline resistance ribosomal protection p...
300   2e-89
AMP51655.1 elongation factor Tu domain 2 [uncultured bacterium]
285   2e-89
WP_087804965.1 tetracycline resistance ribosomal protection p...
300   3e-89
WP_018684401.1 GTP-binding protein [Actinokineospora enzanensis]
299   3e-89
AOS89058.1 truncated TetM [Lactococcus garvieae]
284   3e-89
KII54090.1 tetracycline resistance protein tetM [Enterococcus...
287   3e-89
ASQ96433.1 GTP-binding protein [Streptomyces sp. 11-1-2]
300   3e-89
WP_046256897.1 tetracycline resistance ribosomal protection p...
299   4e-89

WP_100806474.1 tetracycline resistance ribosomal protection p...
299   5e-89
WP_055421184.1 tetracycline resistance ribosomal protection p...
298   9e-89
WP_086667986.1 GTP-binding protein [Lentzea kentuckyensis]
295   1e-88
WP_107019526.1 tetracycline resistance ribosomal protection p...
298   1e-88
WP_014910024.1 GTP-binding protein [Nocardiopsis alba]
298   2e-88
EGG58291.1 GTP-binding domain protein [Enterococcus faecalis ...
282   2e-88
WP_103631420.1 GTP-binding protein [Bacillus thuringiensis]
291   2e-88
AEA35204.1 tetracycline resistance protein class S [unculture...
283   2e-88
WP_013229872.1 GTP-binding protein [Amycolatopsis mediterranei]
296   3e-88
WP_078643040.1 tetracycline resistance ribosomal protection p...
297   3e-88
WP_037348098.1 GTP-binding protein [Sciscionella sp. SE31]
295   3e-88
ADI48098.1 tetracycline-resistance protein [Clostridium perfr...
285   3e-88
KWW51894.1 tetracycline resistance protein [Bacillus cereus]
290   3e-88
WP_083107992.1 tetracycline resistance ribosomal protection p...
297   3e-88
PJN97650.1 elongation factor G [Enterococcus faecium]
281   3e-88
AEA35208.1 tetracycline resistance protein class S [unculture...
283   3e-88
WP_079258644.1 tetracycline resistance ribosomal protection p...
297   4e-88
WP_017533353.1 GTP-binding protein [Nocardiopsis alba]
297   4e-88
CJH79444.1 tetracycline resistance protein TetM [Streptococcu...
280   4e-88
WP_095583585.1 GTP-binding protein [Streptomyces albireticuli]
296   5e-88
WP_102937569.1 tetracycline resistance ribosomal protection p...
296   7e-88
WP_057327374.1 tetracycline resistance protein tetQ [Parabact...
290   9e-88
WP_079457349.1 hypothetical protein [Streptococcus agalactiae]
279   1e-87
CUQ43171.1 Tetracycline resistance protein tetM [Parabacteroi...
289   1e-87
KII55329.1 tetracycline resistance protein tetM [Enterococcus...
283   1e-87
WP_076972834.1 tetracycline resistance ribosomal protection p...
295   1e-87
CIV86160.1 tetracycline resistance protein TetM [Streptococcu...
279   2e-87

WP_020427483.1 GTP-binding protein [Paenibacillus riograndensis]
289   2e-87
AEA35206.1 tetracycline resistance protein class S [unculture...
281   2e-87
WP_046089663.1 GTP-binding protein [Streptomyces antioxidans]
296   2e-87
WP_104634297.1 tetracycline resistance ribosomal protection p...
295   2e-87
WP_100772166.1 GTP-binding protein [Bacillus cereus]
292   3e-87
WP_053210483.1 tetracycline resistance ribosomal protection p...
295   3e-87
WP_072418866.1 MULTISPECIES: GTP-binding protein [Ruminococcus]
300   3e-87
WP_059148850.1 GTP-binding protein [Streptomyces violaceusniger]
295   6e-87
WP_067443428.1 tetracycline resistance ribosomal protection p...
293   7e-87
PLX12895.1 GTP-binding protein [Marinilabiliales bacterium]
288   7e-87
WP_086710200.1 GTP-binding protein [Streptomyces castelarensis]
294   7e-87
WP_082404254.1 GTP-binding protein [Saccharothrix sp. NRRL B-...
294   7e-87
WP_059194672.1 tetracycline resistance ribosomal protection p...
293   8e-87
WP_033278915.1 tetracycline resistance ribosomal protection p...
293   9e-87
WP_053140772.1 tetracycline resistance ribosomal protection p...
293   9e-87
PIS15418.1 elongation factor G [Candidatus Shapirobacteria ba...
295   1e-86
AMP56071.1 elongation factor Tu GTP binding domain protein [u...
278   1e-86
SFP48736.1 ribosomal protection tetracycline resistance prote...
291   1e-86
WP_007389370.1 tetracycline resistance ribosomal protection p...
292   2e-86
WP_071422986.1 GTP-binding protein [Ruminococcaceae bacterium...
292   3e-86
WP_062133520.1 tetracycline resistance ribosomal protection p...
291   3e-86
KII52668.1 tetracycline resistance protein tetM [Enterococcus...
275   3e-86
WP_030043927.1 tetracycline resistance ribosomal protection p...
291   4e-86
WP_063483863.1 tetracycline resistance ribosomal protection p...
291   4e-86
WP_099014622.1 tetracycline resistance ribosomal protection p...
291   4e-86
WP_069455734.1 GTP-binding protein [Streptomyces griseus]
290   8e-86
CJH78357.1 tetracycline resistance protein TetM [Streptococcu...
277   8e-86

WP_077850345.1 GTP-binding protein [Enterococcus faecium]
276   8e-86
WP_043670310.1 tetracycline resistance ribosomal protection p...
290   1e-85
WP_069864988.1 tetracycline resistance ribosomal protection p...
290   1e-85
WP_059296825.1 tetracycline resistance ribosomal protection p...
290   1e-85
KPC76460.1 GTP-binding protein [Streptomyces sp. NRRL WC-3753]
290   1e-85
WP_079150242.1 GTP-binding protein [Streptomyces sp. NBRC 109...
292   1e-85
WP_049060605.1 GTP-binding protein [Enterococcus faecium]
275   2e-85
KOX19037.1 GTP-binding protein [Saccharothrix sp. NRRL B-16348]
290   2e-85
WP_051818878.1 tetracycline resistance ribosomal protection p...
290   2e-85
WP_064731132.1 tetracycline resistance ribosomal protection p...
288   3e-85
WP_092561486.1 GTP-binding protein [[Clostridium] populeti]
289   4e-85
CDE42291.1 tetracycline resistance protein TetO [Clostridium ...
272   6e-85
WP_087788470.1 tetracycline resistance ribosomal protection p...
288   6e-85
PJP85164.1 tetracycline resistance ribosomal protection prote...
273   6e-85
WP_066464840.1 GTP-binding protein [Caryophanon latum]
288   8e-85
AMP51281.1 hypothetical protein [uncultured bacterium]
272   8e-85
WP_079118662.1 GTP-binding protein [Streptococcus pneumoniae]
272   8e-85
WP_072830040.1 GTP-binding protein [Clostridium collagenovorans]
288   1e-84
WP_006380594.1 MULTISPECIES: tetracycline resistance ribosoma...
287   1e-84
WP_103337124.1 GTP-binding protein [Amycolatopsis sp. CA-126428]
286   1e-84
SCJ71024.1 Tetracycline resistance protein tetM from transpos...
271   1e-84
WP_066028268.1 GTP-binding protein [Streptomyces hygroscopicus]
290   1e-84
WP_087161275.1 GTP-binding protein [Lachnoclostridium sp. An169]
294   1e-84
WP_096270147.1 GTP-binding protein [Paucisalibacillus globulus]
288   1e-84
OWW57409.1 tetracycline resistance protein tetM [Enterococcus...
271   2e-84
WP_086851149.1 GTP-binding protein [Amycolatopsis kentuckyensis]
285   2e-84
WP_088775802.1 GTP-binding protein [Enterococcus hirae]
270   3e-84

WP_085803961.1 GTP-binding protein [Enterococcus faecium]
271   3e-84
WP_031015919.1 tetracycline resistance ribosomal protection p...
286   4e-84
AMP55647.1 elongation factor Tu GTP binding domain protein [u...
270   4e-84
AGU02289.1 TetO [Streptococcus uberis]
270   7e-84
WP_013624993.1 GTP-binding protein [Syntrophobotulus glycolicus]
286   7e-84
KKP61449.1 Elongation factor G [Candidatus Roizmanbacteria ba...
286   8e-84
PLQ02832.1 hypothetical protein CYQ99_15070 [Enterococcus fae...
269   8e-84
ANS48646.1 tetracycline resistance protein TetP [Bacillus thu...
283   1e-83
WP_089509560.1 GTP-binding protein [Streptomyces sp. NBS 14/10]
287   1e-83
ABK81676.1 TetS [Streptococcus uberis]
271   1e-83
WP_014054313.1 GTP-binding protein [Streptomyces violaceusniger]
286   1e-83
WP_028510304.1 GTP-binding protein [Ruminococcus sp. NK3A76]
290   1e-83
WP_013781759.1 elongation factor G [Mahella australiensis]
286   1e-83
ONI44112.1 hypothetical protein AN641_08275 [Epulopiscium sp....
290   1e-83
APO26817.1 Elongation factor G C-terminus [uncultured bacterium]
267   2e-83
WP_086875633.1 tetracycline resistance ribosomal protection p...
284   2e-83
WP_019327708.1 tetracycline resistance ribosomal protection p...
283   3e-83
PIV01874.1 elongation factor G [Candidatus Shapirobacteria ba...
285   4e-83
WP_100797582.1 GTP-binding protein [Viridibacillus sp. OK051]
283   5e-83
WP_105969624.1 tetracycline resistance ribosomal protection p...
283   5e-83
AEA35213.1 tetracycline resistance protein class O [unculture...
267   6e-83
PQW15828.1 elongation factor G [Enterococcus faecium]
267   7e-83
OGD96528.1 translation elongation factor G [Candidatus Curtis...
285   7e-83
WP_019057737.1 tetracycline resistance ribosomal protection p...
282   8e-83
GBD10978.1 Elongation factor G [bacterium HR23]
284   8e-83
WP_096918739.1 GTP-binding protein [Eubacterium sp. YI]
283   8e-83
WP_099204091.1 GTP-binding protein [Neglecta sp. Marseille-P3...
288   1e-82

OGE13101.1 translation elongation factor G [Candidatus Curtis...
284   1e-82
KKR58682.1 Elongation factor G [Candidatus Curtissbacteria ba...
284   1e-82
WP_088800563.1 MULTISPECIES: tetracycline resistance ribosoma...
282   1e-82
WP_002352610.1 GTP-binding protein [Enterococcus faecium]
267   1e-82
AGM39434.1 TetO [Staphylococcus aureus]
266   2e-82
WP_071312686.1 GTP-binding protein [Anaerobacillus arsenicise...
282   2e-82
WP_093491555.1 tetracycline resistance ribosomal protection p...
282   2e-82
WP_049932630.1 GTP-binding protein [Lachnospiraceae bacterium...
287   2e-82
ETP71420.1 small GTP-binding domain-containing protein [Lachn...
287   2e-82
WP_003059844.1 GTP-binding protein [Amycolatopsis vancoresmyc...
278   3e-82
WP_093643011.1 tetracycline resistance ribosomal protection p...
281   3e-82
ONI44590.1 hypothetical protein AN642_02200 [Epulopiscium sp....
286   3e-82
OGK18226.1 translation elongation factor G [Candidatus Roizma...
282   3e-82
WP_008860288.1 elongation factor G [Dialister succinatiphilus]
282   4e-82
PIZ64024.1 elongation factor G [Candidatus Roizmanbacteria ba...
282   4e-82
WP_058696196.1 GTP-binding protein [Eubacterium limosum]
281   5e-82
WP_074616473.1 GTP-binding protein [Eubacterium limosum]
281   6e-82
WP_092354504.1 GTP-binding protein [[Clostridium] cocleatum]
285   1e-81
KKQ01599.1 Elongation factor G [Candidatus Roizmanbacteria ba...
281   1e-81
WP_093623495.1 tetracycline resistance ribosomal protection p...
279   1e-81
WP_006420036.1 elongation factor G [delta proteobacterium Nap...
280   1e-81
WP_018659661.1 elongation factor G [Allofustis seminis]
281   2e-81
WP_031046532.1 tetracycline resistance ribosomal protection p...
279   2e-81
WP_102691995.1 GTP-binding protein [Rummeliibacillus pycnus]
279   2e-81
WP_093648431.1 tetracycline resistance ribosomal protection p...
279   2e-81
WP_094792268.1 tetracycline resistance ribosomal protection p...
280   2e-81
OGE71612.1 translation elongation factor G [Candidatus Davies...
280   3e-81

WP_108308693.1 elongation factor G [Thermodesulfobium acidiph...
280   3e-81
ABK81674.1 TetO [Streptococcus uberis]
263   3e-81
GBD28583.1 Elongation factor G [bacterium HR31]
280   3e-81
WP_029199713.1 GTP-binding protein [Oribacterium sp. NK2B42]
284   3e-81
PTQ51252.1 Translation elongation factor G [Brockia lithotrop...
280   3e-81
WP_093392979.1 elongation factor G [Thermodesulforhabdus norv...
280   3e-81
AAD48368.1 tetracycline resistance protein [Neisseria gonorrh...
266   3e-81
WP_087302683.1 GTP-binding protein [Erysipelatoclostridium sp...
283   3e-81
EKD84761.1 hypothetical protein ACD_38C00165G0018 [uncultured...
280   3e-81
WP_054645867.1 elongation factor G [Lactobacillus lindneri]
280   3e-81
WP_022777853.1 GTP-binding protein [Butyrivibrio sp. AE3009]
284   4e-81
WP_068719878.1 elongation factor G [Veillonellaceae bacterium...
280   4e-81
WP_052230178.1 GTP-binding protein [Streptomyces sp. CT34]
279   4e-81
WP_013755869.1 elongation factor G [Thermodesulfobium narugense]
280   4e-81
KII55114.1 tetracycline resistance protein tetM [Enterococcus...
262   4e-81
WP_072889071.1 GTP-binding protein [Ornithinibacillus halophi...
278   5e-81
CRH93428.1 Protein translation elongation factor G (EF-G) [Ch...
271   5e-81
WP_055148632.1 GTP-binding protein [[Ruminococcus] torques]
284   5e-81
KMP74408.1 tetracycline resistance protein [Bacillus cereus]
271   5e-81
WP_054405439.1 GTP-binding protein [Paenibacillus solani]
278   5e-81
WP_052856795.1 tetracycline resistance ribosomal protection p...
278   6e-81
WP_010098752.1 GTP-binding protein [Ornithinibacillus scaphar...
278   6e-81
WP_007070676.1 elongation factor G [Dialister invisus]
279   6e-81
WP_027088750.1 GTP-binding protein [[Clostridium] saccharogumia]
283   6e-81
CUQ91200.1 Tetracycline resistance protein tetM from transpos...
283   7e-81
WP_017262646.1 elongation factor G [Lactobacillus rossiae]
279   8e-81
WP_055173065.1 GTP-binding protein [[Ruminococcus] torques]
284   8e-81

WP_052848087.1 GTP-binding protein [Streptomyces avicenniae]
278   8e-81
WP_051733642.1 tetracycline resistance ribosomal protection p...
278   9e-81
WP_057880298.1 GTP-binding protein [Lactobacillus kimchiensis]
278   9e-81
CCZ53729.1 elongation factor G [Dialister invisus CAG:218]
278   9e-81
OGH10512.1 translation elongation factor G [Candidatus Levyba...
279   1e-80
WP_007391713.1 MULTISPECIES: elongation factor G [Megasphaera]
278   1e-80
WP_019068508.1 tetracycline resistance ribosomal protection p...
276   1e-80
WP_062106466.1 GTP-binding protein [Bacillus niameyensis]
277   1e-80
AIL24707.1 tetracycline-resistance protein TetO [Nocardia vet...
261   1e-80
WP_069679706.1 GTP-binding protein [Clostridium taeniosporum]
277   2e-80
WP_046325358.1 elongation factor G [Lactobacillus melliventris]
278   2e-80
WP_041101244.1 GTP-binding protein [Bacillus badius]
276   2e-80
WP_024722797.1 GTP-binding protein [Clostridiales bacterium V...
281   2e-80
WP_038353213.1 GTP-binding protein [Eubacterium limosum]
276   2e-80
WP_072536333.1 elongation factor G [Anaerococcus mediterranee...
278   2e-80
PIZ27478.1 elongation factor G [Candidatus Berkelbacteria bac...
278   2e-80
WP_072211623.1 elongation factor G [Candidatus Kryptobacter t...
278   2e-80
WP_075062243.1 elongation factor G [Ornatilinea apprima]
278   2e-80
WP_063442095.1 GTP-binding protein [Bacillus badius]
276   2e-80
WP_056979033.1 MULTISPECIES: elongation factor G [Lactobacillus]
278   2e-80
WP_090837612.1 elongation factor G [Paraburkholderia hospita]
277   2e-80
WP_013740885.1 elongation factor G [Selenomonas sputigena]
277   3e-80
WP_034572629.1 GTP-binding protein [Clostridiales bacterium o...
276   3e-80
WP_087285613.1 GTP-binding protein [[Clostridium] spiroforme]
281   3e-80
WP_042833882.1 elongation factor G [Thermoanaerobacter sp. YS13]
277   3e-80
CDC18888.1 putative uncharacterized protein [Eubacterium sp. ...
276   3e-80
WP_097073121.1 GTP-binding protein [Lysinibacillus xyleni]
276   3e-80

WP_066929768.1 GTP-binding protein [Streptomyces sp. NBRC 110...
277   3e-80
WP_099871253.1 tetracycline resistance ribosomal protection p...
276   3e-80
WP_057706172.1 GTP-binding protein [Lactobacillus xiangfangen...
276   3e-80
CDC15180.1 small GTP-binding protein domain [Ruminococcus sp....
282   3e-80
KKP60975.1 Elongation factor G [Candidatus Roizmanbacteria ba...
277   3e-80
WP_055157681.1 GTP-binding protein [[Ruminococcus] torques]
282   4e-80
WP_073386739.1 GTP-binding protein [Butyrivibrio fibrisolvens]
281   4e-80
WP_073346338.1 elongation factor G [Caldanaerobius fijiensis]
276   4e-80
AEA35217.1 tetracycline resistance protein class O [unculture...
260   4e-80
WP_010022706.1 elongation factor G [Lactobacillus fructivorans]
277   5e-80
WP_060203648.1 GTP-binding protein [Sporosarcina koreensis]
275   5e-80
WP_077193787.1 tetracycline resistance ribosomal protection p...
276   5e-80
KKQ59843.1 Elongation factor G [Candidatus Daviesbacteria bac...
276   5e-80
WP_008791700.1 GTP-binding protein [Coprobacillus sp. 8_2_54B...
280   5e-80
WP_093988072.1 GTP-binding protein [Massilimaliae timonensis]
275   6e-80
WP_090411765.1 GTP-binding protein [Eubacterium callanderi]
275   7e-80
WP_042148661.1 GTP-binding protein [Paucisalibacillus sp. EB02]
275   7e-80
ERI89773.1 putative translation elongation factor G [Clostrid...
275   7e-80
WP_053491222.1 GTP-binding protein [Bacillus sp. FJAT-18019]
275   7e-80
AIW80581.1 elongation factor Tu GTP binding domain protein [u...
261   7e-80
AUD72267.1 ribosomal protection tetracycline resistance prote...
260   8e-80
OGF26485.1 translation elongation factor G [Candidatus Falkow...
276   8e-80
WP_026486974.1 elongation factor G [Caldanaerobius polysaccha...
276   8e-80
WP_072892239.1 GTP-binding protein [Clostridium fallax]
275   8e-80
SCH35930.1 Tetracycline resistance protein tetM from transpos...
275   8e-80
WP_057888472.1 GTP-binding protein [Lactobacillus mindensis]
275   8e-80
WP_004609887.1 GTP-binding protein [[Clostridium] spiroforme]
280   8e-80

WP_081832666.1 GTP-binding protein [Oribacterium sp. P6A1]
280   9e-80
KKR72666.1 Elongation factor G [Candidatus Roizmanbacteria ba...
276   9e-80
WP_105326397.1 elongation factor G [Acidaminococcus sp. Marse...
276   9e-80
WP_087255436.1 GTP-binding protein [[Clostridium] spiroforme]
279   1e-79
SKC92731.1 translation elongation factor 2 (EF-2/EF-G) [Maled...
275   1e-79
WP_041114214.1 GTP-binding protein [Bacillus badius]
275   1e-79
AEA35215.1 tetracycline resistance protein class O [unculture...
259   1e-79
WP_078035884.1 GTP-binding protein [Oribacterium sp. C9]
280   1e-79
WP_079496076.1 elongation factor G [Maledivibacter halophilus]
276   1e-79
WP_087373948.1 GTP-binding protein [Erysipelatoclostridium sp...
279   1e-79
WP_009301056.1 GTP-binding protein [Coprobacillus sp. 3_3_56FAA]
279   2e-79
WP_009645153.1 elongation factor G [Selenomonas sp. CM52]
275   2e-79
OGE18986.1 translation elongation factor G [Candidatus Davies...
276   2e-79
WP_090567578.1 GTP-binding protein [Paenisporosarcina quisqui...
274   2e-79
WP_021283040.1 GTP-binding protein [Clostridium sp. BL8]
273   2e-79
PIR27235.1 elongation factor G [Candidatus Berkelbacteria bac...
275   2e-79
WP_009009888.1 GTP-binding protein [Coprobacillus sp. D7]
279   2e-79
WP_047133619.1 elongation factor G [Candidatus Kryptonium tho...
275   2e-79
WP_015164461.1 elongation factor G [Pseudanabaena sp. PCC 7367]
275   2e-79
WP_062189630.1 elongation factor G [Anaerolinea thermolimosa]
275   2e-79
CUU09042.1 translation elongation factor 2 (EF-2/EF-G) [Candi...
275   2e-79
WP_106779097.1 GTP-binding protein [Lysinibacillus sp. Marsei...
274   2e-79
OIN88790.1 translation elongation factor G [Candidatus Berkel...
275   2e-79
ABI95379.1 TetS [Lactococcus sp. LP-P1-2]
259   3e-79
CDC22708.1 putative uncharacterized protein [Clostridium nexi...
279   3e-79
WP_068550218.1 elongation factor G [Thermosulfidibacter takaii]
275   3e-79
OLA06806.1 translation elongation factor G [Eubacterium sp. 3...
279   3e-79

AEA35224.1 tetracycline resistance protein class O [unculture...
258   3e-79
WP_077847757.1 GTP-binding protein [Clostridium puniceum]
274   3e-79
WP_084092933.1 elongation factor G [Andreprevotia lacus]
274   3e-79
WP_074950875.1 elongation factor G [Alicyclobacillus macrospo...
274   4e-79
WP_096231733.1 elongation factor G [Thermoanaerobacterium sp....
274   4e-79
WP_031390650.1 GTP-binding protein [Kineothrix alysoides]
278   4e-79
WP_104438723.1 GTP-binding protein [Bacteroides xylanolyticus]
278   4e-79
WP_065547771.1 MULTISPECIES: GTP-binding protein [Lachnoclost...
279   5e-79
WP_009656098.1 elongation factor G [Selenomonas sp. FOBRC6]
274   5e-79
CBL41369.1 small GTP-binding protein domain [butyrate-produci...
279   5e-79
WP_044916722.1 GTP-binding protein [Oribacterium sp. FC2011]
278   5e-79
WP_045313550.1 GTP-binding protein [Lechevalieria aerocolonig...
269   5e-79
WP_029421883.1 elongation factor G [Alicyclobacillus macrospo...
274   5e-79
WP_037358720.1 elongation factor G [Selenomonas sp. oral taxo...
274   5e-79
OMG45220.1 translation elongation factor G [Paenibacillus mac...
272   5e-79
CBL25338.1 small GTP-binding protein domain [Ruminococcus tor...
278   6e-79
WP_049675302.1 elongation factor G [Desulfocarbo indianensis]
273   6e-79
WP_013382676.1 MULTISPECIES: GTP-binding protein [Clostridiales]
273   6e-79
WP_071120016.1 GTP-binding protein [Romboutsia timonensis]
273   6e-79
OJV65739.1 translation elongation factor G [Clostridiales bac...
273   6e-79
KKR33664.1 Elongation factor G [Candidatus Gottesmanbacteria ...
274   7e-79
WP_074663369.1 GTP-binding protein [[Clostridium] clostridiof...
278   7e-79
AEA35223.1 tetracycline resistance protein class O [unculture...
256   7e-79
AEA35218.1 tetracycline resistance protein class O [unculture...
256   7e-79
WP_047986014.1 GTP-binding protein [Ornithinibacillus califor...
272   8e-79
WP_069126086.1 elongation factor G [Candidatus Thiodiazotroph...
273   8e-79
WP_003537696.1 GTP-binding protein [Erysipelatoclostridium ra...
277   8e-79

ANC47979.1 TetO [Veillonella parvula]
256   8e-79
WP_084774746.1 tetracycline resistance ribosomal protection p...
272   8e-79
CDC10905.1 putative uncharacterized protein [Roseburia sp. CA...
277   9e-79
WP_063384009.1 GTP-binding protein [Bacillus badius]
272   9e-79
SFG35042.1 small GTP-binding protein domain-containing protei...
278   9e-79
WP_009166711.1 elongation factor G [Lactobacillus florum]
273   9e-79
WP_004612550.1 MULTISPECIES: GTP-binding protein [Tyzzerella]
278   1e-78
WP_091265309.1 elongation factor G [Alkalibacterium thalassium]
273   1e-78
WP_087740010.1 elongation factor G [Paraburkholderia piptaden...
273   1e-78
WP_007556212.1 elongation factor G [Dialister micraerophilus]
273   1e-78
WP_018088912.1 tetracycline resistance ribosomal protection p...
272   1e-78
PLQ10613.1 elongation factor G [Enterococcus faecium]
256   1e-78
WP_076326457.1 GTP-binding protein [Paenibacillus lautus]
272   1e-78
WP_009756995.1 GTP-binding protein [Lachnospiraceae bacterium...
277   1e-78
KRT69044.1 fusA-3, elongation factor G, elongation factor G [...
273   1e-78
WP_040330363.1 GTP-binding protein [Clostridium ihumii]
272   1e-78
WP_093495490.1 GTP-binding protein [Psychrobacillus psychrodu...
271   1e-78
WP_022201621.1 GTP-binding protein [Clostridium sp. C105KSO14]
278   1e-78
WP_008300674.1 GTP-binding protein [Bhargavaea cecembensis]
271   1e-78
AMP46962.1 elongation factor Tu domain 2 [uncultured bacterium]
259   1e-78
WP_002587585.1 GTP-binding protein [[Clostridium] clostridiof...
278   1e-78
WP_067204196.1 GTP-binding protein [Sporosarcina psychrophila]
271   1e-78
WP_006555650.1 elongation factor G [Veillonella seminalis]
273   1e-78
WP_057571551.1 GTP-binding protein [[Clostridium] clostridiof...
278   1e-78
WP_091205828.1 GTP-binding protein [Oribacterium sp. WCC10]
277   1e-78
WP_003525381.1 GTP-binding protein [[Clostridium] clostridiof...
278   1e-78
WP_027640156.1 GTP-binding protein [Clostridium cadaveris]
271   1e-78

OFW63305.1 translation elongation factor G [Actinobacteria ba...
273   2e-78
WP_057805226.1 GTP-binding protein [Pediococcus ethanolidurans]
271   2e-78
WP_039957433.1 elongation factor G [Selenomonas infelix]
272   2e-78
WP_057800125.1 elongation factor G [Lactobacillus kalixensis]
273   2e-78
APO66172.1 translation elongation factor/tetracycline resista...
269   2e-78
WP_069181131.1 elongation factor G [Selenomonas sp. oral taxo...
272   2e-78
WP_013705453.1 elongation factor G [Desulfobacca acetoxidans]
272   2e-78
WP_090815323.1 GTP-binding protein [Oribacterium sp. KHPX15]
276   2e-78
WP_021638349.1 MULTISPECIES: GTP-binding protein [Clostridiales]
277   2e-78
WP_071354284.1 GTP-binding protein [Bacillus sp. MUM 116]
271   2e-78
WP_059075190.1 GTP-binding protein [Lactobacillus farciminis]
271   2e-78
WP_028043497.1 GTP-binding protein [Candidatus Stoquefichus m...
276   2e-78
WP_006307576.1 elongation factor G [Centipeda periodontii]
272   3e-78
WP_066846696.1 elongation factor G [Selenomonas sp. oral taxo...
272   3e-78
WP_026886519.1 GTP-binding protein [Clostridium beijerinckii]
271   3e-78
SCJ80828.1 Tetracycline resistance protein tetM from transpos...
276   3e-78
AKP03759.1 elongation factor G [Lactobacillus farciminis]
271   3e-78
WP_054648241.1 elongation factor G [Desulfovibrio brasiliensis]
271   3e-78
WP_006213006.1 GTP-binding protein [Paenibacillus vortex]
271   3e-78
WP_022753643.1 GTP-binding protein [Butyrivibrio fibrisolvens]
276   3e-78
WP_062353716.1 GTP-binding protein [Bacillus kwashiorkori]
271   3e-78
AMP50723.1 elongation factor G, domain IV [uncultured bacterium]
263   3e-78
WP_024544870.1 MULTISPECIES: elongation factor G [Synechococcus]
272   3e-78
WP_077533798.1 GTP-binding protein [Massilimaliae massiliensis]
271   3e-78
WP_102889573.1 elongation factor G [Megasphaera genomosp. typ...
272   3e-78
CDD80643.1 elongation factor G [Dialister sp. CAG:357]
272   3e-78
WP_093062393.1 GTP-binding protein [Psychrobacillus sp. OK028]
271   3e-78

WP_007555316.1 elongation factor G [Dialister micraerophilus]
272   3e-78
WP_100540888.1 GTP-binding protein [Paenibacillus sp. GM2FR]
271   3e-78
WP_002564819.1 GTP-binding protein [[Clostridium] bolteae]
276   3e-78
WP_024728790.1 GTP-binding protein [Anaerostipes sp. BG01]
276   3e-78
WP_002572577.1 GTP-binding protein [[Clostridium] bolteae]
276   3e-78
KKS84649.1 Elongation factor G [Candidatus Gottesmanbacteria ...
272   3e-78
WP_006695836.1 elongation factor G [Selenomonas noxia]
271   3e-78
AMP54817.1 elongation factor G, domain IV [uncultured bacterium]
263   4e-78
WP_027714011.1 elongation factor G [Desulfuromonas sp. TF]
271   4e-78
OGG05096.1 translation elongation factor G [Candidatus Gottes...
271   4e-78
SCH47786.1 Tetracycline resistance protein tetM [uncultured R...
275   4e-78
WP_007392634.1 elongation factor G [Megasphaera sp. UPII 135-E]
271   4e-78
GBC85303.1 Elongation factor G [bacterium HR11]
271   4e-78
OGD92110.1 translation elongation factor G [Candidatus Curtis...
272   4e-78
WP_022757233.1 GTP-binding protein [Butyrivibrio fibrisolvens]
276   4e-78
WP_059364073.1 elongation factor G [Veillonella tobetsuensis]
271   4e-78
WP_011668502.1 elongation factor G [Lactobacillus brevis]
271   4e-78
WP_027415326.1 elongation factor G [Aneurinibacillus terranov...
271   4e-78
OIO06013.1 translation elongation factor G [Candidatus Falkow...
271   4e-78
WP_061776008.1 elongation factor G [Lactobacillus senmaizukei]
271   4e-78
WP_074925007.1 GTP-binding protein [[Clostridium] clostridiof...
276   4e-78
OGL05271.1 translation elongation factor G [Candidatus Rokuba...
271   4e-78
CDC81560.1 small GTP-binding protein domain [Clostridium sp. ...
275   4e-78
WP_106626491.1 elongation factor G [Selenomonas massiliensis]
271   4e-78
AMP55462.1 elongation factor Tu GTP binding domain protein [u...
254   4e-78
SCI32144.1 Tetracycline resistance protein tetM from transpos...
276   4e-78
WP_093042785.1 GTP-binding protein [Ruminococcaceae bacterium...
275   4e-78

WP_034491140.1 GTP-binding protein [Butyrivibrio fibrisolvens]
276   4e-78
WP_108477457.1 elongation factor G [Lactobacillus brevis]
271   5e-78
WP_067403399.1 GTP-binding protein [Sporosarcina sp. HYO08]
270   5e-78
WP_035913850.1 GTP-binding protein [Lechevalieria aerocolonig...
267   5e-78
CUM79245.1 Tetracycline resistance protein tetM [[Ruminococcu...
276   5e-78
OGE25985.1 translation elongation factor G [Candidatus Davies...
272   5e-78
WP_076153330.1 GTP-binding protein [Paenibacillus glucanolyti...
270   5e-78
WP_034489465.1 GTP-binding protein [Butyrivibrio fibrisolvens]
276   5e-78
WP_106459859.1 elongation factor G [Anaerococcus sp. Marseill...
271   6e-78
WP_009729158.1 elongation factor G [Selenomonas sp. F0473]
271   6e-78
WP_074758725.1 GTP-binding protein [Butyrivibrio fibrisolvens]
276   6e-78
WP_079413367.1 elongation factor G [[Clostridium] thermoalcal...
271   6e-78
WP_063477964.1 GTP-binding protein [Paenibacillus glucanolyti...
270   6e-78
OLA04425.1 translation elongation factor G [Clostridiales bac...
276   6e-78
EJX57124.1 elongation factor G [Enterococcus faecium R497]
253   6e-78
OGG07316.1 translation elongation factor G [Candidatus Gottes...
271   6e-78
WP_055109419.1 GTP-binding protein [Paenibacillus ihumii]
270   7e-78
WP_084113261.1 GTP-binding protein [Clostridium acidisoli]
270   7e-78
WP_015009809.1 GTP-binding protein [Amphibacillus xylanus]
270   7e-78
WP_057815988.1 GTP-binding protein [Lactobacillus futsaii]
270   7e-78
WP_075759551.1 GTP-binding protein [Anaerostipes sp. 494a]
275   7e-78
OGG12299.1 translation elongation factor G [Candidatus Gottes...
271   7e-78
WP_107925658.1 GTP-binding protein [Lysinibacillus parviboron...
270   7e-78
WP_034484646.1 GTP-binding protein [Butyrivibrio fibrisolvens]
275   7e-78
OGK23396.1 translation elongation factor G [Candidatus Roizma...
271   8e-78
SCH85645.1 Vegetative protein 19 [uncultured Eubacterium sp.]
275   8e-78
WP_056831315.1 GTP-binding protein [Psychrobacillus sp. FJAT-...
269   8e-78

OON97347.1 hypothetical protein ATN31_08270 [Epulopiscium sp....
274   8e-78
WP_027216775.1 GTP-binding protein [Butyrivibrio fibrisolvens]
275   8e-78
WP_035792592.1 GTP-binding protein [Clostridium botulinum]
270   8e-78
WP_024526561.1 elongation factor G [Lactobacillus brevis]
271   1e-77
WP_021742678.1 elongation factor G [Lactobacillus brevis]
271   1e-77
ABB70063.1 TetS [Lactococcus lactis]
255   1e-77
OGG33099.1 translation elongation factor G [Candidatus Gottes...
271   1e-77
SLL32007.1 translation elongation factor 2 (EF-2/EF-G) [Mycob...
269   1e-77
WP_087094260.1 elongation factor G [Lactobacillus brevis]
270   1e-77
WP_043022653.1 elongation factor G [Lactobacillus brevis]
270   1e-77
PIQ72936.1 elongation factor G [Candidatus Roizmanbacteria ba...
270   1e-77
WP_044505176.1 elongation factor G [Megasphaera massiliensis]
270   1e-77
PUU86428.1 elongation factor G [Halanaerobium sp.]
270   1e-77
WP_066721154.1 GTP-binding protein [Clostridium sp. Marseille...
274   1e-77
PIR20472.1 elongation factor G [Deltaproteobacteria bacterium...
270   1e-77
WP_035586554.1 elongation factor G [Hippea jasoniae]
270   1e-77
WP_091481990.1 elongation factor G [Alkalibacterium pelagium]
270   1e-77
WP_073297585.1 elongation factor G [Atopostipes suicloacalis]
270   1e-77
WP_033159693.1 elongation factor G [Mycoplasma alvi]
270   1e-77
WP_010619587.1 elongation factor G [Lactobacillus maleferment...
270   1e-77
WP_081834113.1 GTP-binding protein [Lachnospiraceae bacterium...
274   1e-77
WP_056966148.1 elongation factor G [Lactobacillus ozensis]
270   1e-77
OKY72037.1 translation elongation factor G [Limnothrix sp. CA...
270   1e-77
WP_015797864.1 elongation factor G [Acidimicrobium ferrooxidans]
270   2e-77
WP_072621274.1 elongation factor G [Spirulina major]
270   2e-77
WP_062413030.1 elongation factor G [Megasphaera sp. MJR8396C]
270   2e-77
WP_036639869.1 MULTISPECIES: GTP-binding protein [Paenibacillus]
269   2e-77

WP_066678229.1 GTP-binding protein [Clostridium septicum]
269   2e-77
WP_090164669.1 elongation factor G [Eubacterium pyruvativorans]
270   2e-77
OGJ88160.1 translation elongation factor G [Candidatus Raymon...
270   2e-77
WP_019228643.1 GTP-binding protein [Sedimentibacter sp. B4]
269   2e-77
WP_092055083.1 GTP-binding protein [Bhargavaea ginsengi]
268   2e-77
WP_011375452.1 elongation factor G [Lactobacillus sakei]
270   2e-77
OGE62981.1 translation elongation factor G [Candidatus Davies...
270   2e-77
EEL50311.1 GTP-binding elongation factor protein, TetM/TetO [...
258   2e-77
EFM70282.1 GTP-binding domain protein [Enterococcus faecalis ...
252   2e-77
KXA66863.1 translation elongation factor G [Megasphaera sp. M...
270   2e-77
WP_099320988.1 elongation factor G [Anaerococcus sp. Marseill...
270   2e-77
WP_065360576.1 elongation factor G [Megasphaera sp. DISK 18]
270   2e-77
WP_105092679.1 elongation factor G [Veillonella tobetsuensis]
270   2e-77
WP_044922697.1 GTP-binding protein [Lachnospiraceae bacterium...
273   2e-77
WP_078766419.1 GTP-binding protein [Eubacterium uniforme]
274   2e-77
CDB18463.1 putative translation elongation factor G [Eubacter...
274   2e-77
WP_036487778.1 elongation factor G [Myxosarcina sp. GI1]
270   2e-77
WP_014256908.1 GTP-binding protein [[Clostridium] clariflavum]
273   2e-77
WP_044149654.1 GTP-binding protein [Epulopiscium sp. 'N.t. mo...
273   2e-77
WP_106056138.1 elongation factor G [Mogibacterium diversum]
270   2e-77
WP_013537013.1 elongation factor G [Thermovibrio ammonificans]
270   2e-77
OGU19087.1 translation elongation factor G [Ignavibacteria ba...
270   2e-77
WP_049660171.1 GTP-binding protein [Bacillus sp. FJAT-27231]
268   2e-77
WP_050638909.1 GTP-binding protein [Eubacterium sp. SB2]
274   2e-77
WP_107950795.1 GTP-binding protein [Lysinibacillus parviboron...
268   2e-77
WP_093537945.1 GTP-binding protein [Psychrobacillus psychroto...
268   2e-77
WP_055155184.1 GTP-binding protein [Faecalicatena contorta]
274   2e-77

WP_057829630.1 elongation factor G [Lactobacillus cacaonum]
270   2e-77
WP_098548384.1 GTP-binding protein [Bacillus megaterium]
268   2e-77
WP_092613280.1 elongation factor G [Raineyella antarctica]
270   3e-77
EEX77972.1 translation elongation factor G [Selenomonas sputi...
269   3e-77
WP_037346671.1 elongation factor G [Selenomonas sp. oral taxo...
269   3e-77
WP_068658173.1 GTP-binding protein [Paenibacillus crassostreae]
268   3e-77
WP_077391271.1 elongation factor G [Mobilibacterium timonense]
269   3e-77
KKS45459.1 Elongation factor G [Candidatus Gottesmanbacteria ...
270   3e-77
WP_060929288.1 elongation factor G [Anaerococcus tetradius]
269   3e-77
WP_004837180.1 elongation factor G [Anaerococcus tetradius]
269   3e-77
WP_035779163.1 GTP-binding protein [Butyrivibrio sp. MC2013]
274   3e-77
WP_035781202.1 GTP-binding protein [Butyrivibrio sp. LB2008]
274   3e-77
WP_009353908.1 MULTISPECIES: elongation factor G [Veillonella]
269   3e-77
WP_016265721.1 elongation factor G [Lactobacillus sakei]
269   3e-77
WP_096212298.1 tetracycline resistance ribosomal protection p...
268   3e-77
WP_049458103.1 elongation factor G [Stenotrophomonas maltophi...
269   3e-77
WP_026905861.1 GTP-binding protein [Paucisalibacillus globulus]
268   3e-77
WP_057886466.1 elongation factor G [Lactobacillus nagelii]
269   3e-77
WP_105083569.1 MULTISPECIES: elongation factor G [Veillonella]
269   4e-77
OIO97028.1 translation elongation factor G [Anaerolineae bact...
269   4e-77
WP_004828288.1 elongation factor G [Anaerococcus lactolyticus]
269   4e-77
OQY07155.1 elongation factor G [Desulfobacteraceae bacterium ...
268   4e-77
KRM90069.1 elongation factor G [Lactobacillus cacaonum DSM 21...
269   4e-77
WP_013559426.1 elongation factor G [Anaerolinea thermophila]
269   4e-77
WP_067978349.1 elongation factor G [Aerococcus urinaehominis]
269   4e-77
WP_009753871.1 MULTISPECIES: elongation factor G [Selenomonas]
269   4e-77
WP_029739039.1 MULTISPECIES: GTP-binding protein [Enterococcus]
251   4e-77

WP_092041823.1 GTP-binding protein [Butyrivibrio sp. TB]
273   4e-77
WP_057769089.1 GTP-binding protein [Lactobacillus selangorensis]
268   4e-77
WP_099798388.1 elongation factor G [Synechococcus lividus]
269   4e-77
WP_038153000.1 elongation factor G [Veillonella montpellieren...
269   4e-77
WP_086043510.1 elongation factor G [Macrococcus canis]
269   4e-77
WP_015393117.1 GTP-binding protein [Clostridium saccharoperbu...
268   4e-77
WP_053591227.1 GTP-binding protein [Bacillus sp. FJAT-22090]
267   4e-77
OGC60812.1 translation elongation factor G [candidate divisio...
269   5e-77
OUW95936.1 elongation factor G [Chloroflexi bacterium TMED230]
269   5e-77
WP_084139632.1 GTP-binding protein [Lachnospiraceae bacterium...
273   5e-77
KKS76370.1 Elongation factor G [Candidatus Gottesmanbacteria ...
270   5e-77
WP_016476503.1 elongation factor G [Veillonella sp. HPA0037]
268   5e-77
WP_004270213.1 elongation factor G [Lactobacillus curvatus]
269   5e-77
WP_093485991.1 MULTISPECIES: tetracycline resistance ribosoma...
267   5e-77
WP_066390093.1 elongation factor G [Bacillus mesonae]
268   5e-77
WP_094673072.1 elongation factor G [Hydrocoleum sp. CS-953]
269   5e-77
WP_025086707.1 elongation factor G [Lactobacillus apodemi]
268   5e-77
WP_075577029.1 GTP-binding protein [Olsenella sp. Marseille-P...
268   5e-77
WP_098749161.1 GTP-binding protein [Paenibacillus sp. EZ-K15]
267   5e-77
WP_023965562.1 GTP-binding protein [Paenibacillus sp. JCM 10914]
267   5e-77
WP_014431694.1 elongation factor G [Caldilinea aerophila]
268   5e-77
WP_015739499.1 elongation factor G [Ammonifex degensii]
268   5e-77
WP_105176556.1 GTP-binding protein [Clostridium sp. Marseille...
267   6e-77
PIS25265.1 elongation factor G [bacterium (Candidatus Stahlba...
268   6e-77
WP_072968773.1 elongation factor G [Caminicella sporogenes]
268   6e-77
WP_087238449.1 GTP-binding protein [Anaeromassilibacillus sp....
268   6e-77
WP_029332256.1 MULTISPECIES: elongation factor G [Exiguobacte...
268   6e-77

WP_056945156.1 GTP-binding protein [Lactobacillus farciminis]
267   6e-77
WP_005377031.1 MULTISPECIES: elongation factor G [Veillonella]
268   6e-77
SCI33380.1 Vegetative protein 19 [uncultured Eubacterium sp.]
272   6e-77
WP_057810434.1 elongation factor G [Lactobacillus siliginis]
268   6e-77
WP_010019535.1 GTP-binding protein [Lactobacillus farciminis]
267   6e-77
PJE63980.1 elongation factor G [Candidatus Roizmanbacteria ba...
269   6e-77
WP_099532980.1 elongation factor G [Limnothrix sp. PR1529]
268   6e-77
WP_010580238.1 elongation factor G [Lactobacillus vini]
268   6e-77
WP_098277897.1 GTP-binding protein [Bacillus megaterium]
267   6e-77
WP_011916517.1 elongation factor G [Caldicellulosiruptor sacc...
268   7e-77
WP_086662059.1 elongation factor G [Lentzea kentuckyensis]
268   7e-77
WP_013433112.1 MULTISPECIES: elongation factor G [Caldicellul...
268   7e-77
WP_009016333.1 MULTISPECIES: elongation factor G [Acidaminoco...
268   7e-77
EEU99562.1 putative translation elongation factor G [Roseburi...
271   7e-77
WP_045165672.1 elongation factor G [Caldicellulosiruptor naga...
268   7e-77
WP_013411632.1 elongation factor G [Caldicellulosiruptor owen...
268   7e-77
WP_096077876.1 elongation factor G [Macrococcus sp. IME1552]
268   7e-77
WP_098600495.1 GTP-binding protein [Bacillus megaterium]
267   8e-77
WP_065713057.1 elongation factor G [Synechococcus sp. PCC 7003]
268   8e-77
WP_006567674.1 MULTISPECIES: GTP-binding protein [Anaerostipes]
272   8e-77
WP_023859682.1 elongation factor G [Lactobacillus equi]
268   8e-77
WP_005345641.1 GTP-binding protein [[Eubacterium] hallii]
272   8e-77
WP_038038870.1 elongation factor G [Thermorudis peleae]
268   8e-77
AMJ37807.1 elongation factor Tu GTP binding domain protein [u...
250   8e-77
WP_097010996.1 elongation factor G [Pseudodesulfovibrio profu...
268   8e-77
AMP55644.1 elongation factor G C-terminus [uncultured bacterium]
249   8e-77
OPX33626.1 translation elongation factor G [Desulfobacteracea...
268   8e-77

KUK84206.1 translation elongation factor G [Microgenomates ba...
268   8e-77
WP_066547691.1 GTP-binding protein [Caryophanon tenue]
267   8e-77
WP_095299362.1 GTP-binding protein [Bacillus sp. 7586-K]
267   8e-77
WP_091363128.1 elongation factor G [Allisonella histaminiform...
268   9e-77
WP_025306585.1 elongation factor G [Thermocrinis ruber]
268   9e-77
CUN86194.1 Vegetative protein 19 [[Eubacterium] hallii]
272   9e-77
PKN81822.1 elongation factor G [Chloroflexi bacterium HGW-Chl...
268   9e-77
WP_057908165.1 elongation factor G [Lactobacillus graminis]
268   9e-77
WP_015225447.1 elongation factor G [Halothece sp. PCC 7418]
268   1e-76
WP_095132221.1 GTP-binding protein [Anaeromicrobium sediminis]
266   1e-76
KXB86033.1 translation elongation factor G [Veillonella dispar]
268   1e-76
WP_013430774.1 elongation factor G [Caldicellulosiruptor kron...
268   1e-76
WP_005385490.1 elongation factor G [Veillonella dispar]
268   1e-76
WP_098901042.1 GTP-binding protein [Bacillus megaterium]
267   1e-76
WP_092246829.1 GTP-binding protein [Butyrivibrio sp. INlla21]
272   1e-76
WP_040773669.1 elongation factor G [Megasphaera micronuciformis]
268   1e-76
WP_091761011.1 elongation factor G [Marinilactibacillus psych...
268   1e-76
WP_068781520.1 GTP-binding protein [Paenibacillus sp. GM2]
267   1e-76
WP_021876179.1 GTP-binding protein [Clostridium chauvoei]
266   1e-76
PIY93887.1 elongation factor G [Candidatus Levybacteria bacte...
268   1e-76
WP_035354112.1 elongation factor G [Bacillus ginsengihumi]
268   1e-76
WP_012422849.1 GTP-binding protein [Clostridium botulinum]
266   1e-76
WP_039235715.1 GTP-binding protein [Quasibacillus thermotoler...
266   1e-76
PIV11069.1 elongation factor G [Candidatus Roizmanbacteria ba...
268   1e-76
WP_096145335.1 GTP-binding protein [Clostridium chauvoei]
266   1e-76
CCX55647.1 elongation factor G [Veillonella sp. CAG:933]
268   1e-76
SCH92033.1 Tetracycline resistance protein tetM from transpos...
273   1e-76

KXB84505.1 translation elongation factor G [Veillonella parvula]
268   1e-76
WP_099577392.1 elongation factor G [Macrococcus goetzii]
268   1e-76
SCI31423.1 Tetracycline resistance protein tetM [uncultured C...
272   1e-76
WP_077363227.1 GTP-binding protein [Clostridium saccharoperbu...
267   1e-76
WP_015907356.1 elongation factor G [Caldicellulosiruptor bescii]
267   1e-76
WP_068623488.1 MULTISPECIES: elongation factor G [Trichococcus]
268   1e-76
WP_069176737.1 elongation factor G [Dialister pneumosintes]
267   1e-76
WP_004693557.1 MULTISPECIES: elongation factor G [Veillonella]
267   1e-76
WP_077839457.1 GTP-binding protein [Clostridium beijerinckii]
267   1e-76
CDA57645.1 small GTP-binding protein domain [Roseburia intest...
272   1e-76
WP_009657363.1 MULTISPECIES: elongation factor G [Selenomonas]
267   1e-76
WP_015737677.1 GTP-binding protein [Paenibacillus sp. Y412MC10]
266   1e-76
CED94179.1 Tetracycline resistance protein TetP [Romboutsia i...
266   2e-76
WP_008714945.1 elongation factor G [Veillonella sp. 3_1_44]
267   2e-76
WP_083624950.1 GTP-binding protein [Roseburia sp. 831b]
271   2e-76
WP_067846524.1 elongation factor G [Alicyclobacillus mali]
267   2e-76
WP_077842625.1 GTP-binding protein [Clostridium beijerinckii]
267   2e-76
PIP74576.1 elongation factor G [Candidatus Levybacteria bacte...
267   2e-76
WP_071274296.1 GTP-binding protein [Bacillus aryabhattai]
266   2e-76
WP_098325311.1 GTP-binding protein [Bacillus megaterium]
266   2e-76
ARF99847.1 elongation factor G [Veillonella atypica]
267   2e-76
WP_045574719.1 GTP-binding protein [Desulfosporosinus sp. I2]
271   2e-76
WP_087058384.1 elongation factor G [Marinilactibacillus psych...
267   2e-76
CBL14198.1 small GTP-binding protein domain [Roseburia intest...
271   2e-76
OLR40953.1 translation elongation factor G [Roseburia sp. 831b]
271   2e-76
CUN04340.1 Vegetative protein 19 [[Eubacterium] hallii]
271   2e-76
WP_070430142.1 MULTISPECIES: elongation factor G [Aerococcus]
267   2e-76

OGL66124.1 translation elongation factor G [Candidatus Uhrbac...
267   2e-76
WP_039307741.1 GTP-binding protein [Clostridium botulinum]
266   2e-76
WP_055182960.1 GTP-binding protein [[Eubacterium] hallii]
271   2e-76
CNJ03978.1 tetracycline resistance protein TetM [Streptococcu...
249   2e-76
WP_028257523.1 elongation factor G [Veillonella montpellieren...
267   2e-76
WP_038092251.1 elongation factor G [Tumebacillus flagellatus]
267   2e-76
WP_031434859.1 elongation factor G [Methylomarinum vadi]
267   2e-76
PKM75866.1 elongation factor G [Firmicutes bacterium HGW-Firm...
267   2e-76
WP_086439865.1 elongation factor G [Lactobacillus fermentum]
267   2e-76
WP_012065444.1 elongation factor G [Alkaliphilus metalliredig...
267   2e-76
WP_020310057.1 MULTISPECIES: elongation factor G [Megasphaera]
267   2e-76
WP_008976776.1 GTP-binding protein [Lachnospiraceae bacterium...
271   2e-76
WP_026573398.1 GTP-binding protein [Bacillus sp. UNC438CL73Ts...
266   2e-76
WP_090096306.1 elongation factor G [Lentzea jiangxiensis]
266   2e-76
WP_069998086.1 GTP-binding protein [Cellulosilyticum sp. I15G...
271   2e-76
WP_086335294.1 elongation factor G [Enterococcus mundtii]
267   2e-76
WP_025270584.1 elongation factor G [Hippea sp. KM1]
267   2e-76
WP_010859228.1 GTP-binding protein [Lysinibacillus sphaericus]
266   2e-76
WP_107933357.1 GTP-binding protein [Lysinibacillus chungkukja...
266   2e-76
WP_045317523.1 elongation factor G [Lechevalieria aerocolonig...
266   2e-76
WP_015639813.1 GTP-binding protein [Lactobacillus plantarum]
266   2e-76
SCG94959.1 Tetracycline resistance protein tetM from transpos...
271   2e-76
WP_017352507.1 GTP-binding protein [Clostridium botulinum]
266   2e-76
OGK57427.1 translation elongation factor G [Candidatus Roizma...
267   2e-76
WP_073152663.1 elongation factor G [Paramaledivibacter camini...
266   2e-76
OGD26070.1 translation elongation factor G [Candidatus Aminic...
267   2e-76
OGK19650.1 translation elongation factor G [Candidatus Roizma...
267   2e-76

WP_095066420.1 elongation factor G [Veillonella rodentium]
266   2e-76
WP_057243523.1 GTP-binding protein [Bacillus sp. Root239]
266   2e-76
GBD22370.1 Elongation factor G [bacterium HR28]
267   2e-76
ABO87316.1 TetO [Streptococcus pyogenes]
252   2e-76
WP_003870266.1 MULTISPECIES: elongation factor G [Thermoanaer...
266   3e-76
SDZ76817.1 small GTP-binding protein domain-containing protei...
271   3e-76
WP_022792003.1 elongation factor G [Weissella halotolerans]
267   3e-76
WP_055193477.1 GTP-binding protein [Roseburia intestinalis]
271   3e-76
WP_007122710.1 elongation factor G [Lactobacillus gastricus]
267   3e-76
OPX30908.1 translation elongation factor G [Candidatus Omnitr...
266   3e-76
OGE25349.1 translation elongation factor G [Candidatus Davies...
267   3e-76
WP_004402356.1 elongation factor G [Thermoanaerobacter thermo...
266   3e-76
WP_055739678.1 GTP-binding protein [Bacillus shackletonii]
266   3e-76
PIE26444.1 elongation factor G [Micrococcales bacterium]
267   3e-76
WP_088460070.1 elongation factor G [Lactobacillus fermentum]
266   3e-76
WP_025729841.1 elongation factor G [Bacillus ginsengihumi]
266   3e-76
ABO87322.1 TetM [Streptococcus pyogenes]
251   3e-76
WP_078081863.1 GTP-binding protein [Bacillus megaterium]
265   3e-76
WP_070618524.1 MULTISPECIES: elongation factor G [Aerococcus]
266   3e-76
WP_013403742.1 elongation factor G [Caldicellulosiruptor hydr...
266   3e-76
WP_082141799.1 GTP-binding protein [Clostridium cylindrosporum]
269   3e-76
WP_076149115.1 elongation factor G [Lactobacillus murinus]
266   3e-76
WP_014063493.1 MULTISPECIES: elongation factor G [Thermoanaer...
266   3e-76
SCJ86555.1 Tetracycline resistance protein tetM from transpos...
270   3e-76
WP_073598145.1 elongation factor G [Hydrococcus rivularis]
266   3e-76
WP_103434485.1 elongation factor G [Lactobacillus sanfrancisc...
266   3e-76
WP_015359647.1 GTP-binding protein [[Clostridium] stercorarium]
270   3e-76

WP_065715831.1 elongation factor G [Synechococcus sp. PCC 8807]
266   3e-76
WP_038116211.1 MULTISPECIES: elongation factor G [Veillonella]
266   3e-76
WP_066128796.1 elongation factor G [Gemella asaccharolytica]
266   3e-76
WP_054025331.1 elongation factor G [Bacillus sp. FJAT-28004]
266   3e-76
WP_025083315.1 elongation factor G [Lactobacillus fuchuensis]
266   3e-76
WP_066162473.1 GTP-binding protein [[Bacillus] sp. KCTC 13219]
265   3e-76
WP_017763331.1 tetracycline resistance protein [Bacillus thur...
263   3e-76
WP_077368848.1 elongation factor G [Anaerosalibacter sp. Mars...
266   3e-76
WP_034993732.1 elongation factor G [Lactobacillus vini]
266   4e-76
WP_045174337.1 elongation factor G [Caldicellulosiruptor dani...
266   4e-76
WP_029321921.1 MULTISPECIES: GTP-binding protein [Bacillus]
265   4e-76
EXY76255.1 small GTP-binding domain protein [Bacteroides frag...
254   4e-76
WP_008406632.1 MULTISPECIES: GTP-binding protein [Solibacillus]
265   4e-76
WP_089940407.1 GTP-binding protein [Lachnospiraceae bacterium...
271   4e-76
WP_022670822.1 elongation factor G [Hippea alviniae]
266   4e-76
WP_076546208.1 elongation factor G [Halanaerobium kushneri]
266   4e-76
WP_066354480.1 elongation factor G [Fervidicola ferrireducens]
266   4e-76
WP_057864746.1 elongation factor G [Lactobacillus diolivorans]
266   4e-76
WP_099448720.1 GTP-binding protein [Lachnospiraceae bacterium]
270   4e-76
WP_012963495.1 elongation factor G [Hydrogenobacter thermophi...
266   4e-76
WP_028255445.1 elongation factor G [Veillonella magna]
266   4e-76
KRT71003.1 elongation factor G, elongation factor G [candidat...
266   4e-76
WP_014082380.1 elongation factor G [Lactobacillus sanfrancisc...
266   4e-76
WP_098563177.1 GTP-binding protein [Bacillus megaterium]
265   4e-76
ABO87314.1 TetO [Streptococcus pyogenes]
251   4e-76
WP_057273225.1 GTP-binding protein [Bacillus sp. Soil531]
265   4e-76
WP_034819253.1 elongation factor G [[Eubacterium] nodatum]
266   4e-76

WP_053984608.1 GTP-binding protein [Niameybacter massiliensis]
270   4e-76
OGH39059.1 translation elongation factor G [Candidatus Levyba...
266   4e-76
WP_015144239.1 elongation factor G [Pleurocapsa minor]
266   4e-76
WP_087975039.1 elongation factor G [Oceanobacillus rekensis]
266   5e-76
OEU50646.1 translation elongation factor G [Desulfobacterales...
266   5e-76
WP_013787147.1 elongation factor G [Thermoanaerobacterium xyl...
266   5e-76
WP_072745447.1 elongation factor G [Sporanaerobacter acetigenes]
266   5e-76
WP_005214458.1 GTP-binding protein [Clostridium celatum]
265   5e-76
PIR57075.1 elongation factor G [Parcubacteria group bacterium...
266   5e-76
PIU28529.1 elongation factor G [Actinobacteria bacterium CG08...
266   5e-76
WP_098196519.1 GTP-binding protein [Bacillus megaterium]
265   5e-76
WP_013057167.1 MULTISPECIES: GTP-binding protein [Bacillus]
265   5e-76
WP_061858888.1 MULTISPECIES: GTP-binding protein [Clostridium]
265   5e-76
KYH28346.1 tetracycline resistance protein TetO [Clostridium ...
265   5e-76
WP_076712804.1 elongation factor G [Desulfurobacterium indicum]
266   5e-76
WP_062305129.1 elongation factor G [Alicyclobacillus sendaien...
266   5e-76
WP_008265364.1 elongation factor G [Stenotrophomonas sp. SKA14]
265   5e-76
WP_088217580.1 elongation factor G [Caldicellulosiruptor bescii]
266   6e-76
WP_027895363.1 elongation factor G [Megasphaera elsdenii]
266   6e-76
WP_047242126.1 elongation factor G [Enterococcus cecorum]
266   6e-76
WP_053509024.1 elongation factor G [Stenotrophomonas maltophi...
265   6e-76
WP_036203202.1 elongation factor G [Megasphaera elsdenii]
266   6e-76
WP_014015732.1 elongation factor G [Megasphaera elsdenii]
266   6e-76
WP_024867029.1 GTP-binding protein [Butyrivibrio sp. FCS014]
270   6e-76
WP_034989692.1 elongation factor G [Lactobacillus sucicola]
266   6e-76
WP_029594429.1 elongation factor G [Exiguobacterium chiriqhucha]
266   6e-76
WP_071219757.1 GTP-binding protein [Paenibacillus sp. LC231]
265   6e-76

WP_019502051.1 elongation factor G [Pseudanabaena sp. PCC 6802]
266   6e-76
WP_013083280.1 GTP-binding protein [Bacillus megaterium]
265   6e-76
WP_097643724.1 elongation factor G [Chloroflexi bacterium Kir...
266   6e-76
WP_039041593.1 GTP-binding protein [Sporosarcina sp. ZBG7A]
265   6e-76
WP_066394237.1 GTP-binding protein [Bacillus horneckiae]
265   6e-76
WP_017751846.1 GTP-binding protein [Clostridium tyrobutyricum]
265   6e-76
WP_076271434.1 elongation factor G [Paenibacillus sp. FSL A5-...
266   6e-76
WP_101646634.1 GTP-binding protein [Bacillus cucumis]
265   6e-76
ABO87317.1 TetO [Streptococcus pyogenes]
251   6e-76
WP_049685823.1 elongation factor G [Thermoanaerobacter kivui]
265   6e-76
WP_010694173.1 elongation factor G [Treponema denticola]
265   6e-76
WP_087227048.1 GTP-binding protein [Olsenella sp. An270]
265   7e-76
WP_096368910.1 elongation factor G [Lactococcus garvieae]
266   7e-76
ERJ95606.1 translation elongation factor G [Selenomonas sp. o...
265   7e-76
OGE41585.1 translation elongation factor G [Candidatus Davies...
266   7e-76
WP_061859810.1 GTP-binding protein [Bacillus megaterium]
265   7e-76
WP_025028109.1 elongation factor G [Bacillus mannanilyticus]
265   7e-76
WP_066894529.1 GTP-binding protein [Clostridium nigeriense]
265   7e-76
WP_098314956.1 GTP-binding protein [Bacillus megaterium]
265   7e-76
WP_017370008.1 elongation factor G [Lactococcus garvieae]
266   7e-76
WP_056942092.1 elongation factor G [Lactobacillus kimchicus]
266   7e-76
OGH04541.1 translation elongation factor G [Candidatus Levyba...
266   7e-76
WP_052168199.1 elongation factor G [Desulfurobacterium sp. TC...
266   7e-76
WP_033934656.1 elongation factor G [Lactobacillus mucosae]
265   7e-76
WP_018125648.1 elongation factor G [Desulfovibrio oxyclinae]
265   7e-76
WP_025751824.1 MULTISPECIES: GTP-binding protein [Bacillus]
265   7e-76
WP_026964707.1 elongation factor G [Alicyclobacillus pomorum]
265   7e-76

WP_013290054.1 elongation factor G [Caldicellulosiruptor obsi...
265   7e-76
WP_017295627.1 elongation factor G [Geminocystis herdmanii]
266   7e-76
WP_066505595.1 GTP-binding protein [Clostridiales bacterium M...
264   8e-76
OGW93077.1 translation elongation factor G [Omnitrophica bact...
265   8e-76
WP_019637176.1 GTP-binding protein [Paenibacillus fonticola]
265   8e-76
WP_039767328.1 elongation factor G [Caldicellulosiruptor sp. ...
265   8e-76
SCH62478.1 Tetracycline resistance protein tetM from transpos...
270   8e-76
KXK09097.1 Elongation factor G [Microgenomates bacterium OLB22]
266   8e-76
OJV64300.1 translation elongation factor G [Clostridiales bac...
270   8e-76
WP_093587804.1 GTP-binding protein [Lentzea waywayandensis]
261   8e-76
WP_024372179.1 MULTISPECIES: elongation factor G [Exiguobacte...
265   8e-76
WP_027643561.1 GTP-binding protein [[Clostridium] clostridiof...
270   8e-76
WP_015136214.1 elongation factor G [Leptolyngbya sp. PCC 7376]
265   8e-76
WP_044665946.1 elongation factor G [Syntrophaceticus schinkii]
265   8e-76
WP_063671653.1 MULTISPECIES: GTP-binding protein [Bacillus]
264   8e-76
WP_057756444.1 GTP-binding protein [Lactobacillus dextrinicus]
265   8e-76
WP_056959057.1 elongation factor G [Lactobacillus murinus]
265   8e-76
GBD15522.1 Elongation factor G [bacterium HR26]
265   9e-76
WP_083429811.1 GTP-binding protein [Blautia sp. Marseille-P32...
269   9e-76
WP_066444786.1 GTP-binding protein [Candidatus Stoquefichus s...
269   9e-76
ABO87326.1 TetM [Streptococcus pyogenes]
250   9e-76
WP_007048812.1 elongation factor G [Anaerofustis stercorihomi...
265   9e-76
OGW77013.1 translation elongation factor G [Omnitrophica bact...
265   9e-76
WP_098238777.1 GTP-binding protein [Bacillus megaterium]
264   9e-76
WP_091526921.1 elongation factor G [Microlunatus soli]
265   9e-76
WP_075727553.1 elongation factor G [Tissierella creatinophila]
265   9e-76
WP_012511014.1 elongation factor G [Stenotrophomonas maltophi...
265   1e-75

KPK87120.1 elongation factor G [bacterium SM23_31]
265   1e-75
WP_016764228.1 GTP-binding protein [Bacillus megaterium]
264   1e-75
ABO87360.1 TetM [Streptococcus pyogenes]
250   1e-75
WP_063724116.1 GTP-binding protein [Lactobacillus plantarum]
265   1e-75
WP_068453776.1 GTP-binding protein [Kurthia sp. 11kri321]
264   1e-75
WP_090871934.1 GTP-binding protein [Oceanobacillus limi]
264   1e-75
OGW99723.1 translation elongation factor G [Omnitrophica bact...
265   1e-75
AMP47122.1 elongation factor Tu GTP binding domain protein [u...
248   1e-75
CBL09694.1 small GTP-binding protein domain [Roseburia intest...
269   1e-75
WP_040467015.1 elongation factor G [[Eubacterium] brachy]
265   1e-75
OGP77075.1 translation elongation factor G [Deltaproteobacter...
265   1e-75
WP_002681223.1 elongation factor G [Treponema denticola]
265   1e-75
ABO87319.1 TetM [Streptococcus pyogenes]
250   1e-75
APO27240.1 Elongation factor G C-terminus [uncultured bacterium]
247   1e-75
WP_014562616.1 elongation factor G [Lactobacillus fermentum]
265   1e-75
WP_074592834.1 elongation factor G [Thermoanaerobacter thermo...
265   1e-75
ABO87318.1 TetO [Streptococcus pyogenes]
250   1e-75
OLA53452.1 translation elongation factor G [Roseburia intesti...
269   1e-75
WP_026962529.1 elongation factor G [Alicyclobacillus herbarius]
265   1e-75
WP_079439476.1 GTP-binding protein [Clostridium chromiireducens]
265   1e-75
WP_098980117.1 GTP-binding protein [Bacillus megaterium]
264   1e-75
KPK98538.1 elongation factor G [Omnitrophica WOR_2 bacterium ...
265   1e-75
WP_097004485.1 GTP-binding protein [[Clostridium] amygdalinum]
269   1e-75
WP_075718252.1 GTP-binding protein [Roseburia sp. 499]
269   1e-75
KPJ81771.1 elongation factor G [Gemmatimonas sp. SG8_23]
265   1e-75
WP_047932425.1 GTP-binding protein [Bacillus megaterium]
264   1e-75
EGC78697.1 elongation factor G 1 [Treponema denticola F0402]
265   1e-75

WP_087374817.1 GTP-binding protein [Butyricicoccus pullicaeco...
268   1e-75
WP_106172571.1 elongation factor G [filamentous cyanobacteriu...
265   1e-75
WP_086874619.1 elongation factor G [Lactobacillus johnsonii]
265   1e-75
ESE29699.1 translation elongation factor G [Eubacterium brach...
265   1e-75
WP_087284777.1 elongation factor G [Lactobacillus johnsonii]
265   1e-75
WP_072693480.1 MULTISPECIES: elongation factor G [Marinilacti...
265   1e-75
WP_034813846.1 elongation factor G [[Eubacterium] sulci]
265   1e-75
WP_098804724.1 GTP-binding protein [Bacillus megaterium]
264   1e-75
WP_003681566.1 MULTISPECIES: elongation factor G [Lactobacillus]
265   1e-75
WP_084067091.1 elongation factor G [Desulfobacterium vacuolatum]
265   1e-75
WP_091651387.1 elongation factor G [Megasphaera paucivorans]
265   1e-75
WP_059075693.1 MULTISPECIES: elongation factor G [Veillonella...
265   1e-75
WP_067532962.1 elongation factor G [Emergencia timonensis]
265   1e-75
OPX41791.1 translation elongation factor G [Desulfobacteracea...
265   1e-75
OGW94332.1 translation elongation factor G [Omnitrophica bact...
265   1e-75
WP_051654590.1 GTP-binding protein [Lachnospiraceae bacterium...
268   1e-75
WP_056937303.1 elongation factor G [Lactobacillus gastricus]
265   1e-75
OGB64916.1 translation elongation factor G [Caldithrix sp. RB...
265   1e-75
WP_034804017.1 MULTISPECIES: elongation factor G [Exiguobacte...
265   1e-75
PDO11306.1 translation elongation factor G [Paenibacillaceae ...
265   1e-75
KPU26423.1 elongation factor G [Caloranaerobacter sp. TR13]
265   1e-75
WP_036223427.1 elongation factor G [Mesoaciditoga lauensis]
265   1e-75
WP_002608208.1 GTP-binding protein [[Clostridium] innocuum]
268   1e-75
WP_105412275.1 MULTISPECIES: GTP-binding protein [Clostridium]
264   1e-75
WP_016147750.1 GTP-binding protein [Butyricicoccus pullicaeco...
268   1e-75
WP_072978060.1 elongation factor G [Tissierella praeacuta]
265   2e-75
GBD24541.1 Elongation factor G [bacterium HR29]
265   2e-75

WP_068335040.1 elongation factor G [Photobacterium jeanii]
265   2e-75
WP_104878538.1 elongation factor G [Lactobacillus fermentum]
265   2e-75
WP_097812491.1 GTP-binding protein [Bacillus megaterium]
263   2e-75
WP_066713831.1 elongation factor G [Clostridiales bacterium K...
265   2e-75
WP_076345527.1 elongation factor G [Alicyclobacillus vulcanalis]
265   2e-75
WP_005998591.1 elongation factor G [[Eubacterium] infirmum]
265   2e-75
WP_039658496.1 GTP-binding protein [Clostridium tyrobutyricum]
263   2e-75
WP_009269507.1 GTP-binding protein [Erysipelotrichaceae bacte...
268   2e-75
WP_008755239.1 GTP-binding protein [Lachnoanaerobaculum sabur...
268   2e-75
KKR86526.1 Elongation factor G [Candidatus Curtissbacteria ba...
265   2e-75
WP_072338030.1 MULTISPECIES: elongation factor G [Paenibacillus]
265   2e-75
WP_066546913.1 GTP-binding protein [Clostridium sp. AT4]
269   2e-75
SNR92387.1 translation elongation factor 2 (EF-2/EF-G) [Desul...
265   2e-75
WP_056995813.1 GTP-binding protein [Lactobacillus equigenerosi]
263   2e-75
WP_069775732.1 elongation factor G [Lactobacillus fermentum]
265   2e-75
OQX65653.1 elongation factor G [Desulfococcus sp. 4484_242]
264   2e-75
WP_004050332.1 MULTISPECIES: elongation factor G [Lactobacillus]
265   2e-75
ABO87345.1 TetM [Streptococcus pyogenes]
249   2e-75
EQC59548.1 elongation factor P [Lactobacillus fermentum MTCC ...
265   2e-75
OGH47673.1 translation elongation factor G [Candidatus Levyba...
265   2e-75
OGH24286.1 translation elongation factor G [Candidatus Levyba...
265   2e-75
WP_057313455.1 elongation factor G [Paenibacillus sp. Soil766]
264   2e-75
WP_011697909.1 elongation factor G [Syntrophobacter fumaroxid...
265   2e-75
WP_050636613.1 GTP-binding protein [Candidatus Stoquefichus s...
268   2e-75
WP_079653813.1 elongation factor G [Thermocrinis minervae]
265   2e-75
WP_025786283.1 GTP-binding protein [Sporosarcina sp. D27]
263   2e-75
WP_012991662.1 elongation factor G [Thermocrinis albus]
265   2e-75

AOR74003.1 Elongation factor G [Lactobacillus fermentum]
265   2e-75
WP_004831805.1 elongation factor G [Parvimonas micra]
264   2e-75
KKS04740.1 Elongation factor G [Candidatus Curtissbacteria ba...
265   2e-75
WP_048735482.1 elongation factor G [Lactobacillus koreensis]
265   2e-75
WP_003373544.1 GTP-binding protein [Clostridium botulinum]
263   2e-75
WP_095183405.1 elongation factor G [Lactobacillus johnsonii]
265   2e-75
WP_044351937.1 elongation factor G [Dethiosulfatarculus sandi...
264   2e-75
WP_092337349.1 GTP-binding protein [Butyrivibrio sp. M55]
268   2e-75
WP_053487289.1 GTP-binding protein [Bacillus sp. FJAT-21351]
263   2e-75
WP_055260322.1 GTP-binding protein [Coprococcus comes]
269   2e-75
WP_077568371.1 GTP-binding protein [Paenibacillus ihbetae]
263   2e-75
WP_014465736.1 elongation factor G [Alicyclobacillus acidocal...
264   2e-75
WP_012081649.1 elongation factor G [Nitratiruptor sp. SB155-2]
264   2e-75
WP_013682368.1 elongation factor G [Hippea maritima]
264   2e-75
WP_058953796.1 GTP-binding protein [Clostridium tyrobutyricum]
262   2e-75
EHG20589.1 elongation factor G [Selenomonas infelix ATCC 43532]
264   2e-75
XP_001513499.2 PREDICTED: ribosome-releasing factor 2, mitoch...
266   2e-75
WP_009052835.1 MULTISPECIES: elongation factor G [Thermoanaer...
264   2e-75
WP_038269301.1 GTP-binding protein [[Clostridium] clostridiof...
269   2e-75
WP_070329247.1 elongation factor G [Exiguobacterium aurantiacum]
264   2e-75
GBC90903.1 Elongation factor G [bacterium HR14]
264   2e-75
WP_006308033.1 elongation factor G [Caldisalinibacter kiritim...
264   2e-75
WP_085098709.1 elongation factor G [Desulfovibrio sp. K3S]
264   2e-75
WP_012305975.1 MULTISPECIES: elongation factor G [Synechococcus]
264   2e-75
WP_057194486.1 elongation factor G [Lactobacillus fermentum]
264   2e-75
WP_091064689.1 GTP-binding protein [Paenibacillus sp. NFR01]
263   2e-75
WP_057872464.1 elongation factor G [Lactobacillus ghanensis]
264   2e-75

WP_049164662.1 GTP-binding protein [Bacillus megaterium]
263   2e-75
PIQ80104.1 elongation factor G [Parcubacteria group bacterium...
264   2e-75
WP_002684885.1 elongation factor G [Treponema denticola]
264   2e-75
WP_049150646.1 elongation factor G [Lactobacillus gasseri]
264   2e-75
WP_103328834.1 elongation factor G [Staphylococcus petrasii]
264   2e-75
WP_011161520.1 elongation factor G [Lactobacillus johnsonii]
264   3e-75
WP_003549022.1 elongation factor G [Lactobacillus acidophilus]
264   3e-75
WP_096239236.1 GTP-binding protein [[Eubacterium] hallii]
268   3e-75
WP_046401283.1 elongation factor G [Lactococcus garvieae]
264   3e-75
WP_065198605.1 elongation factor G [Stenotrophomonas maltophi...
264   3e-75
WP_056987470.1 elongation factor G [Lactobacillus paracollino...
264   3e-75
WP_012845640.1 MULTISPECIES: elongation factor G [Lactobacillus]
264   3e-75
WP_014727708.1 elongation factor G [Streptococcus thermophilus]
264   3e-75
WP_012480150.1 elongation factor G [Stenotrophomonas maltophi...
264   3e-75
WP_013907996.1 elongation factor G [Thermodesulfatator indicus]
264   3e-75
OGY18853.1 translation elongation factor G [Candidatus Chisho...
265   3e-75
WP_065937620.1 elongation factor G [Lactobacillus paracollino...
264   3e-75
EFF66634.1 translation elongation factor G [Selenomonas noxia...
263   3e-75
WP_065183791.1 elongation factor G [Stenotrophomonas maltophi...
263   3e-75
WP_096494589.1 elongation factor G [Lactobacillus fermentum]
264   3e-75
WP_040347119.1 elongation factor G [Anaeroglobus geminatus]
264   3e-75
KPH03387.1 elongation factor G [Lactobacillus fermentum]
264   3e-75
WP_054464837.1 elongation factor G [Planktothricoides sp. SR001]
264   3e-75
WP_098287531.1 GTP-binding protein [Bacillus megaterium]
263   3e-75
WP_054871841.1 elongation factor G [Caloranaerobacter sp. TR13]
264   3e-75
WP_060931721.1 GTP-binding protein [Lachnoanaerobaculum sabur...
268   3e-75
CDA53410.1 putative translation elongation factor G [Clostrid...
268   3e-75

WP_003868704.1 elongation factor G [Thermoanaerobacter ethano...
264   3e-75
PIR71184.1 elongation factor G [Candidatus Nealsonbacteria ba...
264   3e-75
WP_087253572.1 GTP-binding protein [Pseudoflavonifractor sp. ...
267   3e-75
WP_056991606.1 elongation factor G [Lactobacillus mali]
264   3e-75
WP_038283151.1 GTP-binding protein [[Clostridium] bolteae]
268   3e-75
WP_035992322.1 elongation factor G [Leptolyngbya sp. KIOST-1]
264   3e-75
WP_070508652.1 MULTISPECIES: elongation factor G [Streptococcus]
264   3e-75
CDF13081.1 elongation factor G [Eubacterium sp. CAG:581]
264   3e-75
OGC93730.1 translation elongation factor G [candidate divisio...
264   3e-75
WP_098399462.1 GTP-binding protein [Bacillus megaterium]
263   3e-75
EXZ30955.1 elongation factor G, domain IV family protein [Bac...
254   3e-75
WP_075526621.1 elongation factor G [Lysinibacillus sphaericus]
264   3e-75
WP_027627768.1 GTP-binding protein [[Clostridium] cellobioparum]
263   3e-75
OGG38429.1 translation elongation factor G [Candidatus Gottes...
262   3e-75
WP_077303338.1 elongation factor G [Virgibacillus pantothenti...
264   3e-75
WP_029196464.1 elongation factor G [Paenibacillus alginolyticus]
264   3e-75
ABO87344.1 TetM [Streptococcus pyogenes]
249   3e-75
ABO87333.1 TetM [Streptococcus pyogenes]
249   3e-75
WP_028937642.1 elongation factor G [Pseudonocardia spinosispora]
264   3e-75
WP_026693319.1 elongation factor G [Bacillus kribbensis]
264   3e-75
WP_019292792.1 elongation factor G [Lactococcus garvieae]
264   3e-75
WP_043982583.1 GTP-binding protein [Bacillus aryabhattai]
263   3e-75
WP_035630030.1 GTP-binding protein [Lachnospiraceae bacterium...
267   3e-75
WP_090246804.1 elongation factor G [Eubacterium barkeri]
264   3e-75
ABO87324.1 TetM [Streptococcus pyogenes]
249   3e-75
WP_015943930.1 GTP-binding protein [Desulfitobacterium hafnie...
268   3e-75
WP_089323718.1 elongation factor G [Desulfurobacterium atlant...
264   3e-75

WP_004895883.1 elongation factor G [Lactobacillus johnsonii]
264   4e-75
WP_102116081.1 elongation factor G [Lactobacillus fermentum]
264   4e-75
WP_014567086.1 elongation factor G [Lactobacillus johnsonii]
264   4e-75
WP_031497869.1 elongation factor G [Bryobacter aggregatus]
264   4e-75
WP_005358645.1 GTP-binding protein [Eubacterium ventriosum]
267   4e-75
KRN27818.1 elongation factor G [Lactobacillus mali]
264   4e-75
WP_028125025.1 elongation factor G [Eremococcus coleocola]
264   4e-75
WP_006417914.1 elongation factor G [Eremococcus coleocola]
264   4e-75
EFM71690.1 elongation factor Tu domain 2 [Enterococcus faecal...
250   4e-75
WP_003647836.1 elongation factor G [Lactobacillus gasseri]
264   4e-75
WP_017895410.1 GTP-binding protein [Clostridium tyrobutyricum]
262   4e-75
WP_069876426.1 elongation factor G [Fusibacter sp. 3D3]
263   4e-75
WP_085322635.1 GTP-binding protein [Clostridium botulinum]
263   4e-75
WP_094906133.1 elongation factor G [Salinicoccus roseus]
263   4e-75
WP_040106275.1 elongation factor G [Salinicoccus roseus]
263   4e-75
WP_034842828.1 GTP-binding protein [[Clostridium] stercorarium]
268   4e-75
OIP73706.1 translation elongation factor G [Candidatus Paceba...
264   4e-75
WP_057897069.1 MULTISPECIES: elongation factor G [Aneurinibac...
263   4e-75
WP_089151025.1 GTP-binding protein [Lactobacillus crispatus]
262   4e-75
WP_065217061.1 GTP-binding protein [Solibacillus silvestris]
262   4e-75
WP_056948924.1 elongation factor G [Lactobacillus odoratitofui]
264   4e-75
WP_075364416.1 elongation factor G [Desulfosporosinus sp. OL]
263   4e-75
WP_056834176.1 MULTISPECIES: elongation factor G [Paenibacillus]
263   4e-75
EKE12321.1 hypothetical protein ACD_13C00245G0001 [uncultured...
262   4e-75
WP_033610868.1 GTP-binding protein [Lactobacillus paraplantarum]
263   4e-75
WP_061313343.1 GTP-binding protein [Clostridium botulinum]
263   4e-75
ABO87320.1 TetM [Streptococcus pyogenes]
249   4e-75

WP_062050705.1 elongation factor G [Bacillus sp. JCM 19034]
263   4e-75
WP_009449112.1 elongation factor G [Parvimonas sp. oral taxon...
263   4e-75
CDB88539.1 yacP-like NYN domain protein [Firmicutes bacterium...
267   4e-75
AAP88028.1 tetracycline resistance protein B [Clostridium per...
257   4e-75
WP_098044260.1 GTP-binding protein [Lactobacillus sp. UMNPBX4]
262   4e-75
WP_090003744.1 elongation factor G [Lentzea violacea]
263   4e-75
WP_009263154.1 GTP-binding protein [Lachnospiraceae bacterium...
268   4e-75
WP_106799300.1 GTP-binding protein [Desulfosporosinus sp. SbF1]
267   4e-75
WP_057756708.1 elongation factor G [Lactobacillus dextrinicus]
263   4e-75
WP_003865303.1 GTP-binding protein [Holdemanella biformis]
266   4e-75
WP_065723259.1 elongation factor G [Stenotrophomonas maltophi...
263   4e-75
PHR92397.1 elongation factor G [Robiginitomaculum sp.]
263   4e-75
WP_053451742.1 elongation factor G [Exiguobacterium sp. BMC-KP]
263   4e-75
WP_072726381.1 elongation factor G [Tepidibacter thalassicus]
263   4e-75
WP_026826915.1 elongation factor G [Exiguobacterium sibiricum]
263   4e-75
WP_013274959.1 elongation factor G [Thermosediminibacter oceani]
263   4e-75
WP_033579056.1 GTP-binding protein [Bacillus aryabhattai]
262   4e-75
PIP21698.1 elongation factor G [Candidatus Nealsonbacteria ba...
263   4e-75
OQX75958.1 hypothetical protein B6D64_10735 [Bacteroidetes ba...
260   4e-75
WP_024726337.1 GTP-binding protein [[Clostridium] bolteae]
268   4e-75
WP_057737822.1 elongation factor G [Lactobacillus uvarum]
263   4e-75
WP_072969619.1 elongation factor G [Thermoanaerobacter uzonen...
263   5e-75
WP_102412536.1 GTP-binding protein [Clostridiales bacterium M...
263   5e-75
WP_103107982.1 GTP-binding protein [Brevibacillus reuszeri]
262   5e-75
OQY51919.1 elongation factor G [Desulfobacteraceae bacterium ...
263   5e-75
AED99117.1 tetracycline resistance protein [Streptococcus pyo...
246   5e-75
WP_057717990.1 elongation factor G [Lactobacillus taiwanensis]
263   5e-75

WP_026834302.1 elongation factor G [Exiguobacterium undae]
263   5e-75
WP_043662060.1 GTP-binding protein [Clostridium butyricum]
262   5e-75
KRN09665.1 elongation factor G [Lactobacillus mali KCTC 3596 ...
263   5e-75
WP_108534903.1 elongation factor G [Paenibacillus elgii]
263   5e-75
WP_041895779.1 GTP-binding protein [Clostridium beijerinckii]
263   5e-75
KUK96311.1 Elongation factor G [Anaerolineaceae bacterium 46_22]
263   5e-75
WP_050349641.1 MULTISPECIES: elongation factor G [Virgibacillus]
263   5e-75
WP_003656622.1 elongation factor G [Lactobacillus gasseri]
263   5e-75
WP_059034415.1 elongation factor G [Stenotrophomonas maltophi...
263   5e-75
WP_107230510.1 MULTISPECIES: elongation factor G [Stenotropho...
263   5e-75
OGY92350.1 translation elongation factor G [Candidatus Komeil...
263   5e-75
WP_107510334.1 elongation factor G [Staphylococcus equorum]
263   5e-75
WP_058295656.1 GTP-binding protein [Clostridium neonatale]
263   5e-75
WP_098034917.1 GTP-binding protein [Lactobacillus sp. UMNPBX1]
262   5e-75
WP_087617633.1 GTP-binding protein [Solibacillus kalamii]
262   5e-75
WP_017549967.1 elongation factor G [Salinicoccus carnicancri]
263   5e-75
WP_061915233.1 elongation factor G [Bellilinea caldifistulae]
263   5e-75
ABO87325.1 TetM [Streptococcus pyogenes]
248   6e-75
WP_048020793.1 GTP-binding protein [Bacillus aryabhattai]
262   6e-75
WP_010498882.1 elongation factor G [Paenibacillus elgii]
263   6e-75
WP_055296506.1 GTP-binding protein [Coprococcus comes]
268   6e-75
WP_099531224.1 elongation factor G [Stenotrophomonas maltophi...
263   6e-75
WP_017208934.1 GTP-binding protein [Clostridium beijerinckii]
263   6e-75
WP_015921950.1 elongation factor G [Thermomicrobium roseum]
263   6e-75
WP_047683196.1 elongation factor G [Paenibacillus chondroitinus]
263   6e-75
WP_012995916.1 elongation factor G [Thermoanaerobacter italicus]
263   6e-75
WP_021730390.1 GTP-binding protein [Lactobacillus plantarum]
263   6e-75

WP_011969066.1 GTP-binding protein [Clostridium beijerinckii]
263   6e-75
CDB21767.1 putative uncharacterized protein [Blautia sp. CAG:52]
268   6e-75
WP_003518959.1 GTP-binding protein [Ruminiclostridium thermoc...
267   6e-75
WP_107942884.1 GTP-binding protein [Lysinibacillus fluoroglyc...
262   6e-75
WP_019335264.1 MULTISPECIES: elongation factor G [Lactococcus]
263   6e-75
WP_002441110.1 MULTISPECIES: elongation factor G [Staphylococ...
263   6e-75
WP_056618302.1 elongation factor G [Paenibacillus sp. Soil750]
263   6e-75
WP_066228088.1 GTP-binding protein [Bacillus fastidiosus]
262   6e-75
WP_100443335.1 elongation factor G [Stenotrophomonas maltophi...
263   6e-75
WP_003511944.1 GTP-binding protein [Ruminiclostridium thermoc...
267   6e-75
CDA14281.1 putative tetracycline resistance protein TetP [Fir...
267   6e-75
PIR96914.1 elongation factor G [Candidatus Doudnabacteria bac...
263   6e-75
WP_014621968.1 elongation factor G [Streptococcus thermophilus]
263   6e-75
WP_026518150.1 GTP-binding protein [Butyrivibrio sp. MC2021]
267   6e-75
WP_090826709.1 elongation factor G [Paenibacillus sp. yr247]
263   6e-75
WP_100434613.1 elongation factor G [Stenotrophomonas maltophi...
263   6e-75
WP_055155459.1 GTP-binding protein [Coprococcus comes]
268   6e-75
SCH43387.1 Tetracycline resistance protein tetM [uncultured C...
262   6e-75
SJZ37248.1 small GTP-binding protein domain-containing protei...
263   6e-75
WP_067718287.1 elongation factor G [Acidibacillus ferrooxidans]
263   6e-75
WP_003689449.1 elongation factor G [Lactobacillus mali]
263   6e-75
WP_062490494.1 elongation factor G [Paenibacillus sp. 32O-W]
263   6e-75
OGY15888.1 translation elongation factor G [Candidatus Chisho...
263   6e-75
WP_102812763.1 GTP-binding protein [Bacillus megaterium]
262   6e-75
OHD56776.1 translation elongation factor G [Spirochaetes bact...
263   7e-75
WP_065902678.1 elongation factor G [Lactobacillus paracollino...
263   7e-75
OGC56693.1 translation elongation factor G [candidate divisio...
263   7e-75

KMK74861.1 elongation factor G [Bacillus pseudalcaliphilus]
263   7e-75
WP_072874897.1 elongation factor G [Alicyclobacillus montanus]
263   7e-75
WP_019146776.1 elongation factor G [Timonella senegalensis]
263   7e-75
WP_035761642.1 GTP-binding protein [Clostridium butyricum]
262   7e-75
WP_003136105.1 elongation factor G [Lactococcus garvieae]
263   7e-75
WP_004094855.1 elongation factor G [Acetonema longum]
263   7e-75
WP_017826325.1 GTP-binding protein [Clostridium botulinum]
262   7e-75
AFY23863.1 tetracycline resistance protein [Campylobacter coli]
246   7e-75
WP_054761515.1 elongation factor G [Lactobacillus collinoides]
263   7e-75
WP_073049726.1 GTP-binding protein [Dethiosulfatibacter amino...
262   7e-75
WP_100468420.1 elongation factor G [Stenotrophomonas maltophi...
263   7e-75
WP_061279896.1 GTP-binding protein [Clostridium botulinum]
262   7e-75
WP_071398660.1 elongation factor G [Exiguobacterium sp. SH31]
263   7e-75
WP_023073714.1 elongation factor G [Leptolyngbya sp. Heron Is...
263   7e-75
WP_034433733.1 elongation factor G [Clostridiales bacterium S...
263   7e-75
WP_040985103.1 elongation factor G [Oceanobacillus jeddahense]
263   7e-75
WP_003428100.1 MULTISPECIES: GTP-binding protein [Clostridium]
262   7e-75
WP_031545908.1 elongation factor G [Salinicoccus luteus]
263   7e-75
WP_009587903.1 MULTISPECIES: GTP-binding protein [Firmicutes]
266   7e-75
WP_002508564.1 MULTISPECIES: elongation factor G [Staphylococ...
263   7e-75
WP_074897346.1 GTP-binding protein [Bacillus megaterium]
262   8e-75
WP_050613629.1 elongation factor G [Bacillus testis]
263   8e-75
WP_068661876.1 elongation factor G [Paenibacillus sp. 1ZS3-15]
263   8e-75
WP_019294137.1 MULTISPECIES: elongation factor G [Lactococcus]
263   8e-75
SCI87271.1 Tetracycline resistance protein tetM [uncultured C...
262   8e-75
WP_002951942.1 elongation factor G [Streptococcus thermophilus]
263   8e-75
WP_085295485.1 GTP-binding protein [Clostridium botulinum]
262   8e-75

WP_040968303.1 GTP-binding protein [Clostridium botulinum]
262   8e-75
WP_054975994.1 elongation factor G [Paenibacillus sp. A3]
263   8e-75
WP_098823480.1 GTP-binding protein [Lactobacillus silagincola]
261   8e-75
WP_099601094.1 elongation factor G [Stenotrophomonas maltophi...
262   8e-75
WP_006351934.1 elongation factor G [Lactobacillus amylolyticus]
263   8e-75
WP_099523875.1 elongation factor G [Stenotrophomonas maltophi...
262   8e-75
WP_034266336.1 GTP-binding protein [Bacillus sp. Aph1]
262   8e-75
SCJ87272.1 Tetracycline resistance protein tetM from transpos...
267   8e-75
WP_056993361.1 elongation factor G [Lactobacillus saniviri]
263   8e-75
WP_087701440.1 GTP-binding protein [Clostridium diolis]
262   8e-75
WP_096658567.1 elongation factor G [Calothrix parasitica]
263   8e-75
WP_018248770.1 GTP-binding protein [Orenia marismortui]
261   8e-75
BAY50755.1 translation elongation factor EF-G [Thermosynechoc...
263   8e-75
WP_092098076.1 GTP-binding protein [Bhargavaea beijingensis]
261   9e-75
OGG24279.1 translation elongation factor G [Candidatus Gottes...
263   9e-75
WP_067561546.1 elongation factor G [Acidibacillus ferrooxidans]
263   9e-75
WP_013177533.1 elongation factor G [Truepera radiovictrix]
263   9e-75
SKC00846.1 small GTP-binding protein domain-containing protei...
267   9e-75
WP_075419827.1 GTP-binding protein [Bacillus megaterium]
261   9e-75
WP_017867216.1 elongation factor G [Lactobacillus pobuzihii]
263   9e-75
WP_099305471.1 elongation factor G [Bacillus sp. Marseille-P3...
263   9e-75
PSB64547.1 elongation factor G [filamentous cyanobacterium CCP1]
263   9e-75
WP_019413740.1 GTP-binding protein [Paenisporosarcina sp. TG20]
261   9e-75
WP_082309139.1 elongation factor G [Streptococcus thermophilus]
263   9e-75
WP_013921165.1 elongation factor G [Paenibacillus mucilaginosus]
263   9e-75
WP_101353545.1 GTP-binding protein [Bacillus camelliae]
261   9e-75
WP_035782898.1 GTP-binding protein [Clostridium botulinum]
261   9e-75

WP_068559000.1 MULTISPECIES: elongation factor G [Trichococcus]
263   9e-75
KPK99944.1 elongation factor G [candidate division Zixibacter...
263   9e-75
KUK34306.1 Elongation factor G [Caldanaerobacter subterraneus]
262   9e-75
WP_049460032.1 elongation factor G [Stenotrophomonas maltophi...
262   9e-75
WP_073282333.1 GTP-binding protein [Anaerosporobacter mobilis]
266   9e-75
SHJ32519.1 small GTP-binding protein domain-containing protei...
262   9e-75
EKD65191.1 hypothetical protein ACD_50C00164G0002 [uncultured...
263   9e-75
WP_075980313.1 elongation factor G [Bacillus massilionigerien...
263   9e-75
WP_040379534.1 GTP-binding protein [Clostridiales bacterium 1...
267   1e-74
BBA80144.1 elongation factor G [cyanobacterium endosymbiont o...
263   1e-74
WP_106871514.1 elongation factor G [Campylobacter blaseri]
262   1e-74
KRK10112.1 elongation factor G [Lactobacillus pobuzihii E1003...
263   1e-74
CDF14599.1 small GTP-binding protein domain [Eubacterium sp. ...
266   1e-74
WP_058141875.1 GTP-binding protein [Clostridium butyricum]
261   1e-74
WP_069194438.1 MULTISPECIES: GTP-binding protein [Clostridium]
266   1e-74
WP_017646832.1 elongation factor G [Streptococcus agalactiae]
262   1e-74
WP_012811934.1 elongation factor G [Alicyclobacillus acidocal...
262   1e-74
WP_068225480.1 elongation factor G [Lactobacillus backii]
263   1e-74
WP_067973080.1 MULTISPECIES: elongation factor G [Aerococcus]
263   1e-74
WP_035413156.1 MULTISPECIES: elongation factor G [Exiguobacte...
262   1e-74
WP_045486728.1 MULTISPECIES: elongation factor G [Bacillus]
262   1e-74
WP_045170267.1 elongation factor G [Caldicellulosiruptor morg...
262   1e-74
WP_099466826.1 GTP-binding protein [Lachnospiraceae bacterium...
266   1e-74
WP_094884259.1 GTP-binding protein [Virgibacillus indicus]
261   1e-74
WP_057727553.1 elongation factor G [Lactobacillus fermentum]
262   1e-74
WP_003133544.1 elongation factor G [Lactococcus garvieae]
263   1e-74
WP_066787354.1 GTP-binding protein [Rummeliibacillus stabekisii]
261   1e-74

WP_101891288.1 GTP-binding protein [Lactobacillus crispatus]
261   1e-74
WP_035386105.1 elongation factor G [Exiguobacterium sp. S17]
262   1e-74
WP_023466625.1 MULTISPECIES: elongation factor G [Exiguobacte...
262   1e-74
WP_060380914.1 MULTISPECIES: elongation factor G [Stenotropho...
262   1e-74
WP_066460172.1 GTP-binding protein [Anaerotruncus rubiinfantis]
266   1e-74
WP_103672970.1 GTP-binding protein [Clostridium sp. 3-3]
261   1e-74
OGI21072.1 translation elongation factor G [Candidatus Melain...
262   1e-74
WP_094093158.1 GTP-binding protein [Paenibacillus physcomitre...
262   1e-74
EXZ31087.1 small GTP-binding domain protein [Bacteroides frag...
253   1e-74
WP_086987667.1 MULTISPECIES: elongation factor G [Trichococcus]
262   1e-74
WP_015783324.1 elongation factor G [Cyanothece sp. PCC 8802]
262   1e-74
ABO87349.1 TetM [Streptococcus pyogenes]
248   1e-74
WP_013239112.1 MULTISPECIES: GTP-binding protein [Clostridium]
261   1e-74
CCZ04874.1 elongation factor Tu GTP binding domain-containing...
266   1e-74
WP_062607001.1 elongation factor G [Stenotrophomonas maltophi...
262   1e-74
OGG11871.1 translation elongation factor G [Candidatus Gottes...
262   1e-74
WP_053478267.1 MULTISPECIES: elongation factor G [Bacillus]
262   1e-74
WP_070558374.1 MULTISPECIES: elongation factor G [Aerococcus]
262   1e-74
PJA02744.1 elongation factor G [Gammaproteobacteria bacterium...
262   1e-74
WP_002579813.1 GTP-binding protein [Clostridium butyricum]
261   1e-74
WP_099478291.1 GTP-binding protein [Paenibacillus ihbetae]
261   1e-74
WP_027339550.1 elongation factor G [Halonatronum saccharophilum]
262   1e-74
WP_012594128.1 elongation factor G [Cyanothece sp. PCC 8801]
262   1e-74
KUK09995.1 Elongation factor G [Clostridia bacterium 41_269]
262   1e-74
WP_078697797.1 elongation factor G [Caloramator quimbayensis]
262   1e-74
WP_080849043.1 elongation factor G [Bacillus gottheilii]
262   1e-74
WP_084275206.1 elongation factor G [Nitratiruptor tergarcus]
262   1e-74

WP_097823348.1 GTP-binding protein [Bacillus megaterium]
261   1e-74
WP_087803519.1 elongation factor G [Stenotrophomonas maltophi...
262   1e-74
WP_026860144.1 elongation factor G [Jeotgalicoccus psychrophi...
262   1e-74
WP_067290882.1 elongation factor G [Marinobacterium profundum]
262   1e-74
WP_098526477.1 GTP-binding protein [Bacillus megaterium]
261   1e-74
KUJ90102.1 elongation factor G [Thermoanaerobacter thermocopr...
262   1e-74
WP_087415003.1 GTP-binding protein [Butyricicoccus pullicaeco...
265   1e-74
WP_012369006.1 elongation factor G [Exiguobacterium sibiricum]
262   1e-74
WP_065411733.1 GTP-binding protein [Bacillus wudalianchiensis]
261   1e-74
OOL50811.1 hypothetical protein B1P89_04185 [Enterococcus fae...
244   1e-74
WP_042353186.1 elongation factor G [Bacillus massiliogorillae]
262   1e-74
WP_046513157.1 elongation factor G [Salinicoccus sediminis]
262   1e-74
WP_038475780.1 MULTISPECIES: elongation factor G [Bacillus]
262   1e-74
WP_009550873.1 elongation factor G [Lactobacillus saerimneri]
262   1e-74
WP_009354890.1 elongation factor G [Parvimonas sp. oral taxon...
262   1e-74
KKS32913.1 Elongation factor G [Candidatus Collierbacteria ba...
262   1e-74
WP_098626533.1 GTP-binding protein [Bacillus aryabhattai]
261   1e-74
OGC08211.1 translation elongation factor G [candidate divisio...
262   1e-74
WP_027636511.1 GTP-binding protein [Clostridium butyricum]
261   1e-74
PLX20623.1 elongation factor G [Candidatus Parcubacteria bact...
262   1e-74
WP_071982459.1 GTP-binding protein [Clostridium butyricum]
261   1e-74
WP_103297570.1 elongation factor G [Staphylococcus petrasii]
262   1e-74
WP_011226574.1 elongation factor G [Streptococcus thermophilus]
262   1e-74
WP_011837737.1 GTP-binding protein [Ruminiclostridium thermoc...
266   2e-74
WP_036137722.1 elongation factor G [Luteibacter sp. 9135]
262   2e-74
WP_048514604.1 elongation factor G [Megasphaera cerevisiae]
262   2e-74
WP_042219518.1 elongation factor G [Lactococcus garvieae]
262   2e-74

WP_086942423.1 elongation factor G [Trichococcus pasteurii]
262   2e-74
WP_098322386.1 GTP-binding protein [Bacillus megaterium]
261   2e-74
WP_010010483.1 GTP-binding protein [Lactobacillus coryniformis]
261   2e-74
WP_108031820.1 elongation factor G [Trichococcus patagoniensis]
262   2e-74
WP_018663734.1 elongation factor G [Thermobrachium celere]
262   2e-74
WP_077859443.1 GTP-binding protein [Clostridium sp. BL-8]
261   2e-74
PNW37915.1 translation elongation factor G [Euhalothece sp. K...
262   2e-74
WP_090009365.1 GTP-binding protein [Lachnospiraceae bacterium...
266   2e-74
PMQ01883.1 elongation factor G [Dictyoglomus sp. NZ13-RE01]
262   2e-74
WP_096600416.1 elongation factor G [Hydrogenobacter hydrogeno...
262   2e-74
WP_035395642.1 elongation factor G [Bacillus sp. JCM 19047]
262   2e-74
WP_011057586.1 elongation factor G [Thermosynechococcus elong...
262   2e-74
WP_008373340.1 GTP-binding protein [Coprococcus comes]
266   2e-74
WP_098432881.1 GTP-binding protein [Bacillus megaterium]
261   2e-74
ABO87354.1 TetM [Streptococcus pyogenes]
247   2e-74
WP_023062232.1 GTP-binding protein [Ruminiclostridium thermoc...
266   2e-74
WP_011026341.1 elongation factor G [Caldanaerobacter subterra...
262   2e-74
WP_044978293.1 elongation factor G [Treponema putidum]
261   2e-74
WP_069424280.1 GTP-binding protein [Eisenbergiella tayi]
266   2e-74
WP_090910000.1 GTP-binding protein [Paenibacillus sp. cl141a]
261   2e-74
WP_014969147.1 MULTISPECIES: elongation factor G [Exiguobacte...
262   2e-74
ABO87323.1 TetM [Streptococcus pyogenes]
247   2e-74
WP_008418050.1 GTP-binding protein [Clostridium sp. LS]
261   2e-74
CDB02480.1 elongation factor G [Firmicutes bacterium CAG:145]
261   2e-74
WP_056988326.1 GTP-binding protein [Lactobacillus paraplantarum]
261   2e-74
WP_021771529.1 elongation factor G [Mitsuokella sp. oral taxo...
262   2e-74
WP_084826148.1 elongation factor G [Streptococcus thermophilus]
262   2e-74

WP_006963924.1 elongation factor G [Desulfotignum phosphitoxi...
261   2e-74
WP_031586621.1 elongation factor G [Selenomonas bovis]
261   2e-74
WP_011712523.1 elongation factor G [Magnetococcus marinus]
261   2e-74
WP_028992520.1 elongation factor G [Thermoanaerobacter thermo...
261   2e-74
WP_062904580.1 elongation factor G [Pediococcus damnosus]
262   2e-74
WP_053449713.1 elongation factor G [Stenotrophomonas maltophi...
261   2e-74
ABO87334.1 TetM [Streptococcus pyogenes]
247   2e-74
WP_074462231.1 GTP-binding protein [Butyrivibrio hungatei]
266   2e-74
OIO03475.1 translation elongation factor G [Candidatus Desant...
261   2e-74
WP_008356523.1 elongation factor G [Bacillus xiamenensis]
261   2e-74
WP_005488212.1 elongation factor G [Halanaerobium saccharolyt...
261   2e-74
WP_020225530.1 GTP-binding protein [Holdemania massiliensis]
265   2e-74
WP_084829867.1 elongation factor G [Streptococcus thermophilus]
261   2e-74
ENZ14888.1 small GTP-binding protein domain protein [[[Clost...
266   2e-74
WP_074751979.1 elongation factor G [Lactococcus garvieae]
262   2e-74
WP_002511359.1 elongation factor G [Staphylococcus equorum]
262   2e-74
WP_010014492.1 GTP-binding protein [Lactobacillus coryniformis]
261   2e-74
WP_011983243.1 elongation factor G [Bacillus cytotoxicus]
261   2e-74
WP_103699173.1 GTP-binding protein [Clostridium sp. 2-1]
261   2e-74
WP_055247467.1 GTP-binding protein [Coprococcus comes]
266   2e-74
WP_069716742.1 GTP-binding protein [Bacillus solimangrovi]
261   2e-74
WP_050202073.1 elongation factor G [Streptococcus agalactiae]
261   2e-74
WP_098525018.1 GTP-binding protein [Bacillus megaterium]
261   2e-74
WP_102272412.1 elongation factor G [Bacillus massiliogabonensis]
261   2e-74
WP_051594264.1 GTP-binding protein [Butyrivibrio sp. AE3003]
266   2e-74
WP_057151906.1 elongation factor G [Lactobacillus similis]
261   2e-74
WP_005409615.1 elongation factor G [Stenotrophomonas maltophi...
261   2e-74

WP_003322606.1 elongation factor G [Bacillus alcalophilus]
261   2e-74
WP_050690129.1 GTP-binding protein [Bacillus flexus]
261   2e-74
WP_028573681.1 elongation factor G [Desulfonatronovibrio hydr...
261   2e-74
OGH13690.1 translation elongation factor G [Candidatus Levyba...
262   2e-74
WP_076338907.1 elongation factor G [Paenibacillus sp. FSL H8-...
261   2e-74
WP_061326597.1 GTP-binding protein [Clostridium botulinum]
260   2e-74
WP_013669335.1 MULTISPECIES: elongation factor G [Aerococcus]
261   2e-74
WP_099484206.1 elongation factor G [Stenotrophomonas maltophi...
261   2e-74
WP_099473137.1 elongation factor G [Stenotrophomonas maltophi...
261   2e-74
WP_028405464.1 GTP-binding protein [Bacillus sp. J13]
260   2e-74
WP_020700448.1 elongation factor G [Selenomonas bovis]
261   2e-74
WP_015228369.1 elongation factor G [Dactylococcopsis salina]
261   2e-74
WP_072773019.1 GTP-binding protein [Desulfitobacterium chloro...
265   2e-74
WP_057904119.1 elongation factor G [Lactobacillus bifermentans]
261   2e-74
KPK98721.1 elongation factor G [Omnitrophica WOR_2 bacterium ...
261   2e-74
PSP19610.1 elongation factor G [Cyanobacteria bacterium QS_8_...
261   2e-74
WP_090306579.1 elongation factor G [Eubacterium aggregans]
261   2e-74
WP_108586567.1 GTP-binding protein [Paenisporosarcina sp. OV554]
260   2e-74
WP_095379751.1 GTP-binding protein [Bacillus megaterium]
260   2e-74
WP_041105661.1 elongation factor G [Bacillus pumilus]
261   2e-74
WP_016441059.1 GTP-binding protein [Coprococcus sp. HPP0048]
266   2e-74
WP_090013927.1 GTP-binding protein [Clostridium sp. DSM 8431]
260   2e-74
OLD68252.1 translation elongation factor G [Acidobacteria bac...
261   2e-74
WP_089145613.1 GTP-binding protein [Lactobacillus crispatus]
260   2e-74
WP_036691423.1 MULTISPECIES: elongation factor G [Paenibacillus]
261   2e-74
WP_054735143.1 GTP-binding protein [Lactobacillus similis]
261   2e-74
KKQ74836.1 Elongation factor G [Candidatus Woesebacteria bact...
262   2e-74

WP_012449900.1 GTP-binding protein [Clostridium botulinum]
260   2e-74
WP_029343088.1 MULTISPECIES: elongation factor G [Exiguobacte...
261   2e-74
WP_079418025.1 elongation factor G [Paenibacillus ferrarius]
261   2e-74
WP_099479667.1 elongation factor G [Stenotrophomonas maltophi...
261   2e-74
WP_088428737.1 elongation factor G [Stenotrophomonas maltophi...
261   2e-74
WP_078393391.1 elongation factor G [Bacillus patagoniensis]
261   2e-74
EKE02213.1 hypothetical protein ACD_20C00411G0014 [uncultured...
261   2e-74
OLC14124.1 translation elongation factor G [Candidatus Rokuba...
261   2e-74
WP_091976048.1 elongation factor G [Peptostreptococcus russel...
261   2e-74
OLC37343.1 translation elongation factor G [Acidobacteria bac...
261   2e-74
WP_050677142.1 MULTISPECIES: elongation factor G [Exiguobacte...
261   2e-74
WP_090144866.1 GTP-binding protein [Lachnospiraceae bacterium...
265   2e-74
OFV98154.1 translation elongation factor G [Acidobacteria bac...
261   3e-74
WP_093625107.1 elongation factor G [Lactobacillus gorillae]
261   3e-74
WP_024425594.1 MULTISPECIES: elongation factor G [Bacillus]
261   3e-74
ENZ43482.1 small GTP-binding protein domain protein [[[Clost...
266   3e-74
WP_075621308.1 elongation factor G [Bacillus safensis]
261   3e-74
WP_057767199.1 elongation factor G [Bacillus praedii]
261   3e-74
WP_018366302.1 elongation factor G [Streptococcus didelphis]
261   3e-74
SCK03025.1 Vegetative protein 19 [uncultured Eubacterium sp.]
261   3e-74
WP_058353273.1 GTP-binding protein [Acetivibrio ethanolgignens]
265   3e-74
WP_106789845.1 GTP-binding protein [Mordavella sp. Marseille-...
265   3e-74
AFZ47282.1 translation elongation factor 2 (EF-2/EF-G) [Cyano...
261   3e-74
PKM39504.1 elongation factor G [Firmicutes bacterium HGW-Firm...
261   3e-74
WP_003664573.1 elongation factor G [Lactobacillus reuteri]
261   3e-74
WP_087150906.1 GTP-binding protein [Lachnoclostridium sp. An298]
266   3e-74
WP_023347697.1 GTP-binding protein [Firmicutes bacterium ASF500]
265   3e-74

KKQ79244.1 Elongation factor G [Parcubacteria group bacterium...
261   3e-74
WP_108674725.1 elongation factor G [Bacillus megaterium]
260   3e-74
WP_098573691.1 elongation factor G [Bacillus sp. AFS073361]
261   3e-74
WP_000090325.1 MULTISPECIES: elongation factor G [Streptococcus]
261   3e-74
EKX96305.1 translation elongation factor G [Selenomonas sp. o...
261   3e-74
WP_069155327.1 MULTISPECIES: GTP-binding protein [Lachnospira...
265   3e-74
WP_025656647.1 GTP-binding protein [Clostridiales bacterium V...
265   3e-74
WP_025488908.1 GTP-binding protein [Clostridiales bacterium V...
266   3e-74
WP_034781711.1 elongation factor G [Exiguobacterium mexicanum]
261   3e-74
WP_018663964.1 elongation factor G [Bacillus acidiproducens]
261   3e-74
WP_065418349.1 GTP-binding protein [Clostridium beijerinckii]
261   3e-74
WP_022588777.1 elongation factor G [Caldanaerobacter subterra...
261   3e-74
KRL92316.1 tetracycline resistance protein Tet (M) [Lactobaci...
260   3e-74
WP_105097400.1 elongation factor G [Veillonella sp. S13053-19]
261   3e-74
WP_095456182.1 elongation factor G [Streptococcus agalactiae]
261   3e-74
WP_019206166.1 elongation factor G [Lactobacillus ingluviei]
261   3e-74
WP_089109067.1 elongation factor G [Lactobacillus mixtipabuli]
261   3e-74
SEL33874.1 small GTP-binding protein domain-containing protei...
261   3e-74
CDA30096.1 putative translation elongation factor G [Eubacter...
265   3e-74
WP_071227414.1 elongation factor G [Stenotrophomonas maltophi...
261   3e-74
WP_032970205.1 elongation factor G [Stenotrophomonas maltophi...
261   3e-74
WP_084727520.1 GTP-binding protein [Faecalicatena fissicatena]
265   3e-74
ENZ51586.1 small GTP-binding protein domain protein [[[Clost...
265   3e-74
WP_047750913.1 GTP-binding protein [Bacillus aryabhattai]
260   3e-74
OCW82736.1 elongation factor G [Pelagibacteraceae bacterium G...
261   3e-74
WP_024124793.1 elongation factor G [Thermosynechococcus sp. N...
261   3e-74
WP_089120474.1 elongation factor G [Lactobacillus pentosiphilus]
261   3e-74

WP_015770877.1 elongation factor G [Jonesia denitrificans]
261   3e-74
CCZ93301.1 putative translation elongation factor G [Coprococ...
266   3e-74
WP_009170479.1 GTP-binding protein [Clostridium sp. DL-VIII]
260   3e-74
WP_100070891.1 elongation factor G [Lactobacillus backii]
261   3e-74
WP_078115248.1 GTP-binding protein [Clostridium beijerinckii]
261   3e-74
WP_098249656.1 GTP-binding protein [Bacillus megaterium]
260   3e-74
OLA15355.1 translation elongation factor G [Coprococcus sp. 4...
266   3e-74
WP_107519012.1 elongation factor G [Staphylococcus equorum]
261   3e-74
WP_085436174.1 elongation factor G [unicellular cyanobacteriu...
261   3e-74
WP_033647865.1 elongation factor G [Bacillus anthracis]
261   3e-74
WP_092087994.1 elongation factor G [Peptostreptococcaceae bac...
261   3e-74
WP_015122190.1 elongation factor G [Rivularia sp. PCC 7116]
261   3e-74
WP_037595560.1 elongation factor G [Streptococcus phocae]
261   3e-74
EEQ47756.1 translation elongation factor G [Selenomonas flueg...
261   3e-74
WP_007466141.1 elongation factor G [Photobacterium marinum]
261   3e-74
OZV96347.1 elongation factor G [Bacillus coagulans]
261   3e-74
WP_006508157.1 elongation factor G [Xenococcus sp. PCC 7305]
261   3e-74
WP_087236200.1 GTP-binding protein [Lactobacillus gallinarum]
259   3e-74
OJX04134.1 translation elongation factor G [Caedibacter sp. 3...
261   3e-74
WP_008819190.1 GTP-binding protein [[Clostridium] innocuum]
265   4e-74
WP_073037067.1 elongation factor G [Desulfacinum infernum]
261   4e-74
WP_086627523.1 elongation factor G [Sedimentibacter sp. SX930]
261   4e-74
WP_057749912.1 GTP-binding protein [Pediococcus cellicola]
260   4e-74
WP_026478440.1 GTP-binding protein [Alkaliphilus transvaalensis]
260   4e-74
WP_098045902.1 GTP-binding protein [Lactobacillus sp. UMNPBX17]
259   4e-74
WP_090674751.1 elongation factor G [Paenibacillus tianmuensis]
261   4e-74
WP_019535588.1 elongation factor G [Paenibacillus ginsengihumi]
261   4e-74

WP_054732081.1 elongation factor G [Lactobacillus parafarragi...
261   4e-74
WP_102815792.1 elongation factor G [Lactobacillus reuteri]
261   4e-74
SCH40167.1 Tetracycline resistance protein tetM [uncultured C...
265   4e-74
KUK12912.1 Elongation factor G [Moorella sp. 60_41]
261   4e-74
KKU35927.1 Elongation factor G [Candidatus Beckwithbacteria b...
261   4e-74
WP_087176800.1 GTP-binding protein [Lactobacillus gallinarum]
259   4e-74
OQY01315.1 elongation factor G [Desulfobacteraceae bacterium ...
260   4e-74
WP_026528241.1 GTP-binding protein [Butyrivibrio sp. VCD2006]
265   4e-74
WP_078009230.1 elongation factor G [Lactobacillus reuteri]
261   4e-74
WP_044481390.1 GTP-binding protein [Paenibacillus antibiotico...
260   4e-74
WP_078787009.1 GTP-binding protein [Eubacterium ruminantium]
265   4e-74
WP_099552242.1 elongation factor G [Stenotrophomonas maltophi...
260   4e-74
WP_028408440.1 MULTISPECIES: GTP-binding protein [Bacillus]
260   4e-74
WP_085650556.1 elongation factor G [Lactobacillus reuteri]
261   4e-74
WP_013226314.1 elongation factor G [Amycolatopsis mediterranei]
260   4e-74
WP_027309282.1 elongation factor G [Caloramator sp. ALD01]
261   4e-74
WP_049429298.1 elongation factor G [Stenotrophomonas maltophi...
260   4e-74
WP_057974644.1 elongation factor G [Lactobacillus algidus]
261   4e-74
WP_038263716.1 GTP-binding protein [Peptoclostridium litorale]
265   4e-74
WP_055214899.1 GTP-binding protein [Dorea longicatena]
265   4e-74
WP_087033330.1 elongation factor G [Trichococcus palustris]
261   4e-74
WP_101540199.1 elongation factor G [Anaerococcus octavius]
261   4e-74
WP_049397259.1 elongation factor G [Stenotrophomonas maltophi...
260   4e-74
WP_099560486.1 elongation factor G [Stenotrophomonas maltophi...
260   4e-74
WP_036201395.1 GTP-binding protein [Lysinibacillus sinduriensis]
259   4e-74
WP_069429054.1 GTP-binding protein [Eisenbergiella tayi]
265   4e-74
WP_024355278.1 elongation factor G [Brevundimonas naejangsane...
261   4e-74

AOT56860.1 Elongation factor [Weissella soli]
261   4e-74
WP_035771038.1 GTP-binding protein [Butyrivibrio sp. FCS006]
265   4e-74
OGR59452.1 translation elongation factor G [Elusimicrobia bac...
261   4e-74
WP_047199443.1 elongation factor G [Streptococcus agalactiae]
261   4e-74
KKW00863.1 Elongation factor G [Parcubacteria group bacterium...
261   4e-74
OUX65179.1 elongation factor G [Pelagibacteraceae bacterium T...
261   4e-74
WP_042347237.1 GTP-binding protein [Bacillus massiliogorillae]
259   5e-74
WP_063247958.1 GTP-binding protein [Bacillus aryabhattai]
259   5e-74
WP_026974605.1 elongation factor G [Alicyclobacillus contamin...
261   5e-74
WP_030468686.1 elongation factor G [Lechevalieria aerocolonig...
260   5e-74
WP_014824113.1 GTP-binding protein [Solibacillus silvestris]
259   5e-74
OGI05073.1 translation elongation factor G [Candidatus Melain...
261   5e-74
WP_016429288.1 GTP-binding protein [Paenisporosarcina sp. HGH...
259   5e-74
WP_057236454.1 GTP-binding protein [Bacillus sp. Root147]
259   5e-74
WP_014099980.1 elongation factor G [Chloracidobacterium therm...
261   5e-74
WP_091414457.1 elongation factor G [Friedmanniella luteola]
261   5e-74
WP_106468005.1 elongation factor G [Stenotrophomonas maltophi...
260   5e-74
WP_016439669.1 GTP-binding protein [Coprococcus sp. HPP0074]
265   5e-74
OGC46606.1 translation elongation factor G [candidate divisio...
261   5e-74
WP_039291576.1 GTP-binding protein [Paenibacillus sp. IHB B 3...
259   5e-74
KKQ67441.1 Elongation factor G [Candidatus Daviesbacteria bac...
261   5e-74
WP_060459197.1 elongation factor G [Lactobacillus acetotolerans]
261   5e-74
WP_086340780.1 elongation factor G [Enterococcus sp. 6C8_DIV0...
261   5e-74
WP_004851356.1 GTP-binding protein [Coprococcus eutactus]
265   5e-74
GBE17144.1 elongation factor G [bacterium BMS3Abin15]
261   5e-74
WP_054748732.1 elongation factor G [Veillonella rogosae]
260   5e-74
WP_008825032.1 elongation factor G [Haloplasma contractile]
260   5e-74

PKO23793.1 elongation factor G [Chloroflexi bacterium HGW-Chl...
261   5e-74
OGJ69373.1 translation elongation factor G [Candidatus Periba...
261   5e-74
WP_019554402.1 MULTISPECIES: elongation factor G [Propionispira]
260   5e-74
OJV60435.1 translation elongation factor G [Cellulomonas sp. ...
261   5e-74
WP_089982126.1 GTP-binding protein [Lachnospiraceae bacterium...
265   5e-74
SCH65513.1 Elongation factor G [uncultured Clostridium sp.]
265   5e-74
OUW10205.1 elongation factor G [Cellvibrionales bacterium TME...
260   5e-74
WP_084830785.1 elongation factor G [Streptococcus thermophilus]
260   5e-74
WP_025748458.1 MULTISPECIES: elongation factor G [Caldicoprob...
260   5e-74
WP_098333134.1 GTP-binding protein [Bacillus megaterium]
259   5e-74
WP_101576226.1 MULTISPECIES: elongation factor G [Bacillus]
260   5e-74
WP_039175978.1 MULTISPECIES: elongation factor G [Bacillus]
260   5e-74
WP_027130944.1 elongation factor G [Gemella cuniculi]
260   5e-74
WP_064604823.1 elongation factor G [Photobacterium sp. J15]
260   5e-74
WP_021168691.1 MULTISPECIES: elongation factor G [Sporomusa]
260   5e-74
WP_015076456.1 elongation factor G [Carnobacterium maltaromat...
260   6e-74
WP_024377775.1 elongation factor G [Streptococcus suis]
260   6e-74
WP_100403556.1 GTP-binding protein [Bacillus sp. FJAT-42315]
259   6e-74
WP_026684118.1 elongation factor G [Bacillus coagulans]
260   6e-74
ETI90689.1 Tetracycline resistance protein TetP, containing G...
259   6e-74
WP_065530896.1 GTP-binding protein [[Clostridium] innocuum]
264   6e-74
WP_010734473.1 MULTISPECIES: elongation factor G [Enterococcus]
260   6e-74
WP_020682432.1 elongation factor G [Marinobacterium rhizophilum]
260   6e-74
WP_056969525.1 elongation factor G [Lactobacillus acetotolerans]
261   6e-74
WP_060697843.1 elongation factor G [Bacillus australimaris]
260   6e-74
GBD83803.1 elongation factor G [bacterium BMS3Abin02]
261   6e-74
WP_089088838.1 elongation factor G [Lactobacillus pentosiphilus]
261   6e-74

WP_074199243.1 elongation factor G [Acetomicrobium flavidum]
260   6e-74
WP_073155167.1 elongation factor G [Seinonella peptonophila]
260   6e-74
OGJ67201.1 translation elongation factor G [Candidatus Periba...
260   6e-74
WP_050198658.1 elongation factor G [Streptococcus agalactiae]
260   6e-74
WP_000090326.1 elongation factor G [Streptococcus agalactiae]
260   6e-74
WP_105143417.1 elongation factor G [Streptococcus suis]
260   6e-74
KMK72471.1 elongation factor G [Bacillus pumilus]
260   6e-74
WP_046323140.1 elongation factor G [Bifidobacterium asteroides]
261   6e-74
WP_061086384.1 elongation factor G [Bacillus coagulans]
260   6e-74
WP_048240166.1 MULTISPECIES: elongation factor G [Bacillus]
260   6e-74
WP_089150527.1 GTP-binding protein [Lactobacillus crispatus]
259   6e-74
CDA19413.1 putative translation elongation factor G [Ruminoco...
263   6e-74
WP_061793001.1 elongation factor G [Bacillus firmus]
260   6e-74
OGC45478.1 translation elongation factor G [candidate divisio...
260   6e-74
WP_106187109.1 elongation factor G [Umezawaea tangerina]
260   6e-74
WP_028557498.1 elongation factor G [Paenibacillus sp. URHA0014]
260   6e-74
WP_098823979.1 elongation factor G [Lactobacillus silagincola]
260   6e-74
WP_010767912.1 elongation factor G [Enterococcus phoeniculicola]
260   6e-74
WP_089525808.1 elongation factor G [Paenibacillus herberti]
260   6e-74
WP_075269191.1 elongation factor G [Weissella jogaejeotgali]
261   6e-74
WP_014806363.1 elongation factor G [Acetomicrobium mobile]
260   6e-74
WP_042682353.1 elongation factor G [Anaerosalibacter massilie...
260   6e-74
OGH41476.1 translation elongation factor G [Candidatus Levyba...
260   6e-74
WP_049467693.1 MULTISPECIES: elongation factor G [Stenotropho...
260   6e-74
WP_071396719.1 elongation factor G [Bacillus tuaregi]
260   6e-74
WP_013382421.1 MULTISPECIES: elongation factor G [Clostridiales]
260   6e-74
WP_074681142.1 GTP-binding protein [Bacillus aryabhattai]
259   7e-74

WP_076759851.1 GTP-binding protein [Edaphobacillus lindanitol...
259   7e-74
WP_095333387.1 GTP-binding protein [Bacillus circulans]
259   7e-74
WP_099643033.1 elongation factor G [Pseudoalteromonas piscicida]
260   7e-74
WP_009555174.1 elongation factor G [Oscillatoriales cyanobact...
260   7e-74
KKR30282.1 Elongation factor G [Candidatus Woesebacteria bact...
261   7e-74
EFQ04402.1 translation elongation factor G [Megasphaera micro...
260   7e-74
WP_025976279.1 elongation factor G [Brevundimonas naejangsane...
260   7e-74
WP_019006422.1 elongation factor G [Cohnella laeviribosi]
260   7e-74
WP_098484328.1 elongation factor G [Georgenia soli]
260   7e-74
SIT92670.1 small GTP-binding protein domain-containing protei...
259   7e-74
WP_061800844.1 GTP-binding protein [Bacillus circulans]
259   7e-74
WP_088849920.1 elongation factor G [Streptococcus pneumoniae]
260   7e-74
OGD50788.1 translation elongation factor G [Candidatus Beckwi...
261   7e-74
WP_068125554.1 elongation factor G [Rhodothermaceae bacterium...
260   7e-74
WP_016250284.1 elongation factor G [Enterococcus cecorum]
260   7e-74
WP_075333891.1 elongation factor G [Pseudonocardia sp. Ae717_...
260   7e-74
WP_010053699.1 elongation factor G [Carnobacterium maltaromat...
260   7e-74
WP_026072930.1 elongation factor G [Nodosilinea nodulosa]
260   7e-74
WP_098658366.1 GTP-binding protein [Bacillus megaterium]
259   7e-74
WP_079427565.1 GTP-binding protein [Clostridium oryzae]
259   7e-74
OQW63950.1 translation elongation factor G [Nitrospira sp. ST...
260   7e-74
WP_008232939.1 elongation factor G [Richelia intracellularis]
260   7e-74
WP_100445630.1 elongation factor G [Stenotrophomonas maltophi...
260   7e-74
WP_061566913.1 elongation factor G [Bacillus coagulans]
260   7e-74
WP_000090324.1 elongation factor G [Streptococcus agalactiae]
260   7e-74
WP_098112650.1 GTP-binding protein [Bacillus aryabhattai]
259   7e-74
WP_027106710.1 elongation factor G [Lactobacillus ceti]
260   7e-74

WP_050254854.1 elongation factor G [Streptococcus pneumoniae]
260   7e-74
WP_019722053.1 elongation factor G [Bacillus coagulans]
260   7e-74
WP_027718943.1 elongation factor G [Desulfovirgula thermocuni...
260   8e-74
WP_098792218.1 GTP-binding protein [Bacillus megaterium]
259   8e-74
SCH36046.1 Elongation factor G [uncultured Clostridium sp.]
265   8e-74
WP_043033549.1 elongation factor G [Stenotrophomonas maltophi...
259   8e-74
WP_020876986.1 elongation factor G [Desulfococcus multivorans]
259   8e-74
SME87918.1 elongation factor G [Pseudobacteriovorax antillogo...
260   8e-74
WP_027399002.1 GTP-binding protein [Anaerovorax odorimutans]
264   8e-74
WP_000090323.1 MULTISPECIES: elongation factor G [Streptococcus]
260   8e-74
WP_100212789.1 GTP-binding protein [Pediococcus damnosus]
259   8e-74
WP_044538539.1 MULTISPECIES: elongation factor G [Bradyrhizob...
260   8e-74
WP_003678160.1 GTP-binding protein [Lactobacillus coryniformis]
259   8e-74
OHA64696.1 translation elongation factor G [Candidatus Wilder...
260   8e-74
WP_068474483.1 elongation factor G [Parvimonas sp. KA00067]
260   8e-74
WP_027018760.1 elongation factor G [Corynebacterium massiliense]
260   8e-74
WP_017554004.1 elongation factor G [Bacillus coagulans]
260   8e-74
WP_063556439.1 GTP-binding protein [Clostridium ljungdahlii]
259   8e-74
WP_089120419.1 GTP-binding protein [Lactobacillus pentosiphilus]
259   8e-74
WP_098999352.1 GTP-binding protein [Bacillus aryabhattai]
259   8e-74
WP_047154550.1 elongation factor G [Aneurinibacillus tyrosini...
260   8e-74
CDC06074.1 elongation factor G 1 [Clostridium sp. CAG:343]
260   8e-74
AKQ03427.1 elongation factor G, elongation factor G [uncultur...
260   8e-74
WP_045500513.1 elongation factor G [Streptococcus cristatus]
260   8e-74
SCH28555.1 Vegetative protein 19 [uncultured Eubacterium sp.]
260   8e-74
WP_041971696.1 elongation factor G [Geobacter sp. OR-1]
260   8e-74
CCY57876.1 putative tetracycline resistance protein TetP [Clo...
264   8e-74

WP_066330430.1 elongation factor G [Bacillus litoralis]
260   8e-74
WP_061565127.1 elongation factor G [Bacillus coagulans]
260   8e-74
WP_086987888.1 elongation factor G [Trichococcus flocculiformis]
260   8e-74
ASS41726.1 translation elongation factor G [Eubacterium minut...
260   8e-74
WP_061574734.1 elongation factor G [Bacillus coagulans]
260   9e-74
WP_065078130.1 GTP-binding protein [Clostridium ragsdalei]
259   9e-74
KKT26658.1 Elongation factor G [Parcubacteria group bacterium...
260   9e-74
WP_084829113.1 elongation factor G [Streptococcus thermophilus]
260   9e-74
WP_025006117.1 GTP-binding protein [Lactobacillus gallinarum]
259   9e-74
SCI37977.1 Tetracycline resistance protein tetM from transpos...
265   9e-74
WP_049436395.1 elongation factor G [Stenotrophomonas maltophi...
259   9e-74
CDC58361.1 elongation factor G [Lactobacillus ruminis CAG:367]
260   9e-74
WP_107431864.1 elongation factor G [[Pseudomonas] geniculata]
259   9e-74
WP_003672254.1 MULTISPECIES: elongation factor G [Lactobacillus]
260   9e-74
WP_078811129.1 elongation factor G [Selenihalanaerobacter shr...
260   9e-74
WP_009243369.1 GTP-binding protein [Lachnospiraceae bacterium...
261   9e-74
WP_084578494.1 elongation factor G [Sporomusa malonica]
260   9e-74
WP_028413267.1 GTP-binding protein [Bacillus sp. 278922_107]
259   9e-74
WP_075688634.1 GTP-binding protein [Bacillus sp. MRMR6]
259   9e-74
WP_005965298.1 MULTISPECIES: elongation factor G [sulfur-oxid...
260   9e-74
EJQ50665.1 small GTP-binding protein domain protein [Bacillus...
249   9e-74
WP_097049066.1 elongation factor G [Stenotrophomonas sp. CC12...
259   9e-74
WP_011010149.1 GTP-binding protein [Clostridium perfringens]
259   9e-74
WP_013458254.1 elongation factor G [Oceanithermus profundus]
260   9e-74
OIO14240.1 translation elongation factor G [Candidatus Gottes...
260   9e-74
CCQ77858.1 translation elongation factor G [Streptococcus aga...
260   9e-74
WP_073025172.1 GTP-binding protein [Lutispora thermophila]
264   9e-74

WP_063507518.1 elongation factor G [Lactobacillus acetotolerans]
260   9e-74
WP_089145850.1 MULTISPECIES: GTP-binding protein [Lactobacillus]
258   9e-74
WP_099561323.1 elongation factor G [Stenotrophomonas maltophi...
259   9e-74
WP_008751070.1 GTP-binding protein [Lachnoanaerobaculum sabur...
263   1e-73
WP_078686713.1 GTP-binding protein [Olsenella sp. KH1P3]
259   1e-73
WP_046464998.1 elongation factor G [Staphylococcus equorum]
260   1e-73
WP_025851027.1 elongation factor G [Paenibacillus ehimensis]
259   1e-73
WP_017551221.1 elongation factor G [Bacillus coagulans]
259   1e-73
WP_000090322.1 elongation factor G [Streptococcus agalactiae]
259   1e-73
WP_099331020.1 GTP-binding protein [Bacillus aryabhattai]
259   1e-73
WP_088025936.1 elongation factor G [Stenotrophomonas maltophi...
259   1e-73
WP_024957368.1 MULTISPECIES: elongation factor G [Stenotropho...
259   1e-73
WP_046871422.1 GTP-binding protein [Pediococcus damnosus]
259   1e-73
WP_010496686.1 elongation factor G [Lactobacillus acidipiscis]
260   1e-73
OKZ36168.1 translation elongation factor G [Butyrivibrio cros...
263   1e-73
WP_089143186.1 GTP-binding protein [Lactobacillus crispatus]
258   1e-73
OGW43049.1 translation elongation factor G [Nitrospirae bacte...
259   1e-73
SER28428.1 small GTP-binding protein domain-containing protei...
259   1e-73
WP_016290443.1 GTP-binding protein [Lachnospiraceae bacterium...
264   1e-73
WP_041219825.1 GTP-binding protein [Desulfitobacterium dichlo...
263   1e-73
WP_075366530.1 GTP-binding protein [Desulfosporosinus sp. OL]
263   1e-73
WP_014096503.1 elongation factor G [Bacillus coagulans]
259   1e-73
WP_063187735.1 elongation factor G [Paenibacillus elgii]
259   1e-73
CDX01055.1 Tetracycline resistance protein TetP [Desulfitobac...
263   1e-73
WP_014295289.1 MULTISPECIES: elongation factor G [Streptococcus]
259   1e-73
OGY28492.1 translation elongation factor G [Candidatus Woykeb...
260   1e-73
WP_040537650.1 elongation factor G [Lactobacillus parafarragi...
259   1e-73

EEQ56873.1 putative translation elongation factor G [Clostrid...
264   1e-73
WP_095313560.1 elongation factor G [Paenibacillus sp. 7884-2]
259   1e-73
WP_103282370.1 elongation factor G [Stenotrophomonas pavanii]
259   1e-73
WP_016184212.1 elongation factor G [Enterococcus columbae]
259   1e-73
KRM30943.1 elongation factor G [Lactobacillus acidipiscis DSM...
260   1e-73
WP_092073795.1 elongation factor G [Dendrosporobacter quercic...
259   1e-73
WP_009336567.1 MULTISPECIES: elongation factor G [Bacillus]
259   1e-73
WP_004824930.1 elongation factor G [Peptoniphilus lacrimalis]
259   1e-73
WP_009469203.1 elongation factor G [Roseibium sp. TrichSKD4]
259   1e-73
WP_073339058.1 GTP-binding protein [Clostridium grantii]
258   1e-73
EFW29716.1 translation elongation factor G [Selenomonas artem...
259   1e-73
WP_014072856.1 elongation factor G [Lactobacillus ruminis]
259   1e-73
WP_090175459.1 elongation factor G [Luteibacter sp. UNC138MFC...
259   1e-73
WP_071140202.1 elongation factor G [Sporanaerobacter sp. PP17...
259   1e-73
WP_075843423.1 GTP-binding protein [Collinsella bouchesdurhon...
259   1e-73
AWD63000.1 elongation factor P [Lactobacillus reuteri]
259   1e-73
CDA68907.1 putative uncharacterized protein [Clostridium sp. ...
263   1e-73
WP_044106831.1 elongation factor G [cyanobacterium endosymbio...
259   1e-73
WP_071659927.1 elongation factor G [Streptococcus agalactiae]
259   1e-73
OUM99086.1 translation elongation factor G [Paenibacillaceae ...
259   1e-73
OLC30207.1 translation elongation factor G [Armatimonadetes b...
259   1e-73
OGI25903.1 translation elongation factor G [Candidatus Moranb...
259   1e-73
WP_034665610.1 MULTISPECIES: elongation factor G [Bacillus]
259   1e-73
WP_041139494.1 GTP-binding protein [Beduini massiliensis]
263   1e-73
WP_098036905.1 MULTISPECIES: GTP-binding protein [Lactobacillus]
258   1e-73
OQY27113.1 elongation factor G [Candidatus Cloacimonetes bact...
259   1e-73
WP_035172214.1 elongation factor G [Lactobacillus ruminis]
259   1e-73

WP_085719857.1 elongation factor G [Lactobacillus reuteri]
259   1e-73
WP_106031908.1 elongation factor G [Bacillus sp. NMCC4]
259   1e-73
WP_093589776.1 elongation factor G [Lentzea waywayandensis]
259   1e-73
WP_076768484.1 elongation factor G [Jeotgalibaca sp. PTS2502]
259   1e-73
WP_007496301.1 MULTISPECIES: elongation factor G [Bacillus]
259   1e-73
WP_015219284.1 elongation factor G [Cyanobacterium aponinum]
259   1e-73
WP_010750805.1 elongation factor G [Enterococcus villorum]
259   1e-73
WP_092597975.1 elongation factor G [Jeotgalicoccus halophilus]
259   1e-73
WP_003692839.1 elongation factor G [Lactobacillus ruminis]
259   1e-73
WP_050197374.1 elongation factor G [Streptococcus agalactiae]
259   1e-73
WP_062916619.1 GTP-binding protein [Pediococcus damnosus]
258   1e-73
WP_050168154.1 elongation factor G [Streptococcus pneumoniae]
259   1e-73
WP_016623869.1 elongation factor G [Enterococcus faecalis]
259   1e-73
WP_090776231.1 elongation factor G [Bacillus lonarensis]
259   1e-73
OGJ64412.1 translation elongation factor G [Candidatus Periba...
259   1e-73
WP_056949311.1 elongation factor G [Lactobacillus kisonensis]
259   1e-73
WP_090572474.1 GTP-binding protein [Paenibacillus sp. OV219]
258   1e-73
KXK05746.1 Elongation factor EF-G [Chlorobi bacterium OLB4]
259   1e-73
WP_066347212.1 elongation factor G [Geminocystis sp. NIES-3708]
259   1e-73
WP_003674892.1 elongation factor G [Lactobacillus reuteri]
259   1e-73
KGM17565.1 elongation factor G [Actinotalea fermentans ATCC 4...
259   1e-73
WP_003656857.1 GTP-binding protein [Lactobacillus gasseri]
258   1e-73
SCH70892.1 Tetracycline resistance protein tetM [uncultured C...
263   1e-73
WP_047036237.1 GTP-binding protein [Lactobacillus salivarius]
258   1e-73
WP_087476557.1 elongation factor G [Nitrospira cf. moscoviens...
259   1e-73
KKU57286.1 Elongation factor G [Candidatus Amesbacteria bacte...
260   1e-73
WP_098388715.1 elongation factor G [Corynebacterium renale]
259   1e-73

EFR60752.1 translation elongation factor G [Veillonella sp. o...
259   1e-73
AGA69022.1 small GTP-binding protein domain protein [Desulfit...
263   1e-73
WP_043063754.1 elongation factor G [Aneurinibacillus migulanus]
259   1e-73
WP_095136212.1 elongation factor G [Anaeromicrobium sediminis]
259   1e-73
WP_047946815.1 elongation factor G [Bacillus altitudinis]
259   1e-73
OJX09652.1 translation elongation factor G [Caedibacter sp. 3...
259   1e-73
WP_007132255.1 GTP-binding protein [Paenibacillus lactis]
258   1e-73
WP_081115156.1 elongation factor G [Enterococcus hirae]
259   1e-73
WP_100542026.1 GTP-binding protein [Lysinibacillus xylanilyti...
258   2e-73
WP_093335349.1 elongation factor G [Pseudonocardia ammonioxyd...
259   2e-73
WP_049450242.1 elongation factor G [Stenotrophomonas maltophi...
259   2e-73
CDD84703.1 putative translation elongation factor G [Collinse...
258   2e-73
WP_000090327.1 elongation factor G [Streptococcus agalactiae]
259   2e-73
WP_048669813.1 elongation factor G [Candidatus Competibacter ...
259   2e-73
WP_055193481.1 GTP-binding protein [Dorea longicatena]
263   2e-73
EHL99178.1 translation elongation factor G [Lactobacillus par...
259   2e-73
WP_105166615.1 elongation factor G [Pseudoalteromonas sp. T1l...
259   2e-73
WP_034323229.1 MULTISPECIES: elongation factor G [Bacillus]
259   2e-73
WP_103976500.1 GTP-binding protein [Lysinibacillus sphaericus]
258   2e-73
WP_097018994.1 GTP-binding protein [Orenia metallireducens]
258   2e-73
WP_057001039.1 elongation factor G [Carnobacterium maltaromat...
259   2e-73
CDC80071.1 elongation factor G 1 [Clostridium sp. CAG:465]
259   2e-73
WP_027868399.1 elongation factor G [Eubacterium sp. AB3007]
259   2e-73
WP_093728582.1 elongation factor G [Terribacillus halophilus]
259   2e-73
WP_046176545.1 elongation factor G [Domibacillus indicus]
259   2e-73
OGN61498.1 translation elongation factor G [Chlamydiae bacter...
259   2e-73
WP_005545160.1 GTP-binding protein [Paenibacillus alvei]
258   2e-73

WP_020585502.1 elongation factor G [Desulfobacter curvatus]
259   2e-73
WP_039121816.1 elongation factor G [Lactobacillus ruminis]
259   2e-73
PKN33574.1 elongation factor G [Deltaproteobacteria bacterium...
259   2e-73
WP_100442003.1 elongation factor G [Stenotrophomonas maltophi...
259   2e-73
WP_024532235.1 elongation factor G [Streptococcus suis]
259   2e-73
APO31209.1 feoB: ferrous iron transport protein [uncultured b...
241   2e-73
WP_022415881.1 GTP-binding protein [Dorea longicatena]
263   2e-73
WP_017380687.1 GTP-binding protein [Paenisporosarcina sp. TG-14]
258   2e-73
WP_071452225.1 elongation factor G [Bacillus coagulans]
259   2e-73
WP_068724550.1 GTP-binding protein [Paenibacillus sp. DMB5]
258   2e-73
WP_098036108.1 MULTISPECIES: GTP-binding protein [Lactobacillus]
258   2e-73
WP_093275166.1 GTP-binding protein [Psychrobacillus sp. OK032]
258   2e-73
WP_046213349.1 elongation factor G [Paenibacillus wulumuqiensis]
259   2e-73
WP_034926817.1 elongation factor G [Erysipelotrichaceae bacte...
259   2e-73
WP_063600566.1 GTP-binding protein [Clostridium coskatii]
258   2e-73
WP_045091291.1 GTP-binding protein [Clostridium sp. FS41]
263   2e-73
WP_107668160.1 elongation factor G [Cyanothece sp. BG0011]
259   2e-73
WP_101702517.1 elongation factor G [Carnobacterium maltaromat...
259   2e-73
WP_009344724.1 MULTISPECIES: elongation factor G [Peptoniphilus]
259   2e-73
CCX47210.1 elongation factor G [Firmicutes bacterium CAG:103]
259   2e-73
CCZ85265.1 elongation factor G [Firmicutes bacterium CAG:631]
259   2e-73
ABO87328.1 TetM [Streptococcus pyogenes]
244   2e-73
GBE52227.1 elongation factor G [bacterium BMS3Bbin14]
259   2e-73
WP_039649463.1 elongation factor G [Campylobacter insulaenigrae]
259   2e-73
EGM49933.1 elongation factor G [Lactobacillus ruminis SPM0211]
259   2e-73
WP_053415842.1 GTP-binding protein [Viridibacillus arvi]
258   2e-73
WP_005603474.1 GTP-binding protein [Butyrivibrio crossotus]
262   2e-73

KRT77453.1 translation elongation factor G, elongation factor...
259   2e-73
AVM22448.1 elongation factor G [Bacillus pumilus]
259   2e-73
WP_055267945.1 GTP-binding protein [Clostridium disporicum]
258   2e-73
CDA89546.1 putative translation elongation factor G [Ruminoco...
262   2e-73
WP_069697081.1 elongation factor G [Enterococcus rivorum]
259   2e-73
WP_026522632.1 GTP-binding protein [Butyrivibrio sp. VCB2001]
263   2e-73
WP_053418538.1 elongation factor G [Viridibacillus arvi]
259   2e-73
WP_019253132.1 elongation factor G [Lactobacillus reuteri]
259   2e-73
WP_088270042.1 elongation factor G [Enterococcus wangshanyuanii]
259   2e-73
WP_060528235.1 elongation factor G [endosymbiont of Ridgeia p...
259   2e-73
WP_026583279.1 elongation factor G [Bacillus sp. J33]
259   2e-73
WP_090779784.1 elongation factor G [Paenibacillaceae bacteriu...
259   2e-73
CDA92133.1 elongation factor G [Firmicutes bacterium CAG:238]
259   2e-73
CDD59178.1 elongation factor G [Eggerthella sp. CAG:298]
259   2e-73
WP_066226667.1 elongation factor G [Bacillus sporothermodurans]
259   2e-73
WP_070372669.1 elongation factor G [Acetobacterium wieringae]
259   2e-73
WP_012008700.1 elongation factor G [Bacillus pumilus]
259   2e-73
WP_057942043.1 elongation factor G [Lysobacter gummosus]
258   2e-73
WP_014037168.1 elongation factor G [Stenotrophomonas maltophi...
258   2e-73
WP_014067553.1 elongation factor G [Rhodothermus marinus]
259   2e-73
WP_040203233.1 MULTISPECIES: elongation factor G [Bacillus]
259   2e-73
WP_085070577.1 elongation factor G [Candidatus Pelagibacter s...
259   2e-73
WP_045516743.1 GTP-binding protein [Bacillus niacini]
258   2e-73
WP_062913227.1 GTP-binding protein [Pediococcus damnosus]
258   2e-73
WP_083806164.1 GTP-binding protein [Clostridium sp. D5]
263   2e-73
EGB93079.1 putative tetracycline resistance protein TetP [Clo...
263   2e-73
SCJ13374.1 Elongation factor G [uncultured Flavonifractor sp.]
262   2e-73

WP_041084119.1 GTP-binding protein [Clostridium botulinum]
258   2e-73
WP_016466269.1 elongation factor G [Streptococcus sp. HPH0090]
259   2e-73
WP_088116363.1 elongation factor G [Bacillus atrophaeus]
259   2e-73
WP_017327001.1 elongation factor G [Synechococcus sp. PCC 7336]
259   2e-73
CDA30508.1 elongation factor G 1 [Clostridium sp. CAG:492]
259   2e-73
WP_075397861.1 elongation factor G [Domibacillus antri]
259   2e-73
AIL12567.1 elongation factor G [Candidatus Paracaedimonas aca...
259   2e-73
WP_062484676.1 elongation factor G [Candidatus Nitrospira ino...
259   2e-73
WP_027969352.1 elongation factor G [Streptococcus castoreus]
259   2e-73
WP_092034889.1 elongation factor G [Bradyrhizobium sp. OK095]
259   2e-73
WP_091075644.1 elongation factor G [Friedmanniella sagamihare...
259   2e-73
PMP62232.1 elongation factor G [Sulfurihydrogenibium sp.]
259   2e-73
PQC52936.1 hypothetical protein CUN09_04880 [Enterococcus fae...
241   2e-73
KZK73747.1 elongation factor G [Pelodictyon luteolum]
259   2e-73
WP_006515832.1 elongation factor G [Leptolyngbya sp. PCC 7375]
259   2e-73
WP_026233180.1 MULTISPECIES: elongation factor G [Bradyrhizob...
259   2e-73
WP_056978569.1 elongation factor G [Lactobacillus senioris]
259   2e-73
KFD41249.1 elongation factor G [Peptococcaceae bacterium SCAD...
259   2e-73
WP_047000990.1 GTP-binding protein [Clostridium sp. C8]
258   2e-73
WP_049439864.1 elongation factor G [Stenotrophomonas maltophi...
258   2e-73
EHL72128.1 small GTP-binding protein domain protein [Bacillus...
247   2e-73
EJX54514.1 elongation factor G [Enterococcus faecium R497]
241   2e-73
PLX46385.1 elongation factor G [Desulfobulbaceae bacterium]
259   2e-73
WP_050341610.1 MULTISPECIES: elongation factor G [Selenomonas]
259   2e-73
SEH36212.1 small GTP-binding protein domain-containing protei...
258   2e-73
WP_071062267.1 elongation factor G [Andreesenia angusta]
258   2e-73
WP_098623170.1 GTP-binding protein [Bacillus aryabhattai]
258   3e-73

WP_075712786.1 elongation factor G [Eubacterium sp. 68-3-10]
258   3e-73
WP_065178578.1 elongation factor G [Stenotrophomonas maltophi...
258   3e-73
WP_021623682.1 elongation factor G [Aneurinibacillus aneurini...
259   3e-73
WP_005814361.1 GTP-binding protein [Desulfitobacterium hafnie...
262   3e-73
WP_056635998.1 elongation factor G [Paenibacillus sp. Soil522]
258   3e-73
WP_007505930.1 elongation factor G [Caldalkalibacillus therma...
258   3e-73
AMP51642.1 elongation factor G, domain IV [uncultured bacterium]
243   3e-73
WP_018399485.1 elongation factor G [filamentous cyanobacteriu...
258   3e-73
WP_016288260.1 GTP-binding protein [Lachnospiraceae bacterium...
263   3e-73
WP_071542801.1 GTP-binding protein [Lactobacillus plantarum]
258   3e-73
WP_034905182.1 elongation factor G [Campylobacter sp. MIT 97-...
258   3e-73
WP_003464697.1 GTP-binding protein [Clostridium perfringens]
258   3e-73
WP_099435239.1 elongation factor G [Cyanobacterium aponinum]
259   3e-73
WP_074725105.1 elongation factor G [Stenotrophomonas pavanii]
258   3e-73
WP_049399339.1 elongation factor G [Stenotrophomonas maltophi...
258   3e-73
OKZ87125.1 translation elongation factor G [Clostridium sp. C...
259   3e-73
WP_057776535.1 elongation factor G [Bacillus sp. FJAT-25496]
258   3e-73
WP_038179055.1 MULTISPECIES: elongation factor G [Viridibacil...
258   3e-73
WP_018364994.1 elongation factor G [Streptococcus caballi]
258   3e-73
WP_018380518.1 elongation factor G [Streptococcus thoraltensis]
258   3e-73
OGM04931.1 translation elongation factor G [Candidatus Woeseb...
259   3e-73
WP_082375085.1 elongation factor G [Pseudonocardia sp. HH1306...
259   3e-73
WP_066397956.1 GTP-binding protein [Bacillus mesonae]
258   3e-73
WP_010720460.1 MULTISPECIES: elongation factor G [Enterococcus]
258   3e-73
WP_061643205.1 elongation factor G [Streptococcus pneumoniae]
258   3e-73
WP_053505299.1 elongation factor G [Stenotrophomonas maltophi...
258   3e-73
WP_076996498.1 elongation factor G [Streptococcus azizii]
258   3e-73

WP_048690854.1 MULTISPECIES: elongation factor G [Streptococcus]
258   3e-73
WP_007203554.1 elongation factor G [Fictibacillus macauensis]
258   3e-73
WP_067407706.1 elongation factor G [Sporosarcina sp. HYO08]
258   3e-73
WP_019337460.1 elongation factor G [Stenotrophomonas maltophi...
258   3e-73
WP_048789970.1 elongation factor G [Streptococcus sp. 1171_SSPC]
258   3e-73
WP_013837835.1 elongation factor G [Isoptericola variabilis]
259   3e-73
EFM69780.1 elongation factor Tu domain 2 [Enterococcus faecal...
245   3e-73
WP_057746997.1 elongation factor G [Weissella viridescens]
259   3e-73
CCY77601.1 tetracycline resistance protein TetP [Butyrivibrio...
262   3e-73
WP_106052145.1 MULTISPECIES: elongation factor G [Bacillus]
258   3e-73
WP_099679359.1 elongation factor G [Bacillus pumilus]
258   3e-73
WP_095224640.1 elongation factor G [Virgibacillus sp. 7505]
258   3e-73
WP_048930200.1 GTP-binding protein [[Clostridium] citroniae]
263   3e-73
WP_024626602.1 elongation factor G [Bifidobacterium sp. A11]
259   3e-73
WP_094001402.1 elongation factor G [Stenotrophomonas sp. CC12...
258   3e-73
WP_057779933.1 GTP-binding protein [Lactobacillus vaccinoster...
258   3e-73
WP_081184005.1 elongation factor G [Enterococcus villorum]
258   3e-73
WP_017815360.1 MULTISPECIES: elongation factor G [Paenibacillus]
258   3e-73
WP_064238509.1 elongation factor G [Stenotrophomonas maltophi...
258   3e-73
CDF02722.1 small GTP-binding protein domain [Ruminococcus sp....
262   3e-73
WP_083424310.1 GTP-binding protein [[Clostridium] citroniae]
263   3e-73
WP_087178859.1 GTP-binding protein [Lachnoclostridium sp. An76]
263   3e-73
WP_014647099.1 elongation factor G [Stenotrophomonas maltophi...
258   3e-73
WP_050114591.1 elongation factor G [Streptococcus pneumoniae]
258   3e-73
WP_049665521.1 GTP-binding protein [Lysinibacillus xylanilyti...
257   3e-73
WP_028863565.1 elongation factor G [Psychromonas aquimarina]
258   3e-73
WP_047876990.1 elongation factor G [Photobacterium aquae]
258   3e-73

KKS33278.1 Elongation factor G [Candidatus Amesbacteria bacte...
259   3e-73
WP_050237858.1 elongation factor G [Streptococcus pneumoniae]
258   3e-73
WP_027518591.1 MULTISPECIES: elongation factor G [Bradyrhizob...
258   3e-73
WP_089862848.1 elongation factor G [Halanaerobium salsuginis]
258   3e-73
WP_080021671.1 GTP-binding protein [Clostridium thermobutyricum]
257   3e-73
PFG27250.1 translation elongation factor 2 (EF-2/EF-G) [Coryn...
259   3e-73
OGF83144.1 translation elongation factor G [Candidatus Giovan...
259   3e-73
WP_009764296.1 elongation factor G [Microvirga lotononidis]
258   3e-73
WP_022946271.1 elongation factor G [Pseudoalteromonas ruthenica]
258   3e-73
OGD20006.1 translation elongation factor G [Candidatus Aminic...
258   3e-73
WP_105145607.1 elongation factor G [Streptococcus suis]
258   3e-73
WP_018372174.1 elongation factor G [Streptococcus massiliensis]
258   3e-73
WP_057494214.1 elongation factor G [Stenotrophomonas pavanii]
258   3e-73
WP_000090351.1 elongation factor G [Streptococcus pneumoniae]
258   3e-73
WP_019157083.1 elongation factor G [Bacillus massiliosenegale...
258   3e-73
WP_089146917.1 GTP-binding protein [Lactobacillus crispatus]
257   3e-73
WP_092651835.1 elongation factor G [Isobaculum melis]
258   3e-73
WP_026565519.1 elongation factor G [Bacillus sp. UNC41MFS5]
258   3e-73
WP_099728192.1 elongation factor G [Bacillus pumilus]
258   3e-73
WP_063995933.1 elongation factor G [Bradyrhizobium sp.]
258   3e-73
WP_054663815.1 elongation factor G [Lactobacillus camelliae]
258   3e-73
WP_053463179.1 elongation factor G [Stenotrophomonas maltophi...
258   3e-73
WP_100666547.1 elongation factor G [Kyrpidia sp. EA-1]
258   3e-73
WP_057162622.1 elongation factor G [Lysobacter sp. Root916]
258   3e-73
WP_020613353.1 elongation factor G [Sediminispirochaeta bajac...
258   3e-73
WP_105114182.1 elongation factor G [Streptococcus suis]
258   3e-73
WP_101887225.1 GTP-binding protein [Lactobacillus crispatus]
257   3e-73

WP_026395054.1 elongation factor G [Acetobacterium dehalogenans]
258   3e-73
WP_060791163.1 GTP-binding protein [Lactobacillus gasseri]
257   4e-73
WP_007603010.1 elongation factor G [Bradyrhizobium sp. WSM1253]
258   4e-73
WP_092516563.1 elongation factor G [Afipia sp. GAS231]
258   4e-73
WP_044906613.1 GTP-binding protein [[Clostridium] innocuum]
262   4e-73
WP_070990078.1 elongation factor G [Pseudoalteromonas byunsan...
258   4e-73
WP_100450026.1 elongation factor G [Stenotrophomonas maltophi...
258   4e-73
WP_086284177.1 elongation factor G [Enterococcus sp. 10A9_DIV...
258   4e-73
WP_099811378.1 elongation factor G [Streptococcus suis]
258   4e-73
WP_011356943.1 elongation factor G [Pelodictyon luteolum]
258   4e-73
WP_105302359.1 elongation factor G [Anaerotruncus sp. Marseil...
258   4e-73
WP_014459970.1 GTP-binding protein [Bacillus megaterium]
257   4e-73
WP_012843348.1 elongation factor G [Rhodothermus marinus]
258   4e-73
WP_048764138.1 elongation factor G [Streptococcus cristatus]
258   4e-73
WP_089748259.1 MULTISPECIES: elongation factor G [Candidatus ...
258   4e-73
CCX51027.1 small GTP-binding protein domain [Clostridium sp. ...
262   4e-73
CDA59535.1 elongation factor G [Clostridium sp. CAG:245]
258   4e-73
OGP34241.1 translation elongation factor G [Deltaproteobacter...
258   4e-73
WP_039663069.1 elongation factor G [Campylobacter subantarcti...
258   4e-73
WP_031551436.1 elongation factor G [Gemella sanguinis]
258   4e-73
WP_089088067.1 GTP-binding protein [Lactobacillus pentosiphilus]
257   4e-73
WP_065858854.1 elongation factor G [Paenibacillus pectinilyti...
258   4e-73
WP_006455436.1 elongation factor G [Synechococcus sp. PCC 7335]
258   4e-73
CCZ90208.1 putative uncharacterized protein [Clostridium sp. ...
261   4e-73
WP_074828473.1 MULTISPECIES: elongation factor G [Bradyrhizob...
258   4e-73
WP_054171727.1 elongation factor G [Stenotrophomonas maltophi...
258   4e-73
WP_043395678.1 elongation factor G [Stenotrophomonas maltophi...
258   4e-73

WP_060536436.1 elongation factor G [Paenibacillus bovis]
258   4e-73
WP_043277197.1 MULTISPECIES: elongation factor G [Pseudonocar...
258   4e-73
WP_006902830.1 elongation factor G [Thermaerobacter subterran...
258   4e-73
OGI29469.1 translation elongation factor G [Candidatus Moranb...
258   4e-73
WP_031450709.1 elongation factor G [Desulfobacula sp. TS]
258   4e-73
WP_057788353.1 elongation factor G [Weissella minor]
258   4e-73
WP_027119723.1 elongation factor G [Mycoplasma testudinis]
258   4e-73
WP_006037952.1 elongation factor G [Paenibacillus curdlanolyt...
258   4e-73
WP_069662406.1 elongation factor G [Enterococcus termitis]
258   4e-73
WP_036660355.1 GTP-binding protein [Paenibacillus sp. FSL H8-...
257   4e-73
WP_077869462.1 GTP-binding protein [Clostridium beijerinckii]
258   4e-73
WP_004634174.1 elongation factor G [Gemella morbillorum]
258   4e-73
WP_089282911.1 elongation factor G [Anaerovirgula multivorans]
258   4e-73
WP_075521567.1 elongation factor G [Candidatus Pelagibacter u...
258   4e-73
WP_063489172.1 GTP-binding protein [Lactobacillus plantarum]
258   4e-73
OIP57978.1 translation elongation factor G [Candidatus Levyba...
258   4e-73
WP_091757741.1 GTP-binding protein [Butyrivibrio sp. INlla18]
262   4e-73
WP_094496403.1 GTP-binding protein [Lactobacillus taiwanensis]
257   4e-73
WP_073274859.1 GTP-binding protein [Anaerocolumna jejuensis]
262   4e-73
WP_099596737.1 elongation factor G [Stenotrophomonas maltophi...
258   4e-73
WP_049412827.1 elongation factor G [Stenotrophomonas maltophi...
258   4e-73
WP_049618591.1 elongation factor G [Streptococcus sp. X13SY08]
258   4e-73
WP_040636155.1 elongation factor G [Mitsuokella multacida]
258   4e-73
AMP54214.1 elongation factor G, domain IV [uncultured bacterium]
243   4e-73
WP_068698246.1 elongation factor G [Paenibacillus yonginensis]
258   4e-73
WP_047399143.1 elongation factor G [Bacillus anthracis]
258   4e-73
WP_007611610.1 elongation factor G [Bradyrhizobium sp. WSM471]
258   4e-73

OIP03492.1 translation elongation factor G [Candidatus Beckwi...
258   4e-73
WP_013355092.1 MULTISPECIES: GTP-binding protein [Lactobacillus]
258   5e-73
WP_095322600.1 GTP-binding protein [Bacillus circulans]
257   5e-73
WP_075572878.1 elongation factor G [Colibacter massiliensis]
258   5e-73
WP_077714674.1 GTP-binding protein [Desulfotomaculum ferrired...
262   5e-73
WP_068105954.1 elongation factor G [Nocardioides dokdonensis]
258   5e-73
WP_023606956.1 elongation factor G [Vagococcus lutrae]
258   5e-73
WP_067618685.1 elongation factor G [Dissulfuribacter thermoph...
258   5e-73
WP_048695560.1 elongation factor G [Catenovulum maritimum]
258   5e-73
WP_099435968.1 elongation factor G [Cyanobacterium aponinum]
258   5e-73
WP_045924656.1 elongation factor G [Bifidobacterium asteroides]
258   5e-73
GBD01351.1 Elongation factor G [bacterium HR18]
258   5e-73
CDN15846.1 Translation elongation factor G [Richelia intracel...
258   5e-73
WP_079561098.1 MULTISPECIES: GTP-binding protein [Lysinibacil...
257   5e-73
WP_047876445.1 elongation factor G [Photobacterium aphoticum]
258   5e-73
WP_088555345.1 elongation factor G [Calderihabitans maritimus]
258   5e-73
WP_101640209.1 elongation factor G [Bacillus deserti]
258   5e-73
WP_058015803.1 elongation factor G [Bacillus pumilus]
258   5e-73
WP_047401463.1 elongation factor G [Bacillus anthracis]
258   5e-73
OWV27159.1 GTP-binding protein [Fibrobacter sp. UWB1]
240   5e-73
WP_093797719.1 elongation factor G [Sporomusa acidovorans]
258   5e-73
WP_003648118.1 GTP-binding protein [Lactobacillus gasseri]
256   5e-73
AVK85207.1 elongation factor G [Lysinibacillus sp. B2A1]
257   5e-73
WP_105109217.1 elongation factor G [Streptococcus suis]
258   5e-73
WP_057138417.1 GTP-binding protein [Lactobacillus plantarum]
257   5e-73
WP_095246001.1 elongation factor G [Bacillus sp. 7894-2]
258   5e-73
WP_022782312.1 GTP-binding protein [Lachnospiraceae bacterium...
261   5e-73

WP_072152530.1 elongation factor G [Weissella viridescens]
258   5e-73
WP_090076423.1 GTP-binding protein [Lachnospiraceae bacterium...
262   5e-73
WP_066192178.1 MULTISPECIES: elongation factor G [Bacillus]
258   5e-73
WP_012993096.1 elongation factor G [Mageeibacillus indolicus]
258   5e-73
WP_075075974.1 elongation factor G [Stenotrophomonas maltophi...
258   5e-73
WP_090003432.1 elongation factor G [Candidatus Frackibacter s...
258   5e-73
WP_044963795.1 GTP-binding protein [Clostridiaceae bacterium ...
262   5e-73
WP_003495873.1 GTP-binding protein [Clostridium sporogenes]
257   5e-73
WP_100478086.1 elongation factor G [Stenotrophomonas maltophi...
258   5e-73
WP_063851738.1 GTP-binding protein [Lactobacillus plantarum]
257   5e-73
WP_087370265.1 GTP-binding protein [Pseudoflavonifractor sp. ...
261   5e-73
WP_098533578.1 MULTISPECIES: GTP-binding protein [Bacillus]
257   5e-73
WP_024333613.1 elongation factor G [Desulfotignum balticum]
258   5e-73
ALP53439.1 elongation factor G [Candidatus Tenderia electroph...
258   5e-73
WP_066578407.1 GTP-binding protein [Clostridium sp. Marseille...
262   5e-73
WP_041823244.1 GTP-binding protein [Lactobacillus salivarius]
256   5e-73
WP_053994762.1 GTP-binding protein [Lysinibacillus macroides]
256   5e-73
WP_088784845.1 elongation factor G [Streptococcus pneumoniae]
258   5e-73
WP_002940034.1 elongation factor G [Streptococcus suis]
258   5e-73
WP_096721131.1 elongation factor G [Chondrocystis sp. NIES-4102]
258   5e-73
WP_049463343.1 elongation factor G [Stenotrophomonas maltophi...
258   5e-73
AKI05344.1 Tetracycline resistance protein [Lactobacillus sal...
256   5e-73
WP_087378537.1 GTP-binding protein [Anaeromassilibacillus sp....
257   5e-73
WP_049402997.1 elongation factor G [Stenotrophomonas maltophi...
257   5e-73
WP_063205721.1 GTP-binding protein [Lactobacillus plantarum]
257   5e-73
OCQ92869.1 translation elongation factor G [Nostoc sp. MBR 210]
258   5e-73
WP_099605732.1 elongation factor G [Stenotrophomonas maltophi...
257   5e-73

WP_061647819.1 elongation factor G [Streptococcus pneumoniae]
258   5e-73
WP_027306276.1 elongation factor G [Campylobacter cuniculorum]
258   5e-73
ADE56759.1 translation elongation factor G [Aminobacterium co...
258   5e-73
SCB31424.1 ribosomal protection tetracycline resistance prote...
255   6e-73
WP_049421977.1 elongation factor G [Stenotrophomonas maltophi...
257   6e-73
WP_057704896.1 GTP-binding protein [Lactobacillus plantarum]
257   6e-73
WP_104968575.1 elongation factor G [Streptococcus pluranimalium]
258   6e-73
WP_044139654.1 elongation factor G [Bacillus pumilus]
258   6e-73
WP_034574717.1 elongation factor G [Mageeibacillus indolicus]
258   6e-73
WP_099495648.1 elongation factor G [Stenotrophomonas maltophi...
257   6e-73
PIR01470.1 elongation factor G [Nitrospinae bacterium CG11_bi...
258   6e-73
WP_089150635.1 GTP-binding protein [Lactobacillus crispatus]
256   6e-73
WP_045980256.1 MULTISPECIES: elongation factor G [Pseudoalter...
258   6e-73
WP_055069751.1 GTP-binding protein [Clostridium sp. ND2]
257   6e-73
WP_067393995.1 elongation factor G [Enterococcus canis]
258   6e-73
WP_012057172.1 elongation factor G [Thermosipho melanesiensis]
258   6e-73
SCJ31224.1 Elongation factor G [uncultured Clostridium sp.]
262   6e-73
WP_010998476.1 MULTISPECIES: elongation factor G [Nostocaceae]
258   6e-73
WP_006526016.1 elongation factor G [Solobacterium moorei]
258   6e-73
WP_047941806.1 GTP-binding protein [Bacillus circulans]
256   6e-73
WP_087922518.1 elongation factor G [Stenotrophomonas sp. WZN-1]
257   6e-73
WP_066462912.1 elongation factor G [Caryophanon latum]
258   6e-73
WP_105452529.1 elongation factor G [Lactobacillus sp. CBA3605]
258   6e-73
WP_068131188.1 MULTISPECIES: elongation factor G [Nosocomiico...
258   6e-73
WP_041459518.1 elongation factor G [Aminobacterium colombiense]
257   6e-73
WP_075485272.1 elongation factor G [Candidatus Pelagibacter u...
258   6e-73
WP_049452888.1 elongation factor G [Stenotrophomonas maltophi...
257   6e-73

WP_050945820.1 elongation factor G [Bacillus pumilus]
258   6e-73
WP_071132294.1 elongation factor G [Ileibacterium massiliense]
258   6e-73
WP_071854647.1 elongation factor G [Enterococcus ratti]
258   6e-73
EFP63302.1 putative translation elongation factor G [Erysipel...
261   6e-73
WP_084947309.1 elongation factor G [Streptococcus mitis]
258   6e-73
WP_073076115.1 elongation factor G [Sporobacter termitidis]
258   6e-73
WP_069129668.1 elongation factor G [Streptococcus pneumoniae]
258   6e-73
WP_019244373.1 elongation factor G [Bacillus massilioanorexius]
258   6e-73
WP_107742108.1 elongation factor G [Opitutaceae bacterium EW11]
258   6e-73
OGX17584.1 translation elongation factor G [Omnitrophica WOR_...
258   6e-73
WP_055282914.1 GTP-binding protein [Dorea longicatena]
262   6e-73
WP_088805962.1 elongation factor G [Streptococcus pneumoniae]
258   7e-73
WP_013074221.1 elongation factor G [Kyrpidia tusciae]
258   7e-73
WP_007858906.1 GTP-binding protein [[Clostridium] citroniae]
262   7e-73
OGV91364.1 translation elongation factor G [Microgenomates gr...
258   7e-73
WP_102170194.1 elongation factor G [Streptococcus sp. UMB1385]
258   7e-73
WP_010249729.1 GTP-binding protein [Acetivibrio cellulolyticus]
261   7e-73
WP_087323053.1 GTP-binding protein [Faecalibacterium sp. An58]
261   7e-73
WP_075230781.1 elongation factor G [Streptococcus sp. HMSC071...
258   7e-73
OGC47285.1 translation elongation factor G [candidate divisio...
258   7e-73
WP_048011519.1 elongation factor G [Bacillus firmus]
258   7e-73
WP_029690723.1 elongation factor G [Streptococcus hyovaginalis]
258   7e-73
WP_003649710.1 GTP-binding protein [Lactobacillus gasseri]
256   7e-73
WP_013085625.1 GTP-binding protein [Lactobacillus crispatus]
256   7e-73
WP_009253943.1 GTP-binding protein [Lachnospiraceae bacterium...
261   7e-73
WP_079571025.1 elongation factor G [Bradyrhizobium erythrophlei]
257   7e-73
WP_102210835.1 elongation factor G [Streptococcus sp. UMB0029]
258   7e-73

WP_050307884.1 elongation factor G [Streptococcus pneumoniae]
258   7e-73
WP_022374856.1 GTP-binding protein [Anaerostipes hadrus]
261   7e-73
WP_048603305.1 elongation factor G [Enterococcus massiliensis]
258   7e-73
WP_045613413.1 elongation factor G [Streptococcus infantis]
258   7e-73
WP_084002095.1 elongation factor G [Streptococcus pneumoniae]
258   7e-73
WP_050222825.1 elongation factor G [Streptococcus pneumoniae]
258   7e-73
WP_000090345.1 MULTISPECIES: elongation factor G [Streptococcus]
258   7e-73
WP_045008218.1 MULTISPECIES: elongation factor G [Bradyrhizob...
257   7e-73
WP_075674500.1 elongation factor G [Stenotrophomonas sp. TD3]
257   7e-73
WP_085114381.1 elongation factor G [Candidatus Pelagibacter s...
257   7e-73
WP_086950531.1 elongation factor G [Vagococcus fluvialis]
258   7e-73
COO07927.1 elongation factor G [Streptococcus pneumoniae]
257   7e-73
WP_026678037.1 elongation factor G [Fictibacillus gelatini]
257   7e-73
PIU55416.1 elongation factor G [Chloroflexi bacterium CG07_la...
257   7e-73
PID40615.1 elongation factor G [Proteobacteria bacterium]
257   7e-73
WP_075105326.1 elongation factor G [Streptococcus cuniculi]
257   7e-73
WP_072935384.1 elongation factor G [Schwartzia succinivorans]
257   7e-73
WP_027092004.1 elongation factor G [Cohnella thermotolerans]
257   7e-73
WP_087642000.1 elongation factor G [Enterococcus sp. 9D6_DIV0...
257   7e-73
WP_049543655.1 elongation factor G [Streptococcus pseudopneum...
257   7e-73
WP_000090355.1 MULTISPECIES: elongation factor G [Streptococcus]
257   7e-73
WP_060745564.1 GTP-binding protein [Bacillus megaterium]
256   7e-73
WP_015022139.1 elongation factor G [Bifidobacterium asteroides]
258   7e-73
WP_033679614.1 MULTISPECIES: elongation factor G [Streptococcus]
257   7e-73
WP_000090348.1 MULTISPECIES: elongation factor G [Streptococcus]
257   7e-73
KRK48933.1 elongation factor G [Lactobacillus kimchicus JCM 1...
257   7e-73
WP_003646156.1 GTP-binding protein [Lactobacillus plantarum]
257   8e-73

WP_068507460.1 elongation factor G [Leptolyngbya sp. O-77]
258   8e-73
WP_034998433.1 GTP-binding protein [Lactobacillus salivarius]
256   8e-73
WP_089147968.1 MULTISPECIES: GTP-binding protein [Lactobacillus]
256   8e-73
WP_054435075.1 elongation factor G [Streptococcus pneumoniae]
257   8e-73
OQY33846.1 elongation factor G [Anaerolineaceae bacterium 457...
257   8e-73
KOS31159.1 elongation factor G [Bacillus anthracis]
257   8e-73
WP_040286092.1 GTP-binding protein [Sporosarcina koreensis]
256   8e-73
WP_062436629.1 elongation factor G [Synechococcus sp. PCC 73109]
257   8e-73
WP_090117919.1 elongation factor G [Cohnella sp. OV330]
257   8e-73
WP_009201490.1 elongation factor G [Acetomicrobium hydrogenif...
257   8e-73
WP_000090339.1 MULTISPECIES: elongation factor G [Streptococcus]
257   8e-73
WP_032077838.1 GTP-binding protein [Clostridium drakei]
261   8e-73
WP_015466081.1 elongation factor G [Psychromonas sp. CNPT3]
257   8e-73
KFI37745.1 elongation factor G [Peptococcaceae bacterium SCAD...
257   8e-73
WP_088813858.1 elongation factor G [Streptococcus pneumoniae]
257   8e-73
WP_000090350.1 MULTISPECIES: elongation factor G [Streptococcus]
257   8e-73
WP_045612723.1 MULTISPECIES: elongation factor G [Streptococcus]
257   8e-73
WP_000090357.1 MULTISPECIES: elongation factor G [Streptococcus]
257   8e-73
WP_000090354.1 MULTISPECIES: elongation factor G [Streptococcus]
257   8e-73
WP_101432624.1 elongation factor G [Bifidobacterium asteroides]
258   8e-73
WP_075891010.1 elongation factor G [Limnothrix rosea]
257   8e-73
WP_053496663.1 MULTISPECIES: elongation factor G [Stenotropho...
257   8e-73
WP_048802540.1 elongation factor G [Staphylococcus pasteuri]
257   8e-73
WP_105450095.1 elongation factor G [Lactobacillus sp. CBA3606]
257   8e-73
WP_050216661.1 elongation factor G [Streptococcus pneumoniae]
257   8e-73
WP_003217255.1 elongation factor G [Bacillus pumilus]
257   8e-73
WP_034467410.1 GTP-binding protein [Butyrivibrio proteoclasti...
261   8e-73

WP_063992118.1 elongation factor G [Streptococcus pneumoniae]
257   8e-73
WP_072524777.1 GTP-binding protein [Clostridium sp. Marseille...
262   8e-73
WP_087391517.1 GTP-binding protein [Pseudoflavonifractor sp. ...
260   8e-73
WP_050247722.1 elongation factor G [Streptococcus pneumoniae]
257   9e-73
ODT62745.1 translation elongation factor G [Phenylobacterium ...
257   9e-73
WP_050074157.1 elongation factor G [Streptococcus pneumoniae]
257   9e-73
WP_108072413.1 elongation factor G [Bacillus sporothermodurans]
257   9e-73
WP_088811705.1 elongation factor G [Streptococcus pneumoniae]
257   9e-73
WP_042981369.1 elongation factor G [Bacillus mycoides]
257   9e-73
AEJ38522.1 translation elongation factor G [Sulfobacillus aci...
257   9e-73
WP_063490702.1 GTP-binding protein [Lactobacillus plantarum]
257   9e-73
WP_000090358.1 elongation factor G [Streptococcus pneumoniae]
257   9e-73
WP_017068221.1 elongation factor G [Vibrio crassostreae]
257   9e-73
WP_006364511.1 elongation factor G [Gemella sanguinis]
257   9e-73
WP_006155877.1 elongation factor G [Streptococcus infantis]
257   9e-73
WP_004275220.1 elongation factor G [Campylobacter upsaliensis]
257   9e-73
WP_100733100.1 GTP-binding protein [Lactobacillus crispatus]
256   9e-73
WP_088849708.1 elongation factor G [Streptococcus pneumoniae]
257   9e-73
WP_054363045.1 elongation factor G [Streptococcus pneumoniae]
257   9e-73
WP_000090349.1 MULTISPECIES: elongation factor G [Streptococcus]
257   9e-73
WP_021152596.1 elongation factor G [Streptococcus salivarius]
257   9e-73
WP_028559475.1 elongation factor G [Paenibacillus pinihumi]
257   9e-73
WP_003061460.1 elongation factor G [Streptococcus dysgalactiae]
257   9e-73
WP_022667421.1 elongation factor G [Desulfospira joergensenii]
257   9e-73
KRL26843.1 elongation factor EF2 [Lactobacillus mucosae DSM 1...
257   9e-73
WP_087583697.1 elongation factor G [Campylobacter concisus]
257   9e-73
WP_077620213.1 elongation factor G [Bacillus sinesaloumensis]
257   9e-73

WP_011955948.1 elongation factor G [Roseiflexus sp. RS-1]
257   9e-73
WP_045151885.1 elongation factor G [Photobacterium angustum]
257   9e-73
WP_072587180.1 GTP-binding protein [Clostridium botulinum]
256   9e-73
WP_073073405.1 elongation factor G [Thermosipho atlanticus]
257   9e-73
WP_054747205.1 elongation factor G [Lactobacillus rapi]
257   9e-73
WP_081014872.1 elongation factor G [Propionibacterium freuden...
257   9e-73
WP_013160482.1 elongation factor G [Propionibacterium freuden...
257   9e-73
WP_005828865.1 MULTISPECIES: elongation factor G [Bacillales]
257   9e-73
KXK53096.1 elongation factor EF-G [Chlorobi bacterium OLB7]
257   1e-72
WP_000090332.1 MULTISPECIES: elongation factor G [Streptococcus]
257   1e-72
WP_039668128.1 elongation factor G [Campylobacter lari]
257   1e-72
SCH53814.1 Elongation factor G [uncultured Clostridium sp.]
261   1e-72
WP_050150886.1 elongation factor G [Streptococcus agalactiae]
257   1e-72
WP_079543870.1 elongation factor G [Bradyrhizobium lablabi]
257   1e-72
WP_020090162.1 GTP-binding protein [Lactobacillus parabrevis]
256   1e-72
WP_005727117.1 GTP-binding protein [Lactobacillus crispatus]
256   1e-72
WP_017054076.1 elongation factor G [Vibrio genomosp. F6]
257   1e-72
SCJ81780.1 Tetracycline resistance protein tetM [uncultured E...
260   1e-72
WP_018374688.1 elongation factor G [Streptococcus merionis]
257   1e-72
WP_079907273.1 elongation factor G [Carboxydocella sp. JDF658]
257   1e-72
WP_085912590.1 elongation factor G [Pseudonocardia autotrophica]
257   1e-72
WP_081367762.1 GTP-binding protein [Ruminococcus flavefaciens]
260   1e-72
WP_061826366.1 elongation factor G [Streptococcus pneumoniae]
257   1e-72
WP_000090335.1 MULTISPECIES: elongation factor G [Streptococcus]
257   1e-72
WP_084974052.1 elongation factor G [Streptococcus oralis]
257   1e-72
WP_042453948.1 elongation factor G [Bacillus dielmoensis]
257   1e-72
WP_004277750.1 elongation factor G [Campylobacter upsaliensis]
257   1e-72

WP_023920905.1 GTP-binding protein [Ruminococcus lactaris]
261   1e-72
WP_072910152.1 elongation factor G [Malonomonas rubra]
257   1e-72
WP_003328292.1 MULTISPECIES: elongation factor G [Bacillus su...
257   1e-72
WP_091008012.1 GTP-binding protein [Olsenella umbonata]
256   1e-72
WP_077894121.1 GTP-binding protein [Clostridium felsineum]
256   1e-72
WP_003058029.1 elongation factor G [Streptococcus dysgalactiae]
257   1e-72
WP_019506413.1 elongation factor G [Pleurocapsa sp. PCC 7319]
257   1e-72
WP_062470812.1 elongation factor G [Jeotgalibaca dankookensis]
257   1e-72
WP_023853698.1 elongation factor G [Candidatus Pelagibacter u...
257   1e-72
KUK58938.1 Elongation factor G [Synergistales bacterium 53_16]
257   1e-72
WP_056172417.1 elongation factor G [Lysobacter sp. Root604]
257   1e-72
CBK81843.1 small GTP-binding protein domain [Coprococcus catu...
262   1e-72
WP_097850867.1 elongation factor G [Propionibacterium freuden...
257   1e-72
WP_057726854.1 GTP-binding protein [Lactobacillus crispatus]
256   1e-72
WP_071442000.1 GTP-binding protein [Traorella massiliensis]
260   1e-72
WP_061568474.1 elongation factor G [Caldibacillus debilis]
257   1e-72
WP_087245441.1 GTP-binding protein [Faecalibacterium sp. An77]
260   1e-72
WP_075980746.1 GTP-binding protein [Bacillus massilionigerien...
256   1e-72
CDD01808.1 putative uncharacterized protein [Clostridium sp. ...
261   1e-72
WP_024753212.1 elongation factor G [Treponema phagedenis]
257   1e-72
WP_041092028.1 GTP-binding protein [Lactobacillus hokkaidonen...
256   1e-72
WP_068708485.1 elongation factor G [Tetragenococcus solitarius]
257   1e-72
WP_082199267.1 elongation factor G [Campylobacter helveticus]
257   1e-72
WP_065970328.1 elongation factor G [Acidiferrobacter thiooxyd...
257   1e-72
WP_069790140.1 elongation factor G [Cyanobacterium sp. IPPAS ...
257   1e-72
WP_087190456.1 elongation factor G [Gordonibacter urolithinfa...
257   1e-72
OQY48703.1 elongation factor G [Desulfobacteraceae bacterium ...
256   1e-72

WP_070535927.1 elongation factor G [Streptococcus sp. HMSC077...
257   1e-72
WP_000090347.1 MULTISPECIES: elongation factor G [Streptococcus]
257   1e-72
WP_044727750.1 elongation factor G [Streptococcus pneumoniae]
257   1e-72
WP_000090331.1 MULTISPECIES: elongation factor G [Streptococcus]
257   1e-72
WP_088780524.1 elongation factor G [Streptococcus pneumoniae]
257   1e-72
CAQ63495.1 tetracycline resistance protein Tet(S) [Streptococ...
242   1e-72
WP_025640187.1 elongation factor G [[Clostridium] ultunense]
257   1e-72
WP_002845990.1 elongation factor G [Peptostreptococcus anaero...
257   1e-72
WP_049039725.1 GTP-binding protein [Clostridium perfringens]
256   1e-72
WP_061644257.1 elongation factor G [Streptococcus pneumoniae]
257   1e-72
WP_002889328.1 elongation factor G [Streptococcus salivarius]
257   1e-72
WP_084945608.1 elongation factor G [Streptococcus oralis]
257   1e-72
WP_076764338.1 elongation factor G [Domibacillus epiphyticus]
257   1e-72
WP_063846677.1 GTP-binding protein [Lactobacillus plantarum]
256   1e-72
WP_050086604.1 elongation factor G [Streptococcus pneumoniae]
257   1e-72
WP_045617709.1 elongation factor G [Streptococcus oralis]
257   1e-72
WP_100332788.1 elongation factor G [Bacillus sp. FJAT-46582]
257   1e-72
WP_096605351.1 elongation factor G [Calothrix sp. NIES-2100]
257   1e-72
CCQ95441.1 elongation factor G [[Clostridium] ultunense Esp]
257   1e-72
EGV03539.1 translation elongation factor G [Streptococcus inf...
257   1e-72
WP_087366411.1 GTP-binding protein [Faecalibacterium sp. An192]
260   1e-72
WP_096071607.1 GTP-binding protein [Clostridium perfringens]
256   1e-72
WP_107379573.1 elongation factor G [Stenotrophomonas maltophi...
256   1e-72
WP_027304870.1 elongation factor G [Campylobacter upsaliensis]
257   1e-72
WP_009319930.1 MULTISPECIES: GTP-binding protein [unclassifie...
261   1e-72
WP_019617175.1 elongation factor G [Psychromonas ossibalaenae]
257   1e-72
WP_056969368.1 GTP-binding protein [Lactobacillus thailandensis]
256   1e-72

WP_099476086.1 elongation factor G [Stenotrophomonas maltophi...
256   1e-72
WP_012727681.1 MULTISPECIES: elongation factor G [Exiguobacte...
257   1e-72
WP_053494712.1 elongation factor G [Stenotrophomonas maltophi...
256   1e-72
WP_057894291.1 elongation factor G [Lactobacillus brantae]
257   1e-72
WP_022940178.1 elongation factor G [Psychromonas hadalis]
257   1e-72
WP_057890202.1 GTP-binding protein [Lactobacillus oligofermen...
256   1e-72
WP_046388351.1 elongation factor G [Streptococcus uberis]
257   1e-72
WP_068387248.1 elongation factor G [Leptolyngbya sp. NIES-3755]
256   1e-72
WP_041977005.1 elongation factor G [Pyrinomonas methylaliphat...
257   1e-72
WP_005609896.1 GTP-binding protein [Ruminococcus lactaris]
261   1e-72
WP_105202637.1 elongation factor G [Streptococcus suis]
257   1e-72
WP_023511459.1 elongation factor G [Sporolactobacillus laevol...
257   1e-72
WP_106342792.1 elongation factor G [Laceyella sediminis]
257   1e-72
WP_003641688.1 MULTISPECIES: GTP-binding protein [Lactobacillus]
256   1e-72
WP_029068954.1 elongation factor G [Jonesia quinghaiensis]
257   1e-72
WP_061421415.1 elongation factor G [Streptococcus oralis]
257   1e-72
WP_049535536.1 elongation factor G [Streptococcus pseudopneum...
257   1e-72
WP_050208495.1 elongation factor G [Streptococcus pneumoniae]
257   1e-72
WP_095371227.1 elongation factor G [Bacillus kochii]
257   1e-72
WP_046791256.1 elongation factor G [Salinicoccus halodurans]
257   1e-72
WP_057541402.1 elongation factor G [Streptococcus pneumoniae]
257   1e-72
WP_092910280.1 elongation factor G [Hydrogenimonas thermophila]
257   1e-72
WP_063488688.1 GTP-binding protein [Lactobacillus plantarum]
256   1e-72
OZB48273.1 elongation factor G [Cellulomonas sp. 14-74-6]
257   1e-72
WP_084672283.1 elongation factor G [Streptococcus pneumoniae]
257   1e-72
WP_055158730.1 GTP-binding protein [[Ruminococcus] torques]
261   1e-72
WP_002844198.1 elongation factor G [Peptostreptococcus anaero...
257   1e-72

OQX01858.1 translation elongation factor G [Desulfobacteracea...
256   1e-72
WP_012344663.1 GTP-binding protein [Clostridium botulinum]
256   1e-72
WP_068805403.1 elongation factor G [Pediococcus parvulus]
257   1e-72
WP_068816985.1 elongation factor G [Phormidesmis priestleyi]
257   1e-72
WP_060795457.1 GTP-binding protein [Clostridium perfringens]
256   1e-72
WP_057231358.1 GTP-binding protein [Clostridium perfringens]
256   1e-72
WP_024833279.1 GTP-binding protein [[Clostridium] josui]
261   1e-72
WP_063721126.1 GTP-binding protein [Lactobacillus plantarum]
256   1e-72
PIT88850.1 elongation factor G [Candidatus Levybacteria bacte...
257   1e-72
OGO41753.1 translation elongation factor G [Chloroflexi bacte...
257   1e-72
WP_075218816.1 elongation factor G [Streptococcus pneumoniae]
257   1e-72
WP_069155479.1 GTP-binding protein [Eisenbergiella tayi]
261   1e-72
WP_011592075.1 GTP-binding protein [Clostridium perfringens]
256   1e-72
WP_078666624.1 MULTISPECIES: elongation factor G [Carboxydoce...
257   1e-72
OGX26546.1 translation elongation factor G [Omnitrophica WOR_...
257   1e-72
WP_021746413.1 elongation factor G [Leptotrichia wadei]
257   1e-72
WP_032127341.1 elongation factor G [Stenotrophomonas maltophi...
256   1e-72
WP_088784084.1 elongation factor G [Streptococcus pneumoniae]
257   1e-72
WP_003094680.1 MULTISPECIES: elongation factor G [Streptococcus]
257   1e-72
WP_087542695.1 elongation factor G [Nostocales cyanobacterium...
257   1e-72
WP_003146926.1 elongation factor G [Gemella haemolysans]
257   1e-72
WP_105128343.1 elongation factor G [Streptococcus suis]
257   1e-72
WP_106287279.1 elongation factor G [Merismopedia glauca]
257   1e-72
CDB45226.1 elongation factor G [Phascolarctobacterium sp. CAG...
257   1e-72
WP_000090353.1 elongation factor G [Streptococcus pneumoniae]
257   1e-72
WP_040928643.1 elongation factor G [Nosocomiicoccus massilien...
257   1e-72
WP_084233660.1 elongation factor G [Papillibacter cinnamivorans]
257   1e-72

WP_107630402.1 GTP-binding protein [Intestinibacillus sp. Mar...
260   1e-72
WP_050223623.1 elongation factor G [Streptococcus pneumoniae]
257   1e-72
WP_000090352.1 elongation factor G [Streptococcus sp. oral ta...
257   1e-72
WP_021357392.1 GTP-binding protein [Lactobacillus plantarum]
256   1e-72
WP_025434532.1 elongation factor G [Peptoclostridium acidamin...
256   1e-72
WP_010485812.1 elongation factor G [[Pseudomonas] geniculata]
256   1e-72
WP_070458525.1 elongation factor G [Streptococcus sp. HMSC074...
257   1e-72
WP_032462601.1 elongation factor G [Streptococcus pyogenes]
256   1e-72
WP_006445772.1 MULTISPECIES: elongation factor G [Alicyclobac...
256   1e-72
OIN96725.1 translation elongation factor G [Deltaproteobacter...
256   1e-72
WP_087233304.1 GTP-binding protein [Pseudoflavonifractor sp. ...
260   1e-72
KPK86886.1 elongation factor G [Deltaproteobacteria bacterium...
256   1e-72
WP_050279077.1 elongation factor G [Streptococcus pneumoniae]
256   1e-72
WP_101356114.1 elongation factor G [Bacillus camelliae]
256   1e-72
WP_046865358.1 elongation factor G [Microvirga massiliensis]
256   1e-72
WP_094834521.1 GTP-binding protein [Bacillaceae bacterium SAS...
256   1e-72
WP_092276970.1 elongation factor G [Brevibacillus sp. OK042]
256   1e-72
WP_042213600.1 GTP-binding protein [Paenibacillus borealis]
256   1e-72
WP_096411629.1 elongation factor G [Thermus thermophilus]
256   1e-72
WP_088795084.1 elongation factor G [Streptococcus pneumoniae]
256   1e-72
WP_038538444.1 elongation factor G [Bacillus sp. X1(2014)]
256   1e-72
WP_094369384.1 GTP-binding protein [Romboutsia weinsteinii]
256   1e-72
WP_029178041.1 elongation factor G [Streptococcus suis]
256   1e-72
WP_075503646.1 elongation factor G [Candidatus Pelagibacter u...
256   1e-72
WP_070109889.1 GTP-binding protein [Clostridium acetireducens]
256   1e-72
WP_051635853.1 GTP-binding protein [Lachnospiraceae bacterium...
260   1e-72
WP_057906205.1 elongation factor G [Lactobacillus aviarius]
257   1e-72

WP_071306134.1 elongation factor G [Stenotrophomonas maltophi...
256   1e-72
WP_061390990.1 elongation factor G [Streptococcus pneumoniae]
256   1e-72
WP_015911930.1 elongation factor G [Streptococcus uberis]
256   1e-72
CCY47774.1 elongation factor G [Peptostreptococcus anaerobius...
256   1e-72
WP_099297988.1 elongation factor G [Corynebacterium sp. Marse...
257   1e-72
WP_057983436.1 GTP-binding protein [Bacillus cecembensis]
255   1e-72
WP_000090346.1 elongation factor G [Streptococcus pneumoniae]
256   1e-72
KRN65994.1 translation elongation factor G [Pediococcus celli...
257   1e-72
WP_048715725.1 elongation factor G [Streptococcus sp. 343_SSPC]
256   1e-72
WP_021084241.1 elongation factor G [Campylobacter concisus]
256   2e-72
WP_018376715.1 elongation factor G [Streptococcus orisratti]
256   2e-72
KRK35077.1 translation elongation factor (GTPase) [Lactobacil...
256   2e-72
WP_091184632.1 elongation factor G [Friedmanniella flava]
256   2e-72
WP_101786175.1 elongation factor G [Streptococcus mitis]
256   2e-72
WP_050227729.1 elongation factor G [Streptococcus pneumoniae]
256   2e-72
WP_057522493.1 elongation factor G [Streptococcus pneumoniae]
256   2e-72
WP_068984136.1 GTP-binding protein [Lysinibacillus xylanilyti...
255   2e-72
WP_046391624.1 elongation factor G [Streptococcus uberis]
256   2e-72
WP_075904455.1 elongation factor G [Moorea bouillonii]
256   2e-72
WP_069512387.1 GTP-binding protein [Lysinibacillus sphaericus]
255   2e-72
WP_083474341.1 elongation factor G [Pseudonocardia sp. AL0410...
257   2e-72
EFX35860.1 translation elongation factor G [Streptococcus inf...
257   2e-72
WP_011197193.1 elongation factor G [Symbiobacterium thermophi...
256   2e-72
WP_049403886.1 elongation factor G [Stenotrophomonas maltophi...
256   2e-72
WP_000090334.1 elongation factor G [Streptococcus mitis]
256   2e-72
WP_094752520.1 elongation factor G [Psychromonas sp. CD1]
256   2e-72
WP_000090344.1 elongation factor G [Streptococcus pneumoniae]
256   2e-72

WP_095367452.1 elongation factor G [Pseudoalteromonas sp. NBT...
256   2e-72
WP_080066149.1 GTP-binding protein [[Clostridium] hungatei]
260   2e-72
WP_010285039.1 elongation factor G [Bacillus timonensis]
256   2e-72
WP_088783115.1 elongation factor G [Streptococcus pneumoniae]
256   2e-72
WP_047208063.1 elongation factor G [Campylobacter lari]
256   2e-72
EEM41248.1 GTP-binding elongation factor protein, TetM/TetO [...
245   2e-72
WP_024402277.1 elongation factor G [Streptococcus suis]
256   2e-72
WP_078233635.1 GTP-binding protein [Clostridium perfringens]
255   2e-72
WP_025845254.1 elongation factor G [Brevibacillus agri]
256   2e-72
WP_057826626.1 elongation factor G [Lactobacillus aviarius]
256   2e-72
WP_032510536.1 elongation factor G [Streptococcus suis]
256   2e-72
WP_037592623.1 elongation factor G [Streptococcus uberis]
256   2e-72
WP_002596948.1 GTP-binding protein [Clostridium colicanis]
255   2e-72
OGS22190.1 translation elongation factor G [Elusimicrobia bac...
256   2e-72
WP_065527575.1 elongation factor G [Planococcus donghaensis]
256   2e-72
WP_035602222.1 elongation factor G [Haloferula sp. BvORR071]
256   2e-72
WP_088791654.1 elongation factor G [Streptococcus pneumoniae]
256   2e-72
WP_000090329.1 elongation factor G [Streptococcus sp. SK643]
256   2e-72
WP_067621080.1 elongation factor G [Alicyclobacillus acidiphi...
256   2e-72
WP_103339151.1 elongation factor G [Amycolatopsis sp. CA-126428]
255   2e-72
WP_057751135.1 elongation factor G [Pediococcus cellicola]
256   2e-72
WP_088780110.1 elongation factor G [Streptococcus pneumoniae]
256   2e-72
WP_086142281.1 elongation factor G [Lactobacillus reuteri]
256   2e-72
WP_050283644.1 elongation factor G [Streptococcus pneumoniae]
256   2e-72
WP_049552296.1 elongation factor G [Streptococcus pseudopneum...
256   2e-72
WP_036377666.1 elongation factor G [Mitsuokella jalaludinii]
256   2e-72
WP_057257754.1 GTP-binding protein [Clostridium perfringens]
255   2e-72

WP_050242575.1 elongation factor G [Streptococcus pneumoniae]
256   2e-72
ESA45171.1 translation elongation factor G [Streptococcus pyo...
256   2e-72
WP_049532147.1 elongation factor G [Streptococcus pseudopneum...
256   2e-72
WP_087228095.1 GTP-binding protein [Faecalibacterium sp. An121]
259   2e-72
WP_088776626.1 elongation factor G [Streptococcus pneumoniae]
256   2e-72
WP_084947294.1 elongation factor G [Streptococcus oralis]
256   2e-72
WP_099156799.1 elongation factor G [Virgibacillus ndiopensis]
256   2e-72
WP_066441588.1 elongation factor G [Bacillus gottheilii]
256   2e-72
KKR08521.1 Elongation factor G [Parcubacteria group bacterium...
256   2e-72
WP_003712477.1 GTP-binding protein [Lactobacillus oris]
255   2e-72
WP_053596052.1 elongation factor G [Lysinibacillus sp. FJAT-1...
256   2e-72
WP_022852742.1 elongation factor G [Thermodesulfatator atlant...
256   2e-72
KJS78732.1 elongation factor G [Desulfosporosinus sp. BICA1-9]
256   2e-72
PIE61635.1 elongation factor G [Desulfobacterales bacterium]
256   2e-72
WP_073069226.1 elongation factor G [Phormidesmis priestleyi]
256   2e-72
WP_070982843.1 MULTISPECIES: elongation factor G [Pseudoalter...
256   2e-72
OFX12900.1 translation elongation factor G [Armatimonadetes b...
256   2e-72
WP_021090006.1 elongation factor G [Campylobacter concisus]
256   2e-72
WP_003045330.1 elongation factor G [Streptococcus canis]
256   2e-72
WP_091459799.1 elongation factor G [Acetomicrobium thermoterr...
256   2e-72
WP_028598195.1 elongation factor G [Paenibacillus pasadenensis]
256   2e-72
WP_107297717.1 elongation factor G [Photobacterium rosenbergii]
256   2e-72
WP_017095473.1 elongation factor G [Vibrio splendidus]
256   2e-72
WP_006150857.1 elongation factor G [Streptococcus infantis]
256   2e-72
WP_008535635.1 elongation factor G [Streptococcus sp. C150]
256   2e-72
WP_075535975.1 elongation factor G [Candidatus Pelagibacter u...
256   2e-72
WP_012560440.1 elongation factor G [Streptococcus pyogenes]
256   2e-72

WP_029678608.1 elongation factor G [Bifidobacterium sp. 7101]
256   2e-72
WP_066777308.1 elongation factor G [Campylobacter hepaticus]
256   2e-72
WP_037614817.1 elongation factor G [Streptococcus sinensis]
256   2e-72
WP_057496360.1 elongation factor G [Stenotrophomonas maltophi...
256   2e-72
WP_045935561.1 elongation factor G [Bifidobacterium asteroides]
256   2e-72
WP_102458232.1 elongation factor G [Vibrio splendidus]
256   2e-72
WP_092326181.1 GTP-binding protein [Butyrivibrio sp. YAB3001]
260   2e-72
WP_013656980.1 GTP-binding protein [Cellulosilyticum lentocel...
260   2e-72
OUW07943.1 elongation factor G [Cellvibrionales bacterium TME...
256   2e-72
WP_068990483.1 elongation factor G [Streptococcus himalayensis]
256   2e-72
WP_053592771.1 GTP-binding protein [Lysinibacillus sp. FJAT-1...
255   2e-72
WP_049151171.1 GTP-binding protein [Lactobacillus gasseri]
255   2e-72
WP_105105711.1 elongation factor G [Streptococcus suis]
256   2e-72
WP_009754780.1 elongation factor G [Streptococcus sp. oral ta...
256   2e-72
WP_103636832.1 elongation factor G [Campylobacter concisus]
256   2e-72
WP_103608424.1 MULTISPECIES: elongation factor G [Campylobacter]
256   2e-72
PSD19797.1 elongation factor G [Stenotrophomonas maltophilia]
256   2e-72
WP_075506811.1 elongation factor G [Candidatus Pelagibacter u...
256   2e-72
WP_030035366.1 GTP-binding protein [Clostridium botulinum]
255   2e-72
WP_057545214.1 GTP-binding protein [Paeniclostridium sordellii]
256   2e-72
WP_090991416.1 GTP-binding protein [Olsenella umbonata]
255   2e-72
WP_075565740.1 MULTISPECIES: elongation factor G [Streptococcus]
256   2e-72
WP_077832478.1 GTP-binding protein [Clostridium roseum]
255   2e-72
WP_092139224.1 elongation factor G [Cupriavidus sp. YR651]
256   2e-72
WP_012678553.1 elongation factor G [Streptococcus equi]
256   2e-72
WP_040225102.1 GTP-binding protein [Bhargavaea cecembensis]
255   2e-72
WP_096421647.1 elongation factor G [Streptococcus oralis]
256   2e-72

WP_014735306.1 elongation factor G [Streptococcus suis]
256   2e-72
WP_108207788.1 elongation factor G [Vibrio splendidus]
256   2e-72
WP_091234486.1 elongation factor G [Fontibacillus panacisegetis]
256   2e-72
WP_062107672.1 elongation factor G [Bacillus niameyensis]
256   2e-72
WP_039617755.1 MULTISPECIES: elongation factor G [Campylobacter]
256   2e-72
WP_087951331.1 MULTISPECIES: elongation factor G [Bacillus ce...
256   2e-72
WP_070395710.1 elongation factor G [Moorea producens]
256   2e-72
WP_053566374.1 GTP-binding protein [Lactobacillus plantarum]
255   2e-72
WP_058919506.1 elongation factor G [Carnobacterium sp. CP1]
256   2e-72
WP_002938501.1 elongation factor G [Streptococcus suis]
256   2e-72
WP_002986045.1 elongation factor G [Streptococcus pyogenes]
256   2e-72
WP_011888592.1 elongation factor G [Streptococcus pyogenes]
256   2e-72
WP_012514927.1 elongation factor G [Streptococcus equi]
256   2e-72
WP_045763095.1 elongation factor G [Streptococcus infantis]
256   2e-72
WP_074032427.1 elongation factor G [Exiguobacterium sp. AT1b]
256   2e-72
PIZ00262.1 elongation factor G [bacterium (Candidatus Howlett...
256   2e-72
OEW17830.1 translation elongation factor G [Campylobacter sp....
256   2e-72
WP_013637808.1 elongation factor G [Desulfurobacterium thermo...
256   2e-72
WP_005368615.1 MULTISPECIES: elongation factor G [Photobacter...
256   2e-72
OYT70504.1 elongation factor G [Chloracidobacterium sp. CP2_5A]
256   2e-72
WP_000090342.1 MULTISPECIES: elongation factor G [Streptococcus]
256   2e-72
WP_018978371.1 elongation factor G [Saccharibacillus kuerlensis]
256   2e-72
WP_102444415.1 elongation factor G [Vibrio splendidus]
256   2e-72
WP_004734469.1 MULTISPECIES: elongation factor G [Vibrio]
256   2e-72
WP_000090364.1 MULTISPECIES: elongation factor G [Bacillus]
256   2e-72
WP_066006856.1 elongation factor G [Campylobacter ornithocola]
256   2e-72
GAO62847.1 elongation factor G [Bacillus anthracis]
256   2e-72

PIP33490.1 elongation factor G [Candidatus Falkowbacteria bac...
256   2e-72
WP_045605422.1 elongation factor G [Streptococcus mitis]
256   2e-72
WP_029032356.1 elongation factor G [Salinarimonas rosea]
256   2e-72
WP_042236339.1 GTP-binding protein [Paenibacillus sp. FSL R5-...
255   2e-72
WP_057873827.1 elongation factor G [Lactobacillus rennini]
256   2e-72
WP_049455047.1 elongation factor G [Stenotrophomonas maltophi...
256   2e-72
WP_057766370.1 elongation factor G [Lactobacillus tucceti]
256   2e-72
WP_049409834.1 elongation factor G [Stenotrophomonas maltophi...
256   2e-72
WP_003102218.1 elongation factor G [Streptococcus iniae]
256   2e-72
WP_054760433.1 GTP-binding protein [Lactobacillus collinoides]
255   2e-72
PIR93683.1 elongation factor G [Candidatus Falkowbacteria bac...
256   2e-72
OHA08988.1 translation elongation factor G [Candidatus Sungba...
256   2e-72
WP_101168954.1 elongation factor G [Bacillus sp. SN10]
256   2e-72
WP_032117908.1 GTP-binding protein [Clostridium sp. CL-2]
255   2e-72
CUO52751.1 Elongation factor G [Dorea longicatena]
260   2e-72
WP_068615394.1 elongation factor G [Woeseia oceani]
256   2e-72
WP_105104941.1 elongation factor G [Streptococcus suis]
256   2e-72
WP_074389611.1 elongation factor G [Streptococcus suis]
256   2e-72
WP_000090363.1 elongation factor G [Bacillus anthracis]
256   2e-72
KLU39779.1 elongation factor G [Peptococcaceae bacterium 1109]
256   2e-72
KPJ57322.1 elongation factor G [Parcubacteria bacterium DG_74_2]
256   2e-72
WP_000196384.1 MULTISPECIES: elongation factor G [Bacillus]
256   2e-72
WP_060914141.1 elongation factor G [Gemella haemolysans]
256   2e-72
WP_014703768.1 elongation factor G [Methylophaga frappieri]
256   2e-72
OYV59010.1 elongation factor G [Actinobacteria bacterium 21-7...
256   2e-72
WP_045593079.1 elongation factor G [Streptococcus oralis]
256   2e-72
WP_007518588.1 elongation factor G [Streptococcus oralis]
256   2e-72

WP_049439636.1 elongation factor G [Stenotrophomonas maltophi...
256   2e-72
WP_008432482.1 elongation factor G [Planococcus donghaensis]
256   2e-72
WP_039462474.1 elongation factor G [Photobacterium gaetbulicola]
256   2e-72
WP_066916838.1 elongation factor G [Streptococcus sp. DD12]
256   2e-72
WP_009294502.1 MULTISPECIES: elongation factor G [Campylobacter]
256   2e-72
WP_002921034.1 elongation factor G [Campylobacter jejuni]
256   2e-72
WP_036973554.1 MULTISPECIES: elongation factor G [Pseudoalter...
256   2e-72
WP_093998891.1 elongation factor G [Streptococcus canis]
256   2e-72
WP_013296974.1 elongation factor G [Thermoanaerobacterium the...
256   2e-72
WP_074863934.1 elongation factor G [Lysobacter enzymogenes]
256   2e-72
WP_061418724.1 elongation factor G [Streptococcus oralis]
256   2e-72
WP_029752531.1 elongation factor G [Streptococcus suis]
256   2e-72
WP_018379168.1 elongation factor G [Streptococcus ovis]
256   2e-72
WP_050182399.1 elongation factor G [Domibacillus robiginosus]
256   2e-72
WP_077718345.1 elongation factor G [Novibacillus thermophilus]
256   2e-72
WP_083992459.1 elongation factor G [Streptococcus pneumoniae]
256   2e-72
WP_053442993.1 elongation factor G [Stenotrophomonas maltophi...
256   2e-72
WP_049481260.1 elongation factor G [Stenotrophomonas maltophi...
256   2e-72
WP_103572176.1 elongation factor G [Campylobacter concisus]
256   2e-72
WP_071458939.1 elongation factor G [Bacillus mediterraneensis]
256   2e-72
WP_008090094.1 elongation factor G [Streptococcus ictaluri]
256   2e-72
WP_044777415.1 elongation factor G [Streptococcus suis]
256   2e-72
EGM49699.1 Tetracycline resistance protein [Lactobacillus sal...
255   2e-72
WP_055666952.1 GTP-binding protein [Desnuesiella massiliensis]
255   2e-72
PLT44147.1 Translation elongation factor G [Paenibacillus pas...
256   2e-72
WP_044760201.1 elongation factor G [Streptococcus suis]
256   2e-72
WP_029174305.1 elongation factor G [Streptococcus suis]
256   2e-72

WP_098842438.1 elongation factor G [Bacillus toyonensis]
256   2e-72
WP_006828916.1 elongation factor G [Planococcus antarcticus]
256   2e-72
WP_093674103.1 elongation factor G [Sporolactobacillus nakaya...
256   2e-72
WP_032463626.1 elongation factor G [Streptococcus pyogenes]
256   2e-72
WP_015218285.1 elongation factor G [Cyanobacterium aponinum]
256   2e-72
WP_070174027.1 MULTISPECIES: elongation factor G [Bacillus ce...
256   3e-72
WP_080831988.1 elongation factor G [Cohnella sp. 6021052837]
256   3e-72
OHB26365.1 translation elongation factor G [Desulfuromonadace...
256   3e-72
WP_062341106.1 GTP-binding protein [Herbidospora sakaeratensis]
253   3e-72
WP_009854983.1 elongation factor G [Streptococcus gallolyticus]
256   3e-72
WP_000196385.1 MULTISPECIES: elongation factor G [Bacillus]
256   3e-72
WP_087246660.1 MULTISPECIES: elongation factor G [Faecalibact...
256   3e-72
WP_053267572.1 GTP-binding protein [Lactobacillus plantarum]
255   3e-72
WP_006440493.1 elongation factor G [[Clostridium] hiranonis]
256   3e-72
WP_042513813.1 elongation factor G [Bacillus anthracis]
256   3e-72
KKR48488.1 Elongation factor G [Candidatus Magasanikbacteria ...
256   3e-72
WP_000196383.1 MULTISPECIES: elongation factor G [Bacillus]
256   3e-72
WP_087381609.1 elongation factor G [Faecalibacterium sp. An122]
256   3e-72
OGD13074.1 translation elongation factor G [Candidatus Aminic...
256   3e-72
EEL25061.1 Elongation factor G 2 [Bacillus cereus Rock1-3]
256   3e-72
WP_102543666.1 elongation factor G [Vibrio lentus]
256   3e-72
WP_022605150.1 elongation factor G [Rubidibacter lacunae]
256   3e-72
WP_045505313.1 elongation factor G [Streptococcus gordonii]
256   3e-72
GBC83022.1 Elongation factor G [bacterium HR10]
256   3e-72
WP_070088120.1 GTP-binding protein [Merdimonas faecis]
260   3e-72
WP_057488777.1 elongation factor G [Streptococcus oralis]
256   3e-72
WP_024398330.1 elongation factor G [Streptococcus suis]
256   3e-72

OGZ61534.1 translation elongation factor G [Candidatus Specht...
256   3e-72
WP_057962321.1 elongation factor G [Lactobacillus parakefiri]
256   3e-72
WP_028516650.1 GTP-binding protein [Ruminococcus flavefaciens]
259   3e-72
WP_047672460.1 GTP-binding protein [Lactobacillus plantarum]
255   3e-72
WP_105122116.1 elongation factor G [Streptococcus suis]
256   3e-72
WP_054380198.1 elongation factor G [Streptococcus pneumoniae]
256   3e-72
WP_002891917.1 MULTISPECIES: elongation factor G [Streptococcus]
256   3e-72
WP_095378011.1 elongation factor G [Stenotrophomonas maltophi...
256   3e-72
WP_037291591.1 elongation factor G [Saccharibacillus sacchari]
256   3e-72
WP_102189299.1 elongation factor G [Gemella sanguinis]
256   3e-72
WP_055276772.1 GTP-binding protein [Turicibacter sanguinis]
255   3e-72
WP_103346558.1 elongation factor G [Streptococcus parauberis]
256   3e-72
WP_090222001.1 elongation factor G [Fervidobacterium changbai...
256   3e-72
WP_099030921.1 elongation factor G [Pseudoalteromonas sp. GCY]
256   3e-72
WP_053827661.1 elongation factor G [Helicobacter heilmannii]
256   3e-72
CDA40702.1 elongation factor EF-G [Eubacterium eligens CAG:72]
259   3e-72
WP_018166348.1 elongation factor G [Streptococcus minor]
256   3e-72
WP_071516762.1 elongation factor G [Geitlerinema sp. PCC 9228]
256   3e-72
WP_017293370.1 elongation factor G [Geminocystis herdmanii]
256   3e-72
KUK84876.1 Elongation factor G [Synergistales bacterium 58_81]
256   3e-72
WP_057160002.1 elongation factor G [Lysobacter sp. Root983]
255   3e-72
WP_094517122.1 GTP-binding protein [Lactobacillus taiwanensis]
254   3e-72
WP_011173716.1 elongation factor G [Thermus thermophilus]
256   3e-72
WP_057502830.1 elongation factor G [[Pseudomonas] geniculata]
255   3e-72
WP_103612738.1 elongation factor G [Campylobacter concisus]
256   3e-72
WP_076634174.1 elongation factor G [Streptococcus pyogenes]
256   3e-72
WP_071793999.1 elongation factor G [Streptococcus bovimastiti...
256   3e-72

WP_074581692.1 elongation factor G [Streptococcus gallolyticus]
256   3e-72
WP_049694536.1 elongation factor G [Planococcus sp. L10.15]
256   3e-72
WP_003052842.1 elongation factor G [Streptococcus dysgalactiae]
256   3e-72
WP_069137793.1 elongation factor G [Streptococcus pneumoniae]
256   3e-72
CRR21017.1 Elongation factor G [Pseudomonas aeruginosa]
255   3e-72
WP_084058381.1 elongation factor G [Desulfacinum hydrothermale]
256   3e-72
WP_000090365.1 elongation factor G [Bacillus cereus]
256   3e-72
WP_004101432.1 elongation factor G [Thermosipho africanus]
256   3e-72
WP_063731188.1 GTP-binding protein [Lactobacillus plantarum]
255   3e-72
WP_055304762.1 GTP-binding protein [Turicibacter sanguinis]
255   3e-72
WP_038701886.1 elongation factor G [Planococcus sp. PAMC 21323]
256   3e-72
EEM43904.1 Elongation factor G 2 [Bacillus thuringiensis sero...
256   3e-72
WP_105142578.1 elongation factor G [Streptococcus suis]
256   3e-72
WP_018213139.1 GTP-binding protein [Desulfitobacterium hafnie...
259   3e-72
WP_098927043.1 GTP-binding protein [Clostridium sporogenes]
255   3e-72
WP_095480827.1 GTP-binding protein [Bacillaceae bacterium SAO...
255   3e-72
WP_054376407.1 elongation factor G [Streptococcus pneumoniae]
256   3e-72
OUW94389.1 elongation factor G [Pelagibacteraceae bacterium T...
256   3e-72
WP_094549421.1 elongation factor G [Rubricoccus marinus]
256   3e-72
WP_099950267.1 elongation factor G [Ezakiella peruensis]
256   3e-72
AUC62127.1 translation elongation factor G [Cyanobacterium sp...
256   3e-72
WP_017072746.1 elongation factor G [Vibrio splendidus]
256   3e-72
GAL24930.1 translation elongation factor G paralog [Vibrio va...
256   3e-72
GAD59891.1 LOW QUALITY PROTEIN: translation elongation factor...
256   3e-72
WP_102312451.1 elongation factor G [Vibrio lentus]
256   3e-72
WP_056018022.1 MULTISPECIES: elongation factor G [Phenylobact...
256   3e-72
WP_105111026.1 elongation factor G [Streptococcus suis]
256   3e-72

WP_061592470.1 elongation factor G [Streptococcus sanguinis]
256   3e-72
WP_107856669.1 elongation factor G [Campylobacter concisus]
256   3e-72
WP_103593121.1 elongation factor G [Campylobacter concisus]
256   3e-72
WP_071351396.1 elongation factor G [Bacillus sp. MUM 13]
256   3e-72
WP_049449507.1 elongation factor G [Stenotrophomonas maltophi...
255   3e-72
WP_012175037.1 elongation factor G [Desulfococcus oleovorans]
255   3e-72
WP_103580806.1 elongation factor G [Campylobacter concisus]
256   3e-72
WP_098508947.1 elongation factor G [Bacillus cereus]
256   3e-72
WP_065182206.1 elongation factor G [Stenotrophomonas maltophi...
255   3e-72
WP_065991122.1 GTP-binding protein [Lactobacillus crispatus]
254   3e-72
WP_057717355.1 GTP-binding protein [Lactobacillus plantarum]
255   3e-72
WP_061329513.1 GTP-binding protein [Clostridium botulinum]
254   3e-72
WP_035759237.1 MULTISPECIES: elongation factor G [Arthrospira]
256   3e-72
WP_048767366.1 elongation factor G [Streptococcus gordonii]
256   3e-72
WP_018370313.1 elongation factor G [Streptococcus marimammalium]
256   3e-72
EEM31001.1 Elongation factor G 2 [Bacillus thuringiensis Bt407]
256   3e-72
WP_105134156.1 elongation factor G [Streptococcus suis]
256   3e-72
CDB00896.1 small GTP-binding protein domain [Firmicutes bacte...
259   3e-72
WP_053828665.1 elongation factor G [Helicobacter heilmannii]
256   3e-72
WP_012803380.1 elongation factor G [Cryptobacterium curtum]
256   3e-72
WP_061101958.1 elongation factor G [Peptostreptococcus anaero...
256   3e-72
OGM28139.1 translation elongation factor G [Candidatus Woeseb...
256   3e-72
WP_029160211.1 GTP-binding protein [Clostridium scatologenes]
259   3e-72
WP_070678707.1 elongation factor G [Streptococcus sp. HMSC062...
256   3e-72
WP_000090356.1 elongation factor G [Streptococcus pneumoniae]
256   3e-72
WP_097043544.1 elongation factor G [Terribacillus aidingensis]
256   3e-72
WP_102468413.1 MULTISPECIES: elongation factor G [Vibrio]
256   3e-72

WP_084669694.1 elongation factor G [Streptococcus pneumoniae]
256   3e-72
WP_042969299.1 elongation factor G [Bacillus thuringiensis]
256   3e-72
WP_014355309.1 elongation factor G [Acetobacterium woodii]
256   3e-72
WP_075526715.1 GTP-binding protein [Lysinibacillus sphaericus]
254   3e-72
KRM87208.1 elongation factor g [Lactobacillus thailandensis D...
254   3e-72
SCI39602.1 Tetracycline resistance protein tetM [uncultured B...
259   3e-72
WP_049863889.1 elongation factor G [Pseudoalteromonas rubra]
256   3e-72
WP_023939638.1 elongation factor G [Streptococcus pseudopneum...
256   3e-72
WP_069651096.1 elongation factor G [Caloranaerobacter ferrire...
256   3e-72
WP_050323827.1 elongation factor G [Streptococcus equi]
256   3e-72
WP_042514117.1 elongation factor G [Bacillus anthracis]
256   3e-72
WP_003456407.1 GTP-binding protein [Clostridium perfringens]
254   3e-72
WP_036756654.1 elongation factor G [Streptococcus infantis]
256   3e-72
WP_103606020.1 elongation factor G [Campylobacter concisus]
256   3e-72
WP_023487862.1 GTP-binding protein [Lactobacillus crispatus]
254   3e-72
WP_061642981.1 elongation factor G [Streptococcus pneumoniae]
256   3e-72
WP_004250723.1 elongation factor G [Streptococcus infantis]
256   3e-72
WP_098689191.1 elongation factor G [Bacillus toyonensis]
256   3e-72
WP_074781722.1 GTP-binding protein [Sharpea azabuensis]
259   3e-72
WP_061750250.1 elongation factor G [Streptococcus pneumoniae]
256   3e-72
EFI63952.1 elongation factor G [Bacillus cereus SJ1]
256   3e-72
WP_105131489.1 elongation factor G [Streptococcus suis]
256   4e-72
WP_073525552.1 MULTISPECIES: elongation factor G [Bacillus]
256   4e-72
WP_004233287.1 elongation factor G [Streptococcus equinus]
256   4e-72
PNV86669.1 elongation factor G [Desulfobacteraceae bacterium]
256   4e-72
OLD17045.1 translation elongation factor G [Acidobacteriales ...
255   4e-72
WP_038182716.1 MULTISPECIES: GTP-binding protein [Viridibacil...
254   4e-72

OOL50878.1 elongation factor G [Enterococcus faecium]
241   4e-72
WP_041521587.1 elongation factor G [Gilvimarinus agarilyticus]
256   4e-72
WP_032495869.1 elongation factor G [Streptococcus pneumoniae]
256   4e-72
WP_061478675.1 elongation factor G [Stenotrophomonas sp. DDT-1]
255   4e-72
ABK83520.1 translation elongation factor 2 (EF-2/EF-G) [Bacil...
256   4e-72
WP_024399880.1 elongation factor G [Streptococcus suis]
256   4e-72
WP_069328351.1 elongation factor G [Paenibacillus sp. TI45-13ar]
255   4e-72
WP_074628068.1 elongation factor G [Streptococcus gallolyticus]
255   4e-72
WP_092614272.1 elongation factor G [Jiangella sp. DSM 45060]
256   4e-72
OEV42019.1 translation elongation factor G [Campylobacter jej...
255   4e-72
OUU06786.1 elongation factor G [Gammaproteobacteria bacterium...
256   4e-72
CCZ78719.1 translation elongation factor G [Roseburia sp. CAG...
259   4e-72
WP_003448683.1 GTP-binding protein [Clostridium perfringens]
254   4e-72
WP_033260776.1 elongation factor G [Amycolatopsis vancoresmyc...
254   4e-72
SIA40766.1 translation elongation factor 2 (EF-2/EF-G) [Mycob...
255   4e-72
WP_103576219.1 elongation factor G [Campylobacter concisus]
255   4e-72
OGO10837.1 translation elongation factor G [Chloroflexi bacte...
255   4e-72
WP_072539434.1 GTP-binding protein [Lactobacillus plantarum]
255   4e-72
WP_060670222.1 GTP-binding protein [Clostridium perfringens]
254   4e-72
WP_088850796.1 elongation factor G [Streptococcus pneumoniae]
255   4e-72
WP_100878740.1 elongation factor G [Pseudonocardia alni]
256   4e-72
WP_028515623.1 GTP-binding protein [Ruminococcus flavefaciens]
259   4e-72
OUU76318.1 elongation factor G [Chloroflexi bacterium TMED70]
255   4e-72
WP_087936601.1 GTP-binding protein [Massiliomicrobiota sp. An80]
259   4e-72
WP_049445332.1 elongation factor G [Stenotrophomonas maltophi...
255   4e-72
WP_025772185.1 elongation factor G [Thioalkalivibrio sp. HK1]
256   4e-72
WP_086330793.1 elongation factor G [Enterococcus sp. 4G2_DIV0...
255   4e-72

WP_007638037.1 elongation factor G [Paraglaciecola psychrophila]
256   4e-72
WP_050973908.1 elongation factor G [Streptococcus pneumoniae]
255   4e-72
WP_071520323.1 elongation factor G [Moorella thermoacetica]
255   4e-72
WP_061599806.1 elongation factor G [Streptococcus gordonii]
255   4e-72
WP_014793657.1 GTP-binding protein [Desulfitobacterium dehalo...
259   4e-72
WP_106495536.1 elongation factor G [Lentibacillus sp. Marseil...
255   4e-72
WP_098268761.1 elongation factor G [Bacillus cereus]
255   4e-72
WP_004457970.1 GTP-binding protein [Clostridium perfringens]
254   4e-72
WP_063364933.1 elongation factor G [Pseudoalteromonas luteovi...
255   4e-72
WP_105993384.1 elongation factor G [Staphylococcus simulans]
255   4e-72
WP_053941528.1 elongation factor G [Helicobacter ailurogastri...
255   4e-72
WP_073272147.1 elongation factor G [Alkalibacter saccharoferm...
255   4e-72
WP_087245653.1 GTP-binding protein [Massiliomicrobiota sp. An...
258   4e-72
WP_094676224.1 elongation factor G [Hydrocoleum sp. CS-953]
255   4e-72
WP_035984836.1 elongation factor G [Leptolyngbya sp. KIOST-1]
255   4e-72
WP_017084234.1 elongation factor G [Vibrio splendidus]
255   4e-72
WP_088835701.1 elongation factor G [Streptococcus pneumoniae]
255   4e-72
4JUW_Y Chain Y, Crystal Structure Of The Ribosome Bound To El...
255   4e-72
WP_041051295.1 elongation factor G [Paenibacillus sp. VKM B-2...
255   4e-72
WP_011503582.1 elongation factor G [Rhodopseudomonas palustris]
255   4e-72
EHM41558.1 translation elongation factor G [Anaeroglobus gemi...
255   4e-72
WP_074732147.1 GTP-binding protein [Sharpea azabuensis]
258   4e-72
WP_028488538.1 elongation factor G [Thiothrix lacustris]
255   4e-72
WP_087845241.1 elongation factor G [Photobacterium sp. CECT 9...
255   4e-72
WP_063357342.1 elongation factor G [Pseudoalteromonas luteovi...
255   4e-72
OGT32796.1 translation elongation factor G [Gammaproteobacter...
256   4e-72
WP_098477718.1 elongation factor G [Bacillus cereus]
255   4e-72

GBC78339.1 Elongation factor G [bacterium HR08]
256   4e-72
WP_029184949.1 elongation factor G [Streptococcus suis]
255   4e-72
WP_090991240.1 elongation factor G [Bacillus sp. OV322]
255   4e-72
WP_010528984.1 elongation factor G [Lentibacillus jeotgali]
255   4e-72
WP_040537261.1 GTP-binding protein [Lactobacillus pasteurii]
254   4e-72
WP_073249886.1 elongation factor G [Caloramator proteoclasticus]
255   4e-72
WP_074898876.1 elongation factor G [Stenotrophomonas maltophi...
255   4e-72
4M1K_A Chain A, Crystal Structure Of Elongation Factor G (efg)
255   4e-72
WP_055215704.1 GTP-binding protein [[Eubacterium] eligens]
259   4e-72
WP_048685829.1 GTP-binding protein [Lactobacillus gasseri]
254   4e-72
WP_097658738.1 elongation factor G [Bradyrhizobium sp. Y36]
255   4e-72
WP_060552331.1 elongation factor G [Staphylococcus agnetis]
255   4e-72
WP_098545541.1 elongation factor G [Bacillus cereus]
255   4e-72
WP_068813080.1 GTP-binding protein [Lactobacillus crispatus]
254   4e-72
WP_094523670.1 GTP-binding protein [Lactobacillus taiwanensis]
254   4e-72
WP_093649739.1 elongation factor G [Streptococcus varani]
255   4e-72
WP_087368078.1 GTP-binding protein [Lactobacillus salivarius]
254   4e-72
WP_100621688.1 elongation factor G [Campylobacter jejuni]
255   4e-72
WP_033161875.1 GTP-binding protein [Sharpea azabuensis]
258   4e-72
WP_017106964.1 MULTISPECIES: elongation factor G [Vibrio]
255   4e-72
WP_098262329.1 elongation factor G [Bacillus sp. AFS006103]
255   4e-72
WP_075231889.1 elongation factor G [Streptococcus sp. HMSC034...
255   4e-72
WP_061452096.1 elongation factor G [Streptococcus oralis]
255   4e-72
OLA20580.1 translation elongation factor G [[Eubacterium] eli...
259   5e-72
WP_014629062.1 elongation factor G [Thermus thermophilus]
255   5e-72
WP_007788540.1 elongation factor G [Peptostreptococcus stomatis]
255   5e-72
PID60000.1 elongation factor G [Gammaproteobacteria bacterium]
255   5e-72

OQY60116.1 elongation factor G [Desulfobacteraceae bacterium ...
255   5e-72
WP_070256354.1 elongation factor G [Campylobacter lari]
255   5e-72
WP_010279586.1 elongation factor G [Paenibacillus senegalensis]
255   5e-72
EEL42445.1 Elongation factor G 2 [Bacillus cereus Rock3-29]
256   5e-72
WP_105922062.1 GTP-binding protein [Lactobacillus pentosus]
254   5e-72
WP_017150124.1 elongation factor G [Bacillus bingmayongensis]
255   5e-72
WP_000090360.1 elongation factor G [Bacillus cereus]
255   5e-72
WP_068695603.1 GTP-binding protein [Paenibacillus yonginensis]
255   5e-72
KKT13510.1 Elongation factor G [Candidatus Falkowbacteria bac...
255   5e-72
WP_002091512.1 elongation factor G [Bacillus cereus]
255   5e-72
4MYT_A Chain A, Crystal Structure Of Elongation Factor G (efg)
255   5e-72
WP_005544561.1 elongation factor G [Paenibacillus alvei]
255   5e-72
WP_021201794.1 elongation factor G [Stenotrophomonas maltophi...
255   5e-72
WP_054205307.1 MULTISPECIES: elongation factor G [Pseudoalter...
255   5e-72
WP_088876750.1 elongation factor G [Vibrio shilonii]
255   5e-72
WP_067087679.1 elongation factor G [Streptococcus marmotae]
255   5e-72
A7H4P5.1 RecName: Full=Elongation factor G; Short=EF-G
255   5e-72
WP_029505054.1 GTP-binding protein [Lachnoclostridium phytofe...
259   5e-72
WP_073585891.1 elongation factor G [Vibrio quintilis]
255   5e-72
WP_061595327.1 elongation factor G [Streptococcus sanguinis]
255   5e-72
WP_049548396.1 elongation factor G [Streptococcus pseudopneum...
255   5e-72
PKM61604.1 elongation factor G [Firmicutes bacterium HGW-Firm...
255   5e-72
ADJ79897.1 Tetracycline resistance protein [Lactobacillus sal...
254   5e-72
WP_057536678.1 GTP-binding protein [Paeniclostridium sordellii]
254   5e-72
CDE84203.1 elongation factor G [Clostridium sp. CAG:273]
255   5e-72
WP_024859676.1 GTP-binding protein [Ruminococcus flavefaciens]
258   5e-72
WP_094942215.1 elongation factor G [Tetzosporium hominis]
255   5e-72

WP_005416500.1 elongation factor G [Stenotrophomonas maltophi...
255   5e-72
WP_065918448.1 elongation factor G [Lentzea guizhouensis]
254   5e-72
WP_060629211.1 MULTISPECIES: elongation factor G [Bacillus ce...
255   5e-72
EEL52497.1 Elongation factor G 2 [Bacillus cereus Rock3-44]
255   5e-72
WP_009543829.1 MULTISPECIES: elongation factor G [Cyanothece]
255   5e-72
KXJ40776.1 elongation factor G [Methylothermaceae bacteria B42]
255   5e-72
WP_049736641.1 GTP-binding protein [Brevibacillus reuszeri]
254   5e-72
WP_053872511.1 MULTISPECIES: elongation factor G [Campylobacter]
255   5e-72
WP_004193818.1 elongation factor G [Streptococcus sanguinis]
255   5e-72
WP_002884537.1 MULTISPECIES: elongation factor G [Streptococcus]
255   5e-72
WP_098347744.1 elongation factor G [Bacillus cereus]
255   5e-72
WP_003066533.1 MULTISPECIES: elongation factor G [Streptococcus]
255   5e-72
AMP52955.1 elongation factor G, domain IV [uncultured bacterium]
246   5e-72
WP_076634743.1 GTP-binding protein [Lactobacillus plantarum]
254   5e-72
WP_003105809.1 elongation factor G [Streptococcus parauberis]
255   5e-72
WP_062711497.1 elongation factor G [Streptococcus halotolerans]
255   5e-72
WP_045851850.1 elongation factor G [Domibacillus enclensis]
255   5e-72
OLA40292.1 translation elongation factor G [Firmicutes bacter...
255   5e-72
WP_011228848.1 elongation factor G [Thermus thermophilus]
255   5e-72
WP_092085687.1 elongation factor G [Dolosicoccus paucivorans]
255   5e-72
WP_099807131.1 elongation factor G [Streptococcus suis]
255   5e-72
WP_049527297.1 MULTISPECIES: elongation factor G [Streptococcus]
255   5e-72
WP_057807139.1 elongation factor G [Pediococcus ethanolidurans]
255   5e-72
WP_016315398.1 elongation factor G [Anaerotruncus sp. G3(2012)]
255   5e-72
WP_101695959.1 GTP-binding protein [Dorea sp. Marseille-P4003]
259   5e-72
WP_077852098.1 GTP-binding protein [Clostridium aurantibutyri...
254   5e-72
OFW75518.1 translation elongation factor G [Alicyclobacillus ...
255   6e-72

WP_039666716.1 elongation factor G [Campylobacter sp. RM16704]
255   6e-72
CCI84652.1 Elongation factor G [Lactobacillus pasteurii DSM 2...
254   6e-72
4KBT_Y Chain Y, 70s Ribosome Translocation Intermediate Gdpnp...
255   6e-72
PKQ18919.1 elongation factor G [Actinobacteria bacterium HGW-...
255   6e-72
WP_013661465.1 elongation factor G [Marinomonas mediterranea]
255   6e-72
WP_088795322.1 elongation factor G [Streptococcus pneumoniae]
255   6e-72
WP_061650205.1 elongation factor G [Streptococcus pneumoniae]
255   6e-72
WP_069657633.1 elongation factor G [Vulcanibacillus modestica...
255   6e-72
WP_019659660.1 elongation factor G [Stenotrophomonas maltophi...
254   6e-72
PDM38615.1 elongation factor G [Bacillus cereus]
255   6e-72
WP_038225468.1 MULTISPECIES: elongation factor G [Vibrio]
255   6e-72
KUK46625.1 Elongation factor G [Anaerolinea thermophila]
255   6e-72
PID49263.1 elongation factor G [Proteobacteria bacterium]
255   6e-72
WP_015431346.1 elongation factor G [Paraglaciecola psychrophila]
255   6e-72
WP_061318050.1 GTP-binding protein [Clostridium botulinum]
254   6e-72
WP_065792759.1 elongation factor G [Pseudoalteromonas luteovi...
255   6e-72
WP_075999087.1 MULTISPECIES: elongation factor G [Halioglobus]
255   6e-72
WP_029998358.1 elongation factor G [Streptococcus suis]
255   6e-72
WP_003089490.1 elongation factor G [Streptococcus ratti]
255   6e-72
WP_105563626.1 MULTISPECIES: elongation factor G [Brevundimonas]
255   6e-72
WP_047421075.1 elongation factor G [Bacillus anthracis]
255   6e-72
WP_092685302.1 elongation factor G [Rhodopseudomonas pseudopa...
255   6e-72
WP_012225296.1 elongation factor G [Candidatus Microthrix par...
255   6e-72
WP_074741931.1 GTP-binding protein [Ruminococcus flavefaciens]
258   6e-72
WP_099983236.1 elongation factor G [Streptococcus canis]
255   6e-72
WP_076360227.1 MULTISPECIES: elongation factor G [Paenibacillus]
255   6e-72
OJY35522.1 translation elongation factor G [Rhizobiales bacte...
255   6e-72

WP_004819166.1 elongation factor G [Peptoniphilus indolicus]
255   6e-72
WP_005723577.1 GTP-binding protein [Lactobacillus crispatus]
254   6e-72
WP_027868964.1 GTP-binding protein [Eubacterium sp. AB3007]
258   6e-72
WP_003107724.1 elongation factor G [Streptococcus parauberis]
255   6e-72
1EFG_A Chain A, The Crystal Structure Of Elongation Factor G ...
255   6e-72
WP_057771854.1 elongation factor G [Pediococcus inopinatus]
255   6e-72
PJN96892.1 tetracycline resistance ribosomal protection prote...
241   6e-72
WP_044623083.1 elongation factor G [Photobacterium gaetbulicola]
255   6e-72
WP_102227411.1 elongation factor G [Dolosicoccus paucivorans]
255   6e-72
WP_098307104.1 elongation factor G [Bacillus cereus]
255   6e-72
WP_073254188.1 elongation factor G [Caldanaerovirga acetigign...
255   6e-72
WP_015847667.1 GTP-binding protein [Paenibacillus sp. JDR-2]
254   6e-72
OLA51608.1 translation elongation factor G [Oscillibacter sp....
258   6e-72
WP_004185285.1 MULTISPECIES: elongation factor G [Streptococcus]
255   6e-72
WP_045798441.1 elongation factor G [Streptococcus equinus]
255   6e-72
CDB43580.1 elongation factor G [Firmicutes bacterium CAG:240]
255   6e-72
WP_039877408.1 elongation factor G [Paenibacillus sp. FSL R7-...
255   6e-72
OFW62561.1 translation elongation factor G [Actinobacteria ba...
255   6e-72
WP_038855191.1 elongation factor G [Campylobacter coli]
255   6e-72
WP_095670193.1 GTP-binding protein [Lactobacillus gasseri]
254   6e-72
WP_098338758.1 elongation factor G [Bacillus cereus]
255   6e-72
WP_020155388.1 elongation factor G [Caldibacillus debilis]
255   6e-72
WP_070768186.1 elongation factor G [Corynebacterium sp. HMSC0...
255   6e-72
WP_099352738.1 elongation factor G [Bacillus onubensis]
255   6e-72
WP_097898937.1 MULTISPECIES: elongation factor G [Bacillus]
255   6e-72
WP_078111072.1 GTP-binding protein [Bacillus oleronius]
254   6e-72
WP_006531903.1 elongation factor G [Streptococcus infantarius]
255   6e-72

WP_021143166.1 elongation factor G [Streptococcus equinus]
255   6e-72
WP_048743618.1 MULTISPECIES: elongation factor G [Paenibacillus]
255   6e-72
WP_063378556.1 elongation factor G [Pseudoalteromonas luteovi...
255   6e-72
CDB13241.1 putative translation elongation factor G [Eubacter...
258   6e-72
WP_011664988.1 elongation factor G [Rhodopseudomonas palustris]
255   6e-72
WP_083562721.1 elongation factor G [Oceanococcus atlanticus]
255   6e-72
WP_059283708.1 elongation factor G [Bacillus coahuilensis]
255   6e-72
WP_000090343.1 MULTISPECIES: elongation factor G [Streptococcus]
255   6e-72
WP_103594975.1 elongation factor G [Campylobacter concisus]
255   6e-72
OLP63406.1 Elongation factor G [Bacillus pumilus]
255   6e-72
WP_073458720.1 elongation factor G [Pseudonocardia thermophila]
255   7e-72
WP_018767347.1 MULTISPECIES: elongation factor G [Bacillus]
255   7e-72
WP_020379542.1 elongation factor G [Candidatus Microthrix par...
255   7e-72
WP_038602382.1 elongation factor G [Rickettsiales bacterium A...
255   7e-72
WP_004347288.1 elongation factor G [Streptococcus parauberis]
255   7e-72
APO35069.1 Elongation factor G C-terminus [uncultured bacterium]
236   7e-72
WP_087724213.1 elongation factor G [Campylobacter jejuni]
255   7e-72
WP_012084985.1 elongation factor G [Kineococcus radiotolerans]
255   7e-72
WP_101774014.1 elongation factor G [Peptostreptococcus sp. Ma...
254   7e-72
WP_021085492.1 elongation factor G [Campylobacter concisus]
255   7e-72
OGO88484.1 translation elongation factor G [Clostridiales bac...
255   7e-72
WP_092593487.1 elongation factor G [Acidaminobacter hydrogeno...
254   7e-72
WP_035799555.1 elongation factor G [Cyanothece sp. CCY0110]
255   7e-72
WP_050149143.1 elongation factor G [Streptococcus pneumoniae]
255   7e-72
OLA86974.1 translation elongation factor G [Desulfovibrio piger]
255   7e-72
WP_040195945.1 GTP-binding protein [Candidatus Soleaferrea ma...
254   7e-72
COO08007.1 elongation factor G [Streptococcus pneumoniae]
255   7e-72

WP_016397201.1 elongation factor G [Streptococcus pneumoniae]
255   7e-72
WP_071542395.1 elongation factor G [Moorella thermoacetica]
255   7e-72
WP_027972176.1 elongation factor G [Streptococcus plurextorum]
255   7e-72
WP_008179642.1 elongation factor G [Moorea producens]
255   7e-72
WP_087582021.1 elongation factor G [Campylobacter concisus]
255   7e-72
OQY25033.1 elongation factor G [Anaerolineaceae bacterium 457...
255   7e-72
WP_000090369.1 MULTISPECIES: elongation factor G [Bacillus ce...
255   7e-72
OUX46199.1 elongation factor G [Candidatus Pelagibacter sp. T...
254   7e-72
WP_023482173.1 elongation factor G [Paenibacillus larvae]
254   7e-72
OGZ24602.1 translation elongation factor G [Candidatus Nealso...
255   7e-72
WP_073602552.1 elongation factor G [Vibrio aerogenes]
255   7e-72
WP_050235237.1 elongation factor G [Streptococcus pneumoniae]
255   7e-72
PKM79620.1 elongation factor G [Firmicutes bacterium HGW-Firm...
254   7e-72
WP_002797112.1 MULTISPECIES: elongation factor G [Campylobacter]
254   7e-72
PSD25296.1 elongation factor G [Stenotrophomonas maltophilia]
254   7e-72
WP_049161894.1 GTP-binding protein [Lactobacillus gasseri]
253   7e-72
WP_045518748.1 GTP-binding protein [Clostridium sporogenes]
254   7e-72
WP_000532231.1 MULTISPECIES: elongation factor G [Bacillus]
254   7e-72
WP_025149625.1 MULTISPECIES: elongation factor G [Bacillus]
254   7e-72
WP_060809643.1 elongation factor G [Campylobacter jejuni]
254   7e-72
WP_057096452.1 elongation factor G [Campylobacter jejuni]
254   7e-72
WP_014510797.1 elongation factor G [Thermus thermophilus]
254   7e-72
WP_002916556.1 MULTISPECIES: elongation factor G [Streptococcus]
254   7e-72
WP_077996268.1 elongation factor G [Paenibacillus larvae]
254   7e-72
OLB33661.1 translation elongation factor G [Ktedonobacter sp....
254   7e-72
WP_024118743.1 elongation factor G [Thermus thermophilus]
255   7e-72
WP_028977337.1 elongation factor G [Sporolactobacillus terrae]
254   7e-72

PID47937.1 elongation factor G [Proteobacteria bacterium]
254   7e-72
WP_053591311.1 MULTISPECIES: elongation factor G [Bacillaceae]
254   7e-72
WP_086278224.1 MULTISPECIES: elongation factor G [Enterococcus]
255   7e-72
WP_060468519.1 elongation factor G [Vibrio toranzoniae]
255   7e-72
WP_073612297.1 elongation factor G [Desulfopila aestuarii]
254   7e-72
WP_049164945.1 GTP-binding protein [Lactobacillus salivarius]
253   7e-72
CCZ55841.1 elongation factor G 2 [Clostridium sp. CAG:1219]
254   7e-72
WP_006428250.1 GTP-binding protein [Dorea longicatena]
259   8e-72
EAZ90564.1 elongation factor G [Cyanothece sp. CCY0110]
254   8e-72
AMP51886.1 elongation factor G, domain IV [uncultured bacterium]
245   8e-72
WP_049497248.1 elongation factor G [Streptococcus pseudopneum...
254   8e-72
WP_002139943.1 MULTISPECIES: elongation factor G [Bacillus ce...
254   8e-72
WP_002852973.1 GTP-binding protein [Ruminococcus albus]
258   8e-72
WP_054325840.1 GTP-binding protein [Clostridia bacterium UC5....
258   8e-72
WP_076631172.1 GTP-binding protein [Lactobacillus plantarum]
254   8e-72
WP_104401491.1 elongation factor G [Vibrio penaeicida]
254   8e-72
WP_094094352.1 elongation factor G [Paenibacillus physcomitre...
254   8e-72
WP_091236329.1 GTP-binding protein [Fontibacillus panacisegetis]
254   8e-72
WP_055244610.1 GTP-binding protein [Turicibacter sanguinis]
254   8e-72
WP_014637332.1 elongation factor G [Streptococcus suis]
254   8e-72
WP_071679104.1 elongation factor G [Bacillus cereus]
254   8e-72
WP_024386346.1 elongation factor G [Streptococcus suis]
254   8e-72
WP_002899105.1 elongation factor G [Streptococcus sanguinis]
254   8e-72
WP_078174992.1 elongation factor G [Bacillus cereus]
254   8e-72
WP_071711513.1 elongation factor G [Bacillus sp. TD41]
254   8e-72
PKN67404.1 elongation factor G [Deltaproteobacteria bacterium...
254   8e-72
WP_056940300.1 GTP-binding protein [Lactobacillus amylovorus]
253   8e-72

WP_103628906.1 elongation factor G [Campylobacter concisus]
254   8e-72
EEM49935.1 Elongation factor G 2 [Bacillus thuringiensis sero...
255   8e-72
SDG49401.1 small GTP-binding protein domain-containing protei...
254   8e-72
WP_024406062.1 elongation factor G [Streptococcus suis]
254   8e-72
WP_011393940.1 elongation factor G [Moorella thermoacetica]
254   8e-72
WP_071106975.1 elongation factor G [Moorea producens]
254   8e-72
EEM07335.1 Elongation factor G 2 [Bacillus mycoides Rock1-4]
255   8e-72
PIT97940.1 elongation factor G [Candidatus Andersenbacteria b...
255   8e-72
WP_099566357.1 elongation factor G [Bacillus velezensis]
254   8e-72
WP_011921743.1 elongation factor G [Streptococcus suis]
254   8e-72
WP_050254439.1 elongation factor G [Streptococcus pneumoniae]
254   8e-72
WP_087591976.1 elongation factor G [unicellular cyanobacteriu...
254   8e-72
WP_087228914.1 elongation factor G [Anaeromassilibacillus sp....
254   8e-72
WP_024419398.1 elongation factor G [Streptococcus suis]
254   8e-72
WP_061678372.1 MULTISPECIES: elongation factor G [Bacillus]
254   8e-72
WP_100647413.1 elongation factor G [Campylobacter jejuni]
254   8e-72
WP_057542258.1 GTP-binding protein [Paeniclostridium sordellii]
254   8e-72
WP_067194981.1 elongation factor G [Streptococcus sp. DD10]
254   8e-72
WP_055342125.1 GTP-binding protein [Paeniclostridium sordellii]
254   8e-72
WP_054324208.1 elongation factor G [Clostridia bacterium UC5....
254   8e-72
WP_015123965.1 elongation factor G [Synechococcus sp. PCC 6312]
254   8e-72
WP_044756315.1 elongation factor G [Streptococcus suis]
254   9e-72
WP_100625448.1 elongation factor G [Campylobacter jejuni]
254   9e-72
WP_053373894.1 MULTISPECIES: elongation factor G [Bacillales]
254   9e-72
WP_039458084.1 elongation factor G [Thermus sp. 2.9]
254   9e-72
WP_014757921.1 MULTISPECIES: elongation factor G [Thermoanaer...
254   9e-72
PDH18310.1 elongation factor G [Pelagibacterales bacterium ME...
254   9e-72

WP_073576472.1 elongation factor G [Pseudonocardia autotrophica]
254   9e-72
WP_094201702.1 elongation factor G [Oceanimonas doudoroffii]
254   9e-72
EDK21959.1 elongation factor G [Campylobacter jejuni subsp. j...
254   9e-72
KPK75066.1 elongation factor G [candidate division Zixibacter...
254   9e-72
WP_067064300.1 elongation factor G [Streptococcus pantholopis]
254   9e-72
WP_033153375.1 elongation factor G [Streptococcus equinus]
254   9e-72
EEL72892.1 Elongation factor G 2 [Bacillus cereus AH603]
254   9e-72
WP_050338009.1 GTP-binding protein [Lactobacillus pentosus]
254   9e-72
WP_016220410.1 GTP-binding protein [Dorea sp. 5-2]
258   9e-72
WP_050105611.1 elongation factor G [Streptococcus pneumoniae]
254   9e-72
WP_025782819.1 elongation factor G [Sporosarcina sp. D27]
254   9e-72
WP_091543805.1 GTP-binding protein [Alkaliphilus peptidiferme...
258   9e-72
WP_104708270.1 elongation factor G [Helicobacter ailurogastri...
254   9e-72
WP_078385506.1 elongation factor G [Bacillus cereus]
254   9e-72
WP_073197409.1 elongation factor G [Caloranaerobacter azorensis]
254   9e-72
WP_050230042.1 elongation factor G [Streptococcus pneumoniae]
254   9e-72
OUV44660.1 elongation factor G [Candidatus Pelagibacter sp. T...
254   9e-72
PMP69921.1 elongation factor G [Mesoaciditoga sp.]
254   9e-72
WP_064783202.1 MULTISPECIES: elongation factor G [Staphylococ...
254   9e-72
WP_002159606.1 MULTISPECIES: elongation factor G [Bacillus ce...
254   9e-72
WP_002204575.1 elongation factor G [Bacillus cereus]
254   9e-72
WP_068758759.1 GTP-binding protein [Turicibacter sp. H121]
254   9e-72
WP_033521145.1 elongation factor G [Bifidobacterium bohemicum]
255   9e-72
WP_105138339.1 elongation factor G [Streptococcus suis]
254   9e-72
WP_042473637.1 elongation factor G [Vibrio maritimus]
254   9e-72
WP_024389892.1 elongation factor G [Streptococcus suis]
254   9e-72
WP_053945301.1 elongation factor G [Helicobacter ailurogastri...
254   9e-72

WP_103580605.1 elongation factor G [Campylobacter concisus]
254   9e-72
WP_088841067.1 elongation factor G [Streptococcus pneumoniae]
254   9e-72
WP_067102864.1 elongation factor G [Streptococcus sp. DD13]
254   9e-72
WP_054366279.1 elongation factor G [Streptococcus pneumoniae]
254   9e-72
WP_104850488.1 elongation factor G [Bacillaceae bacterium EAG3]
254   9e-72
WP_007146546.1 elongation factor G [Methylophaga aminisulfidi...
254   9e-72
GAF25699.1 translation elongation factors [Moorella thermoace...
254   9e-72
WP_085533004.1 elongation factor G [Anaeromassilibacillus sp....
254   9e-72
WP_061408636.1 elongation factor G [Streptococcus lutetiensis]
254   1e-71
WP_089609466.1 elongation factor G [Dehalobacterium formicoac...
254   1e-71
WP_102509359.1 elongation factor G [Sanguibacter massileinsis]
254   1e-71
WP_014314628.1 GTP-binding protein [Clostridium sp. BNL1100]
258   1e-71
WP_068678652.1 GTP-binding protein [Paenibacillus oryzae]
253   1e-71
WP_090714122.1 GTP-binding protein [Paenibacillus typhae]
253   1e-71
WP_000090370.1 MULTISPECIES: elongation factor G [Bacillus]
254   1e-71
WP_012661189.1 elongation factor G [Campylobacter lari]
254   1e-71
WP_094397428.1 elongation factor G [Thermoanaerobacterium the...
254   1e-71
WP_077412573.1 elongation factor G [Marinicella sp. F2]
254   1e-71
WP_078546666.1 GTP-binding protein [Bacillus alkalitelluris]
253   1e-71
WP_046354275.1 elongation factor G [Pseudoalteromonas luteovi...
254   1e-71
WP_058386735.1 MULTISPECIES: elongation factor G [Planococcus]
254   1e-71
WP_044598456.1 elongation factor G [Campylobacter peloridis]
254   1e-71
WP_106777015.1 elongation factor G [Peptostreptococcus russel...
254   1e-71
AMP46856.1 elongation factor G C-terminus [uncultured bacterium]
237   1e-71
WP_098933841.1 elongation factor G [Bacillus sp. AFS031507]
254   1e-71
WP_035160602.1 elongation factor G [Campylobacter concisus]
254   1e-71
WP_028609661.1 elongation factor G [Paenibacillus harenae]
254   1e-71

WP_071130202.1 elongation factor G [Enterococcus timonensis]
254   1e-71
WP_105156390.1 elongation factor G [Streptococcus suis]
254   1e-71
WP_005720614.1 GTP-binding protein [Lactobacillus crispatus]
253   1e-71
WP_071176635.1 GTP-binding protein [Butyrivibrio hungatei]
258   1e-71
WP_075279662.1 GTP-binding protein [Olsenella provencensis]
254   1e-71
WP_072372467.1 GTP-binding protein [Olsenella mediterranea]
254   1e-71
WP_058031342.1 elongation factor G [Pseudoalteromonas phenolica]
254   1e-71
WP_010606886.1 MULTISPECIES: elongation factor G [Pseudoalter...
254   1e-71
WP_107686541.1 elongation factor G [Campylobacter concisus]
254   1e-71
WP_103582595.1 elongation factor G [Campylobacter concisus]
254   1e-71
WP_000090371.1 elongation factor G [Bacillus cereus]
254   1e-71
WP_090793756.1 elongation factor G [Paenibacillus sp. GP183]
254   1e-71
WP_010376483.1 elongation factor G [Pseudoalteromonas piscicida]
254   1e-71
WP_039493397.1 MULTISPECIES: elongation factor G [Pseudoalter...
254   1e-71
WP_052788463.1 elongation factor G [Campylobacter jejuni]
254   1e-71
WP_090697395.1 elongation factor G [Beijerinckia sp. 28-YEA-48]
254   1e-71
WP_037620074.1 elongation factor G [Streptococcus parauberis]
254   1e-71
CDE10470.1 elongation factor G 1 [Clostridium sp. CAG:354]
254   1e-71
KRK07648.1 translation elongation factor G [Lactobacillus pas...
253   1e-71
WP_103604832.1 elongation factor G [Campylobacter concisus]
254   1e-71
WP_072668834.1 elongation factor G [Vibrio sp. M12-1144]
254   1e-71
WP_011757351.1 MULTISPECIES: elongation factor G [Nocardioides]
254   1e-71
EXZ31133.1 small GTP-binding domain protein [Bacteroides frag...
242   1e-71
WP_042162251.1 GTP-binding protein [Paenibacillus gorillae]
253   1e-71
WP_090033659.1 GTP-binding protein [Lachnospiraceae bacterium...
258   1e-71
WP_012739643.1 GTP-binding protein [[Eubacterium] eligens]
258   1e-71
WP_108672018.1 elongation factor G [Bacillus sp. XY-L28]
254   1e-71

WP_019850220.1 GTP-binding protein [Desulfitobacterium sp. PCE1]
258   1e-71
WP_002914093.1 elongation factor G [Streptococcus sanguinis]
254   1e-71
WP_107969067.1 elongation factor G [Campylobacter concisus]
254   1e-71
WP_033192027.1 elongation factor G [Fervidobacterium islandicum]
254   1e-71
OQW96570.1 translation elongation factor G [Desulfobacteracea...
254   1e-71
WP_018498506.1 elongation factor G [Leptotrichia wadei]
254   1e-71
WP_103600667.1 elongation factor G [Campylobacter concisus]
254   1e-71
WP_103570830.1 elongation factor G [Campylobacter concisus]
254   1e-71
WP_087386393.1 elongation factor G [Megamonas hypermegale]
254   1e-71
WP_085113705.1 elongation factor G [Thermoanaerobacterium sp....
254   1e-71
WP_035642117.1 GTP-binding protein [Lachnospiraceae bacterium...
259   1e-71
AMP52937.1 elongation factor G C-terminus [uncultured bacterium]
236   1e-71
CDD47051.1 small GTP-binding protein [Firmicutes bacterium CA...
258   1e-71
WP_054052256.1 elongation factor G [Alloactinosynnema sp. L-07]
254   1e-71
WP_061292963.1 GTP-binding protein [Clostridium botulinum]
253   1e-71
WP_058005491.1 GTP-binding protein [Bacillus farraginis]
253   1e-71
WP_042137141.1 GTP-binding protein [Paenibacillus sp. FSL P4-...
253   1e-71
WP_103624494.1 elongation factor G [Campylobacter concisus]
254   1e-71
WP_072760487.1 elongation factor G [Fervidobacterium gondwane...
254   1e-71
KXU26434.1 elongation factor G [Campylobacter coli]
254   1e-71
WP_002825107.1 MULTISPECIES: elongation factor G [Campylobacter]
254   1e-71
WP_063656138.1 elongation factor G [Aliivibrio fischeri]
254   1e-71
WP_035622374.1 GTP-binding protein [Lachnospiraceae bacterium...
258   1e-71
WP_061427625.1 GTP-binding protein [Clostridium perfringens]
253   1e-71
WP_010026424.1 elongation factor G [Sporolactobacillus inulinus]
254   1e-71
WP_033797762.1 elongation factor G [Bacillus mycoides]
254   1e-71
WP_103651466.1 elongation factor G [Campylobacter concisus]
254   1e-71

PJB18244.1 elongation factor G [Candidatus Falkowbacteria bac...
254   1e-71
WP_066360521.1 GTP-binding protein [Herbidospora mongoliensis]
252   1e-71
PJE69771.1 elongation factor G [Candidatus Staskawiczbacteria...
254   1e-71
WP_060917606.1 elongation factor G [Leptotrichia wadei]
254   1e-71
WP_000090341.1 elongation factor G [Streptococcus oralis]
254   1e-71
WP_078185653.1 elongation factor G [Bacillus mycoides]
254   1e-71
KEP24076.1 elongation factor G [Georgenia sp. SUBG003]
254   1e-71
WP_075432286.1 elongation factor G [Campylobacter coli]
254   1e-71
WP_070761044.1 elongation factor G [Corynebacterium sp. HMSC2...
254   1e-71
WP_105323437.1 elongation factor G [Corynebacterium sp. J010B...
254   1e-71
CDC10759.1 elongation factor G [Clostridium sp. CAG:413]
254   1e-71
WP_088265997.1 elongation factor G [Bacillus mycoides]
254   1e-71
OLE13756.1 translation elongation factor G [Acidobacteria bac...
254   1e-71
WP_088788544.1 elongation factor G [Streptococcus pneumoniae]
254   1e-71
WP_072074650.1 elongation factor G [Streptococcus sanguinis]
254   1e-71
WP_002905422.1 elongation factor G [Streptococcus sanguinis]
254   1e-71
WP_066728907.1 elongation factor G [Sneathia sanguinegens]
254   1e-71
WP_061654309.1 elongation factor G [Streptococcus pneumoniae]
254   1e-71
WP_088232925.1 MULTISPECIES: elongation factor G [Bacillus]
254   1e-71
WP_015605812.1 elongation factor G [Streptococcus cristatus]
254   1e-71
WP_028868773.1 elongation factor G [Psychromonas arctica]
254   1e-71
WP_004740609.1 elongation factor G [Vibrio splendidus]
254   1e-71
WP_073033080.1 GTP-binding protein [Desulfosporosinus lacus]
258   1e-71
WP_104741914.1 elongation factor G [Helicobacter ailurogastri...
254   1e-71
WP_029377926.1 elongation factor G [Staphylococcus xylosus]
254   1e-71
WP_002893862.1 elongation factor G [Streptococcus sanguinis]
254   1e-71
WP_075470353.1 elongation factor G [Campylobacter coli]
254   1e-71

WP_025563168.1 elongation factor G [Psychromonas sp. SP041]
254   1e-71
WP_005721932.1 GTP-binding protein [Lactobacillus crispatus]
253   1e-71
WP_071677257.1 elongation factor G [Thermus brockianus]
254   1e-71
EEK52837.1 Elongation factor G 2 [Bacillus cereus ATCC 10876]
254   1e-71
WP_059066257.1 GTP-binding protein [Ruminococcus sp. AT10]
258   1e-71
WP_088532065.1 elongation factor G [Pseudoalteromonas piscicida]
254   1e-71
WP_066036837.1 GTP-binding protein [Lysinibacillus sp. AR18-8]
253   1e-71
WP_107627406.1 GTP-binding protein [Lachnospiraceae bacterium...
258   1e-71
WP_105171162.1 elongation factor G [Pseudoalteromonas sp. T1l...
254   1e-71
WP_049672550.1 elongation factor G [Bacillus sp. FJAT-27916]
254   1e-71
WP_053433188.1 elongation factor G [Sporosarcina globispora]
254   1e-71
WP_000196382.1 MULTISPECIES: elongation factor G [Bacillus ce...
254   1e-71
WP_065184866.1 elongation factor G [Stenotrophomonas maltophi...
254   1e-71
CDC09170.1 small GTP-binding protein domain protein [Clostrid...
257   1e-71
WP_010959892.1 elongation factor G [Methylococcus capsulatus]
254   1e-71
KJS82355.1 translation elongation factor G [Desulfosporosinus...
258   1e-71
WP_002922484.1 elongation factor G [Streptococcus sanguinis]
254   1e-71
WP_029540491.1 elongation factor G [Selenomonas ruminantium]
254   1e-71
WP_070311315.1 elongation factor G [Campylobacter jejuni]
254   1e-71
PIE54531.1 elongation factor G [Dethiosulfovibrio peptidovorans]
254   1e-71
WP_103851630.1 GTP-binding protein [Lactobacillus plantarum]
254   1e-71
SDI89845.1 small GTP-binding protein domain-containing protei...
254   1e-71
WP_056987502.1 GTP-binding protein [Lactobacillus paracollino...
253   1e-71
WP_075887607.1 GTP-binding protein [Lactobacillus crustorum]
253   1e-71
CCZ33757.1 putative uncharacterized protein [Firmicutes bacte...
258   1e-71
WP_097858990.1 elongation factor G [Bacillus wiedmannii]
254   1e-71
WP_034990588.1 GTP-binding protein [Lactobacillus salivarius]
253   1e-71

WP_040220028.1 GTP-binding protein [Collinsella sp. MS5]
253   1e-71
WP_075492050.1 elongation factor G [Campylobacter coli]
254   1e-71
WP_053583250.1 GTP-binding protein [Lysinibacillus contaminans]
253   1e-71
CCY72824.1 putative translation elongation factor G [Eubacter...
257   1e-71
PKN26279.1 elongation factor G [Deltaproteobacteria bacterium...
254   1e-71
WP_002928022.1 elongation factor G [Streptococcus sanguinis]
254   1e-71
WP_049727444.1 GTP-binding protein [Dorea sp. D27]
258   1e-71
WP_087557086.1 elongation factor G [Rhodococcus sp. NCIMB 12038]
254   1e-71
WP_020975134.1 elongation factor G [Campylobacter coli]
254   1e-71
WP_099589527.1 elongation factor G [Stenotrophomonas maltophi...
254   1e-71
OGF52990.1 translation elongation factor G [Candidatus Fraser...
254   1e-71
WP_100062070.1 elongation factor G [Bacillus toyonensis]
254   1e-71
WP_099346413.1 GTP-binding protein [Clostridium tertium]
253   1e-71
WP_027973851.1 elongation factor G [Streptococcus porci]
254   1e-71
WP_093912298.1 elongation factor G [Succiniclasticum ruminis]
254   1e-71
WP_012459027.1 elongation factor G [Sulfurihydrogenibium sp. ...
254   1e-71
WP_098417451.1 elongation factor G [Bacillus cereus]
254   1e-71
WP_012688821.1 elongation factor G [Rhodococcus opacus]
254   1e-71
WP_015656269.1 elongation factor G [Streptomyces davaonensis]
254   1e-71
WP_015310892.1 elongation factor G [Thermoanaerobacterium the...
254   1e-71
WP_105920163.1 GTP-binding protein [Lactobacillus pentosus]
253   1e-71
WP_006071965.1 elongation factor G [Vibrio shilonii]
254   1e-71
WP_070140533.1 MULTISPECIES: elongation factor G [Bacillus ce...
254   1e-71
WP_000196381.1 MULTISPECIES: elongation factor G [Bacillus]
254   1e-71
WP_061598603.1 MULTISPECIES: elongation factor G [Streptococcus]
254   1e-71
AVP65986.1 elongation factor G [Clostridium botulinum]
253   1e-71
WP_016962445.1 elongation factor G [Enterovibrio norvegicus]
254   1e-71

WP_042496632.1 elongation factor G [Vibrio maritimus]
254   1e-71
WP_015106397.1 elongation factor G [Helicobacter heilmannii]
254   1e-71
WP_007908604.1 elongation factor G [Ktedonobacter racemifer]
254   1e-71
AMP50096.1 elongation factor G, domain IV [uncultured bacterium]
244   1e-71
WP_053310827.1 elongation factor G [Vibrio alginolyticus]
254   1e-71
WP_077610847.1 GTP-binding protein [Clostridium sp. Marseille...
253   1e-71
OLA25410.1 translation elongation factor G [Dorea sp. 42_8]
258   1e-71
WP_044107440.1 elongation factor G [cyanobacterium endosymbio...
254   1e-71
SCH18337.1 Elongation factor G [uncultured Clostridium sp.]
258   1e-71
WP_100912400.1 elongation factor G [Pseudoalteromonas spongiae]
254   1e-71
WP_052853052.1 elongation factor G [Campylobacter jejuni]
254   1e-71
WP_087297347.1 GTP-binding protein [Massiliomicrobiota sp. An...
257   1e-71
PIZ89365.1 elongation factor G [Candidatus Nealsonbacteria ba...
254   1e-71
WP_037566321.1 MULTISPECIES: elongation factor G [Staphylococ...
254   1e-71
WP_106497863.1 GTP-binding protein [Lentibacillus sp. Marseil...
253   1e-71
WP_074560918.1 elongation factor G [Streptococcus equinus]
254   1e-71
WP_000196380.1 MULTISPECIES: elongation factor G [Bacillus]
254   1e-71
WP_017485509.1 elongation factor G [Methylobacterium sp. MB200]
254   1e-71
WP_076542643.1 elongation factor G [Bacillus cereus]
254   1e-71
WP_075475057.1 elongation factor G [Campylobacter coli]
254   1e-71
EEM01438.1 Elongation factor G 2 [Bacillus mycoides DSM 2048]
254   1e-71
AMP47996.1 elongation factor G C-terminus [uncultured bacterium]
236   1e-71
WP_036747762.1 elongation factor G [Photobacterium galatheae]
254   1e-71
WP_021338309.1 GTP-binding protein [Lactobacillus plantarum]
253   1e-71
WP_087066601.1 elongation factor G [Intestinibacillus massili...
254   1e-71
WP_073519016.1 elongation factor G [Bacillus cereus]
254   1e-71
EEK64167.1 Elongation factor G 2 [Bacillus cereus 172560W]
254   1e-71

WP_090056473.1 elongation factor G [Luteibacter sp. 329MFSha]
254   1e-71
WP_012874664.1 elongation factor G [Thermobaculum terrenum]
254   1e-71
AMM40290.1 elongation factor P [Candidatus Desulfofervidus au...
254   1e-71
WP_044685446.1 elongation factor G [Streptococcus suis]
254   1e-71
WP_058300192.1 elongation factor G [Gorillibacterium timonense]
254   1e-71
WP_052779069.1 elongation factor G [Campylobacter coli]
254   1e-71
WP_024295458.1 GTP-binding protein [[Clostridium] indolis]
253   1e-71
WP_061657391.1 elongation factor G [Bacillus cereus]
254   1e-71
WP_038401063.1 elongation factor G [Campylobacter jejuni]
254   1e-71
WP_087382620.1 GTP-binding protein [Faecalibacterium sp. An122]
257   2e-71
WP_100620556.1 elongation factor G [Campylobacter jejuni]
254   2e-71
WP_026593163.1 MULTISPECIES: elongation factor G [Bacillus]
254   2e-71
WP_002169326.1 MULTISPECIES: elongation factor G [Bacillus]
254   2e-71
WP_002868787.1 MULTISPECIES: elongation factor G [Campylobacter]
254   2e-71
WP_085660288.1 elongation factor G [Prosthecochloris sp. HL-1...
254   2e-71
WP_071878739.1 elongation factor G [Enterococcus silesiacus]
254   2e-71
WP_002920299.1 elongation factor G [Streptococcus sanguinis]
254   2e-71
WP_086048833.1 elongation factor G [Vibrio alginolyticus]
254   2e-71
WP_010562292.1 elongation factor G [Pseudoalteromonas spongiae]
254   2e-71
WP_008218763.1 elongation factor G [Vibrio splendidus]
254   2e-71
WP_023626434.1 elongation factor G [Bacillus sp. 17376]
254   2e-71
WP_019713054.1 elongation factor G [Bacillus subtilis]
254   2e-71
KPJ85412.1 elongation factor G [Parcubacteria bacterium SG8_24]
253   2e-71
WP_065168251.1 elongation factor G [Photobacterium aquimaris]
254   2e-71
WP_066238100.1 elongation factor G [Anaerosporomusa subterranea]
254   2e-71
KXT70772.1 Translation elongation factor G [Streptococcus gor...
254   2e-71
WP_060971223.1 elongation factor G [Streptococcus sp. CCH8-G7]
254   2e-71

WP_043012759.1 elongation factor G [Campylobacter jejuni]
254   2e-71
WP_038814876.1 elongation factor G [Campylobacter jejuni]
254   2e-71
OGX43041.1 translation elongation factor G [Omnitrophica WOR_...
254   2e-71
EEK75388.1 Elongation factor G 2 [Bacillus cereus AH621]
254   2e-71
WP_061736704.1 elongation factor G [Staphylococcus aureus]
254   2e-71
EGV11803.1 translation elongation factor G [Streptococcus inf...
254   2e-71
WP_054611831.1 GTP-binding protein [Lysinibacillus sp. ZYM-1]
253   2e-71
WP_002113318.1 MULTISPECIES: elongation factor G [Bacillus]
254   2e-71
KUK03224.1 Elongation factor G [Thermotoga sp. 50_64]
254   2e-71
WP_075098544.1 elongation factor G [Streptococcus acidominimus]
254   2e-71
WP_011999744.1 elongation factor G [Streptococcus gordonii]
254   2e-71
WP_063524498.1 elongation factor G [Vibrio sp. HI00D65]
254   2e-71
WP_102695763.1 GTP-binding protein [Lactobacillus gasseri]
253   2e-71
WP_103158763.1 elongation factor G [Staphylococcus chromogenes]
254   2e-71
WP_102180336.1 elongation factor G [Fischerella thermalis]
254   2e-71
WP_070286226.1 MULTISPECIES: elongation factor G [Campylobacter]
254   2e-71
EEK69719.1 Elongation factor G 2 [Bacillus cereus MM3]
254   2e-71
OGE32777.1 translation elongation factor G [Candidatus Davies...
253   2e-71
WP_017035766.1 elongation factor G [Vibrio genomosp. F10]
254   2e-71
WP_047380866.1 MULTISPECIES: elongation factor G [Cetobacterium]
254   2e-71
WP_045634430.1 MULTISPECIES: elongation factor G [Streptococcus]
254   2e-71
WP_107793179.1 elongation factor G [Campylobacter concisus]
254   2e-71
WP_002788669.1 MULTISPECIES: elongation factor G [Campylobacter]
254   2e-71
WP_021137656.1 elongation factor G [Campylobacter jejuni]
254   2e-71
WP_009726264.1 elongation factor G [Methylophaga lonarensis]
254   2e-71
WP_094325237.1 elongation factor G [Campylobacter avium]
254   2e-71
WP_075499254.1 elongation factor G [Campylobacter coli]
254   2e-71

WP_076315699.1 MULTISPECIES: elongation factor G [Paenibacillus]
254   2e-71
WP_002779472.1 MULTISPECIES: elongation factor G [Campylobacter]
254   2e-71
WP_002888146.1 MULTISPECIES: elongation factor G [Campylobacter]
254   2e-71
WP_048612295.1 MULTISPECIES: elongation factor G [Vibrio]
254   2e-71
WP_017056696.1 MULTISPECIES: elongation factor G [Vibrio]
254   2e-71
WP_075440179.1 elongation factor G [Campylobacter coli]
254   2e-71
WP_060794071.1 elongation factor G [Campylobacter coli]
254   2e-71
WP_058982731.1 elongation factor G [Stenotrophomonas maltophi...
253   2e-71
WP_013496670.1 elongation factor G [Thermaerobacter marianensis]
254   2e-71
WP_104814208.1 elongation factor G [Kitasatospora sp. MMS16-B...
253   2e-71
WP_090038778.1 GTP-binding protein [Clostridium frigidicarnis]
253   2e-71
WP_099364818.1 elongation factor G [Bacillus onubensis]
254   2e-71
WP_095301353.1 elongation factor G [Bacillus sp. 7586-K]
254   2e-71
WP_021090828.1 elongation factor G [Campylobacter concisus]
254   2e-71
1FNM_A Chain A, STRUCTURE OF THERMUS THERMOPHILUS EF-G H573A
254   2e-71
WP_015016434.1 elongation factor G [Campylobacter jejuni]
254   2e-71
CUQ81810.1 Tetracycline resistance protein tetM [[Eubacterium...
257   2e-71
WP_049495285.1 elongation factor G [Streptococcus pseudopneum...
254   2e-71
WP_085148194.1 elongation factor G [Candidatus Pelagibacter s...
254   2e-71
ORI08667.1 elongation factor G [Campylobacter concisus]
254   2e-71
WP_092871023.1 GTP-binding protein [Acetitomaculum ruminis]
257   2e-71
WP_069994188.1 elongation factor G [Staphylococcus saprophyti...
254   2e-71
WP_065576732.1 elongation factor G [Vibrio genomosp. F10]
254   2e-71
WP_063181721.1 GTP-binding protein [Bhargavaea cecembensis]
253   2e-71
WP_071027939.1 elongation factor G [Peptoniphilus sp. KHD4]
254   2e-71
KEF42352.1 elongation factor G [Cyanobium sp. CACIAM 14]
254   2e-71
WP_025869855.1 MULTISPECIES: elongation factor G [Enterococcus]
254   2e-71

OLD83814.1 translation elongation factor G [Chloroflexi bacte...
254   2e-71
WP_014516949.1 elongation factor G [Campylobacter jejuni]
254   2e-71
CDC02792.1 elongation factor G [Eubacterium sp. CAG:202]
254   2e-71
WP_062201135.1 elongation factor G [Streptococcus sp. DD04]
254   2e-71
WP_026786433.1 MULTISPECIES: elongation factor G [Planktothrix]
254   2e-71
WP_025168770.1 elongation factor G [Streptococcus peroris]
254   2e-71
WP_042493691.1 elongation factor G [Weissella hellenica]
254   2e-71
WP_059167460.1 elongation factor G [Campylobacter jejuni]
254   2e-71
WP_087023977.1 elongation factor G [Thaumasiovibrio subtropicus]
254   2e-71
WP_060461905.1 GTP-binding protein [Lactobacillus crispatus]
253   2e-71
WP_055286271.1 GTP-binding protein [[Eubacterium] eligens]
257   2e-71
WP_074365396.1 GTP-binding protein [Clostridium sp. N3C]
257   2e-71
WP_087578605.1 elongation factor G [Campylobacter concisus]
254   2e-71
APU71244.1 hypothetical protein BI355_0925 [Lactobacillus cru...
253   2e-71
WP_091351167.1 elongation factor G [Anaerobranca gottschalkii]
254   2e-71
WP_039611345.1 elongation factor G [Pseudoalteromonas luteovi...
254   2e-71
WP_034551700.1 elongation factor G [Carnobacterium funditum]
254   2e-71
OGH91849.1 translation elongation factor G [Candidatus Magasa...
254   2e-71
WP_026623979.1 GTP-binding protein [Eggerthia catenaformis]
256   2e-71
WP_084231603.1 elongation factor G [Peptoniphilus asaccharoly...
253   2e-71
3IZP_E Chain E, Conformation Of Ef-G During Translocation
253   2e-71
WP_007410399.1 MULTISPECIES: elongation factor G [Bacillus]
254   2e-71
WP_020917479.1 MULTISPECIES: elongation factor G [Streptococcus]
254   2e-71
WP_065438820.1 GTP-binding protein [Bifidobacterium pseudocat...
253   2e-71
WP_054387161.1 elongation factor G [Streptococcus pneumoniae]
254   2e-71
WP_023024498.1 elongation factor G [Streptococcus sp. I-P16]
254   2e-71
WP_087449157.1 GTP-binding protein [Lactobacillus salivarius]
252   2e-71

WP_009731747.1 elongation factor G [Streptococcus sp. F0442]
254   2e-71
WP_066267946.1 elongation factor G [Bacillus acidicola]
253   2e-71
WP_072235689.1 elongation factor G [Campylobacter coli]
253   2e-71
WP_070310361.1 elongation factor G [Campylobacter jejuni]
253   2e-71
EEL90000.1 Elongation factor G 2 [Bacillus cereus AH1272]
254   2e-71
KGG79789.1 elongation factor G [Caloranaerobacter azorensis H...
253   2e-71
WP_019819832.1 MULTISPECIES: elongation factor G [Vibrio]
254   2e-71
SCI37962.1 Elongation factor G [uncultured Flavonifractor sp.]
257   2e-71
WP_046442670.1 elongation factor G [Catabacter hongkongensis]
253   2e-71
WP_003156450.1 elongation factor G [Bacillus velezensis]
253   2e-71
WP_084803765.1 elongation factor G [Bradyrhizobium sp. NAS80.1]
253   2e-71
WP_106904638.1 GTP-binding protein [Lactobacillus plantarum]
253   2e-71
WP_017629642.1 elongation factor G [Vibrio gigantis]
254   2e-71
WP_046005822.1 elongation factor G [Pseudoalteromonas rubra]
254   2e-71
WP_091189528.1 GTP-binding protein [Paenibacillus catalpae]
253   2e-71
WP_048733566.1 GTP-binding protein [Lactobacillus koreensis]
253   2e-71
4MYU_A Chain A, Crystal Structure Of Elongation Factor G Muta...
253   2e-71
WP_075977314.1 elongation factor G [Actinokineospora bangkoke...
254   2e-71
WP_068715991.1 elongation factor G [Orenia metallireducens]
253   2e-71
WP_031503257.1 elongation factor G [Pseudothermotoga hypogea]
253   2e-71
OGP97989.1 translation elongation factor G [Deltaproteobacter...
253   2e-71
WP_106348931.1 elongation factor G [Antricoccus suffuscus]
254   2e-71
WP_087579882.1 elongation factor G [Campylobacter concisus]
253   2e-71
WP_084040680.1 elongation factor G [Campylobacter concisus]
253   2e-71
WP_026731491.1 elongation factor G [Fischerella sp. PCC 9605]
253   2e-71
ATX83338.1 elongation factor G [Bacillus velezensis]
253   2e-71
WP_082307260.1 elongation factor G [Streptococcus gordonii]
253   2e-71

WP_098917209.1 elongation factor G [Bacillus pseudomycoides]
253   2e-71
KRN23882.1 elongation factor EF2 [Lactobacillus similis DSM 2...
253   2e-71
WP_107896344.1 GTP-binding protein [Lysinibacillus mangiferih...
252   2e-71
EEK80974.1 Elongation factor G 2 [Bacillus cereus R309803]
254   2e-71
WP_029584565.1 elongation factor G [Bradyrhizobium sp. URHD0069]
253   2e-71
SCJ38971.1 Tetracycline resistance protein tetM [uncultured E...
257   2e-71
WP_040953128.1 elongation factor G [Gorillibacterium massilie...
253   2e-71
WP_068556780.1 elongation factor G [Thermotalea metallivorans]
253   2e-71
WP_077959294.1 GTP-binding protein [Lactobacillus gasseri]
252   2e-71
WP_107523267.1 elongation factor G [Staphylococcus chromogenes]
253   2e-71
WP_107788972.1 elongation factor G [Campylobacter concisus]
253   2e-71
WP_020816094.1 GTP-binding protein [[Clostridium] papyrosolvens]
257   2e-71
WP_076540504.1 elongation factor G [Shewanella sp. UCD-KL21]
253   2e-71
WP_085558996.1 elongation factor G [Carnobacterium iners]
253   2e-71
WP_066066399.1 elongation factor G [Candidatus Desulfofervidu...
253   2e-71
WP_085492754.1 GTP-binding protein [Paenibacillus aquistagni]
253   2e-71
WP_035288968.1 GTP-binding protein [Clostridium sp. KNHs214]
253   2e-71
PLX25714.1 elongation factor G [Candidatus Parcubacteria bact...
253   2e-71
KJS32633.1 elongation factor G [Desulfatitalea sp. BRH_c12]
253   2e-71
WP_034785410.1 GTP-binding protein [Eubacterium xylanophilum]
257   2e-71
WP_005258137.1 MULTISPECIES: elongation factor G [Rhodococcus]
254   2e-71
WP_085657556.1 elongation factor G [Campylobacter concisus]
253   2e-71
WP_084886809.1 elongation factor G [Vibrio sp. qd031]
253   2e-71
WP_008497423.1 elongation factor G [Planococcus halocryophilus]
253   2e-71
WP_021252462.1 MULTISPECIES: elongation factor G [Paenibacillus]
253   2e-71
OGM21318.1 translation elongation factor G [Candidatus Woeseb...
253   2e-71
PJA84140.1 elongation factor G [Candidatus Nealsonbacteria ba...
254   2e-71

WP_013977664.1 GTP-binding protein [Clostridium sp. SY8519]
258   2e-71
KKR55630.1 Elongation factor G, partial [Candidatus Curtissba...
252   2e-71
OGW88111.1 translation elongation factor G [Omnitrophica bact...
254   2e-71
WP_036835587.1 elongation factor G [Pontibacillus litoralis]
253   2e-71
WP_026824828.1 elongation factor G [Exiguobacterium marinum]
253   2e-71
WP_002912722.1 elongation factor G [Campylobacter jejuni]
253   2e-71
WP_007475267.1 elongation factor G [Caminibacter mediatlanticus]
253   2e-71
WP_107646074.1 GTP-binding protein [Bifidobacterium adolescen...
253   2e-71
KKU12456.1 Elongation factor G, partial [Candidatus Azambacte...
253   2e-71
OGX31513.1 translation elongation factor G [Omnitrophica WOR_...
253   2e-71
WP_028588273.1 elongation factor G [Desulfocurvus vexinensis]
253   2e-71
WP_077059563.1 elongation factor G [Streptomyces sp. MP131-18]
254   2e-71
WP_091190697.1 elongation factor G [Paenibacillus catalpae]
253   2e-71
WP_096657893.1 elongation factor G [Calothrix parasitica]
253   2e-71
WP_059110443.1 elongation factor G [Campylobacter coli]
253   2e-71
WP_072700414.1 GTP-binding protein [Butyrivibrio hungatei]
257   2e-71
WP_017768900.1 MULTISPECIES: elongation factor G [Streptococcus]
253   2e-71
WP_014516708.1 MULTISPECIES: elongation factor G [Campylobacter]
253   2e-71
WP_101515081.1 GTP-binding protein [Clostridium botulinum]
252   2e-71
WP_029816480.1 elongation factor G [Vibrio parahaemolyticus]
253   2e-71
WP_039725069.1 elongation factor G [Lyngbya confervoides]
253   2e-71
WP_071975969.1 GTP-binding protein [Bacillus obstructivus]
252   2e-71
WP_084588754.1 elongation factor G [Streptococcus pneumoniae]
253   2e-71
OUV01792.1 elongation factor G [Acidimicrobiaceae bacterium T...
253   2e-71
WP_065528392.1 elongation factor G [Planococcus halocryophilus]
253   2e-71
2J7K_A Chain A, Crystal Structure Of The T84a Mutant Ef-G:gdp...
253   2e-71
WP_031537190.1 GTP-binding protein [Bacillus sp. MB2021]
252   2e-71

WP_063371444.1 elongation factor G [Pseudoalteromonas luteovi...
253   2e-71
WP_032592207.1 elongation factor G [Campylobacter jejuni]
253   2e-71
WP_058016031.1 GTP-binding protein [Clostridium sporogenes]
252   2e-71
WP_013861986.1 elongation factor G [Microlunatus phosphovorus]
253   2e-71
WP_073997420.1 elongation factor G [Anaerococcus sp. Marseill...
253   2e-71
WP_044413371.1 elongation factor G [Rhodopseudomonas palustris]
253   2e-71
WP_072540309.1 GTP-binding protein [Lactobacillus plantarum]
253   2e-71
WP_066301541.1 elongation factor G [Bacillus sp. FJAT-29937]
253   2e-71
WP_075438389.1 elongation factor G [Campylobacter coli]
253   2e-71
WP_075516906.1 elongation factor G [Moorella thermoacetica]
253   2e-71
KRK43966.1 translation elongation factor (GTPase) [Lactobacil...
253   2e-71
WP_048605637.1 MULTISPECIES: elongation factor G [Vibrio]
253   2e-71
WP_004801076.1 GTP-binding protein [Eggerthia catenaformis]
256   2e-71
WP_055331316.1 GTP-binding protein [Paeniclostridium sordellii]
253   2e-71
WP_045964730.1 elongation factor G [Pseudoalteromonas piscicida]
253   2e-71
WP_017003174.1 elongation factor G [Enterovibrio norvegicus]
253   2e-71
WP_023051097.1 elongation factor G [Cetobacterium somerae]
253   2e-71
WP_006597107.1 elongation factor G [Streptococcus australis]
253   2e-71
WP_106267321.1 MULTISPECIES: elongation factor G [Isoptericola]
253   2e-71
CDB41834.1 elongation factor G [Ruminococcus sp. CAG:177]
253   2e-71
WP_102423091.1 elongation factor G [Vibrio sp. 10N.261.52.A1]
253   2e-71
WP_017058825.1 MULTISPECIES: elongation factor G [Vibrio]
253   2e-71
WP_006269211.1 elongation factor G [Streptococcus constellatus]
253   3e-71
OUM90565.1 translation elongation factor G [Caldibacillus deb...
253   3e-71
WP_035433719.1 MULTISPECIES: elongation factor G [Bacillus]
253   3e-71
WP_085381719.1 GTP-binding protein [Bifidobacterium adolescen...
253   3e-71
WP_004219610.1 GTP-binding protein [Bifidobacterium pseudocat...
253   3e-71

WP_009474623.1 MULTISPECIES: elongation factor G [Rhodococcus]
253   3e-71
WP_014514871.1 elongation factor G [Thermus sp. CCB_US3_UF1]
253   3e-71
WP_024027562.1 GTP-binding protein [Bacillus vireti]
252   3e-71
CNJ14268.1 tetracycline resistance protein TetM [Streptococcu...
236   3e-71
WP_107698475.1 elongation factor G [Campylobacter concisus]
253   3e-71
WP_098262645.1 GTP-binding protein [Bacillus sp. AFS006103]
252   3e-71
WP_105923455.1 GTP-binding protein [Lactobacillus pentosus]
253   3e-71
WP_038836141.1 elongation factor G [Campylobacter coli]
253   3e-71
OAI42641.1 elongation factor G [Verrucomicrobia bacterium SCG...
254   3e-71
WP_087584812.1 elongation factor G [Campylobacter concisus]
253   3e-71
WP_048570056.1 elongation factor G [Clostridium cylindrosporum]
253   3e-71
CPR56879.1 Protein translation elongation factor G (EF-G) [Ch...
252   3e-71
WP_102341921.1 elongation factor G [Lactomassilus timonensis]
253   3e-71
WP_038638964.1 elongation factor G [Pseudoalteromonas piratica]
253   3e-71
WP_097849711.1 elongation factor G [Bacillus pseudomycoides]
253   3e-71
WP_100400587.1 elongation factor G [Bacillus sp. FJAT-44742]
253   3e-71
WP_027889347.1 elongation factor G [Megamonas hypermegale]
253   3e-71
WP_068225892.1 GTP-binding protein [Lactobacillus backii]
253   3e-71
WP_026079934.1 elongation factor G [Spirulina subsalsa]
253   3e-71
WP_025366241.1 GTP-binding protein [Paenibacillus polymyxa]
252   3e-71
WP_069937431.1 elongation factor G [Domibacillus iocasae]
253   3e-71
WP_055737799.1 elongation factor G [Bacillus shackletonii]
253   3e-71
WP_040108420.1 GTP-binding protein [Clostridium botulinum]
252   3e-71
WP_023400238.1 elongation factor G [Pseudoalteromonas luteovi...
253   3e-71
WP_069988515.1 GTP-binding protein [Massilioclostridium coli]
257   3e-71
OGP16054.1 translation elongation factor G [Deltaproteobacter...
253   3e-71
SCN23737.1 Tetracycline resistance protein TetO [Clostridium ...
257   3e-71

WP_061754072.1 elongation factor G [Streptococcus pneumoniae]
253   3e-71
WP_048792369.1 elongation factor G [Staphylococcus saprophyti...
253   3e-71
PIR71737.1 elongation factor G [Candidatus Nealsonbacteria ba...
253   3e-71
WP_105985710.1 elongation factor G [Staphylococcus chromogenes]
253   3e-71
WP_071350900.1 GTP-binding protein [Bacillus sp. MUM 13]
252   3e-71
WP_054520521.1 elongation factor G [Thermanaerothrix daxensis]
253   3e-71
WP_037572918.1 elongation factor G [Staphylococcus chromogenes]
253   3e-71
WP_061906081.1 GTP-binding protein [Clostridium sporogenes]
252   3e-71
WP_041501327.1 elongation factor G [Lactobacillus heilongjian...
253   3e-71
WP_106240562.1 elongation factor G [Pleurocapsa sp. CCALA 161]
253   3e-71
WP_090833517.1 MULTISPECIES: elongation factor G [Bacillus]
253   3e-71
WP_050633924.1 MULTISPECIES: elongation factor G [Vibrio]
253   3e-71
WP_053830004.1 elongation factor G [Helicobacter heilmannii]
253   3e-71
WP_033677182.1 elongation factor G [Bacillus gaemokensis]
253   3e-71
WP_057866805.1 GTP-binding protein [Lactobacillus crustorum]
252   3e-71
WP_086349653.1 elongation factor G [Enterococcus sp. 9E7_DIV0...
253   3e-71
WP_094833971.1 elongation factor G [Bacillaceae bacterium SAS...
253   3e-71
WP_088789748.1 elongation factor G [Streptococcus pneumoniae]
253   3e-71
WP_052045301.1 elongation factor G [Caloranaerobacter azorensis]
253   3e-71
WP_064666775.1 elongation factor G [Pseudoalteromonas prydzen...
253   3e-71
WP_100486451.1 elongation factor G [Sporolactobacillus pectin...
253   3e-71
WP_072594869.1 elongation factor G [Campylobacter concisus]
253   3e-71
WP_030427390.1 elongation factor G [Allokutzneria albata]
253   3e-71
WP_053597966.1 GTP-binding protein [Bacillus sp. FJAT-18017]
252   3e-71
WP_066067323.1 elongation factor G [[Clostridium] paradoxum]
253   3e-71
WP_012311095.1 elongation factor G [Thermotoga sp. RQ2]
253   3e-71
WP_107543886.1 elongation factor G [Staphylococcus simulans]
253   3e-71

WP_003070974.1 MULTISPECIES: elongation factor G [Streptococcus]
253   3e-71
WP_099460289.1 GTP-binding protein [Lactobacillus salivarius]
252   3e-71
OQX73336.1 elongation factor G [Campylobacteraceae bacterium ...
253   3e-71
WP_066399772.1 elongation factor G [Bacillus horneckiae]
253   3e-71
WP_041072014.1 elongation factor G [Bacillus sp. OxB-1]
253   3e-71
OGD64112.1 translation elongation factor G [Candidatus Berkel...
253   3e-71
EFX40038.1 translation elongation factor G [Streptococcus per...
254   3e-71
WP_039043317.1 elongation factor G [Sporosarcina sp. ZBG7A]
253   3e-71
WP_017315324.1 elongation factor G [Mastigocladopsis repens]
253   3e-71
WP_070299952.1 elongation factor G [Campylobacter jejuni]
253   3e-71
WP_091712835.1 elongation factor G [Methylophaga sulfidovorans]
253   3e-71
WP_084971853.1 elongation factor G [Streptococcus oralis]
253   3e-71
OGV08888.1 translation elongation factor G [Ignavibacteria ba...
253   3e-71
WP_059107363.1 elongation factor G [Staphylococcus auricularis]
253   3e-71
WP_050221618.1 elongation factor G [Streptococcus pneumoniae]
253   3e-71
OCW79078.1 elongation factor G [Pelagibacteraceae bacterium G...
253   3e-71
WP_105126475.1 elongation factor G [Streptococcus suis]
253   3e-71
WP_088491087.1 elongation factor G [Saccharibacillus sp. O23]
253   3e-71
WP_003351430.1 elongation factor G [Bacillus methanolicus]
253   3e-71
WP_069875985.1 GTP-binding protein [Fusibacter sp. 3D3]
257   3e-71
PIU08838.1 elongation factor G [Candidatus Moranbacteria bact...
253   3e-71
OQX88415.1 elongation factor G [Candidatus Omnitrophica bacte...
253   3e-71
WP_099099762.1 elongation factor G [Nostoc sp. 'Peltigera mal...
253   3e-71
WP_079546909.1 elongation factor G [Christensenella massilien...
253   3e-71
WP_102991327.1 elongation factor G [Laceyella sp. FBKL4.010]
253   3e-71
WP_086746069.1 elongation factor G [Pseudoalteromonas ulvae]
253   3e-71
WP_043721502.1 elongation factor G [Kutzneria sp. 744]
253   3e-71

WP_056967763.1 GTP-binding protein [Lactobacillus paralimenta...
252   3e-71
WP_025649133.1 GTP-binding protein [Clostridium perfringens]
252   3e-71
WP_070466788.1 MULTISPECIES: elongation factor G [Aerococcus]
253   3e-71
KKT06370.1 Elongation factor G [Parcubacteria group bacterium...
253   3e-71
WP_044765841.1 elongation factor G [Streptococcus suis]
253   3e-71
WP_075496205.1 elongation factor G [Campylobacter coli]
253   3e-71
WP_061295623.1 GTP-binding protein [Clostridium botulinum]
252   3e-71
WP_073007701.1 GTP-binding protein [Clostridium amylolyticum]
257   3e-71
WP_023353785.1 GTP-binding protein [Catonella morbi]
256   3e-71
WP_010761694.1 elongation factor G [Enterococcus haemoperoxidus]
253   3e-71
WP_070684312.1 elongation factor G [Staphylococcus sp. HMSC05...
253   3e-71
OLE52404.1 translation elongation factor G [Acidobacteria bac...
253   3e-71
WP_040209029.1 MULTISPECIES: GTP-binding protein [Bacillus]
252   3e-71
WP_028773858.1 elongation factor G [Shewanella waksmanii]
253   3e-71
WP_002481336.1 MULTISPECIES: elongation factor G [Staphylococ...
253   3e-71
OOX94452.1 translation elongation factor G [Campylobacter coli]
253   3e-71
PJE76032.1 elongation factor G [Candidatus Uhrbacteria bacter...
253   3e-71
WP_098034820.1 MULTISPECIES: GTP-binding protein [Lactobacillus]
251   3e-71
WP_042394861.1 MULTISPECIES: GTP-binding protein [Clostridium]
252   3e-71
CDB27238.1 putative translation elongation factor G [Oscillib...
256   4e-71
WP_049476300.1 elongation factor G [Streptococcus intermedius]
253   4e-71
OCW78413.1 elongation factor G [Pelagibacteraceae bacterium G...
253   4e-71
WP_002902612.1 elongation factor G [Campylobacter jejuni]
253   4e-71
OGY21926.1 translation elongation factor G [Candidatus Woykeb...
253   4e-71
OGD25259.1 translation elongation factor G [Candidatus Azamba...
253   4e-71
WP_107604679.1 elongation factor G [Staphylococcus simulans]
253   4e-71
WP_023027302.1 elongation factor G [Streptococcus sp. I-G2]
253   4e-71

WP_091499758.1 elongation factor G [Amphibacillus marinus]
253   4e-71
WP_087576841.1 elongation factor G [Campylobacter concisus]
253   4e-71
WP_023929237.1 elongation factor G [Helicobacter canis]
253   4e-71
KKT52920.1 Elongation factor G [candidate division Kazan bact...
253   4e-71
WP_074427544.1 elongation factor G [Weissella hellenica]
253   4e-71
WP_022133117.1 GTP-binding protein [Negativibacillus massilie...
252   4e-71
WP_103576751.1 elongation factor G [Campylobacter concisus]
253   4e-71
WP_011590618.1 GTP-binding protein [Clostridium perfringens]
252   4e-71
WP_076248298.1 GTP-binding protein [Paenibacillus sp. FSL H8-...
252   4e-71
WP_062520336.1 elongation factor G [Paenibacillus jilunlii]
253   4e-71
OUX06207.1 elongation factor G [Flavobacteriales bacterium TM...
253   4e-71
WP_025015532.1 GTP-binding protein [Lactobacillus kitasatonis]
251   4e-71
WP_055680228.1 GTP-binding protein [Bifidobacterium adolescen...
252   4e-71
WP_009249298.1 GTP-binding protein [Lachnospiraceae bacterium...
256   4e-71
WP_027121844.1 elongation factor G [Mycoplasma imitans]
253   4e-71
WP_008808227.1 MULTISPECIES: elongation factor G [Streptococcus]
253   4e-71
WP_006172308.1 elongation factor G [Synechococcus sp. WH 5701]
253   4e-71
WP_072233564.1 elongation factor G [Campylobacter coli]
253   4e-71
WP_052784768.1 elongation factor G [Campylobacter coli]
253   4e-71
SHE50703.1 small GTP-binding protein domain-containing protei...
244   4e-71
WP_020427073.1 MULTISPECIES: elongation factor G [Paenibacill...
253   4e-71
WP_098462744.1 elongation factor G [Isoptericola jiangsuensis]
253   4e-71
WP_047017551.1 elongation factor G [Streptomyces sp. CNQ-509]
253   4e-71
KIM01911.1 GTP-binding protein [Bifidobacterium adolescentis]
252   4e-71
OGJ50629.1 translation elongation factor G [Candidatus Peregr...
253   4e-71
AMS07852.1 elongation factor G [Lactobacillus oris]
252   4e-71
WP_091847160.1 elongation factor G [Bifidobacterium commune]
253   4e-71

WP_087358550.1 GTP-binding protein [Massiliomicrobiota timone...
256   4e-71
WP_002270330.1 elongation factor G [Streptococcus mutans]
253   4e-71
WP_017718048.1 elongation factor G [Oscillatoria sp. PCC 10802]
253   4e-71
WP_028339317.1 elongation factor G [Bradyrhizobium elkanii]
253   4e-71
WP_061296664.1 GTP-binding protein [Herbidospora cretacea]
250   4e-71
WP_026797907.1 MULTISPECIES: elongation factor G [Planktothrix]
253   4e-71
OUV52511.1 elongation factor G [Actinomycetales bacterium TME...
253   4e-71
WP_027185835.1 elongation factor G [Desulfovibrio inopinatus]
253   4e-71
WP_044428641.1 GTP-binding protein [Lactobacillus plantarum]
252   4e-71
OIO08593.1 translation elongation factor G [Candidatus Falkow...
253   4e-71
WP_058840389.1 elongation factor G [Bacillus sp. G3(2015)]
253   4e-71
WP_085392909.1 GTP-binding protein [Bifidobacterium adolescen...
252   4e-71
WP_059108824.1 elongation factor G [Catabacter hongkongensis]
253   4e-71
WP_061855461.1 MULTISPECIES: elongation factor G [Staphylococ...
253   4e-71
WP_017242553.1 elongation factor G [Vibrio breoganii]
253   4e-71
WP_045516547.1 elongation factor G [Bacillus niacini]
253   4e-71
WP_014451931.1 elongation factor G [Fervidobacterium pennivor...
253   4e-71
WP_074350999.1 elongation factor G [Proteiniborus sp. DW1]
253   4e-71
WP_056467398.1 elongation factor G [Bacillus sp. FJAT-25509]
253   4e-71
WP_019508352.1 elongation factor G [Pleurocapsa sp. PCC 7319]
253   4e-71
WP_100077816.1 elongation factor G [Pediococcus damnosus]
253   4e-71
AVP62348.1 elongation factor G [Clostridium botulinum]
252   4e-71
WP_104977679.1 elongation factor G [Sorangium cellulosum]
253   4e-71
WP_010770616.1 elongation factor G [Enterococcus caccae]
253   4e-71
WP_106257756.1 elongation factor G [Leptolyngbya frigida]
253   4e-71
WP_002780804.1 MULTISPECIES: elongation factor G [Campylobacter]
253   4e-71
WP_105960937.1 GTP-binding protein [Lactobacillus pentosus]
252   4e-71

WP_026567234.1 GTP-binding protein [Bacillus sp. UNC41MFS5]
252   4e-71
WP_002907926.1 elongation factor G [Streptococcus sanguinis]
253   4e-71
KIE50096.1 elongation factor G [marine actinobacterium MedAci...
253   4e-71
WP_009617604.1 elongation factor G [Desulfosporosinus sp. OT]
253   4e-71
WP_005591660.1 elongation factor G [Streptococcus cristatus]
253   4e-71
KKS96536.1 Elongation factor G [Candidatus Giovannonibacteria...
253   4e-71
WP_088412308.1 elongation factor G [Bacillus velezensis]
253   4e-71
OGH20388.1 translation elongation factor G [Candidatus Levyba...
253   4e-71
SCJ05046.1 Tetracycline resistance protein tetM [uncultured C...
252   4e-71
WP_061908706.1 elongation factor G [Streptococcus cristatus]
253   4e-71
WP_002850108.1 elongation factor G [Campylobacter fetus]
253   4e-71
EKD33072.1 hypothetical protein ACD_76C00094G0003 [uncultured...
253   4e-71
WP_103640703.1 elongation factor G [Campylobacter concisus]
253   4e-71
WP_103082777.1 GTP-binding protein [[Clostridium] thermosucci...
256   4e-71
WP_102196923.1 GTP-binding protein [Lactobacillus sp. SYF10-1a]
252   4e-71
WP_019190852.1 MULTISPECIES: elongation factor G [Firmicutes]
253   4e-71
KUO61924.1 elongation factor G [Gracilibacter sp. BRH_c7a]
251   4e-71
KKT38648.1 Elongation factor G [Candidatus Gottesmanbacteria ...
253   4e-71
WP_090016930.1 GTP-binding protein [Lachnospiraceae bacterium...
257   4e-71
WP_063606025.1 elongation factor G [Vibrio barjaei]
253   4e-71
WP_107541522.1 elongation factor G [Staphylococcus xylosus]
253   4e-71
PIR73202.1 elongation factor G [Candidatus Moranbacteria bact...
253   4e-71
WP_102284761.1 elongation factor G [Vibrio lentus]
253   4e-71
WP_035020884.1 elongation factor G [Carnobacterium sp. WN1374]
253   4e-71
WP_065175697.1 elongation factor G [Stenotrophomonas maltophi...
252   5e-71
WP_002699433.1 elongation factor G [Treponema phagedenis]
252   5e-71
WP_011318126.1 elongation factor G [Trichormus variabilis]
253   5e-71

EFV99831.1 translation elongation factor G [Streptococcus aus...
253   5e-71
WP_068963099.1 elongation factor G [Desulfosporosinus sp. BG]
253   5e-71
WP_023558744.1 elongation factor G [Brevibacillus panacihumi]
253   5e-71
WP_004395904.1 elongation factor G [Vibrio metschnikovii]
253   5e-71
WP_098862512.1 elongation factor G [Bacillus sp. AFS053548]
253   5e-71
KKQ70085.1 Elongation factor G [Candidatus Shapirobacteria ba...
253   5e-71
WP_073022427.1 GTP-binding protein [Clostridium intestinale]
251   5e-71
WP_010268957.1 elongation factor G [Corynebacterium bovis]
253   5e-71
WP_065440599.1 GTP-binding protein [Bifidobacterium pseudocat...
252   5e-71
WP_065540596.1 MULTISPECIES: GTP-binding protein [Blautia]
256   5e-71
OGE78260.1 translation elongation factor G [Candidatus Doudna...
253   5e-71
WP_017028876.1 elongation factor G [Vibrio breoganii]
253   5e-71
WP_003474778.1 GTP-binding protein [Clostridium perfringens]
251   5e-71
WP_085280077.1 GTP-binding protein [Clostridium botulinum]
251   5e-71
WP_088429180.1 elongation factor G [Halomicronema hongdechloris]
253   5e-71
WP_027554151.1 MULTISPECIES: elongation factor G [Bradyrhizob...
253   5e-71
WP_035399359.1 elongation factor G [Exiguobacterium sp. OS-77]
251   5e-71
WP_044677676.1 elongation factor G [Streptococcus suis]
253   5e-71
WP_014372959.1 elongation factor G [Melissococcus plutonius]
253   5e-71
WP_091752408.1 elongation factor G [Propionispora vibrioides]
253   5e-71
OGE32266.1 translation elongation factor G [Candidatus Davies...
251   5e-71
WP_106421105.1 elongation factor G [Staphylococcus simulans]
253   5e-71
WP_013773175.1 elongation factor G [Melissococcus plutonius]
253   5e-71
WP_072685389.1 elongation factor G [Holdemania sp. Marseille-...
253   5e-71
OGT92554.1 translation elongation factor G [Gemmatimonadetes ...
253   5e-71
WP_102124596.1 GTP-binding protein [Lactobacillus plantarum]
252   5e-71
WP_077453910.1 MULTISPECIES: elongation factor G [Enterococcus]
253   5e-71

WP_042152460.1 elongation factor G [Planktothrix agardhii]
253   5e-71
WP_003003071.1 MULTISPECIES: elongation factor G [Streptococcus]
253   5e-71
KXT86849.1 Translation elongation factor G [Streptococcus par...
253   5e-71
WP_003074701.1 MULTISPECIES: elongation factor G [Streptococcus]
253   5e-71
WP_095842292.1 GTP-binding protein [Lactobacillus salivarius]
251   5e-71
WP_019774991.1 elongation factor G [Streptococcus sobrinus]
253   5e-71
WP_044482525.1 elongation factor G [Paenibacillus antibiotico...
253   5e-71
CCZ53164.1 small GTP-binding protein domain [Clostridium sp. ...
256   5e-71
WP_028303818.1 elongation factor G [Oceanospirillum maris]
253   5e-71
WP_011994301.1 elongation factor G [Fervidobacterium nodosum]
252   5e-71
WP_020807260.1 GTP-binding protein [Lactobacillus gasseri]
251   5e-71
WP_076175560.1 GTP-binding protein [Clostridium botulinum]
251   5e-71
WP_025387078.1 MULTISPECIES: elongation factor G [Corynebacte...
253   5e-71
WP_028949098.1 elongation factor G [Synechocystis sp. PCC 6714]
253   5e-71
WP_049489304.1 elongation factor G [Streptococcus parasanguinis]
252   5e-71
WP_094050632.1 MULTISPECIES: elongation factor G [Cohnella]
252   5e-71
WP_014185997.1 GTP-binding protein [Desulfosporosinus orientis]
256   5e-71
AMP50241.1 elongation factor G, domain IV [uncultured bacterium]
242   5e-71
WP_084877396.1 elongation factor G [Streptococcus oralis]
252   5e-71
WP_012664234.1 elongation factor G [Staphylococcus carnosus]
252   5e-71
WP_025403594.1 elongation factor G [Corynebacterium falsenii]
253   5e-71
WP_040015934.1 elongation factor G [Desulfobacter postgatei]
252   5e-71
CDE55990.1 elongation factor G [Clostridium sp. CAG:269]
252   5e-71
WP_063204863.1 elongation factor G [Bdellovibrio bacteriovorus]
253   5e-71
WP_073712286.1 elongation factor G [Domibacillus mangrovi]
252   5e-71
WP_027536821.1 elongation factor G [Bradyrhizobium sp. URHA0002]
252   5e-71
EGL98081.1 ribosome protection-type tetracycline resistance r...
251   5e-71

WP_057576598.1 GTP-binding protein [Paeniclostridium sordellii]
252   5e-71
WP_092190621.1 elongation factor G [Desulfomicrobium norvegicum]
252   5e-71
WP_103118067.1 GTP-binding protein [Lysinibacillus sp. YS11]
251   5e-71
WP_036187802.1 GTP-binding protein [Lysinibacillus manganicus]
251   5e-71
OGO77665.1 translation elongation factor G [Clostridiales bac...
254   6e-71
WP_010385001.1 elongation factor G [Pseudoalteromonas rubra]
252   6e-71
WP_094349486.1 elongation factor G [Nostoc sp. 'Peltigera mem...
252   6e-71
OGF21011.1 translation elongation factor G [Candidatus Falkow...
252   6e-71
OIQ00561.1 translation elongation factor G [Candidatus Wirthb...
252   6e-71
WP_003028662.1 MULTISPECIES: elongation factor G [Streptococcus]
252   6e-71
WP_027771072.1 elongation factor G [Streptomyces sp. CNQ329]
253   6e-71
WP_047423155.1 elongation factor G [Bacillus anthracis]
252   6e-71
WP_014692622.1 MULTISPECIES: elongation factor G [Actinoplanes]
251   6e-71
KKU95892.1 Elongation factor G [Candidatus Gottesmanbacteria ...
253   6e-71
WP_011742953.1 GTP-binding protein [Bifidobacterium adolescen...
251   6e-71
WP_070758474.1 elongation factor G [Corynebacterium sp. HMSC2...
253   6e-71
WP_096379480.1 elongation factor G [Lysobacter enzymogenes]
252   6e-71
WP_075864472.1 elongation factor G [Carboxydothermus islandicus]
252   6e-71
WP_023599012.1 GTP-binding protein [Lactobacillus johnsonii]
251   6e-71
WP_061204989.1 GTP-binding protein [Lactobacillus crispatus]
251   6e-71
OGD66091.1 translation elongation factor G [Candidatus Berkel...
253   6e-71
WP_031923805.1 elongation factor G [Staphylococcus aureus]
252   6e-71
WP_007579829.1 elongation factor G [Pseudoalteromonas sp. BSi...
252   6e-71
SCI32276.1 Vegetative protein 19 [uncultured Clostridium sp.]
252   6e-71
WP_069278751.1 elongation factor G [Bradyrhizobium elkanii]
252   6e-71
PHS32164.1 elongation factor G [Methylophaga sp.]
252   6e-71
WP_024364106.1 GTP-binding protein [Lysinibacillus sphaericus]
251   6e-71

WP_015891263.1 GTP-binding protein [Brevibacillus brevis]
251   6e-71
WP_078925631.1 elongation factor G [Vibrio cincinnatiensis]
252   6e-71
WP_065599698.1 elongation factor G [Vibrio breoganii]
252   6e-71
COG93254.1 elongation factor G [Streptococcus pneumoniae]
253   6e-71
WP_005252201.1 MULTISPECIES: elongation factor G [Rhodococcus]
253   6e-71
WP_040953945.1 elongation factor G [Virgibacillus sp. SK37]
252   6e-71
WP_026276090.1 MULTISPECIES: elongation factor G [Streptomyces]
253   6e-71
WP_037567135.1 elongation factor G [Streptococcus anginosus]
252   6e-71
WP_026335546.1 elongation factor G [Acidobacteriaceae bacteri...
252   6e-71
OJJ16882.1 translation elongation factor G [Roseofilum reptot...
252   6e-71
WP_052849177.1 elongation factor G [Streptomyces avicenniae]
253   6e-71
WP_019768574.1 elongation factor G [Streptococcus sobrinus]
252   6e-71
WP_006738928.1 elongation factor G [Streptococcus urinalis]
252   6e-71
WP_002712304.1 elongation factor G [Afipia clevelandensis]
252   6e-71
KKT28843.1 Elongation factor G [Parcubacteria group bacterium...
252   6e-71
AMP50072.1 elongation factor G, domain IV [uncultured bacterium]
243   6e-71
WP_015006842.1 MULTISPECIES: elongation factor G [Cycloclasti...
252   6e-71
WP_105992166.1 elongation factor G [Staphylococcus chromogenes]
252   6e-71
WP_104690681.1 elongation factor G [Helicobacter heilmannii]
252   6e-71
WP_009624947.1 GTP-binding protein [Desulfosporosinus sp. OT]
256   6e-71
WP_025085026.1 GTP-binding protein [Lactobacillus paralimenta...
251   6e-71
WP_007729153.1 GTP-binding protein [Brevibacillus sp. BC25]
251   6e-71
WP_010766236.1 elongation factor G [Enterococcus moraviensis]
252   6e-71
WP_006245076.1 elongation factor G [Mycobacterium tusciae]
252   6e-71
WP_049474640.1 elongation factor G [Streptococcus parasanguinis]
252   6e-71
WP_088014607.1 elongation factor G [Bacillus acidiceler]
252   6e-71
KKS90154.1 Elongation factor G [Microgenomates group bacteriu...
253   6e-71

WP_099080208.1 elongation factor G [Vibrio sp. PID17_43]
252   6e-71
WP_102426766.1 elongation factor G [Vibrio breoganii]
252   6e-71
PJE68745.1 elongation factor G [Candidatus Shapirobacteria ba...
253   6e-71
WP_061320868.1 GTP-binding protein [Clostridium botulinum]
251   6e-71
WP_010874145.1 MULTISPECIES: elongation factor G [Synechocystis]
252   6e-71
WP_086273290.1 elongation factor G [Enterococcus sp. 8G7_MSG3...
252   6e-71
WP_009218305.1 GTP-binding protein [Lachnospiraceae bacterium...
256   6e-71
WP_003026447.1 MULTISPECIES: elongation factor G [Streptococcus]
252   6e-71
WP_054981439.1 elongation factor G [Pseudoalteromonas sp. P1-9]
252   6e-71
KKQ31333.1 Elongation factor G [Candidatus Shapirobacteria ba...
252   6e-71
WP_022765010.1 GTP-binding protein [Butyrivibrio sp. XPD2006]
256   6e-71
WP_046967036.1 elongation factor G [Luteibacter rhizovicinus]
252   6e-71
WP_036350591.1 elongation factor G [Microvirga sp. BSC39]
252   6e-71
WP_087531296.1 elongation factor G [Pseudoalteromonas sp. A601]
252   7e-71
WP_034287981.1 elongation factor G [Bacillus sp. 72]
252   7e-71
WP_094407680.1 elongation factor G [Elstera cyanobacteriorum]
252   7e-71
WP_072986233.1 GTP-binding protein [Clostridium cavendishii]
251   7e-71
OGT25679.1 translation elongation factor G [Gammaproteobacter...
252   7e-71
WP_004227233.1 elongation factor G [Streptococcus criceti]
252   7e-71
OQY79343.1 elongation factor G [Ignavibacteriales bacterium U...
252   7e-71
SFC68788.1 small GTP-binding protein domain-containing protei...
251   7e-71
WP_065209511.1 elongation factor G [Vibrio breoganii]
252   7e-71
WP_055678481.1 elongation factor G [Labrenzia alba]
252   7e-71
SCJ74497.1 Tetracycline resistance protein tetM from transpos...
255   7e-71
WP_095656289.1 GTP-binding protein [Virgibacillus profundi]
251   7e-71
WP_008170635.1 elongation factor G [Pseudoalteromonas sp. BSi...
252   7e-71
WP_098428740.1 elongation factor G [Bacillus sp. AFS088145]
252   7e-71

OIP12915.1 translation elongation factor G [Caldisericum sp. ...
252   7e-71
WP_089108435.1 GTP-binding protein [Lactobacillus mixtipabuli]
251   7e-71
WP_066086986.1 GTP-binding protein [Bacillus novalis]
251   7e-71
WP_069137231.1 GTP-binding protein [Lactobacillus plantarum]
251   7e-71
PKO13242.1 elongation factor G [Chloroflexi bacterium HGW-Chl...
252   7e-71
ASY51282.1 elongation factor G [Clostridium perfringens]
251   7e-71
WP_035794855.1 GTP-binding protein [Butyrivibrio sp. WCD3002]
256   7e-71
WP_070888825.1 elongation factor G [Streptococcus sp. HMSC34B10]
252   7e-71
WP_057032889.1 elongation factor G [Campylobacter jejuni]
252   7e-71
WP_108030281.1 GTP-binding protein [Lysinibacillus tabacifolii]
251   7e-71
WP_003477672.1 GTP-binding protein [Clostridium perfringens]
251   7e-71
OGZ18768.1 translation elongation factor G [Candidatus Nealso...
252   7e-71
WP_069641180.1 elongation factor G [Enterococcus ureilyticus]
252   7e-71
WP_084108344.1 elongation factor G [Campylobacter concisus]
252   7e-71
OUR64580.1 translation elongation factor G [Methylophaga sp. ...
252   7e-71
WP_000951555.1 elongation factor G [Streptococcus agalactiae]
252   7e-71
PIP62100.1 elongation factor G [Candidatus Roizmanbacteria ba...
252   7e-71
WP_069635477.1 MULTISPECIES: elongation factor G [Enterococcus]
252   7e-71
WP_010392256.1 MULTISPECIES: elongation factor G [Pseudoalter...
252   7e-71
WP_096991833.1 elongation factor G [Vibrio thalassae]
252   7e-71
WP_088003790.1 elongation factor G [Bacillus solisilvae]
252   7e-71
WP_070273999.1 MULTISPECIES: elongation factor G [Campylobacter]
252   7e-71
WP_057537562.1 GTP-binding protein [Paeniclostridium sordellii]
251   7e-71
WP_055063097.1 GTP-binding protein [Bifidobacterium pseudocat...
251   7e-71
WP_005504794.1 elongation factor G [Grimontia hollisae]
252   7e-71
WP_006793851.1 MULTISPECIES: elongation factor G [Pseudoalter...
252   7e-71
WP_085379386.1 GTP-binding protein [Bifidobacterium adolescen...
251   7e-71

WP_062692218.1 elongation factor G [Photobacterium sanguinica...
252   7e-71
WP_004615927.1 GTP-binding protein [[Clostridium] papyrosolvens]
256   7e-71
WP_107588164.1 elongation factor G [Staphylococcus simulans]
252   7e-71
SNT51645.1 translation elongation factor 2 (EF-2/EF-G) [Rhodo...
252   7e-71
WP_068783694.1 elongation factor G [Paenibacillus phocaensis]
252   7e-71
WP_041156133.1 elongation factor G [Vibrio mytili]
252   7e-71
WP_107375279.1 elongation factor G [Staphylococcus chromogenes]
252   7e-71
WP_080863515.1 GTP-binding protein [Bifidobacterium adolescen...
251   7e-71
WP_094956782.1 elongation factor G [Photobacterium sanguinica...
252   7e-71
WP_002908654.1 MULTISPECIES: elongation factor G [Streptococcus]
252   7e-71
WP_006288640.1 elongation factor G [Parascardovia denticolens]
252   7e-71
CAA46277.1 elongation factor G [Synechocystis sp. PCC 6803]
252   8e-71
GBD34879.1 Elongation factor G [bacterium HR35]
252   8e-71
WP_000090336.1 elongation factor G [Streptococcus oralis]
252   8e-71
WP_024578570.1 MULTISPECIES: elongation factor G [Bradyrhizob...
252   8e-71
WP_017723032.1 elongation factor G [Staphylococcus xylosus]
252   8e-71
WP_009226635.1 elongation factor G [Paenibacillus sp. oral ta...
252   8e-71
WP_055068323.1 GTP-binding protein [Roseburia faecis]
256   8e-71
WP_029276482.1 elongation factor G [Carnobacterium jeotgali]
252   8e-71
WP_100795235.1 elongation factor G [Viridibacillus sp. OK051]
252   8e-71
WP_025117220.1 GTP-binding protein [Lysinibacillus fusiformis]
251   8e-71
WP_010554859.1 MULTISPECIES: elongation factor G [Pseudoalter...
252   8e-71
WP_070278906.1 MULTISPECIES: elongation factor G [Campylobacter]
252   8e-71
PID83138.1 elongation factor G [Candidatus Campbellbacteria b...
253   8e-71
WP_085380244.1 GTP-binding protein [Bifidobacterium adolescen...
251   8e-71
WP_054251760.1 elongation factor G [Neofamilia massiliensis]
252   8e-71
WP_087801954.1 elongation factor G [Saccharibacillus sp. O16]
252   8e-71

WP_003085284.1 MULTISPECIES: elongation factor G [Streptococcus]
252   8e-71
WP_004184294.1 elongation factor G [Streptococcus sanguinis]
252   8e-71
WP_089252708.1 elongation factor G [Rhodococcus kyotonensis]
252   8e-71
WP_033499211.1 GTP-binding protein [Bifidobacterium adolescen...
251   8e-71
WP_053818937.1 GTP-binding protein [Clostridium sporogenes]
251   8e-71
WP_026971002.1 elongation factor G [Aliagarivorans marinus]
252   8e-71
WP_055105069.1 elongation factor G [Paenibacillus ihumii]
252   8e-71
WP_006598935.1 elongation factor G [Pseudoramibacter alactoly...
252   8e-71
WP_013119205.1 MULTISPECIES: elongation factor G [Thermincola]
252   8e-71
WP_088770300.1 GTP-binding protein [Lactobacillus pentosus]
251   8e-71
WP_016709925.1 MULTISPECIES: elongation factor G [Pseudoalter...
252   8e-71
WP_019375537.1 elongation factor G [Virgibacillus halodenitri...
252   8e-71
WP_012809248.1 elongation factor G [Atopobium parvulum]
252   8e-71
WP_053518559.1 elongation factor G [Stenotrophomonas maltophi...
251   8e-71
WP_089977310.1 elongation factor G [Carnobacterium viridans]
252   8e-71
ABW36896.1 mutant TetW [Bifidobacterium adolescentis]
235   9e-71
WP_026196347.1 elongation factor G [Corynebacterium lubricantis]
252   9e-71
WP_075345743.1 elongation factor G [Streptococcus sp. 'caviae']
252   9e-71
WP_034572145.1 elongation factor G [Carnobacterium divergens]
252   9e-71
WP_096023697.1 elongation factor G [Campylobacter lanienae]
252   9e-71
KKT24107.1 Elongation factor G [candidate division TM6 bacter...
252   9e-71
WP_076661284.1 GTP-binding protein [Lactobacillus plantarum]
251   9e-71
WP_085382424.1 GTP-binding protein [Bifidobacterium adolescen...
251   9e-71
WP_075619034.1 GTP-binding protein [Paenisporosarcina indica]
251   9e-71
WP_094241614.1 elongation factor G [Fusobacterium sp. oral ta...
252   9e-71
WP_038804156.1 elongation factor G [Streptococcus oralis]
252   9e-71
WP_023943516.1 elongation factor G [Streptococcus mitis]
252   9e-71

WP_095479275.1 elongation factor G [Bacillaceae bacterium SAO...
252   9e-71
WP_021296613.1 elongation factor G [Alicyclobacillus acidoter...
252   9e-71
KPP97540.1 translation elongation factor G [Bacteroidetes bac...
252   9e-71
AMJ37435.1 elongation factor Tu domain 2 [uncultured bacterium]
238   9e-71
EDP20009.1 translation elongation factor G [Faecalibacterium ...
252   9e-71
WP_107551125.1 elongation factor G [Staphylococcus xylosus]
252   9e-71
PIE57963.1 elongation factor G [Desulfobulbus propionicus]
252   9e-71
WP_106767170.1 elongation factor G [Paenibacillus sp. Marseil...
252   9e-71
OGF59968.1 translation elongation factor G [Candidatus Fische...
252   9e-71
WP_101890880.1 GTP-binding protein [Lactobacillus gasseri]
251   9e-71
OGY24172.1 translation elongation factor G [Candidatus Woykeb...
252   9e-71
WP_035820762.1 elongation factor G [Cupriavidus sp. SK-4]
252   9e-71
OIP81195.1 translation elongation factor G [Parcubacteria gro...
252   9e-71
WP_087585694.1 elongation factor G [Campylobacter concisus]
252   9e-71
WP_063964484.1 elongation factor G [Domibacillus aminovorans]
252   9e-71
PVE16865.1 elongation factor G [Clostridium perfringens]
251   9e-71
WP_072715194.1 elongation factor G [Rhodothermus profundi]
252   9e-71
WP_074716985.1 GTP-binding protein [Lachnobacterium bovis]
256   9e-71
WP_003715495.1 GTP-binding protein [Lactobacillus oris]
251   9e-71
WP_084662971.1 elongation factor G [Thermanaeromonas toyohensis]
252   9e-71
WP_066390663.1 elongation factor G [Fictibacillus phosphorivo...
252   9e-71
WP_013499485.1 GTP-binding protein [Ruminococcus albus]
255   9e-71
WP_029081774.1 elongation factor G [Bradyrhizobium sp. th.b2]
252   9e-71
WP_021081659.1 elongation factor G [Bradyrhizobium sp. MOS004]
252   9e-71
WP_010436214.1 MULTISPECIES: elongation factor G [Vibrio]
252   9e-71
WP_025024850.1 GTP-binding protein [Lactobacillus nodensis]
251   9e-71
WP_039774894.1 GTP-binding protein [Bifidobacterium adolescen...
251   9e-71

WP_058404706.1 elongation factor G [Pseudoalteromonas sp. H71]
252   1e-70
WP_107530606.1 elongation factor G [Staphylococcus xylosus]
252   1e-70
SHJ84602.1 elongation factor G [Dethiosulfatibacter aminovora...
251   1e-70
WP_016860904.1 elongation factor G [Fischerella muscicola]
252   1e-70
WP_085333414.1 GTP-binding protein [Clostridium sporogenes]
251   1e-70
WP_106988943.1 elongation factor G [Lachnoclostridium sp. SNU...
251   1e-70
WP_087478714.1 MULTISPECIES: elongation factor G [Megasphaera]
251   1e-70
WP_078485552.1 GTP-binding protein [Solemya elarraichensis gi...
251   1e-70
ARO65866.1 Elongation factor G [Bacillus cereus]
251   1e-70
WP_032089390.1 elongation factor G [bacterium LF-3]
251   1e-70
WP_022660810.1 elongation factor G [Desulfovibrio longus]
251   1e-70
WP_090990084.1 GTP-binding protein [Bacillus sp. OV322]
251   1e-70
WP_025219608.1 GTP-binding protein [Lysinibacillus varians]
251   1e-70
WP_107690991.1 elongation factor G [Campylobacter concisus]
251   1e-70
WP_095267535.1 MULTISPECIES: elongation factor G [Paenibacillus]
251   1e-70
OFZ24020.1 translation elongation factor G [Bdellovibrionales...
251   1e-70
WP_068731500.1 elongation factor G [Tardiphaga robiniae]
251   1e-70
WP_028650464.1 elongation factor G [Nocardioides halotolerans]
252   1e-70
WP_021276168.1 elongation factor G [Bacteriovorax sp. DB6_IX]
252   1e-70
WP_000090340.1 MULTISPECIES: elongation factor G [Streptococcus]
251   1e-70
OUV77783.1 elongation factor G [Acidimicrobiaceae bacterium T...
251   1e-70
WP_085345852.1 GTP-binding protein [Bifidobacterium adolescen...
251   1e-70
WP_088186608.1 GTP-binding protein [Desulfosporosinus sp. FKA]
255   1e-70
WP_010623587.1 GTP-binding protein [Lactobacillus versmoldensis]
250   1e-70
WP_088921737.1 elongation factor G [Granulosicoccus antarcticus]
252   1e-70
WP_071311376.1 elongation factor G [Anaerobacillus arsenicise...
251   1e-70
WP_069033608.1 MULTISPECIES: elongation factor G [Bacillus]
251   1e-70

WP_046328180.1 elongation factor G [Sneathia amnii]
251   1e-70
WP_010558083.1 elongation factor G [Pseudoalteromonas marina]
251   1e-70
WP_049514482.1 elongation factor G [Streptococcus anginosus]
251   1e-70
CDF11252.1 elongation factor EF-G [Eubacterium sp. CAG:76]
255   1e-70
WP_072235837.1 elongation factor G [Campylobacter coli]
251   1e-70
OJJ08974.1 translation elongation factor G [Alphaproteobacter...
251   1e-70
WP_035009914.1 GTP-binding protein [Bifidobacterium adolescen...
251   1e-70
AJO24893.1 translation elongation factor G [Bacillus coagulans]
251   1e-70
WP_101875390.1 GTP-binding protein [Lachnoclostridium edouardi]
250   1e-70
PKQ36618.1 elongation factor G [Actinobacteria bacterium HGW-...
251   1e-70
WP_038806425.1 elongation factor G [Streptococcus oralis]
251   1e-70
WP_080979556.1 GTP-binding protein [Lactobacillus salivarius]
250   1e-70
WP_009847514.1 MULTISPECIES: elongation factor G [Vibrio]
251   1e-70
EKD91562.1 hypothetical protein ACD_30C00001G0003 [uncultured...
252   1e-70
WP_106006348.1 elongation factor G [Moorella humiferrea]
251   1e-70
WP_070956728.1 GTP-binding protein [Clostridium perfringens]
250   1e-70
WP_000090337.1 MULTISPECIES: elongation factor G [Streptococcus]
251   1e-70
WP_088369560.1 elongation factor G [endosymbiont 'TC1' of Tri...
251   1e-70
WP_027745160.1 MULTISPECIES: elongation factor G [Streptomyces]
252   1e-70
WP_048925245.1 elongation factor G [Coprobacillus sp. 8_1_38FAA]
251   1e-70
WP_013263377.1 elongation factor G [gamma proteobacterium HdN1]
251   1e-70
WP_019323886.1 elongation factor G [Streptococcus mutans]
251   1e-70
WP_003808506.1 GTP-binding protein [Bifidobacterium adolescen...
251   1e-70
CDC66931.1 small GTP-binding protein [Ruminococcus sp. CAG:57]
254   1e-70
WP_019322034.1 elongation factor G [Streptococcus mutans]
251   1e-70
WP_002302260.1 elongation factor G [Streptococcus mutans]
251   1e-70
WP_101422298.1 elongation factor G [Streptomyces sp. CMB-StM0...
252   1e-70

WP_066237971.1 elongation factor G [Fictibacillus phosphorivo...
251   1e-70
WP_075652249.1 elongation factor G [Vibrio ponticus]
251   1e-70
AMP49715.1 elongation factor G, domain IV [uncultured bacterium]
242   1e-70
WP_018395152.1 MULTISPECIES: elongation factor G [Bacillaceae]
251   1e-70
WP_070122193.1 GTP-binding protein [Bifidobacterium adolescen...
251   1e-70
WP_107558153.1 elongation factor G [Staphylococcus xylosus]
251   1e-70
WP_099090501.1 elongation factor G [Staphylococcus edaphicus]
251   1e-70
WP_102391247.1 elongation factor G [Enterovibrio norvegicus]
251   1e-70
OCH56848.1 translation elongation factor G [Vibrio cyclitroph...
251   1e-70
WP_069110683.1 elongation factor G [Jiangella alba]
252   1e-70
PIY96364.1 elongation factor G [Candidatus Kerfeldbacteria ba...
251   1e-70
WP_006823211.1 elongation factor G [Corynebacterium casei]
252   1e-70
OGZ26655.1 translation elongation factor G [Candidatus Nealso...
252   1e-70
OGM29594.1 translation elongation factor G [Candidatus Woeseb...
252   1e-70
WP_102436739.1 MULTISPECIES: elongation factor G [Shewanella]
251   1e-70
WP_069654067.1 elongation factor G [Enterococcus plantarum]
251   1e-70
WP_035013251.1 elongation factor G [Catenovulum agarivorans]
251   1e-70
WP_007546161.1 elongation factor G [Sulfurihydrogenibium yell...
251   1e-70
PJC40345.1 elongation factor G [Parcubacteria group bacterium...
249   1e-70
OGR23469.1 translation elongation factor G [Desulfobacterales...
251   1e-70
WP_096260121.1 elongation factor G [Lebetimonas natsushimae]
251   1e-70
WP_054254526.1 elongation factor G [Clostridiales bacterium S...
251   1e-70
WP_033676064.1 elongation factor G [Streptococcus mitis]
251   1e-70
WP_100899835.1 elongation factor G [Nostoc flagelliforme]
251   1e-70
WP_060681362.1 elongation factor G [Virgibacillus halodenitri...
251   1e-70
WP_057560284.1 GTP-binding protein [Paeniclostridium sordellii]
251   1e-70
WP_057569863.1 GTP-binding protein [Paeniclostridium sordellii]
251   1e-70

WP_024613216.1 GTP-binding protein [Clostridium sp. Ade.TY]
250   1e-70
2BM1_A Chain A, Ribosomal Elongation Factor G (Ef-G) Fusidic ...
251   1e-70
WP_036763261.1 elongation factor G [Peptostreptococcus sp. MV1]
251   1e-70
KUK33840.1 Elongation factor G [Thermotoga sp. 47_83]
251   1e-70
WP_096463196.1 elongation factor G [Aneurinibacillus soli]
251   1e-70
WP_015924569.1 GTP-binding protein [[Clostridium] cellulolyti...
255   1e-70
WP_096013469.1 elongation factor G [Campylobacter lanienae]
251   1e-70
WP_104612845.1 elongation factor G [Thermobifida fusca]
252   1e-70
WP_101214614.1 elongation factor G [Alteromonadales bacterium...
251   1e-70
WP_019322324.1 elongation factor G [Streptococcus mutans]
251   1e-70
WP_100404156.1 elongation factor G [Bacillus sp. FJAT-42315]
251   1e-70
PKN09561.1 elongation factor G [Deltaproteobacteria bacterium...
251   1e-70
WP_024599704.1 elongation factor G [Pseudoalteromonas sp. TAE56]
251   1e-70
WP_095106859.1 elongation factor G [Staphylococcus pisciferme...
251   1e-70
WP_096024343.1 elongation factor G [Campylobacter lanienae]
251   1e-70
CDE82161.1 small GTP-binding protein domain [Ruminococcus sp....
254   1e-70
WP_021106895.1 GTP-binding protein [Clostridium botulinum]
250   1e-70
WP_089271662.1 elongation factor G [Desulfovibrio mexicanus]
251   1e-70
WP_086293699.1 elongation factor G [Campylobacter sp. P0024]
251   1e-70
WP_101216228.1 MULTISPECIES: elongation factor G [Pseudoalter...
251   1e-70
CDD30971.1 gTP-binding domain protein [Firmicutes bacterium C...
255   1e-70
WP_086242591.1 elongation factor G [Campylobacter sp. P0124]
251   1e-70
KXK13560.1 elongation factor G [Chloroflexi bacterium OLB14]
251   1e-70
WP_002715366.1 elongation factor G [Afipia felis]
251   1e-70
WP_076925899.1 elongation factor G [Pseudoalteromonas sp. EB27]
251   1e-70
WP_108305976.1 elongation factor G [Lysinibacillus jejuensis]
251   1e-70
WP_075483600.1 elongation factor G [Candidatus Pelagibacter u...
251   1e-70

WP_079492322.1 GTP-binding protein [Maledivibacter halophilus]
250   1e-70
WP_016798306.1 elongation factor G [Vibrio cyclitrophicus]
251   1e-70
WP_094344972.1 elongation factor G [Nostoc sp. 'Peltigera mem...
251   1e-70
WP_096021118.1 elongation factor G [Campylobacter lanienae]
251   1e-70
WP_075573969.1 elongation factor G [Ezakiella massiliensis]
251   1e-70
WP_056487561.1 GTP-binding protein [Brevibacillus sp. Leaf182]
250   1e-70
WP_087018979.1 elongation factor G [Butyricicoccus porcorum]
251   1e-70
WP_075501283.1 elongation factor G [Candidatus Pelagibacter u...
251   1e-70
WP_072732030.1 elongation factor G [Paenibacillus sp. NAIST15-1]
251   1e-70
WP_067541854.1 GTP-binding protein [Emergencia timonensis]
254   1e-70
WP_080545323.1 elongation factor G [Faecalibacterium prausnit...
252   1e-70
WP_019932693.1 elongation factor G [Nocardia sp. BMG111209]
251   1e-70
WP_069633186.1 elongation factor G [Campylobacter pinnipediorum]
251   1e-70
WP_107348263.1 elongation factor G [Photobacterium lutimaris]
251   1e-70
WP_063696477.1 GTP-binding protein [Pediococcus inopinatus]
250   1e-70
WP_024593825.1 elongation factor G [Pseudoalteromonas sp. TB13]
251   1e-70
WP_002307411.1 elongation factor G [Streptococcus mutans]
251   1e-70
WP_093062482.1 elongation factor G [Psychrobacillus sp. OK028]
251   1e-70
PSR00624.1 elongation factor G [Bacteroidetes bacterium QS_9_...
251   1e-70
WP_046180691.1 elongation factor G [Domibacillus tundrae]
251   1e-70
WP_096016432.1 elongation factor G [Campylobacter lanienae]
251   1e-70
WP_074961081.1 GTP-binding protein [Ruminococcus albus]
254   1e-70
WP_045518156.1 GTP-binding protein [Clostridium sporogenes]
250   1e-70
WP_068099558.1 MULTISPECIES: elongation factor G [Rhodococcus]
251   1e-70
WP_074276035.1 elongation factor G [Bradyrhizobium erythrophlei]
251   1e-70
WP_091984585.1 elongation factor G [Pseudoalteromonas denitri...
251   1e-70
WP_071644101.1 GTP-binding protein [Paenibacillus polymyxa]
250   1e-70

PAY32988.1 elongation factor G [Lactobacillus salivarius]
250   1e-70
WP_002806559.1 elongation factor G [Campylobacter coli]
251   1e-70
WP_011473878.1 elongation factor G [Rhodopseudomonas palustris]
251   1e-70
WP_089868516.1 GTP-binding protein [Lachnospiraceae bacterium...
254   1e-70
WP_002351865.1 elongation factor G [Streptococcus mutans]
251   1e-70
WP_107695337.1 elongation factor G [Campylobacter concisus]
251   1e-70
WP_024955257.1 elongation factor G [Sulfurospirillum arcachon...
251   1e-70
SCI16473.1 Tetracycline resistance protein tetM from transpos...
255   1e-70
WP_106809337.1 elongation factor G [Candidatus Sulfotelmatoba...
251   1e-70
WP_072534233.1 GTP-binding protein [Lactobacillus plantarum]
251   1e-70
WP_008105597.1 elongation factor G [Verrucomicrobiae bacteriu...
251   1e-70
WP_043032008.1 GTP-binding protein [Clostridium botulinum]
250   1e-70
WP_078424308.1 elongation factor G [Campylobacter pinnipediorum]
251   1e-70
WP_100417512.1 elongation factor G [Bradyrhizobium lablabi]
251   1e-70
WP_086994389.1 elongation factor G [Pseudoalteromonas sp. JB197]
251   1e-70
WP_002277579.1 elongation factor G [Streptococcus mutans]
251   1e-70
WP_027187298.1 elongation factor G [Desulfovibrio cuneatus]
251   1e-70
WP_030456365.1 GTP-binding protein [Herbidospora cretacea]
249   1e-70
WP_006703606.1 elongation factor G [Granulicatella elegans]
251   1e-70
WP_097773654.1 elongation factor G [Faecalibacterium prausnit...
251   1e-70
WP_020007569.1 elongation factor G [Salinicoccus albus]
251   1e-70
CDD57279.1 putative uncharacterized protein [Bacteroides pect...
255   1e-70
WP_101080903.1 elongation factor G [Psychromonas sp. Urea-02u...
251   1e-70
CUO62749.1 Tetracycline resistance protein tetM from transpos...
254   1e-70
WP_050355665.1 elongation factor G [Gottschalkia purinilyticum]
251   1e-70
WP_107282919.1 elongation factor G [Photobacterium lipolyticum]
251   1e-70
WP_105200389.1 MULTISPECIES: elongation factor G [Pseudoalter...
251   1e-70

WP_041511338.1 elongation factor G [Shewanella sp. cp20]
251   1e-70
WP_088547371.1 GTP-binding protein [Paenibacillus sp. St-s]
250   1e-70
WP_020620135.1 elongation factor G [Paenibacillus daejeonensis]
251   2e-70
WP_011293072.1 elongation factor G [Thermobifida fusca]
251   2e-70
EKE16153.1 hypothetical protein ACD_11C00029G0033 [uncultured...
251   2e-70
WP_086201018.1 GTP-binding protein [Lactobacillus salivarius]
250   2e-70
OKZ56369.1 translation elongation factor G [Clostridium sp. 2...
251   2e-70
WP_049523435.1 elongation factor G [Streptococcus parasanguinis]
251   2e-70
WP_002268028.1 elongation factor G [Streptococcus mutans]
251   2e-70
WP_094331157.1 MULTISPECIES: elongation factor G [Nostoc]
251   2e-70
WP_088070812.1 elongation factor G [Bacillus luciferensis]
251   2e-70
WP_032551575.1 MULTISPECIES: elongation factor G [Vibrio]
251   2e-70
WP_107399949.1 elongation factor G [Staphylococcus chromogenes]
251   2e-70
WP_061388951.1 elongation factor G [Staphylococcus aureus]
251   2e-70
WP_023385633.1 elongation factor G [Campylobacter fetus]
251   2e-70
KKW17595.1 Elongation factor G [Candidatus Magasanikbacteria ...
251   2e-70
WP_102315855.1 elongation factor G [Enterovibrio norvegicus]
251   2e-70
CCY30688.1 putative uncharacterized protein [Roseburia inulin...
255   2e-70
OGY26099.1 translation elongation factor G [Candidatus Woykeb...
251   2e-70
WP_104148140.1 elongation factor G [Campylobacter hyointestin...
251   2e-70
WP_021259837.1 MULTISPECIES: elongation factor G [Paenibacillus]
251   2e-70
WP_049483638.1 elongation factor G [Streptococcus parasanguinis]
251   2e-70
WP_078387411.1 elongation factor G [Campylobacter pinnipediorum]
251   2e-70
WP_074126944.1 elongation factor G [Bradyrhizobium sp. NAS96.2]
251   2e-70
WP_039068588.1 elongation factor G [Staphylococcus xylosus]
251   2e-70
WP_003637329.1 GTP-binding protein [Lactobacillus pentosus]
250   2e-70
WP_047811629.1 GTP-binding protein [Desulfosporosinus acididu...
255   2e-70

WP_058430294.1 MULTISPECIES: elongation factor G [Pseudoalter...
251   2e-70
WP_075522351.1 elongation factor G [Candidatus Pelagibacter u...
251   2e-70
WP_015847229.1 elongation factor G [Paenibacillus sp. JDR-2]
251   2e-70
WP_101104080.1 elongation factor G [Staphylococcus xylosus]
251   2e-70
WP_066731180.1 GTP-binding protein [Bariatricus massiliensis]
255   2e-70
WP_002262490.1 elongation factor G [Streptococcus mutans]
251   2e-70
WP_086237864.1 elongation factor G [Campylobacter sp. P0078]
251   2e-70
WP_070297985.1 elongation factor G [Campylobacter coli]
251   2e-70
WP_038034548.1 elongation factor G [Thermotoga sp. Xyl54]
251   2e-70
AVQ40527.1 elongation factor G [Clostridium botulinum]
250   2e-70
WP_011986616.1 GTP-binding protein [Clostridium botulinum]
250   2e-70
OXE35226.1 elongation factor G [Phenylobacterium zucineum]
251   2e-70
WP_055302926.1 GTP-binding protein [Roseburia inulinivorans]
255   2e-70
WP_095720292.1 elongation factor G [Calothrix elsteri]
251   2e-70
WP_028543505.1 elongation factor G [Paenibacillus taiwanensis]
251   2e-70
WP_017311595.1 MULTISPECIES: elongation factor G [Hapalosipho...
251   2e-70
WP_066118241.1 elongation factor G [Geminocystis sp. NIES-3709]
251   2e-70
WP_006530855.1 elongation factor G [Gloeocapsa sp. PCC 73106]
251   2e-70
WP_025023859.1 elongation factor G [Lactobacillus nodensis]
251   2e-70
WP_011345195.1 MULTISPECIES: elongation factor G [Carboxydoth...
251   2e-70
SCI52053.1 Vegetative protein 19 [uncultured Clostridium sp.]
251   2e-70
SCI55470.1 Vegetative protein 19 [uncultured Butyricicoccus sp.]
251   2e-70
WP_028848710.1 elongation factor G [Thermocrispum agreste]
251   2e-70
PPR42115.1 Elongation factor G [Alphaproteobacteria bacterium...
251   2e-70
EEU97609.1 translation elongation factor G [Faecalibacterium ...
251   2e-70
WP_066114993.1 elongation factor G [Geminocystis sp. NIES-3709]
251   2e-70
OGL14180.1 translation elongation factor G [Candidatus Rokuba...
251   2e-70

OOL50865.1 tetracycline resistance ribosomal protection prote...
237   2e-70
WP_104629501.1 elongation factor G [Helicobacter bizzozeronii]
251   2e-70
WP_086297046.1 elongation factor G [Campylobacter sp. RM6137]
251   2e-70
SCH92935.1 Elongation factor G [uncultured Blautia sp.]
256   2e-70
KKP79782.1 Elongation factor G [Candidatus Moranbacteria bact...
251   2e-70
WP_024610804.1 elongation factor G [Pseudoalteromonas sp. TB64]
251   2e-70
WP_074402168.1 elongation factor G [Carnobacterium divergens]
251   2e-70
WP_098798327.1 elongation factor G [Bacillus sp. AFS040349]
251   2e-70
WP_086827468.1 elongation factor G [Allokutzneria sp. NRRL B-...
251   2e-70
WP_091382666.1 elongation factor G [Alloactinosynnema album]
251   2e-70
AUC60456.1 translation elongation factor G [Cyanobacterium sp...
251   2e-70
WP_056035778.1 MULTISPECIES: elongation factor G [Paenibacillus]
251   2e-70
WP_011943783.1 MULTISPECIES: elongation factor G [Thermotoga]
251   2e-70
WP_048033133.1 GTP-binding protein [Brevibacillus brevis]
250   2e-70
AVV97781.1 elongation factor G [Lactobacillus plantarum]
251   2e-70
WP_012410238.1 elongation factor G [Nostoc punctiforme]
251   2e-70
WP_088740334.1 elongation factor G [Haliangium sp. UPWRP_2]
251   2e-70
WP_035130122.1 GTP-binding protein [Clostridium sulfidigenes]
250   2e-70
WP_048395714.1 GTP-binding protein [Lysinibacillus sp. LK3]
250   2e-70
OGW58930.1 translation elongation factor G [Nitrospirae bacte...
251   2e-70
WP_015381229.1 elongation factor G [Corynebacterium urealyticum]
251   2e-70
WP_024782052.1 elongation factor G [Streptococcus mutans]
251   2e-70
WP_100041987.1 GTP-binding protein [[Clostridium] sphenoides]
250   2e-70
WP_004459119.1 GTP-binding protein [Clostridium perfringens]
250   2e-70
WP_068537333.1 elongation factor G [Paenibacillus glacialis]
251   2e-70
WP_046234220.1 elongation factor G [Paenibacillus algorifonti...
251   2e-70
SFC35877.1 small GTP-binding protein domain-containing protei...
254   2e-70

WP_069645951.1 elongation factor G [Enterococcus ureasiticus]
251   2e-70
WP_057799805.1 GTP-binding protein [Lactobacillus kalixensis]
250   2e-70
2BM0_A Chain A, Ribosomal elongation factor G (EF-G) Fusidic ...
251   2e-70
WP_071979286.1 MULTISPECIES: elongation factor G [Alteromonas]
251   2e-70
WP_097018169.1 elongation factor G [Orenia metallireducens]
251   2e-70
OJY13277.1 translation elongation factor G [Rhizobiales bacte...
251   2e-70
SCV02387.1 protein chain elongation factor EF-G, GTP-binding ...
251   2e-70
OGP14672.1 translation elongation factor G [Deltaproteobacter...
251   2e-70
WP_015134470.1 elongation factor G [Leptolyngbya sp. PCC 7376]
251   2e-70
WP_105309632.1 GTP-binding protein [Dorea sp. Marseille-P4042]
255   2e-70
WP_080804146.1 elongation factor G [Desulfamplus magnetovalli...
251   2e-70
WP_077605126.1 elongation factor G [Oceanobacillus sojae]
251   2e-70
CCY18773.1 putative translation elongation factor G [Bifidoba...
250   2e-70
OGS46668.1 translation elongation factor G [Elusimicrobia bac...
251   2e-70
WP_024533824.1 GTP-binding protein [Sporosarcina sp. EUR3 2.2.2]
250   2e-70
WP_098121853.1 MULTISPECIES: elongation factor G [Bacillus]
251   2e-70
WP_006292610.1 elongation factor G [Scardovia inopinata]
251   2e-70
WP_012604685.1 elongation factor G [Vibrio tasmaniensis]
251   2e-70
WP_018591139.1 MULTISPECIES: GTP-binding protein [Peptostrept...
250   2e-70
WP_098574641.1 GTP-binding protein [Bacillus sp. AFS073361]
250   2e-70
WP_059430723.1 MULTISPECIES: elongation factor G [Campylobacter]
251   2e-70
WP_006916610.1 elongation factor G [Lactobacillus coleohominis]
251   2e-70
WP_054549242.1 GTP-binding protein [Lysinibacillus sphaericus]
250   2e-70
PHX97285.1 elongation factor G [Gemmatimonadetes bacterium]
251   2e-70
WP_070051991.1 elongation factor G [Staphylococcus xylosus]
251   2e-70
WP_016294151.1 GTP-binding protein [Lachnospiraceae bacterium...
255   2e-70
WP_077538889.1 elongation factor G [Pseudoalteromonas aliena]
251   2e-70

WP_103091247.1 GTP-binding protein [[Clostridium] thermosucci...
254   2e-70
WP_096515876.1 GTP-binding protein [Clostridium perfringens]
250   2e-70
WP_075520593.1 elongation factor G [Candidatus Pelagibacter u...
251   2e-70
WP_012506609.1 elongation factor G [Prosthecochloris aestuarii]
251   2e-70
OGH19088.1 translation elongation factor G [Candidatus Levyba...
251   2e-70
WP_075858210.1 elongation factor G [Carboxydothermus pertinax]
251   2e-70
WP_053364767.1 elongation factor G [Bacillus sp. FJAT-27245]
251   2e-70
WP_044763254.1 elongation factor G [Streptococcus suis]
251   2e-70
WP_018272141.1 elongation factor G [Bradyrhizobium elkanii]
251   2e-70
KYC65035.1 hypothetical protein B4100_1251 [Bacillus coagulans]
251   2e-70
WP_107740916.1 GTP-binding protein [Lactobacillus sp. PFC-70]
250   2e-70
WP_008136532.1 elongation factor G [Pseudoalteromonas sp. BSi...
251   2e-70
WP_024785878.1 elongation factor G [Streptococcus mutans]
251   2e-70
WP_028510794.1 elongation factor G [Ruminococcus sp. NK3A76]
251   2e-70
WP_108066792.1 elongation factor G [Vitiosangium sp. GDMCC 1....
251   2e-70
WP_072581463.1 elongation factor G [Bacillus weihaiensis]
251   2e-70
KRO56217.1 elongation factor G [Pelagibacteraceae bacterium B...
251   2e-70
WP_068727197.1 elongation factor G [Tepidibacillus decaturensis]
251   2e-70
WP_008633428.1 elongation factor G [Thermus parvatiensis]
251   2e-70
WP_017218327.1 elongation factor G [Pseudoalteromonas sp. NJ631]
251   2e-70
WP_033059782.1 GTP-binding protein [Clostridium sporogenes]
250   2e-70
WP_055040468.1 GTP-binding protein [Roseburia inulinivorans]
255   2e-70
KRL88797.1 translation elongation factor G [Lactobacillus kal...
249   2e-70
WP_040198065.1 elongation factor G [Candidatus Soleaferrea ma...
251   2e-70
WP_013255109.1 elongation factor G [Sediminispirochaeta smara...
250   2e-70
WP_020062290.1 elongation factor G [Bacillus sp. 123MFChir2]
251   2e-70
OKZ61310.1 translation elongation factor G [Clostridium sp. 4...
254   2e-70

WP_046999129.1 GTP-binding protein [Bifidobacterium adolescen...
250   2e-70
WP_073203332.1 elongation factor G [Gracilibacillus kekensis]
251   2e-70
WP_036580031.1 elongation factor G [Paenibacillus darwinianus]
251   2e-70
WP_050335477.1 elongation factor G [Campylobacter ureolyticus]
251   2e-70
WP_087611366.1 elongation factor G [Marinomonas sp. QM202]
251   2e-70
WP_053340906.1 GTP-binding protein [Clostridium botulinum]
250   2e-70
OGI26932.1 translation elongation factor G [Candidatus Moranb...
251   2e-70
WP_008291355.1 elongation factor G [Methylophaga thiooxydans]
251   2e-70
WP_098809316.1 elongation factor G [Bacillus sp. AFS041924]
251   2e-70
WP_095526879.1 elongation factor G [Actinobacteria bacterium ...
251   2e-70
WP_057565429.1 GTP-binding protein [Paeniclostridium sordellii]
250   2e-70
WP_012359570.1 elongation factor G [Corynebacterium urealyticum]
251   2e-70
WP_058548791.1 MULTISPECIES: elongation factor G [Pseudoalter...
251   2e-70
WP_027975825.1 elongation factor G [Streptococcus devriesei]
251   2e-70
WP_021801216.1 GTP-binding protein [Clostridium intestinale]
249   2e-70
OUW26578.1 elongation factor G [Rhizobiales bacterium TMED168]
251   2e-70
WP_008116254.1 GTP-binding protein [[Bacteroides] pectinophilus]
253   2e-70
WP_082210757.1 elongation factor G [Faecalibacterium prausnit...
251   2e-70
OGT41876.1 translation elongation factor G [Gammaproteobacter...
251   2e-70
CUQ52805.1 Tetracycline resistance protein tetM [[Ruminococcu...
256   2e-70
WP_043787610.1 elongation factor G [Amycolatopsis rifamycinica]
249   2e-70
PIT97614.1 elongation factor G [Candidatus Berkelbacteria bac...
251   2e-70
PIV65098.1 elongation factor G [Candidatus Nealsonbacteria ba...
251   2e-70
OGF52474.1 translation elongation factor G [Candidatus Giovan...
251   2e-70
WP_012501436.1 elongation factor G [Chlorobaculum parvum]
251   2e-70
WP_016843742.1 elongation factor G [Bradyrhizobium elkanii]
251   2e-70
BBD67779.1 protein synthesis factor, GTP-binding [Nostoc comm...
251   2e-70

WP_010872942.1 MULTISPECIES: elongation factor G [Synechocystis]
251   2e-70
WP_043408975.1 elongation factor G [Archangium violaceum]
251   2e-70
WP_012454024.1 elongation factor G [Methylobacterium populi]
251   2e-70
WP_033538392.1 elongation factor G [Shewanella sp. ECSMB14101]
251   2e-70
WP_107548585.1 elongation factor G [Staphylococcus xylosus]
251   2e-70
WP_104660478.1 elongation factor G [Enterococcus durans]
251   2e-70
WP_072233266.1 elongation factor G [Campylobacter coli]
251   2e-70
WP_048782836.1 elongation factor G [Streptococcus sp. 263_SSPC]
251   2e-70
WP_069637770.1 elongation factor G [Campylobacter pinnipediorum]
251   2e-70
WP_098958586.1 elongation factor G [Pseudonocardia sp. N23]
251   2e-70
WP_106318322.1 elongation factor G [Rhizobium tropici]
251   2e-70
WP_079603860.1 elongation factor G [Bradyrhizobium erythrophlei]
251   2e-70
WP_004208726.1 MULTISPECIES: elongation factor G [Sphingobium]
251   2e-70
WP_006442249.1 GTP-binding protein [[Clostridium] hylemonae]
254   2e-70
WP_089762176.1 elongation factor G [Halarsenatibacter silverm...
251   2e-70
WP_063485540.1 GTP-binding protein [Lactobacillus plantarum]
250   2e-70
WP_050625740.1 MULTISPECIES: elongation factor G [Bradyrhizob...
251   2e-70
WP_046202860.1 elongation factor G [Corynebacterium kroppenst...
251   2e-70
KPJ99869.1 elongation factor G [Gemmatimonas sp. SG8_28]
251   2e-70
WP_053206287.1 elongation factor G [Jiangella muralis]
251   2e-70
WP_016310712.1 MULTISPECIES: elongation factor G [Paenibacillus]
251   2e-70
WP_054016093.1 elongation factor G [Pseudoalteromonas sp. R3]
251   3e-70
WP_028050106.1 elongation factor G [Cellulomonas sp. URHD0024]
251   3e-70
WP_062904125.1 elongation factor G [Pediococcus damnosus]
251   3e-70
WP_085498984.1 MULTISPECIES: elongation factor G [Paenibacillus]
251   3e-70
OLC52098.1 translation elongation factor G [Acidobacteria bac...
251   3e-70
KKU34116.1 Elongation factor G [Candidatus Uhrbacteria bacter...
251   3e-70

WP_096606049.1 elongation factor G [Staphylococcus delphini]
251   3e-70
WP_107904032.1 elongation factor G [Bacillus sp. OV186]
251   3e-70
WP_036619500.1 elongation factor G [Paenibacillus macerans]
251   3e-70
WP_061038674.1 elongation factor G [Vibrio splendidus]
251   3e-70
WP_078121310.1 elongation factor G [Chromatiaceae bacterium 2...
251   3e-70
WP_057489966.1 elongation factor G [Streptococcus orisasini]
251   3e-70
WP_018164835.1 elongation factor G [Streptococcus henryi]
251   3e-70
WP_020559519.1 elongation factor G [Thiothrix flexilis]
251   3e-70
WP_066217567.1 elongation factor G [Balneola sp. EhC07]
251   3e-70
WP_069811010.1 elongation factor G [Chlorobaculum limnaeum]
251   3e-70
WP_044056756.1 elongation factor G [Alteromonas australica]
251   3e-70
WP_027822280.1 GTP-binding protein [Lactobacillus plantarum]
250   3e-70
AID38888.1 Tetracycline resistance protein TetM [Staphylococc...
234   3e-70
PIV39162.1 elongation factor G [Candidatus Omnitrophica bacte...
250   3e-70
KXB49586.1 translation elongation factor G [Corynebacterium k...
251   3e-70
WP_016116950.1 MULTISPECIES: elongation factor G [Bacillus ce...
250   3e-70
WP_012863405.1 elongation factor G [Sebaldella termitidis]
250   3e-70
WP_057539884.1 GTP-binding protein [Paeniclostridium sordellii]
250   3e-70
WP_041628919.1 elongation factor G [Corynebacterium kroppenst...
251   3e-70
WP_045101297.1 elongation factor G [Aliivibrio wodanis]
251   3e-70
WP_063998570.1 elongation factor G [Campylobacter hyointestin...
250   3e-70
KKT20529.1 Elongation factor G [Candidatus Giovannonibacteria...
251   3e-70
WP_090567786.1 MULTISPECIES: elongation factor G [Bacillales]
250   3e-70
WP_102115657.1 GTP-binding protein [Lactobacillus plantarum]
250   3e-70
WP_014276030.1 elongation factor G [Arthrospira platensis]
251   3e-70
KKR44767.1 Elongation factor G [Candidatus Moranbacteria bact...
251   3e-70
WP_074486077.1 elongation factor G [Streptococcus henryi]
250   3e-70

WP_053830265.1 elongation factor G [Helicobacter heilmannii]
250   3e-70
WP_087444230.1 elongation factor G [Paenibacillus thiaminolyt...
250   3e-70
PLX89747.1 elongation factor G [Desulfuromonas sp.]
250   3e-70
KPJ62621.1 elongation factor G [Planctomycetes bacterium DG_23]
250   3e-70
WP_029369151.1 MULTISPECIES: elongation factor G [Mycobacterium]
251   3e-70
WP_036652489.1 elongation factor G [Paenibacillus pini]
250   3e-70
WP_024786228.1 elongation factor G [Streptococcus mutans]
250   3e-70
WP_028926946.1 elongation factor G [Pseudonocardia acaciae]
251   3e-70
WP_053485949.1 GTP-binding protein [Lysinibacillus sp. FJAT-1...
249   3e-70
WP_105050919.1 elongation factor G [Psychrosphaera saromensis]
251   3e-70
KKQ86027.1 Elongation factor G [Candidatus Woesebacteria bact...
251   3e-70
WP_050347838.1 elongation factor G [Arsenicicoccus sp. oral t...
251   3e-70
WP_104640465.1 elongation factor G [Helicobacter bizzozeronii]
250   3e-70
WP_024932789.1 GTP-binding protein [Clostridium botulinum]
249   3e-70
WP_047914092.1 elongation factor G [Paenibacillus sp. TCA20]
250   3e-70
WP_071864206.1 elongation factor G [Enterococcus canintestini]
250   3e-70
WP_041326296.1 elongation factor G [Ruminococcus champanellen...
250   3e-70
WP_057734983.1 GTP-binding protein [Lactobacillus hammesii]
249   3e-70
WP_069799562.1 elongation factor G [Tepidibacillus sp. HK-1]
250   3e-70
KGM07401.1 Translation elongation factor G-like [Methylophaga...
251   3e-70
WP_022721914.1 elongation factor G [Rhodopseudomonas sp. B29]
250   3e-70
PHY12003.1 elongation factor G [Acidobacterium sp.]
251   3e-70
WP_039446224.1 elongation factor G [Thermotoga sp. Cell2]
250   3e-70
WP_064011015.1 elongation factor G [Fervidobacterium pennivor...
250   3e-70
WP_058305735.1 elongation factor G [Gracilibacillus massilien...
250   3e-70
WP_107240870.1 elongation factor G [Photobacterium frigidiphi...
250   3e-70
WP_104907513.1 elongation factor G [Nostoc sp. 'Lobaria pulmo...
250   3e-70

WP_062665456.1 elongation factor G [Grimontia celer]
250   3e-70
WP_083124560.1 elongation factor G [Mycobacterium tusciae]
251   3e-70
WP_003643647.1 GTP-binding protein [Lactobacillus plantarum]
250   3e-70
KTD47461.1 Elongation factor G 2 [Legionella quinlivanii]
251   3e-70
WP_038444250.1 GTP-binding protein [Bifidobacterium adolescen...
249   3e-70
WP_106233012.1 elongation factor G [Pleurocapsa sp. CCALA 161]
250   3e-70
WP_024782939.1 elongation factor G [Streptococcus mutans]
250   3e-70
WP_057878163.1 GTP-binding protein [Lactobacillus paucivorans]
249   3e-70
WP_061468158.1 GTP-binding protein [Lactobacillus plantarum]
250   3e-70
WP_107851444.1 elongation factor G [Oceanimonas sp. 102-Na3]
250   3e-70
WP_085461830.1 GTP-binding protein [Bifidobacterium adolescen...
249   3e-70
WP_016176307.1 elongation factor G [Enterococcus saccharolyti...
250   3e-70
WP_085408224.1 GTP-binding protein [Bifidobacterium adolescen...
249   3e-70
WP_046871291.1 elongation factor G [Pediococcus damnosus]
250   3e-70
OMG46883.1 translation elongation factor G [Paenibacillus mac...
250   3e-70
WP_106780177.1 GTP-binding protein [Brevibacillus sp. NRRL NR...
249   3e-70
WP_087350364.1 GTP-binding protein [Brevibacillus brevis]
249   3e-70
WP_103998014.1 elongation factor G [Paenibacillus sp. UNC499MF]
250   3e-70
WP_097770297.1 elongation factor G [Faecalibacterium prausnit...
251   3e-70
WP_096652072.1 elongation factor G [Staphylococcus delphini]
250   3e-70
PDH18649.1 elongation factor G [Pelagibacterales bacterium ME...
250   3e-70
WP_056945046.1 GTP-binding protein [Lactobacillus namurensis]
249   3e-70
WP_068591148.1 elongation factor G [Paenibacillus macquariensis]
250   3e-70
WP_015117214.1 elongation factor G [Rivularia sp. PCC 7116]
250   3e-70
EHQ89774.1 small GTP-binding protein domain protein [Desulfos...
254   3e-70
AAB19927.2 EF-G [Thermotoga maritima]
250   3e-70
WP_038811054.1 elongation factor G [Enterococcus faecium]
250   3e-70

PQB55767.1 elongation factor G [Enterococcus faecium]
233   3e-70
WP_100070640.1 GTP-binding protein [Lactobacillus backii]
249   3e-70
WP_016423664.1 elongation factor G [Paenibacillus sp. HGH0039]
250   3e-70
WP_076632829.1 GTP-binding protein [Lactobacillus plantarum]
250   3e-70
WP_028766989.1 elongation factor G [Shewanella fidelis]
250   3e-70
WP_058508473.1 elongation factor G [Legionella quinlivanii]
250   3e-70
WP_013712038.1 elongation factor G [Carnobacterium sp. 17-4]
250   3e-70
WP_107764198.1 elongation factor G [Coprothermobacter proteol...
250   3e-70
WP_074685289.1 GTP-binding protein [Kandleria vitulina]
253   3e-70
WP_038312209.1 elongation factor G [bacterium YEK0313]
250   3e-70
WP_073032198.1 elongation factor G [Desulfosporosinus lacus]
250   3e-70
WP_075534966.1 elongation factor G [Candidatus Pelagibacter u...
250   3e-70
WP_028352166.1 MULTISPECIES: elongation factor G [Bradyrhizob...
250   3e-70
WP_008400114.1 GTP-binding protein [Clostridium sp. L2-50]
254   3e-70
WP_057985531.1 MULTISPECIES: elongation factor G [Bacillaceae]
250   3e-70
WP_091312368.1 elongation factor G [Amycolatopsis tolypomycina]
249   3e-70
WP_062713376.1 elongation factor G [Streptomyces regalis]
250   3e-70
WP_085380801.1 GTP-binding protein [Bifidobacterium adolescen...
249   3e-70
WP_019136322.1 elongation factor G [Cellulomonas massiliensis]
250   3e-70
WP_042614102.1 elongation factor G [Stenotrophomonas maltophi...
250   3e-70
WP_061975666.1 elongation factor G [Fictibacillus enclensis]
250   3e-70
WP_040372536.1 elongation factor G [Bacillus psychrosaccharol...
250   3e-70
WP_019227366.1 elongation factor G [Sedimentibacter sp. B4]
250   3e-70
WP_009768832.1 elongation factor G [Oscillatoriales cyanobact...
250   3e-70
WP_107690279.1 elongation factor G [Coprothermobacter proteol...
250   3e-70
WP_092114996.1 elongation factor G [Bradyrhizobium erythrophlei]
250   4e-70
WP_095728810.1 MULTISPECIES: elongation factor G [Pseudoalter...
250   4e-70

WP_088084982.1 elongation factor G [Bacillus sp. OV166]
250   4e-70
WP_063723168.1 GTP-binding protein [Lactobacillus plantarum]
249   4e-70
WP_076235089.1 GTP-binding protein [Clostridium botulinum]
249   4e-70
KFF41940.1 translation elongation factor 2 (EF-2/EF-G) [Candi...
250   4e-70
WP_063700970.1 MULTISPECIES: elongation factor G [Pseudoalter...
250   4e-70
WP_106452634.1 elongation factor G [Zobellella taiwanensis]
250   4e-70
WP_042339699.1 GTP-binding protein [Desulfosporosinus youngiae]
254   4e-70
WP_056986280.1 elongation factor G [Pediococcus damnosus]
250   4e-70
WP_015523525.1 GTP-binding protein [Ruminococcus bromii]
254   4e-70
WP_107272270.1 elongation factor G [Photobacterium sanctipauli]
250   4e-70
WP_043930383.1 GTP-binding protein [Bacillus sp. EB01]
249   4e-70
WP_057019548.1 MULTISPECIES: elongation factor G [Bradyrhizob...
250   4e-70
WP_062430832.1 GTP-binding protein [Herbidospora daliensis]
248   4e-70
CDC05662.1 elongation factor G [Clostridium leptum CAG:27]
250   4e-70
WP_041101789.1 elongation factor G [Bacillus badius]
250   4e-70
WP_042235509.1 elongation factor G [Paenibacillus chitinolyti...
250   4e-70
KKU05206.1 Elongation factor G [Candidatus Giovannonibacteria...
250   4e-70
WP_066503524.1 elongation factor G [Clostridiales bacterium M...
250   4e-70
WP_003929600.1 elongation factor G [Mycobacterium vaccae]
250   4e-70
WP_068650943.1 elongation factor G [Paenibacillus antarcticus]
250   4e-70
WP_048313319.1 elongation factor G [Anaerobacillus macyae]
250   4e-70
WP_036122389.1 GTP-binding protein [Lysinibacillus sphaericus]
249   4e-70
OGH49897.1 translation elongation factor G [Candidatus Levyba...
250   4e-70
WP_100752919.1 elongation factor G [Vibrio salilacus]
250   4e-70
WP_013176383.1 elongation factor G [Syntrophothermus lipocali...
250   4e-70
WP_026958960.1 elongation factor G [Aliagarivorans taiwanensis]
250   4e-70
WP_020162682.1 elongation factor G [Cycloclasticus pugetii]
250   4e-70

WP_063440045.1 elongation factor G [Bacillus badius]
250   4e-70
WP_050402249.1 elongation factor G [Bradyrhizobium embrapense]
250   4e-70
WP_079346409.1 elongation factor G [Vagococcus martis]
250   4e-70
WP_005880821.1 MULTISPECIES: elongation factor G [Enterococcus]
250   4e-70
WP_035793477.1 GTP-binding protein [Butyrivibrio sp. AE3006]
254   4e-70
KXK00782.1 elongation factor G [Acidobacteria bacterium OLB17]
250   4e-70
WP_062350794.1 GTP-binding protein [Herbidospora yilanensis]
248   4e-70
WP_070123547.1 elongation factor G [Alteromonas confluentis]
250   4e-70
WP_068806355.1 GTP-binding protein [Pediococcus parvulus]
249   4e-70
WP_011100870.1 GTP-binding protein [Lactobacillus plantarum]
249   4e-70
WP_004334977.1 MULTISPECIES: elongation factor G [Pseudoalter...
250   4e-70
WP_070379202.1 elongation factor G [Rhodococcus sp. WMMA185]
250   4e-70
WP_062567513.1 elongation factor G [Pseudoalteromonas arabien...
250   4e-70
WP_092978358.1 elongation factor G [Ruminococcus bromii]
250   4e-70
WP_084099224.1 elongation factor G [Aerococcus suis]
250   4e-70
WP_056984447.1 GTP-binding protein [Lactobacillus oris]
249   4e-70
WP_057891254.1 elongation factor G [Lactobacillus nantensis]
250   4e-70
WP_009383850.1 elongation factor G [Staphylococcus massiliensis]
250   4e-70
WP_093523464.1 elongation factor G [Pseudovibrio ascidiaceicola]
250   4e-70
WP_095266985.1 GTP-binding protein [Paenibacillus campinasensis]
249   4e-70
WP_073586817.1 GTP-binding protein [Anaerocolumna xylanovorans]
254   4e-70
WP_054717766.1 elongation factor G [Lactobacillus manihotivor...
250   4e-70
WP_049520835.1 elongation factor G [Streptococcus oralis]
250   4e-70
WP_046860327.1 elongation factor G [Sedimenticola thiotaurini]
250   4e-70
WP_094585986.1 elongation factor G [Synechococcus sp. BO 8801]
250   4e-70
WP_044895190.1 elongation factor G [Bacillus alveayuensis]
250   4e-70
WP_101526608.1 GTP-binding protein [Clostridium botulinum]
249   4e-70

WP_106491940.1 GTP-binding protein [Blautia sp. SG-772]
254   4e-70
WP_066249390.1 elongation factor G [Bacillus drentensis]
250   4e-70
WP_096674901.1 elongation factor G [Fischerella sp. NIES-4106]
250   4e-70
WP_057773323.1 GTP-binding protein [Pediococcus inopinatus]
249   4e-70
CBK99301.1 translation elongation factor 2 (EF-2/EF-G) [Faeca...
250   4e-70
CCX89585.1 small GTP-binding protein domain [Clostridium sp. ...
253   4e-70
WP_038175031.1 elongation factor G [Vibrio pacinii]
250   4e-70
WP_070083183.1 GTP-binding protein [Lactobacillus plantarum]
249   4e-70
WP_044929289.1 GTP-binding protein [[Clostridium] leptum]
249   4e-70
WP_045540125.1 GTP-binding protein [Clostridium botulinum]
249   4e-70
WP_068777674.1 elongation factor G [Paenibacillus sp. FJAT-26...
250   4e-70
EEX68059.1 translation elongation factor G [Mitsuokella multa...
249   4e-70
WP_017262522.1 GTP-binding protein [Lactobacillus rossiae]
249   4e-70
WP_059121472.1 elongation factor G [Vibrio sp. MEBiC08052]
250   4e-70
WP_057556228.1 GTP-binding protein [Paeniclostridium sordellii]
249   4e-70
OGD05207.1 translation elongation factor G [Candidatus Amesba...
250   5e-70
WP_085438849.1 GTP-binding protein [Lactobacillus plantarum]
249   5e-70
WP_010731719.1 MULTISPECIES: elongation factor G [Enterococcus]
250   5e-70
WP_063845867.1 GTP-binding protein [Lactobacillus plantarum]
249   5e-70
WP_101898194.1 GTP-binding protein [Lactobacillus salivarius]
249   5e-70
PHS70748.1 elongation factor G [Methylophaga sp.]
250   5e-70
WP_042151970.1 MULTISPECIES: elongation factor G [Pseudoalter...
250   5e-70
WP_031200974.1 GTP-binding protein [Eubacterium sp. 14-2]
254   5e-70
KUK72726.1 Translation elongation factor G [Clostridiales bac...
250   5e-70
WP_023493334.1 elongation factor G [Methyloglobulus morosus]
250   5e-70
WP_103222495.1 elongation factor G [Labrenzia marina]
250   5e-70
WP_059432139.1 MULTISPECIES: elongation factor G [Campylobacter]
250   5e-70

WP_002309841.1 MULTISPECIES: elongation factor G [Enterococcus]
250   5e-70
WP_033162374.1 elongation factor G [Sharpea azabuensis]
250   5e-70
WP_011363347.1 elongation factor G [Synechococcus sp. CC9605]
250   5e-70
WP_010299428.1 elongation factor G [Candidatus Odyssella thes...
250   5e-70
WP_070085100.1 GTP-binding protein [Lactobacillus plantarum]
249   5e-70
WP_076546111.1 GTP-binding protein [Halanaerobium kushneri]
249   5e-70
WP_042363298.1 elongation factor G [Staphylococcus xylosus]
250   5e-70
WP_096575159.1 MULTISPECIES: elongation factor G [Nostocales]
250   5e-70
WP_074732210.1 elongation factor G [Sharpea azabuensis]
250   5e-70
WP_076655321.1 GTP-binding protein [Lactobacillus plantarum]
249   5e-70
WP_008523087.1 MULTISPECIES: elongation factor G [Jonquetella]
250   5e-70
SCH04273.1 Elongation factor G [uncultured Dorea sp.]
254   5e-70
WP_065763756.1 elongation factor G [Enterococcus faecium]
250   5e-70
WP_027400067.1 elongation factor G [Anaerovorax odorimutans]
249   5e-70
WP_102522177.1 elongation factor G [Vibrio tapetis]
250   5e-70
OGO34996.1 translation elongation factor G [Chloroflexi bacte...
250   5e-70
WP_029134277.1 elongation factor G [Sedimenticola selenatired...
250   5e-70
PIP71038.1 elongation factor G [Nitrospirae bacterium CG22_co...
250   5e-70
WP_077968977.1 elongation factor G [Streptomyces tsukubensis]
250   5e-70
WP_002902195.1 MULTISPECIES: elongation factor G [Streptococcus]
250   5e-70
OUW97985.1 elongation factor G [Pelagibacteraceae bacterium T...
250   5e-70
WP_040192560.1 GTP-binding protein [Clostridium sp. CL-6]
249   5e-70
WP_104878287.1 GTP-binding protein [Lactobacillus fermentum]
249   5e-70
ACR18569.1 elongation factor EF-G [Corynebacterium kroppenste...
250   5e-70
EGN47930.1 hypothetical protein HMPREF0991_01845 [Lachnospira...
251   5e-70
AEI79100.1 elongation factor G 2 [Cupriavidus necator N-1]
250   5e-70
WP_034562425.1 elongation factor G [Carnobacterium gallinarum]
250   5e-70

WP_063384342.1 elongation factor G [Bacillus badius]
250   5e-70
WP_046826835.1 elongation factor G [Afipia massiliensis]
249   5e-70
PIR43983.1 elongation factor G [Candidatus Wolfebacteria bact...
250   5e-70
KUO58209.1 elongation factor G [Gracilibacter sp. BRH_c7a]
250   5e-70
WP_062913106.1 elongation factor G [Pediococcus damnosus]
250   5e-70
WP_062909695.1 elongation factor G [Pediococcus damnosus]
250   5e-70
WP_090087883.1 GTP-binding protein [Clostridium uliginosum]
249   5e-70
WP_092724720.1 GTP-binding protein [Romboutsia lituseburensis]
249   5e-70
WP_096555032.1 elongation factor G [Staphylococcus delphini]
250   5e-70
WP_019166642.1 elongation factor G [Staphylococcus delphini]
250   5e-70
WP_104758762.1 elongation factor G [Helicobacter bizzozeronii]
250   5e-70
CBL03058.1 translation elongation factor 2 (EF-2/EF-G) [Faeca...
250   5e-70
WP_094372042.1 GTP-binding protein [Lactobacillus salivarius]
248   5e-70
WP_035236495.1 elongation factor G [Desulfobacter vibrioformis]
249   5e-70
PKL72271.1 elongation factor G [Candidatus Kuenenbacteria bac...
249   5e-70
WP_086248558.1 elongation factor G [Campylobacter sp. RM8965]
249   5e-70
WP_006715015.1 GTP-binding protein [Desulfitobacterium metall...
253   5e-70
OIP06852.1 translation elongation factor G [Candidatus Berkel...
250   5e-70
WP_041228508.1 elongation factor G [Cupriavidus necator]
250   5e-70
OUX91508.1 elongation factor G [Alteromonas sp. TMED35]
250   5e-70
WP_087842112.1 GTP-binding protein [Lactobacillus plantarum]
249   5e-70
OTF89903.1 elongation factor G [Lactobacillus salivarius]
249   5e-70
WP_021260870.1 GTP-binding protein [Paenibacillus alvei]
249   5e-70
WP_025254791.1 elongation factor G [Alteromonas sp. ALT199]
249   5e-70
WP_095227357.1 elongation factor G [Helicobacter sp. 13S00401-1]
249   5e-70
WP_104066610.1 elongation factor G [Campylobacter hyointestin...
249   5e-70
WP_018712744.1 elongation factor G [Campylobacter ureolyticus]
249   5e-70

WP_006619094.1 elongation factor G [Arthrospira platensis]
250   5e-70
WP_036125633.1 MULTISPECIES: GTP-binding protein [Lysinibacil...
249   5e-70
WP_107554887.1 elongation factor G [Staphylococcus xylosus]
250   5e-70
WP_015919483.1 elongation factor G [Thermotoga neapolitana]
250   5e-70
WP_084943301.1 elongation factor G [Streptococcus oralis]
249   5e-70
OGC93020.1 translation elongation factor G [Candidatus Amesba...
250   5e-70
WP_041966527.1 elongation factor G [Bacillus selenatarsenatis]
249   5e-70
PIT94988.1 elongation factor G [Candidatus Falkowbacteria bac...
249   5e-70
WP_076922352.1 elongation factor G [Pseudoalteromonas sp. SK20]
249   5e-70
WP_087310882.1 GTP-binding protein [Lactobacillus salivarius]
248   6e-70
OJF16397.1 translation elongation factor G [Bacillaceae bacte...
249   6e-70
WP_069790791.1 elongation factor G [Cyanobacterium sp. IPPAS ...
249   6e-70
OLC23543.1 translation elongation factor G [Chloroflexi bacte...
249   6e-70
WP_049552869.1 elongation factor G [Streptococcus sanguinis]
249   6e-70
WP_091161592.1 elongation factor G [Paenibacillus sp. 453mf]
249   6e-70
WP_006676487.1 elongation factor G [Paenibacillus dendritifor...
249   6e-70
WP_063489839.1 GTP-binding protein [Lactobacillus plantarum]
249   6e-70
WP_046037859.1 GTP-binding protein [Lactobacillus plantarum]
249   6e-70
WP_071457769.1 elongation factor G [Vagococcus teuberi]
249   6e-70
WP_006881028.1 elongation factor G [Vibrio brasiliensis]
249   6e-70
SCJ02929.1 Tetracycline resistance protein tetM [uncultured R...
253   6e-70
WP_008679825.1 MULTISPECIES: GTP-binding protein [Clostridium]
249   6e-70
WP_036145897.1 GTP-binding protein [Lysinibacillus fusiformis]
248   6e-70
WP_104706391.1 elongation factor G [Helicobacter bizzozeronii]
249   6e-70
WP_098445971.1 elongation factor G [Bacillus sp. es.036]
249   6e-70
WP_096364375.1 GTP-binding protein [Lysinibacillus fusiformis]
248   6e-70
PIP28423.1 elongation factor G [Candidatus Moranbacteria bact...
249   6e-70

WP_038352020.1 elongation factor G [Eubacterium limosum]
249   6e-70
PKL04290.1 elongation factor G [Synergistetes bacterium HGW-S...
249   6e-70
KPA11652.1 elongation factor P [Candidatus Magnetomorum sp. H...
249   6e-70
PIW90299.1 elongation factor G [Nitrospirae bacterium CG_4_8_...
249   6e-70
WP_007378013.1 MULTISPECIES: elongation factor G [Pseudoalter...
249   6e-70
WP_045213697.1 elongation factor G [Desulfonatronovibrio magnus]
249   6e-70
ESQ08206.1 hypothetical protein N839_06715 [uncultured Desulf...
249   6e-70
KJR41087.1 translation elongation factor 2 (EF-2/EF-G) [Candi...
249   6e-70
WP_028520478.1 GTP-binding protein [Ruminococcus flavefaciens]
253   6e-70
OIP28623.1 translation elongation factor G [Dehalococcoidia b...
249   6e-70
WP_016390898.1 MULTISPECIES: elongation factor G [Cycloclasti...
249   6e-70
WP_096647868.1 elongation factor G [Staphylococcus delphini]
249   6e-70
WP_008195163.1 elongation factor G [Labrenzia alexandrii]
249   6e-70
OGK77830.1 translation elongation factor G [Candidatus Rokuba...
249   6e-70
WP_101052791.1 elongation factor G [Shewanella chilikensis]
249   6e-70
WP_075663213.1 elongation factor G [Corynebacterium frankenfo...
250   6e-70
WP_086305830.1 MULTISPECIES: elongation factor G [Enterococcus]
249   6e-70
WP_066058583.1 elongation factor G [Bacillus korlensis]
249   6e-70
WP_026719867.1 elongation factor G [Fischerella sp. PCC 9431]
249   6e-70
WP_006059338.1 GTP-binding protein [Holdemania filiformis]
253   6e-70
WP_066834240.1 GTP-binding protein [Collinsella ihuae]
249   6e-70
WP_044010299.1 GTP-binding protein [Lactobacillus wasatchensis]
249   6e-70
KKQ26886.1 Elongation factor G [Candidatus Levybacteria bacte...
249   6e-70
BBA79599.1 elongation factor G [cyanobacterium endosymbiont o...
249   6e-70
CCY60805.1 putative translation elongation factor G [Clostrid...
253   6e-70
WP_002290441.1 MULTISPECIES: elongation factor G [Enterococcus]
249   6e-70
WP_074029531.1 GTP-binding protein [Lactobacillus plantarum]
249   6e-70

WP_042202211.1 GTP-binding protein [Paenibacillus camerounensis]
249   6e-70
OFV82485.1 translation elongation factor G [Acidobacteria bac...
249   7e-70
WP_010782065.1 elongation factor G [Enterococcus gilvus]
249   7e-70
WP_071315268.1 elongation factor G [Anaerobacillus isosacchar...
249   7e-70
WP_028472763.1 elongation factor G [Nocardioides alkalitolerans]
249   7e-70
OLA47208.1 translation elongation factor G [Ruminococcus bici...
253   7e-70
WP_104638210.1 elongation factor G [Helicobacter bizzozeronii]
249   7e-70
WP_062139721.1 elongation factor G [Caedimonas varicaedens]
249   7e-70
SHK06230.1 elongation factor G [Geosporobacter subterraneus D...
249   7e-70
WP_081529514.1 GTP-binding protein [Lactobacillus salivarius]
248   7e-70
WP_097802192.1 elongation factor G [Faecalibacterium prausnit...
250   7e-70
SFE44034.1 small GTP-binding protein domain-containing protei...
253   7e-70
KKS94328.1 Elongation factor G [Parcubacteria group bacterium...
249   7e-70
WP_045111328.1 elongation factor G [Moritella viscosa]
249   7e-70
WP_037472406.1 MULTISPECIES: elongation factor G [Sphingobium]
249   7e-70
WP_010754773.1 elongation factor G [Enterococcus asini]
249   7e-70
WP_007990371.1 MULTISPECIES: elongation factor G [Alteromonad...
249   7e-70
WP_017040336.1 elongation factor G [Vibrio genomosp. F10]
249   7e-70
WP_024781486.1 elongation factor G [Streptococcus mutans]
249   7e-70
WP_063872934.1 elongation factor G [Nodularia spumigena]
249   7e-70
WP_059435429.1 MULTISPECIES: elongation factor G [Campylobacter]
249   7e-70
WP_015109450.1 MULTISPECIES: elongation factor G [Cyanobacteria]
249   7e-70
WP_036943533.1 elongation factor G [Pseudobacteroides cellulo...
249   7e-70
WP_055161229.1 GTP-binding protein [Anaerostipes hadrus]
253   7e-70
WP_057766019.1 GTP-binding protein [Lactobacillus tucceti]
248   7e-70
WP_018963297.1 elongation factor G [Coprothermobacter platensis]
249   7e-70
WP_100382420.1 elongation factor G [Afipia broomeae]
249   7e-70

WP_057849130.1 elongation factor G [Bradyrhizobium valentinum]
249   7e-70
WP_008174819.1 GTP-binding protein [Bacillus sp. B14905]
248   7e-70
SCJ21485.1 Elongation factor G [uncultured Ruminococcus sp.]
253   7e-70
WP_013156108.1 elongation factor G [Nocardiopsis dassonvillei]
249   7e-70
WP_081028376.1 elongation factor G [Faecalibacterium prausnit...
249   7e-70
OIO37618.1 translation elongation factor G [Candidatus Omnitr...
249   7e-70
WP_010814028.1 MULTISPECIES: elongation factor G [Cupriavidus]
249   7e-70
WP_072690598.1 elongation factor G [Rhodococcus marinonascens]
249   7e-70
WP_066542794.1 elongation factor G [Caryophanon tenue]
249   7e-70
WP_103354190.1 elongation factor G [Amycolatopsis sp. CA-128772]
248   7e-70
WP_073185569.1 elongation factor G [Anaerosphaera aminiphila]
249   7e-70
PID31125.1 elongation factor G [Candidatus Cloacimonetes bact...
249   7e-70
OQX65622.1 elongation factor G [Anaerolinea sp. 4484_236]
249   7e-70
WP_003079725.1 elongation factor G [Streptococcus macacae]
249   7e-70
WP_004606698.1 GTP-binding protein [[Clostridium] scindens]
253   7e-70
CUQ74317.1 Tetracycline resistance protein tetM from transpos...
253   7e-70
WP_045953930.1 elongation factor G [Vibrio galatheae]
249   7e-70
KKU92971.1 Elongation factor G [Candidatus Yanofskybacteria b...
249   7e-70
WP_008521539.1 elongation factor G [Jonquetella anthropi]
249   7e-70
OGK55061.1 translation elongation factor G [Candidatus Roizma...
249   7e-70
OHC97444.1 translation elongation factor G [Sphingomonadales ...
249   7e-70
WP_069065536.1 elongation factor G [Sphingobium sp. RAC03]
249   7e-70
WP_075476842.1 elongation factor G [Moritella viscosa]
249   8e-70
CCZ94516.1 elongation factor G 1 [Corallococcus sp. CAG:1435]
249   8e-70
WP_071662577.1 elongation factor G [Candidatus Rickettsiella ...
249   8e-70
KKR11571.1 Elongation factor G [Candidatus Woesebacteria bact...
250   8e-70
WP_071160258.1 elongation factor G [Methylomonas sp. LWB]
249   8e-70

CDA62318.1 elongation factor G [Clostridium sp. CAG:169]
249   8e-70
OGO44787.1 translation elongation factor G [Chloroflexi bacte...
249   8e-70
WP_066626632.1 GTP-binding protein [Clostridium magnum]
253   8e-70
PDH41716.1 elongation factor G [OM182 bacterium MED-G24]
249   8e-70
WP_027754780.1 elongation factor G [Streptomyces sp. CNH099]
249   8e-70
WP_068600247.1 elongation factor G [Paenibacillus macquariensis]
249   8e-70
WP_006196401.1 elongation factor G [Nodularia spumigena]
249   8e-70
WP_055015545.1 MULTISPECIES: elongation factor G [Pseudoalter...
249   8e-70
WP_046772614.1 elongation factor G [Jiangella alkaliphila]
249   8e-70
WP_029211822.1 elongation factor G [Arsenicicoccus bolidensis]
249   8e-70
WP_083624165.1 elongation factor G [Planktothrix serta]
249   8e-70
WP_033608170.1 GTP-binding protein [Lactobacillus plantarum]
249   8e-70
ALE81903.1 elongation factor G [Pseudonocardia sp. HH130629-09]
249   8e-70
WP_099329811.1 GTP-binding protein [Clostridium paraputrificum]
248   8e-70
WP_088535214.1 elongation factor G [Geobacter sp. DSM 9736]
249   8e-70
WP_013890395.1 elongation factor G [Helicobacter bizzozeronii]
249   8e-70
WP_101636951.1 elongation factor G [Campylobacter ureolyticus]
249   8e-70
WP_088535038.1 elongation factor G [Geobacter sp. DSM 9736]
249   8e-70
SDR99885.1 translation elongation factor 2 (EF-2/EF-G) [Brady...
249   8e-70
WP_015774942.1 elongation factor G [Desulfomicrobium baculatum]
249   8e-70
WP_107989431.1 elongation factor G [Breoghania corrubedonensis]
249   8e-70
OUU67074.1 elongation factor G [Candidatus Pelagibacter sp. T...
249   8e-70
WP_097792477.1 elongation factor G [Faecalibacterium prausnit...
249   8e-70
WP_004625701.1 GTP-binding protein [[Clostridium] termitidis]
249   8e-70
PSD20233.1 elongation factor G [Stenotrophomonas maltophilia]
249   8e-70
WP_099295182.1 elongation factor G [Pseudoalteromonas sp. 3D05]
249   8e-70
WP_097780182.1 elongation factor G [Faecalibacterium prausnit...
249   8e-70

WP_074831076.1 GTP-binding protein [Ruminococcus albus]
253   8e-70
WP_081531099.1 GTP-binding protein [Lactobacillus salivarius]
248   8e-70
OGG20547.1 translation elongation factor G [Candidatus Gottes...
249   8e-70
WP_024111006.1 elongation factor G [Propionimicrobium sp. BV2F7]
249   8e-70
OQX01800.1 translation elongation factor G [Thiothrix lacustris]
249   8e-70
WP_013770162.1 elongation factor G [Cellulomonas fimi]
249   8e-70
WP_068367602.1 elongation factor G [Peptoniphilus coxii]
249   8e-70
WP_104740689.1 elongation factor G [Helicobacter bizzozeronii]
249   8e-70
WP_103202272.1 GTP-binding protein [Herbinix hemicellulosilyt...
253   8e-70
WP_037288051.1 GTP-binding protein [Ruminococcus albus]
252   8e-70
WP_081511418.1 GTP-binding protein [Lactobacillus salivarius]
248   8e-70
OUU45851.1 elongation factor G [Gammaproteobacteria bacterium...
249   8e-70
WP_024962193.1 elongation factor G [Campylobacter ureolyticus]
249   8e-70
CDD40661.1 putative uncharacterized protein [Collinsella sp. ...
249   9e-70
WP_089984469.1 MULTISPECIES: GTP-binding protein [Lysinibacil...
248   9e-70
WP_056938376.1 GTP-binding protein [Lactobacillus hayakitensis]
248   9e-70
CCX83865.1 small GTP-binding protein domain [Ruminococcus sp....
253   9e-70
WP_055339794.1 GTP-binding protein [Paeniclostridium sordellii]
248   9e-70
WP_014614745.1 MULTISPECIES: elongation factor G [Staphylococ...
249   9e-70
WP_078769102.1 GTP-binding protein [Eubacterium coprostanolig...
252   9e-70
WP_066254567.1 GTP-binding protein [Bacillus drentensis]
248   9e-70
PIR13025.1 elongation factor G [Candidatus Falkowbacteria bac...
249   9e-70
WP_034963282.1 MULTISPECIES: elongation factor G [Campylobacter]
249   9e-70
WP_014162797.1 elongation factor G [Thermovirga lienii]
249   9e-70
WP_057775778.1 GTP-binding protein [Bacillus sp. FJAT-25496]
248   9e-70
OGV63651.1 translation elongation factor G [Lentisphaerae bac...
249   9e-70
WP_013784676.1 elongation factor G [Alteromonas naphthalenivo...
249   9e-70

WP_008538886.1 MULTISPECIES: elongation factor G [Megamonas]
249   9e-70
WP_021682584.1 elongation factor G [Ruminococcus callidus]
249   9e-70
WP_064025317.1 elongation factor G [Methylomonas koyamae]
249   9e-70
WP_105307868.1 GTP-binding protein [Lactobacillus plantarum]
249   9e-70
WP_099034312.1 elongation factor G [Lacimicrobium alkaliphilum]
249   9e-70
WP_023648424.1 elongation factor G [Candidatus Pelagibacter u...
249   9e-70
WP_042176629.1 GTP-binding protein [Paenibacillus sp. FSL R7-...
248   9e-70
PIY68848.1 elongation factor G [Candidatus Roizmanbacteria ba...
248   9e-70
WP_102280580.1 elongation factor G [Lactobacillus timonensis]
249   9e-70
WP_011992520.1 elongation factor G [Campylobacter curvus]
249   9e-70
WP_015780022.1 elongation factor G [Kytococcus sedentarius]
249   9e-70
WP_057894745.1 GTP-binding protein [Lactobacillus brantae]
248   9e-70
CDD54103.1 elongation factor G [Ruminococcus sp. CAG:379]
249   9e-70
WP_027699449.1 elongation factor G [Weissella oryzae]
249   9e-70
WP_069294410.1 elongation factor G [Pelagibacteraceae bacteri...
249   9e-70
WP_028657518.1 MULTISPECIES: elongation factor G [Novosphingo...
249   9e-70
WP_097148707.1 GTP-binding protein [Lysinibacillus acetophenoni]
248   9e-70
OGH23609.1 translation elongation factor G [Candidatus Levyba...
249   9e-70
OUX09743.1 elongation factor G [Candidatus Pelagibacter sp. T...
249   9e-70
WP_016147576.1 elongation factor G [Butyricicoccus pullicaeco...
249   9e-70
WP_072696614.1 elongation factor G [Desulfovibrio litoralis]
249   9e-70
OHC90987.1 translation elongation factor G [Sphingomonadales ...
249   1e-69
WP_054115690.1 elongation factor G [Enterococcus sp. RIT-PI-f]
249   1e-69
PIS34601.1 elongation factor G [Parcubacteria group bacterium...
249   1e-69
PIR60535.1 elongation factor G [Candidatus Pacebacteria bacte...
249   1e-69
PIE00625.1 elongation factor G [Thiothrix nivea]
249   1e-69
OUW44189.1 elongation factor G [Acidimicrobiaceae bacterium T...
249   1e-69

WP_056948563.1 GTP-binding protein [Lactobacillus odoratitofui]
248   1e-69
WP_087325872.1 GTP-binding protein [Clostridium perfringens]
248   1e-69
WP_015336998.1 elongation factor G [Desulfovibrio hydrotherma...
249   1e-69
OGE11689.1 translation elongation factor G [Candidatus Curtis...
248   1e-69
WP_024346648.1 GTP-binding protein [[Clostridium] methoxybenz...
248   1e-69
WP_074956966.1 elongation factor G [Myxococcus fulvus]
249   1e-69
WP_011558403.1 MULTISPECIES: elongation factor G [Mycobacterium]
249   1e-69
WP_075724661.1 elongation factor G [Corynebacterium aquilae]
249   1e-69
WP_103272442.1 elongation factor G [Achromobacter sp. AONIH1]
249   1e-69
WP_077335154.1 elongation factor G [Vibrio ruber]
249   1e-69
WP_002294317.1 MULTISPECIES: elongation factor G [Enterococcus]
249   1e-69
PQC13294.1 tetracycline resistance ribosomal protection prote...
235   1e-69
PHX89143.1 elongation factor G [Pelagibacteraceae bacterium]
249   1e-69
WP_076232058.1 GTP-binding protein [Clostridium botulinum]
248   1e-69
SEA58371.1 small GTP-binding protein domain-containing protei...
252   1e-69
WP_091079412.1 elongation factor G [Micromonospora nigra]
249   1e-69
KPJ83599.1 elongation factor G [Spirochaetes bacterium DG_61]
248   1e-69
WP_032808409.1 elongation factor G [Oenococcus oeni]
249   1e-69
WP_058843194.1 GTP-binding protein [Lysinibacillus sp. F5]
248   1e-69
WP_054202747.1 MULTISPECIES: elongation factor G [Pseudoalter...
249   1e-69
WP_008108561.1 MULTISPECIES: elongation factor G [Pseudoalter...
249   1e-69
WP_100391429.1 elongation factor G [Desulfofaba hansenii]
248   1e-69
WP_071188297.1 elongation factor G [Trichormus sp. NMC-1]
249   1e-69
WP_063573678.1 elongation factor G [Luteibacter rhizovicinus]
249   1e-69
OIP71221.1 translation elongation factor G [Oscillatoriales c...
249   1e-69
WP_069070113.1 elongation factor G [Nostoc sp. KVJ20]
249   1e-69
SCJ97430.1 Elongation factor G [uncultured Clostridium sp.]
248   1e-69

WP_095759015.1 GTP-binding protein [Lactobacillus salivarius]
248   1e-69
OGY98914.1 translation elongation factor G [Candidatus Lipton...
249   1e-69
WP_059105433.1 elongation factor G [Bacillus shacheensis]
249   1e-69
WP_044878722.1 elongation factor G [Paenibacillus sp. IHBB 10...
249   1e-69
WP_012594863.1 MULTISPECIES: elongation factor G [Cyanothece]
249   1e-69
OUX52285.1 elongation factor G [Candidatus Pelagibacter sp. T...
249   1e-69
EJX83305.1 elongation factor G [Enterococcus faecium ERV99]
231   1e-69
WP_058552757.1 elongation factor G [Pseudoalteromonas sp. 10-33]
249   1e-69
WP_057792668.1 MULTISPECIES: elongation factor G [Alteromonas]
249   1e-69
KJS49200.1 elongation factor G [Peptococcaceae bacterium BRH_...
248   1e-69
WP_007099834.1 elongation factor G [Synechococcus sp. RS9916]
249   1e-69
WP_025729602.1 elongation factor G [Atopobacter phocae]
249   1e-69
WP_049530761.1 elongation factor G [Streptococcus pseudopneum...
249   1e-69
WP_003539659.1 MULTISPECIES: elongation factor G [Erysipelotr...
249   1e-69
WP_072823591.1 elongation factor G [Bradyrhizobium erythrophlei]
249   1e-69
WP_072956113.1 elongation factor G [Vibrio gazogenes]
249   1e-69
WP_079217923.1 MULTISPECIES: elongation factor G [Herbaspiril...
249   1e-69
WP_101070252.1 GTP-binding protein [Ruminococcus bromii]
252   1e-69
WP_027089792.1 elongation factor G [[Clostridium] saccharogumia]
249   1e-69
WP_012543752.1 elongation factor G [Coprothermobacter proteol...
249   1e-69
WP_024575000.1 MULTISPECIES: elongation factor G [Afipia]
249   1e-69
WP_046497492.1 elongation factor G [Syntrophomonas zehnderi]
248   1e-69
WP_025633017.1 elongation factor G [Vibrio parahaemolyticus]
249   1e-69
KIT57219.1 elongation factor P [Vibrio parahaemolyticus EN970...
249   1e-69
WP_086254135.1 MULTISPECIES: elongation factor G [Campylobacter]
249   1e-69
WP_019911111.1 GTP-binding protein [Paenibacillus sp. HW567]
248   1e-69
KPJ54903.1 elongation factor G [Parcubacteria bacterium DG_72]
249   1e-69

WP_066523481.1 elongation factor G [Christensenella minuta]
249   1e-69
EXY76976.1 elongation factor G, domain IV family protein [Bac...
239   1e-69
WP_023179574.1 elongation factor G [Carnobacterium inhibens]
249   1e-69
WP_099037226.1 elongation factor G [Mycobacterium sp. CECT 8778]
249   1e-69
OGZ71491.1 translation elongation factor G [Candidatus Staska...
249   1e-69
OLA52029.1 translation elongation factor G [Ruminococcus sp. ...
252   1e-69
OED34175.1 translation elongation factor G [PVC group bacteri...
249   1e-69
WP_053530524.1 GTP-binding protein [Clostridium botulinum]
248   1e-69
WP_014209179.1 elongation factor G [Mycobacterium rhodesiae]
249   1e-69
WP_041276493.1 GTP-binding protein [Desulfosporosinus acidiph...
253   1e-69
WP_006021071.1 elongation factor G [Afipia broomeae]
249   1e-69
WP_018018649.1 elongation factor G [Corynebacterium ciconiae]
249   1e-69
WP_086874541.1 GTP-binding protein [Lactobacillus johnsonii]
248   1e-69
WP_087378737.1 elongation factor G [Anaeromassilibacillus sp....
249   1e-69
WP_105368582.1 GTP-binding protein [Herbivorax saccincola]
252   1e-69
CDB68227.1 elongation factor EF-G [Eubacterium sp. CAG:252]
252   1e-69
OGO46660.1 translation elongation factor G [Chloroflexi bacte...
248   1e-69
WP_049179017.1 GTP-binding protein [Clostridium botulinum]
248   1e-69
WP_009801140.1 elongation factor G [Oceanicaulis sp. HTCC2633]
249   1e-69
WP_059427707.1 elongation factor G [Campylobacter fetus]
249   1e-69
WP_089268268.1 MULTISPECIES: elongation factor G [Tardiphaga]
249   1e-69
WP_024858492.1 GTP-binding protein [Ruminococcus albus]
252   1e-69
WP_056585864.1 elongation factor G [Cellulomonas sp. Leaf334]
249   1e-69
CIV74790.1 elongation factor G [Streptococcus pneumoniae]
248   1e-69
OGT40086.1 translation elongation factor G [Gammaproteobacter...
249   1e-69
WP_086432893.1 GTP-binding protein [Eggerthia catenaformis]
251   1e-69
WP_103649433.1 elongation factor G [Campylobacter concisus]
249   1e-69

WP_089752379.1 elongation factor G [Halobacillus alkaliphilus]
249   1e-69
SCI86862.1 Tetracycline resistance protein tetM from transpos...
248   1e-69
KKP94068.1 Elongation factor G [Candidatus Moranbacteria bact...
249   1e-69
WP_097838602.1 elongation factor G [Faecalibacterium prausnit...
249   1e-69
WP_046713543.1 elongation factor G [Myxococcus fulvus]
249   1e-69
WP_092267337.1 GTP-binding protein [Brevibacillus sp. OK042]
248   1e-69
WP_021257364.1 MULTISPECIES: GTP-binding protein [Paenibacillus]
248   1e-69
WP_064662704.1 elongation factor G [Pseudoalteromonas sp. MQS...
249   1e-69
WP_057564590.1 GTP-binding protein [Paeniclostridium sordellii]
248   1e-69
OUT94900.1 elongation factor G [Rhizobiales bacterium TMED29]
249   1e-69
PLS76064.1 elongation factor G [Actinobacteria bacterium]
249   1e-69
WP_045575451.1 elongation factor G [Desulfosporosinus sp. I2]
249   1e-69
WP_063718162.1 elongation factor G [Leptolyngbya valderiana]
249   1e-69
WP_088330454.1 elongation factor G [Lacimicrobium sp. SS2-24]
249   1e-69
WP_006959822.1 elongation factor G [Vibrio coralliilyticus]
249   1e-69
OQX02366.1 translation elongation factor G [Desulfobacteracea...
248   1e-69
WP_035796504.1 GTP-binding protein [Butyrivibrio sp. MB2005]
252   1e-69
WP_088044562.1 elongation factor G [Bacillus sp. EAC]
249   1e-69
WP_090649059.1 elongation factor G [Paenibacillus sp. UNC496MF]
249   1e-69
WP_016173509.1 elongation factor G [Enterococcus dispar]
249   1e-69
SEK52715.1 small GTP-binding protein domain-containing protei...
252   1e-69
AFM41654.1 small GTP-binding protein domain protein [Desulfos...
252   1e-69
WP_020879814.1 elongation factor G [Desulfovibrio sp. X2]
248   1e-69
WP_074686019.1 elongation factor G [Kandleria vitulina]
249   1e-69
SCJ73837.1 Elongation factor G [uncultured Blautia sp.]
253   1e-69
WP_021356239.1 GTP-binding protein [Lactobacillus plantarum]
248   1e-69
PIS42277.1 elongation factor G [Candidatus Kerfeldbacteria ba...
249   1e-69

WP_018998535.1 elongation factor G [Megamonas rupellensis]
249   1e-69
WP_075672342.1 GTP-binding protein [Anaerostipes sp. 992a]
252   1e-69
WP_014426761.1 elongation factor G [Rubrivivax gelatinosus]
248   1e-69
KKS16856.1 Elongation factor G [candidate division WWE3 bacte...
249   1e-69
WP_016646699.1 elongation factor G [Campylobacter ureolyticus]
248   1e-69
WP_028953573.1 elongation factor G [Synechococcus sp. CC9616]
248   1e-69
WP_060776013.1 elongation factor G [Enterococcus faecium]
249   1e-69
WP_103205496.1 GTP-binding protein [Lactobacillus fermentum]
248   1e-69
WP_067040324.1 elongation factor G [Moritella sp. JT01]
249   1e-69
OGQ44180.1 translation elongation factor G [Deltaproteobacter...
248   1e-69
EFO54203.1 translation elongation factor G [Streptococcus inf...
249   1e-69
WP_014641418.1 elongation factor G [Halobacillus halophilus]
248   1e-69
WP_094555933.1 elongation factor G [Synechococcus sp. 1G10]
248   1e-69
OUX22435.1 elongation factor G [Candidatus Pelagibacter sp. T...
248   1e-69
WP_007058952.1 GTP-binding protein [Clostridium carboxidivorans]
252   1e-69
WP_055176974.1 GTP-binding protein [[Clostridium] symbiosum]
253   1e-69
WP_039873047.1 GTP-binding protein [Paenibacillus sp. FSL R7-...
248   1e-69
WP_066911120.1 elongation factor G [Millisia brevis]
249   1e-69
WP_092952638.1 elongation factor G [Sphingobium faniae]
248   1e-69
WP_104685343.1 elongation factor G [Helicobacter bizzozeronii]
248   1e-69
WP_017144235.1 GTP-binding protein [Coprobacillus sp. D6]
252   1e-69
WP_005270083.1 elongation factor G [Arthrobacter crystallopoi...
249   1e-69
WP_035165777.1 GTP-binding protein [Lactobacillus salivarius]
247   1e-69
SCI41324.1 Elongation factor G [uncultured Ruminococcus sp.]
252   1e-69
WP_106874159.1 elongation factor G [Candidatus Phycorickettsi...
248   1e-69
WP_055232789.1 GTP-binding protein [Anaerostipes hadrus]
252   1e-69
WP_075868551.1 elongation factor G [Kytococcus sp. CUA-901]
249   1e-69

WP_069665551.1 elongation factor G [Vibrio europaeus]
249   1e-69
WP_068151643.1 elongation factor G [Rhodococcus corynebacteri...
249   1e-69
WP_056963289.1 GTP-binding protein [Lactobacillus spicheri]
248   1e-69
WP_086478991.1 elongation factor G [Oceanospirillum sanctuarii]
248   1e-69
WP_014949028.1 elongation factor G [Alteromonas macleodii]
248   1e-69
WP_091072718.1 elongation factor G [Paenibacillus sp. NFR01]
248   1e-69
WP_096140981.1 elongation factor G [Shewanella algae]
249   1e-69
WP_040285479.1 elongation factor G [Sporosarcina koreensis]
248   1e-69
OUV13197.1 elongation factor G [Rhizobiales bacterium TMED94]
248   1e-69
WP_011618353.1 elongation factor G [Synechococcus sp. CC9311]
248   1e-69
WP_020330549.1 MULTISPECIES: elongation factor G [Vibrio]
248   1e-69
WP_012155042.1 elongation factor G [Shewanella pealeana]
248   1e-69
WP_009371285.1 GTP-binding protein [Lysinibacillus fusiformis]
247   2e-69
WP_053457133.1 elongation factor G [Hapalosiphon sp. MRB220]
248   2e-69
WP_095563684.1 elongation factor G [Plantactinospora sp. KBS50]
249   2e-69
WP_024722188.1 elongation factor G [Clostridiales bacterium V...
248   2e-69
WP_070853701.1 elongation factor G [Enterococcus sp. HMSC070F12]
248   2e-69
WP_005456593.1 MULTISPECIES: elongation factor G [Vibrio]
248   2e-69
WP_004747811.1 elongation factor G [Vibrio tubiashii]
248   2e-69
KRT72345.1 translation elongation factor G, elongation factor...
248   2e-69
PIQ00517.1 elongation factor G [Shewanella sp. CG18_big_fil_W...
248   2e-69
WP_009298315.1 GTP-binding protein [Clostridium sp. 7_3_54FAA]
252   2e-69
WP_077275940.1 elongation factor G [Vagococcus penaei]
248   2e-69
WP_055286657.1 GTP-binding protein [Collinsella aerofaciens]
248   2e-69
WP_040225897.1 elongation factor G [Bhargavaea cecembensis]
248   2e-69
WP_019138506.1 MULTISPECIES: elongation factor G [Peptoniphilus]
248   2e-69
WP_043152131.1 elongation factor G [Sphingobium sp. Ant17]
248   2e-69

WP_094926676.1 elongation factor G [Pseudonocardia sp. MH-G8]
249   2e-69
WP_044924825.1 GTP-binding protein [Dorea longicatena]
253   2e-69
WP_094083403.1 elongation factor G [Prosthecochloris sp. GSB1]
249   2e-69
WP_011133137.1 elongation factor G [Prochlorococcus marinus]
248   2e-69
WP_082970461.1 elongation factor G [Mycobacterium sp. E2989]
249   2e-69
WP_021641362.1 GTP-binding protein [[Clostridium] symbiosum]
252   2e-69
OFZ27597.1 translation elongation factor G [Bdellovibrionales...
248   2e-69
WP_104431741.1 elongation factor G [Kineococcus xinjiangensis]
248   2e-69
WP_074688462.1 GTP-binding protein [Kandleria vitulina]
251   2e-69
PIU56687.1 elongation factor G [Chloroflexi bacterium CG07_la...
248   2e-69
XP_017783014.1 PREDICTED: ribosome-releasing factor 2, mitoch...
249   2e-69
KKP89730.1 translation elongation factor 2 (EF-2/EF-G), elong...
248   2e-69
PIP26382.1 elongation factor G [Candidatus Moranbacteria bact...
248   2e-69
WP_060779254.1 elongation factor G [Enterococcus faecium]
248   2e-69
CBL24633.1 small GTP-binding protein domain [Blautia obeum A2...
253   2e-69
WP_036983338.1 MULTISPECIES: elongation factor G [Pseudoalter...
248   2e-69
EOT26916.1 small GTP-binding protein domain protein [Eubacter...
253   2e-69
WP_024739161.1 GTP-binding protein [[Clostridium] symbiosum]
252   2e-69
SCI65233.1 Elongation factor G [uncultured Blautia sp.]
252   2e-69
WP_101225871.1 elongation factor G [Bacillus sp. BA3]
248   2e-69
WP_082499865.1 MULTISPECIES: elongation factor G [Nocardioides]
248   2e-69
WP_086158610.1 elongation factor G [Streptomyces sp. SCSIO 03...
249   2e-69
WP_057579018.1 GTP-binding protein [Paeniclostridium sordellii]
248   2e-69
OHA70131.1 translation elongation factor G [Candidatus Wilder...
248   2e-69
WP_047131408.1 MULTISPECIES: elongation factor G [Staphylococ...
248   2e-69
WP_048342520.1 elongation factor G [Cellulomonas sp. A375-1]
248   2e-69
WP_067754976.1 elongation factor G [Orrella dioscoreae]
248   2e-69

WP_072411396.1 elongation factor G [Chryseobacterium limigeni...
248   2e-69
WP_031590095.1 GTP-binding protein [Kandleria vitulina]
251   2e-69
OFZ22279.1 translation elongation factor G [Bdellovibrionales...
248   2e-69
WP_041844250.1 elongation factor G [Thermotoga sp. RQ7]
248   2e-69
WP_003501284.1 GTP-binding protein [[Clostridium] symbiosum]
252   2e-69
SMF68770.1 translation elongation factor 2 (EF-2/EF-G) [Paeni...
248   2e-69
WP_077568290.1 elongation factor G [Paenibacillus ihbetae]
248   2e-69
WP_014955028.1 elongation factor G [alpha proteobacterium HIM...
248   2e-69
WP_008544337.1 elongation factor G [Candidatus Pelagibacter s...
248   2e-69
WP_096043726.1 GTP-binding protein [Clostridium botulinum]
247   2e-69
WP_072729192.1 GTP-binding protein [Paenibacillus sp. NAIST15-1]
247   2e-69
OYU70662.1 elongation factor G [Alphaproteobacteria bacterium...
248   2e-69
WP_005607488.1 elongation factor G [Granulicatella adiacens]
248   2e-69
OUU09056.1 elongation factor G [Gammaproteobacteria bacterium...
248   2e-69
OGH83201.1 translation elongation factor G [Candidatus Magasa...
248   2e-69
KRL15289.1 translation elongation factor G [Lactobacillus rap...
248   2e-69
WP_097230381.1 elongation factor G [Streptomyces zhaozhouensis]
248   2e-69
CCY92015.1 elongation factor G [Eubacterium sp. CAG:180]
248   2e-69
WP_053544131.1 elongation factor G [Corynebacterium deserti]
249   2e-69
WP_067961230.1 elongation factor G [Mycobacterium sp. NAZ190054]
248   2e-69
WP_102050585.1 GTP-binding protein [Pygmaiobacter massiliensis]
251   2e-69
WP_025008927.1 MULTISPECIES: elongation factor G [Shewanella]
248   2e-69
OGV19947.1 translation elongation factor G [Lentisphaerae bac...
248   2e-69
WP_054405487.1 elongation factor G [Paenibacillus solani]
248   2e-69
WP_053537044.1 MULTISPECIES: elongation factor G [Bacillus]
248   2e-69
PIR52658.1 elongation factor G [Candidatus Peregrinibacteria ...
248   2e-69
WP_010383884.1 elongation factor G [Leuconostoc gelidum]
248   2e-69

WP_107110782.1 elongation factor G [Shewanella algae]
248   2e-69
WP_085323021.1 elongation factor G [Mycobacterium sp. TBL 120...
248   2e-69
WP_049555348.1 MULTISPECIES: elongation factor G [Granulicate...
248   2e-69
WP_018970385.1 elongation factor G [Rubritalea marina]
249   2e-69
WP_097778123.1 elongation factor G [Faecalibacterium prausnit...
248   2e-69
WP_059432755.1 elongation factor G [Campylobacter fetus]
248   2e-69
WP_070439188.1 elongation factor G [Granulicatella sp. HMSC30...
248   2e-69
WP_063278646.1 elongation factor G [Staphylococcus pseudinter...
248   2e-69
WP_081537620.1 GTP-binding protein [Lactobacillus salivarius]
247   2e-69
WP_046860157.1 GTP-binding protein [Sedimenticola thiotaurini]
248   2e-69
WP_062422021.1 elongation factor G [Leptolinea tardivitalis]
248   2e-69
WP_104752948.1 elongation factor G [Helicobacter baculiformis]
248   2e-69
OUM87993.1 translation elongation factor G [Bacillus thermoze...
248   2e-69
SCJ12023.1 Elongation factor G [uncultured Ruminococcus sp.]
251   2e-69
WP_009607065.1 GTP-binding protein [Turicibacter sp. HGF1]
247   2e-69
WP_081514384.1 GTP-binding protein [Lactobacillus salivarius]
247   2e-69
OHA64028.1 translation elongation factor G [Candidatus Wilder...
248   2e-69
WP_015650382.1 elongation factor G [Corynebacterium callunae]
248   2e-69
WP_026489331.1 GTP-binding protein [Butyrivibrio sp. XBB1001]
252   2e-69
WP_106539050.1 elongation factor G [Haloactinopolyspora alba]
248   2e-69
WP_082694104.1 elongation factor G [Mycobacterium sp. GA-1379]
248   2e-69
CDC56630.1 putative uncharacterized protein [Dorea formicigen...
252   2e-69
WP_108602785.1 elongation factor G [Catenovulum sp. CCB-QB4]
248   2e-69
WP_029732548.1 GTP-binding protein [Dorea sp. AGR2135]
252   2e-69
SHE13651.1 elongation factor G [Chlamydia abortus]
248   2e-69
WP_015325926.1 elongation factor G [Halobacteroides halobius]
248   2e-69
XP_004651446.1 PREDICTED: ribosome-releasing factor 2, mitoch...
249   2e-69

WP_045353254.1 GTP-binding protein [Lactobacillus plantarum]
248   2e-69
WP_090495157.1 elongation factor G [Pseudoalteromonas sp. DSM...
248   2e-69
WP_017011740.1 MULTISPECIES: elongation factor G [Enterovibrio]
248   2e-69
WP_049042317.1 GTP-binding protein [Clostridium sporogenes]
247   2e-69
WP_036143371.1 elongation factor G [Lysinibacillus sp. BF-4]
248   2e-69
WP_056293072.1 elongation factor G [Afipia sp. Root123D2]
248   2e-69
PNR89753.1 elongation factor P [Petrotoga sp. 9T1HF07.CasAA.8.2]
248   2e-69
KPJ73344.1 elongation factor G [Parcubacteria bacterium DG_74_1]
248   2e-69
WP_042513903.1 GTP-binding protein [Lactobacillus fermentum]
247   2e-69
WP_006058546.1 elongation factor G [Holdemania filiformis]
248   2e-69
WP_106730048.1 elongation factor G [Zobellella sp. 59N8]
248   2e-69
WP_038671903.1 GTP-binding protein [Ruminococcus bicirculans]
251   2e-69
WP_027697576.1 elongation factor G [Vibrio litoralis]
248   2e-69
WP_056088903.1 elongation factor G [Cellulomonas sp. Root137]
248   2e-69
WP_100066090.1 elongation factor G [Miniphocibacter massilien...
248   2e-69
KWX85856.1 elongation factor G [Paenibacillus riograndensis]
248   2e-69
WP_025531155.1 GTP-binding protein [Hungatella hathewayi]
252   2e-69
WP_088907582.1 GTP-binding protein [Brevibacillus formosus]
247   2e-69
WP_048823006.1 elongation factor G [Bacillus sp. B-jedd]
248   2e-69
WP_066561482.1 GTP-binding protein [Clostridiales bacterium K...
252   2e-69
WP_081511106.1 GTP-binding protein [Lactobacillus salivarius]
247   2e-69
WP_042171460.1 elongation factor G [Paenibacillus gorillae]
248   2e-69
PRD25058.1 fusA2 [Nephila clavipes]
248   2e-69
WP_098749101.1 elongation factor G [Paenibacillus sp. EZ-K15]
248   2e-69
CUM73615.1 Tetracycline resistance protein tetM [Roseburia in...
252   2e-69
WP_095276567.1 elongation factor G [Helicobacter sp. 11S02629-2]
248   2e-69
WP_077150365.1 elongation factor G [Enterococcus faecium]
248   2e-69

WP_006030775.1 elongation factor G [Moritella sp. PE36]
248   2e-69
WP_070711184.1 elongation factor G [Corynebacterium sp. HMSC0...
248   2e-69
WP_099019991.1 elongation factor G [Marinicella litoralis]
248   2e-69
WP_013777319.1 elongation factor G [Tepidanaerobacter acetato...
248   2e-69
WP_065109809.1 elongation factor G [Shewanella sp. UCD-FRSSP1...
248   2e-69
WP_096016825.1 elongation factor G [Campylobacter lanienae]
248   2e-69
WP_070645245.1 MULTISPECIES: elongation factor G [Corynebacte...
248   2e-69
WP_016455360.1 elongation factor G [Propionimicrobium lymphop...
248   2e-69
WP_088225433.1 GTP-binding protein [Desulfosporosinus sp. FKB]
251   2e-69
PKN53464.1 elongation factor G [Deltaproteobacteria bacterium...
248   2e-69
WP_005332210.1 GTP-binding protein [Dorea formicigenerans]
252   2e-69
WP_087170488.1 elongation factor G [Gemmiger sp. An120]
248   2e-69
CEK29441.1 small GTP-binding protein [[Clostridium] sordellii]
247   2e-69
WP_072322089.1 elongation factor G [Luteibacter sp. UNCMF366T...
248   2e-69
WP_069293242.1 elongation factor G [Fervidobacterium thailand...
248   2e-69
WP_087037533.1 elongation factor G [Oceanisphaera profunda]
248   2e-69
SCH06144.1 Tetracycline resistance protein tetM from transpos...
247   2e-69
WP_053491292.1 elongation factor G [Bacillus sp. FJAT-18019]
248   2e-69
OGM91224.1 translation elongation factor G [Candidatus Wolfeb...
248   2e-69
KRL62944.1 translation elongation factor G [Lactobacillus par...
248   2e-69
WP_094869399.1 GTP-binding protein [Paenibacillus sp. VTT E-1...
247   2e-69
SCI37537.1 Vegetative protein 19 [uncultured Butyricicoccus sp.]
248   2e-69
WP_058155543.1 elongation factor G [Pseudoalteromonas issache...
248   2e-69
PIU77808.1 elongation factor G [Candidatus Moranbacteria bact...
248   2e-69
WP_027365854.1 elongation factor G [Desulfotomaculum alcoholi...
248   2e-69
WP_029072394.1 GTP-binding protein [Kandleria vitulina]
251   2e-69
WP_009431661.1 elongation factor G [Peptoniphilus sp. oral ta...
248   2e-69

WP_021316768.1 elongation factor G [Sphingobium ummariense]
248   2e-69
WP_073293852.1 GTP-binding protein [Libanicoccus massiliensis]
247   2e-69
WP_043737735.1 elongation factor G [Lactococcus lactis]
248   2e-69
WP_094498792.1 GTP-binding protein [Lactobacillus taiwanensis]
247   2e-69
WP_062419017.1 elongation factor G [Levilinea saccharolytica]
248   2e-69
OGM73149.1 translation elongation factor G [Candidatus Woeseb...
248   3e-69
OUX06851.1 elongation factor G [Acidimicrobiaceae bacterium T...
248   3e-69
WP_092777657.1 elongation factor G [Actinokineospora terrae]
248   3e-69
WP_036719580.1 elongation factor G [Paenibacillus sp. JCM 10914]
248   3e-69
WP_007132571.1 elongation factor G [Paenibacillus lactis]
248   3e-69
WP_084171901.1 GTP-binding protein [Blautia schinkii]
252   3e-69
WP_098924940.1 elongation factor G [Faecalibacterium prausnit...
248   3e-69
WP_061311090.1 GTP-binding protein [Clostridium sporogenes]
247   3e-69
WP_101889179.1 GTP-binding protein [Lactobacillus fermentum]
247   3e-69
WP_019196579.1 MULTISPECIES: elongation factor G [Bradyrhizob...
248   3e-69
WP_045085422.1 GTP-binding protein [[Clostridium] cellulosi]
251   3e-69
WP_095547648.1 elongation factor G [Corynebacterium sp. NML00...
248   3e-69
PIR49261.1 elongation factor G [Candidatus Peregrinibacteria ...
248   3e-69
WP_099208953.1 elongation factor G [Urinacoccus sp. Marseille...
248   3e-69
WP_091319669.1 elongation factor G [Geodermatophilus ruber]
248   3e-69
WP_015172868.1 elongation factor G [Geitlerinema sp. PCC 7407]
248   3e-69
WP_002600766.1 GTP-binding protein [Hungatella hathewayi]
252   3e-69
WP_082703132.1 elongation factor G [Mycobacterium sp. IS-1496]
248   3e-69
WP_018414176.1 elongation factor G [Candidatus Pelagibacter u...
248   3e-69
WP_066640572.1 elongation factor G [Desulfotomaculum intricatum]
248   3e-69
OUU87489.1 elongation factor G [Verrucomicrobia bacterium TME...
248   3e-69
WP_066866703.1 GTP-binding protein [Neglecta timonensis]
247   3e-69

WP_053997227.1 elongation factor G [Lysinibacillus macroides]
248   3e-69
WP_022133012.1 elongation factor G [Negativibacillus massilie...
248   3e-69
WP_011836173.1 elongation factor G [Lactococcus lactis]
248   3e-69
WP_099478352.1 elongation factor G [Paenibacillus ihbetae]
248   3e-69
WP_081561844.1 GTP-binding protein [Lactobacillus salivarius]
246   3e-69
WP_087282172.1 GTP-binding protein [Eubacterium sp. An3]
253   3e-69
WP_012070050.1 elongation factor G [Marinomonas sp. MWYL1]
248   3e-69
WP_038892244.1 MULTISPECIES: elongation factor G [Vibrio harv...
248   3e-69
WP_098865955.1 elongation factor G [Bacillus sp. AFS043905]
248   3e-69
PCJ94271.1 elongation factor G [Porticoccaceae bacterium]
248   3e-69
WP_099990051.1 elongation factor G [Fusobacterium periodonticum]
248   3e-69
WP_090849678.1 elongation factor G [Nocardioides lianchengensis]
248   3e-69
WP_055657295.1 GTP-binding protein [Hungatella hathewayi]
251   3e-69
WP_050739796.1 elongation factor G [Acetobacterium bakii]
248   3e-69
OGM21843.1 translation elongation factor G [Candidatus Woeseb...
248   3e-69
PHS55323.1 elongation factor G [Alteromonas sp.]
248   3e-69
WP_065594310.1 MULTISPECIES: elongation factor G [Aliivibrio]
248   3e-69
WP_014573452.1 elongation factor G [Lactococcus lactis]
248   3e-69
GBE15735.1 elongation factor G [bacterium BMS3Abin14]
247   3e-69
EHJ62365.1 elongation factor EF-G [Novosphingobium pentaromat...
248   3e-69
WP_005338921.1 GTP-binding protein [Dorea formicigenerans]
252   3e-69
WP_010687963.1 elongation factor G [Leuconostoc gelidum]
248   3e-69
OIO47803.1 translation elongation factor G [Parcubacteria gro...
248   3e-69
WP_075599487.1 elongation factor G [Leptolyngbya sp. 'hensonii']
248   3e-69
KKR16649.1 Elongation factor G [Candidatus Levybacteria bacte...
248   3e-69
WP_102775847.1 elongation factor G [Achromobacter pulmonis]
248   3e-69
WP_006996809.1 elongation factor G [Candidatus Pelagibacter u...
248   3e-69

WP_104371772.1 elongation factor G [Desulfocucumis palustris]
248   3e-69
WP_062279922.1 elongation factor G [Flexilinea flocculi]
248   3e-69
AMP49024.1 elongation factor G C-terminus [uncultured bacterium]
229   3e-69
WP_067440747.1 elongation factor G [Nocardioides jensenii]
248   3e-69
WP_010189691.1 elongation factor G [Corynebacterium aurimucosum]
248   3e-69
EET59252.1 putative translation elongation factor G [Marvinbr...
252   3e-69
WP_063494232.1 GTP-binding protein [Lactobacillus plantarum]
247   3e-69
WP_033120245.1 GTP-binding protein [Oscillibacter sp. ER4]
251   3e-69
WP_068620422.1 elongation factor G [Paenibacillus tuaregi]
248   3e-69
WP_086963738.1 elongation factor G [Oceanisphaera sp. AMac2203]
248   3e-69
WP_044039391.1 elongation factor G [Clostridium bornimense]
248   3e-69
WP_027700851.1 elongation factor G [Clostridioides mangenotii]
248   3e-69
OGX34967.1 translation elongation factor G [Omnitrophica WOR_...
248   3e-69
KLR49785.1 tetracycline resistance protein [[Clostridium] sor...
247   3e-69
CDC04553.1 putative translation elongation factor G [Clostrid...
246   3e-69
WP_082695780.1 elongation factor G [Mycobacterium sp. IS-1742]
248   3e-69
WP_010621089.1 GTP-binding protein [Lactobacillus suebicus]
247   3e-69
WP_086302477.1 MULTISPECIES: elongation factor G [Campylobacter]
248   3e-69
WP_068788210.1 elongation factor G [Phormidium willei]
248   3e-69
WP_033100618.1 elongation factor G [Thermoactinomyces daqus]
248   3e-69
WP_070444764.1 elongation factor G [Corynebacterium sp. HMSC0...
248   3e-69
PKN88871.1 elongation factor G [Deltaproteobacteria bacterium...
247   3e-69
WP_024621754.1 elongation factor G [Clostridioides mangenotii]
248   3e-69
WP_004587465.1 elongation factor G [Pseudoalteromonas agarivo...
248   3e-69
WP_003533720.1 GTP-binding protein [[Clostridium] leptum]
248   3e-69
PIR17307.1 elongation factor G [Deltaproteobacteria bacterium...
248   3e-69
WP_071407912.1 elongation factor G [Globicatella sp. HMSC072A10]
248   3e-69

AMP51607.1 elongation factor Tu GTP binding domain protein [u...
230   3e-69
WP_048677880.1 MULTISPECIES: elongation factor G [Bacillus]
248   3e-69
WP_086983205.1 elongation factor G [Vibrio aphrogenes]
248   3e-69
WP_015077775.1 elongation factor G [Carnobacterium maltaromat...
248   3e-69
WP_099837837.1 GTP-binding protein [[Eubacterium] combesii]
247   3e-69
WP_091502465.1 elongation factor G [Fructobacillus durionis]
248   3e-69
WP_101100465.1 MULTISPECIES: elongation factor G [Stenotropho...
247   3e-69
KXT85474.1 Translation elongation factor G [Streptococcus sp....
248   3e-69
WP_003854224.1 elongation factor G [Corynebacterium glutamicum]
248   3e-69
SCJ41157.1 Tetracycline resistance protein tetM from transpos...
251   3e-69
WP_040784682.1 GTP-binding protein [Marvinbryantia formatexig...
251   3e-69
WP_102979764.1 elongation factor G [Chryseobacterium scophtha...
248   3e-69
ALA96902.1 elongation factor G [Candidatus Carsonella ruddii]
247   3e-69
WP_084203484.1 GTP-binding protein [Ruminococcaceae bacterium...
251   3e-69
WP_038206129.1 elongation factor G [Vibrio tubiashii]
248   3e-69
WP_093073172.1 elongation factor G [Salisediminibacterium hal...
248   3e-69
WP_044841782.1 elongation factor G [Thalassomonas viridans]
248   3e-69
WP_088904699.1 elongation factor G [Shewanella marisflavi]
248   3e-69
WP_057551220.1 GTP-binding protein [Paeniclostridium sordellii]
247   3e-69
WP_039035538.1 MULTISPECIES: elongation factor G [Shewanella]
248   3e-69
WP_076372873.1 elongation factor G [Bacillus simplex]
248   3e-69
OUV82761.1 elongation factor G [Verrucomicrobiaceae bacterium...
248   3e-69
WP_071661110.1 elongation factor G [Rickettsiella grylli]
248   3e-69
WP_089988585.1 GTP-binding protein [Clostridium sp. C105KSO15]
246   3e-69
KKP23308.1 Elongation factor G [Candidatus Woesebacteria bact...
248   3e-69
WP_029467916.1 GTP-binding protein [Clostridiales bacterium V...
251   3e-69
WP_087480343.1 elongation factor G [Vibrio mangrovi]
248   3e-69

WP_090126295.1 GTP-binding protein [Eubacteriaceae bacterium ...
252   3e-69
WP_013466210.1 MULTISPECIES: elongation factor G [Alteromonad...
248   3e-69
WP_026866141.1 elongation factor G [Jeotgalicoccus marinus]
248   3e-69
WP_100509847.1 GTP-binding protein [Bifidobacterium sp. TRE 1]
246   3e-69
WP_089798425.1 elongation factor G [Cellulomonas sp. KH9]
248   3e-69
KKU18131.1 Elongation factor G 1 [Candidatus Azambacteria bac...
248   3e-69
WP_058000123.1 elongation factor G [Bacillus humi]
248   3e-69
WP_085216078.1 elongation factor G [Methylomagnum ishizawai]
248   3e-69
WP_082693965.1 MULTISPECIES: elongation factor G [Mycobacterium]
248   3e-69
WP_045381403.1 elongation factor G [Vibrio campbellii]
248   3e-69
KXJ50055.1 elongation factor G [Cycloclasticus sp. Phe_18]
248   3e-69
OGO59604.1 translation elongation factor G [Chloroflexi bacte...
248   3e-69
PAY49108.1 elongation factor G [Lactobacillus salivarius]
246   3e-69
WP_071238444.1 elongation factor G [Shewanella algae]
248   4e-69
WP_011865670.1 elongation factor G [Shewanella loihica]
248   4e-69
PIV24964.1 elongation factor G [Deltaproteobacteria bacterium...
248   4e-69
WP_080914906.1 elongation factor G [Shewanella japonica]
248   4e-69
WP_070705858.1 MULTISPECIES: elongation factor G [Kytococcus]
248   4e-69
WP_045451315.1 elongation factor G [Vibrio campbellii]
248   4e-69
WP_075133147.1 elongation factor G [Actinophytocola xinjiange...
248   4e-69
WP_076326227.1 elongation factor G [Paenibacillus lautus]
248   4e-69
WP_066790937.1 elongation factor G [Rummeliibacillus stabekisii]
248   4e-69
WP_024790286.1 elongation factor G [Lebetimonas sp. JH292]
248   4e-69
WP_082968344.1 elongation factor G [Mycobacterium scrofulaceum]
248   4e-69
WP_012778325.1 GTP-binding protein [Lactobacillus plantarum]
247   4e-69
WP_005518274.1 MULTISPECIES: elongation factor G [Rhodococcus]
248   4e-69
WP_032095015.1 MULTISPECIES: elongation factor G [Alteromonas]
248   4e-69

WP_096597799.1 elongation factor G [Staphylococcus delphini]
248   4e-69
WP_072622119.1 elongation factor G [Spirulina major]
248   4e-69
WP_010933845.1 elongation factor G [Chlorobaculum tepidum]
248   4e-69
WP_094180759.1 elongation factor G [Cellulomonas sp. PSBB021]
248   4e-69
WP_097149273.1 elongation factor G [Lysinibacillus acetophenoni]
247   4e-69
WP_066344832.1 elongation factor G [Geminocystis sp. NIES-3708]
247   4e-69
OQY33857.1 elongation factor G [Spirochaetaceae bacterium 457...
247   4e-69
WP_079914986.1 elongation factor G [Paenibacillus sp. 32352]
247   4e-69
WP_099186378.1 elongation factor G [Sphingobium fuliginis]
247   4e-69
WP_072683803.1 GTP-binding protein [Holdemania sp. Marseille-...
251   4e-69
WP_095535281.1 elongation factor G [Corynebacterium sp. NML93...
248   4e-69
WP_086290433.1 elongation factor G [Campylobacter sp. P0107]
247   4e-69
WP_033827291.1 elongation factor G [Bacillus andreraoultii]
247   4e-69
WP_058586096.1 elongation factor G [Pseudoalteromonas sp. XI10]
248   4e-69
WP_009650482.1 elongation factor G [Campylobacter sp. FOBRC14]
247   4e-69
WP_025725066.1 elongation factor G [Acholeplasma granularum]
247   4e-69
WP_104639521.1 elongation factor G [Helicobacter bizzozeronii]
247   4e-69
WP_068263811.1 elongation factor G [Rubripirellula obstinata]
247   4e-69
WP_068541847.1 elongation factor G [Thermodesulfatator autotr...
247   4e-69
WP_094279351.1 elongation factor G [Oceanimonas baumannii]
248   4e-69
WP_044661334.1 MULTISPECIES: elongation factor G [Sphingomona...
247   4e-69
WP_088244843.1 elongation factor G [Chlamydiales bacterium SC...
247   4e-69
WP_074624614.1 GTP-binding protein [Kandleria vitulina]
250   4e-69
WP_102404804.1 elongation factor G [Vibrio sp. 10N.261.55.A7]
247   4e-69
EGJ47372.2 hypothetical protein HMPREF0866_00312 [Ruminococca...
251   4e-69
WP_068688275.1 elongation factor G [Thermobifida halotolerans]
248   4e-69
WP_029072788.1 elongation factor G [Kandleria vitulina]
247   4e-69

WP_076116953.1 GTP-binding protein [Paenibacillus odorifer]
246   4e-69
WP_069865220.1 MULTISPECIES: elongation factor G [Streptomyces]
248   4e-69
KUJ55211.1 elongation factor G [Chryseobacterium greenlandense]
247   4e-69
WP_095394237.1 elongation factor G [Bacillus simplex]
247   4e-69
WP_055022652.1 elongation factor G [Shewanella sp. P1-14-1]
247   4e-69
WP_013832929.1 MULTISPECIES: elongation factor G [Novosphingo...
247   4e-69
WP_088133504.1 elongation factor G [Vibrio gazogenes]
247   4e-69
WP_097015759.1 GTP-binding protein [Anaerocolumna aminovalerica]
246   4e-69
WP_101966630.1 GTP-binding protein [Lysinibacillus fusiformis]
246   4e-69
WP_087908232.1 GTP-binding protein [Lactobacillus fermentum]
246   4e-69
WP_101878485.1 GTP-binding protein [[Ruminococcus] gnavus]
251   4e-69
WP_028761942.1 elongation factor G [Shewanella colwelliana]
247   4e-69
WP_029056350.1 MULTISPECIES: elongation factor G [Staphylococ...
247   4e-69
WP_006212928.1 MULTISPECIES: elongation factor G [Paenibacillus]
247   4e-69
WP_054955719.1 GTP-binding protein [Paenibacillus sp. FF9]
246   4e-69
WP_014973682.1 elongation factor G [Leuconostoc carnosum]
248   4e-69
WP_088239611.1 elongation factor G [Calothrix rhizosoleniae]
247   4e-69
WP_031590161.1 elongation factor G [Kandleria vitulina]
247   4e-69
WP_058693314.1 MULTISPECIES: elongation factor G [Eubacterium]
247   4e-69
WP_081560183.1 GTP-binding protein [Lactobacillus salivarius]
246   4e-69
WP_032081770.1 elongation factor G [Vibrio fluvialis]
247   4e-69
WP_032112440.1 elongation factor G [Candidatus Paracaedibacte...
247   4e-69
PCI72420.1 elongation factor G [Gammaproteobacteria bacterium]
247   4e-69
WP_002317544.1 elongation factor G [Enterococcus faecium]
247   4e-69
WP_036712558.1 elongation factor G [Paenibacillus sp. P1XP2]
247   4e-69
WP_031874895.1 elongation factor G [Staphylococcus aureus]
247   4e-69
WP_090583219.1 elongation factor G [Paenibacillus sp. OV219]
247   4e-69

WP_101870444.1 GTP-binding protein [[Ruminococcus] gnavus]
251   4e-69
WP_010365214.1 elongation factor G [Pseudoalteromonas citrea]
247   4e-69
OUR69082.1 translation elongation factor G [Cycloclasticus sp...
247   4e-69
WP_035279515.1 elongation factor G [Actinokineospora sphecios...
247   5e-69
WP_103868489.1 GTP-binding protein [Butyrivibrio sp. Su6]
251   5e-69
WP_058012587.1 GTP-binding protein [Clostridium sporogenes]
246   5e-69
KPH03658.1 elongation factor G [Lactobacillus fermentum]
246   5e-69
WP_019441928.1 elongation factor G [Moritella marina]
247   5e-69
WP_091224752.1 GTP-binding protein [Paenibacillus sp. BC26]
246   5e-69
WP_106655330.1 GTP-binding protein [Brevibacillus brevis]
246   5e-69
WP_015957947.1 GTP-binding protein [Clostridium botulinum]
246   5e-69
WP_036080630.1 GTP-binding protein [Lysinibacillus boronitole...
246   5e-69
WP_007882802.1 GTP-binding protein [Roseburia inulinivorans]
251   5e-69
WP_050343628.1 elongation factor G [Olsenella sp. oral taxon ...
247   5e-69
WP_076858260.1 elongation factor G [Bradyrhizobium mercantei]
247   5e-69
WP_086256469.1 MULTISPECIES: elongation factor G [Campylobacter]
247   5e-69
OLC18577.1 translation elongation factor G [Chloroflexi bacte...
247   5e-69
WP_007622453.1 elongation factor G [Paraglaciecola arctica]
247   5e-69
WP_063516483.1 MULTISPECIES: GTP-binding protein [Lactobacillus]
246   5e-69
WP_002828927.1 elongation factor G [Weissella paramesenteroides]
248   5e-69
WP_040966969.1 elongation factor G [Corynebacterium glutamicum]
248   5e-69
WP_029231668.1 GTP-binding protein [Butyrivibrio sp. VCB2006]
251   5e-69
WP_101176310.1 elongation factor G [Bacillus nealsonii]
247   5e-69
WP_079678030.1 elongation factor G [Planktothrix sp. PCC 11201]
247   5e-69
WP_082961933.1 elongation factor G [Mycobacterium sp. 852014-...
247   5e-69
WP_066453276.1 elongation factor G [Helicobacter sp. CLO-3]
247   5e-69
WP_029071096.1 elongation factor G [Kandleria vitulina]
247   5e-69

WP_076585918.1 elongation factor G [Vibrio ostreicida]
247   5e-69
WP_012208620.1 elongation factor G [Petrotoga mobilis]
247   5e-69
WP_038862710.1 elongation factor G [Vibrio campbellii]
247   5e-69
WP_090986339.1 elongation factor G [Paenibacillus sp. CF384]
247   5e-69
WP_098848452.1 elongation factor G [Bacillus sp. AFS055030]
247   5e-69
KRM19169.1 elongation factor g [Lactobacillus hayakitensis DS...
246   5e-69
CDD75159.1 elongation factor Tu GTP binding domain-containing...
251   5e-69
WP_028400854.1 elongation factor G [Bacillus panaciterrae]
247   5e-69
WP_006234536.1 GTP-binding protein [Collinsella aerofaciens]
246   5e-69
WP_106574729.1 GTP-binding protein [Clostridium botulinum]
246   5e-69
WP_041066946.1 elongation factor G [Cohnella kolymensis]
247   5e-69
WP_090980676.1 GTP-binding protein [Paenibacillus sp. CF384]
246   5e-69
WP_016525534.1 elongation factor G [Treponema maltophilum]
247   5e-69
WP_066020389.1 MULTISPECIES: GTP-binding protein [Clostridium]
246   5e-69
WP_104749642.1 elongation factor G [Helicobacter cynogastricus]
247   5e-69
WP_080325204.1 elongation factor G [Anaerovibrio lipolyticus]
247   5e-69
OGS19446.1 translation elongation factor G [Elusimicrobia bac...
247   5e-69
WP_101029414.1 GTP-binding protein [Ruminococcus bromii]
250   5e-69
WP_055167402.1 GTP-binding protein [Roseburia inulinivorans]
251   5e-69
WP_108467057.1 elongation factor G [Paenibacillus sp. CAA11]
247   5e-69
WP_087432357.1 elongation factor G [Paenibacillus apiarius]
247   5e-69
WP_075666000.1 MULTISPECIES: elongation factor G [Thermosipho]
247   5e-69
WP_084049985.1 elongation factor G [Deinococcus hopiensis]
247   5e-69
WP_017637540.1 MULTISPECIES: elongation factor G [Staphylococ...
247   5e-69
OGZ44143.1 translation elongation factor G [Candidatus Ryanba...
247   5e-69
CDB51547.1 elongation factor G [Clostridium sp. CAG:217]
247   5e-69
WP_061141101.1 elongation factor G [Bacillus simplex]
247   5e-69

KJS16771.1 elongation factor G [Peptococcaceae bacterium BRH_...
247   5e-69
WP_091475270.1 elongation factor G [Aliicoccus persicus]
247   5e-69
WP_057562080.1 GTP-binding protein [Paeniclostridium sordellii]
246   5e-69
WP_026877500.1 elongation factor G [Jiangella gansuensis]
247   5e-69
WP_012301375.1 elongation factor G [Candidatus Desulforudis a...
247   5e-69
WP_005530296.1 elongation factor G [Vibrio campbellii]
247   5e-69
OGS33889.1 translation elongation factor G [Elusimicrobia bac...
247   5e-69
KEA46180.1 elongation factor G [Campylobacter mucosalis]
247   5e-69
WP_014953361.1 elongation factor G [alpha proteobacterium HIMB5]
247   5e-69
WP_089905529.1 GTP-binding protein [Lachnospiraceae bacterium...
251   5e-69
WP_076613695.1 elongation factor G [Lactobacillus allii]
247   5e-69
WP_010621623.1 elongation factor G [Lactobacillus suebicus]
247   5e-69
WP_048018158.1 elongation factor G [Synechococcus sp. GFB01]
247   5e-69
WP_054510534.1 elongation factor G [Chryseobacterium sp. ERMR...
247   5e-69
WP_006035680.1 elongation factor G [Rickettsiella grylli]
247   5e-69
WP_006678336.1 GTP-binding protein [Paenibacillus dendritifor...
246   5e-69
WP_079286646.1 GTP-binding protein [Clostridium baratii]
246   5e-69
EJQ06209.1 small GTP-binding protein domain protein [Bacillus...
235   5e-69
WP_003549264.1 GTP-binding protein [Lactobacillus acidophilus]
246   5e-69
CDA85526.1 small GTP-binding protein domain [Clostridium sp. ...
250   5e-69
WP_028505882.1 elongation factor G [Ruminococcus sp. FC2018]
247   5e-69
WP_038582668.1 elongation factor G [Corynebacterium glutamicum]
247   5e-69
WP_091587318.1 MULTISPECIES: elongation factor G [Bacillaceae]
247   5e-69
WP_086240165.1 elongation factor G [Campylobacter sp. P0222]
247   5e-69
PIR58172.1 elongation factor G [Parcubacteria group bacterium...
247   5e-69
WP_096816557.1 elongation factor G [Lactococcus lactis]
247   5e-69
WP_011896755.1 elongation factor G [Corynebacterium glutamicum]
247   5e-69

WP_078502828.1 GTP-binding protein [Paenibacillus selenitired...
246   5e-69
WP_011254014.1 GTP-binding protein [Lactobacillus acidophilus]
246   6e-69
WP_024791559.1 elongation factor G [Lebetimonas sp. JS032]
247   6e-69
WP_059040318.1 elongation factor G [Paenibacillus rubinfantis]
247   6e-69
WP_031556120.1 GTP-binding protein [Lachnospira multipara]
250   6e-69
WP_030986137.1 elongation factor G [Streptomyces sp. NRRL S-1...
247   6e-69
WP_058561089.1 elongation factor G [Pseudoalteromonas sp. H105]
247   6e-69
WP_046680957.1 elongation factor G [Paenibacillus sp. DMB20]
247   6e-69
KUO75065.1 translation elongation factor G [Clostridia bacter...
251   6e-69
KPJ56345.1 elongation factor G [Parcubacteria bacterium DG_74_2]
246   6e-69
WP_082961854.1 MULTISPECIES: elongation factor G [Mycobacterium]
247   6e-69
WP_011217975.1 elongation factor G [Photobacterium profundum]
247   6e-69
KKT21559.1 Elongation factor G 1 [Parcubacteria group bacteri...
247   6e-69
WP_067425779.1 elongation factor G [Erysipelotrichaceae bacte...
247   6e-69
WP_070957642.1 MULTISPECIES: elongation factor G [Helicobacter]
247   6e-69
WP_087709679.1 elongation factor G [Phormidium sp. HE10JO]
247   6e-69
WP_078232521.1 elongation factor G [Candidatus Synechococcus ...
247   6e-69
OUV08801.1 elongation factor G [Gammaproteobacteria bacterium...
247   6e-69
WP_057749458.1 GTP-binding protein [Lactobacillus frumenti]
246   6e-69
PPD50900.1 elongation factor G [Methylobacter sp.]
247   6e-69
WP_088891814.1 elongation factor G [Leptolyngbya ohadii]
247   6e-69
OGC49993.1 translation elongation factor G [candidate divisio...
247   6e-69
WP_013735956.1 elongation factor G [Verrucosispora maris]
247   6e-69
WP_012100086.1 GTP-binding protein [Clostridium botulinum]
246   6e-69
WP_004081841.1 MULTISPECIES: elongation factor G [Thermotoga]
247   6e-69
P38525.2 RecName: Full=Elongation factor G; Short=EF-G
247   6e-69
WP_005038108.1 elongation factor G [Holophaga foetida]
247   6e-69

EFK79546.1 putative translation elongation factor G [Lactobac...
246   6e-69
WP_068448947.1 elongation factor G [Caviibacter abscessus]
247   6e-69
WP_069972671.1 elongation factor G [Enterococcus faecium]
247   6e-69
WP_050004476.1 elongation factor G [Ruthenibacterium lactatif...
247   6e-69
WP_036224605.1 GTP-binding protein [Lysinibacillus sphaericus]
246   6e-69
WP_069567066.1 elongation factor G [Streptomyces lydicus]
247   6e-69
WP_076459818.1 GTP-binding protein [Lactobacillus sp. Marseil...
246   6e-69
WP_076919855.1 elongation factor G [Pseudoalteromonas sp. SK18]
247   6e-69
WP_093537868.1 elongation factor G [Psychrobacillus psychroto...
247   6e-69
PSO57437.1 elongation factor G [Cyanobacteria bacterium QH_7_...
247   6e-69
WP_094590888.1 elongation factor G [Vulcanococcus limneticus]
247   6e-69
KYF73885.1 elongation factor G [Sorangium cellulosum]
247   6e-69
CDZ99446.1 Elongation factor G [Lysinibacillus saudimassilien...
247   6e-69
WP_073619363.1 elongation factor G [Calothrix sp. HK-06]
247   6e-69
WP_028406467.1 elongation factor G [Bacillus sp. J13]
247   6e-69
WP_101703856.1 elongation factor G [Carnobacterium maltaromat...
247   6e-69
WP_061485411.1 elongation factor G [Alteromonas macleodii]
247   6e-69
WP_055078615.1 elongation factor G [Lagierella massiliensis]
247   6e-69
WP_058258076.1 GTP-binding protein [Herbinix luporum]
251   6e-69
WP_053361315.1 elongation factor G [Bacillus sp. FJAT-27251]
247   6e-69
PIX66748.1 elongation factor G [Sphingomonadales bacterium CG...
247   6e-69
OKZ69635.1 translation elongation factor G [Clostridiales bac...
251   6e-69
WP_003683291.1 GTP-binding protein [Lactobacillus fermentum]
246   6e-69
WP_062566304.1 elongation factor G [Pseudoalteromonas shioyas...
247   6e-69
WP_104636977.1 elongation factor G [Helicobacter felis]
247   7e-69
WP_010320574.1 elongation factor G [Vibrio ordalii]
247   7e-69
WP_076598030.1 elongation factor G [Corynebacterium appendicis]
247   7e-69

WP_107716004.1 elongation factor G [Novosphingobium sp. GV064]
247   7e-69
WP_090023216.1 GTP-binding protein [Lachnospiraceae bacterium...
251   7e-69
OPL08158.1 elongation factor G [delta proteobacterium ML8_F1]
246   7e-69
KKR17911.1 Elongation factor G [candidate division CPR2 bacte...
247   7e-69
WP_057881423.1 elongation factor G [Lactobacillus kimchiensis]
247   7e-69
WP_036168156.1 GTP-binding protein [Lysinibacillus sphaericus]
246   7e-69
WP_034529340.1 GTP-binding protein [Lactobacillus oryzae]
246   7e-69
WP_004413001.1 elongation factor G [Vibrio orientalis]
247   7e-69
OGC56648.1 translation elongation factor G [candidate divisio...
246   7e-69
WP_029166025.1 elongation factor G [Aminiphilus circumscriptus]
246   7e-69
WP_012475666.1 elongation factor G [Chlorobium phaeobacteroides]
247   7e-69
WP_086253047.1 MULTISPECIES: elongation factor G [Campylobacter]
247   7e-69
WP_057587634.1 GTP-binding protein [Paeniclostridium sordellii]
246   7e-69
WP_085146491.1 elongation factor G [Mycobacterium wolinskyi]
247   7e-69
WP_045806608.1 GTP-binding protein [Lactobacillus spicheri]
246   7e-69
WP_023923948.1 GTP-binding protein [[Ruminococcus] gnavus]
251   7e-69
WP_100931931.1 GTP-binding protein [Clostridium sp. HMb25]
251   7e-69
WP_014291652.1 elongation factor G [Oceanimonas sp. GK1]
247   7e-69
WP_103986871.1 GTP-binding protein [Eubacterium ruminantium]
251   7e-69
WP_077587634.1 elongation factor G [Planococcus maritimus]
247   7e-69
WP_073641191.1 elongation factor G [Nostoc calcicola]
247   7e-69
WP_015113496.1 elongation factor G [Nostoc sp. PCC 7107]
247   7e-69
WP_092462759.1 elongation factor G [Weissella bombi]
247   7e-69
WP_092478327.1 GTP-binding protein [[Clostridium] polysacchar...
250   7e-69
WP_086233968.1 elongation factor G [Campylobacter sp. P0138]
246   7e-69
WP_004843565.1 GTP-binding protein [[Ruminococcus] gnavus]
251   7e-69
WP_007686589.1 MULTISPECIES: elongation factor G [Sphingomona...
246   7e-69

WP_029840350.1 elongation factor G [Vibrio parahaemolyticus]
247   7e-69
WP_005661692.1 elongation factor G [Dethiosulfovibrio peptido...
246   7e-69
OIP49481.1 translation elongation factor G [Desulfobacterales...
247   7e-69
WP_062099009.1 MULTISPECIES: elongation factor G [Caulobacter]
246   7e-69
PDH20689.1 elongation factor G [Pelagibacterales bacterium ME...
246   7e-69
WP_048716430.1 GTP-binding protein [Bacillus sp. 522_BSPC]
246   7e-69
WP_014903742.1 GTP-binding protein [Desulfosporosinus meridiei]
250   7e-69
WP_054327682.1 elongation factor G [Intestinibacillus sp. Mar...
247   7e-69
KKU21596.1 Elongation factor G [Microgenomates group bacteriu...
247   7e-69
WP_082462561.1 MULTISPECIES: elongation factor G [Mycobacterium]
247   7e-69
ODS56336.1 translation elongation factor G [Acidobacteria bac...
247   7e-69
WP_026303430.1 elongation factor G [Gordonia kroppenstedtii]
247   7e-69
WP_095277979.1 MULTISPECIES: elongation factor G [Corynebacte...
247   7e-69
WP_048340079.1 GTP-binding protein [Lactobacillus fermentum]
246   7e-69
Q8FS85.2 RecName: Full=Elongation factor G; Short=EF-G
247   7e-69
WP_011889494.1 elongation factor G [Chlorobium phaeovibrioides]
247   7e-69
PIQ83805.1 elongation factor G [Candidatus Omnitrophica bacte...
247   7e-69
WP_094450770.1 elongation factor G [Propionibacteriaceae bact...
247   7e-69
WP_062207502.1 elongation factor G [Streptomyces sp. NBRC 109...
247   7e-69
CDC79368.1 elongation factor G [Clostridium sp. CAG:964]
247   7e-69
WP_093268261.1 elongation factor G [Vibrio xiamenensis]
247   7e-69
WP_073962993.1 GTP-binding protein [[Ruminococcus] gnavus]
250   7e-69
WP_087157477.1 GTP-binding protein [Lachnoclostridium sp. An181]
250   7e-69
OHA73496.1 translation elongation factor G [Candidatus Wilder...
247   7e-69
WP_068522690.1 elongation factor G [Tsukamurella tyrosinosolv...
247   7e-69
WP_058965812.1 elongation factor G [Fournierella massiliensis]
247   8e-69
ETI86395.1 Elongation factor G 2 [Negativicoccus succinicivor...
246   8e-69

WP_034665191.1 elongation factor G [Corynebacterium tuscaniense]
247   8e-69
WP_082973944.1 elongation factor G [Mycobacterium sp. E740]
247   8e-69
WP_012294343.1 GTP-binding protein [Lysinibacillus sphaericus]
246   8e-69
ALE79120.1 elongation factor G [Pseudonocardia sp. AL041005-10]
246   8e-69
WP_067483552.1 MULTISPECIES: elongation factor G [Bacilli]
246   8e-69
WP_026895848.1 elongation factor G [Clostridiisalibacter pauc...
246   8e-69
WP_064462538.1 elongation factor G [Bacillus muralis]
246   8e-69
WP_015404279.1 elongation factor G [Desulfocapsa sulfexigens]
246   8e-69
PIE98424.1 elongation factor G [Treponema sp.]
246   8e-69
WP_035149480.1 GTP-binding protein [Lactobacillus salivarius]
245   8e-69
WP_003860540.1 MULTISPECIES: elongation factor G [Corynebacte...
247   8e-69
WP_019635766.1 elongation factor G [Paenibacillus fonticola]
246   8e-69
WP_012258228.1 MULTISPECIES: elongation factor G [Chloroflexus]
247   8e-69
WP_066732546.1 elongation factor G [Cupriavidus sp. D384]
247   8e-69
WP_068450115.1 elongation factor G [Kurthia sp. 11kri321]
246   8e-69
WP_101677706.1 elongation factor G [Corynebacterium pyruvicip...
247   8e-69
WP_013558076.1 elongation factor G [Deinococcus maricopensis]
246   8e-69
OFX30824.1 translation elongation factor G [Armatimonadetes b...
247   8e-69
WP_094455569.1 elongation factor G [Propionibacteriaceae bact...
246   8e-69
2BV3_A Chain A, Crystal structure of a mutant elongation fact...
246   8e-69
WP_093315608.1 elongation factor G [Tindallia californiensis]
246   8e-69
PHS16774.1 elongation factor G [Kangiella sp.]
246   8e-69
WP_081562363.1 GTP-binding protein [Lactobacillus salivarius]
245   8e-69
WP_091225559.1 elongation factor G [Paenibacillus sp. BC26]
246   8e-69
WP_062955414.1 elongation factor G [Thalassospira sp. MCCC 1A...
246   8e-69
PIP49052.1 elongation factor G [Chloroflexi bacterium CG23_co...
246   8e-69
WP_066859667.1 elongation factor G [Sphingobium sp. TCM1]
246   8e-69

WP_025233063.1 GTP-binding protein [Clostridium sp. ASBs410]
246   8e-69
PJA40817.1 elongation factor G [candidate division WWE3 bacte...
247   8e-69
WP_081516132.1 GTP-binding protein [Lactobacillus salivarius]
245   8e-69
OGP07081.1 translation elongation factor G [Deltaproteobacter...
246   8e-69
OUU79583.1 elongation factor G [Gammaproteobacteria bacterium...
246   8e-69
OUR82142.1 translation elongation factor G [Cycloclasticus sp...
246   8e-69
WP_029772901.1 elongation factor G [Pseudoalteromonas sp. TB51]
246   8e-69
WP_027397546.1 elongation factor G [Anaerovibrio lipolyticus]
246   8e-69
WP_045670701.1 elongation factor G [Paenibacillus beijingensis]
246   8e-69
WP_043605541.1 elongation factor G [Cellulomonas carbonis]
246   8e-69
WP_010233058.1 GTP-binding protein [Clostridium arbusti]
245   8e-69
WP_096379402.1 elongation factor G [Stanieria sp. NIES-3757]
246   9e-69
WP_072805663.1 elongation factor G [Rhodococcus yunnanensis]
246   9e-69
WP_024299905.1 elongation factor G [Methylosarcina lacus]
246   9e-69
WP_003836931.1 GTP-binding protein [Bifidobacterium dentium]
246   9e-69
WP_026223772.1 elongation factor G [Methylosarcina fibrata]
246   9e-69
WP_004803428.1 elongation factor G [Eggerthia catenaformis]
246   9e-69
WP_094868179.1 elongation factor G [Enterococcus faecium]
246   9e-69
WP_055168495.1 GTP-binding protein [[Ruminococcus] gnavus]
250   9e-69
WP_104728426.1 elongation factor G [Helicobacter felis]
246   9e-69
WP_076560436.1 elongation factor G [Salimicrobium flavidum]
246   9e-69
WP_013468808.1 elongation factor G [Helicobacter felis]
246   9e-69
WP_008687388.1 GTP-binding protein [Eubacterium sp. 3_1_31]
249   9e-69
WP_063595514.1 elongation factor G [Bacillus simplex]
246   9e-69
WP_052915590.1 elongation factor G [Mycobacterium haemophilum]
246   9e-69
WP_071221021.1 elongation factor G [Paenibacillus sp. LC231]
246   9e-69
WP_067842054.1 elongation factor G [Mycobacterium wolinskyi]
246   9e-69

WP_019678641.1 GTP-binding protein [Ruminococcus flavefaciens]
249   9e-69
OGW60531.1 translation elongation factor G [Nitrospirae bacte...
246   9e-69
AIV03404.1 elongation factor G [Candidatus Mycoplasma girerdii]
246   9e-69
WP_058475903.1 elongation factor G [Legionella steigerwaltii]
246   9e-69
OGJ81494.1 translation elongation factor G [Candidatus Periba...
246   9e-69
WP_065466918.1 GTP-binding protein [Bifidobacterium dentium]
246   9e-69
WP_096181020.1 elongation factor G [Effusibacillus lacus]
246   9e-69
WP_009323180.1 MULTISPECIES: elongation factor G [Ruminococca...
247   9e-69
ETP68263.1 hypothetical protein G159_13165 [Planomicrobium gl...
245   9e-69
WP_020743283.1 elongation factor G [Alteromonas mediterranea]
246   9e-69
WP_024501010.1 GTP-binding protein [Lactobacillus fermentum]
245   9e-69
WP_077198341.1 MULTISPECIES: elongation factor G [Thermosipho]
246   9e-69
WP_022774988.1 GTP-binding protein [Butyrivibrio sp. AE2015]
250   9e-69
WP_057809621.1 GTP-binding protein [Lactobacillus intestinalis]
245   9e-69
WP_006645265.1 elongation factor G [Photobacterium sp. SKA34]
246   9e-69
WP_102067749.1 elongation factor G [Ralstonia pickettii]
246   1e-68
WP_094600181.1 elongation factor G [Paenibacillus sp. XY044]
246   1e-68
WP_033165746.1 GTP-binding protein [Clostridium sp. KNHs205]
250   1e-68
PID51844.1 elongation factor G [Candidatus Moranbacteria bact...
246   1e-68
WP_004230228.1 GTP-binding protein [Lysinibacillus fusiformis]
245   1e-68
WP_031561192.1 GTP-binding protein [Ruminococcus flavefaciens]
249   1e-68
WP_083506436.1 elongation factor G [Chryseobacterium greenlan...
247   1e-68
OUT72414.1 elongation factor G [Synechococcus sp. TMED20]
246   1e-68
WP_055072866.1 GTP-binding protein [Anaerostipes hadrus]
250   1e-68
PKQ26694.1 elongation factor G [Actinobacteria bacterium HGW-...
246   1e-68
KIF37701.1 elongation factor G [Hassallia byssoidea VB512170]
246   1e-68
KKR10201.1 Elongation factor G [Parcubacteria group bacterium...
246   1e-68

WP_054880671.1 elongation factor G [Mycobacterium haemophilum]
246   1e-68
KUO69923.1 elongation factor G [Clostridia bacterium BRH_c25]
246   1e-68
WP_049153568.1 GTP-binding protein [Lactobacillus salivarius]
245   1e-68
PIS22264.1 elongation factor G [candidate division WWE3 bacte...
246   1e-68
SCI99378.1 Elongation factor G [uncultured Clostridium sp.]
251   1e-68
WP_035109769.1 elongation factor G [Corynebacterium efficiens]
246   1e-68
WP_066159547.1 elongation factor G [Bacillus krulwichiae]
246   1e-68
KKQ23478.1 Elongation factor G [Parcubacteria group bacterium...
246   1e-68
WP_024033968.1 MULTISPECIES: elongation factor G [Pseudoalter...
246   1e-68
WP_042126435.1 GTP-binding protein [Paenibacillus sp. FSL R5-...
246   1e-68
OUT81872.1 elongation factor G [Roseibacillus sp. TMED18]
247   1e-68
GAK54321.1 elongation factor G 2 [Candidatus Moduliflexus flo...
246   1e-68
EPJ43720.1 FusA protein [Osedax symbiont Rs1]
246   1e-68
WP_070740307.1 MULTISPECIES: elongation factor G [Corynebacte...
246   1e-68
WP_015174048.1 elongation factor G [Oscillatoria nigro-viridis]
246   1e-68
WP_082310695.1 elongation factor G [Nonomuraea sp. SBT364]
246   1e-68
WP_070525756.1 MULTISPECIES: elongation factor G [Corynebacte...
246   1e-68
OUV41745.1 elongation factor G [Candidatus Marinimicrobia bac...
246   1e-68
PSO53265.1 elongation factor G [Cyanobacteria bacterium QH_10...
246   1e-68
APR82482.1 Translation elongation factor G [Minicystis rosea]
246   1e-68
WP_015210287.1 elongation factor G [Cylindrospermum stagnale]
246   1e-68
WP_107562447.1 elongation factor G [Staphylococcus xylosus]
246   1e-68
WP_027421686.1 GTP-binding protein [Lachnobacterium bovis]
250   1e-68
WP_058210165.1 elongation factor G [Lactococcus lactis]
246   1e-68
WP_066780101.1 elongation factor G [Coriobacteriales bacteriu...
246   1e-68
WP_095537560.1 elongation factor G [Corynebacterium sp. NML99...
246   1e-68
EDK72571.1 translation elongation factor G [candidate divisio...
246   1e-68

WP_093274824.1 elongation factor G [Psychrobacillus sp. OK032]
246   1e-68
KKS20484.1 translation elongation factor g, elongation factor...
246   1e-68
WP_068756717.1 elongation factor G [Thermobifida cellulosilyt...
246   1e-68
AKQ03851.1 translation elongation factor G, elongation factor...
247   1e-68
OGP12848.1 translation elongation factor G [Deltaproteobacter...
246   1e-68
WP_048347870.1 elongation factor G [Synechococcus sp. WH 8020]
246   1e-68
WP_045986127.1 elongation factor G [Vibrio coralliilyticus]
246   1e-68
WP_042475827.1 GTP-binding protein [Bacillus ndiopicus]
245   1e-68
WP_024788193.1 MULTISPECIES: elongation factor G [Lebetimonas]
246   1e-68
WP_034519115.1 GTP-binding protein [Bifidobacterium sp. MSTE12]
246   1e-68
WP_099045870.1 elongation factor G [Mycobacterium grossiae]
246   1e-68
WP_038164967.1 elongation factor G [Vibrio sp. B183]
246   1e-68
PIW92013.1 elongation factor G [Candidatus Nealsonbacteria ba...
246   1e-68
WP_013103429.1 MULTISPECIES: elongation factor G [Leuconostoc]
246   1e-68
WP_065740688.1 elongation factor G [Vibrio coralliilyticus]
246   1e-68
WP_072940144.1 MULTISPECIES: elongation factor G [Rhodococcus]
246   1e-68
OGC93271.1 translation elongation factor G [candidate divisio...
246   1e-68
WP_044924115.1 GTP-binding protein [Anaerostipes hadrus]
249   1e-68
KXS24737.1 GTP-binding protein [Bifidobacterium dentium]
246   1e-68
WP_105258484.1 elongation factor G [Pseudoalteromonas sp. T1l...
246   1e-68
WP_100658327.1 elongation factor G [Alteromonas sp. P0211]
246   1e-68
WP_089932187.1 MULTISPECIES: GTP-binding protein [Lysinibacil...
245   1e-68
WP_015838551.1 elongation factor G [Geobacter sp. M21]
246   1e-68
WP_101086185.1 elongation factor G [Shewanella sp. ALD9]
246   1e-68
WP_011636038.1 MULTISPECIES: elongation factor G [Shewanella]
246   1e-68
WP_036808106.1 GTP-binding protein [Planomicrobium glaciei]
245   1e-68
WP_082946878.1 elongation factor G [Mycobacterium sp. ACS4054]
246   1e-68

WP_076041670.1 GTP-binding protein [Clostridium botulinum]
245   1e-68
WP_105189512.1 elongation factor G [Pseudoalteromonas sp. T1l...
246   1e-68
WP_015639178.1 GTP-binding protein [Lactobacillus fermentum]
245   1e-68
WP_033490412.1 MULTISPECIES: elongation factor G [Bifidobacte...
246   1e-68
WP_091275148.1 elongation factor G [Alteribacillus persepolen...
246   1e-68
WP_071471524.1 elongation factor G [Oceanisphaera psychrotole...
246   1e-68
WP_018313741.1 elongation factor G [Cupriavidus sp. UYPR2.512]
246   1e-68
KKQ26274.1 Elongation factor G [Microgenomates group bacteriu...
247   1e-68
WP_075839235.1 elongation factor G [Rhodococcus sp. CUA-806]
246   1e-68
WP_092793421.1 elongation factor G [Rheinheimera pacifica]
246   1e-68
WP_086224031.1 MULTISPECIES: elongation factor G [Campylobacter]
246   1e-68
WP_069304992.1 elongation factor G [Pelagibacteraceae bacteri...
246   1e-68
WP_085544297.1 elongation factor G [Dethiosulfovibrio salsugi...
246   1e-68
WP_080539651.1 elongation factor G [Leuconostoc pseudomesente...
246   1e-68
WP_053095175.1 elongation factor G [Moorella thermoacetica]
246   1e-68
WP_070156848.1 elongation factor G [Sphingobium phenoxybenzoa...
246   1e-68
WP_094376139.1 GTP-binding protein [Lachnotalea glycerini]
249   1e-68
OTO43979.1 tetracycline resistance protein TetM transposon [E...
229   1e-68
WP_100142503.1 elongation factor G [Shewanella carassii]
246   1e-68
WP_070955009.1 elongation factor G [Candidatus Rhodoluna plan...
246   1e-68
WP_101177902.1 GTP-binding protein [Bacillus nealsonii]
245   1e-68
WP_020207797.1 elongation factor G [Gilvimarinus chinensis]
246   1e-68
WP_068230957.1 elongation factor G [Rheinheimera sp. EpRS3]
246   1e-68
WP_081027624.1 elongation factor G [Faecalibacterium prausnit...
246   1e-68
WP_077287537.1 elongation factor G [Thermosipho sp. 1074]
246   1e-68
WP_019124176.1 elongation factor G [Peptoniphilus grossensis]
246   1e-68
WP_054552334.1 MULTISPECIES: elongation factor G [Pseudoalter...
246   1e-68

OHA49498.1 translation elongation factor G [Candidatus Terryb...
246   1e-68
WP_047258941.1 elongation factor G [Corynebacterium uterequi]
246   1e-68
WP_008392300.1 GTP-binding protein [Clostridium sp. SS2/1]
249   1e-68
WP_070766532.1 elongation factor G [Fusobacterium sp. HMSC064...
246   1e-68
WP_005286785.1 elongation factor G [Corynebacterium genitalium]
246   1e-68
WP_097245167.1 elongation factor G [Nocardia amikacinitolerans]
246   1e-68
WP_006784720.1 GTP-binding protein [Turicibacter sanguinis]
245   1e-68
WP_089348968.1 elongation factor G [Pseudoalteromonas espejiana]
246   1e-68
WP_092118156.1 elongation factor G [Desulfonatronum thiosulfa...
246   1e-68
WP_037299095.1 GTP-binding protein [Ruminococcus flavefaciens]
249   1e-68
WP_101302453.1 GTP-binding protein [Herbivorax saccincola]
249   1e-68
WP_011128993.1 elongation factor G [Synechococcus sp. WH 8102]
246   1e-68
WP_086439761.1 GTP-binding protein [Lactobacillus fermentum]
245   1e-68
WP_027639825.1 elongation factor G [Clostridium cadaveris]
246   1e-68
WP_027407294.1 elongation factor G [Anaerovibrio sp. RM50]
246   1e-68
OUV61208.1 elongation factor G [Candidatus Pelagibacter sp. T...
246   1e-68
WP_055258388.1 GTP-binding protein [Anaerostipes hadrus]
249   1e-68
WP_034440937.1 elongation factor G [Tissierellia bacterium S5...
246   1e-68
WP_102152576.1 elongation factor G [Fischerella thermalis]
246   1e-68
WP_011895900.1 MULTISPECIES: elongation factor G [Mycobacterium]
246   1e-68
WP_072841939.1 MULTISPECIES: elongation factor G [Marinomonas]
246   1e-68
WP_096623314.1 elongation factor G [Calothrix sp. NIES-3974]
246   1e-68
WP_105452008.1 GTP-binding protein [Lactobacillus sp. CBA3605]
245   1e-68
WP_060810328.1 elongation factor G [Enterococcus faecium]
246   1e-68
WP_024605154.1 MULTISPECIES: elongation factor G [Pseudoalter...
246   1e-68
OFJ54343.1 translation elongation factor G [Mycobacterium gro...
246   1e-68
WP_094693546.1 elongation factor G [Bifidobacterium aquikefiri]
246   1e-68

WP_059048876.1 elongation factor G [Paenibacillus senegalimas...
246   1e-68
OHA02990.1 translation elongation factor G [Candidatus Sungba...
246   1e-68
WP_084229215.1 elongation factor G [Mycobacterium sherrisii]
246   1e-68
WP_084008197.1 elongation factor G [Planomonospora sphaerica]
246   1e-68
WP_095295600.1 elongation factor G [Helicobacter sp. 13S00482-2]
246   1e-68
WP_022789475.1 GTP-binding protein [Faecalicoccus pleomorphus]
249   1e-68
WP_105265668.1 elongation factor G [Pseudoalteromonas sp. T1l...
246   1e-68
WP_039225640.1 MULTISPECIES: elongation factor G [Alteromonas]
246   1e-68
ASJ88926.1 elongation factor G [Candidatus Mycoplasma girerdii]
246   1e-68
PIP20271.1 elongation factor G [Candidatus Omnitrophica bacte...
246   1e-68
CDF08721.1 putative uncharacterized protein [Firmicutes bacte...
249   1e-68
WP_094503094.1 GTP-binding protein [Lactobacillus taiwanensis]
244   1e-68
WP_102178138.1 elongation factor G [Fischerella thermalis]
246   1e-68
WP_014582831.1 elongation factor G [Mycoplasma haemofelis]
246   1e-68
WP_019274079.1 elongation factor G [Vibrio coralliilyticus]
246   1e-68
WP_056023402.1 elongation factor G [Cellulomonas sp. Leaf395]
246   1e-68
KKT11769.1 Elongation factor G [candidate division WWE3 bacte...
246   1e-68
WP_090892778.1 elongation factor G [Bacillus caseinilyticus]
246   1e-68
WP_019935950.1 elongation factor G [Oceanimonas smirnovii]
246   1e-68
OGZ45935.1 translation elongation factor G [Candidatus Ryanba...
246   1e-68
WP_065962965.1 elongation factor G [Streptomyces sparsogenes]
246   1e-68
WP_066669698.1 elongation factor G [Desulfotomaculum copahuen...
246   1e-68
CCZ45504.1 elongation factor G [Firmicutes bacterium CAG:129]
246   1e-68
WP_087434758.1 GTP-binding protein [Paenibacillus apiarius]
245   1e-68
WP_086246770.1 elongation factor G [Campylobacter sp. S0112]
246   1e-68
WP_068381533.1 elongation factor G [Rhodococcus sp. EPR-157]
246   1e-68
WP_105559256.1 elongation factor G [Achromobacter sp. MYb9]
246   1e-68

OUW21593.1 elongation factor G [Candidatus Pelagibacter sp. T...
246   1e-68
WP_075763385.1 elongation factor G [Photobacterium proteolyti...
246   1e-68
WP_089743879.1 GTP-binding protein [Gracilibacillus ureilyticus]
245   1e-68
KKR79240.1 Elongation factor G [Parcubacteria group bacterium...
246   1e-68
WP_072469149.1 elongation factor G [Urinacoccus massiliensis]
246   1e-68
WP_049572478.1 elongation factor G [Streptomyces sp. SBT349]
246   1e-68
WP_009456045.1 MULTISPECIES: elongation factor G [Fischerella]
246   1e-68
WP_101197482.1 elongation factor G [Paraglaciecola sp. MB-3u-78]
246   1e-68
WP_102866715.1 elongation factor G [Nesiotobacter exalbescens]
246   1e-68
WP_102412314.1 elongation factor G [Clostridiales bacterium M...
246   1e-68
EIC28588.1 translation elongation factor EF-G [Methylomicrobi...
246   1e-68
WP_038543042.1 elongation factor G [Synechococcus sp. KORDI-100]
246   1e-68
WP_104718098.1 elongation factor G [Helicobacter trogontum]
246   1e-68
OFW15761.1 translation elongation factor G [Acidobacteria bac...
246   1e-68
WP_028482324.1 elongation factor G [Nesiotobacter exalbescens]
246   1e-68
WP_067409071.1 elongation factor G [Mycobacterium sp. 1423905.2]
246   1e-68
WP_060992420.1 MULTISPECIES: elongation factor G [Aliivibrio]
246   1e-68
XP_021795150.1 ribosome-releasing factor 2, mitochondrial iso...
247   2e-68
WP_095546811.1 elongation factor G [Corynebacterium sp. NML 1...
246   2e-68
WP_057583755.1 elongation factor G [Paeniclostridium sordellii]
246   2e-68
WP_061612077.1 elongation factor G [Sorangium cellulosum]
246   2e-68
KGA04794.1 elongation factor EF-G [actinobacterium acMicro-4]
246   2e-68
AWE07172.1 elongation factor G [Lysinibacillus sp. 2017]
244   2e-68
WP_027829467.1 GTP-binding protein [Lactobacillus harbinensis]
245   2e-68
BBA71284.1 Elongation factor G [Geobacter sulfurreducens]
246   2e-68
WP_018367584.1 elongation factor G [Streptococcus entericus]
246   2e-68
WP_086243888.1 MULTISPECIES: elongation factor G [Campylobacter]
246   2e-68

WP_009204669.1 GTP-binding protein [Anaerostipes hadrus]
249   2e-68
WP_083171591.1 elongation factor G [Mycobacterium paraseoulense]
246   2e-68
WP_010052042.1 elongation factor G [Carnobacterium maltaromat...
246   2e-68
WP_034437241.1 elongation factor G [Tissierellia bacterium S5...
246   2e-68
EQC59314.1 elongation factor G [Lactobacillus fermentum MTCC ...
244   2e-68
PHS24936.1 elongation factor G [Methylophaga sp.]
246   2e-68
ODV42482.1 elongation factor G [Cupriavidus sp. UYMMa02A]
246   2e-68
WP_028036889.1 elongation factor G [Candidatus Pelagibacter u...
246   2e-68
WP_103953376.1 GTP-binding protein [Lachnospira multipara]
249   2e-68
WP_066744963.1 elongation factor G [Sphingomonadales bacteriu...
246   2e-68
WP_012324824.1 MULTISPECIES: elongation factor G [Shewanella]
246   2e-68
WP_104710312.1 elongation factor G [Helicobacter felis]
246   2e-68
WP_075608999.1 MULTISPECIES: elongation factor G [Alteromonad...
246   2e-68
PIR02060.1 elongation factor G [Candidatus Nealsonbacteria ba...
246   2e-68
PQM54370.1 elongation factor G [Deltaproteobacteria bacterium]
246   2e-68
WP_011274173.1 elongation factor G [Corynebacterium jeikeium]
246   2e-68
BAC17326.1 putative translation elongation factor EF-G [Coryn...
246   2e-68
PCJ48891.1 elongation factor G [Gammaproteobacteria bacterium]
246   2e-68
PIU40606.1 elongation factor G [Candidatus Omnitrophica bacte...
246   2e-68
WP_089856846.1 GTP-binding protein [Lachnospiraceae bacterium...
249   2e-68
WP_099805172.1 GTP-binding protein [Lysinibacillus sphaericus]
244   2e-68
WP_105441901.1 elongation factor G [Paenibacillus sp. AR247]
246   2e-68
WP_033068676.1 elongation factor G [Thalassospira australica]
246   2e-68
WP_048416413.1 MULTISPECIES: elongation factor G [Mycobacteri...
246   2e-68
WP_100590511.1 elongation factor G [Campylobacter lanienae]
246   2e-68
WP_089702885.1 elongation factor G [Clostridiales bacterium C...
246   2e-68
OHA46224.1 translation elongation factor G [Candidatus Terryb...
246   2e-68

WP_036216060.1 GTP-binding protein [Lysinibacillus sphaericus]
244   2e-68
WP_104686260.1 elongation factor G [Helicobacter felis]
246   2e-68
WP_047252301.1 elongation factor G [Corynebacterium testudino...
246   2e-68
WP_075705098.1 GTP-binding protein [Intestinimonas butyricipr...
249   2e-68
AJK67994.1 Elongation factor G [Corynebacterium marinum DSM 4...
246   2e-68
PIQ81735.1 elongation factor G [Candidatus Omnitrophica bacte...
246   2e-68
WP_087180357.1 MULTISPECIES: elongation factor G [Gemmiger]
246   2e-68
WP_081662175.1 MULTISPECIES: elongation factor G [Mycobacterium]
246   2e-68
OGP19597.1 translation elongation factor G [Deltaproteobacter...
245   2e-68
WP_095664581.1 elongation factor G [Vibrio coralliilyticus]
246   2e-68
WP_096025764.1 elongation factor G [Campylobacter lanienae]
246   2e-68
WP_104697455.1 MULTISPECIES: elongation factor G [Helicobacter]
246   2e-68
WP_094536345.1 elongation factor G [Pseudanabaena sp. SR411]
246   2e-68
WP_015193497.1 elongation factor G [Stanieria cyanosphaera]
246   2e-68
WP_021382502.1 elongation factor G [Clostridioides difficile]
246   2e-68
WP_086226549.1 MULTISPECIES: elongation factor G [Campylobacter]
246   2e-68
WP_088184257.1 elongation factor G [Sphingobium sp. Z007]
246   2e-68
WP_083405430.1 elongation factor G [Mycobacterium rutilum]
246   2e-68
WP_101759827.1 elongation factor G [Oceanicoccus sp. KOV_DT_Chl]
246   2e-68
PKM20389.1 elongation factor G [Gammaproteobacteria bacterium...
246   2e-68
WP_020429795.1 elongation factor G [Vibrio fluvialis]
246   2e-68
SHK36771.1 translation elongation factor 2 (EF-2/EF-G) [Rubri...
246   2e-68
KKR46592.1 Elongation factor G [Parcubacteria group bacterium...
246   2e-68
WP_079425267.1 elongation factor G [Clostridium oryzae]
245   2e-68
AGS06674.1 elongation factor G [Candidatus Carsonella ruddii DC]
245   2e-68
WP_069481220.1 GTP-binding protein [Lysinibacillus fusiformis]
244   2e-68
WP_020226031.1 elongation factor G [Holdemania massiliensis]
246   2e-68

WP_038183339.1 elongation factor G [Vibrio rhizosphaerae]
246   2e-68
PSP02877.1 elongation factor G [Cyanobacteria bacterium SW_7_...
246   2e-68
WP_065046089.1 elongation factor G [Mycobacterium gordonae]
246   2e-68
PIR66622.1 elongation factor G [Parcubacteria group bacterium...
246   2e-68
WP_056992603.1 GTP-binding protein [Lactobacillus saniviri]
244   2e-68
OGO19430.1 translation elongation factor G [Chloroflexi bacte...
246   2e-68
WP_004911302.1 MULTISPECIES: elongation factor G [Leuconostoc]
246   2e-68
WP_029455111.1 elongation factor G [Candidatus Pelagibacter u...
246   2e-68
CDD35508.1 yacP-like NYN domain protein [Roseburia sp. CAG:309]
249   2e-68
WP_054562931.1 MULTISPECIES: elongation factor G [Pseudoalter...
246   2e-68
WP_014205711.1 elongation factor G [Vibrio furnissii]
246   2e-68
WP_092969157.1 elongation factor G [Ruminococcaceae bacterium...
246   2e-68
WP_030489584.1 elongation factor G [Micromonospora chokoriensis]
246   2e-68
WP_020888149.1 elongation factor G [Desulfovibrio alkalitoler...
245   2e-68
WP_046227954.1 GTP-binding protein [Paenibacillus dauci]
244   2e-68
WP_034246519.1 GTP-binding protein [Lachnospira multipara]
249   2e-68
OON94631.1 translation elongation factor G [Epulopiscium sp. ...
246   2e-68
WP_019783950.1 elongation factor G [Streptococcus sobrinus]
246   2e-68
WP_072948577.1 GTP-binding protein [Ruminococcus flavefaciens]
249   2e-68
WP_094819858.1 elongation factor G [Bordetella genomosp. 4]
246   2e-68
WP_066751818.1 elongation factor G [Chryseobacterium glaciei]
245   2e-68
WP_042620702.1 elongation factor G [Corynebacterium marinum]
246   2e-68
WP_071243061.1 elongation factor G [Enterococcus faecium]
246   2e-68
WP_044833186.1 elongation factor G [Thalassomonas actiniarum]
246   2e-68
WP_074493471.1 elongation factor G [Corynebacterium glutamicum]
246   2e-68
WP_011329565.1 elongation factor G [Pseudoalteromonas halopla...
245   2e-68
WP_012549434.1 elongation factor G [Aliivibrio salmonicida]
245   2e-68

SCH50014.1 Vegetative protein 19 [uncultured Ruminococcus sp.]
245   2e-68
WP_026374651.1 elongation factor G [Aestuariibacter salexigens]
246   2e-68
WP_066062948.1 GTP-binding protein [Bacillus soli]
244   2e-68
WP_052890672.1 elongation factor G [Thermogemmatispora carbox...
245   2e-68
WP_060945186.1 elongation factor G [Streptomyces hygroscopicus]
246   2e-68
WP_046948261.1 GTP-binding protein [Lactobacillus fermentum]
244   2e-68
WP_059012852.1 elongation factor G [Streptomyces specialis]
246   2e-68
WP_034929935.1 GTP-binding protein [Erysipelotrichaceae bacte...
248   2e-68
OGD71119.1 translation elongation factor G [Candidatus Collie...
246   2e-68
WP_030074773.1 elongation factor G [Streptomyces decoyicus]
246   2e-68
WP_082950935.1 elongation factor G [Mycobacterium sp. 1164985.4]
246   2e-68
WP_073885922.1 elongation factor G [Saccharothrix sp. CB00851]
246   2e-68
SCH21196.1 Elongation factor G [uncultured Blautia sp.]
250   2e-68
WP_101113196.1 elongation factor G [Vibrio sp. vnigr-6D03]
245   2e-68
WP_029070269.1 GTP-binding protein [Kandleria vitulina]
248   2e-68
WP_017220942.1 elongation factor G [Moritella dasanensis]
245   2e-68
WP_055912141.1 elongation factor G [Cellulomonas sp. Root485]
246   2e-68
WP_027678874.1 elongation factor G [Ralstonia sp. UNC404CL21Col]
246   2e-68
WP_082600175.1 MULTISPECIES: elongation factor G [Nocardioides]
246   2e-68
OKZ78058.1 translation elongation factor G [Clostridium sp. 2...
245   2e-68
AHF24165.1 elongation factor G [uncultured bacterium Contig1831]
245   2e-68
ADL04589.1 small GTP-binding protein [[Clostridium] saccharol...
244   2e-68
PIW75152.1 elongation factor G [Candidatus Portnoybacteria ba...
245   2e-68
WP_102171786.1 elongation factor G [Fischerella thermalis]
245   2e-68
WP_105059898.1 elongation factor G [Photobacterium angustum]
245   2e-68
WP_057778655.1 elongation factor G [Lactobacillus vaccinoster...
246   2e-68
WP_106534190.1 GTP-binding protein [Planomicrobium soli]
244   2e-68

WP_101508999.1 GTP-binding protein [Paeniclostridium sordellii]
245   2e-68
WP_087570304.1 elongation factor G [Paenibacillus sp. MY03]
245   2e-68
WP_034585995.1 elongation factor G [Clostridium acetobutylicum]
245   2e-68
WP_011530994.1 elongation factor G [Deinococcus geothermalis]
245   2e-68
PIS05441.1 elongation factor G [Candidatus Buchananbacteria b...
245   2e-68
WP_008218496.1 elongation factor G [Rheinheimera nanhaiensis]
245   2e-68
WP_049283640.1 elongation factor G [Staphylococcus aureus]
245   2e-68
WP_087117141.1 elongation factor G [Corynebacterium urinapleo...
246   2e-68
WP_054968801.1 elongation factor G [Alicyclobacillus ferrooxy...
245   2e-68
WP_087440373.1 GTP-binding protein [Paenibacillus thiaminolyt...
244   2e-68
WP_091102550.1 elongation factor G [Micromonospora citrea]
245   2e-68
WP_072415168.1 GTP-binding protein [Collinsella phocaeensis]
245   2e-68
WP_009341972.1 MULTISPECIES: elongation factor G [Aphanizomen...
245   2e-68
WP_016865166.1 elongation factor G [Fischerella muscicola]
245   2e-68
WP_061957648.1 elongation factor G [Cupriavidus pauculus]
246   2e-68
WP_019000481.1 elongation factor G [Succinimonas amylolytica]
245   2e-68
KKQ68261.1 translation elongation factor G, elongation factor...
245   2e-68
EPR43748.1 translation elongation factor G [Desulfovibrio sp....
245   2e-68
OYZ09057.1 elongation factor G [Novosphingobium sp. 28-62-57]
245   2e-68
OUV05624.1 elongation factor G [bacterium TMED80]
245   2e-68
WP_090031897.1 elongation factor G [Cellulomonas marina]
245   2e-68
WP_033428573.1 elongation factor G [Saccharothrix syringae]
245   2e-68
SDY28014.1 translation elongation factor 2 (EF-2/EF-G) [Tessa...
245   2e-68
WP_087220019.1 GTP-binding protein [Blautia sp. An249]
249   2e-68
WP_060624300.1 GTP-binding protein [Paenibacillus etheri]
244   2e-68
GBE31571.1 elongation factor G [bacterium BMS3Bbin05]
245   2e-68
WP_054399532.1 GTP-binding protein [Lactobacillus plantarum]
245   2e-68

WP_014182789.1 elongation factor G [Desulfosporosinus orientis]
245   2e-68
WP_035040696.1 elongation factor G [Desulfovibrio sp. X2]
245   2e-68
WP_064883868.1 elongation factor G [Mycobacterium colombiense]
245   2e-68
WP_012705401.1 GTP-binding protein [Clostridium botulinum]
244   2e-68
KKU07330.1 Elongation factor G [Candidatus Magasanikbacteria ...
245   2e-68
ERJ13284.1 Elongation factor G protein [Haloplasma contractil...
249   2e-68
WP_038865922.1 MULTISPECIES: elongation factor G [Vibrio harv...
245   2e-68
WP_089368884.1 elongation factor G [Pseudoalteromonas nigrifa...
245   2e-68
OGY80431.1 translation elongation factor G [Candidatus Kerfel...
245   2e-68
WP_066818169.1 elongation factor G [Mycobacterium sp. 1100029.7]
245   2e-68
WP_034546946.1 elongation factor G [Carnobacterium alterfundi...
245   2e-68
OGC82986.1 translation elongation factor G [Candidatus Abawac...
245   2e-68
WP_099431355.1 GTP-binding protein [Collinsella aerofaciens]
244   2e-68
WP_031928956.1 elongation factor G [Staphylococcus aureus]
245   2e-68
WP_008825975.1 GTP-binding protein [Haloplasma contractile]
249   2e-68
WP_027087346.1 elongation factor G [Cohnella panacarvi]
245   2e-68
WP_055310009.1 GTP-binding protein [Collinsella aerofaciens]
244   2e-68
EKD94753.1 hypothetical protein ACD_25C00239G0001 [uncultured...
245   2e-68
WP_104696027.1 elongation factor G [Helicobacter salomonis]
245   2e-68
WP_104689667.1 elongation factor G [Helicobacter felis]
245   2e-68
WP_091663700.1 elongation factor G [Alteribacillus iranensis]
245   2e-68
CDC36938.1 putative uncharacterized protein [Butyrivibrio sp....
248   2e-68
WP_058007700.1 GTP-binding protein [Clostridium sporogenes]
244   2e-68
EFQ07439.1 translation elongation factor G [Faecalibacterium ...
245   3e-68
WP_076176733.1 MULTISPECIES: elongation factor G [Paenibacillus]
245   3e-68
WP_062838529.1 elongation factor G [Achromobacter xylosoxidans]
245   3e-68
KGI66984.1 elongation factor G [Mycobacterium rufum]
245   3e-68

WP_017815037.1 GTP-binding protein [Paenibacillus sp. A9]
244   3e-68
WP_066453355.1 elongation factor G [Anaerotruncus rubiinfantis]
245   3e-68
OGP25054.1 translation elongation factor G [Deltaproteobacter...
245   3e-68
WP_090300277.1 GTP-binding protein [Pseudobutyrivibrio sp. AC...
248   3e-68
WP_085240535.1 elongation factor G [Mycobacterium europaeum]
245   3e-68
OLA26091.1 translation elongation factor G [Firmicutes bacter...
248   3e-68
WP_107029414.1 GTP-binding protein [Lachnoclostridium sp. SNU...
248   3e-68
WP_034296159.1 elongation factor G [Herbaspirillum sp. RV1423]
245   3e-68
WP_087199853.1 GTP-binding protein [Collinsella sp. An271]
245   3e-68
WP_068606583.1 elongation factor G [Paenibacillus swuensis]
245   3e-68
ABE11443.1 elongation factor G [uncultured Prochlorococcus ma...
245   3e-68
WP_006632195.1 elongation factor G [Microcoleus vaginatus]
245   3e-68
SCI51730.1 Tetracycline resistance protein tetM [uncultured R...
248   3e-68
WP_047069426.1 GTP-binding protein [Brevibacillus formosus]
244   3e-68
Q8NT19.1 RecName: Full=Elongation factor G; Short=EF-G
245   3e-68
KKS03467.1 Elongation factor G [candidate division WWE3 bacte...
245   3e-68
WP_097786264.1 elongation factor G [Faecalibacterium prausnit...
245   3e-68
WP_024835884.1 GTP-binding protein [Clostridium sp. 12(A)]
244   3e-68
WP_068657356.1 elongation factor G [Paenibacillus crassostreae]
245   3e-68
WP_034580137.1 elongation factor G [Helicobacter bilis]
245   3e-68
WP_087118838.1 GTP-binding protein [Lactobacillus salivarius]
244   3e-68
WP_068284507.1 elongation factor G [Mycobacterium sp. E2462]
245   3e-68
WP_073608944.1 elongation factor G [Phormidium tenue]
245   3e-68
WP_055306333.1 GTP-binding protein [Lachnospira pectinoschiza]
248   3e-68
ORU94926.1 elongation factor G [Cycloclasticus sp. symbiont o...
245   3e-68
WP_010966416.1 MULTISPECIES: elongation factor G [Clostridium]
245   3e-68
WP_006042263.1 elongation factor G [Synechococcus sp. WH 7805]
245   3e-68

WP_011476678.1 GTP-binding protein [Lactobacillus salivarius]
244   3e-68
WP_030912936.1 elongation factor G [Streptosporangium amethys...
245   3e-68
WP_077388463.1 elongation factor G [Helicobacter bilis]
245   3e-68
WP_082946167.1 elongation factor G [Mycobacterium sp. E1747]
245   3e-68
WP_065161808.1 elongation factor G [Mycobacterium gordonae]
245   3e-68
WP_075752818.1 elongation factor G [Sporomusa sphaeroides]
245   3e-68
WP_031892102.1 elongation factor G [Staphylococcus aureus]
245   3e-68
WP_055668486.1 elongation factor G [Desnuesiella massiliensis]
245   3e-68
OPL12776.1 elongation factor G [delta proteobacterium ML8_D]
244   3e-68
WP_060949304.1 MULTISPECIES: elongation factor G [Streptomyces]
245   3e-68
PIZ69817.1 elongation factor G [Candidatus Portnoybacteria ba...
245   3e-68
WP_027964791.1 elongation factor G [Halalkalibacillus halophi...
245   3e-68
WP_017185244.1 elongation factor G [Alkalibacillus haloalkali...
245   3e-68
WP_102794239.1 elongation factor G [Bowmanella denitrificans]
245   3e-68
WP_100184098.1 GTP-binding protein [Lactobacillus fermentum]
244   3e-68
WP_025738358.1 elongation factor G [Mycobacterium genavense]
245   3e-68
CDA36477.1 putative translation elongation factor G [Collinse...
244   3e-68
OYV98551.1 elongation factor G [Acidobacteria bacterium 21-70...
245   3e-68
WP_022841852.1 elongation factor G [Acidobacteriaceae bacteri...
245   3e-68
WP_055262561.1 GTP-binding protein [Roseburia faecis]
249   3e-68
EEJ73406.1 putative translation elongation factor G [Lactobac...
244   3e-68
WP_091198337.1 elongation factor G [Nocardioides alpinus]
245   3e-68
WP_068679378.1 elongation factor G [Paenibacillus oryzae]
245   3e-68
WP_087234713.1 GTP-binding protein [Anaeromassilibacillus sp....
248   3e-68
XP_024646553.1 ribosome-releasing factor 2, mitochondrial iso...
246   3e-68
WP_073087989.1 elongation factor G [Selenomonas ruminantium]
245   3e-68
WP_042529272.1 MULTISPECIES: elongation factor G [Corynebacte...
245   3e-68

WP_034210477.1 GTP-binding protein [Lachnospira multipara]
248   3e-68
WP_012277564.1 elongation factor G [Shewanella halifaxensis]
245   3e-68
WP_033269710.1 MULTISPECIES: elongation factor G [Streptomyces]
245   3e-68
WP_057507246.1 elongation factor G [Stenotrophomonas chelatip...
244   3e-68
WP_082210838.1 elongation factor G [Faecalibacterium prausnit...
245   3e-68
WP_106457273.1 elongation factor G [Aphanothece hegewaldii]
245   3e-68
WP_018753996.1 elongation factor G [Paenibacillus sanguinis]
245   3e-68
WP_053833003.1 GTP-binding protein [Candidatus Dorea massilie...
244   3e-68
WP_097774838.1 elongation factor G [Faecalibacterium prausnit...
245   3e-68
OGZ53646.1 translation elongation factor G [Candidatus Ryanba...
245   3e-68
CCX93731.1 elongation factor G [Firmicutes bacterium CAG:110]
245   3e-68
PQC52044.1 elongation factor G [Enterococcus faecium]
228   3e-68
WP_107836224.1 elongation factor G [Opitutus sp. ER46]
246   3e-68
WP_030538850.1 elongation factor G [Sphingobium sp. DC-2]
245   3e-68
WP_092476095.1 elongation factor G [Desulfotomaculum arcticum]
245   3e-68
WP_075667605.1 GTP-binding protein [Lactobacillus fermentum]
244   3e-68
WP_007167814.1 MULTISPECIES: elongation factor G [Mycobacterium]
245   3e-68
KXS56389.1 elongation factor G [Candidatus Adiutrix intracell...
244   3e-68
WP_009221950.1 elongation factor G [Peptoniphilus sp. oral ta...
245   3e-68
PIR96442.1 elongation factor G [Candidatus Doudnabacteria bac...
245   3e-68
WP_061342282.1 GTP-binding protein [Clostridium botulinum]
244   3e-68
WP_040199028.1 elongation factor G [Geoalkalibacter subterran...
245   3e-68
OUW65355.1 elongation factor G [Opitutales bacterium TMED207]
245   3e-68
OGZ64530.1 translation elongation factor G [Candidatus Staska...
245   3e-68
WP_102168401.1 GTP-binding protein [Gardnerella vaginalis]
244   3e-68
WP_082953480.1 MULTISPECIES: elongation factor G [Mycobacterium]
245   3e-68
WP_006231309.1 elongation factor G [Photobacterium profundum]
245   3e-68

OKZ58792.1 translation elongation factor G [Clostridiales bac...
250   3e-68
WP_012391404.1 GTP-binding protein [Lactobacillus fermentum]
244   3e-68
WP_087377186.1 GTP-binding protein [Flavonifractor sp. An135]
248   3e-68
WP_093373932.1 elongation factor G [Tindallia magadiensis]
245   3e-68
WP_064199425.1 GTP-binding protein [Brevibacillus brevis]
244   3e-68
WP_037354388.1 elongation factor G [Selenomonas sp. FC4001]
245   3e-68
WP_069775629.1 GTP-binding protein [Lactobacillus fermentum]
244   3e-68
WP_085532706.1 GTP-binding protein [Anaeromassilibacillus sp....
248   3e-68
WP_101696352.1 elongation factor G [Clostridium minihomine]
245   3e-68
WP_060564020.1 MULTISPECIES: elongation factor G [Corynebacte...
245   3e-68
WP_019528764.1 elongation factor G [Dasania marina]
245   3e-68
KHF32742.1 Tetracycline resistance protein TetO [Paenibacillu...
236   3e-68
WP_099032433.1 GTP-binding protein [Lactobacillus fermentum]
244   3e-68
WP_070465712.1 elongation factor G [Corynebacterium sp. HMSC2...
245   3e-68
WP_012950485.1 elongation factor G [Geodermatophilus obscurus]
245   3e-68
WP_009783218.1 elongation factor G [Lyngbya sp. PCC 8106]
245   3e-68
WP_021244942.1 elongation factor G [Sphingobium baderi]
245   3e-68
WP_095506429.1 elongation factor G [Paraferrimonas sedimentic...
245   3e-68
WP_019787844.1 elongation factor G [Streptococcus sobrinus]
245   3e-68
WP_056327895.1 MULTISPECIES: elongation factor G [Achromobacter]
245   3e-68
WP_041709088.1 GTP-binding protein [[Clostridium] saccharolyt...
244   3e-68
WP_021239766.1 MULTISPECIES: elongation factor G [Sphingomona...
245   3e-68
WP_011013694.1 elongation factor G [Corynebacterium glutamicum]
245   3e-68
ODU67999.1 translation elongation factor G [Novosphingobium s...
245   3e-68
WP_066606494.1 elongation factor G [Sphingobium czechense]
245   3e-68
WP_057738094.1 GTP-binding protein [Lactobacillus alimentarius]
244   4e-68
WP_107736073.1 elongation factor G [Desulfonatronum sp. SC1]
245   4e-68

OLA01899.1 translation elongation factor G [Clostridium sp. C...
248   4e-68
KGO32739.1 elongation factor G [Desulfobulbus sp. Tol-SR]
245   4e-68
WP_021673417.1 elongation factor G [Streptococcus sobrinus]
245   4e-68
WP_034548151.1 elongation factor G [Tissierellia bacterium S7...
244   4e-68
PKV09661.1 GTP-binding protein [Bifidobacterium asteroides]
244   4e-68
WP_105253877.1 elongation factor G [Pseudoalteromonas sp. T1l...
245   4e-68
WP_020647437.1 elongation factor G [Amycolatopsis balhimycina]
244   4e-68
WP_085022938.1 elongation factor G [Anaerovibrio sp. JC8]
245   4e-68
WP_004729572.1 elongation factor G [Vibrio furnissii]
245   4e-68
WP_072842736.1 elongation factor G [Rhodococcus tukisamuensis]
245   4e-68
WP_076045379.1 GTP-binding protein [Clostridium botulinum]
244   4e-68
WP_023870153.1 elongation factor G [Mycobacterium avium]
245   4e-68
KPJ80449.1 elongation factor G [Gemmatimonas sp. SG8_23]
243   4e-68
WP_066816168.1 elongation factor G [Sphingomonas mali]
245   4e-68
WP_066103001.1 elongation factor G [Bacillus sp. FJAT-27986]
245   4e-68
WP_079526343.1 GTP-binding protein [Solibacillus isronensis]
244   4e-68
WP_084293953.1 elongation factor G [Mycobacterium flavescens]
245   4e-68
KOO14310.1 elongation factor G [Vibrio xuii]
245   4e-68
WP_045085724.1 elongation factor G [[Clostridium] cellulosi]
244   4e-68
AKQ02576.1 elongation factor G, elongation factor G [uncultur...
245   4e-68
WP_016458990.1 elongation factor G [Corynebacterium pyruvicip...
245   4e-68
OUT73407.1 elongation factor G [Synechococcus sp. TMED19]
244   4e-68
WP_014423832.1 elongation factor G [Selenomonas ruminantium]
244   4e-68
WP_067698013.1 elongation factor G [Actinoplanes awajinensis]
243   4e-68
KYF55148.1 elongation factor G [Sorangium cellulosum]
245   4e-68
WP_041903691.1 elongation factor G [Bacillus thermoamylovorans]
244   4e-68
GAO53952.1 translation elongation factor G [Novosphingobium s...
244   4e-68

PON16893.1 elongation factor G [Candidatus Entotheonella serta]
244   4e-68
OLA68527.1 translation elongation factor G [Ruminococcus sp. ...
248   4e-68
PAW68718.1 elongation factor G [Opitutae bacterium Tous-C1TDCM]
245   4e-68
WP_105219138.1 elongation factor G [Gloeocapsopsis sp. AAB1 =...
244   4e-68
WP_057475288.1 elongation factor G [Rhodococcus sp. Leaf278]
245   4e-68
WP_071800893.1 elongation factor G [Synechococcus sp. SynAce01]
244   4e-68
WP_043694757.1 elongation factor G [Synechococcus sp. KORDI-49]
244   4e-68
PIS17150.1 elongation factor G [Candidatus Portnoybacteria ba...
244   4e-68
WP_025559539.1 elongation factor G [Vibrio parahaemolyticus]
244   4e-68
WP_071441958.1 elongation factor G [Traorella massiliensis]
244   4e-68
SOX52210.1 elongation factor G [Mycobacterium sp. AFP003]
245   4e-68
WP_035136992.1 GTP-binding protein [Collinsella sp. 4_8_47FAA]
244   4e-68
OIO18322.1 translation elongation factor G [Candidatus Magasa...
244   4e-68
WP_094335332.1 GTP-binding protein [[Collinsella] massiliensis]
244   4e-68
APO35553.1 Elongation factor G C-terminus [uncultured bacterium]
227   4e-68
KKS39502.1 Elongation factor G [candidate division WWE3 bacte...
244   4e-68
WP_059174125.1 elongation factor G [Bacillus sp. FJAT-27445]
244   4e-68
WP_074572785.1 elongation factor G [Selenomonas ruminantium]
244   4e-68
OKZ60537.1 translation elongation factor G [Clostridium sp. C...
244   4e-68
KKS68685.1 Elongation factor G [candidate division TM6 bacter...
244   4e-68
WP_031790566.1 elongation factor G [Staphylococcus aureus]
244   4e-68
AOR73671.1 Elongation factor G [Lactobacillus fermentum]
243   4e-68
WP_066644163.1 elongation factor G [Clostridium sp. W14A]
244   4e-68
WP_011015672.1 elongation factor G [Fusobacterium nucleatum]
244   4e-68
WP_022937639.1 GTP-binding protein [Dielma fastidiosa]
248   4e-68
WP_049692528.1 elongation factor G [Synechococcus sp. WH 8103]
244   4e-68
WP_092317288.1 elongation factor G [Brevinema andersonii]
244   4e-68

WP_068950020.1 elongation factor G [Achromobacter xylosoxidans]
245   4e-68
WP_066168852.1 MULTISPECIES: elongation factor G [Lysinibacil...
244   4e-68
WP_006564712.1 elongation factor G [Helicobacter suis]
244   4e-68
WP_029066822.1 GTP-binding protein [Lachnobacterium bovis]
248   4e-68
CUM95510.1 Tetracycline resistance protein tetM [Roseburia fa...
249   4e-68
WP_031430851.1 elongation factor G [Methylomicrobium agile]
244   4e-68
WP_078543149.1 elongation factor G [Bacillus alkalitelluris]
244   4e-68
WP_058116901.1 GTP-binding protein [Intestinimonas butyricipr...
248   4e-68
WP_030483039.1 elongation factor G [Marmoricola aequoreus]
245   4e-68
WP_019225209.1 MULTISPECIES: elongation factor G [Dehalobacter]
244   4e-68
WP_090174528.1 GTP-binding protein [Eubacterium oxidoreducens]
248   4e-68
WP_010943489.1 elongation factor G [Geobacter sulfurreducens]
244   4e-68
CCZ65986.1 elongation factor Tu GTP binding domain-containing...
248   4e-68
WP_107318559.1 elongation factor G [Kandleria vitulina]
244   4e-68
WP_096284733.1 elongation factor G [Mycobacterium sp. AFP003]
245   4e-68
WP_058374410.1 elongation factor G [Pseudoalteromonas translu...
244   4e-68
WP_042273869.1 GTP-binding protein [[Clostridium] dakarense]
244   4e-68
WP_060534717.1 GTP-binding protein [Paenibacillus bovis]
244   4e-68
WP_022749606.1 GTP-binding protein [Lachnobacterium bovis]
248   4e-68
WP_105903237.1 elongation factor G [Vibrio gangliei]
244   4e-68
WP_084792338.1 elongation factor G [Mycobacterium sp. E136]
244   4e-68
WP_019420693.1 elongation factor G [Paenibacillus sp. OSY-SE]
244   4e-68
WP_057566245.1 GTP-binding protein [Paeniclostridium sordellii]
244   4e-68
WP_094359687.1 MULTISPECIES: elongation factor G [unclassifie...
244   4e-68
WP_008645473.1 MULTISPECIES: elongation factor G [Cupriavidus]
244   4e-68
WP_013889248.1 elongation factor G [Corynebacterium resistens]
245   5e-68
WP_039315141.1 GTP-binding protein [Clostridium baratii]
243   5e-68

WP_035883047.1 elongation factor G [Cupriavidus metallidurans]
244   5e-68
KPK56915.1 elongation factor G [Gammaproteobacteria bacterium...
244   5e-68
WP_047768514.1 GTP-binding protein [Lactobacillus panis]
243   5e-68
WP_011758702.1 elongation factor G [Shewanella amazonensis]
244   5e-68
WP_009591969.1 MULTISPECIES: elongation factor G [Paenibacillus]
244   5e-68
WP_055462931.1 elongation factor G [Marinomonas fungiae]
244   5e-68
SDB31324.1 small GTP-binding protein domain-containing protei...
248   5e-68
WP_100541551.1 elongation factor G [Paenibacillus sp. GM2FR]
244   5e-68
WP_005903498.1 elongation factor G [Fusobacterium nucleatum]
244   5e-68
WP_016741900.1 MULTISPECIES: GTP-binding protein [Bacillales]
243   5e-68
WP_004086491.1 MULTISPECIES: elongation factor G [Helicobacter]
244   5e-68
WP_052205409.1 elongation factor G [Corynebacterium riegelii]
245   5e-68
CDE12658.1 elongation factor G [Ruminococcus sp. CAG:330]
244   5e-68
WP_005993017.1 elongation factor G [Desulfovibrio fructosivor...
244   5e-68
WP_038885134.1 elongation factor G [Vibrio rotiferianus]
244   5e-68
WP_100641770.1 elongation factor G [Alteromonas sp. P0213]
244   5e-68
WP_090914106.1 elongation factor G [Paenibacillus sp. cl141a]
244   5e-68
WP_060677507.1 GTP-binding protein [Lactobacillus plantarum]
244   5e-68
WP_008979488.1 GTP-binding protein [Erysipelotrichaceae bacte...
248   5e-68
WP_102373094.1 GTP-binding protein [Enorma phocaeensis]
244   5e-68
WP_006284341.1 elongation factor G [Paenibacillus popilliae]
244   5e-68
WP_037191357.1 MULTISPECIES: elongation factor G [Rhodococcus]
244   5e-68
WP_029298547.1 elongation factor G [Chryseobacterium hispalense]
244   5e-68
WP_082964035.1 elongation factor G [Mycobacterium sp. 852002-...
244   5e-68
WP_076518814.1 elongation factor G [Achromobacter sp. MFA1 R4]
244   5e-68
WP_010649885.1 elongation factor G [Vibrio campbellii]
244   5e-68
KUO77668.1 elongation factor G [Desulfosporosinus sp. BRH_c37]
244   5e-68

CDB15270.1 tetracycline resistant protein [Clostridium sp. CA...
243   5e-68
WP_071044914.1 elongation factor G [Microbacteriaceae bacterium]
244   5e-68
WP_034584073.1 GTP-binding protein [Clostridium acetobutylicum]
243   5e-68
WP_007746012.1 elongation factor G [Oenococcus kitaharae]
244   5e-68
OGM78636.1 translation elongation factor G [Candidatus Woeseb...
245   5e-68
WP_025803092.1 elongation factor G [Campylobacter corcagiensis]
244   5e-68
WP_092093197.1 elongation factor G [Bhargavaea beijingensis]
244   5e-68
WP_067143264.1 elongation factor G [Oceanivirga salmonicida]
244   5e-68
WP_016838551.1 elongation factor G [Ureibacillus thermosphaer...
244   5e-68
WP_083176311.1 elongation factor G [Mycobacterium scrofulaceum]
244   5e-68
WP_034223457.1 elongation factor G [Actinotalea ferrariae]
244   5e-68
PIX21343.1 elongation factor G [Deltaproteobacteria bacterium...
244   5e-68
OGR17939.1 translation elongation factor G [Desulfobacterales...
244   5e-68
WP_074890836.1 GTP-binding protein [Butyrivibrio proteoclasti...
248   5e-68
OGY79639.1 translation elongation factor G [Candidatus Kerfel...
244   5e-68
WP_053195442.1 elongation factor G [Herbaspirillum hiltneri]
244   5e-68
WP_034563002.1 elongation factor G [Helicobacter bilis]
244   5e-68
WP_066769652.1 elongation factor G [Sphingobium sp. CCH11-B1]
244   5e-68
WP_003421177.1 elongation factor G [Clostridioides difficile]
244   5e-68
WP_074716388.1 GTP-binding protein [Ruminococcus flavefaciens]
247   5e-68
WP_065133603.1 elongation factor G [Mycobacterium gordonae]
244   5e-68
WP_027326827.1 elongation factor G [Helicobacter pametensis]
244   5e-68
WP_013190148.1 elongation factor G [Trichormus azollae]
244   5e-68
PVC65768.1 elongation factor G [Bacillus megaterium]
243   5e-68
WP_037952877.1 MULTISPECIES: elongation factor G [Streptomyces]
244   5e-68
GBD06721.1 Elongation factor G [bacterium HR21]
244   5e-68
WP_053735725.1 GTP-binding protein [Nocardia sp. NRRL S-836]
240   5e-68

WP_035168161.1 elongation factor G [Lactobacillus curieae]
244   5e-68
WP_006287293.1 elongation factor G [Corynebacterium crenatum]
244   5e-68
OIN89808.1 translation elongation factor G [Anaerolineae bact...
244   5e-68
WP_102565207.1 elongation factor G [Vibrio sp. 10N.286.49.B3]
244   5e-68
KKS46040.1 Elongation factor G [Candidatus Giovannonibacteria...
244   5e-68
WP_008245809.1 elongation factor G [Sulfurovum sp. AR]
244   6e-68
WP_017021085.1 elongation factor G [Aliivibrio logei]
244   6e-68
WP_055328393.1 GTP-binding protein [Paeniclostridium sordellii]
244   6e-68
WP_066803620.1 elongation factor G [Sphingomonas asaccharolyt...
244   6e-68
KGP77163.1 elongation factor G [Desulfosporosinus sp. Tol-M]
244   6e-68
WP_101513899.1 elongation factor G [Thermotoga sp. SG1]
244   6e-68
WP_024854148.1 GTP-binding protein [[Ruminococcus] gnavus]
248   6e-68
WP_011138410.1 elongation factor G [Wolinella succinogenes]
244   6e-68
PIY93680.1 elongation factor G [Candidatus Magasanikbacteria ...
244   6e-68
WP_074371755.1 elongation factor G [Vibrio spartinae]
244   6e-68
WP_094875059.1 GTP-binding protein [Paenibacillus sp. VTT E-1...
243   6e-68
OIP79329.1 translation elongation factor G [Parcubacteria gro...
244   6e-68
WP_049588862.1 elongation factor G [Alteromonas macleodii]
244   6e-68
OGW19973.1 translation elongation factor G [Nitrospirae bacte...
244   6e-68
WP_086241096.1 MULTISPECIES: elongation factor G [Campylobacter]
244   6e-68
OUX63481.1 elongation factor G [bacterium TMED274]
244   6e-68
OLA67985.1 translation elongation factor G [Subdoligranulum s...
244   6e-68
WP_011497385.1 elongation factor G [Shewanella denitrificans]
244   6e-68
PIR64030.1 elongation factor G [Candidatus Pacebacteria bacte...
244   6e-68
WP_007042500.1 elongation factor G [Thiorhodococcus drewsii]
244   6e-68
WP_007287142.1 GTP-binding protein [Intestinibacter bartlettii]
244   6e-68
ORU92083.1 elongation factor G [Cycloclasticus sp. symbiont o...
244   6e-68

WP_069844958.1 GTP-binding protein [Brevibacillus brevis]
243   6e-68
WP_076178215.1 GTP-binding protein [Clostridium botulinum]
243   6e-68
WP_093291979.1 elongation factor G [Thermoactinomyces sp. DSM...
244   6e-68
OGZ63543.1 translation elongation factor G [Candidatus Staska...
244   6e-68
WP_006854775.1 elongation factor G [Synechococcus sp. WH 8016]
244   6e-68
SCJ45626.1 Elongation factor G [uncultured Oscillibacter sp.]
248   6e-68
WP_012570652.1 elongation factor G [Coxiella burnetii]
244   6e-68
WP_089984061.1 GTP-binding protein [Clostridium sp. C105KSO13]
248   6e-68
WP_087217727.1 GTP-binding protein [Lachnoclostridium sp. An14]
243   6e-68
WP_011573387.1 elongation factor G [Pseudoalteromonas atlantica]
244   6e-68
WP_018090905.1 MULTISPECIES: elongation factor G [Streptomyces]
244   6e-68
WP_083549590.1 elongation factor G [Stappia sp. 22II-S9-Z10]
244   6e-68
WP_073880591.1 MULTISPECIES: elongation factor G [Mycobacterium]
244   6e-68
PIZ73645.1 elongation factor G [Candidatus Peregrinibacteria ...
244   6e-68
WP_015737625.1 MULTISPECIES: elongation factor G [Paenibacillus]
244   6e-68
WP_091451947.1 elongation factor G [Micromonospora inyonensis]
244   6e-68
WP_087294413.1 elongation factor G [Gemmiger sp. An194]
244   6e-68
WP_057763001.1 GTP-binding protein [Bacillus praedii]
243   6e-68
WP_028600708.1 GTP-binding protein [Paenibacillus pasadenensis]
243   6e-68
WP_106589952.1 elongation factor G [Salsuginibacillus halophi...
244   6e-68
WP_101032891.1 elongation factor G [Shewanella sp. Actino-tra...
244   6e-68
OHB89872.1 translation elongation factor G [Planctomycetes ba...
244   6e-68
WP_067869481.1 elongation factor G [Neptuniibacter marinus]
244   6e-68
WP_088976127.1 elongation factor G [Micromonospora coxensis]
244   6e-68
WP_044393517.1 elongation factor G [Bacillus subterraneus]
244   6e-68
WP_099702727.1 elongation factor G [Chroococcales cyanobacter...
244   6e-68
CDA90447.1 translation elongation factor G [Ruminococcus sp. ...
244   6e-68

OUS25514.1 translation elongation factor G ['Osedax' symbiont...
244   7e-68
WP_100201506.1 elongation factor G [Streptomyces sp. TRM SA0054]
244   7e-68
WP_091454090.1 elongation factor G [Alloactinosynnema iranicum]
244   7e-68
WP_068072882.1 elongation factor G [Novosphingobium lentum]
244   7e-68
WP_067913269.1 elongation factor G [Novosphingobium fuchskuhl...
244   7e-68
PSQ93581.1 elongation factor G [Bacteroidetes bacterium SW_4_...
244   7e-68
WP_088798410.1 MULTISPECIES: elongation factor G [Streptomyces]
244   7e-68
WP_006012393.1 elongation factor G [Glaciecola pallidula]
244   7e-68
WP_084463567.1 elongation factor G [Microtetraspora fusca]
244   7e-68
WP_046806157.1 MULTISPECIES: elongation factor G [Achromobacter]
244   7e-68
WP_066340191.1 elongation factor G [Helicobacter sp. MIT 01-6...
244   7e-68
WP_107206249.1 MULTISPECIES: elongation factor G [Photobacter...
244   7e-68
WP_023042726.1 elongation factor G [Enterococcus faecalis]
244   7e-68
WP_046086841.1 elongation factor G [Streptomyces antioxidans]
244   7e-68
WP_023946738.1 elongation factor G [Helicobacter fennelliae]
244   7e-68
WP_058469444.1 elongation factor G [Fluoribacter gormanii]
244   7e-68
WP_013171090.1 elongation factor G [[Bacillus] selenitireducens]
244   7e-68
WP_084771812.1 elongation factor G [Streptomyces sp. MOE7]
244   7e-68
WP_030677047.1 elongation factor G [Streptomyces rimosus]
244   7e-68
WP_008547931.1 elongation factor G [Pseudovibrio sp. JE062]
244   7e-68
WP_077040758.1 elongation factor G [Rhodococcus sp. MTM3W5.2]
244   7e-68
WP_045976608.1 elongation factor G [Vibrio neptunius]
244   7e-68
WP_021418072.1 elongation factor G [Clostridioides difficile]
244   7e-68
WP_082972724.1 MULTISPECIES: elongation factor G [Mycobacterium]
244   7e-68
DAB39805.1 TPA: elongation factor G [Sulfurovum sp. UBA12169]
244   7e-68
WP_003842473.1 GTP-binding protein [Bifidobacterium dentium]
244   7e-68
WP_089336458.1 elongation factor G [Blastococcus sp. DSM 44272]
244   7e-68

PKL90459.1 elongation factor G [Candidatus Goldbacteria bacte...
244   7e-68
WP_108410443.1 elongation factor G [Chryseobacterium sp. HMWF...
244   7e-68
WP_040392698.1 elongation factor G [Cellvibrio sp. BR]
244   7e-68
CCY67652.1 elongation factor G [Clostridium sp. CAG:678]
244   7e-68
WP_006604871.1 elongation factor G [Streptomyces auratus]
244   7e-68
WP_055578872.1 elongation factor G [Mycobacterium gordonae]
244   7e-68
WP_051234077.1 GTP-binding protein [Butyrivibrio sp. NC3005]
248   7e-68
WP_039334378.1 MULTISPECIES: elongation factor G [Novosphingo...
244   7e-68
WP_087147668.1 elongation factor G [Crenothrix polyspora]
244   7e-68
WP_057199780.1 elongation factor G [Acidovorax sp. Root217]
244   7e-68
WP_104694833.1 elongation factor G [Clostridioides difficile]
244   7e-68
WP_063216043.1 elongation factor G [Rhodococcus sp. PBTS 2]
244   7e-68
WP_001930725.1 elongation factor G [Helicobacter pylori]
244   8e-68
CCQ11337.1 Translation elongation factor G paralog [Pseudoalt...
244   8e-68
WP_092335404.1 GTP-binding protein [Desulfosporosinus hippei]
247   8e-68
WP_073768997.1 elongation factor G [Streptomyces sp. CB02923]
244   8e-68
SCI31826.1 Elongation factor G [uncultured Ruminococcus sp.]
247   8e-68
WP_036630144.1 MULTISPECIES: GTP-binding protein [Oscillibacter]
248   8e-68
OIP27214.1 translation elongation factor G [Dehalococcoidia b...
244   8e-68
WP_036466679.1 elongation factor G [Mycobacterium triplex]
244   8e-68
EKO40379.1 translation elongation factor EF-G [Desulfovibrio ...
244   8e-68
WP_024546583.1 elongation factor G [Synechococcus sp. NKBG150...
244   8e-68
WP_075219907.1 elongation factor G [Acuticoccus yangtzensis]
244   8e-68
WP_067936567.1 elongation factor G [Mycobacterium sp. E2479]
244   8e-68
WP_032407315.1 elongation factor G [Rhodococcus fascians]
244   8e-68
WP_027498159.1 MULTISPECIES: elongation factor G [Rhodococcus]
244   8e-68
WP_101879361.1 GTP-binding protein [[Ruminococcus] gnavus]
248   8e-68

ALIGNMENTS
>WP_063856423.1 MULTISPECIES: tetracycline resistance ribosomal protection protein
Tet(W) [Clostridiales]
 CAO78565.1 tetracycline resistance protein [Clostridioides difficile]
 OUP67538.1 tetracycline resistance ribosomal protection protein [Anaerotruncus
colihominis]
 OUP71768.1 tetracycline resistance ribosomal protection protein [Anaerotruncus
colihominis]
Length=639

 Score = 1313 bits (3398), Expect = 0.0, Method: Compositional matrix adjust.
 Identities = 639/639 (100%), Positives = 639/639 (100%), Gaps = 0/639 (0%)

Query 1
MKIINIGILAHVDAGKTTLTESLLYASGAISEPGSVEKGTTRTDTMFLERQRGITIQAAV 60

MKIINIGILAHVDAGKTTLTESLLYASGAISEPGSVEKGTTRTDTMFLERQRGITIQAAV
Sbjct 1
MKIINIGILAHVDAGKTTLTESLLYASGAISEPGSVEKGTTRTDTMFLERQRGITIQAAV 60

Query 61
TSFQWHRCKVNIVDTPGHMDFLAEVYRSLAVLDGAILVISAKDGVQAQTRILFHALRKMN 120

TSFQWHRCKVNIVDTPGHMDFLAEVYRSLAVLDGAILVISAKDGVQAQTRILFHALRKMN
Sbjct 61
TSFQWHRCKVNIVDTPGHMDFLAEVYRSLAVLDGAILVISAKDGVQAQTRILFHALRKMN 120

Query 121
IPTVIFINKIDQAGVDLQSVVQSVRDKLSADIIIKQTVSLSPEIVLEENTDIEAWDAVIE 180

IPTVIFINKIDQAGVDLQSVVQSVRDKLSADIIIKQTVSLSPEIVLEENTDIEAWDAVIE
Sbjct 121
IPTVIFINKIDQAGVDLQSVVQSVRDKLSADIIIKQTVSLSPEIVLEENTDIEAWDAVIE 180

Query 181
NNDKLLEKYIAGEPISREKLVREEQRRVQDASLFPVYYGSAKKGLGIQPLMDAVTGLFQP 240

NNDKLLEKYIAGEPISREKLVREEQRRVQDASLFPVYYGSAKKGLGIQPLMDAVTGLFQP
Sbjct 181
NNDKLLEKYIAGEPISREKLVREEQRRVQDASLFPVYYGSAKKGLGIQPLMDAVTGLFQP 240

Query 241
IGEQGSAALCGSVFKVEYTDCGQRRVYLRLYSGTLRLRDTVALAGREKLKITEMRIPSKG 300

IGEQGSAALCGSVFKVEYTDCGQRRVYLRLYSGTLRLRDTVALAGREKLKITEMRIPSKG
Sbjct 241
IGEQGSAALCGSVFKVEYTDCGQRRVYLRLYSGTLRLRDTVALAGREKLKITEMRIPSKG 300

Query 301
EIVRTDTAYPGEIVILPSDSVRLNDVLGDPTRLPRKRWREDPLPMLRTTIAPKTAAQRER 360

EIVRTDTAYPGEIVILPSDSVRLNDVLGDPTRLPRKRWREDPLPMLRTTIAPKTAAQRER
Sbjct 301
EIVRTDTAYPGEIVILPSDSVRLNDVLGDPTRLPRKRWREDPLPMLRTTIAPKTAAQRER 360

Query 361
LLDALTQLADTDPLLRCEVDSITHEIILSFLGRVQLEVVSALLSEKYKLETVVKEPSVIY 420

LLDALTQLADTDPLLRCEVDSITHEIILSFLGRVQLEVVSALLSEKYKLETVVKEPSVIY
Sbjct 361
LLDALTQLADTDPLLRCEVDSITHEIILSFLGRVQLEVVSALLSEKYKLETVVKEPSVIY 420

Query 421
MERPLKAASHTIHIEVPPNPFWASIGLSVTPLSLGSGVQYESRVSLGYLNQSFQNAVRDG 480

MERPLKAASHTIHIEVPPNPFWASIGLSVTPLSLGSGVQYESRVSLGYLNQSFQNAVRDG
Sbjct 421
MERPLKAASHTIHIEVPPNPFWASIGLSVTPLSLGSGVQYESRVSLGYLNQSFQNAVRDG 480

Query 481
IRYGLEQGLFGWNVTDCKICFEYGLYYSPVSTPADFRSLAPIVLEQALKESGTQLLEPYL 540

IRYGLEQGLFGWNVTDCKICFEYGLYYSPVSTPADFRSLAPIVLEQALKESGTQLLEPYL
Sbjct 481
IRYGLEQGLFGWNVTDCKICFEYGLYYSPVSTPADFRSLAPIVLEQALKESGTQLLEPYL 540

Query 541
SFILYAPQEYLSRAYHDAPKYCATIETAQVKKDEVVFTGEIPARCIQAYRTDLAFYTNGR 600

SFILYAPQEYLSRAYHDAPKYCATIETAQVKKDEVVFTGEIPARCIQAYRTDLAFYTNGR
Sbjct 541
SFILYAPQEYLSRAYHDAPKYCATIETAQVKKDEVVFTGEIPARCIQAYRTDLAFYTNGR 600

Query 601 SVCLTELKGYQAAVGQPVIQPRRPNSRLDKVRHMFQKVM 639
            SVCLTELKGYQAAVGQPVIQPRRPNSRLDKVRHMFQKVM
Sbjct 601 SVCLTELKGYQAAVGQPVIQPRRPNSRLDKVRHMFQKVM 639

>WP_075877655.1 TetM/TetW/TetO/TetS family tetracycline resistance ribosomal
protection protein [Merdibacter massiliensis]
Length=639

Score = 1311 bits (3394), Expect = 0.0, Method: Compositional matrix adjust.
Identities = 638/639 (99%), Positives = 639/639 (100%), Gaps = 0/639 (0%)

Query 1
MKIINIGILAHVDAGKTTLTESLLYASGAISEPGSVEKGTTRTDTMFLERQRGITIQAAV 60

MKIINIGILAHVDAGKTTLTESLLYASGAISEPGSVEKGTTRTDTMFLERQRGITIQAAV
Sbjct 1
MKIINIGILAHVDAGKTTLTESLLYASGAISEPGSVEKGTTRTDTMFLERQRGITIQAAV 60

Query 61
TSFQWHRCKVNIVDTPGHMDFLAEVYRSLAVLDGAILVISAKDGVQAQTRILFHALRKMN 120

TSFQWHRCKVNIVDTPGHMDFLAEVYRSLAVLDGAILVISAKDGVQAQTRILFHALRKMN
Sbjct 61
TSFQWHRCKVNIVDTPGHMDFLAEVYRSLAVLDGAILVISAKDGVQAQTRILFHALRKMN 120

Query 121
IPTVIFINKIDQAGVDLQSVVQSVRDKLSADIIIKQTVSLSPEIVLEENTDIEAWDAVIE 180

IPTVIFINKIDQAGVDLQSVVQSVRDKLSADIIIKQTVSLSPEIVLEENTDIEAWDAVIE
Sbjct 121
IPTVIFINKIDQAGVDLQSVVQSVRDKLSADIIIKQTVSLSPEIVLEENTDIEAWDAVIE 180

Query 181
NNDKLLEKYIAGEPISREKLVREEQRRVQDASLFPVYYGSAKKGLGIQPLMDAVTGLFQP 240

NNDKLLEKYIAGEPISREKLVREEQRRVQDASLFPVYYGSAKKGLGIQPLMDAVTGLFQP
Sbjct 181
NNDKLLEKYIAGEPISREKLVREEQRRVQDASLFPVYYGSAKKGLGIQPLMDAVTGLFQP 240

Query 241
IGEQGSAALCGSVFKVEYTDCGQRRVYLRLYSGTLRLRDTVALAGREKLKITEMRIPSKG 300

IGEQGSAALCGSVFKVEYTDCGQRRVYLRLYSGTLRLRDTVALAGREKLKITEMRIPSKG
Sbjct 241
IGEQGSAALCGSVFKVEYTDCGQRRVYLRLYSGTLRLRDTVALAGREKLKITEMRIPSKG 300

Query 301
EIVRTDTAYPGEIVILPSDSVRLNDVLGDPTRLPRKRWREDPLPMLRTTIAPKTAAQRER 360
EIVRTDTAYPGEIVILPSDSVRLNDVLGDPTRLPRKRWREDPLPMLRT
+IAPKTAAQRER
Sbjct 301
EIVRTDTAYPGEIVILPSDSVRLNDVLGDPTRLPRKRWREDPLPMLRTSIAPKTAAQRER 360

Query 361
LLDALTQLADTDPLLRCEVDSITHEIILSFLGRVQLEVVSALLSEKYKLETVVKEPSVIY 420

LLDALTQLADTDPLLRCEVDSITHEIILSFLGRVQLEVVSALLSEKYKLETVVKEPSVIY
Sbjct 361
LLDALTQLADTDPLLRCEVDSITHEIILSFLGRVQLEVVSALLSEKYKLETVVKEPSVIY 420

Query 421
MERPLKAASHTIHIEVPPNPFWASIGLSVTPLSLGSGVQYESRVSLGYLNQSFQNAVRDG 480

MERPLKAASHTIHIEVPPNPFWASIGLSVTPLSLGSGVQYESRVSLGYLNQSFQNAVRDG
Sbjct 421
MERPLKAASHTIHIEVPPNPFWASIGLSVTPLSLGSGVQYESRVSLGYLNQSFQNAVRDG 480

Query 481

IRYGLEQGLFGWNVTDCKICFEYGLYYSPVSTPADFRSLAPIVLEQALKESGTQLLEPYL 540

IRYGLEQGLFGWNVTDCKICFEYGLYYSPVSTPADFRSLAPIVLEQALKESGTQLLEPYL
Sbjct 481
IRYGLEQGLFGWNVTDCKICFEYGLYYSPVSTPADFRSLAPIVLEQALKESGTQLLEPYL 540

Query 541
SFILYAPQEYLSRAYHDAPKYCATIETAQVKKDEVVFTGEIPARCIQAYRTDLAFYTNGR 600

SFILYAPQEYLSRAYHDAPKYCATIETAQVKKDEVVFTGEIPARCIQAYRTDLAFYTNGR
Sbjct 541
SFILYAPQEYLSRAYHDAPKYCATIETAQVKKDEVVFTGEIPARCIQAYRTDLAFYTNGR 600

Query 601 SVCLTELKGYQAAVGQPVIQPRRPNSRLDKVRHMFQKVM 639
            SVCLTELKGYQAAVGQPVIQPRRPNSRLDKVRHMFQKVM
Sbjct 601 SVCLTELKGYQAAVGQPVIQPRRPNSRLDKVRHMFQKVM 639

>WP_087325445.1 TetM/TetW/TetO/TetS family tetracycline resistance ribosomal
protection protein [Faecalibacterium sp. An58]
 OUN67604.1 tetracycline resistance ribosomal protection protein [Faecalibacterium
sp. An58]
Length=639

 Score = 1310 bits (3389), Expect = 0.0, Method: Compositional matrix adjust.
 Identities = 638/639 (99%), Positives = 638/639 (99%), Gaps = 0/639 (0%)

Query 1
MKIINIGILAHVDAGKTTLTESLLYASGAISEPGSVEKGTTRTDTMFLERQRGITIQAAV 60

MKIINIGILAHVDAGKTTLTESLLYASGAISEPGSVEKGTTRTDTMFLERQRGITIQAAV
Sbjct 1
MKIINIGILAHVDAGKTTLTESLLYASGAISEPGSVEKGTTRTDTMFLERQRGITIQAAV 60

Query 61
TSFQWHRCKVNIVDTPGHMDFLAEVYRSLAVLDGAILVISAKDGVQAQTRILFHALRKMN 120

TSFQWHRCKVNIVDTPGHMDFLAEVYRSLAVLDGAILVISAKDGVQAQTRILFHALRKMN
Sbjct 61
TSFQWHRCKVNIVDTPGHMDFLAEVYRSLAVLDGAILVISAKDGVQAQTRILFHALRKMN 120

Query 121
IPTVIFINKIDQAGVDLQSVVQSVRDKLSADIIIKQTVSLSPEIVLEENTDIEAWDAVIE 180

IPTVIFINKIDQAGVDLQSVVQSVRDKLSADIIIKQTVSLSPEIVLEENTDIEAWDAVIE
Sbjct 121
IPTVIFINKIDQAGVDLQSVVQSVRDKLSADIIIKQTVSLSPEIVLEENTDIEAWDAVIE 180

Query 181
NNDKLLEKYIAGEPISREKLVREEQRRVQDASLFPVYYGSAKKGLGIQPLMDAVTGLFQP 240

NNDKLLEKYIAGEPISREKLVREEQRRVQDASLFPVYYGSAKKGLGIQPLMDAVTGLFQP
Sbjct 181
NNDKLLEKYIAGEPISREKLVREEQRRVQDASLFPVYYGSAKKGLGIQPLMDAVTGLFQP 240

Query 241
IGEQGSAALCGSVFKVEYTDCGQRRVYLRLYSGTLRLRDTVALAGREKLKITEMRIPSKG 300

IGEQGSAALCGSVFKVEYTDCGQRRVYLRLYSGTLRLRDTVALAGREKLKITEMRIPSKG
Sbjct 241
IGEQGSAALCGSVFKVEYTDCGQRRVYLRLYSGTLRLRDTVALAGREKLKITEMRIPSKG 300

Query 301
EIVRTDTAYPGEIVILPSDSVRLNDVLGDPTRLPRKRWREDPLPMLRTTIAPKTAAQRER 360
EIVRTDTAYPGEIVILPSDSVRLNDVLGD
TRLPRKRWREDPLPMLRTTIAPKTAAQRER
Sbjct 301
EIVRTDTAYPGEIVILPSDSVRLNDVLGDQTRLPRKRWREDPLPMLRTTIAPKTAAQRER 360

Query 361
LLDALTQLADTDPLLRCEVDSITHEIILSFLGRVQLEVVSALLSEKYKLETVVKEPSVIY 420

LLDALTQLADTDPLLRCEVDSITHEIILSFLGRVQLEVVSALLSEKYKLETVVKEPSVIY
Sbjct 361
LLDALTQLADTDPLLRCEVDSITHEIILSFLGRVQLEVVSALLSEKYKLETVVKEPSVIY 420

Query 421
MERPLKAASHTIHIEVPPNPFWASIGLSVTPLSLGSGVQYESRVSLGYLNQSFQNAVRDG 480

MERPLKAASHTIHIEVPPNPFWASIGLSVTPLSLGSGVQYESRVSLGYLNQSFQNAVRDG
Sbjct 421
MERPLKAASHTIHIEVPPNPFWASIGLSVTPLSLGSGVQYESRVSLGYLNQSFQNAVRDG 480

Query 481
IRYGLEQGLFGWNVTDCKICFEYGLYYSPVSTPADFRSLAPIVLEQALKESGTQLLEPYL 540

IRYGLEQGLFGWNVTDCKICFEYGLYYSPVSTPADFRSLAPIVLEQALKESGTQLLEPYL
Sbjct 481
IRYGLEQGLFGWNVTDCKICFEYGLYYSPVSTPADFRSLAPIVLEQALKESGTQLLEPYL 540

Query 541
SFILYAPQEYLSRAYHDAPKYCATIETAQVKKDEVVFTGEIPARCIQAYRTDLAFYTNGR 600

SFILYAPQEYLSRAYHDAPKYCATIETAQVKKDEVVFTGEIPARCIQAYRTDLAFYTNGR
Sbjct 541
SFILYAPQEYLSRAYHDAPKYCATIETAQVKKDEVVFTGEIPARCIQAYRTDLAFYTNGR 600

Query 601 SVCLTELKGYQAAVGQPVIQPRRPNSRLDKVRHMFQKVM 639
SVCLTELKGYQAAVGQPVIQPRRPNSRLDKVRHMFQKVM
Sbjct 601 SVCLTELKGYQAAVGQPVIQPRRPNSRLDKVRHMFQKVM 639

>AMP42199.1 tetracycline resistance protein Tet(W) [uncultured bacterium

IN-04]
Length=639

 Score = 1308 bits (3385), Expect = 0.0, Method: Compositional matrix adjust.
 Identities = 637/639 (99%), Positives = 638/639 (99%), Gaps = 0/639 (0%)

Query 1
MKIINIGILAHVDAGKTTLTESLLYASGAISEPGSVEKGTTRTDTMFLERQRGITIQAAV 60

MKIINIGILAHVDAGKTTLTESLLYASGAISEPGSVEKGTTRTDTMFLERQRGITIQAAV
Sbjct 1
MKIINIGILAHVDAGKTTLTESLLYASGAISEPGSVEKGTTRTDTMFLERQRGITIQAAV 60

Query 61
TSFQWHRCKVNIVDTPGHMDFLAEVYRSLAVLDGAILVISAKDGVQAQTRILFHALRKMN 120

TSFQWHRCKVNIVDTPGHMDFLAEVYRSLAVLDGAILVISAKDGVQAQTRILFHALRKMN
Sbjct 61
TSFQWHRCKVNIVDTPGHMDFLAEVYRSLAVLDGAILVISAKDGVQAQTRILFHALRKMN 120

Query 121
IPTVIFINKIDQAGVDLQSVVQSVRDKLSADIIIKQTVSLSPEIVLEENTDIEAWDAVIE 180

IPTVIFINKIDQAGVDLQSVVQSVRDKLSADIIIKQTVSLSPEIVLEENTDIEAWDAVIE
Sbjct 121
IPTVIFINKIDQAGVDLQSVVQSVRDKLSADIIIKQTVSLSPEIVLEENTDIEAWDAVIE 180

Query 181
NNDKLLEKYIAGEPISREKLVREEQRRVQDASLFPVYYGSAKKGLGIQPLMDAVTGLFQP 240

NNDKLLEKYIAGEPISREKLVREEQRRVQDASLFPVYYGSAKKGLGIQPLMDAVTGLFQP
Sbjct 181
NNDKLLEKYIAGEPISREKLVREEQRRVQDASLFPVYYGSAKKGLGIQPLMDAVTGLFQP 240

Query 241
IGEQGSAALCGSVFKVEYTDCGQRRVYLRLYSGTLRLRDTVALAGREKLKITEMRIPSKG 300

IGEQGSAALCGSVFKVEYTDCGQRRVYLRLYSGTLRLRDTVALAGREKLKITEMRIPSKG
Sbjct 241
IGEQGSAALCGSVFKVEYTDCGQRRVYLRLYSGTLRLRDTVALAGREKLKITEMRIPSKG 300

Query 301
EIVRTDTAYPGEIVILPSDSVRLNDVLGDPTRLPRKRWREDPLPMLRTTIAPKTAAQRER 360

EIVRTDTAYPGEIVILPSDSVRLNDVLGDPTRLPRKRWREDPLPMLRTTIAPKTAAQRER
Sbjct 301
EIVRTDTAYPGEIVILPSDSVRLNDVLGDPTRLPRKRWREDPLPMLRTTIAPKTAAQRER 360

Query 361
LLDALTQLADTDPLLRCEVDSITHEIILSFLGRVQLEVVSALLSEKYKLETVVKEPSVIY 420

LLDALTQLADTDPLLRCEVDSITHEIILSFLGRVQLEVVSALLSEKYKLETVVKEPSVIY

```
Sbjct  361
LLDALTQLADTDPLLRCEVDSITHEIILSFLGRVQLEVVSALLSEKYKLETVVKEPSVIY  420

Query  421
MERPLKAASHTIHIEVPPNPFWASIGLSVTPLSLGSGVQYESRVSLGYLNQSFQNAVRDG  480

MERPLKAASHTIHIEVPPNPFWASIGLSVTPLSLGSGVQYESRVSLGYLNQSFQNAVRDG
Sbjct  421
MERPLKAASHTIHIEVPPNPFWASIGLSVTPLSLGSGVQYESRVSLGYLNQSFQNAVRDG  480

Query  481
IRYGLEQGLFGWNVTDCKICFEYGLYYSPVSTPADFRSLAPIVLEQALKESGTQLLEPYL  540

IRYGLEQGLFGWNVTDCKICFEYGLYYSPVSTPADFRSLAPIVLEQALKESGTQLLEPYL
Sbjct  481
IRYGLEQGLFGWNVTDCKICFEYGLYYSPVSTPADFRSLAPIVLEQALKESGTQLLEPYL  540

Query  541
SFILYAPQEYLSRAYHDAPKYCATIETAQVKKDEVVFTGEIPARCIQAYRTDLAFYTNGR  600
            SF LYAP
+EYLSRAYHDAPKYCATIETAQVKKDEVVFTGEIPARCIQAYRTDLAFYTNGR
Sbjct  541
SFTLYAPREYLSRAYHDAPKYCATIETAQVKKDEVVFTGEIPARCIQAYRTDLAFYTNGR  600

Query  601  SVCLTELKGYQAAVGQPVIQPRRPNSRLDKVRHMFQKVM  639
            SVCLTELKGYQAAVGQPVIQPRRPNSRLDKVRHMFQKVM
Sbjct  601  SVCLTELKGYQAAVGQPVIQPRRPNSRLDKVRHMFQKVM  639


>WP_043170315.1 TetM/TetW/TetO/TetS family tetracycline resistance
ribosomal
protection protein [Bifidobacterium pullorum]
Length=639

 Score = 1308 bits (3384),  Expect = 0.0, Method: Compositional
matrix adjust.
 Identities = 636/639 (99%), Positives = 638/639 (99%), Gaps = 0/639
(0%)

Query  1
MKIINIGILAHVDAGKTTLTESLLYASGAISEPGSVEKGTTRTDTMFLERQRGITIQAAV  60

MKIINIGILAHVDAGKTTLTESLLYASGAISEPGSVEKGTTRTDTMFLERQRGITIQAAV
Sbjct  1
MKIINIGILAHVDAGKTTLTESLLYASGAISEPGSVEKGTTRTDTMFLERQRGITIQAAV  60

Query  61
TSFQWHRCKVNIVDTPGHMDFLAEVYRSLAVLDGAILVISAKDGVQAQTRILFHALRKMN  120

TSFQWHRCKVNIVDTPGHMDFLAEVYRSLAVLDGAILVISAKDGVQAQTRILFHALRKMN
Sbjct  61
TSFQWHRCKVNIVDTPGHMDFLAEVYRSLAVLDGAILVISAKDGVQAQTRILFHALRKMN  120

Query  121
```

```
IPTVIFINKIDQAGVDLQSVVQSVRDKLSADIIIKQTVSLSPEIVLEENTDIEAWDAVIE  180

IPTVIFINKIDQAGVDLQSVVQSVRDKLSADIIIKQTVSLSPEIVLEENTDIEAWDAVIE
Sbjct  121
IPTVIFINKIDQAGVDLQSVVQSVRDKLSADIIIKQTVSLSPEIVLEENTDIEAWDAVIE  180

Query  181
NNDKLLEKYIAGEPISREKLVREEQRRVQDASLFPVYYGSAKKGLGIQPLMDAVTGLFQP  240
               NND+LLEKYIAGEPISREKL
REEQRRVQDASLFPVYYGSAKKGLGIQPLMDAVTGLFQP
Sbjct  181
NNDELLEKYIAGEPISREKLAREEQRRVQDASLFPVYYGSAKKGLGIQPLMDAVTGLFQP  240

Query  241
IGEQGSAALCGSVFKVEYTDCGQRRVYLRLYSGTLRLRDTVALAGREKLKITEMRIPSKG  300

IGEQGSAALCGSVFKVEYTDCGQRRVYLRLYSGTLRLRDTVALAGREKLKITEMRIPSKG
Sbjct  241
IGEQGSAALCGSVFKVEYTDCGQRRVYLRLYSGTLRLRDTVALAGREKLKITEMRIPSKG  300

Query  301
EIVRTDTAYPGEIVILPSDSVRLNDVLGDPTRLPRKRWREDPLPMLRTTIAPKTAAQRER  360
               EIVRTDTAYPGEIVILPSDSVRLNDVLGDPTRLPRKRWREDPLPMLRT
+IAPKTAAQRER
Sbjct  301
EIVRTDTAYPGEIVILPSDSVRLNDVLGDPTRLPRKRWREDPLPMLRTSIAPKTAAQRER  360

Query  361
LLDALTQLADTDPLLRCEVDSITHEIILSFLGRVQLEVVSALLSEKYKLETVVKEPSVIY  420

LLDALTQLADTDPLLRCEVDSITHEIILSFLGRVQLEVVSALLSEKYKLETVVKEPSVIY
Sbjct  361
LLDALTQLADTDPLLRCEVDSITHEIILSFLGRVQLEVVSALLSEKYKLETVVKEPSVIY  420

Query  421
MERPLKAASHTIHIEVPPNPFWASIGLSVTPLSLGSGVQYESRVSLGYLNQSFQNAVRDG  480

MERPLKAASHTIHIEVPPNPFWASIGLSVTPLSLGSGVQYESRVSLGYLNQSFQNAVRDG
Sbjct  421
MERPLKAASHTIHIEVPPNPFWASIGLSVTPLSLGSGVQYESRVSLGYLNQSFQNAVRDG  480

Query  481
IRYGLEQGLFGWNVTDCKICFEYGLYYSPVSTPADFRSLAPIVLEQALKESGTQLLEPYL  540

IRYGLEQGLFGWNVTDCKICFEYGLYYSPVSTPADFRSLAPIVLEQALKESGTQLLEPYL
Sbjct  481
IRYGLEQGLFGWNVTDCKICFEYGLYYSPVSTPADFRSLAPIVLEQALKESGTQLLEPYL  540

Query  541
SFILYAPQEYLSRAYHDAPKYCATIETAQVKKDEVVFTGEIPARCIQAYRTDLAFYTNGR  600

SFILYAPQEYLSRAYHDAPKYCATIETAQVKKDEVVFTGEIPARCIQAYRTDLAFYTNGR
Sbjct  541
SFILYAPQEYLSRAYHDAPKYCATIETAQVKKDEVVFTGEIPARCIQAYRTDLAFYTNGR  600
```

```
Query  601  SVCLTELKGYQAAVGQPVIQPRRPNSRLDKVRHMFQKVM  639
            SVCLTELKGYQAAVGQPVIQPRRPNSRLDKVRHMFQKVM
Sbjct  601  SVCLTELKGYQAAVGQPVIQPRRPNSRLDKVRHMFQKVM  639
```

>WP_033126273.1 MULTISPECIES: TetM/TetW/TetO/TetS family tetracycline resistance
ribosomal protection protein [Clostridiales]
OUN72156.1 TetM/TetW/TetO/TetS family tetracycline resistance ribosomal
protection protein [Drancourtella sp. An57]
Length=639

Score = 1307 bits (3383), Expect = 0.0, Method: Compositional matrix adjust.
Identities = 636/639 (99%), Positives = 638/639 (99%), Gaps = 0/639 (0%)

```
Query  1
MKIINIGILAHVDAGKTTLTESLLYASGAISEPGSVEKGTTRTDTMFLERQRGITIQAAV  60

MKIINIGILAHVDAGKTTLTESLLYASGAISEPGSVEKGTTRTDTMFLERQRGITIQAAV
Sbjct  1
MKIINIGILAHVDAGKTTLTESLLYASGAISEPGSVEKGTTRTDTMFLERQRGITIQAAV  60

Query  61
TSFQWHRCKVNIVDTPGHMDFLAEVYRSLAVLDGAILVISAKDGVQAQTRILFHALRKMN  120

TSFQWHRCKVNIVDTPGHMDFLAEVYRSLAVLDGAILVISAKDGVQAQTRILFHALRKMN
Sbjct  61
TSFQWHRCKVNIVDTPGHMDFLAEVYRSLAVLDGAILVISAKDGVQAQTRILFHALRKMN  120

Query  121
IPTVIFINKIDQAGVDLQSVVQSVRDKLSADIIIKQTVSLSPEIVLEENTDIEAWDAVIE  180

IPTVIFINKIDQAGVDLQSVVQSVRDKLSADIIIKQTVSLSPEIVLEENTDIEAWDAVIE
Sbjct  121
IPTVIFINKIDQAGVDLQSVVQSVRDKLSADIIIKQTVSLSPEIVLEENTDIEAWDAVIE  180

Query  181
NNDKLLEKYIAGEPISREKLVREEQRRVQDASLFPVYYGSAKKGLGIQPLMDAVTGLFQP  240

NNDKLLEKYIAGEPISREKLVREEQRRVQDASLFPVYYGSAKKGLGIQPLMDAVTGLFQP
Sbjct  181
NNDKLLEKYIAGEPISREKLVREEQRRVQDASLFPVYYGSAKKGLGIQPLMDAVTGLFQP  240

Query  241
IGEQGSAALCGSVFKVEYTDCGQRRVYLRLYSGTLRLRDTVALAGREKLKITEMRIPSKG  300

IGEQGSAALCGSVFKVEYTDCGQRRVYLRLYSGTLRLRDTVALAGREKLKITEMRIPSKG
Sbjct  241
IGEQGSAALCGSVFKVEYTDCGQRRVYLRLYSGTLRLRDTVALAGREKLKITEMRIPSKG  300
```

```
Query  301
EIVRTDTAYPGEIVILPSDSVRLNDVLGDPTRLPRKRWREDPLPMLRTTIAPKTAAQRER  360
            EIVRTDTAYPGEIVILPSDSVRLNDVLGDPTRLPRKRWREDPLPMLRT
+IAPKTAAQRER
Sbjct  301
EIVRTDTAYPGEIVILPSDSVRLNDVLGDPTRLPRKRWREDPLPMLRTSIAPKTAAQRER  360

Query  361
LLDALTQLADTDPLLRCEVDSITHEIILSFLGRVQLEVVSALLSEKYKLETVVKEPSVIY  420
            LLDALTQLADTDPLLRCEVDSITHEIILSFLGRVQLEVVSALLSEKYKLETVVKEP
+VIY
Sbjct  361
LLDALTQLADTDPLLRCEVDSITHEIILSFLGRVQLEVVSALLSEKYKLETVVKEPTVIY  420

Query  421
MERPLKAASHTIHIEVPPNPFWASIGLSVTPLSLGSGVQYESRVSLGYLNQSFQNAVRDG  480
            MERPLKAASHTIHIEVPPNPFWASIGLSVTPL
LGSGVQYESRVSLGYLNQSFQNAVRDG
Sbjct  421
MERPLKAASHTIHIEVPPNPFWASIGLSVTPLPLGSGVQYESRVSLGYLNQSFQNAVRDG  480

Query  481
IRYGLEQGLFGWNVTDCKICFEYGLYYSPVSTPADFRSLAPIVLEQALKESGTQLLEPYL  540

IRYGLEQGLFGWNVTDCKICFEYGLYYSPVSTPADFRSLAPIVLEQALKESGTQLLEPYL
Sbjct  481
IRYGLEQGLFGWNVTDCKICFEYGLYYSPVSTPADFRSLAPIVLEQALKESGTQLLEPYL  540

Query  541
SFILYAPQEYLSRAYHDAPKYCATIETAQVKKDEVVFTGEIPARCIQAYRTDLAFYTNGR  600

SFILYAPQEYLSRAYHDAPKYCATIETAQVKKDEVVFTGEIPARCIQAYRTDLAFYTNGR
Sbjct  541
SFILYAPQEYLSRAYHDAPKYCATIETAQVKKDEVVFTGEIPARCIQAYRTDLAFYTNGR  600

Query  601  SVCLTELKGYQAAVGQPVIQPRRPNSRLDKVRHMFQKVM  639
            SVCLTELKGYQAAVGQPVIQPRRPNSRLDKVRHMFQKVM
Sbjct  601  SVCLTELKGYQAAVGQPVIQPRRPNSRLDKVRHMFQKVM  639


>WP_078934043.1 TetM/TetW/TetO/TetS family tetracycline resistance
ribosomal
protection protein [Treponema porcinum]
 SJZ73215.1 ribosomal protection tetracycline resistance protein
[Treponema
porcinum]
Length=639

 Score = 1305 bits (3377),  Expect = 0.0, Method: Compositional
matrix adjust.
 Identities = 636/639 (99%), Positives = 636/639 (99%), Gaps = 0/639
(0%)

Query  1
```

```
MKIINIGILAHVDAGKTTLTESLLYASGAISEPGSVEKGTTRTDTMFLERQRGITIQAAV  60
            MKIINIGIL
HVDAGKTTLTESLLYASGAISEPGSVEKGTTRTDTMFLERQRGITIQAAV
Sbjct  1
MKIINIGILGHVDAGKTTLTESLLYASGAISEPGSVEKGTTRTDTMFLERQRGITIQAAV  60

Query  61
TSFQWHRCKVNIVDTPGHMDFLAEVYRSLAVLDGAILVISAKDGVQAQTRILFHALRKMN  120

TSFQWHRCKVNIVDTPGHMDFLAEVYRSLAVLDGAILVISAKDGVQAQTRILFHALRKMN
Sbjct  61
TSFQWHRCKVNIVDTPGHMDFLAEVYRSLAVLDGAILVISAKDGVQAQTRILFHALRKMN  120

Query  121
IPTVIFINKIDQAGVDLQSVVQSVRDKLSADIIIKQTVSLSPEIVLEENTDIEAWDAVIE  180

IPTVIFINKIDQAGVDLQSVVQSVRDKLSADIIIKQTVSLSPEIVLEENTDIEAWDAVIE
Sbjct  121
IPTVIFINKIDQAGVDLQSVVQSVRDKLSADIIIKQTVSLSPEIVLEENTDIEAWDAVIE  180

Query  181
NNDKLLEKYIAGEPISREKLVREEQRRVQDASLFPVYYGSAKKGLGIQPLMDAVTGLFQP  240

NNDKLLEKYIAGEPISREKLVREEQRRVQDASLFPVYYGSAKKGLGIQPLMDAVTGLFQP
Sbjct  181
NNDKLLEKYIAGEPISREKLVREEQRRVQDASLFPVYYGSAKKGLGIQPLMDAVTGLFQP  240

Query  241
IGEQGSAALCGSVFKVEYTDCGQRRVYLRLYSGTLRLRDTVALAGREKLKITEMRIPSKG  300

IGEQGSAALCGSVFKVEYTDCGQRRVYLRLYSGTLRLRDTVALAGREKLKITEMRIPSKG
Sbjct  241
IGEQGSAALCGSVFKVEYTDCGQRRVYLRLYSGTLRLRDTVALAGREKLKITEMRIPSKG  300

Query  301
EIVRTDTAYPGEIVILPSDSVRLNDVLGDPTRLPRKRWREDPLPMLRTTIAPKTAAQRER  360
            EIVRTDTAYPGEIVILPSDSVRLNDVLGD
TRLPRKRWREDPLPMLRTTIAPKTAAQRER
Sbjct  301
EIVRTDTAYPGEIVILPSDSVRLNDVLGDQTRLPRKRWREDPLPMLRTTIAPKTAAQRER  360

Query  361
LLDALTQLADTDPLLRCEVDSITHEIILSFLGRVQLEVVSALLSEKYKLETVVKEPSVIY  420

LLDALTQLADTDPLLRCEVDSITHEIILSFLGRVQLEVVSALLSEKYKLETVVKEPSVIY
Sbjct  361
LLDALTQLADTDPLLRCEVDSITHEIILSFLGRVQLEVVSALLSEKYKLETVVKEPSVIY  420

Query  421
MERPLKAASHTIHIEVPPNPFWASIGLSVTPLSLGSGVQYESRVSLGYLNQSFQNAVRDG  480
            MERPLKAASHTIHIEVPPNPFWASIGLSVTPL
LGSGVQYESRVSLGYLNQSFQNAVRDG
Sbjct  421
MERPLKAASHTIHIEVPPNPFWASIGLSVTPLPLGSGVQYESRVSLGYLNQSFQNAVRDG  480
```

Query 481
IRYGLEQGLFGWNVTDCKICFEYGLYYSPVSTPADFRSLAPIVLEQALKESGTQLLEPYL 540

IRYGLEQGLFGWNVTDCKICFEYGLYYSPVSTPADFRSLAPIVLEQALKESGTQLLEPYL
Sbjct 481
IRYGLEQGLFGWNVTDCKICFEYGLYYSPVSTPADFRSLAPIVLEQALKESGTQLLEPYL 540

Query 541
SFILYAPQEYLSRAYHDAPKYCATIETAQVKKDEVVFTGEIPARCIQAYRTDLAFYTNGR 600

SFILYAPQEYLSRAYHDAPKYCATIETAQVKKDEVVFTGEIPARCIQAYRTDLAFYTNGR
Sbjct 541
SFILYAPQEYLSRAYHDAPKYCATIETAQVKKDEVVFTGEIPARCIQAYRTDLAFYTNGR 600

Query 601 SVCLTELKGYQAAVGQPVIQPRRPNSRLDKVRHMFQKVM 639
SVCLTELKGYQAAVGQPVIQPRRPNSRLDKVRHMFQKVM
Sbjct 601 SVCLTELKGYQAAVGQPVIQPRRPNSRLDKVRHMFQKVM 639

>WP_063856418.1 tetracycline resistance ribosomal protection protein Tet(W) [Bifidobacterium
longum]
ABC18266.1 TetW [Bifidobacterium longum subsp. longum F8]
Length=639

Score = 1305 bits (3376), Expect = 0.0, Method: Compositional matrix adjust.
Identities = 634/639 (99%), Positives = 637/639 (99%), Gaps = 0/639 (0%)

Query 1
MKIINIGILAHVDAGKTTLTESLLYASGAISEPGSVEKGTTRTDTMFLERQRGITIQAAV 60

MKIINIGILAHVDAGKTTLTESLLYASGAISEPGSVEKGTTRTDTMFLERQRGITIQAAV
Sbjct 1
MKIINIGILAHVDAGKTTLTESLLYASGAISEPGSVEKGTTRTDTMFLERQRGITIQAAV 60

Query 61
TSFQWHRCKVNIVDTPGHMDFLAEVYRSLAVLDGAILVISAKDGVQAQTRILFHALRKMN 120

TSFQWHRCKVNIVDTPGHMDFLAEVYRSLAVLDGAILVISAKDGVQAQTRILFHALRKMN
Sbjct 61
TSFQWHRCKVNIVDTPGHMDFLAEVYRSLAVLDGAILVISAKDGVQAQTRILFHALRKMN 120

Query 121
IPTVIFINKIDQAGVDLQSVVQSVRDKLSADIIIKQTVSLSPEIVLEENTDIEAWDAVIE 180

IPTVIFINKIDQAGVDLQSVVQSVRDKLSADIIIKQTVSLSPEIVLEENTDIEAWDAVIE
Sbjct 121
IPTVIFINKIDQAGVDLQSVVQSVRDKLSADIIIKQTVSLSPEIVLEENTDIEAWDAVIE 180

Query 181
NNDKLLEKYIAGEPISREKLVREEQRRVQDASLFPVYYGSAKKGLGIQPLMDAVTGLFQP 240

```
NNDKLLEKYIAGEPISREKLVREEQRRVQDASLFPVYYGSAKKGLGIQPLMDAVTGLFQP
Sbjct  181
NNDKLLEKYIAGEPISREKLVREEQRRVQDASLFPVYYGSAKKGLGIQPLMDAVTGLFQP  240

Query  241
IGEQGSAALCGSVFKVEYTDCGQRRVYLRLYSGTLRLRDTVALAGREKLKITEMRIPSKG  300

IGEQGSAALCGSVFKVEYTDCGQRRVYLRLYSGTLRLRDTVALAGREKLKITEMRIPSKG
Sbjct  241
IGEQGSAALCGSVFKVEYTDCGQRRVYLRLYSGTLRLRDTVALAGREKLKITEMRIPSKG  300

Query  301
EIVRTDTAYPGEIVILPSDSVRLNDVLGDPTRLPRKRWREDPLPMLRTTIAPKTAAQRER  360
           EIVRTDTAYPGEIVILPSDSVRLNDVLGDPTRLPRKRWREDPLPMLRT
+IAPKTAAQRER
Sbjct  301
EIVRTDTAYPGEIVILPSDSVRLNDVLGDPTRLPRKRWREDPLPMLRTSIAPKTAAQRER  360

Query  361
LLDALTQLADTDPLLRCEVDSITHEIILSFLGRVQLEVVSALLSEKYKLETVVKEPSVIY  420
           LLDALTQLADTDPLLRCEVDSITHEIILSFLGRVQLEVVSALLSEKYKLETVVKEP
+VIY
Sbjct  361
LLDALTQLADTDPLLRCEVDSITHEIILSFLGRVQLEVVSALLSEKYKLETVVKEPTVIY  420

Query  421
MERPLKAASHTIHIEVPPNPFWASIGLSVTPLSLGSGVQYESRVSLGYLNQSFQNAVRDG  480

MERPLKAASHTIHIEVPPNPFWASIGLSVTPLSLGSGVQYESRVSLGYLNQSFQNAVRDG
Sbjct  421
MERPLKAASHTIHIEVPPNPFWASIGLSVTPLSLGSGVQYESRVSLGYLNQSFQNAVRDG  480

Query  481
IRYGLEQGLFGWNVTDCKICFEYGLYYSPVSTPADFRSLAPIVLEQALKESGTQLLEPYL  540

IRYGLEQGLFGWNVTDCKICFEYGLYYSPVSTPADFRSLAPIVLEQALKESGTQLLEPYL
Sbjct  481
IRYGLEQGLFGWNVTDCKICFEYGLYYSPVSTPADFRSLAPIVLEQALKESGTQLLEPYL  540

Query  541
SFILYAPQEYLSRAYHDAPKYCATIETAQVKKDEVVFTGEIPARCIQAYRTDLAFYTNGR  600
           SF LYAP+EYLSRAYHDAPKYCATIET
QVKKDEVVFTGEIPARCIQAYRTDLAFYTNGR
Sbjct  541
SFTLYAPREYLSRAYHDAPKYCATIETVQVKKDEVVFTGEIPARCIQAYRTDLAFYTNGR  600

Query  601  SVCLTELKGYQAAVGQPVIQPRRPNSRLDKVRHMFQKVM  639
            SVCLTELKGYQAAVGQPVIQPRRPNSRLDKVRHMFQKVM
Sbjct  601  SVCLTELKGYQAAVGQPVIQPRRPNSRLDKVRHMFQKVM  639
```

>WP_087193190.1 TetM/TetW/TetO/TetS family tetracycline resistance ribosomal

protection protein [Gordonibacter sp. An230]
 OUO91052.1 tetracycline resistance ribosomal protection protein
[Gordonibacter
sp. An230]
Length=639

 Score = 1302 bits (3369), Expect = 0.0, Method: Compositional
matrix adjust.
 Identities = 633/639 (99%), Positives = 636/639 (99%), Gaps = 0/639
(0%)

Query 1
MKIINIGILAHVDAGKTTLTESLLYASGAISEPGSVEKGTTRTDTMFLERQRGITIQAAV 60

MKIINIGILAHVDAGKTTLTESLLYASGAISEPGSVEKGTTRTDTMFLERQRGITIQAAV
Sbjct 1
MKIINIGILAHVDAGKTTLTESLLYASGAISEPGSVEKGTTRTDTMFLERQRGITIQAAV 60

Query 61
TSFQWHRCKVNIVDTPGHMDFLAEVYRSLAVLDGAILVISAKDGVQAQTRILFHALRKMN 120

TSFQWHRCKVNIVDTPGHMDFLAEVYRSLAVLDGAILVISAKDGVQAQTRILFHALRKMN
Sbjct 61
TSFQWHRCKVNIVDTPGHMDFLAEVYRSLAVLDGAILVISAKDGVQAQTRILFHALRKMN 120

Query 121
IPTVIFINKIDQAGVDLQSVVQSVRDKLSADIIIKQTVSLSPEIVLEENTDIEAWDAVIE 180

IPTVIFINKIDQAGVDLQSVVQSVRDKLSADIIIKQTVSLSPEIVLEENTDIEAWDAVIE
Sbjct 121
IPTVIFINKIDQAGVDLQSVVQSVRDKLSADIIIKQTVSLSPEIVLEENTDIEAWDAVIE 180

Query 181
NNDKLLEKYIAGEPISREKLVREEQRRVQDASLFPVYYGSAKKGLGIQPLMDAVTGLFQP 240

NNDKLLEKYIAGEPISREKLVREEQRRVQDASLFPVYYGSAKKGLGIQPLMDAVTGLFQP
Sbjct 181
NNDKLLEKYIAGEPISREKLVREEQRRVQDASLFPVYYGSAKKGLGIQPLMDAVTGLFQP 240

Query 241
IGEQGSAALCGSVFKVEYTDCGQRRVYLRLYSGTLRLRDTVALAGREKLKITEMRIPSKG 300

IGEQGSAALCGSVFKVEYTDCGQRRVYLRLYSGTLRLRDTVALAGREKLKITEMRIPSKG
Sbjct 241
IGEQGSAALCGSVFKVEYTDCGQRRVYLRLYSGTLRLRDTVALAGREKLKITEMRIPSKG 300

Query 301
EIVRTDTAYPGEIVILPSDSVRLNDVLGDPTRLPRKRWREDPLPMLRTTIAPKTAAQRER 360
            EIVRTDTAYPGEIVILPSDSVRLNDVLGDPTRLPRKRWREDPLPMLRT
+IAPKTAAQRER
Sbjct 301
EIVRTDTAYPGEIVILPSDSVRLNDVLGDPTRLPRKRWREDPLPMLRTSIAPKTAAQRER 360

Query 361

LLDALTQLADTDPLLRCEVDSITHEIILSFLGRVQLEVVSALLSEKYKLETVVKEPSVIY 420
            LLDALTQLADTDPLLRCEVDSITHEIILSFLGRVQLEVVSALLSEKYKLETVVKEP
+VIY
Sbjct 361
LLDALTQLADTDPLLRCEVDSITHEIILSFLGRVQLEVVSALLSEKYKLETVVKEPTVIY 420

Query 421
MERPLKAASHTIHIEVPPNPFWASIGLSVTPLSLGSGVQYESRVSLGYLNQSFQNAVRDG 480
            MERPLKAASHTIHIEVPPNPFWASIGLSVTPL
LGSGVQYESRVSLGYLNQSFQNAVRDG
Sbjct 421
MERPLKAASHTIHIEVPPNPFWASIGLSVTPLPLGSGVQYESRVSLGYLNQSFQNAVRDG 480

Query 481
IRYGLEQGLFGWNVTDCKICFEYGLYYSPVSTPADFRSLAPIVLEQALKESGTQLLEPYL 540

IRYGLEQGLFGWNVTDCKICFEYGLYYSPVSTPADFRSLAPIVLEQALKESGTQLLEPYL
Sbjct 481
IRYGLEQGLFGWNVTDCKICFEYGLYYSPVSTPADFRSLAPIVLEQALKESGTQLLEPYL 540

Query 541
SFILYAPQEYLSRAYHDAPKYCATIETAQVKKDEVVFTGEIPARCIQAYRTDLAFYTNGR 600
            SF LYAP+EYLSRAYHDAPKYCATIET
QVKKDEVVFTGEIPARCIQAYRTDLAFYTNGR
Sbjct 541
SFTLYAPREYLSRAYHDAPKYCATIETVQVKKDEVVFTGEIPARCIQAYRTDLAFYTNGR 600

Query 601 SVCLTELKGYQAAVGQPVIQPRRPNSRLDKVRHMFQKVM 639
            SVCLTELKGYQAAVGQPVIQPRRPNSRLDKVRHMFQKVM
Sbjct 601 SVCLTELKGYQAAVGQPVIQPRRPNSRLDKVRHMFQKVM 639

>WP_032746258.1 TetM/TetW/TetO/TetS family tetracycline resistance
ribosomal
protection protein [Bifidobacterium longum]
KEY22021.1 tetracycline resistance protein tetM [Bifidobacterium
longum
subsp. longum 17-1B]
Length=639

Score = 1302 bits (3369), Expect = 0.0, Method: Compositional
matrix adjust.
Identities = 633/639 (99%), Positives = 637/639 (99%), Gaps = 0/639
(0%)

Query 1
MKIINIGILAHVDAGKTTLTESLLYASGAISEPGSVEKGTTRTDTMFLERQRGITIQAAV 60

MKIINIGILAHVDAGKTTLTESLLYASGAISEPGSVEKGTTRTDTMFLERQRGITIQAAV
Sbjct 1
MKIINIGILAHVDAGKTTLTESLLYASGAISEPGSVEKGTTRTDTMFLERQRGITIQAAV 60

Query 61
TSFQWHRCKVNIVDTPGHMDFLAEVYRSLAVLDGAILVISAKDGVQAQTRILFHALRKMN 120

TSFQWHRCKVNIVDTPGHMDFLAEVYRSLAVLDGAILVISAKDGVQAQTRILFHALRKMN
Sbjct 61
TSFQWHRCKVNIVDTPGHMDFLAEVYRSLAVLDGAILVISAKDGVQAQTRILFHALRKMN 120

Query 121
IPTVIFINKIDQAGVDLQSVVQSVRDKLSADIIIKQTVSLSPEIVLEENTDIEAWDAVIE 180

IPTVIFINKIDQAGVDLQSVVQSVRDKLSADIIIKQTVSLSPEIVLEENTDIEAWDAVIE
Sbjct 121
IPTVIFINKIDQAGVDLQSVVQSVRDKLSADIIIKQTVSLSPEIVLEENTDIEAWDAVIE 180

Query 181
NNDKLLEKYIAGEPISREKLVREEQRRVQDASLFPVYYGSAKKGLGIQPLMDAVTGLFQP 240
NNDKLLEKYIAGEPISREKLVREEQ
+RVQDASLFPVYYGSAKKGLGIQPLMDAVTGLFQP
Sbjct 181
NNDKLLEKYIAGEPISREKLVREEQQRVQDASLFPVYYGSAKKGLGIQPLMDAVTGLFQP 240

Query 241
IGEQGSAALCGSVFKVEYTDCGQRRVYLRLYSGTLRLRDTVALAGREKLKITEMRIPSKG 300

IGEQGSAALCGSVFKVEYTDCGQRRVYLRLYSGTLRLRDTVALAGREKLKITEMRIPSKG
Sbjct 241
IGEQGSAALCGSVFKVEYTDCGQRRVYLRLYSGTLRLRDTVALAGREKLKITEMRIPSKG 300

Query 301
EIVRTDTAYPGEIVILPSDSVRLNDVLGDPTRLPRKRWREDPLPMLRTTIAPKTAAQRER 360
EIVRTDTAYPGEIVILPSDSVRLNDVLGDPTRLPRKRWREDPLPMLRT
+IAPKTAAQRER
Sbjct 301
EIVRTDTAYPGEIVILPSDSVRLNDVLGDPTRLPRKRWREDPLPMLRTSIAPKTAAQRER 360

Query 361
LLDALTQLADTDPLLRCEVDSITHEIILSFLGRVQLEVVSALLSEKYKLETVVKEPSVIY 420
LLDALTQLADTDPLLRCEVDSITHEIILSFLGRVQLEVVSALLSEKYKLETVVKEP
+VIY
Sbjct 361
LLDALTQLADTDPLLRCEVDSITHEIILSFLGRVQLEVVSALLSEKYKLETVVKEPTVIY 420

Query 421
MERPLKAASHTIHIEVPPNPFWASIGLSVTPLSLGSGVQYESRVSLGYLNQSFQNAVRDG 480
MERPLKAASHTIHIEVPPNPFWASIGLSVTPL
LGSGVQYESRVSLGYLNQSFQNAVRDG
Sbjct 421
MERPLKAASHTIHIEVPPNPFWASIGLSVTPLPLGSGVQYESRVSLGYLNQSFQNAVRDG 480

Query 481
IRYGLEQGLFGWNVTDCKICFEYGLYYSPVSTPADFRSLAPIVLEQALKESGTQLLEPYL 540

IRYGLEQGLFGWNVTDCKICFEYGLYYSPVSTPADFRSLAPIVLEQALKESGTQLLEPYL
Sbjct 481
IRYGLEQGLFGWNVTDCKICFEYGLYYSPVSTPADFRSLAPIVLEQALKESGTQLLEPYL 540

```
Query  541
SFILYAPQEYLSRAYHDAPKYCATIETAQVKKDEVVFTGEIPARCIQAYRTDLAFYTNGR  600
            SF LYAP
+EYLSRAYHDAPKYCATIETAQVKKDEVVFTGEIPARCIQAYRTDLAFYTNGR
Sbjct  541
SFTLYAPREYLSRAYHDAPKYCATIETAQVKKDEVVFTGEIPARCIQAYRTDLAFYTNGR  600

Query  601  SVCLTELKGYQAAVGQPVIQPRRPNSRLDKVRHMFQKVM  639
            SVCLTELKGYQAAVGQPVIQPRRPNSRLDKVRHMFQKVM
Sbjct  601  SVCLTELKGYQAAVGQPVIQPRRPNSRLDKVRHMFQKVM  639


>WP_063856417.1 tetracycline resistance ribosomal protection protein
Tet(W) [Selenomonas
ruminantium]
 ABC18245.1 TetW [Selenomonas ruminantium]
 ABC18250.1 TetW [Selenomonas ruminantium]
 ABC18255.1 TetW [Selenomonas ruminantium]
Length=639

 Score = 1301 bits (3368),  Expect = 0.0, Method: Compositional
matrix adjust.
 Identities = 632/639 (99%), Positives = 637/639 (99%), Gaps = 0/639
(0%)

Query  1
MKIINIGILAHVDAGKTTLTESLLYASGAISEPGSVEKGTTRTDTMFLERQRGITIQAAV  60

MKIINIGILAHVDAGKTTLTESLLYASGAISEPGSVEKGTTRTDTMFLERQRGITIQAAV
Sbjct  1
MKIINIGILAHVDAGKTTLTESLLYASGAISEPGSVEKGTTRTDTMFLERQRGITIQAAV  60

Query  61
TSFQWHRCKVNIVDTPGHMDFLAEVYRSLAVLDGAILVISAKDGVQAQTRILFHALRKMN  120

TSFQWHRCKVNIVDTPGHMDFLAEVYRSLAVLDGAILVISAKDGVQAQTRILFHALRKMN
Sbjct  61
TSFQWHRCKVNIVDTPGHMDFLAEVYRSLAVLDGAILVISAKDGVQAQTRILFHALRKMN  120

Query  121
IPTVIFINKIDQAGVDLQSVVQSVRDKLSADIIIKQTVSLSPEIVLEENTDIEAWDAVIE  180

IPTVIFINKIDQAGVDLQSVVQSVRDKLSADIIIKQTVSLSPEIVLEENTDIEAWDAVIE
Sbjct  121
IPTVIFINKIDQAGVDLQSVVQSVRDKLSADIIIKQTVSLSPEIVLEENTDIEAWDAVIE  180

Query  181
NNDKLLEKYIAGEPISREKLVREEQRRVQDASLFPVYYGSAKKGLGIQPLMDAVTGLFQP  240

NNDKLLEKYIAGEPISREKLVREEQRRVQDASLFPVYYGSAKKGLGIQPLMDAVTGLFQP
Sbjct  181
NNDKLLEKYIAGEPISREKLVREEQRRVQDASLFPVYYGSAKKGLGIQPLMDAVTGLFQP  240

Query  241
```

```
IGEQGSAALCGSVFKVEYTDCGQRRVYLRLYSGTLRLRDTVALAGREKLKITEMRIPSKG  300

IGEQGSAALCGSVFKVEYTDCGQRRVYLRLYSGTLRLRDTVALAGREKLKITEMRIPSKG
Sbjct  241
IGEQGSAALCGSVFKVEYTDCGQRRVYLRLYSGTLRLRDTVALAGREKLKITEMRIPSKG  300

Query  301
EIVRTDTAYPGEIVILPSDSVRLNDVLGDPTRLPRKRWREDPLPMLRTTIAPKTAAQRER  360
            EIVRTDTAYPGEIVILPSDSVRLNDVLGDPTRLPRKRWREDPLPMLRT
+IAPKTAAQRER
Sbjct  301
EIVRTDTAYPGEIVILPSDSVRLNDVLGDPTRLPRKRWREDPLPMLRTSIAPKTAAQRER  360

Query  361
LLDALTQLADTDPLLRCEVDSITHEIILSFLGRVQLEVVSALLSEKYKLETVVKEPSVIY  420
            LLDALTQLADTDPLLRCEVDSITHEIILSFLGRVQLEVVSALLSEKYKLETVVKEP
+VIY
Sbjct  361
LLDALTQLADTDPLLRCEVDSITHEIILSFLGRVQLEVVSALLSEKYKLETVVKEPTVIY  420

Query  421
MERPLKAASHTIHIEVPPNPFWASIGLSVTPLSLGSGVQYESRVSLGYLNQSFQNAVRDG  480

MERPLKAASHTIHIEVPPNPFWASIGLSVTPLSLGSGVQYESRVSLGYLNQSFQNAVRDG
Sbjct  421
MERPLKAASHTIHIEVPPNPFWASIGLSVTPLSLGSGVQYESRVSLGYLNQSFQNAVRDG  480

Query  481
IRYGLEQGLFGWNVTDCKICFEYGLYYSPVSTPADFRSLAPIVLEQALKESGTQLLEPYL  540

IRYGLEQGLFGWNVTDCKICFEYGLYYSPVSTPADFRSLAPIVLEQALKESGTQLLEPYL
Sbjct  481
IRYGLEQGLFGWNVTDCKICFEYGLYYSPVSTPADFRSLAPIVLEQALKESGTQLLEPYL  540

Query  541
SFILYAPQEYLSRAYHDAPKYCATIETAQVKKDEVVFTGEIPARCIQAYRTDLAFYTNGR  600
            SF LYAP+EYLSRAYHDAPKYCATIET
QVKKDEVVFTGEIPARCIQAYRTDLAFYTNG+
Sbjct  541
SFTLYAPREYLSRAYHDAPKYCATIETVQVKKDEVVFTGEIPARCIQAYRTDLAFYTNGQ  600

Query  601  SVCLTELKGYQAAVGQPVIQPRRPNSRLDKVRHMFQKVM  639
            SVCLTELKGYQAAVG+PVIQPRRPNSRLDKVRHMFQKVM
Sbjct  601  SVCLTELKGYQAAVGKPVIQPRRPNSRLDKVRHMFQKVM  639
```

>WP_005876003.1 TetM/TetW/TetO/TetS family tetracycline resistance ribosomal
protection protein [Oxalobacter formigenes]
 EEO27145.1 tetracycline resistance protein tetW [Oxalobacter formigenes
HOxBLS]
Length=639

Score = 1301 bits (3368), Expect = 0.0, Method: Compositional matrix adjust.
Identities = 632/639 (99%), Positives = 637/639 (99%), Gaps = 0/639 (0%)

```
Query  1
MKIINIGILAHVDAGKTTLTESLLYASGAISEPGSVEKGTTRTDTMFLERQRGITIQAAV  60

MKIINIGILAHVDAGKTTLTESLLYASGAISEPGSVEKGTTRTDTMFLERQRGITIQAAV
Sbjct  1
MKIINIGILAHVDAGKTTLTESLLYASGAISEPGSVEKGTTRTDTMFLERQRGITIQAAV  60

Query  61
TSFQWHRCKVNIVDTPGHMDFLAEVYRSLAVLDGAILVISAKDGVQAQTRILFHALRKMN  120

TSFQWHRCKVNIVDTPGHMDFLAEVYRSLAVLDGAILVISAKDGVQAQTRILFHALRKMN
Sbjct  61
TSFQWHRCKVNIVDTPGHMDFLAEVYRSLAVLDGAILVISAKDGVQAQTRILFHALRKMN  120

Query  121
IPTVIFINKIDQAGVDLQSVVQSVRDKLSADIIIKQTVSLSPEIVLEENTDIEAWDAVIE  180

IPTVIFINKIDQAGVDLQSVVQSVRDKLSADIIIKQTVSLSPEIVLEENTDIEAWDAVIE
Sbjct  121
IPTVIFINKIDQAGVDLQSVVQSVRDKLSADIIIKQTVSLSPEIVLEENTDIEAWDAVIE  180

Query  181
NNDKLLEKYIAGEPISREKLVREEQRRVQDASLFPVYYGSAKKGLGIQPLMDAVTGLFQP  240

NNDKLLEKYIAGEPISREKLVREEQRRVQDASLFPVYYGSAKKGLGIQPLMDAVTGLFQP
Sbjct  181
NNDKLLEKYIAGEPISREKLVREEQRRVQDASLFPVYYGSAKKGLGIQPLMDAVTGLFQP  240

Query  241
IGEQGSAALCGSVFKVEYTDCGQRRVYLRLYSGTLRLRDTVALAGREKLKITEMRIPSKG  300

IGEQGSAALCGSVFKVEYTDCGQRRVYLRLYSGTLRLRDTVALAGREKLKITEMRIPSKG
Sbjct  241
IGEQGSAALCGSVFKVEYTDCGQRRVYLRLYSGTLRLRDTVALAGREKLKITEMRIPSKG  300

Query  301
EIVRTDTAYPGEIVILPSDSVRLNDVLGDPTRLPRKRWREDPLPMLRTTIAPKTAAQRER  360
            EIVRTDTAYPGEIVILPSDSVRLNDVLGDPTRLPRKRWREDPLPMLRT
+IAPKTAAQRER
Sbjct  301
EIVRTDTAYPGEIVILPSDSVRLNDVLGDPTRLPRKRWREDPLPMLRTSIAPKTAAQRER  360

Query  361
LLDALTQLADTDPLLRCEVDSITHEIILSFLGRVQLEVVSALLSEKYKLETVVKEPSVIY  420
            LLDALTQLADTDPLLRCEVDSITHEIILSFLGRVQLEVVSALLSEKYK+ETVVKEP
+VIY
Sbjct  361
LLDALTQLADTDPLLRCEVDSITHEIILSFLGRVQLEVVSALLSEKYKIETVVKEPTVIY  420
```

```
Query  421
MERPLKAASHTIHIEVPPNPFWASIGLSVTPLSLGSGVQYESRVSLGYLNQSFQNAVRDG  480
            MERPLKAASHTIHIEVPPNPFWASIGLSVTPL
LGSGVQYESRVSLGYLNQSFQNAVRDG
Sbjct  421
MERPLKAASHTIHIEVPPNPFWASIGLSVTPLPLGSGVQYESRVSLGYLNQSFQNAVRDG  480

Query  481
IRYGLEQGLFGWNVTDCKICFEYGLYYSPVSTPADFRSLAPIVLEQALKESGTQLLEPYL  540

IRYGLEQGLFGWNVTDCKICFEYGLYYSPVSTPADFRSLAPIVLEQALKESGTQLLEPYL
Sbjct  481
IRYGLEQGLFGWNVTDCKICFEYGLYYSPVSTPADFRSLAPIVLEQALKESGTQLLEPYL  540

Query  541
SFILYAPQEYLSRAYHDAPKYCATIETAQVKKDEVVFTGEIPARCIQAYRTDLAFYTNGR  600
            SF
LYAPQEYLSRAYHDAPKYCATIETAQVKKDEVVFTGEIPARCIQAYRTDLAFYTNG+
Sbjct  541
SFTLYAPQEYLSRAYHDAPKYCATIETAQVKKDEVVFTGEIPARCIQAYRTDLAFYTNGQ  600

Query  601  SVCLTELKGYQAAVGQPVIQPRRPNSRLDKVRHMFQKVM  639
            SVCLTELKGYQAAVGQPVIQPRRPN+RLDKVRHMFQKVM
Sbjct  601  SVCLTELKGYQAAVGQPVIQPRRPNNRLDKVRHMFQKVM  639


>WP_033523205.1 MULTISPECIES: TetM/TetW/TetO/TetS family
tetracycline resistance
ribosomal protection protein [Actinobacteria]
 CVH77936.1 Tetracycline resistance protein TetO [Coriobacteriaceae
bacterium
CHKCI002]
 SHE70123.1 ribosomal protection tetracycline resistance protein
[Bifidobacterium
merycicum DSM 6492]
 OUO95347.1 TetM/TetW/TetO/TetS family tetracycline resistance
ribosomal
protection protein [Gordonibacter sp. An232A]
 PNV66097.1 TetM/TetW/TetO/TetS family tetracycline resistance
ribosomal
protection protein [Rubneribacter badeniensis]
Length=639

 Score = 1301 bits (3366),  Expect = 0.0, Method: Compositional
matrix adjust.
 Identities = 632/639 (99%), Positives = 636/639 (99%), Gaps = 0/639
(0%)

Query  1
MKIINIGILAHVDAGKTTLTESLLYASGAISEPGSVEKGTTRTDTMFLERQRGITIQAAV  60

MKIINIGILAHVDAGKTTLTESLLYASGAISEPGSVEKGTTRTDTMFLERQRGITIQAAV
Sbjct  1
MKIINIGILAHVDAGKTTLTESLLYASGAISEPGSVEKGTTRTDTMFLERQRGITIQAAV  60
```

Query 61
TSFQWHRCKVNIVDTPGHMDFLAEVYRSLAVLDGAILVISAKDGVQAQTRILFHALRKMN 120

TSFQWHRCKVNIVDTPGHMDFLAEVYRSLAVLDGAILVISAKDGVQAQTRILFHALRKMN
Sbjct 61
TSFQWHRCKVNIVDTPGHMDFLAEVYRSLAVLDGAILVISAKDGVQAQTRILFHALRKMN 120

Query 121
IPTVIFINKIDQAGVDLQSVVQSVRDKLSADIIIKQTVSLSPEIVLEENTDIEAWDAVIE 180

IPTVIFINKIDQAGVDLQSVVQSVRDKLSADIIIKQTVSLSPEIVLEENTDIEAWDAVIE
Sbjct 121
IPTVIFINKIDQAGVDLQSVVQSVRDKLSADIIIKQTVSLSPEIVLEENTDIEAWDAVIE 180

Query 181
NNDKLLEKYIAGEPISREKLVREEQRRVQDASLFPVYYGSAKKGLGIQPLMDAVTGLFQP 240

NNDKLLEKYIAGEPISREKLVREEQRRVQDASLFPVYYGSAKKGLGIQPLMDAVTGLFQP
Sbjct 181
NNDKLLEKYIAGEPISREKLVREEQRRVQDASLFPVYYGSAKKGLGIQPLMDAVTGLFQP 240

Query 241
IGEQGSAALCGSVFKVEYTDCGQRRVYLRLYSGTLRLRDTVALAGREKLKITEMRIPSKG 300

IGEQGSAALCGSVFKVEYTDCGQRRVYLRLYSGTLRLRDTVALAGREKLKITEMRIPSKG
Sbjct 241
IGEQGSAALCGSVFKVEYTDCGQRRVYLRLYSGTLRLRDTVALAGREKLKITEMRIPSKG 300

Query 301
EIVRTDTAYPGEIVILPSDSVRLNDVLGDPTRLPRKRWREDPLPMLRTTIAPKTAAQRER 360
EIVRTDTAYPGEIVILPSDSVRLNDVLGDPTRLPRKRWREDPLPMLRT
+IAPKTAAQRER
Sbjct 301
EIVRTDTAYPGEIVILPSDSVRLNDVLGDPTRLPRKRWREDPLPMLRTSIAPKTAAQRER 360

Query 361
LLDALTQLADTDPLLRCEVDSITHEIILSFLGRVQLEVVSALLSEKYKLETVVKEPSVIY 420
LLDALTQLADTDPLLRCEVDSITHEIILSFLGRVQLEVVSALLSEKYKLETVVKEP
+VIY
Sbjct 361
LLDALTQLADTDPLLRCEVDSITHEIILSFLGRVQLEVVSALLSEKYKLETVVKEPTVIY 420

Query 421
MERPLKAASHTIHIEVPPNPFWASIGLSVTPLSLGSGVQYESRVSLGYLNQSFQNAVRDG 480
MERPLKAASHTIHIEVPPNPFWASIGLSVTPL
LGSGVQYESRVSLGYLNQSFQNAVRDG
Sbjct 421
MERPLKAASHTIHIEVPPNPFWASIGLSVTPLPLGSGVQYESRVSLGYLNQSFQNAVRDG 480

Query 481
IRYGLEQGLFGWNVTDCKICFEYGLYYSPVSTPADFRSLAPIVLEQALKESGTQLLEPYL 540

IRYGLEQGLFGWNVTDCKICFEYGLYYSPVSTPADFRSLAPIVLEQALKESGTQLLEPYL

Sbjct 481
IRYGLEQGLFGWNVTDCKICFEYGLYYSPVSTPADFRSLAPIVLEQALKESGTQLLEPYL 540

Query 541
SFILYAPQEYLSRAYHDAPKYCATIETAQVKKDEVVFTGEIPARCIQAYRTDLAFYTNGR 600
            SF LYAP+EYLSRAYHDAPKYCATIET
QVKKDEVVFTGEIPARCIQAYRTDLAFYTNG+
Sbjct 541
SFTLYAPREYLSRAYHDAPKYCATIETVQVKKDEVVFTGEIPARCIQAYRTDLAFYTNGQ 600

Query 601 SVCLTELKGYQAAVGQPVIQPRRPNSRLDKVRHMFQKVM 639
            SVCLTELKGYQAAVGQPVIQPRRPNSRLDKVRHMFQKVM
Sbjct 601 SVCLTELKGYQAAVGQPVIQPRRPNSRLDKVRHMFQKVM 639

>DAB17102.1 TPA: tetracycline resistance ribosomal protection protein [Candidatus
Gastranaerophilales bacterium HUM_19]
Length=639

Score = 1300 bits (3364), Expect = 0.0, Method: Compositional matrix adjust.
Identities = 632/639 (99%), Positives = 636/639 (99%), Gaps = 0/639 (0%)

Query 1
MKIINIGILAHVDAGKTTLTESLLYASGAISEPGSVEKGTTRTDTMFLERQRGITIQAAV 60

MKIINIGILAHVDAGKTTLTESLLYASGAISEPGSVEKGTTRTDTMFLERQRGITIQAAV
Sbjct 1
MKIINIGILAHVDAGKTTLTESLLYASGAISEPGSVEKGTTRTDTMFLERQRGITIQAAV 60

Query 61
TSFQWHRCKVNIVDTPGHMDFLAEVYRSLAVLDGAILVISAKDGVQAQTRILFHALRKMN 120

TSFQWHRCKVNIVDTPGHMDFLAEVYRSLAVLDGAILVISAKDGVQAQTRILFHALRKMN
Sbjct 61
TSFQWHRCKVNIVDTPGHMDFLAEVYRSLAVLDGAILVISAKDGVQAQTRILFHALRKMN 120

Query 121
IPTVIFINKIDQAGVDLQSVVQSVRDKLSADIIIKQTVSLSPEIVLEENTDIEAWDAVIE 180

IPTVIFINKIDQAGVDLQSVVQSVRDKLSADIIIKQTVSLSPEIVLEENTDIEAWDAVIE
Sbjct 121
IPTVIFINKIDQAGVDLQSVVQSVRDKLSADIIIKQTVSLSPEIVLEENTDIEAWDAVIE 180

Query 181
NNDKLLEKYIAGEPISREKLVREEQRRVQDASLFPVYYGSAKKGLGIQPLMDAVTGLFQP 240

NNDKLLEKYIAGEPISREKLVREEQRRVQDASLFPVYYGSAKKGLGIQPLMDAVTGLFQP
Sbjct 181
NNDKLLEKYIAGEPISREKLVREEQRRVQDASLFPVYYGSAKKGLGIQPLMDAVTGLFQP 240

Query 241

```
IGEQGSAALCGSVFKVEYTDCGQRRVYLRLYSGTLRLRDTVALAGREKLKITEMRIPSKG  300

IGEQGSAALCGSVFKVEYTDCGQRRVYLRLYSGTLRLRDTVALAGREKLKITEMRIPSKG
Sbjct  241
IGEQGSAALCGSVFKVEYTDCGQRRVYLRLYSGTLRLRDTVALAGREKLKITEMRIPSKG  300

Query  301
EIVRTDTAYPGEIVILPSDSVRLNDVLGDPTRLPRKRWREDPLPMLRTTIAPKTAAQRER  360
            EIVRTDTAYPGEIVILPSDSVRLNDVLGDPTRLPRKRWREDPLPMLRT
+IAPKTAAQRER
Sbjct  301
EIVRTDTAYPGEIVILPSDSVRLNDVLGDPTRLPRKRWREDPLPMLRTSIAPKTAAQRER  360

Query  361
LLDALTQLADTDPLLRCEVDSITHEIILSFLGRVQLEVVSALLSEKYKLETVVKEPSVIY  420

LLDALTQLADTDPLLRCEVDSITHEIILSFLGRVQLEVVSALLSEKYKLETVVKEPSVIY
Sbjct  361
LLDALTQLADTDPLLRCEVDSITHEIILSFLGRVQLEVVSALLSEKYKLETVVKEPSVIY  420

Query  421
MERPLKAASHTIHIEVPPNPFWASIGLSVTPLSLGSGVQYESRVSLGYLNQSFQNAVRDG  480
            MERPLKAASHTIHIEVPPNPFWASIGLSVTPL
LGSGVQYESRVSLGYLNQSFQNAVRDG
Sbjct  421
MERPLKAASHTIHIEVPPNPFWASIGLSVTPLPLGSGVQYESRVSLGYLNQSFQNAVRDG  480

Query  481
IRYGLEQGLFGWNVTDCKICFEYGLYYSPVSTPADFRSLAPIVLEQALKESGTQLLEPYL  540

IRYGLEQGLFGWNVTDCKICFEYGLYYSPVSTPADFRSLAPIVLEQALKESGTQLLEPYL
Sbjct  481
IRYGLEQGLFGWNVTDCKICFEYGLYYSPVSTPADFRSLAPIVLEQALKESGTQLLEPYL  540

Query  541
SFILYAPQEYLSRAYHDAPKYCATIETAQVKKDEVVFTGEIPARCIQAYRTDLAFYTNGR  600
            SF LYAP+EYLSRAYHDAPKYCATIET
QVKKDEVVFTGEIPARCIQAYRTDLAFYTNGR
Sbjct  541
SFTLYAPREYLSRAYHDAPKYCATIETVQVKKDEVVFTGEIPARCIQAYRTDLAFYTNGR  600

Query  601  SVCLTELKGYQAAVGQPVIQPRRPNSRLDKVRHMFQKVM  639
            SVCLTELKGYQAAVGQPVIQPRRPNSRLDKVR+MFQK+M
Sbjct  601  SVCLTELKGYQAAVGQPVIQPRRPNSRLDKVRYMFQKIM  639


>WP_066647691.1 TetM/TetW/TetO/TetS family tetracycline resistance
ribosomal
protection protein [Christensenella timonensis]
Length=639

 Score = 1300 bits (3363),  Expect = 0.0, Method: Compositional
matrix adjust.
 Identities = 632/639 (99%), Positives = 636/639 (99%), Gaps = 0/639
```

(0%)

Query 1
MKIININIGILAHVDAGKTTLTESLLYASGAISEPGSVEKGTTRTDTMFLERQRGITIQAAV 60

MKIININIGILAHVDAGKTTLTESLLYASGAISEPGSVEKGTTRTDTMFLERQRGITIQAAV
Sbjct 1
MKIININIGILAHVDAGKTTLTESLLYASGAISEPGSVEKGTTRTDTMFLERQRGITIQAAV 60

Query 61
TSFQWHRCKVNIVDTPGHMDFLAEVYRSLAVLDGAILVISAKDGVQAQTRILFHALRKMN 120

TSFQWHRCKVNIVDTPGHMDFLAEVYRSLAVLDGAILVISAKDGVQAQTRILFHALRKMN
Sbjct 61
TSFQWHRCKVNIVDTPGHMDFLAEVYRSLAVLDGAILVISAKDGVQAQTRILFHALRKMN 120

Query 121
IPTVIFINKIDQAGVDLQSVVQSVRDKLSADIIIKQTVSLSPEIVLEENTDIEAWDAVIE 180

IPTVIFINKIDQAGVDLQSVVQSVRDKLSADIIIKQTVSLSPEIVLEENTDIEAWDAVIE
Sbjct 121
IPTVIFINKIDQAGVDLQSVVQSVRDKLSADIIIKQTVSLSPEIVLEENTDIEAWDAVIE 180

Query 181
NNDKLLEKYIAGEPISREKLVREEQRRVQDASLFPVYYGSAKKGLGIQPLMDAVTGLFQP 240

NNDKLLEKYIAGEPISREKLVREEQRRVQDASLFPVYYGSAKKGLGIQPLMDAVTGLFQP
Sbjct 181
NNDKLLEKYIAGEPISREKLVREEQRRVQDASLFPVYYGSAKKGLGIQPLMDAVTGLFQP 240

Query 241
IGEQGSAALCGSVFKVEYTDCGQRRVYLRLYSGTLRLRDTVALAGREKLKITEMRIPSKG 300

IGEQGSAALCGSVFKVEYTDCGQRRVYLRLYSGTLRLRDTVALAGREKLKITEMRIPSKG
Sbjct 241
IGEQGSAALCGSVFKVEYTDCGQRRVYLRLYSGTLRLRDTVALAGREKLKITEMRIPSKG 300

Query 301
EIVRTDTAYPGEIVILPSDSVRLNDVLGDPTRLPRKRWREDPLPMLRTTIAPKTAAQRER 360
EIVRTDTAYPGEIVILPSDSVRLNDVLGDPTRLPRKRWREDPLPMLRT
+IAPKTAAQRER
Sbjct 301
EIVRTDTAYPGEIVILPSDSVRLNDVLGDPTRLPRKRWREDPLPMLRTSIAPKTAAQRER 360

Query 361
LLDALTQLADTDPLLRCEVDSITHEIILSFLGRVQLEVVSALLSEKYKLETVVKEPSVIY 420
LLDALTQLADTDPLLRCEVDSITHEIILSFLGRVQLEVVSALLSEKYKLETVVKEP
+VIY
Sbjct 361
LLDALTQLADTDPLLRCEVDSITHEIILSFLGRVQLEVVSALLSEKYKLETVVKEPTVIY 420

Query 421
MERPLKAASHTIHIEVPPNPFWASIGLSVTPLSLGSGVQYESRVSLGYLNQSFQNAVRDG 480
MERPLKAASHTIHIEVPPNPFWASIGLSVTPL

```
LGSGVQYESRVSLGYLNQSFQNAVRDG
Sbjct  421
MERPLKAASHTIHIEVPPNPFWASIGLSVTPLPLGSGVQYESRVSLGYLNQSFQNAVRDG  480

Query  481
IRYGLEQGLFGWNVTDCKICFEYGLYYSPVSTPADFRSLAPIVLEQALKESGTQLLEPYL  540

IRYGLEQGLFGWNVTDCKICFEYGLYYSPVSTPADFRSLAPIVLEQALKESGTQLLEPYL
Sbjct  481
IRYGLEQGLFGWNVTDCKICFEYGLYYSPVSTPADFRSLAPIVLEQALKESGTQLLEPYL  540

Query  541
SFILYAPQEYLSRAYHDAPKYCATIETAQVKKDEVVFTGEIPARCIQAYRTDLAFYTNGR  600
            SFILYAPQEYLSRAYHDAPKYCATIET Q
KKDEVVFTGEIPARCIQAYRTDLAFYTNG+
Sbjct  541
SFILYAPQEYLSRAYHDAPKYCATIETVQEKKDEVVFTGEIPARCIQAYRTDLAFYTNGQ  600

Query  601  SVCLTELKGYQAAVGQPVIQPRRPNSRLDKVRHMFQKVM  639
            SVCLTELKGYQAAVG+PVIQPRRPNSRLDKVRHMFQKVM
Sbjct  601  SVCLTELKGYQAAVGKPVIQPRRPNSRLDKVRHMFQKVM  639


>WP_005924790.1 TetM/TetW/TetO/TetS family tetracycline resistance
ribosomal
protection protein [Faecalibacterium prausnitzii]
 EDP21237.1 putative translation elongation factor G
[Faecalibacterium prausnitzii
M21/2]
 PDX83922.1 TetM/TetW/TetO/TetS family tetracycline resistance
ribosomal
protection protein [Faecalibacterium prausnitzii]
Length=639

 Score = 1299 bits (3362),  Expect = 0.0, Method: Compositional
matrix adjust.
 Identities = 631/639 (99%), Positives = 636/639 (99%), Gaps = 0/639
(0%)

Query  1
MKIINIGILAHVDAGKTTLTESLLYASGAISEPGSVEKGTTRTDTMFLERQRGITIQAAV  60

MKIINIGILAHVDAGKTTLTESLLYASGAISEPGSVEKGTTRTDTMFLERQRGITIQAAV
Sbjct  1
MKIINIGILAHVDAGKTTLTESLLYASGAISEPGSVEKGTTRTDTMFLERQRGITIQAAV  60

Query  61
TSFQWHRCKVNIVDTPGHMDFLAEVYRSLAVLDGAILVISAKDGVQAQTRILFHALRKMN  120

TSFQWHRCKVNIVDTPGHMDFLAEVYRSLAVLDGAILVISAKDGVQAQTRILFHALRKMN
Sbjct  61
TSFQWHRCKVNIVDTPGHMDFLAEVYRSLAVLDGAILVISAKDGVQAQTRILFHALRKMN  120

Query  121
```

IPTVIFINKIDQAGVDLQSVVQSVRDKLSADIIIKQTVSLSPEIVLEENTDIEAWDAVIE 180

IPTVIFINKIDQAGVDLQSVVQSVRDKLSADIIIKQTVSLSPEIVLEENTDIEAWDAVIE
Sbjct 121
IPTVIFINKIDQAGVDLQSVVQSVRDKLSADIIIKQTVSLSPEIVLEENTDIEAWDAVIE 180

Query 181
NNDKLLEKYIAGEPISREKLVREEQRRVQDASLFPVYYGSAKKGLGIQPLMDAVTGLFQP 240

NNDKLLEKYIAGEPISREKLVREEQRRVQDASLFPVYYGSAKKGLGIQPLMDAVTGLFQP
Sbjct 181
NNDKLLEKYIAGEPISREKLVREEQRRVQDASLFPVYYGSAKKGLGIQPLMDAVTGLFQP 240

Query 241
IGEQGSAALCGSVFKVEYTDCGQRRVYLRLYSGTLRLRDTVALAGREKLKITEMRIPSKG 300

IGEQGSAALCGSVFKVEYTDCGQRRVYLRLYSGTLRLRDTVALAGREKLKITEMRIPSKG
Sbjct 241
IGEQGSAALCGSVFKVEYTDCGQRRVYLRLYSGTLRLRDTVALAGREKLKITEMRIPSKG 300

Query 301
EIVRTDTAYPGEIVILPSDSVRLNDVLGDPTRLPRKRWREDPLPMLRTTIAPKTAAQRER 360
EIVRTDTAYPGEIVILPSDSVRLNDVLGDPTRLPRKRWREDPLPMLRT
+IAPKTAAQRER
Sbjct 301
EIVRTDTAYPGEIVILPSDSVRLNDVLGDPTRLPRKRWREDPLPMLRTSIAPKTAAQRER 360

Query 361
LLDALTQLADTDPLLRCEVDSITHEIILSFLGRVQLEVVSALLSEKYKLETVVKEPSVIY 420
LLDALTQLADTDPLLRCEVDSITHEIILSFLGRVQLEVVSALLSEKYKLETVVKEP
+VIY
Sbjct 361
LLDALTQLADTDPLLRCEVDSITHEIILSFLGRVQLEVVSALLSEKYKLETVVKEPTVIY 420

Query 421
MERPLKAASHTIHIEVPPNPFWASIGLSVTPLSLGSGVQYESRVSLGYLNQSFQNAVRDG 480
MERPLKAASHTIHIEVPPNPFWASIGLSVTPL
LGSGVQYESRVSLGYLNQSFQNAVRDG
Sbjct 421
MERPLKAASHTIHIEVPPNPFWASIGLSVTPLPLGSGVQYESRVSLGYLNQSFQNAVRDG 480

Query 481
IRYGLEQGLFGWNVTDCKICFEYGLYYSPVSTPADFRSLAPIVLEQALKESGTQLLEPYL 540

IRYGLEQGLFGWNVTDCKICFEYGLYYSPVSTPADFRSLAPIVLEQALKESGTQLLEPYL
Sbjct 481
IRYGLEQGLFGWNVTDCKICFEYGLYYSPVSTPADFRSLAPIVLEQALKESGTQLLEPYL 540

Query 541
SFILYAPQEYLSRAYHDAPKYCATIETAQVKKDEVVFTGEIPARCIQAYRTDLAFYTNGR 600
SF LYAP+EYLSRAYHDAPKYCATIET
QVKKDEVVFTGEIPARCIQAYRTDLAFYTNG+
Sbjct 541
SFTLYAPREYLSRAYHDAPKYCATIETVQVKKDEVVFTGEIPARCIQAYRTDLAFYTNGQ 600

```
Query  601  SVCLTELKGYQAAVGQPVIQPRRPNSRLDKVRHMFQKVM  639
            SVCLTELKGYQAAVG+PVIQPRRPNSRLDKVRHMFQKVM
Sbjct  601  SVCLTELKGYQAAVGKPVIQPRRPNSRLDKVRHMFQKVM  639
```

>WP_063856414.1 tetracycline resistance ribosomal protection protein Tet(W) [Bifidobacterium
sp. IS03519]
 AAL30847.1 TetW [Bifidobacterium sp. IS03519]
Length=639

 Score = 1298 bits (3360), Expect = 0.0, Method: Compositional matrix adjust.
 Identities = 630/639 (99%), Positives = 637/639 (99%), Gaps = 0/639 (0%)

```
Query  1
MKIINIGILAHVDAGKTTLTESLLYASGAISEPGSVEKGTTRTDTMFLERQRGITIQAAV  60

MKIINIGILAHVDAGKTTLTESLLYASGAISEPGSVEKGTTRTDTMFLERQRGITIQAAV
Sbjct  1
MKIINIGILAHVDAGKTTLTESLLYASGAISEPGSVEKGTTRTDTMFLERQRGITIQAAV  60

Query  61
TSFQWHRCKVNIVDTPGHMDFLAEVYRSLAVLDGAILVISAKDGVQAQTRILFHALRKMN  120

TSFQWHRCKVNIVDTPGHMDFLAEVYRSLAVLDGAILVISAKDGVQAQTRILFHALRKMN
Sbjct  61
TSFQWHRCKVNIVDTPGHMDFLAEVYRSLAVLDGAILVISAKDGVQAQTRILFHALRKMN  120

Query  121
IPTVIFINKIDQAGVDLQSVVQSVRDKLSADIIIKQTVSLSPEIVLEENTDIEAWDAVIE  180

IPTVIFINKIDQAGVDLQSVVQSVRDKLSADIIIKQTVSLSPEIVLEENTDIEAWDAVIE
Sbjct  121
IPTVIFINKIDQAGVDLQSVVQSVRDKLSADIIIKQTVSLSPEIVLEENTDIEAWDAVIE  180

Query  181
NNDKLLEKYIAGEPISREKLVREEQRRVQDASLFPVYYGSAKKGLGIQPLMDAVTGLFQP  240

NNDKLLEKYIAGEPISREKLVREEQRRVQDASLFPVYYGSAKKGLGIQPLMDAVTGLFQP
Sbjct  181
NNDKLLEKYIAGEPISREKLVREEQRRVQDASLFPVYYGSAKKGLGIQPLMDAVTGLFQP  240

Query  241
IGEQGSAALCGSVFKVEYTDCGQRRVYLRLYSGTLRLRDTVALAGREKLKITEMRIPSKG  300

IGEQGSAALCGSVFKVEYTDCGQRRVYLRLYSGTLRLRDTVALAGREKLKITEMRIPSKG
Sbjct  241
IGEQGSAALCGSVFKVEYTDCGQRRVYLRLYSGTLRLRDTVALAGREKLKITEMRIPSKG  300

Query  301
EIVRTDTAYPGEIVILPSDSVRLNDVLGDPTRLPRKRWREDPLPMLRTTIAPKTAAQRER  360
```

EIVRTDTAYPGEIVILPSDSVRLNDVLGDPTRLPRKRWREDPLPMLRT
+IAPKTAAQRER
Sbjct 301
EIVRTDTAYPGEIVILPSDSVRLNDVLGDPTRLPRKRWREDPLPMLRTSIAPKTAAQRER 360

Query 361
LLDALTQLADTDPLLRCEVDSITHEIILSFLGRVQLEVVSALLSEKYKLETVVKEPSVIY 420
LLDALTQLADTDPLLRCEVDSITHEIILSFLGRVQLEVVSALLSEKYKLETVVKEP
+VIY
Sbjct 361
LLDALTQLADTDPLLRCEVDSITHEIILSFLGRVQLEVVSALLSEKYKLETVVKEPTVIY 420

Query 421
MERPLKAASHTIHIEVPPNPFWASIGLSVTPLSLGSGVQYESRVSLGYLNQSFQNAVRDG 480

MERPLKAASHTIHIEVPPNPFWASIGLSVTPLSLGSGVQYESRVSLGYLNQSFQNAVRDG
Sbjct 421
MERPLKAASHTIHIEVPPNPFWASIGLSVTPLSLGSGVQYESRVSLGYLNQSFQNAVRDG 480

Query 481
IRYGLEQGLFGWNVTDCKICFEYGLYYSPVSTPADFRSLAPIVLEQALKESGTQLLEPYL 540

IRYGLEQGLFGWNVTDCKICFEYGLYYSPVSTPADFRSLAPIVLEQALKESGTQLLEPYL
Sbjct 481
IRYGLEQGLFGWNVTDCKICFEYGLYYSPVSTPADFRSLAPIVLEQALKESGTQLLEPYL 540

Query 541
SFILYAPQEYLSRAYHDAPKYCATIETAQVKKDEVVFTGEIPARCIQAYRTDLAFYTNGR 600
SF LYAP+EYLSRAYHDAPKYCATIET
QVKKDEVVFTGEIPARCIQAYRTDLAFYTNG+
Sbjct 541
SFTLYAPREYLSRAYHDAPKYCATIETVQVKKDEVVFTGEIPARCIQAYRTDLAFYTNGQ 600

Query 601 SVCLTELKGYQAAVGQPVIQPRRPNSRLDKVRHMFQKVM 639
SVCLTELKGYQAAVG+PVIQPRRPNSRLDKVR+MFQK+M
Sbjct 601 SVCLTELKGYQAAVGKPVIQPRRPNSRLDKVRYMFQKIM 639

>APO35064.1 ferrous iron transport protein B [uncultured bacterium]
Length=639

Score = 1298 bits (3358), Expect = 0.0, Method: Compositional
matrix adjust.
Identities = 631/639 (99%), Positives = 637/639 (99%), Gaps = 0/639
(0%)

Query 1
MKIINIGILAHVDAGKTTLTESLLYASGAISEPGSVEKGTTRTDTMFLERQRGITIQAAV 60

MKIINIGILAHVDAGKTTLTESLLYASGAISEPGSVEKGTTRTDTMFLERQRGITIQAAV
Sbjct 1
MKIINIGILAHVDAGKTTLTESLLYASGAISEPGSVEKGTTRTDTMFLERQRGITIQAAV 60

Query 61

TSFQWHRCKVNIVDTPGHMDFLAEVYRSLAVLDGAILVISAKDGVQAQTRILFHALRKMN 120

TSFQWHRCKVNIVDTPGHMDFLAEVYRSLAVLDGAILVISAKDGVQAQTRILFHALRKMN
Sbjct 61
TSFQWHRCKVNIVDTPGHMDFLAEVYRSLAVLDGAILVISAKDGVQAQTRILFHALRKMN 120

Query 121
IPTVIFINKIDQAGVDLQSVVQSVRDKLSADIIIKQTVSLSPEIVLEENTDIEAWDAVIE 180

IPTVIFINKIDQAGVDLQSVVQSVRDKLSADIIIKQTVSLSPEIVLEENTDIEAWDAVIE
Sbjct 121
IPTVIFINKIDQAGVDLQSVVQSVRDKLSADIIIKQTVSLSPEIVLEENTDIEAWDAVIE 180

Query 181
NNDKLLEKYIAGEPISREKLVREEQRRVQDASLFPVYYGSAKKGLGIQPLMDAVTGLFQP 240
NNDKLLEKYIAGEPISREKLVREEQRRVQDASLFPVY+GSAK
GLGIQPLMDAVTGLFQP
Sbjct 181
NNDKLLEKYIAGEPISREKLVREEQRRVQDASLFPVYHGSAKNGLGIQPLMDAVTGLFQP 240

Query 241
IGEQGSAALCGSVFKVEYTDCGQRRVYLRLYSGTLRLRDTVALAGREKLKITEMRIPSKG 300

IGEQGSAALCGSVFKVEYTDCGQRRVYLRLYSGTLRLRDTVALAGREKLKITEMRIPSKG
Sbjct 241
IGEQGSAALCGSVFKVEYTDCGQRRVYLRLYSGTLRLRDTVALAGREKLKITEMRIPSKG 300

Query 301
EIVRTDTAYPGEIVILPSDSVRLNDVLGDPTRLPRKRWREDPLPMLRTTIAPKTAAQRER 360
EIVRTDTAYPGEIVILPSDSVRLNDVLGDPTRLPRKRWREDPLPMLRT
+IAPKTAAQRER
Sbjct 301
EIVRTDTAYPGEIVILPSDSVRLNDVLGDPTRLPRKRWREDPLPMLRTSIAPKTAAQRER 360

Query 361
LLDALTQLADTDPLLRCEVDSITHEIILSFLGRVQLEVVSALLSEKYKLETVVKEPSVIY 420
LLDALTQLADTDPLLRCEVDSITHEIILSFLGRVQLEVVSALLSEKYKLETVVKEP
+VIY
Sbjct 361
LLDALTQLADTDPLLRCEVDSITHEIILSFLGRVQLEVVSALLSEKYKLETVVKEPTVIY 420

Query 421
MERPLKAASHTIHIEVPPNPFWASIGLSVTPLSLGSGVQYESRVSLGYLNQSFQNAVRDG 480
MERPLKAASHTIHIEVPPNPFWASIGLSVTPL
LGSGVQYESRVSLGYLNQSFQNAVRDG
Sbjct 421
MERPLKAASHTIHIEVPPNPFWASIGLSVTPLPLGSGVQYESRVSLGYLNQSFQNAVRDG 480

Query 481
IRYGLEQGLFGWNVTDCKICFEYGLYYSPVSTPADFRSLAPIVLEQALKESGTQLLEPYL 540

IRYGLEQGLFGWNVTDCKICFEYGLYYSPVSTPADFRSLAPIVLEQALKESGTQLLEPYL
Sbjct 481
IRYGLEQGLFGWNVTDCKICFEYGLYYSPVSTPADFRSLAPIVLEQALKESGTQLLEPYL 540

Query 541
SFILYAPQEYLSRAYHDAPKYCATIETAQVKKDEVVFTGEIPARCIQAYRTDLAFYTNGR 600
SFILYAP
+EYLSRAYHDAPKYCATIETAQVKKDEVVFTGEIPARCIQAYRTDLAFYTNGR
Sbjct 541
SFILYAPREYLSRAYHDAPKYCATIETAQVKKDEVVFTGEIPARCIQAYRTDLAFYTNGR 600

Query 601 SVCLTELKGYQAAVGQPVIQPRRPNSRLDKVRHMFQKVM 639
SVCLTELKGYQAAVG+PVIQPRRPNSRLDKVR+MFQKVM
Sbjct 601 SVCLTELKGYQAAVGKPVIQPRRPNSRLDKVRYMFQKVM 639

>AMP53489.1 TetM-TetW-TetO-TetS [uncultured bacterium]
Length=639

Score = 1298 bits (3358), Expect = 0.0, Method: Compositional matrix adjust.
Identities = 632/639 (99%), Positives = 635/639 (99%), Gaps = 0/639 (0%)

Query 1
MKIINIGILAHVDAGKTTLTESLLYASGAISEPGSVEKGTTRTDTMFLERQRGITIQAAV 60

MKIINIGILAHVDAGKTTLTESLLYASGAISEPGSVEKGTTRTDTMFLERQRGITIQAAV
Sbjct 1
MKIINIGILAHVDAGKTTLTESLLYASGAISEPGSVEKGTTRTDTMFLERQRGITIQAAV 60

Query 61
TSFQWHRCKVNIVDTPGHMDFLAEVYRSLAVLDGAILVISAKDGVQAQTRILFHALRKMN 120

TSFQWHRCKVNIVDTPGHMDFLAEVYRSLAVLDGAILVISAKDGVQAQTRILFHALRKMN
Sbjct 61
TSFQWHRCKVNIVDTPGHMDFLAEVYRSLAVLDGAILVISAKDGVQAQTRILFHALRKMN 120

Query 121
IPTVIFINKIDQAGVDLQSVVQSVRDKLSADIIIKQTVSLSPEIVLEENTDIEAWDAVIE 180

IPTVIFINKIDQAGVDLQSVVQSVRDKLSADIIIKQTVSLSPEIVLEENTDIEAWDAVIE
Sbjct 121
IPTVIFINKIDQAGVDLQSVVQSVRDKLSADIIIKQTVSLSPEIVLEENTDIEAWDAVIE 180

Query 181
NNDKLLEKYIAGEPISREKLVREEQRRVQDASLFPVYYGSAKKGLGIQPLMDAVTGLFQP 240
NND+LLEKYIAGEPISREKL
REEQRRVQDASLFPVYYGSAKKGLGIQPLMDAVTGLFQP
Sbjct 181
NNDELLEKYIAGEPISREKLAREEQRRVQDASLFPVYYGSAKKGLGIQPLMDAVTGLFQP 240

Query 241
IGEQGSAALCGSVFKVEYTDCGQRRVYLRLYSGTLRLRDTVALAGREKLKITEMRIPSKG 300
IGEQG
AALCGSVFKVEYTDCGQRRVYLRLYSGTLRLRDTVALAGREKLKITEMRIPSKG
Sbjct 241

IGEQGGAALCGSVFKVEYTDCGQRRVYLRLYSGTLRLRDTVALAGREKLKITEMRIPSKG 300

Query 301
EIVRTDTAYPGEIVILPSDSVRLNDVLGDPTRLPRKRWREDPLPMLRTTIAPKTAAQRER 360
            EIVRTDTAY GEIVILPSDSVRLNDVLGD
TRLPRKRWREDPLPMLRTTIAPKTAAQRER
Sbjct 301
EIVRTDTAYQGEIVILPSDSVRLNDVLGDQTRLPRKRWREDPLPMLRTTIAPKTAAQRER 360

Query 361
LLDALTQLADTDPLLRCEVDSITHEIILSFLGRVQLEVVSALLSEKYKLETVVKEPSVIY 420

LLDALTQLADTDPLLRCEVDSITHEIILSFLGRVQLEVVSALLSEKYKLETVVKEPSVIY
Sbjct 361
LLDALTQLADTDPLLRCEVDSITHEIILSFLGRVQLEVVSALLSEKYKLETVVKEPSVIY 420

Query 421
MERPLKAASHTIHIEVPPNPFWASIGLSVTPLSLGSGVQYESRVSLGYLNQSFQNAVRDG 480

MERPLKAASHTIHIEVPPNPFWASIGLSVTPLSLGSGVQYESRVSLGYLNQSFQNAVRDG
Sbjct 421
MERPLKAASHTIHIEVPPNPFWASIGLSVTPLSLGSGVQYESRVSLGYLNQSFQNAVRDG 480

Query 481
IRYGLEQGLFGWNVTDCKICFEYGLYYSPVSTPADFRSLAPIVLEQALKESGTQLLEPYL 540

IRYGLEQGLFGWNVTDCKICFEYGLYYSPVSTPADFRSLAPIVLEQALKESGTQLLEPYL
Sbjct 481
IRYGLEQGLFGWNVTDCKICFEYGLYYSPVSTPADFRSLAPIVLEQALKESGTQLLEPYL 540

Query 541
SFILYAPQEYLSRAYHDAPKYCATIETAQVKKDEVVFTGEIPARCIQAYRTDLAFYTNGR 600

SFILYAPQEYLSRAYHDAPKYCATIETAQVKKDEVVFTGEIPARCIQAYRTDLAFYTNGR
Sbjct 541
SFILYAPQEYLSRAYHDAPKYCATIETAQVKKDEVVFTGEIPARCIQAYRTDLAFYTNGR 600

Query 601 SVCLTELKGYQAAVGQPVIQPRRPNSRLDKVRHMFQKVM 639
            SVCLTELKGYQAAVGQPVIQPRRPNSRLDKVR+MFQK+M
Sbjct 601 SVCLTELKGYQAAVGQPVIQPRRPNSRLDKVRYMFQKIM 639

>AMP51295.1 TetM-TetW-TetO-TetS [uncultured bacterium]
Length=639

Score = 1298 bits (3358), Expect = 0.0, Method: Compositional matrix adjust.
Identities = 631/639 (99%), Positives = 635/639 (99%), Gaps = 0/639 (0%)

Query 1
MKIINIGILAHVDAGKTTLTESLLYASGAISEPGSVEKGTTRTDTMFLERQRGITIQAAV 60

MKIINIGILAHVDAGKTTLTESLLYASGAISEPGSVEKGTTRTDTMFLERQRGITIQAAV

Sbjct 1
MKIINIGILAHVDAGKTTLTESLLYASGAISEPGSVEKGTTRTDTMFLERQRGITIQAAV 60

Query 61
TSFQWHRCKVNIVDTPGHMDFLAEVYRSLAVLDGAILVISAKDGVQAQTRILFHALRKMN 120

TSFQWHRCKVNIVDTPGHMDFLAEVYRSLAVLDGAILVISAKDGVQAQTRILFHALRKMN
Sbjct 61
TSFQWHRCKVNIVDTPGHMDFLAEVYRSLAVLDGAILVISAKDGVQAQTRILFHALRKMN 120

Query 121
IPTVIFINKIDQAGVDLQSVVQSVRDKLSADIIIKQTVSLSPEIVLEENTDIEAWDAVIE 180

IPTVIFINKIDQAGVDLQSVVQSVRDKLSADIIIKQTVSLSPEIVLEENTDIEAWDAVIE
Sbjct 121
IPTVIFINKIDQAGVDLQSVVQSVRDKLSADIIIKQTVSLSPEIVLEENTDIEAWDAVIE 180

Query 181
NNDKLLEKYIAGEPISREKLVREEQRRVQDASLFPVYYGSAKKGLGIQPLMDAVTGLFQP 240
NND+LLEKYIAGEPISREKL REEQ+RVQDASLFPVY+GSAK
GLGIQPLMDAVTGLFQP
Sbjct 181
NNDELLEKYIAGEPISREKLAREEQQRVQDASLFPVYHGSAKNGLGIQPLMDAVTGLFQP 240

Query 241
IGEQGSAALCGSVFKVEYTDCGQRRVYLRLYSGTLRLRDTVALAGREKLKITEMRIPSKG 300
IGEQG
AALCGSVFKVEYTDCGQRRVYLRLYSGTLRLRDTVALAGREKLKITEMRIPSKG
Sbjct 241
IGEQGGAALCGSVFKVEYTDCGQRRVYLRLYSGTLRLRDTVALAGREKLKITEMRIPSKG 300

Query 301
EIVRTDTAYPGEIVILPSDSVRLNDVLGDPTRLPRKRWREDPLPMLRTTIAPKTAAQRER 360
EIVRTDTAY
GEIVILPSDSVRLNDVLGDPTRLPRKRWREDPLPMLRTTIAPKTAAQRER
Sbjct 301
EIVRTDTAYQGEIVILPSDSVRLNDVLGDPTRLPRKRWREDPLPMLRTTIAPKTAAQRER 360

Query 361
LLDALTQLADTDPLLRCEVDSITHEIILSFLGRVQLEVVSALLSEKYKLETVVKEPSVIY 420

LLDALTQLADTDPLLRCEVDSITHEIILSFLGRVQLEVVSALLSEKYKLETVVKEPSVIY
Sbjct 361
LLDALTQLADTDPLLRCEVDSITHEIILSFLGRVQLEVVSALLSEKYKLETVVKEPSVIY 420

Query 421
MERPLKAASHTIHIEVPPNPFWASIGLSVTPLSLGSGVQYESRVSLGYLNQSFQNAVRDG 480

MERPLKAASHTIHIEVPPNPFWASIGLSVTPLSLGSGVQYESRVSLGYLNQSFQNAVRDG
Sbjct 421
MERPLKAASHTIHIEVPPNPFWASIGLSVTPLSLGSGVQYESRVSLGYLNQSFQNAVRDG 480

Query 481
IRYGLEQGLFGWNVTDCKICFEYGLYYSPVSTPADFRSLAPIVLEQALKESGTQLLEPYL 540

IRYGLEQGLFGWNVTDCKICFEYGLYYSPVSTPADFRSLAPIVLEQALKESGTQLLEPYL
Sbjct 481
IRYGLEQGLFGWNVTDCKICFEYGLYYSPVSTPADFRSLAPIVLEQALKESGTQLLEPYL 540

Query 541
SFILYAPQEYLSRAYHDAPKYCATIETAQVKKDEVVFTGEIPARCIQAYRTDLAFYTNGR 600

SFILYAPQEYLSRAYHDAPKYCATIETAQVKKDEVVFTGEIPARCIQAYRTDLAFYTNGR
Sbjct 541
SFILYAPQEYLSRAYHDAPKYCATIETAQVKKDEVVFTGEIPARCIQAYRTDLAFYTNGR 600

```
Query  601  SVCLTELKGYQAAVGQPVIQPRRPNSRLDKVRHMFQKVM  639
            SVCLTELKGYQAAVGQPVIQPRRPNSRLDKVRHMFQK+M
Sbjct  601  SVCLTELKGYQAAVGQPVIQPRRPNSRLDKVRHMFQKIM  639
```

>WP_035010862.1 TetM/TetW/TetO/TetS family tetracycline resistance ribosomal
protection protein [Bifidobacterium adolescentis]
 OSH02963.1 tetracycline resistance protein tetM [Bifidobacterium adolescentis]
Length=639

 Score = 1298 bits (3358), Expect = 0.0, Method: Compositional matrix adjust.
 Identities = 632/639 (99%), Positives = 635/639 (99%), Gaps = 0/639 (0%)

Query 1
MKIINIGILAHVDAGKTTLTESLLYASGAISEPGSVEKGTTRTDTMFLERQRGITIQAAV 60

MKIINIGILAHVDAGKTTLTESLLYASGAISEPGSVEKGTTRTDTMFLERQRGITIQAAV
Sbjct 1
MKIINIGILAHVDAGKTTLTESLLYASGAISEPGSVEKGTTRTDTMFLERQRGITIQAAV 60

Query 61
TSFQWHRCKVNIVDTPGHMDFLAEVYRSLAVLDGAILVISAKDGVQAQTRILFHALRKMN 120

TSFQWHRCKVNIVDTPGHMDFLAEVYRSLAVLDGAILVISAKDGVQAQTRILFHALRKMN
Sbjct 61
TSFQWHRCKVNIVDTPGHMDFLAEVYRSLAVLDGAILVISAKDGVQAQTRILFHALRKMN 120

Query 121
IPTVIFINKIDQAGVDLQSVVQSVRDKLSADIIIKQTVSLSPEIVLEENTDIEAWDAVIE 180

IPTVIFINKIDQAGVDLQSVVQSVRDKLSADIIIKQTVSLSPEIVLEENTDIEAWDAVIE
Sbjct 121
IPTVIFINKIDQAGVDLQSVVQSVRDKLSADIIIKQTVSLSPEIVLEENTDIEAWDAVIE 180

Query 181
NNDKLLEKYIAGEPISREKLVREEQRRVQDASLFPVYYGSAKKGLGIQPLMDAVTGLFQP 240

NNDKLLEKYIAGEPISREKLVREEQRRVQDASLFPVYYGSAKKGLGIQPLMDAVTGLFQP

```
Sbjct  181
NNDKLLEKYIAGEPISREKLVREEQRRVQDASLFPVYYGSAKKGLGIQPLMDAVTGLFQP  240

Query  241
IGEQGSAALCGSVFKVEYTDCGQRRVYLRLYSGTLRLRDTVALAGREKLKITEMRIPSKG  300

IGEQGSAALCGSVFKVEYTDCGQRRVYLRLYSGTLRLRDTVALAGREKLKITEMRIPSKG
Sbjct  241
IGEQGSAALCGSVFKVEYTDCGQRRVYLRLYSGTLRLRDTVALAGREKLKITEMRIPSKG  300

Query  301
EIVRTDTAYPGEIVILPSDSVRLNDVLGDPTRLPRKRWREDPLPMLRTTIAPKTAAQRER  360
            EIVRTDTAYPGEIVILPSDSVRLNDVLGD
TRLPRKRWREDPLPMLRTTIAPKTAAQRER
Sbjct  301
EIVRTDTAYPGEIVILPSDSVRLNDVLGDQTRLPRKRWREDPLPMLRTTIAPKTAAQRER  360

Query  361
LLDALTQLADTDPLLRCEVDSITHEIILSFLGRVQLEVVSALLSEKYKLETVVKEPSVIY  420
            LLDALTQLADTDPLLRCEVDSITHEIILSFLGRVQLEVVSALLSEKYKLETVVKEP
+VIY
Sbjct  361
LLDALTQLADTDPLLRCEVDSITHEIILSFLGRVQLEVVSALLSEKYKLETVVKEPTVIY  420

Query  421
MERPLKAASHTIHIEVPPNPFWASIGLSVTPLSLGSGVQYESRVSLGYLNQSFQNAVRDG  480
            MERPLKAASHTIHIEVPPNPFWASIGLSVTPL
LGSGVQYESRVSLGYLNQSFQNAVRDG
Sbjct  421
MERPLKAASHTIHIEVPPNPFWASIGLSVTPLPLGSGVQYESRVSLGYLNQSFQNAVRDG  480

Query  481
IRYGLEQGLFGWNVTDCKICFEYGLYYSPVSTPADFRSLAPIVLEQALKESGTQLLEPYL  540

IRYGLEQGLFGWNVTDCKICFEYGLYYSPVSTPADFRSLAPIVLEQALKESGTQLLEPYL
Sbjct  481
IRYGLEQGLFGWNVTDCKICFEYGLYYSPVSTPADFRSLAPIVLEQALKESGTQLLEPYL  540

Query  541
SFILYAPQEYLSRAYHDAPKYCATIETAQVKKDEVVFTGEIPARCIQAYRTDLAFYTNGR  600
            SF LYAP+EYLSRAYHDAPKYCATIET
QVKKDEVVFTGEIPARCIQAYRTDLAFYTNGR
Sbjct  541
SFTLYAPREYLSRAYHDAPKYCATIETVQVKKDEVVFTGEIPARCIQAYRTDLAFYTNGR  600

Query  601  SVCLTELKGYQAAVGQPVIQPRRPNSRLDKVRHMFQKVM  639
            SVCLTELKGYQAAVGQPVIQPRRPNSRLDKVR+MFQKVM
Sbjct  601  SVCLTELKGYQAAVGQPVIQPRRPNSRLDKVRYMFQKVM  639


>CBL36335.1 small GTP-binding protein domain [butyrate-producing
bacterium
SM4/1]
Length=639
```

Score = 1298 bits (3358), Expect = 0.0, Method: Compositional matrix adjust.
Identities = 630/639 (99%), Positives = 636/639 (99%), Gaps = 0/639 (0%)

```
Query  1
MKIINIGILAHVDAGKTTLTESLLYASGAISEPGSVEKGTTRTDTMFLERQRGITIQAAV  60

MKIINIGILAHVDAGKTTLTESLLYASGAISEPGSVEKGTTRTDTMFLERQRGITIQAAV
Sbjct  1
MKIINIGILAHVDAGKTTLTESLLYASGAISEPGSVEKGTTRTDTMFLERQRGITIQAAV  60

Query  61
TSFQWHRCKVNIVDTPGHMDFLAEVYRSLAVLDGAILVISAKDGVQAQTRILFHALRKMN  120

TSFQWHRCKVNIVDTPGHMDFLAEVYRSLAVLDGAILVISAKDGVQAQTRILFHALRKMN
Sbjct  61
TSFQWHRCKVNIVDTPGHMDFLAEVYRSLAVLDGAILVISAKDGVQAQTRILFHALRKMN  120

Query  121
IPTVIFINKIDQAGVDLQSVVQSVRDKLSADIIIKQTVSLSPEIVLEENTDIEAWDAVIE  180

IPTVIFINKIDQAGVDLQSVVQSVRDKLSADIIIKQTVSLSPEIVLEENTDIEAWDAVIE
Sbjct  121
IPTVIFINKIDQAGVDLQSVVQSVRDKLSADIIIKQTVSLSPEIVLEENTDIEAWDAVIE  180

Query  181
NNDKLLEKYIAGEPISREKLVREEQRRVQDASLFPVYYGSAKKGLGIQPLMDAVTGLFQP  240
            NND+LLEKYIAGEPISREKL REEQ+RVQDASLFPVY+GSAK
GLGIQPLMDAVTGLFQP
Sbjct  181
NNDELLEKYIAGEPISREKLAREEQQRVQDASLFPVYHGSAKNGLGIQPLMDAVTGLFQP  240

Query  241
IGEQGSAALCGSVFKVEYTDCGQRRVYLRLYSGTLRLRDTVALAGREKLKITEMRIPSKG  300

IGEQGSAALCGSVFKVEYTDCGQRRVYLRLYSGTLRLRDTVALAGREKLKITEMRIPSKG
Sbjct  241
IGEQGSAALCGSVFKVEYTDCGQRRVYLRLYSGTLRLRDTVALAGREKLKITEMRIPSKG  300

Query  301
EIVRTDTAYPGEIVILPSDSVRLNDVLGDPTRLPRKRWREDPLPMLRTTIAPKTAAQRER  360
            EIVRTDTAYPGEIVILPSDSVRLNDVLGDPTRLPRKRWREDPLPMLRT
+IAPKTAAQRER
Sbjct  301
EIVRTDTAYPGEIVILPSDSVRLNDVLGDPTRLPRKRWREDPLPMLRTSIAPKTAAQRER  360

Query  361
LLDALTQLADTDPLLRCEVDSITHEIILSFLGRVQLEVVSALLSEKYKLETVVKEPSVIY  420
            LLDALTQLADTDPLLRCEVDSITHEIILSFLGRVQLEVVSALLSEKYKLETVVKEP
+VIY
Sbjct  361
LLDALTQLADTDPLLRCEVDSITHEIILSFLGRVQLEVVSALLSEKYKLETVVKEPTVIY  420
```

Query 421
MERPLKAASHTIHIEVPPNPFWASIGLSVTPLSLGSGVQYESRVSLGYLNQSFQNAVRDG 480
            MERPLKAASHTIHIEVPPNPFWASIGLSVTPL
LGSGVQYESRVSLGYLNQSFQNAVRDG
Sbjct 421
MERPLKAASHTIHIEVPPNPFWASIGLSVTPLPLGSGVQYESRVSLGYLNQSFQNAVRDG 480

Query 481
IRYGLEQGLFGWNVTDCKICFEYGLYYSPVSTPADFRSLAPIVLEQALKESGTQLLEPYL 540

IRYGLEQGLFGWNVTDCKICFEYGLYYSPVSTPADFRSLAPIVLEQALKESGTQLLEPYL
Sbjct 481
IRYGLEQGLFGWNVTDCKICFEYGLYYSPVSTPADFRSLAPIVLEQALKESGTQLLEPYL 540

Query 541
SFILYAPQEYLSRAYHDAPKYCATIETAQVKKDEVVFTGEIPARCIQAYRTDLAFYTNGR 600

SFILYAPQEYLSRAYHDAPKYCATIETAQVKKDEVVFTGEIPARCIQAYRTDLAFYTNGR
Sbjct 541
SFILYAPQEYLSRAYHDAPKYCATIETAQVKKDEVVFTGEIPARCIQAYRTDLAFYTNGR 600

Query 601 SVCLTELKGYQAAVGQPVIQPRRPNSRLDKVRHMFQKVM 639
            SVCLTELKGYQAAVGQPVIQPRRPNSRLDKVRHMFQ+VM
Sbjct 601 SVCLTELKGYQAAVGQPVIQPRRPNSRLDKVRHMFQEVM 639

>AMP47980.1 TetM-TetW-TetO-TetS [uncultured bacterium]
Length=639

 Score = 1297 bits (3357), Expect = 0.0, Method: Compositional
matrix adjust.
 Identities = 632/639 (99%), Positives = 635/639 (99%), Gaps = 0/639
(0%)

Query 1
MKIINIGILAHVDAGKTTLTESLLYASGAISEPGSVEKGTTRTDTMFLERQRGITIQAAV 60

MKIINIGILAHVDAGKTTLTESLLYASGAISEPGSVEKGTTRTDTMFLERQRGITIQAAV
Sbjct 1
MKIINIGILAHVDAGKTTLTESLLYASGAISEPGSVEKGTTRTDTMFLERQRGITIQAAV 60

Query 61
TSFQWHRCKVNIVDTPGHMDFLAEVYRSLAVLDGAILVISAKDGVQAQTRILFHALRKMN 120

TSFQWHRCKVNIVDTPGHMDFLAEVYRSLAVLDGAILVISAKDGVQAQTRILFHALRKMN
Sbjct 61
TSFQWHRCKVNIVDTPGHMDFLAEVYRSLAVLDGAILVISAKDGVQAQTRILFHALRKMN 120

Query 121
IPTVIFINKIDQAGVDLQSVVQSVRDKLSADIIIKQTVSLSPEIVLEENTDIEAWDAVIE 180

IPTVIFINKIDQAGVDLQSVVQSVRDKLSADIIIKQTVSLSPEIVLEENTDIEAWDAVIE
Sbjct 121

IPTVIFINKIDQAGVDLQSVVQSVRDKLSADIIIKQTVSLSPEIVLEENTDIEAWDAVIE 180

```
Query  181
NNDKLLEKYIAGEPISREKLVREEQRRVQDASLFPVYYGSAKKGLGIQPLMDAVTGLFQP  240
            NND+LLEKYIAGEPISREKLVREEQ+RVQDASLFPVY+GSAK
GLGIQPLMDAVTGLFQP
Sbjct  181
NNDELLEKYIAGEPISREKLVREEQQRVQDASLFPVYHGSAKNGLGIQPLMDAVTGLFQP  240

Query  241
IGEQGSAALCGSVFKVEYTDCGQRRVYLRLYSGTLRLRDTVALAGREKLKITEMRIPSKG  300
            IGEQG
AALCGSVFKVEYTDCGQRRVYLRLYSGTLRLRDTVALAGREKLKITEMRIPSKG
Sbjct  241
IGEQGGAALCGSVFKVEYTDCGQRRVYLRLYSGTLRLRDTVALAGREKLKITEMRIPSKG  300

Query  301
EIVRTDTAYPGEIVILPSDSVRLNDVLGDPTRLPRKRWREDPLPMLRTTIAPKTAAQRER  360
            EIVRTDTAY GEIVILPSDSVRLNDVLGD
TRLPRKRWREDPLPMLRTTIAPKTAAQRER
Sbjct  301
EIVRTDTAYQGEIVILPSDSVRLNDVLGDQTRLPRKRWREDPLPMLRTTIAPKTAAQRER  360

Query  361
LLDALTQLADTDPLLRCEVDSITHEIILSFLGRVQLEVVSALLSEKYKLETVVKEPSVIY  420

LLDALTQLADTDPLLRCEVDSITHEIILSFLGRVQLEVVSALLSEKYKLETVVKEPSVIY
Sbjct  361
LLDALTQLADTDPLLRCEVDSITHEIILSFLGRVQLEVVSALLSEKYKLETVVKEPSVIY  420

Query  421
MERPLKAASHTIHIEVPPNPFWASIGLSVTPLSLGSGVQYESRVSLGYLNQSFQNAVRDG  480

MERPLKAASHTIHIEVPPNPFWASIGLSVTPLSLGSGVQYESRVSLGYLNQSFQNAVRDG
Sbjct  421
MERPLKAASHTIHIEVPPNPFWASIGLSVTPLSLGSGVQYESRVSLGYLNQSFQNAVRDG  480

Query  481
IRYGLEQGLFGWNVTDCKICFEYGLYYSPVSTPADFRSLAPIVLEQALKESGTQLLEPYL  540

IRYGLEQGLFGWNVTDCKICFEYGLYYSPVSTPADFRSLAPIVLEQALKESGTQLLEPYL
Sbjct  481
IRYGLEQGLFGWNVTDCKICFEYGLYYSPVSTPADFRSLAPIVLEQALKESGTQLLEPYL  540

Query  541
SFILYAPQEYLSRAYHDAPKYCATIETAQVKKDEVVFTGEIPARCIQAYRTDLAFYTNGR  600

SFILYAPQEYLSRAYHDAPKYCATIETAQVKKDEVVFTGEIPARCIQAYRTDLAFYTNGR
Sbjct  541
SFILYAPQEYLSRAYHDAPKYCATIETAQVKKDEVVFTGEIPARCIQAYRTDLAFYTNGR  600

Query  601  SVCLTELKGYQAAVGQPVIQPRRPNSRLDKVRHMFQKVM  639
            SVCLTELKGYQAAVGQPVIQPRRPNSRLDKVRHMFQKVM
Sbjct  601  SVCLTELKGYQAAVGQPVIQPRRPNSRLDKVRHMFQKVM  639
```

>WP_013702080.1 TetM/TetW/TetO/TetS family tetracycline resistance ribosomal
protection protein [Treponema succinifaciens]
 AEB14821.1 small GTP-binding protein [Treponema succinifaciens DSM 2489]
Length=639

 Score = 1297 bits (3357), Expect = 0.0, Method: Compositional matrix adjust.
 Identities = 629/639 (98%), Positives = 637/639 (99%), Gaps = 0/639 (0%)

Query 1
MKIINIGILAHVDAGKTTLTESLLYASGAISEPGSVEKGTTRTDTMFLERQRGITIQAAV 60

MKIINIGILAHVDAGKTTLTESLLYASGAISEPGSVEKGTTRTDTMFLERQRGITIQAAV
Sbjct 1
MKIINIGILAHVDAGKTTLTESLLYASGAISEPGSVEKGTTRTDTMFLERQRGITIQAAV 60

Query 61
TSFQWHRCKVNIVDTPGHMDFLAEVYRSLAVLDGAILVISAKDGVQAQTRILFHALRKMN 120

TSFQWHRCKVNIVDTPGHMDFLAEVYRSLAVLDGAILVISAKDGVQAQTRILFHALRKMN
Sbjct 61
TSFQWHRCKVNIVDTPGHMDFLAEVYRSLAVLDGAILVISAKDGVQAQTRILFHALRKMN 120

Query 121
IPTVIFINKIDQAGVDLQSVVQSVRDKLSADIIIKQTVSLSPEIVLEENTDIEAWDAVIE 180

IPTVIFINKIDQAGVDLQSVVQSVRDKLSADIIIKQTVSLSPEIVLEENTDIEAWDAVIE
Sbjct 121
IPTVIFINKIDQAGVDLQSVVQSVRDKLSADIIIKQTVSLSPEIVLEENTDIEAWDAVIE 180

Query 181
NNDKLLEKYIAGEPISREKLVREEQRRVQDASLFPVYYGSAKKGLGIQPLMDAVTGLFQP 240

NNDKLLEKYIAGEPISREKLVREEQRRVQDASLFPVYYGSAKKGLGIQPLMDAVTGLFQP
Sbjct 181
NNDKLLEKYIAGEPISREKLVREEQRRVQDASLFPVYYGSAKKGLGIQPLMDAVTGLFQP 240

Query 241
IGEQGSAALCGSVFKVEYTDCGQRRVYLRLYSGTLRLRDTVALAGREKLKITEMRIPSKG 300

IGEQGSAALCGSVFKVEYTDCGQRRVYLRLYSGTLRLRDTVALAGREKLKITEMRIPSKG
Sbjct 241
IGEQGSAALCGSVFKVEYTDCGQRRVYLRLYSGTLRLRDTVALAGREKLKITEMRIPSKG 300

Query 301
EIVRTDTAYPGEIVILPSDSVRLNDVLGDPTRLPRKRWREDPLPMLRTTIAPKTAAQRER 360
            EIVRTDTAYPGEIVILPSDSVRLNDVLGDPTRLPRKRWREDPLPMLRT
+IAPKTAAQRER
Sbjct 301

EIVRTDTAYPGEIVILPSDSVRLNDVLGDPTRLPRKRWREDPLPMLRTSIAPKTAAQRER 360

Query 361
LLDALTQLADTDPLLRCEVDSITHEIILSFLGRVQLEVVSALLSEKYKLETVVKEPSVIY 420
            LLDALTQLADTDPLLRCEVDSITHEIILSFLGRVQLEVVSALLSEKYKLETVVKEP
++IY
Sbjct 361
LLDALTQLADTDPLLRCEVDSITHEIILSFLGRVQLEVVSALLSEKYKLETVVKEPTIIY 420

Query 421
MERPLKAASHTIHIEVPPNPFWASIGLSVTPLSLGSGVQYESRVSLGYLNQSFQNAVRDG 480
            MERPLKAASHTIHIEVPPNPFWASIGLSVTPL
LGSGVQYESRVSLGYLNQSFQNAVRDG
Sbjct 421
MERPLKAASHTIHIEVPPNPFWASIGLSVTPLPLGSGVQYESRVSLGYLNQSFQNAVRDG 480

Query 481
IRYGLEQGLFGWNVTDCKICFEYGLYYSPVSTPADFRSLAPIVLEQALKESGTQLLEPYL 540

IRYGLEQGLFGWNVTDCKICFEYGLYYSPVSTPADFRSLAPIVLEQALKESGTQLLEPYL
Sbjct 481
IRYGLEQGLFGWNVTDCKICFEYGLYYSPVSTPADFRSLAPIVLEQALKESGTQLLEPYL 540

Query 541
SFILYAPQEYLSRAYHDAPKYCATIETAQVKKDEVVFTGEIPARCIQAYRTDLAFYTNGR 600
            SF
LYAPQEYLSRAYHDAPKYCATIETAQVKKDEVVFTGEIPARCIQAYRTDLAFYTNG+
Sbjct 541
SFTLYAPQEYLSRAYHDAPKYCATIETAQVKKDEVVFTGEIPARCIQAYRTDLAFYTNGQ 600

Query 601 SVCLTELKGYQAAVGQPVIQPRRPNSRLDKVRHMFQKVM 639
            SVCLT+LKGYQAAVG+PVIQPRRPNSRLDKVR+MFQK+M
Sbjct 601 SVCLTKLKGYQAAVGKPVIQPRRPNSRLDKVRYMFQKIM 639

>WP_033493020.1 MULTISPECIES: tetracycline resistance ribosomal
protection protein
Tet(W) [Bifidobacterium]
 ACA23187.1 TetW [Bifidobacterium longum]
 ACA23189.1 TetW [Bifidobacterium thermophilum]
Length=639

 Score = 1297 bits (3356), Expect = 0.0, Method: Compositional
matrix adjust.
 Identities = 630/639 (99%), Positives = 636/639 (99%), Gaps = 0/639
(0%)

Query 1
MKIINIGILAHVDAGKTTLTESLLYASGAISEPGSVEKGTTRTDTMFLERQRGITIQAAV 60

MKIINIGILAHVDAGKTTLTESLLYASGAISEPGSVEKGTTRTDTMFLERQRGITIQAAV
Sbjct 1
MKIINIGILAHVDAGKTTLTESLLYASGAISEPGSVEKGTTRTDTMFLERQRGITIQAAV 60

Query 61
TSFQWHRCKVNIVDTPGHMDFLAEVYRSLAVLDGAILVISAKDGVQAQTRILFHALRKMN 120

TSFQWHRCKVNIVDTPGHMDFLAEVYRSLAVLDGAILVISAKDGVQAQTRILFHALRKMN
Sbjct 61
TSFQWHRCKVNIVDTPGHMDFLAEVYRSLAVLDGAILVISAKDGVQAQTRILFHALRKMN 120

Query 121
IPTVIFINKIDQAGVDLQSVVQSVRDKLSADIIIKQTVSLSPEIVLEENTDIEAWDAVIE 180

IPTVIFINKIDQAGVDLQSVVQSVRDKLSADIIIKQTVSLSPEIVLEENTDIEAWDAVIE
Sbjct 121
IPTVIFINKIDQAGVDLQSVVQSVRDKLSADIIIKQTVSLSPEIVLEENTDIEAWDAVIE 180

Query 181
NNDKLLEKYIAGEPISREKLVREEQRRVQDASLFPVYYGSAKKGLGIQPLMDAVTGLFQP 240
NND
+LLEKYIAGEPISREKLVREEQRRVQDASLFPVYYGSAKKGLGIQPLMDAVTGLFQP
Sbjct 181
NNDELLEKYIAGEPISREKLVREEQRRVQDASLFPVYYGSAKKGLGIQPLMDAVTGLFQP 240

Query 241
IGEQGSAALCGSVFKVEYTDCGQRRVYLRLYSGTLRLRDTVALAGREKLKITEMRIPSKG 300

IGEQGSAALCGSVFKVEYTDCGQRRVYLRLYSGTLRLRDTVALAGREKLKITEMRIPSKG
Sbjct 241
IGEQGSAALCGSVFKVEYTDCGQRRVYLRLYSGTLRLRDTVALAGREKLKITEMRIPSKG 300

Query 301
EIVRTDTAYPGEIVILPSDSVRLNDVLGDPTRLPRKRWREDPLPMLRTTIAPKTAAQRER 360
EIVRTDTAYPGEIVILPSDSVRLNDVLGDPTRLPRKRWREDPLPMLRT
+IAPKTAAQRER
Sbjct 301
EIVRTDTAYPGEIVILPSDSVRLNDVLGDPTRLPRKRWREDPLPMLRTSIAPKTAAQRER 360

Query 361
LLDALTQLADTDPLLRCEVDSITHEIILSFLGRVQLEVVSALLSEKYKLETVVKEPSVIY 420
LLDALTQLADTDPLLRCEVDSITHEIILSFLGRVQLEVVSALLSEKYKLETVVKEP
+VIY
Sbjct 361
LLDALTQLADTDPLLRCEVDSITHEIILSFLGRVQLEVVSALLSEKYKLETVVKEPTVIY 420

Query 421
MERPLKAASHTIHIEVPPNPFWASIGLSVTPLSLGSGVQYESRVSLGYLNQSFQNAVRDG 480
MERPLKAASHTIHIEVPPNPFWASIGLSVTPL
LGSGVQYESRVSLGYLNQSFQNAVRDG
Sbjct 421
MERPLKAASHTIHIEVPPNPFWASIGLSVTPLPLGSGVQYESRVSLGYLNQSFQNAVRDG 480

Query 481
IRYGLEQGLFGWNVTDCKICFEYGLYYSPVSTPADFRSLAPIVLEQALKESGTQLLEPYL 540

IRYGLEQGLFGWNVTDCKICFEYGLYYSPVSTPADFRSLAPIVLEQALKESGTQLLEPYL
Sbjct 481

IRYGLEQGLFGWNVTDCKICFEYGLYYSPVSTPADFRSLAPIVLEQALKESGTQLLEPYL 540

Query 541
SFILYAPQEYLSRAYHDAPKYCATIETAQVKKDEVVFTGEIPARCIQAYRTDLAFYTNGR 600
            SF LYAP+EYLSRAYHDAPKYCATIET
QVKKDEVVFTGEIPARCIQAYRTDLAFYTNG+
Sbjct 541
SFTLYAPREYLSRAYHDAPKYCATIETVQVKKDEVVFTGEIPARCIQAYRTDLAFYTNGQ 600

Query 601 SVCLTELKGYQAAVGQPVIQPRRPNSRLDKVRHMFQKVM 639
            SVCLTELKGYQAAVG+PVIQPRRPNSRLDKVRHMFQKVM
Sbjct 601 SVCLTELKGYQAAVGKPVIQPRRPNSRLDKVRHMFQKVM 639

>APO28712.1 ferrous iron transport protein B [uncultured bacterium]
Length=639

Score = 1297 bits (3356), Expect = 0.0, Method: Compositional matrix adjust.
Identities = 632/639 (99%), Positives = 635/639 (99%), Gaps = 0/639 (0%)

Query 1
MKIINIGILAHVDAGKTTLTESLLYASGAISEPGSVEKGTTRTDTMFLERQRGITIQAAV 60

MKIINIGILAHVDAGKTTLTESLLYASGAISEPGSVEKGTTRTDTMFLERQRGITIQAAV
Sbjct 1
MKIINIGILAHVDAGKTTLTESLLYASGAISEPGSVEKGTTRTDTMFLERQRGITIQAAV 60

Query 61
TSFQWHRCKVNIVDTPGHMDFLAEVYRSLAVLDGAILVISAKDGVQAQTRILFHALRKMN 120

TSFQWHRCKVNIVDTPGHMDFLAEVYRSLAVLDGAILVISAKDGVQAQTRILFHALRKMN
Sbjct 61
TSFQWHRCKVNIVDTPGHMDFLAEVYRSLAVLDGAILVISAKDGVQAQTRILFHALRKMN 120

Query 121
IPTVIFINKIDQAGVDLQSVVQSVRDKLSADIIIKQTVSLSPEIVLEENTDIEAWDAVIE 180

IPTVIFINKIDQAGVDLQSVVQSVRDKLSADIIIKQTVSLSPEIVLEENTDIEAWDAVIE
Sbjct 121
IPTVIFINKIDQAGVDLQSVVQSVRDKLSADIIIKQTVSLSPEIVLEENTDIEAWDAVIE 180

Query 181
NNDKLLEKYIAGEPISREKLVREEQRRVQDASLFPVYYGSAKKGLGIQPLMDAVTGLFQP 240
            NND+LLEKYIAGEPISREKL REEQ
+RVQDASLFPVYYGSAKKGLGIQPLMDAVTGLFQP
Sbjct 181
NNDELLEKYIAGEPISREKLAREEQQRVQDASLFPVYYGSAKKGLGIQPLMDAVTGLFQP 240

Query 241
IGEQGSAALCGSVFKVEYTDCGQRRVYLRLYSGTLRLRDTVALAGREKLKITEMRIPSKG 300
            IGEQG
AALCGSVFKVEYTDCGQRRVYLRLYSGTLRLRDTVALAGREKLKITEMRIPSKG

```
Sbjct  241
IGEQGGAALCGSVFKVEYTDCGQRRVYLRLYSGTLRLRDTVALAGREKLKITEMRIPSKG  300

Query  301
EIVRTDTAYPGEIVILPSDSVRLNDVLGDPTRLPRKRWREDPLPMLRTTIAPKTAAQRER  360
            EIVRTDTAY GEIVILPSDSVRLNDVLGD
TRLPRKRWREDPLPMLRTTIAPKTAAQRER
Sbjct  301
EIVRTDTAYQGEIVILPSDSVRLNDVLGDQTRLPRKRWREDPLPMLRTTIAPKTAAQRER  360

Query  361
LLDALTQLADTDPLLRCEVDSITHEIILSFLGRVQLEVVSALLSEKYKLETVVKEPSVIY  420

LLDALTQLADTDPLLRCEVDSITHEIILSFLGRVQLEVVSALLSEKYKLETVVKEPSVIY
Sbjct  361
LLDALTQLADTDPLLRCEVDSITHEIILSFLGRVQLEVVSALLSEKYKLETVVKEPSVIY  420

Query  421
MERPLKAASHTIHIEVPPNPFWASIGLSVTPLSLGSGVQYESRVSLGYLNQSFQNAVRDG  480

MERPLKAASHTIHIEVPPNPFWASIGLSVTPLSLGSGVQYESRVSLGYLNQSFQNAVRDG
Sbjct  421
MERPLKAASHTIHIEVPPNPFWASIGLSVTPLSLGSGVQYESRVSLGYLNQSFQNAVRDG  480

Query  481
IRYGLEQGLFGWNVTDCKICFEYGLYYSPVSTPADFRSLAPIVLEQALKESGTQLLEPYL  540

IRYGLEQGLFGWNVTDCKICFEYGLYYSPVSTPADFRSLAPIVLEQALKESGTQLLEPYL
Sbjct  481
IRYGLEQGLFGWNVTDCKICFEYGLYYSPVSTPADFRSLAPIVLEQALKESGTQLLEPYL  540

Query  541
SFILYAPQEYLSRAYHDAPKYCATIETAQVKKDEVVFTGEIPARCIQAYRTDLAFYTNGR  600

SFILYAPQEYLSRAYHDAPKYCATIETAQVKKDEVVFTGEIPARCIQAYRTDLAFYTNGR
Sbjct  541
SFILYAPQEYLSRAYHDAPKYCATIETAQVKKDEVVFTGEIPARCIQAYRTDLAFYTNGR  600

Query  601  SVCLTELKGYQAAVGQPVIQPRRPNSRLDKVRHMFQKVM  639
            SVCLTELKGYQAAVGQPVIQPRRPNSRL+KVRHMFQKVM
Sbjct  601  SVCLTELKGYQAAVGQPVIQPRRPNSRLNKVRHMFQKVM  639


>AMJ37860.1 TetM-TetW-TetO-TetS [uncultured bacterium]
Length=639

 Score = 1297 bits (3356),  Expect = 0.0, Method: Compositional
matrix adjust.
 Identities = 630/639 (99%), Positives = 635/639 (99%), Gaps = 0/639
(0%)

Query  1
MKIINIGILAHVDAGKTTLTESLLYASGAISEPGSVEKGTTRTDTMFLERQRGITIQAAV  60
```

MKIINIGILAHVDAGKTTLTESLLYASGAISEPGSVEKGTTRTDTMFLERQRGITIQAAV
Sbjct 1
MKIINIGILAHVDAGKTTLTESLLYASGAISEPGSVEKGTTRTDTMFLERQRGITIQAAV 60

Query 61
TSFQWHRCKVNIVDTPGHMDFLAEVYRSLAVLDGAILVISAKDGVQAQTRILFHALRKMN 120

TSFQWHRCKVNIVDTPGHMDFLAEVYRSLAVLDGAILVISAKDGVQAQTRILFHALRKMN
Sbjct 61
TSFQWHRCKVNIVDTPGHMDFLAEVYRSLAVLDGAILVISAKDGVQAQTRILFHALRKMN 120

Query 121
IPTVIFINKIDQAGVDLQSVVQSVRDKLSADIIIKQTVSLSPEIVLEENTDIEAWDAVIE 180

IPTVIFINKIDQAGVDLQSVVQSVRDKLSADIIIKQTVSLSPEIVLEENTDIEAWDAVIE
Sbjct 121
IPTVIFINKIDQAGVDLQSVVQSVRDKLSADIIIKQTVSLSPEIVLEENTDIEAWDAVIE 180

Query 181
NNDKLLEKYIAGEPISREKLVREEQRRVQDASLFPVYYGSAKKGLGIQPLMDAVTGLFQP 240

NNDKLLEKYIAGEPISREKLVREEQRRVQDASLFPVYYGSAKKGLGIQPLMDAVTGLFQP
Sbjct 181
NNDKLLEKYIAGEPISREKLVREEQRRVQDASLFPVYYGSAKKGLGIQPLMDAVTGLFQP 240

Query 241
IGEQGSAALCGSVFKVEYTDCGQRRVYLRLYSGTLRLRDTVALAGREKLKITEMRIPSKG 300

IGEQGSAALCGSVFKVEYTDCGQRRVYLRLYSGTLRLRDTVALAGREKLKITEMRIPSKG
Sbjct 241
IGEQGSAALCGSVFKVEYTDCGQRRVYLRLYSGTLRLRDTVALAGREKLKITEMRIPSKG 300

Query 301
EIVRTDTAYPGEIVILPSDSVRLNDVLGDPTRLPRKRWREDPLPMLRTTIAPKTAAQRER 360
EIVRTDTAYPGEIVILPSDSVRLNDVLGDPTRLPRKRWREDPLPMLRT
+IAPKTAAQRER
Sbjct 301
EIVRTDTAYPGEIVILPSDSVRLNDVLGDPTRLPRKRWREDPLPMLRTSIAPKTAAQRER 360

Query 361
LLDALTQLADTDPLLRCEVDSITHEIILSFLGRVQLEVVSALLSEKYKLETVVKEPSVIY 420
LLDALTQLADTDPLLRCEVDSITHEIILSFLGRVQLEVVSALLSEKYKLETVVKEP
+VIY
Sbjct 361
LLDALTQLADTDPLLRCEVDSITHEIILSFLGRVQLEVVSALLSEKYKLETVVKEPTVIY 420

Query 421
MERPLKAASHTIHIEVPPNPFWASIGLSVTPLSLGSGVQYESRVSLGYLNQSFQNAVRDG 480
MERPLKAASHTIHIEVPPNPFWASIGLSVTPL
LGSGVQYESRVSLGYLNQSFQNAVRDG
Sbjct 421
MERPLKAASHTIHIEVPPNPFWASIGLSVTPLPLGSGVQYESRVSLGYLNQSFQNAVRDG 480

Query 481

IRYGLEQGLFGWNVTDCKICFEYGLYYSPVSTPADFRSLAPIVLEQALKESGTQLLEPYL 540

IRYGLEQGLFGWNVTDCKICFEYGLYYSPVSTPADFRSLAPIVLEQALKESGTQLLEPYL
Sbjct 481
IRYGLEQGLFGWNVTDCKICFEYGLYYSPVSTPADFRSLAPIVLEQALKESGTQLLEPYL 540

Query 541
SFILYAPQEYLSRAYHDAPKYCATIETAQVKKDEVVFTGEIPARCIQAYRTDLAFYTNGR 600
SF LYAP+EYLSRAYHDAPKYCATIET
QVKKDEVVFTGEIPARCIQAYRTDLAFYTNG+
Sbjct 541
SFTLYAPREYLSRAYHDAPKYCATIETVQVKKDEVVFTGEIPARCIQAYRTDLAFYTNGQ 600

Query 601 SVCLTELKGYQAAVGQPVIQPRRPNSRLDKVRHMFQKVM 639
SVCLTELKGYQAAVG+PVIQPRRPNSRLDKVRHM QKVM
Sbjct 601 SVCLTELKGYQAAVGKPVIQPRRPNSRLDKVRHMLQKVM 639

>WP_013411012.1 TetM/TetW/TetO/TetS family tetracycline resistance ribosomal
protection protein [Bifidobacterium longum]
ADQ03530.1 Hypothetical protein BBMN68_786 [Bifidobacterium longum subsp.
longum BBMN68]
AMP46864.1 TetM-TetW-TetO-TetS [uncultured bacterium]
AMP53769.1 TetM-TetW-TetO-TetS [uncultured bacterium]
Length=639

Score = 1297 bits (3356), Expect = 0.0, Method: Compositional matrix adjust.
Identities = 632/639 (99%), Positives = 635/639 (99%), Gaps = 0/639 (0%)

Query 1
MKIINIGILAHVDAGKTTLTESLLYASGAISEPGSVEKGTTRTDTMFLERQRGITIQAAV 60

MKIINIGILAHVDAGKTTLTESLLYASGAISEPGSVEKGTTRTDTMFLERQRGITIQAAV
Sbjct 1
MKIINIGILAHVDAGKTTLTESLLYASGAISEPGSVEKGTTRTDTMFLERQRGITIQAAV 60

Query 61
TSFQWHRCKVNIVDTPGHMDFLAEVYRSLAVLDGAILVISAKDGVQAQTRILFHALRKMN 120

TSFQWHRCKVNIVDTPGHMDFLAEVYRSLAVLDGAILVISAKDGVQAQTRILFHALRKMN
Sbjct 61
TSFQWHRCKVNIVDTPGHMDFLAEVYRSLAVLDGAILVISAKDGVQAQTRILFHALRKMN 120

Query 121
IPTVIFINKIDQAGVDLQSVVQSVRDKLSADIIIKQTVSLSPEIVLEENTDIEAWDAVIE 180

IPTVIFINKIDQAGVDLQSVVQSVRDKLSADIIIKQTVSLSPEIVLEENTDIEAWDAVIE
Sbjct 121
IPTVIFINKIDQAGVDLQSVVQSVRDKLSADIIIKQTVSLSPEIVLEENTDIEAWDAVIE 180

```
Query  181
NNDKLLEKYIAGEPISREKLVREEQRRVQDASLFPVYYGSAKKGLGIQPLMDAVTGLFQP  240
             NND+LLEKYIAGEPISREKL REEQ+RVQDASLFPVY
+GSAKKGLGIQPLMDAVTGLFQP
Sbjct  181
NNDELLEKYIAGEPISREKLAREEQQRVQDASLFPVYHGSAKKGLGIQPLMDAVTGLFQP  240

Query  241
IGEQGSAALCGSVFKVEYTDCGQRRVYLRLYSGTLRLRDTVALAGREKLKITEMRIPSKG  300
             IGEQG
AALCGSVFKVEYTDCGQRRVYLRLYSGTLRLRDTVALAGREKLKITEMRIPSKG
Sbjct  241
IGEQGGAALCGSVFKVEYTDCGQRRVYLRLYSGTLRLRDTVALAGREKLKITEMRIPSKG  300

Query  301
EIVRTDTAYPGEIVILPSDSVRLNDVLGDPTRLPRKRWREDPLPMLRTTIAPKTAAQRER  360
             EIVRTDTAY GEIVILPSDSVRLNDVLGD
TRLPRKRWREDPLPMLRTTIAPKTAAQRER
Sbjct  301
EIVRTDTAYQGEIVILPSDSVRLNDVLGDQTRLPRKRWREDPLPMLRTTIAPKTAAQRER  360

Query  361
LLDALTQLADTDPLLRCEVDSITHEIILSFLGRVQLEVVSALLSEKYKLETVVKEPSVIY  420

LLDALTQLADTDPLLRCEVDSITHEIILSFLGRVQLEVVSALLSEKYKLETVVKEPSVIY
Sbjct  361
LLDALTQLADTDPLLRCEVDSITHEIILSFLGRVQLEVVSALLSEKYKLETVVKEPSVIY  420

Query  421
MERPLKAASHTIHIEVPPNPFWASIGLSVTPLSLGSGVQYESRVSLGYLNQSFQNAVRDG  480

MERPLKAASHTIHIEVPPNPFWASIGLSVTPLSLGSGVQYESRVSLGYLNQSFQNAVRDG
Sbjct  421
MERPLKAASHTIHIEVPPNPFWASIGLSVTPLSLGSGVQYESRVSLGYLNQSFQNAVRDG  480

Query  481
IRYGLEQGLFGWNVTDCKICFEYGLYYSPVSTPADFRSLAPIVLEQALKESGTQLLEPYL  540

IRYGLEQGLFGWNVTDCKICFEYGLYYSPVSTPADFRSLAPIVLEQALKESGTQLLEPYL
Sbjct  481
IRYGLEQGLFGWNVTDCKICFEYGLYYSPVSTPADFRSLAPIVLEQALKESGTQLLEPYL  540

Query  541
SFILYAPQEYLSRAYHDAPKYCATIETAQVKKDEVVFTGEIPARCIQAYRTDLAFYTNGR  600

SFILYAPQEYLSRAYHDAPKYCATIETAQVKKDEVVFTGEIPARCIQAYRTDLAFYTNGR
Sbjct  541
SFILYAPQEYLSRAYHDAPKYCATIETAQVKKDEVVFTGEIPARCIQAYRTDLAFYTNGR  600

Query  601  SVCLTELKGYQAAVGQPVIQPRRPNSRLDKVRHMFQKVM  639
            SVCLTELKGYQAAVGQPVIQPRRPNSRLDKVRHMFQKVM
Sbjct  601  SVCLTELKGYQAAVGQPVIQPRRPNSRLDKVRHMFQKVM  639
```

>APO30252.1 Ferrous iron transport protein B [uncultured bacterium]
Length=639

Score = 1296 bits (3355), Expect = 0.0, Method: Compositional matrix adjust.
Identities = 631/639 (99%), Positives = 634/639 (99%), Gaps = 0/639 (0%)

```
Query  1
MKIINIGILAHVDAGKTTLTESLLYASGAISEPGSVEKGTTRTDTMFLERQRGITIQAAV  60

MKIINIGILAHVDAGKTTLTESLLYASGAISEPGSVEKGTTRTDTMFLERQRGITIQAAV
Sbjct  1
MKIINIGILAHVDAGKTTLTESLLYASGAISEPGSVEKGTTRTDTMFLERQRGITIQAAV  60

Query  61
TSFQWHRCKVNIVDTPGHMDFLAEVYRSLAVLDGAILVISAKDGVQAQTRILFHALRKMN  120

TSFQWHRCKVNIVDTPGHMDFLAEVYRSLAVLDGAILVISAKDGVQAQTRILFHALRKMN
Sbjct  61
TSFQWHRCKVNIVDTPGHMDFLAEVYRSLAVLDGAILVISAKDGVQAQTRILFHALRKMN  120

Query  121
IPTVIFINKIDQAGVDLQSVVQSVRDKLSADIIIKQTVSLSPEIVLEENTDIEAWDAVIE  180

IPTVIFINKIDQAGVDLQSVVQSVRDKLSADIIIKQTVSLSPEIVLEENTDIEAWDAVIE
Sbjct  121
IPTVIFINKIDQAGVDLQSVVQSVRDKLSADIIIKQTVSLSPEIVLEENTDIEAWDAVIE  180

Query  181
NNDKLLEKYIAGEPISREKLVREEQRRVQDASLFPVYYGSAKKGLGIQPLMDAVTGLFQP  240
            NND+LLEKYIAGEPISREKL REEQ+RVQDASLFPVY+GSAK
GLGIQPLMDAVTGLFQP
Sbjct  181
NNDELLEKYIAGEPISREKLAREEQQRVQDASLFPVYHGSAKNGLGIQPLMDAVTGLFQP  240

Query  241
IGEQGSAALCGSVFKVEYTDCGQRRVYLRLYSGTLRLRDTVALAGREKLKITEMRIPSKG  300
            IGEQG
AALCGSVFKVEYTDCGQRRVYLRLYSGTLRLRDTVALAGREKLKITEMRIPSKG
Sbjct  241
IGEQGGAALCGSVFKVEYTDCGQRRVYLRLYSGTLRLRDTVALAGREKLKITEMRIPSKG  300

Query  301
EIVRTDTAYPGEIVILPSDSVRLNDVLGDPTRLPRKRWREDPLPMLRTTIAPKTAAQRER  360
            EIVRTDTAYPGEIVILPSDSVRLNDVLGD
TRLPRKRWREDPLPMLRTTIAPKTAAQRER
Sbjct  301
EIVRTDTAYPGEIVILPSDSVRLNDVLGDQTRLPRKRWREDPLPMLRTTIAPKTAAQRER  360

Query  361
LLDALTQLADTDPLLRCEVDSITHEIILSFLGRVQLEVVSALLSEKYKLETVVKEPSVIY  420

LLDALTQLADTDPLLRCEVDSITHEIILSFLGRVQLEVVSALLSEKYKLETVVKEPSVIY
```

```
Sbjct  361
LLDALTQLADTDPLLRCEVDSITHEIILSFLGRVQLEVVSALLSEKYKLETVVKEPSVIY  420

Query  421
MERPLKAASHTIHIEVPPNPFWASIGLSVTPLSLGSGVQYESRVSLGYLNQSFQNAVRDG  480

MERPLKAASHTIHIEVPPNPFWASIGLSVTPLSLGSGVQYESRVSLGYLNQSFQNAVRDG
Sbjct  421
MERPLKAASHTIHIEVPPNPFWASIGLSVTPLSLGSGVQYESRVSLGYLNQSFQNAVRDG  480

Query  481
IRYGLEQGLFGWNVTDCKICFEYGLYYSPVSTPADFRSLAPIVLEQALKESGTQLLEPYL  540

IRYGLEQGLFGWNVTDCKICFEYGLYYSPVSTPADFRSLAPIVLEQALKESGTQLLEPYL
Sbjct  481
IRYGLEQGLFGWNVTDCKICFEYGLYYSPVSTPADFRSLAPIVLEQALKESGTQLLEPYL  540

Query  541
SFILYAPQEYLSRAYHDAPKYCATIETAQVKKDEVVFTGEIPARCIQAYRTDLAFYTNGR  600

SFILYAPQEYLSRAYHDAPKYCATIETAQVKKDEVVFTGEIPARCIQAYRTDLAFYTNGR
Sbjct  541
SFILYAPQEYLSRAYHDAPKYCATIETAQVKKDEVVFTGEIPARCIQAYRTDLAFYTNGR  600

Query  601  SVCLTELKGYQAAVGQPVIQPRRPNSRLDKVRHMFQKVM  639
            SVCLTELKGYQAAVGQPVIQPRRPNSRLDKVRHM QKVM
Sbjct  601  SVCLTELKGYQAAVGQPVIQPRRPNSRLDKVRHMIQKVM  639


>WP_090548148.1 MULTISPECIES: TetM/TetW/TetO/TetS family
tetracycline resistance
ribosomal protection protein [Terrabacteria group]
 AET35523.1 TetW [uncultured bacterium]
 SDR79377.1 ribosomal protection tetracycline resistance protein
[Olsenella
umbonata]
 SFH97142.1 ribosomal protection tetracycline resistance protein
[Pseudobutyrivibrio
sp. OR37]
Length=639

 Score = 1296 bits (3355),  Expect = 0.0, Method: Compositional
matrix adjust.
 Identities = 630/639 (99%), Positives = 636/639 (99%), Gaps = 0/639
(0%)

Query  1
MKIINIGILAHVDAGKTTLTESLLYASGAISEPGSVEKGTTRTDTMFLERQRGITIQAAV  60

MKIINIGILAHVDAGKTTLTESLLYASGAISEPGSVEKGTTRTDTMFLERQRGITIQAAV
Sbjct  1
MKIINIGILAHVDAGKTTLTESLLYASGAISEPGSVEKGTTRTDTMFLERQRGITIQAAV  60

Query  61
```

TSFQWHRCKVNIVDTPGHMDFLAEVYRSLAVLDGAILVISAKDGVQAQTRILFHALRKMN 120

TSFQWHRCKVNIVDTPGHMDFLAEVYRSLAVLDGAILVISAKDGVQAQTRILFHALRKMN
Sbjct 61
TSFQWHRCKVNIVDTPGHMDFLAEVYRSLAVLDGAILVISAKDGVQAQTRILFHALRKMN 120

Query 121
IPTVIFINKIDQAGVDLQSVVQSVRDKLSADIIIKQTVSLSPEIVLEENTDIEAWDAVIE 180

IPTVIFINKIDQAGVDLQSVVQSVRDKLSADIIIKQTVSLSPEIVLEENTDIEAWDAVIE
Sbjct 121
IPTVIFINKIDQAGVDLQSVVQSVRDKLSADIIIKQTVSLSPEIVLEENTDIEAWDAVIE 180

Query 181
NNDKLLEKYIAGEPISREKLVREEQRRVQDASLFPVYYGSAKKGLGIQPLMDAVTGLFQP 240

NNDKLLEKYIAGEPISREKLVREEQRRVQDASLFPVYYGSAKKGLGIQPLMDAVTGLFQP
Sbjct 181
NNDKLLEKYIAGEPISREKLVREEQRRVQDASLFPVYYGSAKKGLGIQPLMDAVTGLFQP 240

Query 241
IGEQGSAALCGSVFKVEYTDCGQRRVYLRLYSGTLRLRDTVALAGREKLKITEMRIPSKG 300

IGEQGSAALCGSVFKVEYTDCGQRRVYLRLYSGTLRLRDTVALAGREKLKITEMRIPSKG
Sbjct 241
IGEQGSAALCGSVFKVEYTDCGQRRVYLRLYSGTLRLRDTVALAGREKLKITEMRIPSKG 300

Query 301
EIVRTDTAYPGEIVILPSDSVRLNDVLGDPTRLPRKRWREDPLPMLRTTIAPKTAAQRER 360
EIVRTDTAYPGEIVILPSDSVRLNDVLGDPTRLPRKRWREDPLPMLRT
+IAPKTAAQRER
Sbjct 301
EIVRTDTAYPGEIVILPSDSVRLNDVLGDPTRLPRKRWREDPLPMLRTSIAPKTAAQRER 360

Query 361
LLDALTQLADTDPLLRCEVDSITHEIILSFLGRVQLEVVSALLSEKYKLETVVKEPSVIY 420
LLDALTQLADTDPLLRCEVDSITHEIILSFLGRVQLEVVSALLSEKYKLETVVKEP
+VIY
Sbjct 361
LLDALTQLADTDPLLRCEVDSITHEIILSFLGRVQLEVVSALLSEKYKLETVVKEPTVIY 420

Query 421
MERPLKAASHTIHIEVPPNPFWASIGLSVTPLSLGSGVQYESRVSLGYLNQSFQNAVRDG 480
MERPLKAASHTIHIEVPPNPFWASIGLSVTPL
LGSGVQYESRVSLGYLNQSFQNAVRDG
Sbjct 421
MERPLKAASHTIHIEVPPNPFWASIGLSVTPLPLGSGVQYESRVSLGYLNQSFQNAVRDG 480

Query 481
IRYGLEQGLFGWNVTDCKICFEYGLYYSPVSTPADFRSLAPIVLEQALKESGTQLLEPYL 540

IRYGLEQGLFGWNVTDCKICFEYGLYYSPVSTPADFRSLAPIVLEQALKESGTQLLEPYL
Sbjct 481
IRYGLEQGLFGWNVTDCKICFEYGLYYSPVSTPADFRSLAPIVLEQALKESGTQLLEPYL 540

```
Query  541
SFILYAPQEYLSRAYHDAPKYCATIETAQVKKDEVVFTGEIPARCIQAYRTDLAFYTNGR  600
            SF LYAP+EYLSRAYHDAPKYCATIET
QVKKDEVVFTGEIPARCIQAYRTDLAFYTNG+
Sbjct  541
SFTLYAPREYLSRAYHDAPKYCATIETVQVKKDEVVFTGEIPARCIQAYRTDLAFYTNGQ  600

Query  601  SVCLTELKGYQAAVGQPVIQPRRPNSRLDKVRHMFQKVM  639
            SVCLTELKGYQAAVG+PVIQPRRPNSRLDKVR+MFQKVM
Sbjct  601  SVCLTELKGYQAAVGKPVIQPRRPNSRLDKVRYMFQKVM  639
```

>WP_000691721.1 MULTISPECIES: tetracycline resistance ribosomal
protection protein
Tet(W) [Bacteria]
 ABF68767.1 TetW [Trueperella pyogenes]
 EDN00027.1 putative translation elongation factor G
[Pseudoflavonifractor
capillosus ATCC 29799]
 ACL81257.1 tetracycline resistance protein [Lactobacillus reuteri]
 EEI28000.1 putative translation elongation factor G
[Corynebacterium glucuronolyticum
ATCC 51867]
 EEI64008.1 putative translation elongation factor G
[Corynebacterium glucuronolyticum
ATCC 51866]
 CAZ39314.1 tetracycline resistance protein [Streptococcus suis]
 ADE30941.1 TetW [Streptococcus suis GZ1]
 ADH00696.1 translation elongation factor G [Bifidobacterium longum
subsp.
longum JDM301]
 CBR26881.1 tetracycline resistance protein [Streptococcus phage
phi-SsUD.1]
 EGN34188.1 tetracycline resistance protein tetW [Lachnospiraceae
bacterium
5_1_57FAA]
 AEX48439.1 tetracycline resistance protein [Corynebacterium
diphtheriae
BH8]
 AGH40332.1 TetW [Bifidobacterium thermophilum RBL67]
 EPV34625.1 tetracycline resistance protein tetM [Streptococcus
agalactiae
GB00679]
 ERI88045.1 putative translation elongation factor G
[Bifidobacterium breve
JCP7499]
 ERI98526.1 putative translation elongation factor G [Clostridium
sp. ATCC
29733]
 AGW28779.1 translation elongation factors (GTPases) [uncultured
bacterium
EB3]
 ERS77806.1 tetracycline resistance protein tetW [Corynebacterium

sp. KPL1859]
 AIF90764.1 tetracycline resistance protein tetM [Bifidobacterium longum]
 KFI47265.1 tetracycline resistance protein TetW [Bifidobacterium boum]
 KFI72919.1 TetW [Bifidobacterium longum subsp. suis]
 KFI98584.1 TetW [Bifidobacterium stercoris JCM 15918]
 KGI73145.1 tetracycline resistance protein tetW [Eggerthella lenta 1_1_60AFAA]
 AIW80578.1 TetM-TetW-TetO-TetS [uncultured bacterium]
 CQD12654.1 Tetracycline resistance protein TetW [Corynebacterium striatum]
 KOA45972.1 tetracycline resistance protein tetM [Bifidobacterium breve MCC
0121]
 CUQ23447.1 Tetracycline resistance protein tetM from transposon Tn916 [Anaerotruncus
colihominis]
 KRK07180.1 hypothetical protein FC52_GL001084 [Lactobacillus pasteurii DSM
23907 = CRBIP 24.76]
 KRL24550.1 hypothetical protein FC37_GL000387 [Lactobacillus gallinarum
DSM 10532 = JCM 2011]
 KRL92160.1 hypothetical protein FC43_GL000093 [Lactobacillus ingluviei DSM
15946]
 KRN89434.1 hypothetical protein IV44_GL001046 [Lactobacillus amylovorus
DSM 16698]
 CUR39809.1 Tetracycline resistance protein TetW [Lactobacillus reuteri]
 CYV18322.1 TetW [Streptococcus suis]
 AMJ36429.1 TetM-TetW-TetO-TetS [uncultured bacterium]
 KXN76983.1 tetracycline resistance protein tetM [Lactobacillus johnsonii]
 AMP55527.1 TetM-TetW-TetO-TetS [uncultured bacterium]
 CVI74148.1 Tetracycline resistance protein TetO [Eubacteriaceae bacterium
CHKCI004]
 OCX48011.1 tetracycline resistance ribosomal protection protein [Lactobacillus
animalis]
 SCH65814.1 Tetracycline resistance protein tetM from transposon Tn916 [uncultured
Collinsella sp.]
 OFK29245.1 tetracycline resistance ribosomal protection protein [Turicella
sp. HMSC062E11]
 SDO68498.1 ribosomal protection tetracycline resistance protein [Bifidobacterium
longum]
 OHO81140.1 tetracycline resistance ribosomal protection protein [Corynebacterium

sp. HMSC036E10]
 OIR43477.1 tetracycline resistance ribosomal protection protein [Corynebacterium
sp. NML140438]
 SHG43262.1 ribosomal protection tetracycline resistance protein [Clostridium
sp. DSM 4029]
 APO27241.1 ferrous iron transport protein B [uncultured bacterium]
 OLR56544.1 tetracycline resistance ribosomal protection protein [Eubacterium
sp. 68-3-10]
 SKA90423.1 ribosomal protection tetracycline resistance protein [Gemmiger
formicilis]
 SKA89138.1 ribosomal protection tetracycline resistance protein [Gemmiger
formicilis]
 SKA84017.1 ribosomal protection tetracycline resistance protein [Gemmiger
formicilis]
 SKA82527.1 ribosomal protection tetracycline resistance protein [Gemmiger
formicilis]
 OUN43347.1 tetracycline resistance ribosomal protection protein Tet(W) [Enorma
massiliensis]
 OUN87158.1 tetracycline resistance ribosomal protection protein Tet(W) [Gemmiger
sp. An50]
 OUN89233.1 tetracycline resistance ribosomal protection protein Tet(W) [[Collinsella]
massiliensis]
 OUO28178.1 tetracycline resistance ribosomal protection protein Tet(W) [Lachnoclostridium
sp. An298]
 OUO65884.1 tetracycline resistance ribosomal protection protein Tet(W) [Anaerotruncus
colihominis]
 OWM42766.1 tetracycline resistance ribosomal protection protein [Corynebacterium
diphtheriae]
 OWN45067.1 tetracycline resistance protein tetM [Corynebacterium
diphtheriae
bv. mitis]
 OWN64369.1 tetracycline resistance protein tetM [Corynebacterium
diphtheriae
bv. mitis]
 OZG55310.1 tetracycline resistance protein [Aeriscardovia
aeriphila]
 PAK16797.1 tetracycline resistance ribosomal protection protein Tet(W) [Bifidobacterium
longum]
 DAB26354.1 TPA: tetracycline resistance ribosomal protection
protein Tet(W)

[Candidatus Gastranaerophilales bacterium HUM_23]
 ATZ09232.1 tetracycline resistance ribosomal protection protein Tet(W) [Corynebacterium
striatum]
 AUD98523.1 ribosomal protection tetracycline resistance protein [Bifidobacterium
breve]
 AUX16243.1 tetracycline resistance protein tetM [Lactobacillus amylovorus]
 AVK72381.1 tetracycline resistance ribosomal protection protein Tet(W) [Corynebacterium
jeikeium]
 AVM43940.1 tetracycline resistance ribosomal protection protein Tet(W) [Victivallales
bacterium CCUG 44730]
Length=639

 Score = 1296 bits (3354), Expect = 0.0, Method: Compositional matrix adjust.
 Identities = 629/639 (98%), Positives = 636/639 (99%), Gaps = 0/639 (0%)

```
Query  1
MKIINIGILAHVDAGKTTLTESLLYASGAISEPGSVEKGTTRTDTMFLERQRGITIQAAV  60

MKIINIGILAHVDAGKTTLTESLLYASGAISEPGSVEKGTTRTDTMFLERQRGITIQAAV
Sbjct  1
MKIINIGILAHVDAGKTTLTESLLYASGAISEPGSVEKGTTRTDTMFLERQRGITIQAAV  60

Query  61
TSFQWHRCKVNIVDTPGHMDFLAEVYRSLAVLDGAILVISAKDGVQAQTRILFHALRKMN  120

TSFQWHRCKVNIVDTPGHMDFLAEVYRSLAVLDGAILVISAKDGVQAQTRILFHALRKMN
Sbjct  61
TSFQWHRCKVNIVDTPGHMDFLAEVYRSLAVLDGAILVISAKDGVQAQTRILFHALRKMN  120

Query  121
IPTVIFINKIDQAGVDLQSVVQSVRDKLSADIIIKQTVSLSPEIVLEENTDIEAWDAVIE  180

IPTVIFINKIDQAGVDLQSVVQSVRDKLSADIIIKQTVSLSPEIVLEENTDIEAWDAVIE
Sbjct  121
IPTVIFINKIDQAGVDLQSVVQSVRDKLSADIIIKQTVSLSPEIVLEENTDIEAWDAVIE  180

Query  181
NNDKLLEKYIAGEPISREKLVREEQRRVQDASLFPVYYGSAKKGLGIQPLMDAVTGLFQP  240

NNDKLLEKYIAGEPISREKLVREEQRRVQDASLFPVYYGSAKKGLGIQPLMDAVTGLFQP
Sbjct  181
NNDKLLEKYIAGEPISREKLVREEQRRVQDASLFPVYYGSAKKGLGIQPLMDAVTGLFQP  240

Query  241
IGEQGSAALCGSVFKVEYTDCGQRRVYLRLYSGTLRLRDTVALAGREKLKITEMRIPSKG  300

IGEQGSAALCGSVFKVEYTDCGQRRVYLRLYSGTLRLRDTVALAGREKLKITEMRIPSKG
```

```
Sbjct  241
IGEQGSAALCGSVFKVEYTDCGQRRVYLRLYSGTLRLRDTVALAGREKLKITEMRIPSKG  300

Query  301
EIVRTDTAYPGEIVILPSDSVRLNDVLGDPTRLPRKRWREDPLPMLRTTIAPKTAAQRER  360
            EIVRTDTAYPGEIVILPSDSVRLNDVLGDPTRLPRKRWREDPLPMLRT
+IAPKTAAQRER
Sbjct  301
EIVRTDTAYPGEIVILPSDSVRLNDVLGDPTRLPRKRWREDPLPMLRTSIAPKTAAQRER  360

Query  361
LLDALTQLADTDPLLRCEVDSITHEIILSFLGRVQLEVVSALLSEKYKLETVVKEPSVIY  420
            LLDALTQLADTDPLLRCEVDSITHEIILSFLGRVQLEVVSALLSEKYKLETVVKEP
+VIY
Sbjct  361
LLDALTQLADTDPLLRCEVDSITHEIILSFLGRVQLEVVSALLSEKYKLETVVKEPTVIY  420

Query  421
MERPLKAASHTIHIEVPPNPFWASIGLSVTPLSLGSGVQYESRVSLGYLNQSFQNAVRDG  480
            MERPLKAASHTIHIEVPPNPFWASIGLSVTPL
LGSGVQYESRVSLGYLNQSFQNAVRDG
Sbjct  421
MERPLKAASHTIHIEVPPNPFWASIGLSVTPLPLGSGVQYESRVSLGYLNQSFQNAVRDG  480

Query  481
IRYGLEQGLFGWNVTDCKICFEYGLYYSPVSTPADFRSLAPIVLEQALKESGTQLLEPYL  540

IRYGLEQGLFGWNVTDCKICFEYGLYYSPVSTPADFRSLAPIVLEQALKESGTQLLEPYL
Sbjct  481
IRYGLEQGLFGWNVTDCKICFEYGLYYSPVSTPADFRSLAPIVLEQALKESGTQLLEPYL  540

Query  541
SFILYAPQEYLSRAYHDAPKYCATIETAQVKKDEVVFTGEIPARCIQAYRTDLAFYTNGR  600
            SF LYAP+EYLSRAYHDAPKYCATIET
QVKKDEVVFTGEIPARCIQAYRTDLAFYTNG+
Sbjct  541
SFTLYAPREYLSRAYHDAPKYCATIETVQVKKDEVVFTGEIPARCIQAYRTDLAFYTNGQ  600

Query  601  SVCLTELKGYQAAVGQPVIQPRRPNSRLDKVRHMFQKVM  639
            SVCLTELKGYQAAVG+PVIQPRRPNSRLDKVR+MFQK+M
Sbjct  601  SVCLTELKGYQAAVGKPVIQPRRPNSRLDKVRYMFQKIM  639


>APO34064.1 ferrous iron transport protein B [uncultured bacterium]
Length=639

 Score = 1296 bits (3353),  Expect = 0.0, Method: Compositional
matrix adjust.
 Identities = 630/639 (99%), Positives = 636/639 (99%), Gaps = 0/639
(0%)

Query  1
MKIINIGILAHVDAGKTTLTESLLYASGAISEPGSVEKGTTRTDTMFLERQRGITIQAAV  60
```

MKIINIGILAHVDAGKTTLTESLLYASGAISEPGSVEKGTTRTDTMFLERQRGITIQAAV
Sbjct 1
MKIINIGILAHVDAGKTTLTESLLYASGAISEPGSVEKGTTRTDTMFLERQRGITIQAAV 60

Query 61
TSFQWHRCKVNIVDTPGHMDFLAEVYRSLAVLDGAILVISAKDGVQAQTRILFHALRKMN 120

TSFQWHRCKVNIVDTPGHMDFLAEVYRSLAVLDGAILVISAKDGVQAQTRILFHALRKMN
Sbjct 61
TSFQWHRCKVNIVDTPGHMDFLAEVYRSLAVLDGAILVISAKDGVQAQTRILFHALRKMN 120

Query 121
IPTVIFINKIDQAGVDLQSVVQSVRDKLSADIIIKQTVSLSPEIVLEENTDIEAWDAVIE 180

IPTVIFINKIDQAGVDLQSVVQSVRDKLSADIIIKQTVSLSPEIVLEENTDIEAWDAVIE
Sbjct 121
IPTVIFINKIDQAGVDLQSVVQSVRDKLSADIIIKQTVSLSPEIVLEENTDIEAWDAVIE 180

Query 181
NNDKLLEKYIAGEPISREKLVREEQRRVQDASLFPVYYGSAKKGLGIQPLMDAVTGLFQP 240

NNDKLLEKYIAGEPISREKLVREEQRRVQDASLFPVYYGSAKKGLGIQPLMDAVTGLFQP
Sbjct 181
NNDKLLEKYIAGEPISREKLVREEQRRVQDASLFPVYYGSAKKGLGIQPLMDAVTGLFQP 240

Query 241
IGEQGSAALCGSVFKVEYTDCGQRRVYLRLYSGTLRLRDTVALAGREKLKITEMRIPSKG 300
IGEQGSAALCGSVFKVEYTDCGQR
VYLRLYSGTLRLRDTVALAGREKLKITEMRIPSKG
Sbjct 241
IGEQGSAALCGSVFKVEYTDCGQRLVYLRLYSGTLRLRDTVALAGREKLKITEMRIPSKG 300

Query 301
EIVRTDTAYPGEIVILPSDSVRLNDVLGDPTRLPRKRWREDPLPMLRTTIAPKTAAQRER 360
EIVRTDTAYPGEIVILPSDSVRLNDVLGD TRLPRKRWREDPLPMLRT
+IAPKTAAQRER
Sbjct 301
EIVRTDTAYPGEIVILPSDSVRLNDVLGDKTRLPRKRWREDPLPMLRTSIAPKTAAQRER 360

Query 361
LLDALTQLADTDPLLRCEVDSITHEIILSFLGRVQLEVVSALLSEKYKLETVVKEPSVIY 420
LLDALTQLADTDPLLRCEVDSITHEIILSFLGRVQLEVVSALLSEKYKLETVVKEP
+VIY
Sbjct 361
LLDALTQLADTDPLLRCEVDSITHEIILSFLGRVQLEVVSALLSEKYKLETVVKEPTVIY 420

Query 421
MERPLKAASHTIHIEVPPNPFWASIGLSVTPLSLGSGVQYESRVSLGYLNQSFQNAVRDG 480
MERPLKAASHTIHIEVPPNPFWASIGLSVTPL
LGSGVQYESRVSLGYLNQSFQNAVRDG
Sbjct 421
MERPLKAASHTIHIEVPPNPFWASIGLSVTPLPLGSGVQYESRVSLGYLNQSFQNAVRDG 480

Query 481

```
IRYGLEQGLFGWNVTDCKICFEYGLYYSPVSTPADFRSLAPIVLEQALKESGTQLLEPYL  540

IRYGLEQGLFGWNVTDCKICFEYGLYYSPVSTPADFRSLAPIVLEQALKESGTQLLEPYL
Sbjct  481
IRYGLEQGLFGWNVTDCKICFEYGLYYSPVSTPADFRSLAPIVLEQALKESGTQLLEPYL  540

Query  541
SFILYAPQEYLSRAYHDAPKYCATIETAQVKKDEVVFTGEIPARCIQAYRTDLAFYTNGR  600

SFILYAPQEYLSRAYHDAPKYCATIETAQVKKDEVVFTGEIPARCIQAYRTDLAFYTNG+
Sbjct  541
SFILYAPQEYLSRAYHDAPKYCATIETAQVKKDEVVFTGEIPARCIQAYRTDLAFYTNGQ  600

Query  601  SVCLTELKGYQAAVGQPVIQPRRPNSRLDKVRHMFQKVM  639
            SVCLTELKGYQAAVG+PVIQPRRPNSRLDKVR+MFQK+M
Sbjct  601  SVCLTELKGYQAAVGKPVIQPRRPNSRLDKVRYMFQKIM  639


>APO32472.1 ferrous iron transport protein B [uncultured bacterium]
Length=639

 Score = 1296 bits (3353),  Expect = 0.0, Method: Compositional
matrix adjust.
 Identities = 631/639 (99%), Positives = 635/639 (99%), Gaps = 0/639
(0%)

Query  1
MKIINIGILAHVDAGKTTLTESLLYASGAISEPGSVEKGTTRTDTMFLERQRGITIQAAV  60

MKIINIGILAHVDAGKTTLTESLLYASGAISEPGSVEKGTTRTDTMFLERQRGITIQAAV
Sbjct  1
MKIINIGILAHVDAGKTTLTESLLYASGAISEPGSVEKGTTRTDTMFLERQRGITIQAAV  60

Query  61
TSFQWHRCKVNIVDTPGHMDFLAEVYRSLAVLDGAILVISAKDGVQAQTRILFHALRKMN  120

TSFQWHRCKVNIVDTPGHMDFLAEVYRSLAVLDGAILVISAKDGVQAQTRILFHALRKMN
Sbjct  61
TSFQWHRCKVNIVDTPGHMDFLAEVYRSLAVLDGAILVISAKDGVQAQTRILFHALRKMN  120

Query  121
IPTVIFINKIDQAGVDLQSVVQSVRDKLSADIIIKQTVSLSPEIVLEENTDIEAWDAVIE  180

IPTVIFINKIDQAGVDLQSVVQSVRDKLSADIIIKQTVSLSPEIVLEENTDIEAWDAVIE
Sbjct  121
IPTVIFINKIDQAGVDLQSVVQSVRDKLSADIIIKQTVSLSPEIVLEENTDIEAWDAVIE  180

Query  181
NNDKLLEKYIAGEPISREKLVREEQRRVQDASLFPVYYGSAKKGLGIQPLMDAVTGLFQP  240
            NND+LLEKYIAGEPISREKL REEQ+RVQDASLFPVY
+GSAKKGLGIQPLMDAVTGLFQP
Sbjct  181
NNDELLEKYIAGEPISREKLAREEQQRVQDASLFPVYHGSAKKGLGIQPLMDAVTGLFQP  240
```

```
Query  241
IGEQGSAALCGSVFKVEYTDCGQRRVYLRLYSGTLRLRDTVALAGREKLKITEMRIPSKG  300
           IGEQG
AALCGSVFKVEYTDCGQRRVYLRLYSGTLRLRDTVALAGREKLKITEMRIPSKG
Sbjct  241
IGEQGGAALCGSVFKVEYTDCGQRRVYLRLYSGTLRLRDTVALAGREKLKITEMRIPSKG  300

Query  301
EIVRTDTAYPGEIVILPSDSVRLNDVLGDPTRLPRKRWREDPLPMLRTTIAPKTAAQRER  360
           EIVRTDTAY GEIVILPSDSVRLNDVLGD
TRLPRKRWREDPLPMLRTTIAPKTAAQRER
Sbjct  301
EIVRTDTAYQGEIVILPSDSVRLNDVLGDQTRLPRKRWREDPLPMLRTTIAPKTAAQRER  360

Query  361
LLDALTQLADTDPLLRCEVDSITHEIILSFLGRVQLEVVSALLSEKYKLETVVKEPSVIY  420

LLDALTQLADTDPLLRCEVDSITHEIILSFLGRVQLEVVSALLSEKYKLETVVKEPSVIY
Sbjct  361
LLDALTQLADTDPLLRCEVDSITHEIILSFLGRVQLEVVSALLSEKYKLETVVKEPSVIY  420

Query  421
MERPLKAASHTIHIEVPPNPFWASIGLSVTPLSLGSGVQYESRVSLGYLNQSFQNAVRDG  480

MERPLKAASHTIHIEVPPNPFWASIGLSVTPLSLGSGVQYESRVSLGYLNQSFQNAVRDG
Sbjct  421
MERPLKAASHTIHIEVPPNPFWASIGLSVTPLSLGSGVQYESRVSLGYLNQSFQNAVRDG  480

Query  481
IRYGLEQGLFGWNVTDCKICFEYGLYYSPVSTPADFRSLAPIVLEQALKESGTQLLEPYL  540

IRYGLEQGLFGWNVTDCKICFEYGLYYSPVSTPADFRSLAPIVLEQALKESGTQLLEPYL
Sbjct  481
IRYGLEQGLFGWNVTDCKICFEYGLYYSPVSTPADFRSLAPIVLEQALKESGTQLLEPYL  540

Query  541
SFILYAPQEYLSRAYHDAPKYCATIETAQVKKDEVVFTGEIPARCIQAYRTDLAFYTNGR  600

SFILYAPQEYLSRAYHDAPKYCATIETAQVKKDEVVFTGEIPARCIQAYRTDLAFYTNGR
Sbjct  541
SFILYAPQEYLSRAYHDAPKYCATIETAQVKKDEVVFTGEIPARCIQAYRTDLAFYTNGR  600

Query  601  SVCLTELKGYQAAVGQPVIQPRRPNSRLDKVRHMFQKVM  639
           SVCLTELKGYQAAVGQPVIQPRRPNSRL+KVRHMFQKVM
Sbjct  601  SVCLTELKGYQAAVGQPVIQPRRPNSRLEKVRHMFQKVM  639


>SCJ49094.1 Tetracycline resistance protein tetM from transposon
Tn916 [uncultured
Clostridium sp.]
Length=639

 Score = 1296 bits (3353),  Expect = 0.0, Method: Compositional
matrix adjust.
```

Identities = 631/639 (99%), Positives = 634/639 (99%), Gaps = 0/639 (0%)

Query 1
MKIINIGILAHVDAGKTTLTESLLYASGAISEPGSVEKGTTRTDTMFLERQRGITIQAAV 60

MKIINIGILAHVDAGKTTLTESLLYASGAISEPGSVEKGTTRTDTMFLERQRGITIQAAV
Sbjct 1
MKIINIGILAHVDAGKTTLTESLLYASGAISEPGSVEKGTTRTDTMFLERQRGITIQAAV 60

Query 61
TSFQWHRCKVNIVDTPGHMDFLAEVYRSLAVLDGAILVISAKDGVQAQTRILFHALRKMN 120

TSFQWHRCKVNIVDTPGHMDFLAEVYRSLAVLDGAILVISAKDGVQAQTRILFHALRKMN
Sbjct 61
TSFQWHRCKVNIVDTPGHMDFLAEVYRSLAVLDGAILVISAKDGVQAQTRILFHALRKMN 120

Query 121
IPTVIFINKIDQAGVDLQSVVQSVRDKLSADIIIKQTVSLSPEIVLEENTDIEAWDAVIE 180

IPTVIFINKIDQAGVDLQSVVQSVRDKLSADIIIKQTVSLSPEIVLEENTDIEAWDAVIE
Sbjct 121
IPTVIFINKIDQAGVDLQSVVQSVRDKLSADIIIKQTVSLSPEIVLEENTDIEAWDAVIE 180

Query 181
NNDKLLEKYIAGEPISREKLVREEQRRVQDASLFPVYYGSAKKGLGIQPLMDAVTGLFQP 240
NND+LLEKYIAGEPISREKL REEQ+RVQDASLFPVYYGSAK
GLGIQPLMDAVTGLFQP
Sbjct 181
NNDELLEKYIAGEPISREKLAREEQQRVQDASLFPVYYGSAKNGLGIQPLMDAVTGLFQP 240

Query 241
IGEQGSAALCGSVFKVEYTDCGQRRVYLRLYSGTLRLRDTVALAGREKLKITEMRIPSKG 300
IGEQG
AALCGSVFKVEYTDCGQRRVYLRLYSGTLRLRDTVALAGREKLKITEMRIPSKG
Sbjct 241
IGEQGGAALCGSVFKVEYTDCGQRRVYLRLYSGTLRLRDTVALAGREKLKITEMRIPSKG 300

Query 301
EIVRTDTAYPGEIVILPSDSVRLNDVLGDPTRLPRKRWREDPLPMLRTTIAPKTAAQRER 360
EIVRTDTAY GEIVILPSDSVRLNDVLGD
TRLPRKRWREDPLPMLRTTIAPKTAAQRER
Sbjct 301
EIVRTDTAYQGEIVILPSDSVRLNDVLGDQTRLPRKRWREDPLPMLRTTIAPKTAAQRER 360

Query 361
LLDALTQLADTDPLLRCEVDSITHEIILSFLGRVQLEVVSALLSEKYKLETVVKEPSVIY 420
LLDALTQLADTDPLLRCEVDSITHEIILSFLGRVQLEVVSALLSEKYKLETVVKEP
+VIY
Sbjct 361
LLDALTQLADTDPLLRCEVDSITHEIILSFLGRVQLEVVSALLSEKYKLETVVKEPTVIY 420

Query 421
MERPLKAASHTIHIEVPPNPFWASIGLSVTPLSLGSGVQYESRVSLGYLNQSFQNAVRDG 480

MERPLKAASHTIHIEVPPNPFWASIGLSVTPLSLGSGVQYESRVSLGYLNQSFQNAVRDG
Sbjct 421
MERPLKAASHTIHIEVPPNPFWASIGLSVTPLSLGSGVQYESRVSLGYLNQSFQNAVRDG 480

Query 481
IRYGLEQGLFGWNVTDCKICFEYGLYYSPVSTPADFRSLAPIVLEQALKESGTQLLEPYL 540

IRYGLEQGLFGWNVTDCKICFEYGLYYSPVSTPADFRSLAPIVLEQALKESGTQLLEPYL
Sbjct 481
IRYGLEQGLFGWNVTDCKICFEYGLYYSPVSTPADFRSLAPIVLEQALKESGTQLLEPYL 540

Query 541
SFILYAPQEYLSRAYHDAPKYCATIETAQVKKDEVVFTGEIPARCIQAYRTDLAFYTNGR 600

SFILYAPQEYLSRAYHDAPKYCATIETAQVKKDEVVFTGEIPARCIQAYRTDLAFYTNGR
Sbjct 541
SFILYAPQEYLSRAYHDAPKYCATIETAQVKKDEVVFTGEIPARCIQAYRTDLAFYTNGR 600

Query 601 SVCLTELKGYQAAVGQPVIQPRRPNSRLDKVRHMFQKVM 639
            SVCLTELKGYQAAVGQPVIQPRRPNSRLDKVRHMFQKVM
Sbjct 601 SVCLTELKGYQAAVGQPVIQPRRPNSRLDKVRHMFQKVM 639

>WP_058985878.1 TetM/TetW/TetO/TetS family tetracycline resistance ribosomal
protection protein [Eggerthellaceae bacterium AT8]
Length=639

Score = 1296 bits (3353), Expect = 0.0, Method: Compositional matrix adjust.
Identities = 629/639 (98%), Positives = 635/639 (99%), Gaps = 0/639 (0%)

Query 1
MKIINIGILAHVDAGKTTLTESLLYASGAISEPGSVEKGTTRTDTMFLERQRGITIQAAV 60
            MKIINIGILAHVDAGKTTLTESLLYASGAISEPGSVEKGTTRTDTM
LERQRGITIQAAV
Sbjct 1
MKIINIGILAHVDAGKTTLTESLLYASGAISEPGSVEKGTTRTDTMLLERQRGITIQAAV 60

Query 61
TSFQWHRCKVNIVDTPGHMDFLAEVYRSLAVLDGAILVISAKDGVQAQTRILFHALRKMN 120

TSFQWHRCKVNIVDTPGHMDFLAEVYRSLAVLDGAILVISAKDGVQAQTRILFHALRKMN
Sbjct 61
TSFQWHRCKVNIVDTPGHMDFLAEVYRSLAVLDGAILVISAKDGVQAQTRILFHALRKMN 120

Query 121
IPTVIFINKIDQAGVDLQSVVQSVRDKLSADIIIKQTVSLSPEIVLEENTDIEAWDAVIE 180

IPTVIFINKIDQAGVDLQSVVQSVRDKLSADIIIKQTVSLSPEIVLEENTDIEAWDAVIE
Sbjct 121
IPTVIFINKIDQAGVDLQSVVQSVRDKLSADIIIKQTVSLSPEIVLEENTDIEAWDAVIE 180

```
Query  181
NNDKLLEKYIAGEPISREKLVREEQRRVQDASLFPVYYGSAKKGLGIQPLMDAVTGLFQP  240

NNDKLLEKYIAGEPISREKLVREEQRRVQDASLFPVYYGSAKKGLGIQPLMDAVTGLFQP
Sbjct  181
NNDKLLEKYIAGEPISREKLVREEQRRVQDASLFPVYYGSAKKGLGIQPLMDAVTGLFQP  240

Query  241
IGEQGSAALCGSVFKVEYTDCGQRRVYLRLYSGTLRLRDTVALAGREKLKITEMRIPSKG  300

IGEQGSAALCGSVFKVEYTDCGQRRVYLRLYSGTLRLRDTVALAGREKLKITEMRIPSKG
Sbjct  241
IGEQGSAALCGSVFKVEYTDCGQRRVYLRLYSGTLRLRDTVALAGREKLKITEMRIPSKG  300

Query  301
EIVRTDTAYPGEIVILPSDSVRLNDVLGDPTRLPRKRWREDPLPMLRTTIAPKTAAQRER  360
            EIVRTDTAYPGEIVILPSDSVRLNDVLGDPTRLPRKRWREDPLPMLRT
+IAPKTAAQRER
Sbjct  301
EIVRTDTAYPGEIVILPSDSVRLNDVLGDPTRLPRKRWREDPLPMLRTSIAPKTAAQRER  360

Query  361
LLDALTQLADTDPLLRCEVDSITHEIILSFLGRVQLEVVSALLSEKYKLETVVKEPSVIY  420
            LLDALTQLADTDPLLRCEVDSITHEIILSFLGRVQLEVVSALL+EKYKLETVVKEP
+VIY
Sbjct  361
LLDALTQLADTDPLLRCEVDSITHEIILSFLGRVQLEVVSALLAEKYKLETVVKEPTVIY  420

Query  421
MERPLKAASHTIHIEVPPNPFWASIGLSVTPLSLGSGVQYESRVSLGYLNQSFQNAVRDG  480
            MERPLKAASHTIHIEVPPNPFWASIGLSVTPL
LGSGVQYESRVSLGYLNQSFQNAVRDG
Sbjct  421
MERPLKAASHTIHIEVPPNPFWASIGLSVTPLPLGSGVQYESRVSLGYLNQSFQNAVRDG  480

Query  481
IRYGLEQGLFGWNVTDCKICFEYGLYYSPVSTPADFRSLAPIVLEQALKESGTQLLEPYL  540

IRYGLEQGLFGWNVTDCKICFEYGLYYSPVSTPADFRSLAPIVLEQALKESGTQLLEPYL
Sbjct  481
IRYGLEQGLFGWNVTDCKICFEYGLYYSPVSTPADFRSLAPIVLEQALKESGTQLLEPYL  540

Query  541
SFILYAPQEYLSRAYHDAPKYCATIETAQVKKDEVVFTGEIPARCIQAYRTDLAFYTNGR  600
            SF LYAP+EYLSRAYHDAPKYCATIET
QVKKDEVVFTGEIPARCIQAYRTDLAFYTNG+
Sbjct  541
SFTLYAPREYLSRAYHDAPKYCATIETVQVKKDEVVFTGEIPARCIQAYRTDLAFYTNGQ  600

Query  601  SVCLTELKGYQAAVGQPVIQPRRPNSRLDKVRHMFQKVM  639
            SVCLTELKGYQAAVG+PVIQPRRPNSRLDKVRHMFQKVM
Sbjct  601  SVCLTELKGYQAAVGKPVIQPRRPNSRLDKVRHMFQKVM  639
```

>WP_058966934.1 TetM/TetW/TetO/TetS family tetracycline resistance ribosomal
protection protein [Fournierella massiliensis]
Length=639

Score = 1296 bits (3353), Expect = 0.0, Method: Compositional matrix adjust.
Identities = 631/639 (99%), Positives = 634/639 (99%), Gaps = 0/639 (0%)

```
Query  1
MKIINIGILAHVDAGKTTLTESLLYASGAISEPGSVEKGTTRTDTMFLERQRGITIQAAV  60

MKIINIGILAHVDAGKTTLTESLLYASGAISEPGSVEKGTTRTDTMFLERQRGITIQAAV
Sbjct  1
MKIINIGILAHVDAGKTTLTESLLYASGAISEPGSVEKGTTRTDTMFLERQRGITIQAAV  60

Query  61
TSFQWHRCKVNIVDTPGHMDFLAEVYRSLAVLDGAILVISAKDGVQAQTRILFHALRKMN  120

TSFQWHRCKVNIVDTPGHMDFLAEVYRSLAVLDGAILVISAKDGVQAQTRILFHALRKMN
Sbjct  61
TSFQWHRCKVNIVDTPGHMDFLAEVYRSLAVLDGAILVISAKDGVQAQTRILFHALRKMN  120

Query  121
IPTVIFINKIDQAGVDLQSVVQSVRDKLSADIIIKQTVSLSPEIVLEENTDIEAWDAVIE  180

IPTVIFINKIDQAGVDLQSVVQSVRDKLSADIIIKQTVSLSPEIVLEENTDIEAWDAVIE
Sbjct  121
IPTVIFINKIDQAGVDLQSVVQSVRDKLSADIIIKQTVSLSPEIVLEENTDIEAWDAVIE  180

Query  181
NNDKLLEKYIAGEPISREKLVREEQRRVQDASLFPVYYGSAKKGLGIQPLMDAVTGLFQP  240
            NND+LLEKYIAGEPISREKL REEQ+RVQDASLFPVY+GSAK
GLGIQPLMDAVTGLFQP
Sbjct  181
NNDELLEKYIAGEPISREKLAREEQQRVQDASLFPVYHGSAKNGLGIQPLMDAVTGLFQP  240

Query  241
IGEQGSAALCGSVFKVEYTDCGQRRVYLRLYSGTLRLRDTVALAGREKLKITEMRIPSKG  300
            IGEQGSA
LCGSVFKVEYTDCGQRRVYLRLYSGTLRLRDTVALAGREKLKITEMRIPSKG
Sbjct  241
IGEQGSATLCGSVFKVEYTDCGQRRVYLRLYSGTLRLRDTVALAGREKLKITEMRIPSKG  300

Query  301
EIVRTDTAYPGEIVILPSDSVRLNDVLGDPTRLPRKRWREDPLPMLRTTIAPKTAAQRER  360
            EIVRTDTAYPGEIVILPSDSVRLNDVLGD
TRLPRKRWREDPLPMLRTTIAPKTAAQRER
Sbjct  301
EIVRTDTAYPGEIVILPSDSVRLNDVLGDQTRLPRKRWREDPLPMLRTTIAPKTAAQRER  360

Query  361
```

```
LLDALTQLADTDPLLRCEVDSITHEIILSFLGRVQLEVVSALLSEKYKLETVVKEPSVIY  420

LLDALTQLADTDPLLRCEVDSITHEIILSFLGRVQLEVVSALLSEKYKLETVVKEPSVIY
Sbjct  361
LLDALTQLADTDPLLRCEVDSITHEIILSFLGRVQLEVVSALLSEKYKLETVVKEPSVIY  420

Query  421
MERPLKAASHTIHIEVPPNPFWASIGLSVTPLSLGSGVQYESRVSLGYLNQSFQNAVRDG  480
            MERPLKAASHTI
IEVPPNPFWASIGLSVTPLSLGSGVQYESRVSLGYLNQSFQNAVRDG
Sbjct  421
MERPLKAASHTIDIEVPPNPFWASIGLSVTPLSLGSGVQYESRVSLGYLNQSFQNAVRDG  480

Query  481
IRYGLEQGLFGWNVTDCKICFEYGLYYSPVSTPADFRSLAPIVLEQALKESGTQLLEPYL  540

IRYGLEQGLFGWNVTDCKICFEYGLYYSPVSTPADFRSLAPIVLEQALKESGTQLLEPYL
Sbjct  481
IRYGLEQGLFGWNVTDCKICFEYGLYYSPVSTPADFRSLAPIVLEQALKESGTQLLEPYL  540

Query  541
SFILYAPQEYLSRAYHDAPKYCATIETAQVKKDEVVFTGEIPARCIQAYRTDLAFYTNGR  600

SFILYAPQEYLSRAYHDAPKYCATIETAQVKKDEVVFTGEIPARCIQAYRTDLAFYTNGR
Sbjct  541
SFILYAPQEYLSRAYHDAPKYCATIETAQVKKDEVVFTGEIPARCIQAYRTDLAFYTNGR  600

Query  601  SVCLTELKGYQAAVGQPVIQPRRPNSRLDKVRHMFQKVM  639
            SVCLTELKGYQAAVGQPVIQPRRPNSRLDKVRHMFQKVM
Sbjct  601  SVCLTELKGYQAAVGQPVIQPRRPNSRLDKVRHMFQKVM  639


>WP_033163194.1 TetM/TetW/TetO/TetS family tetracycline resistance
ribosomal
protection protein [Sharpea azabuensis]
Length=639

 Score = 1295 bits (3352),  Expect = 0.0, Method: Compositional
matrix adjust.
 Identities = 629/639 (98%), Positives = 636/639 (99%), Gaps = 0/639
(0%)

Query  1
MKIINIGILAHVDAGKTTLTESLLYASGAISEPGSVEKGTTRTDTMFLERQRGITIQAAV  60

MKIINIGILAHVDAGKTTLTESLLYASGAISEPGSVEKGTTRTDTMFLERQRGITIQAAV
Sbjct  1
MKIINIGILAHVDAGKTTLTESLLYASGAISEPGSVEKGTTRTDTMFLERQRGITIQAAV  60

Query  61
TSFQWHRCKVNIVDTPGHMDFLAEVYRSLAVLDGAILVISAKDGVQAQTRILFHALRKMN  120

TSFQWHRCKVNIVDTPGHMDFLAEVYRSLAVLDGAILVISAKDGVQAQTRILFHALRKMN
Sbjct  61
```

TSFQWHRCKVNIVDTPGHMDFLAEVYRSLAVLDGAILVISAKDGVQAQTRILFHALRKMN 120

Query 121
IPTVIFINKIDQAGVDLQSVVQSVRDKLSADIIIKQTVSLSPEIVLEENTDIEAWDAVIE 180

IPTVIFINKIDQAGVDLQSVVQSVRDKLSADIIIKQTVSLSPEIVLEENTDIEAWDAVIE
Sbjct 121
IPTVIFINKIDQAGVDLQSVVQSVRDKLSADIIIKQTVSLSPEIVLEENTDIEAWDAVIE 180

Query 181
NNDKLLEKYIAGEPISREKLVREEQRRVQDASLFPVYYGSAKKGLGIQPLMDAVTGLFQP 240

NNDKLLEKYIAGEPISREKLVREEQRRVQDASLFPVYYGSAKKGLGIQPLMDAVTGLFQP
Sbjct 181
NNDKLLEKYIAGEPISREKLVREEQRRVQDASLFPVYYGSAKKGLGIQPLMDAVTGLFQP 240

Query 241
IGEQGSAALCGSVFKVEYTDCGQRRVYLRLYSGTLRLRDTVALAGREKLKITEMRIPSKG 300

IGEQGSAALCGSVFKVEYTDCGQRRVYLRLYSGTLRLRDTVALAGREKLKITEMRIPSKG
Sbjct 241
IGEQGSAALCGSVFKVEYTDCGQRRVYLRLYSGTLRLRDTVALAGREKLKITEMRIPSKG 300

Query 301
EIVRTDTAYPGEIVILPSDSVRLNDVLGDPTRLPRKRWREDPLPMLRTTIAPKTAAQRER 360
EIVRTDTAYPGEIVILPSDSVRLNDVLGDPTRLPRKRWREDPLPMLRT
+IAPKTAAQRER
Sbjct 301
EIVRTDTAYPGEIVILPSDSVRLNDVLGDPTRLPRKRWREDPLPMLRTSIAPKTAAQRER 360

Query 361
LLDALTQLADTDPLLRCEVDSITHEIILSFLGRVQLEVVSALLSEKYKLETVVKEPSVIY 420
LLDALTQLADTDPLLRCEVDSITHEIILSFLGRVQLEVVSALLSEKYKLETVVKEP
+VIY
Sbjct 361
LLDALTQLADTDPLLRCEVDSITHEIILSFLGRVQLEVVSALLSEKYKLETVVKEPAVIY 420

Query 421
MERPLKAASHTIHIEVPPNPFWASIGLSVTPLSLGSGVQYESRVSLGYLNQSFQNAVRDG 480
MERPLKAASHTIHIEVPPNPFWASIGLSVTPL
LGSGVQYESRVSLGYLNQSFQNAVRDG
Sbjct 421
MERPLKAASHTIHIEVPPNPFWASIGLSVTPLPLGSGVQYESRVSLGYLNQSFQNAVRDG 480

Query 481
IRYGLEQGLFGWNVTDCKICFEYGLYYSPVSTPADFRSLAPIVLEQALKESGTQLLEPYL 540

IRYGLEQGLFGWNVTDCKICFEYGLYYSPVSTPADFRSLAPIVLEQALKESGTQLLEPYL
Sbjct 481
IRYGLEQGLFGWNVTDCKICFEYGLYYSPVSTPADFRSLAPIVLEQALKESGTQLLEPYL 540

Query 541
SFILYAPQEYLSRAYHDAPKYCATIETAQVKKDEVVFTGEIPARCIQAYRTDLAFYTNGR 600
SF LYAP+EYLSRAYHDAPKYCATIET

```
QVKKDEVVFTGEIPARCIQAYRTDLAFYTNG+
Sbjct  541
SFTLYAPREYLSRAYHDAPKYCATIETVQVKKDEVVFTGEIPARCIQAYRTDLAFYTNGQ  600

Query  601  SVCLTELKGYQAAVGQPVIQPRRPNSRLDKVRHMFQKVM  639
            SVCLTELKGYQAAVG+PVIQPRRPNSRLDKVR+MFQK+M
Sbjct  601  SVCLTELKGYQAAVGKPVIQPRRPNSRLDKVRYMFQKIM  639


>WP_002586627.1 MULTISPECIES: tetracycline resistance ribosomal
protection protein
Tet(W) [Bacteria]
 O52836.1 RecName: Full=Tetracycline resistance protein TetW;
Short=Tet(W)
 CAA10975.1 tetracycline resistance protein [Butyrivibrio
fibrisolvens]
 CAD20560.1 TetW protein [Butyrivibrio fibrisolvens]
 CAD20561.1 TetW protein [Mitsuokella multacida]
 AAT12289.1 tetracycline resistance protein [Mitsuokella multacida]
 AAT27386.1 tetracycline resistance protein [Clostridiaceae
bacterium K10]
 ACA23197.1 TetW [Bifidobacterium longum]
 EEG28895.1 putative translation elongation factor G
[ [[Clostridium] methylpentosum
DSM 5476]
 EEG88154.1 putative translation elongation factor G [Coprococcus
comes ATCC
27758]
 EEI80537.1 putative translation elongation factor G
[Bifidobacterium longum
subsp. longum ATCC 55813]
 ACT97607.1 tetracycline resistance protein tetW [uncultured
organism]
 EFB76893.1 putative translation elongation factor G
[Subdoligranulum variabile
DSM 15176]
 EFD93357.1 putative translation elongation factor G [Megasphaera
genomosp.
type_1 str. 28L]
 EFE13268.1 putative translation elongation factor G [Clostridium
sp. M62/1]
 CBK70657.1 small GTP-binding protein domain [Bifidobacterium longum
subsp.
longum F8]
 CBL34482.1 small GTP-binding protein domain [ [[Eubacterium]
siraeum V10Sc8a]
 CBK76449.1 small GTP-binding protein domain [ [[Clostridium] cf.
saccharolyticum
K10]
 CBK98114.1 small GTP-binding protein domain [Faecalibacterium
prausnitzii
L2-6]
 EGG90070.1 tetracycline resistance protein tetW [Lachnospiraceae
oral taxon
```

107 str. F0167]
 EGJ47164.1 tetracycline resistance protein tetW [Ruminococcaceae bacterium
D16]
 CCA30602.1 tetracycline resistance protein [Clostridioides difficile]
 BAK46968.1 hypothetical protein CXIVA_10010 [Clostridium sp. SY8519]
 EHE98624.1 tetracycline resistance protein tetW [[[Clostridium] citroniae
WAL-17108]
 EHG30528.1 tetracycline resistance protein tetW [[[Clostridium] clostridioforme
2_1_49FAA]
 EHI55192.1 tetracycline resistance protein tetW [Johnsonella ignava ATCC
51276]
 EHL10185.1 tetracycline resistance protein tetW [Oribacterium parvum ACB1]
 EHL67737.1 tetracycline resistance protein tetW [Subdoligranulum sp. 4_3_54A2FAA]
 EHO15637.1 tetracycline resistance protein tetW [Stomatobaculum longum]
 EHO32061.1 tetracycline resistance protein tetW [Lachnospiraceae bacterium
7_1_58FAA]
 EJF14102.1 tetracycline resistance protein TetM [Oribacterium parvum ACB8]
 EJG15429.1 tetracycline resistance protein TetM [Actinomyces sp. ICM47]
 EKU79042.1 tetracycline resistance protein tetW [Veillonella seminalis ACS-216-V-Col6b]
 ENY94381.1 tetracycline resistance protein TetM [[[Clostridium] clostridioforme
CM201]
 ENZ04096.1 tetracycline resistance protein TetM [[[Clostridium] clostridioforme
90B1]
 ENZ19503.1 tetracycline resistance protein TetM [[[Clostridium] clostridioforme
90A3]
 ENZ22372.1 tetracycline resistance protein TetM [[[Clostridium] clostridioforme
90A1]
 ENZ24342.1 tetracycline resistance protein TetM [[[Clostridium] clostridioforme
90A1]
 ENZ63897.1 tetracycline resistance protein TetM [[[Clostridium] clostridioforme
90A6]
 EOQ38030.1 tetracycline resistance protein tetW [Butyricicoccus pullicaecorum
1.2]
 ERI71263.1 putative translation elongation factor G [Clostridium

sp. KLE
1755]
 ERK58323.1 putative translation elongation factor G [Oscillibacter
sp. KLE
1728]
 ERK62770.1 putative translation elongation factor G [Oscillibacter
sp. KLE
1745]
 AHH55323.1 tetracycline resistance protein [Streptococcus suis]
 AHN92780.1 hypothetical protein BIFPSEUDO_03502 [uncultured
bacterium DCM001Tet01]
 AHN92785.1 elongation factor Tu GTP binding domain-containing
protein [uncultured
bacterium DCM004Tet02]
 KMW11759.1 tetracycline resistance protein tetW [[Clostridium]
clostridioforme
WAL-7855]
 KOA62526.1 tetracycline resistance protein tetM [Bifidobacterium
breve MCC
1604]
 CUQ85797.1 Tetracycline resistance protein tetM from transposon
Tn916 [[Eubacterium]
siraeum]
 CUQ28597.1 Tetracycline resistance protein tetM from transposon
Tn916 [Fusicatenibacter
saccharivorans]
 CUO95857.1 Tetracycline resistance protein tetM from transposon
Tn916 [Fusicatenibacter
saccharivorans]
 CUN09955.1 Tetracycline resistance protein tetM from transposon
Tn916 [Roseburia
inulinivorans]
 CUQ44819.1 Tetracycline resistance protein tetM from transposon
Tn916 [Flavonifractor
plautii]
 CUO03890.1 small GTP-binding protein domain [[Clostridium]
symbiosum]
 CUQ59147.1 small GTP-binding protein domain [Flavonifractor
plautii]
 ALP95157.1 Tetracycline resistance protein TetW [Intestinimonas
butyriciproducens]
 KSV60166.1 tetracycline resistance protein tetM [Acetivibrio
ethanolgignens]
 KXA69444.1 putative translation elongation factor G [Megasphaera
sp. MJR8396C]
 CYW11284.1 TetW [Streptococcus suis]
 KXK66666.1 putative translation elongation factor G
[Christensenella minuta]
 AMP46940.1 TetM-TetW-TetO-TetS [uncultured bacterium]
 AMP47001.1 TetM-TetW-TetO-TetS [uncultured bacterium]
 AMP49318.1 TetM-TetW-TetO-TetS [uncultured bacterium]
 AMP49706.1 TetM-TetW-TetO-TetS [uncultured bacterium]
 AMP50702.1 TetM-TetW-TetO-TetS [uncultured bacterium]
 AMP51311.1 TetM-TetW-TetO-TetS [uncultured bacterium]

AMP51381.1 TetM-TetW-TetO-TetS [uncultured bacterium]
AMP52917.1 TetM-TetW-TetO-TetS [uncultured bacterium]
AMP53033.1 TetM-TetW-TetO-TetS [uncultured bacterium]
AMP53886.1 TetM-TetW-TetO-TetS [uncultured bacterium]
AMP55128.1 TetM-TetW-TetO-TetS [uncultured bacterium]
AMP55335.1 TetM-TetW-TetO-TetS [uncultured bacterium]
CVH75252.1 Tetracycline resistance protein TetO [Clostridiales bacterium
CHKCI006]
OAQ36904.1 tetracycline resistance ribosomal protection protein [Christensenella
minuta]
SCI00736.1 Tetracycline resistance protein tetM from transposon Tn916 [uncultured
Megasphaera sp.]
SCI45558.1 Tetracycline resistance protein tetM from transposon Tn916 [uncultured
Flavonifractor sp.]
SCI17219.1 Tetracycline resistance protein tetM from transposon Tn916 [uncultured
Oscillibacter sp.]
SCJ58395.1 Tetracycline resistance protein tetM from transposon Tn916 [uncultured
Butyricicoccus sp.]
SCJ30909.1 Tetracycline resistance protein tetM from transposon Tn916 [uncultured
Oscillibacter sp.]
SCI80665.1 Tetracycline resistance protein tetM from transposon Tn916 [uncultured
Flavonifractor sp.]
SCJ65430.1 Tetracycline resistance protein tetM from transposon Tn916 [uncultured
Clostridium sp.]
SCG93933.1 Tetracycline resistance protein tetM from transposon Tn916 [uncultured
Clostridium sp.]
SCI58288.1 Tetracycline resistance protein tetM from transposon Tn916 [uncultured
Flavonifractor sp.]
SCJ93079.1 Tetracycline resistance protein tetM from transposon Tn916 [uncultured
Eubacterium sp.]
SCI94326.1 Tetracycline resistance protein tetM from transposon Tn916 [uncultured
Clostridium sp.]
SCJ98308.1 Tetracycline resistance protein tetM from transposon Tn916 [uncultured
Clostridium sp.]
SCJ58203.1 Tetracycline resistance protein tetM from transposon Tn916 [uncultured
Ruminococcus sp.]
ODR34013.1 tetracycline resistance ribosomal protection protein Tet(W) [Eisenbergiella
tayi]

OFK71604.1 tetracycline resistance ribosomal protection protein [Rothia
sp. HMSC065G12]
SCY22593.1 ribosomal protection tetracycline resistance protein [Blautia
sp. SF-50]
SDF66147.1 ribosomal protection tetracycline resistance protein [Eubacterium
pyruvativorans]
SDK54921.1 ribosomal protection tetracycline resistance protein [Bifidobacterium
breve]
SEU21916.1 ribosomal protection tetracycline resistance protein [[Clostridium]
lavalense]
SFH77745.1 ribosomal protection tetracycline resistance protein [Megasphaera
elsdenii]
SFR66562.1 ribosomal protection tetracycline resistance protein [Pseudobutyrivibrio
sp. NOR37]
SFR80718.1 ribosomal protection tetracycline resistance protein [[Clostridium]
aminophilum]
SFS18891.1 ribosomal protection tetracycline resistance protein [[Clostridium]
citroniae]
OIN61705.1 tetracycline resistance ribosomal protection protein Tet(W) [Bifidobacterium
longum subsp. suis]
OIN62141.1 tetracycline resistance ribosomal protection protein Tet(W) [Bifidobacterium
longum subsp. infantis]
SHN53919.1 ribosomal protection tetracycline resistance protein [Butyrivibrio
hungatei DSM 14810]
SHK36119.1 ribosomal protection tetracycline resistance protein [[Clostridium]
lactatifermentans DSM 14214]
SHE91933.1 ribosomal protection tetracycline resistance protein [Lactonifactor
longoviformis DSM 17459]
APO27584.1 ferrous iron transport protein B [uncultured bacterium]
APO32222.1 ferrous iron transport protein B [uncultured bacterium]
APO32229.1 ferrous iron transport protein B [uncultured bacterium]
SKA55164.1 ribosomal protection tetracycline resistance protein [Butyricicoccus
pullicaecorum DSM 23266]
OUC50682.1 tetracycline resistance ribosomal protection protein Tet(W) [Eggerthia
catenaformis]
OUM19307.1 tetracycline resistance ribosomal protection protein Tet(W) [Butyricicoccus
porcorum]

OUN09345.1 tetracycline resistance ribosomal protection protein Tet(W) [Flavonifractor
sp. An91]
OUN15889.1 tetracycline resistance ribosomal protection protein Tet(W) [Gemmiger
sp. An87]
OUN33894.1 tetracycline resistance ribosomal protection protein Tet(W) [Lachnoclostridium
sp. An76]
OUN96285.1 tetracycline resistance ribosomal protection protein Tet(W) [Pseudoflavonifractor
sp. An44]
OUO12324.1 tetracycline resistance ribosomal protection protein Tet(W) [Flavonifractor
sp. An4]
OUO32606.1 tetracycline resistance ribosomal protection protein Tet(W) [Flavonifractor
sp. An306]
OUO73126.1 tetracycline resistance ribosomal protection protein Tet(W) [Anaeromassilibacillus
sp. An250]
OUP27183.1 tetracycline resistance ribosomal protection protein Tet(W) [Faecalibacterium
sp. An192]
OUP56995.1 tetracycline resistance ribosomal protection protein Tet(W) [Butyricicoccus
pullicaecorum]
OUP87131.1 tetracycline resistance ribosomal protection protein Tet(W) [[Clostridium]
saccharolyticum]
OUQ14510.1 tetracycline resistance ribosomal protection protein Tet(W) [Lachnoclostridium
sp. An14]
OUQ44861.1 tetracycline resistance ribosomal protection protein Tet(W) [Drancourtella
sp. An12]
OUQ60136.1 tetracycline resistance ribosomal protection protein Tet(W) [Flavonifractor
sp. An112]
OUQ67004.1 tetracycline resistance ribosomal protection protein Tet(W) [Eubacterium
sp. An11]
ATO39299.1 tetracycline resistance ribosomal protection protein Tet(W) [Bifidobacterium
pseudolongum subsp. globosum DSM 20092]
PHU37324.1 tetracycline resistance ribosomal protection protein Tet(W) [Agathobacter
ruminis]
PKD29120.1 Tetracycline resistance protein TetO [Ruminococcus
bromii]
PNC55690.1 tetracycline resistance ribosomal protection protein Tet(W) [Akkermansia
muciniphila]
PNC69561.1 tetracycline resistance ribosomal protection protein

Tet(W) [Akkermansia
muciniphila]
 PNC80700.1 tetracycline resistance ribosomal protection protein
Tet(W) [Akkermansia
muciniphila]
 POO04379.1 Tetracycline resistance protein TetW [Bifidobacterium
breve]
 POP32879.1 tetracycline resistance ribosomal protection protein
Tet(W) [Lactonifactor
longoviformis]
Length=639

 Score = 1295 bits (3351), Expect = 0.0, Method: Compositional
matrix adjust.
 Identities = 631/639 (99%), Positives = 634/639 (99%), Gaps = 0/639
(0%)

Query 1
MKIINIGILAHVDAGKTTLTESLLYASGAISEPGSVEKGTTRTDTMFLERQRGITIQAAV 60

MKIINIGILAHVDAGKTTLTESLLYASGAISEPGSVEKGTTRTDTMFLERQRGITIQAAV
Sbjct 1
MKIINIGILAHVDAGKTTLTESLLYASGAISEPGSVEKGTTRTDTMFLERQRGITIQAAV 60

Query 61
TSFQWHRCKVNIVDTPGHMDFLAEVYRSLAVLDGAILVISAKDGVQAQTRILFHALRKMN 120

TSFQWHRCKVNIVDTPGHMDFLAEVYRSLAVLDGAILVISAKDGVQAQTRILFHALRKMN
Sbjct 61
TSFQWHRCKVNIVDTPGHMDFLAEVYRSLAVLDGAILVISAKDGVQAQTRILFHALRKMN 120

Query 121
IPTVIFINKIDQAGVDLQSVVQSVRDKLSADIIIKQTVSLSPEIVLEENTDIEAWDAVIE 180

IPTVIFINKIDQAGVDLQSVVQSVRDKLSADIIIKQTVSLSPEIVLEENTDIEAWDAVIE
Sbjct 121
IPTVIFINKIDQAGVDLQSVVQSVRDKLSADIIIKQTVSLSPEIVLEENTDIEAWDAVIE 180

Query 181
NNDKLLEKYIAGEPISREKLVREEQRRVQDASLFPVYYGSAKKGLGIQPLMDAVTGLFQP 240
            NND+LLEKYIAGEPISREKL REEQ+RVQDASLFPVY+GSAK
GLGIQPLMDAVTGLFQP
Sbjct 181
NNDELLEKYIAGEPISREKLAREEQQRVQDASLFPVYHGSAKNGLGIQPLMDAVTGLFQP 240

Query 241
IGEQGSAALCGSVFKVEYTDCGQRRVYLRLYSGTLRLRDTVALAGREKLKITEMRIPSKG 300
            IGEQG
AALCGSVFKVEYTDCGQRRVYLRLYSGTLRLRDTVALAGREKLKITEMRIPSKG
Sbjct 241
IGEQGGAALCGSVFKVEYTDCGQRRVYLRLYSGTLRLRDTVALAGREKLKITEMRIPSKG 300

Query 301
EIVRTDTAYPGEIVILPSDSVRLNDVLGDPTRLPRKRWREDPLPMLRTTIAPKTAAQRER 360

            EIVRTDTAY GEIVILPSDSVRLNDVLGD
TRLPRKRWREDPLPMLRTTIAPKTAAQRER
Sbjct 301
EIVRTDTAYQGEIVILPSDSVRLNDVLGDQTRLPRKRWREDPLPMLRTTIAPKTAAQRER 360

Query 361
LLDALTQLADTDPLLRCEVDSITHEIILSFLGRVQLEVVSALLSEKYKLETVVKEPSVIY 420

LLDALTQLADTDPLLRCEVDSITHEIILSFLGRVQLEVVSALLSEKYKLETVVKEPSVIY
Sbjct 361
LLDALTQLADTDPLLRCEVDSITHEIILSFLGRVQLEVVSALLSEKYKLETVVKEPSVIY 420

Query 421
MERPLKAASHTIHIEVPPNPFWASIGLSVTPLSLGSGVQYESRVSLGYLNQSFQNAVRDG 480

MERPLKAASHTIHIEVPPNPFWASIGLSVTPLSLGSGVQYESRVSLGYLNQSFQNAVRDG
Sbjct 421
MERPLKAASHTIHIEVPPNPFWASIGLSVTPLSLGSGVQYESRVSLGYLNQSFQNAVRDG 480

Query 481
IRYGLEQGLFGWNVTDCKICFEYGLYYSPVSTPADFRSLAPIVLEQALKESGTQLLEPYL 540

IRYGLEQGLFGWNVTDCKICFEYGLYYSPVSTPADFRSLAPIVLEQALKESGTQLLEPYL
Sbjct 481
IRYGLEQGLFGWNVTDCKICFEYGLYYSPVSTPADFRSLAPIVLEQALKESGTQLLEPYL 540

Query 541
SFILYAPQEYLSRAYHDAPKYCATIETAQVKKDEVVFTGEIPARCIQAYRTDLAFYTNGR 600

SFILYAPQEYLSRAYHDAPKYCATIETAQVKKDEVVFTGEIPARCIQAYRTDLAFYTNGR
Sbjct 541
SFILYAPQEYLSRAYHDAPKYCATIETAQVKKDEVVFTGEIPARCIQAYRTDLAFYTNGR 600

Query 601 SVCLTELKGYQAAVGQPVIQPRRPNSRLDKVRHMFQKVM 639
            SVCLTELKGYQAAVGQPVIQPRRPNSRLDKVRHMFQKVM
Sbjct 601 SVCLTELKGYQAAVGQPVIQPRRPNSRLDKVRHMFQKVM 639

>WP_039689012.1 TetM/TetW/TetO/TetS family tetracycline resistance ribosomal
protection protein [Coriobacteriaceae bacterium 68-1-3]
 AJC12014.1 tetracycline resistance protein tetM [Coriobacteriaceae bacterium
68-1-3]
Length=639

 Score = 1295 bits (3351), Expect = 0.0, Method: Compositional matrix adjust.
 Identities = 629/639 (98%), Positives = 635/639 (99%), Gaps = 0/639 (0%)

Query 1
MKIINIGILAHVDAGKTTLTESLLYASGAISEPGSVEKGTTRTDTMFLERQRGITIQAAV 60

MKIINIGILAHVDAGKTTLTESLLYASGAISEPGSVEKGTTRTDTMFLERQRGITIQAAV
Sbjct 1
MKIINIGILAHVDAGKTTLTESLLYASGAISEPGSVEKGTTRTDTMFLERQRGITIQAAV 60

Query 61
TSFQWHRCKVNIVDTPGHMDFLAEVYRSLAVLDGAILVISAKDGVQAQTRILFHALRKMN 120

TSFQWHRCKVNIVDTPGHMDFLAEVYRSLAVLDGAILVISAKDGVQAQTRILFHALRKMN
Sbjct 61
TSFQWHRCKVNIVDTPGHMDFLAEVYRSLAVLDGAILVISAKDGVQAQTRILFHALRKMN 120

Query 121
IPTVIFINKIDQAGVDLQSVVQSVRDKLSADIIIKQTVSLSPEIVLEENTDIEAWDAVIE 180

IPTVIFINKIDQAGVDLQSVVQSVRDKLSADIIIKQTVSLSPEIVLEENTDIEAWDAVIE
Sbjct 121
IPTVIFINKIDQAGVDLQSVVQSVRDKLSADIIIKQTVSLSPEIVLEENTDIEAWDAVIE 180

Query 181
NNDKLLEKYIAGEPISREKLVREEQRRVQDASLFPVYYGSAKKGLGIQPLMDAVTGLFQP 240
NND+LLEKYIAGEPISREKL REEQ+RVQDASLFPVY+GSAK
GLGIQPLMDAVTGLFQP
Sbjct 181
NNDELLEKYIAGEPISREKLAREEQQRVQDASLFPVYHGSAKNGLGIQPLMDAVTGLFQP 240

Query 241
IGEQGSAALCGSVFKVEYTDCGQRRVYLRLYSGTLRLRDTVALAGREKLKITEMRIPSKG 300

IGEQGSAALCGSVFKVEYTDCGQRRVYLRLYSGTLRLRDTVALAGREKLKITEMRIPSKG
Sbjct 241
IGEQGSAALCGSVFKVEYTDCGQRRVYLRLYSGTLRLRDTVALAGREKLKITEMRIPSKG 300

Query 301
EIVRTDTAYPGEIVILPSDSVRLNDVLGDPTRLPRKRWREDPLPMLRTTIAPKTAAQRER 360
EIVRTDTAYPGEIVILPSDSVRLNDVLGDPTRLPRKRWREDPLPMLRT
+IAPKTAAQRER
Sbjct 301
EIVRTDTAYPGEIVILPSDSVRLNDVLGDPTRLPRKRWREDPLPMLRTSIAPKTAAQRER 360

Query 361
LLDALTQLADTDPLLRCEVDSITHEIILSFLGRVQLEVVSALLSEKYKLETVVKEPSVIY 420
LLDALTQLADTDPLLRCEVDSITHEIILSFLGRVQLEVVSALLSEKYKLETVVKEP
+VIY
Sbjct 361
LLDALTQLADTDPLLRCEVDSITHEIILSFLGRVQLEVVSALLSEKYKLETVVKEPTVIY 420

Query 421
MERPLKAASHTIHIEVPPNPFWASIGLSVTPLSLGSGVQYESRVSLGYLNQSFQNAVRDG 480
MERPLKAASHTIHIEVPPNPFWASIGLSVTPL
LGSGVQYESRVSLGYLNQSFQNAVRDG
Sbjct 421
MERPLKAASHTIHIEVPPNPFWASIGLSVTPLPLGSGVQYESRVSLGYLNQSFQNAVRDG 480

Query 481

```
IRYGLEQGLFGWNVTDCKICFEYGLYYSPVSTPADFRSLAPIVLEQALKESGTQLLEPYL  540

IRYGLEQGLFGWNVTDCKICFEYGLYYSPVSTPADFRSLAPIVLEQALKESGTQLLEPYL
Sbjct  481
IRYGLEQGLFGWNVTDCKICFEYGLYYSPVSTPADFRSLAPIVLEQALKESGTQLLEPYL  540

Query  541
SFILYAPQEYLSRAYHDAPKYCATIETAQVKKDEVVFTGEIPARCIQAYRTDLAFYTNGR  600
           SF LYAP
+EYLSRAYHDAPKYCATIETAQVKKDEVVFTGEIPARCIQAYRTDLAFYTNGR
Sbjct  541
SFTLYAPREYLSRAYHDAPKYCATIETAQVKKDEVVFTGEIPARCIQAYRTDLAFYTNGR  600

Query  601  SVCLTELKGYQAAVGQPVIQPRRPNSRLDKVRHMFQKVM  639
            SVCLTELKGYQAAVGQPVIQPRRPNSRLDKVRHMFQKVM
Sbjct  601  SVCLTELKGYQAAVGQPVIQPRRPNSRLDKVRHMFQKVM  639


>CCA30603.1 tetracycline resistance protein [Clostridioides
difficile]
Length=639

 Score = 1295 bits (3351),  Expect = 0.0, Method: Compositional
matrix adjust.
 Identities = 629/639 (98%), Positives = 635/639 (99%), Gaps = 0/639
(0%)

Query  1
MKIINIGILAHVDAGKTTLTESLLYASGAISEPGSVEKGTTRTDTMFLERQRGITIQAAV  60

MKIINIGILAHVDAGKTTLTESLLYASGAISEPGSVEKGTTRTDTMFLERQRGITIQAAV
Sbjct  1
MKIINIGILAHVDAGKTTLTESLLYASGAISEPGSVEKGTTRTDTMFLERQRGITIQAAV  60

Query  61
TSFQWHRCKVNIVDTPGHMDFLAEVYRSLAVLDGAILVISAKDGVQAQTRILFHALRKMN  120

TSFQWHRCKVNIVDTPGHMDFLAEVYRSLAVLDGAILVISAKDGVQAQTRILFHALRKMN
Sbjct  61
TSFQWHRCKVNIVDTPGHMDFLAEVYRSLAVLDGAILVISAKDGVQAQTRILFHALRKMN  120

Query  121
IPTVIFINKIDQAGVDLQSVVQSVRDKLSADIIIKQTVSLSPEIVLEENTDIEAWDAVIE  180

IPTVIFINKIDQAGVDLQSVVQSVRDKLSADIIIKQTVSLSPEIVLEENTDIEAWDAVIE
Sbjct  121
IPTVIFINKIDQAGVDLQSVVQSVRDKLSADIIIKQTVSLSPEIVLEENTDIEAWDAVIE  180

Query  181
NNDKLLEKYIAGEPISREKLVREEQRRVQDASLFPVYYGSAKKGLGIQPLMDAVTGLFQP  240
           NND+LLEKYIAGEPISREKL
REEQRRVQDASLFPVYYGSAKKGLGIQPLMDAVTGLFQP
Sbjct  181
NNDELLEKYIAGEPISREKLAREEQRRVQDASLFPVYYGSAKKGLGIQPLMDAVTGLFQP  240
```

```
Query  241
IGEQGSAALCGSVFKVEYTDCGQRRVYLRLYSGTLRLRDTVALAGREKLKITEMRIPSKG  300

IGEQGSAALCGSVFKVEYTDCGQRRVYLRLYSGTLRLRDTVALAGREKLKITEMRIPSKG
Sbjct  241
IGEQGSAALCGSVFKVEYTDCGQRRVYLRLYSGTLRLRDTVALAGREKLKITEMRIPSKG  300

Query  301
EIVRTDTAYPGEIVILPSDSVRLNDVLGDPTRLPRKRWREDPLPMLRTTIAPKTAAQRER  360
            EIVRTDTAYPGEIVILPSDSVRLNDVLGDPTRLPRKRWREDPLPMLRT
+IAPKTAAQRER
Sbjct  301
EIVRTDTAYPGEIVILPSDSVRLNDVLGDPTRLPRKRWREDPLPMLRTSIAPKTAAQRER  360

Query  361
LLDALTQLADTDPLLRCEVDSITHEIILSFLGRVQLEVVSALLSEKYKLETVVKEPSVIY  420
            LLDALTQLADTDPLLRCEVDSITHEIILSFLGRVQLEVVSALLSEKYKLETVVKEP
+VIY
Sbjct  361
LLDALTQLADTDPLLRCEVDSITHEIILSFLGRVQLEVVSALLSEKYKLETVVKEPTVIY  420

Query  421
MERPLKAASHTIHIEVPPNPFWASIGLSVTPLSLGSGVQYESRVSLGYLNQSFQNAVRDG  480
            MERPLKAASHTIHIEVPPNPFWASIGLSVTPL
LGSGVQYESRVSLGYLNQSFQNAVRDG
Sbjct  421
MERPLKAASHTIHIEVPPNPFWASIGLSVTPLPLGSGVQYESRVSLGYLNQSFQNAVRDG  480

Query  481
IRYGLEQGLFGWNVTDCKICFEYGLYYSPVSTPADFRSLAPIVLEQALKESGTQLLEPYL  540

IRYGLEQGLFGWNVTDCKICFEYGLYYSPVSTPADFRSLAPIVLEQALKESGTQLLEPYL
Sbjct  481
IRYGLEQGLFGWNVTDCKICFEYGLYYSPVSTPADFRSLAPIVLEQALKESGTQLLEPYL  540

Query  541
SFILYAPQEYLSRAYHDAPKYCATIETAQVKKDEVVFTGEIPARCIQAYRTDLAFYTNGR  600
            SF LYAP+EYLSRAYHDAPKYCATIET
QVKKDEVVFTGEIPARCIQAYRTDLAFYTNG+
Sbjct  541
SFTLYAPREYLSRAYHDAPKYCATIETVQVKKDEVVFTGEIPARCIQAYRTDLAFYTNGQ  600

Query  601  SVCLTELKGYQAAVGQPVIQPRRPNSRLDKVRHMFQKVM  639
            SVCLTELKGYQAAVG+PVIQPRRPNSRLDKVRHMFQKVM
Sbjct  601  SVCLTELKGYQAAVGKPVIQPRRPNSRLDKVRHMFQKVM  639
```

>WP_087988639.1 TetM/TetW/TetO/TetS family tetracycline resistance ribosomal
protection protein [Anaerotignum lactatifermentans]
 OUN45235.1 tetracycline resistance ribosomal protection protein Tet(W) [[Clostridium]
lactatifermentans]

Length=639

 Score = 1295 bits (3350), Expect = 0.0, Method: Compositional matrix adjust.
 Identities = 630/639 (99%), Positives = 634/639 (99%), Gaps = 0/639 (0%)

```
Query  1
MKIINIGILAHVDAGKTTLTESLLYASGAISEPGSVEKGTTRTDTMFLERQRGITIQAAV  60

MKIINIGILAHVDAGKTTLTESLLYASGAISEPGSVEKGTTRTDTMFLERQRGITIQAAV
Sbjct  1
MKIINIGILAHVDAGKTTLTESLLYASGAISEPGSVEKGTTRTDTMFLERQRGITIQAAV  60

Query  61
TSFQWHRCKVNIVDTPGHMDFLAEVYRSLAVLDGAILVISAKDGVQAQTRILFHALRKMN  120

TSFQWHRCKVNIVDTPGHMDFLAEVYRSLAVLDGAILVISAKDGVQAQTRILFHALRKMN
Sbjct  61
TSFQWHRCKVNIVDTPGHMDFLAEVYRSLAVLDGAILVISAKDGVQAQTRILFHALRKMN  120

Query  121
IPTVIFINKIDQAGVDLQSVVQSVRDKLSADIIIKQTVSLSPEIVLEENTDIEAWDAVIE  180

IPTVIFINKIDQAGVDLQSVVQSVRDKLSADIIIKQTVSLSPEIVLEENTDIEAWDAVIE
Sbjct  121
IPTVIFINKIDQAGVDLQSVVQSVRDKLSADIIIKQTVSLSPEIVLEENTDIEAWDAVIE  180

Query  181
NNDKLLEKYIAGEPISREKLVREEQRRVQDASLFPVYYGSAKKGLGIQPLMDAVTGLFQP  240
            NND+LLEKYIAGEPISREKL REEQ+RVQDASLFPVY+GSAK
GLGIQPLMDAVTGLFQP
Sbjct  181
NNDELLEKYIAGEPISREKLAREEQQRVQDASLFPVYHGSAKNGLGIQPLMDAVTGLFQP  240

Query  241
IGEQGSAALCGSVFKVEYTDCGQRRVYLRLYSGTLRLRDTVALAGREKLKITEMRIPSKG  300
            IGEQG
+ALCGSVFKVEYTDCGQRRVYLRLYSGTLRLRDTVALAGREKLKITEMRIPSKG
Sbjct  241
IGEQGGSALCGSVFKVEYTDCGQRRVYLRLYSGTLRLRDTVALAGREKLKITEMRIPSKG  300

Query  301
EIVRTDTAYPGEIVILPSDSVRLNDVLGDPTRLPRKRWREDPLPMLRTTIAPKTAAQRER  360
            EIVRTDTAY GEIVILPSDSVRLNDVLGD
TRLPRKRWREDPLPMLRTTIAPKTAAQRER
Sbjct  301
EIVRTDTAYQGEIVILPSDSVRLNDVLGDQTRLPRKRWREDPLPMLRTTIAPKTAAQRER  360

Query  361
LLDALTQLADTDPLLRCEVDSITHEIILSFLGRVQLEVVSALLSEKYKLETVVKEPSVIY  420

LLDALTQLADTDPLLRCEVDSITHEIILSFLGRVQLEVVSALLSEKYKLETVVKEPSVIY
Sbjct  361
```

```
LLDALTQLADTDPLLRCEVDSITHEIILSFLGRVQLEVVSALLSEKYKLETVVKEPSVIY  420

Query  421
MERPLKAASHTIHIEVPPNPFWASIGLSVTPLSLGSGVQYESRVSLGYLNQSFQNAVRDG  480

MERPLKAASHTIHIEVPPNPFWASIGLSVTPLSLGSGVQYESRVSLGYLNQSFQNAVRDG
Sbjct  421
MERPLKAASHTIHIEVPPNPFWASIGLSVTPLSLGSGVQYESRVSLGYLNQSFQNAVRDG  480

Query  481
IRYGLEQGLFGWNVTDCKICFEYGLYYSPVSTPADFRSLAPIVLEQALKESGTQLLEPYL  540

IRYGLEQGLFGWNVTDCKICFEYGLYYSPVSTPADFRSLAPIVLEQALKESGTQLLEPYL
Sbjct  481
IRYGLEQGLFGWNVTDCKICFEYGLYYSPVSTPADFRSLAPIVLEQALKESGTQLLEPYL  540

Query  541
SFILYAPQEYLSRAYHDAPKYCATIETAQVKKDEVVFTGEIPARCIQAYRTDLAFYTNGR  600

SFILYAPQEYLSRAYHDAPKYCATIETAQVKKDEVVFTGEIPARCIQAYRTDLAFYTNGR
Sbjct  541
SFILYAPQEYLSRAYHDAPKYCATIETAQVKKDEVVFTGEIPARCIQAYRTDLAFYTNGR  600

Query  601  SVCLTELKGYQAAVGQPVIQPRRPNSRLDKVRHMFQKVM  639
            SVCLTELKGYQAAVGQPVIQPRRPNSRLDKVRHMFQKVM
Sbjct  601  SVCLTELKGYQAAVGQPVIQPRRPNSRLDKVRHMFQKVM  639


>WP_075576245.1 TetM/TetW/TetO/TetS family tetracycline resistance
ribosomal
protection protein [Olsenella sp. Marseille-P2300]
Length=639

 Score = 1295 bits (3350),  Expect = 0.0, Method: Compositional
matrix adjust.
 Identities = 628/639 (98%), Positives = 635/639 (99%), Gaps = 0/639
(0%)

Query  1
MKIINIGILAHVDAGKTTLTESLLYASGAISEPGSVEKGTTRTDTMFLERQRGITIQAAV  60

MKIINIGILAHVDAGKTTLTESLLYASGAISEPGSVEKGTTRTDTMFLERQRGITIQAAV
Sbjct  1
MKIINIGILAHVDAGKTTLTESLLYASGAISEPGSVEKGTTRTDTMFLERQRGITIQAAV  60

Query  61
TSFQWHRCKVNIVDTPGHMDFLAEVYRSLAVLDGAILVISAKDGVQAQTRILFHALRKMN  120

TSFQWHRCKVNIVDTPGHMDFLAEVYRSLAVLDGAILVISAKDGVQAQTRILFHALRKMN
Sbjct  61
TSFQWHRCKVNIVDTPGHMDFLAEVYRSLAVLDGAILVISAKDGVQAQTRILFHALRKMN  120

Query  121
IPTVIFINKIDQAGVDLQSVVQSVRDKLSADIIIKQTVSLSPEIVLEENTDIEAWDAVIE  180
```

IPTVIFINKIDQAGVDLQSVVQSVRDKLSADIIIKQTVSLSPEIVLEENTDIEAWDAVIE
Sbjct 121
IPTVIFINKIDQAGVDLQSVVQSVRDKLSADIIIKQTVSLSPEIVLEENTDIEAWDAVIE 180

Query 181
NNDKLLEKYIAGEPISREKLVREEQRRVQDASLFPVYYGSAKKGLGIQPLMDAVTGLFQP 240
NNDK
LEKYIAGEPISREKLVREEQRRVQDASLFPVYYGSAKKGLGIQPLMDAVTGLFQP
Sbjct 181
NNDKFLEKYIAGEPISREKLVREEQRRVQDASLFPVYYGSAKKGLGIQPLMDAVTGLFQP 240

Query 241
IGEQGSAALCGSVFKVEYTDCGQRRVYLRLYSGTLRLRDTVALAGREKLKITEMRIPSKG 300

IGEQGSAALCGSVFKVEYTDCGQRRVYLRLYSGTLRLRDTVALAGREKLKITEMRIPSKG
Sbjct 241
IGEQGSAALCGSVFKVEYTDCGQRRVYLRLYSGTLRLRDTVALAGREKLKITEMRIPSKG 300

Query 301
EIVRTDTAYPGEIVILPSDSVRLNDVLGDPTRLPRKRWREDPLPMLRTTIAPKTAAQRER 360
EIVRTDTAYPGEIVILPSDSVRLNDVLGDPTRLPRKRWREDPLPMLRT
+IAPKTAAQRER
Sbjct 301
EIVRTDTAYPGEIVILPSDSVRLNDVLGDPTRLPRKRWREDPLPMLRTSIAPKTAAQRER 360

Query 361
LLDALTQLADTDPLLRCEVDSITHEIILSFLGRVQLEVVSALLSEKYKLETVVKEPSVIY 420
LLDALTQLADTDPLLRCEVDSITHEIILSFLGRVQLEVVSALLSEKYKLETVVKEP
+VIY
Sbjct 361
LLDALTQLADTDPLLRCEVDSITHEIILSFLGRVQLEVVSALLSEKYKLETVVKEPTVIY 420

Query 421
MERPLKAASHTIHIEVPPNPFWASIGLSVTPLSLGSGVQYESRVSLGYLNQSFQNAVRDG 480
MERPLKAASHTIHIEVPPNPFWASIGLSVTPL
LGSGVQYESRVSLGYLNQSFQNAVRDG
Sbjct 421
MERPLKAASHTIHIEVPPNPFWASIGLSVTPLPLGSGVQYESRVSLGYLNQSFQNAVRDG 480

Query 481
IRYGLEQGLFGWNVTDCKICFEYGLYYSPVSTPADFRSLAPIVLEQALKESGTQLLEPYL 540

IRYGLEQGLFGWNVTDCKICFEYGLYYSPVSTPADFRSLAPIVLEQALKESGTQLLEPYL
Sbjct 481
IRYGLEQGLFGWNVTDCKICFEYGLYYSPVSTPADFRSLAPIVLEQALKESGTQLLEPYL 540

Query 541
SFILYAPQEYLSRAYHDAPKYCATIETAQVKKDEVVFTGEIPARCIQAYRTDLAFYTNGR 600
SF LYAP+EYLSRAYHDAPKYCATIET
QVKKDEVVFTGEIPARCIQAYRTDLAFYTNG+
Sbjct 541
SFTLYAPREYLSRAYHDAPKYCATIETVQVKKDEVVFTGEIPARCIQAYRTDLAFYTNGQ 600

```
Query  601  SVCLTELKGYQAAVGQPVIQPRRPNSRLDKVRHMFQKVM  639
            SVCLTELKGYQAAVG+PVIQPRRPNSRLDKVR+MFQK+M
Sbjct  601  SVCLTELKGYQAAVGKPVIQPRRPNSRLDKVRYMFQKIM  639
```

>SCI66756.1 Tetracycline resistance protein tetM from transposon Tn916 [uncultured Clostridium sp.]
Length=639

Score = 1295 bits (3350), Expect = 0.0, Method: Compositional matrix adjust.
Identities = 628/639 (98%), Positives = 636/639 (99%), Gaps = 0/639 (0%)

```
Query  1
MKIINIGILAHVDAGKTTLTESLLYASGAISEPGSVEKGTTRTDTMFLERQRGITIQAAV  60

MKIINIGILAHVDAGKTTLTESLLYASGAISEPGSVEKGTTRTDTMFLERQRGITIQAAV
Sbjct  1
MKIINIGILAHVDAGKTTLTESLLYASGAISEPGSVEKGTTRTDTMFLERQRGITIQAAV  60

Query  61
TSFQWHRCKVNIVDTPGHMDFLAEVYRSLAVLDGAILVISAKDGVQAQTRILFHALRKMN  120

TSFQWHRCKVNIVDTPGHMDFLAEVYRSLAVLDGAILVISAKDGVQAQTRILFHALRKMN
Sbjct  61
TSFQWHRCKVNIVDTPGHMDFLAEVYRSLAVLDGAILVISAKDGVQAQTRILFHALRKMN  120

Query  121
IPTVIFINKIDQAGVDLQSVVQSVRDKLSADIIIKQTVSLSPEIVLEENTDIEAWDAVIE  180

IPTVIFINKIDQAGVDLQSVVQSVRDKLSADIIIKQTVSLSPEIVLEENTDIEAWDAVIE
Sbjct  121
IPTVIFINKIDQAGVDLQSVVQSVRDKLSADIIIKQTVSLSPEIVLEENTDIEAWDAVIE  180

Query  181
NNDKLLEKYIAGEPISREKLVREEQRRVQDASLFPVYYGSAKKGLGIQPLMDAVTGLFQP  240

NNDKLLEKYIAGEPISREKLVREEQRRVQDASLFPVYYGSAKKGLGIQPLMDAVTGLFQP
Sbjct  181
NNDKLLEKYIAGEPISREKLVREEQRRVQDASLFPVYYGSAKKGLGIQPLMDAVTGLFQP  240

Query  241
IGEQGSAALCGSVFKVEYTDCGQRRVYLRLYSGTLRLRDTVALAGREKLKITEMRIPSKG  300

IGEQGSAALCGSVFKVEYTDCGQRRVYLRLYSGTLRLRDTVALAGREKLKITEMRIPSKG
Sbjct  241
IGEQGSAALCGSVFKVEYTDCGQRRVYLRLYSGTLRLRDTVALAGREKLKITEMRIPSKG  300

Query  301
EIVRTDTAYPGEIVILPSDSVRLNDVLGDPTRLPRKRWREDPLPMLRTTIAPKTAAQRER  360
            EIVRTDTAYPGEIVILPSDSVRLNDVLGDPTRLPRKRWREDPLPMLRT
+IAPKTAAQRER
```

Sbjct 301
EIVRTDTAYPGEIVILPSDSVRLNDVLGDPTRLPRKRWREDPLPMLRTSIAPKTAAQRER 360

Query 361
LLDALTQLADTDPLLRCEVDSITHEIILSFLGRVQLEVVSALLSEKYKLETVVKEPSVIY 420
            LLDALTQLADTDPLLRCEVDSITHEIILSFLGRVQLEVVSALLSEKYKLETVVKEP
+VIY
Sbjct 361
LLDALTQLADTDPLLRCEVDSITHEIILSFLGRVQLEVVSALLSEKYKLETVVKEPTVIY 420

Query 421
MERPLKAASHTIHIEVPPNPFWASIGLSVTPLSLGSGVQYESRVSLGYLNQSFQNAVRDG 480
            MERPLKAASHTIHIEVPPNPFWASIGLSVTPL
LGSGVQYESRVSLGYLNQSFQNAVRDG
Sbjct 421
MERPLKAASHTIHIEVPPNPFWASIGLSVTPLPLGSGVQYESRVSLGYLNQSFQNAVRDG 480

Query 481
IRYGLEQGLFGWNVTDCKICFEYGLYYSPVSTPADFRSLAPIVLEQALKESGTQLLEPYL 540

IRYGLEQGLFGWNVTDCKICFEYGLYYSPVSTPADFRSLAPIVLEQALKESGTQLLEPYL
Sbjct 481
IRYGLEQGLFGWNVTDCKICFEYGLYYSPVSTPADFRSLAPIVLEQALKESGTQLLEPYL 540

Query 541
SFILYAPQEYLSRAYHDAPKYCATIETAQVKKDEVVFTGEIPARCIQAYRTDLAFYTNGR 600
            SF LYAP+EYLSRAYHDAPKYCATIET QVKKDEVVFTGE
+PARCIQAYRTDLAFYTNG+
Sbjct 541
SFTLYAPREYLSRAYHDAPKYCATIETVQVKKDEVVFTGEMPARCIQAYRTDLAFYTNGQ 600

Query 601 SVCLTELKGYQAAVGQPVIQPRRPNSRLDKVRHMFQKVM 639
            SVCLTELKGYQAAVG+PVIQPRRPNSRLDKVR+MFQK+M
Sbjct 601 SVCLTELKGYQAAVGKPVIQPRRPNSRLDKVRYMFQKIM 639

>SCH27787.1 Tetracycline resistance protein tetM from transposon
Tn916 [uncultured
Clostridium sp.]
 SCI77658.1 Tetracycline resistance protein tetM from transposon
Tn916 [uncultured
Clostridium sp.]
Length=639

 Score = 1295 bits (3350), Expect = 0.0, Method: Compositional
matrix adjust.
 Identities = 629/639 (98%), Positives = 635/639 (99%), Gaps = 0/639
(0%)

Query 1
MKIINIGILAHVDAGKTTLTESLLYASGAISEPGSVEKGTTRTDTMFLERQRGITIQAAV 60
            MKIINIGILAHVDAGKTTLTESLLY SGAI+E G+V+KGTTRTDTM LE
+QRGITIQAAV
Sbjct 1

MKIINIGILAHVDAGKTTLTESLLYTSGAIAEQGNVDKGTTRTDTMILEQQRGITIQAAV 60

Query 61
TSFQWHRCKVNIVDTPGHMDFLAEVYRSLAVLDGAILVISAKDGVQAQTRILFHALRKMN 120

TSFQWHRCKVNIVDTPGHMDFLAEVYRSLAVLDGAILVISAKDGVQAQTRILFHALRKMN
Sbjct 61
TSFQWHRCKVNIVDTPGHMDFLAEVYRSLAVLDGAILVISAKDGVQAQTRILFHALRKMN 120

Query 121
IPTVIFINKIDQAGVDLQSVVQSVRDKLSADIIIKQTVSLSPEIVLEENTDIEAWDAVIE 180

IPTVIFINKIDQAGVDLQSVVQSVRDKLSADIIIKQTVSLSPEIVLEENTDIEAWDAVIE
Sbjct 121
IPTVIFINKIDQAGVDLQSVVQSVRDKLSADIIIKQTVSLSPEIVLEENTDIEAWDAVIE 180

Query 181
NNDKLLEKYIAGEPISREKLVREEQRRVQDASLFPVYYGSAKKGLGIQPLMDAVTGLFQP 240

NNDKLLEKYIAGEPISREKLVREEQRRVQDASLFPVYYGSAKKGLGIQPLMDAVTGLFQP
Sbjct 181
NNDKLLEKYIAGEPISREKLVREEQRRVQDASLFPVYYGSAKKGLGIQPLMDAVTGLFQP 240

Query 241
IGEQGSAALCGSVFKVEYTDCGQRRVYLRLYSGTLRLRDTVALAGREKLKITEMRIPSKG 300

IGEQGSAALCGSVFKVEYTDCGQRRVYLRLYSGTLRLRDTVALAGREKLKITEMRIPSKG
Sbjct 241
IGEQGSAALCGSVFKVEYTDCGQRRVYLRLYSGTLRLRDTVALAGREKLKITEMRIPSKG 300

Query 301
EIVRTDTAYPGEIVILPSDSVRLNDVLGDPTRLPRKRWREDPLPMLRTTIAPKTAAQRER 360
EIVRTDTAYPGEIVILPSDSVRLNDVLGDPTRLPRKRWREDPLPMLRT
+IAPKTAAQRER
Sbjct 301
EIVRTDTAYPGEIVILPSDSVRLNDVLGDPTRLPRKRWREDPLPMLRTSIAPKTAAQRER 360

Query 361
LLDALTQLADTDPLLRCEVDSITHEIILSFLGRVQLEVVSALLSEKYKLETVVKEPSVIY 420
LLDALTQLADTDPLLRCEVDSITHEIILSFLGRVQLEVVSALLSEKYKLETVVKEP
+VIY
Sbjct 361
LLDALTQLADTDPLLRCEVDSITHEIILSFLGRVQLEVVSALLSEKYKLETVVKEPTVIY 420

Query 421
MERPLKAASHTIHIEVPPNPFWASIGLSVTPLSLGSGVQYESRVSLGYLNQSFQNAVRDG 480
MERPLKAASHTIHIEVPPNPFWASIGLSVTPL
LGSGVQYESRVSLGYLNQSFQNAVRDG
Sbjct 421
MERPLKAASHTIHIEVPPNPFWASIGLSVTPLPLGSGVQYESRVSLGYLNQSFQNAVRDG 480

Query 481
IRYGLEQGLFGWNVTDCKICFEYGLYYSPVSTPADFRSLAPIVLEQALKESGTQLLEPYL 540

IRYGLEQGLFGWNVTDCKICFEYGLYYSPVSTPADFRSLAPIVLEQALKESGTQLLEPYL
Sbjct 481
IRYGLEQGLFGWNVTDCKICFEYGLYYSPVSTPADFRSLAPIVLEQALKESGTQLLEPYL 540

Query 541
SFILYAPQEYLSRAYHDAPKYCATIETAQVKKDEVVFTGEIPARCIQAYRTDLAFYTNGR 600

SFILYAPQEYLSRAYHDAPKYCATIETAQVKKDEVVFTGEIPARCIQAYRTDLAFYTNGR
Sbjct 541
SFILYAPQEYLSRAYHDAPKYCATIETAQVKKDEVVFTGEIPARCIQAYRTDLAFYTNGR 600

Query 601 SVCLTELKGYQAAVGQPVIQPRRPNSRLDKVRHMFQKVM 639
            SVCLTELKGYQAAVGQPVIQPRRPNSRLDKVRHMFQKVM
Sbjct 601 SVCLTELKGYQAAVGQPVIQPRRPNSRLDKVRHMFQKVM 639

>AMP49021.1 TetM-TetW-TetO-TetS [uncultured bacterium]
Length=639

Score = 1295 bits (3350), Expect = 0.0, Method: Compositional matrix adjust.
Identities = 630/639 (99%), Positives = 635/639 (99%), Gaps = 0/639 (0%)

Query 1
MKIINIGILAHVDAGKTTLTESLLYASGAISEPGSVEKGTTRTDTMFLERQRGITIQAAV 60

MKIINIGILAHVDAGKTTLTESLLYASGAISEPGSVEKGTTRTDTMFLERQRGITIQAAV
Sbjct 1
MKIINIGILAHVDAGKTTLTESLLYASGAISEPGSVEKGTTRTDTMFLERQRGITIQAAV 60

Query 61
TSFQWHRCKVNIVDTPGHMDFLAEVYRSLAVLDGAILVISAKDGVQAQTRILFHALRKMN 120

TSFQWHRCKVNIVDTPGHMDFLAEVYRSLAVLDGAILVISAKDGVQAQTRILFHALRKMN
Sbjct 61
TSFQWHRCKVNIVDTPGHMDFLAEVYRSLAVLDGAILVISAKDGVQAQTRILFHALRKMN 120

Query 121
IPTVIFINKIDQAGVDLQSVVQSVRDKLSADIIIKQTVSLSPEIVLEENTDIEAWDAVIE 180

IPTVIFINKIDQAGVDLQSVVQSVRDKLSADIIIKQTVSLSPEIVLEENTDIEAWDAVIE
Sbjct 121
IPTVIFINKIDQAGVDLQSVVQSVRDKLSADIIIKQTVSLSPEIVLEENTDIEAWDAVIE 180

Query 181
NNDKLLEKYIAGEPISREKLVREEQRRVQDASLFPVYYGSAKKGLGIQPLMDAVTGLFQP 240
            NND+LLEKYIAGEPISREKL REEQ+RVQDASLFPVY+GSAK
GLGIQPLMDAVTGLFQP
Sbjct 181
NNDELLEKYIAGEPISREKLAREEQQRVQDASLFPVYHGSAKNGLGIQPLMDAVTGLFQP 240

Query 241
IGEQGSAALCGSVFKVEYTDCGQRRVYLRLYSGTLRLRDTVALAGREKLKITEMRIPSKG 300

IGEQG
AALCGSVFKVEYTDCGQRRVYLRLYSGTLRLRDTVALAGREKLKITEMRIPSKG
Sbjct 241
IGEQGGAALCGSVFKVEYTDCGQRRVYLRLYSGTLRLRDTVALAGREKLKITEMRIPSKG 300

Query 301
EIVRTDTAYPGEIVILPSDSVRLNDVLGDPTRLPRKRWREDPLPMLRTTIAPKTAAQRER 360
EIVRTDTAYPGEIVILPSDSVRLNDVLGD
TRLPRKRWREDPLPMLRTTIAPKTAAQRER
Sbjct 301
EIVRTDTAYPGEIVILPSDSVRLNDVLGDQTRLPRKRWREDPLPMLRTTIAPKTAAQRER 360

Query 361
LLDALTQLADTDPLLRCEVDSITHEIILSFLGRVQLEVVSALLSEKYKLETVVKEPSVIY 420

LLDALTQLADTDPLLRCEVDSITHEIILSFLGRVQLEVVSALLSEKYKLETVVKEPSVIY
Sbjct 361
LLDALTQLADTDPLLRCEVDSITHEIILSFLGRVQLEVVSALLSEKYKLETVVKEPSVIY 420

Query 421
MERPLKAASHTIHIEVPPNPFWASIGLSVTPLSLGSGVQYESRVSLGYLNQSFQNAVRDG 480

MERPLKAASHTIHIEVPPNPFWASIGLSVTPLSLGSGVQYESRVSLGYLNQSFQNAVRDG
Sbjct 421
MERPLKAASHTIHIEVPPNPFWASIGLSVTPLSLGSGVQYESRVSLGYLNQSFQNAVRDG 480

Query 481
IRYGLEQGLFGWNVTDCKICFEYGLYYSPVSTPADFRSLAPIVLEQALKESGTQLLEPYL 540

IRYGLEQGLFGWNVTDCKICFEYGLYYSPVSTPADFRSLAPIVLEQALKESGTQLLEPYL
Sbjct 481
IRYGLEQGLFGWNVTDCKICFEYGLYYSPVSTPADFRSLAPIVLEQALKESGTQLLEPYL 540

Query 541
SFILYAPQEYLSRAYHDAPKYCATIETAQVKKDEVVFTGEIPARCIQAYRTDLAFYTNGR 600

SFILYAPQEYLSRAYHDAPKYCATIETAQVKKDEVVFTGEIPARCIQAYRTDLAFYTNGR
Sbjct 541
SFILYAPQEYLSRAYHDAPKYCATIETAQVKKDEVVFTGEIPARCIQAYRTDLAFYTNGR 600

Query 601 SVCLTELKGYQAAVGQPVIQPRRPNSRLDKVRHMFQKVM 639
SVCLTELKGYQAAVGQPVIQPRRPNSRLDKVR+MFQK+M
Sbjct 601 SVCLTELKGYQAAVGQPVIQPRRPNSRLDKVRYMFQKIM 639

>WP_060941960.1 TetM/TetW/TetO/TetS family tetracycline resistance ribosomal
protection protein [Corynebacterium kroppenstedtii]
KXB50177.1 putative translation elongation factor G
[Corynebacterium kroppenstedtii]
Length=639

Score = 1295 bits (3350), Expect = 0.0, Method: Compositional matrix adjust.

Identities = 628/639 (98%), Positives = 636/639 (99%), Gaps = 0/639 (0%)

```
Query  1
MKIINIGILAHVDAGKTTLTESLLYASGAISEPGSVEKGTTRTDTMFLERQRGITIQAAV  60

MKIINIGILAHVDAGKTTLTESLLYASGAISEPGSVEKGTTRTDTMFLERQRGITIQAAV
Sbjct  1
MKIINIGILAHVDAGKTTLTESLLYASGAISEPGSVEKGTTRTDTMFLERQRGITIQAAV  60

Query  61
TSFQWHRCKVNIVDTPGHMDFLAEVYRSLAVLDGAILVISAKDGVQAQTRILFHALRKMN  120

TSFQWHRCKVNIVDTPGHMDFLAEVYRSLAVLDGAILVISAKDGVQAQTRILFHALRKMN
Sbjct  61
TSFQWHRCKVNIVDTPGHMDFLAEVYRSLAVLDGAILVISAKDGVQAQTRILFHALRKMN  120

Query  121
IPTVIFINKIDQAGVDLQSVVQSVRDKLSADIIIKQTVSLSPEIVLEENTDIEAWDAVIE  180

IPTVIFINKIDQAGVDLQSVVQSVRDKLSADIIIKQTVSLSPEIVLEENTDIEAWDAVIE
Sbjct  121
IPTVIFINKIDQAGVDLQSVVQSVRDKLSADIIIKQTVSLSPEIVLEENTDIEAWDAVIE  180

Query  181
NNDKLLEKYIAGEPISREKLVREEQRRVQDASLFPVYYGSAKKGLGIQPLMDAVTGLFQP  240

NNDKLLEKYIAGEPISREKLVREEQRRVQDASLFPVYYGSAKKGLGIQPLMDAVTGLFQP
Sbjct  181
NNDKLLEKYIAGEPISREKLVREEQRRVQDASLFPVYYGSAKKGLGIQPLMDAVTGLFQP  240

Query  241
IGEQGSAALCGSVFKVEYTDCGQRRVYLRLYSGTLRLRDTVALAGREKLKITEMRIPSKG  300

IGEQGSAALCGSVFKVEYTDCGQRRVYLRLYSGTLRLRDTVALAGREKLKITEMRIPSKG
Sbjct  241
IGEQGSAALCGSVFKVEYTDCGQRRVYLRLYSGTLRLRDTVALAGREKLKITEMRIPSKG  300

Query  301
EIVRTDTAYPGEIVILPSDSVRLNDVLGDPTRLPRKRWREDPLPMLRTTIAPKTAAQRER  360
            EIVRTDTAYPGEIVILPSDSVRLNDVLGDPTRLPRKRWREDPLPMLRT
+IAPKTAAQRER
Sbjct  301
EIVRTDTAYPGEIVILPSDSVRLNDVLGDPTRLPRKRWREDPLPMLRTSIAPKTAAQRER  360

Query  361
LLDALTQLADTDPLLRCEVDSITHEIILSFLGRVQLEVVSALLSEKYKLETVVKEPSVIY  420
            LLDALTQLADTDPLLRCEVDSITHEIILSFLGRVQLEVVSALLSEKYKLETVVKEP
+VIY
Sbjct  361
LLDALTQLADTDPLLRCEVDSITHEIILSFLGRVQLEVVSALLSEKYKLETVVKEPTVIY  420

Query  421
MERPLKAASHTIHIEVPPNPFWASIGLSVTPLSLGSGVQYESRVSLGYLNQSFQNAVRDG  480
```

            MERPLKAASHTIHIEVPPNPFWASIGLSVTPL
LGSGVQYESRVSLGYLNQSFQNAVRDG
Sbjct 421
MERPLKAASHTIHIEVPPNPFWASIGLSVTPLPLGSGVQYESRVSLGYLNQSFQNAVRDG 480

Query 481
IRYGLEQGLFGWNVTDCKICFEYGLYYSPVSTPADFRSLAPIVLEQALKESGTQLLEPYL 540

IRYGLEQGLFGWNVTDCKICFEYGLYYSPVSTPADFRSLAPIVLEQALKESGTQLLEPYL
Sbjct 481
IRYGLEQGLFGWNVTDCKICFEYGLYYSPVSTPADFRSLAPIVLEQALKESGTQLLEPYL 540

Query 541
SFILYAPQEYLSRAYHDAPKYCATIETAQVKKDEVVFTGEIPARCIQAYRTDLAFYTNGR 600
            SF LYAP+EYLSRAYHDAPKYCATIET QVKKDEVVFTGEIPARC
+QAYRTDLAFYTNG+
Sbjct 541
SFTLYAPREYLSRAYHDAPKYCATIETVQVKKDEVVFTGEIPARCMQAYRTDLAFYTNGQ 600

Query 601 SVCLTELKGYQAAVGQPVIQPRRPNSRLDKVRHMFQKVM 639
            SVCLTELKGYQAAVG+PVIQPRRPNSRLDKVR+MFQK+M
Sbjct 601 SVCLTELKGYQAAVGKPVIQPRRPNSRLDKVRYMFQKIM 639

>CUN67961.1 Tetracycline resistance protein tetM from transposon
Tn916 [Flavonifractor
plautii]
Length=639

 Score = 1295 bits (3350), Expect = 0.0, Method: Compositional
matrix adjust.
 Identities = 629/639 (98%), Positives = 634/639 (99%), Gaps = 0/639
(0%)

Query 1
MKIINIGILAHVDAGKTTLTESLLYASGAISEPGSVEKGTTRTDTMFLERQRGITIQAAV 60

MKIINIGILAHVDAGKTTLTESLLYASGAISEPGSVEKGTTRTDTMFLERQRGITIQAAV
Sbjct 1
MKIINIGILAHVDAGKTTLTESLLYASGAISEPGSVEKGTTRTDTMFLERQRGITIQAAV 60

Query 61
TSFQWHRCKVNIVDTPGHMDFLAEVYRSLAVLDGAILVISAKDGVQAQTRILFHALRKMN 120

TSFQWHRCKVNIVDTPGHMDFLAEVYRSLAVLDGAILVISAKDGVQAQTRILFHALRKMN
Sbjct 61
TSFQWHRCKVNIVDTPGHMDFLAEVYRSLAVLDGAILVISAKDGVQAQTRILFHALRKMN 120

Query 121
IPTVIFINKIDQAGVDLQSVVQSVRDKLSADIIIKQTVSLSPEIVLEENTDIEAWDAVIE 180

IPTVIFINKIDQAGVDLQSVVQSVRDKLSADIIIKQTVSLSPEIVLEENTDIEAWDAVIE
Sbjct 121
IPTVIFINKIDQAGVDLQSVVQSVRDKLSADIIIKQTVSLSPEIVLEENTDIEAWDAVIE 180

```
Query  181
NNDKLLEKYIAGEPISREKLVREEQRRVQDASLFPVYYGSAKKGLGIQPLMDAVTGLFQP  240
            NND+LLEKYIAGEPISREKL REEQ+RVQDASLFPVY+GSAK
GLGIQPLMDAVTGLFQP
Sbjct  181
NNDELLEKYIAGEPISREKLAREEQQRVQDASLFPVYHGSAKNGLGIQPLMDAVTGLFQP  240

Query  241
IGEQGSAALCGSVFKVEYTDCGQRRVYLRLYSGTLRLRDTVALAGREKLKITEMRIPSKG  300
            IGEQG
AALCGSVFKVEYTDCGQRRVYLRLYSGTLRLRDTVALAGREKLKITEMRIPSKG
Sbjct  241
IGEQGGAALCGSVFKVEYTDCGQRRVYLRLYSGTLRLRDTVALAGREKLKITEMRIPSKG  300

Query  301
EIVRTDTAYPGEIVILPSDSVRLNDVLGDPTRLPRKRWREDPLPMLRTTIAPKTAAQRER  360
            EIVRTDTAY GEIVILPSDSVRLNDVLGD
TRLPRKRWREDPLPMLRTTIAPKTAAQRER
Sbjct  301
EIVRTDTAYQGEIVILPSDSVRLNDVLGDQTRLPRKRWREDPLPMLRTTIAPKTAAQRER  360

Query  361
LLDALTQLADTDPLLRCEVDSITHEIILSFLGRVQLEVVSALLSEKYKLETVVKEPSVIY  420

LLDALTQLADTDPLLRCEVDSITHEIILSFLGRVQLEVVSALLSEKYKLETVVKEPSVIY
Sbjct  361
LLDALTQLADTDPLLRCEVDSITHEIILSFLGRVQLEVVSALLSEKYKLETVVKEPSVIY  420

Query  421
MERPLKAASHTIHIEVPPNPFWASIGLSVTPLSLGSGVQYESRVSLGYLNQSFQNAVRDG  480

MERPLKAASHTIHIEVPPNPFWASIGLSVTPLSLGSGVQYESRVSLGYLNQSFQNAVRDG
Sbjct  421
MERPLKAASHTIHIEVPPNPFWASIGLSVTPLSLGSGVQYESRVSLGYLNQSFQNAVRDG  480

Query  481
IRYGLEQGLFGWNVTDCKICFEYGLYYSPVSTPADFRSLAPIVLEQALKESGTQLLEPYL  540

IRYGLEQGLFGWNVTDCKICFEYGLYYSPVSTPADFRSLAPIVLEQALKESGTQLLEPYL
Sbjct  481
IRYGLEQGLFGWNVTDCKICFEYGLYYSPVSTPADFRSLAPIVLEQALKESGTQLLEPYL  540

Query  541
SFILYAPQEYLSRAYHDAPKYCATIETAQVKKDEVVFTGEIPARCIQAYRTDLAFYTNGR  600

SFILYAPQEYLSRAYHDAPKYCATIETAQVKKDEVVFTGEIPARCIQAYRTDLAFYTNGR
Sbjct  541
SFILYAPQEYLSRAYHDAPKYCATIETAQVKKDEVVFTGEIPARCIQAYRTDLAFYTNGR  600

Query  601  SVCLTELKGYQAAVGQPVIQPRRPNSRLDKVRHMFQKVM  639
            SVCLTELKGYQAAVGQPVIQPRRPNSRLDK+RHMFQK+M
Sbjct  601  SVCLTELKGYQAAVGQPVIQPRRPNSRLDKIRHMFQKIM  639
```

>WP_039199418.1 MULTISPECIES: TetM/TetW/TetO/TetS family
tetracycline resistance
ribosomal protection protein [Bifidobacterium]
 AIZ15232.1 tetracycline resistance protein tetM [Bifidobacterium
kashiwanohense
PV20-2]
 PIB84403.1 TetM/TetW/TetO/TetS family tetracycline resistance
ribosomal
protection protein [Bifidobacterium sp. N4G05]
Length=639

 Score = 1295 bits (3350), Expect = 0.0, Method: Compositional
matrix adjust.
 Identities = 629/639 (98%), Positives = 635/639 (99%), Gaps = 0/639
(0%)

Query 1
MKIINIGILAHVDAGKTTLTESLLYASGAISEPGSVEKGTTRTDTMFLERQRGITIQAAV 60

MKIINIGILAHVDAGKTTLTESLLYASGAISEPGSVEKGTTRTDTMFLERQRGITIQAAV
Sbjct 1
MKIINIGILAHVDAGKTTLTESLLYASGAISEPGSVEKGTTRTDTMFLERQRGITIQAAV 60

Query 61
TSFQWHRCKVNIVDTPGHMDFLAEVYRSLAVLDGAILVISAKDGVQAQTRILFHALRKMN 120

TSFQWHRCKVNIVDTPGHMDFLAEVYRSLAVLDGAILVISAKDGVQAQTRILFHALRKMN
Sbjct 61
TSFQWHRCKVNIVDTPGHMDFLAEVYRSLAVLDGAILVISAKDGVQAQTRILFHALRKMN 120

Query 121
IPTVIFINKIDQAGVDLQSVVQSVRDKLSADIIIKQTVSLSPEIVLEENTDIEAWDAVIE 180

IPTVIFINKIDQAGVDLQSVVQSVRDKLSADIIIKQTVSLSPEIVLEENTDIEAWDAVIE
Sbjct 121
IPTVIFINKIDQAGVDLQSVVQSVRDKLSADIIIKQTVSLSPEIVLEENTDIEAWDAVIE 180

Query 181
NNDKLLEKYIAGEPISREKLVREEQRRVQDASLFPVYYGSAKKGLGIQPLMDAVTGLFQP 240
            NND+LLEKYIAGEPISREKL REEQ
+RVQDASLFPVYYGSAKKGLGIQPLMDAVTGLFQP
Sbjct 181
NNDELLEKYIAGEPISREKLAREEQQRVQDASLFPVYYGSAKKGLGIQPLMDAVTGLFQP 240

Query 241
IGEQGSAALCGSVFKVEYTDCGQRRVYLRLYSGTLRLRDTVALAGREKLKITEMRIPSKG 300

IGEQGSAALCGSVFKVEYTDCGQRRVYLRLYSGTLRLRDTVALAGREKLKITEMRIPSKG
Sbjct 241
IGEQGSAALCGSVFKVEYTDCGQRRVYLRLYSGTLRLRDTVALAGREKLKITEMRIPSKG 300

Query 301
EIVRTDTAYPGEIVILPSDSVRLNDVLGDPTRLPRKRWREDPLPMLRTTIAPKTAAQRER 360

```
            EIVRTDTAYPGEIVILPSDSVRLNDVLGDPTRLPRKRWREDPLPMLRT
+IAPKTAAQRER
Sbjct  301
EIVRTDTAYPGEIVILPSDSVRLNDVLGDPTRLPRKRWREDPLPMLRTSIAPKTAAQRER  360

Query  361
LLDALTQLADTDPLLRCEVDSITHEIILSFLGRVQLEVVSALLSEKYKLETVVKEPSVIY  420
            LLDALTQLADTDPLLRCEVDSITHEIILSFLGRVQLEVVSALLSEKYKLETVVKEP
+VIY
Sbjct  361
LLDALTQLADTDPLLRCEVDSITHEIILSFLGRVQLEVVSALLSEKYKLETVVKEPTVIY  420

Query  421
MERPLKAASHTIHIEVPPNPFWASIGLSVTPLSLGSGVQYESRVSLGYLNQSFQNAVRDG  480
            MERPLKAASHTIHIEVPPNPFWASIGLSVTPL
LGSGVQYESRVSLGYLNQSFQNAVRDG
Sbjct  421
MERPLKAASHTIHIEVPPNPFWASIGLSVTPLPLGSGVQYESRVSLGYLNQSFQNAVRDG  480

Query  481
IRYGLEQGLFGWNVTDCKICFEYGLYYSPVSTPADFRSLAPIVLEQALKESGTQLLEPYL  540

IRYGLEQGLFGWNVTDCKICFEYGLYYSPVSTPADFRSLAPIVLEQALKESGTQLLEPYL
Sbjct  481
IRYGLEQGLFGWNVTDCKICFEYGLYYSPVSTPADFRSLAPIVLEQALKESGTQLLEPYL  540

Query  541
SFILYAPQEYLSRAYHDAPKYCATIETAQVKKDEVVFTGEIPARCIQAYRTDLAFYTNGR  600
            SF LYAP+EYLSRAYHDAPKYCATIET
QVKKDEVVFTGEIPARCIQAYRTDLAFYTNG+
Sbjct  541
SFTLYAPREYLSRAYHDAPKYCATIETVQVKKDEVVFTGEIPARCIQAYRTDLAFYTNGQ  600

Query  601  SVCLTELKGYQAAVGQPVIQPRRPNSRLDKVRHMFQKVM  639
            SVCLTELKGYQAAVGQPVIQPRRPNSRLDKVRHMFQKVM
Sbjct  601  SVCLTELKGYQAAVGQPVIQPRRPNSRLDKVRHMFQKVM  639


>WP_063856424.1 tetracycline resistance ribosomal protection protein
Tet(W) [Bifidobacterium
longum]
 ACA23185.1 TetW [Bifidobacterium longum]
Length=639

 Score = 1294 bits (3349),  Expect = 0.0, Method: Compositional
matrix adjust.
 Identities = 630/639 (99%), Positives = 633/639 (99%), Gaps = 0/639
(0%)

Query  1
MKIINIGILAHVDAGKTTLTESLLYASGAISEPGSVEKGTTRTDTMFLERQRGITIQAAV  60

MKIINIGILAHVDAGKTTLTESLLYASGAISEPGSVEKGTTRTDTMFLERQRGITIQAAV
Sbjct  1
```

MKIINIGILAHVDAGKTTLTESLLYASGAISEPGSVEKGTTRTDTMFLERQRGITIQAAV 60

Query 61
TSFQWHRCKVNIVDTPGHMDFLAEVYRSLAVLDGAILVISAKDGVQAQTRILFHALRKMN 120

TSFQWHRCKVNIVDTPGHMDFLAEVYRSLAVLDGAILVISAKDGVQAQTRILFHALRKMN
Sbjct 61
TSFQWHRCKVNIVDTPGHMDFLAEVYRSLAVLDGAILVISAKDGVQAQTRILFHALRKMN 120

Query 121
IPTVIFINKIDQAGVDLQSVVQSVRDKLSADIIIKQTVSLSPEIVLEENTDIEAWDAVIE 180

IPTVIFINKIDQAGVDLQSVVQSVRDKLSADIIIKQTVSLSPEIVLEENTDIEAWDAVIE
Sbjct 121
IPTVIFINKIDQAGVDLQSVVQSVRDKLSADIIIKQTVSLSPEIVLEENTDIEAWDAVIE 180

Query 181
NNDKLLEKYIAGEPISREKLVREEQRRVQDASLFPVYYGSAKKGLGIQPLMDAVTGLFQP 240
NND+LLEKYIAGEPISREKL REEQ+RVQDASLFPVY+GSAK
GLGIQPLMDAVTGLFQP
Sbjct 181
NNDELLEKYIAGEPISREKLAREEQQRVQDASLFPVYHGSAKNGLGIQPLMDAVTGLFQP 240

Query 241
IGEQGSAALCGSVFKVEYTDCGQRRVYLRLYSGTLRLRDTVALAGREKLKITEMRIPSKG 300
IGEQG AALCGSVFKVEYTDCGQRRVYLRLYSGTLRLRDTVAL
GREKLKITEMRIPSKG
Sbjct 241
IGEQGGAALCGSVFKVEYTDCGQRRVYLRLYSGTLRLRDTVALGGREKLKITEMRIPSKG 300

Query 301
EIVRTDTAYPGEIVILPSDSVRLNDVLGDPTRLPRKRWREDPLPMLRTTIAPKTAAQRER 360
EIVRTDTAY GEIVILPSDSVRLNDVLGD
TRLPRKRWREDPLPMLRTTIAPKTAAQRER
Sbjct 301
EIVRTDTAYQGEIVILPSDSVRLNDVLGDQTRLPRKRWREDPLPMLRTTIAPKTAAQRER 360

Query 361
LLDALTQLADTDPLLRCEVDSITHEIILSFLGRVQLEVVSALLSEKYKLETVVKEPSVIY 420

LLDALTQLADTDPLLRCEVDSITHEIILSFLGRVQLEVVSALLSEKYKLETVVKEPSVIY
Sbjct 361
LLDALTQLADTDPLLRCEVDSITHEIILSFLGRVQLEVVSALLSEKYKLETVVKEPSVIY 420

Query 421
MERPLKAASHTIHIEVPPNPFWASIGLSVTPLSLGSGVQYESRVSLGYLNQSFQNAVRDG 480

MERPLKAASHTIHIEVPPNPFWASIGLSVTPLSLGSGVQYESRVSLGYLNQSFQNAVRDG
Sbjct 421
MERPLKAASHTIHIEVPPNPFWASIGLSVTPLSLGSGVQYESRVSLGYLNQSFQNAVRDG 480

Query 481
IRYGLEQGLFGWNVTDCKICFEYGLYYSPVSTPADFRSLAPIVLEQALKESGTQLLEPYL 540

IRYGLEQGLFGWNVTDCKICFEYGLYYSPVSTPADFRSLAPIVLEQALKESGTQLLEPYL
Sbjct 481
IRYGLEQGLFGWNVTDCKICFEYGLYYSPVSTPADFRSLAPIVLEQALKESGTQLLEPYL 540

Query 541
SFILYAPQEYLSRAYHDAPKYCATIETAQVKKDEVVFTGEIPARCIQAYRTDLAFYTNGR 600

SFILYAPQEYLSRAYHDAPKYCATIETAQVKKDEVVFTGEIPARCIQAYRTDLAFYTNGR
Sbjct 541
SFILYAPQEYLSRAYHDAPKYCATIETAQVKKDEVVFTGEIPARCIQAYRTDLAFYTNGR 600

Query 601 SVCLTELKGYQAAVGQPVIQPRRPNSRLDKVRHMFQKVM 639
SVCLTELKGYQAAVGQPVIQPRRPNSRLDKVRHMFQKVM
Sbjct 601 SVCLTELKGYQAAVGQPVIQPRRPNSRLDKVRHMFQKVM 639

>APO30266.1 Ferrous iron transport protein B [uncultured bacterium]
Length=639

Score = 1294 bits (3349), Expect = 0.0, Method: Compositional matrix adjust.
Identities = 630/639 (99%), Positives = 633/639 (99%), Gaps = 0/639 (0%)

Query 1
MKIINIGILAHVDAGKTTLTESLLYASGAISEPGSVEKGTTRTDTMFLERQRGITIQAAV 60

MKIINIGILAHVDAGKTTLTESLLYASGAISEPGSVEKGTTRTDTMFLERQRGITIQAAV
Sbjct 1
MKIINIGILAHVDAGKTTLTESLLYASGAISEPGSVEKGTTRTDTMFLERQRGITIQAAV 60

Query 61
TSFQWHRCKVNIVDTPGHMDFLAEVYRSLAVLDGAILVISAKDGVQAQTRILFHALRKMN 120

TSFQWHRCKVNIVDTPGHMDFLAEVYRSLAVLDGAILVISAKDGVQAQTRILFHALRKMN
Sbjct 61
TSFQWHRCKVNIVDTPGHMDFLAEVYRSLAVLDGAILVISAKDGVQAQTRILFHALRKMN 120

Query 121
IPTVIFINKIDQAGVDLQSVVQSVRDKLSADIIIKQTVSLSPEIVLEENTDIEAWDAVIE 180

IPTVIFINKIDQAGVDLQSVVQSVRDKLSADIIIKQTVSLSPEIVLEENTDIEAWDAVIE
Sbjct 121
IPTVIFINKIDQAGVDLQSVVQSVRDKLSADIIIKQTVSLSPEIVLEENTDIEAWDAVIE 180

Query 181
NNDKLLEKYIAGEPISREKLVREEQRRVQDASLFPVYYGSAKKGLGIQPLMDAVTGLFQP 240
NND+LLEKYIAGEPISREKL REEQ+RVQDASLFPVY+GSAK
GLGIQPLMDAVTGLFQP
Sbjct 181
NNDELLEKYIAGEPISREKLAREEQQRVQDASLFPVYHGSAKNGLGIQPLMDAVTGLFQP 240

Query 241
IGEQGSAALCGSVFKVEYTDCGQRRVYLRLYSGTLRLRDTVALAGREKLKITEMRIPSKG 300

```
            IGEQG
AALCGSVFKVEYTDCGQRRVYLRLYSGTLRLRDTVALAGREKLKITEMRIPSKG
Sbjct  241
IGEQGGAALCGSVFKVEYTDCGQRRVYLRLYSGTLRLRDTVALAGREKLKITEMRIPSKG  300

Query  301
EIVRTDTAYPGEIVILPSDSVRLNDVLGDPTRLPRKRWREDPLPMLRTTIAPKTAAQRER  360
            EIVRTDTAY GEIVILPSDSVRLNDVLGD
TRLPRKRWREDPLPMLRTTIAPKTAAQRER
Sbjct  301
EIVRTDTAYQGEIVILPSDSVRLNDVLGDQTRLPRKRWREDPLPMLRTTIAPKTAAQRER  360

Query  361
LLDALTQLADTDPLLRCEVDSITHEIILSFLGRVQLEVVSALLSEKYKLETVVKEPSVIY  420

LLDALTQLADTDPLLRCEVDSITHEIILSFLGRVQLEVVSALLSEKYKLETVVKEPSVIY
Sbjct  361
LLDALTQLADTDPLLRCEVDSITHEIILSFLGRVQLEVVSALLSEKYKLETVVKEPSVIY  420

Query  421
MERPLKAASHTIHIEVPPNPFWASIGLSVTPLSLGSGVQYESRVSLGYLNQSFQNAVRDG  480

MERPLKAASHTIHIEVPPNPFWASIGLSVTPLSLGSGVQYESRVSLGYLNQSFQNAVRDG
Sbjct  421
MERPLKAASHTIHIEVPPNPFWASIGLSVTPLSLGSGVQYESRVSLGYLNQSFQNAVRDG  480

Query  481
IRYGLEQGLFGWNVTDCKICFEYGLYYSPVSTPADFRSLAPIVLEQALKESGTQLLEPYL  540

IRYGLEQGLFGWNVTDCKICFEYGLYYSPVSTPADFRSLAPIVLEQALKESGTQLLEPYL
Sbjct  481
IRYGLEQGLFGWNVTDCKICFEYGLYYSPVSTPADFRSLAPIVLEQALKESGTQLLEPYL  540

Query  541
SFILYAPQEYLSRAYHDAPKYCATIETAQVKKDEVVFTGEIPARCIQAYRTDLAFYTNGR  600

SFILYAPQEYLSRAYHDAPKYCATIETAQVKKDEVVFTGEIPARCIQAYRTDLAFYTNGR
Sbjct  541
SFILYAPQEYLSRAYHDAPKYCATIETAQVKKDEVVFTGEIPARCIQAYRTDLAFYTNGR  600

Query  601  SVCLTELKGYQAAVGQPVIQPRRPNSRLDKVRHMFQKVM  639
            SVCLTELKGYQAAVGQPVIQPRRPNSRLDK RHMFQKVM
Sbjct  601  SVCLTELKGYQAAVGQPVIQPRRPNSRLDKARHMFQKVM  639


>WP_070570683.1 TetM/TetW/TetO/TetS family tetracycline resistance
ribosomal
protection protein [Corynebacterium sp. HMSC055D05]
 OFL92063.1 tetracycline resistance ribosomal protection protein
[Corynebacterium
sp. HMSC055D05]
Length=639

 Score = 1294 bits (3349),  Expect = 0.0, Method: Compositional
```

matrix adjust.
 Identities = 628/639 (98%), Positives = 635/639 (99%), Gaps = 0/639 (0%)

Query 1
MKIININIGILAHVDAGKTTLTESLLYASGAISEPGSVEKGTTRTDTMFLERQRGITIQAAV 60

MKIINIGILAHVDAGKTTLTESLLYASGAISEPGSVEKGTTRTDTMFLERQRGITIQAAV
Sbjct 1
MKIINIGILAHVDAGKTTLTESLLYASGAISEPGSVEKGTTRTDTMFLERQRGITIQAAV 60

Query 61
TSFQWHRCKVNIVDTPGHMDFLAEVYRSLAVLDGAILVISAKDGVQAQTRILFHALRKMN 120

TSFQWHRCKVNIVDTPGHMDFLAEVYRSLAVLDGAILVISAKDGVQAQTRILFHALRKMN
Sbjct 61
TSFQWHRCKVNIVDTPGHMDFLAEVYRSLAVLDGAILVISAKDGVQAQTRILFHALRKMN 120

Query 121
IPTVIFINKIDQAGVDLQSVVQSVRDKLSADIIIKQTVSLSPEIVLEENTDIEAWDAVIE 180

IPTVIFINKIDQAGVDLQSVVQSVRDKLSADIIIKQTVSLSPEIVLEENTDIEAWDAVIE
Sbjct 121
IPTVIFINKIDQAGVDLQSVVQSVRDKLSADIIIKQTVSLSPEIVLEENTDIEAWDAVIE 180

Query 181
NNDKLLEKYIAGEPISREKLVREEQRRVQDASLFPVYYGSAKKGLGIQPLMDAVTGLFQP 240

NNDKLLEKYIAGEPISREKLVREEQRRVQDASLFPVYYGSAKKGLGIQPLMDAVTGLFQP
Sbjct 181
NNDKLLEKYIAGEPISREKLVREEQRRVQDASLFPVYYGSAKKGLGIQPLMDAVTGLFQP 240

Query 241
IGEQGSAALCGSVFKVEYTDCGQRRVYLRLYSGTLRLRDTVALAGREKLKITEMRIPSKG 300

IGEQGSAALCGSVFKVEYTDCGQRRVYLRLYSGTLRLRDTVALAGREKLKITEMRIPSKG
Sbjct 241
IGEQGSAALCGSVFKVEYTDCGQRRVYLRLYSGTLRLRDTVALAGREKLKITEMRIPSKG 300

Query 301
EIVRTDTAYPGEIVILPSDSVRLNDVLGDPTRLPRKRWREDPLPMLRTTIAPKTAAQRER 360
            EIVRTDTAYPGEIVILPSDSVRLNDVLGDPTRLPRKRWREDPLPMLRT
+IAPKTAAQRER
Sbjct 301
EIVRTDTAYPGEIVILPSDSVRLNDVLGDPTRLPRKRWREDPLPMLRTSIAPKTAAQRER 360

Query 361
LLDALTQLADTDPLLRCEVDSITHEIILSFLGRVQLEVVSALLSEKYKLETVVKEPSVIY 420
            LLDALTQLADTDPLLRCEVDSITHEIILSFLGRVQLEVVSALLSEKYKLETVVKEP
+VIY
Sbjct 361
LLDALTQLADTDPLLRCEVDSITHEIILSFLGRVQLEVVSALLSEKYKLETVVKEPTVIY 420

Query 421

MERPLKAASHTIHIEVPPNPFWASIGLSVTPLSLGSGVQYESRVSLGYLNQSFQNAVRDG 480
            MERPLKAASHTIHIEVPPNPFWASIGLS TPL
LGSGVQYESRVSLGYLNQSFQNAVRDG
Sbjct 421
MERPLKAASHTIHIEVPPNPFWASIGLSATPLPLGSGVQYESRVSLGYLNQSFQNAVRDG 480

Query 481
IRYGLEQGLFGWNVTDCKICFEYGLYYSPVSTPADFRSLAPIVLEQALKESGTQLLEPYL 540

IRYGLEQGLFGWNVTDCKICFEYGLYYSPVSTPADFRSLAPIVLEQALKESGTQLLEPYL
Sbjct 481
IRYGLEQGLFGWNVTDCKICFEYGLYYSPVSTPADFRSLAPIVLEQALKESGTQLLEPYL 540

Query 541
SFILYAPQEYLSRAYHDAPKYCATIETAQVKKDEVVFTGEIPARCIQAYRTDLAFYTNGR 600
            SF LYAP+EYLSRAYHDAPKYCATIET
QVKKDEVVFTGEIPARCIQAYRTDLAFYTNG+
Sbjct 541
SFTLYAPREYLSRAYHDAPKYCATIETVQVKKDEVVFTGEIPARCIQAYRTDLAFYTNGQ 600

Query 601 SVCLTELKGYQAAVGQPVIQPRRPNSRLDKVRHMFQKVM 639
            SVCLTELKGYQAAVG+PVIQPRRPNSRLDKVR+MFQK+M
Sbjct 601 SVCLTELKGYQAAVGKPVIQPRRPNSRLDKVRYMFQKIM 639

>AMP52575.1 TetM-TetW-TetO-TetS [uncultured bacterium]
Length=639

Score = 1294 bits (3349), Expect = 0.0, Method: Compositional matrix adjust.
Identities = 630/639 (99%), Positives = 634/639 (99%), Gaps = 0/639 (0%)

Query 1
MKIINIGILAHVDAGKTTLTESLLYASGAISEPGSVEKGTTRTDTMFLERQRGITIQAAV 60

MKIINIGILAHVDAGKTTLTESLLYASGAISEPGSVEKGTTRTDTMFLERQRGITIQAAV
Sbjct 1
MKIINIGILAHVDAGKTTLTESLLYASGAISEPGSVEKGTTRTDTMFLERQRGITIQAAV 60

Query 61
TSFQWHRCKVNIVDTPGHMDFLAEVYRSLAVLDGAILVISAKDGVQAQTRILFHALRKMN 120

TSFQWHRCKVNIVDTPGHMDFLAEVYRSLAVLDGAILVISAKDGVQAQTRILFHALRKMN
Sbjct 61
TSFQWHRCKVNIVDTPGHMDFLAEVYRSLAVLDGAILVISAKDGVQAQTRILFHALRKMN 120

Query 121
IPTVIFINKIDQAGVDLQSVVQSVRDKLSADIIIKQTVSLSPEIVLEENTDIEAWDAVIE 180

IPTVIFINKIDQAGVDLQSVVQSVRDKLSADIIIKQTVSLSPEIVLEENTDIEAWDAVIE
Sbjct 121
IPTVIFINKIDQAGVDLQSVVQSVRDKLSADIIIKQTVSLSPEIVLEENTDIEAWDAVIE 180

Query 181
NNDKLLEKYIAGEPISREKLVREEQRRVQDASLFPVYYGSAKKGLGIQPLMDAVTGLFQP 240
NND+LLEKYIAGEPISREKL REEQ+RVQDASLFPVY+GSAK
GLGIQPLMDAVTGLFQP
Sbjct 181
NNDELLEKYIAGEPISREKLAREEQQRVQDASLFPVYHGSAKNGLGIQPLMDAVTGLFQP 240

Query 241
IGEQGSAALCGSVFKVEYTDCGQRRVYLRLYSGTLRLRDTVALAGREKLKITEMRIPSKG 300
IGEQG
AALCGSVFKVEYTDCGQRRVYLRLYSGTLRLRDTVALAGREKLKITEMRIPSKG
Sbjct 241
IGEQGGAALCGSVFKVEYTDCGQRRVYLRLYSGTLRLRDTVALAGREKLKITEMRIPSKG 300

Query 301
EIVRTDTAYPGEIVILPSDSVRLNDVLGDPTRLPRKRWREDPLPMLRTTIAPKTAAQRER 360
EIVRTDTAY GEIVILPSDSVRLNDVLGD
TRLPRKRWREDPLPMLRTTIAPKTAAQRER
Sbjct 301
EIVRTDTAYQGEIVILPSDSVRLNDVLGDQTRLPRKRWREDPLPMLRTTIAPKTAAQRER 360

Query 361
LLDALTQLADTDPLLRCEVDSITHEIILSFLGRVQLEVVSALLSEKYKLETVVKEPSVIY 420

LLDALTQLADTDPLLRCEVDSITHEIILSFLGRVQLEVVSALLSEKYKLETVVKEPSVIY
Sbjct 361
LLDALTQLADTDPLLRCEVDSITHEIILSFLGRVQLEVVSALLSEKYKLETVVKEPSVIY 420

Query 421
MERPLKAASHTIHIEVPPNPFWASIGLSVTPLSLGSGVQYESRVSLGYLNQSFQNAVRDG 480

MERPLKAASHTIHIEVPPNPFWASIGLSVTPLSLGSGVQYESRVSLGYLNQSFQNAVRDG
Sbjct 421
MERPLKAASHTIHIEVPPNPFWASIGLSVTPLSLGSGVQYESRVSLGYLNQSFQNAVRDG 480

Query 481
IRYGLEQGLFGWNVTDCKICFEYGLYYSPVSTPADFRSLAPIVLEQALKESGTQLLEPYL 540

IRYGLEQGLFGWNVTDCKICFEYGLYYSPVSTPADFRSLAPIVLEQALKESGTQLLEPYL
Sbjct 481
IRYGLEQGLFGWNVTDCKICFEYGLYYSPVSTPADFRSLAPIVLEQALKESGTQLLEPYL 540

Query 541
SFILYAPQEYLSRAYHDAPKYCATIETAQVKKDEVVFTGEIPARCIQAYRTDLAFYTNGR 600

SFILYAPQEYLSRAYHDAPKYCATIETAQVKKDEVVFTGEIPARCIQAYRTDLAFYTNGR
Sbjct 541
SFILYAPQEYLSRAYHDAPKYCATIETAQVKKDEVVFTGEIPARCIQAYRTDLAFYTNGR 600

Query 601 SVCLTELKGYQAAVGQPVIQPRRPNSRLDKVRHMFQKVM 639
SVCLTELKGYQAAVGQPVIQPRRPNSRLDK+RHMFQKVM
Sbjct 601 SVCLTELKGYQAAVGQPVIQPRRPNSRLDKMRHMFQKVM 639

>AMP49255.1 TetM-TetW-TetO-TetS [uncultured bacterium]
 AMP49268.1 TetM-TetW-TetO-TetS [uncultured bacterium]
Length=639

 Score = 1294 bits (3349), Expect = 0.0, Method: Compositional matrix adjust.
 Identities = 630/639 (99%), Positives = 634/639 (99%), Gaps = 0/639 (0%)

Query 1
MKIINIGILAHVDAGKTTLTESLLYASGAISEPGSVEKGTTRTDTMFLERQRGITIQAAV 60

MKIINIGILAHVDAGKTTLTESLLYASGAISEPGSVEKGTTRTDTMFLERQRGITIQAAV
Sbjct 1
MKIINIGILAHVDAGKTTLTESLLYASGAISEPGSVEKGTTRTDTMFLERQRGITIQAAV 60

Query 61
TSFQWHRCKVNIVDTPGHMDFLAEVYRSLAVLDGAILVISAKDGVQAQTRILFHALRKMN 120

TSFQWHRCKVNIVDTPGHMDFLAEVYRSLAVLDGAILVISAKDGVQAQTRILFHALRKMN
Sbjct 61
TSFQWHRCKVNIVDTPGHMDFLAEVYRSLAVLDGAILVISAKDGVQAQTRILFHALRKMN 120

Query 121
IPTVIFINKIDQAGVDLQSVVQSVRDKLSADIIIKQTVSLSPEIVLEENTDIEAWDAVIE 180

IPTVIFINKIDQAGVDLQSVVQSVRDKLSADIIIKQTVSLSPEIVLEENTDIEAWDAVIE
Sbjct 121
IPTVIFINKIDQAGVDLQSVVQSVRDKLSADIIIKQTVSLSPEIVLEENTDIEAWDAVIE 180

Query 181
NNDKLLEKYIAGEPISREKLVREEQRRVQDASLFPVYYGSAKKGLGIQPLMDAVTGLFQP 240
            NND+LLEKYIAGEPISREKL REEQ+RVQDASLFPVY+GSAK GLGIQPLMDAVTGLFQP
Sbjct 181
NNDELLEKYIAGEPISREKLAREEQQRVQDASLFPVYHGSAKNGLGIQPLMDAVTGLFQP 240

Query 241
IGEQGSAALCGSVFKVEYTDCGQRRVYLRLYSGTLRLRDTVALAGREKLKITEMRIPSKG 300
            IGEQG AALCGSVFKVEYTDCGQRRVYLRLYSGTLRLRDTVALAGREKLKITEMRIPSKG
Sbjct 241
IGEQGGAALCGSVFKVEYTDCGQRRVYLRLYSGTLRLRDTVALAGREKLKITEMRIPSKG 300

Query 301
EIVRTDTAYPGEIVILPSDSVRLNDVLGDPTRLPRKRWREDPLPMLRTTIAPKTAAQRER 360
            EIVRTDTAY GEIVILPSDSVRLNDVLGD TRLPRKRWREDPLPMLRTTIAPKTAAQRER
Sbjct 301
EIVRTDTAYQGEIVILPSDSVRLNDVLGDQTRLPRKRWREDPLPMLRTTIAPKTAAQRER 360

Query 361
LLDALTQLADTDPLLRCEVDSITHEIILSFLGRVQLEVVSALLSEKYKLETVVKEPSVIY 420

LLDALTQLADTDPLLRCEVDSITHEIILSFLGRVQLEVVSALLSEKYKLETVVKEPSVIY
Sbjct 361
LLDALTQLADTDPLLRCEVDSITHEIILSFLGRVQLEVVSALLSEKYKLETVVKEPSVIY 420

Query 421
MERPLKAASHTIHIEVPPNPFWASIGLSVTPLSLGSGVQYESRVSLGYLNQSFQNAVRDG 480

MERPLKAASHTIHIEVPPNPFWASIGLSVTPLSLGSGVQYESRVSLGYLNQSFQNAVRDG
Sbjct 421
MERPLKAASHTIHIEVPPNPFWASIGLSVTPLSLGSGVQYESRVSLGYLNQSFQNAVRDG 480

Query 481
IRYGLEQGLFGWNVTDCKICFEYGLYYSPVSTPADFRSLAPIVLEQALKESGTQLLEPYL 540

IRYGLEQGLFGWNVTDCKICFEYGLYYSPVSTPADFRSLAPIVLEQALKESGTQLLEPYL
Sbjct 481
IRYGLEQGLFGWNVTDCKICFEYGLYYSPVSTPADFRSLAPIVLEQALKESGTQLLEPYL 540

Query 541
SFILYAPQEYLSRAYHDAPKYCATIETAQVKKDEVVFTGEIPARCIQAYRTDLAFYTNGR 600

SFILYAPQEYLSRAYHDAPKYCATIETAQVKKDEVVFTGEIPARCIQAYRTDLAFYTNGR
Sbjct 541
SFILYAPQEYLSRAYHDAPKYCATIETAQVKKDEVVFTGEIPARCIQAYRTDLAFYTNGR 600

```
Query  601  SVCLTELKGYQAAVGQPVIQPRRPNSRLDKVRHMFQKVM  639
            SVCLTELKGYQAAVGQPVIQPRRPNSRLD+VRHMFQKVM
Sbjct  601  SVCLTELKGYQAAVGQPVIQPRRPNSRLDRVRHMFQKVM  639
```

>WP_060812403.1 TetM/TetW/TetO/TetS family tetracycline resistance ribosomal
protection protein [Bifidobacterium breve]
 KWZ85953.1 putative translation elongation factor G [Bifidobacterium breve]
Length=639

 Score = 1294 bits (3349), Expect = 0.0, Method: Compositional matrix adjust.
 Identities = 628/639 (98%), Positives = 636/639 (99%), Gaps = 0/639 (0%)

Query 1
MKIINIGILAHVDAGKTTLTESLLYASGAISEPGSVEKGTTRTDTMFLERQRGITIQAAV 60

MKIINIGILAHVDAGKTTLTESLLYASGAISEPGSVEKGTTRTDTMFLERQRGITIQAAV
Sbjct 1
MKIINIGILAHVDAGKTTLTESLLYASGAISEPGSVEKGTTRTDTMFLERQRGITIQAAV 60

Query 61
TSFQWHRCKVNIVDTPGHMDFLAEVYRSLAVLDGAILVISAKDGVQAQTRILFHALRKMN 120

TSFQWHRCKVNIVDTPGHMDFLAEVYRSLAVLDGAILVISAKDGVQAQTRILFHALRKMN
Sbjct 61

TSFQWHRCKVNIVDTPGHMDFLAEVYRSLAVLDGAILVISAKDGVQAQTRILFHALRKMN 120

Query 121
IPTVIFINKIDQAGVDLQSVVQSVRDKLSADIIIKQTVSLSPEIVLEENTDIEAWDAVIE 180

IPTVIFINKIDQAGVDLQSVVQSVRDKLSADIIIKQTVSLSPEIVLEENTDIEAWDAVIE
Sbjct 121
IPTVIFINKIDQAGVDLQSVVQSVRDKLSADIIIKQTVSLSPEIVLEENTDIEAWDAVIE 180

Query 181
NNDKLLEKYIAGEPISREKLVREEQRRVQDASLFPVYYGSAKKGLGIQPLMDAVTGLFQP 240

NNDKLLEKYIAGEPISREKLVREEQRRVQDASLFPVYYGSAKKGLGIQPLMDAVTGLFQP
Sbjct 181
NNDKLLEKYIAGEPISREKLVREEQRRVQDASLFPVYYGSAKKGLGIQPLMDAVTGLFQP 240

Query 241
IGEQGSAALCGSVFKVEYTDCGQRRVYLRLYSGTLRLRDTVALAGREKLKITEMRIPSKG 300

IGEQGSAALCGSVFKVEYTDCGQRRVYLRLYSGTLRLRDTVALAGREKLKITEMRIPSKG
Sbjct 241
IGEQGSAALCGSVFKVEYTDCGQRRVYLRLYSGTLRLRDTVALAGREKLKITEMRIPSKG 300

Query 301
EIVRTDTAYPGEIVILPSDSVRLNDVLGDPTRLPRKRWREDPLPMLRTTIAPKTAAQRER 360
EIVRTDTAYPGEIVILPSDSVRLNDVLGDPTRLPRKRWREDPLPMLRT
+IAPKTAAQRER
Sbjct 301
EIVRTDTAYPGEIVILPSDSVRLNDVLGDPTRLPRKRWREDPLPMLRTSIAPKTAAQRER 360

Query 361
LLDALTQLADTDPLLRCEVDSITHEIILSFLGRVQLEVVSALLSEKYKLETVVKEPSVIY 420
LLDALTQLADTDPLLRCEVDSITHEIILSFLGRVQLEVVSALLSEKYKLETVVK+P
+VIY
Sbjct 361
LLDALTQLADTDPLLRCEVDSITHEIILSFLGRVQLEVVSALLSEKYKLETVVKDPTVIY 420

Query 421
MERPLKAASHTIHIEVPPNPFWASIGLSVTPLSLGSGVQYESRVSLGYLNQSFQNAVRDG 480
MERPLKAASHTIHIEVPPNPFWASIGLSVTPL
LGSGVQYESRVSLGYLNQSFQNAVRDG
Sbjct 421
MERPLKAASHTIHIEVPPNPFWASIGLSVTPLPLGSGVQYESRVSLGYLNQSFQNAVRDG 480

Query 481
IRYGLEQGLFGWNVTDCKICFEYGLYYSPVSTPADFRSLAPIVLEQALKESGTQLLEPYL 540

IRYGLEQGLFGWNVTDCKICFEYGLYYSPVSTPADFRSLAPIVLEQALKESGTQLLEPYL
Sbjct 481
IRYGLEQGLFGWNVTDCKICFEYGLYYSPVSTPADFRSLAPIVLEQALKESGTQLLEPYL 540

Query 541
SFILYAPQEYLSRAYHDAPKYCATIETAQVKKDEVVFTGEIPARCIQAYRTDLAFYTNGR 600
SF LYAP+EYLSRAYHDAPKYCATIET

QVKKDEVVFTGEIPARCIQAYRTDLAFYTNG+
Sbjct 541
SFTLYAPREYLSRAYHDAPKYCATIETVQVKKDEVVFTGEIPARCIQAYRTDLAFYTNGQ 600

Query 601 SVCLTELKGYQAAVGQPVIQPRRPNSRLDKVRHMFQKVM 639
            SVCLTELKGYQAAVG+PVIQPRRPNSRLDKVR+MFQK+M
Sbjct 601 SVCLTELKGYQAAVGKPVIQPRRPNSRLDKVRYMFQKIM 639

>WP_056994958.1 MULTISPECIES: TetM/TetW/TetO/TetS family
tetracycline resistance
ribosomal protection protein [Lactobacillus]
 KRN43123.1 hypothetical protein IV41_GL001882 [Lactobacillus
ingluviei]
 OTA84831.1 tetracycline resistance ribosomal protection protein
[Lactobacillus
reuteri]
 OUN47902.1 tetracycline resistance ribosomal protection protein
[Lactobacillus
reuteri]
 OUP88538.1 tetracycline resistance ribosomal protection protein
[Lactobacillus
reuteri]
Length=639

 Score = 1294 bits (3349), Expect = 0.0, Method: Compositional
matrix adjust.
 Identities = 628/639 (98%), Positives = 636/639 (99%), Gaps = 0/639
(0%)

Query 1
MKIINIGILAHVDAGKTTLTESLLYASGAISEPGSVEKGTTRTDTMFLERQRGITIQAAV 60
            MKIINIGILAHVDAGKTTLTESLLYASGAISEPGSV
+KGTTRTDTMFLERQRGITIQAAV
Sbjct 1
MKIINIGILAHVDAGKTTLTESLLYASGAISEPGSVKKGTTRTDTMFLERQRGITIQAAV 60

Query 61
TSFQWHRCKVNIVDTPGHMDFLAEVYRSLAVLDGAILVISAKDGVQAQTRILFHALRKMN 120

TSFQWHRCKVNIVDTPGHMDFLAEVYRSLAVLDGAILVISAKDGVQAQTRILFHALRKMN
Sbjct 61
TSFQWHRCKVNIVDTPGHMDFLAEVYRSLAVLDGAILVISAKDGVQAQTRILFHALRKMN 120

Query 121
IPTVIFINKIDQAGVDLQSVVQSVRDKLSADIIIKQTVSLSPEIVLEENTDIEAWDAVIE 180

IPTVIFINKIDQAGVDLQSVVQSVRDKLSADIIIKQTVSLSPEIVLEENTDIEAWDAVIE
Sbjct 121
IPTVIFINKIDQAGVDLQSVVQSVRDKLSADIIIKQTVSLSPEIVLEENTDIEAWDAVIE 180

Query 181
NNDKLLEKYIAGEPISREKLVREEQRRVQDASLFPVYYGSAKKGLGIQPLMDAVTGLFQP 240

```
NNDKLLEKYIAGEPISREKLVREEQRRVQDASLFPVYYGSAKKGLGIQPLMDAVTGLFQP
Sbjct  181
NNDKLLEKYIAGEPISREKLVREEQRRVQDASLFPVYYGSAKKGLGIQPLMDAVTGLFQP  240

Query  241
IGEQGSAALCGSVFKVEYTDCGQRRVYLRLYSGTLRLRDTVALAGREKLKITEMRIPSKG  300

IGEQGSAALCGSVFKVEYTDCGQRRVYLRLYSGTLRLRDTVALAGREKLKITEMRIPSKG
Sbjct  241
IGEQGSAALCGSVFKVEYTDCGQRRVYLRLYSGTLRLRDTVALAGREKLKITEMRIPSKG  300

Query  301
EIVRTDTAYPGEIVILPSDSVRLNDVLGDPTRLPRKRWREDPLPMLRTTIAPKTAAQRER  360
            EIVRTDTAYPGEIVILPSDSVRLNDVLGDPTRLPRKRWREDPLPMLRT
+IAPKTAAQRER
Sbjct  301
EIVRTDTAYPGEIVILPSDSVRLNDVLGDPTRLPRKRWREDPLPMLRTSIAPKTAAQRER  360

Query  361
LLDALTQLADTDPLLRCEVDSITHEIILSFLGRVQLEVVSALLSEKYKLETVVKEPSVIY  420
            LLDALTQLADTDPLLRCEVDSITHEIILSFLGRVQLEVVSALLSEKYKLETVVKEP
+VIY
Sbjct  361
LLDALTQLADTDPLLRCEVDSITHEIILSFLGRVQLEVVSALLSEKYKLETVVKEPTVIY  420

Query  421
MERPLKAASHTIHIEVPPNPFWASIGLSVTPLSLGSGVQYESRVSLGYLNQSFQNAVRDG  480
            MERPLKAASHTIHIEVPPNPFWASIGLSVTPL
LGSGVQYESRVSLGYLNQSFQNAVRDG
Sbjct  421
MERPLKAASHTIHIEVPPNPFWASIGLSVTPLPLGSGVQYESRVSLGYLNQSFQNAVRDG  480

Query  481
IRYGLEQGLFGWNVTDCKICFEYGLYYSPVSTPADFRSLAPIVLEQALKESGTQLLEPYL  540

IRYGLEQGLFGWNVTDCKICFEYGLYYSPVSTPADFRSLAPIVLEQALKESGTQLLEPYL
Sbjct  481
IRYGLEQGLFGWNVTDCKICFEYGLYYSPVSTPADFRSLAPIVLEQALKESGTQLLEPYL  540

Query  541
SFILYAPQEYLSRAYHDAPKYCATIETAQVKKDEVVFTGEIPARCIQAYRTDLAFYTNGR  600
            SF LYAP+EYLSRAYHDAPKYCATIET
QVKKDEVVFTGEIPARCIQAYRTDLAFYTNG+
Sbjct  541
SFTLYAPREYLSRAYHDAPKYCATIETVQVKKDEVVFTGEIPARCIQAYRTDLAFYTNGQ  600

Query  601  SVCLTELKGYQAAVGQPVIQPRRPNSRLDKVRHMFQKVM  639
            SVCLTELKGYQAAVG+PVIQPRRPNSRLDKVR+MFQK+M
Sbjct  601  SVCLTELKGYQAAVGKPVIQPRRPNSRLDKVRYMFQKIM  639


>WP_013136942.1 TetM/TetW/TetO/TetS family tetracycline resistance
ribosomal
protection protein [Corynebacterium resistens]
```

YP_003657308.1 tetracycline resistance protein (plasmid)
[Corynebacterium resistens
DSM 45100]
CBL95078.1 tetracycline resistance protein (plasmid)
[Corynebacterium resistens
DSM 45100]
Length=639

Score = 1294 bits (3349), Expect = 0.0, Method: Compositional
matrix adjust.
Identities = 628/639 (98%), Positives = 635/639 (99%), Gaps = 0/639
(0%)

```
Query  1
MKIINIGILAHVDAGKTTLTESLLYASGAISEPGSVEKGTTRTDTMFLERQRGITIQAAV  60

MKIINIGILAHVDAGKTTLTESLLYASGAISEPGSVEKGTTRTDTMFLERQRGITIQAAV
Sbjct  1
MKIINIGILAHVDAGKTTLTESLLYASGAISEPGSVEKGTTRTDTMFLERQRGITIQAAV  60

Query  61
TSFQWHRCKVNIVDTPGHMDFLAEVYRSLAVLDGAILVISAKDGVQAQTRILFHALRKMN  120

TSFQWHRCKVNIVDTPGHMDFLAEVYRSLAVLDGAILVISAKDGVQAQTRILFHALRKMN
Sbjct  61
TSFQWHRCKVNIVDTPGHMDFLAEVYRSLAVLDGAILVISAKDGVQAQTRILFHALRKMN  120

Query  121
IPTVIFINKIDQAGVDLQSVVQSVRDKLSADIIIKQTVSLSPEIVLEENTDIEAWDAVIE  180

IPTVIFINKIDQAGVDLQSVVQSVRDKLSADIIIKQTVSLSPEIVLEENTDIEAWDAVIE
Sbjct  121
IPTVIFINKIDQAGVDLQSVVQSVRDKLSADIIIKQTVSLSPEIVLEENTDIEAWDAVIE  180

Query  181
NNDKLLEKYIAGEPISREKLVREEQRRVQDASLFPVYYGSAKKGLGIQPLMDAVTGLFQP  240

NNDKLLEKYIAGEPISREKLVREEQRRVQDASLFPVYYGSAKKGLGIQPLMDAVTGLFQP
Sbjct  181
NNDKLLEKYIAGEPISREKLVREEQRRVQDASLFPVYYGSAKKGLGIQPLMDAVTGLFQP  240

Query  241
IGEQGSAALCGSVFKVEYTDCGQRRVYLRLYSGTLRLRDTVALAGREKLKITEMRIPSKG  300

IGEQGSAALCGSVFKVEYTDCGQRRVYLRLYSGTLRLRDTVALAGREKLKITEMRIPSKG
Sbjct  241
IGEQGSAALCGSVFKVEYTDCGQRRVYLRLYSGTLRLRDTVALAGREKLKITEMRIPSKG  300

Query  301
EIVRTDTAYPGEIVILPSDSVRLNDVLGDPTRLPRKRWREDPLPMLRTTIAPKTAAQRER  360
            EIVRTDTAYPGEIVILPSDSVRLNDVLGDPTRLPRKRWREDPLPMLRT
+IAPKTAAQRER
Sbjct  301
EIVRTDTAYPGEIVILPSDSVRLNDVLGDPTRLPRKRWREDPLPMLRTSIAPKTAAQRER  360
```

```
Query  361
LLDALTQLADTDPLLRCEVDSITHEIILSFLGRVQLEVVSALLSEKYKLETVVKEPSVIY  420
            LLDALTQLADTDPLLRCEVDSITHEIILSFLGRVQLEVVS LLSEKYKLETVVKEP
+VIY
Sbjct  361
LLDALTQLADTDPLLRCEVDSITHEIILSFLGRVQLEVVSTLLSEKYKLETVVKEPTVIY  420

Query  421
MERPLKAASHTIHIEVPPNPFWASIGLSVTPLSLGSGVQYESRVSLGYLNQSFQNAVRDG  480
            MERPLKAASHTIHIEVPPNPFWASIGLSVTPL
LGSGVQYESRVSLGYLNQSFQNAVRDG
Sbjct  421
MERPLKAASHTIHIEVPPNPFWASIGLSVTPLPLGSGVQYESRVSLGYLNQSFQNAVRDG  480

Query  481
IRYGLEQGLFGWNVTDCKICFEYGLYYSPVSTPADFRSLAPIVLEQALKESGTQLLEPYL  540

IRYGLEQGLFGWNVTDCKICFEYGLYYSPVSTPADFRSLAPIVLEQALKESGTQLLEPYL
Sbjct  481
IRYGLEQGLFGWNVTDCKICFEYGLYYSPVSTPADFRSLAPIVLEQALKESGTQLLEPYL  540

Query  541
SFILYAPQEYLSRAYHDAPKYCATIETAQVKKDEVVFTGEIPARCIQAYRTDLAFYTNGR  600
            SF LYAP+EYLSRAYHDAPKYCATIET
QVKKDEVVFTGEIPARCIQAYRTDLAFYTNG+
Sbjct  541
SFTLYAPREYLSRAYHDAPKYCATIETVQVKKDEVVFTGEIPARCIQAYRTDLAFYTNGQ  600

Query  601  SVCLTELKGYQAAVGQPVIQPRRPNSRLDKVRHMFQKVM  639
            SVCLTELKGYQAAVG+PVIQPRRPNSRLDKVR+MFQK+M
Sbjct  601  SVCLTELKGYQAAVGKPVIQPRRPNSRLDKVRYMFQKIM  639


>WP_008863485.1 TetM/TetW/TetO/TetS family tetracycline resistance
ribosomal
protection protein [Parasutterella excrementihominis]
 EGG57129.1 putative translation elongation factor G [Parasutterella
excrementihominis
YIT 11859]
Length=639

 Score = 1294 bits (3349),  Expect = 0.0, Method: Compositional
matrix adjust.
 Identities = 628/639 (98%), Positives = 636/639 (99%), Gaps = 0/639
(0%)

Query  1
MKIINIGILAHVDAGKTTLTESLLYASGAISEPGSVEKGTTRTDTMFLERQRGITIQAAV  60

MKIINIGILAHVDAGKTTLTESLLYASGAISEPGSVEKGTTRTDTMFLERQRGITIQAAV
Sbjct  1
MKIINIGILAHVDAGKTTLTESLLYASGAISEPGSVEKGTTRTDTMFLERQRGITIQAAV  60
```

Query 61
TSFQWHRCKVNIVDTPGHMDFLAEVYRSLAVLDGAILVISAKDGVQAQTRILFHALRKMN 120

TSFQWHRCKVNIVDTPGHMDFLAEVYRSLAVLDGAILVISAKDGVQAQTRILFHALRKMN
Sbjct 61
TSFQWHRCKVNIVDTPGHMDFLAEVYRSLAVLDGAILVISAKDGVQAQTRILFHALRKMN 120

Query 121
IPTVIFINKIDQAGVDLQSVVQSVRDKLSADIIIKQTVSLSPEIVLEENTDIEAWDAVIE 180

IPTVIFINKIDQAGVDLQSVVQSVRDKLSADIIIKQTVSLSPEIVLEENTDIEAWDAVIE
Sbjct 121
IPTVIFINKIDQAGVDLQSVVQSVRDKLSADIIIKQTVSLSPEIVLEENTDIEAWDAVIE 180

Query 181
NNDKLLEKYIAGEPISREKLVREEQRRVQDASLFPVYYGSAKKGLGIQPLMDAVTGLFQP 240

NNDKLLEKYIAGEPISREKLVREEQRRVQDASLFPVYYGSAKKGLGIQPLMDAVTGLFQP
Sbjct 181
NNDKLLEKYIAGEPISREKLVREEQRRVQDASLFPVYYGSAKKGLGIQPLMDAVTGLFQP 240

Query 241
IGEQGSAALCGSVFKVEYTDCGQRRVYLRLYSGTLRLRDTVALAGREKLKITEMRIPSKG 300

IGEQGSAALCGSVFKVEYTDCGQRRVYLRLYSGTLRLRDTVALAGREKLKITEMRIPSKG
Sbjct 241
IGEQGSAALCGSVFKVEYTDCGQRRVYLRLYSGTLRLRDTVALAGREKLKITEMRIPSKG 300

Query 301
EIVRTDTAYPGEIVILPSDSVRLNDVLGDPTRLPRKRWREDPLPMLRTTIAPKTAAQRER 360
EIVRTDTAYPGEIVILPSDSVRLNDVLGDPTRLPRKRWREDPLPMLRT
+IAPKTAAQRER
Sbjct 301
EIVRTDTAYPGEIVILPSDSVRLNDVLGDPTRLPRKRWREDPLPMLRTSIAPKTAAQRER 360

Query 361
LLDALTQLADTDPLLRCEVDSITHEIILSFLGRVQLEVVSALLSEKYKLETVVKEPSVIY 420
LLDALTQLADTDPLLRCEVDSITHEIILSFLGRVQLEVVSALLSEKYKLETVVKEP
+VIY
Sbjct 361
LLDALTQLADTDPLLRCEVDSITHEIILSFLGRVQLEVVSALLSEKYKLETVVKEPTVIY 420

Query 421
MERPLKAASHTIHIEVPPNPFWASIGLSVTPLSLGSGVQYESRVSLGYLNQSFQNAVRDG 480
MERPLKAASHTIHIEVPPNPFWASIGLSVTPL LGSGVQ
+ESRVSLGYLNQSFQNAVRDG
Sbjct 421
MERPLKAASHTIHIEVPPNPFWASIGLSVTPLPLGSGVQHESRVSLGYLNQSFQNAVRDG 480

Query 481
IRYGLEQGLFGWNVTDCKICFEYGLYYSPVSTPADFRSLAPIVLEQALKESGTQLLEPYL 540

IRYGLEQGLFGWNVTDCKICFEYGLYYSPVSTPADFRSLAPIVLEQALKESGTQLLEPYL
Sbjct 481

IRYGLEQGLFGWNVTDCKICFEYGLYYSPVSTPADFRSLAPIVLEQALKESGTQLLEPYL 540

Query 541
SFILYAPQEYLSRAYHDAPKYCATIETAQVKKDEVVFTGEIPARCIQAYRTDLAFYTNGR 600
           SF LYAP+EYLSRAYHDAPKYCATIET
QVKKDEVVFTGEIPARCIQAYRTDLAFYTNG+
Sbjct 541
SFTLYAPREYLSRAYHDAPKYCATIETVQVKKDEVVFTGEIPARCIQAYRTDLAFYTNGQ 600

Query 601 SVCLTELKGYQAAVGQPVIQPRRPNSRLDKVRHMFQKVM 639
            SVCLTELKGYQAAVG+PVIQPRRPNSRLDKVR+MFQK+M
Sbjct 601 SVCLTELKGYQAAVGKPVIQPRRPNSRLDKVRYMFQKIM 639

>ABF72129.1 TetW [Trueperella pyogenes]
Length=639

Score = 1294 bits (3349), Expect = 0.0, Method: Compositional matrix adjust.
Identities = 628/639 (98%), Positives = 636/639 (99%), Gaps = 0/639 (0%)

Query 1
MKIINIGILAHVDAGKTTLTESLLYASGAISEPGSVEKGTTRTDTMFLERQRGITIQAAV 60

MKIINIGILAHVDAGKTTLTESLLYASGAISEPGSVEKGTTRTDTMFLERQRGITIQAAV
Sbjct 1
MKIINIGILAHVDAGKTTLTESLLYASGAISEPGSVEKGTTRTDTMFLERQRGITIQAAV 60

Query 61
TSFQWHRCKVNIVDTPGHMDFLAEVYRSLAVLDGAILVISAKDGVQAQTRILFHALRKMN 120

TSFQWHRCKVNIVDTPGHMDFLAEVYRSLAVLDGAILVISAKDGVQAQTRILFHALRKMN
Sbjct 61
TSFQWHRCKVNIVDTPGHMDFLAEVYRSLAVLDGAILVISAKDGVQAQTRILFHALRKMN 120

Query 121
IPTVIFINKIDQAGVDLQSVVQSVRDKLSADIIIKQTVSLSPEIVLEENTDIEAWDAVIE 180

IPTVIFINKIDQAGVDLQSVVQSVRDKLSADIIIKQTVSLSPEIVLEENTDIEAWDAVIE
Sbjct 121
IPTVIFINKIDQAGVDLQSVVQSVRDKLSADIIIKQTVSLSPEIVLEENTDIEAWDAVIE 180

Query 181
NNDKLLEKYIAGEPISREKLVREEQRRVQDASLFPVYYGSAKKGLGIQPLMDAVTGLFQP 240
           NNDKLLEKYIAGEPISREKLVREEQRRVQ
+ASLFPVYYGSAKKGLGIQPLMDAVTGLFQP
Sbjct 181
NNDKLLEKYIAGEPISREKLVREEQRRVQEASLFPVYYGSAKKGLGIQPLMDAVTGLFQP 240

Query 241
IGEQGSAALCGSVFKVEYTDCGQRRVYLRLYSGTLRLRDTVALAGREKLKITEMRIPSKG 300

IGEQGSAALCGSVFKVEYTDCGQRRVYLRLYSGTLRLRDTVALAGREKLKITEMRIPSKG

```
Sbjct  241
IGEQGSAALCGSVFKVEYTDCGQRRVYLRLYSGTLRLRDTVALAGREKLKITEMRIPSKG  300

Query  301
EIVRTDTAYPGEIVILPSDSVRLNDVLGDPTRLPRKRWREDPLPMLRTTIAPKTAAQRER  360
            EIVRTDTAYPGEIVILPSDSVRLNDVLGDPTRLPRKRWREDPLPMLRT
+IAPKTAAQRER
Sbjct  301
EIVRTDTAYPGEIVILPSDSVRLNDVLGDPTRLPRKRWREDPLPMLRTSIAPKTAAQRER  360

Query  361
LLDALTQLADTDPLLRCEVDSITHEIILSFLGRVQLEVVSALLSEKYKLETVVKEPSVIY  420
            LLDALTQLADTDPLLRCEVDSITHEIILSFLGRVQLEVVSALLSEKYKLETVVKEP
+VIY
Sbjct  361
LLDALTQLADTDPLLRCEVDSITHEIILSFLGRVQLEVVSALLSEKYKLETVVKEPTVIY  420

Query  421
MERPLKAASHTIHIEVPPNPFWASIGLSVTPLSLGSGVQYESRVSLGYLNQSFQNAVRDG  480
            MERPLKAASHTIHIEVPPNPFWASIGLSVTPL
LGSGVQYESRVSLGYLNQSFQNAVRDG
Sbjct  421
MERPLKAASHTIHIEVPPNPFWASIGLSVTPLPLGSGVQYESRVSLGYLNQSFQNAVRDG  480

Query  481
IRYGLEQGLFGWNVTDCKICFEYGLYYSPVSTPADFRSLAPIVLEQALKESGTQLLEPYL  540

IRYGLEQGLFGWNVTDCKICFEYGLYYSPVSTPADFRSLAPIVLEQALKESGTQLLEPYL
Sbjct  481
IRYGLEQGLFGWNVTDCKICFEYGLYYSPVSTPADFRSLAPIVLEQALKESGTQLLEPYL  540

Query  541
SFILYAPQEYLSRAYHDAPKYCATIETAQVKKDEVVFTGEIPARCIQAYRTDLAFYTNGR  600
            SF LYAP+EYLSRAYHDAPKYCATIET
QVKKDEVVFTGEIPARCIQAYRTDLAFYTNG+
Sbjct  541
SFTLYAPREYLSRAYHDAPKYCATIETVQVKKDEVVFTGEIPARCIQAYRTDLAFYTNGQ  600

Query  601  SVCLTELKGYQAAVGQPVIQPRRPNSRLDKVRHMFQKVM  639
            SVCLTELKGYQAAVG+PVIQPRRPNSRLDKVR+MFQK+M
Sbjct  601  SVCLTELKGYQAAVGKPVIQPRRPNSRLDKVRYMFQKIM  639


>WP_102343532.1 TetM/TetW/TetO/TetS family tetracycline resistance
ribosomal
protection protein [Lactomassilus timonensis]
Length=639

 Score = 1294 bits (3348),  Expect = 0.0, Method: Compositional
matrix adjust.
 Identities = 629/639 (98%), Positives = 634/639 (99%), Gaps = 0/639
(0%)

Query  1
```

MKIINIGILAHVDAGKTTLTESLLYASGAISEPGSVEKGTTRTDTMFLERQRGITIQAAV 60

MKIINIGILAHVDAGKTTLTESLLYASGAISEPGSVEKGTTRTDTMFLERQRGITIQAAV
Sbjct 1
MKIINIGILAHVDAGKTTLTESLLYASGAISEPGSVEKGTTRTDTMFLERQRGITIQAAV 60

Query 61
TSFQWHRCKVNIVDTPGHMDFLAEVYRSLAVLDGAILVISAKDGVQAQTRILFHALRKMN 120

TSFQWHRCKVNIVDTPGHMDFLAEVYRSLAVLDGAILVISAKDGVQAQTRILFHALRKMN
Sbjct 61
TSFQWHRCKVNIVDTPGHMDFLAEVYRSLAVLDGAILVISAKDGVQAQTRILFHALRKMN 120

Query 121
IPTVIFINKIDQAGVDLQSVVQSVRDKLSADIIIKQTVSLSPEIVLEENTDIEAWDAVIE 180

IPTVIFINKIDQAGVDLQSVVQSVRDKLSADIIIKQTVSLSPEIVLEENTDIEAWDAVIE
Sbjct 121
IPTVIFINKIDQAGVDLQSVVQSVRDKLSADIIIKQTVSLSPEIVLEENTDIEAWDAVIE 180

Query 181
NNDKLLEKYIAGEPISREKLVREEQRRVQDASLFPVYYGSAKKGLGIQPLMDAVTGLFQP 240
NND LLEKYIAGEPIS+EKL
REEQRRVQDASLFPVYYGSAKKGLGIQPLMDAVTGLFQP
Sbjct 181
NNDALLEKYIAGEPISQEKLAREEQRRVQDASLFPVYYGSAKKGLGIQPLMDAVTGLFQP 240

Query 241
IGEQGSAALCGSVFKVEYTDCGQRRVYLRLYSGTLRLRDTVALAGREKLKITEMRIPSKG 300

IGEQGSAALCGSVFKVEYTDCGQRRVYLRLYSGTLRLRDTVALAGREKLKITEMRIPSKG
Sbjct 241
IGEQGSAALCGSVFKVEYTDCGQRRVYLRLYSGTLRLRDTVALAGREKLKITEMRIPSKG 300

Query 301
EIVRTDTAYPGEIVILPSDSVRLNDVLGDPTRLPRKRWREDPLPMLRTTIAPKTAAQRER 360
EIVRTDTAYPGEIVILPSDSVRLNDVLGDPTRLPRKRWREDPLPMLRT
+IAPKTAAQRER
Sbjct 301
EIVRTDTAYPGEIVILPSDSVRLNDVLGDPTRLPRKRWREDPLPMLRTSIAPKTAAQRER 360

Query 361
LLDALTQLADTDPLLRCEVDSITHEIILSFLGRVQLEVVSALLSEKYKLETVVKEPSVIY 420
LLDALTQLADTDPLLRCEVDSITHEIILSFLGRVQLEVVSALLSEKYKLETVVKEP
+VIY
Sbjct 361
LLDALTQLADTDPLLRCEVDSITHEIILSFLGRVQLEVVSALLSEKYKLETVVKEPTVIY 420

Query 421
MERPLKAASHTIHIEVPPNPFWASIGLSVTPLSLGSGVQYESRVSLGYLNQSFQNAVRDG 480
MERPLKAASHTIHIEVPPNPFWASIGLSVTPL
LGSGVQYESRVSLGYLNQSFQNAVRDG
Sbjct 421
MERPLKAASHTIHIEVPPNPFWASIGLSVTPLPLGSGVQYESRVSLGYLNQSFQNAVRDG 480

Query 481
IRYGLEQGLFGWNVTDCKICFEYGLYYSPVSTPADFRSLAPIVLEQALKESGTQLLEPYL 540

IRYGLEQGLFGWNVTDCKICFEYGLYYSPVSTPADFRSLAPIVLEQALKESGTQLLEPYL
Sbjct 481
IRYGLEQGLFGWNVTDCKICFEYGLYYSPVSTPADFRSLAPIVLEQALKESGTQLLEPYL 540

Query 541
SFILYAPQEYLSRAYHDAPKYCATIETAQVKKDEVVFTGEIPARCIQAYRTDLAFYTNGR 600
            SF LYAPQEYLSRAYHDAPKYCATIETAQVKKDEVVFTGEIPARCIQAYRTDLA
YTNG+
Sbjct 541
SFTLYAPQEYLSRAYHDAPKYCATIETAQVKKDEVVFTGEIPARCIQAYRTDLASYTNGQ 600

Query 601 SVCLTELKGYQAAVGQPVIQPRRPNSRLDKVRHMFQKVM 639
            SVCLTELKGYQAAVGQPVIQPRRPN+RLDKVRHMFQKVM
Sbjct 601 SVCLTELKGYQAAVGQPVIQPRRPNNRLDKVRHMFQKVM 639

>WP_097800170.1 TetM/TetW/TetO/TetS family tetracycline resistance ribosomal
protection protein [Faecalibacterium prausnitzii]
 PDX62079.1 tetracycline resistance ribosomal protection protein Tet(W) [Faecalibacterium
prausnitzii]
Length=639

 Score = 1294 bits (3348), Expect = 0.0, Method: Compositional matrix adjust.
 Identities = 630/639 (99%), Positives = 633/639 (99%), Gaps = 0/639 (0%)

Query 1
MKIINIGILAHVDAGKTTLTESLLYASGAISEPGSVEKGTTRTDTMFLERQRGITIQAAV 60

MKIINIGILAHVDAGKTTLTESLLYASGAISEPGSVEKGTTRTDTMFLERQRGITIQAAV
Sbjct 1
MKIINIGILAHVDAGKTTLTESLLYASGAISEPGSVEKGTTRTDTMFLERQRGITIQAAV 60

Query 61
TSFQWHRCKVNIVDTPGHMDFLAEVYRSLAVLDGAILVISAKDGVQAQTRILFHALRKMN 120

TSFQWHRCKVNIVDTPGHMDFLAEVYRSLAVLDGAILVISAKDGVQAQTRILFHALRKMN
Sbjct 61
TSFQWHRCKVNIVDTPGHMDFLAEVYRSLAVLDGAILVISAKDGVQAQTRILFHALRKMN 120

Query 121
IPTVIFINKIDQAGVDLQSVVQSVRDKLSADIIIKQTVSLSPEIVLEENTDIEAWDAVIE 180

IPTVIFINKIDQAGVDLQSVVQSVRDKLSADIIIKQTVSLSPEIVLEENTDIEAWDAVIE
Sbjct 121
IPTVIFINKIDQAGVDLQSVVQSVRDKLSADIIIKQTVSLSPEIVLEENTDIEAWDAVIE 180

```
Query  181
NNDKLLEKYIAGEPISREKLVREEQRRVQDASLFPVYYGSAKKGLGIQPLMDAVTGLFQP  240
             NND+LLEKYIAGEPISREKL REEQ+RVQDASLFPVY+GSAK
GLGIQPLMDAVTGLFQP
Sbjct  181
NNDELLEKYIAGEPISREKLAREEQQRVQDASLFPVYHGSAKNGLGIQPLMDAVTGLFQP  240

Query  241
IGEQGSAALCGSVFKVEYTDCGQRRVYLRLYSGTLRLRDTVALAGREKLKITEMRIPSKG  300
             IGEQG
AALCGSVFKVEYTDCGQRRVYLRLYSGTLRLRDTVALAGREKLKITEMRIPSKG
Sbjct  241
IGEQGGAALCGSVFKVEYTDCGQRRVYLRLYSGTLRLRDTVALAGREKLKITEMRIPSKG  300

Query  301
EIVRTDTAYPGEIVILPSDSVRLNDVLGDPTRLPRKRWREDPLPMLRTTIAPKTAAQRER  360
             EIVRTDTAY GEIVILPSDSVRLNDVLGD
TRLPRKRWREDPLPMLRTTIAPKTAAQRER
Sbjct  301
EIVRTDTAYQGEIVILPSDSVRLNDVLGDQTRLPRKRWREDPLPMLRTTIAPKTAAQRER  360

Query  361
LLDALTQLADTDPLLRCEVDSITHEIILSFLGRVQLEVVSALLSEKYKLETVVKEPSVIY  420

LLDALTQLADTDPLLRCEVDSITHEIILSFLGRVQLEVVSALLSEKYKLETVVKEPSVIY
Sbjct  361
LLDALTQLADTDPLLRCEVDSITHEIILSFLGRVQLEVVSALLSEKYKLETVVKEPSVIY  420

Query  421
MERPLKAASHTIHIEVPPNPFWASIGLSVTPLSLGSGVQYESRVSLGYLNQSFQNAVRDG  480

MERPLKAASHTIHIEVPPNPFWASIGLSVTPLSLGSGVQYESRVSLGYLNQSFQNAVRDG
Sbjct  421
MERPLKAASHTIHIEVPPNPFWASIGLSVTPLSLGSGVQYESRVSLGYLNQSFQNAVRDG  480

Query  481
IRYGLEQGLFGWNVTDCKICFEYGLYYSPVSTPADFRSLAPIVLEQALKESGTQLLEPYL  540

IRYGLEQGLFGWNVTDCKICFEYGLYYSPVSTPADFRSLAPIVLEQALKESGTQLLEPYL
Sbjct  481
IRYGLEQGLFGWNVTDCKICFEYGLYYSPVSTPADFRSLAPIVLEQALKESGTQLLEPYL  540

Query  541
SFILYAPQEYLSRAYHDAPKYCATIETAQVKKDEVVFTGEIPARCIQAYRTDLAFYTNGR  600

SFILYAPQEYLSRAYHDAPKYCATIETAQVKKDEVVFTGEIPARCIQAYRTDLAFYTNGR
Sbjct  541
SFILYAPQEYLSRAYHDAPKYCATIETAQVKKDEVVFTGEIPARCIQAYRTDLAFYTNGR  600

Query  601  SVCLTELKGYQAAVGQPVIQPRRPNSRLDKVRHMFQKVM  639
            SVCLTELKGYQA VGQPVIQPRRPNSRLDKVRHMFQKVM
Sbjct  601  SVCLTELKGYQAVVGQPVIQPRRPNSRLDKVRHMFQKVM  639
```

>WP_087248211.1 TetM/TetW/TetO/TetS family tetracycline resistance ribosomal
protection protein [Faecalibacterium sp. An77]
 OUN38164.1 tetracycline resistance ribosomal protection protein Tet(W) [Faecalibacterium
sp. An77]
Length=639

 Score = 1294 bits (3348), Expect = 0.0, Method: Compositional matrix adjust.
 Identities = 630/639 (99%), Positives = 634/639 (99%), Gaps = 0/639 (0%)

```
Query  1    MKIINIGILAHVDAGKTTLTESLLYASGAISEPGSVEKGTTRTDTMFLERQRGITIQAAV  60
            MKIINIGILAHVDAGKTTLTESLLYASGAISEPGSVEKGTTRTDTMFLERQRGITIQAAV
Sbjct  1    MKIINIGILAHVDAGKTTLTESLLYASGAISEPGSVEKGTTRTDTMFLERQRGITIQAAV  60

Query  61   TSFQWHRCKVNIVDTPGHMDFLAEVYRSLAVLDGAILVISAKDGVQAQTRILFHALRKMN  120
            TSFQWHRCKVNIVDTPGHMDFLAEVYRSLAVLDGAILVISAKDGVQAQTRILFHALRKMN
Sbjct  61   TSFQWHRCKVNIVDTPGHMDFLAEVYRSLAVLDGAILVISAKDGVQAQTRILFHALRKMN  120

Query  121  IPTVIFINKIDQAGVDLQSVVQSVRDKLSADIIIKQTVSLSPEIVLEENTDIEAWDAVIE  180
            IPTVIFINKIDQAGVDLQSVVQSVRDKLSADIIIKQTVSLSPEIVLEENTDIEAWDAVIE
Sbjct  121  IPTVIFINKIDQAGVDLQSVVQSVRDKLSADIIIKQTVSLSPEIVLEENTDIEAWDAVIE  180

Query  181  NNDKLLEKYIAGEPISREKLVREEQRRVQDASLFPVYYGSAKKGLGIQPLMDAVTGLFQP  240
            NND+LLEKYIAGEPISREKL REEQ+RVQDASLFPVY+GSAK GLGIQPLMDAVTGLFQP
Sbjct  181  NNDELLEKYIAGEPISREKLAREEQQRVQDASLFPVYHGSAKNGLGIQPLMDAVTGLFQP  240

Query  241  IGEQGSAALCGSVFKVEYTDCGQRRVYLRLYSGTLRLRDTVALAGREKLKITEMRIPSKG  300
            IGEQG AALCGSVFKVEYTDCGQRRVYLRLYSGTLRLRDTVALAGREKLKITEMRIPSKG
Sbjct  241  IGEQGGAALCGSVFKVEYTDCGQRRVYLRLYSGTLRLRDTVALAGREKLKITEMRIPSKG  300

Query  301  EIVRTDTAYPGEIVILPSDSVRLNDVLGDPTRLPRKRWREDPLPMLRTTIAPKTAAQRER  360
            EIVRTDTAY GEIVILPSDSVRLNDVLGD TRLPRKRWREDPLPMLRTTIAPKTAAQRER
Sbjct  301  EIVRTDTAYQGEIVILPSDSVRLNDVLGDQTRLPRKRWREDPLPMLRTTIAPKTAAQRER  360
```

```
Query  361  
LLDALTQLADTDPLLRCEVDSITHEIILSFLGRVQLEVVSALLSEKYKLETVVKEPSVIY  420
            
LLDALTQLADTDPLLRCEVDSITHEIILSFLGRVQLEVVSALLSEKYKLETVVKEPSVIY
Sbjct  361  
LLDALTQLADTDPLLRCEVDSITHEIILSFLGRVQLEVVSALLSEKYKLETVVKEPSVIY  420

Query  421  
MERPLKAASHTIHIEVPPNPFWASIGLSVTPLSLGSGVQYESRVSLGYLNQSFQNAVRDG  480
            
MERPLKAASHTIHIEVPPNPFWASIGLSVTPLSLGSGVQYESRVSLGYLNQSFQNAVRDG
Sbjct  421  
MERPLKAASHTIHIEVPPNPFWASIGLSVTPLSLGSGVQYESRVSLGYLNQSFQNAVRDG  480

Query  481  
IRYGLEQGLFGWNVTDCKICFEYGLYYSPVSTPADFRSLAPIVLEQALKESGTQLLEPYL  540
            
IRYGLEQGLFGWNVTDCKICFEYGLYYSPVSTPADFRSLAPIVLEQALKESGTQLLEPYL
Sbjct  481  
IRYGLEQGLFGWNVTDCKICFEYGLYYSPVSTPADFRSLAPIVLEQALKESGTQLLEPYL  540

Query  541  
SFILYAPQEYLSRAYHDAPKYCATIETAQVKKDEVVFTGEIPARCIQAYRTDLAFYTNGR  600
            SFILYAP
+EYLSRAYHDAPKYCATIETAQVKKDEVVFTGEIPARCIQAYRTDLAFYTNGR
Sbjct  541  
SFILYAPREYLSRAYHDAPKYCATIETAQVKKDEVVFTGEIPARCIQAYRTDLAFYTNGR  600

Query  601  SVCLTELKGYQAAVGQPVIQPRRPNSRLDKVRHMFQKVM  639
            SVCLTELKGYQAAVGQPVIQPRRPNSRLDKVRHMFQKVM
Sbjct  601  SVCLTELKGYQAAVGQPVIQPRRPNSRLDKVRHMFQKVM  639


>WP_078931819.1 TetM/TetW/TetO/TetS family tetracycline resistance 
ribosomal
protection protein [Treponema berlinense]
 SKA06552.1 ribosomal protection tetracycline resistance protein 
[Treponema
berlinense]
Length=639

 Score = 1294 bits (3348),  Expect = 0.0, Method: Compositional 
matrix adjust.
 Identities = 629/639 (98%), Positives = 635/639 (99%), Gaps = 0/639 
(0%)

Query  1    
MKIINIGILAHVDAGKTTLTESLLYASGAISEPGSVEKGTTRTDTMFLERQRGITIQAAV  60
            
MKIINIGILAHVDAGKTTLTESLLYASGAISEPGSVEKGTTRTDTMFLERQRGITIQAAV
Sbjct  1    
MKIINIGILAHVDAGKTTLTESLLYASGAISEPGSVEKGTTRTDTMFLERQRGITIQAAV  60
```

Query 61
TSFQWHRCKVNIVDTPGHMDFLAEVYRSLAVLDGAILVISAKDGVQAQTRILFHALRKMN 120

TSFQWHRCKVNIVDTPGHMDFLAEVYRSLAVLDGAILVISAKDGVQAQTRILFHALRKMN
Sbjct 61
TSFQWHRCKVNIVDTPGHMDFLAEVYRSLAVLDGAILVISAKDGVQAQTRILFHALRKMN 120

Query 121
IPTVIFINKIDQAGVDLQSVVQSVRDKLSADIIIKQTVSLSPEIVLEENTDIEAWDAVIE 180

IPTVIFINKIDQAGVDLQSVVQSVRDKLSADIIIKQTVSLSPEIVLEENTDIEAWDAVIE
Sbjct 121
IPTVIFINKIDQAGVDLQSVVQSVRDKLSADIIIKQTVSLSPEIVLEENTDIEAWDAVIE 180

Query 181
NNDKLLEKYIAGEPISREKLVREEQRRVQDASLFPVYYGSAKKGLGIQPLMDAVTGLFQP 240
NND
+LLEKYIAGEPISREKLVREEQRRVQDASLFPVYYGSAKKGLGIQPLMDAVTGLFQP
Sbjct 181
NNDELLEKYIAGEPISREKLVREEQRRVQDASLFPVYYGSAKKGLGIQPLMDAVTGLFQP 240

Query 241
IGEQGSAALCGSVFKVEYTDCGQRRVYLRLYSGTLRLRDTVALAGREKLKITEMRIPSKG 300

IGEQGSAALCGSVFKVEYTDCGQRRVYLRLYSGTLRLRDTVALAGREKLKITEMRIPSKG
Sbjct 241
IGEQGSAALCGSVFKVEYTDCGQRRVYLRLYSGTLRLRDTVALAGREKLKITEMRIPSKG 300

Query 301
EIVRTDTAYPGEIVILPSDSVRLNDVLGDPTRLPRKRWREDPLPMLRTTIAPKTAAQRER 360
EIVRTDTAYPGEIVILPSDSVRLNDVLGDPTRLPRKRWREDPLPMLRT
+IAPKTAAQRER
Sbjct 301
EIVRTDTAYPGEIVILPSDSVRLNDVLGDPTRLPRKRWREDPLPMLRTSIAPKTAAQRER 360

Query 361
LLDALTQLADTDPLLRCEVDSITHEIILSFLGRVQLEVVSALLSEKYKLETVVKEPSVIY 420
LLDALTQLADTDPLLRCEVDSITHEIILSFLGRVQLEVVSALLSEKYKLETVVKEP
+VIY
Sbjct 361
LLDALTQLADTDPLLRCEVDSITHEIILSFLGRVQLEVVSALLSEKYKLETVVKEPTVIY 420

Query 421
MERPLKAASHTIHIEVPPNPFWASIGLSVTPLSLGSGVQYESRVSLGYLNQSFQNAVRDG 480
MERPLKAASHTIHIEVPPNPFWASIGLSVTPL
LGSGVQYESRVSLGYLNQSFQNAVRDG
Sbjct 421
MERPLKAASHTIHIEVPPNPFWASIGLSVTPLPLGSGVQYESRVSLGYLNQSFQNAVRDG 480

Query 481
IRYGLEQGLFGWNVTDCKICFEYGLYYSPVSTPADFRSLAPIVLEQALKESGTQLLEPYL 540

IRYGLEQGLFGWNVTDCKICFEYGLYYSPVSTPADFRSLAPIVLEQALKESGTQLLEPYL
Sbjct 481

IRYGLEQGLFGWNVTDCKICFEYGLYYSPVSTPADFRSLAPIVLEQALKESGTQLLEPYL 540

Query 541
SFILYAPQEYLSRAYHDAPKYCATIETAQVKKDEVVFTGEIPARCIQAYRTDLAFYTNGR 600
            SF LYAP+EYLSRAYHDAPKYCATIET QVKKDEVVFTGEIPARCIQAYRTDLA
YTNGR
Sbjct 541
SFTLYAPREYLSRAYHDAPKYCATIETVQVKKDEVVFTGEIPARCIQAYRTDLASYTNGR 600

Query 601 SVCLTELKGYQAAVGQPVIQPRRPNSRLDKVRHMFQKVM 639
            SVCLTELKGYQAAVGQPVIQPRRPNSRLDKVR+MFQK+M
Sbjct 601 SVCLTELKGYQAAVGQPVIQPRRPNSRLDKVRYMFQKIM 639

>APO29840.1 Ferrous iron transport protein B [uncultured bacterium]
Length=639

Score = 1294 bits (3348), Expect = 0.0, Method: Compositional
matrix adjust.
Identities = 629/639 (98%), Positives = 633/639 (99%), Gaps = 0/639
(0%)

Query 1
MKIINIGILAHVDAGKTTLTESLLYASGAISEPGSVEKGTTRTDTMFLERQRGITIQAAV 60

MKIINIGILAHVDAGKTTLTESLLYASGAISEPGSVEKGTTRTDTMFLERQRGITIQAAV
Sbjct 1
MKIINIGILAHVDAGKTTLTESLLYASGAISEPGSVEKGTTRTDTMFLERQRGITIQAAV 60

Query 61
TSFQWHRCKVNIVDTPGHMDFLAEVYRSLAVLDGAILVISAKDGVQAQTRILFHALRKMN 120

TSFQWHRCKVNIVDTPGHMDFLAEVYRSLAVLDGAILVISAKDGVQAQTRILFHALRKMN
Sbjct 61
TSFQWHRCKVNIVDTPGHMDFLAEVYRSLAVLDGAILVISAKDGVQAQTRILFHALRKMN 120

Query 121
IPTVIFINKIDQAGVDLQSVVQSVRDKLSADIIIKQTVSLSPEIVLEENTDIEAWDAVIE 180

IPTVIFINKIDQAGVDLQSVVQSVRDKLSADIIIKQTVSLSPEIVLEENTDIEAWDAVIE
Sbjct 121
IPTVIFINKIDQAGVDLQSVVQSVRDKLSADIIIKQTVSLSPEIVLEENTDIEAWDAVIE 180

Query 181
NNDKLLEKYIAGEPISREKLVREEQRRVQDASLFPVYYGSAKKGLGIQPLMDAVTGLFQP 240
            NNDKLLEKYIAGEPISREKL REEQ+RVQDASLFPVY+GSAK
GLGIQPLMDAVTGLFQP
Sbjct 181
NNDKLLEKYIAGEPISREKLAREEQQRVQDASLFPVYHGSAKNGLGIQPLMDAVTGLFQP 240

Query 241
IGEQGSAALCGSVFKVEYTDCGQRRVYLRLYSGTLRLRDTVALAGREKLKITEMRIPSKG 300
            IGEQG
AALCGSVFKVEYTDCGQRRVYLRLYSGTLRLRDTVALAGREKLKITEMRIPSKG

```
Sbjct  241
IGEQGGAALCGSVFKVEYTDCGQRRVYLRLYSGTLRLRDTVALAGREKLKITEMRIPSKG  300

Query  301
EIVRTDTAYPGEIVILPSDSVRLNDVLGDPTRLPRKRWREDPLPMLRTTIAPKTAAQRER  360
           EIVRTDTAY GEIVILPSDSVRLNDVLGDPTRLPRKRWREDPLPMLRT
+IAPKTAAQRER
Sbjct  301
EIVRTDTAYQGEIVILPSDSVRLNDVLGDPTRLPRKRWREDPLPMLRTSIAPKTAAQRER  360

Query  361
LLDALTQLADTDPLLRCEVDSITHEIILSFLGRVQLEVVSALLSEKYKLETVVKEPSVIY  420

LLDALTQLADTDPLLRCEVDSITHEIILSFLGRVQLEVVSALLSEKYKLETVVKEPSVIY
Sbjct  361
LLDALTQLADTDPLLRCEVDSITHEIILSFLGRVQLEVVSALLSEKYKLETVVKEPSVIY  420

Query  421
MERPLKAASHTIHIEVPPNPFWASIGLSVTPLSLGSGVQYESRVSLGYLNQSFQNAVRDG  480

MERPLKAASHTIHIEVPPNPFWASIGLSVTPLSLGSGVQYESRVSLGYLNQSFQNAVRDG
Sbjct  421
MERPLKAASHTIHIEVPPNPFWASIGLSVTPLSLGSGVQYESRVSLGYLNQSFQNAVRDG  480

Query  481
IRYGLEQGLFGWNVTDCKICFEYGLYYSPVSTPADFRSLAPIVLEQALKESGTQLLEPYL  540

IRYGLEQGLFGWNVTDCKICFEYGLYYSPVSTPADFRSLAPIVLEQALKESGTQLLEPYL
Sbjct  481
IRYGLEQGLFGWNVTDCKICFEYGLYYSPVSTPADFRSLAPIVLEQALKESGTQLLEPYL  540

Query  541
SFILYAPQEYLSRAYHDAPKYCATIETAQVKKDEVVFTGEIPARCIQAYRTDLAFYTNGR  600
           SF LYAP+EYLSRAYHDAPKYCATIET
QVKKDEVVFTGEIPARCIQAYRTDLAFYTNGR
Sbjct  541
SFTLYAPREYLSRAYHDAPKYCATIETVQVKKDEVVFTGEIPARCIQAYRTDLAFYTNGR  600

Query  601  SVCLTELKGYQAAVGQPVIQPRRPNSRLDKVRHMFQKVM  639
            SVCLTELKGYQAAVGQPVIQPRRPNSRLDKVRHMFQKVM
Sbjct  601  SVCLTELKGYQAAVGQPVIQPRRPNSRLDKVRHMFQKVM  639


>WP_070739220.1 MULTISPECIES: TetM/TetW/TetO/TetS family
tetracycline resistance
ribosomal protection protein [Rothia]
 OFO77233.1 tetracycline resistance ribosomal protection protein
[Rothia
sp. HMSC065D02]
 PLA17928.1 TetM/TetW/TetO/TetS family tetracycline resistance
ribosomal
protection protein [Rothia dentocariosa]
Length=639
```

```
 Score = 1294 bits (3348),  Expect = 0.0, Method: Compositional
matrix adjust.
 Identities = 630/639 (99%), Positives = 634/639 (99%), Gaps = 0/639
(0%)

Query  1
MKIINIGILAHVDAGKTTLTESLLYASGAISEPGSVEKGTTRTDTMFLERQRGITIQAAV  60

MKIINIGILAHVDAGKTTLTESLLYASGAISEPGSVEKGTTRTDTMFLERQRGITIQAAV
Sbjct  1
MKIINIGILAHVDAGKTTLTESLLYASGAISEPGSVEKGTTRTDTMFLERQRGITIQAAV  60

Query  61
TSFQWHRCKVNIVDTPGHMDFLAEVYRSLAVLDGAILVISAKDGVQAQTRILFHALRKMN  120

TSFQWHRCKVNIVDTPGHMDFLAEVYRSLAVLDGAILVISAKDGVQAQTRILFHALRKMN
Sbjct  61
TSFQWHRCKVNIVDTPGHMDFLAEVYRSLAVLDGAILVISAKDGVQAQTRILFHALRKMN  120

Query  121
IPTVIFINKIDQAGVDLQSVVQSVRDKLSADIIIKQTVSLSPEIVLEENTDIEAWDAVIE  180

IPTVIFINKIDQAGVDLQSVVQSVRDKLSADIIIKQTVSLSPEIVLEENTDIEAWDAVIE
Sbjct  121
IPTVIFINKIDQAGVDLQSVVQSVRDKLSADIIIKQTVSLSPEIVLEENTDIEAWDAVIE  180

Query  181
NNDKLLEKYIAGEPISREKLVREEQRRVQDASLFPVYYGSAKKGLGIQPLMDAVTGLFQP  240
            NND+LLEKYIAGEPISREKL REEQ+RVQDASLFPVY+GSAK
GLGIQPLMDAVTGLFQP
Sbjct  181
NNDELLEKYIAGEPISREKLAREEQQRVQDASLFPVYHGSAKNGLGIQPLMDAVTGLFQP  240

Query  241
IGEQGSAALCGSVFKVEYTDCGQRRVYLRLYSGTLRLRDTVALAGREKLKITEMRIPSKG  300
            IGEQG
AALCGSVFKVEYTDCGQRRVYLRLYSGTLRLRDTVALAGREKLKITEMRIPSKG
Sbjct  241
IGEQGGAALCGSVFKVEYTDCGQRRVYLRLYSGTLRLRDTVALAGREKLKITEMRIPSKG  300

Query  301
EIVRTDTAYPGEIVILPSDSVRLNDVLGDPTRLPRKRWREDPLPMLRTTIAPKTAAQRER  360
            EIVRTDTAY GEIVILPSDSVRLNDVLGD
TRLPRKRWREDPLPMLRTTIAPKTAAQRER
Sbjct  301
EIVRTDTAYQGEIVILPSDSVRLNDVLGDQTRLPRKRWREDPLPMLRTTIAPKTAAQRER  360

Query  361
LLDALTQLADTDPLLRCEVDSITHEIILSFLGRVQLEVVSALLSEKYKLETVVKEPSVIY  420

LLDALTQLADTDPLLRCEVDSITHEIILSFLGRVQLEVVSALLSEKYKLETVVKEPSVIY
Sbjct  361
LLDALTQLADTDPLLRCEVDSITHEIILSFLGRVQLEVVSALLSEKYKLETVVKEPSVIY  420
```

Query 421
MERPLKAASHTIHIEVPPNPFWASIGLSVTPLSLGSGVQYESRVSLGYLNQSFQNAVRDG 480

MERPLKAASHTIHIEVPPNPFWASIGLSVTPLSLGSGVQYESRVSLGYLNQSFQNAVRDG
Sbjct 421
MERPLKAASHTIHIEVPPNPFWASIGLSVTPLSLGSGVQYESRVSLGYLNQSFQNAVRDG 480

Query 481
IRYGLEQGLFGWNVTDCKICFEYGLYYSPVSTPADFRSLAPIVLEQALKESGTQLLEPYL 540

IRYGLEQGLFGWNVTDCKICFEYGLYYSPVSTPADFRSLAPIVLEQALKESGTQLLEPYL
Sbjct 481
IRYGLEQGLFGWNVTDCKICFEYGLYYSPVSTPADFRSLAPIVLEQALKESGTQLLEPYL 540

Query 541
SFILYAPQEYLSRAYHDAPKYCATIETAQVKKDEVVFTGEIPARCIQAYRTDLAFYTNGR 600
SFILYAPQEYLSRAYHDAPKYCATIETAQVKKD
+VVFTGEIPARCIQAYRTDLAFYTNGR
Sbjct 541
SFILYAPQEYLSRAYHDAPKYCATIETAQVKKDKVVFTGEIPARCIQAYRTDLAFYTNGR 600

Query 601 SVCLTELKGYQAAVGQPVIQPRRPNSRLDKVRHMFQKVM 639
SVCLTELKGYQAAVGQPVIQPRRPNSRLDKVRHMFQKVM
Sbjct 601 SVCLTELKGYQAAVGQPVIQPRRPNSRLDKVRHMFQKVM 639

>AMP47119.1 TetM-TetW-TetO-TetS [uncultured bacterium]
Length=639

Score = 1294 bits (3348), Expect = 0.0, Method: Compositional matrix adjust.
Identities = 630/639 (99%), Positives = 634/639 (99%), Gaps = 0/639 (0%)

Query 1
MKIINIGILAHVDAGKTTLTESLLYASGAISEPGSVEKGTTRTDTMFLERQRGITIQAAV 60

MKIINIGILAHVDAGKTTLTESLLYASGAISEPGSVEKGTTRTDTMFLERQRGITIQAAV
Sbjct 1
MKIINIGILAHVDAGKTTLTESLLYASGAISEPGSVEKGTTRTDTMFLERQRGITIQAAV 60

Query 61
TSFQWHRCKVNIVDTPGHMDFLAEVYRSLAVLDGAILVISAKDGVQAQTRILFHALRKMN 120

TSFQWHRCKVNIVDTPGHMDFLAEVYRSLAVLDGAILVISAKDGVQAQTRILFHALRKMN
Sbjct 61
TSFQWHRCKVNIVDTPGHMDFLAEVYRSLAVLDGAILVISAKDGVQAQTRILFHALRKMN 120

Query 121
IPTVIFINKIDQAGVDLQSVVQSVRDKLSADIIIKQTVSLSPEIVLEENTDIEAWDAVIE 180

IPTVIFINKIDQAGVDLQSVVQSVRDKLSADIIIKQTVSLSPEIVLEENTDIEAWDAVIE
Sbjct 121
IPTVIFINKIDQAGVDLQSVVQSVRDKLSADIIIKQTVSLSPEIVLEENTDIEAWDAVIE 180

```
Query  181
NNDKLLEKYIAGEPISREKLVREEQRRVQDASLFPVYYGSAKKGLGIQPLMDAVTGLFQP  240
            NND+LLEKYIAGEPISREKL REEQ+RVQDASLFPVY+GSAK
GLGIQPLMDAVTGLFQP
Sbjct  181
NNDELLEKYIAGEPISREKLAREEQQRVQDASLFPVYHGSAKNGLGIQPLMDAVTGLFQP  240

Query  241
IGEQGSAALCGSVFKVEYTDCGQRRVYLRLYSGTLRLRDTVALAGREKLKITEMRIPSKG  300
            IGEQG
AALCGSVFKVEYTDCGQRRVYLRLYSGTLRLRDTVALAGREKLKITEMRIPSKG
Sbjct  241
IGEQGGAALCGSVFKVEYTDCGQRRVYLRLYSGTLRLRDTVALAGREKLKITEMRIPSKG  300

Query  301
EIVRTDTAYPGEIVILPSDSVRLNDVLGDPTRLPRKRWREDPLPMLRTTIAPKTAAQRER  360
            EIVRTDTAY GEIVILPSDSVRLNDVLGD TRLPRKRWREDPLPMLRT
+IAPKTAAQRER
Sbjct  301
EIVRTDTAYQGEIVILPSDSVRLNDVLGDQTRLPRKRWREDPLPMLRTSIAPKTAAQRER  360

Query  361
LLDALTQLADTDPLLRCEVDSITHEIILSFLGRVQLEVVSALLSEKYKLETVVKEPSVIY  420

LLDALTQLADTDPLLRCEVDSITHEIILSFLGRVQLEVVSALLSEKYKLETVVKEPSVIY
Sbjct  361
LLDALTQLADTDPLLRCEVDSITHEIILSFLGRVQLEVVSALLSEKYKLETVVKEPSVIY  420

Query  421
MERPLKAASHTIHIEVPPNPFWASIGLSVTPLSLGSGVQYESRVSLGYLNQSFQNAVRDG  480

MERPLKAASHTIHIEVPPNPFWASIGLSVTPLSLGSGVQYESRVSLGYLNQSFQNAVRDG
Sbjct  421
MERPLKAASHTIHIEVPPNPFWASIGLSVTPLSLGSGVQYESRVSLGYLNQSFQNAVRDG  480

Query  481
IRYGLEQGLFGWNVTDCKICFEYGLYYSPVSTPADFRSLAPIVLEQALKESGTQLLEPYL  540

IRYGLEQGLFGWNVTDCKICFEYGLYYSPVSTPADFRSLAPIVLEQALKESGTQLLEPYL
Sbjct  481
IRYGLEQGLFGWNVTDCKICFEYGLYYSPVSTPADFRSLAPIVLEQALKESGTQLLEPYL  540

Query  541
SFILYAPQEYLSRAYHDAPKYCATIETAQVKKDEVVFTGEIPARCIQAYRTDLAFYTNGR  600

SFILYAPQEYLSRAYHDAPKYCATIETAQVKKDEVVFTGEIPARCIQAYRTDLAFYTNGR
Sbjct  541
SFILYAPQEYLSRAYHDAPKYCATIETAQVKKDEVVFTGEIPARCIQAYRTDLAFYTNGR  600

Query  601  SVCLTELKGYQAAVGQPVIQPRRPNSRLDKVRHMFQKVM  639
            SVCLTELKGYQAAVGQPVIQPRRPNSRLDKVRHMFQKVM
Sbjct  601  SVCLTELKGYQAAVGQPVIQPRRPNSRLDKVRHMFQKVM  639
```

>WP_014078528.1 MULTISPECIES: tetracycline resistance ribosomal protection protein
Tet(W) [Clostridiales]
 CAD13485.1 TetW protein [Roseburia sp. A2-183]
 AEN95456.1 hypothetical protein RHOM_01670 [Roseburia hominis A2-183]
 AUO18771.1 translation elongation and release factors (GTPases) [Monoglobus
pectinilyticus]
Length=639

 Score = 1294 bits (3348), Expect = 0.0, Method: Compositional matrix adjust.
 Identities = 630/639 (99%), Positives = 634/639 (99%), Gaps = 0/639 (0%)

```
Query  1
MKIINIGILAHVDAGKTTLTESLLYASGAISEPGSVEKGTTRTDTMFLERQRGITIQAAV  60

MKIINIGILAHVDAGKTTLTESLLYASGAISEPGSVEKGTTRTDTMFLERQRGITIQAAV
Sbjct  1
MKIINIGILAHVDAGKTTLTESLLYASGAISEPGSVEKGTTRTDTMFLERQRGITIQAAV  60

Query  61
TSFQWHRCKVNIVDTPGHMDFLAEVYRSLAVLDGAILVISAKDGVQAQTRILFHALRKMN  120

TSFQWHRCKVNIVDTPGHMDFLAEVYRSLAVLDGAILVISAKDGVQAQTRILFHALRKMN
Sbjct  61
TSFQWHRCKVNIVDTPGHMDFLAEVYRSLAVLDGAILVISAKDGVQAQTRILFHALRKMN  120

Query  121
IPTVIFINKIDQAGVDLQSVVQSVRDKLSADIIIKQTVSLSPEIVLEENTDIEAWDAVIE  180

IPTVIFINKIDQAGVDLQSVVQSVRDKLSADIIIKQTVSLSPEIVLEENTDIEAWDAVIE
Sbjct  121
IPTVIFINKIDQAGVDLQSVVQSVRDKLSADIIIKQTVSLSPEIVLEENTDIEAWDAVIE  180

Query  181
NNDKLLEKYIAGEPISREKLVREEQRRVQDASLFPVYYGSAKKGLGIQPLMDAVTGLFQP  240
            NND+LLEKYIAGEPISR+KL REEQ+RVQDASLFPVY+GSAK
GLGIQPLMDAVTGLFQP
Sbjct  181
NNDELLEKYIAGEPISRKKLAREEQQRVQDASLFPVYHGSAKNGLGIQPLMDAVTGLFQP  240

Query  241
IGEQGSAALCGSVFKVEYTDCGQRRVYLRLYSGTLRLRDTVALAGREKLKITEMRIPSKG  300
            IGEQG
AALCGSVFKVEYTDCGQRRVYLRLYSGTLRLRDTVALAGREKLKITEMRIPSKG
Sbjct  241
IGEQGGAALCGSVFKVEYTDCGQRRVYLRLYSGTLRLRDTVALAGREKLKITEMRIPSKG  300

Query  301
EIVRTDTAYPGEIVILPSDSVRLNDVLGDPTRLPRKRWREDPLPMLRTTIAPKTAAQRER  360
```

```
            EIVRTDTAY GEIVILPSDSVRLNDVLGD
TRLPRKRWREDPLPMLRTTIAPKTAAQRER
Sbjct  301
EIVRTDTAYQGEIVILPSDSVRLNDVLGDQTRLPRKRWREDPLPMLRTTIAPKTAAQRER  360

Query  361
LLDALTQLADTDPLLRCEVDSITHEIILSFLGRVQLEVVSALLSEKYKLETVVKEPSVIY  420

LLDALTQLADTDPLLRCEVDSITHEIILSFLGRVQLEVVSALLSEKYKLETVVKEPSVIY
Sbjct  361
LLDALTQLADTDPLLRCEVDSITHEIILSFLGRVQLEVVSALLSEKYKLETVVKEPSVIY  420

Query  421
MERPLKAASHTIHIEVPPNPFWASIGLSVTPLSLGSGVQYESRVSLGYLNQSFQNAVRDG  480

MERPLKAASHTIHIEVPPNPFWASIGLSVTPLSLGSGVQYESRVSLGYLNQSFQNAVRDG
Sbjct  421
MERPLKAASHTIHIEVPPNPFWASIGLSVTPLSLGSGVQYESRVSLGYLNQSFQNAVRDG  480

Query  481
IRYGLEQGLFGWNVTDCKICFEYGLYYSPVSTPADFRSLAPIVLEQALKESGTQLLEPYL  540

IRYGLEQGLFGWNVTDCKICFEYGLYYSPVSTPADFRSLAPIVLEQALKESGTQLLEPYL
Sbjct  481
IRYGLEQGLFGWNVTDCKICFEYGLYYSPVSTPADFRSLAPIVLEQALKESGTQLLEPYL  540

Query  541
SFILYAPQEYLSRAYHDAPKYCATIETAQVKKDEVVFTGEIPARCIQAYRTDLAFYTNGR  600

SFILYAPQEYLSRAYHDAPKYCATIETAQVKKDEVVFTGEIPARCIQAYRTDLAFYTNGR
Sbjct  541
SFILYAPQEYLSRAYHDAPKYCATIETAQVKKDEVVFTGEIPARCIQAYRTDLAFYTNGR  600

Query  601  SVCLTELKGYQAAVGQPVIQPRRPNSRLDKVRHMFQKVM  639
            SVCLTELKGYQAAVGQPVIQPRRPNSRLDKVRHMFQKVM
Sbjct  601  SVCLTELKGYQAAVGQPVIQPRRPNSRLDKVRHMFQKVM  639


>AIW80467.1 TetM-TetW-TetO-TetS [uncultured bacterium]
Length=639

 Score = 1294 bits (3348),  Expect = 0.0, Method: Compositional
matrix adjust.
 Identities = 630/639 (99%), Positives = 633/639 (99%), Gaps = 0/639
(0%)

Query  1
MKIINIGILAHVDAGKTTLTESLLYASGAISEPGSVEKGTTRTDTMFLERQRGITIQAAV  60

MKIINIGILAHVDAGKTTLTESLLYASGAISEPGSVEKGTTRTDTMFLERQRGITIQAAV
Sbjct  1
MKIINIGILAHVDAGKTTLTESLLYASGAISEPGSVEKGTTRTDTMFLERQRGITIQAAV  60

Query  61
```

TSFQWHRCKVNIVDTPGHMDFLAEVYRSLAVLDGAILVISAKDGVQAQTRILFHALRKMN 120

TSFQWHRCKVNIVDTPGHMDFLAEVYRSLAVLDGAILVISAKDGVQAQTRILFHALRKMN
Sbjct 61
TSFQWHRCKVNIVDTPGHMDFLAEVYRSLAVLDGAILVISAKDGVQAQTRILFHALRKMN 120

Query 121
IPTVIFINKIDQAGVDLQSVVQSVRDKLSADIIIKQTVSLSPEIVLEENTDIEAWDAVIE 180

IPTVIFINKIDQAGVDLQSVVQSVRDKLSADIIIKQTVSLSPEIVLEENTDIEAWDAVIE
Sbjct 121
IPTVIFINKIDQAGVDLQSVVQSVRDKLSADIIIKQTVSLSPEIVLEENTDIEAWDAVIE 180

Query 181
NNDKLLEKYIAGEPISREKLVREEQRRVQDASLFPVYYGSAKKGLGIQPLMDAVTGLFQP 240
NND+LLEKYIAGEPISREKL REEQ+RVQDASLFPVY+GSAK
GLGIQPLMDAVTGLFQP
Sbjct 181
NNDELLEKYIAGEPISREKLAREEQQRVQDASLFPVYHGSAKNGLGIQPLMDAVTGLFQP 240

Query 241
IGEQGSAALCGSVFKVEYTDCGQRRVYLRLYSGTLRLRDTVALAGREKLKITEMRIPSKG 300
IGEQG
AALCGSVFKVEYTDCGQRRVYLRLYSGTLRLRDTVALAGREKLKITEMRIPSKG
Sbjct 241
IGEQGGAALCGSVFKVEYTDCGQRRVYLRLYSGTLRLRDTVALAGREKLKITEMRIPSKG 300

Query 301
EIVRTDTAYPGEIVILPSDSVRLNDVLGDPTRLPRKRWREDPLPMLRTTIAPKTAAQRER 360
EIVRTDTAY GEIVILPSDSVRLNDVLGD
TRLPRKRWREDPLPMLRTTIAPKTAAQRER
Sbjct 301
EIVRTDTAYQGEIVILPSDSVRLNDVLGDQTRLPRKRWREDPLPMLRTTIAPKTAAQRER 360

Query 361
LLDALTQLADTDPLLRCEVDSITHEIILSFLGRVQLEVVSALLSEKYKLETVVKEPSVIY 420

LLDALTQLADTDPLLRCEVDSITHEIILSFLGRVQLEVVSALLSEKYKLETVVKEPSVIY
Sbjct 361
LLDALTQLADTDPLLRCEVDSITHEIILSFLGRVQLEVVSALLSEKYKLETVVKEPSVIY 420

Query 421
MERPLKAASHTIHIEVPPNPFWASIGLSVTPLSLGSGVQYESRVSLGYLNQSFQNAVRDG 480

MERPLKAASHTIHIEVPPNPFWASIGLSVTPLSLGSGVQYESRVSLGYLNQSFQNAVRDG
Sbjct 421
MERPLKAASHTIHIEVPPNPFWASIGLSVTPLSLGSGVQYESRVSLGYLNQSFQNAVRDG 480

Query 481
IRYGLEQGLFGWNVTDCKICFEYGLYYSPVSTPADFRSLAPIVLEQALKESGTQLLEPYL 540

IRYGLEQGLFGWNVTDCKICFEYGLYYSPVSTPADFRSLAPIVLEQALKESGTQLLEPYL
Sbjct 481
IRYGLEQGLFGWNVTDCKICFEYGLYYSPVSTPADFRSLAPIVLEQALKESGTQLLEPYL 540

```
Query  541  
SFILYAPQEYLSRAYHDAPKYCATIETAQVKKDEVVFTGEIPARCIQAYRTDLAFYTNGR  600

SFILYAPQEYLSRAYHDAPKYCATIETAQVKKDEVVFTGEIPARCIQAYRTDLAFYTNGR
Sbjct  541  
SFILYAPQEYLSRAYHDAPKYCATIETAQVKKDEVVFTGEIPARCIQAYRTDLAFYTNGR  600

Query  601  SVCLTELKGYQAAVGQPVIQPRRPNSRLDKVRHMFQKVM  639
            SVCLTELKGYQAAVGQPVIQPRRPNSRLD VRHMFQKVM
Sbjct  601  SVCLTELKGYQAAVGQPVIQPRRPNSRLDNVRHMFQKVM  639


>ACT97610.1 TetW [uncultured organism]
Length=639

 Score = 1294 bits (3348),  Expect = 0.0, Method: Compositional 
matrix adjust.
 Identities = 630/639 (99%), Positives = 634/639 (99%), Gaps = 0/639 
(0%)

Query  1    
MKIINIGILAHVDAGKTTLTESLLYASGAISEPGSVEKGTTRTDTMFLERQRGITIQAAV  60

MKIINIGILAHVDAGKTTLTESLLYASGAISEPGSVEKGTTRTDTMFLERQRGITIQAAV
Sbjct  1    
MKIINIGILAHVDAGKTTLTESLLYASGAISEPGSVEKGTTRTDTMFLERQRGITIQAAV  60

Query  61   
TSFQWHRCKVNIVDTPGHMDFLAEVYRSLAVLDGAILVISAKDGVQAQTRILFHALRKMN  120

TSFQWHRCKVNIVDTPGHMDFLAEVYRSLAVLDGAILVISAKDGVQAQTRILFHALRKMN
Sbjct  61   
TSFQWHRCKVNIVDTPGHMDFLAEVYRSLAVLDGAILVISAKDGVQAQTRILFHALRKMN  120

Query  121  
IPTVIFINKIDQAGVDLQSVVQSVRDKLSADIIIKQTVSLSPEIVLEENTDIEAWDAVIE  180

IPTVIFINKIDQAGVDLQSVVQSVRDKLSADIIIKQTVSLSPEIVLEENTDIEAWDAVIE
Sbjct  121  
IPTVIFINKIDQAGVDLQSVVQSVRDKLSADIIIKQTVSLSPEIVLEENTDIEAWDAVIE  180

Query  181  
NNDKLLEKYIAGEPISREKLVREEQRRVQDASLFPVYYGSAKKGLGIQPLMDAVTGLFQP  240
            NND+LLEKYIAGEPISREKL REEQ+RVQDASLFPVY+GSAK 
GLGIQPLMDAVTGLFQP
Sbjct  181  
NNDELLEKYIAGEPISREKLAREEQQRVQDASLFPVYHGSAKNGLGIQPLMDAVTGLFQP  240

Query  241  
IGEQGSAALCGSVFKVEYTDCGQRRVYLRLYSGTLRLRDTVALAGREKLKITEMRIPSKG  300
            IGEQG
AALCGSVFKVEYTDCGQRRVYLRLYSGTLRLRDTVALAGREKLKITEMRIPSKG
Sbjct  241
```

```
IGEQGGAALCGSVFKVEYTDCGQRRVYLRLYSGTLRLRDTVALAGREKLKITEMRIPSKG  300

Query  301
EIVRTDTAYPGEIVILPSDSVRLNDVLGDPTRLPRKRWREDPLPMLRTTIAPKTAAQRER  360
            EIVRTDTAY GEIVILPSDSVRLNDVLGD
TRLPRKRWREDPLPMLRTTIAPKTAAQRER
Sbjct  301
EIVRTDTAYQGEIVILPSDSVRLNDVLGDQTRLPRKRWREDPLPMLRTTIAPKTAAQRER  360

Query  361
LLDALTQLADTDPLLRCEVDSITHEIILSFLGRVQLEVVSALLSEKYKLETVVKEPSVIY  420

LLDALTQLADTDPLLRCEVDSITHEIILSFLGRVQLEVVSALLSEKYKLETVVKEPSVIY
Sbjct  361
LLDALTQLADTDPLLRCEVDSITHEIILSFLGRVQLEVVSALLSEKYKLETVVKEPSVIY  420

Query  421
MERPLKAASHTIHIEVPPNPFWASIGLSVTPLSLGSGVQYESRVSLGYLNQSFQNAVRDG  480

MERPLKAASHTIHIEVPPNPFWASIGLSVTPLSLGSGVQYESRVSLGYLNQSFQNAVRDG
Sbjct  421
MERPLKAASHTIHIEVPPNPFWASIGLSVTPLSLGSGVQYESRVSLGYLNQSFQNAVRDG  480

Query  481
IRYGLEQGLFGWNVTDCKICFEYGLYYSPVSTPADFRSLAPIVLEQALKESGTQLLEPYL  540

IRYGLEQGLFGWNVTDCKICFEYGLYYSPVSTPADFRSLAPIVLEQALKESGTQLLEPYL
Sbjct  481
IRYGLEQGLFGWNVTDCKICFEYGLYYSPVSTPADFRSLAPIVLEQALKESGTQLLEPYL  540

Query  541
SFILYAPQEYLSRAYHDAPKYCATIETAQVKKDEVVFTGEIPARCIQAYRTDLAFYTNGR  600
            SFILYAP
+EYLSRAYHDAPKYCATIETAQVKKDEVVFTGEIPARCIQAYRTDLAFYTNGR
Sbjct  541
SFILYAPKEYLSRAYHDAPKYCATIETAQVKKDEVVFTGEIPARCIQAYRTDLAFYTNGR  600

Query  601  SVCLTELKGYQAAVGQPVIQPRRPNSRLDKVRHMFQKVM  639
            SVCLTELKGYQAAVGQPVIQPRRPNSRLDKVRHMFQKVM
Sbjct  601  SVCLTELKGYQAAVGQPVIQPRRPNSRLDKVRHMFQKVM  639


>WP_097782597.1 TetM/TetW/TetO/TetS family tetracycline resistance
ribosomal
protection protein [Faecalibacterium prausnitzii]
 PDX73438.1 tetracycline resistance ribosomal protection protein
Tet(W) [Faecalibacterium
prausnitzii]
Length=639

 Score = 1293 bits (3347),  Expect = 0.0, Method: Compositional
matrix adjust.
 Identities = 630/639 (99%), Positives = 633/639 (99%), Gaps = 0/639
(0%)
```

Query 1
MKIINIGILAHVDAGKTTLTESLLYASGAISEPGSVEKGTTRTDTMFLERQRGITIQAAV 60

MKIINIGILAHVDAGKTTLTESLLYASGAISEPGSVEKGTTRTDTMFLERQRGITIQAAV
Sbjct 1
MKIINIGILAHVDAGKTTLTESLLYASGAISEPGSVEKGTTRTDTMFLERQRGITIQAAV 60

Query 61
TSFQWHRCKVNIVDTPGHMDFLAEVYRSLAVLDGAILVISAKDGVQAQTRILFHALRKMN 120

TSFQWHRCKVNIVDTPGHMDFLAEVYRSLAVLDGAILVISAKDGVQAQTRILFHALRKMN
Sbjct 61
TSFQWHRCKVNIVDTPGHMDFLAEVYRSLAVLDGAILVISAKDGVQAQTRILFHALRKMN 120

Query 121
IPTVIFINKIDQAGVDLQSVVQSVRDKLSADIIIKQTVSLSPEIVLEENTDIEAWDAVIE 180

IPTVIFINKIDQAGVDLQSVVQSVRDKLSADIIIKQTVSLSPEIVLEENTDIEAWDAVIE
Sbjct 121
IPTVIFINKIDQAGVDLQSVVQSVRDKLSADIIIKQTVSLSPEIVLEENTDIEAWDAVIE 180

Query 181
NNDKLLEKYIAGEPISREKLVREEQRRVQDASLFPVYYGSAKKGLGIQPLMDAVTGLFQP 240
NND+LLEKYIAGEPISREKL REEQ+RVQDASLFPVY+GSAK
GLGIQPLMDAVTGLFQP
Sbjct 181
NNDELLEKYIAGEPISREKLAREEQQRVQDASLFPVYHGSAKNGLGIQPLMDAVTGLFQP 240

Query 241
IGEQGSAALCGSVFKVEYTDCGQRRVYLRLYSGTLRLRDTVALAGREKLKITEMRIPSKG 300
IGEQG
AALCGSVFKVEYTDCGQRRVYLRLYSGTLRLRDTVALAGREKLKITEMRIPSKG
Sbjct 241
IGEQGGAALCGSVFKVEYTDCGQRRVYLRLYSGTLRLRDTVALAGREKLKITEMRIPSKG 300

Query 301
EIVRTDTAYPGEIVILPSDSVRLNDVLGDPTRLPRKRWREDPLPMLRTTIAPKTAAQRER 360
EIVRTDTAY GEIVI PSDSVRLNDVLGD
TRLPRKRWREDPLPMLRTTIAPKTAAQRER
Sbjct 301
EIVRTDTAYQGEIVIFPSDSVRLNDVLGDQTRLPRKRWREDPLPMLRTTIAPKTAAQRER 360

Query 361
LLDALTQLADTDPLLRCEVDSITHEIILSFLGRVQLEVVSALLSEKYKLETVVKEPSVIY 420

LLDALTQLADTDPLLRCEVDSITHEIILSFLGRVQLEVVSALLSEKYKLETVVKEPSVIY
Sbjct 361
LLDALTQLADTDPLLRCEVDSITHEIILSFLGRVQLEVVSALLSEKYKLETVVKEPSVIY 420

Query 421
MERPLKAASHTIHIEVPPNPFWASIGLSVTPLSLGSGVQYESRVSLGYLNQSFQNAVRDG 480

MERPLKAASHTIHIEVPPNPFWASIGLSVTPLSLGSGVQYESRVSLGYLNQSFQNAVRDG

```
Sbjct  421
MERPLKAASHTIHIEVPPNPFWASIGLSVTPLSLGSGVQYESRVSLGYLNQSFQNAVRDG  480

Query  481
IRYGLEQGLFGWNVTDCKICFEYGLYYSPVSTPADFRSLAPIVLEQALKESGTQLLEPYL  540

IRYGLEQGLFGWNVTDCKICFEYGLYYSPVSTPADFRSLAPIVLEQALKESGTQLLEPYL
Sbjct  481
IRYGLEQGLFGWNVTDCKICFEYGLYYSPVSTPADFRSLAPIVLEQALKESGTQLLEPYL  540

Query  541
SFILYAPQEYLSRAYHDAPKYCATIETAQVKKDEVVFTGEIPARCIQAYRTDLAFYTNGR  600

SFILYAPQEYLSRAYHDAPKYCATIETAQVKKDEVVFTGEIPARCIQAYRTDLAFYTNGR
Sbjct  541
SFILYAPQEYLSRAYHDAPKYCATIETAQVKKDEVVFTGEIPARCIQAYRTDLAFYTNGR  600

Query  601  SVCLTELKGYQAAVGQPVIQPRRPNSRLDKVRHMFQKVM  639
            SVCLTELKGYQAAVGQPVIQPRRPNSRLDKVRHMFQKVM
Sbjct  601  SVCLTELKGYQAAVGQPVIQPRRPNSRLDKVRHMFQKVM  639


>WP_074754411.1 TetM/TetW/TetO/TetS family tetracycline resistance
ribosomal
protection protein [Butyrivibrio fibrisolvens]
 SER26224.1 ribosomal protection tetracycline resistance protein
[Butyrivibrio
fibrisolvens]
Length=639

 Score = 1293 bits (3347),  Expect = 0.0, Method: Compositional
matrix adjust.
 Identities = 630/639 (99%), Positives = 633/639 (99%), Gaps = 0/639
(0%)

Query  1
MKIINIGILAHVDAGKTTLTESLLYASGAISEPGSVEKGTTRTDTMFLERQRGITIQAAV  60

MKIINIGILAHVDAGKTTLTESLLYASGAISEPGSVEKGTTRTDTMFLERQRGITIQAAV
Sbjct  1
MKIINIGILAHVDAGKTTLTESLLYASGAISEPGSVEKGTTRTDTMFLERQRGITIQAAV  60

Query  61
TSFQWHRCKVNIVDTPGHMDFLAEVYRSLAVLDGAILVISAKDGVQAQTRILFHALRKMN  120

TSFQWHRCKVNIVDTPGHMDFLAEVYRSLAVLDGAILVISAKDGVQAQTRILFHALRKMN
Sbjct  61
TSFQWHRCKVNIVDTPGHMDFLAEVYRSLAVLDGAILVISAKDGVQAQTRILFHALRKMN  120

Query  121
IPTVIFINKIDQAGVDLQSVVQSVRDKLSADIIIKQTVSLSPEIVLEENTDIEAWDAVIE  180

IPTVIFINKIDQAGVDLQSVVQSVRDKLSADIIIKQTVSLSPEIVLEENTDIEAWDAVIE
Sbjct  121
```

IPTVIFINKIDQAGVDLQSVVQSVRDKLSADIIIKQTVSLSPEIVLEENTDIEAWDAVIE 180

Query 181
NNDKLLEKYIAGEPISREKLVREEQRRVQDASLFPVYYGSAKKGLGIQPLMDAVTGLFQP 240
            NND+LLEKYIAGEPISREKL REEQ+RVQDASLFPVY+GSAK
GLGIQPLMDAVTGLFQP
Sbjct 181
NNDELLEKYIAGEPISREKLAREEQQRVQDASLFPVYHGSAKNGLGIQPLMDAVTGLFQP 240

Query 241
IGEQGSAALCGSVFKVEYTDCGQRRVYLRLYSGTLRLRDTVALAGREKLKITEMRIPSKG 300
            IGEQG AALCGSVFKVEYTDCGQR
VYLRLYSGTLRLRDTVALAGREKLKITEMRIPSKG
Sbjct 241
IGEQGGAALCGSVFKVEYTDCGQRSVYLRLYSGTLRLRDTVALAGREKLKITEMRIPSKG 300

Query 301
EIVRTDTAYPGEIVILPSDSVRLNDVLGDPTRLPRKRWREDPLPMLRTTIAPKTAAQRER 360
            EIVRTDTAY GEIVILPSDSVRLNDVLGD
TRLPRKRWREDPLPMLRTTIAPKTAAQRER
Sbjct 301
EIVRTDTAYQGEIVILPSDSVRLNDVLGDQTRLPRKRWREDPLPMLRTTIAPKTAAQRER 360

Query 361
LLDALTQLADTDPLLRCEVDSITHEIILSFLGRVQLEVVSALLSEKYKLETVVKEPSVIY 420

LLDALTQLADTDPLLRCEVDSITHEIILSFLGRVQLEVVSALLSEKYKLETVVKEPSVIY
Sbjct 361
LLDALTQLADTDPLLRCEVDSITHEIILSFLGRVQLEVVSALLSEKYKLETVVKEPSVIY 420

Query 421
MERPLKAASHTIHIEVPPNPFWASIGLSVTPLSLGSGVQYESRVSLGYLNQSFQNAVRDG 480

MERPLKAASHTIHIEVPPNPFWASIGLSVTPLSLGSGVQYESRVSLGYLNQSFQNAVRDG
Sbjct 421
MERPLKAASHTIHIEVPPNPFWASIGLSVTPLSLGSGVQYESRVSLGYLNQSFQNAVRDG 480

Query 481
IRYGLEQGLFGWNVTDCKICFEYGLYYSPVSTPADFRSLAPIVLEQALKESGTQLLEPYL 540

IRYGLEQGLFGWNVTDCKICFEYGLYYSPVSTPADFRSLAPIVLEQALKESGTQLLEPYL
Sbjct 481
IRYGLEQGLFGWNVTDCKICFEYGLYYSPVSTPADFRSLAPIVLEQALKESGTQLLEPYL 540

Query 541
SFILYAPQEYLSRAYHDAPKYCATIETAQVKKDEVVFTGEIPARCIQAYRTDLAFYTNGR 600

SFILYAPQEYLSRAYHDAPKYCATIETAQVKKDEVVFTGEIPARCIQAYRTDLAFYTNGR
Sbjct 541
SFILYAPQEYLSRAYHDAPKYCATIETAQVKKDEVVFTGEIPARCIQAYRTDLAFYTNGR 600

Query 601 SVCLTELKGYQAAVGQPVIQPRRPNSRLDKVRHMFQKVM 639
            SVCLTELKGYQAAVGQPVIQPRRPNSRLDKVRHMFQKVM
Sbjct 601 SVCLTELKGYQAAVGQPVIQPRRPNSRLDKVRHMFQKVM 639

>WP_071129837.1 TetM/TetW/TetO/TetS family tetracycline resistance ribosomal
protection protein [Varibaculum sp. Marseille-P2802]
Length=639

Score = 1293 bits (3347), Expect = 0.0, Method: Compositional matrix adjust.
Identities = 628/639 (98%), Positives = 635/639 (99%), Gaps = 0/639 (0%)

Query 1
MKIINIGILAHVDAGKTTLTESLLYASGAISEPGSVEKGTTRTDTMFLERQRGITIQAAV 60

MKIINIGILAHVDAGKTTLTESLLYASGAISEPGSVEKGTTRTDTMFLERQRGITIQAAV
Sbjct 1
MKIINIGILAHVDAGKTTLTESLLYASGAISEPGSVEKGTTRTDTMFLERQRGITIQAAV 60

Query 61
TSFQWHRCKVNIVDTPGHMDFLAEVYRSLAVLDGAILVISAKDGVQAQTRILFHALRKMN 120

TSFQWHRCKVNIVDTPGHMDFLAEVYRSLAVLDGAILVISAKDGVQAQTRILFHALRKMN
Sbjct 61
TSFQWHRCKVNIVDTPGHMDFLAEVYRSLAVLDGAILVISAKDGVQAQTRILFHALRKMN 120

Query 121
IPTVIFINKIDQAGVDLQSVVQSVRDKLSADIIIKQTVSLSPEIVLEENTDIEAWDAVIE 180

IPTVIFINKIDQAGVDLQSVVQSVRDKLSADIIIKQTVSLSPEIVLEENTDIEAWDAVIE
Sbjct 121
IPTVIFINKIDQAGVDLQSVVQSVRDKLSADIIIKQTVSLSPEIVLEENTDIEAWDAVIE 180

Query 181
NNDKLLEKYIAGEPISREKLVREEQRRVQDASLFPVYYGSAKKGLGIQPLMDAVTGLFQP 240

NNDKLLEKYIAGEPISREKLVREEQRRVQDASLFPVYYGSAKKGLGIQPLMDAVTGLFQP
Sbjct 181
NNDKLLEKYIAGEPISREKLVREEQRRVQDASLFPVYYGSAKKGLGIQPLMDAVTGLFQP 240

Query 241
IGEQGSAALCGSVFKVEYTDCGQRRVYLRLYSGTLRLRDTVALAGREKLKITEMRIPSKG 300

IGEQGSAALCGSVFKVEYTDCGQRRVYLRLYSGTLRLRDTVALAGREKLKITEMRIPSKG
Sbjct 241
IGEQGSAALCGSVFKVEYTDCGQRRVYLRLYSGTLRLRDTVALAGREKLKITEMRIPSKG 300

Query 301
EIVRTDTAYPGEIVILPSDSVRLNDVLGDPTRLPRKRWREDPLPMLRTTIAPKTAAQRER 360
EIVRTDTAYPGEIVILPSDSVRLNDVLGDPTRLPRKRWREDPLPMLRT
+IAPKTAAQRER
Sbjct 301
EIVRTDTAYPGEIVILPSDSVRLNDVLGDPTRLPRKRWREDPLPMLRTSIAPKTAAQRER 360

Query 361
LLDALTQLADTDPLLRCEVDSITHEIILSFLGRVQLEVVSALLSEKYKLETVVKEPSVIY 420
            LLDALTQLADTDPLLRCEVDSITHEIILSFLGRVQLEVVSALLSEKYKLETVVKEP
+VIY
Sbjct 361
LLDALTQLADTDPLLRCEVDSITHEIILSFLGRVQLEVVSALLSEKYKLETVVKEPTVIY 420

Query 421
MERPLKAASHTIHIEVPPNPFWASIGLSVTPLSLGSGVQYESRVSLGYLNQSFQNAVRDG 480
            MERPLKAASHTIHIEVPPNPFWASIGLSVTPL
LGSGVQYESRVSLGYLNQSFQNAVRDG
Sbjct 421
MERPLKAASHTIHIEVPPNPFWASIGLSVTPLPLGSGVQYESRVSLGYLNQSFQNAVRDG 480

Query 481
IRYGLEQGLFGWNVTDCKICFEYGLYYSPVSTPADFRSLAPIVLEQALKESGTQLLEPYL 540

IRYGLEQGLFGWNVTDCKICFEYGLYYSPVSTPADFRSLAPIVLEQALKESGTQLLEPYL
Sbjct 481
IRYGLEQGLFGWNVTDCKICFEYGLYYSPVSTPADFRSLAPIVLEQALKESGTQLLEPYL 540

Query 541
SFILYAPQEYLSRAYHDAPKYCATIETAQVKKDEVVFTGEIPARCIQAYRTDLAFYTNGR 600
            SF LYAP+EYLSRAYHDAPKYCATIET QVKKDEVVFTGEIPARCIQAYRTD
AFYTNG+
Sbjct 541
SFTLYAPREYLSRAYHDAPKYCATIETVQVKKDEVVFTGEIPARCIQAYRTDQAFYTNGQ 600

Query 601 SVCLTELKGYQAAVGQPVIQPRRPNSRLDKVRHMFQKVM 639
            SVCLTELKGYQAAVG+PVIQPRRPNSRLDKVR+MFQK+M
Sbjct 601 SVCLTELKGYQAAVGKPVIQPRRPNSRLDKVRYMFQKIM 639

>WP_061870345.1 MULTISPECIES: TetM/TetW/TetO/TetS family
tetracycline resistance
ribosomal protection protein [Actinobacteria]
 KYJ81078.1 tetracycline resistance protein tetM [Bifidobacterium
longum]
Length=639

 Score = 1293 bits (3347), Expect = 0.0, Method: Compositional
matrix adjust.
 Identities = 630/639 (99%), Positives = 634/639 (99%), Gaps = 0/639
(0%)

Query 1
MKIINIGILAHVDAGKTTLTESLLYASGAISEPGSVEKGTTRTDTMFLERQRGITIQAAV 60

MKIINIGILAHVDAGKTTLTESLLYASGAISEPGSVEKGTTRTDTMFLERQRGITIQAAV
Sbjct 1
MKIINIGILAHVDAGKTTLTESLLYASGAISEPGSVEKGTTRTDTMFLERQRGITIQAAV 60

Query 61
TSFQWHRCKVNIVDTPGHMDFLAEVYRSLAVLDGAILVISAKDGVQAQTRILFHALRKMN 120

TSFQWHRCKVNIVDTPGHMDFLAEVYRSLAVLDGAILVISAKDGVQAQTRILFHALRKMN
Sbjct 61
TSFQWHRCKVNIVDTPGHMDFLAEVYRSLAVLDGAILVISAKDGVQAQTRILFHALRKMN 120

Query 121
IPTVIFINKIDQAGVDLQSVVQSVRDKLSADIIIKQTVSLSPEIVLEENTDIEAWDAVIE 180

IPTVIFINKIDQAGVDLQSVVQSVRDKLSADIIIKQTVSLSPEIVLEENTDIEAWDAVIE
Sbjct 121
IPTVIFINKIDQAGVDLQSVVQSVRDKLSADIIIKQTVSLSPEIVLEENTDIEAWDAVIE 180

Query 181
NNDKLLEKYIAGEPISREKLVREEQRRVQDASLFPVYYGSAKKGLGIQPLMDAVTGLFQP 240
NND+LLEKYIAGEPISREKL REEQ+RVQDASLFPVY+GSAK
GLGIQPLMDAVTGLFQP
Sbjct 181
NNDELLEKYIAGEPISREKLAREEQQRVQDASLFPVYHGSAKNGLGIQPLMDAVTGLFQP 240

Query 241
IGEQGSAALCGSVFKVEYTDCGQRRVYLRLYSGTLRLRDTVALAGREKLKITEMRIPSKG 300
IGEQG
AALCGSVFKVEYTDCGQRRVYLRLYSGTLRLRDTVALAGREKLKITEMRIPSKG
Sbjct 241
IGEQGGAALCGSVFKVEYTDCGQRRVYLRLYSGTLRLRDTVALAGREKLKITEMRIPSKG 300

Query 301
EIVRTDTAYPGEIVILPSDSVRLNDVLGDPTRLPRKRWREDPLPMLRTTIAPKTAAQRER 360
EIVRTDTAY GEIVILPSDSVRLNDVLGD
TRLPRKRWREDPLPMLRTTIAPKTAAQRER
Sbjct 301
EIVRTDTAYQGEIVILPSDSVRLNDVLGDQTRLPRKRWREDPLPMLRTTIAPKTAAQRER 360

Query 361
LLDALTQLADTDPLLRCEVDSITHEIILSFLGRVQLEVVSALLSEKYKLETVVKEPSVIY 420

LLDALTQLADTDPLLRCEVDSITHEIILSFLGRVQLEVVSALLSEKYKLETVVKEPSVIY
Sbjct 361
LLDALTQLADTDPLLRCEVDSITHEIILSFLGRVQLEVVSALLSEKYKLETVVKEPSVIY 420

Query 421
MERPLKAASHTIHIEVPPNPFWASIGLSVTPLSLGSGVQYESRVSLGYLNQSFQNAVRDG 480

MERPLKAASHTIHIEVPPNPFWASIGLSVTPLSLGSGVQYESRVSLGYLNQSFQNAVRDG
Sbjct 421
MERPLKAASHTIHIEVPPNPFWASIGLSVTPLSLGSGVQYESRVSLGYLNQSFQNAVRDG 480

Query 481
IRYGLEQGLFGWNVTDCKICFEYGLYYSPVSTPADFRSLAPIVLEQALKESGTQLLEPYL 540

IRYGLEQGLFGWNVTDCKICFEYGLYYSPVSTPADFRSLAPIVLEQALKESGTQLLEPYL
Sbjct 481
IRYGLEQGLFGWNVTDCKICFEYGLYYSPVSTPADFRSLAPIVLEQALKESGTQLLEPYL 540

```
Query  541
SFILYAPQEYLSRAYHDAPKYCATIETAQVKKDEVVFTGEIPARCIQAYRTDLAFYTNGR  600

SFILYAPQEYLSRAYHDAPKYCATIETAQVKKDEVVFTGEIPARCIQAYRTDLAFYT+GR
Sbjct  541
SFILYAPQEYLSRAYHDAPKYCATIETAQVKKDEVVFTGEIPARCIQAYRTDLAFYTSGR  600

Query  601  SVCLTELKGYQAAVGQPVIQPRRPNSRLDKVRHMFQKVM  639
            SVCLTELKGYQAAVGQPVIQPRRPNSRLDKVRHMFQKVM
Sbjct  601  SVCLTELKGYQAAVGQPVIQPRRPNSRLDKVRHMFQKVM  639


>AMP57701.1 TetM-TetW-TetO-TetS [uncultured bacterium]
Length=639

 Score = 1293 bits (3347),  Expect = 0.0, Method: Compositional
matrix adjust.
 Identities = 630/639 (99%), Positives = 633/639 (99%), Gaps = 0/639
(0%)

Query  1
MKIINIGILAHVDAGKTTLTESLLYASGAISEPGSVEKGTTRTDTMFLERQRGITIQAAV  60

MKIINIGILAHVDAGKTTLTESLLYASGAISEPGSVEKGTTRTDTMFLERQRGITIQAAV
Sbjct  1
MKIINIGILAHVDAGKTTLTESLLYASGAISEPGSVEKGTTRTDTMFLERQRGITIQAAV  60

Query  61
TSFQWHRCKVNIVDTPGHMDFLAEVYRSLAVLDGAILVISAKDGVQAQTRILFHALRKMN  120

TSFQWHRCKVNIVDTPGHMDFLAEVYRSLAVLDGAILVISAKDGVQAQTRILFHALRKMN
Sbjct  61
TSFQWHRCKVNIVDTPGHMDFLAEVYRSLAVLDGAILVISAKDGVQAQTRILFHALRKMN  120

Query  121
IPTVIFINKIDQAGVDLQSVVQSVRDKLSADIIIKQTVSLSPEIVLEENTDIEAWDAVIE  180

IPTVIFINKIDQAGVDLQSVVQSVRDKLSADIIIKQTVSLSPEIVLEENTDIEAWDAVIE
Sbjct  121
IPTVIFINKIDQAGVDLQSVVQSVRDKLSADIIIKQTVSLSPEIVLEENTDIEAWDAVIE  180

Query  181
NNDKLLEKYIAGEPISREKLVREEQRRVQDASLFPVYYGSAKKGLGIQPLMDAVTGLFQP  240
            NND+LLEKYIAGEPISREKL REEQ+RVQDASLFPVY+GSAK
GLGIQPLMDAVTGLFQP
Sbjct  181
NNDELLEKYIAGEPISREKLAREEQQRVQDASLFPVYHGSAKNGLGIQPLMDAVTGLFQP  240

Query  241
IGEQGSAALCGSVFKVEYTDCGQRRVYLRLYSGTLRLRDTVALAGREKLKITEMRIPSKG  300
            IGEQG
AALCGSVFKVEYTDCGQRRVYLRLYSGTLRLRDTVALAGREKLKITEMRIPSKG
Sbjct  241
IGEQGGAALCGSVFKVEYTDCGQRRVYLRLYSGTLRLRDTVALAGREKLKITEMRIPSKG  300
```

Query 301
EIVRTDTAYPGEIVILPSDSVRLNDVLGDPTRLPRKRWREDPLPMLRTTIAPKTAAQRER 360
            EIVRTDTAY GEIVILPSDSVRLNDVLGD
TRLPRKRWREDPLPMLRTTIAPKTAAQRER
Sbjct 301
EIVRTDTAYQGEIVILPSDSVRLNDVLGDQTRLPRKRWREDPLPMLRTTIAPKTAAQRER 360

Query 361
LLDALTQLADTDPLLRCEVDSITHEIILSFLGRVQLEVVSALLSEKYKLETVVKEPSVIY 420

LLDALTQLADTDPLLRCEVDSITHEIILSFLGRVQLEVVSALLSEKYKLETVVKEPSVIY
Sbjct 361
LLDALTQLADTDPLLRCEVDSITHEIILSFLGRVQLEVVSALLSEKYKLETVVKEPSVIY 420

Query 421
MERPLKAASHTIHIEVPPNPFWASIGLSVTPLSLGSGVQYESRVSLGYLNQSFQNAVRDG 480

MERPLKAASHTIHIEVPPNPFWASIGLSVTPLSLGSGVQYESRVSLGYLNQSFQNAVRDG
Sbjct 421
MERPLKAASHTIHIEVPPNPFWASIGLSVTPLSLGSGVQYESRVSLGYLNQSFQNAVRDG 480

Query 481
IRYGLEQGLFGWNVTDCKICFEYGLYYSPVSTPADFRSLAPIVLEQALKESGTQLLEPYL 540

IRYGLEQGLFGWNVTDCKICFEYGLYYSPVSTPADFRSLAPIVLEQALKESGTQLLEPYL
Sbjct 481
IRYGLEQGLFGWNVTDCKICFEYGLYYSPVSTPADFRSLAPIVLEQALKESGTQLLEPYL 540

Query 541
SFILYAPQEYLSRAYHDAPKYCATIETAQVKKDEVVFTGEIPARCIQAYRTDLAFYTNGR 600

SFILYAPQEYLSRAYHDAPKYCATIETAQVKKDEVVFTGEIPARCIQAYRTDLAFYTNGR
Sbjct 541
SFILYAPQEYLSRAYHDAPKYCATIETAQVKKDEVVFTGEIPARCIQAYRTDLAFYTNGR 600

Query 601 SVCLTELKGYQAAVGQPVIQPRRPNSRLDKVRHMFQKVM 639
            SVCLTELKGYQAAVGQPVIQPRRPNSRLDKV HMFQKVM
Sbjct 601 SVCLTELKGYQAAVGQPVIQPRRPNSRLDKVSHMFQKVM 639

>AMJ35140.1 TetM-TetW-TetO-TetS [uncultured bacterium]
Length=639

Score = 1293 bits (3347), Expect = 0.0, Method: Compositional matrix adjust.
Identities = 628/639 (98%), Positives = 635/639 (99%), Gaps = 0/639 (0%)

Query 1
MKIINIGILAHVDAGKTTLTESLLYASGAISEPGSVEKGTTRTDTMFLERQRGITIQAAV 60

MKIINIGILAHVDAGKTTLTESLLYASGAISEPGSVEKGTTRTDTMFLERQRGITIQAAV
Sbjct 1

MKIINIGILAHVDAGKTTLTESLLYASGAISEPGSVEKGTTRTDTMFLERQRGITIQAAV 60

Query 61
TSFQWHRCKVNIVDTPGHMDFLAEVYRSLAVLDGAILVISAKDGVQAQTRILFHALRKMN 120

TSFQWHRCKVNIVDTPGHMDFLAEVYRSLAVLDGAILVISAKDGVQAQTRILFHALRKMN
Sbjct 61
TSFQWHRCKVNIVDTPGHMDFLAEVYRSLAVLDGAILVISAKDGVQAQTRILFHALRKMN 120

Query 121
IPTVIFINKIDQAGVDLQSVVQSVRDKLSADIIIKQTVSLSPEIVLEENTDIEAWDAVIE 180

IPTVIFINKIDQAGVDLQSVVQSVRDKLSADIIIKQTVSLSPEIVLEENTDIEAWDAVIE
Sbjct 121
IPTVIFINKIDQAGVDLQSVVQSVRDKLSADIIIKQTVSLSPEIVLEENTDIEAWDAVIE 180

Query 181
NNDKLLEKYIAGEPISREKLVREEQRRVQDASLFPVYYGSAKKGLGIQPLMDAVTGLFQP 240

NNDKLLEKYIAGEPISREKLVREEQRRVQDASLFPVYYGSAKKGLGIQPLMDAVTGLFQP
Sbjct 181
NNDKLLEKYIAGEPISREKLVREEQRRVQDASLFPVYYGSAKKGLGIQPLMDAVTGLFQP 240

Query 241
IGEQGSAALCGSVFKVEYTDCGQRRVYLRLYSGTLRLRDTVALAGREKLKITEMRIPSKG 300

IGEQGSAALCGSVFKVEYTDCGQRRVYLRLYSGTLRLRDTVALAGREKLKITEMRIPSKG
Sbjct 241
IGEQGSAALCGSVFKVEYTDCGQRRVYLRLYSGTLRLRDTVALAGREKLKITEMRIPSKG 300

Query 301
EIVRTDTAYPGEIVILPSDSVRLNDVLGDPTRLPRKRWREDPLPMLRTTIAPKTAAQRER 360
EIVRT TAYPGEIVILPSDSVRLNDVLGDPTRLPRKRWREDPLPMLRT
+IAPKTAAQRER
Sbjct 301
EIVRTHTAYPGEIVILPSDSVRLNDVLGDPTRLPRKRWREDPLPMLRTSIAPKTAAQRER 360

Query 361
LLDALTQLADTDPLLRCEVDSITHEIILSFLGRVQLEVVSALLSEKYKLETVVKEPSVIY 420
LLDALTQLADTDPLLRCEVDSITHEIILSFLGRVQLEVVSALLSEKYKLETVVKEP
+VIY
Sbjct 361
LLDALTQLADTDPLLRCEVDSITHEIILSFLGRVQLEVVSALLSEKYKLETVVKEPTVIY 420

Query 421
MERPLKAASHTIHIEVPPNPFWASIGLSVTPLSLGSGVQYESRVSLGYLNQSFQNAVRDG 480
MERPLKAASHTIHIEVPPNPFWASIGLSVTPL
LGSGVQYESRVSLGYLNQSFQNAVRDG
Sbjct 421
MERPLKAASHTIHIEVPPNPFWASIGLSVTPLPLGSGVQYESRVSLGYLNQSFQNAVRDG 480

Query 481
IRYGLEQGLFGWNVTDCKICFEYGLYYSPVSTPADFRSLAPIVLEQALKESGTQLLEPYL 540

```
IRYGLEQGLFGWNVTDCKICFEYGLYYSPVSTPADFRSLAPIVLEQALKESGTQLLEPYL
Sbjct  481  
IRYGLEQGLFGWNVTDCKICFEYGLYYSPVSTPADFRSLAPIVLEQALKESGTQLLEPYL  540

Query  541  
SFILYAPQEYLSRAYHDAPKYCATIETAQVKKDEVVFTGEIPARCIQAYRTDLAFYTNGR  600
            SF LYAP+EYLSRAYHDAPKYCATIET 
QVKKDEVVFTGEIPARCIQAYRTDLAFYTNG+
Sbjct  541  
SFTLYAPREYLSRAYHDAPKYCATIETVQVKKDEVVFTGEIPARCIQAYRTDLAFYTNGQ  600

Query  601  SVCLTELKGYQAAVGQPVIQPRRPNSRLDKVRHMFQKVM  639
            SVCLTELKGYQAAVG+PVIQPRRPNSRLDKVR+MFQK+M
Sbjct  601  SVCLTELKGYQAAVGKPVIQPRRPNSRLDKVRYMFQKIM  639


>WP_052792087.1 TetM/TetW/TetO/TetS family tetracycline resistance 
ribosomal 
protection protein [Bifidobacterium breve]
 KOA52309.1 tetracycline resistance protein tetM [Bifidobacterium 
breve MCC 
1340]
Length=639

 Score = 1293 bits (3347),  Expect = 0.0, Method: Compositional 
matrix adjust.
 Identities = 628/638 (98%), Positives = 632/638 (99%), Gaps = 0/638 
(0%)

Query  1    
MKIINIGILAHVDAGKTTLTESLLYASGAISEPGSVEKGTTRTDTMFLERQRGITIQAAV  60
            M 
IINIGILAHVDAGKTTLTESLLYASGAISEPGSVEKGTTRTDTMFLERQRGITIQAAV
Sbjct  1    
MNIINIGILAHVDAGKTTLTESLLYASGAISEPGSVEKGTTRTDTMFLERQRGITIQAAV  60

Query  61   
TSFQWHRCKVNIVDTPGHMDFLAEVYRSLAVLDGAILVISAKDGVQAQTRILFHALRKMN  120
            
TSFQWHRCKVNIVDTPGHMDFLAEVYRSLAVLDGAILVISAKDGVQAQTRILFHALRKMN
Sbjct  61   
TSFQWHRCKVNIVDTPGHMDFLAEVYRSLAVLDGAILVISAKDGVQAQTRILFHALRKMN  120

Query  121  
IPTVIFINKIDQAGVDLQSVVQSVRDKLSADIIIKQTVSLSPEIVLEENTDIEAWDAVIE  180
            
IPTVIFINKIDQAGVDLQSVVQSVRDKLSADIIIKQTVSLSPEIVLEENTDIEAWDAVIE
Sbjct  121  
IPTVIFINKIDQAGVDLQSVVQSVRDKLSADIIIKQTVSLSPEIVLEENTDIEAWDAVIE  180

Query  181  
NNDKLLEKYIAGEPISREKLVREEQRRVQDASLFPVYYGSAKKGLGIQPLMDAVTGLFQP  240
            
NNDKLLEKYIAGEPISREKLVREEQRRVQDASLFPVYYGSAKKGLGIQPLMDAVTGLFQP
```

```
Sbjct  181
NNDKLLEKYIAGEPISREKLVREEQRRVQDASLFPVYYGSAKKGLGIQPLMDAVTGLFQP  240

Query  241
IGEQGSAALCGSVFKVEYTDCGQRRVYLRLYSGTLRLRDTVALAGREKLKITEMRIPSKG  300

IGEQGSAALCGSVFKVEYTDCGQRRVYLRLYSGTLRLRDTVALAGREKLKITEMRIPSKG
Sbjct  241
IGEQGSAALCGSVFKVEYTDCGQRRVYLRLYSGTLRLRDTVALAGREKLKITEMRIPSKG  300

Query  301
EIVRTDTAYPGEIVILPSDSVRLNDVLGDPTRLPRKRWREDPLPMLRTTIAPKTAAQRER  360
            EIVRTDTAYPGEIVILPSDSVRLNDVLGDPTRLPRKRWREDPLPMLRT
+IAPKTAAQRER
Sbjct  301
EIVRTDTAYPGEIVILPSDSVRLNDVLGDPTRLPRKRWREDPLPMLRTSIAPKTAAQRER  360

Query  361
LLDALTQLADTDPLLRCEVDSITHEIILSFLGRVQLEVVSALLSEKYKLETVVKEPSVIY  420
            LLDALTQLADTDPLLRCEVDSITHEIILSFLGRVQLEVVSALLSEKYKLETVVKEP
+VIY
Sbjct  361
LLDALTQLADTDPLLRCEVDSITHEIILSFLGRVQLEVVSALLSEKYKLETVVKEPTVIY  420

Query  421
MERPLKAASHTIHIEVPPNPFWASIGLSVTPLSLGSGVQYESRVSLGYLNQSFQNAVRDG  480
            MERPLKAASHTIHIEVPPNPFWASIGLSVTPL
LGSGVQYESRVSLGYLNQSFQNAVRDG
Sbjct  421
MERPLKAASHTIHIEVPPNPFWASIGLSVTPLPLGSGVQYESRVSLGYLNQSFQNAVRDG  480

Query  481
IRYGLEQGLFGWNVTDCKICFEYGLYYSPVSTPADFRSLAPIVLEQALKESGTQLLEPYL  540

IRYGLEQGLFGWNVTDCKICFEYGLYYSPVSTPADFRSLAPIVLEQALKESGTQLLEPYL
Sbjct  481
IRYGLEQGLFGWNVTDCKICFEYGLYYSPVSTPADFRSLAPIVLEQALKESGTQLLEPYL  540

Query  541
SFILYAPQEYLSRAYHDAPKYCATIETAQVKKDEVVFTGEIPARCIQAYRTDLAFYTNGR  600
            SF LYAPQEYLSRAYHDAPKYCATIETAQVKKDEVVFTGEIP
RCIQAYRTDLAFYTNGR
Sbjct  541
SFTLYAPQEYLSRAYHDAPKYCATIETAQVKKDEVVFTGEIPVRCIQAYRTDLAFYTNGR  600

Query  601  SVCLTELKGYQAAVGQPVIQPRRPNSRLDKVRHMFQKV  638
            SVCLTELKGYQA VG+PVIQPRRPNSRLDKVRHMF K+
Sbjct  601  SVCLTELKGYQATVGEPVIQPRRPNSRLDKVRHMFSKI  638


>WP_052829461.1 TetM/TetW/TetO/TetS family tetracycline resistance
ribosomal
protection protein [Bifidobacterium breve]
 KOA39499.1 tetracycline resistance protein tetM [Bifidobacterium
```

breve MCC
1094]
Length=639

Score = 1293 bits (3347), Expect = 0.0, Method: Compositional matrix adjust.
Identities = 630/639 (99%), Positives = 634/639 (99%), Gaps = 0/639 (0%)

Query 1
MKIINIGILAHVDAGKTTLTESLLYASGAISEPGSVEKGTTRTDTMFLERQRGITIQAAV 60

MKIINIGILAHVDAGKTTLTESLLYASGAISEPGSVEKGTTRTDTMFLERQRGITIQAAV
Sbjct 1
MKIINIGILAHVDAGKTTLTESLLYASGAISEPGSVEKGTTRTDTMFLERQRGITIQAAV 60

Query 61
TSFQWHRCKVNIVDTPGHMDFLAEVYRSLAVLDGAILVISAKDGVQAQTRILFHALRKMN 120

TSFQWHRCKVNIVDTPGHMDFLAEVYRSLAVLDGAILVISAKDGVQAQTRILFHALRKMN
Sbjct 61
TSFQWHRCKVNIVDTPGHMDFLAEVYRSLAVLDGAILVISAKDGVQAQTRILFHALRKMN 120

Query 121
IPTVIFINKIDQAGVDLQSVVQSVRDKLSADIIIKQTVSLSPEIVLEENTDIEAWDAVIE 180

IPTVIFINKIDQAGVDLQSVVQSVRDKLSADIIIKQTVSLSPEIVLEENTDIEAWDAVIE
Sbjct 121
IPTVIFINKIDQAGVDLQSVVQSVRDKLSADIIIKQTVSLSPEIVLEENTDIEAWDAVIE 180

Query 181
NNDKLLEKYIAGEPISREKLVREEQRRVQDASLFPVYYGSAKKGLGIQPLMDAVTGLFQP 240
NND+LLEKYIAGEPISREKL REEQ+RVQDASLFPVY+GSAK
GLGIQPLMDAVTGLFQP
Sbjct 181
NNDELLEKYIAGEPISREKLAREEQQRVQDASLFPVYHGSAKNGLGIQPLMDAVTGLFQP 240

Query 241
IGEQGSAALCGSVFKVEYTDCGQRRVYLRLYSGTLRLRDTVALAGREKLKITEMRIPSKG 300
IGEQG
AALCGSVFKVEYTDCGQRRVYLRLYSGTLRLRDTVALAGREKLKITEMRIPSKG
Sbjct 241
IGEQGGAALCGSVFKVEYTDCGQRRVYLRLYSGTLRLRDTVALAGREKLKITEMRIPSKG 300

Query 301
EIVRTDTAYPGEIVILPSDSVRLNDVLGDPTRLPRKRWREDPLPMLRTTIAPKTAAQRER 360
EIVRTDTAY GEIVILPSDSVRLNDVLGD
TRLPRKRWREDPLPMLRTTIAPKTAAQRER
Sbjct 301
EIVRTDTAYQGEIVILPSDSVRLNDVLGDQTRLPRKRWREDPLPMLRTTIAPKTAAQRER 360

Query 361
LLDALTQLADTDPLLRCEVDSITHEIILSFLGRVQLEVVSALLSEKYKLETVVKEPSVIY 420

LLDALTQLADTDPLLRCEVDSITHEIILSFLGRVQLEVVSALLSEKYKLETVVKEPSVIY
Sbjct 361
LLDALTQLADTDPLLRCEVDSITHEIILSFLGRVQLEVVSALLSEKYKLETVVKEPSVIY 420

Query 421
MERPLKAASHTIHIEVPPNPFWASIGLSVTPLSLGSGVQYESRVSLGYLNQSFQNAVRDG 480

MERPLKAASHTIHIEVPPNPFWASIGLSVTPLSLGSGVQYESRVSLGYLNQSFQNAVRDG
Sbjct 421
MERPLKAASHTIHIEVPPNPFWASIGLSVTPLSLGSGVQYESRVSLGYLNQSFQNAVRDG 480

Query 481
IRYGLEQGLFGWNVTDCKICFEYGLYYSPVSTPADFRSLAPIVLEQALKESGTQLLEPYL 540

IRYGLEQGLFGWNVTDCKICFEYGLYYSPVSTPADFRSLAPIVLEQALKESGTQLLEP+L
Sbjct 481
IRYGLEQGLFGWNVTDCKICFEYGLYYSPVSTPADFRSLAPIVLEQALKESGTQLLEPHL 540

Query 541
SFILYAPQEYLSRAYHDAPKYCATIETAQVKKDEVVFTGEIPARCIQAYRTDLAFYTNGR 600

SFILYAPQEYLSRAYHDAPKYCATIETAQVKKDEVVFTGEIPARCIQAYRTDLAFYTNGR
Sbjct 541
SFILYAPQEYLSRAYHDAPKYCATIETAQVKKDEVVFTGEIPARCIQAYRTDLAFYTNGR 600

Query 601 SVCLTELKGYQAAVGQPVIQPRRPNSRLDKVRHMFQKVM 639
            SVCLTELKGYQAAVGQPVIQPRRPNSRLDKVRHMFQKVM
Sbjct 601 SVCLTELKGYQAAVGQPVIQPRRPNSRLDKVRHMFQKVM 639

>WP_034502607.1 TetM/TetW/TetO/TetS family tetracycline resistance ribosomal
protection protein [Actinomyces sp. S6-Spd3]
KGE99081.1 tetracycline resistance protein tetM [Actinomyces sp. S6-Spd3]
Length=639

Score = 1293 bits (3347), Expect = 0.0, Method: Compositional matrix adjust.
Identities = 630/639 (99%), Positives = 634/639 (99%), Gaps = 0/639 (0%)

Query 1
MKIINIGILAHVDAGKTTLTESLLYASGAISEPGSVEKGTTRTDTMFLERQRGITIQAAV 60

MKIINIGILAHVDAGKTTLTESLLYASGAISEPGSVEKGTTRTDTMFLERQRGITIQAAV
Sbjct 1
MKIINIGILAHVDAGKTTLTESLLYASGAISEPGSVEKGTTRTDTMFLERQRGITIQAAV 60

Query 61
TSFQWHRCKVNIVDTPGHMDFLAEVYRSLAVLDGAILVISAKDGVQAQTRILFHALRKMN 120

TSFQWHRCKVNIVDTPGHMDFLAEVYRSLAVLDGAILVISAKDGVQAQTRILFHALRKMN
Sbjct 61

TSFQWHRCKVNIVDTPGHMDFLAEVYRSLAVLDGAILVISAKDGVQAQTRILFHALRKMN 120

Query 121
IPTVIFINKIDQAGVDLQSVVQSVRDKLSADIIIKQTVSLSPEIVLEENTDIEAWDAVIE 180
            IPTVIFINKI
+QAGVDLQSVVQSVRDKLSADIIIKQTVSLSPEIVLEENTDIEAWDAVIE
Sbjct 121
IPTVIFINKINQAGVDLQSVVQSVRDKLSADIIIKQTVSLSPEIVLEENTDIEAWDAVIE 180

Query 181
NNDKLLEKYIAGEPISREKLVREEQRRVQDASLFPVYYGSAKKGLGIQPLMDAVTGLFQP 240
            NND+LLEKYIAGEPISREKL REEQ+RVQDASLFPVY+GSAK
GLGIQPLMDAVTGLFQP
Sbjct 181
NNDELLEKYIAGEPISREKLAREEQQRVQDASLFPVYHGSAKNGLGIQPLMDAVTGLFQP 240

Query 241
IGEQGSAALCGSVFKVEYTDCGQRRVYLRLYSGTLRLRDTVALAGREKLKITEMRIPSKG 300
            IGEQG
AALCGSVFKVEYTDCGQRRVYLRLYSGTLRLRDTVALAGREKLKITEMRIPSKG
Sbjct 241
IGEQGGAALCGSVFKVEYTDCGQRRVYLRLYSGTLRLRDTVALAGREKLKITEMRIPSKG 300

Query 301
EIVRTDTAYPGEIVILPSDSVRLNDVLGDPTRLPRKRWREDPLPMLRTTIAPKTAAQRER 360
            EIVRTDTAY GEIVILPSDSVRLNDVLGD
TRLPRKRWREDPLPMLRTTIAPKTAAQRER
Sbjct 301
EIVRTDTAYQGEIVILPSDSVRLNDVLGDQTRLPRKRWREDPLPMLRTTIAPKTAAQRER 360

Query 361
LLDALTQLADTDPLLRCEVDSITHEIILSFLGRVQLEVVSALLSEKYKLETVVKEPSVIY 420

LLDALTQLADTDPLLRCEVDSITHEIILSFLGRVQLEVVSALLSEKYKLETVVKEPSVIY
Sbjct 361
LLDALTQLADTDPLLRCEVDSITHEIILSFLGRVQLEVVSALLSEKYKLETVVKEPSVIY 420

Query 421
MERPLKAASHTIHIEVPPNPFWASIGLSVTPLSLGSGVQYESRVSLGYLNQSFQNAVRDG 480

MERPLKAASHTIHIEVPPNPFWASIGLSVTPLSLGSGVQYESRVSLGYLNQSFQNAVRDG
Sbjct 421
MERPLKAASHTIHIEVPPNPFWASIGLSVTPLSLGSGVQYESRVSLGYLNQSFQNAVRDG 480

Query 481
IRYGLEQGLFGWNVTDCKICFEYGLYYSPVSTPADFRSLAPIVLEQALKESGTQLLEPYL 540

IRYGLEQGLFGWNVTDCKICFEYGLYYSPVSTPADFRSLAPIVLEQALKESGTQLLEPYL
Sbjct 481
IRYGLEQGLFGWNVTDCKICFEYGLYYSPVSTPADFRSLAPIVLEQALKESGTQLLEPYL 540

Query 541
SFILYAPQEYLSRAYHDAPKYCATIETAQVKKDEVVFTGEIPARCIQAYRTDLAFYTNGR 600

SFILYAPQEYLSRAYHDAPKYCATIETAQVKKDEVVFTGEIPARCIQAYRTDLAFYTNGR
Sbjct 541
SFILYAPQEYLSRAYHDAPKYCATIETAQVKKDEVVFTGEIPARCIQAYRTDLAFYTNGR 600

Query 601 SVCLTELKGYQAAVGQPVIQPRRPNSRLDKVRHMFQKVM 639
            SVCLTELKGYQAAVGQPVIQPRRPNSRLDKVRHMFQKVM
Sbjct 601 SVCLTELKGYQAAVGQPVIQPRRPNSRLDKVRHMFQKVM 639

>WP_021612625.1 TetM/TetW/TetO/TetS family tetracycline resistance ribosomal
protection protein [Actinomyces sp. oral taxon 172]
 ERH21819.1 putative translation elongation factor G [Actinomyces sp. oral
taxon 172 str. F0311]
Length=639

 Score = 1293 bits (3347), Expect = 0.0, Method: Compositional matrix adjust.
 Identities = 630/639 (99%), Positives = 634/639 (99%), Gaps = 0/639 (0%)

Query 1
MKIINIGILAHVDAGKTTLTESLLYASGAISEPGSVEKGTTRTDTMFLERQRGITIQAAV 60

MKIINIGILAHVDAGKTTLTESLLYASGAISEPGSVEKGTTRTDTMFLERQRGITIQAAV
Sbjct 1
MKIINIGILAHVDAGKTTLTESLLYASGAISEPGSVEKGTTRTDTMFLERQRGITIQAAV 60

Query 61
TSFQWHRCKVNIVDTPGHMDFLAEVYRSLAVLDGAILVISAKDGVQAQTRILFHALRKMN 120

TSFQWHRCKVNIVDTPGHMDFLAEVYRSLAVLDGAILVISAKDGVQAQTRILFHALRKMN
Sbjct 61
TSFQWHRCKVNIVDTPGHMDFLAEVYRSLAVLDGAILVISAKDGVQAQTRILFHALRKMN 120

Query 121
IPTVIFINKIDQAGVDLQSVVQSVRDKLSADIIIKQTVSLSPEIVLEENTDIEAWDAVIE 180

IPTVIFINKIDQAGVDLQSVVQSVRDKLSADIIIKQTVSLSPEIVLEENTDIEAWDAVIE
Sbjct 121
IPTVIFINKIDQAGVDLQSVVQSVRDKLSADIIIKQTVSLSPEIVLEENTDIEAWDAVIE 180

Query 181
NNDKLLEKYIAGEPISREKLVREEQRRVQDASLFPVYYGSAKKGLGIQPLMDAVTGLFQP 240
            NND+LLEKYIAGEPISREKL REEQ+RVQDASLFPVY+GSAK
GLGIQPLMDAVTGLFQP
Sbjct 181
NNDELLEKYIAGEPISREKLAREEQQRVQDASLFPVYHGSAKNGLGIQPLMDAVTGLFQP 240

Query 241
IGEQGSAALCGSVFKVEYTDCGQRRVYLRLYSGTLRLRDTVALAGREKLKITEMRIPSKG 300
            IGEQG
AALCGSVFKVEYTDCGQRRVYLRLYSGTLRLRDTVALAGREKLKITEMRIPSKG

```
Sbjct  241
IGEQGGAALCGSVFKVEYTDCGQRRVYLRLYSGTLRLRDTVALAGREKLKITEMRIPSKG  300

Query  301
EIVRTDTAYPGEIVILPSDSVRLNDVLGDPTRLPRKRWREDPLPMLRTTIAPKTAAQRER  360
            EIVRTDTAY GEIVILPSDSVRLNDVLGD
TRLPRKRWREDPLPMLRTTIAPKTAAQRER
Sbjct  301
EIVRTDTAYQGEIVILPSDSVRLNDVLGDQTRLPRKRWREDPLPMLRTTIAPKTAAQRER  360

Query  361
LLDALTQLADTDPLLRCEVDSITHEIILSFLGRVQLEVVSALLSEKYKLETVVKEPSVIY  420
            LLDALTQLADTDPLLRCEV
+SITHEIILSFLGRVQLEVVSALLSEKYKLETVVKEPSVIY
Sbjct  361
LLDALTQLADTDPLLRCEVNSITHEIILSFLGRVQLEVVSALLSEKYKLETVVKEPSVIY  420

Query  421
MERPLKAASHTIHIEVPPNPFWASIGLSVTPLSLGSGVQYESRVSLGYLNQSFQNAVRDG  480

MERPLKAASHTIHIEVPPNPFWASIGLSVTPLSLGSGVQYESRVSLGYLNQSFQNAVRDG
Sbjct  421
MERPLKAASHTIHIEVPPNPFWASIGLSVTPLSLGSGVQYESRVSLGYLNQSFQNAVRDG  480

Query  481
IRYGLEQGLFGWNVTDCKICFEYGLYYSPVSTPADFRSLAPIVLEQALKESGTQLLEPYL  540

IRYGLEQGLFGWNVTDCKICFEYGLYYSPVSTPADFRSLAPIVLEQALKESGTQLLEPYL
Sbjct  481
IRYGLEQGLFGWNVTDCKICFEYGLYYSPVSTPADFRSLAPIVLEQALKESGTQLLEPYL  540

Query  541
SFILYAPQEYLSRAYHDAPKYCATIETAQVKKDEVVFTGEIPARCIQAYRTDLAFYTNGR  600

SFILYAPQEYLSRAYHDAPKYCATIETAQVKKDEVVFTGEIPARCIQAYRTDLAFYTNGR
Sbjct  541
SFILYAPQEYLSRAYHDAPKYCATIETAQVKKDEVVFTGEIPARCIQAYRTDLAFYTNGR  600

Query  601  SVCLTELKGYQAAVGQPVIQPRRPNSRLDKVRHMFQKVM  639
            SVCLTELKGYQAAVGQPVIQPRRPNSRLDKVRHMFQKVM
Sbjct  601  SVCLTELKGYQAAVGQPVIQPRRPNSRLDKVRHMFQKVM  639


>WP_098924880.1 TetM/TetW/TetO/TetS family tetracycline resistance
ribosomal
protection protein [Faecalibacterium prausnitzii]
 ATL91090.1 tetracycline resistance ribosomal protection protein
Tet(W) [Faecalibacterium
prausnitzii]
Length=639

 Score = 1293 bits (3346),  Expect = 0.0, Method: Compositional
matrix adjust.
 Identities = 630/639 (99%), Positives = 633/639 (99%), Gaps = 0/639
```

(0%)

Query 1
MKIINIGILAHVDAGKTTLTESLLYASGAISEPGSVEKGTTRTDTMFLERQRGITIQAAV 60

MKIINIGILAHVDAGKTTLTESLLYASGAISEPGSVEKGTTRTDTMFLERQRGITIQAAV
Sbjct 1
MKIINIGILAHVDAGKTTLTESLLYASGAISEPGSVEKGTTRTDTMFLERQRGITIQAAV 60

Query 61
TSFQWHRCKVNIVDTPGHMDFLAEVYRSLAVLDGAILVISAKDGVQAQTRILFHALRKMN 120

TSFQWHRCKVNIVDTPGHMDFLAEVYRSLAVLDGAILVISAKDGVQAQTRILFHALRKMN
Sbjct 61
TSFQWHRCKVNIVDTPGHMDFLAEVYRSLAVLDGAILVISAKDGVQAQTRILFHALRKMN 120

Query 121
IPTVIFINKIDQAGVDLQSVVQSVRDKLSADIIIKQTVSLSPEIVLEENTDIEAWDAVIE 180

IPTVIFINKIDQAGVDLQSVVQSVRDKLSADIIIKQTVSLSPEIVLEENTDIEAWDAVIE
Sbjct 121
IPTVIFINKIDQAGVDLQSVVQSVRDKLSADIIIKQTVSLSPEIVLEENTDIEAWDAVIE 180

Query 181
NNDKLLEKYIAGEPISREKLVREEQRRVQDASLFPVYYGSAKKGLGIQPLMDAVTGLFQP 240
NND+LLEKYIAGEPISREKL REEQ+RVQDASLFPVY+GSAK
GLGIQPLMDAVTGLFQP
Sbjct 181
NNDELLEKYIAGEPISREKLAREEQQRVQDASLFPVYHGSAKNGLGIQPLMDAVTGLFQP 240

Query 241
IGEQGSAALCGSVFKVEYTDCGQRRVYLRLYSGTLRLRDTVALAGREKLKITEMRIPSKG 300
IGEQG
AALCGSVFKVEYTDCGQRRVYLRLYSGTLRLRDTVALAGREKLKITEMRIPSKG
Sbjct 241
IGEQGGAALCGSVFKVEYTDCGQRRVYLRLYSGTLRLRDTVALAGREKLKITEMRIPSKG 300

Query 301
EIVRTDTAYPGEIVILPSDSVRLNDVLGDPTRLPRKRWREDPLPMLRTTIAPKTAAQRER 360
EIVRTDTAYPGEIVILPSDSVRLNDVLGD
TRLPRKRWREDPLPMLRTTIAPKTAAQRER
Sbjct 301
EIVRTDTAYPGEIVILPSDSVRLNDVLGDQTRLPRKRWREDPLPMLRTTIAPKTAAQRER 360

Query 361
LLDALTQLADTDPLLRCEVDSITHEIILSFLGRVQLEVVSALLSEKYKLETVVKEPSVIY 420

LLDALTQLADTDPLLRCEVDSITHEIILSFLGRVQLEVVSALLSEKYKLETVVKEPSVIY
Sbjct 361
LLDALTQLADTDPLLRCEVDSITHEIILSFLGRVQLEVVSALLSEKYKLETVVKEPSVIY 420

Query 421
MERPLKAASHTIHIEVPPNPFWASIGLSVTPLSLGSGVQYESRVSLGYLNQSFQNAVRDG 480
MERPLKAASHTIHIEVPPNPFWASIGLSVTPL

LGSGVQYESRVSLGYLNQSFQNAVRDG
Sbjct 421
MERPLKAASHTIHIEVPPNPFWASIGLSVTPLPLGSGVQYESRVSLGYLNQSFQNAVRDG 480

Query 481
IRYGLEQGLFGWNVTDCKICFEYGLYYSPVSTPADFRSLAPIVLEQALKESGTQLLEPYL 540

IRYGLEQGLFGWNVTDCKICFEYGLYYSPVSTPADFRSLAPIVLEQALKESGTQLLEPYL
Sbjct 481
IRYGLEQGLFGWNVTDCKICFEYGLYYSPVSTPADFRSLAPIVLEQALKESGTQLLEPYL 540

Query 541
SFILYAPQEYLSRAYHDAPKYCATIETAQVKKDEVVFTGEIPARCIQAYRTDLAFYTNGR 600
SFILYAPQEYLSRAYHDAPKYCATIETAQVKKDEVV
TGEIPARCIQAYRTDLAFYTNGR
Sbjct 541
SFILYAPQEYLSRAYHDAPKYCATIETAQVKKDEVVITGEIPARCIQAYRTDLAFYTNGR 600

Query 601 SVCLTELKGYQAAVGQPVIQPRRPNSRLDKVRHMFQKVM 639
SVCLTELKGYQAAVGQPVIQPRRPNSRLDKVRHMFQKVM
Sbjct 601 SVCLTELKGYQAAVGQPVIQPRRPNSRLDKVRHMFQKVM 639

>WP_070363278.1 TetM/TetW/TetO/TetS family tetracycline resistance ribosomal
protection protein [Corynebacterium sp. NML98-0116]
AOX06437.1 tetracycline resistance ribosomal protection protein [Corynebacterium
sp. NML98-0116]
Length=639

Score = 1293 bits (3346), Expect = 0.0, Method: Compositional matrix adjust.
Identities = 628/639 (98%), Positives = 635/639 (99%), Gaps = 0/639 (0%)

Query 1
MKIINIGILAHVDAGKTTLTESLLYASGAISEPGSVEKGTTRTDTMFLERQRGITIQAAV 60

MKIINIGILAHVDAGKTTLTESLLYASGAISEPGSVEKGTTRTDTMFLERQRGITIQAAV
Sbjct 1
MKIINIGILAHVDAGKTTLTESLLYASGAISEPGSVEKGTTRTDTMFLERQRGITIQAAV 60

Query 61
TSFQWHRCKVNIVDTPGHMDFLAEVYRSLAVLDGAILVISAKDGVQAQTRILFHALRKMN 120

TSFQWHRCKVNIVDTPGHMDFLAEVYRSLAVLDGAILVISAKDGVQAQTRILFHALRKMN
Sbjct 61
TSFQWHRCKVNIVDTPGHMDFLAEVYRSLAVLDGAILVISAKDGVQAQTRILFHALRKMN 120

Query 121
IPTVIFINKIDQAGVDLQSVVQSVRDKLSADIIIKQTVSLSPEIVLEENTDIEAWDAVIE 180

IPTVIFINKIDQAGVDLQSVVQSVRDKLSADIIIKQTVSLSPEIVLEENTDIEAWDAVIE

Sbjct 121
IPTVIFINKIDQAGVDLQSVVQSVRDKLSADIIIKQTVSLSPEIVLEENTDIEAWDAVIE 180

Query 181
NNDKLLEKYIAGEPISREKLVREEQRRVQDASLFPVYYGSAKKGLGIQPLMDAVTGLFQP 240

NNDKLLEKYIAGEPISREKLVREEQRRVQDASLFPVYYGSAKKGLGIQPLMDAVTGLFQP
Sbjct 181
NNDKLLEKYIAGEPISREKLVREEQRRVQDASLFPVYYGSAKKGLGIQPLMDAVTGLFQP 240

Query 241
IGEQGSAALCGSVFKVEYTDCGQRRVYLRLYSGTLRLRDTVALAGREKLKITEMRIPSKG 300

IGEQGSAALCGSVFKVEYTDCGQRRVYLRLYSGTLRLRDTVALAGREKLKITEMRIPSKG
Sbjct 241
IGEQGSAALCGSVFKVEYTDCGQRRVYLRLYSGTLRLRDTVALAGREKLKITEMRIPSKG 300

Query 301
EIVRTDTAYPGEIVILPSDSVRLNDVLGDPTRLPRKRWREDPLPMLRTTIAPKTAAQRER 360
EIVRTDTAYPGEIVILPSDSVRLNDVLGDPTRLPRKRWREDPLPMLRT
+IAPKTAAQRER
Sbjct 301
EIVRTDTAYPGEIVILPSDSVRLNDVLGDPTRLPRKRWREDPLPMLRTSIAPKTAAQRER 360

Query 361
LLDALTQLADTDPLLRCEVDSITHEIILSFLGRVQLEVVSALLSEKYKLETVVKEPSVIY 420
LLDALTQLADTDPLLRCEVDSITHEIILSFLGRVQLEVVSALLSEKYKLETVVKEP
+VIY
Sbjct 361
LLDALTQLADTDPLLRCEVDSITHEIILSFLGRVQLEVVSALLSEKYKLETVVKEPTVIY 420

Query 421
MERPLKAASHTIHIEVPPNPFWASIGLSVTPLSLGSGVQYESRVSLGYLNQSFQNAVRDG 480
MERPLKAASHTIHIEVPPNPFWASIGLSVTPL
LGSGVQYESRVSLGYLNQSFQNAVRDG
Sbjct 421
MERPLKAASHTIHIEVPPNPFWASIGLSVTPLPLGSGVQYESRVSLGYLNQSFQNAVRDG 480

Query 481
IRYGLEQGLFGWNVTDCKICFEYGLYYSPVSTPADFRSLAPIVLEQALKESGTQLLEPYL 540

IRYGLEQGLFGWNVTDCKICFEYGLYYSPVSTPADFRSLAPIVLEQALKESGTQLLEPYL
Sbjct 481
IRYGLEQGLFGWNVTDCKICFEYGLYYSPVSTPADFRSLAPIVLEQALKESGTQLLEPYL 540

Query 541
SFILYAPQEYLSRAYHDAPKYCATIETAQVKKDEVVFTGEIPARCIQAYRTDLAFYTNGR 600
SF LYAP+EYLSRAYHDAPKYCATIET QVKKDEVVFTGEI
ARCIQAYRTDLAFYTNG+
Sbjct 541
SFTLYAPREYLSRAYHDAPKYCATIETVQVKKDEVVFTGEISARCIQAYRTDLAFYTNGQ 600

Query 601 SVCLTELKGYQAAVGQPVIQPRRPNSRLDKVRHMFQKVM 639
SVCLTELKGYQAAVG+PVIQPRRPNSRLDKVR+MFQK+M

Sbjct 601 SVCLTELKGYQAAVGKPVIQPRRPNSRLDKVRYMFQKIM 639

>WP_066860834.1 TetM/TetW/TetO/TetS family tetracycline resistance ribosomal
protection protein [Neglecta timonensis]
 SCJ20334.1 Tetracycline resistance protein tetM from transposon Tn916 [uncultured
Ruminococcus sp.]
Length=639

 Score = 1293 bits (3346), Expect = 0.0, Method: Compositional matrix adjust.
 Identities = 628/639 (98%), Positives = 635/639 (99%), Gaps = 0/639 (0%)

Query 1
MKIINIGILAHVDAGKTTLTESLLYASGAISEPGSVEKGTTRTDTMFLERQRGITIQAAV 60

MKIINIGILAHVDAGKTTLTESLLYASGAISEPGSVEKGTTRTDTMFLERQRGITIQAAV
Sbjct 1
MKIINIGILAHVDAGKTTLTESLLYASGAISEPGSVEKGTTRTDTMFLERQRGITIQAAV 60

Query 61
TSFQWHRCKVNIVDTPGHMDFLAEVYRSLAVLDGAILVISAKDGVQAQTRILFHALRKMN 120

TSFQWHRCKVNIVDTPGHMDFLAEVYRSLAVLDGAILVISAKDGVQAQTRILFHALRKMN
Sbjct 61
TSFQWHRCKVNIVDTPGHMDFLAEVYRSLAVLDGAILVISAKDGVQAQTRILFHALRKMN 120

Query 121
IPTVIFINKIDQAGVDLQSVVQSVRDKLSADIIIKQTVSLSPEIVLEENTDIEAWDAVIE 180

IPTVIFINKIDQAGVDLQSVVQSVRDKLSADIIIKQTVSLSPEIVLEENTDIEAWDAVIE
Sbjct 121
IPTVIFINKIDQAGVDLQSVVQSVRDKLSADIIIKQTVSLSPEIVLEENTDIEAWDAVIE 180

Query 181
NNDKLLEKYIAGEPISREKLVREEQRRVQDASLFPVYYGSAKKGLGIQPLMDAVTGLFQP 240

NNDKLLEKYIAGEPISREKLVREEQRRVQDASLFPVYYGSAKKGLGIQPLMDAVTGLFQP
Sbjct 181
NNDKLLEKYIAGEPISREKLVREEQRRVQDASLFPVYYGSAKKGLGIQPLMDAVTGLFQP 240

Query 241
IGEQGSAALCGSVFKVEYTDCGQRRVYLRLYSGTLRLRDTVALAGREKLKITEMRIPSKG 300

IGEQGSAALCGSVFKVEYTDCGQRRVYLRLYSGTLRLRDTVALAGREKLKITEMRIPSKG
Sbjct 241
IGEQGSAALCGSVFKVEYTDCGQRRVYLRLYSGTLRLRDTVALAGREKLKITEMRIPSKG 300

Query 301
EIVRTDTAYPGEIVILPSDSVRLNDVLGDPTRLPRKRWREDPLPMLRTTIAPKTAAQRER 360
            EIVRTDTAYPGEIVILPSDSVRLNDVLGDPTRLPRKRWREDPLPMLRT

+IAPKTAAQRER
Sbjct 301
EIVRTDTAYPGEIVILPSDSVRLNDVLGDPTRLPRKRWREDPLPMLRTSIAPKTAAQRER 360

Query 361
LLDALTQLADTDPLLRCEVDSITHEIILSFLGRVQLEVVSALLSEKYKLETVVKEPSVIY 420
            LLDALTQLADTDPLLRCEVDSITHEIILSFLGRVQLEVVSALLSEKYKLETVVKEP
+VIY
Sbjct 361
LLDALTQLADTDPLLRCEVDSITHEIILSFLGRVQLEVVSALLSEKYKLETVVKEPTVIY 420

Query 421
MERPLKAASHTIHIEVPPNPFWASIGLSVTPLSLGSGVQYESRVSLGYLNQSFQNAVRDG 480
            MERPLKAASHTIHIEVPPNPFWASIGLSVTPL
LGSGVQYESRVSLGYLNQSFQNAVRDG
Sbjct 421
MERPLKAASHTIHIEVPPNPFWASIGLSVTPLPLGSGVQYESRVSLGYLNQSFQNAVRDG 480

Query 481
IRYGLEQGLFGWNVTDCKICFEYGLYYSPVSTPADFRSLAPIVLEQALKESGTQLLEPYL 540

IRYGLEQGLFGWNVTDCKICFEYGLYYSPVSTPADFRSLAPIVLEQALKESGTQLLEPYL
Sbjct 481
IRYGLEQGLFGWNVTDCKICFEYGLYYSPVSTPADFRSLAPIVLEQALKESGTQLLEPYL 540

Query 541
SFILYAPQEYLSRAYHDAPKYCATIETAQVKKDEVVFTGEIPARCIQAYRTDLAFYTNGR 600
            SF LYAP+EYLSRAYHDAPKYCATIET Q
KKDEVVFTGEIPARCIQAYRTDLAFYTNG+
Sbjct 541
SFTLYAPREYLSRAYHDAPKYCATIETVQEKKDEVVFTGEIPARCIQAYRTDLAFYTNGQ 600

Query 601 SVCLTELKGYQAAVGQPVIQPRRPNSRLDKVRHMFQKVM 639
            SVCLTELKGYQAAVG+PVIQPRRPNSRLDKVR+MFQK+M
Sbjct 601 SVCLTELKGYQAAVGKPVIQPRRPNSRLDKVRYMFQKIM 639

>WP_033507052.1 TetM/TetW/TetO/TetS family tetracycline resistance ribosomal
protection protein [Bifidobacterium thermacidophilum]
Length=639

 Score = 1293 bits (3346), Expect = 0.0, Method: Compositional matrix adjust.
 Identities = 628/639 (98%), Positives = 635/639 (99%), Gaps = 0/639 (0%)

Query 1
MKIINIGILAHVDAGKTTLTESLLYASGAISEPGSVEKGTTRTDTMFLERQRGITIQAAV 60

MKIINIGILAHVDAGKTTLTESLLYASGAISEPGSVEKGTTRTDTMFLERQRGITIQAAV
Sbjct 1
MKIINIGILAHVDAGKTTLTESLLYASGAISEPGSVEKGTTRTDTMFLERQRGITIQAAV 60

Query 61
TSFQWHRCKVNIVDTPGHMDFLAEVYRSLAVLDGAILVISAKDGVQAQTRILFHALRKMN 120

TSFQWHRCKVNIVDTPGHMDFLAEVYRSLAVLDGAILVISAKDGVQAQTRILFHALRKMN
Sbjct 61
TSFQWHRCKVNIVDTPGHMDFLAEVYRSLAVLDGAILVISAKDGVQAQTRILFHALRKMN 120

Query 121
IPTVIFINKIDQAGVDLQSVVQSVRDKLSADIIIKQTVSLSPEIVLEENTDIEAWDAVIE 180

IPTVIFINKIDQAGVDLQSVVQSVRDKLSADIIIKQTVSLSPEIVLEENTDIEAWDAVIE
Sbjct 121
IPTVIFINKIDQAGVDLQSVVQSVRDKLSADIIIKQTVSLSPEIVLEENTDIEAWDAVIE 180

Query 181
NNDKLLEKYIAGEPISREKLVREEQRRVQDASLFPVYYGSAKKGLGIQPLMDAVTGLFQP 240
NNDKLLEKYIAGEPISR
KLVREEQRRVQDASLFPVYYGSAKKGLGIQPLMDAVTGLFQP
Sbjct 181
NNDKLLEKYIAGEPISRAKLVREEQRRVQDASLFPVYYGSAKKGLGIQPLMDAVTGLFQP 240

Query 241
IGEQGSAALCGSVFKVEYTDCGQRRVYLRLYSGTLRLRDTVALAGREKLKITEMRIPSKG 300

IGEQGSAALCGSVFKVEYTDCGQRRVYLRLYSGTLRLRDTVALAGREKLKITEMRIPSKG
Sbjct 241
IGEQGSAALCGSVFKVEYTDCGQRRVYLRLYSGTLRLRDTVALAGREKLKITEMRIPSKG 300

Query 301
EIVRTDTAYPGEIVILPSDSVRLNDVLGDPTRLPRKRWREDPLPMLRTTIAPKTAAQRER 360
EIVRTDTAYPGEIVILPSDSVRLNDVLGDPTRLPRKRWREDPLPMLRT
+IAPKTAAQRER
Sbjct 301
EIVRTDTAYPGEIVILPSDSVRLNDVLGDPTRLPRKRWREDPLPMLRTSIAPKTAAQRER 360

Query 361
LLDALTQLADTDPLLRCEVDSITHEIILSFLGRVQLEVVSALLSEKYKLETVVKEPSVIY 420
LLDALTQLADTDPLLRCEVDSITHEIILSFLGRVQLEVVSALLSEKYKLETVVKEP
+VIY
Sbjct 361
LLDALTQLADTDPLLRCEVDSITHEIILSFLGRVQLEVVSALLSEKYKLETVVKEPTVIY 420

Query 421
MERPLKAASHTIHIEVPPNPFWASIGLSVTPLSLGSGVQYESRVSLGYLNQSFQNAVRDG 480
MERPLKAASHTIHIEVPPNPFWASIGLSVTPL
LGSGVQYESRVSLGYLNQSFQNAVRDG
Sbjct 421
MERPLKAASHTIHIEVPPNPFWASIGLSVTPLPLGSGVQYESRVSLGYLNQSFQNAVRDG 480

Query 481
IRYGLEQGLFGWNVTDCKICFEYGLYYSPVSTPADFRSLAPIVLEQALKESGTQLLEPYL 540

IRYGLEQGLFGWNVTDCKICFEYGLYYSPVSTPADFRSLAPIVLEQALKESGTQLLEPYL
Sbjct 481

IRYGLEQGLFGWNVTDCKICFEYGLYYSPVSTPADFRSLAPIVLEQALKESGTQLLEPYL 540

Query 541
SFILYAPQEYLSRAYHDAPKYCATIETAQVKKDEVVFTGEIPARCIQAYRTDLAFYTNGR 600
            SF LYAP+EYLSRAYHDAPKYCATIET
QVKKDEVVFTGEIPARCIQAYRTDLAFYTNG+
Sbjct 541
SFTLYAPREYLSRAYHDAPKYCATIETVQVKKDEVVFTGEIPARCIQAYRTDLAFYTNGQ 600

Query 601 SVCLTELKGYQAAVGQPVIQPRRPNSRLDKVRHMFQKVM 639
            SVCLTELKGYQAAVG+PVIQPRRPNSRLDKVR+MFQK+M
Sbjct 601 SVCLTELKGYQAAVGKPVIQPRRPNSRLDKVRYMFQKIM 639

>WP_009305318.1 MULTISPECIES: tetracycline resistance ribosomal
protection protein
Tet(W) [Actinobacteria]
AAY62597.1 TetW [Bifidobacterium longum]
EFV33626.1 elongation factor Tu GTP binding domain-containing
protein [Eggerthella
sp. 1_3_56FAA]
EGC89372.1 putative translation elongation factor G [Eggerthella
sp. HGA1]
AHN92782.1 elongation factor Tu GTP binding domain-containing
protein [uncultured
bacterium DCM003Tet07]
AMP54158.1 TetM-TetW-TetO-TetS [uncultured bacterium]
PNV67184.1 tetracycline resistance ribosomal protection protein
Tet(W) [Enteroscipio
rubneri]
Length=639

Score = 1293 bits (3346), Expect = 0.0, Method: Compositional
matrix adjust.
Identities = 630/639 (99%), Positives = 633/639 (99%), Gaps = 0/639
(0%)

Query 1
MKIINIGILAHVDAGKTTLTESLLYASGAISEPGSVEKGTTRTDTMFLERQRGITIQAAV 60

MKIINIGILAHVDAGKTTLTESLLYASGAISEPGSVEKGTTRTDTMFLERQRGITIQAAV
Sbjct 1
MKIINIGILAHVDAGKTTLTESLLYASGAISEPGSVEKGTTRTDTMFLERQRGITIQAAV 60

Query 61
TSFQWHRCKVNIVDTPGHMDFLAEVYRSLAVLDGAILVISAKDGVQAQTRILFHALRKMN 120

TSFQWHRCKVNIVDTPGHMDFLAEVYRSLAVLDGAILVISAKDGVQAQTRILFHALRKMN
Sbjct 61
TSFQWHRCKVNIVDTPGHMDFLAEVYRSLAVLDGAILVISAKDGVQAQTRILFHALRKMN 120

Query 121
IPTVIFINKIDQAGVDLQSVVQSVRDKLSADIIIKQTVSLSPEIVLEENTDIEAWDAVIE 180

IPTVIFINKIDQAGVDLQSVVQSVRDKLSADIIIKQTVSLSPEIVLEENTDIEAWDAVIE
Sbjct 121
IPTVIFINKIDQAGVDLQSVVQSVRDKLSADIIIKQTVSLSPEIVLEENTDIEAWDAVIE 180

Query 181
NNDKLLEKYIAGEPISREKLVREEQRRVQDASLFPVYYGSAKKGLGIQPLMDAVTGLFQP 240
NND+LLEKYIAGEPISREKL REEQ+RVQDASLFPVY+GSAK
GLGIQPLMDAVTGLFQP
Sbjct 181
NNDELLEKYIAGEPISREKLAREEQQRVQDASLFPVYHGSAKNGLGIQPLMDAVTGLFQP 240

Query 241
IGEQGSAALCGSVFKVEYTDCGQRRVYLRLYSGTLRLRDTVALAGREKLKITEMRIPSKG 300
IGEQG
AALCGSVFKVEYTDCGQRRVYLRLYSGTLRLRDTVALAGREKLKITEMRIPSKG
Sbjct 241
IGEQGGAALCGSVFKVEYTDCGQRRVYLRLYSGTLRLRDTVALAGREKLKITEMRIPSKG 300

Query 301
EIVRTDTAYPGEIVILPSDSVRLNDVLGDPTRLPRKRWREDPLPMLRTTIAPKTAAQRER 360
EIVRTDTAY GEIVILPSDSVRLNDVLGD TRLPRKRWREDPLPMLRT
IAPKTAAQRER
Sbjct 301
EIVRTDTAYQGEIVILPSDSVRLNDVLGDQTRLPRKRWREDPLPMLRTAIAPKTAAQRER 360

Query 361
LLDALTQLADTDPLLRCEVDSITHEIILSFLGRVQLEVVSALLSEKYKLETVVKEPSVIY 420

LLDALTQLADTDPLLRCEVDSITHEIILSFLGRVQLEVVSALLSEKYKLETVVKEPSVIY
Sbjct 361
LLDALTQLADTDPLLRCEVDSITHEIILSFLGRVQLEVVSALLSEKYKLETVVKEPSVIY 420

Query 421
MERPLKAASHTIHIEVPPNPFWASIGLSVTPLSLGSGVQYESRVSLGYLNQSFQNAVRDG 480

MERPLKAASHTIHIEVPPNPFWASIGLSVTPLSLGSGVQYESRVSLGYLNQSFQNAVRDG
Sbjct 421
MERPLKAASHTIHIEVPPNPFWASIGLSVTPLSLGSGVQYESRVSLGYLNQSFQNAVRDG 480

Query 481
IRYGLEQGLFGWNVTDCKICFEYGLYYSPVSTPADFRSLAPIVLEQALKESGTQLLEPYL 540

IRYGLEQGLFGWNVTDCKICFEYGLYYSPVSTPADFRSLAPIVLEQALKESGTQLLEPYL
Sbjct 481
IRYGLEQGLFGWNVTDCKICFEYGLYYSPVSTPADFRSLAPIVLEQALKESGTQLLEPYL 540

Query 541
SFILYAPQEYLSRAYHDAPKYCATIETAQVKKDEVVFTGEIPARCIQAYRTDLAFYTNGR 600

SFILYAPQEYLSRAYHDAPKYCATIETAQVKKDEVVFTGEIPARCIQAYRTDLAFYTNGR
Sbjct 541
SFILYAPQEYLSRAYHDAPKYCATIETAQVKKDEVVFTGEIPARCIQAYRTDLAFYTNGR 600

Query 601 SVCLTELKGYQAAVGQPVIQPRRPNSRLDKVRHMFQKVM 639

```
            SVCLTELKGYQAAVGQPVIQPRRPNSRLDKVRHMFQKVM
Sbjct  601  SVCLTELKGYQAAVGQPVIQPRRPNSRLDKVRHMFQKVM  639


>WP_087376933.1 TetM/TetW/TetO/TetS family tetracycline resistance 
ribosomal 
protection protein [Flavonifractor sp. An135]
 OUQ26342.1 tetracycline resistance ribosomal protection protein 
Tet(W) [Flavonifractor 
sp. An135]
Length=639

 Score = 1293 bits (3345),  Expect = 0.0, Method: Compositional 
matrix adjust.
 Identities = 629/639 (98%), Positives = 633/639 (99%), Gaps = 0/639 
(0%)

Query  1    
MKIINIGILAHVDAGKTTLTESLLYASGAISEPGSVEKGTTRTDTMFLERQRGITIQAAV  60
            M IINIGILAHVDAGKTTLTESLLYASGAISEPGSVEKGTTRTDT
+FLERQRGITIQAAV
Sbjct  1    
MNIINIGILAHVDAGKTTLTESLLYASGAISEPGSVEKGTTRTDTLFLERQRGITIQAAV  60

Query  61   
TSFQWHRCKVNIVDTPGHMDFLAEVYRSLAVLDGAILVISAKDGVQAQTRILFHALRKMN  120
            
TSFQWHRCKVNIVDTPGHMDFLAEVYRSLAVLDGAILVISAKDGVQAQTRILFHALRKMN
Sbjct  61   
TSFQWHRCKVNIVDTPGHMDFLAEVYRSLAVLDGAILVISAKDGVQAQTRILFHALRKMN  120

Query  121  
IPTVIFINKIDQAGVDLQSVVQSVRDKLSADIIIKQTVSLSPEIVLEENTDIEAWDAVIE  180
            
IPTVIFINKIDQAGVDLQSVVQSVRDKLSADIIIKQTVSLSPEIVLEENTDIEAWDAVIE
Sbjct  121  
IPTVIFINKIDQAGVDLQSVVQSVRDKLSADIIIKQTVSLSPEIVLEENTDIEAWDAVIE  180

Query  181  
NNDKLLEKYIAGEPISREKLVREEQRRVQDASLFPVYYGSAKKGLGIQPLMDAVTGLFQP  240
            NND+LLEKYIAGEPISREKL REEQ+RVQDASLFPVY+GSAK 
GLGIQPLMDAVTGLFQP
Sbjct  181  
NNDELLEKYIAGEPISREKLAREEQQRVQDASLFPVYHGSAKNGLGIQPLMDAVTGLFQP  240

Query  241  
IGEQGSAALCGSVFKVEYTDCGQRRVYLRLYSGTLRLRDTVALAGREKLKITEMRIPSKG  300
            IGEQG 
AALCGSVFKVEYTDCGQRRVYLRLYSGTLRLRDTVALAGREKLKITEMRIPSKG
Sbjct  241  
IGEQGGAALCGSVFKVEYTDCGQRRVYLRLYSGTLRLRDTVALAGREKLKITEMRIPSKG  300

Query  301  
EIVRTDTAYPGEIVILPSDSVRLNDVLGDPTRLPRKRWREDPLPMLRTTIAPKTAAQRER  360
```

            EIVRTDTAY GEIVILPSDSVRLNDVLGD
TRLPRKRWREDPLPMLRTTIAPKTAAQRER
Sbjct 301
EIVRTDTAYQGEIVILPSDSVRLNDVLGDQTRLPRKRWREDPLPMLRTTIAPKTAAQRER 360

Query 361
LLDALTQLADTDPLLRCEVDSITHEIILSFLGRVQLEVVSALLSEKYKLETVVKEPSVIY 420

LLDALTQLADTDPLLRCEVDSITHEIILSFLGRVQLEVVSALLSEKYKLETVVKEPSVIY
Sbjct 361
LLDALTQLADTDPLLRCEVDSITHEIILSFLGRVQLEVVSALLSEKYKLETVVKEPSVIY 420

Query 421
MERPLKAASHTIHIEVPPNPFWASIGLSVTPLSLGSGVQYESRVSLGYLNQSFQNAVRDG 480

MERPLKAASHTIHIEVPPNPFWASIGLSVTPLSLGSGVQYESRVSLGYLNQSFQNAVRDG
Sbjct 421
MERPLKAASHTIHIEVPPNPFWASIGLSVTPLSLGSGVQYESRVSLGYLNQSFQNAVRDG 480

Query 481
IRYGLEQGLFGWNVTDCKICFEYGLYYSPVSTPADFRSLAPIVLEQALKESGTQLLEPYL 540

IRYGLEQGLFGWNVTDCKICFEYGLYYSPVSTPADFRSLAPIVLEQALKESGTQLLEPYL
Sbjct 481
IRYGLEQGLFGWNVTDCKICFEYGLYYSPVSTPADFRSLAPIVLEQALKESGTQLLEPYL 540

Query 541
SFILYAPQEYLSRAYHDAPKYCATIETAQVKKDEVVFTGEIPARCIQAYRTDLAFYTNGR 600

SFILYAPQEYLSRAYHDAPKYCATIETAQVKKDEVVFTGEIPARCIQAYRTDLAFYTNGR
Sbjct 541
SFILYAPQEYLSRAYHDAPKYCATIETAQVKKDEVVFTGEIPARCIQAYRTDLAFYTNGR 600

Query 601 SVCLTELKGYQAAVGQPVIQPRRPNSRLDKVRHMFQKVM 639
            SVCLTELKGYQAAVGQPVIQPRRPNSRLDKVRHMFQKVM
Sbjct 601 SVCLTELKGYQAAVGQPVIQPRRPNSRLDKVRHMFQKVM 639

>WP_090529887.1 TetM/TetW/TetO/TetS family tetracycline resistance ribosomal
protection protein [Pseudobutyrivibrio sp. UC1225]
 SFO16568.1 ribosomal protection tetracycline resistance protein [Pseudobutyrivibrio
sp. UC1225]
Length=639

 Score = 1293 bits (3345), Expect = 0.0, Method: Compositional matrix adjust.
 Identities = 630/639 (99%), Positives = 633/639 (99%), Gaps = 0/639 (0%)

Query 1
MKIINIGILAHVDAGKTTLTESLLYASGAISEPGSVEKGTTRTDTMFLERQRGITIQAAV 60

MKIINIGILAHVDAGKTTLTESLLYASGAISEPGSVEKGTTRTDTMFLERQRGITIQAAV
Sbjct 1
MKIINIGILAHVDAGKTTLTESLLYASGAISEPGSVEKGTTRTDTMFLERQRGITIQAAV 60

Query 61
TSFQWHRCKVNIVDTPGHMDFLAEVYRSLAVLDGAILVISAKDGVQAQTRILFHALRKMN 120

TSFQWHRCKVNIVDTPGHMDFLAEVYRSLAVLDGAILVISAKDGVQAQTRILFHALRKMN
Sbjct 61
TSFQWHRCKVNIVDTPGHMDFLAEVYRSLAVLDGAILVISAKDGVQAQTRILFHALRKMN 120

Query 121
IPTVIFINKIDQAGVDLQSVVQSVRDKLSADIIIKQTVSLSPEIVLEENTDIEAWDAVIE 180

IPTVIFINKIDQAGVDLQSVVQSVRDKLSADIIIKQTVSLSPEIVLEENTDIEAWDAVIE
Sbjct 121
IPTVIFINKIDQAGVDLQSVVQSVRDKLSADIIIKQTVSLSPEIVLEENTDIEAWDAVIE 180

Query 181
NNDKLLEKYIAGEPISREKLVREEQRRVQDASLFPVYYGSAKKGLGIQPLMDAVTGLFQP 240
NND+LLEKYIAGEPISREKL REEQ+RVQDASLFPVY+GSAK GLGIQPLMDAVTGLFQP
Sbjct 181
NNDELLEKYIAGEPISREKLAREEQQRVQDASLFPVYHGSAKNGLGIQPLMDAVTGLFQP 240

Query 241
IGEQGSAALCGSVFKVEYTDCGQRRVYLRLYSGTLRLRDTVALAGREKLKITEMRIPSKG 300
IGEQG AALCGSVFKVEYTDCGQRRVYLRLYSGTLRLRDTVALAGREKLKITEMRIPSKG
Sbjct 241
IGEQGGAALCGSVFKVEYTDCGQRRVYLRLYSGTLRLRDTVALAGREKLKITEMRIPSKG 300

Query 301
EIVRTDTAYPGEIVILPSDSVRLNDVLGDPTRLPRKRWREDPLPMLRTTIAPKTAAQRER 360
EIVRTDTAY GEIVILPSDSVRLNDVLGD TRLPRKRWREDPLPMLRTTIAPKTAAQRER
Sbjct 301
EIVRTDTAYQGEIVILPSDSVRLNDVLGDQTRLPRKRWREDPLPMLRTTIAPKTAAQRER 360

Query 361
LLDALTQLADTDPLLRCEVDSITHEIILSFLGRVQLEVVSALLSEKYKLETVVKEPSVIY 420

LLDALTQLADTDPLLRCEVDSITHEIILSFLGRVQLEVVSALLSEKYKLETVVKEPSVIY
Sbjct 361
LLDALTQLADTDPLLRCEVDSITHEIILSFLGRVQLEVVSALLSEKYKLETVVKEPSVIY 420

Query 421
MERPLKAASHTIHIEVPPNPFWASIGLSVTPLSLGSGVQYESRVSLGYLNQSFQNAVRDG 480

MERPLKAASHTIHIEVPPNPFWASIGLSVTPLSLGSGVQYESRVSLGYLNQSFQNAVRDG
Sbjct 421
MERPLKAASHTIHIEVPPNPFWASIGLSVTPLSLGSGVQYESRVSLGYLNQSFQNAVRDG 480

Query 481

IRYGLEQGLFGWNVTDCKICFEYGLYYSPVSTPADFRSLAPIVLEQALKESGTQLLEPYL 540

IRYGLEQGLFGWNVTDCKICFEYGLYYSPVSTPADFRSLAPIVLEQALKESGTQLLEPYL
Sbjct 481
IRYGLEQGLFGWNVTDCKICFEYGLYYSPVSTPADFRSLAPIVLEQALKESGTQLLEPYL 540

Query 541
SFILYAPQEYLSRAYHDAPKYCATIETAQVKKDEVVFTGEIPARCIQAYRTDLAFYTNGR 600
SFILYAPQEYLSRAY
DAPKYCATIETAQVKKDEVVFTGEIPARCIQAYRTDLAFYTNGR
Sbjct 541
SFILYAPQEYLSRAYQDAPKYCATIETAQVKKDEVVFTGEIPARCIQAYRTDLAFYTNGR 600

Query 601 SVCLTELKGYQAAVGQPVIQPRRPNSRLDKVRHMFQKVM 639
SVCLTELKGYQAAVGQPVIQPRRPNSRLDKVRHMFQKVM
Sbjct 601 SVCLTELKGYQAAVGQPVIQPRRPNSRLDKVRHMFQKVM 639

>WP_090152943.1 TetM/TetW/TetO/TetS family tetracycline resistance ribosomal
protection protein [Pseudobutyrivibrio sp. AR14]
SCY08685.1 ribosomal protection tetracycline resistance protein [Pseudobutyrivibrio
sp. AR14]
Length=639

Score = 1293 bits (3345), Expect = 0.0, Method: Compositional matrix adjust.
Identities = 628/639 (98%), Positives = 635/639 (99%), Gaps = 0/639 (0%)

Query 1
MKIINIGILAHVDAGKTTLTESLLYASGAISEPGSVEKGTTRTDTMFLERQRGITIQAAV 60

MKIINIGILAHVDAGKTTLTESLLYASGAISEPGSVEKGTTRTDTMFLERQRGITIQAAV
Sbjct 1
MKIINIGILAHVDAGKTTLTESLLYASGAISEPGSVEKGTTRTDTMFLERQRGITIQAAV 60

Query 61
TSFQWHRCKVNIVDTPGHMDFLAEVYRSLAVLDGAILVISAKDGVQAQTRILFHALRKMN 120

TSFQWHRCKVNIVDTPGHMDFLAEVYRSLAVLDGAILVISAKDGVQAQTRILFHALRKMN
Sbjct 61
TSFQWHRCKVNIVDTPGHMDFLAEVYRSLAVLDGAILVISAKDGVQAQTRILFHALRKMN 120

Query 121
IPTVIFINKIDQAGVDLQSVVQSVRDKLSADIIIKQTVSLSPEIVLEENTDIEAWDAVIE 180

IPTVIFINKIDQAGVDLQSVVQSVRDKLSADIIIKQTVSLSPEIVLEENTDIEAWDAVIE
Sbjct 121
IPTVIFINKIDQAGVDLQSVVQSVRDKLSADIIIKQTVSLSPEIVLEENTDIEAWDAVIE 180

Query 181
NNDKLLEKYIAGEPISREKLVREEQRRVQDASLFPVYYGSAKKGLGIQPLMDAVTGLFQP 240

```
NNDKLLEKYIAGEPISREKLVREEQRRVQDASLFPVYYGSAKKGLGIQPLMDAVTGLFQP
Sbjct  181  
NNDKLLEKYIAGEPISREKLVREEQRRVQDASLFPVYYGSAKKGLGIQPLMDAVTGLFQP  240

Query  241  
IGEQGSAALCGSVFKVEYTDCGQRRVYLRLYSGTLRLRDTVALAGREKLKITEMRIPSKG  300
            
IGEQGSAALCGSVFKVEYTDCGQRRVYLRLYSGTLRLRDTVALAGREKLKITEMRIPSKG
Sbjct  241  
IGEQGSAALCGSVFKVEYTDCGQRRVYLRLYSGTLRLRDTVALAGREKLKITEMRIPSKG  300

Query  301  
EIVRTDTAYPGEIVILPSDSVRLNDVLGDPTRLPRKRWREDPLPMLRTTIAPKTAAQRER  360
            EIVRTDTAYPGEIVILPSDSVRLNDVLGDPTRLPRKRWREDPLPMLRT
+IAPKTAAQRER
Sbjct  301  
EIVRTDTAYPGEIVILPSDSVRLNDVLGDPTRLPRKRWREDPLPMLRTSIAPKTAAQRER  360

Query  361  
LLDALTQLADTDPLLRCEVDSITHEIILSFLGRVQLEVVSALLSEKYKLETVVKEPSVIY  420
            LLDALTQLADTDPLLRCEVDSITHEIILSFLGRVQLEVVSALLSEKYKLETVVKEP
+VIY
Sbjct  361  
LLDALTQLADTDPLLRCEVDSITHEIILSFLGRVQLEVVSALLSEKYKLETVVKEPTVIY  420

Query  421  
MERPLKAASHTIHIEVPPNPFWASIGLSVTPLSLGSGVQYESRVSLGYLNQSFQNAVRDG  480
            MERPLKAASHTIHIEVPPNPFWASIGLSVTPL
LGSGVQYESRVSLGYLNQSFQNAVRDG
Sbjct  421  
MERPLKAASHTIHIEVPPNPFWASIGLSVTPLPLGSGVQYESRVSLGYLNQSFQNAVRDG  480

Query  481  
IRYGLEQGLFGWNVTDCKICFEYGLYYSPVSTPADFRSLAPIVLEQALKESGTQLLEPYL  540
            
IRYGLEQGLFGWNVTDCKICFEYGLYYSPVSTPADFRSLAPIVLEQALKESGTQLLEPYL
Sbjct  481  
IRYGLEQGLFGWNVTDCKICFEYGLYYSPVSTPADFRSLAPIVLEQALKESGTQLLEPYL  540

Query  541  
SFILYAPQEYLSRAYHDAPKYCATIETAQVKKDEVVFTGEIPARCIQAYRTDLAFYTNGR  600
            SF LYAP+EYLSRAYHDAPKYCATIET
QVKKDEVVFTGEIPARCIQAYRTDLAFYTNG+
Sbjct  541  
SFTLYAPREYLSRAYHDAPKYCATIETVQVKKDEVVFTGEIPARCIQAYRTDLAFYTNGQ  600

Query  601  SVCLTELKGYQAAVGQPVIQPRRPNSRLDKVRHMFQKVM  639
            SVCLTELKGYQAAVG+PVIQPRRPNS LDKVR+MFQK+M
Sbjct  601  SVCLTELKGYQAAVGKPVIQPRRPNSLLDKVRYMFQKIM  639


>AMP57752.1 TetM-TetW-TetO-TetS [uncultured bacterium]
Length=639
```

 Score = 1293 bits (3345), Expect = 0.0, Method: Compositional matrix adjust.
 Identities = 629/639 (98%), Positives = 634/639 (99%), Gaps = 0/639 (0%)

```
Query  1
MKIINIGILAHVDAGKTTLTESLLYASGAISEPGSVEKGTTRTDTMFLERQRGITIQAAV  60

MKIINIGILAHVDAGKTTLTESLLYASGAISEPGSVEKGTTRTDTMFLERQRGITIQAAV
Sbjct  1
MKIINIGILAHVDAGKTTLTESLLYASGAISEPGSVEKGTTRTDTMFLERQRGITIQAAV  60

Query  61
TSFQWHRCKVNIVDTPGHMDFLAEVYRSLAVLDGAILVISAKDGVQAQTRILFHALRKMN  120

TSFQWHRCKVNIVDTPGHMDFLAEVYRSLAVLDGAILVISAKDGVQAQTRILFHALRKMN
Sbjct  61
TSFQWHRCKVNIVDTPGHMDFLAEVYRSLAVLDGAILVISAKDGVQAQTRILFHALRKMN  120

Query  121
IPTVIFINKIDQAGVDLQSVVQSVRDKLSADIIIKQTVSLSPEIVLEENTDIEAWDAVIE  180

IPTVIFINKIDQAGVDLQSVVQSVRDKLSADIIIKQTVSLSPEIVLEENTDIEAWDAVIE
Sbjct  121
IPTVIFINKIDQAGVDLQSVVQSVRDKLSADIIIKQTVSLSPEIVLEENTDIEAWDAVIE  180

Query  181
NNDKLLEKYIAGEPISREKLVREEQRRVQDASLFPVYYGSAKKGLGIQPLMDAVTGLFQP  240
            NND+LLEKYIAGEPISREKL REEQ+RVQDASLFPVY+GSAK
GLGIQPLMDAVTGLFQP
Sbjct  181
NNDELLEKYIAGEPISREKLAREEQQRVQDASLFPVYHGSAKNGLGIQPLMDAVTGLFQP  240

Query  241
IGEQGSAALCGSVFKVEYTDCGQRRVYLRLYSGTLRLRDTVALAGREKLKITEMRIPSKG  300
            IGEQG
AALCGSVFKVEYTDCGQRRVYLRLYSGTLRLRDTVALAGREKLKITEMRIPSKG
Sbjct  241
IGEQGGAALCGSVFKVEYTDCGQRRVYLRLYSGTLRLRDTVALAGREKLKITEMRIPSKG  300

Query  301
EIVRTDTAYPGEIVILPSDSVRLNDVLGDPTRLPRKRWREDPLPMLRTTIAPKTAAQRER  360
            EIVRTDTAY GEIVILPSDSVRLNDVLGD
TRLPRKRWREDPLPMLRTTIAPKTAAQRER
Sbjct  301
EIVRTDTAYQGEIVILPSDSVRLNDVLGDQTRLPRKRWREDPLPMLRTTIAPKTAAQRER  360

Query  361
LLDALTQLADTDPLLRCEVDSITHEIILSFLGRVQLEVVSALLSEKYKLETVVKEPSVIY  420

LLDALTQLADTDPLLRCEVDSITHEIILSFLGRVQLEVVSALLSEKYKLETVVKEPSVIY
Sbjct  361
LLDALTQLADTDPLLRCEVDSITHEIILSFLGRVQLEVVSALLSEKYKLETVVKEPSVIY  420
```

Query 421
MERPLKAASHTIHIEVPPNPFWASIGLSVTPLSLGSGVQYESRVSLGYLNQSFQNAVRDG 480

MERPLKAASHTIHIEVPPNPFWASIGLSVTPLSLGSGVQYESRVSLGYLNQSFQNAVRDG
Sbjct 421
MERPLKAASHTIHIEVPPNPFWASIGLSVTPLSLGSGVQYESRVSLGYLNQSFQNAVRDG 480

Query 481
IRYGLEQGLFGWNVTDCKICFEYGLYYSPVSTPADFRSLAPIVLEQALKESGTQLLEPYL 540

IRYGLEQGLFGWNVTDCKICFEYGLYYSPVSTPADFRSLAPIVLEQALKESGTQLLEPYL
Sbjct 481
IRYGLEQGLFGWNVTDCKICFEYGLYYSPVSTPADFRSLAPIVLEQALKESGTQLLEPYL 540

Query 541
SFILYAPQEYLSRAYHDAPKYCATIETAQVKKDEVVFTGEIPARCIQAYRTDLAFYTNGR 600
SFILYAPQE
+LSRAYHDAPKYCATIETAQVKKDEVVFTGEIPARCIQAYRTDLAFYTNGR
Sbjct 541
SFILYAPQEFLSRAYHDAPKYCATIETAQVKKDEVVFTGEIPARCIQAYRTDLAFYTNGR 600

Query 601 SVCLTELKGYQAAVGQPVIQPRRPNSRLDKVRHMFQKVM 639
SVCLTELKGYQAAVGQPVIQPRRPNSRLDKVRHMF+KVM
Sbjct 601 SVCLTELKGYQAAVGQPVIQPRRPNSRLDKVRHMFKKVM 639

>AMP53595.1 TetM-TetW-TetO-TetS [uncultured bacterium]
Length=639

Score = 1293 bits (3345), Expect = 0.0, Method: Compositional matrix adjust.
Identities = 629/639 (98%), Positives = 633/639 (99%), Gaps = 0/639 (0%)

Query 1
MKIINIGILAHVDAGKTTLTESLLYASGAISEPGSVEKGTTRTDTMFLERQRGITIQAAV 60

MKIINIGILAHVDAGKTTLTESLLYASGAISEPGSVEKGTTRTDTMFLERQRGITIQAAV
Sbjct 1
MKIINIGILAHVDAGKTTLTESLLYASGAISEPGSVEKGTTRTDTMFLERQRGITIQAAV 60

Query 61
TSFQWHRCKVNIVDTPGHMDFLAEVYRSLAVLDGAILVISAKDGVQAQTRILFHALRKMN 120

TSFQWHRCKVNIVDTPGHMDFLAEVYRSLAVLDGAILVISAKDGVQAQTRILFHALRKMN
Sbjct 61
TSFQWHRCKVNIVDTPGHMDFLAEVYRSLAVLDGAILVISAKDGVQAQTRILFHALRKMN 120

Query 121
IPTVIFINKIDQAGVDLQSVVQSVRDKLSADIIIKQTVSLSPEIVLEENTDIEAWDAVIE 180

IPTVIFINKIDQAGVDLQSVVQSVRDKLSADIIIKQTVSLSPEIVLEENTDIEAWDAVIE
Sbjct 121

IPTVIFINKIDQAGVDLQSVVQSVRDKLSADIIIKQTVSLSPEIVLEENTDIEAWDAVIE 180

Query 181
NNDKLLEKYIAGEPISREKLVREEQRRVQDASLFPVYYGSAKKGLGIQPLMDAVTGLFQP 240
NND+LLEKYIAGEPISREKL REEQ+RVQDASLFPVY+GSAK
GLGIQPLMDAVTGLFQP
Sbjct 181
NNDELLEKYIAGEPISREKLAREEQQRVQDASLFPVYHGSAKNGLGIQPLMDAVTGLFQP 240

Query 241
IGEQGSAALCGSVFKVEYTDCGQRRVYLRLYSGTLRLRDTVALAGREKLKITEMRIPSKG 300
IGEQG
AALCGSVFKVEYTDCGQRRVYLRLYSGTLRLRDTVALAGREKLKITEMRIPSKG
Sbjct 241
IGEQGGAALCGSVFKVEYTDCGQRRVYLRLYSGTLRLRDTVALAGREKLKITEMRIPSKG 300

Query 301
EIVRTDTAYPGEIVILPSDSVRLNDVLGDPTRLPRKRWREDPLPMLRTTIAPKTAAQRER 360
EIVRTDTAY GEIVILPSDSVRLNDVLGD
TRLPRKRWREDPLPMLRTTIAPKTAAQRER
Sbjct 301
EIVRTDTAYQGEIVILPSDSVRLNDVLGDQTRLPRKRWREDPLPMLRTTIAPKTAAQRER 360

Query 361
LLDALTQLADTDPLLRCEVDSITHEIILSFLGRVQLEVVSALLSEKYKLETVVKEPSVIY 420

LLDALTQLADTDPLLRCEVDSITHEIILSFLGRVQLEVVSALLSEKYKLETVVKEPSVIY
Sbjct 361
LLDALTQLADTDPLLRCEVDSITHEIILSFLGRVQLEVVSALLSEKYKLETVVKEPSVIY 420

Query 421
MERPLKAASHTIHIEVPPNPFWASIGLSVTPLSLGSGVQYESRVSLGYLNQSFQNAVRDG 480

MERPLKAASHTIHIEVPPNPFWASIGLSVTPLSLGSGVQYESRVSLGYLNQSFQNAVRDG
Sbjct 421
MERPLKAASHTIHIEVPPNPFWASIGLSVTPLSLGSGVQYESRVSLGYLNQSFQNAVRDG 480

Query 481
IRYGLEQGLFGWNVTDCKICFEYGLYYSPVSTPADFRSLAPIVLEQALKESGTQLLEPYL 540

IRYGLEQGLFGWNVTDCKICFEYGLYYSPVSTPADFRSLAPIVLEQALKESGTQLLEPYL
Sbjct 481
IRYGLEQGLFGWNVTDCKICFEYGLYYSPVSTPADFRSLAPIVLEQALKESGTQLLEPYL 540

Query 541
SFILYAPQEYLSRAYHDAPKYCATIETAQVKKDEVVFTGEIPARCIQAYRTDLAFYTNGR 600

SFILYAPQEYLSRAYHDAPKYCATIETAQVKKDEVVFTGEIPARCIQAYRTDLAFYTNGR
Sbjct 541
SFILYAPQEYLSRAYHDAPKYCATIETAQVKKDEVVFTGEIPARCIQAYRTDLAFYTNGR 600

Query 601 SVCLTELKGYQAAVGQPVIQPRRPNSRLDKVRHMFQKVM 639
SVCLTELKGYQAAVGQPVIQPRRPNSRLDKVRHM QK+M
Sbjct 601 SVCLTELKGYQAAVGQPVIQPRRPNSRLDKVRHMLQKIM 639

>AMP48954.1 TetM-TetW-TetO-TetS [uncultured bacterium]
Length=639

Score = 1293 bits (3345), Expect = 0.0, Method: Compositional matrix adjust.
Identities = 630/639 (99%), Positives = 634/639 (99%), Gaps = 0/639 (0%)

```
Query  1
MKIINIGILAHVDAGKTTLTESLLYASGAISEPGSVEKGTTRTDTMFLERQRGITIQAAV  60

MKIINIGILAHVDAGKTTLTESLLYASGAISEPGSVEKGTTRTDTMFLERQRGITIQAAV
Sbjct  1
MKIINIGILAHVDAGKTTLTESLLYASGAISEPGSVEKGTTRTDTMFLERQRGITIQAAV  60

Query  61
TSFQWHRCKVNIVDTPGHMDFLAEVYRSLAVLDGAILVISAKDGVQAQTRILFHALRKMN  120

TSFQWHRCKVNIVDTPGHMDFLAEVYRSLAVLDGAILVISAKDGVQAQTRILFHALRKMN
Sbjct  61
TSFQWHRCKVNIVDTPGHMDFLAEVYRSLAVLDGAILVISAKDGVQAQTRILFHALRKMN  120

Query  121
IPTVIFINKIDQAGVDLQSVVQSVRDKLSADIIIKQTVSLSPEIVLEENTDIEAWDAVIE  180

IPTVIFINKIDQAGVDLQSVVQSVRDKLSADIIIKQTVSLSPEIVLEENTDIEAWDAVIE
Sbjct  121
IPTVIFINKIDQAGVDLQSVVQSVRDKLSADIIIKQTVSLSPEIVLEENTDIEAWDAVIE  180

Query  181
NNDKLLEKYIAGEPISREKLVREEQRRVQDASLFPVYYGSAKKGLGIQPLMDAVTGLFQP  240
            NND+LLEKYIAGEPISREKL REEQ+RVQDASLFPVY+GSAK
GLGIQPLMDAVTGLFQP
Sbjct  181
NNDELLEKYIAGEPISREKLAREEQQRVQDASLFPVYHGSAKNGLGIQPLMDAVTGLFQP  240

Query  241
IGEQGSAALCGSVFKVEYTDCGQRRVYLRLYSGTLRLRDTVALAGREKLKITEMRIPSKG  300
            IGEQG
AALCGSVFKVEYTDCGQRRVYLRLYSGTLRLRDTVALAGREKLKITEMRIPSKG
Sbjct  241
IGEQGGAALCGSVFKVEYTDCGQRRVYLRLYSGTLRLRDTVALAGREKLKITEMRIPSKG  300

Query  301
EIVRTDTAYPGEIVILPSDSVRLNDVLGDPTRLPRKRWREDPLPMLRTTIAPKTAAQRER  360
            EIVRTDTAY GEIVILPSDSVRLNDVLGD
TRLPRKRWREDPLPMLRTTIAPKTAAQRER
Sbjct  301
EIVRTDTAYQGEIVILPSDSVRLNDVLGDQTRLPRKRWREDPLPMLRTTIAPKTAAQRER  360

Query  361
LLDALTQLADTDPLLRCEVDSITHEIILSFLGRVQLEVVSALLSEKYKLETVVKEPSVIY  420
```

LLDALTQLADTDPLLRCEVDSITHEIILSFLGRVQLEVVSALLSEKYKLETVVKEPSVIY
Sbjct 361
LLDALTQLADTDPLLRCEVDSITHEIILSFLGRVQLEVVSALLSEKYKLETVVKEPSVIY 420

Query 421
MERPLKAASHTIHIEVPPNPFWASIGLSVTPLSLGSGVQYESRVSLGYLNQSFQNAVRDG 480

MERPLKAASHTIHIEVPPNPFWASIGLSVTPLSLGSGVQYESRVSLGYLNQSFQNAVRDG
Sbjct 421
MERPLKAASHTIHIEVPPNPFWASIGLSVTPLSLGSGVQYESRVSLGYLNQSFQNAVRDG 480

Query 481
IRYGLEQGLFGWNVTDCKICFEYGLYYSPVSTPADFRSLAPIVLEQALKESGTQLLEPYL 540

IRYGLEQGLFGWNVTDCKICFEYGLYYSPVSTPADFRSLAPIVLEQALKESGTQLLEPYL
Sbjct 481
IRYGLEQGLFGWNVTDCKICFEYGLYYSPVSTPADFRSLAPIVLEQALKESGTQLLEPYL 540

Query 541
SFILYAPQEYLSRAYHDAPKYCATIETAQVKKDEVVFTGEIPARCIQAYRTDLAFYTNGR 600

SFILYAPQEYLSRAYHDAPKYCATIETAQVKKDEVVFTGEIPARCIQAYRTDLAFYTNGR
Sbjct 541
SFILYAPQEYLSRAYHDAPKYCATIETAQVKKDEVVFTGEIPARCIQAYRTDLAFYTNGR 600

Query 601 SVCLTELKGYQAAVGQPVIQPRRPNSRLDKVRHMFQKVM 639
            SVCLTELKGYQAAVGQPVIQPRRPNSRLDKVR+MFQKVM
Sbjct 601 SVCLTELKGYQAAVGQPVIQPRRPNSRLDKVRYMFQKVM 639

>AHH55322.1 tetracycline resistance protein [Streptococcus suis]
Length=639

Score = 1293 bits (3345), Expect = 0.0, Method: Compositional matrix adjust.
Identities = 628/639 (98%), Positives = 635/639 (99%), Gaps = 0/639 (0%)

Query 1
MKIINIGILAHVDAGKTTLTESLLYASGAISEPGSVEKGTTRTDTMFLERQRGITIQAAV 60

MKIINIGILAHVDAGKTTLTESLLYASGAISEPGSVEKGTTRTDTMFLERQRGITIQAAV
Sbjct 1
MKIINIGILAHVDAGKTTLTESLLYASGAISEPGSVEKGTTRTDTMFLERQRGITIQAAV 60

Query 61
TSFQWHRCKVNIVDTPGHMDFLAEVYRSLAVLDGAILVISAKDGVQAQTRILFHALRKMN 120

TSFQWHRCKVNIVDTPGHMDFLAEVYRSLAVLDGAILVISAKDGVQAQTRILFHALRKMN
Sbjct 61
TSFQWHRCKVNIVDTPGHMDFLAEVYRSLAVLDGAILVISAKDGVQAQTRILFHALRKMN 120

Query 121

IPTVIFINKIDQAGVDLQSVVQSVRDKLSADIIIKQTVSLSPEIVLEENTDIEAWDAVIE 180

IPTVIFINKIDQAGVDLQSVVQSVRDKLSADIIIKQTVSLSPEIVLEENTDIEAWDAVIE
Sbjct 121
IPTVIFINKIDQAGVDLQSVVQSVRDKLSADIIIKQTVSLSPEIVLEENTDIEAWDAVIE 180

Query 181
NNDKLLEKYIAGEPISREKLVREEQRRVQDASLFPVYYGSAKKGLGIQPLMDAVTGLFQP 240

NNDKLLEKYIAGEPISREKLVREEQRRVQDASLFPVYYGSAKKGLGIQPLMDAVTGLFQP
Sbjct 181
NNDKLLEKYIAGEPISREKLVREEQRRVQDASLFPVYYGSAKKGLGIQPLMDAVTGLFQP 240

Query 241
IGEQGSAALCGSVFKVEYTDCGQRRVYLRLYSGTLRLRDTVALAGREKLKITEMRIPSKG 300

IGEQGSAALCGSVFKVEYTDCGQRRVYLRLYSGTLRLRDTVALAGREKLKITEMRIPSKG
Sbjct 241
IGEQGSAALCGSVFKVEYTDCGQRRVYLRLYSGTLRLRDTVALAGREKLKITEMRIPSKG 300

Query 301
EIVRTDTAYPGEIVILPSDSVRLNDVLGDPTRLPRKRWREDPLPMLRTTIAPKTAAQRER 360
EIVRTDTAYPGEIVILPSDSV LNDVLGDPTRLPRKRWREDPLPMLRT
+IAPKTAAQRER
Sbjct 301
EIVRTDTAYPGEIVILPSDSVGLNDVLGDPTRLPRKRWREDPLPMLRTSIAPKTAAQRER 360

Query 361
LLDALTQLADTDPLLRCEVDSITHEIILSFLGRVQLEVVSALLSEKYKLETVVKEPSVIY 420
LLDALTQLADTDPLLRCEVDSITHEIILSFLGRVQLEVVSALLSEKYKLETVVKEP
+VIY
Sbjct 361
LLDALTQLADTDPLLRCEVDSITHEIILSFLGRVQLEVVSALLSEKYKLETVVKEPTVIY 420

Query 421
MERPLKAASHTIHIEVPPNPFWASIGLSVTPLSLGSGVQYESRVSLGYLNQSFQNAVRDG 480
MERPLKAASHTIHIEVPPNPFWASIGLSVTPL
LGSGVQYESRVSLGYLNQSFQNAVRDG
Sbjct 421
MERPLKAASHTIHIEVPPNPFWASIGLSVTPLPLGSGVQYESRVSLGYLNQSFQNAVRDG 480

Query 481
IRYGLEQGLFGWNVTDCKICFEYGLYYSPVSTPADFRSLAPIVLEQALKESGTQLLEPYL 540

IRYGLEQGLFGWNVTDCKICFEYGLYYSPVSTPADFRSLAPIVLEQALKESGTQLLEPYL
Sbjct 481
IRYGLEQGLFGWNVTDCKICFEYGLYYSPVSTPADFRSLAPIVLEQALKESGTQLLEPYL 540

Query 541
SFILYAPQEYLSRAYHDAPKYCATIETAQVKKDEVVFTGEIPARCIQAYRTDLAFYTNGR 600
SF LYAP+EYLSRAYHDAPKYCATIET
QVKKDEVVFTGEIPARCIQAYRTDLAFYTNG+
Sbjct 541
SFTLYAPREYLSRAYHDAPKYCATIETVQVKKDEVVFTGEIPARCIQAYRTDLAFYTNGQ 600

```
Query  601  SVCLTELKGYQAAVGQPVIQPRRPNSRLDKVRHMFQKVM  639
            SVCLTELKGYQAAVG+PVIQPRRPNSRLDKVR+MFQK+M
Sbjct  601  SVCLTELKGYQAAVGKPVIQPRRPNSRLDKVRYMFQKIM  639
```

>ASQ15577.1 TetW [Bifidobacterium longum subsp. longum]
 ASQ15579.1 TetW [Bifidobacterium longum subsp. longum]
 ASQ15581.1 TetW [Bifidobacterium bifidum]
 ASQ15583.1 TetW [Bifidobacterium bifidum]
 ASQ15585.1 TetW [Bifidobacterium pseudocatenulatum]
 ASQ15587.1 TetW [Bifidobacterium pseudocatenulatum]
 ASQ15589.1 TetW [Bifidobacterium pseudocatenulatum]
 ASQ15591.1 TetW [Bifidobacterium pseudocatenulatum]
 ASQ15593.1 TetW [Bifidobacterium animalis subsp. lactis]
 ASQ15595.1 TetW [Bifidobacterium animalis subsp. lactis]
 ASQ15597.1 TetW [Bifidobacterium animalis subsp. lactis]
 ASQ15599.1 TetW [Bifidobacterium animalis subsp. lactis]
 ASQ15601.1 TetW [Bifidobacterium animalis subsp. lactis]
 ASQ15603.1 TetW [Bifidobacterium animalis subsp. lactis]
 ASQ15605.1 TetW [Bifidobacterium animalis subsp. lactis]
 ASQ15607.1 TetW [Bifidobacterium animalis subsp. lactis]
 ASQ15609.1 TetW [Bifidobacterium animalis subsp. lactis]
 ASQ15611.1 TetW [Bifidobacterium animalis subsp. lactis]
 ASQ15613.1 TetW [Bifidobacterium animalis subsp. lactis]
 ASQ15615.1 TetW [Bifidobacterium animalis subsp. lactis]
 ASU06799.1 TetW [Bifidobacterium animalis subsp. lactis]
Length=639

 Score = 1292 bits (3344), Expect = 0.0, Method: Compositional matrix adjust.
 Identities = 627/639 (98%), Positives = 635/639 (99%), Gaps = 0/639 (0%)

```
Query  1
MKIINIGILAHVDAGKTTLTESLLYASGAISEPGSVEKGTTRTDTMFLERQRGITIQAAV  60
            MKIINIGILAHVDAGKTTLTESLLYASGAISEPGSVEKGTTRTDTM
LERQRGITIQAAV
Sbjct  1
MKIINIGILAHVDAGKTTLTESLLYASGAISEPGSVEKGTTRTDTMLLERQRGITIQAAV  60

Query  61
TSFQWHRCKVNIVDTPGHMDFLAEVYRSLAVLDGAILVISAKDGVQAQTRILFHALRKMN  120

TSFQWHRCKVNIVDTPGHMDFLAEVYRSLAVLDGAILVISAKDGVQAQTRILFHALRKMN
Sbjct  61
TSFQWHRCKVNIVDTPGHMDFLAEVYRSLAVLDGAILVISAKDGVQAQTRILFHALRKMN  120

Query  121
IPTVIFINKIDQAGVDLQSVVQSVRDKLSADIIIKQTVSLSPEIVLEENTDIEAWDAVIE  180

IPTVIFINKIDQAGVDLQSVVQSVRDKLSADIIIKQTVSLSPEIVLEENTDIEAWDAVIE
Sbjct  121
IPTVIFINKIDQAGVDLQSVVQSVRDKLSADIIIKQTVSLSPEIVLEENTDIEAWDAVIE  180
```

```
Query  181
NNDKLLEKYIAGEPISREKLVREEQRRVQDASLFPVYYGSAKKGLGIQPLMDAVTGLFQP  240

NNDKLLEKYIAGEPISREKLVREEQRRVQDASLFPVYYGSAKKGLGIQPLMDAVTGLFQP
Sbjct  181
NNDKLLEKYIAGEPISREKLVREEQRRVQDASLFPVYYGSAKKGLGIQPLMDAVTGLFQP  240

Query  241
IGEQGSAALCGSVFKVEYTDCGQRRVYLRLYSGTLRLRDTVALAGREKLKITEMRIPSKG  300

IGEQGSAALCGSVFKVEYTDCGQRRVYLRLYSGTLRLRDTVALAGREKLKITEMRIPSKG
Sbjct  241
IGEQGSAALCGSVFKVEYTDCGQRRVYLRLYSGTLRLRDTVALAGREKLKITEMRIPSKG  300

Query  301
EIVRTDTAYPGEIVILPSDSVRLNDVLGDPTRLPRKRWREDPLPMLRTTIAPKTAAQRER  360
            EIVRTDTAYPGEIVILPSDSVRLNDVLGDPTRLPRKRWREDPLPMLRT
+IAPKTAAQRER
Sbjct  301
EIVRTDTAYPGEIVILPSDSVRLNDVLGDPTRLPRKRWREDPLPMLRTSIAPKTAAQRER  360

Query  361
LLDALTQLADTDPLLRCEVDSITHEIILSFLGRVQLEVVSALLSEKYKLETVVKEPSVIY  420
            LLDALTQLADTDPLLRCEVDSITHEIILSFLGRVQLEVVSALLSEKYKLETVVKEP
+VIY
Sbjct  361
LLDALTQLADTDPLLRCEVDSITHEIILSFLGRVQLEVVSALLSEKYKLETVVKEPTVIY  420

Query  421
MERPLKAASHTIHIEVPPNPFWASIGLSVTPLSLGSGVQYESRVSLGYLNQSFQNAVRDG  480
            MERPLKAASHTIHIEVPPNPFWASIGLSVTPL LGSGVQY
+SRVSLGYLNQSFQNAVRDG
Sbjct  421
MERPLKAASHTIHIEVPPNPFWASIGLSVTPLPLGSGVQYKSRVSLGYLNQSFQNAVRDG  480

Query  481
IRYGLEQGLFGWNVTDCKICFEYGLYYSPVSTPADFRSLAPIVLEQALKESGTQLLEPYL  540

IRYGLEQGLFGWNVTDCKICFEYGLYYSPVSTPADFRSLAPIVLEQALKESGTQLLEPYL
Sbjct  481
IRYGLEQGLFGWNVTDCKICFEYGLYYSPVSTPADFRSLAPIVLEQALKESGTQLLEPYL  540

Query  541
SFILYAPQEYLSRAYHDAPKYCATIETAQVKKDEVVFTGEIPARCIQAYRTDLAFYTNGR  600
            SF LYAP+EYLSRAYHDAPKYCATIET
QVKKDEVVFTGEIPARCIQAYRTDLAFYTNG+
Sbjct  541
SFTLYAPREYLSRAYHDAPKYCATIETVQVKKDEVVFTGEIPARCIQAYRTDLAFYTNGQ  600

Query  601  SVCLTELKGYQAAVGQPVIQPRRPNSRLDKVRHMFQKVM  639
            SVCLTELKGYQAAVG+PVIQPRRPNSRLDKVR+MFQK+M
Sbjct  601  SVCLTELKGYQAAVGKPVIQPRRPNSRLDKVRYMFQKIM  639
```

>WP_087253332.1 TetM/TetW/TetO/TetS family tetracycline resistance ribosomal
protection protein [Pseudoflavonifractor sp. An85]
 OUN26073.1 tetracycline resistance ribosomal protection protein [Pseudoflavonifractor
sp. An85]
Length=639

 Score = 1292 bits (3344), Expect = 0.0, Method: Compositional matrix adjust.
 Identities = 629/639 (98%), Positives = 633/639 (99%), Gaps = 0/639 (0%)

```
Query  1
MKIINIGILAHVDAGKTTLTESLLYASGAISEPGSVEKGTTRTDTMFLERQRGITIQAAV  60
            M IINIGILAHVDAGKTTLTESLLYASGAISEPGSVEKGTTRTDT
+FLERQRGITIQAAV
Sbjct  1
MNIINIGILAHVDAGKTTLTESLLYASGAISEPGSVEKGTTRTDTLFLERQRGITIQAAV  60

Query  61
TSFQWHRCKVNIVDTPGHMDFLAEVYRSLAVLDGAILVISAKDGVQAQTRILFHALRKMN  120

TSFQWHRCKVNIVDTPGHMDFLAEVYRSLAVLDGAILVISAKDGVQAQTRILFHALRKMN
Sbjct  61
TSFQWHRCKVNIVDTPGHMDFLAEVYRSLAVLDGAILVISAKDGVQAQTRILFHALRKMN  120

Query  121
IPTVIFINKIDQAGVDLQSVVQSVRDKLSADIIIKQTVSLSPEIVLEENTDIEAWDAVIE  180

IPTVIFINKIDQAGVDLQSVVQSVRDKLSADIIIKQTVSLSPEIVLEENTDIEAWDAVIE
Sbjct  121
IPTVIFINKIDQAGVDLQSVVQSVRDKLSADIIIKQTVSLSPEIVLEENTDIEAWDAVIE  180

Query  181
NNDKLLEKYIAGEPISREKLVREEQRRVQDASLFPVYYGSAKKGLGIQPLMDAVTGLFQP  240
            NND LLEKYIAGEPIS+EKL REEQRRVQ+ASLFPVY
+GSAKKGLGIQPLMDAVTGLFQP
Sbjct  181
NNDALLEKYIAGEPISQEKLAREEQRRVQEASLFPVYHGSAKKGLGIQPLMDAVTGLFQP  240

Query  241
IGEQGSAALCGSVFKVEYTDCGQRRVYLRLYSGTLRLRDTVALAGREKLKITEMRIPSKG  300
            IGEQG
AALCGSVFKVEYTDCGQRRVYLRLYSGTLRLRDTVALAGREKLKITEMRIPSKG
Sbjct  241
IGEQGGAALCGSVFKVEYTDCGQRRVYLRLYSGTLRLRDTVALAGREKLKITEMRIPSKG  300

Query  301
EIVRTDTAYPGEIVILPSDSVRLNDVLGDPTRLPRKRWREDPLPMLRTTIAPKTAAQRER  360
            EIVRTDTAY GEIVILPSDSVRLNDVLGD
TRLPRKRWREDPLPMLRTTIAPKTAAQRER
Sbjct  301
```

EIVRTDTAYQGEIVILPSDSVRLNDVLGDQTRLPRKRWREDPLPMLRTTIAPKTAAQRER 360

Query 361
LLDALTQLADTDPLLRCEVDSITHEIILSFLGRVQLEVVSALLSEKYKLETVVKEPSVIY 420

LLDALTQLADTDPLLRCEVDSITHEIILSFLGRVQLEVVSALLSEKYKLETVVKEPSVIY
Sbjct 361
LLDALTQLADTDPLLRCEVDSITHEIILSFLGRVQLEVVSALLSEKYKLETVVKEPSVIY 420

Query 421
MERPLKAASHTIHIEVPPNPFWASIGLSVTPLSLGSGVQYESRVSLGYLNQSFQNAVRDG 480

MERPLKAASHTIHIEVPPNPFWASIGLSVTPLSLGSGVQYESRVSLGYLNQSFQNAVRDG
Sbjct 421
MERPLKAASHTIHIEVPPNPFWASIGLSVTPLSLGSGVQYESRVSLGYLNQSFQNAVRDG 480

Query 481
IRYGLEQGLFGWNVTDCKICFEYGLYYSPVSTPADFRSLAPIVLEQALKESGTQLLEPYL 540

IRYGLEQGLFGWNVTDCKICFEYGLYYSPVSTPADFRSLAPIVLEQALKESGTQLLEPYL
Sbjct 481
IRYGLEQGLFGWNVTDCKICFEYGLYYSPVSTPADFRSLAPIVLEQALKESGTQLLEPYL 540

Query 541
SFILYAPQEYLSRAYHDAPKYCATIETAQVKKDEVVFTGEIPARCIQAYRTDLAFYTNGR 600

SFILYAPQEYLSRAYHDAPKYCATIETAQVKKDEVVFTGEIPARCIQAYRTDLAFYTNGR
Sbjct 541
SFILYAPQEYLSRAYHDAPKYCATIETAQVKKDEVVFTGEIPARCIQAYRTDLAFYTNGR 600

Query 601 SVCLTELKGYQAAVGQPVIQPRRPNSRLDKVRHMFQKVM 639
            SVCLTELKGYQAAVGQPVIQPRRPNSRLDKVRHMFQKVM
Sbjct 601 SVCLTELKGYQAAVGQPVIQPRRPNSRLDKVRHMFQKVM 639

>WP_087384078.1 TetM/TetW/TetO/TetS family tetracycline resistance ribosomal
protection protein [Faecalibacterium sp. An122]
AMP50091.1 TetM-TetW-TetO-TetS [uncultured bacterium]
OUQ38223.1 tetracycline resistance ribosomal protection protein Tet(W) [Faecalibacterium
sp. An122]
Length=639

Score = 1292 bits (3344), Expect = 0.0, Method: Compositional matrix adjust.
Identities = 629/639 (98%), Positives = 634/639 (99%), Gaps = 0/639 (0%)

Query 1
MKIINIGILAHVDAGKTTLTESLLYASGAISEPGSVEKGTTRTDTMFLERQRGITIQAAV 60

MKIINIGILAHVDAGKTTLTESLLYASGAISEPGSVEKGTTRTDTMFLERQRGITIQAAV
Sbjct 1

MKIINIGILAHVDAGKTTLTESLLYASGAISEPGSVEKGTTRTDTMFLERQRGITIQAAV 60

Query 61
TSFQWHRCKVNIVDTPGHMDFLAEVYRSLAVLDGAILVISAKDGVQAQTRILFHALRKMN 120

TSFQWHRCKVNIVDTPGHMDFLAEVYRSLAVLDGAILVISAKDGVQAQTRILFHALRKMN
Sbjct 61
TSFQWHRCKVNIVDTPGHMDFLAEVYRSLAVLDGAILVISAKDGVQAQTRILFHALRKMN 120

Query 121
IPTVIFINKIDQAGVDLQSVVQSVRDKLSADIIIKQTVSLSPEIVLEENTDIEAWDAVIE 180

IPTVIFINKIDQAGVDLQSVVQSVRDKLSADIIIKQTVSLSPEIVLEENTDIEAWDAVIE
Sbjct 121
IPTVIFINKIDQAGVDLQSVVQSVRDKLSADIIIKQTVSLSPEIVLEENTDIEAWDAVIE 180

Query 181
NNDKLLEKYIAGEPISREKLVREEQRRVQDASLFPVYYGSAKKGLGIQPLMDAVTGLFQP 240
NND+LLEKYIAGEPISREKL REEQ+RVQDASLFPVY+GSAK
GLGIQPLMDAVTGLFQP
Sbjct 181
NNDELLEKYIAGEPISREKLAREEQQRVQDASLFPVYHGSAKNGLGIQPLMDAVTGLFQP 240

Query 241
IGEQGSAALCGSVFKVEYTDCGQRRVYLRLYSGTLRLRDTVALAGREKLKITEMRIPSKG 300
IGEQG
AALCGSVFKVEYTDCGQRRVYLRLYSGTLRLRDTVALAGREKLKITEMRIPSKG
Sbjct 241
IGEQGGAALCGSVFKVEYTDCGQRRVYLRLYSGTLRLRDTVALAGREKLKITEMRIPSKG 300

Query 301
EIVRTDTAYPGEIVILPSDSVRLNDVLGDPTRLPRKRWREDPLPMLRTTIAPKTAAQRER 360
EIVRTDTAY GEIVILPSDSVRLNDVLGD
TRLPRKRWREDPLPMLRTTIAPKTAAQRER
Sbjct 301
EIVRTDTAYQGEIVILPSDSVRLNDVLGDQTRLPRKRWREDPLPMLRTTIAPKTAAQRER 360

Query 361
LLDALTQLADTDPLLRCEVDSITHEIILSFLGRVQLEVVSALLSEKYKLETVVKEPSVIY 420

LLDALTQLADTDPLLRCEVDSITHEIILSFLGRVQLEVVSALLSEKYKLETVVKEPSVIY
Sbjct 361
LLDALTQLADTDPLLRCEVDSITHEIILSFLGRVQLEVVSALLSEKYKLETVVKEPSVIY 420

Query 421
MERPLKAASHTIHIEVPPNPFWASIGLSVTPLSLGSGVQYESRVSLGYLNQSFQNAVRDG 480

MERPLKAASHTIHIEVPPNPFWASIGLSVTPLSLGSGVQYESRVSLGYLNQSFQNAVRDG
Sbjct 421
MERPLKAASHTIHIEVPPNPFWASIGLSVTPLSLGSGVQYESRVSLGYLNQSFQNAVRDG 480

Query 481
IRYGLEQGLFGWNVTDCKICFEYGLYYSPVSTPADFRSLAPIVLEQALKESGTQLLEPYL 540

IRYGLEQGLFGWNVTDCKICFEYGLYYSPVSTPADFRSLAPIVLEQALKESGTQLLEPYL
Sbjct 481
IRYGLEQGLFGWNVTDCKICFEYGLYYSPVSTPADFRSLAPIVLEQALKESGTQLLEPYL 540

Query 541
SFILYAPQEYLSRAYHDAPKYCATIETAQVKKDEVVFTGEIPARCIQAYRTDLAFYTNGR 600

SFILYAPQEYLSRAYHDAPKYCATIETAQVKKDEVVFTGEIPARCIQAYRTDLAFYTNGR
Sbjct 541
SFILYAPQEYLSRAYHDAPKYCATIETAQVKKDEVVFTGEIPARCIQAYRTDLAFYTNGR 600

Query 601 SVCLTELKGYQAAVGQPVIQPRRPNSRLDKVRHMFQKVM 639
SVCLTELKGYQAAVGQPVIQPRRPNSRLDKVR+MFQK+M
Sbjct 601 SVCLTELKGYQAAVGQPVIQPRRPNSRLDKVRYMFQKIM 639

>AMP49401.1 TetM-TetW-TetO-TetS [uncultured bacterium]
Length=639

Score = 1292 bits (3344), Expect = 0.0, Method: Compositional matrix adjust.
Identities = 629/639 (98%), Positives = 634/639 (99%), Gaps = 0/639 (0%)

Query 1
MKIINIGILAHVDAGKTTLTESLLYASGAISEPGSVEKGTTRTDTMFLERQRGITIQAAV 60

MKIINIGILAHVDAGKTTLTESLLYASGAISEPGSVEKGTTRTDTMFLERQRGITIQAAV
Sbjct 1
MKIINIGILAHVDAGKTTLTESLLYASGAISEPGSVEKGTTRTDTMFLERQRGITIQAAV 60

Query 61
TSFQWHRCKVNIVDTPGHMDFLAEVYRSLAVLDGAILVISAKDGVQAQTRILFHALRKMN 120

TSFQWHRCKVNIVDTPGHMDFLAEVYRSLAVLDGAILVISAKDGVQAQTRILFHALRKMN
Sbjct 61
TSFQWHRCKVNIVDTPGHMDFLAEVYRSLAVLDGAILVISAKDGVQAQTRILFHALRKMN 120

Query 121
IPTVIFINKIDQAGVDLQSVVQSVRDKLSADIIIKQTVSLSPEIVLEENTDIEAWDAVIE 180
IPTVIFINKIDQAGVDLQSVVQS
RDKLSADIIIKQTVSLSPEIVLEENTDIEAWDAVIE
Sbjct 121
IPTVIFINKIDQAGVDLQSVVQSARDKLSADIIIKQTVSLSPEIVLEENTDIEAWDAVIE 180

Query 181
NNDKLLEKYIAGEPISREKLVREEQRRVQDASLFPVYYGSAKKGLGIQPLMDAVTGLFQP 240
NND+LLEKYIAGEPISREKLVREEQRRVQDASLFPVY+GSAK
GLGIQPLMDAVTGLFQP
Sbjct 181
NNDELLEKYIAGEPISREKLVREEQRRVQDASLFPVYHGSAKNGLGIQPLMDAVTGLFQP 240

Query 241
IGEQGSAALCGSVFKVEYTDCGQRRVYLRLYSGTLRLRDTVALAGREKLKITEMRIPSKG 300

```
            IGEQG
AALCGSVFKVEYTDCGQRRVYLRLYSGTLRLRDTVALAGREKLKITEMRIPSKG
Sbjct  241
IGEQGGAALCGSVFKVEYTDCGQRRVYLRLYSGTLRLRDTVALAGREKLKITEMRIPSKG  300

Query  301
EIVRTDTAYPGEIVILPSDSVRLNDVLGDPTRLPRKRWREDPLPMLRTTIAPKTAAQRER  360
            EIVRTDTAY GEIVILPSDSVRLNDVLGD
TRLPRKRWREDPLPMLRTTIAPKTAAQRER
Sbjct  301
EIVRTDTAYQGEIVILPSDSVRLNDVLGDQTRLPRKRWREDPLPMLRTTIAPKTAAQRER  360

Query  361
LLDALTQLADTDPLLRCEVDSITHEIILSFLGRVQLEVVSALLSEKYKLETVVKEPSVIY  420

LLDALTQLADTDPLLRCEVDSITHEIILSFLGRVQLEVVSALLSEKYKLETVVKEPSVIY
Sbjct  361
LLDALTQLADTDPLLRCEVDSITHEIILSFLGRVQLEVVSALLSEKYKLETVVKEPSVIY  420

Query  421
MERPLKAASHTIHIEVPPNPFWASIGLSVTPLSLGSGVQYESRVSLGYLNQSFQNAVRDG  480

MERPLKAASHTIHIEVPPNPFWASIGLSVTPLSLGSGVQYESRVSLGYLNQSFQNAVRDG
Sbjct  421
MERPLKAASHTIHIEVPPNPFWASIGLSVTPLSLGSGVQYESRVSLGYLNQSFQNAVRDG  480

Query  481
IRYGLEQGLFGWNVTDCKICFEYGLYYSPVSTPADFRSLAPIVLEQALKESGTQLLEPYL  540

IRYGLEQGLFGWNVTDCKICFEYGLYYSPVSTPADFRSLAPIVLEQALKESGTQLLEPYL
Sbjct  481
IRYGLEQGLFGWNVTDCKICFEYGLYYSPVSTPADFRSLAPIVLEQALKESGTQLLEPYL  540

Query  541
SFILYAPQEYLSRAYHDAPKYCATIETAQVKKDEVVFTGEIPARCIQAYRTDLAFYTNGR  600

SFILYAPQEYLSRAYHDAPKYCATIETAQVKKDEVVFTGEIPARCIQAYRTDLAFYTNG+
Sbjct  541
SFILYAPQEYLSRAYHDAPKYCATIETAQVKKDEVVFTGEIPARCIQAYRTDLAFYTNGQ  600

Query  601  SVCLTELKGYQAAVGQPVIQPRRPNSRLDKVRHMFQKVM  639
            SVCLTELKGYQAAVGQPVIQPRRPNSRLDKVR+MFQK+M
Sbjct  601  SVCLTELKGYQAAVGQPVIQPRRPNSRLDKVRYMFQKIM  639


>WP_025299865.1 TetM/TetW/TetO/TetS family tetracycline resistance
ribosomal
protection protein [Bifidobacterium breve]
 AHJ15785.1 tetracycline resistance protein tetW [Bifidobacterium
breve 12L]
Length=639

 Score = 1292 bits (3344),  Expect = 0.0, Method: Compositional
matrix adjust.
```

Identities = 630/639 (99%), Positives = 633/639 (99%), Gaps = 0/639 (0%)

```
Query  1
MKIINIGILAHVDAGKTTLTESLLYASGAISEPGSVEKGTTRTDTMFLERQRGITIQAAV  60

MKIINIGILAHVDAGKTTLTESLLYASGAISEPGSVEKGTTRTDTMFLERQRGITIQAAV
Sbjct  1
MKIINIGILAHVDAGKTTLTESLLYASGAISEPGSVEKGTTRTDTMFLERQRGITIQAAV  60

Query  61
TSFQWHRCKVNIVDTPGHMDFLAEVYRSLAVLDGAILVISAKDGVQAQTRILFHALRKMN  120

TSFQWHRCKVNIVDTPGHMDFLAEVYRSLAVLDGAILVISAKDGVQAQTRILFHALRKMN
Sbjct  61
TSFQWHRCKVNIVDTPGHMDFLAEVYRSLAVLDGAILVISAKDGVQAQTRILFHALRKMN  120

Query  121
IPTVIFINKIDQAGVDLQSVVQSVRDKLSADIIIKQTVSLSPEIVLEENTDIEAWDAVIE  180

IPTVIFINKIDQAGVDLQSVVQSVRDKLSADIIIKQTVSLSPEIVLEENTDIEAWDAVIE
Sbjct  121
IPTVIFINKIDQAGVDLQSVVQSVRDKLSADIIIKQTVSLSPEIVLEENTDIEAWDAVIE  180

Query  181
NNDKLLEKYIAGEPISREKLVREEQRRVQDASLFPVYYGSAKKGLGIQPLMDAVTGLFQP  240
            NND+LLEKYIAGEPISREKL REEQ+RVQDASLFPVY+GSAK
GLGIQPLMDAVTGLFQP
Sbjct  181
NNDELLEKYIAGEPISREKLAREEQQRVQDASLFPVYHGSAKNGLGIQPLMDAVTGLFQP  240

Query  241
IGEQGSAALCGSVFKVEYTDCGQRRVYLRLYSGTLRLRDTVALAGREKLKITEMRIPSKG  300
            IGEQG
AALCGSVFKVEYTDCGQRRVYLRLYSGTLRLRDTVALAGREKLKITEMRIPSKG
Sbjct  241
IGEQGGAALCGSVFKVEYTDCGQRRVYLRLYSGTLRLRDTVALAGREKLKITEMRIPSKG  300

Query  301
EIVRTDTAYPGEIVILPSDSVRLNDVLGDPTRLPRKRWREDPLPMLRTTIAPKTAAQRER  360
            EIVRTDTAY GEIVILPSDSVRLNDVLGD
TRLPRKRWREDPLPMLRTTIAPKTAAQRER
Sbjct  301
EIVRTDTAYQGEIVILPSDSVRLNDVLGDQTRLPRKRWREDPLPMLRTTIAPKTAAQRER  360

Query  361
LLDALTQLADTDPLLRCEVDSITHEIILSFLGRVQLEVVSALLSEKYKLETVVKEPSVIY  420

LLDALTQLADTDPLLRCEVDSITHEIILSFLGRVQLEVVSALLSEKYKLETVVKEPSVIY
Sbjct  361
LLDALTQLADTDPLLRCEVDSITHEIILSFLGRVQLEVVSALLSEKYKLETVVKEPSVIY  420

Query  421
MERPLKAASHTIHIEVPPNPFWASIGLSVTPLSLGSGVQYESRVSLGYLNQSFQNAVRDG  480
```

MERPLKAASHTIHIEVPPNPFWASIGLSVTPLSLGSGVQYESRVSLGYLNQSFQNAVRDG
Sbjct 421
MERPLKAASHTIHIEVPPNPFWASIGLSVTPLSLGSGVQYESRVSLGYLNQSFQNAVRDG 480

Query 481
IRYGLEQGLFGWNVTDCKICFEYGLYYSPVSTPADFRSLAPIVLEQALKESGTQLLEPYL 540

IRYGLEQGLFGWNVTDCKICFEYGLYYSPVSTPADFRSLAPIVLEQALKESGTQLLEPYL
Sbjct 481
IRYGLEQGLFGWNVTDCKICFEYGLYYSPVSTPADFRSLAPIVLEQALKESGTQLLEPYL 540

Query 541
SFILYAPQEYLSRAYHDAPKYCATIETAQVKKDEVVFTGEIPARCIQAYRTDLAFYTNGR 600

SFILYAPQEYLSRAYHDAPKYCATIETAQVKKDEVVFTGEIPARCIQAYRTDLAFYTNGR
Sbjct 541
SFILYAPQEYLSRAYHDAPKYCATIETAQVKKDEVVFTGEIPARCIQAYRTDLAFYTNGR 600

```
Query  601  SVCLTELKGYQAAVGQPVIQPRRPNSRLDKVRHMFQKVM  639
            SVCLTELK YQAAVGQPVIQPRRPNSRLDKVRHMFQKVM
Sbjct  601  SVCLTELKRYQAAVGQPVIQPRRPNSRLDKVRHMFQKVM  639
```

>WP_063856416.1 tetracycline resistance ribosomal protection protein Tet(W) [Megasphaera
elsdenii]
 AAR29972.1 tetracycline resistance protein [Megasphaera elsdenii]
Length=639

 Score = 1292 bits (3344), Expect = 0.0, Method: Compositional matrix adjust.
 Identities = 630/639 (99%), Positives = 633/639 (99%), Gaps = 0/639 (0%)

Query 1
MKIINIGILAHVDAGKTTLTESLLYASGAISEPGSVEKGTTRTDTMFLERQRGITIQAAV 60

MKIINIGILAHVDAGKTTLTESLLYASGAISEPGSVEKGTTRTDTMFLERQRGITIQAAV
Sbjct 1
MKIINIGILAHVDAGKTTLTESLLYASGAISEPGSVEKGTTRTDTMFLERQRGITIQAAV 60

Query 61
TSFQWHRCKVNIVDTPGHMDFLAEVYRSLAVLDGAILVISAKDGVQAQTRILFHALRKMN 120

TSFQWHRCKVNIVDTPGHMDFLAEVYRSLAVLDGAILVISAKDGVQAQTRILFHALRKMN
Sbjct 61
TSFQWHRCKVNIVDTPGHMDFLAEVYRSLAVLDGAILVISAKDGVQAQTRILFHALRKMN 120

Query 121
IPTVIFINKIDQAGVDLQSVVQSVRDKLSADIIIKQTVSLSPEIVLEENTDIEAWDAVIE 180

IPTVIFINKIDQAGVDLQSVVQSVRDKLSADIIIKQTVSLSPEIVLEENTDIEAWDAVIE
Sbjct 121

```
IPTVIFINKIDQAGVDLQSVVQSVRDKLSADIIIKQTVSLSPEIVLEENTDIEAWDAVIE  180

Query  181
NNDKLLEKYIAGEPISREKLVREEQRRVQDASLFPVYYGSAKKGLGIQPLMDAVTGLFQP  240
            NND+LLEKYIAGEPISREKL REEQ+RVQDASLFPVY+GSAK
GLGIQPLMDAVTGLFQP
Sbjct  181
NNDELLEKYIAGEPISREKLAREEQQRVQDASLFPVYHGSAKNGLGIQPLMDAVTGLFQP  240

Query  241
IGEQGSAALCGSVFKVEYTDCGQRRVYLRLYSGTLRLRDTVALAGREKLKITEMRIPSKG  300
            IGEQG
AALCGSVFKVEYTDCGQRRVYLRLYSGTLRLRDTVALAGREKLKITEMRIPSKG
Sbjct  241
IGEQGGAALCGSVFKVEYTDCGQRRVYLRLYSGTLRLRDTVALAGREKLKITEMRIPSKG  300

Query  301
EIVRTDTAYPGEIVILPSDSVRLNDVLGDPTRLPRKRWREDPLPMLRTTIAPKTAAQRER  360
            EIVRTDTAY GEIVILPSDSVRLNDVLGD
TRLPRKRWREDPLPMLRTTIAPKTAAQRER
Sbjct  301
EIVRTDTAYQGEIVILPSDSVRLNDVLGDQTRLPRKRWREDPLPMLRTTIAPKTAAQRER  360

Query  361
LLDALTQLADTDPLLRCEVDSITHEIILSFLGRVQLEVVSALLSEKYKLETVVKEPSVIY  420

LLDALTQLADTDPLLRCEVDSITHEIILSFLGRVQLEVVSALLSEKYKLETVVKEPSVIY
Sbjct  361
LLDALTQLADTDPLLRCEVDSITHEIILSFLGRVQLEVVSALLSEKYKLETVVKEPSVIY  420

Query  421
MERPLKAASHTIHIEVPPNPFWASIGLSVTPLSLGSGVQYESRVSLGYLNQSFQNAVRDG  480

MERPLKAASHTIHIEVPPNPFWASIGLSVTPLSLGSGVQYESRVSLGYLNQSFQNAVRDG
Sbjct  421
MERPLKAASHTIHIEVPPNPFWASIGLSVTPLSLGSGVQYESRVSLGYLNQSFQNAVRDG  480

Query  481
IRYGLEQGLFGWNVTDCKICFEYGLYYSPVSTPADFRSLAPIVLEQALKESGTQLLEPYL  540
            IRYGLEQGLFGW
VTDCKICFEYGLYYSPVSTPADFRSLAPIVLEQALKESGTQLLEPYL
Sbjct  481
IRYGLEQGLFGWXVTDCKICFEYGLYYSPVSTPADFRSLAPIVLEQALKESGTQLLEPYL  540

Query  541
SFILYAPQEYLSRAYHDAPKYCATIETAQVKKDEVVFTGEIPARCIQAYRTDLAFYTNGR  600

SFILYAPQEYLSRAYHDAPKYCATIETAQVKKDEVVFTGEIPARCIQAYRTDLAFYTNGR
Sbjct  541
SFILYAPQEYLSRAYHDAPKYCATIETAQVKKDEVVFTGEIPARCIQAYRTDLAFYTNGR  600

Query  601  SVCLTELKGYQAAVGQPVIQPRRPNSRLDKVRHMFQKVM  639
            SVCLTELKGYQAAVGQPVIQPRRPNSRLDKVRHMFQKVM
Sbjct  601  SVCLTELKGYQAAVGQPVIQPRRPNSRLDKVRHMFQKVM  639
```

>APO30931.1 ferrous iron transport protein B [uncultured bacterium]
 APO30936.1 ferrous iron transport protein B [uncultured bacterium]
Length=639

 Score = 1292 bits (3343), Expect = 0.0, Method: Compositional
matrix adjust.
 Identities = 629/639 (98%), Positives = 633/639 (99%), Gaps = 0/639
(0%)

Query 1
MKIINIGILAHVDAGKTTLTESLLYASGAISEPGSVEKGTTRTDTMFLERQRGITIQAAV 60

MKIINIGILAHVDAGKTTLTESLLYASGAISEPGSVEKGTTRTDTMFLERQRGITIQAAV
Sbjct 1
MKIINIGILAHVDAGKTTLTESLLYASGAISEPGSVEKGTTRTDTMFLERQRGITIQAAV 60

Query 61
TSFQWHRCKVNIVDTPGHMDFLAEVYRSLAVLDGAILVISAKDGVQAQTRILFHALRKMN 120

TSFQWHRCKVNIVDTPGHMDFLAEVYRSLAVLDGAILVISAKDGVQAQTRILFHALRKMN
Sbjct 61
TSFQWHRCKVNIVDTPGHMDFLAEVYRSLAVLDGAILVISAKDGVQAQTRILFHALRKMN 120

Query 121
IPTVIFINKIDQAGVDLQSVVQSVRDKLSADIIIKQTVSLSPEIVLEENTDIEAWDAVIE 180

IPTVIFINKIDQAGVDLQSVVQSVRDKLSADIIIKQTVSLSPEIVLEENTDIEAWDAVIE
Sbjct 121
IPTVIFINKIDQAGVDLQSVVQSVRDKLSADIIIKQTVSLSPEIVLEENTDIEAWDAVIE 180

Query 181
NNDKLLEKYIAGEPISREKLVREEQRRVQDASLFPVYYGSAKKGLGIQPLMDAVTGLFQP 240
            NND+LLEKYIAGEPISREKL REEQ+RVQDASLFPVY+GSAK
GLGIQPLMDAVTGLFQP
Sbjct 181
NNDELLEKYIAGEPISREKLAREEQQRVQDASLFPVYHGSAKNGLGIQPLMDAVTGLFQP 240

Query 241
IGEQGSAALCGSVFKVEYTDCGQRRVYLRLYSGTLRLRDTVALAGREKLKITEMRIPSKG 300
            IGEQG
AALCGSVFKVEYTDCGQRRVYLRLYSGTLRLRDTVALAGREKLKITEMRIPSKG
Sbjct 241
IGEQGGAALCGSVFKVEYTDCGQRRVYLRLYSGTLRLRDTVALAGREKLKITEMRIPSKG 300

Query 301
EIVRTDTAYPGEIVILPSDSVRLNDVLGDPTRLPRKRWREDPLPMLRTTIAPKTAAQRER 360
            EIVRTDTAY GEIVILPSDSVRLNDVLGD TRLPRKRWREDPLPMLRT
IAPKTAAQRER
Sbjct 301
EIVRTDTAYQGEIVILPSDSVRLNDVLGDQTRLPRKRWREDPLPMLRTAIAPKTAAQRER 360

Query 361

```
LLDALTQLADTDPLLRCEVDSITHEIILSFLGRVQLEVVSALLSEKYKLETVVKEPSVIY  420

LLDALTQLADTDPLLRCEVDSITHEIILSFLGRVQLEVVSALLSEKYKLETVVKEPSVIY
Sbjct  361
LLDALTQLADTDPLLRCEVDSITHEIILSFLGRVQLEVVSALLSEKYKLETVVKEPSVIY  420

Query  421
MERPLKAASHTIHIEVPPNPFWASIGLSVTPLSLGSGVQYESRVSLGYLNQSFQNAVRDG  480

MERPLKAASHTIHIEVPPNPFWASIGLSVTPLSLGSGVQYESRVSLGYLNQSFQNAVRDG
Sbjct  421
MERPLKAASHTIHIEVPPNPFWASIGLSVTPLSLGSGVQYESRVSLGYLNQSFQNAVRDG  480

Query  481
IRYGLEQGLFGWNVTDCKICFEYGLYYSPVSTPADFRSLAPIVLEQALKESGTQLLEPYL  540

IRYGLEQGLFGWNVTDCKICFEYGLYYSPVSTPADFRSLAPIVLEQALKESGTQLLEPYL
Sbjct  481
IRYGLEQGLFGWNVTDCKICFEYGLYYSPVSTPADFRSLAPIVLEQALKESGTQLLEPYL  540

Query  541
SFILYAPQEYLSRAYHDAPKYCATIETAQVKKDEVVFTGEIPARCIQAYRTDLAFYTNGR  600

SFILYAPQEYLSRAYHDAPKYCATIETAQVKKDEVVFTGEIPARCIQAYRTDLAFYTNGR
Sbjct  541
SFILYAPQEYLSRAYHDAPKYCATIETAQVKKDEVVFTGEIPARCIQAYRTDLAFYTNGR  600

Query  601  SVCLTELKGYQAAVGQPVIQPRRPNSRLDKVRHMFQKVM  639
            SVCLTELKGYQAAVGQPVIQPRRPNSRL+KVRHMFQKVM
Sbjct  601  SVCLTELKGYQAAVGQPVIQPRRPNSRLNKVRHMFQKVM  639


>WP_066661399.1 TetM/TetW/TetO/TetS family tetracycline resistance
ribosomal
protection protein [Denitrobacterium detoxificans]
 ANE22345.1 tetracycline resistance protein tetM [Denitrobacterium
detoxificans]
 SEP02471.1 ribosomal protection tetracycline resistance protein
[Denitrobacterium
detoxificans]
Length=639

 Score = 1292 bits (3343),  Expect = 0.0, Method: Compositional
matrix adjust.
 Identities = 627/639 (98%), Positives = 634/639 (99%), Gaps = 0/639
(0%)

Query  1
MKIINIGILAHVDAGKTTLTESLLYASGAISEPGSVEKGTTRTDTMFLERQRGITIQAAV  60
            M IINIGILAHVDAGKTTLTESLLYASGAISEPGSVEKGTTRTDTM
LERQRGITIQAAV
Sbjct  1
MNIINIGILAHVDAGKTTLTESLLYASGAISEPGSVEKGTTRTDTMLLERQRGITIQAAV  60
```

Query 61
TSFQWHRCKVNIVDTPGHMDFLAEVYRSLAVLDGAILVISAKDGVQAQTRILFHALRKMN 120

TSFQWHRCKVNIVDTPGHMDFLAEVYRSLAVLDGAILVISAKDGVQAQTRILFHALRKMN
Sbjct 61
TSFQWHRCKVNIVDTPGHMDFLAEVYRSLAVLDGAILVISAKDGVQAQTRILFHALRKMN 120

Query 121
IPTVIFINKIDQAGVDLQSVVQSVRDKLSADIIIKQTVSLSPEIVLEENTDIEAWDAVIE 180

IPTVIFINKIDQAGVDLQSVVQSVRDKLSADIIIKQTVSLSPEIVLEENTDIEAWDAVIE
Sbjct 121
IPTVIFINKIDQAGVDLQSVVQSVRDKLSADIIIKQTVSLSPEIVLEENTDIEAWDAVIE 180

Query 181
NNDKLLEKYIAGEPISREKLVREEQRRVQDASLFPVYYGSAKKGLGIQPLMDAVTGLFQP 240

NNDKLLEKYIAGEPISREKLVREEQRRVQDASLFPVYYGSAKKGLGIQPLMDAVTGLFQP
Sbjct 181
NNDKLLEKYIAGEPISREKLVREEQRRVQDASLFPVYYGSAKKGLGIQPLMDAVTGLFQP 240

Query 241
IGEQGSAALCGSVFKVEYTDCGQRRVYLRLYSGTLRLRDTVALAGREKLKITEMRIPSKG 300

IGEQGSAALCGSVFKVEYTDCGQRRVYLRLYSGTLRLRDTVALAGREKLKITEMRIPSKG
Sbjct 241
IGEQGSAALCGSVFKVEYTDCGQRRVYLRLYSGTLRLRDTVALAGREKLKITEMRIPSKG 300

Query 301
EIVRTDTAYPGEIVILPSDSVRLNDVLGDPTRLPRKRWREDPLPMLRTTIAPKTAAQRER 360
EIVRTDTAYPGEIVILPSDSVRLNDVLGDPTRLPRKRWREDPLPMLRT
+IAPKTAAQRER
Sbjct 301
EIVRTDTAYPGEIVILPSDSVRLNDVLGDPTRLPRKRWREDPLPMLRTSIAPKTAAQRER 360

Query 361
LLDALTQLADTDPLLRCEVDSITHEIILSFLGRVQLEVVSALLSEKYKLETVVKEPSVIY 420
LLDALTQLADTDPLLRCEVDSITHEIILSFLGRVQLEVVSALLSEKYKLETVVKEP
+VIY
Sbjct 361
LLDALTQLADTDPLLRCEVDSITHEIILSFLGRVQLEVVSALLSEKYKLETVVKEPTVIY 420

Query 421
MERPLKAASHTIHIEVPPNPFWASIGLSVTPLSLGSGVQYESRVSLGYLNQSFQNAVRDG 480
MERPLKAASHTIHIEVPPNPFWASIGLSVTPL
LGSGVQYESRVSLGYLNQSFQNAVRDG
Sbjct 421
MERPLKAASHTIHIEVPPNPFWASIGLSVTPLPLGSGVQYESRVSLGYLNQSFQNAVRDG 480

Query 481
IRYGLEQGLFGWNVTDCKICFEYGLYYSPVSTPADFRSLAPIVLEQALKESGTQLLEPYL 540

IRYGLEQGLFGWNVTDCKICFEYGLYYSPVSTPADFRSLAPIVLEQALKESGTQLLEPYL
Sbjct 481

IRYGLEQGLFGWNVTDCKICFEYGLYYSPVSTPADFRSLAPIVLEQALKESGTQLLEPYL 540

Query 541
SFILYAPQEYLSRAYHDAPKYCATIETAQVKKDEVVFTGEIPARCIQAYRTDLAFYTNGR 600
            SF LYAP+EYLSRAYHDAPKYCATIET
QVKKDEVVFTGEIPARCIQAYRTDLAFYTNG+
Sbjct 541
SFTLYAPREYLSRAYHDAPKYCATIETVQVKKDEVVFTGEIPARCIQAYRTDLAFYTNGQ 600

Query 601 SVCLTELKGYQAAVGQPVIQPRRPNSRLDKVRHMFQKVM 639
            SVCLTELKGYQAAVG+PVIQPRRPNSRLDKVR+MFQK+M
Sbjct 601 SVCLTELKGYQAAVGKPVIQPRRPNSRLDKVRYMFQKIM 639

>WP_022542861.1 TetM/TetW/TetO/TetS family tetracycline resistance ribosomal
protection protein [Bifidobacterium animalis]
 AGW85165.1 ribosomal protection tetracycline resistance protein [Bifidobacterium
animalis subsp. lactis ATCC 27673]
 KOA47472.1 tetracycline resistance protein tetM [Bifidobacterium animalis
subsp. lactis ATCC 27673]
Length=639

 Score = 1292 bits (3343), Expect = 0.0, Method: Compositional matrix adjust.
 Identities = 627/638 (98%), Positives = 633/638 (99%), Gaps = 0/638 (0%)

Query 1
MKIINIGILAHVDAGKTTLTESLLYASGAISEPGSVEKGTTRTDTMFLERQRGITIQAAV 60
            MKIINIGILAHVDAGKTTLTESLLYASGAISEPGSVEKGTTRTDTM
LERQRGITIQAAV
Sbjct 1
MKIINIGILAHVDAGKTTLTESLLYASGAISEPGSVEKGTTRTDTMLLERQRGITIQAAV 60

Query 61
TSFQWHRCKVNIVDTPGHMDFLAEVYRSLAVLDGAILVISAKDGVQAQTRILFHALRKMN 120

TSFQWHRCKVNIVDTPGHMDFLAEVYRSLAVLDGAILVISAKDGVQAQTRILFHALRKMN
Sbjct 61
TSFQWHRCKVNIVDTPGHMDFLAEVYRSLAVLDGAILVISAKDGVQAQTRILFHALRKMN 120

Query 121
IPTVIFINKIDQAGVDLQSVVQSVRDKLSADIIIKQTVSLSPEIVLEENTDIEAWDAVIE 180

IPTVIFINKIDQAGVDLQSVVQSVRDKLSADIIIKQTVSLSPEIVLEENTDIEAWDAVIE
Sbjct 121
IPTVIFINKIDQAGVDLQSVVQSVRDKLSADIIIKQTVSLSPEIVLEENTDIEAWDAVIE 180

Query 181
NNDKLLEKYIAGEPISREKLVREEQRRVQDASLFPVYYGSAKKGLGIQPLMDAVTGLFQP 240

NNDKLLEKYIAGEPISREKLVREEQRRVQDASLFPVYYGSAKKGLGIQPLMDAVTGLFQP
Sbjct 181
NNDKLLEKYIAGEPISREKLVREEQRRVQDASLFPVYYGSAKKGLGIQPLMDAVTGLFQP 240

Query 241
IGEQGSAALCGSVFKVEYTDCGQRRVYLRLYSGTLRLRDTVALAGREKLKITEMRIPSKG 300

IGEQGSAALCGSVFKVEYTDCGQRRVYLRLYSGTLRLRDTVALAGREKLKITEMRIPSKG
Sbjct 241
IGEQGSAALCGSVFKVEYTDCGQRRVYLRLYSGTLRLRDTVALAGREKLKITEMRIPSKG 300

Query 301
EIVRTDTAYPGEIVILPSDSVRLNDVLGDPTRLPRKRWREDPLPMLRTTIAPKTAAQRER 360
EIVRTDTAYPGEIVILPSDSVRLNDVLGDPTRLPRKRWREDPLPMLRT
+IAPKTAAQRER
Sbjct 301
EIVRTDTAYPGEIVILPSDSVRLNDVLGDPTRLPRKRWREDPLPMLRTSIAPKTAAQRER 360

Query 361
LLDALTQLADTDPLLRCEVDSITHEIILSFLGRVQLEVVSALLSEKYKLETVVKEPSVIY 420
LLDALTQLADTDPLLRCEVDSITHEIILSFLGRVQLEVVSALLSEKYKLETVVKEP
+VIY
Sbjct 361
LLDALTQLADTDPLLRCEVDSITHEIILSFLGRVQLEVVSALLSEKYKLETVVKEPTVIY 420

Query 421
MERPLKAASHTIHIEVPPNPFWASIGLSVTPLSLGSGVQYESRVSLGYLNQSFQNAVRDG 480
MERPLKAASHTIHIEVPPNPFWASIGLSVTPL
LGSGVQYESRVSLGYLNQSFQNAVRDG
Sbjct 421
MERPLKAASHTIHIEVPPNPFWASIGLSVTPLPLGSGVQYESRVSLGYLNQSFQNAVRDG 480

Query 481
IRYGLEQGLFGWNVTDCKICFEYGLYYSPVSTPADFRSLAPIVLEQALKESGTQLLEPYL 540

IRYGLEQGLFGWNVTDCKICFEYGLYYSPVSTPADFRSLAPIVLEQALKESGTQLLEPYL
Sbjct 481
IRYGLEQGLFGWNVTDCKICFEYGLYYSPVSTPADFRSLAPIVLEQALKESGTQLLEPYL 540

Query 541
SFILYAPQEYLSRAYHDAPKYCATIETAQVKKDEVVFTGEIPARCIQAYRTDLAFYTNGR 600
SF LYAP+EYLSRAYHDAPKYCATIET
QVKKDEVVFTGEIPARCIQAYRTDLAFYTNG+
Sbjct 541
SFTLYAPREYLSRAYHDAPKYCATIETVQVKKDEVVFTGEIPARCIQAYRTDLAFYTNGQ 600

Query 601 SVCLTELKGYQAAVGQPVIQPRRPNSRLDKVRHMFQKV 638
SVCLTELKGYQAAVG+PVIQPRRPNSRLDKVRHMF K+
Sbjct 601 SVCLTELKGYQAAVGKPVIQPRRPNSRLDKVRHMFSKI 638

>APO28864.1 Ferrous iron transport protein B, partial [uncultured bacterium]
Length=643

Score = 1291 bits (3342), Expect = 0.0, Method: Compositional matrix adjust.
Identities = 629/639 (98%), Positives = 632/639 (99%), Gaps = 0/639 (0%)

```
Query  1
MKIINIGILAHVDAGKTTLTESLLYASGAISEPGSVEKGTTRTDTMFLERQRGITIQAAV  60

MKIINIGILAHVDAGKTTLTESLLYASGAISEPGSVEKGTTRTDTMFLERQRGITIQAAV
Sbjct  5
MKIINIGILAHVDAGKTTLTESLLYASGAISEPGSVEKGTTRTDTMFLERQRGITIQAAV  64

Query  61
TSFQWHRCKVNIVDTPGHMDFLAEVYRSLAVLDGAILVISAKDGVQAQTRILFHALRKMN  120

TSFQWHRCKVNIVDTPGHMDFLAEVYRSLAVLDGAILVISAKDGVQAQTRILFHALRKMN
Sbjct  65
TSFQWHRCKVNIVDTPGHMDFLAEVYRSLAVLDGAILVISAKDGVQAQTRILFHALRKMN  124

Query  121
IPTVIFINKIDQAGVDLQSVVQSVRDKLSADIIIKQTVSLSPEIVLEENTDIEAWDAVIE  180

IPTVIFINKIDQAGVDLQSVVQSVRDKLSADIIIKQTVSLSPEIVLEENTDIEAWDAVIE
Sbjct  125
IPTVIFINKIDQAGVDLQSVVQSVRDKLSADIIIKQTVSLSPEIVLEENTDIEAWDAVIE  184

Query  181
NNDKLLEKYIAGEPISREKLVREEQRRVQDASLFPVYYGSAKKGLGIQPLMDAVTGLFQP  240
            NND+LLEKYIAGEPISREKL REEQ+RVQDASLFPVY+GSAK
GLGIQPLMDAVTGLFQP
Sbjct  185
NNDELLEKYIAGEPISREKLAREEQQRVQDASLFPVYHGSAKNGLGIQPLMDAVTGLFQP  244

Query  241
IGEQGSAALCGSVFKVEYTDCGQRRVYLRLYSGTLRLRDTVALAGREKLKITEMRIPSKG  300
            IGEQG
AALCGSVFKVEYTDCGQRRVYLRLYSGTLRLRDTVALAGREKLKITEMRIPSKG
Sbjct  245
IGEQGGAALCGSVFKVEYTDCGQRRVYLRLYSGTLRLRDTVALAGREKLKITEMRIPSKG  304

Query  301
EIVRTDTAYPGEIVILPSDSVRLNDVLGDPTRLPRKRWREDPLPMLRTTIAPKTAAQRER  360
            EIVRTDTAY GEIVILPSDSVRLNDVLGD TRLPRKRWREDPLPMLRT
IAPKTAAQRER
Sbjct  305
EIVRTDTAYQGEIVILPSDSVRLNDVLGDQTRLPRKRWREDPLPMLRTAIAPKTAAQRER  364

Query  361
LLDALTQLADTDPLLRCEVDSITHEIILSFLGRVQLEVVSALLSEKYKLETVVKEPSVIY  420

LLDALTQLADTDPLLRCEVDSITHEIILSFLGRVQLEVVSALLSEKYKLETVVKEPSVIY
Sbjct  365
LLDALTQLADTDPLLRCEVDSITHEIILSFLGRVQLEVVSALLSEKYKLETVVKEPSVIY  424
```

Query 421
MERPLKAASHTIHIEVPPNPFWASIGLSVTPLSLGSGVQYESRVSLGYLNQSFQNAVRDG 480

MERPLKAASHTIHIEVPPNPFWASIGLSVTPLSLGSGVQYESRVSLGYLNQSFQNAVRDG
Sbjct 425
MERPLKAASHTIHIEVPPNPFWASIGLSVTPLSLGSGVQYESRVSLGYLNQSFQNAVRDG 484

Query 481
IRYGLEQGLFGWNVTDCKICFEYGLYYSPVSTPADFRSLAPIVLEQALKESGTQLLEPYL 540

IRYGLEQGLFGWNVTDCKICFEYGLYYSPVSTPADFRSLAPIVLEQALKESGTQLLEPYL
Sbjct 485
IRYGLEQGLFGWNVTDCKICFEYGLYYSPVSTPADFRSLAPIVLEQALKESGTQLLEPYL 544

Query 541
SFILYAPQEYLSRAYHDAPKYCATIETAQVKKDEVVFTGEIPARCIQAYRTDLAFYTNGR 600

SFILYAPQEYLSRAYHDAPKYCATIETAQVKKDEVVFTGEIPARCIQAYRTDLAFYTNGR
Sbjct 545
SFILYAPQEYLSRAYHDAPKYCATIETAQVKKDEVVFTGEIPARCIQAYRTDLAFYTNGR 604

```
Query  601  SVCLTELKGYQAAVGQPVIQPRRPNSRLDKVRHMFQKVM  639
            SVCLTELKGYQAAVGQPVIQPRRPNSRLDKVRHM QKVM
Sbjct  605  SVCLTELKGYQAAVGQPVIQPRRPNSRLDKVRHMSQKVM  643
```

>WP_072574872.1 TetM/TetW/TetO/TetS family tetracycline resistance ribosomal
protection protein [Lactobacillus reuteri]
 OJI11592.1 tetracycline resistance ribosomal protection protein [Lactobacillus
reuteri]
 OJI11598.1 tetracycline resistance ribosomal protection protein [Lactobacillus
reuteri]
Length=644

 Score = 1291 bits (3342), Expect = 0.0, Method: Compositional matrix adjust.
 Identities = 627/639 (98%), Positives = 636/639 (99%), Gaps = 0/639 (0%)

Query 1
MKIINIGILAHVDAGKTTLTESLLYASGAISEPGSVEKGTTRTDTMFLERQRGITIQAAV 60
            MKIINIGILAHVDAGKTTLTESLLYASGAISEPGSV
+KGTTRTDTMFLERQRGITIQAAV
Sbjct 1
MKIINIGILAHVDAGKTTLTESLLYASGAISEPGSVKKGTTRTDTMFLERQRGITIQAAV 60

Query 61
TSFQWHRCKVNIVDTPGHMDFLAEVYRSLAVLDGAILVISAKDGVQAQTRILFHALRKMN 120

TSFQWHRCKVNIVDTPGHMDFLAEVYRSLAVLDGAILVISAKDGVQAQTRILFHALRKMN

Sbjct 61
TSFQWHRCKVNIVDTPGHMDFLAEVYRSLAVLDGAILVISAKDGVQAQTRILFHALRKMN 120

Query 121
IPTVIFINKIDQAGVDLQSVVQSVRDKLSADIIIKQTVSLSPEIVLEENTDIEAWDAVIE 180

IPTVIFINKIDQAGVDLQSVVQSVRDKLSADIIIKQTVSLSPEIVLEENTDIEAWDAVIE
Sbjct 121
IPTVIFINKIDQAGVDLQSVVQSVRDKLSADIIIKQTVSLSPEIVLEENTDIEAWDAVIE 180

Query 181
NNDKLLEKYIAGEPISREKLVREEQRRVQDASLFPVYYGSAKKGLGIQPLMDAVTGLFQP 240

NNDKLLEKYIAGEPISREKLVREEQRRVQDASLFPVYYGSAKKGLGIQPLMDAVTGLFQP
Sbjct 181
NNDKLLEKYIAGEPISREKLVREEQRRVQDASLFPVYYGSAKKGLGIQPLMDAVTGLFQP 240

Query 241
IGEQGSAALCGSVFKVEYTDCGQRRVYLRLYSGTLRLRDTVALAGREKLKITEMRIPSKG 300

IGEQGSAALCGSVFKVEYTDCGQRRVYLRLYSGTLRLRDTVALAGREKLKITEMRIPSKG
Sbjct 241
IGEQGSAALCGSVFKVEYTDCGQRRVYLRLYSGTLRLRDTVALAGREKLKITEMRIPSKG 300

Query 301
EIVRTDTAYPGEIVILPSDSVRLNDVLGDPTRLPRKRWREDPLPMLRTTIAPKTAAQRER 360
EIVRTDTAYPGEIVILPSDSVRLNDVLGDPTRLPRKRWREDPLPMLRT
+IAPKTAAQRER
Sbjct 301
EIVRTDTAYPGEIVILPSDSVRLNDVLGDPTRLPRKRWREDPLPMLRTSIAPKTAAQRER 360

Query 361
LLDALTQLADTDPLLRCEVDSITHEIILSFLGRVQLEVVSALLSEKYKLETVVKEPSVIY 420
LLDALTQLADTDPLLRCEVDSITHEIILSFLGRVQLEVVSALLSEKYKLETVVKEP
+VIY
Sbjct 361
LLDALTQLADTDPLLRCEVDSITHEIILSFLGRVQLEVVSALLSEKYKLETVVKEPTVIY 420

Query 421
MERPLKAASHTIHIEVPPNPFWASIGLSVTPLSLGSGVQYESRVSLGYLNQSFQNAVRDG 480
MERPLKAASHTIHIEVPPNPFWASIGLSVTPL
LGSGVQYESRVSLGYLNQSFQNAVRDG
Sbjct 421
MERPLKAASHTIHIEVPPNPFWASIGLSVTPLPLGSGVQYESRVSLGYLNQSFQNAVRDG 480

Query 481
IRYGLEQGLFGWNVTDCKICFEYGLYYSPVSTPADFRSLAPIVLEQALKESGTQLLEPYL 540

IRYGLEQGLFGWNVTDCKICFEYGLYYSPVSTPADFRSLAPIVLEQALKESGTQLLEPYL
Sbjct 481
IRYGLEQGLFGWNVTDCKICFEYGLYYSPVSTPADFRSLAPIVLEQALKESGTQLLEPYL 540

Query 541
SFILYAPQEYLSRAYHDAPKYCATIETAQVKKDEVVFTGEIPARCIQAYRTDLAFYTNGR 600

            SF LYAP+EYLSRAYHDAPKYCATIET
QVKKDEVVFTGEIPARCIQAYRTDLAFYTNG+
Sbjct 541
SFTLYAPREYLSRAYHDAPKYCATIETVQVKKDEVVFTGEIPARCIQAYRTDLAFYTNGQ 600

Query 601 SVCLTELKGYQAAVGQPVIQPRRPNSRLDKVRHMFQKVM 639
            SVCLTELKGYQAAVG+PVIQPRRPNSRLDKVR+MFQK++
Sbjct 601 SVCLTELKGYQAAVGKPVIQPRRPNSRLDKVRYMFQKIL 639

>AMP56620.1 TetM-TetW-TetO-TetS, partial [uncultured bacterium]
Length=640

 Score = 1291 bits (3342), Expect = 0.0, Method: Compositional
matrix adjust.
 Identities = 629/637 (99%), Positives = 632/637 (99%), Gaps = 0/637
(0%)

Query 1
MKIINIGILAHVDAGKTTLTESLLYASGAISEPGSVEKGTTRTDTMFLERQRGITIQAAV 60

MKIINIGILAHVDAGKTTLTESLLYASGAISEPGSVEKGTTRTDTMFLERQRGITIQAAV
Sbjct 1
MKIINIGILAHVDAGKTTLTESLLYASGAISEPGSVEKGTTRTDTMFLERQRGITIQAAV 60

Query 61
TSFQWHRCKVNIVDTPGHMDFLAEVYRSLAVLDGAILVISAKDGVQAQTRILFHALRKMN 120

TSFQWHRCKVNIVDTPGHMDFLAEVYRSLAVLDGAILVISAKDGVQAQTRILFHALRKMN
Sbjct 61
TSFQWHRCKVNIVDTPGHMDFLAEVYRSLAVLDGAILVISAKDGVQAQTRILFHALRKMN 120

Query 121
IPTVIFINKIDQAGVDLQSVVQSVRDKLSADIIIKQTVSLSPEIVLEENTDIEAWDAVIE 180

IPTVIFINKIDQAGVDLQSVVQSVRDKLSADIIIKQTVSLSPEIVLEENTDIEAWDAVIE
Sbjct 121
IPTVIFINKIDQAGVDLQSVVQSVRDKLSADIIIKQTVSLSPEIVLEENTDIEAWDAVIE 180

Query 181
NNDKLLEKYIAGEPISREKLVREEQRRVQDASLFPVYYGSAKKGLGIQPLMDAVTGLFQP 240
            NND+LLEKYIAGEPISREKL REEQ+RVQDASLFPVY+GSAK
GLGIQPLMDAVTGLFQP
Sbjct 181
NNDELLEKYIAGEPISREKLAREEQQRVQDASLFPVYHGSAKNGLGIQPLMDAVTGLFQP 240

Query 241
IGEQGSAALCGSVFKVEYTDCGQRRVYLRLYSGTLRLRDTVALAGREKLKITEMRIPSKG 300
            IGEQG
AALCGSVFKVEYTDCGQRRVYLRLYSGTLRLRDTVALAGREKLKITEMRIPSKG
Sbjct 241
IGEQGGAALCGSVFKVEYTDCGQRRVYLRLYSGTLRLRDTVALAGREKLKITEMRIPSKG 300

Query 301

EIVRTDTAYPGEIVILPSDSVRLNDVLGDPTRLPRKRWREDPLPMLRTTIAPKTAAQRER 360
            EIVRTDTAY GEIVILPSDSVRLNDVLGD
TRLPRKRWREDPLPMLRTTIAPKTAAQRER
Sbjct 301
EIVRTDTAYQGEIVILPSDSVRLNDVLGDQTRLPRKRWREDPLPMLRTTIAPKTAAQRER 360

Query 361
LLDALTQLADTDPLLRCEVDSITHEIILSFLGRVQLEVVSALLSEKYKLETVVKEPSVIY 420

LLDALTQLADTDPLLRCEVDSITHEIILSFLGRVQLEVVSALLSEKYKLETVVKEPSVIY
Sbjct 361
LLDALTQLADTDPLLRCEVDSITHEIILSFLGRVQLEVVSALLSEKYKLETVVKEPSVIY 420

Query 421
MERPLKAASHTIHIEVPPNPFWASIGLSVTPLSLGSGVQYESRVSLGYLNQSFQNAVRDG 480

MERPLKAASHTIHIEVPPNPFWASIGLSVTPLSLGSGVQYESRVSLGYLNQSFQNAVRDG
Sbjct 421
MERPLKAASHTIHIEVPPNPFWASIGLSVTPLSLGSGVQYESRVSLGYLNQSFQNAVRDG 480

Query 481
IRYGLEQGLFGWNVTDCKICFEYGLYYSPVSTPADFRSLAPIVLEQALKESGTQLLEPYL 540

IRYGLEQGLFGWNVTDCKICFEYGLYYSPVSTPADFRSLAPIVLEQALKESGTQLLEPYL
Sbjct 481
IRYGLEQGLFGWNVTDCKICFEYGLYYSPVSTPADFRSLAPIVLEQALKESGTQLLEPYL 540

Query 541
SFILYAPQEYLSRAYHDAPKYCATIETAQVKKDEVVFTGEIPARCIQAYRTDLAFYTNGR 600

SFILYAPQEYLSRAYHDAPKYCATIETAQVKKDEVVFTGEIPARCIQAYRTDLAFYTNGR
Sbjct 541
SFILYAPQEYLSRAYHDAPKYCATIETAQVKKDEVVFTGEIPARCIQAYRTDLAFYTNGR 600

Query 601 SVCLTELKGYQAAVGQPVIQPRRPNSRLDKVRHMFQK 637
            SVCLTELKGYQAAVGQPVIQPRRPNSRLDKVRHMFQK
Sbjct 601 SVCLTELKGYQAAVGQPVIQPRRPNSRLDKVRHMFQK 637

>WP_024729649.1 TetM/TetW/TetO/TetS family tetracycline resistance
ribosomal
protection protein [Clostridiales bacterium VE202-13]
Length=639

Score = 1291 bits (3342), Expect = 0.0, Method: Compositional
matrix adjust.
Identities = 630/639 (99%), Positives = 633/639 (99%), Gaps = 0/639
(0%)

Query 1
MKIINIGILAHVDAGKTTLTESLLYASGAISEPGSVEKGTTRTDTMFLERQRGITIQAAV 60

MKIINIGILAHVDAGKTTLTESLLYASGAISEPGSVEKGTTRTDTMFLERQRGITIQAAV
Sbjct 1

```
MKIINIGILAHVDAGKTTLTESLLYASGAISEPGSVEKGTTRTDTMFLERQRGITIQAAV  60

Query  61
TSFQWHRCKVNIVDTPGHMDFLAEVYRSLAVLDGAILVISAKDGVQAQTRILFHALRKMN  120

TSFQWHRCKVNIVDTPGHMDFLAEVYRSLAVLDGAILVISAKDGVQAQTRILFHALRKMN
Sbjct  61
TSFQWHRCKVNIVDTPGHMDFLAEVYRSLAVLDGAILVISAKDGVQAQTRILFHALRKMN  120

Query  121
IPTVIFINKIDQAGVDLQSVVQSVRDKLSADIIIKQTVSLSPEIVLEENTDIEAWDAVIE  180

IPTVIFINKIDQAGVDLQSVVQSVRDKLSADIIIKQTVSLSPEIVLEENTDIEAWDAVIE
Sbjct  121
IPTVIFINKIDQAGVDLQSVVQSVRDKLSADIIIKQTVSLSPEIVLEENTDIEAWDAVIE  180

Query  181
NNDKLLEKYIAGEPISREKLVREEQRRVQDASLFPVYYGSAKKGLGIQPLMDAVTGLFQP  240
            NND+LLEKYIAGEPISREKL REEQ+RVQDASLFPVY+GSAK
GLGIQPLMDAVTGLFQP
Sbjct  181
NNDELLEKYIAGEPISREKLAREEQQRVQDASLFPVYHGSAKNGLGIQPLMDAVTGLFQP  240

Query  241
IGEQGSAALCGSVFKVEYTDCGQRRVYLRLYSGTLRLRDTVALAGREKLKITEMRIPSKG  300
            IGEQG
AALCGSVFKVEYTDCGQRRVYLRLYSGTLRLRDTVALAGREKLKITEMRIPSKG
Sbjct  241
IGEQGGAALCGSVFKVEYTDCGQRRVYLRLYSGTLRLRDTVALAGREKLKITEMRIPSKG  300

Query  301
EIVRTDTAYPGEIVILPSDSVRLNDVLGDPTRLPRKRWREDPLPMLRTTIAPKTAAQRER  360
            EIVRTDTAY GEIVILPSDSVRLNDVLGD
TRLPRKRWREDPLPMLRTTIAPKTAAQRER
Sbjct  301
EIVRTDTAYQGEIVILPSDSVRLNDVLGDQTRLPRKRWREDPLPMLRTTIAPKTAAQRER  360

Query  361
LLDALTQLADTDPLLRCEVDSITHEIILSFLGRVQLEVVSALLSEKYKLETVVKEPSVIY  420

LLDALTQLADTDPLLRCEVDSITHEIILSFLGRVQLEVVSALLSEKYKLETVVKEPSVIY
Sbjct  361
LLDALTQLADTDPLLRCEVDSITHEIILSFLGRVQLEVVSALLSEKYKLETVVKEPSVIY  420

Query  421
MERPLKAASHTIHIEVPPNPFWASIGLSVTPLSLGSGVQYESRVSLGYLNQSFQNAVRDG  480
            MERPLKAASHTI
IEVPPNPFWASIGLSVTPLSLGSGVQYESRVSLGYLNQSFQNAVRDG
Sbjct  421
MERPLKAASHTIDIEVPPNPFWASIGLSVTPLSLGSGVQYESRVSLGYLNQSFQNAVRDG  480

Query  481
IRYGLEQGLFGWNVTDCKICFEYGLYYSPVSTPADFRSLAPIVLEQALKESGTQLLEPYL  540
```

```
IRYGLEQGLFGWNVTDCKICFEYGLYYSPVSTPADFRSLAPIVLEQALKESGTQLLEPYL
Sbjct  481
IRYGLEQGLFGWNVTDCKICFEYGLYYSPVSTPADFRSLAPIVLEQALKESGTQLLEPYL  540

Query  541
SFILYAPQEYLSRAYHDAPKYCATIETAQVKKDEVVFTGEIPARCIQAYRTDLAFYTNGR  600

SFILYAPQEYLSRAYHDAPKYCATIETAQVKKDEVVFTGEIPARCIQAYRTDLAFYTNGR
Sbjct  541
SFILYAPQEYLSRAYHDAPKYCATIETAQVKKDEVVFTGEIPARCIQAYRTDLAFYTNGR  600

Query  601  SVCLTELKGYQAAVGQPVIQPRRPNSRLDKVRHMFQKVM  639
            SVCLTELKGYQAAVGQPVIQPRRPNSRLDKVRHMFQKVM
Sbjct  601  SVCLTELKGYQAAVGQPVIQPRRPNSRLDKVRHMFQKVM  639


>WP_087236340.1 TetM/TetW/TetO/TetS family tetracycline resistance
ribosomal
protection protein [Lactobacillus gallinarum]
 OUP98695.1 tetracycline resistance ribosomal protection protein
[Lactobacillus
gallinarum]
Length=641

 Score = 1291 bits (3341),  Expect = 0.0, Method: Compositional
matrix adjust.
 Identities = 628/639 (98%), Positives = 635/639 (99%), Gaps = 0/639
(0%)

Query  1
MKIINIGILAHVDAGKTTLTESLLYASGAISEPGSVEKGTTRTDTMFLERQRGITIQAAV  60
            MKIINIGILAHVDAGKTTLT
+SLLYASGAISEPGSVEKGTTRTDTMFLERQRGITIQAAV
Sbjct  1
MKIINIGILAHVDAGKTTLTDSLLYASGAISEPGSVEKGTTRTDTMFLERQRGITIQAAV  60

Query  61
TSFQWHRCKVNIVDTPGHMDFLAEVYRSLAVLDGAILVISAKDGVQAQTRILFHALRKMN  120

TSFQWHRCKVNIVDTPGHMDFLAEVYRSLAVLDGAILVISAKDGVQAQTRILFHALRKMN
Sbjct  61
TSFQWHRCKVNIVDTPGHMDFLAEVYRSLAVLDGAILVISAKDGVQAQTRILFHALRKMN  120

Query  121
IPTVIFINKIDQAGVDLQSVVQSVRDKLSADIIIKQTVSLSPEIVLEENTDIEAWDAVIE  180

IPTVIFINKIDQAGVDLQSVVQSVRDKLSADIIIKQTVSLSPEIVLEENTDIEAWDAVIE
Sbjct  121
IPTVIFINKIDQAGVDLQSVVQSVRDKLSADIIIKQTVSLSPEIVLEENTDIEAWDAVIE  180

Query  181
NNDKLLEKYIAGEPISREKLVREEQRRVQDASLFPVYYGSAKKGLGIQPLMDAVTGLFQP  240

NNDKLLEKYIAGEPISREKLVREEQRRVQDASLFPVYYGSAKKGLGIQPLMDAVTGLFQP
```

```
Sbjct  181
NNDKLLEKYIAGEPISREKLVREEQRRVQDASLFPVYYGSAKKGLGIQPLMDAVTGLFQP  240

Query  241
IGEQGSAALCGSVFKVEYTDCGQRRVYLRLYSGTLRLRDTVALAGREKLKITEMRIPSKG  300

IGEQGSAALCGSVFKVEYTDCGQRRVYLRLYSGTLRLRDTVALAGREKLKITEMRIPSKG
Sbjct  241
IGEQGSAALCGSVFKVEYTDCGQRRVYLRLYSGTLRLRDTVALAGREKLKITEMRIPSKG  300

Query  301
EIVRTDTAYPGEIVILPSDSVRLNDVLGDPTRLPRKRWREDPLPMLRTTIAPKTAAQRER  360
            EIVRTDTAYPGEIVILPSDSVRLNDVLGDPTRLPRKRWREDPLPMLRT
+IAPKTAAQRER
Sbjct  301
EIVRTDTAYPGEIVILPSDSVRLNDVLGDPTRLPRKRWREDPLPMLRTSIAPKTAAQRER  360

Query  361
LLDALTQLADTDPLLRCEVDSITHEIILSFLGRVQLEVVSALLSEKYKLETVVKEPSVIY  420

LLDALTQLADTDPLLRCEVDSITHEIILSFLGRVQLEVVSALLSEKYKLETVVKEPSVIY
Sbjct  361
LLDALTQLADTDPLLRCEVDSITHEIILSFLGRVQLEVVSALLSEKYKLETVVKEPSVIY  420

Query  421
MERPLKAASHTIHIEVPPNPFWASIGLSVTPLSLGSGVQYESRVSLGYLNQSFQNAVRDG  480
            MERPLKAASHTIHIEVPPNPFWASIGLSVTPL
LGSGVQYESRVSLGYLNQSFQNAVRDG
Sbjct  421
MERPLKAASHTIHIEVPPNPFWASIGLSVTPLPLGSGVQYESRVSLGYLNQSFQNAVRDG  480

Query  481
IRYGLEQGLFGWNVTDCKICFEYGLYYSPVSTPADFRSLAPIVLEQALKESGTQLLEPYL  540

IRYGLEQGLFGWNVTDCKICFEYGLYYSPVSTPADFRSLAPIVLEQALKESGTQLLEPYL
Sbjct  481
IRYGLEQGLFGWNVTDCKICFEYGLYYSPVSTPADFRSLAPIVLEQALKESGTQLLEPYL  540

Query  541
SFILYAPQEYLSRAYHDAPKYCATIETAQVKKDEVVFTGEIPARCIQAYRTDLAFYTNGR  600
            SF LYAP+EYLSRAYHDAPKYCATIET QVKKDEVVFTGEIPARCIQAYRTDLA
YTNG+
Sbjct  541
SFTLYAPREYLSRAYHDAPKYCATIETVQVKKDEVVFTGEIPARCIQAYRTDLASYTNGQ  600

Query  601  SVCLTELKGYQAAVGQPVIQPRRPNSRLDKVRHMFQKVM  639
            SVCLTELKGYQAAVG+PVIQPRRPNSRLDKVR+MFQK+M
Sbjct  601  SVCLTELKGYQAAVGKPVIQPRRPNSRLDKVRYMFQKIM  639
```

>WP_086131634.1 TetM/TetW/TetO/TetS family tetracycline resistance
ribosomal
protection protein [Lactobacillus reuteri]
OTA59508.1 tetracycline resistance ribosomal protection protein

[Lactobacillus
reuteri]
Length=641

 Score = 1291 bits (3341), Expect = 0.0, Method: Compositional
matrix adjust.
 Identities = 627/638 (98%), Positives = 635/638 (99%), Gaps = 0/638
(0%)

```
Query  1
MKIINIGILAHVDAGKTTLTESLLYASGAISEPGSVEKGTTRTDTMFLERQRGITIQAAV  60
            MKIINIGILAHVDAGKTTLTESLLYASGAISEPGSV
+KGTTRTDTMFLERQRGITIQAAV
Sbjct  1
MKIINIGILAHVDAGKTTLTESLLYASGAISEPGSVKKGTTRTDTMFLERQRGITIQAAV  60

Query  61
TSFQWHRCKVNIVDTPGHMDFLAEVYRSLAVLDGAILVISAKDGVQAQTRILFHALRKMN  120

TSFQWHRCKVNIVDTPGHMDFLAEVYRSLAVLDGAILVISAKDGVQAQTRILFHALRKMN
Sbjct  61
TSFQWHRCKVNIVDTPGHMDFLAEVYRSLAVLDGAILVISAKDGVQAQTRILFHALRKMN  120

Query  121
IPTVIFINKIDQAGVDLQSVVQSVRDKLSADIIIKQTVSLSPEIVLEENTDIEAWDAVIE  180

IPTVIFINKIDQAGVDLQSVVQSVRDKLSADIIIKQTVSLSPEIVLEENTDIEAWDAVIE
Sbjct  121
IPTVIFINKIDQAGVDLQSVVQSVRDKLSADIIIKQTVSLSPEIVLEENTDIEAWDAVIE  180

Query  181
NNDKLLEKYIAGEPISREKLVREEQRRVQDASLFPVYYGSAKKGLGIQPLMDAVTGLFQP  240

NNDKLLEKYIAGEPISREKLVREEQRRVQDASLFPVYYGSAKKGLGIQPLMDAVTGLFQP
Sbjct  181
NNDKLLEKYIAGEPISREKLVREEQRRVQDASLFPVYYGSAKKGLGIQPLMDAVTGLFQP  240

Query  241
IGEQGSAALCGSVFKVEYTDCGQRRVYLRLYSGTLRLRDTVALAGREKLKITEMRIPSKG  300

IGEQGSAALCGSVFKVEYTDCGQRRVYLRLYSGTLRLRDTVALAGREKLKITEMRIPSKG
Sbjct  241
IGEQGSAALCGSVFKVEYTDCGQRRVYLRLYSGTLRLRDTVALAGREKLKITEMRIPSKG  300

Query  301
EIVRTDTAYPGEIVILPSDSVRLNDVLGDPTRLPRKRWREDPLPMLRTTIAPKTAAQRER  360
            EIVRTDTAYPGEIVILPSDSVRLNDVLGDPTRLPRKRWREDPLPMLRT
+IAPKTAAQRER
Sbjct  301
EIVRTDTAYPGEIVILPSDSVRLNDVLGDPTRLPRKRWREDPLPMLRTSIAPKTAAQRER  360

Query  361
LLDALTQLADTDPLLRCEVDSITHEIILSFLGRVQLEVVSALLSEKYKLETVVKEPSVIY  420
            LLDALTQLADTDPLLRCEVDSITHEIILSFLGRVQLEVVSALLSEKYKLETVVKEP
```

+VIY
Sbjct 361
LLDALTQLADTDPLLRCEVDSITHEIILSFLGRVQLEVVSALLSEKYKLETVVKEPTVIY 420

Query 421
MERPLKAASHTIHIEVPPNPFWASIGLSVTPLSLGSGVQYESRVSLGYLNQSFQNAVRDG 480
            MERPLKAASHTIHIEVPPNPFWASIGLSVTPL
LGSGVQYESRVSLGYLNQSFQNAVRDG
Sbjct 421
MERPLKAASHTIHIEVPPNPFWASIGLSVTPLPLGSGVQYESRVSLGYLNQSFQNAVRDG 480

Query 481
IRYGLEQGLFGWNVTDCKICFEYGLYYSPVSTPADFRSLAPIVLEQALKESGTQLLEPYL 540

IRYGLEQGLFGWNVTDCKICFEYGLYYSPVSTPADFRSLAPIVLEQALKESGTQLLEPYL
Sbjct 481
IRYGLEQGLFGWNVTDCKICFEYGLYYSPVSTPADFRSLAPIVLEQALKESGTQLLEPYL 540

Query 541
SFILYAPQEYLSRAYHDAPKYCATIETAQVKKDEVVFTGEIPARCIQAYRTDLAFYTNGR 600
            SF LYAP+EYLSRAYHDAPKYCATIET
QVKKDEVVFTGEIPARCIQAYRTDLAFYTNG+
Sbjct 541
SFTLYAPREYLSRAYHDAPKYCATIETVQVKKDEVVFTGEIPARCIQAYRTDLAFYTNGQ 600

Query 601 SVCLTELKGYQAAVGQPVIQPRRPNSRLDKVRHMFQKV 638
            SVCLTELKGYQAAVG+PVIQPRRPNSRLDKVR+MFQK+
Sbjct 601 SVCLTELKGYQAAVGKPVIQPRRPNSRLDKVRYMFQKI 638

>WP_091695423.1 TetM/TetW/TetO/TetS family tetracycline resistance ribosomal
protection protein [Megamonas sp. Calf98-2]
 SEN71291.1 ribosomal protection tetracycline resistance protein [Megamonas
sp. Calf98-2]
Length=646

 Score = 1291 bits (3341), Expect = 0.0, Method: Compositional matrix adjust.
 Identities = 629/637 (99%), Positives = 632/637 (99%), Gaps = 0/637 (0%)

Query 1
MKIINIGILAHVDAGKTTLTESLLYASGAISEPGSVEKGTTRTDTMFLERQRGITIQAAV 60

MKIINIGILAHVDAGKTTLTESLLYASGAISEPGSVEKGTTRTDTMFLERQRGITIQAAV
Sbjct 1
MKIINIGILAHVDAGKTTLTESLLYASGAISEPGSVEKGTTRTDTMFLERQRGITIQAAV 60

Query 61
TSFQWHRCKVNIVDTPGHMDFLAEVYRSLAVLDGAILVISAKDGVQAQTRILFHALRKMN 120

TSFQWHRCKVNIVDTPGHMDFLAEVYRSLAVLDGAILVISAKDGVQAQTRILFHALRKMN

```
Sbjct  61
TSFQWHRCKVNIVDTPGHMDFLAEVYRSLAVLDGAILVISAKDGVQAQTRILFHALRKMN  120

Query  121
IPTVIFINKIDQAGVDLQSVVQSVRDKLSADIIIKQTVSLSPEIVLEENTDIEAWDAVIE  180

IPTVIFINKIDQAGVDLQSVVQSVRDKLSADIIIKQTVSLSPEIVLEENTDIEAWDAVIE
Sbjct  121
IPTVIFINKIDQAGVDLQSVVQSVRDKLSADIIIKQTVSLSPEIVLEENTDIEAWDAVIE  180

Query  181
NNDKLLEKYIAGEPISREKLVREEQRRVQDASLFPVYYGSAKKGLGIQPLMDAVTGLFQP  240
            NND+LLEKYIAGEPISREKL REEQ+RVQDASLFPVY+GSAK
GLGIQPLMDAVTGLFQP
Sbjct  181
NNDELLEKYIAGEPISREKLAREEQQRVQDASLFPVYHGSAKNGLGIQPLMDAVTGLFQP  240

Query  241
IGEQGSAALCGSVFKVEYTDCGQRRVYLRLYSGTLRLRDTVALAGREKLKITEMRIPSKG  300
            IGEQG
AALCGSVFKVEYTDCGQRRVYLRLYSGTLRLRDTVALAGREKLKITEMRIPSKG
Sbjct  241
IGEQGGAALCGSVFKVEYTDCGQRRVYLRLYSGTLRLRDTVALAGREKLKITEMRIPSKG  300

Query  301
EIVRTDTAYPGEIVILPSDSVRLNDVLGDPTRLPRKRWREDPLPMLRTTIAPKTAAQRER  360
            EIVRTDTAY GEIVILPSDSVRLNDVLGD
TRLPRKRWREDPLPMLRTTIAPKTAAQRER
Sbjct  301
EIVRTDTAYQGEIVILPSDSVRLNDVLGDQTRLPRKRWREDPLPMLRTTIAPKTAAQRER  360

Query  361
LLDALTQLADTDPLLRCEVDSITHEIILSFLGRVQLEVVSALLSEKYKLETVVKEPSVIY  420

LLDALTQLADTDPLLRCEVDSITHEIILSFLGRVQLEVVSALLSEKYKLETVVKEPSVIY
Sbjct  361
LLDALTQLADTDPLLRCEVDSITHEIILSFLGRVQLEVVSALLSEKYKLETVVKEPSVIY  420

Query  421
MERPLKAASHTIHIEVPPNPFWASIGLSVTPLSLGSGVQYESRVSLGYLNQSFQNAVRDG  480

MERPLKAASHTIHIEVPPNPFWASIGLSVTPLSLGSGVQYESRVSLGYLNQSFQNAVRDG
Sbjct  421
MERPLKAASHTIHIEVPPNPFWASIGLSVTPLSLGSGVQYESRVSLGYLNQSFQNAVRDG  480

Query  481
IRYGLEQGLFGWNVTDCKICFEYGLYYSPVSTPADFRSLAPIVLEQALKESGTQLLEPYL  540

IRYGLEQGLFGWNVTDCKICFEYGLYYSPVSTPADFRSLAPIVLEQALKESGTQLLEPYL
Sbjct  481
IRYGLEQGLFGWNVTDCKICFEYGLYYSPVSTPADFRSLAPIVLEQALKESGTQLLEPYL  540

Query  541
SFILYAPQEYLSRAYHDAPKYCATIETAQVKKDEVVFTGEIPARCIQAYRTDLAFYTNGR  600
```

SFILYAPQEYLSRAYHDAPKYCATIETAQVKKDEVVFTGEIPARCIQAYRTDLAFYTNGR
Sbjct 541
SFILYAPQEYLSRAYHDAPKYCATIETAQVKKDEVVFTGEIPARCIQAYRTDLAFYTNGR 600

Query 601 SVCLTELKGYQAAVGQPVIQPRRPNSRLDKVRHMFQK 637
            SVCLTELKGYQAAVGQPVIQPRRPNSRLDKVRHMFQK
Sbjct 601 SVCLTELKGYQAAVGQPVIQPRRPNSRLDKVRHMFQK 637

>AMP49509.1 TetM-TetW-TetO-TetS [uncultured bacterium]
Length=639

Score = 1291 bits (3341), Expect = 0.0, Method: Compositional matrix adjust.
Identities = 627/639 (98%), Positives = 634/639 (99%), Gaps = 0/639 (0%)

Query 1
MKIINIGILAHVDAGKTTLTESLLYASGAISEPGSVEKGTTRTDTMFLERQRGITIQAAV 60

MKIINIGILAHVDAGKTTLTESLLYASGAISEPGSVEKGTTRTDTMFLERQRGITIQAAV
Sbjct 1
MKIINIGILAHVDAGKTTLTESLLYASGAISEPGSVEKGTTRTDTMFLERQRGITIQAAV 60

Query 61
TSFQWHRCKVNIVDTPGHMDFLAEVYRSLAVLDGAILVISAKDGVQAQTRILFHALRKMN 120

TSFQWHRCKVNIVDTPGHMDFLAEVYRSLAVLDGAILVISAKDGVQAQTRILFHALRKMN
Sbjct 61
TSFQWHRCKVNIVDTPGHMDFLAEVYRSLAVLDGAILVISAKDGVQAQTRILFHALRKMN 120

Query 121
IPTVIFINKIDQAGVDLQSVVQSVRDKLSADIIIKQTVSLSPEIVLEENTDIEAWDAVIE 180

IPTVIFINKIDQAGVDLQSVVQSVRDKLSADIIIKQTVSLSPEIVLEENTDIEAWDAVIE
Sbjct 121
IPTVIFINKIDQAGVDLQSVVQSVRDKLSADIIIKQTVSLSPEIVLEENTDIEAWDAVIE 180

Query 181
NNDKLLEKYIAGEPISREKLVREEQRRVQDASLFPVYYGSAKKGLGIQPLMDAVTGLFQP 240
            NNDKLLEKYIAGEPISREKLVREEQRRVQDASLFPVYYGSAK
GLGIQPLMDAVTGLFQP
Sbjct 181
NNDKLLEKYIAGEPISREKLVREEQRRVQDASLFPVYYGSAKNGLGIQPLMDAVTGLFQP 240

Query 241
IGEQGSAALCGSVFKVEYTDCGQRRVYLRLYSGTLRLRDTVALAGREKLKITEMRIPSKG 300

IGEQGSAALCGSVFKVEYTDCGQRRVYLRLYSGTLRLRDTVALAGREKLKITEMRIPSKG
Sbjct 241
IGEQGSAALCGSVFKVEYTDCGQRRVYLRLYSGTLRLRDTVALAGREKLKITEMRIPSKG 300

Query 301

```
EIVRTDTAYPGEIVILPSDSVRLNDVLGDPTRLPRKRWREDPLPMLRTTIAPKTAAQRER  360
            EIVRTDTAYPGEIVILPSDSVRLNDVLGDPTRLPRKRWREDPLPMLRT
+IAPKTAAQRER
Sbjct  301
EIVRTDTAYPGEIVILPSDSVRLNDVLGDPTRLPRKRWREDPLPMLRTSIAPKTAAQRER  360

Query  361
LLDALTQLADTDPLLRCEVDSITHEIILSFLGRVQLEVVSALLSEKYKLETVVKEPSVIY  420
            LLDALTQLADTDPLLRCEVDSITHEIILSFLGRVQLEVVSALLSEKYKLETVVKEP
+VIY
Sbjct  361
LLDALTQLADTDPLLRCEVDSITHEIILSFLGRVQLEVVSALLSEKYKLETVVKEPTVIY  420

Query  421
MERPLKAASHTIHIEVPPNPFWASIGLSVTPLSLGSGVQYESRVSLGYLNQSFQNAVRDG  480
            MERPLKAASHTIHIEVPPNPFWASIGLSVTPL
LGSGVQYESRVSLGYLNQSFQNAVRDG
Sbjct  421
MERPLKAASHTIHIEVPPNPFWASIGLSVTPLPLGSGVQYESRVSLGYLNQSFQNAVRDG  480

Query  481
IRYGLEQGLFGWNVTDCKICFEYGLYYSPVSTPADFRSLAPIVLEQALKESGTQLLEPYL  540

IRYGLEQGLFGWNVTDCKICFEYGLYYSPVSTPADFRSLAPIVLEQALKESGTQLLEPYL
Sbjct  481
IRYGLEQGLFGWNVTDCKICFEYGLYYSPVSTPADFRSLAPIVLEQALKESGTQLLEPYL  540

Query  541
SFILYAPQEYLSRAYHDAPKYCATIETAQVKKDEVVFTGEIPARCIQAYRTDLAFYTNGR  600
            SF LYAP+EYLSRAYHDAPKYCATIET Q
KKDEVVFTGEIPARCIQAYRTDLAFYTNG+
Sbjct  541
SFTLYAPREYLSRAYHDAPKYCATIETVQEKKDEVVFTGEIPARCIQAYRTDLAFYTNGQ  600

Query  601  SVCLTELKGYQAAVGQPVIQPRRPNSRLDKVRHMFQKVM  639
            SVCLTELKGYQAAVG+PVIQPRRPNSRLDKVR+MFQK+M
Sbjct  601  SVCLTELKGYQAAVGKPVIQPRRPNSRLDKVRYMFQKIM  639


>WP_003672618.1 tetracycline resistance ribosomal protection protein
Tet(W) [Lactobacillus
reuteri]
 YP_001840820.1 tetracycline resistance protein (plasmid)
[Lactobacillus reuteri]
 ABO43851.1 TetW [Lactobacillus reuteri]
 ACB86596.1 tetracycline resistance protein (plasmid) [Lactobacillus
reuteri]
 EEI65626.1 putative translation elongation factor G [Lactobacillus
reuteri
CF48-3A]
 AEI58466.1 tetracycline resistance protein TetP (plasmid)
[Lactobacillus
reuteri SD2112]
Length=639
```

 Score = 1291 bits (3341), Expect = 0.0, Method: Compositional
matrix adjust.
 Identities = 627/639 (98%), Positives = 635/639 (99%), Gaps = 0/639
(0%)

```
Query  1
MKIINIGILAHVDAGKTTLTESLLYASGAISEPGSVEKGTTRTDTMFLERQRGITIQAAV  60
            MKIINIGILAHVDAGKTTLTESLLYASGAISEPGSV
+KGTTRTDTMFLERQRGITIQAAV
Sbjct  1
MKIINIGILAHVDAGKTTLTESLLYASGAISEPGSVKKGTTRTDTMFLERQRGITIQAAV  60

Query  61
TSFQWHRCKVNIVDTPGHMDFLAEVYRSLAVLDGAILVISAKDGVQAQTRILFHALRKMN  120

TSFQWHRCKVNIVDTPGHMDFLAEVYRSLAVLDGAILVISAKDGVQAQTRILFHALRKMN
Sbjct  61
TSFQWHRCKVNIVDTPGHMDFLAEVYRSLAVLDGAILVISAKDGVQAQTRILFHALRKMN  120

Query  121
IPTVIFINKIDQAGVDLQSVVQSVRDKLSADIIIKQTVSLSPEIVLEENTDIEAWDAVIE  180

IPTVIFINKIDQAGVDLQSVVQSVRDKLSADIIIKQTVSLSPEIVLEENTDIEAWDAVIE
Sbjct  121
IPTVIFINKIDQAGVDLQSVVQSVRDKLSADIIIKQTVSLSPEIVLEENTDIEAWDAVIE  180

Query  181
NNDKLLEKYIAGEPISREKLVREEQRRVQDASLFPVYYGSAKKGLGIQPLMDAVTGLFQP  240

NNDKLLEKYIAGEPISREKLVREEQRRVQDASLFPVYYGSAKKGLGIQPLMDAVTGLFQP
Sbjct  181
NNDKLLEKYIAGEPISREKLVREEQRRVQDASLFPVYYGSAKKGLGIQPLMDAVTGLFQP  240

Query  241
IGEQGSAALCGSVFKVEYTDCGQRRVYLRLYSGTLRLRDTVALAGREKLKITEMRIPSKG  300

IGEQGSAALCGSVFKVEYTDCGQRRVYLRLYSGTLRLRDTVALAGREKLKITEMRIPSKG
Sbjct  241
IGEQGSAALCGSVFKVEYTDCGQRRVYLRLYSGTLRLRDTVALAGREKLKITEMRIPSKG  300

Query  301
EIVRTDTAYPGEIVILPSDSVRLNDVLGDPTRLPRKRWREDPLPMLRTTIAPKTAAQRER  360
            EIVRTDTAYPGEIVILPSDSVRLNDVLGDPTRLPRKRWREDPLPMLRT
+IAPKTAAQRER
Sbjct  301
EIVRTDTAYPGEIVILPSDSVRLNDVLGDPTRLPRKRWREDPLPMLRTSIAPKTAAQRER  360

Query  361
LLDALTQLADTDPLLRCEVDSITHEIILSFLGRVQLEVVSALLSEKYKLETVVKEPSVIY  420
            LLDALTQLADTDPLLRCEVDSITHEIILSFLGRVQLEVVSALLSEKYKLETVVKEP
+VIY
Sbjct  361
LLDALTQLADTDPLLRCEVDSITHEIILSFLGRVQLEVVSALLSEKYKLETVVKEPTVIY  420
```

```
Query  421
MERPLKAASHTIHIEVPPNPFWASIGLSVTPLSLGSGVQYESRVSLGYLNQSFQNAVRDG  480
            MERPLKAASHTIHIEVPPNPFWASIGLSVTPL
LGSGVQYESRVSLGYLNQSFQNAVRDG
Sbjct  421
MERPLKAASHTIHIEVPPNPFWASIGLSVTPLPLGSGVQYESRVSLGYLNQSFQNAVRDG  480

Query  481
IRYGLEQGLFGWNVTDCKICFEYGLYYSPVSTPADFRSLAPIVLEQALKESGTQLLEPYL  540

IRYGLEQGLFGWNVTDCKICFEYGLYYSPVSTPADFRSLAPIVLEQALKESGTQLLEPYL
Sbjct  481
IRYGLEQGLFGWNVTDCKICFEYGLYYSPVSTPADFRSLAPIVLEQALKESGTQLLEPYL  540

Query  541
SFILYAPQEYLSRAYHDAPKYCATIETAQVKKDEVVFTGEIPARCIQAYRTDLAFYTNGR  600
            SF LYAP+EYLSRAYHDAPKYCATIET QVKKDEVVFTGEIPARCIQAYRTDLA
YTNG+
Sbjct  541
SFTLYAPREYLSRAYHDAPKYCATIETVQVKKDEVVFTGEIPARCIQAYRTDLASYTNGQ  600

Query  601  SVCLTELKGYQAAVGQPVIQPRRPNSRLDKVRHMFQKVM  639
            SVCLTELKGYQAAVG+PVIQPRRPNSRLDKVR+MFQK+M
Sbjct  601  SVCLTELKGYQAAVGKPVIQPRRPNSRLDKVRYMFQKIM  639


>WP_003819032.1 TetM/TetW/TetO/TetS family tetracycline resistance
ribosomal
protection protein [Bifidobacterium bifidum]
 EKF16735.1 TetW [Bifidobacterium bifidum IPLA 20015]
 KLN83356.1 translation elongation factor G [Bifidobacterium
bifidum]
Length=639

 Score = 1291 bits (3341),  Expect = 0.0, Method: Compositional
matrix adjust.
 Identities = 629/639 (98%), Positives = 634/639 (99%), Gaps = 0/639
(0%)

Query  1
MKIINIGILAHVDAGKTTLTESLLYASGAISEPGSVEKGTTRTDTMFLERQRGITIQAAV  60

MKIINIGILAHVDAGKTTLTESLLYASGAISEPGSVEKGTTRTDTMFLERQRGITIQAAV
Sbjct  1
MKIINIGILAHVDAGKTTLTESLLYASGAISEPGSVEKGTTRTDTMFLERQRGITIQAAV  60

Query  61
TSFQWHRCKVNIVDTPGHMDFLAEVYRSLAVLDGAILVISAKDGVQAQTRILFHALRKMN  120

TSFQWHRCKVNIVDTPGHMDFLAEVYRSLAVLDGAILVISAKDGVQAQTRILFHALRKMN
Sbjct  61
TSFQWHRCKVNIVDTPGHMDFLAEVYRSLAVLDGAILVISAKDGVQAQTRILFHALRKMN  120
```

Query 121
IPTVIFINKIDQAGVDLQSVVQSVRDKLSADIIIKQTVSLSPEIVLEENTDIEAWDAVIE 180

IPTVIFINKIDQAGVDLQSVVQSVRDKLSADIIIKQTVSLSPEIVLEENTDIEAWDAVIE
Sbjct 121
IPTVIFINKIDQAGVDLQSVVQSVRDKLSADIIIKQTVSLSPEIVLEENTDIEAWDAVIE 180

Query 181
NNDKLLEKYIAGEPISREKLVREEQRRVQDASLFPVYYGSAKKGLGIQPLMDAVTGLFQP 240

NNDKLLEKYIAGEPISREKLVREEQRRVQDASLFPVYYGSAKKGLGIQPLMDAVTGLFQP
Sbjct 181
NNDKLLEKYIAGEPISREKLVREEQRRVQDASLFPVYYGSAKKGLGIQPLMDAVTGLFQP 240

Query 241
IGEQGSAALCGSVFKVEYTDCGQRRVYLRLYSGTLRLRDTVALAGREKLKITEMRIPSKG 300

IGEQGSAALCGSVFKVEYTDCGQRRVYLRLYSGTLRLRDTVALAGREKLKITEMRIPSKG
Sbjct 241
IGEQGSAALCGSVFKVEYTDCGQRRVYLRLYSGTLRLRDTVALAGREKLKITEMRIPSKG 300

Query 301
EIVRTDTAYPGEIVILPSDSVRLNDVLGDPTRLPRKRWREDPLPMLRTTIAPKTAAQRER 360
EIVRTDTAY GEIVILPSDSVRLNDVLGD
TRLPRKRWREDPLPMLRTTIAPKTAAQRER
Sbjct 301
EIVRTDTAYQGEIVILPSDSVRLNDVLGDQTRLPRKRWREDPLPMLRTTIAPKTAAQRER 360

Query 361
LLDALTQLADTDPLLRCEVDSITHEIILSFLGRVQLEVVSALLSEKYKLETVVKEPSVIY 420
LLDALTQLADTDPLLRCEVDSITHEIILSFLGRVQLEVVSALLSEKYKLETVVKEP
+VIY
Sbjct 361
LLDALTQLADTDPLLRCEVDSITHEIILSFLGRVQLEVVSALLSEKYKLETVVKEPTVIY 420

Query 421
MERPLKAASHTIHIEVPPNPFWASIGLSVTPLSLGSGVQYESRVSLGYLNQSFQNAVRDG 480
MERPLKAASHTIHIEVPPNPFWASIGLSVTPL
LGSGVQYESRVSLGYLNQSFQNAVRDG
Sbjct 421
MERPLKAASHTIHIEVPPNPFWASIGLSVTPLPLGSGVQYESRVSLGYLNQSFQNAVRDG 480

Query 481
IRYGLEQGLFGWNVTDCKICFEYGLYYSPVSTPADFRSLAPIVLEQALKESGTQLLEPYL 540

IRYGLEQGLFGWNVTDCKICFEYGLYYSPVSTPADFRSLAPIVLEQALKESGTQLLEPYL
Sbjct 481
IRYGLEQGLFGWNVTDCKICFEYGLYYSPVSTPADFRSLAPIVLEQALKESGTQLLEPYL 540

Query 541
SFILYAPQEYLSRAYHDAPKYCATIETAQVKKDEVVFTGEIPARCIQAYRTDLAFYTNGR 600
SF LYAP+EYLSRAYHDAPKYCATIET
QVKKDEVVFTGEIPARCIQAYRTDLAFYTNG+
Sbjct 541

SFTLYAPREYLSRAYHDAPKYCATIETVQVKKDEVVFTGEIPARCIQAYRTDLAFYTNGQ 600

Query 601 SVCLTELKGYQAAVGQPVIQPRRPNSRLDKVRHMFQKVM 639
             SVCLTELKGYQAAVG+PVIQPRRPNSRLDKVR+MFQKVM
Sbjct 601 SVCLTELKGYQAAVGKPVIQPRRPNSRLDKVRYMFQKVM 639

>WP_105305034.1 TetM/TetW/TetO/TetS family tetracycline resistance ribosomal
protection protein [Anaerotruncus sp. Marseille-P4302]
Length=639

Score = 1291 bits (3340), Expect = 0.0, Method: Compositional matrix adjust.
Identities = 629/639 (98%), Positives = 634/639 (99%), Gaps = 0/639 (0%)

Query 1
MKIINIGILAHVDAGKTTLTESLLYASGAISEPGSVEKGTTRTDTMFLERQRGITIQAAV 60

MKIINIGILAHVDAGKTTLTESLLYASGAISEPGSVEKGTTRTDTMFLERQRGITIQAAV
Sbjct 1
MKIINIGILAHVDAGKTTLTESLLYASGAISEPGSVEKGTTRTDTMFLERQRGITIQAAV 60

Query 61
TSFQWHRCKVNIVDTPGHMDFLAEVYRSLAVLDGAILVISAKDGVQAQTRILFHALRKMN 120

TSFQWHRCKVNIVDTPGHMDFLAEVYRSLAVLDGAILVISAKDGVQAQTRILFHALRKMN
Sbjct 61
TSFQWHRCKVNIVDTPGHMDFLAEVYRSLAVLDGAILVISAKDGVQAQTRILFHALRKMN 120

Query 121
IPTVIFINKIDQAGVDLQSVVQSVRDKLSADIIIKQTVSLSPEIVLEENTDIEAWDAVIE 180

IPTVIFINKIDQAGVDLQSVVQSVRDKLSADIIIKQTVSLSPEIVLEENTDIEAWDAVIE
Sbjct 121
IPTVIFINKIDQAGVDLQSVVQSVRDKLSADIIIKQTVSLSPEIVLEENTDIEAWDAVIE 180

Query 181
NNDKLLEKYIAGEPISREKLVREEQRRVQDASLFPVYYGSAKKGLGIQPLMDAVTGLFQP 240
             NND+LLEKYIAGEPISREKL REEQ
+RVQDASLFPVYYGSAKKGLGIQPLMDAVTGLFQP
Sbjct 181
NNDELLEKYIAGEPISREKLAREEQQRVQDASLFPVYYGSAKKGLGIQPLMDAVTGLFQP 240

Query 241
IGEQGSAALCGSVFKVEYTDCGQRRVYLRLYSGTLRLRDTVALAGREKLKITEMRIPSKG 300

IGEQGSAALCGSVFKVEYTDCGQRRVYLRLYSGTLRLRDTVALAGREKLKITEMRIPSKG
Sbjct 241
IGEQGSAALCGSVFKVEYTDCGQRRVYLRLYSGTLRLRDTVALAGREKLKITEMRIPSKG 300

Query 301
EIVRTDTAYPGEIVILPSDSVRLNDVLGDPTRLPRKRWREDPLPMLRTTIAPKTAAQRER 360

EIVRTDTAYPGEIVILPSDSVRLNDVLGDPTRLPRKRWREDPLPMLRT
+IAPKTAAQRER
Sbjct 301
EIVRTDTAYPGEIVILPSDSVRLNDVLGDPTRLPRKRWREDPLPMLRTSIAPKTAAQRER 360

Query 361
LLDALTQLADTDPLLRCEVDSITHEIILSFLGRVQLEVVSALLSEKYKLETVVKEPSVIY 420
LLDALTQLADTDPLLR EVDSITHEIILSFLGRVQLEVVSALLSEKYK+ETVVKEP
+VIY
Sbjct 361
LLDALTQLADTDPLLRYEVDSITHEIILSFLGRVQLEVVSALLSEKYKIETVVKEPTVIY 420

Query 421
MERPLKAASHTIHIEVPPNPFWASIGLSVTPLSLGSGVQYESRVSLGYLNQSFQNAVRDG 480
MERPLKAASHTIHIEVPPNPFWASIGLSVTPL
LGSGVQYESRVSLGYLNQSFQNAVRDG
Sbjct 421
MERPLKAASHTIHIEVPPNPFWASIGLSVTPLPLGSGVQYESRVSLGYLNQSFQNAVRDG 480

Query 481
IRYGLEQGLFGWNVTDCKICFEYGLYYSPVSTPADFRSLAPIVLEQALKESGTQLLEPYL 540

IRYGLEQGLFGWNVTDCKICFEYGLYYSPVSTPADFRSLAPIVLEQALKESGTQLLEPYL
Sbjct 481
IRYGLEQGLFGWNVTDCKICFEYGLYYSPVSTPADFRSLAPIVLEQALKESGTQLLEPYL 540

Query 541
SFILYAPQEYLSRAYHDAPKYCATIETAQVKKDEVVFTGEIPARCIQAYRTDLAFYTNGR 600
SF LYAPQEYLSRAYHDAPKYCATIETAQVKKDEVVFTGEIPARCIQAYRTDLA
YTNGR
Sbjct 541
SFTLYAPQEYLSRAYHDAPKYCATIETAQVKKDEVVFTGEIPARCIQAYRTDLASYTNGR 600

Query 601 SVCLTELKGYQAAVGQPVIQPRRPNSRLDKVRHMFQKVM 639
SVCLTELKGYQAAVGQPVIQPRRPNSRLDKVRHMFQKVM
Sbjct 601 SVCLTELKGYQAAVGQPVIQPRRPNSRLDKVRHMFQKVM 639

>WP_099570919.1 TetM/TetW/TetO/TetS family tetracycline resistance
ribosomal
protection protein [Bifidobacterium sp. N5G01]
PIB82403.1 tetracycline resistance ribosomal protection protein
Tet(W) [Bifidobacterium
sp. N5G01]
Length=639

Score = 1291 bits (3340), Expect = 0.0, Method: Compositional
matrix adjust.
Identities = 629/639 (98%), Positives = 633/639 (99%), Gaps = 0/639
(0%)

Query 1
MKIINIGILAHVDAGKTTLTESLLYASGAISEPGSVEKGTTRTDTMFLERQRGITIQAAV 60

MKIINIGILAHVDAGKTTLTESLLYASGAISEPGSVEKGTTRTDTMFLERQRGITIQAAV
Sbjct 1
MKIINIGILAHVDAGKTTLTESLLYASGAISEPGSVEKGTTRTDTMFLERQRGITIQAAV 60

Query 61
TSFQWHRCKVNIVDTPGHMDFLAEVYRSLAVLDGAILVISAKDGVQAQTRILFHALRKMN 120

TSFQWHRCKVNIVDTPGHMDFLAEVYRSLAVLDGAILVISAKDGVQAQTRILFHALRKMN
Sbjct 61
TSFQWHRCKVNIVDTPGHMDFLAEVYRSLAVLDGAILVISAKDGVQAQTRILFHALRKMN 120

Query 121
IPTVIFINKIDQAGVDLQSVVQSVRDKLSADIIIKQTVSLSPEIVLEENTDIEAWDAVIE 180

IPTVIFINKIDQAGVDLQSVVQSVRDKLSADIIIKQTVSLSPEIVLEENTDIEAWDAVIE
Sbjct 121
IPTVIFINKIDQAGVDLQSVVQSVRDKLSADIIIKQTVSLSPEIVLEENTDIEAWDAVIE 180

Query 181
NNDKLLEKYIAGEPISREKLVREEQRRVQDASLFPVYYGSAKKGLGIQPLMDAVTGLFQP 240
NND+LLEKYIAGEPISREKL REEQ+RVQDASLFPVY+GSAK GLGIQPLMDAVTGLFQP
Sbjct 181
NNDELLEKYIAGEPISREKLAREEQQRVQDASLFPVYHGSAKNGLGIQPLMDAVTGLFQP 240

Query 241
IGEQGSAALCGSVFKVEYTDCGQRRVYLRLYSGTLRLRDTVALAGREKLKITEMRIPSKG 300
IGEQG AALCGSVFKVEYTDCGQRRVYLRLYSGTLRLRDTVALAGREKLKITEMRIPSKG
Sbjct 241
IGEQGGAALCGSVFKVEYTDCGQRRVYLRLYSGTLRLRDTVALAGREKLKITEMRIPSKG 300

Query 301
EIVRTDTAYPGEIVILPSDSVRLNDVLGDPTRLPRKRWREDPLPMLRTTIAPKTAAQRER 360
EIVRTDTAY GEIVILPSDSVRLNDVLGD TRLPRKRWREDPLPMLRT +IAPKTAAQRER
Sbjct 301
EIVRTDTAYQGEIVILPSDSVRLNDVLGDQTRLPRKRWREDPLPMLRTSIAPKTAAQRER 360

Query 361
LLDALTQLADTDPLLRCEVDSITHEIILSFLGRVQLEVVSALLSEKYKLETVVKEPSVIY 420

LLDALTQLADTDPLLRCEVDSITHEIILSFLGRVQLEVVSALLSEKYKLETVVKEPSVIY
Sbjct 361
LLDALTQLADTDPLLRCEVDSITHEIILSFLGRVQLEVVSALLSEKYKLETVVKEPSVIY 420

Query 421
MERPLKAASHTIHIEVPPNPFWASIGLSVTPLSLGSGVQYESRVSLGYLNQSFQNAVRDG 480

MERPLKAASHTIHIEVPPNPFWASIGLSVTPLSLGSGVQYESRVSLGYLNQSFQNAVRDG
Sbjct 421
MERPLKAASHTIHIEVPPNPFWASIGLSVTPLSLGSGVQYESRVSLGYLNQSFQNAVRDG 480

Query 481

IRYGLEQGLFGWNVTDCKICFEYGLYYSPVSTPADFRSLAPIVLEQALKESGTQLLEPYL 540

IRYGLEQGLFGWNVTDCKICFEYGLYYSPVSTPADFRSLAPIVLEQALKESGTQLLEPYL
Sbjct 481
IRYGLEQGLFGWNVTDCKICFEYGLYYSPVSTPADFRSLAPIVLEQALKESGTQLLEPYL 540

Query 541
SFILYAPQEYLSRAYHDAPKYCATIETAQVKKDEVVFTGEIPARCIQAYRTDLAFYTNGR 600
SFILYAPQEYLSRAYHDA
KYCATIETAQVKKDEVVFTGEIPARCIQAYRTDLAFYTNGR
Sbjct 541
SFILYAPQEYLSRAYHDALKYCATIETAQVKKDEVVFTGEIPARCIQAYRTDLAFYTNGR 600

Query 601 SVCLTELKGYQAAVGQPVIQPRRPNSRLDKVRHMFQKVM 639
SVCLTELKGYQAAVGQPVIQPRRPNSRLDKVRHMFQKVM
Sbjct 601 SVCLTELKGYQAAVGQPVIQPRRPNSRLDKVRHMFQKVM 639

>WP_063856425.1 tetracycline resistance ribosomal protection protein Tet(W) [Bifidobacterium
bifidum]
ACA23195.1 TetW [Bifidobacterium bifidum]
Length=639

Score = 1291 bits (3340), Expect = 0.0, Method: Compositional matrix adjust.
Identities = 630/639 (99%), Positives = 633/639 (99%), Gaps = 0/639 (0%)

Query 1
MKIINIGILAHVDAGKTTLTESLLYASGAISEPGSVEKGTTRTDTMFLERQRGITIQAAV 60

MKIINIGILAHVDAGKTTLTESLLYASGAISEPGSVEKGTTRTDTMFLERQRGITIQAAV
Sbjct 1
MKIINIGILAHVDAGKTTLTESLLYASGAISEPGSVEKGTTRTDTMFLERQRGITIQAAV 60

Query 61
TSFQWHRCKVNIVDTPGHMDFLAEVYRSLAVLDGAILVISAKDGVQAQTRILFHALRKMN 120

TSFQWHRCKVNIVDTPGHMDFLAEVYRSLAVLDGAILVISAKDGVQAQTRILFHALRKMN
Sbjct 61
TSFQWHRCKVNIVDTPGHMDFLAEVYRSLAVLDGAILVISAKDGVQAQTRILFHALRKMN 120

Query 121
IPTVIFINKIDQAGVDLQSVVQSVRDKLSADIIIKQTVSLSPEIVLEENTDIEAWDAVIE 180

IPTVIFINKIDQAGVDLQSVVQSVRDKLSADIIIKQTVSLSPEIVLEENTDIEAWDAVIE
Sbjct 121
IPTVIFINKIDQAGVDLQSVVQSVRDKLSADIIIKQTVSLSPEIVLEENTDIEAWDAVIE 180

Query 181
NNDKLLEKYIAGEPISREKLVREEQRRVQDASLFPVYYGSAKKGLGIQPLMDAVTGLFQP 240
NND+LLEKYIAGEPISREKL REEQ+RVQDASLFPVY+GSAK
GLGIQPLMDAVTGLFQP

Sbjct 181
NNDELLEKYIAGEPISREKLAREEQQRVQDASLFPVYHGSAKNGLGIQPLMDAVTGLFQP 240

Query 241
IGEQGSAALCGSVFKVEYTDCGQRRVYLRLYSGTLRLRDTVALAGREKLKITEMRIPSKG 300
            IGEQG
AALCGSVFKVEYTDCGQRRVYLRLYSGTLRLRDTVALAGREKLKITEMRIPSKG
Sbjct 241
IGEQGGAALCGSVFKVEYTDCGQRRVYLRLYSGTLRLRDTVALAGREKLKITEMRIPSKG 300

Query 301
EIVRTDTAYPGEIVILPSDSVRLNDVLGDPTRLPRKRWREDPLPMLRTTIAPKTAAQRER 360
            EIVRTDTAY GEIVILPSDSVRLNDVLGD
TRLPRKRWREDPLPMLRTTIAPKTAAQRER
Sbjct 301
EIVRTDTAYQGEIVILPSDSVRLNDVLGDQTRLPRKRWREDPLPMLRTTIAPKTAAQRER 360

Query 361
LLDALTQLADTDPLLRCEVDSITHEIILSFLGRVQLEVVSALLSEKYKLETVVKEPSVIY 420

LLDALTQLADTDPLLRCEVDSITHEIILSFLGRVQLEVVSALLSEKYKLETVVKEPSVIY
Sbjct 361
LLDALTQLADTDPLLRCEVDSITHEIILSFLGRVQLEVVSALLSEKYKLETVVKEPSVIY 420

Query 421
MERPLKAASHTIHIEVPPNPFWASIGLSVTPLSLGSGVQYESRVSLGYLNQSFQNAVRDG 480
            MERPLKAAS
TIHIEVPPNPFWASIGLSVTPLSLGSGVQYESRVSLGYLNQSFQNAVRDG
Sbjct 421
MERPLKAASPTIHIEVPPNPFWASIGLSVTPLSLGSGVQYESRVSLGYLNQSFQNAVRDG 480

Query 481
IRYGLEQGLFGWNVTDCKICFEYGLYYSPVSTPADFRSLAPIVLEQALKESGTQLLEPYL 540

IRYGLEQGLFGWNVTDCKICFEYGLYYSPVSTPADFRSLAPIVLEQALKESGTQLLEPYL
Sbjct 481
IRYGLEQGLFGWNVTDCKICFEYGLYYSPVSTPADFRSLAPIVLEQALKESGTQLLEPYL 540

Query 541
SFILYAPQEYLSRAYHDAPKYCATIETAQVKKDEVVFTGEIPARCIQAYRTDLAFYTNGR 600

SFILYAPQEYLSRAYHDAPKYCATIETAQVKKDEVVFTGEIPARCIQAYRTDLAFYTNGR
Sbjct 541
SFILYAPQEYLSRAYHDAPKYCATIETAQVKKDEVVFTGEIPARCIQAYRTDLAFYTNGR 600

Query 601 SVCLTELKGYQAAVGQPVIQPRRPNSRLDKVRHMFQKVM 639
            SVCLTELKGYQAAVGQPVIQPRRPNSRLDKVRHMFQKVM
Sbjct 601 SVCLTELKGYQAAVGQPVIQPRRPNSRLDKVRHMFQKVM 639

>APO35541.1 ferrous iron transport protein B [uncultured bacterium]
 APO35546.1 ferrous iron transport protein B Edit: TetW tetracycline
resistance
protein [uncultured bacterium]

Length=639

 Score = 1291 bits (3340), Expect = 0.0, Method: Compositional matrix adjust.
 Identities = 628/639 (98%), Positives = 634/639 (99%), Gaps = 0/639 (0%)

```
Query  1    MKIINIGILAHVDAGKTTLTESLLYASGAISEPGSVEKGTTRTDTMFLERQRGITIQAAV  60
            MKIINIGILAHVDAGKTTLTESLLYASGAISEPGSVEKGTTRTDTMFLERQRGITIQAAV
Sbjct  1    MKIINIGILAHVDAGKTTLTESLLYASGAISEPGSVEKGTTRTDTMFLERQRGITIQAAV  60

Query  61   TSFQWHRCKVNIVDTPGHMDFLAEVYRSLAVLDGAILVISAKDGVQAQTRILFHALRKMN  120
            TSFQWHRCKVNIVDTPGHMDFLAEVYRSLAVLDGAILVISAKDGVQAQTRILFHALRKMN
Sbjct  61   TSFQWHRCKVNIVDTPGHMDFLAEVYRSLAVLDGAILVISAKDGVQAQTRILFHALRKMN  120

Query  121  IPTVIFINKIDQAGVDLQSVVQSVRDKLSADIIIKQTVSLSPEIVLEENTDIEAWDAVIE  180
            IPTVIFINKIDQAGVDLQSVVQSVRDKLSADIIIKQTVSLSPEIVLEENTDIEAWDAVIE
Sbjct  121  IPTVIFINKIDQAGVDLQSVVQSVRDKLSADIIIKQTVSLSPEIVLEENTDIEAWDAVIE  180

Query  181  NNDKLLEKYIAGEPISREKLVREEQRRVQDASLFPVYYGSAKKGLGIQPLMDAVTGLFQP  240
            NND+LLEKYIAGEPISREKL REEQ +RVQDASLFPVYYGSAKKGLGIQPLMDAVTGLFQP
Sbjct  181  NNDELLEKYIAGEPISREKLAREEQQRVQDASLFPVYYGSAKKGLGIQPLMDAVTGLFQP  240

Query  241  IGEQGSAALCGSVFKVEYTDCGQRRVYLRLYSGTLRLRDTVALAGREKLKITEMRIPSKG  300
            IGEQG AALCGSVFKVEYTDCGQRRVYLRLYSGTLRLRDTVALAGREKLKITEMRIPSKG
Sbjct  241  IGEQGGAALCGSVFKVEYTDCGQRRVYLRLYSGTLRLRDTVALAGREKLKITEMRIPSKG  300

Query  301  EIVRTDTAYPGEIVILPSDSVRLNDVLGDPTRLPRKRWREDPLPMLRTTIAPKTAAQRER  360
            EIVRTDTAY GEIVILPSDSVRLNDVLGD TRLPRKRWREDPLPMLRT +IAPKTAAQRER
Sbjct  301  EIVRTDTAYQGEIVILPSDSVRLNDVLGDQTRLPRKRWREDPLPMLRTSIAPKTAAQRER  360

Query  361  LLDALTQLADTDPLLRCEVDSITHEIILSFLGRVQLEVVSALLSEKYKLETVVKEPSVIY  420
            LLDALTQLADTDPLLRCEVDSITHEIILSFLGRVQLEVVSALLSEKY LETVVKEPSVIY
Sbjct  361
```

LLDALTQLADTDPLLRCEVDSITHEIILSFLGRVQLEVVSALLSEKYNLETVVKEPSVIY 420

Query 421
MERPLKAASHTIHIEVPPNPFWASIGLSVTPLSLGSGVQYESRVSLGYLNQSFQNAVRDG 480

MERPLKAASHTIHIEVPPNPFWASIGLSVTPLSLGSGVQYESRVSLGYLNQSFQNAVRDG
Sbjct 421
MERPLKAASHTIHIEVPPNPFWASIGLSVTPLSLGSGVQYESRVSLGYLNQSFQNAVRDG 480

Query 481
IRYGLEQGLFGWNVTDCKICFEYGLYYSPVSTPADFRSLAPIVLEQALKESGTQLLEPYL 540

IRYGLEQGLFGWNVTDCKICFEYGLYYSPVSTPADFRSLAPIVLEQALKESGTQLLEPYL
Sbjct 481
IRYGLEQGLFGWNVTDCKICFEYGLYYSPVSTPADFRSLAPIVLEQALKESGTQLLEPYL 540

Query 541
SFILYAPQEYLSRAYHDAPKYCATIETAQVKKDEVVFTGEIPARCIQAYRTDLAFYTNGR 600

SFILYAPQEYLSRAYHDAPKYCATIETAQVKKDEVVFTGEIPARCIQAYRTDLAFYTNG+
Sbjct 541
SFILYAPQEYLSRAYHDAPKYCATIETAQVKKDEVVFTGEIPARCIQAYRTDLAFYTNGQ 600

Query 601 SVCLTELKGYQAAVGQPVIQPRRPNSRLDKVRHMFQKVM 639
            SVCLTELKGYQAAVGQPVIQPRRPNSRLDKVR+MFQK+M
Sbjct 601 SVCLTELKGYQAAVGQPVIQPRRPNSRLDKVRYMFQKIM 639

>AMP56769.1 TetM-TetW-TetO-TetS [uncultured bacterium]
 AMP56785.1 TetM-TetW-TetO-TetS [uncultured bacterium]
Length=639

 Score = 1291 bits (3340), Expect = 0.0, Method: Compositional matrix adjust.
 Identities = 628/639 (98%), Positives = 634/639 (99%), Gaps = 0/639 (0%)

Query 1
MKIINIGILAHVDAGKTTLTESLLYASGAISEPGSVEKGTTRTDTMFLERQRGITIQAAV 60

MKIINIGILAHVDAGKTTLTESLLYASGAISEPGSVEKGTTRTDTMFLERQRGITIQAAV
Sbjct 1
MKIINIGILAHVDAGKTTLTESLLYASGAISEPGSVEKGTTRTDTMFLERQRGITIQAAV 60

Query 61
TSFQWHRCKVNIVDTPGHMDFLAEVYRSLAVLDGAILVISAKDGVQAQTRILFHALRKMN 120

TSFQWHRCKVNIVDTPGHMDFLAEVYRSLAVLDGAILVISAKDGVQAQTRILFHALRKMN
Sbjct 61
TSFQWHRCKVNIVDTPGHMDFLAEVYRSLAVLDGAILVISAKDGVQAQTRILFHALRKMN 120

Query 121
IPTVIFINKIDQAGVDLQSVVQSVRDKLSADIIIKQTVSLSPEIVLEENTDIEAWDAVIE 180

IPTVIFINKIDQAGVDLQSVVQSVRDKLSADIIIKQTVSLSPEIVLEENTDIEAWDAVIE
Sbjct 121
IPTVIFINKIDQAGVDLQSVVQSVRDKLSADIIIKQTVSLSPEIVLEENTDIEAWDAVIE 180

Query 181
NNDKLLEKYIAGEPISREKLVREEQRRVQDASLFPVYYGSAKKGLGIQPLMDAVTGLFQP 240
NND+LLEKYIAGEPISREKL REEQ+RVQDASLFPVY+GSAK
GLGIQPLMDAVTGLFQP
Sbjct 181
NNDELLEKYIAGEPISREKLAREEQQRVQDASLFPVYHGSAKNGLGIQPLMDAVTGLFQP 240

Query 241
IGEQGSAALCGSVFKVEYTDCGQRRVYLRLYSGTLRLRDTVALAGREKLKITEMRIPSKG 300
IGEQG
AALCGSVFKVEYTDCGQRRVYLRLYSGTLRLRDTVALAGREKLKITEMRIPSKG
Sbjct 241
IGEQGGAALCGSVFKVEYTDCGQRRVYLRLYSGTLRLRDTVALAGREKLKITEMRIPSKG 300

Query 301
EIVRTDTAYPGEIVILPSDSVRLNDVLGDPTRLPRKRWREDPLPMLRTTIAPKTAAQRER 360
EIVRTDTAY GEIVILPSDSVRLNDVLGD TRLPRKRWREDPLPMLRT
+IAPKTAAQRER
Sbjct 301
EIVRTDTAYQGEIVILPSDSVRLNDVLGDQTRLPRKRWREDPLPMLRTSIAPKTAAQRER 360

Query 361
LLDALTQLADTDPLLRCEVDSITHEIILSFLGRVQLEVVSALLSEKYKLETVVKEPSVIY 420

LLDALTQLADTDPLLRCEVDSITHEIILSFLGRVQLEVVSALLSEKYKLETVVKEPSVIY
Sbjct 361
LLDALTQLADTDPLLRCEVDSITHEIILSFLGRVQLEVVSALLSEKYKLETVVKEPSVIY 420

Query 421
MERPLKAASHTIHIEVPPNPFWASIGLSVTPLSLGSGVQYESRVSLGYLNQSFQNAVRDG 480

MERPLKAASHTIHIEVPPNPFWASIGLSVTPLSLGSGVQYESRVSLGYLNQSFQNAVRDG
Sbjct 421
MERPLKAASHTIHIEVPPNPFWASIGLSVTPLSLGSGVQYESRVSLGYLNQSFQNAVRDG 480

Query 481
IRYGLEQGLFGWNVTDCKICFEYGLYYSPVSTPADFRSLAPIVLEQALKESGTQLLEPYL 540

IRYGLEQGLFGWNVTDCKICFEYGLYYSPVSTPADFRSLAPIVLEQALKESGTQLLEPYL
Sbjct 481
IRYGLEQGLFGWNVTDCKICFEYGLYYSPVSTPADFRSLAPIVLEQALKESGTQLLEPYL 540

Query 541
SFILYAPQEYLSRAYHDAPKYCATIETAQVKKDEVVFTGEIPARCIQAYRTDLAFYTNGR 600

SFILYAPQEYLSRAYHDAPKYCATIETAQVKKDEVVFTGEIPARCIQAYRTDLAFYTNGR
Sbjct 541
SFILYAPQEYLSRAYHDAPKYCATIETAQVKKDEVVFTGEIPARCIQAYRTDLAFYTNGR 600

Query 601 SVCLTELKGYQAAVGQPVIQPRRPNSRLDKVRHMFQKVM 639

            SVCLTELKGYQAAVGQPVIQPRRPNSRLDKVR+MFQK+M
Sbjct 601 SVCLTELKGYQAAVGQPVIQPRRPNSRLDKVRYMFQKIM 639

>AMP56061.1 TetM-TetW-TetO-TetS [uncultured bacterium]
Length=639

 Score = 1291 bits (3340), Expect = 0.0, Method: Compositional matrix adjust.
 Identities = 630/639 (99%), Positives = 633/639 (99%), Gaps = 0/639 (0%)

Query 1
MKIINIGILAHVDAGKTTLTESLLYASGAISEPGSVEKGTTRTDTMFLERQRGITIQAAV 60

MKIINIGILAHVDAGKTTLTESLLYASGAISEPGSVEKGTTRTDTMFLERQRGITIQAAV
Sbjct 1
MKIINIGILAHVDAGKTTLTESLLYASGAISEPGSVEKGTTRTDTMFLERQRGITIQAAV 60

Query 61
TSFQWHRCKVNIVDTPGHMDFLAEVYRSLAVLDGAILVISAKDGVQAQTRILFHALRKMN 120

TSFQWHRCKVNIVDTPGHMDFLAEVYRSLAVLDGAILVISAKDGVQAQTRILFHALRKMN
Sbjct 61
TSFQWHRCKVNIVDTPGHMDFLAEVYRSLAVLDGAILVISAKDGVQAQTRILFHALRKMN 120

Query 121
IPTVIFINKIDQAGVDLQSVVQSVRDKLSADIIIKQTVSLSPEIVLEENTDIEAWDAVIE 180

IPTVIFINKIDQAGVDLQSVVQSVRDKLSADIIIKQTVSLSPEIVLEENTDIEAWDAVIE
Sbjct 121
IPTVIFINKIDQAGVDLQSVVQSVRDKLSADIIIKQTVSLSPEIVLEENTDIEAWDAVIE 180

Query 181
NNDKLLEKYIAGEPISREKLVREEQRRVQDASLFPVYYGSAKKGLGIQPLMDAVTGLFQP 240
            NND+LLEKYIAGEPISREKL REEQ+RVQDASLFPVY+GSAK
GLGIQPLMDAVTGLFQP
Sbjct 181
NNDELLEKYIAGEPISREKLAREEQQRVQDASLFPVYHGSAKNGLGIQPLMDAVTGLFQP 240

Query 241
IGEQGSAALCGSVFKVEYTDCGQRRVYLRLYSGTLRLRDTVALAGREKLKITEMRIPSKG 300
            IGEQG
AALCGSVFKVEYTDCGQRRVYLRLYSGTLRLRDTVALAGREKLKITEMRIPSKG
Sbjct 241
IGEQGGAALCGSVFKVEYTDCGQRRVYLRLYSGTLRLRDTVALAGREKLKITEMRIPSKG 300

Query 301
EIVRTDTAYPGEIVILPSDSVRLNDVLGDPTRLPRKRWREDPLPMLRTTIAPKTAAQRER 360
            EIVRTDTAY GEIVILPSDSVRLNDVLGD
TRLPRKRWREDPLPMLRTTIAPKTAAQRER
Sbjct 301
EIVRTDTAYQGEIVILPSDSVRLNDVLGDQTRLPRKRWREDPLPMLRTTIAPKTAAQRER 360

```
Query  361
LLDALTQLADTDPLLRCEVDSITHEIILSFLGRVQLEVVSALLSEKYKLETVVKEPSVIY  420
            LLDALTQLADTDPLLR
EVDSITHEIILSFLGRVQLEVVSALLSEKYKLETVVKEPSVIY
Sbjct  361
LLDALTQLADTDPLLRYEVDSITHEIILSFLGRVQLEVVSALLSEKYKLETVVKEPSVIY  420

Query  421
MERPLKAASHTIHIEVPPNPFWASIGLSVTPLSLGSGVQYESRVSLGYLNQSFQNAVRDG  480

MERPLKAASHTIHIEVPPNPFWASIGLSVTPLSLGSGVQYESRVSLGYLNQSFQNAVRDG
Sbjct  421
MERPLKAASHTIHIEVPPNPFWASIGLSVTPLSLGSGVQYESRVSLGYLNQSFQNAVRDG  480

Query  481
IRYGLEQGLFGWNVTDCKICFEYGLYYSPVSTPADFRSLAPIVLEQALKESGTQLLEPYL  540

IRYGLEQGLFGWNVTDCKICFEYGLYYSPVSTPADFRSLAPIVLEQALKESGTQLLEPYL
Sbjct  481
IRYGLEQGLFGWNVTDCKICFEYGLYYSPVSTPADFRSLAPIVLEQALKESGTQLLEPYL  540

Query  541
SFILYAPQEYLSRAYHDAPKYCATIETAQVKKDEVVFTGEIPARCIQAYRTDLAFYTNGR  600

SFILYAPQEYLSRAYHDAPKYCATIETAQVKKDEVVFTGEIPARCIQAYRTDLAFYTNGR
Sbjct  541
SFILYAPQEYLSRAYHDAPKYCATIETAQVKKDEVVFTGEIPARCIQAYRTDLAFYTNGR  600

Query  601  SVCLTELKGYQAAVGQPVIQPRRPNSRLDKVRHMFQKVM  639
            SVCLTELKGYQAAVGQPVIQPRRPNSRLDKVRHMFQKVM
Sbjct  601  SVCLTELKGYQAAVGQPVIQPRRPNSRLDKVRHMFQKVM  639


>AMP51613.1 TetM-TetW-TetO-TetS [uncultured bacterium]
Length=639

 Score = 1291 bits (3340),  Expect = 0.0, Method: Compositional
matrix adjust.
 Identities = 628/639 (98%), Positives = 633/639 (99%), Gaps = 0/639
(0%)

Query  1
MKIINIGILAHVDAGKTTLTESLLYASGAISEPGSVEKGTTRTDTMFLERQRGITIQAAV  60
            M
IINIGILAHVDAGKTTLTESLLYASGAISEPGSVEKGTTRTDTMFLERQRGITIQAAV
Sbjct  1
MNIINIGILAHVDAGKTTLTESLLYASGAISEPGSVEKGTTRTDTMFLERQRGITIQAAV  60

Query  61
TSFQWHRCKVNIVDTPGHMDFLAEVYRSLAVLDGAILVISAKDGVQAQTRILFHALRKMN  120

TSFQWHRCKVNIVDTPGHMDFLAEVYRSLAVLDGAILVISAKDGVQAQTRILFHALRKMN
Sbjct  61
TSFQWHRCKVNIVDTPGHMDFLAEVYRSLAVLDGAILVISAKDGVQAQTRILFHALRKMN  120
```

```
Query  121
IPTVIFINKIDQAGVDLQSVVQSVRDKLSADIIIKQTVSLSPEIVLEENTDIEAWDAVIE  180

IPTVIFINKIDQAGVDLQSVVQSVRDKLSADIIIKQTVSLSPEIVLEENTDIEAWDAVIE
Sbjct  121
IPTVIFINKIDQAGVDLQSVVQSVRDKLSADIIIKQTVSLSPEIVLEENTDIEAWDAVIE  180

Query  181
NNDKLLEKYIAGEPISREKLVREEQRRVQDASLFPVYYGSAKKGLGIQPLMDAVTGLFQP  240
            NND+LLEKYIAGEPISREKL REEQ+RVQDASLFPVY
+GSAKKGLGIQPLMDAVTGLFQP
Sbjct  181
NNDELLEKYIAGEPISREKLAREEQQRVQDASLFPVYHGSAKKGLGIQPLMDAVTGLFQP  240

Query  241
IGEQGSAALCGSVFKVEYTDCGQRRVYLRLYSGTLRLRDTVALAGREKLKITEMRIPSKG  300
            IGEQG AALCGSVFKVEYTDCGQR
+YLRLYSGTLRLRDTVALAGREKLKITEMRIPSKG
Sbjct  241
IGEQGGAALCGSVFKVEYTDCGQRLIYLRLYSGTLRLRDTVALAGREKLKITEMRIPSKG  300

Query  301
EIVRTDTAYPGEIVILPSDSVRLNDVLGDPTRLPRKRWREDPLPMLRTTIAPKTAAQRER  360
            EIVRTDTAY GEIVILPSDSVRLNDVLGD
TRLPRKRWREDPLPMLRTTIAPKTAAQRER
Sbjct  301
EIVRTDTAYQGEIVILPSDSVRLNDVLGDQTRLPRKRWREDPLPMLRTTIAPKTAAQRER  360

Query  361
LLDALTQLADTDPLLRCEVDSITHEIILSFLGRVQLEVVSALLSEKYKLETVVKEPSVIY  420

LLDALTQLADTDPLLRCEVDSITHEIILSFLGRVQLEVVSALLSEKYKLETVVKEPSVIY
Sbjct  361
LLDALTQLADTDPLLRCEVDSITHEIILSFLGRVQLEVVSALLSEKYKLETVVKEPSVIY  420

Query  421
MERPLKAASHTIHIEVPPNPFWASIGLSVTPLSLGSGVQYESRVSLGYLNQSFQNAVRDG  480

MERPLKAASHTIHIEVPPNPFWASIGLSVTPLSLGSGVQYESRVSLGYLNQSFQNAVRDG
Sbjct  421
MERPLKAASHTIHIEVPPNPFWASIGLSVTPLSLGSGVQYESRVSLGYLNQSFQNAVRDG  480

Query  481
IRYGLEQGLFGWNVTDCKICFEYGLYYSPVSTPADFRSLAPIVLEQALKESGTQLLEPYL  540

IRYGLEQGLFGWNVTDCKICFEYGLYYSPVSTPADFRSLAPIVLEQALKESGTQLLEPYL
Sbjct  481
IRYGLEQGLFGWNVTDCKICFEYGLYYSPVSTPADFRSLAPIVLEQALKESGTQLLEPYL  540

Query  541
SFILYAPQEYLSRAYHDAPKYCATIETAQVKKDEVVFTGEIPARCIQAYRTDLAFYTNGR  600

SFILYAPQEYLSRAYHDAPKYCATIETAQVKKDEVVFTGEIPARCIQAYRTDLAFYTNGR
```

```
Sbjct  541
SFILYAPQEYLSRAYHDAPKYCATIETAQVKKDEVVFTGEIPARCIQAYRTDLAFYTNGR  600

Query  601  SVCLTELKGYQAAVGQPVIQPRRPNSRLDKVRHMFQKVM  639
            SVCLTELKGYQAAVGQPVIQPRRPNSRL+KVRHMFQKVM
Sbjct  601  SVCLTELKGYQAAVGQPVIQPRRPNSRLNKVRHMFQKVM  639


>WP_004217777.1 tetracycline resistance ribosomal protection protein
Tet(W) [Bifidobacterium
animalis]
 ACA23192.1 TetW [Bifidobacterium animalis subsp. lactis]
 EDT89751.1 ribosomal protection tetracycline resistance protein
[Bifidobacterium
animalis subsp. lactis HN019]
 ACS46328.1 ribosomal protection tetracycline resistance protein
[Bifidobacterium
animalis subsp. lactis Bl-04]
 ACS47895.1 ribosomal protection tetracycline resistance protein
[Bifidobacterium
animalis subsp. lactis DSM 10140]
 ADC85983.1 TetW [Bifidobacterium animalis subsp. lactis BB-12]
 ADG33520.1 ribosomal protection tetracycline resistance protein
[Bifidobacterium
animalis subsp. lactis V9]
 ADG64869.1 tetracycline resistance protein [Bifidobacterium
animalis subsp.
lactis]
 AEK30420.1 Tetracycline resistance protein tetW [Bifidobacterium
animalis
subsp. lactis CNCM I-2494]
 AEN76701.1 ribosomal protection tetracycline resistance protein
[Bifidobacterium
animalis subsp. lactis BLC1]
 EHN17670.1 ribosomal protection tetracycline resistance protein
[Bifidobacterium
animalis subsp. lactis BS 01]
 AFJ16693.1 Tetracycline resistance protein TetW [Bifidobacterium
animalis
subsp. lactis B420]
 AFJ18274.1 Tetracycline resistance protein TetW [Bifidobacterium
animalis
subsp. lactis Bi-07]
 AGO52421.1 ribosomal protection tetracycline resistance protein
[Bifidobacterium
animalis subsp. lactis Bl12]
 CDL71416.1 tetracycline resistance protein tetM from transposon
Tn916 [Bifidobacterium
animalis subsp. lactis CECT 8145]
 AIA32969.1 tetracycline resistance protein tetM [Bifidobacterium
animalis]
 AJC76652.1 tetracycline resistance protein tetM [Bifidobacterium
animalis
subsp. lactis KLDS2.0603]
```

AJD34077.1 ribosomal protection tetracycline resistance protein [Bifidobacterium
animalis]
AJD88694.1 tetracycline resistance protein tetM [Bifidobacterium animalis
subsp. lactis]
KOA46237.1 tetracycline resistance protein tetM [Bifidobacterium animalis
subsp. lactis ATCC 27536]
KOA52581.1 tetracycline resistance protein tetM [Bifidobacterium animalis
subsp. lactis ATCC 27674]
OJS85673.1 tetracycline resistance protein [Bifidobacterium animalis subsp.
lactis]
ASL77806.1 tetracycline resistance ribosomal protection protein [Bifidobacterium
animalis]
PIN32171.1 tetracycline resistance ribosomal protection protein Tet(W) [Bifidobacterium
animalis subsp. lactis BB-12]
PJP06261.1 tetracycline resistance ribosomal protection protein Tet(W) [Bifidobacterium
animalis subsp. lactis]
POO06789.1 Tetracycline resistance protein TetW [Bifidobacterium animalis
subsp. lactis]
POO07579.1 Tetracycline resistance protein TetW [Bifidobacterium animalis
subsp. lactis]
Length=639

Score = 1290 bits (3339), Expect = 0.0, Method: Compositional matrix adjust.
Identities = 626/638 (98%), Positives = 633/638 (99%), Gaps = 0/638 (0%)

```
Query  1
MKIINIGILAHVDAGKTTLTESLLYASGAISEPGSVEKGTTRTDTMFLERQRGITIQAAV  60
            MKIINIGILAHVDAGKTTLTESLLYASGAISEPGSVEKGTTRTDTM
LERQRGITIQAAV
Sbjct  1
MKIINIGILAHVDAGKTTLTESLLYASGAISEPGSVEKGTTRTDTMLLERQRGITIQAAV  60

Query  61
TSFQWHRCKVNIVDTPGHMDFLAEVYRSLAVLDGAILVISAKDGVQAQTRILFHALRKMN  120

TSFQWHRCKVNIVDTPGHMDFLAEVYRSLAVLDGAILVISAKDGVQAQTRILFHALRKMN
Sbjct  61
TSFQWHRCKVNIVDTPGHMDFLAEVYRSLAVLDGAILVISAKDGVQAQTRILFHALRKMN  120

Query  121
IPTVIFINKIDQAGVDLQSVVQSVRDKLSADIIIKQTVSLSPEIVLEENTDIEAWDAVIE  180
```

IPTVIFINKIDQAGVDLQSVVQSVRDKLSADIIIKQTVSLSPEIVLEENTDIEAWDAVIE
Sbjct 121
IPTVIFINKIDQAGVDLQSVVQSVRDKLSADIIIKQTVSLSPEIVLEENTDIEAWDAVIE 180

Query 181
NNDKLLEKYIAGEPISREKLVREEQRRVQDASLFPVYYGSAKKGLGIQPLMDAVTGLFQP 240

NNDKLLEKYIAGEPISREKLVREEQRRVQDASLFPVYYGSAKKGLGIQPLMDAVTGLFQP
Sbjct 181
NNDKLLEKYIAGEPISREKLVREEQRRVQDASLFPVYYGSAKKGLGIQPLMDAVTGLFQP 240

Query 241
IGEQGSAALCGSVFKVEYTDCGQRRVYLRLYSGTLRLRDTVALAGREKLKITEMRIPSKG 300

IGEQGSAALCGSVFKVEYTDCGQRRVYLRLYSGTLRLRDTVALAGREKLKITEMRIPSKG
Sbjct 241
IGEQGSAALCGSVFKVEYTDCGQRRVYLRLYSGTLRLRDTVALAGREKLKITEMRIPSKG 300

Query 301
EIVRTDTAYPGEIVILPSDSVRLNDVLGDPTRLPRKRWREDPLPMLRTTIAPKTAAQRER 360
EIVRTDTAYPGEIVILPSDSVRLNDVLGDPTRLPRKRWREDPLPMLRT
+IAPKTAAQRER
Sbjct 301
EIVRTDTAYPGEIVILPSDSVRLNDVLGDPTRLPRKRWREDPLPMLRTSIAPKTAAQRER 360

Query 361
LLDALTQLADTDPLLRCEVDSITHEIILSFLGRVQLEVVSALLSEKYKLETVVKEPSVIY 420
LLDALTQLADTDPLLRCEVDSITHEIILSFLGRVQLEVVSALLSEKYKLETVVKEP
+VIY
Sbjct 361
LLDALTQLADTDPLLRCEVDSITHEIILSFLGRVQLEVVSALLSEKYKLETVVKEPTVIY 420

Query 421
MERPLKAASHTIHIEVPPNPFWASIGLSVTPLSLGSGVQYESRVSLGYLNQSFQNAVRDG 480
MERPLKAASHTIHIEVPPNPFWASIGLSVTPL LGSGVQY
+SRVSLGYLNQSFQNAVRDG
Sbjct 421
MERPLKAASHTIHIEVPPNPFWASIGLSVTPLPLGSGVQYKSRVSLGYLNQSFQNAVRDG 480

Query 481
IRYGLEQGLFGWNVTDCKICFEYGLYYSPVSTPADFRSLAPIVLEQALKESGTQLLEPYL 540

IRYGLEQGLFGWNVTDCKICFEYGLYYSPVSTPADFRSLAPIVLEQALKESGTQLLEPYL
Sbjct 481
IRYGLEQGLFGWNVTDCKICFEYGLYYSPVSTPADFRSLAPIVLEQALKESGTQLLEPYL 540

Query 541
SFILYAPQEYLSRAYHDAPKYCATIETAQVKKDEVVFTGEIPARCIQAYRTDLAFYTNGR 600
SF LYAP+EYLSRAYHDAPKYCATIET
QVKKDEVVFTGEIPARCIQAYRTDLAFYTNG+
Sbjct 541
SFTLYAPREYLSRAYHDAPKYCATIETVQVKKDEVVFTGEIPARCIQAYRTDLAFYTNGQ 600

Query 601 SVCLTELKGYQAAVGQPVIQPRRPNSRLDKVRHMFQKV 638

            SVCLTELKGYQAAVG+PVIQPRRPNSRLDKVRHMF K+
Sbjct 601 SVCLTELKGYQAAVGKPVIQPRRPNSRLDKVRHMFSKI 638

>WP_085380940.1 TetM/TetW/TetO/TetS family tetracycline resistance ribosomal
protection protein [Bifidobacterium adolescentis]
 OSG96521.1 mosaic tetracycline-resistance protein [Bifidobacterium adolescentis]
 OSG99968.1 mosaic tetracycline-resistance protein [Bifidobacterium adolescentis]
Length=639

 Score = 1290 bits (3338), Expect = 0.0, Method: Compositional matrix adjust.
 Identities = 627/639 (98%), Positives = 632/639 (99%), Gaps = 0/639 (0%)

Query 1
MKIINIGILAHVDAGKTTLTESLLYASGAISEPGSVEKGTTRTDTMFLERQRGITIQAAV 60

MKIINIGILAHVDAGKTTLTESLLYASGAISEPGSVEKGTTRTDTMFLERQRGITIQAAV
Sbjct 1
MKIINIGILAHVDAGKTTLTESLLYASGAISEPGSVEKGTTRTDTMFLERQRGITIQAAV 60

Query 61
TSFQWHRCKVNIVDTPGHMDFLAEVYRSLAVLDGAILVISAKDGVQAQTRILFHALRKMN 120

TSFQWHRCKVNIVDTPGHMDFLAEVYRSLAVLDGAILVISAKDGVQAQTRILFHALRKMN
Sbjct 61
TSFQWHRCKVNIVDTPGHMDFLAEVYRSLAVLDGAILVISAKDGVQAQTRILFHALRKMN 120

Query 121
IPTVIFINKIDQAGVDLQSVVQSVRDKLSADIIIKQTVSLSPEIVLEENTDIEAWDAVIE 180

IPTVIFINKIDQAGVDLQSVVQSVRDKLSADIIIKQTVSLSPEIVLEENTDIEAWDAVIE
Sbjct 121
IPTVIFINKIDQAGVDLQSVVQSVRDKLSADIIIKQTVSLSPEIVLEENTDIEAWDAVIE 180

Query 181
NNDKLLEKYIAGEPISREKLVREEQRRVQDASLFPVYYGSAKKGLGIQPLMDAVTGLFQP 240
            NND+LLEKYIAGEPISREKL REEQ+RVQDASLFPVYYGSAK
GLGIQPLMDAVTGLFQP
Sbjct 181
NNDELLEKYIAGEPISREKLAREEQQRVQDASLFPVYYGSAKNGLGIQPLMDAVTGLFQP 240

Query 241
IGEQGSAALCGSVFKVEYTDCGQRRVYLRLYSGTLRLRDTVALAGREKLKITEMRIPSKG 300
            IGEQG
AALCGSVFKVEYTDCGQRRVYLRLYSGTLRLRDTVALAGREKLKITEMRIPSKG
Sbjct 241
IGEQGGAALCGSVFKVEYTDCGQRRVYLRLYSGTLRLRDTVALAGREKLKITEMRIPSKG 300

Query 301

```
EIVRTDTAYPGEIVILPSDSVRLNDVLGDPTRLPRKRWREDPLPMLRTTIAPKTAAQRER  360
            EIVRTDTAYPGEIVILPSDSVRLNDVLGD TRLPRKRWREDPLPMLRT
+IAPKTAAQRER
Sbjct  301
EIVRTDTAYPGEIVILPSDSVRLNDVLGDQTRLPRKRWREDPLPMLRTSIAPKTAAQRER  360

Query  361
LLDALTQLADTDPLLRCEVDSITHEIILSFLGRVQLEVVSALLSEKYKLETVVKEPSVIY  420
            LLDALTQLADTDPLLRCEVDSITHEIILSFLGRVQLEVVSALLSEKYKLETVVKEP
+VIY
Sbjct  361
LLDALTQLADTDPLLRCEVDSITHEIILSFLGRVQLEVVSALLSEKYKLETVVKEPTVIY  420

Query  421
MERPLKAASHTIHIEVPPNPFWASIGLSVTPLSLGSGVQYESRVSLGYLNQSFQNAVRDG  480
            MERPLKAASHTIHIEVPPNPFWASIGLSVTPL
LGSGVQYESRVSLGYLNQSFQNAVRDG
Sbjct  421
MERPLKAASHTIHIEVPPNPFWASIGLSVTPLPLGSGVQYESRVSLGYLNQSFQNAVRDG  480

Query  481
IRYGLEQGLFGWNVTDCKICFEYGLYYSPVSTPADFRSLAPIVLEQALKESGTQLLEPYL  540

IRYGLEQGLFGWNVTDCKICFEYGLYYSPVSTPADFRSLAPIVLEQALKESGTQLLEPYL
Sbjct  481
IRYGLEQGLFGWNVTDCKICFEYGLYYSPVSTPADFRSLAPIVLEQALKESGTQLLEPYL  540

Query  541
SFILYAPQEYLSRAYHDAPKYCATIETAQVKKDEVVFTGEIPARCIQAYRTDLAFYTNGR  600
            SF LYAP+EYLSRAYHDAPKYCATIET
QVKKDEVVFTGEIPARCIQAYRTDLAFYTNGR
Sbjct  541
SFTLYAPREYLSRAYHDAPKYCATIETVQVKKDEVVFTGEIPARCIQAYRTDLAFYTNGR  600

Query  601  SVCLTELKGYQAAVGQPVIQPRRPNSRLDKVRHMFQKVM  639
            SVCLTELKGYQAAVGQPVIQPRRPNSRLDKVRHMFQKVM
Sbjct  601  SVCLTELKGYQAAVGQPVIQPRRPNSRLDKVRHMFQKVM  639


>AMP55046.1 TetM-TetW-TetO-TetS [uncultured bacterium]
 AMP55053.1 TetM-TetW-TetO-TetS [uncultured bacterium]
Length=639

 Score = 1290 bits (3338),  Expect = 0.0, Method: Compositional
matrix adjust.
 Identities = 628/639 (98%), Positives = 632/639 (99%), Gaps = 0/639
(0%)

Query  1
MKIINIGILAHVDAGKTTLTESLLYASGAISEPGSVEKGTTRTDTMFLERQRGITIQAAV  60

MKIINIGILAHVDAGKTTLTESLLYASGAISEPGSVEKGTTRTDTMFLERQRGITIQAAV
Sbjct  1
MKIINIGILAHVDAGKTTLTESLLYASGAISEPGSVEKGTTRTDTMFLERQRGITIQAAV  60
```

```
Query  61
TSFQWHRCKVNIVDTPGHMDFLAEVYRSLAVLDGAILVISAKDGVQAQTRILFHALRKMN  120

TSFQWHRCKVNIVDTPGHMDFLAEVYRSLAVLDGAILVISAKDGVQAQTRILFHALRKMN
Sbjct  61
TSFQWHRCKVNIVDTPGHMDFLAEVYRSLAVLDGAILVISAKDGVQAQTRILFHALRKMN  120

Query  121
IPTVIFINKIDQAGVDLQSVVQSVRDKLSADIIIKQTVSLSPEIVLEENTDIEAWDAVIE  180

IPTVIFINKIDQAGVDLQSVVQSVRDKLSADIIIKQTVSLSPEIVLEENTDIEAWDAVIE
Sbjct  121
IPTVIFINKIDQAGVDLQSVVQSVRDKLSADIIIKQTVSLSPEIVLEENTDIEAWDAVIE  180

Query  181
NNDKLLEKYIAGEPISREKLVREEQRRVQDASLFPVYYGSAKKGLGIQPLMDAVTGLFQP  240
            NND+LLEKYIAGEPISREKL REEQ+RVQDASLFPVY+GSAK
GLGIQPLMDAVTGLFQP
Sbjct  181
NNDELLEKYIAGEPISREKLAREEQQRVQDASLFPVYHGSAKNGLGIQPLMDAVTGLFQP  240

Query  241
IGEQGSAALCGSVFKVEYTDCGQRRVYLRLYSGTLRLRDTVALAGREKLKITEMRIPSKG  300
            IGEQG
AALCGSVFKVEYTDCGQRRVYLRLYSGTLRLRDTVALAGREKLKITEMRIPSKG
Sbjct  241
IGEQGGAALCGSVFKVEYTDCGQRRVYLRLYSGTLRLRDTVALAGREKLKITEMRIPSKG  300

Query  301
EIVRTDTAYPGEIVILPSDSVRLNDVLGDPTRLPRKRWREDPLPMLRTTIAPKTAAQRER  360
            EIVRTDTAY GEIVILPSDSVRLNDVLGD
TRLPRKRWREDPLPMLRTTIAPKTAAQRER
Sbjct  301
EIVRTDTAYQGEIVILPSDSVRLNDVLGDQTRLPRKRWREDPLPMLRTTIAPKTAAQRER  360

Query  361
LLDALTQLADTDPLLRCEVDSITHEIILSFLGRVQLEVVSALLSEKYKLETVVKEPSVIY  420

LLDALTQLADTDPLLRCEVDSITHEIILSFLGRVQLEVVSALLSEKYKLETVVKEPSVIY
Sbjct  361
LLDALTQLADTDPLLRCEVDSITHEIILSFLGRVQLEVVSALLSEKYKLETVVKEPSVIY  420

Query  421
MERPLKAASHTIHIEVPPNPFWASIGLSVTPLSLGSGVQYESRVSLGYLNQSFQNAVRDG  480

MERPLKAASHTIHIEVPPNPFWASIGLSVTPLSLGSGVQYESRVSLGYLNQSFQNAVRDG
Sbjct  421
MERPLKAASHTIHIEVPPNPFWASIGLSVTPLSLGSGVQYESRVSLGYLNQSFQNAVRDG  480

Query  481
IRYGLEQGLFGWNVTDCKICFEYGLYYSPVSTPADFRSLAPIVLEQALKESGTQLLEPYL  540

IRYGLEQGLFGWNVTDCKICFEYGLYYSPVSTPADFRSLAPIVLEQALKESGTQLLEPYL
```

Sbjct 481
IRYGLEQGLFGWNVTDCKICFEYGLYYSPVSTPADFRSLAPIVLEQALKESGTQLLEPYL 540

Query 541
SFILYAPQEYLSRAYHDAPKYCATIETAQVKKDEVVFTGEIPARCIQAYRTDLAFYTNGR 600
            SF LYAP+EYLSRAYHDAPKYCATIET
QVKKDEVVFTGEIPARCIQAYRTDLAFYTNGR
Sbjct 541
SFTLYAPREYLSRAYHDAPKYCATIETVQVKKDEVVFTGEIPARCIQAYRTDLAFYTNGR 600

Query 601 SVCLTELKGYQAAVGQPVIQPRRPNSRLDKVRHMFQKVM 639
            SVCLTELKGYQAAVGQPVIQPRRPNSRLDKVRHMFQKVM
Sbjct 601 SVCLTELKGYQAAVGQPVIQPRRPNSRLDKVRHMFQKVM 639

>WP_098045504.1 MULTISPECIES: TetM/TetW/TetO/TetS family
tetracycline resistance
ribosomal protection protein [Lactobacillus]
 PEH03599.1 tetracycline resistance ribosomal protection protein
[Lactobacillus
sp. UMNPBX5]
 PEH08097.1 tetracycline resistance ribosomal protection protein
[Lactobacillus
sp. UMNPBX3]
Length=639

 Score = 1290 bits (3337), Expect = 0.0, Method: Compositional
matrix adjust.
 Identities = 627/639 (98%), Positives = 635/639 (99%), Gaps = 0/639
(0%)

Query 1
MKIINIGILAHVDAGKTTLTESLLYASGAISEPGSVEKGTTRTDTMFLERQRGITIQAAV 60
            MKIINIGILAHVDAGKTTLTESLLYASGAISEPGSV
+KGTTRTDTMFLERQRGITIQAAV
Sbjct 1
MKIINIGILAHVDAGKTTLTESLLYASGAISEPGSVKKGTTRTDTMFLERQRGITIQAAV 60

Query 61
TSFQWHRCKVNIVDTPGHMDFLAEVYRSLAVLDGAILVISAKDGVQAQTRILFHALRKMN 120

TSFQWHRCKVNIVDTPGHMDFLAEVYRSLAVLDGAILVISAKDGVQAQTRILFHALRKMN
Sbjct 61
TSFQWHRCKVNIVDTPGHMDFLAEVYRSLAVLDGAILVISAKDGVQAQTRILFHALRKMN 120

Query 121
IPTVIFINKIDQAGVDLQSVVQSVRDKLSADIIIKQTVSLSPEIVLEENTDIEAWDAVIE 180

IPTVIFINKIDQAGVDLQSVVQSVRDKLSADIIIKQTVSLSPEIVLEENTDIEAWDAVIE
Sbjct 121
IPTVIFINKIDQAGVDLQSVVQSVRDKLSADIIIKQTVSLSPEIVLEENTDIEAWDAVIE 180

Query 181
NNDKLLEKYIAGEPISREKLVREEQRRVQDASLFPVYYGSAKKGLGIQPLMDAVTGLFQP 240

```
NNDKLLEKYIAGEPISREKLVREEQRRVQDASLFPVYYGSAKKGLGIQPLMDAVTGLFQP
Sbjct  181
NNDKLLEKYIAGEPISREKLVREEQRRVQDASLFPVYYGSAKKGLGIQPLMDAVTGLFQP  240

Query  241
IGEQGSAALCGSVFKVEYTDCGQRRVYLRLYSGTLRLRDTVALAGREKLKITEMRIPSKG  300

IGEQGSAALCGSVFKVEYTDCGQRRVYLRLYSGTLRLRDTVALAGREKLKITEMRIPSKG
Sbjct  241
IGEQGSAALCGSVFKVEYTDCGQRRVYLRLYSGTLRLRDTVALAGREKLKITEMRIPSKG  300

Query  301
EIVRTDTAYPGEIVILPSDSVRLNDVLGDPTRLPRKRWREDPLPMLRTTIAPKTAAQRER  360
            EIVRTDTAYPGEIVILPSDSVRLNDVLGDPTRLPRKRWREDPLPMLRT
+IAPKTAAQRER
Sbjct  301
EIVRTDTAYPGEIVILPSDSVRLNDVLGDPTRLPRKRWREDPLPMLRTSIAPKTAAQRER  360

Query  361
LLDALTQLADTDPLLRCEVDSITHEIILSFLGRVQLEVVSALLSEKYKLETVVKEPSVIY  420
            LLDALTQLADTDPLLR EVDSITHEIILSFLGRVQLEVVSALLSEKYKLETVVKEP
+VIY
Sbjct  361
LLDALTQLADTDPLLRYEVDSITHEIILSFLGRVQLEVVSALLSEKYKLETVVKEPTVIY  420

Query  421
MERPLKAASHTIHIEVPPNPFWASIGLSVTPLSLGSGVQYESRVSLGYLNQSFQNAVRDG  480
            MERPLKAASHTIHIEVPPNPFWASIGLSVTPL
LGSGVQYESRVSLGYLNQSFQNAVRDG
Sbjct  421
MERPLKAASHTIHIEVPPNPFWASIGLSVTPLPLGSGVQYESRVSLGYLNQSFQNAVRDG  480

Query  481
IRYGLEQGLFGWNVTDCKICFEYGLYYSPVSTPADFRSLAPIVLEQALKESGTQLLEPYL  540

IRYGLEQGLFGWNVTDCKICFEYGLYYSPVSTPADFRSLAPIVLEQALKESGTQLLEPYL
Sbjct  481
IRYGLEQGLFGWNVTDCKICFEYGLYYSPVSTPADFRSLAPIVLEQALKESGTQLLEPYL  540

Query  541
SFILYAPQEYLSRAYHDAPKYCATIETAQVKKDEVVFTGEIPARCIQAYRTDLAFYTNGR  600
            SF LYAP+EYLSRAYHDAPKYCATIET
QVKKDEVVFTGEIPARCIQAYRTDLAFYTNG+
Sbjct  541
SFTLYAPREYLSRAYHDAPKYCATIETVQVKKDEVVFTGEIPARCIQAYRTDLAFYTNGQ  600

Query  601  SVCLTELKGYQAAVGQPVIQPRRPNSRLDKVRHMFQKVM  639
            SVCLTELKGYQAAVG+PVIQPRRPNSRLDKVR+MFQK+M
Sbjct  601  SVCLTELKGYQAAVGKPVIQPRRPNSRLDKVRYMFQKIM  639
```

>WP_098036734.1 MULTISPECIES: TetM/TetW/TetO/TetS family
tetracycline resistance

ribosomal protection protein [Lactobacillus]
 PEG86928.1 tetracycline resistance ribosomal protection protein
[Lactobacillus
sp. UMNPBX14]
 PEH02477.1 tetracycline resistance ribosomal protection protein
[Lactobacillus
sp. UMNPBX6]
 PEH06372.1 tetracycline resistance ribosomal protection protein
[Lactobacillus
sp. UMNPBX4]
Length=641

 Score = 1290 bits (3337), Expect = 0.0, Method: Compositional
matrix adjust.
 Identities = 627/639 (98%), Positives = 634/639 (99%), Gaps = 0/639
(0%)

Query 1
MKIINIGILAHVDAGKTTLTESLLYASGAISEPGSVEKGTTRTDTMFLERQRGITIQAAV 60
            MKIINIGILAHVDAGKTTLTESLLYASGAISEPGSVEKGTT
TDTMFLERQRGITIQAAV
Sbjct 1
MKIINIGILAHVDAGKTTLTESLLYASGAISEPGSVEKGTTSTDTMFLERQRGITIQAAV 60

Query 61
TSFQWHRCKVNIVDTPGHMDFLAEVYRSLAVLDGAILVISAKDGVQAQTRILFHALRKMN 120

TSFQWHRCKVNIVDTPGHMDFLAEVYRSLAVLDGAILVISAKDGVQAQTRILFHALRKMN
Sbjct 61
TSFQWHRCKVNIVDTPGHMDFLAEVYRSLAVLDGAILVISAKDGVQAQTRILFHALRKMN 120

Query 121
IPTVIFINKIDQAGVDLQSVVQSVRDKLSADIIIKQTVSLSPEIVLEENTDIEAWDAVIE 180

IPTVIFINKIDQAGVDLQSVVQSVRDKLSADIIIKQTVSLSPEIVLEENTDIEAWDAVIE
Sbjct 121
IPTVIFINKIDQAGVDLQSVVQSVRDKLSADIIIKQTVSLSPEIVLEENTDIEAWDAVIE 180

Query 181
NNDKLLEKYIAGEPISREKLVREEQRRVQDASLFPVYYGSAKKGLGIQPLMDAVTGLFQP 240

NNDKLLEKYIAGEPISREKLVREEQRRVQDASLFPVYYGSAKKGLGIQPLMDAVTGLFQP
Sbjct 181
NNDKLLEKYIAGEPISREKLVREEQRRVQDASLFPVYYGSAKKGLGIQPLMDAVTGLFQP 240

Query 241
IGEQGSAALCGSVFKVEYTDCGQRRVYLRLYSGTLRLRDTVALAGREKLKITEMRIPSKG 300

IGEQGSAALCGSVFKVEYTDCGQRRVYLRLYSGTLRLRDTVALAGREKLKITEMRIPSKG
Sbjct 241
IGEQGSAALCGSVFKVEYTDCGQRRVYLRLYSGTLRLRDTVALAGREKLKITEMRIPSKG 300

Query 301
EIVRTDTAYPGEIVILPSDSVRLNDVLGDPTRLPRKRWREDPLPMLRTTIAPKTAAQRER 360

```
            EIVRTDTAYPGEIVILPSDSVRLNDVLGDPTRLPRKRWREDPLPMLRT
+IAPKTAAQRER
Sbjct  301
EIVRTDTAYPGEIVILPSDSVRLNDVLGDPTRLPRKRWREDPLPMLRTSIAPKTAAQRER  360

Query  361
LLDALTQLADTDPLLRCEVDSITHEIILSFLGRVQLEVVSALLSEKYKLETVVKEPSVIY  420
            LLDALTQLADTDPLLRCEVDSITHEIILSFLGRVQLEVVSALLSEKYKLETVVKEP
+VIY
Sbjct  361
LLDALTQLADTDPLLRCEVDSITHEIILSFLGRVQLEVVSALLSEKYKLETVVKEPTVIY  420

Query  421
MERPLKAASHTIHIEVPPNPFWASIGLSVTPLSLGSGVQYESRVSLGYLNQSFQNAVRDG  480
            MERPLKAASHTIHIEVPPNPFWASIGLSVTPL
LGSGVQYESRVSLGYLNQSFQNAVRDG
Sbjct  421
MERPLKAASHTIHIEVPPNPFWASIGLSVTPLPLGSGVQYESRVSLGYLNQSFQNAVRDG  480

Query  481
IRYGLEQGLFGWNVTDCKICFEYGLYYSPVSTPADFRSLAPIVLEQALKESGTQLLEPYL  540

IRYGLEQGLFGWNVTDCKICFEYGLYYSPVSTPADFRSLAPIVLEQALKESGTQLLEPYL
Sbjct  481
IRYGLEQGLFGWNVTDCKICFEYGLYYSPVSTPADFRSLAPIVLEQALKESGTQLLEPYL  540

Query  541
SFILYAPQEYLSRAYHDAPKYCATIETAQVKKDEVVFTGEIPARCIQAYRTDLAFYTNGR  600
            SF LYAP+EYLSRAYHDAPKYCATIET QVKKDEVVFTGEIPARCIQAYRTDLA
YTNG+
Sbjct  541
SFTLYAPREYLSRAYHDAPKYCATIETVQVKKDEVVFTGEIPARCIQAYRTDLASYTNGQ  600

Query  601  SVCLTELKGYQAAVGQPVIQPRRPNSRLDKVRHMFQKVM  639
            SVCLTELKGYQAAVG+PVIQPRRPNSRLDKVR+MFQK+M
Sbjct  601  SVCLTELKGYQAAVGKPVIQPRRPNSRLDKVRYMFQKIM  639


>WP_087236767.1 TetM/TetW/TetO/TetS family tetracycline resistance
ribosomal
protection protein [Flavonifractor sp. An10]
 OUQ83729.1 tetracycline resistance ribosomal protection protein
[Flavonifractor
sp. An10]
Length=639

 Score = 1290 bits (3337),  Expect = 0.0, Method: Compositional
matrix adjust.
 Identities = 626/639 (98%), Positives = 634/639 (99%), Gaps = 0/639
(0%)

Query  1
MKIINIGILAHVDAGKTTLTESLLYASGAISEPGSVEKGTTRTDTMFLERQRGITIQAAV  60
            M IINIGILAHVDAGKTTLTESLLYASGAISEPGSVEKGTTRTDT
```

+FLERQRGITIQAAV
Sbjct 1
MNIINIGILAHVDAGKTTLTESLLYASGAISEPGSVEKGTTRTDTLFLERQRGITIQAAV 60

Query 61
TSFQWHRCKVNIVDTPGHMDFLAEVYRSLAVLDGAILVISAKDGVQAQTRILFHALRKMN 120

TSFQWHRCKVNIVDTPGHMDFLAEVYRSLAVLDGAILVISAKDGVQAQTRILFHALRKMN
Sbjct 61
TSFQWHRCKVNIVDTPGHMDFLAEVYRSLAVLDGAILVISAKDGVQAQTRILFHALRKMN 120

Query 121
IPTVIFINKIDQAGVDLQSVVQSVRDKLSADIIIKQTVSLSPEIVLEENTDIEAWDAVIE 180

IPTVIFINKIDQAGVDLQSVVQSVRDKLSADIIIKQTVSLSPEIVLEENTDIEAWDAVIE
Sbjct 121
IPTVIFINKIDQAGVDLQSVVQSVRDKLSADIIIKQTVSLSPEIVLEENTDIEAWDAVIE 180

Query 181
NNDKLLEKYIAGEPISREKLVREEQRRVQDASLFPVYYGSAKKGLGIQPLMDAVTGLFQP 240
NND LLEKYIAGEPIS+EKL REEQRRVQ+ASLFPVY
+GSAKKGLGIQPLMDAVTGLFQP
Sbjct 181
NNDALLEKYIAGEPISQEKLAREEQRRVQEASLFPVYHGSAKKGLGIQPLMDAVTGLFQP 240

Query 241
IGEQGSAALCGSVFKVEYTDCGQRRVYLRLYSGTLRLRDTVALAGREKLKITEMRIPSKG 300

IGEQGSAALCGSVFKVEYTDCGQRRVYLRLYSGTLRLRDTVALAGREKLKITEMRIPSKG
Sbjct 241
IGEQGSAALCGSVFKVEYTDCGQRRVYLRLYSGTLRLRDTVALAGREKLKITEMRIPSKG 300

Query 301
EIVRTDTAYPGEIVILPSDSVRLNDVLGDPTRLPRKRWREDPLPMLRTTIAPKTAAQRER 360
EIVRTDTAYPGEIVILPSDSVRLNDVLGDPTRLPRKRWREDPLPMLRT
+IAPKTAAQRER
Sbjct 301
EIVRTDTAYPGEIVILPSDSVRLNDVLGDPTRLPRKRWREDPLPMLRTSIAPKTAAQRER 360

Query 361
LLDALTQLADTDPLLRCEVDSITHEIILSFLGRVQLEVVSALLSEKYKLETVVKEPSVIY 420
LLDALTQLADTDPLLRCEVDSITHEIILSFLGRVQLEVVSALLSEKYKLETVVKEP
+VIY
Sbjct 361
LLDALTQLADTDPLLRCEVDSITHEIILSFLGRVQLEVVSALLSEKYKLETVVKEPTVIY 420

Query 421
MERPLKAASHTIHIEVPPNPFWASIGLSVTPLSLGSGVQYESRVSLGYLNQSFQNAVRDG 480
MERPLKAASHTIHIEVPPNPFWASIGLSVTPL
LGSGVQYESRVSLGYLNQSFQNAVRDG
Sbjct 421
MERPLKAASHTIHIEVPPNPFWASIGLSVTPLPLGSGVQYESRVSLGYLNQSFQNAVRDG 480

Query 481

IRYGLEQGLFGWNVTDCKICFEYGLYYSPVSTPADFRSLAPIVLEQALKESGTQLLEPYL 540

IRYGLEQGLFGWNVTDCKICFEYGLYYSPVSTPADFRSLAPIVLEQALKESGTQLLEPYL
Sbjct 481
IRYGLEQGLFGWNVTDCKICFEYGLYYSPVSTPADFRSLAPIVLEQALKESGTQLLEPYL 540

Query 541
SFILYAPQEYLSRAYHDAPKYCATIETAQVKKDEVVFTGEIPARCIQAYRTDLAFYTNGR 600
           SF
LYAPQEYLSRAYHDAPKYCATIETAQVKKDEVVFTGEIPARCIQAYRTDLAFYTNG+
Sbjct 541
SFTLYAPQEYLSRAYHDAPKYCATIETAQVKKDEVVFTGEIPARCIQAYRTDLAFYTNGQ 600

Query 601 SVCLTELKGYQAAVGQPVIQPRRPNSRLDKVRHMFQKVM 639
            SVCLTELKGYQAAVGQPVIQPRRPN+RLDKVRHMFQKVM
Sbjct 601 SVCLTELKGYQAAVGQPVIQPRRPNNRLDKVRHMFQKVM 639

>WP_087363777.1 TetM/TetW/TetO/TetS family tetracycline resistance ribosomal
protection protein [Lactobacillus gallinarum]
OUQ45948.1 tetracycline resistance ribosomal protection protein [Lactobacillus
gallinarum]
Length=641

Score = 1290 bits (3337), Expect = 0.0, Method: Compositional matrix adjust.
Identities = 627/639 (98%), Positives = 635/639 (99%), Gaps = 0/639 (0%)

Query 1
MKIINIGILAHVDAGKTTLTESLLYASGAISEPGSVEKGTTRTDTMFLERQRGITIQAAV 60

MKIINIGILAHVDAGKTTLTESLLYASGAISEPGSVEKGTTRTDTMFLERQRGITIQAAV
Sbjct 1
MKIINIGILAHVDAGKTTLTESLLYASGAISEPGSVEKGTTRTDTMFLERQRGITIQAAV 60

Query 61
TSFQWHRCKVNIVDTPGHMDFLAEVYRSLAVLDGAILVISAKDGVQAQTRILFHALRKMN 120

TSFQWHRCKVNIVDTPGHMDFLAEVYRSLAVLDGAILVISAKDGVQAQTRILFHALRKMN
Sbjct 61
TSFQWHRCKVNIVDTPGHMDFLAEVYRSLAVLDGAILVISAKDGVQAQTRILFHALRKMN 120

Query 121
IPTVIFINKIDQAGVDLQSVVQSVRDKLSADIIIKQTVSLSPEIVLEENTDIEAWDAVIE 180
           IPTVIFINKIDQAGVDLQSVVQSVRD
+LSADIIIKQTVSLSPEIVLEENTDIEAWDAVIE
Sbjct 121
IPTVIFINKIDQAGVDLQSVVQSVRDRLSADIIIKQTVSLSPEIVLEENTDIEAWDAVIE 180

Query 181
NNDKLLEKYIAGEPISREKLVREEQRRVQDASLFPVYYGSAKKGLGIQPLMDAVTGLFQP 240

NNDKLLEKYIAGEPISREKLVREEQRRVQDASLFPVYYGSAKKGLGIQPLMDAVTGLFQP
Sbjct 181
NNDKLLEKYIAGEPISREKLVREEQRRVQDASLFPVYYGSAKKGLGIQPLMDAVTGLFQP 240

Query 241
IGEQGSAALCGSVFKVEYTDCGQRRVYLRLYSGTLRLRDTVALAGREKLKITEMRIPSKG 300

IGEQGSAALCGSVFKVEYTDCGQRRVYLRLYSGTLRLRDTVALAGREKLKITEMRIPSKG
Sbjct 241
IGEQGSAALCGSVFKVEYTDCGQRRVYLRLYSGTLRLRDTVALAGREKLKITEMRIPSKG 300

Query 301
EIVRTDTAYPGEIVILPSDSVRLNDVLGDPTRLPRKRWREDPLPMLRTTIAPKTAAQRER 360
EIVRTDTAYPGEIVILPSDSVRLNDVLGDPTRLPRKRWREDPLPMLRT
+IAPKTAAQRER
Sbjct 301
EIVRTDTAYPGEIVILPSDSVRLNDVLGDPTRLPRKRWREDPLPMLRTSIAPKTAAQRER 360

Query 361
LLDALTQLADTDPLLRCEVDSITHEIILSFLGRVQLEVVSALLSEKYKLETVVKEPSVIY 420
LLDALTQLADTDPLLR EVDSITHEIILSFLGRVQLEVVSALLSEKYKLETVVKEP
+VIY
Sbjct 361
LLDALTQLADTDPLLRYEVDSITHEIILSFLGRVQLEVVSALLSEKYKLETVVKEPTVIY 420

Query 421
MERPLKAASHTIHIEVPPNPFWASIGLSVTPLSLGSGVQYESRVSLGYLNQSFQNAVRDG 480
MERPLKAASHTIHIEVPPNPFWASIGLSVTPL
LGSGVQYESRVSLGYLNQSFQNAVRDG
Sbjct 421
MERPLKAASHTIHIEVPPNPFWASIGLSVTPLPLGSGVQYESRVSLGYLNQSFQNAVRDG 480

Query 481
IRYGLEQGLFGWNVTDCKICFEYGLYYSPVSTPADFRSLAPIVLEQALKESGTQLLEPYL 540

IRYGLEQGLFGWNVTDCKICFEYGLYYSPVSTPADFRSLAPIVLEQALKESGTQLLEPYL
Sbjct 481
IRYGLEQGLFGWNVTDCKICFEYGLYYSPVSTPADFRSLAPIVLEQALKESGTQLLEPYL 540

Query 541
SFILYAPQEYLSRAYHDAPKYCATIETAQVKKDEVVFTGEIPARCIQAYRTDLAFYTNGR 600
SF LYAP+EYLSRAYHDAPKYCATIET
QVKKDEVVFTGEIPARCIQAYRTDLAFYTNG+
Sbjct 541
SFTLYAPREYLSRAYHDAPKYCATIETVQVKKDEVVFTGEIPARCIQAYRTDLAFYTNGQ 600

Query 601 SVCLTELKGYQAAVGQPVIQPRRPNSRLDKVRHMFQKVM 639
SVCLTELKGYQAAVG+PVIQPRRPNSRLDKVR+MFQK+M
Sbjct 601 SVCLTELKGYQAAVGKPVIQPRRPNSRLDKVRYMFQKIM 639

>AMJ37421.1 TetM–TetW–TetO–TetS [uncultured bacterium]
Length=639

```
 Score = 1290 bits (3337),  Expect = 0.0, Method: Compositional 
matrix adjust.
 Identities = 628/639 (98%), Positives = 634/639 (99%), Gaps = 0/639 
(0%)

Query  1    
MKIINIGILAHVDAGKTTLTESLLYASGAISEPGSVEKGTTRTDTMFLERQRGITIQAAV  60

MKIINIGILAHVDAGKTTLTESLLYASGAISEPGSVEKGTTRTDTMFLERQRGITIQAAV
Sbjct  1    
MKIINIGILAHVDAGKTTLTESLLYASGAISEPGSVEKGTTRTDTMFLERQRGITIQAAV  60

Query  61   
TSFQWHRCKVNIVDTPGHMDFLAEVYRSLAVLDGAILVISAKDGVQAQTRILFHALRKMN  120

TSFQWHRCKVNIVDTPGHMDFLAEVYRSLAVLDGAILVISAKDGVQAQTRILFHALRKMN
Sbjct  61   
TSFQWHRCKVNIVDTPGHMDFLAEVYRSLAVLDGAILVISAKDGVQAQTRILFHALRKMN  120

Query  121  
IPTVIFINKIDQAGVDLQSVVQSVRDKLSADIIIKQTVSLSPEIVLEENTDIEAWDAVIE  180

IPTVIFINKIDQAGVDLQSVVQSVRDKLSADIIIKQTVSLSPEIVLEENTDIEAWDAVIE
Sbjct  121  
IPTVIFINKIDQAGVDLQSVVQSVRDKLSADIIIKQTVSLSPEIVLEENTDIEAWDAVIE  180

Query  181  
NNDKLLEKYIAGEPISREKLVREEQRRVQDASLFPVYYGSAKKGLGIQPLMDAVTGLFQP  240
            NNDKLLEKYIAGEPISREKLVREEQRRVQDASLFPVY
+GSAKKGLGIQPLMDAVTGLFQP
Sbjct  181  
NNDKLLEKYIAGEPISREKLVREEQRRVQDASLFPVYHGSAKKGLGIQPLMDAVTGLFQP  240

Query  241  
IGEQGSAALCGSVFKVEYTDCGQRRVYLRLYSGTLRLRDTVALAGREKLKITEMRIPSKG  300

IGEQGSAALCGSVFKVEYTDCGQRRVYLRLYSGTLRLRDTVALAGREKLKITEMRIPSKG
Sbjct  241  
IGEQGSAALCGSVFKVEYTDCGQRRVYLRLYSGTLRLRDTVALAGREKLKITEMRIPSKG  300

Query  301  
EIVRTDTAYPGEIVILPSDSVRLNDVLGDPTRLPRKRWREDPLPMLRTTIAPKTAAQRER  360
            EIVRTDTAY GEIVILPSDSVRLNDVLGD 
TRLPRKRWREDPLPMLRTTIAPKTAAQRER
Sbjct  301  
EIVRTDTAYQGEIVILPSDSVRLNDVLGDQTRLPRKRWREDPLPMLRTTIAPKTAAQRER  360

Query  361  
LLDALTQLADTDPLLRCEVDSITHEIILSFLGRVQLEVVSALLSEKYKLETVVKEPSVIY  420
            LLDALTQLADTDPLLRCEVDSITHEIILSFLGRVQLEVVSALLSEKYKLETVVKEP
+VIY
Sbjct  361  
LLDALTQLADTDPLLRCEVDSITHEIILSFLGRVQLEVVSALLSEKYKLETVVKEPTVIY  420
```

```
Query  421  
MERPLKAASHTIHIEVPPNPFWASIGLSVTPLSLGSGVQYESRVSLGYLNQSFQNAVRDG  480
            MERPLKAASHTIHIEVPPNPFWASIGLSVTPL
LGSGVQYESRVSLGYLNQSFQNAVRDG
Sbjct  421  
MERPLKAASHTIHIEVPPNPFWASIGLSVTPLPLGSGVQYESRVSLGYLNQSFQNAVRDG  480

Query  481  
IRYGLEQGLFGWNVTDCKICFEYGLYYSPVSTPADFRSLAPIVLEQALKESGTQLLEPYL  540
            
IRYGLEQGLFGWNVTDCKICFEYGLYYSPVSTPADFRSLAPIVLEQALKESGTQLLEPYL
Sbjct  481  
IRYGLEQGLFGWNVTDCKICFEYGLYYSPVSTPADFRSLAPIVLEQALKESGTQLLEPYL  540

Query  541  
SFILYAPQEYLSRAYHDAPKYCATIETAQVKKDEVVFTGEIPARCIQAYRTDLAFYTNGR  600
            SF LYAP+EYLSRAYHDAPKYCATIET
QVKKDEVVFTGEIPARCIQAYRTDLAFYTNG+
Sbjct  541  
SFTLYAPREYLSRAYHDAPKYCATIETVQVKKDEVVFTGEIPARCIQAYRTDLAFYTNGQ  600

Query  601  SVCLTELKGYQAAVGQPVIQPRRPNSRLDKVRHMFQKVM  639
            SVCLTELKGYQAAVG+PVIQPRRPNSRLDKVR+MFQKVM
Sbjct  601  SVCLTELKGYQAAVGKPVIQPRRPNSRLDKVRYMFQKVM  639


>WP_080907810.1 TetM/TetW/TetO/TetS family tetracycline resistance 
ribosomal
protection protein [Pseudoflavonifractor sp. Marseille-P3106]
Length=639

 Score = 1289 bits (3336),  Expect = 0.0, Method: Compositional 
matrix adjust.
 Identities = 628/638 (98%), Positives = 632/638 (99%), Gaps = 0/638 
(0%)

Query  1    
MKIINIGILAHVDAGKTTLTESLLYASGAISEPGSVEKGTTRTDTMFLERQRGITIQAAV  60
            
MKIINIGILAHVDAGKTTLTESLLYASGAISEPGSVEKGTTRTDTMFLERQRGITIQAAV
Sbjct  1    
MKIINIGILAHVDAGKTTLTESLLYASGAISEPGSVEKGTTRTDTMFLERQRGITIQAAV  60

Query  61   
TSFQWHRCKVNIVDTPGHMDFLAEVYRSLAVLDGAILVISAKDGVQAQTRILFHALRKMN  120
            
TSFQWHRCKVNIVDTPGHMDFLAEVYRSLAVLDGAILVISAKDGVQAQTRILFHALRKMN
Sbjct  61   
TSFQWHRCKVNIVDTPGHMDFLAEVYRSLAVLDGAILVISAKDGVQAQTRILFHALRKMN  120

Query  121  
IPTVIFINKIDQAGVDLQSVVQSVRDKLSADIIIKQTVSLSPEIVLEENTDIEAWDAVIE  180
```

```
IPTVIFINKIDQAGVDLQSVVQSVRDKLSADIIIKQTVSLSPEIVLEENTDIEAWDAVIE
Sbjct  121
IPTVIFINKIDQAGVDLQSVVQSVRDKLSADIIIKQTVSLSPEIVLEENTDIEAWDAVIE  180

Query  181
NNDKLLEKYIAGEPISREKLVREEQRRVQDASLFPVYYGSAKKGLGIQPLMDAVTGLFQP  240
            NND+LLEKYIAGEPISREKL REEQ+RVQDASLFPVY+GSAK
GLGIQPLMDAVTGLFQP
Sbjct  181
NNDELLEKYIAGEPISREKLAREEQQRVQDASLFPVYHGSAKNGLGIQPLMDAVTGLFQP  240

Query  241
IGEQGSAALCGSVFKVEYTDCGQRRVYLRLYSGTLRLRDTVALAGREKLKITEMRIPSKG  300
            IGEQG
AALCGSVFKVEYTDCGQRRVYLRLYSGTLRLRDTVALAGREKLKITEMRIPSKG
Sbjct  241
IGEQGGAALCGSVFKVEYTDCGQRRVYLRLYSGTLRLRDTVALAGREKLKITEMRIPSKG  300

Query  301
EIVRTDTAYPGEIVILPSDSVRLNDVLGDPTRLPRKRWREDPLPMLRTTIAPKTAAQRER  360
            EIVRTDTAY GEIVILPSDSVRLNDVLGD
TRLPRKRWREDPLPMLRTTIAPKTAAQRER
Sbjct  301
EIVRTDTAYQGEIVILPSDSVRLNDVLGDQTRLPRKRWREDPLPMLRTTIAPKTAAQRER  360

Query  361
LLDALTQLADTDPLLRCEVDSITHEIILSFLGRVQLEVVSALLSEKYKLETVVKEPSVIY  420

LLDALTQLADTDPLLRCEVDSITHEIILSFLGRVQLEVVSALLSEKYKLETVVKEPSVIY
Sbjct  361
LLDALTQLADTDPLLRCEVDSITHEIILSFLGRVQLEVVSALLSEKYKLETVVKEPSVIY  420

Query  421
MERPLKAASHTIHIEVPPNPFWASIGLSVTPLSLGSGVQYESRVSLGYLNQSFQNAVRDG  480

MERPLKAASHTIHIEVPPNPFWASIGLSVTPLSLGSGVQYESRVSLGYLNQSFQNAVRDG
Sbjct  421
MERPLKAASHTIHIEVPPNPFWASIGLSVTPLSLGSGVQYESRVSLGYLNQSFQNAVRDG  480

Query  481
IRYGLEQGLFGWNVTDCKICFEYGLYYSPVSTPADFRSLAPIVLEQALKESGTQLLEPYL  540

IRYGLEQGLFGWNVTDCKICFEYGLYYSPVSTPADFRSLAPIVLEQALKESGTQLLEPYL
Sbjct  481
IRYGLEQGLFGWNVTDCKICFEYGLYYSPVSTPADFRSLAPIVLEQALKESGTQLLEPYL  540

Query  541
SFILYAPQEYLSRAYHDAPKYCATIETAQVKKDEVVFTGEIPARCIQAYRTDLAFYTNGR  600

SFILYAPQEYLSRAYHDAPKYCATIETAQVKKDEVVFTGEIPARCIQAYRTDLAFYTNGR
Sbjct  541
SFILYAPQEYLSRAYHDAPKYCATIETAQVKKDEVVFTGEIPARCIQAYRTDLAFYTNGR  600

Query  601  SVCLTELKGYQAAVGQPVIQPRRPNSRLDKVRHMFQKV  638
```

```
            SVCLTELKGYQAAVGQPVIQPRRPNSRLDKVRHMF K+
Sbjct  601  SVCLTELKGYQAAVGQPVIQPRRPNSRLDKVRHMFSKI  638


>WP_071573697.1 TetM/TetW/TetO/TetS family tetracycline resistance
ribosomal
protection protein [Corynebacterium sp. NML120713]
 OIR41933.1 tetracycline resistance ribosomal protection protein
[Corynebacterium
sp. NML120713]
Length=643

 Score = 1289 bits (3336),  Expect = 0.0, Method: Compositional
matrix adjust.
 Identities = 626/638 (98%), Positives = 634/638 (99%), Gaps = 0/638
(0%)

Query  2
KIINIGILAHVDAGKTTLTESLLYASGAISEPGSVEKGTTRTDTMFLERQRGITIQAAVT  61

KIINIGILAHVDAGKTTLTESLLYASGAISEPGSVEKGTTRTDTMFLERQRGITIQAAVT
Sbjct  6
KIINIGILAHVDAGKTTLTESLLYASGAISEPGSVEKGTTRTDTMFLERQRGITIQAAVT  65

Query  62
SFQWHRCKVNIVDTPGHMDFLAEVYRSLAVLDGAILVISAKDGVQAQTRILFHALRKMNI  121
            SFQWHRCKVNIVDTPGHMDFLA
VYRSLAVLDGAILVISAKDGVQAQTRILFHALRKMNI
Sbjct  66
SFQWHRCKVNIVDTPGHMDFLAAVYRSLAVLDGAILVISAKDGVQAQTRILFHALRKMNI  125

Query  122
PTVIFINKIDQAGVDLQSVVQSVRDKLSADIIIKQTVSLSPEIVLEENTDIEAWDAVIEN  181

PTVIFINKIDQAGVDLQSVVQSVRDKLSADIIIKQTVSLSPEIVLEENTDIEAWDAVIEN
Sbjct  126
PTVIFINKIDQAGVDLQSVVQSVRDKLSADIIIKQTVSLSPEIVLEENTDIEAWDAVIEN  185

Query  182
NDKLLEKYIAGEPISREKLVREEQRRVQDASLFPVYYGSAKKGLGIQPLMDAVTGLFQPI  241

NDKLLEKYIAGEPISREKLVREEQRRVQDASLFPVYYGSAKKGLGIQPLMDAVTGLFQPI
Sbjct  186
NDKLLEKYIAGEPISREKLVREEQRRVQDASLFPVYYGSAKKGLGIQPLMDAVTGLFQPI  245

Query  242
GEQGSAALCGSVFKVEYTDCGQRRVYLRLYSGTLRLRDTVALAGREKLKITEMRIPSKGE  301
            GEQGSAALCGSVFKVEYT
+CGQRRVYLRLYSGTLRLRDTVALAGREKLKITEMRIPSKGE
Sbjct  246
GEQGSAALCGSVFKVEYTNCGQRRVYLRLYSGTLRLRDTVALAGREKLKITEMRIPSKGE  305

Query  302
IVRTDTAYPGEIVILPSDSVRLNDVLGDPTRLPRKRWREDPLPMLRTTIAPKTAAQRERL  361
```

```
            IVRTDTAYPGEIVILPSDSVRLNDVLGDPTRLPRKRWREDPLPMLRT
+IAPKTAAQRERL
Sbjct  306  
IVRTDTAYPGEIVILPSDSVRLNDVLGDPTRLPRKRWREDPLPMLRTSIAPKTAAQRERL  365

Query  362  
LDALTQLADTDPLLRCEVDSITHEIILSFLGRVQLEVVSALLSEKYKLETVVKEPSVIYM  421
            LDALTQLADTDPLLRCEVDSITHEIILSFLGRVQLEVVSALLSEKYKLETVVKEP
+VIYM
Sbjct  366  
LDALTQLADTDPLLRCEVDSITHEIILSFLGRVQLEVVSALLSEKYKLETVVKEPTVIYM  425

Query  422  
ERPLKAASHTIHIEVPPNPFWASIGLSVTPLSLGSGVQYESRVSLGYLNQSFQNAVRDGI  481
            ERPLKAASHTIHIEVPPNPFWASIGLSVTPL
LGSGVQYESRVSLGYLNQSFQNAVRDGI
Sbjct  426  
ERPLKAASHTIHIEVPPNPFWASIGLSVTPLPLGSGVQYESRVSLGYLNQSFQNAVRDGI  485

Query  482  
RYGLEQGLFGWNVTDCKICFEYGLYYSPVSTPADFRSLAPIVLEQALKESGTQLLEPYLS  541
            
RYGLEQGLFGWNVTDCKICFEYGLYYSPVSTPADFRSLAPIVLEQALKESGTQLLEPYLS
Sbjct  486  
RYGLEQGLFGWNVTDCKICFEYGLYYSPVSTPADFRSLAPIVLEQALKESGTQLLEPYLS  545

Query  542  
FILYAPQEYLSRAYHDAPKYCATIETAQVKKDEVVFTGEIPARCIQAYRTDLAFYTNGRS  601
            F LYAP+EYLSRAYHDAPKYCATIET
QVKKDEVVFTGEIPARCIQAYRTDLAFYTNG+S
Sbjct  546  
FTLYAPREYLSRAYHDAPKYCATIETVQVKKDEVVFTGEIPARCIQAYRTDLAFYTNGQS  605

Query  602  VCLTELKGYQAAVGQPVIQPRRPNSRLDKVRHMFQKVM  639
            VCLTELKGYQAAVG+PVIQPRRPNSRLDKVR+MFQK+M
Sbjct  606  VCLTELKGYQAAVGKPVIQPRRPNSRLDKVRYMFQKIM  643


>WP_069988272.1 MULTISPECIES: TetM/TetW/TetO/TetS family 
tetracycline resistance
ribosomal protection protein [Clostridiaceae]
Length=639

 Score = 1289 bits (3336),  Expect = 0.0, Method: Compositional 
matrix adjust.
 Identities = 627/638 (98%), Positives = 632/638 (99%), Gaps = 0/638 
(0%)

Query  1    
MKIINIGILAHVDAGKTTLTESLLYASGAISEPGSVEKGTTRTDTMFLERQRGITIQAAV  60
            
MKIINIGILAHVDAGKTTLTESLLYASGAISEPGSVEKGTTRTDTMFLERQRGITIQAAV
Sbjct  1    
MKIINIGILAHVDAGKTTLTESLLYASGAISEPGSVEKGTTRTDTMFLERQRGITIQAAV  60
```

Query 61
TSFQWHRCKVNIVDTPGHMDFLAEVYRSLAVLDGAILVISAKDGVQAQTRILFHALRKMN 120

TSFQWHRCKVNIVDTPGHMDFLAEVYRSLAVLDGAILVISAKDGVQAQTRILFHALRKMN
Sbjct 61
TSFQWHRCKVNIVDTPGHMDFLAEVYRSLAVLDGAILVISAKDGVQAQTRILFHALRKMN 120

Query 121
IPTVIFINKIDQAGVDLQSVVQSVRDKLSADIIIKQTVSLSPEIVLEENTDIEAWDAVIE 180

IPTVIFINKIDQAGVDLQSVVQSVRDKLSADIIIKQTVSLSPEIVLEENTDIEAWDAVIE
Sbjct 121
IPTVIFINKIDQAGVDLQSVVQSVRDKLSADIIIKQTVSLSPEIVLEENTDIEAWDAVIE 180

Query 181
NNDKLLEKYIAGEPISREKLVREEQRRVQDASLFPVYYGSAKKGLGIQPLMDAVTGLFQP 240
NND+LLEKYIAGEPISREKL REEQ+RVQDASLFPVY+GSAK
GLGIQPLMDAVTGLFQP
Sbjct 181
NNDELLEKYIAGEPISREKLAREEQQRVQDASLFPVYHGSAKNGLGIQPLMDAVTGLFQP 240

Query 241
IGEQGSAALCGSVFKVEYTDCGQRRVYLRLYSGTLRLRDTVALAGREKLKITEMRIPSKG 300
IGEQG
AALCGSVFKVEYTDCGQRRVYLRLYSGTLRLRDTVALAGREKLKITEMRIPSKG
Sbjct 241
IGEQGGAALCGSVFKVEYTDCGQRRVYLRLYSGTLRLRDTVALAGREKLKITEMRIPSKG 300

Query 301
EIVRTDTAYPGEIVILPSDSVRLNDVLGDPTRLPRKRWREDPLPMLRTTIAPKTAAQRER 360
EIVRTDTAY GEIVILPSDSVRLNDVLGD
TRLPRKRWREDPLPMLRTTIAPKTAAQRER
Sbjct 301
EIVRTDTAYQGEIVILPSDSVRLNDVLGDQTRLPRKRWREDPLPMLRTTIAPKTAAQRER 360

Query 361
LLDALTQLADTDPLLRCEVDSITHEIILSFLGRVQLEVVSALLSEKYKLETVVKEPSVIY 420

LLDALTQLADTDPLLRCEVDSITHEIILSFLGRVQLEVVSALLSEKYKLETVVKEPSVIY
Sbjct 361
LLDALTQLADTDPLLRCEVDSITHEIILSFLGRVQLEVVSALLSEKYKLETVVKEPSVIY 420

Query 421
MERPLKAASHTIHIEVPPNPFWASIGLSVTPLSLGSGVQYESRVSLGYLNQSFQNAVRDG 480

MERPLKAASHTIHIEVPPNPFWASIGLSVTPLSLGSGVQYESRVSLGYLNQSFQNAVRDG
Sbjct 421
MERPLKAASHTIHIEVPPNPFWASIGLSVTPLSLGSGVQYESRVSLGYLNQSFQNAVRDG 480

Query 481
IRYGLEQGLFGWNVTDCKICFEYGLYYSPVSTPADFRSLAPIVLEQALKESGTQLLEPYL 540

IRYGLEQGLFGWNVTDCKICFEYGLYYSPVSTPADFRSLAPIVLEQALKESGTQLLEPYL

```
Sbjct  481
IRYGLEQGLFGWNVTDCKICFEYGLYYSPVSTPADFRSLAPIVLEQALKESGTQLLEPYL  540

Query  541
SFILYAPQEYLSRAYHDAPKYCATIETAQVKKDEVVFTGEIPARCIQAYRTDLAFYTNGR  600

SFILYAPQEYLSRAYHDAPKYCATIETAQVKKDEVVFTGEIPARCIQAYRTDLAFYTNGR
Sbjct  541
SFILYAPQEYLSRAYHDAPKYCATIETAQVKKDEVVFTGEIPARCIQAYRTDLAFYTNGR  600

Query  601  SVCLTELKGYQAAVGQPVIQPRRPNSRLDKVRHMFQKV  638
            SVCLTELKGYQAAVGQPVIQPRRPNSR+DKVRHMF K+
Sbjct  601  SVCLTELKGYQAAVGQPVIQPRRPNSRIDKVRHMFHKL  638


>SCI17595.1 Tetracycline resistance protein tetM from transposon
Tn916 [uncultured
Ruminococcus sp.]
Length=639

 Score = 1289 bits (3336),  Expect = 0.0, Method: Compositional
matrix adjust.
 Identities = 627/639 (98%), Positives = 633/639 (99%), Gaps = 0/639
(0%)

Query  1
MKIINIGILAHVDAGKTTLTESLLYASGAISEPGSVEKGTTRTDTMFLERQRGITIQAAV  60

MKIINIGILAHVDAGKTTLTESLLYASGAISEPGSVEKGTTRTDTMFLERQRGITIQAAV
Sbjct  1
MKIINIGILAHVDAGKTTLTESLLYASGAISEPGSVEKGTTRTDTMFLERQRGITIQAAV  60

Query  61
TSFQWHRCKVNIVDTPGHMDFLAEVYRSLAVLDGAILVISAKDGVQAQTRILFHALRKMN  120

TSFQWHRCKVNIVDTPGHMDFLAEVYRSLAVLDGAILVISAKDGVQAQTRILFHALRKMN
Sbjct  61
TSFQWHRCKVNIVDTPGHMDFLAEVYRSLAVLDGAILVISAKDGVQAQTRILFHALRKMN  120

Query  121
IPTVIFINKIDQAGVDLQSVVQSVRDKLSADIIIKQTVSLSPEIVLEENTDIEAWDAVIE  180

IPTVIFINKIDQAGVDLQSVVQSVRDKLSADIIIKQTVSLSPEIVLEENTDIEAWDAVIE
Sbjct  121
IPTVIFINKIDQAGVDLQSVVQSVRDKLSADIIIKQTVSLSPEIVLEENTDIEAWDAVIE  180

Query  181
NNDKLLEKYIAGEPISREKLVREEQRRVQDASLFPVYYGSAKKGLGIQPLMDAVTGLFQP  240
            NND LLEKYIAGEPIS+EKL REEQRRVQ+ASLFPVY
+GSAKKGLGIQPLMDAVTGLFQP
Sbjct  181
NNDALLEKYIAGEPISQEKLAREEQRRVQEASLFPVYHGSAKKGLGIQPLMDAVTGLFQP  240

Query  241
```

IGEQGSAALCGSVFKVEYTDCGQRRVYLRLYSGTLRLRDTVALAGREKLKITEMRIPSKG 300
            IGEQGSA
LCGSVFKVEYTDCGQRRVYLRLYSGTLRLRDTVALAGREKLKITEMRIPSKG
Sbjct 241
IGEQGSATLCGSVFKVEYTDCGQRRVYLRLYSGTLRLRDTVALAGREKLKITEMRIPSKG 300

Query 301
EIVRTDTAYPGEIVILPSDSVRLNDVLGDPTRLPRKRWREDPLPMLRTTIAPKTAAQRER 360
            EIVRTDTAYPGEIVILPSDSVRLNDVLGD
TRLPRKRWREDPLPMLRTTIAPKTAAQRER
Sbjct 301
EIVRTDTAYPGEIVILPSDSVRLNDVLGDQTRLPRKRWREDPLPMLRTTIAPKTAAQRER 360

Query 361
LLDALTQLADTDPLLRCEVDSITHEIILSFLGRVQLEVVSALLSEKYKLETVVKEPSVIY 420
            LLDALTQLADTDPLLRCEVDSITHEIILSFLGRVQLEVVSALLSEKYKLETVVKEP
+VIY
Sbjct 361
LLDALTQLADTDPLLRCEVDSITHEIILSFLGRVQLEVVSALLSEKYKLETVVKEPTVIY 420

Query 421
MERPLKAASHTIHIEVPPNPFWASIGLSVTPLSLGSGVQYESRVSLGYLNQSFQNAVRDG 480
            MERPLKAASHTIHIEVPPNPFWASIGLSVTPL
LGSGVQYESRVSLGYLNQSFQNAVRDG
Sbjct 421
MERPLKAASHTIHIEVPPNPFWASIGLSVTPLPLGSGVQYESRVSLGYLNQSFQNAVRDG 480

Query 481
IRYGLEQGLFGWNVTDCKICFEYGLYYSPVSTPADFRSLAPIVLEQALKESGTQLLEPYL 540

IRYGLEQGLFGWNVTDCKICFEYGLYYSPVSTPADFRSLAPIVLEQALKESGTQLLEPYL
Sbjct 481
IRYGLEQGLFGWNVTDCKICFEYGLYYSPVSTPADFRSLAPIVLEQALKESGTQLLEPYL 540

Query 541
SFILYAPQEYLSRAYHDAPKYCATIETAQVKKDEVVFTGEIPARCIQAYRTDLAFYTNGR 600
            SF
LYAPQEYLSRAYHDAPKYCATIETAQVKKDEVVFTGEIPARCIQAYRTDLAFYTNG+
Sbjct 541
SFTLYAPQEYLSRAYHDAPKYCATIETAQVKKDEVVFTGEIPARCIQAYRTDLAFYTNGQ 600

Query 601 SVCLTELKGYQAAVGQPVIQPRRPNSRLDKVRHMFQKVM 639
            SVCLTELKGYQAAVGQPVIQPRRPN+RLDKVRHMFQKVM
Sbjct 601 SVCLTELKGYQAAVGQPVIQPRRPNNRLDKVRHMFQKVM 639

>ACT97618.1 tetracycline resistance protein tetW [uncultured organism]
Length=639

Score = 1289 bits (3336), Expect = 0.0, Method: Compositional matrix adjust.
Identities = 629/639 (98%), Positives = 632/639 (99%), Gaps = 0/639 (0%)

Query 1
MKIINIGILAHVDAGKTTLTESLLYASGAISEPGSVEKGTTRTDTMFLERQRGITIQAAV 60
            MKIINIGILAHV
GKTTLTESLLYASGAISEPGSVEKGTTRTDTMFLERQRGITIQAAV
Sbjct 1
MKIINIGILAHVFFGKTTLTESLLYASGAISEPGSVEKGTTRTDTMFLERQRGITIQAAV 60

Query 61
TSFQWHRCKVNIVDTPGHMDFLAEVYRSLAVLDGAILVISAKDGVQAQTRILFHALRKMN 120

TSFQWHRCKVNIVDTPGHMDFLAEVYRSLAVLDGAILVISAKDGVQAQTRILFHALRKMN
Sbjct 61
TSFQWHRCKVNIVDTPGHMDFLAEVYRSLAVLDGAILVISAKDGVQAQTRILFHALRKMN 120

Query 121
IPTVIFINKIDQAGVDLQSVVQSVRDKLSADIIIKQTVSLSPEIVLEENTDIEAWDAVIE 180

IPTVIFINKIDQAGVDLQSVVQSVRDKLSADIIIKQTVSLSPEIVLEENTDIEAWDAVIE
Sbjct 121
IPTVIFINKIDQAGVDLQSVVQSVRDKLSADIIIKQTVSLSPEIVLEENTDIEAWDAVIE 180

Query 181
NNDKLLEKYIAGEPISREKLVREEQRRVQDASLFPVYYGSAKKGLGIQPLMDAVTGLFQP 240
            NND+LLEKYIAGEPISREKL REEQ+RVQDASLFPVY+GSAK
GLGIQPLMDAVTGLFQP
Sbjct 181
NNDELLEKYIAGEPISREKLAREEQQRVQDASLFPVYHGSAKNGLGIQPLMDAVTGLFQP 240

Query 241
IGEQGSAALCGSVFKVEYTDCGQRRVYLRLYSGTLRLRDTVALAGREKLKITEMRIPSKG 300
            IGEQG
AALCGSVFKVEYTDCGQRRVYLRLYSGTLRLRDTVALAGREKLKITEMRIPSKG
Sbjct 241
IGEQGGAALCGSVFKVEYTDCGQRRVYLRLYSGTLRLRDTVALAGREKLKITEMRIPSKG 300

Query 301
EIVRTDTAYPGEIVILPSDSVRLNDVLGDPTRLPRKRWREDPLPMLRTTIAPKTAAQRER 360
            EIVRTDTAY GEIVILPSDSVRLNDVLGD
TRLPRKRWREDPLPMLRTTIAPKTAAQRER
Sbjct 301
EIVRTDTAYQGEIVILPSDSVRLNDVLGDQTRLPRKRWREDPLPMLRTTIAPKTAAQRER 360

Query 361
LLDALTQLADTDPLLRCEVDSITHEIILSFLGRVQLEVVSALLSEKYKLETVVKEPSVIY 420

LLDALTQLADTDPLLRCEVDSITHEIILSFLGRVQLEVVSALLSEKYKLETVVKEPSVIY
Sbjct 361
LLDALTQLADTDPLLRCEVDSITHEIILSFLGRVQLEVVSALLSEKYKLETVVKEPSVIY 420

Query 421
MERPLKAASHTIHIEVPPNPFWASIGLSVTPLSLGSGVQYESRVSLGYLNQSFQNAVRDG 480

MERPLKAASHTIHIEVPPNPFWASIGLSVTPLSLGSGVQYESRVSLGYLNQSFQNAVRDG

Sbjct 421
MERPLKAASHTIHIEVPPNPFWASIGLSVTPLSLGSGVQYESRVSLGYLNQSFQNAVRDG 480

Query 481
IRYGLEQGLFGWNVTDCKICFEYGLYYSPVSTPADFRSLAPIVLEQALKESGTQLLEPYL 540

IRYGLEQGLFGWNVTDCKICFEYGLYYSPVSTPADFRSLAPIVLEQALKESGTQLLEPYL
Sbjct 481
IRYGLEQGLFGWNVTDCKICFEYGLYYSPVSTPADFRSLAPIVLEQALKESGTQLLEPYL 540

Query 541
SFILYAPQEYLSRAYHDAPKYCATIETAQVKKDEVVFTGEIPARCIQAYRTDLAFYTNGR 600

SFILYAPQEYLSRAYHDAPKYCATIETAQVKKDEVVFTGEIPARCIQAYRTDLAFYTNGR
Sbjct 541
SFILYAPQEYLSRAYHDAPKYCATIETAQVKKDEVVFTGEIPARCIQAYRTDLAFYTNGR 600

Query 601 SVCLTELKGYQAAVGQPVIQPRRPNSRLDKVRHMFQKVM 639
            SVCLTELKGYQAAVGQPVIQPRRPNSRLDKVRHMFQKVM
Sbjct 601 SVCLTELKGYQAAVGQPVIQPRRPNSRLDKVRHMFQKVM 639

>ACT97617.1 tetracycline resistance protein tetW [uncultured organism]
Length=639

Score = 1289 bits (3336), Expect = 0.0, Method: Compositional matrix adjust.
Identities = 628/639 (98%), Positives = 632/639 (99%), Gaps = 0/639 (0%)

Query 1
MKIINIGILAHVDAGKTTLTESLLYASGAISEPGSVEKGTTRTDTMFLERQRGITIQAAV 60
            MKIINIGILAH
+GKTTLTESLLYASGAISEPGSVEKGTTRTDTMFLERQRGITIQAAV
Sbjct 1
MKIINIGILAHFFSGKTTLTESLLYASGAISEPGSVEKGTTRTDTMFLERQRGITIQAAV 60

Query 61
TSFQWHRCKVNIVDTPGHMDFLAEVYRSLAVLDGAILVISAKDGVQAQTRILFHALRKMN 120

TSFQWHRCKVNIVDTPGHMDFLAEVYRSLAVLDGAILVISAKDGVQAQTRILFHALRKMN
Sbjct 61
TSFQWHRCKVNIVDTPGHMDFLAEVYRSLAVLDGAILVISAKDGVQAQTRILFHALRKMN 120

Query 121
IPTVIFINKIDQAGVDLQSVVQSVRDKLSADIIIKQTVSLSPEIVLEENTDIEAWDAVIE 180

IPTVIFINKIDQAGVDLQSVVQSVRDKLSADIIIKQTVSLSPEIVLEENTDIEAWDAVIE
Sbjct 121
IPTVIFINKIDQAGVDLQSVVQSVRDKLSADIIIKQTVSLSPEIVLEENTDIEAWDAVIE 180

Query 181
NNDKLLEKYIAGEPISREKLVREEQRRVQDASLFPVYYGSAKKGLGIQPLMDAVTGLFQP 240

NND+LLEKYIAGEPISREKL REEQ+RVQDASLFPVY+GSAK
GLGIQPLMDAVTGLFQP
Sbjct 181
NNDELLEKYIAGEPISREKLAREEQQRVQDASLFPVYHGSAKNGLGIQPLMDAVTGLFQP 240

Query 241
IGEQGSAALCGSVFKVEYTDCGQRRVYLRLYSGTLRLRDTVALAGREKLKITEMRIPSKG 300
IGEQG
AALCGSVFKVEYTDCGQRRVYLRLYSGTLRLRDTVALAGREKLKITEMRIPSKG
Sbjct 241
IGEQGGAALCGSVFKVEYTDCGQRRVYLRLYSGTLRLRDTVALAGREKLKITEMRIPSKG 300

Query 301
EIVRTDTAYPGEIVILPSDSVRLNDVLGDPTRLPRKRWREDPLPMLRTTIAPKTAAQRER 360
EIVRTDTAY GEIVILPSDSVRLNDVLGD
TRLPRKRWREDPLPMLRTTIAPKTAAQRER
Sbjct 301
EIVRTDTAYQGEIVILPSDSVRLNDVLGDQTRLPRKRWREDPLPMLRTTIAPKTAAQRER 360

Query 361
LLDALTQLADTDPLLRCEVDSITHEIILSFLGRVQLEVVSALLSEKYKLETVVKEPSVIY 420

LLDALTQLADTDPLLRCEVDSITHEIILSFLGRVQLEVVSALLSEKYKLETVVKEPSVIY
Sbjct 361
LLDALTQLADTDPLLRCEVDSITHEIILSFLGRVQLEVVSALLSEKYKLETVVKEPSVIY 420

Query 421
MERPLKAASHTIHIEVPPNPFWASIGLSVTPLSLGSGVQYESRVSLGYLNQSFQNAVRDG 480

MERPLKAASHTIHIEVPPNPFWASIGLSVTPLSLGSGVQYESRVSLGYLNQSFQNAVRDG
Sbjct 421
MERPLKAASHTIHIEVPPNPFWASIGLSVTPLSLGSGVQYESRVSLGYLNQSFQNAVRDG 480

Query 481
IRYGLEQGLFGWNVTDCKICFEYGLYYSPVSTPADFRSLAPIVLEQALKESGTQLLEPYL 540

IRYGLEQGLFGWNVTDCKICFEYGLYYSPVSTPADFRSLAPIVLEQALKESGTQLLEPYL
Sbjct 481
IRYGLEQGLFGWNVTDCKICFEYGLYYSPVSTPADFRSLAPIVLEQALKESGTQLLEPYL 540

Query 541
SFILYAPQEYLSRAYHDAPKYCATIETAQVKKDEVVFTGEIPARCIQAYRTDLAFYTNGR 600

SFILYAPQEYLSRAYHDAPKYCATIETAQVKKDEVVFTGEIPARCIQAYRTDLAFYTNGR
Sbjct 541
SFILYAPQEYLSRAYHDAPKYCATIETAQVKKDEVVFTGEIPARCIQAYRTDLAFYTNGR 600

Query 601 SVCLTELKGYQAAVGQPVIQPRRPNSRLDKVRHMFQKVM 639
SVCLTELKGYQAAVGQPVIQPRRPNSRLDKVRHMFQKVM
Sbjct 601 SVCLTELKGYQAAVGQPVIQPRRPNSRLDKVRHMFQKVM 639

>APO28173.1 ferrous iron transport protein B [uncultured bacterium]
Length=639

Score = 1289 bits (3335), Expect = 0.0, Method: Compositional matrix adjust.
Identities = 627/639 (98%), Positives = 634/639 (99%), Gaps = 0/639 (0%)

```
Query  1
MKIINIGILAHVDAGKTTLTESLLYASGAISEPGSVEKGTTRTDTMFLERQRGITIQAAV  60

MKIINIGILAHVDAGKTTLTESLLYASGAISEPGSVEKGTTRTDTMFLERQRGITIQAAV
Sbjct  1
MKIINIGILAHVDAGKTTLTESLLYASGAISEPGSVEKGTTRTDTMFLERQRGITIQAAV  60

Query  61
TSFQWHRCKVNIVDTPGHMDFLAEVYRSLAVLDGAILVISAKDGVQAQTRILFHALRKMN  120

TSFQWHRCKVNIVDTPGHMDFLAEVYRSLAVLDGAILVISAKDGVQAQTRILFHALRKMN
Sbjct  61
TSFQWHRCKVNIVDTPGHMDFLAEVYRSLAVLDGAILVISAKDGVQAQTRILFHALRKMN  120

Query  121
IPTVIFINKIDQAGVDLQSVVQSVRDKLSADIIIKQTVSLSPEIVLEENTDIEAWDAVIE  180

IPTVIFINKIDQAGVDLQSVVQSVRDKLSADIIIKQTVSLSPEIVLEENTDIEAWDAVIE
Sbjct  121
IPTVIFINKIDQAGVDLQSVVQSVRDKLSADIIIKQTVSLSPEIVLEENTDIEAWDAVIE  180

Query  181
NNDKLLEKYIAGEPISREKLVREEQRRVQDASLFPVYYGSAKKGLGIQPLMDAVTGLFQP  240
            NND+LLEKYIAGEPISREKL REEQ+RVQDASLFPVY+GSAK
GLGIQPLMDAVTGLFQP
Sbjct  181
NNDELLEKYIAGEPISREKLAREEQQRVQDASLFPVYHGSAKNGLGIQPLMDAVTGLFQP  240

Query  241
IGEQGSAALCGSVFKVEYTDCGQRRVYLRLYSGTLRLRDTVALAGREKLKITEMRIPSKG  300
            IGEQG
AALCGSVFKVEYTDCGQRRVYLRLYSGTLRLRDTVALAGREKLKITEMRIPSKG
Sbjct  241
IGEQGGAALCGSVFKVEYTDCGQRRVYLRLYSGTLRLRDTVALAGREKLKITEMRIPSKG  300

Query  301
EIVRTDTAYPGEIVILPSDSVRLNDVLGDPTRLPRKRWREDPLPMLRTTIAPKTAAQRER  360
            EIVRTDTAY GEIVILPSDSVRLNDVLGD
TRLPRKRWREDPLPMLRTTIAPKTAAQRER
Sbjct  301
EIVRTDTAYQGEIVILPSDSVRLNDVLGDQTRLPRKRWREDPLPMLRTTIAPKTAAQRER  360

Query  361
LLDALTQLADTDPLLRCEVDSITHEIILSFLGRVQLEVVSALLSEKYKLETVVKEPSVIY  420

LLDALTQLADTDPLLRCEVDSITHEIILSFLGRVQLEVVSALLSEKYKLETVVKEPSVIY
Sbjct  361
LLDALTQLADTDPLLRCEVDSITHEIILSFLGRVQLEVVSALLSEKYKLETVVKEPSVIY  420
```

```
Query  421
MERPLKAASHTIHIEVPPNPFWASIGLSVTPLSLGSGVQYESRVSLGYLNQSFQNAVRDG  480

MERPLKAASHTIHIEVPPNPFWASIGLSVTPLSLGSGVQYESRVSLGYLNQSFQNAVRDG
Sbjct  421
MERPLKAASHTIHIEVPPNPFWASIGLSVTPLSLGSGVQYESRVSLGYLNQSFQNAVRDG  480

Query  481
IRYGLEQGLFGWNVTDCKICFEYGLYYSPVSTPADFRSLAPIVLEQALKESGTQLLEPYL  540

IRYGLEQGLFGWNVTDCKICFEYGLYYSPVSTPADFRSLAPIVLEQALKESGTQLLEPYL
Sbjct  481
IRYGLEQGLFGWNVTDCKICFEYGLYYSPVSTPADFRSLAPIVLEQALKESGTQLLEPYL  540

Query  541
SFILYAPQEYLSRAYHDAPKYCATIETAQVKKDEVVFTGEIPARCIQAYRTDLAFYTNGR  600

SFILYAPQEYLSRAYHDAPKYCATIETAQVKKDEVVFTGEIPARCIQAYRTDLAFYTNG+
Sbjct  541
SFILYAPQEYLSRAYHDAPKYCATIETAQVKKDEVVFTGEIPARCIQAYRTDLAFYTNGQ  600

Query  601  SVCLTELKGYQAAVGQPVIQPRRPNSRLDKVRHMFQKVM  639
            SVCLTELKGYQAAVG+PVIQPRRPNSRLDKVR+MFQK+M
Sbjct  601  SVCLTELKGYQAAVGKPVIQPRRPNSRLDKVRYMFQKIM  639


>WP_072369412.1 TetM/TetW/TetO/TetS family tetracycline resistance
ribosomal
protection protein [Olsenella mediterranea]
Length=639

 Score = 1289 bits (3335),  Expect = 0.0, Method: Compositional
matrix adjust.
 Identities = 626/639 (98%), Positives = 634/639 (99%), Gaps = 0/639
(0%)

Query  1
MKIINIGILAHVDAGKTTLTESLLYASGAISEPGSVEKGTTRTDTMFLERQRGITIQAAV  60

MKIINIGILAHVDAGKTTLTESLLYASGAISEPGSVEKGTTRTDTMFLERQRGITIQAAV
Sbjct  1
MKIINIGILAHVDAGKTTLTESLLYASGAISEPGSVEKGTTRTDTMFLERQRGITIQAAV  60

Query  61
TSFQWHRCKVNIVDTPGHMDFLAEVYRSLAVLDGAILVISAKDGVQAQTRILFHALRKMN  120

TSFQWHRCKVNIVDTPGHMDFLAEVYRSLAVLDGAILVISAKDGVQAQTRILFHALRKMN
Sbjct  61
TSFQWHRCKVNIVDTPGHMDFLAEVYRSLAVLDGAILVISAKDGVQAQTRILFHALRKMN  120

Query  121
IPTVIFINKIDQAGVDLQSVVQSVRDKLSADIIIKQTVSLSPEIVLEENTDIEAWDAVIE  180
```

IPTVIFINKIDQAGVDLQSVVQSVRDKLSADIIIKQTVSLSPEIVLEENTDIEAWDAVIE
Sbjct 121
IPTVIFINKIDQAGVDLQSVVQSVRDKLSADIIIKQTVSLSPEIVLEENTDIEAWDAVIE 180

Query 181
NNDKLLEKYIAGEPISREKLVREEQRRVQDASLFPVYYGSAKKGLGIQPLMDAVTGLFQP 240
NND+LLEKYIAGEPISREKL REEQ+RVQDASLFPVYYGSAK
GLGIQPLMDAVTGLFQP
Sbjct 181
NNDELLEKYIAGEPISREKLAREEQQRVQDASLFPVYYGSAKNGLGIQPLMDAVTGLFQP 240

Query 241
IGEQGSAALCGSVFKVEYTDCGQRRVYLRLYSGTLRLRDTVALAGREKLKITEMRIPSKG 300

IGEQGSAALCGSVFKVEYTDCGQRRVYLRLYSGTLRLRDTVALAGREKLKITEMRIPSKG
Sbjct 241
IGEQGSAALCGSVFKVEYTDCGQRRVYLRLYSGTLRLRDTVALAGREKLKITEMRIPSKG 300

Query 301
EIVRTDTAYPGEIVILPSDSVRLNDVLGDPTRLPRKRWREDPLPMLRTTIAPKTAAQRER 360
EIVRTDTAYPGEIVILPSDSVRLNDVLGDPTRLPRKRWREDPLPMLRT
+IAPKTAAQRER
Sbjct 301
EIVRTDTAYPGEIVILPSDSVRLNDVLGDPTRLPRKRWREDPLPMLRTSIAPKTAAQRER 360

Query 361
LLDALTQLADTDPLLRCEVDSITHEIILSFLGRVQLEVVSALLSEKYKLETVVKEPSVIY 420
LLDALTQLADTDPLLRCEVDSITHEIILSFLGRVQLEVVSALLSEKYKLETVVKEP
+VIY
Sbjct 361
LLDALTQLADTDPLLRCEVDSITHEIILSFLGRVQLEVVSALLSEKYKLETVVKEPTVIY 420

Query 421
MERPLKAASHTIHIEVPPNPFWASIGLSVTPLSLGSGVQYESRVSLGYLNQSFQNAVRDG 480
MERPLKAASHTIHIEVPPNPFWASIGLSVTPL
LGSGVQYESRVSLGYLNQSFQNAVRDG
Sbjct 421
MERPLKAASHTIHIEVPPNPFWASIGLSVTPLPLGSGVQYESRVSLGYLNQSFQNAVRDG 480

Query 481
IRYGLEQGLFGWNVTDCKICFEYGLYYSPVSTPADFRSLAPIVLEQALKESGTQLLEPYL 540

IRYGLEQGLFGWNVTDCKICFEYGLYYSPVSTPADFRSLAPIVLEQALKESGTQLLEPYL
Sbjct 481
IRYGLEQGLFGWNVTDCKICFEYGLYYSPVSTPADFRSLAPIVLEQALKESGTQLLEPYL 540

Query 541
SFILYAPQEYLSRAYHDAPKYCATIETAQVKKDEVVFTGEIPARCIQAYRTDLAFYTNGR 600
SF LYAP+EYLSRAYHDAPKYCATIET
QVKKDEVVFTGEIPARCIQAYRTDLAFYTNG+
Sbjct 541
SFTLYAPREYLSRAYHDAPKYCATIETVQVKKDEVVFTGEIPARCIQAYRTDLAFYTNGQ 600

Query 601 SVCLTELKGYQAAVGQPVIQPRRPNSRLDKVRHMFQKVM 639

SVCLTELKGYQAAVG+PVIQPRRPNSRLDKVR+MFQKVM
Sbjct 601 SVCLTELKGYQAAVGKPVIQPRRPNSRLDKVRYMFQKVM 639

>WP_063164311.1 TetM/TetW/TetO/TetS family tetracycline resistance ribosomal
protection protein [Lactobacillus reuteri]
AMY14099.1 tetracycline resistance ribosomal protection protein [Lactobacillus
reuteri]
Length=639

Score = 1289 bits (3335), Expect = 0.0, Method: Compositional matrix adjust.
Identities = 627/639 (98%), Positives = 634/639 (99%), Gaps = 0/639 (0%)

Query 1
MKIINIGILAHVDAGKTTLTESLLYASGAISEPGSVEKGTTRTDTMFLERQRGITIQAAV 60
MKIINIGILAHVDAGKTTLTESLLYASGAISEPGSV
KGTTRTDTMFLERQRGITIQAAV
Sbjct 1
MKIINIGILAHVDAGKTTLTESLLYASGAISEPGSVAKGTTRTDTMFLERQRGITIQAAV 60

Query 61
TSFQWHRCKVNIVDTPGHMDFLAEVYRSLAVLDGAILVISAKDGVQAQTRILFHALRKMN 120

TSFQWHRCKVNIVDTPGHMDFLAEVYRSLAVLDGAILVISAKDGVQAQTRILFHALRKMN
Sbjct 61
TSFQWHRCKVNIVDTPGHMDFLAEVYRSLAVLDGAILVISAKDGVQAQTRILFHALRKMN 120

Query 121
IPTVIFINKIDQAGVDLQSVVQSVRDKLSADIIIKQTVSLSPEIVLEENTDIEAWDAVIE 180

IPTVIFINKIDQAGVDLQSVVQSVRDKLSADIIIKQTVSLSPEIVLEENTDIEAWDAVIE
Sbjct 121
IPTVIFINKIDQAGVDLQSVVQSVRDKLSADIIIKQTVSLSPEIVLEENTDIEAWDAVIE 180

Query 181
NNDKLLEKYIAGEPISREKLVREEQRRVQDASLFPVYYGSAKKGLGIQPLMDAVTGLFQP 240

NNDKLLEKYIAGEPISREKLVREEQRRVQDASLFPVYYGSAKKGLGIQPLMDAVTGLFQP
Sbjct 181
NNDKLLEKYIAGEPISREKLVREEQRRVQDASLFPVYYGSAKKGLGIQPLMDAVTGLFQP 240

Query 241
IGEQGSAALCGSVFKVEYTDCGQRRVYLRLYSGTLRLRDTVALAGREKLKITEMRIPSKG 300

IGEQGSAALCGSVFKVEYTDCGQRRVYLRLYSGTLRLRDTVALAGREKLKITEMRIPSKG
Sbjct 241
IGEQGSAALCGSVFKVEYTDCGQRRVYLRLYSGTLRLRDTVALAGREKLKITEMRIPSKG 300

Query 301
EIVRTDTAYPGEIVILPSDSVRLNDVLGDPTRLPRKRWREDPLPMLRTTIAPKTAAQRER 360

EIVRTDTAYPGEIVILPSDSVRLNDVLGDPTRLPRKRWREDPLPMLRT
+IAPKTAAQRER
Sbjct 301
EIVRTDTAYPGEIVILPSDSVRLNDVLGDPTRLPRKRWREDPLPMLRTSIAPKTAAQRER 360

Query 361
LLDALTQLADTDPLLRCEVDSITHEIILSFLGRVQLEVVSALLSEKYKLETVVKEPSVIY 420
LLDALTQLADTDPLLR EVDSITHEIILSFLGRVQLEVVSALLSEKYKLETVVKEP
+VIY
Sbjct 361
LLDALTQLADTDPLLRYEVDSITHEIILSFLGRVQLEVVSALLSEKYKLETVVKEPTVIY 420

Query 421
MERPLKAASHTIHIEVPPNPFWASIGLSVTPLSLGSGVQYESRVSLGYLNQSFQNAVRDG 480
MERPLKAASHTIHIEVPPNPFWASIGLSVTPL
LGSGVQYESRVSLGYLNQSFQNAVRDG
Sbjct 421
MERPLKAASHTIHIEVPPNPFWASIGLSVTPLPLGSGVQYESRVSLGYLNQSFQNAVRDG 480

Query 481
IRYGLEQGLFGWNVTDCKICFEYGLYYSPVSTPADFRSLAPIVLEQALKESGTQLLEPYL 540

IRYGLEQGLFGWNVTDCKICFEYGLYYSPVSTPADFRSLAPIVLEQALKESGTQLLEPYL
Sbjct 481
IRYGLEQGLFGWNVTDCKICFEYGLYYSPVSTPADFRSLAPIVLEQALKESGTQLLEPYL 540

Query 541
SFILYAPQEYLSRAYHDAPKYCATIETAQVKKDEVVFTGEIPARCIQAYRTDLAFYTNGR 600
SF LYAP+EYLSRAYHDAPKYCATIET
QVKKDEVVFTGEIPARCIQAYRTDLAFYTNG+
Sbjct 541
SFTLYAPREYLSRAYHDAPKYCATIETVQVKKDEVVFTGEIPARCIQAYRTDLAFYTNGQ 600

Query 601 SVCLTELKGYQAAVGQPVIQPRRPNSRLDKVRHMFQKVM 639
SVCLTELKGYQAAVG+PVIQPRRPNSRLDKVR+MFQK+M
Sbjct 601 SVCLTELKGYQAAVGKPVIQPRRPNSRLDKVRYMFQKIM 639

>AMP53586.1 TetM-TetW-TetO-TetS [uncultured bacterium]
Length=646

Score = 1289 bits (3335), Expect = 0.0, Method: Compositional
matrix adjust.
Identities = 628/637 (99%), Positives = 631/637 (99%), Gaps = 0/637
(0%)

Query 1
MKIINIGILAHVDAGKTTLTESLLYASGAISEPGSVEKGTTRTDTMFLERQRGITIQAAV 60

MKIINIGILAHVDAGKTTLTESLLYASGAISEPGSVEKGTTRTDTMFLERQRGITIQAAV
Sbjct 1
MKIINIGILAHVDAGKTTLTESLLYASGAISEPGSVEKGTTRTDTMFLERQRGITIQAAV 60

Query 61

TSFQWHRCKVNIVDTPGHMDFLAEVYRSLAVLDGAILVISAKDGVQAQTRILFHALRKMN 120

TSFQWHRCKVNIVDTPGHMDFLAEVYRSLAVLDGAILVISAKDGVQAQTRILFHALRKMN
Sbjct 61
TSFQWHRCKVNIVDTPGHMDFLAEVYRSLAVLDGAILVISAKDGVQAQTRILFHALRKMN 120

Query 121
IPTVIFINKIDQAGVDLQSVVQSVRDKLSADIIIKQTVSLSPEIVLEENTDIEAWDAVIE 180

IPTVIFINKIDQAGVDLQSVVQSVRDKLSADIIIKQTVSLSPEIVLEENTDIEAWDAVIE
Sbjct 121
IPTVIFINKIDQAGVDLQSVVQSVRDKLSADIIIKQTVSLSPEIVLEENTDIEAWDAVIE 180

Query 181
NNDKLLEKYIAGEPISREKLVREEQRRVQDASLFPVYYGSAKKGLGIQPLMDAVTGLFQP 240
NND+LLEKYIAGEPISREKL REEQ+RVQDASLFPVY+GSAK
GLGIQPLMDAVTGLFQP
Sbjct 181
NNDELLEKYIAGEPISREKLAREEQQRVQDASLFPVYHGSAKNGLGIQPLMDAVTGLFQP 240

Query 241
IGEQGSAALCGSVFKVEYTDCGQRRVYLRLYSGTLRLRDTVALAGREKLKITEMRIPSKG 300
IGEQG
AALCGSVFKVEYTDCGQRRVYLRLYSGTLRLRDTVALAGREKLKITEMRIPSKG
Sbjct 241
IGEQGGAALCGSVFKVEYTDCGQRRVYLRLYSGTLRLRDTVALAGREKLKITEMRIPSKG 300

Query 301
EIVRTDTAYPGEIVILPSDSVRLNDVLGDPTRLPRKRWREDPLPMLRTTIAPKTAAQRER 360
EIVRTDTAY GEIVILPSDSVRLNDVLGD
TRLPRKRWREDPLPMLRTTIAPKTAAQRER
Sbjct 301
EIVRTDTAYQGEIVILPSDSVRLNDVLGDQTRLPRKRWREDPLPMLRTTIAPKTAAQRER 360

Query 361
LLDALTQLADTDPLLRCEVDSITHEIILSFLGRVQLEVVSALLSEKYKLETVVKEPSVIY 420

LLDALTQLADTDPLLRCEVDSITHEIILSFLGRVQLEVVSALLSEKYKLETVVKEPSVIY
Sbjct 361
LLDALTQLADTDPLLRCEVDSITHEIILSFLGRVQLEVVSALLSEKYKLETVVKEPSVIY 420

Query 421
MERPLKAASHTIHIEVPPNPFWASIGLSVTPLSLGSGVQYESRVSLGYLNQSFQNAVRDG 480

MERPLKAASHTIHIEVPPNPFWASIGLSVTPLSLGSGVQYESRVSLGYLNQSFQNAVRDG
Sbjct 421
MERPLKAASHTIHIEVPPNPFWASIGLSVTPLSLGSGVQYESRVSLGYLNQSFQNAVRDG 480

Query 481
IRYGLEQGLFGWNVTDCKICFEYGLYYSPVSTPADFRSLAPIVLEQALKESGTQLLEPYL 540

IRYGLEQGLFGWNVTDCKICFEYGLYYSPVSTPADFRSLAPIVLEQALKESGTQLLEPYL
Sbjct 481
IRYGLEQGLFGWNVTDCKICFEYGLYYSPVSTPADFRSLAPIVLEQALKESGTQLLEPYL 540

```
Query  541  
SFILYAPQEYLSRAYHDAPKYCATIETAQVKKDEVVFTGEIPARCIQAYRTDLAFYTNGR  600
            
SFILYAPQEYLSRAYHDAPKYCATIETAQVKKDEVVFTGEIPARCIQAYRTDLAFYTNGR
Sbjct  541  
SFILYAPQEYLSRAYHDAPKYCATIETAQVKKDEVVFTGEIPARCIQAYRTDLAFYTNGR  600

Query  601  SVCLTELKGYQAAVGQPVIQPRRPNSRLDKVRHMFQK  637
            SVCLTELKGYQAAVGQPVIQPRRPNSRLDKVRHM QK
Sbjct  601  SVCLTELKGYQAAVGQPVIQPRRPNSRLDKVRHMLQK  637


>CUR43792.1 Tetracycline resistance protein TetW [Lactobacillus 
reuteri]
Length=639

 Score = 1289 bits (3335),  Expect = 0.0, Method: Compositional 
matrix adjust.
 Identities = 626/639 (98%), Positives = 634/639 (99%), Gaps = 0/639 
(0%)

Query  1    
MKIINIGILAHVDAGKTTLTESLLYASGAISEPGSVEKGTTRTDTMFLERQRGITIQAAV  60
            MKIINIGILAHVDAGKTTLTESLLYASGAISEPG 
+VEKGTTRTDTMFLERQRGITIQAAV
Sbjct  1    
MKIINIGILAHVDAGKTTLTESLLYASGAISEPGNVEKGTTRTDTMFLERQRGITIQAAV  60

Query  61   
TSFQWHRCKVNIVDTPGHMDFLAEVYRSLAVLDGAILVISAKDGVQAQTRILFHALRKMN  120
            
TSFQWHRCKVNIVDTPGHMDFLAEVYRSLAVLDGAILVISAKDGVQAQTRILFHALRKMN
Sbjct  61   
TSFQWHRCKVNIVDTPGHMDFLAEVYRSLAVLDGAILVISAKDGVQAQTRILFHALRKMN  120

Query  121  
IPTVIFINKIDQAGVDLQSVVQSVRDKLSADIIIKQTVSLSPEIVLEENTDIEAWDAVIE  180
            
IPTVIFINKIDQAGVDLQSVVQSVRDKLSADIIIKQTVSLSPEIVLEENTDIEAWDAVIE
Sbjct  121  
IPTVIFINKIDQAGVDLQSVVQSVRDKLSADIIIKQTVSLSPEIVLEENTDIEAWDAVIE  180

Query  181  
NNDKLLEKYIAGEPISREKLVREEQRRVQDASLFPVYYGSAKKGLGIQPLMDAVTGLFQP  240
            NNDKLLEKYIAGEPISREKLVREEQRRVQDASLFPVYYGSAKK 
LGIQPLMDAVTGLFQP
Sbjct  181  
NNDKLLEKYIAGEPISREKLVREEQRRVQDASLFPVYYGSAKKDLGIQPLMDAVTGLFQP  240

Query  241  
IGEQGSAALCGSVFKVEYTDCGQRRVYLRLYSGTLRLRDTVALAGREKLKITEMRIPSKG  300
            
IGEQGSAALCGSVFKVEYTDCGQRRVYLRLYSGTLRLRDTVALAGREKLKITEMRIPSKG
```

```
Sbjct  241
IGEQGSAALCGSVFKVEYTDCGQRRVYLRLYSGTLRLRDTVALAGREKLKITEMRIPSKG  300

Query  301
EIVRTDTAYPGEIVILPSDSVRLNDVLGDPTRLPRKRWREDPLPMLRTTIAPKTAAQRER  360
            EIVRTDTAYPGEIVILPSDSVRLNDVLGDPTRLPRKRWREDPLPMLRT
+IAPKTAAQRER
Sbjct  301
EIVRTDTAYPGEIVILPSDSVRLNDVLGDPTRLPRKRWREDPLPMLRTSIAPKTAAQRER  360

Query  361
LLDALTQLADTDPLLRCEVDSITHEIILSFLGRVQLEVVSALLSEKYKLETVVKEPSVIY  420
            LLDALTQLADTDPLLRCEVDSITHEIILSFLGRVQLEVVSALLSEKYKLETVVKEP
+VIY
Sbjct  361
LLDALTQLADTDPLLRCEVDSITHEIILSFLGRVQLEVVSALLSEKYKLETVVKEPTVIY  420

Query  421
MERPLKAASHTIHIEVPPNPFWASIGLSVTPLSLGSGVQYESRVSLGYLNQSFQNAVRDG  480
            MERPLKAASHTIHIEVPPNPFWASIGLSVTPL
LGSGVQYESRVSLGYLNQSFQNAVRDG
Sbjct  421
MERPLKAASHTIHIEVPPNPFWASIGLSVTPLPLGSGVQYESRVSLGYLNQSFQNAVRDG  480

Query  481
IRYGLEQGLFGWNVTDCKICFEYGLYYSPVSTPADFRSLAPIVLEQALKESGTQLLEPYL  540

IRYGLEQGLFGWNVTDCKICFEYGLYYSPVSTPADFRSLAPIVLEQALKESGTQLLEPYL
Sbjct  481
IRYGLEQGLFGWNVTDCKICFEYGLYYSPVSTPADFRSLAPIVLEQALKESGTQLLEPYL  540

Query  541
SFILYAPQEYLSRAYHDAPKYCATIETAQVKKDEVVFTGEIPARCIQAYRTDLAFYTNGR  600
            SF LYAP+EYLSRAYHDAPKYCATIET QVKKDEVVFTGEIPARCIQAYRTDLA
YTNG+
Sbjct  541
SFTLYAPREYLSRAYHDAPKYCATIETVQVKKDEVVFTGEIPARCIQAYRTDLASYTNGQ  600

Query  601  SVCLTELKGYQAAVGQPVIQPRRPNSRLDKVRHMFQKVM  639
            SVCLTELKGYQAAVG+PVIQPRRPNSRLDKVR+MFQK+M
Sbjct  601  SVCLTELKGYQAAVGKPVIQPRRPNSRLDKVRYMFQKIM  639


>WP_087284258.1 TetM/TetW/TetO/TetS family tetracycline resistance
ribosomal
protection protein [Lactobacillus johnsonii]
 OUP16886.1 tetracycline resistance ribosomal protection protein
[Lactobacillus
johnsonii]
Length=639

 Score = 1288 bits (3334),  Expect = 0.0, Method: Compositional
matrix adjust.
 Identities = 628/639 (98%), Positives = 634/639 (99%), Gaps = 0/639
```

(0%)

Query 1
MKIINIGILAHVDAGKTTLTESLLYASGAISEPGSVEKGTTRTDTMFLERQRGITIQAAV 60
            MKIINIGILAHVDAGKTTLTESLLYASGAISE
GSVEKGTTRTDTMFLERQRGITIQAAV
Sbjct 1
MKIINIGILAHVDAGKTTLTESLLYASGAISERGSVEKGTTRTDTMFLERQRGITIQAAV 60

Query 61
TSFQWHRCKVNIVDTPGHMDFLAEVYRSLAVLDGAILVISAKDGVQAQTRILFHALRKMN 120

TSFQWHRCKVNIVDTPGHMDFLAEVYRSLAVLDGAILVISAKDGVQAQTRILFHALRKMN
Sbjct 61
TSFQWHRCKVNIVDTPGHMDFLAEVYRSLAVLDGAILVISAKDGVQAQTRILFHALRKMN 120

Query 121
IPTVIFINKIDQAGVDLQSVVQSVRDKLSADIIIKQTVSLSPEIVLEENTDIEAWDAVIE 180

IPTVIFINKIDQAGVDLQSVVQSVRDKLSADIIIKQTVSLSPEIVLEENTDIEAWDAVIE
Sbjct 121
IPTVIFINKIDQAGVDLQSVVQSVRDKLSADIIIKQTVSLSPEIVLEENTDIEAWDAVIE 180

Query 181
NNDKLLEKYIAGEPISREKLVREEQRRVQDASLFPVYYGSAKKGLGIQPLMDAVTGLFQP 240

NNDKLLEKYIAGEPISREKLVREEQRRVQDASLFPVYYGSAKKGLGIQPLMDAVTGLFQP
Sbjct 181
NNDKLLEKYIAGEPISREKLVREEQRRVQDASLFPVYYGSAKKGLGIQPLMDAVTGLFQP 240

Query 241
IGEQGSAALCGSVFKVEYTDCGQRRVYLRLYSGTLRLRDTVALAGREKLKITEMRIPSKG 300

IGEQGSAALCGSVFKVEYTDCGQRRVYLRLYSGTLRLRDTVALAGREKLKITEMRIPSKG
Sbjct 241
IGEQGSAALCGSVFKVEYTDCGQRRVYLRLYSGTLRLRDTVALAGREKLKITEMRIPSKG 300

Query 301
EIVRTDTAYPGEIVILPSDSVRLNDVLGDPTRLPRKRWREDPLPMLRTTIAPKTAAQRER 360
            EIVRTDTAYPGEIVILPSDSVRLNDVLGDPTRLPRKRWREDPLPMLRT
+IAPKTAAQRER
Sbjct 301
EIVRTDTAYPGEIVILPSDSVRLNDVLGDPTRLPRKRWREDPLPMLRTSIAPKTAAQRER 360

Query 361
LLDALTQLADTDPLLRCEVDSITHEIILSFLGRVQLEVVSALLSEKYKLETVVKEPSVIY 420
            LLDALTQLADTDPLLR
EVDSITHEIILSFLGRVQLEVVSALLSEKYKLETVVKEPSVIY
Sbjct 361
LLDALTQLADTDPLLRYEVDSITHEIILSFLGRVQLEVVSALLSEKYKLETVVKEPSVIY 420

Query 421
MERPLKAASHTIHIEVPPNPFWASIGLSVTPLSLGSGVQYESRVSLGYLNQSFQNAVRDG 480
            MERPLKAASHTIHIEVPPNPFWASIGLSVTPL

LGSGVQYESRVSLGYLNQSFQNAVRDG
Sbjct 421
MERPLKAASHTIHIEVPPNPFWASIGLSVTPLPLGSGVQYESRVSLGYLNQSFQNAVRDG 480

Query 481
IRYGLEQGLFGWNVTDCKICFEYGLYYSPVSTPADFRSLAPIVLEQALKESGTQLLEPYL 540

IRYGLEQGLFGWNVTDCKICFEYGLYYSPVSTPADFRSLAPIVLEQALKESGTQLLEPYL
Sbjct 481
IRYGLEQGLFGWNVTDCKICFEYGLYYSPVSTPADFRSLAPIVLEQALKESGTQLLEPYL 540

Query 541
SFILYAPQEYLSRAYHDAPKYCATIETAQVKKDEVVFTGEIPARCIQAYRTDLAFYTNGR 600
SF LYAP+EYLSRAYHDAPKYCATIET
QVKKDEVVFTGEIPARCIQAYRTDLAFYTNG+
Sbjct 541
SFTLYAPREYLSRAYHDAPKYCATIETVQVKKDEVVFTGEIPARCIQAYRTDLAFYTNGQ 600

Query 601 SVCLTELKGYQAAVGQPVIQPRRPNSRLDKVRHMFQKVM 639
SVCLTELKGYQAAVG+PVIQPRRPNSRLDKVR+MFQK+M
Sbjct 601 SVCLTELKGYQAAVGKPVIQPRRPNSRLDKVRYMFQKIM 639

>ACA23181.1 TetW [Bifidobacterium longum]
ACA23183.1 TetW [Bifidobacterium longum]
Length=639

Score = 1288 bits (3334), Expect = 0.0, Method: Compositional matrix adjust.
Identities = 629/639 (98%), Positives = 633/639 (99%), Gaps = 0/639 (0%)

Query 1
MKIINIGILAHVDAGKTTLTESLLYASGAISEPGSVEKGTTRTDTMFLERQRGITIQAAV 60

MKIINIGILAHVDAGKTTLTESLLYASGAISEPGSVEKGTTRTDTMFLERQRGITIQAAV
Sbjct 1
MKIINIGILAHVDAGKTTLTESLLYASGAISEPGSVEKGTTRTDTMFLERQRGITIQAAV 60

Query 61
TSFQWHRCKVNIVDTPGHMDFLAEVYRSLAVLDGAILVISAKDGVQAQTRILFHALRKMN 120

TSFQWHRCKVNIVDTPGHMDFLAEVYRSLAVLDGAILVISAKDGVQAQTRILFHALRKMN
Sbjct 61
TSFQWHRCKVNIVDTPGHMDFLAEVYRSLAVLDGAILVISAKDGVQAQTRILFHALRKMN 120

Query 121
IPTVIFINKIDQAGVDLQSVVQSVRDKLSADIIIKQTVSLSPEIVLEENTDIEAWDAVIE 180

IPTVIFINKIDQAGVDLQSVVQSVRDKLSADIIIKQTVSLSPEIVLEENTDIEAWDAVIE
Sbjct 121
IPTVIFINKIDQAGVDLQSVVQSVRDKLSADIIIKQTVSLSPEIVLEENTDIEAWDAVIE 180

Query 181

```
NNDKLLEKYIAGEPISREKLVREEQRRVQDASLFPVYYGSAKKGLGIQPLMDAVTGLFQP  240
            NND+LLEKYIAGEPISREKL REEQ+RVQDASLFPVY+GSAK
GLGIQPLMDAVTGLFQP
Sbjct  181
NNDELLEKYIAGEPISREKLAREEQQRVQDASLFPVYHGSAKNGLGIQPLMDAVTGLFQP  240

Query  241
IGEQGSAALCGSVFKVEYTDCGQRRVYLRLYSGTLRLRDTVALAGREKLKITEMRIPSKG  300
            IGEQG
AALCGSVFKVEYTDCGQRRVYLRLYSGTLRLRDTVALAGREKLKITEMRIPSKG
Sbjct  241
IGEQGGAALCGSVFKVEYTDCGQRRVYLRLYSGTLRLRDTVALAGREKLKITEMRIPSKG  300

Query  301
EIVRTDTAYPGEIVILPSDSVRLNDVLGDPTRLPRKRWREDPLPMLRTTIAPKTAAQRER  360
            EIVRTDTAY GEIVILPSDSVRLNDVLGD
TRLPRKRWREDPLPMLRTTIAPKTAAQRER
Sbjct  301
EIVRTDTAYQGEIVILPSDSVRLNDVLGDQTRLPRKRWREDPLPMLRTTIAPKTAAQRER  360

Query  361
LLDALTQLADTDPLLRCEVDSITHEIILSFLGRVQLEVVSALLSEKYKLETVVKEPSVIY  420

LLDALTQLADTDPLLRCEVDSITHEIILSFLGRVQLEVVSALLSEKYKLETVVKEPSVIY
Sbjct  361
LLDALTQLADTDPLLRCEVDSITHEIILSFLGRVQLEVVSALLSEKYKLETVVKEPSVIY  420

Query  421
MERPLKAASHTIHIEVPPNPFWASIGLSVTPLSLGSGVQYESRVSLGYLNQSFQNAVRDG  480
            MERPLKAAS
TIHIEVPPNPFWASIGLSVTPLSLGSGVQYESRVSLGYLNQSFQNAVRDG
Sbjct  421
MERPLKAASPTIHIEVPPNPFWASIGLSVTPLSLGSGVQYESRVSLGYLNQSFQNAVRDG  480

Query  481
IRYGLEQGLFGWNVTDCKICFEYGLYYSPVSTPADFRSLAPIVLEQALKESGTQLLEPYL  540

IRYGLEQGLFGWNVTDCKICFEYGLYYSPVSTPADFRSLAPIVLEQALKESGTQLLEPYL
Sbjct  481
IRYGLEQGLFGWNVTDCKICFEYGLYYSPVSTPADFRSLAPIVLEQALKESGTQLLEPYL  540

Query  541
SFILYAPQEYLSRAYHDAPKYCATIETAQVKKDEVVFTGEIPARCIQAYRTDLAFYTNGR  600

SFILYAPQEYLSRAYHDAPKYCATIETAQVKKDEVVFTGEIPARCIQAYRTDLAFYT+GR
Sbjct  541
SFILYAPQEYLSRAYHDAPKYCATIETAQVKKDEVVFTGEIPARCIQAYRTDLAFYTSGR  600

Query  601  SVCLTELKGYQAAVGQPVIQPRRPNSRLDKVRHMFQKVM  639
            SVCLTELKGYQAAVGQPVIQPRRPNSRLDKVRHMFQKVM
Sbjct  601  SVCLTELKGYQAAVGQPVIQPRRPNSRLDKVRHMFQKVM  639


>WP_070691289.1 TetM/TetW/TetO/TetS family tetracycline resistance
```

ribosomal
protection protein [Rothia sp. HMSC076D04]
 OFP58510.1 tetracycline resistance ribosomal protection protein
[Rothia
sp. HMSC076D04]
Length=639

 Score = 1288 bits (3334), Expect = 0.0, Method: Compositional
matrix adjust.
 Identities = 626/639 (98%), Positives = 633/639 (99%), Gaps = 0/639
(0%)

Query 1
MKIINIGILAHVDAGKTTLTESLLYASGAISEPGSVEKGTTRTDTMFLERQRGITIQAAV 60

MKIINIGILAHVDAGKTTLTESLLYASGAISEPGSVEKGTTRTDTMFLERQRGITIQAAV
Sbjct 1
MKIINIGILAHVDAGKTTLTESLLYASGAISEPGSVEKGTTRTDTMFLERQRGITIQAAV 60

Query 61
TSFQWHRCKVNIVDTPGHMDFLAEVYRSLAVLDGAILVISAKDGVQAQTRILFHALRKMN 120
            TSFQWHRCKVNIVDTPGHMDFLAEVYRSLAVLDGAILVISAKDGVQAQTRILF
+ALRKMN
Sbjct 61
TSFQWHRCKVNIVDTPGHMDFLAEVYRSLAVLDGAILVISAKDGVQAQTRILFNALRKMN 120

Query 121
IPTVIFINKIDQAGVDLQSVVQSVRDKLSADIIIKQTVSLSPEIVLEENTDIEAWDAVIE 180

IPTVIFINKIDQAGVDLQSVVQSVRDKLSADIIIKQTVSLSPEIVLEENTDIEAWDAVIE
Sbjct 121
IPTVIFINKIDQAGVDLQSVVQSVRDKLSADIIIKQTVSLSPEIVLEENTDIEAWDAVIE 180

Query 181
NNDKLLEKYIAGEPISREKLVREEQRRVQDASLFPVYYGSAKKGLGIQPLMDAVTGLFQP 240
            NND+LLEKYIAGEPISREKL REEQ+RVQDASLFPVY+GSAK
GLGIQPLMDAVTGLFQP
Sbjct 181
NNDELLEKYIAGEPISREKLAREEQQRVQDASLFPVYHGSAKNGLGIQPLMDAVTGLFQP 240

Query 241
IGEQGSAALCGSVFKVEYTDCGQRRVYLRLYSGTLRLRDTVALAGREKLKITEMRIPSKG 300
            IGEQG
AALCGSVFKVEYTDCGQRRVYLRLYSGTLRLRDTVALAGREKLKITEMRIPSKG
Sbjct 241
IGEQGGAALCGSVFKVEYTDCGQRRVYLRLYSGTLRLRDTVALAGREKLKITEMRIPSKG 300

Query 301
EIVRTDTAYPGEIVILPSDSVRLNDVLGDPTRLPRKRWREDPLPMLRTTIAPKTAAQRER 360
            EIVRTDTAYPGEIVILPSDSVRLNDVLGDPTRLPRKRWREDPLPMLRT
+IAPKTAAQRER
Sbjct 301
EIVRTDTAYPGEIVILPSDSVRLNDVLGDPTRLPRKRWREDPLPMLRTSIAPKTAAQRER 360

Query 361
LLDALTQLADTDPLLRCEVDSITHEIILSFLGRVQLEVVSALLSEKYKLETVVKEPSVIY 420
            LLDALTQLADTDPLLRCEVDSITHEIILSFLGRVQLEVVSALLSEKYKLETVVKEP
+VIY
Sbjct 361
LLDALTQLADTDPLLRCEVDSITHEIILSFLGRVQLEVVSALLSEKYKLETVVKEPTVIY 420

Query 421
MERPLKAASHTIHIEVPPNPFWASIGLSVTPLSLGSGVQYESRVSLGYLNQSFQNAVRDG 480
            MERPLKAASHTIHIEVPPNPFWASIGLSVTPL
LGSGVQYESRVSLGYLNQSFQNAVRDG
Sbjct 421
MERPLKAASHTIHIEVPPNPFWASIGLSVTPLPLGSGVQYESRVSLGYLNQSFQNAVRDG 480

Query 481
IRYGLEQGLFGWNVTDCKICFEYGLYYSPVSTPADFRSLAPIVLEQALKESGTQLLEPYL 540

IRYGLEQGLFGWNVTDCKICFEYGLYYSPVSTPADFRSLAPIVLEQALKESGTQLLEPYL
Sbjct 481
IRYGLEQGLFGWNVTDCKICFEYGLYYSPVSTPADFRSLAPIVLEQALKESGTQLLEPYL 540

Query 541
SFILYAPQEYLSRAYHDAPKYCATIETAQVKKDEVVFTGEIPARCIQAYRTDLAFYTNGR 600
            SF LYAP+EYLSRAYHDAPKYCATIET
QVKKDEVVFTGEIPARCIQAYRTDLAFYTNGR
Sbjct 541
SFTLYAPREYLSRAYHDAPKYCATIETVQVKKDEVVFTGEIPARCIQAYRTDLAFYTNGR 600

Query 601 SVCLTELKGYQAAVGQPVIQPRRPNSRLDKVRHMFQKVM 639
            SVCLTELKGYQAAVGQPVIQPRRPNSRLDKVRHMFQKVM
Sbjct 601 SVCLTELKGYQAAVGQPVIQPRRPNSRLDKVRHMFQKVM 639

>AMP58041.1 TetM-TetW-TetO-TetS [uncultured bacterium]
Length=639

Score = 1288 bits (3334), Expect = 0.0, Method: Compositional
matrix adjust.
Identities = 629/639 (98%), Positives = 632/639 (99%), Gaps = 0/639
(0%)

Query 1
MKIINIGILAHVDAGKTTLTESLLYASGAISEPGSVEKGTTRTDTMFLERQRGITIQAAV 60

MKIINIGILAHVDAGKTTLTESLLYASGAISEPGSVEKGTTRTDTMFLERQRGITIQAAV
Sbjct 1
MKIINIGILAHVDAGKTTLTESLLYASGAISEPGSVEKGTTRTDTMFLERQRGITIQAAV 60

Query 61
TSFQWHRCKVNIVDTPGHMDFLAEVYRSLAVLDGAILVISAKDGVQAQTRILFHALRKMN 120

TSFQWHRCKVNIVDTPGHMDFLAEVYRSLAVLDGAILVISAKDGVQAQTRILFHALRKMN
Sbjct 61
TSFQWHRCKVNIVDTPGHMDFLAEVYRSLAVLDGAILVISAKDGVQAQTRILFHALRKMN 120

```
Query  121
IPTVIFINKIDQAGVDLQSVVQSVRDKLSADIIIKQTVSLSPEIVLEENTDIEAWDAVIE  180

IPTVIFINKIDQAGVDLQSVVQSVRDKLSADIIIKQTVSLSPEIVLEENTDIEAWDAVIE
Sbjct  121
IPTVIFINKIDQAGVDLQSVVQSVRDKLSADIIIKQTVSLSPEIVLEENTDIEAWDAVIE  180

Query  181
NNDKLLEKYIAGEPISREKLVREEQRRVQDASLFPVYYGSAKKGLGIQPLMDAVTGLFQP  240
            NND+LLEKYIAGEPISREKL REEQ+RVQDASLFPVY+GSAK
GLGIQPLMDAVTGLFQP
Sbjct  181
NNDELLEKYIAGEPISREKLAREEQQRVQDASLFPVYHGSAKNGLGIQPLMDAVTGLFQP  240

Query  241
IGEQGSAALCGSVFKVEYTDCGQRRVYLRLYSGTLRLRDTVALAGREKLKITEMRIPSKG  300
            IGEQG AALCGSVFKVEYTDCGQRRVYLRLYSGTLRLR
TVALAGREKLKITEMRIPSKG
Sbjct  241
IGEQGGAALCGSVFKVEYTDCGQRRVYLRLYSGTLRLRGTVALAGREKLKITEMRIPSKG  300

Query  301
EIVRTDTAYPGEIVILPSDSVRLNDVLGDPTRLPRKRWREDPLPMLRTTIAPKTAAQRER  360
            EIVRTDTAY GEIVILPSDSVRLNDVLGD
TRLPRKRWREDPLPMLRTTIAPKTAAQRER
Sbjct  301
EIVRTDTAYQGEIVILPSDSVRLNDVLGDQTRLPRKRWREDPLPMLRTTIAPKTAAQRER  360

Query  361
LLDALTQLADTDPLLRCEVDSITHEIILSFLGRVQLEVVSALLSEKYKLETVVKEPSVIY  420

LLDALTQLADTDPLLRCEVDSITHEIILSFLGRVQLEVVSALLSEKYKLETVVKEPSVIY
Sbjct  361
LLDALTQLADTDPLLRCEVDSITHEIILSFLGRVQLEVVSALLSEKYKLETVVKEPSVIY  420

Query  421
MERPLKAASHTIHIEVPPNPFWASIGLSVTPLSLGSGVQYESRVSLGYLNQSFQNAVRDG  480

MERPLKAASHTIHIEVPPNPFWASIGLSVTPLSLGSGVQYESRVSLGYLNQSFQNAVRDG
Sbjct  421
MERPLKAASHTIHIEVPPNPFWASIGLSVTPLSLGSGVQYESRVSLGYLNQSFQNAVRDG  480

Query  481
IRYGLEQGLFGWNVTDCKICFEYGLYYSPVSTPADFRSLAPIVLEQALKESGTQLLEPYL  540

IRYGLEQGLFGWNVTDCKICFEYGLYYSPVSTPADFRSLAPIVLEQALKESGTQLLEPYL
Sbjct  481
IRYGLEQGLFGWNVTDCKICFEYGLYYSPVSTPADFRSLAPIVLEQALKESGTQLLEPYL  540

Query  541
SFILYAPQEYLSRAYHDAPKYCATIETAQVKKDEVVFTGEIPARCIQAYRTDLAFYTNGR  600

SFILYAPQEYLSRAYHDAPKYCATIETAQVKKDEVVFTGEIPARCIQAYRTDLAFYTNGR
```

Sbjct 541
SFILYAPQEYLSRAYHDAPKYCATIETAQVKKDEVVFTGEIPARCIQAYRTDLAFYTNGR 600

Query 601 SVCLTELKGYQAAVGQPVIQPRRPNSRLDKVRHMFQKVM 639
            SVCLTELKGYQAAVGQPVIQPRRPNSRL KVRHMFQKVM
Sbjct 601 SVCLTELKGYQAAVGQPVIQPRRPNSRLYKVRHMFQKVM 639

>AMP55465.1 TetM-TetW-TetO-TetS [uncultured bacterium]
Length=639

Score = 1288 bits (3334), Expect = 0.0, Method: Compositional
matrix adjust.
Identities = 626/639 (98%), Positives = 633/639 (99%), Gaps = 0/639
(0%)

Query 1
MKIINIGILAHVDAGKTTLTESLLYASGAISEPGSVEKGTTRTDTMFLERQRGITIQAAV 60

MKIINIGILAHVDAGKTTLTESLLYASGAISEPGSVEKGTTRTDTMFLERQRGITIQAAV
Sbjct 1
MKIINIGILAHVDAGKTTLTESLLYASGAISEPGSVEKGTTRTDTMFLERQRGITIQAAV 60

Query 61
TSFQWHRCKVNIVDTPGHMDFLAEVYRSLAVLDGAILVISAKDGVQAQTRILFHALRKMN 120

TSFQWHRCKVNIVDTPGHMDFLAEVYRSLAVLDGAILVISAKDGVQAQTRILFHALRKMN
Sbjct 61
TSFQWHRCKVNIVDTPGHMDFLAEVYRSLAVLDGAILVISAKDGVQAQTRILFHALRKMN 120

Query 121
IPTVIFINKIDQAGVDLQSVVQSVRDKLSADIIIKQTVSLSPEIVLEENTDIEAWDAVIE 180

IPTVIFINKIDQAGVDLQSVVQSVRDKLSADIIIKQTVSLSPEIVLEENTDIEAWDAVIE
Sbjct 121
IPTVIFINKIDQAGVDLQSVVQSVRDKLSADIIIKQTVSLSPEIVLEENTDIEAWDAVIE 180

Query 181
NNDKLLEKYIAGEPISREKLVREEQRRVQDASLFPVYYGSAKKGLGIQPLMDAVTGLFQP 240
            NNDKLLEKYIAGEPISREKL REEQ+RVQDASLFPVY+GSAK
GLGIQPLMDAVTGLFQP
Sbjct 181
NNDKLLEKYIAGEPISREKLAREEQQRVQDASLFPVYHGSAKNGLGIQPLMDAVTGLFQP 240

Query 241
IGEQGSAALCGSVFKVEYTDCGQRRVYLRLYSGTLRLRDTVALAGREKLKITEMRIPSKG 300

IGEQGSAALCGSVFKVEYTDCGQRRVYLRLYSGTLRLRDTVALAGREKLKITEMRIPSKG
Sbjct 241
IGEQGSAALCGSVFKVEYTDCGQRRVYLRLYSGTLRLRDTVALAGREKLKITEMRIPSKG 300

Query 301
EIVRTDTAYPGEIVILPSDSVRLNDVLGDPTRLPRKRWREDPLPMLRTTIAPKTAAQRER 360
            EIVRTDTAYPGEIVILPSDSVRLNDVLGDPTRLPRKRWREDPLPMLRT

```
+IAPKTAAQRER
Sbjct  301  
EIVRTDTAYPGEIVILPSDSVRLNDVLGDPTRLPRKRWREDPLPMLRTSIAPKTAAQRER  360

Query  361  
LLDALTQLADTDPLLRCEVDSITHEIILSFLGRVQLEVVSALLSEKYKLETVVKEPSVIY  420
            LLDALTQLADTDPLLRCEVDSITHEIILSFLGRVQLEVVSALLSEKYKLETVVKEP
+VIY
Sbjct  361  
LLDALTQLADTDPLLRCEVDSITHEIILSFLGRVQLEVVSALLSEKYKLETVVKEPTVIY  420

Query  421  
MERPLKAASHTIHIEVPPNPFWASIGLSVTPLSLGSGVQYESRVSLGYLNQSFQNAVRDG  480
            MERPLKAASHTIHIEVPPNPFWASIGLSVTPL
LGSGVQYESRVSLGYLNQSFQNAVRDG
Sbjct  421  
MERPLKAASHTIHIEVPPNPFWASIGLSVTPLPLGSGVQYESRVSLGYLNQSFQNAVRDG  480

Query  481  
IRYGLEQGLFGWNVTDCKICFEYGLYYSPVSTPADFRSLAPIVLEQALKESGTQLLEPYL  540

IRYGLEQGLFGWNVTDCKICFEYGLYYSPVSTPADFRSLAPIVLEQALKESGTQLLEPYL
Sbjct  481  
IRYGLEQGLFGWNVTDCKICFEYGLYYSPVSTPADFRSLAPIVLEQALKESGTQLLEPYL  540

Query  541  
SFILYAPQEYLSRAYHDAPKYCATIETAQVKKDEVVFTGEIPARCIQAYRTDLAFYTNGR  600
            SF LYAP+EYLSRAYHDAPKYCATIET Q
KKDEVVFTGEIPARCIQAYRTDLAFYTNG+
Sbjct  541  
SFTLYAPREYLSRAYHDAPKYCATIETVQEKKDEVVFTGEIPARCIQAYRTDLAFYTNGQ  600

Query  601  SVCLTELKGYQAAVGQPVIQPRRPNSRLDKVRHMFQKVM  639
            SVCLTELKGYQAAVG+PVIQPRRPNSRLDKVRHMFQKVM
Sbjct  601  SVCLTELKGYQAAVGKPVIQPRRPNSRLDKVRHMFQKVM  639


>WP_046921744.1 TetM/TetW/TetO/TetS family tetracycline resistance 
ribosomal 
protection protein [Lactobacillus ruminis]
 KLA43507.1 hypothetical protein LRP_1002 [Lactobacillus ruminis]
Length=640

 Score = 1288 bits (3334),  Expect = 0.0, Method: Compositional 
matrix adjust.
 Identities = 627/638 (98%), Positives = 633/638 (99%), Gaps = 0/638 
(0%)

Query  1    
MKIINIGILAHVDAGKTTLTESLLYASGAISEPGSVEKGTTRTDTMFLERQRGITIQAAV  60
            MKIINIGILAHVDAGKTTLTESLLYASGAISE
GSVEKGTTRTDTMFLERQRGITIQAAV
Sbjct  1    
MKIINIGILAHVDAGKTTLTESLLYASGAISERGSVEKGTTRTDTMFLERQRGITIQAAV  60
```

Query 61
TSFQWHRCKVNIVDTPGHMDFLAEVYRSLAVLDGAILVISAKDGVQAQTRILFHALRKMN 120

TSFQWHRCKVNIVDTPGHMDFLAEVYRSLAVLDGAILVISAKDGVQAQTRILFHALRKMN
Sbjct 61
TSFQWHRCKVNIVDTPGHMDFLAEVYRSLAVLDGAILVISAKDGVQAQTRILFHALRKMN 120

Query 121
IPTVIFINKIDQAGVDLQSVVQSVRDKLSADIIIKQTVSLSPEIVLEENTDIEAWDAVIE 180

IPTVIFINKIDQAGVDLQSVVQSVRDKLSADIIIKQTVSLSPEIVLEENTDIEAWDAVIE
Sbjct 121
IPTVIFINKIDQAGVDLQSVVQSVRDKLSADIIIKQTVSLSPEIVLEENTDIEAWDAVIE 180

Query 181
NNDKLLEKYIAGEPISREKLVREEQRRVQDASLFPVYYGSAKKGLGIQPLMDAVTGLFQP 240

NNDKLLEKYIAGEPISREKLVREEQRRVQDASLFPVYYGSAKKGLGIQPLMDAVTGLFQP
Sbjct 181
NNDKLLEKYIAGEPISREKLVREEQRRVQDASLFPVYYGSAKKGLGIQPLMDAVTGLFQP 240

Query 241
IGEQGSAALCGSVFKVEYTDCGQRRVYLRLYSGTLRLRDTVALAGREKLKITEMRIPSKG 300

IGEQGSAALCGSVFKVEYTDCGQRRVYLRLYSGTLRLRDTVALAGREKLKITEMRIPSKG
Sbjct 241
IGEQGSAALCGSVFKVEYTDCGQRRVYLRLYSGTLRLRDTVALAGREKLKITEMRIPSKG 300

Query 301
EIVRTDTAYPGEIVILPSDSVRLNDVLGDPTRLPRKRWREDPLPMLRTTIAPKTAAQRER 360
EIVRTDTAYPGEIVILPSDSVRLNDVLGDPTRLPRKRWREDPLPMLRT
+IAPKTAAQRER
Sbjct 301
EIVRTDTAYPGEIVILPSDSVRLNDVLGDPTRLPRKRWREDPLPMLRTSIAPKTAAQRER 360

Query 361
LLDALTQLADTDPLLRCEVDSITHEIILSFLGRVQLEVVSALLSEKYKLETVVKEPSVIY 420
LLDALTQLADTDPLLRCEVDSITHEIILSFLGRVQLEVVSALLSEKYKLETVVKEP
+VIY
Sbjct 361
LLDALTQLADTDPLLRCEVDSITHEIILSFLGRVQLEVVSALLSEKYKLETVVKEPTVIY 420

Query 421
MERPLKAASHTIHIEVPPNPFWASIGLSVTPLSLGSGVQYESRVSLGYLNQSFQNAVRDG 480
MERPLKAASHTIHIEVPPNPFWASIGLSVTPL
LGSGVQYESRVSLGYLNQSFQNAVRDG
Sbjct 421
MERPLKAASHTIHIEVPPNPFWASIGLSVTPLPLGSGVQYESRVSLGYLNQSFQNAVRDG 480

Query 481
IRYGLEQGLFGWNVTDCKICFEYGLYYSPVSTPADFRSLAPIVLEQALKESGTQLLEPYL 540

IRYGLEQGLFGWNVTDCKICFEYGLYYSPVSTPADFRSLAPIVLEQALKESGTQLLEPYL

```
Sbjct  481  
IRYGLEQGLFGWNVTDCKICFEYGLYYSPVSTPADFRSLAPIVLEQALKESGTQLLEPYL  540

Query  541  
SFILYAPQEYLSRAYHDAPKYCATIETAQVKKDEVVFTGEIPARCIQAYRTDLAFYTNGR  600
            SF LYAP+EYLSRAYHDAPKYCATIE 
QVKKDEVVFTGEIPARCIQAYRTDLAFYTNG+
Sbjct  541  
SFTLYAPREYLSRAYHDAPKYCATIEPVQVKKDEVVFTGEIPARCIQAYRTDLAFYTNGQ  600

Query  601  SVCLTELKGYQAAVGQPVIQPRRPNSRLDKVRHMFQKV  638
            SVCLTELKGYQAAVG+PVIQPRRPNSRLDKVRHMFQK+
Sbjct  601  SVCLTELKGYQAAVGKPVIQPRRPNSRLDKVRHMFQKM  638


>WP_098045951.1 TetM/TetW/TetO/TetS family tetracycline resistance 
ribosomal 
protection protein [Lactobacillus sp. UMNPBX17]
 PEG79967.1 tetracycline resistance ribosomal protection protein 
[Lactobacillus 
sp. UMNPBX17]
Length=641

 Score = 1288 bits (3333),  Expect = 0.0, Method: Compositional 
matrix adjust.
 Identities = 626/639 (98%), Positives = 633/639 (99%), Gaps = 0/639 
(0%)

Query  1    
MKIINIGILAHVDAGKTTLTESLLYASGAISEPGSVEKGTTRTDTMFLERQRGITIQAAV  60
            MKIINIGILAHVDAGKTTLTESLLYASGAISEPGSVEKGTT 
TDTMFLERQRGITIQAAV
Sbjct  1    
MKIINIGILAHVDAGKTTLTESLLYASGAISEPGSVEKGTTSTDTMFLERQRGITIQAAV  60

Query  61   
TSFQWHRCKVNIVDTPGHMDFLAEVYRSLAVLDGAILVISAKDGVQAQTRILFHALRKMN  120
            
TSFQWHRCKVNIVDTPGHMDFLAEVYRSLAVLDGAILVISAKDGVQAQTRILFHALRKMN
Sbjct  61   
TSFQWHRCKVNIVDTPGHMDFLAEVYRSLAVLDGAILVISAKDGVQAQTRILFHALRKMN  120

Query  121  
IPTVIFINKIDQAGVDLQSVVQSVRDKLSADIIIKQTVSLSPEIVLEENTDIEAWDAVIE  180
            
IPTVIFINKIDQAGVDLQSVVQSVRDKLSADIIIKQTVSLSPEIVLEENTDIEAWDAVIE
Sbjct  121  
IPTVIFINKIDQAGVDLQSVVQSVRDKLSADIIIKQTVSLSPEIVLEENTDIEAWDAVIE  180

Query  181  
NNDKLLEKYIAGEPISREKLVREEQRRVQDASLFPVYYGSAKKGLGIQPLMDAVTGLFQP  240
            
NNDKLLEKYIAGEPISREKLVREEQRRVQDASLFPVYYGSAKKGLGIQPLMDAVTGLFQP
Sbjct  181  
```

```
NNDKLLEKYIAGEPISREKLVREEQRRVQDASLFPVYYGSAKKGLGIQPLMDAVTGLFQP  240

Query  241
IGEQGSAALCGSVFKVEYTDCGQRRVYLRLYSGTLRLRDTVALAGREKLKITEMRIPSKG  300

IGEQGSAALCGSVFKVEYTDCGQRRVYLRLYSGTLRLRDTVALAGREKLKITEMRIPSKG
Sbjct  241
IGEQGSAALCGSVFKVEYTDCGQRRVYLRLYSGTLRLRDTVALAGREKLKITEMRIPSKG  300

Query  301
EIVRTDTAYPGEIVILPSDSVRLNDVLGDPTRLPRKRWREDPLPMLRTTIAPKTAAQRER  360
            EIVRTDTAYPGEIVILPSDSVRLNDVLGDP RLPRKRWREDPLPMLRT
+IAPKTAAQRER
Sbjct  301
EIVRTDTAYPGEIVILPSDSVRLNDVLGDPNRLPRKRWREDPLPMLRTSIAPKTAAQRER  360

Query  361
LLDALTQLADTDPLLRCEVDSITHEIILSFLGRVQLEVVSALLSEKYKLETVVKEPSVIY  420
            LLDALTQLADTDPLLRCEVDSITHEIILSFLGRVQLEVVSALLSEKYKLETVVKEP
+VIY
Sbjct  361
LLDALTQLADTDPLLRCEVDSITHEIILSFLGRVQLEVVSALLSEKYKLETVVKEPTVIY  420

Query  421
MERPLKAASHTIHIEVPPNPFWASIGLSVTPLSLGSGVQYESRVSLGYLNQSFQNAVRDG  480
            MERPLKAASHTIHIEVPPNPFWASIGLSVTPL
LGSGVQYESRVSLGYLNQSFQNAVRDG
Sbjct  421
MERPLKAASHTIHIEVPPNPFWASIGLSVTPLPLGSGVQYESRVSLGYLNQSFQNAVRDG  480

Query  481
IRYGLEQGLFGWNVTDCKICFEYGLYYSPVSTPADFRSLAPIVLEQALKESGTQLLEPYL  540

IRYGLEQGLFGWNVTDCKICFEYGLYYSPVSTPADFRSLAPIVLEQALKESGTQLLEPYL
Sbjct  481
IRYGLEQGLFGWNVTDCKICFEYGLYYSPVSTPADFRSLAPIVLEQALKESGTQLLEPYL  540

Query  541
SFILYAPQEYLSRAYHDAPKYCATIETAQVKKDEVVFTGEIPARCIQAYRTDLAFYTNGR  600
            SF LYAP+EYLSRAYHDAPKYCATIET QVKKDEVVFTGEIPARCIQAYRTDLA
YTNG+
Sbjct  541
SFTLYAPREYLSRAYHDAPKYCATIETVQVKKDEVVFTGEIPARCIQAYRTDLASYTNGQ  600

Query  601  SVCLTELKGYQAAVGQPVIQPRRPNSRLDKVRHMFQKVM  639
            SVCLTELKGYQAAVG+PVIQPRRPNSRLDKVR+MFQK+M
Sbjct  601  SVCLTELKGYQAAVGKPVIQPRRPNSRLDKVRYMFQKIM  639


>AMP54790.1 TetM-TetW-TetO-TetS [uncultured bacterium]
Length=639

 Score = 1288 bits (3333),  Expect = 0.0, Method: Compositional
matrix adjust.
```

Identities = 625/639 (98%), Positives = 634/639 (99%), Gaps = 0/639 (0%)

Query 1
MKIINIGILAHVDAGKTTLTESLLYASGAISEPGSVEKGTTRTDTMFLERQRGITIQAAV 60

MKIINIGILAHVDAGKTTLTESLLYASGAISEPGSVEKGTTRTDTMFLERQRGITIQAAV
Sbjct 1
MKIINIGILAHVDAGKTTLTESLLYASGAISEPGSVEKGTTRTDTMFLERQRGITIQAAV 60

Query 61
TSFQWHRCKVNIVDTPGHMDFLAEVYRSLAVLDGAILVISAKDGVQAQTRILFHALRKMN 120

TSFQWHRCKVNIVDTPGHMDFLAEVYRSLAVLDGAILVISAKDGVQAQTRILFHALRKMN
Sbjct 61
TSFQWHRCKVNIVDTPGHMDFLAEVYRSLAVLDGAILVISAKDGVQAQTRILFHALRKMN 120

Query 121
IPTVIFINKIDQAGVDLQSVVQSVRDKLSADIIIKQTVSLSPEIVLEENTDIEAWDAVIE 180

IPTVIFINKIDQAGVDLQSVVQSVRDKLSADIIIKQTVSLSPEIVLEENTDIEAWDAVIE
Sbjct 121
IPTVIFINKIDQAGVDLQSVVQSVRDKLSADIIIKQTVSLSPEIVLEENTDIEAWDAVIE 180

Query 181
NNDKLLEKYIAGEPISREKLVREEQRRVQDASLFPVYYGSAKKGLGIQPLMDAVTGLFQP 240
NND+LLEKYIAGEPISREKLVREEQRRVQDASLFPVY+GSAK
GLGIQPLMDAVTGLFQP
Sbjct 181
NNDELLEKYIAGEPISREKLVREEQRRVQDASLFPVYHGSAKNGLGIQPLMDAVTGLFQP 240

Query 241
IGEQGSAALCGSVFKVEYTDCGQRRVYLRLYSGTLRLRDTVALAGREKLKITEMRIPSKG 300

IGEQGSAALCGSVFKVEYTDCGQRRVYLRLYSGTLRLRDTVALAGREKLKITEMRIPSKG
Sbjct 241
IGEQGSAALCGSVFKVEYTDCGQRRVYLRLYSGTLRLRDTVALAGREKLKITEMRIPSKG 300

Query 301
EIVRTDTAYPGEIVILPSDSVRLNDVLGDPTRLPRKRWREDPLPMLRTTIAPKTAAQRER 360
EIVRTDTAYPGEIVILPSDSVRLNDVLGDPTRLPRKRWREDPLPMLRT
+IAPKTAAQRER
Sbjct 301
EIVRTDTAYPGEIVILPSDSVRLNDVLGDPTRLPRKRWREDPLPMLRTSIAPKTAAQRER 360

Query 361
LLDALTQLADTDPLLRCEVDSITHEIILSFLGRVQLEVVSALLSEKYKLETVVKEPSVIY 420
LLDALTQLADTDPLLRCEVDSITHEIILSFLGRVQLEVVSALLSEKYKLETVVKEP
+VIY
Sbjct 361
LLDALTQLADTDPLLRCEVDSITHEIILSFLGRVQLEVVSALLSEKYKLETVVKEPTVIY 420

Query 421
MERPLKAASHTIHIEVPPNPFWASIGLSVTPLSLGSGVQYESRVSLGYLNQSFQNAVRDG 480

MERPLKAASHTIHIEVPPNPFWASIGLSVTPL
LGSGVQYESRVSLGYLNQSFQNAVRDG
Sbjct 421
MERPLKAASHTIHIEVPPNPFWASIGLSVTPLPLGSGVQYESRVSLGYLNQSFQNAVRDG 480

Query 481
IRYGLEQGLFGWNVTDCKICFEYGLYYSPVSTPADFRSLAPIVLEQALKESGTQLLEPYL 540

IRYGLEQGLFGWNVTDCKICFEYGLYYSPVSTPADFRSLAPIVLEQALKESGTQLLEPYL
Sbjct 481
IRYGLEQGLFGWNVTDCKICFEYGLYYSPVSTPADFRSLAPIVLEQALKESGTQLLEPYL 540

Query 541
SFILYAPQEYLSRAYHDAPKYCATIETAQVKKDEVVFTGEIPARCIQAYRTDLAFYTNGR 600
SF LYAP+EYLSRAYHDAPKYCATIET Q
KKDEVVFTGEIPARCIQAYRTDLAFYTNG+
Sbjct 541
SFTLYAPREYLSRAYHDAPKYCATIETVQEKKDEVVFTGEIPARCIQAYRTDLAFYTNGQ 600

Query 601 SVCLTELKGYQAAVGQPVIQPRRPNSRLDKVRHMFQKVM 639
SVCLTELKGYQAAVG+PVIQPRRPNSRLDKVR+MFQK+M
Sbjct 601 SVCLTELKGYQAAVGKPVIQPRRPNSRLDKVRYMFQKIM 639

>AMP53110.1 TetM-TetW-TetO-TetS, partial [uncultured bacterium]
Length=636

Score = 1288 bits (3333), Expect = 0.0, Method: Compositional matrix adjust.
Identities = 628/636 (99%), Positives = 631/636 (99%), Gaps = 0/636 (0%)

Query 1
MKIINIGILAHVDAGKTTLTESLLYASGAISEPGSVEKGTTRTDTMFLERQRGITIQAAV 60

MKIINIGILAHVDAGKTTLTESLLYASGAISEPGSVEKGTTRTDTMFLERQRGITIQAAV
Sbjct 1
MKIINIGILAHVDAGKTTLTESLLYASGAISEPGSVEKGTTRTDTMFLERQRGITIQAAV 60

Query 61
TSFQWHRCKVNIVDTPGHMDFLAEVYRSLAVLDGAILVISAKDGVQAQTRILFHALRKMN 120

TSFQWHRCKVNIVDTPGHMDFLAEVYRSLAVLDGAILVISAKDGVQAQTRILFHALRKMN
Sbjct 61
TSFQWHRCKVNIVDTPGHMDFLAEVYRSLAVLDGAILVISAKDGVQAQTRILFHALRKMN 120

Query 121
IPTVIFINKIDQAGVDLQSVVQSVRDKLSADIIIKQTVSLSPEIVLEENTDIEAWDAVIE 180
IPTVIFINKIDQAG
+DLQSVVQSVRDKLSADIIIKQTVSLSPEIVLEENTDIEAWDAVIE
Sbjct 121
IPTVIFINKIDQAGIDLQSVVQSVRDKLSADIIIKQTVSLSPEIVLEENTDIEAWDAVIE 180

Query 181

NNDKLLEKYIAGEPISREKLVREEQRRVQDASLFPVYYGSAKKGLGIQPLMDAVTGLFQP 240
            NND+LLEKYIAGEPISREKL REEQ
+RVQDASLFPVYYGSAKKGLGIQPLMDAVTGLFQP
Sbjct 181
NNDELLEKYIAGEPISREKLAREEQQRVQDASLFPVYYGSAKKGLGIQPLMDAVTGLFQP 240

Query 241
IGEQGSAALCGSVFKVEYTDCGQRRVYLRLYSGTLRLRDTVALAGREKLKITEMRIPSKG 300
            IGEQG
AALCGSVFKVEYTDCGQRRVYLRLYSGTLRLRDTVALAGREKLKITEMRIPSKG
Sbjct 241
IGEQGGAALCGSVFKVEYTDCGQRRVYLRLYSGTLRLRDTVALAGREKLKITEMRIPSKG 300

Query 301
EIVRTDTAYPGEIVILPSDSVRLNDVLGDPTRLPRKRWREDPLPMLRTTIAPKTAAQRER 360
            EIVRTDTAY GEIVILPSDSVRLNDVLGD
TRLPRKRWREDPLPMLRTTIAPKTAAQRER
Sbjct 301
EIVRTDTAYQGEIVILPSDSVRLNDVLGDQTRLPRKRWREDPLPMLRTTIAPKTAAQRER 360

Query 361
LLDALTQLADTDPLLRCEVDSITHEIILSFLGRVQLEVVSALLSEKYKLETVVKEPSVIY 420
            LLDALTQLADTDPLLR
EVDSITHEIILSFLGRVQLEVVSALLSEKYKLETVVKEPSVIY
Sbjct 361
LLDALTQLADTDPLLRYEVDSITHEIILSFLGRVQLEVVSALLSEKYKLETVVKEPSVIY 420

Query 421
MERPLKAASHTIHIEVPPNPFWASIGLSVTPLSLGSGVQYESRVSLGYLNQSFQNAVRDG 480

MERPLKAASHTIHIEVPPNPFWASIGLSVTPLSLGSGVQYESRVSLGYLNQSFQNAVRDG
Sbjct 421
MERPLKAASHTIHIEVPPNPFWASIGLSVTPLSLGSGVQYESRVSLGYLNQSFQNAVRDG 480

Query 481
IRYGLEQGLFGWNVTDCKICFEYGLYYSPVSTPADFRSLAPIVLEQALKESGTQLLEPYL 540

IRYGLEQGLFGWNVTDCKICFEYGLYYSPVSTPADFRSLAPIVLEQALKESGTQLLEPYL
Sbjct 481
IRYGLEQGLFGWNVTDCKICFEYGLYYSPVSTPADFRSLAPIVLEQALKESGTQLLEPYL 540

Query 541
SFILYAPQEYLSRAYHDAPKYCATIETAQVKKDEVVFTGEIPARCIQAYRTDLAFYTNGR 600

SFILYAPQEYLSRAYHDAPKYCATIETAQVKKDEVVFTGEIPARCIQAYRTDLAFYTNGR
Sbjct 541
SFILYAPQEYLSRAYHDAPKYCATIETAQVKKDEVVFTGEIPARCIQAYRTDLAFYTNGR 600

Query 601 SVCLTELKGYQAAVGQPVIQPRRPNSRLDKVRHMFQ 636
            SVCLTELKGYQAAVGQPVIQPRRPNSRLDKVRHMFQ
Sbjct 601 SVCLTELKGYQAAVGQPVIQPRRPNSRLDKVRHMFQ 636

>AMP52280.1 TetM-TetW-TetO-TetS [uncultured bacterium]

```
Length=639

 Score = 1288 bits (3333),  Expect = 0.0, Method: Compositional
matrix adjust.
 Identities = 627/639 (98%), Positives = 633/639 (99%), Gaps = 0/639
(0%)

Query  1
MKIINIGILAHVDAGKTTLTESLLYASGAISEPGSVEKGTTRTDTMFLERQRGITIQAAV  60

MKIINIGILAHVDAGKTTLTESLLYASGAISEPGSVEKGTTRTDTMFLERQRGITIQAAV
Sbjct  1
MKIINIGILAHVDAGKTTLTESLLYASGAISEPGSVEKGTTRTDTMFLERQRGITIQAAV  60

Query  61
TSFQWHRCKVNIVDTPGHMDFLAEVYRSLAVLDGAILVISAKDGVQAQTRILFHALRKMN  120

TSFQWHRCKVNIVDTPGHMDFLAEVYRSLAVLDGAILVISAKDGVQAQTRILFHALRKMN
Sbjct  61
TSFQWHRCKVNIVDTPGHMDFLAEVYRSLAVLDGAILVISAKDGVQAQTRILFHALRKMN  120

Query  121
IPTVIFINKIDQAGVDLQSVVQSVRDKLSADIIIKQTVSLSPEIVLEENTDIEAWDAVIE  180
            IPTVIFINKIDQAGVDLQSVVQSVRDKLSADIIIKQTVSLSPEIVLEENT
IEAWDAVIE
Sbjct  121
IPTVIFINKIDQAGVDLQSVVQSVRDKLSADIIIKQTVSLSPEIVLEENTGIEAWDAVIE  180

Query  181
NNDKLLEKYIAGEPISREKLVREEQRRVQDASLFPVYYGSAKKGLGIQPLMDAVTGLFQP  240
            NND+LLEKYIAGEPISREKL REEQ+RVQDASLFPVY+GSAK
GLGIQPLMDAVTGLFQP
Sbjct  181
NNDELLEKYIAGEPISREKLAREEQQRVQDASLFPVYHGSAKNGLGIQPLMDAVTGLFQP  240

Query  241
IGEQGSAALCGSVFKVEYTDCGQRRVYLRLYSGTLRLRDTVALAGREKLKITEMRIPSKG  300
            IGEQG
AALCGSVFKVEYTDCGQRRVYLRLYSGTLRLRDTVALAGREKLKITEMRIPSKG
Sbjct  241
IGEQGGAALCGSVFKVEYTDCGQRRVYLRLYSGTLRLRDTVALAGREKLKITEMRIPSKG  300

Query  301
EIVRTDTAYPGEIVILPSDSVRLNDVLGDPTRLPRKRWREDPLPMLRTTIAPKTAAQRER  360
            EIVRTDTAY GEIVILPSDSVRLNDVLGD
TRLPRKRWREDPLPMLRTTIAPKTAAQRER
Sbjct  301
EIVRTDTAYQGEIVILPSDSVRLNDVLGDQTRLPRKRWREDPLPMLRTTIAPKTAAQRER  360

Query  361
LLDALTQLADTDPLLRCEVDSITHEIILSFLGRVQLEVVSALLSEKYKLETVVKEPSVIY  420
            LLDALTQLADTDPLLRCEVDSITHEIILSFLGRVQLEVVSALLSEKYKLETVVKEP
+VIY
Sbjct  361
```

```
LLDALTQLADTDPLLRCEVDSITHEIILSFLGRVQLEVVSALLSEKYKLETVVKEPTVIY  420

Query  421
MERPLKAASHTIHIEVPPNPFWASIGLSVTPLSLGSGVQYESRVSLGYLNQSFQNAVRDG  480

MERPLKAASHTIHIEVPPNPFWASIGLSVTPLSLGSGVQYESRVSLGYLNQSFQNAVRDG
Sbjct  421
MERPLKAASHTIHIEVPPNPFWASIGLSVTPLSLGSGVQYESRVSLGYLNQSFQNAVRDG  480

Query  481
IRYGLEQGLFGWNVTDCKICFEYGLYYSPVSTPADFRSLAPIVLEQALKESGTQLLEPYL  540

IRYGLEQGLFGWNVTDCKICFEYGLYYSPVSTPADFRSLAPIVLEQALKESGTQLLEPYL
Sbjct  481
IRYGLEQGLFGWNVTDCKICFEYGLYYSPVSTPADFRSLAPIVLEQALKESGTQLLEPYL  540

Query  541
SFILYAPQEYLSRAYHDAPKYCATIETAQVKKDEVVFTGEIPARCIQAYRTDLAFYTNGR  600

SFILYAPQEYLSRAYHDAPKYCATIETAQVKKDEVVFTGEIPARCIQAYRTDLAFYTNGR
Sbjct  541
SFILYAPQEYLSRAYHDAPKYCATIETAQVKKDEVVFTGEIPARCIQAYRTDLAFYTNGR  600

Query  601  SVCLTELKGYQAAVGQPVIQPRRPNSRLDKVRHMFQKVM  639
            SVCLTELKGYQAAVGQPVIQPRRPNSRLDKVR+MFQK+M
Sbjct  601  SVCLTELKGYQAAVGQPVIQPRRPNSRLDKVRYMFQKIM  639


>WP_014566327.1 TetM/TetW/TetO/TetS family tetracycline resistance
ribosomal
protection protein [Lactobacillus amylovorus]
 AEA32895.1 translation elongation factor G (plasmid) [Lactobacillus
amylovorus
GRL1118]
Length=642

 Score = 1288 bits (3333),  Expect = 0.0, Method: Compositional
matrix adjust.
 Identities = 627/638 (98%), Positives = 634/638 (99%), Gaps = 0/638
(0%)

Query  1
MKIINIGILAHVDAGKTTLTESLLYASGAISEPGSVEKGTTRTDTMFLERQRGITIQAAV  60

MKIINIGILAHVDAGKTTLTESLLYASGAISEPGSVEKGTTRTDTMFLERQRGITIQAAV
Sbjct  1
MKIINIGILAHVDAGKTTLTESLLYASGAISEPGSVEKGTTRTDTMFLERQRGITIQAAV  60

Query  61
TSFQWHRCKVNIVDTPGHMDFLAEVYRSLAVLDGAILVISAKDGVQAQTRILFHALRKMN  120

TSFQWHRCKVNIVDTPGHMDFLAEVYRSLAVLDGAILVISAKDGVQAQTRILFHALRKMN
Sbjct  61
TSFQWHRCKVNIVDTPGHMDFLAEVYRSLAVLDGAILVISAKDGVQAQTRILFHALRKMN  120
```

Query 121
IPTVIFINKIDQAGVDLQSVVQSVRDKLSADIIIKQTVSLSPEIVLEENTDIEAWDAVIE 180

IPTVIFINKIDQAGVDLQSVVQSVRDKLSADIIIKQTVSLSPEIVLEENTDIEAWDAVIE
Sbjct 121
IPTVIFINKIDQAGVDLQSVVQSVRDKLSADIIIKQTVSLSPEIVLEENTDIEAWDAVIE 180

Query 181
NNDKLLEKYIAGEPISREKLVREEQRRVQDASLFPVYYGSAKKGLGIQPLMDAVTGLFQP 240

NNDKLLEKYIAGEPISREKLVREEQRRVQDASLFPVYYGSAKKGLGIQPLMDAVTGLFQP
Sbjct 181
NNDKLLEKYIAGEPISREKLVREEQRRVQDASLFPVYYGSAKKGLGIQPLMDAVTGLFQP 240

Query 241
IGEQGSAALCGSVFKVEYTDCGQRRVYLRLYSGTLRLRDTVALAGREKLKITEMRIPSKG 300

IGEQGSAALCGSVFKVEYTDCGQRRVYLRLYSGTLRLRDTVALAGREKLKITEMRIPSKG
Sbjct 241
IGEQGSAALCGSVFKVEYTDCGQRRVYLRLYSGTLRLRDTVALAGREKLKITEMRIPSKG 300

Query 301
EIVRTDTAYPGEIVILPSDSVRLNDVLGDPTRLPRKRWREDPLPMLRTTIAPKTAAQRER 360
EIVRTDTAYPGEIVILPSDSVRLNDVLGDPTRLPRKRWREDPLPMLRT
+IAPKTAAQRER
Sbjct 301
EIVRTDTAYPGEIVILPSDSVRLNDVLGDPTRLPRKRWREDPLPMLRTSIAPKTAAQRER 360

Query 361
LLDALTQLADTDPLLRCEVDSITHEIILSFLGRVQLEVVSALLSEKYKLETVVKEPSVIY 420
LLDALTQLADTDPLLR EVDSITHEIILSFLGRVQLEVVSALLSEKYKLETVVKEP
+VIY
Sbjct 361
LLDALTQLADTDPLLRYEVDSITHEIILSFLGRVQLEVVSALLSEKYKLETVVKEPTVIY 420

Query 421
MERPLKAASHTIHIEVPPNPFWASIGLSVTPLSLGSGVQYESRVSLGYLNQSFQNAVRDG 480
MERPLKAASHTIHIEVPPNPFWASIGLSVTPL
LGSGVQYESRVSLGYLNQSFQNAVRDG
Sbjct 421
MERPLKAASHTIHIEVPPNPFWASIGLSVTPLPLGSGVQYESRVSLGYLNQSFQNAVRDG 480

Query 481
IRYGLEQGLFGWNVTDCKICFEYGLYYSPVSTPADFRSLAPIVLEQALKESGTQLLEPYL 540

IRYGLEQGLFGWNVTDCKICFEYGLYYSPVSTPADFRSLAPIVLEQALKESGTQLLEPYL
Sbjct 481
IRYGLEQGLFGWNVTDCKICFEYGLYYSPVSTPADFRSLAPIVLEQALKESGTQLLEPYL 540

Query 541
SFILYAPQEYLSRAYHDAPKYCATIETAQVKKDEVVFTGEIPARCIQAYRTDLAFYTNGR 600
SF LYAP+EYLSRAYHDAPKYCATIET
QVKKDEVVFTGEIPARCIQAYRTDLAFYTNG+

Sbjct 541
SFTLYAPREYLSRAYHDAPKYCATIETVQVKKDEVVFTGEIPARCIQAYRTDLAFYTNGQ 600

Query 601 SVCLTELKGYQAAVGQPVIQPRRPNSRLDKVRHMFQKV 638
            SVCLTELKGYQAAVG+PVIQPRRPNSRLDKVR+MFQK+
Sbjct 601 SVCLTELKGYQAAVGKPVIQPRRPNSRLDKVRYMFQKI 638

>WP_107208979.1 TetM/TetW/TetO/TetS family tetracycline resistance
ribosomal
protection protein [Streptococcus suis]
 PTB15716.1 tetracycline resistance ribosomal protection protein
Tet(W) [Streptococcus
suis]
Length=639

 Score = 1288 bits (3332), Expect = 0.0, Method: Compositional
matrix adjust.
 Identities = 627/638 (98%), Positives = 632/638 (99%), Gaps = 0/638
(0%)

Query 1
MKIINIGILAHVDAGKTTLTESLLYASGAISEPGSVEKGTTRTDTMFLERQRGITIQAAV 60

MKIINIGILAHVDAGKTTLTESLLYASGAISEPGSVEKGTTRTDTMFLERQRGITIQAAV
Sbjct 1
MKIINIGILAHVDAGKTTLTESLLYASGAISEPGSVEKGTTRTDTMFLERQRGITIQAAV 60

Query 61
TSFQWHRCKVNIVDTPGHMDFLAEVYRSLAVLDGAILVISAKDGVQAQTRILFHALRKMN 120

TSFQWHRCKVNIVDTPGHMDFLAEVYRSLAVLDGAILVISAKDGVQAQTRILFHALRKMN
Sbjct 61
TSFQWHRCKVNIVDTPGHMDFLAEVYRSLAVLDGAILVISAKDGVQAQTRILFHALRKMN 120

Query 121
IPTVIFINKIDQAGVDLQSVVQSVRDKLSADIIIKQTVSLSPEIVLEENTDIEAWDAVIE 180

IPTVIFINKIDQAGVDLQSVVQSVRDKLSADIIIKQTVSLSPEIVLEENTDIEAWDAVIE
Sbjct 121
IPTVIFINKIDQAGVDLQSVVQSVRDKLSADIIIKQTVSLSPEIVLEENTDIEAWDAVIE 180

Query 181
NNDKLLEKYIAGEPISREKLVREEQRRVQDASLFPVYYGSAKKGLGIQPLMDAVTGLFQP 240
            NND+LLEKYIAGEPISREKL REEQ+RVQDASLFPVY+GSAK
GLGIQPLMDAVTGLFQP
Sbjct 181
NNDELLEKYIAGEPISREKLAREEQQRVQDASLFPVYHGSAKNGLGIQPLMDAVTGLFQP 240

Query 241
IGEQGSAALCGSVFKVEYTDCGQRRVYLRLYSGTLRLRDTVALAGREKLKITEMRIPSKG 300
            IGEQG
AALCGSVFKVEYTDCGQRRVYLRLYSGTLRLRDTVALAGREKLKITEMRIPSKG
Sbjct 241

IGEQGGAALCGSVFKVEYTDCGQRRVYLRLYSGTLRLRDTVALAGREKLKITEMRIPSKG 300

Query 301
EIVRTDTAYPGEIVILPSDSVRLNDVLGDPTRLPRKRWREDPLPMLRTTIAPKTAAQRER 360
            EIVRTDTAY GEIVILPSDSVRLNDVLGD
TRLPRKRWREDPLPMLRTTIAPKTAAQRER
Sbjct 301
EIVRTDTAYQGEIVILPSDSVRLNDVLGDQTRLPRKRWREDPLPMLRTTIAPKTAAQRER 360

Query 361
LLDALTQLADTDPLLRCEVDSITHEIILSFLGRVQLEVVSALLSEKYKLETVVKEPSVIY 420

LLDALTQLADTDPLLRCEVDSITHEIILSFLGRVQLEVVSALLSEKYKLETVVKEPSVIY
Sbjct 361
LLDALTQLADTDPLLRCEVDSITHEIILSFLGRVQLEVVSALLSEKYKLETVVKEPSVIY 420

Query 421
MERPLKAASHTIHIEVPPNPFWASIGLSVTPLSLGSGVQYESRVSLGYLNQSFQNAVRDG 480

MERPLKAASHTIHIEVPPNPFWASIGLSVTPLSLGSGVQYESRVSLGYLNQSFQNAVRDG
Sbjct 421
MERPLKAASHTIHIEVPPNPFWASIGLSVTPLSLGSGVQYESRVSLGYLNQSFQNAVRDG 480

Query 481
IRYGLEQGLFGWNVTDCKICFEYGLYYSPVSTPADFRSLAPIVLEQALKESGTQLLEPYL 540

IRYGLEQGLFGWNVTDCKICFEYGLYYSPVSTPADFRSLAPIVLEQALKESGTQLLEPYL
Sbjct 481
IRYGLEQGLFGWNVTDCKICFEYGLYYSPVSTPADFRSLAPIVLEQALKESGTQLLEPYL 540

Query 541
SFILYAPQEYLSRAYHDAPKYCATIETAQVKKDEVVFTGEIPARCIQAYRTDLAFYTNGR 600

SFILYAPQEYLSRAYHDAPKYCATIETAQVKKDEVVFTGEIPARCIQAYRTDLAFYTNGR
Sbjct 541
SFILYAPQEYLSRAYHDAPKYCATIETAQVKKDEVVFTGEIPARCIQAYRTDLAFYTNGR 600

Query 601 SVCLTELKGYQAAVGQPVIQPRRPNSRLDKVRHMFQKV 638
            SVCLTELKGYQAAVG+PVIQPRRPNSRLDKVRHMF K+
Sbjct 601 SVCLTELKGYQAAVGKPVIQPRRPNSRLDKVRHMFSKI 638

>WP_101693181.1 TetM/TetW/TetO/TetS family tetracycline resistance
ribosomal
protection protein [Clostridium sp. SN20]
Length=639

Score = 1288 bits (3332), Expect = 0.0, Method: Compositional
matrix adjust.
Identities = 624/638 (98%), Positives = 632/638 (99%), Gaps = 0/638
(0%)

Query 1
MKIINIGILAHVDAGKTTLTESLLYASGAISEPGSVEKGTTRTDTMFLERQRGITIQAAV 60

MKIINIGILAHVDAGKTTLTESLLYASGAISEPGSVEKGTTRTDTMFLERQRGITIQAAV
Sbjct 1
MKIINIGILAHVDAGKTTLTESLLYASGAISEPGSVEKGTTRTDTMFLERQRGITIQAAV 60

Query 61
TSFQWHRCKVNIVDTPGHMDFLAEVYRSLAVLDGAILVISAKDGVQAQTRILFHALRKMN 120

TSFQWHRCKVNIVDTPGHMDFLAEVYRSLAVLDGAILVISAKDGVQAQTRILFHALRKMN
Sbjct 61
TSFQWHRCKVNIVDTPGHMDFLAEVYRSLAVLDGAILVISAKDGVQAQTRILFHALRKMN 120

Query 121
IPTVIFINKIDQAGVDLQSVVQSVRDKLSADIIIKQTVSLSPEIVLEENTDIEAWDAVIE 180

IPTVIFINKIDQAGVDLQSVVQSVRDKLSADIIIKQTVSLSPEIVLEENTDIEAWDAVIE
Sbjct 121
IPTVIFINKIDQAGVDLQSVVQSVRDKLSADIIIKQTVSLSPEIVLEENTDIEAWDAVIE 180

Query 181
NNDKLLEKYIAGEPISREKLVREEQRRVQDASLFPVYYGSAKKGLGIQPLMDAVTGLFQP 240
NND+LLEKYIAGEPISREKL REEQ+RVQDASLFPVY+GSAK
GLGIQPLMDAVTGLFQP
Sbjct 181
NNDELLEKYIAGEPISREKLAREEQQRVQDASLFPVYHGSAKNGLGIQPLMDAVTGLFQP 240

Query 241
IGEQGSAALCGSVFKVEYTDCGQRRVYLRLYSGTLRLRDTVALAGREKLKITEMRIPSKG 300
IGEQG
AALCGSVFKVEYTDCGQRRVYLRLYSGTLRLRDTVALAGREKLKITEMRIPSKG
Sbjct 241
IGEQGGAALCGSVFKVEYTDCGQRRVYLRLYSGTLRLRDTVALAGREKLKITEMRIPSKG 300

Query 301
EIVRTDTAYPGEIVILPSDSVRLNDVLGDPTRLPRKRWREDPLPMLRTTIAPKTAAQRER 360
EIVRTDTAYPGEIVILPSDSVRLNDVLGDPTRLPRKRWREDPLPMLRT
+IAPKTAAQRER
Sbjct 301
EIVRTDTAYPGEIVILPSDSVRLNDVLGDPTRLPRKRWREDPLPMLRTSIAPKTAAQRER 360

Query 361
LLDALTQLADTDPLLRCEVDSITHEIILSFLGRVQLEVVSALLSEKYKLETVVKEPSVIY 420
LLDALTQLADTDPLLRCEVDSITHEIILSFLGRVQLEVVSALLSEKYKLETVVKEP
+VIY
Sbjct 361
LLDALTQLADTDPLLRCEVDSITHEIILSFLGRVQLEVVSALLSEKYKLETVVKEPTVIY 420

Query 421
MERPLKAASHTIHIEVPPNPFWASIGLSVTPLSLGSGVQYESRVSLGYLNQSFQNAVRDG 480
MERPLKAASHTIHIEVPPNPFWASIGLSVTPL
LGSGVQYESRVSLGYLNQSFQNAVRDG
Sbjct 421
MERPLKAASHTIHIEVPPNPFWASIGLSVTPLPLGSGVQYESRVSLGYLNQSFQNAVRDG 480

Query 481
IRYGLEQGLFGWNVTDCKICFEYGLYYSPVSTPADFRSLAPIVLEQALKESGTQLLEPYL 540

IRYGLEQGLFGWNVTDCKICFEYGLYYSPVSTPADFRSLAPIVLEQALKESGTQLLEPYL
Sbjct 481
IRYGLEQGLFGWNVTDCKICFEYGLYYSPVSTPADFRSLAPIVLEQALKESGTQLLEPYL 540

Query 541
SFILYAPQEYLSRAYHDAPKYCATIETAQVKKDEVVFTGEIPARCIQAYRTDLAFYTNGR 600
SF
LYAPQEYLSRAYHDAPKYCATIETAQVKKDEVVFTGEIPARCIQAYRTDLAFYTNG+
Sbjct 541
SFTLYAPQEYLSRAYHDAPKYCATIETAQVKKDEVVFTGEIPARCIQAYRTDLAFYTNGQ 600

Query 601 SVCLTELKGYQAAVGQPVIQPRRPNSRLDKVRHMFQKV 638
SVCLTELKGYQAAVG+PVIQPRRPNSRLDKVRHMF K+
Sbjct 601 SVCLTELKGYQAAVGEPVIQPRRPNSRLDKVRHMFSKI 638

>WP_098047066.1 TetM/TetW/TetO/TetS family tetracycline resistance
ribosomal
protection protein [Lactobacillus sp. UMNPBX10]
PEG94235.1 tetracycline resistance ribosomal protection protein
[Lactobacillus
sp. UMNPBX10]
Length=641

Score = 1288 bits (3332), Expect = 0.0, Method: Compositional
matrix adjust.
Identities = 626/638 (98%), Positives = 634/638 (99%), Gaps = 0/638
(0%)

Query 1
MKIINIGILAHVDAGKTTLTESLLYASGAISEPGSVEKGTTRTDTMFLERQRGITIQAAV 60
MKIINIGILAHVDAGKTTLTESLLYASGAISEPGSV
+KGTTRTDTMFLERQRGITIQAAV
Sbjct 1
MKIINIGILAHVDAGKTTLTESLLYASGAISEPGSVKKGTTRTDTMFLERQRGITIQAAV 60

Query 61
TSFQWHRCKVNIVDTPGHMDFLAEVYRSLAVLDGAILVISAKDGVQAQTRILFHALRKMN 120

TSFQWHRCKVNIVDTPGHMDFLAEVYRSLAVLDGAILVISAKDGVQAQTRILFHALRKMN
Sbjct 61
TSFQWHRCKVNIVDTPGHMDFLAEVYRSLAVLDGAILVISAKDGVQAQTRILFHALRKMN 120

Query 121
IPTVIFINKIDQAGVDLQSVVQSVRDKLSADIIIKQTVSLSPEIVLEENTDIEAWDAVIE 180

IPTVIFINKIDQAGVDLQSVVQSVRDKLSADIIIKQTVSLSPEIVLEENTDIEAWDAVIE
Sbjct 121
IPTVIFINKIDQAGVDLQSVVQSVRDKLSADIIIKQTVSLSPEIVLEENTDIEAWDAVIE 180

Query 181

```
NNDKLLEKYIAGEPISREKLVREEQRRVQDASLFPVYYGSAKKGLGIQPLMDAVTGLFQP  240

NNDKLLEKYIAGEPISREKLVREEQRRVQDASLFPVYYGSAKKGLGIQPLMDAVTGLFQP
Sbjct  181
NNDKLLEKYIAGEPISREKLVREEQRRVQDASLFPVYYGSAKKGLGIQPLMDAVTGLFQP  240

Query  241
IGEQGSAALCGSVFKVEYTDCGQRRVYLRLYSGTLRLRDTVALAGREKLKITEMRIPSKG  300

IGEQGSAALCGSVFKVEYTDCGQRRVYLRLYSGTLRLRDTVALAGREKLKITEMRIPSKG
Sbjct  241
IGEQGSAALCGSVFKVEYTDCGQRRVYLRLYSGTLRLRDTVALAGREKLKITEMRIPSKG  300

Query  301
EIVRTDTAYPGEIVILPSDSVRLNDVLGDPTRLPRKRWREDPLPMLRTTIAPKTAAQRER  360
            EIVRTDTAYPGEIVILPSDSVRLNDVLGDPTRLPRKRWREDPLPMLRT
+IAPKTAAQRER
Sbjct  301
EIVRTDTAYPGEIVILPSDSVRLNDVLGDPTRLPRKRWREDPLPMLRTSIAPKTAAQRER  360

Query  361
LLDALTQLADTDPLLRCEVDSITHEIILSFLGRVQLEVVSALLSEKYKLETVVKEPSVIY  420
            LLDALTQLADTDPLLRCEVDSITHEIILSFLGRVQLEVVSALLSEKYKLETVVKEP
+VIY
Sbjct  361
LLDALTQLADTDPLLRCEVDSITHEIILSFLGRVQLEVVSALLSEKYKLETVVKEPTVIY  420

Query  421
MERPLKAASHTIHIEVPPNPFWASIGLSVTPLSLGSGVQYESRVSLGYLNQSFQNAVRDG  480
            MERPLKAASHTIHIEVPPNPFWASIGLSVTPL
LGSGVQYESRVSLGYLNQSFQNAVRDG
Sbjct  421
MERPLKAASHTIHIEVPPNPFWASIGLSVTPLPLGSGVQYESRVSLGYLNQSFQNAVRDG  480

Query  481
IRYGLEQGLFGWNVTDCKICFEYGLYYSPVSTPADFRSLAPIVLEQALKESGTQLLEPYL  540

IRYGLEQGLFGWNVTDCKICFEYGLYYSPVSTPADFRSLAPIVLEQALKESGTQLLEP L
Sbjct  481
IRYGLEQGLFGWNVTDCKICFEYGLYYSPVSTPADFRSLAPIVLEQALKESGTQLLEPCL  540

Query  541
SFILYAPQEYLSRAYHDAPKYCATIETAQVKKDEVVFTGEIPARCIQAYRTDLAFYTNGR  600
            SF LYAP+EYLSRAYHDAPKYCATIET
QVKKDEVVFTGEIPARCIQAYRTDLAFYTNG+
Sbjct  541
SFTLYAPREYLSRAYHDAPKYCATIETVQVKKDEVVFTGEIPARCIQAYRTDLAFYTNGQ  600

Query  601  SVCLTELKGYQAAVGQPVIQPRRPNSRLDKVRHMFQKV  638
            SVCLTELKGYQAAVG+PVIQPRRPNSRLDKVR+MFQK+
Sbjct  601  SVCLTELKGYQAAVGKPVIQPRRPNSRLDKVRYMFQKI  638


>WP_087713428.1 MULTISPECIES: TetM/TetW/TetO/TetS family
```

tetracycline resistance
ribosomal protection protein [Lactobacillus]
 ARW75805.1 tetracycline resistance ribosomal protection protein
[Lactobacillus
johnsonii]
 ARW76235.1 tetracycline resistance ribosomal protection protein
[Lactobacillus
johnsonii]
 PAB43314.1 tetracycline resistance ribosomal protection protein
[Lactobacillus
johnsonii]
 PAB52008.1 tetracycline resistance ribosomal protection protein
[Lactobacillus
johnsonii]
 PAB53400.1 tetracycline resistance ribosomal protection protein
[Lactobacillus
johnsonii]
 PEG66900.1 TetM/TetW/TetO/TetS family tetracycline resistance
ribosomal
protection protein [Lactobacillus johnsonii]
 PEG76973.1 TetM/TetW/TetO/TetS family tetracycline resistance
ribosomal
protection protein [Lactobacillus sp. UMNPBX19]
Length=641

 Score = 1288 bits (3332), Expect = 0.0, Method: Compositional
matrix adjust.
 Identities = 626/638 (98%), Positives = 633/638 (99%), Gaps = 0/638
(0%)

Query 1
MKIINIGILAHVDAGKTTLTESLLYASGAISEPGSVEKGTTRTDTMFLERQRGITIQAAV 60
            MKIINIGILAHVDAGKTTLTESLLYASGAISEPGSVEKGTT
TDTMFLERQRGITIQAAV
Sbjct 1
MKIINIGILAHVDAGKTTLTESLLYASGAISEPGSVEKGTTSTDTMFLERQRGITIQAAV 60

Query 61
TSFQWHRCKVNIVDTPGHMDFLAEVYRSLAVLDGAILVISAKDGVQAQTRILFHALRKMN 120

TSFQWHRCKVNIVDTPGHMDFLAEVYRSLAVLDGAILVISAKDGVQAQTRILFHALRKMN
Sbjct 61
TSFQWHRCKVNIVDTPGHMDFLAEVYRSLAVLDGAILVISAKDGVQAQTRILFHALRKMN 120

Query 121
IPTVIFINKIDQAGVDLQSVVQSVRDKLSADIIIKQTVSLSPEIVLEENTDIEAWDAVIE 180

IPTVIFINKIDQAGVDLQSVVQSVRDKLSADIIIKQTVSLSPEIVLEENTDIEAWDAVIE
Sbjct 121
IPTVIFINKIDQAGVDLQSVVQSVRDKLSADIIIKQTVSLSPEIVLEENTDIEAWDAVIE 180

Query 181
NNDKLLEKYIAGEPISREKLVREEQRRVQDASLFPVYYGSAKKGLGIQPLMDAVTGLFQP 240

```
NNDKLLEKYIAGEPISREKLVREEQRRVQDASLFPVYYGSAKKGLGIQPLMDAVTGLFQP
Sbjct  181
NNDKLLEKYIAGEPISREKLVREEQRRVQDASLFPVYYGSAKKGLGIQPLMDAVTGLFQP  240

Query  241
IGEQGSAALCGSVFKVEYTDCGQRRVYLRLYSGTLRLRDTVALAGREKLKITEMRIPSKG  300

IGEQGSAALCGSVFKVEYTDCGQRRVYLRLYSGTLRLRDTVALAGREKLKITEMRIPSKG
Sbjct  241
IGEQGSAALCGSVFKVEYTDCGQRRVYLRLYSGTLRLRDTVALAGREKLKITEMRIPSKG  300

Query  301
EIVRTDTAYPGEIVILPSDSVRLNDVLGDPTRLPRKRWREDPLPMLRTTIAPKTAAQRER  360
            EIVRTDTAYPGEIVILPSDSVRLNDVLGDPTRLPRKRWREDPLPMLRT
+IAPKTAAQRER
Sbjct  301
EIVRTDTAYPGEIVILPSDSVRLNDVLGDPTRLPRKRWREDPLPMLRTSIAPKTAAQRER  360

Query  361
LLDALTQLADTDPLLRCEVDSITHEIILSFLGRVQLEVVSALLSEKYKLETVVKEPSVIY  420
            LLDALTQLADTDPLLRCEVDSITHEIILSFLGRVQLEVVSALLSEKYKLETVVKEP
+VIY
Sbjct  361
LLDALTQLADTDPLLRCEVDSITHEIILSFLGRVQLEVVSALLSEKYKLETVVKEPTVIY  420

Query  421
MERPLKAASHTIHIEVPPNPFWASIGLSVTPLSLGSGVQYESRVSLGYLNQSFQNAVRDG  480
            MERPLKAASHTIHIEVPPNPFWASIGLSVTPL
LGSGVQYESRVSLGYLNQSFQNAVRDG
Sbjct  421
MERPLKAASHTIHIEVPPNPFWASIGLSVTPLPLGSGVQYESRVSLGYLNQSFQNAVRDG  480

Query  481
IRYGLEQGLFGWNVTDCKICFEYGLYYSPVSTPADFRSLAPIVLEQALKESGTQLLEPYL  540

IRYGLEQGLFGWNVTDCKICFEYGLYYSPVSTPADFRSLAPIVLEQALKESGTQLLEPYL
Sbjct  481
IRYGLEQGLFGWNVTDCKICFEYGLYYSPVSTPADFRSLAPIVLEQALKESGTQLLEPYL  540

Query  541
SFILYAPQEYLSRAYHDAPKYCATIETAQVKKDEVVFTGEIPARCIQAYRTDLAFYTNGR  600
            SF LYAP+EYLSRAYHDAPKYCATIET QVKKDEVVFTGEIPARCIQAYRTDLA
YTNG+
Sbjct  541
SFTLYAPREYLSRAYHDAPKYCATIETVQVKKDEVVFTGEIPARCIQAYRTDLASYTNGQ  600

Query  601  SVCLTELKGYQAAVGQPVIQPRRPNSRLDKVRHMFQKV  638
            SVCLTELKGYQAAVG+PVIQPRRPNSRLDKVR+MFQK+
Sbjct  601  SVCLTELKGYQAAVGKPVIQPRRPNSRLDKVRYMFQKI  638


>WP_087190428.1 TetM/TetW/TetO/TetS family tetracycline resistance
ribosomal
protection protein [Gordonibacter urolithinfaciens]
```

OUO88223.1 tetracycline resistance ribosomal protection protein Tet(W) [Gordonibacter urolithinfaciens]
Length=639

Score = 1288 bits (3332), Expect = 0.0, Method: Compositional matrix adjust.
Identities = 629/639 (98%), Positives = 632/639 (99%), Gaps = 0/639 (0%)

```
Query  1
MKIINIGILAHVDAGKTTLTESLLYASGAISEPGSVEKGTTRTDTMFLERQRGITIQAAV  60

MKIINIGILAHVDAGKTTLTESLLYASGAISEPGSVEKGTTRTDTMFLERQRGITIQAAV
Sbjct  1
MKIINIGILAHVDAGKTTLTESLLYASGAISEPGSVEKGTTRTDTMFLERQRGITIQAAV  60

Query  61
TSFQWHRCKVNIVDTPGHMDFLAEVYRSLAVLDGAILVISAKDGVQAQTRILFHALRKMN  120

TSFQWHRCKVNIVDTPGHMDFLAEVYRSLAVLDGAILVISAKDGVQAQTRILFHALRKMN
Sbjct  61
TSFQWHRCKVNIVDTPGHMDFLAEVYRSLAVLDGAILVISAKDGVQAQTRILFHALRKMN  120

Query  121
IPTVIFINKIDQAGVDLQSVVQSVRDKLSADIIIKQTVSLSPEIVLEENTDIEAWDAVIE  180

IPTVIFINKIDQAGVDLQSVVQSVRDKLSADIIIKQTVSLSPEIVLEENTDIEAWDAVIE
Sbjct  121
IPTVIFINKIDQAGVDLQSVVQSVRDKLSADIIIKQTVSLSPEIVLEENTDIEAWDAVIE  180

Query  181
NNDKLLEKYIAGEPISREKLVREEQRRVQDASLFPVYYGSAKKGLGIQPLMDAVTGLFQP  240
            NND+LLEKYIAGEPISREKL REEQ+RVQDASLFPVY+GSAK
GLGIQPLMDAVTGLFQP
Sbjct  181
NNDELLEKYIAGEPISREKLAREEQQRVQDASLFPVYHGSAKNGLGIQPLMDAVTGLFQP  240

Query  241
IGEQGSAALCGSVFKVEYTDCGQRRVYLRLYSGTLRLRDTVALAGREKLKITEMRIPSKG  300
            IGEQG
AALCGSVFKVEYTDCGQRRVYLRLYSGTLRLRDTVALAGREKLKITEMRIPSKG
Sbjct  241
IGEQGGAALCGSVFKVEYTDCGQRRVYLRLYSGTLRLRDTVALAGREKLKITEMRIPSKG  300

Query  301
EIVRTDTAYPGEIVILPSDSVRLNDVLGDPTRLPRKRWREDPLPMLRTTIAPKTAAQRER  360
            EIVRTDTAY GEIVILPSDSVRLNDVLGD
TRLPRKRWREDPLPMLRTTIAPKTAAQRER
Sbjct  301
EIVRTDTAYQGEIVILPSDSVRLNDVLGDQTRLPRKRWREDPLPMLRTTIAPKTAAQRER  360

Query  361
LLDALTQLADTDPLLRCEVDSITHEIILSFLGRVQLEVVSALLSEKYKLETVVKEPSVIY  420
```

            LLDALTQLADTDPLLR
EVDSITHEIILSFLGRVQLEVVSALLSEKYKLETVVKEPSVIY
Sbjct 361
LLDALTQLADTDPLLRYEVDSITHEIILSFLGRVQLEVVSALLSEKYKLETVVKEPSVIY 420

Query 421
MERPLKAASHTIHIEVPPNPFWASIGLSVTPLSLGSGVQYESRVSLGYLNQSFQNAVRDG 480

MERPLKAASHTIHIEVPPNPFWASIGLSVTPLSLGSGVQYESRVSLGYLNQSFQNAVRDG
Sbjct 421
MERPLKAASHTIHIEVPPNPFWASIGLSVTPLSLGSGVQYESRVSLGYLNQSFQNAVRDG 480

Query 481
IRYGLEQGLFGWNVTDCKICFEYGLYYSPVSTPADFRSLAPIVLEQALKESGTQLLEPYL 540

IRYGLEQGLFGWNVTDCKICFEYGLYYSPVSTPADFRSLAPIVLEQALKESGTQLLEPYL
Sbjct 481
IRYGLEQGLFGWNVTDCKICFEYGLYYSPVSTPADFRSLAPIVLEQALKESGTQLLEPYL 540

Query 541
SFILYAPQEYLSRAYHDAPKYCATIETAQVKKDEVVFTGEIPARCIQAYRTDLAFYTNGR 600
            SFILYAPQEYLSRAYHDAPKYCATIETAQVKKDEVVFTGEIPARCIQAYRTDLA
YTNGR
Sbjct 541
SFILYAPQEYLSRAYHDAPKYCATIETAQVKKDEVVFTGEIPARCIQAYRTDLASYTNGR 600

Query 601 SVCLTELKGYQAAVGQPVIQPRRPNSRLDKVRHMFQKVM 639
            SVCLTELKGYQAAVGQPVIQPRRPNSRLDKVRHMFQKVM
Sbjct 601 SVCLTELKGYQAAVGQPVIQPRRPNSRLDKVRHMFQKVM 639

>AMP54855.1 TetM-TetW-TetO-TetS [uncultured bacterium]
Length=639

 Score = 1288 bits (3332), Expect = 0.0, Method: Compositional
matrix adjust.
 Identities = 627/639 (98%), Positives = 632/639 (99%), Gaps = 0/639
(0%)

Query 1
MKIINIGILAHVDAGKTTLTESLLYASGAISEPGSVEKGTTRTDTMFLERQRGITIQAAV 60

MKIINIGILAHVDAGKTTLTESLLYASGAISEPGSVEKGTTRTDTMFLERQRGITIQAAV
Sbjct 1
MKIINIGILAHVDAGKTTLTESLLYASGAISEPGSVEKGTTRTDTMFLERQRGITIQAAV 60

Query 61
TSFQWHRCKVNIVDTPGHMDFLAEVYRSLAVLDGAILVISAKDGVQAQTRILFHALRKMN 120

TSFQWHRCKVNIVDTPGHMDFLAEVYRSLAVLDGAILVISAKDGVQAQTRILFHALRKMN
Sbjct 61
TSFQWHRCKVNIVDTPGHMDFLAEVYRSLAVLDGAILVISAKDGVQAQTRILFHALRKMN 120

Query 121

```
IPTVIFINKIDQAGVDLQSVVQSVRDKLSADIIIKQTVSLSPEIVLEENTDIEAWDAVIE  180

IPTVIFINKIDQAGVDLQSVVQSVRDKLSADIIIKQTVSLSPEIVLEENTDIEAWDAVIE
Sbjct  121
IPTVIFINKIDQAGVDLQSVVQSVRDKLSADIIIKQTVSLSPEIVLEENTDIEAWDAVIE  180

Query  181
NNDKLLEKYIAGEPISREKLVREEQRRVQDASLFPVYYGSAKKGLGIQPLMDAVTGLFQP  240
            NND+LLEKYIAGEPISREKL REEQ+RVQDASLFPVY+GSAK
GLGIQPLMDAVTGLFQP
Sbjct  181
NNDELLEKYIAGEPISREKLAREEQQRVQDASLFPVYHGSAKNGLGIQPLMDAVTGLFQP  240

Query  241
IGEQGSAALCGSVFKVEYTDCGQRRVYLRLYSGTLRLRDTVALAGREKLKITEMRIPSKG  300
            IGEQG
AALCGSVFKVEYTDCGQRRVYLRLYSGTLRLRDTVALAGREKLKITEMRIPSKG
Sbjct  241
IGEQGGAALCGSVFKVEYTDCGQRRVYLRLYSGTLRLRDTVALAGREKLKITEMRIPSKG  300

Query  301
EIVRTDTAYPGEIVILPSDSVRLNDVLGDPTRLPRKRWREDPLPMLRTTIAPKTAAQRER  360
            EIVRTDTAY GEIVILPSDSVRLNDVLGD
TRLPRKRWREDPLPMLRTTIAPKTAAQRER
Sbjct  301
EIVRTDTAYQGEIVILPSDSVRLNDVLGDQTRLPRKRWREDPLPMLRTTIAPKTAAQRER  360

Query  361
LLDALTQLADTDPLLRCEVDSITHEIILSFLGRVQLEVVSALLSEKYKLETVVKEPSVIY  420

LLDALTQLADTDPLLRCEVDSITHEIILSFLGRVQLEVVSALLSEKYKLETVVKEPSVIY
Sbjct  361
LLDALTQLADTDPLLRCEVDSITHEIILSFLGRVQLEVVSALLSEKYKLETVVKEPSVIY  420

Query  421
MERPLKAASHTIHIEVPPNPFWASIGLSVTPLSLGSGVQYESRVSLGYLNQSFQNAVRDG  480

MERPLKAASHTIHIEVPPNPFWASIGLSVTPLSLGSGVQYESRVSLGYLNQSFQNAVRDG
Sbjct  421
MERPLKAASHTIHIEVPPNPFWASIGLSVTPLSLGSGVQYESRVSLGYLNQSFQNAVRDG  480

Query  481
IRYGLEQGLFGWNVTDCKICFEYGLYYSPVSTPADFRSLAPIVLEQALKESGTQLLEPYL  540

IRYGLEQGLFGWNVTDCKICFEYGLYYSPVSTPADFRSLAPIVLEQALKESGTQLLEPYL
Sbjct  481
IRYGLEQGLFGWNVTDCKICFEYGLYYSPVSTPADFRSLAPIVLEQALKESGTQLLEPYL  540

Query  541
SFILYAPQEYLSRAYHDAPKYCATIETAQVKKDEVVFTGEIPARCIQAYRTDLAFYTNGR  600
            SF LYAP+EYLSRAYHDAPKYCATIET
QVKKDEVVFTGEIPARCIQAYRTDLAFYTNG+
Sbjct  541
SFTLYAPREYLSRAYHDAPKYCATIETVQVKKDEVVFTGEIPARCIQAYRTDLAFYTNGQ  600
```

```
Query  601  SVCLTELKGYQAAVGQPVIQPRRPNSRLDKVRHMFQKVM  639
            SVCLTELKGYQAAVGQPVIQPRRPNSRLDKVRHMFQKVM
Sbjct  601  SVCLTELKGYQAAVGQPVIQPRRPNSRLDKVRHMFQKVM  639
```

>WP_098035490.1 MULTISPECIES: TetM/TetW/TetO/TetS family
tetracycline resistance
ribosomal protection protein [Lactobacillus]
 PEG88374.1 tetracycline resistance ribosomal protection protein
[Lactobacillus
sp. UMNPBX13]
 PEH00967.1 tetracycline resistance ribosomal protection protein
[Lactobacillus
sp. UMNPBX7]
Length=639

 Score = 1287 bits (3331), Expect = 0.0, Method: Compositional
matrix adjust.
 Identities = 626/639 (98%), Positives = 634/639 (99%), Gaps = 0/639
(0%)

```
Query  1
MKIINIGILAHVDAGKTTLTESLLYASGAISEPGSVEKGTTRTDTMFLERQRGITIQAAV  60
            MKIINIGILAHVDAGKTTLTESLLYASGAISEPGSV
+KGTTRTDTMFLERQRGITIQAAV
Sbjct  1
MKIINIGILAHVDAGKTTLTESLLYASGAISEPGSVKKGTTRTDTMFLERQRGITIQAAV  60

Query  61
TSFQWHRCKVNIVDTPGHMDFLAEVYRSLAVLDGAILVISAKDGVQAQTRILFHALRKMN  120

TSFQWHRCKVNIVDTPGHMDFLAEVYRSLAVLDGAILVISAKDGVQAQTRILFHALRKMN
Sbjct  61
TSFQWHRCKVNIVDTPGHMDFLAEVYRSLAVLDGAILVISAKDGVQAQTRILFHALRKMN  120

Query  121
IPTVIFINKIDQAGVDLQSVVQSVRDKLSADIIIKQTVSLSPEIVLEENTDIEAWDAVIE  180

IPTVIFINKIDQAGVDLQSVVQSVRDKLSADIIIKQTVSLSPEIVLEENTDIEAWDAVIE
Sbjct  121
IPTVIFINKIDQAGVDLQSVVQSVRDKLSADIIIKQTVSLSPEIVLEENTDIEAWDAVIE  180

Query  181
NNDKLLEKYIAGEPISREKLVREEQRRVQDASLFPVYYGSAKKGLGIQPLMDAVTGLFQP  240

NNDKLLEKYIAGEPISREKLVREEQRRVQDASLFPVYYGSAKKGLGIQPLMDAVTGLFQP
Sbjct  181
NNDKLLEKYIAGEPISREKLVREEQRRVQDASLFPVYYGSAKKGLGIQPLMDAVTGLFQP  240

Query  241
IGEQGSAALCGSVFKVEYTDCGQRRVYLRLYSGTLRLRDTVALAGREKLKITEMRIPSKG  300

IGEQGSAALCGSVFKVEYTDCGQRRVYLRLYSGTLRLRDTVALAGREKLKITEMRIPSKG
```

Sbjct 241
IGEQGSAALCGSVFKVEYTDCGQRRVYLRLYSGTLRLRDTVALAGREKLKITEMRIPSKG 300

Query 301
EIVRTDTAYPGEIVILPSDSVRLNDVLGDPTRLPRKRWREDPLPMLRTTIAPKTAAQRER 360
EIVRTDTAYPGEIVILPSDSVRLNDVLGDPTRLPRKRWREDPLPMLRT
+IAPKTAAQRER
Sbjct 301
EIVRTDTAYPGEIVILPSDSVRLNDVLGDPTRLPRKRWREDPLPMLRTSIAPKTAAQRER 360

Query 361
LLDALTQLADTDPLLRCEVDSITHEIILSFLGRVQLEVVSALLSEKYKLETVVKEPSVIY 420
LLDALTQLADTDPLLR EVDSITHEIILSFLGRVQLEVVSALLSEKYKLETVVKEP
+VIY
Sbjct 361
LLDALTQLADTDPLLRFEVDSITHEIILSFLGRVQLEVVSALLSEKYKLETVVKEPTVIY 420

Query 421
MERPLKAASHTIHIEVPPNPFWASIGLSVTPLSLGSGVQYESRVSLGYLNQSFQNAVRDG 480
MERPLKAASHTIHIEVPPNPFWASIGLSVTPL
LGSGVQYESRVSLGYLNQSFQNAVRDG
Sbjct 421
MERPLKAASHTIHIEVPPNPFWASIGLSVTPLPLGSGVQYESRVSLGYLNQSFQNAVRDG 480

Query 481
IRYGLEQGLFGWNVTDCKICFEYGLYYSPVSTPADFRSLAPIVLEQALKESGTQLLEPYL 540

IRYGLEQGLFGWNVTDCKICFEYGLYYSPVSTPADFRSLAPIVLEQALKESGTQLLEPYL
Sbjct 481
IRYGLEQGLFGWNVTDCKICFEYGLYYSPVSTPADFRSLAPIVLEQALKESGTQLLEPYL 540

Query 541
SFILYAPQEYLSRAYHDAPKYCATIETAQVKKDEVVFTGEIPARCIQAYRTDLAFYTNGR 600
SF LYAP+EYLSRAYHDAPKYCATIE
QVKKDEVVFTGEIPARCIQAYRTDLAFYTNG+
Sbjct 541
SFTLYAPREYLSRAYHDAPKYCATIEPVQVKKDEVVFTGEIPARCIQAYRTDLAFYTNGQ 600

Query 601 SVCLTELKGYQAAVGQPVIQPRRPNSRLDKVRHMFQKVM 639
SVCLTELKGYQAAVG+PVIQPRRPNSRLDKVR+MFQK+M
Sbjct 601 SVCLTELKGYQAAVGKPVIQPRRPNSRLDKVRYMFQKIM 639

>WP_086118050.1 TetM/TetW/TetO/TetS family tetracycline resistance
ribosomal
protection protein [Lactobacillus reuteri]
OTA47671.1 tetracycline resistance ribosomal protection protein
[Lactobacillus
reuteri]
OTA92969.1 tetracycline resistance ribosomal protection protein
[Lactobacillus
reuteri]
Length=639

Score = 1287 bits (3331), Expect = 0.0, Method: Compositional matrix adjust.
Identities = 626/639 (98%), Positives = 634/639 (99%), Gaps = 0/639 (0%)

```
Query  1
MKIINIGILAHVDAGKTTLTESLLYASGAISEPGSVEKGTTRTDTMFLERQRGITIQAAV  60
            MKIINIGILAHVDAGKTTLTESLLYASGAISE GSV
+KGTTRTDTMFLERQRGITIQAAV
Sbjct  1
MKIINIGILAHVDAGKTTLTESLLYASGAISERGSVKKGTTRTDTMFLERQRGITIQAAV  60

Query  61
TSFQWHRCKVNIVDTPGHMDFLAEVYRSLAVLDGAILVISAKDGVQAQTRILFHALRKMN  120

TSFQWHRCKVNIVDTPGHMDFLAEVYRSLAVLDGAILVISAKDGVQAQTRILFHALRKMN
Sbjct  61
TSFQWHRCKVNIVDTPGHMDFLAEVYRSLAVLDGAILVISAKDGVQAQTRILFHALRKMN  120

Query  121
IPTVIFINKIDQAGVDLQSVVQSVRDKLSADIIIKQTVSLSPEIVLEENTDIEAWDAVIE  180

IPTVIFINKIDQAGVDLQSVVQSVRDKLSADIIIKQTVSLSPEIVLEENTDIEAWDAVIE
Sbjct  121
IPTVIFINKIDQAGVDLQSVVQSVRDKLSADIIIKQTVSLSPEIVLEENTDIEAWDAVIE  180

Query  181
NNDKLLEKYIAGEPISREKLVREEQRRVQDASLFPVYYGSAKKGLGIQPLMDAVTGLFQP  240

NNDKLLEKYIAGEPISREKLVREEQRRVQDASLFPVYYGSAKKGLGIQPLMDAVTGLFQP
Sbjct  181
NNDKLLEKYIAGEPISREKLVREEQRRVQDASLFPVYYGSAKKGLGIQPLMDAVTGLFQP  240

Query  241
IGEQGSAALCGSVFKVEYTDCGQRRVYLRLYSGTLRLRDTVALAGREKLKITEMRIPSKG  300

IGEQGSAALCGSVFKVEYTDCGQRRVYLRLYSGTLRLRDTVALAGREKLKITEMRIPSKG
Sbjct  241
IGEQGSAALCGSVFKVEYTDCGQRRVYLRLYSGTLRLRDTVALAGREKLKITEMRIPSKG  300

Query  301
EIVRTDTAYPGEIVILPSDSVRLNDVLGDPTRLPRKRWREDPLPMLRTTIAPKTAAQRER  360
            EIVRTDTAYPGEIVILPSDSVRLNDVLGDPTRLPRKRWREDPLPMLRT
+IAPKTAAQRER
Sbjct  301
EIVRTDTAYPGEIVILPSDSVRLNDVLGDPTRLPRKRWREDPLPMLRTSIAPKTAAQRER  360

Query  361
LLDALTQLADTDPLLRCEVDSITHEIILSFLGRVQLEVVSALLSEKYKLETVVKEPSVIY  420
            LLDALTQLADTDPLLRCEVDSITHEIILSFLGRVQLEVVSALLSEKYKLETVVKEP
+VIY
Sbjct  361
LLDALTQLADTDPLLRCEVDSITHEIILSFLGRVQLEVVSALLSEKYKLETVVKEPTVIY  420
```

Query 421
MERPLKAASHTIHIEVPPNPFWASIGLSVTPLSLGSGVQYESRVSLGYLNQSFQNAVRDG 480
            MERPLKAASHTIHIEVPPNPFWASIGLSVTPL
LGSGVQYESRVSLGYLNQSFQNAVRDG
Sbjct 421
MERPLKAASHTIHIEVPPNPFWASIGLSVTPLPLGSGVQYESRVSLGYLNQSFQNAVRDG 480

Query 481
IRYGLEQGLFGWNVTDCKICFEYGLYYSPVSTPADFRSLAPIVLEQALKESGTQLLEPYL 540

IRYGLEQGLFGWNVTDCKICFEYGLYYSPVSTPADFRSLAPIVLEQALKESGTQLLEPYL
Sbjct 481
IRYGLEQGLFGWNVTDCKICFEYGLYYSPVSTPADFRSLAPIVLEQALKESGTQLLEPYL 540

Query 541
SFILYAPQEYLSRAYHDAPKYCATIETAQVKKDEVVFTGEIPARCIQAYRTDLAFYTNGR 600
            SF LYAP+EYLSRAYHDAPKYCATIET QVKKDEVVFTGEIPARCIQAYRTDLA
YTNG+
Sbjct 541
SFTLYAPREYLSRAYHDAPKYCATIETVQVKKDEVVFTGEIPARCIQAYRTDLASYTNGQ 600

Query 601 SVCLTELKGYQAAVGQPVIQPRRPNSRLDKVRHMFQKVM 639
            SVCLTELKGYQAAVG+PVIQPRRPNSRLDKVR+MFQK+M
Sbjct 601 SVCLTELKGYQAAVGKPVIQPRRPNSRLDKVRYMFQKIM 639

>AMP52265.1 TetM-TetW-TetO-TetS [uncultured bacterium]
Length=639

Score = 1287 bits (3331), Expect = 0.0, Method: Compositional matrix adjust.
Identities = 627/639 (98%), Positives = 633/639 (99%), Gaps = 0/639 (0%)

Query 1
MKIINIGILAHVDAGKTTLTESLLYASGAISEPGSVEKGTTRTDTMFLERQRGITIQAAV 60

MKIINIGILAHVDAGKTTLTESLLYASGAISEPGSVEKGTTRTDTMFLERQRGITIQAAV
Sbjct 1
MKIINIGILAHVDAGKTTLTESLLYASGAISEPGSVEKGTTRTDTMFLERQRGITIQAAV 60

Query 61
TSFQWHRCKVNIVDTPGHMDFLAEVYRSLAVLDGAILVISAKDGVQAQTRILFHALRKMN 120

TSFQWHRCKVNIVDTPGHMDFLAEVYRSLAVLDGAILVISAKDGVQAQTRILFHALRKMN
Sbjct 61
TSFQWHRCKVNIVDTPGHMDFLAEVYRSLAVLDGAILVISAKDGVQAQTRILFHALRKMN 120

Query 121
IPTVIFINKIDQAGVDLQSVVQSVRDKLSADIIIKQTVSLSPEIVLEENTDIEAWDAVIE 180

IPTVIFINKIDQAGVDLQSVVQSVRDKLSADIIIKQTVSLSPEIVLEENTDIEAWDAVIE
Sbjct 121
IPTVIFINKIDQAGVDLQSVVQSVRDKLSADIIIKQTVSLSPEIVLEENTDIEAWDAVIE 180

```
Query  181
NNDKLLEKYIAGEPISREKLVREEQRRVQDASLFPVYYGSAKKGLGIQPLMDAVTGLFQP  240
            NND+LLEKYIAGEPISREKL REEQ+RVQDASLFPVY+GSAK
GLGIQPLMDAVTGLFQP
Sbjct  181
NNDELLEKYIAGEPISREKLAREEQQRVQDASLFPVYHGSAKNGLGIQPLMDAVTGLFQP  240

Query  241
IGEQGSAALCGSVFKVEYTDCGQRRVYLRLYSGTLRLRDTVALAGREKLKITEMRIPSKG  300
            IGEQG
AALCGSVFKVEYTDCGQRRVYLRLYSGTLRLRDTVALAGREKLKITEMRIPSKG
Sbjct  241
IGEQGGAALCGSVFKVEYTDCGQRRVYLRLYSGTLRLRDTVALAGREKLKITEMRIPSKG  300

Query  301
EIVRTDTAYPGEIVILPSDSVRLNDVLGDPTRLPRKRWREDPLPMLRTTIAPKTAAQRER  360
            EIVRTDTAY GEIVILPSDSVRLNDVLGD
TRLPRKRWREDPLPMLRTTIAPKTAAQRER
Sbjct  301
EIVRTDTAYQGEIVILPSDSVRLNDVLGDQTRLPRKRWREDPLPMLRTTIAPKTAAQRER  360

Query  361
LLDALTQLADTDPLLRCEVDSITHEIILSFLGRVQLEVVSALLSEKYKLETVVKEPSVIY  420

LLDALTQLADTDPLLRCEVDSITHEIILSFLGRVQLEVVSALLSEKYKLETVVKEPSVIY
Sbjct  361
LLDALTQLADTDPLLRCEVDSITHEIILSFLGRVQLEVVSALLSEKYKLETVVKEPSVIY  420

Query  421
MERPLKAASHTIHIEVPPNPFWASIGLSVTPLSLGSGVQYESRVSLGYLNQSFQNAVRDG  480

MERPLKAASHTIHIEVPPNPFWASIGLSVTPLSLGSGVQYESRVSLGYLNQSFQNAVRDG
Sbjct  421
MERPLKAASHTIHIEVPPNPFWASIGLSVTPLSLGSGVQYESRVSLGYLNQSFQNAVRDG  480

Query  481
IRYGLEQGLFGWNVTDCKICFEYGLYYSPVSTPADFRSLAPIVLEQALKESGTQLLEPYL  540
            IRYGLEQGLFGWNVTDCKICFEYGLYYSPVSTPADFRSLAPIVLEQALK
+SGTQLLEPYL
Sbjct  481
IRYGLEQGLFGWNVTDCKICFEYGLYYSPVSTPADFRSLAPIVLEQALKKSGTQLLEPYL  540

Query  541
SFILYAPQEYLSRAYHDAPKYCATIETAQVKKDEVVFTGEIPARCIQAYRTDLAFYTNGR  600
            SFILYAPQEYLSRAYHDAPKYCATIE
AQVKKDEVVFTGEIPARCIQAYRTDLAFYTNGR
Sbjct  541
SFILYAPQEYLSRAYHDAPKYCATIEPAQVKKDEVVFTGEIPARCIQAYRTDLAFYTNGR  600

Query  601  SVCLTELKGYQAAVGQPVIQPRRPNSRLDKVRHMFQKVM  639
            SVCLTELKGYQAAVGQPVIQPRRPNSRLDKVR+MFQK+M
Sbjct  601  SVCLTELKGYQAAVGQPVIQPRRPNSRLDKVRYMFQKIM  639
```

>WP_105303201.1 TetM/TetW/TetO/TetS family tetracycline resistance ribosomal
protection protein [Anaerotruncus sp. Marseille-P4302]
Length=639

Score = 1287 bits (3330), Expect = 0.0, Method: Compositional matrix adjust.
Identities = 625/638 (98%), Positives = 632/638 (99%), Gaps = 0/638 (0%)

```
Query  1
MKIINIGILAHVDAGKTTLTESLLYASGAISEPGSVEKGTTRTDTMFLERQRGITIQAAV  60
            M IINIGILAHVDAGKTTLTESLLYASGAISEPGSVEKGTTRTDTM
LERQRGITIQAAV
Sbjct  1
MNIINIGILAHVDAGKTTLTESLLYASGAISEPGSVEKGTTRTDTMLLERQRGITIQAAV  60

Query  61
TSFQWHRCKVNIVDTPGHMDFLAEVYRSLAVLDGAILVISAKDGVQAQTRILFHALRKMN  120

TSFQWHRCKVNIVDTPGHMDFLAEVYRSLAVLDGAILVISAKDGVQAQTRILFHALRKMN
Sbjct  61
TSFQWHRCKVNIVDTPGHMDFLAEVYRSLAVLDGAILVISAKDGVQAQTRILFHALRKMN  120

Query  121
IPTVIFINKIDQAGVDLQSVVQSVRDKLSADIIIKQTVSLSPEIVLEENTDIEAWDAVIE  180

IPTVIFINKIDQAGVDLQSVVQSVRDKLSADIIIKQTVSLSPEIVLEENTDIEAWDAVIE
Sbjct  121
IPTVIFINKIDQAGVDLQSVVQSVRDKLSADIIIKQTVSLSPEIVLEENTDIEAWDAVIE  180

Query  181
NNDKLLEKYIAGEPISREKLVREEQRRVQDASLFPVYYGSAKKGLGIQPLMDAVTGLFQP  240

NNDKLLEKYIAGEPISREKLVREEQRRVQDASLFPVYYGSAKKGLGIQPLMDAVTGLFQP
Sbjct  181
NNDKLLEKYIAGEPISREKLVREEQRRVQDASLFPVYYGSAKKGLGIQPLMDAVTGLFQP  240

Query  241
IGEQGSAALCGSVFKVEYTDCGQRRVYLRLYSGTLRLRDTVALAGREKLKITEMRIPSKG  300

IGEQGSAALCGSVFKVEYTDCGQRRVYLRLYSGTLRLRDTVALAGREKLKITEMRIPSKG
Sbjct  241
IGEQGSAALCGSVFKVEYTDCGQRRVYLRLYSGTLRLRDTVALAGREKLKITEMRIPSKG  300

Query  301
EIVRTDTAYPGEIVILPSDSVRLNDVLGDPTRLPRKRWREDPLPMLRTTIAPKTAAQRER  360
            EIVRTDTAYPGEIVILPSDSVRLNDVLGDPTRLPRKRWREDPLPMLRT
+IAPKTAAQRER
Sbjct  301
EIVRTDTAYPGEIVILPSDSVRLNDVLGDPTRLPRKRWREDPLPMLRTSIAPKTAAQRER  360

Query  361
```

```
LLDALTQLADTDPLLRCEVDSITHEIILSFLGRVQLEVVSALLSEKYKLETVVKEPSVIY  420
            LLDALTQLADTDPLLRCEVDSITHEIILSFLGRVQLEVVSALLSEKYKLETVVKEP
+VIY
Sbjct  361
LLDALTQLADTDPLLRCEVDSITHEIILSFLGRVQLEVVSALLSEKYKLETVVKEPTVIY  420

Query  421
MERPLKAASHTIHIEVPPNPFWASIGLSVTPLSLGSGVQYESRVSLGYLNQSFQNAVRDG  480
            MERPLKAASHTIHIEVPPNPFWASIGLSVTPL
LGSGVQYESRVSLGYLNQSFQNAVRDG
Sbjct  421
MERPLKAASHTIHIEVPPNPFWASIGLSVTPLPLGSGVQYESRVSLGYLNQSFQNAVRDG  480

Query  481
IRYGLEQGLFGWNVTDCKICFEYGLYYSPVSTPADFRSLAPIVLEQALKESGTQLLEPYL  540

IRYGLEQGLFGWNVTDCKICFEYGLYYSPVSTPADFRSLAPIVLEQALKESGTQLLEPYL
Sbjct  481
IRYGLEQGLFGWNVTDCKICFEYGLYYSPVSTPADFRSLAPIVLEQALKESGTQLLEPYL  540

Query  541
SFILYAPQEYLSRAYHDAPKYCATIETAQVKKDEVVFTGEIPARCIQAYRTDLAFYTNGR  600
            SF LYAPQEYLSRAYHDAPKYCATIETAQVKKDEVVFTGEIP
RCIQAYRTDLAFYT+G+
Sbjct  541
SFTLYAPQEYLSRAYHDAPKYCATIETAQVKKDEVVFTGEIPVRCIQAYRTDLAFYTSGQ  600

Query  601  SVCLTELKGYQAAVGQPVIQPRRPNSRLDKVRHMFQKV  638
            SVCLTELKGYQAAVG+PVIQPRRPNSRLDKVR+MF K+
Sbjct  601  SVCLTELKGYQAAVGKPVIQPRRPNSRLDKVRYMFSKI  638


>WP_006500706.1 TetM/TetW/TetO/TetS family tetracycline resistance
ribosomal
protection protein [Lactobacillus mucosae]
 AJT49830.1 tetracycline resistance protein tetM [Lactobacillus
mucosae LM1]
Length=639

 Score = 1287 bits (3330),  Expect = 0.0, Method: Compositional
matrix adjust.
 Identities = 626/639 (98%), Positives = 633/639 (99%), Gaps = 0/639
(0%)

Query  1
MKIINIGILAHVDAGKTTLTESLLYASGAISEPGSVEKGTTRTDTMFLERQRGITIQAAV  60
            MKIINIGILAHVDAGKTTLTESLLYASGAISEPGSV
KGTTRTDTMFLERQRGITIQAAV
Sbjct  1
MKIINIGILAHVDAGKTTLTESLLYASGAISEPGSVAKGTTRTDTMFLERQRGITIQAAV  60

Query  61
TSFQWHRCKVNIVDTPGHMDFLAEVYRSLAVLDGAILVISAKDGVQAQTRILFHALRKMN  120
```

TSFQWHRCKVNIVDTPGHMDFLAEVYRSLAVLDGAILVISAKDGVQAQTRILFHALRKMN
Sbjct 61
TSFQWHRCKVNIVDTPGHMDFLAEVYRSLAVLDGAILVISAKDGVQAQTRILFHALRKMN 120

Query 121
IPTVIFINKIDQAGVDLQSVVQSVRDKLSADIIIKQTVSLSPEIVLEENTDIEAWDAVIE 180

IPTVIFINKIDQAGVDLQSVVQSVRDKLSADIIIKQTVSLSPEIVLEENTDIEAWDAVIE
Sbjct 121
IPTVIFINKIDQAGVDLQSVVQSVRDKLSADIIIKQTVSLSPEIVLEENTDIEAWDAVIE 180

Query 181
NNDKLLEKYIAGEPISREKLVREEQRRVQDASLFPVYYGSAKKGLGIQPLMDAVTGLFQP 240

NNDKLLEKYIAGEPISREKLVREEQRRVQDASLFPVYYGSAKKGLGIQPLMDAVTGLFQP
Sbjct 181
NNDKLLEKYIAGEPISREKLVREEQRRVQDASLFPVYYGSAKKGLGIQPLMDAVTGLFQP 240

Query 241
IGEQGSAALCGSVFKVEYTDCGQRRVYLRLYSGTLRLRDTVALAGREKLKITEMRIPSKG 300

IGEQGSAALCGSVFKVEYTDCGQRRVYLRLYSGTLRLRDTVALAGREKLKITEMRIPSKG
Sbjct 241
IGEQGSAALCGSVFKVEYTDCGQRRVYLRLYSGTLRLRDTVALAGREKLKITEMRIPSKG 300

Query 301
EIVRTDTAYPGEIVILPSDSVRLNDVLGDPTRLPRKRWREDPLPMLRTTIAPKTAAQRER 360
EIVRTDTAYPGEIVILPSDSVRLNDVLGDPTRLPRKRWREDPLPMLRT
+IAPKTAAQRER
Sbjct 301
EIVRTDTAYPGEIVILPSDSVRLNDVLGDPTRLPRKRWREDPLPMLRTSIAPKTAAQRER 360

Query 361
LLDALTQLADTDPLLRCEVDSITHEIILSFLGRVQLEVVSALLSEKYKLETVVKEPSVIY 420
LLDALTQLADTDPLLR EVDSITHEIILSFLGRVQLEVVSALLSEKYKLETVVKEP
+VIY
Sbjct 361
LLDALTQLADTDPLLRYEVDSITHEIILSFLGRVQLEVVSALLSEKYKLETVVKEPTVIY 420

Query 421
MERPLKAASHTIHIEVPPNPFWASIGLSVTPLSLGSGVQYESRVSLGYLNQSFQNAVRDG 480
MERPLKAASHTIHIEVPPNPFWASIGLSVTPL LGSGVQYESRVSLGYLNQ
FQNAVRDG
Sbjct 421
MERPLKAASHTIHIEVPPNPFWASIGLSVTPLPLGSGVQYESRVSLGYLNQGFQNAVRDG 480

Query 481
IRYGLEQGLFGWNVTDCKICFEYGLYYSPVSTPADFRSLAPIVLEQALKESGTQLLEPYL 540

IRYGLEQGLFGWNVTDCKICFEYGLYYSPVSTPADFRSLAPIVLEQALKESGTQLLEPYL
Sbjct 481
IRYGLEQGLFGWNVTDCKICFEYGLYYSPVSTPADFRSLAPIVLEQALKESGTQLLEPYL 540

Query 541

```
SFILYAPQEYLSRAYHDAPKYCATIETAQVKKDEVVFTGEIPARCIQAYRTDLAFYTNGR  600
            SF LYAP+EYLSRAYHDAPKYCATIET
QVKKDEVVFTGEIPARCIQAYRTDLAFYTNG+
Sbjct  541
SFTLYAPREYLSRAYHDAPKYCATIETVQVKKDEVVFTGEIPARCIQAYRTDLAFYTNGQ  600

Query  601  SVCLTELKGYQAAVGQPVIQPRRPNSRLDKVRHMFQKVM  639
            SVCLTELKGYQAAVG+PVIQPRRPNSRLDKVR+MFQK+M
Sbjct  601  SVCLTELKGYQAAVGKPVIQPRRPNSRLDKVRYMFQKIM  639


>WP_013642473.1 TetM/TetW/TetO/TetS family tetracycline resistance
ribosomal
protection protein [Lactobacillus amylovorus]
 ADZ08019.1 translation elongation factor G [Lactobacillus
amylovorus]
Length=640

 Score = 1287 bits (3330),  Expect = 0.0, Method: Compositional
matrix adjust.
 Identities = 626/638 (98%), Positives = 634/638 (99%), Gaps = 0/638
(0%)

Query  1
MKIINIGILAHVDAGKTTLTESLLYASGAISEPGSVEKGTTRTDTMFLERQRGITIQAAV  60
            MKIINIGILAHVDAGKTTLT
+SLLYASGAISEPGSVEKGTTRTDTMFLERQRGITIQAAV
Sbjct  1
MKIINIGILAHVDAGKTTLTDSLLYASGAISEPGSVEKGTTRTDTMFLERQRGITIQAAV  60

Query  61
TSFQWHRCKVNIVDTPGHMDFLAEVYRSLAVLDGAILVISAKDGVQAQTRILFHALRKMN  120

TSFQWHRCKVNIVDTPGHMDFLAEVYRSLAVLDGAILVISAKDGVQAQTRILFHALRKMN
Sbjct  61
TSFQWHRCKVNIVDTPGHMDFLAEVYRSLAVLDGAILVISAKDGVQAQTRILFHALRKMN  120

Query  121
IPTVIFINKIDQAGVDLQSVVQSVRDKLSADIIIKQTVSLSPEIVLEENTDIEAWDAVIE  180

IPTVIFINKIDQAGVDLQSVVQSVRDKLSADIIIKQTVSLSPEIVLEENTDIEAWDAVIE
Sbjct  121
IPTVIFINKIDQAGVDLQSVVQSVRDKLSADIIIKQTVSLSPEIVLEENTDIEAWDAVIE  180

Query  181
NNDKLLEKYIAGEPISREKLVREEQRRVQDASLFPVYYGSAKKGLGIQPLMDAVTGLFQP  240

NNDKLLEKYIAGEPISREKLVREEQRRVQDASLFPVYYGSAKKGLGIQPLMDAVTGLFQP
Sbjct  181
NNDKLLEKYIAGEPISREKLVREEQRRVQDASLFPVYYGSAKKGLGIQPLMDAVTGLFQP  240

Query  241
IGEQGSAALCGSVFKVEYTDCGQRRVYLRLYSGTLRLRDTVALAGREKLKITEMRIPSKG  300
```

```
IGEQGSAALCGSVFKVEYTDCGQRRVYLRLYSGTLRLRDTVALAGREKLKITEMRIPSKG
Sbjct  241
IGEQGSAALCGSVFKVEYTDCGQRRVYLRLYSGTLRLRDTVALAGREKLKITEMRIPSKG  300

Query  301
EIVRTDTAYPGEIVILPSDSVRLNDVLGDPTRLPRKRWREDPLPMLRTTIAPKTAAQRER  360
            EIVRTDTAYPGEIVILPSDSVRLNDVLGDPTRLPRKRWREDPLPMLRT
+IAPKTAAQRER
Sbjct  301
EIVRTDTAYPGEIVILPSDSVRLNDVLGDPTRLPRKRWREDPLPMLRTSIAPKTAAQRER  360

Query  361
LLDALTQLADTDPLLRCEVDSITHEIILSFLGRVQLEVVSALLSEKYKLETVVKEPSVIY  420
            LLDALTQLADTDPLLR EVDSITHEIILSFLGRVQLEVVSALLSEKYKLETVVKEP
+VIY
Sbjct  361
LLDALTQLADTDPLLRYEVDSITHEIILSFLGRVQLEVVSALLSEKYKLETVVKEPTVIY  420

Query  421
MERPLKAASHTIHIEVPPNPFWASIGLSVTPLSLGSGVQYESRVSLGYLNQSFQNAVRDG  480
            MERPLKAASHTIHIEVPPNPFWASIGLSVTPL
LGSGVQYESRVSLGYLNQSFQNAVRDG
Sbjct  421
MERPLKAASHTIHIEVPPNPFWASIGLSVTPLPLGSGVQYESRVSLGYLNQSFQNAVRDG  480

Query  481
IRYGLEQGLFGWNVTDCKICFEYGLYYSPVSTPADFRSLAPIVLEQALKESGTQLLEPYL  540

IRYGLEQGLFGWNVTDCKICFEYGLYYSPVSTPADFRSLAPIVLEQALKESGTQLLEPYL
Sbjct  481
IRYGLEQGLFGWNVTDCKICFEYGLYYSPVSTPADFRSLAPIVLEQALKESGTQLLEPYL  540

Query  541
SFILYAPQEYLSRAYHDAPKYCATIETAQVKKDEVVFTGEIPARCIQAYRTDLAFYTNGR  600
            SF LYAP+EYLSRAYHDAPKYCATIET
QVKKDEVVFTGEIPARCIQAYRTDLAFYTNG+
Sbjct  541
SFTLYAPREYLSRAYHDAPKYCATIETVQVKKDEVVFTGEIPARCIQAYRTDLAFYTNGQ  600

Query  601  SVCLTELKGYQAAVGQPVIQPRRPNSRLDKVRHMFQKV  638
            SVCLTELKGYQAAVG+PVIQPRRPNSRLDKVR+MFQK+
Sbjct  601  SVCLTELKGYQAAVGKPVIQPRRPNSRLDKVRYMFQKI  638


>WP_098039738.1 TetM/TetW/TetO/TetS family tetracycline resistance
ribosomal
protection protein [Lactobacillus sp. UMNPBX18]
 PEG78674.1 tetracycline resistance ribosomal protection protein
[Lactobacillus
sp. UMNPBX18]
Length=639

 Score = 1286 bits (3329),  Expect = 0.0, Method: Compositional
matrix adjust.
```

Identities = 626/639 (98%), Positives = 634/639 (99%), Gaps = 0/639 (0%)

```
Query  1
MKIINIGILAHVDAGKTTLTESLLYASGAISEPGSVEKGTTRTDTMFLERQRGITIQAAV  60
            MKIINIGILAHVDAGKTTLTESLLYASGAISEPGSV
+KGTTRTDTMFLERQRGITIQAAV
Sbjct  1
MKIINIGILAHVDAGKTTLTESLLYASGAISEPGSVKKGTTRTDTMFLERQRGITIQAAV  60

Query  61
TSFQWHRCKVNIVDTPGHMDFLAEVYRSLAVLDGAILVISAKDGVQAQTRILFHALRKMN  120

TSFQWHRCKVNIVDTPGHMDFLAEVYRSLAVLDGAILVISAKDGVQAQTRILFHALRKMN
Sbjct  61
TSFQWHRCKVNIVDTPGHMDFLAEVYRSLAVLDGAILVISAKDGVQAQTRILFHALRKMN  120

Query  121
IPTVIFINKIDQAGVDLQSVVQSVRDKLSADIIIKQTVSLSPEIVLEENTDIEAWDAVIE  180

IPTVIFINKIDQAGVDLQSVVQSVRDKLSADIIIKQTVSLSPEIVLEENTDIEAWDAVIE
Sbjct  121
IPTVIFINKIDQAGVDLQSVVQSVRDKLSADIIIKQTVSLSPEIVLEENTDIEAWDAVIE  180

Query  181
NNDKLLEKYIAGEPISREKLVREEQRRVQDASLFPVYYGSAKKGLGIQPLMDAVTGLFQP  240

NNDKLLEKYIAGEPISREKLVREEQRRVQDASLFPVYYGSAKKGLGIQPLMDAVTGLFQP
Sbjct  181
NNDKLLEKYIAGEPISREKLVREEQRRVQDASLFPVYYGSAKKGLGIQPLMDAVTGLFQP  240

Query  241
IGEQGSAALCGSVFKVEYTDCGQRRVYLRLYSGTLRLRDTVALAGREKLKITEMRIPSKG  300

IGEQGSAALCGSVFKVEYTDCGQRRVYLRLYSGTLRLRDTVALAGREKLKITEMRIPSKG
Sbjct  241
IGEQGSAALCGSVFKVEYTDCGQRRVYLRLYSGTLRLRDTVALAGREKLKITEMRIPSKG  300

Query  301
EIVRTDTAYPGEIVILPSDSVRLNDVLGDPTRLPRKRWREDPLPMLRTTIAPKTAAQRER  360
            EIVRTDTAYPGEIVILPSDSVRLNDVLGDPTRLPRKRWREDPLPMLRT
+IAPKTAAQRER
Sbjct  301
EIVRTDTAYPGEIVILPSDSVRLNDVLGDPTRLPRKRWREDPLPMLRTSIAPKTAAQRER  360

Query  361
LLDALTQLADTDPLLRCEVDSITHEIILSFLGRVQLEVVSALLSEKYKLETVVKEPSVIY  420
            LLDALTQLADTDPLLR EVDSITHEIILSFLGRVQLEVVSALLSEKYKLETVVKEP
+VIY
Sbjct  361
LLDALTQLADTDPLLRYEVDSITHEIILSFLGRVQLEVVSALLSEKYKLETVVKEPTVIY  420

Query  421
MERPLKAASHTIHIEVPPNPFWASIGLSVTPLSLGSGVQYESRVSLGYLNQSFQNAVRDG  480
```

MERPLKAASHTIHIEVPPNPFWASIGLSVTPL
LGSGVQYESRVSLGYLNQSFQNAVRDG
Sbjct 421
MERPLKAASHTIHIEVPPNPFWASIGLSVTPLPLGSGVQYESRVSLGYLNQSFQNAVRDG 480

Query 481
IRYGLEQGLFGWNVTDCKICFEYGLYYSPVSTPADFRSLAPIVLEQALKESGTQLLEPYL 540

IRYGLEQGLFGWNVTDCKICFEYGLYYSPVSTPADFRSLAPIVLEQALKESGTQLLEPYL
Sbjct 481
IRYGLEQGLFGWNVTDCKICFEYGLYYSPVSTPADFRSLAPIVLEQALKESGTQLLEPYL 540

Query 541
SFILYAPQEYLSRAYHDAPKYCATIETAQVKKDEVVFTGEIPARCIQAYRTDLAFYTNGR 600
SF LYAP+EYLSRAYHDAPKYCATIET
QVKKDEVVFTGEIPARCIQAYRTDLAFYTNG+
Sbjct 541
SFTLYAPREYLSRAYHDAPKYCATIETVQVKKDEVVFTGEIPARCIQAYRTDLAFYTNGQ 600

Query 601 SVCLTELKGYQAAVGQPVIQPRRPNSRLDKVRHMFQKVM 639
SVCLTELKGYQAA G+PVIQPRRPNSRLDKVR+MFQK+M
Sbjct 601 SVCLTELKGYQAADGKPVIQPRRPNSRLDKVRYMFQKIM 639

>WP_095183509.1 TetM/TetW/TetO/TetS family tetracycline resistance
ribosomal
protection protein [Lactobacillus johnsonii]
PAB55068.1 tetracycline resistance ribosomal protection protein
[Lactobacillus
johnsonii]
PEG68642.1 TetM/TetW/TetO/TetS family tetracycline resistance
ribosomal
protection protein [Lactobacillus johnsonii]
Length=641

Score = 1286 bits (3329), Expect = 0.0, Method: Compositional
matrix adjust.
Identities = 626/638 (98%), Positives = 633/638 (99%), Gaps = 0/638
(0%)

Query 1
MKIINIGILAHVDAGKTTLTESLLYASGAISEPGSVEKGTTRTDTMFLERQRGITIQAAV 60
MKIINIGILAHVDAGKTTLTESLLYASGAIS
PGSVEKGTTRTDTMFLERQRGITIQAAV
Sbjct 1
MKIINIGILAHVDAGKTTLTESLLYASGAISGPGSVEKGTTRTDTMFLERQRGITIQAAV 60

Query 61
TSFQWHRCKVNIVDTPGHMDFLAEVYRSLAVLDGAILVISAKDGVQAQTRILFHALRKMN 120

TSFQWHRCKVNIVDTPGHMDFLAEVYRSLAVLDGAILVISAKDGVQAQTRILFHALRKMN
Sbjct 61
TSFQWHRCKVNIVDTPGHMDFLAEVYRSLAVLDGAILVISAKDGVQAQTRILFHALRKMN 120

Query 121
IPTVIFINKIDQAGVDLQSVVQSVRDKLSADIIIKQTVSLSPEIVLEENTDIEAWDAVIE 180

IPTVIFINKIDQAGVDLQSVVQSVRDKLSADIIIKQTVSLSPEIVLEENTDIEAWDAVIE
Sbjct 121
IPTVIFINKIDQAGVDLQSVVQSVRDKLSADIIIKQTVSLSPEIVLEENTDIEAWDAVIE 180

Query 181
NNDKLLEKYIAGEPISREKLVREEQRRVQDASLFPVYYGSAKKGLGIQPLMDAVTGLFQP 240

NNDKLLEKYIAGEPISREKLVREEQRRVQDASLFPVYYGSAKKGLGIQPLMDAVTGLFQP
Sbjct 181
NNDKLLEKYIAGEPISREKLVREEQRRVQDASLFPVYYGSAKKGLGIQPLMDAVTGLFQP 240

Query 241
IGEQGSAALCGSVFKVEYTDCGQRRVYLRLYSGTLRLRDTVALAGREKLKITEMRIPSKG 300

IGEQGSAALCGSVFKVEYTDCGQRRVYLRLYSGTLRLRDTVALAGREKLKITEMRIPSKG
Sbjct 241
IGEQGSAALCGSVFKVEYTDCGQRRVYLRLYSGTLRLRDTVALAGREKLKITEMRIPSKG 300

Query 301
EIVRTDTAYPGEIVILPSDSVRLNDVLGDPTRLPRKRWREDPLPMLRTTIAPKTAAQRER 360
EIVRTDTAYPGEIVILPSDSVRLNDVLGDPTRLPRKRWREDPLPMLRT
+IAPKTAAQRER
Sbjct 301
EIVRTDTAYPGEIVILPSDSVRLNDVLGDPTRLPRKRWREDPLPMLRTSIAPKTAAQRER 360

Query 361
LLDALTQLADTDPLLRCEVDSITHEIILSFLGRVQLEVVSALLSEKYKLETVVKEPSVIY 420
LLDALTQLADTDPLLRCEVDSITHEIILSFLGRVQLEVVSALLSEKYKLETVVKEP
+VIY
Sbjct 361
LLDALTQLADTDPLLRCEVDSITHEIILSFLGRVQLEVVSALLSEKYKLETVVKEPTVIY 420

Query 421
MERPLKAASHTIHIEVPPNPFWASIGLSVTPLSLGSGVQYESRVSLGYLNQSFQNAVRDG 480
MERPLKAASHTIHIEVPPNPFWASIGLSVTPL
LGSGVQYESRVSLGYLNQSFQNAVRDG
Sbjct 421
MERPLKAASHTIHIEVPPNPFWASIGLSVTPLPLGSGVQYESRVSLGYLNQSFQNAVRDG 480

Query 481
IRYGLEQGLFGWNVTDCKICFEYGLYYSPVSTPADFRSLAPIVLEQALKESGTQLLEPYL 540

IRYGLEQGLFGWNVTDCKICFEYGLYYSPVSTPADFRSLAPIVLEQALKESGTQLLEPYL
Sbjct 481
IRYGLEQGLFGWNVTDCKICFEYGLYYSPVSTPADFRSLAPIVLEQALKESGTQLLEPYL 540

Query 541
SFILYAPQEYLSRAYHDAPKYCATIETAQVKKDEVVFTGEIPARCIQAYRTDLAFYTNGR 600
SF LYAP+EYLSRAYHDAPKYCATIET QVKKDEVVFTGEIPARCIQAYRTDLA
YTNG+
Sbjct 541

```
SFTLYAPREYLSRAYHDAPKYCATIETVQVKKDEVVFTGEIPARCIQAYRTDLASYTNGQ  600

Query  601  SVCLTELKGYQAAVGQPVIQPRRPNSRLDKVRHMFQKV  638
            SVCLTELKGYQAAVG+PVIQPRRPNSRLDKVR+MFQK+
Sbjct  601  SVCLTELKGYQAAVGKPVIQPRRPNSRLDKVRYMFQKI  638


>WP_089145870.1 MULTISPECIES: TetM/TetW/TetO/TetS family
tetracycline resistance
ribosomal protection protein [Lactobacillus]
 OXC26114.1 tetracycline resistance ribosomal protection protein
[Lactobacillus
crispatus]
 OXC31357.1 tetracycline resistance ribosomal protection protein
[Lactobacillus
crispatus]
 OXC39436.1 tetracycline resistance ribosomal protection protein
[Lactobacillus
crispatus]
 OXC41134.1 tetracycline resistance ribosomal protection protein
[Lactobacillus
crispatus]
 OXC42054.1 tetracycline resistance ribosomal protection protein
[Lactobacillus
crispatus]
 OXC44575.1 tetracycline resistance ribosomal protection protein
[Lactobacillus
crispatus]
 PEG82365.1 TetM/TetW/TetO/TetS family tetracycline resistance
ribosomal
protection protein [Lactobacillus sp. UMNPBX16]
 PEG97953.1 TetM/TetW/TetO/TetS family tetracycline resistance
ribosomal
protection protein [Lactobacillus sp. UMNPBX8]
Length=640

 Score = 1286 bits (3329),  Expect = 0.0, Method: Compositional
matrix adjust.
 Identities = 625/638 (98%), Positives = 634/638 (99%), Gaps = 0/638
(0%)

Query  1
MKIINIGILAHVDAGKTTLTESLLYASGAISEPGSVEKGTTRTDTMFLERQRGITIQAAV  60
            MKIINIGILAHVDAGKTTLTESLLYASGAISEPGSVEKGTTRTD
+MFLERQRGITIQAAV
Sbjct  1
MKIINIGILAHVDAGKTTLTESLLYASGAISEPGSVEKGTTRTDSMFLERQRGITIQAAV  60

Query  61
TSFQWHRCKVNIVDTPGHMDFLAEVYRSLAVLDGAILVISAKDGVQAQTRILFHALRKMN  120

TSFQWHRCKVNIVDTPGHMDFLAEVYRSLAVLDGAILVISAKDGVQAQTRILFHALRKMN
Sbjct  61
TSFQWHRCKVNIVDTPGHMDFLAEVYRSLAVLDGAILVISAKDGVQAQTRILFHALRKMN  120
```

Query 121
IPTVIFINKIDQAGVDLQSVVQSVRDKLSADIIIKQTVSLSPEIVLEENTDIEAWDAVIE 180

IPTVIFINKIDQAGVDLQSVVQSVRDKLSADIIIKQTVSLSPEIVLEENTDIEAWDAVIE
Sbjct 121
IPTVIFINKIDQAGVDLQSVVQSVRDKLSADIIIKQTVSLSPEIVLEENTDIEAWDAVIE 180

Query 181
NNDKLLEKYIAGEPISREKLVREEQRRVQDASLFPVYYGSAKKGLGIQPLMDAVTGLFQP 240

NNDKLLEKYIAGEPISREKLVREEQRRVQDASLFPVYYGSAKKGLGIQPLMDAVTGLFQP
Sbjct 181
NNDKLLEKYIAGEPISREKLVREEQRRVQDASLFPVYYGSAKKGLGIQPLMDAVTGLFQP 240

Query 241
IGEQGSAALCGSVFKVEYTDCGQRRVYLRLYSGTLRLRDTVALAGREKLKITEMRIPSKG 300

IGEQGSAALCGSVFKVEYTDCGQRRVYLRLYSGTLRLRDTVALAGREKLKITEMRIPSKG
Sbjct 241
IGEQGSAALCGSVFKVEYTDCGQRRVYLRLYSGTLRLRDTVALAGREKLKITEMRIPSKG 300

Query 301
EIVRTDTAYPGEIVILPSDSVRLNDVLGDPTRLPRKRWREDPLPMLRTTIAPKTAAQRER 360
+IVRTDTAYPGEIVILPSDSVRLNDVLGDPTRLPRKRWREDPLPMLRT
+IAPKTAAQRER
Sbjct 301
KIVRTDTAYPGEIVILPSDSVRLNDVLGDPTRLPRKRWREDPLPMLRTSIAPKTAAQRER 360

Query 361
LLDALTQLADTDPLLRCEVDSITHEIILSFLGRVQLEVVSALLSEKYKLETVVKEPSVIY 420
LLDALTQLADTDPLLRCEVDSITHEIILSFLGRVQLEVVSALLSEKYKLETVVKEP
+VIY
Sbjct 361
LLDALTQLADTDPLLRCEVDSITHEIILSFLGRVQLEVVSALLSEKYKLETVVKEPTVIY 420

Query 421
MERPLKAASHTIHIEVPPNPFWASIGLSVTPLSLGSGVQYESRVSLGYLNQSFQNAVRDG 480
MERPLKAASHTIHIEVPPNPFWASIGLSVTPL
LGSGVQYESRVSLGYLNQSFQNAVRDG
Sbjct 421
MERPLKAASHTIHIEVPPNPFWASIGLSVTPLPLGSGVQYESRVSLGYLNQSFQNAVRDG 480

Query 481
IRYGLEQGLFGWNVTDCKICFEYGLYYSPVSTPADFRSLAPIVLEQALKESGTQLLEPYL 540

IRYGLEQGLFGWNVTDCKICFEYGLYYSPVSTPADFRSLAPIVLEQALKESGTQLLEPYL
Sbjct 481
IRYGLEQGLFGWNVTDCKICFEYGLYYSPVSTPADFRSLAPIVLEQALKESGTQLLEPYL 540

Query 541
SFILYAPQEYLSRAYHDAPKYCATIETAQVKKDEVVFTGEIPARCIQAYRTDLAFYTNGR 600
SF LYAP+EYLSRAYHDAPKYCATIET QVKKDEVVFTGEIPARCIQAYRTDLA
YTNG+

Sbjct 541
SFTLYAPREYLSRAYHDAPKYCATIETVQVKKDEVVFTGEIPARCIQAYRTDLASYTNGQ 600

Query 601 SVCLTELKGYQAAVGQPVIQPRRPNSRLDKVRHMFQKV 638
            SVCLTELKGYQAAVG+PVIQPRRPNSRLDKVR+MFQK+
Sbjct 601 SVCLTELKGYQAAVGKPVIQPRRPNSRLDKVRYMFQKI 638

>WP_089150173.1 TetM/TetW/TetO/TetS family tetracycline resistance ribosomal
protection protein [Lactobacillus crispatus]
 OXC13285.1 tetracycline resistance ribosomal protection protein [Lactobacillus
crispatus]
 OXC17662.1 tetracycline resistance ribosomal protection protein [Lactobacillus
crispatus]
 OXC19085.1 tetracycline resistance ribosomal protection protein [Lactobacillus
crispatus]
Length=640

 Score = 1286 bits (3328), Expect = 0.0, Method: Compositional matrix adjust.
 Identities = 625/638 (98%), Positives = 634/638 (99%), Gaps = 0/638 (0%)

Query 1
MKIINIGILAHVDAGKTTLTESLLYASGAISEPGSVEKGTTRTDTMFLERQRGITIQAAV 60
            MKIINIGILAHVDAGKTTLTESLLYASGAISEPGSVEKGTTRTD
+MFLERQRGITIQAAV
Sbjct 1
MKIINIGILAHVDAGKTTLTESLLYASGAISEPGSVEKGTTRTDSMFLERQRGITIQAAV 60

Query 61
TSFQWHRCKVNIVDTPGHMDFLAEVYRSLAVLDGAILVISAKDGVQAQTRILFHALRKMN 120

TSFQWHRCKVNIVDTPGHMDFLAEVYRSLAVLDGAILVISAKDGVQAQTRILFHALRKMN
Sbjct 61
TSFQWHRCKVNIVDTPGHMDFLAEVYRSLAVLDGAILVISAKDGVQAQTRILFHALRKMN 120

Query 121
IPTVIFINKIDQAGVDLQSVVQSVRDKLSADIIIKQTVSLSPEIVLEENTDIEAWDAVIE 180

IPTVIFINKIDQAGVDLQSVVQSVRDKLSADIIIKQTVSLSPEIVLEENTDIEAWDAVIE
Sbjct 121
IPTVIFINKIDQAGVDLQSVVQSVRDKLSADIIIKQTVSLSPEIVLEENTDIEAWDAVIE 180

Query 181
NNDKLLEKYIAGEPISREKLVREEQRRVQDASLFPVYYGSAKKGLGIQPLMDAVTGLFQP 240

NNDKLLEKYIAGEPISREKLVREEQRRVQDASLFPVYYGSAKKGLGIQPLMDAVTGLFQP
Sbjct 181
NNDKLLEKYIAGEPISREKLVREEQRRVQDASLFPVYYGSAKKGLGIQPLMDAVTGLFQP 240

```
Query  241
IGEQGSAALCGSVFKVEYTDCGQRRVYLRLYSGTLRLRDTVALAGREKLKITEMRIPSKG  300

IGEQGSAALCGSVFKVEYTDCGQRRVYLRLYSGTLRLRDTVALAGREKLKITEMRIPSKG
Sbjct  241
IGEQGSAALCGSVFKVEYTDCGQRRVYLRLYSGTLRLRDTVALAGREKLKITEMRIPSKG  300

Query  301
EIVRTDTAYPGEIVILPSDSVRLNDVLGDPTRLPRKRWREDPLPMLRTTIAPKTAAQRER  360
            +IVRTDTAYPGEIVILPSDSVRLNDVLGDPTRLPRKRWREDPLPMLRT
+IAPKTAAQRER
Sbjct  301
KIVRTDTAYPGEIVILPSDSVRLNDVLGDPTRLPRKRWREDPLPMLRTSIAPKTAAQRER  360

Query  361
LLDALTQLADTDPLLRCEVDSITHEIILSFLGRVQLEVVSALLSEKYKLETVVKEPSVIY  420
            LLDALTQLADTDPLLRCEVDSITHEIILSFLGRVQLEVVSALLSEKYKLETVVKEP
+VIY
Sbjct  361
LLDALTQLADTDPLLRCEVDSITHEIILSFLGRVQLEVVSALLSEKYKLETVVKEPNVIY  420

Query  421
MERPLKAASHTIHIEVPPNPFWASIGLSVTPLSLGSGVQYESRVSLGYLNQSFQNAVRDG  480
            MERPLKAASHTIHIEVPPNPFWASIGLSVTPL
LGSGVQYESRVSLGYLNQSFQNAVRDG
Sbjct  421
MERPLKAASHTIHIEVPPNPFWASIGLSVTPLPLGSGVQYESRVSLGYLNQSFQNAVRDG  480

Query  481
IRYGLEQGLFGWNVTDCKICFEYGLYYSPVSTPADFRSLAPIVLEQALKESGTQLLEPYL  540

IRYGLEQGLFGWNVTDCKICFEYGLYYSPVSTPADFRSLAPIVLEQALKESGTQLLEPYL
Sbjct  481
IRYGLEQGLFGWNVTDCKICFEYGLYYSPVSTPADFRSLAPIVLEQALKESGTQLLEPYL  540

Query  541
SFILYAPQEYLSRAYHDAPKYCATIETAQVKKDEVVFTGEIPARCIQAYRTDLAFYTNGR  600
            SF LYAP+EYLSRAYHDAPKYCATIET QVKKDEVVFTGEIPARCIQAYRTDLA
YTNG+
Sbjct  541
SFTLYAPREYLSRAYHDAPKYCATIETVQVKKDEVVFTGEIPARCIQAYRTDLASYTNGQ  600

Query  601  SVCLTELKGYQAAVGQPVIQPRRPNSRLDKVRHMFQKV  638
            SVCLTELKGYQAAVG+PVIQPRRPNSRLDKVR+MFQK+
Sbjct  601  SVCLTELKGYQAAVGKPVIQPRRPNSRLDKVRYMFQKI  638


>WP_052543100.1 tetracycline resistance ribosomal protection protein
Tet(W) [Lactobacillus
sp. OTU4228]
 ACI02041.1 tetracycline resistance protein [uncultured bacterium]
Length=639
```

Score = 1286 bits (3328), Expect = 0.0, Method: Compositional matrix adjust.
Identities = 626/639 (98%), Positives = 633/639 (99%), Gaps = 0/639 (0%)

```
Query  1
MKIINIGILAHVDAGKTTLTESLLYASGAISEPGSVEKGTTRTDTMFLERQRGITIQAAV  60
            MKIINIGILAHVDAGKTTLTESLLYASGA
SEPGSVEKGTTRTDTMFLERQRGITIQAAV
Sbjct  1
MKIINIGILAHVDAGKTTLTESLLYASGATSEPGSVEKGTTRTDTMFLERQRGITIQAAV  60

Query  61
TSFQWHRCKVNIVDTPGHMDFLAEVYRSLAVLDGAILVISAKDGVQAQTRILFHALRKMN  120

TSFQWHRCKVNIVDTPGHMDFLAEVYRSLAVLDGAILVISAKDGVQAQTRILFHALRKMN
Sbjct  61
TSFQWHRCKVNIVDTPGHMDFLAEVYRSLAVLDGAILVISAKDGVQAQTRILFHALRKMN  120

Query  121
IPTVIFINKIDQAGVDLQSVVQSVRDKLSADIIIKQTVSLSPEIVLEENTDIEAWDAVIE  180

IPTVIFINKIDQAGVDLQSVVQSVRDKLSADIIIKQTVSLSPEIVLEENTDIEAWDAVIE
Sbjct  121
IPTVIFINKIDQAGVDLQSVVQSVRDKLSADIIIKQTVSLSPEIVLEENTDIEAWDAVIE  180

Query  181
NNDKLLEKYIAGEPISREKLVREEQRRVQDASLFPVYYGSAKKGLGIQPLMDAVTGLFQP  240

NNDKLLEKYIAGEPISREKLVREEQRRVQDASLFPVYYGSAKKGLGIQPLMDAVTGLFQP
Sbjct  181
NNDKLLEKYIAGEPISREKLVREEQRRVQDASLFPVYYGSAKKGLGIQPLMDAVTGLFQP  240

Query  241
IGEQGSAALCGSVFKVEYTDCGQRRVYLRLYSGTLRLRDTVALAGREKLKITEMRIPSKG  300

IGEQGSAALCGSVFKVEYTDCGQRRVYLRLYSGTLRLRDTVALAGREKLKITEMRIPSKG
Sbjct  241
IGEQGSAALCGSVFKVEYTDCGQRRVYLRLYSGTLRLRDTVALAGREKLKITEMRIPSKG  300

Query  301
EIVRTDTAYPGEIVILPSDSVRLNDVLGDPTRLPRKRWREDPLPMLRTTIAPKTAAQRER  360
            EIVRTDTAYPGEIVILPSDSVRLNDVLGDPTRLPRKRWREDPLPMLRT
+IAPKTAAQRER
Sbjct  301
EIVRTDTAYPGEIVILPSDSVRLNDVLGDPTRLPRKRWREDPLPMLRTSIAPKTAAQRER  360

Query  361
LLDALTQLADTDPLLRCEVDSITHEIILSFLGRVQLEVVSALLSEKYKLETVVKEPSVIY  420
            LLDALTQLADTDPLLR EVDSITHEIILSFLGRVQLEVVSALLSEKYKLETVVKEP
+VIY
Sbjct  361
LLDALTQLADTDPLLRFEVDSITHEIILSFLGRVQLEVVSALLSEKYKLETVVKEPTVIY  420
```

Query 421
MERPLKAASHTIHIEVPPNPFWASIGLSVTPLSLGSGVQYESRVSLGYLNQSFQNAVRDG 480
            MERPLKAASHTIHIEVPPNPFWASIGLSVTPL
LGSGVQYESRVSLGYLNQSFQNAVRDG
Sbjct 421
MERPLKAASHTIHIEVPPNPFWASIGLSVTPLPLGSGVQYESRVSLGYLNQSFQNAVRDG 480

Query 481
IRYGLEQGLFGWNVTDCKICFEYGLYYSPVSTPADFRSLAPIVLEQALKESGTQLLEPYL 540

IRYGLEQGLFGWNVTDCKICFEYGLYYSPVSTPADFRSLAPIVLEQALKESGTQLLEPYL
Sbjct 481
IRYGLEQGLFGWNVTDCKICFEYGLYYSPVSTPADFRSLAPIVLEQALKESGTQLLEPYL 540

Query 541
SFILYAPQEYLSRAYHDAPKYCATIETAQVKKDEVVFTGEIPARCIQAYRTDLAFYTNGR 600
            SF LYAP+EYLSRAYHDAPKYCATIET QVKKDEVVFTGEIPARCIQAYRTDLA
YTNG+
Sbjct 541
SFTLYAPREYLSRAYHDAPKYCATIETVQVKKDEVVFTGEIPARCIQAYRTDLASYTNGQ 600

Query 601 SVCLTELKGYQAAVGQPVIQPRRPNSRLDKVRHMFQKVM 639
            SVCLTELKGYQAAVG+PVIQPRRPNSRLDKVR+MFQK+M
Sbjct 601 SVCLTELKGYQAAVGKPVIQPRRPNSRLDKVRYMFQKIM 639

>WP_065991006.1 TetM/TetW/TetO/TetS family tetracycline resistance ribosomal
protection protein [Lactobacillus crispatus]
OCX09023.1 tetracycline resistance ribosomal protection protein [Lactobacillus
crispatus]
Length=643

Score = 1286 bits (3327), Expect = 0.0, Method: Compositional matrix adjust.
Identities = 625/638 (98%), Positives = 634/638 (99%), Gaps = 0/638 (0%)

Query 1
MKIINIGILAHVDAGKTTLTESLLYASGAISEPGSVEKGTTRTDTMFLERQRGITIQAAV 60
            MKIINIGILAHVDAGKTTLTESLLYASGAISEPGSVEKGTTRTD
+MFLERQRGITIQAAV
Sbjct 1
MKIINIGILAHVDAGKTTLTESLLYASGAISEPGSVEKGTTRTDSMFLERQRGITIQAAV 60

Query 61
TSFQWHRCKVNIVDTPGHMDFLAEVYRSLAVLDGAILVISAKDGVQAQTRILFHALRKMN 120

TSFQWHRCKVNIVDTPGHMDFLAEVYRSLAVLDGAILVISAKDGVQAQTRILFHALRKMN
Sbjct 61
TSFQWHRCKVNIVDTPGHMDFLAEVYRSLAVLDGAILVISAKDGVQAQTRILFHALRKMN 120

Query 121

```
IPTVIFINKIDQAGVDLQSVVQSVRDKLSADIIIKQTVSLSPEIVLEENTDIEAWDAVIE  180

IPTVIFINKIDQAGVDLQSVVQSVRDKLSADIIIKQTVSLSPEIVLEENTDIEAWDAVIE
Sbjct  121
IPTVIFINKIDQAGVDLQSVVQSVRDKLSADIIIKQTVSLSPEIVLEENTDIEAWDAVIE  180

Query  181
NNDKLLEKYIAGEPISREKLVREEQRRVQDASLFPVYYGSAKKGLGIQPLMDAVTGLFQP  240

NNDKLLEKYIAGEPISREKLVREEQRRVQDASLFPVYYGSAKKGLGIQPLMDAVTGLFQP
Sbjct  181
NNDKLLEKYIAGEPISREKLVREEQRRVQDASLFPVYYGSAKKGLGIQPLMDAVTGLFQP  240

Query  241
IGEQGSAALCGSVFKVEYTDCGQRRVYLRLYSGTLRLRDTVALAGREKLKITEMRIPSKG  300

IGEQGSAALCGSVFKVEYTDCGQRRVYLRLYSGTLRLRDTVALAGREKLKITEMRIPSKG
Sbjct  241
IGEQGSAALCGSVFKVEYTDCGQRRVYLRLYSGTLRLRDTVALAGREKLKITEMRIPSKG  300

Query  301
EIVRTDTAYPGEIVILPSDSVRLNDVLGDPTRLPRKRWREDPLPMLRTTIAPKTAAQRER  360
            +IVRTDTAYPGEIVILPSDSVRLNDVLGDPTRLPRKRWREDPLPMLRT
+IAPKTAAQRER
Sbjct  301
KIVRTDTAYPGEIVILPSDSVRLNDVLGDPTRLPRKRWREDPLPMLRTSIAPKTAAQRER  360

Query  361
LLDALTQLADTDPLLRCEVDSITHEIILSFLGRVQLEVVSALLSEKYKLETVVKEPSVIY  420
            LLDALTQLADTDPLLRCEVDSITHEIILSFLGRVQLEVVSALLSEKYKLETVVKEP
+VIY
Sbjct  361
LLDALTQLADTDPLLRCEVDSITHEIILSFLGRVQLEVVSALLSEKYKLETVVKEPTVIY  420

Query  421
MERPLKAASHTIHIEVPPNPFWASIGLSVTPLSLGSGVQYESRVSLGYLNQSFQNAVRDG  480
            MERPLKAASHTIHIEVPPNPFWASIGLSVTPL
LGSGVQYESRVSLGYLNQSFQNAVRDG
Sbjct  421
MERPLKAASHTIHIEVPPNPFWASIGLSVTPLPLGSGVQYESRVSLGYLNQSFQNAVRDG  480

Query  481
IRYGLEQGLFGWNVTDCKICFEYGLYYSPVSTPADFRSLAPIVLEQALKESGTQLLEPYL  540

IRYGLEQGLFGWNVTDCKICFEYGLYYSPVSTPADFRSLAPIVLEQALKESGTQLLEPYL
Sbjct  481
IRYGLEQGLFGWNVTDCKICFEYGLYYSPVSTPADFRSLAPIVLEQALKESGTQLLEPYL  540

Query  541
SFILYAPQEYLSRAYHDAPKYCATIETAQVKKDEVVFTGEIPARCIQAYRTDLAFYTNGR  600
            SF LYAP+EYLSRAYHDAPKYCATIET QVKKDEVVFTGEIPARCIQAYRTDLA
YTNG+
Sbjct  541
SFTLYAPREYLSRAYHDAPKYCATIETVQVKKDEVVFTGEIPARCIQAYRTDLASYTNGQ  600
```

```
Query  601  SVCLTELKGYQAAVGQPVIQPRRPNSRLDKVRHMFQKV  638
            SVCLTELKGYQAAVG+PVIQPRRPNSRLDKVR+MFQK+
Sbjct  601  SVCLTELKGYQAAVGKPVIQPRRPNSRLDKVRYMFQKI  638
```

>WP_063856415.1 tetracycline resistance ribosomal protection protein Tet(W) [Megasphaera
elsdenii]
AAR29971.1 tetracycline resistance protein [Megasphaera elsdenii]
Length=639

Score = 1285 bits (3326), Expect = 0.0, Method: Compositional matrix adjust.
Identities = 625/639 (98%), Positives = 632/639 (99%), Gaps = 0/639 (0%)

```
Query  1
MKIINIGILAHVDAGKTTLTESLLYASGAISEPGSVEKGTTRTDTMFLERQRGITIQAAV  60

MKIINIGILAHVDAGKTTLTESLLYASGAISEPGSVEKGTTRTDTMFLERQRGITIQAAV
Sbjct  1
MKIINIGILAHVDAGKTTLTESLLYASGAISEPGSVEKGTTRTDTMFLERQRGITIQAAV  60

Query  61
TSFQWHRCKVNIVDTPGHMDFLAEVYRSLAVLDGAILVISAKDGVQAQTRILFHALRKMN  120

TSFQWHRCKVNIVDTPGHMDFLAEVYRSLAVLDGAILVISAKDGVQAQTRILFHALRKMN
Sbjct  61
TSFQWHRCKVNIVDTPGHMDFLAEVYRSLAVLDGAILVISAKDGVQAQTRILFHALRKMN  120

Query  121
IPTVIFINKIDQAGVDLQSVVQSVRDKLSADIIIKQTVSLSPEIVLEENTDIEAWDAVIE  180

IPTVIFINKIDQAGVDLQSVVQSVRDKLSADIIIKQTVSLSPEIVLEENTDIEAWDAVIE
Sbjct  121
IPTVIFINKIDQAGVDLQSVVQSVRDKLSADIIIKQTVSLSPEIVLEENTDIEAWDAVIE  180

Query  181
NNDKLLEKYIAGEPISREKLVREEQRRVQDASLFPVYYGSAKKGLGIQPLMDAVTGLFQP  240
            NND LLEKYIAGEPIS+EKL REEQRRVQ+ASLFPVY
+GSAKKGLGIQPLMDAVTGLFQP
Sbjct  181
NNDALLEKYIAGEPISQEKLAREEQRRVQEASLFPVYHGSAKKGLGIQPLMDAVTGLFQP  240

Query  241
IGEQGSAALCGSVFKVEYTDCGQRRVYLRLYSGTLRLRDTVALAGREKLKITEMRIPSKG  300
            IGEQGSA
LCGSVFKVEYTDCGQRRVYLRLYSGTLRLRDTVALAGREKLKITEMRIPSKG
Sbjct  241
IGEQGSATLCGSVFKVEYTDCGQRRVYLRLYSGTLRLRDTVALAGREKLKITEMRIPSKG  300

Query  301
EIVRTDTAYPGEIVILPSDSVRLNDVLGDPTRLPRKRWREDPLPMLRTTIAPKTAAQRER  360
```

EIVRTDTAYPGEIVILPSDSVRLNDVLGD TRLPRKRWRE
PLPMLRTTIAPKTAAQRER
Sbjct 301
EIVRTDTAYPGEIVILPSDSVRLNDVLGDQTRLPRKRWREAPLPMLRTTIAPKTAAQRER 360

Query 361
LLDALTQLADTDPLLRCEVDSITHEIILSFLGRVQLEVVSALLSEKYKLETVVKEPSVIY 420
LLDALTQLADTDPLLRCEVDSITHEIILSFLGRVQLEVVSALLSEKYK+ETVVKEP
+VIY
Sbjct 361
LLDALTQLADTDPLLRCEVDSITHEIILSFLGRVQLEVVSALLSEKYKIETVVKEPTVIY 420

Query 421
MERPLKAASHTIHIEVPPNPFWASIGLSVTPLSLGSGVQYESRVSLGYLNQSFQNAVRDG 480
MERPLKAASHTIHIEVPPNPFWASIGLSVTPL
LGSGVQYESRVSLGYLNQSFQNAVRDG
Sbjct 421
MERPLKAASHTIHIEVPPNPFWASIGLSVTPLPLGSGVQYESRVSLGYLNQSFQNAVRDG 480

Query 481
IRYGLEQGLFGWNVTDCKICFEYGLYYSPVSTPADFRSLAPIVLEQALKESGTQLLEPYL 540

IRYGLEQGLFGWNVTDCKICFEYGLYYSPVSTPADFRSLAPIVLEQALKESGTQLLEPYL
Sbjct 481
IRYGLEQGLFGWNVTDCKICFEYGLYYSPVSTPADFRSLAPIVLEQALKESGTQLLEPYL 540

Query 541
SFILYAPQEYLSRAYHDAPKYCATIETAQVKKDEVVFTGEIPARCIQAYRTDLAFYTNGR 600
SF
LYAPQEYLSRAYHDAPKYCATIETAQVKKDEVVFTGEIPARCIQAYRTDLAFYTNG+
Sbjct 541
SFTLYAPQEYLSRAYHDAPKYCATIETAQVKKDEVVFTGEIPARCIQAYRTDLAFYTNGQ 600

Query 601 SVCLTELKGYQAAVGQPVIQPRRPNSRLDKVRHMFQKVM 639
SVCLTELKGYQAAVGQPVIQPRRPN+RLDKVRHMFQKVM
Sbjct 601 SVCLTELKGYQAAVGQPVIQPRRPNNRLDKVRHMFQKVM 639

>WP_016497304.1 TetM/TetW/TetO/TetS family tetracycline resistance ribosomal
protection protein [Lactobacillus reuteri]
AGO00228.1 TetW (plasmid) [Lactobacillus reuteri I5007]
Length=639

Score = 1285 bits (3325), Expect = 0.0, Method: Compositional matrix adjust.
Identities = 625/639 (98%), Positives = 633/639 (99%), Gaps = 0/639 (0%)

Query 1
MKIINIGILAHVDAGKTTLTESLLYASGAISEPGSVEKGTTRTDTMFLERQRGITIQAAV 60
MKIINIGILAHVDAGKTTLTESLLYASGAISEPGSV
+KGTTRTDTMFLERQRGITIQAAV
Sbjct 1

```
MKIINIGILAHVDAGKTTLTESLLYASGAISEPGSVKKGTTRTDTMFLERQRGITIQAAV  60

Query  61
TSFQWHRCKVNIVDTPGHMDFLAEVYRSLAVLDGAILVISAKDGVQAQTRILFHALRKMN  120

TSFQWHRCKVNIVDTPGHMDFLAEVYRSLAVLDGAILVISAKDGVQAQTRILFHALRKMN
Sbjct  61
TSFQWHRCKVNIVDTPGHMDFLAEVYRSLAVLDGAILVISAKDGVQAQTRILFHALRKMN  120

Query  121
IPTVIFINKIDQAGVDLQSVVQSVRDKLSADIIIKQTVSLSPEIVLEENTDIEAWDAVIE  180

IPTVIFINKIDQAGVDLQSVVQSVRDKLSADIIIKQTVSLSPEIVLEENTDIEAWDAVIE
Sbjct  121
IPTVIFINKIDQAGVDLQSVVQSVRDKLSADIIIKQTVSLSPEIVLEENTDIEAWDAVIE  180

Query  181
NNDKLLEKYIAGEPISREKLVREEQRRVQDASLFPVYYGSAKKGLGIQPLMDAVTGLFQP  240

NNDKLLEKYIAGEPISREKLVREEQRRVQDASLFPVYYGSAKKGLGIQPLMDAVTGLFQP
Sbjct  181
NNDKLLEKYIAGEPISREKLVREEQRRVQDASLFPVYYGSAKKGLGIQPLMDAVTGLFQP  240

Query  241
IGEQGSAALCGSVFKVEYTDCGQRRVYLRLYSGTLRLRDTVALAGREKLKITEMRIPSKG  300

IGEQGSAALCGSVFKVEYTDCGQRRVYLRLYSGTLRLRDTVALAGREKLKITEMRIPSKG
Sbjct  241
IGEQGSAALCGSVFKVEYTDCGQRRVYLRLYSGTLRLRDTVALAGREKLKITEMRIPSKG  300

Query  301
EIVRTDTAYPGEIVILPSDSVRLNDVLGDPTRLPRKRWREDPLPMLRTTIAPKTAAQRER  360
            EIVRTDTAYPGEIVILPSDSVRLNDVLGDPTRLPRKRWREDPLPMLRT
+IAPKTAAQRER
Sbjct  301
EIVRTDTAYPGEIVILPSDSVRLNDVLGDPTRLPRKRWREDPLPMLRTSIAPKTAAQRER  360

Query  361
LLDALTQLADTDPLLRCEVDSITHEIILSFLGRVQLEVVSALLSEKYKLETVVKEPSVIY  420
            LLDALTQLADTDPLLRCEVDSITHEIILSFLGRVQLEVVSALLSEKYKLETVVKEP
+VIY
Sbjct  361
LLDALTQLADTDPLLRCEVDSITHEIILSFLGRVQLEVVSALLSEKYKLETVVKEPTVIY  420

Query  421
MERPLKAASHTIHIEVPPNPFWASIGLSVTPLSLGSGVQYESRVSLGYLNQSFQNAVRDG  480
             ERPLKAASHTIHIEVPPNPFWASIGLSVTPL
LGSGVQYESRVSLGYLNQSFQNAVRDG
Sbjct  421
KERPLKAASHTIHIEVPPNPFWASIGLSVTPLPLGSGVQYESRVSLGYLNQSFQNAVRDG  480

Query  481
IRYGLEQGLFGWNVTDCKICFEYGLYYSPVSTPADFRSLAPIVLEQALKESGTQLLEPYL  540
```

IRYGLEQGLFGWNVTDCKICFEYGLYYSPVSTPADFRSLAPIVLEQALKESGTQLLEPYL
Sbjct 481
IRYGLEQGLFGWNVTDCKICFEYGLYYSPVSTPADFRSLAPIVLEQALKESGTQLLEPYL 540

Query 541
SFILYAPQEYLSRAYHDAPKYCATIETAQVKKDEVVFTGEIPARCIQAYRTDLAFYTNGR 600
            SF LYAP+EYLSRAYHDAPKYCATIET Q
KKDEVVFTGEIPARCIQAYRTDLAFYTNG+
Sbjct 541
SFTLYAPREYLSRAYHDAPKYCATIETVQEKKDEVVFTGEIPARCIQAYRTDLAFYTNGQ 600

Query 601 SVCLTELKGYQAAVGQPVIQPRRPNSRLDKVRHMFQKVM 639
            SVCLTELKGYQAA G+PVIQPRRPNSRLDKVR+MFQK+M
Sbjct 601 SVCLTELKGYQAADGKPVIQPRRPNSRLDKVRYMFQKIM 639

>WP_090265483.1 TetM/TetW/TetO/TetS family tetracycline resistance ribosomal
protection protein [Eubacteriaceae bacterium CHKCI005]
 CZT56114.1 Tetracycline resistance protein TetO [Eubacteriaceae bacterium
CHKCI005]
Length=639

 Score = 1285 bits (3324), Expect = 0.0, Method: Compositional matrix adjust.
 Identities = 624/639 (98%), Positives = 632/639 (99%), Gaps = 0/639 (0%)

Query 1
MKIINIGILAHVDAGKTTLTESLLYASGAISEPGSVEKGTTRTDTMFLERQRGITIQAAV 60

MKIINIGILAHVDAGKTTLTESLLYASGAISEPGSVEKGTTRTDTMFLERQRGITIQAAV
Sbjct 1
MKIINIGILAHVDAGKTTLTESLLYASGAISEPGSVEKGTTRTDTMFLERQRGITIQAAV 60

Query 61
TSFQWHRCKVNIVDTPGHMDFLAEVYRSLAVLDGAILVISAKDGVQAQTRILFHALRKMN 120

TSFQWHRCKVNIVDTPGHMDFLAEVYRSLAVLDGAILVISAKDGVQAQTRILFHALRKMN
Sbjct 61
TSFQWHRCKVNIVDTPGHMDFLAEVYRSLAVLDGAILVISAKDGVQAQTRILFHALRKMN 120

Query 121
IPTVIFINKIDQAGVDLQSVVQSVRDKLSADIIIKQTVSLSPEIVLEENTDIEAWDAVIE 180

IPTVIFINKIDQAGVDLQSVVQSVRDKLSADIIIKQTVSLSPEIVLEENTDIEAWDAVIE
Sbjct 121
IPTVIFINKIDQAGVDLQSVVQSVRDKLSADIIIKQTVSLSPEIVLEENTDIEAWDAVIE 180

Query 181
NNDKLLEKYIAGEPISREKLVREEQRRVQDASLFPVYYGSAKKGLGIQPLMDAVTGLFQP 240
            NND+LLEKYIAGEPISREKL REEQ+RVQDASLFPVY+GSAK
GLGIQPLMDAVTGLFQP

```
Sbjct  181
NNDELLEKYIAGEPISREKLAREEQQRVQDASLFPVYHGSAKNGLGIQPLMDAVTGLFQP  240

Query  241
IGEQGSAALCGSVFKVEYTDCGQRRVYLRLYSGTLRLRDTVALAGREKLKITEMRIPSKG  300
           IGEQG
AALCGSVFKVEYTDCGQRRVYLRLYSGTLRLRDTVALAGREKLKITEMRIPSKG
Sbjct  241
IGEQGGAALCGSVFKVEYTDCGQRRVYLRLYSGTLRLRDTVALAGREKLKITEMRIPSKG  300

Query  301
EIVRTDTAYPGEIVILPSDSVRLNDVLGDPTRLPRKRWREDPLPMLRTTIAPKTAAQRER  360
           EIVRTDTAY GEIVILPSDSVRLNDVLGDPTRLPRKRWREDPLPMLRT
+IAPKTAAQRER
Sbjct  301
EIVRTDTAYQGEIVILPSDSVRLNDVLGDPTRLPRKRWREDPLPMLRTSIAPKTAAQRER  360

Query  361
LLDALTQLADTDPLLRCEVDSITHEIILSFLGRVQLEVVSALLSEKYKLETVVKEPSVIY  420
           LLDALTQLADTDPLLRCEVDSITHEIILSFLGRVQLEVVSALLSEKYKLETVVKEP
+VIY
Sbjct  361
LLDALTQLADTDPLLRCEVDSITHEIILSFLGRVQLEVVSALLSEKYKLETVVKEPTVIY  420

Query  421
MERPLKAASHTIHIEVPPNPFWASIGLSVTPLSLGSGVQYESRVSLGYLNQSFQNAVRDG  480
           MERPLKAASHTIHIEVPPNPFWASIGLSVTPL
LGSGVQYESRVSLGYLNQSFQNAVRDG
Sbjct  421
MERPLKAASHTIHIEVPPNPFWASIGLSVTPLPLGSGVQYESRVSLGYLNQSFQNAVRDG  480

Query  481
IRYGLEQGLFGWNVTDCKICFEYGLYYSPVSTPADFRSLAPIVLEQALKESGTQLLEPYL  540

IRYGLEQGLFGWNVTDCKICFEYGLYYSPVSTPADFRSLAPIVLEQALKESGTQLLEPYL
Sbjct  481
IRYGLEQGLFGWNVTDCKICFEYGLYYSPVSTPADFRSLAPIVLEQALKESGTQLLEPYL  540

Query  541
SFILYAPQEYLSRAYHDAPKYCATIETAQVKKDEVVFTGEIPARCIQAYRTDLAFYTNGR  600
           SF LYAP+EYLSRAYHDAPKYCATIET
QVKKDEVVFTGEIPARCIQAYRTDLAFYTNG+
Sbjct  541
SFTLYAPREYLSRAYHDAPKYCATIETVQVKKDEVVFTGEIPARCIQAYRTDLAFYTNGQ  600

Query  601  SVCLTELKGYQAAVGQPVIQPRRPNSRLDKVRHMFQKVM  639
            SVCLTELKGYQAAVG+PVIQPRRPNSRLDKVRHMFQKVM
Sbjct  601  SVCLTELKGYQAAVGKPVIQPRRPNSRLDKVRHMFQKVM  639


>AMP50237.1 TetM-TetW-TetO-TetS [uncultured bacterium]
Length=639

 Score = 1285 bits (3324),  Expect = 0.0, Method: Compositional
```

matrix adjust.
 Identities = 623/639 (97%), Positives = 633/639 (99%), Gaps = 0/639
(0%)

Query 1
MKIINIGILAHVDAGKTTLTESLLYASGAISEPGSVEKGTTRTDTMFLERQRGITIQAAV 60

MKIINIGILAHVDAGKTTLTESLLYASGAISEPGSVEKGTTRTDTMFLERQRGITIQAAV
Sbjct 1
MKIINIGILAHVDAGKTTLTESLLYASGAISEPGSVEKGTTRTDTMFLERQRGITIQAAV 60

Query 61
TSFQWHRCKVNIVDTPGHMDFLAEVYRSLAVLDGAILVISAKDGVQAQTRILFHALRKMN 120

TSFQWHRCKVNIVDTPGHMDFLAEVYRSLAVLDGAILVISAKDGVQAQTRILFHALRKMN
Sbjct 61
TSFQWHRCKVNIVDTPGHMDFLAEVYRSLAVLDGAILVISAKDGVQAQTRILFHALRKMN 120

Query 121
IPTVIFINKIDQAGVDLQSVVQSVRDKLSADIIIKQTVSLSPEIVLEENTDIEAWDAVIE 180

IPTVIFINKIDQAGVDLQSVVQSVRDKLSADIIIKQTVSLSPEIVLEENTDIEAWDAVIE
Sbjct 121
IPTVIFINKIDQAGVDLQSVVQSVRDKLSADIIIKQTVSLSPEIVLEENTDIEAWDAVIE 180

Query 181
NNDKLLEKYIAGEPISREKLVREEQRRVQDASLFPVYYGSAKKGLGIQPLMDAVTGLFQP 240
            NND+LLEKYIAGEPISREKL REEQ+RVQDASLFPVY+GSAK
GLGIQPLMDAVTGLFQP
Sbjct 181
NNDELLEKYIAGEPISREKLAREEQQRVQDASLFPVYHGSAKNGLGIQPLMDAVTGLFQP 240

Query 241
IGEQGSAALCGSVFKVEYTDCGQRRVYLRLYSGTLRLRDTVALAGREKLKITEMRIPSKG 300
            IGEQG
AALCGSVFKVEYTDCGQRRVYLRLYSGTLRLRDTVALAGREKLKITEMRIPSKG
Sbjct 241
IGEQGGAALCGSVFKVEYTDCGQRRVYLRLYSGTLRLRDTVALAGREKLKITEMRIPSKG 300

Query 301
EIVRTDTAYPGEIVILPSDSVRLNDVLGDPTRLPRKRWREDPLPMLRTTIAPKTAAQRER 360
            EIVRTDTAYPGEIVILPSDSVRLNDVLGDPTRLPRKRWREDPLPMLRT
+IAPKTAAQRER
Sbjct 301
EIVRTDTAYPGEIVILPSDSVRLNDVLGDPTRLPRKRWREDPLPMLRTSIAPKTAAQRER 360

Query 361
LLDALTQLADTDPLLRCEVDSITHEIILSFLGRVQLEVVSALLSEKYKLETVVKEPSVIY 420
            LLDALTQLADTDPLLRCEVDSITHEIILSFLGRVQLEVVSALLSEKYKLETVVKEP
+VIY
Sbjct 361
LLDALTQLADTDPLLRCEVDSITHEIILSFLGRVQLEVVSALLSEKYKLETVVKEPTVIY 420

Query 421

MERPLKAASHTIHIEVPPNPFWASIGLSVTPLSLGSGVQYESRVSLGYLNQSFQNAVRDG 480
            MERPLKAASHTIHIEVPPNPFWASIGLSVTPL
LGSGVQYESRVSLGYLNQSFQNAVRDG
Sbjct 421
MERPLKAASHTIHIEVPPNPFWASIGLSVTPLPLGSGVQYESRVSLGYLNQSFQNAVRDG 480

Query 481
IRYGLEQGLFGWNVTDCKICFEYGLYYSPVSTPADFRSLAPIVLEQALKESGTQLLEPYL 540

IRYGLEQGLFGWNVTDCKICFEYGLYYSPVSTPADFRSLAPIVLEQALKESGTQLLEPYL
Sbjct 481
IRYGLEQGLFGWNVTDCKICFEYGLYYSPVSTPADFRSLAPIVLEQALKESGTQLLEPYL 540

Query 541
SFILYAPQEYLSRAYHDAPKYCATIETAQVKKDEVVFTGEIPARCIQAYRTDLAFYTNGR 600
            SF LYAP+EYLSRAYHDAPKYCATIET
QVKKDEVVFTGEIPARCIQAYRTDLAFYTNG+
Sbjct 541
SFTLYAPREYLSRAYHDAPKYCATIETVQVKKDEVVFTGEIPARCIQAYRTDLAFYTNGQ 600

Query 601 SVCLTELKGYQAAVGQPVIQPRRPNSRLDKVRHMFQKVM 639
            SVCLTELKGYQAAVG+PVIQPRRPNSRLDKVR+MFQK+M
Sbjct 601 SVCLTELKGYQAAVGKPVIQPRRPNSRLDKVRYMFQKIM 639

>WP_086143532.1 TetM/TetW/TetO/TetS family tetracycline resistance ribosomal
protection protein [Lactobacillus reuteri]
 CUR37509.1 Tetracycline resistance protein TetW [Lactobacillus reuteri]
 OTA44014.1 tetracycline resistance ribosomal protection protein [Lactobacillus
reuteri]
Length=641

 Score = 1285 bits (3324), Expect = 0.0, Method: Compositional matrix adjust.
 Identities = 625/638 (98%), Positives = 633/638 (99%), Gaps = 0/638 (0%)

Query 1
MKIINIGILAHVDAGKTTLTESLLYASGAISEPGSVEKGTTRTDTMFLERQRGITIQAAV 60
            MKIINIGILAHVDAGKTTLTESLLYASGAISE GSV
+KGTTRTDTMFLERQRGITIQAAV
Sbjct 1
MKIINIGILAHVDAGKTTLTESLLYASGAISERGSVKKGTTRTDTMFLERQRGITIQAAV 60

Query 61
TSFQWHRCKVNIVDTPGHMDFLAEVYRSLAVLDGAILVISAKDGVQAQTRILFHALRKMN 120

TSFQWHRCKVNIVDTPGHMDFLAEVYRSLAVLDGAILVISAKDGVQAQTRILFHALRKMN
Sbjct 61
TSFQWHRCKVNIVDTPGHMDFLAEVYRSLAVLDGAILVISAKDGVQAQTRILFHALRKMN 120

Query 121
IPTVIFINKIDQAGVDLQSVVQSVRDKLSADIIIKQTVSLSPEIVLEENTDIEAWDAVIE 180

IPTVIFINKIDQAGVDLQSVVQSVRDKLSADIIIKQTVSLSPEIVLEENTDIEAWDAVIE
Sbjct 121
IPTVIFINKIDQAGVDLQSVVQSVRDKLSADIIIKQTVSLSPEIVLEENTDIEAWDAVIE 180

Query 181
NNDKLLEKYIAGEPISREKLVREEQRRVQDASLFPVYYGSAKKGLGIQPLMDAVTGLFQP 240

NNDKLLEKYIAGEPISREKLVREEQRRVQDASLFPVYYGSAKKGLGIQPLMDAVTGLFQP
Sbjct 181
NNDKLLEKYIAGEPISREKLVREEQRRVQDASLFPVYYGSAKKGLGIQPLMDAVTGLFQP 240

Query 241
IGEQGSAALCGSVFKVEYTDCGQRRVYLRLYSGTLRLRDTVALAGREKLKITEMRIPSKG 300

IGEQGSAALCGSVFKVEYTDCGQRRVYLRLYSGTLRLRDTVALAGREKLKITEMRIPSKG
Sbjct 241
IGEQGSAALCGSVFKVEYTDCGQRRVYLRLYSGTLRLRDTVALAGREKLKITEMRIPSKG 300

Query 301
EIVRTDTAYPGEIVILPSDSVRLNDVLGDPTRLPRKRWREDPLPMLRTTIAPKTAAQRER 360
EIVRTDTAYPGEIVILPSDSVRLNDVLGDPTRLPRKRWREDPLPMLRT
+IAPKTAAQRER
Sbjct 301
EIVRTDTAYPGEIVILPSDSVRLNDVLGDPTRLPRKRWREDPLPMLRTSIAPKTAAQRER 360

Query 361
LLDALTQLADTDPLLRCEVDSITHEIILSFLGRVQLEVVSALLSEKYKLETVVKEPSVIY 420
LLDALTQLADTDPLLRCEVDSITHEIILSFLGRVQLEVVSALLSEKYKLETVVKEP
+VIY
Sbjct 361
LLDALTQLADTDPLLRCEVDSITHEIILSFLGRVQLEVVSALLSEKYKLETVVKEPTVIY 420

Query 421
MERPLKAASHTIHIEVPPNPFWASIGLSVTPLSLGSGVQYESRVSLGYLNQSFQNAVRDG 480
MERPLKAASHTIHIEVPPNPFWASIGLSVTPL
LGSGVQYESRVSLGYLNQSFQNAVRDG
Sbjct 421
MERPLKAASHTIHIEVPPNPFWASIGLSVTPLPLGSGVQYESRVSLGYLNQSFQNAVRDG 480

Query 481
IRYGLEQGLFGWNVTDCKICFEYGLYYSPVSTPADFRSLAPIVLEQALKESGTQLLEPYL 540

IRYGLEQGLFGWNVTDCKICFEYGLYYSPVSTPADFRSLAPIVLEQALKESGTQLLEPYL
Sbjct 481
IRYGLEQGLFGWNVTDCKICFEYGLYYSPVSTPADFRSLAPIVLEQALKESGTQLLEPYL 540

Query 541
SFILYAPQEYLSRAYHDAPKYCATIETAQVKKDEVVFTGEIPARCIQAYRTDLAFYTNGR 600
SF LYAP+EYLSRAYHDAPKYCATIET QVKKDEVVFTGEIPARCIQAYRTDLA
YTNG+
Sbjct 541

```
SFTLYAPREYLSRAYHDAPKYCATIETVQVKKDEVVFTGEIPARCIQAYRTDLASYTNGQ  600

Query  601  SVCLTELKGYQAAVGQPVIQPRRPNSRLDKVRHMFQKV  638
            SVCLTELKGYQAAVG+PVIQPRRPNSRLDKVR+MFQK+
Sbjct  601  SVCLTELKGYQAAVGKPVIQPRRPNSRLDKVRYMFQKI  638


>WP_003695455.1 TetM/TetW/TetO/TetS family tetracycline resistance
ribosomal
protection protein [Lactobacillus ruminis]
 EGM51752.1 hypothetical protein LRU_01266 [Lactobacillus ruminis
SPM0211]
Length=639

 Score = 1285 bits (3324),  Expect = 0.0, Method: Compositional
matrix adjust.
 Identities = 622/639 (97%), Positives = 632/639 (99%), Gaps = 0/639
(0%)

Query  1
MKIINIGILAHVDAGKTTLTESLLYASGAISEPGSVEKGTTRTDTMFLERQRGITIQAAV  60
            MKIINIGILAHVDAGKTTLTESLLY SGAI+E G+V+KGTTRTDTM
LERQRGITIQAAV
Sbjct  1
MKIINIGILAHVDAGKTTLTESLLYTSGAIAEQGNVDKGTTRTDTMILERQRGITIQAAV  60

Query  61
TSFQWHRCKVNIVDTPGHMDFLAEVYRSLAVLDGAILVISAKDGVQAQTRILFHALRKMN  120

TSFQWHRCKVNIVDTPGHMDFLAEVYRSLAVLDGAILVISAKDGVQAQTRILFHALRKMN
Sbjct  61
TSFQWHRCKVNIVDTPGHMDFLAEVYRSLAVLDGAILVISAKDGVQAQTRILFHALRKMN  120

Query  121
IPTVIFINKIDQAGVDLQSVVQSVRDKLSADIIIKQTVSLSPEIVLEENTDIEAWDAVIE  180

IPTVIFINKIDQAGVDLQSVVQSVRDKLSADIIIKQTVSLSPEIVLEENTDIEAWDAVIE
Sbjct  121
IPTVIFINKIDQAGVDLQSVVQSVRDKLSADIIIKQTVSLSPEIVLEENTDIEAWDAVIE  180

Query  181
NNDKLLEKYIAGEPISREKLVREEQRRVQDASLFPVYYGSAKKGLGIQPLMDAVTGLFQP  240

NNDKLLEKYIAGEPISREKLVREEQRRVQDASLFPVYYGSAKKGLGIQPLMDAVTGLFQP
Sbjct  181
NNDKLLEKYIAGEPISREKLVREEQRRVQDASLFPVYYGSAKKGLGIQPLMDAVTGLFQP  240

Query  241
IGEQGSAALCGSVFKVEYTDCGQRRVYLRLYSGTLRLRDTVALAGREKLKITEMRIPSKG  300

IGEQGSAALCGSVFKVEYTDCGQRRVYLRLYSGTLRLRDTVALAGREKLKITEMRIPSKG
Sbjct  241
IGEQGSAALCGSVFKVEYTDCGQRRVYLRLYSGTLRLRDTVALAGREKLKITEMRIPSKG  300
```

Query 301
EIVRTDTAYPGEIVILPSDSVRLNDVLGDPTRLPRKRWREDPLPMLRTTIAPKTAAQRER 360
            EIVRTDTAYPGEIVILPSDSVRLNDVLGDPTRLPRKRWREDPLPMLR
+IAPKTAAQRER
Sbjct 301
EIVRTDTAYPGEIVILPSDSVRLNDVLGDPTRLPRKRWREDPLPMLRASIAPKTAAQRER 360

Query 361
LLDALTQLADTDPLLRCEVDSITHEIILSFLGRVQLEVVSALLSEKYKLETVVKEPSVIY 420
            LLDALTQLADTDPLLRCEVDSITHEIILSFLGRVQLEVVSALLSEKYKLETVVKEP
+VIY
Sbjct 361
LLDALTQLADTDPLLRCEVDSITHEIILSFLGRVQLEVVSALLSEKYKLETVVKEPTVIY 420

Query 421
MERPLKAASHTIHIEVPPNPFWASIGLSVTPLSLGSGVQYESRVSLGYLNQSFQNAVRDG 480
            MERPLKAASHTIHIEVPPNPFWASIGLSVTPL
LGSGVQYESRVSLGYLNQSFQNAVRDG
Sbjct 421
MERPLKAASHTIHIEVPPNPFWASIGLSVTPLPLGSGVQYESRVSLGYLNQSFQNAVRDG 480

Query 481
IRYGLEQGLFGWNVTDCKICFEYGLYYSPVSTPADFRSLAPIVLEQALKESGTQLLEPYL 540

IRYGLEQGLFGWNVTDCKICFEYGLYYSPVSTPADFRSLAPIVLEQALKESGTQLLEPYL
Sbjct 481
IRYGLEQGLFGWNVTDCKICFEYGLYYSPVSTPADFRSLAPIVLEQALKESGTQLLEPYL 540

Query 541
SFILYAPQEYLSRAYHDAPKYCATIETAQVKKDEVVFTGEIPARCIQAYRTDLAFYTNGR 600
            SF LYAP+EYLSRAYHDAPKYCATIET
QVKKDEVVFTGEIPARCIQAYRTDLAFYTNG+
Sbjct 541
SFTLYAPREYLSRAYHDAPKYCATIETVQVKKDEVVFTGEIPARCIQAYRTDLAFYTNGQ 600

Query 601 SVCLTELKGYQAAVGQPVIQPRRPNSRLDKVRHMFQKVM 639
            SVCLTELKGYQAAVG+PVIQPRRPNSRLDK+RHMFQK+M
Sbjct 601 SVCLTELKGYQAAVGKPVIQPRRPNSRLDKIRHMFQKIM 639

>WP_089150426.1 TetM/TetW/TetO/TetS family tetracycline resistance
ribosomal
protection protein [Lactobacillus crispatus]
 OXC25414.1 tetracycline resistance ribosomal protection protein
[Lactobacillus
crispatus]
Length=640

 Score = 1284 bits (3323), Expect = 0.0, Method: Compositional
matrix adjust.
 Identities = 624/638 (98%), Positives = 633/638 (99%), Gaps = 0/638
(0%)

Query 1

MKIINIGILAHVDAGKTTLTESLLYASGAISEPGSVEKGTTRTDTMFLERQRGITIQAAV 60
MKIINIGILAHVDAGKTTLTESLLYASGAISEPGSVEKGTTRTD
+MFLERQRGITIQAAV
Sbjct 1
MKIINIGILAHVDAGKTTLTESLLYASGAISEPGSVEKGTTRTDSMFLERQRGITIQAAV 60

Query 61
TSFQWHRCKVNIVDTPGHMDFLAEVYRSLAVLDGAILVISAKDGVQAQTRILFHALRKMN 120

TSFQWHRCKVNIVDTPGHMDFLAEVYRSLAVLDGAILVISAKDGVQAQTRILFHALRKMN
Sbjct 61
TSFQWHRCKVNIVDTPGHMDFLAEVYRSLAVLDGAILVISAKDGVQAQTRILFHALRKMN 120

Query 121
IPTVIFINKIDQAGVDLQSVVQSVRDKLSADIIIKQTVSLSPEIVLEENTDIEAWDAVIE 180

IPTVIFINKIDQAGVDLQSVVQSVRDKLSADIIIKQTVSLSPEIVLEENTDIEAWDAVIE
Sbjct 121
IPTVIFINKIDQAGVDLQSVVQSVRDKLSADIIIKQTVSLSPEIVLEENTDIEAWDAVIE 180

Query 181
NNDKLLEKYIAGEPISREKLVREEQRRVQDASLFPVYYGSAKKGLGIQPLMDAVTGLFQP 240

NNDKLLEKYIAGEPISREKLVREEQRRVQDASLFPVYYGSAKKGLGIQPLMDAVTGLFQP
Sbjct 181
NNDKLLEKYIAGEPISREKLVREEQRRVQDASLFPVYYGSAKKGLGIQPLMDAVTGLFQP 240

Query 241
IGEQGSAALCGSVFKVEYTDCGQRRVYLRLYSGTLRLRDTVALAGREKLKITEMRIPSKG 300

IGEQGSAALCGSVFKVEYTDCGQRRVYLRLYSGTLRLRDTVALAGREKLKITEMRIPSKG
Sbjct 241
IGEQGSAALCGSVFKVEYTDCGQRRVYLRLYSGTLRLRDTVALAGREKLKITEMRIPSKG 300

Query 301
EIVRTDTAYPGEIVILPSDSVRLNDVLGDPTRLPRKRWREDPLPMLRTTIAPKTAAQRER 360
+IVRTDTAYPGEIVILPSDSVRLNDVLGDPTRLPRKRWREDPLPMLRT
+IAPKTAAQRER
Sbjct 301
KIVRTDTAYPGEIVILPSDSVRLNDVLGDPTRLPRKRWREDPLPMLRTSIAPKTAAQRER 360

Query 361
LLDALTQLADTDPLLRCEVDSITHEIILSFLGRVQLEVVSALLSEKYKLETVVKEPSVIY 420
LLDALTQLADTDPLLRCEVDSITHEIILSFLGRVQLEVVSALLSEKYKLETVVKEP
+VIY
Sbjct 361
LLDALTQLADTDPLLRCEVDSITHEIILSFLGRVQLEVVSALLSEKYKLETVVKEPTVIY 420

Query 421
MERPLKAASHTIHIEVPPNPFWASIGLSVTPLSLGSGVQYESRVSLGYLNQSFQNAVRDG 480
MERPLKAASHTIHIEVPPNPFWASIGLSVTPL
LGSGVQYESRVSLGYLNQSFQNAVRDG
Sbjct 421
MERPLKAASHTIHIEVPPNPFWASIGLSVTPLPLGSGVQYESRVSLGYLNQSFQNAVRDG 480

Query 481
IRYGLEQGLFGWNVTDCKICFEYGLYYSPVSTPADFRSLAPIVLEQALKESGTQLLEPYL 540

IRYGLEQGLFGWNVTDCKICFEYGLYYSPVSTPADFRSLAPIVLEQALKESGTQLLEPYL
Sbjct 481
IRYGLEQGLFGWNVTDCKICFEYGLYYSPVSTPADFRSLAPIVLEQALKESGTQLLEPYL 540

Query 541
SFILYAPQEYLSRAYHDAPKYCATIETAQVKKDEVVFTGEIPARCIQAYRTDLAFYTNGR 600
            SF LYAP+EYLSRAYHDAPKYCATIET QVKKDEVVFTGEIPARCIQAYRTDLA
YTNG+
Sbjct 541
SFTLYAPREYLSRAYHDAPKYCATIETVQVKKDEVVFTGEIPARCIQAYRTDLASYTNGQ 600

Query 601 SVCLTELKGYQAAVGQPVIQPRRPNSRLDKVRHMFQKV 638
            SVCLTELKGYQAAVG+PVIQPRRPN RLDKVR+MFQK+
Sbjct 601 SVCLTELKGYQAAVGKPVIQPRRPNRRLDKVRYMFQKI 638

>WP_086120764.1 TetM/TetW/TetO/TetS family tetracycline resistance
ribosomal
protection protein [Lactobacillus reuteri]
 OTA53930.1 tetracycline resistance ribosomal protection protein
[Lactobacillus
reuteri]
 OTA71053.1 tetracycline resistance ribosomal protection protein
[Lactobacillus
reuteri]
 OTA71405.1 tetracycline resistance ribosomal protection protein
[Lactobacillus
reuteri]
Length=645

 Score = 1284 bits (3323), Expect = 0.0, Method: Compositional
matrix adjust.
 Identities = 624/638 (98%), Positives = 633/638 (99%), Gaps = 0/638
(0%)

Query 1
MKIINIGILAHVDAGKTTLTESLLYASGAISEPGSVEKGTTRTDTMFLERQRGITIQAAV 60
            MKIINIGILAHVDAGKTTLTESLLYASGAISEPGSV
+KGTTRTDTMFLERQRGITIQAAV
Sbjct 1
MKIINIGILAHVDAGKTTLTESLLYASGAISEPGSVKKGTTRTDTMFLERQRGITIQAAV 60

Query 61
TSFQWHRCKVNIVDTPGHMDFLAEVYRSLAVLDGAILVISAKDGVQAQTRILFHALRKMN 120

TSFQWHRCKVNIVDTPGHMDFLAEVYRSLAVLDGAILVISAKDGVQAQTRILFHALRKMN
Sbjct 61
TSFQWHRCKVNIVDTPGHMDFLAEVYRSLAVLDGAILVISAKDGVQAQTRILFHALRKMN 120

Query 121

IPTVIFINKIDQAGVDLQSVVQSVRDKLSADIIIKQTVSLSPEIVLEENTDIEAWDAVIE 180

IPTVIFINKIDQAGVDLQSVVQSVRDKLSADIIIKQTVSLSPEIVLEENTDIEAWDAVIE
Sbjct 121
IPTVIFINKIDQAGVDLQSVVQSVRDKLSADIIIKQTVSLSPEIVLEENTDIEAWDAVIE 180

Query 181
NNDKLLEKYIAGEPISREKLVREEQRRVQDASLFPVYYGSAKKGLGIQPLMDAVTGLFQP 240

NNDKLLEKYIAGEPISREKLVREEQRRVQDASLFPVYYGSAKKGLGIQPLMDAVTGLFQP
Sbjct 181
NNDKLLEKYIAGEPISREKLVREEQRRVQDASLFPVYYGSAKKGLGIQPLMDAVTGLFQP 240

Query 241
IGEQGSAALCGSVFKVEYTDCGQRRVYLRLYSGTLRLRDTVALAGREKLKITEMRIPSKG 300

IGEQGSAALCGSVFKVEYTDCGQRRVYLRLYSGTLRLRDTVALAGREKLKITEMRIPSKG
Sbjct 241
IGEQGSAALCGSVFKVEYTDCGQRRVYLRLYSGTLRLRDTVALAGREKLKITEMRIPSKG 300

Query 301
EIVRTDTAYPGEIVILPSDSVRLNDVLGDPTRLPRKRWREDPLPMLRTTIAPKTAAQRER 360
EIVRTDTAYPGEIVILPSDSVRLNDVLGDPTRLPRKRWREDPLPMLRT
+IAPKTAAQRER
Sbjct 301
EIVRTDTAYPGEIVILPSDSVRLNDVLGDPTRLPRKRWREDPLPMLRTSIAPKTAAQRER 360

Query 361
LLDALTQLADTDPLLRCEVDSITHEIILSFLGRVQLEVVSALLSEKYKLETVVKEPSVIY 420
LLDALTQLADTDPLLRCEVDSITHEIILSFLGRVQLEVVSALLSEKYKLETVVKEP
+VIY
Sbjct 361
LLDALTQLADTDPLLRCEVDSITHEIILSFLGRVQLEVVSALLSEKYKLETVVKEPTVIY 420

Query 421
MERPLKAASHTIHIEVPPNPFWASIGLSVTPLSLGSGVQYESRVSLGYLNQSFQNAVRDG 480
MERPLKAASHTIHIEVPPNPFWASIGLSVTPL
LGSGVQYESRVSLGYLNQSFQNAVRDG
Sbjct 421
MERPLKAASHTIHIEVPPNPFWASIGLSVTPLPLGSGVQYESRVSLGYLNQSFQNAVRDG 480

Query 481
IRYGLEQGLFGWNVTDCKICFEYGLYYSPVSTPADFRSLAPIVLEQALKESGTQLLEPYL 540

IRYGLEQGLFGWNVTDCKICFEYGLYYSPVSTPADFRSLAPIVLEQALKESGTQLLEPYL
Sbjct 481
IRYGLEQGLFGWNVTDCKICFEYGLYYSPVSTPADFRSLAPIVLEQALKESGTQLLEPYL 540

Query 541
SFILYAPQEYLSRAYHDAPKYCATIETAQVKKDEVVFTGEIPARCIQAYRTDLAFYTNGR 600
SF LYAP+EYLSRAYHDAP+YCATIET QVKKDEVVFTGEIPARCIQAYRTDLA
YTNG+
Sbjct 541
SFTLYAPREYLSRAYHDAPEYCATIETVQVKKDEVVFTGEIPARCIQAYRTDLASYTNGQ 600

```
Query  601  SVCLTELKGYQAAVGQPVIQPRRPNSRLDKVRHMFQKV  638
            SVCLTELKGYQAA G+PVIQPRRPNSRLDKVR+MFQK+
Sbjct  601  SVCLTELKGYQAADGKPVIQPRRPNSRLDKVRYMFQKI  638
```

>WP_090862004.1 TetM/TetW/TetO/TetS family tetracycline resistance ribosomal
protection protein [Olsenella umbonata]
 SDR72479.1 ribosomal protection tetracycline resistance protein [Olsenella
umbonata]
Length=639

 Score = 1284 bits (3323), Expect = 0.0, Method: Compositional matrix adjust.
 Identities = 624/639 (98%), Positives = 631/639 (99%), Gaps = 0/639 (0%)

```
Query  1
MKIINIGILAHVDAGKTTLTESLLYASGAISEPGSVEKGTTRTDTMFLERQRGITIQAAV  60
            M IINIGILAHVDAGKTTLTESLLYASGAISEPGSVEKGTTRTDT
+FLERQRGITIQAAV
Sbjct  1
MNIINIGILAHVDAGKTTLTESLLYASGAISEPGSVEKGTTRTDTLFLERQRGITIQAAV  60

Query  61
TSFQWHRCKVNIVDTPGHMDFLAEVYRSLAVLDGAILVISAKDGVQAQTRILFHALRKMN  120

TSFQWHRCKVNIVDTPGHMDFLAEVYRSLAVLDGAILVISAKDGVQAQTRILFHALRKMN
Sbjct  61
TSFQWHRCKVNIVDTPGHMDFLAEVYRSLAVLDGAILVISAKDGVQAQTRILFHALRKMN  120

Query  121
IPTVIFINKIDQAGVDLQSVVQSVRDKLSADIIIKQTVSLSPEIVLEENTDIEAWDAVIE  180

IPTVIFINKIDQAGVDLQSVVQSVRDKLSADIIIKQTVSLSPEIVLEENTDIEAWDAVIE
Sbjct  121
IPTVIFINKIDQAGVDLQSVVQSVRDKLSADIIIKQTVSLSPEIVLEENTDIEAWDAVIE  180

Query  181
NNDKLLEKYIAGEPISREKLVREEQRRVQDASLFPVYYGSAKKGLGIQPLMDAVTGLFQP  240
            NND LLEKYIAGEPIS+EKL REEQRRVQ+ASLFPVY
+GSAKKGLGIQPLMDAVTGLFQP
Sbjct  181
NNDALLEKYIAGEPISQEKLAREEQRRVQEASLFPVYHGSAKKGLGIQPLMDAVTGLFQP  240

Query  241
IGEQGSAALCGSVFKVEYTDCGQRRVYLRLYSGTLRLRDTVALAGREKLKITEMRIPSKG  300
            IGEQGSA
LCGSVFKVEYTDCGQRRVYLRLYSGTLRLRDTVALAGREKLKITEMRIPSKG
Sbjct  241
IGEQGSATLCGSVFKVEYTDCGQRRVYLRLYSGTLRLRDTVALAGREKLKITEMRIPSKG  300
```

```
Query  301
EIVRTDTAYPGEIVILPSDSVRLNDVLGDPTRLPRKRWREDPLPMLRTTIAPKTAAQRER  360
            EIVRTDTAYPGEIVILPSDSVRLNDVLGD TRLPRKRWRE
PLPMLRTTIAPKTAAQRER
Sbjct  301
EIVRTDTAYPGEIVILPSDSVRLNDVLGDQTRLPRKRWREAPLPMLRTTIAPKTAAQRER  360

Query  361
LLDALTQLADTDPLLRCEVDSITHEIILSFLGRVQLEVVSALLSEKYKLETVVKEPSVIY  420
            LLDALTQLADTDPLLRCEVDSITHEIILS+LGRVQLEVVSALLSEKYK+ETVVKEP
+VIY
Sbjct  361
LLDALTQLADTDPLLRCEVDSITHEIILSYLGRVQLEVVSALLSEKYKIETVVKEPTVIY  420

Query  421
MERPLKAASHTIHIEVPPNPFWASIGLSVTPLSLGSGVQYESRVSLGYLNQSFQNAVRDG  480
            MERPLKAASHTIHIEVPPNPFWASIGLSVTPL
LGSGVQYESRVSLGYLNQSFQNAVRDG
Sbjct  421
MERPLKAASHTIHIEVPPNPFWASIGLSVTPLPLGSGVQYESRVSLGYLNQSFQNAVRDG  480

Query  481
IRYGLEQGLFGWNVTDCKICFEYGLYYSPVSTPADFRSLAPIVLEQALKESGTQLLEPYL  540

IRYGLEQGLFGWNVTDCKICFEYGLYYSPVSTPADFRSLAPIVLEQALKESGTQLLEPYL
Sbjct  481
IRYGLEQGLFGWNVTDCKICFEYGLYYSPVSTPADFRSLAPIVLEQALKESGTQLLEPYL  540

Query  541
SFILYAPQEYLSRAYHDAPKYCATIETAQVKKDEVVFTGEIPARCIQAYRTDLAFYTNGR  600
            SF
LYAPQEYLSRAYHDAPKYCATIETAQVKKDEVVFTGEIPARCIQAYRTDLAFYTNGR
Sbjct  541
SFTLYAPQEYLSRAYHDAPKYCATIETAQVKKDEVVFTGEIPARCIQAYRTDLAFYTNGR  600

Query  601  SVCLTELKGYQAAVGQPVIQPRRPNSRLDKVRHMFQKVM  639
            SVCLTELKGYQAAVGQPVIQPRRPNSRLDKVRHMFQKVM
Sbjct  601  SVCLTELKGYQAAVGQPVIQPRRPNSRLDKVRHMFQKVM  639


>WP_070628949.1 TetM/TetW/TetO/TetS family tetracycline resistance
ribosomal
protection protein [Rothia sp. HMSC065C12]
 OFJ97827.1 tetracycline resistance ribosomal protection protein
[Rothia
sp. HMSC065C12]
Length=639

 Score = 1284 bits (3323),  Expect = 0.0, Method: Compositional
matrix adjust.
 Identities = 623/639 (97%), Positives = 633/639 (99%), Gaps = 0/639
(0%)

Query  1
```

MKIINIGILAHVDAGKTTLTESLLYASGAISEPGSVEKGTTRTDTMFLERQRGITIQAAV 60

MKIINIGILAHVDAGKTTLTESLLYASGAISEPGSVEKGTTRTDTMFLERQRGITIQAAV
Sbjct 1
MKIINIGILAHVDAGKTTLTESLLYASGAISEPGSVEKGTTRTDTMFLERQRGITIQAAV 60

Query 61
TSFQWHRCKVNIVDTPGHMDFLAEVYRSLAVLDGAILVISAKDGVQAQTRILFHALRKMN 120
TSFQWHRCKVNIVDTPGHMDFLAEVYRSLAVLDGAILVISAKDGVQAQTRILF
+ALRKMN
Sbjct 61
TSFQWHRCKVNIVDTPGHMDFLAEVYRSLAVLDGAILVISAKDGVQAQTRILFNALRKMN 120

Query 121
IPTVIFINKIDQAGVDLQSVVQSVRDKLSADIIIKQTVSLSPEIVLEENTDIEAWDAVIE 180

IPTVIFINKIDQAGVDLQSVVQSVRDKLSADIIIKQTVSLSPEIVLEENTDIEAWDAVIE
Sbjct 121
IPTVIFINKIDQAGVDLQSVVQSVRDKLSADIIIKQTVSLSPEIVLEENTDIEAWDAVIE 180

Query 181
NNDKLLEKYIAGEPISREKLVREEQRRVQDASLFPVYYGSAKKGLGIQPLMDAVTGLFQP 240
NND+LLEKYIAGEPISREKL REEQ+RVQDASLFPVY+GSAK
GLGIQPLMDAVTGLFQP
Sbjct 181
NNDELLEKYIAGEPISREKLAREEQQRVQDASLFPVYHGSAKNGLGIQPLMDAVTGLFQP 240

Query 241
IGEQGSAALCGSVFKVEYTDCGQRRVYLRLYSGTLRLRDTVALAGREKLKITEMRIPSKG 300
IGEQG
AALCGSVFKVEYTDCGQRRVYLRLYSGTLRLRDTVALAGREKLKITEMRIPSKG
Sbjct 241
IGEQGGAALCGSVFKVEYTDCGQRRVYLRLYSGTLRLRDTVALAGREKLKITEMRIPSKG 300

Query 301
EIVRTDTAYPGEIVILPSDSVRLNDVLGDPTRLPRKRWREDPLPMLRTTIAPKTAAQRER 360
EIVRTDTAYPGEIVILPSDSVRLNDVLGDPTRLPRKRWREDPLPMLRT
+IAPKTAAQRER
Sbjct 301
EIVRTDTAYPGEIVILPSDSVRLNDVLGDPTRLPRKRWREDPLPMLRTSIAPKTAAQRER 360

Query 361
LLDALTQLADTDPLLRCEVDSITHEIILSFLGRVQLEVVSALLSEKYKLETVVKEPSVIY 420
LLDALTQLADTDPLLRCEVDSITHEIILSFLGRVQLEVVSALLSEKYKLETVVKEP
+VIY
Sbjct 361
LLDALTQLADTDPLLRCEVDSITHEIILSFLGRVQLEVVSALLSEKYKLETVVKEPTVIY 420

Query 421
MERPLKAASHTIHIEVPPNPFWASIGLSVTPLSLGSGVQYESRVSLGYLNQSFQNAVRDG 480
MERPLKAASHTIHIEVPPNPFWASIGLSVTPL
LGSGVQYESRVSLGYLNQSFQNAVRDG
Sbjct 421
MERPLKAASHTIHIEVPPNPFWASIGLSVTPLPLGSGVQYESRVSLGYLNQSFQNAVRDG 480

Query 481
IRYGLEQGLFGWNVTDCKICFEYGLYYSPVSTPADFRSLAPIVLEQALKESGTQLLEPYL 540

IRYGLEQGLFGWNVTDCKICFEYGLYYSPVSTPADFRSLAPIVLEQALKESGTQLLEPYL
Sbjct 481
IRYGLEQGLFGWNVTDCKICFEYGLYYSPVSTPADFRSLAPIVLEQALKESGTQLLEPYL 540

Query 541
SFILYAPQEYLSRAYHDAPKYCATIETAQVKKDEVVFTGEIPARCIQAYRTDLAFYTNGR 600
SF LYAP+EYLSRAYHDAPKYCATIET
QVKKDEVVFTGEIPARCIQAYRTDLAFYTNG+
Sbjct 541
SFTLYAPREYLSRAYHDAPKYCATIETVQVKKDEVVFTGEIPARCIQAYRTDLAFYTNGQ 600

Query 601 SVCLTELKGYQAAVGQPVIQPRRPNSRLDKVRHMFQKVM 639
SVCLTELKGYQAAVG+PVIQPRRPN+RLDKVRHMFQKVM
Sbjct 601 SVCLTELKGYQAAVGKPVIQPRRPNNRLDKVRHMFQKVM 639

>WP_086132636.1 TetM/TetW/TetO/TetS family tetracycline resistance ribosomal
protection protein [Lactobacillus reuteri]
OTA72357.1 tetracycline resistance ribosomal protection protein [Lactobacillus
reuteri]
Length=645

Score = 1284 bits (3322), Expect = 0.0, Method: Compositional matrix adjust.
Identities = 624/638 (98%), Positives = 632/638 (99%), Gaps = 0/638 (0%)

Query 1
MKIINIGILAHVDAGKTTLTESLLYASGAISEPGSVEKGTTRTDTMFLERQRGITIQAAV 60
MKIINIGILAHVDAGKTTLTESLLYASGAISEPGSV
+KGTTRTDTMFLERQRGITIQAAV
Sbjct 1
MKIINIGILAHVDAGKTTLTESLLYASGAISEPGSVKKGTTRTDTMFLERQRGITIQAAV 60

Query 61
TSFQWHRCKVNIVDTPGHMDFLAEVYRSLAVLDGAILVISAKDGVQAQTRILFHALRKMN 120

TSFQWHRCKVNIVDTPGHMDFLAEVYRSLAVLDGAILVISAKDGVQAQTRILFHALRKMN
Sbjct 61
TSFQWHRCKVNIVDTPGHMDFLAEVYRSLAVLDGAILVISAKDGVQAQTRILFHALRKMN 120

Query 121
IPTVIFINKIDQAGVDLQSVVQSVRDKLSADIIIKQTVSLSPEIVLEENTDIEAWDAVIE 180

IPTVIFINKIDQAGVDLQSVVQSVRDKLSADIIIKQTVSLSPEIVLEENTDIEAWDAVIE
Sbjct 121
IPTVIFINKIDQAGVDLQSVVQSVRDKLSADIIIKQTVSLSPEIVLEENTDIEAWDAVIE 180

```
Query  181
NNDKLLEKYIAGEPISREKLVREEQRRVQDASLFPVYYGSAKKGLGIQPLMDAVTGLFQP  240

NNDKLLEKYIAGEPISREKLVREEQRRVQDASLFPVYYGSAKKGLGIQPLMDAVTGLFQP
Sbjct  181
NNDKLLEKYIAGEPISREKLVREEQRRVQDASLFPVYYGSAKKGLGIQPLMDAVTGLFQP  240

Query  241
IGEQGSAALCGSVFKVEYTDCGQRRVYLRLYSGTLRLRDTVALAGREKLKITEMRIPSKG  300

IGEQGSAALCGSVFKVEYTDCGQRRVYLRLYSGTLRLRDTVALAGREKLKITEMRIPSKG
Sbjct  241
IGEQGSAALCGSVFKVEYTDCGQRRVYLRLYSGTLRLRDTVALAGREKLKITEMRIPSKG  300

Query  301
EIVRTDTAYPGEIVILPSDSVRLNDVLGDPTRLPRKRWREDPLPMLRTTIAPKTAAQRER  360
            EIVRTDTAYPGEIVILPSDSVRLNDVLGDPTRLPRKRWREDPLPMLRT
+IAPKTAAQRER
Sbjct  301
EIVRTDTAYPGEIVILPSDSVRLNDVLGDPTRLPRKRWREDPLPMLRTSIAPKTAAQRER  360

Query  361
LLDALTQLADTDPLLRCEVDSITHEIILSFLGRVQLEVVSALLSEKYKLETVVKEPSVIY  420
            LLDALTQLADTDPLLRCEVDSITHEIILSFLGR QLEVVSALLSEKYKLETVVKEP
+VIY
Sbjct  361
LLDALTQLADTDPLLRCEVDSITHEIILSFLGRTQLEVVSALLSEKYKLETVVKEPTVIY  420

Query  421
MERPLKAASHTIHIEVPPNPFWASIGLSVTPLSLGSGVQYESRVSLGYLNQSFQNAVRDG  480
            MERPLKAASHTIHIEVPPNPFWASIGLSVTPL
LGSGVQYESRVSLGYLNQSFQNAVRDG
Sbjct  421
MERPLKAASHTIHIEVPPNPFWASIGLSVTPLPLGSGVQYESRVSLGYLNQSFQNAVRDG  480

Query  481
IRYGLEQGLFGWNVTDCKICFEYGLYYSPVSTPADFRSLAPIVLEQALKESGTQLLEPYL  540

IRYGLEQGLFGWNVTDCKICFEYGLYYSPVSTPADFRSLAPIVLEQALKESGTQLLEPYL
Sbjct  481
IRYGLEQGLFGWNVTDCKICFEYGLYYSPVSTPADFRSLAPIVLEQALKESGTQLLEPYL  540

Query  541
SFILYAPQEYLSRAYHDAPKYCATIETAQVKKDEVVFTGEIPARCIQAYRTDLAFYTNGR  600
            SF LYAP+EYLSRAYHDAPKYCATIET QVKKDEVVFTGEIPARCIQAYRTDLA
YTNG+
Sbjct  541
SFTLYAPREYLSRAYHDAPKYCATIETVQVKKDEVVFTGEIPARCIQAYRTDLASYTNGQ  600

Query  601  SVCLTELKGYQAAVGQPVIQPRRPNSRLDKVRHMFQKV  638
            SVCLTELKGYQAA G+PVIQPRRPNSRLDKVR+MFQK+
Sbjct  601  SVCLTELKGYQAADGKPVIQPRRPNSRLDKVRYMFQKI  638
```

```
>ABF72138.1 TetW [Trueperella pyogenes]
Length=639

 Score = 1284 bits (3322),  Expect = 0.0, Method: Compositional
matrix adjust.
 Identities = 623/639 (97%), Positives = 632/639 (99%), Gaps = 0/639
(0%)

Query  1
MKIINIGILAHVDAGKTTLTESLLYASGAISEPGSVEKGTTRTDTMFLERQRGITIQAAV  60

MKIINIGILAHVDAGKTTLTESLLYASGAISEPGSVEKGTTRTDTMFLERQRGITIQAAV
Sbjct  1
MKIINIGILAHVDAGKTTLTESLLYASGAISEPGSVEKGTTRTDTMFLERQRGITIQAAV  60

Query  61
TSFQWHRCKVNIVDTPGHMDFLAEVYRSLAVLDGAILVISAKDGVQAQTRILFHALRKMN  120

TSFQWHRCKVNIVDTPGHMDFLAEVYRSLAVLDGAILVISAKDGVQAQTRILFHALRKMN
Sbjct  61
TSFQWHRCKVNIVDTPGHMDFLAEVYRSLAVLDGAILVISAKDGVQAQTRILFHALRKMN  120

Query  121
IPTVIFINKIDQAGVDLQSVVQSVRDKLSADIIIKQTVSLSPEIVLEENTDIEAWDAVIE  180

IPTVIFINKIDQAGVDLQSVVQSVRDKLSADIIIKQTVSLSPEIVLEENTDIEAWDAVIE
Sbjct  121
IPTVIFINKIDQAGVDLQSVVQSVRDKLSADIIIKQTVSLSPEIVLEENTDIEAWDAVIE  180

Query  181
NNDKLLEKYIAGEPISREKLVREEQRRVQDASLFPVYYGSAKKGLGIQPLMDAVTGLFQP  240
            NND+LLEKYIAGEPISREKL REEQRRVQDASLFPVY+GSAK
GLGIQPLMDAVTGLFQP
Sbjct  181
NNDELLEKYIAGEPISREKLAREEQRRVQDASLFPVYHGSAKNGLGIQPLMDAVTGLFQP  240

Query  241
IGEQGSAALCGSVFKVEYTDCGQRRVYLRLYSGTLRLRDTVALAGREKLKITEMRIPSKG  300
            IGEQG
AALCGSVFKVEYTDCGQRRVYLRLYSGTLRLRDTVALAGREKLKITEMRIPSKG
Sbjct  241
IGEQGGAALCGSVFKVEYTDCGQRRVYLRLYSGTLRLRDTVALAGREKLKITEMRIPSKG  300

Query  301
EIVRTDTAYPGEIVILPSDSVRLNDVLGDPTRLPRKRWREDPLPMLRTTIAPKTAAQRER  360
            EIVRTDTAY GEIVILPSDSVRLNDVLGDPTRLPRKRWREDPLPMLRT
+IAPKTAAQRER
Sbjct  301
EIVRTDTAYQGEIVILPSDSVRLNDVLGDPTRLPRKRWREDPLPMLRTSIAPKTAAQRER  360

Query  361
LLDALTQLADTDPLLRCEVDSITHEIILSFLGRVQLEVVSALLSEKYKLETVVKEPSVIY  420
            LLDALTQLADTDPLLRCEVDSITHEIILSFLGRVQLEVVSALLSEKYKLETVVKEP
+VIY
```

Sbjct 361
LLDALTQLADTDPLLRCEVDSITHEIILSFLGRVQLEVVSALLSEKYKLETVVKEPTVIY 420

Query 421
MERPLKAASHTIHIEVPPNPFWASIGLSVTPLSLGSGVQYESRVSLGYLNQSFQNAVRDG 480
            MERPLKAASHTIHIEVPPNPFWASIGLSVTPL
LGSGVQYESRVSLGYLNQSFQNAVRDG
Sbjct 421
MERPLKAASHTIHIEVPPNPFWASIGLSVTPLPLGSGVQYESRVSLGYLNQSFQNAVRDG 480

Query 481
IRYGLEQGLFGWNVTDCKICFEYGLYYSPVSTPADFRSLAPIVLEQALKESGTQLLEPYL 540

IRYGLEQGLFGWNVTDCKICFEYGLYYSPVSTPADFRSLAPIVLEQALKESGTQLLEPYL
Sbjct 481
IRYGLEQGLFGWNVTDCKICFEYGLYYSPVSTPADFRSLAPIVLEQALKESGTQLLEPYL 540

Query 541
SFILYAPQEYLSRAYHDAPKYCATIETAQVKKDEVVFTGEIPARCIQAYRTDLAFYTNGR 600
            SF LYAP+EYLSRAYHDAPKYCATIET
QVKKDEVVFTGEIPARCIQAYRTDLAFYTNG+
Sbjct 541
SFTLYAPREYLSRAYHDAPKYCATIETVQVKKDEVVFTGEIPARCIQAYRTDLAFYTNGQ 600

Query 601 SVCLTELKGYQAAVGQPVIQPRRPNSRLDKVRHMFQKVM 639
            SVCLTELKGYQAAVG+PVIQPRRPNSRLDKVR+MFQK+M
Sbjct 601 SVCLTELKGYQAAVGKPVIQPRRPNSRLDKVRYMFQKIM 639

>WP_089147928.1 MULTISPECIES: TetM/TetW/TetO/TetS family
tetracycline resistance
ribosomal protection protein [Lactobacillus]
 OXC47181.1 tetracycline resistance ribosomal protection protein
[Lactobacillus
crispatus]
 OXC50041.1 tetracycline resistance ribosomal protection protein
[Lactobacillus
crispatus]
 OXC51127.1 tetracycline resistance ribosomal protection protein
[Lactobacillus
crispatus]
 OXC54411.1 tetracycline resistance ribosomal protection protein
[Lactobacillus
crispatus]
 OXC56772.1 tetracycline resistance ribosomal protection protein
[Lactobacillus
crispatus]
 OXC57334.1 tetracycline resistance ribosomal protection protein
[Lactobacillus
crispatus]
 PEG85303.1 TetM/TetW/TetO/TetS family tetracycline resistance
ribosomal
protection protein [Lactobacillus sp. UMNPBX15]
 PEG90300.1 TetM/TetW/TetO/TetS family tetracycline resistance

ribosomal
protection protein [Lactobacillus sp. UMNPBX12]
 PEG92214.1 TetM/TetW/TetO/TetS family tetracycline resistance
ribosomal
protection protein [Lactobacillus sp. UMNPBX11]
Length=640

 Score = 1283 bits (3321), Expect = 0.0, Method: Compositional
matrix adjust.
 Identities = 624/638 (98%), Positives = 633/638 (99%), Gaps = 0/638
(0%)

```
Query  1
MKIINIGILAHVDAGKTTLTESLLYASGAISEPGSVEKGTTRTDTMFLERQRGITIQAAV  60
            MKIINIGILAHVDAGKTTLTESLLYASGAISEPGSVEKGTTRTD
+MFLERQRGITIQAAV
Sbjct  1
MKIINIGILAHVDAGKTTLTESLLYASGAISEPGSVEKGTTRTDSMFLERQRGITIQAAV  60

Query  61
TSFQWHRCKVNIVDTPGHMDFLAEVYRSLAVLDGAILVISAKDGVQAQTRILFHALRKMN  120

TSFQWHRCKVNIVDTPGHMDFLAEVYRSLAVLDGAILVISAKDGVQAQTRILFHALRKMN
Sbjct  61
TSFQWHRCKVNIVDTPGHMDFLAEVYRSLAVLDGAILVISAKDGVQAQTRILFHALRKMN  120

Query  121
IPTVIFINKIDQAGVDLQSVVQSVRDKLSADIIIKQTVSLSPEIVLEENTDIEAWDAVIE  180

IPTVIFINKIDQAGVDLQSVVQSVRDKLSADIIIKQTVSLSPEIVLEENTDIEAWDAVIE
Sbjct  121
IPTVIFINKIDQAGVDLQSVVQSVRDKLSADIIIKQTVSLSPEIVLEENTDIEAWDAVIE  180

Query  181
NNDKLLEKYIAGEPISREKLVREEQRRVQDASLFPVYYGSAKKGLGIQPLMDAVTGLFQP  240

NNDKLLEKYIAGEPISREKLVREEQRRVQDASLFPVYYGSAKKGLGIQPLMDAVTGLFQP
Sbjct  181
NNDKLLEKYIAGEPISREKLVREEQRRVQDASLFPVYYGSAKKGLGIQPLMDAVTGLFQP  240

Query  241
IGEQGSAALCGSVFKVEYTDCGQRRVYLRLYSGTLRLRDTVALAGREKLKITEMRIPSKG  300

IGEQGSAALCGSVFKVEYTDCGQRRVYLRLYSGTLRLRDTVALAGREKLKITEMRIPSKG
Sbjct  241
IGEQGSAALCGSVFKVEYTDCGQRRVYLRLYSGTLRLRDTVALAGREKLKITEMRIPSKG  300

Query  301
EIVRTDTAYPGEIVILPSDSVRLNDVLGDPTRLPRKRWREDPLPMLRTTIAPKTAAQRER  360
            +IVRTDTAYPGEIVILPSDSVRLNDVLGDPTRLPRKRWRE PLPMLRT
+IAPKTAAQRER
Sbjct  301
KIVRTDTAYPGEIVILPSDSVRLNDVLGDPTRLPRKRWREAPLPMLRTSIAPKTAAQRER  360
```

```
Query  361
LLDALTQLADTDPLLRCEVDSITHEIILSFLGRVQLEVVSALLSEKYKLETVVKEPSVIY  420
            LLDALTQLADTDPLLRCEVDSITHEIILSFLGRVQLEVVSALLSEKYKLETVVKEP
+VIY
Sbjct  361
LLDALTQLADTDPLLRCEVDSITHEIILSFLGRVQLEVVSALLSEKYKLETVVKEPNVIY  420

Query  421
MERPLKAASHTIHIEVPPNPFWASIGLSVTPLSLGSGVQYESRVSLGYLNQSFQNAVRDG  480
            MERPLKAASHTIHIEVPPNPFWASIGLSVTPL
LGSGVQYESRVSLGYLNQSFQNAVRDG
Sbjct  421
MERPLKAASHTIHIEVPPNPFWASIGLSVTPLPLGSGVQYESRVSLGYLNQSFQNAVRDG  480

Query  481
IRYGLEQGLFGWNVTDCKICFEYGLYYSPVSTPADFRSLAPIVLEQALKESGTQLLEPYL  540

IRYGLEQGLFGWNVTDCKICFEYGLYYSPVSTPADFRSLAPIVLEQALKESGTQLLEPYL
Sbjct  481
IRYGLEQGLFGWNVTDCKICFEYGLYYSPVSTPADFRSLAPIVLEQALKESGTQLLEPYL  540

Query  541
SFILYAPQEYLSRAYHDAPKYCATIETAQVKKDEVVFTGEIPARCIQAYRTDLAFYTNGR  600
            SF LYAP+EYLSRAYHDAPKYCATIET QVKKDEVVFTGEIPARCIQAYRTDLA
YTNG+
Sbjct  541
SFTLYAPREYLSRAYHDAPKYCATIETVQVKKDEVVFTGEIPARCIQAYRTDLASYTNGQ  600

Query  601  SVCLTELKGYQAAVGQPVIQPRRPNSRLDKVRHMFQKV  638
            SVCLTELKGYQAAVG+PVIQPRRPNSRLDKVR+MFQK+
Sbjct  601  SVCLTELKGYQAAVGKPVIQPRRPNSRLDKVRYMFQKI  638


>WP_065436612.1 TetM/TetW/TetO/TetS family tetracycline resistance
ribosomal
protection protein [Bifidobacterium longum]
Length=634

 Score = 1283 bits (3321),  Expect = 0.0, Method: Compositional
matrix adjust.
 Identities = 624/634 (98%), Positives = 628/634 (99%), Gaps = 0/634
(0%)

Query  6
IGILAHVDAGKTTLTESLLYASGAISEPGSVEKGTTRTDTMFLERQRGITIQAAVTSFQW  65

+GILAHVDAGKTTLTESLLYASGAISEPGSVEKGTTRTDTMFLERQRGITIQAAVTSFQW
Sbjct  1
MGILAHVDAGKTTLTESLLYASGAISEPGSVEKGTTRTDTMFLERQRGITIQAAVTSFQW  60

Query  66
HRCKVNIVDTPGHMDFLAEVYRSLAVLDGAILVISAKDGVQAQTRILFHALRKMNIPTVI  125

HRCKVNIVDTPGHMDFLAEVYRSLAVLDGAILVISAKDGVQAQTRILFHALRKMNIPTVI
```

Sbjct 61
HRCKVNIVDTPGHMDFLAEVYRSLAVLDGAILVISAKDGVQAQTRILFHALRKMNIPTVI 120

Query 126
FINKIDQAGVDLQSVVQSVRDKLSADIIIKQTVSLSPEIVLEENTDIEAWDAVIENNDKL 185

FINKIDQAGVDLQSVVQSVRDKLSADIIIKQTVSLSPEIVLEENTDIEAWDAVIENND+L
Sbjct 121
FINKIDQAGVDLQSVVQSVRDKLSADIIIKQTVSLSPEIVLEENTDIEAWDAVIENNDEL 180

Query 186
LEKYIAGEPISREKLVREEQRRVQDASLFPVYYGSAKKGLGIQPLMDAVTGLFQPIGEQG 245
LEKYIAGEPISREKL REEQ+RVQDASLFPVY+GSAK
GLGIQPLMDAVTGLFQPIGEQG
Sbjct 181
LEKYIAGEPISREKLAREEQQRVQDASLFPVYHGSAKNGLGIQPLMDAVTGLFQPIGEQG 240

Query 246
SAALCGSVFKVEYTDCGQRRVYLRLYSGTLRLRDTVALAGREKLKITEMRIPSKGEIVRT 305

AALCGSVFKVEYTDCGQRRVYLRLYSGTLRLRDTVALAGREKLKITEMRIPSKGEIVRT
Sbjct 241
GAALCGSVFKVEYTDCGQRRVYLRLYSGTLRLRDTVALAGREKLKITEMRIPSKGEIVRT 300

Query 306
DTAYPGEIVILPSDSVRLNDVLGDPTRLPRKRWREDPLPMLRTTIAPKTAAQRERLLDAL 365
DTAY GEIVILPSDSVRLNDVLGD TRLPRKRWREDPLPMLRT
IAPKTAAQRERLLDAL
Sbjct 301
DTAYQGEIVILPSDSVRLNDVLGDQTRLPRKRWREDPLPMLRTAIAPKTAAQRERLLDAL 360

Query 366
TQLADTDPLLRCEVDSITHEIILSFLGRVQLEVVSALLSEKYKLETVVKEPSVIYMERPL 425

TQLADTDPLLRCEVDSITHEIILSFLGRVQLEVVSALLSEKYKLETVVKEPSVIYMERPL
Sbjct 361
TQLADTDPLLRCEVDSITHEIILSFLGRVQLEVVSALLSEKYKLETVVKEPSVIYMERPL 420

Query 426
KAASHTIHIEVPPNPFWASIGLSVTPLSLGSGVQYESRVSLGYLNQSFQNAVRDGIRYGL 485

KAASHTIHIEVPPNPFWASIGLSVTPLSLGSGVQYESRVSLGYLNQSFQNAVRDGIRYGL
Sbjct 421
KAASHTIHIEVPPNPFWASIGLSVTPLSLGSGVQYESRVSLGYLNQSFQNAVRDGIRYGL 480

Query 486
EQGLFGWNVTDCKICFEYGLYYSPVSTPADFRSLAPIVLEQALKESGTQLLEPYLSFILY 545

EQGLFGWNVTDCKICFEYGLYYSPVSTPADFRSLAPIVLEQALKESGTQLLEPYLSFILY
Sbjct 481
EQGLFGWNVTDCKICFEYGLYYSPVSTPADFRSLAPIVLEQALKESGTQLLEPYLSFILY 540

Query 546
APQEYLSRAYHDAPKYCATIETAQVKKDEVVFTGEIPARCIQAYRTDLAFYTNGRSVCLT 605

APQEYLSRAYHDAPKYCATIETAQVKKDEVVFTGEIPARCIQAYRTDLAFYTNGRSVCLT
Sbjct 541
APQEYLSRAYHDAPKYCATIETAQVKKDEVVFTGEIPARCIQAYRTDLAFYTNGRSVCLT 600

Query 606 ELKGYQAAVGQPVIQPRRPNSRLDKVRHMFQKVM 639
            ELKGYQAAVGQPVIQPRRPNSRLDKVRHMFQKVM
Sbjct 601 ELKGYQAAVGQPVIQPRRPNSRLDKVRHMFQKVM 634

>WP_089143419.1 MULTISPECIES: TetM/TetW/TetO/TetS family
tetracycline resistance
ribosomal protection protein [Lactobacillus]
 OXC13377.1 tetracycline resistance ribosomal protection protein
[Lactobacillus
crispatus]
 OXC17726.1 tetracycline resistance ribosomal protection protein
[Lactobacillus
crispatus]
 OXC22163.1 tetracycline resistance ribosomal protection protein
[Lactobacillus
crispatus]
 OXC24768.1 tetracycline resistance ribosomal protection protein
[Lactobacillus
crispatus]
 OXC30722.1 tetracycline resistance ribosomal protection protein
[Lactobacillus
crispatus]
 OXC33259.1 tetracycline resistance ribosomal protection protein
[Lactobacillus
crispatus]
 OXC35321.1 tetracycline resistance ribosomal protection protein
[Lactobacillus
crispatus]
 OXC36330.1 tetracycline resistance ribosomal protection protein
[Lactobacillus
crispatus]
 PEH12424.1 TetM/TetW/TetO/TetS family tetracycline resistance
ribosomal
protection protein [Lactobacillus sp. UMNPBX1]
Length=640

 Score = 1283 bits (3320), Expect = 0.0, Method: Compositional
matrix adjust.
 Identities = 624/638 (98%), Positives = 633/638 (99%), Gaps = 0/638
(0%)

Query 1
MKIINIGILAHVDAGKTTLTESLLYASGAISEPGSVEKGTTRTDTMFLERQRGITIQAAV 60
            MKIINIGILAHVDAGKTTLTESLLYASGAISEPGSVEKGTTRTD
+MFLERQRGITIQAAV
Sbjct 1
MKIINIGILAHVDAGKTTLTESLLYASGAISEPGSVEKGTTRTDSMFLERQRGITIQAAV 60

Query 61
TSFQWHRCKVNIVDTPGHMDFLAEVYRSLAVLDGAILVISAKDGVQAQTRILFHALRKMN 120

TSFQWHRCKVNIVDTPGHMDFLAEVYRSLAVLDGAILVISAKDGVQAQTRILFHALRKMN
Sbjct 61
TSFQWHRCKVNIVDTPGHMDFLAEVYRSLAVLDGAILVISAKDGVQAQTRILFHALRKMN 120

Query 121
IPTVIFINKIDQAGVDLQSVVQSVRDKLSADIIIKQTVSLSPEIVLEENTDIEAWDAVIE 180

IPTVIFINKIDQAGVDLQSVVQSVRDKLSADIIIKQTVSLSPEIVLEENTDIEAWDAVIE
Sbjct 121
IPTVIFINKIDQAGVDLQSVVQSVRDKLSADIIIKQTVSLSPEIVLEENTDIEAWDAVIE 180

Query 181
NNDKLLEKYIAGEPISREKLVREEQRRVQDASLFPVYYGSAKKGLGIQPLMDAVTGLFQP 240

NNDKLLEKYIAGEPISREKLVREEQRRVQDASLFPVYYGSAKKGLGIQPLMDAVTGLFQP
Sbjct 181
NNDKLLEKYIAGEPISREKLVREEQRRVQDASLFPVYYGSAKKGLGIQPLMDAVTGLFQP 240

Query 241
IGEQGSAALCGSVFKVEYTDCGQRRVYLRLYSGTLRLRDTVALAGREKLKITEMRIPSKG 300

IGEQGSAALCGSVFKVEYTDCGQRRVYLRLYSGTLRLRDTVALAGREKLKITEMRIPSKG
Sbjct 241
IGEQGSAALCGSVFKVEYTDCGQRRVYLRLYSGTLRLRDTVALAGREKLKITEMRIPSKG 300

Query 301
EIVRTDTAYPGEIVILPSDSVRLNDVLGDPTRLPRKRWREDPLPMLRTTIAPKTAAQRER 360
+IVRTDTAYPGEIVILPSDSVRLNDVLGDPTRLPRKRWRE PLPMLRT
+IAPKTAAQRER
Sbjct 301
KIVRTDTAYPGEIVILPSDSVRLNDVLGDPTRLPRKRWREAPLPMLRTSIAPKTAAQRER 360

Query 361
LLDALTQLADTDPLLRCEVDSITHEIILSFLGRVQLEVVSALLSEKYKLETVVKEPSVIY 420
LLDALTQLADTDPLLRCEVDSITHEIILSFLGRVQLEVVSALLSEKYKLETVVKEP
+VIY
Sbjct 361
LLDALTQLADTDPLLRCEVDSITHEIILSFLGRVQLEVVSALLSEKYKLETVVKEPTVIY 420

Query 421
MERPLKAASHTIHIEVPPNPFWASIGLSVTPLSLGSGVQYESRVSLGYLNQSFQNAVRDG 480
MERPLKAASHTIHIEVPPNPFWASIGLSVTPL
LGSGVQYESRVSLGYLNQSFQNAVRDG
Sbjct 421
MERPLKAASHTIHIEVPPNPFWASIGLSVTPLPLGSGVQYESRVSLGYLNQSFQNAVRDG 480

Query 481
IRYGLEQGLFGWNVTDCKICFEYGLYYSPVSTPADFRSLAPIVLEQALKESGTQLLEPYL 540

IRYGLEQGLFGWNVTDCKICFEYGLYYSPVSTPADFRSLAPIVLEQALKESGTQLLEPYL
Sbjct 481

IRYGLEQGLFGWNVTDCKICFEYGLYYSPVSTPADFRSLAPIVLEQALKESGTQLLEPYL 540

Query 541
SFILYAPQEYLSRAYHDAPKYCATIETAQVKKDEVVFTGEIPARCIQAYRTDLAFYTNGR 600
            SF LYAP+EYLSRAYHDAPKYCATIET QVKKDEVVFTGEIPARCIQAYRTDLA
YTNG+
Sbjct 541
SFTLYAPREYLSRAYHDAPKYCATIETVQVKKDEVVFTGEIPARCIQAYRTDLASYTNGQ 600

Query 601 SVCLTELKGYQAAVGQPVIQPRRPNSRLDKVRHMFQKV 638
            SVCLTELKGYQAAVG+PVIQPRRPNSRLDKVR+MFQK+
Sbjct 601 SVCLTELKGYQAAVGKPVIQPRRPNSRLDKVRYMFQKI 638

>WP_086141728.1 TetM/TetW/TetO/TetS family tetracycline resistance
ribosomal
protection protein [Lactobacillus reuteri]
 OTA76215.1 tetracycline resistance ribosomal protection protein
[Lactobacillus
reuteri]
Length=641

 Score = 1283 bits (3320), Expect = 0.0, Method: Compositional
matrix adjust.
 Identities = 624/638 (98%), Positives = 633/638 (99%), Gaps = 0/638
(0%)

Query 1
MKIINIGILAHVDAGKTTLTESLLYASGAISEPGSVEKGTTRTDTMFLERQRGITIQAAV 60
            MKIINIGILAHVDAGKTTLTESLLYASGAISE GSV
+KGTTRTDTMFLERQRGITIQAAV
Sbjct 1
MKIINIGILAHVDAGKTTLTESLLYASGAISERGSVKKGTTRTDTMFLERQRGITIQAAV 60

Query 61
TSFQWHRCKVNIVDTPGHMDFLAEVYRSLAVLDGAILVISAKDGVQAQTRILFHALRKMN 120

TSFQWHRCKVNIVDTPGHMDFLAEVYRSLAVLDGAILVISAKDGVQAQTRILFHALRKMN
Sbjct 61
TSFQWHRCKVNIVDTPGHMDFLAEVYRSLAVLDGAILVISAKDGVQAQTRILFHALRKMN 120

Query 121
IPTVIFINKIDQAGVDLQSVVQSVRDKLSADIIIKQTVSLSPEIVLEENTDIEAWDAVIE 180

IPTVIFINKIDQAGVDLQSVVQSVRDKLSADIIIKQTVSLSPEIVLEENTDIEAWDAVIE
Sbjct 121
IPTVIFINKIDQAGVDLQSVVQSVRDKLSADIIIKQTVSLSPEIVLEENTDIEAWDAVIE 180

Query 181
NNDKLLEKYIAGEPISREKLVREEQRRVQDASLFPVYYGSAKKGLGIQPLMDAVTGLFQP 240

NNDKLLEKYIAGEPISREKLVREEQRRVQDASLFPVYYGSAKKGLGIQPLMDAVTGLFQP
Sbjct 181
NNDKLLEKYIAGEPISREKLVREEQRRVQDASLFPVYYGSAKKGLGIQPLMDAVTGLFQP 240

Query 241
IGEQGSAALCGSVFKVEYTDCGQRRVYLRLYSGTLRLRDTVALAGREKLKITEMRIPSKG 300

IGEQGSAALCGSVFKVEYTDCGQRRVYLRLYSGTLRLRDTVALAGREKLKITEMRIPSKG
Sbjct 241
IGEQGSAALCGSVFKVEYTDCGQRRVYLRLYSGTLRLRDTVALAGREKLKITEMRIPSKG 300

Query 301
EIVRTDTAYPGEIVILPSDSVRLNDVLGDPTRLPRKRWREDPLPMLRTTIAPKTAAQRER 360
EIVRTDTAYPGEIVILPSDSVRLNDVLGDPTRLPRKRWREDPLPMLRT
+IAPKTAAQRER
Sbjct 301
EIVRTDTAYPGEIVILPSDSVRLNDVLGDPTRLPRKRWREDPLPMLRTSIAPKTAAQRER 360

Query 361
LLDALTQLADTDPLLRCEVDSITHEIILSFLGRVQLEVVSALLSEKYKLETVVKEPSVIY 420
LLDALTQLADTDPLLRCEVDSITHEIILSFLGRVQLEVVSALLSEKYKLETVV+EP
+VIY
Sbjct 361
LLDALTQLADTDPLLRCEVDSITHEIILSFLGRVQLEVVSALLSEKYKLETVVEEPTVIY 420

Query 421
MERPLKAASHTIHIEVPPNPFWASIGLSVTPLSLGSGVQYESRVSLGYLNQSFQNAVRDG 480
MERPLKAASHTIHIEVPPNPFWASIGLSVTPL
LGSGVQYESRVSLGYLNQSFQNAVRDG
Sbjct 421
MERPLKAASHTIHIEVPPNPFWASIGLSVTPLPLGSGVQYESRVSLGYLNQSFQNAVRDG 480

Query 481
IRYGLEQGLFGWNVTDCKICFEYGLYYSPVSTPADFRSLAPIVLEQALKESGTQLLEPYL 540

IRYGLEQGLFGWNVTDCKICFEYGLYYSPVSTPADFRSLAPIVLEQALKESGTQLLEPYL
Sbjct 481
IRYGLEQGLFGWNVTDCKICFEYGLYYSPVSTPADFRSLAPIVLEQALKESGTQLLEPYL 540

Query 541
SFILYAPQEYLSRAYHDAPKYCATIETAQVKKDEVVFTGEIPARCIQAYRTDLAFYTNGR 600
SF LYAP+EYLSRAYHDAPKYCATIET QVKKDEVVFTGEIPARCIQAYRTDLA
YTNG+
Sbjct 541
SFTLYAPREYLSRAYHDAPKYCATIETVQVKKDEVVFTGEIPARCIQAYRTDLASYTNGQ 600

Query 601 SVCLTELKGYQAAVGQPVIQPRRPNSRLDKVRHMFQKV 638
SVCLTELKGYQAAVG+PVIQPRRPNSRLDKVR+MFQK+
Sbjct 601 SVCLTELKGYQAAVGKPVIQPRRPNSRLDKVRYMFQKI 638

>WP_086141437.1 TetM/TetW/TetO/TetS family tetracycline resistance
ribosomal
protection protein [Lactobacillus reuteri]
OTA48382.1 tetracycline resistance ribosomal protection protein
[Lactobacillus
reuteri]

Length=641

 Score = 1283 bits (3320), Expect = 0.0, Method: Compositional
matrix adjust.
 Identities = 624/638 (98%), Positives = 633/638 (99%), Gaps = 0/638
(0%)

```
Query  1
MKIINIGILAHVDAGKTTLTESLLYASGAISEPGSVEKGTTRTDTMFLERQRGITIQAAV  60
            MKIINIGILAHVDAGKTTLTESLLYASGAISE GSV
+KGTTRTDTMFLERQRGITIQAAV
Sbjct  1
MKIINIGILAHVDAGKTTLTESLLYASGAISERGSVKKGTTRTDTMFLERQRGITIQAAV  60

Query  61
TSFQWHRCKVNIVDTPGHMDFLAEVYRSLAVLDGAILVISAKDGVQAQTRILFHALRKMN  120

TSFQWHRCKVNIVDTPGHMDFLAEVYRSLAVLDGAILVISAKDGVQAQTRILFHALRKMN
Sbjct  61
TSFQWHRCKVNIVDTPGHMDFLAEVYRSLAVLDGAILVISAKDGVQAQTRILFHALRKMN  120

Query  121
IPTVIFINKIDQAGVDLQSVVQSVRDKLSADIIIKQTVSLSPEIVLEENTDIEAWDAVIE  180

IPTVIFINKIDQAGVDLQSVVQSVRDKLSADIIIKQTVSLSPEIVLEENTDIEAWDAVIE
Sbjct  121
IPTVIFINKIDQAGVDLQSVVQSVRDKLSADIIIKQTVSLSPEIVLEENTDIEAWDAVIE  180

Query  181
NNDKLLEKYIAGEPISREKLVREEQRRVQDASLFPVYYGSAKKGLGIQPLMDAVTGLFQP  240

NNDKLLEKYIAGEPISREKLVREEQRRVQDASLFPVYYGSAKKGLGIQPLMDAVTGLFQP
Sbjct  181
NNDKLLEKYIAGEPISREKLVREEQRRVQDASLFPVYYGSAKKGLGIQPLMDAVTGLFQP  240

Query  241
IGEQGSAALCGSVFKVEYTDCGQRRVYLRLYSGTLRLRDTVALAGREKLKITEMRIPSKG  300

IGEQGSAALCGSVFKVEYTDCGQRRVYLRLYSGTLRLRDTVALAGREKLKITEMRIPSKG
Sbjct  241
IGEQGSAALCGSVFKVEYTDCGQRRVYLRLYSGTLRLRDTVALAGREKLKITEMRIPSKG  300

Query  301
EIVRTDTAYPGEIVILPSDSVRLNDVLGDPTRLPRKRWREDPLPMLRTTIAPKTAAQRER  360
            EIVRTDTAYPGEIVILPSDSVRLNDVLGDPTRLPRKRWREDPLPMLRT
+IAPKTAAQRER
Sbjct  301
EIVRTDTAYPGEIVILPSDSVRLNDVLGDPTRLPRKRWREDPLPMLRTSIAPKTAAQRER  360

Query  361
LLDALTQLADTDPLLRCEVDSITHEIILSFLGRVQLEVVSALLSEKYKLETVVKEPSVIY  420
            LLDALTQLADTDPLLRCEVDSITHEIILSFLGRVQLEVVSALLSEKYKLETVVKEP
+VIY
Sbjct  361
```

LLDALTQLADTDPLLRCEVDSITHEIILSFLGRVQLEVVSALLSEKYKLETVVKEPTVIY 420

Query 421
MERPLKAASHTIHIEVPPNPFWASIGLSVTPLSLGSGVQYESRVSLGYLNQSFQNAVRDG 480
            MERPLKAASHTIHIEVPPNPFWASIGLSVTPL
LGSGVQYESRVSLGYLNQSFQNAVRDG
Sbjct 421
MERPLKAASHTIHIEVPPNPFWASIGLSVTPLPLGSGVQYESRVSLGYLNQSFQNAVRDG 480

Query 481
IRYGLEQGLFGWNVTDCKICFEYGLYYSPVSTPADFRSLAPIVLEQALKESGTQLLEPYL 540

IRYGLEQGLFGWNVTDCKICFEYGLYYSPVSTPADFRSLAPIVLEQALKESGTQLLEPYL
Sbjct 481
IRYGLEQGLFGWNVTDCKICFEYGLYYSPVSTPADFRSLAPIVLEQALKESGTQLLEPYL 540

Query 541
SFILYAPQEYLSRAYHDAPKYCATIETAQVKKDEVVFTGEIPARCIQAYRTDLAFYTNGR 600
            SF LYAP+EYLSRAYHDAP+YCATIET QVKKDEVVFTGEIPARCIQAYRTDLA
YTNG+
Sbjct 541
SFTLYAPREYLSRAYHDAPEYCATIETVQVKKDEVVFTGEIPARCIQAYRTDLASYTNGQ 600

Query 601 SVCLTELKGYQAAVGQPVIQPRRPNSRLDKVRHMFQKV 638
            SVCLTELKGYQAAVG+PVIQPRRPNSRLDKVR+MFQK+
Sbjct 601 SVCLTELKGYQAAVGKPVIQPRRPNSRLDKVRYMFQKI 638

>APO34595.1 ferrous iron transport protein B Edit: TetW [uncultured bacterium]
Length=639

 Score = 1283 bits (3320), Expect = 0.0, Method: Compositional matrix adjust.
 Identities = 624/639 (98%), Positives = 633/639 (99%), Gaps = 0/639 (0%)

Query 1
MKIINIGILAHVDAGKTTLTESLLYASGAISEPGSVEKGTTRTDTMFLERQRGITIQAAV 60

MKIINIGILAHVDAGKTTLTESLLYASGAISEPGSVEKGTTRTDTMFLERQRGITIQAAV
Sbjct 1
MKIINIGILAHVDAGKTTLTESLLYASGAISEPGSVEKGTTRTDTMFLERQRGITIQAAV 60

Query 61
TSFQWHRCKVNIVDTPGHMDFLAEVYRSLAVLDGAILVISAKDGVQAQTRILFHALRKMN 120

TSFQWHRCKVNIVDTPGHMDFLAEVYRSLAVLDGAILVISAKDGVQAQTRILFHALRKMN
Sbjct 61
TSFQWHRCKVNIVDTPGHMDFLAEVYRSLAVLDGAILVISAKDGVQAQTRILFHALRKMN 120

Query 121
IPTVIFINKIDQAGVDLQSVVQSVRDKLSADIIIKQTVSLSPEIVLEENTDIEAWDAVIE 180

IPTVIFINKIDQAGVDLQSVVQSVRDKLSADIIIKQTVSLSPEIVLEENTDIEAWDAVIE
Sbjct 121
IPTVIFINKIDQAGVDLQSVVQSVRDKLSADIIIKQTVSLSPEIVLEENTDIEAWDAVIE 180

Query 181
NNDKLLEKYIAGEPISREKLVREEQRRVQDASLFPVYYGSAKKGLGIQPLMDAVTGLFQP 240
NND+LLEKYIAGEPISREKL +EEQRRVQDASLFPVY
+GSAKKGLGIQPLMDAVTGLFQP
Sbjct 181
NNDELLEKYIAGEPISREKLAQEEQRRVQDASLFPVYHGSAKKGLGIQPLMDAVTGLFQP 240

Query 241
IGEQGSAALCGSVFKVEYTDCGQRRVYLRLYSGTLRLRDTVALAGREKLKITEMRIPSKG 300
IGEQGSAALCGSVFKVEYTDCGQR
VYLRLYSGTLRLRDTVALAGREKLKITEMRIPSKG
Sbjct 241
IGEQGSAALCGSVFKVEYTDCGQRLVYLRLYSGTLRLRDTVALAGREKLKITEMRIPSKG 300

Query 301
EIVRTDTAYPGEIVILPSDSVRLNDVLGDPTRLPRKRWREDPLPMLRTTIAPKTAAQRER 360
EIVRTDTAYPGEIVILPSDS+RLND+LGD T+LPR+ WREDPLPMLRT
+IAPKTAAQRER
Sbjct 301
EIVRTDTAYPGEIVILPSDSLRLNDILGDKTQLPREMWREDPLPMLRTSIAPKTAAQRER 360

Query 361
LLDALTQLADTDPLLRCEVDSITHEIILSFLGRVQLEVVSALLSEKYKLETVVKEPSVIY 420

LLDALTQLADTDPLLRCEVDSITHEIILSFLGRVQLEVVSALLSEKYKLETVVKEPSVIY
Sbjct 361
LLDALTQLADTDPLLRCEVDSITHEIILSFLGRVQLEVVSALLSEKYKLETVVKEPSVIY 420

Query 421
MERPLKAASHTIHIEVPPNPFWASIGLSVTPLSLGSGVQYESRVSLGYLNQSFQNAVRDG 480
MERPLKAASHTI IEVPPNPFWASIGLSVTPL
LGSGVQYESRVSLGYLNQSFQNAVRDG
Sbjct 421
MERPLKAASHTIDIEVPPNPFWASIGLSVTPLPLGSGVQYESRVSLGYLNQSFQNAVRDG 480

Query 481
IRYGLEQGLFGWNVTDCKICFEYGLYYSPVSTPADFRSLAPIVLEQALKESGTQLLEPYL 540

IRYGLEQGLFGWNVTDCKICFEYGLYYSPVSTPADFRSLAPIVLEQALKESGTQLLEPYL
Sbjct 481
IRYGLEQGLFGWNVTDCKICFEYGLYYSPVSTPADFRSLAPIVLEQALKESGTQLLEPYL 540

Query 541
SFILYAPQEYLSRAYHDAPKYCATIETAQVKKDEVVFTGEIPARCIQAYRTDLAFYTNGR 600

SFILYAPQEYLSRAYHDAPKYCATIETAQVKKDEVVFTGEIPARCIQAYRTDLAFYTNGR
Sbjct 541
SFILYAPQEYLSRAYHDAPKYCATIETAQVKKDEVVFTGEIPARCIQAYRTDLAFYTNGR 600

Query 601 SVCLTELKGYQAAVGQPVIQPRRPNSRLDKVRHMFQKVM 639

SVCLTELKGYQAAVGQPVIQPRRPNSRLD+VRHMFQKVM
Sbjct 601 SVCLTELKGYQAAVGQPVIQPRRPNSRLDEVRHMFQKVM 639

>WP_033524005.1 TetM/TetW/TetO/TetS family tetracycline resistance ribosomal
protection protein [Bifidobacterium pseudocatenulatum]
 KFI75787.1 tetracycline resistance protein TetW [Bifidobacterium pseudocatenulatum
DSM 20438 = JCM 1200 = LMG 10505]
 BAR03454.1 ribosomal protection tetracycline resistance protein [Bifidobacterium
pseudocatenulatum DSM 20438 = JCM 1200 = LMG 10505]
Length=639

 Score = 1283 bits (3319), Expect = 0.0, Method: Compositional matrix adjust.
 Identities = 623/638 (98%), Positives = 630/638 (99%), Gaps = 0/638 (0%)

Query 1
MKIINIGILAHVDAGKTTLTESLLYASGAISEPGSVEKGTTRTDTMFLERQRGITIQAAV 60
            M IINIGILAHVDAGKTTLTESLLYASGAISEPGSVEKGTTRTDTM LERQRGITIQAAV
Sbjct 1
MNIINIGILAHVDAGKTTLTESLLYASGAISEPGSVEKGTTRTDTMLLERQRGITIQAAV 60

Query 61
TSFQWHRCKVNIVDTPGHMDFLAEVYRSLAVLDGAILVISAKDGVQAQTRILFHALRKMN 120

TSFQWHRCKVNIVDTPGHMDFLAEVYRSLAVLDGAILVISAKDGVQAQTRILFHALRKMN
Sbjct 61
TSFQWHRCKVNIVDTPGHMDFLAEVYRSLAVLDGAILVISAKDGVQAQTRILFHALRKMN 120

Query 121
IPTVIFINKIDQAGVDLQSVVQSVRDKLSADIIIKQTVSLSPEIVLEENTDIEAWDAVIE 180

IPTVIFINKIDQAGVDLQSVVQSVRDKLSADIIIKQTVSLSPEIVLEENTDIEAWDAVIE
Sbjct 121
IPTVIFINKIDQAGVDLQSVVQSVRDKLSADIIIKQTVSLSPEIVLEENTDIEAWDAVIE 180

Query 181
NNDKLLEKYIAGEPISREKLVREEQRRVQDASLFPVYYGSAKKGLGIQPLMDAVTGLFQP 240

NNDKLLEKYIAGEPISREKLVREEQRRVQDASLFPVYYGSAKKGLGIQPLMDAVTGLFQP
Sbjct 181
NNDKLLEKYIAGEPISREKLVREEQRRVQDASLFPVYYGSAKKGLGIQPLMDAVTGLFQP 240

Query 241
IGEQGSAALCGSVFKVEYTDCGQRRVYLRLYSGTLRLRDTVALAGREKLKITEMRIPSKG 300
            IGEQGSAALCGSVFKVEYTDC QR +YLRLYSGTLRLRDTVALAGREKLKITEMRIPSKG
Sbjct 241
IGEQGSAALCGSVFKVEYTDCDQRLIYLRLYSGTLRLRDTVALAGREKLKITEMRIPSKG 300

Query 301
EIVRTDTAYPGEIVILPSDSVRLNDVLGDPTRLPRKRWREDPLPMLRTTIAPKTAAQRER 360
            EIVRTDTAYPGEIVILPSDSVRLNDVLGDPTRLPRKRWREDPLPMLRT
+IAPKTAAQRER
Sbjct 301
EIVRTDTAYPGEIVILPSDSVRLNDVLGDPTRLPRKRWREDPLPMLRTSIAPKTAAQRER 360

Query 361
LLDALTQLADTDPLLRCEVDSITHEIILSFLGRVQLEVVSALLSEKYKLETVVKEPSVIY 420
            LLDALTQLADTDPLLRCEVDSITHEIILSFLGRVQLEVVSALLSEKYKLETVVKEP
+VIY
Sbjct 361
LLDALTQLADTDPLLRCEVDSITHEIILSFLGRVQLEVVSALLSEKYKLETVVKEPTVIY 420

Query 421
MERPLKAASHTIHIEVPPNPFWASIGLSVTPLSLGSGVQYESRVSLGYLNQSFQNAVRDG 480
            MERPLKAASHTIHIEVPPNPFWASIGLSVTPL
LGSGVQYESRVSLGYLNQSFQNAVRDG
Sbjct 421
MERPLKAASHTIHIEVPPNPFWASIGLSVTPLPLGSGVQYESRVSLGYLNQSFQNAVRDG 480

Query 481
IRYGLEQGLFGWNVTDCKICFEYGLYYSPVSTPADFRSLAPIVLEQALKESGTQLLEPYL 540

IRYGLEQGLFGWNVTDCKICFEYGLYYSPVSTPADFRSLAPIVLEQALKESGTQLLEPYL
Sbjct 481
IRYGLEQGLFGWNVTDCKICFEYGLYYSPVSTPADFRSLAPIVLEQALKESGTQLLEPYL 540

Query 541
SFILYAPQEYLSRAYHDAPKYCATIETAQVKKDEVVFTGEIPARCIQAYRTDLAFYTNGR 600
            SF LYAP+EYLSRAYHDAPKYCATIET
QVKKDEVVFTGEIPARCIQAYRTDLAFYTNG+
Sbjct 541
SFTLYAPREYLSRAYHDAPKYCATIETVQVKKDEVVFTGEIPARCIQAYRTDLAFYTNGQ 600

Query 601 SVCLTELKGYQAAVGQPVIQPRRPNSRLDKVRHMFQKV 638
            SVCLTELKGYQAAVG+PVIQPRRPNSRLDKVRHMF K+
Sbjct 601 SVCLTELKGYQAAVGKPVIQPRRPNSRLDKVRHMFSKI 638

>APO33185.1 ferrous iron transport protein B [uncultured bacterium]
Length=639

 Score = 1283 bits (3319), Expect = 0.0, Method: Compositional
matrix adjust.
 Identities = 624/639 (98%), Positives = 632/639 (99%), Gaps = 0/639
(0%)

Query 1
MKIINIGILAHVDAGKTTLTESLLYASGAISEPGSVEKGTTRTDTMFLERQRGITIQAAV 60

MKIINIGILAHVDAGKTTLTESLLYASGAISEPGSVEKGTTRTDTMFLERQRGITIQAAV
Sbjct 1

MKIINIGILAHVDAGKTTLTESLLYASGAISEPGSVEKGTTRTDTMFLERQRGITIQAAV 60

Query 61
TSFQWHRCKVNIVDTPGHMDFLAEVYRSLAVLDGAILVISAKDGVQAQTRILFHALRKMN 120

TSFQWHRCKVNIVDTPGHMDFLAEVYRSLAVLDGAILVISAKDGVQAQTRILFHALRKMN
Sbjct 61
TSFQWHRCKVNIVDTPGHMDFLAEVYRSLAVLDGAILVISAKDGVQAQTRILFHALRKMN 120

Query 121
IPTVIFINKIDQAGVDLQSVVQSVRDKLSADIIIKQTVSLSPEIVLEENTDIEAWDAVIE 180

IPTVIFINKIDQAGVDLQSVVQSVRDKLSADIIIKQTVSLSPEIVLEENTDIEAWDAVIE
Sbjct 121
IPTVIFINKIDQAGVDLQSVVQSVRDKLSADIIIKQTVSLSPEIVLEENTDIEAWDAVIE 180

Query 181
NNDKLLEKYIAGEPISREKLVREEQRRVQDASLFPVYYGSAKKGLGIQPLMDAVTGLFQP 240
NND+LLEKYIAGEPISREKL +EEQRRVQDASLFPVY
+GSAKKGLGIQPLMDAVTGLFQP
Sbjct 181
NNDELLEKYIAGEPISREKLAQEEQRRVQDASLFPVYHGSAKKGLGIQPLMDAVTGLFQP 240

Query 241
IGEQGSAALCGSVFKVEYTDCGQRRVYLRLYSGTLRLRDTVALAGREKLKITEMRIPSKG 300
IGEQGSAALCGSVFKVEYTDCGQR
VYLRLYSGTLRLRDTVALAGREKLKITEMRIPSKG
Sbjct 241
IGEQGSAALCGSVFKVEYTDCGQRLVYLRLYSGTLRLRDTVALAGREKLKITEMRIPSKG 300

Query 301
EIVRTDTAYPGEIVILPSDSVRLNDVLGDPTRLPRKRWREDPLPMLRTTIAPKTAAQRER 360
EIVRTDTAYPGEIVILPSDS+RLND+LGD T+LPR+ WREDPLPMLRT
+IAPKTAAQRER
Sbjct 301
EIVRTDTAYPGEIVILPSDSLRLNDILGDKTQLPREMWREDPLPMLRTSIAPKTAAQRER 360

Query 361
LLDALTQLADTDPLLRCEVDSITHEIILSFLGRVQLEVVSALLSEKYKLETVVKEPSVIY 420

LLDALTQLADTDPLLRCEVDSITHEIILSFLGRVQLEVVSALLSEKYKLETVVKEPSVIY
Sbjct 361
LLDALTQLADTDPLLRCEVDSITHEIILSFLGRVQLEVVSALLSEKYKLETVVKEPSVIY 420

Query 421
MERPLKAASHTIHIEVPPNPFWASIGLSVTPLSLGSGVQYESRVSLGYLNQSFQNAVRDG 480
MERPLKAASHTI IEVPPNPFWASIGLSVTPL
LGSGVQYESRVSLGYLNQSFQNAVRDG
Sbjct 421
MERPLKAASHTIDIEVPPNPFWASIGLSVTPLPLGSGVQYESRVSLGYLNQSFQNAVRDG 480

Query 481
IRYGLEQGLFGWNVTDCKICFEYGLYYSPVSTPADFRSLAPIVLEQALKESGTQLLEPYL 540

IRYGLEQGLFGWNVTDCKICFEYGLYYSPVSTPADFRSLAPIVLEQALKESGTQLLEPYL
Sbjct 481
IRYGLEQGLFGWNVTDCKICFEYGLYYSPVSTPADFRSLAPIVLEQALKESGTQLLEPYL 540

Query 541
SFILYAPQEYLSRAYHDAPKYCATIETAQVKKDEVVFTGEIPARCIQAYRTDLAFYTNGR 600

SFILYAPQEYLSRAYHDAPKYCATIETAQVKKDEVVFTGEIPARCIQAYRTDLAFYTNGR
Sbjct 541
SFILYAPQEYLSRAYHDAPKYCATIETAQVKKDEVVFTGEIPARCIQAYRTDLAFYTNGR 600

```
Query  601  SVCLTELKGYQAAVGQPVIQPRRPNSRLDKVRHMFQKVM  639
            SVCLTELKGYQAAVGQPVIQPRRPNSRLDKVRHMF KVM
Sbjct  601  SVCLTELKGYQAAVGQPVIQPRRPNSRLDKVRHMFHKVM  639
```

>WP_033510437.1 TetM/TetW/TetO/TetS family tetracycline resistance ribosomal
protection protein, partial [Bifidobacterium saeculare]
Length=632

Score = 1282 bits (3318), Expect = 0.0, Method: Compositional matrix adjust.
Identities = 624/632 (99%), Positives = 629/632 (99%), Gaps = 0/632 (0%)

Query 1
MKIINIGILAHVDAGKTTLTESLLYASGAISEPGSVEKGTTRTDTMFLERQRGITIQAAV 60

MKIINIGILAHVDAGKTTLTESLLYASGAISEPGSVEKGTTRTDTMFLERQRGITIQAAV
Sbjct 1
MKIINIGILAHVDAGKTTLTESLLYASGAISEPGSVEKGTTRTDTMFLERQRGITIQAAV 60

Query 61
TSFQWHRCKVNIVDTPGHMDFLAEVYRSLAVLDGAILVISAKDGVQAQTRILFHALRKMN 120

TSFQWHRCKVNIVDTPGHMDFLAEVYRSLAVLDGAILVISAKDGVQAQTRILFHALRKMN
Sbjct 61
TSFQWHRCKVNIVDTPGHMDFLAEVYRSLAVLDGAILVISAKDGVQAQTRILFHALRKMN 120

Query 121
IPTVIFINKIDQAGVDLQSVVQSVRDKLSADIIIKQTVSLSPEIVLEENTDIEAWDAVIE 180

IPTVIFINKIDQAGVDLQSVVQSVRDKLSADIIIKQTVSLSPEIVLEENTDIEAWDAVIE
Sbjct 121
IPTVIFINKIDQAGVDLQSVVQSVRDKLSADIIIKQTVSLSPEIVLEENTDIEAWDAVIE 180

Query 181
NNDKLLEKYIAGEPISREKLVREEQRRVQDASLFPVYYGSAKKGLGIQPLMDAVTGLFQP 240

NNDKLLEKYIAGEPISREKLVREEQRRVQDASLFPVYYGSAKKGLGIQPLMDAVTGLFQP
Sbjct 181
NNDKLLEKYIAGEPISREKLVREEQRRVQDASLFPVYYGSAKKGLGIQPLMDAVTGLFQP 240

Query 241
IGEQGSAALCGSVFKVEYTDCGQRRVYLRLYSGTLRLRDTVALAGREKLKITEMRIPSKG 300

IGEQGSAALCGSVFKVEYTDCGQRRVYLRLYSGTLRLRDTVALAGREKLKITEMRIPSKG
Sbjct 241
IGEQGSAALCGSVFKVEYTDCGQRRVYLRLYSGTLRLRDTVALAGREKLKITEMRIPSKG 300

Query 301
EIVRTDTAYPGEIVILPSDSVRLNDVLGDPTRLPRKRWREDPLPMLRTTIAPKTAAQRER 360
EIVRTDTAYPGEIVILPSDSVRLNDVLGDPTRLPRKRWREDPLPMLRT
+IAPKTAAQRER
Sbjct 301
EIVRTDTAYPGEIVILPSDSVRLNDVLGDPTRLPRKRWREDPLPMLRTSIAPKTAAQRER 360

Query 361
LLDALTQLADTDPLLRCEVDSITHEIILSFLGRVQLEVVSALLSEKYKLETVVKEPSVIY 420
LLDALTQLADTDPLLRCEVDSITHEIILSFLGRVQLEVVSALLSEKYKLETVVKEP
+VIY
Sbjct 361
LLDALTQLADTDPLLRCEVDSITHEIILSFLGRVQLEVVSALLSEKYKLETVVKEPTVIY 420

Query 421
MERPLKAASHTIHIEVPPNPFWASIGLSVTPLSLGSGVQYESRVSLGYLNQSFQNAVRDG 480
MERPLKAASHTIHIEVPPNPFWASIGLSVTPL
LGSGVQYESRVSLGYLNQSFQNAVRDG
Sbjct 421
MERPLKAASHTIHIEVPPNPFWASIGLSVTPLPLGSGVQYESRVSLGYLNQSFQNAVRDG 480

Query 481
IRYGLEQGLFGWNVTDCKICFEYGLYYSPVSTPADFRSLAPIVLEQALKESGTQLLEPYL 540

IRYGLEQGLFGWNVTDCKICFEYGLYYSPVSTPADFRSLAPIVLEQALKESGTQLLEPYL
Sbjct 481
IRYGLEQGLFGWNVTDCKICFEYGLYYSPVSTPADFRSLAPIVLEQALKESGTQLLEPYL 540

Query 541
SFILYAPQEYLSRAYHDAPKYCATIETAQVKKDEVVFTGEIPARCIQAYRTDLAFYTNGR 600
SF LYAP+EYLSRAYHDAPKYCATIET
QVKKDEVVFTGEIPARCIQAYRTDLAFYTNG+
Sbjct 541
SFTLYAPREYLSRAYHDAPKYCATIETVQVKKDEVVFTGEIPARCIQAYRTDLAFYTNGQ 600

Query 601 SVCLTELKGYQAAVGQPVIQPRRPNSRLDKVR 632
SVCLTELKGYQAAVG+PVIQPRRPNSRLDKVR
Sbjct 601 SVCLTELKGYQAAVGKPVIQPRRPNSRLDKVR 632

>WP_031474227.1 MULTISPECIES: TetM/TetW/TetO/TetS family tetracycline resistance
ribosomal protection protein [Clostridiales]
KUE74776.1 tetracycline resistance protein tetM [Ruthenibacterium lactatiformans]
SCI58526.1 Tetracycline resistance protein tetM from transposon Tn916 [uncultured

Flavonifractor sp.]
 SCJ03778.1 Tetracycline resistance protein tetM from transposon
Tn916 [uncultured
Flavonifractor sp.]
 SCJ93164.1 Tetracycline resistance protein tetM from transposon
Tn916 [uncultured
Clostridium sp.]
 SFQ17752.1 ribosomal protection tetracycline resistance protein
[Oscillibacter
sp. PC13]
 OUN08774.1 TetM/TetW/TetO/TetS family tetracycline resistance
ribosomal
protection protein [Flavonifractor sp. An92]
 OUN10306.1 TetM/TetW/TetO/TetS family tetracycline resistance
ribosomal
protection protein [Flavonifractor sp. An9]
 OUN20964.1 TetM/TetW/TetO/TetS family tetracycline resistance
ribosomal
protection protein [Flavonifractor sp. An82]
 OUN83256.1 TetM/TetW/TetO/TetS family tetracycline resistance
ribosomal
protection protein [Flavonifractor sp. An52]
 OUP59312.1 TetM/TetW/TetO/TetS family tetracycline resistance
ribosomal
protection protein [Pseudoflavonifractor sp. An184]
 GBF70292.1 hypothetical protein P3BBH22_3023 [Lawsonibacter
asaccharolyticus]
Length=639

 Score = 1282 bits (3318), Expect = 0.0, Method: Compositional
matrix adjust.
 Identities = 623/639 (97%), Positives = 631/639 (99%), Gaps = 0/639
(0%)

Query 1
MKIINIGILAHVDAGKTTLTESLLYASGAISEPGSVEKGTTRTDTMFLERQRGITIQAAV 60
            M IINIGILAHVDAGKTTLTESLLYASGAISEPGSVEKGTTRTDT
+FLERQRGITIQAAV
Sbjct 1
MNIINIGILAHVDAGKTTLTESLLYASGAISEPGSVEKGTTRTDTLFLERQRGITIQAAV 60

Query 61
TSFQWHRCKVNIVDTPGHMDFLAEVYRSLAVLDGAILVISAKDGVQAQTRILFHALRKMN 120

TSFQWHRCKVNIVDTPGHMDFLAEVYRSLAVLDGAILVISAKDGVQAQTRILFHALRKMN
Sbjct 61
TSFQWHRCKVNIVDTPGHMDFLAEVYRSLAVLDGAILVISAKDGVQAQTRILFHALRKMN 120

Query 121
IPTVIFINKIDQAGVDLQSVVQSVRDKLSADIIIKQTVSLSPEIVLEENTDIEAWDAVIE 180

IPTVIFINKIDQAGVDLQSVVQSVRDKLSADIIIKQTVSLSPEIVLEENTDIEAWDAVIE
Sbjct 121
IPTVIFINKIDQAGVDLQSVVQSVRDKLSADIIIKQTVSLSPEIVLEENTDIEAWDAVIE 180

```
Query  181
NNDKLLEKYIAGEPISREKLVREEQRRVQDASLFPVYYGSAKKGLGIQPLMDAVTGLFQP  240
            NND LLEKYIAGEPIS+EKL REEQRRVQ+ASLFPVY
+GSAKKGLGIQPLMDAVTGLFQP
Sbjct  181
NNDALLEKYIAGEPISQEKLAREEQRRVQEASLFPVYHGSAKKGLGIQPLMDAVTGLFQP  240

Query  241
IGEQGSAALCGSVFKVEYTDCGQRRVYLRLYSGTLRLRDTVALAGREKLKITEMRIPSKG  300
            IGEQGSA
LCGSVFKVEYTDCGQRRVYLRLYSGTLRLRDTVALAGREKLKITEMRIPSKG
Sbjct  241
IGEQGSATLCGSVFKVEYTDCGQRRVYLRLYSGTLRLRDTVALAGREKLKITEMRIPSKG  300

Query  301
EIVRTDTAYPGEIVILPSDSVRLNDVLGDPTRLPRKRWREDPLPMLRTTIAPKTAAQRER  360
            EIVRTDTAYPGEIVILPSDSVRLNDVLGD TRLPRKRWRE
PLPMLRTTIAPKTAAQRER
Sbjct  301
EIVRTDTAYPGEIVILPSDSVRLNDVLGDQTRLPRKRWREAPLPMLRTTIAPKTAAQRER  360

Query  361
LLDALTQLADTDPLLRCEVDSITHEIILSFLGRVQLEVVSALLSEKYKLETVVKEPSVIY  420
            LLDALTQLADTDPLLRCEVDSITHEIILSFLGRVQLEVVSALLSEKYK+ETVVKEP
+VIY
Sbjct  361
LLDALTQLADTDPLLRCEVDSITHEIILSFLGRVQLEVVSALLSEKYKIETVVKEPTVIY  420

Query  421
MERPLKAASHTIHIEVPPNPFWASIGLSVTPLSLGSGVQYESRVSLGYLNQSFQNAVRDG  480
            MERPLKAASHTIHIEVPPNPFWASIGLSVTPL
LGSGVQYESRVSLGYLNQSFQNAVRDG
Sbjct  421
MERPLKAASHTIHIEVPPNPFWASIGLSVTPLPLGSGVQYESRVSLGYLNQSFQNAVRDG  480

Query  481
IRYGLEQGLFGWNVTDCKICFEYGLYYSPVSTPADFRSLAPIVLEQALKESGTQLLEPYL  540

IRYGLEQGLFGWNVTDCKICFEYGLYYSPVSTPADFRSLAPIVLEQALKESGTQLLEPYL
Sbjct  481
IRYGLEQGLFGWNVTDCKICFEYGLYYSPVSTPADFRSLAPIVLEQALKESGTQLLEPYL  540

Query  541
SFILYAPQEYLSRAYHDAPKYCATIETAQVKKDEVVFTGEIPARCIQAYRTDLAFYTNGR  600
            SF
LYAPQEYLSRAYHDAPKYCATIETAQVKKDEVVFTGEIPARCIQAYRTDLAFYTNG+
Sbjct  541
SFTLYAPQEYLSRAYHDAPKYCATIETAQVKKDEVVFTGEIPARCIQAYRTDLAFYTNGQ  600

Query  601  SVCLTELKGYQAAVGQPVIQPRRPNSRLDKVRHMFQKVM  639
            SVCLTELKGYQAAVGQPVIQPRRPN+RLDKVRHMFQKVM
Sbjct  601  SVCLTELKGYQAAVGQPVIQPRRPNNRLDKVRHMFQKVM  639
```

>WP_049342005.1 TetM/TetW/TetO/TetS family tetracycline resistance ribosomal
protection protein [Rothia mucilaginosa]
Length=640

Score = 1282 bits (3317), Expect = 0.0, Method: Compositional matrix adjust.
Identities = 622/638 (97%), Positives = 632/638 (99%), Gaps = 0/638 (0%)

```
Query  1
MKIINIGILAHVDAGKTTLTESLLYASGAISEPGSVEKGTTRTDTMFLERQRGITIQAAV  60

MKIINIGILAHVDAGKTTLTESLLYASGAISEPGSVEKGTTRTDTMFLERQRGITIQAAV
Sbjct  1
MKIINIGILAHVDAGKTTLTESLLYASGAISEPGSVEKGTTRTDTMFLERQRGITIQAAV  60

Query  61
TSFQWHRCKVNIVDTPGHMDFLAEVYRSLAVLDGAILVISAKDGVQAQTRILFHALRKMN  120
            TSFQWHRCKVNIVDTPGHMDFLAEVYRSLAVLDGAILVISAKDGVQAQTRILF
+ALRKMN
Sbjct  61
TSFQWHRCKVNIVDTPGHMDFLAEVYRSLAVLDGAILVISAKDGVQAQTRILFNALRKMN  120

Query  121
IPTVIFINKIDQAGVDLQSVVQSVRDKLSADIIIKQTVSLSPEIVLEENTDIEAWDAVIE  180

IPTVIFINKIDQAGVDLQSVVQSVRDKLSADIIIKQTVSLSPEIVLEENTDIEAWDAVIE
Sbjct  121
IPTVIFINKIDQAGVDLQSVVQSVRDKLSADIIIKQTVSLSPEIVLEENTDIEAWDAVIE  180

Query  181
NNDKLLEKYIAGEPISREKLVREEQRRVQDASLFPVYYGSAKKGLGIQPLMDAVTGLFQP  240
            NND+LLEKYIAGEPISRE+L REEQRRVQDASLFPVY
+GSAKKGLGIQPLMDAVTGLFQP
Sbjct  181
NNDELLEKYIAGEPISREELAREEQRRVQDASLFPVYHGSAKKGLGIQPLMDAVTGLFQP  240

Query  241
IGEQGSAALCGSVFKVEYTDCGQRRVYLRLYSGTLRLRDTVALAGREKLKITEMRIPSKG  300
            IGEQG
AALCGSVFKVEYTDCGQRRVYLRLYSGTLRLRDTVALAGREKLKITEMRIPSKG
Sbjct  241
IGEQGGAALCGSVFKVEYTDCGQRRVYLRLYSGTLRLRDTVALAGREKLKITEMRIPSKG  300

Query  301
EIVRTDTAYPGEIVILPSDSVRLNDVLGDPTRLPRKRWREDPLPMLRTTIAPKTAAQRER  360
            EIVRTDTAYPGEIVILPSDSVRLNDVLGDPTRLPRKRWREDPLPMLRT
+IAPKTAAQRER
Sbjct  301
EIVRTDTAYPGEIVILPSDSVRLNDVLGDPTRLPRKRWREDPLPMLRTSIAPKTAAQRER  360

Query  361
```

LLDALTQLADTDPLLRCEVDSITHEIILSFLGRVQLEVVSALLSEKYKLETVVKEPSVIY 420
            LLDALTQLADTDPLLRCEVDSITHEIILSFLGRVQLEVVSALLSEKYKLETVVKEP
+VIY
Sbjct 361
LLDALTQLADTDPLLRCEVDSITHEIILSFLGRVQLEVVSALLSEKYKLETVVKEPTVIY 420

Query 421
MERPLKAASHTIHIEVPPNPFWASIGLSVTPLSLGSGVQYESRVSLGYLNQSFQNAVRDG 480
            MERPLKAASHTIHIEVPPNPFWASIGLSVTPL
LGSGVQYESRVSLGYLNQSFQNAVRDG
Sbjct 421
MERPLKAASHTIHIEVPPNPFWASIGLSVTPLPLGSGVQYESRVSLGYLNQSFQNAVRDG 480

Query 481
IRYGLEQGLFGWNVTDCKICFEYGLYYSPVSTPADFRSLAPIVLEQALKESGTQLLEPYL 540

IRYGLEQGLFGWNVTDCKICFEYGLYYSPVSTPADFRSLAPIVLEQALKESGTQLLEPYL
Sbjct 481
IRYGLEQGLFGWNVTDCKICFEYGLYYSPVSTPADFRSLAPIVLEQALKESGTQLLEPYL 540

Query 541
SFILYAPQEYLSRAYHDAPKYCATIETAQVKKDEVVFTGEIPARCIQAYRTDLAFYTNGR 600
            SF LYAP+EYLSRAYHDAPKYCATIET
QVKKDEVVFTGEIPARCIQAYRTDLAFYTNG+
Sbjct 541
SFTLYAPREYLSRAYHDAPKYCATIETVQVKKDEVVFTGEIPARCIQAYRTDLAFYTNGQ 600

Query 601 SVCLTELKGYQAAVGQPVIQPRRPNSRLDKVRHMFQKV 638
            SVCLTELKGYQAAVG+PVIQPRRPNSRLDKVRHMF K+
Sbjct 601 SVCLTELKGYQAAVGKPVIQPRRPNSRLDKVRHMFSKI 638

>AMP58106.1 TetM-TetW-TetO-TetS [uncultured bacterium]
Length=629

Score = 1281 bits (3316), Expect = 0.0, Method: Compositional
matrix adjust.
Identities = 625/629 (99%), Positives = 626/629 (99%), Gaps = 0/629
(0%)

Query 1
MKIINIGILAHVDAGKTTLTESLLYASGAISEPGSVEKGTTRTDTMFLERQRGITIQAAV 60

MKIINIGILAHVDAGKTTLTESLLYASGAISEPGSVEKGTTRTDTMFLERQRGITIQAAV
Sbjct 1
MKIINIGILAHVDAGKTTLTESLLYASGAISEPGSVEKGTTRTDTMFLERQRGITIQAAV 60

Query 61
TSFQWHRCKVNIVDTPGHMDFLAEVYRSLAVLDGAILVISAKDGVQAQTRILFHALRKMN 120

TSFQWHRCKVNIVDTPGHMDFLAEVYRSLAVLDGAILVISAKDGVQAQTRILFHALRKMN
Sbjct 61
TSFQWHRCKVNIVDTPGHMDFLAEVYRSLAVLDGAILVISAKDGVQAQTRILFHALRKMN 120

Query 121
IPTVIFINKIDQAGVDLQSVVQSVRDKLSADIIIKQTVSLSPEIVLEENTDIEAWDAVIE 180

IPTVIFINKIDQAGVDLQSVVQSVRDKLSADIIIKQTVSLSPEIVLEENTDIEAWDAVIE
Sbjct 121
IPTVIFINKIDQAGVDLQSVVQSVRDKLSADIIIKQTVSLSPEIVLEENTDIEAWDAVIE 180

Query 181
NNDKLLEKYIAGEPISREKLVREEQRRVQDASLFPVYYGSAKKGLGIQPLMDAVTGLFQP 240
NND+LLEKYIAGEPISREKL
REEQRRVQDASLFPVYYGSAKKGLGIQPLMDAVTGLFQP
Sbjct 181
NNDELLEKYIAGEPISREKLAREEQRRVQDASLFPVYYGSAKKGLGIQPLMDAVTGLFQP 240

Query 241
IGEQGSAALCGSVFKVEYTDCGQRRVYLRLYSGTLRLRDTVALAGREKLKITEMRIPSKG 300
IGEQG
AALCGSVFKVEYTDCGQRRVYLRLYSGTLRLRDTVALAGREKLKITEMRIPSKG
Sbjct 241
IGEQGGAALCGSVFKVEYTDCGQRRVYLRLYSGTLRLRDTVALAGREKLKITEMRIPSKG 300

Query 301
EIVRTDTAYPGEIVILPSDSVRLNDVLGDPTRLPRKRWREDPLPMLRTTIAPKTAAQRER 360
EIVRTDTAY
GEIVILPSDSVRLNDVLGDPTRLPRKRWREDPLPMLRTTIAPKTAAQRER
Sbjct 301
EIVRTDTAYQGEIVILPSDSVRLNDVLGDPTRLPRKRWREDPLPMLRTTIAPKTAAQRER 360

Query 361
LLDALTQLADTDPLLRCEVDSITHEIILSFLGRVQLEVVSALLSEKYKLETVVKEPSVIY 420

LLDALTQLADTDPLLRCEVDSITHEIILSFLGRVQLEVVSALLSEKYKLETVVKEPSVIY
Sbjct 361
LLDALTQLADTDPLLRCEVDSITHEIILSFLGRVQLEVVSALLSEKYKLETVVKEPSVIY 420

Query 421
MERPLKAASHTIHIEVPPNPFWASIGLSVTPLSLGSGVQYESRVSLGYLNQSFQNAVRDG 480

MERPLKAASHTIHIEVPPNPFWASIGLSVTPLSLGSGVQYESRVSLGYLNQSFQNAVRDG
Sbjct 421
MERPLKAASHTIHIEVPPNPFWASIGLSVTPLSLGSGVQYESRVSLGYLNQSFQNAVRDG 480

Query 481
IRYGLEQGLFGWNVTDCKICFEYGLYYSPVSTPADFRSLAPIVLEQALKESGTQLLEPYL 540

IRYGLEQGLFGWNVTDCKICFEYGLYYSPVSTPADFRSLAPIVLEQALKESGTQLLEPYL
Sbjct 481
IRYGLEQGLFGWNVTDCKICFEYGLYYSPVSTPADFRSLAPIVLEQALKESGTQLLEPYL 540

Query 541
SFILYAPQEYLSRAYHDAPKYCATIETAQVKKDEVVFTGEIPARCIQAYRTDLAFYTNGR 600

SFILYAPQEYLSRAYHDAPKYCATIETAQVKKDEVVFTGEIPARCIQAYRTDLAFYTNGR
Sbjct 541

```
SFILYAPQEYLSRAYHDAPKYCATIETAQVKKDEVVFTGEIPARCIQAYRTDLAFYTNGR  600

Query  601  SVCLTELKGYQAAVGQPVIQPRRPNSRLD  629
            SVCLTELKGYQAAVGQPVIQPRRPNSRLD
Sbjct  601  SVCLTELKGYQAAVGQPVIQPRRPNSRLD  629


>AMP55886.1 TetM-TetW-TetO-TetS, partial [uncultured bacterium]
Length=635

 Score = 1281 bits (3316),  Expect = 0.0, Method: Compositional 
matrix adjust.
 Identities = 624/635 (98%), Positives = 627/635 (99%), Gaps = 0/635 
(0%)

Query  1    
MKIINIGILAHVDAGKTTLTESLLYASGAISEPGSVEKGTTRTDTMFLERQRGITIQAAV  60
            
MKIINIGILAHVDAGKTTLTESLLYASGAISEPGSVEKGTTRTDTMFLERQRGITIQAAV
Sbjct  1    
MKIINIGILAHVDAGKTTLTESLLYASGAISEPGSVEKGTTRTDTMFLERQRGITIQAAV  60

Query  61   
TSFQWHRCKVNIVDTPGHMDFLAEVYRSLAVLDGAILVISAKDGVQAQTRILFHALRKMN  120
            
TSFQWHRCKVNIVDTPGHMDFLAEVYRSLAVLDGAILVISAKDGVQAQTRILFHALRKMN
Sbjct  61   
TSFQWHRCKVNIVDTPGHMDFLAEVYRSLAVLDGAILVISAKDGVQAQTRILFHALRKMN  120

Query  121  
IPTVIFINKIDQAGVDLQSVVQSVRDKLSADIIIKQTVSLSPEIVLEENTDIEAWDAVIE  180
            IPTVIFINKIDQAGVDLQSVVQSVRDKLSADIIIKQTVSLSPEIVLEEN DIE 
WDAVIE
Sbjct  121  
IPTVIFINKIDQAGVDLQSVVQSVRDKLSADIIIKQTVSLSPEIVLEENNDIEEWDAVIE  180

Query  181  
NNDKLLEKYIAGEPISREKLVREEQRRVQDASLFPVYYGSAKKGLGIQPLMDAVTGLFQP  240
            NND+LLEKYIAGEPISREKL REEQ+RVQDASLFPVY+GSAK 
GLGIQPLMDAVTGLFQP
Sbjct  181  
NNDELLEKYIAGEPISREKLAREEQQRVQDASLFPVYHGSAKNGLGIQPLMDAVTGLFQP  240

Query  241  
IGEQGSAALCGSVFKVEYTDCGQRRVYLRLYSGTLRLRDTVALAGREKLKITEMRIPSKG  300
            IGEQG 
AALCGSVFKVEYTDCGQRRVYLRLYSGTLRLRDTVALAGREKLKITEMRIPSKG
Sbjct  241  
IGEQGGAALCGSVFKVEYTDCGQRRVYLRLYSGTLRLRDTVALAGREKLKITEMRIPSKG  300

Query  301  
EIVRTDTAYPGEIVILPSDSVRLNDVLGDPTRLPRKRWREDPLPMLRTTIAPKTAAQRER  360
            EIVRTDTAY GEIVILPSDSVRLNDVLGD 
TRLPRKRWREDPLPMLRTTIAPKTAAQRER
```

```
Sbjct  301
EIVRTDTAYQGEIVILPSDSVRLNDVLGDQTRLPRKRWREDPLPMLRTTIAPKTAAQRER  360

Query  361
LLDALTQLADTDPLLRCEVDSITHEIILSFLGRVQLEVVSALLSEKYKLETVVKEPSVIY  420

LLDALTQLADTDPLLRCEVDSITHEIILSFLGRVQLEVVSALLSEKYKLETVVKEPSVIY
Sbjct  361
LLDALTQLADTDPLLRCEVDSITHEIILSFLGRVQLEVVSALLSEKYKLETVVKEPSVIY  420

Query  421
MERPLKAASHTIHIEVPPNPFWASIGLSVTPLSLGSGVQYESRVSLGYLNQSFQNAVRDG  480

MERPLKAASHTIHIEVPPNPFWASIGLSVTPLSLGSGVQYESRVSLGYLNQSFQNAVRDG
Sbjct  421
MERPLKAASHTIHIEVPPNPFWASIGLSVTPLSLGSGVQYESRVSLGYLNQSFQNAVRDG  480

Query  481
IRYGLEQGLFGWNVTDCKICFEYGLYYSPVSTPADFRSLAPIVLEQALKESGTQLLEPYL  540

IRYGLEQGLFGWNVTDCKICFEYGLYYSPVSTPADFRSLAPIVLEQALKESGTQLLEPYL
Sbjct  481
IRYGLEQGLFGWNVTDCKICFEYGLYYSPVSTPADFRSLAPIVLEQALKESGTQLLEPYL  540

Query  541
SFILYAPQEYLSRAYHDAPKYCATIETAQVKKDEVVFTGEIPARCIQAYRTDLAFYTNGR  600
            SFILYAPQEYLSRAYHDAPKYCATIETAQVKKDEVVFTGEIP
RCIQAYRTDLAFYTNGR
Sbjct  541
SFILYAPQEYLSRAYHDAPKYCATIETAQVKKDEVVFTGEIPDRCIQAYRTDLAFYTNGR  600

Query  601  SVCLTELKGYQAAVGQPVIQPRRPNSRLDKVRHMF  635
            SVCLTELKGYQAAVGQPVIQPRRPNSRLDKVRHMF
Sbjct  601  SVCLTELKGYQAAVGQPVIQPRRPNSRLDKVRHMF  635


>WP_050618045.1 MULTISPECIES: tetracycline resistance ribosomal
protection protein
Tet(W) [Firmicutes]
 CAX85496.1 tetracycline resistance protein [Streptococcus suis]
 CAZ39277.1 tetracycline resistance protein [Streptococcus suis]
 CAZ39293.1 tetracycline resistance protein [Streptococcus suis]
 APO31165.1 Ferrous iron transport protein B [uncultured bacterium]
 AUS90889.1 TetW [Streptococcus suis]
Length=639

 Score = 1281 bits (3316),  Expect = 0.0, Method: Compositional
matrix adjust.
 Identities = 623/639 (97%), Positives = 631/639 (99%), Gaps = 0/639
(0%)

Query  1
MKIINIGILAHVDAGKTTLTESLLYASGAISEPGSVEKGTTRTDTMFLERQRGITIQAAV  60
```

MKIINIGILAHVDAGKTTLTESLLYASGAISEPGSVEKGTTRTDTMFLERQRGITIQAAV
Sbjct 1
MKIINIGILAHVDAGKTTLTESLLYASGAISEPGSVEKGTTRTDTMFLERQRGITIQAAV 60

Query 61
TSFQWHRCKVNIVDTPGHMDFLAEVYRSLAVLDGAILVISAKDGVQAQTRILFHALRKMN 120

TSFQWHRCKVNIVDTPGHMDFLAEVYRSLAVLDGAILVISAKDGVQAQTRILFHALRKMN
Sbjct 61
TSFQWHRCKVNIVDTPGHMDFLAEVYRSLAVLDGAILVISAKDGVQAQTRILFHALRKMN 120

Query 121
IPTVIFINKIDQAGVDLQSVVQSVRDKLSADIIIKQTVSLSPEIVLEENTDIEAWDAVIE 180

IPTVIFINKIDQAGVDLQSVVQSVRDKLSADIIIKQTVSLSPEIVLEENTDIEAWDAVIE
Sbjct 121
IPTVIFINKIDQAGVDLQSVVQSVRDKLSADIIIKQTVSLSPEIVLEENTDIEAWDAVIE 180

Query 181
NNDKLLEKYIAGEPISREKLVREEQRRVQDASLFPVYYGSAKKGLGIQPLMDAVTGLFQP 240
NND+LLEKYIAGEPISREKL REEQ+RVQDASLFPVYYGSAK
GLGIQPLMDAVTGLFQP
Sbjct 181
NNDELLEKYIAGEPISREKLAREEQQRVQDASLFPVYYGSAKNGLGIQPLMDAVTGLFQP 240

Query 241
IGEQGSAALCGSVFKVEYTDCGQRRVYLRLYSGTLRLRDTVALAGREKLKITEMRIPSKG 300
IGEQG
AALCGSVFKVEYTDCGQRRVYLRLYSGTLRLRDTVALAGREKLKITEMRIPSKG
Sbjct 241
IGEQGGAALCGSVFKVEYTDCGQRRVYLRLYSGTLRLRDTVALAGREKLKITEMRIPSKG 300

Query 301
EIVRTDTAYPGEIVILPSDSVRLNDVLGDPTRLPRKRWREDPLPMLRTTIAPKTAAQRER 360
EIVRTDTAY GEIVILPSDSVRLNDVLGD
TRLPRKRWREDPLPMLRTTIAPKTAAQRER
Sbjct 301
EIVRTDTAYQGEIVILPSDSVRLNDVLGDQTRLPRKRWREDPLPMLRTTIAPKTAAQRER 360

Query 361
LLDALTQLADTDPLLRCEVDSITHEIILSFLGRVQLEVVSALLSEKYKLETVVKEPSVIY 420
LLDALTQLADTDPLLRCEVDSITHEIILSFLGRVQLEVVSALLSEKYKLETVVKEP
+VIY
Sbjct 361
LLDALTQLADTDPLLRCEVDSITHEIILSFLGRVQLEVVSALLSEKYKLETVVKEPTVIY 420

Query 421
MERPLKAASHTIHIEVPPNPFWASIGLSVTPLSLGSGVQYESRVSLGYLNQSFQNAVRDG 480
MERPLKAASHTIHIEVPPNPFWASIGLSVTPL
LGSGVQYESRVSLGYLNQSFQNAVRDG
Sbjct 421
MERPLKAASHTIHIEVPPNPFWASIGLSVTPLPLGSGVQYESRVSLGYLNQSFQNAVRDG 480

Query 481

IRYGLEQGLFGWNVTDCKICFEYGLYYSPVSTPADFRSLAPIVLEQALKESGTQLLEPYL 540

IRYGLEQGLFGWNVTDCKICFEYGLYYSPVSTPADFRSLAPIVLEQALKESGTQLLEPYL
Sbjct 481
IRYGLEQGLFGWNVTDCKICFEYGLYYSPVSTPADFRSLAPIVLEQALKESGTQLLEPYL 540

Query 541
SFILYAPQEYLSRAYHDAPKYCATIETAQVKKDEVVFTGEIPARCIQAYRTDLAFYTNGR 600
SF LYAP+EYLSRAYHDAPKYCATIET
QVKKDEVVFTGEIPARCIQAYRTDLAFYTNG+
Sbjct 541
SFTLYAPREYLSRAYHDAPKYCATIETVQVKKDEVVFTGEIPARCIQAYRTDLAFYTNGQ 600

Query 601 SVCLTELKGYQAAVGQPVIQPRRPNSRLDKVRHMFQKVM 639
SVCLTELKGYQAAVG+PVIQPRRPNSRLDKVR+MFQK+M
Sbjct 601 SVCLTELKGYQAAVGKPVIQPRRPNSRLDKVRYMFQKIM 639

>WP_086143304.1 TetM/TetW/TetO/TetS family tetracycline resistance ribosomal
protection protein [Lactobacillus reuteri]
OTA58516.1 tetracycline resistance ribosomal protection protein [Lactobacillus
reuteri]
Length=645

Score = 1281 bits (3315), Expect = 0.0, Method: Compositional matrix adjust.
Identities = 624/638 (98%), Positives = 632/638 (99%), Gaps = 0/638 (0%)

Query 1
MKIINIGILAHVDAGKTTLTESLLYASGAISEPGSVEKGTTRTDTMFLERQRGITIQAAV 60
MKIINIGILAHVDAGKTTLTESLLYASGAISEPGSV
+KGTTRTDTMFLERQRGITIQAAV
Sbjct 1
MKIINIGILAHVDAGKTTLTESLLYASGAISEPGSVKKGTTRTDTMFLERQRGITIQAAV 60

Query 61
TSFQWHRCKVNIVDTPGHMDFLAEVYRSLAVLDGAILVISAKDGVQAQTRILFHALRKMN 120

TSFQWHRCKVNIVDTPGHMDFLAEVYRSLAVLDGAILVISAKDGVQAQTRILFHALRKMN
Sbjct 61
TSFQWHRCKVNIVDTPGHMDFLAEVYRSLAVLDGAILVISAKDGVQAQTRILFHALRKMN 120

Query 121
IPTVIFINKIDQAGVDLQSVVQSVRDKLSADIIIKQTVSLSPEIVLEENTDIEAWDAVIE 180

IPTVIFINKIDQAGVDLQSVVQSVRDKLSADIIIKQTVSLSPEIVLEENTDIEAWDAVIE
Sbjct 121
IPTVIFINKIDQAGVDLQSVVQSVRDKLSADIIIKQTVSLSPEIVLEENTDIEAWDAVIE 180

Query 181
NNDKLLEKYIAGEPISREKLVREEQRRVQDASLFPVYYGSAKKGLGIQPLMDAVTGLFQP 240

```
NNDKLLEKYIAGEPISREKLVREEQRRVQDASLFPVYYGSAKKGLGIQPLMDAVTGLFQP
Sbjct  181  
NNDKLLEKYIAGEPISREKLVREEQRRVQDASLFPVYYGSAKKGLGIQPLMDAVTGLFQP  240

Query  241  
IGEQGSAALCGSVFKVEYTDCGQRRVYLRLYSGTLRLRDTVALAGREKLKITEMRIPSKG  300
            
IGEQGSAALCGSVFKVEYTDCGQRRVYLRLYSGTLRLRDTVALAGREKLKITEMRIPSKG
Sbjct  241  
IGEQGSAALCGSVFKVEYTDCGQRRVYLRLYSGTLRLRDTVALAGREKLKITEMRIPSKG  300

Query  301  
EIVRTDTAYPGEIVILPSDSVRLNDVLGDPTRLPRKRWREDPLPMLRTTIAPKTAAQRER  360
            EIVRTDTAYPGEIVILPSDSVRLNDVLGDPTRLPRKR REDPLPMLRT 
+IAPKTAAQRER
Sbjct  301  
EIVRTDTAYPGEIVILPSDSVRLNDVLGDPTRLPRKRLREDPLPMLRTSIAPKTAAQRER  360

Query  361  
LLDALTQLADTDPLLRCEVDSITHEIILSFLGRVQLEVVSALLSEKYKLETVVKEPSVIY  420
            LLDALTQLADTDPLLRCEVDSITHEIILSFLGRVQLEVVSALLSEKYKLETVVKEP 
+VIY
Sbjct  361  
LLDALTQLADTDPLLRCEVDSITHEIILSFLGRVQLEVVSALLSEKYKLETVVKEPTVIY  420

Query  421  
MERPLKAASHTIHIEVPPNPFWASIGLSVTPLSLGSGVQYESRVSLGYLNQSFQNAVRDG  480
            MERPLKAASHTIHIEVPPNPFWASIGLSVTPL 
LGSGVQYESRVSLGYLNQSFQNAVRDG
Sbjct  421  
MERPLKAASHTIHIEVPPNPFWASIGLSVTPLPLGSGVQYESRVSLGYLNQSFQNAVRDG  480

Query  481  
IRYGLEQGLFGWNVTDCKICFEYGLYYSPVSTPADFRSLAPIVLEQALKESGTQLLEPYL  540
            
IRYGLEQGLFGWNVTDCKICFEYGLYYSPVSTPADFRSLAPIVLEQALKESGTQLLEPYL
Sbjct  481  
IRYGLEQGLFGWNVTDCKICFEYGLYYSPVSTPADFRSLAPIVLEQALKESGTQLLEPYL  540

Query  541  
SFILYAPQEYLSRAYHDAPKYCATIETAQVKKDEVVFTGEIPARCIQAYRTDLAFYTNGR  600
            SF LYAP+EYLSRAYHDAPKYCATIET QVKKDEVVFTGEIPARCIQAYRTDLA 
YTNG+
Sbjct  541  
SFTLYAPREYLSRAYHDAPKYCATIETVQVKKDEVVFTGEIPARCIQAYRTDLASYTNGQ  600

Query  601  SVCLTELKGYQAAVGQPVIQPRRPNSRLDKVRHMFQKV  638
            SVCLTELKGYQAA G+PVIQPRRPNSRLDKVR+MFQK+
Sbjct  601  SVCLTELKGYQAADGKPVIQPRRPNSRLDKVRYMFQKI  638
```

>WP_004010384.1 MULTISPECIES: TetM/TetW/TetO/TetS family tetracycline resistance

ribosomal protection protein [Mobiluncus]
 EEZ91703.1 putative translation elongation factor G [Mobiluncus mulieris
28-1]
 EFU82924.1 putative translation elongation factor G [Mobiluncus curtisii
subsp. holmesii ATCC 35242]
Length=639

 Score = 1281 bits (3314), Expect = 0.0, Method: Compositional matrix adjust.
 Identities = 622/639 (97%), Positives = 631/639 (99%), Gaps = 0/639 (0%)

```
Query  1
MKIINIGILAHVDAGKTTLTESLLYASGAISEPGSVEKGTTRTDTMFLERQRGITIQAAV  60
            M IINIGILAHVDAGKTTLTESLLYASGAISEPGSVEKGTTRTDT
+FLERQRGITIQAAV
Sbjct  1
MNIINIGILAHVDAGKTTLTESLLYASGAISEPGSVEKGTTRTDTLFLERQRGITIQAAV  60

Query  61
TSFQWHRCKVNIVDTPGHMDFLAEVYRSLAVLDGAILVISAKDGVQAQTRILFHALRKMN  120

TSFQWHRCKVNIVDTPGHMDFLAEVYRSLAVLDGAILVISAKDGVQAQTRILFHALRKMN
Sbjct  61
TSFQWHRCKVNIVDTPGHMDFLAEVYRSLAVLDGAILVISAKDGVQAQTRILFHALRKMN  120

Query  121
IPTVIFINKIDQAGVDLQSVVQSVRDKLSADIIIKQTVSLSPEIVLEENTDIEAWDAVIE  180

IPTVIFINKIDQAGVDLQSVVQSVRDKLSADIIIKQTVSLSPEIVLEENTDIEAWDAVIE
Sbjct  121
IPTVIFINKIDQAGVDLQSVVQSVRDKLSADIIIKQTVSLSPEIVLEENTDIEAWDAVIE  180

Query  181
NNDKLLEKYIAGEPISREKLVREEQRRVQDASLFPVYYGSAKKGLGIQPLMDAVTGLFQP  240
            NND LLEKYIAGEPIS+EKL REEQRRVQ+ASLFPVY
+GSAKKGLGIQPLMDAVTGLFQP
Sbjct  181
NNDALLEKYIAGEPISQEKLAREEQRRVQEASLFPVYHGSAKKGLGIQPLMDAVTGLFQP  240

Query  241
IGEQGSAALCGSVFKVEYTDCGQRRVYLRLYSGTLRLRDTVALAGREKLKITEMRIPSKG  300
            IGEQGSA
LCGSVFKVEYTDCGQRRVYLRLYSGTLRLRDTVALAGREKLKITEMRIPSKG
Sbjct  241
IGEQGSATLCGSVFKVEYTDCGQRRVYLRLYSGTLRLRDTVALAGREKLKITEMRIPSKG  300

Query  301
EIVRTDTAYPGEIVILPSDSVRLNDVLGDPTRLPRKRWREDPLPMLRTTIAPKTAAQRER  360
            EIVRTDTAYPGEIVILPSDSVRLNDVLGD TRLPRKRWRE
PLPMLRTTIAPKTAAQRER
Sbjct  301
```

```
EIVRTDTAYPGEIVILPSDSVRLNDVLGDQTRLPRKRWREAPLPMLRTTIAPKTAAQRER  360

Query  361
LLDALTQLADTDPLLRCEVDSITHEIILSFLGRVQLEVVSALLSEKYKLETVVKEPSVIY  420
            LLDALTQLADTDPLLRCEVDSITHEIILSFLGRVQLEVVSALLSEKYK+ETVVKEP
+VIY
Sbjct  361
LLDALTQLADTDPLLRCEVDSITHEIILSFLGRVQLEVVSALLSEKYKIETVVKEPTVIY  420

Query  421
MERPLKAASHTIHIEVPPNPFWASIGLSVTPLSLGSGVQYESRVSLGYLNQSFQNAVRDG  480
            MERPLKAASHTIHIEVPPNPFWASIGLSVTPL
LGSGVQYESRVSLGYLNQSFQNAVRDG
Sbjct  421
MERPLKAASHTIHIEVPPNPFWASIGLSVTPLPLGSGVQYESRVSLGYLNQSFQNAVRDG  480

Query  481
IRYGLEQGLFGWNVTDCKICFEYGLYYSPVSTPADFRSLAPIVLEQALKESGTQLLEPYL  540

IRYGLEQGLFGWNVTDCKICFEYGLYYSPVSTPADFRSLAPIVLEQALKESGTQLLEPYL
Sbjct  481
IRYGLEQGLFGWNVTDCKICFEYGLYYSPVSTPADFRSLAPIVLEQALKESGTQLLEPYL  540

Query  541
SFILYAPQEYLSRAYHDAPKYCATIETAQVKKDEVVFTGEIPARCIQAYRTDLAFYTNGR  600
            SF LYAPQEYLSRAYH
+APKYCATIETAQVKKDEVVFTGEIPARCIQAYRTDLAFYTNG+
Sbjct  541
SFTLYAPQEYLSRAYHNAPKYCATIETAQVKKDEVVFTGEIPARCIQAYRTDLAFYTNGQ  600

Query  601  SVCLTELKGYQAAVGQPVIQPRRPNSRLDKVRHMFQKVM  639
            SVCLTELKGYQAAVGQPVIQPRRPN+RLDKVRHMFQKVM
Sbjct  601  SVCLTELKGYQAAVGQPVIQPRRPNNRLDKVRHMFQKVM  639


>WP_024532698.1 TetM/TetW/TetO/TetS family tetracycline resistance
ribosomal
protection protein [Streptococcus suis]
Length=639

 Score = 1280 bits (3312),  Expect = 0.0, Method: Compositional
matrix adjust.
 Identities = 621/638 (97%), Positives = 631/638 (99%), Gaps = 0/638
(0%)

Query  1
MKIINIGILAHVDAGKTTLTESLLYASGAISEPGSVEKGTTRTDTMFLERQRGITIQAAV  60
            MKIINIGILAHVDAGKTTLTESLLY SGAI+E G+V+KGTTRTDTM LE
+QRGITIQAAV
Sbjct  1
MKIINIGILAHVDAGKTTLTESLLYTSGAIAEQGNVDKGTTRTDTMILEQQRGITIQAAV  60

Query  61
TSFQWHRCKVNIVDTPGHMDFLAEVYRSLAVLDGAILVISAKDGVQAQTRILFHALRKMN  120
```

TSFQWHRCKVNIVDTPGHMDFLAEVYRSLAVLDGAILVISAKDGVQAQTRILFHALRKMN
Sbjct 61
TSFQWHRCKVNIVDTPGHMDFLAEVYRSLAVLDGAILVISAKDGVQAQTRILFHALRKMN 120

Query 121
IPTVIFINKIDQAGVDLQSVVQSVRDKLSADIIIKQTVSLSPEIVLEENTDIEAWDAVIE 180

IPTVIFINKIDQAGVDLQSVVQSVRDKLSADIIIKQTVSLSPEIVLEENTDIEAWDAVIE
Sbjct 121
IPTVIFINKIDQAGVDLQSVVQSVRDKLSADIIIKQTVSLSPEIVLEENTDIEAWDAVIE 180

Query 181
NNDKLLEKYIAGEPISREKLVREEQRRVQDASLFPVYYGSAKKGLGIQPLMDAVTGLFQP 240

NNDKLLEKYIAGEPISREKLVREEQRRVQDASLFPVYYGSAKKGLGIQPLMDAVTGLFQP
Sbjct 181
NNDKLLEKYIAGEPISREKLVREEQRRVQDASLFPVYYGSAKKGLGIQPLMDAVTGLFQP 240

Query 241
IGEQGSAALCGSVFKVEYTDCGQRRVYLRLYSGTLRLRDTVALAGREKLKITEMRIPSKG 300

IGEQGSAALCGSVFKVEYTDCGQRRVYLRLYSGTLRLRDTVALAGREKLKITEMRIPSKG
Sbjct 241
IGEQGSAALCGSVFKVEYTDCGQRRVYLRLYSGTLRLRDTVALAGREKLKITEMRIPSKG 300

Query 301
EIVRTDTAYPGEIVILPSDSVRLNDVLGDPTRLPRKRWREDPLPMLRTTIAPKTAAQRER 360
EIVRTDTAYPGEIVILPSDSVRLNDVLGDPTRLPRKRWREDPLPMLRT
+IAPKTAAQRER
Sbjct 301
EIVRTDTAYPGEIVILPSDSVRLNDVLGDPTRLPRKRWREDPLPMLRTSIAPKTAAQRER 360

Query 361
LLDALTQLADTDPLLRCEVDSITHEIILSFLGRVQLEVVSALLSEKYKLETVVKEPSVIY 420
LLDALTQLADTDPLLRCEVDSITHEIILSFLGRVQLEVVSALLSEKYKLETVVKEP
+VIY
Sbjct 361
LLDALTQLADTDPLLRCEVDSITHEIILSFLGRVQLEVVSALLSEKYKLETVVKEPTVIY 420

Query 421
MERPLKAASHTIHIEVPPNPFWASIGLSVTPLSLGSGVQYESRVSLGYLNQSFQNAVRDG 480
MERPLKAASHTIHIEVPPNPFWASIGLSVTPL
LGSGVQYESRVSLGYLNQSFQNAVRDG
Sbjct 421
MERPLKAASHTIHIEVPPNPFWASIGLSVTPLPLGSGVQYESRVSLGYLNQSFQNAVRDG 480

Query 481
IRYGLEQGLFGWNVTDCKICFEYGLYYSPVSTPADFRSLAPIVLEQALKESGTQLLEPYL 540

IRYGLEQGLFGWNVTDCKICFEYGLYYSPVSTPADFRSLAPIVLEQALKESGTQLLEPYL
Sbjct 481
IRYGLEQGLFGWNVTDCKICFEYGLYYSPVSTPADFRSLAPIVLEQALKESGTQLLEPYL 540

Query 541
SFILYAPQEYLSRAYHDAPKYCATIETAQVKKDEVVFTGEIPARCIQAYRTDLAFYTNGR 600
            SF LYAP+EYLSRAYHDAPKYCATIET
QVKKDEVVFTGEIPARCIQAYRTDLAFYTNG+
Sbjct 541
SFTLYAPREYLSRAYHDAPKYCATIETVQVKKDEVVFTGEIPARCIQAYRTDLAFYTNGQ 600

Query 601 SVCLTELKGYQAAVGQPVIQPRRPNSRLDKVRHMFQKV 638
            SVCLTELKGYQAAVG+PVIQPRRPNSRLDKVRHMF K+
Sbjct 601 SVCLTELKGYQAAVGKPVIQPRRPNSRLDKVRHMFSKI 638

>WP_049362095.1 MULTISPECIES: TetM/TetW/TetO/TetS family
tetracycline resistance
ribosomal protection protein [Rothia]
 OFM21067.1 tetracycline resistance ribosomal protection protein
[Rothia
sp. HMSC069D01]
 OFN70303.1 tetracycline resistance ribosomal protection protein
[Rothia
sp. HMSC078H08]
 OFQ29149.1 tetracycline resistance ribosomal protection protein
[Rothia
sp. HMSC072E10]
 OFR47435.1 tetracycline resistance ribosomal protection protein
[Rothia
sp. HMSC073B08]
 ATF63696.1 TetM/TetW/TetO/TetS family tetracycline resistance
ribosomal
protection protein [Rothia mucilaginosa]
Length=640

 Score = 1279 bits (3309), Expect = 0.0, Method: Compositional
matrix adjust.
 Identities = 621/638 (97%), Positives = 631/638 (99%), Gaps = 0/638
(0%)

Query 1
MKIINIGILAHVDAGKTTLTESLLYASGAISEPGSVEKGTTRTDTMFLERQRGITIQAAV 60

MKIINIGILAHVDAGKTTLTESLLYASGAISEPGSVEKGTTRTDTMFLERQRGITIQAAV
Sbjct 1
MKIINIGILAHVDAGKTTLTESLLYASGAISEPGSVEKGTTRTDTMFLERQRGITIQAAV 60

Query 61
TSFQWHRCKVNIVDTPGHMDFLAEVYRSLAVLDGAILVISAKDGVQAQTRILFHALRKMN 120
            TSFQWHRCKVNIVDTPGHMDFLAEVYRSLAVLDGAILVISAKDGVQAQTRILF
+ALRKMN
Sbjct 61
TSFQWHRCKVNIVDTPGHMDFLAEVYRSLAVLDGAILVISAKDGVQAQTRILFNALRKMN 120

Query 121
IPTVIFINKIDQAGVDLQSVVQSVRDKLSADIIIKQTVSLSPEIVLEENTDIEAWDAVIE 180

IPTVIFINKIDQAGVDLQSVVQSVRDKLSADIIIKQTVSLSPEIVLEENTDIEAWDAVIE
Sbjct 121
IPTVIFINKIDQAGVDLQSVVQSVRDKLSADIIIKQTVSLSPEIVLEENTDIEAWDAVIE 180

Query 181
NNDKLLEKYIAGEPISREKLVREEQRRVQDASLFPVYYGSAKKGLGIQPLMDAVTGLFQP 240
            NND+LLEKYIAGEPISREKL REEQ+RVQDASLFPVY+GSAK
GLGIQPLMDAVTGLFQP
Sbjct 181
NNDELLEKYIAGEPISREKLAREEQQRVQDASLFPVYHGSAKNGLGIQPLMDAVTGLFQP 240

Query 241
IGEQGSAALCGSVFKVEYTDCGQRRVYLRLYSGTLRLRDTVALAGREKLKITEMRIPSKG 300
            IGEQG
AALCGSVFKVEYTDCGQRRVYLRLYSGTLRLRDTVALAGREKLKITEMRIPSKG
Sbjct 241
IGEQGGAALCGSVFKVEYTDCGQRRVYLRLYSGTLRLRDTVALAGREKLKITEMRIPSKG 300

Query 301
EIVRTDTAYPGEIVILPSDSVRLNDVLGDPTRLPRKRWREDPLPMLRTTIAPKTAAQRER 360
            EIVRTDTAYPGEIVILPSDSVRLNDVLGDPTRLPRKRWREDPLPMLRT
+IAPKTAAQRER
Sbjct 301
EIVRTDTAYPGEIVILPSDSVRLNDVLGDPTRLPRKRWREDPLPMLRTSIAPKTAAQRER 360

Query 361
LLDALTQLADTDPLLRCEVDSITHEIILSFLGRVQLEVVSALLSEKYKLETVVKEPSVIY 420
            LLDALTQLADTDPLLRCEVDSITHEIILSFLGRVQLEVVSALLSEKYKLETVVKEP
+VIY
Sbjct 361
LLDALTQLADTDPLLRCEVDSITHEIILSFLGRVQLEVVSALLSEKYKLETVVKEPTVIY 420

Query 421
MERPLKAASHTIHIEVPPNPFWASIGLSVTPLSLGSGVQYESRVSLGYLNQSFQNAVRDG 480
            MERPLKAASHTIHIEVPPNPFWASIGLSVTPL
LGSGVQYESRVSLGYLNQSFQNAVRDG
Sbjct 421
MERPLKAASHTIHIEVPPNPFWASIGLSVTPLPLGSGVQYESRVSLGYLNQSFQNAVRDG 480

Query 481
IRYGLEQGLFGWNVTDCKICFEYGLYYSPVSTPADFRSLAPIVLEQALKESGTQLLEPYL 540

IRYGLEQGLFGWNVTDCKICFEYGLYYSPVSTPADFRSLAPIVLEQALKESGTQLLEPYL
Sbjct 481
IRYGLEQGLFGWNVTDCKICFEYGLYYSPVSTPADFRSLAPIVLEQALKESGTQLLEPYL 540

Query 541
SFILYAPQEYLSRAYHDAPKYCATIETAQVKKDEVVFTGEIPARCIQAYRTDLAFYTNGR 600
            SF LYAP+EYLSRAYHDAPKYCATIET
QVKKDEVVFTGEIPARCIQAYRTDLAFYTNG+
Sbjct 541
SFTLYAPREYLSRAYHDAPKYCATIETVQVKKDEVVFTGEIPARCIQAYRTDLAFYTNGQ 600

Query 601 SVCLTELKGYQAAVGQPVIQPRRPNSRLDKVRHMFQKV 638

SVCLTELKGYQAAVG+PVIQPRRPNSRLDKVRHMF K+
Sbjct 601 SVCLTELKGYQAAVGKPVIQPRRPNSRLDKVRHMFSKI 638

>SCI53441.1 Tetracycline resistance protein tetM from transposon Tn916 [uncultured
Roseburia sp.]
Length=639

Score = 1278 bits (3308), Expect = 0.0, Method: Compositional matrix adjust.
Identities = 621/639 (97%), Positives = 632/639 (99%), Gaps = 0/639 (0%)

Query 1
MKIINIGILAHVDAGKTTLTESLLYASGAISEPGSVEKGTTRTDTMFLERQRGITIQAAV 60
MKIINIGILAHVDAGKTTLTESLLY SGAI+E G+V+KGTTRTDTM LE
+QRGITIQAAV
Sbjct 1
MKIINIGILAHVDAGKTTLTESLLYTSGAIAEQGNVDKGTTRTDTMILEQQRGITIQAAV 60

Query 61
TSFQWHRCKVNIVDTPGHMDFLAEVYRSLAVLDGAILVISAKDGVQAQTRILFHALRKMN 120

TSFQWHRCKVNIVDTPGHMDFLAEVYRSLAVLDGAILVISAKDGVQAQTRILFHALRKMN
Sbjct 61
TSFQWHRCKVNIVDTPGHMDFLAEVYRSLAVLDGAILVISAKDGVQAQTRILFHALRKMN 120

Query 121
IPTVIFINKIDQAGVDLQSVVQSVRDKLSADIIIKQTVSLSPEIVLEENTDIEAWDAVIE 180

IPTVIFINKIDQAGVDLQSVVQSVRDKLSADIIIKQTVSLSPEIVLEENTDIEAWDAVIE
Sbjct 121
IPTVIFINKIDQAGVDLQSVVQSVRDKLSADIIIKQTVSLSPEIVLEENTDIEAWDAVIE 180

Query 181
NNDKLLEKYIAGEPISREKLVREEQRRVQDASLFPVYYGSAKKGLGIQPLMDAVTGLFQP 240

NNDKLLEKYIAGEPISREKLVREEQRRVQDASLFPVYYGSAKKGLGIQPLMDAVTGLFQP
Sbjct 181
NNDKLLEKYIAGEPISREKLVREEQRRVQDASLFPVYYGSAKKGLGIQPLMDAVTGLFQP 240

Query 241
IGEQGSAALCGSVFKVEYTDCGQRRVYLRLYSGTLRLRDTVALAGREKLKITEMRIPSKG 300

IGEQGSAALCGSVFKVEYTDCGQRRVYLRLYSGTLRLRDTVALAGREKLKITEMRIPSKG
Sbjct 241
IGEQGSAALCGSVFKVEYTDCGQRRVYLRLYSGTLRLRDTVALAGREKLKITEMRIPSKG 300

Query 301
EIVRTDTAYPGEIVILPSDSVRLNDVLGDPTRLPRKRWREDPLPMLRTTIAPKTAAQRER 360
EIVRTDTAYPGEIVILPSDSVRL DVLGDPTRLPRKRWREDPLPMLRT
+IAPKTAAQRER
Sbjct 301

EIVRTDTAYPGEIVILPSDSVRLFDVLGDPTRLPRKRWREDPLPMLRTSIAPKTAAQRER 360

Query 361
LLDALTQLADTDPLLRCEVDSITHEIILSFLGRVQLEVVSALLSEKYKLETVVKEPSVIY 420
            LLDALTQLADTDPLLRCEVDSITHEIILSFLGRVQLEVVSALLSEKYKLETVVKEP
+VIY
Sbjct 361
LLDALTQLADTDPLLRCEVDSITHEIILSFLGRVQLEVVSALLSEKYKLETVVKEPTVIY 420

Query 421
MERPLKAASHTIHIEVPPNPFWASIGLSVTPLSLGSGVQYESRVSLGYLNQSFQNAVRDG 480
            MERPLKAASHTIHIEVPPNPFWASIGLSVTPL
LGSGVQYESRVSLGYLNQSFQNAVRDG
Sbjct 421
MERPLKAASHTIHIEVPPNPFWASIGLSVTPLPLGSGVQYESRVSLGYLNQSFQNAVRDG 480

Query 481
IRYGLEQGLFGWNVTDCKICFEYGLYYSPVSTPADFRSLAPIVLEQALKESGTQLLEPYL 540

IRYGLEQGLFGWNVTDCKICFEYGLYYSPVSTPADFRSLAPIVLEQALKESGTQLLEPYL
Sbjct 481
IRYGLEQGLFGWNVTDCKICFEYGLYYSPVSTPADFRSLAPIVLEQALKESGTQLLEPYL 540

Query 541
SFILYAPQEYLSRAYHDAPKYCATIETAQVKKDEVVFTGEIPARCIQAYRTDLAFYTNGR 600
            SF LYAP+EYLSRAYHDAPKYCATIET
QVKKDEVVFTGEIPARCIQAYRTDLAFYTNG+
Sbjct 541
SFTLYAPREYLSRAYHDAPKYCATIETVQVKKDEVVFTGEIPARCIQAYRTDLAFYTNGQ 600

Query 601 SVCLTELKGYQAAVGQPVIQPRRPNSRLDKVRHMFQKVM 639
            SVCLTELKGYQAAVG+PVIQPRRPNSRLDKVR+MFQK+M
Sbjct 601 SVCLTELKGYQAAVGKPVIQPRRPNSRLDKVRYMFQKIM 639

>AMP54005.1 TetM-TetW-TetO-TetS [uncultured bacterium]
Length=639

Score = 1278 bits (3308), Expect = 0.0, Method: Compositional
matrix adjust.
Identities = 621/639 (97%), Positives = 630/639 (99%), Gaps = 0/639
(0%)

Query 1
MKIINIGILAHVDAGKTTLTESLLYASGAISEPGSVEKGTTRTDTMFLERQRGITIQAAV 60
            M IINIGILAHVDAGKTTLTESLLYASGAISEPGSVEKGTTRTDTM
LERQRGITIQAAV
Sbjct 1
MNIINIGILAHVDAGKTTLTESLLYASGAISEPGSVEKGTTRTDTMLLERQRGITIQAAV 60

Query 61
TSFQWHRCKVNIVDTPGHMDFLAEVYRSLAVLDGAILVISAKDGVQAQTRILFHALRKMN 120

TSFQWHRCKVNIVDTPGHMDFLAEVYRSLAVLDGAILVISAKDGVQAQTRILFHALRKMN

Sbjct 61
TSFQWHRCKVNIVDTPGHMDFLAEVYRSLAVLDGAILVISAKDGVQAQTRILFHALRKMN 120

Query 121
IPTVIFINKIDQAGVDLQSVVQSVRDKLSADIIIKQTVSLSPEIVLEENTDIEAWDAVIE 180
IPTVIFINKIDQAGVDLQ V QSVRDKLSADIIIKQTVSLSPEIVLEENT
IEAWDAVIE
Sbjct 121
IPTVIFINKIDQAGVDLQGVYQSVRDKLSADIIIKQTVSLSPEIVLEENTGIEAWDAVIE 180

Query 181
NNDKLLEKYIAGEPISREKLVREEQRRVQDASLFPVYYGSAKKGLGIQPLMDAVTGLFQP 240
NNDKLLEKYIAGEPISRE+L REEQRRVQDASLFPVY
+GSAKKGLGIQPLMDAVTGLFQP
Sbjct 181
NNDKLLEKYIAGEPISREELAREEQRRVQDASLFPVYHGSAKKGLGIQPLMDAVTGLFQP 240

Query 241
IGEQGSAALCGSVFKVEYTDCGQRRVYLRLYSGTLRLRDTVALAGREKLKITEMRIPSKG 300

IGEQGSAALCGSVFKVEYTDCGQRRVYLRLYSGTLRLRDTVALAGREKLKITEMRIPSKG
Sbjct 241
IGEQGSAALCGSVFKVEYTDCGQRRVYLRLYSGTLRLRDTVALAGREKLKITEMRIPSKG 300

Query 301
EIVRTDTAYPGEIVILPSDSVRLNDVLGDPTRLPRKRWREDPLPMLRTTIAPKTAAQRER 360
EIVRTDTAYPGEIVILPSDSVRLNDVLGDPTRLPRKRWREDPLPMLRT
+IAPKTAAQRER
Sbjct 301
EIVRTDTAYPGEIVILPSDSVRLNDVLGDPTRLPRKRWREDPLPMLRTSIAPKTAAQRER 360

Query 361
LLDALTQLADTDPLLRCEVDSITHEIILSFLGRVQLEVVSALLSEKYKLETVVKEPSVIY 420
LLDALTQLADTDPLLRCEVDSITHEIILSFLGRVQLEVVSALLSEKYKLETVVKEP
+VIY
Sbjct 361
LLDALTQLADTDPLLRCEVDSITHEIILSFLGRVQLEVVSALLSEKYKLETVVKEPTVIY 420

Query 421
MERPLKAASHTIHIEVPPNPFWASIGLSVTPLSLGSGVQYESRVSLGYLNQSFQNAVRDG 480
MERPLKAASHTIHIEVPPNPFWASIGLSVTPL
LGSGVQYESRVSLGYLNQSFQNAVRDG
Sbjct 421
MERPLKAASHTIHIEVPPNPFWASIGLSVTPLPLGSGVQYESRVSLGYLNQSFQNAVRDG 480

Query 481
IRYGLEQGLFGWNVTDCKICFEYGLYYSPVSTPADFRSLAPIVLEQALKESGTQLLEPYL 540

IRYGLEQGLFGWNVTDCKICFEYGLYYSPVSTPADFRSLAPIVLEQALKESGTQLLEPYL
Sbjct 481
IRYGLEQGLFGWNVTDCKICFEYGLYYSPVSTPADFRSLAPIVLEQALKESGTQLLEPYL 540

Query 541
SFILYAPQEYLSRAYHDAPKYCATIETAQVKKDEVVFTGEIPARCIQAYRTDLAFYTNGR 600

SF LYAP+EYLSRAYHDAPKYCATIET
QVKKDEVVFTGEIPARCIQAYRTDLAFYTNG+
Sbjct 541
SFTLYAPREYLSRAYHDAPKYCATIETVQVKKDEVVFTGEIPARCIQAYRTDLAFYTNGQ 600

Query 601 SVCLTELKGYQAAVGQPVIQPRRPNSRLDKVRHMFQKVM 639
SVCLTELKGYQAAVG+PVIQPRRPNSRLDKVR+MFQK+M
Sbjct 601 SVCLTELKGYQAAVGKPVIQPRRPNSRLDKVRYMFQKIM 639

>WP_055251078.1 TetM/TetW/TetO/TetS family tetracycline resistance ribosomal
protection protein [Collinsella aerofaciens]
CUO98060.1 Tetracycline resistance protein tetM from transposon Tn916 [Collinsella
aerofaciens]
Length=639

Score = 1278 bits (3308), Expect = 0.0, Method: Compositional matrix adjust.
Identities = 621/639 (97%), Positives = 630/639 (99%), Gaps = 0/639 (0%)

Query 1
MKIINIGILAHVDAGKTTLTESLLYASGAISEPGSVEKGTTRTDTMFLERQRGITIQAAV 60
M IINIGILAHVDAGKTTLTESLLYASGAISEPGSVEKGTTRTDTM
LERQRGITIQAAV
Sbjct 1
MNIINIGILAHVDAGKTTLTESLLYASGAISEPGSVEKGTTRTDTMLLERQRGITIQAAV 60

Query 61
TSFQWHRCKVNIVDTPGHMDFLAEVYRSLAVLDGAILVISAKDGVQAQTRILFHALRKMN 120

TSFQWHRCKVNIVDTPGHMDFLAEVYRSLAVLDGAILVISAKDGVQAQTRILFHALRKM+
Sbjct 61
TSFQWHRCKVNIVDTPGHMDFLAEVYRSLAVLDGAILVISAKDGVQAQTRILFHALRKMD 120

Query 121
IPTVIFINKIDQAGVDLQSVVQSVRDKLSADIIIKQTVSLSPEIVLEENTDIEAWDAVIE 180
IPTVIFINKIDQAGVDLQ V
QSVRDKLSADIIIKQTVSLSPEIVLEENTDIEAWDAVIE
Sbjct 121
IPTVIFINKIDQAGVDLQGVYQSVRDKLSADIIIKQTVSLSPEIVLEENTDIEAWDAVIE 180

Query 181
NNDKLLEKYIAGEPISREKLVREEQRRVQDASLFPVYYGSAKKGLGIQPLMDAVTGLFQP 240
NND+LLEKYIAGEPISRE+L +EEQRRVQDASLFPVY
+GSAKKGLGIQPLMDAVTGLFQP
Sbjct 181
NNDELLEKYIAGEPISREELAQEEQRRVQDASLFPVYHGSAKKGLGIQPLMDAVTGLFQP 240

Query 241
IGEQGSAALCGSVFKVEYTDCGQRRVYLRLYSGTLRLRDTVALAGREKLKITEMRIPSKG 300
IGEQGS ALCGSVFKVEYTDCGQR

```
+YLRLYSGTLRLRDTVALAGREKLKITEMRIPSKG
Sbjct  241
IGEQGSDALCGSVFKVEYTDCGQRLIYLRLYSGTLRLRDTVALAGREKLKITEMRIPSKG  300

Query  301
EIVRTDTAYPGEIVILPSDSVRLNDVLGDPTRLPRKRWREDPLPMLRTTIAPKTAAQRER  360
            EIVRTDTAYPGEIVILPSDSVRLNDVLGDPTRLPRKRWREDPLPMLRT
+IAPKTAAQRER
Sbjct  301
EIVRTDTAYPGEIVILPSDSVRLNDVLGDPTRLPRKRWREDPLPMLRTSIAPKTAAQRER  360

Query  361
LLDALTQLADTDPLLRCEVDSITHEIILSFLGRVQLEVVSALLSEKYKLETVVKEPSVIY  420
            LLDALTQLADTDPLLRCEVDSITHEIILSFLGRVQLEVVSALLSEKYKLETVVKEP
+VIY
Sbjct  361
LLDALTQLADTDPLLRCEVDSITHEIILSFLGRVQLEVVSALLSEKYKLETVVKEPTVIY  420

Query  421
MERPLKAASHTIHIEVPPNPFWASIGLSVTPLSLGSGVQYESRVSLGYLNQSFQNAVRDG  480
            MERPLKAASHTIHIEVPPNPFWASIGLSVTPL
LGSGVQYESRVSLGYLNQSFQNAVRDG
Sbjct  421
MERPLKAASHTIHIEVPPNPFWASIGLSVTPLPLGSGVQYESRVSLGYLNQSFQNAVRDG  480

Query  481
IRYGLEQGLFGWNVTDCKICFEYGLYYSPVSTPADFRSLAPIVLEQALKESGTQLLEPYL  540

IRYGLEQGLFGWNVTDCKICFEYGLYYSPVSTPADFRSLAPIVLEQALKESGTQLLEPYL
Sbjct  481
IRYGLEQGLFGWNVTDCKICFEYGLYYSPVSTPADFRSLAPIVLEQALKESGTQLLEPYL  540

Query  541
SFILYAPQEYLSRAYHDAPKYCATIETAQVKKDEVVFTGEIPARCIQAYRTDLAFYTNGR  600
            SFILYAPQEYLSRAYHDAPKYCATIETAQVKKDEVV
TGEIPARCIQAYRTDLAFYTNGR
Sbjct  541
SFILYAPQEYLSRAYHDAPKYCATIETAQVKKDEVVITGEIPARCIQAYRTDLAFYTNGR  600

Query  601  SVCLTELKGYQAAVGQPVIQPRRPNSRLDKVRHMFQKVM  639
            SVCLTELKGYQAAVGQPVIQPRRPNSRLDKVRHMFQK+M
Sbjct  601  SVCLTELKGYQAAVGQPVIQPRRPNSRLDKVRHMFQKIM  639


>WP_070648983.1 TetM/TetW/TetO/TetS family tetracycline resistance
ribosomal
protection protein [Rothia sp. HMSC068F09]
 OFR65982.1 tetracycline resistance ribosomal protection protein
[Rothia
sp. HMSC068F09]
Length=640

 Score = 1278 bits (3307),  Expect = 0.0, Method: Compositional
matrix adjust.
```

Identities = 621/638 (97%), Positives = 630/638 (99%), Gaps = 0/638 (0%)

Query 1
MKIININGILAHVDAGKTTLTESLLYASGAISEPGSVEKGTTRTDTMFLERQRGITIQAAV 60

MKIINIGILAHVDAGKTTLTESLLYASGAISEPGSVEKGTTRTDTMFLERQRGITIQAAV
Sbjct 1
MKIINIGILAHVDAGKTTLTESLLYASGAISEPGSVEKGTTRTDTMFLERQRGITIQAAV 60

Query 61
TSFQWHRCKVNIVDTPGHMDFLAEVYRSLAVLDGAILVISAKDGVQAQTRILFHALRKMN 120
TSFQWHRCKVNIVDTPGHMDFLAEVYRSLAVLDGAILVISAKDGVQAQTRILF
+ALRKMN
Sbjct 61
TSFQWHRCKVNIVDTPGHMDFLAEVYRSLAVLDGAILVISAKDGVQAQTRILFNALRKMN 120

Query 121
IPTVIFINKIDQAGVDLQSVVQSVRDKLSADIIIKQTVSLSPEIVLEENTDIEAWDAVIE 180

IPTVIFINKIDQAGVDLQSVVQSVRDKLSADIIIKQTVSLSPEIVLEENTDIEAWDAVIE
Sbjct 121
IPTVIFINKIDQAGVDLQSVVQSVRDKLSADIIIKQTVSLSPEIVLEENTDIEAWDAVIE 180

Query 181
NNDKLLEKYIAGEPISREKLVREEQRRVQDASLFPVYYGSAKKGLGIQPLMDAVTGLFQP 240
NND+LLEKYIAGEPISREKL REEQ+RVQDASLFPVY+GSAK
GLGIQPLMDAVTGLFQP
Sbjct 181
NNDELLEKYIAGEPISREKLAREEQQRVQDASLFPVYHGSAKNGLGIQPLMDAVTGLFQP 240

Query 241
IGEQGSAALCGSVFKVEYTDCGQRRVYLRLYSGTLRLRDTVALAGREKLKITEMRIPSKG 300
IGEQG
AALCGSVFKVEYTDCGQRRVYLRLYSGTLRLRDTVALAGREKLKITEMRIPSKG
Sbjct 241
IGEQGGAALCGSVFKVEYTDCGQRRVYLRLYSGTLRLRDTVALAGREKLKITEMRIPSKG 300

Query 301
EIVRTDTAYPGEIVILPSDSVRLNDVLGDPTRLPRKRWREDPLPMLRTTIAPKTAAQRER 360
EIVRTDTAYPGEIVILPSDSVRLNDVLGDPTRLPRKRWREDPLPMLRT
+IAPKTAAQRER
Sbjct 301
EIVRTDTAYPGEIVILPSDSVRLNDVLGDPTRLPRKRWREDPLPMLRTSIAPKTAAQRER 360

Query 361
LLDALTQLADTDPLLRCEVDSITHEIILSFLGRVQLEVVSALLSEKYKLETVVKEPSVIY 420
LLDALTQLADTDPLLRCEVDSITHEIILSFLGRVQLEVVSALLSEKYKLETVVKEP
VIY
Sbjct 361
LLDALTQLADTDPLLRCEVDSITHEIILSFLGRVQLEVVSALLSEKYKLETVVKEPPVIY 420

Query 421
MERPLKAASHTIHIEVPPNPFWASIGLSVTPLSLGSGVQYESRVSLGYLNQSFQNAVRDG 480

```
            MERPLKAASHTIHIEVPPNPFWASIGLSVTPL
LGSGVQYESRVSLGYLNQSFQNAVRDG
Sbjct  421  
MERPLKAASHTIHIEVPPNPFWASIGLSVTPLPLGSGVQYESRVSLGYLNQSFQNAVRDG  480

Query  481  
IRYGLEQGLFGWNVTDCKICFEYGLYYSPVSTPADFRSLAPIVLEQALKESGTQLLEPYL  540
            
IRYGLEQGLFGWNVTDCKICFEYGLYYSPVSTPADFRSLAPIVLEQALKESGTQLLEPYL
Sbjct  481  
IRYGLEQGLFGWNVTDCKICFEYGLYYSPVSTPADFRSLAPIVLEQALKESGTQLLEPYL  540

Query  541  
SFILYAPQEYLSRAYHDAPKYCATIETAQVKKDEVVFTGEIPARCIQAYRTDLAFYTNGR  600
            SF LYAP+EYLSRAYHDAPKYCATIET 
QVKKDEVVFTGEIPARCIQAYRTDLAFYTNG+
Sbjct  541  
SFTLYAPREYLSRAYHDAPKYCATIETVQVKKDEVVFTGEIPARCIQAYRTDLAFYTNGQ  600

Query  601  SVCLTELKGYQAAVGQPVIQPRRPNSRLDKVRHMFQKV  638
            SVCLTELKGYQAAVG+PVIQPRRPNSRLDKVRHMF K+
Sbjct  601  SVCLTELKGYQAAVGKPVIQPRRPNSRLDKVRHMFSKI  638


>WP_070593944.1 TetM/TetW/TetO/TetS family tetracycline resistance 
ribosomal 
protection protein [Rothia sp. HMSC075F09]
 OFL75663.1 tetracycline resistance ribosomal protection protein 
[Rothia 
sp. HMSC075F09]
Length=640

 Score = 1277 bits (3305),  Expect = 0.0, Method: Compositional 
matrix adjust.
 Identities = 619/638 (97%), Positives = 629/638 (99%), Gaps = 0/638 
(0%)

Query  1    
MKIINIGILAHVDAGKTTLTESLLYASGAISEPGSVEKGTTRTDTMFLERQRGITIQAAV  60
            M IINIGILAHVDAGKTTLTESLLYASGAISEPGSVEKGTTRTDT
+FLERQRGITIQAAV
Sbjct  1    
MNIINIGILAHVDAGKTTLTESLLYASGAISEPGSVEKGTTRTDTLFLERQRGITIQAAV  60

Query  61   
TSFQWHRCKVNIVDTPGHMDFLAEVYRSLAVLDGAILVISAKDGVQAQTRILFHALRKMN  120
            
TSFQWHRCKVNIVDTPGHMDFLAEVYRSLAVLDGAILVISAKDGVQAQTRILFHALRKMN
Sbjct  61   
TSFQWHRCKVNIVDTPGHMDFLAEVYRSLAVLDGAILVISAKDGVQAQTRILFHALRKMN  120

Query  121  
IPTVIFINKIDQAGVDLQSVVQSVRDKLSADIIIKQTVSLSPEIVLEENTDIEAWDAVIE  180
```

IPTVIFINKIDQAGVDLQSVVQSVRDKLSADIIIKQTVSLSPEIVLEENTDIEAWDAVIE
Sbjct 121
IPTVIFINKIDQAGVDLQSVVQSVRDKLSADIIIKQTVSLSPEIVLEENTDIEAWDAVIE 180

Query 181
NNDKLLEKYIAGEPISREKLVREEQRRVQDASLFPVYYGSAKKGLGIQPLMDAVTGLFQP 240
NND LLEKYIAGEPIS+EKL REEQRRVQ+ SLFPVY
+GSAKKGLGIQPLMDAVTGLFQP
Sbjct 181
NNDALLEKYIAGEPISQEKLAREEQRRVQEVSLFPVYHGSAKKGLGIQPLMDAVTGLFQP 240

Query 241
IGEQGSAALCGSVFKVEYTDCGQRRVYLRLYSGTLRLRDTVALAGREKLKITEMRIPSKG 300
IGEQGSA
LCGSVFKVEYTDCGQRRVYLRLYSGTLRLRDTVALAGREKLKITEMRIPSKG
Sbjct 241
IGEQGSATLCGSVFKVEYTDCGQRRVYLRLYSGTLRLRDTVALAGREKLKITEMRIPSKG 300

Query 301
EIVRTDTAYPGEIVILPSDSVRLNDVLGDPTRLPRKRWREDPLPMLRTTIAPKTAAQRER 360
EIVRTDTAYPGEIVILPSDSVRLNDVLGDPTRLPRKRWREDPLPMLRT
+IAPKTAAQRER
Sbjct 301
EIVRTDTAYPGEIVILPSDSVRLNDVLGDPTRLPRKRWREDPLPMLRTSIAPKTAAQRER 360

Query 361
LLDALTQLADTDPLLRCEVDSITHEIILSFLGRVQLEVVSALLSEKYKLETVVKEPSVIY 420
LLDALTQLADTDPLLRCEVDSITHEIILSFLGRVQLEVVSALLSEKYKLETVVKEP
+VIY
Sbjct 361
LLDALTQLADTDPLLRCEVDSITHEIILSFLGRVQLEVVSALLSEKYKLETVVKEPTVIY 420

Query 421
MERPLKAASHTIHIEVPPNPFWASIGLSVTPLSLGSGVQYESRVSLGYLNQSFQNAVRDG 480
MERPLKAASHTIHIEVPPNPFWASIGLSVTPL
LGSGVQYESRVSLGYLNQSFQNAVRDG
Sbjct 421
MERPLKAASHTIHIEVPPNPFWASIGLSVTPLPLGSGVQYESRVSLGYLNQSFQNAVRDG 480

Query 481
IRYGLEQGLFGWNVTDCKICFEYGLYYSPVSTPADFRSLAPIVLEQALKESGTQLLEPYL 540

IRYGLEQGLFGWNVTDCKICFEYGLYYSPVSTPADFRSLAPIVLEQALKESGTQLLEPYL
Sbjct 481
IRYGLEQGLFGWNVTDCKICFEYGLYYSPVSTPADFRSLAPIVLEQALKESGTQLLEPYL 540

Query 541
SFILYAPQEYLSRAYHDAPKYCATIETAQVKKDEVVFTGEIPARCIQAYRTDLAFYTNGR 600
SF LYAP+EYLSRAYHDAPKYCATIET
QVKKDEVVFTGEIPARCIQAYRTDLAFYTNG+
Sbjct 541
SFTLYAPREYLSRAYHDAPKYCATIETVQVKKDEVVFTGEIPARCIQAYRTDLAFYTNGQ 600

Query 601 SVCLTELKGYQAAVGQPVIQPRRPNSRLDKVRHMFQKV 638

SVCLTELKGYQAAVG+PVIQPRRPNSRLDKVRHMF K+
Sbjct 601 SVCLTELKGYQAAVGKPVIQPRRPNSRLDKVRHMFSKI 638

>WP_049353247.1 TetM/TetW/TetO/TetS family tetracycline resistance ribosomal
protection protein [Rothia mucilaginosa]
Length=640

Score = 1277 bits (3305), Expect = 0.0, Method: Compositional matrix adjust.
Identities = 621/638 (97%), Positives = 628/638 (98%), Gaps = 0/638 (0%)

Query 1
MKIINIGILAHVDAGKTTLTESLLYASGAISEPGSVEKGTTRTDTMFLERQRGITIQAAV 60
M IINIGILAHVDAGKTTLTESLLYASGAISEPGSVEKGTTRTDT
+FLERQRGITIQAAV
Sbjct 1
MNIINIGILAHVDAGKTTLTESLLYASGAISEPGSVEKGTTRTDTLFLERQRGITIQAAV 60

Query 61
TSFQWHRCKVNIVDTPGHMDFLAEVYRSLAVLDGAILVISAKDGVQAQTRILFHALRKMN 120

TSFQWHRCKVNIVDTPGHMDFLAEVYRSLAVLDGAILVISAKDGVQAQTRILFHALRKMN
Sbjct 61
TSFQWHRCKVNIVDTPGHMDFLAEVYRSLAVLDGAILVISAKDGVQAQTRILFHALRKMN 120

Query 121
IPTVIFINKIDQAGVDLQSVVQSVRDKLSADIIIKQTVSLSPEIVLEENTDIEAWDAVIE 180

IPTVIFINKIDQAGVDLQSVVQSVRDKLSADIIIKQTVSLSPEIVLEENTDIEAWDAVIE
Sbjct 121
IPTVIFINKIDQAGVDLQSVVQSVRDKLSADIIIKQTVSLSPEIVLEENTDIEAWDAVIE 180

Query 181
NNDKLLEKYIAGEPISREKLVREEQRRVQDASLFPVYYGSAKKGLGIQPLMDAVTGLFQP 240
NND LLEKYIAGEPIS+EKL REEQRRVQDASLFPVY
+GSAKKGLGIQPLMDAVTGLFQP
Sbjct 181
NNDALLEKYIAGEPISQEKLAREEQRRVQDASLFPVYHGSAKKGLGIQPLMDAVTGLFQP 240

Query 241
IGEQGSAALCGSVFKVEYTDCGQRRVYLRLYSGTLRLRDTVALAGREKLKITEMRIPSKG 300
IGEQGSA
LCGSVFKVEYTDCGQRRVYLRLYSGTLRLRDTVALAGREKLKITEMRIPSKG
Sbjct 241
IGEQGSATLCGSVFKVEYTDCGQRRVYLRLYSGTLRLRDTVALAGREKLKITEMRIPSKG 300

Query 301
EIVRTDTAYPGEIVILPSDSVRLNDVLGDPTRLPRKRWREDPLPMLRTTIAPKTAAQRER 360
EIVRTDTAY GEIVILPSDSVRLNDVLGD
TRLPRKRWREDPLPMLRTTIAPKTAAQRER
Sbjct 301

EIVRTDTAYQGEIVILPSDSVRLNDVLGDQTRLPRKRWREDPLPMLRTTIAPKTAAQRER 360

Query 361
LLDALTQLADTDPLLRCEVDSITHEIILSFLGRVQLEVVSALLSEKYKLETVVKEPSVIY 420

LLDALTQLADTDPLLRCEVDSITHEIILSFLGRVQLEVVSALLSEKYKLETVVKEPSVIY
Sbjct 361
LLDALTQLADTDPLLRCEVDSITHEIILSFLGRVQLEVVSALLSEKYKLETVVKEPSVIY 420

Query 421
MERPLKAASHTIHIEVPPNPFWASIGLSVTPLSLGSGVQYESRVSLGYLNQSFQNAVRDG 480
MERPLKAASHTIHIEVPPNPFWASIGLSVTPL
LGSGVQYESRVSLGYLNQSFQNAVRDG
Sbjct 421
MERPLKAASHTIHIEVPPNPFWASIGLSVTPLPLGSGVQYESRVSLGYLNQSFQNAVRDG 480

Query 481
IRYGLEQGLFGWNVTDCKICFEYGLYYSPVSTPADFRSLAPIVLEQALKESGTQLLEPYL 540

IRYGLEQGLFGWNVTDCKICFEYGLYYSPVSTPADFRSLAPIVLEQALKESGTQLLEPYL
Sbjct 481
IRYGLEQGLFGWNVTDCKICFEYGLYYSPVSTPADFRSLAPIVLEQALKESGTQLLEPYL 540

Query 541
SFILYAPQEYLSRAYHDAPKYCATIETAQVKKDEVVFTGEIPARCIQAYRTDLAFYTNGR 600
SF LYAP+EYLSRAYHDAPKYCATIET
QVKKDEVVFTGEIPARCIQAYRTDLAFYTNG+
Sbjct 541
SFTLYAPREYLSRAYHDAPKYCATIETVQVKKDEVVFTGEIPARCIQAYRTDLAFYTNGQ 600

Query 601 SVCLTELKGYQAAVGQPVIQPRRPNSRLDKVRHMFQKV 638
SVCLTELKGYQAAVG+PVIQPRRPNSRLDKVRHMF K+
Sbjct 601 SVCLTELKGYQAAVGKPVIQPRRPNSRLDKVRHMFSKI 638

>WP_070643840.1 TetM/TetW/TetO/TetS family tetracycline resistance
ribosomal
protection protein [Rothia sp. HMSC069C10]
OFJ78480.1 tetracycline resistance ribosomal protection protein
[Rothia
sp. HMSC069C10]
Length=640

Score = 1276 bits (3302), Expect = 0.0, Method: Compositional
matrix adjust.
Identities = 619/638 (97%), Positives = 630/638 (99%), Gaps = 0/638
(0%)

Query 1
MKIINIGILAHVDAGKTTLTESLLYASGAISEPGSVEKGTTRTDTMFLERQRGITIQAAV 60
M IINIGILAHVDAGKTTLTESLLYASGAISEPGSVEKGTTRTDT
+FLERQRGITIQAAV
Sbjct 1
MNIINIGILAHVDAGKTTLTESLLYASGAISEPGSVEKGTTRTDTLFLERQRGITIQAAV 60

Query 61
TSFQWHRCKVNIVDTPGHMDFLAEVYRSLAVLDGAILVISAKDGVQAQTRILFHALRKMN 120
TSFQWHRCKVNIVDTPGHMDFLAEVYRSLAVLDGAILVISAKDGVQAQTRILF
+ALRKMN
Sbjct 61
TSFQWHRCKVNIVDTPGHMDFLAEVYRSLAVLDGAILVISAKDGVQAQTRILFNALRKMN 120

Query 121
IPTVIFINKIDQAGVDLQSVVQSVRDKLSADIIIKQTVSLSPEIVLEENTDIEAWDAVIE 180

IPTVIFINKIDQAGVDLQSVVQSVRDKLSADIIIKQTVSLSPEIVLEENTDIEAWDAVIE
Sbjct 121
IPTVIFINKIDQAGVDLQSVVQSVRDKLSADIIIKQTVSLSPEIVLEENTDIEAWDAVIE 180

Query 181
NNDKLLEKYIAGEPISREKLVREEQRRVQDASLFPVYYGSAKKGLGIQPLMDAVTGLFQP 240
NND+LLEKYIAGEPISREKL REEQ+RVQDASLFPVY+GSAK
GLGIQPLMDAVTGLFQP
Sbjct 181
NNDELLEKYIAGEPISREKLAREEQQRVQDASLFPVYHGSAKNGLGIQPLMDAVTGLFQP 240

Query 241
IGEQGSAALCGSVFKVEYTDCGQRRVYLRLYSGTLRLRDTVALAGREKLKITEMRIPSKG 300
IGEQG
AALCGSVFKVEYTDCGQRRVYLRLYSGTLRLRDTVALAGREKLKITEMRIPSKG
Sbjct 241
IGEQGGAALCGSVFKVEYTDCGQRRVYLRLYSGTLRLRDTVALAGREKLKITEMRIPSKG 300

Query 301
EIVRTDTAYPGEIVILPSDSVRLNDVLGDPTRLPRKRWREDPLPMLRTTIAPKTAAQRER 360
EIVRTDTAYPGEIVILPSDSVRLNDVLGDPTRLPRKRWREDPLPMLRT
+IAPKTAAQRER
Sbjct 301
EIVRTDTAYPGEIVILPSDSVRLNDVLGDPTRLPRKRWREDPLPMLRTSIAPKTAAQRER 360

Query 361
LLDALTQLADTDPLLRCEVDSITHEIILSFLGRVQLEVVSALLSEKYKLETVVKEPSVIY 420
LLDALTQLADTDPLLRCEVDSITHEIILSFLGRVQLEVVSALLSEKYKLETVVKEP
+VIY
Sbjct 361
LLDALTQLADTDPLLRCEVDSITHEIILSFLGRVQLEVVSALLSEKYKLETVVKEPTVIY 420

Query 421
MERPLKAASHTIHIEVPPNPFWASIGLSVTPLSLGSGVQYESRVSLGYLNQSFQNAVRDG 480
MERPLKAASHTIHIEVPPNPFWASIGLSVTPL
LGSGVQYESRVSLGYLNQSFQNAVRDG
Sbjct 421
MERPLKAASHTIHIEVPPNPFWASIGLSVTPLPLGSGVQYESRVSLGYLNQSFQNAVRDG 480

Query 481
IRYGLEQGLFGWNVTDCKICFEYGLYYSPVSTPADFRSLAPIVLEQALKESGTQLLEPYL 540

IRYGLEQGLFGWNVTDCKICFEYGLYYSPVSTPADFRSLAPIVLEQALKESGTQLLEPYL

Sbjct 481
IRYGLEQGLFGWNVTDCKICFEYGLYYSPVSTPADFRSLAPIVLEQALKESGTQLLEPYL 540

Query 541
SFILYAPQEYLSRAYHDAPKYCATIETAQVKKDEVVFTGEIPARCIQAYRTDLAFYTNGR 600
            SF LYAP+EYLSRAYHDAPKYCATIET
QVKKDEVVFTGEIPARCIQAYRTDLAFYTNG+
Sbjct 541
SFTLYAPREYLSRAYHDAPKYCATIETVQVKKDEVVFTGEIPARCIQAYRTDLAFYTNGQ 600

Query 601 SVCLTELKGYQAAVGQPVIQPRRPNSRLDKVRHMFQKV 638
            SVCLTELKGYQAAVG+PVIQPRRPNSRLDKVRHMF K+
Sbjct 601 SVCLTELKGYQAAVGKPVIQPRRPNSRLDKVRHMFSKI 638

>WP_019541681.1 TetM/TetW/TetO/TetS family tetracycline resistance
ribosomal
protection protein [Selenomonas bovis]
Length=639

 Score = 1276 bits (3301), Expect = 0.0, Method: Compositional
matrix adjust.
 Identities = 619/638 (97%), Positives = 628/638 (98%), Gaps = 0/638
(0%)

Query 1
MKIINIGILAHVDAGKTTLTESLLYASGAISEPGSVEKGTTRTDTMFLERQRGITIQAAV 60
            M IINIGILAHVDAGKTTLTESLLYASGAISEPGSVEKGTTRTDT
+FLERQRGITIQAAV
Sbjct 1
MNIINIGILAHVDAGKTTLTESLLYASGAISEPGSVEKGTTRTDTLFLERQRGITIQAAV 60

Query 61
TSFQWHRCKVNIVDTPGHMDFLAEVYRSLAVLDGAILVISAKDGVQAQTRILFHALRKMN 120

TSFQWHRCKVNIVDTPGHMDFLAEVYRSLAVLDGAILVISAKDGVQAQTRILFHALRKMN
Sbjct 61
TSFQWHRCKVNIVDTPGHMDFLAEVYRSLAVLDGAILVISAKDGVQAQTRILFHALRKMN 120

Query 121
IPTVIFINKIDQAGVDLQSVVQSVRDKLSADIIIKQTVSLSPEIVLEENTDIEAWDAVIE 180

IPTVIFINKIDQAGVDLQSVVQSVRDKLSADIIIKQTVSLSPEIVLEENTDIEAWDAVIE
Sbjct 121
IPTVIFINKIDQAGVDLQSVVQSVRDKLSADIIIKQTVSLSPEIVLEENTDIEAWDAVIE 180

Query 181
NNDKLLEKYIAGEPISREKLVREEQRRVQDASLFPVYYGSAKKGLGIQPLMDAVTGLFQP 240
            NND LLEKYIAGEPIS+EKL REEQRRVQ+ASLFPVY
+GSAKKGLGIQPLMDAVTGLFQP
Sbjct 181
NNDALLEKYIAGEPISQEKLAREEQRRVQEASLFPVYHGSAKKGLGIQPLMDAVTGLFQP 240

Query 241

```
IGEQGSAALCGSVFKVEYTDCGQRRVYLRLYSGTLRLRDTVALAGREKLKITEMRIPSKG  300
            IGEQGSA
LCGSVFKVEYTDCGQRRVYLRLYSGTLRLRDTVALAGREKLKITEMRIPSKG
Sbjct  241
IGEQGSATLCGSVFKVEYTDCGQRRVYLRLYSGTLRLRDTVALAGREKLKITEMRIPSKG  300

Query  301
EIVRTDTAYPGEIVILPSDSVRLNDVLGDPTRLPRKRWREDPLPMLRTTIAPKTAAQRER  360
            EIVRTDTAYPGEIVILPSDSVRLNDVLGD TRLPRKRWRE
PLPMLRTTIAPKTAAQRER
Sbjct  301
EIVRTDTAYPGEIVILPSDSVRLNDVLGDQTRLPRKRWREAPLPMLRTTIAPKTAAQRER  360

Query  361
LLDALTQLADTDPLLRCEVDSITHEIILSFLGRVQLEVVSALLSEKYKLETVVKEPSVIY  420
            LLDALTQLADTDPLLRCEVDSITHEIILSFLGRVQLEVVSALLSEKYK+ETVVKEP
+VIY
Sbjct  361
LLDALTQLADTDPLLRCEVDSITHEIILSFLGRVQLEVVSALLSEKYKIETVVKEPTVIY  420

Query  421
MERPLKAASHTIHIEVPPNPFWASIGLSVTPLSLGSGVQYESRVSLGYLNQSFQNAVRDG  480
            MERPLKAASHTIHIEVPPNPFWASIGLSVTPL
LGSGVQYESRVSLGYLNQSFQNAVRDG
Sbjct  421
MERPLKAASHTIHIEVPPNPFWASIGLSVTPLPLGSGVQYESRVSLGYLNQSFQNAVRDG  480

Query  481
IRYGLEQGLFGWNVTDCKICFEYGLYYSPVSTPADFRSLAPIVLEQALKESGTQLLEPYL  540

IRYGLEQGLFGWNVTDCKICFEYGLYYSPVSTPADFRSLAPIVLEQALKESGTQLLEPYL
Sbjct  481
IRYGLEQGLFGWNVTDCKICFEYGLYYSPVSTPADFRSLAPIVLEQALKESGTQLLEPYL  540

Query  541
SFILYAPQEYLSRAYHDAPKYCATIETAQVKKDEVVFTGEIPARCIQAYRTDLAFYTNGR  600
            SF
LYAPQEYLSRAYHDAPKYCATIETAQVKKDEVVFTGEIPARCIQAYRTDLAFYTNG+
Sbjct  541
SFTLYAPQEYLSRAYHDAPKYCATIETAQVKKDEVVFTGEIPARCIQAYRTDLAFYTNGQ  600

Query  601  SVCLTELKGYQAAVGQPVIQPRRPNSRLDKVRHMFQKV  638
            SVCLTELKGYQA VG+PVIQPRRPNSRLDKVRHMF K+
Sbjct  601  SVCLTELKGYQATVGEPVIQPRRPNSRLDKVRHMFSKI  638


>AMP54009.1 TetM-TetW-TetO-TetS [uncultured bacterium]
Length=639

 Score = 1275 bits (3300),  Expect = 0.0, Method: Compositional
matrix adjust.
 Identities = 619/639 (97%), Positives = 628/639 (98%), Gaps = 0/639
(0%)
```

Query 1
MKIINIGILAHVDAGKTTLTESLLYASGAISEPGSVEKGTTRTDTMFLERQRGITIQAAV 60
            M IINIGILAHVDAGKTTLTESLLYASG
ISEPGSVEKGTTRTDTMFLERQRGITIQ AV
Sbjct 1
MNIINIGILAHVDAGKTTLTESLLYASGTISEPGSVEKGTTRTDTMFLERQRGITIQTAV 60

Query 61
TSFQWHRCKVNIVDTPGHMDFLAEVYRSLAVLDGAILVISAKDGVQAQTRILFHALRKMN 120
            TSFQWH
CKVNIVDTPGHMDFLAEVYRSLAVLDGAILVISAKDGVQAQTRILFHALRKMN
Sbjct 61
TSFQWHSCKVNIVDTPGHMDFLAEVYRSLAVLDGAILVISAKDGVQAQTRILFHALRKMN 120

Query 121
IPTVIFINKIDQAGVDLQSVVQSVRDKLSADIIIKQTVSLSPEIVLEENTDIEAWDAVIE 180
            IPTVIFINKIDQAGVDLQ V QSVRDKLSADIIIKQTVSLSPEIVLEENT
IEAWDAVIE
Sbjct 121
IPTVIFINKIDQAGVDLQGVYQSVRDKLSADIIIKQTVSLSPEIVLEENTGIEAWDAVIE 180

Query 181
NNDKLLEKYIAGEPISREKLVREEQRRVQDASLFPVYYGSAKKGLGIQPLMDAVTGLFQP 240
            NNDKLLEKYIAGEPISRE+L REEQRRVQDASLFPVY
+GSAKKGLGIQPLMDAVTGLFQP
Sbjct 181
NNDKLLEKYIAGEPISREELAREEQRRVQDASLFPVYHGSAKKGLGIQPLMDAVTGLFQP 240

Query 241
IGEQGSAALCGSVFKVEYTDCGQRRVYLRLYSGTLRLRDTVALAGREKLKITEMRIPSKG 300

IGEQGSAALCGSVFKVEYTDCGQRRVYLRLYSGTLRLRDTVALAGREKLKITEMRIPSKG
Sbjct 241
IGEQGSAALCGSVFKVEYTDCGQRRVYLRLYSGTLRLRDTVALAGREKLKITEMRIPSKG 300

Query 301
EIVRTDTAYPGEIVILPSDSVRLNDVLGDPTRLPRKRWREDPLPMLRTTIAPKTAAQRER 360
            EIVRTDTAYPGEIVILPSDSVRLNDVLGDPTRLPRKRWREDPLPMLRT
+IAPKTAAQRER
Sbjct 301
EIVRTDTAYPGEIVILPSDSVRLNDVLGDPTRLPRKRWREDPLPMLRTSIAPKTAAQRER 360

Query 361
LLDALTQLADTDPLLRCEVDSITHEIILSFLGRVQLEVVSALLSEKYKLETVVKEPSVIY 420
            LLDALTQLADTDPLLRCEVDSITHEIILSFLGRVQLEVVSALLSEKYKLETVVKEP
+VIY
Sbjct 361
LLDALTQLADTDPLLRCEVDSITHEIILSFLGRVQLEVVSALLSEKYKLETVVKEPTVIY 420

Query 421
MERPLKAASHTIHIEVPPNPFWASIGLSVTPLSLGSGVQYESRVSLGYLNQSFQNAVRDG 480
            MERPLKAASHTIHIEVPPNPFWASIGLSVTPL
LGSGVQYESRVSLGYLNQSFQNAVRDG
Sbjct 421

```
MERPLKAASHTIHIEVPPNPFWASIGLSVTPLPLGSGVQYESRVSLGYLNQSFQNAVRDG  480

Query  481
IRYGLEQGLFGWNVTDCKICFEYGLYYSPVSTPADFRSLAPIVLEQALKESGTQLLEPYL  540

IRYGLEQGLFGWNVTDCKICFEYGLYYSPVSTPADFRSLAPIVLEQALKESGTQLLEPYL
Sbjct  481
IRYGLEQGLFGWNVTDCKICFEYGLYYSPVSTPADFRSLAPIVLEQALKESGTQLLEPYL  540

Query  541
SFILYAPQEYLSRAYHDAPKYCATIETAQVKKDEVVFTGEIPARCIQAYRTDLAFYTNGR  600
            SF LYAP+EYLSRAYHDAPKYCATIET
QVKKDEVVFTGEIPARCIQAYRTDLAFYTNG+
Sbjct  541
SFTLYAPREYLSRAYHDAPKYCATIETVQVKKDEVVFTGEIPARCIQAYRTDLAFYTNGQ  600

Query  601  SVCLTELKGYQAAVGQPVIQPRRPNSRLDKVRHMFQKVM  639
            SVCLTELKGYQAAVG+PVIQPRRPNSRLDKVR+MFQK+M
Sbjct  601  SVCLTELKGYQAAVGKPVIQPRRPNSRLDKVRYMFQKIM  639


>WP_070824660.1 MULTISPECIES: TetM/TetW/TetO/TetS family
tetracycline resistance
ribosomal protection protein [Rothia]
 OHP74004.1 tetracycline resistance ribosomal protection protein
[Rothia
sp. HMSC062F03]
 OHQ19053.1 tetracycline resistance ribosomal protection protein
[Rothia
sp. HMSC065C03]
Length=640

 Score = 1274 bits (3297),  Expect = 0.0, Method: Compositional
matrix adjust.
 Identities = 618/638 (97%), Positives = 628/638 (98%), Gaps = 0/638
(0%)

Query  1
MKIINIGILAHVDAGKTTLTESLLYASGAISEPGSVEKGTTRTDTMFLERQRGITIQAAV  60
            M IINIGILAHVDAGKTTLTESLLYASGAISEPGSVEKGTTRTDT
+FLERQRGITIQAAV
Sbjct  1
MNIINIGILAHVDAGKTTLTESLLYASGAISEPGSVEKGTTRTDTLFLERQRGITIQAAV  60

Query  61
TSFQWHRCKVNIVDTPGHMDFLAEVYRSLAVLDGAILVISAKDGVQAQTRILFHALRKMN  120

TSFQWHRCKVNIVDTPGHMDFLAEVYRSLAVLDGAILVISAKDGVQAQTRILFHALRKMN
Sbjct  61
TSFQWHRCKVNIVDTPGHMDFLAEVYRSLAVLDGAILVISAKDGVQAQTRILFHALRKMN  120

Query  121
IPTVIFINKIDQAGVDLQSVVQSVRDKLSADIIIKQTVSLSPEIVLEENTDIEAWDAVIE  180
```

IPTVIFINKIDQAGVDLQSVVQSVRDKLSADIIIKQTVSLSPEIVLEENTDIEAWDAVIE
Sbjct 121
IPTVIFINKIDQAGVDLQSVVQSVRDKLSADIIIKQTVSLSPEIVLEENTDIEAWDAVIE 180

Query 181
NNDKLLEKYIAGEPISREKLVREEQRRVQDASLFPVYYGSAKKGLGIQPLMDAVTGLFQP 240
NND LLEKYIAGEPIS+EKL REEQRRVQ+ SLFPVY
+GSAKKGLGIQPLMDAVTGLFQP
Sbjct 181
NNDALLEKYIAGEPISQEKLAREEQRRVQEVSLFPVYHGSAKKGLGIQPLMDAVTGLFQP 240

Query 241
IGEQGSAALCGSVFKVEYTDCGQRRVYLRLYSGTLRLRDTVALAGREKLKITEMRIPSKG 300
IGEQGSA
LCGSVFKVEYTDCGQRRVYLRLYSGTLRLRDTVALAGREKLKITEMRIPSKG
Sbjct 241
IGEQGSATLCGSVFKVEYTDCGQRRVYLRLYSGTLRLRDTVALAGREKLKITEMRIPSKG 300

Query 301
EIVRTDTAYPGEIVILPSDSVRLNDVLGDPTRLPRKRWREDPLPMLRTTIAPKTAAQRER 360
EIVRTDTAY GEIVILPSDSVRLNDVLGDPTRLPRKRWREDPLPMLRT
+IAPKTAAQRER
Sbjct 301
EIVRTDTAYQGEIVILPSDSVRLNDVLGDPTRLPRKRWREDPLPMLRTSIAPKTAAQRER 360

Query 361
LLDALTQLADTDPLLRCEVDSITHEIILSFLGRVQLEVVSALLSEKYKLETVVKEPSVIY 420
LLDALTQLADTDPLLRCEVDSITHEIILSFLGRVQLEVVSALLSEKYKLETVVKEP
+VIY
Sbjct 361
LLDALTQLADTDPLLRCEVDSITHEIILSFLGRVQLEVVSALLSEKYKLETVVKEPTVIY 420

Query 421
MERPLKAASHTIHIEVPPNPFWASIGLSVTPLSLGSGVQYESRVSLGYLNQSFQNAVRDG 480
MERPLKAASHTIHIEVPPNPFWASIGLSVTPL
LGSGVQYESRVSLGYLNQSFQNAVRDG
Sbjct 421
MERPLKAASHTIHIEVPPNPFWASIGLSVTPLPLGSGVQYESRVSLGYLNQSFQNAVRDG 480

Query 481
IRYGLEQGLFGWNVTDCKICFEYGLYYSPVSTPADFRSLAPIVLEQALKESGTQLLEPYL 540

IRYGLEQGLFGWNVTDCKICFEYGLYYSPVSTPADFRSLAPIVLEQALKESGTQLLEPYL
Sbjct 481
IRYGLEQGLFGWNVTDCKICFEYGLYYSPVSTPADFRSLAPIVLEQALKESGTQLLEPYL 540

Query 541
SFILYAPQEYLSRAYHDAPKYCATIETAQVKKDEVVFTGEIPARCIQAYRTDLAFYTNGR 600
SF LYAP+EYLSRAYHDAPKYCATIET
QVKKDEVVFTGEIPARCIQAYRTDLAFYTNG+
Sbjct 541
SFTLYAPREYLSRAYHDAPKYCATIETVQVKKDEVVFTGEIPARCIQAYRTDLAFYTNGQ 600

Query 601 SVCLTELKGYQAAVGQPVIQPRRPNSRLDKVRHMFQKV 638

```
            SVCLTELKGYQAAVG+PVIQPRRPNSRLDKVRHMF K+
Sbjct  601  SVCLTELKGYQAAVGKPVIQPRRPNSRLDKVRHMFSKI  638


>WP_066305045.1 TetM/TetW/TetO/TetS family tetracycline resistance
ribosomal
protection protein [Atopobium deltae]
 KXB34974.1 putative translation elongation factor G [Atopobium
deltae]
Length=639

 Score = 1274 bits (3297),  Expect = 0.0, Method: Compositional
matrix adjust.
 Identities = 619/638 (97%), Positives = 627/638 (98%), Gaps = 0/638
(0%)

Query  1
MKIINIGILAHVDAGKTTLTESLLYASGAISEPGSVEKGTTRTDTMFLERQRGITIQAAV  60
            M IINIGILAHVDAGKTTLTESLLYASGAISEPGSVEKGTTRTDTM
LERQRGITIQAAV
Sbjct  1
MNIINIGILAHVDAGKTTLTESLLYASGAISEPGSVEKGTTRTDTMLLERQRGITIQAAV  60

Query  61
TSFQWHRCKVNIVDTPGHMDFLAEVYRSLAVLDGAILVISAKDGVQAQTRILFHALRKMN  120

TSFQWHRCKVNIVDTPGHMDFLAEVYRSLAVLDGAILVISAKDGVQAQTRILFHALRKMN
Sbjct  61
TSFQWHRCKVNIVDTPGHMDFLAEVYRSLAVLDGAILVISAKDGVQAQTRILFHALRKMN  120

Query  121
IPTVIFINKIDQAGVDLQSVVQSVRDKLSADIIIKQTVSLSPEIVLEENTDIEAWDAVIE  180

IPTVIFINKIDQAGVDLQSVVQSVRDKLSADIIIKQTVSLSPEIVLEENTDIEAWDAVIE
Sbjct  121
IPTVIFINKIDQAGVDLQSVVQSVRDKLSADIIIKQTVSLSPEIVLEENTDIEAWDAVIE  180

Query  181
NNDKLLEKYIAGEPISREKLVREEQRRVQDASLFPVYYGSAKKGLGIQPLMDAVTGLFQP  240
            NND LLEKYIAGEPIS+EKL REEQRRVQ+ASLFPVY
+GSAKKGLGIQPLMDAVTGLFQP
Sbjct  181
NNDALLEKYIAGEPISQEKLAREEQRRVQEASLFPVYHGSAKKGLGIQPLMDAVTGLFQP  240

Query  241
IGEQGSAALCGSVFKVEYTDCGQRRVYLRLYSGTLRLRDTVALAGREKLKITEMRIPSKG  300
            IGEQGSA
LCGSVFKVEYTDCGQRRVYLRLYSGTLRLRDTVALAGREKLKITEMRIPSKG
Sbjct  241
IGEQGSATLCGSVFKVEYTDCGQRRVYLRLYSGTLRLRDTVALAGREKLKITEMRIPSKG  300

Query  301
EIVRTDTAYPGEIVILPSDSVRLNDVLGDPTRLPRKRWREDPLPMLRTTIAPKTAAQRER  360
            EIVRTDTAYPGEIVILPSDSVRLNDVLGD TRLPRKRWRE
```

PLPMLRTTIAPKTAAQRER
Sbjct 301
EIVRTDTAYPGEIVILPSDSVRLNDVLGDQTRLPRKRWREAPLPMLRTTIAPKTAAQRER 360

Query 361
LLDALTQLADTDPLLRCEVDSITHEIILSFLGRVQLEVVSALLSEKYKLETVVKEPSVIY 420
            LLDALTQLADTDPLLRCEVDSITHEIILSFLGRVQLEVVSALLSEKYK+ETVVKEP
++IY
Sbjct 361
LLDALTQLADTDPLLRCEVDSITHEIILSFLGRVQLEVVSALLSEKYKIETVVKEPTLIY 420

Query 421
MERPLKAASHTIHIEVPPNPFWASIGLSVTPLSLGSGVQYESRVSLGYLNQSFQNAVRDG 480
            MERPLKAASHTIHIEVPPNPFWASIGLSVTPL
LGSGVQYESRVSLGYLNQSFQNAVRDG
Sbjct 421
MERPLKAASHTIHIEVPPNPFWASIGLSVTPLPLGSGVQYESRVSLGYLNQSFQNAVRDG 480

Query 481
IRYGLEQGLFGWNVTDCKICFEYGLYYSPVSTPADFRSLAPIVLEQALKESGTQLLEPYL 540

IRYGLEQGLFGWNVTDCKICFEYGLYYSPVSTPADFRSLAPIVLEQALKESGTQLLEPYL
Sbjct 481
IRYGLEQGLFGWNVTDCKICFEYGLYYSPVSTPADFRSLAPIVLEQALKESGTQLLEPYL 540

Query 541
SFILYAPQEYLSRAYHDAPKYCATIETAQVKKDEVVFTGEIPARCIQAYRTDLAFYTNGR 600
            SF LYAPQEYLSRAYHDAPKYCATIETAQVKKDEVVFTGEIPAR
IQAYRTDLAFYTNGR
Sbjct 541
SFTLYAPQEYLSRAYHDAPKYCATIETAQVKKDEVVFTGEIPARYIQAYRTDLAFYTNGR 600

Query 601 SVCLTELKGYQAAVGQPVIQPRRPNSRLDKVRHMFQKV 638
            SVCLTELKGYQAAVG+PVIQPRRPNSRLDKVRHMF K+
Sbjct 601 SVCLTELKGYQAAVGEPVIQPRRPNSRLDKVRHMFSKI 638

>WP_042433210.1 TetM/TetW/TetO/TetS family tetracycline resistance
ribosomal
protection protein [Senegalimassilia anaerobia]
Length=639

 Score = 1274 bits (3296), Expect = 0.0, Method: Compositional
matrix adjust.
 Identities = 619/639 (97%), Positives = 630/639 (99%), Gaps = 0/639
(0%)

Query 1
MKIINIGILAHVDAGKTTLTESLLYASGAISEPGSVEKGTTRTDTMFLERQRGITIQAAV 60
            M IINIGILAHVDAGKTTLTESLLYASGAISEPGSVEKGTTRTDTM
LERQRGITIQAAV
Sbjct 1
MNIINIGILAHVDAGKTTLTESLLYASGAISEPGSVEKGTTRTDTMLLERQRGITIQAAV 60

Query 61
TSFQWHRCKVNIVDTPGHMDFLAEVYRSLAVLDGAILVISAKDGVQAQTRILFHALRKMN 120

TSFQWHRCKVNIVDTPGHMDFLAEVYRSLAVLDGAILVISAKDGVQAQTRILFHALRKM+
Sbjct 61
TSFQWHRCKVNIVDTPGHMDFLAEVYRSLAVLDGAILVISAKDGVQAQTRILFHALRKMD 120

Query 121
IPTVIFINKIDQAGVDLQSVVQSVRDKLSADIIIKQTVSLSPEIVLEENTDIEAWDAVIE 180
IPTVIFINKIDQAGVDLQ V
QSVRDKLSADIIIKQTVSLSPEIVLEENTDIEAWDAVIE
Sbjct 121
IPTVIFINKIDQAGVDLQGVYQSVRDKLSADIIIKQTVSLSPEIVLEENTDIEAWDAVIE 180

Query 181
NNDKLLEKYIAGEPISREKLVREEQRRVQDASLFPVYYGSAKKGLGIQPLMDAVTGLFQP 240
NND+LLEKYIAGEPISRE+L +EEQRRVQDASLFPVY
+GSAKKGLGIQPLMDAVTGLFQP
Sbjct 181
NNDELLEKYIAGEPISREELAQEEQRRVQDASLFPVYHGSAKKGLGIQPLMDAVTGLFQP 240

Query 241
IGEQGSAALCGSVFKVEYTDCGQRRVYLRLYSGTLRLRDTVALAGREKLKITEMRIPSKG 300
IGEQGS ALCGSVFKVEYTDCGQR
+YLRLYSGTLRLRDTVALAGREKLKITEMRIPSKG
Sbjct 241
IGEQGSDALCGSVFKVEYTDCGQRLIYLRLYSGTLRLRDTVALAGREKLKITEMRIPSKG 300

Query 301
EIVRTDTAYPGEIVILPSDSVRLNDVLGDPTRLPRKRWREDPLPMLRTTIAPKTAAQRER 360
EIVRTDTAYPGEIVILPSDSVRLNDVLGDPTRLPRKRWREDPLPMLRT
+IAPKTAAQRER
Sbjct 301
EIVRTDTAYPGEIVILPSDSVRLNDVLGDPTRLPRKRWREDPLPMLRTSIAPKTAAQRER 360

Query 361
LLDALTQLADTDPLLRCEVDSITHEIILSFLGRVQLEVVSALLSEKYKLETVVKEPSVIY 420
LLDALTQLADTDPLLRCEVDSITHEIILSFLGRVQLEVVSALLSEKYKLETVVKEP
+VIY
Sbjct 361
LLDALTQLADTDPLLRCEVDSITHEIILSFLGRVQLEVVSALLSEKYKLETVVKEPTVIY 420

Query 421
MERPLKAASHTIHIEVPPNPFWASIGLSVTPLSLGSGVQYESRVSLGYLNQSFQNAVRDG 480
MERPLKAASHTIHIEVPPNPFWASIGLSVTPL
LGSGVQYESRVSLGYLNQSFQNAVRDG
Sbjct 421
MERPLKAASHTIHIEVPPNPFWASIGLSVTPLPLGSGVQYESRVSLGYLNQSFQNAVRDG 480

Query 481
IRYGLEQGLFGWNVTDCKICFEYGLYYSPVSTPADFRSLAPIVLEQALKESGTQLLEPYL 540

IRYGLEQGLFGWNVTDCKICFEYGLYYSPVSTPADFRSLAPIVLEQALKESGTQLLEPYL
Sbjct 481

IRYGLEQGLFGWNVTDCKICFEYGLYYSPVSTPADFRSLAPIVLEQALKESGTQLLEPYL 540

Query 541
SFILYAPQEYLSRAYHDAPKYCATIETAQVKKDEVVFTGEIPARCIQAYRTDLAFYTNGR 600
            SF LYAP
+EYLSRAYHDAPKYCATIETAQVKKDEVVFTGEIPARCIQAYRTDLAFYTNG+
Sbjct 541
SFTLYAPREYLSRAYHDAPKYCATIETAQVKKDEVVFTGEIPARCIQAYRTDLAFYTNGQ 600

Query 601 SVCLTELKGYQAAVGQPVIQPRRPNSRLDKVRHMFQKVM 639
            SVCLTELKGYQAAVG+PVIQPRRPNSRLDKVRHMFQKVM
Sbjct 601 SVCLTELKGYQAAVGKPVIQPRRPNSRLDKVRHMFQKVM 639

>WP_070738148.1 TetM/TetW/TetO/TetS family tetracycline resistance ribosomal
protection protein [Rothia sp. HMSC061C12]
 OFO23565.1 tetracycline resistance ribosomal protection protein [Rothia
sp. HMSC061C12]
Length=640

 Score = 1273 bits (3293), Expect = 0.0, Method: Compositional matrix adjust.
 Identities = 618/638 (97%), Positives = 627/638 (98%), Gaps = 0/638 (0%)

Query 1
MKIINIGILAHVDAGKTTLTESLLYASGAISEPGSVEKGTTRTDTMFLERQRGITIQAAV 60
            M IINIGILAHVDAGKTTLTESLLYASGAISEPGSVEKGTTRTDT
+FLERQRGITIQAAV
Sbjct 1
MNIINIGILAHVDAGKTTLTESLLYASGAISEPGSVEKGTTRTDTLFLERQRGITIQAAV 60

Query 61
TSFQWHRCKVNIVDTPGHMDFLAEVYRSLAVLDGAILVISAKDGVQAQTRILFHALRKMN 120

TSFQWHRCKVNIVDTPGHMDFLAEVYRSLAVLDGAILVISAKDGVQAQTRILFHALRKMN
Sbjct 61
TSFQWHRCKVNIVDTPGHMDFLAEVYRSLAVLDGAILVISAKDGVQAQTRILFHALRKMN 120

Query 121
IPTVIFINKIDQAGVDLQSVVQSVRDKLSADIIIKQTVSLSPEIVLEENTDIEAWDAVIE 180

IPTVIFINKIDQAGVDLQSVVQSVRDKLSADIIIKQTVSLSPEIVLEENTDIEAWDAVIE
Sbjct 121
IPTVIFINKIDQAGVDLQSVVQSVRDKLSADIIIKQTVSLSPEIVLEENTDIEAWDAVIE 180

Query 181
NNDKLLEKYIAGEPISREKLVREEQRRVQDASLFPVYYGSAKKGLGIQPLMDAVTGLFQP 240
            NND LLEKYIAGEPIS+EKL REEQRRVQ+ SLFPVY
+GSAKKGLGIQPLMDAVTGLFQP
Sbjct 181
NNDALLEKYIAGEPISQEKLAREEQRRVQEVSLFPVYHGSAKKGLGIQPLMDAVTGLFQP 240

Query 241
IGEQGSAALCGSVFKVEYTDCGQRRVYLRLYSGTLRLRDTVALAGREKLKITEMRIPSKG 300
IGEQGSA
LCGSVFKVEYTDCGQRRVYLRLYSGTLRLRDTVALAGREKLKITEMRIPSKG
Sbjct 241
IGEQGSATLCGSVFKVEYTDCGQRRVYLRLYSGTLRLRDTVALAGREKLKITEMRIPSKG 300

Query 301
EIVRTDTAYPGEIVILPSDSVRLNDVLGDPTRLPRKRWREDPLPMLRTTIAPKTAAQRER 360
EIVRTDTAY GEIVILPSDSVRLNDVLGDPTRLPRKRWREDPLPMLRT
+IAPKTAAQRER
Sbjct 301
EIVRTDTAYQGEIVILPSDSVRLNDVLGDPTRLPRKRWREDPLPMLRTSIAPKTAAQRER 360

Query 361
LLDALTQLADTDPLLRCEVDSITHEIILSFLGRVQLEVVSALLSEKYKLETVVKEPSVIY 420
LLDALTQLADTDPLLRCEVDSITHEIILSFLGRVQLEVVSALLSEKYKLETVVKEP
VIY
Sbjct 361
LLDALTQLADTDPLLRCEVDSITHEIILSFLGRVQLEVVSALLSEKYKLETVVKEPPVIY 420

Query 421
MERPLKAASHTIHIEVPPNPFWASIGLSVTPLSLGSGVQYESRVSLGYLNQSFQNAVRDG 480
MERPLKAASHTIHIEVPPNPFWASIGLSVTPL
LGSGVQYESRVSLGYLNQSFQNAVRDG
Sbjct 421
MERPLKAASHTIHIEVPPNPFWASIGLSVTPLPLGSGVQYESRVSLGYLNQSFQNAVRDG 480

Query 481
IRYGLEQGLFGWNVTDCKICFEYGLYYSPVSTPADFRSLAPIVLEQALKESGTQLLEPYL 540

IRYGLEQGLFGWNVTDCKICFEYGLYYSPVSTPADFRSLAPIVLEQALKESGTQLLEPYL
Sbjct 481
IRYGLEQGLFGWNVTDCKICFEYGLYYSPVSTPADFRSLAPIVLEQALKESGTQLLEPYL 540

Query 541
SFILYAPQEYLSRAYHDAPKYCATIETAQVKKDEVVFTGEIPARCIQAYRTDLAFYTNGR 600
SF LYAP+EYLSRAYHDAPKYCATIET
QVKKDEVVFTGEIPARCIQAYRTDLAFYTNG+
Sbjct 541
SFTLYAPREYLSRAYHDAPKYCATIETVQVKKDEVVFTGEIPARCIQAYRTDLAFYTNGQ 600

Query 601 SVCLTELKGYQAAVGQPVIQPRRPNSRLDKVRHMFQKV 638
SVCLTELKGYQAAVG+PVIQPRRPNSRLDKVRHMF K+
Sbjct 601 SVCLTELKGYQAAVGKPVIQPRRPNSRLDKVRHMFSKI 638

>WP_075843486.1 MULTISPECIES: TetM/TetW/TetO/TetS family
tetracycline resistance
ribosomal protection protein [Actinobacteria]
PLA22237.1 TetM/TetW/TetO/TetS family tetracycline resistance
ribosomal
protection protein [Corynebacterium pyruviciproducens]

Length=639

 Score = 1272 bits (3292), Expect = 0.0, Method: Compositional matrix adjust.
 Identities = 618/639 (97%), Positives = 629/639 (98%), Gaps = 0/639 (0%)

```
Query  1
MKIINIGILAHVDAGKTTLTESLLYASGAISEPGSVEKGTTRTDTMFLERQRGITIQAAV  60
            M IINIGILAHVDAGKTTLTESLLYASGAISEPGSVEKGTTRTDTM
LERQRGITIQAAV
Sbjct  1
MNIINIGILAHVDAGKTTLTESLLYASGAISEPGSVEKGTTRTDTMLLERQRGITIQAAV  60

Query  61
TSFQWHRCKVNIVDTPGHMDFLAEVYRSLAVLDGAILVISAKDGVQAQTRILFHALRKMN  120

TSFQWHRCKVNIVDTPGHMDFLAEVYRSLAVLDGAILVISAKDGVQAQTRILFHALRKM+
Sbjct  61
TSFQWHRCKVNIVDTPGHMDFLAEVYRSLAVLDGAILVISAKDGVQAQTRILFHALRKMD  120

Query  121
IPTVIFINKIDQAGVDLQSVVQSVRDKLSADIIIKQTVSLSPEIVLEENTDIEAWDAVIE  180
            IPTVIFINKIDQAGVDLQ V
QSVRDKLSADIIIKQTVSLSPEIVLEENTDIEAWDAVIE
Sbjct  121
IPTVIFINKIDQAGVDLQGVYQSVRDKLSADIIIKQTVSLSPEIVLEENTDIEAWDAVIE  180

Query  181
NNDKLLEKYIAGEPISREKLVREEQRRVQDASLFPVYYGSAKKGLGIQPLMDAVTGLFQP  240
            NND+LLEKYIAGEPISRE+L +EEQRRVQDASLFPVY
+GSAKKGLGIQPLMDAVTGLFQP
Sbjct  181
NNDELLEKYIAGEPISREELAQEEQRRVQDASLFPVYHGSAKKGLGIQPLMDAVTGLFQP  240

Query  241
IGEQGSAALCGSVFKVEYTDCGQRRVYLRLYSGTLRLRDTVALAGREKLKITEMRIPSKG  300
            IGEQGS ALCGSVFKVEYTDCGQR
+YLRLYSGTLRLRDTVALAGREKLKITEMRIPSKG
Sbjct  241
IGEQGSDALCGSVFKVEYTDCGQRLIYLRLYSGTLRLRDTVALAGREKLKITEMRIPSKG  300

Query  301
EIVRTDTAYPGEIVILPSDSVRLNDVLGDPTRLPRKRWREDPLPMLRTTIAPKTAAQRER  360
            EIVRTDTAYPGEIVILPSDSVRLNDVLGDPTRLPRKRWREDPLPMLRT
+IAPKTAAQRER
Sbjct  301
EIVRTDTAYPGEIVILPSDSVRLNDVLGDPTRLPRKRWREDPLPMLRTSIAPKTAAQRER  360

Query  361
LLDALTQLADTDPLLRCEVDSITHEIILSFLGRVQLEVVSALLSEKYKLETVVKEPSVIY  420
            LLDALTQLADTDPLLRCEVDSITHEIILSFLGRVQLEVVSALLSEKYKLETVVKEP
+VIY
Sbjct  361
```

LLDALTQLADTDPLLRCEVDSITHEIILSFLGRVQLEVVSALLSEKYKLETVVKEPTVIY 420

Query 421
MERPLKAASHTIHIEVPPNPFWASIGLSVTPLSLGSGVQYESRVSLGYLNQSFQNAVRDG 480
            MERPLKAASHTIHIEVPPNPFWASIGLSVTPL
LGSGVQYESRVSLGYLNQSFQNAVRDG
Sbjct 421
MERPLKAASHTIHIEVPPNPFWASIGLSVTPLPLGSGVQYESRVSLGYLNQSFQNAVRDG 480

Query 481
IRYGLEQGLFGWNVTDCKICFEYGLYYSPVSTPADFRSLAPIVLEQALKESGTQLLEPYL 540

IRYGLEQGLFGWNVTDCKICFEYGLYYSPVSTPADFRSLAPIVLEQALKESGTQLLEPYL
Sbjct 481
IRYGLEQGLFGWNVTDCKICFEYGLYYSPVSTPADFRSLAPIVLEQALKESGTQLLEPYL 540

Query 541
SFILYAPQEYLSRAYHDAPKYCATIETAQVKKDEVVFTGEIPARCIQAYRTDLAFYTNGR 600
            SF LYAP+EYLSRAYHDAPKYCATIET
QVKKDEVVFTGEIPARCIQAYRTDLAFYTNG+
Sbjct 541
SFTLYAPREYLSRAYHDAPKYCATIETVQVKKDEVVFTGEIPARCIQAYRTDLAFYTNGQ 600

Query 601 SVCLTELKGYQAAVGQPVIQPRRPNSRLDKVRHMFQKVM 639
            SVCLTELKGYQAAVG+PVIQPRRPNSRLDKVRHMFQKVM
Sbjct 601 SVCLTELKGYQAAVGKPVIQPRRPNSRLDKVRHMFQKVM 639

>AMP42228.1 tetracycline resistance protein Tet(W) [uncultured
bacterium
IN-05]
Length=639

 Score = 1272 bits (3291), Expect = 0.0, Method: Compositional
matrix adjust.
 Identities = 619/639 (97%), Positives = 628/639 (98%), Gaps = 0/639
(0%)

Query 1
MKIINIGILAHVDAGKTTLTESLLYASGAISEPGSVEKGTTRTDTMFLERQRGITIQAAV 60
            M IINIGILAHVDAGKTTLTESLLYASGAISEPGSVEKGTTRTDTM
LERQRGITIQAAV
Sbjct 1
MNIINIGILAHVDAGKTTLTESLLYASGAISEPGSVEKGTTRTDTMLLERQRGITIQAAV 60

Query 61
TSFQWHRCKVNIVDTPGHMDFLAEVYRSLAVLDGAILVISAKDGVQAQTRILFHALRKMN 120

TSFQWHRCKVNIVDTPGHMDFLAEVYRSLAVLDGAILVISAKDGVQAQTRILFHALRKM+
Sbjct 61
TSFQWHRCKVNIVDTPGHMDFLAEVYRSLAVLDGAILVISAKDGVQAQTRILFHALRKMD 120

Query 121
IPTVIFINKIDQAGVDLQSVVQSVRDKLSADIIIKQTVSLSPEIVLEENTDIEAWDAVIE 180

IPTVIFINKIDQAGVDLQ V
QSVRDKLSADIIIKQTVSLSPEIVLEENTDIEAWDAVIE
Sbjct 121
IPTVIFINKIDQAGVDLQGVYQSVRDKLSADIIIKQTVSLSPEIVLEENTDIEAWDAVIE 180

Query 181
NNDKLLEKYIAGEPISREKLVREEQRRVQDASLFPVYYGSAKKGLGIQPLMDAVTGLFQP 240
NND+LLEKYIAGEPISRE+L +EEQ+RVQDASLFPVY+GSAK
GLGIQPLMDAVTGLFQP
Sbjct 181
NNDELLEKYIAGEPISREELAQEEQQRVQDASLFPVYHGSAKNGLGIQPLMDAVTGLFQP 240

Query 241
IGEQGSAALCGSVFKVEYTDCGQRRVYLRLYSGTLRLRDTVALAGREKLKITEMRIPSKG 300
IGEQGSAALCGSVFKVEYTDC QR
+YLRLYSGTLRLRDTVALAGREKLKITEMRIPSKG
Sbjct 241
IGEQGSAALCGSVFKVEYTDCDQRLIYLRLYSGTLRLRDTVALAGREKLKITEMRIPSKG 300

Query 301
EIVRTDTAYPGEIVILPSDSVRLNDVLGDPTRLPRKRWREDPLPMLRTTIAPKTAAQRER 360
EIVRTDTAYPGEIVILPSDSVRLNDVLGDPTRLPRKRWREDPLPMLRT
+IAPKTAAQRER
Sbjct 301
EIVRTDTAYPGEIVILPSDSVRLNDVLGDPTRLPRKRWREDPLPMLRTSIAPKTAAQRER 360

Query 361
LLDALTQLADTDPLLRCEVDSITHEIILSFLGRVQLEVVSALLSEKYKLETVVKEPSVIY 420
LLDALTQLADTDPLLR
EVDSITHEIILSFLGRVQLEVVSALLSEKYKLETVVKEPSVIY
Sbjct 361
LLDALTQLADTDPLLRYEVDSITHEIILSFLGRVQLEVVSALLSEKYKLETVVKEPSVIY 420

Query 421
MERPLKAASHTIHIEVPPNPFWASIGLSVTPLSLGSGVQYESRVSLGYLNQSFQNAVRDG 480
MERPLKAASHTIHIEVPPNPFWASIGLSVTPL
LGSGVQYESRVSLGYLNQSFQNAVRDG
Sbjct 421
MERPLKAASHTIHIEVPPNPFWASIGLSVTPLPLGSGVQYESRVSLGYLNQSFQNAVRDG 480

Query 481
IRYGLEQGLFGWNVTDCKICFEYGLYYSPVSTPADFRSLAPIVLEQALKESGTQLLEPYL 540

IRYGLEQGLFGWNVTDCKICFEYGLYYSPVSTPADFRSLAPIVLEQALKESGTQLLEPYL
Sbjct 481
IRYGLEQGLFGWNVTDCKICFEYGLYYSPVSTPADFRSLAPIVLEQALKESGTQLLEPYL 540

Query 541
SFILYAPQEYLSRAYHDAPKYCATIETAQVKKDEVVFTGEIPARCIQAYRTDLAFYTNGR 600

SFILYAPQEYLSRAYHDAPKYCATIETAQVKKDEVVFTGEIPARCIQAYRTDLAFYTNGR
Sbjct 541
SFILYAPQEYLSRAYHDAPKYCATIETAQVKKDEVVFTGEIPARCIQAYRTDLAFYTNGR 600

```
Query  601  SVCLTELKGYQAAVGQPVIQPRRPNSRLDKVRHMFQKVM  639
            SVCLTELKGYQA VG+PVIQPRRPNSRLDKVRHMFQKVM
Sbjct  601  SVCLTELKGYQATVGEPVIQPRRPNSRLDKVRHMFQKVM  639


>WP_009141881.1 TetM/TetW/TetO/TetS family tetracycline resistance
ribosomal
protection protein [Collinsella tanakaei]
 EGX69294.1 tetracycline resistance protein tetW [Collinsella
tanakaei YIT
12063]
Length=639

 Score = 1272 bits (3291),  Expect = 0.0, Method: Compositional
matrix adjust.
 Identities = 619/639 (97%), Positives = 626/639 (98%), Gaps = 0/639
(0%)

Query  1
MKIINIGILAHVDAGKTTLTESLLYASGAISEPGSVEKGTTRTDTMFLERQRGITIQAAV  60
            M IINIGILAHVDAGKTTLTESLLYASGAISEPGSVEKGTTRTDT
+FLERQRGITIQAAV
Sbjct  1
MNIINIGILAHVDAGKTTLTESLLYASGAISEPGSVEKGTTRTDTLFLERQRGITIQAAV  60

Query  61
TSFQWHRCKVNIVDTPGHMDFLAEVYRSLAVLDGAILVISAKDGVQAQTRILFHALRKMN  120

TSFQWHRCKVNIVDTPGHMDFLAEVYRSLAVLDGAILVISAKDGVQAQTRILFHALRKMN
Sbjct  61
TSFQWHRCKVNIVDTPGHMDFLAEVYRSLAVLDGAILVISAKDGVQAQTRILFHALRKMN  120

Query  121
IPTVIFINKIDQAGVDLQSVVQSVRDKLSADIIIKQTVSLSPEIVLEENTDIEAWDAVIE  180

IPTVIFINKIDQAGVDLQSVVQSVRDKLSADIIIKQTVSLSPEIVLEENTDIEAWDAVIE
Sbjct  121
IPTVIFINKIDQAGVDLQSVVQSVRDKLSADIIIKQTVSLSPEIVLEENTDIEAWDAVIE  180

Query  181
NNDKLLEKYIAGEPISREKLVREEQRRVQDASLFPVYYGSAKKGLGIQPLMDAVTGLFQP  240
            NND LLEKYIAGEPIS+EKL REEQRRVQ+ASLFPVY
+GSAKKGLGIQPLMDAVTGLFQP
Sbjct  181
NNDALLEKYIAGEPISQEKLAREEQRRVQEASLFPVYHGSAKKGLGIQPLMDAVTGLFQP  240

Query  241
IGEQGSAALCGSVFKVEYTDCGQRRVYLRLYSGTLRLRDTVALAGREKLKITEMRIPSKG  300
            IGEQGSA
LCGSVFKVEYTDCGQRRVYLRLYSGTLRLRDTVALAGREKLKITEMRIPSKG
Sbjct  241
IGEQGSATLCGSVFKVEYTDCGQRRVYLRLYSGTLRLRDTVALAGREKLKITEMRIPSKG  300

Query  301
```

EIVRTDTAYPGEIVILPSDSVRLNDVLGDPTRLPRKRWREDPLPMLRTTIAPKTAAQRER 360
            EIVRTDTAYPGEIVILPSDSVRLNDVLGD TRLPRKRWRE PLPMLRT
IAPKTAAQRER
Sbjct 301
EIVRTDTAYPGEIVILPSDSVRLNDVLGDQTRLPRKRWREAPLPMLRTAIAPKTAAQRER 360

Query 361
LLDALTQLADTDPLLRCEVDSITHEIILSFLGRVQLEVVSALLSEKYKLETVVKEPSVIY 420
            LLDALTQLADTDPLLRCEVDSITHEIILSFLGRVQLEVVSALLSEKYK+ETVVKEP
+VIY
Sbjct 361
LLDALTQLADTDPLLRCEVDSITHEIILSFLGRVQLEVVSALLSEKYKIETVVKEPTVIY 420

Query 421
MERPLKAASHTIHIEVPPNPFWASIGLSVTPLSLGSGVQYESRVSLGYLNQSFQNAVRDG 480
            MERPLKAASHTIHIEVPPNPFWASIGLSVTPL
LGSGVQYESRVSLGYLNQSFQNAV DG
Sbjct 421
MERPLKAASHTIHIEVPPNPFWASIGLSVTPLPLGSGVQYESRVSLGYLNQSFQNAVMDG 480

Query 481
IRYGLEQGLFGWNVTDCKICFEYGLYYSPVSTPADFRSLAPIVLEQALKESGTQLLEPYL 540
            IRYGLEQGL
GWNVTDCKICFEYGLYYSPVSTPADFRSLAPIVLEQALKESGTQLLEPYL
Sbjct 481
IRYGLEQGLCGWNVTDCKICFEYGLYYSPVSTPADFRSLAPIVLEQALKESGTQLLEPYL 540

Query 541
SFILYAPQEYLSRAYHDAPKYCATIETAQVKKDEVVFTGEIPARCIQAYRTDLAFYTNGR 600
            SF LYAPQEYLSRAY
DAPKYCATIETAQVKKDEVVFTGEIPARCIQAYRTDLAFYTNGR
Sbjct 541
SFTLYAPQEYLSRAYRDAPKYCATIETAQVKKDEVVFTGEIPARCIQAYRTDLAFYTNGR 600

Query 601 SVCLTELKGYQAAVGQPVIQPRRPNSRLDKVRHMFQKVM 639
            SVCLTELKGYQA VG+PVIQPRRPNSRLDKVRHMFQKVM
Sbjct 601 SVCLTELKGYQATVGEPVIQPRRPNSRLDKVRHMFQKVM 639

>WP_075279296.1 TetM/TetW/TetO/TetS family tetracycline resistance
ribosomal
protection protein [Olsenella provencensis]
Length=639

 Score = 1271 bits (3290), Expect = 0.0, Method: Compositional
matrix adjust.
 Identities = 618/639 (97%), Positives = 629/639 (98%), Gaps = 0/639
(0%)

Query 1
MKIINIGILAHVDAGKTTLTESLLYASGAISEPGSVEKGTTRTDTMFLERQRGITIQAAV 60
            M IINIGILAHVDAGKTTLTESLLYASGAISEPGSVEKGTTRTDTM
LERQRGITIQAAV
Sbjct 1

```
MNIINIGILAHVDAGKTTLTESLLYASGAISEPGSVEKGTTRTDTMLLERQRGITIQAAV  60

Query  61
TSFQWHRCKVNIVDTPGHMDFLAEVYRSLAVLDGAILVISAKDGVQAQTRILFHALRKMN  120

TSFQWHRCKVNIVDTPGHMDFLAEVYRSLAVLDGAILVISAKDGVQAQTRILFHALRKM+
Sbjct  61
TSFQWHRCKVNIVDTPGHMDFLAEVYRSLAVLDGAILVISAKDGVQAQTRILFHALRKMD  120

Query  121
IPTVIFINKIDQAGVDLQSVVQSVRDKLSADIIIKQTVSLSPEIVLEENTDIEAWDAVIE  180
            IPTVIFINKIDQAGVDLQ V
QSVRDKLSADIIIKQTVSLSPEIVLEENTDIEAWDAVIE
Sbjct  121
IPTVIFINKIDQAGVDLQGVYQSVRDKLSADIIIKQTVSLSPEIVLEENTDIEAWDAVIE  180

Query  181
NNDKLLEKYIAGEPISREKLVREEQRRVQDASLFPVYYGSAKKGLGIQPLMDAVTGLFQP  240
            NND+LLEKYIAGEPISRE+L +EEQRRVQDASLFPVY
+GSAKKGLGIQPLMDAVTGLFQP
Sbjct  181
NNDELLEKYIAGEPISREELAQEEQRRVQDASLFPVYHGSAKKGLGIQPLMDAVTGLFQP  240

Query  241
IGEQGSAALCGSVFKVEYTDCGQRRVYLRLYSGTLRLRDTVALAGREKLKITEMRIPSKG  300
            IGEQGS ALCGSVFKVEYTDCGQR
+YLRLYSGTLRLRDTVALAGREKLKITEMRIPSKG
Sbjct  241
IGEQGSDALCGSVFKVEYTDCGQRLIYLRLYSGTLRLRDTVALAGREKLKITEMRIPSKG  300

Query  301
EIVRTDTAYPGEIVILPSDSVRLNDVLGDPTRLPRKRWREDPLPMLRTTIAPKTAAQRER  360
            EIVRTDTAYPGEIVILPSDSVRLNDVLGDPTRLPRKRWREDPLPMLRT
+IAPKTAAQRER
Sbjct  301
EIVRTDTAYPGEIVILPSDSVRLNDVLGDPTRLPRKRWREDPLPMLRTSIAPKTAAQRER  360

Query  361
LLDALTQLADTDPLLRCEVDSITHEIILSFLGRVQLEVVSALLSEKYKLETVVKEPSVIY  420
            LLDALTQLADTDPLLRCEVDSITHEIILSFLGRVQLEVVSALLSEKYKLETVVKEP
+VIY
Sbjct  361
LLDALTQLADTDPLLRCEVDSITHEIILSFLGRVQLEVVSALLSEKYKLETVVKEPAVIY  420

Query  421
MERPLKAASHTIHIEVPPNPFWASIGLSVTPLSLGSGVQYESRVSLGYLNQSFQNAVRDG  480
            MERPLKAASHTIHIEVPPNPFWASIGLSVTPL
LGSGVQYESRVSLGYLNQSFQNAVRDG
Sbjct  421
MERPLKAASHTIHIEVPPNPFWASIGLSVTPLPLGSGVQYESRVSLGYLNQSFQNAVRDG  480

Query  481
IRYGLEQGLFGWNVTDCKICFEYGLYYSPVSTPADFRSLAPIVLEQALKESGTQLLEPYL  540
```

IRYGLEQGLFGWNVTDCKICFEYGLYYSPVSTPADFRSLAPIVLEQALKESGTQLLEPYL
Sbjct 481
IRYGLEQGLFGWNVTDCKICFEYGLYYSPVSTPADFRSLAPIVLEQALKESGTQLLEPYL 540

Query 541
SFILYAPQEYLSRAYHDAPKYCATIETAQVKKDEVVFTGEIPARCIQAYRTDLAFYTNGR 600
            SF LYAP+EYLSRAYHDAPKYCATIET
QVKKDEVVFTGEIPARCIQAYRTDLAFYTNG+
Sbjct 541
SFTLYAPREYLSRAYHDAPKYCATIETVQVKKDEVVFTGEIPARCIQAYRTDLAFYTNGQ 600

Query 601 SVCLTELKGYQAAVGQPVIQPRRPNSRLDKVRHMFQKVM 639
            SVCLTELKGYQAAVG+PVIQPRRPNSRLDKVRHMFQKVM
Sbjct 601 SVCLTELKGYQAAVGKPVIQPRRPNSRLDKVRHMFQKVM 639

>WP_070633855.1 TetM/TetW/TetO/TetS family tetracycline resistance ribosomal
protection protein [Rothia sp. HMSC065B04]
 OFJ74226.1 tetracycline resistance ribosomal protection protein [Rothia
sp. HMSC065B04]
Length=640

 Score = 1271 bits (3288), Expect = 0.0, Method: Compositional matrix adjust.
 Identities = 616/638 (97%), Positives = 628/638 (98%), Gaps = 0/638 (0%)

Query 1
MKIINIGILAHVDAGKTTLTESLLYASGAISEPGSVEKGTTRTDTMFLERQRGITIQAAV 60
            MKIINIGILAHVDAGKTTLTESLLYASGAISEPGSVEKGTTRTDTM
LERQRGITIQAAV
Sbjct 1
MKIINIGILAHVDAGKTTLTESLLYASGAISEPGSVEKGTTRTDTMLLERQRGITIQAAV 60

Query 61
TSFQWHRCKVNIVDTPGHMDFLAEVYRSLAVLDGAILVISAKDGVQAQTRILFHALRKMN 120

TSFQWHRCKVNIVDTPGHMDFLAEVYRSLAVLDGAILVISAKDGVQAQTRILFHALRKM+
Sbjct 61
TSFQWHRCKVNIVDTPGHMDFLAEVYRSLAVLDGAILVISAKDGVQAQTRILFHALRKMD 120

Query 121
IPTVIFINKIDQAGVDLQSVVQSVRDKLSADIIIKQTVSLSPEIVLEENTDIEAWDAVIE 180
            IPTVIFINKIDQAGVDLQ V
QSVRDKLSADIIIKQTVSLSPEIVLEENTDIEAWDAVIE
Sbjct 121
IPTVIFINKIDQAGVDLQGVYQSVRDKLSADIIIKQTVSLSPEIVLEENTDIEAWDAVIE 180

Query 181
NNDKLLEKYIAGEPISREKLVREEQRRVQDASLFPVYYGSAKKGLGIQPLMDAVTGLFQP 240
            NND+LLEKYIAGEPISRE+L +EEQRRVQDASLFPVY
+GSAKKGLGIQPLMDAVTGLFQP

```
Sbjct  181
NNDELLEKYIAGEPISREELAQEEQRRVQDASLFPVYHGSAKKGLGIQPLMDAVTGLFQP  240

Query  241
IGEQGSAALCGSVFKVEYTDCGQRRVYLRLYSGTLRLRDTVALAGREKLKITEMRIPSKG  300
            IGEQGS ALCGSVFKVEYTDCGQR
+YLRLYSGTLRLRDTVALAGREKLKITEMRIPSKG
Sbjct  241
IGEQGSDALCGSVFKVEYTDCGQRLIYLRLYSGTLRLRDTVALAGREKLKITEMRIPSKG  300

Query  301
EIVRTDTAYPGEIVILPSDSVRLNDVLGDPTRLPRKRWREDPLPMLRTTIAPKTAAQRER  360
            EIVRTDTAYPGEIVILPSDSVRLNDVLGDPTRLPRKRWREDPLPMLRT
+IAPKTAAQRER
Sbjct  301
EIVRTDTAYPGEIVILPSDSVRLNDVLGDPTRLPRKRWREDPLPMLRTSIAPKTAAQRER  360

Query  361
LLDALTQLADTDPLLRCEVDSITHEIILSFLGRVQLEVVSALLSEKYKLETVVKEPSVIY  420
            LLDALTQLADTDPLLRCEVDSITHEIILSFLGRVQLEVVSALLSEKYKLETVVKEP
+VIY
Sbjct  361
LLDALTQLADTDPLLRCEVDSITHEIILSFLGRVQLEVVSALLSEKYKLETVVKEPTVIY  420

Query  421
MERPLKAASHTIHIEVPPNPFWASIGLSVTPLSLGSGVQYESRVSLGYLNQSFQNAVRDG  480
            MERPLKAASHTIHIEVPPNPFWASIGLSVTPL
LGSGVQYESRVSLGYLNQSFQNAVRDG
Sbjct  421
MERPLKAASHTIHIEVPPNPFWASIGLSVTPLPLGSGVQYESRVSLGYLNQSFQNAVRDG  480

Query  481
IRYGLEQGLFGWNVTDCKICFEYGLYYSPVSTPADFRSLAPIVLEQALKESGTQLLEPYL  540

IRYGLEQGLFGWNVTDCKICFEYGLYYSPVSTPADFRSLAPIVLEQALKESGTQLLEPYL
Sbjct  481
IRYGLEQGLFGWNVTDCKICFEYGLYYSPVSTPADFRSLAPIVLEQALKESGTQLLEPYL  540

Query  541
SFILYAPQEYLSRAYHDAPKYCATIETAQVKKDEVVFTGEIPARCIQAYRTDLAFYTNGR  600
            SF LYAP+EYLSRAYHDAPKYCATIET
QVKKDEVVFTGEIPARCIQAYRTDLAFYTNG+
Sbjct  541
SFTLYAPREYLSRAYHDAPKYCATIETVQVKKDEVVFTGEIPARCIQAYRTDLAFYTNGQ  600

Query  601  SVCLTELKGYQAAVGQPVIQPRRPNSRLDKVRHMFQKV  638
            SVCLTELKGYQAAVG+PVIQPRRPNSRLDKVRHMF K+
Sbjct  601  SVCLTELKGYQAAVGKPVIQPRRPNSRLDKVRHMFSKI  638


>WP_099432720.1 TetM/TetW/TetO/TetS family tetracycline resistance
ribosomal
protection protein [Collinsella aerofaciens]
 ATP54798.1 tetracycline resistance ribosomal protection protein
```

[Collinsella
aerofaciens]
Length=639

 Score = 1270 bits (3287), Expect = 0.0, Method: Compositional
matrix adjust.
 Identities = 617/639 (97%), Positives = 629/639 (98%), Gaps = 0/639
(0%)

```
Query  1
MKIINIGILAHVDAGKTTLTESLLYASGAISEPGSVEKGTTRTDTMFLERQRGITIQAAV  60
            M IINIGILAHVDAGKTTLTESLLYASGAISEPGSVEKGTTRTDTM
LERQRGITIQAAV
Sbjct  1
MNIINIGILAHVDAGKTTLTESLLYASGAISEPGSVEKGTTRTDTMLLERQRGITIQAAV  60

Query  61
TSFQWHRCKVNIVDTPGHMDFLAEVYRSLAVLDGAILVISAKDGVQAQTRILFHALRKMN  120

TSFQWHRCKVNIVDTPGHMDFLAEVYRSLAVLDGAILVISAKDGVQAQTRILFHALRKM+
Sbjct  61
TSFQWHRCKVNIVDTPGHMDFLAEVYRSLAVLDGAILVISAKDGVQAQTRILFHALRKMD  120

Query  121
IPTVIFINKIDQAGVDLQSVVQSVRDKLSADIIIKQTVSLSPEIVLEENTDIEAWDAVIE  180
            IPTVIFINKIDQAGVDLQ V
QSVRDKLSADIIIKQTVSLSPEIVLEENTDIEAWDAVIE
Sbjct  121
IPTVIFINKIDQAGVDLQGVYQSVRDKLSADIIIKQTVSLSPEIVLEENTDIEAWDAVIE  180

Query  181
NNDKLLEKYIAGEPISREKLVREEQRRVQDASLFPVYYGSAKKGLGIQPLMDAVTGLFQP  240
            NND+LLEKYIAGEPISRE+L +EEQRRVQDASLFPVY
+GSAKKGLGIQPLMDAVTGLFQP
Sbjct  181
NNDELLEKYIAGEPISREELAQEEQRRVQDASLFPVYHGSAKKGLGIQPLMDAVTGLFQP  240

Query  241
IGEQGSAALCGSVFKVEYTDCGQRRVYLRLYSGTLRLRDTVALAGREKLKITEMRIPSKG  300
            IGEQGS ALCGSVFKVEYTDCGQR
+YLRLYSGTLRLRDTVALAGREKLKITEMRIPSKG
Sbjct  241
IGEQGSDALCGSVFKVEYTDCGQRLIYLRLYSGTLRLRDTVALAGREKLKITEMRIPSKG  300

Query  301
EIVRTDTAYPGEIVILPSDSVRLNDVLGDPTRLPRKRWREDPLPMLRTTIAPKTAAQRER  360
            EIVRTDTAYPGEIVILPSDSVRLNDVLGDPTRLPRKRWREDPLPMLRT
+IAPKTAAQRER
Sbjct  301
EIVRTDTAYPGEIVILPSDSVRLNDVLGDPTRLPRKRWREDPLPMLRTSIAPKTAAQRER  360

Query  361
LLDALTQLADTDPLLRCEVDSITHEIILSFLGRVQLEVVSALLSEKYKLETVVKEPSVIY  420
            LLDALTQLADTDPLLRCEVDSITHEIILSFLGRVQLEVVSALLSEKYKLETVVKEP
```

```
+VIY
Sbjct  361
LLDALTQLADTDPLLRCEVDSITHEIILSFLGRVQLEVVSALLSEKYKLETVVKEPTVIY  420

Query  421
MERPLKAASHTIHIEVPPNPFWASIGLSVTPLSLGSGVQYESRVSLGYLNQSFQNAVRDG  480
           MERPLKAASHTIHIEVPPNPFWASIGLSVTPL
LGSGVQYESRVSLGYLNQSFQNAVRDG
Sbjct  421
MERPLKAASHTIHIEVPPNPFWASIGLSVTPLPLGSGVQYESRVSLGYLNQSFQNAVRDG  480

Query  481
IRYGLEQGLFGWNVTDCKICFEYGLYYSPVSTPADFRSLAPIVLEQALKESGTQLLEPYL  540

IRYGLEQGLFGWNVTDCKICFEYGLYYSPVSTPADFRSLAPIVLEQALKESGTQLLEP+L
Sbjct  481
IRYGLEQGLFGWNVTDCKICFEYGLYYSPVSTPADFRSLAPIVLEQALKESGTQLLEPHL  540

Query  541
SFILYAPQEYLSRAYHDAPKYCATIETAQVKKDEVVFTGEIPARCIQAYRTDLAFYTNGR  600
           SF LYAP+EYLSRAYHDAPKYCATIET
QVKKDEVVFTGEIPARCIQAYRTDLAFYTNG+
Sbjct  541
SFTLYAPREYLSRAYHDAPKYCATIETVQVKKDEVVFTGEIPARCIQAYRTDLAFYTNGQ  600

Query  601  SVCLTELKGYQAAVGQPVIQPRRPNSRLDKVRHMFQKVM  639
            SVCLTELKGYQAAVG+PVIQPRRPNSRLDKVRHMFQKVM
Sbjct  601  SVCLTELKGYQAAVGKPVIQPRRPNSRLDKVRHMFQKVM  639


>WP_068539726.1 MULTISPECIES: TetM/TetW/TetO/TetS family
tetracycline resistance
ribosomal protection protein [Coriobacteriales]
Length=639

 Score = 1270 bits (3287),  Expect = 0.0, Method: Compositional
matrix adjust.
 Identities = 616/638 (97%), Positives = 627/638 (98%), Gaps = 0/638
(0%)

Query  1
MKIINIGILAHVDAGKTTLTESLLYASGAISEPGSVEKGTTRTDTMFLERQRGITIQAAV  60
           M IINIGILAHVDAGKTTLTESLLYASGAISEPGSVEKGTTRTDTM
LERQRGITIQAAV
Sbjct  1
MNIINIGILAHVDAGKTTLTESLLYASGAISEPGSVEKGTTRTDTMLLERQRGITIQAAV  60

Query  61
TSFQWHRCKVNIVDTPGHMDFLAEVYRSLAVLDGAILVISAKDGVQAQTRILFHALRKMN  120

TSFQWHRCKVNIVDTPGHMDFLAEVYRSLAVLDGAILVISAKDGVQAQTRILFHALRKM+
Sbjct  61
TSFQWHRCKVNIVDTPGHMDFLAEVYRSLAVLDGAILVISAKDGVQAQTRILFHALRKMD  120
```

Query 121
IPTVIFINKIDQAGVDLQSVVQSVRDKLSADIIIKQTVSLSPEIVLEENTDIEAWDAVIE 180
            IPTVIFINKIDQAGVDLQ V
QSVRDKLSADIIIKQTVSLSPEIVLEENTDIEAWDAVIE
Sbjct 121
IPTVIFINKIDQAGVDLQGVYQSVRDKLSADIIIKQTVSLSPEIVLEENTDIEAWDAVIE 180

Query 181
NNDKLLEKYIAGEPISREKLVREEQRRVQDASLFPVYYGSAKKGLGIQPLMDAVTGLFQP 240
            NND+LLEKYIAGEPISRE+L +EEQRRVQDASLFPVY
+GSAKKGLGIQPLMDAVTGLFQP
Sbjct 181
NNDELLEKYIAGEPISREELAQEEQRRVQDASLFPVYHGSAKKGLGIQPLMDAVTGLFQP 240

Query 241
IGEQGSAALCGSVFKVEYTDCGQRRVYLRLYSGTLRLRDTVALAGREKLKITEMRIPSKG 300
            IGEQGS ALCGSVFKVEYTDCGQR
+YLRLYSGTLRLRDTVALAGREKLKITEMRIPSKG
Sbjct 241
IGEQGSDALCGSVFKVEYTDCGQRLIYLRLYSGTLRLRDTVALAGREKLKITEMRIPSKG 300

Query 301
EIVRTDTAYPGEIVILPSDSVRLNDVLGDPTRLPRKRWREDPLPMLRTTIAPKTAAQRER 360
            EIVRTDTAYPGEIVILPSDSVRLNDVLGDPTRLPRKRWREDPLPMLRT
+IAPKTAAQRER
Sbjct 301
EIVRTDTAYPGEIVILPSDSVRLNDVLGDPTRLPRKRWREDPLPMLRTSIAPKTAAQRER 360

Query 361
LLDALTQLADTDPLLRCEVDSITHEIILSFLGRVQLEVVSALLSEKYKLETVVKEPSVIY 420
            LLDALTQLADTDPLLRCEVDSITHEIILSFLGRVQLEVVSALLSEKYKLETVVKEP
+VIY
Sbjct 361
LLDALTQLADTDPLLRCEVDSITHEIILSFLGRVQLEVVSALLSEKYKLETVVKEPTVIY 420

Query 421
MERPLKAASHTIHIEVPPNPFWASIGLSVTPLSLGSGVQYESRVSLGYLNQSFQNAVRDG 480
            MERPLKAASHTIHIEVPPNPFWASIGLSVTPL
LGSGVQYESRVSLGYLNQSFQNAVRDG
Sbjct 421
MERPLKAASHTIHIEVPPNPFWASIGLSVTPLPLGSGVQYESRVSLGYLNQSFQNAVRDG 480

Query 481
IRYGLEQGLFGWNVTDCKICFEYGLYYSPVSTPADFRSLAPIVLEQALKESGTQLLEPYL 540

IRYGLEQGLFGWNVTDCKICFEYGLYYSPVSTPADFRSLAPIVLEQALKESGTQLLEPYL
Sbjct 481
IRYGLEQGLFGWNVTDCKICFEYGLYYSPVSTPADFRSLAPIVLEQALKESGTQLLEPYL 540

Query 541
SFILYAPQEYLSRAYHDAPKYCATIETAQVKKDEVVFTGEIPARCIQAYRTDLAFYTNGR 600
            SF LYAP+EYLSRAYHDAPKYCATIET
QVKKDEVVFTGEIPARCIQAYRTDLAFYTNGR
Sbjct 541

```
SFTLYAPREYLSRAYHDAPKYCATIETVQVKKDEVVFTGEIPARCIQAYRTDLAFYTNGR  600

Query  601  SVCLTELKGYQAAVGQPVIQPRRPNSRLDKVRHMFQKV  638
            SVCLTELKGYQAAVG+PVIQPRRPNSRLDKVRHMF K+
Sbjct  601  SVCLTELKGYQAAVGEPVIQPRRPNSRLDKVRHMFSKI  638


>WP_005987406.1 TetM/TetW/TetO/TetS family tetracycline resistance
ribosomal
protection protein [Actinomyces graevenitzii]
 EHM87253.1 tetracycline resistance protein tetW [Actinomyces
graevenitzii
C83]
Length=640

 Score = 1270 bits (3287),  Expect = 0.0, Method: Compositional
matrix adjust.
 Identities = 616/638 (97%), Positives = 629/638 (99%), Gaps = 0/638
(0%)

Query  1
MKIINIGILAHVDAGKTTLTESLLYASGAISEPGSVEKGTTRTDTMFLERQRGITIQAAV  60
            MKIINIGILAHVDAGKTTLTESLLYASGAISEPGSVEKGTTRTDTM
LERQRGITIQAAV
Sbjct  1
MKIINIGILAHVDAGKTTLTESLLYASGAISEPGSVEKGTTRTDTMLLERQRGITIQAAV  60

Query  61
TSFQWHRCKVNIVDTPGHMDFLAEVYRSLAVLDGAILVISAKDGVQAQTRILFHALRKMN  120

TSFQWHRCKVNIVDTPGHMDFLAEVYRSLAVLDGAILVISAKDGVQAQTRILFHALRKM+
Sbjct  61
TSFQWHRCKVNIVDTPGHMDFLAEVYRSLAVLDGAILVISAKDGVQAQTRILFHALRKMD  120

Query  121
IPTVIFINKIDQAGVDLQSVVQSVRDKLSADIIIKQTVSLSPEIVLEENTDIEAWDAVIE  180
            IPTVIFINKIDQAGVDLQ
VVQSVRDKLSADIIIKQTVSLSPEIVLEENTDIEAWDAVIE
Sbjct  121
IPTVIFINKIDQAGVDLQGVVQSVRDKLSADIIIKQTVSLSPEIVLEENTDIEAWDAVIE  180

Query  181
NNDKLLEKYIAGEPISREKLVREEQRRVQDASLFPVYYGSAKKGLGIQPLMDAVTGLFQP  240
            NND+LLE+YIAGEPISRE+L +EEQRRVQDASLFPVY
+GSAKKGLGIQPLMDAVTGLFQP
Sbjct  181
NNDELLEEYIAGEPISREELAQEEQRRVQDASLFPVYHGSAKKGLGIQPLMDAVTGLFQP  240

Query  241
IGEQGSAALCGSVFKVEYTDCGQRRVYLRLYSGTLRLRDTVALAGREKLKITEMRIPSKG  300
            IGEQGS ALCGSVFKVEYTDCGQR
+YLRLYSGTLRLRDTVALAGREKLKITEMRIPSKG
Sbjct  241
IGEQGSDALCGSVFKVEYTDCGQRLIYLRLYSGTLRLRDTVALAGREKLKITEMRIPSKG  300
```

Query 301
EIVRTDTAYPGEIVILPSDSVRLNDVLGDPTRLPRKRWREDPLPMLRTTIAPKTAAQRER 360
            EIVRTDTAYPGEIVILPSDSVRLNDVLGDPTRLPRKRWREDPLPMLRT
+IAPKTAAQRER
Sbjct 301
EIVRTDTAYPGEIVILPSDSVRLNDVLGDPTRLPRKRWREDPLPMLRTSIAPKTAAQRER 360

Query 361
LLDALTQLADTDPLLRCEVDSITHEIILSFLGRVQLEVVSALLSEKYKLETVVKEPSVIY 420
            LLDALTQLADTDPLLRCEVDSITHEIILSFLGRVQLEVVSALLSEKYKLETVVKEP
+VIY
Sbjct 361
LLDALTQLADTDPLLRCEVDSITHEIILSFLGRVQLEVVSALLSEKYKLETVVKEPTVIY 420

Query 421
MERPLKAASHTIHIEVPPNPFWASIGLSVTPLSLGSGVQYESRVSLGYLNQSFQNAVRDG 480
            MERPLKAASHTIHIEVPPNPFWASIGLSVTPL
LGSGVQYESRVSLGYLNQSFQNAVRDG
Sbjct 421
MERPLKAASHTIHIEVPPNPFWASIGLSVTPLPLGSGVQYESRVSLGYLNQSFQNAVRDG 480

Query 481
IRYGLEQGLFGWNVTDCKICFEYGLYYSPVSTPADFRSLAPIVLEQALKESGTQLLEPYL 540

IRYGLEQGLFGWNVTDCKICFEYGLYYSPVSTPADFRSLAPIVLEQALKESGTQLLEPYL
Sbjct 481
IRYGLEQGLFGWNVTDCKICFEYGLYYSPVSTPADFRSLAPIVLEQALKESGTQLLEPYL 540

Query 541
SFILYAPQEYLSRAYHDAPKYCATIETAQVKKDEVVFTGEIPARCIQAYRTDLAFYTNGR 600
            SF LYAP+EYLSRAYHDAPKYCATIET
QVKKDEVVFTGEIPARCIQAYRTDLAFYTNG+
Sbjct 541
SFTLYAPREYLSRAYHDAPKYCATIETVQVKKDEVVFTGEIPARCIQAYRTDLAFYTNGQ 600

Query 601 SVCLTELKGYQAAVGQPVIQPRRPNSRLDKVRHMFQKV 638
            SVCLTELKGYQAAVG+PVIQPRRPNSRLDKVRHMF K+
Sbjct 601 SVCLTELKGYQAAVGKPVIQPRRPNSRLDKVRHMFSKI 638

>APO33657.1 ferrous iron transport protein B [uncultured bacterium]
Length=678

 Score = 1269 bits (3285), Expect = 0.0, Method: Compositional
matrix adjust.
 Identities = 621/635 (98%), Positives = 625/635 (98%), Gaps = 0/635
(0%)

Query 1
MKIINIGILAHVDAGKTTLTESLLYASGAISEPGSVEKGTTRTDTMFLERQRGITIQAAV 60
            MKIINIGILAHVDAGKTTLTESLLYASGAISEPGSVEKGTTRTDTM
LERQRGITIQAAV
Sbjct 1

MKIINIGILAHVDAGKTTLTESLLYASGAISEPGSVEKGTTRTDTMLLERQRGITIQAAV 60

Query 61
TSFQWHRCKVNIVDTPGHMDFLAEVYRSLAVLDGAILVISAKDGVQAQTRILFHALRKMN 120

TSFQWHRCKVNIVDTPGHMDFLAEVYRSLAVLDGAILVISAKDGVQAQTRILFHALRKMN
Sbjct 61
TSFQWHRCKVNIVDTPGHMDFLAEVYRSLAVLDGAILVISAKDGVQAQTRILFHALRKMN 120

Query 121
IPTVIFINKIDQAGVDLQSVVQSVRDKLSADIIIKQTVSLSPEIVLEENTDIEAWDAVIE 180
IPTVIFINKIDQAGVDLQSV
QSVRDKLSADIIIKQTVSLSPEIVLEENTDIEAWDAVIE
Sbjct 121
IPTVIFINKIDQAGVDLQSVDQSVRDKLSADIIIKQTVSLSPEIVLEENTDIEAWDAVIE 180

Query 181
NNDKLLEKYIAGEPISREKLVREEQRRVQDASLFPVYYGSAKKGLGIQPLMDAVTGLFQP 240
NND+LLEKYIAGEPISREKL REEQ+RVQDASLFPVY+GSAK
GLGIQPLMDAVTGLFQP
Sbjct 181
NNDELLEKYIAGEPISREKLAREEQQRVQDASLFPVYHGSAKNGLGIQPLMDAVTGLFQP 240

Query 241
IGEQGSAALCGSVFKVEYTDCGQRRVYLRLYSGTLRLRDTVALAGREKLKITEMRIPSKG 300
IGEQGSAALCGSVFKVEYTDCGQR
+YLRLYSGTLRLRDTVALAGREKLKITEMRIPSKG
Sbjct 241
IGEQGSAALCGSVFKVEYTDCGQRLIYLRLYSGTLRLRDTVALAGREKLKITEMRIPSKG 300

Query 301
EIVRTDTAYPGEIVILPSDSVRLNDVLGDPTRLPRKRWREDPLPMLRTTIAPKTAAQRER 360
EIVRTDTAY GEIVILPSDSVRLNDVLGD
TRLPRKRWREDPLPMLRTTIAPKTAAQRER
Sbjct 301
EIVRTDTAYQGEIVILPSDSVRLNDVLGDQTRLPRKRWREDPLPMLRTTIAPKTAAQRER 360

Query 361
LLDALTQLADTDPLLRCEVDSITHEIILSFLGRVQLEVVSALLSEKYKLETVVKEPSVIY 420

LLDALTQLADTDPLLRCEVDSITHEIILSFLGRVQLEVVSALLSEKYKLETVVKEPSVIY
Sbjct 361
LLDALTQLADTDPLLRCEVDSITHEIILSFLGRVQLEVVSALLSEKYKLETVVKEPSVIY 420

Query 421
MERPLKAASHTIHIEVPPNPFWASIGLSVTPLSLGSGVQYESRVSLGYLNQSFQNAVRDG 480
MERPLKAASHTIHIEVPPNPFWASIGLSVTPL
LGSGVQYESRVSLGYLNQSFQNAVRDG
Sbjct 421
MERPLKAASHTIHIEVPPNPFWASIGLSVTPLPLGSGVQYESRVSLGYLNQSFQNAVRDG 480

Query 481
IRYGLEQGLFGWNVTDCKICFEYGLYYSPVSTPADFRSLAPIVLEQALKESGTQLLEPYL 540

IRYGLEQGLFGWNVTDCKICFEYGLYYSPVSTPADFRSLAPIVLEQALKESGTQLLEPYL
Sbjct 481
IRYGLEQGLFGWNVTDCKICFEYGLYYSPVSTPADFRSLAPIVLEQALKESGTQLLEPYL 540

Query 541
SFILYAPQEYLSRAYHDAPKYCATIETAQVKKDEVVFTGEIPARCIQAYRTDLAFYTNGR 600
            SF
LYAPQEYLSRAYHDAPKYCATIETAQVKKDEVVFTGEIPARCIQAYRTDLAFYTNGR
Sbjct 541
SFTLYAPQEYLSRAYHDAPKYCATIETAQVKKDEVVFTGEIPARCIQAYRTDLAFYTNGR 600

Query 601 SVCLTELKGYQAAVGQPVIQPRRPNSRLDKVRHMF 635
            SVCLTELKGYQAAVGQPVIQPRRPNSRL KVRHMF
Sbjct 601 SVCLTELKGYQAAVGQPVIQPRRPNSRLYKVRHMF 635

>WP_102240720.1 TetM/TetW/TetO/TetS family tetracycline resistance ribosomal
protection protein [Actinomyces graevenitzii]
PMC91340.1 tetracycline resistance ribosomal protection protein [Actinomyces
graevenitzii]
Length=640

Score = 1269 bits (3283), Expect = 0.0, Method: Compositional matrix adjust.
Identities = 616/638 (97%), Positives = 628/638 (98%), Gaps = 0/638 (0%)

Query 1
MKIINIGILAHVDAGKTTLTESLLYASGAISEPGSVEKGTTRTDTMFLERQRGITIQAAV 60
            MKIINIGILAHVDAGKTTLTESLLYASGAISEPGSVEKGTTRTDTM
LERQRGITIQAAV
Sbjct 1
MKIINIGILAHVDAGKTTLTESLLYASGAISEPGSVEKGTTRTDTMLLERQRGITIQAAV 60

Query 61
TSFQWHRCKVNIVDTPGHMDFLAEVYRSLAVLDGAILVISAKDGVQAQTRILFHALRKMN 120

TSFQWHRCKVNIVDTPGHMDFLAEVYRSLAVLDGAILVISAKDGVQAQTRILFHALRKM+
Sbjct 61
TSFQWHRCKVNIVDTPGHMDFLAEVYRSLAVLDGAILVISAKDGVQAQTRILFHALRKMD 120

Query 121
IPTVIFINKIDQAGVDLQSVVQSVRDKLSADIIIKQTVSLSPEIVLEENTDIEAWDAVIE 180
            IPTVIFINKIDQAGVDLQ
VVQSVRDKLSADIIIKQTVSLSPEIVLEENTDIEAWDAVIE
Sbjct 121
IPTVIFINKIDQAGVDLQGVVQSVRDKLSADIIIKQTVSLSPEIVLEENTDIEAWDAVIE 180

Query 181
NNDKLLEKYIAGEPISREKLVREEQRRVQDASLFPVYYGSAKKGLGIQPLMDAVTGLFQP 240
            NND+LLEKYIAGEPISRE+L +EEQRRVQDASLFPVY
+GSAKKGLGIQPLMDAVTGLFQP

```
Sbjct  181
NNDELLEKYIAGEPISREELAQEEQRRVQDASLFPVYHGSAKKGLGIQPLMDAVTGLFQP  240

Query  241
IGEQGSAALCGSVFKVEYTDCGQRRVYLRLYSGTLRLRDTVALAGREKLKITEMRIPSKG  300
           IGEQGS ALCGSVFKVEYTD GQR
+YLRLYSGTLRLRDTVALAGREKLKITEMRIPSKG
Sbjct  241
IGEQGSDALCGSVFKVEYTDYGQRLIYLRLYSGTLRLRDTVALAGREKLKITEMRIPSKG  300

Query  301
EIVRTDTAYPGEIVILPSDSVRLNDVLGDPTRLPRKRWREDPLPMLRTTIAPKTAAQRER  360
           EIVRTDTAYPGEIVILPSDSVRLNDVLGDPTRLPRKRWREDPLPMLRT
+IAPKTAAQRER
Sbjct  301
EIVRTDTAYPGEIVILPSDSVRLNDVLGDPTRLPRKRWREDPLPMLRTSIAPKTAAQRER  360

Query  361
LLDALTQLADTDPLLRCEVDSITHEIILSFLGRVQLEVVSALLSEKYKLETVVKEPSVIY  420
           LLDALTQLADTDPLLRCEVDSITHEIILSFLGRVQLEVVSALLSEKYKLETVVKEP
+VIY
Sbjct  361
LLDALTQLADTDPLLRCEVDSITHEIILSFLGRVQLEVVSALLSEKYKLETVVKEPTVIY  420

Query  421
MERPLKAASHTIHIEVPPNPFWASIGLSVTPLSLGSGVQYESRVSLGYLNQSFQNAVRDG  480
           MERPLKAASHTIHIEVPPNPFWASIGLSVTPL
LGSGVQYESRVSLGYLNQSFQNAVRDG
Sbjct  421
MERPLKAASHTIHIEVPPNPFWASIGLSVTPLPLGSGVQYESRVSLGYLNQSFQNAVRDG  480

Query  481
IRYGLEQGLFGWNVTDCKICFEYGLYYSPVSTPADFRSLAPIVLEQALKESGTQLLEPYL  540

IRYGLEQGLFGWNVTDCKICFEYGLYYSPVSTPADFRSLAPIVLEQALKESGTQLLEPYL
Sbjct  481
IRYGLEQGLFGWNVTDCKICFEYGLYYSPVSTPADFRSLAPIVLEQALKESGTQLLEPYL  540

Query  541
SFILYAPQEYLSRAYHDAPKYCATIETAQVKKDEVVFTGEIPARCIQAYRTDLAFYTNGR  600
           SF LYAP+EYLSRAYHDAPKYCATIET
QVKKDEVVFTGEIPARCIQAYRTDLAFYTNG+
Sbjct  541
SFTLYAPREYLSRAYHDAPKYCATIETVQVKKDEVVFTGEIPARCIQAYRTDLAFYTNGQ  600

Query  601  SVCLTELKGYQAAVGQPVIQPRRPNSRLDKVRHMFQKV  638
            SVCLTELKGYQAAVG+PVIQPRRPNSRLDKVRHMF K+
Sbjct  601  SVCLTELKGYQAAVGKPVIQPRRPNSRLDKVRHMFSKI  638
```

>APO35550.1 ferrous iron transport protein B, partial [uncultured bacterium]
Length=627

Score = 1269 bits (3283), Expect = 0.0, Method: Compositional matrix adjust.
Identities = 618/627 (99%), Positives = 622/627 (99%), Gaps = 0/627 (0%)

```
Query  1
MKIINIGILAHVDAGKTTLTESLLYASGAISEPGSVEKGTTRTDTMFLERQRGITIQAAV  60

MKIINIGILAHVDAGKTTLTESLLYASGAISEPGSVEKGTTRTDTMFLERQRGITIQAAV
Sbjct  1
MKIINIGILAHVDAGKTTLTESLLYASGAISEPGSVEKGTTRTDTMFLERQRGITIQAAV  60

Query  61
TSFQWHRCKVNIVDTPGHMDFLAEVYRSLAVLDGAILVISAKDGVQAQTRILFHALRKMN  120

TSFQWHRCKVNIVDTPGHMDFLAEVYRSLAVLDGAILVISAKDGVQAQTRILFHALRKMN
Sbjct  61
TSFQWHRCKVNIVDTPGHMDFLAEVYRSLAVLDGAILVISAKDGVQAQTRILFHALRKMN  120

Query  121
IPTVIFINKIDQAGVDLQSVVQSVRDKLSADIIIKQTVSLSPEIVLEENTDIEAWDAVIE  180

IPTVIFINKIDQAGVDLQSVVQSVRDKLSADIIIKQTVSLSPEIVLEENTDIEAWDAVIE
Sbjct  121
IPTVIFINKIDQAGVDLQSVVQSVRDKLSADIIIKQTVSLSPEIVLEENTDIEAWDAVIE  180

Query  181
NNDKLLEKYIAGEPISREKLVREEQRRVQDASLFPVYYGSAKKGLGIQPLMDAVTGLFQP  240
            NND+LLEKYIAGEPISREKL REEQ
+RVQDASLFPVYYGSAKKGLGIQPLMDAVTGLFQP
Sbjct  181
NNDELLEKYIAGEPISREKLAREEQQRVQDASLFPVYYGSAKKGLGIQPLMDAVTGLFQP  240

Query  241
IGEQGSAALCGSVFKVEYTDCGQRRVYLRLYSGTLRLRDTVALAGREKLKITEMRIPSKG  300
            IGEQG
AALCGSVFKVEYTDCGQRRVYLRLYSGTLRLRDTVALAGREKLKITEMRIPSKG
Sbjct  241
IGEQGGAALCGSVFKVEYTDCGQRRVYLRLYSGTLRLRDTVALAGREKLKITEMRIPSKG  300

Query  301
EIVRTDTAYPGEIVILPSDSVRLNDVLGDPTRLPRKRWREDPLPMLRTTIAPKTAAQRER  360
            EIVRTDTAY GEIVILPSDSVRLNDVLGD TRLPRKRWREDPLPMLRT
+IAPKTAAQRER
Sbjct  301
EIVRTDTAYQGEIVILPSDSVRLNDVLGDQTRLPRKRWREDPLPMLRTSIAPKTAAQRER  360

Query  361
LLDALTQLADTDPLLRCEVDSITHEIILSFLGRVQLEVVSALLSEKYKLETVVKEPSVIY  420
            LLDALTQLADTDPLLRCEVDSITHEIILSFLGRVQLEVVSALLSEKY
LETVVKEPSVIY
Sbjct  361
LLDALTQLADTDPLLRCEVDSITHEIILSFLGRVQLEVVSALLSEKYNLETVVKEPSVIY  420
```

Query 421
MERPLKAASHTIHIEVPPNPFWASIGLSVTPLSLGSGVQYESRVSLGYLNQSFQNAVRDG 480

MERPLKAASHTIHIEVPPNPFWASIGLSVTPLSLGSGVQYESRVSLGYLNQSFQNAVRDG
Sbjct 421
MERPLKAASHTIHIEVPPNPFWASIGLSVTPLSLGSGVQYESRVSLGYLNQSFQNAVRDG 480

Query 481
IRYGLEQGLFGWNVTDCKICFEYGLYYSPVSTPADFRSLAPIVLEQALKESGTQLLEPYL 540

IRYGLEQGLFGWNVTDCKICFEYGLYYSPVSTPADFRSLAPIVLEQALKESGTQLLEPYL
Sbjct 481
IRYGLEQGLFGWNVTDCKICFEYGLYYSPVSTPADFRSLAPIVLEQALKESGTQLLEPYL 540

Query 541
SFILYAPQEYLSRAYHDAPKYCATIETAQVKKDEVVFTGEIPARCIQAYRTDLAFYTNGR 600

SFILYAPQEYLSRAYHDAPKYCATIETAQVKKDEVVFTGEIPARCIQAYRTDLAFYTNG+
Sbjct 541
SFILYAPQEYLSRAYHDAPKYCATIETAQVKKDEVVFTGEIPARCIQAYRTDLAFYTNGQ 600

Query 601 SVCLTELKGYQAAVGQPVIQPRRPNSR 627
            SVCLTELKGYQAAVGQPVIQPRRPNSR
Sbjct 601 SVCLTELKGYQAAVGQPVIQPRRPNSR 627

>WP_063856422.1 MULTISPECIES: tetracycline resistance ribosomal
protection protein
Tet(W) [Rothia]
 ABN80187.1 tet(W) [Rothia sp. T40-1]
 OFM96490.1 tetracycline resistance ribosomal protection protein
[Rothia
sp. HMSC072B03]
 OFQ61728.1 tetracycline resistance ribosomal protection protein
[Rothia
sp. HMSC072B04]
 OFQ65278.1 tetracycline resistance ribosomal protection protein
[Rothia
sp. HMSC061E04]
 OFR62370.1 tetracycline resistance ribosomal protection protein
[Rothia
sp. HMSC069C04]
Length=640

 Score = 1268 bits (3281), Expect = 0.0, Method: Compositional
matrix adjust.
 Identities = 615/638 (96%), Positives = 627/638 (98%), Gaps = 0/638
(0%)

Query 1
MKIINIGILAHVDAGKTTLTESLLYASGAISEPGSVEKGTTRTDTMFLERQRGITIQAAV 60
            M IINIGILAHVDAGKTTLTESLLYASGAISEPGSVEKGTTRTDTM
LERQRGITIQAAV
Sbjct 1

MNIINIGILAHVDAGKTTLTESLLYASGAISEPGSVEKGTTRTDTMLLERQRGITIQAAV 60

Query 61
TSFQWHRCKVNIVDTPGHMDFLAEVYRSLAVLDGAILVISAKDGVQAQTRILFHALRKMN 120

TSFQWHRCKVNIVDTPGHMDFLAEVYRSLAVLDGAILVISAKDGVQAQTRILFHALRKM+
Sbjct 61
TSFQWHRCKVNIVDTPGHMDFLAEVYRSLAVLDGAILVISAKDGVQAQTRILFHALRKMD 120

Query 121
IPTVIFINKIDQAGVDLQSVVQSVRDKLSADIIIKQTVSLSPEIVLEENTDIEAWDAVIE 180
IPTVIFINKIDQAGVDLQ V
QSVRDKLSADIIIKQTVSLSPEIVLEENTDIEAWDAVIE
Sbjct 121
IPTVIFINKIDQAGVDLQGVYQSVRDKLSADIIIKQTVSLSPEIVLEENTDIEAWDAVIE 180

Query 181
NNDKLLEKYIAGEPISREKLVREEQRRVQDASLFPVYYGSAKKGLGIQPLMDAVTGLFQP 240
NND+LLEKYIAGEPISRE+L +EEQRRVQDASLFPVY
+GSAKKGLGIQPLMDAVTGLFQP
Sbjct 181
NNDELLEKYIAGEPISREELAQEEQRRVQDASLFPVYHGSAKKGLGIQPLMDAVTGLFQP 240

Query 241
IGEQGSAALCGSVFKVEYTDCGQRRVYLRLYSGTLRLRDTVALAGREKLKITEMRIPSKG 300
IGEQGS ALCGSVFKVEYTDCGQR
+YLRLYSGTLRLRDTVALAGREKLKITEMRIPSKG
Sbjct 241
IGEQGSDALCGSVFKVEYTDCGQRLIYLRLYSGTLRLRDTVALAGREKLKITEMRIPSKG 300

Query 301
EIVRTDTAYPGEIVILPSDSVRLNDVLGDPTRLPRKRWREDPLPMLRTTIAPKTAAQRER 360
EIVRTDTAYPGEIVILPSDSVRLNDVLGDPTRLPRKRWREDPLPMLRT
+IAPKTAAQRER
Sbjct 301
EIVRTDTAYPGEIVILPSDSVRLNDVLGDPTRLPRKRWREDPLPMLRTSIAPKTAAQRER 360

Query 361
LLDALTQLADTDPLLRCEVDSITHEIILSFLGRVQLEVVSALLSEKYKLETVVKEPSVIY 420
LLDALTQLADTDPLLRCEVDSITHEIILSFLGRVQLEVVSALLSEKYKLETVVKEP
+VIY
Sbjct 361
LLDALTQLADTDPLLRCEVDSITHEIILSFLGRVQLEVVSALLSEKYKLETVVKEPTVIY 420

Query 421
MERPLKAASHTIHIEVPPNPFWASIGLSVTPLSLGSGVQYESRVSLGYLNQSFQNAVRDG 480
MERPLKAASHTIHIEVPPNPFWASIGLSVTPL
LGSGVQYESRVSLGYLNQSFQNAVRDG
Sbjct 421
MERPLKAASHTIHIEVPPNPFWASIGLSVTPLPLGSGVQYESRVSLGYLNQSFQNAVRDG 480

Query 481
IRYGLEQGLFGWNVTDCKICFEYGLYYSPVSTPADFRSLAPIVLEQALKESGTQLLEPYL 540

IRYGLEQGLFGWNVTDCKICFEYGLYYSPVSTPADFRSLAPIVLEQALKESGTQLLEPYL
Sbjct 481
IRYGLEQGLFGWNVTDCKICFEYGLYYSPVSTPADFRSLAPIVLEQALKESGTQLLEPYL 540

Query 541
SFILYAPQEYLSRAYHDAPKYCATIETAQVKKDEVVFTGEIPARCIQAYRTDLAFYTNGR 600
           SF LYAP+EYLSRAYHDAPKYCATIET
QVKKDEVVFTGEIPARCIQAYRTDLAFYTNG+
Sbjct 541
SFTLYAPREYLSRAYHDAPKYCATIETVQVKKDEVVFTGEIPARCIQAYRTDLAFYTNGQ 600

Query 601 SVCLTELKGYQAAVGQPVIQPRRPNSRLDKVRHMFQKV 638
            SVCLTELKGYQAAVG+PVIQPRRPNSRLDKVRHMF K+
Sbjct 601 SVCLTELKGYQAAVGKPVIQPRRPNSRLDKVRHMFSKI 638

>AMP49825.1 TetM-TetW-TetO-TetS [uncultured bacterium]
Length=639

Score = 1268 bits (3280), Expect = 0.0, Method: Compositional
matrix adjust.
Identities = 616/639 (96%), Positives = 626/639 (98%), Gaps = 0/639
(0%)

Query 1
MKIINIGILAHVDAGKTTLTESLLYASGAISEPGSVEKGTTRTDTMFLERQRGITIQAAV 60

MKIINIGILAHVDAGKTTLTESLLYASGAISEPGSVEKGTTRTDTMFLERQRGITIQAAV
Sbjct 1
MKIINIGILAHVDAGKTTLTESLLYASGAISEPGSVEKGTTRTDTMFLERQRGITIQAAV 60

Query 61
TSFQWHRCKVNIVDTPGHMDFLAEVYRSLAVLDGAILVISAKDGVQAQTRILFHALRKMN 120

TSFQWHRCKVNIVDTPGHMDFLAEVYRSLAVLDGAILVISAKDGVQAQTRILFHALRKMN
Sbjct 61
TSFQWHRCKVNIVDTPGHMDFLAEVYRSLAVLDGAILVISAKDGVQAQTRILFHALRKMN 120

Query 121
IPTVIFINKIDQAGVDLQSVVQSVRDKLSADIIIKQTVSLSPEIVLEENTDIEAWDAVIE 180

IPTVIFINKIDQAGVDLQSVVQSVRDKLSADIIIKQTVSLSPEIVLEENTDIEAWDAVIE
Sbjct 121
IPTVIFINKIDQAGVDLQSVVQSVRDKLSADIIIKQTVSLSPEIVLEENTDIEAWDAVIE 180

Query 181
NNDKLLEKYIAGEPISREKLVREEQRRVQDASLFPVYYGSAKKGLGIQPLMDAVTGLFQP 240
           NND+LLEKYIAGEPISREKL REEQ+RVQDASLFPVY+GSAK
GLGIQPLMDAVTGLFQP
Sbjct 181
NNDELLEKYIAGEPISREKLAREEQQRVQDASLFPVYHGSAKNGLGIQPLMDAVTGLFQP 240

Query 241
IGEQGSAALCGSVFKVEYTDCGQRRVYLRLYSGTLRLRDTVALAGREKLKITEMRIPSKG 300

IGEQG
AALCGSVFKVEYTDCGQRRVYLRLYSGTLRLRDTVALAGREKLKITEMRIPSKG
Sbjct 241
IGEQGGAALCGSVFKVEYTDCGQRRVYLRLYSGTLRLRDTVALAGREKLKITEMRIPSKG 300

Query 301
EIVRTDTAYPGEIVILPSDSVRLNDVLGDPTRLPRKRWREDPLPMLRTTIAPKTAAQRER 360
EIVRTDTA+ GEIVI PSDSV LNDVLGD TRLPR+RWR+
PLPMLRTTIAPKTAAQRER
Sbjct 301
EIVRTDTAHKGEIVIFPSDSVGLNDVLGDQTRLPRERWRDAPLPMLRTTIAPKTAAQRER 360

Query 361
LLDALTQLADTDPLLRCEVDSITHEIILSFLGRVQLEVVSALLSEKYKLETVVKEPSVIY 420
LLDALTQ+ADTDPLLR EVDSITHEIILSFLGRVQLEVVSALL+EKYK+ET VKEP
+VIY
Sbjct 361
LLDALTQIADTDPLLRYEVDSITHEIILSFLGRVQLEVVSALLAEKYKIETAVKEPTVIY 420

Query 421
MERPLKAASHTIHIEVPPNPFWASIGLSVTPLSLGSGVQYESRVSLGYLNQSFQNAVRDG 480
MERPLKAASHTIHIEVPPNPFWASIGLSVTPL
LGSGVQYESRVSLGYLNQSFQNAVRDG
Sbjct 421
MERPLKAASHTIHIEVPPNPFWASIGLSVTPLPLGSGVQYESRVSLGYLNQSFQNAVRDG 480

Query 481
IRYGLEQGLFGWNVTDCKICFEYGLYYSPVSTPADFRSLAPIVLEQALKESGTQLLEPYL 540

IRYGLEQGLFGWNVTDCKICFEYGLYYSPVSTPADFRSLAPIVLEQALKESGTQLLEPYL
Sbjct 481
IRYGLEQGLFGWNVTDCKICFEYGLYYSPVSTPADFRSLAPIVLEQALKESGTQLLEPYL 540

Query 541
SFILYAPQEYLSRAYHDAPKYCATIETAQVKKDEVVFTGEIPARCIQAYRTDLAFYTNGR 600
SF
LYAPQEYLSRAYHDAPKYCATIETAQVKKDEVVFTGEIPARCIQAYRTDLAFYTNGR
Sbjct 541
SFTLYAPQEYLSRAYHDAPKYCATIETAQVKKDEVVFTGEIPARCIQAYRTDLAFYTNGR 600

Query 601 SVCLTELKGYQAAVGQPVIQPRRPNSRLDKVRHMFQKVM 639
SVCLTELKGYQAAVGQPVIQPRRPNSRLDK RHMFQKVM
Sbjct 601 SVCLTELKGYQAAVGQPVIQPRRPNSRLDKARHMFQKVM 639

>WP_029731081.1 TetM/TetW/TetO/TetS family tetracycline resistance
ribosomal
protection protein [Dorea sp. AGR2135]
Length=639

Score = 1267 bits (3278), Expect = 0.0, Method: Compositional
matrix adjust.
Identities = 614/638 (96%), Positives = 626/638 (98%), Gaps = 0/638
(0%)

Query 1
MKIINIGILAHVDAGKTTLTESLLYASGAISEPGSVEKGTTRTDTMFLERQRGITIQAAV 60

MKIINIGILAHVDAGKTTLTESLLYASGAISEPGSVEKGTTRTDTMFLERQRGITIQAAV
Sbjct 1
MKIINIGILAHVDAGKTTLTESLLYASGAISEPGSVEKGTTRTDTMFLERQRGITIQAAV 60

Query 61
TSFQWHRCKVNIVDTPGHMDFLAEVYRSLAVLDGAILVISAKDGVQAQTRILFHALRKMN 120

TSFQWHRCKVNIVDTPGHMDFLAEVYRSLAVLDGAILVISAKDGVQAQTRILFHALRKMN
Sbjct 61
TSFQWHRCKVNIVDTPGHMDFLAEVYRSLAVLDGAILVISAKDGVQAQTRILFHALRKMN 120

Query 121
IPTVIFINKIDQAGVDLQSVVQSVRDKLSADIIIKQTVSLSPEIVLEENTDIEAWDAVIE 180

IPTVIFINKIDQAGVDLQSVVQSVRDKLSADIIIKQTVSLSPEIVLEENTDIEAWDAVIE
Sbjct 121
IPTVIFINKIDQAGVDLQSVVQSVRDKLSADIIIKQTVSLSPEIVLEENTDIEAWDAVIE 180

Query 181
NNDKLLEKYIAGEPISREKLVREEQRRVQDASLFPVYYGSAKKGLGIQPLMDAVTGLFQP 240

NNDKLLEKYIAGEPISREKLVREEQRRVQDASLFPVYYGSAKKGLGIQPLMDAVTGLFQP
Sbjct 181
NNDKLLEKYIAGEPISREKLVREEQRRVQDASLFPVYYGSAKKGLGIQPLMDAVTGLFQP 240

Query 241
IGEQGSAALCGSVFKVEYTDCGQRRVYLRLYSGTLRLRDTVALAGREKLKITEMRIPSKG 300

IGEQGSAALCGSVFKVEYTDCGQRRVYLRLYSGTLRLRDTVALAGREKLKITEMRIPSKG
Sbjct 241
IGEQGSAALCGSVFKVEYTDCGQRRVYLRLYSGTLRLRDTVALAGREKLKITEMRIPSKG 300

Query 301
EIVRTDTAYPGEIVILPSDSVRLNDVLGDPTRLPRKRWREDPLPMLRTTIAPKTAAQRER 360
EIVRTDTAYPGEIVILPSDSVRLND+LGD T+LPR+ W + P PMLRTTI PKTA
QR+R
Sbjct 301
EIVRTDTAYPGEIVILPSDSVRLNDILGDKTQLPREMWSDVPFPMLRTTITPKTAEQRDR 360

Query 361
LLDALTQLADTDPLLRCEVDSITHEIILSFLGRVQLEVVSALLSEKYKLETVVKEPSVIY 420
LLDALTQLADTDPLLRCEVDSITHEIILSFLGRVQLEVVSALL+EKYK+ET VKEP
+VIY
Sbjct 361
LLDALTQLADTDPLLRCEVDSITHEIILSFLGRVQLEVVSALLTEKYKIETAVKEPTVIY 420

Query 421
MERPLKAASHTIHIEVPPNPFWASIGLSVTPLSLGSGVQYESRVSLGYLNQSFQNAVRDG 480
MERPLKAASHTIHIEVPPNPFWASIGLSVTPL
LGSGVQYESRVSLGYLNQSFQNAVRDG

Sbjct 421
MERPLKAASHTIHIEVPPNPFWASIGLSVTPLPLGSGVQYESRVSLGYLNQSFQNAVRDG 480

Query 481
IRYGLEQGLFGWNVTDCKICFEYGLYYSPVSTPADFRSLAPIVLEQALKESGTQLLEPYL 540

IRYGLEQGLFGWNVTDCKICFEYGLYYSPVSTPADFRSLAPIVLEQALKESGTQLLEPYL
Sbjct 481
IRYGLEQGLFGWNVTDCKICFEYGLYYSPVSTPADFRSLAPIVLEQALKESGTQLLEPYL 540

Query 541
SFILYAPQEYLSRAYHDAPKYCATIETAQVKKDEVVFTGEIPARCIQAYRTDLAFYTNGR 600
            SF LYAP+EYLSRAYHDAPKYCATIET
QVKKDEVVFTGEIPARCIQAYRTDLAFYTNG+
Sbjct 541
SFTLYAPREYLSRAYHDAPKYCATIETVQVKKDEVVFTGEIPARCIQAYRTDLAFYTNGQ 600

Query 601 SVCLTELKGYQAAVGQPVIQPRRPNSRLDKVRHMFQKV 638
            SVCLTELKGYQAAVG+PVIQPRRPNSRLDKVRHMF K+
Sbjct 601 SVCLTELKGYQAAVGKPVIQPRRPNSRLDKVRHMFSKI 638

>WP_073324048.1 TetM/TetW/TetO/TetS family tetracycline resistance
ribosomal
protection protein [Fibrobacter sp. UWOV1]
 SHL88965.1 ribosomal protection tetracycline resistance protein
[Fibrobacter
sp. UWOV1]
Length=639

 Score = 1266 bits (3277), Expect = 0.0, Method: Compositional
matrix adjust.
 Identities = 616/639 (96%), Positives = 626/639 (98%), Gaps = 0/639
(0%)

Query 1
MKIINIGILAHVDAGKTTLTESLLYASGAISEPGSVEKGTTRTDTMFLERQRGITIQAAV 60
            MKIIN+GILAHVDAGKTTLTESLLY SGAI+E GSV++GTTRTDTM
LERQRGITIQ AV
Sbjct 1
MKIINLGILAHVDAGKTTLTESLLYTSGAIAELGSVDEGTTRTDTMNLERQRGITIQTAV 60

Query 61
TSFQWHRCKVNIVDTPGHMDFLAEVYRSLAVLDGAILVISAKDGVQAQTRILFHALRKMN 120
            TSFQW   KVNI
+DTPGHMDFLAEVYRSLAVLDGAILVISAKDGVQAQTRILFHALRKMN
Sbjct 61
TSFQWEDVKVNIIDTPGHMDFLAEVYRSLAVLDGAILVISAKDGVQAQTRILFHALRKMN 120

Query 121
IPTVIFINKIDQAGVDLQSVVQSVRDKLSADIIIKQTVSLSPEIVLEENTDIEAWDAVIE 180
            IPTVIFINKIDQAGVDLQSVVQSVRDKLSADIIIKQTVSLSPEIVLEENTDIEAW
+AVIE
Sbjct 121

```
IPTVIFINKIDQAGVDLQSVVQSVRDKLSADIIIKQTVSLSPEIVLEENTDIEAWNAVIE  180

Query  181
NNDKLLEKYIAGEPISREKLVREEQRRVQDASLFPVYYGSAKKGLGIQPLMDAVTGLFQP  240
            NNDKLLEKYIAGEPISREKL REEQ+RVQDASLFPVY+GSAK
GLGIQPLMDAVTGLFQP
Sbjct  181
NNDKLLEKYIAGEPISREKLAREEQQRVQDASLFPVYHGSAKNGLGIQPLMDAVTGLFQP  240

Query  241
IGEQGSAALCGSVFKVEYTDCGQRRVYLRLYSGTLRLRDTVALAGREKLKITEMRIPSKG  300
            IGEQG
AALCGSVFKVEYTDCGQRRVYLRLYSGTLRLRDTVALAGREKLKITEMRIPSKG
Sbjct  241
IGEQGGAALCGSVFKVEYTDCGQRRVYLRLYSGTLRLRDTVALAGREKLKITEMRIPSKG  300

Query  301
EIVRTDTAYPGEIVILPSDSVRLNDVLGDPTRLPRKRWREDPLPMLRTTIAPKTAAQRER  360
            EIVRTDTAYPGEIVILPSDSVRLNDVLGD
TRLPRKRWREDPLPMLRTTIAPKTAAQRER
Sbjct  301
EIVRTDTAYPGEIVILPSDSVRLNDVLGDQTRLPRKRWREDPLPMLRTTIAPKTAAQRER  360

Query  361
LLDALTQLADTDPLLRCEVDSITHEIILSFLGRVQLEVVSALLSEKYKLETVVKEPSVIY  420

LLDALTQLADTDPLLRCEVDSITHEIILSFLGRVQLEVVSALLSEKYKLETVVKEPSVIY
Sbjct  361
LLDALTQLADTDPLLRCEVDSITHEIILSFLGRVQLEVVSALLSEKYKLETVVKEPSVIY  420

Query  421
MERPLKAASHTIHIEVPPNPFWASIGLSVTPLSLGSGVQYESRVSLGYLNQSFQNAVRDG  480
            MERPLKAASHTIHIEVPPNPFWASIGLSVTPL
LGSGVQYESRVSLGYLNQSFQNAVRDG
Sbjct  421
MERPLKAASHTIHIEVPPNPFWASIGLSVTPLPLGSGVQYESRVSLGYLNQSFQNAVRDG  480

Query  481
IRYGLEQGLFGWNVTDCKICFEYGLYYSPVSTPADFRSLAPIVLEQALKESGTQLLEPYL  540

IRYGLEQGLFGWNVTDCKICFEYGLYYSPVSTPADFRSLAPIVLEQALKESGTQLLEPYL
Sbjct  481
IRYGLEQGLFGWNVTDCKICFEYGLYYSPVSTPADFRSLAPIVLEQALKESGTQLLEPYL  540

Query  541
SFILYAPQEYLSRAYHDAPKYCATIETAQVKKDEVVFTGEIPARCIQAYRTDLAFYTNGR  600
            SF LYAP
+EYLSRAYHDAPKYCATIETAQVKKDEVVFTGEIPARCIQAYRTDLAFYTNGR
Sbjct  541
SFTLYAPREYLSRAYHDAPKYCATIETAQVKKDEVVFTGEIPARCIQAYRTDLAFYTNGR  600

Query  601  SVCLTELKGYQAAVGQPVIQPRRPNSRLDKVRHMFQKVM  639
            SVCLTELKGYQAAVGQPVIQPRRPNSRLDKVRHMFQK+M
Sbjct  601  SVCLTELKGYQAAVGQPVIQPRRPNSRLDKVRHMFQKIM  639
```

>APO33653.1 ferrous iron transport protein B [uncultured bacterium]
Length=678

Score = 1262 bits (3266), Expect = 0.0, Method: Compositional matrix adjust.
Identities = 616/635 (97%), Positives = 624/635 (98%), Gaps = 0/635 (0%)

Query 1
MKIINIGILAHVDAGKTTLTESLLYASGAISEPGSVEKGTTRTDTMFLERQRGITIQAAV 60
MKIINIGILAHVDAGKTTLTESLLYASGAISEPGSVEKGTTRTDTM LERQRGITIQAAV
Sbjct 1
MKIINIGILAHVDAGKTTLTESLLYASGAISEPGSVEKGTTRTDTMLLERQRGITIQAAV 60

Query 61
TSFQWHRCKVNIVDTPGHMDFLAEVYRSLAVLDGAILVISAKDGVQAQTRILFHALRKMN 120
TSFQWHRCKVNIVDTPGHMDFLAEVYRSLAVLDGAILVISAKDGVQAQTRILFHALRKMN
Sbjct 61
TSFQWHRCKVNIVDTPGHMDFLAEVYRSLAVLDGAILVISAKDGVQAQTRILFHALRKMN 120

Query 121
IPTVIFINKIDQAGVDLQSVVQSVRDKLSADIIIKQTVSLSPEIVLEENTDIEAWDAVIE 180
IPTVIFINKIDQAGVDLQSV QSVRDKLSADIIIKQTVSLSPEIVLEENTDIEAWDAVIE
Sbjct 121
IPTVIFINKIDQAGVDLQSVDQSVRDKLSADIIIKQTVSLSPEIVLEENTDIEAWDAVIE 180

Query 181
NNDKLLEKYIAGEPISREKLVREEQRRVQDASLFPVYYGSAKKGLGIQPLMDAVTGLFQP 240
NND+LLEKYIAGEPISREKL REEQ+RVQDASLFPVY+GSAK GLGIQPLMDAVTGLFQP
Sbjct 181
NNDELLEKYIAGEPISREKLAREEQQRVQDASLFPVYHGSAKNGLGIQPLMDAVTGLFQP 240

Query 241
IGEQGSAALCGSVFKVEYTDCGQRRVYLRLYSGTLRLRDTVALAGREKLKITEMRIPSKG 300
IGEQGSAALCGSVFKVEYTDCGQR +YLRLYSGTLRLRDTVALAGREKLKITEMRIPSKG
Sbjct 241
IGEQGSAALCGSVFKVEYTDCGQRLIYLRLYSGTLRLRDTVALAGREKLKITEMRIPSKG 300

Query 301
EIVRTDTAYPGEIVILPSDSVRLNDVLGDPTRLPRKRWREDPLPMLRTTIAPKTAAQRER 360
EIVRTDTAY GEIVILPSDSVRLNDVLGD TRLPRKRWREDPLPMLRTTIAPKTAAQRER
Sbjct 301
EIVRTDTAYQGEIVILPSDSVRLNDVLGDQTRLPRKRWREDPLPMLRTTIAPKTAAQRER 360

Query 361
LLDALTQLADTDPLLRCEVDSITHEIILSFLGRVQLEVVSALLSEKYKLETVVKEPSVIY 420

            LLDALTQLADTDPLLRCEVDSITHEIILSFLGRVQLEVVSALL+EKYK+ETVVKEP
+VIY
Sbjct 361
LLDALTQLADTDPLLRCEVDSITHEIILSFLGRVQLEVVSALLAEKYKIETVVKEPTVIY 420

Query 421
MERPLKAASHTIHIEVPPNPFWASIGLSVTPLSLGSGVQYESRVSLGYLNQSFQNAVRDG 480
            +ERPLK ASHTIHIEVPPNPFWASIGLSVTPL
LGSGVQYESRVSLGYLNQSFQNAVRDG
Sbjct 421
LERPLKVASHTIHIEVPPNPFWASIGLSVTPLPLGSGVQYESRVSLGYLNQSFQNAVRDG 480

Query 481
IRYGLEQGLFGWNVTDCKICFEYGLYYSPVSTPADFRSLAPIVLEQALKESGTQLLEPYL 540

IRYGLEQGLFGWNVTDCKICFEYGLYYSPVSTPADFRSLAPIVLEQALKESGTQLLEPYL
Sbjct 481
IRYGLEQGLFGWNVTDCKICFEYGLYYSPVSTPADFRSLAPIVLEQALKESGTQLLEPYL 540

Query 541
SFILYAPQEYLSRAYHDAPKYCATIETAQVKKDEVVFTGEIPARCIQAYRTDLAFYTNGR 600
            SF
LYAPQEYLSRAYHDAPKYCATIETAQVKKDEVVFTGEIPARCIQAYRTDLAFYTNGR
Sbjct 541
SFTLYAPQEYLSRAYHDAPKYCATIETAQVKKDEVVFTGEIPARCIQAYRTDLAFYTNGR 600

Query 601 SVCLTELKGYQAAVGQPVIQPRRPNSRLDKVRHMF 635
            SVCLTELKGYQAAVGQPVIQPRRPNSRL KVRHMF
Sbjct 601 SVCLTELKGYQAAVGQPVIQPRRPNSRLYKVRHMF 635

>AAR29969.1 mosaic tetracycline-resistance protein [Megasphaera elsdenii]
 AAR29973.1 mosaic tetracycline-resistance protein [Megasphaera elsdenii]
Length=639

 Score = 1261 bits (3264), Expect = 0.0, Method: Compositional matrix adjust.
 Identities = 613/639 (96%), Positives = 625/639 (98%), Gaps = 0/639 (0%)

Query 1
MKIINIGILAHVDAGKTTLTESLLYASGAISEPGSVEKGTTRTDTMFLERQRGITIQAAV 60
            MKIIN+GILAHVDAGKTTLTESLLY SGAI+E GSV++GTTRTDTM
LERQRGITIQ AV
Sbjct 1
MKIINLGILAHVDAGKTTLTESLLYTSGAIAELGSVDEGTTRTDTMNLERQRGITIQTAV 60

Query 61
TSFQWHRCKVNIVDTPGHMDFLAEVYRSLAVLDGAILVISAKDGVQAQTRILFHALRKMN 120
            TSFQW   KVNI
+DTPGHMDFLAEVYRSLAVLDGAILVISAKDGVQAQTRILFHALRKMN
Sbjct 61

TSFQWEDVKVNIIDTPGHMDFLAEVYRSLAVLDGAILVISAKDGVQAQTRILFHALRKMN 120

Query 121
IPTVIFINKIDQAGVDLQSVVQSVRDKLSADIIIKQTVSLSPEIVLEENTDIEAWDAVIE 180

IPTVIFINKIDQAGVDLQSVVQSVRDKLSADIIIKQTVSLSPEIVLEENTDIEAWDAVIE
Sbjct 121
IPTVIFINKIDQAGVDLQSVVQSVRDKLSADIIIKQTVSLSPEIVLEENTDIEAWDAVIE 180

Query 181
NNDKLLEKYIAGEPISREKLVREEQRRVQDASLFPVYYGSAKKGLGIQPLMDAVTGLFQP 240
NND LLEKYIAGEPIS+EKL REEQRRVQ+ASLFPVY
+GSAKKGLGIQPLMDAVTGLFQP
Sbjct 181
NNDALLEKYIAGEPISQEKLAREEQRRVQEASLFPVYHGSAKKGLGIQPLMDAVTGLFQP 240

Query 241
IGEQGSAALCGSVFKVEYTDCGQRRVYLRLYSGTLRLRDTVALAGREKLKITEMRIPSKG 300
IGEQGSA
LCGSVFKVEYTDCGQRRVYLRLYSGTLRLRDTVALAGREKLKITEMRIPSKG
Sbjct 241
IGEQGSATLCGSVFKVEYTDCGQRRVYLRLYSGTLRLRDTVALAGREKLKITEMRIPSKG 300

Query 301
EIVRTDTAYPGEIVILPSDSVRLNDVLGDPTRLPRKRWREDPLPMLRTTIAPKTAAQRER 360
EIVRTDTAYPGEIVILPSDSVRLNDVLGD TRLPRKRWRE
PLPMLRTTIAPKTAAQRER
Sbjct 301
EIVRTDTAYPGEIVILPSDSVRLNDVLGDQTRLPRKRWREAPLPMLRTTIAPKTAAQRER 360

Query 361
LLDALTQLADTDPLLRCEVDSITHEIILSFLGRVQLEVVSALLSEKYKLETVVKEPSVIY 420
LLDALTQLADTDPLLRCEVDSITHEIILSFLGRVQLEVVSALLSEKYK+ETVVKEP
+VIY
Sbjct 361
LLDALTQLADTDPLLRCEVDSITHEIILSFLGRVQLEVVSALLSEKYKIETVVKEPTVIY 420

Query 421
MERPLKAASHTIHIEVPPNPFWASIGLSVTPLSLGSGVQYESRVSLGYLNQSFQNAVRDG 480
MERPLKAASHTIHIEVPPNPFWASIGLSVTPL
LGSGVQYESRVSLGYLNQSFQNAVRDG
Sbjct 421
MERPLKAASHTIHIEVPPNPFWASIGLSVTPLPLGSGVQYESRVSLGYLNQSFQNAVRDG 480

Query 481
IRYGLEQGLFGWNVTDCKICFEYGLYYSPVSTPADFRSLAPIVLEQALKESGTQLLEPYL 540

IRYGLEQGLFGWNVTDCKICFEYGLYYSPVSTPADFRSLAPIVLEQALKESGTQLLEPYL
Sbjct 481
IRYGLEQGLFGWNVTDCKICFEYGLYYSPVSTPADFRSLAPIVLEQALKESGTQLLEPYL 540

Query 541
SFILYAPQEYLSRAYHDAPKYCATIETAQVKKDEVVFTGEIPARCIQAYRTDLAFYTNGR 600
SF

LYAPQEYLSRAYHDAPKYCATIETAQVKKDEVVFTGEIPARCIQAYRTDLAFYTNG+
Sbjct 541
SFTLYAPQEYLSRAYHDAPKYCATIETAQVKKDEVVFTGEIPARCIQAYRTDLAFYTNGQ 600

Query 601 SVCLTELKGYQAAVGQPVIQPRRPNSRLDKVRHMFQKVM 639
            SVCLTELKGYQAAVGQPVIQPRRPN+RLDKVRHMFQKVM
Sbjct 601 SVCLTELKGYQAAVGQPVIQPRRPNNRLDKVRHMFQKVM 639

>WP_066743970.1 TetM/TetW/TetO/TetS family tetracycline resistance ribosomal
protection protein [Cloacibacillus porcorum]
 ANZ44645.1 tetracycline resistance ribosomal protection protein [Cloacibacillus
porcorum]
Length=639

 Score = 1259 bits (3259), Expect = 0.0, Method: Compositional matrix adjust.
 Identities = 608/638 (95%), Positives = 622/638 (97%), Gaps = 0/638 (0%)

Query 1
MKIINIGILAHVDAGKTTLTESLLYASGAISEPGSVEKGTTRTDTMFLERQRGITIQAAV 60
            M IINIGILAHVDAGKTTLTESLLYASG
ISEPGSVEKGTTRTDTMFLERQRGITIQ AV
Sbjct 1
MNIINIGILAHVDAGKTTLTESLLYASGTISEPGSVEKGTTRTDTMFLERQRGITIQTAV 60

Query 61
TSFQWHRCKVNIVDTPGHMDFLAEVYRSLAVLDGAILVISAKDGVQAQTRILFHALRKMN 120
            TSFQWH CKVNIVDTPGHMDFLAEVYRSLAVLDGAILV+SAKDGVQAQTR
+LFHALRK+N
Sbjct 61
TSFQWHSCKVNIVDTPGHMDFLAEVYRSLAVLDGAILVLSAKDGVQAQTRVLFHALRKLN 120

Query 121
IPTVIFINKIDQAGVDLQSVVQSVRDKLSADIIIKQTVSLSPEIVLEENTDIEAWDAVIE 180
            IPT+IFINKIDQ +DL+ V
QSVRDKLSADIIIKQTVSLSPEIVLEENTDIEAWDAVIE
Sbjct 121
IPTIIFINKIDQVDIDLEGVYQSVRDKLSADIIIKQTVSLSPEIVLEENTDIEAWDAVIE 180

Query 181
NNDKLLEKYIAGEPISREKLVREEQRRVQDASLFPVYYGSAKKGLGIQPLMDAVTGLFQP 240
            NND LLEKYIAGEPIS+EKL REEQRRVQ+ASLFPVY
+GSAKKGLGIQPLMDAVTGLFQP
Sbjct 181
NNDALLEKYIAGEPISQEKLAREEQRRVQEASLFPVYHGSAKKGLGIQPLMDAVTGLFQP 240

Query 241
IGEQGSAALCGSVFKVEYTDCGQRRVYLRLYSGTLRLRDTVALAGREKLKITEMRIPSKG 300
            IGEQGSA
LCGSVFKVEYTDCGQRRVYLRLYSGTLRLRDTVALAGREKLKITEMRIPSKG

Sbjct 241
IGEQGSATLCGSVFKVEYTDCGQRRVYLRLYSGTLRLRDTVALAGREKLKITEMRIPSKG 300

Query 301
EIVRTDTAYPGEIVILPSDSVRLNDVLGDPTRLPRKRWREDPLPMLRTTIAPKTAAQRER 360
EIVRTDTAYPGEIVILPSDSVRLNDVLGD TRLPRKRWRE
PLPMLRTTIAPKTAAQRER
Sbjct 301
EIVRTDTAYPGEIVILPSDSVRLNDVLGDQTRLPRKRWREAPLPMLRTTIAPKTAAQRER 360

Query 361
LLDALTQLADTDPLLRCEVDSITHEIILSFLGRVQLEVVSALLSEKYKLETVVKEPSVIY 420
LLDALTQLADTDPLLRCEVDSITHEIILSFLGRVQLEVVSALLSEKYK+ETVVKEP
+VIY
Sbjct 361
LLDALTQLADTDPLLRCEVDSITHEIILSFLGRVQLEVVSALLSEKYKIETVVKEPTVIY 420

Query 421
MERPLKAASHTIHIEVPPNPFWASIGLSVTPLSLGSGVQYESRVSLGYLNQSFQNAVRDG 480
MERPLKAASHTIHIEVPPNPFWASIGLSVTPL
LGSGVQYESRVSLGYLNQSFQNAVRDG
Sbjct 421
MERPLKAASHTIHIEVPPNPFWASIGLSVTPLPLGSGVQYESRVSLGYLNQSFQNAVRDG 480

Query 481
IRYGLEQGLFGWNVTDCKICFEYGLYYSPVSTPADFRSLAPIVLEQALKESGTQLLEPYL 540

IRYGLEQGLFGWNVTDCKICFEYGLYYSPVSTPADFRSLAPIVLEQALKESGTQLLEPYL
Sbjct 481
IRYGLEQGLFGWNVTDCKICFEYGLYYSPVSTPADFRSLAPIVLEQALKESGTQLLEPYL 540

Query 541
SFILYAPQEYLSRAYHDAPKYCATIETAQVKKDEVVFTGEIPARCIQAYRTDLAFYTNGR 600
SF
LYAPQEYLSRAYHDAPKYCATIETAQVKKDEVVFTGEIPARCIQAYRTDLAFYTNG+
Sbjct 541
SFTLYAPQEYLSRAYHDAPKYCATIETAQVKKDEVVFTGEIPARCIQAYRTDLAFYTNGQ 600

Query 601 SVCLTELKGYQAAVGQPVIQPRRPNSRLDKVRHMFQKV 638
SVCLTELKGYQAAVGQPVIQPRRPN+RLDKVRHMF K+
Sbjct 601 SVCLTELKGYQAAVGQPVIQPRRPNNRLDKVRHMFSKI 638

>AMP53584.1 TetM-TetW-TetO-TetS, partial [uncultured bacterium]
AMP53593.1 TetM-TetW-TetO-TetS, partial [uncultured bacterium]
Length=625

Score = 1248 bits (3229), Expect = 0.0, Method: Compositional
matrix adjust.
Identities = 608/616 (99%), Positives = 611/616 (99%), Gaps = 0/616
(0%)

Query 22
SLLYASGAISEPGSVEKGTTRTDTMFLERQRGITIQAAVTSFQWHRCKVNIVDTPGHMDF 81

SLLYASGAISEPGSVEKGTTRTDTMFLERQRGITIQAAVTSFQWHRCKVNIVDTPGHMDF
Sbjct 1
SLLYASGAISEPGSVEKGTTRTDTMFLERQRGITIQAAVTSFQWHRCKVNIVDTPGHMDF 60

Query 82
LAEVYRSLAVLDGAILVISAKDGVQAQTRILFHALRKMNIPTVIFINKIDQAGVDLQSVV 141

LAEVYRSLAVLDGAILVISAKDGVQAQTRILFHALRKMNIPTVIFINKIDQAGVDLQSVV
Sbjct 61
LAEVYRSLAVLDGAILVISAKDGVQAQTRILFHALRKMNIPTVIFINKIDQAGVDLQSVV 120

Query 142
QSVRDKLSADIIIKQTVSLSPEIVLEENTDIEAWDAVIENNDKLLEKYIAGEPISREKLV 201
QSVRDKLSADIIIKQTVSLSPEIVLEENTDIEAWDAVIENND
+LLEKYIAGEPISREKL
Sbjct 121
QSVRDKLSADIIIKQTVSLSPEIVLEENTDIEAWDAVIENNDELLEKYIAGEPISREKLA 180

Query 202
REEQRRVQDASLFPVYYGSAKKGLGIQPLMDAVTGLFQPIGEQGSAALCGSVFKVEYTDC 261
REEQ+RVQDASLFPVY+GSAK GLGIQPLMDAVTGLFQPIGEQG
AALCGSVFKVEYTDC
Sbjct 181
REEQQRVQDASLFPVYHGSAKNGLGIQPLMDAVTGLFQPIGEQGGAALCGSVFKVEYTDC 240

Query 262
GQRRVYLRLYSGTLRLRDTVALAGREKLKITEMRIPSKGEIVRTDTAYPGEIVILPSDSV 321
GQRRVYLRLYSGTLRLRDTVALAGREKLKITEMRIPSKGEIVRTDTAY
GEIVILPSDSV
Sbjct 241
GQRRVYLRLYSGTLRLRDTVALAGREKLKITEMRIPSKGEIVRTDTAYQGEIVILPSDSV 300

Query 322
RLNDVLGDPTRLPRKRWREDPLPMLRTTIAPKTAAQRERLLDALTQLADTDPLLRCEVDS 381
RLNDVLGD
TRLPRKRWREDPLPMLRTTIAPKTAAQRERLLDALTQLADTDPLLRCEVDS
Sbjct 301
RLNDVLGDQTRLPRKRWREDPLPMLRTTIAPKTAAQRERLLDALTQLADTDPLLRCEVDS 360

Query 382
ITHEIILSFLGRVQLEVVSALLSEKYKLETVVKEPSVIYMERPLKAASHTIHIEVPPNPF 441

ITHEIILSFLGRVQLEVVSALLSEKYKLETVVKEPSVIYMERPLKAASHTIHIEVPPNPF
Sbjct 361
ITHEIILSFLGRVQLEVVSALLSEKYKLETVVKEPSVIYMERPLKAASHTIHIEVPPNPF 420

Query 442
WASIGLSVTPLSLGSGVQYESRVSLGYLNQSFQNAVRDGIRYGLEQGLFGWNVTDCKICF 501

WASIGLSVTPLSLGSGVQYESRVSLGYLNQSFQNAVRDGIRYGLEQGLFGWNVTDCKICF
Sbjct 421
WASIGLSVTPLSLGSGVQYESRVSLGYLNQSFQNAVRDGIRYGLEQGLFGWNVTDCKICF 480

Query 502
EYGLYYSPVSTPADFRSLAPIVLEQALKESGTQLLEPYLSFILYAPQEYLSRAYHDAPKY 561

EYGLYYSPVSTPADFRSLAPIVLEQALKESGTQLLEPYLSFILYAPQEYLSRAYHDAPKY
Sbjct 481
EYGLYYSPVSTPADFRSLAPIVLEQALKESGTQLLEPYLSFILYAPQEYLSRAYHDAPKY 540

Query 562
CATIETAQVKKDEVVFTGEIPARCIQAYRTDLAFYTNGRSVCLTELKGYQAAVGQPVIQP 621

CATIETAQVKKDEVVFTGEIPARCIQAYRTDLAFYTNGRSVCLTELKGYQAAVGQPVIQP
Sbjct 541
CATIETAQVKKDEVVFTGEIPARCIQAYRTDLAFYTNGRSVCLTELKGYQAAVGQPVIQP 600

Query 622 RRPNSRLDKVRHMFQK 637
RRPNSRLDKVRHMFQK
Sbjct 601 RRPNSRLDKVRHMFQK 616

>CAP09895.1 mosaic tetracycline resistance protein, partial
[Bifidobacterium
thermophilum]
Length=629

Score = 1247 bits (3226), Expect = 0.0, Method: Compositional
matrix adjust.
Identities = 607/629 (97%), Positives = 619/629 (98%), Gaps = 0/629
(0%)

Query 1
MKIINIGILAHVDAGKTTLTESLLYASGAISEPGSVEKGTTRTDTMFLERQRGITIQAAV 60
MKIIN+GILAHVDAGKTTLTESLLY SGAI+E GSV++GTTRTDTM
LERQRGITIQ AV
Sbjct 1
MKIINLGILAHVDAGKTTLTESLLYTSGAIAELGSVDEGTTRTDTMNLERQRGITIQTAV 60

Query 61
TSFQWHRCKVNIVDTPGHMDFLAEVYRSLAVLDGAILVISAKDGVQAQTRILFHALRKMN 120
TSFQW   KVNI
+DTPGHMDFLAEVYRSLAVLDGAILVISAKDGVQAQTRILFHALRKMN
Sbjct 61
TSFQWEDVKVNIIDTPGHMDFLAEVYRSLAVLDGAILVISAKDGVQAQTRILFHALRKMN 120

Query 121
IPTVIFINKIDQAGVDLQSVVQSVRDKLSADIIIKQTVSLSPEIVLEENTDIEAWDAVIE 180

IPTVIFINKIDQAGVDLQSVVQSVRDKLSADIIIKQTVSLSPEIVLEENTDIEAWDAVIE
Sbjct 121
IPTVIFINKIDQAGVDLQSVVQSVRDKLSADIIIKQTVSLSPEIVLEENTDIEAWDAVIE 180

Query 181
NNDKLLEKYIAGEPISREKLVREEQRRVQDASLFPVYYGSAKKGLGIQPLMDAVTGLFQP 240

NNDKLLEKYIAGEPISREKLVREEQRRVQDASLFPVYYGSAKKGLGIQPLMDAVTGLFQP

```
Sbjct  181
NNDKLLEKYIAGEPISREKLVREEQRRVQDASLFPVYYGSAKKGLGIQPLMDAVTGLFQP  240

Query  241
IGEQGSAALCGSVFKVEYTDCGQRRVYLRLYSGTLRLRDTVALAGREKLKITEMRIPSKG  300

IGEQGSAALCGSVFKVEYTDCGQRRVYLRLYSGTLRLRDTVALAGREKLKITEMRIPSKG
Sbjct  241
IGEQGSAALCGSVFKVEYTDCGQRRVYLRLYSGTLRLRDTVALAGREKLKITEMRIPSKG  300

Query  301
EIVRTDTAYPGEIVILPSDSVRLNDVLGDPTRLPRKRWREDPLPMLRTTIAPKTAAQRER  360
            EIVRTDTAYPGEIVILPSDSVRLNDVLGDPTRLPRKRWREDPLPMLRT
+IAPKTAAQRER
Sbjct  301
EIVRTDTAYPGEIVILPSDSVRLNDVLGDPTRLPRKRWREDPLPMLRTSIAPKTAAQRER  360

Query  361
LLDALTQLADTDPLLRCEVDSITHEIILSFLGRVQLEVVSALLSEKYKLETVVKEPSVIY  420
            LLDALTQLADTDPLLRCEVDSITHEIILSFLGRVQLEVVSALLSEKYKLETVVKEP
+VIY
Sbjct  361
LLDALTQLADTDPLLRCEVDSITHEIILSFLGRVQLEVVSALLSEKYKLETVVKEPTVIY  420

Query  421
MERPLKAASHTIHIEVPPNPFWASIGLSVTPLSLGSGVQYESRVSLGYLNQSFQNAVRDG  480
            +ERPLKAASHTIHIEVPPNPFWASIGLSVTPL
LGSGVQYESRVSLGYLNQSFQNAVRDG
Sbjct  421
IERPLKAASHTIHIEVPPNPFWASIGLSVTPLPLGSGVQYESRVSLGYLNQSFQNAVRDG  480

Query  481
IRYGLEQGLFGWNVTDCKICFEYGLYYSPVSTPADFRSLAPIVLEQALKESGTQLLEPYL  540

IRYGLEQGLFGWNVTDCKICFEYGLYYSPVSTPADFRSLAPIVLEQALKESGTQLLEPYL
Sbjct  481
IRYGLEQGLFGWNVTDCKICFEYGLYYSPVSTPADFRSLAPIVLEQALKESGTQLLEPYL  540

Query  541
SFILYAPQEYLSRAYHDAPKYCATIETAQVKKDEVVFTGEIPARCIQAYRTDLAFYTNGR  600
            SF LYAP+EYLSRAYHDAPKYCATIET
QVKKDEVVFTGEIPARCIQAYRTDLAFYTNG+
Sbjct  541
SFTLYAPREYLSRAYHDAPKYCATIETVQVKKDEVVFTGEIPARCIQAYRTDLAFYTNGQ  600

Query  601  SVCLTELKGYQAAVGQPVIQPRRPNSRLD  629
            SVCLTELKGYQAAVG+PVIQPRRPN+RLD
Sbjct  601  SVCLTELKGYQAAVGKPVIQPRRPNNRLD  629


>CAP09892.1 mosaic tetracycline resistance protein, partial
[Bifidobacterium
thermophilum]
Length=629
```

Score = 1246 bits (3223), Expect = 0.0, Method: Compositional matrix adjust.
Identities = 606/629 (96%), Positives = 619/629 (98%), Gaps = 0/629 (0%)

```
Query  1
MKIINIGILAHVDAGKTTLTESLLYASGAISEPGSVEKGTTRTDTMFLERQRGITIQAAV  60
            MKIIN+GILAHVDAGKTTLTESLLY SGAI+E GSV++GTTRTDTM
LERQRGITIQ AV
Sbjct  1
MKIINLGILAHVDAGKTTLTESLLYTSGAIAELGSVDEGTTRTDTMNLERQRGITIQTAV  60

Query  61
TSFQWHRCKVNIVDTPGHMDFLAEVYRSLAVLDGAILVISAKDGVQAQTRILFHALRKMN  120
            TSFQW   KVNI
+DTPGHMDFLAEVYRSLAVLDGAILVISAKDGVQAQTRILFHALRKMN
Sbjct  61
TSFQWEDVKVNIIDTPGHMDFLAEVYRSLAVLDGAILVISAKDGVQAQTRILFHALRKMN  120

Query  121
IPTVIFINKIDQAGVDLQSVVQSVRDKLSADIIIKQTVSLSPEIVLEENTDIEAWDAVIE  180

IPTVIFINKIDQAGVDLQSVVQSVRDKLSADIIIKQTVSLSPEIVLEENTDIEAWDAVIE
Sbjct  121
IPTVIFINKIDQAGVDLQSVVQSVRDKLSADIIIKQTVSLSPEIVLEENTDIEAWDAVIE  180

Query  181
NNDKLLEKYIAGEPISREKLVREEQRRVQDASLFPVYYGSAKKGLGIQPLMDAVTGLFQP  240

NNDKLLEKYIAGEPISREKLVREEQRRVQDASLFPVYYGSAKKGLGIQPLMDAVTGLFQP
Sbjct  181
NNDKLLEKYIAGEPISREKLVREEQRRVQDASLFPVYYGSAKKGLGIQPLMDAVTGLFQP  240

Query  241
IGEQGSAALCGSVFKVEYTDCGQRRVYLRLYSGTLRLRDTVALAGREKLKITEMRIPSKG  300

IGEQGSAALCGSVFKVEYTDCGQRRVYLRLYSGTLRLRDTVALAGREKLKITEMRIPSKG
Sbjct  241
IGEQGSAALCGSVFKVEYTDCGQRRVYLRLYSGTLRLRDTVALAGREKLKITEMRIPSKG  300

Query  301
EIVRTDTAYPGEIVILPSDSVRLNDVLGDPTRLPRKRWREDPLPMLRTTIAPKTAAQRER  360
            EIVRTDTAYPGEIVILPSDSVRL+DVLGDPTRLPRKRWREDPLPMLRT
+IAPKTAAQRER
Sbjct  301
EIVRTDTAYPGEIVILPSDSVRLSDVLGDPTRLPRKRWREDPLPMLRTSIAPKTAAQRER  360

Query  361
LLDALTQLADTDPLLRCEVDSITHEIILSFLGRVQLEVVSALLSEKYKLETVVKEPSVIY  420
            LL+ALTQLADTDPLLRCEVDSITHEIILSFLGRVQLEVVSALLSEKYKLETVVKEP
+VIY
Sbjct  361
LLNALTQLADTDPLLRCEVDSITHEIILSFLGRVQLEVVSALLSEKYKLETVVKEPTVIY  420
```

Query 421
MERPLKAASHTIHIEVPPNPFWASIGLSVTPLSLGSGVQYESRVSLGYLNQSFQNAVRDG 480
            MERPLKAASHTIHIEVPPNPFWASIGLSVTPL
LGSGVQYESRVSLGYLNQSFQNAVRDG
Sbjct 421
MERPLKAASHTIHIEVPPNPFWASIGLSVTPLPLGSGVQYESRVSLGYLNQSFQNAVRDG 480

Query 481
IRYGLEQGLFGWNVTDCKICFEYGLYYSPVSTPADFRSLAPIVLEQALKESGTQLLEPYL 540

IRYGLEQGLFGWNVTDCKICFEYGLYYSPVSTPADFRSLAPIVLEQALKESGTQLLEPYL
Sbjct 481
IRYGLEQGLFGWNVTDCKICFEYGLYYSPVSTPADFRSLAPIVLEQALKESGTQLLEPYL 540

Query 541
SFILYAPQEYLSRAYHDAPKYCATIETAQVKKDEVVFTGEIPARCIQAYRTDLAFYTNGR 600
            SF LYAP+EYLSRAYHDAPKYCATIET
QVKKDEVVFTGEIPARCIQAYRTDLAFYTNG+
Sbjct 541
SFTLYAPREYLSRAYHDAPKYCATIETVQVKKDEVVFTGEIPARCIQAYRTDLAFYTNGQ 600

Query 601 SVCLTELKGYQAAVGQPVIQPRRPNSRLD 629
            SVCLTELKGYQAAVG+PVIQPRRPN+RLD
Sbjct 601 SVCLTELKGYQAAVGKPVIQPRRPNNRLD 629

>AMP42147.1 tetracycline resistance protein Tet(W/N/W) [uncultured bacterium
IN-02]
Length=639

 Score = 1242 bits (3214), Expect = 0.0, Method: Compositional matrix adjust.
 Identities = 602/638 (94%), Positives = 618/638 (97%), Gaps = 0/638 (0%)

Query 1
MKIINIGILAHVDAGKTTLTESLLYASGAISEPGSVEKGTTRTDTMFLERQRGITIQAAV 60

MKIINIGILAHVDAGKTTLTESLLYASGAISEPGSVEKGTTRTDTMFLERQRGITIQAAV
Sbjct 1
MKIINIGILAHVDAGKTTLTESLLYASGAISEPGSVEKGTTRTDTMFLERQRGITIQAAV 60

Query 61
TSFQWHRCKVNIVDTPGHMDFLAEVYRSLAVLDGAILVISAKDGVQAQTRILFHALRKMN 120

TSFQWHRCKVNIVDTPGHMDFLAEVYRSLAVLDGAILVISAKDGVQAQTRILFHALRKMN
Sbjct 61
TSFQWHRCKVNIVDTPGHMDFLAEVYRSLAVLDGAILVISAKDGVQAQTRILFHALRKMN 120

Query 121
IPTVIFINKIDQAGVDLQSVVQSVRDKLSADIIIKQTVSLSPEIVLEENTDIEAWDAVIE 180
            IPTVIFINKIDQAGVDLQSV

QSVRDKLSADIIIKQTVSLSPEIVLEENTDIEAWDAVIE
Sbjct 121
IPTVIFINKIDQAGVDLQSVYQSVRDKLSADIIIKQTVSLSPEIVLEENTDIEAWDAVIE 180

Query 181
NNDKLLEKYIAGEPISREKLVREEQRRVQDASLFPVYYGSAKKGLGIQPLMDAVTGLFQP 240
NND+LLEKYIAGEPISREKL REEQ+RVQDASLFPVY+GSAK
GLGIQPLMDAVTGLFQP
Sbjct 181
NNDELLEKYIAGEPISREKLAREEQQRVQDASLFPVYHGSAKNGLGIQPLMDAVTGLFQP 240

Query 241
IGEQGSAALCGSVFKVEYTDCGQRRVYLRLYSGTLRLRDTVALAGREKLKITEMRIPSKG 300
IGEQG AALCGSVFKVEYTDCGQR
VYLRLYSGTLRLRDTVALAGREKLKITEMRIPSKG
Sbjct 241
IGEQGGAALCGSVFKVEYTDCGQRLVYLRLYSGTLRLRDTVALAGREKLKITEMRIPSKG 300

Query 301
EIVRTDTAYPGEIVILPSDSVRLNDVLGDPTRLPRKRWREDPLPMLRTTIAPKTAAQRER 360
EIVRTDTA+ GEIVILPSDS+RLND+LGD T+LPR+ W + P PMLRTTI PKTA
QR+R
Sbjct 301
EIVRTDTAHKGEIVILPSDSLRLNDILGDKTQLPREMWSDVPFPMLRTTITPKTAEQRDR 360

Query 361
LLDALTQLADTDPLLRCEVDSITHEIILSFLGRVQLEVVSALLSEKYKLETVVKEPSVIY 420
LLDALTQ+ADTDPLL EVDSITHEIILSFLGRVQLEVVSALLSEKYKLETVVKEP
+VIY
Sbjct 361
LLDALTQIADTDPLLHYEVDSITHEIILSFLGRVQLEVVSALLSEKYKLETVVKEPTVIY 420

Query 421
MERPLKAASHTIHIEVPPNPFWASIGLSVTPLSLGSGVQYESRVSLGYLNQSFQNAVRDG 480
MERPLKAASHTIHIEVPPNPFWASIGLSVTPL
LGSGVQYESRVSLGYLNQSFQNAVRDG
Sbjct 421
MERPLKAASHTIHIEVPPNPFWASIGLSVTPLPLGSGVQYESRVSLGYLNQSFQNAVRDG 480

Query 481
IRYGLEQGLFGWNVTDCKICFEYGLYYSPVSTPADFRSLAPIVLEQALKESGTQLLEPYL 540

IRYGLEQGLFGWNVTDCKICFEYGLYYSPVSTPADFRSLAPIVLEQALKESGTQLLEPYL
Sbjct 481
IRYGLEQGLFGWNVTDCKICFEYGLYYSPVSTPADFRSLAPIVLEQALKESGTQLLEPYL 540

Query 541
SFILYAPQEYLSRAYHDAPKYCATIETAQVKKDEVVFTGEIPARCIQAYRTDLAFYTNGR 600
SF LYAP+EYLSRAYHDAPKYCATIET
QVKKDEVVFTGEIPARCIQAYRTDLAFYTNGR
Sbjct 541
SFTLYAPREYLSRAYHDAPKYCATIETVQVKKDEVVFTGEIPARCIQAYRTDLAFYTNGR 600

Query 601 SVCLTELKGYQAAVGQPVIQPRRPNSRLDKVRHMFQKV 638

            SVCLTELKGYQA VG+P+IQPRRPNSRLDKVRHMF K+
Sbjct 601 SVCLTELKGYQATVGEPIIQPRRPNSRLDKVRHMFSKI 638

>AMP51298.1 TetM–TetW–TetO–TetS [uncultured bacterium]
Length=639

 Score = 1242 bits (3213), Expect = 0.0, Method: Compositional matrix adjust.
 Identities = 600/639 (94%), Positives = 617/639 (97%), Gaps = 0/639 (0%)

Query 1
MKIINIGILAHVDAGKTTLTESLLYASGAISEPGSVEKGTTRTDTMFLERQRGITIQAAV 60

MKIINIGILAHVDAGKTTLTESLLYASGAISEPGSVEKGTTRTDTMFLERQRGITIQAAV
Sbjct 1
MKIINIGILAHVDAGKTTLTESLLYASGAISEPGSVEKGTTRTDTMFLERQRGITIQAAV 60

Query 61
TSFQWHRCKVNIVDTPGHMDFLAEVYRSLAVLDGAILVISAKDGVQAQTRILFHALRKMN 120
            TSFQWHRCKVNIVDTPGHMDFLAEVYRSLAVLDGAILV+SAKDGVQAQTR
+LFHALRK+N
Sbjct 61
TSFQWHRCKVNIVDTPGHMDFLAEVYRSLAVLDGAILVLSAKDGVQAQTRVLFHALRKLN 120

Query 121
IPTVIFINKIDQAGVDLQSVVQSVRDKLSADIIIKQTVSLSPEIVLEENTDIEAWDAVIE 180
            IPT+IFINKIDQ G+DL+ V QSVRDKLSADIIIKQTVSLS +I L ENT E WD
+VIE
Sbjct 121
IPTIIFINKIDQVGIDLEGVYQSVRDKLSADIIIKQTVSLSSKITLTENTSAEVWDSVIE 180

Query 181
NNDKLLEKYIAGEPISREKLVREEQRRVQDASLFPVYYGSAKKGLGIQPLMDAVTGLFQP 240
            NND+LL KYIAGE IS+++L +EEQRRVQDASL PVY+GSAK GLGIQ
LMDAVTGLFQ
Sbjct 181
NNDELLAKYIAGESISQKELAQEEQRRVQDASLLPVYHGSAKNGLGIQQLMDAVTGLFQS 240

Query 241
IGEQGSAALCGSVFKVEYTDCGQRRVYLRLYSGTLRLRDTVALAGREKLKITEMRIPSKG 300
              EQGSAALCG VFKVEYTDCGQR
VYLRLYSGTLRLRDTVALAGREKLKITEMRIPSKG
Sbjct 241
TKEQGSAALCGRVFKVEYTDCGQRLVYLRLYSGTLRLRDTVALAGREKLKITEMRIPSKG 300

Query 301
EIVRTDTAYPGEIVILPSDSVRLNDVLGDPTRLPRKRWREDPLPMLRTTIAPKTAAQRER 360
            EIVRTDTA+ GEIVILPSDS+RLNDVLGD
TRLPRKRWREDPLPMLRTTIAPKTAAQRER
Sbjct 301
EIVRTDTAHKGEIVILPSDSLRLNDVLGDQTRLPRKRWREDPLPMLRTTIAPKTAAQRER 360

```
Query  361
LLDALTQLADTDPLLRCEVDSITHEIILSFLGRVQLEVVSALLSEKYKLETVVKEPSVIY  420

LLDALTQLADTDPLLRCEVDSITHEIILSFLGRVQLEVVSALLSEKYKLETVVKEPSVIY
Sbjct  361
LLDALTQLADTDPLLRCEVDSITHEIILSFLGRVQLEVVSALLSEKYKLETVVKEPSVIY  420

Query  421
MERPLKAASHTIHIEVPPNPFWASIGLSVTPLSLGSGVQYESRVSLGYLNQSFQNAVRDG  480

MERPLKAASHTIHIEVPPNPFWASIGLSVTPLSLGSGVQYESRVSLGYLNQSFQNAVRDG
Sbjct  421
MERPLKAASHTIHIEVPPNPFWASIGLSVTPLSLGSGVQYESRVSLGYLNQSFQNAVRDG  480

Query  481
IRYGLEQGLFGWNVTDCKICFEYGLYYSPVSTPADFRSLAPIVLEQALKESGTQLLEPYL  540

IRYGLEQGLFGWNVTDCKICFEYGLYYSPVSTPADFRSLAPIVLEQALKESGTQLLEPYL
Sbjct  481
IRYGLEQGLFGWNVTDCKICFEYGLYYSPVSTPADFRSLAPIVLEQALKESGTQLLEPYL  540

Query  541
SFILYAPQEYLSRAYHDAPKYCATIETAQVKKDEVVFTGEIPARCIQAYRTDLAFYTNGR  600

SFILYAPQEYLSRAYHDAPKYCATIETAQVKKDEVVFTGEIPARCIQAYRTDLAFYTNGR
Sbjct  541
SFILYAPQEYLSRAYHDAPKYCATIETAQVKKDEVVFTGEIPARCIQAYRTDLAFYTNGR  600

Query  601  SVCLTELKGYQAAVGQPVIQPRRPNSRLDKVRHMFQKVM  639
            SVCLTELKGYQAAVGQPVIQPRRPNSRLDKVRHMFQK+M
Sbjct  601  SVCLTELKGYQAAVGQPVIQPRRPNSRLDKVRHMFQKIM  639


>CAP09894.1 mosaic tetracycline resistance protein, partial
[Bifidobacterium
thermophilum]
Length=629

 Score = 1242 bits (3213),  Expect = 0.0, Method: Compositional
matrix adjust.
 Identities = 605/629 (96%), Positives = 616/629 (98%), Gaps = 0/629
(0%)

Query  1
MKIINIGILAHVDAGKTTLTESLLYASGAISEPGSVEKGTTRTDTMFLERQRGITIQAAV  60
            MKIIN+GILAHVDAGKTTLTESLLY SGAI+E GSV++GTTRTDTM
LERQRGITIQ AV
Sbjct  1
MKIINLGILAHVDAGKTTLTESLLYTSGAIAELGSVDEGTTRTDTMNLERQRGITIQTAV  60

Query  61
TSFQWHRCKVNIVDTPGHMDFLAEVYRSLAVLDGAILVISAKDGVQAQTRILFHALRKMN  120
            TSFQW   KVNI
+DTPGHMDFLAEVYRSLAVLDGAILVISAKDGVQAQTRILFHALRKMN
```

Sbjct 61
TSFQWEDVKVNIIDTPGHMDFLAEVYRSLAVLDGAILVISAKDGVQAQTRILFHALRKMN 120

Query 121
IPTVIFINKIDQAGVDLQSVVQSVRDKLSADIIIKQTVSLSPEIVLEENTDIEAWDAVIE 180

IPTVIFINKIDQAGVDLQSVVQSVRDKLSADIIIKQTVSLSPEIVLEENTDIEAWDAVIE
Sbjct 121
IPTVIFINKIDQAGVDLQSVVQSVRDKLSADIIIKQTVSLSPEIVLEENTDIEAWDAVIE 180

Query 181
NNDKLLEKYIAGEPISREKLVREEQRRVQDASLFPVYYGSAKKGLGIQPLMDAVTGLFQP 240
NNDKLLEKYIAGEPISREKL
REEQRRVQDASLFPVYYGSAKKGLGIQPLMDAVTGLFQP
Sbjct 181
NNDKLLEKYIAGEPISREKLAREEQRRVQDASLFPVYYGSAKKGLGIQPLMDAVTGLFQP 240

Query 241
IGEQGSAALCGSVFKVEYTDCGQRRVYLRLYSGTLRLRDTVALAGREKLKITEMRIPSKG 300

IGEQGSAALCGSVFKVEYTDCGQRRVYLRLYSGTLRLRDTVALAGREKLKITEMRIPSKG
Sbjct 241
IGEQGSAALCGSVFKVEYTDCGQRRVYLRLYSGTLRLRDTVALAGREKLKITEMRIPSKG 300

Query 301
EIVRTDTAYPGEIVILPSDSVRLNDVLGDPTRLPRKRWREDPLPMLRTTIAPKTAAQRER 360
EIVRTDTAYPGEIVILPSDSVRLNDVLGDPTRLPRKRWREDPLPMLRT
+IAPKTAAQR R
Sbjct 301
EIVRTDTAYPGEIVILPSDSVRLNDVLGDPTRLPRKRWREDPLPMLRTSIAPKTAAQRAR 360

Query 361
LLDALTQLADTDPLLRCEVDSITHEIILSFLGRVQLEVVSALLSEKYKLETVVKEPSVIY 420
LLDALTQLADTDPLLRCEVDSITHEIILSFLGRVQLEVVSALLSEKYKLETVVKEP
+VIY
Sbjct 361
LLDALTQLADTDPLLRCEVDSITHEIILSFLGRVQLEVVSALLSEKYKLETVVKEPTVIY 420

Query 421
MERPLKAASHTIHIEVPPNPFWASIGLSVTPLSLGSGVQYESRVSLGYLNQSFQNAVRDG 480
MERPLKAASHTIHIEVPPNPFWASIGLSVTPL
GSGVQYESRVSLGYLNQSFQNAVRDG
Sbjct 421
MERPLKAASHTIHIEVPPNPFWASIGLSVTPLPPGSGVQYESRVSLGYLNQSFQNAVRDG 480

Query 481
IRYGLEQGLFGWNVTDCKICFEYGLYYSPVSTPADFRSLAPIVLEQALKESGTQLLEPYL 540

IRYGLEQGLFGWNVTDCKICFEYGLYYSPVSTPADFRSLAPIVLEQALKESGTQLLEPYL
Sbjct 481
IRYGLEQGLFGWNVTDCKICFEYGLYYSPVSTPADFRSLAPIVLEQALKESGTQLLEPYL 540

Query 541
SFILYAPQEYLSRAYHDAPKYCATIETAQVKKDEVVFTGEIPARCIQAYRTDLAFYTNGR 600

            SF LYAP+EYLSRAYHDAPKYCATIET
QVKKDEVVFTGEIPARCIQAYRTDLAFYTNG+
Sbjct 541
SFTLYAPREYLSRAYHDAPKYCATIETVQVKKDEVVFTGEIPARCIQAYRTDLAFYTNGQ 600

Query 601 SVCLTELKGYQAAVGQPVIQPRRPNSRLD 629
            SVCLTELKGYQAAVG+PVIQPRRPN+RLD
Sbjct 601 SVCLTELKGYQAAVGKPVIQPRRPNNRLD 629

>ALE37170.1 tetracycline resistance protein TetW [Bifidobacterium longum]
 OQM49593.1 tetracycline resistance protein [Bifidobacterium longum]
Length=693

 Score = 1241 bits (3211), Expect = 0.0, Method: Compositional matrix adjust.
 Identities = 609/627 (97%), Positives = 614/627 (98%), Gaps = 3/627 (0%)

Query 1
MKIINIGILAHVDAGKTTLTESLLYASGAISEPGSVEKGTTRTDTMFLERQRGITIQAAV 60

MKIINIGILAHVDAGKTTLTESLLYASGAISEPGSVEKGTTRTDTMFLERQRGITIQAAV
Sbjct 1
MKIINIGILAHVDAGKTTLTESLLYASGAISEPGSVEKGTTRTDTMFLERQRGITIQAAV 60

Query 61
TSFQWHRCKVNIVDTPGHMDFLAEVYRSLAVLDGAILVISAKDGVQAQTRILFHALRKMN 120

TSFQWHRCKVNIVDTPGHMDFLAEVYRSLAVLDGAILVISAKDGVQAQTRILFHALRKMN
Sbjct 61
TSFQWHRCKVNIVDTPGHMDFLAEVYRSLAVLDGAILVISAKDGVQAQTRILFHALRKMN 120

Query 121
IPTVIFINKIDQAGVDLQSVVQSVRDKLSADIIIKQTVSLSPEIVLEENTDIEAWDAVIE 180

IPTVIFINKIDQAGVDLQSVVQSVRDKLSADIIIKQTVSLSPEIVLEENTDIEAWDAVIE
Sbjct 121
IPTVIFINKIDQAGVDLQSVVQSVRDKLSADIIIKQTVSLSPEIVLEENTDIEAWDAVIE 180

Query 181
NNDKLLEKYIAGEPISREKLVREEQRRVQDASLFPVYYGSAKKGLGIQPLMDAVTGLFQP 240

NNDKLLEKYIAGEPISREKLVREEQRRVQDASLFPVYYGSAKKGLGIQPLMDAVTGLFQP
Sbjct 181
NNDKLLEKYIAGEPISREKLVREEQRRVQDASLFPVYYGSAKKGLGIQPLMDAVTGLFQP 240

Query 241
IGEQGSAALCGSVFKVEYTDCGQRRVYLRLYSGTLRLRDTVALAGREKLKITEMRIPSKG 300

IGEQGSAALCGSVFKVEYTDCGQRRVYLRLYSGTLRLRDTVALAGREKLKITEMRIPSKG
Sbjct 241
IGEQGSAALCGSVFKVEYTDCGQRRVYLRLYSGTLRLRDTVALAGREKLKITEMRIPSKG 300

```
Query  301
EIVRTDTAYPGEIVILPSDSVRLNDVLGDPTRLPRKRWREDPLPMLRTTIAPKTAAQRER  360
            EIVRTDTAYPGEIVILPSDSVRLNDVLGDPTRLPRKRWREDPLPMLRT
+IAPKTAAQRER
Sbjct  301
EIVRTDTAYPGEIVILPSDSVRLNDVLGDPTRLPRKRWREDPLPMLRTSIAPKTAAQRER  360

Query  361
LLDALTQLADTDPLLRCEVDSITHEIILSFLGRVQLEVVSALLSEKYKLETVVKEPSVIY  420

LLDALTQLADTDPLLRCEVDSITHEIILSFLGRVQLEVVSALLSEKYKLETVVKEPSVIY
Sbjct  361
LLDALTQLADTDPLLRCEVDSITHEIILSFLGRVQLEVVSALLSEKYKLETVVKEPSVIY  420

Query  421
MERPLKAASHTIHIEVPPNPFWASIGLSVTPLSLGSGVQYESRVSLGYLNQSFQNAVRDG  480

MERPLKAASHTIHIEVPPNPFWASIGLSVTPLSLGSGVQYESRVSLGYLNQSFQNAVRDG
Sbjct  421
MERPLKAASHTIHIEVPPNPFWASIGLSVTPLSLGSGVQYESRVSLGYLNQSFQNAVRDG  480

Query  481
IRYGLEQGLFGWNVTDCKICFEYGLYYSPVSTPADFRSLAPIVLEQALKESGTQLLEPYL  540

IRYGLEQGLFGWNVTDCKICFEYGLYYSPVSTPADFRSLAPIVLEQALKESGTQLLEPYL
Sbjct  481
IRYGLEQGLFGWNVTDCKICFEYGLYYSPVSTPADFRSLAPIVLEQALKESGTQLLEPYL  540

Query  541
SFILYAPQEYLSRAYHDAPKYCATIETAQVKKDEVVFTGEIPARCIQAYRTDLAFYTNGR  600

SFILYAPQEYLSRAYHDAPKYCATIETAQVKKDEVVFTGEIPARCIQAYRTDLAFYTNGR
Sbjct  541
SFILYAPQEYLSRAYHDAPKYCATIETAQVKKDEVVFTGEIPARCIQAYRTDLAFYTNGR  600

Query  601  SVCLTE---LKGYQAAVGQPVIQPRRP  624
            SVCLTE   + G   + G P    ++P
Sbjct  601  SVCLTEPERISGRCRSAGHPAPPSKQP  627


>CAP09896.1 mosaic tetracycline resistance protein, partial
[Bifidobacterium
thermophilum]
Length=629

 Score = 1241 bits (3211),  Expect = 0.0, Method: Compositional
matrix adjust.
 Identities = 606/629 (96%), Positives = 617/629 (98%), Gaps = 0/629
(0%)

Query  1
MKIINIGILAHVDAGKTTLTESLLYASGAISEPGSVEKGTTRTDTMFLERQRGITIQAAV  60
            MKIIN+GI AHVDAGKTTLTESLLY SGAI+E GSV++GTTRTDTM
```

LERQRGITIQ AV
Sbjct 1
MKIINLGIPAHVDAGKTTLTESLLYTSGAIAELGSVDEGTTRTDTMNLERQRGITIQTAV 60

Query 61
TSFQWHRCKVNIVDTPGHMDFLAEVYRSLAVLDGAILVISAKDGVQAQTRILFHALRKMN 120
TSFQW KVNI
+DTPGHMDFLAEVYRSLAVLDGAILVISAKDGVQAQTRILFHALRKMN
Sbjct 61
TSFQWEDVKVNIIDTPGHMDFLAEVYRSLAVLDGAILVISAKDGVQAQTRILFHALRKMN 120

Query 121
IPTVIFINKIDQAGVDLQSVVQSVRDKLSADIIIKQTVSLSPEIVLEENTDIEAWDAVIE 180

IPTVIFINKIDQAGVDLQSVVQSVRDKLSADIIIKQTVSLSPEIVLEENTDIEAWDAVIE
Sbjct 121
IPTVIFINKIDQAGVDLQSVVQSVRDKLSADIIIKQTVSLSPEIVLEENTDIEAWDAVIE 180

Query 181
NNDKLLEKYIAGEPISREKLVREEQRRVQDASLFPVYYGSAKKGLGIQPLMDAVTGLFQP 240

NNDKLLEKYIAGEPISREKLVREEQRRVQDASLFPVYYGSAKKGLGIQPLMDAVTGLFQP
Sbjct 181
NNDKLLEKYIAGEPISREKLVREEQRRVQDASLFPVYYGSAKKGLGIQPLMDAVTGLFQP 240

Query 241
IGEQGSAALCGSVFKVEYTDCGQRRVYLRLYSGTLRLRDTVALAGREKLKITEMRIPSKG 300

IGEQGSAALCGSVFKVEYTDCGQRRVYLRLYSGTLRLRDTVALAGREKLKITEMRIPSKG
Sbjct 241
IGEQGSAALCGSVFKVEYTDCGQRRVYLRLYSGTLRLRDTVALAGREKLKITEMRIPSKG 300

Query 301
EIVRTDTAYPGEIVILPSDSVRLNDVLGDPTRLPRKRWREDPLPMLRTTIAPKTAAQRER 360
EIVRTDTAYPGEIVILPSDSVRLNDVLGDPTRLPRKR REDPLPMLRT
+IAPKTAAQRER
Sbjct 301
EIVRTDTAYPGEIVILPSDSVRLNDVLGDPTRLPRKRRREDPLPMLRTSIAPKTAAQRER 360

Query 361
LLDALTQLADTDPLLRCEVDSITHEIILSFLGRVQLEVVSALLSEKYKLETVVKEPSVIY 420
LLDALTQLADTDPLLRCEVDSITHEIILSFLGRVQLEVVSALLSEKYKLETVVKEP
+VIY
Sbjct 361
LLDALTQLADTDPLLRCEVDSITHEIILSFLGRVQLEVVSALLSEKYKLETVVKEPTVIY 420

Query 421
MERPLKAASHTIHIEVPPNPFWASIGLSVTPLSLGSGVQYESRVSLGYLNQSFQNAVRDG 480
MERPLKAASHTIHIEVPPNPFWASIGLSVTPL
LGSGVQYESRVSLGYLNQSFQNAVRDG
Sbjct 421
MERPLKAASHTIHIEVPPNPFWASIGLSVTPLPLGSGVQYESRVSLGYLNQSFQNAVRDG 480

Query 481

```
IRYGLEQGLFGWNVTDCKICFEYGLYYSPVSTPADFRSLAPIVLEQALKESGTQLLEPYL  540

IRYGLEQGLFGWNVTDCKICFEYGLYYSPVSTPADFRSLAPIVLEQALKESGTQLLEPYL
Sbjct  481
IRYGLEQGLFGWNVTDCKICFEYGLYYSPVSTPADFRSLAPIVLEQALKESGTQLLEPYL  540

Query  541
SFILYAPQEYLSRAYHDAPKYCATIETAQVKKDEVVFTGEIPARCIQAYRTDLAFYTNGR  600
            SF LYAP+EYLSRAYHDAPKYCATIET
QVKKDEVVFTGEIPARCIQAYRTDLAFYTNG+
Sbjct  541
SFTLYAPREYLSRAYHDAPKYCATIETVQVKKDEVVFTGEIPARCIQAYRTDLAFYTNGQ  600

Query  601  SVCLTELKGYQAAVGQPVIQPRRPNSRLD  629
            SVCLTELKGYQAAVG+PVIQPRRPN+RLD
Sbjct  601  SVCLTELKGYQAAVGKPVIQPRRPNNRLD  629


>AMP55635.1 TetM-TetW-TetO-TetS [uncultured bacterium]
 AMP55659.1 TetM-TetW-TetO-TetS [uncultured bacterium]
Length=639

 Score = 1239 bits (3207),  Expect = 0.0, Method: Compositional
matrix adjust.
 Identities = 603/639 (94%), Positives = 619/639 (97%), Gaps = 0/639
(0%)

Query  1
MKIINIGILAHVDAGKTTLTESLLYASGAISEPGSVEKGTTRTDTMFLERQRGITIQAAV  60

MKIINIGILAHVDAGKTTLTESLLYASGAISEPGSVEKGTTRTDTMFLERQRGITIQAAV
Sbjct  1
MKIINIGILAHVDAGKTTLTESLLYASGAISEPGSVEKGTTRTDTMFLERQRGITIQAAV  60

Query  61
TSFQWHRCKVNIVDTPGHMDFLAEVYRSLAVLDGAILVISAKDGVQAQTRILFHALRKMN  120

TSFQWHRCKVNIVDTPGHMDFLAEVYRSLAVLDGAILVISAKDGVQAQTRILFHALRKMN
Sbjct  61
TSFQWHRCKVNIVDTPGHMDFLAEVYRSLAVLDGAILVISAKDGVQAQTRILFHALRKMN  120

Query  121
IPTVIFINKIDQAGVDLQSVVQSVRDKLSADIIIKQTVSLSPEIVLEENTDIEAWDAVIE  180
            IPTVIFINKIDQAGVDLQSV
QSVRDKLSADIIIKQTVSLSPEIVLEENTDIEAWDAVIE
Sbjct  121
IPTVIFINKIDQAGVDLQSVYQSVRDKLSADIIIKQTVSLSPEIVLEENTDIEAWDAVIE  180

Query  181
NNDKLLEKYIAGEPISREKLVREEQRRVQDASLFPVYYGSAKKGLGIQPLMDAVTGLFQP  240
            NND+LL KYIAGE IS+++L +EEQRRVQDASL PVY+GSAK GLGIQ LMDAV
GLFQ
Sbjct  181
NNDELLAKYIAGESISQKELAQEEQRRVQDASLLPVYHGSAKNGLGIQQLMDAVIGLFQS  240
```

Query 241
IGEQGSAALCGSVFKVEYTDCGQRRVYLRLYSGTLRLRDTVALAGREKLKITEMRIPSKG 300
              EQGSAALCG VFKVEYTDCGQR
VYLRLYSGTLRLRDTVALAGREKLKITEMRIPSKG
Sbjct 241
TKEQGSAALCGRVFKVEYTDCGQRLVYLRLYSGTLRLRDTVALAGREKLKITEMRIPSKG 300

Query 301
EIVRTDTAYPGEIVILPSDSVRLNDVLGDPTRLPRKRWREDPLPMLRTTIAPKTAAQRER 360
            EIVRTDTA+ GEIVILPSDS+RLND+LGD TRLPR+RWR+
PLPMLRTTIAPKTAAQRER
Sbjct 301
EIVRTDTAHKGEIVILPSDSLRLNDILGDNTRLPRERWRDAPLPMLRTTIAPKTAAQRER 360

Query 361
LLDALTQLADTDPLLRCEVDSITHEIILSFLGRVQLEVVSALLSEKYKLETVVKEPSVIY 420
            LLDALTQLADTDPLLR EVDSITHEIILSFLGRVQLEVVSALLSEKYK+ET VKEP
+VIY
Sbjct 361
LLDALTQLADTDPLLRYEVDSITHEIILSFLGRVQLEVVSALLSEKYKIETAVKEPTVIY 420

Query 421
MERPLKAASHTIHIEVPPNPFWASIGLSVTPLSLGSGVQYESRVSLGYLNQSFQNAVRDG 480
            +ERPLK ASHTIHIEVPPNPFWASIGLSVTPL LGSGV
+YESRVSLGYLNQSFQNAVRDG
Sbjct 421
LERPLKVASHTIHIEVPPNPFWASIGLSVTPLPLGSGVKYESRVSLGYLNQSFQNAVRDG 480

Query 481
IRYGLEQGLFGWNVTDCKICFEYGLYYSPVSTPADFRSLAPIVLEQALKESGTQLLEPYL 540

IRYGLEQGLFGWNVTDCKICFEYGLYYSPVSTPADFRSLAPIVLEQALKESGTQLLEPYL
Sbjct 481
IRYGLEQGLFGWNVTDCKICFEYGLYYSPVSTPADFRSLAPIVLEQALKESGTQLLEPYL 540

Query 541
SFILYAPQEYLSRAYHDAPKYCATIETAQVKKDEVVFTGEIPARCIQAYRTDLAFYTNGR 600

SFILYAPQEYLSRAYHDAPKYCATIETAQVKKDEVVFTGEIPARCIQAYRTDLAFYTNGR
Sbjct 541
SFILYAPQEYLSRAYHDAPKYCATIETAQVKKDEVVFTGEIPARCIQAYRTDLAFYTNGR 600

Query 601 SVCLTELKGYQAAVGQPVIQPRRPNSRLDKVRHMFQKVM 639
            SVCLTELKGYQAAVGQPVIQPRRPNSRLDK+RHMFQKVM
Sbjct 601 SVCLTELKGYQAAVGQPVIQPRRPNSRLDKMRHMFQKVM 639

>WP_080908661.1 TetM/TetW/TetO/TetS family tetracycline resistance ribosomal
protection protein [Pseudoflavonifractor sp. Marseille-P3106]
Length=639

Score = 1239 bits (3206), Expect = 0.0, Method: Compositional

matrix adjust.
 Identities = 603/638 (95%), Positives = 618/638 (97%), Gaps = 0/638 (0%)

Query 1
MKIINIGILAHVDAGKTTLTESLLYASGAISEPGSVEKGTTRTDTMFLERQRGITIQAAV 60
            MKIINIGILAHVDAGKTTLTESLLYASGAISEPGSVEKGTTRTDTM LERQRGITIQAAV
Sbjct 1
MKIINIGILAHVDAGKTTLTESLLYASGAISEPGSVEKGTTRTDTMLLERQRGITIQAAV 60

Query 61
TSFQWHRCKVNIVDTPGHMDFLAEVYRSLAVLDGAILVISAKDGVQAQTRILFHALRKMN 120

TSFQWHRCKVNIVDTPGHMDFLAEVYRSLAVLDGAILVISAKDGVQAQTRILFHALRKM+
Sbjct 61
TSFQWHRCKVNIVDTPGHMDFLAEVYRSLAVLDGAILVISAKDGVQAQTRILFHALRKMD 120

Query 121
IPTVIFINKIDQAGVDLQSVVQSVRDKLSADIIIKQTVSLSPEIVLEENTDIEAWDAVIE 180
            IPTVIFINKIDQ GVDLQ V QSVRDKLSADIIIKQTVSLSPEIVLEENTDIEAWDAVIE
Sbjct 121
IPTVIFINKIDQTGVDLQGVYQSVRDKLSADIIIKQTVSLSPEIVLEENTDIEAWDAVIE 180

Query 181
NNDKLLEKYIAGEPISREKLVREEQRRVQDASLFPVYYGSAKKGLGIQPLMDAVTGLFQP 240
            NND+LLEKYIAGEPISRE+L REEQRRVQDASLFPVY +GSAKKGLGIQPLMDAVTGLFQP
Sbjct 181
NNDELLEKYIAGEPISREELAREEQRRVQDASLFPVYHGSAKKGLGIQPLMDAVTGLFQP 240

Query 241
IGEQGSAALCGSVFKVEYTDCGQRRVYLRLYSGTLRLRDTVALAGREKLKITEMRIPSKG 300
            IGEQGSAALCGSVFKVEYTDC QR +YLRLYSGTLRLRDTVALAGREKLKITEMRIPSKG
Sbjct 241
IGEQGSAALCGSVFKVEYTDCDQRLIYLRLYSGTLRLRDTVALAGREKLKITEMRIPSKG 300

Query 301
EIVRTDTAYPGEIVILPSDSVRLNDVLGDPTRLPRKRWREDPLPMLRTTIAPKTAAQRER 360
            EIVRTDTA+ GEIVILPSDSV LNDVLGD TRLPR+RWR+ PLPMLRTTIAPKTAAQRER
Sbjct 301
EIVRTDTAHKGEIVILPSDSVGLNDVLGDDTRLPRERWRDAPLPMLRTTIAPKTAAQRER 360

Query 361
LLDALTQLADTDPLLRCEVDSITHEIILSFLGRVQLEVVSALLSEKYKLETVVKEPSVIY 420
            LLDALTQ+ADTDPLLR EVDSITHEIILSFLGRVQLEVVSALL+EKYK+ET VKEP +VIY
Sbjct 361
LLDALTQIADTDPLLRYEVDSITHEIILSFLGRVQLEVVSALLAEKYKIETAVKEPTVIY 420

Query 421

MERPLKAASHTIHIEVPPNPFWASIGLSVTPLSLGSGVQYESRVSLGYLNQSFQNAVRDG 480
            +ERPLK ASHTIHIEVPPNPFWASIGLSVTPL
LGSGVQYESRVSLGYLNQSFQNAV DG
Sbjct 421
LERPLKVASHTIHIEVPPNPFWASIGLSVTPLPLGSGVQYESRVSLGYLNQSFQNAVMDG 480

Query 481
IRYGLEQGLFGWNVTDCKICFEYGLYYSPVSTPADFRSLAPIVLEQALKESGTQLLEPYL 540
            IRYGLEQGL
GWNVTDCKICFEYGLYYSPVSTPADFRSLAPIVLEQALKESGTQLLEPYL
Sbjct 481
IRYGLEQGLCGWNVTDCKICFEYGLYYSPVSTPADFRSLAPIVLEQALKESGTQLLEPYL 540

Query 541
SFILYAPQEYLSRAYHDAPKYCATIETAQVKKDEVVFTGEIPARCIQAYRTDLAFYTNGR 600
            SF LYAPQEYLSRAYHDAPKYCATIETAQVKKDEVVFTGEIP
RCIQAYRTDLAFYTNGR
Sbjct 541
SFTLYAPQEYLSRAYHDAPKYCATIETAQVKKDEVVFTGEIPVRCIQAYRTDLAFYTNGR 600

Query 601 SVCLTELKGYQAAVGQPVIQPRRPNSRLDKVRHMFQKV 638
            SVCLTELKGYQAAVG+PVIQPRRPNSRLDKVRHMF K+
Sbjct 601 SVCLTELKGYQAAVGEPVIQPRRPNSRLDKVRHMFSKI 638

>WP_102815896.1 TetM/TetW/TetO/TetS family tetracycline resistance ribosomal
protection protein [Lactobacillus reuteri]
 CUR40217.1 Tetracycline resistance protein TetM [Lactobacillus reuteri]
Length=639

 Score = 1238 bits (3204), Expect = 0.0, Method: Compositional matrix adjust.
 Identities = 599/638 (94%), Positives = 618/638 (97%), Gaps = 0/638 (0%)

Query 1
MKIINIGILAHVDAGKTTLTESLLYASGAISEPGSVEKGTTRTDTMFLERQRGITIQAAV 60
            MKIINIGILAHVDAGKTTLTESLLYASGAISEPGSV
+KGTTRTDTMFLERQRGITIQAAV
Sbjct 1
MKIINIGILAHVDAGKTTLTESLLYASGAISEPGSVKKGTTRTDTMFLERQRGITIQAAV 60

Query 61
TSFQWHRCKVNIVDTPGHMDFLAEVYRSLAVLDGAILVISAKDGVQAQTRILFHALRKMN 120

TSFQWHRCKVNIVDTPGHMDFLAEVYRSLAVLDGAILVISAKDGVQAQTRILFHALRKMN
Sbjct 61
TSFQWHRCKVNIVDTPGHMDFLAEVYRSLAVLDGAILVISAKDGVQAQTRILFHALRKMN 120

Query 121
IPTVIFINKIDQAGVDLQSVVQSVRDKLSADIIIKQTVSLSPEIVLEENTDIEAWDAVIE 180

IPTVIFINKIDQAGVDLQSVVQSVRDKLSADIIIKQTVSLSPEIVLEENTDIEAWDAVIE
Sbjct 121
IPTVIFINKIDQAGVDLQSVVQSVRDKLSADIIIKQTVSLSPEIVLEENTDIEAWDAVIE 180

Query 181
NNDKLLEKYIAGEPISREKLVREEQRRVQDASLFPVYYGSAKKGLGIQPLMDAVTGLFQP 240

NNDKLLEKYIAGEPISREKLVREEQRRVQDASLFPVYYGSAKKGLGIQPLMDAVTGLFQP
Sbjct 181
NNDKLLEKYIAGEPISREKLVREEQRRVQDASLFPVYYGSAKKGLGIQPLMDAVTGLFQP 240

Query 241
IGEQGSAALCGSVFKVEYTDCGQRRVYLRLYSGTLRLRDTVALAGREKLKITEMRIPSKG 300

IGEQGSAALCGSVFKVEYTDCGQRRVYLRLYSGTLRLRDTVALAGREKLKITEMRIPSKG
Sbjct 241
IGEQGSAALCGSVFKVEYTDCGQRRVYLRLYSGTLRLRDTVALAGREKLKITEMRIPSKG 300

Query 301
EIVRTDTAYPGEIVILPSDSVRLNDVLGDPTRLPRKRWREDPLPMLRTTIAPKTAAQRER 360
EIVRTDTAYPGEIVILPSDSVRLNDVLGDPTRLPRKRWREDPLPMLRT
+IAPKTAAQRER
Sbjct 301
EIVRTDTAYPGEIVILPSDSVRLNDVLGDPTRLPRKRWREDPLPMLRTSIAPKTAAQRER 360

Query 361
LLDALTQLADTDPLLRCEVDSITHEIILSFLGRVQLEVVSALLSEKYKLETVVKEPSVIY 420
LLDALTQLADTDPLLR EVDSITHEIILSFLGRVQLEVVSALLSEKYKLETVVKEP
+VIY
Sbjct 361
LLDALTQLADTDPLLRYEVDSITHEIILSFLGRVQLEVVSALLSEKYKLETVVKEPTVIY 420

Query 421
MERPLKAASHTIHIEVPPNPFWASIGLSVTPLSLGSGVQYESRVSLGYLNQSFQNAVRDG 480
MERPLKAASHTIHIEVPPNPFWASIGLSVTPL
LGSGVQYESRVSLGYLNQSFQNAVRDG
Sbjct 421
MERPLKAASHTIHIEVPPNPFWASIGLSVTPLPLGSGVQYESRVSLGYLNQSFQNAVRDG 480

Query 481
IRYGLEQGLFGWNVTDCKICFEYGLYYSPVSTPADFRSLAPIVLEQALKESGTQLLEPYL 540
IRYGLEQGLFGWNVTDCKICF+YGLYYSPVSTPADFR LAPIVLEQ LK++GT
+LLEPYL
Sbjct 481
IRYGLEQGLFGWNVTDCKICFKYGLYYSPVSTPADFRMLAPIVLEQVLKKAGTELLEPYL 540

Query 541
SFILYAPQEYLSRAYHDAPKYCATIETAQVKKDEVVFTGEIPARCIQAYRTDLAFYTNGR 600
SF +YAPQEYLSRAY+DAPKYCA I Q+K +EV+ +GEIPARCIQ YR+DL
+TNGR
Sbjct 541
SFKIYAPQEYLSRAYNDAPKYCANIVDTQLKNNEVILSGEIPARCIQEYRSDLTSFTNGR 600

Query 601 SVCLTELKGYQAAVGQPVIQPRRPNSRLDKVRHMFQKV 638

```
             SVCLTELKGY    G+PV QPRRPNSR+DKVR+MF K+
Sbjct  601  SVCLTELKGYHVTTGEPVCQPRRPNSRIDKVRYMFNKI  638


>WP_052822407.1 TetM/TetW/TetO/TetS family tetracycline resistance
ribosomal
protection protein [Bifidobacterium breve]
 KOA37393.1 tetracycline resistance protein tetM [Bifidobacterium
breve MCC
0476]
Length=639

 Score = 1237 bits (3201),  Expect = 0.0, Method: Compositional
matrix adjust.
 Identities = 602/638 (94%), Positives = 616/638 (97%), Gaps = 0/638
(0%)

Query  1
MKIINIGILAHVDAGKTTLTESLLYASGAISEPGSVEKGTTRTDTMFLERQRGITIQAAV  60
            M IINIGILAHVDAGKTTLTESLLYASGAISEPGSVEKGTTRTDTM
LERQRGITIQAAV
Sbjct  1
MNIINIGILAHVDAGKTTLTESLLYASGAISEPGSVEKGTTRTDTMLLERQRGITIQAAV  60

Query  61
TSFQWHRCKVNIVDTPGHMDFLAEVYRSLAVLDGAILVISAKDGVQAQTRILFHALRKMN  120

TSFQWHRCKVNIVDTPGHMDFLAEVYRSLAVLDGAILVISAKDGVQAQTRILFHALRKMN
Sbjct  61
TSFQWHRCKVNIVDTPGHMDFLAEVYRSLAVLDGAILVISAKDGVQAQTRILFHALRKMN  120

Query  121
IPTVIFINKIDQAGVDLQSVVQSVRDKLSADIIIKQTVSLSPEIVLEENTDIEAWDAVIE  180
            IPTVIFINKIDQAGVDLQ V QSVRDKLS DIIIKQTVSL
PEIVLEENTDIEAWDAVIE
Sbjct  121
IPTVIFINKIDQAGVDLQGVYQSVRDKLSVDIIIKQTVSLPPEIVLEENTDIEAWDAVIE  180

Query  181
NNDKLLEKYIAGEPISREKLVREEQRRVQDASLFPVYYGSAKKGLGIQPLMDAVTGLFQP  240
            NND+LLEKYIAGEPISRE+LVREEQRRVQDASLFPVY
+GSAKKGLGIQPLMDAVTGLFQP
Sbjct  181
NNDELLEKYIAGEPISREELVREEQRRVQDASLFPVYHGSAKKGLGIQPLMDAVTGLFQP  240

Query  241
IGEQGSAALCGSVFKVEYTDCGQRRVYLRLYSGTLRLRDTVALAGREKLKITEMRIPSKG  300
            IGEQGSAALCGSVFKVEYTDCGQR +YLRLYSGTL
LRDTVALAGREKLKITEMRIPSKG
Sbjct  241
IGEQGSAALCGSVFKVEYTDCGQRLIYLRLYSGTLHLRDTVALAGREKLKITEMRIPSKG  300

Query  301
EIVRTDTAYPGEIVILPSDSVRLNDVLGDPTRLPRKRWREDPLPMLRTTIAPKTAAQRER  360
```

EIVRTDTA+ GEIVILPSDSV LNDVLGD TRLPR+RWR+
PLPMLRTTIAPKTAAQRER
Sbjct 301
EIVRTDTAHKGEIVILPSDSVGLNDVLGDDTRLPRERWRDAPLPMLRTTIAPKTAAQRER 360

Query 361
LLDALTQLADTDPLLRCEVDSITHEIILSFLGRVQLEVVSALLSEKYKLETVVKEPSVIY 420
LLDALTQ+ADTDPLLR EVDSITHEIILSFLGRVQLEVVSALL+EKYK+ET VKEP
+VIY
Sbjct 361
LLDALTQIADTDPLLRYEVDSITHEIILSFLGRVQLEVVSALLAEKYKIETAVKEPTVIY 420

Query 421
MERPLKAASHTIHIEVPPNPFWASIGLSVTPLSLGSGVQYESRVSLGYLNQSFQNAVRDG 480
+ERPLK ASHTIHIEVPPNPFWASIGLSVTPL
LGSGVQYESRVSLGYLNQSFQNAV DG
Sbjct 421
LERPLKVASHTIHIEVPPNPFWASIGLSVTPLPLGSGVQYESRVSLGYLNQSFQNAVMDG 480

Query 481
IRYGLEQGLFGWNVTDCKICFEYGLYYSPVSTPADFRSLAPIVLEQALKESGTQLLEPYL 540
IRYGLEQGL
GWNVTDCKICFEYGLYYSPVSTPADFRSLAPIVLEQALKESGTQLLEPYL
Sbjct 481
IRYGLEQGLCGWNVTDCKICFEYGLYYSPVSTPADFRSLAPIVLEQALKESGTQLLEPYL 540

Query 541
SFILYAPQEYLSRAYHDAPKYCATIETAQVKKDEVVFTGEIPARCIQAYRTDLAFYTNGR 600
SF LYAPQEYLSRAYHDAPKYCATIETAQVKKDEVVFTGEIP
RCIQAYRTDLAFYTNGR
Sbjct 541
SFTLYAPQEYLSRAYHDAPKYCATIETAQVKKDEVVFTGEIPVRCIQAYRTDLAFYTNGR 600

Query 601 SVCLTELKGYQAAVGQPVIQPRRPNSRLDKVRHMFQKV 638
SVCLTELKGYQA VG+PVIQPRRPNSRLDKVRHMF K+
Sbjct 601 SVCLTELKGYQAVVGEPVIQPRRPNSRLDKVRHMFSKI 638

>WP_075704061.1 TetM/TetW/TetO/TetS family tetracycline resistance
ribosomal
protection protein [Intestinimonas butyriciproducens]
OLR66990.1 tetracycline resistance ribosomal protection protein
[Intestinimonas
butyriciproducens]
Length=639

Score = 1237 bits (3200), Expect = 0.0, Method: Compositional
matrix adjust.
Identities = 602/638 (94%), Positives = 617/638 (97%), Gaps = 0/638
(0%)

Query 1
MKIINIGILAHVDAGKTTLTESLLYASGAISEPGSVEKGTTRTDTMFLERQRGITIQAAV 60
M IINIGILAHVDAGKTTLTESLLYASGAISEPGSVEKGTTRTDTM

LERQRGITIQAAV
Sbjct 1
MNIINIGILAHVDAGKTTLTESLLYASGAISEPGSVEKGTTRTDTMLLERQRGITIQAAV 60

Query 61
TSFQWHRCKVNIVDTPGHMDFLAEVYRSLAVLDGAILVISAKDGVQAQTRILFHALRKMN 120

TSFQWHRCKVNIVDTPGHMDFLAEVYRSLAVLDGAILVISAKDGVQAQTRILFHALRKM+
Sbjct 61
TSFQWHRCKVNIVDTPGHMDFLAEVYRSLAVLDGAILVISAKDGVQAQTRILFHALRKMD 120

Query 121
IPTVIFINKIDQAGVDLQSVVQSVRDKLSADIIIKQTVSLSPEIVLEENTDIEAWDAVIE 180
IPTVIFINKIDQ GVDLQ V
QSVRDKLSADIIIKQTVSLSPEIVLEENTDIEAWDAVIE
Sbjct 121
IPTVIFINKIDQTGVDLQGVYQSVRDKLSADIIIKQTVSLSPEIVLEENTDIEAWDAVIE 180

Query 181
NNDKLLEKYIAGEPISREKLVREEQRRVQDASLFPVYYGSAKKGLGIQPLMDAVTGLFQP 240
NND+LLEKYIAGEPISRE+L REEQRRVQDASLFPVY
+GSAKKGLGIQPLMDAVTGLFQP
Sbjct 181
NNDELLEKYIAGEPISREELAREEQRRVQDASLFPVYHGSAKKGLGIQPLMDAVTGLFQP 240

Query 241
IGEQGSAALCGSVFKVEYTDCGQRRVYLRLYSGTLRLRDTVALAGREKLKITEMRIPSKG 300
IGEQGSAALCGSVFKVEYTDC QR
+YLRLYSGTLRLRDTVALAGREKLKITEMRIPSKG
Sbjct 241
IGEQGSAALCGSVFKVEYTDCDQRLIYLRLYSGTLRLRDTVALAGREKLKITEMRIPSKG 300

Query 301
EIVRTDTAYPGEIVILPSDSVRLNDVLGDPTRLPRKRWREDPLPMLRTTIAPKTAAQRER 360
EIVRTDTA+ GEIVILPSDSV LNDVLGD TRLPR+RWR+
PLPMLRTTIAPKTAAQRER
Sbjct 301
EIVRTDTAHKGEIVILPSDSVGLNDVLGDDTRLPRERWRDAPLPMLRTTIAPKTAAQRER 360

Query 361
LLDALTQLADTDPLLRCEVDSITHEIILSFLGRVQLEVVSALLSEKYKLETVVKEPSVIY 420
LLDALTQ+ADTDPLLR EVDSITHEIILSFLGRVQLEVVSALL+EKYK+ET VKEP
+VIY
Sbjct 361
LLDALTQIADTDPLLRYEVDSITHEIILSFLGRVQLEVVSALLAEKYKIETAVKEPTVIY 420

Query 421
MERPLKAASHTIHIEVPPNPFWASIGLSVTPLSLGSGVQYESRVSLGYLNQSFQNAVRDG 480
+ERPLK ASHTIHIEVPPNPFWASIGLSVTPL
LGSGVQYESRVSLGYLNQSFQNAV DG
Sbjct 421
LERPLKVASHTIHIEVPPNPFWASIGLSVTPLPLGSGVQYESRVSLGYLNQSFQNAVMDG 480

Query 481

```
IRYGLEQGLFGWNVTDCKICFEYGLYYSPVSTPADFRSLAPIVLEQALKESGTQLLEPYL  540
            IRYGLEQGL
GWNVTDCKICFEYGLYYSPVSTPADFRSLAPIVLEQALKESGTQLLEPYL
Sbjct  481
IRYGLEQGLCGWNVTDCKICFEYGLYYSPVSTPADFRSLAPIVLEQALKESGTQLLEPYL  540

Query  541
SFILYAPQEYLSRAYHDAPKYCATIETAQVKKDEVVFTGEIPARCIQAYRTDLAFYTNGR  600
            SF LYAPQEYLSRAYHDAPKYCATIETAQVKKDEVVFTGEIP
RCIQAYRTDLAFYTNGR
Sbjct  541
SFTLYAPQEYLSRAYHDAPKYCATIETAQVKKDEVVFTGEIPVRCIQAYRTDLAFYTNGR  600

Query  601  SVCLTELKGYQAAVGQPVIQPRRPNSRLDKVRHMFQKV  638
            SVCLTELKGYQAAVG+PVIQPRRPNSRLDKVRHMF K+
Sbjct  601  SVCLTELKGYQAAVGEPVIQPRRPNSRLDKVRHMFSKI  638


>WP_055244167.1 MULTISPECIES: TetM/TetW/TetO/TetS family
tetracycline resistance
ribosomal protection protein [Bacteria]
 CUP41015.1 Tetracycline resistance protein tetM from transposon
Tn916 [Anaerotruncus
colihominis]
 OVA26478.1 tetracycline resistance protein TetW [Klebsiella
pneumoniae]
Length=639

 Score = 1237 bits (3200),  Expect = 0.0, Method: Compositional
matrix adjust.
 Identities = 601/638 (94%), Positives = 615/638 (96%), Gaps = 0/638
(0%)

Query  1
MKIINIGILAHVDAGKTTLTESLLYASGAISEPGSVEKGTTRTDTMFLERQRGITIQAAV  60
            M IINIGILAHVDAGKTTLTESLLYASGAISEPGSVEKGTTRTDTM
LERQRGITIQAAV
Sbjct  1
MNIINIGILAHVDAGKTTLTESLLYASGAISEPGSVEKGTTRTDTMLLERQRGITIQAAV  60

Query  61
TSFQWHRCKVNIVDTPGHMDFLAEVYRSLAVLDGAILVISAKDGVQAQTRILFHALRKMN  120

TSFQWHRCKVNIVDTPGHMDFLAEVYRSLAVLDGAILVISAKDGVQAQTRILFHALRKMN
Sbjct  61
TSFQWHRCKVNIVDTPGHMDFLAEVYRSLAVLDGAILVISAKDGVQAQTRILFHALRKMN  120

Query  121
IPTVIFINKIDQAGVDLQSVVQSVRDKLSADIIIKQTVSLSPEIVLEENTDIEAWDAVIE  180
            IPTVIFINKIDQAGVDLQ V QSVRDKLS DIIIKQTVSL
PEIVLEENTDIEAWDAVIE
Sbjct  121
IPTVIFINKIDQAGVDLQGVYQSVRDKLSVDIIIKQTVSLPPEIVLEENTDIEAWDAVIE  180
```

Query 181
NNDKLLEKYIAGEPISREKLVREEQRRVQDASLFPVYYGSAKKGLGIQPLMDAVTGLFQP 240
NND+LLEKYIAGEPISRE+L REEQRRVQDASLFPVY
+GSAKKGLGIQPLMDAVTGLFQP
Sbjct 181
NNDELLEKYIAGEPISREELAREEQRRVQDASLFPVYHGSAKKGLGIQPLMDAVTGLFQP 240

Query 241
IGEQGSAALCGSVFKVEYTDCGQRRVYLRLYSGTLRLRDTVALAGREKLKITEMRIPSKG 300
IGEQGSAALCGSVFKVEYTDCGQR +YLRLYSGTL
LRDTVALAGREKLKITEMRIPSKG
Sbjct 241
IGEQGSAALCGSVFKVEYTDCGQRLIYLRLYSGTLHLRDTVALAGREKLKITEMRIPSKG 300

Query 301
EIVRTDTAYPGEIVILPSDSVRLNDVLGDPTRLPRKRWREDPLPMLRTTIAPKTAAQRER 360
EIVRTDTA+ GEIVILPSDSV LNDVLGD TRLPR+RWR+
PLPMLRTTIAPKTAAQRER
Sbjct 301
EIVRTDTAHKGEIVILPSDSVGLNDVLGDDTRLPRERWRDAPLPMLRTTIAPKTAAQRER 360

Query 361
LLDALTQLADTDPLLRCEVDSITHEIILSFLGRVQLEVVSALLSEKYKLETVVKEPSVIY 420
LLDALTQ+ADTDPLLR EVDSITHEIILSFLGRVQLEVVSALL+EKYK+ET VKEP
+VIY
Sbjct 361
LLDALTQIADTDPLLRYEVDSITHEIILSFLGRVQLEVVSALLAEKYKIETAVKEPTVIY 420

Query 421
MERPLKAASHTIHIEVPPNPFWASIGLSVTPLSLGSGVQYESRVSLGYLNQSFQNAVRDG 480
+ERPLK ASHTIHIEVPPNPFWASIGLSVTPL
LGSGVQYESRVSLGYLNQSFQNAV DG
Sbjct 421
LERPLKVASHTIHIEVPPNPFWASIGLSVTPLPLGSGVQYESRVSLGYLNQSFQNAVMDG 480

Query 481
IRYGLEQGLFGWNVTDCKICFEYGLYYSPVSTPADFRSLAPIVLEQALKESGTQLLEPYL 540
IRYGLEQGL
GWNVTDCKICFEYGLYYSPVSTPADFRSLAPIVLEQALKESGTQLLEPYL
Sbjct 481
IRYGLEQGLCGWNVTDCKICFEYGLYYSPVSTPADFRSLAPIVLEQALKESGTQLLEPYL 540

Query 541
SFILYAPQEYLSRAYHDAPKYCATIETAQVKKDEVVFTGEIPARCIQAYRTDLAFYTNGR 600
SF LYAPQEYLSRAYHDAPKYCATIETAQVKKDEVVFTGEIP
RCIQAYRTDLAFYTNGR
Sbjct 541
SFTLYAPQEYLSRAYHDAPKYCATIETAQVKKDEVVFTGEIPVRCIQAYRTDLAFYTNGR 600

Query 601 SVCLTELKGYQAAVGQPVIQPRRPNSRLDKVRHMFQKV 638
SVCLTELKGYQA VG+PVIQPRRPNSRLDKVRHMF K+
Sbjct 601 SVCLTELKGYQAVVGEPVIQPRRPNSRLDKVRHMFSKI 638

>WP_070637913.1 TetM/TetW/TetO/TetS family tetracycline resistance ribosomal
protection protein [Olsenella sp. HMSC062G07]
 OFK23027.1 tetracycline resistance ribosomal protection protein [Olsenella
sp. HMSC062G07]
Length=639

 Score = 1235 bits (3195), Expect = 0.0, Method: Compositional matrix adjust.
 Identities = 601/638 (94%), Positives = 616/638 (97%), Gaps = 0/638 (0%)

```
Query  1
MKIINIGILAHVDAGKTTLTESLLYASGAISEPGSVEKGTTRTDTMFLERQRGITIQAAV  60
            M IINIGILAHVDAGKTTLTESLLYASGAISEPGSVEKGTTRTDTM
LERQRGITIQAAV
Sbjct  1
MNIINIGILAHVDAGKTTLTESLLYASGAISEPGSVEKGTTRTDTMLLERQRGITIQAAV  60

Query  61
TSFQWHRCKVNIVDTPGHMDFLAEVYRSLAVLDGAILVISAKDGVQAQTRILFHALRKMN  120

TSFQWHRCKVNIVDTPGHMDFLAEVYRSLAVLDGAILVISAKDGVQAQTRILFHALRKM+
Sbjct  61
TSFQWHRCKVNIVDTPGHMDFLAEVYRSLAVLDGAILVISAKDGVQAQTRILFHALRKMD  120

Query  121
IPTVIFINKIDQAGVDLQSVVQSVRDKLSADIIIKQTVSLSPEIVLEENTDIEAWDAVIE  180
            IPTVIFINKIDQ GVDLQ V
QSVRDKLSADIIIKQTVSLSPEIVLEENTDIEAWDAVIE
Sbjct  121
IPTVIFINKIDQTGVDLQGVYQSVRDKLSADIIIKQTVSLSPEIVLEENTDIEAWDAVIE  180

Query  181
NNDKLLEKYIAGEPISREKLVREEQRRVQDASLFPVYYGSAKKGLGIQPLMDAVTGLFQP  240
            NND+LLEKYIAGEPISRE+L REEQRRVQDASLFPVY
+GSAKKGLGIQPLMDAVTGLFQP
Sbjct  181
NNDELLEKYIAGEPISREELAREEQRRVQDASLFPVYHGSAKKGLGIQPLMDAVTGLFQP  240

Query  241
IGEQGSAALCGSVFKVEYTDCGQRRVYLRLYSGTLRLRDTVALAGREKLKITEMRIPSKG  300
            IGEQGSAALCGSVFKVEYTDC QR
+YLRLYSGTLRLRDTVALAGREKLKITEMRIPSKG
Sbjct  241
IGEQGSAALCGSVFKVEYTDCDQRLIYLRLYSGTLRLRDTVALAGREKLKITEMRIPSKG  300

Query  301
EIVRTDTAYPGEIVILPSDSVRLNDVLGDPTRLPRKRWREDPLPMLRTTIAPKTAAQRER  360
            EIVRTDTA+ GEIVILPSDSV LNDVLGD TRLPR+RWR+ PLPMLRT
IAPKTAAQRER
Sbjct  301
EIVRTDTAHKGEIVILPSDSVGLNDVLGDDTRLPRERWRDAPLPMLRTAIAPKTAAQRER  360
```

Query 361
LLDALTQLADTDPLLRCEVDSITHEIILSFLGRVQLEVVSALLSEKYKLETVVKEPSVIY 420
            LLDALTQ+ADTDPLLR EVDSITHEIILSFLGRVQLEVVSALL+EKYK+ET VKEP
+VIY
Sbjct 361
LLDALTQIADTDPLLRYEVDSITHEIILSFLGRVQLEVVSALLAEKYKIETAVKEPTVIY 420

Query 421
MERPLKAASHTIHIEVPPNPFWASIGLSVTPLSLGSGVQYESRVSLGYLNQSFQNAVRDG 480
            +ERPLK ASHTIHIEVPPNPFWASIGLSVTPL
LGSGVQYESRVSLGYLNQSFQNAV DG
Sbjct 421
LERPLKVASHTIHIEVPPNPFWASIGLSVTPLPLGSGVQYESRVSLGYLNQSFQNAVMDG 480

Query 481
IRYGLEQGLFGWNVTDCKICFEYGLYYSPVSTPADFRSLAPIVLEQALKESGTQLLEPYL 540
            IRYGLEQGL
GWNVTDCKICFEYGLYYSPVSTPADFRSLAPIVLEQALKESGTQLLEPYL
Sbjct 481
IRYGLEQGLCGWNVTDCKICFEYGLYYSPVSTPADFRSLAPIVLEQALKESGTQLLEPYL 540

Query 541
SFILYAPQEYLSRAYHDAPKYCATIETAQVKKDEVVFTGEIPARCIQAYRTDLAFYTNGR 600
            SF LYAPQEYLSRAYHDAPKYCATIETAQVKKDEVVFTGEIP
RCIQAYRTDLAFYTNGR
Sbjct 541
SFTLYAPQEYLSRAYHDAPKYCATIETAQVKKDEVVFTGEIPVRCIQAYRTDLAFYTNGR 600

Query 601 SVCLTELKGYQAAVGQPVIQPRRPNSRLDKVRHMFQKV 638
            SVCLTELKGYQAAVG+PVIQPRRPNSRLDKVRHMF K+
Sbjct 601 SVCLTELKGYQAAVGEPVIQPRRPNSRLDKVRHMFSKI 638

>CAP09893.1 mosaic tetracycline resistance protein, partial
[Bifidobacterium
thermophilum]
Length=629

 Score = 1234 bits (3194), Expect = 0.0, Method: Compositional
matrix adjust.
 Identities = 603/629 (96%), Positives = 615/629 (98%), Gaps = 0/629
(0%)

Query 1
MKIINIGILAHVDAGKTTLTESLLYASGAISEPGSVEKGTTRTDTMFLERQRGITIQAAV 60
            MKIIN+GILAHVDAGKTTLTESLLY SGAI+E GSV++GTTRTDTM
LERQRGITIQ AV
Sbjct 1
MKIINLGILAHVDAGKTTLTESLLYTSGAIAELGSVDEGTTRTDTMNLERQRGITIQTAV 60

Query 61
TSFQWHRCKVNIVDTPGHMDFLAEVYRSLAVLDGAILVISAKDGVQAQTRILFHALRKMN 120
            TSFQW   KVNI

+DTPGHMDFLAEVYRSLAVLDGAILVISAKDGVQAQTRILFHALRKMN
Sbjct 61
TSFQWEDVKVNIIDTPGHMDFLAEVYRSLAVLDGAILVISAKDGVQAQTRILFHALRKMN 120

Query 121
IPTVIFINKIDQAGVDLQSVVQSVRDKLSADIIIKQTVSLSPEIVLEENTDIEAWDAVIE 180

IPTVIFINKIDQAGVDLQSVVQSVRDKLSADIIIKQTVSLSPEIVLEENTDIEAWDAVIE
Sbjct 121
IPTVIFINKIDQAGVDLQSVVQSVRDKLSADIIIKQTVSLSPEIVLEENTDIEAWDAVIE 180

Query 181
NNDKLLEKYIAGEPISREKLVREEQRRVQDASLFPVYYGSAKKGLGIQPLMDAVTGLFQP 240
NNDKLL
KYIAGEPISREKLVREEQRRVQDASLFPVYYGSAKKGLGIQPLMDAVTGLFQP
Sbjct 181
NNDKLLGKYIAGEPISREKLVREEQRRVQDASLFPVYYGSAKKGLGIQPLMDAVTGLFQP 240

Query 241
IGEQGSAALCGSVFKVEYTDCGQRRVYLRLYSGTLRLRDTVALAGREKLKITEMRIPSKG 300
IGEQGSAALCG
VFKVEYTDCGQRRVYLRLYSGTLRLRDTVALAGREKLKITEMRIPSKG
Sbjct 241
IGEQGSAALCGRVFKVEYTDCGQRRVYLRLYSGTLRLRDTVALAGREKLKITEMRIPSKG 300

Query 301
EIVRTDTAYPGEIVILPSDSVRLNDVLGDPTRLPRKRWREDPLPMLRTTIAPKTAAQRER 360
EIVRTDTAYPGEIVILPSDSVRLNDVLGDPTRLPRKRWREDPLPMLRT
+IAPKTAAQRER
Sbjct 301
EIVRTDTAYPGEIVILPSDSVRLNDVLGDPTRLPRKRWREDPLPMLRTSIAPKTAAQRER 360

Query 361
LLDALTQLADTDPLLRCEVDSITHEIILSFLGRVQLEVVSALLSEKYKLETVVKEPSVIY 420
LL+ALTQLADTDPLLRCEVDSITHEIILSFLGRVQLEVVSALLSEKYKLETVVKEP
+VIY
Sbjct 361
LLNALTQLADTDPLLRCEVDSITHEIILSFLGRVQLEVVSALLSEKYKLETVVKEPTVIY 420

Query 421
MERPLKAASHTIHIEVPPNPFWASIGLSVTPLSLGSGVQYESRVSLGYLNQSFQNAVRDG 480
MERPLKAAS TIHIEVPPNPFWASIGLSVTPL
LGSGVQYESRVSLGYLNQSFQNAVRDG
Sbjct 421
MERPLKAASRTIHIEVPPNPFWASIGLSVTPLPLGSGVQYESRVSLGYLNQSFQNAVRDG 480

Query 481
IRYGLEQGLFGWNVTDCKICFEYGLYYSPVSTPADFRSLAPIVLEQALKESGTQLLEPYL 540

IRYGLEQGLFGWNVTDCKICFEYGLYYSPVSTPADFRSLAPIVLEQALKESGTQLLEPYL
Sbjct 481
IRYGLEQGLFGWNVTDCKICFEYGLYYSPVSTPADFRSLAPIVLEQALKESGTQLLEPYL 540

Query 541

SFILYAPQEYLSRAYHDAPKYCATIETAQVKKDEVVFTGEIPARCIQAYRTDLAFYTNGR 600
            SF LYAP+EYLSRAYHDAPKYCATIET QVKKDEVVFTGEIPAR
IQAYRTDLAFYTNG+
Sbjct 541
SFTLYAPREYLSRAYHDAPKYCATIETVQVKKDEVVFTGEIPARRIQAYRTDLAFYTNGQ 600

Query 601 SVCLTELKGYQAAVGQPVIQPRRPNSRLD 629
            SVCLTELKGYQAAVG+PVIQPRRPN+RLD
Sbjct 601 SVCLTELKGYQAAVGKPVIQPRRPNNRLD 629

>WP_101549584.1 TetM/TetW/TetO/TetS family tetracycline resistance ribosomal
protection protein [Anaerotruncus sp. AT3]
Length=639

 Score = 1229 bits (3180), Expect = 0.0, Method: Compositional matrix adjust.
 Identities = 598/638 (94%), Positives = 614/638 (96%), Gaps = 0/638 (0%)

Query 1
MKIINIGILAHVDAGKTTLTESLLYASGAISEPGSVEKGTTRTDTMFLERQRGITIQAAV 60
            M IINIGILAHVDAGKTTLTESLLYASGAISEPGSVEKGTTRTDTM
LERQRGITIQAAV
Sbjct 1
MNIINIGILAHVDAGKTTLTESLLYASGAISEPGSVEKGTTRTDTMLLERQRGITIQAAV 60

Query 61
TSFQWHRCKVNIVDTPGHMDFLAEVYRSLAVLDGAILVISAKDGVQAQTRILFHALRKMN 120

TSFQWHRCKVNIVDTPGHMDFLAEVYRSLAVLDGAILVISAKDGVQAQTRILFHALRKM+
Sbjct 61
TSFQWHRCKVNIVDTPGHMDFLAEVYRSLAVLDGAILVISAKDGVQAQTRILFHALRKMD 120

Query 121
IPTVIFINKIDQAGVDLQSVVQSVRDKLSADIIIKQTVSLSPEIVLEENTDIEAWDAVIE 180
            IPTVIFINKIDQ GVDLQ V
QSVRDKLSADIIIKQTVSLSPEIVLEENTDIEAWDAVIE
Sbjct 121
IPTVIFINKIDQTGVDLQGVYQSVRDKLSADIIIKQTVSLSPEIVLEENTDIEAWDAVIE 180

Query 181
NNDKLLEKYIAGEPISREKLVREEQRRVQDASLFPVYYGSAKKGLGIQPLMDAVTGLFQP 240
            NND+LLEKYIAGEPISRE+L REEQRRVQDASLFPVY
+GSAKKGLGIQPLMDAVTGLFQP
Sbjct 181
NNDELLEKYIAGEPISREELAREEQRRVQDASLFPVYHGSAKKGLGIQPLMDAVTGLFQP 240

Query 241
IGEQGSAALCGSVFKVEYTDCGQRRVYLRLYSGTLRLRDTVALAGREKLKITEMRIPSKG 300
            IGEQGSAALCGSVFKVEYTDC QR +YLRLYSGTLRLRDTVALAGREKLKITE
+RIP KG
Sbjct 241

IGEQGSAALCGSVFKVEYTDCDQRLIYLRLYSGTLRLRDTVALAGREKLKITEIRIPFKG 300

Query 301
EIVRTDTAYPGEIVILPSDSVRLNDVLGDPTRLPRKRWREDPLPMLRTTIAPKTAAQRER 360
            EIVRTDTA+ GEIVILPSDSV LNDVLGD TRLPR+RWR+ PLPMLRTTIAPKTA
QRER
Sbjct 301
EIVRTDTAHKGEIVILPSDSVGLNDVLGDDTRLPRERWRDAPLPMLRTTIAPKTATQRER 360

Query 361
LLDALTQLADTDPLLRCEVDSITHEIILSFLGRVQLEVVSALLSEKYKLETVVKEPSVIY 420
            LLDALTQ+ADTDPLLR EVDSITHEIILSFLGRVQLEVVSALL+EKYK+ET VKEP
+VIY
Sbjct 361
LLDALTQIADTDPLLRYEVDSITHEIILSFLGRVQLEVVSALLAEKYKIETAVKEPTVIY 420

Query 421
MERPLKAASHTIHIEVPPNPFWASIGLSVTPLSLGSGVQYESRVSLGYLNQSFQNAVRDG 480
            +ERPLK ASHTIHIEVPPNPFWASIGLSVTPL
LGSGVQYESRVSLGYLNQSFQNAV DG
Sbjct 421
LERPLKVASHTIHIEVPPNPFWASIGLSVTPLPLGSGVQYESRVSLGYLNQSFQNAVMDG 480

Query 481
IRYGLEQGLFGWNVTDCKICFEYGLYYSPVSTPADFRSLAPIVLEQALKESGTQLLEPYL 540
            IRYGLEQGL
GWNVTDCKICFEYGLYYSPVSTPADFRSLAPIVLEQALKESGTQLLEPYL
Sbjct 481
IRYGLEQGLCGWNVTDCKICFEYGLYYSPVSTPADFRSLAPIVLEQALKESGTQLLEPYL 540

Query 541
SFILYAPQEYLSRAYHDAPKYCATIETAQVKKDEVVFTGEIPARCIQAYRTDLAFYTNGR 600
            SF LYAPQEYLSRAYHDAPKYCATIETAQVKKDEVVF GEIP
RCIQAYRTDLAFYTNGR
Sbjct 541
SFTLYAPQEYLSRAYHDAPKYCATIETAQVKKDEVVFIGEIPVRCIQAYRTDLAFYTNGR 600

Query 601 SVCLTELKGYQAAVGQPVIQPRRPNSRLDKVRHMFQKV 638
            SVCLTELKGYQAAVG+PVIQPRRPNSRLDKVRHMF K+
Sbjct 601 SVCLTELKGYQAAVGEPVIQPRRPNSRLDKVRHMFSKI 638

>WP_026385517.1 TetM/TetW/TetO/TetS family tetracycline resistance ribosomal
protection protein [Acidaminococcus intestini]
Length=639

 Score = 1222 bits (3162), Expect = 0.0, Method: Compositional matrix adjust.
 Identities = 597/638 (94%), Positives = 615/638 (96%), Gaps = 0/638 (0%)

Query 1
MKIINIGILAHVDAGKTTLTESLLYASGAISEPGSVEKGTTRTDTMFLERQRGITIQAAV 60

M IINIGILAHVDAGKTTLTESLLYASGAISEPGSVEKGTTRTDTM
LERQRGITIQAAV
Sbjct 1
MNIINIGILAHVDAGKTTLTESLLYASGAISEPGSVEKGTTRTDTMLLERQRGITIQAAV 60

Query 61
TSFQWHRCKVNIVDTPGHMDFLAEVYRSLAVLDGAILVISAKDGVQAQTRILFHALRKMN 120
TSFQWHRCKVNIVDTPGHMDFLAEVYRSLAVLDGAILVISAKDGVQAQTRILF
+ALRKMN
Sbjct 61
TSFQWHRCKVNIVDTPGHMDFLAEVYRSLAVLDGAILVISAKDGVQAQTRILFYALRKMN 120

Query 121
IPTVIFINKIDQAGVDLQSVVQSVRDKLSADIIIKQTVSLSPEIVLEENTDIEAWDAVIE 180
IPTVIFINKIDQAGVDLQ V QSVRDKLSADIIIKQTVSLSPEIVLEENT
IEAWDAVIE
Sbjct 121
IPTVIFINKIDQAGVDLQGVYQSVRDKLSADIIIKQTVSLSPEIVLEENTGIEAWDAVIE 180

Query 181
NNDKLLEKYIAGEPISREKLVREEQRRVQDASLFPVYYGSAKKGLGIQPLMDAVTGLFQP 240
NND LLEKYIAGEPIS+E+L +EEQRRVQ ASLFPVY
+GSAKKGLGIQPLMDAVTGLFQP
Sbjct 181
NNDGLLEKYIAGEPISQEELAQEEQRRVQAASLFPVYHGSAKKGLGIQPLMDAVTGLFQP 240

Query 241
IGEQGSAALCGSVFKVEYTDCGQRRVYLRLYSGTLRLRDTVALAGREKLKITEMRIPSKG 300
IGEQGSAALCGSVFKVEYTDCGQR +YLRLYSGTL
LRDTVALAGREKLKITEMRIPSKG
Sbjct 241
IGEQGSAALCGSVFKVEYTDCGQRLIYLRLYSGTLCLRDTVALAGREKLKITEMRIPSKG 300

Query 301
EIVRTDTAYPGEIVILPSDSVRLNDVLGDPTRLPRKRWREDPLPMLRTTIAPKTAAQRER 360
EIVRTDTA+ GEIVI PSDSV LNDVLGD TRLPR+RWR+
PLPMLRTTIAPKTAAQRER
Sbjct 301
EIVRTDTAHKGEIVIFPSDSVGLNDVLGDQTRLPRERWRDAPLPMLRTTIAPKTAAQRER 360

Query 361
LLDALTQLADTDPLLRCEVDSITHEIILSFLGRVQLEVVSALLSEKYKLETVVKEPSVIY 420
LLDALTQ+ADTDPLLR EVDSITHEIILSFLGRVQLEVVSALL+EKYK+ET VKEP
+VIY
Sbjct 361
LLDALTQIADTDPLLRYEVDSITHEIILSFLGRVQLEVVSALLAEKYKIETAVKEPTVIY 420

Query 421
MERPLKAASHTIHIEVPPNPFWASIGLSVTPLSLGSGVQYESRVSLGYLNQSFQNAVRDG 480
+ERPLKAA+HTIHIEVPPNPFWASIGLSVTPL
LGSGVQYESRVSLGYLNQSFQNAV DG
Sbjct 421
LERPLKAANHTIHIEVPPNPFWASIGLSVTPLPLGSGVQYESRVSLGYLNQSFQNAVMDG 480

Query 481
IRYGLEQGLFGWNVTDCKICFEYGLYYSPVSTPADFRSLAPIVLEQALKESGTQLLEPYL 540
            IRYGLEQGL GWNVTDCKICFEYGLYYSPVSTPADFRSLAPIVLEQALK
+SGTQLLEPYL
Sbjct 481
IRYGLEQGLCGWNVTDCKICFEYGLYYSPVSTPADFRSLAPIVLEQALKKSGTQLLEPYL 540

Query 541
SFILYAPQEYLSRAYHDAPKYCATIETAQVKKDEVVFTGEIPARCIQAYRTDLAFYTNGR 600
            SF LYAPQEYLSRAYHDAPKYCATIETAQVKKDEVVFTGEIPAR
IQAYRTDLAFYTNGR
Sbjct 541
SFTLYAPQEYLSRAYHDAPKYCATIETAQVKKDEVVFTGEIPARYIQAYRTDLAFYTNGR 600

Query 601 SVCLTELKGYQAAVGQPVIQPRRPNSRLDKVRHMFQKV 638
            SVCLTELKGYQAAVG+PVIQPRRPNSRLDKVRHMF K+
Sbjct 601 SVCLTELKGYQAAVGEPVIQPRRPNSRLDKVRHMFSKI 638

>KFI80889.1 tetracycline resistance protein [Bifidobacterium
pullorum]
Length=594

 Score = 1219 bits (3154), Expect = 0.0, Method: Compositional
matrix adjust.
 Identities = 591/594 (99%), Positives = 593/594 (99%), Gaps = 0/594
(0%)

Query 46
MFLERQRGITIQAAVTSFQWHRCKVNIVDTPGHMDFLAEVYRSLAVLDGAILVISAKDGV 105

MFLERQRGITIQAAVTSFQWHRCKVNIVDTPGHMDFLAEVYRSLAVLDGAILVISAKDGV
Sbjct 1
MFLERQRGITIQAAVTSFQWHRCKVNIVDTPGHMDFLAEVYRSLAVLDGAILVISAKDGV 60

Query 106
QAQTRILFHALRKMNIPTVIFINKIDQAGVDLQSVVQSVRDKLSADIIIKQTVSLSPEIV 165

QAQTRILFHALRKMNIPTVIFINKIDQAGVDLQSVVQSVRDKLSADIIIKQTVSLSPEIV
Sbjct 61
QAQTRILFHALRKMNIPTVIFINKIDQAGVDLQSVVQSVRDKLSADIIIKQTVSLSPEIV 120

Query 166
LEENTDIEAWDAVIENNDKLLEKYIAGEPISREKLVREEQRRVQDASLFPVYYGSAKKGL 225
            LEENTDIEAWDAVIENND+LLEKYIAGEPISREKL
REEQRRVQDASLFPVYYGSAKKGL
Sbjct 121
LEENTDIEAWDAVIENNDELLEKYIAGEPISREKLAREEQRRVQDASLFPVYYGSAKKGL 180

Query 226
GIQPLMDAVTGLFQPIGEQGSAALCGSVFKVEYTDCGQRRVYLRLYSGTLRLRDTVALAG 285

GIQPLMDAVTGLFQPIGEQGSAALCGSVFKVEYTDCGQRRVYLRLYSGTLRLRDTVALAG
Sbjct 181

GIQPLMDAVTGLFQPIGEQGSAALCGSVFKVEYTDCGQRRVYLRLYSGTLRLRDTVALAG 240

Query 286
REKLKITEMRIPSKGEIVRTDTAYPGEIVILPSDSVRLNDVLGDPTRLPRKRWREDPLPM 345

REKLKITEMRIPSKGEIVRTDTAYPGEIVILPSDSVRLNDVLGDPTRLPRKRWREDPLPM
Sbjct 241
REKLKITEMRIPSKGEIVRTDTAYPGEIVILPSDSVRLNDVLGDPTRLPRKRWREDPLPM 300

Query 346
LRTTIAPKTAAQRERLLDALTQLADTDPLLRCEVDSITHEIILSFLGRVQLEVVSALLSE 405
LRT
+IAPKTAAQRERLLDALTQLADTDPLLRCEVDSITHEIILSFLGRVQLEVVSALLSE
Sbjct 301
LRTSIAPKTAAQRERLLDALTQLADTDPLLRCEVDSITHEIILSFLGRVQLEVVSALLSE 360

Query 406
KYKLETVVKEPSVIYMERPLKAASHTIHIEVPPNPFWASIGLSVTPLSLGSGVQYESRVS 465

KYKLETVVKEPSVIYMERPLKAASHTIHIEVPPNPFWASIGLSVTPLSLGSGVQYESRVS
Sbjct 361
KYKLETVVKEPSVIYMERPLKAASHTIHIEVPPNPFWASIGLSVTPLSLGSGVQYESRVS 420

Query 466
LGYLNQSFQNAVRDGIRYGLEQGLFGWNVTDCKICFEYGLYYSPVSTPADFRSLAPIVLE 525

LGYLNQSFQNAVRDGIRYGLEQGLFGWNVTDCKICFEYGLYYSPVSTPADFRSLAPIVLE
Sbjct 421
LGYLNQSFQNAVRDGIRYGLEQGLFGWNVTDCKICFEYGLYYSPVSTPADFRSLAPIVLE 480

Query 526
QALKESGTQLLEPYLSFILYAPQEYLSRAYHDAPKYCATIETAQVKKDEVVFTGEIPARC 585

QALKESGTQLLEPYLSFILYAPQEYLSRAYHDAPKYCATIETAQVKKDEVVFTGEIPARC
Sbjct 481
QALKESGTQLLEPYLSFILYAPQEYLSRAYHDAPKYCATIETAQVKKDEVVFTGEIPARC 540

Query 586 IQAYRTDLAFYTNGRSVCLTELKGYQAAVGQPVIQPRRPNSRLDKVRHMFQKVM
639
IQAYRTDLAFYTNGRSVCLTELKGYQAAVGQPVIQPRRPNSRLDKVRHMFQKVM
Sbjct 541 IQAYRTDLAFYTNGRSVCLTELKGYQAAVGQPVIQPRRPNSRLDKVRHMFQKVM
594

>WP_055161581.1 MULTISPECIES: TetM/TetW/TetO/TetS family
tetracycline resistance
ribosomal protection protein [Firmicutes]
CUN75580.1 Tetracycline resistance protein tetM from transposon
Tn916 [Mitsuokella
jalaludinii]
PST30064.1 TetM/TetW/TetO/TetS family tetracycline resistance
ribosomal
protection protein [[Clostridium] lavalense]
Length=639

Score = 1217 bits (3148), Expect = 0.0, Method: Compositional matrix adjust.
Identities = 595/638 (93%), Positives = 614/638 (96%), Gaps = 0/638 (0%)

```
Query  1
MKIINIGILAHVDAGKTTLTESLLYASGAISEPGSVEKGTTRTDTMFLERQRGITIQAAV  60
            M IINIGILAHVDAGKTTLTESLLYASGAISEPGSVEKGTTRTDTM
LERQRGITIQAAV
Sbjct  1
MNIINIGILAHVDAGKTTLTESLLYASGAISEPGSVEKGTTRTDTMLLERQRGITIQAAV  60

Query  61
TSFQWHRCKVNIVDTPGHMDFLAEVYRSLAVLDGAILVISAKDGVQAQTRILFHALRKMN  120
            TSF W RCKVNIVDTPGHMDFLAEVYRSLAVLDGAILVISAKDGVQAQTRILF
+ALRKMN
Sbjct  61
TSFLWRRCKVNIVDTPGHMDFLAEVYRSLAVLDGAILVISAKDGVQAQTRILFYALRKMN  120

Query  121
IPTVIFINKIDQAGVDLQSVVQSVRDKLSADIIIKQTVSLSPEIVLEENTDIEAWDAVIE  180
            IPTVIFINKIDQAGVDLQ V QSVRDKLSADIIIKQTVSLSPEIVL
+ENTDIEAWDAVIE
Sbjct  121
IPTVIFINKIDQAGVDLQGVYQSVRDKLSADIIIKQTVSLSPEIVLDENTDIEAWDAVIE  180

Query  181
NNDKLLEKYIAGEPISREKLVREEQRRVQDASLFPVYYGSAKKGLGIQPLMDAVTGLFQP  240
            NND+LLEKYIAGEPISRE+L +EEQRRVQDASLFPVY
+GSAKKGLGIQPLMDAVTGLFQP
Sbjct  181
NNDELLEKYIAGEPISREELAQEEQRRVQDASLFPVYHGSAKKGLGIQPLMDAVTGLFQP  240

Query  241
IGEQGSAALCGSVFKVEYTDCGQRRVYLRLYSGTLRLRDTVALAGREKLKITEMRIPSKG  300
            IGEQGSAALCG+VFKVEYTDC QR
+YLRLYSGTLRLRDTVALAGREKLKITEMRIPSKG
Sbjct  241
IGEQGSAALCGNVFKVEYTDCDQRLIYLRLYSGTLRLRDTVALAGREKLKITEMRIPSKG  300

Query  301
EIVRTDTAYPGEIVILPSDSVRLNDVLGDPTRLPRKRWREDPLPMLRTTIAPKTAAQRER  360
            EIVRTDTA+ GEIVILPSDSV LNDVLGD TRLPR+RW + PLPMLRTTI
PKTAAQRER
Sbjct  301
EIVRTDTAHKGEIVILPSDSVGLNDVLGDNTRLPRERWCDAPLPMLRTTITPKTAAQRER  360

Query  361
LLDALTQLADTDPLLRCEVDSITHEIILSFLGRVQLEVVSALLSEKYKLETVVKEPSVIY  420
            LLDALTQ+ADTDPLLR EVDSITHEIILSFLGRVQLEVVSALL+EKYK+ET VKEP
+VIY
Sbjct  361
LLDALTQIADTDPLLRYEVDSITHEIILSFLGRVQLEVVSALLAEKYKIETAVKEPTVIY  420
```

Query 421
MERPLKAASHTIHIEVPPNPFWASIGLSVTPLSLGSGVQYESRVSLGYLNQSFQNAVRDG 480
            +ERPLKAASHTIHIEVPPNPFWASIGLSVTPL
LGSGVQYESRVSLGYLNQSFQNAV DG
Sbjct 421
LERPLKAASHTIHIEVPPNPFWASIGLSVTPLPLGSGVQYESRVSLGYLNQSFQNAVMDG 480

Query 481
IRYGLEQGLFGWNVTDCKICFEYGLYYSPVSTPADFRSLAPIVLEQALKESGTQLLEPYL 540
            IRYGLEQGL GWNVTDCKICFEYGLYYSPVSTPADFRSLAPIVLEQALK
+SGTQLLEPYL
Sbjct 481
IRYGLEQGLCGWNVTDCKICFEYGLYYSPVSTPADFRSLAPIVLEQALKKSGTQLLEPYL 540

Query 541
SFILYAPQEYLSRAYHDAPKYCATIETAQVKKDEVVFTGEIPARCIQAYRTDLAFYTNGR 600
            SF LYAPQEYLSRAYHDAPKYCA IETAQVKKDEVVFTGEIPAR
IQAYRTDLAFYTNGR
Sbjct 541
SFTLYAPQEYLSRAYHDAPKYCAAIETAQVKKDEVVFTGEIPARYIQAYRTDLAFYTNGR 600

Query 601 SVCLTELKGYQAAVGQPVIQPRRPNSRLDKVRHMFQKV 638
            SVCLTELKGYQAAVG+PVIQPRRPNSRLDKVRHMF K+
Sbjct 601 SVCLTELKGYQAAVGEPVIQPRRPNSRLDKVRHMFSKI 638

>WP_077597359.1 TetM/TetW/TetO/TetS family tetracycline resistance ribosomal
protection protein [Olsenella urininfantis]
Length=639

Score = 1216 bits (3146), Expect = 0.0, Method: Compositional matrix adjust.
Identities = 594/638 (93%), Positives = 613/638 (96%), Gaps = 0/638 (0%)

Query 1
MKIINIGILAHVDAGKTTLTESLLYASGAISEPGSVEKGTTRTDTMFLERQRGITIQAAV 60
            M IINIGILAHVDAGKTTLTESLLYASGAISEPGSVEKGTTRTDTM
LERQRGITIQAAV
Sbjct 1
MNIINIGILAHVDAGKTTLTESLLYASGAISEPGSVEKGTTRTDTMLLERQRGITIQAAV 60

Query 61
TSFQWHRCKVNIVDTPGHMDFLAEVYRSLAVLDGAILVISAKDGVQAQTRILFHALRKMN 120
            TSF W RCKVNIVDTPGHMDFLAEVYRSLAVLDGAILVISAKDGVQAQTRILF
+ALRKM+
Sbjct 61
TSFLWRRCKVNIVDTPGHMDFLAEVYRSLAVLDGAILVISAKDGVQAQTRILFYALRKMD 120

Query 121
IPTVIFINKIDQAGVDLQSVVQSVRDKLSADIIIKQTVSLSPEIVLEENTDIEAWDAVIE 180
            IPTVIFINKIDQAGVDLQ V QSVRDKLSADIIIKQTVSLSPEIVL

+ENTNTDIEAWDAVIE
Sbjct 121
IPTVIFINKIDQAGVDLQGVYQSVRDKLSADIIIKQTVSLSPEIVLDENTDIEAWDAVIE 180

Query 181
NNDKLLEKYIAGEPISREKLVREEQRRVQDASLFPVYYGSAKKGLGIQPLMDAVTGLFQP 240
NND+LLEKYIAGEPISRE+L +EEQRRVQDASLFPVY
+GSAKKGLGIQPLMDAVTGLFQP
Sbjct 181
NNDELLEKYIAGEPISREELAQEEQRRVQDASLFPVYHGSAKKGLGIQPLMDAVTGLFQP 240

Query 241
IGEQGSAALCGSVFKVEYTDCGQRRVYLRLYSGTLRLRDTVALAGREKLKITEMRIPSKG 300
IGEQGSAALCG+VFKVEYTDC QR
+YLRLYSGTLRLRDTVALAGREKLKITEMRIPSKG
Sbjct 241
IGEQGSAALCGNVFKVEYTDCDQRLIYLRLYSGTLRLRDTVALAGREKLKITEMRIPSKG 300

Query 301
EIVRTDTAYPGEIVILPSDSVRLNDVLGDPTRLPRKRWREDPLPMLRTTIAPKTAAQRER 360
EIVRTDTA+ GEIVILPSDSV LNDVLGD TRLPR+RW + PLPMLRT I
PKTAAQRER
Sbjct 301
EIVRTDTAHKGEIVILPSDSVGLNDVLGDNTRLPRERWCDAPLPMLRTAITPKTAAQRER 360

Query 361
LLDALTQLADTDPLLRCEVDSITHEIILSFLGRVQLEVVSALLSEKYKLETVVKEPSVIY 420
LLDALTQ+ADTDPLLR EVDSITHEIILSFLGRVQLEVVSALL+EKYK+ET VKEP
+VIY
Sbjct 361
LLDALTQIADTDPLLRYEVDSITHEIILSFLGRVQLEVVSALLAEKYKIETAVKEPTVIY 420

Query 421
MERPLKAASHTIHIEVPPNPFWASIGLSVTPLSLGSGVQYESRVSLGYLNQSFQNAVRDG 480
MERPLKAASHTIHIEVPPNPFWASIGLSVTPL
LGSGVQYESRVSLGYLNQSFQNAV DG
Sbjct 421
MERPLKAASHTIHIEVPPNPFWASIGLSVTPLPLGSGVQYESRVSLGYLNQSFQNAVMDG 480

Query 481
IRYGLEQGLFGWNVTDCKICFEYGLYYSPVSTPADFRSLAPIVLEQALKESGTQLLEPYL 540
IRYGLEQGL GWNVTDCKICFEYGLYYSPVSTPADFRSLAPIVLEQALK
+SGTQLLEPYL
Sbjct 481
IRYGLEQGLCGWNVTDCKICFEYGLYYSPVSTPADFRSLAPIVLEQALKKSGTQLLEPYL 540

Query 541
SFILYAPQEYLSRAYHDAPKYCATIETAQVKKDEVVFTGEIPARCIQAYRTDLAFYTNGR 600
SF LYAPQEYLSRAYHDAPKYCA IETAQVKKDEVVFTGEIPAR
IQAYRTDLAFYTNGR
Sbjct 541
SFTLYAPQEYLSRAYHDAPKYCAAIETAQVKKDEVVFTGEIPARYIQAYRTDLAFYTNGR 600

Query 601 SVCLTELKGYQAAVGQPVIQPRRPNSRLDKVRHMFQKV 638

            SVCLTELKGYQAAVG+PVIQPRRPNSRLDKVRHMF K+
Sbjct 601 SVCLTELKGYQAAVGEPVIQPRRPNSRLDKVRHMFSKI 638

>WP_054336482.1 tetracycline resistance ribosomal protection protein Tet(W) [Megasphaera
elsdenii]
 AAO42740.1 tetracycline resistance protein [Megasphaera elsdenii]
 ALG42441.1 tetracycline resistance protein tetM [Megasphaera
elsdenii 14-14]
Length=639

 Score = 1215 bits (3143), Expect = 0.0, Method: Compositional
matrix adjust.
 Identities = 588/638 (92%), Positives = 610/638 (96%), Gaps = 0/638
(0%)

Query 1
MKIINIGILAHVDAGKTTLTESLLYASGAISEPGSVEKGTTRTDTMFLERQRGITIQAAV 60
            MKIIN+GILAHVDAGKTTLTESLLY SGAI+E GSV++GTTRTDTM
LERQRGITIQ AV
Sbjct 1
MKIINLGILAHVDAGKTTLTESLLYTSGAIAELGSVDEGTTRTDTMNLERQRGITIQTAV 60

Query 61
TSFQWHRCKVNIVDTPGHMDFLAEVYRSLAVLDGAILVISAKDGVQAQTRILFHALRKMN 120
            TSFQW   KVNI
+DTPGHMDFLAEVYRSLAVLDGAILVISAKDGVQAQTRILFHALRKMN
Sbjct 61
TSFQWEDVKVNIIDTPGHMDFLAEVYRSLAVLDGAILVISAKDGVQAQTRILFHALRKMN 120

Query 121
IPTVIFINKIDQAGVDLQSVVQSVRDKLSADIIIKQTVSLSPEIVLEENTDIEAWDAVIE 180

IPTVIFINKIDQAGVDLQSVVQSVRDKLSADIIIKQTVSLSPEIVLEENTDIEAWDAVIE
Sbjct 121
IPTVIFINKIDQAGVDLQSVVQSVRDKLSADIIIKQTVSLSPEIVLEENTDIEAWDAVIE 180

Query 181
NNDKLLEKYIAGEPISREKLVREEQRRVQDASLFPVYYGSAKKGLGIQPLMDAVTGLFQP 240
            NND LLEKYIAGEPIS+EKL REEQRRVQ+ASLFPVY
+GSAKKGLGIQPLMDAVTGLFQP
Sbjct 181
NNDALLEKYIAGEPISQEKLAREEQRRVQEASLFPVYHGSAKKGLGIQPLMDAVTGLFQP 240

Query 241
IGEQGSAALCGSVFKVEYTDCGQRRVYLRLYSGTLRLRDTVALAGREKLKITEMRIPSKG 300
            IGEQGSA
LCGSVFKVEYTDCGQRRVYLRLYSGTLRLRDTVALAGREKLKITEMRIPSKG
Sbjct 241
IGEQGSATLCGSVFKVEYTDCGQRRVYLRLYSGTLRLRDTVALAGREKLKITEMRIPSKG 300

Query 301
EIVRTDTAYPGEIVILPSDSVRLNDVLGDPTRLPRKRWREDPLPMLRTTIAPKTAAQRER 360

EIVRTDTAYPGEIVILPSDSVRLNDVLGD TRLPRKRWRE
PLPMLRTTIAPKTAAQRER
Sbjct 301
EIVRTDTAYPGEIVILPSDSVRLNDVLGDQTRLPRKRWREAPLPMLRTTIAPKTAAQRER 360

Query 361
LLDALTQLADTDPLLRCEVDSITHEIILSFLGRVQLEVVSALLSEKYKLETVVKEPSVIY 420
LLDALTQLADTDPLLRCEVDSITHEIILSFLGRVQLEVVSALLSEKYK+ETVVKEP
+VIY
Sbjct 361
LLDALTQLADTDPLLRCEVDSITHEIILSFLGRVQLEVVSALLSEKYKIETVVKEPTVIY 420

Query 421
MERPLKAASHTIHIEVPPNPFWASIGLSVTPLSLGSGVQYESRVSLGYLNQSFQNAVRDG 480
MERPLKAASHTIHIEVPPNPFWASIGLSVTPL
LGSGVQYESRVSLGYLNQSFQNAVRDG
Sbjct 421
MERPLKAASHTIHIEVPPNPFWASIGLSVTPLPLGSGVQYESRVSLGYLNQSFQNAVRDG 480

Query 481
IRYGLEQGLFGWNVTDCKICFEYGLYYSPVSTPADFRSLAPIVLEQALKESGTQLLEPYL 540

IRYGLEQGLFGWNVTDCKICFEYGLYYSPVSTPADFRSLAPIVLEQALKESGTQLLEPYL
Sbjct 481
IRYGLEQGLFGWNVTDCKICFEYGLYYSPVSTPADFRSLAPIVLEQALKESGTQLLEPYL 540

Query 541
SFILYAPQEYLSRAYHDAPKYCATIETAQVKKDEVVFTGEIPARCIQAYRTDLAFYTNGR 600
SF LYAPQEYLSRAYHDAP+YCA I + Q+K DEV+ GEIPARCIQ YR DL +
+TNG+
Sbjct 541
SFTLYAPQEYLSRAYHDAPRYCADIVSTQIKNDEVILKGEIPARCIQEYRNDLTYFTNGQ 600

Query 601 SVCLTELKGYQAAVGQPVIQPRRPNSRLDKVRHMFQKV 638
VCLTELKGYQ A+G+ + QPRRPNSR+DKVRHMF K+
Sbjct 601 GVCLTELKGYQPAIGKFICQPRRPNSRIDKVRHMFHKL 638

>PKU93883.1 tetracycline resistance protein [Bifidobacterium
pseudolongum
subsp. globosum]
PKV01677.1 tetracycline resistance protein [Bifidobacterium
pseudolongum
subsp. globosum]
Length=594

Score = 1210 bits (3131), Expect = 0.0, Method: Compositional
matrix adjust.
Identities = 587/594 (99%), Positives = 591/594 (99%), Gaps = 0/594
(0%)

Query 46
MFLERQRGITIQAAVTSFQWHRCKVNIVDTPGHMDFLAEVYRSLAVLDGAILVISAKDGV 105

MFLERQRGITIQAAVTSFQWHRCKVNIVDTPGHMDFLAEVYRSLAVLDGAILVISAKDGV
Sbjct 1
MFLERQRGITIQAAVTSFQWHRCKVNIVDTPGHMDFLAEVYRSLAVLDGAILVISAKDGV 60

Query 106
QAQTRILFHALRKMNIPTVIFINKIDQAGVDLQSVVQSVRDKLSADIIIKQTVSLSPEIV 165

QAQTRILFHALRKMNIPTVIFINKIDQAGVDLQSVVQSVRDKLSADIIIKQTVSLSPEIV
Sbjct 61
QAQTRILFHALRKMNIPTVIFINKIDQAGVDLQSVVQSVRDKLSADIIIKQTVSLSPEIV 120

Query 166
LEENTDIEAWDAVIENNDKLLEKYIAGEPISREKLVREEQRRVQDASLFPVYYGSAKKGL 225

LEENTDIEAWDAVIENNDKLLEKYIAGEPISREKLVREEQRRVQDASLFPVYYGSAKKGL
Sbjct 121
LEENTDIEAWDAVIENNDKLLEKYIAGEPISREKLVREEQRRVQDASLFPVYYGSAKKGL 180

Query 226
GIQPLMDAVTGLFQPIGEQGSAALCGSVFKVEYTDCGQRRVYLRLYSGTLRLRDTVALAG 285

GIQPLMDAVTGLFQPIGEQGSAALCGSVFKVEYTDCGQRRVYLRLYSGTLRLRDTVALAG
Sbjct 181
GIQPLMDAVTGLFQPIGEQGSAALCGSVFKVEYTDCGQRRVYLRLYSGTLRLRDTVALAG 240

Query 286
REKLKITEMRIPSKGEIVRTDTAYPGEIVILPSDSVRLNDVLGDPTRLPRKRWREDPLPM 345

REKLKITEMRIPSKGEIVRTDTAYPGEIVILPSDSVRLNDVLGDPTRLPRKRWREDPLPM
Sbjct 241
REKLKITEMRIPSKGEIVRTDTAYPGEIVILPSDSVRLNDVLGDPTRLPRKRWREDPLPM 300

Query 346
LRTTIAPKTAAQRERLLDALTQLADTDPLLRCEVDSITHEIILSFLGRVQLEVVSALLSE 405
LRT
+IAPKTAAQRERLLDALTQLADTDPLLRCEVDSITHEIILSFLGRVQLEVVSALLSE
Sbjct 301
LRTSIAPKTAAQRERLLDALTQLADTDPLLRCEVDSITHEIILSFLGRVQLEVVSALLSE 360

Query 406
KYKLETVVKEPSVIYMERPLKAASHTIHIEVPPNPFWASIGLSVTPLSLGSGVQYESRVS 465
KYKLETVVKEP+VIYMERPLKAASHTIHIEVPPNPFWASIGLSVTPL
LGSGVQYESRVS
Sbjct 361
KYKLETVVKEPTVIYMERPLKAASHTIHIEVPPNPFWASIGLSVTPLPLGSGVQYESRVS 420

Query 466
LGYLNQSFQNAVRDGIRYGLEQGLFGWNVTDCKICFEYGLYYSPVSTPADFRSLAPIVLE 525

LGYLNQSFQNAVRDGIRYGLEQGLFGWNVTDCKICFEYGLYYSPVSTPADFRSLAPIVLE
Sbjct 421
LGYLNQSFQNAVRDGIRYGLEQGLFGWNVTDCKICFEYGLYYSPVSTPADFRSLAPIVLE 480

Query 526

QALKESGTQLLEPYLSFILYAPQEYLSRAYHDAPKYCATIETAQVKKDEVVFTGEIPARC 585
            QALKESGTQLLEPYLSF LYAP+EYLSRAYHDAPKYCATIET
QVKKDEVVFTGEIPARC
Sbjct 481
QALKESGTQLLEPYLSFTLYAPREYLSRAYHDAPKYCATIETVQVKKDEVVFTGEIPARC 540

Query 586 IQAYRTDLAFYTNGRSVCLTELKGYQAAVGQPVIQPRRPNSRLDKVRHMFQKVM
639
            IQAYRTDLAFYTNG+SVCLTELKGYQAAVGQPVIQPRRPNSRLDKVRHMFQKVM
Sbjct 541 IQAYRTDLAFYTNGQSVCLTELKGYQAAVGQPVIQPRRPNSRLDKVRHMFQKVM
594

>WP_063856421.1 tetracycline resistance ribosomal protection protein Tet(W) [uncultured
bacterium]
 ABN79417.1 mosaic tetracycline resistance protein [uncultured
bacterium]
Length=639

 Score = 1207 bits (3123), Expect = 0.0, Method: Compositional
matrix adjust.
 Identities = 585/638 (92%), Positives = 607/638 (95%), Gaps = 0/638
(0%)

Query 1
MKIINIGILAHVDAGKTTLTESLLYASGAISEPGSVEKGTTRTDTMFLERQRGITIQAAV 60
            MKIIN+GILAHVDAGKTTLTESLLY SGAI+E GSV++GTTRTDTM
LERQRGITIQ AV
Sbjct 1
MKIINLGILAHVDAGKTTLTESLLYTSGAIAELGSVDEGTTRTDTMNLERQRGITIQTAV 60

Query 61
TSFQWHRCKVNIVDTPGHMDFLAEVYRSLAVLDGAILVISAKDGVQAQTRILFHALRKMN 120
            TSFQW   KVNI+DTPGHMDFLAEVYRSLAVLDGAILVISAKDGVQAQTRIL
HALRKMN
Sbjct 61
TSFQWEDVKVNIIDTPGHMDFLAEVYRSLAVLDGAILVISAKDGVQAQTRILLHALRKMN 120

Query 121
IPTVIFINKIDQAGVDLQSVVQSVRDKLSADIIIKQTVSLSPEIVLEENTDIEAWDAVIE 180

IPTVIFINKIDQAGVDLQSVVQSVRDKLSADIIIKQTVSLSPEIVLEENTDIEAWDAVIE
Sbjct 121
IPTVIFINKIDQAGVDLQSVVQSVRDKLSADIIIKQTVSLSPEIVLEENTDIEAWDAVIE 180

Query 181
NNDKLLEKYIAGEPISREKLVREEQRRVQDASLFPVYYGSAKKGLGIQPLMDAVTGLFQP 240
            NND LLEKYIAGEPIS+EKL REEQRRVQ+ASLFPVY
+GSAKKGLGIQPLMDAVTGLFQP
Sbjct 181
NNDALLEKYIAGEPISQEKLAREEQRRVQEASLFPVYHGSAKKGLGIQPLMDAVTGLFQP 240

Query 241

IGEQGSAALCGSVFKVEYTDCGQRRVYLRLYSGTLRLRDTVALAGREKLKITEMRIPSKG 300
            IGEQG
AALCGSVFKVEYTDCGQRRVYLRLYSGTLRLRDTVALAGREKLKITEMRIPSKG
Sbjct 241
IGEQGGAALCGSVFKVEYTDCGQRRVYLRLYSGTLRLRDTVALAGREKLKITEMRIPSKG 300

Query 301
EIVRTDTAYPGEIVILPSDSVRLNDVLGDPTRLPRKRWREDPLPMLRTTIAPKTAAQRER 360
            EIVRTDTAYPGEIVILPSDSVRLNDVLGD TRLPRKRWRE
PLPMLRTTIAPKTAAQRER
Sbjct 301
EIVRTDTAYPGEIVILPSDSVRLNDVLGDQTRLPRKRWREAPLPMLRTTIAPKTAAQRER 360

Query 361
LLDALTQLADTDPLLRCEVDSITHEIILSFLGRVQLEVVSALLSEKYKLETVVKEPSVIY 420
            LLDALTQLADTDPLLRCEVDSITHEIILSFLGRVQLEVVSALLSEKYK+ETVVKEP
+VIY
Sbjct 361
LLDALTQLADTDPLLRCEVDSITHEIILSFLGRVQLEVVSALLSEKYKIETVVKEPTVIY 420

Query 421
MERPLKAASHTIHIEVPPNPFWASIGLSVTPLSLGSGVQYESRVSLGYLNQSFQNAVRDG 480
             ERPLKAASHTIHIEVPPNPFWASIGLSVTPL
LGSGVQYESRVSLGYLNQSFQNAVRDG
Sbjct 421
TERPLKAASHTIHIEVPPNPFWASIGLSVTPLPLGSGVQYESRVSLGYLNQSFQNAVRDG 480

Query 481
IRYGLEQGLFGWNVTDCKICFEYGLYYSPVSTPADFRSLAPIVLEQALKESGTQLLEPYL 540

IRYGLEQGLFGWNVTDCKICFEYGLYYSPVSTPADFRSLAPIVLEQALKESGTQLLEPYL
Sbjct 481
IRYGLEQGLFGWNVTDCKICFEYGLYYSPVSTPADFRSLAPIVLEQALKESGTQLLEPYL 540

Query 541
SFILYAPQEYLSRAYHDAPKYCATIETAQVKKDEVVFTGEIPARCIQAYRTDLAFYTNGR 600
            SF LYAPQEYLSRAYHDAP+YCA I + Q+K DEV+ GEIPARCIQ YR DL +
+TN +
Sbjct 541
SFTLYAPQEYLSRAYHDAPRYCADIVSTQIKNDEVILKGEIPARCIQEYRNDLTYFTNEQ 600

Query 601 SVCLTELKGYQAAVGQPVIQPRRPNSRLDKVRHMFQKV 638
             VCLTELKGYQ A+G+ + QPRRPNSR+DKVRHMF K+
Sbjct 601 GVCLTELKGYQPAIGKFICQPRRPNSRIDKVRHMFHKL 638

>PKU99030.1 tetracycline resistance protein [Bifidobacterium
pseudolongum
subsp. pseudolongum]
 PKV06547.1 tetracycline resistance protein [Bifidobacterium
pseudolongum
subsp. pseudolongum]
Length=594

Score = 1206 bits (3120), Expect = 0.0, Method: Compositional matrix adjust.
Identities = 584/594 (98%), Positives = 591/594 (99%), Gaps = 0/594 (0%)

```
Query  46
MFLERQRGITIQAAVTSFQWHRCKVNIVDTPGHMDFLAEVYRSLAVLDGAILVISAKDGV  105

MFLERQRGITIQAAVTSFQWHRCKVNIVDTPGHMDFLAEVYRSLAVLDGAILVISAKDGV
Sbjct  1
MFLERQRGITIQAAVTSFQWHRCKVNIVDTPGHMDFLAEVYRSLAVLDGAILVISAKDGV  60

Query  106
QAQTRILFHALRKMNIPTVIFINKIDQAGVDLQSVVQSVRDKLSADIIIKQTVSLSPEIV  165

QAQTRILFHALRKMNIPTVIFINKIDQAGVDLQSVVQSVRDKLSADIIIKQTVSLSPEIV
Sbjct  61
QAQTRILFHALRKMNIPTVIFINKIDQAGVDLQSVVQSVRDKLSADIIIKQTVSLSPEIV  120

Query  166
LEENTDIEAWDAVIENNDKLLEKYIAGEPISREKLVREEQRRVQDASLFPVYYGSAKKGL  225

LEENTDIEAWDAVIENNDKLLEKYIAGEPISREKLVREEQRRVQDASLFPVYYGSAKKGL
Sbjct  121
LEENTDIEAWDAVIENNDKLLEKYIAGEPISREKLVREEQRRVQDASLFPVYYGSAKKGL  180

Query  226
GIQPLMDAVTGLFQPIGEQGSAALCGSVFKVEYTDCGQRRVYLRLYSGTLRLRDTVALAG  285

GIQPLMDAVTGLFQPIGEQGSAALCGSVFKVEYTDCGQRRVYLRLYSGTLRLRDTVALAG
Sbjct  181
GIQPLMDAVTGLFQPIGEQGSAALCGSVFKVEYTDCGQRRVYLRLYSGTLRLRDTVALAG  240

Query  286
REKLKITEMRIPSKGEIVRTDTAYPGEIVILPSDSVRLNDVLGDPTRLPRKRWREDPLPM  345

REKLKITEMRIPSKGEIVRTDTAYPGEIVILPSDSVRLNDVLGDPTRLPRKRWREDPLPM
Sbjct  241
REKLKITEMRIPSKGEIVRTDTAYPGEIVILPSDSVRLNDVLGDPTRLPRKRWREDPLPM  300

Query  346
LRTTIAPKTAAQRERLLDALTQLADTDPLLRCEVDSITHEIILSFLGRVQLEVVSALLSE  405
            LRT
+IAPKTAAQRERLLDALTQLADTDPLLRCEVDSITHEIILSFLGRVQLEVVSALLSE
Sbjct  301
LRTSIAPKTAAQRERLLDALTQLADTDPLLRCEVDSITHEIILSFLGRVQLEVVSALLSE  360

Query  406
KYKLETVVKEPSVIYMERPLKAASHTIHIEVPPNPFWASIGLSVTPLSLGSGVQYESRVS  465
            KYKLETVVKEP+VIYMERPLKAASHTIHIEVPPNPFWASIGLSVTPL
LGSGVQYESRVS
Sbjct  361
KYKLETVVKEPTVIYMERPLKAASHTIHIEVPPNPFWASIGLSVTPLPLGSGVQYESRVS  420
```

```
Query  466
LGYLNQSFQNAVRDGIRYGLEQGLFGWNVTDCKICFEYGLYYSPVSTPADFRSLAPIVLE  525

LGYLNQSFQNAVRDGIRYGLEQGLFGWNVTDCKICFEYGLYYSPVSTPADFRSLAPIVLE
Sbjct  421
LGYLNQSFQNAVRDGIRYGLEQGLFGWNVTDCKICFEYGLYYSPVSTPADFRSLAPIVLE  480

Query  526
QALKESGTQLLEPYLSFILYAPQEYLSRAYHDAPKYCATIETAQVKKDEVVFTGEIPARC  585
            QALKESGTQLLEPYLSF LYAP+EYLSRAYHDAPKYCATIET
QVKKDEVVFTGEIPARC
Sbjct  481
QALKESGTQLLEPYLSFTLYAPREYLSRAYHDAPKYCATIETVQVKKDEVVFTGEIPARC  540

Query  586  IQAYRTDLAFYTNGRSVCLTELKGYQAAVGQPVIQPRRPNSRLDKVRHMFQKVM
639
            IQAYRTDLAFYTNG+SVCLTELKGYQAAVG+PVIQPRRPNSRLDKVR+MFQK+M
Sbjct  541  IQAYRTDLAFYTNGQSVCLTELKGYQAAVGKPVIQPRRPNSRLDKVRYMFQKIM
594


>KOP61953.1 tetracycline resistance protein [Bifidobacterium longum
subsp.
longum]
Length=594

 Score = 1206 bits (3119),  Expect = 0.0, Method: Compositional
matrix adjust.
 Identities = 586/594 (99%), Positives = 589/594 (99%), Gaps = 0/594
(0%)

Query  46
MFLERQRGITIQAAVTSFQWHRCKVNIVDTPGHMDFLAEVYRSLAVLDGAILVISAKDGV  105

MFLERQRGITIQAAVTSFQWHRCKVNIVDTPGHMDFLAEVYRSLAVLDGAILVISAKDGV
Sbjct  1
MFLERQRGITIQAAVTSFQWHRCKVNIVDTPGHMDFLAEVYRSLAVLDGAILVISAKDGV  60

Query  106
QAQTRILFHALRKMNIPTVIFINKIDQAGVDLQSVVQSVRDKLSADIIIKQTVSLSPEIV  165

QAQTRILFHALRKMNIPTVIFINKIDQAGVDLQSVVQSVRDKLSADIIIKQTVSLSPEIV
Sbjct  61
QAQTRILFHALRKMNIPTVIFINKIDQAGVDLQSVVQSVRDKLSADIIIKQTVSLSPEIV  120

Query  166
LEENTDIEAWDAVIENNDKLLEKYIAGEPISREKLVREEQRRVQDASLFPVYYGSAKKGL  225
            LEENTDIEAWDAVIENND+LLEKYIAGEPISREKL REEQ+RVQDASLFPVY
+GSAK GL
Sbjct  121
LEENTDIEAWDAVIENNDELLEKYIAGEPISREKLAREEQQRVQDASLFPVYHGSAKNGL  180

Query  226
GIQPLMDAVTGLFQPIGEQGSAALCGSVFKVEYTDCGQRRVYLRLYSGTLRLRDTVALAG  285
```

GIQPLMDAVTGLFQPIGEQG
AALCGSVFKVEYTDCGQRRVYLRLYSGTLRLRDTVALAG
Sbjct 181
GIQPLMDAVTGLFQPIGEQGGAALCGSVFKVEYTDCGQRRVYLRLYSGTLRLRDTVALAG 240

Query 286
REKLKITEMRIPSKGEIVRTDTAYPGEIVILPSDSVRLNDVLGDPTRLPRKRWREDPLPM 345
REKLKITEMRIPSKGEIVRTDTAY GEIVILPSDSVRLNDVLGD
TRLPRKRWREDPLPM
Sbjct 241
REKLKITEMRIPSKGEIVRTDTAYQGEIVILPSDSVRLNDVLGDQTRLPRKRWREDPLPM 300

Query 346
LRTTIAPKTAAQRERLLDALTQLADTDPLLRCEVDSITHEIILSFLGRVQLEVVSALLSE 405

LRTTIAPKTAAQRERLLDALTQLADTDPLLRCEVDSITHEIILSFLGRVQLEVVSALLSE
Sbjct 301
LRTTIAPKTAAQRERLLDALTQLADTDPLLRCEVDSITHEIILSFLGRVQLEVVSALLSE 360

Query 406
KYKLETVVKEPSVIYMERPLKAASHTIHIEVPPNPFWASIGLSVTPLSLGSGVQYESRVS 465

KYKLETVVKEPSVIYMERPLKAASHTIHIEVPPNPFWASIGLSVTPLSLGSGVQYESRVS
Sbjct 361
KYKLETVVKEPSVIYMERPLKAASHTIHIEVPPNPFWASIGLSVTPLSLGSGVQYESRVS 420

Query 466
LGYLNQSFQNAVRDGIRYGLEQGLFGWNVTDCKICFEYGLYYSPVSTPADFRSLAPIVLE 525

LGYLNQSFQNAVRDGIRYGLEQGLFGWNVTDCKICFEYGLYYSPVSTPADFRSLAPIVLE
Sbjct 421
LGYLNQSFQNAVRDGIRYGLEQGLFGWNVTDCKICFEYGLYYSPVSTPADFRSLAPIVLE 480

Query 526
QALKESGTQLLEPYLSFILYAPQEYLSRAYHDAPKYCATIETAQVKKDEVVFTGEIPARC 585

QALKESGTQLLEPYLSFILYAPQEYLSRAYHDAPKYCATIETAQVKKDEVVFTGEIPARC
Sbjct 481
QALKESGTQLLEPYLSFILYAPQEYLSRAYHDAPKYCATIETAQVKKDEVVFTGEIPARC 540

Query 586 IQAYRTDLAFYTNGRSVCLTELKGYQAAVGQPVIQPRRPNSRLDKVRHMFQKVM
639
IQAYRTDLAFYTNGRSVCLTELKGYQAAVGQPVIQPRRPNSRLDKVRHMFQKVM
Sbjct 541 IQAYRTDLAFYTNGRSVCLTELKGYQAAVGQPVIQPRRPNSRLDKVRHMFQKVM
594

>WP_086129278.1 TetM/TetW/TetO/TetS family tetracycline resistance
ribosomal
protection protein, partial [Lactobacillus reuteri]
OTA48603.1 tetracycline resistance ribosomal protection protein,
partial
[Lactobacillus reuteri]
Length=591

Score = 1197 bits (3098), Expect = 0.0, Method: Compositional matrix adjust.
Identities = 583/591 (99%), Positives = 588/591 (99%), Gaps = 0/591 (0%)

```
Query  1
MKIINIGILAHVDAGKTTLTESLLYASGAISEPGSVEKGTTRTDTMFLERQRGITIQAAV  60
            MKIINIGILAHVDAGKTTLTESLLYASGAISEPGSV
+KGTTRTDTMFLERQRGITIQAAV
Sbjct  1
MKIINIGILAHVDAGKTTLTESLLYASGAISEPGSVKKGTTRTDTMFLERQRGITIQAAV  60

Query  61
TSFQWHRCKVNIVDTPGHMDFLAEVYRSLAVLDGAILVISAKDGVQAQTRILFHALRKMN  120

TSFQWHRCKVNIVDTPGHMDFLAEVYRSLAVLDGAILVISAKDGVQAQTRILFHALRKMN
Sbjct  61
TSFQWHRCKVNIVDTPGHMDFLAEVYRSLAVLDGAILVISAKDGVQAQTRILFHALRKMN  120

Query  121
IPTVIFINKIDQAGVDLQSVVQSVRDKLSADIIIKQTVSLSPEIVLEENTDIEAWDAVIE  180

IPTVIFINKIDQAGVDLQSVVQSVRDKLSADIIIKQTVSLSPEIVLEENTDIEAWDAVIE
Sbjct  121
IPTVIFINKIDQAGVDLQSVVQSVRDKLSADIIIKQTVSLSPEIVLEENTDIEAWDAVIE  180

Query  181
NNDKLLEKYIAGEPISREKLVREEQRRVQDASLFPVYYGSAKKGLGIQPLMDAVTGLFQP  240

NNDKLLEKYIAGEPISREKLVREEQRRVQDASLFPVYYGSAKKGLGIQPLMDAVTGLFQP
Sbjct  181
NNDKLLEKYIAGEPISREKLVREEQRRVQDASLFPVYYGSAKKGLGIQPLMDAVTGLFQP  240

Query  241
IGEQGSAALCGSVFKVEYTDCGQRRVYLRLYSGTLRLRDTVALAGREKLKITEMRIPSKG  300

IGEQGSAALCGSVFKVEYTDCGQRRVYLRLYSGTLRLRDTVALAGREKLKITEMRIPSKG
Sbjct  241
IGEQGSAALCGSVFKVEYTDCGQRRVYLRLYSGTLRLRDTVALAGREKLKITEMRIPSKG  300

Query  301
EIVRTDTAYPGEIVILPSDSVRLNDVLGDPTRLPRKRWREDPLPMLRTTIAPKTAAQRER  360
            EIVRTDTAYPGEIVILPSDSVRLNDVLGDPTRLPRKRWREDPLPMLRT
+IAPKTAAQRER
Sbjct  301
EIVRTDTAYPGEIVILPSDSVRLNDVLGDPTRLPRKRWREDPLPMLRTSIAPKTAAQRER  360

Query  361
LLDALTQLADTDPLLRCEVDSITHEIILSFLGRVQLEVVSALLSEKYKLETVVKEPSVIY  420
            LLDALTQLADTDPLLRCEVDSITHEIILSFLGRVQLEVVSALLSEKYKLETVVKEP
+VIY
Sbjct  361
LLDALTQLADTDPLLRCEVDSITHEIILSFLGRVQLEVVSALLSEKYKLETVVKEPTVIY  420
```

Query 421
MERPLKAASHTIHIEVPPNPFWASIGLSVTPLSLGSGVQYESRVSLGYLNQSFQNAVRDG 480
            MERPLKAASHTIHIEVPPNPFWASIGLSVTPL
LGSGVQYESRVSLGYLNQSFQNAVRDG
Sbjct 421
MERPLKAASHTIHIEVPPNPFWASIGLSVTPLPLGSGVQYESRVSLGYLNQSFQNAVRDG 480

Query 481
IRYGLEQGLFGWNVTDCKICFEYGLYYSPVSTPADFRSLAPIVLEQALKESGTQLLEPYL 540

IRYGLEQGLFGWNVTDCKICFEYGLYYSPVSTPADFRSLAPIVLEQALKESGTQLLEPYL
Sbjct 481
IRYGLEQGLFGWNVTDCKICFEYGLYYSPVSTPADFRSLAPIVLEQALKESGTQLLEPYL 540

Query 541 SFILYAPQEYLSRAYHDAPKYCATIETAQVKKDEVVFTGEIPARCIQAYRT 591
            SF LYAP+EYLSRAYHDAP+YCATIET QVKKDEVVFTGEIPARCIQAYRT
Sbjct 541 SFTLYAPREYLSRAYHDAPEYCATIETVQVKKDEVVFTGEIPARCIQAYRT 591

>WP_024963478.1 tetracycline resistance ribosomal protection protein Tet(W) [Trueperella
pyogenes]
 AAL09826.1 ribosomal protection tetracycline resistance protein [Trueperella
pyogenes]
 AHU89330.1 tetracycline resistance protein tetM [Trueperella
pyogenes]
 AWA43289.1 tetracycline resistance ribosomal protection protein Tet(W) [Trueperella
pyogenes]
Length=639

 Score = 1189 bits (3076), Expect = 0.0, Method: Compositional matrix adjust.
 Identities = 577/639 (90%), Positives = 600/639 (94%), Gaps = 0/639 (0%)

Query 1
MKIINIGILAHVDAGKTTLTESLLYASGAISEPGSVEKGTTRTDTMFLERQRGITIQAAV 60
            M IINIGILAHVDAGKTTLTESLLYASG
ISEPGSVEKGTTRTDTMFLERQRGITIQ AV
Sbjct 1
MNIINIGILAHVDAGKTTLTESLLYASGTISEPGSVEKGTTRTDTMFLERQRGITIQTAV 60

Query 61
TSFQWHRCKVNIVDTPGHMDFLAEVYRSLAVLDGAILVISAKDGVQAQTRILFHALRKMN 120
            TSFQWH CKVNIVDTPGHMDFLAEVYRSLAVLDGAILV+SA+DGVQAQTR
+LFHALRK+N
Sbjct 61
TSFQWHSCKVNIVDTPGHMDFLAEVYRSLAVLDGAILVLSARDGVQAQTRVLFHALRKLN 120

Query 121
IPTVIFINKIDQAGVDLQSVVQSVRDKLSADIIIKQTVSLSPEIVLEENTDIEAWDAVIE 180

IPT+IFINKIDQ +DL+ V
QSVRDKLSADIIIKQTVSLSPEIVLEENTDIEAWDAVIE
Sbjct 121
IPTIIFINKIDQVDIDLEGVYQSVRDKLSADIIIKQTVSLSPEIVLEENTDIEAWDAVIE 180

Query 181
NNDKLLEKYIAGEPISREKLVREEQRRVQDASLFPVYYGSAKKGLGIQPLMDAVTGLFQP 240
NND LLEKYIAGEPISRE+L REEQRRVQ ASLFPVY+GSAK GLGIQ LMDAV
GLFQP
Sbjct 181
NNDGLLEKYIAGEPISREELAREEQRRVQAASLFPVYHGSAKNGLGIQRLMDAVIGLFQP 240

Query 241
IGEQGSAALCGSVFKVEYTDCGQRRVYLRLYSGTLRLRDTVALAGREKLKITEMRIPSKG 300
EQG ALCGSVFKVEYTDCGQR
VYLRLYSGTLRLRDTVALAGREKLKITEMRIPSKG
Sbjct 241
TKEQGRTALCGSVFKVEYTDCGQRLVYLRLYSGTLRLRDTVALAGREKLKITEMRIPSKG 300

Query 301
EIVRTDTAYPGEIVILPSDSVRLNDVLGDPTRLPRKRWREDPLPMLRTTIAPKTAAQRER 360
EIVRTDTA+ GEIVILPSDS+RLND+LGD T+LPR+ W + P MLRTTI PKTA
QR+R
Sbjct 301
EIVRTDTAHKGEIVILPSDSLRLNDILGDKTQLPREMWSDAPFSMLRTTITPKTAEQRDR 360

Query 361
LLDALTQLADTDPLLRCEVDSITHEIILSFLGRVQLEVVSALLSEKYKLETVVKEPSVIY 420
LLDALTQ+ADTDPLL EVDSIT EIILSFLGRVQLEVVSALL+EKYK+ET VKEP
+VIY
Sbjct 361
LLDALTQIADTDPLLCYEVDSITQEIILSFLGRVQLEVVSALLAEKYKIETAVKEPTVIY 420

Query 421
MERPLKAASHTIHIEVPPNPFWASIGLSVTPLSLGSGVQYESRVSLGYLNQSFQNAVRDG 480
+ERPLK ASHTIHIEVPPNPFWASIGLSVTPL
LGSGVQYESRVSLGYLNQSFQNAV DG
Sbjct 421
LERPLKVASHTIHIEVPPNPFWASIGLSVTPLPLGSGVQYESRVSLGYLNQSFQNAVMDG 480

Query 481
IRYGLEQGLFGWNVTDCKICFEYGLYYSPVSTPADFRSLAPIVLEQALKESGTQLLEPYL 540
IRYGLEQGL GWNVTDCKICFEYGLYYSPVSTPADFRSLAPIVLE
ALKESGTQLLEPYL
Sbjct 481
IRYGLEQGLCGWNVTDCKICFEYGLYYSPVSTPADFRSLAPIVLELALKESGTQLLEPYL 540

Query 541
SFILYAPQEYLSRAYHDAPKYCATIETAQVKKDEVVFTGEIPARCIQAYRTDLAFYTNGR 600
SF LYAPQEYLSRAYHDAPKYCATIETAQ+KKDEVVFTGEIPARCIQAYRTDLA
YTNGR
Sbjct 541
SFTLYAPQEYLSRAYHDAPKYCATIETAQIKKDEVVFTGEIPARCIQAYRTDLASYTNGR 600

```
Query  601  SVCLTELKGYQAAVGQPVIQPRRPNSRLDKVRHMFQKVM  639
            SVCLTELKGYQAAVGQPVIQPRRPNSRLD+VRHMFQKVM
Sbjct  601  SVCLTELKGYQAAVGQPVIQPRRPNSRLDRVRHMFQKVM  639
```

>AAO42741.1 tetracycline resistance protein [Megasphaera elsdenii]
Length=639

Score = 1184 bits (3063), Expect = 0.0, Method: Compositional matrix adjust.
Identities = 570/638 (89%), Positives = 603/638 (95%), Gaps = 0/638 (0%)

```
Query  1
MKIINIGILAHVDAGKTTLTESLLYASGAISEPGSVEKGTTRTDTMFLERQRGITIQAAV  60
            MKIIN+GILAHVDAGKTTLTESLLY SGAI+E GSV++GTTRTDTM
LERQRGITIQ AV
Sbjct  1
MKIINLGILAHVDAGKTTLTESLLYTSGAIAELGSVDEGTTRTDTMNLERQRGITIQTAV  60

Query  61
TSFQWHRCKVNIVDTPGHMDFLAEVYRSLAVLDGAILVISAKDGVQAQTRILFHALRKMN  120
            TSFQW   KVNI
+DTPGHMDFLAEVYRSLAVLDGAILVISAKDGVQAQTRILFHALRKMN
Sbjct  61
TSFQWEDVKVNIIDTPGHMDFLAEVYRSLAVLDGAILVISAKDGVQAQTRILFHALRKMN  120

Query  121
IPTVIFINKIDQAGVDLQSVVQSVRDKLSADIIIKQTVSLSPEIVLEENTDIEAWDAVIE  180

IPTVIFINKIDQAGVDLQSVVQSVRDKLSADIIIKQTVSLSPEIVLEENTDIEAWDAVIE
Sbjct  121
IPTVIFINKIDQAGVDLQSVVQSVRDKLSADIIIKQTVSLSPEIVLEENTDIEAWDAVIE  180

Query  181
NNDKLLEKYIAGEPISREKLVREEQRRVQDASLFPVYYGSAKKGLGIQPLMDAVTGLFQP  240
            NND LLEKYIAGEPIS+EKL REEQRRVQ+ASLFPVY
+GSAKKGLGIQPLMDAVTGLFQP
Sbjct  181
NNDALLEKYIAGEPISQEKLAREEQRRVQEASLFPVYHGSAKKGLGIQPLMDAVTGLFQP  240

Query  241
IGEQGSAALCGSVFKVEYTDCGQRRVYLRLYSGTLRLRDTVALAGREKLKITEMRIPSKG  300
            IGEQGSA
LCGSVFKVEYTDCGQRRVYLRLYSGTLRLRDTVALAGREKLKITEMRIPSKG
Sbjct  241
IGEQGSATLCGSVFKVEYTDCGQRRVYLRLYSGTLRLRDTVALAGREKLKITEMRIPSKG  300

Query  301
EIVRTDTAYPGEIVILPSDSVRLNDVLGDPTRLPRKRWREDPLPMLRTTIAPKTAAQRER  360
            EIVRTDTAYPGEIVILPSDSVRLNDVLGD TRLPRKRWRE
PLPMLRTTIAPKTAAQRER
Sbjct  301
EIVRTDTAYPGEIVILPSDSVRLNDVLGDQTRLPRKRWREAPLPMLRTTIAPKTAAQRER  360
```

```
Query  361
LLDALTQLADTDPLLRCEVDSITHEIILSFLGRVQLEVVSALLSEKYKLETVVKEPSVIY  420
            LLDALTQLADTDPLLRCEVDSITHEIILSFLGRVQLEVVSALLSEKYK+ETVVKEP
+VIY
Sbjct  361
LLDALTQLADTDPLLRCEVDSITHEIILSFLGRVQLEVVSALLSEKYKIETVVKEPTVIY  420

Query  421
MERPLKAASHTIHIEVPPNPFWASIGLSVTPLSLGSGVQYESRVSLGYLNQSFQNAVRDG  480
            MERPLKAASHTIHIEVPPNPFWAS+GLS+ PL
+GSGVQYESRVSLGYLNQSFQNAV +G
Sbjct  421
MERPLKAASHTIHIEVPPNPFWASVGLSIEPLPIGSGVQYESRVSLGYLNQSFQNAVMEG  480

Query  481
IRYGLEQGLFGWNVTDCKICFEYGLYYSPVSTPADFRSLAPIVLEQALKESGTQLLEPYL  540
            + YG EQGL+GW VTDCKICFEYGLYYSPVSTPADFR L+PIVLEQALK++GT
+LLEPYL
Sbjct  481
VLYGCEQGLYGWKVTDCKICFEYGLYYSPVSTPADFRLLSPIVLEQALKKAGTELLEPYL  540

Query  541
SFILYAPQEYLSRAYHDAPKYCATIETAQVKKDEVVFTGEIPARCIQAYRTDLAFYTNGR  600
             F +YAPQEYLSRAYHDAP+YCA I + Q+K DEV+  GEIPARCIQ YR DL +
+TNG+
Sbjct  541
HFEIYAPQEYLSRAYHDAPRYCADIVSTQIKNDEVILKGEIPARCIQEYRNDLTYFTNGQ  600

Query  601  SVCLTELKGYQAAVGQPVIQPRRPNSRLDKVRHMFQKV  638
             VCLTELKGYQ A+G+ + QPRRPNSR+DKVRHMF K+
Sbjct  601  GVCLTELKGYQPAIGKFICQPRRPNSRIDKVRHMFNKL  638


>WP_016230557.1 TetM/TetW/TetO/TetS family tetracycline resistance
ribosomal
protection protein, partial [Lachnospiraceae bacterium 10-1]
 EOS72341.1 tetracycline resistance protein tetW, partial
[Lachnospiraceae
bacterium 10-1]
Length=582

 Score = 1174 bits (3036),  Expect = 0.0, Method: Compositional
matrix adjust.
 Identities = 574/582 (99%), Positives = 577/582 (99%), Gaps = 0/582
(0%)

Query  1
MKIINIGILAHVDAGKTTLTESLLYASGAISEPGSVEKGTTRTDTMFLERQRGITIQAAV  60

MKIINIGILAHVDAGKTTLTESLLYASGAISEPGSVEKGTTRTDTMFLERQRGITIQAAV
Sbjct  1
MKIINIGILAHVDAGKTTLTESLLYASGAISEPGSVEKGTTRTDTMFLERQRGITIQAAV  60
```

```
Query  61
TSFQWHRCKVNIVDTPGHMDFLAEVYRSLAVLDGAILVISAKDGVQAQTRILFHALRKMN  120

TSFQWHRCKVNIVDTPGHMDFLAEVYRSLAVLDGAILVISAKDGVQAQTRILFHALRKMN
Sbjct  61
TSFQWHRCKVNIVDTPGHMDFLAEVYRSLAVLDGAILVISAKDGVQAQTRILFHALRKMN  120

Query  121
IPTVIFINKIDQAGVDLQSVVQSVRDKLSADIIIKQTVSLSPEIVLEENTDIEAWDAVIE  180

IPTVIFINKIDQAGVDLQSVVQSVRDKLSADIIIKQTVSLSPEIVLEENTDIEAWDAVIE
Sbjct  121
IPTVIFINKIDQAGVDLQSVVQSVRDKLSADIIIKQTVSLSPEIVLEENTDIEAWDAVIE  180

Query  181
NNDKLLEKYIAGEPISREKLVREEQRRVQDASLFPVYYGSAKKGLGIQPLMDAVTGLFQP  240
            NND+LLEKYIAGEPISREKL REEQ+RVQDASLFPVY+GSAK
GLGIQPLMDAVTGLFQP
Sbjct  181
NNDELLEKYIAGEPISREKLAREEQQRVQDASLFPVYHGSAKNGLGIQPLMDAVTGLFQP  240

Query  241
IGEQGSAALCGSVFKVEYTDCGQRRVYLRLYSGTLRLRDTVALAGREKLKITEMRIPSKG  300
            IGEQG
AALCGSVFKVEYTDCGQRRVYLRLYSGTLRLRDTVALAGREKLKITEMRIPSKG
Sbjct  241
IGEQGGAALCGSVFKVEYTDCGQRRVYLRLYSGTLRLRDTVALAGREKLKITEMRIPSKG  300

Query  301
EIVRTDTAYPGEIVILPSDSVRLNDVLGDPTRLPRKRWREDPLPMLRTTIAPKTAAQRER  360
            EIVRTDTAY GEIVILPSDSVRLNDVLGD
TRLPRKRWREDPLPMLRTTIAPKTAAQRER
Sbjct  301
EIVRTDTAYQGEIVILPSDSVRLNDVLGDQTRLPRKRWREDPLPMLRTTIAPKTAAQRER  360

Query  361
LLDALTQLADTDPLLRCEVDSITHEIILSFLGRVQLEVVSALLSEKYKLETVVKEPSVIY  420

LLDALTQLADTDPLLRCEVDSITHEIILSFLGRVQLEVVSALLSEKYKLETVVKEPSVIY
Sbjct  361
LLDALTQLADTDPLLRCEVDSITHEIILSFLGRVQLEVVSALLSEKYKLETVVKEPSVIY  420

Query  421
MERPLKAASHTIHIEVPPNPFWASIGLSVTPLSLGSGVQYESRVSLGYLNQSFQNAVRDG  480

MERPLKAASHTIHIEVPPNPFWASIGLSVTPLSLGSGVQYESRVSLGYLNQSFQNAVRDG
Sbjct  421
MERPLKAASHTIHIEVPPNPFWASIGLSVTPLSLGSGVQYESRVSLGYLNQSFQNAVRDG  480

Query  481
IRYGLEQGLFGWNVTDCKICFEYGLYYSPVSTPADFRSLAPIVLEQALKESGTQLLEPYL  540

IRYGLEQGLFGWNVTDCKICFEYGLYYSPVSTPADFRSLAPIVLEQALKESGTQLLEPYL
Sbjct  481
```

IRYGLEQGLFGWNVTDCKICFEYGLYYSPVSTPADFRSLAPIVLEQALKESGTQLLEPYL 540

Query 541 SFILYAPQEYLSRAYHDAPKYCATIETAQVKKDEVVFTGEIP 582
            SFILYAPQEYLSRAYHDAPKYCATIETAQVKKDEVVFTGEIP
Sbjct 541 SFILYAPQEYLSRAYHDAPKYCATIETAQVKKDEVVFTGEIP 582

>WP_031583918.1 TetM/TetW/TetO/TetS family tetracycline resistance ribosomal
protection protein [Selenomonas bovis]
Length=639

Score = 1172 bits (3031), Expect = 0.0, Method: Compositional matrix adjust.
Identities = 562/638 (88%), Positives = 595/638 (93%), Gaps = 0/638 (0%)

Query 1
MKIINIGILAHVDAGKTTLTESLLYASGAISEPGSVEKGTTRTDTMFLERQRGITIQAAV 60
            M IINIGILAHVDAGKTTLTESLLYASG
ISEPGSVEKGTTRTDTMFLERQRGITIQ AV
Sbjct 1
MNIINIGILAHVDAGKTTLTESLLYASGTISEPGSVEKGTTRTDTMFLERQRGITIQTAV 60

Query 61
TSFQWHRCKVNIVDTPGHMDFLAEVYRSLAVLDGAILVISAKDGVQAQTRILFHALRKMN 120
            TSFQWH CKVNIVDTPGHMDFLAEVYRSLAVLDGAILV+SAKDGVQAQTR
+LFHALRK+N
Sbjct 61
TSFQWHSCKVNIVDTPGHMDFLAEVYRSLAVLDGAILVLSAKDGVQAQTRVLFHALRKLN 120

Query 121
IPTVIFINKIDQAGVDLQSVVQSVRDKLSADIIIKQTVSLSPEIVLEENTDIEAWDAVIE 180
            IPT+IFINKIDQ G+DL+SV QSVRDKLSADIIIKQTVSLS +I L ENT E WD
+VIE
Sbjct 121
IPTIIFINKIDQVGIDLESVYQSVRDKLSADIIIKQTVSLSSKITLTENTSAEVWDSVIE 180

Query 181
NNDKLLEKYIAGEPISREKLVREEQRRVQDASLFPVYYGSAKKGLGIQPLMDAVTGLFQP 240
            NND+LL KYIAGE IS+++L +EEQRRVQDASL PVY+GSAK GLGIQ LMDAV
GLFQ
Sbjct 181
NNDELLAKYIAGESISQKELAQEEQRRVQDASLLPVYHGSAKNGLGIQQLMDAVIGLFQS 240

Query 241
IGEQGSAALCGSVFKVEYTDCGQRRVYLRLYSGTLRLRDTVALAGREKLKITEMRIPSKG 300
              EQGSAALCG VFKVEYTDCGQR
VYLRLYSGTLRLRDTVALAGREKLKITEMRIPSKG
Sbjct 241
TKEQGSAALCGRVFKVEYTDCGQRLVYLRLYSGTLRLRDTVALAGREKLKITEMRIPSKG 300

Query 301
EIVRTDTAYPGEIVILPSDSVRLNDVLGDPTRLPRKRWREDPLPMLRTTIAPKTAAQRER 360

EIVRTDTA+ GEIVILPSDS+RLND+LGD T+LPR+ W + P PMLRTTI PKTA
QR+R
Sbjct 301
EIVRTDTAHKGEIVILPSDSLRLNDILGDKTQLPREMWSDVPFPMLRTTITPKTAEQRDR 360

Query 361
LLDALTQLADTDPLLRCEVDSITHEIILSFLGRVQLEVVSALLSEKYKLETVVKEPSVIY 420
LLDALTQ+ADTDPLL EVDS THEIILSFLGR+QLEVVSALL+EKYK+ET VKEP
+VIY
Sbjct 361
LLDALTQIADTDPLLHYEVDSTTHEIILSFLGRMQLEVVSALLTEKYKIETAVKEPTVIY 420

Query 421
MERPLKAASHTIHIEVPPNPFWASIGLSVTPLSLGSGVQYESRVSLGYLNQSFQNAVRDG 480
+ERPLK ASHTIHIEVPPNPFWASIGLSVTPL LGSGV
+YESRVSLGYLNQSFQNAV DG
Sbjct 421
LERPLKVASHTIHIEVPPNPFWASIGLSVTPLPLGSGVKYESRVSLGYLNQSFQNAVMDG 480

Query 481
IRYGLEQGLFGWNVTDCKICFEYGLYYSPVSTPADFRSLAPIVLEQALKESGTQLLEPYL 540
IRYGL QGL+GWNVTDCKICFEYGLYYSPVSTPADFRSLAPIVLEQALK
+SGTQLLEPYL
Sbjct 481
IRYGLGQGLYGWNVTDCKICFEYGLYYSPVSTPADFRSLAPIVLEQALKKSGTQLLEPYL 540

Query 541
SFILYAPQEYLSRAYHDAPKYCATIETAQVKKDEVVFTGEIPARCIQAYRTDLAFYTNGR 600
SF LYAPQEYLSRAYHDAPKYCATIETAQ
+KKDEVVFTGEIPARCIQAYRTDLAFYTNGR
Sbjct 541
SFTLYAPQEYLSRAYHDAPKYCATIETAQIKKDEVVFTGEIPARCIQAYRTDLAFYTNGR 600

Query 601 SVCLTELKGYQAAVGQPVIQPRRPNSRLDKVRHMFQKV 638
SVCLTELKGYQA VG+PVIQPRRPNSRLDKVRHMF K+
Sbjct 601 SVCLTELKGYQATVGEPVIQPRRPNSRLDKVRHMFSKI 638

>WP_050625029.1 MULTISPECIES: TetM/TetW/TetO/TetS family
tetracycline resistance
ribosomal protection protein [Clostridiales]
Length=639

Score = 1170 bits (3026), Expect = 0.0, Method: Compositional
matrix adjust.
Identities = 562/638 (88%), Positives = 594/638 (93%), Gaps = 0/638
(0%)

Query 1
MKIINIGILAHVDAGKTTLTESLLYASGAISEPGSVEKGTTRTDTMFLERQRGITIQAAV 60
M IINIGILAHVDAGKTTLTESLLYASG
ISEPGSVEKGTTRTDTMFLERQRGITIQ AV
Sbjct 1
MNIINIGILAHVDAGKTTLTESLLYASGTISEPGSVEKGTTRTDTMFLERQRGITIQTAV 60

Query 61
TSFQWHRCKVNIVDTPGHMDFLAEVYRSLAVLDGAILVISAKDGVQAQTRILFHALRKMN 120
            TSFQWH CKVNIVDTPGHMDFLAEVYRSLAVLDGAILV+SAKDGVQAQTR
+LFHALRK+N
Sbjct 61
TSFQWHSCKVNIVDTPGHMDFLAEVYRSLAVLDGAILVLSAKDGVQAQTRVLFHALRKLN 120

Query 121
IPTVIFINKIDQAGVDLQSVVQSVRDKLSADIIIKQTVSLSPEIVLEENTDIEAWDAVIE 180
            IPT+IFINKIDQ G+DL+SV QSVRDKLSADIIIKQTVSLS +I L ENT E WD
+VIE
Sbjct 121
IPTIIFINKIDQVGIDLESVYQSVRDKLSADIIIKQTVSLSSKITLTENTSAEVWDSVIE 180

Query 181
NNDKLLEKYIAGEPISREKLVREEQRRVQDASLFPVYYGSAKKGLGIQPLMDAVTGLFQP 240
            NND+LL KYIAGE IS+++L +EEQRRVQDASL PVY+GSAK GLGIQ LMDAV
GLFQ
Sbjct 181
NNDELLAKYIAGESISQKELAQEEQRRVQDASLLPVYHGSAKNGLGIQQLMDAVIGLFQS 240

Query 241
IGEQGSAALCGSVFKVEYTDCGQRRVYLRLYSGTLRLRDTVALAGREKLKITEMRIPSKG 300
              EQGSAALCG VFKVEYTDCGQR
VYLRLYSGTLRLRDTVALAGREKLKITEMRIPSKG
Sbjct 241
TKEQGSAALCGRVFKVEYTDCGQRLVYLRLYSGTLRLRDTVALAGREKLKITEMRIPSKG 300

Query 301
EIVRTDTAYPGEIVILPSDSVRLNDVLGDPTRLPRKRWREDPLPMLRTTIAPKTAAQRER 360
            EIVRTDTA+ GEIVILPSDS+RLND+LGD T+LPR+ W + P PMLRTTI PKTA
QR+R
Sbjct 301
EIVRTDTAHKGEIVILPSDSLRLNDILGDKTQLPREMWSDVPFPMLRTTITPKTAEQRDR 360

Query 361
LLDALTQLADTDPLLRCEVDSITHEIILSFLGRVQLEVVSALLSEKYKLETVVKEPSVIY 420
            LLDALTQ+ADTDPLL EVDS THEIILSFLGR+QLEVVSALL+EKYK+ET VKEP
+VIY
Sbjct 361
LLDALTQIADTDPLLHYEVDSTTHEIILSFLGRMQLEVVSALLTEKYKIETAVKEPTVIY 420

Query 421
MERPLKAASHTIHIEVPPNPFWASIGLSVTPLSLGSGVQYESRVSLGYLNQSFQNAVRDG 480
            +ERPLK ASHTIHIEVPPNPFWASIGLSVTPL LGSGV
+YESRVSLGYLNQSFQNAV DG
Sbjct 421
LERPLKVASHTIHIEVPPNPFWASIGLSVTPLPLGSGVKYESRVSLGYLNQSFQNAVMDG 480

Query 481
IRYGLEQGLFGWNVTDCKICFEYGLYYSPVSTPADFRSLAPIVLEQALKESGTQLLEPYL 540
            IRYGL QGL GWNVTDCKICFEYGLYYSPVSTPADFRSLAPIVLEQALK
+SGTQLLEPYL

```
Sbjct  481
IRYGLGQGLCGWNVTDCKICFEYGLYYSPVSTPADFRSLAPIVLEQALKKSGTQLLEPYL  540

Query  541
SFILYAPQEYLSRAYHDAPKYCATIETAQVKKDEVVFTGEIPARCIQAYRTDLAFYTNGR  600
            SF LYAPQEYLSRAYHDAPKYCATIETAQ
+KKDEVVFTGEIPARCIQAYRTDLAFYTNGR
Sbjct  541
SFTLYAPQEYLSRAYHDAPKYCATIETAQIKKDEVVFTGEIPARCIQAYRTDLAFYTNGR  600

Query  601  SVCLTELKGYQAAVGQPVIQPRRPNSRLDKVRHMFQKV  638
            SVCLTELKGYQA VG+PVIQPRRPNSRLDKVRHMF K+
Sbjct  601  SVCLTELKGYQATVGEPVIQPRRPNSRLDKVRHMFSKI  638


>WP_011526261.1 MULTISPECIES: tetracycline resistance ribosomal
protection protein
Tet(W) [Bacteria]
 CAJ54235.1 ribosomal protection tetracycline resistance protein
[Lawsonia
intracellularis PHE/MN1-00]
 AGC49582.1 ribosomal protection tetracycline resistance protein
[Lawsonia
intracellularis N343]
Length=639

 Score = 1169 bits (3023),  Expect = 0.0, Method: Compositional
matrix adjust.
 Identities = 561/638 (88%), Positives = 594/638 (93%), Gaps = 0/638
(0%)

Query  1
MKIINIGILAHVDAGKTTLTESLLYASGAISEPGSVEKGTTRTDTMFLERQRGITIQAAV  60
            M IINIGILAHVDAGKTTLTESLLYASG
ISEPGSVEKGTTRTDTMFLERQRGITIQ AV
Sbjct  1
MNIINIGILAHVDAGKTTLTESLLYASGTISEPGSVEKGTTRTDTMFLERQRGITIQTAV  60

Query  61
TSFQWHRCKVNIVDTPGHMDFLAEVYRSLAVLDGAILVISAKDGVQAQTRILFHALRKMN  120
            TSFQWH CKVNIVDTPGHMDFLAEVYRSLAVLDGAILV+SAKDGVQAQTR
+LFHALRK+N
Sbjct  61
TSFQWHSCKVNIVDTPGHMDFLAEVYRSLAVLDGAILVLSAKDGVQAQTRVLFHALRKLN  120

Query  121
IPTVIFINKIDQAGVDLQSVVQSVRDKLSADIIIKQTVSLSPEIVLEENTDIEAWDAVIE  180
            IPT+IFINKIDQ G+DL+SV QSVRDKLSADIIIKQTVSLS +I L ENT  E WD
+VIE
Sbjct  121
IPTIIFINKIDQVGIDLESVYQSVRDKLSADIIIKQTVSLSSKITLTENTSAEVWDSVIE  180

Query  181
NNDKLLEKYIAGEPISREKLVREEQRRVQDASLFPVYYGSAKKGLGIQPLMDAVTGLFQP  240
```

NND+LL KYIAGE IS+++L +EEQRRVQDASL PVY+GSAK GLGIQ LMDAV
GLFQ
Sbjct 181
NNDELLAKYIAGESISQKELAQEEQRRVQDASLLPVYHGSAKNGLGIQQLMDAVIGLFQS 240

Query 241
IGEQGSAALCGSVFKVEYTDCGQRRVYLRLYSGTLRLRDTVALAGREKLKITEMRIPSKG 300
EQGSAALCG VFKVEYTDCGQR
VYLRLYSGTLRLRDTVALAGREKLKITEMRIPSKG
Sbjct 241
TKEQGSAALCGRVFKVEYTDCGQRLVYLRLYSGTLRLRDTVALAGREKLKITEMRIPSKG 300

Query 301
EIVRTDTAYPGEIVILPSDSVRLNDVLGDPTRLPRKRWREDPLPMLRTTIAPKTAAQRER 360
EIVRTDTA+ GEIVILPSDS+RLND+LGD T+LPR+ W + P PMLRTTI PKTA
QR+R
Sbjct 301
EIVRTDTAHKGEIVILPSDSLRLNDILGDKTQLPREMWSDVPFPMLRTTITPKTAEQRDR 360

Query 361
LLDALTQLADTDPLLRCEVDSITHEIILSFLGRVQLEVVSALLSEKYKLETVVKEPSVIY 420
LLDALTQ+ADTDPLL EVDS THEIILSFLGR+QLEVVSALL+EKYK+ET VKEP
+VIY
Sbjct 361
LLDALTQIADTDPLLHYEVDSTTHEIILSFLGRMQLEVVSALLTEKYKIETAVKEPTVIY 420

Query 421
MERPLKAASHTIHIEVPPNPFWASIGLSVTPLSLGSGVQYESRVSLGYLNQSFQNAVRDG 480
+ERPLK ASHTIHIEVPPNPFWASIGLSVTPL LGSGV
+YESRVSLGYLNQSFQNAV DG
Sbjct 421
LERPLKVASHTIHIEVPPNPFWASIGLSVTPLPLGSGVKYESRVSLGYLNQSFQNAVMDG 480

Query 481
IRYGLEQGLFGWNVTDCKICFEYGLYYSPVSTPADFRSLAPIVLEQALKESGTQLLEPYL 540
IRYGL QGL GWNVTDCKICFEYGLYYSPVSTPADFRSLAPIVLEQALK
+SGTQLLEPYL
Sbjct 481
IRYGLGQGLCGWNVTDCKICFEYGLYYSPVSTPADFRSLAPIVLEQALKKSGTQLLEPYL 540

Query 541
SFILYAPQEYLSRAYHDAPKYCATIETAQVKKDEVVFTGEIPARCIQAYRTDLAFYTNGR 600
SF LYAPQEYLSRAYHDAPKYCATIETAQ
+KKDEVVFTGEIPARCIQAYRTDLAFYTNGR
Sbjct 541
SFTLYAPQEYLSRAYHDAPKYCATIETAQIKKDEVVFTGEIPARCIQAYRTDLAFYTNGR 600

Query 601 SVCLTELKGYQAAVGQPVIQPRRPNSRLDKVRHMFQKV 638
SVCLTELKGYQA VG+P+IQPRRPNSRLDKVRHMF K+
Sbjct 601 SVCLTELKGYQATVGEPIIQPRRPNSRLDKVRHMFSKI 638

>WP_070835237.1 TetM/TetW/TetO/TetS family tetracycline resistance
ribosomal

protection protein, partial [Actinomyces sp. HMSC035G02]
 OHR17901.1 tetracycline resistance ribosomal protection protein, partial
[Actinomyces sp. HMSC035G02]
Length=598

 Score = 1166 bits (3016), Expect = 0.0, Method: Compositional matrix adjust.
 Identities = 564/585 (96%), Positives = 576/585 (98%), Gaps = 0/585 (0%)

Query 54
ITIQAAVTSFQWHRCKVNIVDTPGHMDFLAEVYRSLAVLDGAILVISAKDGVQAQTRILF 113

ITIQAAVTSFQWHRCKVNIVDTPGHMDFLAEVYRSLAVLDGAILVISAKDGVQAQTRILF
Sbjct 12
ITIQAAVTSFQWHRCKVNIVDTPGHMDFLAEVYRSLAVLDGAILVISAKDGVQAQTRILF 71

Query 114
HALRKMNIPTVIFINKIDQAGVDLQSVVQSVRDKLSADIIIKQTVSLSPEIVLEENTDIE 173
            HALRKM+IPTVIFINKIDQAGVDLQ V
QSVRDKLSADIIIKQTVSLSPEIVLEENTDIE
Sbjct 72
HALRKMDIPTVIFINKIDQAGVDLQGVYQSVRDKLSADIIIKQTVSLSPEIVLEENTDIE 131

Query 174
AWDAVIENNDKLLEKYIAGEPISREKLVREEQRRVQDASLFPVYYGSAKKGLGIQPLMDA 233
            AWDAVIENND+LLEKYIAGEPISRE+L +EEQRRVQDASLFPVY
+GSAKKGLGIQPLMDA
Sbjct 132
AWDAVIENNDELLEKYIAGEPISREELAQEEQRRVQDASLFPVYHGSAKKGLGIQPLMDA 191

Query 234
VTGLFQPIGEQGSAALCGSVFKVEYTDCGQRRVYLRLYSGTLRLRDTVALAGREKLKITE 293
            VTGLFQPIGEQGS ALCGSVFKVEYTDCGQR
+YLRLYSGTLRLRDTVALAGREKLKITE
Sbjct 192
VTGLFQPIGEQGSDALCGSVFKVEYTDCGQRLIYLRLYSGTLRLRDTVALAGREKLKITE 251

Query 294
MRIPSKGEIVRTDTAYPGEIVILPSDSVRLNDVLGDPTRLPRKRWREDPLPMLRTTIAPK 353
            MRIPSKGEIVRTDTAYPGEIVILPSDSVRLNDVLGDPTRLPRKRWREDPLPMLRT
+IAPK
Sbjct 252
MRIPSKGEIVRTDTAYPGEIVILPSDSVRLNDVLGDPTRLPRKRWREDPLPMLRTSIAPK 311

Query 354
TAAQRERLLDALTQLADTDPLLRCEVDSITHEIILSFLGRVQLEVVSALLSEKYKLETVV 413

TAAQRERLLDALTQLADTDPLLRCEVDSITHEIILSFLGRVQLEVVSALLSEKYKLETVV
Sbjct 312
TAAQRERLLDALTQLADTDPLLRCEVDSITHEIILSFLGRVQLEVVSALLSEKYKLETVV 371

Query 414

KEPSVIYMERPLKAASHTIHIEVPPNPFWASIGLSVTPLSLGSGVQYESRVSLGYLNQSF 473
            KEP+VIYMERPLKAASHTIHIEVPPNPFWASIGLSVTPL
LGSGVQYESRVSLGYLNQSF
Sbjct 372
KEPTVIYMERPLKAASHTIHIEVPPNPFWASIGLSVTPLPLGSGVQYESRVSLGYLNQSF 431

Query 474
QNAVRDGIRYGLEQGLFGWNVTDCKICFEYGLYYSPVSTPADFRSLAPIVLEQALKESGT 533

QNAVRDGIRYGLEQGLFGWNVTDCKICFEYGLYYSPVSTPADFRSLAPIVLEQALKESGT
Sbjct 432
QNAVRDGIRYGLEQGLFGWNVTDCKICFEYGLYYSPVSTPADFRSLAPIVLEQALKESGT 491

Query 534
QLLEPYLSFILYAPQEYLSRAYHDAPKYCATIETAQVKKDEVVFTGEIPARCIQAYRTDL 593
            QLLEPYLSF LYAP+EYLSRAYHDAPKYCATIET
QVKKDEVVFTGEIPARCIQAYRTDL
Sbjct 492
QLLEPYLSFTLYAPREYLSRAYHDAPKYCATIETVQVKKDEVVFTGEIPARCIQAYRTDL 551

Query 594 AFYTNGRSVCLTELKGYQAAVGQPVIQPRRPNSRLDKVRHMFQKV 638
            AFYTNG+SVCLTELKGYQAAVG+PVIQPRRPNSRLDKVRHMF K+
Sbjct 552 AFYTNGQSVCLTELKGYQAAVGKPVIQPRRPNSRLDKVRHMFSKI 596

>WP_096920879.1 TetM/TetW/TetO/TetS family tetracycline resistance ribosomal
protection protein, partial [Eubacterium sp. YI]
 PCS24331.1 tetracycline resistance ribosomal protection protein Tet(W),
partial [Eubacterium sp. YI]
Length=577

 Score = 1165 bits (3015), Expect = 0.0, Method: Compositional matrix adjust.
 Identities = 568/577 (98%), Positives = 571/577 (99%), Gaps = 0/577 (0%)

Query 23
LLYASGAISEPGSVEKGTTRTDTMFLERQRGITIQAAVTSFQWHRCKVNIVDTPGHMDFL 82

LLYASGAISEPGSVEKGTTRTDTMFLERQRGITIQAAVTSFQWHRCKVNIVDTPGHMDFL
Sbjct 1
LLYASGAISEPGSVEKGTTRTDTMFLERQRGITIQAAVTSFQWHRCKVNIVDTPGHMDFL 60

Query 83
AEVYRSLAVLDGAILVISAKDGVQAQTRILFHALRKMNIPTVIFINKIDQAGVDLQSVVQ 142

AEVYRSLAVLDGAILVISAKDGVQAQTRILFHALRKMNIPTVIFINKIDQAGVDLQSVVQ
Sbjct 61
AEVYRSLAVLDGAILVISAKDGVQAQTRILFHALRKMNIPTVIFINKIDQAGVDLQSVVQ 120

Query 143
SVRDKLSADIIIKQTVSLSPEIVLEENTDIEAWDAVIENNDKLLEKYIAGEPISREKLVR 202

SVRDKLSADIIIKQTVSLSPEIVLEENTDIEAWDAVIENND
+LLEKYIAGEPISREKL R
Sbjct 121
SVRDKLSADIIIKQTVSLSPEIVLEENTDIEAWDAVIENNDELLEKYIAGEPISREKLAR 180

Query 203
EEQRRVQDASLFPVYYGSAKKGLGIQPLMDAVTGLFQPIGEQGSAALCGSVFKVEYTDCG 262
EEQ+RVQDASLFPVY+GSAK GLGIQPLMDAVTGLFQPIGEQG
AALCGSVFKVEYTDCG
Sbjct 181
EEQQRVQDASLFPVYHGSAKNGLGIQPLMDAVTGLFQPIGEQGGAALCGSVFKVEYTDCG 240

Query 263
QRRVYLRLYSGTLRLRDTVALAGREKLKITEMRIPSKGEIVRTDTAYPGEIVILPSDSVR 322
QRRVYLRLYSGTLRLRDTVALAGREKLKITEMRIPSKGEIVRTDTAY
GEIVILPSDSVR
Sbjct 241
QRRVYLRLYSGTLRLRDTVALAGREKLKITEMRIPSKGEIVRTDTAYQGEIVILPSDSVR 300

Query 323
LNDVLGDPTRLPRKRWREDPLPMLRTTIAPKTAAQRERLLDALTQLADTDPLLRCEVDSI 382
LNDVLGD
TRLPRKRWREDPLPMLRTTIAPKTAAQRERLLDALTQLADTDPLLRCEVDSI
Sbjct 301
LNDVLGDQTRLPRKRWREDPLPMLRTTIAPKTAAQRERLLDALTQLADTDPLLRCEVDSI 360

Query 383
THEIILSFLGRVQLEVVSALLSEKYKLETVVKEPSVIYMERPLKAASHTIHIEVPPNPFW 442

THEIILSFLGRVQLEVVSALLSEKYKLETVVKEPSVIYMERPLKAASHTIHIEVPPNPFW
Sbjct 361
THEIILSFLGRVQLEVVSALLSEKYKLETVVKEPSVIYMERPLKAASHTIHIEVPPNPFW 420

Query 443
ASIGLSVTPLSLGSGVQYESRVSLGYLNQSFQNAVRDGIRYGLEQGLFGWNVTDCKICFE 502

ASIGLSVTPLSLGSGVQYESRVSLGYLNQSFQNAVRDGIRYGLEQGLFGWNVTDCKICFE
Sbjct 421
ASIGLSVTPLSLGSGVQYESRVSLGYLNQSFQNAVRDGIRYGLEQGLFGWNVTDCKICFE 480

Query 503
YGLYYSPVSTPADFRSLAPIVLEQALKESGTQLLEPYLSFILYAPQEYLSRAYHDAPKYC 562
YGLYYSPVSTPADFRSLAPIVLEQALKESGTQLLEPYLSF
LYAPQEYLSRAYHDAPKYC
Sbjct 481
YGLYYSPVSTPADFRSLAPIVLEQALKESGTQLLEPYLSFTLYAPQEYLSRAYHDAPKYC 540

Query 563 ATIETAQVKKDEVVFTGEIPARCIQAYRTDLAFYTNG 599
ATIETAQVKKDEVVFTGEIPARCIQAYRTDLAFYTNG
Sbjct 541 ATIETAQVKKDEVVFTGEIPARCIQAYRTDLAFYTNG 577

>ACX71625.1 tetracycline resistance protein, partial [Streptococcus suis]

Length=575

 Score = 1162 bits (3006), Expect = 0.0, Method: Compositional matrix adjust.
 Identities = 567/575 (99%), Positives = 570/575 (99%), Gaps = 0/575 (0%)

```
Query  28
GAISEPGSVEKGTTRTDTMFLERQRGITIQAAVTSFQWHRCKVNIVDTPGHMDFLAEVYR  87

GAISEPGSVEKGTTRTDTMFLERQRGITIQAAVTSFQWHRCKVNIVDTPGHMDFLAEVYR
Sbjct  1
GAISEPGSVEKGTTRTDTMFLERQRGITIQAAVTSFQWHRCKVNIVDTPGHMDFLAEVYR  60

Query  88
SLAVLDGAILVISAKDGVQAQTRILFHALRKMNIPTVIFINKIDQAGVDLQSVVQSVRDK  147

SLAVLDGAILVISAKDGVQAQTRILFHALRKMNIPTVIFINKIDQAGVDLQSVVQSVRDK
Sbjct  61
SLAVLDGAILVISAKDGVQAQTRILFHALRKMNIPTVIFINKIDQAGVDLQSVVQSVRDK  120

Query  148
LSADIIIKQTVSLSPEIVLEENTDIEAWDAVIENNDKLLEKYIAGEPISREKLVREEQRR  207
            LSADIIIKQTVSLSPEIVLEENTDIEAWDAVIENND+LLEKYIAGEPISREKL
REEQ+R
Sbjct  121
LSADIIIKQTVSLSPEIVLEENTDIEAWDAVIENNDELLEKYIAGEPISREKLAREEQQR  180

Query  208
VQDASLFPVYYGSAKKGLGIQPLMDAVTGLFQPIGEQGSAALCGSVFKVEYTDCGQRRVY  267
            VQDASLFPVY+GSAK GLGIQPLMDAVTGLFQPIGEQG
AALCGSVFKVEYTDCGQRRVY
Sbjct  181
VQDASLFPVYHGSAKNGLGIQPLMDAVTGLFQPIGEQGGAALCGSVFKVEYTDCGQRRVY  240

Query  268
LRLYSGTLRLRDTVALAGREKLKITEMRIPSKGEIVRTDTAYPGEIVILPSDSVRLNDVL  327
            LRLYSGTLRLRDTVALAGREKLKITEMRIPSKGEIVRTDTAY
GEIVILPSDSVRLNDVL
Sbjct  241
LRLYSGTLRLRDTVALAGREKLKITEMRIPSKGEIVRTDTAYQGEIVILPSDSVRLNDVL  300

Query  328
GDPTRLPRKRWREDPLPMLRTTIAPKTAAQRERLLDALTQLADTDPLLRCEVDSITHEII  387
            GD
TRLPRKRWREDPLPMLRTTIAPKTAAQRERLLDALTQLADTDPLLRCEVDSITHEII
Sbjct  301
GDQTRLPRKRWREDPLPMLRTTIAPKTAAQRERLLDALTQLADTDPLLRCEVDSITHEII  360

Query  388
LSFLGRVQLEVVSALLSEKYKLETVVKEPSVIYMERPLKAASHTIHIEVPPNPFWASIGL  447

LSFLGRVQLEVVSALLSEKYKLETVVKEPSVIYMERPLKAASHTIHIEVPPNPFWASIGL
Sbjct  361
```

LSFLGRVQLEVVSALLSEKYKLETVVKEPSVIYMERPLKAASHTIHIEVPPNPFWASIGL 420

Query 448
SVTPLSLGSGVQYESRVSLGYLNQSFQNAVRDGIRYGLEQGLFGWNVTDCKICFEYGLYY 507

SVTPLSLGSGVQYESRVSLGYLNQSFQNAVRDGIRYGLEQGLFGWNVTDCKICFEYGLYY
Sbjct 421
SVTPLSLGSGVQYESRVSLGYLNQSFQNAVRDGIRYGLEQGLFGWNVTDCKICFEYGLYY 480

Query 508
SPVSTPADFRSLAPIVLEQALKESGTQLLEPYLSFILYAPQEYLSRAYHDAPKYCATIET 567

SPVSTPADFRSLAPIVLEQALKESGTQLLEPYLSFILYAPQEYLSRAYHDAPKYCATIET
Sbjct 481
SPVSTPADFRSLAPIVLEQALKESGTQLLEPYLSFILYAPQEYLSRAYHDAPKYCATIET 540

Query 568 AQVKKDEVVFTGEIPARCIQAYRTDLAFYTNGRSV 602
AQVKKDEVVFTGEIPARCIQAYRTDLAFYTNGRSV
Sbjct 541 AQVKKDEVVFTGEIPARCIQAYRTDLAFYTNGRSV 575

>CBK96064.1 small GTP-binding protein domain [[[Eubacterium] siraeum 70/3]
Length=650

Score = 1159 bits (2998), Expect = 0.0, Method: Compositional matrix adjust.
Identities = 566/574 (99%), Positives = 569/574 (99%), Gaps = 0/574 (0%)

Query 66
HRCKVNIVDTPGHMDFLAEVYRSLAVLDGAILVISAKDGVQAQTRILFHALRKMNIPTVI 125

HRCKVNIVDTPGHMDFLAEVYRSLAVLDGAILVISAKDGVQAQTRILFHALRKMNIPTVI
Sbjct 77
HRCKVNIVDTPGHMDFLAEVYRSLAVLDGAILVISAKDGVQAQTRILFHALRKMNIPTVI 136

Query 126
FINKIDQAGVDLQSVVQSVRDKLSADIIIKQTVSLSPEIVLEENTDIEAWDAVIENNDKL 185

FINKIDQAGVDLQSVVQSVRDKLSADIIIKQTVSLSPEIVLEENTDIEAWDAVIENND+L
Sbjct 137
FINKIDQAGVDLQSVVQSVRDKLSADIIIKQTVSLSPEIVLEENTDIEAWDAVIENNDEL 196

Query 186
LEKYIAGEPISREKLVREEQRRVQDASLFPVYYGSAKKGLGIQPLMDAVTGLFQPIGEQG 245
LEKYIAGEPISREKL REEQ+RVQDASLFPVY+GSAK
GLGIQPLMDAVTGLFQPIGEQG
Sbjct 197
LEKYIAGEPISREKLAREEQQRVQDASLFPVYHGSAKNGLGIQPLMDAVTGLFQPIGEQG 256

Query 246
SAALCGSVFKVEYTDCGQRRVYLRLYSGTLRLRDTVALAGREKLKITEMRIPSKGEIVRT 305

AALCGSVFKVEYTDCGQRRVYLRLYSGTLRLRDTVALAGREKLKITEMRIPSKGEIVRT
Sbjct 257
GAALCGSVFKVEYTDCGQRRVYLRLYSGTLRLRDTVALAGREKLKITEMRIPSKGEIVRT 316

Query 306
DTAYPGEIVILPSDSVRLNDVLGDPTRLPRKRWREDPLPMLRTTIAPKTAAQRERLLDAL 365
DTAY GEIVILPSDSVRLNDVLGD
TRLPRKRWREDPLPMLRTTIAPKTAAQRERLLDAL
Sbjct 317
DTAYQGEIVILPSDSVRLNDVLGDQTRLPRKRWREDPLPMLRTTIAPKTAAQRERLLDAL 376

Query 366
TQLADTDPLLRCEVDSITHEIILSFLGRVQLEVVSALLSEKYKLETVVKEPSVIYMERPL 425

TQLADTDPLLRCEVDSITHEIILSFLGRVQLEVVSALLSEKYKLETVVKEPSVIYMERPL
Sbjct 377
TQLADTDPLLRCEVDSITHEIILSFLGRVQLEVVSALLSEKYKLETVVKEPSVIYMERPL 436

Query 426
KAASHTIHIEVPPNPFWASIGLSVTPLSLGSGVQYESRVSLGYLNQSFQNAVRDGIRYGL 485

KAASHTIHIEVPPNPFWASIGLSVTPLSLGSGVQYESRVSLGYLNQSFQNAVRDGIRYGL
Sbjct 437
KAASHTIHIEVPPNPFWASIGLSVTPLSLGSGVQYESRVSLGYLNQSFQNAVRDGIRYGL 496

Query 486
EQGLFGWNVTDCKICFEYGLYYSPVSTPADFRSLAPIVLEQALKESGTQLLEPYLSFILY 545

EQGLFGWNVTDCKICFEYGLYYSPVSTPADFRSLAPIVLEQALKESGTQLLEPYLSFILY
Sbjct 497
EQGLFGWNVTDCKICFEYGLYYSPVSTPADFRSLAPIVLEQALKESGTQLLEPYLSFILY 556

Query 546
APQEYLSRAYHDAPKYCATIETAQVKKDEVVFTGEIPARCIQAYRTDLAFYTNGRSVCLT 605

APQEYLSRAYHDAPKYCATIETAQVKKDEVVFTGEIPARCIQAYRTDLAFYTNGRSVCLT
Sbjct 557
APQEYLSRAYHDAPKYCATIETAQVKKDEVVFTGEIPARCIQAYRTDLAFYTNGRSVCLT 616

Query 606 ELKGYQAAVGQPVIQPRRPNSRLDKVRHMFQKVM 639
ELKGYQAAVGQPVIQPRRPNSRLDKVRHMFQKVM
Sbjct 617 ELKGYQAAVGQPVIQPRRPNSRLDKVRHMFQKVM 650

>AMP53220.1 TetM-TetW-TetO-TetS, partial [uncultured bacterium]
Length=596

Score = 1142 bits (2954), Expect = 0.0, Method: Compositional matrix adjust.
Identities = 555/596 (93%), Positives = 574/596 (96%), Gaps = 0/596 (0%)

Query 1
MKIINIGILAHVDAGKTTLTESLLYASGAISEPGSVEKGTTRTDTMFLERQRGITIQAAV 60

MKIINIGILAHVDAGKTTLTESLLYASGAISEPGSVEKGTTRTDTMFLERQRGITIQAAV
Sbjct 1
MKIINIGILAHVDAGKTTLTESLLYASGAISEPGSVEKGTTRTDTMFLERQRGITIQAAV 60

Query 61
TSFQWHRCKVNIVDTPGHMDFLAEVYRSLAVLDGAILVISAKDGVQAQTRILFHALRKMN 120

TSFQWHRCKVNIVDTPGHMDFLAEVYRSLAVLDGAILVISAKDGVQAQTRILFHALRKMN
Sbjct 61
TSFQWHRCKVNIVDTPGHMDFLAEVYRSLAVLDGAILVISAKDGVQAQTRILFHALRKMN 120

Query 121
IPTVIFINKIDQAGVDLQSVVQSVRDKLSADIIIKQTVSLSPEIVLEENTDIEAWDAVIE 180

IPTVIFINKIDQAGVDLQSVVQSVRDKLSADIIIKQTVSLSPEIVLEENTDIEAWDAVIE
Sbjct 121
IPTVIFINKIDQAGVDLQSVVQSVRDKLSADIIIKQTVSLSPEIVLEENTDIEAWDAVIE 180

Query 181
NNDKLLEKYIAGEPISREKLVREEQRRVQDASLFPVYYGSAKKGLGIQPLMDAVTGLFQP 240
NND LLEKYIAGEPIS+EKL REEQ+RVQDASLFPVY
+GSAKKGLGIQPLMDAVTGLFQP
Sbjct 181
NNDALLEKYIAGEPISQEKLAREEQQRVQDASLFPVYHGSAKKGLGIQPLMDAVTGLFQP 240

Query 241
IGEQGSAALCGSVFKVEYTDCGQRRVYLRLYSGTLRLRDTVALAGREKLKITEMRIPSKG 300
IGEQG AALCGSVFKVEYTDCGQR
VYLRLYSGTLRLRDTVALAGREKLKITEMRIPSKG
Sbjct 241
IGEQGGAALCGSVFKVEYTDCGQRLVYLRLYSGTLRLRDTVALAGREKLKITEMRIPSKG 300

Query 301
EIVRTDTAYPGEIVILPSDSVRLNDVLGDPTRLPRKRWREDPLPMLRTTIAPKTAAQRER 360
EIVRTDTA+ GEIVILPSDS+RLND+LGD T+LPR+ W + P PMLRTTI PKTA
QR+R
Sbjct 301
EIVRTDTAHKGEIVILPSDSLRLNDILGDKTQLPREMWSDVPFPMLRTTITPKTAEQRDR 360

Query 361
LLDALTQLADTDPLLRCEVDSITHEIILSFLGRVQLEVVSALLSEKYKLETVVKEPSVIY 420
LLDALTQ+ADTDPLL EVDS THEIILSFLGR+QLEVVSALL+EKYK+ET VKEP
+VIY
Sbjct 361
LLDALTQIADTDPLLHYEVDSTTHEIILSFLGRMQLEVVSALLTEKYKIETAVKEPTVIY 420

Query 421
MERPLKAASHTIHIEVPPNPFWASIGLSVTPLSLGSGVQYESRVSLGYLNQSFQNAVRDG 480
+ERPLK ASHTIHIEVPPNPFWASIGLSVTPL LGSGV
+YESRVSLGYLNQSFQNAV DG
Sbjct 421
LERPLKVASHTIHIEVPPNPFWASIGLSVTPLPLGSGVKYESRVSLGYLNQSFQNAVMDG 480

```
Query  481  
IRYGLEQGLFGWNVTDCKICFEYGLYYSPVSTPADFRSLAPIVLEQALKESGTQLLEPYL  540
            IRYGL QGL GWNVTDCKICFEYGLYYSPVSTPADFRSLAPIVLEQALK 
+SGTQLLEPYL
Sbjct  481  
IRYGLGQGLCGWNVTDCKICFEYGLYYSPVSTPADFRSLAPIVLEQALKKSGTQLLEPYL  540

Query  541  SFILYAPQEYLSRAYHDAPKYCATIETAQVKKDEVVFTGEIPARCIQAYRTDLAFY  
596
            SF LYAPQEYLSRAYHDAPKYCATIETAQ+KKDEVVFTGEIPARCIQAYRTDLAFY
Sbjct  541  SFTLYAPQEYLSRAYHDAPKYCATIETAQIKKDEVVFTGEIPARCIQAYRTDLAFY  
596


>EQI16708.1 tetracycline resistance protein tetM [Clostridioides 
difficile 
F501]
Length=567

 Score = 1141 bits (2952),  Expect = 0.0, Method: Compositional 
matrix adjust.
 Identities = 556/567 (98%), Positives = 561/567 (99%), Gaps = 0/567 
(0%)

Query  73   
VDTPGHMDFLAEVYRSLAVLDGAILVISAKDGVQAQTRILFHALRKMNIPTVIFINKIDQ  132
            
+DTPGHMDFLAEVYRSLAVLDGAILVISAKDGVQAQTRILFHALRKMNIPTVIFINKIDQ
Sbjct  1    
MDTPGHMDFLAEVYRSLAVLDGAILVISAKDGVQAQTRILFHALRKMNIPTVIFINKIDQ  60

Query  133  
AGVDLQSVVQSVRDKLSADIIIKQTVSLSPEIVLEENTDIEAWDAVIENNDKLLEKYIAG  192
            AGVDLQSVVQSVRDKLSADIIIKQTVSLSPEIVLEENTDIEAWDAVIENND 
+LLEKYIAG
Sbjct  61   
AGVDLQSVVQSVRDKLSADIIIKQTVSLSPEIVLEENTDIEAWDAVIENNDELLEKYIAG  120

Query  193  
EPISREKLVREEQRRVQDASLFPVYYGSAKKGLGIQPLMDAVTGLFQPIGEQGSAALCGS  252
            EPISREKL REEQ+RVQDASLFPVY+GSAK GLGIQPLMDAVTGLFQPIGEQG 
AALCGS
Sbjct  121  
EPISREKLAREEQQRVQDASLFPVYHGSAKNGLGIQPLMDAVTGLFQPIGEQGGAALCGS  180

Query  253  
VFKVEYTDCGQRRVYLRLYSGTLRLRDTVALAGREKLKITEMRIPSKGEIVRTDTAYPGE  312
            
VFKVEYTDCGQRRVYLRLYSGTLRLRDTVALAGREKLKITEMRIPSKGEIVRTDTAY GE
Sbjct  181  
VFKVEYTDCGQRRVYLRLYSGTLRLRDTVALAGREKLKITEMRIPSKGEIVRTDTAYQGE  240

Query  313  
IVILPSDSVRLNDVLGDPTRLPRKRWREDPLPMLRTTIAPKTAAQRERLLDALTQLADTD  372
```

            IVILPSDSVRLNDVLGD TRLPRKRWREDPLPMLRT
IAPKTAAQRERLLDALTQLADTD
Sbjct 241
IVILPSDSVRLNDVLGDQTRLPRKRWREDPLPMLRTAIAPKTAAQRERLLDALTQLADTD 300

Query 373
PLLRCEVDSITHEIILSFLGRVQLEVVSALLSEKYKLETVVKEPSVIYMERPLKAASHTI 432

PLLRCEVDSITHEIILSFLGRVQLEVVSALLSEKYKLETVVKEPSVIYMERPLKAASHTI
Sbjct 301
PLLRCEVDSITHEIILSFLGRVQLEVVSALLSEKYKLETVVKEPSVIYMERPLKAASHTI 360

Query 433
HIEVPPNPFWASIGLSVTPLSLGSGVQYESRVSLGYLNQSFQNAVRDGIRYGLEQGLFGW 492

HIEVPPNPFWASIGLSVTPLSLGSGVQYESRVSLGYLNQSFQNAVRDGIRYGLEQGLFGW
Sbjct 361
HIEVPPNPFWASIGLSVTPLSLGSGVQYESRVSLGYLNQSFQNAVRDGIRYGLEQGLFGW 420

Query 493
NVTDCKICFEYGLYYSPVSTPADFRSLAPIVLEQALKESGTQLLEPYLSFILYAPQEYLS 552
            NVTDCKICFEYGLYYSPVSTPADFRSLAPIVLEQALKESGTQLLEP
+LSFILYAPQEYLS
Sbjct 421
NVTDCKICFEYGLYYSPVSTPADFRSLAPIVLEQALKESGTQLLEPHLSFILYAPQEYLS 480

Query 553
RAYHDAPKYCATIETAQVKKDEVVFTGEIPARCIQAYRTDLAFYTNGRSVCLTELKGYQA 612

RAYHDAPKYCATIETAQVKKDEVVFTGEIPARCIQAYRTDLAFYTNGRSVCLTELKGYQA
Sbjct 481
RAYHDAPKYCATIETAQVKKDEVVFTGEIPARCIQAYRTDLAFYTNGRSVCLTELKGYQA 540

Query 613 AVGQPVIQPRRPNSRLDKVRHMFQKVM 639
            AVGQPVIQPRRPNSRLDKVRHMFQKVM
Sbjct 541 AVGQPVIQPRRPNSRLDKVRHMFQKVM 567

>CAM97383.1 mosaic tetracycline resistance protein [Bifidobacterium thermophilum]
 CAM97386.1 mosaic tetracycline resistance protein [Bifidobacterium thermophilum]
 CAM97389.1 mosaic tetracycline resistance protein [Bifidobacterium thermophilum]
 CAM97390.1 mosaic tetracycline resistance protein [Bifidobacterium thermophilum]
 CAM97380.1 mosaic tetracycline resistance protein [Bifidobacterium thermophilum]
Length=639

 Score = 1134 bits (2932), Expect = 0.0, Method: Compositional matrix adjust.
 Identities = 540/638 (85%), Positives = 589/638 (92%), Gaps = 0/638 (0%)

Query 1
MKIINIGILAHVDAGKTTLTESLLYASGAISEPGSVEKGTTRTDTMFLERQRGITIQAAV 60

MKIINIGILAHVDAGKTTLTESLLYASGAISEPGSVEKGTTRTDTMFLERQRGITIQAAV
Sbjct 1
MKIINIGILAHVDAGKTTLTESLLYASGAISEPGSVEKGTTRTDTMFLERQRGITIQAAV 60

Query 61
TSFQWHRCKVNIVDTPGHMDFLAEVYRSLAVLDGAILVISAKDGVQAQTRILFHALRKMN 120

TSFQWHRCKVNIVDTPGHMDFLAEVYRSLAVLDGAILVISAKDGVQAQTRILFHALRKMN
Sbjct 61
TSFQWHRCKVNIVDTPGHMDFLAEVYRSLAVLDGAILVISAKDGVQAQTRILFHALRKMN 120

Query 121
IPTVIFINKIDQAGVDLQSVVQSVRDKLSADIIIKQTVSLSPEIVLEENTDIEAWDAVIE 180

IPTVIFINKIDQAGVDLQSVVQSVRDKLSADIIIKQTVSLSPEIVLEENTDIEAWDAVIE
Sbjct 121
IPTVIFINKIDQAGVDLQSVVQSVRDKLSADIIIKQTVSLSPEIVLEENTDIEAWDAVIE 180

Query 181
NNDKLLEKYIAGEPISREKLVREEQRRVQDASLFPVYYGSAKKGLGIQPLMDAVTGLFQP 240

NNDKLLEKYIAGEPISREKLVREEQRRVQDASLFPVYYGSAKKGLGIQPLMDAVTGLFQP
Sbjct 181
NNDKLLEKYIAGEPISREKLVREEQRRVQDASLFPVYYGSAKKGLGIQPLMDAVTGLFQP 240

Query 241
IGEQGSAALCGSVFKVEYTDCGQRRVYLRLYSGTLRLRDTVALAGREKLKITEMRIPSKG 300

IGEQGSAALCGSVFKVEYTDCGQRRVYLRLYSGTLRLRDTVALAGREKLKITEMRIPSKG
Sbjct 241
IGEQGSAALCGSVFKVEYTDCGQRRVYLRLYSGTLRLRDTVALAGREKLKITEMRIPSKG 300

Query 301
EIVRTDTAYPGEIVILPSDSVRLNDVLGDPTRLPRKRWREDPLPMLRTTIAPKTAAQRER 360
EIVRTDTAYPGEIVIL D+++LND+LG+   LP K   ++P+P+LRTT+ P+
QRE
Sbjct 301
EIVRTDTAYPGEIVILADDTLKLNDILGNEKLLPHKTRIDNPMPLLRTTVEPQKPEQREA 360

Query 361
LLDALTQLADTDPLLRCEVDSITHEIILSFLGRVQLEVVSALLSEKYKLETVVKEPSVIY 420
LL+AL ++ADTDPLL ++D++THEI+LSFLG+VQLEV+ +LL EKY +
+KEPSVIY
Sbjct 361
LLNALAEIADTDPLLHFDIDTVTHEIMLSFLGKVQLEVICSLLEEKYHVGVAMKEPSVIY 420

Query 421
MERPLKAASHTIHIEVPPNPFWASIGLSVTPLSLGSGVQYESRVSLGYLNQSFQNAVRDG 480
+ERPL+ A +TIHIEVPPNPFWAS+GLS+ PL
+GSGVQYESRVSLGYLNQSFQNAV +G

Sbjct 421
LERPLRKAEYTIHIEVPPNPFWASVGLSIEPLPIGSGVQYESRVSLGYLNQSFQNAVMEG 480

Query 481
IRYGLEQGLFGWNVTDCKICFEYGLYYSPVSTPADFRSLAPIVLEQALKESGTQLLEPYL 540
            + YG EQGL+GW VTDCKICFEYGLYYSPVSTPADFR L+PIVLEQALK++GT
+LLEPYL
Sbjct 481
VLYGCEQGLYGWKVTDCKICFEYGLYYSPVSTPADFRLLSPIVLEQALKKAGTELLEPYL 540

Query 541
SFILYAPQEYLSRAYHDAPKYCATIETAQVKKDEVVFTGEIPARCIQAYRTDLAFYTNGR 600
             F +YAPQEYLSRAYHDAP+YCA I + Q+K DEV+ GEIPARCIQ YR DL
+TNG+
Sbjct 541
HFEIYAPQEYLSRAYHDAPRYCADIVSTQIKNDEVILKGEIPARCIQEYRNDLTNFTNGQ 600

Query 601 SVCLTELKGYQAAVGQPVIQPRRPNSRLDKVRHMFQKV 638
             VCLTELKGYQ A+G+ + QPRRPNSR+DKVRHMF K+
Sbjct 601 GVCLTELKGYQPAIGKFICQPRRPNSRIDKVRHMFHKL 638

>APO27506.1 EF-G: translation elongation factor [uncultured
bacterium]
Length=561

 Score = 1125 bits (2910), Expect = 0.0, Method: Compositional
matrix adjust.
 Identities = 548/561 (98%), Positives = 555/561 (99%), Gaps = 0/561
(0%)

Query 79
MDFLAEVYRSLAVLDGAILVISAKDGVQAQTRILFHALRKMNIPTVIFINKIDQAGVDLQ 138

MDFLAEVYRSLAVLDGAILVISAKDGVQAQTRILFHALRKMNIPTVIFINKIDQAGVDLQ
Sbjct 1
MDFLAEVYRSLAVLDGAILVISAKDGVQAQTRILFHALRKMNIPTVIFINKIDQAGVDLQ 60

Query 139
SVVQSVRDKLSADIIIKQTVSLSPEIVLEENTDIEAWDAVIENNDKLLEKYIAGEPISRE 198

SVVQSVRDKLSADIIIKQTVSLSPEIVLEENTDIEAWDAVIENNDKLLEKYIAGEPISRE
Sbjct 61
SVVQSVRDKLSADIIIKQTVSLSPEIVLEENTDIEAWDAVIENNDKLLEKYIAGEPISRE 120

Query 199
KLVREEQRRVQDASLFPVYYGSAKKGLGIQPLMDAVTGLFQPIGEQGSAALCGSVFKVEY 258

KLVREEQRRVQDASLFPVYYGSAKKGLGIQPLMDAVTGLFQPIGEQGSAALCGSVFKVEY
Sbjct 121
KLVREEQRRVQDASLFPVYYGSAKKGLGIQPLMDAVTGLFQPIGEQGSAALCGSVFKVEY 180

Query 259
TDCGQRRVYLRLYSGTLRLRDTVALAGREKLKITEMRIPSKGEIVRTDTAYPGEIVILPS 318

```
TDCGQRRVYLRLYSGTLRLRDTVALAGREKLKITEMRIPSKGEIVRTDTAYPGEIVILPS
Sbjct  181
TDCGQRRVYLRLYSGTLRLRDTVALAGREKLKITEMRIPSKGEIVRTDTAYPGEIVILPS  240

Query  319
DSVRLNDVLGDPTRLPRKRWREDPLPMLRTTIAPKTAAQRERLLDALTQLADTDPLLRCE  378
            DSVRLNDVLGDPTRLPRKRWREDPLPMLRT
+IAPKTAAQRERLLDALTQLADTDPLLRCE
Sbjct  241
DSVRLNDVLGDPTRLPRKRWREDPLPMLRTSIAPKTAAQRERLLDALTQLADTDPLLRCE  300

Query  379
VDSITHEIILSFLGRVQLEVVSALLSEKYKLETVVKEPSVIYMERPLKAASHTIHIEVPP  438
            VDSITHEIILSFLGRVQLEVVSALLSEKYKLETVVKEP
+VIYMERPLKAASHTIHIEVPP
Sbjct  301
VDSITHEIILSFLGRVQLEVVSALLSEKYKLETVVKEPTVIYMERPLKAASHTIHIEVPP  360

Query  439
NPFWASIGLSVTPLSLGSGVQYESRVSLGYLNQSFQNAVRDGIRYGLEQGLFGWNVTDCK  498
            NPFWASIGLSVTPL
LGSGVQYESRVSLGYLNQSFQNAVRDGIRYGLEQGLFGWNVTDCK
Sbjct  361
NPFWASIGLSVTPLPLGSGVQYESRVSLGYLNQSFQNAVRDGIRYGLEQGLFGWNVTDCK  420

Query  499
ICFEYGLYYSPVSTPADFRSLAPIVLEQALKESGTQLLEPYLSFILYAPQEYLSRAYHDA  558
            ICFEYGLYYSPVSTPADFRSLAP   EQALKESGTQLLEPYLSF LYAP
+EYLSRAYHDA
Sbjct  421
ICFEYGLYYSPVSTPADFRSLAPSGWEQALKESGTQLLEPYLSFTLYAPREYLSRAYHDA  480

Query  559
PKYCATIETAQVKKDEVVFTGEIPARCIQAYRTDLAFYTNGRSVCLTELKGYQAAVGQPV  618
            PKYCATIET QVKKDEVVFTGEIPARCIQAYRTDLAFYTNG
+SVCLTELKGYQAAVG+PV
Sbjct  481
PKYCATIETVQVKKDEVVFTGEIPARCIQAYRTDLAFYTNGQSVCLTELKGYQAAVGKPV  540

Query  619  IQPRRPNSRLDKVRHMFQKVM  639
            IQPRRPNSRLDKVR+MFQK+M
Sbjct  541  IQPRRPNSRLDKVRYMFQKIM  561


>WP_063856419.1 tetracycline resistance ribosomal protection protein
Tet(W) [Lactobacillus
johnsonii]
 ABF69686.1 tetracycline resistance protein [Lactobacillus
johnsonii]
Length=639

 Score = 1122 bits (2902),  Expect = 0.0, Method: Compositional
matrix adjust.
```

Identities = 537/638 (84%), Positives = 586/638 (92%), Gaps = 0/638 (0%)

```
Query  1
MKIINIGILAHVDAGKTTLTESLLYASGAISEPGSVEKGTTRTDTMFLERQRGITIQAAV  60
            MKIIN+GILAHVDAGKTTLTESLLY SGAI+E GSV++GTTRTDTM
LERQRGITIQ AV
Sbjct  1
MKIINLGILAHVDAGKTTLTESLLYTSGAIAELGSVDEGTTRTDTMNLERQRGITIQTAV  60

Query  61
TSFQWHRCKVNIVDTPGHMDFLAEVYRSLAVLDGAILVISAKDGVQAQTRILFHALRKMN  120
            TSFQW   KVNI
+DTPGHMDFLAEVYRSLAVLDGAILVISAKDGVQAQTRILFHALRKMN
Sbjct  61
TSFQWEDVKVNIIDTPGHMDFLAEVYRSLAVLDGAILVISAKDGVQAQTRILFHALRKMN  120

Query  121
IPTVIFINKIDQAGVDLQSVVQSVRDKLSADIIIKQTVSLSPEIVLEENTDIEAWDAVIE  180

IPTVIFINKIDQAGVDLQSVVQSVRDKLSADIIIKQTVSLSPEIVLEENTDIEAWDAVIE
Sbjct  121
IPTVIFINKIDQAGVDLQSVVQSVRDKLSADIIIKQTVSLSPEIVLEENTDIEAWDAVIE  180

Query  181
NNDKLLEKYIAGEPISREKLVREEQRRVQDASLFPVYYGSAKKGLGIQPLMDAVTGLFQP  240

NNDKLLEKYIAGEPISREKLVREEQRRVQDASLFPVYYGSAKKGLGIQPLMDAVTGLFQP
Sbjct  181
NNDKLLEKYIAGEPISREKLVREEQRRVQDASLFPVYYGSAKKGLGIQPLMDAVTGLFQP  240

Query  241
IGEQGSAALCGSVFKVEYTDCGQRRVYLRLYSGTLRLRDTVALAGREKLKITEMRIPSKG  300

IGEQGSAALCGSVFKVEYTDCGQRRVYLRLYSGTLRLRDTVALAGREKLKITEMRIPSKG
Sbjct  241
IGEQGSAALCGSVFKVEYTDCGQRRVYLRLYSGTLRLRDTVALAGREKLKITEMRIPSKG  300

Query  301
EIVRTDTAYPGEIVILPSDSVRLNDVLGDPTRLPRKRWREDPLPMLRTTIAPKTAAQRER  360
            EIVRTDTAYPGEIVIL  D+++LND+LG+   LP K   ++P+P+LRTT+ P+
QRE
Sbjct  301
EIVRTDTAYPGEIVILADDTLKLNDILGNEKLLPHKTRIDNPMPLLRTTVEPQKPEQREA  360

Query  361
LLDALTQLADTDPLLRCEVDSITHEIILSFLGRVQLEVVSALLSEKYKLETVVKEPSVIY  420
            LL+AL ++ADTDPLL  ++D++THEI+LSFLG+VQLEV+ +LL EKY +
+KEPSVIY
Sbjct  361
LLNALAEIADTDPLLHFDIDTVTHEIMLSFLGKVQLEVICSLLEEKYHVGVAMKEPSVIY  420

Query  421
MERPLKAASHTIHIEVPPNPFWASIGLSVTPLSLGSGVQYESRVSLGYLNQSFQNAVRDG  480
```

+ERPL+ A +TIHIEVPPNPFWASIGLSVTPL
LGSGVQYESRVSLGYLNQSFQNAVRDG
Sbjct 421
LERPLRKAEYTIHIEVPPNPFWASIGLSVTPLPLGSGVQYESRVSLGYLNQSFQNAVRDG 480

Query 481
IRYGLEQGLFGWNVTDCKICFEYGLYYSPVSTPADFRSLAPIVLEQALKESGTQLLEPYL 540
IRYGLEQGL+GW VTDCKICFEYGLYYSPVSTPADFR L+PIVLEQALK++GT
+LLEPYL
Sbjct 481
IRYGLEQGLYGWKVTDCKICFEYGLYYSPVSTPADFRLLSPIVLEQALKKAGTELLEPYL 540

Query 541
SFILYAPQEYLSRAYHDAPKYCATIETAQVKKDEVVFTGEIPARCIQAYRTDLAFYTNGR 600
F +YAPQEYLSRAYHDAP+YCA I + Q+K DEV+ GEIPARCIQ YR DL
+TNG+
Sbjct 541
HFEIYAPQEYLSRAYHDAPRYCADIVSTQIKNDEVILKGEIPARCIQEYRNDLTNFTNGQ 600

Query 601 SVCLTELKGYQAAVGQPVIQPRRPNSRLDKVRHMFQKV 638
VCLTELKGYQ A+G+ + QPRRPNSR+DKVRHMF K+
Sbjct 601 GVCLTELKGYQPAIGKFICQPRRPNSRIDKVRHMFHKL 638

>KFI71207.1 TetW [Bifidobacterium merycicum]
KFI89186.1 tetracycline resistance protein [Bifidobacterium
saeculare DSM
6531 = LMG 14934]
Length=569

Score = 1120 bits (2898), Expect = 0.0, Method: Compositional
matrix adjust.
Identities = 548/553 (99%), Positives = 551/553 (99%), Gaps = 0/553
(0%)

Query 1
MKIINIGILAHVDAGKTTLTESLLYASGAISEPGSVEKGTTRTDTMFLERQRGITIQAAV 60

MKIINIGILAHVDAGKTTLTESLLYASGAISEPGSVEKGTTRTDTMFLERQRGITIQAAV
Sbjct 1
MKIINIGILAHVDAGKTTLTESLLYASGAISEPGSVEKGTTRTDTMFLERQRGITIQAAV 60

Query 61
TSFQWHRCKVNIVDTPGHMDFLAEVYRSLAVLDGAILVISAKDGVQAQTRILFHALRKMN 120

TSFQWHRCKVNIVDTPGHMDFLAEVYRSLAVLDGAILVISAKDGVQAQTRILFHALRKMN
Sbjct 61
TSFQWHRCKVNIVDTPGHMDFLAEVYRSLAVLDGAILVISAKDGVQAQTRILFHALRKMN 120

Query 121
IPTVIFINKIDQAGVDLQSVVQSVRDKLSADIIIKQTVSLSPEIVLEENTDIEAWDAVIE 180

IPTVIFINKIDQAGVDLQSVVQSVRDKLSADIIIKQTVSLSPEIVLEENTDIEAWDAVIE
Sbjct 121

```
IPTVIFINKIDQAGVDLQSVVQSVRDKLSADIIIKQTVSLSPEIVLEENTDIEAWDAVIE  180

Query  181
NNDKLLEKYIAGEPISREKLVREEQRRVQDASLFPVYYGSAKKGLGIQPLMDAVTGLFQP  240

NNDKLLEKYIAGEPISREKLVREEQRRVQDASLFPVYYGSAKKGLGIQPLMDAVTGLFQP
Sbjct  181
NNDKLLEKYIAGEPISREKLVREEQRRVQDASLFPVYYGSAKKGLGIQPLMDAVTGLFQP  240

Query  241
IGEQGSAALCGSVFKVEYTDCGQRRVYLRLYSGTLRLRDTVALAGREKLKITEMRIPSKG  300

IGEQGSAALCGSVFKVEYTDCGQRRVYLRLYSGTLRLRDTVALAGREKLKITEMRIPSKG
Sbjct  241
IGEQGSAALCGSVFKVEYTDCGQRRVYLRLYSGTLRLRDTVALAGREKLKITEMRIPSKG  300

Query  301
EIVRTDTAYPGEIVILPSDSVRLNDVLGDPTRLPRKRWREDPLPMLRTTIAPKTAAQRER  360
            EIVRTDTAYPGEIVILPSDSVRLNDVLGDPTRLPRKRWREDPLPMLRT
+IAPKTAAQRER
Sbjct  301
EIVRTDTAYPGEIVILPSDSVRLNDVLGDPTRLPRKRWREDPLPMLRTSIAPKTAAQRER  360

Query  361
LLDALTQLADTDPLLRCEVDSITHEIILSFLGRVQLEVVSALLSEKYKLETVVKEPSVIY  420
            LLDALTQLADTDPLLRCEVDSITHEIILSFLGRVQLEVVSALLSEKYKLETVVKEP
+VIY
Sbjct  361
LLDALTQLADTDPLLRCEVDSITHEIILSFLGRVQLEVVSALLSEKYKLETVVKEPTVIY  420

Query  421
MERPLKAASHTIHIEVPPNPFWASIGLSVTPLSLGSGVQYESRVSLGYLNQSFQNAVRDG  480
            MERPLKAASHTIHIEVPPNPFWASIGLSVTPL
LGSGVQYESRVSLGYLNQSFQNAVRDG
Sbjct  421
MERPLKAASHTIHIEVPPNPFWASIGLSVTPLPLGSGVQYESRVSLGYLNQSFQNAVRDG  480

Query  481
IRYGLEQGLFGWNVTDCKICFEYGLYYSPVSTPADFRSLAPIVLEQALKESGTQLLEPYL  540

IRYGLEQGLFGWNVTDCKICFEYGLYYSPVSTPADFRSLAPIVLEQALKESGTQLLEPYL
Sbjct  481
IRYGLEQGLFGWNVTDCKICFEYGLYYSPVSTPADFRSLAPIVLEQALKESGTQLLEPYL  540

Query  541  SFILYAPQEYLSR  553
            SF LYAP+EYLSR
Sbjct  541  SFTLYAPREYLSR  553


>WP_063856420.1 tetracycline resistance ribosomal protection protein
Tet(W) [uncultured
bacterium]
 ABG81258.1 tetracycline resistance protein [uncultured bacterium]
Length=639
```

Score = 1119 bits (2895), Expect = 0.0, Method: Compositional matrix adjust.
Identities = 536/638 (84%), Positives = 585/638 (92%), Gaps = 0/638 (0%)

```
Query  1
MKIINIGILAHVDAGKTTLTESLLYASGAISEPGSVEKGTTRTDTMFLERQRGITIQAAV  60
            MKIIN+GILAHVDAGKTTLTESLLY SGAI+E GSV++GTTRTDTM 
LERQRGITIQ AV
Sbjct  1
MKIINLGILAHVDAGKTTLTESLLYTSGAIAELGSVDEGTTRTDTMNLERQRGITIQTAV  60

Query  61
TSFQWHRCKVNIVDTPGHMDFLAEVYRSLAVLDGAILVISAKDGVQAQTRILFHALRKMN  120
            TSFQW   KVNI
+DTPGHMDFLAEVYRSLAVLDGAILVISAKDGVQAQTRILFHALRKMN
Sbjct  61
TSFQWEDVKVNIIDTPGHMDFLAEVYRSLAVLDGAILVISAKDGVQAQTRILFHALRKMN  120

Query  121
IPTVIFINKIDQAGVDLQSVVQSVRDKLSADIIIKQTVSLSPEIVLEENTDIEAWDAVIE  180

IPTVIFINKIDQAGVDLQSVVQSVRDKLSADIIIKQTVSLSPEIVLEENTDIEAWDAVIE
Sbjct  121
IPTVIFINKIDQAGVDLQSVVQSVRDKLSADIIIKQTVSLSPEIVLEENTDIEAWDAVIE  180

Query  181
NNDKLLEKYIAGEPISREKLVREEQRRVQDASLFPVYYGSAKKGLGIQPLMDAVTGLFQP  240

NNDKLLEKYIAGEPISREKLVREEQRRVQDASLFPVYYGSAKKGLGIQPLMDAVTGLFQP
Sbjct  181
NNDKLLEKYIAGEPISREKLVREEQRRVQDASLFPVYYGSAKKGLGIQPLMDAVTGLFQP  240

Query  241
IGEQGSAALCGSVFKVEYTDCGQRRVYLRLYSGTLRLRDTVALAGREKLKITEMRIPSKG  300

IGEQGSAALCGSVFKVEYTDCGQRRVYLRLYSGTLRLRDTVALAGREKLKITEMRIPSKG
Sbjct  241
IGEQGSAALCGSVFKVEYTDCGQRRVYLRLYSGTLRLRDTVALAGREKLKITEMRIPSKG  300

Query  301
EIVRTDTAYPGEIVILPSDSVRLNDVLGDPTRLPRKRWREDPLPMLRTTIAPKTAAQRER  360
            EIVRTDTAYPGEIVIL  D+++LND+LG+   LP K   ++P+P+LRTT+ P+
QRE
Sbjct  301
EIVRTDTAYPGEIVILADDTLKLNDILGNEKLLPHKTRIDNPMPLLRTTVEPQKPEQREA  360

Query  361
LLDALTQLADTDPLLRCEVDSITHEIILSFLGRVQLEVVSALLSEKYKLETVVKEPSVIY  420
            LL+AL ++ADTDPLL  ++D++THEI+LSFLG+VQLEV+ +LL EKY +
+KEPSVIY
Sbjct  361
LLNALAEIADTDPLLHFDIDTVTHEIMLSFLGKVQLEVICSLLEEKYHVGVAMKEPSVIY  420
```

Query 421
MERPLKAASHTIHIEVPPNPFWASIGLSVTPLSLGSGVQYESRVSLGYLNQSFQNAVRDG 480
            +ERPL+ A +TIHIEVPPNPFWASIGLSVTPL LGSGVQYESRVSLGYLNQS
QNAVRDG
Sbjct 421
LERPLRKAEYTIHIEVPPNPFWASIGLSVTPLPLGSGVQYESRVSLGYLNQSSQNAVRDG 480

Query 481
IRYGLEQGLFGWNVTDCKICFEYGLYYSPVSTPADFRSLAPIVLEQALKESGTQLLEPYL 540
            IRYGLEQGL+GW VTDCKICFEYGLYYSPVSTPADFR L+PIVLEQALK++GT
+LLEPYL
Sbjct 481
IRYGLEQGLYGWKVTDCKICFEYGLYYSPVSTPADFRLLSPIVLEQALKKAGTELLEPYL 540

Query 541
SFILYAPQEYLSRAYHDAPKYCATIETAQVKKDEVVFTGEIPARCIQAYRTDLAFYTNGR 600
             F +YAPQEYLSRAYHDAP+YCA I + Q+K DEV+ GEIPARCIQ YR DL
+TNG+
Sbjct 541
HFEIYAPQEYLSRAYHDAPRYCADIVSTQIKNDEVILKGEIPARCIQEYRNDLTNFTNGQ 600

Query 601 SVCLTELKGYQAAVGQPVIQPRRPNSRLDKVRHMFQKV 638
             VCLTELKGYQ A+G+ + QPRRPNSR+DKVRHMF K+
Sbjct 601 GVCLTELKGYQPAIGKFIYQPRRPNSRIDKVRHMFHKL 638

>WP_044674422.1 TetM/TetW/TetO/TetS family tetracycline resistance
ribosomal
protection protein [Streptococcus suis]
 AHH55326.1 tetracycline resistance protein [Streptococcus suis]
 CYU93660.1 putative tetracycline resistance protein tet(O/W/32/O)
[Streptococcus
suis]
Length=639

 Score = 1116 bits (2886), Expect = 0.0, Method: Compositional
matrix adjust.
 Identities = 530/638 (83%), Positives = 584/638 (92%), Gaps = 0/638
(0%)

Query 1
MKIINIGILAHVDAGKTTLTESLLYASGAISEPGSVEKGTTRTDTMFLERQRGITIQAAV 60
            MKIIN+GILAHVDAGKTTLTESLLY SGAI+EPGSV+KGTTRTDTM
LERQRGITIQ AV
Sbjct 1
MKIINLGILAHVDAGKTTLTESLLYTSGAIAEPGSVDKGTTRTDTMNLERQRGITIQTAV 60

Query 61
TSFQWHRCKVNIVDTPGHMDFLAEVYRSLAVLDGAILVISAKDGVQAQTRILFHALRKMN 120
            TSFQW   KVNI
+DTPGHMDFLAEVYRSLAVLDGAILVISAKDGVQAQTRILFHALRKMN
Sbjct 61
TSFQWEDVKVNIIDTPGHMDFLAEVYRSLAVLDGAILVISAKDGVQAQTRILFHALRKMN 120

Query 121
IPTVIFINKIDQAGVDLQSVVQSVRDKLSADIIIKQTVSLSPEIVLEENTDIEAWDAVIE 180

IPTVIFINKIDQAGVDLQSVVQSVRDKLSADIIIKQTVSLSPEIVLEENTDIEAWDAVIE
Sbjct 121
IPTVIFINKIDQAGVDLQSVVQSVRDKLSADIIIKQTVSLSPEIVLEENTDIEAWDAVIE 180

Query 181
NNDKLLEKYIAGEPISREKLVREEQRRVQDASLFPVYYGSAKKGLGIQPLMDAVTGLFQP 240

NNDKLLEKYIAGEPISREKLVREEQRRVQDASLFPVYYGSAKKGLGIQPLMDAVTGLFQP
Sbjct 181
NNDKLLEKYIAGEPISREKLVREEQRRVQDASLFPVYYGSAKKGLGIQPLMDAVTGLFQP 240

Query 241
IGEQGSAALCGSVFKVEYTDCGQRRVYLRLYSGTLRLRDTVALAGREKLKITEMRIPSKG 300

IGEQGSAALCGSVFKVEYTDCGQRRVYLRLYSGTLRLRDTVALAGREKLKITEMRIPSKG
Sbjct 241
IGEQGSAALCGSVFKVEYTDCGQRRVYLRLYSGTLRLRDTVALAGREKLKITEMRIPSKG 300

Query 301
EIVRTDTAYPGEIVILPSDSVRLNDVLGDPTRLPRKRWREDPLPMLRTTIAPKTAAQRER 360
EIVRTDTAYPGEIVIL D+++LND+LG+   LP K   ++P+P+LRTT+ P+
QRE
Sbjct 301
EIVRTDTAYPGEIVILADDTLKLNDILGNEKLLPHKTRIDNPMPLLRTTVEPQKPEQREA 360

Query 361
LLDALTQLADTDPLLRCEVDSITHEIILSFLGRVQLEVVSALLSEKYKLETVVKEPSVIY 420
LL+AL ++ADTDPLL ++D++THEI+LSFLG+VQLEV+ +LL EKY +
+KEPSVIY
Sbjct 361
LLNALAEIADTDPLLHFDIDTVTHEIMLSFLGKVQLEVICSLLEEKYHVGVAMKEPSVIY 420

Query 421
MERPLKAASHTIHIEVPPNPFWASIGLSVTPLSLGSGVQYESRVSLGYLNQSFQNAVRDG 480
+ERPL+ A +TIHIEVPPNPFWAS+GLS+ PL
+GSGVQYESRVSLGYLNQSFQNAV +G
Sbjct 421
LERPLRKAEYTIHIEVPPNPFWASVGLSIEPLPIGSGVQYESRVSLGYLNQSFQNAVMEG 480

Query 481
IRYGLEQGLFGWNVTDCKICFEYGLYYSPVSTPADFRSLAPIVLEQALKESGTQLLEPYL 540
+ YG EQGL+GW VTDCKICFEYGLYYSPVSTPADFR L+PIVLEQALK++GT
+LLEPYL
Sbjct 481
VLYGCEQGLYGWKVTDCKICFEYGLYYSPVSTPADFRLLSPIVLEQALKKAGTELLEPYL 540

Query 541
SFILYAPQEYLSRAYHDAPKYCATIETAQVKKDEVVFTGEIPARCIQAYRTDLAFYTNGR 600
F +YAPQEYLSRAYHDAP+YCA I + Q+K DEV+ GEIPARCIQ YR DL +
+TNG+

Sbjct 541
HFEIYAPQEYLSRAYHDAPRYCADIVSTQIKNDEVILKGEIPARCIQEYRNDLTYFTNGQ 600

Query 601 SVCLTELKGYQAAVGQPVIQPRRPNSRLDKVRHMFQKV 638
             VCLTELKGYQ A+G+ + QPRRPNSR+DKVRHMF K+
Sbjct 601 GVCLTELKGYQPAIGKFICQPRRPNSRIDKVRHMFHKL 638

>ACJ38544.1 tetracycline resistance protein, partial [Trueperella pyogenes]
Length=600

 Score = 1116 bits (2886), Expect = 0.0, Method: Compositional matrix adjust.
 Identities = 540/600 (90%), Positives = 563/600 (94%), Gaps = 0/600 (0%)

Query 40
TTRTDTMFLERQRGITIQAAVTSFQWHRCKVNIVDTPGHMDFLAEVYRSLAVLDGAILVI 99
            TTRTDTMFLERQRGITIQ AVTSFQWH
CKVNIVDTPGHMDFLAEVYRSLAVLDGAILV+
Sbjct 1
TTRTDTMFLERQRGITIQTAVTSFQWHSCKVNIVDTPGHMDFLAEVYRSLAVLDGAILVL 60

Query 100
SAKDGVQAQTRILFHALRKMNIPTVIFINKIDQAGVDLQSVVQSVRDKLSADIIIKQTVS 159
            SA+DGVQAQTR+LFHALRK+NIPT+IFINKIDQ +DL+ V
QSVRDKLSADIIIKQTVS
Sbjct 61
SARDGVQAQTRVLFHALRKLNIPTIIFINKIDQVDIDLEGVYQSVRDKLSADIIIKQTVS 120

Query 160
LSPEIVLEENTDIEAWDAVIENNDKLLEKYIAGEPISREKLVREEQRRVQDASLFPVYYG 219
            LSPEIVLEENTDIEAWDAVIENND LLEKYIAGEPISRE+L REEQRRVQ
ASLFPVY+G
Sbjct 121
LSPEIVLEENTDIEAWDAVIENNDGLLEKYIAGEPISREELAREEQRRVQAASLFPVYHG 180

Query 220
SAKKGLGIQPLMDAVTGLFQPIGEQGSAALCGSVFKVEYTDCGQRRVYLRLYSGTLRLRD 279
            SAK GLGIQ LMDAV GLFQP EQG ALCGSVFKVEYTDCGQR
VYLRLYSGTLRLRD
Sbjct 181
SAKNGLGIQRLMDAVIGLFQPTKEQGRTALCGSVFKVEYTDCGQRLVYLRLYSGTLRLRD 240

Query 280
TVALAGREKLKITEMRIPSKGEIVRTDTAYPGEIVILPSDSVRLNDVLGDPTRLPRKRWR 339
            TVALAGREKLKITEMRIPSKGEIVRTDTA+ GEIVILPSDS+RLND+LGD T+LPR
+ W
Sbjct 241
TVALAGREKLKITEMRIPSKGEIVRTDTAHKGEIVILPSDSLRLNDILGDKTQLPREMWS 300

Query 340
EDPLPMLRTTIAPKTAAQRERLLDALTQLADTDPLLRCEVDSITHEIILSFLGRVQLEVV 399

+ P MLRTTI PKTA QR+RLLDALTQ+ADTDPLL EVDSIT
EIILSFLGRVQLEVV
Sbjct 301
DAPFSMLRTTITPKTAEQRDRLLDALTQIADTDPLLCYEVDSITQEIILSFLGRVQLEVV 360

Query 400
SALLSEKYKLETVVKEPSVIYMERPLKAASHTIHIEVPPNPFWASIGLSVTPLSLGSGVQ 459
SALL+EKYK+ET VKEP+VIY+ERPLK ASHTIHIEVPPNPFWASIGLSVTPL
LGSGVQ
Sbjct 361
SALLAEKYKIETAVKEPTVIYLERPLKVASHTIHIEVPPNPFWASIGLSVTPLPLGSGVQ 420

Query 460
YESRVSLGYLNQSFQNAVRDGIRYGLEQGLFGWNVTDCKICFEYGLYYSPVSTPADFRSL 519
YESRVSLGYLNQSFQNAV DGIRYGLEQGL
GWNVTDCKICFEYGLYYSPVSTPADFRSL
Sbjct 421
YESRVSLGYLNQSFQNAVMDGIRYGLEQGLCGWNVTDCKICFEYGLYYSPVSTPADFRSL 480

Query 520
APIVLEQALKESGTQLLEPYLSFILYAPQEYLSRAYHDAPKYCATIETAQVKKDEVVFTG 579
APIVLE ALKESGTQLLEPYLSF LYAPQEYLSRAYHDAPKYCATIETAQ
+KKDEVVFTG
Sbjct 481
APIVLELALKESGTQLLEPYLSFTLYAPQEYLSRAYHDAPKYCATIETAQIKKDEVVFTG 540

Query 580
EIPARCIQAYRTDLAFYTNGRSVCLTELKGYQAAVGQPVIQPRRPNSRLDKVRHMFQKVM 639
EIPARCIQAYRTDLA YTNGRSVCLTELKGYQAAVGQPVIQPRRPNSRLD
+VRHMFQKVM
Sbjct 541
EIPARCIQAYRTDLASYTNGRSVCLTELKGYQAAVGQPVIQPRRPNSRLDRVRHMFQKVM 600

>WP_105136801.1 TetM/TetW/TetO/TetS family tetracycline resistance ribosomal
protection protein [Streptococcus suis]
Length=639

Score = 1115 bits (2883), Expect = 0.0, Method: Compositional matrix adjust.
Identities = 530/638 (83%), Positives = 584/638 (92%), Gaps = 0/638 (0%)

Query 1
MKIINIGILAHVDAGKTTLTESLLYASGAISEPGSVEKGTTRTDTMFLERQRGITIQAAV 60
MKIIN+GILAHVDAGKTTLTESLLY SGAI+EPGSV+KGTTRTDTM
LERQRGITIQ AV
Sbjct 1
MKIINLGILAHVDAGKTTLTESLLYTSGAIAEPGSVDKGTTRTDTMNLERQRGITIQTAV 60

Query 61
TSFQWHRCKVNIVDTPGHMDFLAEVYRSLAVLDGAILVISAKDGVQAQTRILFHALRKMN 120
TSFQW   KVNI

+DTPGHMDFLAEVYRSLAVLDGAILVISAKDGVQAQTRILFHALRKMN
Sbjct 61
TSFQWEDVKVNIIDTPGHMDFLAEVYRSLAVLDGAILVISAKDGVQAQTRILFHALRKMN 120

Query 121
IPTVIFINKIDQAGVDLQSVVQSVRDKLSADIIIKQTVSLSPEIVLEENTDIEAWDAVIE 180

IPTVIFINKIDQAGVDLQSVVQSVRDKLSADIIIKQTVSLSPEIVLEENTDIEAWDAVIE
Sbjct 121
IPTVIFINKIDQAGVDLQSVVQSVRDKLSADIIIKQTVSLSPEIVLEENTDIEAWDAVIE 180

Query 181
NNDKLLEKYIAGEPISREKLVREEQRRVQDASLFPVYYGSAKKGLGIQPLMDAVTGLFQP 240

NNDKLLEKYIAGEPISREKLVREEQRRVQDASLFPVYYGSAKKGLGIQPLMDAVTGLFQP
Sbjct 181
NNDKLLEKYIAGEPISREKLVREEQRRVQDASLFPVYYGSAKKGLGIQPLMDAVTGLFQP 240

Query 241
IGEQGSAALCGSVFKVEYTDCGQRRVYLRLYSGTLRLRDTVALAGREKLKITEMRIPSKG 300

IGEQGSAALCGSVFKVEYTDCGQRRVYLRLYSGTLRLRDTVALAGREKLKITEMRIPSKG
Sbjct 241
IGEQGSAALCGSVFKVEYTDCGQRRVYLRLYSGTLRLRDTVALAGREKLKITEMRIPSKG 300

Query 301
EIVRTDTAYPGEIVILPSDSVRLNDVLGDPTRLPRKRWREDPLPMLRTTIAPKTAAQRER 360
EIVRTDTAYPGEIVIL D+++LND+LG+ LP K ++P+P+LRTT+ P+
QRE
Sbjct 301
EIVRTDTAYPGEIVILADDTLKLNDILGNEKLLPHKTRIDNPMPLLRTTVEPQKPEQREA 360

Query 361
LLDALTQLADTDPLLRCEVDSITHEIILSFLGRVQLEVVSALLSEKYKLETVVKEPSVIY 420
LL+AL ++ADTDPLL ++D++THEI+LSFLG+VQLEV+ +LL EKY ++
+KEPSVIY
Sbjct 361
LLNALAEIADTDPLLHFDIDTVTHEIMLSFLGKVQLEVICSLLEEKYHVDVAMKEPSVIY 420

Query 421
MERPLKAASHTIHIEVPPNPFWASIGLSVTPLSLGSGVQYESRVSLGYLNQSFQNAVRDG 480
+ERPL+ A +TIHIEVPPNPFWAS+GLS+ PL
+GSGVQYESRVSLGYLNQSFQNAV +G
Sbjct 421
LERPLRKAEYTIHIEVPPNPFWASVGLSIEPLPIGSGVQYESRVSLGYLNQSFQNAVMEG 480

Query 481
IRYGLEQGLFGWNVTDCKICFEYGLYYSPVSTPADFRSLAPIVLEQALKESGTQLLEPYL 540
+ YG EQGL+GW VTDCKICFEYGLYYSPVSTPADFR L+PIVLEQALK++GT
+LLEPYL
Sbjct 481
VLYGCEQGLYGWKVTDCKICFEYGLYYSPVSTPADFRLLSPIVLEQALKKAGTELLEPYL 540

Query 541

```
SFILYAPQEYLSRAYHDAPKYCATIETAQVKKDEVVFTGEIPARCIQAYRTDLAFYTNGR  600
             F +YAPQEYLSRAYHDAP+YCA I + Q+K DEV+  GEIPARCIQ YR DL
+TNG+
Sbjct  541
HFEIYAPQEYLSRAYHDAPRYCADIVSTQIKNDEVILKGEIPARCIQEYRNDLTNFTNGQ  600

Query  601  SVCLTELKGYQAAVGQPVIQPRRPNSRLDKVRHMFQKV  638
             VCLTELKGYQ A+G+ + QPRRPNSR+DKVRHMF K+
Sbjct  601  GVCLTELKGYQPAIGKFICQPRRPNSRIDKVRHMFHKL  638


>WP_027971259.1 MULTISPECIES: TetM/TetW/TetO/TetS family
tetracycline resistance
ribosomal protection protein [Streptococcus]
 AFK31669.1 tetracycline resistance protein O/W/32/O [Streptococcus
suis]
 AHH29650.1 Tet(O/W/32/O) [Streptococcus suis]
 CYU71212.1 putative tetracycline resistance protein tet(O/W/32/O)
[Streptococcus
suis]
Length=639

 Score = 1114 bits (2882),  Expect = 0.0, Method: Compositional
matrix adjust.
 Identities = 530/638 (83%), Positives = 583/638 (91%), Gaps = 0/638
(0%)

Query  1
MKIINIGILAHVDAGKTTLTESLLYASGAISEPGSVEKGTTRTDTMFLERQRGITIQAAV  60
            MKIIN+GILAHVDAGKTTLTESLLY SGAI+EPGSV+KGTTRTDTM
LERQRGITIQ AV
Sbjct  1
MKIINLGILAHVDAGKTTLTESLLYTSGAIAEPGSVDKGTTRTDTMNLERQRGITIQTAV  60

Query  61
TSFQWHRCKVNIVDTPGHMDFLAEVYRSLAVLDGAILVISAKDGVQAQTRILFHALRKMN  120
            TSFQW   KVNI
+DTPGHMDFLAEVYRSLAVLDGAILVISAKDGVQAQTRILFHALRKMN
Sbjct  61
TSFQWEDVKVNIIDTPGHMDFLAEVYRSLAVLDGAILVISAKDGVQAQTRILFHALRKMN  120

Query  121
IPTVIFINKIDQAGVDLQSVVQSVRDKLSADIIIKQTVSLSPEIVLEENTDIEAWDAVIE  180

IPTVIFINKIDQAGVDLQSVVQSVRDKLSADIIIKQTVSLSPEIVLEENTDIEAWDAVIE
Sbjct  121
IPTVIFINKIDQAGVDLQSVVQSVRDKLSADIIIKQTVSLSPEIVLEENTDIEAWDAVIE  180

Query  181
NNDKLLEKYIAGEPISREKLVREEQRRVQDASLFPVYYGSAKKGLGIQPLMDAVTGLFQP  240

NNDKLLEKYIAGEPISREKLVREEQRRVQDASLFPVYYGSAKKGLGIQPLMDAVTGLFQP
Sbjct  181
NNDKLLEKYIAGEPISREKLVREEQRRVQDASLFPVYYGSAKKGLGIQPLMDAVTGLFQP  240
```

```
Query  241  
IGEQGSAALCGSVFKVEYTDCGQRRVYLRLYSGTLRLRDTVALAGREKLKITEMRIPSKG  300

IGEQGSAALCGSVFKVEYTDCGQRRVYLRLYSGTLRLRDTVALAGREKLKITEMRIPSKG
Sbjct  241  
IGEQGSAALCGSVFKVEYTDCGQRRVYLRLYSGTLRLRDTVALAGREKLKITEMRIPSKG  300

Query  301  
EIVRTDTAYPGEIVILPSDSVRLNDVLGDPTRLPRKRWREDPLPMLRTTIAPKTAAQRER  360
            EIVRTDTAYPGEIVIL  D+++LND+LG+   LP K   ++P+P+LRTT+ P+
QRE
Sbjct  301  
EIVRTDTAYPGEIVILADDTLKLNDILGNEKLLPHKTRIDNPMPLLRTTVEPQKPEQREA  360

Query  361  
LLDALTQLADTDPLLRCEVDSITHEIILSFLGRVQLEVVSALLSEKYKLETVVKEPSVIY  420
            LL+AL ++ADTDPLL  ++D++THEI+LSFLG+VQLEV+ +LL EKY +
+KEPSVIY
Sbjct  361  
LLNALAEIADTDPLLHFDIDTVTHEIMLSFLGKVQLEVICSLLEEKYHVGVAMKEPSVIY  420

Query  421  
MERPLKAASHTIHIEVPPNPFWASIGLSVTPLSLGSGVQYESRVSLGYLNQSFQNAVRDG  480
            +ERPL+ A +TIHIEVPPNPFWAS+GLS+ PL
+GSGVQYESRVSLGYLNQSFQNAV +G
Sbjct  421  
LERPLRKAEYTIHIEVPPNPFWASVGLSIEPLPIGSGVQYESRVSLGYLNQSFQNAVMEG  480

Query  481  
IRYGLEQGLFGWNVTDCKICFEYGLYYSPVSTPADFRSLAPIVLEQALKESGTQLLEPYL  540
            + YG EQGL+GW VTDCKICFEYGLYYSPVSTPADFR L+PIVLEQALK++GT
+LLEPYL
Sbjct  481  
VLYGCEQGLYGWKVTDCKICFEYGLYYSPVSTPADFRLLSPIVLEQALKKAGTELLEPYL  540

Query  541  
SFILYAPQEYLSRAYHDAPKYCATIETAQVKKDEVVFTGEIPARCIQAYRTDLAFYTNGR  600
             F +YAPQEYLSRAYHDAP+YCA I + Q+K DEV+  GEIPARCIQ YR DL
+TNG+
Sbjct  541  
HFEIYAPQEYLSRAYHDAPRYCADIVSTQIKNDEVILKGEIPARCIQEYRNDLTNFTNGQ  600

Query  601  SVCLTELKGYQAAVGQPVIQPRRPNSRLDKVRHMFQKV  638
             VCLTELKGYQ A+G+ + QPRRPNSR+DKVRHMF K+
Sbjct  601  GVCLTELKGYQPAIGKFICQPRRPNSRIDKVRHMFHKL  638
```

>KFI69516.1 Tetracycline resistance protein tetW [Bifidobacterium magnum]
Length=569

Score = 1112 bits (2876), Expect = 0.0, Method: Compositional matrix adjust.

Identities = 545/553 (99%), Positives = 548/553 (99%), Gaps = 0/553 (0%)

```
Query  1
MKIINIGILAHVDAGKTTLTESLLYASGAISEPGSVEKGTTRTDTMFLERQRGITIQAAV  60

MKIINIGILAHVDAGKTTLTESLLYASGAISEPGSVEKGTTRTDTMFLERQRGITIQAAV
Sbjct  1
MKIINIGILAHVDAGKTTLTESLLYASGAISEPGSVEKGTTRTDTMFLERQRGITIQAAV  60

Query  61
TSFQWHRCKVNIVDTPGHMDFLAEVYRSLAVLDGAILVISAKDGVQAQTRILFHALRKMN  120

TSFQWHRCKVNIVDTPGHMDFLAEVYRSLAVLDGAILVISAKDGVQAQTRILFHALRKMN
Sbjct  61
TSFQWHRCKVNIVDTPGHMDFLAEVYRSLAVLDGAILVISAKDGVQAQTRILFHALRKMN  120

Query  121
IPTVIFINKIDQAGVDLQSVVQSVRDKLSADIIIKQTVSLSPEIVLEENTDIEAWDAVIE  180

IPTVIFINKIDQAGVDLQSVVQSVRDKLSADIIIKQTVSLSPEIVLEENTDIEAWDAVIE
Sbjct  121
IPTVIFINKIDQAGVDLQSVVQSVRDKLSADIIIKQTVSLSPEIVLEENTDIEAWDAVIE  180

Query  181
NNDKLLEKYIAGEPISREKLVREEQRRVQDASLFPVYYGSAKKGLGIQPLMDAVTGLFQP  240
            NND+LLEKYIAGEPISREKL REEQ+RVQDASLFPVY+GSAK
GLGIQPLMDAVTGLFQP
Sbjct  181
NNDELLEKYIAGEPISREKLAREEQQRVQDASLFPVYHGSAKNGLGIQPLMDAVTGLFQP  240

Query  241
IGEQGSAALCGSVFKVEYTDCGQRRVYLRLYSGTLRLRDTVALAGREKLKITEMRIPSKG  300
            IGEQG
AALCGSVFKVEYTDCGQRRVYLRLYSGTLRLRDTVALAGREKLKITEMRIPSKG
Sbjct  241
IGEQGGAALCGSVFKVEYTDCGQRRVYLRLYSGTLRLRDTVALAGREKLKITEMRIPSKG  300

Query  301
EIVRTDTAYPGEIVILPSDSVRLNDVLGDPTRLPRKRWREDPLPMLRTTIAPKTAAQRER  360
            EIVRTDTAY GEIVILPSDSVRLNDVLGD
TRLPRKRWREDPLPMLRTTIAPKTAAQRER
Sbjct  301
EIVRTDTAYQGEIVILPSDSVRLNDVLGDQTRLPRKRWREDPLPMLRTTIAPKTAAQRER  360

Query  361
LLDALTQLADTDPLLRCEVDSITHEIILSFLGRVQLEVVSALLSEKYKLETVVKEPSVIY  420

LLDALTQLADTDPLLRCEVDSITHEIILSFLGRVQLEVVSALLSEKYKLETVVKEPSVIY
Sbjct  361
LLDALTQLADTDPLLRCEVDSITHEIILSFLGRVQLEVVSALLSEKYKLETVVKEPSVIY  420

Query  421
MERPLKAASHTIHIEVPPNPFWASIGLSVTPLSLGSGVQYESRVSLGYLNQSFQNAVRDG  480
```

MERPLKAASHTIHIEVPPNPFWASIGLSVTPLSLGSGVQYESRVSLGYLNQSFQNAVRDG
Sbjct 421
MERPLKAASHTIHIEVPPNPFWASIGLSVTPLSLGSGVQYESRVSLGYLNQSFQNAVRDG 480

Query 481
IRYGLEQGLFGWNVTDCKICFEYGLYYSPVSTPADFRSLAPIVLEQALKESGTQLLEPYL 540

IRYGLEQGLFGWNVTDCKICFEYGLYYSPVSTPADFRSLAPIVLEQALKESGTQLLEPYL
Sbjct 481
IRYGLEQGLFGWNVTDCKICFEYGLYYSPVSTPADFRSLAPIVLEQALKESGTQLLEPYL 540

Query 541 SFILYAPQEYLSR 553
            SFILYAPQEYLSR
Sbjct 541 SFILYAPQEYLSR 553

>WP_029176297.1 TetM/TetW/TetO/TetS family tetracycline resistance ribosomal
protection protein [Streptococcus suis]
 AHH29649.1 Tet(O/W/32/O) [Streptococcus suis]
Length=639

 Score = 1112 bits (2876), Expect = 0.0, Method: Compositional matrix adjust.
 Identities = 529/638 (83%), Positives = 583/638 (91%), Gaps = 0/638 (0%)

Query 1
MKIINIGILAHVDAGKTTLTESLLYASGAISEPGSVEKGTTRTDTMFLERQRGITIQAAV 60
            MKIIN+GILAHVDAGKTTLTESLLY SGAI+EPGSV++GTTRTDTM
LERQRGITIQ AV
Sbjct 1
MKIINLGILAHVDAGKTTLTESLLYTSGAIAEPGSVDEGTTRTDTMNLERQRGITIQTAV 60

Query 61
TSFQWHRCKVNIVDTPGHMDFLAEVYRSLAVLDGAILVISAKDGVQAQTRILFHALRKMN 120
            TSFQW   KVNI
+DTPGHMDFLAEVYRSLAVLDGAILVISAKDGVQAQTRILFHALRKMN
Sbjct 61
TSFQWEDVKVNIIDTPGHMDFLAEVYRSLAVLDGAILVISAKDGVQAQTRILFHALRKMN 120

Query 121
IPTVIFINKIDQAGVDLQSVVQSVRDKLSADIIIKQTVSLSPEIVLEENTDIEAWDAVIE 180

IPTVIFINKIDQAGVDLQSVVQSVRDKLSADIIIKQTVSLSPEIVLEENTDIEAWDAVIE
Sbjct 121
IPTVIFINKIDQAGVDLQSVVQSVRDKLSADIIIKQTVSLSPEIVLEENTDIEAWDAVIE 180

Query 181
NNDKLLEKYIAGEPISREKLVREEQRRVQDASLFPVYYGSAKKGLGIQPLMDAVTGLFQP 240

NNDKLLEKYIAGEPISREKLVREEQRRVQDASLFPVYYGSAKKGLGIQPLMDAVTGLFQP
Sbjct 181

NNDKLLEKYIAGEPISREKLVREEQRRVQDASLFPVYYGSAKKGLGIQPLMDAVTGLFQP 240

Query 241
IGEQGSAALCGSVFKVEYTDCGQRRVYLRLYSGTLRLRDTVALAGREKLKITEMRIPSKG 300

IGEQGSAALCGSVFKVEYTDCGQRRVYLRLYSGTLRLRDTVALAGREKLKITEMRIPSKG
Sbjct 241
IGEQGSAALCGSVFKVEYTDCGQRRVYLRLYSGTLRLRDTVALAGREKLKITEMRIPSKG 300

Query 301
EIVRTDTAYPGEIVILPSDSVRLNDVLGDPTRLPRKRWREDPLPMLRTTIAPKTAAQRER 360
EIVRTDTAYPGEIVIL D+++LND+LG+   LP K   ++P+P+LRTT+ P+
QRE
Sbjct 301
EIVRTDTAYPGEIVILADDTLKLNDILGNEKLLPHKTRIDNPMPLLRTTVEPQKPEQREA 360

Query 361
LLDALTQLADTDPLLRCEVDSITHEIILSFLGRVQLEVVSALLSEKYKLETVVKEPSVIY 420
LL+AL ++ADTDPLL ++D++THEI+LSFLG+VQLEV+ +LL EKY +
+KEPSVIY
Sbjct 361
LLNALAEIADTDPLLHFDIDTVTHEIMLSFLGKVQLEVICSLLEEKYHVGVAMKEPSVIY 420

Query 421
MERPLKAASHTIHIEVPPNPFWASIGLSVTPLSLGSGVQYESRVSLGYLNQSFQNAVRDG 480
+ERPL+ A +TIHIEVPPNPFWAS+GLS+ PL
+GSGVQYESRVSLGYLNQSFQNAV +G
Sbjct 421
LERPLRKAEYTIHIEVPPNPFWASVGLSIEPLPIGSGVQYESRVSLGYLNQSFQNAVMEG 480

Query 481
IRYGLEQGLFGWNVTDCKICFEYGLYYSPVSTPADFRSLAPIVLEQALKESGTQLLEPYL 540
+ YG EQGL+GW VTDCKICFEYGLYYSPVSTPADFR L+PIVLEQALK++GT
+LLEPYL
Sbjct 481
VLYGCEQGLYGWKVTDCKICFEYGLYYSPVSTPADFRLLSPIVLEQALKKAGTELLEPYL 540

Query 541
SFILYAPQEYLSRAYHDAPKYCATIETAQVKKDEVVFTGEIPARCIQAYRTDLAFYTNGR 600
F +YAPQEYLSRAYHDAP+YCA I + Q+K DEV+ GEIPARCIQ YR DL
+TNG+
Sbjct 541
HFEIYAPQEYLSRAYHDAPRYCADIVSTQIKNDEVILKGEIPARCIQEYRNDLTNFTNGQ 600

Query 601 SVCLTELKGYQAAVGQPVIQPRRPNSRLDKVRHMFQKV 638
VCLTELKGYQ A+G+ + QPRRPNSR+DKVRHMF K+
Sbjct 601 GVCLTELKGYQPAIGKFICQPRRPNSRIDKVRHMFHKL 638

>WP_038817059.1 MULTISPECIES: TetM/TetW/TetO/TetS family
tetracycline resistance
ribosomal protection protein [Campylobacter]
Length=639

```
 Score = 1112 bits (2875),  Expect = 0.0, Method: Compositional
matrix adjust.
 Identities = 529/638 (83%), Positives = 582/638 (91%), Gaps = 0/638
(0%)

Query  1
MKIINIGILAHVDAGKTTLTESLLYASGAISEPGSVEKGTTRTDTMFLERQRGITIQAAV  60
            MKIIN+GILAHVDAGKTTLTESLLY SGAI+EPGSV++GTTRTDTM
LERQRGITIQ AV
Sbjct  1
MKIINLGILAHVDAGKTTLTESLLYTSGAIAEPGSVDEGTTRTDTMNLERQRGITIQTAV  60

Query  61
TSFQWHRCKVNIVDTPGHMDFLAEVYRSLAVLDGAILVISAKDGVQAQTRILFHALRKMN  120
            TSFQW   KVNI
+DTPGHMDFLAEVYRSLAVLDGAILVISAKDGVQAQTRILFHALRKMN
Sbjct  61
TSFQWEDVKVNIIDTPGHMDFLAEVYRSLAVLDGAILVISAKDGVQAQTRILFHALRKMN  120

Query  121
IPTVIFINKIDQAGVDLQSVVQSVRDKLSADIIIKQTVSLSPEIVLEENTDIEAWDAVIE  180

IPTVIFINKIDQAGVDLQSVVQSVRDKLSADIIIKQTVSLSPEIVLEENTDIEAWDAVIE
Sbjct  121
IPTVIFINKIDQAGVDLQSVVQSVRDKLSADIIIKQTVSLSPEIVLEENTDIEAWDAVIE  180

Query  181
NNDKLLEKYIAGEPISREKLVREEQRRVQDASLFPVYYGSAKKGLGIQPLMDAVTGLFQP  240

NNDKLLEKYIAGEPISREKLVREEQRRVQDASLFPVYYGSAKKGLGIQPLMDAVTGLFQP
Sbjct  181
NNDKLLEKYIAGEPISREKLVREEQRRVQDASLFPVYYGSAKKGLGIQPLMDAVTGLFQP  240

Query  241
IGEQGSAALCGSVFKVEYTDCGQRRVYLRLYSGTLRLRDTVALAGREKLKITEMRIPSKG  300

IGEQGSAALCGSVFKVEYTDCGQRRVYLRLYSGTLRLRDTVALAGREKLKITEMRIPSKG
Sbjct  241
IGEQGSAALCGSVFKVEYTDCGQRRVYLRLYSGTLRLRDTVALAGREKLKITEMRIPSKG  300

Query  301
EIVRTDTAYPGEIVILPSDSVRLNDVLGDPTRLPRKRWREDPLPMLRTTIAPKTAAQRER  360
            EIVRTDTAYPGEIVIL  D+++LND+LG+   LP K   ++P+P+LRTT+ P+
QRE
Sbjct  301
EIVRTDTAYPGEIVILADDTLKLNDILGNEKLLPHKTRIDNPMPLLRTTVEPQKPEQREA  360

Query  361
LLDALTQLADTDPLLRCEVDSITHEIILSFLGRVQLEVVSALLSEKYKLETVVKEPSVIY  420
            LL+AL ++ADTDPLL  ++D++THEI+LSFLG+VQLEV+ +LL EKY +
+KEPSVIY
Sbjct  361
LLNALAEIADTDPLLHFDIDTVTHEIMLSFLGKVQLEVICSLLEEKYHVGVAMKEPSVIY  420
```

```
Query  421
MERPLKAASHTIHIEVPPNPFWASIGLSVTPLSLGSGVQYESRVSLGYLNQSFQNAVRDG  480
            +ERPL+ A +TIHIEVPPNPFWAS+GLS+ PL
+GSGVQYESRVSLGYLNQSFQNAV  G
Sbjct  421
LERPLRKAEYTIHIEVPPNPFWASVGLSIEPLPIGSGVQYESRVSLGYLNQSFQNAVMKG  480

Query  481
IRYGLEQGLFGWNVTDCKICFEYGLYYSPVSTPADFRSLAPIVLEQALKESGTQLLEPYL  540
            + YG EQGL+GW VTDCKICFEYGLYYSPVSTPADFR L+PIVLEQALK++GT
+LLEPYL
Sbjct  481
VLYGCEQGLYGWKVTDCKICFEYGLYYSPVSTPADFRLLSPIVLEQALKKAGTELLEPYL  540

Query  541
SFILYAPQEYLSRAYHDAPKYCATIETAQVKKDEVVFTGEIPARCIQAYRTDLAFYTNGR  600
             F +YAPQEYLSRAYHDAP+YCA I + Q+K DEV+  GEIPARCIQ YR DL
+TNG+
Sbjct  541
HFEIYAPQEYLSRAYHDAPRYCADIVSTQIKNDEVILKGEIPARCIQEYRNDLTNFTNGQ  600

Query  601  SVCLTELKGYQAAVGQPVIQPRRPNSRLDKVRHMFQKV  638
             VCLTELKGYQ A+G+ + QPRRPNSR+DKVRHMF K+
Sbjct  601  GVCLTELKGYQPAIGKFICQPRRPNSRIDKVRHMFHKL  638


>WP_029177869.1 TetM/TetW/TetO/TetS family tetracycline resistance
ribosomal
protection protein [Streptococcus suis]
Length=639

 Score = 1112 bits (2875),  Expect = 0.0, Method: Compositional
matrix adjust.
 Identities = 529/638 (83%), Positives = 582/638 (91%), Gaps = 0/638
(0%)

Query  1
MKIININIGILAHVDAGKTTLTESLLYASGAISEPGSVEKGTTRTDTMFLERQRGITIQAAV  60
            MKIIN+GILAHVDAGKTTLTESLLY SGAI+EPGSV+KGTTRTDTM
LERQRGITIQ AV
Sbjct  1
MKIINLGILAHVDAGKTTLTESLLYTSGAIAEPGSVDKGTTRTDTMNLERQRGITIQTAV  60

Query  61
TSFQWHRCKVNIVDTPGHMDFLAEVYRSLAVLDGAILVISAKDGVQAQTRILFHALRKMN  120
            TSFQW   KVNI
+DTPGHMDFLAEVYRSLAVLDGAILVISAKDGVQAQTRILFHALRKMN
Sbjct  61
TSFQWEDVKVNIIDTPGHMDFLAEVYRSLAVLDGAILVISAKDGVQAQTRILFHALRKMN  120

Query  121
IPTVIFINKIDQAGVDLQSVVQSVRDKLSADIIIKQTVSLSPEIVLEENTDIEAWDAVIE  180

IPTVIFINKIDQAGVDLQSVVQSVRDKLSADIIIKQTVSLSPEIVLEENTDIEAWDAVIE
```

Sbjct 121
IPTVIFINKIDQAGVDLQSVVQSVRDKLSADIIIKQTVSLSPEIVLEENTDIEAWDAVIE 180

Query 181
NNDKLLEKYIAGEPISREKLVREEQRRVQDASLFPVYYGSAKKGLGIQPLMDAVTGLFQP 240

NNDKLLEKYIAGEPISREKLVREEQRRVQDASLFPVYYGSAKKGLGIQPLMDAVTGLFQP
Sbjct 181
NNDKLLEKYIAGEPISREKLVREEQRRVQDASLFPVYYGSAKKGLGIQPLMDAVTGLFQP 240

Query 241
IGEQGSAALCGSVFKVEYTDCGQRRVYLRLYSGTLRLRDTVALAGREKLKITEMRIPSKG 300
IGEQGSAALCGSVFKVEYTDCGQRRVYLRLYSGTL
LRDTVALAGREKLKITEMRIPSKG
Sbjct 241
IGEQGSAALCGSVFKVEYTDCGQRRVYLRLYSGTLHLRDTVALAGREKLKITEMRIPSKG 300

Query 301
EIVRTDTAYPGEIVILPSDSVRLNDVLGDPTRLPRKRWREDPLPMLRTTIAPKTAAQRER 360
EIVRTDTAYPGEIVIL D+++LND+LG+   LP K   ++P+P+LRTT+ P+
QRE
Sbjct 301
EIVRTDTAYPGEIVILADDTLKLNDILGNEKLLPHKTRIDNPMPLLRTTVEPQKPEQREA 360

Query 361
LLDALTQLADTDPLLRCEVDSITHEIILSFLGRVQLEVVSALLSEKYKLETVVKEPSVIY 420
LL+AL ++ADTDPLL ++D++THEI+LSFLG+VQLEV+ +LL EKY +
+KEPSVIY
Sbjct 361
LLNALAEIADTDPLLHFDIDTVTHEIMLSFLGKVQLEVICSLLEEKYHVGVAMKEPSVIY 420

Query 421
MERPLKAASHTIHIEVPPNPFWASIGLSVTPLSLGSGVQYESRVSLGYLNQSFQNAVRDG 480
+ERPL+ A +TIHIEVPPNPFWAS+GLS+ PL
+GSGVQYESRVSLGYLNQSFQNAV +G
Sbjct 421
LERPLRKAEYTIHIEVPPNPFWASVGLSIEPLPIGSGVQYESRVSLGYLNQSFQNAVMEG 480

Query 481
IRYGLEQGLFGWNVTDCKICFEYGLYYSPVSTPADFRSLAPIVLEQALKESGTQLLEPYL 540
+ YG EQGL+GW VTDCKICFEYGLYYSPVSTPADFR L+PIVLEQALK++GT
+LLEPYL
Sbjct 481
VLYGCEQGLYGWKVTDCKICFEYGLYYSPVSTPADFRLLSPIVLEQALKKAGTELLEPYL 540

Query 541
SFILYAPQEYLSRAYHDAPKYCATIETAQVKKDEVVFTGEIPARCIQAYRTDLAFYTNGR 600
F +YAPQEYLSRAYHDAP+YCA I + Q+K DEV+ GEIPARCIQ YR DL
+TNG+
Sbjct 541
HFEIYAPQEYLSRAYHDAPRYCADIVSTQIKNDEVILKGEIPARCIQEYRNDLTNFTNGQ 600

Query 601 SVCLTELKGYQAAVGQPVIQPRRPNSRLDKVRHMFQKV 638
VCLTELKGYQ A+G+ + QPRRPNSR+DKVRHMF K+

```
Sbjct  601  GVCLTELKGYQPAIGKFICQPRRPNSRIDKVRHMFHKL  638


>WP_105207356.1 TetM/TetW/TetO/TetS family tetracycline resistance
ribosomal
protection protein [Streptococcus suis]
Length=639

 Score = 1111 bits (2874),  Expect = 0.0, Method: Compositional
matrix adjust.
 Identities = 529/638 (83%), Positives = 582/638 (91%), Gaps = 0/638
(0%)

Query  1
MKIINIGILAHVDAGKTTLTESLLYASGAISEPGSVEKGTTRTDTMFLERQRGITIQAAV  60
            MKIIN+GILAHVDAGKTTLTESLLY SGAI+EPGSV+KGTTRTDTM
LERQRGITIQ AV
Sbjct  1
MKIINLGILAHVDAGKTTLTESLLYTSGAIAEPGSVDKGTTRTDTMNLERQRGITIQTAV  60

Query  61
TSFQWHRCKVNIVDTPGHMDFLAEVYRSLAVLDGAILVISAKDGVQAQTRILFHALRKMN  120
            TSFQW   KVNI
+DTPGHMDFLAEVYRSLAVLDGAILVISAKDGVQAQTRILFHALRKMN
Sbjct  61
TSFQWEDVKVNIIDTPGHMDFLAEVYRSLAVLDGAILVISAKDGVQAQTRILFHALRKMN  120

Query  121
IPTVIFINKIDQAGVDLQSVVQSVRDKLSADIIIKQTVSLSPEIVLEENTDIEAWDAVIE  180

IPTVIFINKIDQAGVDLQSVVQSVRDKLSADIIIKQTVSLSPEIVLEENTDIEAWDAVIE
Sbjct  121
IPTVIFINKIDQAGVDLQSVVQSVRDKLSADIIIKQTVSLSPEIVLEENTDIEAWDAVIE  180

Query  181
NNDKLLEKYIAGEPISREKLVREEQRRVQDASLFPVYYGSAKKGLGIQPLMDAVTGLFQP  240

NNDKLLEKYIAGEPISREKLVREEQRRVQDASLFPVYYGSAKKGLGIQPLMDAVTGLFQP
Sbjct  181
NNDKLLEKYIAGEPISREKLVREEQRRVQDASLFPVYYGSAKKGLGIQPLMDAVTGLFQP  240

Query  241
IGEQGSAALCGSVFKVEYTDCGQRRVYLRLYSGTLRLRDTVALAGREKLKITEMRIPSKG  300
            IGEQGSAALCG
VFKVEYTDCGQRRVYLRLYSGTLRLRDTVALAGREKLKITEMRIPSKG
Sbjct  241
IGEQGSAALCGGVFKVEYTDCGQRRVYLRLYSGTLRLRDTVALAGREKLKITEMRIPSKG  300

Query  301
EIVRTDTAYPGEIVILPSDSVRLNDVLGDPTRLPRKRWREDPLPMLRTTIAPKTAAQRER  360
            EIVRTDTAYPGEIVIL  D+++LND+LG+   LP K   ++P+P+LRTT+ P+
QRE
Sbjct  301
EIVRTDTAYPGEIVILADDTLKLNDILGNEKLLPHKTRIDNPMPLLRTTVEPQKPEQREA  360
```

```
Query  361
LLDALTQLADTDPLLRCEVDSITHEIILSFLGRVQLEVVSALLSEKYKLETVVKEPSVIY  420
            LL+AL ++ADTDPLL  ++D++THEI+LSFLG+VQLEV+ +LL EKY +
+KEPSVIY
Sbjct  361
LLNALAEIADTDPLLHFDIDTVTHEIMLSFLGKVQLEVICSLLEEKYHVGVAMKEPSVIY  420

Query  421
MERPLKAASHTIHIEVPPNPFWASIGLSVTPLSLGSGVQYESRVSLGYLNQSFQNAVRDG  480
            +ERPL+ A +TIHIEVPPNPFWAS+GLS+ PL
+GSGVQYESRVSLGYLNQSFQNAV +G
Sbjct  421
LERPLRKAEYTIHIEVPPNPFWASVGLSIEPLPIGSGVQYESRVSLGYLNQSFQNAVMEG  480

Query  481
IRYGLEQGLFGWNVTDCKICFEYGLYYSPVSTPADFRSLAPIVLEQALKESGTQLLEPYL  540
            + YG EQGL+GW VTDCKICFEYGLYYSPVSTPADFR L+PIVLEQALK++GT
+LLEPYL
Sbjct  481
VLYGCEQGLYGWKVTDCKICFEYGLYYSPVSTPADFRLLSPIVLEQALKKAGTELLEPYL  540

Query  541
SFILYAPQEYLSRAYHDAPKYCATIETAQVKKDEVVFTGEIPARCIQAYRTDLAFYTNGR  600
             F +YAPQEYLSRAYHDAP+YCA I + Q+K DEV+  GEIPARCIQ YR DL
+TNG+
Sbjct  541
HFEIYAPQEYLSRAYHDAPRYCADIVSTQIKNDEVILKGEIPARCIQEYRNDLTNFTNGQ  600

Query  601  SVCLTELKGYQAAVGQPVIQPRRPNSRLDKVRHMFQKV  638
             VCLTELKGYQ A+G+ + QPRRPNSR+DKVRHMF K+
Sbjct  601  GVCLTELKGYQPAIGKFICQPRRPNSRIDKVRHMFHKL  638


>WP_107209023.1 TetM/TetW/TetO/TetS family tetracycline resistance
ribosomal
protection protein [Streptococcus suis]
 PTB14715.1 tetracycline resistance ribosomal protection protein
[Streptococcus
suis]
Length=639

 Score = 1110 bits (2871),  Expect = 0.0, Method: Compositional
matrix adjust.
 Identities = 528/638 (83%), Positives = 582/638 (91%), Gaps = 0/638
(0%)

Query  1
MKIINIGILAHVDAGKTTLTESLLYASGAISEPGSVEKGTTRTDTMFLERQRGITIQAAV  60
            MKIIN+GILAHVDAGKTTLTESLLY SGAI+EPGSV++GTTRTDTM
LERQRGITIQ AV
Sbjct  1
MKIINLGILAHVDAGKTTLTESLLYTSGAIAEPGSVDEGTTRTDTMNLERQRGITIQTAV  60
```

Query 61
TSFQWHRCKVNIVDTPGHMDFLAEVYRSLAVLDGAILVISAKDGVQAQTRILFHALRKMN 120
            TSFQW   KVNI
+DTPGHMDFLAEVYRSLAVLDGAILVISAKDGVQAQTRILFHALRKMN
Sbjct 61
TSFQWEDVKVNIIDTPGHMDFLAEVYRSLAVLDGAILVISAKDGVQAQTRILFHALRKMN 120

Query 121
IPTVIFINKIDQAGVDLQSVVQSVRDKLSADIIIKQTVSLSPEIVLEENTDIEAWDAVIE 180

IPTVIFINKIDQAGVDLQSVVQSVRDKLSADIIIKQTVSLSPEIVLEENTDIEAWDAVIE
Sbjct 121
IPTVIFINKIDQAGVDLQSVVQSVRDKLSADIIIKQTVSLSPEIVLEENTDIEAWDAVIE 180

Query 181
NNDKLLEKYIAGEPISREKLVREEQRRVQDASLFPVYYGSAKKGLGIQPLMDAVTGLFQP 240
            NNDKLLEKYIAGEPISREKLVREEQRRV
DASLFPVYYGSAKKGLGIQPLMDAVTGLFQP
Sbjct 181
NNDKLLEKYIAGEPISREKLVREEQRRVHDASLFPVYYGSAKKGLGIQPLMDAVTGLFQP 240

Query 241
IGEQGSAALCGSVFKVEYTDCGQRRVYLRLYSGTLRLRDTVALAGREKLKITEMRIPSKG 300

IGEQGSAALCGSVFKVEYTDCGQRRVYLRLYSGTLRLRDTVALAGREKLKITEMRIPSKG
Sbjct 241
IGEQGSAALCGSVFKVEYTDCGQRRVYLRLYSGTLRLRDTVALAGREKLKITEMRIPSKG 300

Query 301
EIVRTDTAYPGEIVILPSDSVRLNDVLGDPTRLPRKRWREDPLPMLRTTIAPKTAAQRER 360
            EIVRTDTAYPGEIVIL D+++LND+LG+   LP K   ++P+P+LRTT+ P+
QRE
Sbjct 301
EIVRTDTAYPGEIVILADDTLKLNDILGNEKLLPHKTRIDNPMPLLRTTVEPQKPEQREA 360

Query 361
LLDALTQLADTDPLLRCEVDSITHEIILSFLGRVQLEVVSALLSEKYKLETVVKEPSVIY 420
            LL+AL ++ADTDPLL ++D++THEI+LSFLG+VQLEV+ +LL EKY +
+KEPSVIY
Sbjct 361
LLNALAEIADTDPLLHFDIDTVTHEIMLSFLGKVQLEVICSLLEEKYHVGVAMKEPSVIY 420

Query 421
MERPLKAASHTIHIEVPPNPFWASIGLSVTPLSLGSGVQYESRVSLGYLNQSFQNAVRDG 480
            +ERPL+ A +TIHIEVPPNPFWAS+GLS+ PL
+GSGVQYESRVSLGYLNQSFQNAV +G
Sbjct 421
LERPLRKAEYTIHIEVPPNPFWASVGLSIEPLPIGSGVQYESRVSLGYLNQSFQNAVMEG 480

Query 481
IRYGLEQGLFGWNVTDCKICFEYGLYYSPVSTPADFRSLAPIVLEQALKESGTQLLEPYL 540
            + YG EQGL+GW VTDCKICFEYGLYYSPVSTPADFR L+PIVLEQALK++GT
+LLEPYL
Sbjct 481

VLYGCEQGLYGWKVTDCKICFEYGLYYSPVSTPADFRLLSPIVLEQALKKAGTELLEPYL 540

Query 541
SFILYAPQEYLSRAYHDAPKYCATIETAQVKKDEVVFTGEIPARCIQAYRTDLAFYTNGR 600
             F +YAPQEYLSRAYHDAP+YCA I + Q+K DEV+ GEIPARCIQ YR DL
+TNG+
Sbjct 541
HFEIYAPQEYLSRAYHDAPRYCADIVSTQIKNDEVILKGEIPARCIQEYRNDLTNFTNGQ 600

Query 601 SVCLTELKGYQAAVGQPVIQPRRPNSRLDKVRHMFQKV 638
             VCLTELKGYQ A+G+ + QPRRPNSR+DKVRHMF K+
Sbjct 601 GVCLTELKGYQPAIGKFICQPRRPNSRIDKVRHMFHKL 638

>WP_024408858.1 TetM/TetW/TetO/TetS family tetracycline resistance
ribosomal
protection protein [Streptococcus suis]
Length=639

 Score = 1109 bits (2869), Expect = 0.0, Method: Compositional
matrix adjust.
 Identities = 525/638 (82%), Positives = 583/638 (91%), Gaps = 0/638
(0%)

Query 1
MKIINIGILAHVDAGKTTLTESLLYASGAISEPGSVEKGTTRTDTMFLERQRGITIQAAV 60
            MKIIN+GILAHVDAGKTTLTESLLY SGAI+EPGSV+KGTTRTDTM
LERQRGITIQ AV
Sbjct 1
MKIINLGILAHVDAGKTTLTESLLYTSGAIAEPGSVDKGTTRTDTMNLERQRGITIQTAV 60

Query 61
TSFQWHRCKVNIVDTPGHMDFLAEVYRSLAVLDGAILVISAKDGVQAQTRILFHALRKMN 120
            TSFQW   KVNI+DTPGHMDFLAEVYRSL+VLDGA+L++SAKDG
+QAQTRILFHALRKMN
Sbjct 61
TSFQWEDVKVNIIDTPGHMDFLAEVYRSLSVLDGAVLLVSAKDGIQAQTRILFHALRKMN 120

Query 121
IPTVIFINKIDQAGVDLQSVVQSVRDKLSADIIIKQTVSLSPEIVLEENTDIEAWDAVIE 180

IPTVIFINKIDQAGVDLQSVVQSVRDKLSADIIIKQTVSLSPEIVLEENTDIEAWDAVIE
Sbjct 121
IPTVIFINKIDQAGVDLQSVVQSVRDKLSADIIIKQTVSLSPEIVLEENTDIEAWDAVIE 180

Query 181
NNDKLLEKYIAGEPISREKLVREEQRRVQDASLFPVYYGSAKKGLGIQPLMDAVTGLFQP 240

NNDKLLEKYIAGEPISREKLVREEQRRVQDASLFPVYYGSAKKGLGIQPLMDAVTGLFQP
Sbjct 181
NNDKLLEKYIAGEPISREKLVREEQRRVQDASLFPVYYGSAKKGLGIQPLMDAVTGLFQP 240

Query 241
IGEQGSAALCGSVFKVEYTDCGQRRVYLRLYSGTLRLRDTVALAGREKLKITEMRIPSKG 300

IGEQGSAALCGSVFKVEYTDCGQRRVYLRLYSGTLRLRDTVALAGREKLKITEMRIPSKG
Sbjct 241
IGEQGSAALCGSVFKVEYTDCGQRRVYLRLYSGTLRLRDTVALAGREKLKITEMRIPSKG 300

Query 301
EIVRTDTAYPGEIVILPSDSVRLNDVLGDPTRLPRKRWREDPLPMLRTTIAPKTAAQRER 360
            EIVRTDTAYPGEIVIL D+++LND+LG+   LP K   ++P+P+LRTT+ P+
QRE
Sbjct 301
EIVRTDTAYPGEIVILADDTLKLNDILGNEKLLPHKTRIDNPMPLLRTTVEPQKPEQREA 360

Query 361
LLDALTQLADTDPLLRCEVDSITHEIILSFLGRVQLEVVSALLSEKYKLETVVKEPSVIY 420
            LL+AL ++ADTDPLL ++D++THEI+LSFLG+VQLEV+ +LL EKY +
+KEPSVIY
Sbjct 361
LLNALAEIADTDPLLHFDIDTVTHEIMLSFLGKVQLEVICSLLEEKYHVGVAMKEPSVIY 420

Query 421
MERPLKAASHTIHIEVPPNPFWASIGLSVTPLSLGSGVQYESRVSLGYLNQSFQNAVRDG 480
            +ERPL+ A +TIHIEVPPNPFWAS+GLS+ PL
+GSGVQYESRVSLGYLNQSFQNAV +G
Sbjct 421
LERPLRKAEYTIHIEVPPNPFWASVGLSIEPLPIGSGVQYESRVSLGYLNQSFQNAVMEG 480

Query 481
IRYGLEQGLFGWNVTDCKICFEYGLYYSPVSTPADFRSLAPIVLEQALKESGTQLLEPYL 540
            + YG EQGL+GW VTDCKICFEYGLYYSPVSTPADFR L+PIVLEQALK++GT
+LLEPYL
Sbjct 481
VLYGCEQGLYGWKVTDCKICFEYGLYYSPVSTPADFRLLSPIVLEQALKKAGTELLEPYL 540

Query 541
SFILYAPQEYLSRAYHDAPKYCATIETAQVKKDEVVFTGEIPARCIQAYRTDLAFYTNGR 600
             F +YAPQEYLSRAYHDAP+YCA I + Q+K DEV+ GEIPARCIQ YR DL
+TNG+
Sbjct 541
HFEIYAPQEYLSRAYHDAPRYCADIVSTQIKNDEVILKGEIPARCIQEYRNDLTNFTNGQ 600

Query 601 SVCLTELKGYQAAVGQPVIQPRRPNSRLDKVRHMFQKV 638
             VCLTELKGYQ A+G+ + QPRRPNSR+DKVRHMF K+
Sbjct 601 GVCLTELKGYQPAIGKFICQPRRPNSRIDKVRHMFHKL 638

>WP_013646121.1 MULTISPECIES: TetM/TetW/TetO/TetS family
tetracycline resistance
ribosomal protection protein [Streptococcus]
 CAQ58207.1 tetracycline resistance protein [Streptococcus suis]
 CBZ49489.1 putative tetracycline resistance protein tet(O/W/32/O)
(plasmid)
[Streptococcus gallolyticus subsp. gallolyticus ATCC BAA-2069]
 CBZ42067.1 ribosomal protection tetracycline resistance protein
[Streptococcus

suis]
 KHD42989.1 tetracycline resistance protein tetM [Streptococcus
uberis]
 CZA80273.1 putative tetracycline resistance protein tet(O/W/32/O)
[Streptococcus
suis]
 CZA97916.1 putative tetracycline resistance protein tet(O/W/32/O)
[Streptococcus
suis]
 CZA83529.1 putative tetracycline resistance protein tet(O/W/32/O)
[Streptococcus
suis]
 CZB11747.1 putative tetracycline resistance protein tet(O/W/32/O)
[Streptococcus
suis]
 CYU43540.1 putative tetracycline resistance protein tet(O/W/32/O)
[Streptococcus
suis]
 CYV25871.1 putative tetracycline resistance protein tet(O/W/32/O)
[Streptococcus
suis]
 CYU95779.1 putative tetracycline resistance protein tet(O/W/32/O)
[Streptococcus
suis]
 AMP42276.1 tetracycline resistance protein Tet(O/W/32/O)
[uncultured bacterium
IN-07]
 AMP42313.1 tetracycline resistance protein Tet(O/W/32/O)
[uncultured bacterium
IN-08]
 ARS43256.1 Tet(O/W/32/O) (plasmid) [Streptococcus suis]
Length=639

 Score = 1108 bits (2867), Expect = 0.0, Method: Compositional
matrix adjust.
 Identities = 528/638 (83%), Positives = 582/638 (91%), Gaps = 0/638
(0%)

```
Query  1
MKIINIGILAHVDAGKTTLTESLLYASGAISEPGSVEKGTTRTDTMFLERQRGITIQAAV  60
            MKIIN+GILAHVDAGKTTLTESLLY SGAI+E GSV++GTTRTDTM
LERQRGITIQ AV
Sbjct  1
MKIINLGILAHVDAGKTTLTESLLYTSGAIAELGSVDEGTTRTDTMNLERQRGITIQTAV  60

Query  61
TSFQWHRCKVNIVDTPGHMDFLAEVYRSLAVLDGAILVISAKDGVQAQTRILFHALRKMN  120
            TSFQW   KVNI
+DTPGHMDFLAEVYRSLAVLDGAILVISAKDGVQAQTRILFHALRKMN
Sbjct  61
TSFQWEDVKVNIIDTPGHMDFLAEVYRSLAVLDGAILVISAKDGVQAQTRILFHALRKMN  120

Query  121
IPTVIFINKIDQAGVDLQSVVQSVRDKLSADIIIKQTVSLSPEIVLEENTDIEAWDAVIE  180
```

IPTVIFINKIDQAGVDLQSVVQSVRDKLSADIIIKQTVSLSPEIVLEENTDIEAWDAVIE
Sbjct 121
IPTVIFINKIDQAGVDLQSVVQSVRDKLSADIIIKQTVSLSPEIVLEENTDIEAWDAVIE 180

Query 181
NNDKLLEKYIAGEPISREKLVREEQRRVQDASLFPVYYGSAKKGLGIQPLMDAVTGLFQP 240

NNDKLLEKYIAGEPISREKLVREEQRRVQDASLFPVYYGSAKKGLGIQPLMDAVTGLFQP
Sbjct 181
NNDKLLEKYIAGEPISREKLVREEQRRVQDASLFPVYYGSAKKGLGIQPLMDAVTGLFQP 240

Query 241
IGEQGSAALCGSVFKVEYTDCGQRRVYLRLYSGTLRLRDTVALAGREKLKITEMRIPSKG 300

IGEQGSAALCGSVFKVEYTDCGQRRVYLRLYSGTLRLRDTVALAGREKLKITEMRIPSKG
Sbjct 241
IGEQGSAALCGSVFKVEYTDCGQRRVYLRLYSGTLRLRDTVALAGREKLKITEMRIPSKG 300

Query 301
EIVRTDTAYPGEIVILPSDSVRLNDVLGDPTRLPRKRWREDPLPMLRTTIAPKTAAQRER 360
EIVRTDTAYPGEIVIL D+++LND+LG+   LP K   ++P+P+LRTT+ P+
QRE
Sbjct 301
EIVRTDTAYPGEIVILADDTLKLNDILGNEKLLPHKTRIDNPMPLLRTTVEPQKPEQREA 360

Query 361
LLDALTQLADTDPLLRCEVDSITHEIILSFLGRVQLEVVSALLSEKYKLETVVKEPSVIY 420
LL+AL ++ADTDPLL ++D++THEI+LSFLG+VQLEV+ +LL EKY +
+KEPSVIY
Sbjct 361
LLNALAEIADTDPLLHFDIDTVTHEIMLSFLGKVQLEVICSLLEEKYHVGVAMKEPSVIY 420

Query 421
MERPLKAASHTIHIEVPPNPFWASIGLSVTPLSLGSGVQYESRVSLGYLNQSFQNAVRDG 480
+ERPL+ A +TIHIEVPPNPFWAS+GLS+ PL
+GSGVQYESRVSLGYLNQSFQNAV +G
Sbjct 421
LERPLRKAEYTIHIEVPPNPFWASVGLSIEPLPIGSGVQYESRVSLGYLNQSFQNAVMEG 480

Query 481
IRYGLEQGLFGWNVTDCKICFEYGLYYSPVSTPADFRSLAPIVLEQALKESGTQLLEPYL 540
+ YG EQGL+GW VTDCKICFEYGLYYSPVSTPADFR L+PIVLEQALK++GT
+LLEPYL
Sbjct 481
VLYGCEQGLYGWKVTDCKICFEYGLYYSPVSTPADFRLLSPIVLEQALKKAGTELLEPYL 540

Query 541
SFILYAPQEYLSRAYHDAPKYCATIETAQVKKDEVVFTGEIPARCIQAYRTDLAFYTNGR 600
F +YAPQEYLSRAYHDAP+YCA I + Q+K DEV+ GEIPARCIQ YR DL
+TNG+
Sbjct 541
HFEIYAPQEYLSRAYHDAPRYCADIVSTQIKNDEVILKGEIPARCIQEYRNDLTNFTNGQ 600

```
Query  601  SVCLTELKGYQAAVGQPVIQPRRPNSRLDKVRHMFQKV  638
             VCLTELKGYQ A+G+ + QPRRPNSR+DKVRHMF K+
Sbjct  601  GVCLTELKGYQPAIGKFICQPRRPNSRIDKVRHMFHKL  638


>ATD53312.1 ribosomal protection tetracycline resistance protein
[Riemerella
anatipestifer]
Length=639

 Score = 1108 bits (2865),  Expect = 0.0, Method: Compositional
matrix adjust.
 Identities = 528/638 (83%), Positives = 582/638 (91%), Gaps = 0/638
(0%)

Query  1
MKIINIGILAHVDAGKTTLTESLLYASGAISEPGSVEKGTTRTDTMFLERQRGITIQAAV  60
            MKIIN+GILAHVDAGKTTLTESLLY SGAI+E GSV++GTTRTDTM
LERQRGITIQ AV
Sbjct  1
MKIINLGILAHVDAGKTTLTESLLYTSGAIAELGSVDEGTTRTDTMNLERQRGITIQTAV  60

Query  61
TSFQWHRCKVNIVDTPGHMDFLAEVYRSLAVLDGAILVISAKDGVQAQTRILFHALRKMN  120
            TSFQW   KVNI
+DTPGHMDFLAEVYRSLAVLDGAILVISAKDGVQAQTRILFHALRKMN
Sbjct  61
TSFQWEDVKVNIIDTPGHMDFLAEVYRSLAVLDGAILVISAKDGVQAQTRILFHALRKMN  120

Query  121
IPTVIFINKIDQAGVDLQSVVQSVRDKLSADIIIKQTVSLSPEIVLEENTDIEAWDAVIE  180

IPTVIFINKIDQAGVDLQSVVQSVRDKLSADIIIKQTVSLSPEIVLEENTDIEAWDAVIE
Sbjct  121
IPTVIFINKIDQAGVDLQSVVQSVRDKLSADIIIKQTVSLSPEIVLEENTDIEAWDAVIE  180

Query  181
NNDKLLEKYIAGEPISREKLVREEQRRVQDASLFPVYYGSAKKGLGIQPLMDAVTGLFQP  240

NNDKLLEKYIAGEPISREKLVREEQRRVQDASLFPVYYGSAKKGLGIQPLMDAVTGLFQP
Sbjct  181
NNDKLLEKYIAGEPISREKLVREEQRRVQDASLFPVYYGSAKKGLGIQPLMDAVTGLFQP  240

Query  241
IGEQGSAALCGSVFKVEYTDCGQRRVYLRLYSGTLRLRDTVALAGREKLKITEMRIPSKG  300

IGEQGSAALCGSVFKVEYTDCGQRRVYLRLYSGTLRLRDTVALAGREKLKITEMRIPSKG
Sbjct  241
IGEQGSAALCGSVFKVEYTDCGQRRVYLRLYSGTLRLRDTVALAGREKLKITEMRIPSKG  300

Query  301
EIVRTDTAYPGEIVILPSDSVRLNDVLGDPTRLPRKRWREDPLPMLRTTIAPKTAAQRER  360
            EIVRTDTAYPGEIVIL  D+++LND+LG+   LP K   ++P+P+LRTT+ P+
QRE
```

Sbjct 301
EIVRTDTAYPGEIVILADDTLKLNDILGNEKLLPHKTRIDNPMPLLRTTVEPQKPEQREA 360

Query 361
LLDALTQLADTDPLLRCEVDSITHEIILSFLGRVQLEVVSALLSEKYKLETVVKEPSVIY 420
            LL+AL ++ADTDPLL ++D++THEI+LSFLG+VQLEV+ +LL EKY +
+KEPSVIY
Sbjct 361
LLNALAEIADTDPLLHFDIDTVTHEIMLSFLGKVQLEVICSLLEEKYYVGVAMKEPSVIY 420

Query 421
MERPLKAASHTIHIEVPPNPFWASIGLSVTPLSLGSGVQYESRVSLGYLNQSFQNAVRDG 480
            +ERPL+ A +TIHIEVPPNPFWAS+GLS+ PL
+GSGVQYESRVSLGYLNQSFQNAV +G
Sbjct 421
LERPLRKAEYTIHIEVPPNPFWASVGLSIEPLPIGSGVQYESRVSLGYLNQSFQNAVMEG 480

Query 481
IRYGLEQGLFGWNVTDCKICFEYGLYYSPVSTPADFRSLAPIVLEQALKESGTQLLEPYL 540
            + YG EQGL+GW VTDCKICFEYGLYYSPVSTPADFR L+PIVLEQALK++GT
+LLEPYL
Sbjct 481
VLYGCEQGLYGWKVTDCKICFEYGLYYSPVSTPADFRLLSPIVLEQALKKAGTELLEPYL 540

Query 541
SFILYAPQEYLSRAYHDAPKYCATIETAQVKKDEVVFTGEIPARCIQAYRTDLAFYTNGR 600
             F +YAPQEYLSRAYHDAP+YCA I + Q+K DEV+ GEIPARCIQ YR DL
+TNG+
Sbjct 541
HFEIYAPQEYLSRAYHDAPRYCADIVSTQIKNDEVILKGEIPARCIQEYRNDLTNFTNGQ 600

Query 601 SVCLTELKGYQAAVGQPVIQPRRPNSRLDKVRHMFQKV 638
             VCLTELKGYQ A+G+ + QPRRPNSR+DKVRHMF K+
Sbjct 601 GVCLTELKGYQPAIGKFICQPRRPNSRIDKVRHMFHKL 638

>WP_105160480.1 TetM/TetW/TetO/TetS family tetracycline resistance
ribosomal
protection protein [Streptococcus suis]
Length=639

 Score = 1107 bits (2862), Expect = 0.0, Method: Compositional
matrix adjust.
 Identities = 527/638 (83%), Positives = 581/638 (91%), Gaps = 0/638
(0%)

Query 1
MKIINIGILAHVDAGKTTLTESLLYASGAISEPGSVEKGTTRTDTMFLERQRGITIQAAV 60
            MKIIN+GILAHVDAGKTTLTESLLY SGAI+E GSV++GTTRTDTM
LERQRGITIQ AV
Sbjct 1
MKIINLGILAHVDAGKTTLTESLLYTSGAIAELGSVDEGTTRTDTMNLERQRGITIQTAV 60

Query 61

TSFQWHRCKVNIVDTPGHMDFLAEVYRSLAVLDGAILVISAKDGVQAQTRILFHALRKMN 120
            TSFQW   KVNI
+DTPGHMDFLAEVYRSLAVLDGAILVISAKDGVQAQTRILFHALRKMN
Sbjct 61
TSFQWEDVKVNIIDTPGHMDFLAEVYRSLAVLDGAILVISAKDGVQAQTRILFHALRKMN 120

Query 121
IPTVIFINKIDQAGVDLQSVVQSVRDKLSADIIIKQTVSLSPEIVLEENTDIEAWDAVIE 180

IPTVIFINKIDQAGVDLQSVVQSVRDKLSADIIIKQTVSLSPEIVLEENTDIEAWDAVIE
Sbjct 121
IPTVIFINKIDQAGVDLQSVVQSVRDKLSADIIIKQTVSLSPEIVLEENTDIEAWDAVIE 180

Query 181
NNDKLLEKYIAGEPISREKLVREEQRRVQDASLFPVYYGSAKKGLGIQPLMDAVTGLFQP 240

NNDKLLEKYIAGEPISREKLVREEQRRVQDASLFPVYYGSAKKGLGIQPLMDAVTGLFQP
Sbjct 181
NNDKLLEKYIAGEPISREKLVREEQRRVQDASLFPVYYGSAKKGLGIQPLMDAVTGLFQP 240

Query 241
IGEQGSAALCGSVFKVEYTDCGQRRVYLRLYSGTLRLRDTVALAGREKLKITEMRIPSKG 300
            IGEQGSAALCGSVFKVEYTDCGQRRVYLRLYSGTL
LRDTVALAGREKLKITEMRIPSKG
Sbjct 241
IGEQGSAALCGSVFKVEYTDCGQRRVYLRLYSGTLHLRDTVALAGREKLKITEMRIPSKG 300

Query 301
EIVRTDTAYPGEIVILPSDSVRLNDVLGDPTRLPRKRWREDPLPMLRTTIAPKTAAQRER 360
            EIVRTDTAYPGEIVIL D+++LND+LG+   LP K   ++P+P+LRTT+ P+
QRE
Sbjct 301
EIVRTDTAYPGEIVILADDTLKLNDILGNEKLLPHKTRIDNPMPLLRTTVEPQKPEQREA 360

Query 361
LLDALTQLADTDPLLRCEVDSITHEIILSFLGRVQLEVVSALLSEKYKLETVVKEPSVIY 420
            LL+AL ++ADTDPLL ++D++THEI+LSFLG+VQLEV+ +LL EKY +
+KEPSVIY
Sbjct 361
LLNALAEIADTDPLLHFDIDTVTHEIMLSFLGKVQLEVICSLLEEKYHVGVAMKEPSVIY 420

Query 421
MERPLKAASHTIHIEVPPNPFWASIGLSVTPLSLGSGVQYESRVSLGYLNQSFQNAVRDG 480
            +ERPL+ A +TIHIEVPPNPFWAS+GLS+ PL
+GSGVQYESRVSLGYLNQSFQNAV +G
Sbjct 421
LERPLRKAEYTIHIEVPPNPFWASVGLSIEPLPIGSGVQYESRVSLGYLNQSFQNAVMEG 480

Query 481
IRYGLEQGLFGWNVTDCKICFEYGLYYSPVSTPADFRSLAPIVLEQALKESGTQLLEPYL 540
            + YG EQGL+GW VTDCKICFEYGLYYSPVSTPADFR L+PIVLEQALK++GT
+LLEPYL
Sbjct 481
VLYGCEQGLYGWKVTDCKICFEYGLYYSPVSTPADFRLLSPIVLEQALKKAGTELLEPYL 540

```
Query  541
SFILYAPQEYLSRAYHDAPKYCATIETAQVKKDEVVFTGEIPARCIQAYRTDLAFYTNGR  600
             F +YAPQEYLSRAYHDAP+YCA I + Q+K DEV+  GEIPARCIQ YR DL
+TNG+
Sbjct  541
HFEIYAPQEYLSRAYHDAPRYCADIVSTQIKNDEVILKGEIPARCIQEYRNDLTNFTNGQ  600

Query  601  SVCLTELKGYQAAVGQPVIQPRRPNSRLDKVRHMFQKV  638
             VCLTELKGYQ A+G+ + QPRRPNSR+DKVRHMF K+
Sbjct  601  GVCLTELKGYQPAIGKFICQPRRPNSRIDKVRHMFHKL  638


>WP_105126631.1 TetM/TetW/TetO/TetS family tetracycline resistance
ribosomal
protection protein [Streptococcus suis]
Length=639

 Score = 1107 bits (2862),  Expect = 0.0, Method: Compositional
matrix adjust.
 Identities = 527/638 (83%), Positives = 581/638 (91%), Gaps = 0/638
(0%)

Query  1
MKIINIGILAHVDAGKTTLTESLLYASGAISEPGSVEKGTTRTDTMFLERQRGITIQAAV  60
            MKIIN+GILAHVDAGKTTLTESLLY SGAI+E GSV++GTTRTDTM
LERQRGITIQ AV
Sbjct  1
MKIINLGILAHVDAGKTTLTESLLYTSGAIAELGSVDEGTTRTDTMNLERQRGITIQTAV  60

Query  61
TSFQWHRCKVNIVDTPGHMDFLAEVYRSLAVLDGAILVISAKDGVQAQTRILFHALRKMN  120
            TSFQW   KVNI
+DTPGHMDFLAEVYRSLAVLDGAILVISAKDGVQAQTRILFHALRKMN
Sbjct  61
TSFQWEDVKVNIIDTPGHMDFLAEVYRSLAVLDGAILVISAKDGVQAQTRILFHALRKMN  120

Query  121
IPTVIFINKIDQAGVDLQSVVQSVRDKLSADIIIKQTVSLSPEIVLEENTDIEAWDAVIE  180

IPTVIFINKIDQAGVDLQSVVQSVRDKLSADIIIKQTVSLSPEIVLEENTDIEAWDAVIE
Sbjct  121
IPTVIFINKIDQAGVDLQSVVQSVRDKLSADIIIKQTVSLSPEIVLEENTDIEAWDAVIE  180

Query  181
NNDKLLEKYIAGEPISREKLVREEQRRVQDASLFPVYYGSAKKGLGIQPLMDAVTGLFQP  240

NNDKLLEKYIAGEPISREKLVREEQRRVQDASLFPVYYGSAKKGLGIQPLMDAVTGLFQP
Sbjct  181
NNDKLLEKYIAGEPISREKLVREEQRRVQDASLFPVYYGSAKKGLGIQPLMDAVTGLFQP  240

Query  241
IGEQGSAALCGSVFKVEYTDCGQRRVYLRLYSGTLRLRDTVALAGREKLKITEMRIPSKG  300
```

IGEQGSAALCGSVFKVEYTDCGQRRVYLRLYSGTLRLRDTVALAGREKLKITEMRIPSKG
Sbjct 241
IGEQGSAALCGSVFKVEYTDCGQRRVYLRLYSGTLRLRDTVALAGREKLKITEMRIPSKG 300

Query 301
EIVRTDTAYPGEIVILPSDSVRLNDVLGDPTRLPRKRWREDPLPMLRTTIAPKTAAQRER 360
            EIVRTDTAYPGEIVIL D+++LND+LG+   LP K   ++P+P+LRTT+ P+
QRE
Sbjct 301
EIVRTDTAYPGEIVILADDTLKLNDILGNEKLLPHKTRIDNPMPLLRTTVEPQKPEQREA 360

Query 361
LLDALTQLADTDPLLRCEVDSITHEIILSFLGRVQLEVVSALLSEKYKLETVVKEPSVIY 420
            LL+AL ++ADTDPLL ++D++THEI+LSFLG+VQLEV+ +LL EKY +
+KEPSVIY
Sbjct 361
LLNALAEIADTDPLLHFDIDTVTHEIMLSFLGKVQLEVICSLLEEKYHVGVAMKEPSVIY 420

Query 421
MERPLKAASHTIHIEVPPNPFWASIGLSVTPLSLGSGVQYESRVSLGYLNQSFQNAVRDG 480
            +ERPL+ A +TIHIEVPPNPFWAS+GLS+ PL
+GSGVQYESRVSLGYLNQSFQNAV +G
Sbjct 421
LERPLRKAEYTIHIEVPPNPFWASVGLSIEPLPIGSGVQYESRVSLGYLNQSFQNAVMEG 480

Query 481
IRYGLEQGLFGWNVTDCKICFEYGLYYSPVSTPADFRSLAPIVLEQALKESGTQLLEPYL 540
            + YG EQGL+GW VTDCKICFEYGLYYSPVSTPADFR L+PIVLEQALK++GT
+LLEPYL
Sbjct 481
VLYGCEQGLYGWKVTDCKICFEYGLYYSPVSTPADFRLLSPIVLEQALKKAGTELLEPYL 540

Query 541
SFILYAPQEYLSRAYHDAPKYCATIETAQVKKDEVVFTGEIPARCIQAYRTDLAFYTNGR 600
             F +YAPQEYLSRAYHDAP+YCA I + +K DEV+ GEIPARCIQ YR DL
+TNG+
Sbjct 541
HFEIYAPQEYLSRAYHDAPRYCADIVSTHIKNDEVILKGEIPARCIQEYRNDLTNFTNGQ 600

Query 601 SVCLTELKGYQAAVGQPVIQPRRPNSRLDKVRHMFQKV 638
             VCLTELKGYQ A+G+ + QPRRPNSR+DKVRHMF K+
Sbjct 601 GVCLTELKGYQPAIGKFICQPRRPNSRIDKVRHMFHKL 638

>WP_105211840.1 TetM/TetW/TetO/TetS family tetracycline resistance ribosomal
protection protein [Streptococcus suis]
Length=639

Score = 1106 bits (2861), Expect = 0.0, Method: Compositional matrix adjust.
Identities = 527/639 (82%), Positives = 582/639 (91%), Gaps = 0/639 (0%)

```
Query  1
MKIINIGILAHVDAGKTTLTESLLYASGAISEPGSVEKGTTRTDTMFLERQRGITIQAAV  60
            MKIIN+GILAHVDAGKTTLTESLLY SGAI+E GSV++GTTRTDTM
LERQRGITIQ AV
Sbjct  1
MKIINLGILAHVDAGKTTLTESLLYTSGAIAELGSVDEGTTRTDTMNLERQRGITIQTAV  60

Query  61
TSFQWHRCKVNIVDTPGHMDFLAEVYRSLAVLDGAILVISAKDGVQAQTRILFHALRKMN  120
            TSFQW   KVNI
+DTPGHMDFLAEVYRSLAVLDGAILVISAKDGVQAQTRILFHALRKMN
Sbjct  61
TSFQWEDVKVNIIDTPGHMDFLAEVYRSLAVLDGAILVISAKDGVQAQTRILFHALRKMN  120

Query  121
IPTVIFINKIDQAGVDLQSVVQSVRDKLSADIIIKQTVSLSPEIVLEENTDIEAWDAVIE  180

IPTVIFINKIDQAGVDLQSVVQSVRDKLSADIIIKQTVSLSPEIVLEENTDIEAWDAVIE
Sbjct  121
IPTVIFINKIDQAGVDLQSVVQSVRDKLSADIIIKQTVSLSPEIVLEENTDIEAWDAVIE  180

Query  181
NNDKLLEKYIAGEPISREKLVREEQRRVQDASLFPVYYGSAKKGLGIQPLMDAVTGLFQP  240

NNDKLLEKYIAGEPISREKLVREEQRRVQDASLFPVYYGSAKKGLGIQPLMDAVTGLFQP
Sbjct  181
NNDKLLEKYIAGEPISREKLVREEQRRVQDASLFPVYYGSAKKGLGIQPLMDAVTGLFQP  240

Query  241
IGEQGSAALCGSVFKVEYTDCGQRRVYLRLYSGTLRLRDTVALAGREKLKITEMRIPSKG  300
            IGEQGSAALCGSVFKVEYTDCGQRRVYLRLYSGTL
LRDTVALAGREKLKITEMRIPSKG
Sbjct  241
IGEQGSAALCGSVFKVEYTDCGQRRVYLRLYSGTLHLRDTVALAGREKLKITEMRIPSKG  300

Query  301
EIVRTDTAYPGEIVILPSDSVRLNDVLGDPTRLPRKRWREDPLPMLRTTIAPKTAAQRER  360
            EIVRTDTAYPGEIVIL  D+++LND+LG+   LP K   ++P+P+LRTT+ P+
QRE
Sbjct  301
EIVRTDTAYPGEIVILADDTLKLNDILGNEKLLPHKTRIDNPMPLLRTTVEPQKPEQREA  360

Query  361
LLDALTQLADTDPLLRCEVDSITHEIILSFLGRVQLEVVSALLSEKYKLETVVKEPSVIY  420
            LL+AL ++ADTDPLL  ++D++THEI+LSFLG+VQLEV+ +LL EKY +
+KEPSVIY
Sbjct  361
LLNALAEIADTDPLLHFDIDTVTHEIMLSFLGKVQLEVICSLLEEKYHVGVAMKEPSVIY  420

Query  421
MERPLKAASHTIHIEVPPNPFWASIGLSVTPLSLGSGVQYESRVSLGYLNQSFQNAVRDG  480
            +ERPL+ A +TIHIEVPPNPFWAS+GLS+ PL
+GSGVQYESRVSLGYLNQSFQNAV +G
Sbjct  421
```

```
LERPLRKAEYTIHIEVPPNPFWASVGLSIEPLPIGSGVQYESRVSLGYLNQSFQNAVMEG  480

Query  481  
IRYGLEQGLFGWNVTDCKICFEYGLYYSPVSTPADFRSLAPIVLEQALKESGTQLLEPYL  540
             + YG EQGL+GW VTDCKICFEYGLYYSPVSTPADFR L+PIVLEQALK++GT
+LLEPYL
Sbjct  481  
VLYGCEQGLYGWKVTDCKICFEYGLYYSPVSTPADFRLLSPIVLEQALKKAGTELLEPYL  540

Query  541  
SFILYAPQEYLSRAYHDAPKYCATIETAQVKKDEVVFTGEIPARCIQAYRTDLAFYTNGR  600
              F +YAPQEYLSRAYHDAP+YCA I + Q+K DEV+  GEIPARCIQ YR DL
+TNG+
Sbjct  541  
HFEIYAPQEYLSRAYHDAPRYCADIVSTQIKNDEVILKGEIPARCIQEYRNDLTNFTNGQ  600

Query  601  SVCLTELKGYQAAVGQPVIQPRRPNSRLDKVRHMFQKVM  639
              VCLTELKGYQ A+G+ + QPRRPNSR+DKVRHMF K++
Sbjct  601  GVCLTELKGYQPAIGKFICQPRRPNSRIDKVRHMFHKLV  639


>ABN79416.1 mosaic tetracycline resistance protein [uncultured 
bacterium]
Length=639

 Score = 1106 bits (2860),  Expect = 0.0, Method: Compositional 
matrix adjust.
 Identities = 527/638 (83%), Positives = 581/638 (91%), Gaps = 0/638 
(0%)

Query  1    
MKIINIGILAHVDAGKTTLTESLLYASGAISEPGSVEKGTTRTDTMFLERQRGITIQAAV  60
            MKIIN+GILAHVDAGKTTLTESLLY SGAI+E GSV++GTTRTDTM
LERQRGITIQ AV
Sbjct  1    
MKIINLGILAHVDAGKTTLTESLLYTSGAIAELGSVDEGTTRTDTMNLERQRGITIQTAV  60

Query  61   
TSFQWHRCKVNIVDTPGHMDFLAEVYRSLAVLDGAILVISAKDGVQAQTRILFHALRKMN  120
            TSFQW   KVNI+DTPGHMDFLAEVYRSLAVLDGAI
VISAKDGVQAQTRILFHALRKMN
Sbjct  61   
TSFQWEDVKVNIIDTPGHMDFLAEVYRSLAVLDGAISVISAKDGVQAQTRILFHALRKMN  120

Query  121  
IPTVIFINKIDQAGVDLQSVVQSVRDKLSADIIIKQTVSLSPEIVLEENTDIEAWDAVIE  180

IPTVIFINKIDQAGVDLQSVVQSVRDKLSADIIIKQTVSLSPEIVLEENTDIEAWDAVIE
Sbjct  121  
IPTVIFINKIDQAGVDLQSVVQSVRDKLSADIIIKQTVSLSPEIVLEENTDIEAWDAVIE  180

Query  181  
NNDKLLEKYIAGEPISREKLVREEQRRVQDASLFPVYYGSAKKGLGIQPLMDAVTGLFQP  240
```

```
NNDKLLEKYIAGEPISREKLVREEQRRVQDASLFPVYYGSAKKGLGIQPLMDAVTGLFQP
Sbjct  181
NNDKLLEKYIAGEPISREKLVREEQRRVQDASLFPVYYGSAKKGLGIQPLMDAVTGLFQP  240

Query  241
IGEQGSAALCGSVFKVEYTDCGQRRVYLRLYSGTLRLRDTVALAGREKLKITEMRIPSKG  300

IGEQGSAALCGSVFKVEYTDCGQRRVYLRLYSGTLRLRDTVALAGREKLKITEMRIPSKG
Sbjct  241
IGEQGSAALCGSVFKVEYTDCGQRRVYLRLYSGTLRLRDTVALAGREKLKITEMRIPSKG  300

Query  301
EIVRTDTAYPGEIVILPSDSVRLNDVLGDPTRLPRKRWREDPLPMLRTTIAPKTAAQRER  360
            EIVRTDTAYPGEIVIL  D+++LND+LG+   LP K   ++P+P+LRTT+ P+
QRE
Sbjct  301
EIVRTDTAYPGEIVILADDTLKLNDILGNEKLLPHKTRIDNPMPLLRTTVEPQKPEQREA  360

Query  361
LLDALTQLADTDPLLRCEVDSITHEIILSFLGRVQLEVVSALLSEKYKLETVVKEPSVIY  420
            LL+AL ++ADTDPLL  ++D++THEI+LSFLG+VQLEV+ +LL EKY +
+KEPSVIY
Sbjct  361
LLNALAEIADTDPLLHFDIDTVTHEIMLSFLGKVQLEVICSLLEEKYHVGVAMKEPSVIY  420

Query  421
MERPLKAASHTIHIEVPPNPFWASIGLSVTPLSLGSGVQYESRVSLGYLNQSFQNAVRDG  480
            +ERPL+ A +TIHIEVPPNPFWAS+GLS+ PL
+GSGVQYESRVSLGYLNQSFQNAV +G
Sbjct  421
LERPLRKAEYTIHIEVPPNPFWASVGLSIEPLPIGSGVQYESRVSLGYLNQSFQNAVMEG  480

Query  481
IRYGLEQGLFGWNVTDCKICFEYGLYYSPVSTPADFRSLAPIVLEQALKESGTQLLEPYL  540
            + YG EQGL+GW VTDCKICFEYGLYYSPVSTPADFR L+PIVLEQALK++GT
+LLEPYL
Sbjct  481
VLYGCEQGLYGWKVTDCKICFEYGLYYSPVSTPADFRLLSPIVLEQALKKAGTELLEPYL  540

Query  541
SFILYAPQEYLSRAYHDAPKYCATIETAQVKKDEVVFTGEIPARCIQAYRTDLAFYTNGR  600
             F +YAPQEYLSRAYHDAP+YCA I + Q+K DEV+  GEIPARCIQ YR DL
+TNG+
Sbjct  541
HFEIYAPQEYLSRAYHDAPRYCADIVSTQIKNDEVILKGEIPARCIQEYRNDLTNFTNGQ  600

Query  601  SVCLTELKGYQAAVGQPVIQPRRPNSRLDKVRHMFQKV  638
             VCLTELKGYQ A+G+ + QPRRPNSR+DKVRHMF K+
Sbjct  601  GVCLTELKGYQPAIGKFICQPRRPNSRIDKVRHMFHKL  638
```

>WP_052777607.1 TetM/TetW/TetO/TetS family tetracycline resistance ribosomal
protection protein [Campylobacter coli]

```
Length=639

 Score = 1105 bits (2858),  Expect = 0.0, Method: Compositional
matrix adjust.
 Identities = 527/638 (83%), Positives = 581/638 (91%), Gaps = 0/638
(0%)

Query  1
MKIINIGILAHVDAGKTTLTESLLYASGAISEPGSVEKGTTRTDTMFLERQRGITIQAAV  60
            MKIIN+GILAHVDAGKTTLTESLLY SGAI+E GSV++GTTRTDTM
LERQRGITIQ AV
Sbjct  1
MKIINLGILAHVDAGKTTLTESLLYTSGAIAELGSVDEGTTRTDTMNLERQRGITIQTAV  60

Query  61
TSFQWHRCKVNIVDTPGHMDFLAEVYRSLAVLDGAILVISAKDGVQAQTRILFHALRKMN  120
            TSFQW   KVNI
+DTPGHMDFLAEVYRSLAVLDGAILVISAKDGVQAQTRILFHALRKMN
Sbjct  61
TSFQWEDVKVNIIDTPGHMDFLAEVYRSLAVLDGAILVISAKDGVQAQTRILFHALRKMN  120

Query  121
IPTVIFINKIDQAGVDLQSVVQSVRDKLSADIIIKQTVSLSPEIVLEENTDIEAWDAVIE  180

IPTVIFINKIDQAGVDLQSVVQSVRDKLSADIIIKQTVSLSPEIVLEENTDIEAWDAVIE
Sbjct  121
IPTVIFINKIDQAGVDLQSVVQSVRDKLSADIIIKQTVSLSPEIVLEENTDIEAWDAVIE  180

Query  181
NNDKLLEKYIAGEPISREKLVREEQRRVQDASLFPVYYGSAKKGLGIQPLMDAVTGLFQP  240

NNDKLLEKYIAGEPISREKLVREEQRRVQDASLFPVYYGSAKKGLGIQPLMDAVTGLFQP
Sbjct  181
NNDKLLEKYIAGEPISREKLVREEQRRVQDASLFPVYYGSAKKGLGIQPLMDAVTGLFQP  240

Query  241
IGEQGSAALCGSVFKVEYTDCGQRRVYLRLYSGTLRLRDTVALAGREKLKITEMRIPSKG  300

IGEQGSAALCGSVFKVEYTDCGQRRVYLRLYSGTLRLRDTVALAGREKLKITEMRIPSKG
Sbjct  241
IGEQGSAALCGSVFKVEYTDCGQRRVYLRLYSGTLRLRDTVALAGREKLKITEMRIPSKG  300

Query  301
EIVRTDTAYPGEIVILPSDSVRLNDVLGDPTRLPRKRWREDPLPMLRTTIAPKTAAQRER  360
            EIVRTDTAYPGEIVIL  D+++LND+LG+   LP K   ++P+P+LRTT+ P+
QRE
Sbjct  301
EIVRTDTAYPGEIVILADDTLKLNDILGNEKLLPHKTRIDNPMPLLRTTVEPQKPEQREA  360

Query  361
LLDALTQLADTDPLLRCEVDSITHEIILSFLGRVQLEVVSALLSEKYKLETVVKEPSVIY  420
            LL+AL ++ADTDPLL  ++D++THEI+LSFLG+VQLEV+ +LL EKY +
+KEPSVIY
Sbjct  361
```

LLNALAEIADTDPLLHFDIDTVTHEIMLSFLGKVQLEVICSLLEEKYHVGVAMKEPSVIY 420

Query 421
MERPLKAASHTIHIEVPPNPFWASIGLSVTPLSLGSGVQYESRVSLGYLNQSFQNAVRDG 480
            +ERPL+ A +TIHIEVPPNPFWAS+GLS+ PL
+GSGVQYESRVSLGYLNQSFQNAV +G
Sbjct 421
LERPLRKAEYTIHIEVPPNPFWASVGLSIEPLPIGSGVQYESRVSLGYLNQSFQNAVMEG 480

Query 481
IRYGLEQGLFGWNVTDCKICFEYGLYYSPVSTPADFRSLAPIVLEQALKESGTQLLEPYL 540
            + YG EQGL+GW VTDCKICFEYGLYYSPVSTPADFR L+PIVLEQALK++GT
+LLEPYL
Sbjct 481
VLYGCEQGLYGWKVTDCKICFEYGLYYSPVSTPADFRLLSPIVLEQALKKAGTELLEPYL 540

Query 541
SFILYAPQEYLSRAYHDAPKYCATIETAQVKKDEVVFTGEIPARCIQAYRTDLAFYTNGR 600
             F + APQEYLSRAYHDAP+YCA I + Q+K DEV+ GEIPARCIQ YR DL
+TNG+
Sbjct 541
HFEICAPQEYLSRAYHDAPRYCADIVSTQIKNDEVILKGEIPARCIQEYRNDLTNFTNGQ 600

Query 601 SVCLTELKGYQAAVGQPVIQPRRPNSRLDKVRHMFQKV 638
             VCLTELKGYQ A+G+ + QPRRPNSR+DKVRHMF K+
Sbjct 601 GVCLTELKGYQPAIGKFICQPRRPNSRIDKVRHMFHKL 638

>AMP53218.1 TetM-TetW-TetO-TetS, partial [uncultured bacterium]
Length=539

 Score = 1091 bits (2821), Expect = 0.0, Method: Compositional
matrix adjust.
 Identities = 531/539 (99%), Positives = 534/539 (99%), Gaps = 0/539
(0%)

Query 101
AKDGVQAQTRILFHALRKMNIPTVIFINKIDQAGVDLQSVVQSVRDKLSADIIIKQTVSL 160

AKDGVQAQTRILFHALRKMNIPTVIFINKIDQAGVDLQSVVQSVRDKLSADIIIKQTVSL
Sbjct 1
AKDGVQAQTRILFHALRKMNIPTVIFINKIDQAGVDLQSVVQSVRDKLSADIIIKQTVSL 60

Query 161
SPEIVLEENTDIEAWDAVIENNDKLLEKYIAGEPISREKLVREEQRRVQDASLFPVYYGS 220
            SPEIVLEENTDIEAWDAVIENND+LLEKYIAGEPISREKL REEQ
+RVQDASLFPVY+GS
Sbjct 61
SPEIVLEENTDIEAWDAVIENNDELLEKYIAGEPISREKLAREEQQRVQDASLFPVYHGS 120

Query 221
AKKGLGIQPLMDAVTGLFQPIGEQGSAALCGSVFKVEYTDCGQRRVYLRLYSGTLRLRDT 280
            AK GLGIQPLMDAVTGLFQPIGEQG
AALCGSVFKVEYTDCGQRRVYLRLYSGTLRLRDT

Sbjct 121
AKNGLGIQPLMDAVTGLFQPIGEQGGAALCGSVFKVEYTDCGQRRVYLRLYSGTLRLRDT 180

Query 281
VALAGREKLKITEMRIPSKGEIVRTDTAYPGEIVILPSDSVRLNDVLGDPTRLPRKRWRE 340
            VALAGREKLKITEMRIPSKGEIVRTDTAY GEIVILPSDSVRLNDVLGD
TRLPRKRWRE
Sbjct 181
VALAGREKLKITEMRIPSKGEIVRTDTAYQGEIVILPSDSVRLNDVLGDQTRLPRKRWRE 240

Query 341
DPLPMLRTTIAPKTAAQRERLLDALTQLADTDPLLRCEVDSITHEIILSFLGRVQLEVVS 400

DPLPMLRTTIAPKTAAQRERLLDALTQLADTDPLLRCEVDSITHEIILSFLGRVQLEVVS
Sbjct 241
DPLPMLRTTIAPKTAAQRERLLDALTQLADTDPLLRCEVDSITHEIILSFLGRVQLEVVS 300

Query 401
ALLSEKYKLETVVKEPSVIYMERPLKAASHTIHIEVPPNPFWASIGLSVTPLSLGSGVQY 460

ALLSEKYKLETVVKEPSVIYMERPLKAASHTIHIEVPPNPFWASIGLSVTPLSLGSGVQY
Sbjct 301
ALLSEKYKLETVVKEPSVIYMERPLKAASHTIHIEVPPNPFWASIGLSVTPLSLGSGVQY 360

Query 461
ESRVSLGYLNQSFQNAVRDGIRYGLEQGLFGWNVTDCKICFEYGLYYSPVSTPADFRSLA 520

ESRVSLGYLNQSFQNAVRDGIRYGLEQGLFGWNVTDCKICFEYGLYYSPVSTPADFRSLA
Sbjct 361
ESRVSLGYLNQSFQNAVRDGIRYGLEQGLFGWNVTDCKICFEYGLYYSPVSTPADFRSLA 420

Query 521
PIVLEQALKESGTQLLEPYLSFILYAPQEYLSRAYHDAPKYCATIETAQVKKDEVVFTGE 580

PIVLEQALKESGTQLLEPYLSFILYAPQEYLSRAYHDAPKYCATIETAQVKKDEVVFTGE
Sbjct 421
PIVLEQALKESGTQLLEPYLSFILYAPQEYLSRAYHDAPKYCATIETAQVKKDEVVFTGE 480

Query 581
IPARCIQAYRTDLAFYTNGRSVCLTELKGYQAAVGQPVIQPRRPNSRLDKVRHMFQKVM 639

IPARCIQAYRTDLAFYTNGRSVCLTELKGYQAAVGQPVIQPRRPNSRLDKVRHMFQKVM
Sbjct 481
IPARCIQAYRTDLAFYTNGRSVCLTELKGYQAAVGQPVIQPRRPNSRLDKVRHMFQKVM 539

>AMP53219.1 TetM-TetW-TetO-TetS, partial [uncultured bacterium]
Length=539

 Score = 1082 bits (2798), Expect = 0.0, Method: Compositional
matrix adjust.
 Identities = 525/538 (98%), Positives = 531/538 (99%), Gaps = 0/538
(0%)

Query 101
AKDGVQAQTRILFHALRKMNIPTVIFINKIDQAGVDLQSVVQSVRDKLSADIIIKQTVSL 160

AKDGVQAQTRILFHALRKMNIPTVIFINKIDQAGVDLQSVVQSVRDKLSADIIIKQTVSL
Sbjct 1
AKDGVQAQTRILFHALRKMNIPTVIFINKIDQAGVDLQSVVQSVRDKLSADIIIKQTVSL 60

Query 161
SPEIVLEENTDIEAWDAVIENNDKLLEKYIAGEPISREKLVREEQRRVQDASLFPVYYGS 220
SPEIVLEENTDIEAWDAVIENND+LLEKYIAGEPISREKL REEQ
+RVQDASLFPVY+GS
Sbjct 61
SPEIVLEENTDIEAWDAVIENNDELLEKYIAGEPISREKLAREEQQRVQDASLFPVYHGS 120

Query 221
AKKGLGIQPLMDAVTGLFQPIGEQGSAALCGSVFKVEYTDCGQRRVYLRLYSGTLRLRDT 280
AK GLGIQPLMDAVTGLFQPIGEQG
AALCGSVFKVEYTDCGQRRVYLRLYSGTLRLRDT
Sbjct 121
AKNGLGIQPLMDAVTGLFQPIGEQGGAALCGSVFKVEYTDCGQRRVYLRLYSGTLRLRDT 180

Query 281
VALAGREKLKITEMRIPSKGEIVRTDTAYPGEIVILPSDSVRLNDVLGDPTRLPRKRWRE 340
VALAGREKLKITEMRIPSKGEIVRTDTAY GEIVILPSDSVRLNDVLGD
TRLPRKRWRE
Sbjct 181
VALAGREKLKITEMRIPSKGEIVRTDTAYQGEIVILPSDSVRLNDVLGDQTRLPRKRWRE 240

Query 341
DPLPMLRTTIAPKTAAQRERLLDALTQLADTDPLLRCEVDSITHEIILSFLGRVQLEVVS 400

DPLPMLRTTIAPKTAAQRERLLDALTQLADTDPLLRCEVDSITHEIILSFLGRVQLEVVS
Sbjct 241
DPLPMLRTTIAPKTAAQRERLLDALTQLADTDPLLRCEVDSITHEIILSFLGRVQLEVVS 300

Query 401
ALLSEKYKLETVVKEPSVIYMERPLKAASHTIHIEVPPNPFWASIGLSVTPLSLGSGVQY 460

ALLSEKYKLETVVKEPSVIYMERPLKAASHTIHIEVPPNPFWASIGLSVTPLSLGSGVQY
Sbjct 301
ALLSEKYKLETVVKEPSVIYMERPLKAASHTIHIEVPPNPFWASIGLSVTPLSLGSGVQY 360

Query 461
ESRVSLGYLNQSFQNAVRDGIRYGLEQGLFGWNVTDCKICFEYGLYYSPVSTPADFRSLA 520

ESRVSLGYLNQSFQNAVRDGIRYGLEQGLFGWNVTDCKICFEYGLYYSPVSTPADFRSLA
Sbjct 361
ESRVSLGYLNQSFQNAVRDGIRYGLEQGLFGWNVTDCKICFEYGLYYSPVSTPADFRSLA 420

Query 521
PIVLEQALKESGTQLLEPYLSFILYAPQEYLSRAYHDAPKYCATIETAQVKKDEVVFTGE 580

PIVLEQALKESGTQLLEPYLSFILYAPQEYLSRAYHDAPKYCATIETAQVKKDEVVFTGE
Sbjct 421

PIVLEQALKESGTQLLEPYLSFILYAPQEYLSRAYHDAPKYCATIETAQVKKDEVVFTGE 480

Query 581
IPARCIQAYRTDLAFYTNGRSVCLTELKGYQAAVGQPVIQPRRPNSRLDKVRHMFQKV 638
            IPARCIQAYRTDLAFYTNGRSVCLTELKGYQA VG+P+IQPRRPNSRLDKVRHMF
K+
Sbjct 481
IPARCIQAYRTDLAFYTNGRSVCLTELKGYQATVGEPIIQPRRPNSRLDKVRHMFSKI 538

>AMP49819.1 TetM-TetW-TetO-TetS, partial [uncultured bacterium]
Length=546

Score = 1077 bits (2785), Expect = 0.0, Method: Compositional matrix adjust.
Identities = 528/546 (97%), Positives = 536/546 (98%), Gaps = 0/546 (0%)

Query 1
MKIINIGILAHVDAGKTTLTESLLYASGAISEPGSVEKGTTRTDTMFLERQRGITIQAAV 60

MKIINIGILAHVDAGKTTLTESLLYASGAISEPGSVEKGTTRTDTMFLERQRGITIQAAV
Sbjct 1
MKIINIGILAHVDAGKTTLTESLLYASGAISEPGSVEKGTTRTDTMFLERQRGITIQAAV 60

Query 61
TSFQWHRCKVNIVDTPGHMDFLAEVYRSLAVLDGAILVISAKDGVQAQTRILFHALRKMN 120

TSFQWHRCKVNIVDTPGHMDFLAEVYRSLAVLDGAILVISAKDGVQAQTRILFHALRKMN
Sbjct 61
TSFQWHRCKVNIVDTPGHMDFLAEVYRSLAVLDGAILVISAKDGVQAQTRILFHALRKMN 120

Query 121
IPTVIFINKIDQAGVDLQSVVQSVRDKLSADIIIKQTVSLSPEIVLEENTDIEAWDAVIE 180

IPTVIFINKIDQAGVDLQSVVQSVRDKLSADIIIKQTVSLSPEIVLEENTDIEAWDAVIE
Sbjct 121
IPTVIFINKIDQAGVDLQSVVQSVRDKLSADIIIKQTVSLSPEIVLEENTDIEAWDAVIE 180

Query 181
NNDKLLEKYIAGEPISREKLVREEQRRVQDASLFPVYYGSAKKGLGIQPLMDAVTGLFQP 240
            NND+LLEKYIAGEPISREKL REEQ+RVQDASLFPVY+GSAK
GLGIQPLMDAVTGLFQP
Sbjct 181
NNDELLEKYIAGEPISREKLAREEQQRVQDASLFPVYHGSAKNGLGIQPLMDAVTGLFQP 240

Query 241
IGEQGSAALCGSVFKVEYTDCGQRRVYLRLYSGTLRLRDTVALAGREKLKITEMRIPSKG 300
            IGEQG
AALCGSVFKVEYTDCGQRRVYLRLYSGTLRLRDTVALAGREKLKITEMRIPSKG
Sbjct 241
IGEQGGAALCGSVFKVEYTDCGQRRVYLRLYSGTLRLRDTVALAGREKLKITEMRIPSKG 300

Query 301

EIVRTDTAYPGEIVILPSDSVRLNDVLGDPTRLPRKRWREDPLPMLRTTIAPKTAAQRER 360
            EIVRTDTA+ GEIVI PSDSV LNDVLGD TRLPR+RWR+
PLPMLRTTIAPKTAAQRER
Sbjct 301
EIVRTDTAHKGEIVIFPSDSVGLNDVLGDQTRLPRERWRDAPLPMLRTTIAPKTAAQRER 360

Query 361
LLDALTQLADTDPLLRCEVDSITHEIILSFLGRVQLEVVSALLSEKYKLETVVKEPSVIY 420
            LLDALTQ+ADTDPLLR EVDSITHEIILSFLGRVQLEVVSALL
+EKYKLETVVKEPSVIY
Sbjct 361
LLDALTQIADTDPLLRYEVDSITHEIILSFLGRVQLEVVSALLAEKYKLETVVKEPSVIY 420

Query 421
MERPLKAASHTIHIEVPPNPFWASIGLSVTPLSLGSGVQYESRVSLGYLNQSFQNAVRDG 480
            MERPLKAASHTIHIEVPPNPFWASIGLSVTPL
LGSGVQYESRVSLGYLNQSFQNAVRDG
Sbjct 421
MERPLKAASHTIHIEVPPNPFWASIGLSVTPLPLGSGVQYESRVSLGYLNQSFQNAVRDG 480

Query 481
IRYGLEQGLFGWNVTDCKICFEYGLYYSPVSTPADFRSLAPIVLEQALKESGTQLLEPYL 540

IRYGLEQGLFGWNVTDCKICFEYGLYYSPVSTPADFRSLAPIVLEQALKESGTQLLEPYL
Sbjct 481
IRYGLEQGLFGWNVTDCKICFEYGLYYSPVSTPADFRSLAPIVLEQALKESGTQLLEPYL 540

Query 541 SFILYA 546
            SFILYA
Sbjct 541 SFILYA 546

>KXB53642.1 elongation factor Tu GTP binding domain protein
[Coriobacteriales
bacterium DNF00809]
Length=561

 Score = 1065 bits (2753), Expect = 0.0, Method: Compositional
matrix adjust.
 Identities = 520/560 (93%), Positives = 539/560 (96%), Gaps = 0/560
(0%)

Query 79
MDFLAEVYRSLAVLDGAILVISAKDGVQAQTRILFHALRKMNIPTVIFINKIDQAGVDLQ 138
            MDFLAEVYRSLAVLDGAILVISAKDGVQAQTRILF
+ALRKMNIPTVIFINKIDQAGVDLQ
Sbjct 1
MDFLAEVYRSLAVLDGAILVISAKDGVQAQTRILFYALRKMNIPTVIFINKIDQAGVDLQ 60

Query 139
SVVQSVRDKLSADIIIKQTVSLSPEIVLEENTDIEAWDAVIENNDKLLEKYIAGEPISRE 198
             V QSVRDKLSADIIIKQTVSLSPEIVL+ENTDIEAWDAVIENND
+LLEKYIAGEPISRE
Sbjct 61

GVYQSVRDKLSADIIIKQTVSLSPEIVLDENTDIEAWDAVIENNDELLEKYIAGEPISRE 120

Query 199
KLVREEQRRVQDASLFPVYYGSAKKGLGIQPLMDAVTGLFQPIGEQGSAALCGSVFKVEY 258
            +L +EEQRRVQDASLFPVY+GSAKKGLGIQPLMDAVTGLFQPIGEQGSAALCG
+VFKVEY
Sbjct 121
ELAQEEQRRVQDASLFPVYHGSAKKGLGIQPLMDAVTGLFQPIGEQGSAALCGNVFKVEY 180

Query 259
TDCGQRRVYLRLYSGTLRLRDTVALAGREKLKITEMRIPSKGEIVRTDTAYPGEIVILPS 318
            TDC QR +YLRLYSGTLRLRDTVALAGREKLKITEMRIPSKGEIVRTDTA+
GEIVILPS
Sbjct 181
TDCDQRLIYLRLYSGTLRLRDTVALAGREKLKITEMRIPSKGEIVRTDTAHKGEIVILPS 240

Query 319
DSVRLNDVLGDPTRLPRKRWREDPLPMLRTTIAPKTAAQRERLLDALTQLADTDPLLRCE 378
            DSV LNDVLGD TRLPR+RW + PLPMLRT I PKTAAQRERLLDALTQ
+ADTDPLLR E
Sbjct 241
DSVGLNDVLGDNTRLPRERWCDAPLPMLRTAITPKTAAQRERLLDALTQIADTDPLLRYE 300

Query 379
VDSITHEIILSFLGRVQLEVVSALLSEKYKLETVVKEPSVIYMERPLKAASHTIHIEVPP 438
            VDSITHEIILSFLGRVQLEVVSALL+EKYK+ET VKEP+VIY
+ERPLKAASHTIHIEVPP
Sbjct 301
VDSITHEIILSFLGRVQLEVVSALLAEKYKIETAVKEPTVIYLERPLKAASHTIHIEVPP 360

Query 439
NPFWASIGLSVTPLSLGSGVQYESRVSLGYLNQSFQNAVRDGIRYGLEQGLFGWNVTDCK 498
            NPFWASIGLSVTPL LGSGVQYESRVSLGYLNQSFQNAV DGIRYGLEQGL
GWNVTDCK
Sbjct 361
NPFWASIGLSVTPLPLGSGVQYESRVSLGYLNQSFQNAVMDGIRYGLEQGLCGWNVTDCK 420

Query 499
ICFEYGLYYSPVSTPADFRSLAPIVLEQALKESGTQLLEPYLSFILYAPQEYLSRAYHDA 558
            ICFEYGLYYSPVSTPADFRSLAPIVLEQALK+SGTQLLEPYLSF
LYAPQEYLSRAYHDA
Sbjct 421
ICFEYGLYYSPVSTPADFRSLAPIVLEQALKKSGTQLLEPYLSFTLYAPQEYLSRAYHDA 480

Query 559
PKYCATIETAQVKKDEVVFTGEIPARCIQAYRTDLAFYTNGRSVCLTELKGYQAAVGQPV 618
            PKYCA IETAQVKKDEVVFTGEIPAR
IQAYRTDLAFYTNGRSVCLTELKGYQAAVG+PV
Sbjct 481
PKYCAAIETAQVKKDEVVFTGEIPARYIQAYRTDLAFYTNGRSVCLTELKGYQAAVGEPV 540

Query 619 IQPRRPNSRLDKVRHMFQKV 638
            IQPRRPNSRLDKVRHMF K+
Sbjct 541 IQPRRPNSRLDKVRHMFSKI 560

>WP_035300359.1 MULTISPECIES: TetM/TetW/TetO/TetS family tetracycline resistance
ribosomal protection protein [Clostridiales]
CUP60463.1 Tetracycline resistance protein tetM from transposon Tn916 [Flavonifractor
plautii]
CUP72037.1 small GTP-binding protein domain [Flavonifractor plautii]
Length=645

Score = 1061 bits (2743), Expect = 0.0, Method: Compositional matrix adjust.
Identities = 502/639 (79%), Positives = 564/639 (88%), Gaps = 0/639 (0%)

```
Query  1
MKIINIGILAHVDAGKTTLTESLLYASGAISEPGSVEKGTTRTDTMFLERQRGITIQAAV  60
            MKIINIGILAHVDAGKTTLTESLLY SGAI  PGSV+
+GTTRTDTMFLERQRGITIQ AV
Sbjct  1
MKIINIGILAHVDAGKTTLTESLLYTSGAIEAPGSVDQGTTRTDTMFLERQRGITIQTAV  60

Query  61
TSFQWHRCKVNIVDTPGHMDFLAEVYRSLAVLDGAILVISAKDGVQAQTRILFHALRKMN  120
            TSFQWH CKVNIVDTPGHMDFLAEVYRSLA+LDGAILV+SAKDGVQAQTRILFHAL
+ M
Sbjct  61
TSFQWHDCKVNIVDTPGHMDFLAEVYRSLAILDGAILVVSAKDGVQAQTRILFHALQVMK  120

Query  121
IPTVIFINKIDQAGVDLQSVVQSVRDKLSADIIIKQTVSLSPEIVLEENTDIEAWDAVIE  180
            IPTVIFINKIDQ G+DL  V QS+RDKLSA++IIKQ V LSP+I + EN  +E WD
VI
Sbjct  121
IPTVIFINKIDQDGIDLPGVYQSIRDKLSANMIIKQNVQLSPDISIMENMGLENWDTVIA  180

Query  181
NNDKLLEKYIAGEPISREKLVREEQRRVQDASLFPVYYGSAKKGLGIQPLMDAVTGLFQP  240
            + D+LLEKYIAGEP+ +E+L+REE RR+Q  SLFPVY+GSAK  LGI+ L++AVT
FQ
Sbjct  181
DCDELLEKYIAGEPMDKEELLREENRRIQSVSLFPVYHGSAKVNLGIRQLIEAVTDTFQS  240

Query  241
IGEQGSAALCGSVFKVEYTDCGQRRVYLRLYSGTLRLRDTVALAGREKLKITEMRIPSKG  300
               Q S+ LCG+VFKVEY +  QR  YLRLYSGTL LRD+VALAG
+EKLKITEMRIPSKG
Sbjct  241
PTGQNSSELCGTVFKVEYANQSQRLAYLRLYSGTLHLRDSVALAGKEKLKITEMRIPSKG  300

Query  301
EIVRTDTAYPGEIVILPSDSVRLNDVLGDPTRLPRKRWREDPLPMLRTTIAPKTAAQRER  360
```

            EIVRT+ A+ GEIVI+P DS+RLNDVLG+   LPR+ W ++PLP+LRTTIAP+
QRER
Sbjct 301
EIVRTEIAHAGEIVIVPCDSLRLNDVLGNKLLLPRETWSDNPLPLLRTTIAPEKPEQRER 360

Query 361
LLDALTQLADTDPLLRCEVDSITHEIILSFLGRVQLEVVSALLSEKYKLETVVKEPSVIY 420
            LL+ALT++ADTDPLLR EVD++THEIILSFLGRVQLE++S LL EKY+L T KEP
+VIY
Sbjct 361
LLNALTEIADTDPLLRYEVDAVTHEIILSFLGRVQLEIISDLLVEKYQLNTTAKEPTVIY 420

Query 421
MERPLKAASHTIHIEVPPNPFWASIGLSVTPLSLGSGVQYESRVSLGYLNQSFQNAVRDG 480
            MERPLKA SHTIHIEVPPNPFWASIGLSVTPL LG+GVQYES+VS
+GYLNQSFQNAV DG
Sbjct 421
MERPLKAVSHTIHIEVPPNPFWASIGLSVTPLPLGTGVQYESKVSVGYLNQSFQNAVLDG 480

Query 481
IRYGLEQGLFGWNVTDCKICFEYGLYYSPVSTPADFRSLAPIVLEQALKESGTQLLEPYL 540
            IRYGLEQG++GW VTDCKICFEYGLYYSPVSTPADFRSLAPIVLEQ LK+
+GTQLLEPYL
Sbjct 481
IRYGLEQGVYGWKVTDCKICFEYGLYYSPVSTPADFRSLAPIVLEQVLKKAGTQLLEPYL 540

Query 541
SFILYAPQEYLSRAYHDAPKYCATIETAQVKKDEVVFTGEIPARCIQAYRTDLAFYTNGR 600
            SF LYAPQEYLSRAYHDAPKYCA+IET QVK EV+FTGEIPARC+Q YR DL
FYTNGR
Sbjct 541
SFTLYAPQEYLSRAYHDAPKYCASIETTQVKNSEVIFTGEIPARCVQEYRNDLTFYTNGR 600

Query 601 SVCLTELKGYQAAVGQPVIQPRRPNSRLDKVRHMFQKVM 639
            SVCLTELKGYQ A G+PV QPRRPN+R+DKVRHMF K++
Sbjct 601 SVCLTELKGYQIASGEPVFQPRRPNTRIDKVRHMFNKIL 639

>ERI78723.1 putative translation elongation factor G [Clostridium
sp. ATCC
BAA-442]
Length=650

 Score = 1060 bits (2742), Expect = 0.0, Method: Compositional
matrix adjust.
 Identities = 502/639 (79%), Positives = 564/639 (88%), Gaps = 0/639
(0%)

Query 1
MKIINIGILAHVDAGKTTLTESLLYASGAISEPGSVEKGTTRTDTMFLERQRGITIQAAV 60
            MKIINIGILAHVDAGKTTLTESLLY SGAI PGSV+
+GTTRTDTMFLERQRGITIQ AV
Sbjct 6
MKIINIGILAHVDAGKTTLTESLLYTSGAIEAPGSVDQGTTRTDTMFLERQRGITIQTAV 65

Query 61
TSFQWHRCKVNIVDTPGHMDFLAEVYRSLAVLDGAILVISAKDGVQAQTRILFHALRKMN 120
            TSFQWH CKVNIVDTPGHMDFLAEVYRSLA+LDGAILV+SAKDGVQAQTRILFHAL
+ M
Sbjct 66
TSFQWHDCKVNIVDTPGHMDFLAEVYRSLAILDGAILVVSAKDGVQAQTRILFHALQVMK 125

Query 121
IPTVIFINKIDQAGVDLQSVVQSVRDKLSADIIIKQTVSLSPEIVLEENTDIEAWDAVIE 180
            IPTVIFINKIDQ G+DL V QS+RDKLSA++IIKQ V LSP+I + EN +E WD
VI
Sbjct 126
IPTVIFINKIDQDGIDLPGVYQSIRDKLSANMIIKQNVQLSPDISIMENMGLENWDTVIA 185

Query 181
NNDKLLEKYIAGEPISREKLVREEQRRVQDASLFPVYYGSAKKGLGIQPLMDAVTGLFQP 240
            + D+LLEKYIAGEP+ +E+L+REE RR+Q SLFPVY+GSAK LGI+ L++AVT
FQ
Sbjct 186
DCDELLEKYIAGEPMDKEELLREENRRIQSVSLFPVYHGSAKVNLGIRQLIEAVTDTFQS 245

Query 241
IGEQGSAALCGSVFKVEYTDCGQRRVYLRLYSGTLRLRDTVALAGREKLKITEMRIPSKG 300
               Q S+ LCG+VFKVEY + QR YLRLYSGTL LRD+VALAG
+EKLKITEMRIPSKG
Sbjct 246
PTGQNSSELCGTVFKVEYANQSQRLAYLRLYSGTLHLRDSVALAGKEKLKITEMRIPSKG 305

Query 301
EIVRTDTAYPGEIVILPSDSVRLNDVLGDPTRLPRKRWREDPLPMLRTTIAPKTAAQRER 360
            EIVRT+ A+ GEIVI+P DS+RLNDVLG+   LPR+ W ++PLP+LRTTIAP+
QRER
Sbjct 306
EIVRTEIAHAGEIVIVPCDSLRLNDVLGNKLLLPRETWSDNPLPLLRTTIAPEKPEQRER 365

Query 361
LLDALTQLADTDPLLRCEVDSITHEIILSFLGRVQLEVVSALLSEKYKLETVVKEPSVIY 420
            LL+ALT++ADTDPLLR EVD++THEIILSFLGRVQLE++S LL EKY+L T KEP
+VIY
Sbjct 366
LLNALTEIADTDPLLRYEVDAVTHEIILSFLGRVQLEIISDLLVEKYQLNTTAKEPTVIY 425

Query 421
MERPLKAASHTIHIEVPPNPFWASIGLSVTPLSLGSGVQYESRVSLGYLNQSFQNAVRDG 480
            MERPLKA SHTIHIEVPPNPFWASIGLSVTPL LG+GVQYES+VS
+GYLNQSFQNAV DG
Sbjct 426
MERPLKAVSHTIHIEVPPNPFWASIGLSVTPLPLGTGVQYESKVSVGYLNQSFQNAVLDG 485

Query 481
IRYGLEQGLFGWNVTDCKICFEYGLYYSPVSTPADFRSLAPIVLEQALKESGTQLLEPYL 540
            IRYGLEQG++GW VTDCKICFEYGLYYSPVSTPADFRSLAPIVLEQ LK+
+GTQLLEPYL

Sbjct 486
IRYGLEQGVYGWKVTDCKICFEYGLYYSPVSTPADFRSLAPIVLEQVLKKAGTQLLEPYL 545

Query 541
SFILYAPQEYLSRAYHDAPKYCATIETAQVKKDEVVFTGEIPARCIQAYRTDLAFYTNGR 600
            SF LYAPQEYLSRAYHDAPKYCA+IET QVK EV+FTGEIPARC+Q YR DL
FYTNGR
Sbjct 546
SFTLYAPQEYLSRAYHDAPKYCASIETTQVKNSEVIFTGEIPARCVQEYRNDLTFYTNGR 605

Query 601 SVCLTELKGYQAAVGQPVIQPRRPNSRLDKVRHMFQKVM 639
            SVCLTELKGYQ A G+PV QPRRPN+R+DKVRHMF K++
Sbjct 606 SVCLTELKGYQIASGEPVFQPRRPNTRIDKVRHMFNKIL 644

>APO34062.1 ferrous iron transport protein B [uncultured bacterium]
Length=650

 Score = 1058 bits (2737), Expect = 0.0, Method: Compositional
matrix adjust.
 Identities = 501/639 (78%), Positives = 564/639 (88%), Gaps = 0/639
(0%)

Query 1
MKIINIGILAHVDAGKTTLTESLLYASGAISEPGSVEKGTTRTDTMFLERQRGITIQAAV 60
            MKIINIGILAHVDAGKTTLTESLLY SGAI PGSV+
+GTTRTDTMFLERQRGITIQ AV
Sbjct 6
MKIINIGILAHVDAGKTTLTESLLYTSGAIEAPGSVDQGTTRTDTMFLERQRGITIQTAV 65

Query 61
TSFQWHRCKVNIVDTPGHMDFLAEVYRSLAVLDGAILVISAKDGVQAQTRILFHALRKMN 120
            TSFQWH CKVNIVDTPGHMDFLAEVYRSLA+LDGAILV+SAKDGVQAQTRILFHAL
+ M
Sbjct 66
TSFQWHDCKVNIVDTPGHMDFLAEVYRSLAILDGAILVVSAKDGVQAQTRILFHALQVMK 125

Query 121
IPTVIFINKIDQAGVDLQSVVQSVRDKLSADIIIKQTVSLSPEIVLEENTDIEAWDAVIE 180
            IPTVIFINKIDQ G+DL V QS+RDKLSA++IIKQ V LSP+I + E+ +E WD
VI
Sbjct 126
IPTVIFINKIDQDGIDLPGVYQSIRDKLSANMIIKQNVQLSPDISIMEDMGLENWDTVIA 185

Query 181
NNDKLLEKYIAGEPISREKLVREEQRRVQDASLFPVYYGSAKKGLGIQPLMDAVTGLFQP 240
            + D+LLEKYIAGEP+ +E+L+REE RR+Q SLFPVY+GSAK LGI+ L++AVT
FQ
Sbjct 186
DCDELLEKYIAGEPMDKEELLREENRRIQSVSLFPVYHGSAKVNLGIRQLIEAVTDTFQS 245

Query 241
IGEQGSAALCGSVFKVEYTDCGQRRVYLRLYSGTLRLRDTVALAGREKLKITEMRIPSKG 300
               Q S+ LCG+VFKVEY + QR YLRLYSGTL LRD+VALAG

+EKLKITEMRIPSKG
Sbjct 246
PTGQNSSELCGTVFKVEYANQSQRLAYLRLYSGTLHLRDSVALAGKEKLKITEMRIPSKG 305

Query 301
EIVRTDTAYPGEIVILPSDSVRLNDVLGDPTRLPRKRWREDPLPMLRTTIAPKTAAQRER 360
            EIVRT+ A+ GEIVI+P DS+RLNDVLG+   LPR+ W ++PLP+LRTTIAP+
QRER
Sbjct 306
EIVRTEIAHAGEIVIVPCDSLRLNDVLGNKLLLPRETWSDNPLPLLRTTIAPEKPEQRER 365

Query 361
LLDALTQLADTDPLLRCEVDSITHEIILSFLGRVQLEVVSALLSEKYKLETVVKEPSVIY 420
            LL+ALT++ADTDPLLR EVD++THEIILSFLGRVQLE++S LL EKY+L T KEP
+VIY
Sbjct 366
LLNALTEIADTDPLLRYEVDAVTHEIILSFLGRVQLEIISDLLVEKYQLNTTAKEPTVIY 425

Query 421
MERPLKAASHTIHIEVPPNPFWASIGLSVTPLSLGSGVQYESRVSLGYLNQSFQNAVRDG 480
            MERPLKA SHTIHIEVPPNPFWASIGLSVTPL LG+GVQYES+VS
+GYLNQSFQNAV DG
Sbjct 426
MERPLKAVSHTIHIEVPPNPFWASIGLSVTPLPLGTGVQYESKVSVGYLNQSFQNAVLDG 485

Query 481
IRYGLEQGLFGWNVTDCKICFEYGLYYSPVSTPADFRSLAPIVLEQALKESGTQLLEPYL 540
            IRYGLEQG++GW VTDCKICFEYGLYYSPVSTPADFRSLAPIVLEQ LK+
+GTQLLEPYL
Sbjct 486
IRYGLEQGVYGWKVTDCKICFEYGLYYSPVSTPADFRSLAPIVLEQVLKKAGTQLLEPYL 545

Query 541
SFILYAPQEYLSRAYHDAPKYCATIETAQVKKDEVVFTGEIPARCIQAYRTDLAFYTNGR 600
            SF LYAPQEYLSRAYHDAPKYCA+IET QVK EV+FTGEIPARC+Q YR DL
FYTNGR
Sbjct 546
SFTLYAPQEYLSRAYHDAPKYCASIETTQVKNSEVIFTGEIPARCVQEYRNDLTFYTNGR 605

Query 601 SVCLTELKGYQAAVGQPVIQPRRPNSRLDKVRHMFQKVM 639
            SVCLTELKGYQ A G+PV QPRRPN+R+DKVRHMF K++
Sbjct 606 SVCLTELKGYQIASGEPVFQPRRPNTRIDKVRHMFNKIL 644

>ALB05595.1 tetracycline resistance protein W, partial
[Bifidobacterium sp.
F21]
Length=520

 Score = 1055 bits (2727), Expect = 0.0, Method: Compositional
matrix adjust.
 Identities = 515/520 (99%), Positives = 518/520 (99%), Gaps = 0/520
(0%)

Query 1
MKIINIGILAHVDAGKTTLTESLLYASGAISEPGSVEKGTTRTDTMFLERQRGITIQAAV 60
            MKIINIGILAHVDAGKTTLTESLLYASGAISEPGSVEKGTTRTDTM
LERQRGITIQAAV
Sbjct 1
MKIINIGILAHVDAGKTTLTESLLYASGAISEPGSVEKGTTRTDTMLLERQRGITIQAAV 60

Query 61
TSFQWHRCKVNIVDTPGHMDFLAEVYRSLAVLDGAILVISAKDGVQAQTRILFHALRKMN 120

TSFQWHRCKVNIVDTPGHMDFLAEVYRSLAVLDGAILVISAKDGVQAQTRILFHALRKMN
Sbjct 61
TSFQWHRCKVNIVDTPGHMDFLAEVYRSLAVLDGAILVISAKDGVQAQTRILFHALRKMN 120

Query 121
IPTVIFINKIDQAGVDLQSVVQSVRDKLSADIIIKQTVSLSPEIVLEENTDIEAWDAVIE 180

IPTVIFINKIDQAGVDLQSVVQSVRDKLSADIIIKQTVSLSPEIVLEENTDIEAWDAVIE
Sbjct 121
IPTVIFINKIDQAGVDLQSVVQSVRDKLSADIIIKQTVSLSPEIVLEENTDIEAWDAVIE 180

Query 181
NNDKLLEKYIAGEPISREKLVREEQRRVQDASLFPVYYGSAKKGLGIQPLMDAVTGLFQP 240

NNDKLLEKYIAGEPISREKLVREEQRRVQDASLFPVYYGSAKKGLGIQPLMDAVTGLFQP
Sbjct 181
NNDKLLEKYIAGEPISREKLVREEQRRVQDASLFPVYYGSAKKGLGIQPLMDAVTGLFQP 240

Query 241
IGEQGSAALCGSVFKVEYTDCGQRRVYLRLYSGTLRLRDTVALAGREKLKITEMRIPSKG 300

IGEQGSAALCGSVFKVEYTDCGQRRVYLRLYSGTLRLRDTVALAGREKLKITEMRIPSKG
Sbjct 241
IGEQGSAALCGSVFKVEYTDCGQRRVYLRLYSGTLRLRDTVALAGREKLKITEMRIPSKG 300

Query 301
EIVRTDTAYPGEIVILPSDSVRLNDVLGDPTRLPRKRWREDPLPMLRTTIAPKTAAQRER 360
            EIVRTDTAYPGEIVILPSDSVRLNDVLGDPTRLPRKRWREDPLPMLRT
+IAPKTAAQRER
Sbjct 301
EIVRTDTAYPGEIVILPSDSVRLNDVLGDPTRLPRKRWREDPLPMLRTSIAPKTAAQRER 360

Query 361
LLDALTQLADTDPLLRCEVDSITHEIILSFLGRVQLEVVSALLSEKYKLETVVKEPSVIY 420
            LLDALTQLADTDPLLRCEVDSITHEIILSFLGRVQLEVVSALLSEKYKLETVVKEP
+VIY
Sbjct 361
LLDALTQLADTDPLLRCEVDSITHEIILSFLGRVQLEVVSALLSEKYKLETVVKEPTVIY 420

Query 421
MERPLKAASHTIHIEVPPNPFWASIGLSVTPLSLGSGVQYESRVSLGYLNQSFQNAVRDG 480
            MERPLKAASHTIHIEVPPNPFWASIGLSVTPL LGSGVQY
+SRVSLGYLNQSFQNAVRDG
Sbjct 421

MERPLKAASHTIHIEVPPNPFWASIGLSVTPLPLGSGVQYKSRVSLGYLNQSFQNAVRDG 480

```
Query  481  IRYGLEQGLFGWNVTDCKICFEYGLYYSPVSTPADFRSLA  520
            IRYGLEQGLFGWNVTDCKICFEYGLYYSPVSTPADFRSLA
Sbjct  481  IRYGLEQGLFGWNVTDCKICFEYGLYYSPVSTPADFRSLA  520
```

>ACX71626.1 tetracycline resistance protein, partial [Streptococcus suis]
Length=600

Score = 1034 bits (2674), Expect = 0.0, Method: Compositional matrix adjust.
Identities = 494/600 (82%), Positives = 545/600 (91%), Gaps = 0/600 (0%)

```
Query  21
ESLLYASGAISEPGSVEKGTTRTDTMFLERQRGITIQAAVTSFQWHRCKVNIVDTPGHMD  80
            ESLLY SGAI+E GSV++GTTRTDTM LERQRGITIQ AVTSFQW   KVNI
+DTPGHMD
Sbjct  1
ESLLYTSGAIAELGSVDEGTTRTDTMNLERQRGITIQTAVTSFQWEDVKVNIIDTPGHMD  60

Query  81
FLAEVYRSLAVLDGAILVISAKDGVQAQTRILFHALRKMNIPTVIFINKIDQAGVDLQSV  140

FLAEVYRSLAVLDGAILVISAKDGVQAQTRILFHALRKMNIPTVIFINKIDQAGVDLQSV
Sbjct  61
FLAEVYRSLAVLDGAILVISAKDGVQAQTRILFHALRKMNIPTVIFINKIDQAGVDLQSV  120

Query  141
VQSVRDKLSADIIIKQTVSLSPEIVLEENTDIEAWDAVIENNDKLLEKYIAGEPISREKL  200

VQSVRDKLSADIIIKQTVSLSPEIVLEENTDIEAWDAVIENNDKLLEKYIAGEPISREKL
Sbjct  121
VQSVRDKLSADIIIKQTVSLSPEIVLEENTDIEAWDAVIENNDKLLEKYIAGEPISREKL  180

Query  201
VREEQRRVQDASLFPVYYGSAKKGLGIQPLMDAVTGLFQPIGEQGSAALCGSVFKVEYTD  260

VREEQRRVQDASLFPVYYGSAKKGLGIQPLMDAVTGLFQPIGEQGSAALCGSVFKVEYTD
Sbjct  181
VREEQRRVQDASLFPVYYGSAKKGLGIQPLMDAVTGLFQPIGEQGSAALCGSVFKVEYTD  240

Query  261
CGQRRVYLRLYSGTLRLRDTVALAGREKLKITEMRIPSKGEIVRTDTAYPGEIVILPSDS  320
            CGQRRVYLRLYSGTLRLRDTVALAGREKLKITEMRIPSKGEIVRTDTAYPGEIVIL
D+
Sbjct  241
CGQRRVYLRLYSGTLRLRDTVALAGREKLKITEMRIPSKGEIVRTDTAYPGEIVILADDT  300

Query  321
VRLNDVLGDPTRLPRKRWREDPLPMLRTTIAPKTAAQRERLLDALTQLADTDPLLRCEVD  380
            ++LND+LG+   LP K   ++P+P+LRTT+ P+   QRE LL+AL ++ADTDPLL
```

++D
Sbjct 301
LKLNDILGNEKLLPHKTRIDNPMPLLRTTVEPQKPEQREALLNALAEIADTDPLLHFDID 360

Query 381
SITHEIILSFLGRVQLEVVSALLSEKYKLETVVKEPSVIYMERPLKAASHTIHIEVPPNP 440
            ++THEI+LSFLG+VQLEV+ +LL EKY +   +KEPSVIY+ERPL+ A
+TIHIEVPPNP
Sbjct 361
TVTHEIMLSFLGKVQLEVICSLLEEKYHVGVAMKEPSVIYLERPLRKAEYTIHIEVPPNP 420

Query 441
FWASIGLSVTPLSLGSGVQYESRVSLGYLNQSFQNAVRDGIRYGLEQGLFGWNVTDCKIC 500
            FWAS+GLS+ PL +GSGVQYESRVSLGYLNQSFQNAV +G+ YG EQGL+GW
VTDCKIC
Sbjct 421
FWASVGLSIEPLPIGSGVQYESRVSLGYLNQSFQNAVMEGVLYGCEQGLYGWKVTDCKIC 480

Query 501
FEYGLYYSPVSTPADFRSLAPIVLEQALKESGTQLLEPYLSFILYAPQEYLSRAYHDAPK 560
            FEYGLYYSPVSTPADFR L+PIVLEQALK++GT+LLEPYL F
+YAPQEYLSRAYHDAP+
Sbjct 481
FEYGLYYSPVSTPADFRLLSPIVLEQALKKAGTELLEPYLHFEIYAPQEYLSRAYHDAPR 540

Query 561
YCATIETAQVKKDEVVFTGEIPARCIQAYRTDLAFYTNGRSVCLTELKGYQAAVGQPVIQ 620
            YCA I + Q+K DEV+ GEIPARCIQ YR DL +TNG+ VCLTELKGYQ A+G+
+ Q
Sbjct 541
YCADIVSTQIKNDEVILKGEIPARCIQEYRNDLTNFTNGQGVCLTELKGYQPAIGKFICQ 600

>AMP50917.1 TetM-TetW-TetO-TetS, partial [uncultured bacterium]
Length=539

 Score = 1025 bits (2651), Expect = 0.0, Method: Compositional
matrix adjust.
 Identities = 494/538 (92%), Positives = 516/538 (96%), Gaps = 0/538
(0%)

Query 101
AKDGVQAQTRILFHALRKMNIPTVIFINKIDQAGVDLQSVVQSVRDKLSADIIIKQTVSL 160

AKDGVQAQTRILFHALRKMNIPTVIFINKIDQAGVDLQSVVQSVRDKLSADIIIKQTVSL
Sbjct 1
AKDGVQAQTRILFHALRKMNIPTVIFINKIDQAGVDLQSVVQSVRDKLSADIIIKQTVSL 60

Query 161
SPEIVLEENTDIEAWDAVIENNDKLLEKYIAGEPISREKLVREEQRRVQDASLFPVYYGS 220
            SPEIVLEENTDIEAWDAVIENND+LLEKYIAGEPISREKL REEQ
+RVQDASLFPVY+GS
Sbjct 61
SPEIVLEENTDIEAWDAVIENNDELLEKYIAGEPISREKLAREEQQRVQDASLFPVYHGS 120

Query 221
AKKGLGIQPLMDAVTGLFQPIGEQGSAALCGSVFKVEYTDCGQRRVYLRLYSGTLRLRDT 280
AK GLGIQPLMDAVTGLFQPIGEQG
AALCGSVFKVEYTDCGQRRVYLRLYSGTLRLRDT
Sbjct 121
AKNGLGIQPLMDAVTGLFQPIGEQGGAALCGSVFKVEYTDCGQRRVYLRLYSGTLRLRDT 180

Query 281
VALAGREKLKITEMRIPSKGEIVRTDTAYPGEIVILPSDSVRLNDVLGDPTRLPRKRWRE 340
VALAGREKLKITEMRIPSKGEIVRTDTA+ GEIVILPSDS+RLND+LGD T+LPR+
W +
Sbjct 181
VALAGREKLKITEMRIPSKGEIVRTDTAHKGEIVILPSDSLRLNDILGDKTQLPREMWSD 240

Query 341
DPLPMLRTTIAPKTAAQRERLLDALTQLADTDPLLRCEVDSITHEIILSFLGRVQLEVVS 400
P PMLRTTI PKTA QR+RLLDALTQ+ADTDPLL EVDS THEIILSFLGR
+QLEVVS
Sbjct 241
VPFPMLRTTITPKTAEQRDRLLDALTQIADTDPLLHYEVDSTTHEIILSFLGRMQLEVVS 300

Query 401
ALLSEKYKLETVVKEPSVIYMERPLKAASHTIHIEVPPNPFWASIGLSVTPLSLGSGVQY 460
ALL+EKYK+ET VKEP+VIY+ERPLK ASHTIHIEVPPNPFWASIGLSVTPL
LGSGV+Y
Sbjct 301
ALLTEKYKIETAVKEPTVIYLERPLKVASHTIHIEVPPNPFWASIGLSVTPLPLGSGVKY 360

Query 461
ESRVSLGYLNQSFQNAVRDGIRYGLEQGLFGWNVTDCKICFEYGLYYSPVSTPADFRSLA 520
ESRVSLGYLNQSFQNAV DGIRYGL QGL
GWNVTDCKICFEYGLYYSPVSTPADFRSLA
Sbjct 361
ESRVSLGYLNQSFQNAVMDGIRYGLGQGLCGWNVTDCKICFEYGLYYSPVSTPADFRSLA 420

Query 521
PIVLEQALKESGTQLLEPYLSFILYAPQEYLSRAYHDAPKYCATIETAQVKKDEVVFTGE 580
PIVLEQALK+SGTQLLEPYLSFILYAPQEYLSRAYHDAPKYCATIETAQ
+KKDEVVFTGE
Sbjct 421
PIVLEQALKKSGTQLLEPYLSFILYAPQEYLSRAYHDAPKYCATIETAQIKKDEVVFTGE 480

Query 581
IPARCIQAYRTDLAFYTNGRSVCLTELKGYQAAVGQPVIQPRRPNSRLDKVRHMFQKV 638
IPARCIQAYRTDLAFYTNGRSVCLTELKGYQA VG+P+IQPRRPNSRLDKVRHMF
K+
Sbjct 481
IPARCIQAYRTDLAFYTNGRSVCLTELKGYQATVGEPIIQPRRPNSRLDKVRHMFSKI 538

>KFI74536.1 tetracycline resistance protein tetW [Bifidobacterium
pseudolongum
subsp. globosum]

Length=524

 Score = 1023 bits (2646), Expect = 0.0, Method: Compositional matrix adjust.
 Identities = 500/508 (98%), Positives = 503/508 (99%), Gaps = 0/508 (0%)

```
Query  46
MFLERQRGITIQAAVTSFQWHRCKVNIVDTPGHMDFLAEVYRSLAVLDGAILVISAKDGV  105

MFLERQRGITIQAAVTSFQWHRCKVNIVDTPGHMDFLAEVYRSLAVLDGAILVISAKDGV
Sbjct  1
MFLERQRGITIQAAVTSFQWHRCKVNIVDTPGHMDFLAEVYRSLAVLDGAILVISAKDGV  60

Query  106
QAQTRILFHALRKMNIPTVIFINKIDQAGVDLQSVVQSVRDKLSADIIIKQTVSLSPEIV  165

QAQTRILFHALRKMNIPTVIFINKIDQAGVDLQSVVQSVRDKLSADIIIKQTVSLSPEIV
Sbjct  61
QAQTRILFHALRKMNIPTVIFINKIDQAGVDLQSVVQSVRDKLSADIIIKQTVSLSPEIV  120

Query  166
LEENTDIEAWDAVIENNDKLLEKYIAGEPISREKLVREEQRRVQDASLFPVYYGSAKKGL  225
            LEENTDIEAWDAVIENND+LLEKYIAGEPISREKL REEQ+RVQDASLFPVY
+GSAK GL
Sbjct  121
LEENTDIEAWDAVIENNDELLEKYIAGEPISREKLAREEQQRVQDASLFPVYHGSAKNGL  180

Query  226
GIQPLMDAVTGLFQPIGEQGSAALCGSVFKVEYTDCGQRRVYLRLYSGTLRLRDTVALAG  285
            GIQPLMDAVTGLFQPIGEQG
AALCGSVFKVEYTDCGQRRVYLRLYSGTLRLRDTVALAG
Sbjct  181
GIQPLMDAVTGLFQPIGEQGGAALCGSVFKVEYTDCGQRRVYLRLYSGTLRLRDTVALAG  240

Query  286
REKLKITEMRIPSKGEIVRTDTAYPGEIVILPSDSVRLNDVLGDPTRLPRKRWREDPLPM  345
            REKLKITEMRIPSKGEIVRTDTAY GEIVILPSDSVRLNDVLGD
TRLPRKRWREDPLPM
Sbjct  241
REKLKITEMRIPSKGEIVRTDTAYQGEIVILPSDSVRLNDVLGDQTRLPRKRWREDPLPM  300

Query  346
LRTTIAPKTAAQRERLLDALTQLADTDPLLRCEVDSITHEIILSFLGRVQLEVVSALLSE  405

LRTTIAPKTAAQRERLLDALTQLADTDPLLRCEVDSITHEIILSFLGRVQLEVVSALLSE
Sbjct  301
LRTTIAPKTAAQRERLLDALTQLADTDPLLRCEVDSITHEIILSFLGRVQLEVVSALLSE  360

Query  406
KYKLETVVKEPSVIYMERPLKAASHTIHIEVPPNPFWASIGLSVTPLSLGSGVQYESRVS  465

KYKLETVVKEPSVIYMERPLKAASHTIHIEVPPNPFWASIGLSVTPLSLGSGVQYESRVS
Sbjct  361
```

KYKLETVVKEPSVIYMERPLKAASHTIHIEVPPNPFWASIGLSVTPLSLGSGVQYESRVS 420

Query 466
LGYLNQSFQNAVRDGIRYGLEQGLFGWNVTDCKICFEYGLYYSPVSTPADFRSLAPIVLE 525

LGYLNQSFQNAVRDGIRYGLEQGLFGWNVTDCKICFEYGLYYSPVSTPADFRSLAPIVLE
Sbjct 421
LGYLNQSFQNAVRDGIRYGLEQGLFGWNVTDCKICFEYGLYYSPVSTPADFRSLAPIVLE 480

Query 526 QALKESGTQLLEPYLSFILYAPQEYLSR 553
            QALKESGTQLLEPYLSFILYAPQEYLSR
Sbjct 481 QALKESGTQLLEPYLSFILYAPQEYLSR 508

>AMP56474.1 TetM-TetW-TetO-TetS, partial [uncultured bacterium]
Length=504

Score = 1013 bits (2618), Expect = 0.0, Method: Compositional
matrix adjust.
Identities = 495/504 (98%), Positives = 499/504 (99%), Gaps = 0/504
(0%)

Query 71
NIVDTPGHMDFLAEVYRSLAVLDGAILVISAKDGVQAQTRILFHALRKMNIPTVIFINKI 130

NIVDTPGHMDFLAEVYRSLAVLDGAILVISAKDGVQAQTRILFHALRKMNIPTVIFINKI
Sbjct 1
NIVDTPGHMDFLAEVYRSLAVLDGAILVISAKDGVQAQTRILFHALRKMNIPTVIFINKI 60

Query 131
DQAGVDLQSVVQSVRDKLSADIIIKQTVSLSPEIVLEENTDIEAWDAVIENNDKLLEKYI 190
            DQAGVDLQSVVQSVRDKLSA+IIIKQTVSLSPEIVLEENTDIEAWDAVIENND
+LLEKYI
Sbjct 61
DQAGVDLQSVVQSVRDKLSANIIIKQTVSLSPEIVLEENTDIEAWDAVIENNDELLEKYI 120

Query 191
AGEPISREKLVREEQRRVQDASLFPVYYGSAKKGLGIQPLMDAVTGLFQPIGEQGSAALC 250
            AGEPISREKL REEQ+RVQDASLFPVY+GSAK GLGIQPLMDAVTGLFQPIGEQG
AALC
Sbjct 121
AGEPISREKLAREEQQRVQDASLFPVYHGSAKNGLGIQPLMDAVTGLFQPIGEQGGAALC 180

Query 251
GSVFKVEYTDCGQRRVYLRLYSGTLRLRDTVALAGREKLKITEMRIPSKGEIVRTDTAYP 310

GSVFKVEYTDCGQRRVYLRLYSGTLRLRDTVALAGREKLKITEMRIPSKGEIVRTDTAY
Sbjct 181
GSVFKVEYTDCGQRRVYLRLYSGTLRLRDTVALAGREKLKITEMRIPSKGEIVRTDTAYQ 240

Query 311
GEIVILPSDSVRLNDVLGDPTRLPRKRWREDPLPMLRTTIAPKTAAQRERLLDALTQLAD 370
            GEIVILPSDSVRLNDVLGD
TRLPRKRWREDPLPMLRTTIAPKTAAQRERLLDALTQLAD

Sbjct 241
GEIVILPSDSVRLNDVLGDQTRLPRKRWREDPLPMLRTTIAPKTAAQRERLLDALTQLAD 300

Query 371
TDPLLRCEVDSITHEIILSFLGRVQLEVVSALLSEKYKLETVVKEPSVIYMERPLKAASH 430

TDPLLRCEVDSITHEIILSFLGRVQLEVVSALLSEKYKLETVVKEPSVIYMERPLKAASH
Sbjct 301
TDPLLRCEVDSITHEIILSFLGRVQLEVVSALLSEKYKLETVVKEPSVIYMERPLKAASH 360

Query 431
TIHIEVPPNPFWASIGLSVTPLSLGSGVQYESRVSLGYLNQSFQNAVRDGIRYGLEQGLF 490

TIHIEVPPNPFWASIGLSVTPLSLGSGVQYESRVSLGYLNQSFQNAVRDGIRYGLEQGLF
Sbjct 361
TIHIEVPPNPFWASIGLSVTPLSLGSGVQYESRVSLGYLNQSFQNAVRDGIRYGLEQGLF 420

Query 491
GWNVTDCKICFEYGLYYSPVSTPADFRSLAPIVLEQALKESGTQLLEPYLSFILYAPQEY 550

GWNVTDCKICFEYGLYYSPVSTPADFRSLAPIVLEQALKESGTQLLEPYLSFILYAPQEY
Sbjct 421
GWNVTDCKICFEYGLYYSPVSTPADFRSLAPIVLEQALKESGTQLLEPYLSFILYAPQEY 480

Query 551 LSRAYHDAPKYCATIETAQVKKDE 574
LSRAYHDAPKYCATIETAQVKKDE
Sbjct 481 LSRAYHDAPKYCATIETAQVKKDE 504

>WP_070087585.1 MULTISPECIES: TetM/TetW/TetO/TetS family
tetracycline resistance
ribosomal protection protein [Bacteria]
CVI73754.1 Tetracycline resistance protein TetO [Eubacteriaceae
bacterium
CHKCI004]
SHK50999.1 ribosomal protection tetracycline resistance protein
[[Clostridium]
lactatifermentans DSM 14214]
OUN91760.1 tetracycline resistance ribosomal protection protein
Tet(32)
[Blautia sp. An46]
OUO24576.1 tetracycline resistance ribosomal protection protein
Tet(32)
[Eubacterium sp. An3]
OUO72196.1 tetracycline resistance ribosomal protection protein
Tet(32)
[Anaeromassilibacillus sp. An250]
OUO81366.1 tetracycline resistance ribosomal protection protein
Tet(32)
[Blautia sp. An249]
OUO82615.1 tetracycline resistance ribosomal protection protein
Tet(32)
[Flavonifractor plautii]
OUQ03730.1 tetracycline resistance ribosomal protection protein

Tet(32)
[[Clostridium] spiroforme]
 OUQ05897.1 tetracycline resistance ribosomal protection protein Tet(32)
[[Clostridium] spiroforme]
 OUQ07079.1 tetracycline resistance ribosomal protection protein Tet(32)
[Erysipelatoclostridium sp. An15]
 OUQ17054.1 tetracycline resistance ribosomal protection protein Tet(32)
[Lachnoclostridium sp. An138]
 OUQ28373.1 tetracycline resistance ribosomal protection protein Tet(32)
[Lachnoclostridium sp. An131]
 OUQ45027.1 tetracycline resistance ribosomal protection protein Tet(32)
[Drancourtella sp. An12]
 OUQ51788.1 tetracycline resistance ribosomal protection protein Tet(32)
[Lachnoclostridium sp. An118]
Length=639

 Score = 972 bits (2512), Expect = 0.0, Method: Compositional matrix adjust.
 Identities = 455/638 (71%), Positives = 541/638 (85%), Gaps = 0/638 (0%)

```
Query  1
MKIINIGILAHVDAGKTTLTESLLYASGAISEPGSVEKGTTRTDTMFLERQRGITIQAAV  60
            MKIINIGILAHVDAGKTTLTESLLY SGA+ E GSV+KGTTRTDTM
LERQRGITIQ AV
Sbjct  1
MKIINIGILAHVDAGKTTLTESLLYTSGAVPELGSVDKGTTRTDTMLLERQRGITIQTAV  60

Query  61
TSFQWHRCKVNIVDTPGHMDFLAEVYRSLAVLDGAILVISAKDGVQAQTRILFHALRKMN  120
            TSF W   K+NIVDTPGHMDFLAEVYRSLAVLDGAILV+SAKDGVQAQTR+LFHAL
+KM
Sbjct  61
TSFGWKNYKINIVDTPGHMDFLAEVYRSLAVLDGAILVVSAKDGVQAQTRVLFHALQKMK  120

Query  121
IPTVIFINKIDQAGVDLQSVVQSVRDKLSADIIIKQTVSLSPEIVLEENTDIEAWDAVIE  180
            IPT+IF+NKIDQ G+DLQSV Q++R+KLS D+++ Q VSL+PE+ L +  DIE WD
++I
Sbjct  121
IPTIIFVNKIDQEGIDLQSVYQNIREKLSDDVMVMQDVSLTPEVSLTDIEDIEKWDSIIA  180

Query  181
NNDKLLEKYIAGEPISREKLVREEQRRVQDASLFPVYYGSAKKGLGIQPLMDAVTGLFQP  240
             ND+LLEKYIAGEP+  + L RE+ RR+Q+ SLFP+Y+GSAK  +G + L++ +
F
Sbjct  181
GNDELLEKYIAGEPLKIQDLQREKCRRMQNGSLFPIYHGSAKNNIGTEKLIEVIAETFTS  240
```

```
Query  241
IGEQGSAALCGSVFKVEYTDCGQRRVYLRLYSGTLRLRDTVALAGREKLKITEMRIPSKG  300
              +   + LCGSVFK+EYTD  +R VYLRLYSGTL LRDT+ L
+KLKITEMRIPS G
Sbjct  241
GADNDQSELCGSVFKIEYTDQKKRLVYLRLYSGTLHLRDTILLPQNQKLKITEMRIPSNG  300

Query  301
EIVRTDTAYPGEIVILPSDSVRLNDVLGDPTRLPRKRWREDPLPMLRTTIAPKTAAQRER  360
            EI+  DTA  GEIVIL +D+++LND LG+   LPRK W ++P+P+LRTT+ P+
QR+
Sbjct  301
EIIPADTACCGEIVILTNDTLKLNDTLGNVELLPRKAWEKNPIPLLRTTVEPQNQEQRDL  360

Query  361
LLDALTQLADTDPLLRCEVDSITHEIILSFLGRVQLEVVSALLSEKYKLETVVKEPSVIY  420
            LL+ALT++ADTDPLL   VD+ITHEII+SFLG+VQLEVV +LL E+Y +   VKEP
+VIY
Sbjct  361
LLNALTEIADTDPLLHYYVDTITHEIIISFLGKVQLEVVCSLLVERYHVNINVKEPTVIY  420

Query  421
MERPLKAASHTIHIEVPPNPFWASIGLSVTPLSLGSGVQYESRVSLGYLNQSFQNAVRDG  480
            +ERPLK AS+TIHIEVPPNPFWASIGL+VTPL  GSG +++S
+VSLGYLNQSFQNAV +G
Sbjct  421
LERPLKTASYTIHIEVPPNPFWASIGLTVTPLPAGSGTRFKSKVSLGYLNQSFQNAVMEG  480

Query  481
IRYGLEQGLFGWNVTDCKICFEYGLYYSPVSTPADFRSLAPIVLEQALKESGTQLLEPYL  540
            +RYG+EQGL+GW VTDC+ICF+YG+YYSPVSTPADFRSLAPIVLEQALK
+GTQLLEPYL
Sbjct  481
VRYGMEQGLYGWEVTDCEICFDYGVYYSPVSTPADFRSLAPIVLEQALKRAGTQLLEPYL  540

Query  541
SFILYAPQEYLSRAYHDAPKYCATIETAQVKKDEVVFTGEIPARCIQAYRTDLAFYTNGR  600
            SF L+APQEY+SRAY+DAPKYCA IE+  +K DEV+FTGEIPARCI  YR DL
FYTNGR
Sbjct  541
SFTLFAPQEYISRAYNDAPKYCAVIESTLLKNDEVIFTGEIPARCIGEYRNDLNFYTNGR  600

Query  601  SVCLTELKGYQAAVGQPVIQPRRPNSRLDKVRHMFQKV  638
            SVCLTELKGYQ   G+PV+QPRRPNSRLDKVRHMFQK+
Sbjct  601  SVCLTELKGYQEISGEPVLQPRRPNSRLDKVRHMFQKI  638
```

>APO27487.1 ferrous iron transport protein B [uncultured bacterium]
 APO27589.1 ferrous iron transport protein B [uncultured bacterium]
Length=644

 Score = 971 bits (2510), Expect = 0.0, Method: Compositional
matrix adjust.

Identities = 455/638 (71%), Positives = 541/638 (85%), Gaps = 0/638 (0%)

Query 1
MKIINIGILAHVDAGKTTLTESLLYASGAISEPGSVEKGTTRTDTMFLERQRGITIQAAV 60
MKIINIGILAHVDAGKTTLTESLLY SGA+ E GSV+KGTTRTDTM
LERQRGITIQ AV
Sbjct 6
MKIINIGILAHVDAGKTTLTESLLYTSGAVPELGSVDKGTTRTDTMLLERQRGITIQTAV 65

Query 61
TSFQWHRCKVNIVDTPGHMDFLAEVYRSLAVLDGAILVISAKDGVQAQTRILFHALRKMN 120
TSF W   K+NIVDTPGHMDFLAEVYRSLAVLDGAILV+SAKDGVQAQTR+LFHAL
+KM
Sbjct 66
TSFGWKNYKINIVDTPGHMDFLAEVYRSLAVLDGAILVVSAKDGVQAQTRVLFHALQKMK 125

Query 121
IPTVIFINKIDQAGVDLQSVVQSVRDKLSADIIIKQTVSLSPEIVLEENTDIEAWDAVIE 180
IPT+IF+NKIDQ G+DLQSV Q++R+KLS D+++ Q VSL+PE+ L + DIE WD
++I
Sbjct 126
IPTIIFVNKIDQEGIDLQSVYQNIREKLSDDVMVMQDVSLTPEVSLTDIEDIEKWDSIIA 185

Query 181
NNDKLLEKYIAGEPISREKLVREEQRRVQDASLFPVYYGSAKKGLGIQPLMDAVTGLFQP 240
ND+LLEKYIAGEP+ + L RE+ RR+Q+ SLFP+Y+GSAK +G + L++ +
F
Sbjct 186
GNDELLEKYIAGEPLKIQDLQREKCRRMQNGSLFPIYHGSAKNNIGTEKLIEVIAETFTS 245

Query 241
IGEQGSAALCGSVFKVEYTDCGQRRVYLRLYSGTLRLRDTVALAGREKLKITEMRIPSKG 300
+   + LCGSVFK+EYTD +R VYLRLYSGTL LRDT+ L
+KLKITEMRIPS G
Sbjct 246
GADNDQSELCGSVFKIEYTDQKKRLVYLRLYSGTLHLRDTILLPQNQKLKITEMRIPSNG 305

Query 301
EIVRTDTAYPGEIVILPSDSVRLNDVLGDPTRLPRKRWREDPLPMLRTTIAPKTAAQRER 360
EI+ DTA GEIVIL +D+++LND LG+   LPRK W ++P+P+LRTT+ P+
QR+
Sbjct 306
EIIPADTACCGEIVILTNDTLKLNDTLGNVELLPRKAWEKNPIPLLRTTVEPQNQEQRDL 365

Query 361
LLDALTQLADTDPLLRCEVDSITHEIILSFLGRVQLEVVSALLSEKYKLETVVKEPSVIY 420
LL+ALT++ADTDPLL   VD+ITHEII+SFLG+VQLEVV +LL E+Y +   VKEP
+VIY
Sbjct 366
LLNALTEIADTDPLLHYYVDTITHEIIISFLGKVQLEVVCSLLVERYHVNINVKEPTVIY 425

Query 421
MERPLKAASHTIHIEVPPNPFWASIGLSVTPLSLGSGVQYESRVSLGYLNQSFQNAVRDG 480

            +ERPLK AS+TIHIEVPPNPFWASIGL+VTPL GSG +++S
+VSLGYLNQSFQNAV +G
Sbjct 426
LERPLKTASYTIHIEVPPNPFWASIGLTVTPLPAGSGTRFKSKVSLGYLNQSFQNAVMEG 485

Query 481
IRYGLEQGLFGWNVTDCKICFEYGLYYSPVSTPADFRSLAPIVLEQALKESGTQLLEPYL 540
            +RYG+EQGL+GW VTDC+ICF+YG+YYSPVSTPADFRSLAPIVLEQALK
+GTQLLEPYL
Sbjct 486
VRYGMEQGLYGWEVTDCEICFDYGVYYSPVSTPADFRSLAPIVLEQALKRAGTQLLEPYL 545

Query 541
SFILYAPQEYLSRAYHDAPKYCATIETAQVKKDEVVFTGEIPARCIQAYRTDLAFYTNGR 600
            SF L+APQEY+SRAY+DAPKYCA IE+ +K DEV+FTGEIPARCI YR DL
FYTNGR
Sbjct 546
SFTLFAPQEYISRAYNDAPKYCAVIESTLLKNDEVIFTGEIPARCIGEYRNDLNFYTNGR 605

Query 601 SVCLTELKGYQAAVGQPVIQPRRPNSRLDKVRHMFQKV 638
            SVCLTELKGYQ   G+PV+QPRRPNSRLDKVRHMFQK+
Sbjct 606 SVCLTELKGYQEISGEPVLQPRRPNSRLDKVRHMFQKI 643

>AMP50126.1 TetM-TetW-TetO-TetS [uncultured bacterium]
Length=482

 Score = 971 bits (2510), Expect = 0.0, Method: Compositional
matrix adjust.
 Identities = 470/482 (98%), Positives = 477/482 (99%), Gaps = 0/482
(0%)

Query 158
VSLSPEIVLEENTDIEAWDAVIENNDKLLEKYIAGEPISREKLVREEQRRVQDASLFPVY 217
            +SLSPEIVLEENTDIEAWDAVIENND+LLEKYIAGEPISREKL
REEQRRVQDASLFPVY
Sbjct 1
MSLSPEIVLEENTDIEAWDAVIENNDELLEKYIAGEPISREKLAREEQRRVQDASLFPVY 60

Query 218
YGSAKKGLGIQPLMDAVTGLFQPIGEQGSAALCGSVFKVEYTDCGQRRVYLRLYSGTLRL 277
            +GSAK GLGIQPLMDAVTGLFQPIGEQG
AALCGSVFKVEYTDCGQRRVYLRLYSGTLRL
Sbjct 61
HGSAKNGLGIQPLMDAVTGLFQPIGEQGGAALCGSVFKVEYTDCGQRRVYLRLYSGTLRL 120

Query 278
RDTVALAGREKLKITEMRIPSKGEIVRTDTAYPGEIVILPSDSVRLNDVLGDPTRLPRKR 337
            RDTVALAGREKLKITEMRIPSKGEIVRTDTAY GEIVILPSDSVRLNDVLGD
TRLPRKR
Sbjct 121
RDTVALAGREKLKITEMRIPSKGEIVRTDTAYQGEIVILPSDSVRLNDVLGDQTRLPRKR 180

Query 338

WREDPLPMLRTTIAPKTAAQRERLLDALTQLADTDPLLRCEVDSITHEIILSFLGRVQLE 397

WREDPLPMLRTTIAPKTAAQRERLLDALTQLADTDPLLRCEVDSITHEIILSFLGRVQLE
Sbjct 181
WREDPLPMLRTTIAPKTAAQRERLLDALTQLADTDPLLRCEVDSITHEIILSFLGRVQLE 240

Query 398
VVSALLSEKYKLETVVKEPSVIYMERPLKAASHTIHIEVPPNPFWASIGLSVTPLSLGSG 457

VVSALLSEKYKLETVVKEPSVIYMERPLKAASHTIHIEVPPNPFWASIGLSVTPLSLGSG
Sbjct 241
VVSALLSEKYKLETVVKEPSVIYMERPLKAASHTIHIEVPPNPFWASIGLSVTPLSLGSG 300

Query 458
VQYESRVSLGYLNQSFQNAVRDGIRYGLEQGLFGWNVTDCKICFEYGLYYSPVSTPADFR 517

VQYESRVSLGYLNQSFQNAVRDGIRYGLEQGLFGWNVTDCKICFEYGLYYSPVSTPADFR
Sbjct 301
VQYESRVSLGYLNQSFQNAVRDGIRYGLEQGLFGWNVTDCKICFEYGLYYSPVSTPADFR 360

Query 518
SLAPIVLEQALKESGTQLLEPYLSFILYAPQEYLSRAYHDAPKYCATIETAQVKKDEVVF 577

SLAPIVLEQALKESGTQLLEPYLSFILYAPQEYLSRAYHDAPKYCATIETAQVKKDEVVF
Sbjct 361
SLAPIVLEQALKESGTQLLEPYLSFILYAPQEYLSRAYHDAPKYCATIETAQVKKDEVVF 420

Query 578
TGEIPARCIQAYRTDLAFYTNGRSVCLTELKGYQAAVGQPVIQPRRPNSRLDKVRHMFQK 637
TGEIPARCIQAYRTDLAFYTNG+SVCLTELKGYQAAVG+PVIQPRRPNSRLDKVR
+MFQK
Sbjct 421
TGEIPARCIQAYRTDLAFYTNGQSVCLTELKGYQAAVGKPVIQPRRPNSRLDKVRYMFQK 480

Query 638 VM 639
+M
Sbjct 481 IM 482

>WP_009266810.1 TetM/TetW/TetO/TetS family tetracycline resistance
ribosomal
protection protein [Lachnospiraceae bacterium 1_4_56FAA]
EGN31145.1 tetracycline resistance protein tetW [Lachnospiraceae
bacterium
1_4_56FAA]
Length=646

Score = 966 bits (2496), Expect = 0.0, Method: Compositional
matrix adjust.
Identities = 453/637 (71%), Positives = 539/637 (85%), Gaps = 0/637
(0%)

Query 1
MKIINIGILAHVDAGKTTLTESLLYASGAISEPGSVEKGTTRTDTMFLERQRGITIQAAV 60

MKIINIGILAHVDAGKTTLTESLLY SGAI E GSV
+KGTTRTDTMFLERQRGITIQAAV
Sbjct 1
MKIINIGILAHVDAGKTTLTESLLYTSGAILELGSVDKGTTRTDTMFLERQRGITIQAAV 60

Query 61
TSFQWHRCKVNIVDTPGHMDFLAEVYRSLAVLDGAILVISAKDGVQAQTRILFHALRKMN 120
TSF W+ K+NIVDTPGHMDFL EVYRSL+VLDGA+LVISAKDGVQAQTRILFHAL
+KMN
Sbjct 61
TSFNWNGYKINIVDTPGHMDFLTEVYRSLSVLDGAVLVISAKDGVQAQTRILFHALQKMN 120

Query 121
IPTVIFINKIDQAGVDLQSVVQSVRDKLSADIIIKQTVSLSPEIVLEENTDIEAWDAVIE 180
IPT+IFINKIDQ G++L + Q++++KLS DIII Q V+L+PE+ LE TD+E
WDAVI
Sbjct 121
IPTIIFINKIDQYGINLHNTYQNIKEKLSTDIIIMQNVTLTPEVSLENITDLEKWDAVIA 180

Query 181
NNDKLLEKYIAGEPISREKLVREEQRRVQDASLFPVYYGSAKKGLGIQPLMDAVTGLFQP 240
ND+LLEKYIAGE ++ ++L +EE RVQ SLFP+Y+GSAK +G Q L++ ++
+F
Sbjct 181
GNDRLLEKYIAGETLTVQELKQEEYGRVQKGSLFPIYHGSAKNNIGTQQLIEVISDIFCS 240

Query 241
IGEQGSAALCGSVFKVEYTDCGQRRVYLRLYSGTLRLRDTVALAGREKLKITEMRIPSKG 300
++ + LCGSVFK+EYTD QR YLRLYSGTL LRDT+ L+ ++K+K+TEM
IPS G
Sbjct 241
EMDKSQSELCGSVFKIEYTDRKQRLAYLRLYSGTLHLRDTIILSEKKKMKLTEMYIPSNG 300

Query 301
EIVRTDTAYPGEIVILPSDSVRLNDVLGDPTRLPRKRWREDPLPMLRTTIAPKTAAQRER 360
E+++T+ A G+IVILP+D ++LND++G+ LP W ++P+PMLRT I P
QRE
Sbjct 301
EMIQTEIARSGDIVILPNDILKLNDIVGNEKMLPCNAWNDNPVPMLRTMIEPIKPEQREY 360

Query 361
LLDALTQLADTDPLLRCEVDSITHEIILSFLGRVQLEVVSALLSEKYKLETVVKEPSVIY 420
LLDALT++ADTDPLLR VD+ITHEII+SFLG VQLEV+ +LL+EKY ++ VKEP
+VIY
Sbjct 361
LLDALTEIADTDPLLRYYVDTITHEIIISFLGTVQLEVICSLLTEKYHIDINVKEPTVIY 420

Query 421
MERPLKAASHTIHIEVPPNPFWASIGLSVTPLSLGSGVQYESRVSLGYLNQSFQNAVRDG 480
+E+PL+ A +TIHIEVPPNPFWASIGLSVTPL +GSG+QYES
+VSLGYLNQSFQNAV DG
Sbjct 421
LEKPLQKADYTIHIEVPPNPFWASIGLSVTPLPIGSGIQYESKVSLGYLNQSFQNAVMDG 480

Query 481
IRYGLEQGLFGWNVTDCKICFEYGLYYSPVSTPADFRSLAPIVLEQALKESGTQLLEPYL 540
            I YGLEQGL+GW VTDCKICFEYG+YYSPVSTPADFR LAPIVLEQ LK+
+GTQLLEPYL
Sbjct 481
IDYGLEQGLYGWKVTDCKICFEYGVYYSPVSTPADFRFLAPIVLEQVLKKTGTQLLEPYL 540

Query 541
SFILYAPQEYLSRAYHDAPKYCATIETAQVKKDEVVFTGEIPARCIQAYRTDLAFYTNGR 600
            SF L+ PQEYLSRAY+DAP+YCA IET+Q K DEV+FTG IP RCI AYR L
YTNG+
Sbjct 541
SFTLFTPQEYLSRAYNDAPQYCAIIETSQPKNDEVIFTGHIPVRCISAYRNALTLYTNGQ 600

Query 601 SVCLTELKGYQAAVGQPVIQPRRPNSRLDKVRHMFQK 637
            +VCLTELKGYQAA +PVIQ RRPNSR+DKVRHMF K
Sbjct 601 AVCLTELKGYQAAACEPVIQSRRPNSRIDKVRHMFNK 637

>AP027459.1 EF-G: translation elongation factor [uncultured
bacterium]
Length=476

 Score = 964 bits (2492), Expect = 0.0, Method: Compositional
matrix adjust.
 Identities = 465/476 (98%), Positives = 473/476 (99%), Gaps = 0/476
(0%)

Query 164
IVLEENTDIEAWDAVIENNDKLLEKYIAGEPISREKLVREEQRRVQDASLFPVYYGSAKK 223

+VLEENTDIEAWDAVIENNDKLLEKYIAGEPISREKLVREEQRRVQDASLFPVYYGSAKK
Sbjct 1
MVLEENTDIEAWDAVIENNDKLLEKYIAGEPISREKLVREEQRRVQDASLFPVYYGSAKK 60

Query 224
GLGIQPLMDAVTGLFQPIGEQGSAALCGSVFKVEYTDCGQRRVYLRLYSGTLRLRDTVAL 283

GLGIQPLMDAVTGLFQPIGEQGSAALCGSVFKVEYTDCGQRRVYLRLYSGTLRLRDTVAL
Sbjct 61
GLGIQPLMDAVTGLFQPIGEQGSAALCGSVFKVEYTDCGQRRVYLRLYSGTLRLRDTVAL 120

Query 284
AGREKLKITEMRIPSKGEIVRTDTAYPGEIVILPSDSVRLNDVLGDPTRLPRKRWREDPL 343

AGREKLKITEMRIPSKGEIVRTDTAYPGEIVILPSDSVRLNDVLGDPTRLPRKRWREDPL
Sbjct 121
AGREKLKITEMRIPSKGEIVRTDTAYPGEIVILPSDSVRLNDVLGDPTRLPRKRWREDPL 180

Query 344
PMLRTTIAPKTAAQRERLLDALTQLADTDPLLRCEVDSITHEIILSFLGRVQLEVVSALL 403
            PMLRT
+IAPKTAAQRERLLDALTQLADTDPLLRCEVDSITHEIILSFLGRVQLEVVSALL
Sbjct 181

PMLRTSIAPKTAAQRERLLDALTQLADTDPLLRCEVDSITHEIILSFLGRVQLEVVSALL 240

Query 404
SEKYKLETVVKEPSVIYMERPLKAASHTIHIEVPPNPFWASIGLSVTPLSLGSGVQYESR 463
            SEKYKLETVVKEP+VIYMERPLKAASHTIHIEVPPNPFWASIGLSVTPL
LGSGVQYESR
Sbjct 241
SEKYKLETVVKEPTVIYMERPLKAASHTIHIEVPPNPFWASIGLSVTPLPLGSGVQYESR 300

Query 464
VSLGYLNQSFQNAVRDGIRYGLEQGLFGWNVTDCKICFEYGLYYSPVSTPADFRSLAPIV 523

VSLGYLNQSFQNAVRDGIRYGLEQGLFGWNVTDCKICFEYGLYYSPVSTPADFRSLAPIV
Sbjct 301
VSLGYLNQSFQNAVRDGIRYGLEQGLFGWNVTDCKICFEYGLYYSPVSTPADFRSLAPIV 360

Query 524
LEQALKESGTQLLEPYLSFILYAPQEYLSRAYHDAPKYCATIETAQVKKDEVVFTGEIPA 583
            LEQALKESGTQLLEPYLSF LYAP+EYLSRAYHDAPKYCATIET
QVKKDEVVFTGEIPA
Sbjct 361
LEQALKESGTQLLEPYLSFTLYAPREYLSRAYHDAPKYCATIETVQVKKDEVVFTGEIPA 420

Query 584 RCIQAYRTDLAFYTNGRSVCLTELKGYQAAVGQPVIQPRRPNSRLDKVRHMFQKVM
639
            RCIQAYRTDLAFYTNG+SVCLTELKGYQAAVG+PVIQPRRPNSRLDKVR+MFQK+M
Sbjct 421 RCIQAYRTDLAFYTNGQSVCLTELKGYQAAVGKPVIQPRRPNSRLDKVRYMFQKIM
476

>WP_059430671.1 TetM/TetW/TetO/TetS family tetracycline resistance
ribosomal
protection protein [Campylobacter fetus]
 CUU82673.1 tetracycline resistance protein [Campylobacter fetus
subsp. fetus]
Length=646

 Score = 963 bits (2490), Expect = 0.0, Method: Compositional
matrix adjust.
 Identities = 451/637 (71%), Positives = 538/637 (84%), Gaps = 0/637
(0%)

Query 1
MKIINIGILAHVDAGKTTLTESLLYASGAISEPGSVEKGTTRTDTMFLERQRGITIQAAV 60
            MKIINIGILAHVDAGKTTLTESLLY SGAI E GSV
+KGTTRTDTMFLERQRGITIQAAV
Sbjct 1
MKIINIGILAHVDAGKTTLTESLLYTSGAILELGSVDKGTTRTDTMFLERQRGITIQAAV 60

Query 61
TSFQWHRCKVNIVDTPGHMDFLAEVYRSLAVLDGAILVISAKDGVQAQTRILFHALRKMN 120
            TSF W+ K+NIVDTPGHMDFL EVYRSL+VLDGA+LVISAKDGVQAQTRILFHAL
+KMN
Sbjct 61

TSFNWNGYKINIVDTPGHMDFLTEVYRSLSVLDGAVLVISAKDGVQAQTRILFHALQKMN 120

Query 121
IPTVIFINKIDQAGVDLQSVVQSVRDKLSADIIIKQTVSLSPEIVLEENTDIEAWDAVIE 180
            IPT+IFINKIDQ G++L + Q++++KLS DIII Q V+L+PE+ LE TD+E
WDAVI
Sbjct 121
IPTIIFINKIDQYGINLHNTYQNIKEKLSTDIIIMQNVTLTPEVSLENITDLEKWDAVIA 180

Query 181
NNDKLLEKYIAGEPISREKLVREEQRRVQDASLFPVYYGSAKKGLGIQPLMDAVTGLFQP 240
             ND+LLEKYI GE ++ ++L +EE +VQ SLFP+Y+GSAK +G Q L++ ++
+F
Sbjct 181
GNDRLLEKYITGETLTVQELKQEEYEQVQKGSLFPIYHGSAKNNIGTQQLIEVISDIFCS 240

Query 241
IGEQGSAALCGSVFKVEYTDCGQRRVYLRLYSGTLRLRDTVALAGREKLKITEMRIPSKG 300
              ++ + LCGSVFK+EYTD QR YLRLYSGTL LRDT+ L+ ++K+K+TEM
IPS G
Sbjct 241
EMDKSQSELCGSVFKIEYTDRKQRLAYLRLYSGTLHLRDTIILSEKKKMKLTEMYIPSNG 300

Query 301
EIVRTDTAYPGEIVILPSDSVRLNDVLGDPTRLPRKRWREDPLPMLRTTIAPKTAAQRER 360
            E+++T+ A G+IVILP+D+++LND++G+   LP   W ++P+PMLRT I P
QRE
Sbjct 301
EMIQTEIACSGDIVILPNDTLKLNDIVGNEKMLPCNAWNDNPVPMLRTMIEPIKPEQREY 360

Query 361
LLDALTQLADTDPLLRCEVDSITHEIILSFLGRVQLEVVSALLSEKYKLETVVKEPSVIY 420
            LLDALT++ADTDPLLR VD+ITHEII+SFLG VQLEV+ +LL+EKY ++ VKEP
+VIY
Sbjct 361
LLDALTEIADTDPLLRYYVDTITHEIIISFLGTVQLEVICSLLTEKYHIDINVKEPTVIY 420

Query 421
MERPLKAASHTIHIEVPPNPFWASIGLSVTPLSLGSGVQYESRVSLGYLNQSFQNAVRDG 480
            +E+PL+ A +TIHIEVPPNPFWASIGLSVTPL +GSG+QYES
+VSLGYLNQSFQNAV DG
Sbjct 421
LEKPLQKADYTIHIEVPPNPFWASIGLSVTPLPIGSGIQYESKVSLGYLNQSFQNAVMDG 480

Query 481
IRYGLEQGLFGWNVTDCKICFEYGLYYSPVSTPADFRSLAPIVLEQALKESGTQLLEPYL 540
            I YGLEQGL+GW VTDCKICFEYG+YYSPVSTPADFR LAPIVLEQ LK+
+GTQLLEPYL
Sbjct 481
IDYGLEQGLYGWKVTDCKICFEYGVYYSPVSTPADFRFLAPIVLEQVLKKTGTQLLEPYL 540

Query 541
SFILYAPQEYLSRAYHDAPKYCATIETAQVKKDEVVFTGEIPARCIQAYRTDLAFYTNGR 600
            SF L PQEYLSRAY+DAP+YCA IET+Q K DEV+FTG IP RCI AYR L

YTNG+
Sbjct 541
SFTLSTPQEYLSRAYNDAPQYCAIIETSQPKNDEVIFTGHIPVRCISAYRNALTLYTNGQ 600

Query 601 SVCLTELKGYQAAVGQPVIQPRRPNSRLDKVRHMFQK 637
            +VCLTELKGYQAA +PVIQ RRPNSR+DKVRHMF K
Sbjct 601 AVCLTELKGYQAAACEPVIQSRRPNSRIDKVRHMFNK 637

>APO28175.1 ferrous iron transport protein B, partial [uncultured bacterium]
Length=633

 Score = 959 bits (2480), Expect = 0.0, Method: Compositional matrix adjust.
 Identities = 449/632 (71%), Positives = 535/632 (85%), Gaps = 0/632 (0%)

Query 7
GILAHVDAGKTTLTESLLYASGAISEPGSVEKGTTRTDTMFLERQRGITIQAAVTSFQWH 66
            GILAHVDAGKTTLTESLLY SGA+ E GSV+KGTTRTDTM LERQRGITIQ
AVTSF W
Sbjct 1
GILAHVDAGKTTLTESLLYTSGAVPELGSVDKGTTRTDTMLLERQRGITIQTAVTSFGWK 60

Query 67
RCKVNIVDTPGHMDFLAEVYRSLAVLDGAILVISAKDGVQAQTRILFHALRKMNIPTVIF 126
              K+NIVDTPGHMDFLAEVYRSLAVLDGAILV+SAKDGVQAQTR+LFHAL+KM
IPT+IF
Sbjct 61
NYKINIVDTPGHMDFLAEVYRSLAVLDGAILVVSAKDGVQAQTRVLFHALQKMKIPTIIF 120

Query 127
INKIDQAGVDLQSVVQSVRDKLSADIIIKQTVSLSPEIVLEENTDIEAWDAVIENNDKLL 186
            +NKIDQ G+DLQSV Q++R+KLS D+++ Q VSL+PE+ L + DIE WD++I
ND+LL
Sbjct 121
VNKIDQEGIDLQSVYQNIREKLSDDVMVMQDVSLTPEVSLTDIEDIEKWDSIIAGNDELL 180

Query 187
EKYIAGEPISREKLVREEQRRVQDASLFPVYYGSAKKGLGIQPLMDAVTGLFQPIGEQGS 246
            EKYIAGEP+ + L RE+ RR+Q+ SLFP+Y+GSAK +G + L++ +   F
+
Sbjct 181
EKYIAGEPLKIQDLQREKCRRMQNGSLFPIYHGSAKNNIGTEKLIEVIAETFTSGADNDQ 240

Query 247
AALCGSVFKVEYTDCGQRRVYLRLYSGTLRLRDTVALAGREKLKITEMRIPSKGEIVRTD 306
            + LCGSVFK+EYTD +R VYLRLYSGTL LRDT+ L   +KLKITEMRIPS GEI
+ D
Sbjct 241
SELCGSVFKIEYTDQKKRLVYLRLYSGTLHLRDTILLPQNQKLKITEMRIPSNGEIIPAD 300

Query 307

```
TAYPGEIVILPSDSVRLNDVLGDPTRLPRKRWREDPLPMLRTTIAPKTAAQRERLLDALT  366
            TA  GEIVIL +D+++LND LG+   LPRK W ++P+P+LRTT+ P+   QR+ LL
+ALT
Sbjct  301
TACCGEIVILTNDTLKLNDTLGNVELLPRKAWEKNPIPLLRTTVEPQNQEQRDLLLNALT  360

Query  367
QLADTDPLLRCEVDSITHEIILSFLGRVQLEVVSALLSEKYKLETVVKEPSVIYMERPLK  426
            ++ADTDPLL   VD+ITHEII+SFLG+VQLEVV +LL E+Y +   VKEP+VIY
+ERPLK
Sbjct  361
EIADTDPLLHYYVDTITHEIIISFLGKVQLEVVCSLLVERYHVNINVKEPTVIYLERPLK  420

Query  427
AASHTIHIEVPPNPFWASIGLSVTPLSLGSGVQYESRVSLGYLNQSFQNAVRDGIRYGLE  486
             AS+TIHIEVPPNPFWASIGL+VTPL  GSG +++S+VSLGYLNQSFQNAV +G
+RYG+E
Sbjct  421
TASYTIHIEVPPNPFWASIGLTVTPLPAGSGTRFKSKVSLGYLNQSFQNAVMEGVRYGME  480

Query  487
QGLFGWNVTDCKICFEYGLYYSPVSTPADFRSLAPIVLEQALKESGTQLLEPYLSFILYA  546
            QGL+GW VTDC+ICF+YG+YYSPVSTPADFRSLAPIVLEQALK +GTQLLEPYLSF
L+A
Sbjct  481
QGLYGWEVTDCEICFDYGVYYSPVSTPADFRSLAPIVLEQALKRAGTQLLEPYLSFTLFA  540

Query  547
PQEYLSRAYHDAPKYCATIETAQVKKDEVVFTGEIPARCIQAYRTDLAFYTNGRSVCLTE  606
            PQEY+SRAY+DAPKYCA IE+  +K DEV+FTGEIPARCI  YR DL
FYTNGRSVCLTE
Sbjct  541
PQEYISRAYNDAPKYCAVIESTLLKNDEVIFTGEIPARCIGEYRNDLNFYTNGRSVCLTE  600

Query  607  LKGYQAAVGQPVIQPRRPNSRLDKVRHMFQKV  638
            LKGYQ   G+PV+QPRRPNSRLDKVRHMFQK+
Sbjct  601  LKGYQEISGEPVLQPRRPNSRLDKVRHMFQKI  632


>SCJ12353.1 Tetracycline resistance protein tetM from transposon
Tn916 [uncultured
Blautia sp.]
 SCJ19923.1 Tetracycline resistance protein tetM from transposon
Tn916 [uncultured
Clostridium sp.]
Length=639

 Score = 945 bits (2442),  Expect = 0.0, Method: Compositional
matrix adjust.
 Identities = 449/639 (70%), Positives = 533/639 (83%), Gaps = 0/639
(0%)

Query  1
MKIINIGILAHVDAGKTTLTESLLYASGAISEPGSVEKGTTRTDTMFLERQRGITIQAAV  60
```

            MKIINIGILAHVDAGKTTLTESLLY SGAI EPGSV+KGTTRTD+M LERQR
ITIQAAV
Sbjct 1
MKIINIGILAHVDAGKTTLTESLLYTSGAIMEPGSVDKGTTRTDSMALERQRKITIQAAV 60

Query 61
TSFQWHRCKVNIVDTPGHMDFLAEVYRSLAVLDGAILVISAKDGVQAQTRILFHALRKMN 120
            TSF+W   KVN+VDTPGHMDFL EV+RSL+VLDGAILV+SAKDGVQAQTR
+LFHALRKM
Sbjct 61
TSFRWKGYKVNLVDTPGHMDFLTEVHRSLSVLDGAILVVSAKDGVQAQTRVLFHALRKMK 120

Query 121
IPTVIFINKIDQAGVDLQSVVQSVRDKLSADIIIKQTVSLSPEIVLEENTDIEAWDAVIE 180
            IPT+IFINKIDQ+GV L+   Q++R+KL+ D+I+ Q V L EI+L + D E
WDAVI
Sbjct 121
IPTIIFINKIDQSGVVLEQTYQNIREKLTEDMIVMQEVHLCSEIILSDVADFEKWDAVIA 180

Query 181
NNDKLLEKYIAGEPISREKLVREEQRRVQDASLFPVYYGSAKKGLGIQPLMDAVTGLFQP 240
             ND +LEKY++G P++ +L +E +RRVQ +LFPVY+GSAK +G++ L++ +T
+F
Sbjct 181
GNDDILEKYLSGVPLTLWELQKEIKRRVQRGTLFPVYHGSAKDNIGVKELLEVITEVFSL 240

Query 241
IGEQGSAALCGSVFKVEYTDCGQRRVYLRLYSGTLRLRDTVALAGREKLKITEMRIPSKG 300
              +   + LCG VFK+EYT+ +RR YLRLYSGTL LR+T+ L+ +EK+KITEM
IP G
Sbjct 241
ETDDSQSELCGYVFKIEYTEQKKRRCYLRLYSGTLCLRETILLSKKEKIKITEMSIPFDG 300

Query 301
EIVRTDTAYPGEIVILPSDSVRLNDVLGDPTRLPRKRWREDPLPMLRTTIAPKTAAQRER 360
            EIV TDTA GEIVIL ++++LNDVLGD   LPRK W + PLP RTTI P A
QRE
Sbjct 301
EIVPTDTADSGEIVILSDNTLKLNDVLGDEKLLPRKAWTDSPLPFFRTTIEPIKAEQREV 360

Query 361
LLDALTQLADTDPLLRCEVDSITHEIILSFLGRVQLEVVSALLSEKYKLETVVKEPSVIY 420
            LLDALT++ADTDPLL   +DSITHEIILSFLGRVQLEVV +LL+EKY +E +KEP
+VIY
Sbjct 361
LLDALTEIADTDPLLHFMIDSITHEIILSFLGRVQLEVVCSLLNEKYGVEVAIKEPTVIY 420

Query 421
MERPLKAASHTIHIEVPPNPFWASIGLSVTPLSLGSGVQYESRVSLGYLNQSFQNAVRDG 480
            +ERP K A +TIHIEVPPNPFWASIGL+VTPL +GSG++YES
+VSLGYLNQSFQNAV +G
Sbjct 421
LERPRKEAHYTIHIEVPPNPFWASIGLAVTPLPVGSGIEYESKVSLGYLNQSFQNAVMEG 480

Query 481
IRYGLEQGLFGWNVTDCKICFEYGLYYSPVSTPADFRSLAPIVLEQALKESGTQLLEPYL 540
            IRYGLEQG++GW VTDC+ICFEYG+YYSPVSTPADFR LAP+VLEQ LK++GTQ
+LEPYL
Sbjct 481
IRYGLEQGVYGWEVTDCRICFEYGVYYSPVSTPADFRFLAPVVLEQVLKKAGTQVLEPYL 540

Query 541
SFILYAPQEYLSRAYHDAPKYCATIETAQVKKDEVVFTGEIPARCIQAYRTDLAFYTNGR 600
            SF L+APQEYLSRAYHDA KY A IET +K +E + TGEIPAR I Y++DL
FYTNGR
Sbjct 541
SFTLFAPQEYLSRAYHDAVKYDAVIETTSLKNNEAILTGEIPARRIGEYKSDLNFYTNGR 600

Query 601 SVCLTELKGYQAAVGQPVIQPRRPNSRLDKVRHMFQKVM 639
            SVCLTELKGYQ   G+PV+QPRRPNSRLDKVRHMFQK+M
Sbjct 601 SVCLTELKGYQETSGEPVVQPRRPNSRLDKVRHMFQKIM 639

>AMP50067.1 TetM-TetW-TetO-TetS [uncultured bacterium]
Length=639

 Score = 942 bits (2436), Expect = 0.0, Method: Compositional
matrix adjust.
 Identities = 439/638 (69%), Positives = 534/638 (84%), Gaps = 0/638
(0%)

Query 1
MKIINIGILAHVDAGKTTLTESLLYASGAISEPGSVEKGTTRTDTMFLERQRGITIQAAV 60
            MKIIN+GILAHVDAGKTTLTESLLY SGAI+EPGSV+KGTTRTDTM
LERQRGITIQ AV
Sbjct 1
MKIINLGILAHVDAGKTTLTESLLYTSGAIAEPGSVDKGTTRTDTMNLERQRGITIQTAV 60

Query 61
TSFQWHRCKVNIVDTPGHMDFLAEVYRSLAVLDGAILVISAKDGVQAQTRILFHALRKMN 120
            TSFQW   KVNI+DTPGHMDFLAEVYRSL+VLDGA+L++SAKDG+QAQTRILFHAL
+ M
Sbjct 61
TSFQWEDVKVNIIDTPGHMDFLAEVYRSLSVLDGAVLLVSAKDGIQAQTRILFHALQTMK 120

Query 121
IPTVIFINKIDQAGVDLQSVVQSVRDKLSADIIIKQTVSLSPEIVLEENTDIEAWDAVIE 180
            IPT+ FINKIDQ G+DL V Q ++ KLS++II+KQ V   P I + +N D+E
WDAVI
Sbjct 121
IPTIFFINKIDQEGIDLPMVYQEMKAKLSSEIIVKQKVGQHPHINVTDNDDMEQWDAVIM 180

Query 181
NNDKLLEKYIAGEPISREKLVREEQRRVQDASLFPVYYGSAKKGLGIQPLMDAVTGLFQP 240
             ND+LLEKY++G+P   +L +EE RR Q+ +LFPVY+GSAK LGI+ L++ +
F
Sbjct 181
GNDELLEKYMSGKPFKMSELEQEENRRFQNGTLFPVYHGSAKNNLGIRQLIEVIASKFYS 240

```
Query  241
IGEQGSAALCGSVFKVEYTDCGQRRVYLRLYSGTLRLRDTVALAGREKLKITEMRIPSKG  300
               +G + LCG VFK+EY++  +R VY+R+YSGTL LRD + ++ +EK+KITEM
+P+ G
Sbjct  241
STPEGQSELCGQVFKIEYSEKRRRFVYVRIYSGTLHLRDVIKISEKEKIKITEMCVPTNG  300

Query  301
EIVRTDTAYPGEIVILPSDSVRLNDVLGDPTRLPRKRWREDPLPMLRTTIAPKTAAQRER  360
            E+  +DTA  G+IVILP+D ++LN +LG+   LP++++ E+PLPML+TTIA K +
QRE
Sbjct  301
ELCSSDTACSGDIVILPNDVLQLNSILGNEILLPQRKFIENPLPMLQTTIAVKKSEQREI  360

Query  361
LLDALTQLADTDPLLRCEVDSITHEIILSFLGRVQLEVVSALLSEKYKLETVVKEPSVIY  420
            LL ALT+++D DPLL+  VD+ THEIILSFLG+VQ+EV+SALL EKY +E  +KEP
+VIY
Sbjct  361
LLGALTEISDGDPLLKYYVDTTTHEIILSFLGKVQMEVISALLQEKYHVEIEIKEPTVIY  420

Query  421
MERPLKAASHTIHIEVPPNPFWASIGLSVTPLSLGSGVQYESRVSLGYLNQSFQNAVRDG  480
            MERPLK A +TIHIEVPPNPFWASIGLSV+PL LGSG+QYES
VSLGYLNQSFQNAV +G
Sbjct  421
MERPLKNAEYTIHIEVPPNPFWASIGLSVSPLPLGSGMQYESSVSLGYLNQSFQNAVMEG  480

Query  481
IRYGLEQGLFGWNVTDCKICFEYGLYYSPVSTPADFRSLAPIVLEQALKESGTQLLEPYL  540
            IRYG EQGL+GWNVTDCKICF+YGLYYSPVSTPADFR LAPIVLEQ LK++GT
+LLEPYL
Sbjct  481
IRYGCEQGLYGWNVTDCKICFKYGLYYSPVSTPADFRMLAPIVLEQVLKKAGTELLEPYL  540

Query  541
SFILYAPQEYLSRAYHDAPKYCATIETAQVKKDEVVFTGEIPARCIQAYRTDLAFYTNGR  600
            SF +YAPQEYLSRAY+DAPKYCA I   Q+K +EV+ +GEIPARCIQ YR+DL F
+TNGR
Sbjct  541
SFKIYAPQEYLSRAYNDAPKYCANIVDTQLKNNEVILSGEIPARCIQEYRSDLTFFTNGR  600

Query  601  SVCLTELKGYQAAVGQPVIQPRRPNSRLDKVRHMFQKV  638
            SVCLTELKGY    G+PV QPRRPNSR+DKVR+MF K+
Sbjct  601  SVCLTELKGYHVTTGEPVCQPRRPNSRIDKVRYMFHKL  638


>WP_074390203.1 TetM/TetW/TetO/TetS family tetracycline resistance
ribosomal
protection protein [Streptococcus suis]
Length=639

 Score = 941 bits (2431),  Expect = 0.0, Method: Compositional
```

matrix adjust.
 Identities = 434/638 (68%), Positives = 529/638 (83%), Gaps = 0/638 (0%)

```
Query  1
MKIINIGILAHVDAGKTTLTESLLYASGAISEPGSVEKGTTRTDTMFLERQRGITIQAAV  60
            MKIIN+GILAHVDAGKTTLTESLLY SGAI+EPGSV+KGTTRTDTM
LERQRGITIQ AV
Sbjct  1
MKIINLGILAHVDAGKTTLTESLLYTSGAIAEPGSVDKGTTRTDTMNLERQRGITIQTAV  60

Query  61
TSFQWHRCKVNIVDTPGHMDFLAEVYRSLAVLDGAILVISAKDGVQAQTRILFHALRKMN  120
            TSFQW   KVNI+DTPGHMDFLAEVYRSL+VLDGA+L++SAKDG+QAQTRILFHAL
+ M
Sbjct  61
TSFQWEDVKVNIIDTPGHMDFLAEVYRSLSVLDGAVLLVSAKDGIQAQTRILFHALQSMK  120

Query  121
IPTVIFINKIDQAGVDLQSVVQSVRDKLSADIIIKQTVSLSPEIVLEENTDIEAWDAVIE  180
            IPT+ FINKIDQ G+DL  V Q ++ KLS++II+KQ V   P I + +N D+E
WDAVI
Sbjct  121
IPTIFFINKIDQEGIDLPMVYQEMKAKLSSEIIVKQKVGQHPHINVTDNDDMEQWDAVIM  180

Query  181
NNDKLLEKYIAGEPISREKLVREEQRRVQDASLFPVYYGSAKKGLGIQPLMDAVTGLFQP  240
             ND+LLEKY++G+P    +L +EE RR Q+ +LFPVY+GSAK  LGI+ L++ +
F
Sbjct  181
GNDELLEKYMSGKPFKMSELEQEENRRFQNGTLFPVYHGSAKNNLGIRQLIEVIASKFYS  240

Query  241
IGEQGSAALCGSVFKVEYTDCGQRRVYLRLYSGTLRLRDTVALAGREKLKITEMRIPSKG  300
               +G + LCG VFK+EY++  +R VY+R+YSGTL LRD + ++ +EK+KITEM
+PS G
Sbjct  241
STPEGQSELCGQVFKIEYSEKRRRFVYVRIYSGTLHLRDVIRISEKEKIKITEMCVPSNG  300

Query  301
EIVRTDTAYPGEIVILPSDSVRLNDVLGDPTRLPRKRWREDPLPMLRTTIAPKTAAQRER  360
            EIV  D A PGEIVIL  D+++LND+LG+   LP K W ++P+P+LRTT+ P+
QRE
Sbjct  301
EIVPADHACPGEIVILADDTLKLNDILGNEKLLPHKTWIDNPMPLLRTTVEPQKPEQREA  360

Query  361
LLDALTQLADTDPLLRCEVDSITHEIILSFLGRVQLEVVSALLSEKYKLETVVKEPSVIY  420
            LL+AL ++ADTDPLL  ++D++THEIILSFLG VQ+EV+ A+L EKY +E  +KEP
+VIY
Sbjct  361
LLNALAEIADTDPLLHFDIDTVTHEIILSFLGNVQMEVICAILEEKYHVEAEIKEPTVIY  420

Query  421
```

MERPLKAASHTIHIEVPPNPFWASIGLSVTPLSLGSGVQYESRVSLGYLNQSFQNAVRDG 480
            MERPL+ A +TIHIEVPPNPFWAS+GLS+ PL
+GSGVQYESRVSLGYLNQSFQNAV +G
Sbjct 421
MERPLRKAEYTIHIEVPPNPFWASVGLSIEPLPIGSGVQYESRVSLGYLNQSFQNAVMEG 480

Query 481
IRYGLEQGLFGWNVTDCKICFEYGLYYSPVSTPADFRSLAPIVLEQALKESGTQLLEPYL 540
            + YG EQGL+GW VTDCKICFEYGLYYSPVSTPADFR L+PIVLEQALK++GT
+LLEPYL
Sbjct 481
VLYGCEQGLYGWKVTDCKICFEYGLYYSPVSTPADFRLLSPIVLEQALKKAGTELLEPYL 540

Query 541
SFILYAPQEYLSRAYHDAPKYCATIETAQVKKDEVVFTGEIPARCIQAYRTDLAFYTNGR 600
             F +YAPQEYLSRAYHDAP+YCA I + QVK DEV+ GEIPARCIQ YR DL +
+TNG+
Sbjct 541
HFEIYAPQEYLSRAYHDAPRYCADIVSTQVKNDEVILKGEIPARCIQEYRNDLTYFTNGQ 600

Query 601 SVCLTELKGYQAAVGQPVIQPRRPNSRLDKVRHMFQKV 638
             VCLTELKGYQ A+G+ + QPRRPNSR+DKVRHMF K+
Sbjct 601 GVCLTELKGYQPAIGKFICQPRRPNSRIDKVRHMFHKL 638

>WP_055195147.1 TetM/TetW/TetO/TetS family tetracycline resistance ribosomal
protection protein [Dorea longicatena]
 CUO42590.1 Tetracycline resistance protein tetM from transposon Tn916 [Dorea
longicatena]
Length=639

 Score = 941 bits (2431), Expect = 0.0, Method: Compositional matrix adjust.
 Identities = 433/638 (68%), Positives = 529/638 (83%), Gaps = 0/638 (0%)

Query 1
MKIINIGILAHVDAGKTTLTESLLYASGAISEPGSVEKGTTRTDTMFLERQRGITIQAAV 60
            MKIIN+GILAHVDAGKTTLTESLLY SGAI+EPGSV+KGTTRTDTM
LERQRGITIQ AV
Sbjct 1
MKIINLGILAHVDAGKTTLTESLLYTSGAIAEPGSVDKGTTRTDTMNLERQRGITIQTAV 60

Query 61
TSFQWHRCKVNIVDTPGHMDFLAEVYRSLAVLDGAILVISAKDGVQAQTRILFHALRKMN 120
            TSFQW   KVNI+DTPGHMDFLAEVYRSL+VLDGA+L++SAKDG+QAQTRILFHAL
+ M
Sbjct 61
TSFQWEDVKVNIIDTPGHMDFLAEVYRSLSVLDGAVLLVSAKDGIQAQTRILFHALQTMK 120

Query 121
IPTVIFINKIDQAGVDLQSVVQSVRDKLSADIIIKQTVSLSPEIVLEENTDIEAWDAVIE 180

IPT+ FINKIDQ G+DL V Q ++ KLS++II+KQ V   P I + +N D+E
WDAVI
Sbjct 121
IPTIFFINKIDQEGIDLPMVYQEMKAKLSSEIIVKQKVGQHPHINVTDNDDMEQWDAVIM 180

Query 181
NNDKLLEKYIAGEPISREKLVREEQRRVQDASLFPVYYGSAKKGLGIQPLMDAVTGLFQP 240
ND+LLEKY++G+P   +L +EE RR Q+ +LFPVY+GSAK LGI+ L++ +
F
Sbjct 181
GNDELLEKYMSGKPFKMSELEQEENRRFQNGTLFPVYHGSAKNNLGIRQLIEVIASKFYS 240

Query 241
IGEQGSAALCGSVFKVEYTDCGQRRVYLRLYSGTLRLRDTVALAGREKLKITEMRIPSKG 300
+G + LCG VFK+EY++ +R VY+R+YSGTL LRD + ++ +EK+KITEM
+PS G
Sbjct 241
STPEGQSELCGQVFKIEYSEKRRRFVYVRIYSGTLHLRDVIKISEKEKIKITEMCVPSNG 300

Query 301
EIVRTDTAYPGEIVILPSDSVRLNDVLGDPTRLPRKRWREDPLPMLRTTIAPKTAAQRER 360
EIV D A PGEIVIL D+++LND+LG+   LP K W ++P+P+LRTT+ P+
QRE
Sbjct 301
EIVPADHACPGEIVILADDTLKLNDILGNEKLLPHKTWIDNPMPLLRTTVEPQKPEQREA 360

Query 361
LLDALTQLADTDPLLRCEVDSITHEIILSFLGRVQLEVVSALLSEKYKLETVVKEPSVIY 420
LL+AL ++ADTDPLL ++D++THEIILSFLG VQ+EV+ A+L EKY +E +KEP
+VIY
Sbjct 361
LLNALAEIADTDPLLHFDIDTVTHEIILSFLGNVQMEVICAILEEKYHVEAEIKEPTVIY 420

Query 421
MERPLKAASHTIHIEVPPNPFWASIGLSVTPLSLGSGVQYESRVSLGYLNQSFQNAVRDG 480
MERPL+ A +TIHIEVPPNPFWAS+GLS+ PL
+GSGVQYESRVSLGYLNQSFQNAV +G
Sbjct 421
MERPLRKAEYTIHIEVPPNPFWASVGLSIEPLPIGSGVQYESRVSLGYLNQSFQNAVMEG 480

Query 481
IRYGLEQGLFGWNVTDCKICFEYGLYYSPVSTPADFRSLAPIVLEQALKESGTQLLEPYL 540
+ YG EQGL+GW VTDCKICFEYGLYYSPVSTPADFR L+PIVLEQALK++GT
+LLEPYL
Sbjct 481
VLYGCEQGLYGWKVTDCKICFEYGLYYSPVSTPADFRLLSPIVLEQALKKAGTELLEPYL 540

Query 541
SFILYAPQEYLSRAYHDAPKYCATIETAQVKKDEVVFTGEIPARCIQAYRTDLAFYTNGR 600
F +YAPQEYLSRAYHDAP+YCA I + Q+K DEV+ GEIPARCIQ YR DL +
+TNG+
Sbjct 541
HFEIYAPQEYLSRAYHDAPRYCADIVSTQIKNDEVILKGEIPARCIQEYRNDLTYFTNGQ 600

```
Query  601  SVCLTELKGYQAAVGQPVIQPRRPNSRLDKVRHMFQKV  638
             VCLTELKGYQ A+G+ + QPRRPNSR+DKVRHMF K+
Sbjct  601  GVCLTELKGYQPAIGKFICQPRRPNSRIDKVRHMFHKL  638
```

>WP_107435213.1 TetM/TetW/TetO/TetS family tetracycline resistance ribosomal
protection protein [[Clostridium] lavalense]
 PST32733.1 tetracycline resistance ribosomal protection protein [[Clostridium]
lavalense]
Length=639

 Score = 940 bits (2430), Expect = 0.0, Method: Compositional matrix adjust.
 Identities = 447/639 (70%), Positives = 532/639 (83%), Gaps = 0/639 (0%)

```
Query  1
MKIINIGILAHVDAGKTTLTESLLYASGAISEPGSVEKGTTRTDTMFLERQRGITIQAAV  60
            MKIINIGILAHVDAGKTTLTESLLY SGAI EPGSV+KGTTRTD+M LERQR
ITIQAAV
Sbjct  1
MKIINIGILAHVDAGKTTLTESLLYTSGAIMEPGSVDKGTTRTDSMALERQRKITIQAAV  60

Query  61
TSFQWHRCKVNIVDTPGHMDFLAEVYRSLAVLDGAILVISAKDGVQAQTRILFHALRKMN  120
            TSF+W   KVN+VDTPGHMDFL EV+RSL+VLDGAILV+SAKDGVQAQTR
+LFHALRKM
Sbjct  61
TSFRWKGYKVNLVDTPGHMDFLTEVHRSLSVLDGAILVVSAKDGVQAQTRVLFHALRKMK  120

Query  121
IPTVIFINKIDQAGVDLQSVVQSVRDKLSADIIIKQTVSLSPEIVLEENTDIEAWDAVIE  180
            IPT+IFINKIDQ+GV L+   Q++R+KL+ D+I+ Q V L  EI+L +  D E
WDAVI
Sbjct  121
IPTIIFINKIDQSGVVLEQTYQNIREKLTEDMIVMQEVHLCSEIILSDVADFEKWDAVIA  180

Query  181
NNDKLLEKYIAGEPISREKLVREEQRRVQDASLFPVYYGSAKKGLGIQPLMDAVTGLFQP  240
             ND +LEKY++G P++  +L +E +RRVQ  +LFPVY+GSAK  +G++ L++ +T
+F
Sbjct  181
GNDDILEKYLSGVPLTLWELQKEIKRRVQRGTLFPVYHGSAKDNIGVKELLEVITEVFSL  240

Query  241
IGEQGSAALCGSVFKVEYTDCGQRRVYLRLYSGTLRLRDTVALAGREKLKITEMRIPSKG  300
              +   + LCG VFK+EYT+  +RR YLRLYSGTL LR+T+ L+ +EK+KITEM
IP  G
Sbjct  241
ETDDSQSELCGYVFKIEYTEQKKRRCYLRLYSGTLCLRETILLSKKEKIKITEMSIPFDG  300

Query  301
```

EIVRTDTAYPGEIVILPSDSVRLNDVLGDPTRLPRKRWREDPLPMLRTTIAPKTAAQRER 360
            EIV TDTA GEIVIL ++++LNDVLGD   LPRK W ++PLP RTTI P A QR
Sbjct 301 EIVPTDTADSGEIVILSDNTLKLNDVLGDEKLLPRKAWTDNPLPFFRTTIEPIKAEQRGV 360

Query 361 LLDALTQLADTDPLLRCEVDSITHEIILSFLGRVQLEVVSALLSEKYKLETVVKEPSVIY 420
            LLDALT++ADTDPLL   +DSITHEIILSFLG+VQLEVV +LL+EKY +E +KEP +VIY
Sbjct 361 LLDALTEIADTDPLLHFMIDSITHEIILSFLGKVQLEVVCSLLNEKYSVEVAIKEPTVIY 420

Query 421 MERPLKAASHTIHIEVPPNPFWASIGLSVTPLSLGSGVQYESRVSLGYLNQSFQNAVRDG 480
            +ERP K A +TIHIEVPPNPFWASIGL+VTPL +GSG +YES +VSLGYLNQSFQNAV +G
Sbjct 421 LERPRKEAHYTIHIEVPPNPFWASIGLAVTPLPVGSGTEYESKVSLGYLNQSFQNAVMEG 480

Query 481 IRYGLEQGLFGWNVTDCKICFEYGLYYSPVSTPADFRSLAPIVLEQALKESGTQLLEPYL 540
            IRYGLEQG++GW VTDC+ICFEYG+YYSPVSTPADFR LAP+VLEQ LK++GTQ +LEPYL
Sbjct 481 IRYGLEQGVYGWEVTDCRICFEYGVYYSPVSTPADFRFLAPVVLEQVLKKAGTQVLEPYL 540

Query 541 SFILYAPQEYLSRAYHDAPKYCATIETAQVKKDEVVFTGEIPARCIQAYRTDLAFYTNGR 600
            SF L+APQEYLSRAYHDA KY A IET +K +E + TGEIPAR I Y++DL FYTNGR
Sbjct 541 SFTLFAPQEYLSRAYHDAVKYDAVIETTSLKNNEAILTGEIPARRIGEYKSDLNFYTNGR 600

Query 601 SVCLTELKGYQAAVGQPVIQPRRPNSRLDKVRHMFQKVM 639
            SVCLTELKGYQ   G+PV+QPRRPNSRLDKVRHMFQK+M
Sbjct 601 SVCLTELKGYQETSGEPVVQPRRPNSRLDKVRHMFQKIM 639

>APO27580.1 ferrous iron transport protein B [uncultured bacterium]
Length=639

Score = 939 bits (2427), Expect = 0.0, Method: Compositional matrix adjust.
Identities = 445/639 (70%), Positives = 526/639 (82%), Gaps = 0/639 (0%)

Query 1 MKIINIGILAHVDAGKTTLTESLLYASGAISEPGSVEKGTTRTDTMFLERQRGITIQAAV 60
            MKIINIGILAHVDAGKTTLTESLLY SGAI EPGSV+KGTTRTD+M LERQRGITIQAAV
Sbjct 1 MKIINIGILAHVDAGKTTLTESLLYTSGAIMEPGSVDKGTTRTDSMALERQRGITIQAAV 60

Query 61
TSFQWHRCKVNIVDTPGHMDFLAEVYRSLAVLDGAILVISAKDGVQAQTRILFHALRKMN 120
TSF W KVN+VDTPGHMDFLAEVYRSL+VLDGAILV+SAKDGVQAQTR
+LFHALRKM
Sbjct 61
TSFHWKEYKVNLVDTPGHMDFLAEVYRSLSVLDGAILVVSAKDGVQAQTRVLFHALRKMK 120

Query 121
IPTVIFINKIDQAGVDLQSVVQSVRDKLSADIIIKQTVSLSPEIVLEENTDIEAWDAVIE 180
IPT+IFINKIDQ G Q++R+KL+ D+++ Q V L E+ L + D E
WDAVI
Sbjct 121
IPTIIFINKIDQEGFVPAHTYQNIREKLTEDMMVMQEVHLFSELTLSDVADFEKWDAVIA 180

Query 181
NNDKLLEKYIAGEPISREKLVREEQRRVQDASLFPVYYGSAKKGLGIQPLMDAVTGLFQP 240
ND LLEKY++G P++ +L E +RRVQ + FPVY+GSAK+ +G + L+D +T
+F
Sbjct 181
GNDDLLEKYLSGAPLTLRELQEEIKRRVQQGTFFPVYHGSAKENIGTKELLDVITDIFSS 240

Query 241
IGEQGSAALCGSVFKVEYTDCGQRRVYLRLYSGTLRLRDTVALAGREKLKITEMRIPSKG 300
+ + LCG VFK+EYT+ +RR Y+RLYSGTL LR+T+ L REK+KITEM
IP KG
Sbjct 241
KTDNCQSVLCGYVFKIEYTEQKKRRCYVRLYSGTLCLRETIFLQKREKIKITEMSIPFKG 300

Query 301
EIVRTDTAYPGEIVILPSDSVRLNDVLGDPTRLPRKRWREDPLPMLRTTIAPKTAAQRER 360
EIV+ DTA GEIVIL ++++LND+LGD LPRK W ++PLP+LRTTI P A
QRE
Sbjct 301
EIVQKDTADSGEIVILSDNTLKLNDILGDEKLLPRKAWTDNPLPLLRTTIEPAKAEQREV 360

Query 361
LLDALTQLADTDPLLRCEVDSITHEIILSFLGRVQLEVVSALLSEKYKLETVVKEPSVIY 420
LLDALT++ADTDPLLR +D +THEIILSFLG+VQLEVV +LL+EKY +E VKEP
+VIY
Sbjct 361
LLDALTEIADTDPLLRFVIDPVTHEIILSFLGKVQLEVVCSLLNEKYGIEAEVKEPTVIY 420

Query 421
MERPLKAASHTIHIEVPPNPFWASIGLSVTPLSLGSGVQYESRVSLGYLNQSFQNAVRDG 480
+ERP K +TIHIEVPPNPFWASIGL+VTPL GSG +YES
+VSLGYLNQSFQNAV +G
Sbjct 421
LERPRKEEHYTIHIEVPPNPFWASIGLAVTPLPAGSGTEYESKVSLGYLNQSFQNAVMEG 480

Query 481
IRYGLEQGLFGWNVTDCKICFEYGLYYSPVSTPADFRSLAPIVLEQALKESGTQLLEPYL 540
I+YGLEQG++GW VTDC+ICFEYG+YYSPVSTPADFR LAP+VLE ALK++GTQ
+LEPYL
Sbjct 481

IQYGLEQGVYGWEVTDCRICFEYGIYYSPVSTPADFRFLAPVVLELALKKAGTQVLEPYL 540

Query 541
SFILYAPQEYLSRAYHDAPKYCATIETAQVKKDEVVFTGEIPARCIQAYRTDLAFYTNGR 600
            SF L+APQEYLSRAYHDA KY A IET +K +E + TGEIPAR I Y++DL
FYTNGR
Sbjct 541
SFTLFAPQEYLSRAYHDAVKYDAVIETTSIKNNEAILTGEIPARRIGEYKSDLNFYTNGR 600

Query 601 SVCLTELKGYQAAVGQPVIQPRRPNSRLDKVRHMFQKVM 639
            SVCLTELKGYQ   G+P +QPRRPNSRLDKVRHMFQK+M
Sbjct 601 SVCLTELKGYQETSGEPAVQPRRPNSRLDKVRHMFQKIM 639

>WP_053865959.1 TetM/TetW/TetO/TetS family tetracycline resistance
ribosomal
protection protein [Streptococcus suis]
 KPA66079.1 tetracycline resistance protein tetM [Streptococcus
suis]
Length=639

 Score = 939 bits (2426), Expect = 0.0, Method: Compositional
matrix adjust.
 Identities = 431/638 (68%), Positives = 529/638 (83%), Gaps = 0/638
(0%)

Query 1
MKIINIGILAHVDAGKTTLTESLLYASGAISEPGSVEKGTTRTDTMFLERQRGITIQAAV 60
            MKIIN+GILAHVDAGKTTLTESLLY SGAI+EPGSV+KGTTRTDTM
LERQRGITIQ AV
Sbjct 1
MKIINLGILAHVDAGKTTLTESLLYTSGAIAEPGSVDKGTTRTDTMNLERQRGITIQTAV 60

Query 61
TSFQWHRCKVNIVDTPGHMDFLAEVYRSLAVLDGAILVISAKDGVQAQTRILFHALRKMN 120
            TSFQW   KVNI+DTPGHMDFLAEVYRSL+VLDGA+L++SAKDG+QAQTRILFHAL
+ M
Sbjct 61
TSFQWEDVKVNIIDTPGHMDFLAEVYRSLSVLDGAVLLVSAKDGIQAQTRILFHALQTMK 120

Query 121
IPTVIFINKIDQAGVDLQSVVQSVRDKLSADIIIKQTVSLSPEIVLEENTDIEAWDAVIE 180
            IPT+ FINKIDQ G+DL V Q ++ KLS++II+KQ V   P I + +N D+E
WDAVI
Sbjct 121
IPTIFFINKIDQEGIDLPMVYQEMKAKLSSEIIVKQKVGQHPHINVTDNDDMEQWDAVIM 180

Query 181
NNDKLLEKYIAGEPISREKLVREEQRRVQDASLFPVYYGSAKKGLGIQPLMDAVTGLFQP 240
             ND+LLEKY++G+P   +L +EE RR Q+ +LFPVY+GSAK LGI+ L++ +
F
Sbjct 181
GNDELLEKYMSGKPFKMSELEQEENRRFQNGTLFPVYHGSAKNNLGIRQLIEVIASKFYS 240

```
Query  241  
IGEQGSAALCGSVFKVEYTDCGQRRVYLRLYSGTLRLRDTVALAGREKLKITEMRIPSKG  300
               +G + LCG VFK+EY++  +R VY+R+YSGTL LRD + ++ +EK+KITEM 
+P+ G
Sbjct  241  
STPEGQSELCGQVFKIEYSEKRRRFVYVRIYSGTLHLRDVIKISEKEKIKITEMYVPTNG  300

Query  301  
EIVRTDTAYPGEIVILPSDSVRLNDVLGDPTRLPRKRWREDPLPMLRTTIAPKTAAQRER  360
            EIV  D A PGEIVIL  D+++LND+LG+   LP K W ++P+P+LRTT+ P+ 
QRE
Sbjct  301  
EIVPADHACPGEIVILADDTLKLNDILGNEKLLPHKTWIDNPMPLLRTTVEPQKPEQREA  360

Query  361  
LLDALTQLADTDPLLRCEVDSITHEIILSFLGRVQLEVVSALLSEKYKLETVVKEPSVIY  420
            LL+AL ++ADTDPLL  ++D++THEI+LSFLG VQ+EV+ A+L EKY +E  +KEP 
+VIY
Sbjct  361  
LLNALAEIADTDPLLHFDIDTVTHEIMLSFLGNVQMEVICAILEEKYHVEAEIKEPTVIY  420

Query  421  
MERPLKAASHTIHIEVPPNPFWASIGLSVTPLSLGSGVQYESRVSLGYLNQSFQNAVRDG  480
            MERPL+ A +TIHIEVPPNPFWAS+GLS+ PL 
+GSGVQYESRVSLGYLNQSFQNAV +G
Sbjct  421  
MERPLRKAEYTIHIEVPPNPFWASVGLSIEPLPIGSGVQYESRVSLGYLNQSFQNAVMEG  480

Query  481  
IRYGLEQGLFGWNVTDCKICFEYGLYYSPVSTPADFRSLAPIVLEQALKESGTQLLEPYL  540
            + YG EQGL+GW VTDCKICFEYGLYYSPVSTPADFR L+PIVLEQALK++GT 
+LLEPYL
Sbjct  481  
VLYGCEQGLYGWKVTDCKICFEYGLYYSPVSTPADFRLLSPIVLEQALKKAGTELLEPYL  540

Query  541  
SFILYAPQEYLSRAYHDAPKYCATIETAQVKKDEVVFTGEIPARCIQAYRTDLAFYTNGR  600
             F +YAPQEYLSRAYHDAP+YCA I + Q+K DEV+  GEIPARCIQ YR DL + 
+TNG+
Sbjct  541  
HFEIYAPQEYLSRAYHDAPRYCADIVSTQIKNDEVILKGEIPARCIQEYRNDLTYFTNGQ  600

Query  601  SVCLTELKGYQAAVGQPVIQPRRPNSRLDKVRHMFQKV  638
             VCLTELKGYQ A+G+ + QPRRPNSR+DKVRHMF K+
Sbjct  601  GVCLTELKGYQPAIGKFICQPRRPNSRIDKVRHMFHKL  638
```

>WP_074924842.1 TetM/TetW/TetO/TetS family tetracycline resistance ribosomal
protection protein [[Clostridium] clostridioforme]
 SFG19940.1 ribosomal protection tetracycline resistance protein [[Clostridium]
clostridioforme]
 APO28178.1 ferrous iron transport protein B [uncultured bacterium]

```
Length=639

 Score = 938 bits (2424),  Expect = 0.0, Method: Compositional
matrix adjust.
 Identities = 445/639 (70%), Positives = 526/639 (82%), Gaps = 0/639
(0%)

Query  1
MKIINIGILAHVDAGKTTLTESLLYASGAISEPGSVEKGTTRTDTMFLERQRGITIQAAV  60
            MKIINIGILAHVDAGKTTLTESLLY SGAI EPGSV+KGTTRTD+M
LERQRGITIQAAV
Sbjct  1
MKIINIGILAHVDAGKTTLTESLLYTSGAIMEPGSVDKGTTRTDSMALERQRGITIQAAV  60

Query  61
TSFQWHRCKVNIVDTPGHMDFLAEVYRSLAVLDGAILVISAKDGVQAQTRILFHALRKMN  120
            TSF W   KVN+VDTPGHMDFLAEVYRSL+VLDGAILV+SAKDGVQAQTR
+LFHALRKM
Sbjct  61
TSFHWKEYKVNLVDTPGHMDFLAEVYRSLSVLDGAILVVSAKDGVQAQTRVLFHALRKMK  120

Query  121
IPTVIFINKIDQAGVDLQSVVQSVRDKLSADIIIKQTVSLSPEIVLEENTDIEAWDAVIE  180
            IPT+IFINKIDQ G       Q++R+KL+ D+++ Q V L  E+ L +  D E
WDAVI
Sbjct  121
IPTIIFINKIDQEGFVPAHTYQNIREKLTEDMMVMQEVHLFSELTLSDVADFEKWDAVIA  180

Query  181
NNDKLLEKYIAGEPISREKLVREEQRRVQDASLFPVYYGSAKKGLGIQPLMDAVTGLFQP  240
             ND LLEKY++G P++  +L  E +RRVQ  + FPVY+GSAK+ +G + L+D +T
+F
Sbjct  181
GNDDLLEKYLSGAPLTLRELQEEIKRRVQQGTFFPVYHGSAKENIGTKELLDVITDIFSS  240

Query  241
IGEQGSAALCGSVFKVEYTDCGQRRVYLRLYSGTLRLRDTVALAGREKLKITEMRIPSKG  300
              +   + LCG VFK+EYT+  +RR Y+RLYSGTL LR+T+ L  REK+KITEM
IP KG
Sbjct  241
KTDNCQSELCGYVFKIEYTEQKKRRCYVRLYSGTLCLRETIFLQKREKIKITEMSIPFKG  300

Query  301
EIVRTDTAYPGEIVILPSDSVRLNDVLGDPTRLPRKRWREDPLPMLRTTIAPKTAAQRER  360
            EIV+ DTA  GEIVIL  ++++LND+LGD   LPRK W ++PLP+LRTTI P  A
QRE
Sbjct  301
EIVQKDTADSGEIVILSDNTLKLNDILGDEKLLPRKAWTDNPLPLLRTTIEPAKAEQREV  360

Query  361
LLDALTQLADTDPLLRCEVDSITHEIILSFLGRVQLEVVSALLSEKYKLETVVKEPSVIY  420
            LLDALT++ADTDPLLR  +D +THEIILSFLG+VQLEVV +LL+EKY +E  VKEP
+VIY
Sbjct  361
```

LLDALTEIADTDPLLRFVIDPVTHEIILSFLGKVQLEVVCSLLNEKYGIEAEVKEPTVIY 420

Query 421
MERPLKAASHTIHIEVPPNPFWASIGLSVTPLSLGSGVQYESRVSLGYLNQSFQNAVRDG 480
            +ERP K   +TIHIEVPPNPFWASIGL+VTPL GSG +YES
+VSLGYLNQSFQNAV +G
Sbjct 421
LERPRKEEHYTIHIEVPPNPFWASIGLAVTPLPAGSGTEYESKVSLGYLNQSFQNAVMEG 480

Query 481
IRYGLEQGLFGWNVTDCKICFEYGLYYSPVSTPADFRSLAPIVLEQALKESGTQLLEPYL 540
            I+YGLEQG++GW VTDC+ICFEYG+YYSPVSTPADFR LAP+VLE ALK++GTQ
+LEPYL
Sbjct 481
IQYGLEQGVYGWEVTDCRICFEYGIYYSPVSTPADFRFLAPVVLELALKKAGTQVLEPYL 540

Query 541
SFILYAPQEYLSRAYHDAPKYCATIETAQVKKDEVVFTGEIPARCIQAYRTDLAFYTNGR 600
            SF L+APQEYLSRAYHDA KY A IET +K +E + TGEIPAR I Y++DL
FYTNGR
Sbjct 541
SFTLFAPQEYLSRAYHDAVKYDAVIETTSIKNNEAILTGEIPARRIGEYKSDLNFYTNGR 600

Query 601 SVCLTELKGYQAAVGQPVIQPRRPNSRLDKVRHMFQKVM 639
            SVCLTELKGYQ   G+P +QPRRPNSRLDKVRHMFQK+M
Sbjct 601 SVCLTELKGYQETSGEPAVQPRRPNSRLDKVRHMFQKIM 639

>WP_105310307.1 TetM/TetW/TetO/TetS family tetracycline resistance ribosomal
protection protein [Dorea sp. Marseille-P4042]
 AMP51623.1 TetM-TetW-TetO-TetS [uncultured bacterium]
 AMP55141.1 TetM-TetW-TetO-TetS [uncultured bacterium]
Length=639

 Score = 937 bits (2423), Expect = 0.0, Method: Compositional matrix adjust.
 Identities = 445/639 (70%), Positives = 526/639 (82%), Gaps = 0/639 (0%)

Query 1
MKIINIGILAHVDAGKTTLTESLLYASGAISEPGSVEKGTTRTDTMFLERQRGITIQAAV 60
            MKIINIGILAHVDAGKTTLTESLLY SGAI EPGSV+KGTTRTD+M
LERQRGITIQAAV
Sbjct 1
MKIINIGILAHVDAGKTTLTESLLYTSGAIIEPGSVDKGTTRTDSMALERQRGITIQAAV 60

Query 61
TSFQWHRCKVNIVDTPGHMDFLAEVYRSLAVLDGAILVISAKDGVQAQTRILFHALRKMN 120
            TSF W   KVN+VDTPGHMDFL EVYRSL+VLDGAILV+SAKDGVQAQTR
+LFHALRKM
Sbjct 61
TSFHWKEYKVNLVDTPGHMDFLTEVYRSLSVLDGAILVVSAKDGVQAQTRVLFHALRKMK 120

```
Query  121
IPTVIFINKIDQAGVDLQSVVQSVRDKLSADIIIKQTVSLSPEIVLEENTDIEAWDAVIE  180
            IPT+IFINKIDQ G       Q++R+KL+ D+++ Q V L  E+ L +  D E
WDAVI
Sbjct  121
IPTIIFINKIDQEGFVPAQTYQNIREKLTEDMMVMQEVHLFSELTLSDVADFEKWDAVIA  180

Query  181
NNDKLLEKYIAGEPISREKLVREEQRRVQDASLFPVYYGSAKKGLGIQPLMDAVTGLFQP  240
             ND LLEKY++G P++  +L  E +RRVQ  + FPVY+GSAK+ +G + L+D +T
+F
Sbjct  181
GNDDLLEKYLSGAPLTLRELQEEIKRRVQQGTFFPVYHGSAKENIGTKELLDVITDIFSS  240

Query  241
IGEQGSAALCGSVFKVEYTDCGQRRVYLRLYSGTLRLRDTVALAGREKLKITEMRIPSKG  300
              +   + LCG VFK+EYT+  +RR Y+RLYSGTL LR+T+ L  +EK+KITEM
IP KG
Sbjct  241
KTDNCQSELCGYVFKIEYTEQKKRRCYVRLYSGTLCLRETIFLQKKEKIKITEMSIPFKG  300

Query  301
EIVRTDTAYPGEIVILPSDSVRLNDVLGDPTRLPRKRWREDPLPMLRTTIAPKTAAQRER  360
            EIV+TDTA  GEIVIL  ++++LND+LGD   LPRK W ++PLP+LRTTI P  A
QRE
Sbjct  301
EIVQTDTADSGEIVILSDNTLKLNDILGDEKLLPRKAWTDNPLPLLRTTIEPAKAEQREV  360

Query  361
LLDALTQLADTDPLLRCEVDSITHEIILSFLGRVQLEVVSALLSEKYKLETVVKEPSVIY  420
            LLDALT++ADTDPLLR  +D +THEIILSFLG+VQLEVV +LL+EKY +E  VKEP
+VIY
Sbjct  361
LLDALTEIADTDPLLRFVIDPVTHEIILSFLGKVQLEVVCSLLNEKYCIEAEVKEPTVIY  420

Query  421
MERPLKAASHTIHIEVPPNPFWASIGLSVTPLSLGSGVQYESRVSLGYLNQSFQNAVRDG  480
            MERP K   +TIHIEVPPNPFWASIGL+VTPL  GSG +YES
+VSLGYLNQSFQNAV +G
Sbjct  421
MERPRKEEHYTIHIEVPPNPFWASIGLAVTPLPAGSGTEYESKVSLGYLNQSFQNAVMEG  480

Query  481
IRYGLEQGLFGWNVTDCKICFEYGLYYSPVSTPADFRSLAPIVLEQALKESGTQLLEPYL  540
            I+YGLEQG++GW VTDC+ICFEYG+YYSPVSTP+DFR LAPIVLE ALK++GTQ
+LEPYL
Sbjct  481
IQYGLEQGVYGWEVTDCRICFEYGIYYSPVSTPSDFRFLAPIVLELALKKAGTQVLEPYL  540

Query  541
SFILYAPQEYLSRAYHDAPKYCATIETAQVKKDEVVFTGEIPARCIQAYRTDLAFYTNGR  600
            SF L+APQEYLSRAYHDA KY A IET  +K +E + TGEIPAR I  Y++DL
FYTNGR
Sbjct  541
```

SFTLFAPQEYLSRAYHDAVKYDAVIETTSIKNNEAILTGEIPARRIGEYKSDLNFYTNGR 600

Query 601 SVCLTELKGYQAAVGQPVIQPRRPNSRLDKVRHMFQKVM 639
            SVCLTELKGYQ   G+P +QPRRPNSRLDKVRHMFQK+M
Sbjct 601 SVCLTELKGYQETSGEPAVQPRRPNSRLDKVRHMFQKIM 639

>WP_078768811.1 TetM/TetW/TetO/TetS family tetracycline resistance ribosomal
protection protein [Eubacterium coprostanoligenes]
 SJZ69450.1 ribosomal protection tetracycline resistance protein [Eubacterium
coprostanoligenes]
Length=639

 Score = 937 bits (2422), Expect = 0.0, Method: Compositional matrix adjust.
 Identities = 433/638 (68%), Positives = 529/638 (83%), Gaps = 0/638 (0%)

Query 1
MKIINIGILAHVDAGKTTLTESLLYASGAISEPGSVEKGTTRTDTMFLERQRGITIQAAV 60
            MKIIN+GILAHVDAGKTTLTESLLY SGAI+EPGSV+KGTTRTDTM
LERQRGITIQ AV
Sbjct 1
MKIINLGILAHVDAGKTTLTESLLYTSGAIAEPGSVDKGTTRTDTMNLERQRGITIQTAV 60

Query 61
TSFQWHRCKVNIVDTPGHMDFLAEVYRSLAVLDGAILVISAKDGVQAQTRILFHALRKMN 120
            TSFQW   KVNI+DTPGHMDFLAEVYRSL+VLDGA+L++SAKDG+QAQTRILFHAL
+ M
Sbjct 61
TSFQWEDVKVNIIDTPGHMDFLAEVYRSLSVLDGAVLLVSAKDGIQAQTRILFHALQTMK 120

Query 121
IPTVIFINKIDQAGVDLQSVVQSVRDKLSADIIIKQTVSLSPEIVLEENTDIEAWDAVIE 180
            IPT+ FINKIDQ G+DL V Q ++ KLS++II+KQ V   P I + +N D+E
WDAVI
Sbjct 121
IPTIFFINKIDQEGIDLPMVYQEMKAKLSSEIIVKQKVGQHPHINVTDNDDMEQWDAVIM 180

Query 181
NNDKLLEKYIAGEPISREKLVREEQRRVQDASLFPVYYGSAKKGLGIQPLMDAVTGLFQP 240
             ND+LLEKY++G+P   +L +EE RR Q+ +LFPVY+GSAK LGI+ L++ +
F
Sbjct 181
GNDELLEKYMSGKPFKMSELEQEENRRFQNGTLFPVYHGSAKNNLGIRQLIEVIASKFYS 240

Query 241
IGEQGSAALCGSVFKVEYTDCGQRRVYLRLYSGTLRLRDTVALAGREKLKITEMRIPSKG 300
              +G + LCG VFK+EY++ +R VY+R+YSGTL LRD + ++ +EK+KITEM
IPS G
Sbjct 241
STPEGQSELCGQVFKIEYSEKRRRFVYVRIYSGTLHLRDVIRISEKEKIKITEMCIPSNG 300

Query 301
EIVRTDTAYPGEIVILPSDSVRLNDVLGDPTRLPRKRWREDPLPMLRTTIAPKTAAQRER 360
            EIV D A PGEIVIL D+++LND+LG+   LP K W ++P+P+LRTT+ P+
QRE
Sbjct 301
EIVPADHACPGEIVILADDTLKLNDILGNEKLLPHKTWIDNPMPLLRTTVEPQKPEQREA 360

Query 361
LLDALTQLADTDPLLRCEVDSITHEIILSFLGRVQLEVVSALLSEKYKLETVVKEPSVIY 420
            LL+AL ++ADTDPLL ++D++THEI+LSFLG+VQLEV+ +LL EKY +
+KEPSVIY
Sbjct 361
LLNALAEIADTDPLLHFDIDTVTHEIMLSFLGKVQLEVICSLLEEKYHVGVAMKEPSVIY 420

Query 421
MERPLKAASHTIHIEVPPNPFWASIGLSVTPLSLGSGVQYESRVSLGYLNQSFQNAVRDG 480
            +ERPL+ A +TIHIEVPPNPFWAS+GLS+ PL
+GSGVQYESRVSLGYLNQSFQNAV +G
Sbjct 421
LERPLRKAEYTIHIEVPPNPFWASVGLSIEPLPIGSGVQYESRVSLGYLNQSFQNAVMEG 480

Query 481
IRYGLEQGLFGWNVTDCKICFEYGLYYSPVSTPADFRSLAPIVLEQALKESGTQLLEPYL 540
            + YG EQGL+GW VTDCKICFEYGLYYSPVSTPADFR L+PIVLEQALK++GT
+LLEPYL
Sbjct 481
VLYGCEQGLYGWKVTDCKICFEYGLYYSPVSTPADFRLLSPIVLEQALKKAGTELLEPYL 540

Query 541
SFILYAPQEYLSRAYHDAPKYCATIETAQVKKDEVVFTGEIPARCIQAYRTDLAFYTNGR 600
             F +YAPQEYLSRAYHDAP+YCA I + Q+K DEV+ GEIPARCIQ YR DL +
+TNG+
Sbjct 541
HFEIYAPQEYLSRAYHDAPRYCADIVSTQIKNDEVILKGEIPARCIQEYRNDLTYFTNGQ 600

Query 601 SVCLTELKGYQAAVGQPVIQPRRPNSRLDKVRHMFQKV 638
             VCLTELKGYQ A+G+ + QPRRPNSR+DKVRHMF K+
Sbjct 601 GVCLTELKGYQPAIGKFICQPRRPNSRIDKVRHMFHKL 638

>APO28015.1 ferrous iron transport protein B [uncultured bacterium]
Length=639

Score = 936 bits (2419), Expect = 0.0, Method: Compositional
matrix adjust.
Identities = 444/639 (69%), Positives = 525/639 (82%), Gaps = 0/639
(0%)

Query 1
MKIINIGILAHVDAGKTTLTESLLYASGAISEPGSVEKGTTRTDTMFLERQRGITIQAAV 60
            MKIINIGILAHVDAGKTTLTESLLY SGAI EPGSV+KGTTRTD+M
LERQRGITIQAAV
Sbjct 1

MKIINIGILAHVDAGKTTLTESLLYTSGAIMEPGSVDKGTTRTDSMALERQRGITIQAAV 60

Query 61
TSFQWHRCKVNIVDTPGHMDFLAEVYRSLAVLDGAILVISAKDGVQAQTRILFHALRKMN 120
TSF W KVN+VDTPGHMDFLAEVYRSL+VLDGAILV+SAKDGVQAQTR
+LFHALRKM
Sbjct 61
TSFHWKEYKVNLVDTPGHMDFLAEVYRSLSVLDGAILVVSAKDGVQAQTRVLFHALRKMK 120

Query 121
IPTVIFINKIDQAGVDLQSVVQSVRDKLSADIIIKQTVSLSPEIVLEENTDIEAWDAVIE 180
IPT+IFINKIDQ G Q++R+KL+ D+++ Q V L E+ L + D E
WDAVI
Sbjct 121
IPTIIFINKIDQEGFVPAHTYQNIREKLTEDMMVMQEVHLFSELTLSDVADFEKWDAVIA 180

Query 181
NNDKLLEKYIAGEPISREKLVREEQRRVQDASLFPVYYGSAKKGLGIQPLMDAVTGLFQP 240
ND LLEKY++G P++ +L E +RRVQ + FPVY+GSAK+ +G + L+D +T
+F
Sbjct 181
GNDDLLEKYLSGAPLTLRELQEEIKRRVQQGTFFPVYHGSAKENIGTKELLDVITDIFSS 240

Query 241
IGEQGSAALCGSVFKVEYTDCGQRRVYLRLYSGTLRLRDTVALAGREKLKITEMRIPSKG 300
+ + LCG VFK+EYT+ +RR Y+RLYSGTL LR+T+ L REK+KITEM
IP KG
Sbjct 241
KTDNCQSELCGYVFKIEYTEQKKRRCYVRLYSGTLCLRETIFLQKREKIKITEMSIPFKG 300

Query 301
EIVRTDTAYPGEIVILPSDSVRLNDVLGDPTRLPRKRWREDPLPMLRTTIAPKTAAQRER 360
EI + DTA GEIVIL ++++LND+LGD LPRK W ++PLP+LRTTI P A
QRE
Sbjct 301
EIFQKDTADSGEIVILSDNTLKLNDILGDEKLLPRKAWTDNPLPLLRTTIEPAKAEQREV 360

Query 361
LLDALTQLADTDPLLRCEVDSITHEIILSFLGRVQLEVVSALLSEKYKLETVVKEPSVIY 420
LLDALT++ADTDPLLR +D +THEIILSFLG+VQLEVV +LL+EKY +E VKEP
+VIY
Sbjct 361
LLDALTEIADTDPLLRFVIDPVTHEIILSFLGKVQLEVVCSLLNEKYGIEAEVKEPTVIY 420

Query 421
MERPLKAASHTIHIEVPPNPFWASIGLSVTPLSLGSGVQYESRVSLGYLNQSFQNAVRDG 480
+ERP K +TIHIEVPPNPFWASIGL+VTPL GSG +YES
+VSLGYLNQSFQNAV +G
Sbjct 421
LERPRKEEHYTIHIEVPPNPFWASIGLAVTPLPAGSGTEYESKVSLGYLNQSFQNAVMEG 480

Query 481
IRYGLEQGLFGWNVTDCKICFEYGLYYSPVSTPADFRSLAPIVLEQALKESGTQLLEPYL 540
I+YGLEQG++GW VTDC+ICFEYG+YYSPVSTPADFR LAP+VLE ALK++GTQ

+LEPYL
Sbjct 481
IQYGLEQGVYGWEVTDCRICFEYGIYYSPVSTPADFRFLAPVVLELALKKAGTQVLEPYL 540

Query 541
SFILYAPQEYLSRAYHDAPKYCATIETAQVKKDEVVFTGEIPARCIQAYRTDLAFYTNGR 600
            SF L+APQEYLSRAYHDA KY A IET +K +E + TGEIPAR I Y++DL
FYTNGR
Sbjct 541
SFTLFAPQEYLSRAYHDAVKYDAVIETTSIKNNEAILTGEIPARRIGEYKSDLNFYTNGR 600

Query 601 SVCLTELKGYQAAVGQPVIQPRRPNSRLDKVRHMFQKVM 639
            SVCLTELKGYQ   G+P +QPRRPNSRLDKVRHMFQK+M
Sbjct 601 SVCLTELKGYQETSGEPAVQPRRPNSRLDKVRHMFQKIM 639

>WP_002602099.1 MULTISPECIES: tetracycline resistance ribosomal protection protein
Tet(32) [Bacteria]
 ABG36114.2 ribosomal protection type tetracycline resistance protein [Streptococcus
salivarius]
 ABV82120.1 Tet32 [Lachnoanaerobaculum saburreum]
 ABV82122.1 Tet32 [Streptococcus salivarius]
 EEF68716.1 putative translation elongation factor G [Holdemania filiformis
DSM 12042]
 EEG50113.1 putative translation elongation factor G [Blautia hydrogenotrophica
DSM 10507]
 EFE91639.1 putative translation elongation factor G [Oribacterium sp. oral
taxon 078 str. F0262]
 AEQ21926.1 conserved hypothetical protein [Acidaminococcus intestini RyC-MR95]
 AET35525.1 Tet32 [uncultured bacterium]
 EHL19320.1 tetracycline resistance protein tetO [Peptoanaerobacter stomatis]
 EHO51309.1 putative translation elongation factor G [Lachnospiraceae bacterium
oral taxon 082 str. F0431]
 EIC95420.1 tetracycline resistance protein TetM [Lachnoanaerobaculum saburreum
F0468]
 EJO23765.1 tetracycline resistance protein TetM [Selenomonas sp. FOBRC6]
 ENY96130.1 tetracycline resistance protein TetM [[Clostridium] hathewayi
12489931]
 AGU73073.1 putative tetracycline resistance protein [Streptococcus constellatus
subsp. pharyngis C232]
 AGU74828.1 putative tetracycline resistance protein [Streptococcus constellatus

subsp. pharyngis C818]
 ERI76192.1 putative translation elongation factor G [Clostridium sp. ATCC
BAA-442]
 ESL02384.1 putative translation elongation factor G [Catonella morbi ATCC
51271]
 KKI51860.1 Tetracycline resistance protein TetM [Catabacter hongkongensis]
 AKO36342.1 tetracycline resistance protein tetM [[Haemophilus] ducreyi]
 AKO37791.1 tetracycline resistance protein tetM [[Haemophilus] ducreyi]
 AKO39349.1 tetracycline resistance protein tetM [[Haemophilus] ducreyi]
 AKO41858.1 tetracycline resistance protein tetM [[Haemophilus] ducreyi]
 AKO42291.1 tetracycline resistance protein tetM [[Haemophilus] ducreyi]
 CUN72612.1 small GTP-binding protein domain [Hungatella hathewayi]
 KXB92909.1 putative translation elongation factor G [Veillonellaceae bacterium
DNF00751]
 OCL29209.1 tetracycline resistance ribosomal protection protein [Criibacterium
bergeronii]
 SCI12731.1 Tetracycline resistance protein tetM from transposon Tn916 [uncultured
Clostridium sp.]
 SCH93921.1 Tetracycline resistance protein tetM from transposon Tn916 [uncultured
Blautia sp.]
 OFT80598.1 tetracycline resistance ribosomal protection protein [Granulicatella
sp. HMSC30F09]
 OKQ24379.1 tetracycline resistance ribosomal protection protein [Streptococcus
pneumoniae]
 PKB54612.1 tetracycline resistance ribosomal protection protein Tet(32)
[Clostridium sp. HMb25]
 PLT52817.1 tetracycline resistance ribosomal protection protein Tet(32)
[[Ruminococcus] gnavus]
 PLT52858.1 tetracycline resistance ribosomal protection protein Tet(32)
[[Ruminococcus] gnavus]
 PNH21646.1 tetracycline resistance ribosomal protection protein Tet(32)
[Megasphaera genomosp. type_2]
 PNV62583.1 tetracycline resistance ribosomal protection protein Tet(32)
[Clostridium sp. chh4-2]
 PSJ02767.1 tetracycline resistance ribosomal protection protein

Tet(32)
[Blautia sp. SG-772]
 PSL10817.1 ribosomal protection tetracycline resistance protein
[Fusobacterium
naviforme]
Length=639

 Score = 936 bits (2418), Expect = 0.0, Method: Compositional
matrix adjust.
 Identities = 439/639 (69%), Positives = 537/639 (84%), Gaps = 0/639
(0%)

```
Query  1
MKIINIGILAHVDAGKTTLTESLLYASGAISEPGSVEKGTTRTDTMFLERQRGITIQAAV  60
            MKIINIGILAHVDAGKTTLTESLLY SGAI+E G+V+KGTTRTDTM
LERQRGITIQ AV
Sbjct  1
MKIINIGILAHVDAGKTTLTESLLYTSGAIAEQGNVDKGTTRTDTMILERQRGITIQTAV  60

Query  61
TSFQWHRCKVNIVDTPGHMDFLAEVYRSLAVLDGAILVISAKDGVQAQTRILFHALRKMN  120
            TSF W+  K+NIVDTPGHMDFL E YRSL+VLDGA+LVISAKDGVQAQTRILFHAL
+KM+
Sbjct  61
TSFCWNDYKINIVDTPGHMDFLTEAYRSLSVLDGAVLVISAKDGVQAQTRILFHALQKMD  120

Query  121
IPTVIFINKIDQAGVDLQSVVQSVRDKLSADIIIKQTVSLSPEIVLEENTDIEAWDAVIE  180
            IPT+IFINKIDQ G+DL+ V QS++DKL++D+I+ Q VSLSP+I + + +D++ WD
+I
Sbjct  121
IPTIIFINKIDQNGIDLRRVYQSIKDKLTSDMIVMQEVSLSPKITMTDISDLDKWDMIIS  180

Query  181
NNDKLLEKYIAGEPISREKLVREEQRRVQDASLFPVYYGSAKKGLGIQPLMDAVTGLFQP  240
             +D+LLE+Y+A + +  ++L  E+ +R +  SLFPVY+GSAK  LG + L++A+T
F
Sbjct  181
GSDELLERYVAEDSLDIQELQYEKCKRTRCCSLFPVYHGSAKDNLGTEKLIEAITETFIT  240

Query  241
IGEQGSAALCGSVFKVEYTDCGQRRVYLRLYSGTLRLRDTVALAGREKLKITEMRIPSKG  300
              +   + LCG VFKVEYT+  +R  YLRLY GTL LRDT+ L+ +EK+KITEM
IPS G
Sbjct  241
ETDDIQSELCGYVFKVEYTERKKRLSYLRLYHGTLHLRDTLLLSKKEKIKITEMCIPSNG  300

Query  301
EIVRTDTAYPGEIVILPSDSVRLNDVLGDPTRLPRKRWREDPLPMLRTTIAPKTAAQRER  360
            EIV  D A PGEIVIL  D+++LND+LG+   LP K   ++P+P+LRTT+ P+
QRE
Sbjct  301
EIVPVDHACPGEIVILADDTLKLNDILGNEKLLPHKTRIDNPMPLLRTTVEPQKPEQREA  360
```

Query 361
LLDALTQLADTDPLLRCEVDSITHEIILSFLGRVQLEVVSALLSEKYKLETVVKEPSVIY 420
            LL+ALT++ADTDPLL ++D++THEIILSFLG+VQLEV+ +LL EKY +
+KEPSVIY
Sbjct 361
LLNALTEIADTDPLLHFDIDTVTHEIILSFLGKVQLEVICSLLEEKYHVGVAMKEPSVIY 420

Query 421
MERPLKAASHTIHIEVPPNPFWASIGLSVTPLSLGSGVQYESRVSLGYLNQSFQNAVRDG 480
            +ERP K AS+TIHIEVPPNPFWASIGL+VTPL +GSG QY+S
VSLGYLNQSFQNAV +G
Sbjct 421
LERPQKKASYTIHIEVPPNPFWASIGLTVTPLPVGSGTQYKSEVSLGYLNQSFQNAVMEG 480

Query 481
IRYGLEQGLFGWNVTDCKICFEYGLYYSPVSTPADFRSLAPIVLEQALKESGTQLLEPYL 540
            +RYG+EQGL+GW VTDC+ICF+YG+YYSPVSTPADFR LAP+VLEQALK+
+GTQLLEPYL
Sbjct 481
VRYGMEQGLYGWGVTDCQICFDYGVYYSPVSTPADFRFLAPVVLEQALKKAGTQLLEPYL 540

Query 541
SFILYAPQEYLSRAYHDAPKYCATIETAQVKKDEVVFTGEIPARCIQAYRTDLAFYTNGR 600
            SF L+APQEYLSRAY+DAPKYCA IE+ +++KDEV+F GEIPARCI YR DL
FYTNGR
Sbjct 541
SFTLFAPQEYLSRAYNDAPKYCAIIESTRLEKDEVIFKGEIPARCIGEYRNDLNFYTNGR 600

Query 601 SVCLTELKGYQAAVGQPVIQPRRPNSRLDKVRHMFQKVM 639
            SVC+TELKGYQ   G+PV QPRRPNSRLDK+RHMFQK+M
Sbjct 601 SVCITELKGYQETSGEPVFQPRRPNSRLDKIRHMFQKIM 639

>WP_044761229.1 TetM/TetW/TetO/TetS family tetracycline resistance ribosomal
protection protein [Streptococcus suis]
 AHH55325.1 tetracycline resistance protein [Streptococcus suis]
 CYU80554.1 tetracycline resistance protein tetM [Streptococcus suis]
Length=639

 Score = 935 bits (2417), Expect = 0.0, Method: Compositional matrix adjust.
 Identities = 432/638 (68%), Positives = 529/638 (83%), Gaps = 0/638 (0%)

Query 1
MKIINIGILAHVDAGKTTLTESLLYASGAISEPGSVEKGTTRTDTMFLERQRGITIQAAV 60
            MKIIN+GILAHVDAGKTTLTESLLY SGAI+EPGSV+KGTTRTDTM
LERQRGITIQ AV
Sbjct 1
MKIINLGILAHVDAGKTTLTESLLYTSGAIAEPGSVDKGTTRTDTMNLERQRGITIQTAV 60

Query 61

TSFQWHRCKVNIVDTPGHMDFLAEVYRSLAVLDGAILVISAKDGVQAQTRILFHALRKMN 120
            TSFQW   KVNI+DTPGHMDFLAEVYRSL+VLDGA+L++SAKDG+QAQTRILFHAL
+ M
Sbjct 61
TSFQWEDVKVNIIDTPGHMDFLAEVYRSLSVLDGAVLLVSAKDGIQAQTRILFHALQIMK 120

Query 121
IPTVIFINKIDQAGVDLQSVVQSVRDKLSADIIIKQTVSLSPEIVLEENTDIEAWDAVIE 180
            IPT+ FINKIDQ G+DL V + ++ KLS++II+KQ V   P I + +N D+E
WDAVI
Sbjct 121
IPTIFFINKIDQEGIDLPMVYREMKAKLSSEIIVKQKVGQHPHINVTDNDDMEQWDAVIM 180

Query 181
NNDKLLEKYIAGEPISREKLVREEQRRVQDASLFPVYYGSAKKGLGIQPLMDAVTGLFQP 240
             ND+LLEKY++G+P   +L +EE RR Q+ +LFPVY+GSAK LGI+ L++ +
F
Sbjct 181
GNDELLEKYMSGKPFKMSELEQEENRRFQNGTLFPVYHGSAKNNLGIRQLIEVIASKFYS 240

Query 241
IGEQGSAALCGSVFKVEYTDCGQRRVYLRLYSGTLRLRDTVALAGREKLKITEMRIPSKG 300
               +G + LCG VFK+EY++ +R VY+R+YSGTL LRD + ++ +EK+KITEM
IPS G
Sbjct 241
STPEGQSELCGQVFKIEYSEKRRRFVYVRIYSGTLHLRDVIRISEKEKIKITEMCIPSNG 300

Query 301
EIVRTDTAYPGEIVILPSDSVRLNDVLGDPTRLPRKRWREDPLPMLRTTIAPKTAAQRER 360
            EIV D A PGEIVIL D+++LND+LG+   LP K W ++P+P+LRTT+ P+
QRE
Sbjct 301
EIVPADHACPGEIVILADDTLKLNDILGNEKLLPHKTWIDNPMPLLRTTVEPQKPEQREA 360

Query 361
LLDALTQLADTDPLLRCEVDSITHEIILSFLGRVQLEVVSALLSEKYKLETVVKEPSVIY 420
            LL+AL ++ADTDPLL ++D++THEI+LSFLG+VQLEV+ +LL EKY +   +KEP
+VIY
Sbjct 361
LLNALAEIADTDPLLHFDIDTVTHEIMLSFLGKVQLEVICSLLEEKYHVGVAMKEPTVIY 420

Query 421
MERPLKAASHTIHIEVPPNPFWASIGLSVTPLSLGSGVQYESRVSLGYLNQSFQNAVRDG 480
            MERPL+ A +TIHIEVPPNPFWAS+GLS+ PL
+GSGVQYESRVSLGYLNQSFQNAV +G
Sbjct 421
MERPLRKAEYTIHIEVPPNPFWASVGLSIEPLPIGSGVQYESRVSLGYLNQSFQNAVMEG 480

Query 481
IRYGLEQGLFGWNVTDCKICFEYGLYYSPVSTPADFRSLAPIVLEQALKESGTQLLEPYL 540
            + YG EQGL+GW VTDCKICFEYGLYYSPVSTPADFR L+PIVLEQALK++GT
+LLEPYL
Sbjct 481
VLYGCEQGLYGWKVTDCKICFEYGLYYSPVSTPADFRLLSPIVLEQALKKAGTELLEPYL 540

Query 541
SFILYAPQEYLSRAYHDAPKYCATIETAQVKKDEVVFTGEIPARCIQAYRTDLAFYTNGR 600
             F +YAPQEYLSRAYHDAP+YCA I + Q+K DEV+ GEIPARCIQ YR DL +
+TNG+
Sbjct 541
HFEIYAPQEYLSRAYHDAPRYCADIVSTQIKNDEVILKGEIPARCIQEYRNDLTYFTNGQ 600

Query 601 SVCLTELKGYQAAVGQPVIQPRRPNSRLDKVRHMFQKV 638
             VCLTELKGYQ A+G+ + QPRRPNSR+DKVRHMF K+
Sbjct 601 GVCLTELKGYQPAIGKFICQPRRPNSRIDKVRHMFHKL 638

>WP_055065785.1 TetM/TetW/TetO/TetS family tetracycline resistance
ribosomal
protection protein [Blautia obeum]
 CUN78436.1 Tetracycline resistance protein tetM from transposon
Tn916 [Blautia
obeum]
Length=639

 Score = 934 bits (2414), Expect = 0.0, Method: Compositional
matrix adjust.
 Identities = 429/638 (67%), Positives = 528/638 (83%), Gaps = 0/638
(0%)

Query 1
MKIINIGILAHVDAGKTTLTESLLYASGAISEPGSVEKGTTRTDTMFLERQRGITIQAAV 60
            MKIIN+GILAHVDAGKTTLTESLLY SGAI+EPGSV+KGTTRTDTM
LERQRGITIQ AV
Sbjct 1
MKIINLGILAHVDAGKTTLTESLLYTSGAIAEPGSVDKGTTRTDTMNLERQRGITIQTAV 60

Query 61
TSFQWHRCKVNIVDTPGHMDFLAEVYRSLAVLDGAILVISAKDGVQAQTRILFHALRKMN 120
            TSFQW   KVNI+DTPGHMDFLAEVYRSL+VLDGA+L++SAKDG+QAQTRILFHAL
+ M
Sbjct 61
TSFQWEDVKVNIIDTPGHMDFLAEVYRSLSVLDGAVLLVSAKDGIQAQTRILFHALQTMK 120

Query 121
IPTVIFINKIDQAGVDLQSVVQSVRDKLSADIIIKQTVSLSPEIVLEENTDIEAWDAVIE 180
            IPT+ FINKIDQ G+DL V + ++ KLS++II+KQ V   P I + +N D+E WD
VI
Sbjct 121
IPTIFFINKIDQEGIDLPMVYREMKAKLSSEIIVKQKVGQHPHINVTDNDDMEQWDTVIM 180

Query 181
NNDKLLEKYIAGEPISREKLVREEQRRVQDASLFPVYYGSAKKGLGIQPLMDAVTGLFQP 240
             ND+LLEKY++G+P   +L +EE RR Q+ +LFPVY+GSAK LGI+ L++ +
F
Sbjct 181
GNDELLEKYMSGKPFKMSELEQEENRRFQNGTLFPVYHGSAKNNLGIRQLIEVIASKFYS 240

```
Query  241
IGEQGSAALCGSVFKVEYTDCGQRRVYLRLYSGTLRLRDTVALAGREKLKITEMRIPSKG  300
               +G + LCG VFK+EY++  +R VY+R+YSGTL LRD + ++ +EK+KITEM
+P+ G
Sbjct  241
STPEGQSELCGQVFKIEYSEKRRRFVYVRIYSGTLHLRDVIRISEKEKIKITEMCVPTNG  300

Query  301
EIVRTDTAYPGEIVILPSDSVRLNDVLGDPTRLPRKRWREDPLPMLRTTIAPKTAAQRER  360
            EIV  D A PGEIVIL  D+++LND+LG+   LP K W ++P+P+LRTT+ P+
QRE
Sbjct  301
EIVPADHACPGEIVILADDTLKLNDILGNEKLLPHKTWIDNPMPLLRTTVEPQKPEQREA  360

Query  361
LLDALTQLADTDPLLRCEVDSITHEIILSFLGRVQLEVVSALLSEKYKLETVVKEPSVIY  420
            LL+AL ++ADTDPLL  ++D++THEI+LSFLG VQ+EV+ A+L EKY +E  +KEP
+VIY
Sbjct  361
LLNALAEIADTDPLLHFDIDTVTHEIMLSFLGNVQMEVICAILEEKYHVEAEIKEPTVIY  420

Query  421
MERPLKAASHTIHIEVPPNPFWASIGLSVTPLSLGSGVQYESRVSLGYLNQSFQNAVRDG  480
            MERPL+ A +TIHIEVPPNPFWAS+GLS+ PL
+GSGVQYESRVSLGYLNQSFQNAV +G
Sbjct  421
MERPLRKAEYTIHIEVPPNPFWASVGLSIEPLPIGSGVQYESRVSLGYLNQSFQNAVMEG  480

Query  481
IRYGLEQGLFGWNVTDCKICFEYGLYYSPVSTPADFRSLAPIVLEQALKESGTQLLEPYL  540
            + YG EQGL+GW VTDCKICFEYGLYYSPVSTPADFR L+PIVLEQALK++GT
+LLEPYL
Sbjct  481
VLYGCEQGLYGWKVTDCKICFEYGLYYSPVSTPADFRLLSPIVLEQALKKAGTELLEPYL  540

Query  541
SFILYAPQEYLSRAYHDAPKYCATIETAQVKKDEVVFTGEIPARCIQAYRTDLAFYTNGR  600
             F +YAPQEYLSRAYHDAP+YCA I + Q+K DEV+  GEIPARCIQ YR DL +
+TNG+
Sbjct  541
HFEIYAPQEYLSRAYHDAPRYCADIVSTQIKNDEVILKGEIPARCIQEYRNDLTYFTNGQ  600

Query  601  SVCLTELKGYQAAVGQPVIQPRRPNSRLDKVRHMFQKV  638
             VCLTELKGYQ A+G+ + QPRRPNSR+DKVRHMF K+
Sbjct  601  GVCLTELKGYQPAIGKFICQPRRPNSRIDKVRHMFHKL  638
```

>WP_002584856.1 MULTISPECIES: TetM/TetW/TetO/TetS family
tetracycline resistance
ribosomal protection protein [Lachnoclostridium]
 EGB18591.1 putative translation elongation factor G
[[[Clostridium] symbiosum
WAL-14673]
 ENZ58503.1 tetracycline resistance protein TetM [[[Clostridium]

clostridioforme
90A6]
 ENZ58640.1 tetracycline resistance protein TetM [[[Clostridium]
clostridioforme
90A4]
 KMW09116.1 tetracycline resistance protein tetO [[Clostridium]
clostridioforme
WAL-7855]
 SFG14178.1 ribosomal protection tetracycline resistance protein
[[Clostridium]
clostridioforme]
Length=639

 Score = 933 bits (2411), Expect = 0.0, Method: Compositional
matrix adjust.
 Identities = 437/639 (68%), Positives = 536/639 (84%), Gaps = 0/639
(0%)

Query 1
MKIINIGILAHVDAGKTTLTESLLYASGAISEPGSVEKGTTRTDTMFLERQRGITIQAAV 60
            MKIINIGILAHVDAGKTTLTESLLY SGAI+E G+V+KGTTRTDTM
LERQRGITIQ AV
Sbjct 1
MKIINIGILAHVDAGKTTLTESLLYTSGAIAEQGNVDKGTTRTDTMILERQRGITIQTAV 60

Query 61
TSFQWHRCKVNIVDTPGHMDFLAEVYRSLAVLDGAILVISAKDGVQAQTRILFHALRKMN 120
            TSF W+ K+NIVDTPGHMDFL E YRSL+VLDGA+LVISAKDGVQAQTRILFHAL
+KM+
Sbjct 61
TSFCWNDYKINIVDTPGHMDFLTEAYRSLSVLDGAVLVISAKDGVQAQTRILFHALQKMD 120

Query 121
IPTVIFINKIDQAGVDLQSVVQSVRDKLSADIIIKQTVSLSPEIVLEENTDIEAWDAVIE 180
            IPT+IFINKIDQ G+DL+ V QS++DKL++D+I+ Q VSLSP+I + + +D++ WD
+I
Sbjct 121
IPTIIFINKIDQNGIDLRRVYQSIKDKLTSDMIVMQEVSLSPKITMTDISDLDKWDMIIS 180

Query 181
NNDKLLEKYIAGEPISREKLVREEQRRVQDASLFPVYYGSAKKGLGIQPLMDAVTGLFQP 240
             +D+LLE+Y+A + + ++L E+ +R + SLFPVY+GSAK LG + L++A+T
F
Sbjct 181
GSDELLERYVAEDSLDIQELQYEKCKRTRCCSLFPVYHGSAKDNLGTEKLIEAITETFIT 240

Query 241
IGEQGSAALCGSVFKVEYTDCGQRRVYLRLYSGTLRLRDTVALAGREKLKITEMRIPSKG 300
             +   + LCG VFKVEYT+ +R YLRLY GTL LRDT+ L+ +EK+KITEM
IPS G
Sbjct 241
ETDDIQSELCGYVFKVEYTERKKRLSYLRLYHGTLHLRDTLLLSKKEKIKITEMCIPSNG 300

Query 301

```
EIVRTDTAYPGEIVILPSDSVRLNDVLGDPTRLPRKRWREDPLPMLRTTIAPKTAAQRER  360
            EIV  D A PGEIVIL  D+++LND+LG+   LP K   ++P+P+LRTT+ P+
QRE
Sbjct  301
EIVPVDHACPGEIVILADDTLKLNDILGNEKLLPHKTRIDNPMPLLRTTVEPQKPEQREA  360

Query  361
LLDALTQLADTDPLLRCEVDSITHEIILSFLGRVQLEVVSALLSEKYKLETVVKEPSVIY  420
            LL+AL ++ADTDPLL  ++D++THEI+LSFLG+VQLEV+ +LL EKY +
+KEPSVIY
Sbjct  361
LLNALAEIADTDPLLHFDIDTVTHEIMLSFLGKVQLEVICSLLEEKYHVGVAMKEPSVIY  420

Query  421
MERPLKAASHTIHIEVPPNPFWASIGLSVTPLSLGSGVQYESRVSLGYLNQSFQNAVRDG  480
            +ERP K AS+TIHIEVPPNPFWASIGL+VTPL +GSG QY+S
VSLGYLNQSFQNAV +G
Sbjct  421
LERPQKKASYTIHIEVPPNPFWASIGLTVTPLPVGSGTQYKSEVSLGYLNQSFQNAVMEG  480

Query  481
IRYGLEQGLFGWNVTDCKICFEYGLYYSPVSTPADFRSLAPIVLEQALKESGTQLLEPYL  540
            +RYG+EQGL+GW VTDC+ICF+YG+YYSPVSTPADFR LAP+VLEQALK+
+GTQLLEPYL
Sbjct  481
VRYGMEQGLYGWGVTDCQICFDYGVYYSPVSTPADFRFLAPVVLEQALKKAGTQLLEPYL  540

Query  541
SFILYAPQEYLSRAYHDAPKYCATIETAQVKKDEVVFTGEIPARCIQAYRTDLAFYTNGR  600
            SF L+APQEYLSRAY+DAPKYCA IE+ +++KDEV+F GEIPARCI  YR DL
FYTNGR
Sbjct  541
SFTLFAPQEYLSRAYNDAPKYCAIIESTRLEKDEVIFKGEIPARCIGEYRNDLNFYTNGR  600

Query  601  SVCLTELKGYQAAVGQPVIQPRRPNSRLDKVRHMFQKVM  639
            SVC+TELKGYQ   G+PV QPRRPNSRLDK+RHMFQK+M
Sbjct  601  SVCITELKGYQETSGEPVFQPRRPNSRLDKIRHMFQKIM  639


>WP_073282779.1 TetM/TetW/TetO/TetS family tetracycline resistance
ribosomal
protection protein [Anaerosporobacter mobilis]
 SHM07585.1 ribosomal protection tetracycline resistance protein
[Anaerosporobacter
mobilis DSM 15930]
Length=639

 Score = 932 bits (2410),  Expect = 0.0, Method: Compositional
matrix adjust.
 Identities = 438/639 (69%), Positives = 535/639 (84%), Gaps = 0/639
(0%)

Query  1
MKIINIGILAHVDAGKTTLTESLLYASGAISEPGSVEKGTTRTDTMFLERQRGITIQAAV  60
```

MKIINIGILAHVDAGKTTLTESLLY SGAI+E G+V+KGTTRTDTM
LERQRGITIQ AV
Sbjct 1
MKIINIGILAHVDAGKTTLTESLLYTSGAIAEQGNVDKGTTRTDTMILERQRGITIQTAV 60

Query 61
TSFQWHRCKVNIVDTPGHMDFLAEVYRSLAVLDGAILVISAKDGVQAQTRILFHALRKMN 120
TSF W+ K+NIVDTPGHMDFL E YRSL+VLDGA+LVISAKDGVQAQTRILFHAL
+KM+
Sbjct 61
TSFCWNDYKINIVDTPGHMDFLTEAYRSLSVLDGAVLVISAKDGVQAQTRILFHALQKMD 120

Query 121
IPTVIFINKIDQAGVDLQSVVQSVRDKLSADIIIKQTVSLSPEIVLEENTDIEAWDAVIE 180
IPT+IFINKIDQ G+DL+ V QS++DKL++D+I+ Q VSLSP+I + + +D++ WD
+I
Sbjct 121
IPTIIFINKIDQNGIDLRRVYQSIKDKLTSDMIVMQEVSLSPKITMTDISDLDKWDMIIS 180

Query 181
NNDKLLEKYIAGEPISREKLVREEQRRVQDASLFPVYYGSAKKGLGIQPLMDAVTGLFQP 240
+D+LLE+Y+A + + ++L E+ +R + SLFPVY+GSAK LG + L++A+T
F
Sbjct 181
GSDELLERYVAEDSLDIQELQYEKCKRTRCCSLFPVYHGSAKDNLGTEKLIEAITETFIT 240

Query 241
IGEQGSAALCGSVFKVEYTDCGQRRVYLRLYSGTLRLRDTVALAGREKLKITEMRIPSKG 300
+ + LCG VFKVEYT+ +R YLRLY GTL LRDT+ L+ +EK+KITEM
IPS G
Sbjct 241
ETDDIQSELCGYVFKVEYTERKKRLSYLRLYHGTLHLRDTLLLSKKEKIKITEMCIPSNG 300

Query 301
EIVRTDTAYPGEIVILPSDSVRLNDVLGDPTRLPRKRWREDPLPMLRTTIAPKTAAQRER 360
EIV D A PGEIVIL D+++LND+LG+ LP K ++P+P+LRTT+ P+
QRE
Sbjct 301
EIVPADHACPGEIVILADDTLKLNDILGNEKLLPHKTRIDNPMPLLRTTVEPQKPEQREA 360

Query 361
LLDALTQLADTDPLLRCEVDSITHEIILSFLGRVQLEVVSALLSEKYKLETVVKEPSVIY 420
LL+AL ++ADTDPLL ++D++THEIILSFLG+VQLEV+ +LL EKY +
+KEPSVIY
Sbjct 361
LLNALAEIADTDPLLHFDIDTVTHEIILSFLGKVQLEVICSLLEEKYHVGVAMKEPSVIY 420

Query 421
MERPLKAASHTIHIEVPPNPFWASIGLSVTPLSLGSGVQYESRVSLGYLNQSFQNAVRDG 480
+ERP K AS TIHIEVPPNPFWASIGL+VTPL +GSG QY+S
VSLGYLNQSFQNAV +G
Sbjct 421
LERPQKKASCTIHIEVPPNPFWASIGLTVTPLPVGSGTQYKSEVSLGYLNQSFQNAVMEG 480

Query 481
IRYGLEQGLFGWNVTDCKICFEYGLYYSPVSTPADFRSLAPIVLEQALKESGTQLLEPYL 540
            +RYG+EQGL+GW VTDC+ICF+YG+YYSPVSTPADFR LAP+VLEQALK+
+GTQLLEPYL
Sbjct 481
VRYGMEQGLYGWGVTDCQICFDYGVYYSPVSTPADFRFLAPVVLEQALKKAGTQLLEPYL 540

Query 541
SFILYAPQEYLSRAYHDAPKYCATIETAQVKKDEVVFTGEIPARCIQAYRTDLAFYTNGR 600
            SF L+APQEYLSRAY+DAPKYCA IE+ +++KDEV+F GEIPARCI YR DL
FYTNGR
Sbjct 541
SFTLFAPQEYLSRAYNDAPKYCAIIESTRLEKDEVIFKGEIPARCIGEYRNDLNFYTNGR 600

Query 601 SVCLTELKGYQAAVGQPVIQPRRPNSRLDKVRHMFQKVM 639
            SVC+TELKGYQ   G+PV QPRRPNSRLDK+RHMFQK+M
Sbjct 601 SVCITELKGYQETSGEPVFQPRRPNSRLDKIRHMFQKIM 639

>WP_063725249.1 TetM/TetW/TetO/TetS family tetracycline resistance
ribosomal
protection protein [Streptococcus suis]
 ANC99818.1 tetracycline resistance ribosomal protection protein
[Streptococcus
suis]
Length=639

 Score = 932 bits (2410), Expect = 0.0, Method: Compositional
matrix adjust.
 Identities = 431/638 (68%), Positives = 531/638 (83%), Gaps = 0/638
(0%)

Query 1
MKIINIGILAHVDAGKTTLTESLLYASGAISEPGSVEKGTTRTDTMFLERQRGITIQAAV 60
            MKIIN+GILAHVDAGKTTLTESLLY SGAI+EPGSV+KGTTRTDTM
LERQRGITIQ AV
Sbjct 1
MKIINLGILAHVDAGKTTLTESLLYTSGAIAEPGSVDKGTTRTDTMNLERQRGITIQTAV 60

Query 61
TSFQWHRCKVNIVDTPGHMDFLAEVYRSLAVLDGAILVISAKDGVQAQTRILFHALRKMN 120
            TSFQW   KVNI+DTPGHMDFLAEVYRSL+VLDGA+L++SAKDG+QAQTRILFHAL
+ M
Sbjct 61
TSFQWEDVKVNIIDTPGHMDFLAEVYRSLSVLDGAVLLVSAKDGIQAQTRILFHALQTMK 120

Query 121
IPTVIFINKIDQAGVDLQSVVQSVRDKLSADIIIKQTVSLSPEIVLEENTDIEAWDAVIE 180
            IPT+ FINKIDQ G+DL V Q ++ KLS++II+KQ V   P I + +N D+E
WDAVI
Sbjct 121
IPTIFFINKIDQEGIDLPMVYQEMKAKLSSEIIVKQKVGQHPHINVTDNDDMEQWDAVIM 180

Query 181

```
NNDKLLEKYIAGEPISREKLVREEQRRVQDASLFPVYYGSAKKGLGIQPLMDAVTGLFQP  240
             ND+LLEKY++G+P    +L +EE RR Q+ +LFPVY+GSAK  LGI+ L++ +
F
Sbjct  181
GNDELLEKYMSGKPFKMSELEQEENRRFQNGTLFPVYHGSAKNNLGIRQLIEVIASKFYS  240

Query  241
IGEQGSAALCGSVFKVEYTDCGQRRVYLRLYSGTLRLRDTVALAGREKLKITEMRIPSKG  300
               +G + LCG VFK+EY++  +R VY+R+YSGTL LRD + ++ +EK+KITEM
+P+ G
Sbjct  241
STPEGQSELCGQVFKIEYSEKRRRFVYVRIYSGTLHLRDVIKISEKEKIKITEMCVPTNG  300

Query  301
EIVRTDTAYPGEIVILPSDSVRLNDVLGDPTRLPRKRWREDPLPMLRTTIAPKTAAQRER  360
            E+  +DTA  G+IVILP+D ++LN +LG+   LP++++ E+PLPML+TTIA K +
QRE
Sbjct  301
ELYSSDTACSGDIVILPNDVLQLNSILGNEMLLPQRKFIENPLPMLQTTIAVKKSEQREI  360

Query  361
LLDALTQLADTDPLLRCEVDSITHEIILSFLGRVQLEVVSALLSEKYKLETVVKEPSVIY  420
            LL+ALT+++D DPLL+  VD+ THEIILSFLG VQ+EV+ A+L EKY +E  +KEP
+VIY
Sbjct  361
LLEALTEISDGDPLLKYYVDTTTHEIILSFLGNVQMEVICAILEEKYHVEAEIKEPTVIY  420

Query  421
MERPLKAASHTIHIEVPPNPFWASIGLSVTPLSLGSGVQYESRVSLGYLNQSFQNAVRDG  480
            MERPL+ A +TIHIEVPPNPFWAS+GLS+ PL
+GSGVQYESRVSLGYLNQSFQNAV +G
Sbjct  421
MERPLRKAEYTIHIEVPPNPFWASVGLSIEPLPIGSGVQYESRVSLGYLNQSFQNAVMEG  480

Query  481
IRYGLEQGLFGWNVTDCKICFEYGLYYSPVSTPADFRSLAPIVLEQALKESGTQLLEPYL  540
            + YG EQGL+GW VTDCKICFEYGLYYSPVSTPADFR L+PIVLEQALK++GT
+LLEPYL
Sbjct  481
VLYGCEQGLYGWKVTDCKICFEYGLYYSPVSTPADFRLLSPIVLEQALKKAGTELLEPYL  540

Query  541
SFILYAPQEYLSRAYHDAPKYCATIETAQVKKDEVVFTGEIPARCIQAYRTDLAFYTNGR  600
             F +YAPQEYLSRAYHDAP+YCA I + Q+K DEV+  GEIPARCIQ YR DL +
+TNG+
Sbjct  541
HFEIYAPQEYLSRAYHDAPRYCADIVSTQIKNDEVILKGEIPARCIQEYRNDLTYFTNGQ  600

Query  601  SVCLTELKGYQAAVGQPVIQPRRPNSRLDKVRHMFQKV  638
             VCLTELKGYQ A+G+ + QPRRPNSR+DKVRHMF K+
Sbjct  601  GVCLTELKGYQPAIGKFICQPRRPNSRIDKVRHMFHKL  638
```

>AMP47017.1 TetM-TetW-TetO-TetS [uncultured bacterium]

Length=639

 Score = 932 bits (2410), Expect = 0.0, Method: Compositional matrix adjust.
 Identities = 434/638 (68%), Positives = 531/638 (83%), Gaps = 0/638 (0%)

```
Query  1
MKIINIGILAHVDAGKTTLTESLLYASGAISEPGSVEKGTTRTDTMFLERQRGITIQAAV  60
            MKIIN+GILAHVDAGKTTLTESLLY SGAI+EPGSV++GTTRTDTM 
LERQRGITIQ AV
Sbjct  1
MKIINLGILAHVDAGKTTLTESLLYTSGAIAEPGSVDEGTTRTDTMNLERQRGITIQTAV  60

Query  61
TSFQWHRCKVNIVDTPGHMDFLAEVYRSLAVLDGAILVISAKDGVQAQTRILFHALRKMN  120
            TSFQW   KVNI+DTPGHMDFL E YR+L+VLDGA+LVISAKDGVQAQTRILFHAL
+KMN
Sbjct  61
TSFQWEDVKVNIIDTPGHMDFLTEAYRALSVLDGAVLVISAKDGVQAQTRILFHALQKMN  120

Query  121
IPTVIFINKIDQAGVDLQSVVQSVRDKLSADIIIKQTVSLSPEIVLEENTDIEAWDAVIE  180
            IPT+IFINKIDQ G+DL  V + ++ KLS++II+KQ V   P I + +N D+E
WDAVI
Sbjct  121
IPTIIFINKIDQEGIDLPMVYREMKAKLSSEIIVKQKVGQHPHINVTDNDDMEQWDAVIM  180

Query  181
NNDKLLEKYIAGEPISREKLVREEQRRVQDASLFPVYYGSAKKGLGIQPLMDAVTGLFQP  240
             ND+LLEKY++G+P    +L +EE RR Q+ +LFPVY+GSAK  LGI+ L++ +
F
Sbjct  181
GNDELLEKYMSGKPFKMSELEQEENRRFQNGTLFPVYHGSAKNNLGIRQLIEVIASKFYS  240

Query  241
IGEQGSAALCGSVFKVEYTDCGQRRVYLRLYSGTLRLRDTVALAGREKLKITEMRIPSKG  300
               +G + LCG VFK+EY++  +R VY+R+YSGTL LRD + ++ +EK+KITEM
IPS G
Sbjct  241
STPEGQSELCGQVFKIEYSEKRRRFVYVRIYSGTLHLRDVIRISEKEKIKITEMCIPSNG  300

Query  301
EIVRTDTAYPGEIVILPSDSVRLNDVLGDPTRLPRKRWREDPLPMLRTTIAPKTAAQRER  360
            E+  +DTA  G+IVILP+D ++LN +LG+   LP++++ E+PLPML+TTIA K +
QRE
Sbjct  301
ELYSSDTACSGDIVILPNDVLQLNSILGNEMLLPQRKFIENPLPMLQTTIAVKKSEQREI  360

Query  361
LLDALTQLADTDPLLRCEVDSITHEIILSFLGRVQLEVVSALLSEKYKLETVVKEPSVIY  420
            LL ALT+++D DPLL+  VD+ THEIILSFLG VQ+EV+ A+L EKY +E  +KEP
+VIY
Sbjct  361
```

LLGALTEISDGDPLLKYYVDTTTHEIILSFLGNVQMEVICAILEEKYHVEAEIKEPTVIY 420

Query 421
MERPLKAASHTIHIEVPPNPFWASIGLSVTPLSLGSGVQYESRVSLGYLNQSFQNAVRDG 480
            MERPL+ A +TIHIEVPPNPFWAS+GLS+ PL
+GSGVQYESRVSLGYLNQSFQNAV +G
Sbjct 421
MERPLRKAEYTIHIEVPPNPFWASVGLSIEPLPIGSGVQYESRVSLGYLNQSFQNAVMEG 480

Query 481
IRYGLEQGLFGWNVTDCKICFEYGLYYSPVSTPADFRSLAPIVLEQALKESGTQLLEPYL 540
            + YG EQGL+GW VTDCKICFEYGLYYSPVSTPADFR L+PIVLEQALK++GT
+LLEPYL
Sbjct 481
VLYGCEQGLYGWKVTDCKICFEYGLYYSPVSTPADFRLLSPIVLEQALKKAGTELLEPYL 540

Query 541
SFILYAPQEYLSRAYHDAPKYCATIETAQVKKDEVVFTGEIPARCIQAYRTDLAFYTNGR 600
             F +YAPQEYLSRAYHDAP+YCA I + Q+K DEV+ GEIPARCIQ YR DL +
+TNG+
Sbjct 541
HFEIYAPQEYLSRAYHDAPRYCADIVSTQIKNDEVILKGEIPARCIQEYRNDLTYFTNGQ 600

Query 601 SVCLTELKGYQAAVGQPVIQPRRPNSRLDKVRHMFQKV 638
             VCLTELKGYQ A+G+ + QPRRPNSR+DKVRHMF K+
Sbjct 601 GVCLTELKGYQPAIGKFICQPRRPNSRIDKVRHMFHKL 638

>WP_002605705.1 TetM/TetW/TetO/TetS family tetracycline resistance
ribosomal
protection protein [Hungatella hathewayi]
 ENY89979.1 tetracycline resistance protein TetM [[Clostridium]
hathewayi
12489931] [Hungatella hathewayi 12489931]
Length=639

 Score = 932 bits (2410), Expect = 0.0, Method: Compositional
matrix adjust.
 Identities = 438/639 (69%), Positives = 536/639 (84%), Gaps = 0/639
(0%)

Query 1
MKIINIGILAHVDAGKTTLTESLLYASGAISEPGSVEKGTTRTDTMFLERQRGITIQAAV 60
            MKIINIGILAHVDAGKTTLTESLLY SGAI+E G+V+KGTTRTDTM
LERQRGITIQ AV
Sbjct 1
MKIINIGILAHVDAGKTTLTESLLYTSGAIAEQGNVDKGTTRTDTMILERQRGITIQTAV 60

Query 61
TSFQWHRCKVNIVDTPGHMDFLAEVYRSLAVLDGAILVISAKDGVQAQTRILFHALRKMN 120
            TSF W+ K+NIVDTPGHMDFL E YRSL+VLDGA+LVISAKDGVQAQTRILFHAL
+KM+
Sbjct 61
TSFCWNDYKINIVDTPGHMDFLTEAYRSLSVLDGAVLVISAKDGVQAQTRILFHALQKMD 120

Query 121
IPTVIFINKIDQAGVDLQSVVQSVRDKLSADIIIKQTVSLSPEIVLEENTDIEAWDAVIE 180
            IPT+IFINKIDQ G+DL+ V QS++DKL++D+I+ Q VSLSP+I + + +D++ WD
+I
Sbjct 121
IPTIIFINKIDQNGIDLRRVYQSIKDKLTSDMIVMQEVSLSPKITMTDISDLDKWDMIIS 180

Query 181
NNDKLLEKYIAGEPISREKLVREEQRRVQDASLFPVYYGSAKKGLGIQPLMDAVTGLFQP 240
             +D+LLE+Y+A + + ++L E+ +R + SLFPVY+GSAK LG + L++A+T
F
Sbjct 181
GSDELLERYVAEDSLDIQELQYEKCKRTRCCSLFPVYHGSAKDNLGTEKLIEAITETFIT 240

Query 241
IGEQGSAALCGSVFKVEYTDCGQRRVYLRLYSGTLRLRDTVALAGREKLKITEMRIPSKG 300
              +   + LCG VFKVEYT+ +R YLRLY GTL LRDT+ L+ +EK+KITEM
IPS G
Sbjct 241
ETDDIQSELCGYVFKVEYTERKKRLSYLRLYHGTLHLRDTLLLSKKEKIKITEMCIPSNG 300

Query 301
EIVRTDTAYPGEIVILPSDSVRLNDVLGDPTRLPRKRWREDPLPMLRTTIAPKTAAQRER 360
            EIV D A PGEIVIL D+++LND+LG+   LP K   ++P+P+LRTT+ P+
QRE
Sbjct 301
EIVPVDHACPGEIVILADDTLKLNDILGNEKLLPHKTRIDNPMPLLRTTVEPQKPEQREA 360

Query 361
LLDALTQLADTDPLLRCEVDSITHEIILSFLGRVQLEVVSALLSEKYKLETVVKEPSVIY 420
            LL+ALT++ADTDPLL ++D++THEIILSFLG+VQLEV+ +LL EKY +
+KEPSVIY
Sbjct 361
LLNALTEIADTDPLLHFDIDTVTHEIILSFLGKVQLEVICSLLEEKYHVGVAMKEPSVIY 420

Query 421
MERPLKAASHTIHIEVPPNPFWASIGLSVTPLSLGSGVQYESRVSLGYLNQSFQNAVRDG 480
            +ERP K AS+TIHIEVPPNPFWASIGL+VTPL +GSG QY+S
VSLGYLNQSFQNAV +G
Sbjct 421
LERPQKKASYTIHIEVPPNPFWASIGLTVTPLPVGSGTQYKSEVSLGYLNQSFQNAVMEG 480

Query 481
IRYGLEQGLFGWNVTDCKICFEYGLYYSPVSTPADFRSLAPIVLEQALKESGTQLLEPYL 540
            +RYG+EQGL+GW TDC+ICF+YG+YYSPVSTPADFR LAP+VLEQALK+
+GTQLLEPYL
Sbjct 481
VRYGMEQGLYGWGGTDCQICFDYGVYYSPVSTPADFRFLAPVVLEQALKKAGTQLLEPYL 540

Query 541
SFILYAPQEYLSRAYHDAPKYCATIETAQVKKDEVVFTGEIPARCIQAYRTDLAFYTNGR 600
            SF L+APQEYLSRAY+DAPKYCA IE+ +++KDEV+F GEIPARCI YR DL
FYTNGR

Sbjct 541
SFTLFAPQEYLSRAYNDAPKYCAIIESTRLEKDEVIFKGEIPARCIGEYRNDLNFYTNGR 600

Query 601 SVCLTELKGYQAAVGQPVIQPRRPNSRLDKVRHMFQKVM 639
            SVC+TELKGYQ   G+PV QPRRPNSRLDK+RHMFQK+M
Sbjct 601 SVCITELKGYQETSGEPVFQPRRPNSRLDKIRHMFQKIM 639

>WP_055257520.1 TetM/TetW/TetO/TetS family tetracycline resistance ribosomal
protection protein [Anaerostipes hadrus]
 CUM70860.1 Tetracycline resistance protein tetM from transposon Tn916 [Anaerostipes
hadrus]
Length=639

 Score = 932 bits (2409), Expect = 0.0, Method: Compositional matrix adjust.
 Identities = 433/638 (68%), Positives = 527/638 (83%), Gaps = 0/638 (0%)

Query 1
MKIINIGILAHVDAGKTTLTESLLYASGAISEPGSVEKGTTRTDTMFLERQRGITIQAAV 60
            MKIIN+GILAHVDAGKTTLTESLLY SGAI+E GSV++GTTRTDTM
LERQRGITIQ AV
Sbjct 1
MKIINLGILAHVDAGKTTLTESLLYTSGAIAELGSVDEGTTRTDTMNLERQRGITIQTAV 60

Query 61
TSFQWHRCKVNIVDTPGHMDFLAEVYRSLAVLDGAILVISAKDGVQAQTRILFHALRKMN 120
            TSFQW   KVNI+DTPGHMDFLAEVYRSL+VLDGA+L++SAKDG+QAQTRILFHAL
+ M
Sbjct 61
TSFQWEDVKVNIIDTPGHMDFLAEVYRSLSVLDGAVLLVSAKDGIQAQTRILFHALQIMK 120

Query 121
IPTVIFINKIDQAGVDLQSVVQSVRDKLSADIIIKQTVSLSPEIVLEENTDIEAWDAVIE 180
            IPT+ FINKIDQ G+DL V + ++ KLS++II+KQ V   P I + +N D+E
WDAVI
Sbjct 121
IPTIFFINKIDQEGIDLPMVYREMKAKLSSEIIVKQKVGQHPHINVTDNDDMEQWDAVIM 180

Query 181
NNDKLLEKYIAGEPISREKLVREEQRRVQDASLFPVYYGSAKKGLGIQPLMDAVTGLFQP 240
             ND+LLEKY++G+P   +L +EE RR Q+ +LFPVY+GSAK LGI+ L++ +
F
Sbjct 181
GNDELLEKYMSGKPFKMSELEQEENRRFQNGTLFPVYHGSAKNNLGIRQLIEVIASKFYS 240

Query 241
IGEQGSAALCGSVFKVEYTDCGQRRVYLRLYSGTLRLRDTVALAGREKLKITEMRIPSKG 300
               +G + LCG VFK+EY++ +R VY+R+YSGTL LRD + ++ +EK+KITEM
IPS G
Sbjct 241

STPEGQSELCGQVFKIEYSEKRRRFVYVRIYSGTLHLRDVIRISEKEKIKITEMCIPSNG 300

Query 301
EIVRTDTAYPGEIVILPSDSVRLNDVLGDPTRLPRKRWREDPLPMLRTTIAPKTAAQRER 360
            EIV D A PGEIVIL D+++LND+LG+   LP K W ++P+P+LRTT+ P+
QRE
Sbjct 301
EIVPADHACPGEIVILADDTLKLNDILGNEKLLPHKTWIDNPMPLLRTTVEPQKPEQREA 360

Query 361
LLDALTQLADTDPLLRCEVDSITHEIILSFLGRVQLEVVSALLSEKYKLETVVKEPSVIY 420
            LL+AL ++ADTDPLL+ VD+ THEIILSFLG VQ+EV+ A+L EKY +E
+KEPSVIY
Sbjct 361
LLNALAEIADTDPLLKYYVDTTTHEIILSFLGNVQMEVICAILEEKYHVEAEIKEPSVIY 420

Query 421
MERPLKAASHTIHIEVPPNPFWASIGLSVTPLSLGSGVQYESRVSLGYLNQSFQNAVRDG 480
            MERPL+ A +TIHIEVPPNPFWAS+GLS+ PL
+GSGVQYESRVSLGYLNQSFQNAV +G
Sbjct 421
MERPLRKAEYTIHIEVPPNPFWASVGLSIEPLPIGSGVQYESRVSLGYLNQSFQNAVMEG 480

Query 481
IRYGLEQGLFGWNVTDCKICFEYGLYYSPVSTPADFRSLAPIVLEQALKESGTQLLEPYL 540
            + YG EQGL+GW VTDCKICFEYGLYYSPVSTPADFR L+PIVLEQALK++GT
+LLEPYL
Sbjct 481
VLYGCEQGLYGWKVTDCKICFEYGLYYSPVSTPADFRLLSPIVLEQALKKAGTELLEPYL 540

Query 541
SFILYAPQEYLSRAYHDAPKYCATIETAQVKKDEVVFTGEIPARCIQAYRTDLAFYTNGR 600
             F +YAPQEYLSRAYHDAP+YCA I + Q+K DEV+ GEIPARCIQ YR DL +
+TNG+
Sbjct 541
HFEIYAPQEYLSRAYHDAPRYCADIVSTQIKNDEVILKGEIPARCIQEYRNDLTYFTNGQ 600

Query 601 SVCLTELKGYQAAVGQPVIQPRRPNSRLDKVRHMFQKV 638
             VCLTELKGYQ A+G+ + QPRRPNSR+DKVRHMF K+
Sbjct 601 GVCLTELKGYQPAIGKFICQPRRPNSRIDKVRHMFHKL 638

>WP_105105938.1 TetM/TetW/TetO/TetS family tetracycline resistance
ribosomal
protection protein [Streptococcus suis]
Length=639

Score = 932 bits (2408), Expect = 0.0, Method: Compositional
matrix adjust.
Identities = 431/638 (68%), Positives = 530/638 (83%), Gaps = 0/638
(0%)

Query 1
MKIINIGILAHVDAGKTTLTESLLYASGAISEPGSVEKGTTRTDTMFLERQRGITIQAAV 60

MKIIN+GILAHVDAGKTTLTESLLY SGAI+EPGSV+KGTTRTDTM
LERQRGITIQ AV
Sbjct 1
MKIINLGILAHVDAGKTTLTESLLYTSGAIAEPGSVDKGTTRTDTMNLERQRGITIQTAV 60

Query 61
TSFQWHRCKVNIVDTPGHMDFLAEVYRSLAVLDGAILVISAKDGVQAQTRILFHALRKMN 120
TSFQW   KVNI+DTPGHMDFLAEVYRSL+VLDGA+L++SAKDG+QAQTRILFHAL
+ M
Sbjct 61
TSFQWEDVKVNIIDTPGHMDFLAEVYRSLSVLDGAVLLVSAKDGIQAQTRILFHALQTMK 120

Query 121
IPTVIFINKIDQAGVDLQSVVQSVRDKLSADIIIKQTVSLSPEIVLEENTDIEAWDAVIE 180
IPT+ FINKIDQ G+DL V Q ++ KLS++II+KQ V   P I + +N D+E
WDAVI
Sbjct 121
IPTIFFINKIDQEGIDLPMVYQEMKAKLSSEIIVKQKVGQHPHINVTDNDDMEQWDAVIM 180

Query 181
NNDKLLEKYIAGEPISREKLVREEQRRVQDASLFPVYYGSAKKGLGIQPLMDAVTGLFQP 240
ND+LLEKY++G+P   +L +EE RR Q+ +LFPVY+GSAK LGI+ L++ +
F
Sbjct 181
GNDELLEKYMSGKPFKMSELEQEENRRFQNGTLFPVYHGSAKNNLGIRQLIEVIASKFYS 240

Query 241
IGEQGSAALCGSVFKVEYTDCGQRRVYLRLYSGTLRLRDTVALAGREKLKITEMRIPSKG 300
+G + LCG VFK+EY++ +R VY+R+YSGTL LRD + ++ +EK+KITEM
+P+ G
Sbjct 241
STPEGQSELCGQVFKIEYSEKRRRFVYVRIYSGTLHLRDVIKISEKEKIKITEMCVPTNG 300

Query 301
EIVRTDTAYPGEIVILPSDSVRLNDVLGDPTRLPRKRWREDPLPMLRTTIAPKTAAQRER 360
E+ +DTA G+IVILP+D ++LN +LG+   LP++++ E+PLPML+TTIA K +
QRE
Sbjct 301
ELYSSDTACSGDIVILPNDVLQLNSILGNEMLLPQRKFIENPLPMLQTTIAVKKSEQREI 360

Query 361
LLDALTQLADTDPLLRCEVDSITHEIILSFLGRVQLEVVSALLSEKYKLETVVKEPSVIY 420
LL ALT+++D DPLL+ VD+ THEIILSFLG VQ+EV+ A+L EKY +E +KEP
+VIY
Sbjct 361
LLGALTEISDCDPLLKYYVDTTTHEIILSFLGNVQMEVICAILEEKYHVEAEIKEPTVIY 420

Query 421
MERPLKAASHTIHIEVPPNPFWASIGLSVTPLSLGSGVQYESRVSLGYLNQSFQNAVRDG 480
MERPL+ A +TIHIEVPPNPFWAS+GLS+ PL
+GSGVQYESRVSLGYLNQSFQNAV +G
Sbjct 421
MERPLRKAEYTIHIEVPPNPFWASVGLSIEPLPIGSGVQYESRVSLGYLNQSFQNAVMEG 480

Query 481
IRYGLEQGLFGWNVTDCKICFEYGLYYSPVSTPADFRSLAPIVLEQALKESGTQLLEPYL 540
            + YG EQGL+GW VTDCKICFEYGLYYSPVSTPADFR L+PIVLEQALK++GT
+LLEPYL
Sbjct 481
VLYGCEQGLYGWKVTDCKICFEYGLYYSPVSTPADFRLLSPIVLEQALKKAGTELLEPYL 540

Query 541
SFILYAPQEYLSRAYHDAPKYCATIETAQVKKDEVVFTGEIPARCIQAYRTDLAFYTNGR 600
             F +YAPQEYLSRAYHDAP+YCA I + Q+K DEV+ GEIPARCIQ YR DL +
+TNG+
Sbjct 541
HFEIYAPQEYLSRAYHDAPRYCADIVSTQIKNDEVILKGEIPARCIQEYRNDLTYFTNGQ 600

Query 601 SVCLTELKGYQAAVGQPVIQPRRPNSRLDKVRHMFQKV 638
             VCLTELKGYQ A+G+ + QPRRPNSR+DKVRHMF K+
Sbjct 601 GVCLTELKGYQPAIGKFICQPRRPNSRIDKVRHMFHKL 638

>AMP50690.1 TetM-TetW-TetO-TetS [uncultured bacterium]
Length=639

 Score = 932 bits (2408), Expect = 0.0, Method: Compositional
matrix adjust.
 Identities = 434/638 (68%), Positives = 530/638 (83%), Gaps = 0/638
(0%)

Query 1
MKIINIGILAHVDAGKTTLTESLLYASGAISEPGSVEKGTTRTDTMFLERQRGITIQAAV 60
            MKIINIGILAHVDAGKTTLTESLLY SGAI+EPGSV+KGTTRTDTM
LERQRGITIQ AV
Sbjct 1
MKIINIGILAHVDAGKTTLTESLLYTSGAIAEPGSVDKGTTRTDTMNLERQRGITIQTAV 60

Query 61
TSFQWHRCKVNIVDTPGHMDFLAEVYRSLAVLDGAILVISAKDGVQAQTRILFHALRKMN 120
            TSFQW   KVNI+DTPGHMDFLAEVYRSL+VLDGA+L++SAKDG+QAQTRILFHAL
+ M
Sbjct 61
TSFQWEDVKVNIIDTPGHMDFLAEVYRSLSVLDGAVLLVSAKDGIQAQTRILFHALQIMK 120

Query 121
IPTVIFINKIDQAGVDLQSVVQSVRDKLSADIIIKQTVSLSPEIVLEENTDIEAWDAVIE 180
            IPT+ FINKIDQ G+DL V + ++ KLS++II+KQ V   P I + +N D+E
WDAVI
Sbjct 121
IPTIFFINKIDQEGIDLPMVYREMKAKLSSEIIVKQKVGQHPHINVTDNDDMEQWDAVIM 180

Query 181
NNDKLLEKYIAGEPISREKLVREEQRRVQDASLFPVYYGSAKKGLGIQPLMDAVTGLFQP 240
             ND+LLEKY++G+P   +L +EE RR Q+ +LFPVY+GSAK LGI+ L++ +
F
Sbjct 181
GNDELLEKYMSGKPFKMSELEQEENRRFQNGTLFPVYHGSAKNNLGIRQLIEVIASKFYS 240

Query 241
IGEQGSAALCGSVFKVEYTDCGQRRVYLRLYSGTLRLRDTVALAGREKLKITEMRIPSKG 300
               +G + LCG VFK+EY++ +R VY+R+YSGTL LRD + ++ +EK+KITEM
+P+ G
Sbjct 241
STPEGQSELCGQVFKIEYSEKRRRFVYVRIYSGTLHLRDVIRISEKEKIKITEMCVPTNG 300

Query 301
EIVRTDTAYPGEIVILPSDSVRLNDVLGDPTRLPRKRWREDPLPMLRTTIAPKTAAQRER 360
            EIV D A PGEIVIL D+++LND+LG+   LP++++ E+PLPML+TTIA K +
QRE
Sbjct 301
EIVPADHACPGEIVILADDTLKLNDILGNEILLPQRKFIENPLPMLQTTIAVKKSEQREI 360

Query 361
LLDALTQLADTDPLLRCEVDSITHEIILSFLGRVQLEVVSALLSEKYKLETVVKEPSVIY 420
            LL ALT+++D DPLL+ VD+ THEIILSFLG VQ+EV+ A+L EKY +E +KEP
+VIY
Sbjct 361
LLGALTEISDGDPLLKYYVDTTTHEIILSFLGNVQMEVICAILEEKYHVEAEIKEPTVIY 420

Query 421
MERPLKAASHTIHIEVPPNPFWASIGLSVTPLSLGSGVQYESRVSLGYLNQSFQNAVRDG 480
            MERPL+ A +TIHIEVPPNPFWAS+GLS+ PL
+GSGVQYESRVSLGYLNQSFQNAV +G
Sbjct 421
MERPLRKAEYTIHIEVPPNPFWASVGLSIEPLPIGSGVQYESRVSLGYLNQSFQNAVMEG 480

Query 481
IRYGLEQGLFGWNVTDCKICFEYGLYYSPVSTPADFRSLAPIVLEQALKESGTQLLEPYL 540
            + YG EQGL+GW VTDCKICFEYGLYYSPVSTPADFR L+PIVLEQALK++GT
+LLEPYL
Sbjct 481
VLYGCEQGLYGWKVTDCKICFEYGLYYSPVSTPADFRLLSPIVLEQALKKAGTELLEPYL 540

Query 541
SFILYAPQEYLSRAYHDAPKYCATIETAQVKKDEVVFTGEIPARCIQAYRTDLAFYTNGR 600
             F +YAPQEYLSRAYHDAP+YCA I + Q+K DEV+ GEIPARCIQ YR DL +
+TNG+
Sbjct 541
HFEIYAPQEYLSRAYHDAPRYCADIVSTQIKNDEVILKGEIPARCIQEYRNDLTYFTNGQ 600

Query 601 SVCLTELKGYQAAVGQPVIQPRRPNSRLDKVRHMFQKV 638
             VCLTELKGYQ A+G+ + QPRRPNSR+DKVRHMF K+
Sbjct 601 GVCLTELKGYQPAIGKFICQPRRPNSRIDKVRHMFHKL 638

>WP_003505402.1 MULTISPECIES: tetracycline resistance ribosomal
protection protein
Tet(32) [Firmicutes]
 ABV82118.1 Tet32 [Streptococcus parasanguinis]
 EDS08798.1 putative translation elongation factor G
[[[Clostridium] scindens

ATCC 35704]
 EDS12311.1 putative translation elongation factor G [Anaerotruncus colihominis
DSM 17241]
 ACH87088.1 Tet(32) [Escherichia coli]
 EGA91509.1 ribosomal protection type tetracycline resistance protein [[[Clostridium]
symbiosum WAL-14163]
 EHL15110.1 tetracycline resistance protein tetO [Peptoanaerobacter stomatis]
 EHO15797.1 small GTP-binding protein domain protein [Stomatobaculum longum]
 EJP24214.1 tetracycline resistance protein TetM [Peptostreptococcaceae bacterium
AS15]
 EJZ70581.1 small GTP-binding protein domain protein [Lachnoanaerobaculum
sp. OBRC5-5]
 AIK77516.1 tetracycline resistance protein tetM [Streptococcus anginosus]
 KMW18068.1 tetracycline resistance protein tetO [[Clostridium] citroniae
WAL-19142]
 KXB52902.1 putative translation elongation factor G [Lachnoanaerobaculum
saburreum]
 AMP53992.1 TetM-TetW-TetO-TetS [uncultured bacterium]
 OFS50791.1 tetracycline resistance ribosomal protection protein [Streptococcus
sp. HMSC072D03]
 OHS92409.1 tetracycline resistance ribosomal protection protein [Streptococcus
sp. HMSC36C04]
 APO28870.1 Ferrous iron transport protein B [uncultured bacterium]
 OUP81678.1 tetracycline resistance ribosomal protection protein Tet(32)
[Lachnoclostridium sp. An169]
 AVM69083.1 tetracycline resistance ribosomal protection protein Tet(32)
[Lachnospiraceae bacterium oral taxon 500]
 PRT78934.1 tetracycline resistance ribosomal protection protein Tet(32)
[Streptococcus anginosus]
Length=639

 Score = 932 bits (2408), Expect = 0.0, Method: Compositional matrix adjust.
 Identities = 437/639 (68%), Positives = 534/639 (84%), Gaps = 0/639 (0%)

Query 1
MKIINIGILAHVDAGKTTLTESLLYASGAISEPGSVEKGTTRTDTMFLERQRGITIQAAV 60
            MKIINIGILAHVDAGKTTLTESLLY SGAI+E G+V+KGTTRTDTM
LERQRGITIQ AV

Sbjct 1
MKIINIGILAHVDAGKTTLTESLLYTSGAIAEQGNVDKGTTRTDTMILERQRGITIQTAV 60

Query 61
TSFQWHRCKVNIVDTPGHMDFLAEVYRSLAVLDGAILVISAKDGVQAQTRILFHALRKMN 120
TSF W+ K+NIVDTPGHMDFL E YRSL+VLDGA+LVISAKDGVQAQTRILFHAL
+KM+
Sbjct 61
TSFYWNDYKINIVDTPGHMDFLTEAYRSLSVLDGAVLVISAKDGVQAQTRILFHALQKMD 120

Query 121
IPTVIFINKIDQAGVDLQSVVQSVRDKLSADIIIKQTVSLSPEIVLEENTDIEAWDAVIE 180
IPT+IFINKIDQ G+DLQ V QS++DKL++D+I+ Q VSLSP+I + + +D++ WD
+I
Sbjct 121
IPTIIFINKIDQNGIDLQCVYQSIKDKLTSDMIVMQEVSLSPKITMTDISDLDKWDMIIS 180

Query 181
NNDKLLEKYIAGEPISREKLVREEQRRVQDASLFPVYYGSAKKGLGIQPLMDAVTGLFQP 240
+D+LLE+Y+A + + ++L E+ +R + SLFPVY+GSAK LG + L++A+
F
Sbjct 181
GSDELLERYVAEDSLDIQELQYEKCKRTRCCSLFPVYHGSAKDNLGTEKLIEAIIETFIT 240

Query 241
IGEQGSAALCGSVFKVEYTDCGQRRVYLRLYSGTLRLRDTVALAGREKLKITEMRIPSKG 300
+   + LCG VFKVEYT+ +R YLRLY GTL LRDT+ L+ +EK+KITEM
IPS G
Sbjct 241
ETDDIQSELCGYVFKVEYTERKKRLSYLRLYHGTLHLRDTLLLSKKEKIKITEMCIPSNG 300

Query 301
EIVRTDTAYPGEIVILPSDSVRLNDVLGDPTRLPRKRWREDPLPMLRTTIAPKTAAQRER 360
EIV D A PGEIVIL D+++LND+LG+   LP K   ++P+P+LRTT+ P+
QRE
Sbjct 301
EIVPADHACPGEIVILADDTLKLNDILGNEKLLPHKTRIDNPMPLLRTTVEPQKPEQREA 360

Query 361
LLDALTQLADTDPLLRCEVDSITHEIILSFLGRVQLEVVSALLSEKYKLETVVKEPSVIY 420
LL+AL ++ADTDPLL ++D++THEI+LSFLG+VQLEV+ +LL EKY +
+KEPSVIY
Sbjct 361
LLNALAEIADTDPLLHFDIDTVTHEIMLSFLGKVQLEVICSLLEEKYHVGVAMKEPSVIY 420

Query 421
MERPLKAASHTIHIEVPPNPFWASIGLSVTPLSLGSGVQYESRVSLGYLNQSFQNAVRDG 480
+ERP K AS TIHIEVPPNPFWASIGL+VTPL +GSG QY+S
VSLGYLNQSFQNAV +G
Sbjct 421
LERPQKKASCTIHIEVPPNPFWASIGLTVTPLPVGSGTQYKSEVSLGYLNQSFQNAVMEG 480

Query 481
IRYGLEQGLFGWNVTDCKICFEYGLYYSPVSTPADFRSLAPIVLEQALKESGTQLLEPYL 540

            +RYG+EQGL+GW VTDC+ICF+YG+YYSPVSTPADFR LAP+VLEQALK+
+GTQLLEPYL
Sbjct 481
VRYGMEQGLYGWGVTDCQICFDYGVYYSPVSTPADFRFLAPVVLEQALKKAGTQLLEPYL 540

Query 541
SFILYAPQEYLSRAYHDAPKYCATIETAQVKKDEVVFTGEIPARCIQAYRTDLAFYTNGR 600
            SF L+APQEYLSRAY+DAPKYCA IE+ +++KDEV+F GEIPARCI YR DL
FYTNGR
Sbjct 541
SFTLFAPQEYLSRAYNDAPKYCAIIESTRLEKDEVIFKGEIPARCIGEYRNDLNFYTNGR 600

Query 601 SVCLTELKGYQAAVGQPVIQPRRPNSRLDKVRHMFQKVM 639
            SVC+TELKGYQ   G+PV QPRRPNSRLDK+RHMFQK+M
Sbjct 601 SVCITELKGYQETSGEPVFQPRRPNSRLDKIRHMFQKIM 639

>WP_105153139.1 TetM/TetW/TetO/TetS family tetracycline resistance
ribosomal
protection protein [Streptococcus suis]
Length=639

 Score = 931 bits (2407), Expect = 0.0, Method: Compositional
matrix adjust.
 Identities = 431/638 (68%), Positives = 530/638 (83%), Gaps = 0/638
(0%)

Query 1
MKIINIGILAHVDAGKTTLTESLLYASGAISEPGSVEKGTTRTDTMFLERQRGITIQAAV 60
            MKIIN+GILAHVDAGKTTLTESLLY SGAI+EPGSV+KGTTRTDTM
LERQRGITIQ AV
Sbjct 1
MKIINLGILAHVDAGKTTLTESLLYTSGAIAEPGSVDKGTTRTDTMNLERQRGITIQTAV 60

Query 61
TSFQWHRCKVNIVDTPGHMDFLAEVYRSLAVLDGAILVISAKDGVQAQTRILFHALRKMN 120
            TSFQW   KVNI+DTPGHMDFLAEVYRSL+VLDGA+L++SAKDG+QAQTRILFHAL
+ M
Sbjct 61
TSFQWEDVKVNIIDTPGHMDFLAEVYRSLSVLDGAVLLVSAKDGIQAQTRILFHALQTMK 120

Query 121
IPTVIFINKIDQAGVDLQSVVQSVRDKLSADIIIKQTVSLSPEIVLEENTDIEAWDAVIE 180
            IPT+ FINKIDQ G+DL V Q ++ KLS++II+KQ V   P I + +N D+E
WDAVI
Sbjct 121
IPTIFFINKIDQEGIDLPMVYQEMKAKLSSEIIVKQKVGQHPHINVTDNDDMEQWDAVIM 180

Query 181
NNDKLLEKYIAGEPISREKLVREEQRRVQDASLFPVYYGSAKKGLGIQPLMDAVTGLFQP 240
             ND+LLEKY++G+P   +L +EE RR Q+ +LFPVY+GSAK LGI+ L++ +
F
Sbjct 181
GNDELLEKYMSGKPFKMSELEQEENRRFQNGTLFPVYHGSAKNNLGIRQLIEVIASKFYS 240

```
Query  241
IGEQGSAALCGSVFKVEYTDCGQRRVYLRLYSGTLRLRDTVALAGREKLKITEMRIPSKG  300
               +G + LCG VFK+EY++  +R VY+R+YSGTL LRD + ++ +EK+KITEM
+P+ G
Sbjct  241
STPEGQSELCGQVFKIEYSEKRRRFVYVRIYSGTLHLRDVIKISEKEKIKITEMCVPTNG  300

Query  301
EIVRTDTAYPGEIVILPSDSVRLNDVLGDPTRLPRKRWREDPLPMLRTTIAPKTAAQRER  360
            E+  +DTA  G+IVILP+D ++LN +LG+   LP++++ E+PLPML+TTIA K +
QRE
Sbjct  301
ELYSSDTACSGDIVILPNDVLQLNSILGNEMLLPQRKFIENPLPMLQTTIAVKKSEQREI  360

Query  361
LLDALTQLADTDPLLRCEVDSITHEIILSFLGRVQLEVVSALLSEKYKLETVVKEPSVIY  420
            LL ALT+++D DPLL+  VD+ THEIILSFLG VQ+EV+ A+L EKY +E  +KEP
+VIY
Sbjct  361
LLGALTEISDGDPLLKYYVDTTTHEIILSFLGNVQMEVICAILEEKYHVEVEIKEPTVIY  420

Query  421
MERPLKAASHTIHIEVPPNPFWASIGLSVTPLSLGSGVQYESRVSLGYLNQSFQNAVRDG  480
            MERPL+ A +TIHIEVPPNPFWAS+GLS+ PL
+GSGVQYESRVSLGYLNQSFQNAV +G
Sbjct  421
MERPLRKAEYTIHIEVPPNPFWASVGLSIEPLPIGSGVQYESRVSLGYLNQSFQNAVMEG  480

Query  481
IRYGLEQGLFGWNVTDCKICFEYGLYYSPVSTPADFRSLAPIVLEQALKESGTQLLEPYL  540
            + YG EQGL+GW VTDCKICFEYGLYYSPVSTPADFR L+PIVLEQALK++GT
+LLEPYL
Sbjct  481
VLYGCEQGLYGWKVTDCKICFEYGLYYSPVSTPADFRLLSPIVLEQALKKAGTELLEPYL  540

Query  541
SFILYAPQEYLSRAYHDAPKYCATIETAQVKKDEVVFTGEIPARCIQAYRTDLAFYTNGR  600
             F +YAPQEYLSRAYHDAP+YCA I + Q+K DEV+  GEIPARCIQ YR DL +
+TNG+
Sbjct  541
HFEIYAPQEYLSRAYHDAPRYCADIVSTQIKNDEVILKGEIPARCIQEYRNDLTYFTNGQ  600

Query  601  SVCLTELKGYQAAVGQPVIQPRRPNSRLDKVRHMFQKV  638
             VCLTELKGYQ A+G+ + QPRRPNSR+DKVRHMF K+
Sbjct  601  GVCLTELKGYQTAIGKFICQPRRPNSRIDKVRHMFHKL  638


>WP_075759481.1 TetM/TetW/TetO/TetS family tetracycline resistance
ribosomal
protection protein [Anaerostipes sp. 494a]
 OLR59286.1 tetracycline resistance ribosomal protection protein
Tet(O) [Anaerostipes
sp. 494a]
```

Length=639

 Score = 931 bits (2407), Expect = 0.0, Method: Compositional
matrix adjust.
 Identities = 432/638 (68%), Positives = 527/638 (83%), Gaps = 0/638
(0%)

Query 1
MKIINIGILAHVDAGKTTLTESLLYASGAISEPGSVEKGTTRTDTMFLERQRGITIQAAV 60
            MKIIN+GILAHVDAGKTTLTESLLY SGAI+E GSV++GTTRTDTM
LERQRGITIQ AV
Sbjct 1
MKIINLGILAHVDAGKTTLTESLLYTSGAIAELGSVDEGTTRTDTMNLERQRGITIQTAV 60

Query 61
TSFQWHRCKVNIVDTPGHMDFLAEVYRSLAVLDGAILVISAKDGVQAQTRILFHALRKMN 120
            TSFQW   KVNI+DTPGHMDFLAEVYRSL+VLDGA+L++SAKDG+QAQTRILFHAL
+ M
Sbjct 61
TSFQWEDVKVNIIDTPGHMDFLAEVYRSLSVLDGAVLLVSAKDGIQAQTRILFHALQIMK 120

Query 121
IPTVIFINKIDQAGVDLQSVVQSVRDKLSADIIIKQTVSLSPEIVLEENTDIEAWDAVIE 180
            IPT+ FINKIDQ G+DL V + ++ KLS++II+KQ V   P I + +N D+E
WDAVI
Sbjct 121
IPTIFFINKIDQEGIDLPMVYREMKAKLSSEIIVKQKVGQHPHINVTDNDDMEQWDAVIM 180

Query 181
NNDKLLEKYIAGEPISREKLVREEQRRVQDASLFPVYYGSAKKGLGIQPLMDAVTGLFQP 240
             ND+LLEKY++G+P   +L +EE RR Q+ +LFPVY+GSAK LGI+ L++ +
F
Sbjct 181
GNDELLEKYMSGKPFKMSELEQEENRRFQNGTLFPVYHGSAKNNLGIRQLIEVIASKFYS 240

Query 241
IGEQGSAALCGSVFKVEYTDCGQRRVYLRLYSGTLRLRDTVALAGREKLKITEMRIPSKG 300
               +G + LCG VFK+EY++ +R VY+R+YSGTL LRD + ++ +EK+KITEM
+PS G
Sbjct 241
STPEGQSELCGQVFKIEYSEKRRRFVYVRIYSGTLHLRDVIRISEKEKIKITEMCVPSNG 300

Query 301
EIVRTDTAYPGEIVILPSDSVRLNDVLGDPTRLPRKRWREDPLPMLRTTIAPKTAAQRER 360
            EIV D A PGEIVIL D+++LND+LG+   LP K W ++P+P+LRTT+ P+
QRE
Sbjct 301
EIVPADHACPGEIVILADDTLKLNDILGNEKLLPHKTWIDNPMPLLRTTVEPQKPEQREA 360

Query 361
LLDALTQLADTDPLLRCEVDSITHEIILSFLGRVQLEVVSALLSEKYKLETVVKEPSVIY 420
            LL+AL ++ADTDPLL+ VD+ THEIILSFLG VQ+EV+ A+L EKY +E
+KEPSVIY
Sbjct 361

LLNALAEIADTDPLLKYYVDTTTHEIILSFLGNVQMEVICAILEEKYHVEAEIKEPSVIY 420

Query 421
MERPLKAASHTIHIEVPPNPFWASIGLSVTPLSLGSGVQYESRVSLGYLNQSFQNAVRDG 480
            MERPL+ A +TIHIEVPPNPFWAS+GLS+ PL
+GSGVQYESRVSLGYLNQSFQNAV +G
Sbjct 421
MERPLRKAEYTIHIEVPPNPFWASVGLSIEPLPIGSGVQYESRVSLGYLNQSFQNAVMEG 480

Query 481
IRYGLEQGLFGWNVTDCKICFEYGLYYSPVSTPADFRSLAPIVLEQALKESGTQLLEPYL 540
            + YG EQGL+GW VTDCKICFEYGLYYSPVSTPADFR L+PIVLEQALK++GT
+LLEPYL
Sbjct 481
VLYGCEQGLYGWKVTDCKICFEYGLYYSPVSTPADFRLLSPIVLEQALKKAGTELLEPYL 540

Query 541
SFILYAPQEYLSRAYHDAPKYCATIETAQVKKDEVVFTGEIPARCIQAYRTDLAFYTNGR 600
             F +YAPQEYLSRAYHDAP+YCA I + Q+K DEV+ GEIPARCIQ YR DL +
+TNG+
Sbjct 541
HFEIYAPQEYLSRAYHDAPRYCADIVSTQIKNDEVILKGEIPARCIQEYRNDLTYFTNGQ 600

Query 601 SVCLTELKGYQAAVGQPVIQPRRPNSRLDKVRHMFQKV 638
             VCLTELKGYQ A+G+ + QPRRPNSR+DKVRHMF K+
Sbjct 601 GVCLTELKGYQPAIGKFICQPRRPNSRIDKVRHMFHKL 638

>APO32499.1 feoB: ferrous iron transport protein, partial
[uncultured bacterium]
Length=635

Score = 931 bits (2407), Expect = 0.0, Method: Compositional
matrix adjust.
Identities = 435/635 (69%), Positives = 533/635 (84%), Gaps = 0/635
(0%)

Query 5
NIGILAHVDAGKTTLTESLLYASGAISEPGSVEKGTTRTDTMFLERQRGITIQAAVTSFQ 64
            NIGILAHVDAGKTTLTESLLY SGAI+E G+V+KGTTRTDTM LERQRGITIQ
AVTSF
Sbjct 1
NIGILAHVDAGKTTLTESLLYTSGAIAEQGNVDKGTTRTDTMILERQRGITIQTAVTSFC 60

Query 65
WHRCKVNIVDTPGHMDFLAEVYRSLAVLDGAILVISAKDGVQAQTRILFHALRKMNIPTV 124
            W+ K+NIVDTPGHMDFL E YRSL+VLDGA+LVISAKDGVQAQTRILFHAL+KM
+IPT+
Sbjct 61
WNDYKINIVDTPGHMDFLTEAYRSLSVLDGAVLVISAKDGVQAQTRILFHALQKMDIPTI 120

Query 125
IFINKIDQAGVDLQSVVQSVRDKLSADIIIKQTVSLSPEIVLEENTDIEAWDAVIENNDK 184
            IFINKIDQ G+DL+ V QS++DKL++D+I+ Q VSLSP+I + + +D++ WD +I

+D+
Sbjct 121
IFINKIDQNGIDLRRVYQSIKDKLTSDMIVMQEVSLSPKITMTDISDLDKWDMIISGSDE 180

Query 185
LLEKYIAGEPISREKLVREEQRRVQDASLFPVYYGSAKKGLGIQPLMDAVTGLFQPIGEQ 244
            LLE+Y+A + + ++L E+ +R + SLFPVY+GSAK LG + L++A+T F
+
Sbjct 181
LLERYVAEDSLDIQELQYEKCKRTRCCSLFPVYHGSAKDNLGTEKLIEAITETFITETDD 240

Query 245
GSAALCGSVFKVEYTDCGQRRVYLRLYSGTLRLRDTVALAGREKLKITEMRIPSKGEIVR 304
              + LCG VFKVEYT+ +R YLRLY GTL LRDT+ L+ +EK+KITEM IPS
GEIV
Sbjct 241
IQSELCGYVFKVEYTERKKRLSYLRLYHGTLHLRDTLLLSKKEKIKITEMCIPSNGEIVP 300

Query 305
TDTAYPGEIVILPSDSVRLNDVLGDPTRLPRKRWREDPLPMLRTTIAPKTAAQRERLLDA 364
             D A PGEIVIL D+++LND+LG+   LP K W ++P+P+LRTT+ P+   QRE
LL+A
Sbjct 301
ADHACPGEIVILADDTLKLNDILGNEKLLPHKTWIDNPMPLLRTTVEPQKPEQREALLNA 360

Query 365
LTQLADTDPLLRCEVDSITHEIILSFLGRVQLEVVSALLSEKYKLETVVKEPSVIYMERP 424
            L ++ADTDPLL ++D++THEIILSFLG+VQLEV+ +LL EKY +   +KEPSVIY
+ERP
Sbjct 361
LAEIADTDPLLHFDIDTVTHEIILSFLGKVQLEVICSLLEEKYHVGVAMKEPSVIYLERP 420

Query 425
LKAASHTIHIEVPPNPFWASIGLSVTPLSLGSGVQYESRVSLGYLNQSFQNAVRDGIRYG 484
             K AS+TIHIEVPPNPFWASIGL+VTPL +GSG QY+S VSLGYLNQSFQNAV +G
+RYG
Sbjct 421
QKKASYTIHIEVPPNPFWASIGLTVTPLPVGSGTQYKSEVSLGYLNQSFQNAVMEGVRYG 480

Query 485
LEQGLFGWNVTDCKICFEYGLYYSPVSTPADFRSLAPIVLEQALKESGTQLLEPYLSFIL 544
            +EQGL+GW VTDC+ICF+YG+YYSPVSTPADFR LAP+VLEQALK+
+GTQLLEPYLSF L
Sbjct 481
MEQGLYGWGVTDCQICFDYGVYYSPVSTPADFRFLAPVVLEQALKKAGTQLLEPYLSFTL 540

Query 545
YAPQEYLSRAYHDAPKYCATIETAQVKKDEVVFTGEIPARCIQAYRTDLAFYTNGRSVCL 604
            +APQEYLSRAY+DAPKYCA IE+ +++KDEV+F GEIPARCI YR DL
FYTNGRSVC+
Sbjct 541
FAPQEYLSRAYNDAPKYCAIIESTRLEKDEVIFKGEIPARCIGEYRNDLNFYTNGRSVCI 600

Query 605 TELKGYQAAVGQPVIQPRRPNSRLDKVRHMFQKVM 639

```
            TELKGYQ   G+PV QPRRPNSRLDK+RHMFQK+M
Sbjct  601  TELKGYQETSGEPVFQPRRPNSRLDKIRHMFQKIM  635


>WP_070432258.1 TetM/TetW/TetO/TetS family tetracycline resistance 
ribosomal 
protection protein [Corynebacterium sp. HMSC11H10]
 OFU53610.1 tetracycline resistance ribosomal protection protein 
[Corynebacterium 
sp. HMSC11H10]
Length=639

 Score = 931 bits (2407),  Expect = 0.0, Method: Compositional 
matrix adjust.
 Identities = 432/638 (68%), Positives = 530/638 (83%), Gaps = 0/638 
(0%)

Query  1    
MKIINIGILAHVDAGKTTLTESLLYASGAISEPGSVEKGTTRTDTMFLERQRGITIQAAV  60
            MKIIN+GILAHVDAGKTTLTESLLY SGAI+EPGSV+KGTTRTDTM  
LERQRGITIQ AV
Sbjct  1    
MKIINLGILAHVDAGKTTLTESLLYTSGAIAEPGSVDKGTTRTDTMNLERQRGITIQTAV  60

Query  61   
TSFQWHRCKVNIVDTPGHMDFLAEVYRSLAVLDGAILVISAKDGVQAQTRILFHALRKMN  120
            TSFQW   KVNI+DTPGHMDFLAEVYRSL+VLDGA+L++SAKDG+QAQTRILFHAL 
+ M
Sbjct  61   
TSFQWEDVKVNIIDTPGHMDFLAEVYRSLSVLDGAVLLVSAKDGIQAQTRILFHALQTMK  120

Query  121  
IPTVIFINKIDQAGVDLQSVVQSVRDKLSADIIIKQTVSLSPEIVLEENTDIEAWDAVIE  180
            IPT+ FINKIDQ G+DL  V Q ++ KLS++II+KQ V   P I + +N D+E 
WDAVI
Sbjct  121  
IPTIFFINKIDQEGIDLPMVYQEMKAKLSSEIIVKQKVGQHPHINVTDNDDMEQWDAVIM  180

Query  181  
NNDKLLEKYIAGEPISREKLVREEQRRVQDASLFPVYYGSAKKGLGIQPLMDAVTGLFQP  240
             ND+LLEKY++G+P    +L +EE RR Q+ +LFPVY+GSAK  LGI+ L++ + 
F
Sbjct  181  
GNDELLEKYMSGKPFKMSELEQEENRRFQNGTLFPVYHGSAKNNLGIRQLIEVIASKFYS  240

Query  241  
IGEQGSAALCGSVFKVEYTDCGQRRVYLRLYSGTLRLRDTVALAGREKLKITEMRIPSKG  300
               +G + LCG VFK+EY++  +R VY+R+YSGTL LRD + ++ +EK+KITEM 
+P+ G
Sbjct  241  
STPEGQSELCGQVFKIEYSEKRRRFVYVRIYSGTLHLRDVIKISEKEKIKITEMCVPTNG  300

Query  301  
EIVRTDTAYPGEIVILPSDSVRLNDVLGDPTRLPRKRWREDPLPMLRTTIAPKTAAQRER  360
```

```
            E+  +DTA  G+IVILP+D ++LN +LG+   LP++++ E+PLPML+TTIA K +
QRE
Sbjct  301
ELYSSDTARSGDIVILPNDVLQLNSILGNEMLLPQRKFIENPLPMLQTTIAVKKSEQREI  360

Query  361
LLDALTQLADTDPLLRCEVDSITHEIILSFLGRVQLEVVSALLSEKYKLETVVKEPSVIY  420
            LL ALT+++D DPLL+  VD+ THEIILSFLG VQ+EV+ A+L EKY +E  +KEP
+VIY
Sbjct  361
LLGALTEISDGDPLLKYYVDTTTHEIILSFLGNVQMEVICAILEEKYHVEAEIKEPTVIY  420

Query  421
MERPLKAASHTIHIEVPPNPFWASIGLSVTPLSLGSGVQYESRVSLGYLNQSFQNAVRDG  480
            MERPL+ A +TIHIEVPPNPFWAS+GLS+ PL
+GSGVQYESRVSLGYLNQSFQNAV +G
Sbjct  421
MERPLRKAEYTIHIEVPPNPFWASVGLSIEPLPIGSGVQYESRVSLGYLNQSFQNAVMEG  480

Query  481
IRYGLEQGLFGWNVTDCKICFEYGLYYSPVSTPADFRSLAPIVLEQALKESGTQLLEPYL  540
            + YG EQGL+GW VTDCKICFEYGLYYSPVSTPADFR L+PIVLEQALK++GT
+LLEPYL
Sbjct  481
VLYGCEQGLYGWKVTDCKICFEYGLYYSPVSTPADFRLLSPIVLEQALKKAGTELLEPYL  540

Query  541
SFILYAPQEYLSRAYHDAPKYCATIETAQVKKDEVVFTGEIPARCIQAYRTDLAFYTNGR  600
             F +YAPQEYLSRAYHDAP+YCA I + QVK DEV+  GEIPARCIQ YR DL +
+TNG+
Sbjct  541
HFEIYAPQEYLSRAYHDAPRYCADIVSTQVKNDEVILKGEIPARCIQEYRNDLTYFTNGQ  600

Query  601  SVCLTELKGYQAAVGQPVIQPRRPNSRLDKVRHMFQKV  638
             VCLTELKGYQ A+G+ + QPRRPNSR+DKVRHMF K+
Sbjct  601  GVCLTELKGYQPAIGKFICQPRRPNSRIDKVRHMFHKL  638


>WP_055159350.1 MULTISPECIES: TetM/TetW/TetO/TetS family
tetracycline resistance
ribosomal protection protein [Clostridiales]
 CUO37964.1 Tetracycline resistance protein tetM from transposon
Tn916 [[Ruminococcus]
torques]
 SCI79103.1 Tetracycline resistance protein tetM from transposon
Tn916 [uncultured
Ruminococcus sp.]
 SCI58003.1 Tetracycline resistance protein tetM from transposon
Tn916 [uncultured
Ruminococcus sp.]
 SHJ73142.1 ribosomal protection tetracycline resistance protein
[Hespellia
stercorisuis DSM 15480]
Length=639
```

Score = 931 bits (2407), Expect = 0.0, Method: Compositional matrix adjust.
Identities = 430/638 (67%), Positives = 528/638 (83%), Gaps = 0/638 (0%)

```
Query  1
MKIINIGILAHVDAGKTTLTESLLYASGAISEPGSVEKGTTRTDTMFLERQRGITIQAAV  60
            MKIIN+GILAHVDAGKTTLTESLLY SGAI+E GSV++GTTRTDTM
LERQRGITIQ AV
Sbjct  1
MKIINLGILAHVDAGKTTLTESLLYTSGAIAELGSVDEGTTRTDTMNLERQRGITIQTAV  60

Query  61
TSFQWHRCKVNIVDTPGHMDFLAEVYRSLAVLDGAILVISAKDGVQAQTRILFHALRKMN  120
            TSFQW   KVNI+DTPGHMDFLAEVYRSL+VLDGA+L++SAKDG+QAQTRILFHAL
+ M
Sbjct  61
TSFQWEDVKVNIIDTPGHMDFLAEVYRSLSVLDGAVLLVSAKDGIQAQTRILFHALQIMK  120

Query  121
IPTVIFINKIDQAGVDLQSVVQSVRDKLSADIIIKQTVSLSPEIVLEENTDIEAWDAVIE  180
            IPT+ FINKIDQ G+DL  V + ++ KLS++II+KQ V   P I + +N D+E
WDAVI
Sbjct  121
IPTIFFINKIDQEGIDLPMVYREMKAKLSSEIIVKQKVGQHPHINVTDNDDMEQWDAVIM  180

Query  181
NNDKLLEKYIAGEPISREKLVREEQRRVQDASLFPVYYGSAKKGLGIQPLMDAVTGLFQP  240
             ND+LLEKY++G+P    +L +EE RR Q+ +LFPVY+GSAK  LGI+ L++ +
F
Sbjct  181
GNDELLEKYMSGKPFKMSELEQEENRRFQNGTLFPVYHGSAKNNLGIRQLIEVIASKFYS  240

Query  241
IGEQGSAALCGSVFKVEYTDCGQRRVYLRLYSGTLRLRDTVALAGREKLKITEMRIPSKG  300
               +G + LCG VFK+EY++  +R VY+R+YSGTL LRD + ++ +EK+KITEM
+PS G
Sbjct  241
STPEGQSELCGQVFKIEYSEKRRRFVYVRIYSGTLHLRDVIRISEKEKIKITEMCVPSNG  300

Query  301
EIVRTDTAYPGEIVILPSDSVRLNDVLGDPTRLPRKRWREDPLPMLRTTIAPKTAAQRER  360
            EIV  D A PGEIVIL  D+++LND+LG+   LP K W ++P+P+LRTT+ P+
QRE
Sbjct  301
EIVPADHACPGEIVILADDTLKLNDILGNEKLLPHKTWIDNPMPLLRTTVEPQKPEQREA  360

Query  361
LLDALTQLADTDPLLRCEVDSITHEIILSFLGRVQLEVVSALLSEKYKLETVVKEPSVIY  420
            LL+AL ++ADTDPLL  ++D++THEIILSFLG VQ+EV+ A+L EKY +E  +KEP
+VIY
Sbjct  361
LLNALAEIADTDPLLHFDIDTVTHEIILSFLGNVQMEVICAILEEKYHVEAEIKEPTVIY  420
```

Query 421
MERPLKAASHTIHIEVPPNPFWASIGLSVTPLSLGSGVQYESRVSLGYLNQSFQNAVRDG 480
            MERPL+ A +TIHIEVPPNPFWAS+GLS+ PL
+GSGVQYESRVSLGYLNQSFQNAV +G
Sbjct 421
MERPLRKAEYTIHIEVPPNPFWASVGLSIEPLPIGSGVQYESRVSLGYLNQSFQNAVMEG 480

Query 481
IRYGLEQGLFGWNVTDCKICFEYGLYYSPVSTPADFRSLAPIVLEQALKESGTQLLEPYL 540
            + YG EQGL+GW VTDCKICFEYGLYYSPVSTPADFR L+PIVLEQALK++GT
+LLEPYL
Sbjct 481
VLYGCEQGLYGWKVTDCKICFEYGLYYSPVSTPADFRLLSPIVLEQALKKAGTELLEPYL 540

Query 541
SFILYAPQEYLSRAYHDAPKYCATIETAQVKKDEVVFTGEIPARCIQAYRTDLAFYTNGR 600
             F +YAPQEYLSRAYHDAP+YCA I + Q+K DEV+ GEIPARCIQ YR DL +
+TNG+
Sbjct 541
HFEIYAPQEYLSRAYHDAPRYCADIVSTQIKNDEVILKGEIPARCIQEYRNDLTYFTNGQ 600

Query 601 SVCLTELKGYQAAVGQPVIQPRRPNSRLDKVRHMFQKV 638
             VCLTELKGYQ A+G+ + QPRRPNSR+DKVRHMF K+
Sbjct 601 GVCLTELKGYQPAIGKFICQPRRPNSRIDKVRHMFHKL 638

>WP_044774256.1 TetM/TetW/TetO/TetS family tetracycline resistance
ribosomal
protection protein [Streptococcus suis]
CYV12551.1 tetracycline resistance protein tetM [Streptococcus
suis]
Length=639

Score = 931 bits (2407), Expect = 0.0, Method: Compositional
matrix adjust.
Identities = 432/638 (68%), Positives = 530/638 (83%), Gaps = 0/638
(0%)

Query 1
MKIINIGILAHVDAGKTTLTESLLYASGAISEPGSVEKGTTRTDTMFLERQRGITIQAAV 60
            MKIIN+GILAHVDAGKTTLTESLLY SGAI+EPGSV+KGTTRTDTM
LERQRGITIQ AV
Sbjct 1
MKIINLGILAHVDAGKTTLTESLLYTSGAIAEPGSVDKGTTRTDTMNLERQRGITIQTAV 60

Query 61
TSFQWHRCKVNIVDTPGHMDFLAEVYRSLAVLDGAILVISAKDGVQAQTRILFHALRKMN 120
            TSFQW   KVNI+DTPGHMDFLAEVYRSL+VLDGA+L++SAKDG+QAQTRILFHAL
+ M
Sbjct 61
TSFQWEDVKVNIIDTPGHMDFLAEVYRSLSVLDGAVLLVSAKDGIQAQTRILFHALQTMK 120

Query 121

```
IPTVIFINKIDQAGVDLQSVVQSVRDKLSADIIIKQTVSLSPEIVLEENTDIEAWDAVIE  180
            IPT+ FINKIDQ G+DL  V Q ++ KLS++II+KQ V   P I + +N D+E
WDAVI
Sbjct  121
IPTIFFINKIDQEGIDLPMVYQEMKAKLSSEIIVKQKVGQHPHINVTDNDDMEQWDAVIM  180

Query  181
NNDKLLEKYIAGEPISREKLVREEQRRVQDASLFPVYYGSAKKGLGIQPLMDAVTGLFQP  240
             ND+LLEKY++G+P    +L +EE RR Q+ +LFPVY+GSAK  LGI+ L++ +
F
Sbjct  181
GNDELLEKYMSGKPFKMSELEQEENRRFQNGTLFPVYHGSAKNNLGIRQLIEVIASKFYS  240

Query  241
IGEQGSAALCGSVFKVEYTDCGQRRVYLRLYSGTLRLRDTVALAGREKLKITEMRIPSKG  300
               +G + LCG VFK+EY++  +R VY+R+YSGTL LRD + ++ +EK+KITEM
+P+ G
Sbjct  241
STPEGQSELCGQVFKIEYSEKRRRFVYVRIYSGTLHLRDVIKISEKEKIKITEMCVPTNG  300

Query  301
EIVRTDTAYPGEIVILPSDSVRLNDVLGDPTRLPRKRWREDPLPMLRTTIAPKTAAQRER  360
            E+  +DTA  G+IVILP+D ++LN +LG+   LP++++ E+PLPML+TTIA K +
QRE
Sbjct  301
ELYSSDTACSGDIVILPNDVLQLNSILGNEILLPQRKFIENPLPMLQTTIAVKKSEQREI  360

Query  361
LLDALTQLADTDPLLRCEVDSITHEIILSFLGRVQLEVVSALLSEKYKLETVVKEPSVIY  420
            LL ALT+++D DPLL+  VD+ THEIILSFLG VQ+EV+ A+L EKY +E  +KEP
+VIY
Sbjct  361
LLGALTEISDGDPLLKYYVDTTTHEIILSFLGNVQMEVICAILEEKYHVEAEIKEPTVIY  420

Query  421
MERPLKAASHTIHIEVPPNPFWASIGLSVTPLSLGSGVQYESRVSLGYLNQSFQNAVRDG  480
            MERPL+ A +TIHIEVPPNPFWAS+GLS+ PL
+GSGVQYESRVSLGYLNQSFQNAV +G
Sbjct  421
MERPLRKAEYTIHIEVPPNPFWASVGLSIEPLPIGSGVQYESRVSLGYLNQSFQNAVMEG  480

Query  481
IRYGLEQGLFGWNVTDCKICFEYGLYYSPVSTPADFRSLAPIVLEQALKESGTQLLEPYL  540
            + YG EQGL+GW VTDCKICFEYGLYYSPVSTPADFR L+PIVLEQALK++GT
+LLEPYL
Sbjct  481
VLYGCEQGLYGWKVTDCKICFEYGLYYSPVSTPADFRLLSPIVLEQALKKAGTELLEPYL  540

Query  541
SFILYAPQEYLSRAYHDAPKYCATIETAQVKKDEVVFTGEIPARCIQAYRTDLAFYTNGR  600
             F +YAPQEYLSRAYHDAP+YCA I + QVK DEV+  GEIPARCIQ YR DL +
+TNG+
Sbjct  541
HFEIYAPQEYLSRAYHDAPRYCADIVSTQVKNDEVILKGEIPARCIQEYRNDLTYFTNGQ  600
```

```
Query  601  SVCLTELKGYQAAVGQPVIQPRRPNSRLDKVRHMFQKV  638
             VCLTELKGYQ A+G+ + QPRRPNSR+DKVRHMF K+
Sbjct  601  GVCLTELKGYQPAIGKFICQPRRPNSRIDKVRHMFHKL  638


>WP_044770894.1 TetM/TetW/TetO/TetS family tetracycline resistance
ribosomal
protection protein [Streptococcus suis]
 CYU74129.1 tetracycline resistance protein tetM [Streptococcus
suis]
Length=639

 Score = 931 bits (2407),  Expect = 0.0, Method: Compositional
matrix adjust.
 Identities = 431/638 (68%), Positives = 530/638 (83%), Gaps = 0/638
(0%)

Query  1
MKIINIGILAHVDAGKTTLTESLLYASGAISEPGSVEKGTTRTDTMFLERQRGITIQAAV  60
            MKIIN+GILAHVDAGKTTLTESLLY SGAI+EPGSV+KGTTRTDTM
LERQRGITIQ AV
Sbjct  1
MKIINLGILAHVDAGKTTLTESLLYTSGAIAEPGSVDKGTTRTDTMNLERQRGITIQTAV  60

Query  61
TSFQWHRCKVNIVDTPGHMDFLAEVYRSLAVLDGAILVISAKDGVQAQTRILFHALRKMN  120
            TSFQW   KVNI+DTPGHMDFLAEVYRSL+VLDGA+L++SAKDG+QAQTRILFHAL
+ M
Sbjct  61
TSFQWEDVKVNIIDTPGHMDFLAEVYRSLSVLDGAVLLVSAKDGIQAQTRILFHALQTMK  120

Query  121
IPTVIFINKIDQAGVDLQSVVQSVRDKLSADIIIKQTVSLSPEIVLEENTDIEAWDAVIE  180
            IPT+ FINKIDQ G+DL  V Q ++ KLS++II+KQ V   P I + +N D+E
WDAVI
Sbjct  121
IPTIFFINKIDQEGIDLPMVYQEMKAKLSSEIIVKQKVGQHPHINVTDNDDMEQWDAVIM  180

Query  181
NNDKLLEKYIAGEPISREKLVREEQRRVQDASLFPVYYGSAKKGLGIQPLMDAVTGLFQP  240
             ND+LLEKY++G+P    +L +EE RR Q+ +LFPVY+GSAK  LGI+ L++ +
F
Sbjct  181
GNDELLEKYMSGKPFKMSELEQEENRRFQNGTLFPVYHGSAKNNLGIRQLIEVIASKFYS  240

Query  241
IGEQGSAALCGSVFKVEYTDCGQRRVYLRLYSGTLRLRDTVALAGREKLKITEMRIPSKG  300
               +G + LCG VFK+EY++  +R VY+R+YSGTL LRD + ++ +EK+KITEM
+P+ G
Sbjct  241
STPEGQSELCGQVFKIEYSEKRRRFVYVRIYSGTLHLRDVIKISEKEKIKITEMCVPTNG  300

Query  301
```

EIVRTDTAYPGEIVILPSDSVRLNDVLGDPTRLPRKRWREDPLPMLRTTIAPKTAAQRER 360
            E+ +DTA G+IVILP+D ++LN +LG+   LP++++ E+PLPML+TTIA K +
QRE
Sbjct 301
ELYSSDTACSGDIVILPNDVLQLNSILGNEMLLPQRKFIENPLPMLQTTIAVKKSEQREI 360

Query 361
LLDALTQLADTDPLLRCEVDSITHEIILSFLGRVQLEVVSALLSEKYKLETVVKEPSVIY 420
            LL ALT+++D DPLL+ VD+ THEIILSFLG VQ+EV+ A+L EKY +E +KEP
+VIY
Sbjct 361
LLGALTEISDGDPLLKYYVDTTTHEIILSFLGNVQMEVICAILEEKYHVEAEIKEPAVIY 420

Query 421
MERPLKAASHTIHIEVPPNPFWASIGLSVTPLSLGSGVQYESRVSLGYLNQSFQNAVRDG 480
            MERPL+ A +TIHIEVPPNPFWAS+GLS+ PL
+GSGVQYESRVSLGYLNQSFQNAV +G
Sbjct 421
MERPLRKAEYTIHIEVPPNPFWASVGLSIEPLPIGSGVQYESRVSLGYLNQSFQNAVMEG 480

Query 481
IRYGLEQGLFGWNVTDCKICFEYGLYYSPVSTPADFRSLAPIVLEQALKESGTQLLEPYL 540
            + YG EQGL+GW VTDCKICFEYGLYYSPVSTPADFR L+PIVLEQALK++GT
+LLEPYL
Sbjct 481
VLYGCEQGLYGWKVTDCKICFEYGLYYSPVSTPADFRLLSPIVLEQALKKAGTELLEPYL 540

Query 541
SFILYAPQEYLSRAYHDAPKYCATIETAQVKKDEVVFTGEIPARCIQAYRTDLAFYTNGR 600
             F +YAPQEYLSRAYHDAP+YCA I + Q+K DEV+ GEIPARCIQ YR DL +
+TNG+
Sbjct 541
HFEIYAPQEYLSRAYHDAPRYCADIVSTQIKNDEVILKGEIPARCIQEYRNDLTYFTNGQ 600

Query 601 SVCLTELKGYQAAVGQPVIQPRRPNSRLDKVRHMFQKV 638
             VCLTELKGYQ A+G+ + QPRRPNSR+DKVRHMF K+
Sbjct 601 GVCLTELKGYQPAIGKFICQPRRPNSRIDKVRHMFHKL 638

>WP_044677300.1 TetM/TetW/TetO/TetS family tetracycline resistance
ribosomal
protection protein [Streptococcus suis]
 CYV10094.1 tetracycline resistance protein tetM [Streptococcus
suis]
Length=639

 Score = 931 bits (2407), Expect = 0.0, Method: Compositional
matrix adjust.
 Identities = 432/638 (68%), Positives = 530/638 (83%), Gaps = 0/638
(0%)

Query 1
MKIINIGILAHVDAGKTTLTESLLYASGAISEPGSVEKGTTRTDTMFLERQRGITIQAAV 60
            MKIIN+GILAHVDAGKTTLTESLLY SGAI+EPGSV+KGTTRTDTM

LERQRGITIQ AV
Sbjct 1
MKIINLGILAHVDAGKTTLTESLLYTSGAIAEPGSVDKGTTRTDTMNLERQRGITIQTAV 60

Query 61
TSFQWHRCKVNIVDTPGHMDFLAEVYRSLAVLDGAILVISAKDGVQAQTRILFHALRKMN 120
            TSFQW   KVNI+DTPGHMDFLAEVYRSL+VLDGA+L++SAKDG+QAQTRILFHAL
+ M
Sbjct 61
TSFQWEDVKVNIIDTPGHMDFLAEVYRSLSVLDGAVLLVSAKDGIQAQTRILFHALQTMK 120

Query 121
IPTVIFINKIDQAGVDLQSVVQSVRDKLSADIIIKQTVSLSPEIVLEENTDIEAWDAVIE 180
            IPT+ FINKIDQ G+DL V Q ++ KLS++II+KQ V   P I + +N D+E
WDAVI
Sbjct 121
IPTIFFINKIDQEGIDLPMVYQEMKAKLSSEIIVKQKVGQHPHINVMDNDDMEQWDAVIM 180

Query 181
NNDKLLEKYIAGEPISREKLVREEQRRVQDASLFPVYYGSAKKGLGIQPLMDAVTGLFQP 240
             ND+LLEKY++G+P   +L +EE RR Q+ +LFPVY+GSAK LGI+ L++ +
F
Sbjct 181
GNDELLEKYMSGKPFKMSELEQEENRRFQNGTLFPVYHGSAKNNLGIRQLIEVIASKFYS 240

Query 241
IGEQGSAALCGSVFKVEYTDCGQRRVYLRLYSGTLRLRDTVALAGREKLKITEMRIPSKG 300
               +G + LCG VFK+EY++ +R VY+R+YSGTL LRD + ++ +EK+KITEM
+P+ G
Sbjct 241
STPEGQSELCGQVFKIEYSEKRRRFVYVRIYSGTLHLRDVIKISEKEKIKITEMCVPTNG 300

Query 301
EIVRTDTAYPGEIVILPSDSVRLNDVLGDPTRLPRKRWREDPLPMLRTTIAPKTAAQRER 360
            E+ +DTA G+IVILP+D ++LN +LG+   LP++++ E+PLPML+TTIA K +
QRE
Sbjct 301
ELYSSDTACSGDIVILPNDVLQLNSILGNEMLLPQRKFIENPLPMLQTTIAVKKSEQREI 360

Query 361
LLDALTQLADTDPLLRCEVDSITHEIILSFLGRVQLEVVSALLSEKYKLETVVKEPSVIY 420
            LL ALT+++D DPLL+ VD+ THEIILSFLG VQ+EV+ A+L EKY +E +KEP
+VIY
Sbjct 361
LLGALTEISDGDPLLKYYVDTTTHEIILSFLGNVQMEVICAILEEKYHVEAEIKEPTVIY 420

Query 421
MERPLKAASHTIHIEVPPNPFWASIGLSVTPLSLGSGVQYESRVSLGYLNQSFQNAVRDG 480
            MERPL+ A +TIHIEVPPNPFWAS+GLS+ PL
+GSGVQYESRVSLGYLNQSFQNAV +G
Sbjct 421
MERPLRKAEYTIHIEVPPNPFWASVGLSIEPLPIGSGVQYESRVSLGYLNQSFQNAVMEG 480

Query 481

IRYGLEQGLFGWNVTDCKICFEYGLYYSPVSTPADFRSLAPIVLEQALKESGTQLLEPYL 540
            + YG EQGL+GW VTDCKICFEYGLYYSPVSTPADFR L+PIVLEQALK++GT
+LLEPYL
Sbjct 481
VLYGCEQGLYGWKVTDCKICFEYGLYYSPVSTPADFRLLSPIVLEQALKKAGTELLEPYL 540

Query 541
SFILYAPQEYLSRAYHDAPKYCATIETAQVKKDEVVFTGEIPARCIQAYRTDLAFYTNGR 600
             F +YAPQEYLSRAYHDAP+YCA I + QVK DEV+ GEIPARCIQ YR DL +
+TNG+
Sbjct 541
HFEIYAPQEYLSRAYHDAPRYCADIVSTQVKNDEVILKGEIPARCIQEYRNDLTYFTNGQ 600

Query 601 SVCLTELKGYQAAVGQPVIQPRRPNSRLDKVRHMFQKV 638
             VCLTELKGYQ A+G+ + QPRRPNSR+DKVRHMF K+
Sbjct 601 GVCLTELKGYQPAIGKFICQPRRPNSRIDKVRHMFHKL 638

>WP_024377848.1 TetM/TetW/TetO/TetS family tetracycline resistance ribosomal
protection protein [Streptococcus suis]
Length=639

 Score = 931 bits (2407), Expect = 0.0, Method: Compositional matrix adjust.
 Identities = 432/638 (68%), Positives = 528/638 (83%), Gaps = 0/638 (0%)

Query 1
MKIINIGILAHVDAGKTTLTESLLYASGAISEPGSVEKGTTRTDTMFLERQRGITIQAAV 60
            MKIIN+GILAHVDAGKTTLTESLLY SGAI+EPGSV+KGTTRTDTM
LERQRGITIQ AV
Sbjct 1
MKIINLGILAHVDAGKTTLTESLLYTSGAIAEPGSVDKGTTRTDTMNLERQRGITIQTAV 60

Query 61
TSFQWHRCKVNIVDTPGHMDFLAEVYRSLAVLDGAILVISAKDGVQAQTRILFHALRKMN 120
            TSFQW   KVNI+DTPGHMDFLAEVYRSL+VLDGA+L++SAKDG+QAQTRILFHAL
+ M
Sbjct 61
TSFQWEDVKVNIIDTPGHMDFLAEVYRSLSVLDGAVLLVSAKDGIQAQTRILFHALQTMK 120

Query 121
IPTVIFINKIDQAGVDLQSVVQSVRDKLSADIIIKQTVSLSPEIVLEENTDIEAWDAVIE 180
            IPT+ FINKIDQ G+DL V Q ++ KLS++II+KQ V   P I + +N D+E
WDAVI
Sbjct 121
IPTIFFINKIDQEGIDLPMVYQEMKAKLSSEIIVKQKVGQHPHINVTDNDDMEQWDAVIM 180

Query 181
NNDKLLEKYIAGEPISREKLVREEQRRVQDASLFPVYYGSAKKGLGIQPLMDAVTGLFQP 240
             ND+LLEKY++G+P   +L +EE RR Q+ +LFPVY+GSAK LGI+ L++ +
F
Sbjct 181

```
GNDELLEKYMSGKPFKMSELEQEENRRFQNGTLFPVYHGSAKNNLGIRQLIEVIASKFYS  240

Query  241  
IGEQGSAALCGSVFKVEYTDCGQRRVYLRLYSGTLRLRDTVALAGREKLKITEMRIPSKG  300
               +G + LCG VFK+EY++  +R VY+RLYSG L  RD V ++ +EK+KITEM 
IP+ G
Sbjct  241  
STPEGQSELCGQVFKIEYSEKRRRFVYVRLYSGALHSRDVVRISEKEKIKITEMSIPTNG  300

Query  301  
EIVRTDTAYPGEIVILPSDSVRLNDVLGDPTRLPRKRWREDPLPMLRTTIAPKTAAQRER  360
            E+   DTAY G+IVILP+D ++LN +LG+   LP++++ E+PLPML+TTI  K + 
QRE
Sbjct  301  
ELCLADTAYSGDIVILPNDVLQLNSILGNEILLPQRKFIENPLPMLQTTIEVKKSEQREV  360

Query  361  
LLDALTQLADTDPLLRCEVDSITHEIILSFLGRVQLEVVSALLSEKYKLETVVKEPSVIY  420
            LL+ALT+++D DPLL+  VD+ THEIILSFLG VQ+EV+ A+L EKY +E  +KEP 
+VIY
Sbjct  361  
LLEALTEISDGDPLLKYYVDTTTHEIILSFLGNVQMEVICAILEEKYHVEAEIKEPTVIY  420

Query  421  
MERPLKAASHTIHIEVPPNPFWASIGLSVTPLSLGSGVQYESRVSLGYLNQSFQNAVRDG  480
            MERPL+ A +TIHIEVPPNPFWAS+GLS+ PL 
+GSGVQYESRVSLGYLNQSFQNAV +G
Sbjct  421  
MERPLRKAEYTIHIEVPPNPFWASVGLSIEPLPIGSGVQYESRVSLGYLNQSFQNAVMEG  480

Query  481  
IRYGLEQGLFGWNVTDCKICFEYGLYYSPVSTPADFRSLAPIVLEQALKESGTQLLEPYL  540
            + YG EQGL+GW VTDCKICFEYGLYYSPVSTPADFR L+PIVLEQALK++GT 
+LLEPYL
Sbjct  481  
VLYGCEQGLYGWKVTDCKICFEYGLYYSPVSTPADFRLLSPIVLEQALKKAGTELLEPYL  540

Query  541  
SFILYAPQEYLSRAYHDAPKYCATIETAQVKKDEVVFTGEIPARCIQAYRTDLAFYTNGR  600
             F +YAPQEYLSRAYHDAP+YCA I + Q+K DEV+  GEIPARCIQ YR DL + 
+TNG+
Sbjct  541  
HFEIYAPQEYLSRAYHDAPRYCADIVSTQIKNDEVILKGEIPARCIQEYRNDLTYFTNGQ  600

Query  601  SVCLTELKGYQAAVGQPVIQPRRPNSRLDKVRHMFQKV  638
             VCLTELKGYQ A+G+ + QPRRPNSR+DKVRHMF K+
Sbjct  601  GVCLTELKGYQPAIGKFICQPRRPNSRIDKVRHMFHKL  638


>WP_024397217.1 TetM/TetW/TetO/TetS family tetracycline resistance 
ribosomal 
protection protein [Streptococcus suis]
Length=639
```

Score = 931 bits (2407), Expect = 0.0, Method: Compositional matrix adjust.
Identities = 432/638 (68%), Positives = 531/638 (83%), Gaps = 0/638 (0%)

```
Query  1
MKIINIGILAHVDAGKTTLTESLLYASGAISEPGSVEKGTTRTDTMFLERQRGITIQAAV  60
            MKIIN+GILAHVDAGKTTLTESLLY SGAI+EPGSV+KGTTRTDTM 
LERQRGITIQ AV
Sbjct  1
MKIINLGILAHVDAGKTTLTESLLYTSGAIAEPGSVDKGTTRTDTMNLERQRGITIQTAV  60

Query  61
TSFQWHRCKVNIVDTPGHMDFLAEVYRSLAVLDGAILVISAKDGVQAQTRILFHALRKMN  120
            TSFQW   KVNI+DTPGHMDFLAEVYRSL+VLDGA+L++SAKDG+QAQTRILFHAL
+ M
Sbjct  61
TSFQWEDVKVNIIDTPGHMDFLAEVYRSLSVLDGAVLLVSAKDGIQAQTRILFHALQTMK  120

Query  121
IPTVIFINKIDQAGVDLQSVVQSVRDKLSADIIIKQTVSLSPEIVLEENTDIEAWDAVIE  180
            IPT+ FINKIDQ G+DL  V Q ++ KLS++II+KQ V   P I + +N D+E
WDAVI
Sbjct  121
IPTIFFINKIDQEGIDLPMVYQEMKAKLSSEIIVKQKVGQHPHINVTDNDDMEQWDAVIM  180

Query  181
NNDKLLEKYIAGEPISREKLVREEQRRVQDASLFPVYYGSAKKGLGIQPLMDAVTGLFQP  240
             ND+LLEKY++G+P+   +L +EE RR Q+ +LFPVY+GSAK  LGI+ L++ +
F
Sbjct  181
GNDELLEKYMSGKPLKMSELEQEENRRFQNGTLFPVYHGSAKNNLGIRQLIEVIASKFYS  240

Query  241
IGEQGSAALCGSVFKVEYTDCGQRRVYLRLYSGTLRLRDTVALAGREKLKITEMRIPSKG  300
               +G + LCG VFK+EY++  +R VY+R+YSGTL LRD + ++ +EK+KITEM
+P+ G
Sbjct  241
STPEGQSELCGQVFKIEYSEKRRRFVYVRIYSGTLHLRDVIKISEKEKIKITEMCVPTNG  300

Query  301
EIVRTDTAYPGEIVILPSDSVRLNDVLGDPTRLPRKRWREDPLPMLRTTIAPKTAAQRER  360
            E+  +DTA  G+IVILP+D ++LN +LG+   LP++++ E+PLPML+TTIA K +
QRE
Sbjct  301
ELYSSDTACSGDIVILPNDVLQLNSILGNEMLLPQRKFIENPLPMLQTTIAVKKSEQREI  360

Query  361
LLDALTQLADTDPLLRCEVDSITHEIILSFLGRVQLEVVSALLSEKYKLETVVKEPSVIY  420
            LL ALT+++D DPLL+  VD+ THEIILSFLG VQ+EV+ A+L EKY +E  +KEP
+VIY
Sbjct  361
LLGALTEISDGDPLLKYYVDTTTHEIILSFLGNVQMEVICAILEEKYHVEAEIKEPTVIY  420
```

Query 421
MERPLKAASHTIHIEVPPNPFWASIGLSVTPLSLGSGVQYESRVSLGYLNQSFQNAVRDG 480
            MERPL+ A +TIHIEVPPNPFWAS+GLS+ PL
+GSGVQYESRVSLGYLNQSFQNAV +G
Sbjct 421
MERPLRKAEYTIHIEVPPNPFWASVGLSIEPLPIGSGVQYESRVSLGYLNQSFQNAVMEG 480

Query 481
IRYGLEQGLFGWNVTDCKICFEYGLYYSPVSTPADFRSLAPIVLEQALKESGTQLLEPYL 540
            + YG EQGL+GW VTDCKICFEYGLYYSPVSTPADFR L+PIVLEQALK++GT
+LLEPYL
Sbjct 481
VLYGCEQGLYGWKVTDCKICFEYGLYYSPVSTPADFRLLSPIVLEQALKKAGTELLEPYL 540

Query 541
SFILYAPQEYLSRAYHDAPKYCATIETAQVKKDEVVFTGEIPARCIQAYRTDLAFYTNGR 600
             F +YAPQEYLSRAYHDAP+YCA I + QVK DEV+ GEIPARCIQ YR DL +
+TNG+
Sbjct 541
HFEIYAPQEYLSRAYHDAPRYCADIVSTQVKNDEVILKGEIPARCIQEYRNDLTYFTNGQ 600

Query 601 SVCLTELKGYQAAVGQPVIQPRRPNSRLDKVRHMFQKV 638
             VCLTELKGYQ A+G+ + QPRRPNSR+DKVRHMF K+
Sbjct 601 GVCLTELKGYQPAIGKFICQPRRPNSRIDKVRHMFHKL 638

>CDD17739.1 small GTP-binding protein domain [Clostridium sp. CAG:
798]
 CDE97032.1 small GTP-binding protein domain [Clostridium sp. CAG:
567]
Length=640

 Score = 931 bits (2407), Expect = 0.0, Method: Compositional
matrix adjust.
 Identities = 432/637 (68%), Positives = 532/637 (84%), Gaps = 0/637
(0%)

Query 1
MKIINIGILAHVDAGKTTLTESLLYASGAISEPGSVEKGTTRTDTMFLERQRGITIQAAV 60
            MKIINIGILAHVDAGKTTLTESLLY SGAI E GSV
+KGTTRTDTMFLERQRGITIQAAV
Sbjct 1
MKIINIGILAHVDAGKTTLTESLLYTSGAILELGSVDKGTTRTDTMFLERQRGITIQAAV 60

Query 61
TSFQWHRCKVNIVDTPGHMDFLAEVYRSLAVLDGAILVISAKDGVQAQTRILFHALRKMN 120
            TSF W+ K+NIVDTPGH DF+ EVYRSL+VLDGAILVISAKDGVQAQTRILFHAL
+KMN
Sbjct 61
TSFNWNDYKINIVDTPGHTDFITEVYRSLSVLDGAILVISAKDGVQAQTRILFHALQKMN 120

Query 121
IPTVIFINKIDQAGVDLQSVVQSVRDKLSADIIIKQTVSLSPEIVLEENTDIEAWDAVIE 180
            IPT+IFINKIDQ G++L ++ Q++++KLS DII+ Q V+L+PEI ++   D++ WD

VI
Sbjct 121
IPTIIFINKIDQYGINLNNIYQNIKEKLSNDIIVMQNVTLTPEISIKNIIDLDEWDPVIS 180

Query 181
NNDKLLEKYIAGEPISREKLVREEQRRVQDASLFPVYYGSAKKGLGIQPLMDAVTGLFQP 240
NDKLL+KYIAGE ++ ++L +EE R V+ SLFP+Y+GSAK +G Q L++A++
LF P
Sbjct 181
KNDKLLKKYIAGEKLTIQELTQEEYRCVKKGSLFPIYHGSAKNNIGTQQLIEAISNLFCP 240

Query 241
IGEQGSAALCGSVFKVEYTDCGQRRVYLRLYSGTLRLRDTVALAGREKLKITEMRIPSKG 300
+ + LCG VFK+EYTD QR VYLRLYSGTL LRDT+ L+ ++K+K+TE+
IPS G
Sbjct 241
EMNKNYSELCGRVFKIEYTDHKQRLVYLRLYSGTLHLRDTIVLSEKKKVKLTEIYIPSNG 300

Query 301
EIVRTDTAYPGEIVILPSDSVRLNDVLGDPTRLPRKRWREDPLPMLRTTIAPKTAAQRER 360
E+++T+   G+I I+P++++RLND++G+   LP   W + P+PMLRT I P
+RE+
Sbjct 301
EMIQTEIVCSGDIFIIPNNTLRLNDIIGNEKILPCNVWNDKPVPMLRTRIEPIKIEEREK 360

Query 361
LLDALTQLADTDPLLRCEVDSITHEIILSFLGRVQLEVVSALLSEKYKLETVVKEPSVIY 420
LLDALT++ADTDPLLR VD+ITHEI++SFLG VQLEV+ +LL EKY +   +++P
+VIY
Sbjct 361
LLDALTEIADTDPLLRYCVDTITHEIVISFLGTVQLEVICSLLIEKYHINIRIEDPTVIY 420

Query 421
MERPLKAASHTIHIEVPPNPFWASIGLSVTPLSLGSGVQYESRVSLGYLNQSFQNAVRDG 480
+E+PL+ A +TIHIEVPPNPFWASIGLS+TPL +GSG+QYES
+VSLGYLNQSFQNAVR+G
Sbjct 421
LEKPLQKADYTIHIEVPPNPFWASIGLSITPLPIGSGIQYESKVSLGYLNQSFQNAVREG 480

Query 481
IRYGLEQGLFGWNVTDCKICFEYGLYYSPVSTPADFRSLAPIVLEQALKESGTQLLEPYL 540
I YGLEQGL+GW VTDCKICFEYG+YYSPVSTP+DFR LAPIVLEQ LK+
+GTQLLEPYL
Sbjct 481
INYGLEQGLYGWEVTDCKICFEYGVYYSPVSTPSDFRFLAPIVLEQTLKKAGTQLLEPYL 540

Query 541
SFILYAPQEYLSRAYHDAPKYCATIETAQVKKDEVVFTGEIPARCIQAYRTDLAFYTNGR 600
SFIL+ PQ YLSRAY+DA K+CA IET+Q K DE++FTG IP RCI YR L
YTNG+
Sbjct 541
SFILFTPQGYLSRAYNDAQKHCAIIETSQSKNDEIIFTGHIPVRCINEYRNTLTLYTNGQ 600

Query 601 SVCLTELKGYQAAVGQPVIQPRRPNSRLDKVRHMFQK 637

```
            +V LTELKGYQ A  +PVIQ RRPN+R+DKVRHMF K
Sbjct  601  AVFLTELKGYQIATCEPVIQSRRPNNRIDKVRHMFNK  637
```

>WP_000691759.1 MULTISPECIES: tetracycline resistance ribosomal protection protein
Tet(O) [Terrabacteria group]
 P72533.1 RecName: Full=Tetracycline resistance protein TetO; Short=Tet(O)
 CAA69103.1 tet(O) [Streptococcus pneumoniae]
 EDP26737.1 putative translation elongation factor G [Coprococcus eutactus
ATCC 27759]
 EDR45408.1 putative translation elongation factor G [Dorea formicigenerans
ATCC 27755]
 EEB62247.1 putative translation elongation factor G [Corynebacterium amycolatum
SK46]
 CBK82584.1 small GTP-binding protein domain [Coprococcus sp. ART55/1]
 EGJ26992.1 putative translation elongation factor G [Streptococcus porcinus
str. Jelinkova 176]
 EPU28317.1 tetracycline resistance protein tetM [Streptococcus agalactiae
MRI Z1-211]
 EPU31912.1 tetracycline resistance protein tetM [Streptococcus agalactiae
MRI Z1-212]
 EPU53095.1 tetracycline resistance protein tetM [Streptococcus agalactiae
GB00003]
 EPV07871.1 tetracycline resistance protein tetM [Streptococcus agalactiae
GB00548]
 EPV30301.1 tetracycline resistance protein tetM [Streptococcus agalactiae
GB00653]
 EPV57823.1 tetracycline resistance protein tetM [Streptococcus agalactiae
GB00922]
 EPV77354.1 tetracycline resistance protein tetM [Streptococcus agalactiae
GB00984]
 EPW16523.1 tetracycline resistance protein tetM [Streptococcus agalactiae
CCUG 29376]
 EPW39142.1 tetracycline resistance protein tetM [Streptococcus agalactiae
CCUG 44104]
 EPX00289.1 tetracycline resistance protein tetM [Streptococcus agalactiae
MRI Z1-209]

EPX24493.1 tetracycline resistance protein tetM [Streptococcus agalactiae
str. Gottschalk 998A]
ERI95065.1 putative translation elongation factor G [Blautia sp.
KLE 1732]
ESV55833.1 tetracycline resistance protein tetM [Streptococcus agalactiae
LMG 14747]
ETJ94231.1 tetracycline resistance protein TetM [Streptococcus agalactiae
BV3L5]
AHF59671.1 Tetracycline resistance protein TetO [Streptococcus suis
05HAS68]
KGF09145.1 tetracycline resistance protein tetM [Clostridiales bacterium
S5-A14a]
KIS12810.1 tetracycline resistance protein tetM [Streptococcus equi subsp.
zooepidemicus SzAM60]
COE25667.1 tetracycline resistance protein tetM [Streptococcus pneumoniae]
KLJ53112.1 tetracycline resistance protein tetM [Streptococcus agalactiae]
KLJ70468.1 tetracycline resistance protein tetM [Streptococcus agalactiae]
KLK01279.1 tetracycline resistance protein tetM [Streptococcus agalactiae]
KLK02612.1 tetracycline resistance protein tetM [Streptococcus agalactiae]
KLK14071.1 tetracycline resistance protein tetM [Streptococcus agalactiae]
KLK35727.1 tetracycline resistance protein tetM [Streptococcus agalactiae]
KLK47555.1 tetracycline resistance protein tetM [Streptococcus agalactiae]
KLL34110.1 tetracycline resistance protein tetM [Streptococcus agalactiae]
KLL59626.1 tetracycline resistance protein tetM [Streptococcus agalactiae]
KLL89077.1 tetracycline resistance protein tetM [Streptococcus agalactiae]
CGG77682.1 tetracycline resistance protein tetM [Streptococcus pneumoniae]
CIS35727.1 tetracycline resistance protein tetM [Streptococcus pneumoniae]
CIS10940.1 tetracycline resistance protein tetM [Streptococcus pneumoniae]
CJI03737.1 tetracycline resistance protein tetM [Streptococcus pneumoniae]
CJH40353.1 tetracycline resistance protein tetM [Streptococcus pneumoniae]
CNJ63018.1 tetracycline resistance protein tetM [Streptococcus agalactiae]
COQ21058.1 tetracycline resistance protein tetM [Streptococcus

pneumoniae]
 COO94127.1 tetracycline resistance protein tetM [Streptococcus pneumoniae]
 CQH67345.1 tetracycline resistance protein tetM [Streptococcus agalactiae]
 ALA28779.1 tetracycline resistance protein tetM [Streptococcus suis]
 KPA55448.1 tetracycline resistance protein tetM [Streptococcus suis]
 KPA58305.1 tetracycline resistance protein tetM [Streptococcus suis]
 KPA61278.1 tetracycline resistance protein tetM [Streptococcus suis]
 CYV68424.1 tetracycline resistance protein tetM [Streptococcus suis]
 CYU35866.1 tetracycline resistance protein tetM [Streptococcus suis]
 CYU60528.1 tetracycline resistance protein tetM [Streptococcus suis]
 CYU04023.1 tetracycline resistance protein tetM [Streptococcus suis]
 CYU72955.1 tetracycline resistance protein tetM [Streptococcus suis]
 CYX11524.1 tetracycline resistance protein tetM [Streptococcus suis]
 CYX67867.1 tetracycline resistance protein tetM [Streptococcus suis]
 CZB01636.1 tetracycline resistance protein tetM [Streptococcus suis]
 CYV13084.1 tetracycline resistance protein tetM [Streptococcus suis]
 CYV59371.1 tetracycline resistance protein tetM [Streptococcus suis]
 CZB26707.1 tetracycline resistance protein tetM [Streptococcus suis]
 CYW19351.1 tetracycline resistance protein tetM [Streptococcus suis]
 CYY19243.1 tetracycline resistance protein tetM [Streptococcus suis]
 CYV10867.1 tetracycline resistance protein tetM [Streptococcus suis]
 CYY23935.1 tetracycline resistance protein tetM [Streptococcus suis]
 CYV11273.1 tetracycline resistance protein tetM [Streptococcus suis]
 CYX10453.1 tetracycline resistance protein tetM [Streptococcus suis]
 CYW39262.1 tetracycline resistance protein tetM [Streptococcus suis]
 CYX57268.1 tetracycline resistance protein tetM [Streptococcus suis]
 CYW67289.1 tetracycline resistance protein tetM [Streptococcus suis]
 CYW60217.1 tetracycline resistance protein tetM [Streptococcus

suis]
 CYX42024.1 tetracycline resistance protein tetM [Streptococcus suis]
 CYX39412.1 tetracycline resistance protein tetM [Streptococcus suis]
 CZB03757.1 tetracycline resistance protein tetM [Streptococcus suis]
 CYX08611.1 tetracycline resistance protein tetM [Streptococcus suis]
 CYT73643.1 tetracycline resistance protein tetM [Streptococcus suis]
 CYW79996.1 tetracycline resistance protein tetM [Streptococcus suis]
 CYV99367.1 tetracycline resistance protein tetM [Streptococcus suis]
 CYV95213.1 tetracycline resistance protein tetM [Streptococcus suis]
 CYX12234.1 tetracycline resistance protein tetM [Streptococcus suis]
 CYV42311.1 tetracycline resistance protein tetM [Streptococcus suis]
 CYZ38604.1 tetracycline resistance protein tetM [Streptococcus suis]
 CYX39695.1 tetracycline resistance protein tetM [Streptococcus suis]
 CYX23843.1 tetracycline resistance protein tetM [Streptococcus suis]
 CYU51550.1 tetracycline resistance protein tetM [Streptococcus suis]
 CYU02839.1 tetracycline resistance protein tetM [Streptococcus suis]
 CYU12990.1 tetracycline resistance protein tetM [Streptococcus suis]
 CZA73126.1 tetracycline resistance protein tetM [Streptococcus suis]
 CYW35162.1 tetracycline resistance protein tetM [Streptococcus suis]
 CYV91632.1 tetracycline resistance protein tetM [Streptococcus suis]
 CYU18540.1 tetracycline resistance protein tetM [Streptococcus suis]
 CZA21454.1 tetracycline resistance protein tetM [Streptococcus suis]
 CYX68174.1 tetracycline resistance protein tetM [Streptococcus suis]
 CYW08979.1 tetracycline resistance protein tetM [Streptococcus suis]
 CYX21421.1 tetracycline resistance protein tetM [Streptococcus suis]
 CYX65547.1 tetracycline resistance protein tetM [Streptococcus suis]
 CZA21682.1 tetracycline resistance protein tetM [Streptococcus suis]
 CYX75905.1 tetracycline resistance protein tetM [Streptococcus

suis]
 CYX59072.1 tetracycline resistance protein tetM [Streptococcus suis]
 CYW78659.1 tetracycline resistance protein tetM [Streptococcus suis]
 CZB10165.1 tetracycline resistance protein tetM [Streptococcus suis]
 CYW43852.1 tetracycline resistance protein tetM [Streptococcus suis]
 CYW17882.1 tetracycline resistance protein tetM [Streptococcus suis]
 CZB46555.1 tetracycline resistance protein tetM [Streptococcus suis]
 CZB17217.1 tetracycline resistance protein tetM [Streptococcus suis]
 CYW40521.1 tetracycline resistance protein tetM [Streptococcus suis]
 CYU63862.1 tetracycline resistance protein tetM [Streptococcus suis]
 CYU46730.1 tetracycline resistance protein tetM [Streptococcus suis]
 CYY44182.1 tetracycline resistance protein tetM [Streptococcus suis]
 CZA90707.1 tetracycline resistance protein tetM [Streptococcus suis]
 CYV98172.1 tetracycline resistance protein tetM [Streptococcus suis]
 CZB07283.1 tetracycline resistance protein tetM [Streptococcus suis]
 CYU31261.1 tetracycline resistance protein tetM [Streptococcus suis]
 CYW41518.1 tetracycline resistance protein tetM [Streptococcus suis]
 CYU72514.1 tetracycline resistance protein tetM [Streptococcus suis]
 CYU40707.1 tetracycline resistance protein tetM [Streptococcus suis]
 CYV46194.1 tetracycline resistance protein tetM [Streptococcus suis]
 CYZ62703.1 tetracycline resistance protein tetM [Streptococcus suis]
 CYU37204.1 tetracycline resistance protein tetM [Streptococcus suis]
 CYU36261.1 tetracycline resistance protein tetM [Streptococcus suis]
 CYV39697.1 tetracycline resistance protein tetM [Streptococcus suis]
 CYU37031.1 tetracycline resistance protein tetM [Streptococcus suis]
 CYT94538.1 tetracycline resistance protein tetM [Streptococcus suis]
 CYV35909.1 tetracycline resistance protein tetM [Streptococcus suis]
 CYU82659.1 tetracycline resistance protein tetM [Streptococcus

suis]
 CYW54868.1 tetracycline resistance protein tetM [Streptococcus
suis]
 CYV74634.1 tetracycline resistance protein tetM [Streptococcus
suis]
 CYV39577.1 tetracycline resistance protein tetM [Streptococcus
suis]
 CYT80511.1 tetracycline resistance protein tetM [Streptococcus
suis]
 CZA90777.1 tetracycline resistance protein tetM [Streptococcus
suis]
 CYX23730.1 tetracycline resistance protein tetM [Streptococcus
suis]
 CYU32071.1 tetracycline resistance protein tetM [Streptococcus
suis]
 CYX32403.1 tetracycline resistance protein tetM [Streptococcus
suis]
 CYV80772.1 tetracycline resistance protein tetM [Streptococcus
suis]
 CYX18961.1 tetracycline resistance protein tetM [Streptococcus
suis]
 CYX39829.1 tetracycline resistance protein tetM [Streptococcus
suis]
 CYW85968.1 tetracycline resistance protein tetM [Streptococcus
suis]
 CYW29482.1 tetracycline resistance protein tetM [Streptococcus
suis]
 CYX16774.1 tetracycline resistance protein tetM [Streptococcus
suis]
 CYW00920.1 tetracycline resistance protein tetM [Streptococcus
suis]
 CYU49844.1 tetracycline resistance protein tetM [Streptococcus
suis]
 CYX29167.1 tetracycline resistance protein tetM [Streptococcus
suis]
 CYV88822.1 tetracycline resistance protein tetM [Streptococcus
suis]
 CYV44287.1 tetracycline resistance protein tetM [Streptococcus
suis]
 CYV53734.1 tetracycline resistance protein tetM [Streptococcus
suis]
 CYV39181.1 tetracycline resistance protein tetM [Streptococcus
suis]
 CYW40525.1 tetracycline resistance protein tetM [Streptococcus
suis]
 CYT66937.1 tetracycline resistance protein tetM [Streptococcus
suis]
 CYX45190.1 tetracycline resistance protein tetM [Streptococcus
suis]
 CYV06419.1 tetracycline resistance protein tetM [Streptococcus
suis]
 CYW18158.1 tetracycline resistance protein tetM [Streptococcus
suis]
 CYW71410.1 tetracycline resistance protein tetM [Streptococcus

suis]
 CYV70921.1 tetracycline resistance protein tetM [Streptococcus
suis]
 AMU80520.1 putative elongation factor G [Streptococcus suis]
 ANJ64109.1 tetracycline resistance protein TetO [Streptococcus
suis]
 OCL60731.1 tetracycline resistance ribosomal protection protein
[Streptococcus
agalactiae]
 OCM51803.1 tetracycline resistance ribosomal protection protein
[Streptococcus
agalactiae]
 OCM61078.1 tetracycline resistance ribosomal protection protein
[Streptococcus
agalactiae]
 OCM64220.1 tetracycline resistance ribosomal protection protein
[Streptococcus
agalactiae]
 SCH35469.1 Tetracycline resistance protein tetM from transposon
Tn916 [uncultured
Blautia sp.]
 AOO94963.1 tetracycline resistance protein TetO [Streptococcus
suis]
 OFN92662.1 tetracycline resistance ribosomal protection protein
[Streptococcus
sp. HMSC074F05]
 OFR64307.1 tetracycline resistance ribosomal protection protein
[Streptococcus
sp. HMSC073A12]
 SFO47853.1 ribosomal protection tetracycline resistance protein
[Eubacterium
callanderi]
 OIX63345.1 tetracycline resistance ribosomal protection protein
[Streptococcus
agalactiae serogroup III]
 OIX64344.1 tetracycline resistance ribosomal protection protein
[Streptococcus
agalactiae serogroup III]
 OIX64959.1 tetracycline resistance ribosomal protection protein
[Streptococcus
agalactiae serogroup III]
 OIX66565.1 tetracycline resistance ribosomal protection protein
[Streptococcus
agalactiae serogroup III]
 OIX68556.1 tetracycline resistance ribosomal protection protein
[Streptococcus
agalactiae serogroup III]
 OIX70206.1 tetracycline resistance ribosomal protection protein
[Streptococcus
agalactiae serogroup III]
 OIX73945.1 tetracycline resistance ribosomal protection protein
[Streptococcus
agalactiae serogroup III]
 OIX76314.1 tetracycline resistance ribosomal protection protein

[Streptococcus
agalactiae serogroup III]
 OIX77737.1 tetracycline resistance ribosomal protection protein
[Streptococcus
agalactiae serogroup III]
 OIX80457.1 tetracycline resistance ribosomal protection protein
[Streptococcus
agalactiae serogroup III]
 OIX83808.1 tetracycline resistance ribosomal protection protein
[Streptococcus
agalactiae serogroup III]
 OIX87362.1 tetracycline resistance ribosomal protection protein
[Streptococcus
agalactiae serogroup III]
 OIX89873.1 tetracycline resistance ribosomal protection protein
[Streptococcus
agalactiae serogroup III]
 OLN15430.1 tetracycline resistance ribosomal protection protein
[Corynebacterium
diphtheriae]
 AQY23908.1 tetracycline resistance ribosomal protection protein
Tet(O) [Streptococcus
agalactiae]
 OQO43522.1 tetracycline resistance ribosomal protection protein
[Enterococcus
hirae]
 OQO55540.1 tetracycline resistance ribosomal protection protein
[Enterococcus
hirae]
 ARS43228.1 TetO (plasmid) [Streptococcus suis]
 ASA79323.1 tetracycline resistance ribosomal protection protein
Tet(O) [Streptococcus
agalactiae]
 ASA89721.1 tetracycline resistance ribosomal protection protein
Tet(O) [Streptococcus
agalactiae]
 OXT30549.1 tetracycline resistance ribosomal protection protein
Tet(O) [Streptococcus
agalactiae]
 OXT46078.1 tetracycline resistance ribosomal protection protein
Tet(O) [Streptococcus
agalactiae]
 PLA54473.1 tetracycline resistance ribosomal protection protein
Tet(O) [Streptococcus
macedonicus]
 AUW26011.1 tetracycline resistance ribosomal protection protein
Tet(O) [Streptococcus
suis]
 PQC42738.1 tetracycline resistance ribosomal protection protein
Tet(O) [Enterococcus
faecium]
Length=639

 Score = 931 bits (2407), Expect = 0.0, Method: Compositional

matrix adjust.
 Identities = 432/638 (68%), Positives = 530/638 (83%), Gaps = 0/638 (0%)

```
Query  1
MKIININGILAHVDAGKTTLTESLLYASGAISEPGSVEKGTTRTDTMFLERQRGITIQAAV  60
            MKIIN+GILAHVDAGKTTLTESLLY SGAI+EPGSV+KGTTRTDTM
LERQRGITIQ AV
Sbjct  1
MKIINLGILAHVDAGKTTLTESLLYTSGAIAEPGSVDKGTTRTDTMNLERQRGITIQTAV  60

Query  61
TSFQWHRCKVNIVDTPGHMDFLAEVYRSLAVLDGAILVISAKDGVQAQTRILFHALRKMN  120
            TSFQW   KVNI+DTPGHMDFLAEVYRSL+VLDGA+L++SAKDG+QAQTRILFHAL
+ M
Sbjct  61
TSFQWEDVKVNIIDTPGHMDFLAEVYRSLSVLDGAVLLVSAKDGIQAQTRILFHALQTMK  120

Query  121
IPTVIFINKIDQAGVDLQSVVQSVRDKLSADIIIKQTVSLSPEIVLEENTDIEAWDAVIE  180
            IPT+ FINKIDQ G+DL  V Q ++ KLS++II+KQ V   P I + +N D+E
WDAVI
Sbjct  121
IPTIFFINKIDQEGIDLPMVYQEMKAKLSSEIIVKQKVGQHPHINVTDNDDMEQWDAVIM  180

Query  181
NNDKLLEKYIAGEPISREKLVREEQRRVQDASLFPVYYGSAKKGLGIQPLMDAVTGLFQP  240
             ND+LLEKY++G+P    +L +EE RR Q+ +LFPVY+GSAK  LGI+ L++ +
F
Sbjct  181
GNDELLEKYMSGKPFKMSELEQEENRRFQNGTLFPVYHGSAKNNLGIRQLIEVIASKFYS  240

Query  241
IGEQGSAALCGSVFKVEYTDCGQRRVYLRLYSGTLRLRDTVALAGREKLKITEMRIPSKG  300
               +G + LCG VFK+EY++  +R VY+R+YSGTL LRD + ++ +EK+KITEM
+P+ G
Sbjct  241
STPEGQSELCGQVFKIEYSEKRRRFVYVRIYSGTLHLRDVIKISEKEKIKITEMCVPTNG  300

Query  301
EIVRTDTAYPGEIVILPSDSVRLNDVLGDPTRLPRKRWREDPLPMLRTTIAPKTAAQRER  360
            E+  +DTA  G+IVILP+D ++LN +LG+   LP++++ E+PLPML+TTIA K +
QRE
Sbjct  301
ELYSSDTACSGDIVILPNDVLQLNSILGNEMLLPQRKFIENPLPMLQTTIAVKKSEQREI  360

Query  361
LLDALTQLADTDPLLRCEVDSITHEIILSFLGRVQLEVVSALLSEKYKLETVVKEPSVIY  420
            LL ALT+++D DPLL+  VD+ THEIILSFLG VQ+EV+ A+L EKY +E  +KEP
+VIY
Sbjct  361
LLGALTEISDGDPLLKYYVDTTTHEIILSFLGNVQMEVICAILEEKYHVEAEIKEPTVIY  420

Query  421
```

MERPLKAASHTIHIEVPPNPFWASIGLSVTPLSLGSGVQYESRVSLGYLNQSFQNAVRDG 480
            MERPL+ A +TIHIEVPPNPFWAS+GLS+ PL
+GSGVQYESRVSLGYLNQSFQNAV +G
Sbjct 421
MERPLRKAEYTIHIEVPPNPFWASVGLSIEPLPIGSGVQYESRVSLGYLNQSFQNAVMEG 480

Query 481
IRYGLEQGLFGWNVTDCKICFEYGLYYSPVSTPADFRSLAPIVLEQALKESGTQLLEPYL 540
            + YG EQGL+GW VTDCKICFEYGLYYSPVSTPADFR L+PIVLEQALK++GT
+LLEPYL
Sbjct 481
VLYGCEQGLYGWKVTDCKICFEYGLYYSPVSTPADFRLLSPIVLEQALKKAGTELLEPYL 540

Query 541
SFILYAPQEYLSRAYHDAPKYCATIETAQVKKDEVVFTGEIPARCIQAYRTDLAFYTNGR 600
             F +YAPQEYLSRAYHDAP+YCA I + QVK DEV+ GEIPARCIQ YR DL +
+TNG+
Sbjct 541
HFEIYAPQEYLSRAYHDAPRYCADIVSTQVKNDEVILKGEIPARCIQEYRNDLTYFTNGQ 600

Query 601 SVCLTELKGYQAAVGQPVIQPRRPNSRLDKVRHMFQKV 638
             VCLTELKGYQ A+G+ + QPRRPNSR+DKVRHMF K+
Sbjct 601 GVCLTELKGYQPAIGKFICQPRRPNSRIDKVRHMFHKL 638

>SCI83194.1 Tetracycline resistance protein tetM from transposon
Tn916 [uncultured
Clostridium sp.]
Length=639

 Score = 931 bits (2406), Expect = 0.0, Method: Compositional
matrix adjust.
 Identities = 431/638 (68%), Positives = 530/638 (83%), Gaps = 0/638
(0%)

Query 1
MKIINIGILAHVDAGKTTLTESLLYASGAISEPGSVEKGTTRTDTMFLERQRGITIQAAV 60
            MKIIN+GILAHVDAGKTTLTESLLY SGAI+EPGSV+KGTTRTDTM
LERQRGITIQ AV
Sbjct 1
MKIINLGILAHVDAGKTTLTESLLYTSGAIAEPGSVDKGTTRTDTMNLERQRGITIQTAV 60

Query 61
TSFQWHRCKVNIVDTPGHMDFLAEVYRSLAVLDGAILVISAKDGVQAQTRILFHALRKMN 120
            TSFQW   KVNI+DTPGHMDFLAEVYRSL+VLDGA+L++SAKDG+QAQTRILFHAL
+ M
Sbjct 61
TSFQWEDVKVNIIDTPGHMDFLAEVYRSLSVLDGAVLLVSAKDGIQAQTRILFHALQTMK 120

Query 121
IPTVIFINKIDQAGVDLQSVVQSVRDKLSADIIIKQTVSLSPEIVLEENTDIEAWDAVIE 180
            IPT+ FINKIDQ G+DL V Q ++ KLS++II+KQ V   P I + +N D+E
WDAVI
Sbjct 121

IPTIFFINKIDQEGIDLPMVYQEMKAKLSSEIIVKQKVGQHPHINVTDNDDMEQWDAVIM 180

Query 181
NNDKLLEKYIAGEPISREKLVREEQRRVQDASLFPVYYGSAKKGLGIQPLMDAVTGLFQP 240
             ND+LLEKY++G+P   +L +EE RR Q+ +LFPVY+GSAK LGI+ L++ +
F
Sbjct 181
GNDELLEKYMSGKPFKMSELEQEENRRFQNGTLFPVYHGSAKNNLGIRQLIEVIASKFYS 240

Query 241
IGEQGSAALCGSVFKVEYTDCGQRRVYLRLYSGTLRLRDTVALAGREKLKITEMRIPSKG 300
               +G + LCG VFK+EY++ +R VY+R+YSGTL LRD + ++ +EK+KITEM
+P+ G
Sbjct 241
STPEGQSELCGQVFKIEYSEKRRRFVYVRIYSGTLHLRDVIKISEKEKIKITEMCVPTNG 300

Query 301
EIVRTDTAYPGEIVILPSDSVRLNDVLGDPTRLPRKRWREDPLPMLRTTIAPKTAAQRER 360
            E+ +DTA G+IVILP+D ++LN +LG+   LP++++ E+PLPML+TTIA K +
QRE
Sbjct 301
ELYSSDTACSGDIVILPNDVLQLNSILGNEMLLPQRKFIENPLPMLQTTIAVKKSEQREI 360

Query 361
LLDALTQLADTDPLLRCEVDSITHEIILSFLGRVQLEVVSALLSEKYKLETVVKEPSVIY 420
            LL ALT+++D DPLL+ VD+ THEIILSFLG VQ+E++ A+L EKY +E +KEP
+VIY
Sbjct 361
LLGALTEISDGDPLLKYYVDTTTHEIILSFLGNVQMEIICAILEEKYHVEAEIKEPTVIY 420

Query 421
MERPLKAASHTIHIEVPPNPFWASIGLSVTPLSLGSGVQYESRVSLGYLNQSFQNAVRDG 480
            MERPL+ A +TIHIEVPPNPFWAS+GLS+ PL
+GSGVQYESRVSLGYLNQSFQNAV +G
Sbjct 421
MERPLRKAEYTIHIEVPPNPFWASVGLSIEPLPIGSGVQYESRVSLGYLNQSFQNAVMEG 480

Query 481
IRYGLEQGLFGWNVTDCKICFEYGLYYSPVSTPADFRSLAPIVLEQALKESGTQLLEPYL 540
            + YG EQGL+GW VTDCKICFEYGLYYSPVSTPADFR L+PIVLEQALK++GT
+LLEPYL
Sbjct 481
VLYGCEQGLYGWKVTDCKICFEYGLYYSPVSTPADFRLLSPIVLEQALKKAGTELLEPYL 540

Query 541
SFILYAPQEYLSRAYHDAPKYCATIETAQVKKDEVVFTGEIPARCIQAYRTDLAFYTNGR 600
             F +YAPQEYLSRAYHDAP+YCA I + QVK DEV+ GEIPARCIQ YR DL +
+TNG+
Sbjct 541
HFEIYAPQEYLSRAYHDAPRYCADIVSTQVKNDEVILKGEIPARCIQEYRNDLTYFTNGQ 600

Query 601 SVCLTELKGYQAAVGQPVIQPRRPNSRLDKVRHMFQKV 638
             VCLTELKGYQ A+G+ + QPRRPNSR+DKVRHMF K+
Sbjct 601 GVCLTELKGYQPAIGKFICQPRRPNSRIDKVRHMFHKL 638

>WP_044991838.1 TetM/TetW/TetO/TetS family tetracycline resistance ribosomal
protection protein [Streptococcus suis]
 CYX92737.1 tetracycline resistance protein tetM [Streptococcus suis]
Length=639

 Score = 931 bits (2406), Expect = 0.0, Method: Compositional matrix adjust.
 Identities = 432/638 (68%), Positives = 530/638 (83%), Gaps = 0/638 (0%)

```
Query  1
MKIINIGILAHVDAGKTTLTESLLYASGAISEPGSVEKGTTRTDTMFLERQRGITIQAAV  60
            MKIIN+GILAHVDAGKTTLTESLLY SGAI+EPGSV+KGTTRTDTM
LERQRGITIQ AV
Sbjct  1
MKIINLGILAHVDAGKTTLTESLLYTSGAIAEPGSVDKGTTRTDTMNLERQRGITIQTAV  60

Query  61
TSFQWHRCKVNIVDTPGHMDFLAEVYRSLAVLDGAILVISAKDGVQAQTRILFHALRKMN  120
            TSFQW   KVNI+DTPGHMDFLAEVYRSL+VLDGA+L++SAKDG+QAQTRILFHAL
+ M
Sbjct  61
TSFQWEDVKVNIIDTPGHMDFLAEVYRSLSVLDGAVLLVSAKDGIQAQTRILFHALQIMK  120

Query  121
IPTVIFINKIDQAGVDLQSVVQSVRDKLSADIIIKQTVSLSPEIVLEENTDIEAWDAVIE  180
            IPT+ FINKIDQ G+DL  V Q ++ KLS++II+KQ V   P I + +N D+E
WDAVI
Sbjct  121
IPTIFFINKIDQEGIDLPMVYQEMKAKLSSEIIVKQKVGQHPHINVTDNDDMEQWDAVIM  180

Query  181
NNDKLLEKYIAGEPISREKLVREEQRRVQDASLFPVYYGSAKKGLGIQPLMDAVTGLFQP  240
             ND+LLEKY++G+P    +L +EE RR Q+ +LFPVY+GSAK  LGI+ L++ +
F
Sbjct  181
GNDELLEKYMSGKPFKMSELEQEENRRFQNGTLFPVYHGSAKNNLGIRQLIEVIASKFYS  240

Query  241
IGEQGSAALCGSVFKVEYTDCGQRRVYLRLYSGTLRLRDTVALAGREKLKITEMRIPSKG  300
               +G + LCG VFK+EY++  +R VY+R+YSGTL LRD + ++ +EK+KITEM
+P+ G
Sbjct  241
STPEGQSELCGQVFKIEYSEKRRRFVYVRIYSGTLHLRDVIKISEKEKIKITEMCVPTNG  300

Query  301
EIVRTDTAYPGEIVILPSDSVRLNDVLGDPTRLPRKRWREDPLPMLRTTIAPKTAAQRER  360
            E+  +DTA  G+IVILP+D ++LN +LG+   LP++++ E+PLPML+TTIA K +
QRE
Sbjct  301
```

ELYSSDTACSGDIVILPNDVLQLNSILGNEMLLPQRKFIENPLPMLQTTIAVKKSEQREI 360

Query 361
LLDALTQLADTDPLLRCEVDSITHEIILSFLGRVQLEVVSALLSEKYKLETVVKEPSVIY 420
            LL ALT+++D DPLL+ VD+ THEIILSFLG VQ+EV+ A+L EKY +E +KEP
+VIY
Sbjct 361
LLGALTEISDGDPLLKYYVDTTTHEIILSFLGNVQMEVICAILEEKYHVEAEIKEPTVIY 420

Query 421
MERPLKAASHTIHIEVPPNPFWASIGLSVTPLSLGSGVQYESRVSLGYLNQSFQNAVRDG 480
            MERPL+ A +TIHIEVPPNPFWAS+GLS+ PL
+GSGVQYESRVSLGYLNQSFQNAV +G
Sbjct 421
MERPLRKAEYTIHIEVPPNPFWASVGLSIEPLPIGSGVQYESRVSLGYLNQSFQNAVMEG 480

Query 481
IRYGLEQGLFGWNVTDCKICFEYGLYYSPVSTPADFRSLAPIVLEQALKESGTQLLEPYL 540
            + YG EQGL+GW VTDCKICFEYGLYYSPVSTPADFR L+PIVLEQALK++GT
+LLEPYL
Sbjct 481
VLYGCEQGLYGWKVTDCKICFEYGLYYSPVSTPADFRLLSPIVLEQALKKAGTELLEPYL 540

Query 541
SFILYAPQEYLSRAYHDAPKYCATIETAQVKKDEVVFTGEIPARCIQAYRTDLAFYTNGR 600
             F +YAPQEYLSRAYHDAP+YCA I + QVK DEV+ GEIPARCIQ YR DL +
+TNG+
Sbjct 541
HFEIYAPQEYLSRAYHDAPRYCADIVSTQVKNDEVILKGEIPARCIQEYRNDLTYFTNGQ 600

Query 601 SVCLTELKGYQAAVGQPVIQPRRPNSRLDKVRHMFQKV 638
             VCLTELKGYQ A+G+ + QPRRPNSR+DKVRHMF K+
Sbjct 601 GVCLTELKGYQPAIGKFICQPRRPNSRIDKVRHMFHKL 638

>WP_006858004.1 TetM/TetW/TetO/TetS family tetracycline resistance
ribosomal
protection protein [Roseburia intestinalis]
 EEV00180.1 putative translation elongation factor G [Roseburia
intestinalis
L1-82]
 APO32451.1 ferrous iron transport protein B [uncultured bacterium]
 APO32461.1 ferrous iron transport protein B [uncultured bacterium]
Length=639

 Score = 931 bits (2406), Expect = 0.0, Method: Compositional
matrix adjust.
 Identities = 431/638 (68%), Positives = 530/638 (83%), Gaps = 0/638
(0%)

Query 1
MKIINIGILAHVDAGKTTLTESLLYASGAISEPGSVEKGTTRTDTMFLERQRGITIQAAV 60
            MKIIN+GILAHVDAGKTTLTESLLY SGAI+EPGSV+KGTTRTDTM
LERQRGITIQ AV

Sbjct 1
MKIINLGILAHVDAGKTTLTESLLYTSGAIAEPGSVDKGTTRTDTMNLERQRGITIQTAV 60

Query 61
TSFQWHRCKVNIVDTPGHMDFLAEVYRSLAVLDGAILVISAKDGVQAQTRILFHALRKMN 120
            TSFQW   KVNI+DTPGHMDFLAEVYRSL+VLDGA+L++SAKDG+QAQTRILFHAL
+ M
Sbjct 61
TSFQWEDVKVNIIDTPGHMDFLAEVYRSLSVLDGAVLLVSAKDGIQAQTRILFHALQTMK 120

Query 121
IPTVIFINKIDQAGVDLQSVVQSVRDKLSADIIIKQTVSLSPEIVLEENTDIEAWDAVIE 180
            IPT+ FINKIDQ G+DL V Q ++ KLS++II+KQ V   P I + +N D+E
WDAVI
Sbjct 121
IPTIFFINKIDQEGIDLPMVYQEMKAKLSSEIIVKQKVGQHPHINVTDNDDMEQWDAVIM 180

Query 181
NNDKLLEKYIAGEPISREKLVREEQRRVQDASLFPVYYGSAKKGLGIQPLMDAVTGLFQP 240
             ND+LLEKY++G+P   +L +EE RR Q+ +LFPVY+GSAK LGI+ L++ +
F
Sbjct 181
GNDELLEKYMSGKPFKMSELEQEENRRFQNGTLFPVYHGSAKNNLGIRQLIEVIASKFYS 240

Query 241
IGEQGSAALCGSVFKVEYTDCGQRRVYLRLYSGTLRLRDTVALAGREKLKITEMRIPSKG 300
               +G + LCG VFK+EY++ +R VY+R+YSGTL LRD + ++ +EK+KITEM
+P+ G
Sbjct 241
STPEGQSELCGQVFKIEYSEKRRRFVYVRIYSGTLHLRDVIKISEKEKIKITEMCVPTNG 300

Query 301
EIVRTDTAYPGEIVILPSDSVRLNDVLGDPTRLPRKRWREDPLPMLRTTIAPKTAAQRER 360
            E+ +DTA G+IVILP+D ++LN +LG+   LP++ + E+PLPML+TTIA K +
QRE
Sbjct 301
ELCSSDTACSGDIVILPNDVLQLNSILGNEMLLPQRTFIENPLPMLQTTIAAKKSEQREI 360

Query 361
LLDALTQLADTDPLLRCEVDSITHEIILSFLGRVQLEVVSALLSEKYKLETVVKEPSVIY 420
            LL ALT+++D DPLL+ VD+ THEIILSFLG+VQ+EV+ A+L EKY +E +KEP
+VIY
Sbjct 361
LLGALTEISDGDPLLKYYVDTTTHEIILSFLGKVQMEVICAILEEKYHVEAEIKEPTVIY 420

Query 421
MERPLKAASHTIHIEVPPNPFWASIGLSVTPLSLGSGVQYESRVSLGYLNQSFQNAVRDG 480
            MERPL+ A +TIHIEVPPNPFWAS+GLS+ PL
+GSGVQYESRVSLGYLNQSFQNAV +G
Sbjct 421
MERPLRKAEYTIHIEVPPNPFWASVGLSIEPLPIGSGVQYESRVSLGYLNQSFQNAVMEG 480

Query 481
IRYGLEQGLFGWNVTDCKICFEYGLYYSPVSTPADFRSLAPIVLEQALKESGTQLLEPYL 540

```
            + YG EQGL+GW VTDCKICFEYGLYYSPVSTPADFR L+PIVLEQALK++GT
+LLEPYL
Sbjct  481
VLYGCEQGLYGWKVTDCKICFEYGLYYSPVSTPADFRLLSPIVLEQALKKAGTELLEPYL  540

Query  541
SFILYAPQEYLSRAYHDAPKYCATIETAQVKKDEVVFTGEIPARCIQAYRTDLAFYTNGR  600
             F +YAPQEYLSRAYHDAP+YCA I + Q+K DEV+  GEIPARCIQ YR DL +
+TNG+
Sbjct  541
HFEIYAPQEYLSRAYHDAPRYCADIVSTQIKNDEVILKGEIPARCIQEYRNDLTYFTNGQ  600

Query  601  SVCLTELKGYQAAVGQPVIQPRRPNSRLDKVRHMFQKV  638
             VCLTELKGYQ A+G+ + QPRRPNSR+DKVRHMF K+
Sbjct  601  GVCLTELKGYQPAIGKFICQPRRPNSRIDKVRHMFHKL  638
```

>WP_002935081.1 TetM/TetW/TetO/TetS family tetracycline resistance ribosomal
protection protein [Streptococcus suis]
 EEF65535.1 small GTP-binding protein [Streptococcus suis 89/1591]
Length=639

 Score = 931 bits (2406), Expect = 0.0, Method: Compositional matrix adjust.
 Identities = 431/638 (68%), Positives = 531/638 (83%), Gaps = 0/638 (0%)

```
Query  1
MKIINIGILAHVDAGKTTLTESLLYASGAISEPGSVEKGTTRTDTMFLERQRGITIQAAV  60
            MKIIN+GILAHVDAGKTTLTESLLY SGAI+EPGSV+KGTTRTDTM
LERQRGITIQ AV
Sbjct  1
MKIINLGILAHVDAGKTTLTESLLYTSGAIAEPGSVDKGTTRTDTMNLERQRGITIQTAV  60

Query  61
TSFQWHRCKVNIVDTPGHMDFLAEVYRSLAVLDGAILVISAKDGVQAQTRILFHALRKMN  120
            TSFQW   KVNI+DTPGHMDFLAEVYRSL+VLDGA+L++SAKDG+QAQTRILFHAL
+ M
Sbjct  61
TSFQWEDVKVNIIDTPGHMDFLAEVYRSLSVLDGAVLLVSAKDGIQAQTRILFHALQTMK  120

Query  121
IPTVIFINKIDQAGVDLQSVVQSVRDKLSADIIIKQTVSLSPEIVLEENTDIEAWDAVIE  180
            IPT+ FINKIDQ G+DL  V Q ++ KLS++II+KQ V   P I + +N D+E
WDAVI
Sbjct  121
IPTIFFINKIDQEGIDLPMVYQEMKAKLSSEIIVKQKVGQHPHINVTDNDDMEQWDAVIM  180

Query  181
NNDKLLEKYIAGEPISREKLVREEQRRVQDASLFPVYYGSAKKGLGIQPLMDAVTGLFQP  240
             ND+LLEKY++G+P+   +L +EE RR Q+ +LFPVY+GSAK  LGI+ L++ +
F
Sbjct  181
```

GNDELLEKYMSGKPLKMSELEQEENRRFQNGTLFPVYHGSAKNNLGIRQLIEVIASKFYS 240

Query 241
IGEQGSAALCGSVFKVEYTDCGQRRVYLRLYSGTLRLRDTVALAGREKLKITEMRIPSKG 300
               +G + LCG VFK+EY++ +R VY+R+YSGTL LRD + ++ +EK+KITEM
+P+ G
Sbjct 241
STPEGQSELCGQVFKIEYSEKRRRFVYVRIYSGTLHLRDVIKISEKEKIKITEMCVPTNG 300

Query 301
EIVRTDTAYPGEIVILPSDSVRLNDVLGDPTRLPRKRWREDPLPMLRTTIAPKTAAQRER 360
            E+ +DTA G+IVILP+D ++LN +LG+   LP++++ E+PLPML+TTIA K +
QRE
Sbjct 301
ELYSSDTACSGDIVILPNDVLQLNSILGNEMLLPQRKFIENPLPMLQTTIAVKKSEQREI 360

Query 361
LLDALTQLADTDPLLRCEVDSITHEIILSFLGRVQLEVVSALLSEKYKLETVVKEPSVIY 420
            LL ALT+++D DPLL+ VD+ THEIILSFLG VQ+EV+ A+L EKY +E +KEP
+VIY
Sbjct 361
LLGALTEISDGDPLLKYYVDTTTHEIILSFLGNVQMEVICAILEEKYHVEAEIKEPTVIY 420

Query 421
MERPLKAASHTIHIEVPPNPFWASIGLSVTPLSLGSGVQYESRVSLGYLNQSFQNAVRDG 480
            MERPL+ A +TIHIEVPPNPFWAS+GLS+ PL
+GSGVQYESRVSLGYLNQSFQNAV +G
Sbjct 421
MERPLRKAEYTIHIEVPPNPFWASVGLSIEPLPIGSGVQYESRVSLGYLNQSFQNAVMEG 480

Query 481
IRYGLEQGLFGWNVTDCKICFEYGLYYSPVSTPADFRSLAPIVLEQALKESGTQLLEPYL 540
            + YG EQGL+GW VTDCKICFEYGLYYSPVSTPADFR L+PIVLEQALK++GT
+LLEPYL
Sbjct 481
VLYGCEQGLYGWKVTDCKICFEYGLYYSPVSTPADFRLLSPIVLEQALKKAGTELLEPYL 540

Query 541
SFILYAPQEYLSRAYHDAPKYCATIETAQVKKDEVVFTGEIPARCIQAYRTDLAFYTNGR 600
             F +YAPQEYLSRAYHDAP+YCA I + Q+K DEV+ GEIPARCIQ YR DL +
+TNG+
Sbjct 541
HFEIYAPQEYLSRAYHDAPRYCADIVSTQIKNDEVILKGEIPARCIQEYRNDLTYFTNGQ 600

Query 601 SVCLTELKGYQAAVGQPVIQPRRPNSRLDKVRHMFQKV 638
             VCLTELKGYQ A+G+ + QPRRPNSR+DKVRHMF K+
Sbjct 601 GVCLTELKGYQPAIGKFICQPRRPNSRIDKVRHMFHKL 638

>WP_009440328.1 TetM/TetW/TetO/TetS family tetracycline resistance ribosomal
protection protein [Selenomonas sp. oral taxon 138]
 EKX98430.1 putative translation elongation factor G [Selenomonas sp. oral

taxon 138 str. F0429]
Length=639

 Score = 931 bits (2406), Expect = 0.0, Method: Compositional
matrix adjust.
 Identities = 438/639 (69%), Positives = 536/639 (84%), Gaps = 0/639
(0%)

Query 1
MKIINIGILAHVDAGKTTLTESLLYASGAISEPGSVEKGTTRTDTMFLERQRGITIQAAV 60
            MKIINIGILAHVDAGKTTLTESLLY SGAI+E G+V+KGTTRTDTM
LERQRGITIQ AV
Sbjct 1
MKIINIGILAHVDAGKTTLTESLLYTSGAIAEQGNVDKGTTRTDTMILERQRGITIQTAV 60

Query 61
TSFQWHRCKVNIVDTPGHMDFLAEVYRSLAVLDGAILVISAKDGVQAQTRILFHALRKMN 120
            TSF W+ K+NIVDTPGHMDFL E YRSL+VLDGA+LVISAKDGVQAQTRILFHAL
+KM+
Sbjct 61
TSFCWNDYKINIVDTPGHMDFLTEAYRSLSVLDGAVLVISAKDGVQAQTRILFHALQKMD 120

Query 121
IPTVIFINKIDQAGVDLQSVVQSVRDKLSADIIIKQTVSLSPEIVLEENTDIEAWDAVIE 180
            IPT+IFINKIDQ G+DL+ V QS++DKL++D+I+ Q VSLSP+I + + +D++ WD
+I
Sbjct 121
IPTIIFINKIDQNGIDLRRVYQSIKDKLTSDMIVMQEVSLSPKITMTDISDLDKWDMIIS 180

Query 181
NNDKLLEKYIAGEPISREKLVREEQRRVQDASLFPVYYGSAKKGLGIQPLMDAVTGLFQP 240
             +D+LLE+Y+A + + ++L E+ +R + SLFPVY+GSAK LG + L++A+T
F
Sbjct 181
GSDELLERYVAEDSLDIQELQYEKCKRTRCCSLFPVYHGSAKDNLGTEKLIEAITETFIT 240

Query 241
IGEQGSAALCGSVFKVEYTDCGQRRVYLRLYSGTLRLRDTVALAGREKLKITEMRIPSKG 300
             +   + LCG VFKVEYT+ +R YLRLY GTL LRDT+ L+ +EK+KITEM
IPS G
Sbjct 241
ETDDIQSELCGYVFKVEYTERKKRLSYLRLYHGTLHLRDTLLLSKKEKIKITEMCIPSNG 300

Query 301
EIVRTDTAYPGEIVILPSDSVRLNDVLGDPTRLPRKRWREDPLPMLRTTIAPKTAAQRER 360
            EIV D A PGEIVIL D+++LND+LG+   LP K   ++P+P+LRTT+ P+
QRE
Sbjct 301
EIVPVDHACPGEIVILADDTLKLNDILGNEKLLPHKTRIDNPMPLLRTTVEPQKPEQREA 360

Query 361
LLDALTQLADTDPLLRCEVDSITHEIILSFLGRVQLEVVSALLSEKYKLETVVKEPSVIY 420
            LL+ALT++ADTDPLL ++D++THEIILSFLG+VQLEV+ +LL EKY +
+KEPSVIY

Sbjct 361
LLNALTEIADTDPLLHFDIDTVTHEIILSFLGKVQLEVICSLLEEKYHVGVAMKEPSVIY 420

Query 421
MERPLKAASHTIHIEVPPNPFWASIGLSVTPLSLGSGVQYESRVSLGYLNQSFQNAVRDG 480
            +ERP K AS+TIHIEVPPNPFWASIGL+VTPL +GSG QY+S
VSLGYLNQSFQNAV +G
Sbjct 421
LERPQKKASYTIHIEVPPNPFWASIGLTVTPLPVGSGTQYKSEVSLGYLNQSFQNAVMEG 480

Query 481
IRYGLEQGLFGWNVTDCKICFEYGLYYSPVSTPADFRSLAPIVLEQALKESGTQLLEPYL 540
            +RYG+EQGL+GW VTDC+I F+YG+YYSPVSTPADFR LAP+VLEQALK+
+GTQLLEPYL
Sbjct 481
VRYGMEQGLYGWGVTDCQIWFDYGVYYSPVSTPADFRFLAPVVLEQALKKAGTQLLEPYL 540

Query 541
SFILYAPQEYLSRAYHDAPKYCATIETAQVKKDEVVFTGEIPARCIQAYRTDLAFYTNGR 600
            SF L+APQEYLSRAY+DAPKYCA IE+ +++KDEV+F GEIPARCI YR DL
FYTNGR
Sbjct 541
SFTLFAPQEYLSRAYNDAPKYCAIIESTRLEKDEVIFKGEIPARCIGEYRNDLNFYTNGR 600

Query 601 SVCLTELKGYQAAVGQPVIQPRRPNSRLDKVRHMFQKVM 639
            SVC+TELKGYQ   G+PV QPRRPNSRLDK+RHMFQK+M
Sbjct 601 SVCITELKGYQETSGEPVFQPRRPNSRLDKIRHMFQKIM 639

>WP_014636938.1 TetM/TetW/TetO/TetS family tetracycline resistance ribosomal
protection protein [Streptococcus suis]
AER17261.1 tetracycline resistance protein TetO [Streptococcus suis D9]
ANJ63996.1 tetracycline resistance protein TetO [Streptococcus suis]
Length=639

Score = 931 bits (2406), Expect = 0.0, Method: Compositional matrix adjust.
Identities = 431/638 (68%), Positives = 530/638 (83%), Gaps = 0/638 (0%)

Query 1
MKIINIGILAHVDAGKTTLTESLLYASGAISEPGSVEKGTTRTDTMFLERQRGITIQAAV 60
            MKIIN+GILAHVDAGKTTLTESLLY SGAI+EPGSV+KGTTRTDTM
LERQRGITIQ AV
Sbjct 1
MKIINLGILAHVDAGKTTLTESLLYTSGAIAEPGSVDKGTTRTDTMNLERQRGITIQTAV 60

Query 61
TSFQWHRCKVNIVDTPGHMDFLAEVYRSLAVLDGAILVISAKDGVQAQTRILFHALRKMN 120
            TSFQW   KVNI+DTPGHMDFLAEVYRSL+VLDGA+L++SAKDG+QAQTRILFHAL
+ M

Sbjct 61
TSFQWEDVKVNIIDTPGHMDFLAEVYRSLSVLDGAVLLVSAKDGIQAQTRILFHALQTMK 120

Query 121
IPTVIFINKIDQAGVDLQSVVQSVRDKLSADIIIKQTVSLSPEIVLEENTDIEAWDAVIE 180
IPT+ FINKIDQ G+DL V Q ++ KLS++II+KQ V   P I + +N D+E
WDAVI
Sbjct 121
IPTIFFINKIDQEGIDLPMVYQEMKAKLSSEIIVKQKVGQHPHINVTDNDDMEQWDAVIM 180

Query 181
NNDKLLEKYIAGEPISREKLVREEQRRVQDASLFPVYYGSAKKGLGIQPLMDAVTGLFQP 240
ND+LLEKY++G+P   +L +EE RR Q+ +LFPVY+GSAK LGI+ L++ +
F
Sbjct 181
GNDELLEKYMSGKPFKMSELEQEENRRFQNGTLFPVYHGSAKNNLGIRQLIEVIASKFYS 240

Query 241
IGEQGSAALCGSVFKVEYTDCGQRRVYLRLYSGTLRLRDTVALAGREKLKITEMRIPSKG 300
+G + LCG VFK+EY++ +R VY+R+YSGTL LRD + ++ +EK+KITEM
+P+ G
Sbjct 241
STPEGQSELCGQVFKIEYSEKRRRFVYVRIYSGTLHLRDVIKISEKEKIKITEMCVPTNG 300

Query 301
EIVRTDTAYPGEIVILPSDSVRLNDVLGDPTRLPRKRWREDPLPMLRTTIAPKTAAQRER 360
E+ +DTA G+IVILP+D ++LN +LG+   LP++++ E+PLPML+TTIA K +
QRE
Sbjct 301
ELYSSDTACSGDIVILPNDVLQLNSILGNEILLPQRKFIENPLPMLQTTIAVKKSEQREI 360

Query 361
LLDALTQLADTDPLLRCEVDSITHEIILSFLGRVQLEVVSALLSEKYKLETVVKEPSVIY 420
LL ALT+++D DPLL+ VD+ THEIILSFLG VQ+EV+ A+L EKY +E +KEP
+VIY
Sbjct 361
LLGALTEISDGDPLLKYYVDTTTHEIILSFLGNVQMEVICAILEEKYHVEAEIKEPTVIY 420

Query 421
MERPLKAASHTIHIEVPPNPFWASIGLSVTPLSLGSGVQYESRVSLGYLNQSFQNAVRDG 480
MERPL+ A +TIHIEVPPNPFWAS+GLS+ PL
+GSGVQYESRVSLGYLNQSFQNAV +G
Sbjct 421
MERPLRKAEYTIHIEVPPNPFWASVGLSIEPLPIGSGVQYESRVSLGYLNQSFQNAVMEG 480

Query 481
IRYGLEQGLFGWNVTDCKICFEYGLYYSPVSTPADFRSLAPIVLEQALKESGTQLLEPYL 540
+ YG EQGL+GW VTDCKICFEYGLYYSPVSTPADFR L+PIVLEQALK++GT
+LLEPYL
Sbjct 481
VLYGCEQGLYGWKVTDCKICFEYGLYYSPVSTPADFRLLSPIVLEQALKKAGTELLEPYL 540

Query 541
SFILYAPQEYLSRAYHDAPKYCATIETAQVKKDEVVFTGEIPARCIQAYRTDLAFYTNGR 600

```
             F +YAPQEYLSRAYHDAP+YCA I + Q+K DEV+  GEIPARCIQ YR DL +
+TNG+
Sbjct  541
HFEIYAPQEYLSRAYHDAPRYCADIVSTQIKNDEVILKGEIPARCIQEYRNDLTYFTNGQ  600

Query  601  SVCLTELKGYQAAVGQPVIQPRRPNSRLDKVRHMFQKV  638
             VCLTELKGYQ A+G+ + QPRRPNSR+DKVRHMF K+
Sbjct  601  GVCLTELKGYQPAIGKFICQPRRPNSRIDKVRHMFHKL  638
```

>WP_014636291.1 MULTISPECIES: tetracycline resistance ribosomal protection protein
Tet(O) [Streptococcus]
 ADO27720.1 tetracycline-resistance protein [Streptococcus suis]
 ADV69685.1 tetracycline resistance protein TetO [Streptococcus suis JS14]
 AET35518.1 Tet(O) [uncultured bacterium]
 AFC34419.2 tetracycline resistance protein TetO [Streptococcus suis]
 AFC34421.2 tetracycline resistance protein TetO [Streptococcus suis]
 AFC34423.2 tetracycline resistance protein TetO [Streptococcus suis]
 AGG64056.1 translation elongation factor (GTPases) [Streptococcus suis SC070731]
 KPA66912.1 tetracycline resistance protein tetM [Streptococcus suis]
 KPA69361.1 tetracycline resistance protein tetM [Streptococcus suis]
 CYX59327.1 tetracycline resistance protein tetM [Streptococcus suis]
 CZA11587.1 tetracycline resistance protein tetM [Streptococcus suis]
 CYZ42026.1 tetracycline resistance protein tetM [Streptococcus suis]
 CYY15736.1 tetracycline resistance protein tetM [Streptococcus suis]
 CYV76857.1 tetracycline resistance protein tetM [Streptococcus suis]
 CYV92832.1 tetracycline resistance protein tetM [Streptococcus suis]
 CYU71737.1 tetracycline resistance protein tetM [Streptococcus suis]
 CYU57389.1 tetracycline resistance protein tetM [Streptococcus suis]
 CYU57789.1 tetracycline resistance protein tetM [Streptococcus suis]
 CYX41575.1 tetracycline resistance protein tetM [Streptococcus suis]
 CYV02921.1 tetracycline resistance protein tetM [Streptococcus suis]
 AMP53470.1 TetM-TetW-TetO-TetS [uncultured bacterium]
 ANJ64642.1 tetracycline resistance protein TetO [Streptococcus suis]

ANJ64768.1 tetracycline resistance protein TetO [Streptococcus
suis]
AOM74538.1 tetracycline resistance ribosomal protection protein
[Streptococcus
suis]
GAW39207.1 tetO [Streptococcus suis]
AUC91423.1 tetracycline resistance ribosomal protection protein
Tet(O) [Streptococcus
suis]
AUC91551.1 tetracycline resistance ribosomal protection protein
Tet(O) [Streptococcus
suis]
AUS90966.1 tetracycline resistance protein tetM [Streptococcus
suis]
AUW23615.1 tetracycline resistance ribosomal protection protein
Tet(O) [Streptococcus
suis]
Length=639

Score = 931 bits (2406), Expect = 0.0, Method: Compositional
matrix adjust.
Identities = 431/638 (68%), Positives = 530/638 (83%), Gaps = 0/638
(0%)

Query 1
MKIINIGILAHVDAGKTTLTESLLYASGAISEPGSVEKGTTRTDTMFLERQRGITIQAAV 60
MKIIN+GILAHVDAGKTTLTESLLY SGAI+EPGSV+KGTTRTDTM
LERQRGITIQ AV
Sbjct 1
MKIINLGILAHVDAGKTTLTESLLYTSGAIAEPGSVDKGTTRTDTMNLERQRGITIQTAV 60

Query 61
TSFQWHRCKVNIVDTPGHMDFLAEVYRSLAVLDGAILVISAKDGVQAQTRILFHALRKMN 120
TSFQW   KVNI+DTPGHMDFLAEVYRSL+VLDGA+L++SAKDG+QAQTRILFHAL
+ M
Sbjct 61
TSFQWEDVKVNIIDTPGHMDFLAEVYRSLSVLDGAVLLVSAKDGIQAQTRILFHALQTMK 120

Query 121
IPTVIFINKIDQAGVDLQSVVQSVRDKLSADIIIKQTVSLSPEIVLEENTDIEAWDAVIE 180
IPT+ FINKIDQ G+DL V Q ++ KLS++II+KQ V   P I + +N D+E
WDAVI
Sbjct 121
IPTIFFINKIDQEGIDLPMVYQEMKAKLSSEIIVKQKVGQHPHINVTDNDDMEQWDAVIM 180

Query 181
NNDKLLEKYIAGEPISREKLVREEQRRVQDASLFPVYYGSAKKGLGIQPLMDAVTGLFQP 240
ND+LLEKY++G+P   +L +EE RR Q+ +LFPVY+GSAK LGI+ L++ +
F
Sbjct 181
GNDELLEKYMSGKPFKMSELEQEENRRFQNGTLFPVYHGSAKNNLGIRQLIEVIASKFYS 240

Query 241
IGEQGSAALCGSVFKVEYTDCGQRRVYLRLYSGTLRLRDTVALAGREKLKITEMRIPSKG 300

```
              +G + LCG VFK+EY++  +R VY+R+YSGTL LRD + ++ +EK+KITEM
+P+ G
Sbjct  241
STPEGQSELCGQVFKIEYSEKRRRFVYVRIYSGTLHLRDVIKISEKEKIKITEMCVPTNG  300

Query  301
EIVRTDTAYPGEIVILPSDSVRLNDVLGDPTRLPRKRWREDPLPMLRTTIAPKTAAQRER  360
           E+  +DTA  G+IVILP+D ++LN +LG+   LP++++ E+PLPML+TTIA K +
QRE
Sbjct  301
ELYSSDTACSGDIVILPNDVLQLNSILGNEMLLPQRKFIENPLPMLQTTIAVKKSEQREI  360

Query  361
LLDALTQLADTDPLLRCEVDSITHEIILSFLGRVQLEVVSALLSEKYKLETVVKEPSVIY  420
           LL ALT+++D DPLL+  VD+ THEIILSFLG VQ+EV+ A+L EKY +E  +KEP
+VIY
Sbjct  361
LLGALTEISDGDPLLKYYVDTTTHEIILSFLGNVQMEVICAILEEKYHVEAEIKEPTVIY  420

Query  421
MERPLKAASHTIHIEVPPNPFWASIGLSVTPLSLGSGVQYESRVSLGYLNQSFQNAVRDG  480
           MERPL+ A +TIHIEVPPNPFWAS+GLS+ PL
+GSGVQYESRVSLGYLNQSFQNAV +G
Sbjct  421
MERPLRKAEYTIHIEVPPNPFWASVGLSIEPLPIGSGVQYESRVSLGYLNQSFQNAVMEG  480

Query  481
IRYGLEQGLFGWNVTDCKICFEYGLYYSPVSTPADFRSLAPIVLEQALKESGTQLLEPYL  540
           + YG EQGL+GW VTDCKICFEYGLYYSPVSTPADFR L+PIVLEQALK++GT
+LLEPYL
Sbjct  481
VLYGCEQGLYGWKVTDCKICFEYGLYYSPVSTPADFRLLSPIVLEQALKKAGTELLEPYL  540

Query  541
SFILYAPQEYLSRAYHDAPKYCATIETAQVKKDEVVFTGEIPARCIQAYRTDLAFYTNGR  600
            F +YAPQEYLSRAYHDAP+YCA I + Q+K DEV+  GEIPARCIQ YR DL +
+TNG+
Sbjct  541
HFEIYAPQEYLSRAYHDAPRYCADIVSTQIKNDEVILKGEIPARCIQEYRNDLTYFTNGQ  600

Query  601  SVCLTELKGYQAAVGQPVIQPRRPNSRLDKVRHMFQKV  638
             VCLTELKGYQ A+G+ + QPRRPNSR+DKVRHMF K+
Sbjct  601  GVCLTELKGYQPAIGKFICQPRRPNSRIDKVRHMFHKL  638
```

>APO28702.1 ferrous iron transport protein B [uncultured bacterium]
 APO31191.1 Ferrous iron transport protein B [uncultured bacterium]
Length=639

 Score = 931 bits (2405), Expect = 0.0, Method: Compositional
matrix adjust.
 Identities = 429/638 (67%), Positives = 528/638 (83%), Gaps = 0/638
(0%)

Query 1
MKIINIGILAHVDAGKTTLTESLLYASGAISEPGSVEKGTTRTDTMFLERQRGITIQAAV 60
            MKIIN+GILAHVDAGKTTLTESLLY SGAI+E GSV++GTTRTDTM
LERQRGITIQ AV
Sbjct 1
MKIINLGILAHVDAGKTTLTESLLYTSGAIAELGSVDEGTTRTDTMNLERQRGITIQTAV 60

Query 61
TSFQWHRCKVNIVDTPGHMDFLAEVYRSLAVLDGAILVISAKDGVQAQTRILFHALRKMN 120
            TSFQW   KVNI+DTPGHMDFLAEVYRSL+VLDGA+L++SAKDG+QAQTRILFHAL
+ M
Sbjct 61
TSFQWEDVKVNIIDTPGHMDFLAEVYRSLSVLDGAVLLVSAKDGIQAQTRILFHALQIMK 120

Query 121
IPTVIFINKIDQAGVDLQSVVQSVRDKLSADIIIKQTVSLSPEIVLEENTDIEAWDAVIE 180
            IPT+ FINKIDQ G+DL V + ++ KLS++II+KQ V   P I + +N D+E
WDAVI
Sbjct 121
IPTIFFINKIDQEGIDLPMVYREMKAKLSSEIIVKQKVGQHPHINVTDNDDMEQWDAVIM 180

Query 181
NNDKLLEKYIAGEPISREKLVREEQRRVQDASLFPVYYGSAKKGLGIQPLMDAVTGLFQP 240
             ND+LLEKY++G+P   +L +EE RR Q+ +LFPVY+GSAK LGI+ L++ +
F
Sbjct 181
GNDELLEKYMSGKPFKMSELEQEENRRFQNGTLFPVYHGSAKNNLGIRQLIEVIASKFYS 240

Query 241
IGEQGSAALCGSVFKVEYTDCGQRRVYLRLYSGTLRLRDTVALAGREKLKITEMRIPSKG 300
               +G + LCG VFK+EY++ +R VY+R+YSGTL LRD + ++ +EK+KITEM
+PS G
Sbjct 241
STPEGQSELCGQVFKIEYSEKRRRFVYVRIYSGTLHLRDVIRISEKEKIKITEMCVPSNG 300

Query 301
EIVRTDTAYPGEIVILPSDSVRLNDVLGDPTRLPRKRWREDPLPMLRTTIAPKTAAQRER 360
            EIV D A PGEIVIL D+++LND+LG+   LP K W ++P+P+LRTT+ P+
QRE
Sbjct 301
EIVPADHACPGEIVILADDTLKLNDILGNEKLLPHKTWIDNPMPLLRTTVEPQKPEQREA 360

Query 361
LLDALTQLADTDPLLRCEVDSITHEIILSFLGRVQLEVVSALLSEKYKLETVVKEPSVIY 420
            LL+AL ++ADTDPLL ++D++THEI+LSFLG VQ+EV+ A+L EKY +E +KEP
+VIY
Sbjct 361
LLNALAEIADTDPLLHFDIDTVTHEIMLSFLGNVQMEVICAILEEKYHVEAEIKEPTVIY 420

Query 421
MERPLKAASHTIHIEVPPNPFWASIGLSVTPLSLGSGVQYESRVSLGYLNQSFQNAVRDG 480
            MERPL+ A +TIHIEVPPNPFWAS+GLS+ PL
+GSGVQYESRVSLGYLNQSFQNAV +G
Sbjct 421

MERPLRKAEYTIHIEVPPNPFWASVGLSIEPLPIGSGVQYESRVSLGYLNQSFQNAVMEG 480

Query 481
IRYGLEQGLFGWNVTDCKICFEYGLYYSPVSTPADFRSLAPIVLEQALKESGTQLLEPYL 540
            + YG EQGL+GW VTDCKICFEYGLYYSPVSTPADFR L+PIVLEQALK++GT
+LLEPYL
Sbjct 481
VLYGCEQGLYGWKVTDCKICFEYGLYYSPVSTPADFRLLSPIVLEQALKKAGTELLEPYL 540

Query 541
SFILYAPQEYLSRAYHDAPKYCATIETAQVKKDEVVFTGEIPARCIQAYRTDLAFYTNGR 600
             F +YAPQEYLSRAYHDAP+YCA I + Q+K DEV+ GEIPARCIQ YR DL +
+TNG+
Sbjct 541
HFEIYAPQEYLSRAYHDAPRYCADIVSTQIKNDEVILKGEIPARCIQEYRNDLTYFTNGQ 600

Query 601 SVCLTELKGYQAAVGQPVIQPRRPNSRLDKVRHMFQKV 638
             VCLTELKGYQ A+G+ + QPRRPNSR+DKVRHMF K+
Sbjct 601 GVCLTELKGYQPAIGKFICQPRRPNSRIDKVRHMFHKL 638

>AMP50099.1 TetM–TetW–TetO–TetS [uncultured bacterium]
Length=639

Score = 931 bits (2405), Expect = 0.0, Method: Compositional
matrix adjust.
Identities = 431/638 (68%), Positives = 530/638 (83%), Gaps = 0/638
(0%)

Query 1
MKIINIGILAHVDAGKTTLTESLLYASGAISEPGSVEKGTTRTDTMFLERQRGITIQAAV 60
            MKIIN+GILAHVDAGKTTLTESLLY SGAI+EPGSV+KGTTRTDTM
LERQRGITIQ AV
Sbjct 1
MKIINLGILAHVDAGKTTLTESLLYTSGAIAEPGSVDKGTTRTDTMNLERQRGITIQTAV 60

Query 61
TSFQWHRCKVNIVDTPGHMDFLAEVYRSLAVLDGAILVISAKDGVQAQTRILFHALRKMN 120
            TSFQW   KVNI+DTPGHMDFLAEVYRSL+VLDGA+L++SAKDG+QAQTRILFHAL
+ M
Sbjct 61
TSFQWEDVKVNIIDTPGHMDFLAEVYRSLSVLDGAVLLVSAKDGIQAQTRILFHALQTMK 120

Query 121
IPTVIFINKIDQAGVDLQSVVQSVRDKLSADIIIKQTVSLSPEIVLEENTDIEAWDAVIE 180
            IPT+ FINKIDQ G+DL V Q ++ KLS++II+KQ V   P I + +N D+E
WDAVI
Sbjct 121
IPTIFFINKIDQEGIDLPMVYQEMKAKLSSEIIVKQKVGQHPHINVTDNDDMEQWDAVIM 180

Query 181
NNDKLLEKYIAGEPISREKLVREEQRRVQDASLFPVYYGSAKKGLGIQPLMDAVTGLFQP 240
             ND+LLEKY++G+P   +L +EE RR Q+ +LFPVY+GSAK LGI+ L++ +
F

Sbjct 181
GNDELLEKYMSGKPFKMSELEQEENRRFQNGTLFPVYHGSAKNNLGIRQLIEVIASKFYS 240

Query 241
IGEQGSAALCGSVFKVEYTDCGQRRVYLRLYSGTLRLRDTVALAGREKLKITEMRIPSKG 300
               +G + LCG VFK+EY++ +R VY+R+YSGTL LRD + ++ +EK+KITEM
+P+ G
Sbjct 241
STPEGQSELCGQVFKIEYSEKRRRFVYVRIYSGTLHLRDVIKISEKEKIKITEMCVPTNG 300

Query 301
EIVRTDTAYPGEIVILPSDSVRLNDVLGDPTRLPRKRWREDPLPMLRTTIAPKTAAQRER 360
            E+ +DTA G+IVILP+D ++LN +LG+   LP++++ E+PLPML+TTIA K +
QRE
Sbjct 301
ELCSSDTACSGDIVILPNDVLQLNSILGNEILLPQRKFIENPLPMLQTTIAVKKSEQREI 360

Query 361
LLDALTQLADTDPLLRCEVDSITHEIILSFLGRVQLEVVSALLSEKYKLETVVKEPSVIY 420
            LL ALT+++D DPLL+ VD+ THEIILSFLG VQ+EV+ A+L EKY +E +KEP
+VIY
Sbjct 361
LLGALTEISDGDPLLKYYVDTTTHEIILSFLGNVQMEVICAILEEKYHVEAEIKEPTVIY 420

Query 421
MERPLKAASHTIHIEVPPNPFWASIGLSVTPLSLGSGVQYESRVSLGYLNQSFQNAVRDG 480
            MERPL+ A +TIHIEVPPNPFWAS+GLS+ PL
+GSGVQYESRVSLGYLNQSFQNAV +G
Sbjct 421
MERPLRKAEYTIHIEVPPNPFWASVGLSIEPLPIGSGVQYESRVSLGYLNQSFQNAVMEG 480

Query 481
IRYGLEQGLFGWNVTDCKICFEYGLYYSPVSTPADFRSLAPIVLEQALKESGTQLLEPYL 540
            + YG EQGL+GW VTDCKICFEYGLYYSPVSTPADFR L+PIVLEQALK++GT
+LLEPYL
Sbjct 481
VLYGCEQGLYGWKVTDCKICFEYGLYYSPVSTPADFRLLSPIVLEQALKKAGTELLEPYL 540

Query 541
SFILYAPQEYLSRAYHDAPKYCATIETAQVKKDEVVFTGEIPARCIQAYRTDLAFYTNGR 600
             F +YAPQEYLSRAYHDAP+YCA I + Q+K DEV+ GEIPARCIQ YR DL +
+TNG+
Sbjct 541
HFEIYAPQEYLSRAYHDAPRYCADIVSTQIKNDEVILKGEIPARCIQEYRNDLTYFTNGQ 600

Query 601 SVCLTELKGYQAAVGQPVIQPRRPNSRLDKVRHMFQKV 638
             VCLTELKGYQ A+G+ + QPRRPNSR+DKVRHMF K+
Sbjct 601 GVCLTELKGYQPAIGKFICQPRRPNSRIDKVRHMFHKL 638

>AMP49403.1 TetM-TetW-TetO-TetS [uncultured bacterium]
Length=639

Score = 931 bits (2405), Expect = 0.0, Method: Compositional

matrix adjust.
 Identities = 432/638 (68%), Positives = 527/638 (83%), Gaps = 0/638 (0%)

Query 1
MKIINIGILAHVDAGKTTLTESLLYASGAISEPGSVEKGTTRTDTMFLERQRGITIQAAV 60
            MKIIN+GILAHVDAGKTTLTESLLY SGAI+E GSV++GTTRTDTM
LERQRGITIQ AV
Sbjct 1
MKIINLGILAHVDAGKTTLTESLLYTSGAIAELGSVDEGTTRTDTMNLERQRGITIQTAV 60

Query 61
TSFQWHRCKVNIVDTPGHMDFLAEVYRSLAVLDGAILVISAKDGVQAQTRILFHALRKMN 120
            TSFQW   KVNI+DTPGHMDFLAEVYRSL+VLDGA+L++SAKDG+QAQTRILFHAL
+ M
Sbjct 61
TSFQWEDVKVNIIDTPGHMDFLAEVYRSLSVLDGAVLLVSAKDGIQAQTRILFHALQIMK 120

Query 121
IPTVIFINKIDQAGVDLQSVVQSVRDKLSADIIIKQTVSLSPEIVLEENTDIEAWDAVIE 180
            IPT+ FINKIDQ G+DL V + ++ KLS++II+KQ V   P I + +N D+E
WDAVI
Sbjct 121
IPTIFFINKIDQEGIDLPMVYREMKAKLSSEIIVKQKVGQHPHINVTDNDDMEQWDAVIM 180

Query 181
NNDKLLEKYIAGEPISREKLVREEQRRVQDASLFPVYYGSAKKGLGIQPLMDAVTGLFQP 240
             ND+LLEKY++G+P   +L +EE RR Q+ +LFPVY+GSAK LGI+ L++ +
F
Sbjct 181
GNDELLEKYMSGKPFKMSELEQEENRRFQNGTLFPVYHGSAKNNLGIRQLIEVIASKFYS 240

Query 241
IGEQGSAALCGSVFKVEYTDCGQRRVYLRLYSGTLRLRDTVALAGREKLKITEMRIPSKG 300
               +G + LCG VFK+EY++ +R VY+R+YSGTL LRD + ++ +EK+KITEM
IPS G
Sbjct 241
STPEGQSELCGQVFKIEYSEKRRRFVYVRIYSGTLHLRDVIRISEKEKIKITEMCIPSNG 300

Query 301
EIVRTDTAYPGEIVILPSDSVRLNDVLGDPTRLPRKRWREDPLPMLRTTIAPKTAAQRER 360
            EIV D A PGEIVIL D+++LND+LG+   LP K W ++P+P+LRTT+ P+
QRE
Sbjct 301
EIVPADHACPGEIVILADDTLKLNDILGNEKLLPHKTWIDNPMPLLRTTVEPQKPEQREA 360

Query 361
LLDALTQLADTDPLLRCEVDSITHEIILSFLGRVQLEVVSALLSEKYKLETVVKEPSVIY 420
            LL+AL ++ADTDPLL+ VD+ THEIILSFLG VQ+EV+ A+L EKY +E +KEP
+VIY
Sbjct 361
LLNALAEIADTDPLLKYYVDTTTHEIILSFLGNVQMEVICAILEEKYHVEAEIKEPTVIY 420

Query 421

MERPLKAASHTIHIEVPPNPFWASIGLSVTPLSLGSGVQYESRVSLGYLNQSFQNAVRDG 480
            MERPL+ A +TIHIEVPPNPFWAS+GLS+ PL
+GSGVQYESRVSLGYLNQSFQNAV +G
Sbjct 421
MERPLRKAEYTIHIEVPPNPFWASVGLSIEPLPIGSGVQYESRVSLGYLNQSFQNAVMEG 480

Query 481
IRYGLEQGLFGWNVTDCKICFEYGLYYSPVSTPADFRSLAPIVLEQALKESGTQLLEPYL 540
            + YG EQGL+GW VTDCKICFEYGLYYSPVSTPADFR L+PIVLEQALK++GT
+LLEPYL
Sbjct 481
VLYGCEQGLYGWKVTDCKICFEYGLYYSPVSTPADFRLLSPIVLEQALKKAGTELLEPYL 540

Query 541
SFILYAPQEYLSRAYHDAPKYCATIETAQVKKDEVVFTGEIPARCIQAYRTDLAFYTNGR 600
             F +YAPQEYLSRAYHDAP+YCA I + Q+K DEV+ GEIPARCIQ YR DL +
+TNG+
Sbjct 541
HFEIYAPQEYLSRAYHDAPRYCADIVSTQIKNDEVILKGEIPARCIQEYRNDLTYFTNGQ 600

Query 601 SVCLTELKGYQAAVGQPVIQPRRPNSRLDKVRHMFQKV 638
             VCLTELKGYQ A+G+ + QPRRPNSR+DKVRHMF K+
Sbjct 601 GVCLTELKGYQPAIGKFICQPRRPNSRIDKVRHMFHKL 638

>WP_055182589.1 TetM/TetW/TetO/TetS family tetracycline resistance ribosomal
protection protein [[Eubacterium] hallii]
 CUM89219.1 Tetracycline resistance protein tetM from transposon Tn916 [[Eubacterium]
hallii]
 AMP55326.1 TetM-TetW-TetO-TetS [uncultured bacterium]
Length=639

 Score = 931 bits (2405), Expect = 0.0, Method: Compositional matrix adjust.
 Identities = 430/638 (67%), Positives = 528/638 (83%), Gaps = 0/638 (0%)

Query 1
MKIINIGILAHVDAGKTTLTESLLYASGAISEPGSVEKGTTRTDTMFLERQRGITIQAAV 60
            MKIIN+GILAHVDAGKTTLTESLLY SGAI+E GSV++GTTRTDTM
LERQRGITIQ AV
Sbjct 1
MKIINLGILAHVDAGKTTLTESLLYTSGAIAELGSVDEGTTRTDTMNLERQRGITIQTAV 60

Query 61
TSFQWHRCKVNIVDTPGHMDFLAEVYRSLAVLDGAILVISAKDGVQAQTRILFHALRKMN 120
            TSFQW   KVNI+DTPGHMDFLAEVYRSL+VLDGA+L++SAKDG+QAQTRILFHAL
+ M
Sbjct 61
TSFQWEDVKVNIIDTPGHMDFLAEVYRSLSVLDGAVLLVSAKDGIQAQTRILFHALQIMK 120

Query 121

IPTVIFINKIDQAGVDLQSVVQSVRDKLSADIIIKQTVSLSPEIVLEENTDIEAWDAVIE 180
IPT+ FINKIDQ G+DL V + ++ KLS++II+KQ V   P I + +N D+E
WDAVI
Sbjct 121
IPTIFFINKIDQEGIDLPMVYREMKAKLSSEIIVKQKVGQHPHINVTDNDDMEQWDAVIM 180

Query 181
NNDKLLEKYIAGEPISREKLVREEQRRVQDASLFPVYYGSAKKGLGIQPLMDAVTGLFQP 240
ND+LLEKY++G+P   +L +EE RR Q+ +LFPVY+GSAK LGI+ L++ +
F
Sbjct 181
GNDELLEKYMSGKPFKMSELEQEENRRFQNGTLFPVYHGSAKNNLGIRQLIEVIASKFYS 240

Query 241
IGEQGSAALCGSVFKVEYTDCGQRRVYLRLYSGTLRLRDTVALAGREKLKITEMRIPSKG 300
+G + LCG VFK+EY++ +R VY+R+YSGTL LRD + ++ +EK+KITEM
IPS G
Sbjct 241
STPEGQSELCGQVFKIEYSEKRRRFVYVRIYSGTLHLRDVIRISEKEKIKITEMCIPSNG 300

Query 301
EIVRTDTAYPGEIVILPSDSVRLNDVLGDPTRLPRKRWREDPLPMLRTTIAPKTAAQRER 360
EIV D A PGEIVIL D+++LND+LG+   LP K W ++P+P+LRTT+ P+
QRE
Sbjct 301
EIVPADHACPGEIVILADDTLKLNDILGNEKLLPHKTWIDNPMPLLRTTVEPQKPEQREA 360

Query 361
LLDALTQLADTDPLLRCEVDSITHEIILSFLGRVQLEVVSALLSEKYKLETVVKEPSVIY 420
LL+AL ++ADTDPLL ++D++THEI+LSFLG VQ+EV+ A+L EKY +E +KEP
+VIY
Sbjct 361
LLNALAEIADTDPLLHFDIDTVTHEIMLSFLGNVQMEVICAILEEKYHVEAEIKEPTVIY 420

Query 421
MERPLKAASHTIHIEVPPNPFWASIGLSVTPLSLGSGVQYESRVSLGYLNQSFQNAVRDG 480
MERPL+ A +TIHIEVPPNPFWAS+GLS+ PL
+GSGVQYESRVSLGYLNQSFQNAV +G
Sbjct 421
MERPLRKAEYTIHIEVPPNPFWASVGLSIEPLPIGSGVQYESRVSLGYLNQSFQNAVMEG 480

Query 481
IRYGLEQGLFGWNVTDCKICFEYGLYYSPVSTPADFRSLAPIVLEQALKESGTQLLEPYL 540
+ YG EQGL+GW VTDCKICFEYGLYYSPVSTPADFR L+PIVLEQALK++GT
+LLEPYL
Sbjct 481
VLYGCEQGLYGWKVTDCKICFEYGLYYSPVSTPADFRLLSPIVLEQALKKAGTELLEPYL 540

Query 541
SFILYAPQEYLSRAYHDAPKYCATIETAQVKKDEVVFTGEIPARCIQAYRTDLAFYTNGR 600
F +YAPQEYLSRAYHDAP+YCA I + Q+K DEV+ GEIPARCIQ YR DL +
+TNG+
Sbjct 541
HFEIYAPQEYLSRAYHDAPRYCADIVSTQIKNDEVILKGEIPARCIQEYRNDLTYFTNGQ 600

```
Query  601  SVCLTELKGYQAAVGQPVIQPRRPNSRLDKVRHMFQKV  638
             VCLTELKGYQ A+G+ + QPRRPNSR+DKVRHMF K+
Sbjct  601  GVCLTELKGYQPAIGKFICQPRRPNSRIDKVRHMFHKL  638
```

>WP_105157116.1 TetM/TetW/TetO/TetS family tetracycline resistance ribosomal
protection protein [Streptococcus suis]
Length=639

Score = 930 bits (2404), Expect = 0.0, Method: Compositional matrix adjust.
Identities = 431/638 (68%), Positives = 530/638 (83%), Gaps = 0/638 (0%)

```
Query  1
MKIINIGILAHVDAGKTTLTESLLYASGAISEPGSVEKGTTRTDTMFLERQRGITIQAAV  60
            MKIIN+GILAHVDAGKTTLTESLLY SGAI+EPGSV+KGTTRTDTM
LERQRGITIQ AV
Sbjct  1
MKIINLGILAHVDAGKTTLTESLLYTSGAIAEPGSVDKGTTRTDTMNLERQRGITIQTAV  60

Query  61
TSFQWHRCKVNIVDTPGHMDFLAEVYRSLAVLDGAILVISAKDGVQAQTRILFHALRKMN  120
            TSFQW   KVNI+DTPGHMDFLAEVYRSL+VLDGA+L++SAKDG+QAQTRILFHAL
+ M
Sbjct  61
TSFQWEDVKVNIIDTPGHMDFLAEVYRSLSVLDGAVLLVSAKDGIQAQTRILFHALQTMK  120

Query  121
IPTVIFINKIDQAGVDLQSVVQSVRDKLSADIIIKQTVSLSPEIVLEENTDIEAWDAVIE  180
            IPT+ FINKIDQ G+DL  V Q ++ KLS++II+KQ V   P I + +N D+E
WDAVI
Sbjct  121
IPTIFFINKIDQEGIDLPMVYQEMKAKLSSEIIVKQKVGQHPHINVTDNDDMEQWDAVIM  180

Query  181
NNDKLLEKYIAGEPISREKLVREEQRRVQDASLFPVYYGSAKKGLGIQPLMDAVTGLFQP  240
             ND+LLEKY++G+P    +L +EE RR Q+ +LFPVY+GSAK  LGI+ L++ +
F
Sbjct  181
GNDELLEKYMSGKPFKMSELEQEENRRFQNGTLFPVYHGSAKNNLGIRQLIEVIASKFYS  240

Query  241
IGEQGSAALCGSVFKVEYTDCGQRRVYLRLYSGTLRLRDTVALAGREKLKITEMRIPSKG  300
               +G + LCG VFK+EY++  +R VY+R+YSGTL LRD + ++ +EK+KITEM
+P+ G
Sbjct  241
STPEGQSELCGQVFKIEYSEKRRRFVYVRIYSGTLHLRDVIRISEKEKIKITEMCVPTNG  300

Query  301
EIVRTDTAYPGEIVILPSDSVRLNDVLGDPTRLPRKRWREDPLPMLRTTIAPKTAAQRER  360
            E+  +DTA  G+IVILP+D ++LN +LG+   LP++++ E+PLPML+TTIA K +
```

QRE
Sbjct 301
ELYSSDTACSGDIVILPNDVLQLNSILGNEMLLPQRKFIENPLPMLQTTIAVKKSEQREI 360

Query 361
LLDALTQLADTDPLLRCEVDSITHEIILSFLGRVQLEVVSALLSEKYKLETVVKEPSVIY 420
LL ALT+++D DPLL+ VD+ THEIILSFLG VQ+EV+ A+L EKY +E +KEP
+VIY
Sbjct 361
LLGALTEISDGDPLLKYYVDTTTHEIILSFLGNVQMEVICAILEEKYHVEVEIKEPTVIY 420

Query 421
MERPLKAASHTIHIEVPPNPFWASIGLSVTPLSLGSGVQYESRVSLGYLNQSFQNAVRDG 480
MERPL+ A +TIHIEVPPNPFWAS+GLS+ PL
+GSGVQYESRVSLGYLNQSFQNAV +G
Sbjct 421
MERPLRKAEYTIHIEVPPNPFWASVGLSIEPLPIGSGVQYESRVSLGYLNQSFQNAVMEG 480

Query 481
IRYGLEQGLFGWNVTDCKICFEYGLYYSPVSTPADFRSLAPIVLEQALKESGTQLLEPYL 540
+ YG EQGL+GW VTDCKICFEYGLYYSPVSTPADFR L+PIVLEQALK++GT
+LLEPYL
Sbjct 481
VLYGCEQGLYGWKVTDCKICFEYGLYYSPVSTPADFRLLSPIVLEQALKKAGTELLEPYL 540

Query 541
SFILYAPQEYLSRAYHDAPKYCATIETAQVKKDEVVFTGEIPARCIQAYRTDLAFYTNGR 600
F +YAPQEYLSRAYHDAP+YCA I + Q+K DEV+ GEIPARCIQ YR DL +
+TNG+
Sbjct 541
HFEIYAPQEYLSRAYHDAPRYCADIVSTQIKNDEVILKGEIPARCIQEYRNDLTYFTNGQ 600

Query 601 SVCLTELKGYQAAVGQPVIQPRRPNSRLDKVRHMFQKV 638
VCLTELKGYQ A+G+ + QPRRPNSR+DKVRHMF K+
Sbjct 601 GVCLTELKGYQPAIGKFICQPRRPNSRIDKVRHMFHKL 638

>WP_105145235.1 TetM/TetW/TetO/TetS family tetracycline resistance
ribosomal
protection protein [Streptococcus suis]
Length=639

Score = 930 bits (2404), Expect = 0.0, Method: Compositional
matrix adjust.
Identities = 432/638 (68%), Positives = 527/638 (83%), Gaps = 0/638
(0%)

Query 1
MKIINIGILAHVDAGKTTLTESLLYASGAISEPGSVEKGTTRTDTMFLERQRGITIQAAV 60
MKIIN+GILAHVDAGKTTLTESLLY SGAI+EPGSV+KGTTRTDTM
LERQRGITIQ AV
Sbjct 1
MKIINLGILAHVDAGKTTLTESLLYTSGAIAEPGSVDKGTTRTDTMNLERQRGITIQTAV 60

Query 61
TSFQWHRCKVNIVDTPGHMDFLAEVYRSLAVLDGAILVISAKDGVQAQTRILFHALRKMN 120
            TSFQW   KVNI+DTPGHMDFLAEVYRSL+VLDGA+L++SAKDG+QAQTRILFHAL
+ M
Sbjct 61
TSFQWEDVKVNIIDTPGHMDFLAEVYRSLSVLDGAVLLVSAKDGIQAQTRILFHALQTMK 120

Query 121
IPTVIFINKIDQAGVDLQSVVQSVRDKLSADIIIKQTVSLSPEIVLEENTDIEAWDAVIE 180
            IPT+ FINKIDQ G+DL V Q ++ KLS++II+KQ V   P I + +N D+E
WDAVI
Sbjct 121
IPTIFFINKIDQEGIDLPMVYQEMKAKLSSEIIVKQKVGQHPHINVTDNDDMEQWDAVIM 180

Query 181
NNDKLLEKYIAGEPISREKLVREEQRRVQDASLFPVYYGSAKKGLGIQPLMDAVTGLFQP 240
             ND+LLEKY++G+P   +L +EE RR Q+ +LFPVY+GSAK LGI+ L++ +
F
Sbjct 181
GNDELLEKYMSGKPFKMSELEQEENRRFQNGTLFPVYHGSAKNNLGIRQLIEVIASKFYS 240

Query 241
IGEQGSAALCGSVFKVEYTDCGQRRVYLRLYSGTLRLRDTVALAGREKLKITEMRIPSKG 300
               +G + LCG VFK+EY++ +R Y+RLYSG L RD V ++ +EK+KITEM
IP+ G
Sbjct 241
STPEGQSELCGQVFKIEYSEKRRRLAYVRLYSGALHSRDVVRISEKEKIKITEMSIPTNG 300

Query 301
EIVRTDTAYPGEIVILPSDSVRLNDVLGDPTRLPRKRWREDPLPMLRTTIAPKTAAQRER 360
            E+   DTAY G+IVILP+D ++LN +LG+   LP++++ E+PLPML+TTI K +
QRE
Sbjct 301
ELCLADTAYSGDIVILPNDVLQLNSILGNEILLPQRKFIENPLPMLQTTIEVKKSEQREV 360

Query 361
LLDALTQLADTDPLLRCEVDSITHEIILSFLGRVQLEVVSALLSEKYKLETVVKEPSVIY 420
            LL+ALT+++D DPLL+ VD+ THEIILSFLG VQ+EV+ A+L EKY +E +KEP
+VIY
Sbjct 361
LLEALTEISDGDPLLKYYVDTTTHEIILSFLGNVQMEVICAILEEKYHVEAEIKEPTVIY 420

Query 421
MERPLKAASHTIHIEVPPNPFWASIGLSVTPLSLGSGVQYESRVSLGYLNQSFQNAVRDG 480
            MERPL+ A +TIHIEVPPNPFWAS+GLS+ PL
+GSGVQYESRVSLGYLNQSFQNAV +G
Sbjct 421
MERPLRKAEYTIHIEVPPNPFWASVGLSIEPLPIGSGVQYESRVSLGYLNQSFQNAVMEG 480

Query 481
IRYGLEQGLFGWNVTDCKICFEYGLYYSPVSTPADFRSLAPIVLEQALKESGTQLLEPYL 540
            + YG EQGL+GW VTDCKICFEYGLYYSPVSTPADFR L+PIVLEQALK+SGT
+LLEPYL
Sbjct 481

```
VLYGCEQGLYGWKVTDCKICFEYGLYYSPVSTPADFRLLSPIVLEQALKKSGTELLEPYL  540

Query  541
SFILYAPQEYLSRAYHDAPKYCATIETAQVKKDEVVFTGEIPARCIQAYRTDLAFYTNGR  600
             F +YAPQEYLSRAYHDAP+YCA I + Q+K DEV+  GEIPARCIQ YR DL +
+TNG+
Sbjct  541
HFEIYAPQEYLSRAYHDAPRYCADIVSTQIKNDEVILKGEIPARCIQEYRNDLTYFTNGQ  600

Query  601  SVCLTELKGYQAAVGQPVIQPRRPNSRLDKVRHMFQKV  638
             VCLTELKGYQ A+G+ + QPRRPNSR+DKVRHMF K+
Sbjct  601  GVCLTELKGYQPAIGKFICQPRRPNSRIDKVRHMFHKL  638


>AUS90925.1 tetracycline resistance protein TetO [Streptococcus
suis]
Length=639

 Score = 930 bits (2404),  Expect = 0.0, Method: Compositional
matrix adjust.
 Identities = 430/638 (67%), Positives = 530/638 (83%), Gaps = 0/638
(0%)

Query  1
MKIINIGILAHVDAGKTTLTESLLYASGAISEPGSVEKGTTRTDTMFLERQRGITIQAAV  60
            MKIIN+GILAHVDAGKTTLTESLLY SGAI+EPGSV+KGTTRTDTM
LERQRGITIQ AV
Sbjct  1
MKIINLGILAHVDAGKTTLTESLLYTSGAIAEPGSVDKGTTRTDTMNLERQRGITIQTAV  60

Query  61
TSFQWHRCKVNIVDTPGHMDFLAEVYRSLAVLDGAILVISAKDGVQAQTRILFHALRKMN  120
            TSFQW   KVNI+DTPGHMDFLAEVYRSL+VLDGA+L++SAKDG+QAQTRILFHAL
+ M
Sbjct  61
TSFQWEDVKVNIIDTPGHMDFLAEVYRSLSVLDGAVLLVSAKDGIQAQTRILFHALQTMK  120

Query  121
IPTVIFINKIDQAGVDLQSVVQSVRDKLSADIIIKQTVSLSPEIVLEENTDIEAWDAVIE  180
            IPT+ FINKIDQ G+DL  V Q ++ KLS++II+KQ V   P I + +N D+E
WDAVI
Sbjct  121
IPTIFFINKIDQEGIDLPMVYQEMKAKLSSEIIVKQKVGQHPHINVTDNDDMEQWDAVIM  180

Query  181
NNDKLLEKYIAGEPISREKLVREEQRRVQDASLFPVYYGSAKKGLGIQPLMDAVTGLFQP  240
             ND+LLEKY++G+P    +L +EE RR Q+ +LFPVY+GSAK  LGI+ L++ +
F
Sbjct  181
GNDELLEKYMSGKPFKMSELEQEENRRFQNGTLFPVYHGSAKNNLGIRQLIEVIASKFYS  240

Query  241
IGEQGSAALCGSVFKVEYTDCGQRRVYLRLYSGTLRLRDTVALAGREKLKITEMRIPSKG  300
               +G + LCG VFK+EY++  +R VY+R+YSGTL LRD + ++ +EK+KITEM
```

+P+ G
Sbjct 241
STPEGQSELCGQVFKIEYSEKRRRFVYVRIYSGTLHLRDVIKISEKEKIKITEMCVPTNG 300

Query 301
EIVRTDTAYPGEIVILPSDSVRLNDVLGDPTRLPRKRWREDPLPMLRTTIAPKTAAQRER 360
            E+ +DTA G+IVILP+D ++LN +LG+   LP++++ E+PLPML+TTI K +
QRE
Sbjct 301
ELYSSDTACSGDIVILPNDVLQLNSILGNEILLPQRKFIENPLPMLQTTIEVKKSEQREV 360

Query 361
LLDALTQLADTDPLLRCEVDSITHEIILSFLGRVQLEVVSALLSEKYKLETVVKEPSVIY 420
            LL+ALT+++D DPLL+ VD+ THEIILSFLG VQ+EV+ A+L EKY +E +KEP
+VIY
Sbjct 361
LLEALTEISDGDPLLKYYVDTTTHEIILSFLGNVQMEVICAILEEKYHVEAEIKEPTVIY 420

Query 421
MERPLKAASHTIHIEVPPNPFWASIGLSVTPLSLGSGVQYESRVSLGYLNQSFQNAVRDG 480
            MERPL+ A +TIHIEVPPNPFWAS+GLS+ PL
+GSGVQYESRVSLGYLNQSFQNAV +G
Sbjct 421
MERPLRKAEYTIHIEVPPNPFWASVGLSIEPLPIGSGVQYESRVSLGYLNQSFQNAVMEG 480

Query 481
IRYGLEQGLFGWNVTDCKICFEYGLYYSPVSTPADFRSLAPIVLEQALKESGTQLLEPYL 540
            + YG EQGL+GW VTDCKICFEYGLYYSPVSTPADFR L+PIVLEQALK++GT
+LLEPYL
Sbjct 481
VLYGCEQGLYGWKVTDCKICFEYGLYYSPVSTPADFRLLSPIVLEQALKKAGTELLEPYL 540

Query 541
SFILYAPQEYLSRAYHDAPKYCATIETAQVKKDEVVFTGEIPARCIQAYRTDLAFYTNGR 600
             F +YAPQEYLSRAYHDAP+YCA I + Q+K DEV+ GEIPARCIQ YR DL +
+TNG+
Sbjct 541
HFEIYAPQEYLSRAYHDAPRYCADIVSTQIKNDEVILKGEIPARCIQEYRNDLTYFTNGQ 600

Query 601 SVCLTELKGYQAAVGQPVIQPRRPNSRLDKVRHMFQKV 638
             VCLTELKGYQ A+G+ + QPRRPNSR+DKVRHMF K+
Sbjct 601 GVCLTELKGYQPAIGKFICQPRRPNSRIDKVRHMFHKL 638

>SCJ84798.1 Tetracycline resistance protein tetM from transposon
Tn916 [uncultured
Lachnospira sp.]
Length=639

 Score = 930 bits (2404), Expect = 0.0, Method: Compositional
matrix adjust.
 Identities = 438/639 (69%), Positives = 535/639 (84%), Gaps = 0/639
(0%)

Query 1
MKIINIGILAHVDAGKTTLTESLLYASGAISEPGSVEKGTTRTDTMFLERQRGITIQAAV 60
            MKIINIGILAHVDAGKTTLTESLLY SGAI+E G+V+KGTTRTDTM
LERQRGITIQ AV
Sbjct 1
MKIINIGILAHVDAGKTTLTESLLYTSGAIAEQGNVDKGTTRTDTMILERQRGITIQTAV 60

Query 61
TSFQWHRCKVNIVDTPGHMDFLAEVYRSLAVLDGAILVISAKDGVQAQTRILFHALRKMN 120
            TSF W+ K+NIVDTPGHMDFL E YRSL+VLDGA+LVISAKDGVQAQTRILF+AL
+KMN
Sbjct 61
TSFCWNDYKINIVDTPGHMDFLTEAYRSLSVLDGAVLVISAKDGVQAQTRILFNALQKMN 120

Query 121
IPTVIFINKIDQAGVDLQSVVQSVRDKLSADIIIKQTVSLSPEIVLEENTDIEAWDAVIE 180
            IPT+IF+NKIDQ G+DL+ V QS++DKL++D+I+ Q V+LSP+I + + +D++ WD
VI
Sbjct 121
IPTIIFVNKIDQNGIDLRRVYQSIKDKLTSDMIVMQEVALSPKITITDISDLDKWDIVIS 180

Query 181
NNDKLLEKYIAGEPISREKLVREEQRRVQDASLFPVYYGSAKKGLGIQPLMDAVTGLFQP 240
             +D+LLE+Y+A +P+ ++L E+ +R + SLFPVY+GSAK LG + L++A+T
F
Sbjct 181
GSDELLERYVAEDPLDIQELQYEKCKRTRCCSLFPVYHGSAKDNLGTEKLIEAITETFIT 240

Query 241
IGEQGSAALCGSVFKVEYTDCGQRRVYLRLYSGTLRLRDTVALAGREKLKITEMRIPSKG 300
              +   + LCG VFKVEYTD +R YLRLY GTL LRD + L+ ++K+KITEM
IP G
Sbjct 241
ETDDIQSELCGYVFKVEYTDRKKRLTYLRLYHGTLHLRDELPLSKKKKIKITEMCIPLNG 300

Query 301
EIVRTDTAYPGEIVILPSDSVRLNDVLGDPTRLPRKRWREDPLPMLRTTIAPKTAAQRER 360
            EIV D A PGEIV+L D+++LND+LG+   LP+K   +DP+P+LRTT+ P+
QRE
Sbjct 301
EIVPADHADPGEIVVLADDTLKLNDILGNEKLLPQKTRIDDPMPLLRTTVEPQKPEQREA 360

Query 361
LLDALTQLADTDPLLRCEVDSITHEIILSFLGRVQLEVVSALLSEKYKLETVVKEPSVIY 420
            LL+AL ++ADTDPLL ++D++THEI+LSFLG+VQLEV+ +LL EKY + V
KEPSVIY
Sbjct 361
LLNALVEIADTDPLLHFDIDTVTHEIMLSFLGKVQLEVICSLLEEKYHVGVVTKEPSVIY 420

Query 421
MERPLKAASHTIHIEVPPNPFWASIGLSVTPLSLGSGVQYESRVSLGYLNQSFQNAVRDG 480
            +ERP K AS TIHIEVPPNPFWASIGL+VTPL +GSG QY+S
VSLGYLNQSFQNAV +G
Sbjct 421

LERPQKKASCTIHIEVPPNPFWASIGLTVTPLPIGSGTQYKSEVSLGYLNQSFQNAVMEG 480

Query 481
IRYGLEQGLFGWNVTDCKICFEYGLYYSPVSTPADFRSLAPIVLEQALKESGTQLLEPYL 540
            +RYG+EQGL+GW VTDC+ICF+YG+YYSPVSTPADFR LAPIVLEQALK+
+GTQLLEPYL
Sbjct 481
VRYGMEQGLYGWGVTDCQICFDYGVYYSPVSTPADFRFLAPIVLEQALKKAGTQLLEPYL 540

Query 541
SFILYAPQEYLSRAYHDAPKYCATIETAQVKKDEVVFTGEIPARCIQAYRTDLAFYTNGR 600
            SF L+APQEYLSRAY+DAPKYCA IE+ +++KDEV+F GEIPARCI YR DL
FYTNGR
Sbjct 541
SFTLFAPQEYLSRAYNDAPKYCAIIESTRLEKDEVIFKGEIPARCIGEYRNDLNFYTNGR 600

Query 601 SVCLTELKGYQAAVGQPVIQPRRPNSRLDKVRHMFQKVM 639
            SVC+TELKGYQ   G+PV QPRRPNSRLDKVRHMFQK+M
Sbjct 601 SVCITELKGYQETSGEPVFQPRRPNSRLDKVRHMFQKIM 639

>AMP57970.1 TetM-TetW-TetO-TetS [uncultured bacterium]
Length=639

 Score = 930 bits (2404), Expect = 0.0, Method: Compositional
matrix adjust.
 Identities = 431/638 (68%), Positives = 530/638 (83%), Gaps = 0/638
(0%)

Query 1
MKIINIGILAHVDAGKTTLTESLLYASGAISEPGSVEKGTTRTDTMFLERQRGITIQAAV 60
            MKIIN+GILAHVDAGKTTLTESLLY SGAI+EPGSV+KGTTRTDTM
LERQRGITIQ AV
Sbjct 1
MKIINLGILAHVDAGKTTLTESLLYTSGAIAEPGSVDKGTTRTDTMNLERQRGITIQTAV 60

Query 61
TSFQWHRCKVNIVDTPGHMDFLAEVYRSLAVLDGAILVISAKDGVQAQTRILFHALRKMN 120
            TSFQW   KVNI+DTPGHMDFLAEVYRSL+VLDGA+L++SAKDG+QAQTRILFHAL
+ M
Sbjct 61
TSFQWEDVKVNIIDTPGHMDFLAEVYRSLSVLDGAVLLVSAKDGIQAQTRILFHALQTMK 120

Query 121
IPTVIFINKIDQAGVDLQSVVQSVRDKLSADIIIKQTVSLSPEIVLEENTDIEAWDAVIE 180
            IPT+ FINKIDQ G+DL V Q ++ KLS++II+KQ V   P I + +N D+E
WDAVI
Sbjct 121
IPTIFFINKIDQEGIDLPMVYQEMKAKLSSEIIVKQKVGQHPHINVTDNDDMEQWDAVIM 180

Query 181
NNDKLLEKYIAGEPISREKLVREEQRRVQDASLFPVYYGSAKKGLGIQPLMDAVTGLFQP 240
             ND+LLEKY++G+P   +L +EE RR Q+ +LFPVY+GSAK LGI+ L++ +
F

```
Sbjct  181
GNDELLEKYMSGKPFKMSELEQEENRRFQNGTLFPVYHGSAKNNLGIRQLIEVIASKFYS  240

Query  241
IGEQGSAALCGSVFKVEYTDCGQRRVYLRLYSGTLRLRDTVALAGREKLKITEMRIPSKG  300
               +G + LCG VFK+EY++  +R VY+R+YSGTL LRD + ++ +EK+KITEM
+P+ G
Sbjct  241
STPEGQSELCGQVFKIEYSEKRRRFVYVRIYSGTLHLRDVIRISEKEKIKITEMCVPTNG  300

Query  301
EIVRTDTAYPGEIVILPSDSVRLNDVLGDPTRLPRKRWREDPLPMLRTTIAPKTAAQRER  360
            E+  +DTA  G+IVILP+D ++LN +LG+   LP++++ E+PLPML+TTIA K +
QRE
Sbjct  301
ELYSSDTACSGDIVILPNDVLQLNSILGNEMLLPQRKFIENPLPMLQTTIAVKKSEQREI  360

Query  361
LLDALTQLADTDPLLRCEVDSITHEIILSFLGRVQLEVVSALLSEKYKLETVVKEPSVIY  420
            LL ALT+++D DPLL+  VD+ THEIILSFLG VQ+EV+ A+L EKY +E  +KEP
+VIY
Sbjct  361
LLGALTEISDGDPLLKYYVDTTTHEIILSFLGNVQMEVICAILEEKYHVEAEIKEPTVIY  420

Query  421
MERPLKAASHTIHIEVPPNPFWASIGLSVTPLSLGSGVQYESRVSLGYLNQSFQNAVRDG  480
            MERPL+ A +TIHIEVPPNPFWAS+GLS+ PL
+GSGVQYESRVSLGYLNQSFQNAV +G
Sbjct  421
MERPLRKAEYTIHIEVPPNPFWASVGLSIEPLPIGSGVQYESRVSLGYLNQSFQNAVMEG  480

Query  481
IRYGLEQGLFGWNVTDCKICFEYGLYYSPVSTPADFRSLAPIVLEQALKESGTQLLEPYL  540
            + YG EQGL+GW VTDCKICFEYGLYYSPVSTPADFR L+PIVLEQALK++GT
+LLEPYL
Sbjct  481
VLYGCEQGLYGWKVTDCKICFEYGLYYSPVSTPADFRLLSPIVLEQALKKAGTELLEPYL  540

Query  541
SFILYAPQEYLSRAYHDAPKYCATIETAQVKKDEVVFTGEIPARCIQAYRTDLAFYTNGR  600
             F +YAPQEYLSRAYHDAP+YCA I + Q+K DEV+  GEIPARCIQ YR DL +
+TNG+
Sbjct  541
HFEIYAPQEYLSRAYHDAPRYCADIVSTQIKNDEVILKGEIPARCIQEYRNDLTYFTNGQ  600

Query  601  SVCLTELKGYQAAVGQPVIQPRRPNSRLDKVRHMFQKV  638
             VCLTELKGYQ A+G+ + QPRRPNSR+DKVRHMF K+
Sbjct  601  GVCLTELKGYQPAIGKFICQPRRPNSRIDKVRHMFHKL  638


>AMP51260.1 TetM-TetW-TetO-TetS [uncultured bacterium]
Length=639

 Score = 930 bits (2404),  Expect = 0.0, Method: Compositional
```

matrix adjust.
 Identities = 432/638 (68%), Positives = 526/638 (82%), Gaps = 0/638 (0%)

```
Query  1
MKIINIGILAHVDAGKTTLTESLLYASGAISEPGSVEKGTTRTDTMFLERQRGITIQAAV  60
            MKIIN+GILAHVDAGKTTLTESLLY SGAI+E GSV++GTTRTDTM
LERQRGITIQ AV
Sbjct  1
MKIINLGILAHVDAGKTTLTESLLYTSGAIAELGSVDEGTTRTDTMNLERQRGITIQTAV  60

Query  61
TSFQWHRCKVNIVDTPGHMDFLAEVYRSLAVLDGAILVISAKDGVQAQTRILFHALRKMN  120
            TSFQW   KVNI+DTPGHMDFLAEVYRSL+VLDGA+L++SAKDG+QAQTRILFHAL
+ M
Sbjct  61
TSFQWEDVKVNIIDTPGHMDFLAEVYRSLSVLDGAVLLVSAKDGIQAQTRILFHALQIMK  120

Query  121
IPTVIFINKIDQAGVDLQSVVQSVRDKLSADIIIKQTVSLSPEIVLEENTDIEAWDAVIE  180
            IPT+ FINKIDQ G+DL  V + ++ KLS++II+KQ V   P I + +N D+E
WDAVI
Sbjct  121
IPTIFFINKIDQEGIDLPMVYREMKAKLSSEIIVKQKVGQHPHINVTDNDDMEQWDAVIM  180

Query  181
NNDKLLEKYIAGEPISREKLVREEQRRVQDASLFPVYYGSAKKGLGIQPLMDAVTGLFQP  240
             ND+LLEKY++G+P    +L +EE RR Q+ +LFPVY+GSAK  LGI+ L++ +
F
Sbjct  181
GNDELLEKYMSGKPFKMSELEQEENRRFQNGTLFPVYHGSAKNNLGIRQLIEVIASKFYS  240

Query  241
IGEQGSAALCGSVFKVEYTDCGQRRVYLRLYSGTLRLRDTVALAGREKLKITEMRIPSKG  300
               +G + LCG VFK+EY++  +R VY+R+YSGTL LRD + ++ +EK+KITEM
IPS G
Sbjct  241
STPEGQSELCGQVFKIEYSEKRRRFVYVRIYSGTLHLRDVIRISEKEKIKITEMCIPSNG  300

Query  301
EIVRTDTAYPGEIVILPSDSVRLNDVLGDPTRLPRKRWREDPLPMLRTTIAPKTAAQRER  360
            EIV  D A PGEIVIL  D+++LND+LG+   LP K W ++P+P+LRTT+ P+
QRE
Sbjct  301
EIVPADHACPGEIVILADDTLKLNDILGNEKLLPHKTWIDNPMPLLRTTVEPQKPEQREA  360

Query  361
LLDALTQLADTDPLLRCEVDSITHEIILSFLGRVQLEVVSALLSEKYKLETVVKEPSVIY  420
            LL+AL ++ADTDPLL+  VD+ THEIILSFLG VQ+EV+ A+L EKY +E
+KEPSVIY
Sbjct  361
LLNALAEIADTDPLLKYYVDTTTHEIILSFLGNVQMEVICAILEEKYHVEAEIKEPSVIY  420

Query  421
```

MERPLKAASHTIHIEVPPNPFWASIGLSVTPLSLGSGVQYESRVSLGYLNQSFQNAVRDG 480
            MERPL+ A +TIHIEVPPNPFWAS+GLS+ PL
+GSGVQYESRVSLGYLNQSFQNAV +G
Sbjct 421
MERPLRKAEYTIHIEVPPNPFWASVGLSIEPLPIGSGVQYESRVSLGYLNQSFQNAVMEG 480

Query 481
IRYGLEQGLFGWNVTDCKICFEYGLYYSPVSTPADFRSLAPIVLEQALKESGTQLLEPYL 540
            + YG EQGL+GW VTDCKICFEYG YYSPVSTPADFR L+PIVLEQALK++GT
+LLEPYL
Sbjct 481
VLYGCEQGLYGWKVTDCKICFEYGFYYSPVSTPADFRLLSPIVLEQALKKAGTELLEPYL 540

Query 541
SFILYAPQEYLSRAYHDAPKYCATIETAQVKKDEVVFTGEIPARCIQAYRTDLAFYTNGR 600
             F +YAPQEYLSRAYHDAP+YCA I + Q+K DEV+ GEIPARCIQ YR DL +
+TNG+
Sbjct 541
HFEIYAPQEYLSRAYHDAPRYCADIVSTQIKNDEVILKGEIPARCIQEYRNDLTYFTNGQ 600

Query 601 SVCLTELKGYQAAVGQPVIQPRRPNSRLDKVRHMFQKV 638
             VCLTELKGYQ A+G+ + QPRRPNSR+DKVRHMF K+
Sbjct 601 GVCLTELKGYQPAIGKFICQPRRPNSRIDKVRHMFHKL 638

>WP_055284540.1 TetM/TetW/TetO/TetS family tetracycline resistance
ribosomal
protection protein [Dorea longicatena]
 CUQ12588.1 Tetracycline resistance protein tetM from transposon
Tn916 [Dorea
longicatena]
Length=639

 Score = 930 bits (2404), Expect = 0.0, Method: Compositional
matrix adjust.
 Identities = 432/638 (68%), Positives = 529/638 (83%), Gaps = 0/638
(0%)

Query 1
MKIINIGILAHVDAGKTTLTESLLYASGAISEPGSVEKGTTRTDTMFLERQRGITIQAAV 60
            MKIIN+GILAHVDAGKTTLTESLLY SGAI+EPGSV+KGTTRTDTM
LERQRGITIQ AV
Sbjct 1
MKIINLGILAHVDAGKTTLTESLLYTSGAIAEPGSVDKGTTRTDTMNLERQRGITIQTAV 60

Query 61
TSFQWHRCKVNIVDTPGHMDFLAEVYRSLAVLDGAILVISAKDGVQAQTRILFHALRKMN 120
            TSFQW   KVNI+DTPGHMDFLAEVYRSL+VLDGA+L++SAKDG+QAQTRILFHAL
+ M
Sbjct 61
TSFQWEDVKVNIIDTPGHMDFLAEVYRSLSVLDGAVLLVSAKDGIQAQTRILFHALQTMK 120

Query 121
IPTVIFINKIDQAGVDLQSVVQSVRDKLSADIIIKQTVSLSPEIVLEENTDIEAWDAVIE 180

```
            IPT+ FINKIDQ G+DL  V Q ++ KLS++II+KQ V   P I + +N D+E
WDAVI
Sbjct  121
IPTIFFINKIDQEGIDLPMVYQEMKAKLSSEIIVKQKVGQHPHINVTDNDDMEQWDAVIM  180

Query  181
NNDKLLEKYIAGEPISREKLVREEQRRVQDASLFPVYYGSAKKGLGIQPLMDAVTGLFQP  240
             ND+LLEKY++G+P    +L +EE RR Q+ +LFPVY+GSAK  LGI+ L++ +
F
Sbjct  181
GNDELLEKYMSGKPFKMSELEQEENRRFQNGTLFPVYHGSAKNNLGIRQLIEVIASKFYS  240

Query  241
IGEQGSAALCGSVFKVEYTDCGQRRVYLRLYSGTLRLRDTVALAGREKLKITEMRIPSKG  300
               +G + LCG VFK+EY++  +R VY+R+YSGTL LRD + ++ +EK+KITEM
+P+ G
Sbjct  241
STPEGQSELCGQVFKIEYSEKRRRFVYVRIYSGTLHLRDVIKISEKEKIKITEMCVPTNG  300

Query  301
EIVRTDTAYPGEIVILPSDSVRLNDVLGDPTRLPRKRWREDPLPMLRTTIAPKTAAQRER  360
            E+  +DTA  G+IVILP+D ++LN +LG+   LP++ + E+PLPML+TTIA K +
QRE
Sbjct  301
ELCSSDTACSGDIVILPNDVLQLNSILGNEMLLPQRTFIENPLPMLQTTIAAKKSEQREI  360

Query  361
LLDALTQLADTDPLLRCEVDSITHEIILSFLGRVQLEVVSALLSEKYKLETVVKEPSVIY  420
            LL ALT+++D DPLL+  VD+ THEIILSFLG+VQ+EV+ A+L EKY +E  +KEP
+VIY
Sbjct  361
LLGALTEISDGDPLLKYYVDTTTHEIILSFLGKVQMEVICAILEEKYHVEAEIKEPTVIY  420

Query  421
MERPLKAASHTIHIEVPPNPFWASIGLSVTPLSLGSGVQYESRVSLGYLNQSFQNAVRDG  480
            MERPL+ A +TIHIEVPPNPFWASIGLS+ PL
+GSGVQYESRVSLGYLNQSFQNAV +G
Sbjct  421
MERPLRKAEYTIHIEVPPNPFWASIGLSIEPLPIGSGVQYESRVSLGYLNQSFQNAVMEG  480

Query  481
IRYGLEQGLFGWNVTDCKICFEYGLYYSPVSTPADFRSLAPIVLEQALKESGTQLLEPYL  540
            + YG EQGL+GW VTDCKICFEYGLYYSPVSTPADFR L+PIVLEQALK++GT
+LLEPYL
Sbjct  481
VLYGCEQGLYGWKVTDCKICFEYGLYYSPVSTPADFRLLSPIVLEQALKKAGTELLEPYL  540

Query  541
SFILYAPQEYLSRAYHDAPKYCATIETAQVKKDEVVFTGEIPARCIQAYRTDLAFYTNGR  600
             F +YAPQEYLSRAYHDAP+YCA I + Q+K DEV+  GEIPARCIQ YR DL +
+TNG+
Sbjct  541
HFEIYAPQEYLSRAYHDAPRYCADIVSTQIKNDEVILKGEIPARCIQEYRNDLTYFTNGQ  600
```

```
Query  601  SVCLTELKGYQAAVGQPVIQPRRPNSRLDKVRHMFQKV  638
             VCLTELKGYQ A G+ + QPRRPNSR+DKVRHMF K+
Sbjct  601  GVCLTELKGYQPATGKLICQPRRPNSRIDKVRHMFHKL  638


>WP_044776625.1 TetM/TetW/TetO/TetS family tetracycline resistance 
ribosomal 
protection protein [Streptococcus suis]
 CYU85363.1 tetracycline resistance protein tetM [Streptococcus 
suis]
Length=639

 Score = 930 bits (2404),  Expect = 0.0, Method: Compositional 
matrix adjust.
 Identities = 431/638 (68%), Positives = 530/638 (83%), Gaps = 0/638 
(0%)

Query  1    
MKIINIGILAHVDAGKTTLTESLLYASGAISEPGSVEKGTTRTDTMFLERQRGITIQAAV  60
            MKIIN+GILAHVDAGKTTLTESLLY SGAI+EPGSV+KGTTRTDTM 
LERQRGITIQ AV
Sbjct  1    
MKIINLGILAHVDAGKTTLTESLLYTSGAIAEPGSVDKGTTRTDTMNLERQRGITIQTAV  60

Query  61   
TSFQWHRCKVNIVDTPGHMDFLAEVYRSLAVLDGAILVISAKDGVQAQTRILFHALRKMN  120
            TSFQW   KVNI+DTPGHMDFLAEVYRSL+VLDGA+L++SAKDG+QAQTRILFHAL 
+ M
Sbjct  61   
TSFQWEDVKVNIIDTPGHMDFLAEVYRSLSVLDGAVLLVSAKDGIQAQTRILFHALQTMK  120

Query  121  
IPTVIFINKIDQAGVDLQSVVQSVRDKLSADIIIKQTVSLSPEIVLEENTDIEAWDAVIE  180
            IPT+ FINKIDQ G+DL  V Q ++ KLS++II+KQ V   P I + +N D+E 
WDAVI
Sbjct  121  
IPTIFFINKIDQEGIDLPMVYQEMKAKLSSEIIVKQKVGQHPHINVTDNHDMEQWDAVIM  180

Query  181  
NNDKLLEKYIAGEPISREKLVREEQRRVQDASLFPVYYGSAKKGLGIQPLMDAVTGLFQP  240
             ND+LLEKY++G+P    +L +EE RR Q+ +LFPVY+GSAK  LGI+ L++ + 
F
Sbjct  181  
GNDELLEKYMSGKPFKMSELEQEENRRFQNGTLFPVYHGSAKNNLGIRQLIEVIASKFYS  240

Query  241  
IGEQGSAALCGSVFKVEYTDCGQRRVYLRLYSGTLRLRDTVALAGREKLKITEMRIPSKG  300
               +G + LCG VFK+EY++  +R VY+R+YSGTL LRD + ++ +EK+KITEM 
+P+ G
Sbjct  241  
STPEGQSELCGQVFKIEYSEKRRRFVYVRIYSGTLHLRDVIRISEKEKIKITEMYVPTNG  300

Query  301  
EIVRTDTAYPGEIVILPSDSVRLNDVLGDPTRLPRKRWREDPLPMLRTTIAPKTAAQRER  360
```

E+ +DTA G+IVILP+D ++LN +LG+   LP++++ E+PLPML+TTIA K + QRE
Sbjct 301 ELYSSDTACSGDIVILPNDVLQLNSILGNEILLPQRKFIENPLPMLQTTIAVKKSEQREI 360

Query 361 LLDALTQLADTDPLLRCEVDSITHEIILSFLGRVQLEVVSALLSEKYKLETVVKEPSVIY 420
LL ALT+++D DPLL+ VD+ THEIILSFLG VQ+EV+ A+L EKY +E +KEP +VIY
Sbjct 361 LLGALTEISDGDPLLKYYVDTTTHEIILSFLGNVQMEVICAILEEKYHVEVEIKEPTVIY 420

Query 421 MERPLKAASHTIHIEVPPNPFWASIGLSVTPLSLGSGVQYESRVSLGYLNQSFQNAVRDG 480
MERPL+ A +TIHIEVPPNPFWAS+GLS+ PL +GSGVQYESRVSLGYLNQSFQNAV +G
Sbjct 421 MERPLRKAEYTIHIEVPPNPFWASVGLSIEPLPIGSGVQYESRVSLGYLNQSFQNAVMEG 480

Query 481 IRYGLEQGLFGWNVTDCKICFEYGLYYSPVSTPADFRSLAPIVLEQALKESGTQLLEPYL 540
+ YG EQGL+GW VTDCKICFEYGLYYSPVSTPADFR L+PIVLEQALK++GT +LLEPYL
Sbjct 481 VLYGCEQGLYGWKVTDCKICFEYGLYYSPVSTPADFRLLSPIVLEQALKKAGTELLEPYL 540

Query 541 SFILYAPQEYLSRAYHDAPKYCATIETAQVKKDEVVFTGEIPARCIQAYRTDLAFYTNGR 600
F +YAPQEYLSRAYHDAP+YCA I + Q+K DEV+ GEIPARCIQ YR DL + +TNG+
Sbjct 541 HFEIYAPQEYLSRAYHDAPRYCADIVSTQIKNDEVILKGEIPARCIQEYRNDLTYFTNGQ 600

Query 601 SVCLTELKGYQAAVGQPVIQPRRPNSRLDKVRHMFQKV 638
VCLTELKGYQ A+G+ + QPRRPNSR+DKVRHMF K+
Sbjct 601 GVCLTELKGYQPAIGKFICQPRRPNSRIDKVRHMFHKL 638

>WP_008399617.1 MULTISPECIES: TetM/TetW/TetO/TetS family tetracycline resistance
ribosomal protection protein [Firmicutes]
EFE10671.1 putative translation elongation factor G [Clostridium sp. M62/1]
CBK88143.1 small GTP-binding protein domain [Faecalitalea cylindroides T2-87]
AGW28732.1 translation elongation factors (GTPases) [uncultured bacterium
EB2]
CUO21707.1 Tetracycline resistance protein tetM from transposon Tn916 [Coprococcus
comes]
CUP42488.1 Tetracycline resistance protein tetM from transposon Tn916 [Coprococcus
comes]

CUQ29424.1 Tetracycline resistance protein tetM from transposon Tn916 [Fusicatenibacter saccharivorans]
CUQ04507.1 Tetracycline resistance protein tetM from transposon Tn916 [Blautia obeum]
CUN87004.1 Tetracycline resistance protein tetM from transposon Tn916 [Blautia wexlerae]
CUP89135.1 Tetracycline resistance protein tetM from transposon Tn916 [Blautia wexlerae]
CUN15740.1 Tetracycline resistance protein tetM from transposon Tn916 [[Ruminococcus] torques]
SCI61689.1 Tetracycline resistance protein tetM from transposon Tn916 [uncultured Clostridium sp.]
SCI86029.1 Tetracycline resistance protein tetM from transposon Tn916 [uncultured Blautia sp.]
PLT59941.1 TetM/TetW/TetO/TetS family tetracycline resistance ribosomal protection protein [[Ruminococcus] gnavus]
Length=639

Score = 930 bits (2404), Expect = 0.0, Method: Compositional matrix adjust.
Identities = 431/638 (68%), Positives = 529/638 (83%), Gaps = 0/638 (0%)

```
Query  1
MKIININIGILAHVDAGKTTLTESLLYASGAISEPGSVEKGTTRTDTMFLERQRGITIQAAV  60
            MKIIN+GILAHVDAGKTTLTESLLY SGAI+EPGSV+KGTTRTDTM
LERQRGITIQ AV
Sbjct  1
MKIINLGILAHVDAGKTTLTESLLYTSGAIAEPGSVDKGTTRTDTMNLERQRGITIQTAV  60

Query  61
TSFQWHRCKVNIVDTPGHMDFLAEVYRSLAVLDGAILVISAKDGVQAQTRILFHALRKMN  120
            TSFQW   KVNI+DTPGHMDFLAEVYRSL+VLDGA+L++SAKDG+QAQTRILFHAL
+ M
Sbjct  61
TSFQWEDVKVNIIDTPGHMDFLAEVYRSLSVLDGAVLLVSAKDGIQAQTRILFHALQTMK  120

Query  121
IPTVIFINKIDQAGVDLQSVVQSVRDKLSADIIIKQTVSLSPEIVLEENTDIEAWDAVIE  180
            IPT+ FINKIDQ G+DL  V Q ++ KLS++II+KQ V   P I + +N D+E
WDAVI
Sbjct  121
IPTIFFINKIDQEGIDLPMVYQEMKAKLSSEIIVKQKVGQHPHINVTDNDDMEQWDAVIM  180

Query  181
NNDKLLEKYIAGEPISREKLVREEQRRVQDASLFPVYYGSAKKGLGIQPLMDAVTGLFQP  240
```

ND+LLEKY++G+P   +L +EE RR Q+ +LFPVY+GSAK LGI+ L++ +
F
Sbjct 181
GNDELLEKYMSGKPFKMSELEQEENRRFQNGTLFPVYHGSAKNNLGIRQLIEVIASKFYS 240

Query 241
IGEQGSAALCGSVFKVEYTDCGQRRVYLRLYSGTLRLRDTVALAGREKLKITEMRIPSKG 300
+G + LCG VFK+EY++ +R VY+R+YSGTL LRD + ++ +EK+KITEM
+P+ G
Sbjct 241
STPEGQSELCGQVFKIEYSEKRRRFVYVRIYSGTLHLRDVIKISEKEKIKITEMCVPTNG 300

Query 301
EIVRTDTAYPGEIVILPSDSVRLNDVLGDPTRLPRKRWREDPLPMLRTTIAPKTAAQRER 360
E+ +DTA G+IVILP+D ++LN +LG+   LP++ + E+PLPML+TTIA K +
QRE
Sbjct 301
ELCSSDTACSGDIVILPNDVLQLNSILGNEMLLPQRTFIENPLPMLQTTIAAKKSEQREI 360

Query 361
LLDALTQLADTDPLLRCEVDSITHEIILSFLGRVQLEVVSALLSEKYKLETVVKEPSVIY 420
LL ALT+++D DPLL+ VD+ THEIILSFLG+VQ+EV+ A+L EKY +E +KEP
+VIY
Sbjct 361
LLGALTEISDGDPLLKYYVDTTTHEIILSFLGKVQMEVICAILEEKYHVEAEIKEPTVIY 420

Query 421
MERPLKAASHTIHIEVPPNPFWASIGLSVTPLSLGSGVQYESRVSLGYLNQSFQNAVRDG 480
MERPL+ A +TIHIEVPPNPFWAS+GLS+ PL
+GSGVQYESRVSLGYLNQSFQNAV +G
Sbjct 421
MERPLRKAEYTIHIEVPPNPFWASVGLSIEPLPIGSGVQYESRVSLGYLNQSFQNAVMEG 480

Query 481
IRYGLEQGLFGWNVTDCKICFEYGLYYSPVSTPADFRSLAPIVLEQALKESGTQLLEPYL 540
+ YG EQGL+GW VTDCKICFEYGLYYSPVSTPADFR L+PIVLEQALK++GT
+LLEPYL
Sbjct 481
VLYGCEQGLYGWKVTDCKICFEYGLYYSPVSTPADFRLLSPIVLEQALKKAGTELLEPYL 540

Query 541
SFILYAPQEYLSRAYHDAPKYCATIETAQVKKDEVVFTGEIPARCIQAYRTDLAFYTNGR 600
F +YAPQEYLSRAYHDAP+YCA I + Q+K DEV+ GEIPARCIQ YR DL +
+TNG+
Sbjct 541
HFEIYAPQEYLSRAYHDAPRYCADIVSTQIKNDEVILKGEIPARCIQEYRNDLTYFTNGQ 600

Query 601 SVCLTELKGYQAAVGQPVIQPRRPNSRLDKVRHMFQKV 638
VCLTELKGYQ A G+ + QPRRPNSR+DKVRHMF K+
Sbjct 601 GVCLTELKGYQPATGKLICQPRRPNSRIDKVRHMFHKL 638

>WP_105158873.1 TetM/TetW/TetO/TetS family tetracycline resistance
ribosomal

protection protein [Streptococcus suis]
Length=639

 Score = 930 bits (2403), Expect = 0.0, Method: Compositional
matrix adjust.
 Identities = 431/638 (68%), Positives = 527/638 (83%), Gaps = 0/638
(0%)

Query 1
MKIINIGILAHVDAGKTTLTESLLYASGAISEPGSVEKGTTRTDTMFLERQRGITIQAAV 60
            MKIIN+GILAHVDAGKTTLTESLLY SGAI+EPGSV+KGTTRTDTM
LERQRGITIQ AV
Sbjct 1
MKIINLGILAHVDAGKTTLTESLLYTSGAIAEPGSVDKGTTRTDTMNLERQRGITIQTAV 60

Query 61
TSFQWHRCKVNIVDTPGHMDFLAEVYRSLAVLDGAILVISAKDGVQAQTRILFHALRKMN 120
            TSFQW   KVNI+DTPGHMDFLAEVYRSL+VLDGA+L++SAKDG+QAQTRILFHAL
+ M
Sbjct 61
TSFQWEDVKVNIIDTPGHMDFLAEVYRSLSVLDGAVLLVSAKDGIQAQTRILFHALQTMK 120

Query 121
IPTVIFINKIDQAGVDLQSVVQSVRDKLSADIIIKQTVSLSPEIVLEENTDIEAWDAVIE 180
            IPT+ FINKIDQ G+DL V Q ++ KLS++II+KQ V   P I + +N D+E WD
VI
Sbjct 121
IPTIFFINKIDQEGIDLPMVYQEMKAKLSSEIIVKQKVGQHPHINVTDNDDMEQWDTVIM 180

Query 181
NNDKLLEKYIAGEPISREKLVREEQRRVQDASLFPVYYGSAKKGLGIQPLMDAVTGLFQP 240
             ND+LLEKY++G+P   +L +EE RR Q+ +LFPVY+GSAK LGI+ L++ +
F
Sbjct 181
GNDELLEKYMSGKPFKMSELEQEENRRFQNGTLFPVYHGSAKNNLGIRQLIEVIASKFYS 240

Query 241
IGEQGSAALCGSVFKVEYTDCGQRRVYLRLYSGTLRLRDTVALAGREKLKITEMRIPSKG 300
               +G + LCG VFK+EY++ +R Y+RLYSG L RD V ++ +EK+KITEM
IP+ G
Sbjct 241
STPEGQSELCGQVFKIEYSEKRRRLAYVRLYSGALHSRDVVRISEKEKIKITEMSIPTNG 300

Query 301
EIVRTDTAYPGEIVILPSDSVRLNDVLGDPTRLPRKRWREDPLPMLRTTIAPKTAAQRER 360
            E+   DTAY G+IVILP+D ++LN +LG+   LP++++ E+PLPML+TTIA K +
QRE
Sbjct 301
ELCLADTAYSGDIVILPNDVLQLNSILGNEMLLPQRKFIENPLPMLQTTIAVKKSEQREV 360

Query 361
LLDALTQLADTDPLLRCEVDSITHEIILSFLGRVQLEVVSALLSEKYKLETVVKEPSVIY 420
            LL+ALT+++D DPLL+ VD+ THEIILSFLG VQ+EV+ A+L EKY +E +KEP
+VIY

Sbjct 361
LLEALTEISDGDPLLKYYVDTTTHEIILSFLGNVQMEVICAILEEKYHVEAEIKEPTVIY 420

Query 421
MERPLKAASHTIHIEVPPNPFWASIGLSVTPLSLGSGVQYESRVSLGYLNQSFQNAVRDG 480
            MERPL+ A +TIHIEVPPNPFWAS+GLS+ PL
+GSGVQYESRVSLGYLNQSFQNAV +G
Sbjct 421
MERPLRKAEYTIHIEVPPNPFWASVGLSIEPLPIGSGVQYESRVSLGYLNQSFQNAVMEG 480

Query 481
IRYGLEQGLFGWNVTDCKICFEYGLYYSPVSTPADFRSLAPIVLEQALKESGTQLLEPYL 540
            + YG EQGL+GW VTDCKICFEYGLYYSPVSTPADFR L+PIVLEQALK++GT
+LLEPYL
Sbjct 481
VLYGCEQGLYGWKVTDCKICFEYGLYYSPVSTPADFRLLSPIVLEQALKKAGTELLEPYL 540

Query 541
SFILYAPQEYLSRAYHDAPKYCATIETAQVKKDEVVFTGEIPARCIQAYRTDLAFYTNGR 600
             F +YAPQEYLSRAYHDAP+YCA I + Q+K DEV+ GEIPARCIQ YR DL +
+TNG+
Sbjct 541
HFEIYAPQEYLSRAYHDAPRYCADIVSTQIKNDEVILKGEIPARCIQEYRNDLTYFTNGQ 600

Query 601 SVCLTELKGYQAAVGQPVIQPRRPNSRLDKVRHMFQKV 638
             VCLTELKGYQ A+G+ + QPRRPNSR+DKVRHMF K+
Sbjct 601 GVCLTELKGYQPAIGKFICQPRRPNSRIDKVRHMFHKL 638

>WP_087378658.1 MULTISPECIES: TetM/TetW/TetO/TetS family tetracycline resistance
ribosomal protection protein [Clostridiales]
 OUP77054.1 tetracycline resistance ribosomal protection protein Tet(O) [Anaeromassilibacillus
sp. An172]
 PST39197.1 TetM/TetW/TetO/TetS family tetracycline resistance ribosomal
protection protein [Lachnoclostridium sp. SNUG30386]
Length=639

 Score = 930 bits (2403), Expect = 0.0, Method: Compositional matrix adjust.
 Identities = 428/638 (67%), Positives = 528/638 (83%), Gaps = 0/638 (0%)

Query 1
MKIINIGILAHVDAGKTTLTESLLYASGAISEPGSVEKGTTRTDTMFLERQRGITIQAAV 60
            MKIIN+GILAHVDAGKTTLTESLLY SGAI+E GSV++GTTRTDTM
LERQRGITIQ AV
Sbjct 1
MKIINLGILAHVDAGKTTLTESLLYTSGAIAELGSVDEGTTRTDTMNLERQRGITIQTAV 60

Query 61
TSFQWHRCKVNIVDTPGHMDFLAEVYRSLAVLDGAILVISAKDGVQAQTRILFHALRKMN 120

TSFQW   KVNI+DTPGHMDFLAEVYRSL+VLDGA+L++SAKDG+QAQTRILFHAL
+ M
Sbjct 61
TSFQWEDVKVNIIDTPGHMDFLAEVYRSLSVLDGAVLLVSAKDGIQAQTRILFHALQIMK 120

Query 121
IPTVIFINKIDQAGVDLQSVVQSVRDKLSADIIIKQTVSLSPEIVLEENTDIEAWDAVIE 180
IPT+ FINKIDQ G+DL V + ++ KLS++II+KQ V   P I + +N D+E
WDAVI
Sbjct 121
IPTIFFINKIDQEGIDLPMVYREMKAKLSSEIIVKQKVGQHPHINVTDNDDMEQWDAVIM 180

Query 181
NNDKLLEKYIAGEPISREKLVREEQRRVQDASLFPVYYGSAKKGLGIQPLMDAVTGLFQP 240
ND+LLEKY++G+P   +L +EE RR Q+ +LFPVY+GSAK LGI+ L++ +
F
Sbjct 181
GNDELLEKYMSGKPFKMSELEQEENRRFQNGTLFPVYHGSAKNNLGIRQLIEVIASKFYS 240

Query 241
IGEQGSAALCGSVFKVEYTDCGQRRVYLRLYSGTLRLRDTVALAGREKLKITEMRIPSKG 300
+G + LCG VFK+EY++ +R VY+R+YSGTL LRD + ++ +EK+KITEM
+P+ G
Sbjct 241
STPEGQSELCGQVFKIEYSEKRRRFVYVRIYSGTLHLRDVIRISEKEKIKITEMYVPTNG 300

Query 301
EIVRTDTAYPGEIVILPSDSVRLNDVLGDPTRLPRKRWREDPLPMLRTTIAPKTAAQRER 360
EIV D A PGEIVIL D+++LND+LG+   LP K W ++P+P+LRTT+ P+
QRE
Sbjct 301
EIVPADHACPGEIVILADDTLKLNDILGNEKLLPHKTWIDNPMPLLRTTVEPQKPEQREA 360

Query 361
LLDALTQLADTDPLLRCEVDSITHEIILSFLGRVQLEVVSALLSEKYKLETVVKEPSVIY 420
LL+AL ++ADTDPLL ++D++THEI+LSFLG VQ+EV+ A+L EKY +E +KEP
+VIY
Sbjct 361
LLNALAEIADTDPLLHFDIDTVTHEIMLSFLGNVQMEVICAILEEKYHVEAEIKEPTVIY 420

Query 421
MERPLKAASHTIHIEVPPNPFWASIGLSVTPLSLGSGVQYESRVSLGYLNQSFQNAVRDG 480
MERPL+ A +TIHIEVPPNPFWAS+GLS+ PL
+GSGVQYESRVSLGYLNQSFQNAV +G
Sbjct 421
MERPLRKAEYTIHIEVPPNPFWASVGLSIEPLPIGSGVQYESRVSLGYLNQSFQNAVMEG 480

Query 481
IRYGLEQGLFGWNVTDCKICFEYGLYYSPVSTPADFRSLAPIVLEQALKESGTQLLEPYL 540
+ YG EQGL+GW VTDCKICFEYGLYYSPVSTPADFR L+PIVLEQALK++GT
+LLEPYL
Sbjct 481
VLYGCEQGLYGWKVTDCKICFEYGLYYSPVSTPADFRLLSPIVLEQALKKAGTELLEPYL 540

Query 541
SFILYAPQEYLSRAYHDAPKYCATIETAQVKKDEVVFTGEIPARCIQAYRTDLAFYTNGR 600
             F +YAPQEYLSRAYHDAP+YCA I + Q+K DEV+ GEIPARCIQ YR DL +
+TNG+
Sbjct 541
HFEIYAPQEYLSRAYHDAPRYCADIVSTQIKNDEVILKGEIPARCIQEYRNDLTYFTNGQ 600

Query 601 SVCLTELKGYQAAVGQPVIQPRRPNSRLDKVRHMFQKV 638
             VCLTELKGYQ A+G+ + QPRRPNSR+DKVRHMF K+
Sbjct 601 GVCLTELKGYQPAIGKFICQPRRPNSRIDKVRHMFHKL 638

>WP_076773831.1 TetM/TetW/TetO/TetS family tetracycline resistance ribosomal
protection protein [Corynebacterium amycolatum]
 OMQ01622.1 tetracycline resistance ribosomal protection protein [Corynebacterium
amycolatum]
Length=639

 Score = 930 bits (2403), Expect = 0.0, Method: Compositional matrix adjust.
 Identities = 431/638 (68%), Positives = 529/638 (83%), Gaps = 0/638 (0%)

Query 1
MKIINIGILAHVDAGKTTLTESLLYASGAISEPGSVEKGTTRTDTMFLERQRGITIQAAV 60
            MKIIN+GILAHVDAGKTTLTESLLY SGAI+EPG V+KGTTRTDTM
LERQRGITIQ AV
Sbjct 1
MKIINLGILAHVDAGKTTLTESLLYTSGAIAEPGGVDKGTTRTDTMNLERQRGITIQTAV 60

Query 61
TSFQWHRCKVNIVDTPGHMDFLAEVYRSLAVLDGAILVISAKDGVQAQTRILFHALRKMN 120
            TSFQW   KVNI+DTPGHMDFLAEVYRSL+VLDGA+L++SAKDG+QAQTRILFHAL
+ M
Sbjct 61
TSFQWEDVKVNIIDTPGHMDFLAEVYRSLSVLDGAVLLVSAKDGIQAQTRILFHALQTMK 120

Query 121
IPTVIFINKIDQAGVDLQSVVQSVRDKLSADIIIKQTVSLSPEIVLEENTDIEAWDAVIE 180
            IPT+ FINKIDQ G+DL V Q ++ KLS++II+KQ V   P I + +N D+E
WDAVI
Sbjct 121
IPTIFFINKIDQEGIDLPMVYQEMKAKLSSEIIVKQKVGQHPHINVTDNDDMEQWDAVIM 180

Query 181
NNDKLLEKYIAGEPISREKLVREEQRRVQDASLFPVYYGSAKKGLGIQPLMDAVTGLFQP 240
             ND+LLEKY++G+P   +L +EE RR Q+ +LFPVY+GSAK LGI+ L++ +
F
Sbjct 181
GNDELLEKYMSGKPFKMSELEQEENRRFQNGTLFPVYHGSAKNNLGIRQLIEVIASKFYS 240

Query 241

IGEQGSAALCGSVFKVEYTDCGQRRVYLRLYSGTLRLRDTVALAGREKLKITEMRIPSKG 300
               +G + LCG VFK+EY++ +R VY+R+YSGTL LRD + ++ +EK+KITEM
+P+ G
Sbjct 241
STPEGQSELCGQVFKIEYSEKRRRFVYVRIYSGTLHLRDVIKISEKEKIKITEMCVPTNG 300

Query 301
EIVRTDTAYPGEIVILPSDSVRLNDVLGDPTRLPRKRWREDPLPMLRTTIAPKTAAQRER 360
            E+ +DTA G+IVILP+D ++LN +LG+   LP++++ E+PLPML+TTIA K +
QRE
Sbjct 301
ELYSSDTACSGDIVILPNDVLQLNSILGNEMLLPQRKFIENPLPMLQTTIAVKKSEQREI 360

Query 361
LLDALTQLADTDPLLRCEVDSITHEIILSFLGRVQLEVVSALLSEKYKLETVVKEPSVIY 420
            LL ALT+++D DPLL+ VD+ THEIILSFLG VQ+EV+ A+L EKY +E +KEP
+VIY
Sbjct 361
LLGALTEISDGDPLLKYYVDTTTHEIILSFLGNVQMEVICAILEEKYHVEAEIKEPTVIY 420

Query 421
MERPLKAASHTIHIEVPPNPFWASIGLSVTPLSLGSGVQYESRVSLGYLNQSFQNAVRDG 480
            MERPL+ A +TIHIEVPPNPFWAS+GLS+ PL
+GSGVQYESRVSLGYLNQSFQNAV +G
Sbjct 421
MERPLRKAEYTIHIEVPPNPFWASVGLSIEPLPIGSGVQYESRVSLGYLNQSFQNAVMEG 480

Query 481
IRYGLEQGLFGWNVTDCKICFEYGLYYSPVSTPADFRSLAPIVLEQALKESGTQLLEPYL 540
            + YG EQGL+GW VTDCKICFEYGLYYSPVSTPADFR L+PIVLEQALK++GT
+LLEPYL
Sbjct 481
VLYGCEQGLYGWKVTDCKICFEYGLYYSPVSTPADFRLLSPIVLEQALKKAGTELLEPYL 540

Query 541
SFILYAPQEYLSRAYHDAPKYCATIETAQVKKDEVVFTGEIPARCIQAYRTDLAFYTNGR 600
             F +YAPQEYLSRAYHDAP+YCA I + QVK DEV+ GEIPARCIQ YR DL +
+TNG+
Sbjct 541
HFEIYAPQEYLSRAYHDAPRYCADIVSTQVKNDEVILKGEIPARCIQEYRNDLTYFTNGQ 600

Query 601 SVCLTELKGYQAAVGQPVIQPRRPNSRLDKVRHMFQKV 638
             VCLTELKGYQ A+G+ + QPRRPNSR+DKVRHMF K+
Sbjct 601 GVCLTELKGYQPAIGKFICQPRRPNSRIDKVRHMFHKL 638

>AMP55043.1 TetM-TetW-TetO-TetS, partial [uncultured bacterium]
Length=643

Score = 930 bits (2403), Expect = 0.0, Method: Compositional
matrix adjust.
Identities = 432/638 (68%), Positives = 531/638 (83%), Gaps = 0/638
(0%)

Query 1
MKIINIGILAHVDAGKTTLTESLLYASGAISEPGSVEKGTTRTDTMFLERQRGITIQAAV 60
MKIINIGILAHVDAGKTTLTESLLY SGAI+E G+V+KGTTRTDTM
LERQRGITIQ AV
Sbjct 5
MKIINIGILAHVDAGKTTLTESLLYTSGAIAEQGNVDKGTTRTDTMILERQRGITIQTAV 64

Query 61
TSFQWHRCKVNIVDTPGHMDFLAEVYRSLAVLDGAILVISAKDGVQAQTRILFHALRKMN 120
TSF W+ K+NIVDTPGHMDFL E YRSL+VLDGA+LVISAKDGVQAQTRILFHAL
+KM+
Sbjct 65
TSFYWNDYKINIVDTPGHMDFLTEAYRSLSVLDGAVLVISAKDGVQAQTRILFHALQKMD 124

Query 121
IPTVIFINKIDQAGVDLQSVVQSVRDKLSADIIIKQTVSLSPEIVLEENTDIEAWDAVIE 180
IPT+IFINKIDQ G+DLQ V QS++DKL++D+I+ Q VSLSP+I + + +D++ WD
+I
Sbjct 125
IPTIIFINKIDQNGIDLQCVYQSIKDKLTSDMIVMQEVSLSPKITMTDISDLDKWDMIIS 184

Query 181
NNDKLLEKYIAGEPISREKLVREEQRRVQDASLFPVYYGSAKKGLGIQPLMDAVTGLFQP 240
+D+LLE+Y+A + + ++L E+ +R + SLFPVY+GSAK LG + L++A+
F
Sbjct 185
GSDELLERYVAEDSLDIQELQYEKCKRTRCCSLFPVYHGSAKDNLGTEKLIEAIIETFIT 244

Query 241
IGEQGSAALCGSVFKVEYTDCGQRRVYLRLYSGTLRLRDTVALAGREKLKITEMRIPSKG 300
+ + LCG VFKVEYT+ +R YLRLY GTL LRDT+ L+ +EK+KITEM
IPS G
Sbjct 245
ETDDIQSELCGYVFKVEYTERKKRLSYLRLYHGTLHLRDTLLLSKKEKIKITEMCIPSNG 304

Query 301
EIVRTDTAYPGEIVILPSDSVRLNDVLGDPTRLPRKRWREDPLPMLRTTIAPKTAAQRER 360
EIV D A PGEIVIL D+++LND+LG+ LP K W ++P+P+LRTT+ P+
QRE
Sbjct 305
EIVPADHACPGEIVILADDTLKLNDILGNEKLLPHKTWIDNPMPLLRTTVEPQKPEQREA 364

Query 361
LLDALTQLADTDPLLRCEVDSITHEIILSFLGRVQLEVVSALLSEKYKLETVVKEPSVIY 420
LL+AL ++ADTDPLL ++D++THEI+LSFLG+VQ+EV+ A+L EKY +E +KEP
+VIY
Sbjct 365
LLNALAEIADTDPLLHFDIDTVTHEIMLSFLGKVQMEVICAILEEKYHVEAEIKEPTVIY 424

Query 421
MERPLKAASHTIHIEVPPNPFWASIGLSVTPLSLGSGVQYESRVSLGYLNQSFQNAVRDG 480
MERPL+ A +TIHIEVPPNPFWAS+GLS+ PL
+GSGVQYESRVSLGYLNQSFQNAV +G
Sbjct 425

MERPLRKAEYTIHIEVPPNPFWASVGLSIEPLPIGSGVQYESRVSLGYLNQSFQNAVMEG 484

Query 481
IRYGLEQGLFGWNVTDCKICFEYGLYYSPVSTPADFRSLAPIVLEQALKESGTQLLEPYL 540
            + YG EQGL+GW VTDCKICFEYGLYYSPVSTPADFR L+PIVLEQALK++GT
+LLEPYL
Sbjct 485
VLYGCEQGLYGWKVTDCKICFEYGLYYSPVSTPADFRLLSPIVLEQALKKAGTELLEPYL 544

Query 541
SFILYAPQEYLSRAYHDAPKYCATIETAQVKKDEVVFTGEIPARCIQAYRTDLAFYTNGR 600
             F +YAPQEYLSRAYHDAP+YCA I + Q+K DEV+ GEIPARCIQ YR DL +
+TNG+
Sbjct 545
HFEIYAPQEYLSRAYHDAPRYCADIVSTQIKNDEVILKGEIPARCIQEYRNDLTYFTNGQ 604

Query 601 SVCLTELKGYQAAVGQPVIQPRRPNSRLDKVRHMFQKV 638
             VCLTELKGYQ A+G+ + QPRRPNSR+DKVRHMF K+
Sbjct 605 GVCLTELKGYQPAIGKFICQPRRPNSRIDKVRHMFHKL 642

>WP_063856401.1 tetracycline resistance ribosomal protection protein
Tet(M) [Streptococcus
pyogenes]
 CAL69903.1 tetracycline resistance protein [Streptococcus pyogenes]
Length=639

 Score = 930 bits (2403), Expect = 0.0, Method: Compositional
matrix adjust.
 Identities = 438/638 (69%), Positives = 519/638 (81%), Gaps = 0/638
(0%)

Query 1
MKIINIGILAHVDAGKTTLTESLLYASGAISEPGSVEKGTTRTDTMFLERQRGITIQAAV 60
            MKIINIG+LAHVDAGKTTLTESLLY SGAI+E GSV+KGTTRTD
LERQRGITIQ +
Sbjct 1
MKIINIGVLAHVDAGKTTLTESLLYTSGAITELGSVDKGTTRTDNTLLERQRGITIQTGI 60

Query 61
TSFQWHRCKVNIVDTPGHMDFLAEVYRSLAVLDGAILVISAKDGVQAQTRILFHALRKMN 120
            TSFQW   KVNI+DTPGHMDFLAEVYRSL+VLDGAIL
+ISAKDGVQAQTRILFHALRKM
Sbjct 61
TSFQWENTKVNIIDTPGHMDFLAEVYRSLSVLDGAILLISAKDGVQAQTRILFHALRKMG 120

Query 121
IPTVIFINKIDQAGVDLQSVVQSVRDKLSADIIIKQTVSLSPEIVLEENTDIEAWDAVIE 180
            IPT+ FINKIDQ G+DL +V Q +++KLSA+I+IKQ V L P + +   T+ E WD
VIE
Sbjct 121
IPTIFFINKIDQNGIDLSTVYQDIKEKLSAEIVIKQKVELYPNMCVTNFTESEQWDTVIE 180

Query 181

```
NNDKLLEKYIAGEPISREKLVREEQRRVQDASLFPVYYGSAKKGLGIQPLMDAVTGLFQP  240
             ND LLEKY++G+ +   +L +EE  R  + SLFPVYYGSAK  +GI  L++ +T
F
Sbjct  181
GNDDLLEKYMSGKSLEALELQQEESIRFHNCSLFPVYYGSAKNNIGIDNLIEVITNKFYS  240

Query  241
IGEQGSAALCGSVFKVEYTDCGQRRVYLRLYSGTLRLRDTVALAGREKLKITEMRIPSKG  300
               +G + LCG+VFK+EY++  QR  Y+RLYSG L LRD+V ++ +EK+KITEM
G
Sbjct  241
STHRGPSELCGNVFKIEYSEKRQRLAYIRLYSGVLHLRDSVRISEKEKIKITEMYTSING  300

Query  301
EIVRTDTAYPGEIVILPSDSVRLNDVLGDPTRLPRKRWREDPLPMLRTTIAPKTAAQRER  360
            E+ + D AY GEIVIL ++ ++LN VLGD   LP++   E+PLP+L+TT+ P
QRE
Sbjct  301
ELCKIDKAYSGEIVILQNEFLKLNSVLGDTKLLPQRERIENPLPLLQTTVEPSKPQQREM  360

Query  361
LLDALTQLADTDPLLRCEVDSITHEIILSFLGRVQLEVVSALLSEKYKLETVVKEPSVIY  420
            LLDAL +++D+DPLLR  VDS THEIILSFLG+VQ+EV  ALL EKY +E  +KEP
+VIY
Sbjct  361
LLDALLEISDSDPLLRYYVDSATHEIILSFLGKVQMEVTCALLQEKYHVEIEIKEPTVIY  420

Query  421
MERPLKAASHTIHIEVPPNPFWASIGLSVTPLSLGSGVQYESRVSLGYLNQSFQNAVRDG  480
            MERPLK A +TIHIEVPPNPFWASIGLSV PL LGSGVQYES
VSLGYLNQSFQNAV +G
Sbjct  421
MERPLKKAEYTIHIEVPPNPFWASIGLSVAPLPLGSGVQYESSVSLGYLNQSFQNAVMEG  480

Query  481
IRYGLEQGLFGWNVTDCKICFEYGLYYSPVSTPADFRSLAPIVLEQALKESGTQLLEPYL  540
            IRYG EQGL+GWNVTDCKICF+YGLYYSPVSTPADFR LAPIVLEQ LK++GT
+LLEPYL
Sbjct  481
IRYGCEQGLYGWNVTDCKICFKYGLYYSPVSTPADFRMLAPIVLEQVLKKAGTELLEPYL  540

Query  541
SFILYAPQEYLSRAYHDAPKYCATIETAQVKKDEVVFTGEIPARCIQAYRTDLAFYTNGR  600
            SF +YAPQEYLSRAY+DAPKYCA I   Q+K +EV+ +GEIPARCIQ YR+DL F
+TNGR
Sbjct  541
SFKIYAPQEYLSRAYNDAPKYCANIVDTQLKNNEVILSGEIPARCIQEYRSDLTFFTNGR  600

Query  601  SVCLTELKGYQAAVGQPVIQPRRPNSRLDKVRHMFQKV  638
            SVCLTELKGY    G+PV QPRRPNSR+DKVR+MF K+
Sbjct  601  SVCLTELKGYHVTTGEPVCQPRRPNSRIDKVRYMFNKI  638
```

>WP_044772610.1 TetM/TetW/TetO/TetS family tetracycline resistance

ribosomal
protection protein [Streptococcus suis]
 CYZ56297.1 tetracycline resistance protein tetM [Streptococcus
suis]
 CYZ54792.1 tetracycline resistance protein tetM [Streptococcus
suis]
 CYZ82313.1 tetracycline resistance protein tetM [Streptococcus
suis]
Length=639

 Score = 930 bits (2403), Expect = 0.0, Method: Compositional
matrix adjust.
 Identities = 431/638 (68%), Positives = 529/638 (83%), Gaps = 0/638
(0%)

Query 1
MKIINIGILAHVDAGKTTLTESLLYASGAISEPGSVEKGTTRTDTMFLERQRGITIQAAV 60
            MKIIN+GILAHVDAGKTTLTESLLY SGAI+EPGSV+KGTTRTDTM
LERQRGITIQ AV
Sbjct 1
MKIINLGILAHVDAGKTTLTESLLYTSGAIAEPGSVDKGTTRTDTMNLERQRGITIQTAV 60

Query 61
TSFQWHRCKVNIVDTPGHMDFLAEVYRSLAVLDGAILVISAKDGVQAQTRILFHALRKMN 120
            TSFQW   KVNI+DTPGHMDFLAEVYRSL+VLDGA+L++SAKDG+QAQTRILFHAL
+ M
Sbjct 61
TSFQWEDVKVNIIDTPGHMDFLAEVYRSLSVLDGAVLLVSAKDGIQAQTRILFHALQTMK 120

Query 121
IPTVIFINKIDQAGVDLQSVVQSVRDKLSADIIIKQTVSLSPEIVLEENTDIEAWDAVIE 180
            IPT+ FINKIDQ G+DL V Q ++ KLS++II+KQ V   P I + +N D+E
WDAVI
Sbjct 121
IPTIFFINKIDQEGIDLPMVYQEMKAKLSSEIIVKQKVGQHPHINVTDNDDMEQWDAVIM 180

Query 181
NNDKLLEKYIAGEPISREKLVREEQRRVQDASLFPVYYGSAKKGLGIQPLMDAVTGLFQP 240
             ND+LLEKY++G+P   +L +EE RR Q+ +LFPVY+GSAK LGI+ L++ +
F
Sbjct 181
GNDELLEKYMSGKPFKMSELEQEENRRFQNGTLFPVYHGSAKNNLGIRQLIEVIASKFYS 240

Query 241
IGEQGSAALCGSVFKVEYTDCGQRRVYLRLYSGTLRLRDTVALAGREKLKITEMRIPSKG 300
               +G + LCG VFK+EY++ +R VY+R+YSGTL LRD + ++ +EK+KITEM
+P+ G
Sbjct 241
STPEGQSELCGQVFKIEYSEKRRRFVYVRIYSGTLHLRDVIKISEKEKIKITEMCVPTNG 300

Query 301
EIVRTDTAYPGEIVILPSDSVRLNDVLGDPTRLPRKRWREDPLPMLRTTIAPKTAAQRER 360
            E+ +DTA G+IVILP+D ++LN +LG+   LP++++ E+PLPML+TTIA K +
QRE

Sbjct 301
ELYSSDTACSGDIVILPNDVLQLNSILGNEMLLPQRKFIENPLPMLQTTIAVKKSEQREI 360

Query 361
LLDALTQLADTDPLLRCEVDSITHEIILSFLGRVQLEVVSALLSEKYKLETVVKEPSVIY 420
            LL ALT+++D DPLL+ VD+ THEIILSFLG VQ+EV+ A+L EKY +E   KEP
+VIY
Sbjct 361
LLGALTEISDGDPLLKYYVDTTTHEIILSFLGNVQMEVICAILEEKYHVEAETKEPTVIY 420

Query 421
MERPLKAASHTIHIEVPPNPFWASIGLSVTPLSLGSGVQYESRVSLGYLNQSFQNAVRDG 480
            MERPL+ A +TIHIEVPPNPFWAS+GLS+ PL
+GSGVQYESRVSLGYLNQSFQNAV +G
Sbjct 421
MERPLRKAEYTIHIEVPPNPFWASVGLSIEPLPIGSGVQYESRVSLGYLNQSFQNAVMEG 480

Query 481
IRYGLEQGLFGWNVTDCKICFEYGLYYSPVSTPADFRSLAPIVLEQALKESGTQLLEPYL 540
            + YG EQGL+GW VTDCKICFEYGLYYSPVSTPADFR L+PIVLEQALK++GT
+LLEPYL
Sbjct 481
VLYGCEQGLYGWKVTDCKICFEYGLYYSPVSTPADFRLLSPIVLEQALKKAGTELLEPYL 540

Query 541
SFILYAPQEYLSRAYHDAPKYCATIETAQVKKDEVVFTGEIPARCIQAYRTDLAFYTNGR 600
             F +YAPQEYLSRAYHDAP+YCA I + Q+K DEV+ GEIPARCIQ YR DL +
+TNG+
Sbjct 541
HFEIYAPQEYLSRAYHDAPRYCADIVSTQIKNDEVILKGEIPARCIQEYRNDLTYFTNGQ 600

Query 601 SVCLTELKGYQAAVGQPVIQPRRPNSRLDKVRHMFQKV 638
             VCLTELKGYQ A+G+ + QPRRPNSR+DKVRHMF K+
Sbjct 601 GVCLTELKGYQPAIGKFICQPRRPNSRIDKVRHMFHKL 638

>WP_044769057.1 TetM/TetW/TetO/TetS family tetracycline resistance
ribosomal
protection protein [Streptococcus suis]
 CYU72128.1 tetracycline resistance protein tetM [Streptococcus
suis]
Length=639

 Score = 930 bits (2403), Expect = 0.0, Method: Compositional
matrix adjust.
 Identities = 432/638 (68%), Positives = 529/638 (83%), Gaps = 0/638
(0%)

Query 1
MKIINIGILAHVDAGKTTLTESLLYASGAISEPGSVEKGTTRTDTMFLERQRGITIQAAV 60
            MKIIN+GILAHVDAGKTTLTESLLY SGAI+EPGSV+KGTTRTDTM
LERQRGITIQ AV
Sbjct 1
MKIINLGILAHVDAGKTTLTESLLYTSGAIAEPGSVDKGTTRTDTMNLERQRGITIQTAV 60

Query 61
TSFQWHRCKVNIVDTPGHMDFLAEVYRSLAVLDGAILVISAKDGVQAQTRILFHALRKMN 120
            TSFQW   KVNI+DTPGHMDFLAEVYRSL+VLDGA+L++SAKDG+QAQTRILFHAL
+ M
Sbjct 61
TSFQWEDVKVNIIDTPGHMDFLAEVYRSLSVLDGAVLLVSAKDGIQAQTRILFHALQTMK 120

Query 121
IPTVIFINKIDQAGVDLQSVVQSVRDKLSADIIIKQTVSLSPEIVLEENTDIEAWDAVIE 180
            IPT+ FINKIDQ G+DL V Q ++ KLS++II+KQ V   P I + +N D+E
WDAVI
Sbjct 121
IPTIFFINKIDQEGIDLPMVYQEMKAKLSSEIIVKQKVGQHPHINVTDNDDMEQWDAVIM 180

Query 181
NNDKLLEKYIAGEPISREKLVREEQRRVQDASLFPVYYGSAKKGLGIQPLMDAVTGLFQP 240
             ND+LLEKY++G+P   +L +EE RR Q+ +LFPVY+GSAK LGI+ L++ +
F
Sbjct 181
GNDELLEKYMSGKPFKMSELEQEENRRFQNGTLFPVYHGSAKNNLGIRQLIEVIASKFYS 240

Query 241
IGEQGSAALCGSVFKVEYTDCGQRRVYLRLYSGTLRLRDTVALAGREKLKITEMRIPSKG 300
               +G + LCG VFK+EY++ +R VY+R+YSGTL LRD + ++ +EK+KITEM
+P+ G
Sbjct 241
STPEGQSELCGQVFKIEYSEKRRRFVYVRIYSGTLHLRDVIKISEKEKIKITEMCVPTNG 300

Query 301
EIVRTDTAYPGEIVILPSDSVRLNDVLGDPTRLPRKRWREDPLPMLRTTIAPKTAAQRER 360
            E+ +DTA G+IVILP+D ++LN +LG+   LP++++ E+PLPML+TTIA K +
QRE
Sbjct 301
ELYSSDTACSGDIVILPNDVLQLNSILGNEMLLPQRKFIENPLPMLQTTIAVKKSEQREI 360

Query 361
LLDALTQLADTDPLLRCEVDSITHEIILSFLGRVQLEVVSALLSEKYKLETVVKEPSVIY 420
            LL ALT+++D DPLL+ VD+ THEIILSFLG VQ+EV+ A+L EKY +E +KEP
+VIY
Sbjct 361
LLGALTEISDGDPLLKYYVDTTTHEIILSFLGNVQMEVICAILEEKYHVEAEIKEPTVIY 420

Query 421
MERPLKAASHTIHIEVPPNPFWASIGLSVTPLSLGSGVQYESRVSLGYLNQSFQNAVRDG 480
            MERPL A +TIHIEVPPNPFWAS+GLS+ PL
+GSGVQYESRVSLGYLNQSFQNAV +G
Sbjct 421
MERPLGKAEYTIHIEVPPNPFWASVGLSIEPLPIGSGVQYESRVSLGYLNQSFQNAVMEG 480

Query 481
IRYGLEQGLFGWNVTDCKICFEYGLYYSPVSTPADFRSLAPIVLEQALKESGTQLLEPYL 540
            + YG EQGL+GW VTDCKICFEYGLYYSPVSTPADFR L+PIVLEQALK++GT
+LLEPYL

```
Sbjct  481  
VLYGCEQGLYGWKVTDCKICFEYGLYYSPVSTPADFRLLSPIVLEQALKKAGTELLEPYL  540

Query  541  
SFILYAPQEYLSRAYHDAPKYCATIETAQVKKDEVVFTGEIPARCIQAYRTDLAFYTNGR  600
             F +YAPQEYLSRAYHDAP+YCA I + QVK DEV+  GEIPARCIQ YR DL +
+TNG+
Sbjct  541  
HFEIYAPQEYLSRAYHDAPRYCADIVSTQVKNDEVILKGEIPARCIQEYRNDLTYFTNGQ  600

Query  601  SVCLTELKGYQAAVGQPVIQPRRPNSRLDKVRHMFQKV  638
             VCLTELKGYQ A+G+ + QPRRPNSR+DKVRHMF K+
Sbjct  601  GVCLTELKGYQPAIGKFICQPRRPNSRIDKVRHMFHKL  638


>WP_044762341.1 TetM/TetW/TetO/TetS family tetracycline resistance 
ribosomal 
protection protein [Streptococcus suis]
 CYU07592.1 tetracycline resistance protein tetM [Streptococcus 
suis]
Length=639

 Score = 930 bits (2403),  Expect = 0.0, Method: Compositional 
matrix adjust.
 Identities = 431/638 (68%), Positives = 530/638 (83%), Gaps = 0/638 
(0%)

Query  1    
MKIINIGILAHVDAGKTTLTESLLYASGAISEPGSVEKGTTRTDTMFLERQRGITIQAAV  60
            MKIIN+GILAHVDAGKTTLTESLLY SGAI+EPGSV+KGTTRTDTM  
LERQRGITIQ AV
Sbjct  1    
MKIINLGILAHVDAGKTTLTESLLYTSGAIAEPGSVDKGTTRTDTMNLERQRGITIQTAV  60

Query  61   
TSFQWHRCKVNIVDTPGHMDFLAEVYRSLAVLDGAILVISAKDGVQAQTRILFHALRKMN  120
            TSFQW   KVNI+DTPGHMDFLAEVYRSL+VLDGA+L++SAKDG+QAQTRILFHAL
+ M
Sbjct  61   
TSFQWEDVKVNIIDTPGHMDFLAEVYRSLSVLDGAVLLVSAKDGIQAQTRILFHALQTMK  120

Query  121  
IPTVIFINKIDQAGVDLQSVVQSVRDKLSADIIIKQTVSLSPEIVLEENTDIEAWDAVIE  180
            IPT+ FINKIDQ G+DL  V Q ++ KLS++II+KQ V   P I + +N D+E
WDAVI
Sbjct  121  
IPTIFFINKIDQEGIDLPMVYQEMKAKLSSEIIVKQKVGQHPHINVTDNDDMEQWDAVIM  180

Query  181  
NNDKLLEKYIAGEPISREKLVREEQRRVQDASLFPVYYGSAKKGLGIQPLMDAVTGLFQP  240
             ND+LLEKY++G+P    +L +EE RR Q+ +LFPVY+GSAK  LGI+ L++ +
F
Sbjct  181  
GNDELLEKYMSGKPFKMSELEQEENRRFQNGTLFPVYHGSAKNNLGIRQLIEVIASKFYS  240
```

```
Query  241
IGEQGSAALCGSVFKVEYTDCGQRRVYLRLYSGTLRLRDTVALAGREKLKITEMRIPSKG  300
               +G + LCG VFK+EY++  +R VY+R+YSGTL LRD + ++ +EK+KITEM
+P+ G
Sbjct  241
STPEGQSELCGQVFKIEYSEKRRRFVYVRIYSGTLHLRDVIKISEKEKIKITEMCVPTNG  300

Query  301
EIVRTDTAYPGEIVILPSDSVRLNDVLGDPTRLPRKRWREDPLPMLRTTIAPKTAAQRER  360
            E+  +DTA  G+IVILP+D ++LN +LG+   LP++++ E+PLPML+TTIA K +
QRE
Sbjct  301
ELYSSDTACSGDIVILPNDVLQLNSILGNEMLLPQRKFIENPLPMLQTTIAVKKSEQREI  360

Query  361
LLDALTQLADTDPLLRCEVDSITHEIILSFLGRVQLEVVSALLSEKYKLETVVKEPSVIY  420
            LL ALT+++D DPLL+  VD+ THEIILSFLG VQ+EV+ A+L EKY +E  +K+P
+VIY
Sbjct  361
LLGALTEISDGDPLLKYYVDTTTHEIILSFLGNVQMEVICAILEEKYHVEAEIKDPTVIY  420

Query  421
MERPLKAASHTIHIEVPPNPFWASIGLSVTPLSLGSGVQYESRVSLGYLNQSFQNAVRDG  480
            MERPL+ A +TIHIEVPPNPFWAS+GLS+ PL
+GSGVQYESRVSLGYLNQSFQNAV +G
Sbjct  421
MERPLRKAEYTIHIEVPPNPFWASVGLSIEPLPIGSGVQYESRVSLGYLNQSFQNAVMEG  480

Query  481
IRYGLEQGLFGWNVTDCKICFEYGLYYSPVSTPADFRSLAPIVLEQALKESGTQLLEPYL  540
            + YG EQGL+GW VTDCKICFEYGLYYSPVSTPADFR L+PIVLEQALK++GT
+LLEPYL
Sbjct  481
VLYGCEQGLYGWKVTDCKICFEYGLYYSPVSTPADFRLLSPIVLEQALKKAGTELLEPYL  540

Query  541
SFILYAPQEYLSRAYHDAPKYCATIETAQVKKDEVVFTGEIPARCIQAYRTDLAFYTNGR  600
             F +YAPQEYLSRAYHDAP+YCA I + QVK DEV+  GEIPARCIQ YR DL +
+TNG+
Sbjct  541
HFEIYAPQEYLSRAYHDAPRYCADIVSTQVKNDEVILKGEIPARCIQEYRNDLTYFTNGQ  600

Query  601  SVCLTELKGYQAAVGQPVIQPRRPNSRLDKVRHMFQKV  638
             VCLTELKGYQ A+G+ + QPRRPNSR+DKVRHMF K+
Sbjct  601  GVCLTELKGYQPAIGKFICQPRRPNSRIDKVRHMFHKL  638


>WP_024413659.1 TetM/TetW/TetO/TetS family tetracycline resistance
ribosomal
protection protein [Streptococcus suis]
 CYZ51529.1 tetracycline resistance protein tetM [Streptococcus
suis]
 CZB07794.1 tetracycline resistance protein tetM [Streptococcus
```

suis]
 CZB00149.1 tetracycline resistance protein tetM [Streptococcus
suis]
 CYY40641.1 tetracycline resistance protein tetM [Streptococcus
suis]
 CYZ15349.1 tetracycline resistance protein tetM [Streptococcus
suis]
 CZA07034.1 tetracycline resistance protein tetM [Streptococcus
suis]
 CZB10861.1 tetracycline resistance protein tetM [Streptococcus
suis]
 CZB11365.1 tetracycline resistance protein tetM [Streptococcus
suis]
Length=639

 Score = 930 bits (2403), Expect = 0.0, Method: Compositional
matrix adjust.
 Identities = 431/638 (68%), Positives = 528/638 (83%), Gaps = 0/638
(0%)

```
Query  1
MKIINIGILAHVDAGKTTLTESLLYASGAISEPGSVEKGTTRTDTMFLERQRGITIQAAV  60
            MKIIN+GILAHVDAGKTTLTESLLY SGAI+EPGSV+KGTTRTDTM 
LERQRGITIQ AV
Sbjct  1
MKIINLGILAHVDAGKTTLTESLLYTSGAIAEPGSVDKGTTRTDTMNLERQRGITIQTAV  60

Query  61
TSFQWHRCKVNIVDTPGHMDFLAEVYRSLAVLDGAILVISAKDGVQAQTRILFHALRKMN  120
            TSFQW   KVNI+DTPGHMDFLAEVYRSL+VLDGA+L++SAKDG+QAQTRILFHAL
+ M
Sbjct  61
TSFQWEDVKVNIIDTPGHMDFLAEVYRSLSVLDGAVLLVSAKDGIQAQTRILFHALQTMK  120

Query  121
IPTVIFINKIDQAGVDLQSVVQSVRDKLSADIIIKQTVSLSPEIVLEENTDIEAWDAVIE  180
            IPT+ FINKIDQ G+DL  V Q ++ KLS++II+KQ V   P I + +N D+E
WDAVI
Sbjct  121
IPTIFFINKIDQEGIDLPMVYQEMKAKLSSEIIVKQKVGQHPHINVTDNDDMEQWDAVIM  180

Query  181
NNDKLLEKYIAGEPISREKLVREEQRRVQDASLFPVYYGSAKKGLGIQPLMDAVTGLFQP  240
             ND+LLEKY++G+P    +L +EE RR Q+ +LFPVY+GSAK  LGI+ L++ +
F
Sbjct  181
GNDELLEKYMSGKPFKMSELEQEENRRFQNGTLFPVYHGSAKNNLGIRQLIEVIASKFYS  240

Query  241
IGEQGSAALCGSVFKVEYTDCGQRRVYLRLYSGTLRLRDTVALAGREKLKITEMRIPSKG  300
               +G + LCG VFK+EY++  +R  Y+RLYSG L  RD V ++ +EK+KITEM
IP+ G
Sbjct  241
STPEGQSELCGQVFKIEYSEKRRRLAYVRLYSGALHSRDVVRISEKEKIKITEMSIPTNG  300
```

Query 301
EIVRTDTAYPGEIVILPSDSVRLNDVLGDPTRLPRKRWREDPLPMLRTTIAPKTAAQRER 360
            E+   DTAY G+IVILP+D ++LN +LG+   LP++++ E+PLPML+TTI K +
QRE
Sbjct 301
ELCLADTAYSGDIVILPNDVLQLNSILGNEILLPQRKFIENPLPMLQTTIEVKKSEQREV 360

Query 361
LLDALTQLADTDPLLRCEVDSITHEIILSFLGRVQLEVVSALLSEKYKLETVVKEPSVIY 420
            LL+ALT+++D DPLL+ VD+ THEIILSFLG+VQ+EV+ A+L EKY +E +KEP
+VIY
Sbjct 361
LLEALTEISDGDPLLKYYVDTTTHEIILSFLGKVQMEVICAILEEKYHVEAEIKEPTVIY 420

Query 421
MERPLKAASHTIHIEVPPNPFWASIGLSVTPLSLGSGVQYESRVSLGYLNQSFQNAVRDG 480
            MERPL+ A +TIHIEVPPNPFWAS+GLS+ PL
+GSGVQYESRVSLGYLNQSFQNAV +G
Sbjct 421
MERPLRKAEYTIHIEVPPNPFWASVGLSIEPLPIGSGVQYESRVSLGYLNQSFQNAVMEG 480

Query 481
IRYGLEQGLFGWNVTDCKICFEYGLYYSPVSTPADFRSLAPIVLEQALKESGTQLLEPYL 540
            + YG EQGL+GW VTDCKICFEYGLYYSPVSTPADFR L+PIVLEQALK++GT
+LLEPYL
Sbjct 481
VLYGCEQGLYGWKVTDCKICFEYGLYYSPVSTPADFRLLSPIVLEQALKKAGTELLEPYL 540

Query 541
SFILYAPQEYLSRAYHDAPKYCATIETAQVKKDEVVFTGEIPARCIQAYRTDLAFYTNGR 600
             F +YAPQEYLSRAYHDAP+YCA I + Q+K DEV+ GEIPARCIQ YR DL +
+TNG+
Sbjct 541
HFEIYAPQEYLSRAYHDAPRYCADIVSTQIKNDEVILKGEIPARCIQEYRNDLTYFTNGQ 600

Query 601 SVCLTELKGYQAAVGQPVIQPRRPNSRLDKVRHMFQKV 638
             VCLTELKGYQ A+G+ + QPRRPNSR+DKVRHMF K+
Sbjct 601 GVCLTELKGYQPAIGKFICQPRRPNSRIDKVRHMFHKL 638

>WP_024402538.1 TetM/TetW/TetO/TetS family tetracycline resistance
ribosomal
protection protein [Streptococcus suis]
Length=639

 Score = 930 bits (2403), Expect = 0.0, Method: Compositional
matrix adjust.
 Identities = 431/638 (68%), Positives = 530/638 (83%), Gaps = 0/638
(0%)

Query 1
MKIINIGILAHVDAGKTTLTESLLYASGAISEPGSVEKGTTRTDTMFLERQRGITIQAAV 60
            MKIIN+GILAHVDAGKTTLTESLLY SGAI+EPGSV+KGTTRTDTM

LERQRGITIQ AV
Sbjct 1
MKIINLGILAHVDAGKTTLTESLLYTSGAIAEPGSVDKGTTRTDTMNLERQRGITIQTAV 60

Query 61
TSFQWHRCKVNIVDTPGHMDFLAEVYRSLAVLDGAILVISAKDGVQAQTRILFHALRKMN 120
TSFQW   KVNI+DTPGHMDFLAEVYRSL+VLDGA+L++SAKDG+QAQTRILFHAL
+ M
Sbjct 61
TSFQWEDVKVNIIDTPGHMDFLAEVYRSLSVLDGAVLLVSAKDGIQAQTRILFHALQTMK 120

Query 121
IPTVIFINKIDQAGVDLQSVVQSVRDKLSADIIIKQTVSLSPEIVLEENTDIEAWDAVIE 180
IPT+ FINKIDQ G+DL V Q ++ KLS++II+KQ V   P I + +N D+E
WDAVI
Sbjct 121
IPTIFFINKIDQEGIDLPMVYQEMKAKLSSEIIVKQKVGQHPHINVTDNDDMEQWDAVIM 180

Query 181
NNDKLLEKYIAGEPISREKLVREEQRRVQDASLFPVYYGSAKKGLGIQPLMDAVTGLFQP 240
ND+LLEKY++G+P   +L +EE RR Q+ +LFPVY+GSAK LGI+ L++ +
F
Sbjct 181
GNDELLEKYMSGKPFKMSELEQEENRRFQNGTLFPVYHGSAKNNLGIRQLIEVIASKFYS 240

Query 241
IGEQGSAALCGSVFKVEYTDCGQRRVYLRLYSGTLRLRDTVALAGREKLKITEMRIPSKG 300
+G + LCG VFK+EY++ +R VY+R+YSGTL LRD + ++ +EK+KITEM
+P+ G
Sbjct 241
STPEGQSELCGQVFKIEYSEKRRRFVYVRIYSGTLHLRDVIRISEKEKIKITEMCVPTNG 300

Query 301
EIVRTDTAYPGEIVILPSDSVRLNDVLGDPTRLPRKRWREDPLPMLRTTIAPKTAAQRER 360
E+ +DTA G+IVILP+D ++LN +LG+   LP++++ E+PLPML+TTIA K +
QRE
Sbjct 301
ELYSSDTACSGDIVILPNDVLQLNSILGNEILLPQRKFIENPLPMLQTTIAVKKSEQREI 360

Query 361
LLDALTQLADTDPLLRCEVDSITHEIILSFLGRVQLEVVSALLSEKYKLETVVKEPSVIY 420
LL ALT+++D DPLL+ VD+ THEIILSFLG VQ+EV+ A+L EKY +E +KEP
+VIY
Sbjct 361
LLGALTEISDGDPLLKYYVDTTTHEIILSFLGNVQMEVICAILEEKYHVEAEIKEPTVIY 420

Query 421
MERPLKAASHTIHIEVPPNPFWASIGLSVTPLSLGSGVQYESRVSLGYLNQSFQNAVRDG 480
MERPL+ A +TIHIEVPPNPFWAS+GLS+ PL
+GSGVQYESRVSLGYLNQSFQNAV +G
Sbjct 421
MERPLRKAEYTIHIEVPPNPFWASVGLSIEPLPIGSGVQYESRVSLGYLNQSFQNAVMEG 480

Query 481

IRYGLEQGLFGWNVTDCKICFEYGLYYSPVSTPADFRSLAPIVLEQALKESGTQLLEPYL 540
            + YG EQGL+GW VTDCKICFEYGLYYSPVSTPADFR L+PIVLEQALK++GT
+LLEPYL
Sbjct 481
VLYGCEQGLYGWKVTDCKICFEYGLYYSPVSTPADFRLLSPIVLEQALKKAGTELLEPYL 540

Query 541
SFILYAPQEYLSRAYHDAPKYCATIETAQVKKDEVVFTGEIPARCIQAYRTDLAFYTNGR 600
             F +YAPQEYLSRAYHDAP+YCA I + Q+K DEV+ GEIPARCIQ YR DL +
+TNG+
Sbjct 541
HFEIYAPQEYLSRAYHDAPRYCADIVSTQIKNDEVILKGEIPARCIQEYRNDLTYFTNGQ 600

Query 601 SVCLTELKGYQAAVGQPVIQPRRPNSRLDKVRHMFQKV 638
             VCLTELKGYQ A+G+ + QPRRPNSR+DKVRHMF K+
Sbjct 601 GVCLTELKGYQPAIGKFICQPRRPNSRIDKVRHMFHKL 638

>WP_105135974.1 TetM/TetW/TetO/TetS family tetracycline resistance ribosomal
protection protein [Streptococcus suis]
Length=639

 Score = 929 bits (2402), Expect = 0.0, Method: Compositional matrix adjust.
 Identities = 430/638 (67%), Positives = 529/638 (83%), Gaps = 0/638 (0%)

Query 1
MKIINIGILAHVDAGKTTLTESLLYASGAISEPGSVEKGTTRTDTMFLERQRGITIQAAV 60
            MKIIN+GILAHVDAGKTTLTESLLY SGAI+EPGSV+KGTTRTDTM
LERQRGITIQ AV
Sbjct 1
MKIINLGILAHVDAGKTTLTESLLYTSGAIAEPGSVDKGTTRTDTMNLERQRGITIQTAV 60

Query 61
TSFQWHRCKVNIVDTPGHMDFLAEVYRSLAVLDGAILVISAKDGVQAQTRILFHALRKMN 120
            TSFQW   KVNI+DTPGHMDFLAEVYRSL+VLDGA+L++SAKDG+QAQTRILFHAL
+ M
Sbjct 61
TSFQWEDVKVNIIDTPGHMDFLAEVYRSLSVLDGAVLLVSAKDGIQAQTRILFHALQTMK 120

Query 121
IPTVIFINKIDQAGVDLQSVVQSVRDKLSADIIIKQTVSLSPEIVLEENTDIEAWDAVIE 180
            IPT+ FINKIDQ G+DL V Q ++ KLS++II+KQ V   P I + +N D+E WD
VI
Sbjct 121
IPTIFFINKIDQEGIDLPMVYQEMKAKLSSEIIVKQKVGQHPHINVTDNDDMEQWDTVIM 180

Query 181
NNDKLLEKYIAGEPISREKLVREEQRRVQDASLFPVYYGSAKKGLGIQPLMDAVTGLFQP 240
             ND+LLEKY++G+P   +L +EE RR Q+ +LFPVY+GSAK LGI+ L++ +
F
Sbjct 181

GNDELLEKYMSGKPFKMSELEQEENRRFQNGTLFPVYHGSAKNNLGIRQLIEVIASKFYS 240

Query 241
IGEQGSAALCGSVFKVEYTDCGQRRVYLRLYSGTLRLRDTVALAGREKLKITEMRIPSKG 300
               +G + LCG VFK+EY++ +R VY+R+YSGTL LRD + ++ +EK+KITEM
+P+ G
Sbjct 241
STPEGQSELCGQVFKIEYSEKRRRFVYVRIYSGTLHLRDVIKISEKEKIKITEMCVPTNG 300

Query 301
EIVRTDTAYPGEIVILPSDSVRLNDVLGDPTRLPRKRWREDPLPMLRTTIAPKTAAQRER 360
            E+ +DTA G+IVILP+D ++LN +LG+   LP++++ E+PLPML+TTIA K +
QRE
Sbjct 301
ELYSSDTACSGDIVILPNDVLQLNSILGNEMLLPQRKFIENPLPMLQTTIAVKKSEQREI 360

Query 361
LLDALTQLADTDPLLRCEVDSITHEIILSFLGRVQLEVVSALLSEKYKLETVVKEPSVIY 420
            LL ALT+++D DPLL+ VD+ THEIILSFLG VQ+EV+ A+L EKY +E +KEP
+VIY
Sbjct 361
LLGALTEISDGDPLLKYYVDTTTHEIILSFLGNVQMEVICAILEEKYHVEAEIKEPTVIY 420

Query 421
MERPLKAASHTIHIEVPPNPFWASIGLSVTPLSLGSGVQYESRVSLGYLNQSFQNAVRDG 480
            MERPL+ A +TIHIEVPPNPFWAS+GLS+ PL
+GSGVQYESRVSLGYLNQSFQNAV +G
Sbjct 421
MERPLRKAEYTIHIEVPPNPFWASVGLSIEPLPIGSGVQYESRVSLGYLNQSFQNAVMEG 480

Query 481
IRYGLEQGLFGWNVTDCKICFEYGLYYSPVSTPADFRSLAPIVLEQALKESGTQLLEPYL 540
            + YG EQGL+GW VTDCKICFEYGLYYSPVSTPADFR L+PIVLEQALK++GT
+LLEPYL
Sbjct 481
VLYGCEQGLYGWKVTDCKICFEYGLYYSPVSTPADFRLLSPIVLEQALKKAGTELLEPYL 540

Query 541
SFILYAPQEYLSRAYHDAPKYCATIETAQVKKDEVVFTGEIPARCIQAYRTDLAFYTNGR 600
             F +YAPQEYLSRAYHDAP+YCA I + Q+K DEV+ GEIPARCIQ YR DL +
+TNG+
Sbjct 541
HFEIYAPQEYLSRAYHDAPRYCADIVSTQIKNDEVILKGEIPARCIQEYRNDLTYFTNGQ 600

Query 601 SVCLTELKGYQAAVGQPVIQPRRPNSRLDKVRHMFQKV 638
             VCLTELKGYQ A+G+ + QPRRPNSR+DKVRHMF K+
Sbjct 601 GVCLTELKGYQPAIGKFICQPRRPNSRIDKVRHMFHKL 638

>WP_099778982.1 TetM/TetW/TetO/TetS family tetracycline resistance
ribosomal
protection protein [Streptococcus suis]
Length=639

Score = 929 bits (2402), Expect = 0.0, Method: Compositional matrix adjust.
 Identities = 435/638 (68%), Positives = 529/638 (83%), Gaps = 0/638 (0%)

```
Query  1
MKIINIGILAHVDAGKTTLTESLLYASGAISEPGSVEKGTTRTDTMFLERQRGITIQAAV  60
            MKIIN+GILAHVDAGKTTLTESLLY SGAI+EPGSV+KGTTRTDTM 
LERQRGITIQ AV
Sbjct  1
MKIINLGILAHVDAGKTTLTESLLYTSGAIAEPGSVDKGTTRTDTMNLERQRGITIQTAV  60

Query  61
TSFQWHRCKVNIVDTPGHMDFLAEVYRSLAVLDGAILVISAKDGVQAQTRILFHALRKMN  120
            TSFQW   KVNI+DTPGHMDFLAEVYRSLAVLDGAILVISAKDGVQAQTRILFHAL
+ M
Sbjct  61
TSFQWEDVKVNIIDTPGHMDFLAEVYRSLAVLDGAILVISAKDGVQAQTRILFHALQTMK  120

Query  121
IPTVIFINKIDQAGVDLQSVVQSVRDKLSADIIIKQTVSLSPEIVLEENTDIEAWDAVIE  180
            IPT+ FINKIDQ G+DL  V + ++ KLS++II+KQ V   P I + +N D+E
WDAVI
Sbjct  121
IPTIFFINKIDQEGIDLPMVYREMKAKLSSEIIVKQKVGQHPHINVTDNDDMEQWDAVIM  180

Query  181
NNDKLLEKYIAGEPISREKLVREEQRRVQDASLFPVYYGSAKKGLGIQPLMDAVTGLFQP  240
             ND+LLEKY++G+P    +L +EE RR Q+ +LFPVY+GSAK  LGI+ L++ +
F
Sbjct  181
GNDELLEKYMSGKPFKMSELEQEENRRFQNGTLFPVYHGSAKNNLGIRQLIEVIASKFYS  240

Query  241
IGEQGSAALCGSVFKVEYTDCGQRRVYLRLYSGTLRLRDTVALAGREKLKITEMRIPSKG  300
               +G + LCG VFK+EY++  +R VY+R+YSGTL LRD + ++ +EK+KITEM
+P+ G
Sbjct  241
STPEGQSELCGQVFKIEYSEKRRRFVYVRIYSGTLHLRDVIRISEKEKIKITEMCVPTNG  300

Query  301
EIVRTDTAYPGEIVILPSDSVRLNDVLGDPTRLPRKRWREDPLPMLRTTIAPKTAAQRER  360
            E+  +DTA  G+IVILP+D ++LN +LG+   LP++++ E+PLPML+TTIA K
QRE
Sbjct  301
ELYPSDTACSGDIVILPNDVLQLNSILGNEMLLPQRKFIENPLPMLQTTIAVKKPEQREI  360

Query  361
LLDALTQLADTDPLLRCEVDSITHEIILSFLGRVQLEVVSALLSEKYKLETVVKEPSVIY  420
            LL ALT+++D DPLL+  VD+ THEIILSFLG VQ+EV+ A+L EKY +E  +KEP
+VIY
Sbjct  361
LLGALTEISDGDPLLKYYVDTTTHEIILSFLGNVQMEVICAILEEKYHVEAEIKEPTVIY  420
```

```
Query  421  
MERPLKAASHTIHIEVPPNPFWASIGLSVTPLSLGSGVQYESRVSLGYLNQSFQNAVRDG  480
            MERPL+ A +TIHIEVPPNPFWAS+GLS+ PL 
+GSGVQYESRVSLGYLNQSFQNAV +G
Sbjct  421  
MERPLRKAEYTIHIEVPPNPFWASVGLSIEPLPIGSGVQYESRVSLGYLNQSFQNAVMEG  480

Query  481  
IRYGLEQGLFGWNVTDCKICFEYGLYYSPVSTPADFRSLAPIVLEQALKESGTQLLEPYL  540
            + YG EQGL+GW VTDCKICFEYGLYYSPVSTPADFR L+PIVLEQALK++GT 
+LLEPYL
Sbjct  481  
VLYGCEQGLYGWKVTDCKICFEYGLYYSPVSTPADFRLLSPIVLEQALKKAGTELLEPYL  540

Query  541  
SFILYAPQEYLSRAYHDAPKYCATIETAQVKKDEVVFTGEIPARCIQAYRTDLAFYTNGR  600
             F +YAPQEYLSRAYHDAP+YCA I + Q+K DEV+  GEIPARCIQ YR DL + 
+TNG+
Sbjct  541  
HFEIYAPQEYLSRAYHDAPRYCADIVSTQIKNDEVILKGEIPARCIQEYRNDLTYFTNGQ  600

Query  601  SVCLTELKGYQAAVGQPVIQPRRPNSRLDKVRHMFQKV  638
             VCLTELKGYQ A+G+ + QPRRPNSR+DKVRHMF K+
Sbjct  601  GVCLTELKGYQPAIGKFICQPRRPNSRIDKVRHMFHKL  638


>WP_074413645.1 TetM/TetW/TetO/TetS family tetracycline resistance 
ribosomal 
protection protein [Streptococcus suis]
Length=639

 Score = 929 bits (2402),  Expect = 0.0, Method: Compositional 
matrix adjust.
 Identities = 431/638 (68%), Positives = 529/638 (83%), Gaps = 0/638 
(0%)

Query  1    
MKIINIGILAHVDAGKTTLTESLLYASGAISEPGSVEKGTTRTDTMFLERQRGITIQAAV  60
            MKIIN+GILAHVDAGKTTLTESLLY SGAI+EPGSV+KGTTR DTM 
LERQRGITIQ AV
Sbjct  1    
MKIINLGILAHVDAGKTTLTESLLYTSGAIAEPGSVDKGTTRIDTMNLERQRGITIQTAV  60

Query  61   
TSFQWHRCKVNIVDTPGHMDFLAEVYRSLAVLDGAILVISAKDGVQAQTRILFHALRKMN  120
            TSFQW   KVNI+DTPGHMDFLAEVYRSL+VLDGA+L++SAKDG+QAQTRILFHAL 
+ M
Sbjct  61   
TSFQWEDVKVNIIDTPGHMDFLAEVYRSLSVLDGAVLLVSAKDGIQAQTRILFHALQTMK  120

Query  121  
IPTVIFINKIDQAGVDLQSVVQSVRDKLSADIIIKQTVSLSPEIVLEENTDIEAWDAVIE  180
            IPT+ FINKIDQ G+DL  V Q ++ KLS++II+KQ V   P I + +N D+E 
WDAVI
```

Sbjct 121
IPTIFFINKIDQEGIDLPMVYQEMKAKLSSEIIVKQKVGQHPHINVTDNDDMEQWDAVIM 180

Query 181
NNDKLLEKYIAGEPISREKLVREEQRRVQDASLFPVYYGSAKKGLGIQPLMDAVTGLFQP 240
ND+LLEKY++G+P +L +EE RR Q+ +LFPVY+GSAK LGI+ L++ +
F
Sbjct 181
GNDELLEKYMSGKPFKMSELEQEENRRFQNGTLFPVYHGSAKNNLGIRQLIEVIASKFYS 240

Query 241
IGEQGSAALCGSVFKVEYTDCGQRRVYLRLYSGTLRLRDTVALAGREKLKITEMRIPSKG 300
+G + LCG VFK+EY++ +R VY+R+YSGTL LRD + ++ +EK+KITEM
+P+ G
Sbjct 241
STPEGQSELCGQVFKIEYSEKRRRFVYVRIYSGTLHLRDVIKISEKEKIKITEMCVPTNG 300

Query 301
EIVRTDTAYPGEIVILPSDSVRLNDVLGDPTRLPRKRWREDPLPMLRTTIAPKTAAQRER 360
E+ +DTA G+IVILP+D ++LN +LG+ LP++++ E+PLPML+TTIA K +
QRE
Sbjct 301
ELYSSDTACSGDIVILPNDVLQLNSILGNEMLLPQRKFIENPLPMLQTTIAVKKSEQREI 360

Query 361
LLDALTQLADTDPLLRCEVDSITHEIILSFLGRVQLEVVSALLSEKYKLETVVKEPSVIY 420
LL ALT+++D DPLL+ VD+ THEIILSFLG VQ+EV+ A+L EKY +E +KEP
+VIY
Sbjct 361
LLGALTEISDGDPLLKYYVDTTTHEIILSFLGNVQMEVICAILEEKYHVEAEIKEPTVIY 420

Query 421
MERPLKAASHTIHIEVPPNPFWASIGLSVTPLSLGSGVQYESRVSLGYLNQSFQNAVRDG 480
MERPL+ A +TIHIEVPPNPFWAS+GLS+ PL
+GSGVQYESRVSLGYLNQSFQNAV +G
Sbjct 421
MERPLRKAEYTIHIEVPPNPFWASVGLSIEPLPIGSGVQYESRVSLGYLNQSFQNAVMEG 480

Query 481
IRYGLEQGLFGWNVTDCKICFEYGLYYSPVSTPADFRSLAPIVLEQALKESGTQLLEPYL 540
+ YG EQGL+GW VTDCKICFEYGLYYSPVSTPADFR L+PIVLEQALK++GT
+LLEPYL
Sbjct 481
VLYGCEQGLYGWKVTDCKICFEYGLYYSPVSTPADFRLLSPIVLEQALKKAGTELLEPYL 540

Query 541
SFILYAPQEYLSRAYHDAPKYCATIETAQVKKDEVVFTGEIPARCIQAYRTDLAFYTNGR 600
F +YAPQEYLSRAYHDAP+YCA I + QVK DEV+ GEIPARCIQ YR DL +
+TNG+
Sbjct 541
HFEIYAPQEYLSRAYHDAPRYCADIVSTQVKNDEVILKGEIPARCIQEYRNDLTYFTNGQ 600

Query 601 SVCLTELKGYQAAVGQPVIQPRRPNSRLDKVRHMFQKV 638
VCLTELKGYQ A+G+ + QPRRPNSR+DKVRHMF K+

```
Sbjct  601  GVCLTELKGYQPAIGKFICQPRRPNSRIDKVRHMFHKL  638


>AMP50505.1 TetM-TetW-TetO-TetS [uncultured bacterium]
Length=639

 Score = 929 bits (2402),  Expect = 0.0, Method: Compositional 
matrix adjust.
 Identities = 430/638 (67%), Positives = 531/638 (83%), Gaps = 0/638 
(0%)

Query  1    
MKIINIGILAHVDAGKTTLTESLLYASGAISEPGSVEKGTTRTDTMFLERQRGITIQAAV  60
            MKIIN+GILAHVDAGKTTLTESLLY SGAI+EPGSV++GTTRTDTM 
LERQRGITIQ AV
Sbjct  1    
MKIINLGILAHVDAGKTTLTESLLYTSGAIAEPGSVDEGTTRTDTMNLERQRGITIQTAV  60

Query  61   
TSFQWHRCKVNIVDTPGHMDFLAEVYRSLAVLDGAILVISAKDGVQAQTRILFHALRKMN  120
            TSFQW   KVNI+DTPGHMDFLAEVYRSL+VLDGA+L++SAKDG+QAQTRILFHAL 
+ M
Sbjct  61   
TSFQWEDVKVNIIDTPGHMDFLAEVYRSLSVLDGAVLLVSAKDGIQAQTRILFHALQTMK  120

Query  121  
IPTVIFINKIDQAGVDLQSVVQSVRDKLSADIIIKQTVSLSPEIVLEENTDIEAWDAVIE  180
            IPT+ FINKIDQ G+DL  V + ++ KLS++II+KQ V   P I + +N D+E 
WDAVI
Sbjct  121  
IPTIFFINKIDQEGIDLPMVYREMKAKLSSEIIVKQKVGQHPHINVTDNDDMEQWDAVIM  180

Query  181  
NNDKLLEKYIAGEPISREKLVREEQRRVQDASLFPVYYGSAKKGLGIQPLMDAVTGLFQP  240
             ND+LLEKY++G+P    +L +EE RR Q+ +LFPVY+GSAK  LGI+ L++ + 
F
Sbjct  181  
GNDELLEKYMSGKPFKMSELEQEENRRFQNGTLFPVYHGSAKNNLGIRQLIEVIASKFYS  240

Query  241  
IGEQGSAALCGSVFKVEYTDCGQRRVYLRLYSGTLRLRDTVALAGREKLKITEMRIPSKG  300
               +G + LCG VFK+EY++  +R VY+R+YSGTL LRD + ++ +EK+KITEMR 
+P+ G
Sbjct  241  
STPEGQSELCGQVFKIEYSEKRRRFVYVRIYSGTLHLRDVIRISEKEKIKITEMRVPTNG  300

Query  301  
EIVRTDTAYPGEIVILPSDSVRLNDVLGDPTRLPRKRWREDPLPMLRTTIAPKTAAQRER  360
            E+  +DTA  G+IVILP+D ++LN +LG+   LP++++ E+PLPML+TTIA K + 
QRE
Sbjct  301  
ELYSSDTACSGDIVILPNDVLQLNSILGNGMLLPQRKFIENPLPMLQTTIAVKKSEQREI  360

Query  361
```

LLDALTQLADTDPLLRCEVDSITHEIILSFLGRVQLEVVSALLSEKYKLETVVKEPSVIY 420
            LL ALT+++D DPLL+ VD+ THEIILSFLG VQ+EV+ A+L EKY +E +KEP
+VIY
Sbjct 361
LLGALTEISDGDPLLKYYVDTTTHEIILSFLGNVQMEVICAILEEKYHVEAEIKEPTVIY 420

Query 421
MERPLKAASHTIHIEVPPNPFWASIGLSVTPLSLGSGVQYESRVSLGYLNQSFQNAVRDG 480
            MERPL+ A +TIHIEVPPNPFWAS+GLS+ PL
+GSGVQYESRVSLGYLNQSFQNAV +G
Sbjct 421
MERPLRKAEYTIHIEVPPNPFWASVGLSIEPLPIGSGVQYESRVSLGYLNQSFQNAVMEG 480

Query 481
IRYGLEQGLFGWNVTDCKICFEYGLYYSPVSTPADFRSLAPIVLEQALKESGTQLLEPYL 540
            + YG EQGL+GW VTDCKICFEYGLYYSPVSTPADFR L+PIVLEQALK++GT
+LLEPYL
Sbjct 481
VLYGCEQGLYGWKVTDCKICFEYGLYYSPVSTPADFRLLSPIVLEQALKKAGTELLEPYL 540

Query 541
SFILYAPQEYLSRAYHDAPKYCATIETAQVKKDEVVFTGEIPARCIQAYRTDLAFYTNGR 600
             F +YAPQEYLSRAYHDAP+YCA I + Q+K DEV+ GEIPARCIQ YR DL +
+TNG+
Sbjct 541
HFEIYAPQEYLSRAYHDAPRYCADIVSTQIKNDEVILKGEIPARCIQEYRNDLTYFTNGQ 600

Query 601 SVCLTELKGYQAAVGQPVIQPRRPNSRLDKVRHMFQKV 638
             VCLTELKGYQ A+G+ + QPRRPNSR+DKVRHMF K+
Sbjct 601 GVCLTELKGYQPAIGKFICQPRRPNSRIDKVRHMFHKL 638

>WP_006427719.1 MULTISPECIES: TetM/TetW/TetO/TetS family
tetracycline resistance
ribosomal protection protein [Lachnospiraceae]
 EDM61985.1 putative translation elongation factor G [Dorea
longicatena DSM
13814]
 CUQ70530.1 Tetracycline resistance protein tetM from transposon
Tn916 [[Ruminococcus]
torques]
 CUO43541.1 Tetracycline resistance protein tetM from transposon
Tn916 [Blautia
obeum]
 AMP51629.1 TetM-TetW-TetO-TetS [uncultured bacterium]
 AMP51651.1 TetM-TetW-TetO-TetS [uncultured bacterium]
 AMP55108.1 TetM-TetW-TetO-TetS [uncultured bacterium]
 SCI14462.1 Tetracycline resistance protein tetM from transposon
Tn916 [uncultured
Ruminococcus sp.]
 SCG91009.1 Tetracycline resistance protein tetM from transposon
Tn916 [uncultured
Blautia sp.]
Length=639

Score = 929 bits (2402), Expect = 0.0, Method: Compositional matrix adjust.
 Identities = 428/638 (67%), Positives = 528/638 (83%), Gaps = 0/638 (0%)

```
Query  1
MKIINIGILAHVDAGKTTLTESLLYASGAISEPGSVEKGTTRTDTMFLERQRGITIQAAV  60
            MKIIN+GILAHVDAGKTTLTESLLY SGAI+E GSV++GTTRTDTM 
LERQRGITIQ AV
Sbjct  1
MKIINLGILAHVDAGKTTLTESLLYTSGAIAELGSVDEGTTRTDTMNLERQRGITIQTAV  60

Query  61
TSFQWHRCKVNIVDTPGHMDFLAEVYRSLAVLDGAILVISAKDGVQAQTRILFHALRKMN  120
            TSFQW   KVNI+DTPGHMDFLAEVYRSL+VLDGA+L++SAKDG+QAQTRILFHAL 
+ M
Sbjct  61
TSFQWEDVKVNIIDTPGHMDFLAEVYRSLSVLDGAVLLVSAKDGIQAQTRILFHALQIMK  120

Query  121
IPTVIFINKIDQAGVDLQSVVQSVRDKLSADIIIKQTVSLSPEIVLEENTDIEAWDAVIE  180
            IPT+ FINKIDQ G+DL  V + ++ KLS++II+KQ V   P I + +N D+E 
WDAVI
Sbjct  121
IPTIFFINKIDQEGIDLPMVYREMKAKLSSEIIVKQKVGQHPHINVTDNDDMEQWDAVIM  180

Query  181
NNDKLLEKYIAGEPISREKLVREEQRRVQDASLFPVYYGSAKKGLGIQPLMDAVTGLFQP  240
             ND+LLEKY++G+P    +L +EE RR Q+ +LFPVY+GSAK  LGI+ L++ + 
F
Sbjct  181
GNDELLEKYMSGKPFKMSELEQEENRRFQNGTLFPVYHGSAKNNLGIRQLIEVIASKFYS  240

Query  241
IGEQGSAALCGSVFKVEYTDCGQRRVYLRLYSGTLRLRDTVALAGREKLKITEMRIPSKG  300
               +G + LCG VFK+EY++  +R VY+R+YSGTL LRD + ++ +EK+KITEM 
+P+ G
Sbjct  241
STPEGQSELCGQVFKIEYSEKRRRFVYVRIYSGTLHLRDVIRISEKEKIKITEMCVPTNG  300

Query  301
EIVRTDTAYPGEIVILPSDSVRLNDVLGDPTRLPRKRWREDPLPMLRTTIAPKTAAQRER  360
            EIV  D A PGEIVIL  D+++LND+LG+   LP K W ++P+P+LRTT+ P+
QRE
Sbjct  301
EIVPADHACPGEIVILADDTLKLNDILGNEKLLPHKTWIDNPMPLLRTTVEPQKPEQREA  360

Query  361
LLDALTQLADTDPLLRCEVDSITHEIILSFLGRVQLEVVSALLSEKYKLETVVKEPSVIY  420
            LL+AL ++ADTDPLL  ++D++THEI+LSFLG VQ+EV+ A+L EKY +E  +KEP
+VIY
Sbjct  361
LLNALAEIADTDPLLHFDIDTVTHEIMLSFLGNVQMEVICAILEEKYHVEAEIKEPTVIY  420
```

```
Query  421
MERPLKAASHTIHIEVPPNPFWASIGLSVTPLSLGSGVQYESRVSLGYLNQSFQNAVRDG  480
            MERPL+ A +TIHIEVPPNPFWAS+GLS+ PL
+GSGVQYESRVSLGYLNQSFQNAV +G
Sbjct  421
MERPLRKAEYTIHIEVPPNPFWASVGLSIEPLPIGSGVQYESRVSLGYLNQSFQNAVMEG  480

Query  481
IRYGLEQGLFGWNVTDCKICFEYGLYYSPVSTPADFRSLAPIVLEQALKESGTQLLEPYL  540
            + YG EQGL+GW VTDCKICFEYGLYYSPVSTPADFR L+PIVLEQALK++GT
+LLEPYL
Sbjct  481
VLYGCEQGLYGWKVTDCKICFEYGLYYSPVSTPADFRLLSPIVLEQALKKAGTELLEPYL  540

Query  541
SFILYAPQEYLSRAYHDAPKYCATIETAQVKKDEVVFTGEIPARCIQAYRTDLAFYTNGR  600
             F +YAPQEYLSRAYHDAP+YCA I + Q+K DEV+  GEIPARCIQ YR DL +
+TNG+
Sbjct  541
HFEIYAPQEYLSRAYHDAPRYCADIVSTQIKNDEVILKGEIPARCIQEYRNDLTYFTNGQ  600

Query  601  SVCLTELKGYQAAVGQPVIQPRRPNSRLDKVRHMFQKV  638
             VCLTELKGYQ A+G+ + QPRRPNSR+DKVRHMF K+
Sbjct  601  GVCLTELKGYQPAIGKFICQPRRPNSRIDKVRHMFHKL  638


>WP_057485305.1 tetracycline resistance protein tetM [Streptococcus
agalactiae]
Length=639

 Score = 929 bits (2402),  Expect = 0.0, Method: Compositional
matrix adjust.
 Identities = 431/638 (68%), Positives = 530/638 (83%), Gaps = 0/638
(0%)

Query  1
MKIINIGILAHVDAGKTTLTESLLYASGAISEPGSVEKGTTRTDTMFLERQRGITIQAAV  60
            MKIIN+GILAHVDAGKTTLTESLLY SGAI+EPGSV+KGTTRTDTM
LERQRGITIQ AV
Sbjct  1
MKIINLGILAHVDAGKTTLTESLLYTSGAIAEPGSVDKGTTRTDTMNLERQRGITIQTAV  60

Query  61
TSFQWHRCKVNIVDTPGHMDFLAEVYRSLAVLDGAILVISAKDGVQAQTRILFHALRKMN  120
            TSFQW   KVNI+DTPGHMDFLAEVYRSL+VLDGA+L++SAKDG+QAQTRILFHAL
+ M
Sbjct  61
TSFQWEDVKVNIIDTPGHMDFLAEVYRSLSVLDGAVLLVSAKDGIQAQTRILFHALQTMK  120

Query  121
IPTVIFINKIDQAGVDLQSVVQSVRDKLSADIIIKQTVSLSPEIVLEENTDIEAWDAVIE  180
            IPT+ FINKIDQ G+DL  V + ++ KLS++II+KQ V   P I + +N D+E
WDAVI
```

Sbjct 121
IPTIFFINKIDQEGIDLPMVYREMKAKLSSEIIVKQKVGQHPHINVTDNDDMEQWDAVIM 180

Query 181
NNDKLLEKYIAGEPISREKLVREEQRRVQDASLFPVYYGSAKKGLGIQPLMDAVTGLFQP 240
ND+LLEKY++G+P   +L +EE RR Q+ +LFPVY+GSAK LGI+ L++ +
F
Sbjct 181
GNDELLEKYMSGKPFKMSELEQEENRRFQNGTLFPVYHGSAKNNLGIRQLIEVIASKFYS 240

Query 241
IGEQGSAALCGSVFKVEYTDCGQRRVYLRLYSGTLRLRDTVALAGREKLKITEMRIPSKG 300
+G + LCG VFK+EY++ +R VY+R+YSGTL LRD + ++ +EK+KITEM
+P+ G
Sbjct 241
STPEGQSELCGQVFKIEYSEKRRRFVYVRIYSGTLHLRDVIKISEKEKIKITEMCVPTNG 300

Query 301
EIVRTDTAYPGEIVILPSDSVRLNDVLGDPTRLPRKRWREDPLPMLRTTIAPKTAAQRER 360
E+ +DTA G+IVILP+D ++LN +LG+   LP++++ E+PLPML+TTIA K +
QRE
Sbjct 301
ELYSSDTACSGDIVILPNDVLQLNSILGNEMLLPQRKFIENPLPMLQTTIAVKKSEQREI 360

Query 361
LLDALTQLADTDPLLRCEVDSITHEIILSFLGRVQLEVVSALLSEKYKLETVVKEPSVIY 420
LL ALT+++D DPLL+ VD+ THEIILSFLG VQ+EV+ A+L EKY +E +KEP
+VIY
Sbjct 361
LLGALTEISDGDPLLKYYVDTTTHEIILSFLGNVQMEVICAILEEKYHVEAEIKEPTVIY 420

Query 421
MERPLKAASHTIHIEVPPNPFWASIGLSVTPLSLGSGVQYESRVSLGYLNQSFQNAVRDG 480
MERPL+ A +TIHIEVPPNPFWAS+GLS+ PL
+GSGVQYESRVSLGYLNQSFQNAV +G
Sbjct 421
MERPLRKAEYTIHIEVPPNPFWASVGLSIEPLPIGSGVQYESRVSLGYLNQSFQNAVMEG 480

Query 481
IRYGLEQGLFGWNVTDCKICFEYGLYYSPVSTPADFRSLAPIVLEQALKESGTQLLEPYL 540
+ YG EQGL+GW VTDCKICFEYGLYYSPVSTPADFR L+PIVLEQALK++GT
+LLEPYL
Sbjct 481
VLYGCEQGLYGWKVTDCKICFEYGLYYSPVSTPADFRLLSPIVLEQALKKAGTELLEPYL 540

Query 541
SFILYAPQEYLSRAYHDAPKYCATIETAQVKKDEVVFTGEIPARCIQAYRTDLAFYTNGR 600
F +YAPQEYLSRAYHDAP+YCA I + QVK DEV+ GEIPARCIQ YR DL +
+TNG+
Sbjct 541
HFEIYAPQEYLSRAYHDAPRYCADIVSTQVKNDEVILKGEIPARCIQEYRNDLTYFTNGQ 600

Query 601 SVCLTELKGYQAAVGQPVIQPRRPNSRLDKVRHMFQKV 638
VCLTELKGYQ A+G+ + QPRRPNSR+DKVRHMF K+

Sbjct 601 GVCLTELKGYQPAIGKFICQPRRPNSRIDKVRHMFHKL 638

>WP_055160143.1 TetM/TetW/TetO/TetS family tetracycline resistance ribosomal
protection protein [Anaerostipes hadrus]
 CUP55278.1 Tetracycline resistance protein tetM from transposon Tn916 [Anaerostipes
hadrus]
Length=639

 Score = 929 bits (2402), Expect = 0.0, Method: Compositional matrix adjust.
 Identities = 431/638 (68%), Positives = 529/638 (83%), Gaps = 0/638 (0%)

Query 1
MKIINIGILAHVDAGKTTLTESLLYASGAISEPGSVEKGTTRTDTMFLERQRGITIQAAV 60
            MKIIN+GILAHVDAGKTTLTESLLY SGAI+EPGSV+KGTTRTDTM
LERQRGITIQ AV
Sbjct 1
MKIINLGILAHVDAGKTTLTESLLYTSGAIAEPGSVDKGTTRTDTMNLERQRGITIQTAV 60

Query 61
TSFQWHRCKVNIVDTPGHMDFLAEVYRSLAVLDGAILVISAKDGVQAQTRILFHALRKMN 120
            TSFQW   KVNI+DTPGHMDFLAEVYRSL+VLDGA+L++SAKDG+QAQTRILFHAL
+ M
Sbjct 61
TSFQWEDVKVNIIDTPGHMDFLAEVYRSLSVLDGAVLLVSAKDGIQAQTRILFHALQTMK 120

Query 121
IPTVIFINKIDQAGVDLQSVVQSVRDKLSADIIIKQTVSLSPEIVLEENTDIEAWDAVIE 180
            IPT+ FINKIDQ G+DL V Q ++ KLS++II+KQ V   P I + +N D+E
WDAVI
Sbjct 121
IPTIFFINKIDQEGIDLPMVYQEMKAKLSSEIIVKQKVGQHPHINVTDNDDMEQWDAVIM 180

Query 181
NNDKLLEKYIAGEPISREKLVREEQRRVQDASLFPVYYGSAKKGLGIQPLMDAVTGLFQP 240
             ND+LLEKY++G+P   +L +EE RR Q+ +LFPVY+GSAK LGI+ L++ +
F
Sbjct 181
GNDELLEKYMSGKPFKMSELEQEENRRFQNGTLFPVYHGSAKNNLGIRQLIEVIASKFYS 240

Query 241
IGEQGSAALCGSVFKVEYTDCGQRRVYLRLYSGTLRLRDTVALAGREKLKITEMRIPSKG 300
               +G + LCG VFK+EY++ +R VY+R+YSGTL LRD + ++ +EK+KITEM
+P+ G
Sbjct 241
STPEGQSELCGQVFKIEYSEKRRRFVYVRIYSGTLHLRDVIKISEKEKIKITEMCVPTNG 300

Query 301
EIVRTDTAYPGEIVILPSDSVRLNDVLGDPTRLPRKRWREDPLPMLRTTIAPKTAAQRER 360
            E+ +DTA G+IVILP+D ++LN +LG+   LP++ + E+PLPML+TTIA K +

QRE
Sbjct 301
ELCSSDTACSGDIVILPNDVLQLNSILGNEMLLPQRTFIENPLPMLQTTIAAKKSEQREI 360

Query 361
LLDALTQLADTDPLLRCEVDSITHEIILSFLGRVQLEVVSALLSEKYKLETVVKEPSVIY 420
            LL ALT+++D DPLL+ VD+ THEIILSFLG+VQ+EV+ A+L EKY +E +KEP
+VIY
Sbjct 361
LLGALTEISDGDPLLKYYVDTTTHEIILSFLGKVQMEVICAILEEKYHVEVEIKEPTVIY 420

Query 421
MERPLKAASHTIHIEVPPNPFWASIGLSVTPLSLGSGVQYESRVSLGYLNQSFQNAVRDG 480
            MERPL+ A +TIHIEVPPNPFWAS+GLS+ PL
+GSGVQYESRVSLGYLNQSFQNAV +G
Sbjct 421
MERPLRKAEYTIHIEVPPNPFWASVGLSIEPLPIGSGVQYESRVSLGYLNQSFQNAVMEG 480

Query 481
IRYGLEQGLFGWNVTDCKICFEYGLYYSPVSTPADFRSLAPIVLEQALKESGTQLLEPYL 540
            + YG EQGL+GW VTDCKICFEYGLYYSPVSTPADFR L+PIVLEQALK++GT
+LLEPYL
Sbjct 481
VLYGCEQGLYGWKVTDCKICFEYGLYYSPVSTPADFRLLSPIVLEQALKKAGTELLEPYL 540

Query 541
SFILYAPQEYLSRAYHDAPKYCATIETAQVKKDEVVFTGEIPARCIQAYRTDLAFYTNGR 600
             F +YAPQEYLSRAYHDAP+YCA I + Q+K DEV+ GEIPARCIQ YR DL +
+TNG+
Sbjct 541
HFEIYAPQEYLSRAYHDAPRYCADIVSTQIKNDEVILKGEIPARCIQEYRNDLTYFTNGQ 600

Query 601 SVCLTELKGYQAAVGQPVIQPRRPNSRLDKVRHMFQKV 638
             VCLTELKGYQ A G+ + QPRRPNSR+DKVRHMF K+
Sbjct 601 GVCLTELKGYQPATGKLICQPRRPNSRIDKVRHMFHKL 638

>WP_029943221.1 TetM/TetW/TetO/TetS family tetracycline resistance
ribosomal
protection protein [Streptococcus suis]
Length=639

 Score = 929 bits (2402), Expect = 0.0, Method: Compositional
matrix adjust.
 Identities = 431/638 (68%), Positives = 529/638 (83%), Gaps = 0/638
(0%)

Query 1
MKIINIGILAHVDAGKTTLTESLLYASGAISEPGSVEKGTTRTDTMFLERQRGITIQAAV 60
            MKIIN+GILAHVDAGKTTLTESLLY SGAI+EPGSV+KGTTRTDTM
LERQRGITIQ AV
Sbjct 1
MKIINLGILAHVDAGKTTLTESLLYTSGAIAEPGSVDKGTTRTDTMNLERQRGITIQTAV 60

Query 61
TSFQWHRCKVNIVDTPGHMDFLAEVYRSLAVLDGAILVISAKDGVQAQTRILFHALRKMN 120
            TSFQW   KVNI+DTPGHMDFLAEVYRSL+VLDGA+L++SAKDG+QAQTRILFHAL
+ M
Sbjct 61
TSFQWEDVKVNIIDTPGHMDFLAEVYRSLSVLDGAVLLVSAKDGIQAQTRILFHALQTMK 120

Query 121
IPTVIFINKIDQAGVDLQSVVQSVRDKLSADIIIKQTVSLSPEIVLEENTDIEAWDAVIE 180
            IPT+ FINKIDQ G+DL V Q ++ KLS++II+KQ V   P I + +N D+E
WDAVI
Sbjct 121
IPTIFFINKIDQEGIDLPMVYQEMKAKLSSEIIVKQKVGQHPHINVTDNDDMEQWDAVIM 180

Query 181
NNDKLLEKYIAGEPISREKLVREEQRRVQDASLFPVYYGSAKKGLGIQPLMDAVTGLFQP 240
             ND+LLEKY++G+P   +L +EE RR Q+ +LFPVY+GSAK LGI+ L++ +
F
Sbjct 181
GNDELLEKYMSGKPFKMSELEQEENRRFQNGTLFPVYHGSAKNNLGIRQLIEVIASKFYS 240

Query 241
IGEQGSAALCGSVFKVEYTDCGQRRVYLRLYSGTLRLRDTVALAGREKLKITEMRIPSKG 300
               +G + LCG VFK+EY++ +R VY+R+YSGTL LRD + ++ +EK+KITEM
+P+ G
Sbjct 241
STPEGQSELCGQVFKIEYSEKRRRFVYVRIYSGTLHLRDVIRISEKEKIKITEMCVPTNG 300

Query 301
EIVRTDTAYPGEIVILPSDSVRLNDVLGDPTRLPRKRWREDPLPMLRTTIAPKTAAQRER 360
            E+ +DTA G+IVILP+D ++LN +LG+   LP++ + E+PLPML+TTIA K +
QRE
Sbjct 301
ELYSSDTACSGDIVILPNDVLQLNSILGNEMLLPQRNFIENPLPMLQTTIAVKKSEQREI 360

Query 361
LLDALTQLADTDPLLRCEVDSITHEIILSFLGRVQLEVVSALLSEKYKLETVVKEPSVIY 420
            LL ALT+++D DPLL+ VD+ THEIILSFLG VQ+EV+ A+L EKY +E +KEP
+VIY
Sbjct 361
LLGALTEISDGDPLLKYYVDTTTHEIILSFLGNVQMEVICAILEEKYHVEAEIKEPTVIY 420

Query 421
MERPLKAASHTIHIEVPPNPFWASIGLSVTPLSLGSGVQYESRVSLGYLNQSFQNAVRDG 480
            MERPL+ A +TIHIEVPPNPFWAS+GLS+ PL
+GSGVQYESRVSLGYLNQSFQNAV +G
Sbjct 421
MERPLRKAEYTIHIEVPPNPFWASVGLSIEPLPIGSGVQYESRVSLGYLNQSFQNAVMEG 480

Query 481
IRYGLEQGLFGWNVTDCKICFEYGLYYSPVSTPADFRSLAPIVLEQALKESGTQLLEPYL 540
            + YG EQGL+GW VTDCKICFEYGLYYSPVSTPADFR L+PIVLEQALK++GT
+LLEPYL
Sbjct 481

VLYGCEQGLYGWKVTDCKICFEYGLYYSPVSTPADFRLLSPIVLEQALKKAGTELLEPYL 540

Query 541
SFILYAPQEYLSRAYHDAPKYCATIETAQVKKDEVVFTGEIPARCIQAYRTDLAFYTNGR 600
             F +YAPQEYLSRAYHDAP+YCA I + Q+K DEV+ GEIPARCIQ YR DL +
+TNG+
Sbjct 541
HFEIYAPQEYLSRAYHDAPRYCADIVSTQIKNDEVILKGEIPARCIQEYRNDLTYFTNGQ 600

Query 601 SVCLTELKGYQAAVGQPVIQPRRPNSRLDKVRHMFQKV 638
             VCLTELKGYQ A+G+ + QPRRPNSR+DKVRHMF K+
Sbjct 601 GVCLTELKGYQPAIGKFICQPRRPNSRIDKVRHMFHKL 638

>WP_014638231.1 TetM/TetW/TetO/TetS family tetracycline resistance ribosomal
protection protein [Streptococcus suis]
 AER19607.1 tetracycline resistance protein TetO [Streptococcus suis D12]
 CYV58350.1 tetracycline resistance protein tetM [Streptococcus suis]
 CYV61753.1 tetracycline resistance protein tetM [Streptococcus suis]
Length=639

 Score = 929 bits (2402), Expect = 0.0, Method: Compositional matrix adjust.
 Identities = 431/638 (68%), Positives = 529/638 (83%), Gaps = 0/638 (0%)

Query 1
MKIINIGILAHVDAGKTTLTESLLYASGAISEPGSVEKGTTRTDTMFLERQRGITIQAAV 60
            MKIIN+GILAHVDAGKTTLTESLLY SGAI+EPGSV+KGTTRTDTM
LERQRGITIQ AV
Sbjct 1
MKIINLGILAHVDAGKTTLTESLLYTSGAIAEPGSVDKGTTRTDTMNLERQRGITIQTAV 60

Query 61
TSFQWHRCKVNIVDTPGHMDFLAEVYRSLAVLDGAILVISAKDGVQAQTRILFHALRKMN 120
            TSFQW   KVNI+DTPGHMDFLAEVYRSL+VLDGA+L++SAKDG+QAQTRILFHAL
+ M
Sbjct 61
TSFQWEDVKVNIIDTPGHMDFLAEVYRSLSVLDGAVLLVSAKDGIQAQTRILFHALQTMK 120

Query 121
IPTVIFINKIDQAGVDLQSVVQSVRDKLSADIIIKQTVSLSPEIVLEENTDIEAWDAVIE 180
            IPT+ FINKIDQ G+DL V Q ++ KLS++II+KQ V   P I + +N D+E
WDAVI
Sbjct 121
IPTIFFINKIDQEGIDLPMVYQEMKAKLSSEIIVKQKVGQHPHINVTDNDDMEQWDAVIM 180

Query 181
NNDKLLEKYIAGEPISREKLVREEQRRVQDASLFPVYYGSAKKGLGIQPLMDAVTGLFQP 240
             ND+LLEKY++G+P   +L +EE RR Q+ +LFPVY+GSAK LGI+ L++ +

F
Sbjct 181
GNDELLEKYMSGKPFKMSELEQEENRRFQNGTLFPVYHGSAKNNLGIRQLIEVIASKFYS 240

Query 241
IGEQGSAALCGSVFKVEYTDCGQRRVYLRLYSGTLRLRDTVALAGREKLKITEMRIPSKG 300
               +G + LCG VFK+EY++ +R VY+R+YSGTL LRD + ++ +EK+KITEM
+P+ G
Sbjct 241
STPEGQSELCGQVFKIEYSEKRRRFVYVRIYSGTLHLRDVIKISEKEKIKITEMCVPTNG 300

Query 301
EIVRTDTAYPGEIVILPSDSVRLNDVLGDPTRLPRKRWREDPLPMLRTTIAPKTAAQRER 360
            E+ +DTA G+IVILP+D ++LN +LG+   LP++++ E+PLPML+TTIA K
QRE
Sbjct 301
ELYSSDTACSGDIVILPNDVLQLNSILGNEMLLPQRKFIENPLPMLQTTIAVKKPEQREI 360

Query 361
LLDALTQLADTDPLLRCEVDSITHEIILSFLGRVQLEVVSALLSEKYKLETVVKEPSVIY 420
            LL ALT+++D DPLL+ VD+ THEIILSFLG VQ+EV+ A+L EKY +E +KEP
+VIY
Sbjct 361
LLGALTEISDGDPLLKYYVDTTTHEIILSFLGNVQMEVICAILEEKYHVEAEIKEPTVIY 420

Query 421
MERPLKAASHTIHIEVPPNPFWASIGLSVTPLSLGSGVQYESRVSLGYLNQSFQNAVRDG 480
            MERPL+ A +TIHIEVPPNPFWAS+GLS+ PL
+GSGVQYESRVSLGYLNQSFQNAV +G
Sbjct 421
MERPLRKAEYTIHIEVPPNPFWASVGLSIEPLPIGSGVQYESRVSLGYLNQSFQNAVMEG 480

Query 481
IRYGLEQGLFGWNVTDCKICFEYGLYYSPVSTPADFRSLAPIVLEQALKESGTQLLEPYL 540
            + YG EQGL+GW VTDCKICFEYGLYYSPVSTPADFR L+PIVLEQALK++GT
+LLEPYL
Sbjct 481
VLYGCEQGLYGWKVTDCKICFEYGLYYSPVSTPADFRLLSPIVLEQALKKAGTELLEPYL 540

Query 541
SFILYAPQEYLSRAYHDAPKYCATIETAQVKKDEVVFTGEIPARCIQAYRTDLAFYTNGR 600
             F +YAPQEYLSRAYHDAP+YCA I + Q+K DEV+ GEIPARCIQ YR DL +
+TNG+
Sbjct 541
HFEIYAPQEYLSRAYHDAPRYCADIVSTQIKNDEVILKGEIPARCIQEYRNDLTYFTNGQ 600

Query 601 SVCLTELKGYQAAVGQPVIQPRRPNSRLDKVRHMFQKV 638
             VCLTELKGYQ A+G+ + QPRRPNSR+DKVRHMF K+
Sbjct 601 GVCLTELKGYQPAIGKFICQPRRPNSRIDKVRHMFHKL 638

>AUS91022.1 tetracycline resistance protein tetM [Streptococcus suis]
Length=639

```
 Score = 929 bits (2401),  Expect = 0.0, Method: Compositional
matrix adjust.
 Identities = 430/638 (67%), Positives = 529/638 (83%), Gaps = 0/638
(0%)

Query  1
MKIINIGILAHVDAGKTTLTESLLYASGAISEPGSVEKGTTRTDTMFLERQRGITIQAAV  60
            MKIIN+GILAHVDAGKTTLTESLLY SGAI+EPGSV+KGTTRTDTM
LERQRGITIQ AV
Sbjct  1
MKIINLGILAHVDAGKTTLTESLLYTSGAIAEPGSVDKGTTRTDTMNLERQRGITIQTAV  60

Query  61
TSFQWHRCKVNIVDTPGHMDFLAEVYRSLAVLDGAILVISAKDGVQAQTRILFHALRKMN  120
            TSFQW   KVNI+DTPGHMDFL EVYRSL+VLDGA+L++SAKDG+QAQTRILFHAL
+ M
Sbjct  61
TSFQWEDVKVNIIDTPGHMDFLVEVYRSLSVLDGAVLLVSAKDGIQAQTRILFHALQTMK  120

Query  121
IPTVIFINKIDQAGVDLQSVVQSVRDKLSADIIIKQTVSLSPEIVLEENTDIEAWDAVIE  180
            IPT+ FINKIDQ G+DL  V Q ++ KLS++II+KQ V   P I + +N D+E
WDAVI
Sbjct  121
IPTIFFINKIDQEGIDLPMVYQEMKAKLSSEIIVKQKVGQHPHINVTDNDDMEQWDAVIM  180

Query  181
NNDKLLEKYIAGEPISREKLVREEQRRVQDASLFPVYYGSAKKGLGIQPLMDAVTGLFQP  240
             ND+LLEKY++G+P    +L +EE RR Q+ +LFPVY+GSAK  LGI+ L++ +
F
Sbjct  181
GNDELLEKYMSGKPFKMSELEQEENRRFQNGTLFPVYHGSAKNNLGIRQLIEVIASKFYS  240

Query  241
IGEQGSAALCGSVFKVEYTDCGQRRVYLRLYSGTLRLRDTVALAGREKLKITEMRIPSKG  300
               +G + LCG VFK+EY++  +R VY+R+YSGTL LRD + ++ +EK+KITEM
+P+ G
Sbjct  241
STPEGQSELCGQVFKIEYSEKRRRFVYVRIYSGTLHLRDVIKISEKEKIKITEMCVPTNG  300

Query  301
EIVRTDTAYPGEIVILPSDSVRLNDVLGDPTRLPRKRWREDPLPMLRTTIAPKTAAQRER  360
            E+  +DTA  G+IVILP+D ++LN +LG+   LP++++ E+PLPML+TTIA K +
QRE
Sbjct  301
ELYSSDTACSGDIVILPNDVLQLNSILGNEMLLPQRKFIENPLPMLQTTIAVKKSEQREI  360

Query  361
LLDALTQLADTDPLLRCEVDSITHEIILSFLGRVQLEVVSALLSEKYKLETVVKEPSVIY  420
            LL ALT+++D DPLL+  VD+ THEIILSFLG VQ+EV+ A+L EKY +E  +KEP
+VIY
Sbjct  361
LLGALTEISDGDPLLKYYVDTTTHEIILSFLGNVQMEVICAILEEKYHVEAEIKEPTVIY  420
```

```
Query  421
MERPLKAASHTIHIEVPPNPFWASIGLSVTPLSLGSGVQYESRVSLGYLNQSFQNAVRDG  480
            MERPL+ A +TIHIEVPPNPFWAS+GLS+ PL
+GSGVQYESRVSLGYLNQSFQNAV +G
Sbjct  421
MERPLRKAEYTIHIEVPPNPFWASVGLSIEPLPIGSGVQYESRVSLGYLNQSFQNAVMEG  480

Query  481
IRYGLEQGLFGWNVTDCKICFEYGLYYSPVSTPADFRSLAPIVLEQALKESGTQLLEPYL  540
            + YG EQGL+GW VTDCKICFEYGLYYSPVSTPADFR L+PIVLEQALK++GT
+LLEPYL
Sbjct  481
VLYGCEQGLYGWKVTDCKICFEYGLYYSPVSTPADFRLLSPIVLEQALKKAGTELLEPYL  540

Query  541
SFILYAPQEYLSRAYHDAPKYCATIETAQVKKDEVVFTGEIPARCIQAYRTDLAFYTNGR  600
             F +YAPQEYLSRAYHDAP+YCA I + Q+K DEV+  GEIPARCIQ YR DL +
+TNG+
Sbjct  541
HFEIYAPQEYLSRAYHDAPRYCADIVSTQIKNDEVILKGEIPARCIQEYRNDLTYFTNGQ  600

Query  601  SVCLTELKGYQAAVGQPVIQPRRPNSRLDKVRHMFQKV  638
             VCLTELKGYQ A+G+ + QPRRPNSR+DKVRHMF K+
Sbjct  601  GVCLTELKGYQPAIGKFICQPRRPNSRIDKVRHMFHKL  638


>WP_099779911.1 TetM/TetW/TetO/TetS family tetracycline resistance
ribosomal
protection protein [Streptococcus suis]
Length=639

 Score = 929 bits (2401),  Expect = 0.0, Method: Compositional
matrix adjust.
 Identities = 431/638 (68%), Positives = 527/638 (83%), Gaps = 0/638
(0%)

Query  1
MKIINIGILAHVDAGKTTLTESLLYASGAISEPGSVEKGTTRTDTMFLERQRGITIQAAV  60
            MKIIN+GILAHVDAGKTTLTESLLY SGAI+EPGSV+KGTTRTDTM
LERQRGITIQ AV
Sbjct  1
MKIINLGILAHVDAGKTTLTESLLYTSGAIAEPGSVDKGTTRTDTMNLERQRGITIQTAV  60

Query  61
TSFQWHRCKVNIVDTPGHMDFLAEVYRSLAVLDGAILVISAKDGVQAQTRILFHALRKMN  120
            TSFQW   KVNI+DTPGHMDFLAEVYRSL+VLDGA+L++SAKDG+QAQTRILFHAL
+ M
Sbjct  61
TSFQWEDVKVNIIDTPGHMDFLAEVYRSLSVLDGAVLLVSAKDGIQAQTRILFHALQTMK  120

Query  121
IPTVIFINKIDQAGVDLQSVVQSVRDKLSADIIIKQTVSLSPEIVLEENTDIEAWDAVIE  180
            IPT+ FINKIDQ G+DL  V Q ++ KLS++II+KQ V   P I + +N D+E
```

```
WDAVI
Sbjct  121  
IPTIFFINKIDQEGIDLPMVYQEMKAKLSSEIIVKQKVGQHPHINVTDNDDMEQWDAVIM  180

Query  181  
NNDKLLEKYIAGEPISREKLVREEQRRVQDASLFPVYYGSAKKGLGIQPLMDAVTGLFQP  240
             ND+LLEKY++G+P    +L +EE RR Q+ +LFPVY+GSAK  LGI+ L++ +
F
Sbjct  181  
GNDELLEKYMSGKPFKMSELEQEENRRFQNGTLFPVYHGSAKNNLGIRQLIEVIASKFYS  240

Query  241  
IGEQGSAALCGSVFKVEYTDCGQRRVYLRLYSGTLRLRDTVALAGREKLKITEMRIPSKG  300
               +G + LCG VFK+EY++  +R  Y+RLYSG L  RD V ++ +EK+KITEM
IP+ G
Sbjct  241  
STPEGQSELCGQVFKIEYSEKRRRLAYVRLYSGALHSRDVVRISEKEKIKITEMSIPTNG  300

Query  301  
EIVRTDTAYPGEIVILPSDSVRLNDVLGDPTRLPRKRWREDPLPMLRTTIAPKTAAQRER  360
            E+   DTAY G+IVILP+D ++LN +LG+   LP++++ E+PLPML+TTI  K +
QRE
Sbjct  301  
ELCLADTAYSGDIVILPNDVLQLNSILGNEILLPQRKFIENPLPMLQTTIEVKKSEQREV  360

Query  361  
LLDALTQLADTDPLLRCEVDSITHEIILSFLGRVQLEVVSALLSEKYKLETVVKEPSVIY  420
            LL+ALT+++D DPLL+  VD+ THEIILSFLG VQ+EV+ A+L EKY +E  +KEP
+VIY
Sbjct  361  
LLEALTEISDGDPLLKYYVDTTTHEIILSFLGNVQMEVICAILEEKYHVEAEIKEPTVIY  420

Query  421  
MERPLKAASHTIHIEVPPNPFWASIGLSVTPLSLGSGVQYESRVSLGYLNQSFQNAVRDG  480
            MERPL+ A +TIHIEVPPNPFWAS+GLS+ PL
+GSGVQYESRVSLGYLNQSFQNAV +G
Sbjct  421  
MERPLRKAEYTIHIEVPPNPFWASVGLSIEPLPIGSGVQYESRVSLGYLNQSFQNAVMEG  480

Query  481  
IRYGLEQGLFGWNVTDCKICFEYGLYYSPVSTPADFRSLAPIVLEQALKESGTQLLEPYL  540
            + YG EQGL+GW VTDCKICFEYGLYYSPVSTPADFR L+PIVLEQALK++GT
+LLEPYL
Sbjct  481  
VLYGCEQGLYGWKVTDCKICFEYGLYYSPVSTPADFRLLSPIVLEQALKKAGTELLEPYL  540

Query  541  
SFILYAPQEYLSRAYHDAPKYCATIETAQVKKDEVVFTGEIPARCIQAYRTDLAFYTNGR  600
             F +YAPQEYLSRAYHDAP+YCA I + Q+K DEV+  GEIPARCIQ YR DL +
+TNG+
Sbjct  541  
HFEIYAPQEYLSRAYHDAPRYCADIVSTQIKNDEVILKGEIPARCIQEYRNDLTYFTNGQ  600

Query  601  SVCLTELKGYQAAVGQPVIQPRRPNSRLDKVRHMFQKV  638
```

VCLTELKGYQ A+G+ + QPRRPNSR+DKVRHMF K+
Sbjct 601 GVCLTELKGYQPAIGKFICQPRRPNSRIDKVRHMFHKL 638

>AFK31665.1 tetracycline resistance protein O/32/O [Streptococcus suis]
Length=639

Score = 929 bits (2401), Expect = 0.0, Method: Compositional matrix adjust.
Identities = 433/638 (68%), Positives = 531/638 (83%), Gaps = 0/638 (0%)

Query 1
MKIINIGILAHVDAGKTTLTESLLYASGAISEPGSVEKGTTRTDTMFLERQRGITIQAAV 60
MKIIN+GILAHVDAGKTTLTESLLY SGAI+E GSV++GTTRTDTM
LERQRGITIQ AV
Sbjct 1
MKIINLGILAHVDAGKTTLTESLLYTSGAIAELGSVDEGTTRTDTMNLERQRGITIQTAV 60

Query 61
TSFQWHRCKVNIVDTPGHMDFLAEVYRSLAVLDGAILVISAKDGVQAQTRILFHALRKMN 120
TSFQW   KVNI+DTPGHMDFL E YR+L+VLDGA+LVISAKDGVQAQTRILFHAL
+KMN
Sbjct 61
TSFQWEDVKVNIIDTPGHMDFLTEAYRALSVLDGAVLVISAKDGVQAQTRILFHALQKMN 120

Query 121
IPTVIFINKIDQAGVDLQSVVQSVRDKLSADIIIKQTVSLSPEIVLEENTDIEAWDAVIE 180
IPT+IFINKIDQ G+DLQ V QS++DKL++D+I+ Q VSLSP+I + + +D++ WD
+I
Sbjct 121
IPTIIFINKIDQNGIDLQRVYQSIKDKLTSDMIVMQEVSLSPQISMTDISDLDKWDMIIS 180

Query 181
NNDKLLEKYIAGEPISREKLVREEQRRVQDASLFPVYYGSAKKGLGIQPLMDAVTGLFQP 240
+D+LLE+Y+A + + ++L E+ +R + SLFPVY+GSAK LG + L++A+T
F
Sbjct 181
GSDELLERYVAEDSLDIQELQYEKCKRTRCCSLFPVYHGSAKDNLGTEKLIEAITETFIT 240

Query 241
IGEQGSAALCGSVFKVEYTDCGQRRVYLRLYSGTLRLRDTVALAGREKLKITEMRIPSKG 300
+   + LCG VFKVEYT+ +R YLRLY GTL LRDT+ L+ +EK+KITEM
IPS G
Sbjct 241
ETDDIQSELCGYVFKVEYTERKKRLSYLRLYHGTLHLRDTLLLSKKEKIKITEMCIPSNG 300

Query 301
EIVRTDTAYPGEIVILPSDSVRLNDVLGDPTRLPRKRWREDPLPMLRTTIAPKTAAQRER 360
EIV D A PGEIVIL D+++LND+LG+   LP K W ++P+P+LRTT+ P+
QRE
Sbjct 301
EIVPADHACPGEIVILADDTLKLNDILGNEKLLPHKTWIDNPMPLLRTTVEPQKPEQREA 360

Query 361
LLDALTQLADTDPLLRCEVDSITHEIILSFLGRVQLEVVSALLSEKYKLETVVKEPSVIY 420
            LL+AL ++ADTDPLL ++D++THEI+LSFLG+VQLEV+ +LL EKY +
+KEPSVIY
Sbjct 361
LLNALAEIADTDPLLHFDIDTVTHEIMLSFLGKVQLEVICSLLEEKYHVGVAMKEPSVIY 420

Query 421
MERPLKAASHTIHIEVPPNPFWASIGLSVTPLSLGSGVQYESRVSLGYLNQSFQNAVRDG 480
            +ERPL+ A +TIHIEVPPNPFWAS+GLS+ PL
+GSGVQYESRVSLGYLNQSFQNAV +G
Sbjct 421
LERPLRKAEYTIHIEVPPNPFWASVGLSIEPLPIGSGVQYESRVSLGYLNQSFQNAVMEG 480

Query 481
IRYGLEQGLFGWNVTDCKICFEYGLYYSPVSTPADFRSLAPIVLEQALKESGTQLLEPYL 540
            + YG EQGL+GW VTDCKICFEYGLYYSPVSTPADFR L+PIVLEQALK++GT
+LLEPYL
Sbjct 481
VLYGCEQGLYGWKVTDCKICFEYGLYYSPVSTPADFRLLSPIVLEQALKKAGTELLEPYL 540

Query 541
SFILYAPQEYLSRAYHDAPKYCATIETAQVKKDEVVFTGEIPARCIQAYRTDLAFYTNGR 600
             F +YAPQEYLSRAYHDAP+YCA I + Q+K DEV+ GEIPARCIQ YR DL +
+TNG+
Sbjct 541
HFEIYAPQEYLSRAYHDAPRYCADIVSTQIKNDEVILKGEIPARCIQEYRNDLTYFTNGQ 600

Query 601 SVCLTELKGYQAAVGQPVIQPRRPNSRLDKVRHMFQKV 638
             VCLTELKGYQ A+G+ + QPRRPNSR+DKVRHMF K+
Sbjct 601 GVCLTELKGYQPAIGKFICQPRRPNSRIDKVRHMFHKL 638

>ALA07062.1 tetracycline resistance protein TetO [Streptococcus phage phiNJ3]
Length=639

Score = 929 bits (2401), Expect = 0.0, Method: Compositional matrix adjust.
Identities = 430/638 (67%), Positives = 529/638 (83%), Gaps = 0/638 (0%)

Query 1
MKIINIGILAHVDAGKTTLTESLLYASGAISEPGSVEKGTTRTDTMFLERQRGITIQAAV 60
            MKIIN+GILAHVDAGKTTLTESLLY SGAI+EPGSV+KGTTRTDTM
LERQRGITIQ AV
Sbjct 1
MKIINLGILAHVDAGKTTLTESLLYTSGAIAEPGSVDKGTTRTDTMNLERQRGITIQTAV 60

Query 61
TSFQWHRCKVNIVDTPGHMDFLAEVYRSLAVLDGAILVISAKDGVQAQTRILFHALRKMN 120
            TSFQW   KVNI+DTPGHMDFLAEVYRSL+VLDGA+L++SAKDG+QAQTRILFHAL
+ M

Sbjct 61
TSFQWEDVKVNIIDTPGHMDFLAEVYRSLSVLDGAVLLVSAKDGIQAQTRILFHALQTMK 120

Query 121
IPTVIFINKIDQAGVDLQSVVQSVRDKLSADIIIKQTVSLSPEIVLEENTDIEAWDAVIE 180
IPT+ FINKIDQ G+DL V Q ++ KLS++II+KQ V   P I + +N D+E
WDAVI
Sbjct 121
IPTIFFINKIDQEGIDLPMVYQEMKAKLSSEIIVKQKVGQHPHINVTDNDDMEQWDAVIM 180

Query 181
NNDKLLEKYIAGEPISREKLVREEQRRVQDASLFPVYYGSAKKGLGIQPLMDAVTGLFQP 240
ND+LLEKY++G+P   +L +EE RR Q+ +LFPVY+GSAK LGI+ L++ +
F
Sbjct 181
GNDELLEKYMSGKPFKMSELEQEENRRFQNGTLFPVYHGSAKNNLGIRQLIEVIASKFYS 240

Query 241
IGEQGSAALCGSVFKVEYTDCGQRRVYLRLYSGTLRLRDTVALAGREKLKITEMRIPSKG 300
+G + LCG VFK+EY++ +R VY+R+YSGTL LRD + ++ +EK+KITEM
+P+ G
Sbjct 241
STPEGQSELCGQVFKIEYSEKRRRFVYVRIYSGTLHLRDVIKISEKEKIKITEMCVPTNG 300

Query 301
EIVRTDTAYPGEIVILPSDSVRLNDVLGDPTRLPRKRWREDPLPMLRTTIAPKTAAQRER 360
E+ +DTA G+IVILP+D ++LN +LG+   LP++++ E+PLPML+TTIA K +
QRE
Sbjct 301
ELYSSDTACSGDIVILPNDVLQLNSILGNEMLLPQRKFIENPLPMLQTTIAVKKSEQREI 360

Query 361
LLDALTQLADTDPLLRCEVDSITHEIILSFLGRVQLEVVSALLSEKYKLETVVKEPSVIY 420
LL ALT+++D DPLL+ VD+ THEIILSFLG VQ+EV+ A+L EKY +E +KEP
+VIY
Sbjct 361
LLGALTEISDGDPLLKYYVDTTTHEIILSFLGNVQMEVICAILEEKYHVEAEIKEPTVIY 420

Query 421
MERPLKAASHTIHIEVPPNPFWASIGLSVTPLSLGSGVQYESRVSLGYLNQSFQNAVRDG 480
MERPL+ A +TIHIEVPPNPFWAS+GLS+ PL
+GSGVQYESRVSLGYLNQSFQNAV +G
Sbjct 421
MERPLRKAEYTIHIEVPPNPFWASVGLSIEPLPIGSGVQYESRVSLGYLNQSFQNAVMEG 480

Query 481
IRYGLEQGLFGWNVTDCKICFEYGLYYSPVSTPADFRSLAPIVLEQALKESGTQLLEPYL 540
+ YG EQGL+GW VTDCKICFEYGLYYSPVSTPADFR L+PIVLEQALK++GT
+LLEPYL
Sbjct 481
VLYGCEQGLYGWKVTDCKICFEYGLYYSPVSTPADFRLLSPIVLEQALKKAGTELLEPYL 540

Query 541
SFILYAPQEYLSRAYHDAPKYCATIETAQVKKDEVVFTGEIPARCIQAYRTDLAFYTNGR 600

F +YAP EYLSRAYHDAP+YCA I + Q+K DEV+ GEIPARCIQ YR DL +
+TNG+
Sbjct 541
HFEIYAPHEYLSRAYHDAPRYCADIVSTQIKNDEVILKGEIPARCIQEYRNDLTYFTNGQ 600

Query 601 SVCLTELKGYQAAVGQPVIQPRRPNSRLDKVRHMFQKV 638
VCLTELKGYQ A+G+ + QPRRPNSR+DKVRHMF K+
Sbjct 601 GVCLTELKGYQPAIGKFICQPRRPNSRIDKVRHMFHKL 638

>AMP55640.1 TetM–TetW–TetO–TetS [uncultured bacterium]
Length=639

Score = 929 bits (2401), Expect = 0.0, Method: Compositional
matrix adjust.
Identities = 432/638 (68%), Positives = 529/638 (83%), Gaps = 0/638
(0%)

Query 1
MKIINIGILAHVDAGKTTLTESLLYASGAISEPGSVEKGTTRTDTMFLERQRGITIQAAV 60
MKIIN+GILAHVDAGKTTLTESLLY SGAI+EPGSV+KGTTRTDTM
LERQRGITIQ AV
Sbjct 1
MKIINLGILAHVDAGKTTLTESLLYTSGAIAEPGSVDKGTTRTDTMNLERQRGITIQTAV 60

Query 61
TSFQWHRCKVNIVDTPGHMDFLAEVYRSLAVLDGAILVISAKDGVQAQTRILFHALRKMN 120
TSFQW   KVNI+DTPGHMDFLAEVYRSL+VLDGA+L++SAKDG+QAQTRILFHAL
+ M
Sbjct 61
TSFQWEDVKVNIIDTPGHMDFLAEVYRSLSVLDGAVLLVSAKDGIQAQTRILFHALQTMK 120

Query 121
IPTVIFINKIDQAGVDLQSVVQSVRDKLSADIIIKQTVSLSPEIVLEENTDIEAWDAVIE 180
IPT+ FINKIDQ G+DL V Q ++ KLS++II+KQ V   P I + +N D+E
WDAVI
Sbjct 121
IPTIFFINKIDQEGIDLPMVYQEMKAKLSSEIIVKQKVGQHPHINVTDNDDMEQWDAVIM 180

Query 181
NNDKLLEKYIAGEPISREKLVREEQRRVQDASLFPVYYGSAKKGLGIQPLMDAVTGLFQP 240
ND+LLEKY++G+P   +L +EE RR Q+ +LFPVY+GSAK LGI+ L++ +
F
Sbjct 181
GNDELLEKYMSGKPFKMSELEQEENRRFQNGTLFPVYHGSAKNNLGIRQLIEVIASKFYS 240

Query 241
IGEQGSAALCGSVFKVEYTDCGQRRVYLRLYSGTLRLRDTVALAGREKLKITEMRIPSKG 300
+G + LCG VFK+EY++ +R VY+R+YSGTL LRD + ++ +EK+KITEM
+P+ G
Sbjct 241
STPEGQSELCGQVFKIEYSEKRRRFVYVRIYSGTLHLRDVIKISEKEKIKITEMCVPTNG 300

Query 301

EIVRTDTAYPGEIVILPSDSVRLNDVLGDPTRLPRKRWREDPLPMLRTTIAPKTAAQRER 360
            E+ +DTA G+IVILP+D ++LN +LG+   LP++++ E+PLPML+TTIA K +
QRE
Sbjct 301
ELYSSDTACSGDIVILPNDVLQLNSILGNEMLLPQRKFIENPLPMLQTTIAVKKSEQREI 360

Query 361
LLDALTQLADTDPLLRCEVDSITHEIILSFLGRVQLEVVSALLSEKYKLETVVKEPSVIY 420
            LL ALT+++D DPLL+ VD+ THEIILSFLG VQ+EV+ A+L EKY +E +KEP
+VIY
Sbjct 361
LLGALTEISDGDPLLKYYVDTTTHEIILSFLGNVQMEVICAILEEKYHVEAEIKEPTVIY 420

Query 421
MERPLKAASHTIHIEVPPNPFWASIGLSVTPLSLGSGVQYESRVSLGYLNQSFQNAVRDG 480
            MERPL+ A +TIHIEVPPNPFWAS+GLS+ PL
+GSGVQYESRVSLGYLNQSFQNAV +G
Sbjct 421
MERPLRKAEYTIHIEVPPNPFWASVGLSIEPLPIGSGVQYESRVSLGYLNQSFQNAVMEG 480

Query 481
IRYGLEQGLFGWNVTDCKICFEYGLYYSPVSTPADFRSLAPIVLEQALKESGTQLLEPYL 540
            + YG EQGL+GW VTDCKICFEYGLYYSPVSTPADFR L+PIVLEQALK++GT
+LLEPYL
Sbjct 481
VLYGCEQGLYGWKVTDCKICFEYGLYYSPVSTPADFRLLSPIVLEQALKKAGTELLEPYL 540

Query 541
SFILYAPQEYLSRAYHDAPKYCATIETAQVKKDEVVFTGEIPARCIQAYRTDLAFYTNGR 600
             F +YAPQEYLSRAYHDAP+YCA I + QVK DEV+ GEIPARCIQ YR DL
+TNG+
Sbjct 541
HFEIYAPQEYLSRAYHDAPRYCADIVSTQVKNDEVILKGEIPARCIQEYRNDLTSFTNGQ 600

Query 601 SVCLTELKGYQAAVGQPVIQPRRPNSRLDKVRHMFQKV 638
             VCLTELKGYQ A+G+ + QPRRPNSR+DKVRHMF K+
Sbjct 601 GVCLTELKGYQPAIGKFICQPRRPNSRIDKVRHMFHKL 638

>WP_049496346.1 TetM/TetW/TetO/TetS family tetracycline resistance ribosomal
protection protein [Streptococcus parasanguinis]
Length=639

Score = 929 bits (2401), Expect = 0.0, Method: Compositional matrix adjust.
Identities = 431/638 (68%), Positives = 529/638 (83%), Gaps = 0/638 (0%)

Query 1
MKIINIGILAHVDAGKTTLTESLLYASGAISEPGSVEKGTTRTDTMFLERQRGITIQAAV 60
            MKIIN+GILAHVDAGKTTLTESLLY SGAI+EPGSV+KGTTRTDTM
LERQRGITIQ AV
Sbjct 1

MKIINLGILAHVDAGKTTLTESLLYTSGAIAEPGSVDKGTTRTDTMNLERQRGITIQTAV 60

Query 61
TSFQWHRCKVNIVDTPGHMDFLAEVYRSLAVLDGAILVISAKDGVQAQTRILFHALRKMN 120
TSFQW KVNI+DTPGHMDFLAEVYRSL+VLDGA+L++SAKDG+QAQTRILFHAL
+ M
Sbjct 61
TSFQWEDVKVNIIDTPGHMDFLAEVYRSLSVLDGAVLLVSAKDGIQAQTRILFHALQTMK 120

Query 121
IPTVIFINKIDQAGVDLQSVVQSVRDKLSADIIIKQTVSLSPEIVLEENTDIEAWDAVIE 180
IPT+ FINKIDQ G+DL V Q ++ KLS++II+KQ V P I + +N D+E
WDAVI
Sbjct 121
IPTIFFINKIDQEGIDLPMVYQEMKAKLSSEIIVKQKVGQHPHINVTDNDDMEQWDAVIM 180

Query 181
NNDKLLEKYIAGEPISREKLVREEQRRVQDASLFPVYYGSAKKGLGIQPLMDAVTGLFQP 240
ND+LLEKY++G+P +L +EE R Q+ +LFPVY+GSAK LGI+ L++ +
F
Sbjct 181
GNDELLEKYMSGKPFKMSELEQEENMRFQNGTLFPVYHGSAKNNLGIRQLIEVIASKFYS 240

Query 241
IGEQGSAALCGSVFKVEYTDCGQRRVYLRLYSGTLRLRDTVALAGREKLKITEMRIPSKG 300
+G + LCG VFK+EY++ +R VY+R+YSGTL LRD + ++ +EK+KITEM
+P+ G
Sbjct 241
STPEGQSELCGQVFKIEYSEKRRRFVYVRIYSGTLHLRDVIKISEKEKIKITEMCVPTNG 300

Query 301
EIVRTDTAYPGEIVILPSDSVRLNDVLGDPTRLPRKRWREDPLPMLRTTIAPKTAAQRER 360
E+ +DTA G+IVILP+D ++LN +LG+ LP++++ E+PLPML+TTIA K +
QRE
Sbjct 301
ELYSSDTACSGDIVILPNDVLQLNSILGNEMLLPQRKFIENPLPMLQTTIAVKKSEQREI 360

Query 361
LLDALTQLADTDPLLRCEVDSITHEIILSFLGRVQLEVVSALLSEKYKLETVVKEPSVIY 420
LL ALT+++D DPLL+ VD+ THEIILSFLG VQ+EV+ A+L EKY +E +KEP
+VIY
Sbjct 361
LLGALTEISDGDPLLKYYVDTTTHEIILSFLGNVQMEVICAILEEKYHVEAEIKEPTVIY 420

Query 421
MERPLKAASHTIHIEVPPNPFWASIGLSVTPLSLGSGVQYESRVSLGYLNQSFQNAVRDG 480
MERPL+ A +TIHIEVPPNPFWAS+GLS+ PL
+GSGVQYESRVSLGYLNQSFQNAV +G
Sbjct 421
MERPLRKAEYTIHIEVPPNPFWASVGLSIEPLPIGSGVQYESRVSLGYLNQSFQNAVMEG 480

Query 481
IRYGLEQGLFGWNVTDCKICFEYGLYYSPVSTPADFRSLAPIVLEQALKESGTQLLEPYL 540
+ YG EQGL+GW VTDCKICFEYGLYYSPVSTPADFR L+PIVLEQALK++GT

+LLEPYL
Sbjct 481
VLYGCEQGLYGWKVTDCKICFEYGLYYSPVSTPADFRLLSPIVLEQALKKAGTELLEPYL 540

Query 541
SFILYAPQEYLSRAYHDAPKYCATIETAQVKKDEVVFTGEIPARCIQAYRTDLAFYTNGR 600
             F +YAPQEYLSRAYHDAP+YCA I + QVK DEV+ GEIPARCIQ YR DL +
+TNG+
Sbjct 541
HFEIYAPQEYLSRAYHDAPRYCADIVSTQVKNDEVILKGEIPARCIQEYRNDLTYFTNGQ 600

Query 601 SVCLTELKGYQAAVGQPVIQPRRPNSRLDKVRHMFQKV 638
             VCLTELKGYQ A+G+ + QPRRPNSR+DKVRHMF K+
Sbjct 601 GVCLTELKGYQPAIGKFICQPRRPNSRIDKVRHMFHKL 638

>WP_028086456.1 MULTISPECIES: TetM/TetW/TetO/TetS family
tetracycline resistance
ribosomal protection protein [Lachnospiraceae]
 SCJ84203.1 Tetracycline resistance protein tetM from transposon
Tn916 [uncultured
Eubacterium sp.]
 OLR63201.1 tetracycline resistance ribosomal protection protein
Tet(O) [Anaerostipes
sp. 992a]
Length=639

 Score = 929 bits (2401), Expect = 0.0, Method: Compositional
matrix adjust.
 Identities = 430/638 (67%), Positives = 528/638 (83%), Gaps = 0/638
(0%)

Query 1
MKIINIGILAHVDAGKTTLTESLLYASGAISEPGSVEKGTTRTDTMFLERQRGITIQAAV 60
            MKIIN+GILAHVDAGKTTLTESLLY SGAI+E GSV++GTTRTDTM
LERQRGITIQ AV
Sbjct 1
MKIINLGILAHVDAGKTTLTESLLYTSGAIAELGSVDEGTTRTDTMNLERQRGITIQTAV 60

Query 61
TSFQWHRCKVNIVDTPGHMDFLAEVYRSLAVLDGAILVISAKDGVQAQTRILFHALRKMN 120
            TSFQW   KVNI+DTPGHMDFLAEVYRSL+VLDGA+L++SAKDG+QAQTRILFHAL
+ M
Sbjct 61
TSFQWEDVKVNIIDTPGHMDFLAEVYRSLSVLDGAVLLVSAKDGIQAQTRILFHALQIMK 120

Query 121
IPTVIFINKIDQAGVDLQSVVQSVRDKLSADIIIKQTVSLSPEIVLEENTDIEAWDAVIE 180
            IPT+ FINKIDQ G+DL V + ++ KLS++II+KQ V   P I + +N D+E
WDAVI
Sbjct 121
IPTIFFINKIDQEGIDLPMVYREMKAKLSSEIIVKQKVGQHPHINVTDNDDMEQWDAVIM 180

Query 181

NNDKLLEKYIAGEPISREKLVREEQRRVQDASLFPVYYGSAKKGLGIQPLMDAVTGLFQP 240
ND+LLEKY++G+P   +L +EE RR Q+ +LFPVY+GSAK LGI+ L++ +
F
Sbjct 181
GNDELLEKYMSGKPFKMSELEQEENRRFQNGTLFPVYHGSAKNNLGIRQLIEVIASKFYS 240

Query 241
IGEQGSAALCGSVFKVEYTDCGQRRVYLRLYSGTLRLRDTVALAGREKLKITEMRIPSKG 300
+G + LCG VFK+EY++ +R VY+R+YSGTL LRD + ++ +EK+KITEM
IPS G
Sbjct 241
STPEGQSELCGQVFKIEYSEKRRRFVYVRIYSGTLHLRDVIRISEKEKIKITEMCIPSNG 300

Query 301
EIVRTDTAYPGEIVILPSDSVRLNDVLGDPTRLPRKRWREDPLPMLRTTIAPKTAAQRER 360
EIV D A PGEIVIL D+++LND+LG+   LP K W ++P+P+LRTT+ P+
QRE
Sbjct 301
EIVPADHACPGEIVILADDTLKLNDILGNEKLLPHKTWIDNPMPLLRTTVEPQKPEQREA 360

Query 361
LLDALTQLADTDPLLRCEVDSITHEIILSFLGRVQLEVVSALLSEKYKLETVVKEPSVIY 420
LL+AL ++ADTDPLL ++D++THEI+LSFLG+VQLEV+ +LL EKY +
+KEPSVIY
Sbjct 361
LLNALAEIADTDPLLHFDIDTVTHEIMLSFLGKVQLEVICSLLEEKYHVGVAMKEPSVIY 420

Query 421
MERPLKAASHTIHIEVPPNPFWASIGLSVTPLSLGSGVQYESRVSLGYLNQSFQNAVRDG 480
+ERPL+ A +TIHIEVPPNPFWAS+GLS+ PL
+GSGVQYESRVSLGYLNQSFQNAV +G
Sbjct 421
LERPLRKAEYTIHIEVPPNPFWASVGLSIEPLPIGSGVQYESRVSLGYLNQSFQNAVMEG 480

Query 481
IRYGLEQGLFGWNVTDCKICFEYGLYYSPVSTPADFRSLAPIVLEQALKESGTQLLEPYL 540
+ YG EQGL+GW VTDCKICFEYGLYYSPVSTPADFR L+PIVLEQALK++GT
+LLEPYL
Sbjct 481
VLYGCEQGLYGWKVTDCKICFEYGLYYSPVSTPADFRLLSPIVLEQALKKAGTELLEPYL 540

Query 541
SFILYAPQEYLSRAYHDAPKYCATIETAQVKKDEVVFTGEIPARCIQAYRTDLAFYTNGR 600
F +YAPQEYLSRAYHDAP+YCA I + Q+K DEV+ GEIPARCIQ YR DL +
+TNG+
Sbjct 541
HFEIYAPQEYLSRAYHDAPRYCADIVSTQIKNDEVILKGEIPARCIQEYRNDLTYFTNGQ 600

Query 601 SVCLTELKGYQAAVGQPVIQPRRPNSRLDKVRHMFQKV 638
VCLTELKGYQ A+G+ + QPRRPNSR+DKVRHMF K+
Sbjct 601 GVCLTELKGYQPAIGKFICQPRRPNSRIDKVRHMFHKL 638

>WP_002594219.1 TetM/TetW/TetO/TetS family tetracycline resistance

ribosomal
protection protein [[Clostridium] clostridioforme]
 ENZ08991.1 tetracycline resistance protein TetM [[[Clostridium]
clostridioforme
90A8]
Length=639

 Score = 929 bits (2401), Expect = 0.0, Method: Compositional
matrix adjust.
 Identities = 431/638 (68%), Positives = 529/638 (83%), Gaps = 0/638
(0%)

Query 1
MKIINIGILAHVDAGKTTLTESLLYASGAISEPGSVEKGTTRTDTMFLERQRGITIQAAV 60
            MKIIN+GILAHVDAGKTTLTESLLY SGAI+EPGSV+KGTTRTDTM
LERQRGITIQ AV
Sbjct 1
MKIINLGILAHVDAGKTTLTESLLYTSGAIAEPGSVDKGTTRTDTMNLERQRGITIQTAV 60

Query 61
TSFQWHRCKVNIVDTPGHMDFLAEVYRSLAVLDGAILVISAKDGVQAQTRILFHALRKMN 120
            TSFQW   KVNI+DTPGHMDFLAEVYRSL+VLDGA+L++SAKDG+QAQTRILFHAL
+ M
Sbjct 61
TSFQWEDVKVNIIDTPGHMDFLAEVYRSLSVLDGAVLLVSAKDGIQAQTRILFHALQTMK 120

Query 121
IPTVIFINKIDQAGVDLQSVVQSVRDKLSADIIIKQTVSLSPEIVLEENTDIEAWDAVIE 180
            IPT+ FINKIDQ G+DL V Q ++ KLS++II+KQ V   P I + +N D+E
WDAVI
Sbjct 121
IPTIFFINKIDQEGIDLPMVYQEMKAKLSSEIIVKQKVGQHPHINVTDNDDMEQWDAVIM 180

Query 181
NNDKLLEKYIAGEPISREKLVREEQRRVQDASLFPVYYGSAKKGLGIQPLMDAVTGLFQP 240
             ND+LLEKY++G+P   +L +EE RR Q+ +LFPVY+GSAK LGI+ L++ +
F
Sbjct 181
GNDELLEKYMSGKPFKMSELEQEENRRFQNGTLFPVYHGSAKNNLGIRQLIEVIASKFYS 240

Query 241
IGEQGSAALCGSVFKVEYTDCGQRRVYLRLYSGTLRLRDTVALAGREKLKITEMRIPSKG 300
               +G + LCG VFK+EY++ +R VY+R+YSGTL LRD + ++ +EK+KITEM
+P+ G
Sbjct 241
STPEGQSELCGQVFKIEYSEKRRRFVYVRIYSGTLHLRDVIKISEKEKIKITEMCVPTNG 300

Query 301
EIVRTDTAYPGEIVILPSDSVRLNDVLGDPTRLPRKRWREDPLPMLRTTIAPKTAAQRER 360
            E+ +DTA G+IVILP+D ++LN +LG+   LP++++ E+PLPML+TTIA K +
QRE
Sbjct 301
ELYSSDTACSGDIVILPNDVLQLNSILGNEMLLPQRKFIENPLPMLQTTIAVKKSEQREI 360

Query 361
LLDALTQLADTDPLLRCEVDSITHEIILSFLGRVQLEVVSALLSEKYKLETVVKEPSVIY 420
            LL AL +++D DPLL+ VD+ THEIILSFLG VQ+EV+ A+L EKY +E +KEP
+VIY
Sbjct 361
LLGALREISDGDPLLKYYVDTTTHEIILSFLGNVQMEVICAILEEKYHVEAEIKEPTVIY 420

Query 421
MERPLKAASHTIHIEVPPNPFWASIGLSVTPLSLGSGVQYESRVSLGYLNQSFQNAVRDG 480
            MERPL+ A +TIHIEVPPNPFWAS+GLS+ PL
+GSGVQYESRVSLGYLNQSFQNAV +G
Sbjct 421
MERPLRKAEYTIHIEVPPNPFWASVGLSIEPLPIGSGVQYESRVSLGYLNQSFQNAVMEG 480

Query 481
IRYGLEQGLFGWNVTDCKICFEYGLYYSPVSTPADFRSLAPIVLEQALKESGTQLLEPYL 540
            + YG EQGL+GW VTDCKICFEYGLYYSPVSTPADFR L+PIVLEQALK++GT
+LLEPYL
Sbjct 481
VLYGCEQGLYGWKVTDCKICFEYGLYYSPVSTPADFRLLSPIVLEQALKKAGTELLEPYL 540

Query 541
SFILYAPQEYLSRAYHDAPKYCATIETAQVKKDEVVFTGEIPARCIQAYRTDLAFYTNGR 600
             F +YAPQEYLSRAYHDAP+YCA I + QVK DEV+ GEIPARCIQ YR DL +
+TNG+
Sbjct 541
HFEIYAPQEYLSRAYHDAPRYCADIVSTQVKNDEVILKGEIPARCIQEYRNDLTYFTNGQ 600

Query 601 SVCLTELKGYQAAVGQPVIQPRRPNSRLDKVRHMFQKV 638
             VCLTELKGYQ A+G+ + QPRRPNSR+DKVRHMF K+
Sbjct 601 GVCLTELKGYQPAIGKFICQPRRPNSRIDKVRHMFHKL 638

>WP_105146473.1 TetM/TetW/TetO/TetS family tetracycline resistance
ribosomal
protection protein [Streptococcus suis]
Length=639

 Score = 929 bits (2400), Expect = 0.0, Method: Compositional
matrix adjust.
 Identities = 431/638 (68%), Positives = 527/638 (83%), Gaps = 0/638
(0%)

Query 1
MKIINIGILAHVDAGKTTLTESLLYASGAISEPGSVEKGTTRTDTMFLERQRGITIQAAV 60
            MKIIN+GILAHVDAGKTTLTESLLY SGAI+EPGSV++GTTRTDTM
LERQRGITIQ AV
Sbjct 1
MKIINLGILAHVDAGKTTLTESLLYTSGAIAEPGSVDEGTTRTDTMNLERQRGITIQTAV 60

Query 61
TSFQWHRCKVNIVDTPGHMDFLAEVYRSLAVLDGAILVISAKDGVQAQTRILFHALRKMN 120
            TSFQW   KVNI+DTPGHMDFLAEVYRSL+VLDGA+L++SAKDG+QAQTRILFHAL
+ M

Sbjct 61
TSFQWEDVKVNIIDTPGHMDFLAEVYRSLSVLDGAVLLVSAKDGIQAQTRILFHALQTMK 120

Query 121
IPTVIFINKIDQAGVDLQSVVQSVRDKLSADIIIKQTVSLSPEIVLEENTDIEAWDAVIE 180
            IPT+ FINKIDQ G+DL V Q ++ KLS++II+KQ V   P I + +N D+E
WDAVI
Sbjct 121
IPTIFFINKIDQEGIDLPMVYQEMKAKLSSEIIVKQKVGQHPHINVTDNDDMEQWDAVIM 180

Query 181
NNDKLLEKYIAGEPISREKLVREEQRRVQDASLFPVYYGSAKKGLGIQPLMDAVTGLFQP 240
             ND+LLEKY++G+P   +L +EE RR Q+ +LFPVY+GSAK LGI+ L++ +
F
Sbjct 181
GNDELLEKYMSGKPFKMSELEQEENRRFQNGTLFPVYHGSAKNNLGIRQLIEVIASKFYS 240

Query 241
IGEQGSAALCGSVFKVEYTDCGQRRVYLRLYSGTLRLRDTVALAGREKLKITEMRIPSKG 300
             +G + LCG VFK+EYT+ +R Y+RLYSG L RD V ++ +EK+KITEM
IP+ G
Sbjct 241
STPEGQSELCGQVFKIEYTEKRRRLAYVRLYSGALHSRDVVRISEKEKIKITEMSIPTNG 300

Query 301
EIVRTDTAYPGEIVILPSDSVRLNDVLGDPTRLPRKRWREDPLPMLRTTIAPKTAAQRER 360
            E+   DTAY G+IVILP+D ++LN +LG+   LP++++ E+PLPML+TTI K +
QRE
Sbjct 301
ELCLADTAYSGDIVILPNDVLQLNSILGNEILLPQRKFIENPLPMLQTTIEVKKSEQREV 360

Query 361
LLDALTQLADTDPLLRCEVDSITHEIILSFLGRVQLEVVSALLSEKYKLETVVKEPSVIY 420
            LL+ALT+++D DPLL+ VD+ THEIILSFLG VQ+EV+ A+L EKY +E +KEP
+VIY
Sbjct 361
LLEALTEISDGDPLLKYYVDTTTHEIILSFLGNVQMEVICAILEEKYHVEVEIKEPTVIY 420

Query 421
MERPLKAASHTIHIEVPPNPFWASIGLSVTPLSLGSGVQYESRVSLGYLNQSFQNAVRDG 480
            MERPL+ A +TIHIEVPPNPFWAS+GLS+ PL
+GSGVQYESRVSLGYLNQSFQNAV +G
Sbjct 421
MERPLRKAEYTIHIEVPPNPFWASVGLSIEPLPIGSGVQYESRVSLGYLNQSFQNAVMEG 480

Query 481
IRYGLEQGLFGWNVTDCKICFEYGLYYSPVSTPADFRSLAPIVLEQALKESGTQLLEPYL 540
            + YG EQGL+GW VTDCKICFEYGLYYSPVSTPADFR L+PIVLEQALK++GT
+LLEPYL
Sbjct 481
VLYGCEQGLYGWKVTDCKICFEYGLYYSPVSTPADFRLLSPIVLEQALKKAGTELLEPYL 540

Query 541
SFILYAPQEYLSRAYHDAPKYCATIETAQVKKDEVVFTGEIPARCIQAYRTDLAFYTNGR 600

F +YAPQEYLSRAYHDAP+YCA I + Q+K DEV+ GEIPARCIQ YR DL +
+TNG+
Sbjct 541
HFEIYAPQEYLSRAYHDAPRYCADIVSTQIKNDEVILKGEIPARCIQEYRNDLTYFTNGQ 600

Query 601 SVCLTELKGYQAAVGQPVIQPRRPNSRLDKVRHMFQKV 638
VCLTELKGYQ A+G+ + QPRRPNSR+DKVRHMF K+
Sbjct 601 GVCLTELKGYQPAIGKFICQPRRPNSRIDKVRHMFHKL 638

>WP_105124962.1 TetM/TetW/TetO/TetS family tetracycline resistance ribosomal
protection protein [Streptococcus suis]
Length=639

Score = 929 bits (2400), Expect = 0.0, Method: Compositional matrix adjust.
Identities = 431/638 (68%), Positives = 527/638 (83%), Gaps = 0/638 (0%)

Query 1
MKIINIGILAHVDAGKTTLTESLLYASGAISEPGSVEKGTTRTDTMFLERQRGITIQAAV 60
MKIIN+GILAHVDAGKTTLTESLLY SGAI+EPGSV+KGTTRTDTM
LERQRGITIQ AV
Sbjct 1
MKIINLGILAHVDAGKTTLTESLLYTSGAIAEPGSVDKGTTRTDTMNLERQRGITIQTAV 60

Query 61
TSFQWHRCKVNIVDTPGHMDFLAEVYRSLAVLDGAILVISAKDGVQAQTRILFHALRKMN 120
TSFQW   KVNI+DTPGHMDFLAEVYRSL+VLDGA+L++SAKDG+QAQTRILFHAL
+ M
Sbjct 61
TSFQWEDVKVNIIDTPGHMDFLAEVYRSLSVLDGAVLLVSAKDGIQAQTRILFHALQTMK 120

Query 121
IPTVIFINKIDQAGVDLQSVVQSVRDKLSADIIIKQTVSLSPEIVLEENTDIEAWDAVIE 180
IPT+ FINKIDQ G+DL V Q ++ KLS++II+KQ V   P I + +N D+E WD
VI
Sbjct 121
IPTIFFINKIDQEGIDLPMVYQEMKAKLSSEIIVKQKVGQHPHINVTDNDDMEQWDTVIM 180

Query 181
NNDKLLEKYIAGEPISREKLVREEQRRVQDASLFPVYYGSAKKGLGIQPLMDAVTGLFQP 240
ND+LLEKY++G+P   +L +EE RR Q+ +LFPVY+GSAK LGI+ L++ +
F
Sbjct 181
GNDELLEKYMSGKPFKMSELEQEENRRFQNGTLFPVYHGSAKNNLGIRQLIEVIASKFYS 240

Query 241
IGEQGSAALCGSVFKVEYTDCGQRRVYLRLYSGTLRLRDTVALAGREKLKITEMRIPSKG 300
+G + LCG VFK+EY++ +R Y+RLYSG L RD V ++ +EK+KITEM
IP+ G
Sbjct 241
STPEGQSELCGQVFKIEYSEKRRRLAYVRLYSGALHSRDVVRISEKEKIKITEMSIPTNG 300

```
Query  301
EIVRTDTAYPGEIVILPSDSVRLNDVLGDPTRLPRKRWREDPLPMLRTTIAPKTAAQRER  360
            E+   DTAY G+IVILP+D ++LN +LG+   LP++++ E+PLPML+TTI  K +
QRE
Sbjct  301
ELCLADTAYSGDIVILPNDVLQLNSILGNEILLPQRKFIENPLPMLQTTIEVKKSEQREV  360

Query  361
LLDALTQLADTDPLLRCEVDSITHEIILSFLGRVQLEVVSALLSEKYKLETVVKEPSVIY  420
            LL+ALT+++D DPLL+  VD+ THEIILSFLG+VQ+EV+ A+L EKY +E  +KEP
+VIY
Sbjct  361
LLEALTEISDGDPLLKYYVDTTTHEIILSFLGKVQMEVICAILEEKYHVEAEIKEPTVIY  420

Query  421
MERPLKAASHTIHIEVPPNPFWASIGLSVTPLSLGSGVQYESRVSLGYLNQSFQNAVRDG  480
            MERPL+ A +TIHIEVPPNPFWAS+GLS+ PL
+GSGVQYESRVSLGYLNQSFQNAV +G
Sbjct  421
MERPLRKAEYTIHIEVPPNPFWASVGLSIEPLPIGSGVQYESRVSLGYLNQSFQNAVMEG  480

Query  481
IRYGLEQGLFGWNVTDCKICFEYGLYYSPVSTPADFRSLAPIVLEQALKESGTQLLEPYL  540
            + YG EQGL+GW VTDCKICFEYGLYYSPVSTPADFR L+PIVLEQALK++GT
+LLEPYL
Sbjct  481
VLYGCEQGLYGWKVTDCKICFEYGLYYSPVSTPADFRLLSPIVLEQALKKAGTELLEPYL  540

Query  541
SFILYAPQEYLSRAYHDAPKYCATIETAQVKKDEVVFTGEIPARCIQAYRTDLAFYTNGR  600
             F +YAPQEYLSRAYHDAP+YCA I + QVK DEV+  GEIPARCIQ YR DL +
+TNG+
Sbjct  541
HFEIYAPQEYLSRAYHDAPRYCADIVSTQVKNDEVILKGEIPARCIQEYRNDLTYFTNGQ  600

Query  601  SVCLTELKGYQAAVGQPVIQPRRPNSRLDKVRHMFQKV  638
             VCLTELKGYQ A+G+ + QPRRPNSR+DKVRHMF K+
Sbjct  601  GVCLTELKGYQPAIGKFICQPRRPNSRIDKVRHMFHKL  638


>WP_105115755.1 TetM/TetW/TetO/TetS family tetracycline resistance
ribosomal
protection protein [Streptococcus suis]
Length=639

 Score = 929 bits (2400),  Expect = 0.0, Method: Compositional
matrix adjust.
 Identities = 430/638 (67%), Positives = 528/638 (83%), Gaps = 0/638
(0%)

Query  1
MKIINIGILAHVDAGKTTLTESLLYASGAISEPGSVEKGTTRTDTMFLERQRGITIQAAV  60
            MKIIN+GILAHVDAGKTTLTESLLY SGAI+EPGSV++GTTRTDTM
```

LERQRGITIQ AV
Sbjct 1
MKIINLGILAHVDAGKTTLTESLLYTSGAIAEPGSVDEGTTRTDTMNLERQRGITIQTAV 60

Query 61
TSFQWHRCKVNIVDTPGHMDFLAEVYRSLAVLDGAILVISAKDGVQAQTRILFHALRKMN 120
TSFQW   KVNI+DTPGHMDFLAEVYRSL+VLDGA+L++SAKDG+QAQTRILFHAL
+ M
Sbjct 61
TSFQWEDVKVNIIDTPGHMDFLAEVYRSLSVLDGAVLLVSAKDGIQAQTRILFHALQTMK 120

Query 121
IPTVIFINKIDQAGVDLQSVVQSVRDKLSADIIIKQTVSLSPEIVLEENTDIEAWDAVIE 180
IPT+ FINKIDQ G+DL V Q ++ KLS++II+KQ V   P I + +N D+E
WDAVI
Sbjct 121
IPTIFFINKIDQEGIDLPMVYQEMKAKLSSEIIVKQKVGQHPHINVTDNDDMEQWDAVIM 180

Query 181
NNDKLLEKYIAGEPISREKLVREEQRRVQDASLFPVYYGSAKKGLGIQPLMDAVTGLFQP 240
ND+LLEKY++G+P   +L +EE RR Q+ +LFPVY+GSAK LGI+ L++ +
F
Sbjct 181
GNDELLEKYMSGKPFKMSELEQEENRRFQNGTLFPVYHGSAKNNLGIRQLIEVIASKFYS 240

Query 241
IGEQGSAALCGSVFKVEYTDCGQRRVYLRLYSGTLRLRDTVALAGREKLKITEMRIPSKG 300
+G + LCG VFK+EY++ +R Y+RLYSG L RD V ++ +EK+KITEM
IP+ G
Sbjct 241
STPEGQSELCGQVFKIEYSEKRRRLAYVRLYSGALHSRDVVRISEKEKIKITEMSIPTNG 300

Query 301
EIVRTDTAYPGEIVILPSDSVRLNDVLGDPTRLPRKRWREDPLPMLRTTIAPKTAAQRER 360
E+   DTAY G+IVILP+D ++LN +LG+   LP++++ E+PLPML+TTI K +
QRE
Sbjct 301
ELCLADTAYSGDIVILPNDVLQLNSILGNEMLLPQRKFIENPLPMLQTTIEVKKSEQREV 360

Query 361
LLDALTQLADTDPLLRCEVDSITHEIILSFLGRVQLEVVSALLSEKYKLETVVKEPSVIY 420
LL+ALT+++D DPLL+ VD+ THEIILSFLG+VQ+EV+ A+L EKY +E +KEP
+VIY
Sbjct 361
LLEALTEISDGDPLLKYYVDTTTHEIILSFLGKVQMEVICAILEEKYHVEAEIKEPTVIY 420

Query 421
MERPLKAASHTIHIEVPPNPFWASIGLSVTPLSLGSGVQYESRVSLGYLNQSFQNAVRDG 480
MERPL+ A +TIHIEVPPNPFWAS+GLS+ PL
+GSGVQYESRVSLGYLNQSFQNAV +G
Sbjct 421
MERPLRKAEYTIHIEVPPNPFWASVGLSIEPLPIGSGVQYESRVSLGYLNQSFQNAVMEG 480

Query 481

IRYGLEQGLFGWNVTDCKICFEYGLYYSPVSTPADFRSLAPIVLEQALKESGTQLLEPYL 540
            + YG EQGL+GW VTDCKICFEYGLYYSPVSTPADFR L+PIVLEQALK++GT
+LLEPYL
Sbjct 481
VLYGCEQGLYGWKVTDCKICFEYGLYYSPVSTPADFRLLSPIVLEQALKKAGTELLEPYL 540

Query 541
SFILYAPQEYLSRAYHDAPKYCATIETAQVKKDEVVFTGEIPARCIQAYRTDLAFYTNGR 600
             F +YAPQEYLSRAYHDAP+YCA I + Q+K DEV+ GEIPARCIQ YR DL +
+TNG+
Sbjct 541
HFEIYAPQEYLSRAYHDAPRYCADIVSTQIKNDEVILKGEIPARCIQEYRNDLTYFTNGQ 600

Query 601 SVCLTELKGYQAAVGQPVIQPRRPNSRLDKVRHMFQKV 638
             VCLTELKGYQ A+G+ + QPRRPNSR+DKVRHMF K+
Sbjct 601 GVCLTELKGYQPAIGKFICQPRRPNSRIDKVRHMFHKL 638

>APO27460.1 ferrous iron transport protein B [uncultured bacterium]
 APO27496.1 ferrous iron transport protein B [uncultured bacterium]
 APO28022.1 ferrous iron transport protein B [uncultured bacterium]
Length=639

 Score = 929 bits (2400), Expect = 0.0, Method: Compositional
matrix adjust.
 Identities = 430/638 (67%), Positives = 531/638 (83%), Gaps = 0/638
(0%)

Query 1
MKIINIGILAHVDAGKTTLTESLLYASGAISEPGSVEKGTTRTDTMFLERQRGITIQAAV 60
            MKIIN+GILAHVDAGKTTLTESLLY SGAI+E GSV++GTTRTDTM
LERQRGITIQ AV
Sbjct 1
MKIINLGILAHVDAGKTTLTESLLYTSGAIAELGSVDEGTTRTDTMNLERQRGITIQTAV 60

Query 61
TSFQWHRCKVNIVDTPGHMDFLAEVYRSLAVLDGAILVISAKDGVQAQTRILFHALRKMN 120
            TSFQW   KVNI+DTPGHMDFLAEVYRSL+VLDGA+L++SAKDG+QAQTRILFHAL
+ M
Sbjct 61
TSFQWEDVKVNIIDTPGHMDFLAEVYRSLSVLDGAVLLVSAKDGIQAQTRILFHALQTMK 120

Query 121
IPTVIFINKIDQAGVDLQSVVQSVRDKLSADIIIKQTVSLSPEIVLEENTDIEAWDAVIE 180
            IPT+ FINKIDQ G+DL V + ++ KLS++II+KQ V   P I + +N D+E
WDAVI
Sbjct 121
IPTIFFINKIDQEGIDLPMVYREMKAKLSSEIIVKQKVGQHPHINVTDNDDMEQWDAVIM 180

Query 181
NNDKLLEKYIAGEPISREKLVREEQRRVQDASLFPVYYGSAKKGLGIQPLMDAVTGLFQP 240
             ND+LLEKY++GEP   +L +EE RR Q+ +LFPVY+GSAK LGI+ L++ +
F
Sbjct 181

GNDELLEKYMSGEPFKMSELEQEENRRFQNGTLFPVYHGSAKNNLGIRQLIEVIASKFYS 240

Query 241
IGEQGSAALCGSVFKVEYTDCGQRRVYLRLYSGTLRLRDTVALAGREKLKITEMRIPSKG 300
               +G + LCG VFK+EY++ +R VY+R+YSGTL LRD + ++ +EK+KITEMR
+P+ G
Sbjct 241
STPEGQSELCGQVFKIEYSEKRRRFVYVRIYSGTLHLRDVIRISEKEKIKITEMRVPTNG 300

Query 301
EIVRTDTAYPGEIVILPSDSVRLNDVLGDPTRLPRKRWREDPLPMLRTTIAPKTAAQRER 360
            E+ +DTA G+IVILP+D ++LN +LG+   LP++++ E+PLPML+TTIA K +
QRE
Sbjct 301
ELYSSDTACSGDIVILPNDVLQLNSILGNEMLLPQRKFIENPLPMLQTTIAVKKSEQREI 360

Query 361
LLDALTQLADTDPLLRCEVDSITHEIILSFLGRVQLEVVSALLSEKYKLETVVKEPSVIY 420
            LL ALT+++D DPLL+ VD+ THEIILSFLG+VQ+EV+ A+L EKY +E +KEP
+VIY
Sbjct 361
LLGALTEISDGDPLLKYYVDTTTHEIILSFLGKVQMEVICAILEEKYHVEAEIKEPTVIY 420

Query 421
MERPLKAASHTIHIEVPPNPFWASIGLSVTPLSLGSGVQYESRVSLGYLNQSFQNAVRDG 480
            MERPL+ A +TIHIEVPPNPFWAS+GLS+ PL
+GSGVQYESRVSLGYLNQSFQNAV +G
Sbjct 421
MERPLRKAEYTIHIEVPPNPFWASVGLSIEPLPIGSGVQYESRVSLGYLNQSFQNAVMEG 480

Query 481
IRYGLEQGLFGWNVTDCKICFEYGLYYSPVSTPADFRSLAPIVLEQALKESGTQLLEPYL 540
            + YG EQGL+GW VTDCKICFEYGLYYSPVSTPADFR L+PIVLEQALK++GT
+LLEPYL
Sbjct 481
VLYGCEQGLYGWKVTDCKICFEYGLYYSPVSTPADFRLLSPIVLEQALKKAGTELLEPYL 540

Query 541
SFILYAPQEYLSRAYHDAPKYCATIETAQVKKDEVVFTGEIPARCIQAYRTDLAFYTNGR 600
             F +YAPQEYLSRAYHDAP+YCA I + Q+K DEV+ GEIPARCIQ YR DL +
+TNG+
Sbjct 541
HFEIYAPQEYLSRAYHDAPRYCADIVSTQIKNDEVILKGEIPARCIQEYRNDLTYFTNGQ 600

Query 601 SVCLTELKGYQAAVGQPVIQPRRPNSRLDKVRHMFQKV 638
             VCLTELKGYQ A+G+ + QPRRPNSR+DKVRHMF K+
Sbjct 601 GVCLTELKGYQPAIGKFICQPRRPNSRIDKVRHMFHKL 638

>AMP50086.1 TetM-TetW-TetO-TetS [uncultured bacterium]
Length=639

 Score = 929 bits (2400), Expect = 0.0, Method: Compositional
matrix adjust.

Identities = 430/638 (67%), Positives = 529/638 (83%), Gaps = 0/638 (0%)

```
Query  1
MKIINIGILAHVDAGKTTLTESLLYASGAISEPGSVEKGTTRTDTMFLERQRGITIQAAV  60
            MKIIN+GILAHVDAGKTTLTESLLY SGAI+EPGSV+KGTTRTDTM
LERQRGITIQ AV
Sbjct  1
MKIINLGILAHVDAGKTTLTESLLYTSGAIAEPGSVDKGTTRTDTMNLERQRGITIQTAV  60

Query  61
TSFQWHRCKVNIVDTPGHMDFLAEVYRSLAVLDGAILVISAKDGVQAQTRILFHALRKMN  120
            TSFQW   KVNI+DTPGHMDFLAEVYRSL+VLDGA+L++SAKDG+QAQTRILFHAL
+ M
Sbjct  61
TSFQWEDVKVNIIDTPGHMDFLAEVYRSLSVLDGAVLLVSAKDGIQAQTRILFHALQTMK  120

Query  121
IPTVIFINKIDQAGVDLQSVVQSVRDKLSADIIIKQTVSLSPEIVLEENTDIEAWDAVIE  180
            IPT+ FINKIDQ G+DL  V Q ++ KLS++II+KQ V   P I + +N D+E
WDAVI
Sbjct  121
IPTIFFINKIDQEGIDLPMVYQEMKAKLSSEIIVKQKVGQHPHINVTDNDDMEQWDAVIM  180

Query  181
NNDKLLEKYIAGEPISREKLVREEQRRVQDASLFPVYYGSAKKGLGIQPLMDAVTGLFQP  240
             ND+LLEKY++G+P    +L +EE RR Q+ +LFPVY+GSAK  LGI+ L++ +
F
Sbjct  181
GNDELLEKYMSGKPFKMSELEQEENRRFQNGTLFPVYHGSAKNNLGIRQLIEVIASKFYS  240

Query  241
IGEQGSAALCGSVFKVEYTDCGQRRVYLRLYSGTLRLRDTVALAGREKLKITEMRIPSKG  300
               +G + LCG VFK+EY++  +R VY+R+YSGTL LRD + ++ +EK+KITEM
+P+ G
Sbjct  241
STPEGQSELCGQVFKIEYSEKRRRFVYVRIYSGTLHLRDVIKISEKEKIKITEMCVPTNG  300

Query  301
EIVRTDTAYPGEIVILPSDSVRLNDVLGDPTRLPRKRWREDPLPMLRTTIAPKTAAQRER  360
            E+  +DTA  G+IVILP+D ++LN +LG+   LP++++ E+PLPML+TTIA K +
QRE
Sbjct  301
ELCSSDTACSGDIVILPNDVLQLNSILGNEILLPQRKFIENPLPMLQTTIAVKKSEQREI  360

Query  361
LLDALTQLADTDPLLRCEVDSITHEIILSFLGRVQLEVVSALLSEKYKLETVVKEPSVIY  420
            LL ALT+++D DPLL+  VD+ THEIILSFLG VQ+EV+ A+L EKY +E  +KEP
+VIY
Sbjct  361
LLGALTEISDGDPLLKYYVDTTTHEIILSFLGNVQMEVICAILEEKYHVEAEIKEPTVIY  420

Query  421
MERPLKAASHTIHIEVPPNPFWASIGLSVTPLSLGSGVQYESRVSLGYLNQSFQNAVRDG  480
```

            MERPL+ A +TIHIEVPPNPFWAS+GLS+ PL
+GSGVQYESRVSLGYLNQSFQNAV +G
Sbjct 421
MERPLRKAEYTIHIEVPPNPFWASVGLSIEPLPIGSGVQYESRVSLGYLNQSFQNAVMEG 480

Query 481
IRYGLEQGLFGWNVTDCKICFEYGLYYSPVSTPADFRSLAPIVLEQALKESGTQLLEPYL 540
            + YG EQGL+GW VTDCKICFEYGLYYSPVSTPADFR L+PIVLEQALK++GT
+LLEPYL
Sbjct 481
VLYGCEQGLYGWKVTDCKICFEYGLYYSPVSTPADFRLLSPIVLEQALKKAGTELLEPYL 540

Query 541
SFILYAPQEYLSRAYHDAPKYCATIETAQVKKDEVVFTGEIPARCIQAYRTDLAFYTNGR 600
             F +YAPQEYLSRAYHDAP+YCA I + Q+K DEV+ GEIPARCIQ YR DL +
+TNG+
Sbjct 541
HFEIYAPQEYLSRAYHDAPRYCADIVSTQIKNDEVILKGEIPARCIQEYRNDLTYFTNGQ 600

Query 601 SVCLTELKGYQAAVGQPVIQPRRPNSRLDKVRHMFQKV 638
             VCLTELKGYQ A+G+ + PRRPNSR+DKVRHMF K+
Sbjct 601 GVCLTELKGYQPAIGKFICHPRRPNSRIDKVRHMFHKL 638

>WP_044977943.1 TetM/TetW/TetO/TetS family tetracycline resistance
ribosomal
protection protein [Streptococcus suis]
 CYY06811.1 tetracycline resistance protein tetM [Streptococcus
suis]
Length=639

 Score = 929 bits (2400), Expect = 0.0, Method: Compositional
matrix adjust.
 Identities = 429/638 (67%), Positives = 529/638 (83%), Gaps = 0/638
(0%)

Query 1
MKIINIGILAHVDAGKTTLTESLLYASGAISEPGSVEKGTTRTDTMFLERQRGITIQAAV 60
            MKIIN+GILAHVDAGKTTLTESLLY SGAI+EPGSV+KGTTRTDTM
LERQRGITIQ AV
Sbjct 1
MKIINLGILAHVDAGKTTLTESLLYTSGAIAEPGSVDKGTTRTDTMNLERQRGITIQTAV 60

Query 61
TSFQWHRCKVNIVDTPGHMDFLAEVYRSLAVLDGAILVISAKDGVQAQTRILFHALRKMN 120
            TSFQW   KVNI+DTPGHMDFLAEVYRSL+VLDGA+L++SAKDG+QAQTRILFHAL
+ M
Sbjct 61
TSFQWEDVKVNIIDTPGHMDFLAEVYRSLSVLDGAVLLVSAKDGIQAQTRILFHALQTMK 120

Query 121
IPTVIFINKIDQAGVDLQSVVQSVRDKLSADIIIKQTVSLSPEIVLEENTDIEAWDAVIE 180
            IPT+ FINKIDQ G+DL V Q ++ KLS++II+KQ V   P I + +N D+E WD
VI

Sbjct 121
IPTIFFINKIDQEGIDLPMVYQEMKAKLSSEIIVKQKVGQHPHINVTDNDDMEQWDTVIM 180

Query 181
NNDKLLEKYIAGEPISREKLVREEQRRVQDASLFPVYYGSAKKGLGIQPLMDAVTGLFQP 240
             ND+LLEKY++G+P   +L +EE RR Q+ +LFPVY+GSAK +GI+ L++ +
F
Sbjct 181
GNDELLEKYMSGKPFKMSELEQEENRRFQNGTLFPVYHGSAKNNMGIRQLIEVIASKFYS 240

Query 241
IGEQGSAALCGSVFKVEYTDCGQRRVYLRLYSGTLRLRDTVALAGREKLKITEMRIPSKG 300
               +G + LCG VFK+EY++ +R VY+R+YSGTL LRD + ++ +EK+KITEM
+P+ G
Sbjct 241
STPEGQSELCGQVFKIEYSEKRRRFVYVRIYSGTLHLRDVIKISEKEKIKITEMCVPTNG 300

Query 301
EIVRTDTAYPGEIVILPSDSVRLNDVLGDPTRLPRKRWREDPLPMLRTTIAPKTAAQRER 360
            E+ +DTA G+IVILP+D ++LN +LG+   LP++++ E+PLPML+TTIA K +
QRE
Sbjct 301
ELYSSDTACSGDIVILPNDVLQLNSILGNEMLLPQRKFIENPLPMLQTTIAVKKSEQREI 360

Query 361
LLDALTQLADTDPLLRCEVDSITHEIILSFLGRVQLEVVSALLSEKYKLETVVKEPSVIY 420
            LL ALT+++D DPLL+ VD+ THEIILSFLG VQ+EV+ A+L EKY +E +KEP
+VIY
Sbjct 361
LLGALTEISDGDPLLKYYVDTTTHEIILSFLGNVQMEVICAILEEKYHVEAEIKEPTVIY 420

Query 421
MERPLKAASHTIHIEVPPNPFWASIGLSVTPLSLGSGVQYESRVSLGYLNQSFQNAVRDG 480
            MERPL+ A +TIHIEVPPNPFWAS+GLS+ PL
+GSGVQYESRVSLGYLNQSFQNAV +G
Sbjct 421
MERPLRKAEYTIHIEVPPNPFWASVGLSIEPLPIGSGVQYESRVSLGYLNQSFQNAVMEG 480

Query 481
IRYGLEQGLFGWNVTDCKICFEYGLYYSPVSTPADFRSLAPIVLEQALKESGTQLLEPYL 540
            + YG EQGL+GW VTDCKICFEYGLYYSPVSTPADFR L+PIVLEQALK++GT
+LLEPYL
Sbjct 481
VLYGCEQGLYGWKVTDCKICFEYGLYYSPVSTPADFRLLSPIVLEQALKKAGTELLEPYL 540

Query 541
SFILYAPQEYLSRAYHDAPKYCATIETAQVKKDEVVFTGEIPARCIQAYRTDLAFYTNGR 600
             F +YAPQEYLSRAYHDAP+YCA I + Q+K DEV+ GEIPARCIQ YR DL +
+TNG+
Sbjct 541
HFEIYAPQEYLSRAYHDAPRYCADIVSTQIKNDEVILKGEIPARCIQEYRNDLTYFTNGQ 600

Query 601 SVCLTELKGYQAAVGQPVIQPRRPNSRLDKVRHMFQKV 638
             VCLTELKGYQ A+G+ + QPRRPNSR+DKVRHMF K+

Sbjct 601 GVCLTELKGYQPAIGKFICQPRRPNSRIDKVRHMFHKL 638

>WP_044681968.1 TetM/TetW/TetO/TetS family tetracycline resistance ribosomal
protection protein [Streptococcus suis]
 CYW93661.1 tetracycline resistance protein tetM [Streptococcus suis]
 CYW46889.1 tetracycline resistance protein tetM [Streptococcus suis]
Length=639

 Score = 929 bits (2400), Expect = 0.0, Method: Compositional matrix adjust.
 Identities = 430/638 (67%), Positives = 530/638 (83%), Gaps = 0/638 (0%)

```
Query  1
MKIINIGILAHVDAGKTTLTESLLYASGAISEPGSVEKGTTRTDTMFLERQRGITIQAAV  60
            MKIIN+GILAHVDAGKTTLTESLLY SGAI+EPGSV+KGTTRTDTM
LERQRGITIQ AV
Sbjct  1
MKIINLGILAHVDAGKTTLTESLLYTSGAIAEPGSVDKGTTRTDTMNLERQRGITIQTAV  60

Query  61
TSFQWHRCKVNIVDTPGHMDFLAEVYRSLAVLDGAILVISAKDGVQAQTRILFHALRKMN  120
            TSFQW   KVNI+DTPGHMDFLAEVYRSL+VLDGA+L++SAKDG+QAQTRILFHAL
+ M
Sbjct  61
TSFQWEDVKVNIIDTPGHMDFLAEVYRSLSVLDGAVLLVSAKDGIQAQTRILFHALQTMK  120

Query  121
IPTVIFINKIDQAGVDLQSVVQSVRDKLSADIIIKQTVSLSPEIVLEENTDIEAWDAVIE  180
            IPT+ FINKIDQ G+DL  V + ++ KLS++II+KQ V   P I + +N D+E
WDAVI
Sbjct  121
IPTIFFINKIDQEGIDLPMVYREMKAKLSSEIIVKQKVGQHPHINVTDNDDMEQWDAVIM  180

Query  181
NNDKLLEKYIAGEPISREKLVREEQRRVQDASLFPVYYGSAKKGLGIQPLMDAVTGLFQP  240
             ND+LLEKY++G+P    +L +EE RR Q+ +LFPVY+GSAK  LGI+ L++ +
F
Sbjct  181
GNDELLEKYMSGKPFKMSELEQEENRRFQNGTLFPVYHGSAKNNLGIRQLIEVIASKFYS  240

Query  241
IGEQGSAALCGSVFKVEYTDCGQRRVYLRLYSGTLRLRDTVALAGREKLKITEMRIPSKG  300
             +G + LCG VFK+EY++  +R VY+R+YSGTL LRD + ++ +EK+KITEM
+P+ G
Sbjct  241
STPEGQSELCGQVFKIEYSEKRRRFVYVRIYSGTLHLRDVIRISEKEKIKITEMYVPTNG  300

Query  301
EIVRTDTAYPGEIVILPSDSVRLNDVLGDPTRLPRKRWREDPLPMLRTTIAPKTAAQRER  360
```

E+ +DTA G+IVILP+D ++LN +LG+ LP++++ E+PLPML+TTIA K +
QRE
Sbjct 301
ELYSSDTACSGDIVILPNDVLQLNSILGNEILLPQRKFIENPLPMLQTTIAVKKSEQREI 360

Query 361
LLDALTQLADTDPLLRCEVDSITHEIILSFLGRVQLEVVSALLSEKYKLETVVKEPSVIY 420
LL ALT+++D DPLL+ VD+ THEIILSFLG VQ+EV+ A+L EKY +E +KEP
+VIY
Sbjct 361
LLGALTEISDGDPLLKYYVDTTTHEIILSFLGNVQMEVICAILEEKYHVEAEIKEPTVIY 420

Query 421
MERPLKAASHTIHIEVPPNPFWASIGLSVTPLSLGSGVQYESRVSLGYLNQSFQNAVRDG 480
MERPL+ A +TIHIEVPPNPFWAS+GLS+ PL
+GSGVQYESRVSLGYLNQSFQNAV +G
Sbjct 421
MERPLRKAEYTIHIEVPPNPFWASVGLSIEPLPIGSGVQYESRVSLGYLNQSFQNAVMEG 480

Query 481
IRYGLEQGLFGWNVTDCKICFEYGLYYSPVSTPADFRSLAPIVLEQALKESGTQLLEPYL 540
+ YG EQGL+GW VTDCKICFEYGLYYSPVSTPADFR L+PIVLEQALK++GT
+LLEPYL
Sbjct 481
VLYGCEQGLYGWKVTDCKICFEYGLYYSPVSTPADFRLLSPIVLEQALKKAGTELLEPYL 540

Query 541
SFILYAPQEYLSRAYHDAPKYCATIETAQVKKDEVVFTGEIPARCIQAYRTDLAFYTNGR 600
F +YAPQEYLSRAYHDAP+YCA I + Q+K DEV+ GEIPARCIQ YR DL +
+TNG+
Sbjct 541
HFEIYAPQEYLSRAYHDAPRYCADIVSTQIKNDEVILKGEIPARCIQEYRNDLTYFTNGQ 600

Query 601 SVCLTELKGYQAAVGQPVIQPRRPNSRLDKVRHMFQKV 638
VCLTELKGYQ A+G+ + QPRRPNSR+DKVRHMF K+
Sbjct 601 GVCLTELKGYQPAIGKFICQPRRPNSRIDKVRHMFHKL 638

>WP_031912846.1 MULTISPECIES: TetM/TetW/TetO/TetS family
tetracycline resistance
ribosomal protection protein [Bacilli]
EZS75590.1 tetracycline resistance protein tetO [Staphylococcus
aureus VET0184R]
EZS77234.1 tetracycline resistance protein tetO [Staphylococcus
aureus VET0183R]
EZT15778.1 tetracycline resistance protein tetO [Staphylococcus
aureus VET0078R]
KAA35719.1 tetracycline resistance protein tetO [Staphylococcus
aureus VET0077R]
KAD04820.1 tetracycline resistance protein tetO [Staphylococcus
aureus VET0283R]
KAD12991.1 tetracycline resistance protein tetO [Staphylococcus
aureus VET0287R]
KAE74568.1 tetracycline resistance protein tetO [Staphylococcus

```
aureus VET0396R]
Length=639

 Score = 929 bits (2400),  Expect = 0.0, Method: Compositional
matrix adjust.
 Identities = 431/638 (68%), Positives = 529/638 (83%), Gaps = 0/638
(0%)

Query  1
MKIINIGILAHVDAGKTTLTESLLYASGAISEPGSVEKGTTRTDTMFLERQRGITIQAAV  60
            MKIIN+GILAHVDAGKTTLTESLLY SGAI+EPGSV+KGTTRTDTM
LERQRGITIQ AV
Sbjct  1
MKIINLGILAHVDAGKTTLTESLLYTSGAIAEPGSVDKGTTRTDTMNLERQRGITIQTAV  60

Query  61
TSFQWHRCKVNIVDTPGHMDFLAEVYRSLAVLDGAILVISAKDGVQAQTRILFHALRKMN  120
            TSFQW   KVNI+DTPGHMDFLAEVYRSL+VLDGA+L++SAKDG+QAQTRILFHAL
+ M
Sbjct  61
TSFQWEDVKVNIIDTPGHMDFLAEVYRSLSVLDGAVLLVSAKDGIQAQTRILFHALQTMK  120

Query  121
IPTVIFINKIDQAGVDLQSVVQSVRDKLSADIIIKQTVSLSPEIVLEENTDIEAWDAVIE  180
            IPT+ FINKIDQ G+DL  V Q ++ KLS++II+KQ V   P I + +N D+E
WDAVI
Sbjct  121
IPTIFFINKIDQEGIDLPMVYQEMKAKLSSEIIVKQKVGQHPHINVTDNDDMEQWDAVIM  180

Query  181
NNDKLLEKYIAGEPISREKLVREEQRRVQDASLFPVYYGSAKKGLGIQPLMDAVTGLFQP  240
             ND+LLEKY++G+P    +L +EE RR Q+ +LFPVY+GSAK  LGI+ L++ +
F
Sbjct  181
GNDELLEKYMSGKPFKMSELEQEENRRFQNGTLFPVYHGSAKNNLGIRQLIEVIASKFYS  240

Query  241
IGEQGSAALCGSVFKVEYTDCGQRRVYLRLYSGTLRLRDTVALAGREKLKITEMRIPSKG  300
               +G + LCG VFK+EY++  +R VY+R+YSGTL LRD + ++ +EK+KITEM
+P+ G
Sbjct  241
STPEGQSELCGQVFKIEYSEKRRRFVYVRIYSGTLHLRDVIKISEKEKIKITEMCVPTNG  300

Query  301
EIVRTDTAYPGEIVILPSDSVRLNDVLGDPTRLPRKRWREDPLPMLRTTIAPKTAAQRER  360
            E+  +DTA  G+IVILP+D ++LN +LG+   LP++++ E+PLPML+TTIA K +
QRE
Sbjct  301
ELYSSDTACSGDIVILPNDVLQLNSILGNEMLLPQRKFIENPLPMLQTTIAVKKSEQREI  360

Query  361
LLDALTQLADTDPLLRCEVDSITHEIILSFLGRVQLEVVSALLSEKYKLETVVKEPSVIY  420
            LL ALT+++D DPLL+  VD+ THEIILSFLG VQ+EV+ A+L EKY +E  +KEP
+VIY
```

```
Sbjct  361
LLGALTEISDGDPLLKYYVDTTTHEIILSFLGNVQMEVICAILEEKYHVEAEIKEPTVIY  420

Query  421
MERPLKAASHTIHIEVPPNPFWASIGLSVTPLSLGSGVQYESRVSLGYLNQSFQNAVRDG  480
            MERPL+ A +TIHIEVPPNPFWAS+GLS+ PL
+GSGVQYESRVSLGYLNQSFQNAV +G
Sbjct  421
MERPLRKAEYTIHIEVPPNPFWASVGLSIEPLPIGSGVQYESRVSLGYLNQSFQNAVMEG  480

Query  481
IRYGLEQGLFGWNVTDCKICFEYGLYYSPVSTPADFRSLAPIVLEQALKESGTQLLEPYL  540
            + YG EQGL+GW VTDCKICFEYGLYYSPVSTPADFR L+PIVLEQALK++GT
+LLEPYL
Sbjct  481
VLYGCEQGLYGWKVTDCKICFEYGLYYSPVSTPADFRLLSPIVLEQALKKAGTELLEPYL  540

Query  541
SFILYAPQEYLSRAYHDAPKYCATIETAQVKKDEVVFTGEIPARCIQAYRTDLAFYTNGR  600
             F +YAPQEYLSRAYHDAP+YCA I + Q+K DEV+  GEIPARCIQ YR DL
+TNG+
Sbjct  541
HFEIYAPQEYLSRAYHDAPRYCADIVSTQIKNDEVILKGEIPARCIQEYRNDLTNFTNGQ  600

Query  601  SVCLTELKGYQAAVGQPVIQPRRPNSRLDKVRHMFQKV  638
             VCLTELKGYQ A+G+ + QPRRPNSR+DKVRHMF K+
Sbjct  601  GVCLTELKGYQPAIGKFICQPRRPNSRIDKVRHMFHKL  638


>WP_009300547.1 MULTISPECIES: TetM/TetW/TetO/TetS family
tetracycline resistance
ribosomal protection protein [Firmicutes]
 EHM90339.1 small GTP-binding protein domain [Coprobacillus sp.
3_3_56FAA]
 OMK36213.1 hypothetical protein BER34_003072 [Clostridioides
difficile]
 OMK63657.1 hypothetical protein BER35_000531 [Clostridioides
difficile]
 SJP39193.1 Tetracycline resistance protein tetM from transposon
Tn916 [Clostridioides
difficile]
 SJV27559.1 small GTP-binding protein domain [Clostridioides
difficile]
 PCD10102.1 tetracycline resistance protein tetM [Clostridioides
difficile]
 PCN55619.1 tetracycline resistance protein tetM [Clostridioides
difficile]
 SHO34401.1 tetracycline resistance ribosomal protection protein
478510:480432
forward MW:72969 [Clostridioides difficile M120]
Length=640

 Score = 929 bits (2400),  Expect = 0.0, Method: Compositional
matrix adjust.
```

Identities = 431/637 (68%), Positives = 531/637 (83%), Gaps = 0/637 (0%)

```
Query  1
MKIINIGILAHVDAGKTTLTESLLYASGAISEPGSVEKGTTRTDTMFLERQRGITIQAAV  60
            MKIINIGILAHVDAGKTTLTESLLY SGAI E GSV
+KGTTRTDTMFLERQRGITIQAAV
Sbjct  1
MKIINIGILAHVDAGKTTLTESLLYTSGAILELGSVDKGTTRTDTMFLERQRGITIQAAV  60

Query  61
TSFQWHRCKVNIVDTPGHMDFLAEVYRSLAVLDGAILVISAKDGVQAQTRILFHALRKMN  120
            TSF W+  K+NIVDTPGH DF+ EVYRSL+VLDGAILVISAKDGVQAQTRILFHAL
+KMN
Sbjct  61
TSFNWNDYKINIVDTPGHTDFITEVYRSLSVLDGAILVISAKDGVQAQTRILFHALQKMN  120

Query  121
IPTVIFINKIDQAGVDLQSVVQSVRDKLSADIIIKQTVSLSPEIVLEENTDIEAWDAVIE  180
            IPT+IFINKIDQ G++L ++ Q++++KLS DII+ Q V+L+PEI ++   D++ WD
VI
Sbjct  121
IPTIIFINKIDQYGINLNNIYQNIKEKLSNDIIVMQNVTLTPEISIKNIIDLDEWDPVIS  180

Query  181
NNDKLLEKYIAGEPISREKLVREEQRRVQDASLFPVYYGSAKKGLGIQPLMDAVTGLFQP  240
             NDKLL+KYIAGE ++ ++L +EE R V+  SLFP+Y+GSAK  +G Q L++A++
LF P
Sbjct  181
KNDKLLKKYIAGEKLTIQELTQEEYRCVKKGSLFPIYHGSAKNNIGTQQLIEAISNLFCP  240

Query  241
IGEQGSAALCGSVFKVEYTDCGQRRVYLRLYSGTLRLRDTVALAGREKLKITEMRIPSKG  300
               +  + LCG VFK+EYTD  QR VYLRLYSGTL LRDT+ L+ ++K+K+TE+
IPS G
Sbjct  241
EMNKNYSELCGRVFKIEYTDHKQRLVYLRLYSGTLHLRDTIVLSEKKKVKLTEIYIPSNG  300

Query  301
EIVRTDTAYPGEIVILPSDSVRLNDVLGDPTRLPRKRWREDPLPMLRTTIAPKTAAQRER  360
            E+++T+    G+I I+P++++RLND++G+   LP   W + P+PMLRT I P
+RE+
Sbjct  301
EMIQTEIVCSGDIFIIPNNTLRLNDIIGNEKILPCNVWNDKPVPMLRTRIEPIKIEEREK  360

Query  361
LLDALTQLADTDPLLRCEVDSITHEIILSFLGRVQLEVVSALLSEKYKLETVVKEPSVIY  420
            LLDALT++ADTDPLLR  VD+ITHEI++SFLG VQLEV+ +LL EKY +   +++P
+VIY
Sbjct  361
LLDALTEIADTDPLLRYCVDTITHEIVISFLGTVQLEVICSLLIEKYHINIRIEDPTVIY  420

Query  421
MERPLKAASHTIHIEVPPNPFWASIGLSVTPLSLGSGVQYESRVSLGYLNQSFQNAVRDG  480
```

+E+PL+ A +TIHIEVPPNPFWASIGLS+TPL +GSG+QYES
+VSLGYLNQSFQNAVR+G
Sbjct 421
LEKPLQKADYTIHIEVPPNPFWASIGLSITPLPIGSGIQYESKVSLGYLNQSFQNAVREG 480

Query 481
IRYGLEQGLFGWNVTDCKICFEYGLYYSPVSTPADFRSLAPIVLEQALKESGTQLLEPYL 540
I YGLEQGL+GW VTDCKICFEYG+YYSPVSTP+DFR LAPIVLEQ LK+
+GTQLLEPYL
Sbjct 481
INYGLEQGLYGWEVTDCKICFEYGVYYSPVSTPSDFRFLAPIVLEQTLKKAGTQLLEPYL 540

Query 541
SFILYAPQEYLSRAYHDAPKYCATIETAQVKKDEVVFTGEIPARCIQAYRTDLAFYTNGR 600
SFIL+ PQ YLSRAY+DA K+CA IET+Q K DE++FTG IP RCI YR L
YTNG+
Sbjct 541
SFILFTPQGYLSRAYNDAQKHCAIIETSQSKNDEIIFTGHIPVRCINEYRNTLTLYTNGQ 600

Query 601 SVCLTELKGYQAAVGQPVIQPRRPNSRLDKVRHMFQK 637
+V LTELK YQ A +PVIQ RRPN+R+DKVRHMF K
Sbjct 601 AVFLTELKDYQIATCEPVIQSRRPNNRIDKVRHMFNK 637

>WP_100664818.1 TetM/TetW/TetO/TetS family tetracycline resistance
ribosomal
protection protein [Streptococcus suis]
ANJ64422.1 tetracycline resistance protein TetO [Streptococcus
suis]
ATZ03648.1 tetracycline resistance ribosomal protection protein
[Streptococcus
suis]
Length=639

Score = 928 bits (2399), Expect = 0.0, Method: Compositional
matrix adjust.
Identities = 432/638 (68%), Positives = 529/638 (83%), Gaps = 0/638
(0%)

Query 1
MKIINIGILAHVDAGKTTLTESLLYASGAISEPGSVEKGTTRTDTMFLERQRGITIQAAV 60
MKIIN+GILAHVDAGKTTLTESLLY SGAI+EPGSV+KGTTRTDTM
LERQRGITIQ AV
Sbjct 1
MKIINLGILAHVDAGKTTLTESLLYTSGAIAEPGSVDKGTTRTDTMNLERQRGITIQTAV 60

Query 61
TSFQWHRCKVNIVDTPGHMDFLAEVYRSLAVLDGAILVISAKDGVQAQTRILFHALRKMN 120
TSFQW   KVNI+DTPGHMDFLAEVYRSL+VLDGA+L++SAKDG+QAQTRILFHAL
+ M
Sbjct 61
TSFQWEDVKVNIIDTPGHMDFLAEVYRSLSVLDGAVLLVSAKDGIQAQTRILFHALQTMK 120

Query 121

IPTVIFINKIDQAGVDLQSVVQSVRDKLSADIIIKQTVSLSPEIVLEENTDIEAWDAVIE 180
            IPT+ FINKIDQ G+DL V Q ++ KLS++II+KQ V   P I + +N D+E
WDAVI
Sbjct 121
IPTIFFINKIDQEGIDLPMVYQEMKAKLSSEIIVKQKVGQHPHINVTDNDDMEQWDAVIM 180

Query 181
NNDKLLEKYIAGEPISREKLVREEQRRVQDASLFPVVYGSAKKGLGIQPLMDAVTGLFQP 240
             ND+LLEKY++G+P   +L +EE RR Q+ +LFPVY+GSAK LGI+ L++ +
F
Sbjct 181
GNDELLEKYMSGKPFKMSELEQEENRRFQNGTLFPVYHGSAKNNLGIRQLIEVIASKFYS 240

Query 241
IGEQGSAALCGSVFKVEYTDCGQRRVYLRLYSGTLRLRDTVALAGREKLKITEMRIPSKG 300
               +G + LCG VFK+EY++ +R VY+R+YSGTL LRD + ++ +EK+KITEM
+P+ G
Sbjct 241
STPEGQSELCGQVFKIEYSEKRRRFVYVRIYSGTLHLRDVIRISEKEKIKITEMCVPTNG 300

Query 301
EIVRTDTAYPGEIVILPSDSVRLNDVLGDPTRLPRKRWREDPLPMLRTTIAPKTAAQRER 360
            E+ +DTA G+IVILP+D ++LN +LG+   LP++++ E+PLPML+TTIA K
QRE
Sbjct 301
ELYPSDTACSGDIVILPNDVLQLNSILGNEMLLPQRKFIENPLPMLQTTIAVKKPEQREI 360

Query 361
LLDALTQLADTDPLLRCEVDSITHEIILSFLGRVQLEVVSALLSEKYKLETVVKEPSVIY 420
            LL ALT+++D DPLL+ VD+ THEIILSFLG VQ+EV+ A+L EKY +E +KEP
+VIY
Sbjct 361
LLGALTEISDGDPLLKYYVDTTTHEIILSFLGNVQMEVICAILEEKYHVEAEIKEPTVIY 420

Query 421
MERPLKAASHTIHIEVPPNPFWASIGLSVTPLSLGSGVQYESRVSLGYLNQSFQNAVRDG 480
            MERPL+ A +TIHIEVPPNPFWAS+GLS+ PL
+GSGVQYESRVSLGYLNQSFQNAV +G
Sbjct 421
MERPLRKAEYTIHIEVPPNPFWASVGLSIEPLPIGSGVQYESRVSLGYLNQSFQNAVMEG 480

Query 481
IRYGLEQGLFGWNVTDCKICFEYGLYYSPVSTPADFRSLAPIVLEQALKESGTQLLEPYL 540
            + YG EQGL+GW VTDCKICFEYGLYYSPVSTPADFR L+PIVLEQALK++GT
+LLEPYL
Sbjct 481
VLYGCEQGLYGWKVTDCKICFEYGLYYSPVSTPADFRLLSPIVLEQALKKAGTELLEPYL 540

Query 541
SFILYAPQEYLSRAYHDAPKYCATIETAQVKKDEVVFTGEIPARCIQAYRTDLAFYTNGR 600
             F +YAPQEYLSRAYHDAP+YCA I + QVK DEV+ GEIPARCIQ YR DL +
+TNG+
Sbjct 541
HFEIYAPQEYLSRAYHDAPRYCADIVSTQVKNDEVILKGEIPARCIQEYRNDLTYFTNGQ 600

```
Query  601  SVCLTELKGYQAAVGQPVIQPRRPNSRLDKVRHMFQKV  638
             VCLTELKGYQ A+G+ + QPRRPNSR+DKVRHMF K+
Sbjct  601  GVCLTELKGYQPAIGKFICQPRRPNSRIDKVRHMFHKL  638


>WP_074392924.1 TetM/TetW/TetO/TetS family tetracycline resistance 
ribosomal 
protection protein [Streptococcus suis]
Length=639

 Score = 928 bits (2399),  Expect = 0.0, Method: Compositional 
matrix adjust.
 Identities = 431/638 (68%), Positives = 529/638 (83%), Gaps = 0/638 
(0%)

Query  1    
MKIINIGILAHVDAGKTTLTESLLYASGAISEPGSVEKGTTRTDTMFLERQRGITIQAAV  60
            MKIIN+GILAHVDAGKTTLTESLLY SGAI+EPGSV+KGTTRTDTM 
LERQRGITIQ AV
Sbjct  1    
MKIINLGILAHVDAGKTTLTESLLYTSGAIAEPGSVDKGTTRTDTMNLERQRGITIQTAV  60

Query  61   
TSFQWHRCKVNIVDTPGHMDFLAEVYRSLAVLDGAILVISAKDGVQAQTRILFHALRKMN  120
            TSFQW   KVNI+DTPGHMDFLAEVYRSL+VLDGA+L++SAKDG+QAQTRILFHAL 
+ M
Sbjct  61   
TSFQWEDVKVNIIDTPGHMDFLAEVYRSLSVLDGAVLLVSAKDGIQAQTRILFHALQTMK  120

Query  121  
IPTVIFINKIDQAGVDLQSVVQSVRDKLSADIIIKQTVSLSPEIVLEENTDIEAWDAVIE  180
            IPT+ FINKIDQ G+DL  V Q ++ KLS++II+KQ V     I + +N D+E 
WDAVI
Sbjct  121  
IPTIFFINKIDQEGIDLPMVYQEMKAKLSSEIIVKQKVGQHSHINVTDNDDMEQWDAVIM  180

Query  181  
NNDKLLEKYIAGEPISREKLVREEQRRVQDASLFPVYYGSAKKGLGIQPLMDAVTGLFQP  240
             ND+LLEKY++G+P    +L +EE RR Q+ +LFPVY+GSAK  LGI+ L++ + 
F
Sbjct  181  
GNDELLEKYMSGKPFKMSELEQEENRRFQNGTLFPVYHGSAKNNLGIRQLIEVIASKFYS  240

Query  241  
IGEQGSAALCGSVFKVEYTDCGQRRVYLRLYSGTLRLRDTVALAGREKLKITEMRIPSKG  300
               +G + LCG VFK+EY++  +R VY+R+YSGTL LRD + ++ +EK+KITEM 
+P+ G
Sbjct  241  
STPEGQSELCGQVFKIEYSEKRRRFVYVRIYSGTLHLRDVIKISEKEKIKITEMCVPTNG  300

Query  301  
EIVRTDTAYPGEIVILPSDSVRLNDVLGDPTRLPRKRWREDPLPMLRTTIAPKTAAQRER  360
            E+  +DTA  G+IVILP+D ++LN +LG+   LP++++ E+PLPML+TTIA K +
```

QRE
Sbjct 301
ELYSSDTACSGDIVILPNDVLQLNSILGNEMLLPQRKFIENPLPMLQTTIAVKKSEQREI 360

Query 361
LLDALTQLADTDPLLRCEVDSITHEIILSFLGRVQLEVVSALLSEKYKLETVVKEPSVIY 420
            LL ALT+++D DPLL+ VD+ THEIILSFLG VQ+EV+ A+L EKY +E +KEP
+VIY
Sbjct 361
LLGALTEISDGDPLLKYYVDTTTHEIILSFLGNVQMEVICAILEEKYHVEAEIKEPTVIY 420

Query 421
MERPLKAASHTIHIEVPPNPFWASIGLSVTPLSLGSGVQYESRVSLGYLNQSFQNAVRDG 480
            MERPL+ A +TIHIEVPPNPFWAS+GLS+ PL
+GSGVQYESRVSLGYLNQSFQNAV +G
Sbjct 421
MERPLRKAEYTIHIEVPPNPFWASVGLSIEPLPIGSGVQYESRVSLGYLNQSFQNAVMEG 480

Query 481
IRYGLEQGLFGWNVTDCKICFEYGLYYSPVSTPADFRSLAPIVLEQALKESGTQLLEPYL 540
            + YG EQGL+GW VTDCKICFEYGLYYSPVSTPADFR L+PIVLEQALK++GT
+LLEPYL
Sbjct 481
VLYGCEQGLYGWKVTDCKICFEYGLYYSPVSTPADFRLLSPIVLEQALKKAGTELLEPYL 540

Query 541
SFILYAPQEYLSRAYHDAPKYCATIETAQVKKDEVVFTGEIPARCIQAYRTDLAFYTNGR 600
             F +YAPQEYLSRAYHDAP+YCA I + QVK DEV+ GEIPARCIQ YR DL +
+TNG+
Sbjct 541
HFEIYAPQEYLSRAYHDAPRYCADIVSTQVKNDEVILKGEIPARCIQEYRNDLTYFTNGQ 600

Query 601 SVCLTELKGYQAAVGQPVIQPRRPNSRLDKVRHMFQKV 638
             VCLTELKGYQ A+G+ + QPRRPNSR+DKVRHMF K+
Sbjct 601 GVCLTELKGYQPAIGKFICQPRRPNSRIDKVRHMFHKL 638

>WP_074392551.1 TetM/TetW/TetO/TetS family tetracycline resistance ribosomal
protection protein [Streptococcus suis]
Length=644

Score = 928 bits (2399), Expect = 0.0, Method: Compositional matrix adjust.
Identities = 431/637 (68%), Positives = 529/637 (83%), Gaps = 0/637 (0%)

Query 2
KIINIGILAHVDAGKTTLTESLLYASGAISEPGSVEKGTTRTDTMFLERQRGITIQAAVT 61
            KIIN+GILAHVDAGKTTLTESLLY SGAI+EPGSV+KGTTRTDTM LERQRGITIQ
AVT
Sbjct 7
KIINLGILAHVDAGKTTLTESLLYTSGAIAEPGSVDKGTTRTDTMNLERQRGITIQTAVT 66

Query 62
SFQWHRCKVNIVDTPGHMDFLAEVYRSLAVLDGAILVISAKDGVQAQTRILFHALRKMNI 121
SFQW KVNI+DTPGHMDFLAEVYRSL+VLDGA+L++SAKDG+QAQTRILFHAL+
M I
Sbjct 67
SFQWEDVKVNIIDTPGHMDFLAEVYRSLSVLDGAVLLVSAKDGIQAQTRILFHALQTMKI 126

Query 122
PTVIFINKIDQAGVDLQSVVQSVRDKLSADIIIKQTVSLSPEIVLEENTDIEAWDAVIEN 181
PT+ FINKIDQ G+DL V Q ++ KLS++II+KQ V P I + +N D+E
WDAVI
Sbjct 127
PTIFFINKIDQEGIDLPMVYQEMKAKLSSEIIVKQKVGQHPHINVTDNDDMEQWDAVIMG 186

Query 182
NDKLLEKYIAGEPISREKLVREEQRRVQDASLFPVYYGSAKKGLGIQPLMDAVTGLFQPI 241
ND+LLEKY++G+P +L +EE RR Q+ +LFPVY+GSAK LGI+ L++ +
F
Sbjct 187
NDELLEKYMSGKPFKMSELEQEENRRFQNGTLFPVYHGSAKNNLGIRQLIEVIASKFYSS 246

Query 242
GEQGSAALCGSVFKVEYTDCGQRRVYLRLYSGTLRLRDTVALAGREKLKITEMRIPSKGE 301
+G + LCG VFK+EY++ +R VY+R+YSGTL LRD + ++ +EK+KITEM +P
+ GE
Sbjct 247
TPEGQSELCGQVFKIEYSEKRRRFVYVRIYSGTLHLRDVIKISEKEKIKITEMCVPTNGE 306

Query 302
IVRTDTAYPGEIVILPSDSVRLNDVLGDPTRLPRKRWREDPLPMLRTTIAPKTAAQRERL 361
+ +DTA G+IVILP+D ++LN +LG+ LP++++ E+PLPML+TTIA K +
QRE L
Sbjct 307
LYSSDTACSGDIVILPNDVLQLNSILGNEMLLPQRKFIENPLPMLQTTIAVKKSEQREIL 366

Query 362
LDALTQLADTDPLLRCEVDSITHEIILSFLGRVQLEVVSALLSEKYKLETVVKEPSVIYM 421
L ALT+++D DPLL+ VD+ THEIILSFLG VQ+EV+ A+L EKY +E +KEP
+VIYM
Sbjct 367
LGALTEISDGDPLLKYYVDTTTHEIILSFLGNVQMEVICAILEEKYHVEAEIKEPTVIYM 426

Query 422
ERPLKAASHTIHIEVPPNPFWASIGLSVTPLSLGSGVQYESRVSLGYLNQSFQNAVRDGI 481
ERPL+ A +TIHIEVPPNPFWAS+GLS+ PL +GSGVQYESRVSLGYLNQSFQNAV
+G+
Sbjct 427
ERPLRKAEYTIHIEVPPNPFWASVGLSIEPLPIGSGVQYESRVSLGYLNQSFQNAVMEGV 486

Query 482
RYGLEQGLFGWNVTDCKICFEYGLYYSPVSTPADFRSLAPIVLEQALKESGTQLLEPYLS 541
YG EQGL+GW VTDCKICFEYGLYYSPVSTPADFR L+PIVLEQALK++GT
+LLEPYL
Sbjct 487

```
LYGCEQGLYGWKVTDCKICFEYGLYYSPVSTPADFRLLSPIVLEQALKKAGTELLEPYLH  546

Query  542  
FILYAPQEYLSRAYHDAPKYCATIETAQVKKDEVVFTGEIPARCIQAYRTDLAFYTNGRS  601
            F +YAPQEYLSRAYHDAP+YCA I + QVK DEV+  GEIPARCIQ YR DL + 
+TNG+
Sbjct  547  
FEIYAPQEYLSRAYHDAPRYCADIVSTQVKNDEVILKGEIPARCIQEYRNDLTYFTNGQG  606

Query  602  VCLTELKGYQAAVGQPVIQPRRPNSRLDKVRHMFQKV  638
            VCLTELKGYQ A+G+ + QPRRPNSR+DKVRHMF K+
Sbjct  607  VCLTELKGYQPAIGKFICQPRRPNSRIDKVRHMFHKL  643


>AMP52239.1 TetM-TetW-TetO-TetS [uncultured bacterium]
Length=639

 Score = 928 bits (2399),  Expect = 0.0, Method: Compositional 
matrix adjust.
 Identities = 430/638 (67%), Positives = 530/638 (83%), Gaps = 0/638 
(0%)

Query  1    
MKIINIGILAHVDAGKTTLTESLLYASGAISEPGSVEKGTTRTDTMFLERQRGITIQAAV  60
            MKIIN+GILAHVDAGKTTLTESLLY SGAI+EPGSV+KGTTRTDTM 
LERQRGITIQ AV
Sbjct  1    
MKIINLGILAHVDAGKTTLTESLLYTSGAIAEPGSVDKGTTRTDTMNLERQRGITIQTAV  60

Query  61   
TSFQWHRCKVNIVDTPGHMDFLAEVYRSLAVLDGAILVISAKDGVQAQTRILFHALRKMN  120
            TSFQW   KVNI+DTPGHMDFLAEVYRSL+VLDGA+L++SAKDG+QAQTRILFHAL
+ M
Sbjct  61   
TSFQWEDVKVNIIDTPGHMDFLAEVYRSLSVLDGAVLLVSAKDGIQAQTRILFHALQIMK  120

Query  121  
IPTVIFINKIDQAGVDLQSVVQSVRDKLSADIIIKQTVSLSPEIVLEENTDIEAWDAVIE  180
            IPT+ FINKIDQ G+DL  V + ++ KLS++II+KQ V   P I + +N D+E 
WDAVI
Sbjct  121  
IPTIFFINKIDQEGIDLPMVYREMKAKLSSEIIVKQKVGQHPHINVTDNDDMEQWDAVIM  180

Query  181  
NNDKLLEKYIAGEPISREKLVREEQRRVQDASLFPVYYGSAKKGLGIQPLMDAVTGLFQP  240
             ND+LLEKY++G+P    +L +EE RR Q+ +LFPVY+GSAK  LGI+ L++ + 
F
Sbjct  181  
GNDELLEKYMSGKPFKMSELEQEENRRFQNGTLFPVYHGSAKNNLGIRQLIEVIASKFYS  240

Query  241  
IGEQGSAALCGSVFKVEYTDCGQRRVYLRLYSGTLRLRDTVALAGREKLKITEMRIPSKG  300
               +G + LCG VFK+EY++  +R VY+R+YSGTL LRD + ++ +EK+KITEM 
+P+ G
```

Sbjct 241
STPEGQSELCGQVFKIEYSEKRRRFVYVRIYSGTLHLRDVIKISEKEKIKITEMCVPTNG 300

Query 301
EIVRTDTAYPGEIVILPSDSVRLNDVLGDPTRLPRKRWREDPLPMLRTTIAPKTAAQRER 360
            E+ +DTA G+IVILP+D ++LN +LG+   LP++++ E+PLPML+TTIA K +
QRE
Sbjct 301
ELYSSDTACSGDIVILPNDVLQLNSILGNEMLLPQRKFIENPLPMLQTTIAVKKSEQREI 360

Query 361
LLDALTQLADTDPLLRCEVDSITHEIILSFLGRVQLEVVSALLSEKYKLETVVKEPSVIY 420
            LL ALT+++D DPLL+ VD+ THEIILSFLG VQ+EV+ A+L EKY +E +KEP
+VIY
Sbjct 361
LLGALTEISDGDPLLKYYVDTTTHEIILSFLGNVQMEVICAILEEKYHVEAEIKEPTVIY 420

Query 421
MERPLKAASHTIHIEVPPNPFWASIGLSVTPLSLGSGVQYESRVSLGYLNQSFQNAVRDG 480
            MERPL+ A +TIHIEVPPNPFWAS+GLS+ PL
+GSGVQYESRVSLGYLNQSFQNAV +G
Sbjct 421
MERPLRKAEYTIHIEVPPNPFWASVGLSIEPLPIGSGVQYESRVSLGYLNQSFQNAVMEG 480

Query 481
IRYGLEQGLFGWNVTDCKICFEYGLYYSPVSTPADFRSLAPIVLEQALKESGTQLLEPYL 540
            + YG EQGL+GW VTDCKICFEYGLYYSPVSTPADFR L+PIVLEQALK++GT
+LLEPYL
Sbjct 481
VLYGCEQGLYGWKVTDCKICFEYGLYYSPVSTPADFRLLSPIVLEQALKKAGTELLEPYL 540

Query 541
SFILYAPQEYLSRAYHDAPKYCATIETAQVKKDEVVFTGEIPARCIQAYRTDLAFYTNGR 600
             F +YAPQEYLSRAYHDAP+YCA I + Q+K DEV+ GEIPARCIQ YR DL +
+TNG+
Sbjct 541
HFEIYAPQEYLSRAYHDAPRYCADIVSTQIKNDEVILKGEIPARCIQEYRNDLTYFTNGQ 600

Query 601 SVCLTELKGYQAAVGQPVIQPRRPNSRLDKVRHMFQKV 638
             VCLTELKGYQ A+G+ + QPRRPNSR+DKVRHMF K+
Sbjct 601 GVCLTELKGYQPAIGKFICQPRRPNSRIDKVRHMFHKL 638

>WP_029180377.1 TetM/TetW/TetO/TetS family tetracycline resistance
ribosomal
protection protein [Streptococcus suis]
Length=639

 Score = 928 bits (2399), Expect = 0.0, Method: Compositional
matrix adjust.
 Identities = 429/638 (67%), Positives = 531/638 (83%), Gaps = 0/638
(0%)

Query 1

MKIINIGILAHVDAGKTTLTESLLYASGAISEPGSVEKGTTRTDTMFLERQRGITIQAAV 60
            MKIIN+GILAHVDAGKTTLTESLLY SGAI+EPGSV+KGTTRTDTM
LERQRGITIQ AV
Sbjct 1
MKIINLGILAHVDAGKTTLTESLLYTSGAIAEPGSVDKGTTRTDTMNLERQRGITIQTAV 60

Query 61
TSFQWHRCKVNIVDTPGHMDFLAEVYRSLAVLDGAILVISAKDGVQAQTRILFHALRKMN 120
            TSFQW   KVNI+DTPGHMDFLAEVYRSL+VLDGA+L++SAKDG+QAQTRILFHAL
+ M
Sbjct 61
TSFQWEDVKVNIIDTPGHMDFLAEVYRSLSVLDGAVLLVSAKDGIQAQTRILFHALQTMK 120

Query 121
IPTVIFINKIDQAGVDLQSVVQSVRDKLSADIIIKQTVSLSPEIVLEENTDIEAWDAVIE 180
            IPT+ FINKIDQ G+DL V + ++ KLS++II+KQ V   P I + +N D+E
WDAVI
Sbjct 121
IPTIFFINKIDQEGIDLPMVYREMKAKLSSEIIVKQKVGQHPHINVTDNDDMEQWDAVIM 180

Query 181
NNDKLLEKYIAGEPISREKLVREEQRRVQDASLFPVYYGSAKKGLGIQPLMDAVTGLFQP 240
             ND+LLEKY++G+P   +L +EE RR Q+ +LFPVY+GSAK LGI+ L++ +
F
Sbjct 181
GNDELLEKYMSGKPFKMSELEQEENRRFQNGTLFPVYHGSAKNNLGIRQLIEVIASKFYS 240

Query 241
IGEQGSAALCGSVFKVEYTDCGQRRVYLRLYSGTLRLRDTVALAGREKLKITEMRIPSKG 300
               +G + LCG VFK+EY++ +R VY+R+YSGTL LRD + ++ +EK+KITEM
+P+ G
Sbjct 241
STPEGQSELCGQVFKIEYSEKRRRFVYVRIYSGTLHLRDVIRISEKEKIKITEMYVPTNG 300

Query 301
EIVRTDTAYPGEIVILPSDSVRLNDVLGDPTRLPRKRWREDPLPMLRTTIAPKTAAQRER 360
            E+ +DTA G+IVILP+D ++LN +LG+   LP++++ E+PLPML+TTIA K +
QRE
Sbjct 301
ELYSSDTACSGDIVILPNDVLQLNSILGNEILLPQRKFIENPLPMLQTTIAVKKSEQREI 360

Query 361
LLDALTQLADTDPLLRCEVDSITHEIILSFLGRVQLEVVSALLSEKYKLETVVKEPSVIY 420
            LL+ALT+++D DPLL+ VD+ THEIILSFLG VQ+EV+ A+L EKY +E +KEP
+VIY
Sbjct 361
LLEALTEISDCDPLLKYYVDTTTHEIILSFLGNVQMEVICAILEEKYHVEAEIKEPTVIY 420

Query 421
MERPLKAASHTIHIEVPPNPFWASIGLSVTPLSLGSGVQYESRVSLGYLNQSFQNAVRDG 480
            MERPL+ A +TIHIEVPPNPFWAS+GLS+ PL
+GSGVQYESRVSLGYLNQSFQNAV +G
Sbjct 421
MERPLRKAEYTIHIEVPPNPFWASVGLSIEPLPIGSGVQYESRVSLGYLNQSFQNAVMEG 480

Query 481
IRYGLEQGLFGWNVTDCKICFEYGLYYSPVSTPADFRSLAPIVLEQALKESGTQLLEPYL 540
            + YG EQGL+GW VTDCKICFEYGLYYSPVSTPADFR L+PIVLEQALK++GT
+LLEPYL
Sbjct 481
VLYGCEQGLYGWKVTDCKICFEYGLYYSPVSTPADFRLLSPIVLEQALKKAGTELLEPYL 540

Query 541
SFILYAPQEYLSRAYHDAPKYCATIETAQVKKDEVVFTGEIPARCIQAYRTDLAFYTNGR 600
             F +YAPQEYLSRAYHDAP+YCA I + Q+K DEV+ GEIPARCIQ YR DL +
+T+G+
Sbjct 541
HFEIYAPQEYLSRAYHDAPRYCADIVSTQIKNDEVILKGEIPARCIQEYRNDLTYFTHGQ 600

Query 601 SVCLTELKGYQAAVGQPVIQPRRPNSRLDKVRHMFQKV 638
             VCLTELKGYQ A+G+ + QPRRPNSR+DKVRHMF K+
Sbjct 601 GVCLTELKGYQPAIGKFICQPRRPNSRIDKVRHMFHKL 638

>WP_024418038.1 TetM/TetW/TetO/TetS family tetracycline resistance
ribosomal
protection protein [Streptococcus suis]
Length=639

 Score = 928 bits (2399), Expect = 0.0, Method: Compositional
matrix adjust.
 Identities = 430/638 (67%), Positives = 529/638 (83%), Gaps = 0/638
(0%)

Query 1
MKIINIGILAHVDAGKTTLTESLLYASGAISEPGSVEKGTTRTDTMFLERQRGITIQAAV 60
            MKIIN+GILAHVDAGKTTLTESLLY SGAI+EPGSV+KGTTRTDTM
LERQRGITIQ AV
Sbjct 1
MKIINLGILAHVDAGKTTLTESLLYTSGAIAEPGSVDKGTTRTDTMNLERQRGITIQTAV 60

Query 61
TSFQWHRCKVNIVDTPGHMDFLAEVYRSLAVLDGAILVISAKDGVQAQTRILFHALRKMN 120
            TSFQW   KVNI+DTPGHMDFLAEVYRSL+VLDGA+L++SAKDG+QAQTRILFHAL
+ M
Sbjct 61
TSFQWEDVKVNIIDTPGHMDFLAEVYRSLSVLDGAVLLVSAKDGIQAQTRILFHALQTMK 120

Query 121
IPTVIFINKIDQAGVDLQSVVQSVRDKLSADIIIKQTVSLSPEIVLEENTDIEAWDAVIE 180
            IPT+ FINKIDQ G+DL V Q ++ KLS++II+KQ V   P I + +N D+E WD
VI
Sbjct 121
IPTIFFINKIDQEGIDLPMVYQEMKAKLSSEIIVKQKVGQHPHINVTDNDDMEQWDTVIM 180

Query 181
NNDKLLEKYIAGEPISREKLVREEQRRVQDASLFPVYYGSAKKGLGIQPLMDAVTGLFQP 240
             ND+LLEKY++G+P   +L +EE RR Q+ +LFPVY+GSAK LGI+ L++ +

F
Sbjct 181
GNDELLEKYMSGKPFKMSELEQEENRRFQNGTLFPVYHGSAKNNLGIRQLIEVIASKFYS 240

Query 241
IGEQGSAALCGSVFKVEYTDCGQRRVYLRLYSGTLRLRDTVALAGREKLKITEMRIPSKG 300
              +G + LCG VFK+EY++ +R VY+R+YSGTL LRD + ++ +EK+KITEM
+P+ G
Sbjct 241
STPEGQSELCGQVFKIEYSEKRRRFVYVRIYSGTLHLRDVIRISEKEKIKITEMCVPTNG 300

Query 301
EIVRTDTAYPGEIVILPSDSVRLNDVLGDPTRLPRKRWREDPLPMLRTTIAPKTAAQRER 360
            E+ +DTA G+IVILP+D ++LN +LG+   LP++++ E+PLPML+TTIA K +
QRE
Sbjct 301
ELYSSDTACSGDIVILPNDVLQLNSILGNEMLLPQRKFIENPLPMLQTTIAVKKSEQREI 360

Query 361
LLDALTQLADTDPLLRCEVDSITHEIILSFLGRVQLEVVSALLSEKYKLETVVKEPSVIY 420
            LL ALT+++D DPLL+ VD+ THEIILSFLG VQ+EV+ A+L EKY +E +KEP
+VIY
Sbjct 361
LLGALTEISDGDPLLKYYVDTTTHEIILSFLGNVQMEVICAILEEKYHVEAEIKEPTVIY 420

Query 421
MERPLKAASHTIHIEVPPNPFWASIGLSVTPLSLGSGVQYESRVSLGYLNQSFQNAVRDG 480
            MERPL+ A +TIHIEVPPNPFWAS+GLS+ PL
+GSGVQYESRVSLGYLNQSFQNAV +G
Sbjct 421
MERPLRKAEYTIHIEVPPNPFWASVGLSIEPLPIGSGVQYESRVSLGYLNQSFQNAVMEG 480

Query 481
IRYGLEQGLFGWNVTDCKICFEYGLYYSPVSTPADFRSLAPIVLEQALKESGTQLLEPYL 540
            + YG EQGL+GW VTDCKICFEYGLYYSPVSTPADFR L+PIVLEQALK++GT
+LLEPYL
Sbjct 481
VLYGCEQGLYGWKVTDCKICFEYGLYYSPVSTPADFRLLSPIVLEQALKKAGTELLEPYL 540

Query 541
SFILYAPQEYLSRAYHDAPKYCATIETAQVKKDEVVFTGEIPARCIQAYRTDLAFYTNGR 600
             F +YAPQEYLSRAYHDAP+YCA I + Q+K DEV+ GEIPARCIQ YR DL +
+TNG+
Sbjct 541
HFEIYAPQEYLSRAYHDAPRYCADIVSTQIKNDEVILKGEIPARCIQEYRNDLTYFTNGQ 600

Query 601 SVCLTELKGYQAAVGQPVIQPRRPNSRLDKVRHMFQKV 638
             VCLTELKGYQ A+G+ + QPRRPNSR+DKVRHMF K+
Sbjct 601 GVCLTELKGYQPAIGKFICQPRRPNSRIDKVRHMFHKL 638

>ACT76129.1 TetO [Streptococcus suis]
Length=639

Score = 928 bits (2399), Expect = 0.0, Method: Compositional matrix adjust.
Identities = 430/638 (67%), Positives = 529/638 (83%), Gaps = 0/638 (0%)

```
Query  1
MKIINIGILAHVDAGKTTLTESLLYASGAISEPGSVEKGTTRTDTMFLERQRGITIQAAV  60
            MKIIN+GILAHVDAGKTTLTESLLY SGAI+EPGSV+KGTTRTDTM
LERQRGITIQ AV
Sbjct  1
MKIINLGILAHVDAGKTTLTESLLYTSGAIAEPGSVDKGTTRTDTMNLERQRGITIQTAV  60

Query  61
TSFQWHRCKVNIVDTPGHMDFLAEVYRSLAVLDGAILVISAKDGVQAQTRILFHALRKMN  120
            TSFQW   KVNI+DTPGHMDFLAEVYRSL+VLDGA+L++SAKDG+QAQTRILFHAL
+ M
Sbjct  61
TSFQWEDVKVNIIDTPGHMDFLAEVYRSLSVLDGAVLLVSAKDGIQAQTRILFHALQTMK  120

Query  121
IPTVIFINKIDQAGVDLQSVVQSVRDKLSADIIIKQTVSLSPEIVLEENTDIEAWDAVIE  180
            IPT+ FINKIDQ G+DL  V Q ++ KLS++II+KQ V   P I + +N D+E
WDAVI
Sbjct  121
IPTIFFINKIDQEGIDLPMVYQEMKAKLSSEIIVKQKVGQHPHINVTDNDDMEQWDAVIM  180

Query  181
NNDKLLEKYIAGEPISREKLVREEQRRVQDASLFPVYYGSAKKGLGIQPLMDAVTGLFQP  240
             ND+LLEKY++G+P    +L +EE RR Q+ +LFPVY+GSAK  LGI+ L++ +
F
Sbjct  181
GNDELLEKYMSGKPFKMSELEQEENRRFQNGTLFPVYHGSAKNNLGIRQLIEVIASKFYS  240

Query  241
IGEQGSAALCGSVFKVEYTDCGQRRVYLRLYSGTLRLRDTVALAGREKLKITEMRIPSKG  300
              +G + LCG VFK+EY++  +R VY+R+YSGTL LRD + ++ +EK+KI EM
+P+ G
Sbjct  241
STPEGQSELCGQVFKIEYSEKRRRFVYVRIYSGTLHLRDVIKISEKEKIKIPEMCVPTNG  300

Query  301
EIVRTDTAYPGEIVILPSDSVRLNDVLGDPTRLPRKRWREDPLPMLRTTIAPKTAAQRER  360
            E+  +DTA  G+IVILP+D ++LN +LG+   LP++++ E+PLPML+TTIA K +
QRE
Sbjct  301
ELYSSDTACSGDIVILPNDVLQLNSILGNEMLLPQRKFIENPLPMLQTTIAVKKSEQREI  360

Query  361
LLDALTQLADTDPLLRCEVDSITHEIILSFLGRVQLEVVSALLSEKYKLETVVKEPSVIY  420
            LL ALT+++D DPLL+  VD+ THEIILSFLG VQ+EV+ A+L EKY +E  +KEP
+VIY
Sbjct  361
LLGALTEISDGDPLLKYYVDTTTHEIILSFLGNVQMEVICAILEEKYHVEAEIKEPTVIY  420
```

```
Query  421
MERPLKAASHTIHIEVPPNPFWASIGLSVTPLSLGSGVQYESRVSLGYLNQSFQNAVRDG  480
            MERPL+ A +TIHIEVPPNPFWAS+GLS+ PL
+GSGVQYESRVSLGYLNQSFQNAV +G
Sbjct  421
MERPLRKAEYTIHIEVPPNPFWASVGLSIEPLPIGSGVQYESRVSLGYLNQSFQNAVMEG  480

Query  481
IRYGLEQGLFGWNVTDCKICFEYGLYYSPVSTPADFRSLAPIVLEQALKESGTQLLEPYL  540
            + YG EQGL+GW VTDCKICFEYGLYYSPVSTPADFR L+PIVLEQALK++GT
+LLEPYL
Sbjct  481
VLYGCEQGLYGWKVTDCKICFEYGLYYSPVSTPADFRLLSPIVLEQALKKAGTELLEPYL  540

Query  541
SFILYAPQEYLSRAYHDAPKYCATIETAQVKKDEVVFTGEIPARCIQAYRTDLAFYTNGR  600
             F +YAPQEYLSRAYHDAP+YCA I + Q+K DEV+  GEIPARCIQ YR DL +
+TNG+
Sbjct  541
HFEIYAPQEYLSRAYHDAPRYCADIVSTQIKNDEVILKGEIPARCIQEYRNDLTYFTNGQ  600

Query  601  SVCLTELKGYQAAVGQPVIQPRRPNSRLDKVRHMFQKV  638
             VCLTELKGYQ A+G+ + QPRRPNSR+DKVRHMF K+
Sbjct  601  GVCLTELKGYQPAIGKFICQPRRPNSRIDKVRHMFHKL  638


>ACT76128.1 TetO [Streptococcus suis]
Length=639

 Score = 928 bits (2399),  Expect = 0.0, Method: Compositional
matrix adjust.
 Identities = 431/638 (68%), Positives = 528/638 (83%), Gaps = 0/638
(0%)

Query  1
MKIINIGILAHVDAGKTTLTESLLYASGAISEPGSVEKGTTRTDTMFLERQRGITIQAAV  60
            MKIIN+GILAHVDAGKTTLTESLLY SGAI+EPGSV+KGTTRTDTM
LERQRGITIQ AV
Sbjct  1
MKIINLGILAHVDAGKTTLTESLLYTSGAIAEPGSVDKGTTRTDTMNLERQRGITIQTAV  60

Query  61
TSFQWHRCKVNIVDTPGHMDFLAEVYRSLAVLDGAILVISAKDGVQAQTRILFHALRKMN  120
            TSFQW   KVNI+DTPGHMDFLAEVYRSL+VLDGA+L++SAKDG+QAQTRILFHAL
+ M
Sbjct  61
TSFQWEDVKVNIIDTPGHMDFLAEVYRSLSVLDGAVLLVSAKDGIQAQTRILFHALQTMK  120

Query  121
IPTVIFINKIDQAGVDLQSVVQSVRDKLSADIIIKQTVSLSPEIVLEENTDIEAWDAVIE  180
            IPT+ FINKIDQ G+DL  V Q ++ KLS++II+KQ V   P I + +N D+E
WDAVI
Sbjct  121
IPTIFFINKIDQEGIDLPMVYQEMKAKLSSEIIVKQKVGQHPHINVTDNDDMEQWDAVIM  180
```

```
Query  181
NNDKLLEKYIAGEPISREKLVREEQRRVQDASLFPVYYGSAKKGLGIQPLMDAVTGLFQP  240
             ND+LLEKY++G+P     L +EE RR Q+ +LFPVY+GSAK  LGI+ L++ +
F
Sbjct  181
GNDELLEKYMSGKPFKMSDLEQEENRRFQNGTLFPVYHGSAKNNLGIRQLIEVIASKFYS  240

Query  241
IGEQGSAALCGSVFKVEYTDCGQRRVYLRLYSGTLRLRDTVALAGREKLKITEMRIPSKG  300
                G + LCG VFK+EY++  +R VY+R+YSGTL LRD + ++ +EK+KITEM
+P+ G
Sbjct  241
STPGGQSELCGQVFKIEYSEKRRRFVYVRIYSGTLHLRDVIKISEKEKIKITEMCVPTNG  300

Query  301
EIVRTDTAYPGEIVILPSDSVRLNDVLGDPTRLPRKRWREDPLPMLRTTIAPKTAAQRER  360
            E+  +DTA  G+IVILP+D ++LN +LG+   LP++++ E+PLPML+TTIA K +
QRE
Sbjct  301
ELYSSDTACSGDIVILPNDVLQLNSILGNEMLLPQRKFIENPLPMLQTTIAVKKSEQREI  360

Query  361
LLDALTQLADTDPLLRCEVDSITHEIILSFLGRVQLEVVSALLSEKYKLETVVKEPSVIY  420
            LL ALT+++D DPLL+  VD+ THEIILSFLG VQ+EV+ A+L EKY +E  +KEP
+VIY
Sbjct  361
LLGALTEISDGDPLLKYYVDTTTHEIILSFLGNVQMEVICAILEEKYHVEAEIKEPTVIY  420

Query  421
MERPLKAASHTIHIEVPPNPFWASIGLSVTPLSLGSGVQYESRVSLGYLNQSFQNAVRDG  480
            MERPL+ A +TIHIEVPPNPFWAS+GLS+ PL
+GSGVQYESRVSLGYLNQSFQNAV +G
Sbjct  421
MERPLRKAEYTIHIEVPPNPFWASVGLSIEPLPIGSGVQYESRVSLGYLNQSFQNAVMEG  480

Query  481
IRYGLEQGLFGWNVTDCKICFEYGLYYSPVSTPADFRSLAPIVLEQALKESGTQLLEPYL  540
            + YG EQGL+GW VTDCKICFEYGLYYSPVSTPADFR L+PIVLEQALK++GT
+LLEPYL
Sbjct  481
VLYGCEQGLYGWKVTDCKICFEYGLYYSPVSTPADFRLLSPIVLEQALKKAGTELLEPYL  540

Query  541
SFILYAPQEYLSRAYHDAPKYCATIETAQVKKDEVVFTGEIPARCIQAYRTDLAFYTNGR  600
             F +YAPQEYLSRAYHDAP+YCA I + Q+K DEV+  GEIPARCIQ YR DL +
+TNG+
Sbjct  541
HFEIYAPQEYLSRAYHDAPRYCADIVSTQIKNDEVILKGEIPARCIQEYRNDLTYFTNGQ  600

Query  601  SVCLTELKGYQAAVGQPVIQPRRPNSRLDKVRHMFQKV  638
             VCLTELKGYQ A+G+ + QPRRPNSR+DKVRHMF K+
Sbjct  601  GVCLTELKGYQPAIGKFICQPRRPNSRIDKVRHMFHKL  638
```

>WP_055169801.1 TetM/TetW/TetO/TetS family tetracycline resistance ribosomal
protection protein [[Ruminococcus] gnavus]
 CUO46767.1 Tetracycline resistance protein tetM from transposon Tn916 [[Ruminococcus]
gnavus]
 SCJ67513.1 Tetracycline resistance protein tetM from transposon Tn916 [uncultured
Ruminococcus sp.]
Length=639

 Score = 928 bits (2398), Expect = 0.0, Method: Compositional matrix adjust.
 Identities = 431/638 (68%), Positives = 529/638 (83%), Gaps = 0/638 (0%)

```
Query  1
MKIINIGILAHVDAGKTTLTESLLYASGAISEPGSVEKGTTRTDTMFLERQRGITIQAAV  60
            MKIIN+GILAHVDAGKTTLTESLLY SGAI+EPGSV+KGTTRTDTM
LERQRGITIQ AV
Sbjct  1
MKIINLGILAHVDAGKTTLTESLLYTSGAIAEPGSVDKGTTRTDTMNLERQRGITIQTAV  60

Query  61
TSFQWHRCKVNIVDTPGHMDFLAEVYRSLAVLDGAILVISAKDGVQAQTRILFHALRKMN  120
            TSFQW   KVNI+DTPGHMDFLAEVYRSL+VLDGA+L++SAKDG+QAQTRILFHAL
+ M
Sbjct  61
TSFQWEDVKVNIIDTPGHMDFLAEVYRSLSVLDGAVLLVSAKDGIQAQTRILFHALQTMK  120

Query  121
IPTVIFINKIDQAGVDLQSVVQSVRDKLSADIIIKQTVSLSPEIVLEENTDIEAWDAVIE  180
            IPT+ FINKIDQ G+DL  V Q ++ KLS++II+KQ V   P I + +N D+E
WDAVI
Sbjct  121
IPTIFFINKIDQEGIDLPMVYQEMKAKLSSEIIVKQKVGQHPHINVTDNDDMEQWDAVIM  180

Query  181
NNDKLLEKYIAGEPISREKLVREEQRRVQDASLFPVYYGSAKKGLGIQPLMDAVTGLFQP  240
             ND+LLEKY++G+P    +L +EE RR Q+ +LFPVY+GSAK  LGI+ L++ +
F
Sbjct  181
GNDELLEKYMSGKPFKMSELEQEENRRFQNGTLFPVYHGSAKNNLGIRQLIEVIASKFYS  240

Query  241
IGEQGSAALCGSVFKVEYTDCGQRRVYLRLYSGTLRLRDTVALAGREKLKITEMRIPSKG  300
              +G + LCG VFK+EY++  +R VY+R+YSGTL LRD + ++ +EK+KITEM
+P+ G
Sbjct  241
STPEGQSELCGQVFKIEYSEKRRRFVYVRIYSGTLHLRDVIKISEKEKIKITEMCVPTNG  300

Query  301
EIVRTDTAYPGEIVILPSDSVRLNDVLGDPTRLPRKRWREDPLPMLRTTIAPKTAAQRER  360
```

```
            E+  +DTA  G+IVILP+D ++LN +LG+   LP++++ E+PLPML+TTIA K + 
QRE
Sbjct  301  
ELYSSDTACSGDIVILPNDVLQLNSILGNEMLLPQRKFIENPLPMLQTTIAVKKSEQREI  360

Query  361  
LLDALTQLADTDPLLRCEVDSITHEIILSFLGRVQLEVVSALLSEKYKLETVVKEPSVIY  420
            LL ALT+++D DPLL+  VD+ THEIILSFLG VQ+EV+ A+L EKY +E  +KEP 
+VIY
Sbjct  361  
LLGALTEISDGDPLLKYYVDTTTHEIILSFLGNVQMEVICAILEEKYHVEAEIKEPTVIY  420

Query  421  
MERPLKAASHTIHIEVPPNPFWASIGLSVTPLSLGSGVQYESRVSLGYLNQSFQNAVRDG  480
            MERPL+ A +TIHIEVPPNPFWAS+GLS+ PL 
+GSGVQYESRVSLGYLNQSFQNAV +G
Sbjct  421  
MERPLRKAEYTIHIEVPPNPFWASVGLSIEPLPIGSGVQYESRVSLGYLNQSFQNAVMEG  480

Query  481  
IRYGLEQGLFGWNVTDCKICFEYGLYYSPVSTPADFRSLAPIVLEQALKESGTQLLEPYL  540
            + YG EQGL+GW VTDCKICFEYGLYYSPVSTPADFR L+PIVLEQALK++GT 
+LLEPYL
Sbjct  481  
VLYGCEQGLYGWKVTDCKICFEYGLYYSPVSTPADFRLLSPIVLEQALKKAGTELLEPYL  540

Query  541  
SFILYAPQEYLSRAYHDAPKYCATIETAQVKKDEVVFTGEIPARCIQAYRTDLAFYTNGR  600
             F +YAPQEYLSRAYHDA +YCA I + QVK DEV+  GEIPARCIQ YR DL + 
+TNG+
Sbjct  541  
HFEIYAPQEYLSRAYHDAQRYCADIVSTQVKNDEVILKGEIPARCIQEYRNDLTYFTNGQ  600

Query  601  SVCLTELKGYQAAVGQPVIQPRRPNSRLDKVRHMFQKV  638
             VCLTELKGYQ A+G+ + QPRRPNSR+DKVRHMF K+
Sbjct  601  GVCLTELKGYQPAIGKFICQPRRPNSRIDKVRHMFHKL  638


>WP_054705475.1 TetM/TetW/TetO/TetS family tetracycline resistance 
ribosomal 
protection protein [[Clostridium] glycyrrhizinilyticum]
Length=639

 Score = 928 bits (2398),  Expect = 0.0, Method: Compositional 
matrix adjust.
 Identities = 430/638 (67%), Positives = 530/638 (83%), Gaps = 0/638 
(0%)

Query  1    
MKIINIGILAHVDAGKTTLTESLLYASGAISEPGSVEKGTTRTDTMFLERQRGITIQAAV  60
            MKIIN+GILAHVDAGKTTLTESLLY SGAI+EPGSV+KGTTRTDTM  
LERQRGITIQ AV
Sbjct  1    
MKIINLGILAHVDAGKTTLTESLLYTSGAIAEPGSVDKGTTRTDTMNLERQRGITIQTAV  60
```

Query 61
TSFQWHRCKVNIVDTPGHMDFLAEVYRSLAVLDGAILVISAKDGVQAQTRILFHALRKMN 120
            TSFQW   KVNI+DTPGHMDFLAEVYRSL+VLDGA+L++SAKDG+QAQTRILFHAL
+ M
Sbjct 61
TSFQWEDVKVNIIDTPGHMDFLAEVYRSLSVLDGAVLLVSAKDGIQAQTRILFHALQIMK 120

Query 121
IPTVIFINKIDQAGVDLQSVVQSVRDKLSADIIIKQTVSLSPEIVLEENTDIEAWDAVIE 180
            IPT+ FINKIDQ G+DL V + ++ KLS++II+KQ V   P I + +N D+E
WDAVI
Sbjct 121
IPTIFFINKIDQEGIDLPMVYREMKAKLSSEIIVKQKVGQHPHINVTDNDDMEQWDAVIM 180

Query 181
NNDKLLEKYIAGEPISREKLVREEQRRVQDASLFPVYYGSAKKGLGIQPLMDAVTGLFQP 240
             ND+LLEKY++G+P   +L +EE RR Q+ +LFPVY+GSAK LGI+ L++ +
F
Sbjct 181
GNDELLEKYMSGKPFKMSELEQEENRRFQNGTLFPVYHGSAKNNLGIRQLIEVIASKFYS 240

Query 241
IGEQGSAALCGSVFKVEYTDCGQRRVYLRLYSGTLRLRDTVALAGREKLKITEMRIPSKG 300
               +G + LCG VFK+EY++ +R VY+R+YSGTL LRD + ++ +EK+KITEM
+P+ G
Sbjct 241
STPEGQSELCGQVFKIEYSEKRRRFVYVRIYSGTLHLRDVIRISEKEKIKITEMCVPTNG 300

Query 301
EIVRTDTAYPGEIVILPSDSVRLNDVLGDPTRLPRKRWREDPLPMLRTTIAPKTAAQRER 360
            E+ +DTA G+IVILP+D ++LN +LG+   LP++++ E+PLPML+TTIA K +
QRE
Sbjct 301
ELYSSDTACSGDIVILPNDVLQLNSILGNEMLLPQRKFIENPLPMLQTTIAVKKSEQREI 360

Query 361
LLDALTQLADTDPLLRCEVDSITHEIILSFLGRVQLEVVSALLSEKYKLETVVKEPSVIY 420
            LL ALT+++D DPLL+ VD+ THEIILSFLG VQ+EV+ A+L EKY +E +KEP
+VIY
Sbjct 361
LLGALTEISDGDPLLKYYVDTTTHEIILSFLGNVQMEVICAILEEKYHVEAEIKEPTVIY 420

Query 421
MERPLKAASHTIHIEVPPNPFWASIGLSVTPLSLGSGVQYESRVSLGYLNQSFQNAVRDG 480
            MERPL+ A +TIHIEVPPNPFWAS+GLS+ PL
+GSGVQYESRVSLGYLNQSFQNAV +G
Sbjct 421
MERPLRKAEYTIHIEVPPNPFWASVGLSIEPLPIGSGVQYESRVSLGYLNQSFQNAVMEG 480

Query 481
IRYGLEQGLFGWNVTDCKICFEYGLYYSPVSTPADFRSLAPIVLEQALKESGTQLLEPYL 540
            + YG EQGL+GW VTDCKICFEYGLYYSPVSTPADFR L+PIVLEQALK++GT
+LLEPYL

Sbjct 481
VLYGCEQGLYGWKVTDCKICFEYGLYYSPVSTPADFRLLSPIVLEQALKKAGTELLEPYL 540

Query 541
SFILYAPQEYLSRAYHDAPKYCATIETAQVKKDEVVFTGEIPARCIQAYRTDLAFYTNGR 600
             F +YAPQEYLSRAYHDAP+YCA I + Q+K DEV+ GEIPARCIQ YR DL +
+TNG+
Sbjct 541
HFEIYAPQEYLSRAYHDAPRYCADIVSTQIKNDEVILKGEIPARCIQEYRNDLTYFTNGQ 600

Query 601 SVCLTELKGYQAAVGQPVIQPRRPNSRLDKVRHMFQKV 638
             VCLTELKGYQ A+G+ + QPRRPNSR+DKVRHMF K+
Sbjct 601 GVCLTELKGYQPAIGKFICQPRRPNSRIDKVRHMFHKL 638

>WP_015573056.1 tetracycline resistance ribosomal protection protein
Tet(32)
[Clostridiaceae bacterium K10]
 CAC41371.2 tetracycline resistance protein [Clostridiaceae
bacterium K10]
 CAM12478.1 Tet(O/32/O) protein [uncultured bacterium]
 CBK76341.1 small GTP-binding protein domain [[[Clostridium] cf.
saccharolyticum
K10]
Length=639

 Score = 928 bits (2398), Expect = 0.0, Method: Compositional
matrix adjust.
 Identities = 435/638 (68%), Positives = 531/638 (83%), Gaps = 0/638
(0%)

Query 1
MKIINIGILAHVDAGKTTLTESLLYASGAISEPGSVEKGTTRTDTMFLERQRGITIQAAV 60
            MKIIN+GILAHVDAGKTTLTESLLY SGAI+E GSV++GTTRTDTM
LERQRGITIQ AV
Sbjct 1
MKIINLGILAHVDAGKTTLTESLLYTSGAIAELGSVDEGTTRTDTMNLERQRGITIQTAV 60

Query 61
TSFQWHRCKVNIVDTPGHMDFLAEVYRSLAVLDGAILVISAKDGVQAQTRILFHALRKMN 120
            TSFQW   KVNI+DTPGHMDFL E YRSL+VLDGA+LVISAKDGVQAQTRILFHAL
+KMN
Sbjct 61
TSFQWEDVKVNIIDTPGHMDFLTEAYRSLSVLDGAVLVISAKDGVQAQTRILFHALQKMN 120

Query 121
IPTVIFINKIDQAGVDLQSVVQSVRDKLSADIIIKQTVSLSPEIVLEENTDIEAWDAVIE 180
            IPT+IFINKIDQ G+DLQ V QS++DKL++D+I+ Q VSLSP+I + + +D++ WD
+I
Sbjct 121
IPTIIFINKIDQNGIDLQRVYQSIKDKLTSDMIVMQEVSLSPKITMTDISDLDKWDMIIS 180

Query 181
NNDKLLEKYIAGEPISREKLVREEQRRVQDASLFPVYYGSAKKGLGIQPLMDAVTGLFQP 240

```
             +D+LLE+Y+A + +  ++L  E+ +R +  SLFPVY+GSAK  LG + L++A+T
F
Sbjct  181
GSDELLERYVAEDSLDIQELQYEKCKRTRCCSLFPVYHGSAKDNLGTEKLIEAITETFIT  240

Query  241
IGEQGSAALCGSVFKVEYTDCGQRRVYLRLYSGTLRLRDTVALAGREKLKITEMRIPSKG  300
              +   + LCG VFKVEYT+  +R  YLRLY GTL LRDT+ L+ +EK+KITEM
IPS G
Sbjct  241
ETDDIQSELCGYVFKVEYTERKKRLSYLRLYHGTLHLRDTLLLSKKEKIKITEMCIPSNG  300

Query  301
EIVRTDTAYPGEIVILPSDSVRLNDVLGDPTRLPRKRWREDPLPMLRTTIAPKTAAQRER  360
            EIV  D A PGEIVIL  D+++LND+LG+   LP K   ++P+P+LRTT+ P+
QRE
Sbjct  301
EIVPVDHACPGEIVILADDTLKLNDILGNEKLLPHKTRIDNPMPLLRTTVEPQKPEQREA  360

Query  361
LLDALTQLADTDPLLRCEVDSITHEIILSFLGRVQLEVVSALLSEKYKLETVVKEPSVIY  420
            LL+ALT++ADTDPLL  ++D++THEIILSFLG+VQLEV+ +LL EKY +
+KEPSVIY
Sbjct  361
LLNALTEIADTDPLLHFDIDTVTHEIILSFLGKVQLEVICSLLEEKYHVGVAMKEPSVIY  420

Query  421
MERPLKAASHTIHIEVPPNPFWASIGLSVTPLSLGSGVQYESRVSLGYLNQSFQNAVRDG  480
            +ERPL+ A +TIHIEVPPNPFWAS+GLS+ PL
+GSGVQYESRVSLGYLNQSFQNAV +G
Sbjct  421
LERPLRKAEYTIHIEVPPNPFWASVGLSIEPLPIGSGVQYESRVSLGYLNQSFQNAVMEG  480

Query  481
IRYGLEQGLFGWNVTDCKICFEYGLYYSPVSTPADFRSLAPIVLEQALKESGTQLLEPYL  540
            + YG EQGL+GW VTDCKICFEYGLYYSPVSTPADFR L+PIVLEQALK++GT
+LLEPYL
Sbjct  481
VLYGCEQGLYGWKVTDCKICFEYGLYYSPVSTPADFRLLSPIVLEQALKKAGTELLEPYL  540

Query  541
SFILYAPQEYLSRAYHDAPKYCATIETAQVKKDEVVFTGEIPARCIQAYRTDLAFYTNGR  600
             F +YAPQEYLSRAYHDAP+YCA I + Q+K DEV+  GEIPARCIQ YR DL +
+TNG+
Sbjct  541
HFEIYAPQEYLSRAYHDAPRYCADIVSTQIKNDEVILKGEIPARCIQEYRNDLTYFTNGQ  600

Query  601  SVCLTELKGYQAAVGQPVIQPRRPNSRLDKVRHMFQKV  638
             VCLTELKGYQ A+G+ + QPRRPNSR+DKVRHMF K+
Sbjct  601  GVCLTELKGYQPAIGKFICQPRRPNSRIDKVRHMFHKL  638
```

>WP_105123813.1 TetM/TetW/TetO/TetS family tetracycline resistance ribosomal

protection protein [Streptococcus suis]
Length=639

 Score = 927 bits (2397), Expect = 0.0, Method: Compositional matrix adjust.
 Identities = 431/638 (68%), Positives = 526/638 (82%), Gaps = 0/638 (0%)

```
Query  1
MKIINIGILAHVDAGKTTLTESLLYASGAISEPGSVEKGTTRTDTMFLERQRGITIQAAV  60
            MKIIN+GILAHVDAGKTTLTESLLY SGAI+EPGSV+KGTTRTDTM
LERQRGITIQ AV
Sbjct  1
MKIINLGILAHVDAGKTTLTESLLYTSGAIAEPGSVDKGTTRTDTMNLERQRGITIQTAV  60

Query  61
TSFQWHRCKVNIVDTPGHMDFLAEVYRSLAVLDGAILVISAKDGVQAQTRILFHALRKMN  120
            TSFQW   KVNI+DTPGHMDFLAEVYRSL+VLDGA+L++SAKDG+QAQTRILFHAL
+ M
Sbjct  61
TSFQWEDVKVNIIDTPGHMDFLAEVYRSLSVLDGAVLLVSAKDGIQAQTRILFHALQTMK  120

Query  121
IPTVIFINKIDQAGVDLQSVVQSVRDKLSADIIIKQTVSLSPEIVLEENTDIEAWDAVIE  180
            IPT+ FINKIDQ G+DL  V Q ++ KLS++II+KQ V   P I + +N D+E WD
VI
Sbjct  121
IPTIFFINKIDQEGIDLPMVYQEMKAKLSSEIIVKQKVGQHPHINVTDNDDMEQWDTVIM  180

Query  181
NNDKLLEKYIAGEPISREKLVREEQRRVQDASLFPVYYGSAKKGLGIQPLMDAVTGLFQP  240
             ND+LLEKY++G+P    +L +EE RR Q+ +LFPVY+GSAK  LGI+ L++ +
F
Sbjct  181
GNDELLEKYMSGKPFKMSELEQEENRRFQNGTLFPVYHGSAKNNLGIRQLIEVIASKFYS  240

Query  241
IGEQGSAALCGSVFKVEYTDCGQRRVYLRLYSGTLRLRDTVALAGREKLKITEMRIPSKG  300
              +G + LCG VFK+EY++  +R  Y+RLYSG L  RD V ++ +EK+KITEM
IP+ G
Sbjct  241
STPEGQSELCGQVFKIEYSEKRRRLAYVRLYSGALHSRDVVRISEKEKIKITEMSIPTNG  300

Query  301
EIVRTDTAYPGEIVILPSDSVRLNDVLGDPTRLPRKRWREDPLPMLRTTIAPKTAAQRER  360
            E+   DTAY G+IVILP+D ++LN +LG+   LP++++ E+PLPML+TTIA K +
QRE
Sbjct  301
ELCLADTAYSGDIVILPNDVLQLNSILGNEILLPQRKFIENPLPMLQTTIAVKKSEQREI  360

Query  361
LLDALTQLADTDPLLRCEVDSITHEIILSFLGRVQLEVVSALLSEKYKLETVVKEPSVIY  420
            LL ALT+++D DPLL+  VD+ THEIILSFLG VQ+EV+ A+L EKY +E  +KEP
+VIY
```

Sbjct 361
LLGALTEISDGDPLLKYYVDTTTHEIILSFLGNVQMEVICAILEEKYHVEAEIKEPTVIY 420

Query 421
MERPLKAASHTIHIEVPPNPFWASIGLSVTPLSLGSGVQYESRVSLGYLNQSFQNAVRDG 480
            MERPL+ A +TIHIEVPPNPFWAS+GLS+ PL
+GSGVQYESRVSLGYLNQSFQNAV +G
Sbjct 421
MERPLRKAEYTIHIEVPPNPFWASVGLSIEPLPIGSGVQYESRVSLGYLNQSFQNAVMEG 480

Query 481
IRYGLEQGLFGWNVTDCKICFEYGLYYSPVSTPADFRSLAPIVLEQALKESGTQLLEPYL 540
            + YG EQGL+GW VTDCKICFEYGLYYSPVSTPADFR L+PIVLEQALK++GT
+LLEPYL
Sbjct 481
VLYGCEQGLYGWKVTDCKICFEYGLYYSPVSTPADFRLLSPIVLEQALKKAGTELLEPYL 540

Query 541
SFILYAPQEYLSRAYHDAPKYCATIETAQVKKDEVVFTGEIPARCIQAYRTDLAFYTNGR 600
             F +YAPQEYLSRAYHDAP+YCA I + Q+K DEV+ GEIPARCIQ YR DL +
+TNG+
Sbjct 541
HFEIYAPQEYLSRAYHDAPRYCADIVSTQIKNDEVILKGEIPARCIQEYRNDLTYFTNGQ 600

Query 601 SVCLTELKGYQAAVGQPVIQPRRPNSRLDKVRHMFQKV 638
             VCLTELKGYQ A+G+ + QPRRPNSR+DKVRHMF K+
Sbjct 601 GVCLTELKGYQPAIGKFICQPRRPNSRIDKVRHMFHKL 638

>WP_105122866.1 TetM/TetW/TetO/TetS family tetracycline resistance ribosomal
protection protein [Streptococcus suis]
Length=639

 Score = 927 bits (2397), Expect = 0.0, Method: Compositional matrix adjust.
 Identities = 430/638 (67%), Positives = 527/638 (83%), Gaps = 0/638 (0%)

Query 1
MKIINIGILAHVDAGKTTLTESLLYASGAISEPGSVEKGTTRTDTMFLERQRGITIQAAV 60
            MKIIN+GILAHVDAGKTTLTESLLY SGAI+EPGSV+KGTTRTDTM
LERQRGITIQ AV
Sbjct 1
MKIINLGILAHVDAGKTTLTESLLYTSGAIAEPGSVDKGTTRTDTMNLERQRGITIQTAV 60

Query 61
TSFQWHRCKVNIVDTPGHMDFLAEVYRSLAVLDGAILVISAKDGVQAQTRILFHALRKMN 120
            TSFQW   KVNI+DTPGHMDFLAEVYRSL+VLDGA+L++SAKDG+QAQTRILFHAL
+ M
Sbjct 61
TSFQWEDVKVNIIDTPGHMDFLAEVYRSLSVLDGAVLLVSAKDGIQAQTRILFHALQTMK 120

Query 121

IPTVIFINKIDQAGVDLQSVVQSVRDKLSADIIIKQTVSLSPEIVLEENTDIEAWDAVIE 180
            IPT+ FINKIDQ G+DL V + ++ KLS++II+KQ V   P I + +N D+E
WDAVI
Sbjct 121
IPTIFFINKIDQEGIDLPMVYREMKAKLSSEIIVKQKVGQHPHINVTDNDDMEQWDAVIM 180

Query 181
NNDKLLEKYIAGEPISREKLVREEQRRVQDASLFPVYYGSAKKGLGIQPLMDAVTGLFQP 240
             ND+LLEKY++G+P   +L +EE RR Q+ +LFPVY+GSAK LGI+ L++ +
F
Sbjct 181
GNDELLEKYMSGKPFKMSELEQEENRRFQNGTLFPVYHGSAKNNLGIRQLIEVIASKFYS 240

Query 241
IGEQGSAALCGSVFKVEYTDCGQRRVYLRLYSGTLRLRDTVALAGREKLKITEMRIPSKG 300
               +G + LCG VFK+EY++ +R Y+RLYSG L RD V ++ +EK+KITEM
IP+ G
Sbjct 241
STPEGQSELCGQVFKIEYSEKRRRLAYVRLYSGALHSRDVVRISEKEKIKITEMSIPTNG 300

Query 301
EIVRTDTAYPGEIVILPSDSVRLNDVLGDPTRLPRKRWREDPLPMLRTTIAPKTAAQRER 360
            E+   DTAY G+IVILP+D ++LN +LG+   LP++++ E+PLPML+TTI K +
QRE
Sbjct 301
ELCLADTAYSGDIVILPNDVLQLNSILGNEILLPQRKFIENPLPMLQTTIEVKKSEQREV 360

Query 361
LLDALTQLADTDPLLRCEVDSITHEIILSFLGRVQLEVVSALLSEKYKLETVVKEPSVIY 420
            LL+ALT+++D DPLL+ VD+ THEIILSFLG VQ+EV+ A+L EKY +E +KEP
+VIY
Sbjct 361
LLEALTEISDGDPLLKYYVDTTTHEIILSFLGNVQMEVICAILEEKYHVEAEIKEPTVIY 420

Query 421
MERPLKAASHTIHIEVPPNPFWASIGLSVTPLSLGSGVQYESRVSLGYLNQSFQNAVRDG 480
            MERPL+ A +TIHIEVPPNPFWAS+GLS+ PL
+GSGVQYESRVSLGYLNQSFQNAV +G
Sbjct 421
MERPLRKAEYTIHIEVPPNPFWASVGLSIEPLPIGSGVQYESRVSLGYLNQSFQNAVMEG 480

Query 481
IRYGLEQGLFGWNVTDCKICFEYGLYYSPVSTPADFRSLAPIVLEQALKESGTQLLEPYL 540
            + YG EQGL+GW VTDCKICFEYGLYYSPVSTPADFR L+PIVLEQALK++GT
+LLEPYL
Sbjct 481
VLYGCEQGLYGWKVTDCKICFEYGLYYSPVSTPADFRLLSPIVLEQALKKAGTELLEPYL 540

Query 541
SFILYAPQEYLSRAYHDAPKYCATIETAQVKKDEVVFTGEIPARCIQAYRTDLAFYTNGR 600
             F +YAPQEYLSRAYHDAP+YCA I + Q+K DEV+ GEIPARCIQ YR DL +
+TNG+
Sbjct 541
HFEIYAPQEYLSRAYHDAPRYCADIVSTQIKNDEVILKGEIPARCIQEYRNDLTYFTNGQ 600

```
Query  601  SVCLTELKGYQAAVGQPVIQPRRPNSRLDKVRHMFQKV  638
             VCLTELKGYQ A+G+ + QPRRPNSR+DKVRHMF K+
Sbjct  601  GVCLTELKGYQPAIGKFICQPRRPNSRIDKVRHMFHKL  638
```

>WP_086301235.1 TetM/TetW/TetO/TetS family tetracycline resistance ribosomal
protection protein [Campylobacter sp. P090]
Length=639

Score = 927 bits (2397), Expect = 0.0, Method: Compositional matrix adjust.
Identities = 432/638 (68%), Positives = 526/638 (82%), Gaps = 0/638 (0%)

```
Query  1
MKIINIGILAHVDAGKTTLTESLLYASGAISEPGSVEKGTTRTDTMFLERQRGITIQAAV  60
            MKIIN+GILAHVDAGKTTLTESLLY SGAI+E GSV++GTTRTDTM
LERQRGITIQ AV
Sbjct  1
MKIINLGILAHVDAGKTTLTESLLYTSGAIAELGSVDEGTTRTDTMNLERQRGITIQTAV  60

Query  61
TSFQWHRCKVNIVDTPGHMDFLAEVYRSLAVLDGAILVISAKDGVQAQTRILFHALRKMN  120
            TSFQW   KVNI+DTPGHMDFLAEVYRSL+VLDGA+L++SAKDG+QAQTRILFHAL
+ M
Sbjct  61
TSFQWEDVKVNIIDTPGHMDFLAEVYRSLSVLDGAVLLVSAKDGIQAQTRILFHALQIMK  120

Query  121
IPTVIFINKIDQAGVDLQSVVQSVRDKLSADIIIKQTVSLSPEIVLEENTDIEAWDAVIE  180
            IPT+ FINKIDQ G+DL  V + ++ KLS++II+KQ V   P I + +N D+E
WDAVI
Sbjct  121
IPTIFFINKIDQEGIDLPMVYREMKAKLSSEIIVKQKVGQHPHINVTDNDDMEQWDAVIM  180

Query  181
NNDKLLEKYIAGEPISREKLVREEQRRVQDASLFPVYYGSAKKGLGIQPLMDAVTGLFQP  240
             ND+LLEKY++G+P    +L +EE RR Q+ +LFPVY+GSAK  LGI+ L++ +
F
Sbjct  181
GNDELLEKYMSGKPFKMSELEQEENRRFQNGTLFPVYHGSAKNNLGIRQLIEVIASKFYS  240

Query  241
IGEQGSAALCGSVFKVEYTDCGQRRVYLRLYSGTLRLRDTVALAGREKLKITEMRIPSKG  300
               +G + LCG VFK+EY++  +R VY+R+YSGTL LRD + ++ +EK+KITEM
IPS G
Sbjct  241
STPEGQSELCGQVFKIEYSEKRRRFVYVRIYSGTLHLRDVIRISEKEKIKITEMCIPSNG  300

Query  301
EIVRTDTAYPGEIVILPSDSVRLNDVLGDPTRLPRKRWREDPLPMLRTTIAPKTAAQRER  360
            EIV  D A PGEIVIL  D+++LND+LG+   LP K W ++P+P+LRTT+ P+
```

QRE
Sbjct 301
EIVPADHACPGEIVILADDTLKLNDILGNEKLLPHKTWIDNPMPLLRTTVEPQKPEQREA 360

Query 361
LLDALTQLADTDPLLRCEVDSITHEIILSFLGRVQLEVVSALLSEKYKLETVVKEPSVIY 420
            LL+AL ++ADTDPLL+ VD+ THEIILSFLG VQ+EV+ A+L EKY +E +KEP
+VIY
Sbjct 361
LLNALAEIADTDPLLKYYVDTTTHEIILSFLGNVQMEVICAILEEKYHVEVEIKEPTVIY 420

Query 421
MERPLKAASHTIHIEVPPNPFWASIGLSVTPLSLGSGVQYESRVSLGYLNQSFQNAVRDG 480
            MERPL+ A +TIHIEVPPNPFWAS+GLS+ PL
+GSGVQYESRVSLGYLNQSFQNAV +G
Sbjct 421
MERPLRKAEYTIHIEVPPNPFWASVGLSIEPLPIGSGVQYESRVSLGYLNQSFQNAVMEG 480

Query 481
IRYGLEQGLFGWNVTDCKICFEYGLYYSPVSTPADFRSLAPIVLEQALKESGTQLLEPYL 540
            + YG EQGL+GW VTDCKICFEYGLYYSPVSTPADFR L+PIVLEQALK++GT
+LLEPYL
Sbjct 481
VLYGCEQGLYGWKVTDCKICFEYGLYYSPVSTPADFRLLSPIVLEQALKKAGTELLEPYL 540

Query 541
SFILYAPQEYLSRAYHDAPKYCATIETAQVKKDEVVFTGEIPARCIQAYRTDLAFYTNGR 600
             F +YAPQEYLSRAYHDAP+YCA I + Q+K DEV+ GEIPARCIQ YR DL
+TNG+
Sbjct 541
HFEIYAPQEYLSRAYHDAPRYCADIVSTQIKNDEVILKGEIPARCIQEYRNDLTCFTNGQ 600

Query 601 SVCLTELKGYQAAVGQPVIQPRRPNSRLDKVRHMFQKV 638
             VCLTELKGYQ A+G+ + QPRRPNSR+DKVRHMF K+
Sbjct 601 GVCLTELKGYQPAIGKFICQPRRPNSRIDKVRHMFHKL 638

>WP_073930560.1 TetM/TetW/TetO/TetS family tetracycline resistance ribosomal
protection protein [[Clostridium] symbiosum]
Length=638

 Score = 927 bits (2397), Expect = 0.0, Method: Compositional matrix adjust.
 Identities = 437/639 (68%), Positives = 534/639 (84%), Gaps = 1/639 (0%)

Query 1
MKIINIGILAHVDAGKTTLTESLLYASGAISEPGSVEKGTTRTDTMFLERQRGITIQAAV 60
            MKIINIGILAHVDAGKTTLTESLLY SGAI+E G+V+KGTTRTDTM
LERQRGITIQ AV
Sbjct 1
MKIINIGILAHVDAGKTTLTESLLYTSGAIAEQGNVDKGTTRTDTMILERQRGITIQTAV 60

Query 61
TSFQWHRCKVNIVDTPGHMDFLAEVYRSLAVLDGAILVISAKDGVQAQTRILFHALRKMN 120
TSF W+ K+NIVDTPGHMDFL E YRSL+VLDGA+LVISAKDGVQAQTRILFHAL
+KM+
Sbjct 61
TSFCWNDYKINIVDTPGHMDFLTEAYRSLSVLDGAVLVISAKDGVQAQTRILFHALQKMD 120

Query 121
IPTVIFINKIDQAGVDLQSVVQSVRDKLSADIIIKQTVSLSPEIVLEENTDIEAWDAVIE 180
IPT+IFINKIDQ G+DL+ V QS++DKL++D+I+ Q VSLSP+I + + +D++ WD
+I
Sbjct 121
IPTIIFINKIDQNGIDLRRVYQSIKDKLTSDMIVMQEVSLSPKITMTDISDLDKWDMIIS 180

Query 181
NNDKLLEKYIAGEPISREKLVREEQRRVQDASLFPVYYGSAKKGLGIQPLMDAVTGLFQP 240
+D+LLE+Y+A + + ++L E+ +R + SLFPVY+GSAK LG + L++A+T
F
Sbjct 181
GSDELLERYVAEDSLDIQELQYEKCKRTRCCSLFPVYHGSAKDNLGTEKLIEAITETFIT 240

Query 241
IGEQGSAALCGSVFKVEYTDCGQRRVYLRLYSGTLRLRDTVALAGREKLKITEMRIPSKG 300
+ + LCG VFKVEYT+ +R YLRLY GTL LRDT+ L+ +EK+KITEM
IPS G
Sbjct 241
ETDDIQSELCGYVFKVEYTERKKRLSYLRLYHGTLHLRDTLLLSKKEKIKITEMCIPSNG 300

Query 301
EIVRTDTAYPGEIVILPSDSVRLNDVLGDPTRLPRKRWREDPLPMLRTTIAPKTAAQRER 360
EIV D A PGEIVIL D+++LND+LG+ LP K ++P+P+LRTT+ P+
QRE
Sbjct 301
EIVPVDHACPGEIVILADDTLKLNDILGNEKLLPHKTRIDNPMPLLRTTVEPQKPEQREA 360

Query 361
LLDALTQLADTDPLLRCEVDSITHEIILSFLGRVQLEVVSALLSEKYKLETVVKEPSVIY 420
L AL ++ADTDPLL ++D++THEIILSFLG+VQLEV+ +LL EKY +
+KEPSVIY
Sbjct 361 LY-
ALAEIADTDPLLHFDIDTVTHEIILSFLGKVQLEVICSLLEEKYHVGVAMKEPSVIY 419

Query 421
MERPLKAASHTIHIEVPPNPFWASIGLSVTPLSLGSGVQYESRVSLGYLNQSFQNAVRDG 480
+ERP K AS+TIHIEVPPNPFWASIGL+VTPL +GSG QY+S
VSLGYLNQSFQNAV +G
Sbjct 420
LERPQKKASYTIHIEVPPNPFWASIGLTVTPLPVGSGTQYKSEVSLGYLNQSFQNAVMEG 479

Query 481
IRYGLEQGLFGWNVTDCKICFEYGLYYSPVSTPADFRSLAPIVLEQALKESGTQLLEPYL 540
+RYG+EQGL+GW VTDC+ICF+YG+YYSPVSTPADFR LAP+VLEQALK+
+GTQLLEPYL
Sbjct 480

```
VRYGMEQGLYGWGVTDCQICFDYGVYYSPVSTPADFRFLAPVVLEQALKKAGTQLLEPYL  539

Query  541
SFILYAPQEYLSRAYHDAPKYCATIETAQVKKDEVVFTGEIPARCIQAYRTDLAFYTNGR  600
            SF L+APQEYLSRAY+DAPKYCA IE+ +++KDEV+F GEIPARCI  YR DL
FYTNGR
Sbjct  540
SFTLFAPQEYLSRAYNDAPKYCAIIESTRLEKDEVIFKGEIPARCIGEYRNDLNFYTNGR  599

Query  601  SVCLTELKGYQAAVGQPVIQPRRPNSRLDKVRHMFQKVM  639
            SVC+TELKGYQ   G+PV QPRRPNSRLDK+RHMFQK+M
Sbjct  600  SVCITELKGYQETSGEPVFQPRRPNSRLDKIRHMFQKIM  638


>WP_073689108.1 tetracycline resistance ribosomal protection protein
Tet(M) [Streptococcus
salivarius]
Length=639

 Score = 927 bits (2397),  Expect = 0.0, Method: Compositional
matrix adjust.
 Identities = 436/638 (68%), Positives = 522/638 (82%), Gaps = 0/638
(0%)

Query  1
MKIINIGILAHVDAGKTTLTESLLYASGAISEPGSVEKGTTRTDTMFLERQRGITIQAAV  60
            MKIINIG+LAHVDAGKTTLTESLLY SGAI+E GSV+KGTTRTD
LERQRGITIQ  +
Sbjct  1
MKIINIGVLAHVDAGKTTLTESLLYNSGAITELGSVDKGTTRTDNTLLERQRGITIQTGI  60

Query  61
TSFQWHRCKVNIVDTPGHMDFLAEVYRSLAVLDGAILVISAKDGVQAQTRILFHALRKMN  120
            TSFQW   KVNI+DTPGHMDFLAEVYRSL+VLDGAIL
+ISAKDGVQAQTRILFHALRKM
Sbjct  61
TSFQWENTKVNIIDTPGHMDFLAEVYRSLSVLDGAILLISAKDGVQAQTRILFHALRKMG  120

Query  121
IPTVIFINKIDQAGVDLQSVVQSVRDKLSADIIIKQTVSLSPEIVLEENTDIEAWDAVIE  180
            IPT+ FINKIDQ G+DL +V Q +++KLSA+I+IKQ V L P + +   T+ E WD
VIE
Sbjct  121
IPTIFFINKIDQNGIDLSTVYQDIKEKLSAEIVIKQKVELYPNMCVTNFTESEQWDTVIE  180

Query  181
NNDKLLEKYIAGEPISREKLVREEQRRVQDASLFPVYYGSAKKGLGIQPLMDAVTGLFQP  240
             ND LLEKY++G+ +   +L +EE  R Q+ SLFP+Y+GSAK  +GI  L++ +T
F
Sbjct  181
GNDDLLEKYMSGKSLEALELAQEESIRFQNCSLFPLYHGSAKSNIGIDNLIEVITNKFYS  240

Query  241
IGEQGSAALCGSVFKVEYTDCGQRRVYLRLYSGTLRLRDTVALAGREKLKITEMRIPSKG  300
```

+G + LCG+VFK+EYT   QR Y+RLYSG L LRD+V ++ +EK+K+TEM
G
Sbjct 241
STHRGPSELCGNVFKIEYTKKRQRLAYIRLYSGVLHLRDSVRVSEKEKIKVTEMYTSING 300

Query 301
EIVRTDTAYPGEIVILPSDSVRLNDVLGDPTRLPRKRWREDPLPMLRTTIAPKTAAQRER 360
E+ + D AY GEIVIL ++ ++LN VLGD   LP+++ E+P P+L+TT+ P
QRE
Sbjct 301
ELCKIDRAYSGEIVILQNEFLKLNSVLGDTKLLPQRKKIENPHPLLQTTVEPSKPEQREM 360

Query 361
LLDALTQLADTDPLLRCEVDSITHEIILSFLGRVQLEVVSALLSEKYKLETVVKEPSVIY 420
LLDAL +++D+DPLLR VDS THEIILSFLG+VQ+EV+SALL EKY +E +KEP
+VIY
Sbjct 361
LLDALLEISDSDPLLRYYVDSTTHEIILSFLGKVQMEVISALLQEKYHVEIELKEPTVIY 420

Query 421
MERPLKAASHTIHIEVPPNPFWASIGLSVTPLSLGSGVQYESRVSLGYLNQSFQNAVRDG 480
MERPLK A +TIHIEVPPNPFWASIGLSV+PL LGSG+QYES
VSLGYLNQSFQNAV +G
Sbjct 421
MERPLKNAEYTIHIEVPPNPFWASIGLSVSPLPLGSGMQYESSVSLGYLNQSFQNAVMEG 480

Query 481
IRYGLEQGLFGWNVTDCKICFEYGLYYSPVSTPADFRSLAPIVLEQALKESGTQLLEPYL 540
IRYG EQGL+GWNVTDCKICF+YGLYYSPVSTPADFR LAPIVLEQ LK++GT
+LLEPYL
Sbjct 481
IRYGCEQGLYGWNVTDCKICFKYGLYYSPVSTPADFRMLAPIVLEQVLKKAGTELLEPYL 540

Query 541
SFILYAPQEYLSRAYHDAPKYCATIETAQVKKDEVVFTGEIPARCIQAYRTDLAFYTNGR 600
SF +YAPQEYLSRAY+DAPKYCA I   Q+K +EV+ +GEIPARCIQ YR+DL F
+TNGR
Sbjct 541
SFKIYAPQEYLSRAYNDAPKYCANIVDTQLKNNEVILSGEIPARCIQEYRSDLTFFTNGR 600

Query 601 SVCLTELKGYQAAVGQPVIQPRRPNSRLDKVRHMFQKV 638
SVCLTELKGY   G+PV QPRRPNSR+DKVR+MF K+
Sbjct 601 SVCLTELKGYHVTTGEPVCQPRRPNSRIDKVRYMFNKI 638

>SCJ39489.1 Tetracycline resistance protein tetM from transposon
Tn916 [uncultured
Flavonifractor sp.]
Length=639

Score = 927 bits (2397), Expect = 0.0, Method: Compositional
matrix adjust.
Identities = 430/638 (67%), Positives = 530/638 (83%), Gaps = 0/638
(0%)

```
Query  1
MKIINIGILAHVDAGKTTLTESLLYASGAISEPGSVEKGTTRTDTMFLERQRGITIQAAV  60
            MKIIN+GILAHVDAGKTTLTESLLY SGAI+EPGSV+KGTTRTDTM
LERQRGITIQ AV
Sbjct  1
MKIINLGILAHVDAGKTTLTESLLYTSGAIAEPGSVDKGTTRTDTMNLERQRGITIQTAV  60

Query  61
TSFQWHRCKVNIVDTPGHMDFLAEVYRSLAVLDGAILVISAKDGVQAQTRILFHALRKMN  120
            TSFQW   KVNI+DTPGHMDFLAEVYRSL+VLDGA+L++SAKDG+QAQTRILFHAL
+ M
Sbjct  61
TSFQWEDVKVNIIDTPGHMDFLAEVYRSLSVLDGAVLLVSAKDGIQAQTRILFHALQTMK  120

Query  121
IPTVIFINKIDQAGVDLQSVVQSVRDKLSADIIIKQTVSLSPEIVLEENTDIEAWDAVIE  180
            IPT+ FINKIDQ G+DL  V + ++ KLS++II+KQ V   P I + +N D+E
WDAVI
Sbjct  121
IPTIFFINKIDQEGIDLPMVYREMKAKLSSEIIVKQKVGQHPHINVTDNDDMEQWDAVIM  180

Query  181
NNDKLLEKYIAGEPISREKLVREEQRRVQDASLFPVYYGSAKKGLGIQPLMDAVTGLFQP  240
             ND+LLEKY++G+P    +L +EE RR Q+ +LFPVY+GSAK  LGI+ L++ +
F
Sbjct  181
GNDELLEKYMSGKPFKMSELEQEENRRFQNGTLFPVYHGSAKNNLGIRQLIEVIASKFYS  240

Query  241
IGEQGSAALCGSVFKVEYTDCGQRRVYLRLYSGTLRLRDTVALAGREKLKITEMRIPSKG  300
               +G + LCG VFK+EY++  +R VY+R+YSGTL LRD + ++ +EK+KITEM
+P+ G
Sbjct  241
STPEGQSELCGQVFKIEYSEKRRRFVYVRIYSGTLHLRDVIRISEKEKIKITEMCVPTNG  300

Query  301
EIVRTDTAYPGEIVILPSDSVRLNDVLGDPTRLPRKRWREDPLPMLRTTIAPKTAAQRER  360
            E+  +DTA  G+IVILP+D ++LN +LG+   LP++++ E+PLPML+TTIA K +
QRE
Sbjct  301
ELYSSDTACSGDIVILPNDVLQLNSILGNEILLPQRKFIENPLPMLQTTIAVKKSEQREI  360

Query  361
LLDALTQLADTDPLLRCEVDSITHEIILSFLGRVQLEVVSALLSEKYKLETVVKEPSVIY  420
            LL ALT+++D DPLL+  VD+ THEIILSFLG VQ+EV+ A+L EKY +E  +KEP
+VIY
Sbjct  361
LLGALTEISDGDPLLKYYVDTTTHEIILSFLGNVQMEVICAILEEKYHVEAEIKEPTVIY  420

Query  421
MERPLKAASHTIHIEVPPNPFWASIGLSVTPLSLGSGVQYESRVSLGYLNQSFQNAVRDG  480
            MERPL+ A +TIHIEVPPNPFWAS+GLS+ PL
+GSGVQYESRVSLGYLNQSFQNAV +G
```

```
Sbjct  421
MERPLRKAEYTIHIEVPPNPFWASVGLSIEPLPIGSGVQYESRVSLGYLNQSFQNAVMEG  480

Query  481
IRYGLEQGLFGWNVTDCKICFEYGLYYSPVSTPADFRSLAPIVLEQALKESGTQLLEPYL  540
            + YG EQGL+GW VTDCKICFEYGLYYSPVSTPADFR L+PIVLEQALK++GT
+LLEPYL
Sbjct  481
VLYGCEQGLYGWKVTDCKICFEYGLYYSPVSTPADFRLLSPIVLEQALKKAGTELLEPYL  540

Query  541
SFILYAPQEYLSRAYHDAPKYCATIETAQVKKDEVVFTGEIPARCIQAYRTDLAFYTNGR  600
             F +YAPQEYLSRAYHDAP+YCA I + Q+K DEV+  GEIPARCIQ YR DL +
+TNG+
Sbjct  541
HFEIYAPQEYLSRAYHDAPRYCADIVSTQIKNDEVILKGEIPARCIQEYRNDLTYFTNGQ  600

Query  601  SVCLTELKGYQAAVGQPVIQPRRPNSRLDKVRHMFQKV  638
             VCLTELKGYQ A+G+ + QPRRPNSR+DKVRHMF K+
Sbjct  601  GVCLTELKGYQPAIGKFICQPRRPNSRIDKVRHMFHKL  638


>WP_024415696.1 TetM/TetW/TetO/TetS family tetracycline resistance
ribosomal
protection protein [Streptococcus suis]
Length=639

 Score = 927 bits (2397),  Expect = 0.0, Method: Compositional
matrix adjust.
 Identities = 430/638 (67%), Positives = 529/638 (83%), Gaps = 0/638
(0%)

Query  1
MKIINIGILAHVDAGKTTLTESLLYASGAISEPGSVEKGTTRTDTMFLERQRGITIQAAV  60
            MKIIN+GILAHVDAGKTTLTESLLY SGAI+EPGSV+KGTTRTDTM
LERQRGITIQ AV
Sbjct  1
MKIINLGILAHVDAGKTTLTESLLYTSGAIAEPGSVDKGTTRTDTMNLERQRGITIQTAV  60

Query  61
TSFQWHRCKVNIVDTPGHMDFLAEVYRSLAVLDGAILVISAKDGVQAQTRILFHALRKMN  120
            TSFQW   KVNI+DTPGHMDFLAEVYRSL+VLDGA+L++SAKDG+QAQTRILFHAL
+ M
Sbjct  61
TSFQWEDVKVNIIDTPGHMDFLAEVYRSLSVLDGAVLLVSAKDGIQAQTRILFHALQTMK  120

Query  121
IPTVIFINKIDQAGVDLQSVVQSVRDKLSADIIIKQTVSLSPEIVLEENTDIEAWDAVIE  180
            IPT+ FINKIDQ G+DL  V Q ++ KLS++II+KQ V   P I + +N D+E
WDAVI
Sbjct  121
IPTIFFINKIDQEGIDLPMVYQEMKAKLSSEIIVKQKVGQHPHINVTDNDDMEQWDAVIM  180

Query  181
```

```
NNDKLLEKYIAGEPISREKLVREEQRRVQDASLFPVYYGSAKKGLGIQPLMDAVTGLFQP  240
             ND+LLEKY++G+P    +L +EE RR Q+ +LFPVY+GSAK  LGI+ L++ +
F
Sbjct  181
GNDELLEKYMSGKPFKMSELEQEENRRFQNGTLFPVYHGSAKNNLGIRQLIEVIASKFYS  240

Query  241
IGEQGSAALCGSVFKVEYTDCGQRRVYLRLYSGTLRLRDTVALAGREKLKITEMRIPSKG  300
               +G + LCG VFK+EY++  +R VY+R+YSGTL LRD + ++ +EK+KITEM
+P+ G
Sbjct  241
STPEGQSELCGQVFKIEYSEKRRRFVYVRIYSGTLHLRDVIKISEKEKIKITEMCVPTNG  300

Query  301
EIVRTDTAYPGEIVILPSDSVRLNDVLGDPTRLPRKRWREDPLPMLRTTIAPKTAAQRER  360
            E+  +DTA  G+IVILP+D ++LN +LG+   LP++++ E+PLPML+TTIA K +
QRE
Sbjct  301
ELYSSDTACSGDIVILPNDVLQLNSILGNEMLLPQRKFIENPLPMLQTTIAVKKSEQREI  360

Query  361
LLDALTQLADTDPLLRCEVDSITHEIILSFLGRVQLEVVSALLSEKYKLETVVKEPSVIY  420
            LL ALT+++D DPLL+  VD+ THEIILSFLG VQ+EV+ A+L EKY +E  +KEP
+VIY
Sbjct  361
LLGALTEISDGDPLLKYYVDTTTHEIILSFLGNVQMEVICAILEEKYHVEAEIKEPTVIY  420

Query  421
MERPLKAASHTIHIEVPPNPFWASIGLSVTPLSLGSGVQYESRVSLGYLNQSFQNAVRDG  480
            ME PL+ A +TIHIEVPPNPFWAS+GLS+ PL
+GSGVQYESRVSLGYLNQSFQNAV +G
Sbjct  421
MEIPLRKAEYTIHIEVPPNPFWASVGLSIEPLPIGSGVQYESRVSLGYLNQSFQNAVMEG  480

Query  481
IRYGLEQGLFGWNVTDCKICFEYGLYYSPVSTPADFRSLAPIVLEQALKESGTQLLEPYL  540
            + YG EQGL+GW VTDCKICFEYGLYYSPVSTPADFR L+PIVLEQALK++GT
+LLEPYL
Sbjct  481
VLYGCEQGLYGWKVTDCKICFEYGLYYSPVSTPADFRLLSPIVLEQALKKAGTELLEPYL  540

Query  541
SFILYAPQEYLSRAYHDAPKYCATIETAQVKKDEVVFTGEIPARCIQAYRTDLAFYTNGR  600
             F +YAPQEYLSRAYHDAP+YCA I + Q+K DEV+  GEIPARCIQ YR DL +
+TNG+
Sbjct  541
HFEIYAPQEYLSRAYHDAPRYCADIVSTQIKNDEVILKGEIPARCIQEYRNDLTYFTNGQ  600

Query  601  SVCLTELKGYQAAVGQPVIQPRRPNSRLDKVRHMFQKV  638
             VCLTELKGYQ A+G+ + QPRRPNSR+DKVRHMF K+
Sbjct  601  GVCLTELKGYQPAIGKFICQPRRPNSRIDKVRHMFHKL  638


>WP_105141148.1 TetM/TetW/TetO/TetS family tetracycline resistance
```

ribosomal
protection protein [Streptococcus suis]
Length=639

 Score = 927 bits (2396), Expect = 0.0, Method: Compositional
matrix adjust.
 Identities = 431/638 (68%), Positives = 526/638 (82%), Gaps = 0/638
(0%)

```
Query  1
MKIINIGILAHVDAGKTTLTESLLYASGAISEPGSVEKGTTRTDTMFLERQRGITIQAAV  60
            MKIIN+GILAHVDAGKTTLTESLLY SGAI+EPGSV+KGTTRTDTM
LERQRGITIQ AV
Sbjct  1
MKIINLGILAHVDAGKTTLTESLLYTSGAIAEPGSVDKGTTRTDTMNLERQRGITIQTAV  60

Query  61
TSFQWHRCKVNIVDTPGHMDFLAEVYRSLAVLDGAILVISAKDGVQAQTRILFHALRKMN  120
            TSFQW   KVNI+DTPGHMDFLAEVYRSL+VLDGA+L++SAKDG+QAQTRILFHAL
+ M
Sbjct  61
TSFQWEDVKVNIIDTPGHMDFLAEVYRSLSVLDGAVLLVSAKDGIQAQTRILFHALQTMK  120

Query  121
IPTVIFINKIDQAGVDLQSVVQSVRDKLSADIIIKQTVSLSPEIVLEENTDIEAWDAVIE  180
            IPT+ FINKIDQ G+DL  V Q ++ KLS++II+KQ V   P I + +N D+E
WDAVI
Sbjct  121
IPTIFFINKIDQEGIDLPMVYQEMKAKLSSEIIVKQKVGQHPHINVTDNDDMEQWDAVIM  180

Query  181
NNDKLLEKYIAGEPISREKLVREEQRRVQDASLFPVYYGSAKKGLGIQPLMDAVTGLFQP  240
             ND+LLEKY++G+P    +L +EE RR Q+ +LFPVY+GSAK  LGI+ L++ +
F
Sbjct  181
GNDELLEKYMSGKPFKMSELEQEENRRFQNGTLFPVYHGSAKNNLGIRQLIEVIASKFYS  240

Query  241
IGEQGSAALCGSVFKVEYTDCGQRRVYLRLYSGTLRLRDTVALAGREKLKITEMRIPSKG  300
             +G + LCG VFK+EY++  +R  Y+RLYSG L  RD V ++ +EK+KITEM
IP+ G
Sbjct  241
STPEGQSELCGQVFKIEYSEKRRRLAYVRLYSGALHSRDVVRISEKEKIKITEMSIPTNG  300

Query  301
EIVRTDTAYPGEIVILPSDSVRLNDVLGDPTRLPRKRWREDPLPMLRTTIAPKTAAQRER  360
            E+   DTAY G+IVILP+D ++LN +LG+   LP++++ E+PLPML+TTI  K +
QRE
Sbjct  301
ELCLADTAYSGDIVILPNDVLQLNSILGNEILLPQRKFIENPLPMLQTTIEVKKSEQREV  360

Query  361
LLDALTQLADTDPLLRCEVDSITHEIILSFLGRVQLEVVSALLSEKYKLETVVKEPSVIY  420
            LL+ALT+++D DPLL+  VD+ THEIILSFLG VQ+EV+ A+L EKY +E  +KEP
```

+VIY
Sbjct 361
LLEALTEISDGDPLLKYYVDTTTHEIILSFLGNVQMEVICAILEEKYHVEAEIKEPTVIY 420

Query 421
MERPLKAASHTIHIEVPPNPFWASIGLSVTPLSLGSGVQYESRVSLGYLNQSFQNAVRDG 480
            MERPL+ A +TIHIEVPPNPFWAS+GLS+ PL
+GSGVQYESRVSLGYLNQSFQNAV +G
Sbjct 421
MERPLRKAEYTIHIEVPPNPFWASVGLSIEPLPIGSGVQYESRVSLGYLNQSFQNAVMEG 480

Query 481
IRYGLEQGLFGWNVTDCKICFEYGLYYSPVSTPADFRSLAPIVLEQALKESGTQLLEPYL 540
            + YG EQGL+GW VTDCKICFEYGLYYSPVSTPADFR L+PIVLEQALK++GT
+LLEPYL
Sbjct 481
VLYGCEQGLYGWKVTDCKICFEYGLYYSPVSTPADFRLLSPIVLEQALKKAGTELLEPYL 540

Query 541
SFILYAPQEYLSRAYHDAPKYCATIETAQVKKDEVVFTGEIPARCIQAYRTDLAFYTNGR 600
             F +YAPQEYLSRAYHDAP+YCA I + Q+K DEV+ GEIPARCIQ YR DL
+TNG+
Sbjct 541
HFEIYAPQEYLSRAYHDAPRYCADIVSTQIKNDEVILKGEIPARCIQEYRNDLTNFTNGQ 600

Query 601 SVCLTELKGYQAAVGQPVIQPRRPNSRLDKVRHMFQKV 638
             VCLTELKGYQ A+G+ + QPRRPNSR+DKVRHMF K+
Sbjct 601 GVCLTELKGYQPAIGKFICQPRRPNSRIDKVRHMFHKL 638

>WP_105130533.1 TetM/TetW/TetO/TetS family tetracycline resistance ribosomal
protection protein [Streptococcus suis]
Length=639

 Score = 927 bits (2396), Expect = 0.0, Method: Compositional matrix adjust.
 Identities = 430/638 (67%), Positives = 529/638 (83%), Gaps = 0/638 (0%)

Query 1
MKIINIGILAHVDAGKTTLTESLLYASGAISEPGSVEKGTTRTDTMFLERQRGITIQAAV 60
            MKIIN+GILAHVDAGKTTLTESLLY SGAI+EPGSV++GTTRTDTM
LERQRGITIQ AV
Sbjct 1
MKIINLGILAHVDAGKTTLTESLLYTSGAIAEPGSVDEGTTRTDTMNLERQRGITIQTAV 60

Query 61
TSFQWHRCKVNIVDTPGHMDFLAEVYRSLAVLDGAILVISAKDGVQAQTRILFHALRKMN 120
            TSFQW   KVNI+DTPGHMDFLAEVYRSL+VLDGA+L++SAKDG+QAQTRILFHAL
+ M
Sbjct 61
TSFQWEDVKVNIIDTPGHMDFLAEVYRSLSVLDGAVLLVSAKDGIQAQTRILFHALQTMK 120

Query 121
IPTVIFINKIDQAGVDLQSVVQSVRDKLSADIIIKQTVSLSPEIVLEENTDIEAWDAVIE 180
IPT+ FINKIDQ G+DL V Q ++ KLS++II+KQ V P I + +N D+E
WDAVI
Sbjct 121
IPTIFFINKIDQEGIDLPMVYQEMKAKLSSEIIVKQKVGQHPHINVTDNDDMEQWDAVIM 180

Query 181
NNDKLLEKYIAGEPISREKLVREEQRRVQDASLFPVYYGSAKKGLGIQPLMDAVTGLFQP 240
ND+LLEKY++G+P +L +EE RR Q+ +LFPVY+GSAK LGI+ L++ +
F
Sbjct 181
GNDELLEKYMSGKPFKMSELEQEENRRFQNGTLFPVYHGSAKNNLGIRQLIEVIASKFYS 240

Query 241
IGEQGSAALCGSVFKVEYTDCGQRRVYLRLYSGTLRLRDTVALAGREKLKITEMRIPSKG 300
+G + LCG VFK+EY++ +R VY+R+YSGTL LRD + ++ +EK+KITEM
+P+ G
Sbjct 241
STPEGQSELCGQVFKIEYSEKRRRFVYVRIYSGTLHLRDVIKISEKEKIKITEMCVPTNG 300

Query 301
EIVRTDTAYPGEIVILPSDSVRLNDVLGDPTRLPRKRWREDPLPMLRTTIAPKTAAQRER 360
E+ +DTA G+IVILP+D ++LN +LG+ LP++++ E+PLPML+TTIA K +
QRE
Sbjct 301
ELYSSDTACSGDIVILPNDVLQLNSILGNEMLLPQRKFIENPLPMLQTTIAVKKSEQREI 360

Query 361
LLDALTQLADTDPLLRCEVDSITHEIILSFLGRVQLEVVSALLSEKYKLETVVKEPSVIY 420
LL ALT+++D DPLL+ VD+ THEIILSFLG VQ+EV+ A+L EKY +E +KEP
+VIY
Sbjct 361
LLGALTEISDGDPLLKYYVDTTTHEIILSFLGNVQMEVICAILEEKYHVEAEIKEPTVIY 420

Query 421
MERPLKAASHTIHIEVPPNPFWASIGLSVTPLSLGSGVQYESRVSLGYLNQSFQNAVRDG 480
MERPL+ A +TIHIEVPPNPFWAS+GLS+ PL
+GSGVQYESRVSLGYLNQSFQNAV +G
Sbjct 421
MERPLRKAEYTIHIEVPPNPFWASVGLSIEPLPIGSGVQYESRVSLGYLNQSFQNAVMEG 480

Query 481
IRYGLEQGLFGWNVTDCKICFEYGLYYSPVSTPADFRSLAPIVLEQALKESGTQLLEPYL 540
+ YG EQGL+GW VTDCKICFEYGLYYSPVSTPADFR L+PIVLEQALK++GT
+LLEPYL
Sbjct 481
VLYGCEQGLYGWKVTDCKICFEYGLYYSPVSTPADFRLLSPIVLEQALKKAGTELLEPYL 540

Query 541
SFILYAPQEYLSRAYHDAPKYCATIETAQVKKDEVVFTGEIPARCIQAYRTDLAFYTNGR 600
F +YAPQEYLSRAYHDAP+YCA I + Q+K DEV+ GEIPARCIQ YR DL
+TNG+
Sbjct 541

```
HFEIYAPQEYLSRAYHDAPRYCADIVSTQIKNDEVILKGEIPARCIQEYRNDLTNFTNGQ  600

Query  601  SVCLTELKGYQAAVGQPVIQPRRPNSRLDKVRHMFQKV  638
             VCLTELKGYQ A+G+ + QPRRPNSR+DKVRHMF K+
Sbjct  601  GVCLTELKGYQPAIGKFICQPRRPNSRIDKVRHMFHKL  638
```

>WP_105095358.1 TetM/TetW/TetO/TetS family tetracycline resistance ribosomal
protection protein [Streptococcus suis]
Length=639

Score = 927 bits (2396), Expect = 0.0, Method: Compositional matrix adjust.
Identities = 431/638 (68%), Positives = 527/638 (83%), Gaps = 0/638 (0%)

```
Query  1
MKIININIGILAHVDAGKTTLTESLLYASGAISEPGSVEKGTTRTDTMFLERQRGITIQAAV  60
            MKIIN+GILAHVDAGKTTLTESLLY SGAI+EPGSV+KGTTRTDTM
LERQRGITIQ AV
Sbjct  1
MKIINLGILAHVDAGKTTLTESLLYTSGAIAEPGSVDKGTTRTDTMNLERQRGITIQTAV  60

Query  61
TSFQWHRCKVNIVDTPGHMDFLAEVYRSLAVLDGAILVISAKDGVQAQTRILFHALRKMN  120
            TSFQW   KVNI+DTPGHMDFLAEVYRSL+VLDGA+L++SAKDG+QAQTRILFHAL
+ M
Sbjct  61
TSFQWEDVKVNIIDTPGHMDFLAEVYRSLSVLDGAVLLVSAKDGIQAQTRILFHALQTMK  120

Query  121
IPTVIFINKIDQAGVDLQSVVQSVRDKLSADIIIKQTVSLSPEIVLEENTDIEAWDAVIE  180
            IPT+ FINKIDQ G+DL  V + ++ KLS++II+KQ V   P I + +N D+E
WDAVI
Sbjct  121
IPTIFFINKIDQEGIDLPMVYREMKAKLSSEIIVKQKVGQHPHINVTDNDDMEQWDAVIM  180

Query  181
NNDKLLEKYIAGEPISREKLVREEQRRVQDASLFPVYYGSAKKGLGIQPLMDAVTGLFQP  240
             ND+LLEKY++G+P    +L +EE RR Q+ +LFPVY+GSAK  LGI+ L++ +
F
Sbjct  181
GNDELLEKYMSGKPFKMSELEQEENRRFQNGTLFPVYHGSAKNNLGIRQLIEVIASKFYS  240

Query  241
IGEQGSAALCGSVFKVEYTDCGQRRVYLRLYSGTLRLRDTVALAGREKLKITEMRIPSKG  300
               +G + LCG VFK+EY++  +R  Y+RLYSG L  RD V ++ +EK+KITEM
IP+ G
Sbjct  241
STPEGQSELCGQVFKIEYSEKRRRLAYVRLYSGALHSRDVVRISEKEKIKITEMSIPTNG  300

Query  301
EIVRTDTAYPGEIVILPSDSVRLNDVLGDPTRLPRKRWREDPLPMLRTTIAPKTAAQRER  360
```

E+   DTAY G+IVILP+D ++LN +LG+   LP++++ E+PLPML+TTIA K +
QRE
Sbjct 301
ELCLADTAYSGDIVILPNDVLQLNSILGNEILLPQRKFIENPLPMLQTTIAVKKSEQREI 360

Query 361
LLDALTQLADTDPLLRCEVDSITHEIILSFLGRVQLEVVSALLSEKYKLETVVKEPSVIY 420
LL ALT+++D DPLL+ VD+ THEIILSFLG VQ+EV+ A+L EKY +E +KEP
+VIY
Sbjct 361
LLGALTEISDGDPLLKYYVDTTTHEIILSFLGNVQMEVICAILEEKYHVEAEIKEPTVIY 420

Query 421
MERPLKAASHTIHIEVPPNPFWASIGLSVTPLSLGSGVQYESRVSLGYLNQSFQNAVRDG 480
MERPL+ A +TIHIEVPPNPFWAS+GLS+ PL
+GSGVQYESRVSLGYLNQSFQNAV +G
Sbjct 421
MERPLRKAEYTIHIEVPPNPFWASVGLSIEPLPIGSGVQYESRVSLGYLNQSFQNAVMEG 480

Query 481
IRYGLEQGLFGWNVTDCKICFEYGLYYSPVSTPADFRSLAPIVLEQALKESGTQLLEPYL 540
+ YG EQGL+GW VTDCKICFEYGLYYSPVSTPADFR L+PIVLEQALK++GT
+LLEPYL
Sbjct 481
VLYGCEQGLYGWKVTDCKICFEYGLYYSPVSTPADFRLLSPIVLEQALKKAGTELLEPYL 540

Query 541
SFILYAPQEYLSRAYHDAPKYCATIETAQVKKDEVVFTGEIPARCIQAYRTDLAFYTNGR 600
F +YAPQEYLSRAYHDAP+YCA I + Q+K DEV+ GEIPARCIQ YR DL +
+TNG+
Sbjct 541
HFEIYAPQEYLSRAYHDAPRYCADIVSTQIKNDEVILKGEIPARCIQEYRNDLTYFTNGQ 600

Query 601 SVCLTELKGYQAAVGQPVIQPRRPNSRLDKVRHMFQKV 638
VCLTELKGYQ A+G+ + QPRRPNSR+DKVRHMF K+
Sbjct 601 GVCLTELKGYQPAIGKFICQPRRPNSRIDKVRHMFHKL 638

>WP_095122938.1 TetM/TetW/TetO/TetS family tetracycline resistance
ribosomal
protection protein [Streptococcus acidominimus]
SNV41999.1 tetracycline resistance protein tetM [Streptococcus
acidominimus]
Length=639

Score = 927 bits (2396), Expect = 0.0, Method: Compositional
matrix adjust.
Identities = 429/638 (67%), Positives = 529/638 (83%), Gaps = 0/638
(0%)

Query 1
MKIINIGILAHVDAGKTTLTESLLYASGAISEPGSVEKGTTRTDTMFLERQRGITIQAAV 60
MKIIN+GILAHVDAGKTTLTESLLY SGAI+EPGSV+KGTTRTDTM
LERQRGITIQ AV

Sbjct 1
MKIINLGILAHVDAGKTTLTESLLYTSGAIAEPGSVDKGTTRTDTMNLERQRGITIQTAV 60

Query 61
TSFQWHRCKVNIVDTPGHMDFLAEVYRSLAVLDGAILVISAKDGVQAQTRILFHALRKMN 120
            TSFQW   KVNI+DTPGHMDFLAEVYRSL+VLDGA+L++SAKDG+QAQTRILFHAL
+ M
Sbjct 61
TSFQWEDVKVNIIDTPGHMDFLAEVYRSLSVLDGAVLLVSAKDGIQAQTRILFHALQTMK 120

Query 121
IPTVIFINKIDQAGVDLQSVVQSVRDKLSADIIIKQTVSLSPEIVLEENTDIEAWDAVIE 180
            IPT+ FINKIDQ G+DL V + ++ KLS++II+KQ V   P I + +N D+E WD
VI
Sbjct 121
IPTIFFINKIDQEGIDLPMVYREMKAKLSSEIIVKQKVGQHPHINVTDNDDMEQWDTVIM 180

Query 181
NNDKLLEKYIAGEPISREKLVREEQRRVQDASLFPVYYGSAKKGLGIQPLMDAVTGLFQP 240
             ND+LLEKY++G+P   +L +EE RR Q+ +LFPVY+GSAK LGI+ L++ +
F
Sbjct 181
GNDELLEKYMSGKPFKMSELEQEENRRFQNGTLFPVYHGSAKNNLGIRQLIEVIASKFYS 240

Query 241
IGEQGSAALCGSVFKVEYTDCGQRRVYLRLYSGTLRLRDTVALAGREKLKITEMRIPSKG 300
               +G + LCG VFK+EY++ +R VY+R+YSGTL LRD + ++ +EK+KITEM
+P+ G
Sbjct 241
STPEGQSELCGQVFKIEYSEKRRRFVYVRIYSGTLHLRDVIRISEKEKIKITEMCVPTNG 300

Query 301
EIVRTDTAYPGEIVILPSDSVRLNDVLGDPTRLPRKRWREDPLPMLRTTIAPKTAAQRER 360
            E+ +DTA G+IVILP+D ++LN +LG+   LP++++ E+PLPML+TTIA K +
QRE
Sbjct 301
ELYSSDTACSGDIVILPNDVLQLNSILGNEMLLPQRKFIENPLPMLQTTIAVKKSEQREI 360

Query 361
LLDALTQLADTDPLLRCEVDSITHEIILSFLGRVQLEVVSALLSEKYKLETVVKEPSVIY 420
            LL ALT+++D DPLL+ VD+ THEIILSFLG VQ+EV+ A+L EKY +E +KEP
+VIY
Sbjct 361
LLGALTEISDGDPLLKYYVDTTTHEIILSFLGNVQMEVICAILEEKYHVEAEIKEPTVIY 420

Query 421
MERPLKAASHTIHIEVPPNPFWASIGLSVTPLSLGSGVQYESRVSLGYLNQSFQNAVRDG 480
            MERPL+ A +TIHIEVPPNPFWAS+GLS+ PL
+GSGVQYESRVSLGYLNQSFQNAV +G
Sbjct 421
MERPLRKAEYTIHIEVPPNPFWASVGLSIEPLPIGSGVQYESRVSLGYLNQSFQNAVMEG 480

Query 481
IRYGLEQGLFGWNVTDCKICFEYGLYYSPVSTPADFRSLAPIVLEQALKESGTQLLEPYL 540

            + YG EQGL+GW VTDCKICFEYGLYYSPVSTPADFR L+PIVLEQALK++GT
+LLEPYL
Sbjct 481
VLYGCEQGLYGWKVTDCKICFEYGLYYSPVSTPADFRLLSPIVLEQALKKAGTELLEPYL 540

Query 541
SFILYAPQEYLSRAYHDAPKYCATIETAQVKKDEVVFTGEIPARCIQAYRTDLAFYTNGR 600
             F +YAPQEYLSRAYHDAP+YCA I + Q+K DEV+ GEIPARCIQ YR DL +
+TNG+
Sbjct 541
HFEIYAPQEYLSRAYHDAPRYCADIVSTQIKNDEVILKGEIPARCIQEYRNDLTYFTNGQ 600

Query 601 SVCLTELKGYQAAVGQPVIQPRRPNSRLDKVRHMFQKV 638
             VCLTELKGYQ A+G+ + QPRRPNSR+DKVRHMF K+
Sbjct 601 GVCLTELKGYQTAIGKFICQPRRPNSRIDKVRHMFHKL 638

>WP_063856404.1 tetracycline resistance ribosomal protection protein
Tet(O) [Streptococcus
pyogenes]
 CAG30567.1 tetracycline resistance protein [Streptococcus pyogenes]
 CAR95429.1 tetracycline-resistance protein [Streptococcus phage
phi-m46.1]
Length=639

 Score = 927 bits (2396), Expect = 0.0, Method: Compositional
matrix adjust.
 Identities = 431/638 (68%), Positives = 527/638 (83%), Gaps = 0/638
(0%)

Query 1
MKIINIGILAHVDAGKTTLTESLLYASGAISEPGSVEKGTTRTDTMFLERQRGITIQAAV 60
            MKIIN+GILAHVDAGKTTLTESLLY SGAI+EPGSV+KGTTRTDTM
LERQRGITIQ AV
Sbjct 1
MKIINLGILAHVDAGKTTLTESLLYTSGAIAEPGSVDKGTTRTDTMNLERQRGITIQTAV 60

Query 61
TSFQWHRCKVNIVDTPGHMDFLAEVYRSLAVLDGAILVISAKDGVQAQTRILFHALRKMN 120
            TSFQW   KVNI+DTPGHMDFLAEVYRSL+VLDGA+L++SAKDG+QAQTRILFHAL
+ M
Sbjct 61
TSFQWEDVKVNIIDTPGHMDFLAEVYRSLSVLDGAVLLVSAKDGIQAQTRILFHALQTMK 120

Query 121
IPTVIFINKIDQAGVDLQSVVQSVRDKLSADIIIKQTVSLSPEIVLEENTDIEAWDAVIE 180
            IPT+ FINKIDQ G+DL V Q ++ KLS++II+KQ V   P I + +N D+E
WDAVI
Sbjct 121
IPTIFFINKIDQEGIDLPMVYQEMKAKLSSEIIVKQKVGQHPHINVTDNDDMEQWDAVIM 180

Query 181
NNDKLLEKYIAGEPISREKLVREEQRRVQDASLFPVYYGSAKKGLGIQPLMDAVTGLFQP 240
             ND+LLEKY++G+P   +L +EE R+ Q +LFPVY+GSAK LGI+ L   +

F
Sbjct 181
GNDELLEKYMSGKPFKMSELEQEENRKFQTGTLFPVYHGSAKNNLGIRQLKKGIASKFYS 240

Query 241
IGEQGSAALCGSVFKVEYTDCGQRRVYLRLYSGTLRLRDTVALAGREKLKITEMRIPSKG 300
              +G + LCG VFK+EY++ +R VY+R+YSGTL LRD + ++ +EK+KITEM
+P+ G
Sbjct 241
STPEGQSELCGQVFKIEYSEKRRRFVYVRIYSGTLHLRDVIKISEKEKIKITEMCVPTNG 300

Query 301
EIVRTDTAYPGEIVILPSDSVRLNDVLGDPTRLPRKRWREDPLPMLRTTIAPKTAAQRER 360
           E+ +DTA G+IVILP+D ++LN +LG+   LP++++ E+PLPML+TTIA K +
QRE
Sbjct 301
ELYSSDTACSGDIVILPNDVLQLNSILGNEMLLPQRKFIENPLPMLQTTIAVKKSEQREI 360

Query 361
LLDALTQLADTDPLLRCEVDSITHEIILSFLGRVQLEVVSALLSEKYKLETVVKEPSVIY 420
           LL ALT+++D DPLL+ VD+ THEIILSFLG VQ+EV+ A+L EKY +E +KEP
+VIY
Sbjct 361
LLGALTEISDGDPLLKYYVDTTTHEIILSFLGNVQMEVICAILEEKYHVEAEIKEPTVIY 420

Query 421
MERPLKAASHTIHIEVPPNPFWASIGLSVTPLSLGSGVQYESRVSLGYLNQSFQNAVRDG 480
           MERPL+ A +TIHIEVPPNPFWAS+GLS+ PL
+GSGVQYESRVSLGYLNQSFQNAV +G
Sbjct 421
MERPLRKAEYTIHIEVPPNPFWASVGLSIEPLPIGSGVQYESRVSLGYLNQSFQNAVMEG 480

Query 481
IRYGLEQGLFGWNVTDCKICFEYGLYYSPVSTPADFRSLAPIVLEQALKESGTQLLEPYL 540
           + YG EQGL+GW VTDCKICFEYGLYYSPVSTPADFR L+PIVLEQALK++GT
+LLEPYL
Sbjct 481
VLYGCEQGLYGWKVTDCKICFEYGLYYSPVSTPADFRLLSPIVLEQALKKAGTELLEPYL 540

Query 541
SFILYAPQEYLSRAYHDAPKYCATIETAQVKKDEVVFTGEIPARCIQAYRTDLAFYTNGR 600
            F +YAPQEYLSRAYHDAP+YCA I + QVK DEV+ GEIPARCIQ YR DL +
+TNG+
Sbjct 541
HFEIYAPQEYLSRAYHDAPRYCADIVSTQVKNDEVILKGEIPARCIQEYRNDLTYFTNGQ 600

Query 601 SVCLTELKGYQAAVGQPVIQPRRPNSRLDKVRHMFQKV 638
            VCLTELKGYQ A+G+ + QPRRPNSR+DKVRHMF K+
Sbjct 601 GVCLTELKGYQPAIGKFICQPRRPNSRIDKVRHMFHKL 638

>AMP46920.1 TetM-TetW-TetO-TetS [uncultured bacterium]
Length=639

```
 Score = 927 bits (2395),  Expect = 0.0, Method: Compositional
matrix adjust.
 Identities = 429/638 (67%), Positives = 530/638 (83%), Gaps = 0/638
(0%)

Query  1
MKIINIGILAHVDAGKTTLTESLLYASGAISEPGSVEKGTTRTDTMFLERQRGITIQAAV  60
            MKIIN+GILAHVDAGKTTLTESLLY SGAI+EPGSV++GTTRTDTM
LERQRGITIQ AV
Sbjct  1
MKIINLGILAHVDAGKTTLTESLLYTSGAIAEPGSVDEGTTRTDTMNLERQRGITIQTAV  60

Query  61
TSFQWHRCKVNIVDTPGHMDFLAEVYRSLAVLDGAILVISAKDGVQAQTRILFHALRKMN  120
            TSFQW   KVNI+DTPGHMDFLAEVYRSL+VLDGA+L++SAKDG+QAQTRILFHAL
+ M
Sbjct  61
TSFQWEDVKVNIIDTPGHMDFLAEVYRSLSVLDGAVLLVSAKDGIQAQTRILFHALQTMK  120

Query  121
IPTVIFINKIDQAGVDLQSVVQSVRDKLSADIIIKQTVSLSPEIVLEENTDIEAWDAVIE  180
            IPT+ FINKIDQ G+DL  V + ++ KLS++II+KQ V   P I + +N D+E
WDAVI
Sbjct  121
IPTIFFINKIDQEGIDLPMVYREMKAKLSSEIIVKQKVGQHPHINVTDNDDMEQWDAVIM  180

Query  181
NNDKLLEKYIAGEPISREKLVREEQRRVQDASLFPVYYGSAKKGLGIQPLMDAVTGLFQP  240
             ND+LLEKY++G+P    +L +EE RR Q+ +LFPVY+GSAK  LGI+ L++ +
F
Sbjct  181
GNDELLEKYMSGKPFKMSELEQEENRRFQNGTLFPVYHGSAKNNLGIRQLIEVIASKFYS  240

Query  241
IGEQGSAALCGSVFKVEYTDCGQRRVYLRLYSGTLRLRDTVALAGREKLKITEMRIPSKG  300
               +G + LCG VFK+EY++  +R VY+R+YSGTL LRD + ++ +EK+KITEM
+P+ G
Sbjct  241
STPEGQSELCGQVFKIEYSEKRRRFVYVRIYSGTLHLRDVIRISEKEKIKITEMCVPTNG  300

Query  301
EIVRTDTAYPGEIVILPSDSVRLNDVLGDPTRLPRKRWREDPLPMLRTTIAPKTAAQRER  360
            E+  +DTA  G+IVILP+D ++LN +LG+   LP++++ E+PLPML+TTIA K +
QRE
Sbjct  301
ELYSSDTACSGDIVILPNDVLQLNSILGNEMLLPQRKFIENPLPMLQTTIAVKKSEQREI  360

Query  361
LLDALTQLADTDPLLRCEVDSITHEIILSFLGRVQLEVVSALLSEKYKLETVVKEPSVIY  420
            LL ALT+++D DPLL+  VD+ THEIILSFLG VQ+EV+ A+L EKY +E  +KEP
+VIY
Sbjct  361
LLGALTEISDGDPLLKYYVDTTTHEIILSFLGNVQMEVICAILEEKYHVEAEIKEPTVIY  420
```

Query 421
MERPLKAASHTIHIEVPPNPFWASIGLSVTPLSLGSGVQYESRVSLGYLNQSFQNAVRDG 480
            MERPL+ A +TIHIEVPPNPFWAS+GLS+ PL
+GSGVQYESRVSLGYLNQSFQNAV +G
Sbjct 421
MERPLRKAEYTIHIEVPPNPFWASVGLSIEPLPIGSGVQYESRVSLGYLNQSFQNAVMEG 480

Query 481
IRYGLEQGLFGWNVTDCKICFEYGLYYSPVSTPADFRSLAPIVLEQALKESGTQLLEPYL 540
            + YG EQGL+GW VTDCKICFEYGLYYSPVSTPADFR L+PIVLEQALK++GT
+LLEPYL
Sbjct 481
VLYGCEQGLYGWKVTDCKICFEYGLYYSPVSTPADFRLLSPIVLEQALKKAGTELLEPYL 540

Query 541
SFILYAPQEYLSRAYHDAPKYCATIETAQVKKDEVVFTGEIPARCIQAYRTDLAFYTNGR 600
             F +YAPQEYLSRAYHDAP+YCA I + Q+K DEV+ GEIPARCIQ YR DL +
+TNG+
Sbjct 541
HFEIYAPQEYLSRAYHDAPRYCADIVSTQIKNDEVILKGEIPARCIQEYRNDLTYFTNGQ 600

Query 601 SVCLTELKGYQAAVGQPVIQPRRPNSRLDKVRHMFQKV 638
             VCLTELKGYQ A+G+ + QPRRPNSR+DKVRHMF K+
Sbjct 601 GVCLTELKGYQPAIGKFICQPRRPNSRIDKVRHMFHKL 638

>WP_047205445.1 tetracycline resistance ribosomal protection protein Tet(M) [Streptococcus
agalactiae]
 KLJ80600.1 tetracycline resistance protein tetM [Streptococcus
agalactiae]
Length=639

 Score = 927 bits (2395), Expect = 0.0, Method: Compositional
matrix adjust.
 Identities = 440/638 (69%), Positives = 522/638 (82%), Gaps = 0/638
(0%)

Query 1
MKIINIGILAHVDAGKTTLTESLLYASGAISEPGSVEKGTTRTDTMFLERQRGITIQAAV 60
            MKIINIG+LAHVDAGKTTLTESLLY SGAI+E GSV+KGTTRTD
LERQRGITIQ +
Sbjct 1
MKIINIGVLAHVDAGKTTLTESLLYNSGAITELGSVDKGTTRTDNTLLERQRGITIQTGI 60

Query 61
TSFQWHRCKVNIVDTPGHMDFLAEVYRSLAVLDGAILVISAKDGVQAQTRILFHALRKMN 120
            TSFQW   KVNI+DTPGHMDFLAEVYRSL+VLDGAIL
+ISAKDGVQAQTRILFHALRKM
Sbjct 61
TSFQWENTKVNIIDTPGHMDFLAEVYRSLSVLDGAILLISAKDGVQAQTRILFHALRKMG 120

Query 121
IPTVIFINKIDQAGVDLQSVVQSVRDKLSADIIIKQTVSLSPEIVLEENTDIEAWDAVIE 180

IPT+ FINKIDQ G+DL +V Q +++KLSA+I+IKQ V L P + + T+ E WD
VIE
Sbjct 121
IPTIFFINKIDQNGIDLSTVYQDIKEKLSAEIVIKQKVELYPNMCVTNFTESEQWDTVIE 180

Query 181
NNDKLLEKYIAGEPISREKLVREEQRRVQDASLFPVYYGSAKKGLGIQPLMDAVTGLFQP 240
ND LLEKY++G+ + +L +EE R Q+ SLFPVY+GSAK +GI L++ +T
F
Sbjct 181
GNDDLLEKYMSGKSLEVLELEQEESIRFQNCSLFPVYHGSAKNNIGIDNLIEVITNKFYS 240

Query 241
IGEQGSAALCGSVFKVEYTDCGQRRVYLRLYSGTLRLRDTVALAGREKLKITEMRIPSKG 300
+G + LCG+VFK+EYT QR Y+RLYSG L LRD+V ++ +EK+KITEM
G
Sbjct 241
STHRGQSELCGNVFKIEYTKKRQRLAYIRLYSGVLHLRDSVRISEKEKIKITEMYTSING 300

Query 301
EIVRTDTAYPGEIVILPSDSVRLNDVLGDPTRLPRKRWREDPLPMLRTTIAPKTAAQRER 360
E+ + D AY GEIVIL ++ ++LN VLGD LP++ E+PLP+L+TT+ P
QRE
Sbjct 301
ELCKIDKAYSGEIVILQNEFLKLNSVLGDTKLLPQRERIENPLPLLQTTVEPSKPQQREM 360

Query 361
LLDALTQLADTDPLLRCEVDSITHEIILSFLGRVQLEVVSALLSEKYKLETVVKEPSVIY 420
LLDAL +++D+DPLLR VDS THEIILSFLG+VQ+EV+SALL EKY +E +KEP
+VIY
Sbjct 361
LLDALLEISDSDPLLRYYVDSTTHEIILSFLGKVQMEVISALLQEKYHVEIELKEPTVIY 420

Query 421
MERPLKAASHTIHIEVPPNPFWASIGLSVTPLSLGSGVQYESRVSLGYLNQSFQNAVRDG 480
MERPLK A +TIHIEVPPNPFWASIGLSV+PL LGSGVQYES
VSLGYLNQSFQNAV +G
Sbjct 421
MERPLKNAEYTIHIEVPPNPFWASIGLSVSPLPLGSGVQYESSVSLGYLNQSFQNAVMEG 480

Query 481
IRYGLEQGLFGWNVTDCKICFEYGLYYSPVSTPADFRSLAPIVLEQALKESGTQLLEPYL 540
IRYG EQGL+GWNVTDCKICF+YGLYYSPVSTPADFR LAPIVLEQ LK++GT
+LLEPYL
Sbjct 481
IRYGCEQGLYGWNVTDCKICFKYGLYYSPVSTPADFRMLAPIVLEQVLKKAGTELLEPYL 540

Query 541
SFILYAPQEYLSRAYHDAPKYCATIETAQVKKDEVVFTGEIPARCIQAYRTDLAFYTNGR 600
SF +YAPQEYLSRAY+DAPKYCA I Q+K +EV+ +GEIPARCIQ YR+DL F
+TNGR
Sbjct 541
SFKIYAPQEYLSRAYNDAPKYCANIVDTQLKNNEVILSGEIPARCIQEYRSDLTFFTNGR 600

```
Query  601  SVCLTELKGYQAAVGQPVIQPRRPNSRLDKVRHMFQKV  638
            SVCLTELKGY    G+PV QPRRPNSR+DKVR+MF K+
Sbjct  601  SVCLTELKGYHVTTGEPVCQPRRPNSRIDKVRYMFNKI  638
```

>WP_024395804.1 TetM/TetW/TetO/TetS family tetracycline resistance ribosomal
protection protein [Streptococcus suis]
Length=639

Score = 927 bits (2395), Expect = 0.0, Method: Compositional matrix adjust.
Identities = 430/638 (67%), Positives = 529/638 (83%), Gaps = 0/638 (0%)

```
Query  1
MKIINIGILAHVDAGKTTLTESLLYASGAISEPGSVEKGTTRTDTMFLERQRGITIQAAV  60
            MKIIN+GILAHVDAGKTTLTESLLY SGAI+EPGSV+KGTTRTDTM
LERQRGITIQ AV
Sbjct  1
MKIINLGILAHVDAGKTTLTESLLYTSGAIAEPGSVDKGTTRTDTMNLERQRGITIQTAV  60

Query  61
TSFQWHRCKVNIVDTPGHMDFLAEVYRSLAVLDGAILVISAKDGVQAQTRILFHALRKMN  120
            TSFQW   KVNI+DTPGHMDFLAEVYRSL+VLDGA+L++SAKDG+QAQTRILFHAL
+ M
Sbjct  61
TSFQWEDVKVNIIDTPGHMDFLAEVYRSLSVLDGAVLLVSAKDGIQAQTRILFHALQTMK  120

Query  121
IPTVIFINKIDQAGVDLQSVVQSVRDKLSADIIIKQTVSLSPEIVLEENTDIEAWDAVIE  180
            IPT+ FINKIDQ G+DL  V Q ++ KLS++II+KQ V   P I + +N D+E
WDAVI
Sbjct  121
IPTIFFINKIDQEGIDLPMVYQEMKAKLSSEIIVKQKVGQHPHINVTDNDDMEQWDAVIM  180

Query  181
NNDKLLEKYIAGEPISREKLVREEQRRVQDASLFPVYYGSAKKGLGIQPLMDAVTGLFQP  240
             ND+LLEKY++G+P    +L +EE RR Q+ +LFPVY+GSAK  LGI+ L++ +
F
Sbjct  181
GNDELLEKYMSGKPFKMSELEQEENRRFQNGTLFPVYHGSAKNNLGIRQLIEVIASKFYS  240

Query  241
IGEQGSAALCGSVFKVEYTDCGQRRVYLRLYSGTLRLRDTVALAGREKLKITEMRIPSKG  300
              +G + LCG VFK+EY++  +R VY+R+YSGTL LRD + ++ +EK+KITEM
+P+ G
Sbjct  241
STPEGQSELCGQVFKIEYSEKRRRFVYVRIYSGTLHLRDVIKISEKEKIKITEMCVPTNG  300

Query  301
EIVRTDTAYPGEIVILPSDSVRLNDVLGDPTRLPRKRWREDPLPMLRTTIAPKTAAQRER  360
            E+  +DTA  G+IVILP+D ++LN +LG+   L ++++ E+PLPML+TTIA K +
QRE
```

Sbjct 301
ELYSSDTACSGDIVILPNDVLQLNSILGNEMLLLQRKFIENPLPMLQTTIAVKKSEQREI 360

Query 361
LLDALTQLADTDPLLRCEVDSITHEIILSFLGRVQLEVVSALLSEKYKLETVVKEPSVIY 420
            LL ALT+++D DPLL+ VD+ THEIILSFLG VQ+EV+ A+L EKY +E +KEP
+VIY
Sbjct 361
LLGALTEISDGDPLLKYYVDTTTHEIILSFLGNVQMEVICAILEEKYHVEAEIKEPTVIY 420

Query 421
MERPLKAASHTIHIEVPPNPFWASIGLSVTPLSLGSGVQYESRVSLGYLNQSFQNAVRDG 480
            MERPL+ A +TIHIEVPPNPFWAS+GLS+ PL
+GSGVQYESRVSLGYLNQSFQNAV +G
Sbjct 421
MERPLRKAEYTIHIEVPPNPFWASVGLSIEPLPIGSGVQYESRVSLGYLNQSFQNAVMEG 480

Query 481
IRYGLEQGLFGWNVTDCKICFEYGLYYSPVSTPADFRSLAPIVLEQALKESGTQLLEPYL 540
            + YG EQGL+GW VTDCKICFEYGLYYSPVSTPADFR L+PIVLEQALK++GT
+LLEPYL
Sbjct 481
VLYGCEQGLYGWKVTDCKICFEYGLYYSPVSTPADFRLLSPIVLEQALKKAGTELLEPYL 540

Query 541
SFILYAPQEYLSRAYHDAPKYCATIETAQVKKDEVVFTGEIPARCIQAYRTDLAFYTNGR 600
             F +YAPQEYLSRAYHDAP+YCA I + Q+K DEV+ GEIPARCIQ YR DL +
+TNG+
Sbjct 541
HFEIYAPQEYLSRAYHDAPRYCADIVSTQIKNDEVILKGEIPARCIQEYRNDLTYFTNGQ 600

Query 601 SVCLTELKGYQAAVGQPVIQPRRPNSRLDKVRHMFQKV 638
             VCLTELKGYQ A+G+ + QPRRPNSR+DKVRHMF K+
Sbjct 601 GVCLTELKGYQPAIGKFICQPRRPNSRIDKVRHMFHKL 638

>WP_017144211.1 TetM/TetW/TetO/TetS family tetracycline resistance
ribosomal
protection protein [Coprobacillus sp. D6]
Length=639

 Score = 927 bits (2395), Expect = 0.0, Method: Compositional
matrix adjust.
 Identities = 435/639 (68%), Positives = 534/639 (84%), Gaps = 0/639
(0%)

Query 1
MKIINIGILAHVDAGKTTLTESLLYASGAISEPGSVEKGTTRTDTMFLERQRGITIQAAV 60
            MKIINIGILAHVDAGKTTLTESLLY SGAI+E G+V+KGTTRTDTM LER
+RGITIQ AV
Sbjct 1
MKIINIGILAHVDAGKTTLTESLLYTSGAIAEQGNVDKGTTRTDTMILERKRGITIQTAV 60

Query 61

TSFQWHRCKVNIVDTPGHMDFLAEVYRSLAVLDGAILVISAKDGVQAQTRILFHALRKMN 120
TSF W+ K+NIVDTPGHMDFL E YRSL+VLDGA+LVISAKDGVQAQTRILFHAL
+KMN
Sbjct 61
TSFCWNDYKINIVDTPGHMDFLTEAYRSLSVLDGAVLVISAKDGVQAQTRILFHALQKMN 120

Query 121
IPTVIFINKIDQAGVDLQSVVQSVRDKLSADIIIKQTVSLSPEIVLEENTDIEAWDAVIE 180
IPT+IF+NKIDQ G+DLQ V QS++DKL++D+I+ Q V+LSP+I + + +D++ WD
+I
Sbjct 121
IPTIIFVNKIDQNGIDLQRVYQSIKDKLTSDMIVMQEVALSPKITITDISDLDKWDIIIS 180

Query 181
NNDKLLEKYIAGEPISREKLVREEQRRVQDASLFPVYYGSAKKGLGIQPLMDAVTGLFQP 240
+D+LLE+Y+A + + ++L E+ +R + SLFPVY+GSAK LG + L++A+T
F
Sbjct 181
GSDELLERYVAEDSLDIQELQYEKCKRTRCCSLFPVYHGSAKDNLGTEKLIEAITETFIT 240

Query 241
IGEQGSAALCGSVFKVEYTDCGQRRVYLRLYSGTLRLRDTVALAGREKLKITEMRIPSKG 300
+ + LCG VFKVEYTD +R YLRLY GTL LRD + L+ ++K+KITEM
IP G
Sbjct 241
ETDDIQSELCGYVFKVEYTDRKKRLSYLRLYHGTLHLRDALPLSKKKKIKITEMCIPLNG 300

Query 301
EIVRTDTAYPGEIVILPSDSVRLNDVLGDPTRLPRKRWREDPLPMLRTTIAPKTAAQRER 360
+IV D AYPGEIVIL D+++LND+LG+ LP+K ++P+P+LRTT+ P+
QRE
Sbjct 301
KIVPADHAYPGEIVILADDTLKLNDILGNEKLLPQKTQIDNPMPLLRTTVEPQKPEQREA 360

Query 361
LLDALTQLADTDPLLRCEVDSITHEIILSFLGRVQLEVVSALLSEKYKLETVVKEPSVIY 420
LL+AL ++ADTDPLL E+D++THEI+LSFLG+VQLEV+ +LL EKY + V
+KEPSVIY
Sbjct 361
LLNALVEIADTDPLLHFEIDTVTHEIMLSFLGKVQLEVICSLLEEKYHVGVVMKEPSVIY 420

Query 421
MERPLKAASHTIHIEVPPNPFWASIGLSVTPLSLGSGVQYESRVSLGYLNQSFQNAVRDG 480
+ERP K AS TIHIEVPPNPFWASIGL+VTPL +GSG QY+S
VSLGYLNQSFQNAV +G
Sbjct 421
LERPQKKASFTIHIEVPPNPFWASIGLTVTPLPVGSGTQYKSEVSLGYLNQSFQNAVMEG 480

Query 481
IRYGLEQGLFGWNVTDCKICFEYGLYYSPVSTPADFRSLAPIVLEQALKESGTQLLEPYL 540
+ YG+EQGL+GW VTDC+ICF+YG+YYSPVSTPADFR LAP+VLEQALK+
+GTQLLEPYL
Sbjct 481
VCYGMEQGLYGWGVTDCQICFDYGVYYSPVSTPADFRFLAPVVLEQALKKAGTQLLEPYL 540

```
Query  541  
SFILYAPQEYLSRAYHDAPKYCATIETAQVKKDEVVFTGEIPARCIQAYRTDLAFYTNGR  600
            SF L+APQEYLSRAY+DA KYCA IE+ +++KDEV+F GEIPARCI  YR DL 
FYTNGR
Sbjct  541  
SFTLFAPQEYLSRAYNDALKYCAIIESTRLEKDEVIFKGEIPARCIGEYRNDLNFYTNGR  600

Query  601  SVCLTELKGYQAAVGQPVIQPRRPNSRLDKVRHMFQKVM  639
            SVC+TELKGYQ   G+PV QPRRPNSRLDKVRHMFQK+M
Sbjct  601  SVCITELKGYQETSGEPVFQPRRPNSRLDKVRHMFQKIM  639


>WP_009321211.1 TetM/TetW/TetO/TetS family tetracycline resistance 
ribosomal
protection protein [Lachnospiraceae bacterium 8_1_57FAA]
 EFV19990.1 hypothetical protein HMPREF1026_00782 [Lachnospiraceae 
bacterium
8_1_57FAA]
Length=639

 Score = 927 bits (2395),  Expect = 0.0, Method: Compositional 
matrix adjust.
 Identities = 435/639 (68%), Positives = 533/639 (83%), Gaps = 0/639 
(0%)

Query  1    
MKIINIGILAHVDAGKTTLTESLLYASGAISEPGSVEKGTTRTDTMFLERQRGITIQAAV  60
            MKIINIGILAHVDAGKTTLTESLLY SGAI+E G+V+KGTTRTDTM 
LERQRGITIQ AV
Sbjct  1    
MKIINIGILAHVDAGKTTLTESLLYTSGAIAEQGNVDKGTTRTDTMILERQRGITIQTAV  60

Query  61   
TSFQWHRCKVNIVDTPGHMDFLAEVYRSLAVLDGAILVISAKDGVQAQTRILFHALRKMN  120
            TSF W+  K+NIVDTPGHMDFL E YRSL+VLDGA+LVISAKDGVQAQTRILFHAL
+KM+
Sbjct  61   
TSFCWNDYKINIVDTPGHMDFLTEAYRSLSVLDGAVLVISAKDGVQAQTRILFHALQKMD  120

Query  121  
IPTVIFINKIDQAGVDLQSVVQSVRDKLSADIIIKQTVSLSPEIVLEENTDIEAWDAVIE  180
            IPT+IFINKIDQ G+DL+ V QS++DKL++D+I+ Q VSLSP+I + + +D++ WD
+I
Sbjct  121  
IPTIIFINKIDQNGIDLRRVYQSIKDKLTSDMIVMQEVSLSPKITMTDISDLDKWDMIIS  180

Query  181  
NNDKLLEKYIAGEPISREKLVREEQRRVQDASLFPVYYGSAKKGLGIQPLMDAVTGLFQP  240
             +D+LLE+Y+A   +  ++L  E+ +R +  SLFPVY+GSAK  LG + L++A+T
F
Sbjct  181  
GSDELLERYVAENSLDIQELQYEKCKRTRCCSLFPVYHGSAKDNLGTEKLIEAITETFIT  240
```

```
Query  241
IGEQGSAALCGSVFKVEYTDCGQRRVYLRLYSGTLRLRDTVALAGREKLKITEMRIPSKG  300
              +   + LCG VFKVEYT+  +R  YLRLY GTL LRDT+ L+ ++K+KITEM
IPS G
Sbjct  241
ETDDIQSELCGYVFKVEYTERKKRLSYLRLYHGTLHLRDTLPLSKKKKIKITEMCIPSNG  300

Query  301
EIVRTDTAYPGEIVILPSDSVRLNDVLGDPTRLPRKRWREDPLPMLRTTIAPKTAAQRER  360
            EIV  D A PGEIVIL  D+++LND+LG+   LP K   ++P+P+LRTT+ P+
QRE
Sbjct  301
EIVPADHACPGEIVILADDTLKLNDILGNEKLLPHKTRIDNPMPLLRTTVEPQKPEQREA  360

Query  361
LLDALTQLADTDPLLRCEVDSITHEIILSFLGRVQLEVVSALLSEKYKLETVVKEPSVIY  420
            LL+AL ++ADTDPLL  ++D++THEI+LSFLG+VQLEV+ +LL EKY +
+KEPSVIY
Sbjct  361
LLNALAEIADTDPLLHFDIDTVTHEIMLSFLGKVQLEVICSLLEEKYHVGVAMKEPSVIY  420

Query  421
MERPLKAASHTIHIEVPPNPFWASIGLSVTPLSLGSGVQYESRVSLGYLNQSFQNAVRDG  480
            +ERP K AS TIHIEVPPNPFWASIGL+VTPL +GSG QY+S
VSLGYLNQSFQNAV +G
Sbjct  421
LERPQKKASCTIHIEVPPNPFWASIGLTVTPLPVGSGTQYKSEVSLGYLNQSFQNAVMEG  480

Query  481
IRYGLEQGLFGWNVTDCKICFEYGLYYSPVSTPADFRSLAPIVLEQALKESGTQLLEPYL  540
            +RYG+EQGL+GW VTDC+ICF+YG+YYSPVSTPADFR LAP+VLEQALK+
+GTQLLEPYL
Sbjct  481
VRYGMEQGLYGWGVTDCQICFDYGVYYSPVSTPADFRFLAPVVLEQALKKAGTQLLEPYL  540

Query  541
SFILYAPQEYLSRAYHDAPKYCATIETAQVKKDEVVFTGEIPARCIQAYRTDLAFYTNGR  600
            SF L+APQEYLSRAY+DAPKYCA IE+ +++KDEV+  GEIPARCI  YR DL
FYTNGR
Sbjct  541
SFTLFAPQEYLSRAYNDAPKYCAIIESTRLEKDEVILKGEIPARCIGEYRNDLNFYTNGR  600

Query  601  SVCLTELKGYQAAVGQPVIQPRRPNSRLDKVRHMFQKVM  639
            SVC+TELKGYQ   G+PV QPRRPNSRLDK+RHMFQK+M
Sbjct  601  SVCITELKGYQETSGEPVFQPRRPNSRLDKIRHMFQKIM  639
```

>WP_012775603.1 tetracycline resistance ribosomal protection protein Tet(O) [Streptococcus
suis]
 YP_003028699.1 tetracycline resistance protein TetO [Streptococcus suis BM407]
 CAZ55807.1 tetracycline resistance protein TetO [Streptococcus suis BM407]

CYY85696.1 tetracycline resistance protein tetM [Streptococcus suis]
CYY47351.1 tetracycline resistance protein tetM [Streptococcus suis]
CYZ60449.1 tetracycline resistance protein tetM [Streptococcus suis]
CZA68630.1 tetracycline resistance protein tetM [Streptococcus suis]
Length=639

Score = 927 bits (2395), Expect = 0.0, Method: Compositional matrix adjust.
Identities = 430/638 (67%), Positives = 528/638 (83%), Gaps = 0/638 (0%)

```
Query  1
MKIINIGILAHVDAGKTTLTESLLYASGAISEPGSVEKGTTRTDTMFLERQRGITIQAAV  60
            MKIIN+GILAHVDAGKTTLTESLLY SGAI+EPGSV+KGTTRTDTM
LERQRGITIQ AV
Sbjct  1
MKIINLGILAHVDAGKTTLTESLLYTSGAIAEPGSVDKGTTRTDTMNLERQRGITIQTAV  60

Query  61
TSFQWHRCKVNIVDTPGHMDFLAEVYRSLAVLDGAILVISAKDGVQAQTRILFHALRKMN  120
            TSFQW   KVNI+DTPGHMDFLAEVYRSL+VLDGA+L++SAKDG+QAQTRILFHAL
+ M
Sbjct  61
TSFQWEDVKVNIIDTPGHMDFLAEVYRSLSVLDGAVLLVSAKDGIQAQTRILFHALQTMK  120

Query  121
IPTVIFINKIDQAGVDLQSVVQSVRDKLSADIIIKQTVSLSPEIVLEENTDIEAWDAVIE  180
            IPT+ FINKIDQ G+DL  V Q ++ KLS++II+KQ V   P I + +N D+E
WDAVI
Sbjct  121
IPTIFFINKIDQEGIDLPMVYQEMKAKLSSEIIVKQKVGQHPHINVTDNDDMEQWDAVIM  180

Query  181
NNDKLLEKYIAGEPISREKLVREEQRRVQDASLFPVYYGSAKKGLGIQPLMDAVTGLFQP  240
             ND+LLEKY++G+P    +L +EE RR Q+ +LFPVY+GSAK  LGI+ L++ +
F
Sbjct  181
GNDELLEKYMSGKPFKMSELEQEENRRFQNGTLFPVYHGSAKNNLGIRQLIEVIASKFYS  240

Query  241
IGEQGSAALCGSVFKVEYTDCGQRRVYLRLYSGTLRLRDTVALAGREKLKITEMRIPSKG  300
               +G + LCG VFK+EY++  +R VY+R+YSGTL LRD + ++ +EK+KITEM
+P+ G
Sbjct  241
STPEGQSELCGQVFKIEYSEKRRRFVYVRIYSGTLHLRDVIKISEKEKIKITEMCVPTNG  300

Query  301
EIVRTDTAYPGEIVILPSDSVRLNDVLGDPTRLPRKRWREDPLPMLRTTIAPKTAAQRER  360
            E+  +DTA  G+IVILP+D ++LN +LG+   LP++++ E+PLPML+TTIA K +
QRE
```

Sbjct 301
ELYSSDTACSGDIVILPNDVLQLNSILGNEMLLPQRKFIENPLPMLQTTIAVKKSEQREI 360

Query 361
LLDALTQLADTDPLLRCEVDSITHEIILSFLGRVQLEVVSALLSEKYKLETVVKEPSVIY 420
            LL ALT+++D DPLL+ VD+ THEIILSFLG VQ+EV+ A+L EKY +E   KE
+VIY
Sbjct 361
LLGALTEISDGDPLLKYYVDTTTHEIILSFLGNVQMEVICAILEEKYHVEAETKESTVIY 420

Query 421
MERPLKAASHTIHIEVPPNPFWASIGLSVTPLSLGSGVQYESRVSLGYLNQSFQNAVRDG 480
            MERPL+ A +TIHIEVPPNPFWAS+GLS+ PL
+GSGVQYESRVSLGYLNQSFQNAV +G
Sbjct 421
MERPLRKAEYTIHIEVPPNPFWASVGLSIEPLPIGSGVQYESRVSLGYLNQSFQNAVMEG 480

Query 481
IRYGLEQGLFGWNVTDCKICFEYGLYYSPVSTPADFRSLAPIVLEQALKESGTQLLEPYL 540
            + YG EQGL+GW VTDCKICFEYGLYYSPVSTPADFR L+PIVLEQALK++GT
+LLEPYL
Sbjct 481
VLYGCEQGLYGWKVTDCKICFEYGLYYSPVSTPADFRLLSPIVLEQALKKAGTELLEPYL 540

Query 541
SFILYAPQEYLSRAYHDAPKYCATIETAQVKKDEVVFTGEIPARCIQAYRTDLAFYTNGR 600
             F +YAPQEYLSRAYHDAP+YCA I + Q+K DEV+ GEIPARCIQ YR DL +
+TNG+
Sbjct 541
HFEIYAPQEYLSRAYHDAPRYCADIVSTQIKNDEVILKGEIPARCIQEYRNDLTYFTNGQ 600

Query 601 SVCLTELKGYQAAVGQPVIQPRRPNSRLDKVRHMFQKV 638
             VCLTELKGYQ A+G+ + QPRRPNSR+DKVRHMF K+
Sbjct 601 GVCLTELKGYQPAIGKFICQPRRPNSRIDKVRHMFHKL 638

>WP_009015508.1 TetM/TetW/TetO/TetS family tetracycline resistance
ribosomal
protection protein [Acidaminococcus sp. D21]
 EEH90688.1 putative translation elongation factor G
[Acidaminococcus sp.
D21]
Length=639

 Score = 927 bits (2395), Expect = 0.0, Method: Compositional
matrix adjust.
 Identities = 438/639 (69%), Positives = 536/639 (84%), Gaps = 0/639
(0%)

Query 1
MKIINIGILAHVDAGKTTLTESLLYASGAISEPGSVEKGTTRTDTMFLERQRGITIQAAV 60
            MKIINIGILAHVDAGKTTLTESLLY SGAI+E G+V+KGTTRTDTM
LERQRGITIQ AV
Sbjct 1

MKIINIGILAHVDAGKTTLTESLLYTSGAIAEQGNVDKGTTRTDTMILERQRGITIQTAV 60

Query 61
TSFQWHRCKVNIVDTPGHMDFLAEVYRSLAVLDGAILVISAKDGVQAQTRILFHALRKMN 120
            TSF W+ K+NIVDTPGHMDFL E YRSL+VLDGA+LVISAKDGVQAQTRILFHAL
+KM+
Sbjct 61
TSFCWNDYKINIVDTPGHMDFLTEAYRSLSVLDGAVLVISAKDGVQAQTRILFHALQKMD 120

Query 121
IPTVIFINKIDQAGVDLQSVVQSVRDKLSADIIIKQTVSLSPEIVLEENTDIEAWDAVIE 180
            IPT+IFINKIDQ G+DL+ V QS++DKL++D+I+ Q VSLSP+I + + +D++ WD
+I
Sbjct 121
IPTIIFINKIDQNGIDLRRVYQSIKDKLTSDMIVMQEVSLSPKITMTDISDLDKWDMIIS 180

Query 181
NNDKLLEKYIAGEPISREKLVREEQRRVQDASLFPVYYGSAKKGLGIQPLMDAVTGLFQP 240
             +D+LLE+Y+A + + ++L E+ +R + SLFPVY+GSAK LG + L++ +T
F
Sbjct 181
GSDELLERYVAEDSLDIQELQYEKCKRTRCCSLFPVYHGSAKDNLGTEKLIETITETFIT 240

Query 241
IGEQGSAALCGSVFKVEYTDCGQRRVYLRLYSGTLRLRDTVALAGREKLKITEMRIPSKG 300
             +   + LCG VFKVEYT+ +R YLRLY GTL LRDT+ L+ +EK+KITEM
IPS G
Sbjct 241
ETDDIQSELCGYVFKVEYTERKKRLSYLRLYHGTLHLRDTLLLSKKEKIKITEMCIPSNG 300

Query 301
EIVRTDTAYPGEIVILPSDSVRLNDVLGDPTRLPRKRWREDPLPMLRTTIAPKTAAQRER 360
            EIV D A PGEIVIL D+++LND+LG+   LP K   ++P+P+LRTT+ P+
QRE
Sbjct 301
EIVPVDHACPGEIVILADDTLKLNDILGNEKLLPHKTRIDNPMPLLRTTVEPQKPEQREA 360

Query 361
LLDALTQLADTDPLLRCEVDSITHEIILSFLGRVQLEVVSALLSEKYKLETVVKEPSVIY 420
            LL+ALT++ADTDPLL ++D++THEIILSFLG+VQLEV+ +LL EKY +
+KEPSVIY
Sbjct 361
LLNALTEIADTDPLLHFDIDTVTHEIILSFLGKVQLEVICSLLEEKYHVGVAMKEPSVIY 420

Query 421
MERPLKAASHTIHIEVPPNPFWASIGLSVTPLSLGSGVQYESRVSLGYLNQSFQNAVRDG 480
            +ERP K AS+TIHIEVPPNPFWASIGL+VTPL +GSG QY+S
VSLGYLNQSFQNAV +G
Sbjct 421
LERPQKKASYTIHIEVPPNPFWASIGLTVTPLPVGSGTQYKSEVSLGYLNQSFQNAVMEG 480

Query 481
IRYGLEQGLFGWNVTDCKICFEYGLYYSPVSTPADFRSLAPIVLEQALKESGTQLLEPYL 540
            +RYG+EQGL+GW VTDC+ICF+YG+YYSPVSTPADFR LAP+VLEQALK+

```
+GTQLLEPYL
Sbjct  481
VRYGMEQGLYGWGVTDCQICFDYGVYYSPVSTPADFRFLAPVVLEQALKKAGTQLLEPYL  540

Query  541
SFILYAPQEYLSRAYHDAPKYCATIETAQVKKDEVVFTGEIPARCIQAYRTDLAFYTNGR  600
            SF L+APQEYLSRAY+DAPKYCA IE+ +++KDEV+F GEIPARCI  YR DL
FYTNGR
Sbjct  541
SFTLFAPQEYLSRAYNDAPKYCAIIESTRLEKDEVIFKGEIPARCIGEYRNDLNFYTNGR  600

Query  601  SVCLTELKGYQAAVGQPVIQPRRPNSRLDKVRHMFQKVM  639
            SVC+TELKGYQ   G+PV QPRRPNSRLDK+RHMFQK+M
Sbjct  601  SVCITELKGYQETSGEPVFQPRRPNSRLDKIRHMFQKIM  639


>WP_000691746.1 MULTISPECIES: tetracycline resistance ribosomal
protection protein
Tet(M) [Firmicutes]
 ABB70047.1 TetA(M) [Enterococcus faecalis]
 EEW61461.1 tetracycline resistance protein tetM transposon
[Enterococcus
faecium C68]
 EOG10042.1 tetracycline resistance protein tetM transposon
[Enterococcus
faecium EnGen0175]
 EOH50046.1 tetracycline resistance protein tetM transposon
[Enterococcus
faecium EnGen0191]
 EOI42401.1 tetracycline resistance protein tetM transposon
[Enterococcus
faecium EnGen0313]
 EOK19530.1 tetracycline resistance protein tetM transposon
[Enterococcus
faecalis EnGen0329]
 EOL21295.1 tetracycline resistance protein tetM transposon
[Enterococcus
faecalis EnGen0331]
 EOL70169.1 tetracycline resistance protein tetM transposon
[Enterococcus
faecium EnGen0305]
 EPV43472.1 tetracycline resistance protein tetM [Streptococcus
agalactiae
GB00901]
 ETT98531.1 tetracycline resistance protein tetM transposon
[Enterococcus
faecalis EnGen0403]
 ETU01495.1 tetracycline resistance protein tetM transposon
[Enterococcus
faecalis EnGen0404]
 ETU06200.1 tetracycline resistance protein tetM transposon
[Enterococcus
faecalis EnGen0405]
 ETU10166.1 tetracycline resistance protein tetM transposon
```

[Enterococcus
faecalis EnGen0406]
 ETU16577.1 tetracycline resistance protein tetM transposon
[Enterococcus
faecalis EnGen0408]
 ETU21302.1 tetracycline resistance protein tetM transposon
[Enterococcus
faecalis EnGen0410]
 ETU24420.1 tetracycline resistance protein tetM transposon
[Enterococcus
faecalis EnGen0412]
 ETU31957.1 tetracycline resistance protein tetM transposon
[Enterococcus
faecalis EnGen0415]
 ETU40575.1 tetracycline resistance protein tetM transposon
[Enterococcus
faecalis EnGen0418]
 EXG73476.1 tetracycline resistance protein tetM transposon
[Enterococcus
faecium C68]
 EXG73517.1 tetracycline resistance protein tetM transposon
[Enterococcus
faecium C68]
 AIF86183.1 tetracycline resistance protein tetM [Streptococcus
agalactiae]
 KJE99800.1 tetracycline resistance protein tetM [Streptococcus
gallolyticus
subsp. gallolyticus]
 AJW72810.1 tetracycline resistance protein [Erysipelothrix
rhusiopathiae]
 CNF32730.1 tetracycline resistance protein TetM [Streptococcus
agalactiae]
 CNE36190.1 tetracycline resistance protein TetM [Streptococcus
agalactiae]
 CNI06148.1 tetracycline resistance protein TetM [Streptococcus
agalactiae]
 KWW53866.1 tetracycline resistance protein tetM [Enterococcus
faecium]
 CWW49546.1 Tetracycline resistance protein tetM from transposon
Tn916 [Listeria
monocytogenes]
 KXW80675.1 tetracycline resistance protein tetM [Enterococcus
faecium C68]
 KXW81194.1 tetracycline resistance protein tetM [Enterococcus
faecium C68]
 KXW82618.1 tetracycline resistance protein tetM [Enterococcus
faecium C68]
 AMS10715.1 tetracycline resistance ribosomal protection protein
[Erysipelothrix
rhusiopathiae]
 OCL38543.1 tetracycline resistance ribosomal protection protein
[Streptococcus
agalactiae]
 OCL47101.1 tetracycline resistance ribosomal protection protein

[Streptococcus
agalactiae]
 OCL62883.1 tetracycline resistance ribosomal protection protein
[Streptococcus
agalactiae]
 OCL66388.1 tetracycline resistance ribosomal protection protein
[Streptococcus
agalactiae]
 OCL67104.1 tetracycline resistance ribosomal protection protein
[Streptococcus
agalactiae]
 OCL73105.1 tetracycline resistance ribosomal protection protein
[Streptococcus
agalactiae]
 OCL73560.1 tetracycline resistance ribosomal protection protein
[Streptococcus
agalactiae]
 OCL76005.1 tetracycline resistance ribosomal protection protein
[Streptococcus
agalactiae]
 OCL76094.1 tetracycline resistance ribosomal protection protein
[Streptococcus
agalactiae]
 OCL81504.1 tetracycline resistance ribosomal protection protein
[Streptococcus
agalactiae]
 OCM06948.1 tetracycline resistance ribosomal protection protein
[Streptococcus
agalactiae]
 OCM09284.1 tetracycline resistance ribosomal protection protein
[Streptococcus
agalactiae]
 OCM16853.1 tetracycline resistance ribosomal protection protein
[Streptococcus
agalactiae]
 OCM20501.1 tetracycline resistance ribosomal protection protein
[Streptococcus
agalactiae]
 OCM26428.1 tetracycline resistance ribosomal protection protein
[Streptococcus
agalactiae]
 OCM31878.1 tetracycline resistance ribosomal protection protein
[Streptococcus
agalactiae]
 OCM34003.1 tetracycline resistance ribosomal protection protein
[Streptococcus
agalactiae]
 OCM34602.1 tetracycline resistance ribosomal protection protein
[Streptococcus
agalactiae]
 OCM40088.1 tetracycline resistance ribosomal protection protein
[Streptococcus
agalactiae]
 OCM50019.1 tetracycline resistance ribosomal protection protein

[Streptococcus
agalactiae]
 OCM55222.1 tetracycline resistance ribosomal protection protein
[Streptococcus
agalactiae]
 OCM59761.1 tetracycline resistance ribosomal protection protein
[Streptococcus
agalactiae]
 OCM61057.1 tetracycline resistance ribosomal protection protein
[Streptococcus
agalactiae]
 OCM64241.1 tetracycline resistance ribosomal protection protein
[Streptococcus
agalactiae]
 OCM67397.1 tetracycline resistance ribosomal protection protein
[Streptococcus
agalactiae]
 OCM70692.1 tetracycline resistance ribosomal protection protein
[Streptococcus
agalactiae]
 OCM75068.1 tetracycline resistance ribosomal protection protein
[Streptococcus
agalactiae]
 OCM82869.1 tetracycline resistance ribosomal protection protein
[Streptococcus
agalactiae]
 OCM87631.1 tetracycline resistance ribosomal protection protein
[Streptococcus
agalactiae]
 OCM93420.1 tetracycline resistance ribosomal protection protein
[Streptococcus
agalactiae]
 OCM93576.1 tetracycline resistance ribosomal protection protein
[Streptococcus
agalactiae]
 SCS89562.1 tetracycline resistance protein TetM [Staphylococcus
aureus]
 ASX97932.1 Tetracycline resistance protein TetM [Erysipelothrix
rhusiopathiae]
 PCF85134.1 tetracycline resistance ribosomal protection protein
Tet(M) [Staphylococcus
delphini]
 PDG14308.1 tetracycline resistance ribosomal protection protein
Tet(M) [Listeria
monocytogenes]
 PDH10276.1 tetracycline resistance ribosomal protection protein
Tet(M) [Listeria
monocytogenes]
 PQC11078.1 tetracycline resistance ribosomal protection protein
Tet(M) [Enterococcus
faecium]
Length=639

 Score = 927 bits (2395), Expect = 0.0, Method: Compositional

```
matrix adjust.
 Identities = 440/638 (69%), Positives = 522/638 (82%), Gaps = 0/638
(0%)

Query  1
MKIINIGILAHVDAGKTTLTESLLYASGAISEPGSVEKGTTRTDTMFLERQRGITIQAAV  60
            MKIINIG+LAHVDAGKTTLTESLLY SGAI+E GSV+KGTTRTD
LERQRGITIQ  +
Sbjct  1
MKIINIGVLAHVDAGKTTLTESLLYNSGAITELGSVDKGTTRTDNTLLERQRGITIQTGI  60

Query  61
TSFQWHRCKVNIVDTPGHMDFLAEVYRSLAVLDGAILVISAKDGVQAQTRILFHALRKMN  120
            TSFQW   KVNI+DTPGHMDFLAEVYRSL+VLDGAIL
+ISAKDGVQAQTRILFHALRKM
Sbjct  61
TSFQWENTKVNIIDTPGHMDFLAEVYRSLSVLDGAILLISAKDGVQAQTRILFHALRKMG  120

Query  121
IPTVIFINKIDQAGVDLQSVVQSVRDKLSADIIIKQTVSLSPEIVLEENTDIEAWDAVIE  180
            IPT+ FINKIDQ G+DL +V Q +++KLSA+I+IKQ V L P + +   T+ E WD
VIE
Sbjct  121
IPTIFFINKIDQNGIDLSTVYQDIKEKLSAEIVIKQKVELYPNMCVTNFTESEQWDTVIE  180

Query  181
NNDKLLEKYIAGEPISREKLVREEQRRVQDASLFPVYYGSAKKGLGIQPLMDAVTGLFQP  240
             ND LLEKY++G+ +   +L +EE  R Q+ SLFPVY+GSAK  +GI  L++ +T
F
Sbjct  181
GNDDLLEKYMSGKSLEALELEQEESIRFQNCSLFPVYHGSAKNNIGIDNLIEVITNKFYS  240

Query  241
IGEQGSAALCGSVFKVEYTDCGQRRVYLRLYSGTLRLRDTVALAGREKLKITEMRIPSKG  300
               +G + LCG+VFK+EYT   QR  Y+RLYSG L LRD+V ++ +EK+KITEM
G
Sbjct  241
STHRGQSELCGNVFKIEYTKKRQRLAYIRLYSGVLHLRDSVRISEKEKIKITEMYTSING  300

Query  301
EIVRTDTAYPGEIVILPSDSVRLNDVLGDPTRLPRKRWREDPLPMLRTTIAPKTAAQRER  360
            E+ + D AY GEIVIL ++ ++LN VLGD   LP++   E+PLP+L+TT+ P
QRE
Sbjct  301
ELCKIDKAYSGEIVILQNEFLKLNSVLGDTKLLPQRERIENPLPLLQTTVEPSKPQQREM  360

Query  361
LLDALTQLADTDPLLRCEVDSITHEIILSFLGRVQLEVVSALLSEKYKLETVVKEPSVIY  420
            LLDAL +++D+DPLLR  VDS THEIILSFLG+VQ+EV+SALL EKY +E  +KEP
+VIY
Sbjct  361
LLDALLEISDSDPLLRYYVDSTTHEIILSFLGKVQMEVISALLQEKYHVEIELKEPTVIY  420

Query  421
```

MERPLKAASHTIHIEVPPNPFWASIGLSVTPLSLGSGVQYESRVSLGYLNQSFQNAVRDG 480
            MERPLK A +TIHIEVPPNPFWASIGLSV+PL LGSGVQYES
VSLGYLNQSFQNAV +G
Sbjct 421
MERPLKNAEYTIHIEVPPNPFWASIGLSVSPLPLGSGVQYESSVSLGYLNQSFQNAVMEG 480

Query 481
IRYGLEQGLFGWNVTDCKICFEYGLYYSPVSTPADFRSLAPIVLEQALKESGTQLLEPYL 540
            IRYG EQGL+GWNVTDCKICF+YGLYYSPVSTPADFR LAPIVLEQ LK++GT
+LLEPYL
Sbjct 481
IRYGCEQGLYGWNVTDCKICFKYGLYYSPVSTPADFRMLAPIVLEQVLKKAGTELLEPYL 540

Query 541
SFILYAPQEYLSRAYHDAPKYCATIETAQVKKDEVVFTGEIPARCIQAYRTDLAFYTNGR 600
            SF +YAPQEYLSRAY+DAPKYCA I   Q+K +EV+ +GEIPARCIQ YR+DL F
+TNGR
Sbjct 541
SFKIYAPQEYLSRAYNDAPKYCANIVDTQLKNNEVILSGEIPARCIQEYRSDLTFFTNGR 600

Query 601 SVCLTELKGYQAAVGQPVIQPRRPNSRLDKVRHMFQKV 638
            SVCLTELKGY   G+PV QPRRPNSR+DKVR+MF K+
Sbjct 601 SVCLTELKGYHVTTGEPVCQPRRPNSRIDKVRYMFNKI 638

>AMP54850.1 TetM−TetW−TetO−TetS [uncultured bacterium]
Length=639

 Score = 926 bits (2394), Expect = 0.0, Method: Compositional
matrix adjust.
 Identities = 432/638 (68%), Positives = 529/638 (83%), Gaps = 0/638
(0%)

Query 1
MKIINIGILAHVDAGKTTLTESLLYASGAISEPGSVEKGTTRTDTMFLERQRGITIQAAV 60
            MKIINIGILAHVDAGKTTLTESLLY SGAI+E GSV++GTTRTDTM
LERQRGITIQ AV
Sbjct 1
MKIINIGILAHVDAGKTTLTESLLYTSGAIAELGSVDEGTTRTDTMNLERQRGITIQTAV 60

Query 61
TSFQWHRCKVNIVDTPGHMDFLAEVYRSLAVLDGAILVISAKDGVQAQTRILFHALRKMN 120
            TSFQW   KVNI+DTPGHMDFLAEVYRSL+VLDGA+L++SAKDG+QAQTRILFHAL
+ M
Sbjct 61
TSFQWEDVKVNIIDTPGHMDFLAEVYRSLSVLDGAVLLVSAKDGIQAQTRILFHALQIMK 120

Query 121
IPTVIFINKIDQAGVDLQSVVQSVRDKLSADIIIKQTVSLSPEIVLEENTDIEAWDAVIE 180
            IPT+ FINKIDQ G+DL V + ++ KLS++II+KQ V   P I + +N D+E
WDAVI
Sbjct 121
IPTIFFINKIDQEGIDLPMVYREMKAKLSSEIIVKQKVGQHPHINVTDNDDMEQWDAVIM 180

```
Query  181
NNDKLLEKYIAGEPISREKLVREEQRRVQDASLFPVYYGSAKKGLGIQPLMDAVTGLFQP  240
             ND+LLEKY++G+P    +L +EE RR Q+ +LFPVY+GSAK  LGI+ L++ +
F
Sbjct  181
GNDELLEKYMSGKPFKMSELEQEENRRFQNGTLFPVYHGSAKNNLGIRQLIEVIASKFYS  240

Query  241
IGEQGSAALCGSVFKVEYTDCGQRRVYLRLYSGTLRLRDTVALAGREKLKITEMRIPSKG  300
               +G + LCG VFK+EY++  +R VY+R+YSGTL LRD + ++ +EK+KITEM
+P+ G
Sbjct  241
STPEGQSELCGQVFKIEYSEKRRRFVYVRIYSGTLHLRDVIRISEKEKIKITEMCVPTNG  300

Query  301
EIVRTDTAYPGEIVILPSDSVRLNDVLGDPTRLPRKRWREDPLPMLRTTIAPKTAAQRER  360
            EIV  D A PGEIVIL  D+++LND+LG+   LP++++ E+PLPML+TTIA K +
QRE
Sbjct  301
EIVPADHACPGEIVILADDTLKLNDILGNEILLPQRKFIENPLPMLQTTIAVKKSEQREI  360

Query  361
LLDALTQLADTDPLLRCEVDSITHEIILSFLGRVQLEVVSALLSEKYKLETVVKEPSVIY  420
            LL ALT+++D DPLL+  VD+ THEIILSFLG VQ+EV+ A+L EKY +E  +KEP
+VIY
Sbjct  361
LLGALTEISDGDPLLKYYVDTTTHEIILSFLGNVQMEVICAILEEKYHVEAEIKEPTVIY  420

Query  421
MERPLKAASHTIHIEVPPNPFWASIGLSVTPLSLGSGVQYESRVSLGYLNQSFQNAVRDG  480
            MERPL+ A +TIHIEVPPNPFWAS+GLS+ PL
+GSGVQYESRVSLGYLNQSFQNAV +G
Sbjct  421
MERPLRKAEYTIHIEVPPNPFWASVGLSIEPLPIGSGVQYESRVSLGYLNQSFQNAVMEG  480

Query  481
IRYGLEQGLFGWNVTDCKICFEYGLYYSPVSTPADFRSLAPIVLEQALKESGTQLLEPYL  540
            + YG EQGL+GW VTDCKICFEYGLYYSPVSTPADFR L+PIVLEQALK++GT
+LLEPYL
Sbjct  481
VLYGCEQGLYGWKVTDCKICFEYGLYYSPVSTPADFRLLSPIVLEQALKKAGTELLEPYL  540

Query  541
SFILYAPQEYLSRAYHDAPKYCATIETAQVKKDEVVFTGEIPARCIQAYRTDLAFYTNGR  600
             F +YAPQEYLSRAYHDAP+YCA I + Q+K DEV+  GEIPARCIQ YR DL +
+TNG+
Sbjct  541
HFEIYAPQEYLSRAYHDAPRYCADIVSTQIKNDEVILKGEIPARCIQEYRNDLTYFTNGQ  600

Query  601  SVCLTELKGYQAAVGQPVIQPRRPNSRLDKVRHMFQKV  638
             VCLTELKGYQ A+G+ + QPRRPNSR+DKVRHMF K+
Sbjct  601  GVCLTELKGYQPAIGKFICQPRRPNSRIDKVRHMFHKL  638
```

```
>EFM82549.1 putative translation elongation factor G [Enterococcus
faecalis
TX4248]
 EJV41397.1 putative translation elongation factor G [Enterococcus
faecalis
R508]
Length=644

 Score = 926 bits (2394),  Expect = 0.0, Method: Compositional
matrix adjust.
 Identities = 440/638 (69%), Positives = 522/638 (82%), Gaps = 0/638
(0%)

Query  1
MKIINIGILAHVDAGKTTLTESLLYASGAISEPGSVEKGTTRTDTMFLERQRGITIQAAV  60
            MKIINIG+LAHVDAGKTTLTESLLY SGAI+E GSV+KGTTRTD
LERQRGITIQ  +
Sbjct  6
MKIINIGVLAHVDAGKTTLTESLLYNSGAITELGSVDKGTTRTDNTLLERQRGITIQTGI  65

Query  61
TSFQWHRCKVNIVDTPGHMDFLAEVYRSLAVLDGAILVISAKDGVQAQTRILFHALRKMN  120
            TSFQW   KVNI+DTPGHMDFLAEVYRSL+VLDGAIL
+ISAKDGVQAQTRILFHALRKM
Sbjct  66
TSFQWENTKVNIIDTPGHMDFLAEVYRSLSVLDGAILLISAKDGVQAQTRILFHALRKMG  125

Query  121
IPTVIFINKIDQAGVDLQSVVQSVRDKLSADIIIKQTVSLSPEIVLEENTDIEAWDAVIE  180
            IPT+ FINKIDQ G+DL +V Q +++KLSA+I+IKQ V L P + +   T+ E WD
VIE
Sbjct  126
IPTIFFINKIDQNGIDLSTVYQDIKEKLSAEIVIKQKVELYPNMCVTNFTESEQWDTVIE  185

Query  181
NNDKLLEKYIAGEPISREKLVREEQRRVQDASLFPVYYGSAKKGLGIQPLMDAVTGLFQP  240
             ND LLEKY++G+ +   +L +EE  R Q+ SLFPVY+GSAK  +GI  L++ +T
F
Sbjct  186
GNDDLLEKYMSGKSLEALELEQEESIRFQNCSLFPVYHGSAKNNIGIDNLIEVITNKFYS  245

Query  241
IGEQGSAALCGSVFKVEYTDCGQRRVYLRLYSGTLRLRDTVALAGREKLKITEMRIPSKG  300
               +G + LCG+VFK+EYT   QR  Y+RLYSG L LRD+V ++ +EK+KITEM
G
Sbjct  246
STHRGQSELCGNVFKIEYTKKRQRLAYIRLYSGVLHLRDSVRISEKEKIKITEMYTSING  305

Query  301
EIVRTDTAYPGEIVILPSDSVRLNDVLGDPTRLPRKRWREDPLPMLRTTIAPKTAAQRER  360
            E+ + D AY GEIVIL ++ ++LN VLGD   LP++   E+PLP+L+TT+ P
QRE
Sbjct  306
ELCKIDKAYSGEIVILQNEFLKLNSVLGDTKLLPQRERIENPLPLLQTTVEPSKPQQREM  365
```

Query 361
LLDALTQLADTDPLLRCEVDSITHEIILSFLGRVQLEVVSALLSEKYKLETVVKEPSVIY 420
LLDAL +++D+DPLLR VDS THEIILSFLG+VQ+EV+SALL EKY +E +KEP
+VIY
Sbjct 366
LLDALLEISDSDPLLRYYVDSTTHEIILSFLGKVQMEVISALLQEKYHVEIELKEPTVIY 425

Query 421
MERPLKAASHTIHIEVPPNPFWASIGLSVTPLSLGSGVQYESRVSLGYLNQSFQNAVRDG 480
MERPLK A +TIHIEVPPNPFWASIGLSV+PL LGSGVQYES
VSLGYLNQSFQNAV +G
Sbjct 426
MERPLKNAEYTIHIEVPPNPFWASIGLSVSPLPLGSGVQYESSVSLGYLNQSFQNAVMEG 485

Query 481
IRYGLEQGLFGWNVTDCKICFEYGLYYSPVSTPADFRSLAPIVLEQALKESGTQLLEPYL 540
IRYG EQGL+GWNVTDCKICF+YGLYYSPVSTPADFR LAPIVLEQ LK++GT
+LLEPYL
Sbjct 486
IRYGCEQGLYGWNVTDCKICFKYGLYYSPVSTPADFRMLAPIVLEQVLKKAGTELLEPYL 545

Query 541
SFILYAPQEYLSRAYHDAPKYCATIETAQVKKDEVVFTGEIPARCIQAYRTDLAFYTNGR 600
SF +YAPQEYLSRAY+DAPKYCA I Q+K +EV+ +GEIPARCIQ YR+DL F
+TNGR
Sbjct 546
SFKIYAPQEYLSRAYNDAPKYCANIVDTQLKNNEVILSGEIPARCIQEYRSDLTFFTNGR 605

Query 601 SVCLTELKGYQAAVGQPVIQPRRPNSRLDKVRHMFQKV 638
SVCLTELKGY G+PV QPRRPNSR+DKVR+MF K+
Sbjct 606 SVCLTELKGYHVTTGEPVCQPRRPNSRIDKVRYMFNKI 643

>ACT76131.1 TetO [Streptococcus suis]
Length=639

Score = 926 bits (2394), Expect = 0.0, Method: Compositional
matrix adjust.
Identities = 430/638 (67%), Positives = 528/638 (83%), Gaps = 0/638
(0%)

Query 1
MKIINIGILAHVDAGKTTLTESLLYASGAISEPGSVEKGTTRTDTMFLERQRGITIQAAV 60
MKIIN+GILAHVDAGKTTLTESLLY SGAI+EPGSV+KGTTRTDTM
LERQRGITIQ AV
Sbjct 1
MKIINLGILAHVDAGKTTLTESLLYTSGAIAEPGSVDKGTTRTDTMNLERQRGITIQTAV 60

Query 61
TSFQWHRCKVNIVDTPGHMDFLAEVYRSLAVLDGAILVISAKDGVQAQTRILFHALRKMN 120
TSFQW KVNI+DTPGHMDFLAEVYRSL+VLDGA+L++SAKDG+QAQTRILFHAL
+ M
Sbjct 61

TSFQWEDVKVNIIDTPGHMDFLAEVYRSLSVLDGAVLLVSAKDGIQAQTRILFHALQTMK 120

Query 121
IPTVIFINKIDQAGVDLQSVVQSVRDKLSADIIIKQTVSLSPEIVLEENTDIEAWDAVIE 180
            IPT+ FINKIDQ G+DL V Q ++ KLS++II+KQ V   P I + +N D+E
WDAVI
Sbjct 121
IPTIFFINKIDQEGIDLPMVYQEMKAKLSSEIIVKQKVGQHPHINVTDNDDMEQWDAVIM 180

Query 181
NNDKLLEKYIAGEPISREKLVREEQRRVQDASLFPVYYGSAKKGLGIQPLMDAVTGLFQP 240
             ND+LLEKY++G+P   +L +EE RR Q+ +LFPVY+GSAK LGI+ L++ +
F
Sbjct 181
GNDELLEKYMSGKPFKMSELEQEENRRFQNGTLFPVYHGSAKNNLGIRQLIEVIASKFYS 240

Query 241
IGEQGSAALCGSVFKVEYTDCGQRRVYLRLYSGTLRLRDTVALAGREKLKITEMRIPSKG 300
                G + LCG VFK++Y++   R VY+R+YSGTL LRD + ++ +EK+KITEM
+P+ G
Sbjct 241
STPGGQSELCGQVFKIDYSEKRPRFVYVRIYSGTLHLRDVIKISEKEKIKITEMCVPTNG 300

Query 301
EIVRTDTAYPGEIVILPSDSVRLNDVLGDPTRLPRKRWREDPLPMLRTTIAPKTAAQRER 360
            E+ +DTA G+IVILP+D ++LN +LG+   LP++++ E+PLPML+TTIA K +
QRE
Sbjct 301
ELYSSDTACSGDIVILPNDVLQLNSILGNEMLLPQRKFIENPLPMLQTTIAVKKSEQREI 360

Query 361
LLDALTQLADTDPLLRCEVDSITHEIILSFLGRVQLEVVSALLSEKYKLETVVKEPSVIY 420
            LL ALT+++D DPLL+ VD+ THEIILSFLG VQ+EV+ A+L EKY +E +KEP
+VIY
Sbjct 361
LLGALTEISDGDPLLKYYVDTTTHEIILSFLGNVQMEVICAILEEKYHVEAEIKEPTVIY 420

Query 421
MERPLKAASHTIHIEVPPNPFWASIGLSVTPLSLGSGVQYESRVSLGYLNQSFQNAVRDG 480
            MERPL+ A +TIHIEVPPNPFWAS+GLS+ PL
+GSGVQYESRVSLGYLNQSFQNAV +G
Sbjct 421
MERPLRKAEYTIHIEVPPNPFWASVGLSIEPLPIGSGVQYESRVSLGYLNQSFQNAVMEG 480

Query 481
IRYGLEQGLFGWNVTDCKICFEYGLYYSPVSTPADFRSLAPIVLEQALKESGTQLLEPYL 540
            + YG EQGL+GW VTDCKICFEYGLYYSPVSTPADFR L+PIVLEQALK++GT
+LLEPYL
Sbjct 481
VLYGCEQGLYGWKVTDCKICFEYGLYYSPVSTPADFRLLSPIVLEQALKKAGTELLEPYL 540

Query 541
SFILYAPQEYLSRAYHDAPKYCATIETAQVKKDEVVFTGEIPARCIQAYRTDLAFYTNGR 600
             F +YAPQEYLSRAYHDAP+YCA I + Q+K DEV+ GEIPARCIQ YR DL +

+TNG+
Sbjct 541
HFEIYAPQEYLSRAYHDAPRYCADIVSTQIKNDEVILKGEIPARCIQEYRNDLTYFTNGQ 600

Query 601 SVCLTELKGYQAAVGQPVIQPRRPNSRLDKVRHMFQKV 638
             VCLTELKGYQ A+G+ + QPRRPNSR+DKVRHMF K+
Sbjct 601 GVCLTELKGYQPAIGKFICQPRRPNSRIDKVRHMFHKL 638

>WP_105155653.1 TetM/TetW/TetO/TetS family tetracycline resistance ribosomal
protection protein [Streptococcus suis]
Length=639

 Score = 926 bits (2393), Expect = 0.0, Method: Compositional matrix adjust.
 Identities = 431/638 (68%), Positives = 529/638 (83%), Gaps = 0/638 (0%)

Query 1
MKIINIGILAHVDAGKTTLTESLLYASGAISEPGSVEKGTTRTDTMFLERQRGITIQAAV 60
            MKIIN+GILAHVDAGKTTLTESLLY SGAI+EPGSV+KGTTRTDTM
LERQRGITIQ AV
Sbjct 1
MKIINLGILAHVDAGKTTLTESLLYTSGAIAEPGSVDKGTTRTDTMNLERQRGITIQTAV 60

Query 61
TSFQWHRCKVNIVDTPGHMDFLAEVYRSLAVLDGAILVISAKDGVQAQTRILFHALRKMN 120
            TSFQW   KVNI+DTPGHMDFLAEVYRSL+VLDGA+L++SAKDG+QAQTRILFHAL
+ M
Sbjct 61
TSFQWEDVKVNIIDTPGHMDFLAEVYRSLSVLDGAVLLVSAKDGIQAQTRILFHALQTMK 120

Query 121
IPTVIFINKIDQAGVDLQSVVQSVRDKLSADIIIKQTVSLSPEIVLEENTDIEAWDAVIE 180
            IPT+ FINKIDQ G+DL V + ++ KLS++II+KQ V   P I + +N D+E
WDAVI
Sbjct 121
IPTIFFINKIDQEGIDLPMVYREMKAKLSSEIIVKQKVGQHPHINVTDNDDMEQWDAVIM 180

Query 181
NNDKLLEKYIAGEPISREKLVREEQRRVQDASLFPVYYGSAKKGLGIQPLMDAVTGLFQP 240
             ND+LLEKY++G+P   +L +EE RR Q+ +LFPVY+GSAK LGI+ L++ +
F
Sbjct 181
GNDELLEKYMSGKPFKMSELEQEENRRFQNGTLFPVYHGSAKNNLGIRQLIEVIASKFYS 240

Query 241
IGEQGSAALCGSVFKVEYTDCGQRRVYLRLYSGTLRLRDTVALAGREKLKITEMRIPSKG 300
              +G + LCG VFK+EY++ +R VY+R+YSGTL LRD + ++ +EK+KITEM
+P+ G
Sbjct 241
STPEGQSELCGQVFKIEYSEKRRRFVYVRIYSGTLHLRDVIRISEKEKIKITEMCVPTNG 300

Query 301
EIVRTDTAYPGEIVILPSDSVRLNDVLGDPTRLPRKRWREDPLPMLRTTIAPKTAAQRER 360
            E+ +DTA G+IVILP+D ++LN +LG+   LP++++ E+PLPML+TTIA K
QRE
Sbjct 301
ELYPSDTACSGDIVILPNDVLQLNSILGNEMLLPQRKFIENPLPMLQTTIAVKKPEQREI 360

Query 361
LLDALTQLADTDPLLRCEVDSITHEIILSFLGRVQLEVVSALLSEKYKLETVVKEPSVIY 420
            LL ALT+++D DPLL+ VD+ THEIILSFLG VQ+EV+ A+L EKY +E +KEP
+VIY
Sbjct 361
LLGALTEISDGDPLLKYYVDTTTHEIILSFLGNVQMEVICAILEEKYHVEAEIKEPTVIY 420

Query 421
MERPLKAASHTIHIEVPPNPFWASIGLSVTPLSLGSGVQYESRVSLGYLNQSFQNAVRDG 480
            MERPL+ A +TIHIEVPPNPFWAS+GLS+ PL
+GSGVQYESRVSLGYLNQSFQNAV +G
Sbjct 421
MERPLRKAEYTIHIEVPPNPFWASVGLSIEPLPIGSGVQYESRVSLGYLNQSFQNAVMEG 480

Query 481
IRYGLEQGLFGWNVTDCKICFEYGLYYSPVSTPADFRSLAPIVLEQALKESGTQLLEPYL 540
            + YG EQGL+GW VTDCKICFEYGLYYSPVSTPADFR L+PIVLEQALK++GT
+LLEPYL
Sbjct 481
VLYGCEQGLYGWKVTDCKICFEYGLYYSPVSTPADFRLLSPIVLEQALKKAGTELLEPYL 540

Query 541
SFILYAPQEYLSRAYHDAPKYCATIETAQVKKDEVVFTGEIPARCIQAYRTDLAFYTNGR 600
             F +YAPQEYLSRAYHDAP+YCA I + QVK DEV+ GEIPARCIQ YR DL +
+TNG+
Sbjct 541
HFEIYAPQEYLSRAYHDAPRYCADIVSTQVKNDEVILKGEIPARCIQEYRNDLTYFTNGQ 600

Query 601 SVCLTELKGYQAAVGQPVIQPRRPNSRLDKVRHMFQKV 638
             VCLTELKGYQ A+G+ + QPRRPNSR+DKVRHMF K+
Sbjct 601 GVCLTELKGYQPAIGKFICQPRRPNSRIDKVRHMFHKL 638

>P23835.1 RecName: Full=Tetracycline resistance protein TetO;
Short=Tet(O)
Length=639

 Score = 926 bits (2393), Expect = 0.0, Method: Compositional
matrix adjust.
 Identities = 431/638 (68%), Positives = 529/638 (83%), Gaps = 0/638
(0%)

Query 1
MKIINIGILAHVDAGKTTLTESLLYASGAISEPGSVEKGTTRTDTMFLERQRGITIQAAV 60
            MKIIN+GILAHVDAGKTTLTESLLY SGAI+EPGSV+KGTTRTDT+
LERQRGITIQ AV
Sbjct 1

```
MKIINLGILAHVDAGKTTLTESLLYTSGAIAEPGSVDKGTTRTDTINLERQRGITIQTAV  60

Query  61
TSFQWHRCKVNIVDTPGHMDFLAEVYRSLAVLDGAILVISAKDGVQAQTRILFHALRKMN  120
            TSFQW   KVNI+DTPGHMDFLAEVYRSL+VLDGA+L++SAKDG+QAQTRILFHAL
+ M
Sbjct  61
TSFQWEDVKVNIIDTPGHMDFLAEVYRSLSVLDGAVLLVSAKDGIQAQTRILFHALQIMK  120

Query  121
IPTVIFINKIDQAGVDLQSVVQSVRDKLSADIIIKQTVSLSPEIVLEENTDIEAWDAVIE  180
            IPT+ FINKIDQ G+DL  V Q ++ KLS++II+KQ V   P I + +N D+E
WDAVI
Sbjct  121
IPTIFFINKIDQEGIDLPMVYQEMKAKLSSEIIVKQKVGQHPHINVTDNDDMEQWDAVIM  180

Query  181
NNDKLLEKYIAGEPISREKLVREEQRRVQDASLFPVYYGSAKKGLGIQPLMDAVTGLFQP  240
             ND+LLEKY++G+P    +L +EE RR Q+ +LFPVY+GSAK  LGI+ L++ +
F
Sbjct  181
GNDELLEKYMSGKPFKMSELEQEENRRFQNGTLFPVYHGSAKNNLGIRQLIEVIASKFYS  240

Query  241
IGEQGSAALCGSVFKVEYTDCGQRRVYLRLYSGTLRLRDTVALAGREKLKITEMRIPSKG  300
               +G + LCG VFK+EY++  +R VY+R+YSGTL LRD + ++ +EK+KITEM
+P+ G
Sbjct  241
STPEGQSELCGQVFKIEYSEKRRRFVYVRIYSGTLHLRDVIRISEKEKIKITEMCVPTNG  300

Query  301
EIVRTDTAYPGEIVILPSDSVRLNDVLGDPTRLPRKRWREDPLPMLRTTIAPKTAAQRER  360
            E+  +DTA  G+IVILP+D ++LN +LG+   LP++++ E+PLPML+TTIA K +
QRE
Sbjct  301
ELYSSDTACSGDIVILPNDVLQLNSILGNEILLPQRKFIENPLPMLQTTIAVKKSEQREI  360

Query  361
LLDALTQLADTDPLLRCEVDSITHEIILSFLGRVQLEVVSALLSEKYKLETVVKEPSVIY  420
            LL ALT+++D DPLL+  VD+ THEIILSFLG VQ+EV+ A+L EKY +E  +KEP
+VIY
Sbjct  361
LLGALTEISDGDPLLKYYVDTTTHEIILSFLGNVQMEVICAILEEKYHVEAEIKEPTVIY  420

Query  421
MERPLKAASHTIHIEVPPNPFWASIGLSVTPLSLGSGVQYESRVSLGYLNQSFQNAVRDG  480
            MERPL+ A +TIHIEVPPNPFWAS+GLS+ PL
LGSGVQYESRVSLGYLNQSFQNAV +G
Sbjct  421
MERPLRKAEYTIHIEVPPNPFWASVGLSIEPLLLGSGVQYESRVSLGYLNQSFQNAVMEG  480

Query  481
IRYGLEQGLFGWNVTDCKICFEYGLYYSPVSTPADFRSLAPIVLEQALKESGTQLLEPYL  540
            + YG EQGL+GW VTDCKICFEYGLYYSPVSTPADFR L+PIVLEQALK++GT
```

```
+LLEPYL
Sbjct  481
VLYGCEQGLYGWKVTDCKICFEYGLYYSPVSTPADFRLLSPIVLEQALKKAGTELLEPYL  540

Query  541
SFILYAPQEYLSRAYHDAPKYCATIETAQVKKDEVVFTGEIPARCIQAYRTDLAFYTNGR  600
             F +YAPQEYLSRAYHDAP+YCA I + Q+K DEV+  GEIPARCIQ YR DL
+TNG+
Sbjct  541
HFEIYAPQEYLSRAYHDAPRYCADIVSTQIKNDEVILKGEIPARCIQEYRNDLTNFTNGQ  600

Query  601  SVCLTELKGYQAAVGQPVIQPRRPNSRLDKVRHMFQKV  638
             VCLTELKGYQ A+G+ + QPRRPNSR+DKVRHMF K+
Sbjct  601  GVCLTELKGYQPAIGKFICQPRRPNSRIDKVRHMFHKL  638


>WP_105129783.1 TetM/TetW/TetO/TetS family tetracycline resistance
ribosomal
protection protein [Streptococcus suis]
Length=639

 Score = 926 bits (2392),  Expect = 0.0, Method: Compositional
matrix adjust.
 Identities = 432/638 (68%), Positives = 524/638 (82%), Gaps = 0/638
(0%)

Query  1
MKIINIGILAHVDAGKTTLTESLLYASGAISEPGSVEKGTTRTDTMFLERQRGITIQAAV  60
            MKIIN+GILAHVDAGKTTLTESLLY SGAI+EPGSV+KGTTRTDTM
LERQRGITIQ AV
Sbjct  1
MKIINLGILAHVDAGKTTLTESLLYTSGAIAEPGSVDKGTTRTDTMNLERQRGITIQTAV  60

Query  61
TSFQWHRCKVNIVDTPGHMDFLAEVYRSLAVLDGAILVISAKDGVQAQTRILFHALRKMN  120
            TSFQW   KVNI+DTPGHMDFLAEVYRSL+VLDGA+L++SAKDG+QAQTRILFHAL
+ M
Sbjct  61
TSFQWEDVKVNIIDTPGHMDFLAEVYRSLSVLDGAVLLVSAKDGIQAQTRILFHALQTMK  120

Query  121
IPTVIFINKIDQAGVDLQSVVQSVRDKLSADIIIKQTVSLSPEIVLEENTDIEAWDAVIE  180
            IPT+ FINKIDQ G+DL  V Q ++ KLS++II+KQ V   P I + +N D+E WD
VI
Sbjct  121
IPTIFFINKIDQEGIDLPMVYQEMKAKLSSEIIVKQKVGQHPHINVTDNDDMEQWDTVIM  180

Query  181
NNDKLLEKYIAGEPISREKLVREEQRRVQDASLFPVYYGSAKKGLGIQPLMDAVTGLFQP  240
             ND+LLEKY++G+P    +L +EE RR Q+ +LFPVY+GSAK  LGI+ L++ +
F
Sbjct  181
GNDELLEKYMSGKPFKMSELEQEENRRFQNGTLFPVYHGSAKNNLGIRQLIEVIASKFYS  240
```

```
Query  241
IGEQGSAALCGSVFKVEYTDCGQRRVYLRLYSGTLRLRDTVALAGREKLKITEMRIPSKG  300
               +G + LCG VFK+EY++  +R  Y+RLYSG L  RD V ++ +EK+KITEM
IP+ G
Sbjct  241
STPEGQSELCGQVFKIEYSEKRRRLAYVRLYSGALHSRDVVRISEKEKIKITEMSIPTNG  300

Query  301
EIVRTDTAYPGEIVILPSDSVRLNDVLGDPTRLPRKRWREDPLPMLRTTIAPKTAAQRER  360
            E+   DTAY GEIVILP+D ++LN +LG+   LP++   E+PLP+L+TTI  K +
QRE
Sbjct  301
ELCLADTAYSGEIVILPNDVLQLNSILGNEILLPQRESIENPLPILQTTIEVKKSEQREV  360

Query  361
LLDALTQLADTDPLLRCEVDSITHEIILSFLGRVQLEVVSALLSEKYKLETVVKEPSVIY  420
            LL+ALT+++D DPLL+  VD+ THEIILSFLG+VQ+EV+ A+L EKY +E  +KEP
+VIY
Sbjct  361
LLEALTEISDGDPLLKYYVDTTTHEIILSFLGKVQMEVICAILEEKYHVEAEIKEPTVIY  420

Query  421
MERPLKAASHTIHIEVPPNPFWASIGLSVTPLSLGSGVQYESRVSLGYLNQSFQNAVRDG  480
            MERPL  A +TIHIEVPPNPFWA+IGLSV PL
LGSGVQYESRVSLGYLNQSFQNAV +G
Sbjct  421
MERPLGKAEYTIHIEVPPNPFWATIGLSVEPLPLGSGVQYESRVSLGYLNQSFQNAVMEG  480

Query  481
IRYGLEQGLFGWNVTDCKICFEYGLYYSPVSTPADFRSLAPIVLEQALKESGTQLLEPYL  540
            + YG EQGL+GW VTDCKICFEYGLYYSPVSTPADFR L+PIVLEQALK++GT
+LLEPYL
Sbjct  481
VLYGCEQGLYGWKVTDCKICFEYGLYYSPVSTPADFRLLSPIVLEQALKKAGTELLEPYL  540

Query  541
SFILYAPQEYLSRAYHDAPKYCATIETAQVKKDEVVFTGEIPARCIQAYRTDLAFYTNGR  600
             F +YAPQEYLSRAYHDAP+YCA I + Q+K DEV+  GEIPARCIQ YR DL +
+TNG+
Sbjct  541
HFEIYAPQEYLSRAYHDAPRYCADIVSTQIKNDEVILKGEIPARCIQEYRNDLTYFTNGQ  600

Query  601  SVCLTELKGYQAAVGQPVIQPRRPNSRLDKVRHMFQKV  638
             VCLTELKGYQ A+G+ + QPRRPNSR+DKVRHMF K+
Sbjct  601  GVCLTELKGYQPAIGKFICQPRRPNSRIDKVRHMFHKL  638
```

>WP_105108102.1 TetM/TetW/TetO/TetS family tetracycline resistance ribosomal
protection protein [Streptococcus suis]
Length=639

Score = 926 bits (2392), Expect = 0.0, Method: Compositional matrix adjust.

Identities = 429/638 (67%), Positives = 526/638 (82%), Gaps = 0/638 (0%)

```
Query  1
MKIINIGILAHVDAGKTTLTESLLYASGAISEPGSVEKGTTRTDTMFLERQRGITIQAAV  60
            MKIIN+GILAHVDAGKTTLTESLLY SGAI+EPGSV+KGTTRTDTM
LERQRGITIQ AV
Sbjct  1
MKIINLGILAHVDAGKTTLTESLLYTSGAIAEPGSVDKGTTRTDTMNLERQRGITIQTAV  60

Query  61
TSFQWHRCKVNIVDTPGHMDFLAEVYRSLAVLDGAILVISAKDGVQAQTRILFHALRKMN  120
            TSFQW   KVNI+DTPGHMDFLAEVYRSL+VLDGA+L++SAKDG+QAQTRILFHAL
+ M
Sbjct  61
TSFQWEDVKVNIIDTPGHMDFLAEVYRSLSVLDGAVLLVSAKDGIQAQTRILFHALQTMK  120

Query  121
IPTVIFINKIDQAGVDLQSVVQSVRDKLSADIIIKQTVSLSPEIVLEENTDIEAWDAVIE  180
            IPT+ FINKIDQ G+DL  V + ++ KLS++II+KQ V   P I + +N D+E WD
VI
Sbjct  121
IPTIFFINKIDQEGIDLPMVYREMKAKLSSEIIVKQKVGQHPHINVTDNDDMEQWDTVIM  180

Query  181
NNDKLLEKYIAGEPISREKLVREEQRRVQDASLFPVYYGSAKKGLGIQPLMDAVTGLFQP  240
             ND+LLEKY++G+P    +L +EE RR Q+ +LFPVY+GSAK  LGI+ L++ +
F
Sbjct  181
GNDELLEKYMSGKPFKMSELEQEENRRFQNGTLFPVYHGSAKNNLGIRQLIEVIASKFYS  240

Query  241
IGEQGSAALCGSVFKVEYTDCGQRRVYLRLYSGTLRLRDTVALAGREKLKITEMRIPSKG  300
               +G + LCG VFK+EY++  +R  Y+RLYSG L  RD V ++ +EK+KITEM
IP+ G
Sbjct  241
STPEGQSELCGQVFKIEYSEKRRRLAYVRLYSGALHSRDVVRISEKEKIKITEMSIPTNG  300

Query  301
EIVRTDTAYPGEIVILPSDSVRLNDVLGDPTRLPRKRWREDPLPMLRTTIAPKTAAQRER  360
            E+   DTAY G+IVILP+D ++LN +LG+   LP++++ E+PLPML+TTI  K +
QRE
Sbjct  301
ELCLADTAYSGDIVILPNDVLQLNSILGNEILLPQRKFIENPLPMLQTTIEVKKSEQREV  360

Query  361
LLDALTQLADTDPLLRCEVDSITHEIILSFLGRVQLEVVSALLSEKYKLETVVKEPSVIY  420
            LL+ALT+++D DPLL+  VD+ THEIILSFLG VQ+EV+ A+L EKY +E  +KEP
+VIY
Sbjct  361
LLEALTEISDGDPLLKYYVDTTTHEIILSFLGNVQMEVICAILEEKYHVEAEIKEPTVIY  420

Query  421
MERPLKAASHTIHIEVPPNPFWASIGLSVTPLSLGSGVQYESRVSLGYLNQSFQNAVRDG  480
```

```
            MERPL+ A +TIHIEVPPNPFWAS+GLS+ PL
+GSGVQYESRVSLGYLNQSFQNAV +G
Sbjct  421
MERPLRKAEYTIHIEVPPNPFWASVGLSIEPLPIGSGVQYESRVSLGYLNQSFQNAVMEG  480

Query  481
IRYGLEQGLFGWNVTDCKICFEYGLYYSPVSTPADFRSLAPIVLEQALKESGTQLLEPYL  540
            + YG EQGL+GW VTDCKICFEYGLYYSPVSTPADFR L+PIVLEQALK++GT
+LLEPYL
Sbjct  481
VLYGCEQGLYGWKVTDCKICFEYGLYYSPVSTPADFRLLSPIVLEQALKKAGTELLEPYL  540

Query  541
SFILYAPQEYLSRAYHDAPKYCATIETAQVKKDEVVFTGEIPARCIQAYRTDLAFYTNGR  600
             F +YAPQEYLSRAYHDAP+YCA I + Q+K DEV+  GEIPARCIQ YR DL +
+TNG+
Sbjct  541
HFEIYAPQEYLSRAYHDAPRYCADIVSTQIKNDEVILKGEIPARCIQEYRNDLTYFTNGQ  600

Query  601  SVCLTELKGYQAAVGQPVIQPRRPNSRLDKVRHMFQKV  638
             VCLTELKGYQ A+G+ + QPRRPNSR+DKVRHMF K+
Sbjct  601  GVCLTELKGYQPAIGKFICQPRRPNSRIDKVRHMFHKL  638


>WP_099806636.1 TetM/TetW/TetO/TetS family tetracycline resistance
ribosomal
protection protein [Streptococcus suis]
Length=639

 Score = 926 bits (2392),  Expect = 0.0, Method: Compositional
matrix adjust.
 Identities = 430/638 (67%), Positives = 528/638 (83%), Gaps = 0/638
(0%)

Query  1
MKIINIGILAHVDAGKTTLTESLLYASGAISEPGSVEKGTTRTDTMFLERQRGITIQAAV  60
            MKIIN+GILAHVDAGKTTLTESLLY SGAI+EPGSV+KGTTRTDTM
LERQRGITIQ AV
Sbjct  1
MKIINLGILAHVDAGKTTLTESLLYTSGAIAEPGSVDKGTTRTDTMNLERQRGITIQTAV  60

Query  61
TSFQWHRCKVNIVDTPGHMDFLAEVYRSLAVLDGAILVISAKDGVQAQTRILFHALRKMN  120
            TSFQW   KVNI+DTPGHMDFLAEVYRSL+VLDGA+L++SAKDG+QAQTRILFHAL
+ M
Sbjct  61
TSFQWEDVKVNIIDTPGHMDFLAEVYRSLSVLDGAVLLVSAKDGIQAQTRILFHALQTMK  120

Query  121
IPTVIFINKIDQAGVDLQSVVQSVRDKLSADIIIKQTVSLSPEIVLEENTDIEAWDAVIE  180
            IPT+ FINKIDQ G+DL  V Q ++ KLS++II+KQ V   P I + +N D+E WD
VI
Sbjct  121
IPTIFFINKIDQEGIDLPMVYQEMKAKLSSEIIVKQKVGQHPHINVTDNDDMEQWDTVIM  180
```

```
Query  181
NNDKLLEKYIAGEPISREKLVREEQRRVQDASLFPVYYGSAKKGLGIQPLMDAVTGLFQP  240
             ND+LLEKY++G+P    +L +EE RR Q+ +LFPVY+GSAK  LGI+ L++ +
F
Sbjct  181
GNDELLEKYMSGKPFKMSELEQEENRRFQNGTLFPVYHGSAKNNLGIRQLIEVIASKFYS  240

Query  241
IGEQGSAALCGSVFKVEYTDCGQRRVYLRLYSGTLRLRDTVALAGREKLKITEMRIPSKG  300
               +G + LCG VFK+EY++  +R VY+R+YSGTL LRD + ++ +EK+KITEM
+P+ G
Sbjct  241
STPEGQSELCGQVFKIEYSEKRRRFVYVRIYSGTLHLRDVIRISEKEKIKITEMCVPTNG  300

Query  301
EIVRTDTAYPGEIVILPSDSVRLNDVLGDPTRLPRKRWREDPLPMLRTTIAPKTAAQRER  360
            E+  +DTA  G+IVILP+D ++LN +LG+   LP++++ E+PLPML+TTIA K
QRE
Sbjct  301
ELYPSDTACSGDIVILPNDVLQLNSILGNEMLLPQRKFIENPLPMLQTTIAVKKPEQREI  360

Query  361
LLDALTQLADTDPLLRCEVDSITHEIILSFLGRVQLEVVSALLSEKYKLETVVKEPSVIY  420
            LL ALT+++D DPLL+  VD+ THEIILSFLG VQ+EV+ A+L EKY +E  +KEP
+VIY
Sbjct  361
LLGALTEISDGDPLLKYYVDTTTHEIILSFLGNVQMEVICAILEEKYHVEAEIKEPTVIY  420

Query  421
MERPLKAASHTIHIEVPPNPFWASIGLSVTPLSLGSGVQYESRVSLGYLNQSFQNAVRDG  480
            MERPL+ A +TIHIEVPPNPFWAS+GLS+ PL
+GSGVQYESRVSLGYLNQSFQNAV +G
Sbjct  421
MERPLRKAEYTIHIEVPPNPFWASVGLSIEPLPIGSGVQYESRVSLGYLNQSFQNAVMEG  480

Query  481
IRYGLEQGLFGWNVTDCKICFEYGLYYSPVSTPADFRSLAPIVLEQALKESGTQLLEPYL  540
            + YG EQGL+GW VTDCKICFEYGLYYSPVSTPADFR L+PIVLEQALK++GT
+LLEPYL
Sbjct  481
VLYGCEQGLYGWKVTDCKICFEYGLYYSPVSTPADFRLLSPIVLEQALKKAGTELLEPYL  540

Query  541
SFILYAPQEYLSRAYHDAPKYCATIETAQVKKDEVVFTGEIPARCIQAYRTDLAFYTNGR  600
             F +YAPQEYLSRAYHDAP+YCA I + Q+K DEV+  GEIPARCIQ YR DL +
+TNG+
Sbjct  541
HFEIYAPQEYLSRAYHDAPRYCADIVSTQIKNDEVILKGEIPARCIQEYRNDLTYFTNGQ  600

Query  601  SVCLTELKGYQAAVGQPVIQPRRPNSRLDKVRHMFQKV  638
             VCLTELKGYQ A+G+ + QPRRPNSR+DKVRHMF K+
Sbjct  601  GVCLTELKGYQPAIGKFICQPRRPNSRIDKVRHMFHKL  638
```

>AMP54781.1 TetM-TetW-TetO-TetS [uncultured bacterium]
Length=639

Score = 926 bits (2392), Expect = 0.0, Method: Compositional matrix adjust.
Identities = 429/638 (67%), Positives = 530/638 (83%), Gaps = 0/638 (0%)

```
Query  1
MKIINIGILAHVDAGKTTLTESLLYASGAISEPGSVEKGTTRTDTMFLERQRGITIQAAV  60
            MKIIN+GILAHVDAGKTTLTESLLY SGAI+EPGSV++GTTRTDTM
LERQRGITIQ AV
Sbjct  1
MKIINLGILAHVDAGKTTLTESLLYTSGAIAEPGSVDEGTTRTDTMNLERQRGITIQTAV  60

Query  61
TSFQWHRCKVNIVDTPGHMDFLAEVYRSLAVLDGAILVISAKDGVQAQTRILFHALRKMN  120
            TSFQW   KVNI+DTPGHMDFLAEVYRSL+VLDGA+L++SAKDG+QAQTRILFHAL
+ M
Sbjct  61
TSFQWEDVKVNIIDTPGHMDFLAEVYRSLSVLDGAVLLVSAKDGIQAQTRILFHALQIMK  120

Query  121
IPTVIFINKIDQAGVDLQSVVQSVRDKLSADIIIKQTVSLSPEIVLEENTDIEAWDAVIE  180
            IPT+ FINKIDQ G+DL  V + ++ KLS++II+KQ V   P I + +N D+E
WDAVI
Sbjct  121
IPTIFFINKIDQEGIDLPMVYREMKAKLSSEIIVKQKVGQHPHINVTDNDDMEQWDAVIM  180

Query  181
NNDKLLEKYIAGEPISREKLVREEQRRVQDASLFPVYYGSAKKGLGIQPLMDAVTGLFQP  240
             ND+LLEKY++G+P    +L +EE RR Q+ +LFPVY+GSAK  LGI+ L++ +
F
Sbjct  181
GNDELLEKYMSGKPFKMSELEQEENRRFQNGTLFPVYHGSAKNNLGIRQLIEVIASKFYS  240

Query  241
IGEQGSAALCGSVFKVEYTDCGQRRVYLRLYSGTLRLRDTVALAGREKLKITEMRIPSKG  300
               +G + LCG VFK+EY++  +R VY+R+YSGTL LRD + ++ +EK+KITEM
+P+ G
Sbjct  241
STPEGQSELCGQVFKIEYSEKRRRFVYVRIYSGTLHLRDVIRISEKEKIKITEMCVPTNG  300

Query  301
EIVRTDTAYPGEIVILPSDSVRLNDVLGDPTRLPRKRWREDPLPMLRTTIAPKTAAQRER  360
            E+  +DTA  G+IVILP+D ++LN +LG+   LP++++ E+PLPML+TTIA K +
QRE
Sbjct  301
ELYSSDTACSGDIVILPNDVLQLNSILGNEILLPQRKFIENPLPMLQTTIAVKKSEQREI  360

Query  361
LLDALTQLADTDPLLRCEVDSITHEIILSFLGRVQLEVVSALLSEKYKLETVVKEPSVIY  420
            LL ALT+++D DPLL+  VD+ THEIILSFLG VQ+EV+ A+L EKY +E  +KEP
```

+VIY
Sbjct 361
LLGALTEISDGDPLLKYYVDTTTHEIILSFLGNVQMEVICAILEEKYHVEAEIKEPTVIY 420

Query 421
MERPLKAASHTIHIEVPPNPFWASIGLSVTPLSLGSGVQYESRVSLGYLNQSFQNAVRDG 480
MERPL+ A +TIHIEVPPNPFWAS+GLS+ PL
+GSGVQYESRVSLGYLNQSFQNAV +G
Sbjct 421
MERPLRKAEYTIHIEVPPNPFWASVGLSIEPLPIGSGVQYESRVSLGYLNQSFQNAVMEG 480

Query 481
IRYGLEQGLFGWNVTDCKICFEYGLYYSPVSTPADFRSLAPIVLEQALKESGTQLLEPYL 540
+ YG EQGL+GW VTDCKICFEYGLYYSPVSTPADFR L+PIVLEQALK++GT
+LLEPYL
Sbjct 481
VLYGCEQGLYGWKVTDCKICFEYGLYYSPVSTPADFRLLSPIVLEQALKKAGTELLEPYL 540

Query 541
SFILYAPQEYLSRAYHDAPKYCATIETAQVKKDEVVFTGEIPARCIQAYRTDLAFYTNGR 600
F +YAPQEYLSRAYHDAP+YCA I + Q+K DEV+ GEIPARCIQ YR DL +
+TNG+
Sbjct 541
HFEIYAPQEYLSRAYHDAPRYCADIVSTQIKNDEVILKGEIPARCIQEYRNDLTYFTNGQ 600

Query 601 SVCLTELKGYQAAVGQPVIQPRRPNSRLDKVRHMFQKV 638
VCLTELKGYQ A+G+ + QPRRPNSR+DKVRHMF K+
Sbjct 601 GVCLTELKGYQPAIGKFICQPRRPNSRIDKVRHMFHKL 638

>WP_044666383.1 MULTISPECIES: tetracycline resistance ribosomal protection protein
Tet(O) [Streptococcus]
P20174.1 RecName: Full=Tetracycline resistance protein TetO; Short=Tet(O)
[truncated: 421,950 more chars]
