# Supplementary material for: Dissemination of antibiotic resistance genes associated with the sporobiota in sediments impacted by wastewater
Source: PeerJ. 2018 Jun 20;6:e4989. doi: 10.7717/peerj.4989 (PMC6015491; doi:10.7717/peerj.4989)
Supplement: Supplemental Information 3 — Summary of the Blastp results for the presence of Sul1 in the genomes of the close relatives to the 10-most positively correlated OTUs in the dataset (Table 2). [file peerj-06-4989-s008.pdf]

BLASTP 2.8.0+
Reference: Stephen F. Altschul, Thomas L. Madden, Alejandro
A. Schaffer, Jinghui Zhang, Zheng Zhang, Webb Miller, and
David J. Lipman (1997), "Gapped BLAST and PSI-BLAST: a new
generation of protein database search programs", Nucleic
Acids Res. 25:3389-3402.

Reference for compositional score matrix adjustment: Stephen
F. Altschul, John C. Wootton, E. Michael Gertz, Richa
Agarwala, Aleksandr Morgulis, Alejandro A. Schaffer, and
Yi-Kuo Yu (2005) "Protein database searches using
compositionally adjusted substitution matrices", FEBS J.
272:5101-5109.

RID: CT2JBWCM01R

Database: All non-redundant GenBank CDS
translations+PDB+SwissProt+PIR+PRF excluding environmental samples
from WGS projects
149,694,506 sequences; 54,860,295,172 total letters
Query= WP_007929940.1 MULTISPECIES: sulfonamide-resistant
dihydropteroate
synthase Sul1 [Actinobacteria]

Length=283

Score     E
Sequences producing significant alignments:
(Bits)  Value

ACA34300.1  dihydrofolate synthase 2 [Leucobacter komagatae]
223     1e-71
CZQ86650.1  dihydropteroate synthase signature 2 [Trichococcus...
126     2e-33
WP_014355480.1  dihydropteroate synthase [Acetobacterium woodii]
124     1e-32
WP_050739642.1  dihydropteroate synthase [Acetobacterium bakii]
124     2e-32
WP_086986002.1  dihydropteroate synthase [Trichococcus collinsii]
122     3e-32
CZQ85205.1  dihydropteroate synthase signature 2 [Trichococcus...
121     1e-31
CZQ89210.1  dihydropteroate synthase signature 2 [Trichococcus...
121     2e-31
SFE72892.1  dihydropteroate synthase [Trichococcus pasteurii]
119     6e-31
WP_069911287.1  dihydropteroate synthase [Devosia insulae]
119     1e-30
WP_057081671.1  dihydropteroate synthase [Bifidobacterium bifi...

119     2e-30
WP_086988369.1  dihydropteroate synthase [Trichococcus floccul...
118     2e-30
WP_013390177.1  dihydropteroate synthase [Bifidobacterium bifi...
119     2e-30
BBA47465.1  dihydropteroate synthase [Bifidobacterium bifidum ...
119     2e-30
WP_083546429.1  MULTISPECIES: dihydropteroate synthase [Tricho...
117     2e-30
WP_013363793.1  dihydropteroate synthase [Bifidobacterium bifi...
118     3e-30
WP_047298337.1  dihydropteroate synthase [Bifidobacterium bifi...
118     4e-30
WP_026394512.1  dihydropteroate synthase [Acetobacterium dehal...
117     4e-30
WP_034876901.1  dihydropteroate synthase [Bifidobacterium mouk...
117     6e-30
WP_101629030.1  dihydropteroate synthase [Bifidobacterium longum]
117     6e-30
WP_010081417.1  dihydropteroate synthase [Bifidobacterium longum]
117     7e-30
WP_032744530.1  dihydropteroate synthase [Bifidobacterium longum]
117     7e-30
WP_033499260.1  dihydropteroate synthase [Bifidobacterium adol...
117     7e-30
WP_034525832.1  dihydropteroate synthase [Bifidobacterium stel...
117     8e-30
WP_003808269.1  dihydropteroate synthase [Bifidobacterium adol...
117     9e-30
WP_086943467.1  dihydropteroate synthase [Trichococcus pasteurii]
116     9e-30
WP_049705597.1  dihydropteroate synthase [Devosia sp. H5989]
117     1e-29
WP_042990425.1  dihydropteroate synthase [Bifidobacterium adol...
117     1e-29
WP_033523224.1  dihydropteroate synthase [Bifidobacterium mery...
117     1e-29
WP_041777256.1  dihydropteroate synthase [Bifidobacterium adol...
116     2e-29
WP_065506906.1  dihydropteroate synthase [Bifidobacterium breve]
116     2e-29
WP_032746349.1  dihydropteroate synthase [Bifidobacterium longum]
116     2e-29
WP_053825171.1  dihydropteroate synthase [Bifidobacterium bifi...
116     2e-29
WP_055881405.1  dihydropteroate synthase [Devosia sp. Root105]
115     2e-29
WP_007053863.1  dihydropteroate synthase [Bifidobacterium longum]
116     2e-29
WP_077385559.1  dihydropteroate synthase [Bifidobacterium longum]
116     2e-29
BAF39192.1  dihydropteroate synthase 1 [Bifidobacterium adoles...
116     2e-29
WP_003814775.1  dihydropteroate synthase [Bifidobacterium bifi...

115     2e-29
WP_065445269.1  dihydropteroate synthase [Bifidobacterium longum]
115     3e-29
WP_065454755.1  dihydropteroate synthase [Bifidobacterium longum]
115     3e-29
AUD75228.1  Dihydropteroate synthase [Bifidobacterium breve]
115     3e-29
WP_100513572.1  dihydropteroate synthase [Bifidobacterium sp. ...
115     3e-29
WP_014760561.1  dihydropteroate synthase [Bifidobacterium bifi...
115     3e-29
WP_039774850.1  dihydropteroate synthase [Bifidobacterium adol...
115     4e-29
WP_032736608.1  dihydropteroate synthase [Bifidobacterium longum]
115     4e-29
WP_033892007.1  dihydropteroate synthase [Bifidobacterium sagu...
115     4e-29
WP_085379343.1  dihydropteroate synthase [Bifidobacterium adol...
115     4e-29
CZQ91691.1  dihydropteroate synthase signature 1 [Trichococcus...
115     4e-29
WP_047290331.1  dihydropteroate synthase [Bifidobacterium bifi...
115     4e-29
BBA55689.1  dihydropteroate synthase [Bifidobacterium bifidum]
115     5e-29
KXS22994.1  dihydropteroate synthase [Bifidobacterium breve]
115     5e-29
WP_080867818.1  dihydropteroate synthase [Bifidobacterium breve]
115     5e-29
WP_032682980.1  dihydropteroate synthase [Bifidobacterium longum]
115     5e-29
EIJ22047.1  dihydropteroate synthase [Bifidobacterium longum s...
115     5e-29
WP_026646441.1  dihydropteroate synthase [Bifidobacterium rumi...
115     6e-29
WP_019728223.1  dihydropteroate synthase [Bifidobacterium breve]
114     6e-29
WP_025222047.1  dihydropteroate synthase [Bifidobacterium breve]
114     6e-29
WP_055996483.1  dihydropteroate synthase [Devosia sp. Root413D1]
114     6e-29
WP_025301383.1  dihydropteroate synthase [Bifidobacterium breve]
114     7e-29
WP_100510016.1  dihydropteroate synthase [Bifidobacterium sp. ...
114     7e-29
AUD91812.1  Dihydropteroate synthase [Bifidobacterium breve]
114     9e-29
WP_085461798.1  dihydropteroate synthase [Bifidobacterium adol...
114     9e-29
WP_065472186.1  dihydropteroate synthase [Bifidobacterium longum]
114     9e-29
CZQ85951.1  dihydropteroate synthase signature 2 [Trichococcus...
114     9e-29
WP_100496241.1  dihydropteroate synthase [Bifidobacterium sp. ...

114     9e-29
WP_015439115.1  dihydropteroate synthase [Bifidobacterium breve]
114     9e-29
WP_029679339.1  dihydropteroate synthase [Bifidobacterium longum]
114     1e-28
WP_021649768.1  dihydropteroate synthase [Bifidobacterium breve]
114     1e-28
WP_080784119.1  dihydropteroate synthase [Bifidobacterium breve]
114     1e-28
WP_077424703.1  dihydropteroate synthase [Bifidobacterium longum]
114     1e-28
WP_094667142.1  dihydropteroate synthase [Bifidobacterium myos...
114     1e-28
WP_070372479.1  dihydropteroate synthase [Acetobacterium wieri...
113     1e-28
WP_070122223.1  dihydropteroate synthase [Bifidobacterium adol...
114     1e-28
WP_094730457.1  dihydropteroate synthase [Bifidobacterium hapali]
114     2e-28
WP_101621332.1  dihydropteroate synthase [Bifidobacterium parmae]
113     2e-28
WP_101626177.1  dihydropteroate synthase [Bifidobacterium impe...
113     2e-28
OXS26090.1  dihydropteroate synthase [Acetobacterium sp. MES1]
113     2e-28
WP_077389951.1  dihydropteroate synthase [Bifidobacterium longum]
113     2e-28
WP_065436770.1  dihydropteroate synthase [Bifidobacterium longum]
113     2e-28
WP_013410667.1  dihydropteroate synthase [Bifidobacterium longum]
113     2e-28
WP_100493538.1  dihydropteroate synthase [Bifidobacterium sp. ...
113     2e-28
WP_044089021.1  dihydropteroate synthase [Bifidobacterium reut...
113     2e-28
WP_085382394.1  dihydropteroate synthase [Bifidobacterium adol...
113     2e-28
WP_085381689.1  dihydropteroate synthase [Bifidobacterium adol...
113     2e-28
PLS29794.1  dihydropteroate synthase [Bifidobacterium parmae]
113     3e-28
WP_060620656.1  dihydropteroate synthase [Bifidobacterium longum]
113     3e-28
KXS24820.1  dihydropteroate synthase [Bifidobacterium dentium]
113     3e-28
WP_056251287.1  dihydropteroate synthase [Devosia sp. Root436]
112     3e-28
WP_092184669.1  dihydropteroate synthase [Devosia sp. YR412]
112     3e-28
WP_100968037.1  dihydropteroate synthase [Bifidobacterium longum]
112     3e-28
ODS95184.1  dihydropteroate synthase [Devosia sp. SCN 66-27]
112     4e-28
WP_034518937.1  MULTISPECIES: dihydropteroate synthase [Bifido...

112     4e-28
WP_003837180.1  dihydropteroate synthase [Bifidobacterium dent...
112     4e-28
WP_058594680.1  dihydropteroate synthase [Leucobacter chromiir...
115     4e-28
WP_003830054.1  dihydropteroate synthase [Bifidobacterium breve]
112     5e-28
KXS29119.1  dihydropteroate synthase [Bifidobacterium longum]
112     6e-28
WP_008782915.1  MULTISPECIES: dihydropteroate synthase [Bifido...
112     6e-28
WP_087033063.1  dihydropteroate synthase [Trichococcus palustris]
111     6e-28
WP_095346990.1  dihydropteroate synthase [Bifidobacterium longum]
112     6e-28
WP_011068790.1  dihydropteroate synthase [Bifidobacterium longum]
112     7e-28
WP_046138407.1  dihydropteroate synthase [Devosia epidermidihi...
112     7e-28

ALIGNMENTS
>ACA34300.1 dihydrofolate synthase 2, partial (plasmid) [Leucobacter komagatae]
Length=241

Score = 223 bits (569),  Expect = 1e-71, Method: Compositional matrix adjust.
Identities = 133/243 (55%), Positives = 169/243 (70%), Gaps = 2/243 (1%)

```
Query  9
FGILNLTEDSFFDESRRLDPAGAVTAAIEMLRVGSDVVDVGPAASHPDARPVSPADEIRR  68
            FGI+N+T DSF D  R L P  A+  A +++  G+DV+D+GPA+S+PDA PVS
EI R
Sbjct  1
FGIVNITSDSFSDGGRYLAPDAAIAQARKLMAEGADVIDLGPASSNPDAAPVSSDTEIAR  60

Query  69
IAPLLDALSDQMHRVSIDSFQPETQRYALKRGVGYLNDIQGFPDPALYPDIAEADCRLVV  128
            IAP+LDAL      VS+DS+QP TQ YAL RGV YLNDI+GFPD A YP +A++
+LVV
Sbjct  61
IAPVLDALKADGIPVSLDSYQPATQAYALSRGVAYLNDIRGFPDAAFYPQLAKSSAKLVV  120

Query  129
MHSAQRDGIATRTGHLRPEDALDEIVRFFEARVSALRRSGVAADRLILDPGMGFFLSPAP  188
            MHS Q DG A R       D +D I  FF+AR++AL  +G+  +RL+LDPGMGFFL
AP
Sbjct  121  MHSVQ-DGQADRR-
EAPAGDIMDHIAAFFDARIAALTGAGIKRNRLVLDPGMGFFLGAAP  178

Query  189
ETSLHVLSNLQKLKSALGLPLLVSVSRKSFLGATVGLPVKDLGPASLAAELHAIGNGADY  248
            ETSL VL+   +L+    LP+L+SVSRKSFL A  G    D+G A+LAAEL A
```

```
GAD+
Sbjct  179  
ETSLSVLARFDELRLRFDLPVLLSVSRKSFLRALTGRGPGDVGAATLAAELAAAAGGADF  238

Query  249  VRT  251
            +RT
Sbjct  239  IRT  241


>CZQ86650.1 dihydropteroate synthase signature 2 [Trichococcus 
collinsii]
 SDZ76153.1 dihydropteroate synthase [Trichococcus collinsii]
Length=277

 Score = 126 bits (316),  Expect = 2e-33, Method: Compositional 
matrix adjust.
 Identities = 89/256 (35%), Positives = 136/256 (53%), Gaps = 19/256 
(7%)

Query  2    
LRSRVTVFGILNLTEDSFFDESRRLDPAGAVTAAIEMLRVGSDVVDVGPAASHPDARPVS  61
            L  R  + GILN+T DSF D         A+  AIEM+  G+D++D+G  ++ P 
+S
Sbjct  12   
LSQRSHIMGILNVTPDSFSDGGEWNTVEKAIAHAIEMVAEGADIIDIGGESTRPGHTQIS  71

Query  62   PADEIRRIAPLLDALSDQMHR-VSIDSFQPETQRYALKRGVGYLNDIQGFP-
DPALYPDI  119
              +EI R+ P+++AL   +   +SID+++    R A + G+  +NDI G   DP +
Sbjct  72   
DEEEIARVVPIIEALKKAVDVPLSIDTYKSAVARAACEAGIDIINDIWGCKYDPEIAAVA  131

Query  120  
AEADCRLVVMHSAQRDGIATRTGHLRPEDALDEIVRFFEARVSALRRSGVAADRLILDPG  179
            AE D  +++MH+ ++   A        ED L ++       VSA    GV  +
+ILDPG
Sbjct  132  AEFDVPIILMHNREKSDYA-----FLIEDMLADLAESVRIAVSA----
GVKRENIILDPG  182

Query  180  MGFFLSPAPETSLHVLSNLQKLKSALGLPLLVSVSRKSFLGATVG-LPVK--
DLGPASLA  236
             GF      E +L+V+ +L++  + LG P+L+  SRK F+G  +G LP K  DLG
A+
Sbjct  183  CGF--GKTYEDNLNVVHHLKRF-
TELGYPVLLGTSRKRFIGTALGDLPFKERDLGTAATT  239

Query  237  AELHAIGNGADYVRTH  252
            A    I NGA   R H
Sbjct  240  A--LGIMNGAQLFRVH  253


>WP_014355480.1 dihydropteroate synthase [Acetobacterium woodii]
 AFA47877.1 dihydropteroate synthase FolP [Acetobacterium woodii DSM 
1030]
```

Length=275

 Score = 124 bits (311),  Expect = 1e-32, Method: Compositional matrix adjust.
 Identities = 82/253 (32%), Positives = 136/253 (54%), Gaps = 14/253 (6%)

```
Query  2
LRSRVTVFGILNLTEDSFFDESRRLDPAGAVTAAIEMLRVGSDVVDVGPAASHPDARPVS  61
            L   V + GILN+T DSF D  +      ++    +M+  G+D++D+G  ++ P
+S
Sbjct  10
LTHEVLIMGILNVTPDSFSDGGKFNTRDTSLKQVEKMITDGADLIDLGGESTRPGHTQIS  69

Query  62   PADEIRRIAPLLDALSDQMHR-VSIDSFQPETQRYALKRGVGYLNDIQGFP-
DPALYPDI  119
             A+EI R+AP+++A+  +    +SID+++      ALK G   +NDI GF  DPAL
Sbjct  70
DAEEIDRVAPMIEAIRQRFDIPISIDTYKSAVGEAALKAGADLINDIWGFKYDPALAKVT  129

Query  120
AEADCRLVVMHSAQRDGIATRTGHLRPEDALDEIVRFFEARVSALRRSGVAADRLILDPG  179
            A+     V+MH+            HL     + ++ R  +  +     +GV+ D
+ILDPG
Sbjct  130  AKYKVPCVLMHNRNN----RNYDHL-----
MTDLNRDLQESIEIAINAGVSQDAIILDPG  180

Query  180
MGFFLSPAPETSLHVLSNLQKLKSALGLPLLVSVSRKSFLGATVGLPVKDLGPASLAAEL  239
            +GF    A   ++ V+++L+ L+ ALG P+L+  SRK F+G T+ LPV +    +
+A  +
Sbjct  181  IGFAKDYAQ--NMAVMNHLETLQ-
ALGYPILLGTSRKGFIGLTLDLPVTERVEGTVATTV  237

Query  240  HAIGNGADYVRTH  252
              I  GA  +R H
Sbjct  238  IGIMKGASIIRVH  250
```

>WP_050739642.1 dihydropteroate synthase [Acetobacterium bakii]
 KNZ42351.1 dihydropteroate synthase [Acetobacterium bakii]
Length=275

 Score = 124 bits (310),  Expect = 2e-32, Method: Compositional matrix adjust.
 Identities = 78/253 (31%), Positives = 138/253 (55%), Gaps = 14/253 (6%)

```
Query  2
LRSRVTVFGILNLTEDSFFDESRRLDPAGAVTAAIEMLRVGSDVVDVGPAASHPDARPVS  61
            L + V + GILN+T DSF D  +      ++    +M+  G+D++D+G  ++ P
PV
Sbjct  10
LDNCVVIMGILNVTPDSFSDGGKFNSLDTSLKHVEKMIHEGADIIDLGGESTRPGHEPVG  69
```

```
Query  62   PADEIRRIAPLLDALSDQMHR-VSIDSFQPETQRYALKRGVGYLNDIQGFP-
DPALYPDI  119
             A EI R+ P+++A+  +    +SID+++ +    A+K G   +ND+ GF  D  L
Sbjct  70
EAQEIDRVVPMIEAIRSRFDIPLSIDTYKSKVGELAIKAGADLINDVWGFKRDLKLAEVT  129

Query  120
AEADCRLVVMHSAQRDGIATRTGHLRPEDALDEIVRFFEARVSALRRSGVAADRLILDPG  179
            A+ +   V+MH+  RD       +   +  + ++    EA ++    +GVA D+
+ILDPG
Sbjct  130  AKYEVPCVLMHN--RD-------
NQNYDHFMIDVCHDLEASIAIAVNAGVARDKIILDPG  180

Query  180
MGFFLSPAPETSLHVLSNLQKLKSALGLPLLVSVSRKSFLGATVGLPVKDLGPASLAAEL  239
            +GF  +   + ++ ++ +L KL+  LG P+L+  SRK F+G T+ LPV +    +
+A  +
Sbjct  181  VGF--AKDYQLNMTMMKHLDKLQD-
LGFPILLGTSRKGFIGITLDLPVTERIEGTVATTV  237

Query  240  HAIGNGADYVRTH  252
              I  GA  +R H
Sbjct  238  MGIMKGASIIRVH  250
```

>WP_086986002.1 dihydropteroate synthase [Trichococcus collinsii]
Length=259

Score = 122 bits (307),  Expect = 3e-32, Method: Compositional matrix adjust.
Identities = 87/249 (35%), Positives = 133/249 (53%), Gaps = 19/249 (8%)

```
Query  9
FGILNLTEDSFFDESRRLDPAGAVTAAIEMLRVGSDVVDVGPAASHPDARPVSPADEIRR  68
             GILN+T DSF D         A+  AIEM+  G+D++D+G  ++ P    +S
+EI R
Sbjct  1
MGILNVTPDSFSDGGEWNTVEKAIAHAIEMVAEGADIIDIGGESTRPGHTQISDEEEIAR  60

Query  69   IAPLLDALSDQMHR-VSIDSFQPETQRYALKRGVGYLNDIQGFP-
DPALYPDIAEADCRL  126
            + P+++AL   +   +SID+++    R A + G+  +NDI G   DP +    AE
D  +
Sbjct  61
VVPIIEALKKAVDVPLSIDTYKSAVARAACEAGIDIINDIWGCKYDPEIAAVAAEFDVPI  120

Query  127
VVMHSAQRDGIATRTGHLRPEDALDEIVRFFEARVSALRRSGVAADRLILDPGMGFFLSP  186
            ++MH+ ++   A        ED L ++       VSA    GV  + +ILDPG GF
Sbjct  121  ILMHNREKSDYA-----FLIEDMLADLAESVRIAVSA----
GVKRENIILDPGCGF--GK  169
```

```
Query  187  APETSLHVLSNLQKLKSALGLPLLVSVSRKSFLGATVG-LPVK--
DLGPASLAAELHAIG  243
              E +L+V+ +L++  + LG P+L+  SRK F+G  +G LP K  DLG A+  A
I
Sbjct  170  TYEDNLNVVHHLKRF-TELGYPVLLGTSRKRFIGTALGDLPFKERDLGTAATTA--
LGIM  226

Query  244  NGADYVRTH  252
            NGA   R H
Sbjct  227  NGAQLFRVH  235


>CZQ85205.1 dihydropteroate synthase signature 2 [Trichococcus
flocculiformis]
 SFH58580.1 dihydropteroate synthase [Trichococcus flocculiformis]
Length=277

 Score = 121 bits (304),  Expect = 1e-31, Method: Compositional
matrix adjust.
 Identities = 90/256 (35%), Positives = 135/256 (53%), Gaps = 19/256
(7%)

Query  2
LRSRVTVFGILNLTEDSFFDESRRLDPAGAVTAAIEMLRVGSDVVDVGPAASHPDARPVS  61
            L  R  + GILN+T DSF D         AV  A+EM+  G+D++D+G  ++ P
+S
Sbjct  12
LSQRSYIMGILNVTPDSFSDGGEWNTVEKAVAHALEMVADGADIIDIGGESTRPGHTQIS  71

Query  62   PADEIRRIAPLLDALSDQMHR-VSIDSFQPETQRYALKRGVGYLNDIQGF-
PDPALYPDI  119
              +EI RI P+++AL   +   +SID+++    R A + GV  +NDI G   DP +
Sbjct  72
DEEEIARIVPVIEALKKAVDVPLSIDTYKSAVARAACEAGVDIINDIWGCRYDPQIAAVA  131

Query  120
AEADCRLVVMHSAQRDGIATRTGHLRPEDALDEIVRFFEARVSALRRSGVAADRLILDPG  179
            A  D  +++MH+ ++   A        ED L ++       VSA    GV  +
+ILDPG
Sbjct  132  ASFDVPIILMHNREKPDYA-----FLIEDMLTDLAESVRIAVSA----
GVKRENIILDPG  182

Query  180  MGFFLSPAPETSLHVLSNLQKLKSALGLPLLVSVSRKSFLGATVG-LPVK--
DLGPASLA  236
             GF      E +L+V+ +L++  + LG PLL+  SRK F+G  +G +P K  DLG
A+
Sbjct  183  CGF--GKTYEDNLNVVHHLKRF-
ADLGYPLLLGTSRKRFIGTALGDIPFKERDLGTAATT  239

Query  237  AELHAIGNGADYVRTH  252
            A    I NGA   R H
Sbjct  240  A--LGIVNGAQIFRVH  253
```

>CZQ89210.1 dihydropteroate synthase signature 2 [Trichococcus sp. ES5]
 SHF49980.1 dihydropteroate synthase [Trichococcus flocculiformis]
Length=277

 Score = 121 bits (303),  Expect = 2e-31, Method: Compositional matrix adjust.
 Identities = 89/256 (35%), Positives = 135/256 (53%), Gaps = 19/256 (7%)

```
Query  2
LRSRVTVFGILNLTEDSFFDESRRLDPAGAVTAAIEMLRVGSDVVDVGPAASHPDARPVS  61
           L  R  + GILN+T DSF D         AV  A+EM+  G+D++D+G  ++ P
+S
Sbjct  12
LSQRSYIMGILNVTPDSFSDGGEWNTVEKAVAHALEMVADGADIIDIGGESTRPGHTQIS  71

Query  62   PADEIRRIAPLLDALSDQMHR-VSIDSFQPETQRYALKRGVGYLNDIQGF-
PDPALYPDI  119
             +EI RI P+++AL   +   +SID+++    R A + G+  +NDI G   DP +
Sbjct  72
DEEEIARIVPVIEALKKAVDVPLSIDTYKSAVARAACEAGIDIINDIWGCRYDPQIAAVA  131

Query  120
AEADCRLVVMHSAQRDGIATRTGHLRPEDALDEIVRFFEARVSALRRSGVAADRLILDPG  179
           A  D  +++MH+ ++   A        ED L ++       VSA    GV  +
+ILDPG
Sbjct  132  ASFDVPIILMHNREKPDYA-----FLIEDMLTDLAESVRIAVSA----
GVKRENIILDPG  182

Query  180  MGFFLSPAPETSLHVLSNLQKLKSALGLPLLVSVSRKSFLGATVG-LPVK--
DLGPASLA  236
             GF      E +L+V+ +L++  + LG PLL+  SRK F+G  +G +P K  DLG
A+
Sbjct  183  CGF--GKTYEDNLNVVHHLKRF-
ADLGYPLLLGTSRKRFIGTALGDIPFKERDLGTAATT  239

Query  237  AELHAIGNGADYVRTH  252
           A    I NGA   R H
Sbjct  240  A--LGIVNGAQIFRVH  253
```

>SFE72892.1 dihydropteroate synthase [Trichococcus pasteurii]
 SLM52671.1 dihydropteroate synthase signature 2 [Trichococcus pasteurii]
Length=277

 Score = 119 bits (299),  Expect = 6e-31, Method: Compositional matrix adjust.
 Identities = 89/256 (35%), Positives = 135/256 (53%), Gaps = 19/256 (7%)

```
Query  2
LRSRVTVFGILNLTEDSFFDESRRLDPAGAVTAAIEMLRVGSDVVDVGPAASHPDARPVS  61
```

L  R  + GILN+T DSF D         AV  A+EM+  G+D++D+G  ++ P
+S
Sbjct  12
LSKRSYIMGILNVTPDSFSDGGEWNTVEKAVAHALEMVADGADIIDIGGESTRPGHTQIS  71

Query  62   PADEIRRIAPLLDALSDQMHR-VSIDSFQPETQRYALKRGVGYLNDIQGFP-
DPALYPDI  119
+EI RI P+++A+   +   +SID+++    R A + GV  +NDI G   DP +
Sbjct  72
DEEEIARIVPVIEAVKKAVDVPLSIDTYKSAVARAACEAGVDIINDIWGCKYDPEIAAVA  131

Query  120
AEADCRLVVMHSAQRDGIATRTGHLRPEDALDEIVRFFEARVSALRRSGVAADRLILDPG  179
A  D  +++MH+ ++   A        ED L ++       VSA    GV  +
+ILDPG
Sbjct  132  AAFDVPIILMHNREKPDYA-----FLIEDMLADLAESVRIAVSA----
GVKRENIILDPG  182

Query  180  MGFFLSPAPETSLHVLSNLQKLKSALGLPLLVSVSRKSFLGATVG-LPVK--
DLGPASLA  236
GF      E +L+V+ +L++  + LG PLL+  SRK F+G  +G +P K  DLG
A+
Sbjct  183  CGF--GKTYEDNLNVVHHLKRF-
ADLGYPLLLGTSRKRFIGTALGDVPFKGRDLGTAATT  239

Query  237  AELHAIGNGADYVRTH  252
A    I NGA   R H
Sbjct  240  A--LGIVNGAQLFRVH  253

>WP_069911287.1 dihydropteroate synthase [Devosia insulae]
OEO29376.1 dihydropteroate synthase [Devosia insulae DS-56]
Length=281

Score = 119 bits (298),  Expect = 1e-30, Method: Compositional
matrix adjust.
Identities = 85/248 (34%), Positives = 132/248 (53%), Gaps = 14/248
(6%)

Query  8
VFGILNLTEDSFFDESRRLDPAGAVTAAIEMLRVGSDVVDVGPAASHPDARPVSPADEIR  67
+ GILN+T DSF D  R      A+  A  ML  G+D++D+G  ++ P+A  VS
ADE+
Sbjct  24
LMGILNVTPDSFSDGGRFDAVPAALAQARLMLAEGADIIDIGGESTRPEASEVSVADELA  83

Query  68   RIAPLLDAL-SDQMHR-VSIDSFQPETQRYALKRGVGYLNDIQGFP-
DPALYPDIAEADC  124
R+ P ++AL +D +   +SID+++ E    A+  G   +ND+ G   +P +
E
Sbjct  84
RVLPAIEALRADGIAAPISIDTYKAEVAERAIAAGATIINDVHGLQREPEVAAVATEHGA  143

Query  125

```
RLVVMHSAQRDGIATRTGHLRPEDALDEIVRFFEARVSALRRSGVAADRLILDPGMGFFL  184
             LVVMH  +    A  TG     D + E+ R+F+  +    ++GVA DR++LDPG
GF
Sbjct  144  PLVVMHWDK----ARDTGR----
DVMAEMARYFDVTLDIADKAGVARDRIVLDPGFGFAK  195

Query  185
SPAPETSLHVLSNLQKLKSALGLPLLVSVSRKSFLGATVGLPVKDLGPASLAAELHAIGN  244
             S +   +  +L  L +L  ALG P+LV  SRKS +G  + +P  +    ++A  +
Sbjct  196  SLS--ENYEILRRLPEL-
VALGFPVLVGTSRKSMIGKLLDVPASERLAGTVATSVLGYTA  252

Query  245  GADYVRTH  252
             GA   R H
Sbjct  253  GAHIFRVH  260


>WP_057081671.1 dihydropteroate synthase [Bifidobacterium bifidum]
 CUN14007.1 dihydropteroate synthase [Bifidobacterium bifidum]
Length=290

 Score = 119 bits (297),  Expect = 2e-30, Method: Compositional
matrix adjust.
 Identities = 90/280 (32%), Positives = 140/280 (50%), Gaps = 19/280
(7%)

Query  8
VFGILNLTEDSFFDESRRLDPAGAVTAAIEMLRVGSDVVDVGPAASHPDARPVSPADEIR  67
            + G+LN+TEDSF D    LDP  A     +ML  G+D++D+G  ++ P A+ VS
DE+
Sbjct  15
LMGVLNITEDSFSDGGLWLDPGKARDHGDDMLHAGADIIDIGAESTRPGAKRVSEKDELD  74

Query  68   RIAPLLDALSDQMHRVSIDSFQPETQRYALKRGVGYLNDIQGFP-
DPALYPDIAEADCRL  126
             R+     AL      VSID+ +    + AL  G   +ND+ G   D  L   +A+
DC
Sbjct  75
RVTGAAKALIAHGAVVSIDTTRSAVAQAALNEGAQIINDVSGGRLDRDLPHVVADHDCLY  134

Query  127  VVMHSAQRDGIATRTGHLRPE--------
DALDEIVRFFEARVSALRRSGVAADRLILDP  178
             +V H   R  +A   G   P+        D L ++     ++V A+ ++GVA +R
+I+DP
Sbjct  135  IVQH--
WRGWLAGSKGGAAPDADTSHYEHDVLKDVYDELMSQVDAVLKAGVAPERVIIDP  192

Query  179  GMGFFLSPAPETSLHVLSNLQKLKSALGLPLLVSVSRKSFLGAT-----VGLP-
VKDLGP  232
             G+GF   P  E +L +L  L   K + G P+L+  SRK F+ A      +G P +
D
Sbjct  193  GLGFS-KPGIEHNLPLLVGLDLFKRS-
GYPVLIGASRKRFVSAALNDAGIGEPTMDDRDN  250
```

```
Query  233  ASLAAELHAIGNGADYVRTHAPGDLRSAITFSETLAKFRS  272
            A+ A       +GA  VR H     R+A+T      ++++
Sbjct  251  ATAALSALCAEHGAWAVRVHDMRRSRAALTIGSLWRQYQN  290


>WP_086988369.1 dihydropteroate synthase [Trichococcus
flocculiformis]
Length=259

 Score = 118 bits (295),  Expect = 2e-30, Method: Compositional
matrix adjust.
 Identities = 88/249 (35%), Positives = 132/249 (53%), Gaps = 19/249
(8%)

Query  9
FGILNLTEDSFFDESRRLDPAGAVTAAIEMLRVGSDVVDVGPAASHPDARPVSPADEIRR  68
             GILN+T DSF D         AV  A+EM+  G+D++D+G  ++ P    +S
+EI R
Sbjct  1
MGILNVTPDSFSDGGEWNTVEKAVAHALEMVADGADIIDIGGESTRPGHTQISDEEEIAR  60

Query  69   IAPLLDALSDQMHR-VSIDSFQPETQRYALKRGVGYLNDIQGF-
PDPALYPDIAEADCRL  126
            I P+++AL   +   +SID+++    R A + GV  +NDI G   DP +    A
D  +
Sbjct  61
IVPVIEALKKAVDVPLSIDTYKSAVARAACEAGVDIINDIWGCRYDPQIAAVAASFDVPI  120

Query  127
VVMHSAQRDGIATRTGHLRPEDALDEIVRFFEARVSALRRSGVAADRLILDPGMGFFLSP  186
            ++MH+ ++   A        ED L ++       VSA    GV  + +ILDPG GF
Sbjct  121  ILMHNREKPDYA-----FLIEDMLTDLAESVRIAVSA----
GVKRENIILDPGCGF--GK  169

Query  187  APETSLHVLSNLQKLKSALGLPLLVSVSRKSFLGATVG-LPVK--
DLGPASLAAELHAIG  243
              E +L+V+ +L++  + LG PLL+  SRK F+G  +G +P K  DLG A+  A
I
Sbjct  170  TYEDNLNVVHHLKRF-ADLGYPLLLGTSRKRFIGTALGDIPFKERDLGTAATTA--
LGIV  226

Query  244  NGADYVRTH  252
            NGA   R H
Sbjct  227  NGAQIFRVH  235


>WP_013390177.1 dihydropteroate synthase [Bifidobacterium bifidum]
 YP_003971513.1 dihydropteroate synthase [Bifidobacterium bifidum
PRL2010]
 ADP36476.1 FolP Dihydropteroate synthase [Bifidobacterium bifidum
PRL2010]
 KLN79105.1 FolP Dihydropteroate synthase [Bifidobacterium bifidum]
 KLN82217.1 FolP dihydropteroate synthase [Bifidobacterium bifidum]
Length=290
```

```
 Score = 119 bits (297),  Expect = 2e-30, Method: Compositional
matrix adjust.
 Identities = 90/280 (32%), Positives = 140/280 (50%), Gaps = 19/280
(7%)

Query  8
VFGILNLTEDSFFDESRRLDPAGAVTAAIEMLRVGSDVVDVGPAASHPDARPVSPADEIR  67
            + G+LN+TEDSF D    LDP  A     +ML  G+D++D+G  ++ P A+ VS
DE+
Sbjct  15
LMGVLNITEDSFSDGGLWLDPGKARDHGDDMLHAGADIIDIGAESTRPGAKRVSEKDELD  74

Query  68   RIAPLLDALSDQMHRVSIDSFQPETQRYALKRGVGYLNDIQGFP-
DPALYPDIAEADCRL  126
            R+     AL      VSID+ +    + AL  G   +ND+ G   D  L   +A+
DC
Sbjct  75
RVTGAAKALIAHGAVVSIDTTRSAVAQAALNEGAQIINDVSGGRLDRDLPHVVADHDCLY  134

Query  127  VVMHSAQRDGIATRTGHLRPE--------
DALDEIVRFFEARVSALRRSGVAADRLILDP  178
            +V H   R  +A   G   P+        D L ++     ++V A+ ++GVA +R
+I+DP
Sbjct  135  IVQH--
WRGWLAGSKGGAAPDADTSHYEHDVLKDVYDELMSQVDAVLKAGVAPERVIIDP  192

Query  179  GMGFFLSPAPETSLHVLSNLQKLKSALGLPLLVSVSRKSFLGAT-----VGLP-
VKDLGP  232
            G+GF   P  E +L +L  L   K + G P+L+  SRK F+ A      +G P +
D
Sbjct  193  GLGFS-KPGIEHNLPLLVGLDLFKRS-
GYPVLIGASRKRFVSAALNDAGIGEPTMDDRDN  250

Query  233  ASLAAELHAIGNGADYVRTHAPGDLRSAITFSETLAKFRS  272
            A+ A       +GA  VR H     R+A+T      ++++
Sbjct  251  ATAALSALCAEHGAWAVRVHDVRRSRAALTIGSLWRQYQN  290


>BBA47465.1 dihydropteroate synthase [Bifidobacterium bifidum LMG
13195]
Length=290

 Score = 119 bits (297),  Expect = 2e-30, Method: Compositional
matrix adjust.
 Identities = 90/280 (32%), Positives = 140/280 (50%), Gaps = 19/280
(7%)

Query  8
VFGILNLTEDSFFDESRRLDPAGAVTAAIEMLRVGSDVVDVGPAASHPDARPVSPADEIR  67
            + G+LN+TEDSF D    LDP  A     +ML  G+D++D+G  ++ P A+ VS
DE+
Sbjct  15
LMGVLNITEDSFSDGGLWLDPGKARDHGDDMLHAGADIIDIGAESTRPGAKRVSEKDELD  74
```

```
Query  68   RIAPLLDALSDQMHRVSIDSFQPETQRYALKRGVGYLNDIQGFP-
DPALYPDIAEADCRL  126
            R+     AL      VSID+ +    + AL  G   +ND+ G   D  L   +A+
DC
Sbjct  75
RVTGAAKALIAHGAVVSIDTTRSAVAQAALDEGAQIINDVSGGRLDRDLPHVVADHDCLY  134

Query  127  VVMHSAQRDGIATRTGHLRPE--------
DALDEIVRFFEARVSALRRSGVAADRLILDP  178
            +V H   R  +A   G   P+        D L ++     ++V A+ ++GVA +R
+I+DP
Sbjct  135  IVQH--
WRGWLAGSKGGAAPDADTSHYEHDVLKDVYDELMSQVDAVLKAGVAPERIIIDP  192

Query  179  GMGFFLSPAPETSLHVLSNLQKLKSALGLPLLVSVSRKSFLGAT-----VGLP-
VKDLGP  232
            G+GF   P  E +L +L  L   K + G P+L+  SRK F+ A      +G P +
D
Sbjct  193  GLGFS-KPGIEHNLPLLVGLDLFKRS-
GYPVLIGASRKRFVSAALNDAGIGEPTMDDRDN  250

Query  233  ASLAAELHAIGNGADYVRTHAPGDLRSAITFSETLAKFRS  272
            A+ A       +GA  VR H     R+A+T      ++++
Sbjct  251  ATAALSALCAEHGAWAVRVHDVRRSRAALTIGSLWRQYQN  290


>WP_083546429.1 MULTISPECIES: dihydropteroate synthase
[Trichococcus]
Length=259

 Score = 117 bits (294),  Expect = 2e-30, Method: Compositional
matrix adjust.
 Identities = 87/249 (35%), Positives = 132/249 (53%), Gaps = 19/249
(8%)

Query  9
FGILNLTEDSFFDESRRLDPAGAVTAAIEMLRVGSDVVDVGPAASHPDARPVSPADEIRR  68
             GILN+T DSF D         AV  A+EM+  G+D++D+G  ++ P    +S
+EI R
Sbjct  1
MGILNVTPDSFSDGGEWNTVEKAVAHALEMVADGADIIDIGGESTRPGHTQISDEEEIAR  60

Query  69   IAPLLDALSDQMHR-VSIDSFQPETQRYALKRGVGYLNDIQGF-
PDPALYPDIAEADCRL  126
            I P+++AL   +   +SID+++    R A + G+  +NDI G   DP +    A
D  +
Sbjct  61
IVPVIEALKKAVDVPLSIDTYKSAVARAACEAGIDIINDIWGCRYDPQIAAVAASFDVPI  120

Query  127
VVMHSAQRDGIATRTGHLRPEDALDEIVRFFEARVSALRRSGVAADRLILDPGMGFFLSP  186
            ++MH+ ++   A        ED L ++       VSA    GV  + +ILDPG GF
Sbjct  121  ILMHNREKPDYA-----FLIEDMLTDLAESVRIAVSA----
```

GVKRENIILDPGCGF--GK  169

Query  187  APETSLHVLSNLQKLKSALGLPLLVSVSRKSFLGATVG-LPVK--
DLGPASLAAELHAIG  243
              E +L+V+ +L++  + LG PLL+  SRK F+G  +G +P K  DLG A+  A
I
Sbjct  170  TYEDNLNVVHHLKRF-ADLGYPLLLGTSRKRFIGTALGDIPFKERDLGTAATTA--
LGIV  226

Query  244  NGADYVRTH  252
            NGA   R H
Sbjct  227  NGAQIFRVH  235

>WP_013363793.1 dihydropteroate synthase [Bifidobacterium bifidum]
 ADO53591.1 Dihydropteroate synthase [Bifidobacterium bifidum S17]
 CDB21893.1 dihydropteroate synthase [Bifidobacterium bifidum CAG:
234]
 ERI83392.1 dihydropteroate synthase [Bifidobacterium bifidum ATCC
29521
= JCM 1255 = DSM 20456]
 KFI43765.1 FolP Dihydropteroate synthase [Bifidobacterium bifidum]
 BAQ98646.1 dihydropteroate synthase [Bifidobacterium bifidum ATCC
29521
= JCM 1255 = DSM 20456]
 KLN76489.1 FolP Dihydropteroate synthase [Bifidobacterium bifidum]
 KLN80026.1 FolP Dihydropteroate synthase [Bifidobacterium bifidum]
 KLN84260.1 FolP Dihydropteroate synthase [Bifidobacterium bifidum]
 KLN88624.1 FolP Dihydropteroate synthase [Bifidobacterium bifidum]
 KLN90835.1 FolP Dihydropteroate synthase [Bifidobacterium bifidum]
 KWZ81377.1 dihydropteroate synthase [Bifidobacterium bifidum]
 KXS27988.1 dihydropteroate synthase [Bifidobacterium bifidum]
 SFB95832.1 Dihydropteroate synthase [Bifidobacterium bifidum]
 OKY90249.1 dihydropteroate synthase [Bifidobacterium sp. 56_9_plus]
 OQM67463.1 dihydropteroate synthase [Bifidobacterium bifidum]
Length=290

 Score = 118 bits (296),  Expect = 3e-30, Method: Compositional
matrix adjust.
 Identities = 90/280 (32%), Positives = 140/280 (50%), Gaps = 19/280
(7%)

Query  8
VFGILNLTEDSFFDESRRLDPAGAVTAAIEMLRVGSDVVDVGPAASHPDARPVSPADEIR  67
            + G+LN+TEDSF D    LDP  A     +ML  G+D++D+G  ++ P A+ VS
DE+
Sbjct  15
LMGVLNITEDSFSDGGLWLDPGKARDHGDDMLHAGADIIDIGAESTRPGAKRVSEKDELD  74

Query  68   RIAPLLDALSDQMHRVSIDSFQPETQRYALKRGVGYLNDIQGFP-
DPALYPDIAEADCRL  126
            R+     AL      VSID+ +    + AL  G   +ND+ G   D  L   +A+
DC
Sbjct  75

```
RVTGAAKALIAHGAVVSIDTTRSAVAQAALDEGAQIINDVSGGRLDRDLPHVVADHDCLY  134

Query  127  VVMHSAQRDGIATRTGHLRPE--------
DALDEIVRFFEARVSALRRSGVAADRLILDP  178
            +V H   R  +A   G   P+        D L ++     ++V A+ ++GVA +R
+I+DP
Sbjct  135  IVQH--
WRGWLAGSKGGAAPDADTSHYEHDVLKDVYDELMSQVDAVLKAGVAPERVIIDP  192

Query  179  GMGFFLSPAPETSLHVLSNLQKLKSALGLPLLVSVSRKSFLGAT-----VGLP-
VKDLGP  232
            G+GF   P  E +L +L  L   K + G P+L+  SRK F+ A      +G P +
D
Sbjct  193  GLGFS-KPGIEHNLPLLVGLDLFKRS-
GYPVLIGASRKRFVSAALNDAGIGEPTMDDRDN  250

Query  233  ASLAAELHAIGNGADYVRTHAPGDLRSAITFSETLAKFRS  272
            A+ A       +GA  VR H     R+A+T      ++++
Sbjct  251  ATAALSALCAEHGAWAVRVHDVRRSRAALTIGSLWRQYQN  290


>WP_047298337.1 dihydropteroate synthase [Bifidobacterium bifidum]
 KLN89647.1 FolP Dihydropteroate synthase [Bifidobacterium bifidum]
Length=290

 Score = 118 bits (295),  Expect = 4e-30, Method: Compositional
matrix adjust.
 Identities = 90/280 (32%), Positives = 140/280 (50%), Gaps = 19/280
(7%)

Query  8
VFGILNLTEDSFFDESRRLDPAGAVTAAIEMLRVGSDVVDVGPAASHPDARPVSPADEIR  67
            + G+LN+TEDSF D    LDP  A     +ML  G+D++D+G  ++ P A+ VS
DE+
Sbjct  15
LMGVLNITEDSFSDGGLWLDPGKARDHGDDMLHAGADIIDIGAESTRPGAKRVSEKDELD  74

Query  68   RIAPLLDALSDQMHRVSIDSFQPETQRYALKRGVGYLNDIQGFP-
DPALYPDIAEADCRL  126
            R+     AL      VSID+ +    + AL  G   +ND+ G   D  L   +A+
DC
Sbjct  75
RVTGAAKALIAYGAVVSIDTTRSAVAQAALDEGAQIINDVSGGRLDRDLPHVVADHDCLY  134

Query  127  VVMHSAQRDGIATRTGHLRPE--------
DALDEIVRFFEARVSALRRSGVAADRLILDP  178
            +V H   R  +A   G   P+        D L ++     ++V A+ ++GVA +R
+I+DP
Sbjct  135  IVQH--
WRGWLAGSKGGAAPDADTSHYEHDVLKDVYDELMSQVDAVLKAGVAPERIIIDP  192

Query  179  GMGFFLSPAPETSLHVLSNLQKLKSALGLPLLVSVSRKSFLGAT-----VGLP-
VKDLGP  232
            G+GF   P  E +L +L  L   K + G P+L+  SRK F+ A      +G P +
```

```
D
Sbjct  193  GLGFS-KPGIEHNLPLLVGLDLFKRS-
GYPVLIGASRKRFVSAALNDAGIGEPTMDDRDN  250

Query  233  ASLAAELHAIGNGADYVRTHAPGDLRSAITFSETLAKFRS  272
            A+ A       +GA  VR H     R+A+T      ++++
Sbjct  251  ATAALSALCAEHGAWAVRVHDVRRSRAALTIGSLWRQYQN  290


>WP_026394512.1 dihydropteroate synthase [Acetobacterium
dehalogenans]
Length=275

 Score = 117 bits (294),  Expect = 4e-30, Method: Compositional
matrix adjust.
 Identities = 77/253 (30%), Positives = 133/253 (53%), Gaps = 14/253
(6%)

Query  2
LRSRVTVFGILNLTEDSFFDESRRLDPAGAVTAAIEMLRVGSDVVDVGPAASHPDARPVS  61
            L +++ + GILN+T DSF D  +  +   ++    +M+  G+D++D+G  ++ P
+S
Sbjct  10
LTNQILIMGILNVTPDSFSDGGKFNNLDASLKQVEKMIADGADLIDLGGESTRPGHTQIS  69

Query  62   PADEIRRIAPLLDALSDQMHR-VSIDSFQPETQRYALKRGVGYLNDIQGFP-
DPALYPDI  119
              +EI RI P++ A+  +    +SID+++      AL+ G   +NDI GF  DP L
Sbjct  70
DEEEIDRIVPMITAIQKRFDIPISIDTYKGPVGAAALQAGADMVNDIWGFKYDPTLADVT  129

Query  120
AEADCRLVVMHSAQRDGIATRTGHLRPEDALDEIVRFFEARVSALRRSGVAADRLILDPG  179
            A      V+MH+            HL     + +I+   +  +    ++G++ D
+ILDPG
Sbjct  130  ARYQVPCVLMHNRNNQNYC----HL-----
ITDIISDLQESIDIALQAGISRDAIILDPG  180

Query  180
MGFFLSPAPETSLHVLSNLQKLKSALGLPLLVSVSRKSFLGATVGLPVKDLGPASLAAEL  239
            +GF    A   ++  + +L+ L+S LG PLL+  SRK F+G T+ LPV +    +
+A  +
Sbjct  181  IGFAKDYA--QNMETMHHLEALQS-
LGYPLLLGTSRKGFIGLTLDLPVTERVEGTVATTV  237

Query  240  HAIGNGADYVRTH  252
              I  GA  +R H
Sbjct  238  IGIMKGASIIRVH  250


>WP_034876901.1 dihydropteroate synthase [Bifidobacterium
moukalabense]
 ETY70958.1 dihydropteroate synthase [Bifidobacterium moukalabense
DSM 27321]
```

```
Length=291

 Score = 117 bits (294),  Expect = 6e-30, Method: Compositional
matrix adjust.
 Identities = 87/278 (31%), Positives = 138/278 (50%), Gaps = 19/278
(7%)

Query  8
VFGILNLTEDSFFDESRRLDPAGAVTAAIEMLRVGSDVVDVGPAASHPDARPVSPADEIR  67
            V G+LN+TEDSF D    LDPA A      M+R G+D++D+G  ++ P A+ VS
DE
Sbjct  16
VMGVLNITEDSFSDGGLWLDPARAKAHGEAMMRQGADIIDIGAESTRPGAKRVSEEDERS  75

Query  68   RIAPLLDALSDQMHRVSIDSFQPETQRYALKRGVGYLNDIQGFPDPALYPDI--
AEADCR  125
            R+   +DAL  +   +SID+ +      AL  G   +ND+ G    A  P +  A
++C
Sbjct  76
RVLGAVDALIPEGAVLSIDTTRASVALAALDHGAQIINDVSGGRLDAELPHVVAAHSECL  135

Query  126  LVVMHSAQRDGIATRTGHLRPEDA-------
LDEIVRFFEARVSALRRSGVAADRLILDP  178
             +V H   R  +A   G +   D        L+++     A+V A+  +GV  ++
+I+DP
Sbjct  136  YIVQH--
WRGWLAGAAGTVPDADTSVYANGVLNDVYDELMAQVDAVLAAGVGPEQVIIDP  193

Query  179  GMGFFLSPAPETSLHVLSNLQKLKSALGLPLLVSVSRKSFLGATVGLP------
VKDLGP  232
            G+GF   P  E +L +L+ L++  +A G P+L+  SRK F+GA +         +
D
Sbjct  194  GLGFS-KPGIEHNLPLLAGLERF-
NATGYPVLIGASRKRFVGAMLAAAGIDEPTMADKDN  251

Query  233  ASLAAELHAIGNGADYVRTHAPGDLRSAITFSETLAKF  270
            A+ A    +  +GA  VR H     R A+        +
Sbjct  252  ATAAISALSAEHGAWAVRVHDVAKTRDAVAIGSAWRAY  289


>WP_101629030.1 dihydropteroate synthase [Bifidobacterium longum]
 PKY77274.1 dihydropteroate synthase [Bifidobacterium longum]
Length=291

 Score = 117 bits (294),  Expect = 6e-30, Method: Compositional
matrix adjust.
 Identities = 93/287 (32%), Positives = 141/287 (49%), Gaps = 27/287
(9%)

Query  4
SRVTVFGILNLTEDSFFDESRRLDPAGAVTAAIEMLRVGSDVVDVGPAASHPDARPVSPA  63
            +   V G+LN+TEDSF D    LDPA AV    +M+  G+D++D+G  ++ P A+
VS A
Sbjct  11
```

THTMVMGVLNITEDSFSDGGLWLDPAKAVQHGRDMMAAGADIIDIGAESTRPGAKRVSEA  70

Query  64   DEIRRIAPLLDALSDQMHRVSIDSFQPETQRYALKRGVGYLNDIQGFPDPALYPD-IAEA  122
            DE+ RI   +  L      +SID+ +      AL   G   +ND+ G    A  P +A+
Sbjct  71   DELARITGAVKTLIPAGAVLSIDTTRASVAAAALSEGAQIINDVSGGTLDAELPHVVADH  130

Query  123  DCRLVVMH---------SAQRDGIATRTGHLRPEDALDEIVRFFEARVSALRRSGVAADR  173
            DC  +V H          A  D   +   H    D  DE++R    +V  +  +GV +R
Sbjct  131  DCLYIVQHWRGWLVGSKGANPDQDTSVYEHGVLTDVHDELMR----QVDGVLAAGVKPER  186

Query  174  LILDPGMGFFLSPAPETSLHVLSNLQKLKSALGLPLLVSVSRKSFLGATV---GLPVKDL  230
            +I+DPG+GF   P  E +L +L+ L+  + A G P+L+  SRK F+ A +   G D
Sbjct  187  IIIDPGLGFS-KPGIEHNLPLLTGLETFR-ATGYPVLIGQSRKRFISAMLTGTGTAGAD-  243

Query  231  GPA-----SLAAELHAIG--NGADYVRTHAPGDLRSAITFSETLAKF  270
            GP       + A L A+   +GA  VR H     R+A+    T  ++
Sbjct  244  GPTMAQRDDVTAALSALSAEHGAWAVRVHDVAKSRAAVIAGNTWREY  290

>WP_010081417.1 dihydropteroate synthase [Bifidobacterium longum]
 ACD99296.1 Dihydropteroate synthase [Bifidobacterium longum DJO10A]
 KWZ92177.1 dihydropteroate synthase [Bifidobacterium longum]
Length=291

 Score = 117 bits (293),  Expect = 7e-30, Method: Compositional matrix adjust.
 Identities = 93/287 (32%), Positives = 141/287 (49%), Gaps = 27/287 (9%)

Query  4    SRVTVFGILNLTEDSFFDESRRLDPAGAVTAAIEMLRVGSDVVDVGPAASHPDARPVSPA  63
            +   V G+LN+TEDSF D    LDPA AV    +M+  G+D++D+G  ++ P A+VS A
Sbjct  11   THTMVMGVLNITEDSFSDGGLWLDPAKAVQHGRDMMAAGADIIDIGAESTRPGAKRVSEA  70

Query  64   DEIRRIAPLLDALSDQMHRVSIDSFQPETQRYALKRGVGYLNDIQGFPDPALYPD-IAEA  122
            DE+ RI   +  L      +SID+ +      AL   G   +ND+ G    A  P +A+
Sbjct  71   DELARITGAVKTLIPAGAVLSIDTTRASVAAAALSEGAQIINDVSGGTLDAELPHVVADH  130

Query  123  DCRLVVMH---------SAQRDGIATRTGHLRPEDALDEIVRFFEARVSALRRSGVAADR  173

```
            DC  +V H          A  D   +   H    D  DE++R    +V  +  +GV
+R
Sbjct  131  DCLYIVQHWRGWLVGSKGANPDQDTSVYEHGVLTDVHDELMR----
QVDGVLAAGVKPER  186

Query  174  LILDPGMGFFLSPAPETSLHVLSNLQKLKSALGLPLLVSVSRKSFLGATV---
GLPVKDL  230
            +I+DPG+GF   P  E +L +L+ L+  + A G P+L+  SRK F+ A +   G
D
Sbjct  187  IIIDPGLGFS-KPGIEHNLPLLTGLETFR-
ATGYPVLIGQSRKRFISAMLTGAGTAGAD-  243

Query  231  GPA-----SLAAELHAIG--NGADYVRTHAPGDLRSAITFSETLAKF  270
            GP       + A L A+   +GA  VR H     R+A+    T  ++
Sbjct  244  GPTMAQRDDVTAALSALSAEHGAWAVRVHDVAKSRAAVIAGNTWREY  290


>WP_032744530.1 dihydropteroate synthase [Bifidobacterium longum]
 KEY29142.1 dihydropteroate synthase [Bifidobacterium longum subsp.
infantis
EK3]
Length=291

 Score = 117 bits (293),  Expect = 7e-30, Method: Compositional
matrix adjust.
 Identities = 94/289 (33%), Positives = 142/289 (49%), Gaps = 31/289
(11%)

Query  4
SRVTVFGILNLTEDSFFDESRRLDPAGAVTAAIEMLRVGSDVVDVGPAASHPDARPVSPA  63
            SR  V G+LN+TEDSF D    LDPA A     +M+  G+D++D+G  ++ P A+
V+ A
Sbjct  11
SRTMVMGVLNITEDSFSDGGLWLDPAKAAQHGRDMMAAGADIIDIGAESTRPGAKRVAEA  70

Query  64   DEIRRIAPLLDALSDQMHRVSIDSFQPETQRYALKRGVGYLNDIQGFPDPALYPD-
IAEA  122
            DE+ RI   +  L      +SID+ +      AL  G   +ND+ G    A  P
+A+
Sbjct  71
DELARITGAVKTLIPAGAILSIDTTRASVAAAALSEGTQIINDVSGGTLDAELPHVVADH  130

Query  123  DCRLVVMHSAQRDGIATRTG-----------
HLRPEDALDEIVRFFEARVSALRRSGVAA  171
            DC  +V H   R  +A   G           H    D  DE++R    +V  +
+GV
Sbjct  131  DCLYIVQH--WRGWLAGSKGANPDQDTSVYEHGVLTDVHDELMR----
QVDGVLAAGVKP  184

Query  172  DRLILDPGMGFFLSPAPETSLHVLSNLQKLKSALGLPLLVSVSRKSFLGATV---
GLPVK  228
            +R+I+DPG+GF   P  E +L +L+ L+  + A G P+L+  SRK F+ A +   G
Sbjct  185  ERIIIDPGLGFS-KPGIEHNLPLLTGLETFR-
ATGYPVLIGQSRKRFISAMLTEAGAAGA  242
```

```
Query  229  DLGPA-----SLAAELHAIG--NGADYVRTHAPGDLRSAITFSETLAKF  270
            D GP       + A L A+   +GA  VR H     R+A+    T  ++
Sbjct  243  D-GPIMAQRDDVTAALSALSAEHGAWAVRVHDVAKSRAAVIAGNTWREY  290
```

>WP_033499260.1 dihydropteroate synthase [Bifidobacterium adolescentis]
 KFI96588.1 dihydropteroate synthase 1 [Bifidobacterium stercoris JCM 15918]
Length=292

 Score = 117 bits (293),  Expect = 7e-30, Method: Compositional matrix adjust.
 Identities = 87/283 (31%), Positives = 136/283 (48%), Gaps = 23/283 (8%)

```
Query  5
RVTVFGILNLTEDSFFDESRRLDPAGAVTAAIEMLRVGSDVVDVGPAASHPDARPVSPAD  64
            R  V G+LN+TEDSF D    L P  A      M++ G+D++D+G  ++ P A+
VS AD
Sbjct  13
RTLVMGVLNITEDSFSDGGLWLAPEAAKAHGEAMMKAGADIIDIGAESTRPGAKRVSEAD  72

Query  65
EIRRIAPLLDALSDQMHRVSIDSFQPETQRYALKRGVGYLNDIQGFPDPALYPDIA--EA  122
            E  R+   +DAL  +   +SID+ +      AL+ G   +ND+ G       P +
+
Sbjct  73
EKARVLGAVDALIPEGAVLSIDTTRASVALAALEHGAQIINDVSGGQLDRELPHVVADHS  132

Query  123  DCRLVVMHSAQR---------
DGIATRTGHLRPEDALDEIVRFFEARVSALRRSGVAADR  173
            DC  +V H             D   +R  H    D  DE++R    +V A+  +GV
A++
Sbjct  133  DCLYIVQHWRGWLAGAAGNVPDADTSRYEHGVVNDVYDELMR----
QVDAVLEAGVQAEQ  188

Query  174  LILDPGMGFFLSPAPETSLHVLSNLQKLKSALGLPLLVSVSRKSFLG---
ATVGLPVKDL  230
            +I+DPG+GF   P  E +L +L+ L +  +A G P+L+  SRK F+G   A  G+
D+
Sbjct  189  VIIDPGLGFS-KPGVEHNLPILAALDRFNAA-
GYPVLIGASRKRFVGSLLAGAGVTEPDM  246

Query  231  GP---ASLAAELHAIGNGADYVRTHAPGDLRSAITFSETLAKF  270
                 A+ A       +G   VR H     R A+       ++
Sbjct  247  ASKDNATAAISALCTEHGVWAVRVHDVAKSRDAVAVGNAWREY  289
```

>WP_034525832.1 dihydropteroate synthase [Bifidobacterium stellenboschense]
 KFJ01301.1 dihydropteroate synthase [Bifidobacterium stellenboschense]

Length=294

 Score = 117 bits (293),  Expect = 8e-30, Method: Compositional
matrix adjust.
 Identities = 92/283 (33%), Positives = 144/283 (51%), Gaps = 30/283
(11%)

```
Query  8
VFGILNLTEDSFFDESRRLDPAGAVTAAIEMLRVGSDVVDVGPAASHPDARPVSPADEIR  67
            V G+LN+TEDSF D    LDP  A      M+  G+D++D+G  ++ P A+ VS
DE+R
Sbjct  20
VMGVLNITEDSFSDGGLWLDPRAAADHGRAMMAAGADIIDIGAESTRPGAKRVSEEDELR  79

Query  68   RIAPLLDALSDQMHRVSIDSFQPETQRYALKRGVGYLNDIQGFP-
DPALYPDIAEADCRL  126
            RI   ++AL      +SID+ +      AL  G   +ND+ G   D  L   +A+
DC
Sbjct  80
RITGAVEALIPAGATLSIDTTRASVAAAALDGGAQIINDVSGGTLDTELPHVVADHDCLY  139

Query  127  VVMHSAQRDGIA-TRTG----------
HLRPEDALDEIVRFFEARVSALRRSGVAADRLI  175
            +V H   R  +A ++TG          H   +D  DE++R    +V A+  +GVA
+R+I
Sbjct  140  IVQH--WRGWLAGSKTGAPDADTSHYEHGVLQDVHDELMR----
QVDAVLAAGVAPERII  193

Query  176  LDPGMGFFLSPAPETSLHVLSNLQKLKSALGLPLLVSVSRKSFLGAT--------
VGLPV  227
            +DPG+GF   P    +L +L+ L + + A G P+L+  SRK F+ A         V
+
Sbjct  194  IDPGLGFS-KPGIAHNLPLLAGLDRFR-
ATGYPVLIGQSRKRFVTAILDKAGFTDVTMAA  251

Query  228  KDLGPASLAAELHAIGNGADYVRTHAPGDLRSAITFSETLAKF  270
            +D   A+ +A      +GA  VR H     R+A+   +T  ++
Sbjct  252  RDDATAAFSALCAE--HGAWAVRVHDVARSRAAVAVGDTWREY  292
```

>WP_003808269.1 dihydropteroate synthase [Bifidobacterium
adolescentis]
 EDN83746.1 dihydropteroate synthase [Bifidobacterium adolescentis
L2-32]
 CCY19839.1 dihydropteroate synthase [Bifidobacterium adolescentis
CAG:119]
 AII75842.1 dihydropteroate synthase 1 [Bifidobacterium
adolescentis]
 KLE28154.1 dihydropteroate synthase [Bifidobacterium adolescentis]
 CUN79986.1 dihydropteroate synthase 1 [Bifidobacterium
adolescentis]
 CUN66354.1 dihydropteroate synthase 1 [Bifidobacterium
adolescentis]
 OQM57688.1 dihydropteroate synthase [Bifidobacterium adolescentis]

```
 OSG94211.1 dihydropteroate synthase [Bifidobacterium adolescentis]
 OSH02390.1 dihydropteroate synthase [Bifidobacterium adolescentis]
 OSH04392.1 dihydropteroate synthase [Bifidobacterium adolescentis]
Length=292

 Score = 117 bits (292),  Expect = 9e-30, Method: Compositional
matrix adjust.
 Identities = 87/283 (31%), Positives = 136/283 (48%), Gaps = 23/283
(8%)

Query  5
RVTVFGILNLTEDSFFDESRRLDPAGAVTAAIEMLRVGSDVVDVGPAASHPDARPVSPAD  64
            R  V G+LN+TEDSF D    L P  A      M++ G+D++D+G  ++ P A+
VS AD
Sbjct  13
RTLVMGVLNITEDSFSDGGLWLAPEAAKAHGEAMMKAGADIIDIGAESTRPGAKRVSEAD  72

Query  65
EIRRIAPLLDALSDQMHRVSIDSFQPETQRYALKRGVGYLNDIQGFPDPALYPDIA--EA  122
            E  R+   +DAL  +   +SID+ +      AL+ G   +ND+ G       P +
+
Sbjct  73
EKARVLGAVDALIPEGAVLSIDTTRASVALAALEHGAQIINDVSGGQLDRELPHVVADHS  132

Query  123  DCRLVVMHSAQR---------
DGIATRTGHLRPEDALDEIVRFFEARVSALRRSGVAADR  173
            DC  +V H             D   +R  H    D  DE++R    +V A+  +GV
A++
Sbjct  133  DCLYIVQHWRGWLAGAAGNVPDADTSRYEHGVVNDVYDELMR----
QVDAVLEAGVQAEQ  188

Query  174  LILDPGMGFFLSPAPETSLHVLSNLQKLKSALGLPLLVSVSRKSFLG---
ATVGLPVKDL  230
            +I+DPG+GF   P  E +L +L+ L +  +A G P+L+  SRK F+G   A  G+
D+
Sbjct  189  VIIDPGLGFS-KPGVEHNLPILAALDRFNAA-
GYPVLIGASRKRFVGSLLAGAGVTEPDM  246

Query  231  GP---ASLAAELHAIGNGADYVRTHAPGDLRSAITFSETLAKF  270
                 A+ A       +G   VR H     R A+       ++
Sbjct  247  ASKDNATAAISALCAEHGVWAVRVHDVAKSRDAVAVGNAWREY  289


>WP_086943467.1 dihydropteroate synthase [Trichococcus pasteurii]
Length=259

 Score = 116 bits (290),  Expect = 9e-30, Method: Compositional
matrix adjust.
 Identities = 87/249 (35%), Positives = 131/249 (53%), Gaps = 19/249
(8%)

Query  9
FGILNLTEDSFFDESRRLDPAGAVTAAIEMLRVGSDVVDVGPAASHPDARPVSPADEIRR  68
             GILN+T DSF D         AV  A+EM+  G+D++D+G  ++ P    +S
```

```
+EI R
Sbjct  1
MGILNVTPDSFSDGGEWNTVEKAVAHALEMVADGADIIDIGGESTRPGHTQISDEEEIAR  60

Query  69   IAPLLDALSDQMHR-VSIDSFQPETQRYALKRGVGYLNDIQGFP-
DPALYPDIAEADCRL  126
            I P+++A+   +   +SID+++    R A + GV  +NDI G   DP +    A
D  +
Sbjct  61
IVPVIEAVKKAVDVPLSIDTYKSAVARAACEAGVDIINDIWGCKYDPEIAAVAAAFDVPI  120

Query  127
VVMHSAQRDGIATRTGHLRPEDALDEIVRFFEARVSALRRSGVAADRLILDPGMGFFLSP  186
            ++MH+ ++   A        ED L ++       VSA    GV  + +ILDPG GF
Sbjct  121  ILMHNREKPDYA-----FLIEDMLADLAESVRIAVSA----
GVKRENIILDPGCGF--GK  169

Query  187  APETSLHVLSNLQKLKSALGLPLLVSVSRKSFLGATVG-LPVK--
DLGPASLAAELHAIG  243
              E +L+V+ +L++    LG PLL+  SRK F+G  +G +P K  DLG A+  A
I
Sbjct  170  TYEDNLNVVHHLKRFAD-LGYPLLLGTSRKRFIGTALGDVPFKGRDLGTAATTA--
LGIV  226

Query  244  NGADYVRTH  252
            NGA   R H
Sbjct  227  NGAQLFRVH  235


>WP_049705597.1 dihydropteroate synthase [Devosia sp. H5989]
 AKR56010.1 Dihydropteroate synthase [Devosia sp. H5989]
Length=287

 Score = 117 bits (292),  Expect = 1e-29, Method: Compositional
matrix adjust.
 Identities = 83/249 (33%), Positives = 129/249 (52%), Gaps = 16/249
(6%)

Query  8
VFGILNLTEDSFFDESRRLDPAGAVTAAIEMLRVGSDVVDVGPAASHPDARPVSPADEIR  67
            V GILN+T DSF D         A+  A EM+  G+D++DVG  ++ P    VS
DE+
Sbjct  27
VMGILNVTPDSFSDGGDFAGVEAALAHAREMVAEGADIIDVGGESTRPGHEEVSVQDELD  86

Query  68   RIAPLLDALSDQM--HRVSIDSFQPETQRYALKRGVGYLNDIQGFP-
DPALYPDIAEADC  124
            R+ P+++AL D      VSID+++      A++ G   +ND+ G   +P +    A
+
Sbjct  87
RVVPVMEALRDAALDTLVSIDTYKALVADQAVQSGAAIINDVYGLQREPEIADVAALHNV  146

Query  125  RLVVMH-
SAQRDGIATRTGHLRPEDALDEIVRFFEARVSALRRSGVAADRLILDPGMGFF  183
```

```
             ++ MH  A RD         RP   +DE++R+FE  +    ++G+A DRLILDPG
GF
Sbjct  147  PVIAMHWDAARDPN-------RP--
LIDEMLRYFERTLEIASKAGIAKDRLILDPGFGF-  196

Query  184
LSPAPETSLHVLSNLQKLKSALGLPLLVSVSRKSFLGATVGLPVKDLGPASLAAELHAIG  243
               + + +  ++    +L   LG P+L+  SRKS +G  +G   K+    +LA  +
A
Sbjct  197  -GKSLQENYRLMQTFGRLHE-
LGAPVLIGTSRKSMIGKLLGNEPKERLAGTLATTILAYA  254

Query  244  NGADYVRTH  252
             GA   R H
Sbjct  255  GGAHIFRVH  263


>WP_042990425.1 dihydropteroate synthase [Bifidobacterium
adolescentis]
 KIM01708.1 dihydropteroate synthase [Bifidobacterium adolescentis]
 OSG87519.1 dihydropteroate synthase [Bifidobacterium adolescentis]
Length=292

 Score = 117 bits (292),  Expect = 1e-29, Method: Compositional
matrix adjust.
 Identities = 87/283 (31%), Positives = 136/283 (48%), Gaps = 23/283
(8%)

Query  5
RVTVFGILNLTEDSFFDESRRLDPAGAVTAAIEMLRVGSDVVDVGPAASHPDARPVSPAD  64
            R  V G+LN+TEDSF D    L P  A      M++ G+D++D+G  ++ P A+
VS AD
Sbjct  13
RTLVMGVLNITEDSFSDGGLWLAPEAAKAHGEAMMKAGADIIDIGAESTRPGAKRVSEAD  72

Query  65
EIRRIAPLLDALSDQMHRVSIDSFQPETQRYALKRGVGYLNDIQGFPDPALYPDIA--EA  122
            E  R+   +DAL  +   +SID+ +      AL+ G   +ND+ G       P +
+
Sbjct  73
EKARVLGAVDALIPEGAVLSIDTTRASVALAALEHGAQIINDVSGGQLDRELPHVVADHS  132

Query  123  DCRLVVMHSAQR---------
DGIATRTGHLRPEDALDEIVRFFEARVSALRRSGVAADR  173
            DC  +V H             D   +R  H    D  DE++R    +V A+  +GV
A++
Sbjct  133  DCLYIVQHWRGWLAGAAGDVPDADTSRYEHGVVNDVYDELMR----
QVDAVLEAGVQAEQ  188

Query  174  LILDPGMGFFLSPAPETSLHVLSNLQKLKSALGLPLLVSVSRKSFLG---
ATVGLPVKDL  230
            +I+DPG+GF   P  E +L +L+ L +  +A G P+L+  SRK F+G   A  G+
D+
Sbjct  189  VIIDPGLGFS-KPGVEHNLPILAALDRFNAA-
```

```
GYPVLIGASRKRFVGSLLAGAGVTEPDM  246

Query  231  GP---ASLAAELHAIGNGADYVRTHAPGDLRSAITFSETLAKF  270
                 A+ A       +G   VR H     R A+       ++
Sbjct  247  ASKDNATAAISALCAEHGVWAVRVHDVAKSRDAVAVGNAWREY  289


>WP_033523224.1 dihydropteroate synthase [Bifidobacterium merycicum]
 KFI71188.1 dihydropteroate synthase [Bifidobacterium merycicum]
 SHE69583.1 dihydropteroate synthase [Bifidobacterium merycicum DSM
6492]
Length=290

 Score = 117 bits (292),  Expect = 1e-29, Method: Compositional
matrix adjust.
 Identities = 88/285 (31%), Positives = 140/285 (49%), Gaps = 22/285
(8%)

Query  5
RVTVFGILNLTEDSFFDESRRLDPAGAVTAAIEMLRVGSDVVDVGPAASHPDARPVSPAD  64
            R  V G+LN+TEDSF D    LDP  A     +M+  G+D++D+G  ++ P A+
VS AD
Sbjct  12
RTLVMGVLNITEDSFSDGGLWLDPGKAAEHGRDMMATGADIIDIGAESTRPGAKRVSEAD  71

Query  65   EIRRIAPLLDALSDQMHRVSIDSFQPETQRYALKRGVGYLNDIQ-
GFPDPALYPDIAEAD  123
            E+ RI   ++AL  +   +SID+ +      AL+ G   +ND+  G  D  L
+A+ D
Sbjct  72
ELARIKGAVEALIPEGAVLSIDTTRSSVAAGALELGAQIINDVSGGMLDKELPHVVADHD  131

Query  124  CRLVVM---------
HSAQRDGIATRTGHLRPEDALDEIVRFFEARVSALRRSGVAADRL  174
            C  +V          H +  D   +R  H    D  DE++R  +A ++A    GV
R+
Sbjct  132  CLYIVQHWRGWLAGSHGSTPDADTSRYEHGVLADVRDELMRQVDAVIAA----
GVDPKRI  187

Query  175  ILDPGMGFFLSPAPETSLHVLSNLQKLKSALGLPLLVSVSRKSFLGATVG------
LPVK  228
            I+DPG+GF   P  E +L +L+ L    +  G P+L+  SRK F+ A +
+ +
Sbjct  188  IIDPGLGFS-KPGVEHNLPLLAGLDAF-
TGTGYPVLIGQSRKRFVSALLADAGFDRIDMD  245

Query  229  DLGPASLAAELHAIGNGADYVRTHAPGDLRSAITFSETLAKFRSR  273
            +   A+ A    +  +GA  VR H     R+A+   +    +  R
Sbjct  246  ERDNATAAISALSAEHGAWAVRVHDVARSRAAVAVGDAWRDYAKR  290


>WP_041777256.1 dihydropteroate synthase [Bifidobacterium
adolescentis]
 OSG98699.1 dihydropteroate synthase [Bifidobacterium adolescentis]
```

Length=292

 Score = 116 bits (291),  Expect = 2e-29, Method: Compositional
matrix adjust.
 Identities = 87/283 (31%), Positives = 136/283 (48%), Gaps = 23/283
(8%)

```
Query  5
RVTVFGILNLTEDSFFDESRRLDPAGAVTAAIEMLRVGSDVVDVGPAASHPDARPVSPAD  64
            R  V G+LN+TEDSF D    L P  A      M++ G+D++D+G  ++ P A+
VS AD
Sbjct  13
RTLVMGVLNITEDSFSDGGLWLAPEAAKAHGEAMMKAGADIIDIGAESTRPGAKRVSEAD  72

Query  65
EIRRIAPLLDALSDQMHRVSIDSFQPETQRYALKRGVGYLNDIQGFPDPALYPDIA--EA  122
            E  R+   +DAL  +   +SID+ +      AL+ G   +ND+ G       P +
+
Sbjct  73
EKARVLGAVDALIPEGAVLSIDTTRASVALAALEHGAQIINDVSGGQLDRELPHVVADHS  132

Query  123  DCRLVVMHSAQR---------
DGIATRTGHLRPEDALDEIVRFFEARVSALRRSGVAADR  173
            DC  +V H             D   +R  H    D  DE++R    +V A+  +GV
A++
Sbjct  133  DCLYIVQHWRGWLAGAAGNVPDADTSRYEHGVVNDVYDELMR----
QVDAVLEAGVQAEQ  188

Query  174  LILDPGMGFFLSPAPETSLHVLSNLQKLKSALGLPLLVSVSRKSFLG---
ATVGLPVKDL  230
            +I+DPG+GF   P  E +L +L+ L +  +A G P+L+  SRK F+G   A  G+
D+
Sbjct  189  VIIDPGLGFS-KPGVEHNLPILAALDRF-
NATGYPVLIGASRKRFVGSLLAGAGVTEPDM  246

Query  231  GP---ASLAAELHAIGNGADYVRTHAPGDLRSAITFSETLAKF  270
                 A+ A       +G   VR H     R A+       ++
Sbjct  247  ASKDNATAAISALCAEHGVWAVRVHDVAKSRDAVAVGNAWREY  289
```

>WP_065506906.1 dihydropteroate synthase [Bifidobacterium breve]
Length=291

 Score = 116 bits (291),  Expect = 2e-29, Method: Compositional
matrix adjust.
 Identities = 93/288 (32%), Positives = 141/288 (49%), Gaps = 29/288
(10%)

```
Query  4
SRVTVFGILNLTEDSFFDESRRLDPAGAVTAAIEMLRVGSDVVDVGPAASHPDARPVSPA  63
            S   V G+LN+TEDSF D    LDPA A     +M+  G+D++D+G  ++ P A+
VS
Sbjct  11
SHTMVMGVLNITEDSFSDGGLWLDPAKAAQHGRDMMAAGADIIDIGAESTRPGAKRVSEE  70
```

```
Query  64   DEIRRIAPLLDALSDQMHRVSIDSFQPETQRYALKRGVGYLNDIQGFPDPALYPD-
IAEA  122
            DE+ RI   ++AL      +SID+ +      AL  G   +ND+ G    A  P
IA+
Sbjct  71
DELARITGAVNALIPAGAVLSIDTTRASVAAAALDGGAQIINDVSGGTLDANLPHVIADH  130

Query  123  DCRLVVMHSAQRDGIATRTG-----------
HLRPEDALDEIVRFFEARVSALRRSGVAA  171
            DC  +V H   R  +A   G           H    D  DE++R    +V  +
+GV
Sbjct  131  DCLYIVQH--WRGWLAGSKGANPDQDTSVYEHGVLTDVHDELMR----
QVDGVLAAGVKP  184

Query  172  DRLILDPGMGFFLSPAPETSLHVLSNLQKLKSALGLPLLVSVSRKSFLGA--
TVGLPVKD  229
            +R+I+DPG+GF   P+ E +L +L+ L+  +   G P+L+  SRK F+ A  T
V +
Sbjct  185  ERIIIDPGLGFS-KPSIEHNLPLLTGLETFRGT-
GYPVLIGQSRKRFISAMLTEAGAVGE  242

Query  230  LGPA-----SLAAELHAIG--NGADYVRTHAPGDLRSAITFSETLAKF  270
             GP       + A L A+   +GA  VR H      +A+    T  ++
Sbjct  243  DGPTMAQRDDVTAALSALSAEHGAWAVRVHDVAKSHAAVIAGNTWRQY  290


>WP_032746349.1 dihydropteroate synthase [Bifidobacterium longum]
 KEY21526.1 dihydropteroate synthase [Bifidobacterium longum subsp.
longum
17-1B]
Length=291

 Score = 116 bits (290),  Expect = 2e-29, Method: Compositional
matrix adjust.
 Identities = 89/286 (31%), Positives = 137/286 (48%), Gaps = 25/286
(9%)

Query  4
SRVTVFGILNLTEDSFFDESRRLDPAGAVTAAIEMLRVGSDVVDVGPAASHPDARPVSPA  63
            +   V G+LN+TEDSF D    LDPA A     +M+  G+D++D+G  ++ P A+
VS A
Sbjct  11
THTMVMGVLNITEDSFSDGGLWLDPAKAAQHGRDMMAAGADIIDIGAESTRPGAKRVSEA  70

Query  64   DEIRRIAPLLDALSDQMHRVSIDSFQPETQRYALKRGVGYLNDIQGFPDPALYPD-
IAEA  122
            DE+ RI   +  L      +SID+ +      AL  G   +ND+ G    A  P
+A+
Sbjct  71
DELARITGAVKTLIPAGAVLSIDTTRASVAAAALSEGAQIINDVSGGTLDAELPHVVADH  130

Query  123  DCRLVVMH---------
SAQRDGIATRTGHLRPEDALDEIVRFFEARVSALRRSGVAADR  173
```

```
            DC  VV H          A  D   +   H    D  DE++R    +V  +  +GV
+R
Sbjct  131  DCLYVVQHWRGWLVGSKGANPDQDTSVYEHGVLTDVHDELMR----
QVDGVLAAGVKPER  186

Query  174
LILDPGMGFFLSPAPETSLHVLSNLQKLKSALGLPLLVSVSRKSFLGATV---------G  224
            +I+DPG+GF   P  E +L +L+ L+  + A G P+L+  SRK F+ A +
G
Sbjct  187  IIIDPGLGFS-KPGIEHNLPLLTGLETFR-
ATGYPVLIGQSRKRFISAMLTGAGTAGADG  244

Query  225  LPVKDLGPASLAAELHAIGNGADYVRTHAPGDLRSAITFSETLAKF  270
            L +      + A    +  +GA  VR H     R+A+    T  ++
Sbjct  245  LTMAQRDDVTAALSALSAEHGAWAVRVHDVAKSRAAVIAGNTWREY  290


>WP_053825171.1 dihydropteroate synthase [Bifidobacterium bifidum]
 ALE11915.1 Dihydropteroate synthase [Bifidobacterium bifidum]
Length=290

 Score = 116 bits (290),  Expect = 2e-29, Method: Compositional
matrix adjust.
 Identities = 89/280 (32%), Positives = 139/280 (50%), Gaps = 19/280
(7%)

Query  8
VFGILNLTEDSFFDESRRLDPAGAVTAAIEMLRVGSDVVDVGPAASHPDARPVSPADEIR  67
            + G+LN+TEDS  D    LDP  A     +ML  G+D++D+G  ++ P A+ VS
DE+
Sbjct  15
LMGVLNITEDSVSDGGLWLDPGKARDHGDDMLHAGADIIDIGAESTRPGAKRVSEKDELD  74

Query  68   RIAPLLDALSDQMHRVSIDSFQPETQRYALKRGVGYLNDIQGFP-
DPALYPDIAEADCRL  126
            R+     AL      VSID+ +    + AL  G   +ND+ G   D  L   +A+
DC
Sbjct  75
RVTGAAKALIAHGAVVSIDTTRSAVAQAALDEGAQIINDVSGGRLDRDLPHVVADHDCLY  134

Query  127  VVMHSAQRDGIATRTGHLRPE--------
DALDEIVRFFEARVSALRRSGVAADRLILDP  178
            +V H   R  +A   G   P+        D L ++     ++V A+ ++GVA +R
+I+DP
Sbjct  135  IVQH--
WRGWLAGSKGGAAPDADTSHYEHDVLKDVYDELMSQVDAVLKAGVAPERIIIDP  192

Query  179  GMGFFLSPAPETSLHVLSNLQKLKSALGLPLLVSVSRKSFLGAT-----VGLP-
VKDLGP  232
            G+GF   P  E +L +L  L   K + G P+L+  SRK F+ A      +G P +
D
Sbjct  193  GLGFS-KPGIEHNLPLLVGLDLFKRS-
GYPVLIGASRKRFVSAALNDAGIGEPTMDDRDN  250
```

```
Query  233  ASLAAELHAIGNGADYVRTHAPGDLRSAITFSETLAKFRS  272
            A+ A       +GA  VR H     R+A+T      ++++
Sbjct  251  ATAALSALCAEHGAWAVRVHDVRRSRAALTIGSLWRQYQN  290
```

>WP_055881405.1 dihydropteroate synthase [Devosia sp. Root105]
 KQU96312.1 dihydropteroate synthase [Devosia sp. Root105]
Length=281

 Score = 115 bits (289),  Expect = 2e-29, Method: Compositional
matrix adjust.
 Identities = 83/248 (33%), Positives = 131/248 (53%), Gaps = 14/248
(6%)

```
Query  8
VFGILNLTEDSFFDESRRLDPAGAVTAAIEMLRVGSDVVDVGPAASHPDARPVSPADEIR  67
            + GILN+T DSF D  R      A+  A  ML  G+D++D+G  ++ P A  VS
ADE+
Sbjct  24
LMGILNVTPDSFSDGGRFDAAPAALAQARLMLAEGADIIDIGGESTRPGAEEVSVADELT  83

Query  68   RIAPLLDAL-SDQMHR-VSIDSFQPETQRYALKRGVGYLNDIQGFP-
DPALYPDIAEADC  124
            R+ P++ AL +D +   +SID+++ E    A+  G   +ND+ G   +P +
AE
Sbjct  84
RVLPVIAALRADGITAPISIDTYKAEVAEQAIAAGATIINDVHGLQREPEVAAVAAEHGT  143

Query  125
RLVVMHSAQRDGIATRTGHLRPEDALDEIVRFFEARVSALRRSGVAADRLILDPGMGFFL  184
             LVVMH  +    A  TG     D + E+ R+F+  +    ++GV  DR++LDPG
GF
Sbjct  144  PLVVMHWDK----ARDTGR----
DVMAEMARYFDVTLDIADKAGVTRDRIVLDPGFGF--  193

Query  185
SPAPETSLHVLSNLQKLKSALGLPLLVSVSRKSFLGATVGLPVKDLGPASLAAELHAIGN  244
            + +   +  +L  L +L  A+G P+LV  SRKS +G  + +P  +    ++A  +
Sbjct  194  AKSLSENYEILRRLPEL-
VAMGFPVLVGTSRKSMIGKLLDMPANERLAGTIATSVLGYTA  252

Query  245  GADYVRTH  252
            GA   R H
Sbjct  253  GAHIFRVH  260
```

>WP_007053863.1 dihydropteroate synthase [Bifidobacterium longum]
 EEI80776.1 dihydropteroate synthase [Bifidobacterium longum subsp.
longum
ATCC 55813]
 BAJ67244.1 dihydropteroate synthase [Bifidobacterium longum subsp.
longum
JCM 1217]
 EPE38573.1 Dihydropteroate synthase [Bifidobacterium longum D2957]

KFI62537.1 dihydropteroate synthase 1 [Bifidobacterium longum subsp. longum]
AIW44191.1 dihydropteroate synthase [Bifidobacterium longum subsp. longum
GT15]
KHD95645.1 dihydropteroate synthase [Bifidobacterium longum subsp. longum]
ALO75193.1 dihydropteroate synthase [Bifidobacterium longum subsp. longum]
KSA08440.1 dihydropteroate synthase [Bifidobacterium longum subsp. longum]
KSA09168.1 dihydropteroate synthase [Bifidobacterium longum subsp. longum]
AOL10901.1 dihydropteroate synthase [Bifidobacterium longum]
SEB29401.1 Dihydropteroate synthase [Bifidobacterium longum]
OJS82496.1 dihydropteroate synthase [Bifidobacterium longum subsp. longum]
PKC83753.1 Dihydropteroate synthase [Bifidobacterium longum]
PKD09814.1 Dihydropteroate synthase [Bifidobacterium longum]
Length=291

Score = 116 bits (290), Expect = 2e-29, Method: Compositional matrix adjust.
Identities = 93/287 (32%), Positives = 140/287 (49%), Gaps = 27/287 (9%)

```
Query  4
SRVTVFGILNLTEDSFFDESRRLDPAGAVTAAIEMLRVGSDVVDVGPAASHPDARPVSPA  63
            +   V G+LN+TEDSF D    LDPA A     +M+  G+D++D+G  ++ P A+
VS A
Sbjct  11
THTMVMGVLNITEDSFSDGGLWLDPAKAAQHGRDMMAAGADIIDIGAESTRPGAKRVSEA  70

Query  64   DEIRRIAPLLDALSDQMHRVSIDSFQPETQRYALKRGVGYLNDIQGFPDPALYPD-
IAEA  122
            DE+ RI   +  L      +SID+ +      AL  G   +ND+ G    A  P
+A+
Sbjct  71
DELARITGAVKTLIPAGAVLSIDTTRASVAAAALSEGAQIINDVSGGTLDAELPHVVADH  130

Query  123  DCRLVVMH---------
SAQRDGIATRTGHLRPEDALDEIVRFFEARVSALRRSGVAADR  173
            DC  VV H          A  D   +   H    D  DE++R    +V  +  +GV
+R
Sbjct  131  DCLYVVQHWRGWLVGSKGANPDQDTSVYEHGVLTDVHDELMR----
QVDGVLAAGVKPER  186

Query  174  LILDPGMGFFLSPAPETSLHVLSNLQKLKSALGLPLLVSVSRKSFLGATV---
GLPVKDL  230
            +I+DPG+GF   P  E +L +L+ L+  + A G P+L+  SRK F+ A +   G
D
Sbjct  187  IIIDPGLGFS-KPGIEHNLPLLTGLETFR-
ATGYPVLIGQSRKRFISAMLTGAGTAGAD-  243
```

```
Query  231  GPA-----SLAAELHAIG--NGADYVRTHAPGDLRSAITFSETLAKF  270
            GP       + A L A+   +GA  VR H     R+A+    T  ++
Sbjct  244  GPTMAQRDDVTAALSALSAEHGAWAVRVHDVAKSRAAVIAGNTWREY  290
```

>WP_077385559.1 dihydropteroate synthase [Bifidobacterium longum]
Length=291

Score = 116 bits (290),  Expect = 2e-29, Method: Compositional matrix adjust.
Identities = 93/287 (32%), Positives = 140/287 (49%), Gaps = 27/287 (9%)

```
Query  4
SRVTVFGILNLTEDSFFDESRRLDPAGAVTAAIEMLRVGSDVVDVGPAASHPDARPVSPA  63
            +   V G+LN+TEDSF D    LDPA A     +M+  G+D++D+G  ++ P A+
VS A
Sbjct  11
THTMVMGVLNITEDSFSDGGLWLDPAKAAQHGRDMMAAGADIIDIGAESTRPGAKRVSEA  70

Query  64   DEIRRIAPLLDALSDQMHRVSIDSFQPETQRYALKRGVGYLNDIQGFPDPALYPD-
IAEA  122
            DE+ RI   +  L      +SID+ +      AL   G   +ND+ G    A  P
+A+
Sbjct  71
DELARITGAVKTLIPAGAVLSIDTTRASVAAAALSEGAQIINDVSGGTLDAELPHVVADH  130

Query  123  DCRLVVMH---------
SAQRDGIATRTGHLRPEDALDEIVRFFEARVSALRRSGVAADR  173
            DC  VV H          A  D   +   H    D  DE++R    +V  +  +GV
+R
Sbjct  131  DCLYVVQHWRGWLVGSKGANPDQDTSVYEHGVLTDVHDELMR----
QVDGVLAAGVKPER  186

Query  174  LILDPGMGFFLSPAPETSLHVLSNLQKLKSALGLPLLVSVSRKSFLGATV---
GLPVKDL  230
            +I+DPG+GF   P  E +L +L+ L+  + A G P+L+  SRK F+ A +   G
D
Sbjct  187  IIIDPGLGFS-KPGIEHNLPLLTGLETFR-
ATGYPVLIGQSRKRFISAMLTGAGTAGAD-  243

Query  231  GPA-----SLAAELHAIG--NGADYVRTHAPGDLRSAITFSETLAKF  270
            GP       + A L A+   +GA  VR H     R+A+    T  ++
Sbjct  244  GPTMAQRDDVTAALSALSAEHGAWAVRVHDVAKSRAAVIAGNTWREY  290
```

>BAF39192.1 dihydropteroate synthase 1 [Bifidobacterium adolescentis ATCC
15703]
Length=299

Score = 116 bits (290),  Expect = 2e-29, Method: Compositional matrix adjust.
Identities = 87/283 (31%), Positives = 136/283 (48%), Gaps = 23/283

(8%)

Query  5
RVTVFGILNLTEDSFFDESRRLDPAGAVTAAIEMLRVGSDVVDVGPAASHPDARPVSPAD  64
            R  V G+LN+TEDSF D    L P  A      M++ G+D++D+G  ++ P A+
VS AD
Sbjct  20
RTLVMGVLNITEDSFSDGGLWLAPEAAKAHGEAMMKAGADIIDIGAESTRPGAKRVSEAD  79

Query  65
EIRRIAPLLDALSDQMHRVSIDSFQPETQRYALKRGVGYLNDIQGFPDPALYPDIA--EA  122
            E  R+   +DAL  +   +SID+ +      AL+ G   +ND+ G       P +
+
Sbjct  80
EKARVLGAVDALIPEGAVLSIDTTRASVALAALEHGAQIINDVSGGQLDRELPHVVADHS  139

Query  123  DCRLVVMHSAQR---------
DGIATRTGHLRPEDALDEIVRFFEARVSALRRSGVAADR  173
            DC  +V H             D   +R  H    D  DE++R    +V A+  +GV
A++
Sbjct  140  DCLYIVQHWRGWLAGAAGNVPDADTSRYEHGVVNDVYDELMR----
QVDAVLEAGVQAEQ  195

Query  174  LILDPGMGFFLSPAPETSLHVLSNLQKLKSALGLPLLVSVSRKSFLG---
ATVGLPVKDL  230
            +I+DPG+GF   P  E +L +L+ L +  +A G P+L+  SRK F+G   A  G+
D+
Sbjct  196  VIIDPGLGFS-KPGVEHNLPILAALDRF-
NATGYPVLIGASRKRFVGSLLAGAGVTEPDM  253

Query  231  GP---ASLAAELHAIGNGADYVRTHAPGDLRSAITFSETLAKF  270
                 A+ A       +G   VR H     R A+       ++
Sbjct  254  ASKDNATAAISALCAEHGVWAVRVHDVAKSRDAVAVGNAWREY  296

>WP_003814775.1 dihydropteroate synthase [Bifidobacterium bifidum]
 EFR51108.1 dihydropteroate synthase [Bifidobacterium bifidum NCIMB 41171]
 EKE50939.1 dihydropteroate synthase [Bifidobacterium bifidum LMG 13195]
 KLN84981.1 FolP Dihydropteroate synthase [Bifidobacterium bifidum LMG 13195]
 KXS26740.1 dihydropteroate synthase [Bifidobacterium bifidum]
 KYJ84132.1 dihydropteroate synthase [Bifidobacterium bifidum]
 OSP26627.1 dihydropteroate synthase [Bifidobacterium bifidum]
Length=290

 Score = 115 bits (289),  Expect = 2e-29, Method: Compositional matrix adjust.
 Identities = 89/280 (32%), Positives = 139/280 (50%), Gaps = 19/280 (7%)

Query  8
VFGILNLTEDSFFDESRRLDPAGAVTAAIEMLRVGSDVVDVGPAASHPDARPVSPADEIR  67

+ G+LN+TEDSF D    LDP  A     +ML  G+D++D+G  ++ P A+ VS
DE+
Sbjct  15
LMGVLNITEDSFSDGGLWLDPGKARDHGDDMLHAGADIIDIGAESTRPGAKRVSEKDELD  74

Query  68   RIAPLLDALSDQMHRVSIDSFQPETQRYALKRGVGYLNDIQGFP-
DPALYPDIAEADCRL  126
            R+     AL      VSID+ +    + AL  G   +ND+ G   D  L   +A+
DC
Sbjct  75
RVTGAAKALIAHGAVVSIDTTRSAVAQAALDEGAQIINDVSGGRLDRDLPHVVADHDCLY  134

Query  127  VVMHSAQRDGIATRTGHLRPE--------
DALDEIVRFFEARVSALRRSGVAADRLILDP  178
            +V H   R  +A   G   P+        D L ++     ++V A+ ++ VA +R
+I+DP
Sbjct  135  IVQH--
WRGWLAGSKGGAAPDADTSHYEHDVLKDVYDELMSQVDAVLKARVAPERIIIDP  192

Query  179  GMGFFLSPAPETSLHVLSNLQKLKSALGLPLLVSVSRKSFLGAT-----VGLP-
VKDLGP  232
            G+GF   P  E +L +L  L   K + G P+L+  SRK F+ A      +G P +
D
Sbjct  193  GLGFS-KPGIEHNLPLLVGLDMFKRS-
GYPVLIGASRKRFVSAALNDAGIGEPTMDDRDN  250

Query  233  ASLAAELHAIGNGADYVRTHAPGDLRSAITFSETLAKFRS  272
            A+ A       +GA  VR H     R+A+T      ++++
Sbjct  251  ATAALSALCAEHGAWAVRVHDVRRSRAALTIGSLWRQYQN  290


>WP_065445269.1 dihydropteroate synthase [Bifidobacterium longum]
Length=291

 Score = 115 bits (289),  Expect = 3e-29, Method: Compositional
matrix adjust.
 Identities = 92/287 (32%), Positives = 140/287 (49%), Gaps = 27/287
(9%)

Query  4
SRVTVFGILNLTEDSFFDESRRLDPAGAVTAAIEMLRVGSDVVDVGPAASHPDARPVSPA  63
            +   V G+LN+TEDSF D    LDPA A     +M+  G+D++D+G  ++ P A+
VS A
Sbjct  11
THTMVMGVLNITEDSFSDGGLWLDPAKAAQRGRDMMAAGADIIDIGAESTRPGAKRVSEA  70

Query  64   DEIRRIAPLLDALSDQMHRVSIDSFQPETQRYALKRGVGYLNDIQGFPDPALYPD-
IAEA  122
            DE+ RI   +  L      +SID+ +      AL  G   +ND+ G    A  P
+A+
Sbjct  71
DELARITGAVKTLIPAGAVLSIDTTRASVATAALSEGAQIINDVSGGTLDAELPHVVADH  130

Query  123  DCRLVVMH---------

```
SAQRDGIATRTGHLRPEDALDEIVRFFEARVSALRRSGVAADR  173
            DC  +V H          A  D   +   H    D  DE++R    +V  +  +GV  
+R
Sbjct  131  DCLYIVQHWRGWLVGSKGANPDQDTSVYEHGVLTDVHDELMR----
QVDGVLAAGVKPER  186

Query  174  LILDPGMGFFLSPAPETSLHVLSNLQKLKSALGLPLLVSVSRKSFLGATV---
GLPVKDL  230
            +I+DPG+GF   P  E +L +L+ L+  + A G P+L+  SRK F+ A +   G  
D
Sbjct  187  IIIDPGLGFS-KPGIEHNLPLLTGLETFR-
ATGYPVLIGQSRKRFISAMLTGAGTAGAD-  243

Query  231  GPA-----SLAAELHAIG--NGADYVRTHAPGDLRSAITFSETLAKF  270
            GP       + A L A+   +GA  VR H     R+A+    T  ++
Sbjct  244  GPTMAQRDDVTAALSALSAEHGAWAVRVHDVAKSRAAVIAGNTWREY  290


>WP_065454755.1 dihydropteroate synthase [Bifidobacterium longum]
Length=291

 Score = 115 bits (289),  Expect = 3e-29, Method: Compositional 
matrix adjust.
 Identities = 93/287 (32%), Positives = 140/287 (49%), Gaps = 27/287 
(9%)

Query  4    
SRVTVFGILNLTEDSFFDESRRLDPAGAVTAAIEMLRVGSDVVDVGPAASHPDARPVSPA  63
            +   V G+LN+TEDSF D    LDPA A     +M+  G+D++D+G  ++ P A+
VS A
Sbjct  11   
THTMVMGVLNITEDSFSDGGLWLDPAKAAQHGRDMMAAGADIIDIGAESTRPGAKRVSEA  70

Query  64   DEIRRIAPLLDALSDQMHRVSIDSFQPETQRYALKRGVGYLNDIQGFPDPALYPD-
IAEA  122
            DE+ RI   +  L      +SID+ +      AL  G   +ND+ G    A  P  
+A+
Sbjct  71   
DELARITGAVKTLIPAGAVLSIDTTRASVAAAALSEGAQIVNDVSGGTLDAELPHVVADH  130

Query  123  DCRLVVMH---------
SAQRDGIATRTGHLRPEDALDEIVRFFEARVSALRRSGVAADR  173
            DC  VV H          A  D   +   H    D  DE++R    +V  +  +GV  
+R
Sbjct  131  DCLYVVQHWRGWLVGSKGANPDQDTSVYEHGVLTDVHDELMR----
QVDGVLAAGVKPER  186

Query  174  LILDPGMGFFLSPAPETSLHVLSNLQKLKSALGLPLLVSVSRKSFLGATV---
GLPVKDL  230
            +I+DPG+GF   P  E +L +L+ L+  + A G P+L+  SRK F+ A +   G  
D
Sbjct  187  IIIDPGLGFS-KPGIEHNLPLLTGLETFR-
ATGYPVLIGQSRKRFISAMLTGAGTAGAD-  243
```

```
Query  231  GPA-----SLAAELHAIG--NGADYVRTHAPGDLRSAITFSETLAKF  270
            GP       + A L A+   +GA  VR H     R+A+    T  ++
Sbjct  244  GPTMAQRDDVTAALSALSAEHGAWAVRVHDVAKSRAAVIAGNTWREY  290
```

>AUD75228.1 Dihydropteroate synthase [Bifidobacterium breve]
 AUD87765.1 Dihydropteroate synthase [Bifidobacterium breve]
Length=291

 Score = 115 bits (288),  Expect = 3e-29, Method: Compositional matrix adjust.
 Identities = 92/288 (32%), Positives = 142/288 (49%), Gaps = 29/288 (10%)

```
Query  4
SRVTVFGILNLTEDSFFDESRRLDPAGAVTAAIEMLRVGSDVVDVGPAASHPDARPVSPA  63
            S   V G+LN+TEDSF D    LDPA A     +M+  G+D++D+G  ++ P A+
VS
Sbjct  11
SHTMVMGVLNITEDSFSDGGLWLDPAKAAQHGRDMMAAGADIIDIGAESTRPGAKRVSEE  70

Query  64   DEIRRIAPLLDALSDQMHRVSIDSFQPETQRYALKRGVGYLNDIQGFPDPALYPD-
IAEA  122
            DE+ RI   ++AL      +SID+ +      AL  G   +ND+ G    A  P
IA+
Sbjct  71
DELARITGAVNALIPAGAVLSIDTTRASVAAAALDGGAQIINDVSGGTLDANLPHVIADH  130

Query  123  DCRLVVMHSAQRDGIATRTG-----------
HLRPEDALDEIVRFFEARVSALRRSGVAA  171
            DC  +V H   R  +A   G           H    D  DE++R    +V  +
+GV
Sbjct  131  DCLYIVQH--WRGWLAGSKGANPDQDTSVYEHGVLTDVHDELMR----
QVDDVLAAGVKP  184

Query  172  DRLILDPGMGFFLSPAPETSLHVLSNLQKLKSALGLPLLVSVSRKSFLGATVGL--
PVKD  229
            +R+++DPG+GF   P+ E +L +L+ L+  + A G P+L+  SRK F+ A +
+
Sbjct  185  ERIVIDPGLGFS-KPSIEHNLPLLTGLETFR-
ATGYPVLIGQSRKRFISAMLAEAGASGE  242

Query  230  LGPA-----SLAAELHAIG--NGADYVRTHAPGDLRSAITFSETLAKF  270
             GP       + A L A+   +GA  VR H     R+A+    T  ++
Sbjct  243  NGPTMDQRDDVTAALSALSAEHGAWAVRVHDVAKSRAAVITGNTWRQY  290
```

>WP_100513572.1 dihydropteroate synthase [Bifidobacterium sp. TRI 7]
 PJM74691.1 dihydropteroate synthase [Bifidobacterium sp. TRI 7]
Length=293

 Score = 115 bits (288),  Expect = 3e-29, Method: Compositional matrix adjust.
 Identities = 92/285 (32%), Positives = 138/285 (48%), Gaps = 28/285

(10%)

```
Query  5
RVTVFGILNLTEDSFFDESRRLDPAGAVTAAIEMLRVGSDVVDVGPAASHPDARPVSPAD  64
            R  V G+LN+TEDSF D    LDPA A      ML  G+D+VD+G  ++ P A+
VS AD
Sbjct  15
RTLVMGVLNITEDSFSDGGLWLDPAKAREHGAAMLDAGADIVDIGAESTRPGAKRVSEAD  74

Query  65   EIRRIAPLLDALSDQMHRVSIDSFQPETQRYALKRGVGYLNDIQGFPDPALYPD-
IAEAD  123
            E+ R+     AL +    +SID+ +      AL  G   +ND+ G    A  P
+A+ D
Sbjct  75
ELARVTGATKALLEHGAVLSIDTTRASVAAAALDEGAQIINDVSGGTLDAELPHVVADHD  134

Query  124  CRLVVMHSAQRDGIATRTGHLRPE------------
DALDEIVRFFEARVSALRRSGVAA  171
            C  +V H   R  +A   G   P+            D  DE++R    +V A+
+GV
Sbjct  135  CLYIVQH--WRGWLAGSKG-ATPDADTSVYHGGVLHDVRDELMR----
QVDAVLAAGVKP  187

Query  172
DRLILDPGMGFFLSPAPETSLHVLSNLQKLKSALGLPLLVSVSRKSFLGATVGL------  225
            +R+I+DPG+GF   P  E +L +L+ L+  + A G P+L+  SRK F+ A +
Sbjct  188  ERIIIDPGLGFS-KPGIEHNLPLLAGLETFR-
ATGYPVLIGQSRKRFVSALLADAGFDEP  245

Query  226  PVKDLGPASLAAELHAIGNGADYVRTHAPGDLRSAITFSETLAKF  270
             + D   A+ A       +GA  VR H     R+A+       ++
Sbjct  246  TMDDRDNATAAFSALCAEHGAWAVRVHDVARSRAAVAVGNVWRRY  290
```

>WP_014760561.1 dihydropteroate synthase [Bifidobacterium bifidum]
 AFL05010.1 dihydropteroate synthase [Bifidobacterium bifidum BGN4]
Length=290

 Score = 115 bits (288),  Expect = 3e-29, Method: Compositional
matrix adjust.
 Identities = 89/280 (32%), Positives = 139/280 (50%), Gaps = 19/280
(7%)

```
Query  8
VFGILNLTEDSFFDESRRLDPAGAVTAAIEMLRVGSDVVDVGPAASHPDARPVSPADEIR  67
            + G+LN+TEDS  D    LDP  A     +ML  G+D++D+G  ++ P A+ VS
DE+
Sbjct  15
LMGVLNITEDSSSDGGLWLDPVKARDHGDDMLHAGADIIDIGAESTRPGAKRVSEKDELD  74

Query  68   RIAPLLDALSDQMHRVSIDSFQPETQRYALKRGVGYLNDIQGFP-
DPALYPDIAEADCRL  126
            R+     AL      VSID+ +    + AL  G   +ND+ G   D  L   +A+
DC
```

```
Sbjct  75
RVTGAAKALIAHGAVVSIDTTRSAVAQAALDEGAQIINDVSGGRLDRDLPHVVADHDCLY  134

Query  127  VVMHSAQRDGIATRTGHLRPE--------
DALDEIVRFFEARVSALRRSGVAADRLILDP  178
            +V H   R  +A   G   P+        D L ++     ++V A+ ++GVA +R
+I+DP
Sbjct  135  IVQH--
WRGWLAGSKGGAEPDADTSHYEHDVLKDVYDELMSQVDAVLKAGVAPERVIIDP  192

Query  179  GMGFFLSPAPETSLHVLSNLQKLKSALGLPLLVSVSRKSFLGAT-----VGLP-
VKDLGP  232
            G+GF   P  E +L +L  L   K + G P+L+  SRK F+ A      +G P +
D
Sbjct  193  GLGFS-KPGIEHNLPLLVGLDLFKRS-
GYPVLIGASRKRFVSAALNVAGIGEPTMDDRDN  250

Query  233  ASLAAELHAIGNGADYVRTHAPGDLRSAITFSETLAKFRS  272
            A+ A       +GA  VR H     R+A+T      ++++
Sbjct  251  ATAALSALCAEHGAWAVRVHDVRRSRAALTIGSLWRQYQN  290


>WP_039774850.1 dihydropteroate synthase [Bifidobacterium
adolescentis]
 AJE05414.1 dihydropteroate synthase 1 [Bifidobacterium
adolescentis]
 CUN53000.1 dihydropteroate synthase 1 [Bifidobacterium
adolescentis]
 OSG95197.1 dihydropteroate synthase [Bifidobacterium adolescentis]
Length=292

 Score = 115 bits (288),  Expect = 4e-29, Method: Compositional
matrix adjust.
 Identities = 87/283 (31%), Positives = 136/283 (48%), Gaps = 23/283
(8%)

Query  5
RVTVFGILNLTEDSFFDESRRLDPAGAVTAAIEMLRVGSDVVDVGPAASHPDARPVSPAD  64
            R  V G+LN+TEDSF D    L P  A      M++ G+D++D+G  ++ P A+
VS AD
Sbjct  13
RTLVMGVLNITEDSFSDGGLWLAPEAAKAHGEAMMKAGADIIDIGAESTRPGAKRVSEAD  72

Query  65
EIRRIAPLLDALSDQMHRVSIDSFQPETQRYALKRGVGYLNDIQGFPDPALYPDIA--EA  122
            E  R+   +DAL  +   +SID+ +      AL+ G   +ND+ G       P +
+
Sbjct  73
EKARVLGAVDALIPEGAVLSIDTTRASVALAALEHGAQIINDVSGGQLDRELPHVVADHS  132

Query  123  DCRLVVMHSAQR---------
DGIATRTGHLRPEDALDEIVRFFEARVSALRRSGVAADR  173
            DC  +V H             D   +R  H    D  DE++R    +V A+  +GV
A++
```

```
Sbjct  133  DCLYIVQHWRGWLAGAAGNVPDADTSRYEHGVVNDVHDELMR----
QVDAVLEAGVRAEQ  188

Query  174  LILDPGMGFFLSPAPETSLHVLSNLQKLKSALGLPLLVSVSRKSFLG---
ATVGLPVKDL  230
            +I+DPG+GF   P  E +L +L+ L +  +A G P+L+  SRK F+G   A  G+
D+
Sbjct  189  VIIDPGLGFS-KPGVEHNLPILAALDRFNAA-
GYPVLIGASRKRFVGSLLAGAGVTEPDM  246

Query  231  GP---ASLAAELHAIGNGADYVRTHAPGDLRSAITFSETLAKF  270
                 A+ A       +G   VR H     R A+       ++
Sbjct  247  ASKDNATAAISALCAEHGVWAVRVHDVAKSRDAVAVGNAWREY  289


>WP_032736608.1 dihydropteroate synthase [Bifidobacterium longum]
Length=291

 Score = 115 bits (288),  Expect = 4e-29, Method: Compositional
matrix adjust.
 Identities = 94/289 (33%), Positives = 141/289 (49%), Gaps = 31/289
(11%)

Query  4
SRVTVFGILNLTEDSFFDESRRLDPAGAVTAAIEMLRVGSDVVDVGPAASHPDARPVSPA  63
            SR  V G+LN+TEDS  D    LDPA A     +M+  G+D++D+G  ++ P A+
VS A
Sbjct  11
SRTMVMGVLNITEDSSSDGGLWLDPAKAAQHGRDMMAAGADIIDIGAESTRPGAKRVSEA  70

Query  64   DEIRRIAPLLDALSDQMHRVSIDSFQPETQRYALKRGVGYLNDIQGFPDPALYPD-
IAEA  122
            DE+ RI   +  L      +SID+ +      AL  G   +ND+ G    A  P
+A+
Sbjct  71
DELARITGAVKTLIPAGAILSIDTTRASVAAAALSEGAQIINDVSGGTLDAELPQVVADH  130

Query  123  DCRLVVMHSAQRDGIATRTG-----------
HLRPEDALDEIVRFFEARVSALRRSGVAA  171
            DC  +V H   R  +A   G           H    D  DE++R    +V  +
+GV
Sbjct  131  DCLYIVQH--WRGWLAGSKGANPDQDTSVYEHGVLTDVHDELMR----
QVDGVLAAGVKP  184

Query  172  DRLILDPGMGFFLSPAPETSLHVLSNLQKLKSALGLPLLVSVSRKSFLGATV---
GLPVK  228
            +R+I+DPG+GF   P  E +L +L+ L+  + A G P+L+  SRK F+ A +   G
Sbjct  185  ERIIIDPGLGFS-KPGIEHNLPLLTGLETFR-
ATGYPVLIGQSRKRFISAMLTEAGAAGA  242

Query  229  DLGPA-----SLAAELHAIG--NGADYVRTHAPGDLRSAITFSETLAKF  270
            D GP       + A L A+   +GA  VR H     R+A+    T  ++
Sbjct  243  D-GPIMAQRDDVTAALSALSAEHGAWAVRVHDVAKSRAAVIAGNTWREY  290
```

>WP_033892007.1 dihydropteroate synthase [Bifidobacterium saguini]
 KFI90997.1 Dihydropteroate synthase [Bifidobacterium saguini DSM
23967]
Length=290

 Score = 115 bits (288),  Expect = 4e-29, Method: Compositional
matrix adjust.
 Identities = 91/286 (32%), Positives = 144/286 (50%), Gaps = 30/286
(10%)

Query  8
VFGILNLTEDSFFDESRRLDPAGAVTAAIEMLRVGSDVVDVGPAASHPDARPVSPADEIR  67
            V G+LN+TEDSF D    LDPA A     +M+  G+D++D+G  ++ P A+ VS
DE++
Sbjct  15
VMGVLNITEDSFSDGGLWLDPAKAAQHGRDMMAAGADIIDIGAESTRPGAKRVSEEDELK  74

Query  68   RIAPLLDALSDQMHRVSIDSFQPETQRYALKRGVGYLNDIQGFP-
DPALYPDIAEADCRL  126
            RI   + AL      +SID+ +      AL+ G   +ND+ G   D  L   +A+
DC
Sbjct  75
RITGAVSALIPAGAVLSIDTTRASVACAALEGGAQIINDVSGGTLDRELPHVVADHDCLY  134

Query  127  VVMHSAQRDGIATRTG-----------
HLRPEDALDEIVRFFEARVSALRRSGVAADRLI  175
            +V H   R  +A   G           H    D  DE++R    +V  +  +GV
+R+I
Sbjct  135  IVQH--WRGWLAGSKGVNPDQDTSVYEHGVLTDVHDELMR----
QVDDVLAAGVKPERII  188

Query  176
LDPGMGFFLSPAPETSLHVLSNLQKLKSALGLPLLVSVSRKSFLGATVG--------LPV  227
            +DPG+GF   P  E +L +L+ L+  + A G P+L+  SRK F+ A +
+
Sbjct  189  IDPGLGFS-KPGIEHNLPLLAGLETFR-
ATGYPVLIGQSRKRFISAMLAEDGIDEPTMAQ  246

Query  228  KDLGPASLAAELHAIGNGADYVRTHAPGDLRSAITFSETLAKFRSR  273
            +D   A+L+A   +  +GA  VR H     R+A+    T  ++ +R
Sbjct  247  RDDVTAALSAL--SAEHGAWAVRVHDVTKSRAAVIAGNTWRQYSAR  290


>WP_085379343.1 dihydropteroate synthase [Bifidobacterium
adolescentis]
 OSG92753.1 dihydropteroate synthase [Bifidobacterium adolescentis]
Length=292

 Score = 115 bits (288),  Expect = 4e-29, Method: Compositional
matrix adjust.
 Identities = 88/285 (31%), Positives = 137/285 (48%), Gaps = 27/285
(9%)

```
Query  5    
RVTVFGILNLTEDSFFDESRRLDPAGAVTAAIEMLRVGSDVVDVGPAASHPDARPVSPAD  64
            R  V G+LN+TEDSF D    L P  A      M++ G+D++D+G  ++ P A+
VS AD
Sbjct  13   
RTLVMGVLNITEDSFSDGGLWLAPEAAKAHGEAMMKAGADIIDIGAESTRPGAKRVSEAD  72

Query  65   
EIRRIAPLLDALSDQMHRVSIDSFQPETQRYALKRGVGYLNDIQGFPDPALYPDIA--EA  122
            E  R+   +DAL  +   +SID+ +      AL+ G   +ND+ G       P +
+
Sbjct  73   
EKARVLGAVDALIPEGAVLSIDTTRASVALAALEHGAQIINDVSGGQLDRELPHVVADHS  132

Query  123  DCRLVVMHSAQRDGIATRTG-----------
HLRPEDALDEIVRFFEARVSALRRSGVAA  171
            DC  +V H   R  +A   G           H    D  DE++R    +V A+
+GV A
Sbjct  133  DCLYIVQH--WRGWLAGAAGNVPDADTSCYEHGVVNDVHDELMR----
QVDAVLEAGVQA  186

Query  172  DRLILDPGMGFFLSPAPETSLHVLSNLQKLKSALGLPLLVSVSRKSFLG---
ATVGLPVK  228
            +++I+DPG+GF   P  E +L +L+ L +  +A G P+L+  SRK F+G   A  G
+
Sbjct  187  EQVIIDPGLGFS-KPGVEHNLPILAALDRFNAA-
GYPVLIGASRKRFVGSLLAGAGVTEP  244

Query  229  DLGP---ASLAAELHAIGNGADYVRTHAPGDLRSAITFSETLAKF  270
            D+     A+ A       +G   VR H     R A+       ++
Sbjct  245  DMASKDNATAAISALCAEHGVWAVRVHDVAKSRDAVAVGNAWREY  289


>CZQ91691.1 dihydropteroate synthase signature 1 [Trichococcus 
palustris]
 SFL04062.1 dihydropteroate synthase [Trichococcus palustris]
Length=277

 Score = 115 bits (287),  Expect = 4e-29, Method: Compositional 
matrix adjust.
 Identities = 82/254 (32%), Positives = 130/254 (51%), Gaps = 15/254 
(6%)

Query  2    
LRSRVTVFGILNLTEDSFFDESRRLDPAGAVTAAIEMLRVGSDVVDVGPAASHPDARPVS  61
            L  R  + GILN+T DSF D         AV  AIEM+R G+D +D+G  ++ P
+S
Sbjct  12   
LSKRSYIMGILNVTPDSFSDGGEWNTLEAAVAHAIEMVRDGADFIDIGGESTRPGHTQIS  71

Query  62   PADEIRRIAPLLDALSDQMHR-VSIDSFQPETQRYALKRGVGYLNDIQGFP-
DPALYPDI  119
              +EI RI P++ A+   +   +S+D+++    R A + G+  +NDI G   DP +
Sbjct  72   
```

```
DEEEIARIVPVIKAVKAAVAVPLSVDTYKSAVARAACEAGIDIINDIWGCKYDPEIAAVA  131

Query  120
AEADCRLVVMHSAQRDGIATRTGHLRPEDALDEIVRFFEARVSALRRSGVAADRLILDPG  179
            AE D  +++MH+ +         +   ED + ++       ++A    GV
+ILDPG
Sbjct  132  AEFDVPIILMHNRE-----AADYNFLIEDMIQDLSESMRIALAA----
GVPRHNIILDPG  182

Query  180  MGFFLSPAPETSLHVLSNLQKLKSALGLPLLVSVSRKSFLGATVG-
LPVKDLGPASLAAE  238
             GF  +   E +L+V+ +L++    LG PLL++ SRK F+G  +G  P K+    +
A
Sbjct  183  CGF--AKTFEDNLNVIHHLRRFAD-
LGYPLLLATSRKRFIGTVLGDAPFKERDEGTAATT  239

Query  239  LHAIGNGADYVRTH  252
               I NGA   R H
Sbjct  240  ALGIMNGAHIFRVH  253


>WP_047290331.1 dihydropteroate synthase [Bifidobacterium bifidum]
 KLN88250.1 FolP Dihydropteroate synthase [Bifidobacterium bifidum]
Length=290

 Score = 115 bits (287),  Expect = 4e-29, Method: Compositional
matrix adjust.
 Identities = 88/280 (31%), Positives = 138/280 (49%), Gaps = 19/280
(7%)

Query  8
VFGILNLTEDSFFDESRRLDPAGAVTAAIEMLRVGSDVVDVGPAASHPDARPVSPADEIR  67
            + G+LN+TEDS  D    LDP  A     +ML  G+D++D+G  ++ P A+ VS
DE+
Sbjct  15
LMGVLNITEDSVSDGGLWLDPGKARDHGDDMLHAGADIIDIGAESTRPGAKRVSEKDELD  74

Query  68   RIAPLLDALSDQMHRVSIDSFQPETQRYALKRGVGYLNDIQGFPDPALYPD-
IAEADCRL  126
            R+     AL      VSID+ +    + AL  G   +ND+ G       P  +A+
DC
Sbjct  75
RVTGAAKALIAHGAVVSIDTTRSAVAQAALNEGAQIINDVSGGRLGRDLPHVVADHDCLY  134

Query  127  VVMHSAQRDGIATRTGHLRPE--------
DALDEIVRFFEARVSALRRSGVAADRLILDP  178
            +V H   R  +A   G   P+        D L ++     ++V A+ ++GVA +R
+I+DP
Sbjct  135  IVQH--
WRGWLAGSKGGGEPDADTSHYEHDVLKDVYDELMSQVDAVLKAGVAPERVIIDP  192

Query  179  GMGFFLSPAPETSLHVLSNLQKLKSALGLPLLVSVSRKSFLGAT-----VGLP-
VKDLGP  232
            G+GF   P  E +L +L  L   K + G P+L+  SRK F+ A      +G P +
```

D
Sbjct  193  GLGFS-KPGIEHNLPLLVGLDLFKRS-
GYPVLIGASRKRFVSAALNDAGIGEPTMDDRDN  250

Query  233  ASLAAELHAIGNGADYVRTHAPGDLRSAITFSETLAKFRS  272
            A+ A       +GA  VR H     R+A+T      ++++
Sbjct  251  ATAALSALCAEHGAWAVRVHDVRRSRAALTIGSLWRQYQN  290


>BBA55689.1 dihydropteroate synthase [Bifidobacterium bifidum]
Length=290

 Score = 115 bits (287),  Expect = 5e-29, Method: Compositional
matrix adjust.
 Identities = 89/280 (32%), Positives = 139/280 (50%), Gaps = 19/280
(7%)

Query  8
VFGILNLTEDSFFDESRRLDPAGAVTAAIEMLRVGSDVVDVGPAASHPDARPVSPADEIR  67
            + G+LN+TEDS  D    LDP  A     +ML  G+D++D+G  ++ P A+ VS
DE+
Sbjct  15
LMGVLNITEDSSSDGGLWLDPGKARDHGDDMLHAGADIIDIGAESTRPGAKRVSEKDELD  74

Query  68   RIAPLLDALSDQMHRVSIDSFQPETQRYALKRGVGYLNDIQGFP-
DPALYPDIAEADCRL  126
            R+     AL      VSID+ +    + AL  G   +ND+ G   D  L   +A+
DC
Sbjct  75
RVTGAAKALIAHGAVVSIDTTRSAVAQAALDEGAQIINDVSGGRLDRDLPHVVADHDCLY  134

Query  127  VVMHSAQRDGIATRTGHLRPE--------
DALDEIVRFFEARVSALRRSGVAADRLILDP  178
            +V H   R  +A   G   P+        D L ++     ++V A+ ++GVA +R
+I+DP
Sbjct  135  IVQH--
WRGWLAGSKGGAAPDADTSHYEHDVLKDVYDELMSQVDAVLKAGVAPERVIIDP  192

Query  179  GMGFFLSPAPETSLHVLSNLQKLKSALGLPLLVSVSRKSFLGAT-----VGLP-
VKDLGP  232
            G+GF   P  E +L +L  L   K + G P+L+  SRK F+ A      +G P +
D
Sbjct  193  GLGFS-KPGIEHNLPLLVGLDLFKRS-
GYPVLIGASRKRFVSAALNDAGIGEPTMDDRDN  250

Query  233  ASLAAELHAIGNGADYVRTHAPGDLRSAITFSETLAKFRS  272
            A+ A       +GA  VR H     R+A+T      ++++
Sbjct  251  ATAALSALCAEHGAWAVRVHDVRRSRAALTIGSLWRQYQN  290


>KXS22994.1 dihydropteroate synthase [Bifidobacterium breve]
Length=291

 Score = 115 bits (287),  Expect = 5e-29, Method: Compositional

```
matrix adjust.
 Identities = 91/288 (32%), Positives = 142/288 (49%), Gaps = 29/288
(10%)

Query  4
SRVTVFGILNLTEDSFFDESRRLDPAGAVTAAIEMLRVGSDVVDVGPAASHPDARPVSPA  63
            S   V G+LN+TEDSF D    LDPA A     +M+  G+D++D+G  ++ P A+
VS
Sbjct  11
SHTMVMGVLNITEDSFSDGGLWLDPAKAAQHGRDMMAAGADIIDIGAESTRPGAKRVSEE  70

Query  64   DEIRRIAPLLDALSDQMHRVSIDSFQPETQRYALKRGVGYLNDIQGFPDPALYPD-
IAEA  122
            DE+ RI   ++AL      +SID+ +      AL   G   +ND+ G    A  P
+A+
Sbjct  71
DELARITGAVNALIPAGAVLSIDTTRASVAAAALDGGAQIINDVSGGTLDANLPHVVADH  130

Query  123  DCRLVVMHSAQRDGIATRTG-----------
HLRPEDALDEIVRFFEARVSALRRSGVAA  171
            DC  +V H   R  +A   G           H    D  DE++R    +V  +
+GV
Sbjct  131  DCLYIVQH--WRGWLAGSKGANPDQDTSVYEHGVLTDVHDELMR----
QVDDVLAAGVKP  184

Query  172  DRLILDPGMGFFLSPAPETSLHVLSNLQKLKSALGLPLLVSVSRKSFLGATVGL--
PVKD  229
            +R+++DPG+GF   P+ E +L +L+ L+  + A G P+L+  SRK F+ A +
+
Sbjct  185  ERIVIDPGLGFS-KPSVEHNLPLLTGLETFR-
ATGYPVLIGQSRKRFISAMLAEAGASGE  242

Query  230  LGPA-----SLAAELHAIG--NGADYVRTHAPGDLRSAITFSETLAKF  270
             GP       + A L A+   +GA  VR H     R+A+    T  ++
Sbjct  243  NGPTMDQRDDVTAALSALSAEHGAWAVRVHDVAKSRAAVITGNTWRQY  290


>WP_080867818.1 dihydropteroate synthase [Bifidobacterium breve]
 OQM65039.1 dihydropteroate synthase [Bifidobacterium breve]
Length=292

 Score = 115 bits (287),  Expect = 5e-29, Method: Compositional
matrix adjust.
 Identities = 93/290 (32%), Positives = 141/290 (49%), Gaps = 29/290
(10%)

Query  4
SRVTVFGILNLTEDSFFDESRRLDPAGAVTAAIEMLRVGSDVVDVGPAASHPDARPVSPA  63
            S   V G+LN+TEDSF D    LDPA A     +M+  G+D++D+G  ++ P A+
VS
Sbjct  11
SHTMVMGVLNITEDSFSDGGLWLDPAKAAQHGRDMMAAGADIIDIGAESTRPGAKRVSEE  70

Query  64   DEIRRIAPLLDALSDQMHRVSIDSFQPETQRYALKRGVGYLNDIQGFPDPALYPD-
```

```
IAEA  122
            DE+ RI   ++AL      +SID+ +      AL  G   +ND+ G    A  P
IA+
Sbjct  71
DELARITGAVNALIPAGAVLSIDTTRASVAAAALDGGAQIINDVSGGTLDANLPHVIADH  130

Query  123  DCRLVVMHSAQRDGIATRTG-----------
HLRPEDALDEIVRFFEARVSALRRSGVAA  171
            DC  +V H   R  +A   G           H    D  DE++R    +V  +
+GV
Sbjct  131  DCLYIVQH--WRGWLAGSKGAIPDQDTSVYEHGVLTDVHDELMR----
QVDGVLAAGVKP  184

Query  172  DRLILDPGMGFFLSPAPETSLHVLSNLQKLKSALGLPLLVSVSRKSFLGA--
TVGLPVKD  229
            +R+I+DPG+GF   P+ E +L +L+ L+  +   G P+L+  SRK F+ A  T
V +
Sbjct  185  ERIIIDPGLGFS-KPSIEHNLPLLTGLETFRGT-
GYPVLIGQSRKRFISAMLTEAGAVGE  242

Query  230  LGPA-----SLAAELHAIG--NGADYVRTHAPGDLRSAITFSETLAKFRS  272
             GP       + A L A+   +GA  VR H      +A+       ++ S
Sbjct  243  DGPTMAQRDDVTAALSALSAEHGAWAVRVHDVAKSHAAVIAGNIWRQYLS  292


>WP_032682980.1 dihydropteroate synthase [Bifidobacterium longum]
 KFI73711.1 dihydropteroate synthase [Bifidobacterium longum subsp.
suis]
 ALO73129.1 dihydropteroate synthase [Bifidobacterium longum subsp.
longum]
 SDO38494.1 Dihydropteroate synthase [Bifidobacterium longum]
 PKD14212.1 Dihydropteroate synthase [Bifidobacterium longum]
Length=291

 Score = 115 bits (287),  Expect = 5e-29, Method: Compositional
matrix adjust.
 Identities = 93/289 (32%), Positives = 141/289 (49%), Gaps = 31/289
(11%)

Query  4
SRVTVFGILNLTEDSFFDESRRLDPAGAVTAAIEMLRVGSDVVDVGPAASHPDARPVSPA  63
            +   V G+LN+TEDSF D    LDPA A     +M+  G+D++D+G  ++ P A+
VS A
Sbjct  11
THTMVMGVLNITEDSFSDGGLWLDPAKAAQHGRDMMAAGADIIDIGAESTRPGAKRVSEA  70

Query  64   DEIRRIAPLLDALSDQMHRVSIDSFQPETQRYALKRGVGYLNDIQGFPDPALYPD-
IAEA  122
            DE+ RI   +  L      +SID+ +      AL  G   +ND+ G    A  P
+A+
Sbjct  71
DELVRITGAVKTLIPAGAILSIDTTRASVAAAALSEGAQIINDVSGGTLDAELPHVVADH  130

Query  123  DCRLVVMHSAQRDGIATRTG-----------
```

HLRPEDALDEIVRFFEARVSALRRSGVAA  171
            DC  +V H   R  +A   G           H    D  DE++R    +V  +
+GV
Sbjct  131  DCLYIVQH--WRGWLAGSKGANPDQDTSVYEHGVLTDVHDELMR----
QVDGVLAAGVKP  184

Query  172  DRLILDPGMGFFLSPAPETSLHVLSNLQKLKSALGLPLLVSVSRKSFLGATV---
GLPVK  228
            +R+I+DPG+GF   P  E +L +L+ L+  + A G P+L+  SRK F+ A +   G
Sbjct  185  ERIIIDPGLGFS-KPGIEHNLPLLTGLETFR-
ATGYPVLIGQSRKRFISAMLTEAGAAGA  242

Query  229  DLGPA-----SLAAELHAIG--NGADYVRTHAPGDLRSAITFSETLAKF  270
            D GP       + A L A+   +GA  VR H     R+A+    T  ++
Sbjct  243  D-GPIMAQRDDVTAALSALSAEHGAWAVRVHDVAKSRAAVIAGNTWREY  290

>EIJ22047.1 dihydropteroate synthase [Bifidobacterium longum subsp.
longum
35B]
 EIJ26900.1 dihydropteroate synthase [Bifidobacterium longum subsp.
longum
2-2B]
Length=296

 Score = 115 bits (287),  Expect = 5e-29, Method: Compositional
matrix adjust.
 Identities = 94/289 (33%), Positives = 141/289 (49%), Gaps = 31/289
(11%)

Query  4
SRVTVFGILNLTEDSFFDESRRLDPAGAVTAAIEMLRVGSDVVDVGPAASHPDARPVSPA  63
            SR  V G+LN+TEDS  D    LDPA A     +M+  G+D++D+G  ++ P A+
VS A
Sbjct  16
SRTMVMGVLNITEDSSSDGGLWLDPAKAAQHGRDMMAAGADIIDIGAESTRPGAKRVSEA  75

Query  64   DEIRRIAPLLDALSDQMHRVSIDSFQPETQRYALKRGVGYLNDIQGFPDPALYPD-
IAEA  122
            DE+ RI   +  L      +SID+ +      AL   G   +ND+ G    A  P
+A+
Sbjct  76
DELARITGAVKTLIPAGAILSIDTTRASVAAAALSEGAQIINDVSGGTLDAELPQVVADH  135

Query  123  DCRLVVMHSAQRDGIATRTG-----------
HLRPEDALDEIVRFFEARVSALRRSGVAA  171
            DC  +V H   R  +A   G           H    D  DE++R    +V  +
+GV
Sbjct  136  DCLYIVQH--WRGWLAGSKGANPDQDTSVYEHGVLTDVHDELMR----
QVDGVLAAGVKP  189

Query  172  DRLILDPGMGFFLSPAPETSLHVLSNLQKLKSALGLPLLVSVSRKSFLGATV---
GLPVK  228
            +R+I+DPG+GF   P  E +L +L+ L+  + A G P+L+  SRK F+ A +   G

```
Sbjct  190  ERIIIDPGLGFS-KPGIEHNLPLLTGLETFR-
ATGYPVLIGQSRKRFISAMLTEAGAAGA  247

Query  229  DLGPA-----SLAAELHAIG--NGADYVRTHAPGDLRSAITFSETLAKF  270
            D GP       + A L A+   +GA  VR H     R+A+    T  ++
Sbjct  248  D-GPIMAQRDDVTAALSALSAEHGAWAVRVHDVAKSRAAVIAGNTWREY  295
```

>WP_026646441.1 dihydropteroate synthase [Bifidobacterium ruminantium]
 KFI87656.1 dihydropteroate synthase 1 [Bifidobacterium ruminantium]
Length=292

 Score = 115 bits (287),  Expect = 6e-29, Method: Compositional matrix adjust.
 Identities = 91/286 (32%), Positives = 140/286 (49%), Gaps = 35/286 (12%)

```
Query  5    
RVTVFGILNLTEDSFFDESRRLDPAGAVTAAIEMLRVGSDVVDVGPAASHPDARPVSPAD  64
            R  V G+LN+TEDSF D    LDPA A      M++ G+D++D+G  ++ P A+ V 
AD
Sbjct  13   
RTLVMGVLNITEDSFSDGGLWLDPAAAKAHGEAMMKDGADIIDIGAESTRPGAKRVGEAD  72

Query  65   EIRRIAPLLDALSDQMHRVSIDSFQPETQRYALKRGVGYLNDIQGF----
PDPALYPDIA  120
            E  R+   +DAL+ +   +SID+ +      AL+ G   +ND+ G       P +  
D A
Sbjct  73   
EKTRVLGAVDALAPEGAVLSIDTTRASVASAALEHGAQIINDVSGGQLDRELPHVVADYA  132

Query  121  EADCRLVVMHSAQRDGIATRTG-----------
HLRPEDALDEIVRFFEARVSALRRSGV  169
            E+    +V H   R  +A   G           H    D  DE++R    +V A+  
+GV
Sbjct  133  ES--LYIVQH--WRGWLAGAAGNVPDADTSVYEHGVVRDVYDELMR----
QVDAVLAAGV  184

Query  170  
AADRLILDPGMGFFLSPAPETSLHVLSNLQKLKSALGLPLLVSVSRKSFLGATVG-----  224
             A+++I+DPG+GF   P  E +L +L  L +  +A G P+L+  SRK F+GA +
Sbjct  185  RAEQIIIDPGLGFS-KPGVEHNLPILKALDRFNAA-
GYPVLIGASRKRFVGALLADAGVD  242

Query  225  ---LPVKDLGPASLAAELHAIGNGADYVRTHAPGDLRSAITFSETL  267
               +  KD   A+++A      +GA  VR H     R A+
Sbjct  243  EPDMASKDNATAAISALCAE--HGAWAVRVHDVAKSRDAVAVGNAW  286
```

>WP_019728223.1 dihydropteroate synthase [Bifidobacterium breve]
 EWH42066.1 Dihydropteroate synthase [Bifidobacterium breve 2L]
 AHJ16152.1 Dihydropteroate synthase [Bifidobacterium breve 12L]
 AUE05841.1 Dihydropteroate synthase [Bifidobacterium breve]

```
 AUE21264.1 Dihydropteroate synthase [Bifidobacterium breve]
Length=291

 Score = 114 bits (286),  Expect = 6e-29, Method: Compositional
matrix adjust.
 Identities = 91/288 (32%), Positives = 142/288 (49%), Gaps = 29/288
(10%)

Query  4
SRVTVFGILNLTEDSFFDESRRLDPAGAVTAAIEMLRVGSDVVDVGPAASHPDARPVSPA  63
            S   V G+LN+TEDSF D    LDPA A     +M+  G+D++D+G  ++ P A+
VS
Sbjct  11
SHTMVMGVLNITEDSFSDGGLWLDPAKAAQHGRDMMAAGADIIDIGAESTRPGAKRVSEE  70

Query  64   DEIRRIAPLLDALSDQMHRVSIDSFQPETQRYALKRGVGYLNDIQGFPDPALYPD-
IAEA  122
            DE+ RI   ++AL      +SID+ +      AL   G   +ND+ G    A  P
+A+
Sbjct  71
DELARITGAVNALIPAGAVLSIDTTRASVAAAALDGGAQIINDVSGGTLDANLPHVVADH  130

Query  123  DCRLVVMHSAQRDGIATRTG-----------
HLRPEDALDEIVRFFEARVSALRRSGVAA  171
            DC  +V H   R  +A   G           H    D  DE++R    +V  +
+GV
Sbjct  131  DCLYIVQH--WRGWLAGSKGANPDQDTSVYEHGVLTDVHDELMR----
QVDDVLAAGVKP  184

Query  172  DRLILDPGMGFFLSPAPETSLHVLSNLQKLKSALGLPLLVSVSRKSFLGATVGL--
PVKD  229
            +R+++DPG+GF   P+ E +L +L+ L+  + A G P+L+  SRK F+ A +
+
Sbjct  185  ERIVIDPGLGFS-KPSIEHNLPLLTGLETFR-
ATGYPVLIGQSRKRFISAMLAEAGASGE  242

Query  230  LGPA-----SLAAELHAIG--NGADYVRTHAPGDLRSAITFSETLAKF  270
             GP       + A L A+   +GA  VR H     R+A+    T  ++
Sbjct  243  NGPTMDQRDDVTAALSALSAEHGAWAVRVHDVAKSRAAVITGNTWRQY  290


>WP_025222047.1 dihydropteroate synthase [Bifidobacterium breve]
 AHJ19861.1 Dihydropteroate synthase [Bifidobacterium breve JCM
7019]
 KOA67036.1 dihydropteroate synthase [Bifidobacterium breve MCC
1605]
 AUD77311.1 Dihydropteroate synthase [Bifidobacterium breve]
 AUE03837.1 Dihydropteroate synthase [Bifidobacterium breve]
Length=292

 Score = 114 bits (286),  Expect = 6e-29, Method: Compositional
matrix adjust.
 Identities = 93/290 (32%), Positives = 141/290 (49%), Gaps = 29/290
(10%)
```

Query  4
SRVTVFGILNLTEDSFFDESRRLDPAGAVTAAIEMLRVGSDVVDVGPAASHPDARPVSPA  63
            S   V G+LN+TEDSF D    LDPA A     +M+  G+D++D+G  ++ P A+
VS
Sbjct  11
SHTMVMGVLNITEDSFSDGGLWLDPAKAAQHGRDMMAAGADIIDIGAESTRPGAKRVSEE  70

Query  64   DEIRRIAPLLDALSDQMHRVSIDSFQPETQRYALKRGVGYLNDIQGFPDPALYPD-
IAEA  122
            DE+ RI   ++AL      +SID+ +      AL   G   +ND+ G    A  P
IA+
Sbjct  71
DELARITGAVNALIPAGAVLSIDTTRASVAAAALDGGAQIINDVSGGTLDANLPHVIADH  130

Query  123  DCRLVVMHSAQRDGIATRTG-----------
HLRPEDALDEIVRFFEARVSALRRSGVAA  171
            DC  +V H   R  +A   G           H    D  DE++R    +V  +
+GV
Sbjct  131  DCLYIVQH--WRGWLAGSKGANPDQDTSVYEHGVLTDVHDELMR----
QVDGVLAAGVKP  184

Query  172  DRLILDPGMGFFLSPAPETSLHVLSNLQKLKSALGLPLLVSVSRKSFLGA--
TVGLPVKD  229
            +R+I+DPG+GF   P+ E +L +L+ L+  +   G P+L+  SRK F+ A  T
V +
Sbjct  185  ERIIIDPGLGFS-KPSIEHNLPLLTGLETFRGT-
GYPVLIGQSRKRFISAMLTEAGAVGE  242

Query  230  LGPA-----SLAAELHAIG--NGADYVRTHAPGDLRSAITFSETLAKFRS  272
             GP       + A L A+   +GA  VR H      +A+       ++ S
Sbjct  243  DGPTMAQRDDVTAALSALSAEHGAWAVRVHDVAKSHAAVIAGNIWRQYLS  292

>WP_055996483.1 dihydropteroate synthase [Devosia sp. Root413D1]
 KQW81595.1 dihydropteroate synthase [Devosia sp. Root413D1]
Length=281

 Score = 114 bits (286),  Expect = 6e-29, Method: Compositional
matrix adjust.
 Identities = 87/261 (33%), Positives = 136/261 (52%), Gaps = 14/261
(5%)

Query  8
VFGILNLTEDSFFDESRRLDPAGAVTAAIEMLRVGSDVVDVGPAASHPDARPVSPADEIR  67
            + GILN+T DSF D  R      A+  A  ML  G+D++D+G  ++ P A  VS
ADE+
Sbjct  24
LMGILNVTPDSFSDGGRFDAAPAALAQARLMLAEGADIIDIGGESTRPGAEEVSVADELT  83

Query  68   RIAPLLDAL-SDQMHR-VSIDSFQPETQRYALKRGVGYLNDIQGFP-
DPALYPDIAEADC  124
            R+ P++ AL +D +   +SID+++ E    A+  G   +ND+ G   +P +
AE

```
Sbjct  84
RVLPVIAALRADGITAPISIDTYKAEVAEQAIAAGATIINDVHGLQREPEVAAVAAEHGT  143

Query  125
RLVVMHSAQRDGIATRTGHLRPEDALDEIVRFFEARVSALRRSGVAADRLILDPGMGFFL  184
             LVVMH  +    A  TG     D + E+ R+F+  +    ++GV  DR++LDPG
GF
Sbjct  144  PLVVMHWDK----ARDTGR----
DVMAEMARYFDVTLDIADKAGVTRDRIVLDPGFGFAK  195

Query  185
SPAPETSLHVLSNLQKLKSALGLPLLVSVSRKSFLGATVGLPVKDLGPASLAAELHAIGN  244
            S +   +  +L  L +L  A+G P+LV  SRKS +G  + +P  +    ++A  +
Sbjct  196  SLS--ENYEILRRLPEL-
VAMGFPVLVGTSRKSMIGKLLDMPPNERLAGTIATSVLGYTA  252

Query  245  GADYVRTHAPGDLRSAITFSE  265
            GA   R H     R A+  +E
Sbjct  253  GAHIFRVHDIRPNRDALRVAE  273


>WP_025301383.1 dihydropteroate synthase [Bifidobacterium breve]
 AHJ17932.1 Dihydropteroate synthase [Bifidobacterium breve JCM
7017]
 KOA56795.1 dihydropteroate synthase [Bifidobacterium breve MCC
1454]
 KOA60024.1 dihydropteroate synthase [Bifidobacterium breve MCC
0305]
 AUD81693.1 Dihydropteroate synthase [Bifidobacterium breve]
 AUD83619.1 Dihydropteroate synthase [Bifidobacterium breve]
 AUD85597.1 Dihydropteroate synthase [Bifidobacterium breve]
 POO09024.1 Dihydropteroate synthase [Bifidobacterium breve]
Length=292

 Score = 114 bits (286),  Expect = 7e-29, Method: Compositional
matrix adjust.
 Identities = 93/290 (32%), Positives = 141/290 (49%), Gaps = 29/290
(10%)

Query  4
SRVTVFGILNLTEDSFFDESRRLDPAGAVTAAIEMLRVGSDVVDVGPAASHPDARPVSPA  63
            S   V G+LN+TEDSF D    LDPA A     +M+  G+D++D+G  ++ P A+
VS
Sbjct  11
SHTMVMGVLNITEDSFSDGGLWLDPAKAAQHGRDMMAAGADIIDIGAESTRPGAKRVSEE  70

Query  64   DEIRRIAPLLDALSDQMHRVSIDSFQPETQRYALKRGVGYLNDIQGFPDPALYPD-
IAEA  122
            DE+ RI   ++AL      +SID+ +      AL  G   +ND+ G    A  P
IA+
Sbjct  71
DELARITGAVNALIPAGAVLSIDTTRASVAAAALDGGAQIINDVSGGTLDANLPHVIADH  130

Query  123  DCRLVVMHSAQRDGIATRTG-----------
```

```
HLRPEDALDEIVRFFEARVSALRRSGVAA  171
            DC  +V H   R  +A   G           H    D  DE++R    +V  +
+GV
Sbjct  131  DCLYIVQH--WRGWLAGSKGANPDQDTSVYEHGVLTDVHDELMR----
QVDGVLAAGVKP  184

Query  172  DRLILDPGMGFFLSPAPETSLHVLSNLQKLKSALGLPLLVSVSRKSFLGA--
TVGLPVKD  229
            +R+I+DPG+GF   P+ E +L +L+ L+  +   G P+L+  SRK F+ A  T
V +
Sbjct  185  ERIIIDPGLGFS-KPSIEHNLPLLTGLETFRGT-
GYPVLIGQSRKRFISAMLTEAGAVGE  242

Query  230  LGPA-----SLAAELHAIG--NGADYVRTHAPGDLRSAITFSETLAKFRS  272
             GP       + A L A+   +GA  VR H      +A+       ++ S
Sbjct  243  DGPTMAQRDDVTAALSALSAEHGAWAVRVHDVAKSHAAVIAGNIWRQYLS  292


>WP_100510016.1 dihydropteroate synthase [Bifidobacterium sp. TRE 1]
 PJM73870.1 dihydropteroate synthase [Bifidobacterium sp. TRE 1]
Length=291

 Score = 114 bits (286),  Expect = 7e-29, Method: Compositional
matrix adjust.
 Identities = 83/232 (36%), Positives = 121/232 (52%), Gaps = 22/232
(9%)

Query  4
SRVTVFGILNLTEDSFFDESRRLDPAGAVTAAIEMLRVGSDVVDVGPAASHPDARPVSPA  63
            SR  V G+LN+TEDSF D    LDPA A     +M+  G+D++D+G  ++ P A+
VS
Sbjct  11
SRTLVMGVLNITEDSFSDGGLWLDPAKARRHGADMMADGADIIDIGAESTRPGAKRVSEE  70

Query  64   DEIRRIAPLLDALSDQMHRV-SIDSFQPETQRYALKRGVGYLNDIQGFP-
DPALYPDIAE  121
            DE+ RI   + AL D    V SID+ +      AL  G   +ND+ G   D  L
+A+
Sbjct  71
DELTRITGAVTALIDHPGAVLSIDTTRASVAAAALDEGAQIINDVSGGRLDKELPHVVAD  130

Query  122  ADCRLVVMHSAQRDGIATRTGHLRPE------------
DALDEIVRFFEARVSALRRSGV  169
             DC  +V H   R  +A   G   P+             D  DE++R  +  ++A
GV
Sbjct  131  HDCLYIVQH--
WRGWLAGSHGGAAPDADTSHYEHGVLNDVRDELMRQVDDVIAA----GV  184

Query  170  AADRLILDPGMGFFLSPAPETSLHVLSNLQKLKSALGLPLLVSVSRKSFLGA
221
              +R+++DPG+GF   P  E +L +L+ L   K A G P+L+  SRK F+GA
Sbjct  185  RPERIVIDPGLGFS-KPGIEHNLPLLAGLAAFK-ATGYPVLIGQSRKRFIGA
234
```

```
>AUD91812.1 Dihydropteroate synthase [Bifidobacterium breve]
 AUE19244.1 Dihydropteroate synthase [Bifidobacterium breve]
Length=291

 Score = 114 bits (286),  Expect = 9e-29, Method: Compositional
matrix adjust.
 Identities = 91/288 (32%), Positives = 142/288 (49%), Gaps = 29/288
(10%)

Query  4
SRVTVFGILNLTEDSFFDESRRLDPAGAVTAAIEMLRVGSDVVDVGPAASHPDARPVSPA  63
           S   V G+LN+TEDSF D    LDPA A     +M+  G+D++D+G  ++ P A+
VS
Sbjct  11
SHTMVMGVLNITEDSFSDGGLWLDPAKAAQHGRDMMAAGADIIDIGAESTRPGAKRVSEE  70

Query  64   DEIRRIAPLLDALSDQMHRVSIDSFQPETQRYALKRGVGYLNDIQGFPDPALYPD-
IAEA  122
            DE+ RI   ++AL      +SID+ +      AL  G   +ND+ G    A  P
+A+
Sbjct  71
DELARITGAVNALIPAGAVLSIDTTRASVAAAALDGGAQIINDVSGGTLDANLPHVVADH  130

Query  123  DCRLVVMHSAQRDGIATRTG-----------
HLRPEDALDEIVRFFEARVSALRRSGVAA  171
            DC  +V H   R  +A   G           H    D  DE++R    +V  +
+GV
Sbjct  131  DCLYIVQH--WRGWLAGSKGANPDQDTSVYEHGVLTDVHDELMR----
QVDDVLAAGVKP  184

Query  172  DRLILDPGMGFFLSPAPETSLHVLSNLQKLKSALGLPLLVSVSRKSFLGATVGL--
PVKD  229
            +R+++DPG+GF   P+ E +L +L+ L+  + A G P+L+  SRK F+ A +
+
Sbjct  185  ERVVIDPGLGFS-KPSIEHNLPLLTGLETFR-
ATGYPVLIGQSRKRFISAMLAEAGASGE  242

Query  230  LGPA-----SLAAELHAIG--NGADYVRTHAPGDLRSAITFSETLAKF  270
             GP       + A L A+   +GA  VR H     R+A+    T  ++
Sbjct  243  NGPTMDQRDDVTAALSALSAEHGAWAVRVHDVAKSRAAVIAGNTWRQY  290


>WP_085461798.1 dihydropteroate synthase [Bifidobacterium
adolescentis]
 OSG90301.1 dihydropteroate synthase [Bifidobacterium adolescentis]
Length=292

 Score = 114 bits (286),  Expect = 9e-29, Method: Compositional
matrix adjust.
 Identities = 86/280 (31%), Positives = 135/280 (48%), Gaps = 23/280
(8%)

Query  8
```

```
VFGILNLTEDSFFDESRRLDPAGAVTAAIEMLRVGSDVVDVGPAASHPDARPVSPADEIR  67
            V G+LN+TEDSF D    L P  A      M++ G+D++D+G  ++ P A+ VS
ADE
Sbjct  16
VMGVLNITEDSFSDGGLWLAPEAAKAHGEAMMKAGADIIDIGAESTRPGAKRVSEADEKA  75

Query  68   RIAPLLDALSDQMHRVSIDSFQPETQRYALKRGVGYLNDIQGFPDPALYPDIA--
EADCR  125
            R+   +DAL  +   +SID+ +      AL+ G   +ND+ G       P +
+DC
Sbjct  76
RVLGAVDALIPEGAVLSIDTTRASVALAALEHGAQIINDVSGGQLDRELPHVVADHSDCL  135

Query  126  LVVMHSAQR---------
DGIATRTGHLRPEDALDEIVRFFEARVSALRRSGVAADRLIL  176
             +V H             D   +R  H    D  DE++R    +V A+  +GV A+
++I+
Sbjct  136  YIVQHWRGWLAGAAGNVPDADTSRYEHGVVNDVHDELMR----
QVDAVLEAGVQAEQVII  191

Query  177  DPGMGFFLSPAPETSLHVLSNLQKLKSALGLPLLVSVSRKSFLG---
ATVGLPVKDLGP-  232
            DPG+GF   P  E +L +L+ L +  +A G P+L+  SRK F+G   A  G+   D
+
Sbjct  192  DPGLGFS-KPGVEHNLPILAALDRFNAA-
GYPVLIGASRKRFVGSLLAGAGVTEPDMASK  249

Query  233  --ASLAAELHAIGNGADYVRTHAPGDLRSAITFSETLAKF  270
              A+ A       +G   VR H     R A+       ++
Sbjct  250  DNATAAISALCAEHGVWAVRVHDVAKSRDAVAVGNAWREY  289


>WP_065472186.1 dihydropteroate synthase [Bifidobacterium longum]
Length=291

 Score = 114 bits (285),  Expect = 9e-29, Method: Compositional
matrix adjust.
 Identities = 93/289 (32%), Positives = 140/289 (48%), Gaps = 31/289
(11%)

Query  4
SRVTVFGILNLTEDSFFDESRRLDPAGAVTAAIEMLRVGSDVVDVGPAASHPDARPVSPA  63
            S   V G+LN+TEDS  D    LDPA A     +M+  G+D++D+G  ++ P A+
VS A
Sbjct  11
SHTMVMGVLNITEDSVSDGGLWLDPAKAAQHGRDMMAAGADIIDIGAESTRPGAKRVSEA  70

Query  64   DEIRRIAPLLDALSDQMHRVSIDSFQPETQRYALKRGVGYLNDIQGFPDPALYPD-
IAEA  122
            DE+ RI   +  L      +SID+ +      AL  G   +ND+ G    A  P
+A+
Sbjct  71
DELARITGAVKTLISAGAILSIDTTRASVAAAALSEGAQIINDVSGGTLDAELPHVVADH  130
```

```
Query  123  DCRLVVMHSAQRDGIATRTG-----------
HLRPEDALDEIVRFFEARVSALRRSGVAA  171
            DC  +V H   R  +A   G           H    D  DE++R    +V  +
+GV
Sbjct  131  DCLYIVQH--WRGWLAGSKGANPDQDTSVYEHGVLTDVHDELMR----
QVDGVLAAGVKP  184

Query  172  DRLILDPGMGFFLSPAPETSLHVLSNLQKLKSALGLPLLVSVSRKSFLGATV---
GLPVK  228
            +R+I+DPG+GF   P  E +L +L+ L+  + A G P+L+  SRK F+ A +   G
Sbjct  185  ERIIIDPGLGFS-KPGIEHNLPLLTGLETFR-
ATGYPVLIGQSRKRFISAMLTEAGAAGA  242

Query  229  DLGPA-----SLAAELHAIG--NGADYVRTHAPGDLRSAITFSETLAKF  270
            D GP       + A L A+   +GA  VR H     R+A+    T  ++
Sbjct  243  D-GPIMAQRDDVTAALSALSAEHGAWAVRVHDVAKSRAAVIAGNTWREY  290


>CZQ85951.1 dihydropteroate synthase signature 2 [Trichococcus
ilyis]
 SEJ69782.1 dihydropteroate synthase [Trichococcus sp. DSM 22150]
Length=277

 Score = 114 bits (284),  Expect = 9e-29, Method: Compositional
matrix adjust.
 Identities = 84/256 (33%), Positives = 133/256 (52%), Gaps = 19/256
(7%)

Query  2
LRSRVTVFGILNLTEDSFFDESRRLDPAGAVTAAIEMLRVGSDVVDVGPAASHPDARPVS  61
            L  R  + GILN T DSF D         AV  A+EM+  G+D++D+G  ++ P
+S
Sbjct  12
LSQRSYIMGILNATPDSFSDGGEWNTAEKAVAHALEMVADGADIIDIGGESTRPGHVQIS  71

Query  62   PADEIRRIAPLLDALSDQMHR-VSIDSFQPETQRYALKRGVGYLNDIQGFP-
DPALYPDI  119
              +EI R+ P++ A+   +   +SID+++    R A + G+  +NDI G   DP +
Sbjct  72
DEEEIARVVPIIKAVKAAVDVPLSIDTYKSAVARAACEAGIDIINDIWGCKYDPEIAVVA  131

Query  120
AEADCRLVVMHSAQRDGIATRTGHLRPEDALDEIVRFFEARVSALRRSGVAADRLILDPG  179
            A  +  +++MH+ ++   A        ED L+++        +A    GV  +
+ILDPG
Sbjct  132  AAFEVPIILMHNREKADYA-----FLIEDMLEDLAESVRIATAA----
GVKRENIILDPG  182

Query  180  MGFFLSPAPETSLHVLSNLQKLKSALGLPLLVSVSRKSFLGATVG-LPVK--
DLGPASLA  236
             GF  +   E +L+V+  L++  + LG PLL+  SRK F+G  +G +P K  DLG
A+
Sbjct  183  CGF--AKTYEDNLNVVHQLKRF-
ADLGYPLLLGTSRKRFIGTALGDIPFKERDLGTAATT  239
```

```
Query  237  AELHAIGNGADYVRTH  252
            A    I NGA   R H
Sbjct  240  A--LGIVNGAQLFRVH  253


>WP_100496241.1 dihydropteroate synthase [Bifidobacterium sp. TRE D]
 PJM79384.1 dihydropteroate synthase [Bifidobacterium sp. TRE D]
Length=290

 Score = 114 bits (285),  Expect = 9e-29, Method: Compositional
matrix adjust.
 Identities = 87/283 (31%), Positives = 140/283 (49%), Gaps = 28/283
(10%)

Query  8
VFGILNLTEDSFFDESRRLDPAGAVTAAIEMLRVGSDVVDVGPAASHPDARPVSPADEIR  67
            V G+LN+T DSF D    LDPA A     +M+  G+D++D+G  ++ P A+ VS
DE+
Sbjct  15
VMGVLNITADSFSDGGLWLDPAKAAQHGRDMMAAGADIIDIGAESTRPGAKRVSETDELT  74

Query  68   RIAPLLDALSDQMHRVSIDSFQPETQRYALKRGVGYLNDIQGFPDPALYPD-
IAEADCRL  126
            RI   ++ L      +SID+ +      AL+RG   +ND+ G    A  P  +A+
DC
Sbjct  75
RITGAVETLLPAGATLSIDTTRASVAAAALERGAQIINDVSGGTLDAELPHVVADYDCLY  134

Query  127  VVMHSAQRDGIATRTGHLRPEDA-------
LDEIVRFFEARVSALRRSGVAADRLILDPG  179
            +V H   R  +A   G    +D        L ++      +V  +  +GV  +R+I
+DPG
Sbjct  135  IVQH--
WRGWLAGSKGATPDQDTSVYEHGVLADVRDELMHQVDGVLAAGVKPERIIIDPG  192

Query  180  MGFFLSPAPETSLHVLSNLQKLKSALGLPLLVSVSRKSFLGATV---
GLPVKDL------  230
            +GF   P  E +L +L+ L+  + A G P+L+  SRK F+ A +   G+   D+
Sbjct  193  LGFS-KPGIEHNLPLLAGLETFR-
ATGYPVLIGQSRKRFISAMLTAGGVADADMADRDNV  250

Query  231  --GPASLAAELHAIGNGADYVRTHAPGDLRSAITFSETLAKFR  271
              G ++L AE     +GA  VR H     R+A+       +++
Sbjct  251  TAGLSALCAE-----HGAWAVRVHDVAKSRAAVIAGNAWRRYQ  288


>WP_015439115.1 dihydropteroate synthase [Bifidobacterium breve]
 ABE96165.1 Dihydropteroate synthase [Bifidobacterium breve UCC2003]
Length=291

 Score = 114 bits (285),  Expect = 9e-29, Method: Compositional
matrix adjust.
 Identities = 91/270 (34%), Positives = 135/270 (50%), Gaps = 29/270
```

```
(11%)

Query  4
SRVTVFGILNLTEDSFFDESRRLDPAGAVTAAIEMLRVGSDVVDVGPAASHPDARPVSPA  63
            S   V G+LN+TEDSF D    LDPA A     +M+  G+D++D+G  ++ P A+
VS
Sbjct  11
SHTMVMGVLNITEDSFSDGGLWLDPAKAAQHGRDMMAAGADIIDIGAESTRPGAKRVSEE  70

Query  64   DEIRRIAPLLDALSDQMHRVSIDSFQPETQRYALKRGVGYLNDIQGFPDPALYPD-
IAEA  122
            DE+ RI   ++AL      +SID+ +      AL   G   +ND+ G    A  P
IA+
Sbjct  71
DELARITGAVNALIPAGAVLSIDTTRASVAAAALDGGAQIINDVSGGTLDANLPHVIADH  130

Query  123  DCRLVVMHSAQRDGIATRTG-----------
HLRPEDALDEIVRFFEARVSALRRSGVAA  171
            DC  +V H   R  +A   G           H    D  DE++R    +V  +
+GV
Sbjct  131  DCLYIVQH--WRGWLAGSKGANPDQDTSVYEHGVLTDVHDELMR----
QVDGVLAAGVKP  184

Query  172  DRLILDPGMGFFLSPAPETSLHVLSNLQKLKSALGLPLLVSVSRKSFLGA--
TVGLPVKD  229
            +R+I+DPG+GF   P+ E +L +L+ L+  +   G P+L+  SRK F+ A  T
V +
Sbjct  185  ERIIIDPGLGFS-KPSIEHNLPLLTGLETFRGT-
GYPVLIGQSRKRFISAMLTEAGAVGE  242

Query  230  LGPA-----SLAAELHAIG--NGADYVRTH  252
             GP       + A L A+   +GA  VR H
Sbjct  243  DGPTMAQRDDVTAALSALSAEHGAWAVRVH  272


>WP_029679339.1 dihydropteroate synthase [Bifidobacterium longum]
Length=291

 Score = 114 bits (285),  Expect = 1e-28, Method: Compositional
matrix adjust.
 Identities = 93/289 (32%), Positives = 141/289 (49%), Gaps = 31/289
(11%)

Query  4
SRVTVFGILNLTEDSFFDESRRLDPAGAVTAAIEMLRVGSDVVDVGPAASHPDARPVSPA  63
            +   V G+LN+TEDSF D    LDPA A     +M+  G+D++D+G  ++ P A+
VS A
Sbjct  11
THTMVMGVLNITEDSFSDGGLWLDPAKAAQHGRDMMAAGADIIDIGAESTRPGAKRVSEA  70

Query  64   DEIRRIAPLLDALSDQMHRVSIDSFQPETQRYALKRGVGYLNDIQGFPDPALYPD-
IAEA  122
            DE+ RI   +  L      +SID+ +      AL   G   +ND+ G    A  P
+A+
```

Sbjct  71
DELVRITGAVKTLIPAGAILSIDTTRASVAAAALSEGAQIINDVSGGTLDAELPHVVADH  130

Query  123  DCRLVVMHSAQRDGIATRTG-----------
HLRPEDALDEIVRFFEARVSALRRSGVAA  171
            DC  +V H   R  +A   G           H    D  DE++R    +V  +
+GV
Sbjct  131  DCLYIVQH--WRGWLAGSKGANPDQDTSVYEHGVLTDVHDELMR----
QVDGVLAAGVKP  184

Query  172  DRLILDPGMGFFLSPAPETSLHVLSNLQKLKSALGLPLLVSVSRKSFLGATV---
GLPVK  228
            +R+I+DPG+GF   P  E +L +L+ L+  + A G P+L+  SRK F+ A +   G
Sbjct  185  ERIIIDPGLGFS-KPGIEHNLPLLTGLETFR-
ATGYPVLIGQSRKRFISAMLTEAGAAGA  242

Query  229  DLGPA-----SLAAELHAIG--NGADYVRTHAPGDLRSAITFSETLAKF  270
            D GP       + A L A+   +GA  VR H     R+A+    T  ++
Sbjct  243  D-GPIMAQRDDVTAVLSALSAEHGAWAVRVHDVAKSRAAVIAGNTWREY  290

>WP_021649768.1 dihydropteroate synthase [Bifidobacterium breve]
 ERI87299.1 dihydropteroate synthase [Bifidobacterium breve JCP7499]
 EWH39267.1 Dihydropteroate synthase [Bifidobacterium breve 31L]
 AHJ23522.1 Dihydropteroate synthase [Bifidobacterium breve 689b]
 AHJ25292.1 Dihydropteroate synthase [Bifidobacterium breve S27]
 KOA39427.1 dihydropteroate synthase [Bifidobacterium breve MCC
1094]
 KOA45225.1 dihydropteroate synthase [Bifidobacterium breve MCC
0121]
 KOA53197.1 dihydropteroate synthase [Bifidobacterium breve MCC
1340]
 KOA65512.1 dihydropteroate synthase [Bifidobacterium breve MCC
1114]
 KWZ84373.1 dihydropteroate synthase [Bifidobacterium breve]
 SDK09910.1 Dihydropteroate synthase [Bifidobacterium breve]
 PAC75530.1 dihydropteroate synthase [Bifidobacterium breve]
 AUD99757.1 Dihydropteroate synthase [Bifidobacterium breve]
 AUE01676.1 Dihydropteroate synthase [Bifidobacterium breve]
Length=292

 Score = 114 bits (285),  Expect = 1e-28, Method: Compositional
matrix adjust.
 Identities = 92/290 (32%), Positives = 141/290 (49%), Gaps = 29/290
(10%)

Query  4
SRVTVFGILNLTEDSFFDESRRLDPAGAVTAAIEMLRVGSDVVDVGPAASHPDARPVSPA  63
            S   V G+LN+TEDSF D    LDPA A     +M+  G+D++D+G  ++ P A+
VS
Sbjct  11
SHTMVMGVLNITEDSFSDGGLWLDPAKAAQHGRDMMAAGADIIDIGAESTRPGAKRVSEE  70

Query  64   DEIRRIAPLLDALSDQMHRVSIDSFQPETQRYALKRGVGYLNDIQGFPDPALYPD-

```
IAEA  122
            DE+ RI   ++AL      +SID+ +      AL  G   +ND+ G    A  P
+A+
Sbjct  71
DELARITGAVNALIPAGAVLSIDTTRASVAAAALDGGAQIINDVSGGTLDANLPHVVADH  130

Query  123  DCRLVVMHSAQRDGIATRTG-----------
HLRPEDALDEIVRFFEARVSALRRSGVAA  171
            DC  +V H   R  +A   G           H    D  DE++R    +V  +
+GV
Sbjct  131  DCLYIVQH--WRGWLAGSKGANPDQDTSVYEHGVLTDVHDELMR----
QVDGVLAAGVKP  184

Query  172  DRLILDPGMGFFLSPAPETSLHVLSNLQKLKSALGLPLLVSVSRKSFLGA--
TVGLPVKD  229
            +R+I+DPG+GF   P+ E +L +L+ L+  +   G P+L+  SRK F+ A  T
V +
Sbjct  185  ERIIIDPGLGFS-KPSIEHNLPLLTGLETFRGT-
GYPVLIGQSRKRFISAMLTEAGAVGE  242

Query  230  LGPA-----SLAAELHAIG--NGADYVRTHAPGDLRSAITFSETLAKFRS  272
             GP       + A L A+   +GA  VR H      +A+       ++ S
Sbjct  243  DGPTMAQRDDVTAALSALSAEHGAWAVRVHDVAKSHAAVIAGNIWRQYLS  292


>WP_080784119.1 dihydropteroate synthase [Bifidobacterium breve]
 OQM47684.1 dihydropteroate synthase [Bifidobacterium breve]
Length=292

 Score = 114 bits (285),  Expect = 1e-28, Method: Compositional
matrix adjust.
 Identities = 92/290 (32%), Positives = 141/290 (49%), Gaps = 29/290
(10%)

Query  4
SRVTVFGILNLTEDSFFDESRRLDPAGAVTAAIEMLRVGSDVVDVGPAASHPDARPVSPA  63
            S   V G+LN+TEDSF D    LDPA A     +M+  G+D++D+G  ++ P A+
VS
Sbjct  11
SHTMVMGVLNITEDSFSDGGLWLDPAKAAQHGRDMMAAGADIIDIGAESTRPGAKRVSEE  70

Query  64   DEIRRIAPLLDALSDQMHRVSIDSFQPETQRYALKRGVGYLNDIQGFPDPALYPD-
IAEA  122
            DE+ RI   ++AL      +SID+ +      AL  G   +ND+ G    A  P
+A+
Sbjct  71
DELARITGAVNALIPAGAVLSIDTTRASVAAAALDGGAQIINDVSGGTLDANLPHVVADH  130

Query  123  DCRLVVMHSAQRDGIATRTG-----------
HLRPEDALDEIVRFFEARVSALRRSGVAA  171
            DC  +V H   R  +A   G           H    D  DE++R    +V  +
+GV
Sbjct  131  DCLYIVQH--WRGWLAGSKGANPDQDTSIYEHGVLTDVHDELMR----
QVDGVLAAGVKP  184
```

```
Query  172  DRLILDPGMGFFLSPAPETSLHVLSNLQKLKSALGLPLLVSVSRKSFLGA--
TVGLPVKD  229
            +R+I+DPG+GF   P+ E +L +L+ L+  +   G P+L+  SRK F+ A  T
V +
Sbjct  185  ERIIIDPGLGFS-KPSIEHNLPLLTGLETFRGT-
GYPVLIGQSRKRFISAMLTEAGAVGE  242

Query  230  LGPA-----SLAAELHAIG--NGADYVRTHAPGDLRSAITFSETLAKFRS  272
             GP       + A L A+   +GA  VR H      +A+       ++ S
Sbjct  243  DGPTMAQRDDVTAALSALSAEHGAWAVRVHDVAKSHAAVIAGNIWRQYLS  292


>WP_077424703.1 dihydropteroate synthase [Bifidobacterium longum]
Length=291

 Score = 114 bits (285),  Expect = 1e-28, Method: Compositional
matrix adjust.
 Identities = 92/289 (32%), Positives = 141/289 (49%), Gaps = 31/289
(11%)

Query  4
SRVTVFGILNLTEDSFFDESRRLDPAGAVTAAIEMLRVGSDVVDVGPAASHPDARPVSPA  63
            +   V G+LN+TEDSF D    LDPA A     +M+  G+D++D+G  ++ P A+
V+ A
Sbjct  11
THTMVMGVLNITEDSFSDGGLWLDPAKAAQHGRDMMAAGADIIDIGAESTRPGAKRVAEA  70

Query  64   DEIRRIAPLLDALSDQMHRVSIDSFQPETQRYALKRGVGYLNDIQGFPDPALYPD-
IAEA  122
            DE+ RI   +  L      +SID+ +      AL  G   +ND+ G    A  P
+A+
Sbjct  71
DELARITGAVKTLIPAGAILSIDTTRASVAAAALSEGAQIINDVSGGTLDAELPHVVADH  130

Query  123  DCRLVVMHSAQRDGIATRTG-----------
HLRPEDALDEIVRFFEARVSALRRSGVAA  171
            DC  +V H   R  +A   G           H    D  DE++R    +V  +
+GV
Sbjct  131  DCLYIVQH--WRGWLAGSKGANPDQDTSVYEHGVLTDVHDELMR----
QVDGVLAAGVKP  184

Query  172  DRLILDPGMGFFLSPAPETSLHVLSNLQKLKSALGLPLLVSVSRKSFLGATV---
GLPVK  228
            +R+I+DPG+GF   P  E +L +L+ L+  + A G P+L+  SRK F+ A +   G
Sbjct  185  ERIIIDPGLGFS-KPGIEHNLPLLTGLETFR-
ATGYPVLIGQSRKRFISAMLTEAGAAGA  242

Query  229  DLGPA-----SLAAELHAIG--NGADYVRTHAPGDLRSAITFSETLAKF  270
            D GP       + A L A+   +GA  VR H     R+A+    T  ++
Sbjct  243  D-GPIMAQRDDVTAALSALSAEHGAWAVRVHDVAKSRAAVIAGNTWREY  290


>WP_094667142.1 dihydropteroate synthase [Bifidobacterium myosotis]
```

OZG60824.1 dihydropteroate synthase [Bifidobacterium myosotis]
Length=294

Score = 114 bits (284), Expect = 1e-28, Method: Compositional matrix adjust.
Identities = 91/288 (32%), Positives = 138/288 (48%), Gaps = 36/288 (13%)

```
Query  8
VFGILNLTEDSFFDESRRLDPAGAVTAAIEMLRVGSDVVDVGPAASHPDARPVSPADEIR  67
            V G+LN+TEDSF D    LDP  A     +M+  G+D++D+G  ++ P A+ VS
DE R
Sbjct  17
VMGVLNITEDSFSDGGLWLDPDKAAQHGRDMMAAGADIIDIGAESTRPGAKRVSEEDEHR  76

Query  68   RIAPLLDALSDQMHRVSIDSFQPETQRYALKRGVGYLNDIQGFPDPALYPD-
IAEADCRL  126
            RI   ++AL      +SID+ +      AL+RG   +ND+ G    A  P  +A+
DC
Sbjct  77
RITGAVEALIPAGAVLSIDTTRASVAAAALERGAQIINDVSGGTLDAELPHVVADHDCLY  136

Query  127  VVMHSAQRDGIATRTG-----------
HLRPEDALDEIVRFFEARVSALRRSGVAADRLI  175
            +V H   R  +A   G           H    D  DE++R    +V  +  +GV
+R+I
Sbjct  137  IVQH--WRGWLAGSKGANPDQDTSVYEHGVLRDVHDELMR----
QVDGVLAAGVRPERII  190

Query  176
LDPGMGFFLSPAPETSLHVLSNLQKLKSALGLPLLVSVSRKSFLGATVGLPVKD------  229
            +DPG+GF   P    +L +L+ L+  + A G P+L+  SRK F+ A +     D
Sbjct  191  IDPGLGFS-KPGIGHNLPLLTGLETFR-
ATGYPVLIGQSRKRFISAMLAEAGVDEPTMAD  248

Query  230  -----LGPASLAAELHAIGNGADYVRTHAPGDLRSAITFSETLAKFRS  272
                  G +++ AE     +GA  VR H     RSA+         +R
Sbjct  249  RDGVTAGLSAMCAE-----HGAWAVRVHDVAASRSAVIAGNVWRAYRQ  291
```

>WP_070372479.1 dihydropteroate synthase [Acetobacterium wieringae]
OFV69371.1 dihydropteroate synthase [Acetobacterium wieringae]
Length=275

Score = 113 bits (283), Expect = 1e-28, Method: Compositional matrix adjust.
Identities = 78/254 (31%), Positives = 134/254 (53%), Gaps = 16/254 (6%)

```
Query  2
LRSRVTVFGILNLTEDSFFDESRRLDPAGAVTAAIEMLRVGSDVVDVGPAASHPDARPVS  61
            L ++V + GILN+T DSF D  +  +   ++    +M+  G+D++D+G  ++ P
+S
Sbjct  10
```

```
LSNQVLIMGILNVTPDSFSDGGKFNNLDASLRQVEKMILDGADIIDLGGESTRPGHTKIS  69

Query  62   PADEIRRIAPLLDALSDQMHR-VSIDSFQPETQRYALKRGVGYLNDIQGFP-
DPALYPDI  119
             A+EI R+ P+++A++ +    +SID+++      AL  G   +NDI GF  DP L
Sbjct  70   
DAEEIERVVPMIEAINQRFDIPISIDTYKGPVGAAALAAGADMVNDIWGFKYDPTLADIT  129

Query  120  
AEADCRLVVMHSAQRDGIATRTGHLRPEDALDEIVRFFEARVSALRRSGVAADRLILDPG  179
            A+     V+MH+        RT      + + +I    +  ++    +G++ D 
+ILDPG
Sbjct  130  AQYQVPCVLMHN--------RTNQ-
NYHNLMTDINSDLQESINIALSAGISRDAIILDPG  180

Query  180  MGFFLSPAPET-
SLHVLSNLQKLKSALGLPLLVSVSRKSFLGATVGLPVKDLGPASLAAE  238
            +GF    A    ++H L +LQ     LG P+L+  SRK F+G T+ LPV +    +
+A
Sbjct  181  IGFAKDYAQNMETMHHLEDLQ----
TLGYPVLLGTSRKGFIGLTLDLPVTERVEGTVATT  236

Query  239  LHAIGNGADYVRTH  252
            +  I  GA  +R H
Sbjct  237  VIGIMKGASIIRVH  250


>WP_070122223.1 dihydropteroate synthase [Bifidobacterium 
adolescentis]
 OFA35872.1 dihydropteroate synthase [Bifidobacterium adolescentis]
 OSG88808.1 Dihydropteroate synthase [Bifidobacterium adolescentis]
 OSG97856.1 Dihydropteroate synthase [Bifidobacterium adolescentis]
Length=292

 Score = 114 bits (284),  Expect = 1e-28, Method: Compositional 
matrix adjust.
 Identities = 86/280 (31%), Positives = 135/280 (48%), Gaps = 23/280 
(8%)

Query  8    
VFGILNLTEDSFFDESRRLDPAGAVTAAIEMLRVGSDVVDVGPAASHPDARPVSPADEIR  67
            V G+LN+TEDSF D    L P  A      M++ G+D++D+G  ++ P A+ VS 
ADE
Sbjct  16   
VMGVLNITEDSFSDGGLWLAPEAAKAHGEAMMKAGADIIDIGAESTRPGAKRVSEADEKA  75

Query  68   RIAPLLDALSDQMHRVSIDSFQPETQRYALKRGVGYLNDIQGFPDPALYPDIA--
EADCR  125
            R+   +DAL  +   +SID+ +      AL+ G   +ND+ G       P + 
+DC
Sbjct  76   
RVLGAVDALIPEGAVLSIDTTRASVALAALEHGAQIINDVSGGQLDRELPHVVADHSDCL  135

Query  126  LVVMHSAQR---------
```

DGIATRTGHLRPEDALDEIVRFFEARVSALRRSGVAADRLIL  176
             +V H             D   +R  H    D  DE++R    +V A+  +GV A+
++I+
Sbjct  136  YIVQHWRGWLAGAAGNVPDADTSRYEHGVVNDVHDELMR----
QVDAVLEAGVRAEQVII  191

Query  177  DPGMGFFLSPAPETSLHVLSNLQKLKSALGLPLLVSVSRKSFLG---
ATVGLPVKDLGP-  232
            DPG+GF   P  E +L +L+ L +  +A G P+L+  SRK F+G   A  G+   D
+
Sbjct  192  DPGLGFS-KPGVEHNLPILAALDRFNAA-
GYPVLIGASRKRFVGSLLAGAGVTEPDMASK  249

Query  233  --ASLAAELHAIGNGADYVRTHAPGDLRSAITFSETLAKF  270
              A+ A       +G   VR H     R A+       ++
Sbjct  250  DNATAAISALCAEHGVWAVRVHDVAKSRDAVAVGNAWREY  289


>WP_094730457.1 dihydropteroate synthase [Bifidobacterium hapali]
 OZG63254.1 dihydropteroate synthase [Bifidobacterium hapali]
Length=288

 Score = 114 bits (284),  Expect = 2e-28, Method: Compositional
matrix adjust.
 Identities = 77/226 (34%), Positives = 118/226 (52%), Gaps = 16/226
(7%)

Query  8
VFGILNLTEDSFFDESRRLDPAGAVTAAIEMLRVGSDVVDVGPAASHPDARPVSPADEIR  67
            V G+LN+TEDSF D    LDPA A    + ML  G+D++D+G  ++ P A+ VS
ADE+
Sbjct  15
VMGVLNITEDSFSDGGLWLDPAKAREHGLAMLNDGADIIDIGAESTRPGAKRVSEADELS  74

Query  68   RIAPLLDALSDQMHRVSIDSFQPETQRYALKRGVGYLNDIQGFP-
DPALYPDIAEADCRL  126
            R+   + AL      +SID+ +      AL+ G   +ND+ G   D  L   +A+
DC
Sbjct  75
RVTGAVKALIGGNTVLSIDTTRALVAAAALEDGAQIINDVSGGTLDKELPHVVADHDCLY  134

Query  127  VVM---------
HSAQRDGIATRTGHLRPEDALDEIVRFFEARVSALRRSGVAADRLILD  177
            +V          H A  D   +   H    D  DE+++    +V A+  +GV  +R
+I+D
Sbjct  135  IVQHWRGWLAGAHGATPDADTSHYEHGVLRDVYDELMQ----
QVDAVLEAGVKPERIIID  190

Query  178  PGMGFFLSPAPETSLHVLSNLQKLKSALGLPLLVSVSRKSFLGATV  223
            PG+GF   P  E +L +L+ L   +   G P+L+  SRK F+ A +
Sbjct  191  PGLGFS-KPGIEHNLPLLAGLSTFEDT-GYPVLIGASRKRFVSAVL  234


>WP_101621332.1 dihydropteroate synthase [Bifidobacterium parmae]

Length=290

 Score = 113 bits (283),  Expect = 2e-28, Method: Compositional matrix adjust.
 Identities = 90/283 (32%), Positives = 142/283 (50%), Gaps = 30/283 (11%)

```
Query  8
VFGILNLTEDSFFDESRRLDPAGAVTAAIEMLRVGSDVVDVGPAASHPDARPVSPADEIR  67
            V G+LN+TEDSF D    LDP  A      M+  G+D++D+G  ++ P A+ VS
DE+
Sbjct  16
VMGVLNITEDSFSDGGLWLDPRAAADHGRAMMAAGADIIDIGAESTRPGAKRVSEEDELT  75

Query  68   RIAPLLDALSDQMHRVSIDSFQPETQRYALKRGVGYLNDIQGFP-
DPALYPDIAEADCRL  126
            RI   ++AL      +SID+ +      AL   G   +ND+ G   D  L   +A+
DC
Sbjct  76
RITGAVEALIPAGATLSIDTTRASVAAAALDGGAQIINDVSGGTLDTELPHVVADHDCLY  135

Query  127  VVMHSAQRDGIA-TRTG----------
HLRPEDALDEIVRFFEARVSALRRSGVAADRLI  175
            +V H   R  +A ++TG          H   +D  DE++R    +V A+  +GVA
+R+I
Sbjct  136  IVQH--WRGWLAGSKTGAPDADTSHYEHGVLQDVHDELMR----
QVDAVLAAGVAPERII  189

Query  176  LDPGMGFFLSPAPETSLHVLSNLQKLKSALGLPLLVSVSRKSFLGAT--------
VGLPV  227
            +DPG+GF   P    +L +L+ L + + A G P+L+  SRK F+ A         +
+
Sbjct  190  IDPGLGFS-KPGIAHNLPLLAGLDRFR-
ATGYPVLIGQSRKRFVTAILDKAGFTDITMDA  247

Query  228  KDLGPASLAAELHAIGNGADYVRTHAPGDLRSAITFSETLAKF  270
            +D   A+ +A      +GA  VR H     R+A+    T  ++
Sbjct  248  RDDATAAFSALCAE--HGAWAVRVHDVARSRAAVAVGNTWREY  288
```

>WP_101626177.1 dihydropteroate synthase [Bifidobacterium imperatoris]
 PLS24383.1 dihydropteroate synthase [Bifidobacterium imperatoris]
Length=290

 Score = 113 bits (283),  Expect = 2e-28, Method: Compositional matrix adjust.
 Identities = 90/290 (31%), Positives = 143/290 (49%), Gaps = 30/290 (10%)

```
Query  4
SRVTVFGILNLTEDSFFDESRRLDPAGAVTAAIEMLRVGSDVVDVGPAASHPDARPVSPA  63
            S   V G+LN+TEDSF D    LDPA A     +M+  G+D++D+G  ++ P A+
VS
```

```
Sbjct  11
SHTLVMGVLNITEDSFSDGGLWLDPAKAAQHGRDMMAAGADIIDIGAESTRPGAKRVSEE  70

Query  64   DEIRRIAPLLDALSDQMHRVSIDSFQPETQRYALKRGVGYLNDIQGFP-
DPALYPDIAEA  122
            DE++RI   ++ L      +SID+ +      AL+ G   +ND+ G   D  L
+A+
Sbjct  71
DELKRITGAVNTLIPAGAVLSIDTTRASVACAALEGGAQIINDVSGGTLDRELPHVVADH  130

Query  123  DCRLVVMHSAQRDGIATRTG-----------
HLRPEDALDEIVRFFEARVSALRRSGVAA  171
            DC  +V H   R  +A   G           H    D  DE++     +V  +
+GV
Sbjct  131  DCLYIVQH--WRGWLAGSKGANPDQDTSVYEHGVLTDVHDELMH----
QVDDVLAAGVKP  184

Query  172
DRLILDPGMGFFLSPAPETSLHVLSNLQKLKSALGLPLLVSVSRKSFLGATVG-------  224
            +R+I+DPG+GF   P  E +L +L+ L+  + A G P+L+  SRK F+ A +
Sbjct  185  ERIIIDPGLGFS-KPGIEHNLPLLAGLETFR-
ATGYPVLIGQSRKRFISAMLAENGIDEP  242

Query  225  -LPVKDLGPASLAAELHAIGNGADYVRTHAPGDLRSAITFSETLAKFRSR  273
             +  +D   A+L+A   +  +GA  VR H     R+A+    T  K+  +
Sbjct  243  TMAQRDDVTAALSAL--SAEHGAWAVRVHDVAKSRAAVIAGNTWRKYSEQ  290


>OXS26090.1 dihydropteroate synthase [Acetobacterium sp. MES1]
Length=275

 Score = 113 bits (282),  Expect = 2e-28, Method: Compositional
matrix adjust.
 Identities = 78/254 (31%), Positives = 134/254 (53%), Gaps = 16/254
(6%)

Query  2
LRSRVTVFGILNLTEDSFFDESRRLDPAGAVTAAIEMLRVGSDVVDVGPAASHPDARPVS  61
            L ++V + GILN+T DSF D  +  +   ++    +M+  G+D++D+G  ++ P
+S
Sbjct  10
LSNQVLIMGILNVTPDSFSDGGKFNNLDASLRQVEKMILDGADIIDLGGESTRPGHTQIS  69

Query  62   PADEIRRIAPLLDALSDQMHR-VSIDSFQPETQRYALKRGVGYLNDIQGFP-
DPALYPDI  119
             A+EI R+ P+++A+  +    +SID+++      AL  G   +NDI GF  DP+L
Sbjct  70
DAEEIERVVPMIEAIRQRFDIPISIDTYKGSVGAAALAAGADMVNDIWGFKYDPSLAEFT  129

Query  120
AEADCRLVVMHSAQRDGIATRTGHLRPEDALDEIVRFFEARVSALRRSGVAADRLILDPG  179
            A+     V+MH+        RT      + + +I    +  ++    +G++ D
+ILDPG
Sbjct  130  AKYQVPCVLMHN--------RTNQ-
```

```
NYHNLMTDINGDLQESINIAVSAGISKDAIILDPG  180

Query  180  MGFFLSPAPET-
SLHVLSNLQKLKSALGLPLLVSVSRKSFLGATVGLPVKDLGPASLAAE  238
            +GF    A    ++H L +LQ     LG P+L+  SRK F+G T+ LPV +    +
+A
Sbjct  181  IGFAKDYAQNMETMHHLEDLQ----
TLGYPVLLGTSRKGFIGLTLDLPVTERVEGTVATT  236

Query  239  LHAIGNGADYVRTH  252
            +  I  GA  +R H
Sbjct  237  VIGIMKGASIIRVH  250


>WP_077389951.1 dihydropteroate synthase [Bifidobacterium longum]
Length=291

 Score = 113 bits (283),  Expect = 2e-28, Method: Compositional
matrix adjust.
 Identities = 91/287 (32%), Positives = 140/287 (49%), Gaps = 27/287
(9%)

Query  4
SRVTVFGILNLTEDSFFDESRRLDPAGAVTAAIEMLRVGSDVVDVGPAASHPDARPVSPA  63
            +   V G+LN+TEDSF D    LDPA A     +M+  G+D++D+G  ++ P A+
V+ A
Sbjct  11
THTMVMGVLNITEDSFSDGGLWLDPAKAAQHGRDMMAAGADIIDIGAESTRPGAKRVAEA  70

Query  64   DEIRRIAPLLDALSDQMHRVSIDSFQPETQRYALKRGVGYLNDIQGFPDPALYPD-
IAEA  122
            DE+ RI   +  L      +SID+ +      AL  G   +ND+ G    A  P
+A+
Sbjct  71
DELARITGAVKTLIPAGAILSIDTTRASVAAAALSEGAQIINDVSGGTLDAELPHVVADH  130

Query  123  DCRLVVMH---------
SAQRDGIATRTGHLRPEDALDEIVRFFEARVSALRRSGVAADR  173
            DC  +V H          A  D   +   H    D  DE++R    +V  +  +GV
+R
Sbjct  131  DCLYIVQHWRGWLVGSKGANPDQDTSVYEHGVLTDVHDELMR----
QVDGVLAAGVKPER  186

Query  174  LILDPGMGFFLSPAPETSLHVLSNLQKLKSALGLPLLVSVSRKSFLGATV---
GLPVKDL  230
            +I+DPG+GF   P  E +L +L+ L+  + A G P+L+  SRK F+ A +   G
D
Sbjct  187  IIIDPGLGFS-KPGIEHNLPLLTGLETFR-
ATGYPVLIGQSRKRFISAMLTEAGAAGAD-  243

Query  231  GPA-----SLAAELHAIG--NGADYVRTHAPGDLRSAITFSETLAKF  270
            GP       + A L A+   +GA  VR H     R+A+    T  ++
Sbjct  244  GPIMAQRDDVTAALSALSAEHGAWAVRVHDVAKSRAAVIAGNTWREY  290
```

```
>WP_065436770.1 dihydropteroate synthase [Bifidobacterium longum]
Length=291

 Score = 113 bits (283),  Expect = 2e-28, Method: Compositional
matrix adjust.
 Identities = 78/230 (34%), Positives = 118/230 (51%), Gaps = 20/230
(9%)

Query  4
SRVTVFGILNLTEDSFFDESRRLDPAGAVTAAIEMLRVGSDVVDVGPAASHPDARPVSPA  63
            +   V G+LN+TEDSF D    LDPA A     +M+  G+D++D+G  ++ P A+
VS A
Sbjct  11
THTMVMGVLNITEDSFSDGGLWLDPAKAAQHGRDMMAAGADIIDIGAESTRPGAKRVSEA  70

Query  64   DEIRRIAPLLDALSDQMHRVSIDSFQPETQRYALKRGVGYLNDIQGFPDPALYPD-
IAEA  122
            DE+ RI   +  L      +SID+ +      AL   G   +ND+ G    A  P
+A+
Sbjct  71
DELARITGAVKTLIPAGAILSIDTTRASVAAAALSEGARIINDVSGGTLDAELPHVVADH  130

Query  123  DCRLVVMHSAQRDGIATRTG-----------
HLRPEDALDEIVRFFEARVSALRRSGVAA  171
            DC  +V H   R  +A   G           H    D  DE++R    +V  +
+GV
Sbjct  131  DCLYIVQH--WRGWLAGSKGANPDQDTSVYEHGVLTDVHDELMR----
QVDGVLAAGVKP  184

Query  172  DRLILDPGMGFFLSPAPETSLHVLSNLQKLKSALGLPLLVSVSRKSFLGA  221
            +R+I+DPG+GF   P  E +L +L+ L+  + A G P+L+  SRK F+ A
Sbjct  185  ERIIIDPGLGFS-KPGIEHNLPLLTGLETFR-ATGYPVLIGQSRKRFISA  232


>WP_013410667.1 dihydropteroate synthase [Bifidobacterium longum]
 ADQ02765.1 FolP [Bifidobacterium longum subsp. longum BBMN68]
Length=291

 Score = 113 bits (283),  Expect = 2e-28, Method: Compositional
matrix adjust.
 Identities = 88/286 (31%), Positives = 136/286 (48%), Gaps = 25/286
(9%)

Query  4
SRVTVFGILNLTEDSFFDESRRLDPAGAVTAAIEMLRVGSDVVDVGPAASHPDARPVSPA  63
            +   V G+LN+TEDS  D    LDPA A     +M+  G+D++D+G  ++ P A+
VS A
Sbjct  11
THTMVMGVLNITEDSVSDGGLWLDPAKAAQHGRDMMAAGADIIDIGAESTRPGAKRVSEA  70

Query  64   DEIRRIAPLLDALSDQMHRVSIDSFQPETQRYALKRGVGYLNDIQGFPDPALYPD-
IAEA  122
            DE+ RI   +  L      +SID+ +      AL   G   +ND+ G    A  P
```

+A+
Sbjct  71
DELARITGAVKTLIPAGAVLSIDTTRASVAAAALSEGAQIINDVSGGTLDAELPHVVADH  130

Query  123  DCRLVVMH---------
SAQRDGIATRTGHLRPEDALDEIVRFFEARVSALRRSGVAADR  173
            DC  VV H          A  D   +   H    D  DE++R    +V  +  +GV
+R
Sbjct  131  DCLYVVQHWRGWLVGSKGANPDQDTSVYEHGVLTDVHDELMR----
QVDGVLAAGVKPER  186

Query  174
LILDPGMGFFLSPAPETSLHVLSNLQKLKSALGLPLLVSVSRKSFLGATV---------G  224
            +I+DPG+GF   P  E +L +L+ L+  + A G P+L+  SRK F+ A +
G
Sbjct  187  IIIDPGLGFS-KPGIEHNLPLLTGLETFR-
ATGYPVLIGQSRKRFISAMLTGAGTAGADG  244

Query  225  LPVKDLGPASLAAELHAIGNGADYVRTHAPGDLRSAITFSETLAKF  270
            L +      + A    +  +GA  VR H     R+A+    T  ++
Sbjct  245  LTMAQRDDVTAALSALSAEHGAWAVRVHDVAKSRAAVIAGNTWREY  290

>WP_100493538.1 dihydropteroate synthase [Bifidobacterium sp. TRE H]
 PJM77959.1 dihydropteroate synthase [Bifidobacterium sp. TRE H]
Length=293

 Score = 113 bits (283),  Expect = 2e-28, Method: Compositional
matrix adjust.
 Identities = 89/293 (30%), Positives = 147/293 (50%), Gaps = 30/293
(10%)

Query  4
SRVTVFGILNLTEDSFFDESRRLDPAGAVTAAIEMLRVGSDVVDVGPAASHPDARPVSPA  63
            S+  V G+LN+TEDSF D    LDP  A     +M+  G+D++D+G  ++ P A+
VS
Sbjct  11
SQTLVMGVLNITEDSFSDGGLWLDPDKAAQHGRDMMAAGADIIDIGAESTRPGAKRVSEE  70

Query  64   DEIRRIAPLLDALSDQMHRVSIDSFQPETQRYALKRGVGYLNDIQGFP-
DPALYPDIAEA  122
            DE++RI   + AL      +SID+ +      AL+ G   +ND+ G   D  L
+A+
Sbjct  71
DELKRITGAVHALVPAGAVLSIDTTRASVAAAALEGGAQIINDVSGGTLDRELPHVVADH  130

Query  123  DCRLVVMHSAQRDGIATRTG-----------
HLRPEDALDEIVRFFEARVSALRRSGVAA  171
            DC  +V H   R  +A   G            H    D  DE++R    +V  +
+G+
Sbjct  131  DCLYIVQH--WRGWLAGSKGANPDQDTSVYEHGVLTDVHDELMR----
QVDDVLAAGIKP  184

Query  172

```
DRLILDPGMGFFLSPAPETSLHVLSNLQKLKSALGLPLLVSVSRKSFLGATVG-------  224
            +R+I+DPG+GF   P  E +L +L+ L+  + A G P+L+  SRK F+ A +
Sbjct  185  ERIIIDPGLGFS-KPGIEHNLPLLAGLETFR-
ATGYPVLIGQSRKRFISAMLADNGITEP  242

Query  225  -LPVKDLGPASLAAELHAIGNGADYVRTHAPGDLRSAITFSETLAKFRSRNAR  
276
             +  +D   A+L+A   +  +GA  VR H     R+A+       ++ +++A+
Sbjct  243  TMAQRDDVTAALSAL--SAEHGAWAVRVHDVAKSRAAVIAGNAWRQYSAQSAQ  
293


>WP_044089021.1 dihydropteroate synthase [Bifidobacterium reuteri]
 KFI86589.1 dihydropteroate synthase 1 [Bifidobacterium reuteri DSM 
23975]
Length=288

 Score = 113 bits (282),  Expect = 2e-28, Method: Compositional 
matrix adjust.
 Identities = 93/283 (33%), Positives = 141/283 (50%), Gaps = 30/283 
(11%)

Query  8    
VFGILNLTEDSFFDESRRLDPAGAVTAAIEMLRVGSDVVDVGPAASHPDARPVSPADEIR  67
            V G+LN+TEDSF D    LDP  A     +M+  G+D++D+G  ++ P A+ VS 
DE R
Sbjct  15   
VMGVLNITEDSFSDGGLWLDPDKAAQHGRDMMAAGADIIDIGAESTRPGAKRVSEEDEHR  74

Query  68   RIAPLLDALSDQMHRVSIDSFQPETQRYALKRGVGYLNDIQGFPDPALYPD-
IAEADCRL  126
            RI   + AL      +SID+ +      AL+ G   +ND+ G    A  P  +A+ 
DC
Sbjct  75   
RITGAVSALIPAGAVLSIDTTRASVAAAALEGGAQIINDVSGGTLDAELPHVVADHDCLY  134

Query  127  VVMHSAQRDGIATRTG-----------
HLRPEDALDEIVRFFEARVSALRRSGVAADRLI  175
            +V H   R  +A   G           H    D  DE++R    +V A+  +GV 
+R+I
Sbjct  135  IVQH--WRGWLAGSKGANPDQDTSVYEHGVLIDVHDELMR----
QVDAVLDAGVKPERII  188

Query  176  
LDPGMGFFLSPAPETSLHVLSNLQKLKSALGLPLLVSVSRKSFLGATVG--------LPV  227
            +DPG+GF   P  E +L +L+ L KL  A G P+L+  SRK F+ A + 
+
Sbjct  189  IDPGLGFS-KPGIEHNLPLLTGL-
KLFRAAGYPVLIGQSRKRFISAMLADAGISEPSMEQ  246

Query  228  KDLGPASLAAELHAIGNGADYVRTHAPGDLRSAITFSETLAKF  270
            +D   A+L+A   +  +GA  VR H     R+A+    T  ++
Sbjct  247  RDDVTAALSAL--SAEHGAWAVRVHDVAKSRAAVIAGNTWRQY  287
```

```
>WP_085382394.1 dihydropteroate synthase [Bifidobacterium
adolescentis]
 OSH06033.1 dihydropteroate synthase [Bifidobacterium adolescentis]
Length=292

 Score = 113 bits (282),  Expect = 2e-28, Method: Compositional
matrix adjust.
 Identities = 86/283 (30%), Positives = 135/283 (48%), Gaps = 23/283
(8%)

Query  5
RVTVFGILNLTEDSFFDESRRLDPAGAVTAAIEMLRVGSDVVDVGPAASHPDARPVSPAD  64
            R  V G+LN+TEDS  D    L P  A      M++ G+D++D+G  ++ P A+
VS AD
Sbjct  13
RTLVMGVLNITEDSSSDGGLWLAPEAAKAHGEAMMKAGADIIDIGAESTRPGAKRVSEAD  72

Query  65
EIRRIAPLLDALSDQMHRVSIDSFQPETQRYALKRGVGYLNDIQGFPDPALYPDIA--EA  122
            E  R+   +DAL  +   +SID+ +      AL+ G   +ND+ G       P +
+
Sbjct  73
EKARVLGAVDALIPEGAVLSIDTTRASVALAALEHGAQIINDVSGGQLDRELPHVVADHS  132

Query  123  DCRLVVMHSAQR---------
DGIATRTGHLRPEDALDEIVRFFEARVSALRRSGVAADR  173
            DC  +V H             D   +R  H    D  DE++R    +V A+  +GV
A++
Sbjct  133  DCLYIVQHWRGWLAGAAGNVPDADTSRYEHGVVNDVYDELMR----
QVDAVLEAGVQAEQ  188

Query  174  LILDPGMGFFLSPAPETSLHVLSNLQKLKSALGLPLLVSVSRKSFLG---
ATVGLPVKDL  230
            +I+DPG+GF   P  E +L +L+ L +  +A G P+L+  SRK F+G   A  G+
D+
Sbjct  189  VIIDPGLGFS-KPGVEHNLPILAALDRFNAA-
GYPVLIGASRKRFVGSLLAGAGVTEPDM  246

Query  231  GP---ASLAAELHAIGNGADYVRTHAPGDLRSAITFSETLAKF  270
                 A+ A       +G   VR H     R A+       ++
Sbjct  247  ASKDNATAAISALCAEHGVWAVRVHDVAKSRDAVAVGNAWREY  289


>WP_085381689.1 dihydropteroate synthase [Bifidobacterium
adolescentis]
 OSH01388.1 dihydropteroate synthase [Bifidobacterium adolescentis]
Length=292

 Score = 113 bits (282),  Expect = 2e-28, Method: Compositional
matrix adjust.
 Identities = 86/283 (30%), Positives = 135/283 (48%), Gaps = 23/283
(8%)
```

```
Query  5
RVTVFGILNLTEDSFFDESRRLDPAGAVTAAIEMLRVGSDVVDVGPAASHPDARPVSPAD  64
            R  V G+LN+TEDS  D    L P  A      M++ G+D++D+G  ++ P A+
VS AD
Sbjct  13
RTLVMGVLNITEDSSSDGGLWLAPEAAKAHGEAMMKAGADIIDIGAESTRPGAKRVSEAD  72

Query  65
EIRRIAPLLDALSDQMHRVSIDSFQPETQRYALKRGVGYLNDIQGFPDPALYPDIA--EA  122
            E  R+   +DAL  +   +SID+ +      AL+ G   +ND+ G       P +
+
Sbjct  73
EKARVLGAVDALIPEGAVLSIDTTRASVALAALEHGAQIINDVSGGQLDRELPHVVADHS  132

Query  123  DCRLVVMHSAQR---------
DGIATRTGHLRPEDALDEIVRFFEARVSALRRSGVAADR  173
            DC  +V H            D   +R  H    D  DE++R    +V A+  +GV
A++
Sbjct  133  DCLYIVQHWRGWLAGAAGDVPDADTSRYEHGVVNDVYDELMR----
QVDAVLEAGVQAEQ  188

Query  174  LILDPGMGFFLSPAPETSLHVLSNLQKLKSALGLPLLVSVSRKSFLG---
ATVGLPVKDL  230
            +I+DPG+GF   P  E +L +L+ L +  +A G P+L+  SRK F+G   A  G+
D+
Sbjct  189  VIIDPGLGFS-KPGVEHNLPILAALDRFNAA-
GYPVLIGASRKRFVGSLLAGAGVTEPDM  246

Query  231  GP---ASLAAELHAIGNGADYVRTHAPGDLRSAITFSETLAKF  270
                 A+ A       +G   VR H     R A+       ++
Sbjct  247  ASKDNATAAISALCAEHGVWAVRVHDVAKSRDAVAVGNAWREY  289
```

>PLS29794.1 dihydropteroate synthase [Bifidobacterium parmae]
Length=311

Score = 113 bits (283), Expect = 3e-28, Method: Compositional matrix adjust.
Identities = 90/283 (32%), Positives = 142/283 (50%), Gaps = 30/283 (11%)

```
Query  8
VFGILNLTEDSFFDESRRLDPAGAVTAAIEMLRVGSDVVDVGPAASHPDARPVSPADEIR  67
            V G+LN+TEDSF D    LDP  A      M+  G+D++D+G  ++ P A+ VS
DE+
Sbjct  37
VMGVLNITEDSFSDGGLWLDPRAAADHGRAMMAAGADIIDIGAESTRPGAKRVSEEDELT  96

Query  68   RIAPLLDALSDQMHRVSIDSFQPETQRYALKRGVGYLNDIQGFP-
DPALYPDIAEADCRL  126
            RI   ++AL      +SID+ +      AL  G   +ND+ G   D  L   +A+
DC
Sbjct  97
RITGAVEALIPAGATLSIDTTRASVAAAALDGGAQIINDVSGGTLDTELPHVVADHDCLY  156
```

```
Query  127  VVMHSAQRDGIA-TRTG----------
HLRPEDALDEIVRFFEARVSALRRSGVAADRLI  175
            +V H   R  +A ++TG          H   +D  DE++R    +V A+  +GVA
+R+I
Sbjct  157  IVQH--WRGWLAGSKTGAPDADTSHYEHGVLQDVHDELMR----
QVDAVLAAGVAPERII  210

Query  176  LDPGMGFFLSPAPETSLHVLSNLQKLKSALGLPLLVSVSRKSFLGAT--------
VGLPV  227
            +DPG+GF   P    +L +L+ L + + A G P+L+  SRK F+ A         +
+
Sbjct  211  IDPGLGFS-KPGIAHNLPLLAGLDRFR-
ATGYPVLIGQSRKRFVTAILDKAGFTDITMDA  268

Query  228  KDLGPASLAAELHAIGNGADYVRTHAPGDLRSAITFSETLAKF  270
            +D   A+ +A      +GA  VR H     R+A+    T  ++
Sbjct  269  RDDATAAFSALCAE--HGAWAVRVHDVARSRAAVAVGNTWREY  309


>WP_060620656.1 dihydropteroate synthase [Bifidobacterium longum]
 ALE09286.1 Dihydropteroate synthase [Bifidobacterium longum subsp.
infantis]
 OQM70287.1 Dihydropteroate synthase [Bifidobacterium longum subsp.
infantis]
Length=291

 Score = 113 bits (282),  Expect = 3e-28, Method: Compositional
matrix adjust.
 Identities = 92/289 (32%), Positives = 140/289 (48%), Gaps = 31/289
(11%)

Query  4
SRVTVFGILNLTEDSFFDESRRLDPAGAVTAAIEMLRVGSDVVDVGPAASHPDARPVSPA  63
            +   V G+LN+TEDS  D    LDPA A     +M+  G+D++D+G  ++ P A+
VS A
Sbjct  11
THTMVMGVLNITEDSVSDGGLWLDPAKAAQHGRDMMAAGADIIDIGAESTRPGAKRVSEA  70

Query  64   DEIRRIAPLLDALSDQMHRVSIDSFQPETQRYALKRGVGYLNDIQGFPDPALYPD-
IAEA  122
            DE+ RI   +  L      +SID+ +      AL  G   +ND+ G    A  P
+A+
Sbjct  71
DELARITGAVKTLIPAGAILSIDTTRASVAAAALSEGAQIINDVSGGTLDAELPHVVADH  130

Query  123  DCRLVVMHSAQRDGIATRTG-----------
HLRPEDALDEIVRFFEARVSALRRSGVAA  171
            DC  +V H   R  +A   G           H    D  DE++R    +V  +
+GV
Sbjct  131  DCLYIVQH--WRGWLAGSKGANPDQDTSVYEHGVLTDVHDELMR----
QVDGVLAAGVKP  184

Query  172  DRLILDPGMGFFLSPAPETSLHVLSNLQKLKSALGLPLLVSVSRKSFLGATV---
```

```
GLPVK  228
            +R+I+DPG+GF   P  E +L +L+ L+  + A G P+L+  SRK F+ A +   G
Sbjct  185  ERIIIDPGLGFS-KPGIEHNLPLLTGLETFR-
ATGYPVLIGQSRKRFISAMLTEAGAAGA  242

Query  229  DLGPA-----SLAAELHAIG--NGADYVRTHAPGDLRSAITFSETLAKF  270
            D GP       + A L A+   +GA  VR H     R+A+    T  ++
Sbjct  243  D-GPIMAQRDDVTAALSALSAEHGAWAVRVHDVAKSRAAVIVGNTWREY  290


>KXS24820.1 dihydropteroate synthase [Bifidobacterium dentium]
Length=292

 Score = 113 bits (282),  Expect = 3e-28, Method: Compositional
matrix adjust.
 Identities = 87/278 (31%), Positives = 137/278 (49%), Gaps = 19/278
(7%)

Query  8
VFGILNLTEDSFFDESRRLDPAGAVTAAIEMLRVGSDVVDVGPAASHPDARPVSPADEIR  67
            V G+LN+TEDSF D    LDPA A T    M++ G+D++D+G  ++ P A+ VS
DE
Sbjct  17
VMGVLNITEDSFSDGGLWLDPAKAKTHGEAMMKDGADIIDIGAESTRPGAKRVSEEDEKS  76

Query  68   RIAPLLDALSDQMHRVSIDSFQPETQRYALKRGVGYLNDIQGFPDPALYPDIA--
EADCR  125
            R+   +DAL  +   +SID+ +      AL+ G   +ND+ G    A  P +
+
Sbjct  77
RVLGAVDALVPEGAILSIDTTRASVASAALEHGAQIINDVSGGRLDAELPHVVADHPESL  136

Query  126  LVVMHSAQRDGIATRTGHLRPEDA-------
LDEIVRFFEARVSALRRSGVAADRLILDP  178
             +V H   R  +A   G++   D        L+++     A+V A+   GV  ++
+I+DP
Sbjct  137  YIVQH--
WRGWLAGAAGNVPDADTSVYANGVLNDVYDELMAQVDAVLSVGVKPEQVIIDP  194

Query  179  GMGFFLSPAPETSLHVLSNLQKLKSALGLPLLVSVSRKSFLGATVGL------
PVKDLGP  232
            G+GF   P  E +L +L+ L++  +A G P+LV  SRK F+GA +         +
D
Sbjct  195  GLGFS-KPGIEHNLPLLAGLERF-
NATGHPVLVGASRKRFIGAVLAAAGVSEPTMADRDN  252

Query  233  ASLAAELHAIGNGADYVRTHAPGDLRSAITFSETLAKF  270
            A+ A    +  +GA  VR H     R AI        +
Sbjct  253  ATAAISALSAEHGAWAVRVHDVARTRDAIAIGSAWRAY  290


>WP_056251287.1 dihydropteroate synthase [Devosia sp. Root436]
 KQX42591.1 dihydropteroate synthase [Devosia sp. Root436]
Length=283
```

Score = 112 bits (281),  Expect = 3e-28, Method: Compositional matrix adjust.
Identities = 88/267 (33%), Positives = 135/267 (51%), Gaps = 25/267 (9%)

```
Query  1
MLRSRVTVFGILNLTEDSFFDESRRLDPAGAVTAAIEMLRVGSDVVDVGPAASHPDARPV  60
            +L  R  V GILN+T DSF D  +    AGA+    +ML  G+D++D+G  ++ P
A PV
Sbjct  16
LLGQRPLVMGILNVTPDSFSDGGQHNRVAGALAHVRQMLAEGADIIDIGGESTRPGAAPV  75

Query  61   SPADEIRRIAPLLDALSDQ--
MHRVSIDSFQPETQRYALKRGVGYLNDIQGFPDPALYPD  118
               +E+ R+ P++DAL  +     +SID+++P     A++ G   +ND+ G
P+
Sbjct  76
GVQEELDRVMPVIDALIAEGITAPISIDTYKPLVADQAIQAGASIINDVHGLQG---APE  132

Query  119  IAEA----DCRLVVMHSAQ-
RDGIATRTGHLRPEDALDEIVRFFEARVSALRRSGVAADR  173
            +AE        ++VMH    RD  A   G          ++ +    ++   R+G+
DR
Sbjct  133  MAEVAALHGAPVIVMHWDNARDPAAPFIG---------
AVMDYLARSIAIAERAGLHRDR  183

Query  174
LILDPGMGFFLSPAPETSLHVLSNLQKLKSALGLPLLVSVSRKSFLGATVGLPVKDLGPA  233
            LILDPG GF  S A    L  L  L +L+ ALGLPLLV  SRKS +G  +    ++
Sbjct  184  LILDPGFGFGKSLAENYQL--LDRLAELQ-
ALGLPLLVGTSRKSMIGKLLDNQPEERLAG  240

Query  234  SLAAELHAIGNGADYVRTHAPGDLRSA  260
            ++A  +     G    R H   D+R+A
Sbjct  241  TIATTVLGYIKGGHIFRVH---DVRAA  264
```

>WP_092184669.1 dihydropteroate synthase [Devosia sp. YR412]
SEQ42521.1 dihydropteroate synthase [Devosia sp. YR412]
Length=287

Score = 112 bits (281),  Expect = 3e-28, Method: Compositional matrix adjust.
Identities = 75/249 (30%), Positives = 128/249 (51%), Gaps = 10/249 (4%)

```
Query  7
TVFGILNLTEDSFFDESRRLDPAGAVTAAIEMLRVGSDVVDVGPAASHPDARPVSPADEI  66
             V GILN+T DSF D  +      A+  A +M+  G+D++D+G  ++ P ++PV
+E+
Sbjct  22
CVMGILNVTPDSFSDGGQHDRVDAAMAHAHQMIAEGADIIDIGGESTRPGSQPVGVQEEL  81
```

```
Query  67   RRIAPLLDAL--
SDQMHRVSIDSFQPETQRYALKRGVGYLNDIQGFPDPALYPDIAEADC  124
             R+ P++ AL  +     +SID+++P     A++ G   +ND+ G        D+A
Sbjct  82
DRVMPVIQALVAAGITTPLSIDTYKPLVADQAIQAGAAIINDVHGLQGADEMADVAALHQ  141

Query  125
RLVVMHSAQRDGIATRTGHLRPEDALDEIVRFFEARVSALRRSGVAADRLILDPGMGFFL  184
              V++   Q+D           P   LD++ R+FE  ++   ++G++ DRLILDPG
GF
Sbjct  142  VPVIVMHWQKD-----RDKFFP--
LLDDMRRYFERSIARAEQAGISRDRLILDPGFGFGK  194

Query  185  SPAPETS-
LHVLSNLQKLKSALGLPLLVSVSRKSFLGATVGLPVKDLGPASLAAELHAIG  243
            + +   + L  +  +Q  +    LPLLV  SRKS +G  +G   ++    ++AA +
Sbjct  195
TLSENYAILQQIGGIQHERWQQKLPLLVGCSRKSMIGKLLGNEPEERLAGTIAANVIGYL  254

Query  244  NGADYVRTH  252
            NG    R H
Sbjct  255  NGGHIFRVH  263


>WP_100968037.1 dihydropteroate synthase [Bifidobacterium longum]
 PKC92892.1 Dihydropteroate synthase [Bifidobacterium longum]
 PKC96401.1 Dihydropteroate synthase [Bifidobacterium longum]
Length=291

 Score = 112 bits (281),  Expect = 3e-28, Method: Compositional
matrix adjust.
 Identities = 92/289 (32%), Positives = 140/289 (48%), Gaps = 31/289
(11%)

Query  4
SRVTVFGILNLTEDSFFDESRRLDPAGAVTAAIEMLRVGSDVVDVGPAASHPDARPVSPA  63
            +   V G+LN+TEDS  D    LDPA A     +M+  G+D++D+G  ++ P A+
VS A
Sbjct  11
THTMVMGVLNITEDSVSDGGLWLDPAKAAQHGRDMMAAGADIIDIGAESTRPGAKRVSEA  70

Query  64   DEIRRIAPLLDALSDQMHRVSIDSFQPETQRYALKRGVGYLNDIQGFPDPALYPD-
IAEA  122
            DE+ RI   +  L      +SID+ +      AL  G   +ND+ G    A  P
+A+
Sbjct  71
DELVRITGAVKTLIPAGAILSIDTTRASVAAAALSEGAQIINDVSGGTLDAELPHVVADH  130

Query  123  DCRLVVMHSAQRDGIATRTG-----------
HLRPEDALDEIVRFFEARVSALRRSGVAA  171
            DC  +V H   R  +A   G           H    D  DE++R    +V  +
+GV
Sbjct  131  DCLYIVQH--WRGWLAGSKGANPDQDTSVYEHGVLTDVHDELMR----
QVDGVLAAGVKP  184
```

```
Query  172  DRLILDPGMGFFLSPAPETSLHVLSNLQKLKSALGLPLLVSVSRKSFLGATV---
GLPVK  228
            +R+I+DPG+GF   P  E +L +L+ L+  + A G P+L+  SRK F+ A +   G
Sbjct  185  ERIIIDPGLGFS-KPGIEHNLPLLTGLETFR-
ATGYPVLIGQSRKRFISAMLTGAGTAGA  242

Query  229  DLGPA-----SLAAELHAIG--NGADYVRTHAPGDLRSAITFSETLAKF  270
            D GP       + A L A+   +GA  VR H     R+A+    T  ++
Sbjct  243  D-GPTMAQRDDVTAALSALSAEHGAWAVRVHDVAKSRAAVIAGNTWREY  290


>ODS95184.1 dihydropteroate synthase [Devosia sp. SCN 66-27]
 OJX26605.1 dihydropteroate synthase [Devosia sp. 66-14]
Length=284

 Score = 112 bits (281),  Expect = 4e-28, Method: Compositional
matrix adjust.
 Identities = 87/255 (34%), Positives = 129/255 (51%), Gaps = 16/255
(6%)

Query  2
LRSRVTVFGILNLTEDSFFDESRRLDPAGAVTAAIEMLRVGSDVVDVGPAASHPDARPVS  61
            L  R  + GILN+T DSF D  R      A+  A  ML  G+D++D+G  ++ P A
VS
Sbjct  18
LGRRARLMGILNVTPDSFSDGGRFDAVPAALGQARRMLAEGADIIDIGGESTRPGAAEVS  77

Query  62   PADEIRRIAPLLDALSDQ--MHRVSIDSFQPETQRYALKRGVGYLNDIQGFP-
DPALYPD  118
             ADE+ R+ P + AL     +  +SID+++ E    A+  G   +ND+ G   +P
L
Sbjct  78
AADELARVLPAIAALRADGIVAPISIDTYKAEVAEQAIAAGATIINDVHGLQREPELAAV  137

Query  119  IAEADCRLVVMHSAQ-
RDGIATRTGHLRPEDALDEIVRFFEARVSALRRSGVAADRLILD  177
             A     LVVMH  + RD  A+R       D + E+ R+FE  +     +GVA
++LD
Sbjct  138  AASRGVPLVVMHWDKARD--ASR-------
DVMAEMARYFEVTLRIADEAGVAPSGIVLD  188

Query  178
PGMGFFLSPAPETSLHVLSNLQKLKSALGLPLLVSVSRKSFLGATVGLPVKDLGPASLAA  237
            PG GF  S +    +  +L  L +L  ALG P+LV  SRKS +G  + +P  +
++A
Sbjct  189  PGFGFAKSLS--ENYEILHRLPEL-
VALGFPVLVGTSRKSMIGRLLDVPADERLAGTVAT  245

Query  238  ELHAIGNGADYVRTH  252
             +     GA   R H
Sbjct  246  SVLGYTAGAHLFRVH  260
```

```
>WP_034518937.1 MULTISPECIES: dihydropteroate synthase
[Bifidobacterium]
 ETO98375.1 dihydropteroate synthase [Bifidobacterium sp. MSTE12]
Length=292

 Score = 112 bits (281),  Expect = 4e-28, Method: Compositional
matrix adjust.
 Identities = 87/281 (31%), Positives = 137/281 (49%), Gaps = 19/281
(7%)

Query  5
RVTVFGILNLTEDSFFDESRRLDPAGAVTAAIEMLRVGSDVVDVGPAASHPDARPVSPAD  64
            R  V G+LN+TEDSF D    LDPA A      M++ G+D++D+G  ++ P A+
VS  D
Sbjct  14
RTLVMGVLNITEDSFSDGGLWLDPAKAKAHGEAMMKDGADIIDIGAESTRPGAKRVSEED  73

Query  65
EIRRIAPLLDALSDQMHRVSIDSFQPETQRYALKRGVGYLNDIQGFPDPALYPDIA--EA  122
            E  R+   +DAL  +   +SID+ +      AL+ G   +ND+ G    A  P +
Sbjct  74
EKSRVLGAVDALVPEGAILSIDTTRASVASVALEHGAQIINDVSGGRLDAELPHVVADHP  133

Query  123  DCRLVVMHSAQRDGIATRTGHLRPEDA-------
LDEIVRFFEARVSALRRSGVAADRLI  175
            +   +V H   R  +A   G++   D        L+++     A+V A+   GV
+++I
Sbjct  134  ESLYIVQH--
WRGWLAGAAGNVPDADTSVYANGVLNDVYDELMAQVDAVLSVGVKPEQVI  191

Query  176  LDPGMGFFLSPAPETSLHVLSNLQKLKSALGLPLLVSVSRKSFLGATVGL------
PVKD  229
            +DPG+GF   P  E +L +L+ L++  +A G P+LV  SRK F+GA +
+ D
Sbjct  192  IDPGLGFS-KPGIEHNLPLLAGLERF-
NATGHPVLVGASRKRFIGAVLAAAGVSEPTMAD  249

Query  230  LGPASLAAELHAIGNGADYVRTHAPGDLRSAITFSETLAKF  270
               A+ A    +  +GA  VR H     R AI        +
Sbjct  250  RDNATAAISALSAEHGAWAVRVHDVARTRDAIAIGSAWRAY  290


>WP_003837180.1 dihydropteroate synthase [Bifidobacterium dentium]
 EDT44426.1 dihydropteroate synthase [Bifidobacterium dentium ATCC
27678]
 ADB09220.1 folP Dihydropteroate synthase [Bifidobacterium dentium
Bd1]
 BAQ26519.1 dihydropteroate synthase [Bifidobacterium dentium JCM
1195 =
DSM 20436]
 SEB76962.1 dihydropteroate synthase [Bifidobacterium dentium JCM
1195 =
DSM 20436]
Length=292
```

```
 Score = 112 bits (281),  Expect = 4e-28, Method: Compositional
matrix adjust.
 Identities = 87/281 (31%), Positives = 137/281 (49%), Gaps = 19/281
(7%)

Query  5
RVTVFGILNLTEDSFFDESRRLDPAGAVTAAIEMLRVGSDVVDVGPAASHPDARPVSPAD  64
            R  V G+LN+TEDSF D    LDPA A      M++ G+D++D+G  ++ P A+
VS  D
Sbjct  14
RTLVMGVLNITEDSFSDGGLWLDPAKAKAHGEAMMKDGADIIDIGAESTRPGAKRVSEED  73

Query  65
EIRRIAPLLDALSDQMHRVSIDSFQPETQRYALKRGVGYLNDIQGFPDPALYPDIA--EA  122
            E  R+   +DAL  +   +SID+ +      AL+ G   +ND+ G    A  P +
Sbjct  74
EKSRVLGAVDALVPEGAILSIDTTRASVASAALEHGAQIINDVSGGRLDAELPHVVADHP  133

Query  123  DCRLVVMHSAQRDGIATRTGHLRPEDA-------
LDEIVRFFEARVSALRRSGVAADRLI  175
            +   +V H   R  +A   G++   D         L+++     A+V A+   GV
+++I
Sbjct  134  ESLYIVQH--
WRGWLAGAAGNVPDADTSVYANGVLNDVYDELMAQVDAVLSVGVKPEQVI  191

Query  176  LDPGMGFFLSPAPETSLHVLSNLQKLKSALGLPLLVSVSRKSFLGATVGL------
PVKD  229
            +DPG+GF   P  E +L +L+ L++  +A G P+LV  SRK F+GA +
+ D
Sbjct  192  IDPGLGFS-KPGIEHNLPLLAGLERF-
NATGHPVLVGASRKRFIGAVLAAAGVSEPTMAD  249

Query  230  LGPASLAAELHAIGNGADYVRTHAPGDLRSAITFSETLAKF  270
               A+ A    +  +GA  VR H     R AI        +
Sbjct  250  RDNATAAISALSAEHGAWAVRVHDVARTRDAIAIGSAWRAY  290


>WP_058594680.1 dihydropteroate synthase [Leucobacter
chromiiresistens]
 KTR81892.1 hypothetical protein NS354_11885 [Leucobacter
chromiiresistens]
Length=424

 Score = 115 bits (287),  Expect = 4e-28, Method: Compositional
matrix adjust.
 Identities = 75/222 (34%), Positives = 123/222 (55%), Gaps = 14/222
(6%)

Query  9
FGILNLTEDSFFDESRRLDPAGAVTAAIEMLRVGSDVVDVGPAASHPDARPVSPADEIRR  68
             G+LN+T DSF D     +   A+   +E+ R+G+D+VDVG  ++ P A PV
+DE  R
Sbjct  1
```

```
MGVLNVTPDSFSDGGAYAEHDAAIAHGVELARLGADLVDVGGESTRPGAAPVRSSDEQER  60

Query  69   IAPLLDALSDQMHRVSIDSFQPETQRYALKRGVGYLNDIQ-
GFPDPALYPDIAEADCR--  125
            + P++ AL+ +   VS+D+   +T   A++ G  ++ND+  G  DP +    A+A
R
Sbjct  61
VLPVIRALASRSIGVSVDTLHADTAHAAVEAGARWINDVSGGLHDPEMLDAAADASRRGA  120

Query  126  --LVVMHSAQRDGIATRTGHLRPE--
DALDEIVRFFEARVSALRRSGVAADRLILDPGMG  181
              L++ H     G+    GHLR +  D + ++ R  + R SA   +GV   R+
+LDPG+G
Sbjct  121  AGLIIGH---WRGVPD-
PGHLRSDYADVVSDVTRALKERASAAVAAGVDPGRIVLDPGLG  176

Query  182  FFLSPAPETSLHVLSNLQKLKSALGLPLLVSVSRKSFLGATV  223
            F  + A      +L+ L +++ ALG P+L+  SRK  LG  +
Sbjct  177  FDKTGA--QGWQLLAGLDEVR-ALGFPVLIGASRKRMLGEAI  215


>WP_003830054.1 dihydropteroate synthase [Bifidobacterium breve]
 EFE88671.1 dihydropteroate synthase [Bifidobacterium breve DSM
20213 = JCM
1192]
 KFI51697.1 Dihydropteroate synthase [Bifidobacterium breve]
 BAR00571.1 dihydropteroate synthase [Bifidobacterium breve DSM
20213 = JCM
1192]
 AUD67752.1 Dihydropteroate synthase [Bifidobacterium breve]
Length=292

 Score = 112 bits (281),  Expect = 5e-28, Method: Compositional
matrix adjust.
 Identities = 92/290 (32%), Positives = 140/290 (48%), Gaps = 29/290
(10%)

Query  4
SRVTVFGILNLTEDSFFDESRRLDPAGAVTAAIEMLRVGSDVVDVGPAASHPDARPVSPA  63
            S   V G+LN+TEDS  D    LDPA A     +M+  G+D++D+G  ++ P A+
VS
Sbjct  11
SHTMVMGVLNITEDSLSDGGLWLDPAKAAQHGRDMMAAGADIIDIGAESTRPGAKRVSEE  70

Query  64   DEIRRIAPLLDALSDQMHRVSIDSFQPETQRYALKRGVGYLNDIQGFPDPALYPD-
IAEA  122
            DE+ RI   ++AL      +SID+ +      AL  G   +ND+ G    A  P
IA+
Sbjct  71
DELARITGAVNALIPAGAVLSIDTTRASVAAAALDGGAQIINDVSGGTLDANLPHVIADH  130

Query  123  DCRLVVMHSAQRDGIATRTG-----------
HLRPEDALDEIVRFFEARVSALRRSGVAA  171
            DC  +V H   R  +A   G           H    D  DE++R    +V  +
```

+GV
Sbjct  131  DCLYIVQH--WRGWLAGSKGANPDQDTSVYEHGVLTDVHDELMR----
QVDGVLAAGVKP  184

Query  172  DRLILDPGMGFFLSPAPETSLHVLSNLQKLKSALGLPLLVSVSRKSFLGA--
TVGLPVKD  229
            +R+I+DPG+GF   P+ E +L +L+ L+  +   G P+L+  SRK F+ A  T
V +
Sbjct  185  ERIIIDPGLGFS-KPSIEHNLPLLTGLETFRGT-
GYPVLIGQSRKRFISAMLTEAGAVGE  242

Query  230  LGPA-----SLAAELHAIG--NGADYVRTHAPGDLRSAITFSETLAKFRS  272
             GP       + A L A+   +GA  VR H      +A+       ++ S
Sbjct  243  DGPTMAQRDDVTAALSALSAEHGAWAVRVHDVAKSHAAVIAGNIWRQYLS  292

>KXS29119.1 dihydropteroate synthase [Bifidobacterium longum]
Length=291

 Score = 112 bits (280),  Expect = 6e-28, Method: Compositional
matrix adjust.
 Identities = 92/289 (32%), Positives = 140/289 (48%), Gaps = 31/289
(11%)

Query  4
SRVTVFGILNLTEDSFFDESRRLDPAGAVTAAIEMLRVGSDVVDVGPAASHPDARPVSPA  63
            +   V G+LN+TEDS  D    LDPA A     +M+  G+D++D+G  ++ P A+
VS A
Sbjct  11
THTMVMGVLNITEDSSSDGGLWLDPAKAAQHGRDMMAAGADIIDIGAESTRPGAKRVSEA  70

Query  64   DEIRRIAPLLDALSDQMHRVSIDSFQPETQRYALKRGVGYLNDIQGFPDPALYPD-
IAEA  122
            DE+ RI   +  L      +SID+ +      AL  G   +ND+ G    A  P
+A+
Sbjct  71
DELVRITGAVKTLIPAGAILSIDTTRASVAAAALSEGAQIINDVSGGTLDAELPHVVADH  130

Query  123  DCRLVVMHSAQRDGIATRTG-----------
HLRPEDALDEIVRFFEARVSALRRSGVAA  171
            DC  +V H   R  +A   G           H    D  DE++R    +V  +
+GV
Sbjct  131  DCLYIVQH--WRGWLAGSKGANPDQDTSVYEHGVLTDVHDELMR----
QVDGVLAAGVKP  184

Query  172  DRLILDPGMGFFLSPAPETSLHVLSNLQKLKSALGLPLLVSVSRKSFLGATV---
GLPVK  228
            +R+I+DPG+GF   P  E +L +L+ L+  + A G P+L+  SRK F+ A +   G
Sbjct  185  ERIIIDPGLGFS-KPGIEHNLPLLTGLETFR-
ATGYPVLIGQSRKRFISAMLTGAGTAGA  242

Query  229  DLGPA-----SLAAELHAIG--NGADYVRTHAPGDLRSAITFSETLAKF  270
            D GP       + A L A+   +GA  VR H     R+A+    T  ++
Sbjct  243  D-GPTMAQRDDVTAALSALSAEHGAWAVRVHDVAKSRAAVIAGNTWREY  290

```
>WP_008782915.1 MULTISPECIES: dihydropteroate synthase
[Bifidobacterium]
 EFV37870.1 dihydropteroate synthase [Bifidobacterium sp.
12_1_47BFAA]
 PKC86636.1 Dihydropteroate synthase [Bifidobacterium longum]
 PKC87768.1 Dihydropteroate synthase [Bifidobacterium longum]
Length=291

 Score = 112 bits (280),  Expect = 6e-28, Method: Compositional
matrix adjust.
 Identities = 92/289 (32%), Positives = 140/289 (48%), Gaps = 31/289
(11%)

Query  4
SRVTVFGILNLTEDSFFDESRRLDPAGAVTAAIEMLRVGSDVVDVGPAASHPDARPVSPA  63
            +   V G+LN+TEDS  D    LDPA A     +M+  G+D++D+G  ++ P A+
VS A
Sbjct  11
THTMVMGVLNITEDSVSDGGLWLDPAKAAQHGRDMMAAGADIIDIGAESTRPGAKRVSEA  70

Query  64   DEIRRIAPLLDALSDQMHRVSIDSFQPETQRYALKRGVGYLNDIQGFPDPALYPD-
IAEA  122
            DE+ RI   +  L      +SID+ +      AL  G   +ND+ G    A  P
+A+
Sbjct  71
DELVRITGAVKTLIPAGAVLSIDTTRASVAAAALSEGAQIINDVSGGTLDAELPHVVADH  130

Query  123  DCRLVVMHSAQRDGIATRTG-----------
HLRPEDALDEIVRFFEARVSALRRSGVAA  171
            DC  +V H   R  +A   G           H    D  DE++R    +V  +
+GV
Sbjct  131  DCLYIVQH--WRGWLAGSKGANPDQDTSVYEHGVLTDVHDELMR----
QVDGVLAAGVKP  184

Query  172  DRLILDPGMGFFLSPAPETSLHVLSNLQKLKSALGLPLLVSVSRKSFLGATV---
GLPVK  228
            +R+I+DPG+GF   P  E +L +L+ L+  + A G P+L+  SRK F+ A +   G
Sbjct  185  ERIIIDPGLGFS-KPGIEHNLPLLTGLETFR-
ATGYPVLIGQSRKRFISAMLTEAGAAGA  242

Query  229  DLGPA-----SLAAELHAIG--NGADYVRTHAPGDLRSAITFSETLAKF  270
            D GP       + A L A+   +GA  VR H     R+A+    T  ++
Sbjct  243  D-GPIMAQRDDVTAALSALSAEHGAWAVRVHDVAKSRAAVIAGNTWREY  290


>WP_087033063.1 dihydropteroate synthase [Trichococcus palustris]
Length=259

 Score = 111 bits (278),  Expect = 6e-28, Method: Compositional
matrix adjust.
 Identities = 80/247 (32%), Positives = 127/247 (51%), Gaps = 15/247
(6%)
```

```
Query  9
FGILNLTEDSFFDESRRLDPAGAVTAAIEMLRVGSDVVDVGPAASHPDARPVSPADEIRR  68
             GILN+T DSF D         AV  AIEM+R G+D +D+G  ++ P    +S
+EI R
Sbjct  1
MGILNVTPDSFSDGGEWNTLEAAVAHAIEMVRDGADFIDIGGESTRPGHTQISDEEEIAR  60

Query  69   IAPLLDALSDQMHR-VSIDSFQPETQRYALKRGVGYLNDIQGFP-
DPALYPDIAEADCRL  126
            I P++ A+   +   +S+D+++    R A + G+  +NDI G   DP +    AE
D  +
Sbjct  61
IVPVIKAVKAAVAVPLSVDTYKSAVARAACEAGIDIINDIWGCKYDPEIAAVAAEFDVPI  120

Query  127
VVMHSAQRDGIATRTGHLRPEDALDEIVRFFEARVSALRRSGVAADRLILDPGMGFFLSP  186
            ++MH+ +         +   ED + ++       ++A    GV    +ILDPG GF
+
Sbjct  121  ILMHNRE-----AADYNFLIEDMIQDLSESMRIALAA----
GVPRHNIILDPGCGF--AK  169

Query  187  APETSLHVLSNLQKLKSALGLPLLVSVSRKSFLGATVG-
LPVKDLGPASLAAELHAIGNG  245
              E +L+V+ +L++    LG PLL++ SRK F+G  +G  P K+    + A
I NG
Sbjct  170  TFEDNLNVIHHLRRFAD-
LGYPLLLATSRKRFIGTVLGDAPFKERDEGTAATTALGIMNG  228

Query  246  ADYVRTH  252
            A   R H
Sbjct  229  AHIFRVH  235


>WP_095346990.1 dihydropteroate synthase [Bifidobacterium longum]
 PAK16983.1 dihydropteroate synthase [Bifidobacterium longum]
Length=291

 Score = 112 bits (279),  Expect = 6e-28, Method: Compositional
matrix adjust.
 Identities = 91/287 (32%), Positives = 139/287 (48%), Gaps = 27/287
(9%)

Query  4
SRVTVFGILNLTEDSFFDESRRLDPAGAVTAAIEMLRVGSDVVDVGPAASHPDARPVSPA  63
            +   V G+LN+TEDS  D    LDPA A     +M+  G+D++D+G  ++ P A+
VS A
Sbjct  11
THTMVMGVLNITEDSSSDGGLWLDPAKAAQHGRDMMAAGADIIDIGAESTRPGAKRVSEA  70

Query  64   DEIRRIAPLLDALSDQMHRVSIDSFQPETQRYALKRGVGYLNDIQGFPDPALYPD-
IAEA  122
            DE+ RI   +  L      +SID+ +      AL  G   +ND+ G    A  P
+A+
```

Sbjct  71
DELARITGAVKTLIPAGAVLSIDTTRASVAAAALSEGAQIINDVSGGTLDAELPHVVADH  130

Query  123  DCRLVVMH---------
SAQRDGIATRTGHLRPEDALDEIVRFFEARVSALRRSGVAADR  173
            DC  +V H         A  D   +   H    D  DE++R    +V  +  +GV
+R
Sbjct  131  DCLYIVQHWRGWLVGSKGANPDQDTSVYEHGVLTDVHDELMR----
QVDGVLAAGVKPER  186

Query  174  LILDPGMGFFLSPAPETSLHVLSNLQKLKSALGLPLLVSVSRKSFLGATV---
GLPVKDL  230
            +I+DPG+GF   P  E +L +L+ L+  + A G P+L+  SRK F+ A +   G
D
Sbjct  187  IIIDPGLGFS-KPGIEHNLPLLTGLETFR-
ATGYPVLIGQSRKRFISAMLTGAGTAGAD-  243

Query  231  GPA-----SLAAELHAIG--NGADYVRTHAPGDLRSAITFSETLAKF  270
            GP       + A L A+   +GA  VR H     R+A+    T  ++
Sbjct  244  GPTMAQRDDVTAALSALSAEHGAWAVRVHDVAKSRAAVIAGNTWREY  290


>WP_011068790.1 dihydropteroate synthase [Bifidobacterium longum]
 NP_696835.1 dihydropteroate synthase 1 [Bifidobacterium longum
NCC2705]
 AAN25471.1 dihydropteroate synthase 1 [Bifidobacterium longum
NCC2705]
 KEY33436.1 dihydropteroate synthase [Bifidobacterium longum subsp.
longum
EK13]
 PKC77810.1 Dihydropteroate synthase [Bifidobacterium longum]
Length=291

 Score = 112 bits (279),  Expect = 7e-28, Method: Compositional
matrix adjust.
 Identities = 91/289 (31%), Positives = 140/289 (48%), Gaps = 31/289
(11%)

Query  4
SRVTVFGILNLTEDSFFDESRRLDPAGAVTAAIEMLRVGSDVVDVGPAASHPDARPVSPA  63
            +   V G+LN+TEDSF D    LDPA A     +M+  G+ ++D+G  ++ P A+
V+ A
Sbjct  11
THTMVMGVLNITEDSFSDGGLWLDPAKAAQHGRDMMAAGAAIIDIGAESTRPGAKRVAEA  70

Query  64   DEIRRIAPLLDALSDQMHRVSIDSFQPETQRYALKRGVGYLNDIQGFPDPALYPD-
IAEA  122
            DE+ RI   +  L      +SID+ +      AL   G   +ND+ G    A  P
+A+
Sbjct  71
DELARITGAVKTLIPAGAVLSIDTTRASVAAAALSEGAQIINDVSGGTLDAELPHVVADH  130

Query  123  DCRLVVMHSAQRDGIATRTG-----------
HLRPEDALDEIVRFFEARVSALRRSGVAA  171

```
            DC  +V H   R  +A   G           H    D  DE++R    +V  +
+GV
Sbjct  131  DCLYIVQH--WRGWLAGSKGANPDQDTSVYEHGVLTDVHDELMR----
QVDGVLAAGVKP  184

Query  172  DRLILDPGMGFFLSPAPETSLHVLSNLQKLKSALGLPLLVSVSRKSFLGATV---
GLPVK  228
            +R+I+DPG+GF   P  E +L +L+ L+  + A G P+L+  SRK F+ A +   G
Sbjct  185  ERIIIDPGLGFS-KPGIEHNLPLLTGLETFR-
ATGYPVLIGQSRKRFISAMLTGAGTAGA  242

Query  229  DLGPA-----SLAAELHAIG--NGADYVRTHAPGDLRSAITFSETLAKF  270
            D GP       + A L A+   +GA  VR H     R+A+    T  ++
Sbjct  243  D-GPTMAQRDDVTAALSALSAEHGAWAVRVHDVAKSRAAVIAGNTWREY  290


>WP_046138407.1 dihydropteroate synthase [Devosia
epidermidihirudinis]
 KKC39364.1 dihydropteroate synthase [Devosia epidermidihirudinis]
Length=283

 Score = 112 bits (279),  Expect = 7e-28, Method: Compositional
matrix adjust.
 Identities = 82/256 (32%), Positives = 131/256 (51%), Gaps = 16/256
(6%)

Query  1
MLRSRVTVFGILNLTEDSFFDESRRLDPAGAVTAAIEMLRVGSDVVDVGPAASHPDARPV  60
            +L  R  V GILN+T DSF D  +      AV  A  M+  G+D++D+G  ++ P
A  V
Sbjct  16
ILGQRALVMGILNVTPDSFSDGGQHNLIENAVAHAQAMIADGADIIDIGGESTRPGAALV  75

Query  61   SPADEIRRIAPLLDAL--
SDQMHRVSIDSFQPETQRYALKRGVGYLNDIQGFPDPALYPD  118
            S  +E+ R+ P++DAL  +     +SIDS++P     A++ G   +ND+ G
P+
Sbjct  76
SVQEELDRVMPVIDALVAAGITTPISIDSYKPLVADQAIQAGASIINDVHGLQGA---PE  132

Query  119  IAEADCRLVVMHSAQRDGIATRTGHLRPEDA--
LDEIVRFFEARVSALRRSGVAADRLIL  176
            +AE    +  +HS     IA     +R E    + E+ R+FE  ++  +  GV
+R+IL
Sbjct  133  MAE----IAALHSVPV--
IAMHWDKVRDEAKPIIAEMARYFETTLALAKAVGVGRERIIL  186

Query  177
DPGMGFFLSPAPETSLHVLSNLQKLKSALGLPLLVSVSRKSFLGATVGLPVKDLGPASLA  236
            DPG GF  S A   +  VL  L +++   G+P+LV  SRKS +G  + +P  +
++A
Sbjct  187  DPGFGFGKSLA--ENYEVLRELTEVQPR-
GMPILVGTSRKSMIGRLLDIPADERLAGTIA  243
```

```
Query  237  AELHAIGNGADYVRTH  252
              +     G    R H
Sbjct  244  TTVAGYLKGGHIFRVH  259


  Database: All non-redundant GenBank CDS translations+PDB+SwissProt
+PIR+PRF
excluding environmental samples from WGS projects
    Posted date:  Mar 26, 2018  2:20 PM
  Number of letters in database: 248,125,090
  Number of sequences in database:  703,403

Lambda      K        H
   0.321    0.137    0.392
Gapped
Lambda      K        H
   0.267   0.0410    0.140
Matrix: BLOSUM62
Gap Penalties: Existence: 11, Extension: 1
Number of Sequences: 703403
Number of Hits to DB: 48261
Number of extensions: 32
Number of successful extensions: 11
Number of sequences better than 100: 4
Number of HSP's better than 100 without gapping: 0
Number of HSP's gapped: 8
Number of HSP's successfully gapped: 4
Length of query: 283
Length of database: 248125090
Length adjustment: 118
Effective length of query: 165
Effective length of database: 165123536
Effective search space: 27245383440
Effective search space used: 27245383440
T: 21
A: 40
X1: 16 (7.4 bits)
X2: 38 (14.6 bits)
X3: 64 (24.7 bits)
S1: 41 (20.4 bits)
S2: 61 (28.1 bits)
ka-blk-alpha gapped: 1.9
ka-blk-alpha ungapped: 0.7916
ka-blk-alpha_v gapped: 42.6028
ka-blk-alpha_v ungapped: 4.96466
ka-blk-sigma gapped: 43.6362
```
